# Insights into pathological mechanisms and interventions revealed by analyzing a mathematical model for cone metabolism

**DOI:** 10.1042/BSR20212457

**Published:** 2022-03-08

**Authors:** Atanaska Dobreva, Erika Tatiana Camacho, Kamila Larripa, Anca Rǎdulescu, Deena R. Schmidt, Imelda Trejo

**Affiliations:** 1 School of Mathematical and Natural Sciences, Arizona State University, Glendale, AZ, U.S.A.; 2 Department of Mathematics, Humboldt State University, Arcata, CA, U.S.A.; 3 Department of Mathematics, State University of New York at New Paltz, New Paltz, NY, U.S.A.; 4 Department of Mathematics and Statistics, University of Nevada, Reno, NV, U.S.A.; 5 Theoretical Biology and Biophysics Group (T-6), Theoretical Division, Los Alamos National Laboratory, Los Alamos, NM, U.S.A.

**Keywords:** Bifurcation Analysis, Metabolism, Oxidation of fatty acids, Photoreceptors, Retina, Sensitivity Analysis

## Abstract

This work analyzes a mathematical model for the metabolic dynamics of a cone photoreceptor, which is the first model to account for energy generation from fatty acids oxidation of shed photoreceptor outer segments (POS). Multiple parameter bifurcation analysis shows that joint variations in external glucose, the efficiency of glucose transporter 1 (GLUT1), lipid utilization for POS renewal, and oxidation of fatty acids affect the cone’s metabolic vitality and its capability to adapt under glucose-deficient conditions. The analysis further reveals that when glucose is scarce, cone viability cannot be sustained by only fueling energy production in the mitochondria, but it also requires supporting anabolic processes to create lipids necessary for cell maintenance and repair. *In silico* experiments are used to investigate how the duration of glucose deprivation impacts the cell without and with a potential GLUT1 or oxidation of fatty acids intervention as well as a dual intervention. The results show that for prolonged duration of glucose deprivation, the cone metabolic system does not recover with higher oxidation of fatty acids and requires greater effectiveness of GLUT1 to recover. Finally, time-varying global sensitivity analysis (GSA) is applied to assess the sensitivity of the model outputs of interest to changes and uncertainty in the parameters at specific times. The results reveal a critical temporal window where there would be more flexibility for interventions to rescue a cone cell from the detrimental consequences of glucose shortage.

## Introduction

Photoreceptors, both cones and rods, are essential in vision, and metabolic dysfunction within photoreceptors can lead to irreversible vision loss and blindness [1]. Photoreceptors are the most metabolically demanding cells in the body and are in constant need of glucose, lipids, and other metabolites for maintenance [2–4]. Glucose is the main energy source for photoreceptors, which is metabolized through aerobic glycolysis [4]. The retinal pigment epithelium (RPE) supplies the rods and cones the glucose it receives from the blood circulation and utilizes it for its own metabolic needs if fuels for its energy production, namely lactate and *β*-hydroxybutyrate (*β-*HB), are scarce. Through a complex that it forms with Basigin-1 (BSG1) at the cone surface, glucose transporter 1 (GLUT1) regulates and facilitates glucose transport from the RPE into the cones. The binding of rod-derived cone viability factor (RdCVF) to the BSG1–GLUT1 complex stimulates the glucose transport activity and accelerates glucose uptake by the cones [2,3,5]. How much RdCVF is synthesized depends on the number of rods present in the retina system [6].

Together, the RPE, cones, and rods act as symbiotes: the RPE provides glucose to the photoreceptors, the rods help the cones via RdCVF, and both rods and cones produce lactate and shed outer segments whose fatty acids are used to fuel the RPE [7,8]. When glucose is scarce, rods and cones can also generate energy from external lactate [9] and oxidized fatty acids [9,10]. Phagocytosis and digestion of photoreceptor outer segments (POS) by the RPE is followed by activation of the fatty acid *β*-oxidation and ketogenesis pathways [10]. The RPE cells metabolize fatty acids derived from shed POS and through *β*-oxidation create ketone bodies in the form of *β*-HB. During glucose starvation, *β*-HB is metabolized and used in the Krebs, or tricarboxylic acid (TCA) cycle, to fuel oxidative phosphorylation (OXPHOS) and adenosine triphosphate (ATP) production in the RPE [8,10] and in photoreceptors [1,9–13].

The POS renewal and shedding are critical to the creation of this alternative substrate for oxidative metabolism [9]. As POS renewal requires lipids, it relies on the diversion of glucose to the Kennedy pathway (KP) to produce glycerol-3-phosphate (G3P), the precursor of glycerol for lipid synthesis [14,15]. A delicate balance of the above processes and mechanisms that drive photoreceptors’ glucose uptake, energy production and structural maintenance is required to keep the photoreceptor cells viable and functioning properly, especially under glucose-deficient conditions.

Additionally, lactate from photoreceptors can be utilized to meet the retina’s energy needs. Pyruvate produced through glycolysis or converted from external lactate can be utilized for photoreceptor OXPHOS and energy production. Lactate dehydrogenase A (LDHA) converts pyruvate into lactate, and lactate dehydrogenase B (LDHB) converts lactate into pyruvate. Experimental findings show that cones can use rod-derived lactate as an alternative fuel when glycolysis is inhibited [16]. Using LDHB, photoreceptors convert lactate into pyruvate which then goes into the mitochondria to facilitate energy production. Other intermediate metabolites could also sustain the TCA cycle in the short run, as shown in *in vitro* experiments where glucose was withdrawn from photoreceptors, but they were able to maintain oxidative metabolism for 90 min if supplemented with pyruvate, malate, glutamine, and leucine [4]. Glucose deprivation beyond 90 min, led to photoreceptors’ death despite this cocktail of intermediate metabolites [4].

When glucose is scarce, the expression of the glycolysis enzyme hexokinase-2 (HK2) increases [1]. HK2 helps photoreceptors to retain glucose for glycolysis [1,2,4]. Cones’ function in mice remains unaffected by HK2 loss, which reveals their ability to utilize alternative intermediate metabolites, such as *β*-HB, and thereby adapt to the inhibition of aerobic glycolysis [16]. In addition, experiments in mice have shown that if GLUT1 expression falls below a certain critical threshold then POS renewal and photoreceptor cell survival are negatively affected, but the impact was found to be greater for rods (reflected by 50% reduction in rod POS length and a 50% rod cell death) than for cones (reflected by 10% reduction in cone POS length and 17% reduction in cone cell number) [17]. This reveals a potential temporary cone resilience and adaptation capability due to potential auxiliary and intermediate metabolites.

It is well-established that photoreceptor degeneration in diseases such as age-related macular degeneration (AMD) and retinitis pigmentosa (RP) is intricately linked to glucose deprivation and alterations in cellular metabolism. As such, understanding the role of metabolites under glucose starvation conditions may elucidate pathways which result in these pathologies. In RP, one widely accepted hypothesis for the secondary wave of cone death is the insufficient glucose uptake by cones, which results from a drastic reduction in RdCVF as the rods degenerate, since RdCVF is only produced by rods [5,11,15]. The significant decrease in glucose uptake leads to a decrease in the length of cone outer segments, and eventually, central blindness [15]. Cones in RP up-regulate GLUT1 and HK2 [18,19], which indicates that the cells are attempting to metabolize more glucose through aerobic glycolysis, perhaps in response to the diminished RdCVF.

Mechanisms that affect photoreceptors’ glucose supply, transport, and uptake as well as POS renewal and phagocytosis affect the supply of auxiliary/alternate metabolites for ATP production, such as *β*-HB and amino acids. All these elements are critical in mitigating photoreceptor and RPE degeneration [12]. For example, in AMD, drusen formation between the RPE and the underlying Bruch’s membrane blocks the transfer of glucose from the choroidal blood supply to the RPE. This deprives photoreceptors of glucose, leading to an imbalance in their cellular metabolic energy demands, similar to what is observed in RP [1]. Without sufficient glucose, cones produce less lactate for the RPE’s metabolism. In addition, AMD is often associated with deficiencies in lactate clearance, which can lead to complications in glucose transport and ultimately impairment of cone function, aerobic glycolysis, and POS renewal [7]. The build-up of lactate in the RPE and/or the inter-photoreceptor matrix counteracts the efflux of lactate from the cones and subsequent lactate transport into the RPE. The reduction in lactate in the RPE drives these cells to use glucose as a substrate for their own metabolism rather than transporting it to the photoreceptors [7]. This further decreases the glucose supply to cones and rods, exacerbating their starvation and inability to effectively meet their energy and structural maintenance demands [1,11]. The development of RP and AMD not only causes a significant decline in the amount of glucose available to the photoreceptors, but also interferes with the transport of auxiliary metabolites, such as *β*-HB, which are instrumental under starvation and glucose-deprived conditions [20]. Disruptions in the renewal or clearance of the POS, largely due to protein build-up or accumulation of fatty lipid-rich deposits and debris, can impair this auxiliary ATP resource response [10].

Retinal metabolic imbalances have been studied with mathematical models including the temporal population dynamics of rods and cones [2,21]. Analyzing the qualitative behavior of such models via bifurcation analysis has helped to identify the one-way interaction from rods to cones via RdCVF, determine the conditions for coexistence of cones and rods, and detect key processes implicated in the progression of RP, including tracking its various stages [5,22–24]. In Camacho et al*.* (2020), we constructed a mathematical model and focused on the modulating effect of external lactate in a single cone cell and the levels of lactate, glucose, and pyruvate [25]. The model incorporates energy production for the cone via oxidation of fatty acids and extracellular lactate—pathways that were not accounted for in previous models. It represents a setting where *β*-HB is only used by the cones, as the pathways for *β*-HB feeding into the rods and RPE are not included. This model design allows to keep the dynamics focused on cone behavior. For the same reason, the model does not incorporate explicitly any RPE and rod compartments, and feedback mechanisms in the cone metabolic pathways mediated by the RPE and rod cells are captured via parameters and proxy terms.

In the current paper, we examine the interplay in the cone cell between glucose modulation by the RPE and energy generation from oxidized fatty acids. In particular, applying multiple parameter bifurcation analysis, we study the effects of joint variations in external glucose availability, the effectiveness of GLUT1, and the utilization of lipids and *β*-HB. The results show that the interactions among these processes impact the cone’s metabolic vitality and its ability to change dynamic course under glucose scarcity. For example, increasing external glucose availability and GLUT1’s efficiency in tandem provides a more effective way to achieve a healthy metabolic state compared with when they are increased independently. Also, for a set of different combinations of external glucose availability and utilization of lipids and *β*-HB, increasing the effectiveness of GLUT1 expands the possibility for an outcome with higher levels of metabolites. The bifurcation analysis further indicates that energy production in the mitochondria is not enough to maintain cone viability under glucose scarcity, but supporting anabolic processes to create lipids necessary for cell maintenance and repair is also required. Empirical evidence suggests that the outcome of cell recovery after glucose starvation heavily depends on the amount of time photoreceptors are deprived of glucose [4]. In light of this, we use the mathematical model to generate computational illustrations of glucose deprivation and short-term rescue of oxidative substrates through supplementation of exogenous energy fuels. Furthermore, we conduct *in silico* experiments to study how different modes of cellular operation, without and with a potential GLUT1 and/or *β*-HB intervention, may affect the longer term recovery of oxidative and glycolytic metabolites as well as of G3P after glucose shutdown. The results show that with higher oxidation of fatty acids, the cone metabolic system does not recover after prolonged glucose deprivation and requires greater GLUT1 efficiency to recover. Additionally, we apply time-varying global sensitivity analysis (GSA) to capture over time key mechanisms defined by the model parameters, whose changes or uncertainty impact the model output. The results show very high sustained sensitivity of the model outputs and their respective variability to changes in the input values of G3P utilization and oxidation of fatty acids and the uncertainty in these inputs. Additionally, the sensitivity analysis reveals the first 100 min of the simulation as a temporal window presenting greater flexibility for interventions to help a cone cell under glucose deprivation, which is consistent with *in vitro* experimental results for photoreceptors in glucose-deprived conditions showing cellular stress and death within 90 min [4]. The ‘Discussion’ section will address the relation of the above results to certain known features characterizing the development of AMD and RP and some potential therapeutic options. However, it should be noted that the present study is not a direct investigation into these pathologies.

## Methods

### Mathematical model

**Figure 1:**
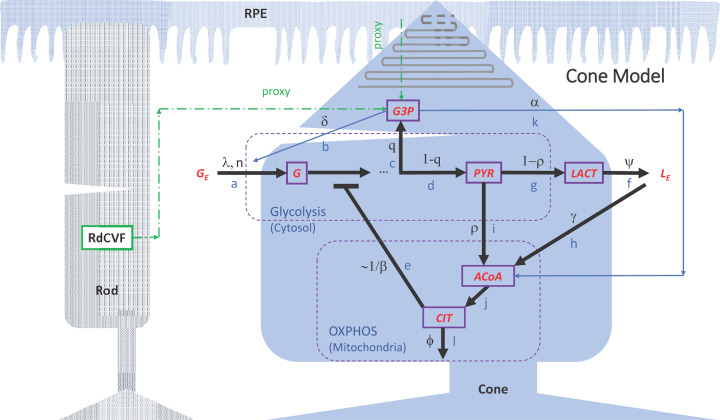
Model diagram of key metabolic pathways within a single cone photoreceptor The diagram was modified from Camacho et al*.* (2020) [25] to show interactions of the cone with the RPE and a rod photoreceptor (dashed green arrows). Parameters α and δ used in the cone model are defined as proxies for interactions with the RPE and rods. To isolate the impact on the cone cell of changes in glucose modulation by the RPE in response to reduction in RPE’s metabolic fuel, the pathways for energy generation in the RPE and rods via β-HB are not included. Model variables are represented by boxes with purple border: glucose (G), G3P, PYR, lactate (LACT), ACoA, and CIT. Metabolic pathways are labeled with blue letters: (**a**,**b**) Glucose transport and uptake (without and with RdCVF); (**c**) G3P synthesis; (**d**) Pyruvate synthesis; (**e**) Glycolysis inhibition; (**f**) Outward lactate transport; (**g**) Lactate synthesis; (**h**) Inward lactate transport for ACoA production; (**i**) Pyruvate conversion into ACoA; (**j**) Citrate synthesis; (**k**) ACoA synthesis from β-HB; (**l**) Diversion of citrate to the cytosol and other pathways. Pathway-associated parameters are labeled with black letters, and external glucose and lactate are denoted by *G_E_* and *L_E_*, respectively. Parameter descriptions are provided in Table A1 in Appendix A.

The flow diagram in Figure 1 represents the metabolic pathways model for a single cone (governing equations shown in Appendix A), which we developed in Camacho et al*.* (2020) [25]. Mass-action, Michaelis–Menten kinetics and allosteric regulations were applied to the relevant parts of the variable interactions, following key features of photoreceptor biochemistry [2,26,27]. The model consists of 25 parameters defining various metabolic kinetic processes that affect the concentrations of internal glucose ([G]), pyruvate ([PYR]), G3P ([G3P]), acetyl coenzyme A ([ACoA]), citrate ([CIT]), and lactate ([LACT]) within a cone cell; see Table A1 in Appendix A for parameter descriptions. Note that we define glycerol-3-phosphate as G3P, which should not be confused with glyceraldehyde-3-phosphate (abbreviated as GAP, G3P, and GA3P in some literature).

External glucose that the RPE makes avaible to the cone is reflected through the parameter *G_E_* in our model, and the parameter λ measures the efficiency of GLUT1 to transport glucose into the cone cell. The RPE and rod cells are not explicitly incorporated in the model, so two intermediate metabolites, namely G3P and ACoA, are considered as sources of feedback mechanisms in the metabolic pathways mediated by the RPE and rod cells. In the model, these two metabolites provide feedback mechanisms for fatty acids transported to and uptaken by the cone cell. The parameter α capturing the utilization of G3P is a proxy for the lipids that are phagocytosed by the RPE as well as the fatty acids that are *β*-oxidized and converted into *β*-HB, which are transported from the RPE to the cone, ultimately contributing to ACoA synthsis in the cone cell. In addition, in our model G3P was used to approximate the rods since the G3P concentration in a healthy cone cell could be considered an indicator for rod abundance, which depends on the POS renewal capacity, namely, lipid creation and utilization. For this approximation, the scaling factor δ was used to reflect that in a healthy retina there is approximately one cone for every 20 rods synthesizing RdCVF [25].

The intermediate metabolite ACoA is produced from pyruvate, via the pathways represented by ρ and γ, as well as from oxidized POS fatty acids, quantified with the parameter α. The parameter ρ reflects the fraction of pyruvate that leaks into the mitochondria by the mitochondrial respiratory chain, while γ captures the process of converting external lactate taken up from the external pool back into pyruvate by LDHB and bringing it into the cone cell to fuel the TCA cycle. The intermediate metabolite CIT provides a self-regulating mechanism through its inhibition of phosphofructokinase (PFK), measured by the parameter *β*. If CIT builds up, it signals the cell that the TCA cycle is backed up and does not need more intermediates to create ATP, slowing down glycolysis [28,29].

### Bifurcation analysis

Building from our previous work in Camacho et al. (2020) [25], in this study we use multiple parameter bifurcation analysis to examine changes in solutions of the system in the long run and changes in their corresponding stability as a set of multiple parameters are varied. We utilize bifurcation analysis to examine changes in the number of the constant (also known as equilibrium) solutions of the system and changes in the stability of these solutions, as one or more parameters are varied while the remaining parameters are fixed at given values (usually at their baseline). In one of the simplest situations, for a given initial condition of the system (representing concentration levels of the various metabolites), the solution will approach a stable equilibrium in the system. Equilibrium solutions can emerge or disappear via a bifurcation, a qualitative change in the system’s dynamics produced by varying the parameter values. When a stable equilibrium solution exists, a saddle node bifurcation is one type of bifurcation that can give rise to a second stable equilibrium whereby a pair of equilibria (one stable and one unstable) are born simultaneously. In the event that two stable equilibrium solutions are present (bistability), the initial condition will determine which equilibrium solution the system will approach. Having bistability regions in our system allows for the possibility of moving the system away from one stable state (e.g., the pathological state) to another stable state (e.g., of higher metabolites’ levels). In tracking the equilibrium solutions, it is important to note the emergence of different regions of bistability in the empirical parameter space where two (or more) stable equilibrium solutions occur. Bifurcation analysis allows us to identify the mechanisms, defined by the parameters, that need to be varied or perturbed to achieve this. Our numerical results identified the existence of two stable solutions that are present for glucose levels corresponding to a healthy state and a pathological state. The bifurcation analysis was performed with the programming and computation software MATLAB (MathWorks Inc., Natick, MA, U.S.A.) using the graphical package MATCONT [30].

While studying the effects of single parameters can be helpful with explaining direct response mechanisms, it is only by considering the joint effects of changing several parameters simultaneously that we gain insight into more complex phenomena. Thus, in the present paper, with bifurcation analysis involving multiple parameters we show how changes in combinations of key processes can transition the cone from a pathological to a healthy regime and *vice versa*. Through this more involved analysis we are able to elucidate aspects of the dynamic consequences of varying α, GLUT1 transport effectiveness (quantified by λ), and *G_E_* that were not evident in the single parameter bifurcation analysis in Camacho et al. (2020) [25].

Since internal glucose is the main fuel for the cone’s operations, and G3P is most essential for its structural maintenance, one would expect the long-term prognosis of cellular viability to be critically affected by the glucose and G3P dynamics. As mentioned earlier, the cone’s internal glucose level is principally controlled by the external glucose modulated by the RPE and the efficiency of GLUT1, the transporter allowing the entry of glucose from the RPE into the cone cell. Thus, in combination *G_E_* and λ reflect how the RPE directly affects the cone’s glucose supply, so we first examine the joint effects of these parameters on the cone’s intracellular glucose ([G]) and G3P concentration ([G3P]). Next, we investigate how the interplay between *G_E_* and the processes responsible for essential alternative metabolites for cone energy production, such as *β*-oxidation of POS fatty acids (incorporated by α), affect [G] and [G3P]. This analysis brought to light (through α by virtue of its connection to G3P and the role of G3P as a proxy for rods) how the cone is affected as the level of rods synthesizing RdCVF changes in response to fluctuations in the glucose modulated by the RPE.

To study the combined effects of *G_E_*, λ, and α using bifurcation analysis, we vary two parameters within their physiological ranges given while setting the third to its baseline in Table 1. We also examine the joint variations of *G_E_* and α for three different values of λ in the parameter set. For the entire analysis, the processes and mechanisms defined by parameters other than *G_E_*, λ and α are specified by their nominal values in Table 1.

**Table 1:** Parameter ranges used in eFAST and PRCC

Par/IC	Baseline	eFAST	PRCC	PRCC
		[G](0) = 0.02 & [G](0) = 2	[G](0) = 0.02	[G](0) = 2
λ	0.0755	[0.062, 0.093]	[0.062, 0.084]	[0.062, 0.093]
*V*_[G__]_	1.2	[0.1, 1.56]	[0.1, 1.47]	[0.1, 1.56]
*K*_[G]_	19	[5, 24.7]	[5, 24.7]	[5, 24.7]
*n*	0.001	[0.0007, 0.0013]	[0.0007, 0.0012]	[0.0007, 0.0013]
*δ*	65	[45.5, 95]	[45.5, 72]	[45.5, 95]
*q*	0.18	[0.04, 0.2]	[0.04, 0.2]	[0.04, 0.2]
*V*_[G3P]_	0.15	[0.12, 0.18]	[0.12, 0.18]	[0.12, 0.18]
*K*_[G3P]_	0.143	[0.02, 0.171]	[0.08, 0.171]	[0.02, 0.171]
*V*_[PYR]_	0.15	[0.0013, 0.3915]	[0.0013, 0.3915]	[0.0013, 0.243]
*K*_[PYR]_	1.7	[0.05, 2.21]	[0.05, 2.21]	[0.66, 2.21]
*β*	1	[0.7, 1.3]	[0.7, 1.3]	[0.7, 1.3]
*α*	0.2	[0.002, 1]	[0.002, 1]	[0.002, 1]
*ρ*	0.05	[0.04, 0.0895]	[0.04, 0.0895]	[0.04, 0.0895]
*V*_[LACT]_	0.14	[0.098, 0.33]	[0.098, 0.33]	[0.098, 0.33]
*K*_[LACT]_	0.125	[0.0875, 10]	[0.0875, 10]	[0.0875, 10]
*V*_[ACoA]_	0.15	[0.105, 0.195]	[0.105, 0.195]	[0.105, 0.195]
*K*_[ACoA]_	0.02	[0.005, 0.026]	[0.005, 0.026]	[0.005, 0.026]
*ϕ*	1	[0.7, 1.3]	[0.7, 1.3]	[0.7, 1.3]
*V*_[CIT]_	0.03	[0.021, 0.039]	[0.03, 0.0326]	[0.021, 0.032]
*K*_[CIT]_	0.0054	[0.0046, 0.00702]	[0.00531, 0.0056]	[0.0046, 0.00702]
ψ	8	[6.4, 9.6]	[6.4, 9.6]	[7.68, 8.57]
κ	10	[7, 13]	[9.84, 10.2]	[7, 13]
γ	1	[0.7, 1.3]	[1, 1.07]	[0.7, 1.3]
*G_E_*	11.5	[5, 20]	[5, 20]	[5, 20]
*L_E_*	10	[5, 22]	[10, 22]	[10, 12.22]
[G]_0_			[0.02 * 0.7, 0.02 * 1.3]	[2 * 0.7, 2 * 1.3]
[G3P]_0_			[0 * 0.7, 10−4]	[0 * 0.7, 10−4]
[PYR]_0_			[0 * 0.7, 10−4]	[0 * 0.7, 10−4]
[LACT]_0_			[9.4 * 0.7, 9.55]	[9.4 * 0.7, 9.4 * 1.3]
[ACoA]_0_			[0 * 0.7, 10−4]	[0 * 0.7, 10−4]
[CIT]_0_			[0 * 0.7, 10^−4^]	[0 * 0.7, 10−4]

Parameter ranges under the eFAST column are the full physiological ranges. Where necessary, parameter ranges were restricted to maintain monotonicity required to implement PRCC. Abbreviations: eFAST, Extended Fourier Amplitude Sensitivity Test; PRCC, partial rank correlation coefficient.

*The upper limit for *K*_[ACoA]_ is 30% above its baseline value, since the baseline is the right end point of the parameter interval in Camacho et al. (2020), and based on further physiological considerations, the upper limit for ρ is larger than the value in Camacho et al. (2020) [25].

### Computational experiments

We use the model to generate computational illustration of glucose deprivation and short-term rescue of CIT and ACoA metabolites through supplementation of exogenous fuels from fatty acids oxidation and external lactate conversion into pyruvate. To simulate glucose deprivation we consider the time frame 0–120 min, which is informed by *in vitro* experimental work on mouse retinas under glucose deprivation [4]. We set the external glucose (*G_E_*) and initial internal glucose ([G](0)) to very low levels, and then we equate *G_E_* to the internal glucose concentration throughout the simulation. The latter enforces no glucose gradient differential, which shuts down glucose uptake and further decreases the internal glucose to zero.

This scenario is first accompanied by scarcity of alternative energy resources, which we simulate through blocking pathways h and k in the model (Figure 1), which blocks the provided substrates for ACoA production and subsequent CIT synthesis via exogenous resources. The h pathway describes converted pyruvate from external lactate (*L_E_*) that enters into the TCA cycle, captured by the parameter γ, where the gradient of lactate flow into the cell is governed by the parameter*, L_E_*. The k pathway represents the dynamics of fatty acids from ingested POS, which are oxidized to make β-HB, captured by the parameter α. *L_E_* is set to a level that prevents lactate uptake by the cell, and thereby energy generation from external lactate. The initial G3P level ([G3P](0)) is set to zero to reflect absence of lipids, precursor of POS and their fatty acids, and consequently blocked β-HB production.

In summary, to illustrate the positive effect of supplementation with external lactate, we simulate the glucose shutdown and blocked k pathway on 0–120 min together with a boost along the h pathway via increasing *L_E_*. This promotes gradient-based flow of lactate into the cell. To illustrate the positive effect of supplementation via fatty acids, we simulate the glucose shutdown and blocked h pathway on 0–120 min together with a boost along the k pathway by an increase in the G3P initial concentration. This provides lipids for POS whose fatty acids undergo oxidation for β-HB synthesis.

In a second set of *in silico* experiments performed with our model, we explore how different operation modes of the cellular system, without and with a potential GLUT1 intervention mitigate the impact of the duration of glucose shutdown. We captured both longer and short-term recuperation of all six metabolites (G, G3P, PYR, LACT, ACoA and CIT) by changing the efficiency of GLUT1 (quantified by λ) and/or the β-oxidation of POS fatty acids (quantified by α). For this, in addition to glucose shutdown throughout the whole time window, we also model temporary glucose shutdown lasting 90 min or less. The 90-min shutdown is achieved by preventing glucose uptake between t = 40 and t = 130 min with external glucose (originally fixed at *G_E_* = 10) equated to the internal glucose concentration, and then restoring *G_E_* to its original level, so glucose uptake can resume. The shorter period of glucose deprivation is simulated by restoring glucose uptake earlier. Source code for both sets of simulation experiments was implemented in and run with numerical ODE solver routines in MATLAB (https://www.mathworks.com/help/matlab/ref/ode45.html; https://www.mathworks.com/help/matlab/ref/ode15s.html).

### GSA

We utilize sensitivity analysis to determine which parameters (and thus the processes they represent) have the greatest impact on the system described by the mathematical model, as well as how the uncertainty in their experimental measurements or mathematical estimations may manifest in the variability of the metabolic outputs of interest. For the reasons stated earlier, we again mainly focus on internal glucose and G3P. We apply two different GSA approaches where all parameters are varied simultaneously: Partial Rank Correlation Coefficient (PRCC) and Extended Fourier Amplitude Sensitivity Test (eFAST). The information provided by PRCC reveals how the output of a model is affected if a parameter is altered, and this is complemented by the variance-based method eFAST that determines the parameters whose uncertainties have the largest impact on output variability [31–33]. In PRCC, in addition to model parameters, initial conditions can also be included as input factors in the analysis. Table 1 shows the ranges over which GSA input factors are varied. Where necessary, ranges were restricted to meet the PRCC’s requirement for a monotonic relationship between changes in the inputs and the output [31].

PRCC values with a magnitude greater than 0.4, i.e., |PRCC| > 0.4 (for our purposes), and statistical significance (small* P*-value) are the criteria for the outcome of interest being sensitive to the input factors [34]. A negative PRCC value means that input increase results in output decrease, and a positive PRCC means that input increase results in output increase [31]. The eFAST method supplies a first-order sensitivity index, which quantifies the contribution to outcome variance of a single parameter on its own, and a total-order sensitivity index, which includes the parameter’s individual contribution to variance as well as contributions due to its higher order interactions with the remaining parameters. A parameter is classified as important if it stands out with a large total-order index relative to other parameters [31–33].

We applied the GSA methodologies over time to capture the transient as well as longer term temporal impact of parameter variations, which reflects the dynamic influence of changes in processes driving the system. The GSA was conducted for low ([G](0) = 0.02 mM) and higher ([G](0) = 2 mM) initial internal glucose level, informed by an experimental study on cone-dense pig retina [35]. [G](0) = 0.02 mM serves to indicate severe nutrient stress conditions, and [G](0) = 2 mM is within a range considered in the experiments as sufficient for cone operations. The temporal window was 0–240 min, in accordance with the interval from experiments where mouse retinas were placed in glucose deprivation conditions for a few hours [4]. The PRCC computations were performed using 200 random samples of input factor values, and Figures 7 and 8 (left) include the PRCC plots for only the inputs that are influential in the window 0–240 min, that is, |PRCC| > 0.4 somewhere during this time interval. Implementing the dynamic eFAST analysis we relied on the most recent developments in the methodology for computing variance-based sensitivity measures when time-varying outcomes of interest are considered [36]. We used 325 random samples of values in the parameter space to calculate the eFAST indices, Figures 7 and 8 (right). The index plot for a parameter was included only if the parameter was deemed influential somewhere in the window 0–240 min, that is, its corresponding index curve stands out and lies above other index curves that are grouped and cannot be distinguished from one another. The GSA codes, which were implemented in and run with MATLAB, use random number generation (https://www.mathworks.com/help/matlab/ref/rand.html), statistics (https://www.mathworks.com/help/stats/tiedrank.html; https://www.mathworks.com/help/stats/regress.html; https://www.mathworks.com/help/stats/corr.html; https://www.mathworks.com/help/matlab/dataanalysis/descriptive-statistics.html) and trigonometry routines (https://www.mathworks.com/help/matlab/trigonometry.html), and were modified and adapted from codes available at http://malthus.micro.med.umich.edu/lab/usadata/ [31]. We wrote source code for eFAST over time using the MATLAB package for trapezoidal numerical integration (https://www.mathworks.com/help/matlab/ref/trapz.html).

## Results

### Combined effects of external glucose availability and the efficiency of GLUT1

**Figure 2:**
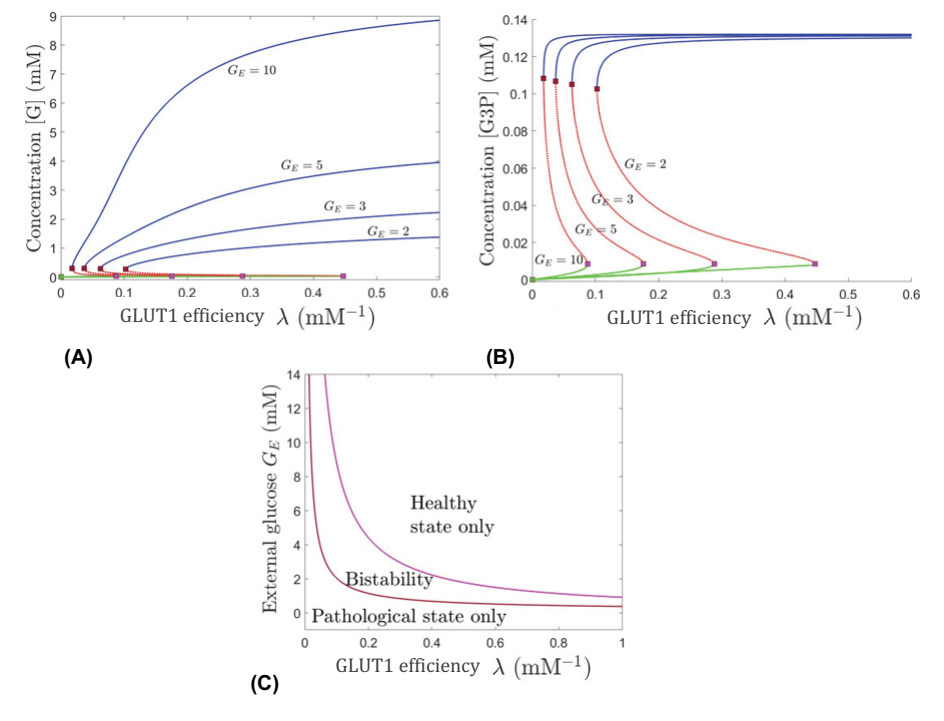
Bifurcations in dynamics with respect to the glucose transport factor λ, which serves as a quantifier for the efficiency of GLUT1, for different levels of the external RPE-mediated glucose *G_E_* Evolution of the [G] (**A**) and [G3P] (**B**) components of the system’s equilibria, as λ is increased; blue (healthy) and green (pathological) curves are stable while red is unstable. Different curves represent the equilibrium curves for different levels of *G_E_*, from right to left: *G_E_* = 2, 3, 5, and 10 mM. Two saddle node transitions occur along each equilibrium curve for all values of *G_E_*. (**C**) Regions of unistability and bistability delimited in the (λ, *G_E_*) slice of the parameter space. The value of α was fixed to 0.2 min^−1^.

Panels (A,B) in Figure 2 contain bifurcation diagrams showing the steady-state concentrations of the cone’s internal glucose, [G], and G3P with respect to λ (which describes the effectiveness of GLUT1) for several different levels of *G_E_*, from 2 to 10 mM. These diagrams illustrate qualitative effects on the long-term metabolic dynamics of the cone, such as changes in the number and stability of steady states, i.e., equilibria, brought about by joint variations in external glucose and the GLUT1 transporter’s efficiency. The system has two connected saddle noddle bifurcations. A pair of a stable equilibrium (represented as the blue or green curve) and an unstable equilibrium (represented as a red curve) undergo a saddle node bifurcation and annihilate each other at the bifurcation point (represented by red or pink square). The blue curve is associated with the healthy state, where glucose, G3P, and the other metabolites of the system are at levels indicative of normal physiological functioning of the cone, and the green curve is associated with the pathological state, where the metabolite levels are not sufficient to support normal cellular operations. Mathematically, either one of these two locally stable equilibria can be attained in the bistability region between two saddle node bifurcation curves in panel (C) in Figure 2. Depending on the initial levels of metabolites, the system will go to either the healthy or pathological state in this bistability region. Panel (C) in Figure 2 illustrates the stability regimes within different regions of the (λ, *G_E_*) parameter plane. Depending on the parameter values, the system may have access to either the healthy state only, to the pathological state only, or to both, as long-term stable states.

Figure 2B shows that for any level of *G_E_*, a decrease in λ in the healthy or pathological state, translates mainly into a slightly lower concentration of G3P. In the bistability region (where both the pathological and healthy states are possible) in Figure 2C, depending on the initial state of the system and the *G_E_* value, the change in G3P can be categorized in one of the four ways. Either the G3P change is drastic or slight, and the achieved steady state is at a viable level of cellular operations (as depicted by the blue curves in Figure 2B), or the achieved steady state is at an extremely low G3P level insufficient to sustain cellular operation (as depicted by the green curves in Figure 2B). For a cone with parameter values in the bistability region, the lower *G_E_*, the more efficient GLUT1 (i.e., larger λ) must be to allow glucose to enter the cell and drive the system to the healthy levels of G3P (corresponding with moving from the bistability region to the healthy state region in Figure 2C). However, the ability to enhance G3P and move into a healthy state by changing the efficiency of GLUT1, categorized by changing λ, is limited. In the long run, the G3P outcome is pretty much the same for all different external glucose levels, as illustrated in the blue or green curves in Figure 2B, indicating that the system will take only as much as it needs to produce new lipids. This is not the case for the internal glucose concentration, [G], where the steady state levels are drastically different for different external glucose levels associated with the healthy state (as illustrated in blue in Figure 2A). These effects are enhanced when the external glucose levels are high (see the curves for *G_E_* = 5 and 10 mM in Figure 2A), and are diminished under low external glucose (see the curves for *G_E_* = 2 and 3 mM).

If the GLUT1 transporter is extremely inefficient, corresponding to a very small λ value, no amount of external glucose will move the system out of the pathological state, as suggested by the presence of the bottom region of Figure 2C. As one would expect, increasing GLUT1’s efficiency to bring glucose into the cell through λ generally promotes a healthy state as long as the level of external glucose is appropriate. If *G_E_* is at an extremely low level (less than approximately 0.3 mM), the system can never leave the pathological state region just by increasing the efficiency of GLUT1 (the magnitude of λ). For *G_E_* between approximately 0.5 and 0.8 mM, the system is able to enter the bistability region but remains there and cannot reach the healthy (only) region even if λ is increased, if all parameters remain fixed. For *G_E_* between approximately 1 and 1.2 mM pushing out of the bistability region to the healthy region is possible but requires a very large increase in λ. For *G_E_* levels 3 mM or above, a small increase in λ can move the system from the pathological (only) region to bistability and potentially to the healthy only regime, if *G_E_* is sufficiently high. Also, as *G_E_* increases, the system is better able to be in the bistability region even when λ becomes very low.

Depending on the system’s initial state or history, a cone in the bistability region can either end up in the pathological or in the healthy state. For levels of external glucose that allow the system to be in the bistability region, making GLUT1 more efficient in transporting glucose into the cone moves the system into a regime of increased internal glucose that guarantees convergence to a healthy state (i.e., blue curve). Therefore, to more effectively push the system from the pathological state to a healthy state, within a prescribed context of fixed processes and mechanisms depicted by the fixed parameter values, we would need to increase *G_E_*, or λ, or both. Increasing just one of these two parameters represents a successful strategy to move the system to the bistability region or the healthy state regime only if the other parameter is already at an adequate level. A system confined by *G_E_* or λ to the bistability region may require tuning of additional processes to ensure that the healthy state is achieved in the long run.

**Figure 3:**
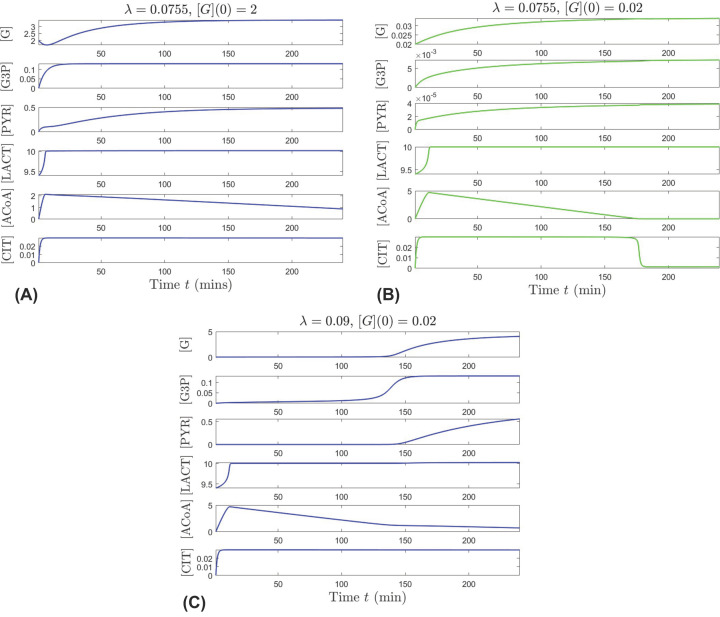
Transition out of the bistability regime, as the glucose transport factor λ that quantifies the efficiency of GLUT1 increases (top panels for λ = 0.0755 vs. bottom panel for λ = 0.09) under high external glucose level *G_E_* = 11.5 mM Each panel shows the simultaneous evolution of all six components for one solution of the system, for initial conditions fixed at [G3P](0) = 0, [PYR](0) = 0, [LACT](0) = 9.4, [ACoA](0) = 0, [CIT](0) = 0 and parameters (other than λ) set to their nominal values given in Table 1. Blue represents solutions which converge to the healthy state, and green represents solutions that converge to the pathological state. For λ = 0.0755 mM^−1^, the system is in the bistability window; it converges to the healthy state when starting from higher internal glucose [G](0) = 2 mM (**A**) and to the pathological state when starting from low internal glucose [G](0) = 0.02 mM (**B**). For λ = 0.09 mM^−1^, the system has a unique healthy equilibrium, and converges to it even when starting from very low internal glucose [G](0) = 0.02 mM (**C**).

In Figure 3 we present temporal evolutions of the system that illustrate transitioning from bistability to either pathological or healthy state, when changing the efficiency of GLUT1 (λ) within its physiological range in Table 1, in the presence of high external glucose (*G_E_* = 11.5 mM). A mid-range value of λ = 0.0755 mM^−1^ places the system in the bistability window, with convergence to the healthy state, if the initial internal glucose is [G](0) = 2 mM, illustrated in Figure 3A, and with convergence to the pathological state, if the initial internal glucose is insufficient ([G](0) = 0.02 mM, as depicted in Figure 3B). If λ is enhanced to approach the higher end of its physiological range (λ = 0.09 mM^−1^), the system exits bistability, and the healthy state is achieved even when the initial internal glucose is insufficient ([G](0) = 0.02, Figure 3C). In this case, metabolites that start with very low levels eventually increase and even though [ACoA] remains low, the system is able to be sustained.

### Combined effects of external glucose, lipid utilization, and oxidation of fatty acids

In our model, the parameter α has a complex role, as it reflects a collection of mechanisms, including lipid utilization for POS renewal, phagocytosis of the shed POS to generate fatty acids, and oxidation of those fatty acids to produce β-HB substrate for ACoA synthesis in the cone cell. A delicate balance among all of these processes ensures the cone’s metabolic vitality. Larger α is associated with greater use of β-HB by the cone.

**Figure 4:**
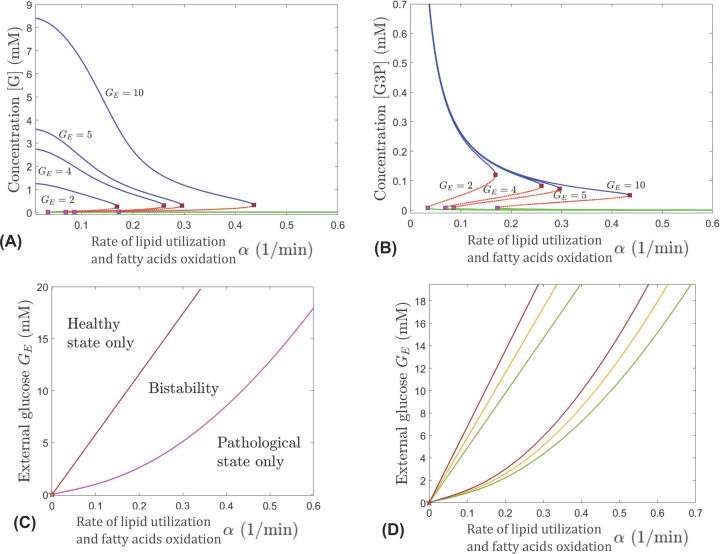
Bifurcations in dynamics with respect to α, for different levels of external RPE-mediated glucose *G_E_* Top panels: Evolution of the [G] (**A**) and [G3P] (**B**) components of the system’s equilibria, as α is increased; blue (healthy) and green (pathological) curves are stable while red is unstable. Different curves represent the equilibrium curves for different levels of *G_E_*, from left to right: *G_E_* = 2, 4, 5, and 10 mM. Two saddle node bifurcations occur along each equilibrium curve for all values of *G_E_*. (**C**) Regions of unistability and bistability delimited (as labeled) in the (α, *G_E_*) parameter slice by two saddle node curves, shown in brown and in pink, meeting at a cusp point near the origin, marked with a star. The value of λ is set to 0.0755 mM^−1^. (**D**) Delimitation between healthy/bistability/pathological regimes in the (α, *G_E_*) parameter plane for three different values of the glucose transport factor: λ = 0.065 mM^−1^ (red curve), λ = 0.075 mM^−1^ (orange curve), and λ = 0.09 mM^−1^ (green curve).

Using bifurcation analysis, we explore how α affects the system over a range of different external glucose levels (2–10 mM). For this, the parameters *G_E_* and α are varied, and λ is set to its nominal value in Table 1. The processes defined by other parameters are specified by their nominal values in Table 1. The results indicate a negative feedback mechanism that leads to the reduction in internal glucose and G3P concentrations when oxidation of fatty acids for β-HB utilization by the TCA cycle only fuels the cone and not the RPE and rods. As illustrated in Figure 4A, in order for the system to achieve viable levels of glucose concentration [G] and a healthy steady state (depicted by the blue curve) as α increases, *G_E_* must also increase; otherwise the pathological steady state (depicted by the green curve) is the only feasible state for large α values. Similar for [G3P], to have viable levels and for the other metabolites in the system to be in a healthy state (blue curve in Figure 4B), as α increases, *G_E_* must also increase. For any combination of α and *G_E_*, the system of metabolites can achieve one of two steady states: healthy (blue) or pathological (green). The red curves in Figure 4A,B represent unstable steady states that live in the bistability region in Figure 4C. The steady state achieved in this region for a given combination of α and *G_E_* depends on the initial state of the system defined by the initial levels of metabolites. For very small or large values of α there is only one stable steady state in the system as represented by only the green or blue curve in Figure 4A,B for a fixed value of α and any *G_E_* value. For example, for α = 0.01, the system converges to the healthy only state. However, values of α less than 0.0025 lead to dangerously high accumulation of G3P, which may indicate impaired production of new POS tips and inefficient phagocytosis of shed POS.

The coupling between the parameters *G_E_* and α in maintaining viability of the cell is illustrated in Figure 4C, which shows the parameter regions corresponding to different dynamic regimes. We see that throughout the range of *G_E_* between 2 and 10 mM, as α is increased the system undergoes two saddle node bifurcations as it crosses the red and pink bifurcations curves, respectively. The system goes from a region where the healthy state is the only stable steady state, to a bistability region, and then to a region where the pathological state is the only stable steady state, as α increases, keeping *G_E_* at a fixed value. The region of bistability is separated from the unistability regions by the two saddle node branches, meeting at a cusp point very close to the origin (depicted by a star in Figure 4C). The regions where the healthy state is the only stable solution and where bistabilty exists get wider for higher values of *G_E_*. This indicates the importance of plentiful external glucose to maintain the viability of cones. If a cone is in the pathological state, a combination of increasing *G_E_* and decreasing α would give the most effective way to get to bistability or a healthy state.

When α is increased beyond the value corresponding to the rightmost saddle node bifurcation in Figure 4C, the system exits bistability, and only the pathological equilibrium (represented by a green curve in Figure 4A,B) remains. This suggests a negative feedback mechanism from higher level of oxidation of fatty acids if these are only fueling the cones (and thus compromising the assistance from the rods and RPE to the cones). High value for α in the absence of fatty acids oxidation pathways feeding into the rods and RPE is reflective of the situation when cones increase their use of β-HB and none is left for the rods and RPE. In response, the RPE would withhold glucose from the photoreceptors to utilize it for its own fuel. The deprivation of β-HB and other nutrients in the rods, subsequently severely diminishes the number of rods providing RdCVF to the cone cell. Since α directly affects the dynamics of G3P, which is the metabolic component serving as a proxy for rods, increasing α translates into lower G3P (Figure 4B), and therefore less rods producing RdCVF. Figure 4A shows that the negative feedback from higher level of oxidation of fatty acids leads to low internal glucose in the cone, due to impaired glucose uptake induced by inadequate RdCVF supply. It is important to notice that as *G_E_* is gradually lowered, the bistability window narrows, and its onset occurs for gradually lower values of α. This suggests that as the RPE passes on less glucose, the cone becomes more reliant on having sufficient RdCVF to ensure effective glucose uptake and be able to maintain healthy internal glucose levels.

### Interplay among external glucose, GLUT1’s effectiveness, lipid utilization, and oxidation of fatty acids

Our analysis incorporates additional information into Figure 4D by coupling the three key physiological parameters *G_E_*, λ, and α in maintaining cellular glucose metabolism. We illustrate the importance of efficient GLUT1 transporter function (i.e., higher λ values) in conjunction with changes in two mechanisms, (i) external glucose, *G_E_*, and (ii) fatty acid oxidation and G3P utilization, which may lead to a healthy metabolic state. The figure shows the partition of the (α, *G_E_*) parameter plane into healthy/bistable/pathological regimes, for three different values of λ, all within the physiological range shown in Table 1. The first value is at the lower end of this range (red curves); the second is in the middle (yellow curves); the third is at the higher end of the range (green curves).

The (α, *G_E_*) range for the healthy regime (where only the healthy steady state is stable) expands, and the range for the pathological regime (where only the pathological steady state is stable) shrinks with improved glucose transport efficiency (as the bifurcation set rotates to the right, when λ is increased). This suggests that when glucose transport into the cell is more efficient, the healthy state can be maintained even at lower external glucose made available by the RPE (lower *G_E_*) and/or at higher G3P utilization and fatty acid oxidation for β-HB metabolism to fuel (only) the cone (higher α). Conversely, if inefficient glucose transport cannot be improved, the cell may require higher *G_E_* and/or lower α in order to maintain healthy metabolic levels. Overall, increasing the GLUT1 efficiency expands the regions in Figure 4C,D of the (α, *G_E_*) bifurcation set corresponding to the healthy only state and bistabiltiy and thus increases the feasibility of maintaining the cone’s metabolite concentrations at a healthy level or bringing them back to a healthy level by altering appropriate mechanisms that improve energy resources and glucose availability and uptake.

### Computational illustration of glucose deprivation and short-term rescue of oxidative metabolism

Photoreceptor mitochondria need a constant supply of acetyl groups from ACoA and oxaloacetate (OAA), provided by pyruvate as well as fatty acids and ketone bodies, to synthesize CIT and sustain the TCA cycle [10,37]. *In vitro* experiments show that photoreceptors under glucose deprivation continue to have functional TCA cycles and survive for roughly 90 min when supplemented with mitochondrion-specific fuels, but cell survival beyond this point is linked to glucose availability [4]. Our model is able to qualitatively capture these experimental findings and provide further insight into the role of alternative metabolites during glucose starvation.

**Figure 5:**
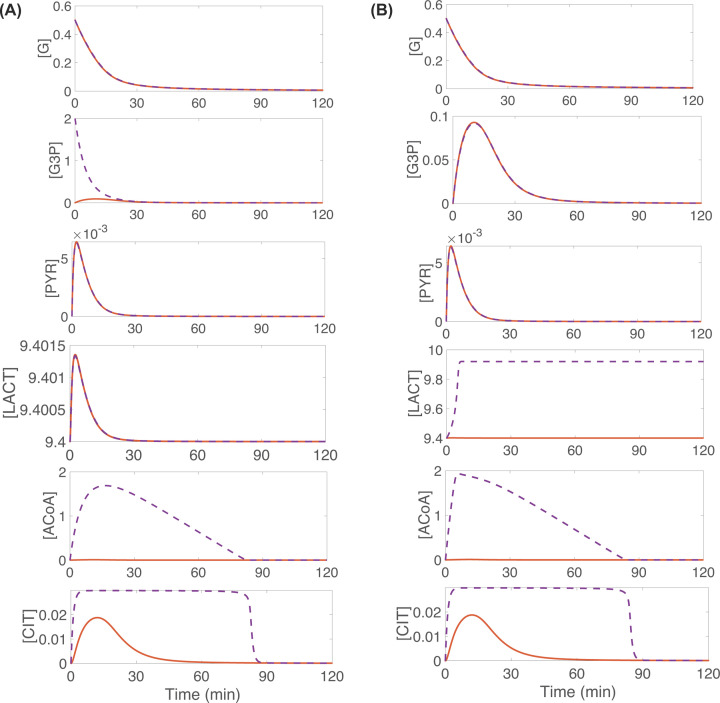
In glucose deprivation conditions, supplementation of exogenous fuels from fatty acids oxidation and lactate conversion into pyruvate prompts short-term rescue of oxidative metabolism (as reflected by the purple curves for CIT and ACoA) For all curves in panels (**A**,**B**), *G_E_* = [G] throughout the simulation, eliminating the glucose gradient differential and thus blocking glucose uptake. Additionally, [G](0) = 0.5, [LACT](0) = 9.4 and different initial concentrations of G3P and external lactate, *L_E_*, are considered; [G3P](0) = 0 and *L_E_* = 9.4 (orange curves in both panels), [G3P](0) = 2 and *L_E_* = 9.4 (purple curves in panel A), and [G3P](0) = 0 and LE = 9.92 (purple curves in panel B). All other initial conditions are set to 0, and all other parameters are kept at the nominal values presented in Table 1.

By conducting *in silico* experiments we simulate: (i) glucose deprivation accompanied by scarcity of auxiliary energy resources and (ii) glucose deprivation and short-term rescue of oxidative metabolism and energy production through supplementation of alternative metabolites or exogenous fuels. The orange curves in Figure 5A,B illustrate the consequences of complete glucose deprivation, where there is no glucose gradient differential and the initial internal glucose ([G](0)) and external glucose (*G_E_*) levels are very low. This situation precludes glucose uptake and further decreases the internal glucose to zero. Additionally, the pathways for energy generation via external lactate and β-HB are shut off. The simulation results show that the glucose shortage leads to insufficient production of pyruvate with oxidation of fatty acids for β-HB and uptake of external lactate also inadequate. As a consequence of this, by 50 min ACoA and CIT decay to extremely low levels (ACoA ≈ 1.5 × 10^−4^ mM, CIT ≈ 8 × 10^−4^ mM). In the context of our model, this implies the detrimental outcome of inability to fuel OXPHOS (orange curves in Figure 5A,B).

The purple curves in Figure 5 reflect sustaining the oxidative metabolism of a glucose-deprived cone through boosting G3P by an increase in its initial concentration (panel A) or through increasing external lactate (*L_E_*) (panel B). Either intervention allows to sustain viable ACoA and CIT concentrations until their levels drop significantly (to approximately 1.3 × 10^−4^ mM for ACoA and approximately 9 × 10^−4^ mM for CIT) at approximately 85 min, a timeline which is consistent with the *in vitro* experimental results for photoreceptors under glucose deprivation [4]. Increasing G3P levels in our model signify increased amount of POS fatty acids that would be oxidized to provide alternative fuel to the cone in the form of β-HB. The increase in external lactate leads to an extracellular lactate gradient differential that promotes the conversion of lactate into pyruvate which then enters into the TCA cycle. The simulation results in Figure 5 indicate that both of these modifications, which essentially provide intermediate metabolites for energy production, allow the cone to maintain its oxidative metabolism (as reflected by ACoA and CIT) in the short run if glucose is scarce, but the supply of these intermediate fuels cannot sustain the cell if glucose shortage is prolonged.

### Impact of glucose shutdown duration and the timing of interventions

Empirical evidence suggests that speed of intervention is crucial to the survival of photoreceptors when glucose is scarce. As discussed earlier, experimental work with mouse retinas showed that severe cellular stress occurs in photoreceptors within 90 min under glucose deprivation conditions. However, cell recovery was achieved by restoring glucose uptake in this time frame [4]. Our *in silico* experiments, through our model, reveal how different conditions of the cellular system may affect the impact that glucose shutdown duration has on recuperation. We also test some potential therapeutic interventions. We model temporary glucose shutdown by equating the level of external glucose (originally fixed at *G_E_* = 10) to the internal glucose concentration to prevent glucose uptake for a time window between *t* = 40 and *t* = 130 min, then restoring *G_E_* to its original value for the remainder of the time.

**Figure 6:**
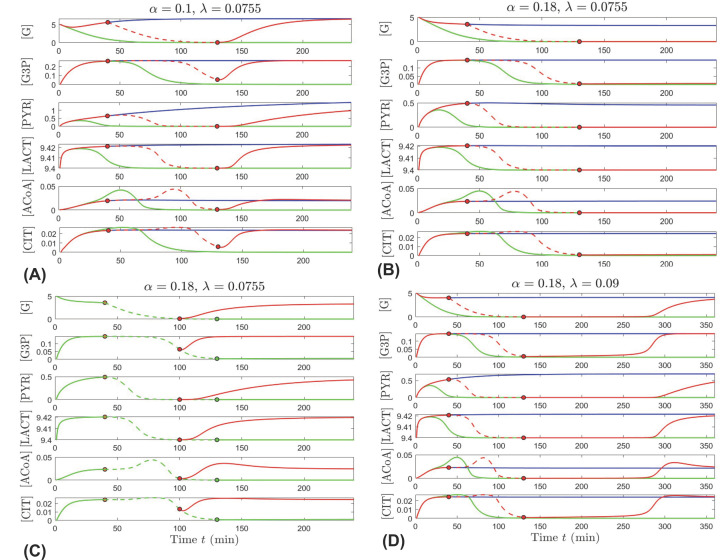
Impact of glucose shutdown duration and interventions (**A**,**B**) Effect of temporarily shutting down glucose uptake. (A) Time evolution of the six metabolites when α = 0.1 and λ = 0.0755, under three different circumstances: *G_E_* = 10 mM (blue curves); *G_E_* = [G] (green curves); *G_E_* = [G] only between *t* = 40 min and *t* = 130 min, and *G_E_* = 10 otherwise (red curves). (B) Similar trajectories to the ones illustrated in (A), for a higher α = 0.18. (**C**,**D**) Strategies for restoring healthy metabolite levels after temporarily shutting down glucose uptake. (C) **Restore*** G_E_*** earlier**. Time evolution of the six metabolites when α = 0.18 and λ = 0.0755, when *G_E_* = [G] between *t* = 40 min and *t* = 130 min (green curve), and when shutdown ends early at *t* = 100 min (red curve). (D) **Increase** λ. Time evolution of the six metabolites when α = 0.18 and λ = 0.09, under the same three conditions as in panels (A,B): *G_E_* = 10 mM (blue curves); *G_E_* = [G] (green curves); *G_E_* = [G] only between *t* = 40 min and *t* = 130 min, and *G_E_* = 10 otherwise (red curves). For all panels, the trigger times are shown as dots along the corresponding curves; *L_E_* = 9.4 mM and the other parameters are kept at their nominal values in Table 1; the initial conditions are [G](0) = 5 mM, [LACT](0) = 9.4 mM, and the other components set to zero.

In panels (A,B) of Figure 6, the cone’s metabolic trajectory for temporary glucose shutdown (red) is displayed together with the trajectory for no shutdown (blue) and the trajectory for glucose shutdown during the whole time (green). No glucose shutdown consists in our model of internal glucose concentration, [G], being lower than external glucose, *G_E_* which is set at 10 mM i.e., 10 = *G_E_* > [G]. Glucose shutdown consists of no glucose gradient differential, such that *G_E_* = [G], which prevents glucose uptake. The period where glucose uptake is temporarily shut off is represented by a dashed curve in Figure 6A–D.

**Figure 7:**
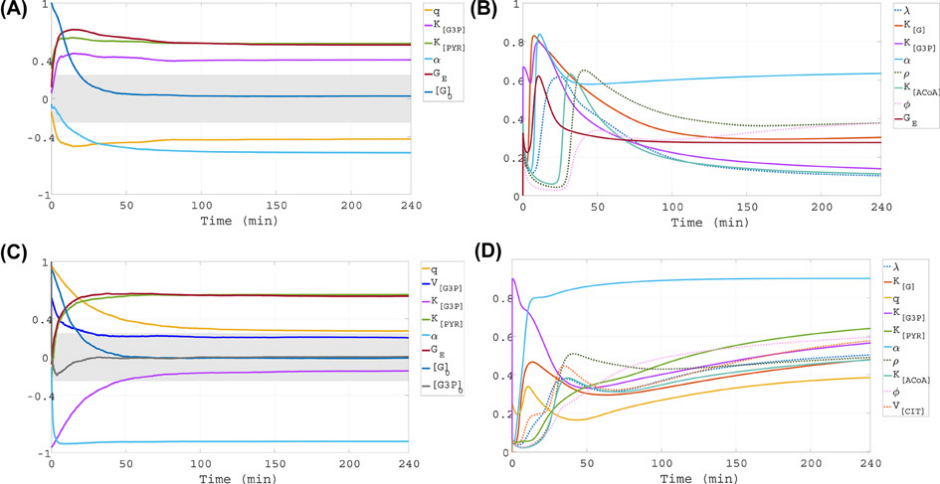
Time-dependent GSA results for low initial glucose level Left panels present time-dependent results of PRCC and right panels present time-dependent results of eFAST, both for initial glucose concentration of [G](0) = 0.02 mM. Two outcomes of interest are considered, presented in rows 1,2: intracellular concentrations of glucose ([G]) and G3P ([G3P]). The gray bands on the left panels indicate PRCC values which are not statistically significant (*P*-value ≥0.001).

**Figure 8:**
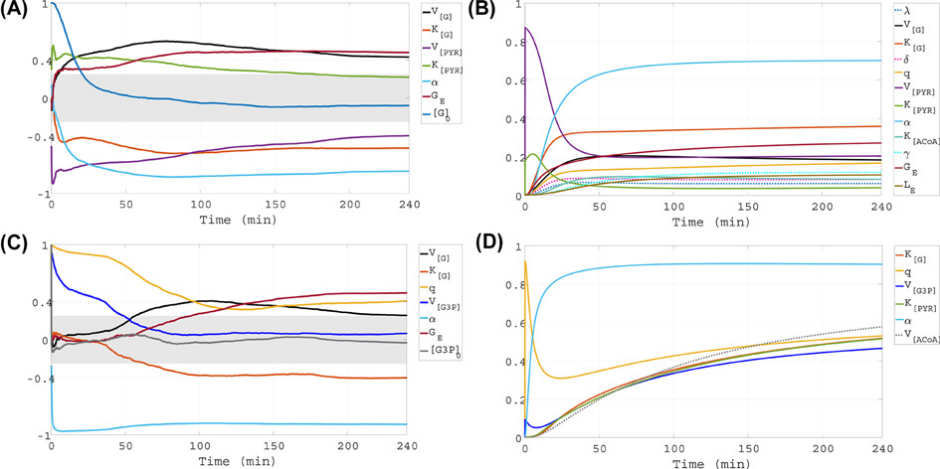
Time-dependent GSA results for higher initial glucose level Left panels present time-dependent results of PRCC and right panels present time-dependent results of eFAST, both for initial glucose concentration of [G](0) = 2 mM. Two outcomes of interest are considered, presented in rows 1,2: intracellular concentrations of glucose ([G]) and G3P ([G3P]). The gray bands on the left panels indicate PRCC values which are not statistically significant (*P*-value ≥0.001).

Figure 6A illustrates the simulated outcome with α = 0.1, a parameter value reflecting moderate use of β-HB by the cone cell. The model predicts cell survival if no shutdown occurs (blue trajectory converges to the healthy state), and cell death when glucose is shut down for the whole time (green trajectory converges to the pathological state). Restoring glucose uptake within 90 min from shutdown prompts recuperation, as the red trajectory slowly builds up to the metabolite concentration levels corresponding to the healthy state. The presence and timing of this recovery coincides with the *in vitro* experimental findings [4]. However, our model further suggests that this recovery is gated by a combination of factors and may differ in its overall effect as well as the time it takes the cell to recover depending on the factors involved, a few of which we analyze below.

While long term the behavior of the cone is not significantly affected when glucose is shut down temporarily for a short time or not shut down at all (red and blue curves in Figure 6A appear almost indistinguishable after 200 min, except for [PYR]), the duration and initiation of reduction in the respective metabolic concentrations varies. This is shorter for [G3P], [ACoA], and [CIT], and the decrease in these concentrations is delayed by approximately 60 min after the shutdown at *t* = 40 min. These results are indicative of the priority in the cone metabolic system to maintain the TCA cycle and the POS renewal functioning as well as the interdependence on oxidation of POS fatty acids for alternative fuel source for the TCA cycle.

Figure 6B shows the predicted outcome for a larger value of α, which illustrates larger utilization of β-HB by the cone that would enhance the negative feedback mechanism leading to reduction in rods producing RdCVF and less glucose going into the cones. While the qualitative behavior of the metabolite concentrations when glucose is always present appear almost indistinguishable (blue curves in Figure 6A,B), the cell’s fate is dramatically impacted in the case of temporary glucose shutdown. The red trajectory in panel (B) leads to the pathological state suggesting that after a 90-min period of glucose deprivation, the negative feedback from higher oxidation of fatty acids fueling only the cones would prevent recovery to healthy levels when the cone’s glucose uptake is restored, as this uptake would not be effectively accelerated due to the significant reduction in rods supplying RdCVF.

Our *in silico* analysis results suggest two factors that could counteract the negative feedback on RdCVF from higher β-oxidation fueling only the cones. Panel (C) in Figure 6 shows that if the duration of glucose shutdown is shorter, then cone recovery is possible as glucose uptake is restored, even if this uptake is not optimally accelerated by RdCVF. Comparing Figure 6B,D, the model simulations reveal that the negative impacts of higher β-oxidation and utilization of β-HB by the cone (α = 0.18 versus α = 0.1) can also be offset by increasing the efficiency of GLUT1, represented by a larger λ value (0.0755 versus 0.09). The recovery time is slow but by sufficiently increasing the efficiency of glucose entry into the cone cell, the metabolic concentrations of the system can be restored to healthy levels. As we see in panel (D), the red trajectory corresponding to temporary glucose shutdown eventually recovers to the blue curve that designates the healthy state, but this recovery occurs after almost 3 h from the shutdown window.

### Model output sensitivity for low initial glucose level

Note that the left panels in Figures 7 and 8 include only the PRCC curves for the input factors to which the model outputs are sensitive in the interval 0–240 min, i.e., |PRCC| > 0.4 somewhere in this time window. The right panels in Figures 7 and 8, include only the sensitivity index curves for the parameters highlighted as important using eFAST somewhere in the window 0–240 min, i.e., their corresponding index curves stand out and lie above the other parameters’ index curves, which are grouped and indistinguishable from one another.

#### PRCC, [G]

The PRCC results with the internal glucose concentration ([G]) as outcome of interest are shown in the top left panel of Figure 7. In the beginning of the simulation, [G] is sensitive to changes in the initial glucose concentration, [G]_0_, but over time this sensitivity disappears as other pathways gain importance. [G] becomes sensitive to changes in the parameters *K*_[PYR]_, *G_E_*, *q* and α. The first two have positive PRCC values, so as they increase, [G] increases. The parameter *K*_[PYR]_ is the substrate concentration that gives the half maximal rate *V*_[PYR]_, which governs the rate at which glucose is converted into pyruvate in our model. Increasing *K*_[PYR]_ essentially keeps the rate of glucose conversion into pyruvate operating at lower levels, allowing for less utilization of internal glucose for aerobic glycolysis and thus large [G]. Higher *G_E_* signifies that the RPE makes more external glucose available to the cone, and as a result the gradient differential that promotes flow of glucose into the cone increases, causing larger [G]. The PRCC values for the parameters *q* and α are negative, meaning that as they increase, [G] decreases. The parameter *q* represents the proportion of internal glucose diverted to create G3P, so bigger value for *q* leads to lower [G]. A larger α is associated with higher fatty acids oxidation and depletion of G3P. Because the G3P concentration, [G3P], with the appropriate scaling factor is used as a proxy for the contribution of RdCVF by rods, lower [G3P] translates into reduced glucose uptake by the cone due to RdCVF, and thereby lower [G]. Another parameter that connects the internal glucose level to the dynamics of [G3P] is *K*_[G3P]_, which is on the boundary of being deemed influential.

#### eFAST, [G]

The top right panel of Figure 7 presents the eFAST results with [G] as outcome of interest, and we see that this methodology also classifies α, *K*_[G3P]_ and *G_E_* as important. The uncertainties of the last two parameters have much greater influence on the variability of [G] before 40 min, as indicated by the peaks in the curves of their corresponding sensitivity indices. Another parameter that is most influential at this early time is *K*_[G]_, the half-limiting value of RdCVF-stimulated glucose uptake. Between 40 and 60 min, peak sensitivity of [G]'s variability to uncertainty in λ, the efficiency of GLUT1, occurs. Toward the end of the simulation, once transient dynamics have settled, the parameters standing out as most important are α, ρ, ϕ, *K*_[G]_, and *G_E_*. These findings indicate that as the system dynamics evolve, the variability of [G] is impacted to the greatest extent by uncertainties in G3P utilization and oxidation of fatty acids, the fraction of pyruvate converted into ACoA, the rate at which CIT is converted into ATP, glucose uptake stimulated by RdCVF, and external glucose modulated by the RPE.

#### PRCC, [G3P]

The PRCC analysis with [G3P] as outcome of interest (Figure 7, bottom left panel) shows that while [G3P] is initially very sensitive to *K*_[G3P]_, *q* and [G]_0_, all of them are no longer influential after 50 min. After the first 25 min, [G3P] becomes sensitive to changes in *G_E_*, *K*_[PYR]_ and α very quickly, and with high PRCC values. This indicates that a small change in the mechanisms defined by these parameters may lead to big changes in [G3P]. The influence of these parameters on [G3P] is sustained throughout the simulation. Increased *G_E_* promotes gradient-based flow of glucose into the cone, resulting in greater amount of internal glucose which is then diverted either to G3P or to pyruvate. Since *K*_[PYR]_ slows down the reaction rate of conversion into pyruvate, more glucose becomes available to be diverted to G3P production. Finally, the greater G3P depletion associated with higher α leads to lower [G3P], as indicated by the negative PRCC value. These results suggest that early on, [G3P] is greatly impacted by the reaction governing G3P synthesis, but as the system behavior evolves over time, other factors become more important, such as the G3P utilization and oxidation of fatty acids, glucose modulated by the RPE, and the half-limiting value of the pyruvate maximum production rate.

#### eFAST, [G3P]

The eFAST results for G3P (Figure 7, bottom right panel) show that the variability of this metabolite is sensitive to uncertainties in *q*, *K*_[G3P]_, *K*_[PYR]_, and α, which were also highlighted as important by PRCC. Additionally, uncertainty in *V*_[CIT]_ (the maximum velocity of CIT production), *K*_[G]_, ρ, *K*_[ACoA]_, ϕ and λ affects [G3P]’s variability. *K*_[G3P]_ has the highest sensitivity index in the very beginning. Then its index exhibits a sharp decline followed by steady increase on 50–240 min. The sensitivities of [G3P] to *K*_[G]_, *q*, *V*_[CIT]_, ρ, *K*_[ACoA]_ and λ rise, then fall in the first 60 min, and afterwards all of them increase with the exception of the sensitivity to ρ which continues to decrease. On the other hand, the indices of *K*_[PYR]_, ϕ and α steadily increase on 0–240 min, with α becoming the dominating parameter after 20 min. This indicates that in the beginning, various pathways drive G3P variability, including GLUT1 transport, RdCVF-stimulated glucose uptake, glucose diversion to G3P, and CIT and ACoA dynamics, but later on these contributions are all exceeded by the influence of G3P utilization and oxidation of fatty acids.

### Model output sensitivity for higher initial glucose level

#### PRCC, [G]

PRCC shows that the internal glucose level is sensitive to *V*_[G]_ (the maximum rate of glucose uptake stimulated by RdCVF), *V*_[PYR]_, *K*_[G]_, α and *G_E_* (Figure 8, top left panel). All of these parameters maintain high level of influence on [G] after 50 min. However, the magnitude of the sensitivity coefficient for *V*_[PYR]_ declines, while the rest increase. These results suggest that early on, the initial glucose level and the pyruvate biochemical reaction impact the internal glucose concentration, but as time continues, other processes exert stronger influence, including G3P utilization and oxidation of fatty acids, glucose modulated by the RPE, and RdCVF-stimulated glucose uptake.

#### eFAST, [G]

The eFAST results for glucose identify as important factors *q*, *K*_[ACoA]_, λ, *L_E_* (external lactate), γ (the maximum velocity of lactate transport contributing to ACoA), and δ (the scaling factor multiplying [G3P] to approximate the RdCVF supply by rods) (Figure 8, top right panel). In addition, eFAST shows that variability in [G] is sensitive to uncertainty in *V*_[G]_, *K*_[G]_, *G_E_*, α, *V*_[PYR]_ and *K*_[PYR]_, which were also classified as influential with PRCC. While the sensitivity indices of all other parameters steadily increase, after an initial rise the indices of *V*_[PYR]_ and *K*_[PYR]_ decrease. *V*_[PYR]_ and α exchange roles as the parameter whose uncertainty drives the most variability in [G], with *V*_[PYR]_ dominating on 0–20 min and α overtaking it shortly after. After ∼75 min, *K*_[G]_ and *G_E_* follow α as the second and third most influential parameters. These findings indicate that initially the uncertainty in processes related to pyruvate production influence [G]’s variability more, but later on the factors of greatest importance become external glucose modulated by the RPE and RdCVF-stimulated glucose uptake together with G3P utilization to produce POS whose fatty acids are *β*-oxidized.

#### PRCC, [G3P]

PRCC shows that the G3P level is sensitive to *V*_[G3P]_, *q*, *G_E_*, *K*_[G]_, *V*_[G]_ and α (Figure 8, bottom left panel). The most dominant factor for the duration of the simulation is α. The parameters *q* and *V*_[G3P]_ are initially very influential, but over time their impact declines until they no longer contribute to significant changes in [G3P], by 40 min for *V*_[G3P]_ and 100 min for *q*. [G3P] is initially insensitive to changes in *K*_[G]_ but becomes borderline sensitive by 100 min. Similarly, [G3P] is initially insensitive to the parameter *V*_[G]_ but becomes borderline sensitive for a short window in the middle of the simulation. The G3P concentration is also initially insensitive to changes in *G_E_*. However, *G_E_* gains influence from 150 min onward, becoming the second parameter that contributes most to changes in [G3P] after α. These results imply that for the first part of the simulation, the pathway from glucose to G3P has a substantial impact on the G3P level, but as the temporal dynamics of the system keep evolving, [G3P] becomes more influenced by other processes, including G3P utilization and oxidation of fatty acids, the glucose modulated by the RPE, and glucose uptake stimulated by RdCVF.

#### eFAST, [G3P]

With eFAST, *K*_[G]_, *q*, *V*_[G3P]_ and α are identified as parameters whose uncertainty strongly affects the variability of [G3P] (Figure 8, bottom right panel), and these were also highlighted by PRCC. In addition, eFAST classifies as important *K*_[PYR]_ and *V*_[ACoA]_, the maximum production rate of ACoA from pyruvate. At first, uncertainty in *q* drives the most variability in [G3P], but by 10 min into the simulation, α takes over the role of the most impactful parameter.

Comparing the GSA results with the two different initial glucose levels, the following should be noted. Both [G] and [G3P] become sensitive to *G_E_* earlier, and the corresponding PRCC is larger with low compared with higher initial glucose (*G_E_* becoming important earlier under low [G](0) was also found for pyruvate using PRCC (supplementary data)). On the other hand, α, whose changes and uncertainties impact the most all the metabolic concentrations of the system, plays a similar role regardless of how much initial glucose is available (the high sensitivity to changes or uncertainties in α according to PRCC or eFAST, respectively, is also exhibited when pyruvate and lactate are considered as outcomes of interest (supplementary data)). Additionally, we see that the internal glucose level is sensitive to the parameter *q* when initial glucose is low, but insensitive when it is higher. In contrast, PRCC and eFAST show that changes and uncertainty in *q* have a greater impact on the level and variability of [G3P] when initial glucose is higher. Furthermore, the eFAST results for both [G] and [G3P] reveal that with low initial glucose, the behavior of almost all sensitivity indices is marked by transient dynamics before 100 min, while after that sensitivities settle to fairly consistent levels (this was also found for pyruvate using eFAST (supplementary data). With the higher initial condition for glucose, the indices of only five parameters exhibit transient dynamics early on: *K*_[LACT]_ (supplementary data), *V*_[PYR]_, *K*_[PYR]_, *q*, and *V*_[G3P]_.

## Discussion

The current study makes use of the equations for cone metabolism developed in Camacho et al. (2020), which constitute the first mathematical model that takes into account the ATP production in the cone cell from β-HB created by the RPE via oxidation of POS fatty acids [25]. To keep the dynamics focused on cone behavior, the model does not include explicit rod and RPE compartments and examines the situation when β-HB is utilized by the system only to fuel the cone’s TCA cycle, without reflecting use of β-HB by the rods and RPE. However, key feedback mechanisms in the cone metabolic pathways mediated by the RPE and rod cells are incorporated through model parameters and proxies. Through time-varying sensitivity analysis and multiple parameter bifurcation analysis of the model, together with computational experiments, the present paper aims to elucidate how interactions between glucose modulation by the RPE, GLUT1 transporter, and energy generation from β-HB affect the cone’s metabolic vitality. With *in silico* experiments and simulation of our model, we explore the possibility for recovery after a period of glucose deprivation. While, throughout this section we will discuss key results of the present study in relation to certain known aspects of AMD and RP development and some potential therapeutic options, our work is not a direct investigation of these retinal diseases. However, as photoreceptor degeneration in diseases like AMD and RP has been linked to glucose deprivation and disruptions in cellular metabolism, our work provides insight into the role of metabolites under glucose starvation conditions, which may elucidate metabolic pathways and disruption in mechanisms resulting in these pathologies.

We used two GSA methods for a more complete picture of the sensitivity of the model outputs to different parameters, and the results reveal very high sensitivity to the parameter α, which plays a complex role, representing the utilization of lipids associated with POS renewal, as well as the shed and phagocytosed POS whose fatty acids are oxidized to produce β-HB for ACoA synthesis in the cone. While all metabolic concentrations are most sensitive to α for the duration of our time-dependent sensitivity analysis, our results also show that most of the metabolites in our system are sensitive to other processes and factors earlier on (approximately within the first 50–100 min) but not afterwards. This highlights the importance of early interventions, as there is more flexibility in which processes to alter in order to impact the metabolic concentrations in the system and the overall functionality of the cone cell. The importance of timing was also confirmed with our *in silico* experiments that reveal how a shorter duration of glucose deprivation can successfully restore the metabolic concentrations in a situation that was destined for the pathological state. The key processes that impact the energy sources and input into the cone emerge in all our analyses, and the flexibility to perturb them to achieve a healthy state is also corroborated by our bifurcation analysis.

With both low and higher initial glucose, the PRCC analysis shows that a factor with considerable impact on the concentrations of internal glucose, G3P, and pyruvate is glucose made available to the cone by the RPE (reflected by the parameter *G_E_*). Moreover, the comparison of results between the two levels of initial glucose shows that the metabolites become sensitive to *G_E_* much earlier, and *G_E_* has higher corresponding PRCC values when initial glucose is low. In agreement with physiological understanding, this implies that the glucose supply from the RPE is of utmost importance for a metabolically stressed cone cell to maintain aerobic glycolysis and lipid creation for POS renewal. The significant impact of external glucose is further supported by our bifurcation analysis, which suggests that as long as *G_E_* does not drop to dangerously low levels for extended periods of time, maintaining metabolic vitality is guaranteed when GLUT1’s effectiveness as well as G3P utilization and fatty acids β-oxidation (represented by λ and α, respectively) are operating within normal ranges.

Our bifurcation analysis shows that very low α is associated with G3P buildup that could be interpreted as ineffective or disrupted POS renewal, which is a distinctive feature of AMD [1]. On the other hand, large α suggests a negative feedback on internal glucose from increasing amounts of β-HB being used up by the cones, while none of it is fueling the RPE and rods. In response to being deprived of β-HB, the RPE would withhold nutrients from the photoreceptors, further reducing the resources for rods, and thereby causing their level to decrease. As a result less rods provide RdCVF, and consequently the cone cell would have a reduction in acceleration of glucose uptake and subsequently low internal glucose. The outcome of reduced RdCVF supply to the cone cell is consistent with what occurs in RP as rods die [15]. Previous studies have indicated that RdCVF and β-HB are most essential for photoreceptors during glucose-deprived conditions and under stress [2,9,10,12,13]. Taken together, the high sensitivity to α and its impact revealed by the bifurcation analysis show that issues with glucose uptake and shed POS processing are important factors that can instigate pathological consequences for cone vitality.

The bifurcation results also show that if α becomes too large especially when glucose made available by the RPE is already insufficient, viable cone metabolism and lipid synthesis cannot be sustained, so ultimately a pathological state would persist, even though larger α also means greater use of β-HB for fueling the cone's TCA cycle. Additionally, the computational illustration of glucose deprivation and short-term rescue of oxidative substrates (namely ACoA and CIT) indicates that supplying auxiliary metabolites for energy production would allow the cone’ s oxidative metabolism to continue for a short time under glucose scarcity, but this cannot prevent cell death from prolonged glucose deprivation. These results support the experimental finding that the long-term survival of photoreceptors is not reliant on just ATP production, but also on the proper functioning of anabolic processes to create components, such as lipids and proteins, needed to repair and maintain the cell [4]. Our sensitivity analysis further alludes to the highly demanding nature of lipid creation through the PRCC finding that the internal glucose concentration is sensitive to the parameter *q* only in the case of low initial internal glucose. Since the process defined by the parameter *q* modulates the diversion of glucose to the KP and thus its use to synthesize lipids for POS renewal, this result shows that the demands of the renewal process create further metabolic stress when the cone cell has experienced insufficiency of intracellular glucose.

The eFAST results suggest that uncertainty in the efficiency of GLUT1 (reflected through parameter λ) drives variability in the concentrations of internal glucose and G3P when the initial glucose level is low. In accordance with physiological knowledge, this indicates that the effective transport of glucose from the RPE into the cell is crucial to maintain stable levels of metabolic resources in the glycolysis and Kennedy pathways. The bifurcation analysis also reveals that the parameters *G_E_* and λ have critical impact on the internal glucose concentration and G3P. Since G3P production is directly linked to the synthesis of lipids for the renewal of POS, these results suggest that inadequate availability and/or transport of glucose, leading to nutrient shortage in the cone, would result in an impaired renewal process. This agrees with experimental findings showing that glucose starvation in cones during the progression of RP and AMD impairs the POS renewal and shedding process and blocks the regeneration of POS [1,11].

The bifurcation analysis highlights important aspects in the context of potential therapeutic interventions to stimulate recovery from a pathological state associated with extremely low levels of glucose and other key metabolites. First, considering external glucose made available by the RPE (*G_E_*) and the efficiency of GLUT1 (λ) as two coupled factors that change simultaneously, our findings indicate that both have to increase for an ailing cone to eventually achieve a state of health and that when these two factors work in tandem, the increase required to achieve a healthy state is less than when they are increased independently. Second, we see that increasing *G_E_* in conjunction with lowering α (to decrease the negative feedback on RdCVF explained earlier) is an effective gateway to bistability or the healthy state. These bifurcation results indicate that halting the progression towards a pathological state might be feasible by perturbing processes that lead to higher *G_E_*. Achieving a healthy state under bistability will depend on the state of the system. Finally, for a set combination of *G_E_* and α, increasing the effectiveness of GLUT1 expands the possibility for a healthy outcome.

Comparing the eFAST results for glucose and G3P with the two different initial internal glucose levels ([G](0)), it is apparent that when [G](0) is low, there are notable transient dynamics of the sensitivity indices for almost all parameters in the first 100 min of the simulation, while for higher [G](0), the indices for the majority of parameters have dynamics with stable evolution over time. This suggests that when initial glucose is closer to adequate, there are less fluctuations in the relative importance that different pathways have in relation to the variability in the early dynamics of internal glucose and G3P. Furthermore, the time frame with transient sensitivity dynamics in the case of low initial glucose presents a critical window with more flexibility for interventions to rescue a cone cell from the detrimental consequences of nutrient scarcity. This time window is consistent with the *in vitro* experimental results for photoreceptors under glucose starvation, which showed cellular stress and death within 90 min [4].

Our *in silico* experiments for the metabolic course under temporary parameter perturbations support the importance of this time frame and further show that the mode of cellular operations plays a critical role in determining how the cone responds to the duration of glucose deprivation. The six key metabolites tracked by the model exhibit essentially the same behavior if glucose shutdown is absent or permanent, regardless of whether the cone makes moderate or heavy use of β-HB. However, the level of β-HB utilization makes a big difference in the case of a 90-min glucose shutdown. While with moderate use of β-HB recovery occurs when glucose uptake is restored, heavy β-HB use, which is associated with stronger negative feedback on the rods, precludes recovery since the cone’s glucose uptake diminishes without sufficient stimulation by RdCVF. Additionally, the simulation experiments suggest that the negative feedback on RdCVF from higher β-oxidation fueling only the cones can be counteracted and recovery achieved if glucose shutdown lasts 60 instead of 90 min, or if the shutdown duration remains 90 min, then recovery requires an intervention to increase glucose transport into the cone by enhancing GLUT1’s efficiency.

The findings discussed above are congruent with current knowledge about pathological mechamisms in RP and AMD, where potential therapeutic interventions are being focused on the early disease stages by trying to prevent photoreceptor glucose starvation, to promote appropriate clearance of POS, and in the case of RP, to also prevent secondary cone death [3,11]. In vitro administration of glucose or RdCVF in cones delayed their loss of function during RP progression [11,38], and injection of RdCVF in animal models of RP prevents the shortening of cone outer segments, which precedes cone loss [3,39]. In AMD, appropriate enhancement of phagocytosis in the RPE, in particular autophagy (self-eating), can be beneficial in terms of clearing cellular waste and providing alternative metabolites (which might be needed if the flux of glucose to the photoreceptors is reduced due to the AMD-associated RPE degeneration). However, excessive autophagy can also be harmful as it causes stress and ultimately results in apoptosis and necrosis [40,41].

## Limitations and future work

A limitation of the current model is that the parameter α amalgamates several different processes each of which is critically important for the metabolism and energy production of photoreceptors. Moving forward, we will pursue a model extension that includes these mechanisms more explicitly. Additionally, in the current model the approximation for the supply of RdCVF is coupled to the G3P model component, which precludes directly observing dysfunction in the metabolic system associated with the build up of lipids due to inefficiency of POS phagocytosis and renewal. This aspect would be addressed in future work by including additional compartments and processes in the model to capture the use of lipids to renew POS as well as the shedding of POS, and incorporating explicit RPE and rod compartments and their interactions with the cone cell (where the rods contribute RdCVF to the cone, and the RPE takes up and digests shed cone POS to produce β-HB). Through these modifications, together with capturing the effects in the cone, we will also have oxidation of fatty acids from shed POS feed back into to the energy production processes in rods and the RPE.

## Supplementary Material

Supplementary Data

## Data Availability

Graphical MATLAB software package MatCont used for bifurcation analysis (https://sourceforge.net/projects/matcont/), MATLAB numerical solver routines used for model simulations (https://www.mathworks.com/help/matlab/ref/ode45.html; https://www.mathworks.com/help/matlab/ref/ode15s.html). The GSA codes we applied to our system use random number generation (https://www.mathworks.com/help/matlab/ref/rand.html), statistics (https://www.mathworks.com/help/stats/tiedrank.html; https://www.mathworks.com/help/stats/regress.html; https://www.mathworks.com/help/stats/corr.html; https://www.mathworks.com/help/matlab/data_analysis/descriptive-statistics.html) and trigonometry MATLAB routines (https://www.mathworks.com/help/matlab/trigonometry.html), and were modified and adapted from codes available at, http://malthus.micro.med.umich.edu/lab/usadata/. We wrote source code to implement eFAST over time using the MATLAB package for trapezoidal numerical integration (https://www.mathworks.com/help/matlab/ref/trapz.html). The PRCC and eFAST data for our model generated during the study is included as part of the supplementary data.

## Appendix A Table A1 and Model Equations

**Table A1:** Parameter descriptions, units and baseline values

Parameter	Description (units)	Baseline
λ	Transport conversion factor (mM^−1^)	0.0755
*V*_[G]_	Maximum transport rate of glucose (mM.min^−1^)	1.2
*K*_[G]_	Substrate concentration that gives half the maximal rate of *V*_[G]_ (mM)	19
*n*	Rate of passive glucose transport in the absence of RdCVF (mM.min^−1^)	0.001
δ	Scaling factor for contribution of RdCVF by rods (no units)	65
*q*	Fraction of G converted into G3P (no units)	0.18
*V*_[G3P]_	Maximum production rate of G3P (mM.min^−1^)	0.15
*K*_[G3P]_	Substrate concentration that gives half the maximal rate of *V*_[G3P]_ (mM)	0.143
*V*_[PYR]_	Maximum production rate of PYR (mM.min^−1^)	0.15
*K*_[PYR]_	Substrate concentration that gives half the maximal rate of *V*_[PYR]_ (mM)	1.7
β	Rate of CIT inhibition of G catabolism, times an appropriate conversion factor (mM^−1^)	1
α	Rate of β-oxidation of ingested POS fatty acids (created from G3P) to generate the β-HB substrate for ACoA (min^−1^)	0.2
ρ	Fraction of PYR converted into ACoA (no units)	0.05
*V*_[LACT]_	Maximum production rate of LACT (mM.min^−1^)	0.14
*K*_[LACT]_	Substrate concentration that gives half the maximal rate of *V*_[LACT]_ (mM)	0.125
*V*_[ACoA]_	Maximum production rate of ACoA (mM.min^−1^)	0.15
*K*_[ACoA]_	Substrate concentration that gives half the maximal rate of *V*_[ACoA]_ (mM)	0.02
ϕ	Rate of CIT converted into ATP (min^−1^)	1
*V*_[CIT]_	Maximum production rate of CIT (mM.min^−1^)	0.03
*K*_[CIT]_	Substrate concentration that gives half the maximal rate of *V*_[CIT]_ (mM)	0.0054
ψ	Maximum velocity of lactate transport (min^−1^)	8
κ	Measurement of binding affinity of lactate transporter (mM^−1^)	10
γ	Maximum velocity of lactate transport contributing to ACoA (min^−1^)	1
*G_E_*	Concentration of glucose outside the cell (mM)	11.5
*L_E_*	Concentration of lactate outside the cell (mM)	10

The descriptions, units and baseline values of parameters are as defined in Camacho et al. (2020) [25].

Model equations (eqns 1-6):

(1)
$$\begin{array}{l} \frac{d[\text{G}]}{dt}=\overset{a}{\overbrace{\lambda (G_{E}-[\text{G}])\;}}\overset{b}{\overbrace{\left(\frac{V_{\left[\text{G}\right]}{\left(\delta [G3\text{P}]\right)}^{2}}{K_{\left[\text{G}\right]}^{2}+{\left(\delta [\text{G}3\text{P}]\right)}^{2}}+n\right)\;}}-\;\\ \left(\overset{c}{\overbrace{\frac{qV_{\left[\text{G}3\text{P}\right]}{\left[\text{G}\right]}^{2}}{K_{\left[\text{G3P}\right]}^{2}+{\left[\text{G}\right]}^{2}}}}+\;\overset{d}{\overbrace{\frac{(1-q)V_{\left[\text{PYR}\right]}{\left[\text{G}\right]}^{2}}{K_{\left[\text{PYR}\right]}^{2}+{\left[\text{G}\right]}^{2}}}}\right)\overset{e}{\overbrace{\left(\frac{1}{1+\beta [\text{CIT}]}\right)}} \end{array}$$

(2)
$$\frac{d[\text{G3P}]}{dt}=\overset{c}{\overbrace{\frac{qV_{\left[\text{G3P}\right]}{\left[\text{G}\right]}^{2}}{K_{\left[\text{G3P}\right]}^{2}+{\left[\text{G}\right]}^{2}}}}\overset{e}{\overbrace{\left(\frac{1}{1+\beta [\text{CIT}]}\right)}}-\overset{k}{\overbrace{\alpha (\text{G}3\text{P})}}$$

(3)
$$\begin{array}{l} \frac{d[\text{PYR}]}{dt}=\overset{d}{\overbrace{\left(\frac{(1-q)V_{\left[\text{PYR}\right]}{\left[\text{G}\right]}^{2}}{K_{\left[\text{PYR}\right]}^{2}+{\left[\text{G}\right]}^{2}}\right)}}\overset{e}{\overbrace{\left(\frac{1}{1+\beta [\text{CIT}]}\right)}}-\overset{g}{\overbrace{\frac{\left(1-\rho )V_{\left[\text{LACT}\right]}[\text{PYR}\right]}{K_{\left[\text{LACT}\right]}+[\text{PYR}]}}}-\\ \overset{i}{\overbrace{\frac{\rho V_{\left[\text{ACoA}\right]}[\text{PYR}]}{K_{\left[\text{ACoA}\right]}+[\text{PYR}]}}} \end{array}$$

(4)
$$\frac{d[\text{LACT}]}{dt}=\overset{g}{\overbrace{\frac{\left(1-\rho )V_{\left[\text{LACT}\right]}[\text{PYR}\right]}{K_{\left[\text{LACT}\right]}+[\text{PYR}]}}}-\overset{f}{\overbrace{f([\text{LACT}])([\text{LACT}]-L_{E})}}$$

(5)
$$\begin{array}{l} \frac{d[\text{ACoA}]}{dt}=\overset{i}{\overset{︷}{\rho V_{\text{[ACoA}]}[\text{PYR}]/K_{\left[\text{ACoA}\right]}+[\text{PYR}]}}+\overset{h}{\overset{︷}{h([\text{LACT}])(L_{E}-\text{[LACT}])}}\\ -\overset{j}{\overset{︷}{V_{\left[\text{CIT}\right]}[\text{ACoA}]/K_{\left[\text{CIT}\right]}+[\text{ACoA}]}}+\overset{k}{\overset{︷}{\alpha [\text{G3P}]}} \end{array}$$

(6)
$$\frac{d[\text{CIT}]}{dt}=\overset{j}{\overbrace{\frac{V_{\left[\text{CIT}\right]}[\text{ACoA}]}{K_{\left[\text{CIT}\right]}+[\text{ACoA}]}}}+\overset{l}{\overbrace{\phi [\text{CIT}]}}$$

## Abbreviations

β-HB: β-hydroxybutyrate
ACoA: acetyl coenzyme A
AMD: age-related macular degeneration
ATP: adenosine triphosphate
BSG1: Basigin-1
CIT: citrate
eFAST: Extended Fourier Amplitude Sensitivity Test
G: glucose
G3P: glycerol-3-phosphate
GLUT1: glucose transporter 1
GSA: global sensitivity analysis
HK2: hexokinase-2
KP: Kennedy pathway
LACT: lactate
LDHA: lactate dehydrogenase A
LDHB: lactate dehydrogenase B
OXPHOS: oxidative phosphorylation
PFK: phosphofructokinase
POS: photoreceptor outer segments
PRCC: partial rank correlation coefficient
PYR: pyruvate
RdCVF: rod-derived cone viability factor
RP: retinitis pigmentosa
RPE: retinal pigment epithelium
TCA: tricarboxylic acid
