# Supplementary material for: Insights into pathological mechanisms and interventions revealed by analyzing a mathematical model for cone metabolism
Source: Biosci Rep. 2022 Mar 8;42(3):BSR20212457. doi: 10.1042/BSR20212457 (PMC8905305; doi:10.1042/BSR20212457)
Supplement: Supplementary Data [file BSR-2021-2457_supp.pdf]

Supplementary material

Supplementary time-dependent global sensitivity analysis (GSA) results from the "Model output sensitivity for low initial glucose level" section in the main article are presented in Figure S1 and explained in "GSA results for low initial glucose level, [G](0)=0.02 mM". Supplementary time-dependent global GSA results from the "Model output sensitivity for higher initial glucose level" section in the main article are presented in Figure S2 and explained in "GSA results for higher initial glucose level, [G](0)=2 mM". PRCC sensitivity analysis time series data generated during this study are presented in Tables S1 through S8. eFAST sensitivity analysis time series data generated during this study are presented in Tables S9 through S16.

GSA results for low initial glucose level, [G](0)=0.02 mM

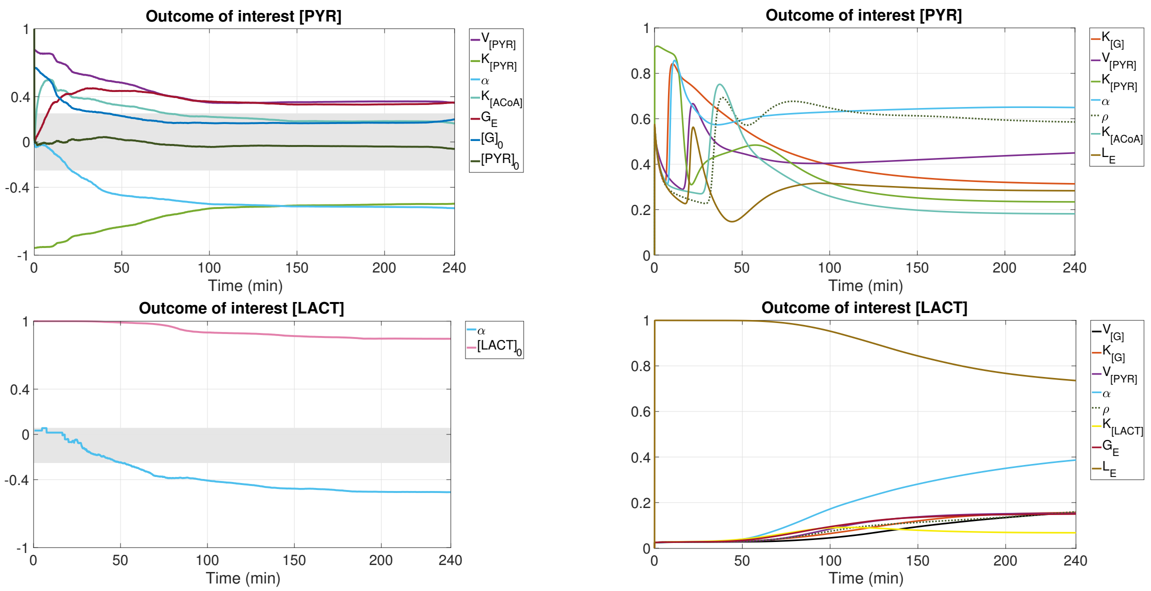

Figure S1: **Time-dependent global sensitivity analysis results for low initial glucose level.** Left panels present time-dependent results of PRCC and right panels present time-dependent results of eFAST, both for initial glucose concentration of $[G](0) = 0.02$ mM. Outcome of interest: row 1 - pyruvate ([PYR]); row 2 - lactate ([LACT]). The gray bands on the left panels indicate PRCC values which are not statistically significant (p-value ≥ 0.001).

PRCC, [PYR]. The PRCC analysis shows that the input factors which significantly affect the pyruvate level initially are $V_{[\mathrm{PYR}]}$, $K_{[\mathrm{PYR}]}$, $K_{[\mathrm{ACoA}]}$, $[\mathrm{G}]_0$, and $[\mathrm{PYR}]_0$ (the initial pyruvate concentration) (Figure 1, top left panel). By approximately 40 minutes the last three are no longer important, and [PYR] becomes sensitive to G_E and α. After about 75 minutes, $K_{[\mathrm{PYR}]}$ and α are the only influential factors. These results indicate that as the temporal dynamics of the metabolic system evolve, the ACoA biochemical reaction, external glucose, the maximal rate of pyruvate production and the initial glucose and pyruvate concentration lose their impact, but the half-limiting value of pyruvate synthesis from glucose as well as G3P utilization and oxidation of fatty acids remain essential for the pyruvate level.

eFAST, [PYR]. Similar to PRCC, eFAST highlights $V_{[\mathrm{PYR}]}$, $K_{[\mathrm{PYR}]}$, $K_{[\mathrm{ACoA}]}$ and α as important (Figure 1, top right panel). Additional factors to which [PYR] is sensitive include $K_{[\mathrm{G}]}$, ρ, and L_E. In the first 100 minutes of the simulation, the sensitivity indices of all parameters exhibit transient dynamics, reaching a peak before 50 minutes. As the indices settle to stable levels, toward the end of the simulation α, ρ and $V_{[\mathrm{PYR}]}$ stand out as the three most important parameters. These findings suggest that RdCVF-stimulated glucose uptake, the half-limiting values of the pyruvate and ACoA biochemical reactions, external lactate, G3P utilization and oxidation of fatty acids, diversion of pyruvate to OXPHOS, and the maximal rate of puruvate production are all influential in the beginning. However, later on the last three processes contribute

most to variability in the pyruvate concentration.

PRCC, [LACT]. PRCC shows that [LACT] is sensitive to just two factors: $[\text{LACT}]_0$, which is influential throughout the whole time window, and α, which becomes influential at about 75 minutes (Figure 1, bottom left). This means that with low initial glucose, only changes in the initial lactate concentration and changes in G3P utilization and oxidation of fatty acids affect the level of internal lactate.

eFAST, [LACT]. For the variability of internal lactate, eFAST identifies as most important L_E, which controls the lactate gradient-based gating mechanisms for flow into and out of the cell (Figure 1, bottom right). After 50 minutes, [LACT] also becomes sensitive to uncertainties other parameters: α, $V_{[\text{G}]}$, $K_{[\text{G}]}$, $V_{[\text{PYR}]}$, ρ, G_E, and $K_{[\text{LACT}]}$ (half-limiting value of lactate production from pyruvate). Among those, α has the most pronounced effect. These findings indicate that as the metabolic system dynamics evolve, in addition to the lactate gating mechanisms, other processes, most notably G3P utilization and oxidation of fatty acids, become important in relation to the variability of the internal lactate concentration.

GSA results for higher initial glucose level, [G](0)=2 mM

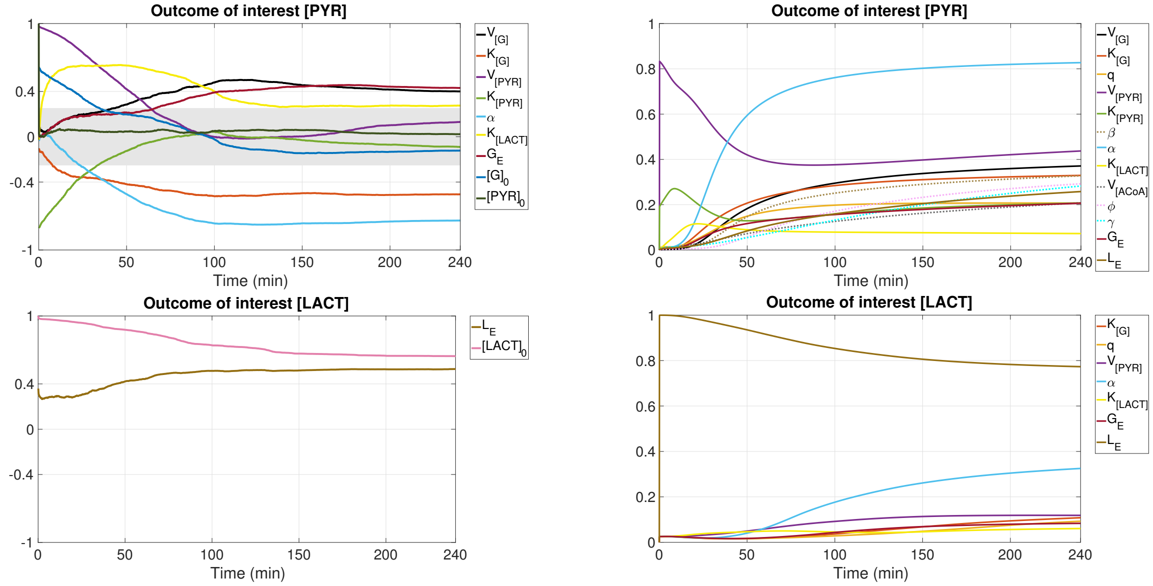

Figure S2: **Time-dependent global sensitivity analysis results for higher initial glucose level.** Left panels present time-dependent results of PRCC and right panels present time-dependent results of eFAST, both for initial glucose concentration of $[\text{G}](0) = 2$ mM. Outcome of interest: row 1 - pyruvate ([PYR]); row 2 - lactate ([LACT]). The gray bands on the left panels indicate PRCC values which are not statistically significant (p-value ≥ 0.001); no gray band means that the PRCC values are statistically significant throughout the whole time frame (left-bottom panel).

PRCC, [PYR]. PRCC reveals that initially the pyruvate level is sensitive to $V_{[\text{PYR}]}$, $K_{[\text{PYR}]}$, $[\text{G}]_0$ and $[\text{PYR}]_0$, but these factors are no longer important after 50 minutes (Figure 2, top left). On the other hand, $K_{[\text{LACT}]}$ has an impact between 5 and 100 minutes. Also, [PYR] is initially insensitive to changes in α, $K_{[\text{G}]}$, $V_{[\text{G}]}$, and G_E, but becomes sensitive at about 40, 50, 75, and 10 minutes, respectively. These results suggest that while in the beginning the pyruvate biochemical dynamics and the initial glucose and pyruvate concentrations influence the pyruvate level the most, as time progresses other factors become dominant, such as lactate synthesis, RdCVF-stimulated glucose uptake, external glucose, and G3P utilization and oxidation of fatty acids.

eFAST, [PYR]. In addition to $V_{[\text{G}]}$, $K_{[\text{G}]}$, $V_{[\text{PYR}]}$, $K_{[\text{PYR}]}$, α, $K_{[\text{LACT}]}$, and G_E, which were highlighted as important with PRCC, eFAST identifies several new parameters as contributing to the variability of pyruvate: q, β (the rate of CIT inhibition of glycolysis), $V_{[\text{ACoA}]}$, ϕ, γ (the maximum velocity of lactate transport contributing to ACoA production), and L_E (Figure 2, top right). Among all of those, the parameters with

the greatest sensitivity indices are $V_{[\mathrm{PYR}]}$, dominating until 40 minutes, and α, dominating from 40 minutes until the end of the simulation. These findings indicate that apart from uncertainty in the production of PYR, uncertainty in other processes, such as external glucose, RdCVF-stimulated glucose uptake, glycolysis inhibition, external lactate, G3P synthesis, LACT and ACoA production, CIT conversion to ATP and G3P utilization and oxidation of fatty acids, also play an important role in the variability of pyruvate.

PRCC, [LACT]. PRCC shows that the internal lactate level is sensitive to only $[\mathrm{LACT}]_0$ and L_E (Figure 2, bottom left). $[\mathrm{LACT}]_0$ is influential throughout the entire simulation window, and L_E becomes influential after 50. This means that with higher initial glucose, only changes in the initial lactate concentration and external lactate affect the level of internal lactate.

eFAST, [LACT]. eFAST shows that L_E has the greatest impact on the variability of internal lactate (Figure 2, bottom right). After 50 minutes, several other parameters, including α, $K_{[\mathrm{G}]}$, q, $V_{[\mathrm{PYR}]}$, $K_{[\mathrm{LACT}]}$, and G_E, emerge as important, but to a lower extent compared to L_E. Among those, α has the most pronounced effect. These findings suggest that over time, apart from uncertainty in external lactate, uncertainties in additional processes, most notably G3P utilization and oxidation of fatty acids, make a sizable contribution to the variability of internal lactate.

Comparing the GSA results with the two different initial glucose levels, we make the following observations. PRCC reveals that when [G](0) is low, G_E plays a role in the level of pyruvate earlier compared to the case of higher [G](0). With PRCC we also see that [PYR] is sensitive to $V_{[\mathrm{PYR}]}$ for both levels of initial glucose, but becomes insensitive faster for higher levels of initial glucose. Additionally, while $K_{[\mathrm{PYR}]}$ is important throughout the whole time frame with low initial glucose, it is only important in the first 25 minutes with higher initial glucose. This indicates that when there is more glucose present initially, the pyruvate level is not strongly affected by the maximal velocity and half-saturation value of the glucose conversion to pyruvate. Finally, with low initial glucose, [PYR] is sensitive to $K_{[\mathrm{ACoA}]}$, but for higher initial glucose, $K_{[\mathrm{LACT}]}$ appears as influential. This suggests that the pyruvate-to-ACoA pathway plays a role for low initial glucose, while the pyruvate-to-lactate pathway gains importance for higher initial glucose.

When intracellular lactate is the outcome of interest, PRCC identifies $[\mathrm{LACT}]_0$ as a factor influencing the lactate level whether [G](0) is low or higher. For low [G](0), [LACT] is sensitive to α, whereas for higher [G](0), [LACT] is sensitive to L_E. With the low [G](0), the cell may not have enough material to produce ample lactate, so the internal lactate concentration will possibly never exceed or only slightly exceed the external lactate level. Since the gating mechanisms for lactate leaving the cell is governed by the gradient between internal and external lactate, the gate would remain closed or be open with very minimal flux, which explains why the internal lactate level is not sensitive to L_E for low [G](0). With higher [G](0), PRCC shows that an increase in L_E will result in higher level of lactate inside the cell. This is because with more initial glucose, the cell can produce sufficient amount of lactate allowing the gate for lactate exit to be open with strong flux. Thus, increasing L_E beyond the intracellular lactate level will cause the gate to close, promoting an increase of lactate inside the cell.

PRCC and eFAST sensitivity analysis data

The rest of this document contains the time series data generated during this study by conducting PRCC and eFAST sensitivity analysis in the temporal window 0–240 minutes with [G](0)=0.02 mM and [G](0)=2 mM. The tables show the time discretization and include only the parameters to which the level and variability of the model outputs are sensitive to. In Tables S1 through S8, the sensitivity analysis data from PRCC for [G], [G3P], [PYR] and [LACT] is presented (PRCC sensitivity indices followed by their corresponding p-values). In Tables S9 through S16, the sensitivity analysis data from eFAST for [G], [G3P], [PYR] and [LACT] is shown.

Table S1: PRCC analysis data for [G] with [G](0)=0.02 mM: PRCC sensitivity indices and their corresponding p-values

Sensitivity analysis data from PRCC for [G] with [G](0)=0.02 mM (only the parameters to which [G]'s level is sensitive to appear)

Time discretization	q	K_{[G3P]}	K_{[PYR]}	alpha	G_E	[G]_0
0	0	0	0	0	0	1
0.1	-0.1390002	0.05532506	0.31412239	-0.0563168	0.11638685	0.99876751
0.2	-0.1641038	0.0864537	0.36244009	-0.0923359	0.19027401	0.99559437
0.3	-0.1830956	0.08845134	0.3740805	-0.0679697	0.20742458	0.99102836
0.4	-0.1892814	0.08820997	0.37069885	-0.081605	0.21047314	0.98536887
0.5	-0.1816553	0.08729013	0.38160484	-0.0815255	0.22811764	0.97885758
0.6	-0.1896545	0.09798131	0.3951706	-0.0871358	0.25316357	0.97408802
0.7	-0.1879903	0.10472405	0.4067644	-0.0885761	0.27782391	0.96920011
0.8	-0.1981118	0.10809428	0.41394194	-0.093241	0.29513104	0.96444609
0.9	-0.2127556	0.1132974	0.42136323	-0.0941419	0.31124996	0.95922739
1	-0.2216911	0.12627525	0.43190348	-0.1010247	0.33353398	0.9544625
1.1	-0.2293361	0.13549306	0.43984488	-0.0965036	0.35374715	0.94913175
1.2	-0.2330976	0.14809543	0.44434071	-0.0935121	0.3641148	0.94407237
1.3	-0.2396698	0.15702451	0.44952099	-0.097403	0.37803043	0.94035317
1.4	-0.2499119	0.1703554	0.46121113	-0.0967871	0.38851027	0.93655716
1.5	-0.2573201	0.17957022	0.4704408	-0.0928125	0.40464942	0.9328587
1.6	-0.2665541	0.18785354	0.4745432	-0.0905133	0.41409429	0.92952705
1.7	-0.2767014	0.19866819	0.48532565	-0.0894958	0.42746371	0.9263291
1.8	-0.2813968	0.2056891	0.49393035	-0.0865033	0.43702402	0.92290096
1.9	-0.2890839	0.21933442	0.50170103	-0.0889793	0.445462	0.91987617
2	-0.2963645	0.23108568	0.50979716	-0.0942998	0.45684444	0.91773234
2.1	-0.3011628	0.23728476	0.51152024	-0.0986124	0.46544297	0.91458666
2.2	-0.3038976	0.2467364	0.51453326	-0.1009624	0.47422841	0.91083975
2.3	-0.3142675	0.25619011	0.52125423	-0.1019903	0.48453309	0.90847737
2.4	-0.3246585	0.26358061	0.52589724	-0.1055187	0.4956123	0.90642766
2.5	-0.3323903	0.27325248	0.52952476	-0.1083725	0.50061658	0.90332172
2.6	-0.3357647	0.28030627	0.53514393	-0.1061688	0.50957727	0.90098053
2.7	-0.3418405	0.28885359	0.54007187	-0.107391	0.51944683	0.89712111
2.8	-0.3522641	0.29792992	0.54370305	-0.1105533	0.52836115	0.89449499
2.9	-0.3584484	0.30356046	0.54933284	-0.1096917	0.53377341	0.89070427
3	-0.3629639	0.31234349	0.55382216	-0.114026	0.5382816	0.88806498
3.1	-0.3675585	0.32039173	0.55422501	-0.1167885	0.54251165	0.88361765
3.2	-0.3741117	0.33155203	0.55894247	-0.1196125	0.54869856	0.88014071
3.3	-0.3775978	0.33693486	0.56289733	-0.1211475	0.55502332	0.87788268
3.4	-0.3825285	0.3469071	0.57074684	-0.1281198	0.56344501	0.87553927
3.5	-0.3843242	0.35300831	0.57217819	-0.1277377	0.56821403	0.87082215
3.6	-0.390783	0.36018292	0.5758853	-0.130925	0.57176301	0.86738364
3.7	-0.3939658	0.36621277	0.58015611	-0.1340837	0.57559832	0.86360283
3.8	-0.4022765	0.37202659	0.58632075	-0.1382233	0.58125586	0.86088147
3.9	-0.4091226	0.37677741	0.59382448	-0.1441053	0.59089743	0.85899229
4	-0.4124565	0.37909753	0.59621729	-0.1517181	0.59495134	0.85560023
4.1	-0.4134389	0.38290335	0.59845102	-0.1531569	0.60051435	0.84969196
4.2	-0.4186373	0.38968152	0.60189291	-0.1566585	0.60607582	0.84648874
4.3	-0.4191081	0.39390956	0.60465095	-0.159404	0.61246559	0.84240378
4.4	-0.4219807	0.39554826	0.60751409	-0.1577176	0.62125253	0.83819246
4.5	-0.4273797	0.39748132	0.60928493	-0.1593389	0.62788779	0.83425501
4.6	-0.4273551	0.40232978	0.61061121	-0.1555432	0.63271284	0.82889841
4.7	-0.4278681	0.40453897	0.60951046	-0.1568287	0.63831518	0.82351407
4.8	-0.4284502	0.40690509	0.60936529	-0.1586198	0.64164078	0.81848131
4.9	-0.4319004	0.41148112	0.61136641	-0.1621619	0.64250894	0.81341084
5	-0.4341663	0.41369869	0.61499316	-0.1638311	0.64415656	0.80872398
5.1	-0.4364011	0.42175532	0.61884253	-0.1672597	0.64769475	0.80357268
5.2	-0.4388561	0.42679068	0.62040001	-0.1664113	0.65152338	0.79847103
5.3	-0.4416209	0.43003922	0.62094243	-0.1697546	0.65319245	0.79405704
5.4	-0.4440435	0.42899322	0.62403593	-0.173478	0.65818357	0.78926109
5.5	-0.4463672	0.42977609	0.62413666	-0.177857	0.66153906	0.78474694
5.6	-0.4494608	0.42983424	0.62476626	-0.1773394	0.66401358	0.77958637
5.7	-0.4471234	0.42863732	0.62519262	-0.1781183	0.66787211	0.77439874
5.8	-0.4501044	0.43076059	0.62667418	-0.1822499	0.66954715	0.76982846
5.9	-0.4501489	0.43327693	0.62744102	-0.1843498	0.6704821	0.76327532
6	-0.4511735	0.43461834	0.62738853	-0.1871848	0.67211176	0.75793059
6.1	-0.4527295	0.43757454	0.62641045	-0.1921246	0.67380383	0.7522069
6.2	-0.4554938	0.43892075	0.62660452	-0.1944016	0.67636327	0.74754054
6.3	-0.4588108	0.43902856	0.62755864	-0.1954729	0.67786327	0.7408894
6.4	-0.4617132	0.44137991	0.62929644	-0.2032341	0.67849003	0.73549557
6.5	-0.4628551	0.44530545	0.62977176	-0.204174	0.68072429	0.72918846
6.6	-0.464458	0.44525627	0.63043775	-0.2059526	0.68232856	0.72274198
6.7	-0.4642094	0.44435217	0.62978997	-0.2092879	0.68346482	0.71779134
6.8	-0.4627237	0.44255753	0.62971013	-0.2114988	0.68335976	0.71059453
6.9	-0.4608445	0.43932278	0.6287603	-0.2123726	0.68311562	0.70543528
7	-0.4619743	0.43902968	0.62809339	-0.2137434	0.68264022	0.69976447
7.1	-0.4607332	0.43583517	0.62619593	-0.2159682	0.68467447	0.69189559
7.2	-0.4601056	0.43483024	0.6263609	-0.2215226	0.68573195	0.68670738
7.3	-0.4624535	0.43683211	0.62869684	-0.2245797	0.68840761	0.68248846
7.4	-0.4623749	0.43692956	0.62708802	-0.2285759	0.68958882	0.67729837
7.5	-0.4621287	0.43559931	0.62585356	-0.2312894	0.69091892	0.67130419
7.6	-0.4613191	0.43560402	0.62454299	-0.2356233	0.69055446	0.66492858
7.7	-0.4607922	0.43486601	0.6237326	-0.23592	0.69226689	0.65940747
7.8	-0.4601491	0.43514594	0.62362431	-0.2380826	0.69358952	0.65397518
7.9	-0.4624892	0.43607111	0.62526467	-0.2389388	0.69466391	0.65018665
8	-0.4643885	0.43853084	0.6254666	-0.2435273	0.6970251	0.64402857
8.1	-0.4655528	0.43988364	0.62653553	-0.2459403	0.69743817	0.63793882
8.2	-0.4677987	0.44023607	0.62702298	-0.2455872	0.69750787	0.63137328
8.3	-0.4689801	0.44229328	0.62643661	-0.2498192	0.69733486	0.6248125
8.4	-0.4719286	0.44252312	0.62695065	-0.2502081	0.69910212	0.62069757
8.5	-0.4723787	0.44184908	0.62697424	-0.2502114	0.69895119	0.61510916
8.6	-0.4733006	0.44276251	0.62662998	-0.2494473	0.69861874	0.61033007
8.7	-0.4726398	0.44550066	0.62799497	-0.2516679	0.70023717	0.60455059
8.8	-0.4757953	0.44653655	0.62903001	-0.2543625	0.70154106	0.59846084
8.9	-0.475243	0.44742855	0.62855893	-0.2578688	0.70233455	0.59374313
9	-0.4742645	0.44707188	0.62777018	-0.2596345	0.70264053	0.5881996
9.1	-0.4733045	0.44774502	0.6276145	-0.2605723	0.70292187	0.58223503
9.2	-0.4757875	0.44858085	0.62803714	-0.2634391	0.70330428	0.57683842
9.3	-0.4742885	0.44859518	0.62783178	-0.2679322	0.70364481	0.5724943
9.4	-0.4754429	0.45004557	0.62944263	-0.2714192	0.7042991	0.56683544
9.5	-0.4750028	0.44852467	0.62805931	-0.272408	0.70440479	0.56082893
9.6	-0.4760175	0.45061283	0.62897546	-0.274752	0.70576929	0.55766126
9.7	-0.4774922	0.45211897	0.62910984	-0.2778297	0.70566664	0.55163251
9.8	-0.4765785	0.45196444	0.62911949	-0.2787073	0.70663621	0.54501175
9.9	-0.4756332	0.45150472	0.62919842	-0.2810877	0.70737882	0.54161745
10	-0.4766957	0.45219766	0.62881017	-0.2808936	0.70776054	0.53494685
10.1	-0.4754745	0.4529011	0.62930422	-0.2838416	0.70814422	0.53059708
10.2	-0.4754407	0.45438223	0.62931861	-0.28472	0.70850147	0.52462676
10.3	-0.4758807	0.45470468	0.62815011	-0.287106	0.70908472	0.5191311
10.4	-0.4773575	0.45487632	0.62764964	-0.2885595	0.70971293	0.51272225
10.5	-0.4784947	0.45590666	0.62875974	-0.2883527	0.71146766	0.50685721
10.6	-0.4796455	0.45514811	0.62886438	-0.2902254	0.71096168	0.50322178
10.7	-0.4806253	0.45807305	0.62953145	-0.2910987	0.71146642	0.49825348
10.8	-0.4822189	0.45967058	0.63040048	-0.2957248	0.71278359	0.49390731
10.9	-0.4835705	0.45912306	0.6308227	-0.298757	0.71345084	0.49076587
11	-0.4847664	0.45979963	0.63054605	-0.2991747	0.71352875	0.48476975
11.1	-0.4847472	0.46009246	0.6298963	-0.2999598	0.71384227	0.47962621
11.2	-0.4835027	0.46112478	0.63013311	-0.3025544	0.71470371	0.47532238
11.3	-0.4845794	0.46282858	0.63019635	-0.3046215	0.71349513	0.46972539
11.4	-0.4861337	0.46275244	0.63062419	-0.3053908	0.71415619	0.46568522
11.5	-0.4863696	0.4619622	0.63016154	-0.3048055	0.71416154	0.46206157
11.6	-0.4870161	0.46218408	0.63061263	-0.3075875	0.7151811	0.45811107
11.7	-0.4889438	0.46324947	0.63149876	-0.309001	0.71559554	0.45342719
11.8	-0.4884965	0.46291691	0.63143805	-0.310216	0.71567462	0.44878708

Time discretization	q	K_{[G3P]}	K_{[PYR]}	alpha	G_E	[G]_0
15	-0.4989683	0.47245973	0.63654515	-0.3450432	0.72228459	0.34156372
15.1	-0.4982164	0.47182741	0.63615457	-0.3458033	0.72239727	0.33949154
15.2	-0.498343	0.47179293	0.63615692	-0.3472641	0.72174835	0.33614223
15.3	-0.498246	0.47146031	0.6354037	-0.347728	0.72137664	0.33371344
15.4	-0.4977097	0.47210284	0.63487927	-0.3487897	0.72097933	0.33127584
15.5	-0.4972228	0.4715201	0.6341256	-0.3505839	0.72096863	0.32705836
15.6	-0.4978591	0.47246838	0.63469471	-0.3526819	0.72096709	0.32344226
15.7	-0.4972261	0.47208264	0.63431369	-0.3536166	0.72084441	0.32015311
15.8	-0.4972896	0.47089228	0.63353936	-0.3524905	0.72072017	0.31811245
15.9	-0.4962713	0.47099377	0.63336943	-0.3538074	0.71963429	0.31435012
16	-0.4962578	0.4702315	0.63241248	-0.3537043	0.71992196	0.30928427
16.1	-0.4959528	0.47044928	0.63256436	-0.3536295	0.71969526	0.30723198
16.2	-0.4956178	0.47095665	0.6335839	-0.3538632	0.72031087	0.30594157
16.3	-0.4966282	0.47122112	0.63335804	-0.3560289	0.72015017	0.30353146
16.4	-0.4962952	0.47145607	0.63361024	-0.3565236	0.72016625	0.302028
16.5	-0.4972312	0.47122786	0.63363633	-0.3579833	0.7197943	0.29985196
16.6	-0.4967903	0.47085881	0.63406993	-0.3573986	0.71989113	0.29846729
16.7	-0.4975118	0.47084021	0.6335771	-0.3595817	0.72042409	0.2961928
16.8	-0.4977241	0.46990621	0.63312422	-0.3611921	0.72095351	0.29467546
16.9	-0.4976346	0.47013669	0.63389805	-0.3623437	0.72127137	0.29289506
17	-0.4970337	0.47035525	0.63383397	-0.3628067	0.72136187	0.29055783
17.1	-0.4961822	0.46988723	0.63353857	-0.3637015	0.72099788	0.28755531
17.2	-0.4947237	0.46838969	0.63301776	-0.3651713	0.72081784	0.28526986
17.3	-0.4948153	0.46837422	0.63252927	-0.3650672	0.720685	0.28261718
17.4	-0.4949777	0.46881191	0.63194714	-0.366674	0.72025292	0.27861115
17.5	-0.4946038	0.46764481	0.6312931	-0.3685255	0.7196883	0.27458482
17.6	-0.4946578	0.46754832	0.63053791	-0.3689104	0.71955295	0.27137961
17.7	-0.495047	0.46720324	0.63124877	-0.3706534	0.71997023	0.26961879
17.8	-0.4953959	0.46868625	0.63208135	-0.3728935	0.72052516	0.26818179
17.9	-0.4963503	0.4685582	0.63241254	-0.3734516	0.72074519	0.26721103
18	-0.4961017	0.46883022	0.63262614	-0.3745494	0.7208108	0.26425589
18.1	-0.4962718	0.46788137	0.63240308	-0.3757328	0.72074252	0.26132396
18.2	-0.4964156	0.46783048	0.63158246	-0.3761492	0.72010233	0.25981464
18.3	-0.4966248	0.46724634	0.63144587	-0.3772043	0.7194042	0.25681138
18.4	-0.4974282	0.46751582	0.63165791	-0.3773467	0.71970847	0.25640171
18.5	-0.4960125	0.46635382	0.63105529	-0.3775755	0.71910199	0.25463263
18.6	-0.4963623	0.46642357	0.63045499	-0.3782898	0.71904337	0.25184319
18.7	-0.4964022	0.46746565	0.63034553	-0.3791908	0.71821776	0.24752876
18.8	-0.4970588	0.46739268	0.63051511	-0.3801934	0.71803261	0.2468762
18.9	-0.4968346	0.46702662	0.63046719	-0.3802784	0.71828417	0.24573854
19	-0.4966478	0.4676496	0.63065971	-0.3819233	0.71810127	0.24308761
19.1	-0.4971813	0.46872032	0.63120233	-0.3835405	0.7179907	0.23990416
19.2	-0.4965919	0.46781277	0.63041071	-0.3849151	0.71776907	0.23870868
19.3	-0.4967599	0.46746996	0.63022445	-0.3858523	0.71781935	0.23581811
19.4	-0.4961118	0.46651527	0.62939634	-0.3871907	0.71758349	0.23384646
19.5	-0.4953596	0.46671843	0.62931982	-0.3881397	0.71738229	0.23326304
19.6	-0.4951908	0.46739343	0.62968458	-0.3889525	0.71725236	0.23076627
19.7	-0.4961441	0.46637777	0.62960594	-0.3911995	0.71719946	0.22778438
19.8	-0.4950738	0.46728424	0.62897022	-0.3913887	0.71656621	0.22594393
19.9	-0.4951921	0.46707628	0.62930351	-0.3917413	0.7159954	0.22590365
20	-0.4950283	0.46650831	0.62806262	-0.3934706	0.71588344	0.22305635
20.1	-0.4952865	0.46651954	0.62856718	-0.3959793	0.71568566	0.22250331
20.2	-0.4953635	0.46630398	0.62834092	-0.3964826	0.71560187	0.2219777
20.3	-0.4952395	0.46644735	0.6282253	-0.3974229	0.71536167	0.22055903
20.4	-0.4945297	0.46695106	0.62805529	-0.3983452	0.71569582	0.21817194
20.5	-0.4934901	0.46734788	0.62762236	-0.3988411	0.71529692	0.21572081
20.6	-0.4918425	0.46670838	0.62668168	-0.3986665	0.71399635	0.21201418
20.7	-0.4928827	0.46537444	0.62594088	-0.3995389	0.71337256	0.20931352
20.8	-0.4926795	0.46506819	0.62512859	-0.399672	0.71282797	0.20733303
20.9	-0.4928411	0.46531952	0.62487385	-0.4000158	0.71297932	0.20580449
21	-0.4922498	0.46518825	0.62485276	-0.4009997	0.71308003	0.20317672
21.1	-0.4925955	0.46538213	0.62460287	-0.4013247	0.71298195	0.20248647
21.2	-0.492899	0.46550284	0.62406039	-0.4032412	0.71323988	0.20140175
21.3	-0.4925344	0.46470109	0.62362881	-0.4043291	0.71239559	0.2010524
21.4	-0.4932652	0.4638835	0.62329093	-0.4051687	0.71185533	0.19889794
21.5	-0.4944337	0.46408549	0.62386659	-0.4070653	0.71100476	0.19747771
21.6	-0.4937927	0.46340964	0.62370508	-0.4074556	0.71077802	0.19607021
21.7	-0.4940348	0.46343537	0.62383195	-0.4086771	0.71084749	0.19506049
21.8	-0.493373	0.46354686	0.62348401	-0.4104053	0.71051018	0.19257766
21.9	-0.4922417	0.46410558	0.6224897	-0.4103618	0.70945224	0.19124014
22	-0.4913362	0.4647217	0.62193426	-0.4098519	0.70909028	0.18973873
22.1	-0.4917988	0.46433825	0.62207229	-0.4105285	0.70910088	0.18796629
22.2	-0.4923139	0.46474722	0.62164302	-0.4118063	0.70862011	0.18718404
22.3	-0.4925291	0.46436872	0.62079707	-0.4116021	0.7082386	0.18519734
22.4	-0.49137	0.46359553	0.6205626	-0.4130015	0.70781224	0.18402196
22.5	-0.4910719	0.46395303	0.62080191	-0.4133574	0.70749697	0.18205196
22.6	-0.4912537	0.46356722	0.62096914	-0.4143122	0.70764139	0.18168213
22.7	-0.4918871	0.46412858	0.62162895	-0.4149881	0.70778796	0.17981216
22.8	-0.491374	0.46434319	0.62130949	-0.4160214	0.70713841	0.17873121
22.9	-0.4908675	0.46328875	0.62092187	-0.4167129	0.70652113	0.17777062
23	-0.4908096	0.46259985	0.62104648	-0.4184176	0.70641734	0.17776838
23.1	-0.4908463	0.46185323	0.62083059	-0.4191585	0.70617255	0.17730392
23.2	-0.4901978	0.46051523	0.62060183	-0.4194467	0.70524439	0.17536356
23.3	-0.4896658	0.46044906	0.6194551	-0.4215406	0.7043767	0.17296599
23.4	-0.4898781	0.45954612	0.61929606	-0.4228518	0.70394687	0.17245686
23.5	-0.4897312	0.45975977	0.61898188	-0.4234127	0.70412963	0.17074538
23.6	-0.489002	0.45927306	0.61896532	-0.4235679	0.70399799	0.17007649
23.7	-0.4887972	0.4591935	0.61864629	-0.4249348	0.70396373	0.16776155
23.8	-0.4878695	0.46000183	0.61850312	-0.4265469	0.70354406	0.16592946
23.9	-0.4886655	0.45989595	0.61922849	-0.428191	0.70334443	0.16465633
24	-0.4883537	0.45882221	0.61872388	-0.4288732	0.70295251	0.16299167
24.1	-0.4878159	0.45861739	0.6190709	-0.4293062	0.70248712	0.15993651
24.2	-0.4881783	0.45834768	0.61893964	-0.431272	0.70175581	0.1595597
24.3	-0.4882133	0.45876247	0.61906353	-0.4315699	0.70130218	0.15868038
24.4	-0.4879365	0.45915608	0.61854146	-0.4315795	0.70093659	0.1571401
24.5	-0.4868294	0.45775	0.61765471	-0.4328983	0.69967319	0.15741119
24.6	-0.4872451	0.45796973	0.61781054	-0.4339926	0.69945973	0.15634156
24.7	-0.48701	0.45781699	0.61751443	-0.434121	0.6988483	0.15542682
24.8	-0.4864503	0.45811073	0.61702725	-0.4336541	0.69847793	0.15441809
24.9	-0.4862618	0.45728776	0.61650134	-0.4340866	0.69770286	0.15307547
25	-0.4853073	0.45711286	0.61533838	-0.4342029	0.69698907	0.15264977
25.1	-0.4843909	0.45680186	0.61478647	-0.4348835	0.6969809	0.15186696
25.2	-0.4844984	0.45645627	0.61515283	-0.4347191	0.69697683	0.15118014
25.3	-0.4830472	0.45535584	0.61410541	-0.4359799	0.69585321	0.15201899
25.4	-0.4828492	0.45487579	0.61399692	-0.4370709	0.69596236	0.15098256
25.5	-0.4820104	0.45468449	0.61286678	-0.4373077	0.6953443	0.15043673
25.6	-0.4817412	0.45466375	0.6130636	-0.4378949	0.69506843	0.14870499
25.7	-0.4815925	0.45575815	0.61369502	-0.4390246	0.69494456	0.14627109
25.8	-0.4809043	0.45522557	0.6132133	-0.4399465	0.69464677	0.14514804
25.9	-0.4799952	0.45471286	0.61222231	-0.4399325	0.69384473	0.14393669
26	-0.4787137	0.45362159	0.61156202	-0.4404444	0.69320367	0.14256875
26.1	-0.4783037	0.45340267	0.61139601	-0.4406626	0.69290049	0.142152
26.2	-0.4794448	0.45312721	0.6113615	-0.4413649	0.69249161	0.14168681
26.3	-0.4790788	0.4523373	0.61146366	-0.4426989	0.69164912	0.14098507
26.4	-0.4805141	0.45148526	0.61107957	-0.4433428	0.69099871	0.14016262
26.5	-0.4801348	0.45098202	0.61070536	-0.4453283	0.68993984	0.13832434
26.6	-0.4802265	0.45098867	0.61041744	-0.4453957	0.68972194	0.13818579
26.7	-0.4799216	0.45052952	0.61035739	-0.4465078	0.68926021	0.13771111
26.8	-0.4793061	0.4490687	0.60995573	-0.4470904	0.68925896	0.13597394

Time discretization	q	K_{[G3P]}	K_{[PYR]}	alpha	G_E	[G]_0
30	-0.4732696	0.44040556	0.60686436	-0.4662202	0.67693529	0.1142849
30.1	-0.4730297	0.44008467	0.60652229	-0.4665125	0.67627601	0.11435909
30.2	-0.4723476	0.43988751	0.60590986	-0.4666645	0.67562399	0.11394857
30.3	-0.4724205	0.44036107	0.60637141	-0.4670019	0.67518546	0.1126609
30.4	-0.4718189	0.44013874	0.60577492	-0.4669339	0.67476487	0.11224983
30.5	-0.47271	0.44021158	0.60592816	-0.4680173	0.67446476	0.1125987
30.6	-0.4725056	0.43974831	0.60556462	-0.4688277	0.67402136	0.11233069
30.7	-0.4717788	0.43899926	0.60523629	-0.4689948	0.67355118	0.11211884
30.8	-0.4710595	0.4387267	0.60523115	-0.4694719	0.67311283	0.11205325
30.9	-0.4704993	0.43880002	0.60499361	-0.470207	0.67332858	0.11154664
31	-0.4695524	0.4379867	0.60455138	-0.4699944	0.67274254	0.11080506
31.1	-0.4690623	0.43660624	0.60402327	-0.4707994	0.67130063	0.11090312
31.2	-0.4690774	0.43630375	0.60382766	-0.4707769	0.67092376	0.1093818
31.3	-0.4691732	0.43660061	0.60407603	-0.4710832	0.67097943	0.10867211
31.4	-0.4688779	0.43577563	0.60379815	-0.471429	0.67015679	0.10852221
31.5	-0.4689559	0.43578388	0.60358231	-0.4718133	0.67010407	0.10836247
31.6	-0.4690044	0.43575258	0.60352326	-0.4716201	0.66961609	0.10825375
31.7	-0.468691	0.4358946	0.60327032	-0.4718218	0.66918488	0.10854267
31.8	-0.468363	0.43593702	0.60297202	-0.4717174	0.66873203	0.10771349
31.9	-0.4687994	0.43589238	0.60325205	-0.4733604	0.66825482	0.10590768
32	-0.4686674	0.43644895	0.60312977	-0.4730746	0.66794739	0.1047628
32.1	-0.4689331	0.43669078	0.60330192	-0.4735745	0.66781589	0.10413031
32.2	-0.4690315	0.43612996	0.60325861	-0.4743963	0.66724184	0.10357145
32.3	-0.4701088	0.43668351	0.6034178	-0.4747758	0.66756441	0.10375221
32.4	-0.4698901	0.43726874	0.60340118	-0.4748463	0.66728554	0.10333152
32.5	-0.4697809	0.4370879	0.60290073	-0.4754615	0.66696377	0.10189579
32.6	-0.4702497	0.43701948	0.60321583	-0.4759724	0.66702974	0.10124717
32.7	-0.4699947	0.4365075	0.60264089	-0.4760778	0.66622937	0.10081713
32.8	-0.4698588	0.43601213	0.60239368	-0.4761112	0.66576743	0.1002853
32.9	-0.469677	0.43590724	0.60221737	-0.4762989	0.66560299	0.09915766
33	-0.4701649	0.43574928	0.60204487	-0.4770366	0.66561869	0.09834125
33.1	-0.4706174	0.4357888	0.60257641	-0.4776738	0.66526227	0.09789315
33.2	-0.4707538	0.43562674	0.60316471	-0.4773237	0.66497439	0.09691702
33.3	-0.4707494	0.43528018	0.60273009	-0.4773306	0.66475083	0.0948017
33.4	-0.4710729	0.43534558	0.60290547	-0.4772165	0.66429475	0.09396382
33.5	-0.4711732	0.4354075	0.60248456	-0.4771808	0.66415915	0.09328128
33.6	-0.470885	0.4351563	0.60206475	-0.4768357	0.66378891	0.09220587
33.7	-0.4706666	0.43489037	0.60163353	-0.4771925	0.6635008	0.0914871
33.8	-0.4699346	0.43372349	0.60159136	-0.4783973	0.66301302	0.0914217
33.9	-0.4696676	0.43341998	0.60141237	-0.4782123	0.66298027	0.09126928
34	-0.4693905	0.43341555	0.60134057	-0.4783861	0.66292392	0.0909728
34.1	-0.4696451	0.43353589	0.60120701	-0.4788608	0.66242402	0.09016905
34.2	-0.4694863	0.43314735	0.60098374	-0.4788504	0.66244787	0.08988461
34.3	-0.4698467	0.4330727	0.60108078	-0.4792243	0.66260383	0.08970897
34.4	-0.4702673	0.43300737	0.60059584	-0.4800133	0.66225754	0.08896883
34.5	-0.4703212	0.43289287	0.60055032	-0.4801928	0.66190418	0.08846847
34.6	-0.4706467	0.43267301	0.60067543	-0.4797922	0.66154647	0.08822413
34.7	-0.4706964	0.43242588	0.60061693	-0.479905	0.66119926	0.08751775
34.8	-0.4702705	0.43221912	0.60069316	-0.4803263	0.66073618	0.08718182
34.9	-0.4699853	0.43194289	0.60065144	-0.4804791	0.66046651	0.08750048
35	-0.4696803	0.43098294	0.60113717	-0.4813149	0.66064553	0.08682384
35.1	-0.4688028	0.43121038	0.60092626	-0.4808736	0.65957415	0.08509559
35.2	-0.4683393	0.43171633	0.60120179	-0.4813844	0.65957893	0.08410288
35.3	-0.4680527	0.43192249	0.60132485	-0.4813928	0.65965174	0.08375286
35.4	-0.4682175	0.43167218	0.60100633	-0.4817651	0.65923275	0.0833495
35.5	-0.4676402	0.43154936	0.6012349	-0.4820112	0.65930609	0.0824902
35.6	-0.4680023	0.43161326	0.60121486	-0.4822149	0.65898717	0.08228795
35.7	-0.4679536	0.43193026	0.60122105	-0.4829952	0.6587794	0.08225414
35.8	-0.4678616	0.43200571	0.60106669	-0.4832163	0.65855191	0.08170622
35.9	-0.4679322	0.43194609	0.60105737	-0.4834057	0.65820694	0.08167193
36	-0.4677167	0.43282628	0.60130271	-0.4836232	0.65826514	0.08136348
36.1	-0.4678808	0.43348961	0.60121583	-0.4833696	0.65824087	0.08104457
36.2	-0.467921	0.43347197	0.60111436	-0.4837619	0.65800884	0.0812194
36.3	-0.4679112	0.43382877	0.6013186	-0.4840892	0.65799856	0.08102406
36.4	-0.4681903	0.4339975	0.60151535	-0.4847816	0.65811915	0.08047973
36.5	-0.4681903	0.4339975	0.60151535	-0.4847816	0.65811915	0.08047973
36.6	-0.467909	0.43357639	0.60143585	-0.4850107	0.65763325	0.07965401
36.7	-0.467909	0.43357639	0.60143585	-0.4850107	0.65763325	0.07965401
36.8	-0.4672769	0.43396235	0.60129281	-0.4848027	0.65711239	0.07890063
36.9	-0.4669137	0.43432232	0.60088872	-0.4849598	0.65651835	0.07911283
37	-0.4669015	0.43432525	0.60076239	-0.4853397	0.65634929	0.07867567
37.1	-0.4666502	0.43431465	0.60065284	-0.4854245	0.65625211	0.07858416
37.2	-0.4666566	0.43449193	0.60095565	-0.48588	0.65633924	0.07825232
37.3	-0.466666	0.43433689	0.60069817	-0.4865977	0.65629758	0.07884813
37.4	-0.4661414	0.43420896	0.60070176	-0.4870696	0.6561026	0.07831074
37.5	-0.4658699	0.43451724	0.60092756	-0.487416	0.65620329	0.07786031
37.6	-0.4658699	0.43451724	0.60092756	-0.487416	0.65620329	0.07786031
37.7	-0.4662415	0.43454037	0.60074595	-0.4872106	0.65609221	0.077622
37.8	-0.4659764	0.43398255	0.60087501	-0.4872433	0.65583112	0.07781039
37.9	-0.4656547	0.43418516	0.60092008	-0.4873325	0.65575899	0.07706988
38	-0.4653612	0.43417412	0.60129138	-0.487629	0.65536363	0.07728265
38.1	-0.4651513	0.43382349	0.60116966	-0.487695	0.65531272	0.07702464
38.2	-0.465304	0.43436641	0.60141746	-0.488244	0.65514883	0.07643424
38.3	-0.4655622	0.43426732	0.60171069	-0.4883986	0.65539959	0.07559248
38.4	-0.4656204	0.43433077	0.60178708	-0.4884763	0.65553102	0.07555603
38.5	-0.4657028	0.43428964	0.60185385	-0.4886328	0.65542031	0.07583784
38.6	-0.4655268	0.43423617	0.60251536	-0.4891066	0.65518662	0.07581106
38.7	-0.4650627	0.43441136	0.60240517	-0.4893277	0.65489189	0.07552496
38.8	-0.4654952	0.43437377	0.60226581	-0.4895087	0.65452323	0.07530051
38.9	-0.4651939	0.43463446	0.60218666	-0.4895511	0.65440276	0.07544633
39	-0.4652559	0.43484334	0.60243056	-0.4900457	0.65397264	0.07391847
39.1	-0.4648821	0.43439058	0.60257429	-0.4901859	0.65379065	0.07427507
39.2	-0.4647073	0.43449634	0.60233366	-0.4901519	0.65371617	0.07391542
39.3	-0.4648697	0.43472548	0.60239929	-0.4902481	0.6536138	0.07337102
39.4	-0.4648726	0.43453689	0.60242986	-0.4905817	0.65352079	0.07311604
39.5	-0.4643257	0.43418293	0.60256096	-0.4909365	0.65304486	0.07329869
39.6	-0.4639672	0.43362669	0.60266485	-0.4905151	0.65280461	0.07321284
39.7	-0.464527	0.43355993	0.60203457	-0.4904639	0.65228041	0.07255522
39.8	-0.4645326	0.43333166	0.60210252	-0.490577	0.65218379	0.07199084
39.9	-0.4639351	0.43323138	0.60145793	-0.4909528	0.65113002	0.07171078
40	-0.4635627	0.43341682	0.60120429	-0.4914711	0.65130366	0.07018714
40.1	-0.4636465	0.43369711	0.60107969	-0.4920359	0.65159579	0.06923496
40.2	-0.4639784	0.43346813	0.60145407	-0.4923406	0.65161213	0.06855206
40.3	-0.4649975	0.43429563	0.60189894	-0.4922693	0.6508912	0.06716413
40.4	-0.465216	0.4347894	0.60233695	-0.4928789	0.6505869	0.06671566
40.5	-0.4648528	0.43492168	0.60264898	-0.4930632	0.65073927	0.06608606
40.6	-0.4647618	0.43513145	0.60275619	-0.4934014	0.65072248	0.06602333
40.7	-0.4651658	0.43522847	0.60287397	-0.494332	0.65080723	0.06574393
40.8	-0.4648715	0.4356641	0.60263005	-0.4942695	0.65025345	0.06534932
40.9	-0.4648288	0.43568667	0.60288967	-0.494374	0.65026201	0.06521503
41	-0.4648439	0.4354573	0.60295635	-0.4949658	0.65033225	0.06435962
41.1	-0.4647803	0.43584456	0.60305986	-0.4955247	0.65030572	0.06350552
41.2	-0.4646412	0.43571536	0.6028082	-0.4957901	0.64992707	0.06403645
41.3	-0.4647942	0.43571868	0.60267201	-0.4957368	0.64971426	0.06386662
41.4	-0.4652321	0.43594145	0.60213534	-0.4961778	0.64952044	0.06201994
41.5	-0.4652319	0.43628284	0.6021843	-0.4962843	0.64934152	0.06204256
41.6	-0.4652085	0.43620999	0.6019628	-0.4957123	0.6492027	0.06153956
41.7	-0.4651714	0.4363934	0.60186513	-0.4958835	0.64916298	0.06172848
41.8	-0.4653298	0.4365701	0.60184222	-0.4962932	0.64897855	0.06138519

Time discretization	q	K_{[G3P]}	K_{[PYR]}	alpha	G_E	[G]_0
45	-0.4614051	0.43282045	0.59873667	-0.5045389	0.64382283	0.05539996
45.1	-0.4610873	0.43259301	0.59882171	-0.504602	0.64377578	0.05575557
45.2	-0.4610873	0.43259301	0.59882171	-0.504602	0.64377578	0.05575557
45.3	-0.4609292	0.4330839	0.59905854	-0.5046369	0.64364848	0.05523655
45.4	-0.4617317	0.43329166	0.59886663	-0.5048733	0.64335066	0.05521942
45.5	-0.46161	0.43349792	0.59873138	-0.5052733	0.64300867	0.0546275
45.6	-0.4612582	0.43348569	0.59851686	-0.5053044	0.64266305	0.05476102
45.7	-0.4612582	0.43348569	0.59851686	-0.5053044	0.64266305	0.05476102
45.8	-0.4616492	0.4335582	0.59869812	-0.505407	0.64265258	0.05469223
45.9	-0.4617333	0.43352046	0.59884196	-0.5061345	0.64282114	0.0544196
46	-0.461882	0.43322851	0.59873535	-0.5062826	0.6426733	0.05336218
46.1	-0.461882	0.43322851	0.59873535	-0.5062826	0.6426733	0.05336218
46.2	-0.461882	0.43322851	0.59873535	-0.5062826	0.6426733	0.05336218
46.3	-0.4611987	0.43383597	0.59832478	-0.5068193	0.64153119	0.05232389
46.4	-0.4610903	0.43372629	0.59826024	-0.5065569	0.64121363	0.05220749
46.5	-0.4612734	0.43389249	0.59827605	-0.5072322	0.64105289	0.05212993
46.6	-0.4612734	0.43389249	0.59827605	-0.5072322	0.64105289	0.05212993
46.7	-0.4613686	0.43406046	0.59826412	-0.5072531	0.64090125	0.05221884
46.8	-0.461469	0.4335548	0.59797528	-0.5072159	0.64082903	0.0518046
46.9	-0.4610508	0.43310717	0.59799239	-0.5073302	0.64051842	0.05193184
47	-0.4608606	0.43308294	0.59792036	-0.5074201	0.64052883	0.0514965
47.1	-0.4607642	0.43294551	0.59784244	-0.5074978	0.64040534	0.05050936
47.2	-0.4607476	0.4327845	0.5977513	-0.5075956	0.64045161	0.05041051
47.3	-0.4607476	0.4327845	0.5977513	-0.5075956	0.64045161	0.05041051
47.4	-0.4607476	0.4327845	0.5977513	-0.5075956	0.64045161	0.05041051
47.5	-0.4607265	0.43296726	0.59783797	-0.5079509	0.64044219	0.05045881
47.6	-0.4607265	0.43296726	0.59783797	-0.5079509	0.64044219	0.05045881
47.7	-0.4607265	0.43296726	0.59783797	-0.5079509	0.64044219	0.05045881
47.8	-0.4603294	0.43253613	0.59801691	-0.5080741	0.63968619	0.05168644
47.9	-0.4596569	0.43240587	0.59759452	-0.507678	0.63925943	0.05179975
48	-0.4595199	0.43172582	0.59774319	-0.5084468	0.63909257	0.05138266
48.1	-0.4596645	0.43162751	0.59786446	-0.5089907	0.63923292	0.0514874
48.2	-0.4593758	0.43169893	0.59778985	-0.5092785	0.6390353	0.05196324
48.3	-0.4593758	0.43169893	0.59778985	-0.5092785	0.6390353	0.05196324
48.4	-0.4598459	0.43149093	0.59821979	-0.5097082	0.63911438	0.05180443
48.5	-0.4597187	0.43099312	0.59812877	-0.5097146	0.63908466	0.05206907
48.6	-0.4600175	0.43125791	0.59831819	-0.5101589	0.63912291	0.05204552
48.7	-0.4597318	0.431474	0.59808687	-0.5104757	0.63881943	0.052188
48.8	-0.4594692	0.43150668	0.59806981	-0.5107989	0.63878857	0.05190845
48.9	-0.4592184	0.43174289	0.59802884	-0.5113611	0.63825578	0.05140269
49	-0.4592184	0.43174289	0.59802884	-0.5113611	0.63825578	0.05140269
49.1	-0.459324	0.43162034	0.59812388	-0.5115706	0.63834419	0.05146302
49.2	-0.4599764	0.43176794	0.59870802	-0.5114224	0.63775351	0.05180673
49.3	-0.4597744	0.43130968	0.59893193	-0.5115607	0.63797104	0.05120103
49.4	-0.4597923	0.43095683	0.59886415	-0.5119986	0.63736652	0.050926
49.5	-0.4598621	0.430843	0.59873859	-0.5117612	0.6372402	0.05010727
49.6	-0.4597852	0.43078265	0.59866348	-0.5118865	0.63709479	0.0504379
49.7	-0.4597852	0.43078265	0.59866348	-0.5118865	0.63709479	0.0504379
49.8	-0.459701	0.43103361	0.59869668	-0.5119873	0.6370915	0.0504793
49.9	-0.4595845	0.43076584	0.59870179	-0.5122185	0.63659813	0.05048559
50	-0.4593744	0.43102896	0.59820912	-0.5121894	0.63598651	0.05006516
50.1	-0.4589425	0.43074531	0.59784925	-0.5122194	0.63578235	0.04963815
50.2	-0.4589425	0.43074531	0.59784925	-0.5122194	0.63578235	0.04963815
50.3	-0.4589425	0.43074531	0.59784925	-0.5122194	0.63578235	0.04963815
50.4	-0.4589215	0.43052863	0.59827662	-0.5127208	0.63564348	0.04975046
50.5	-0.4589215	0.43052863	0.59827662	-0.5127208	0.63564348	0.04975046
50.6	-0.458561	0.43013168	0.59842346	-0.5130691	0.6356194	0.04970285
50.7	-0.4582486	0.43038781	0.59839653	-0.5141499	0.63510375	0.04952881
50.8	-0.4581885	0.43060334	0.59826106	-0.5144283	0.63506248	0.04966704
50.9	-0.458042	0.4304111	0.59809092	-0.5142891	0.63490585	0.04870019
51	-0.4578801	0.43032707	0.59803434	-0.514277	0.63491533	0.0481894
51.1	-0.4579244	0.43043887	0.5979529	-0.5142274	0.63463081	0.04831891
51.2	-0.4581664	0.4308521	0.59813706	-0.514737	0.63452956	0.04841166
51.3	-0.4579925	0.43067441	0.59788704	-0.5147132	0.63422283	0.04781628
51.4	-0.4580183	0.43052656	0.59794803	-0.5149597	0.63396771	0.04823155
51.5	-0.4583499	0.43025624	0.59793216	-0.5153989	0.63374831	0.04822712
51.6	-0.4585809	0.42997143	0.59807283	-0.5154376	0.6336515	0.0478577
51.7	-0.4585861	0.43003738	0.598082	-0.51563	0.63354212	0.04782177
51.8	-0.4582071	0.42948971	0.59795534	-0.5160653	0.63317644	0.04771735
51.9	-0.4585443	0.42924409	0.59821915	-0.5161191	0.63263288	0.04762545
52	-0.4584511	0.42892003	0.59790766	-0.5160572	0.63246814	0.04718215
52.1	-0.4582382	0.42865804	0.59794646	-0.5166172	0.63243941	0.04732858
52.2	-0.4582382	0.42865804	0.59794646	-0.5166172	0.63243941	0.04732858
52.3	-0.4584791	0.42862475	0.59792579	-0.5170317	0.63243057	0.04735863
52.4	-0.4586578	0.42887027	0.59789616	-0.5172599	0.63217431	0.04734058
52.5	-0.4580045	0.42798846	0.59764078	-0.5176549	0.63158351	0.0470663
52.6	-0.4580484	0.42803126	0.5976541	-0.5177532	0.63143809	0.04721063
52.7	-0.4578342	0.42822537	0.59770316	-0.5182776	0.63124415	0.04690325
52.8	-0.4577293	0.42819725	0.59761199	-0.5185481	0.63117356	0.04662122
52.9	-0.4576919	0.42823347	0.59755617	-0.5188013	0.63083805	0.04621141
53	-0.4576919	0.42823347	0.59755617	-0.5188013	0.63083805	0.04621141
53.1	-0.4576919	0.42823347	0.59755617	-0.5188013	0.63083805	0.04621141
53.2	-0.4574251	0.42807505	0.59817467	-0.5194376	0.63093827	0.04534745
53.3	-0.4573299	0.4279714	0.59826491	-0.519465	0.63060787	0.04517248
53.4	-0.4570921	0.42799787	0.59784526	-0.5196503	0.63034668	0.04551348
53.5	-0.4570921	0.42799787	0.59784526	-0.5196503	0.63034668	0.04551348
53.6	-0.4569229	0.42798126	0.59739334	-0.5195248	0.62980191	0.04548726
53.7	-0.4569229	0.42798126	0.59739334	-0.5195248	0.62980191	0.04548726
53.8	-0.4569229	0.42798126	0.59739334	-0.5195248	0.62980191	0.04548726
53.9	-0.4566343	0.42772361	0.59708173	-0.5194884	0.6293294	0.04543574
54	-0.4564875	0.42762115	0.59724297	-0.5195971	0.62925495	0.045289
54.1	-0.4565005	0.42766362	0.5969391	-0.5202483	0.62908069	0.04534467
54.2	-0.45681	0.42775825	0.59684115	-0.520613	0.62897524	0.04526834
54.3	-0.4574723	0.42757504	0.59675779	-0.5207015	0.62873677	0.04526348
54.4	-0.4572984	0.42773788	0.59737201	-0.5213964	0.62867444	0.04419632
54.5	-0.4570338	0.42751112	0.59705231	-0.522034	0.62818813	0.04434321
54.6	-0.4567284	0.42716885	0.59704463	-0.5217457	0.62772886	0.04486124
54.7	-0.4565392	0.42739654	0.59677458	-0.5217316	0.62745395	0.04484707
54.8	-0.456379	0.4272732	0.59674194	-0.5220948	0.62726615	0.04402866
54.9	-0.4563283	0.42724358	0.59668942	-0.5222613	0.62726489	0.04441004
55	-0.456621	0.42680328	0.59668934	-0.5223658	0.62714859	0.04422436
55.1	-0.456602	0.42692512	0.59638017	-0.522365	0.62682076	0.04401328
55.2	-0.4569301	0.42721643	0.59665044	-0.5227236	0.62687513	0.04442443
55.3	-0.456998	0.42692989	0.59660306	-0.5229655	0.62676602	0.04469947
55.4	-0.4569446	0.42661155	0.59647276	-0.5230386	0.62656174	0.04476449
55.5	-0.4571127	0.42681688	0.59657685	-0.5233143	0.62649398	0.04500367
55.6	-0.4574184	0.42724886	0.5966364	-0.523636	0.6268878	0.04456599
55.7	-0.4574165	0.42770231	0.59663268	-0.5238165	0.62638682	0.04406613
55.8	-0.4574165	0.42770231	0.59663268	-0.5238165	0.62638682	0.04406613
55.9	-0.4571182	0.42693498	0.59653652	-0.5240847	0.62590375	0.04361303
56	-0.4576615	0.42646037	0.59655458	-0.5248442	0.62614164	0.04394589
56.1	-0.4581026	0.42631334	0.59617354	-0.5254182	0.62589984	0.04400901
56.2	-0.4581026	0.42631334	0.59617354	-0.5254182	0.62589984	0.04400901
56.3	-0.4581026	0.42631334	0.59617354	-0.5254182	0.62589984	0.04400901
56.4	-0.4581026	0.42631334	0.59617354	-0.5254182	0.62589984	0.04400901
56.5	-0.4583955	0.42644005	0.5963066	-0.5255257	0.62578025	0.04404407
56.6	-0.4583603	0.42640248	0.59653385	-0.5258267	0.62587081	0.04374666
56.7	-0.4582604	0.426754	0.59683875	-0.5263093	0.62591569	0.0436624
56.8	-0.4583076	0.42670469	0.59711675	-0.5266983	0.62592659	0.04337073

11.9	-0.4880503	0.46277706	0.63235306	-0.310374	0.71558433	0.44448372
12	-0.4867328	0.46344432	0.6335003	-0.3103627	0.7165624	0.44002471
12.1	-0.4857481	0.46261293	0.63328126	-0.311076	0.71621702	0.4377163
12.2	-0.4860629	0.46338576	0.63404231	-0.3124309	0.71642129	0.43477944
12.3	-0.4868962	0.46229291	0.63368392	-0.312552	0.71631005	0.43082241
12.4	-0.4882984	0.46265237	0.6342166	-0.3133664	0.71684441	0.42793544
12.5	-0.4877707	0.46289759	0.634747	-0.3141535	0.71679899	0.42394493
12.6	-0.487018	0.46257835	0.63500481	-0.3142175	0.7175849	0.42057823
12.7	-0.4879381	0.46480518	0.63618823	-0.3158267	0.71832329	0.4176562
12.8	-0.489247	0.4643404	0.63561364	-0.3167956	0.71901967	0.41279894
12.9	-0.4892303	0.46428522	0.63483067	-0.3176926	0.71969925	0.40727526
13	-0.4891481	0.46465844	0.6348632	-0.3192082	0.72024992	0.40433178
13.1	-0.4904613	0.46546075	0.63495415	-0.3202428	0.719961	0.40074465
13.2	-0.4921562	0.46677712	0.63567118	-0.3228123	0.72062731	0.39702736
13.3	-0.4926896	0.46791788	0.63536573	-0.3252832	0.72082485	0.39130625
13.4	-0.4935682	0.46831668	0.63628333	-0.3278724	0.72111419	0.38775744
13.5	-0.4941854	0.46877262	0.63735496	-0.3297465	0.72076083	0.38455153
13.6	-0.4939168	0.46880539	0.636641	-0.3301647	0.72070865	0.38120486
13.7	-0.4957925	0.46910551	0.63626999	-0.3323429	0.72098184	0.37922313
13.8	-0.4961309	0.46917762	0.63680244	-0.3322169	0.72148514	0.37624765
13.9	-0.4981913	0.4702275	0.63619307	-0.3319865	0.72159072	0.37318622
14	-0.4985937	0.46953034	0.63612949	-0.3339161	0.72144209	0.36875283
14.1	-0.4991703	0.46998466	0.63625243	-0.333103	0.72115633	0.36567546
14.2	-0.499414	0.47162543	0.63678486	-0.3356518	0.7217195	0.36326467
14.3	-0.4988193	0.47291215	0.63642462	-0.3365085	0.72159728	0.36147781
14.4	-0.498844	0.47224995	0.63677313	-0.336651	0.72084192	0.35704905
14.5	-0.4990879	0.47141228	0.63672897	-0.336908	0.72080831	0.35390813
14.6	-0.4974413	0.47062415	0.63582828	-0.3380083	0.72112644	0.3519655
14.7	-0.4985522	0.47204979	0.63742608	-0.3423859	0.72183484	0.34798266
14.8	-0.4989445	0.47170589	0.63738653	-0.3431543	0.72205533	0.34532122
14.9	-0.4991111	0.47242268	0.6374033	-0.3438775	0.72193125	0.34440253

60	-0.4546996	0.42362857	0.59574376	-0.5291973	0.6212815	0.04077654
60.1	-0.4550585	0.42329996	0.59576278	-0.5290959	0.62120285	0.04077631
60.2	-0.4549999	0.42298936	0.59578757	-0.5294325	0.6209381	0.04073355
60.3	-0.4549999	0.42298936	0.59578757	-0.5294325	0.6209381	0.04073355
60.4	-0.4547052	0.42270354	0.59562486	-0.5294765	0.62057615	0.04100253
60.5	-0.4543609	0.42280615	0.5955409	-0.529772	0.620439	0.04083045
60.6	-0.4540631	0.42204438	0.59511625	-0.5299872	0.61995759	0.04052966
60.7	-0.4536961	0.42180759	0.59516622	-0.5301315	0.61990703	0.04027101
60.8	-0.4536107	0.42103198	0.59480843	-0.5304893	0.6199674	0.04009934
60.9	-0.4536098	0.42092092	0.59497237	-0.5304914	0.6197702	0.03993955
61	-0.4534382	0.42024431	0.59482139	-0.5303076	0.61943424	0.03856843
61.1	-0.4538755	0.42042355	0.59495654	-0.5303992	0.61937709	0.03851623
61.2	-0.4537604	0.42024941	0.59461969	-0.5305256	0.61904689	0.03896276
61.3	-0.4537604	0.42024941	0.59461969	-0.5305256	0.61904689	0.03896276
61.4	-0.4536402	0.41991701	0.59432805	-0.5303763	0.61901851	0.03882488
61.5	-0.4536402	0.41991701	0.59432805	-0.5303763	0.61901851	0.03882488
61.6	-0.4532639	0.41972998	0.59431218	-0.5305841	0.61879912	0.0388127
61.7	-0.4531612	0.41949651	0.59399342	-0.5306302	0.61849069	0.03861037
61.8	-0.4527335	0.41905961	0.59351606	-0.5305209	0.61817065	0.03892509
61.9	-0.4526241	0.41897081	0.59353683	-0.5303885	0.61799489	0.03840604
62	-0.4525681	0.41872517	0.59330105	-0.5304484	0.61772726	0.03833317
62.1	-0.4525683	0.41833723	0.59327655	-0.530554	0.61757424	0.03853646
62.2	-0.4525786	0.417669	0.59329985	-0.5305108	0.61736246	0.03910723
62.3	-0.4525786	0.417669	0.59329985	-0.5305108	0.61736246	0.03910723
62.4	-0.4524071	0.41739606	0.59299571	-0.5305484	0.61708866	0.03899049
62.5	-0.4524071	0.41739606	0.59299571	-0.5305484	0.61708866	0.03899049
62.6	-0.4523022	0.41700871	0.59285151	-0.5304608	0.61672876	0.03892568
62.7	-0.4522194	0.41709584	0.59264907	-0.5304438	0.61654465	0.03893152
62.8	-0.4522194	0.41709584	0.59264907	-0.5304438	0.61654465	0.03893152
62.9	-0.4519348	0.41699112	0.59265133	-0.5304574	0.61636052	0.0393105
63	-0.4519619	0.41684783	0.59251583	-0.5301283	0.616176	0.0393016
63.1	-0.4517432	0.41642978	0.5923054	-0.5301389	0.61574951	0.03940023
63.2	-0.4518222	0.41680741	0.59247993	-0.5301127	0.61565998	0.03927496
63.3	-0.4507993	0.4167594	0.59260892	-0.5300011	0.61531853	0.03918631
63.4	-0.4507993	0.4167594	0.59260892	-0.5300011	0.61531853	0.03918631
63.5	-0.4506627	0.41652626	0.59219921	-0.5301106	0.61509025	0.03927748
63.6	-0.4505658	0.41626164	0.59225299	-0.530308	0.61499128	0.03889563
63.7	-0.4503776	0.41596601	0.59205699	-0.5301756	0.61487626	0.0389444
63.8	-0.4502381	0.4155441	0.59190844	-0.5303165	0.61394295	0.03917349
63.9	-0.4498568	0.41543233	0.5915823	-0.5301568	0.61394463	0.03864906
64	-0.4498568	0.41543233	0.5915823	-0.5301568	0.61394463	0.03864906
64.1	-0.449457	0.41556037	0.59136447	-0.5302847	0.61362149	0.0382788
64.2	-0.449219	0.415341	0.59144274	-0.530501	0.61335271	0.03873708
64.3	-0.4489525	0.4151555	0.59129128	-0.5305025	0.61324012	0.03912151
64.4	-0.448774	0.41482646	0.59130708	-0.5307327	0.61288152	0.03979916
64.5	-0.448774	0.41482646	0.59130708	-0.5307327	0.61288152	0.03979916
64.6	-0.4488474	0.41491495	0.59106428	-0.5314118	0.61292554	0.03919492
64.7	-0.448521	0.41460287	0.59045105	-0.5318796	0.61210117	0.03917516
64.8	-0.4485363	0.41454921	0.59019531	-0.531933	0.61201294	0.03900217
64.9	-0.4485223	0.41478456	0.59024382	-0.5319758	0.61212435	0.03905894
65	-0.4485223	0.41478456	0.59024382	-0.5319758	0.61212435	0.03905894
65.1	-0.4485223	0.41478456	0.59024382	-0.5319758	0.61212435	0.03905894
65.2	-0.4478576	0.41427828	0.58990027	-0.5322965	0.61142101	0.03962613
65.3	-0.447167	0.41336213	0.58959701	-0.5321464	0.61080376	0.03982732
65.4	-0.447167	0.41336213	0.58959701	-0.5321464	0.61080376	0.03982732
65.5	-0.4470975	0.41297378	0.5893115	-0.5322035	0.61012386	0.04014715
65.6	-0.4474607	0.41294424	0.5895253	-0.5325405	0.6100514	0.04024853
65.7	-0.4473729	0.41247387	0.58943113	-0.5328	0.60972112	0.0400547
65.8	-0.4469845	0.41229388	0.5888752	-0.5329792	0.6091163	0.04011275
65.9	-0.4469845	0.41229388	0.5888752	-0.5329792	0.6091163	0.04011275
66	-0.446899	0.41208331	0.58882364	-0.5329683	0.6088426	0.04039107
66.1	-0.4464585	0.41141141	0.58846486	-0.5331321	0.60841726	0.04033602
66.2	-0.4461153	0.4108503	0.58791735	-0.5332459	0.60802529	0.0403915
66.3	-0.4460578	0.41089154	0.587759	-0.5333395	0.60805018	0.04001877
66.4	-0.445196	0.41009617	0.58782402	-0.5341913	0.60758463	0.04010033
66.5	-0.4453685	0.41006427	0.58785105	-0.5345059	0.60723176	0.03986847
66.6	-0.4453434	0.40972373	0.58732017	-0.53444	0.60651283	0.03990063
66.7	-0.4451444	0.40963143	0.58725348	-0.5345254	0.6064558	0.04002865
66.8	-0.4447301	0.40941769	0.58705176	-0.5347442	0.60564519	0.0403624
66.9	-0.4447063	0.40941188	0.58697418	-0.5348568	0.60542822	0.04045544
67	-0.4447063	0.40941188	0.58697418	-0.5348568	0.60542822	0.04045544
67.1	-0.4445762	0.40886253	0.58679303	-0.5348215	0.60524593	0.04021446
67.2	-0.4445348	0.40865087	0.58664816	-0.5347737	0.6047258	0.03973725
67.3	-0.444297	0.40842842	0.58652021	-0.5349973	0.60441416	0.0401425
67.4	-0.4438561	0.40792456	0.58642028	-0.5350855	0.60410227	0.04030411
67.5	-0.4433521	0.40767124	0.58581427	-0.5349841	0.60364818	0.04060958
67.6	-0.443316	0.40755653	0.5856346	-0.5351182	0.60364029	0.04085345
67.7	-0.4433351	0.40759976	0.5858372	-0.5352207	0.60366411	0.04067302
67.8	-0.4427445	0.40797044	0.585806	-0.5356962	0.60345577	0.04062527
67.9	-0.4427187	0.40754226	0.58556211	-0.5356547	0.60348691	0.04096253
68	-0.4429951	0.407557	0.58562021	-0.5359829	0.60344853	0.04061646
68.1	-0.4429951	0.407557	0.58562021	-0.5359829	0.60344853	0.04061646
68.2	-0.4429978	0.40745027	0.58562456	-0.5360762	0.60343545	0.04074381
68.3	-0.4429497	0.40674858	0.58565245	-0.5362697	0.60307147	0.04123742
68.4	-0.4428008	0.40659977	0.58539918	-0.5364174	0.60294559	0.04150559
68.5	-0.4423698	0.40659183	0.58541293	-0.5360883	0.60270844	0.04099525
68.6	-0.4425536	0.40638791	0.58546944	-0.5362343	0.60248935	0.0408506
68.7	-0.4424172	0.4064385	0.58540828	-0.536395	0.60185326	0.04090621
68.8	-0.4422579	0.40643934	0.58508814	-0.5362931	0.60172854	0.04097515
68.9	-0.4424597	0.40655075	0.58519673	-0.5367103	0.60165603	0.04052196

26.9	-0.4797294	0.44890321	0.61031546	-0.4479797	0.68933041	0.13502123
27	-0.4793142	0.44803772	0.61031004	-0.4482278	0.68924347	0.13392503
27.1	-0.47829	0.44785545	0.60977182	-0.4484282	0.68862862	0.1321462
27.2	-0.4783917	0.44779469	0.60933286	-0.4486944	0.68869303	0.13103419
27.3	-0.4783678	0.44693596	0.60920081	-0.449119	0.68836974	0.13043558
27.4	-0.4786459	0.44677855	0.6088308	-0.4489882	0.68821643	0.12930159
27.5	-0.4791963	0.447264	0.60871063	-0.4507655	0.68794326	0.12842978
27.6	-0.4783912	0.44599371	0.60855772	-0.4518408	0.68726712	0.12771469
27.7	-0.4785355	0.44570101	0.60873603	-0.4536547	0.68716422	0.12732401
27.8	-0.4787057	0.4468402	0.60902803	-0.4548087	0.6869424	0.12700988
27.9	-0.4783083	0.44604018	0.60895141	-0.4550559	0.68651517	0.12758412
28	-0.4779163	0.44616488	0.6086238	-0.4552835	0.68618602	0.12640775
28.1	-0.4776019	0.44588334	0.60838169	-0.4554224	0.68592167	0.12579662
28.2	-0.4776014	0.44515306	0.60789969	-0.4557592	0.68550889	0.12575439
28.3	-0.4769639	0.4452546	0.60793556	-0.4564837	0.68525503	0.12577252
28.4	-0.4780295	0.44568776	0.60824077	-0.4571307	0.68499431	0.12478614
28.5	-0.478159	0.4461153	0.60837238	-0.4577218	0.68495384	0.12320641
28.6	-0.4769762	0.44466236	0.60757865	-0.458129	0.68341946	0.12323288
28.7	-0.477176	0.4439142	0.60753581	-0.4588493	0.6832826	0.12234843
28.8	-0.4768357	0.44328002	0.60725295	-0.4591271	0.68257879	0.12118481
28.9	-0.4775098	0.44347717	0.60783573	-0.4589614	0.68265621	0.12058758
29	-0.4774622	0.44359566	0.60808899	-0.4600212	0.68209801	0.11918119
29.1	-0.4774765	0.44359477	0.6080862	-0.460085	0.68180748	0.11823206
29.2	-0.4778822	0.44391972	0.60786102	-0.4608839	0.68144117	0.11745167
29.3	-0.4769892	0.44250466	0.60754128	-0.4628554	0.68025206	0.11647323
29.4	-0.4766272	0.44169723	0.6075734	-0.4632525	0.67961263	0.11653063
29.5	-0.4755878	0.44155647	0.60744965	-0.4635598	0.679312	0.11654259
29.6	-0.4747346	0.44059889	0.60734771	-0.4642034	0.67878692	0.1165121
29.7	-0.4750418	0.44032589	0.6072125	-0.464872	0.67866672	0.11593959
29.8	-0.4739933	0.44003337	0.60630007	-0.4658954	0.67769459	0.11656182
29.9	-0.4738382	0.44056849	0.60649592	-0.4665295	0.67753494	0.11564701

75	-0.4342404	0.39808221	0.58247968	-0.541952	0.59219693	0.03748547
75.1	-0.4342404	0.39808221	0.58247968	-0.541952	0.59219693	0.03748547
75.2	-0.4342404	0.39808221	0.58247968	-0.541952	0.59219693	0.03748547
75.3	-0.4339657	0.39765539	0.58235555	-0.5422417	0.59142473	0.03800456
75.4	-0.4339657	0.39765539	0.58235555	-0.5422417	0.59142473	0.03800456
75.5	-0.434302	0.39769675	0.58244291	-0.542574	0.59129182	0.03795742
75.6	-0.434302	0.39769675	0.58244291	-0.542574	0.59129182	0.03795742
75.7	-0.434302	0.39769675	0.58244291	-0.542574	0.59129182	0.03795742
75.8	-0.434302	0.39769675	0.58244291	-0.542574	0.59129182	0.03795742
75.9	-0.4340192	0.39725768	0.58226313	-0.5424798	0.59079304	0.0381335
76	-0.4340339	0.39739514	0.58227487	-0.542472	0.59068217	0.03813356
76.1	-0.4334679	0.39678897	0.58200628	-0.5423215	0.59021141	0.03845646
76.2	-0.4334679	0.39678897	0.58200628	-0.5423215	0.59021141	0.03845646
76.3	-0.4334679	0.39678897	0.58200628	-0.5423215	0.59021141	0.03845646
76.4	-0.4334679	0.39678897	0.58200628	-0.5423215	0.59021141	0.03845646
76.5	-0.43275	0.39571405	0.58163245	-0.5426576	0.58925364	0.0381257
76.6	-0.432797	0.39594997	0.58184131	-0.5426979	0.58932739	0.0379936
76.7	-0.432797	0.39594997	0.58184131	-0.5426979	0.58932739	0.0379936
76.8	-0.4327088	0.39607303	0.58196544	-0.5426443	0.58934493	0.03757135
76.9	-0.4327088	0.39607303	0.58196544	-0.5426443	0.58934493	0.03757135
77	-0.4324067	0.39553885	0.58184699	-0.5425815	0.58896857	0.0376427
77.1	-0.4324067	0.39553885	0.58184699	-0.5425815	0.58896857	0.0376427
77.2	-0.4312219	0.39502108	0.5815619	-0.542594	0.58822691	0.03742849
77.3	-0.4308381	0.39533348	0.58145974	-0.5428318	0.587581	0.03755709
77.4	-0.4308381	0.39533348	0.58145974	-0.5428318	0.587581	0.03755709
77.5	-0.4308381	0.39533348	0.58145974	-0.5428318	0.587581	0.03755709
77.6	-0.4308381	0.39533348	0.58145974	-0.5428318	0.587581	0.03755709
77.7	-0.4310058	0.39536498	0.58133724	-0.5427803	0.58727696	0.03749
77.8	-0.4305441	0.39495103	0.58119375	-0.5428749	0.58696802	0.03773445
77.9	-0.4305441	0.39495103	0.58119375	-0.5428749	0.58696802	0.03773445
78	-0.4304098	0.39483903	0.58110986	-0.5427817	0.58691274	0.03744513
78.1	-0.4304098	0.39483903	0.58110986	-0.5427817	0.58691274	0.03744513
78.2	-0.4305121	0.39486301	0.58087086	-0.5427391	0.58705943	0.03769094
78.3	-0.4305121	0.39486301	0.58087086	-0.5427391	0.58705943	0.03769094
78.4	-0.4302034	0.39508814	0.58097562	-0.5430022	0.58723857	0.03796922
78.5	-0.4302034	0.39508814	0.58097562	-0.5430022	0.58723857	0.03796922
78.6	-0.4302034	0.39508814	0.58097562	-0.5430022	0.58723857	0.03796922
78.7	-0.4301398	0.39514849	0.58102859	-0.5434038	0.58724568	0.0379192
78.8	-0.4301827	0.3951643	0.58092579	-0.5433644	0.58736217	0.03753194
78.9	-0.4306638	0.39516774	0.58132323	-0.5437959	0.58726644	0.03769504
79	-0.4302818	0.39450128	0.58125405	-0.5442422	0.58639345	0.03757381
79.1	-0.430185	0.3943702	0.58124965	-0.5444974	0.58639805	0.03746298
79.2	-0.430185	0.3943702	0.58124965	-0.5444974	0.58639805	0.03746298
79.3	-0.4302713	0.39446272	0.58131987	-0.5445778	0.58639724	0.03716198
79.4	-0.4302713	0.39446272	0.58131987	-0.5445778	0.58639724	0.03716198
79.5	-0.4302713	0.39446272	0.58131987	-0.5445778	0.58639724	0.03716198
79.6	-0.4302713	0.39446272	0.58131987	-0.5445778	0.58639724	0.03716198
79.7	-0.4302742	0.39434711	0.58142837	-0.5445388	0.58639141	0.03709345
79.8	-0.4296795	0.39440391	0.58124389	-0.5445325	0.58622454	0.03669817
79.9	-0.4296795	0.39440391	0.58124389	-0.5445325	0.58622454	0.03669817
80	-0.4296795	0.39440391	0.58124389	-0.5445325	0.58622454	0.03669817
80.1	-0.4296855	0.39471233	0.58088639	-0.5444472	0.5863025	0.03584525
80.2	-0.429562	0.39459896	0.58045018	-0.5442985	0.5861384	0.03609231
80.3	-0.4290519	0.39452035	0.58041193	-0.5445328	0.58570686	0.03593415
80.4	-0.4290519	0.39452035	0.58041193	-0.5445328	0.58570686	0.03593415
80.5	-0.4288895	0.39440187	0.5804577	-0.5448032	0.585809	0.03547665
80.6	-0.4288895	0.39440187	0.5804577	-0.5448032	0.585809	0.03547665
80.7	-0.4289145	0.3944175	0.5803766	-0.5449257	0.58564464	0.03552509
80.8	-0.4290656	0.39433618	0.5803413	-0.5451233	0.58556419	0.03537068
80.9	-0.4287823	0.39416965	0.58025306	-0.5450677	0.58559369	0.03551754
81	-0.4287823	0.39416965	0.58025306	-0.5450677	0.58559369	0.03551754
81.1	-0.428871	0.39405162	0.5803811	-0.5453354	0.58576896	0.03556719
81.2	-0.4283805	0.39364786	0.58015469	-0.5451892	0.58556978	0.03561547
81.3	-0.4283033	0.39380333	0.58005709	-0.5452055	0.58549366	0.03555471
81.4	-0.4283033	0.39380333	0.58005709	-0.5452055	0.58549366	0.03555471
81.5	-0.4287292	0.39368771	0.5799999	-0.5450917	0.58513762	0.03542756
81.6	-0.4287292	0.39368771	0.5799999	-0.5450917	0.58513762	0.03542756
81.7	-0.4286475	0.39364005	0.57974237	-0.5450672	0.58475408	0.03585821
81.8	-0.4286475	0.39364005	0.57974237	-0.5450672	0.58475408	0.03585821
81.9	-0.4287154	0.39369765	0.57966742	-0.5453315	0.58467148	0.03568626
82	-0.4287154	0.39369765	0.57966742	-0.5453315	0.58467148	0.03568626
82.1	-0.4288706	0.39351113	0.5795414	-0.5452962	0.5846186	0.03552477
82.2	-0.4290182	0.39393832	0.57982195	-0.5454757	0.58444334	0.03552924
82.3	-0.4290182	0.39393832	0.57982195	-0.5454757	0.58444334	0.03552924
82.4	-0.429108	0.39396465	0.58012665	-0.5461764	0.5842293	0.03458955
82.5	-0.429108	0.39396465	0.58012665	-0.5461764	0.5842293	0.03458955
82.6	-0.4294461	0.39418946	0.58002192	-0.5460245	0.58379673	0.03436471
82.7	-0.4294461	0.39418946	0.58002192	-0.5460245	0.58379673	0.03436471
82.8	-0.4294461	0.39418946	0.58002192	-0.5460245	0.58379673	0.03436471
82.9	-0.4294461	0.39418946	0.58002192	-0.5460245	0.58379673	0.03436471
83	-0.4294461	0.39418946	0.58002192	-0.5460245	0.58379673	0.03436471
83.1	-0.4294534	0.39431443	0.5802297	-0.5468621	0.58374518	0.03428149
83.2	-0.4295702	0.39459561	0.58024075	-0.5467715	0.5840604	0.03380958
83.3	-0.4295702	0.39459561	0.58024075	-0.5467715	0.5840604	0.03380958
83.4	-0.4295702	0.39459561	0.58024075	-0.5467715	0.5840604	0.03380958
83.5	-0.4299551	0.39465493	0.5801147	-0.5470195	0.58418279	0.03374447
83.6	-0.4299551	0.39465493	0.5801147	-0.5470195	0.58418279	0.03374447
83.7	-0.4299551	0.39465493	0.5801147	-0.5470195	0.58418279	0.03374447
83.8	-0.4299024	0.39483056	0.58015478	-0.5470795	0.58408192	0.03397234
83.9	-0.4299024	0.39483056	0.58015478	-0.5470795	0.58408192	0.03397234

41.9	-0.4651271	0.43644911	0.60181792	-0.4965339	0.64897392	0.06094729
42	-0.4651271	0.43644911	0.60181792	-0.4965339	0.64897392	0.06094729
42.1	-0.4646831	0.43575309	0.60110564	-0.4961128	0.64823088	0.06032025
42.2	-0.4640766	0.43593224	0.60110541	-0.4965105	0.6478428	0.06025144
42.3	-0.4644216	0.43478394	0.60087883	-0.497005	0.64751882	0.05978822
42.4	-0.4640435	0.43484944	0.60092733	-0.497627	0.64731181	0.05943386
42.5	-0.4641457	0.43485195	0.60080098	-0.4979177	0.64700899	0.05969125
42.6	-0.4642874	0.43478361	0.60049642	-0.4982593	0.64674447	0.05909075
42.7	-0.4643285	0.43474076	0.60040834	-0.4980761	0.64664522	0.05869138
42.8	-0.463275	0.43431666	0.60021323	-0.4979399	0.64602536	0.05839632
42.9	-0.4633005	0.43444476	0.60020673	-0.4981893	0.64611079	0.05804178
43	-0.4632608	0.43432162	0.60014589	-0.4985098	0.64591703	0.0584632
43.1	-0.4635946	0.4345285	0.60034975	-0.499931	0.64608189	0.05783819
43.2	-0.4633046	0.43455229	0.60003455	-0.4997543	0.64599384	0.05780358
43.3	-0.4633001	0.43434976	0.5999072	-0.4998234	0.64592812	0.05760122
43.4	-0.4632672	0.43440036	0.6001404	-0.5007676	0.64614614	0.05709261
43.5	-0.4632672	0.43440036	0.6001404	-0.5007676	0.64614614	0.05709261
43.6	-0.46302	0.43434968	0.59959905	-0.5019702	0.64597802	0.05576223
43.7	-0.4629317	0.43427317	0.59946333	-0.5019179	0.64600611	0.05584986
43.8	-0.4622161	0.43425567	0.5992938	-0.5023327	0.64604551	0.05613415
43.9	-0.4624237	0.43427726	0.59963966	-0.502687	0.64563496	0.05545085
44	-0.4623213	0.43419738	0.59965455	-0.5023956	0.6456638	0.05501836
44.1	-0.4624982	0.43414286	0.59970766	-0.5025194	0.64558925	0.05457065
44.2	-0.4622309	0.43428833	0.59973518	-0.502567	0.64528208	0.05478347
44.3	-0.461742	0.43345263	0.5995392	-0.5030955	0.64499888	0.0551995
44.4	-0.4618747	0.43381978	0.5997603	-0.5036768	0.64517988	0.05459072
44.5	-0.4622707	0.43356301	0.59950509	-0.5036314	0.64490145	0.05437736
44.6	-0.4618149	0.43365236	0.59943272	-0.5040483	0.64432246	0.05483759
44.7	-0.4613824	0.43353429	0.59912044	-0.5039796	0.64390978	0.05522619
44.8	-0.4610203	0.4328477	0.59887855	-0.5042484	0.64384492	0.0552757
44.9	-0.4610203	0.4328477	0.59887855	-0.5042484	0.64384492	0.0552757

90	-0.4304196	0.39672272	0.57903919	-0.5493224	0.58230126	0.03027997
90.1	-0.4304196	0.39672272	0.57903919	-0.5493224	0.58230126	0.03027997
90.2	-0.4304196	0.39672272	0.57903919	-0.5493224	0.58230126	0.03027997
90.3	-0.4304196	0.39672272	0.57903919	-0.5493224	0.58230126	0.03027997
90.4	-0.4304196	0.39672272	0.57903919	-0.5493224	0.58230126	0.03027997
90.5	-0.4302409	0.39688269	0.57895093	-0.5496156	0.5825379	0.03025348
90.6	-0.4302409	0.39688269	0.57895093	-0.5496156	0.5825379	0.03025348
90.7	-0.4300746	0.39714489	0.57894864	-0.5496568	0.58251713	0.03022404
90.8	-0.4300882	0.39725548	0.57889721	-0.5497111	0.58219913	0.02999689
90.9	-0.4300882	0.39725548	0.57889721	-0.5497111	0.58219913	0.02999689
91	-0.4305047	0.39751959	0.578743	-0.5494725	0.58199969	0.02931532
91.1	-0.4305047	0.39751959	0.578743	-0.5494725	0.58199969	0.02931532
91.2	-0.4305849	0.39756631	0.578999	-0.5494959	0.58238348	0.02888152
91.3	-0.4305849	0.39756631	0.578999	-0.5494959	0.58238348	0.02888152
91.4	-0.4305849	0.39756631	0.578999	-0.5494959	0.58238348	0.02888152
91.5	-0.4305849	0.39756631	0.578999	-0.5494959	0.58238348	0.02888152
91.6	-0.4305173	0.39779918	0.57906893	-0.5497126	0.58220894	0.02900642
91.7	-0.4304487	0.39798079	0.57927035	-0.5498622	0.58193573	0.0290451
91.8	-0.4304487	0.39798079	0.57927035	-0.5498622	0.58193573	0.0290451
91.9	-0.4304354	0.39832827	0.57941848	-0.5499039	0.58180574	0.02906823
92	-0.4304354	0.39832827	0.57941848	-0.5499039	0.58180574	0.02906823
92.1	-0.4303264	0.39842826	0.57948219	-0.5501611	0.58187546	0.02983554
92.2	-0.4303264	0.39842826	0.57948219	-0.5501611	0.58187546	0.02983554
92.3	-0.4306215	0.3984611	0.5790736	-0.5503333	0.58170219	0.02920142
92.4	-0.4306215	0.3984611	0.5790736	-0.5503333	0.58170219	0.02920142
92.5	-0.4306215	0.3984611	0.5790736	-0.5503333	0.58170219	0.02920142
92.6	-0.4306215	0.3984611	0.5790736	-0.5503333	0.58170219	0.02920142
92.7	-0.4306215	0.3984611	0.5790736	-0.5503333	0.58170219	0.02920142
92.8	-0.4306215	0.3984611	0.5790736	-0.5503333	0.58170219	0.02920142
92.9	-0.4306215	0.3984611	0.5790736	-0.5503333	0.58170219	0.02920142
93	-0.4306215	0.3984611	0.5790736	-0.5503333	0.58170219	0.02920142
93.1	-0.4306215	0.3984611	0.5790736	-0.5503333	0.58170219	0.02920142
93.2	-0.4306215	0.3984611	0.5790736	-0.5503333	0.58170219	0.02920142
93.3	-0.4306215	0.3984611	0.5790736	-0.5503333	0.58170219	0.02920142
93.4	-0.4305737	0.39874949	0.57912806	-0.550626	0.58136369	0.02890161
93.5	-0.4305737	0.39874949	0.57912806	-0.550626	0.58136369	0.02890161
93.6	-0.4307604	0.39863218	0.57919079	-0.5506782	0.58131717	0.02881933
93.7	-0.4307604	0.39863218	0.57919079	-0.5506782	0.58131717	0.02881933
93.8	-0.4306423	0.39863953	0.57885516	-0.5507486	0.58105939	0.02801807
93.9	-0.4306423	0.39863953	0.57885516	-0.5507486	0.58105939	0.02801807
94	-0.4304668	0.39886498	0.57886399	-0.5509922	0.58099255	0.02810649
94.1	-0.4304668	0.39886498	0.57886399	-0.5509922	0.58099255	0.02810649
94.2	-0.4304668	0.39886498	0.57886399	-0.5509922	0.58099255	0.02810649
94.3	-0.4303903	0.39883488	0.57881782	-0.551157	0.58087391	0.02818138
94.4	-0.4303903	0.39883488	0.57881782	-0.551157	0.58087391	0.02818138
94.5	-0.4303903	0.39883488	0.57881782	-0.551157	0.58087391	0.02818138
94.6	-0.4304753	0.39884471	0.57880345	-0.5512742	0.5808186	0.02850753
94.7	-0.4304753	0.39884471	0.57880345	-0.5512742	0.5808186	0.02850753
94.8	-0.4302904	0.39890124	0.57904177	-0.551621	0.58098111	0.0286361
94.9	-0.4300341	0.39890902	0.57920662	-0.5518097	0.58110574	0.02827631
95	-0.4301428	0.3988465	0.57905693	-0.5518087	0.58092805	0.02865913
95.1	-0.4301428	0.3988465	0.57905693	-0.5518087	0.58092805	0.02865913
95.2	-0.4301428	0.3988465	0.57905693	-0.5518087	0.58092805	0.02865913
95.3	-0.4301428	0.3988465	0.57905693	-0.5518087	0.58092805	0.02865913
95.4	-0.4304936	0.39869876	0.57912405	-0.5519248	0.58123318	0.0285846
95.5	-0.4304936	0.39869876	0.57912405	-0.5519248	0.58123318	0.0285846
95.6	-0.4304076	0.39886221	0.57917475	-0.5519619	0.58144948	0.02818331
95.7	-0.4304076	0.39886221	0.57917475	-0.5519619	0.58144948	0.02818331
95.8	-0.4304076	0.39886221	0.57917475	-0.5519619	0.58144948	0.02818331
95.9	-0.4304076	0.39886221	0.57917475	-0.5519619	0.58144948	0.02818331
96	-0.4303721	0.39901145	0.57905781	-0.5522365	0.58140882	0.02810209
96.1	-0.4302363	0.39905486	0.57914089	-0.5522786	0.58131224	0.02823424
96.2	-0.4302363	0.39905486	0.57914089	-0.5522786	0.58131224	0.02823424
96.3	-0.4302363	0.39905486	0.57914089	-0.5522786	0.58131224	0.02823424
96.4	-0.430205	0.39899117	0.57900896	-0.5523235	0.58096334	0.02820906
96.5	-0.4304054	0.39916923	0.57903788	-0.5525211	0.58066069	0.02814546
96.6	-0.4304054	0.39916923	0.57903788	-0.5525211	0.58066069	0.02814546
96.7	-0.4304054	0.39916923	0.57903788	-0.5525211	0.58066069	0.02814546
96.8	-0.4303198	0.39945071	0.57904162	-0.5527023	0.58048029	0.02811934
96.9	-0.4303198	0.39945071	0.57904162	-0.5527023	0.58048029	0.02811934
97	-0.4303198	0.39945071	0.57904162	-0.5527023	0.58048029	0.02811934
97.1	-0.4303198	0.39945071	0.57904162	-0.5527023	0.58048029	0.02811934
97.2	-0.4301803	0.39950532	0.57906306	-0.5524687	0.58046617	0.02819034
97.3	-0.4301803	0.39950532	0.57906306	-0.5524687	0.58046617	0.02819034
97.4	-0.4300241	0.3997442	0.57885916	-0.5526751	0.58037787	0.02812935
97.5	-0.4300241	0.3997442	0.57885916	-0.5526751	0.58037787	0.02812935
97.6	-0.4300241	0.3997442	0.57885916	-0.5526751	0.58037787	0.02812935
97.7	-0.4300847	0.39953688	0.57881618	-0.5526085	0.58018721	0.02788995
97.8	-0.4300847	0.39953688	0.57881618	-0.5526085	0.58018721	0.02788995
97.9	-0.4300847	0.39953688	0.57881618	-0.5526085	0.58018721	0.02788995
98	-0.4302159	0.39926267	0.5787011	-0.5525425	0.58029553	0.02784594
98.1	-0.4302159	0.39926267	0.5787011	-0.5525425	0.58029553	0.02784594
98.2	-0.4300632	0.39942684	0.57866415	-0.552547	0.58028979	0.0278681
98.3	-0.4300649	0.39967874	0.57870037	-0.5526928	0.58003603	0.02809715
98.4	-0.4300649	0.39967874	0.57870037	-0.5526928	0.58003603	0.02809715
98.5	-0.4300649	0.39967874	0.57870037	-0.5526928	0.58003603	0.02809715
98.6	-0.4300277	0.3996718	0.57850728	-0.5527028	0.58007359	0.02800007
98.7	-0.4300277	0.3996718	0.57850728	-0.5527028	0.58007359	0.02800007
98.8	-0.4299674	0.39968932	0.57841121	-0.5529178	0.58007764	0.02807969
98.9	-0.4299674	0.39968932	0.57841121	-0.5529178	0.58007764	0.02807969

56.9	-0.4580259	0.42610975	0.59698284	-0.5267255	0.62570466	0.04288827
57	-0.4581116	0.42632686	0.59710433	-0.5267991	0.62553531	0.04282266
57.1	-0.4582209	0.42624584	0.59731213	-0.5270214	0.62542244	0.04251894
57.2	-0.4582209	0.42624584	0.59731213	-0.5270214	0.62542244	0.04251894
57.3	-0.4580854	0.426067	0.59707849	-0.5271794	0.62504357	0.04310469
57.4	-0.4581584	0.42594832	0.59710582	-0.5277873	0.62467737	0.04260262
57.5	-0.4580347	0.42567611	0.59724264	-0.5276856	0.62477757	0.04239643
57.6	-0.4575725	0.42545678	0.59699394	-0.5279259	0.62459756	0.04266535
57.7	-0.4574966	0.42519216	0.59664042	-0.5278609	0.62452763	0.04264558
57.8	-0.4572005	0.42544613	0.59653196	-0.5276659	0.62437451	0.0423552
57.9	-0.4572005	0.42544613	0.59653196	-0.5276659	0.62437451	0.0423552
58	-0.4570837	0.42579882	0.59643731	-0.5279515	0.62433859	0.04219308
58.1	-0.4572001	0.42582701	0.59650674	-0.5279753	0.62434563	0.04223715
58.2	-0.4569785	0.42529769	0.59646604	-0.5283398	0.62411771	0.04238881
58.3	-0.4569738	0.42502738	0.59637569	-0.5288714	0.6234186	0.04204363
58.4	-0.4563891	0.42457097	0.59645416	-0.5290309	0.62292379	0.04208895
58.5	-0.4561036	0.4246791	0.59625613	-0.5287665	0.62280066	0.04192837
58.6	-0.4561036	0.4246791	0.59625613	-0.5287665	0.62280066	0.04192837
58.7	-0.4556869	0.42444056	0.59636338	-0.5288441	0.62266306	0.04196057
58.8	-0.4556655	0.42427461	0.59648963	-0.5288	0.62255876	0.04193774
58.9	-0.4556655	0.42427461	0.59648963	-0.5288	0.62255876	0.04193774
59	-0.4553334	0.42397665	0.59650798	-0.5287205	0.62246694	0.04221354
59.1	-0.4552422	0.42435137	0.596678	-0.5286434	0.62248503	0.04109632
59.2	-0.4551116	0.42452053	0.59673854	-0.5285897	0.62244795	0.04099557
59.3	-0.4551116	0.42452053	0.59673854	-0.5285897	0.62244795	0.04099557
59.4	-0.4551116	0.42452053	0.59673854	-0.5285897	0.62244795	0.04099557
59.5	-0.4550293	0.4241926	0.59656446	-0.5286312	0.6223232	0.04136303
59.6	-0.4550293	0.4241926	0.59656446	-0.5286312	0.6223232	0.04136303
59.7	-0.4547264	0.42418741	0.5961757	-0.528658	0.6217781	0.04110477
59.8	-0.4544959	0.42432139	0.59589078	-0.5287575	0.62170375	0.0410472
59.9	-0.4545138	0.42417016	0.59587018	-0.5290911	0.62169511	0.04103338

105	-0.4315035	0.40143931	0.57808418	-0.5559295	0.57785706	0.02770945
105.1	-0.4315035	0.40143931	0.57808418	-0.5559295	0.57785706	0.02770945
105.2	-0.4315035	0.40143931	0.57808418	-0.5559295	0.57785706	0.02770945
105.3	-0.4311543	0.40115342	0.57782873	-0.5558124	0.57750895	0.02763894
105.4	-0.4311446	0.40124475	0.57782228	-0.5559116	0.57735659	0.02786941
105.5	-0.4311446	0.40124475	0.57782228	-0.5559116	0.57735659	0.02786941
105.6	-0.4311446	0.40124475	0.57782228	-0.5559116	0.57735659	0.02786941
105.7	-0.4311446	0.40124475	0.57782228	-0.5559116	0.57735659	0.02786941
105.8	-0.4311446	0.40124475	0.57782228	-0.5559116	0.57735659	0.02786941
105.9	-0.4311479	0.40103152	0.57780688	-0.5556826	0.57704572	0.02792543
106	-0.4313615	0.40089363	0.57775585	-0.5557417	0.57720152	0.02771229
106.1	-0.4313615	0.40089363	0.57775585	-0.5557417	0.57720152	0.02771229
106.2	-0.4313615	0.40089363	0.57775585	-0.5557417	0.57720152	0.02771229
106.3	-0.4313615	0.40089363	0.57775585	-0.5557417	0.57720152	0.02771229
106.4	-0.4313615	0.40089363	0.57775585	-0.5557417	0.57720152	0.02771229
106.5	-0.4313615	0.40089363	0.57775585	-0.5557417	0.57720152	0.02771229
106.6	-0.4313907	0.40105433	0.57773209	-0.5557832	0.57720908	0.02762695
106.7	-0.4313907	0.40105433	0.57773209	-0.5557832	0.57720908	0.02762695
106.8	-0.4313907	0.40105433	0.57773209	-0.5557832	0.57720908	0.02762695
106.9	-0.4313907	0.40105433	0.57773209	-0.5557832	0.57720908	0.02762695
107	-0.4314124	0.40086905	0.57749088	-0.5558567	0.57714523	0.02742106
107.1	-0.4316476	0.40089781	0.57763732	-0.5557806	0.57688124	0.0274387
107.2	-0.4315549	0.40110328	0.57746273	-0.5558594	0.57650058	0.02724206
107.3	-0.4315549	0.40110328	0.57746273	-0.5558594	0.57650058	0.02724206
107.4	-0.4315549	0.40110328	0.57746273	-0.5558594	0.57650058	0.02724206
107.5	-0.4315549	0.40110328	0.57746273	-0.5558594	0.57650058	0.02724206
107.6	-0.4315549	0.40110328	0.57746273	-0.5558594	0.57650058	0.02724206
107.7	-0.4315549	0.40110328	0.57746273	-0.5558594	0.57650058	0.02724206
107.8	-0.4315549	0.40110328	0.57746273	-0.5558594	0.57650058	0.02724206
107.9	-0.4315268	0.40099761	0.57722398	-0.5559461	0.57641052	0.02690673
108	-0.4315268	0.40099761	0.57722398	-0.5559461	0.57641052	0.02690673
108.1	-0.4315268	0.40099761	0.57722398	-0.5559461	0.57641052	0.02690673
108.2	-0.4315268	0.40099761	0.57722398	-0.5559461	0.57641052	0.02690673
108.3	-0.4315268	0.40099761	0.57722398	-0.5559461	0.57641052	0.02690673
108.4	-0.4316147	0.40123726	0.57731459	-0.5561912	0.57627783	0.02710187
108.5	-0.4314005	0.40151256	0.57704412	-0.5561293	0.57583915	0.02717482
108.6	-0.4314005	0.40151256	0.57704412	-0.5561293	0.57583915	0.02717482
108.7	-0.4314005	0.40151256	0.57704412	-0.5561293	0.57583915	0.02717482
108.8	-0.4314005	0.40151256	0.57704412	-0.5561293	0.57583915	0.02717482
108.9	-0.4314005	0.40151256	0.57704412	-0.5561293	0.57583915	0.02717482
109	-0.4312129	0.40179952	0.57691425	-0.5565439	0.57540683	0.02746756
109.1	-0.4310857	0.40180011	0.57683469	-0.5566339	0.57507416	0.02742134
109.2	-0.4310857	0.40180011	0.57683469	-0.5566339	0.57507416	0.02742134
109.3	-0.4310857	0.40180011	0.57683469	-0.5566339	0.57507416	0.02742134
109.4	-0.4310857	0.40180011	0.57683469	-0.5566339	0.57507416	0.02742134
109.5	-0.4310857	0.40180011	0.57683469	-0.5566339	0.57507416	0.02742134
109.6	-0.4310857	0.40180011	0.57683469	-0.5566339	0.57507416	0.02742134
109.7	-0.4310857	0.40180011	0.57683469	-0.5566339	0.57507416	0.02742134
109.8	-0.4310857	0.40180011	0.57683469	-0.5566339	0.57507416	0.02742134
109.9	-0.4310141	0.40196086	0.57711068	-0.556593	0.57481473	0.02719755
110	-0.4310141	0.40196086	0.57711068	-0.556593	0.57481473	0.02719755
110.1	-0.4310141	0.40196086	0.57711068	-0.556593	0.57481473	0.02719755
110.2	-0.4308789	0.40195879	0.57699323	-0.5566991	0.57480122	0.02715236
110.3	-0.4308789	0.40195879	0.57699323	-0.5566991	0.57480122	0.02715236
110.4	-0.4308378	0.40200883	0.57700025	-0.5567136	0.57482837	0.02689694
110.5	-0.4308378	0.40200883	0.57700025	-0.5567136	0.57482837	0.02689694
110.6	-0.4308378	0.40200883	0.57700025	-0.5567136	0.57482837	0.02689694
110.7	-0.4308378	0.40200883	0.57700025	-0.5567136	0.57482837	0.02689694
110.8	-0.4308378	0.40200883	0.57700025	-0.5567136	0.57482837	0.02689694
110.9	-0.4308378	0.40200883	0.57700025	-0.5567136	0.57482837	0.02689694
111	-0.4308378	0.40200883	0.57700025	-0.5567136	0.57482837	0.02689694
111.1	-0.4308378	0.40200883	0.57700025	-0.5567136	0.57482837	0.02689694
111.2	-0.4308378	0.40200883	0.57700025	-0.5567136	0.57482837	0.02689694
111.3	-0.4308378	0.40200883	0.57700025	-0.5567136	0.57482837	0.02689694
111.4	-0.4308378	0.40200883	0.57700025	-0.5567136	0.57482837	0.02689694
111.5	-0.4308378	0.40200883	0.57700025	-0.5567136	0.57482837	0.02689694
111.6	-0.4308921	0.40183839	0.57687738	-0.5566756	0.57454389	0.02679933
111.7	-0.4308921	0.40183839	0.57687738	-0.5566756	0.57454389	0.02679933
111.8	-0.4308921	0.40183839	0.57687738	-0.5566756	0.57454389	0.02679933
111.9	-0.4308921	0.40183839	0.57687738	-0.5566756	0.57454389	0.02679933
112	-0.4310172	0.40200679	0.57690725	-0.5566663	0.57435293	0.02691264
112.1	-0.4310172	0.40200679	0.57690725	-0.5566663	0.57435293	0.02691264
112.2	-0.4310172	0.40200679	0.57690725	-0.5566663	0.57435293	0.02691264
112.3	-0.4310172	0.40200679	0.57690725	-0.5566663	0.57435293	0.02691264
112.4	-0.4310172	0.40200679	0.57690725	-0.5566663	0.57435293	0.02691264
112.5	-0.4310172	0.40200679	0.57690725	-0.5566663	0.57435293	0.02691264
112.6	-0.4311393	0.40210769	0.5766375	-0.5571269	0.57381733	0.0269306
112.7	-0.4311393	0.40210769	0.5766375	-0.5571269	0.57381733	0.0269306
112.8	-0.4311393	0.40210769	0.5766375	-0.5571269	0.57381733	0.0269306
112.9	-0.4311393	0.40210769	0.5766375	-0.5571269	0.57381733	0.0269306
113	-0.4311393	0.40210769	0.5766375	-0.5571269	0.57381733	0.0269306
113.1	-0.4311393	0.40210769	0.5766375	-0.5571269	0.57381733	0.0269306
113.2	-0.4311393	0.40210769	0.5766375	-0.5571269	0.57381733	0.0269306
113.3	-0.4311393	0.40210769	0.5766375	-0.5571269	0.57381733	0.0269306
113.4	-0.4311393	0.40210769	0.5766375	-0.5571269	0.57381733	0.0269306
113.5	-0.4311393	0.40210769	0.5766375	-0.5571269	0.57381733	0.0269306
113.6	-0.4311393	0.40210769	0.5766375	-0.5571269	0.57381733	0.0269306
113.7	-0.4311393	0.40210769	0.5766375	-0.5571269	0.57381733	0.0269306
113.8	-0.4311393	0.40210769	0.5766375	-0.5571269	0.57381733	0.0269306
113.9	-0.4311393	0.40210769	0.5766375	-0.5571269	0.57381733	0.0269306

69	-0.4424597	0.40655075	0.58519673	-0.5361719	0.60165603	0.04052196
69.1	-0.4423057	0.40635378	0.58499833	-0.5363312	0.60144276	0.04037831
69.2	-0.4416607	0.40606855	0.58491643	-0.5365695	0.60097401	0.04068985
69.3	-0.4412348	0.40567548	0.5848089	-0.5365568	0.60056742	0.04082362
69.4	-0.4411217	0.40544781	0.58484546	-0.5365724	0.60038075	0.04058292
69.5	-0.4411217	0.40544781	0.58484546	-0.5365724	0.60038075	0.04058292
69.6	-0.4412176	0.40514502	0.5847291	-0.5370984	0.60003619	0.0406929
69.7	-0.4412176	0.40514502	0.5847291	-0.5370984	0.60003619	0.0406929
69.8	-0.4412176	0.40514502	0.5847291	-0.5370984	0.60003619	0.0406929
69.9	-0.440736	0.40480682	0.58459476	-0.5371973	0.59954392	0.04005333
70	-0.4405888	0.40435795	0.58478673	-0.5374474	0.59924712	0.04014029
70.1	-0.440329	0.40407985	0.58482935	-0.5377712	0.59942288	0.03968831
70.2	-0.4400711	0.40413819	0.58472942	-0.5378183	0.59941747	0.04008044
70.3	-0.4397502	0.40412651	0.58458354	-0.5379167	0.59894994	0.03918923
70.4	-0.4400658	0.4041474	0.5844029	-0.5389304	0.59847662	0.0389956
70.5	-0.4398336	0.40381009	0.58424702	-0.5390691	0.59813845	0.0391871
70.6	-0.4398336	0.40381009	0.58424702	-0.5390691	0.59813845	0.0391871
70.7	-0.4398336	0.40381009	0.58424702	-0.5390691	0.59813845	0.0391871
70.8	-0.4397202	0.40333278	0.58409836	-0.5389896	0.59778833	0.03901927
70.9	-0.4396463	0.40316955	0.58394961	-0.5392248	0.59763766	0.03898234
71	-0.4391394	0.40288328	0.58393644	-0.5393462	0.59746528	0.03875861
71.1	-0.4389551	0.40282736	0.58407175	-0.5395941	0.59780689	0.0388081
71.2	-0.4389551	0.40282736	0.58407175	-0.5395941	0.59780689	0.0388081
71.3	-0.4389551	0.40282736	0.58407175	-0.5395941	0.59780689	0.0388081
71.4	-0.4388983	0.40253216	0.58436598	-0.53952	0.59754498	0.03842513
71.5	-0.4386925	0.4022468	0.58414628	-0.5396917	0.59720342	0.03751006
71.6	-0.4386825	0.4021934	0.58414912	-0.5401784	0.59727825	0.03783324
71.7	-0.4385636	0.4029111	0.58447253	-0.5400402	0.59743647	0.03738076
71.8	-0.437703	0.40223241	0.58418214	-0.5398685	0.59653979	0.03791249
71.9	-0.4374256	0.4017924	0.58434284	-0.5403758	0.59623119	0.038075
72	-0.4374256	0.4017924	0.58434284	-0.5403758	0.59623119	0.038075
72.1	-0.4371335	0.40137703	0.58392123	-0.5404253	0.59587193	0.03819904
72.2	-0.4372386	0.40139167	0.58374158	-0.5404338	0.59566968	0.03806964
72.3	-0.4372386	0.40139167	0.58374158	-0.5404338	0.59566968	0.03806964
72.4	-0.4373213	0.40123655	0.58370117	-0.5405023	0.59548511	0.03788871
72.5	-0.4373213	0.40123655	0.58370117	-0.5405023	0.59548511	0.03788871
72.6	-0.4373213	0.40123655	0.58370117	-0.5405023	0.59548511	0.03788871
72.7	-0.4373603	0.40156371	0.58379661	-0.5406945	0.59533803	0.03786222
72.8	-0.4373603	0.40156371	0.58379661	-0.5406945	0.59533803	0.03786222
72.9	-0.4369173	0.40120564	0.58358641	-0.54064	0.59498628	0.03805156
73	-0.4369173	0.40120564	0.58358641	-0.54064	0.59498628	0.03805156
73.1	-0.4365269	0.40144094	0.58350087	-0.5405946	0.59488687	0.03800516
73.2	-0.4365269	0.40144094	0.58350087	-0.5405946	0.59488687	0.03800516
73.3	-0.4365269	0.40144094	0.58350087	-0.5405946	0.59488687	0.03800516
73.4	-0.4362919	0.40123409	0.58336268	-0.5406562	0.59454275	0.03803726
73.5	-0.4365542	0.40097997	0.58324883	-0.5406717	0.59460684	0.03814182
73.6	-0.4362427	0.40078512	0.58319215	-0.5406786	0.59399021	0.03776202
73.7	-0.4363116	0.400739	0.58344874	-0.5413698	0.59414559	0.03816646
73.8	-0.4363116	0.400739	0.58344874	-0.5413698	0.59414559	0.03816646
73.9	-0.4363116	0.400739	0.58344874	-0.5413698	0.59414559	0.03816646
74	-0.4363604	0.40038817	0.5833085	-0.5412265	0.59397474	0.03816504
74.1	-0.4363604	0.40038817	0.5833085	-0.5412265	0.59397474	0.03816504
74.2	-0.4362168	0.40072284	0.58348018	-0.5412421	0.59418399	0.03798442
74.3	-0.4357972	0.39991413	0.583229	-0.5419449	0.59348507	0.03745345
74.4	-0.4357972	0.39991413	0.583229	-0.5419449	0.59348507	0.03745345
74.5	-0.434887	0.39890313	0.5828743	-0.5417422	0.59277793	0.03767059
74.6	-0.434887	0.39890313	0.5828743	-0.5417422	0.59277793	0.03767059
74.7	-0.434887	0.39890313	0.5828743	-0.5417422	0.59277793	0.03767059
74.8	-0.4343804	0.39831864	0.58253068	-0.5418624	0.59230533	0.0377705
74.9	-0.4343804	0.39831864	0.58253068	-0.5418624	0.59230533	0.0377705
120	-0.4299222	0.40247784	0.57659689	-0.5584595	0.57224866	0.02565021
120.1	-0.4299222	0.40247784	0.57659689	-0.5584595	0.57224866	0.02565021
120.2	-0.4299222	0.40247784	0.57659689	-0.5584595	0.57224866	0.02565021
120.3	-0.4299222	0.40247784	0.57659689	-0.5584595	0.57224866	0.02565021
120.4	-0.4299399	0.40226313	0.57662634	-0.5583905	0.57253441	0.02522419
120.5	-0.4297845	0.40228618	0.57643078	-0.5584266	0.57225058	0.02523336
120.6	-0.4297845	0.40228618	0.57643078	-0.5584266	0.57225058	0.02523336
120.7	-0.4297845	0.40228618	0.57643078	-0.5584266	0.57225058	0.02523336
120.8	-0.4298346	0.40234647	0.57661822	-0.5585226	0.57246972	0.02526045
120.9	-0.4298346	0.40234647	0.57661822	-0.5585226	0.57246972	0.02526045
121	-0.4298346	0.40234647	0.57661822	-0.5585226	0.57246972	0.02526045
121.1	-0.4298346	0.40234647	0.57661822	-0.5585226	0.57246972	0.02526045
121.2	-0.4298346	0.40234647	0.57661822	-0.5585226	0.57246972	0.02526045
121.3	-0.4298346	0.40234647	0.57661822	-0.5585226	0.57246972	0.02526045
121.4	-0.4298346	0.40234647	0.57661822	-0.5585226	0.57246972	0.02526045
121.5	-0.4297198	0.40254207	0.57653195	-0.5585432	0.57215694	0.02506472
121.6	-0.4297198	0.40254207	0.57653195	-0.5585432	0.57215694	0.02506472
121.7	-0.4297198	0.40254207	0.57653195	-0.5585432	0.57215694	0.02506472
121.8	-0.4298567	0.40273553	0.57686106	-0.5586834	0.57233676	0.02466902
121.9	-0.4298567	0.40273553	0.57686106	-0.5586834	0.57233676	0.02466902
122	-0.4298567	0.40273553	0.57686106	-0.5586834	0.57233676	0.02466902
122.1	-0.4298567	0.40273553	0.57686106	-0.5586834	0.57233676	0.02466902
122.2	-0.4298567	0.40273553	0.57686106	-0.5586834	0.57233676	0.02466902
122.3	-0.4298567	0.40273553	0.57686106	-0.5586834	0.57233676	0.02466902
122.4	-0.4298567	0.40273553	0.57686106	-0.5586834	0.57233676	0.02466902
122.5	-0.4298567	0.40273553	0.57686106	-0.5586834	0.57233676	0.02466902
122.6	-0.4298567	0.40273553	0.57686106	-0.5586834	0.57233676	0.02466902
122.7	-0.4298567	0.40273553	0.57686106	-0.5586834	0.57233676	0.02466902
122.8	-0.4298857	0.40286154	0.57709912	-0.558761	0.57243566	0.02462554
122.9	-0.4298857	0.40286154	0.57709912	-0.558761	0.57243566	0.02462554
123	-0.4298857	0.40286154	0.57709912	-0.558761	0.57243566	0.02462554
123.1	-0.4301187	0.40310388	0.57726206	-0.5589143	0.57248349	0.02458667
123.2	-0.4301187	0.40310388	0.57726206	-0.5589143	0.57248349	0.02458667
123.3	-0.4301187	0.40310388	0.57726206	-0.5589143	0.57248349	0.02458667
123.4	-0.4301187	0.40310388	0.57726206	-0.5589143	0.57248349	0.02458667
123.5	-0.4301187	0.40310388	0.57726206	-0.5589143	0.57248349	0.02458667
123.6	-0.4298799	0.40311831	0.5770826	-0.5588371	0.57225768	0.02427077
123.7	-0.4295509	0.40323697	0.57702247	-0.5588608	0.57226065	0.02462548
123.8	-0.4294406	0.40317943	0.57678217	-0.558939	0.57192838	0.02463712
123.9	-0.4294406	0.40317943	0.57678217	-0.558939	0.57192838	0.02463712
124	-0.4294406	0.40317943	0.57678217	-0.558939	0.57192838	0.02463712
124.1	-0.4294406	0.40317943	0.57678217	-0.558939	0.57192838	0.02463712
124.2	-0.4294923	0.40341698	0.57670442	-0.5590775	0.57184479	0.02447945
124.3	-0.4294923	0.40341698	0.57670442	-0.5590775	0.57184479	0.02447945
124.4	-0.4294923	0.40341698	0.57670442	-0.5590775	0.57184479	0.02447945
124.5	-0.4294923	0.40341698	0.57670442	-0.5590775	0.57184479	0.02447945
124.6	-0.4291533	0.4033275	0.57645289	-0.559399	0.57169484	0.02443674
124.7	-0.4291533	0.4033275	0.57645289	-0.559399	0.57169484	0.02443674
124.8	-0.4291419	0.40352774	0.57662215	-0.5594832	0.57196643	0.02487367
124.9	-0.4291419	0.40352774	0.57662215	-0.5594832	0.57196643	0.02487367
125	-0.4291419	0.40352774	0.57662215	-0.5594832	0.57196643	0.02487367
125.1	-0.4291419	0.40352774	0.57662215	-0.5594832	0.57196643	0.02487367
125.2	-0.429143	0.4035848	0.57673622	-0.5597673	0.57189939	0.02473906
125.3	-0.429143	0.4035848	0.57673622	-0.5597673	0.57189939	0.02473906
125.4	-0.429143	0.4035848	0.57673622	-0.5597673	0.57189939	0.02473906
125.5	-0.429072	0.4035079	0.57657234	-0.5598523	0.57160231	0.02487496
125.6	-0.429072	0.4035079	0.57657234	-0.5598523	0.57160231	0.02487496
125.7	-0.429072	0.4035079	0.57657234	-0.5598523	0.57160231	0.02487496
125.8	-0.429072	0.4035079	0.57657234	-0.5598523	0.57160231	0.02487496
125.9	-0.4288552	0.40297766	0.57638135	-0.5598202	0.57105526	0.02515418
126	-0.4286374	0.40279489	0.576304	-0.559731	0.57082553	0.02500267
84	-0.42972	0.39489602	0.58015256	-0.5471328	0.58421146	0.03365237
84.1	-0.42972	0.39489602	0.58015256	-0.5471328	0.58421146	0.03365237
84.2	-0.42972	0.39489602	0.58015256	-0.5471328	0.58421146	0.03365237
84.3	-0.4295923	0.39498766	0.57975489	-0.5470487	0.58450157	0.03328636
84.4	-0.429682	0.39546996	0.57948827	-0.5471252	0.5842625	0.03327281
84.5	-0.429682	0.39546996	0.57948827	-0.5471252	0.5842625	0.03327281
84.6	-0.4301555	0.39601346	0.57972195	-0.5473285	0.58460342	0.03281029
84.7	-0.4301555	0.39601346	0.57972195	-0.5473285	0.58460342	0.03281029
84.8	-0.430138	0.39576816	0.57955377	-0.5475418	0.58458409	0.0320989
84.9	-0.430138	0.39576816	0.57955377	-0.5475418	0.58458409	0.0320989
85	-0.430138	0.39576816	0.57955377	-0.5475418	0.58458409	0.0320989
85.1	-0.430138	0.39576816	0.57955377	-0.5475418	0.58458409	0.0320989
85.2	-0.4299408	0.39600246	0.57933429	-0.5475423	0.58458485	0.0320566
85.3	-0.4299408	0.39600246	0.57933429	-0.5475423	0.58458485	0.0320566
85.4	-0.4299408	0.39600246	0.57933429	-0.5475423	0.58458485	0.0320566
85.5	-0.4299408	0.39600246	0.57933429	-0.5475423	0.58458485	0.0320566
85.6	-0.4299408	0.39600246	0.57933429	-0.5475423	0.58458485	0.0320566
85.7	-0.4303233	0.39600097	0.57950664	-0.5478126	0.58461818	0.03177098
85.8	-0.4302676	0.39621852	0.57924556	-0.5477087	0.5841882	0.031826
85.9	-0.430243	0.39642919	0.57920861	-0.5477723	0.58390249	0.03225209
86	-0.4301071	0.39629137	0.57941056	-0.5482143	0.58374613	0.03134941
86.1	-0.4301071	0.39629137	0.57941056	-0.5482143	0.58374613	0.03134941
86.2	-0.4301535	0.39634638	0.57945452	-0.5481496	0.58375735	0.03147072
86.3	-0.4301188	0.39614996	0.57921137	-0.5480101	0.58350246	0.0314382
86.4	-0.4301188	0.39614996	0.57921137	-0.5480101	0.58350246	0.0314382
86.5	-0.4301751	0.39640783	0.57943112	-0.5480976	0.58360005	0.03143155
86.6	-0.4301751	0.39640783	0.57943112	-0.5480976	0.58360005	0.03143155
86.7	-0.4301751	0.39640783	0.57943112	-0.5480976	0.58360005	0.03143155
86.8	-0.4301751	0.39640783	0.57943112	-0.5480976	0.58360005	0.03143155
86.9	-0.430274	0.39632286	0.57927114	-0.5480506	0.58339944	0.03168698
87	-0.430274	0.39632286	0.57927114	-0.5480506	0.58339944	0.03168698
87.1	-0.4304698	0.39633012	0.57915294	-0.5480688	0.58311038	0.03144309
87.2	-0.4302094	0.39649671	0.57904987	-0.5480259	0.58312029	0.03170611
87.3	-0.4301376	0.3964482	0.57909941	-0.5481353	0.58305955	0.03164355
87.4	-0.4301376	0.3964482	0.57909941	-0.5481353	0.58305955	0.03164355
87.5	-0.4300722	0.39655915	0.57939332	-0.5480972	0.58292433	0.03219393
87.6	-0.4300722	0.39655915	0.57939332	-0.5480972	0.58292433	0.03219393
87.7	-0.4300849	0.39663762	0.57928389	-0.5480872	0.58293868	0.03204898
87.8	-0.4300849	0.39663762	0.57928389	-0.5480872	0.58293868	0.03204898
87.9	-0.4300849	0.39663762	0.57928389	-0.5480872	0.58293868	0.03204898
88	-0.4299756	0.39665491	0.57920918	-0.5480954	0.58294312	0.03174187
88.1	-0.4299756	0.39665491	0.57920918	-0.5480954	0.58294312	0.03174187
88.2	-0.4299756	0.39665491	0.57920918	-0.5480954	0.58294312	0.03174187
88.3	-0.4297474	0.39685735	0.57917877	-0.54844	0.58292235	0.03138554
88.4	-0.4297474	0.39685735	0.57917877	-0.54844	0.58292235	0.03138554
88.5	-0.4298662	0.39683487	0.5790747	-0.5486903	0.58286194	0.03112784
88.6	-0.4298662	0.39683487	0.5790747	-0.5486903	0.58286194	0.03112784
88.7	-0.429952	0.39681562	0.57889414	-0.5488027	0.58250767	0.03104455
88.8	-0.429952	0.39681562	0.57889414	-0.5488027	0.58250767	0.03104455
88.9	-0.429952	0.39681562	0.57889414	-0.5488027	0.58250767	0.03104455
89	-0.4301362	0.3967873	0.57908856	-0.5489646	0.58239844	0.03066105
89.1	-0.4301362	0.3967873	0.57908856	-0.5489646	0.58239844	0.03066105
89.2	-0.4300946	0.39646784	0.5788356	-0.5488395	0.58238952	0.03053214
89.3	-0.4301841	0.39645982	0.57889728	-0.5488506	0.58230601	0.03041572
89.4	-0.4301078	0.39671429	0.57895698	-0.5489034	0.58220204	0.03026991
89.5	-0.4304196	0.39672272	0.57903919	-0.5493224	0.58230126	0.03027997
89.6	-0.4304196	0.39672272	0.57903919	-0.5493224	0.58230126	0.03027997
89.7	-0.4304196	0.39672272	0.57903919	-0.5493224	0.58230126	0.03027997
89.8	-0.4304196	0.39672272	0.57903919	-0.5493224	0.58230126	0.03027997
89.9	-0.4304196	0.39672272	0.57903919	-0.5493224	0.58230126	0.03027997
135	-0.4263835	0.40133155	0.57428879	-0.5601794	0.56604945	0.02570304
135.1	-0.4263835	0.40133155	0.57428879	-0.5601794	0.56604945	0.02570304
135.2	-0.4265692	0.40154319	0.5745131	-0.5602634	0.5660759	0.02548855
135.3	-0.4265692	0.40154319	0.5745131	-0.5602634	0.5660759	0.02548855
135.4	-0.4266509	0.40141671	0.57440757	-0.5604792	0.56595656	0.02569394
135.5	-0.4266509	0.40141671	0.57440757	-0.5604792	0.56595656	0.02569394
135.6	-0.4266013	0.40154761	0.57452914	-0.5603886	0.56581389	0.02574871
135.7	-0.4265508	0.40186776	0.5749017	-0.5604808	0.5660958	0.02536081
135.8	-0.4265508	0.40186776	0.5749017	-0.5604808	0.5660958	0.02536081
135.9	-0.4265508	0.40186776	0.5749017	-0.5604808	0.5660958	0.02536081
136	-0.4265508	0.40186776	0.5749017	-0.5604808	0.5660958	0.02536081
136.1	-0.4265508	0.40186776	0.5749017	-0.5604808	0.5660958	0.02536081
136.2	-0.4267424	0.40208991	0.57520509	-0.5605093	0.56643915	0.02509328
136.3	-0.4267424	0.40208991	0.57520509	-0.5605093	0.56643915	0.02509328
136.4	-0.4266419	0.40204297	0.57518094	-0.560776	0.56617002	0.02485558
136.5	-0.4268838	0.40194457	0.57511149	-0.5607119	0.56623114	0.0249762
136.6	-0.4268838	0.40194457	0.57511149	-0.5607119	0.56623114	0.0249762
136.7	-0.4268838	0.40194457	0.57511149	-0.5607119	0.56623114	0.0249762
136.8	-0.4268838	0.40194457	0.57511149	-0.5607119	0.56623114	0.0249762
136.9	-0.4269607	0.40208796	0.57524353	-0.5607324	0.56617503	0.0251121
137	-0.4269607	0.40208796	0.57524353	-0.5607324	0.56617503	0.0251121
137.1	-0.4269607	0.40208796	0.57524353	-0.5607324	0.56617503	0.0251121
137.2	-0.4269607	0.40208796	0.57524353	-0.5607324	0.56617503	0.0251121
137.3	-0.4269607	0.40208796	0.57524353	-0.5607324	0.56617503	0.0251121
137.4	-0.4269607	0.40208796	0.57524353	-0.5607324	0.56617503	0.0251121
137.5	-0.4269607	0.40208796	0.57524353	-0.5607324	0.56617503	0.0251121
137.6	-0.4269607	0.40208796	0.57524353	-0.5607324	0.56617503	0.0251121
137.7	-0.426703	0.401861	0.57550287	-0.560879	0.56597758	0.02526112
137.8	-0.426703	0.401861	0.57550287	-0.560879	0.56597758	0.02526112
137.9	-0.426703	0.401861	0.57550287	-0.560879	0.56597758	0.02526112
138	-0.426703	0.401861	0.57550287	-0.560879	0.56597758	0.02526112
138.1	-0.426703	0.401861	0.57550287	-0.560879	0.56597758	0.02526112
138.2	-0.426703	0.401861	0.57550287	-0.560879	0.56597758	0.02526112
138.3	-0.426703	0.401861	0.57550287	-0.560879	0.56597758	0.02526112
138.4	-0.426703	0.401861	0.57550287	-0.560879	0.56597758	0.02526112
138.5	-0.426703	0.401861	0.57550287	-0.560879	0.56597758	0.02526112
138.6	-0.426703	0.401861	0.57550287	-0.560879	0.56597758	0.02526112
138.7	-0.426703	0.401861	0.57550287	-0.560879	0.56597758	0.02526112
138.8	-0.4266604	0.40200058	0.57537122	-0.5609144	0.56600247	0.02524215
138.9	-0.4266604	0.40200058	0.57537122	-0.5609144	0.56600247	0.02524215
139	-0.4266604	0.40200058	0.57537122	-0.5609144	0.56600247	0.02524215
139.1	-0.4266604	0.40200058	0.57537122	-0.5609144	0.56600247	0.02524215
139.2	-0.4266604	0.40200058	0.57537122	-0.5609144	0.56600247	0.02524215
139.3	-0.4266604	0.40200058	0.57537122	-0.5609144	0.56600247	0.02524215
139.4	-0.4266604	0.40200058	0.57537122	-0.5609144	0.56600247	0.02524215
139.5	-0.4266604	0.40200058	0.57537122	-0.5609144	0.56600247	0.02524215
139.6	-0.4266604	0.40200058	0.57537122	-0.5609144	0.56600247	0.02524215
139.7	-0.4266604	0.40200058	0.57537122	-0.5609144	0.56600247	0.02524215
139.8	-0.4266604	0.40200058	0.57537122	-0.5609144	0.56600247	0.02524215
139.9	-0.4266604	0.40200058	0.57537122	-0.5609144	0.56600247	0.02524215
140	-0.4266604	0.40200058	0.57537122	-0.5609144	0.56600247	0.02524215
140.1	-0.4266604	0.40200058	0.57537122	-0.5609144	0.56600247	0.02524215
140.2	-0.4266604	0.40200058	0.57537122	-0.5609144	0.56600247	0.02524215
140.3	-0.4266604	0.40200058	0.57537122	-0.5609144	0.56600247	0.02524215
140.4	-0.4266604	0.40200058	0.57537122	-0.5609144	0.56600247	0.02524215
140.5	-0.4266604	0.40200058	0.57537122	-0.5609144	0.56600247	0.02524215
140.6	-0.4266604	0.40200058	0.57537122	-0.5609144	0.56600247	0.02524215
140.7	-0.4265473	0.4020151	0.57543437	-0.5609583	0.5658288	0.02535329
140.8	-0.4265473	0.4020151	0.57543437	-0.5609583	0.5658288	0.02535329
140.9	-0.4265473	0.4020151	0.57543437	-0.5609583	0.5658288	0.02535329
141	-0.4265473	0.4020151	0.57543437	-0.5609583	0.5658288	0.02535329
99	-0.4299674	0.39968932	0.57841121	-0.5529178	0.58007764	0.02807969
99.1	-0.4299674	0.39968932	0.57841121	-0.5529178	0.58007764	0.02807969
99.2	-0.4299674	0.39968932	0.57841121	-0.5529178	0.58007764	0.02807969
99.3	-0.4299674	0.39968932	0.57841121	-0.5529178	0.58007764	0.02807969
99.4	-0.4299674	0.39968932	0.57841121	-0.5529178	0.58007764	0.02807969
99.5	-0.4299674	0.39968932	0.57841121	-0.5529178	0.58007764	0.02807969
99.6	-0.4299674	0.39968932	0.57841121	-0.5529178	0.58007764	0.02807969
99.7	-0.4299738	0.39969051	0.57844701	-0.5528999	0.57975971	0.02807849
99.8	-0.4297435	0.39972071	0.578323	-0.5529552	0.57984088	0.02804267
99.9	-0.4297247	0.3996927	0.57835805	-0.553263	0.57953837	0.02772968
100	-0.4301619	0.39971488	0.57855439	-0.5535349	0.57947113	0.02773816
100.1	-0.4301619	0.39971488	0.57855439	-0.5535349	0.57947113	0.02773816
100.2	-0.4301619	0.39971488	0.57855439	-0.5535349	0.57947113	0.02773816
100.3	-0.4301619	0.39971488	0.57855439	-0.5535349	0.57947113	0.02773816
100.4	-0.4301827	0.39986718	0.57846939	-0.5539486	0.57921611	0.02785913
100.5	-0.4302846	0.39956556	0.57800491	-0.5541206	0.5789164	0.02832856
100.6	-0.430516	0.39990534	0.57843966	-0.5545393	0.57907536	0.02826513
100.7	-0.430516	0.39990534	0.57843966	-0.5545393	0.57907536	0.02826513
100.8	-0.430516	0.39990534	0.57843966	-0.5545393	0.57907536	0.02826513
100.9	-0.430516	0.39990534	0.57843966	-0.5545393	0.57907536	0.02826513
101	-0.430516	0.39990534	0.57843966	-0.5545393	0.57907536	0.02826513
101.1	-0.430516	0.39990534	0.57843966	-0.5545393	0.57907536	0.02826513
101.2	-0.430516	0.39990534	0.57843966	-0.5545393	0.57907536	0.02826513
101.3	-0.430516	0.39990534	0.57843966	-0.5545393	0.57907536	0.02826513
101.4	-0.4308652	0.3998285	0.57841143	-0.5547284	0.57902284	0.02819534
101.5	-0.4307793	0.39982864	0.57802301	-0.554598	0.57869195	0.02822379
101.6	-0.4307793	0.39982864	0.57802301	-0.554598	0.57869195	0.02822379
101.7	-0.4307793	0.39982864	0.57802301	-0.554598	0.57869195	0.02822379
101.8	-0.4307793	0.39982864	0.57802301	-0.554598	0.57869195	0.02822379
101.9	-0.4307793	0.39982864	0.57802301	-0.554598	0.57869195	0.02822379
102	-0.4307793	0.39982864	0.57802301	-0.554598	0.57869195	0.02822379
102.1	-0.4306421	0.40014961	0.57800567	-0.5545577	0.57863803	0.02786207
102.2	-0.4306421	0.40014961	0.57800567	-0.5545577	0.57863803	0.02786207
102.3	-0.4306421	0.40014961	0.57800567	-0.5545577	0.57863803	0.02786207
102.4	-0.4306421	0.40014961	0.57800567	-0.5545577	0.57863803	0.02786207
102.5	-0.4306421	0.40014961	0.57800567	-0.5545577	0.57863803	0.02786207
102.6	-0.4306421	0.40014961	0.57800567	-0.5545577	0.57863803	0.02786207
102.7	-0.4310732	0.40037587	0.57818252	-0.5547445	0.57851267	0.02806774
102.8	-0.4310732	0.40037587	0.57818252	-0.5547445	0.57851267	0.02806774
102.9	-0.4310732	0.40037587	0.57818252	-0.5547445	0.57851267	0.02806774
103	-0.4310732	0.40037587	0.57818252	-0.5547445	0.57851267	0.02806774
103.1	-0.4310732	0.40037587	0.57818252	-0.5547445	0.57851267	0.02806774
103.2	-0.4310732	0.40037587	0.57818252	-0.5547445	0.57851267	0.02806774
103.3	-0.4310732	0.40037587	0.57818252	-0.5547445	0.57851267	0.02806774
103.4	-0.4310732	0.40037587	0.57818252	-0.5547445	0.57851267	0.02806774
103.5	-0.4311777	0.40026918	0.57796769	-0.5547473	0.57825964	0.02791058
103.6	-0.4311777	0.40026918	0.57796769	-0.5547473	0.57825964	0.02791058
103.7	-0.4312309	0.40043292	0.57785871	-0.555056	0.57801475	0.02812855
103.8	-0.4312309	0.40043292	0.57785871	-0.555056	0.57801475	0.02812855
103.9	-0.4312309	0.40043292	0.57785871	-0.555056	0.57801475	0.02812855
104	-0.4312309	0.40043292	0.57785871	-0.555056	0.57801475	0.02812855
104.1	-0.4312309	0.40043292	0.57785871	-0.555056	0.57801475	0.02812855
104.2	-0.4310719	0.40055295	0.577834	-0.555211	0.57808461	0.02830115
104.3	-0.4310719	0.40055295	0.577834	-0.555211	0.57808461	0.02830115
104.4	-0.4309412	0.4006329	0.5780772	-0.5552551	0.5782622	0.02796192
104.5	-0.4309412	0.4006329	0.5780772	-0.5552551	0.5782622	0.02796192
104.6	-0.4309412	0.4006329	0.5780772	-0.5552551	0.5782622	0.02796192
104.7	-0.4313655	0.40128052	0.57828782	-0.5557954	0.57799735	0.02802981
104.8	-0.4313794	0.40125751	0.57799084	-0.555831	0.57794254	0.02758854
104.9	-0.4313794	0.40125751	0.57799084	-0.555831	0.57794254	0.02758854
150	-0.4268098	0.40380855	0.57627889	-0.5617522	0.5654521	0.0241514
150.1	-0.4268098	0.40380855	0.57627889	-0.5617522	0.5654521	0.0241514
150.2	-0.4268098	0.40380855	0.57627889	-0.5617522	0.5654521	0.0241514
150.3	-0.4268098	0.40380855	0.57627889	-0.5617522	0.5654521	0.0241514
150.4	-0.4268098	0.40380855	0.57627889	-0.5617522	0.5654521	0.0241514
150.5	-0.4268098	0.40380855	0.57627889	-0.5617522	0.5654521	0.0241514
150.6	-0.4268098	0.40380855	0.57627889	-0.5617522	0.5654521	0.0241514
150.7	-0.4268098	0.40380855	0.57627889	-0.5617522	0.5654521	0.0241514
150.8	-0.4266367	0.4039699	0.57630751	-0.5617607	0.56533323	0.02452375
150.9	-0.4265705	0.40413011	0.57604989	-0.5619279	0.56512078	0.02431055
151	-0.4265705	0.40413011	0.57604989	-0.5619279	0.56512078	0.02431055
151.1	-0.4265705	0.40413011	0.57604989	-0.5619279	0.56512078	0.02431055
151.2	-0.4265705	0.40413011	0.57604989	-0.5619279	0.56512078	0.02431055
151.3	-0.4265705	0.40413011	0.57604989	-0.5619279	0.56512078	0.02431055
151.4	-0.4262897	0.40417135	0.57594886	-0.5619965	0.56507922	0.02466628
151.5	-0.426202	0.40424435	0.57605853	-0.5623288	0.56522338	0.0243835
151.6	-0.426202	0.40424435	0.57605853	-0.5623288	0.56522338	0.0243835
151.7	-0.426202	0.40424435	0.57605853	-0.5623288	0.56522338	0.0243835
151.8	-0.426202	0.40424435	0.57605853	-0.5623288	0.56522338	0.0243835
151.9	-0.426202	0.40424435	0.57605853	-0.5623288	0.56522338	0.0243835
152	-0.426202	0.40424435	0.57605853	-0.5623288	0.56522338	0.0243835
152.1	-0.426202	0.40424435	0.57605853	-0.5623288	0.56522338	0.0243835
152.2	-0.426202	0.40424435	0.57605853	-0.5623288	0.56522338	0.0243835
152.3	-0.426202	0.40424435	0.57605853	-0.5623288	0.56522338	0.0243835
152.4	-0.426202	0.40424435	0.57605853	-0.5623288	0.56522338	0.0243835
152.5	-0.426202	0.40424435	0.57605853	-0.5623288	0.56522338	0.0243835
152.6	-0.426202	0.40424435	0.57605853	-0.5623288	0.56522338	0.0243835
152.7	-0.426202	0.40424435	0.57605853	-0.5623288	0.56522338	0.0243835
152.8	-0.426202	0.40424435	0.57605853	-0.5623288	0.56522338	0.0243835
152.9	-0.4263447	0.40415079	0.57585525	-0.5624289	0.56488759	0.02421741
153	-0.4263447	0.40415079	0.57585525	-0.5624289	0.56488759	0.02421741
153.1	-0.4263447	0.40415079	0.57585525	-0.5624289	0.56488759	0.02421741
153.2	-0.4263447	0.40415079	0.57585525	-0.5624289	0.56488759	0.02421741
153.3	-0.4263447	0.40415079	0.57585525	-0.5624289	0.56488759	0.02421741
153.4	-0.4263447	0.40415079	0.57585525	-0.5624289	0.56488759	0.02421741
153.5	-0.4263447	0.40415079	0.57585525	-0.5624289	0.56488759	0.02421741
153.6	-0.4263447	0.40415079	0.57585525	-0.5624289	0.56488759	0.02421741
153.7	-0.4263447	0.40415079	0.57585525	-0.5624289	0.56488759	0.02421741
153.8	-0.4263447	0.40415079	0.57585525	-0.5624289	0.56488759	0.02421741
153.9	-0.4263447	0.40415079	0.57585525	-0.5624289	0.56488759	0.02421741
154	-0.4263447	0.40415079	0.57585525	-0.5624289	0.56488759	0.02421741
154.1	-0.4263447	0.40415079	0.57585525	-0.5624289	0.56488759	0.02421741
154.2	-0.4262736	0.40426888	0.57596375	-0.5626326	0.56482946	0.024499
154.3	-0.4262736	0.40426888	0.57596375	-0.5626326	0.56482946	0.024499
154.4	-0.4262736	0.40426888	0.57596375	-0.5626326	0.56482946	0.024499
154.5	-0.4262736	0.40426888	0.57596375	-0.5626326	0.56482946	0.024499
154.6	-0.4262736	0.40426888	0.57596375	-0.5626326	0.56482946	0.024499
154.7	-0.4262736	0.40426888	0.57596375	-0.5626326	0.56482946	0.024499
154.8	-0.4262736	0.40426888	0.57596375	-0.5626326	0.56482946	0.024499
154.9	-0.4262736	0.40426888	0.57596375	-0.5626326	0.56482946	0.024499
155	-0.4262736	0.40426888	0.57596375	-0.5626326	0.56482946	0.024499
155.1	-0.4262736	0.40426888	0.57596375	-0.5626326	0.56482946	0.024499
155.2	-0.4262736	0.40426888	0.57596375	-0.5626326	0.56482946	0.024499
155.3	-0.4262736	0.40426888	0.57596375	-0.5626326	0.56482946	0.024499
155.4	-0.4262935	0.4040716	0.57606454	-0.5626197	0.56482477	0.02466265
155.5	-0.4262935	0.4040716	0.57606454	-0.5626197	0.56482477	0.02466265
155.6	-0.4262935	0.4040716	0.57606454	-0.5626197	0.56482477	0.02466265
155.7	-0.4262935	0.4040716	0.57606454	-0.5626197	0.56482477	0.02466265
155.8	-0.4262935	0.4040716	0.57606454	-0.5626197	0.56482477	0.02466265
155.9	-0.4262935	0.4040716	0.57606454	-0.5626197	0.56482477	0.02466265
156	-0.4262935	0.4040716	0.57606454	-0.5626197	0.56482477	0.02466265
114	-0.4311393	0.40210769	0.5766375	-0.5571269	0.57381733	0.0269306
114.1	-0.4311393	0.40210769	0.5766375	-0.5571269	0.57381733	0.0269306
114.2	-0.4309446	0.40216864	0.57639825	-0.5569972	0.57366399	0.0265774
114.3	-0.4309446	0.40216864	0.57639825	-0.5569972	0.57366399	0.0265774
114.4	-0.4309446	0.40216864	0.57639825	-0.5569972	0.57366399	0.0265774
114.5	-0.4309601	0.40204128	0.57617815	-0.557071	0.57348075	0.02605902
114.6	-0.4308028	0.40227964	0.57618551	-0.5572721	0.57338666	0.02629969
114.7	-0.4308028	0.40227964	0.57618551	-0.5572721	0.57338666	0.02629969
114.8	-0.4307848	0.40226748	0.57606574	-0.5575925	0.57361317	0.02625158
114.9	-0.4308964	0.40235108	0.57609	-0.5578375	0.57370559	0.0264668
115	-0.4308964	0.40235108	0.57609	-0.5578375	0.57370559	0.0264668
115.1	-0.4308964	0.40235108	0.57609	-0.5578375	0.57370559	0.0264668
115.2	-0.4308964	0.40235108	0.57609	-0.5578375	0.57370559	0.0264668
115.3	-0.4308964	0.40235108	0.57609	-0.5578375	0.57370559	0.0264668
115.4	-0.4308964	0.40235108	0.57609	-0.5578375	0.57370559	0.0264668
115.5	-0.4307534	0.40250318	0.57618272	-0.5578798	0.57364887	0.0264458
115.6	-0.4307534	0.40250318	0.57618272	-0.5578798	0.57364887	0.0264458
115.7	-0.4306489	0.40270289	0.5762228	-0.5582545	0.57342745	0.02673038
115.8	-0.4303659	0.40277066	0.57617739	-0.55824	0.57356159	0.02688816
115.9	-0.4303659	0.40277066	0.57617739	-0.55824	0.57356159	0.02688816
116	-0.4303659	0.40277066	0.57617739	-0.55824	0.57356159	0.02688816
116.1	-0.4303659	0.40277066	0.57617739	-0.55824	0.57356159	0.02688816
116.2	-0.4303659	0.40277066	0.57617739	-0.55824	0.57356159	0.02688816
116.3	-0.4303817	0.40273357	0.57618179	-0.5585019	0.57346148	0.02679583
116.4	-0.4305248	0.40285266	0.57615585	-0.5585344	0.57331475	0.02667385
116.5	-0.4305248	0.40285266	0.57615585	-0.5585344	0.57331475	0.02667385
116.6	-0.4305248	0.40285266	0.57615585	-0.5585344	0.57331475	0.02667385
116.7	-0.4304639	0.40271028	0.57610804	-0.5586444	0.57308943	0.02694341
116.8	-0.4304639	0.40271028	0.57610804	-0.5586444	0.57308943	0.02694341
116.9	-0.4304639	0.40271028	0.57610804	-0.5586444	0.57308943	0.02694341
117	-0.4304639	0.40271028	0.57610804	-0.5586444	0.57308943	0.02694341
117.1	-0.4304561	0.40267471	0.57621465	-0.5590023	0.5729693	0.02672548
117.2	-0.4304561	0.40267471	0.57621465	-0.5590023	0.5729693	0.02672548
117.3	-0.4305481	0.40259654	0.57641324	-0.5590158	0.57287894	0.02644425
117.4	-0.4304649	0.40248412	0.57640088	-0.5589008	0.57278689	0.0264964
117.5	-0.4304649	0.40248412	0.57640088	-0.5589008	0.57278689	0.0264964
117.6	-0.4304649	0.40248412	0.57640088	-0.5589008	0.57278689	0.0264964
117.7	-0.4303272	0.40284251	0.57657135	-0.558611	0.57267832	0.02638785
117.8	-0.4303272	0.40284251	0.57657135	-0.558611	0.57267832	0.02638785
117.9	-0.4303272	0.40284251	0.57657135	-0.558611	0.57267832	0.02638785
118	-0.4303272	0.40284251	0.57657135	-0.558611	0.57267832	0.02638785
118.1	-0.4303272	0.40284251	0.57657135	-0.558611	0.57267832	0.02638785
118.2	-0.4303272	0.40284251	0.57657135	-0.558611	0.57267832	0.02638785
118.3	-0.4302113	0.40280908	0.5767295	-0.5585431	0.57262875	0.02645472
118.4	-0.4296409	0.40250645	0.57664159	-0.5583848	0.5724549	0.02634801
118.5	-0.4297487	0.40257385	0.57673251	-0.5584474	0.5725357	0.02599574
118.6	-0.4297487	0.40257385	0.57673251	-0.5584474	0.5725357	0.02599574
118.7	-0.4297487	0.40257385	0.57673251	-0.5584474	0.5725357	0.02599574
118.8	-0.4297487	0.40257385	0.57673251	-0.5584474	0.5725357	0.02599574
118.9	-0.4297487	0.40257385	0.57673251	-0.5584474	0.5725357	0.02599574
119	-0.4297487	0.40257385	0.57673251	-0.5584474	0.5725357	0.02599574
119.1	-0.4297487	0.40257385	0.57673251	-0.5584474	0.5725357	0.02599574
119.2	-0.4297487	0.40257385	0.57673251	-0.5584474	0.5725357	0.02599574
119.3	-0.4297487	0.40257385	0.57673251	-0.5584474	0.5725357	0.02599574
119.4	-0.4297487	0.40257385	0.57673251	-0.5584474	0.5725357	0.02599574
119.5	-0.4297487	0.40257385	0.57673251	-0.5584474	0.5725357	0.02599574
119.6	-0.4297487	0.40257385	0.57673251	-0.5584474	0.5725357	0.02599574
119.7	-0.4297487	0.40257385	0.57673251	-0.5584474	0.5725357	0.02599574
119.8	-0.4297487	0.40257385	0.57673251	-0.5584474	0.5725357	0.02599574
119.9	-0.4297487	0.40257385	0.57673251	-0.5584474	0.5725357	0.02599574
165	-0.4259817	0.40382852	0.57586104	-0.5627932	0.56369996	0.02509621
165.1	-0.4257392	0.40384982	0.57583539	-0.562868	0.56369307	0.02557546
165.2	-0.4257392	0.40384982	0.57583539	-0.562868	0.56369307	0.02557546
165.3	-0.4257392	0.40384982	0.57583539	-0.562868	0.56369307	0.02557546
165.4	-0.4257392	0.40384982	0.57583539	-0.562868	0.56369307	0.02557546
165.5	-0.4257392	0.40384982	0.57583539	-0.562868	0.56369307	0.02557546
165.6	-0.4257392	0.40384982	0.57583539	-0.562868	0.56369307	0.02557546
165.7	-0.4257392	0.40384982	0.57583539	-0.562868	0.56369307	0.02557546
165.8	-0.4257392	0.40384982	0.57583539	-0.562868	0.56369307	0.02557546
165.9	-0.4257392	0.40384982	0.57583539	-0.562868	0.56369307	0.02557546
166	-0.4257392	0.40384982	0.57583539	-0.562868	0.56369307	0.02557546
166.1	-0.4257392	0.40384982	0.57583539	-0.562868	0.56369307	0.02557546
166.2	-0.4257392	0.40384982	0.57583539	-0.562868	0.56369307	0.02557546
166.3	-0.4260402	0.40367144	0.57587851	-0.5629925	0.56370357	0.02539385
166.4	-0.4260402	0.40367144	0.57587851	-0.5629925	0.56370357	0.02539385
166.5	-0.4260402	0.40367144	0.57587851	-0.5629925	0.56370357	0.02539385
166.6	-0.4260402	0.40367144	0.57587851	-0.5629925	0.56370357	0.02539385
166.7	-0.4260402	0.40367144	0.57587851	-0.5629925	0.56370357	0.02539385
166.8	-0.4260402	0.40367144	0.57587851	-0.5629925	0.56370357	0.02539385
166.9	-0.4260402	0.40367144	0.57587851	-0.5629925	0.56370357	0.02539385
167	-0.4260402	0.40367144	0.57587851	-0.5629925	0.56370357	0.02539385
167.1	-0.4260402	0.40367144	0.57587851	-0.5629925	0.56370357	0.02539385
167.2	-0.4260402	0.40367144	0.57587851	-0.5629925	0.56370357	0.02539385
167.3	-0.4260402	0.40367144	0.57587851	-0.5629925	0.56370357	0.02539385
167.4	-0.4260402	0.40367144	0.57587851	-0.5629925	0.56370357	0.02539385
167.5	-0.4260402	0.40367144	0.57587851	-0.5629925	0.56370357	0.02539385
167.6	-0.4260402	0.40367144	0.57587851	-0.5629925	0.56370357	0.02539385
167.7	-0.4260402	0.40367144	0.57587851	-0.5629925	0.56370357	0.02539385
167.8	-0.4260625	0.40383801	0.57606169	-0.5633464	0.56359149	0.02532648
167.9	-0.4260625	0.40383801	0.57606169	-0.5633464	0.56359149	0.02532648
168	-0.4260625	0.40383801	0.57606169	-0.5633464	0.56359149	0.02532648
168.1	-0.4260625	0.40383801	0.57606169	-0.5633464	0.56359149	0.02532648
168.2	-0.4260625	0.40383801	0.57606169	-0.5633464	0.56359149	0.02532648
168.3	-0.4260625	0.40383801	0.57606169	-0.5633464	0.56359149	0.02532648
168.4	-0.4260625	0.40383801	0.57606169	-0.5633464	0.56359149	0.02532648
168.5	-0.4260625	0.40383801	0.57606169	-0.5633464	0.56359149	0.02532648
168.6	-0.4260625	0.40383801	0.57606169	-0.5633464	0.56359149	0.02532648
168.7	-0.4260625	0.40383801	0.57606169	-0.5633464	0.56359149	0.02532648
168.8	-0.4260625	0.40383801	0.57606169	-0.5633464	0.56359149	0.02532648
168.9	-0.4261956	0.40380161	0.57614177	-0.5632842	0.5638359	0.0250668
169	-0.4261956	0.40380161	0.57614177	-0.5632842	0.5638359	0.0250668
169.1	-0.4261956	0.40380161	0.57614177	-0.5632842	0.5638359	0.0250668
169.2	-0.4261956	0.40380161	0.57614177	-0.5632842	0.5638359	0.0250668
169.3	-0.4261956	0.40380161	0.57614177	-0.5632842	0.5638359	0.0250668
169.4	-0.4261956	0.40380161	0.57614177	-0.5632842	0.5638359	0.0250668
169.5	-0.4261956	0.40380161	0.57614177	-0.5632842	0.5638359	0.0250668
169.6	-0.4261956	0.40380161	0.57614177	-0.5632842	0.5638359	0.0250668
169.7	-0.4261956	0.40380161	0.57614177	-0.5632842	0.5638359	0.0250668
169.8	-0.4265311	0.40385193	0.57629076	-0.5636148	0.56389552	0.02470733
169.9	-0.4265311	0.40385193	0.57629076	-0.5636148	0.56389552	0.02470733
170	-0.4265311	0.40385193	0.57629076	-0.5636148	0.56389552	0.02470733
170.1	-0.4265311	0.40385193	0.57629076	-0.5636148	0.56389552	0.02470733
170.2	-0.4265311	0.40385193	0.57629076	-0.5636148	0.56389552	0.02470733
170.3	-0.4265311	0.40385193	0.57629076	-0.5636148	0.56389552	0.02470733
170.4	-0.4265311	0.40385193	0.57629076	-0.5636148	0.56389552	0.02470733
170.5	-0.4265311	0.40385193	0.57629076	-0.5636148	0.56389552	0.02470733
170.6	-0.4265311	0.40385193	0.57629076	-0.5636148	0.56389552	0.02470733
170.7	-0.4265311	0.40385193	0.57629076	-0.5636148	0.56389552	0.02470733
170.8	-0.4265311	0.40385193	0.57629076	-0.5636148	0.56389552	0.02470733
170.9	-0.4265065	0.40375438	0.57634706	-0.5636409	0.56401934	0.02460233
171	-0.4265065	0.40375438	0.57634706	-0.5636409	0.56401934	0.02460233

126.1	-0.4286374	0.40279489	0.576304	-0.559731	0.57082553	0.02500267
126.2	-0.4286374	0.40279489	0.576304	-0.559731	0.57082553	0.02500267
126.3	-0.4286374	0.40279489	0.576304	-0.559731	0.57082553	0.02500267
126.4	-0.4286374	0.40279489	0.576304	-0.559731	0.57082553	0.02500267
126.5	-0.4286374	0.40279489	0.576304	-0.559731	0.57082553	0.02500267
126.6	-0.4286374	0.40279489	0.576304	-0.559731	0.57082553	0.02500267
126.7	-0.4286374	0.40279489	0.576304	-0.559731	0.57082553	0.02500267
126.8	-0.4286374	0.40279489	0.576304	-0.559731	0.57082553	0.02500267
126.9	-0.4286374	0.40279489	0.576304	-0.559731	0.57082553	0.02500267
127	-0.4286374	0.40279489	0.576304	-0.559731	0.57082553	0.02500267
127.1	-0.4286374	0.40279489	0.576304	-0.559731	0.57082553	0.02500267
127.2	-0.4287815	0.40257243	0.57592069	-0.5597133	0.57037202	0.02445712
127.3	-0.4287815	0.40257243	0.57592069	-0.5597133	0.57037202	0.02445712
127.4	-0.4287815	0.40257243	0.57592069	-0.5597133	0.57037202	0.02445712
127.5	-0.4287815	0.40257243	0.57592069	-0.5597133	0.57037202	0.02445712
127.6	-0.4287815	0.40257243	0.57592069	-0.5597133	0.57037202	0.02445712
127.7	-0.4287815	0.40257243	0.57592069	-0.5597133	0.57037202	0.02445712
127.8	-0.4287815	0.40257243	0.57592069	-0.5597133	0.57037202	0.02445712
127.9	-0.4287815	0.40257243	0.57592069	-0.5597133	0.57037202	0.02445712
128	-0.4285079	0.40235343	0.57567162	-0.5596607	0.56997194	0.02460228
128.1	-0.4284269	0.40245293	0.57581054	-0.5596502	0.56999812	0.02444341
128.2	-0.4282353	0.40234523	0.57557275	-0.5597112	0.56968897	0.02453617
128.3	-0.4282353	0.40234523	0.57557275	-0.5597112	0.56968897	0.02453617
128.4	-0.4279343	0.40215775	0.57557275	-0.55965	0.56951815	0.02437407
128.5	-0.4279343	0.40215775	0.57557275	-0.55965	0.56951815	0.02437407
128.6	-0.4279343	0.40215775	0.57557275	-0.55965	0.56951815	0.02437407
128.7	-0.4279343	0.40215775	0.57557275	-0.55965	0.56951815	0.02437407
128.8	-0.4279343	0.40215775	0.57557275	-0.55965	0.56951815	0.02437407
128.9	-0.4279343	0.40215775	0.57557275	-0.55965	0.56951815	0.02437407
129	-0.4279343	0.40215775	0.57557275	-0.55965	0.56951815	0.02437407
129.1	-0.4279343	0.40215775	0.57557275	-0.55965	0.56951815	0.02437407
129.2	-0.4277186	0.40202237	0.57539974	-0.5596718	0.5691644	0.02426755
129.3	-0.4277186	0.40202237	0.57539974	-0.5596718	0.5691644	0.02426755
129.4	-0.4277186	0.40202237	0.57539974	-0.5596718	0.5691644	0.02426755
129.5	-0.4277186	0.40202237	0.57539974	-0.5596718	0.5691644	0.02426755
129.6	-0.4277186	0.40202237	0.57539974	-0.5596718	0.5691644	0.02426755
129.7	-0.4277186	0.40202237	0.57539974	-0.5596718	0.5691644	0.02426755
129.8	-0.4277186	0.40202237	0.57539974	-0.5596718	0.5691644	0.02426755
129.9	-0.4274958	0.40199615	0.57511271	-0.5597256	0.56881266	0.02422957
130	-0.4274958	0.40199615	0.57511271	-0.5597256	0.56881266	0.02422957
130.1	-0.4274958	0.40199615	0.57511271	-0.5597256	0.56881266	0.02422957
130.2	-0.4270863	0.40174077	0.57502094	-0.5596011	0.56855983	0.02441881
130.3	-0.4270863	0.40174077	0.57502094	-0.5596011	0.56855983	0.02441881
130.4	-0.4270863	0.40174077	0.57502094	-0.5596011	0.56855983	0.02441881
130.5	-0.4270863	0.40174077	0.57502094	-0.5596011	0.56855983	0.02441881
130.6	-0.4270863	0.40174077	0.57502094	-0.5596011	0.56855983	0.02441881
130.7	-0.4270863	0.40174077	0.57502094	-0.5596011	0.56855983	0.02441881
130.8	-0.4268107	0.40175285	0.57481343	-0.5595873	0.56826045	0.02457336
130.9	-0.4268107	0.40175285	0.57481343	-0.5595873	0.56826045	0.02457336
131	-0.4268107	0.40175285	0.57481343	-0.5595873	0.56826045	0.02457336
131.1	-0.4268107	0.40175285	0.57481343	-0.5595873	0.56826045	0.02457336
131.2	-0.4268107	0.40175285	0.57481343	-0.5595873	0.56826045	0.02457336
131.3	-0.4268107	0.40175285	0.57481343	-0.5595873	0.56826045	0.02457336
131.4	-0.4266374	0.40172149	0.57468602	-0.5597699	0.56802205	0.0246372
131.5	-0.4266374	0.40172149	0.57468602	-0.5597699	0.56802205	0.0246372
131.6	-0.4266374	0.40172149	0.57468602	-0.5597699	0.56802205	0.0246372
131.7	-0.4266374	0.40172149	0.57468602	-0.5597699	0.56802205	0.0246372
131.8	-0.4266374	0.40172149	0.57468602	-0.5597699	0.56802205	0.0246372
131.9	-0.4266374	0.40172149	0.57468602	-0.5597699	0.56802205	0.0246372
132	-0.4266459	0.4016668	0.57452476	-0.5597712	0.56758808	0.02463469
132.1	-0.4266459	0.4016668	0.57452476	-0.5597712	0.56758808	0.02463469
132.2	-0.4264754	0.40159818	0.57451232	-0.5597411	0.56736877	0.02499697
132.3	-0.4264754	0.40159818	0.57451232	-0.5597411	0.56736877	0.02499697
132.4	-0.4262336	0.40145099	0.57427057	-0.5597687	0.56730485	0.02518743
132.5	-0.4262336	0.40145099	0.57427057	-0.5597687	0.56730485	0.02518743
132.6	-0.4259455	0.4017422	0.5743206	-0.5597779	0.56727947	0.02533637
132.7	-0.4259455	0.4017422	0.5743206	-0.5597779	0.56727947	0.02533637
132.8	-0.4259455	0.4017422	0.5743206	-0.5597779	0.56727947	0.02533637
132.9	-0.4259455	0.4017422	0.5743206	-0.5597779	0.56727947	0.02533637
133	-0.4261188	0.40159274	0.57437583	-0.5598773	0.56731799	0.02550921
133.1	-0.4261188	0.40159274	0.57437583	-0.5598773	0.56731799	0.02550921
133.2	-0.4261033	0.4014407	0.57427158	-0.5599052	0.56700712	0.02540162
133.3	-0.4262968	0.40127617	0.57426985	-0.5597658	0.56673703	0.02534996
133.4	-0.4262968	0.40127617	0.57426985	-0.5597658	0.56673703	0.02534996
133.5	-0.4262968	0.40127617	0.57426985	-0.5597658	0.56673703	0.02534996
133.6	-0.4262968	0.40127617	0.57426985	-0.5597658	0.56673703	0.02534996
133.7	-0.4264415	0.40122027	0.57436709	-0.5597268	0.56649564	0.02524941
133.8	-0.4262998	0.40137613	0.57429557	-0.5600476	0.56625618	0.02537233
133.9	-0.4262998	0.40137613	0.57429557	-0.5600476	0.56625618	0.02537233
134	-0.4262998	0.40137613	0.57429557	-0.5600476	0.56625618	0.02537233
134.1	-0.4262998	0.40137613	0.57429557	-0.5600476	0.56625618	0.02537233
134.2	-0.4263835	0.40133155	0.57428879	-0.5601794	0.56604945	0.02570304
134.3	-0.4263835	0.40133155	0.57428879	-0.5601794	0.56604945	0.02570304
134.4	-0.4263835	0.40133155	0.57428879	-0.5601794	0.56604945	0.02570304
134.5	-0.4263835	0.40133155	0.57428879	-0.5601794	0.56604945	0.02570304
134.6	-0.4263835	0.40133155	0.57428879	-0.5601794	0.56604945	0.02570304
134.7	-0.4263835	0.40133155	0.57428879	-0.5601794	0.56604945	0.02570304
134.8	-0.4263835	0.40133155	0.57428879	-0.5601794	0.56604945	0.02570304
134.9	-0.4263835	0.40133155	0.57428879	-0.5601794	0.56604945	0.02570304

141.1	-0.4264655	0.40170674	0.57536647	-0.5610128	0.56582279	0.02524117
141.2	-0.4264655	0.40170674	0.57536647	-0.5610128	0.56582279	0.02524117
141.3	-0.4264655	0.40170674	0.57536647	-0.5610128	0.56582279	0.02524117
141.4	-0.4264655	0.40170674	0.57536647	-0.5610128	0.56582279	0.02524117
141.5	-0.4264655	0.40170674	0.57536647	-0.5610128	0.56582279	0.02524117
141.6	-0.4264023	0.40187677	0.57541936	-0.561319	0.56584321	0.02506362
141.7	-0.4264023	0.40187677	0.57541936	-0.561319	0.56584321	0.02506362
141.8	-0.4264023	0.40187677	0.57541936	-0.561319	0.56584321	0.02506362
141.9	-0.4264221	0.40187793	0.57530712	-0.5613674	0.56581619	0.02463644
142	-0.4264221	0.40187793	0.57530712	-0.5613674	0.56581619	0.02463644
142.1	-0.4264221	0.40187793	0.57530712	-0.5613674	0.56581619	0.02463644
142.2	-0.4264221	0.40187793	0.57530712	-0.5613674	0.56581619	0.02463644
142.3	-0.4264221	0.40187793	0.57530712	-0.5613674	0.56581619	0.02463644
142.4	-0.4264221	0.40187793	0.57530712	-0.5613674	0.56581619	0.02463644
142.5	-0.4264221	0.40187793	0.57530712	-0.5613674	0.56581619	0.02463644
142.6	-0.4264221	0.40187793	0.57530712	-0.5613674	0.56581619	0.02463644
142.7	-0.4264221	0.40187793	0.57530712	-0.5613674	0.56581619	0.02463644
142.8	-0.4264221	0.40187793	0.57530712	-0.5613674	0.56581619	0.02463644
142.9	-0.4264221	0.40187793	0.57530712	-0.5613674	0.56581619	0.02463644
143	-0.4264221	0.40187793	0.57530712	-0.5613674	0.56581619	0.02463644
143.1	-0.4264221	0.40187793	0.57530712	-0.5613674	0.56581619	0.02463644
143.2	-0.4264221	0.40187793	0.57530712	-0.5613674	0.56581619	0.02463644
143.3	-0.4264221	0.40187793	0.57530712	-0.5613674	0.56581619	0.02463644
143.4	-0.4264221	0.40187793	0.57530712	-0.5613674	0.56581619	0.02463644
143.5	-0.4264221	0.40187793	0.57530712	-0.5613674	0.56581619	0.02463644
143.6	-0.4264221	0.40187793	0.57530712	-0.5613674	0.56581619	0.02463644
143.7	-0.4263992	0.40212913	0.5755343	-0.5617116	0.56576884	0.02425412
143.8	-0.4263992	0.40212913	0.5755343	-0.5617116	0.56576884	0.02425412
143.9	-0.4263992	0.40212913	0.5755343	-0.5617116	0.56576884	0.02425412
144	-0.4263992	0.40212913	0.5755343	-0.5617116	0.56576884	0.02425412
144.1	-0.4263992	0.40212913	0.5755343	-0.5617116	0.56576884	0.02425412
144.2	-0.4263992	0.40212913	0.5755343	-0.5617116	0.56576884	0.02425412
144.3	-0.4263992	0.40212913	0.5755343	-0.5617116	0.56576884	0.02425412
144.4	-0.4263992	0.40212913	0.5755343	-0.5617116	0.56576884	0.02425412
144.5	-0.4263992	0.40212913	0.5755343	-0.5617116	0.56576884	0.02425412
144.6	-0.4263992	0.40212913	0.5755343	-0.5617116	0.56576884	0.02425412
144.7	-0.4263992	0.40212913	0.5755343	-0.5617116	0.56576884	0.02425412
144.8	-0.4263992	0.40212913	0.5755343	-0.5617116	0.56576884	0.02425412
144.9	-0.4270539	0.40243743	0.57577429	-0.5620184	0.56589462	0.02465105
145	-0.4270539	0.40243743	0.57577429	-0.5620184	0.56589462	0.02465105
145.1	-0.4270539	0.40243743	0.57577429	-0.5620184	0.56589462	0.02465105
145.2	-0.4270539	0.40243743	0.57577429	-0.5620184	0.56589462	0.02465105
145.3	-0.4270539	0.40243743	0.57577429	-0.5620184	0.56589462	0.02465105
145.4	-0.4266959	0.40275738	0.57580825	-0.5617385	0.56593768	0.02423429
145.5	-0.4266959	0.40275738	0.57580825	-0.5617385	0.56593768	0.02423429
145.6	-0.4266959	0.40275738	0.57580825	-0.5617385	0.56593768	0.02423429
145.7	-0.4266959	0.40275738	0.57580825	-0.5617385	0.56593768	0.02423429
145.8	-0.4266959	0.40275738	0.57580825	-0.5617385	0.56593768	0.02423429
145.9	-0.4266959	0.40275738	0.57580825	-0.5617385	0.56593768	0.02423429
146	-0.426537	0.4031138	0.5759475	-0.5617746	0.56592261	0.02418327
146.1	-0.426537	0.4031138	0.5759475	-0.5617746	0.56592261	0.02418327
146.2	-0.426537	0.4031138	0.5759475	-0.5617746	0.56592261	0.02418327
146.3	-0.426537	0.4031138	0.5759475	-0.5617746	0.56592261	0.02418327
146.4	-0.426537	0.4031138	0.5759475	-0.5617746	0.56592261	0.02418327
146.5	-0.426537	0.4031138	0.5759475	-0.5617746	0.56592261	0.02418327
146.6	-0.426537	0.4031138	0.5759475	-0.5617746	0.56592261	0.02418327
146.7	-0.4264784	0.40352499	0.57591595	-0.5617299	0.56545237	0.02437233
146.8	-0.4264784	0.40352499	0.57591595	-0.5617299	0.56545237	0.02437233
146.9	-0.4264784	0.40352499	0.57591595	-0.5617299	0.56545237	0.02437233
147	-0.4264784	0.40352499	0.57591595	-0.5617299	0.56545237	0.02437233
147.1	-0.4264784	0.40352499	0.57591595	-0.5617299	0.56545237	0.02437233
147.2	-0.4264784	0.40352499	0.57591595	-0.5617299	0.56545237	0.02437233
147.3	-0.4264784	0.40352499	0.57591595	-0.5617299	0.56545237	0.02437233
147.4	-0.4264784	0.40352499	0.57591595	-0.5617299	0.56545237	0.02437233
147.5	-0.4264784	0.40352499	0.57591595	-0.5617299	0.56545237	0.02437233
147.6	-0.4264784	0.40352499	0.57591595	-0.5617299	0.56545237	0.02437233
147.7	-0.4264784	0.40352499	0.57591595	-0.5617299	0.56545237	0.02437233
147.8	-0.4264784	0.40352499	0.57591595	-0.5617299	0.56545237	0.02437233
147.9	-0.4264784	0.40352499	0.57591595	-0.5617299	0.56545237	0.02437233
148	-0.4264784	0.40352499	0.57591595	-0.5617299	0.56545237	0.02437233
148.1	-0.4264784	0.40352499	0.57591595	-0.5617299	0.56545237	0.02437233
148.2	-0.4264784	0.40352499	0.57591595	-0.5617299	0.56545237	0.02437233
148.3	-0.4264784	0.40352499	0.57591595	-0.5617299	0.56545237	0.02437233
148.4	-0.4264784	0.40352499	0.57591595	-0.5617299	0.56545237	0.02437233
148.5	-0.4264784	0.40352499	0.57591595	-0.5617299	0.56545237	0.02437233
148.6	-0.4264784	0.40352499	0.57591595	-0.5617299	0.56545237	0.02437233
148.7	-0.4264784	0.40352499	0.57591595	-0.5617299	0.56545237	0.02437233
148.8	-0.4264784	0.40352499	0.57591595	-0.5617299	0.56545237	0.02437233
148.9	-0.4264784	0.40352499	0.57591595	-0.5617299	0.56545237	0.02437233
149	-0.4264784	0.40352499	0.57591595	-0.5617299	0.56545237	0.02437233
149.1	-0.4264784	0.40352499	0.57591595	-0.5617299	0.56545237	0.02437233
149.2	-0.4264784	0.40352499	0.57591595	-0.5617299	0.56545237	0.02437233
149.3	-0.4264784	0.40352499	0.57591595	-0.5617299	0.56545237	0.02437233
149.4	-0.426533	0.40375413	0.57610038	-0.5617002	0.5656427	0.02396725
149.5	-0.4268098	0.40380855	0.57627889	-0.5617522	0.5654521	0.0241514
149.6	-0.4268098	0.40380855	0.57627889	-0.5617522	0.5654521	0.0241514
149.7	-0.4268098	0.40380855	0.57627889	-0.5617522	0.5654521	0.0241514
149.8	-0.4268098	0.40380855	0.57627889	-0.5617522	0.5654521	0.0241514
149.9	-0.4268098	0.40380855	0.57627889	-0.5617522	0.5654521	0.0241514

156.1	-0.4262935	0.4040716	0.57606454	-0.5626197	0.56482477	0.02466265
156.2	-0.4265192	0.4041064	0.57634622	-0.5627265	0.56478594	0.02466743
156.3	-0.4265192	0.4041064	0.57634622	-0.5627265	0.56478594	0.02466743
156.4	-0.4265192	0.4041064	0.57634622	-0.5627265	0.56478594	0.02466743
156.5	-0.4265192	0.4041064	0.57634622	-0.5627265	0.56478594	0.02466743
156.6	-0.4265192	0.4041064	0.57634622	-0.5627265	0.56478594	0.02466743
156.7	-0.4265192	0.4041064	0.57634622	-0.5627265	0.56478594	0.02466743
156.8	-0.4265192	0.4041064	0.57634622	-0.5627265	0.56478594	0.02466743
156.9	-0.4265192	0.4041064	0.57634622	-0.5627265	0.56478594	0.02466743
157	-0.4265192	0.4041064	0.57634622	-0.5627265	0.56478594	0.02466743
157.1	-0.4265192	0.4041064	0.57634622	-0.5627265	0.56478594	0.02466743
157.2	-0.4265192	0.4041064	0.57634622	-0.5627265	0.56478594	0.02466743
157.3	-0.4265192	0.4041064	0.57634622	-0.5627265	0.56478594	0.02466743
157.4	-0.4265192	0.4041064	0.57634622	-0.5627265	0.56478594	0.02466743
157.5	-0.4265192	0.4041064	0.57634622	-0.5627265	0.56478594	0.02466743
157.6	-0.4265192	0.4041064	0.57634622	-0.5627265	0.56478594	0.02466743
157.7	-0.4265192	0.4041064	0.57634622	-0.5627265	0.56478594	0.02466743
157.8	-0.4265192	0.4041064	0.57634622	-0.5627265	0.56478594	0.02466743
157.9	-0.4265192	0.4041064	0.57634622	-0.5627265	0.56478594	0.02466743
158	-0.4265192	0.4041064	0.57634622	-0.5627265	0.56478594	0.02466743
158.1	-0.4265192	0.4041064	0.57634622	-0.5627265	0.56478594	0.02466743
158.2	-0.4265192	0.4041064	0.57634622	-0.5627265	0.56478594	0.02466743
158.3	-0.4265192	0.4041064	0.57634622	-0.5627265	0.56478594	0.02466743
158.4	-0.4263294	0.40421477	0.57614421	-0.5626656	0.56454364	0.02453696
158.5	-0.4263294	0.40421477	0.57614421	-0.5626656	0.56454364	0.02453696
158.6	-0.4263294	0.40421477	0.57614421	-0.5626656	0.56454364	0.02453696
158.7	-0.4263294	0.40421477	0.57614421	-0.5626656	0.56454364	0.02453696
158.8	-0.4263294	0.40421477	0.57614421	-0.5626656	0.56454364	0.02453696
158.9	-0.4263061	0.40421726	0.57624688	-0.562762	0.56452318	0.02441164
159	-0.4263061	0.40421726	0.57624688	-0.562762	0.56452318	0.02441164
159.1	-0.4263061	0.40421726	0.57624688	-0.562762	0.56452318	0.02441164
159.2	-0.4263061	0.40421726	0.57624688	-0.562762	0.56452318	0.02441164
159.3	-0.4263061	0.40421726	0.57624688	-0.562762	0.56452318	0.02441164
159.4	-0.4263061	0.40421726	0.57624688	-0.562762	0.56452318	0.02441164
159.5	-0.4263061	0.40421726	0.57624688	-0.562762	0.56452318	0.02441164
159.6	-0.4263061	0.40421726	0.57624688	-0.562762	0.56452318	0.02441164
159.7	-0.425977	0.40420249	0.57609984	-0.5628901	0.56446828	0.02464682
159.8	-0.425977	0.40420249	0.57609984	-0.5628901	0.56446828	0.02464682
159.9	-0.425977	0.40420249	0.57609984	-0.5628901	0.56446828	0.02464682
160	-0.425977	0.40420249	0.57609984	-0.5628901	0.56446828	0.02464682
160.1	-0.425977	0.40420249	0.57609984	-0.5628901	0.56446828	0.02464682
160.2	-0.425977	0.40420249	0.57609984	-0.5628901	0.56446828	0.02464682
160.3	-0.425977	0.40420249	0.57609984	-0.5628901	0.56446828	0.02464682
160.4	-0.425977	0.40420249	0.57609984	-0.5628901	0.56446828	0.02464682
160.5	-0.425977	0.40420249	0.57609984	-0.5628901	0.56446828	0.02464682
160.6	-0.425977	0.40420249	0.57609984	-0.5628901	0.56446828	0.02464682
160.7	-0.425977	0.40420249	0.57609984	-0.5628901	0.56446828	0.02464682
160.8	-0.425977	0.40420249	0.57609984	-0.5628901	0.56446828	0.02464682
160.9	-0.425977	0.40420249	0.57609984	-0.5628901	0.56446828	0.02464682
161	-0.425977	0.40420249	0.57609984	-0.5628901	0.56446828	0.02464682
161.1	-0.425977	0.40420249	0.57609984	-0.5628901	0.56446828	0.02464682
161.2	-0.425977	0.40420249	0.57609984	-0.5628901	0.56446828	0.02464682
161.3	-0.425977	0.40420249	0.57609984	-0.5628901	0.56446828	0.02464682
161.4	-0.425977	0.40420249	0.57609984	-0.5628901	0.56446828	0.02464682
161.5	-0.425977	0.40420249	0.57609984	-0.5628901	0.56446828	0.02464682
161.6	-0.425977	0.40420249	0.57609984	-0.5628901	0.56446828	0.02464682
161.7	-0.425977	0.40420249	0.57609984	-0.5628901	0.56446828	0.02464682
161.8	-0.425977	0.40420249	0.57609984	-0.5628901	0.56446828	0.02464682
161.9	-0.425977	0.40420249	0.57609984	-0.5628901	0.56446828	0.02464682
162	-0.425977	0.40420249	0.57609984	-0.5628901	0.56446828	0.02464682
162.1	-0.425977	0.40420249	0.57609984	-0.5628901	0.56446828	0.02464682
162.2	-0.425977	0.40420249	0.57609984	-0.5628901	0.56446828	0.02464682
162.3	-0.425977	0.40420249	0.57609984	-0.5628901	0.56446828	0.02464682
162.4	-0.425977	0.40420249	0.57609984	-0.5628901	0.56446828	0.02464682
162.5	-0.425977	0.40420249	0.57609984	-0.5628901	0.56446828	0.02464682
162.6	-0.425977	0.40420249	0.57609984	-0.5628901	0.56446828	0.02464682
162.7	-0.425977	0.40420249	0.57609984	-0.5628901	0.56446828	0.02464682
162.8	-0.425977	0.40420249	0.57609984	-0.5628901	0.56446828	0.02464682
162.9	-0.425977	0.40420249	0.57609984	-0.5628901	0.56446828	0.02464682
163	-0.425977	0.40420249	0.57609984	-0.5628901	0.56446828	0.02464682
163.1	-0.425977	0.40420249	0.57609984	-0.5628901	0.56446828	0.02464682
163.2	-0.425977	0.40420249	0.57609984	-0.5628901	0.56446828	0.02464682
163.3	-0.4257681	0.40385914	0.5760554	-0.5627977	0.56408039	0.02488406
163.4	-0.4259717	0.40391784	0.57604555	-0.5629271	0.56382835	0.02493842
163.5	-0.4259717	0.40391784	0.57604555	-0.5629271	0.56382835	0.02493842
163.6	-0.4259717	0.40391784	0.57604555	-0.5629271	0.56382835	0.02493842
163.7	-0.4259717	0.40391784	0.57604555	-0.5629271	0.56382835	0.02493842
163.8	-0.4259717	0.40391784	0.57604555	-0.5629271	0.56382835	0.02493842
163.9	-0.4259717	0.40391784	0.57604555	-0.5629271	0.56382835	0.02493842
164	-0.4259717	0.40391784	0.57604555	-0.5629271	0.56382835	0.02493842
164.1	-0.4259717	0.40391784	0.57604555	-0.5629271	0.56382835	0.02493842
164.2	-0.4259717	0.40391784	0.57604555	-0.5629271	0.56382835	0.02493842
164.3	-0.4259717	0.40391784	0.57604555	-0.5629271	0.56382835	0.02493842
164.4	-0.4259717	0.40391784	0.57604555	-0.5629271	0.56382835	0.02493842
164.5	-0.4259717	0.40391784	0.57604555	-0.5629271	0.56382835	0.02493842
164.6	-0.4259817	0.40382852	0.57586104	-0.5627932	0.56369996	0.02509621
164.7	-0.4259817	0.40382852	0.57586104	-0.5627932	0.56369996	0.02509621
164.8	-0.4259817	0.40382852	0.57586104	-0.5627932	0.56369996	0.02509621
164.9	-0.4259817	0.40382852	0.57586104	-0.5627932	0.56369996	0.02509621

171.1	-0.4265065	0.40375438	0.57634706	-0.5636409	0.56401934	0.02460233
171.2	-0.4265065	0.40375438	0.57634706	-0.5636409	0.56401934	0.02460233
171.3	-0.4265065	0.40375438	0.57634706	-0.5636409	0.56401934	0.02460233
171.4	-0.4265065	0.40375438	0.57634706	-0.5636409	0.56401934	0.02460233
171.5	-0.4265065	0.40375438	0.57634706	-0.5636409	0.56401934	0.02460233
171.6	-0.4269074	0.40410689	0.5765445	-0.5636519	0.5641771	0.02444102
171.7	-0.4269074	0.40410689	0.5765445	-0.5636519	0.5641771	0.02444102
171.8	-0.4269074	0.40410689	0.5765445	-0.5636519	0.5641771	0.02444102
171.9	-0.4269074	0.40410689	0.5765445	-0.5636519	0.5641771	0.02444102
172	-0.4269074	0.40410689	0.5765445	-0.5636519	0.5641771	0.02444102
172.1	-0.4265169	0.40402133	0.57660369	-0.5635528	0.56423786	0.02463421
172.2	-0.4265169	0.40402133	0.57660369	-0.5635528	0.56423786	0.02463421
172.3	-0.4265169	0.40402133	0.57660369	-0.5635528	0.56423786	0.02463421
172.4	-0.426484	0.40379066	0.57653658	-0.5635792	0.56406447	0.02509394
172.5	-0.426484	0.40379066	0.57653658	-0.5635792	0.56406447	0.02509394
172.6	-0.426484	0.40379066	0.57653658	-0.5635792	0.56406447	0.02509394
172.7	-0.426484	0.40379066	0.57653658	-0.5635792	0.56406447	0.02509394
172.8	-0.426484	0.40379066	0.57653658	-0.5635792	0.56406447	0.02509394
172.9	-0.426484	0.40379066	0.57653658	-0.5635792	0.56406447	0.02509394
173	-0.426484	0.40379066	0.57653658	-0.5635792	0.56406447	0.02509394
173.1	-0.426484	0.40379066	0.57653658	-0.5635792	0.56406447	0.02509394
173.2	-0.426484	0.40379066	0.57653658	-0.5635792	0.56406447	0.02509394
173.3	-0.426484	0.40379066	0.57653658	-0.5635792	0.56406447	0.02509394
173.4	-0.426484	0.40379066	0.57653658	-0.5635792	0.56406447	0.02509394
173.5	-0.426484	0.40379066	0.57653658	-0.5635792	0.56406447	0.02509394
173.6	-0.426484	0.40379066	0.57653658	-0.5635792	0.56406447	0.02509394
173.7	-0.4266099	0.40406123	0.57639753	-0.5636666	0.56395259	0.02529323
173.8	-0.4266099	0.40406123	0.57639753	-0.5636666	0.56395259	0.02529323
173.9	-0.4266099	0.40406123	0.57639753	-0.5636666	0.56395259	0.02529323
174	-0.4266099	0.40406123	0.57639753	-0.5636666	0.56395259	0.02529323
174.1	-0.4266099	0.40406123	0.57639753	-0.5636666	0.56395259	0.02529323
174.2	-0.4266099	0.40406123	0.57639753	-0.5636666	0.56395259	0.02529323
174.3	-0.4266099	0.40406123	0.57639753	-0.5636666	0.56395259	0.02529323
174.4	-0.4266099	0.40406123	0.57639753	-0.5636666	0.56395259	0.02529323
174.5	-0.4266099	0.40406123	0.57639753	-0.5636666	0.56395259	0.02529323
174.6	-0.4266099	0.40406123	0.57639753	-0.5636666	0.56395259	0.02529323
174.7	-0.4266099	0.40406123	0.57639753	-0.5636666	0.56395259	0.02529323
174.8	-0.4266099	0.40406123	0.57639753	-0.5636666	0.56395259	0.02529323
174.9	-0.4266099	0.40406123	0.57639753	-0.5636666	0.56395259	0.02529323
175	-0.4265014	0.40407015	0.57653525	-0.5639319	0.5641197	0.025058
175.1	-0.4265014	0.40407015	0.57653525	-0.5639319	0.5641197	0.025058
175.2	-0.4268817	0.40402761	0.57656039	-0.5638928	0.56388077	0.02490733
175.3	-0.4268266	0.40435359	0.57665266	-0.5639621	0.56376997	0.02487874
175.4	-0.4268266	0.40435359	0.57665266	-0.5639621	0.56376997	0.02487874
175.5	-0.4268266	0.40435359	0.57665266	-0.5639621	0.56376997	0.02487874
175.6	-0.4268266	0.40435359	0.57665266	-0.5639621	0.56376997	0.02487874
175.7	-0.4268266	0.40435359	0.57665266	-0.5639621	0.56376997	0.02487874
175.8	-0.4268266	0.40435359	0.57665266	-0.5639621	0.56376997	0.02487874
175.9	-0.4268266	0.40435359	0.57665266	-0.5639621	0.56376997	0.02487874
176	-0.4268266	0.40435359	0.57665266	-0.5639621	0.56376997	0.02487874
176.1	-0.4268266	0.40435359	0.57665266	-0.5639621	0.56376997	0.02487874
176.2	-0.4268266	0.40435359	0.57665266	-0.5639621	0.56376997	0.02487874
176.3	-0.4268266	0.40435359	0.57665266	-0.5639621	0.56376997	0.02487874
176.4	-0.4268266	0.40435359	0.57665266	-0.5639621	0.56376997	0.02487874
176.5	-0.4268266	0.40435359	0.57665266	-0.5639621	0.56376997	0.02487874
176.6	-0.4268266	0.40435359	0.57665266	-0.5639621	0.56376997	0.02487874
176.7	-0.4268266	0.40435359	0.57665266	-0.5639621	0.56376997	0.02487874
176.8	-0.4268266	0.40435359	0.57665266	-0.5639621	0.56376997	0.02487874
176.9	-0.4267617	0.40444288	0.57640907	-0.5639383	0.5634899	0.0246254
177	-0.4266779	0.4045493	0.57649047	-0.5641774	0.56353312	0.02449504
177.1	-0.4266779	0.4045493	0.57649047	-0.5641774	0.56353312	0.02449504
177.2	-0.4266779	0.4045493	0.57649047	-0.5641774	0.56353312	0.02449504
177.3	-0.4266779	0.4045493	0.57649047	-0.5641774	0.56353312	0.02449504
177.4	-0.4266779	0.4045493	0.57649047	-0.5641774	0.56353312	0.02449504
177.5	-0.4266779	0.4045493	0.57649047	-0.5641774	0.56353312	0.02449504
177.6	-0.4266779	0.4045493	0.57649047	-0.5641774	0.56353312	0.02449504
177.7	-0.4266779	0.4045493	0.57649047	-0.5641774	0.56353312	0.02449504
177.8	-0.4266779	0.4045493	0.57649047	-0.5641774	0.56353312	0.02449504
177.9	-0.4266779	0.4045493	0.57649047	-0.5641774	0.56353312	0.02449504
178	-0.4266779	0.4045493	0.57649047	-0.5641774	0.56353312	0.02449504
178.1	-0.4266779	0.4045493	0.57649047	-0.5641774	0.56353312	0.02449504
178.2	-0.4266779	0.4045493	0.57649047	-0.5641774	0.56353312	0.02449504
178.3	-0.4266779	0.4045493	0.57649047	-0.5641774	0.56353312	0.02449504
178.4	-0.4266779	0.4045493	0.57649047	-0.5641774	0.56353312	0.02449504
178.5	-0.4266779	0.4045493	0.57649047	-0.5641774	0.56353312	0.02449504
178.6	-0.42678	0.40445506	0.57629931	-0.5640521	0.5634374	0.02447859
178.7	-0.42678	0.40445506	0.57629931	-0.5640521	0.5634374	0.02447859
178.8	-0.42678	0.40445506	0.57629931	-0.5640521	0.5634374	0.02447859
178.9	-0.42678	0.40445506	0.57629931	-0.5640521	0.5634374	0.02447859
179	-0.42678	0.40445506	0.57629931	-0.5640521	0.5634374	0.02447859
179.1	-0.42678	0.40445506	0.57629931	-0.5640521	0.5634374	0.02447859
179.2	-0.42678	0.40445506	0.57629931	-0.5640521	0.5634374	0.02447859
179.3	-0.4269254	0.40416943	0.57615973	-0.5638589	0.56314896	0.02436658
179.4	-0.4269254	0.40416943	0.57615973	-0.5638589	0.56314896	0.02436658
179.5	-0.4269254	0.40416943	0.57615973	-0.5638589	0.56314896	0.02436658
179.6	-0.4264821	0.40405035	0.57605081	-0.5637989	0.56304064	0.02485557
179.7	-0.4264821	0.40405035	0.57605081	-0.5637989	0.56304064	0.02485557
179.8	-0.4264821	0.40405035	0.57605081	-0.5637989	0.56304064	0.02485557
179.9	-0.4264821	0.40405035	0.57605081	-0.5637989	0.56304064	0.02485557

180	-0.4264821	0.40405035	0.57605081	-0.5637989	0.56304064	0.02485557
180.1	-0.4264821	0.40405035	0.57605081	-0.5637989	0.56304064	0.02485557
180.2	-0.4264821	0.40405035	0.57605081	-0.5637989	0.56304064	0.02485557
180.3	-0.4264821	0.40405035	0.57605081	-0.5637989	0.56304064	0.02485557
180.4	-0.4265589	0.40416243	0.57614885	-0.5640326	0.56299182	0.02463461
180.5	-0.4266049	0.40404006	0.57608975	-0.5641685	0.56290622	0.02504174
180.6	-0.4266049	0.40404006	0.57608975	-0.5641685	0.56290622	0.02504174
180.7	-0.4266049	0.40404006	0.57608975	-0.5641685	0.56290622	0.02504174
180.8	-0.4266065	0.40414263	0.57630099	-0.5644557	0.56281665	0.0250217
180.9	-0.4266065	0.40414263	0.57630099	-0.5644557	0.56281665	0.0250217
181	-0.4266065	0.40414263	0.57630099	-0.5644557	0.56281665	0.0250217
181.1	-0.4266065	0.40414263	0.57630099	-0.5644557	0.56281665	0.0250217
181.2	-0.4266065	0.40414263	0.57630099	-0.5644557	0.56281665	0.0250217
181.3	-0.4266065	0.40414263	0.57630099	-0.5644557	0.56281665	0.0250217
181.4	-0.4266065	0.40414263	0.57630099	-0.5644557	0.56281665	0.0250217
181.5	-0.4266065	0.40414263	0.57630099	-0.5644557	0.56281665	0.0250217
181.6	-0.4266065	0.40414263	0.57630099	-0.5644557	0.56281665	0.0250217
181.7	-0.4266065	0.40414263	0.57630099	-0.5644557	0.56281665	0.0250217
181.8	-0.4266065	0.40414263	0.57630099	-0.5644557	0.56281665	0.0250217
181.9	-0.4266065	0.40414263	0.57630099	-0.5644557	0.56281665	0.0250217
182	-0.4266065	0.40414263	0.57630099	-0.5644557	0.56281665	0.0250217
182.1	-0.4266065	0.40414263	0.57630099	-0.5644557	0.56281665	0.0250217
182.2	-0.4266065	0.40414263	0.57630099	-0.5644557	0.56281665	0.0250217
182.3	-0.4266065	0.40414263	0.57630099	-0.5644557	0.56281665	0.0250217
182.4	-0.4266065	0.40414263	0.57630099	-0.5644557	0.56281665	0.0250217
182.5	-0.4266065	0.40414263	0.57630099	-0.5644557	0.56281665	0.0250217
182.6	-0.4266065	0.40414263	0.57630099	-0.5644557	0.56281665	0.0250217
182.7	-0.4266065	0.40414263	0.57630099	-0.5644557	0.56281665	0.0250217
182.8	-0.4266065	0.40414263	0.57630099	-0.5644557	0.56281665	0.0250217
182.9	-0.4266065	0.40414263	0.57630099	-0.5644557	0.56281665	0.0250217
183	-0.4266065	0.40414263	0.57630099	-0.5644557	0.56281665	0.0250217
183.1	-0.4266065	0.40414263	0.57630099	-0.5644557	0.56281665	0.0250217

195	-0.424269	0.40452794	0.57596209	-0.5644754	0.56234648	0.02610328
195.1	-0.424269	0.40452794	0.57596209	-0.5644754	0.56234648	0.02610328
195.2	-0.424269	0.40452794	0.57596209	-0.5644754	0.56234648	0.02610328
195.3	-0.424269	0.40452794	0.57596209	-0.5644754	0.56234648	0.02610328
195.4	-0.424269	0.40452794	0.57596209	-0.5644754	0.56234648	0.02610328
195.5	-0.424269	0.40452794	0.57596209	-0.5644754	0.56234648	0.02610328
195.6	-0.424269	0.40452794	0.57596209	-0.5644754	0.56234648	0.02610328
195.7	-0.424269	0.40452794	0.57596209	-0.5644754	0.56234648	0.02610328
195.8	-0.424269	0.40452794	0.57596209	-0.5644754	0.56234648	0.02610328
195.9	-0.4239222	0.40459399	0.57597231	-0.5646467	0.56239885	0.02651098
196	-0.4239222	0.40459399	0.57597231	-0.5646467	0.56239885	0.02651098
196.1	-0.4239222	0.40459399	0.57597231	-0.5646467	0.56239885	0.02651098
196.2	-0.4239222	0.40459399	0.57597231	-0.5646467	0.56239885	0.02651098
196.3	-0.4239222	0.40459399	0.57597231	-0.5646467	0.56239885	0.02651098
196.4	-0.4239222	0.40459399	0.57597231	-0.5646467	0.56239885	0.02651098
196.5	-0.4239222	0.40459399	0.57597231	-0.5646467	0.56239885	0.02651098
196.6	-0.4239222	0.40459399	0.57597231	-0.5646467	0.56239885	0.02651098
196.7	-0.4239222	0.40459399	0.57597231	-0.5646467	0.56239885	0.02651098
196.8	-0.4239222	0.40459399	0.57597231	-0.5646467	0.56239885	0.02651098
196.9	-0.4239222	0.40459399	0.57597231	-0.5646467	0.56239885	0.02651098
197	-0.4239222	0.40459399	0.57597231	-0.5646467	0.56239885	0.02651098
197.1	-0.4239222	0.40459399	0.57597231	-0.5646467	0.56239885	0.02651098
197.2	-0.4239222	0.40459399	0.57597231	-0.5646467	0.56239885	0.02651098
197.3	-0.4239222	0.40459399	0.57597231	-0.5646467	0.56239885	0.02651098
197.4	-0.4239222	0.40459399	0.57597231	-0.5646467	0.56239885	0.02651098
197.5	-0.4239222	0.40459399	0.57597231	-0.5646467	0.56239885	0.02651098
197.6	-0.4239222	0.40459399	0.57597231	-0.5646467	0.56239885	0.02651098
197.7	-0.4239222	0.40459399	0.57597231	-0.5646467	0.56239885	0.02651098
197.8	-0.4239222	0.40459399	0.57597231	-0.5646467	0.56239885	0.02651098
197.9	-0.4238099	0.40468951	0.57591818	-0.5645815	0.5622039	0.02631376
198	-0.4238099	0.40468951	0.57591818	-0.5645815	0.5622039	0.02631376
198.1	-0.4238099	0.40468951	0.57591818	-0.5645815	0.5622039	0.02631376

210	-0.4233431	0.40491289	0.57588818	-0.5650268	0.56193358	0.02691138
210.1	-0.4233431	0.40491289	0.57588818	-0.5650268	0.56193358	0.02691138
210.2	-0.4233431	0.40491289	0.57588818	-0.5650268	0.56193358	0.02691138
210.3	-0.4233431	0.40491289	0.57588818	-0.5650268	0.56193358	0.02691138
210.4	-0.4233431	0.40491289	0.57588818	-0.5650268	0.56193358	0.02691138
210.5	-0.4233431	0.40491289	0.57588818	-0.5650268	0.56193358	0.02691138
210.6	-0.4233431	0.40491289	0.57588818	-0.5650268	0.56193358	0.02691138
210.7	-0.4233431	0.40491289	0.57588818	-0.5650268	0.56193358	0.02691138
210.8	-0.4233519	0.405035	0.57590236	-0.5650747	0.56198074	0.02677199
210.9	-0.4233519	0.405035	0.57590236	-0.5650747	0.56198074	0.02677199
211	-0.4233519	0.405035	0.57590236	-0.5650747	0.56198074	0.02677199
211.1	-0.4233519	0.405035	0.57590236	-0.5650747	0.56198074	0.02677199
211.2	-0.4233519	0.405035	0.57590236	-0.5650747	0.56198074	0.02677199
211.3	-0.4233519	0.405035	0.57590236	-0.5650747	0.56198074	0.02677199
211.4	-0.4233519	0.405035	0.57590236	-0.5650747	0.56198074	0.02677199
211.5	-0.4233519	0.405035	0.57590236	-0.5650747	0.56198074	0.02677199
211.6	-0.4233519	0.405035	0.57590236	-0.5650747	0.56198074	0.02677199
211.7	-0.4233519	0.405035	0.57590236	-0.5650747	0.56198074	0.02677199
211.8	-0.4233519	0.405035	0.57590236	-0.5650747	0.56198074	0.02677199
211.9	-0.4233519	0.405035	0.57590236	-0.5650747	0.56198074	0.02677199
212	-0.423077	0.4050855	0.57601523	-0.5653296	0.56200191	0.02687844
212.1	-0.423077	0.4050855	0.57601523	-0.5653296	0.56200191	0.02687844
212.2	-0.423077	0.4050855	0.57601523	-0.5653296	0.56200191	0.02687844
212.3	-0.423077	0.4050855	0.57601523	-0.5653296	0.56200191	0.02687844
212.4	-0.423077	0.4050855	0.57601523	-0.5653296	0.56200191	0.02687844
212.5	-0.423077	0.4050855	0.57601523	-0.5653296	0.56200191	0.02687844
212.6	-0.423077	0.4050855	0.57601523	-0.5653296	0.56200191	0.02687844
212.7	-0.423077	0.4050855	0.57601523	-0.5653296	0.56200191	0.02687844
212.8	-0.423077	0.4050855	0.57601523	-0.5653296	0.56200191	0.02687844
212.9	-0.423077	0.4050855	0.57601523	-0.5653296	0.56200191	0.02687844
213	-0.423077	0.4050855	0.57601523	-0.5653296	0.56200191	0.02687844
213.1	-0.4231417	0.40504031	0.57606718	-0.5655867	0.56198867	0.02725703

225	-0.4232806	0.40506396	0.57623954	-0.5658117	0.56190306	0.02680183
225.1	-0.4232806	0.40506396	0.57623954	-0.5658117	0.56190306	0.02680183
225.2	-0.4232806	0.40506396	0.57623954	-0.5658117	0.56190306	0.02680183
225.3	-0.4232806	0.40506396	0.57623954	-0.5658117	0.56190306	0.02680183
225.4	-0.4232806	0.40506396	0.57623954	-0.5658117	0.56190306	0.02680183
225.5	-0.4232806	0.40506396	0.57623954	-0.5658117	0.56190306	0.02680183
225.6	-0.4232806	0.40506396	0.57623954	-0.5658117	0.56190306	0.02680183
225.7	-0.4232806	0.40506396	0.57623954	-0.5658117	0.56190306	0.02680183
225.8	-0.4232806	0.40506396	0.57623954	-0.5658117	0.56190306	0.02680183
225.9	-0.4232806	0.40506396	0.57623954	-0.5658117	0.56190306	0.02680183
226	-0.4232806	0.40506396	0.57623954	-0.5658117	0.56190306	0.02680183
226.1	-0.4232806	0.40506396	0.57623954	-0.5658117	0.56190306	0.02680183
226.2	-0.4232806	0.40506396	0.57623954	-0.5658117	0.56190306	0.02680183
226.3	-0.4232806	0.40506396	0.57623954	-0.5658117	0.56190306	0.02680183
226.4	-0.4232806	0.40506396	0.57623954	-0.5658117	0.56190306	0.02680183
226.5	-0.4232806	0.40506396	0.57623954	-0.5658117	0.56190306	0.02680183
226.6	-0.4232806	0.40506396	0.57623954	-0.5658117	0.56190306	0.02680183
226.7	-0.4232806	0.40506396	0.57623954	-0.5658117	0.56190306	0.02680183
226.8	-0.4232806	0.40506396	0.57623954	-0.5658117	0.56190306	0.02680183
226.9	-0.4232806	0.40506396	0.57623954	-0.5658117	0.56190306	0.02680183
227	-0.4232806	0.40506396	0.57623954	-0.5658117	0.56190306	0.02680183
227.1	-0.4232806	0.40506396	0.57623954	-0.5658117	0.56190306	0.02680183
227.2	-0.4232806	0.40506396	0.57623954	-0.5658117	0.56190306	0.02680183
227.3	-0.4232806	0.40506396	0.57623954	-0.5658117	0.56190306	0.02680183
227.4	-0.4232806	0.40506396	0.57623954	-0.5658117	0.56190306	0.02680183
227.5	-0.4232806	0.40506396	0.57623954	-0.5658117	0.56190306	0.02680183
227.6	-0.4232806	0.40506396	0.57623954	-0.5658117	0.56190306	0.02680183
227.7	-0.4232806	0.40506396	0.57623954	-0.5658117	0.56190306	0.02680183
227.8	-0.4232806	0.40506396	0.57623954	-0.5658117	0.56190306	0.02680183
227.9	-0.4232806	0.40506396	0.57623954	-0.5658117	0.56190306	0.02680183
228	-0.4232806	0.40506396	0.57623954	-0.5658117	0.56190306	0.02680183
228.1	-0.4232806	0.40506396	0.57623954	-0.5658117	0.56190306	0.02680183

183.2	-0.4266065	0.40414263	0.57630099	-0.5644557	0.56281665	0.0250217
183.3	-0.4262415	0.40413144	0.57619979	-0.5645049	0.56279612	0.02545826
183.4	-0.4262415	0.40413144	0.57619979	-0.5645049	0.56279612	0.02545826
183.5	-0.4262415	0.40413144	0.57619979	-0.5645049	0.56279612	0.02545826
183.6	-0.4262415	0.40413144	0.57619979	-0.5645049	0.56279612	0.02545826
183.7	-0.4263267	0.40412752	0.5763295	-0.5645529	0.56266324	0.02556845
183.8	-0.4263267	0.40412752	0.5763295	-0.5645529	0.56266324	0.02556845
183.9	-0.4263267	0.40412752	0.5763295	-0.5645529	0.56266324	0.02556845
184	-0.4263267	0.40412752	0.5763295	-0.5645529	0.56266324	0.02556845
184.1	-0.4263267	0.40412752	0.5763295	-0.5645529	0.56266324	0.02556845
184.2	-0.4263267	0.40412752	0.5763295	-0.5645529	0.56266324	0.02556845
184.3	-0.4263267	0.40412752	0.5763295	-0.5645529	0.56266324	0.02556845
184.4	-0.4263267	0.40412752	0.5763295	-0.5645529	0.56266324	0.02556845
184.5	-0.4263267	0.40412752	0.5763295	-0.5645529	0.56266324	0.02556845
184.6	-0.4263267	0.40412752	0.5763295	-0.5645529	0.56266324	0.02556845
184.7	-0.4263267	0.40412752	0.5763295	-0.5645529	0.56266324	0.02556845
184.8	-0.4263267	0.40412752	0.5763295	-0.5645529	0.56266324	0.02556845
184.9	-0.4263267	0.40412752	0.5763295	-0.5645529	0.56266324	0.02556845
185	-0.4263267	0.40412752	0.5763295	-0.5645529	0.56266324	0.02556845
185.1	-0.4263267	0.40412752	0.5763295	-0.5645529	0.56266324	0.02556845
185.2	-0.4259155	0.40423855	0.57628722	-0.5644661	0.5627282	0.02559776
185.3	-0.4259155	0.40423855	0.57628722	-0.5644661	0.5627282	0.02559776
185.4	-0.4259155	0.40423855	0.57628722	-0.5644661	0.5627282	0.02559776
185.5	-0.4259155	0.40423855	0.57628722	-0.5644661	0.5627282	0.02559776
185.6	-0.4259155	0.40423855	0.57628722	-0.5644661	0.5627282	0.02559776
185.7	-0.4259155	0.40423855	0.57628722	-0.5644661	0.5627282	0.02559776
185.8	-0.4259155	0.40423855	0.57628722	-0.5644661	0.5627282	0.02559776
185.9	-0.4259155	0.40423855	0.57628722	-0.5644661	0.5627282	0.02559776
186	-0.4259155	0.40423855	0.57628722	-0.5644661	0.5627282	0.02559776
186.1	-0.4259155	0.40423855	0.57628722	-0.5644661	0.5627282	0.02559776
186.2	-0.4259155	0.40423855	0.57628722	-0.5644661	0.5627282	0.02559776
186.3	-0.4259155	0.40423855	0.57628722	-0.5644661	0.5627282	0.02559776
186.4	-0.4258843	0.4041777	0.57598649	-0.5644166	0.5624778	0.02514331
186.5	-0.4258843	0.4041777	0.57598649	-0.5644166	0.5624778	0.02514331
186.6	-0.4254963	0.40414017	0.57595136	-0.5644287	0.56241378	0.02538077
186.7	-0.4254963	0.40414017	0.57595136	-0.5644287	0.56241378	0.02538077
186.8	-0.4254963	0.40414017	0.57595136	-0.5644287	0.56241378	0.02538077
186.9	-0.4254963	0.40414017	0.57595136	-0.5644287	0.56241378	0.02538077
187	-0.4254963	0.40414017	0.57595136	-0.5644287	0.56241378	0.02538077
187.1	-0.4254963	0.40414017	0.57595136	-0.5644287	0.56241378	0.02538077
187.2	-0.4254963	0.40414017	0.57595136	-0.5644287	0.56241378	0.02538077
187.3	-0.4254963	0.40414017	0.57595136	-0.5644287	0.56241378	0.02538077
187.4	-0.4254963	0.40414017	0.57595136	-0.5644287	0.56241378	0.02538077
187.5	-0.4254963	0.40414017	0.57595136	-0.5644287	0.56241378	0.02538077
187.6	-0.4254963	0.40414017	0.57595136	-0.5644287	0.56241378	0.02538077
187.7	-0.4254963	0.40414017	0.57595136	-0.5644287	0.56241378	0.02538077
187.8	-0.4254963	0.40414017	0.57595136	-0.5644287	0.56241378	0.02538077
187.9	-0.4254963	0.40414017	0.57595136	-0.5644287	0.56241378	0.02538077
188	-0.4254963	0.40414017	0.57595136	-0.5644287	0.56241378	0.02538077
188.1	-0.4254963	0.40414017	0.57595136	-0.5644287	0.56241378	0.02538077
188.2	-0.4255144	0.40436029	0.57624627	-0.5644447	0.56248828	0.02536121
188.3	-0.4255144	0.40436029	0.57624627	-0.5644447	0.56248828	0.02536121
188.4	-0.4255144	0.40436029	0.57624627	-0.5644447	0.56248828	0.02536121
188.5	-0.4255144	0.40436029	0.57624627	-0.5644447	0.56248828	0.02536121
188.6	-0.4255377	0.40421174	0.57625337	-0.5645435	0.56235918	0.02556938
188.7	-0.4255377	0.40421174	0.57625337	-0.5645435	0.56235918	0.02556938
188.8	-0.4255377	0.40421174	0.57625337	-0.5645435	0.56235918	0.02556938
188.9	-0.4255377	0.40421174	0.57625337	-0.5645435	0.56235918	0.02556938
189	-0.4255377	0.40421174	0.57625337	-0.5645435	0.56235918	0.02556938
189.1	-0.4252677	0.40432949	0.57628376	-0.5645341	0.56255811	0.0255131
189.2	-0.4252677	0.40432949	0.57628376	-0.5645341	0.56255811	0.0255131
189.3	-0.4252677	0.40432949	0.57628376	-0.5645341	0.56255811	0.0255131
189.4	-0.4252677	0.40432949	0.57628376	-0.5645341	0.56255811	0.0255131
189.5	-0.4252677	0.40432949	0.57628376	-0.5645341	0.56255811	0.0255131
189.6	-0.4252677	0.40432949	0.57628376	-0.5645341	0.56255811	0.0255131
189.7	-0.4252677	0.40432949	0.57628376	-0.5645341	0.56255811	0.0255131
189.8	-0.4252677	0.40432949	0.57628376	-0.5645341	0.56255811	0.0255131
189.9	-0.4252677	0.40432949	0.57628376	-0.5645341	0.56255811	0.0255131
190	-0.4252677	0.40432949	0.57628376	-0.5645341	0.56255811	0.0255131
190.1	-0.4252677	0.40432949	0.57628376	-0.5645341	0.56255811	0.0255131
190.2	-0.4252677	0.40432949	0.57628376	-0.5645341	0.56255811	0.0255131
190.3	-0.4252677	0.40432949	0.57628376	-0.5645341	0.56255811	0.0255131
190.4	-0.4252677	0.40432949	0.57628376	-0.5645341	0.56255811	0.0255131
190.5	-0.4252677	0.40432949	0.57628376	-0.5645341	0.56255811	0.0255131
190.6	-0.4252677	0.40432949	0.57628376	-0.5645341	0.56255811	0.0255131
190.7	-0.4252677	0.40432949	0.57628376	-0.5645341	0.56255811	0.0255131
190.8	-0.4252677	0.40432949	0.57628376	-0.5645341	0.56255811	0.0255131
190.9	-0.4252677	0.40432949	0.57628376	-0.5645341	0.56255811	0.0255131
191	-0.4252677	0.40432949	0.57628376	-0.5645341	0.56255811	0.0255131
191.1	-0.4251689	0.4043022	0.57624904	-0.5644023	0.56261347	0.02529053
191.2	-0.4251689	0.4043022	0.57624904	-0.5644023	0.56261347	0.02529053
191.3	-0.4246122	0.40435591	0.57599778	-0.5645981	0.56252194	0.02578687
191.4	-0.4246122	0.40435591	0.57599778	-0.5645981	0.56252194	0.02578687
191.5	-0.4246122	0.40435591	0.57599778	-0.5645981	0.56252194	0.02578687
191.6	-0.4246122	0.40435591	0.57599778	-0.5645981	0.56252194	0.02578687
191.7	-0.4246122	0.40435591	0.57599778	-0.5645981	0.56252194	0.02578687
191.8	-0.4246122	0.40435591	0.57599778	-0.5645981	0.56252194	0.02578687
191.9	-0.4246122	0.40435591	0.57599778	-0.5645981	0.56252194	0.02578687
192	-0.4246122	0.40435591	0.57599778	-0.5645981	0.56252194	0.02578687
192.1	-0.4246122	0.40435591	0.57599778	-0.5645981	0.56252194	0.02578687
192.2	-0.4246122	0.40435591	0.57599778	-0.5645981	0.56252194	0.02578687
192.3	-0.4246122	0.40435591	0.57599778	-0.5645981	0.56252194	0.02578687
192.4	-0.4246122	0.40435591	0.57599778	-0.5645981	0.56252194	0.02578687
192.5	-0.4246122	0.40435591	0.57599778	-0.5645981	0.56252194	0.02578687
192.6	-0.4246122	0.40435591	0.57599778	-0.5645981	0.56252194	0.02578687
192.7	-0.4246122	0.40435591	0.57599778	-0.5645981	0.56252194	0.02578687
192.8	-0.4246122	0.40435591	0.57599778	-0.5645981	0.56252194	0.02578687
192.9	-0.4246122	0.40435591	0.57599778	-0.5645981	0.56252194	0.02578687
193	-0.4246122	0.40435591	0.57599778	-0.5645981	0.56252194	0.02578687
193.1	-0.4247217	0.40444869	0.57607043	-0.5646192	0.5622641	0.02600179
193.2	-0.4247217	0.40444869	0.57607043	-0.5646192	0.5622641	0.02600179
193.3	-0.4247217	0.40444869	0.57607043	-0.5646192	0.5622641	0.02600179
193.4	-0.4247217	0.40444869	0.57607043	-0.5646192	0.5622641	0.02600179
193.5	-0.4247217	0.40444869	0.57607043	-0.5646192	0.5622641	0.02600179
193.6	-0.4247217	0.40444869	0.57607043	-0.5646192	0.5622641	0.02600179
193.7	-0.4247217	0.40444869	0.57607043	-0.5646192	0.5622641	0.02600179
193.8	-0.4247217	0.40444869	0.57607043	-0.5646192	0.5622641	0.02600179
193.9	-0.4242736	0.40455833	0.57600149	-0.5645966	0.56225625	0.02649995
194	-0.4242736	0.40455833	0.57600149	-0.5645966	0.56225625	0.02649995
194.1	-0.4242736	0.40455833	0.57600149	-0.5645966	0.56225625	0.02649995
194.2	-0.4242736	0.40455833	0.57600149	-0.5645966	0.56225625	0.02649995
194.3	-0.4242736	0.40455833	0.57600149	-0.5645966	0.56225625	0.02649995
194.4	-0.4242736	0.40455833	0.57600149	-0.5645966	0.56225625	0.02649995
194.5	-0.4242736	0.40455833	0.57600149	-0.5645966	0.56225625	0.02649995
194.6	-0.4242736	0.40455833	0.57600149	-0.5645966	0.56225625	0.02649995
194.7	-0.4242736	0.40455833	0.57600149	-0.5645966	0.56225625	0.02649995
194.8	-0.4242736	0.40455833	0.57600149	-0.5645966	0.56225625	0.02649995
194.9	-0.4242736	0.40455833	0.57600149	-0.5645966	0.56225625	0.02649995

198.2	-0.4238099	0.40468951	0.57591818	-0.5645815	0.5622039	0.02631376
198.3	-0.4238099	0.40468951	0.57591818	-0.5645815	0.5622039	0.02631376
198.4	-0.4238099	0.40468951	0.57591818	-0.5645815	0.5622039	0.02631376
198.5	-0.4238099	0.40468951	0.57591818	-0.5645815	0.5622039	0.02631376
198.6	-0.4238099	0.40468951	0.57591818	-0.5645815	0.5622039	0.02631376
198.7	-0.4238099	0.40468951	0.57591818	-0.5645815	0.5622039	0.02631376
198.8	-0.4238099	0.40468951	0.57591818	-0.5645815	0.5622039	0.02631376
198.9	-0.4238099	0.40468951	0.57591818	-0.5645815	0.5622039	0.02631376
199	-0.4237021	0.40475283	0.5760694	-0.5645849	0.56238934	0.02593378
199.1	-0.4237021	0.40475283	0.5760694	-0.5645849	0.56238934	0.02593378
199.2	-0.4237021	0.40475283	0.5760694	-0.5645849	0.56238934	0.02593378
199.3	-0.4237021	0.40475283	0.5760694	-0.5645849	0.56238934	0.02593378
199.4	-0.4237021	0.40475283	0.5760694	-0.5645849	0.56238934	0.02593378
199.5	-0.4237021	0.40475283	0.5760694	-0.5645849	0.56238934	0.02593378
199.6	-0.4237021	0.40475283	0.5760694	-0.5645849	0.56238934	0.02593378
199.7	-0.4237021	0.40475283	0.5760694	-0.5645849	0.56238934	0.02593378
199.8	-0.4236174	0.40484017	0.57593725	-0.5645122	0.56213552	0.02610735
199.9	-0.4236174	0.40484017	0.57593725	-0.5645122	0.56213552	0.02610735
200	-0.4236174	0.40484017	0.57593725	-0.5645122	0.56213552	0.02610735
200.1	-0.4236174	0.40484017	0.57593725	-0.5645122	0.56213552	0.02610735
200.2	-0.4236174	0.40484017	0.57593725	-0.5645122	0.56213552	0.02610735
200.3	-0.4236174	0.40484017	0.57593725	-0.5645122	0.56213552	0.02610735
200.4	-0.4236174	0.40484017	0.57593725	-0.5645122	0.56213552	0.02610735
200.5	-0.4236174	0.40484017	0.57593725	-0.5645122	0.56213552	0.02610735
200.6	-0.4236174	0.40484017	0.57593725	-0.5645122	0.56213552	0.02610735
200.7	-0.4236341	0.40480058	0.57583109	-0.5646426	0.5620094	0.02638361
200.8	-0.4236341	0.40480058	0.57583109	-0.5646426	0.5620094	0.02638361
200.9	-0.4236341	0.40480058	0.57583109	-0.5646426	0.5620094	0.02638361
201	-0.4236341	0.40480058	0.57583109	-0.5646426	0.5620094	0.02638361
201.1	-0.4236341	0.40480058	0.57583109	-0.5646426	0.5620094	0.02638361
201.2	-0.4236341	0.40480058	0.57583109	-0.5646426	0.5620094	0.02638361
201.3	-0.4236341	0.40480058	0.57583109	-0.5646426	0.5620094	0.02638361
201.4	-0.4236341	0.40480058	0.57583109	-0.5646426	0.5620094	0.02638361
201.5	-0.4236341	0.40480058	0.57583109	-0.5646426	0.5620094	0.02638361
201.6	-0.4236341	0.40480058	0.57583109	-0.5646426	0.5620094	0.02638361
201.7	-0.4236341	0.40480058	0.57583109	-0.5646426	0.5620094	0.02638361
201.8	-0.4236341	0.40480058	0.57583109	-0.5646426	0.5620094	0.02638361
201.9	-0.4236341	0.40480058	0.57583109	-0.5646426	0.5620094	0.02638361
202	-0.4236341	0.40480058	0.57583109	-0.5646426	0.5620094	0.02638361
202.1	-0.4236341	0.40480058	0.57583109	-0.5646426	0.5620094	0.02638361
202.2	-0.4236341	0.40480058	0.57583109	-0.5646426	0.5620094	0.02638361
202.3	-0.4236341	0.40480058	0.57583109	-0.5646426	0.5620094	0.02638361
202.4	-0.4236341	0.40480058	0.57583109	-0.5646426	0.5620094	0.02638361
202.5	-0.4236341	0.40480058	0.57583109	-0.5646426	0.5620094	0.02638361
202.6	-0.4236341	0.40480058	0.57583109	-0.5646426	0.5620094	0.02638361
202.7	-0.4236341	0.40480058	0.57583109	-0.5646426	0.5620094	0.02638361
202.8	-0.4236341	0.40480058	0.57583109	-0.5646426	0.5620094	0.02638361
202.9	-0.4236341	0.40480058	0.57583109	-0.5646426	0.5620094	0.02638361
203	-0.4236341	0.40480058	0.57583109	-0.5646426	0.5620094	0.02638361
203.1	-0.4236341	0.40480058	0.57583109	-0.5646426	0.5620094	0.02638361
203.2	-0.4236341	0.40480058	0.57583109	-0.5646426	0.5620094	0.02638361
203.3	-0.4236341	0.40480058	0.57583109	-0.5646426	0.5620094	0.02638361
203.4	-0.4236341	0.40480058	0.57583109	-0.5646426	0.5620094	0.02638361
203.5	-0.4236341	0.40480058	0.57583109	-0.5646426	0.5620094	0.02638361
203.6	-0.4236341	0.40480058	0.57583109	-0.5646426	0.5620094	0.02638361
203.7	-0.4236341	0.40480058	0.57583109	-0.5646426	0.5620094	0.02638361
203.8	-0.4236341	0.40480058	0.57583109	-0.5646426	0.5620094	0.02638361
203.9	-0.4236341	0.40480058	0.57583109	-0.5646426	0.5620094	0.02638361
204	-0.4236341	0.40480058	0.57583109	-0.5646426	0.5620094	0.02638361
204.1	-0.4236341	0.40480058	0.57583109	-0.5646426	0.5620094	0.02638361
204.2	-0.4236341	0.40480058	0.57583109	-0.5646426	0.5620094	0.02638361
204.3	-0.4236341	0.40480058	0.57583109	-0.5646426	0.5620094	0.02638361
204.4	-0.4233797	0.40491444	0.57591522	-0.564963	0.56200686	0.02644262
204.5	-0.4233797	0.40491444	0.57591522	-0.564963	0.56200686	0.02644262
204.6	-0.4233797	0.40491444	0.57591522	-0.564963	0.56200686	0.02644262
204.7	-0.4233797	0.40491444	0.57591522	-0.564963	0.56200686	0.02644262
204.8	-0.4233797	0.40491444	0.57591522	-0.564963	0.56200686	0.02644262
204.9	-0.4233797	0.40491444	0.57591522	-0.564963	0.56200686	0.02644262
205	-0.4233797	0.40491444	0.57591522	-0.564963	0.56200686	0.02644262
205.1	-0.4233797	0.40491444	0.57591522	-0.564963	0.56200686	0.02644262
205.2	-0.4233797	0.40491444	0.57591522	-0.564963	0.56200686	0.02644262
205.3	-0.4233797	0.40491444	0.57591522	-0.564963	0.56200686	0.02644262
205.4	-0.4233797	0.40491444	0.57591522	-0.564963	0.56200686	0.02644262
205.5	-0.4233797	0.40491444	0.57591522	-0.564963	0.56200686	0.02644262
205.6	-0.4233797	0.40491444	0.57591522	-0.564963	0.56200686	0.02644262
205.7	-0.4233797	0.40491444	0.57591522	-0.564963	0.56200686	0.02644262
205.8	-0.4233797	0.40491444	0.57591522	-0.564963	0.56200686	0.02644262
205.9	-0.4233797	0.40491444	0.57591522	-0.564963	0.56200686	0.02644262
206	-0.4233797	0.40491444	0.57591522	-0.564963	0.56200686	0.02644262
206.1	-0.4233797	0.40491444	0.57591522	-0.564963	0.56200686	0.02644262
206.2	-0.4233797	0.40491444	0.57591522	-0.564963	0.56200686	0.02644262
206.3	-0.4233797	0.40491444	0.57591522	-0.564963	0.56200686	0.02644262
206.4	-0.4233797	0.40491444	0.57591522	-0.564963	0.56200686	0.02644262
206.5	-0.4233797	0.40491444	0.57591522	-0.564963	0.56200686	0.02644262
206.6	-0.4233797	0.40491444	0.57591522	-0.564963	0.56200686	0.02644262
206.7	-0.4233797	0.40491444	0.57591522	-0.564963	0.56200686	0.02644262
206.8	-0.4233797	0.40491444	0.57591522	-0.564963	0.56200686	0.02644262
206.9	-0.4233797	0.40491444	0.57591522	-0.564963	0.56200686	0.02644262
207	-0.4233797	0.40491444	0.57591522	-0.564963	0.56200686	0.02644262
207.1	-0.4233797	0.40491444	0.57591522	-0.564963	0.56200686	0.02644262
207.2	-0.4233797	0.40491444	0.57591522	-0.564963	0.56200686	0.02644262
207.3	-0.4233797	0.40491444	0.57591522	-0.564963	0.56200686	0.02644262
207.4	-0.4233797	0.40491444	0.57591522	-0.564963	0.56200686	0.02644262
207.5	-0.4233431	0.40491289	0.57588818	-0.5650268	0.56193358	0.02691138
207.6	-0.4233431	0.40491289	0.57588818	-0.5650268	0.56193358	0.02691138
207.7	-0.4233431	0.40491289	0.57588818	-0.5650268	0.56193358	0.02691138
207.8	-0.4233431	0.40491289	0.57588818	-0.5650268	0.56193358	0.02691138
207.9	-0.4233431	0.40491289	0.57588818	-0.5650268	0.56193358	0.02691138
208	-0.4233431	0.40491289	0.57588818	-0.5650268	0.56193358	0.02691138
208.1	-0.4233431	0.40491289	0.57588818	-0.5650268	0.56193358	0.02691138
208.2	-0.4233431	0.40491289	0.57588818	-0.5650268	0.56193358	0.02691138
208.3	-0.4233431	0.40491289	0.57588818	-0.5650268	0.56193358	0.02691138
208.4	-0.4233431	0.40491289	0.57588818	-0.5650268	0.56193358	0.02691138
208.5	-0.4233431	0.40491289	0.57588818	-0.5650268	0.56193358	0.02691138
208.6	-0.4233431	0.40491289	0.57588818	-0.5650268	0.56193358	0.02691138
208.7	-0.4233431	0.40491289	0.57588818	-0.5650268	0.56193358	0.02691138
208.8	-0.4233431	0.40491289	0.57588818	-0.5650268	0.56193358	0.02691138
208.9	-0.4233431	0.40491289	0.57588818	-0.5650268	0.56193358	0.02691138
209	-0.4233431	0.40491289	0.57588818	-0.5650268	0.56193358	0.02691138
209.1	-0.4233431	0.40491289	0.57588818	-0.5650268	0.56193358	0.02691138
209.2	-0.4233431	0.40491289	0.57588818	-0.5650268	0.56193358	0.02691138
209.3	-0.4233431	0.40491289	0.57588818	-0.5650268	0.56193358	0.02691138
209.4	-0.4233431	0.40491289	0.57588818	-0.5650268	0.56193358	0.02691138
209.5	-0.4233431	0.40491289	0.57588818	-0.5650268	0.56193358	0.02691138
209.6	-0.4233431	0.40491289	0.57588818	-0.5650268	0.56193358	0.02691138
209.7	-0.4233431	0.40491289	0.57588818	-0.5650268	0.56193358	0.02691138
209.8	-0.4233431	0.40491289	0.57588818	-0.5650268	0.56193358	0.02691138
209.9	-0.4233431	0.40491289	0.57588818	-0.5650268	0.56193358	0.02691138

213.2	-0.4231417	0.40504031	0.57606718	-0.5655867	0.56198867	0.02725703
213.3	-0.4231417	0.40504031	0.57606718	-0.5655867	0.56198867	0.02725703
213.4	-0.4231417	0.40504031	0.57606718	-0.5655867	0.56198867	0.02725703
213.5	-0.4231417	0.40504031	0.57606718	-0.5655867	0.56198867	0.02725703
213.6	-0.4231417	0.40504031	0.57606718	-0.5655867	0.56198867	0.02725703
213.7	-0.4231417	0.40504031	0.57606718	-0.5655867	0.56198867	0.02725703
213.8	-0.4231417	0.40504031	0.57606718	-0.5655867	0.56198867	0.02725703
213.9	-0.4231417	0.40504031	0.57606718	-0.5655867	0.56198867	0.02725703
214	-0.4231417	0.40504031	0.57606718	-0.5655867	0.56198867	0.02725703
214.1	-0.4231417	0.40504031	0.57606718	-0.5655867	0.56198867	0.02725703
214.2	-0.4231417	0.40504031	0.57606718	-0.5655867	0.56198867	0.02725703
214.3	-0.4231417	0.40504031	0.57606718	-0.5655867	0.56198867	0.02725703
214.4	-0.4231417	0.40504031	0.57606718	-0.5655867	0.56198867	0.02725703
214.5	-0.4231417	0.40504031	0.57606718	-0.5655867	0.56198867	0.02725703
214.6	-0.4231417	0.40504031	0.57606718	-0.5655867	0.56198867	0.02725703
214.7	-0.4231417	0.40504031	0.57606718	-0.5655867	0.56198867	0.02725703
214.8	-0.4231417	0.40504031	0.57606718	-0.5655867	0.56198867	0.02725703
214.9	-0.4231417	0.40504031	0.57606718	-0.5655867	0.56198867	0.02725703
215	-0.4231417	0.40504031	0.57606718	-0.5655867	0.56198867	0.02725703
215.1	-0.4231417	0.40504031	0.57606718	-0.5655867	0.56198867	0.02725703
215.2	-0.4231417	0.40504031	0.57606718	-0.5655867	0.56198867	0.02725703
215.3	-0.4231417	0.40504031	0.57606718	-0.5655867	0.56198867	0.02725703
215.4	-0.4231417	0.40504031	0.57606718	-0.5655867	0.56198867	0.02725703
215.5	-0.4231417	0.40504031	0.57606718	-0.5655867	0.56198867	0.02725703
215.6	-0.4231417	0.40504031	0.57606718	-0.5655867	0.56198867	0.02725703
215.7	-0.4231417	0.40504031	0.57606718	-0.5655867	0.56198867	0.02725703
215.8	-0.4231417	0.40504031	0.57606718	-0.5655867	0.56198867	0.02725703
215.9	-0.4231417	0.40504031	0.57606718	-0.5655867	0.56198867	0.02725703
216	-0.4231417	0.40504031	0.57606718	-0.5655867	0.56198867	0.02725703
216.1	-0.4231417	0.40504031	0.57606718	-0.5655867	0.56198867	0.02725703
216.2	-0.4231417	0.40504031	0.57606718	-0.5655867	0.56198867	0.02725703
216.3	-0.4231417	0.40504031	0.57606718	-0.5655867	0.56198867	0.02725703
216.4	-0.4231417	0.40504031	0.57606718	-0.5655867	0.56198867	0.02725703
216.5	-0.4231417	0.40504031	0.57606718	-0.5655867	0.56198867	0.02725703
216.6	-0.4231417	0.40504031	0.57606718	-0.5655867	0.56198867	0.02725703
216.7	-0.4231417	0.40504031	0.57606718	-0.5655867	0.56198867	0.02725703
216.8	-0.4231417	0.40504031	0.57606718	-0.5655867	0.56198867	0.02725703
216.9	-0.4231417	0.40504031	0.57606718	-0.5655867	0.56198867	0.02725703
217	-0.4231417	0.40504031	0.57606718	-0.5655867	0.56198867	0.02725703
217.1	-0.4231417	0.40504031	0.57606718	-0.5655867	0.56198867	0.02725703
217.2	-0.4231417	0.40504031	0.57606718	-0.5655867	0.56198867	0.02725703
217.3	-0.4231417	0.40504031	0.57606718	-0.5655867	0.56198867	0.02725703
217.4	-0.4231417	0.40504031	0.57606718	-0.5655867	0.56198867	0.02725703
217.5	-0.4231417	0.40504031	0.57606718	-0.5655867	0.56198867	0.02725703
217.6	-0.4231417	0.40504031	0.57606718	-0.5655867	0.56198867	0.02725703
217.7	-0.4231417	0.40504031	0.57606718	-0.5655867	0.56198867	0.02725703
217.8	-0.4231417	0.40504031	0.57606718	-0.5655867	0.56198867	0.02725703
217.9	-0.4231417	0.40504031	0.57606718	-0.5655867	0.56198867	0.02725703
218	-0.4231417	0.40504031	0.57606718	-0.5655867	0.56198867	0.02725703
218.1	-0.4231417	0.40504031	0.57606718	-0.5655867	0.56198867	0.02725703
218.2	-0.4231417	0.40504031	0.57606718	-0.5655867	0.56198867	0.02725703
218.3	-0.4231417	0.40504031	0.57606718	-0.5655867	0.56198867	0.02725703
218.4	-0.4231417	0.40504031	0.57606718	-0.5655867	0.56198867	0.02725703
218.5	-0.4231417	0.40504031	0.57606718	-0.5655867	0.56198867	0.02725703
218.6	-0.4231417	0.40504031	0.57606718	-0.5655867	0.56198867	0.02725703
218.7	-0.4231417	0.40504031	0.57606718	-0.5655867	0.56198867	0.02725703
218.8	-0.4231417	0.40504031	0.57606718	-0.5655867	0.56198867	0.02725703
218.9	-0.4231417	0.40504031	0.57606718	-0.5655867	0.56198867	0.02725703
219	-0.4231417	0.40504031	0.57606718	-0.5655867	0.56198867	0.02725703
219.1	-0.4231417	0.40504031	0.57606718	-0.5655867	0.56198867	0.02725703
219.2	-0.4231417	0.40504031	0.57606718	-0.5655867	0.56198867	0.02725703
219.3	-0.4231417	0.40504031	0.57606718	-0.5655867	0.56198867	0.02725703
219.4	-0.4231417	0.40504031	0.57606718	-0.5655867	0.56198867	0.02725703
219.5	-0.4231417	0.40504031	0.57606718	-0.5655867	0.56198867	0.02725703
219.6	-0.4231417	0.40504031	0.57606718	-0.5655867	0.56198867	0.02725703
219.7	-0.4231417	0.40504031	0.57606718	-0.5655867	0.56198867	0.02725703
219.8	-0.4231417	0.40504031	0.57606718	-0.5655867	0.56198867	0.02725703
219.9	-0.4231417	0.40504031	0.57606718	-0.5655867	0.56198867	0.02725703
220	-0.4231417	0.40504031	0.57606718	-0.5655867	0.56198867	0.02725703
220.1	-0.4231417	0.40504031	0.57606718	-0.5655867	0.56198867	0.02725703
220.2	-0.4231417	0.40504031	0.57606718	-0.5655867	0.56198867	0.02725703
220.3	-0.4231417	0.40504031	0.57606718	-0.5655867	0.56198867	0.02725703
220.4	-0.4231417	0.40504031	0.57606718	-0.5655867	0.56198867	0.02725703
220.5	-0.4231417	0.40504031	0.57606718	-0.5655867	0.56198867	0.02725703
220.6	-0.4231417	0.40504031	0.57606718	-0.5655867	0.56198867	0.02725703
220.7	-0.4231417	0.40504031	0.57606718	-0.5655867	0.56198867	0.02725703
220.8	-0.4231417	0.40504031	0.57606718	-0.5655867	0.56198867	0.02725703
220.9	-0.4231417	0.40504031	0.57606718	-0.5655867	0.56198867	0.02725703
221	-0.4231417	0.40504031	0.57606718	-0.5655867	0.56198867	0.02725703
221.1	-0.4231417	0.40504031	0.57606718	-0.5655867	0.56198867	0.02725703
221.2	-0.4231417	0.40504031	0.57606718	-0.5655867	0.56198867	0.02725703
221.3	-0.4231417	0.40504031	0.57606718	-0.5655867	0.56198867	0.02725703
221.4	-0.4231417	0.40504031	0.57606718	-0.5655867	0.56198867	0.02725703
221.5	-0.4231417	0.40504031	0.57606718	-0.5655867	0.56198867	0.02725703
221.6	-0.4231417	0.40504031	0.57606718	-0.5655867	0.56198867	0.02725703
221.7	-0.4231417	0.40504031	0.57606718	-0.5655867	0.56198867	0.02725703
221.8	-0.4231417	0.40504031	0.57606718	-0.5655867	0.56198867	0.02725703
221.9	-0.4231417	0.40504031	0.57606718	-0.5655867	0.56198867	0.02725703
222	-0.4231417	0.40504031	0.57606718	-0.5655867	0.56198867	0.02725703
222.1	-0.4231417	0.40504031	0.57606718	-0.5655867	0.56198867	0.02725703
222.2	-0.4231417	0.40504031	0.57606718	-0.5655867	0.56198867	0.02725703
222.3	-0.4231417	0.40504031	0.57606718	-0.5655867	0.56198867	0.02725703
222.4	-0.4231417	0.40504031	0.57606718	-0.5655867	0.56198867	0.02725703
222.5	-0.4231417	0.40504031	0.57606718	-0.5655867	0.56198867	0.02725703
222.6	-0.4231417	0.40504031	0.57606718	-0.5655867	0.56198867	0.02725703
222.7	-0.4231417	0.40504031	0.57606718	-0.5655867	0.56198867	0.02725703
222.8	-0.4231417	0.40504031	0.57606718	-0.5655867	0.56198867	0.02725703
222.9	-0.4231417	0.40504031	0.57606718	-0.5655867	0.56198867	0.02725703
223	-0.4231417	0.40504031	0.57606718	-0.5655867	0.56198867	0.02725703
223.1	-0.4231417	0.40504031	0.57606718	-0.5655867	0.56198867	0.02725703
223.2	-0.4231417	0.40504031	0.57606718	-0.5655867	0.56198867	0.02725703
223.3	-0.4231417	0.40504031	0.57606718	-0.5655867	0.56198867	0.02725703
223.4	-0.4231417	0.40504031	0.57606718	-0.5655867	0.56198867	0.02725703
223.5	-0.4231417	0.40504031	0.57606718	-0.5655867	0.56198867	0.02725703
223.6	-0.4232806	0.40506396	0.57623954	-0.5658117	0.56190306	0.02680183
223.7	-0.4232806	0.40506396	0.57623954	-0.5658117	0.56190306	0.02680183
223.8	-0.4232806	0.40506396	0.57623954	-0.5658117	0.56190306	0.02680183
223.9	-0.4232806	0.40506396	0.57623954	-0.5658117	0.56190306	0.02680183
224	-0.4232806	0.40506396	0.57623954	-0.5658117	0.56190306	0.02680183
224.1	-0.4232806	0.40506396	0.57623954	-0.5658117	0.56190306	0.02680183
224.2	-0.4232806	0.40506396	0.57623954	-0.5658117	0.56190306	0.02680183
224.3	-0.4232806	0.40506396	0.57623954	-0.5658117	0.56190306	0.02680183
224.4	-0.4232806	0.40506396	0.57623954	-0.5658117	0.56190306	0.02680183
224.5	-0.4232806	0.40506396	0.57623954	-0.5658117	0.56190306	0.02680183
224.6	-0.4232806	0.40506396	0.57623954	-0.5658117	0.56190306	0.02680183
224.7	-0.4232806	0.40506396	0.57623954	-0.5658117	0.56190306	0.02680183
224.8	-0.4232806	0.40506396	0.57623954	-0.5658117	0.56190306	0.02680183
224.9	-0.4232806	0.40506396	0.57623954	-0.5658117	0.56190306	0.02680183

228.2	-0.4232806	0.40506396	0.57623954	-0.5658117	0.56190306	0.02680183
228.3	-0.4232806	0.40506396	0.57623954	-0.5658117	0.56190306	0.02680183
228.4	-0.4232806	0.40506396	0.57623954	-0.5658117	0.56190306	0.02680183
228.5	-0.4232806	0.40506396	0.57623954	-0.5658117	0.56190306	0.02680183
228.6	-0.4232806	0.40506396	0.57623954	-0.5658117	0.56190306	0.02680183
228.7	-0.4232806	0.40506396	0.57623954	-0.5658117	0.56190306	0.02680183
228.8	-0.4232806	0.40506396	0.57623954	-0.5658117	0.56190306	0.02680183
228.9	-0.4230906	0.40500816	0.5762711	-0.5658282	0.56188123	0.02703814
229	-0.4230906	0.40500816	0.5762711	-0.5658282	0.56188123	0.02703814
229.1	-0.4230906	0.40500816	0.5762711	-0.5658282	0.56188123	0.02703814
229.2	-0.4230906	0.40500816	0.5762711	-0.5658282	0.56188123	0.02703814
229.3	-0.4230906	0.40500816	0.5762711	-0.5658282	0.56188123	0.02703814
229.4	-0.4230906	0.40500816	0.5762711	-0.5658282	0.56188123	0.02703814
229.5	-0.4230906	0.40500816	0.5762711	-0.5658282	0.56188123	0.02703814
229.6	-0.4230906	0.40500816	0.5762711	-0.5658282	0.56188123	0.02703814
229.7	-0.4230906	0.40500816	0.5762711	-0.5658282	0.56188123	0.02703814
229.8	-0.4230906	0.40500816	0.5762711	-0.5658282	0.56188123	0.02703814
229.9	-0.4230906	0.40500816	0.5762711	-0.5658282	0.56188123	0.02703814
230	-0.4230906	0.40500816	0.5762711	-0.5658282	0.56188123	0.02703814
230.1	-0.4230906	0.40500816	0.5762711	-0.5658282	0.56188123	0.02703814
230.2	-0.4230906	0.40500816	0.5762711	-0.5658282	0.56188123	0.02703814
230.3	-0.4230906	0.40500816	0.5762711	-0.5658282	0.56188123	0.02703814
230.4	-0.4230906	0.40500816	0.5762711	-0.5658282	0.56188123	0.02703814
230.5	-0.4230906	0.40500816	0.5762711	-0.5658282	0.56188123	0.02703814
230.6	-0.4230906	0.40500816	0.5762711	-0.5658282	0.56188123	0.02703814
230.7	-0.4230906	0.40500816	0.5762711	-0.5658282	0.56188123	0.02703814
230.8	-0.4230906	0.40500816	0.5762711	-0.5658282	0.56188123	0.02703814
230.9	-0.4230906	0.40500816	0.5762711	-0.5658282	0.56188123	0.02703814
231	-0.4230906	0.40500816	0.5762711	-0.5658282	0.56188123	0.02703814
231.1	-0.4230906	0.40500816	0.5762711	-0.5658282	0.56188123	0.02703814
231.2	-0.4230906	0.40500816	0.5762711	-0.5658282	0.56188123	0.02703814
231.3	-0.4230906	0.40500816	0.5762711	-0.5658282	0.56188123	0.02703814
231.4	-0.4230906	0.40500816	0.5762711	-0.5658282	0.56188123	0.02703814
231.5	-0.4230906	0.40500816	0.5762711	-0.5658282	0.56188123	0.02703814
231.6	-0.4230906	0.40500816	0.5762711	-0.5658282	0.56188123	0.02703814
231.7	-0.4229351	0.40537732	0.57651146	-0.5657467	0.56191804	0.02684591
231.8	-0.4229351	0.40537732	0.57651146	-0.5657467	0.56191804	0.02684591
231.9	-0.4229351	0.40537732	0.57651146	-0.5657467	0.56191804	0.02684591
232	-0.4229351	0.40537732	0.57651146	-0.5657467	0.56191804	0.02684591
232.1	-0.4229351	0.40537732	0.57651146	-0.5657467	0.56191804	0.02684591
232.2	-0.4229351	0.40537732	0.57651146	-0.5657467	0.56191804	0.02684591
232.3	-0.4229351	0.40537732	0.57651146	-0.5657467	0.56191804	0.02684591
232.4	-0.4229351	0.40537732	0.57651146	-0.5657467	0.56191804	0.02684591
232.5	-0.4229351	0.40537732	0.57651146	-0.5657467	0.56191804	0.02684591
232.6	-0.4229351	0.40537732	0.57651146	-0.5657467	0.56191804	0.02684591
232.7	-0.4229351	0.40537732	0.57651146	-0.5657467	0.56191804	0.02684591
232.8	-0.4229351	0.40537732	0.57651146	-0.5657467	0.56191804	0.02684591
232.9	-0.4229351	0.40537732	0.57651146	-0.5657467	0.56191804	0.02684591
233	-0.4229351	0.40537732	0.57651146	-0.5657467	0.56191804	0.02684591
233.1	-0.4229351	0.40537732	0.57651146	-0.5657467	0.56191804	0.02684591
233.2	-0.4229351	0.40537732	0.57651146	-0.5657467	0.56191804	0.02684591
233.3	-0.4229351	0.40537732	0.57651146	-0.5657467	0.56191804	0.02684591
233.4	-0.4229351	0.40537732	0.57651146	-0.5657467	0.56191804	0.02684591
233.5	-0.4229351	0.40537732	0.57651146	-0.5657467	0.56191804	0.02684591
233.6	-0.4229351	0.40537732	0.57651146	-0.5657467	0.56191804	0.02684591
233.7	-0.4229351	0.40537732	0.57651146	-0.5657467	0.56191804	0.02684591
233.8	-0.4229351	0.40537732	0.57651146	-0.5657467	0.56191804	0.02684591
233.9	-0.4229351	0.40537732	0.57651146	-0.5657467	0.56191804	0.02684591
234	-0.4229351	0.40537732	0.57651146	-0.5657467	0.56191804	0.02684591
234.1	-0.4229351	0.40537732	0.57651146	-0.5657467	0.56191804	0.02684591
234.2	-0.4229351	0.40537732	0.57651146	-0.5657467	0.56191804	0.02684591
234.3	-0.4229351	0.40537732	0.57651146	-0.5657467	0.56191804	0.02684591
234.4	-0.4229351	0.40537732	0.57651146	-0.5657467	0.56191804	0.02684591
234.5	-0.4229351	0.40537732	0.57651146	-0.5657467	0.56191804	0.02684591
234.6	-0.4229351	0.40537732	0.57651146	-0.5657467	0.56191804	0.02684591
234.7	-0.4229351	0.40537732	0.57651146	-0.5657467	0.56191804	0.02684591
234.8	-0.4229351	0.40537732	0.57651146	-0.5657467	0.56191804	0.02684591
234.9	-0.4229351	0.40537732	0.57651146	-0.5657467	0.56191804	0.02684591
235	-0.4229351	0.40537732	0.57651146	-0.5657467	0.56191804	0.02684591
235.1	-0.4229351	0.40537732	0.57651146	-0.5657467	0.56191804	0.02684591
235.2	-0.4229351	0.40537732	0.57651146	-0.5657467	0.56191804	0.02684591
235.3	-0.4229351	0.40537732	0.57651146	-0.5657467	0.56191804	0.02684591
235.4	-0.4229351	0.40537732	0.57651146	-0.5657467	0.56191804	0.02684591
235.5	-0.4229351	0.40537732	0.57651146	-0.5657467	0.56191804	0.02684591
235.6	-0.4229351	0.40537732	0.57651146	-0.5657467	0.56191804	0.02684591
235.7	-0.4229351	0.40537732	0.57651146	-0.5657467	0.56191804	0.02684591
235.8	-0.4229351	0.40537732	0.57651146	-0.5657467	0.56191804	0.02684591
235.9	-0.4229351	0.40537732	0.57651146	-0.5657467	0.56191804	0.02684591
236	-0.4229351	0.40537732	0.57651146	-0.5657467	0.56191804	0.02684591
236.1	-0.4229351	0.40537732	0.57651146	-0.5657467	0.56191804	0.02684591
236.2	-0.4229351	0.40537732	0.57651146	-0.5657467	0.56191804	0.02684591
236.3	-0.4229351	0.40537732	0.57651146	-0.5657467	0.56191804	0.02684591
236.4	-0.4229351	0.40537732	0.57651146	-0.5657467	0.56191804	0.02684591
236.5	-0.4229351	0.40537732	0.57651146	-0.5657467	0.56191804	0.02684591
236.6	-0.4229351	0.40537732	0.57651146	-0.5657467	0.56191804	0.02684591
236.7	-0.4229351	0.40537732	0.57651146	-0.5657467	0.56191804	0.02684591
236.8	-0.4229351	0.40537732	0.57651146	-0.5657467	0.56191804	0.02684591
236.9	-0.4229351	0.40537732	0.57651146	-0.5657467	0.56191804	0.02684591
237	-0.4229351	0.40537732	0.57651146	-0.5657467	0.56191804	0.02684591
237.1	-0.4229351	0.40537732	0.57651146	-0.5657467	0.56191804	0.02684591
237.2	-0.4229351	0.40537732	0.57651146	-0.5657467	0.56191804	0.02684591
237.3	-0.4229351	0.40537732	0.57651146	-0.5657467	0.56191804	0.02684591
237.4	-0.4229351	0.40537732	0.57651146	-0.5657467	0.56191804	0.02684591
237.5	-0.4229351	0.40537732	0.57651146	-0.5657467	0.56191804	0.02684591
237.6	-0.4229351	0.40537732	0.57651146	-0.5657467	0.56191804	0.02684591
237.7	-0.4232831	0.40570818	0.57680974	-0.5658111	0.56222642	0.02650617
237.8	-0.4232831	0.40570818	0.57680974	-0.5658111	0.56222642	0.02650617
237.9	-0.4232831	0.40570818	0.57680974	-0.5658111	0.56222642	0.02650617
238	-0.4232831	0.40570818	0.57680974	-0.5658111	0.56222642	0.02650617
238.1	-0.4232831	0.40570818	0.57680974	-0.5658111	0.56222642	0.02650617
238.2	-0.4232831	0.40570818	0.57680974	-0.5658111	0.56222642	0.02650617
238.3	-0.4232831	0.40570818	0.57680974	-0.5658111	0.56222642	0.02650617
238.4	-0.4234403	0.40571061	0.5769352	-0.5662169	0.56215775	0.02653597
238.5	-0.4234403	0.40571061	0.5769352	-0.5662169	0.56215775	0.02653597
238.6	-0.4234403	0.40571061	0.5769352	-0.5662169	0.56215775	0.02653597
238.7	-0.4234403	0.40571061	0.5769352	-0.5662169	0.56215775	0.02653597
238.8	-0.4234403	0.40571061	0.5769352	-0.5662169	0.56215775	0.02653597
238.9	-0.4234403	0.40571061	0.5769352	-0.5662169	0.56215775	0.02653597
239	-0.4234403	0.40571061	0.5769352	-0.5662169	0.56215775	0.02653597
239.1	-0.4234403	0.40571061	0.5769352	-0.5662169	0.56215775	0.02653597
239.2	-0.4234403	0.40571061	0.5769352	-0.5662169	0.56215775	0.02653597
239.3	-0.4234403	0.40571061	0.5769352	-0.5662169	0.56215775	0.02653597
239.4	-0.4234403	0.40571061	0.5769352	-0.5662169	0.56215775	0.02653597
239.5	-0.4232343	0.40619206	0.57723798	-0.566177	0.56228573	0.0260285
239.6	-0.4232343	0.40619206	0.57723798	-0.566177	0.56228573	0.0260285
239.7	-0.4232343	0.40619206	0.57723798	-0.566177	0.56228573	0.0260285
239.8	-0.4232343	0.40619206	0.57723798	-0.566177	0.56228573	0.0260285
239.9	-0.4232343	0.40619206	0.57723798	-0.566177	0.56228573	0.0260285
240	-0.4232343	0.40619206	0.57723798	-0.566177	0.56228573	0.0260285

Corresponding p-values for PRCC sensitivtiy indices

Time discretization	q	K_{[G3P]}	K_{[PYR]}	alpha	G_E	[G]_0
0	0	0	0	0	0	0
0.1	0.07149007	0.47495803	3.20E-05	0.46706516	0.13182969	9.65E-220
0.2	0.03300758	0.26371615	1.28E-06	0.23247401	0.0132164	1.33E-173
0.3	0.0171836	0.25279935	5.44E-07	0.37990858	0.00680987	6.79E-148
0.4	0.0137121	0.25410159	7.00E-07	0.29153867	0.00602017	2.93E-130
0.5	0.0180938	0.25910664	3.07E-07	0.2920107	0.00285483	5.01E-117
0.6	0.01352391	0.20502825	1.05E-07	0.25995278	0.00089705	9.81E-110
0.7	0.01438115	0.17540556	4.07E-08	0.25212833	0.00025502	1.48E-103
0.8	0.00982344	0.16184335	2.22E-08	0.22790792	9.81E-05	1.95E-98
0.9	0.00548356	0.14246813	1.16E-08	0.22342654	3.81E-05	1.45E-93
1	0.00377077	0.10185002	4.54E-09	0.1912399	9.37E-06	1.21E-89
1.1	0.00270581	0.07901227	2.19E-09	0.21197554	2.38E-06	1.01E-85
1.2	0.00228914	0.05466097	1.43E-09	0.22655248	1.14E-06	2.23E-82
1.3	0.00169841	0.04146659	8.74E-10	0.20772735	4.04E-07	4.13E-80
1.4	0.00104954	0.02680155	2.78E-10	0.21062983	1.79E-07	6.08E-78
1.5	0.00073177	0.01948554	1.09E-10	0.23006158	4.85E-08	5.90E-76
1.6	0.00045991	0.01445368	7.13E-11	0.24186256	2.19E-08	2.93E-74
1.7	0.00027075	0.00961484	2.27E-11	0.24721771	6.78E-09	1.04E-72
1.8	0.00021041	0.00729951	8.85E-12	0.26344123	2.84E-09	4.01E-71
1.9	0.0001379	0.00416801	3.69E-12	0.24996701	1.29E-09	8.77E-70
2	9.14E-05	0.00250414	1.45E-12	0.22264728	4.29E-10	7.26E-69
2.1	6.93E-05	0.00189448	1.19E-12	0.2021116	1.82E-10	1.46E-67
2.2	5.90E-05	0.00122106	8.30E-13	0.19151504	7.37E-11	4.48E-66
2.3	3.17E-05	0.00077368	3.71E-13	0.18700957	2.47E-11	3.60E-65
2.4	1.66E-05	0.00053508	2.10E-13	0.17213463	7.34E-12	2.10E-64
2.5	1.01E-05	0.00032492	1.34E-13	0.16075989	4.18E-12	2.80E-63
2.6	8.09E-06	0.00022319	6.60E-14	0.16949208	1.49E-12	1.87E-62
2.7	5.40E-06	0.00013968	3.51E-14	0.16460663	4.61E-13	3.86E-61
2.8	2.64E-06	8.35E-05	2.19E-14	0.15245422	1.55E-13	2.83E-60
2.9	1.71E-06	6.02E-05	1.04E-14	0.15569605	7.86E-14	4.58E-59
3	1.23E-06	3.56E-05	5.71E-15	0.1399021	4.42E-14	3.00E-58
3.1	8.83E-07	2.17E-05	5.41E-15	0.13049274	2.56E-14	6.40E-57
3.2	5.43E-07	1.07E-05	2.84E-15	0.12138436	1.14E-14	6.44E-56
3.3	4.17E-07	7.49E-06	1.64E-15	0.11664464	4.86E-15	2.77E-55
3.4	2.86E-07	3.83E-06	5.43E-16	0.09690549	1.52E-15	1.22E-54
3.5	2.49E-07	2.51E-06	4.42E-16	0.09791375	7.79E-16	2.23E-53
3.6	1.50E-07	1.51E-06	2.59E-16	0.08975261	4.69E-16	1.72E-52
3.7	1.16E-07	9.75E-07	1.38E-16	0.08220942	2.70E-16	1.53E-51
3.8	5.91E-08	6.35E-07	5.51E-17	0.07310425	1.18E-16	7.05E-51
3.9	3.34E-08	4.44E-07	1.75E-17	0.06159037	2.75E-17	2.00E-50
4	2.52E-08	3.72E-07	1.21E-17	0.04894034	1.47E-17	1.26E-49
4.1	2.31E-08	2.78E-07	8.50E-18	0.0468109	6.14E-18	2.76E-48
4.2	1.48E-08	1.63E-07	4.93E-18	0.04194934	2.52E-18	1.39E-47
4.3	1.42E-08	1.17E-07	3.17E-18	0.03844038	8.87E-19	1.04E-46
4.4	1.10E-08	1.02E-07	2.00E-18	0.04056518	2.03E-19	7.83E-46
4.5	6.83E-09	8.75E-08	1.50E-18	0.03852059	6.46E-20	4.89E-45
4.6	6.84E-09	5.88E-08	1.20E-18	0.04344965	2.76E-20	5.49E-44
4.7	6.53E-09	4.90E-08	1.44E-18	0.04172432	1.01E-20	5.74E-43
4.8	6.20E-09	4.02E-08	1.48E-18	0.0394165	5.50E-21	4.80E-42
4.9	4.54E-09	2.73E-08	1.06E-18	0.03516781	4.69E-21	3.82E-41
5	3.69E-09	2.26E-08	5.83E-19	0.03330398	3.46E-21	2.45E-40
5.1	3.01E-09	1.12E-08	3.06E-19	0.02973712	1.79E-21	1.79E-39
5.2	2.40E-09	7.20E-09	2.35E-19	0.03058803	8.66E-22	1.21E-38
5.3	1.85E-09	5.37E-09	2.14E-19	0.02735105	6.29E-22	6.05E-38
5.4	1.47E-09	5.91E-09	1.26E-19	0.02409526	2.40E-22	3.33E-37
5.5	1.18E-09	5.50E-09	1.24E-19	0.02069748	1.24E-22	1.59E-36
5.6	8.79E-10	5.48E-09	1.11E-19	0.02107629	7.57E-23	9.09E-36
5.7	1.10E-09	6.10E-09	1.03E-19	0.02050853	3.48E-23	5.00E-35
5.8	8.27E-10	5.04E-09	7.98E-20	0.01771309	2.48E-23	2.16E-34
5.9	8.23E-10	4.01E-09	6.98E-20	0.01642364	2.04E-23	1.67E-33
6	7.45E-10	3.54E-09	7.05E-20	0.01481289	1.46E-23	8.41E-33
6.1	6.41E-10	2.70E-09	8.35E-20	0.01233403	1.03E-23	4.54E-32
6.2	4.89E-10	2.38E-09	8.08E-20	0.01131959	6.04E-24	1.74E-31
6.3	3.53E-10	2.36E-09	6.84E-20	0.01086828	4.41E-24	1.12E-30
6.4	2.64E-10	1.89E-09	5.05E-20	0.00804556	3.86E-24	4.86E-30
6.5	2.36E-10	1.31E-09	4.64E-20	0.0077523	2.40E-24	2.58E-29
6.6	2.01E-10	1.31E-09	4.13E-20	0.00722321	1.71E-24	1.36E-28
6.7	2.06E-10	1.43E-09	4.63E-20	0.00631692	1.34E-24	4.72E-28
6.8	2.39E-10	1.70E-09	4.69E-20	0.00577348	1.37E-24	2.75E-27
6.9	2.88E-10	2.29E-09	5.54E-20	0.0055705	1.44E-24	9.41E-27
7	2.58E-10	2.36E-09	6.23E-20	0.00526488	1.59E-24	3.53E-26
7.1	2.92E-10	3.17E-09	8.67E-20	0.00480073	1.03E-24	2.10E-25
7.2	3.10E-10	3.48E-09	8.43E-20	0.00379798	8.17E-25	6.61E-25
7.3	2.45E-10	2.89E-09	5.61E-20	0.00333055	4.55E-25	1.65E-24
7.4	2.47E-10	2.86E-09	7.42E-20	0.00279793	3.51E-25	4.97E-24
7.5	2.54E-10	3.24E-09	9.20E-20	0.00248157	2.61E-25	1.73E-23
7.6	2.75E-10	3.24E-09	1.15E-19	0.002043	2.83E-25	6.30E-23
7.7	2.90E-10	3.46E-09	1.33E-19	0.00201573	1.93E-25	1.88E-22
7.8	3.09E-10	3.38E-09	1.35E-19	0.0018267	1.44E-25	5.42E-22
7.9	2.45E-10	3.10E-09	1.02E-19	0.00175645	1.13E-25	1.12E-21
8	2.02E-10	2.47E-09	9.84E-20	0.00142011	6.61E-26	3.54E-21
8.1	1.80E-10	2.18E-09	8.17E-20	0.0012679	6.02E-26	1.08E-20
8.2	1.43E-10	2.11E-09	7.51E-20	0.0012892	5.92E-26	3.50E-20
8.3	1.27E-10	1.74E-09	8.32E-20	0.00105422	6.16E-26	1.10E-19
8.4	9.35E-11	1.70E-09	7.60E-20	0.00103472	4.11E-26	2.23E-19
8.5	8.93E-11	1.81E-09	7.57E-20	0.00103456	4.26E-26	5.72E-19
8.6	8.11E-11	1.66E-09	8.04E-20	0.00107317	4.59E-26	1.26E-18
8.7	8.69E-11	1.28E-09	6.34E-20	0.00096447	3.17E-26	3.22E-18
8.8	6.25E-11	1.16E-09	5.29E-20	0.00084617	2.34E-26	8.49E-18
8.9	6.63E-11	1.07E-09	5.74E-20	0.00071219	1.95E-26	1.77E-17
9	7.34E-11	1.11E-09	6.59E-20	0.00065238	1.81E-26	4.15E-17
9.1	8.11E-11	1.04E-09	6.77E-20	0.00062253	1.70E-26	1.02E-16
9.2	6.26E-11	9.57E-10	6.29E-20	0.00053892	1.55E-26	2.25E-16
9.3	7.32E-11	9.56E-10	6.52E-20	0.00042849	1.43E-26	4.23E-16
9.4	6.49E-11	8.31E-10	4.92E-20	0.00035765	1.23E-26	9.47E-16
9.5	6.79E-11	9.62E-10	6.27E-20	0.00033964	1.20E-26	2.19E-15
9.6	6.11E-11	7.87E-10	5.34E-20	0.00030024	8.70E-27	3.39E-15
9.7	5.23E-11	6.80E-10	5.21E-20	0.00025494	8.91E-27	7.67E-15
9.8	5.76E-11	6.91E-10	5.21E-20	0.00024323	7.09E-27	1.85E-14
9.9	6.36E-11	7.22E-10	5.13E-20	0.00021396	5.94E-27	2.88E-14
10	5.69E-11	6.75E-10	5.50E-20	0.00021622	5.42E-27	6.77E-14
10.1	6.47E-11	6.31E-10	5.04E-20	0.00018419	4.95E-27	1.17E-13
10.2	6.49E-11	5.46E-10	5.03E-20	0.00017554	4.55E-27	2.46E-13
10.3	6.20E-11	5.29E-10	6.17E-20	0.00015391	3.95E-27	4.79E-13
10.4	5.31E-11	5.20E-10	6.73E-20	0.00014198	3.40E-27	1.03E-12
10.5	4.71E-11	4.70E-10	5.54E-20	0.00014363	2.23E-27	2.04E-12
10.6	4.17E-11	5.06E-10	5.44E-20	0.00012937	2.52E-27	3.10E-12
10.7	3.76E-11	3.80E-10	4.84E-20	0.00012319	2.23E-27	5.46E-12
10.8	3.17E-11	3.24E-10	4.16E-20	9.48E-05	1.62E-27	8.87E-12
10.9	2.74E-11	3.42E-10	3.86E-20	7.96E-05	1.38E-27	1.25E-11
11	2.41E-11	3.20E-10	4.05E-20	7.77E-05	1.35E-27	2.41E-11
11.1	2.42E-11	3.11E-10	4.54E-20	7.43E-05	1.25E-27	4.18E-11
11.2	2.76E-11	2.80E-10	4.36E-20	6.38E-05	1.01E-27	6.57E-11
11.3	2.46E-11	2.36E-10	4.31E-20	5.65E-05	1.36E-27	1.17E-10
11.4	2.08E-11	2.38E-10	3.99E-20	5.40E-05	1.16E-27	1.77E-10
11.5	2.03E-11	2.58E-10	4.33E-20	5.59E-05	1.16E-27	2.55E-10
11.6	1.89E-11	2.52E-10	4.00E-20	4.74E-05	9.00E-28	3.78E-10
11.7	1.53E-11	2.27E-10	3.42E-20	4.36E-05	8.13E-28	5.99E-10
11.8	1.61E-11	2.34E-10	3.46E-20	4.05E-05	7.97E-28	9.38E-10

Time discretization	q	K_{[G3P]}	K_{[PYR]}	alpha	G_E	[G]_0
15	5.03E-12	8.85E-11	1.39E-20	4.35E-06	1.53E-28	5.50E-06
15.1	5.48E-12	9.45E-11	1.49E-20	4.13E-06	1.49E-28	6.32E-06
15.2	5.40E-12	9.48E-11	1.49E-20	3.73E-06	1.75E-28	7.89E-06
15.3	5.46E-12	9.82E-11	1.71E-20	3.62E-06	1.92E-28	9.26E-06
15.4	5.80E-12	9.18E-11	1.88E-20	3.36E-06	2.13E-28	1.09E-05
15.5	6.13E-12	9.75E-11	2.15E-20	2.97E-06	2.13E-28	1.42E-05
15.6	5.70E-12	8.84E-11	1.94E-20	2.57E-06	2.13E-28	1.79E-05
15.7	6.12E-12	9.20E-11	2.07E-20	2.40E-06	2.20E-28	2.20E-05
15.8	6.08E-12	1.04E-10	2.38E-20	2.60E-06	2.27E-28	2.50E-05
15.9	6.81E-12	1.03E-10	2.46E-20	2.37E-06	2.98E-28	3.15E-05
16	6.83E-12	1.11E-10	2.91E-20	2.39E-06	2.77E-28	4.28E-05
16.1	7.06E-12	1.09E-10	2.83E-20	2.40E-06	2.94E-28	4.84E-05
16.2	7.33E-12	1.03E-10	2.36E-20	2.36E-06	2.52E-28	5.23E-05
16.3	6.55E-12	1.01E-10	2.46E-20	2.03E-06	2.62E-28	6.03E-05
16.4	6.80E-12	9.82E-11	2.35E-20	1.96E-06	2.61E-28	6.58E-05
16.5	6.12E-12	1.01E-10	2.34E-20	1.77E-06	2.86E-28	7.47E-05
16.6	6.43E-12	1.04E-10	2.17E-20	1.84E-06	2.80E-28	8.10E-05
16.7	5.93E-12	1.05E-10	2.37E-20	1.58E-06	2.45E-28	9.23E-05
16.8	5.79E-12	1.15E-10	2.57E-20	1.40E-06	2.14E-28	0.00010063
16.9	5.85E-12	1.12E-10	2.23E-20	1.29E-06	1.98E-28	0.00011133
17	6.26E-12	1.10E-10	2.26E-20	1.25E-06	1.93E-28	0.00012699
17.1	6.88E-12	1.15E-10	2.38E-20	1.17E-06	2.12E-28	0.00015013
17.2	8.10E-12	1.35E-10	2.61E-20	1.05E-06	2.21E-28	0.00017032
17.3	8.02E-12	1.35E-10	2.85E-20	1.06E-06	2.29E-28	0.00019691
17.4	7.87E-12	1.29E-10	3.16E-20	9.43E-07	2.55E-28	0.00024449
17.5	8.21E-12	1.45E-10	3.55E-20	8.23E-07	2.94E-28	0.0003029
17.6	8.16E-12	1.47E-10	4.06E-20	8.00E-07	3.04E-28	0.00035839
17.7	7.81E-12	1.52E-10	3.58E-20	7.03E-07	2.74E-28	0.00039275
17.8	7.52E-12	1.31E-10	3.09E-20	5.95E-07	2.38E-28	0.00042302
17.9	6.75E-12	1.32E-10	2.91E-20	5.70E-07	2.26E-28	0.00044468
18	6.95E-12	1.29E-10	2.80E-20	5.25E-07	2.22E-28	0.00051707
18.1	6.81E-12	1.42E-10	2.92E-20	4.80E-07	2.26E-28	0.00059952
18.2	6.71E-12	1.42E-10	3.37E-20	4.66E-07	2.65E-28	0.00064655
18.3	6.55E-12	1.51E-10	3.46E-20	4.30E-07	3.16E-28	0.00075038
18.4	5.99E-12	1.47E-10	3.33E-20	4.25E-07	2.93E-28	0.00076567
18.5	7.02E-12	1.66E-10	3.70E-20	4.18E-07	3.41E-28	0.00083507
18.6	6.75E-12	1.64E-10	4.12E-20	3.96E-07	3.46E-28	0.00095634
18.7	6.72E-12	1.48E-10	4.20E-20	3.69E-07	4.25E-28	0.00117601
18.8	6.24E-12	1.49E-10	4.07E-20	3.42E-07	4.45E-28	0.001213
18.9	6.40E-12	1.55E-10	4.11E-20	3.40E-07	4.18E-28	0.00128003
19	6.53E-12	1.45E-10	3.97E-20	3.00E-07	4.37E-28	0.00144958
19.1	6.15E-12	1.30E-10	3.61E-20	2.64E-07	4.49E-28	0.00168018
19.2	6.57E-12	1.43E-10	4.15E-20	2.38E-07	4.75E-28	0.00177508
19.3	6.45E-12	1.48E-10	4.29E-20	2.21E-07	4.69E-28	0.00202505
19.4	6.94E-12	1.63E-10	4.96E-20	1.99E-07	4.97E-28	0.00221349
19.5	7.55E-12	1.60E-10	5.03E-20	1.85E-07	5.23E-28	0.00227223
19.6	7.69E-12	1.49E-10	4.71E-20	1.73E-07	5.40E-28	0.00253991
19.7	6.91E-12	1.65E-10	4.78E-20	1.45E-07	5.47E-28	0.00289686
19.8	7.79E-12	1.51E-10	5.34E-20	1.43E-07	6.40E-28	0.00313922
19.9	7.69E-12	1.54E-10	5.04E-20	1.39E-07	7.37E-28	0.00314473
20	7.83E-12	1.63E-10	6.26E-20	1.21E-07	7.57E-28	0.00355655
20.1	7.61E-12	1.63E-10	5.73E-20	9.88E-08	7.95E-28	0.00364197
20.2	7.54E-12	1.66E-10	5.97E-20	9.48E-08	8.12E-28	0.00372485
20.3	7.65E-12	1.64E-10	6.09E-20	8.79E-08	8.61E-28	0.00395712
20.4	8.28E-12	1.56E-10	6.27E-20	8.15E-08	7.93E-28	0.00437749
20.5	9.29E-12	1.50E-10	6.76E-20	7.83E-08	8.75E-28	0.00485047
20.6	1.11E-11	1.60E-10	7.97E-20	7.94E-08	1.20E-27	0.00565298
20.7	9.94E-12	1.83E-10	9.06E-20	7.40E-08	1.40E-27	0.00631038
20.8	1.02E-11	1.89E-10	1.04E-19	7.32E-08	1.60E-27	0.00683495
20.9	9.98E-12	1.84E-10	1.09E-19	7.11E-08	1.54E-27	0.00726601
21	1.07E-11	1.86E-10	1.09E-19	6.56E-08	1.51E-27	0.00806379
21.1	1.03E-11	1.83E-10	1.14E-19	6.39E-08	1.54E-27	0.00828584
21.2	9.92E-12	1.81E-10	1.25E-19	5.45E-08	1.45E-27	0.00864574
21.3	1.03E-11	1.96E-10	1.35E-19	4.98E-08	1.78E-27	0.00876457
21.4	9.52E-12	2.13E-10	1.43E-19	4.65E-08	2.03E-27	0.00952985
21.5	8.37E-12	2.08E-10	1.30E-19	3.97E-08	2.49E-27	0.01006606
21.6	8.98E-12	2.23E-10	1.33E-19	3.84E-08	2.63E-27	0.01062357
21.7	8.74E-12	2.22E-10	1.30E-19	3.46E-08	2.59E-27	0.0110401
21.8	9.41E-12	2.20E-10	1.39E-19	2.99E-08	2.81E-27	0.01212605
21.9	1.07E-11	2.08E-10	1.64E-19	3.01E-08	3.62E-27	0.01274907
22	1.18E-11	1.95E-10	1.81E-19	3.14E-08	3.95E-27	0.01348173
22.1	1.12E-11	2.03E-10	1.76E-19	2.96E-08	3.94E-27	0.01439387
22.2	1.06E-11	1.95E-10	1.90E-19	2.66E-08	4.42E-27	0.0148133
22.3	1.03E-11	2.02E-10	2.19E-19	2.70E-08	4.84E-27	0.01592684
22.4	1.17E-11	2.19E-10	2.28E-19	2.40E-08	5.36E-27	0.01661941
22.5	1.21E-11	2.11E-10	2.19E-19	2.33E-08	5.78E-27	0.01783905
22.6	1.19E-11	2.19E-10	2.13E-19	2.15E-08	5.58E-27	0.01807648
22.7	1.11E-11	2.07E-10	1.90E-19	2.02E-08	5.39E-27	0.01931944
22.8	1.17E-11	2.03E-10	2.01E-19	1.85E-08	6.29E-27	0.02007118
22.9	1.24E-11	2.26E-10	2.15E-19	1.75E-08	7.28E-27	0.02076031
23	1.25E-11	2.42E-10	2.10E-19	1.51E-08	7.46E-27	0.02076194
23.1	1.24E-11	2.61E-10	2.18E-19	1.41E-08	7.91E-27	0.02110244
23.2	1.34E-11	2.98E-10	2.27E-19	1.38E-08	9.85E-27	0.02257757
23.3	1.42E-11	3.00E-10	2.75E-19	1.15E-08	1.21E-26	0.02452223
23.4	1.38E-11	3.28E-10	2.83E-19	1.02E-08	1.34E-26	0.02495321
23.5	1.41E-11	3.21E-10	2.98E-19	9.72E-09	1.28E-26	0.02644995
23.6	1.52E-11	3.37E-10	2.99E-19	9.58E-09	1.32E-26	0.02705545
23.7	1.56E-11	3.40E-10	3.16E-19	8.49E-09	1.33E-26	0.02924343
23.8	1.72E-11	3.14E-10	3.24E-19	7.35E-09	1.47E-26	0.03108035
23.9	1.58E-11	3.17E-10	2.86E-19	6.35E-09	1.54E-26	0.03241387
24	1.63E-11	3.53E-10	3.12E-19	5.97E-09	1.69E-26	0.0342305
24.1	1.73E-11	3.60E-10	2.94E-19	5.74E-09	1.88E-26	0.03778921
24.2	1.67E-11	3.70E-10	3.01E-19	4.81E-09	2.23E-26	0.038249
24.3	1.66E-11	3.55E-10	2.94E-19	4.68E-09	2.47E-26	0.03934033
24.4	1.71E-11	3.41E-10	3.22E-19	4.68E-09	2.69E-26	0.0413151
24.5	1.93E-11	3.92E-10	3.73E-19	4.15E-09	3.61E-26	0.04096163
24.6	1.84E-11	3.84E-10	3.64E-19	3.75E-09	3.79E-26	0.04237122
24.7	1.89E-11	3.89E-10	3.82E-19	3.71E-09	4.36E-26	0.04360874
24.8	2.01E-11	3.78E-10	4.15E-19	3.87E-09	4.75E-26	0.04500835
24.9	2.05E-11	4.10E-10	4.53E-19	3.72E-09	5.67E-26	0.04692927
25	2.27E-11	4.17E-10	5.51E-19	3.68E-09	6.67E-26	0.04755244
25.1	2.51E-11	4.30E-10	6.04E-19	3.46E-09	6.68E-26	0.04871633
25.2	2.48E-11	4.45E-10	5.68E-19	3.51E-09	6.69E-26	0.04975693
25.3	2.90E-11	4.96E-10	6.76E-19	3.13E-09	8.63E-26	0.04848845
25.4	2.96E-11	5.20E-10	6.88E-19	2.83E-09	8.42E-26	0.05005969
25.5	3.24E-11	5.30E-10	8.30E-19	2.77E-09	9.69E-26	0.05090403
25.6	3.34E-11	5.31E-10	8.04E-19	2.62E-09	1.03E-25	0.05366144
25.7	3.39E-11	4.77E-10	7.24E-19	2.36E-09	1.06E-25	0.05774474
25.8	3.65E-11	5.03E-10	7.84E-19	2.17E-09	1.13E-25	0.05971337
25.9	4.02E-11	5.28E-10	9.24E-19	2.17E-09	1.36E-25	0.06189829
26	4.60E-11	5.88E-10	1.03E-18	2.07E-09	1.57E-25	0.06444403
26.1	4.80E-11	6.00E-10	1.06E-18	2.03E-09	1.68E-25	0.0652364
26.2	4.26E-11	6.17E-10	1.06E-18	1.90E-09	1.84E-25	0.06613027
26.3	4.43E-11	6.66E-10	1.05E-18	1.67E-09	2.22E-25	0.06749757
26.4	3.80E-11	7.23E-10	1.11E-18	1.57E-09	2.57E-25	0.0691293
26.5	3.96E-11	7.59E-10	1.19E-18	1.31E-09	3.25E-25	0.07289266
26.6	3.92E-11	7.59E-10	1.24E-18	1.30E-09	3.41E-25	0.07318291
26.7	4.05E-11	7.93E-10	1.26E-18	1.17E-09	3.77E-25	0.07418442
26.8	4.32E-11	9.13E-10	1.34E-18	1.10E-09	3.77E-25	0.07794463

Time discretization	q	K_{[G3P]}	K_{[PYR]}	alpha	G_E	[G]_0
30	8.14E-11	2.07E-09	2.22E-18	1.68E-10	5.36E-24	0.13899895
30.1	8.34E-11	2.14E-09	2.35E-18	1.63E-10	6.16E-24	0.13874095
30.2	8.95E-11	2.18E-09	2.59E-18	1.60E-10	7.06E-24	0.14017318
30.3	8.89E-11	2.08E-09	2.40E-18	1.55E-10	7.73E-24	0.14473886
30.4	9.46E-11	2.13E-09	2.65E-18	1.56E-10	8.44E-24	0.14621993
30.5	8.62E-11	2.11E-09	2.58E-18	1.40E-10	8.99E-24	0.14496222
30.6	8.81E-11	2.21E-09	2.74E-18	1.29E-10	9.85E-24	0.14592769
30.7	9.50E-11	2.36E-09	2.89E-18	1.26E-10	1.09E-23	0.14669428
30.8	1.02E-10	2.43E-09	2.89E-18	1.20E-10	1.19E-23	0.14693224
30.9	1.08E-10	2.41E-09	3.00E-18	1.12E-10	1.14E-23	0.14878006
31	1.19E-10	2.60E-09	3.22E-18	1.14E-10	1.28E-23	0.15151656
31.1	1.26E-10	2.95E-09	3.51E-18	1.05E-10	1.73E-23	0.15115256
31.2	1.25E-10	3.03E-09	3.62E-18	1.05E-10	1.87E-23	0.1568747
31.3	1.24E-10	2.95E-09	3.48E-18	1.02E-10	1.85E-23	0.15959909
31.4	1.28E-10	3.19E-09	3.64E-18	9.85E-11	2.19E-23	0.16017904
31.5	1.27E-10	3.18E-09	3.76E-18	9.46E-11	2.21E-23	0.16079879
31.6	1.26E-10	3.19E-09	3.80E-18	9.65E-11	2.44E-23	0.16122162
31.7	1.30E-10	3.15E-09	3.96E-18	9.46E-11	2.67E-23	0.16009979
31.8	1.35E-10	3.14E-09	4.15E-18	9.56E-11	2.92E-23	0.16333516
31.9	1.29E-10	3.15E-09	3.97E-18	8.06E-11	3.22E-23	0.17054992
32	1.31E-10	2.99E-09	4.05E-18	8.30E-11	3.43E-23	0.17524502
32.1	1.27E-10	2.93E-09	3.94E-18	7.88E-11	3.52E-23	0.17787945
32.2	1.26E-10	3.08E-09	3.96E-18	7.24E-11	3.95E-23	0.18023143
32.3	1.13E-10	2.93E-09	3.87E-18	6.96E-11	3.70E-23	0.17946822
32.4	1.15E-10	2.78E-09	3.88E-18	6.91E-11	3.92E-23	0.18124818
32.5	1.17E-10	2.82E-09	4.20E-18	6.48E-11	4.18E-23	0.18742066
32.6	1.11E-10	2.84E-09	3.99E-18	6.14E-11	4.13E-23	0.19025907
32.7	1.14E-10	2.98E-09	4.38E-18	6.07E-11	4.85E-23	0.19215818
32.8	1.16E-10	3.12E-09	4.55E-18	6.05E-11	5.32E-23	0.19452589
32.9	1.18E-10	3.15E-09	4.68E-18	5.93E-11	5.50E-23	0.1996161
33	1.12E-10	3.19E-09	4.81E-18	5.49E-11	5.49E-23	0.20336114
33.1	1.07E-10	3.18E-09	4.42E-18	5.13E-11	5.89E-23	0.20543806
33.2	1.06E-10	3.23E-09	4.02E-18	5.33E-11	6.24E-23	0.21001525
33.3	1.06E-10	3.33E-09	4.31E-18	5.32E-11	6.53E-23	0.2201841
33.4	1.02E-10	3.31E-09	4.20E-18	5.39E-11	7.15E-23	0.22430728
33.5	1.01E-10	3.30E-09	4.49E-18	5.41E-11	7.35E-23	0.22770619
33.6	1.04E-10	3.37E-09	4.80E-18	5.61E-11	7.92E-23	0.23313507
33.7	1.07E-10	3.46E-09	5.14E-18	5.40E-11	8.38E-23	0.23681388
33.8	1.15E-10	3.85E-09	5.17E-18	4.76E-11	9.24E-23	0.2371506
33.9	1.18E-10	3.95E-09	5.32E-18	4.85E-11	9.30E-23	0.23793675
34	1.21E-10	3.96E-09	5.38E-18	4.76E-11	9.41E-23	0.23947103
34.1	1.18E-10	3.91E-09	5.50E-18	4.53E-11	1.04E-22	0.24366519
34.2	1.20E-10	4.05E-09	5.70E-18	4.53E-11	1.03E-22	0.24516168
34.3	1.16E-10	4.08E-09	5.61E-18	4.36E-11	1.00E-22	0.24608892
34.4	1.11E-10	4.11E-09	6.06E-18	4.01E-11	1.07E-22	0.25002299
34.5	1.10E-10	4.15E-09	6.10E-18	3.93E-11	1.15E-22	0.25270716
34.6	1.07E-10	4.23E-09	5.98E-18	4.10E-11	1.24E-22	0.25402507
34.7	1.06E-10	4.33E-09	6.04E-18	4.06E-11	1.32E-22	0.25786185
34.8	1.11E-10	4.41E-09	5.97E-18	3.88E-11	1.45E-22	0.25970036
34.9	1.14E-10	4.52E-09	6.01E-18	3.82E-11	1.53E-22	0.25795613
35	1.18E-10	4.94E-09	5.56E-18	3.49E-11	1.48E-22	0.26166946
35.1	1.29E-10	4.83E-09	5.75E-18	3.66E-11	1.82E-22	0.27131949
35.2	1.35E-10	4.62E-09	5.50E-18	3.46E-11	1.82E-22	0.27697038
35.3	1.39E-10	4.53E-09	5.40E-18	3.46E-11	1.80E-22	0.27898167
35.4	1.37E-10	4.64E-09	5.68E-18	3.33E-11	1.95E-22	0.2813116
35.5	1.45E-10	4.69E-09	5.47E-18	3.24E-11	1.92E-22	0.28631878
35.6	1.40E-10	4.66E-09	5.49E-18	3.17E-11	2.05E-22	0.28750589
35.7	1.41E-10	4.53E-09	5.49E-18	2.92E-11	2.13E-22	0.28770467
35.8	1.42E-10	4.50E-09	5.62E-18	2.85E-11	2.23E-22	0.29093875
35.9	1.41E-10	4.52E-09	5.63E-18	2.79E-11	2.38E-22	0.29114194
36	1.44E-10	4.17E-09	5.42E-18	2.73E-11	2.36E-22	0.29297403
36.1	1.42E-10	3.93E-09	5.49E-18	2.80E-11	2.37E-22	0.29487633
36.2	1.41E-10	3.94E-09	5.58E-18	2.69E-11	2.48E-22	0.29383249
36.3	1.41E-10	3.81E-09	5.40E-18	2.59E-11	2.48E-22	0.29499894
36.4	1.37E-10	3.75E-09	5.24E-18	2.41E-11	2.43E-22	0.29826563
36.5	1.37E-10	3.75E-09	5.24E-18	2.41E-11	2.43E-22	0.29826563
36.6	1.41E-10	3.90E-09	5.30E-18	2.35E-11	2.67E-22	0.30326653
36.7	1.41E-10	3.90E-09	5.30E-18	2.35E-11	2.67E-22	0.30326653
36.8	1.51E-10	3.76E-09	5.42E-18	2.40E-11	2.95E-22	0.30787725
36.9	1.56E-10	3.64E-09	5.78E-18	2.36E-11	3.31E-22	0.30657399
37	1.57E-10	3.64E-09	5.90E-18	2.27E-11	3.42E-22	0.30926288
37.1	1.61E-10	3.64E-09	6.00E-18	2.25E-11	3.49E-22	0.30982772
37.2	1.61E-10	3.58E-09	5.72E-18	2.14E-11	3.43E-22	0.31188155
37.3	1.60E-10	3.64E-09	5.96E-18	1.98E-11	3.46E-22	0.30820029
37.4	1.69E-10	3.68E-09	5.96E-18	1.88E-11	3.59E-22	0.31151932
37.5	1.74E-10	3.58E-09	5.75E-18	1.81E-11	3.52E-22	0.31431926
37.6	1.74E-10	3.58E-09	5.75E-18	1.81E-11	3.52E-22	0.31431926
37.7	1.67E-10	3.57E-09	5.92E-18	1.85E-11	3.60E-22	0.3158072
37.8	1.72E-10	3.76E-09	5.80E-18	1.84E-11	3.79E-22	0.31463057
37.9	1.78E-10	3.69E-09	5.76E-18	1.83E-11	3.84E-22	0.31927216
38	1.83E-10	3.69E-09	5.43E-18	1.77E-11	4.14E-22	0.31793392
38.1	1.87E-10	3.81E-09	5.53E-18	1.76E-11	4.18E-22	0.31955713
38.2	1.84E-10	3.63E-09	5.32E-18	1.65E-11	4.32E-22	0.32329159
38.3	1.79E-10	3.66E-09	5.08E-18	1.63E-11	4.11E-22	0.32866458
38.4	1.78E-10	3.64E-09	5.01E-18	1.61E-11	4.01E-22	0.32889849
38.5	1.77E-10	3.65E-09	4.96E-18	1.59E-11	4.10E-22	0.32709257
38.6	1.80E-10	3.67E-09	4.46E-18	1.51E-11	4.29E-22	0.32726387
38.7	1.89E-10	3.61E-09	4.54E-18	1.47E-11	4.54E-22	0.32909799
38.8	1.81E-10	3.62E-09	4.65E-18	1.44E-11	4.87E-22	0.33054155
38.9	1.86E-10	3.54E-09	4.71E-18	1.43E-11	4.99E-22	0.32960326
39	1.85E-10	3.47E-09	4.53E-18	1.36E-11	5.42E-22	0.33951919
39.1	1.92E-10	3.62E-09	4.42E-18	1.34E-11	5.61E-22	0.33718808
39.2	1.96E-10	3.58E-09	4.60E-18	1.34E-11	5.69E-22	0.33953922
39.3	1.92E-10	3.51E-09	4.55E-18	1.33E-11	5.80E-22	0.34311788
39.4	1.92E-10	3.57E-09	4.53E-18	1.28E-11	5.91E-22	0.34480219
39.5	2.03E-10	3.69E-09	4.43E-18	1.23E-11	6.47E-22	0.34359512
39.6	2.11E-10	3.88E-09	4.36E-18	1.29E-11	6.78E-22	0.34416215
39.7	1.99E-10	3.90E-09	4.82E-18	1.30E-11	7.49E-22	0.34852521
39.8	1.99E-10	3.99E-09	4.77E-18	1.28E-11	7.63E-22	0.35229724
39.9	2.11E-10	4.02E-09	5.28E-18	1.23E-11	9.33E-22	0.35417851
40	2.20E-10	3.96E-09	5.50E-18	1.16E-11	9.03E-22	0.36452324
40.1	2.18E-10	3.86E-09	5.61E-18	1.09E-11	8.54E-22	0.37108201
40.2	2.11E-10	3.94E-09	5.29E-18	1.05E-11	8.51E-22	0.37583036
40.3	1.90E-10	3.65E-09	4.93E-18	1.06E-11	9.77E-22	0.38559487
40.4	1.86E-10	3.49E-09	4.59E-18	9.94E-12	1.03E-21	0.38878249
40.5	1.93E-10	3.45E-09	4.37E-18	9.74E-12	1.01E-21	0.39328434
40.6	1.95E-10	3.38E-09	4.30E-18	9.38E-12	1.01E-21	0.39373459
40.7	1.87E-10	3.35E-09	4.22E-18	8.46E-12	9.92E-22	0.39574376
40.8	1.92E-10	3.22E-09	4.38E-18	8.52E-12	1.10E-21	0.39859177
40.9	1.93E-10	3.21E-09	4.21E-18	8.42E-12	1.10E-21	0.39956381
41	1.93E-10	3.28E-09	4.16E-18	7.88E-12	1.09E-21	0.40578848
41.1	1.94E-10	3.17E-09	4.09E-18	7.41E-12	1.09E-21	0.41206053
41.2	1.97E-10	3.20E-09	4.26E-18	7.19E-12	1.17E-21	0.40815498
41.3	1.94E-10	3.20E-09	4.35E-18	7.23E-12	1.22E-21	0.40940184
41.4	1.86E-10	3.14E-09	4.74E-18	6.89E-12	1.27E-21	0.42310453
41.5	1.86E-10	3.04E-09	4.71E-18	6.80E-12	1.31E-21	0.42293503
41.6	1.86E-10	3.06E-09	4.88E-18	7.25E-12	1.34E-21	0.42671213
41.7	1.87E-10	3.01E-09	4.95E-18	7.12E-12	1.35E-21	0.42529118
41.8	1.84E-10	2.96E-09	4.97E-18	6.80E-12	1.40E-21	0.42787519

Time discretization	q	K_{[G3P]}	K_{[PYR]}	alpha	G_E	[G]_0
45	2.73E-10	4.18E-09	8.13E-18	2.67E-12	3.68E-21	0.47435942
45.1	2.81E-10	4.26E-09	8.02E-18	2.65E-12	3.71E-21	0.47152294
45.2	2.81E-10	4.26E-09	8.02E-18	2.65E-12	3.71E-21	0.47152294
45.3	2.86E-10	4.08E-09	7.73E-18	2.64E-12	3.80E-21	0.47566595
45.4	2.64E-10	4.00E-09	7.96E-18	2.57E-12	4.01E-21	0.47580306
45.5	2.67E-10	3.93E-09	8.13E-18	2.45E-12	4.28E-21	0.48055274
45.6	2.77E-10	3.93E-09	8.41E-18	2.44E-12	4.56E-21	0.47947913
45.7	2.77E-10	3.93E-09	8.41E-18	2.44E-12	4.56E-21	0.47947913
45.8	2.66E-10	3.90E-09	8.18E-18	2.41E-12	4.57E-21	0.48003204
45.9	2.64E-10	3.92E-09	7.99E-18	2.22E-12	4.43E-21	0.48222704
46	2.60E-10	4.02E-09	8.13E-18	2.18E-12	4.55E-21	0.49079121
46.1	2.60E-10	4.02E-09	8.13E-18	2.18E-12	4.55E-21	0.49079121
46.2	2.60E-10	4.02E-09	8.13E-18	2.18E-12	4.55E-21	0.49079121
46.3	2.78E-10	3.81E-09	8.67E-18	2.05E-12	5.61E-21	0.49927859
46.4	2.81E-10	3.84E-09	8.76E-18	2.11E-12	5.95E-21	0.50023495
46.5	2.76E-10	3.79E-09	8.74E-18	1.96E-12	6.13E-21	0.50087266
46.6	2.76E-10	3.79E-09	8.74E-18	1.96E-12	6.13E-21	0.50087266
46.7	2.74E-10	3.73E-09	8.75E-18	1.95E-12	6.30E-21	0.50014163
46.8	2.71E-10	3.91E-09	9.16E-18	1.96E-12	6.38E-21	0.50355228
46.9	2.82E-10	4.07E-09	9.14E-18	1.93E-12	6.76E-21	0.50250338
47	2.88E-10	4.08E-09	9.24E-18	1.91E-12	6.74E-21	0.50609696
47.1	2.91E-10	4.13E-09	9.35E-18	1.90E-12	6.90E-21	0.51429487
47.2	2.91E-10	4.19E-09	9.49E-18	1.87E-12	6.84E-21	0.5151195
47.3	2.91E-10	4.19E-09	9.49E-18	1.87E-12	6.84E-21	0.5151195
47.4	2.91E-10	4.19E-09	9.49E-18	1.87E-12	6.84E-21	0.5151195
47.5	2.92E-10	4.12E-09	9.36E-18	1.80E-12	6.85E-21	0.51471646
47.6	2.92E-10	4.12E-09	9.36E-18	1.80E-12	6.85E-21	0.51471646
47.7	2.92E-10	4.12E-09	9.36E-18	1.80E-12	6.85E-21	0.51471646
47.8	3.04E-10	4.29E-09	9.10E-18	1.77E-12	7.87E-21	0.50452745
47.9	3.25E-10	4.34E-09	9.73E-18	1.86E-12	8.50E-21	0.50359232
48	3.29E-10	4.61E-09	9.50E-18	1.70E-12	8.76E-21	0.50703889
48.1	3.24E-10	4.65E-09	9.32E-18	1.59E-12	8.54E-21	0.50617222
48.2	3.34E-10	4.62E-09	9.43E-18	1.54E-12	8.85E-21	0.50224473
48.3	3.34E-10	4.62E-09	9.43E-18	1.54E-12	8.85E-21	0.50224473
48.4	3.18E-10	4.71E-09	8.82E-18	1.47E-12	8.73E-21	0.50355369
48.5	3.23E-10	4.93E-09	8.94E-18	1.46E-12	8.78E-21	0.50137339
48.6	3.13E-10	4.81E-09	8.68E-18	1.39E-12	8.71E-21	0.50156723
48.7	3.22E-10	4.72E-09	9.00E-18	1.34E-12	9.21E-21	0.50039512
48.8	3.31E-10	4.71E-09	9.03E-18	1.29E-12	9.26E-21	0.50269615
48.9	3.39E-10	4.61E-09	9.08E-18	1.21E-12	1.02E-20	0.50687304
49	3.39E-10	4.61E-09	9.08E-18	1.21E-12	1.02E-20	0.50687304
49.1	3.35E-10	4.66E-09	8.95E-18	1.18E-12	1.00E-20	0.50637389
49.2	3.14E-10	4.60E-09	8.16E-18	1.20E-12	1.12E-20	0.50353473
49.3	3.21E-10	4.79E-09	7.88E-18	1.18E-12	1.07E-20	0.50854361
49.4	3.20E-10	4.95E-09	7.97E-18	1.12E-12	1.20E-20	0.51082644
49.5	3.18E-10	5.00E-09	8.12E-18	1.15E-12	1.23E-20	0.51765357
49.6	3.20E-10	5.03E-09	8.22E-18	1.14E-12	1.26E-20	0.51489093
49.7	3.20E-10	5.03E-09	8.22E-18	1.14E-12	1.26E-20	0.51489093
49.8	3.23E-10	4.91E-09	8.18E-18	1.12E-12	1.26E-20	0.51454555
49.9	3.27E-10	5.03E-09	8.17E-18	1.09E-12	1.38E-20	0.51449306
50	3.34E-10	4.91E-09	8.83E-18	1.10E-12	1.54E-20	0.51800594
50.1	3.48E-10	5.04E-09	9.34E-18	1.09E-12	1.59E-20	0.52158641
50.2	3.48E-10	5.04E-09	9.34E-18	1.09E-12	1.59E-20	0.52158641
50.3	3.48E-10	5.04E-09	9.34E-18	1.09E-12	1.59E-20	0.52158641
50.4	3.49E-10	5.14E-09	8.74E-18	1.03E-12	1.63E-20	0.52064344
50.5	3.49E-10	5.14E-09	8.74E-18	1.03E-12	1.63E-20	0.52064344
50.6	3.62E-10	5.33E-09	8.54E-18	9.88E-13	1.64E-20	0.52104306
50.7	3.73E-10	5.21E-09	8.57E-18	8.69E-13	1.80E-20	0.52250519
50.8	3.75E-10	5.11E-09	8.76E-18	8.41E-13	1.81E-20	0.52134372
50.9	3.81E-10	5.20E-09	9.00E-18	8.55E-13	1.87E-20	0.52949509
51	3.87E-10	5.24E-09	9.08E-18	8.56E-13	1.86E-20	0.53382721
51.1	3.85E-10	5.18E-09	9.19E-18	8.61E-13	1.96E-20	0.53272717
51.2	3.76E-10	4.99E-09	8.93E-18	8.10E-13	2.00E-20	0.53193995
51.3	3.83E-10	5.07E-09	9.29E-18	8.13E-13	2.11E-20	0.53700291
51.4	3.82E-10	5.14E-09	9.20E-18	7.89E-13	2.21E-20	0.53346903
51.5	3.69E-10	5.27E-09	9.22E-18	7.49E-13	2.30E-20	0.53350665
51.6	3.61E-10	5.41E-09	9.02E-18	7.46E-13	2.34E-20	0.5366499
51.7	3.61E-10	5.38E-09	9.01E-18	7.29E-13	2.38E-20	0.5369561
51.8	3.75E-10	5.65E-09	9.19E-18	6.92E-13	2.54E-20	0.53784649
51.9	3.62E-10	5.77E-09	8.82E-18	6.87E-13	2.80E-20	0.53863072
52	3.66E-10	5.95E-09	9.26E-18	6.93E-13	2.88E-20	0.54242169
52.1	3.74E-10	6.09E-09	9.20E-18	6.48E-13	2.90E-20	0.54116797
52.2	3.74E-10	6.09E-09	9.20E-18	6.48E-13	2.90E-20	0.54116797
52.3	3.65E-10	6.11E-09	9.23E-18	6.16E-13	2.90E-20	0.54091088
52.4	3.58E-10	5.97E-09	9.28E-18	6.00E-13	3.04E-20	0.5410653
52.5	3.82E-10	6.46E-09	9.66E-18	5.72E-13	3.37E-20	0.54341449
52.6	3.81E-10	6.44E-09	9.63E-18	5.65E-13	3.46E-20	0.54217768
52.7	3.89E-10	6.33E-09	9.56E-18	5.31E-13	3.58E-20	0.54481345
52.8	3.93E-10	6.34E-09	9.70E-18	5.14E-13	3.63E-20	0.54723728
52.9	3.94E-10	6.32E-09	9.78E-18	4.98E-13	3.85E-20	0.55076874
53	3.94E-10	6.32E-09	9.78E-18	4.98E-13	3.85E-20	0.55076874
53.1	3.94E-10	6.32E-09	9.78E-18	4.98E-13	3.85E-20	0.55076874
53.2	4.05E-10	6.41E-09	8.88E-18	4.62E-13	3.78E-20	0.55824996
53.3	4.09E-10	6.47E-09	8.75E-18	4.60E-13	4.01E-20	0.55977097
53.4	4.18E-10	6.46E-09	9.35E-18	4.50E-13	4.20E-20	0.55680848
53.5	4.18E-10	6.46E-09	9.35E-18	4.50E-13	4.20E-20	0.55680848
53.6	4.25E-10	6.47E-09	1.00E-17	4.57E-13	4.62E-20	0.55703598
53.7	4.25E-10	6.47E-09	1.00E-17	4.57E-13	4.62E-20	0.55703598
53.8	4.25E-10	6.47E-09	1.00E-17	4.57E-13	4.62E-20	0.55703598
53.9	4.38E-10	6.62E-09	1.05E-17	4.59E-13	5.02E-20	0.55748315
54	4.44E-10	6.68E-09	1.03E-17	4.53E-13	5.08E-20	0.5587578
54.1	4.43E-10	6.66E-09	1.08E-17	4.19E-13	5.24E-20	0.55827407
54.2	4.30E-10	6.60E-09	1.09E-17	4.01E-13	5.34E-20	0.55893742
54.3	4.03E-10	6.71E-09	1.11E-17	3.96E-13	5.57E-20	0.55897969
54.4	4.10E-10	6.61E-09	1.01E-17	3.64E-13	5.63E-20	0.56829324
54.5	4.21E-10	6.75E-09	1.06E-17	3.37E-13	6.13E-20	0.56700694
54.6	4.34E-10	6.96E-09	1.06E-17	3.49E-13	6.64E-20	0.56248153
54.7	4.42E-10	6.82E-09	1.11E-17	3.50E-13	6.97E-20	0.5626051
54.8	4.49E-10	6.89E-09	1.11E-17	3.35E-13	7.20E-20	0.56976314
54.9	4.51E-10	6.91E-09	1.12E-17	3.28E-13	7.20E-20	0.56642212
55	4.38E-10	7.19E-09	1.12E-17	3.24E-13	7.35E-20	0.56804762
55.1	4.39E-10	7.11E-09	1.18E-17	3.24E-13	7.78E-20	0.56989806
55.2	4.25E-10	6.93E-09	1.13E-17	3.10E-13	7.70E-20	0.56629627
55.3	4.22E-10	7.11E-09	1.14E-17	3.01E-13	7.85E-20	0.56389286
55.4	4.24E-10	7.31E-09	1.16E-17	2.98E-13	8.14E-20	0.56332545
55.5	4.17E-10	7.18E-09	1.14E-17	2.88E-13	8.23E-20	0.56124035
55.6	4.05E-10	6.91E-09	1.14E-17	2.77E-13	7.69E-20	0.56505866
55.7	4.05E-10	6.63E-09	1.13E-17	2.71E-13	8.39E-20	0.56943454
55.8	4.05E-10	6.63E-09	1.13E-17	2.71E-13	8.39E-20	0.56943454
55.9	4.17E-10	7.10E-09	1.15E-17	2.62E-13	9.12E-20	0.57341479
56	3.95E-10	7.41E-09	1.14E-17	2.39E-13	8.75E-20	0.57048952
56.1	3.79E-10	7.51E-09	1.21E-17	2.23E-13	9.13E-20	0.56993554
56.2	3.79E-10	7.51E-09	1.21E-17	2.23E-13	9.13E-20	0.56993554
56.3	3.79E-10	7.51E-09	1.21E-17	2.23E-13	9.13E-20	0.56993554
56.4	3.79E-10	7.51E-09	1.21E-17	2.23E-13	9.13E-20	0.56993554
56.5	3.68E-10	7.42E-09	1.19E-17	2.20E-13	9.32E-20	0.56962802
56.6	3.69E-10	7.45E-09	1.15E-17	2.12E-13	9.17E-20	0.57223957
56.7	3.73E-10	7.22E-09	1.09E-17	2.00E-13	9.10E-20	0.57298046
56.8	3.71E-10	7.25E-09	1.05E-17	1.90E-13	9.09E-20	0.57554869

11.9	1.69E-11	2.38E-10	2.94E-20	4.01E-05	8.15E-28	1.41E-09
12	1.95E-11	2.22E-10	2.40E-20	4.01E-05	6.41E-28	2.15E-09
12.1	2.17E-11	2.42E-10	2.49E-20	3.85E-05	6.98E-28	2.66E-09
12.2	2.10E-11	2.24E-10	2.18E-20	3.54E-05	6.63E-28	3.49E-09
12.3	1.91E-11	2.49E-10	2.32E-20	3.52E-05	6.82E-28	5.01E-09
12.4	1.64E-11	2.41E-10	2.11E-20	3.35E-05	5.97E-28	6.50E-09
12.5	1.74E-11	2.35E-10	1.92E-20	3.19E-05	6.04E-28	9.27E-09
12.6	1.89E-11	2.42E-10	1.83E-20	3.18E-05	4.97E-28	1.25E-08
12.7	1.71E-11	1.94E-10	1.48E-20	2.88E-05	4.14E-28	1.61E-08
12.8	1.48E-11	2.03E-10	1.64E-20	2.71E-05	3.48E-28	2.44E-08
12.9	1.48E-11	2.04E-10	1.89E-20	2.57E-05	2.93E-28	3.90E-08
13	1.50E-11	1.97E-10	1.88E-20	2.34E-05	2.55E-28	4.98E-08
13.1	1.30E-11	1.81E-10	1.85E-20	2.19E-05	2.75E-28	6.70E-08
13.2	1.08E-11	1.59E-10	1.63E-20	1.86E-05	2.32E-28	9.07E-08
13.3	1.02E-11	1.41E-10	1.72E-20	1.59E-05	2.21E-28	1.44E-07
13.4	9.21E-12	1.36E-10	1.46E-20	1.35E-05	2.05E-28	1.90E-07
13.5	8.60E-12	1.29E-10	1.20E-20	1.20E-05	2.25E-28	2.44E-07
13.6	8.86E-12	1.29E-10	1.37E-20	1.17E-05	2.28E-28	3.17E-07
13.7	7.19E-12	1.25E-10	1.46E-20	1.01E-05	2.12E-28	3.69E-07
13.8	6.92E-12	1.24E-10	1.33E-20	1.02E-05	1.87E-28	4.62E-07
13.9	5.49E-12	1.11E-10	1.48E-20	1.04E-05	1.82E-28	5.82E-07
14	5.25E-12	1.20E-10	1.50E-20	9.13E-06	1.89E-28	8.09E-07
14.1	4.92E-12	1.14E-10	1.47E-20	9.63E-06	2.03E-28	1.01E-06
14.2	4.79E-12	9.65E-11	1.33E-20	8.15E-06	1.76E-28	1.21E-06
14.3	5.12E-12	8.45E-11	1.42E-20	7.70E-06	1.82E-28	1.37E-06
14.4	5.10E-12	9.05E-11	1.33E-20	7.63E-06	2.20E-28	1.89E-06
14.5	4.97E-12	9.86E-11	1.34E-20	7.50E-06	2.22E-28	2.35E-06
14.6	5.98E-12	1.07E-10	1.58E-20	6.97E-06	2.05E-28	2.70E-06
14.7	5.27E-12	9.24E-11	1.19E-20	5.20E-06	1.71E-28	3.55E-06
14.8	5.05E-12	9.57E-11	1.19E-20	4.94E-06	1.62E-28	4.26E-06
14.9	4.95E-12	8.89E-11	1.19E-20	4.70E-06	1.67E-28	4.54E-06

60	5.29E-10	9.53E-09	1.30E-17	1.40E-13	2.02E-19	0.59862431
60.1	5.11E-10	9.81E-09	1.30E-17	1.41E-13	2.05E-19	0.59862645
60.2	5.14E-10	1.01E-08	1.29E-17	1.36E-13	2.14E-19	0.59901025
60.3	5.14E-10	1.01E-08	1.29E-17	1.36E-13	2.14E-19	0.59901025
60.4	5.29E-10	1.03E-08	1.32E-17	1.35E-13	2.28E-19	0.59659772
60.5	5.47E-10	1.02E-08	1.34E-17	1.30E-13	2.33E-19	0.59814059
60.6	5.63E-10	1.10E-08	1.43E-17	1.26E-13	2.53E-19	0.60084192
60.7	5.84E-10	1.12E-08	1.42E-17	1.24E-13	2.55E-19	0.60316904
60.8	5.88E-10	1.20E-08	1.50E-17	1.19E-13	2.53E-19	0.6047159
60.9	5.88E-10	1.21E-08	1.46E-17	1.19E-13	2.61E-19	0.60615725
61	5.98E-10	1.28E-08	1.50E-17	1.21E-13	2.76E-19	0.61858712
61.1	5.73E-10	1.26E-08	1.47E-17	1.20E-13	2.79E-19	0.61906255
61.2	5.80E-10	1.28E-08	1.55E-17	1.18E-13	2.95E-19	0.61500106
61.3	5.80E-10	1.28E-08	1.55E-17	1.18E-13	2.95E-19	0.61500106
61.4	5.87E-10	1.32E-08	1.62E-17	1.20E-13	2.97E-19	0.61625393
61.5	5.87E-10	1.32E-08	1.62E-17	1.20E-13	2.97E-19	0.61625393
61.6	6.09E-10	1.34E-08	1.62E-17	1.17E-13	3.08E-19	0.61636464
61.7	6.15E-10	1.37E-08	1.70E-17	1.17E-13	3.24E-19	0.61820531
61.8	6.41E-10	1.42E-08	1.84E-17	1.18E-13	3.42E-19	0.61534325
61.9	6.48E-10	1.43E-08	1.83E-17	1.20E-13	3.53E-19	0.6200666
62	6.51E-10	1.47E-08	1.90E-17	1.19E-13	3.69E-19	0.62073094
62.1	6.51E-10	1.52E-08	1.90E-17	1.18E-13	3.78E-19	0.61887831
62.2	6.51E-10	1.61E-08	1.90E-17	1.18E-13	3.92E-19	0.61368951
62.3	6.51E-10	1.61E-08	1.90E-17	1.18E-13	3.92E-19	0.61368951
62.4	6.62E-10	1.64E-08	1.99E-17	1.18E-13	4.11E-19	0.61474917
62.5	6.62E-10	1.64E-08	1.99E-17	1.18E-13	4.11E-19	0.61474917
62.6	6.68E-10	1.70E-08	2.03E-17	1.19E-13	4.36E-19	0.61533788
62.7	6.74E-10	1.69E-08	2.10E-17	1.19E-13	4.50E-19	0.61528477
62.8	6.74E-10	1.69E-08	2.10E-17	1.19E-13	4.50E-19	0.61528477
62.9	6.93E-10	1.70E-08	2.10E-17	1.19E-13	4.64E-19	0.6118462
63	6.91E-10	1.72E-08	2.14E-17	1.24E-13	4.79E-19	0.61192685
63.1	7.05E-10	1.79E-08	2.21E-17	1.24E-13	5.14E-19	0.61103322
63.2	7.00E-10	1.73E-08	2.15E-17	1.24E-13	5.22E-19	0.61216831
63.3	7.73E-10	1.74E-08	2.11E-17	1.26E-13	5.52E-19	0.61297205
63.4	7.73E-10	1.74E-08	2.11E-17	1.26E-13	5.52E-19	0.61297205
63.5	7.83E-10	1.77E-08	2.25E-17	1.25E-13	5.74E-19	0.61214542
63.6	7.91E-10	1.81E-08	2.23E-17	1.21E-13	5.83E-19	0.61561089
63.7	8.05E-10	1.86E-08	2.30E-17	1.24E-13	5.95E-19	0.61516778
63.8	8.16E-10	1.93E-08	2.35E-17	1.21E-13	6.95E-19	0.61308831
63.9	8.47E-10	1.95E-08	2.47E-17	1.24E-13	6.94E-19	0.61785318
64	8.47E-10	1.95E-08	2.47E-17	1.24E-13	6.94E-19	0.61785318
64.1	8.80E-10	1.93E-08	2.56E-17	1.22E-13	7.33E-19	0.62122689
64.2	9.00E-10	1.96E-08	2.53E-17	1.19E-13	7.66E-19	0.61705234
64.3	9.23E-10	2.00E-08	2.59E-17	1.19E-13	7.80E-19	0.61355991
64.4	9.39E-10	2.05E-08	2.58E-17	1.15E-13	8.28E-19	0.60742485
64.5	9.39E-10	2.05E-08	2.58E-17	1.15E-13	8.28E-19	0.60742485
64.6	9.33E-10	2.04E-08	2.68E-17	1.06E-13	8.22E-19	0.61289395
64.7	9.62E-10	2.09E-08	2.94E-17	9.98E-14	9.42E-19	0.61307319
64.8	9.61E-10	2.10E-08	3.06E-17	9.91E-14	9.56E-19	0.61464315
64.9	9.62E-10	2.06E-08	3.04E-17	9.86E-14	9.39E-19	0.61412773
65	9.62E-10	2.06E-08	3.04E-17	9.86E-14	9.39E-19	0.61412773
65.1	9.62E-10	2.06E-08	3.04E-17	9.86E-14	9.39E-19	0.61412773
65.2	1.03E-09	2.15E-08	3.20E-17	9.47E-14	1.05E-18	0.60898874
65.3	1.10E-09	2.33E-08	3.35E-17	9.65E-14	1.17E-18	0.60717049
65.4	1.10E-09	2.33E-08	3.35E-17	9.65E-14	1.17E-18	0.60717049
65.5	1.10E-09	2.41E-08	3.50E-17	9.58E-14	1.30E-18	0.60428493
65.6	1.07E-09	2.41E-08	3.39E-17	9.18E-14	1.32E-18	0.6033715
65.7	1.07E-09	2.51E-08	3.44E-17	8.88E-14	1.39E-18	0.60511842
65.8	1.11E-09	2.55E-08	3.74E-17	8.69E-14	1.54E-18	0.60459497
65.9	1.11E-09	2.55E-08	3.74E-17	8.69E-14	1.54E-18	0.60459497
66	1.12E-09	2.60E-08	3.77E-17	8.70E-14	1.61E-18	0.60208837
66.1	1.17E-09	2.75E-08	3.98E-17	8.52E-14	1.72E-18	0.60258377
66.2	1.21E-09	2.88E-08	4.33E-17	8.40E-14	1.84E-18	0.60208443
66.3	1.22E-09	2.87E-08	4.43E-17	8.30E-14	1.83E-18	0.60544245
66.4	1.32E-09	3.07E-08	4.39E-17	7.45E-14	1.97E-18	0.60470699
66.5	1.30E-09	3.08E-08	4.37E-17	7.16E-14	2.09E-18	0.60679889
66.6	1.30E-09	3.17E-08	4.74E-17	7.22E-14	2.35E-18	0.60650855
66.7	1.33E-09	3.20E-08	4.79E-17	7.14E-14	2.37E-18	0.60535334
66.8	1.38E-09	3.26E-08	4.93E-17	6.95E-14	2.70E-18	0.60234639
66.9	1.38E-09	3.26E-08	4.99E-17	6.85E-14	2.80E-18	0.60150923
67	1.38E-09	3.26E-08	4.99E-17	6.85E-14	2.80E-18	0.60150923
67.1	1.40E-09	3.41E-08	5.13E-17	6.88E-14	2.88E-18	0.60367844
67.2	1.41E-09	3.47E-08	5.24E-17	6.92E-14	3.13E-18	0.60798418
67.3	1.44E-09	3.54E-08	5.35E-17	6.73E-14	3.29E-18	0.60432683
67.4	1.50E-09	3.69E-08	5.43E-17	6.65E-14	3.46E-18	0.60287106
67.5	1.57E-09	3.77E-08	5.95E-17	6.74E-14	3.73E-18	0.60012366
67.6	1.58E-09	3.81E-08	6.11E-17	6.63E-14	3.73E-18	0.59793429
67.7	1.58E-09	3.79E-08	5.93E-17	6.54E-14	3.72E-18	0.59955377
67.8	1.67E-09	3.68E-08	5.95E-17	6.16E-14	3.84E-18	0.59998265
67.9	1.67E-09	3.81E-08	6.18E-17	6.19E-14	3.82E-18	0.59695619
68	1.63E-09	3.81E-08	6.12E-17	5.94E-14	3.85E-18	0.60006179
68.1	1.63E-09	3.81E-08	6.12E-17	5.94E-14	3.85E-18	0.60006179
68.2	1.63E-09	3.84E-08	6.12E-17	5.87E-14	3.85E-18	0.59891812
68.3	1.63E-09	4.07E-08	6.09E-17	5.72E-14	4.09E-18	0.59449451
68.4	1.66E-09	4.12E-08	6.33E-17	5.62E-14	4.17E-18	0.5920974
68.5	1.73E-09	4.13E-08	6.32E-17	5.86E-14	4.33E-18	0.59666296
68.6	1.70E-09	4.20E-08	6.26E-17	5.75E-14	4.48E-18	0.59795985
68.7	1.72E-09	4.18E-08	6.32E-17	5.63E-14	4.96E-18	0.59746107
68.8	1.74E-09	4.18E-08	6.63E-17	5.71E-14	5.06E-18	0.59684305
68.9	1.71E-09	4.14E-08	6.53E-17	5.79E-14	5.12E-18	0.60091111

26.9	4.13E-11	9.28E-10	1.26E-18	1.01E-09	3.71E-25	0.08007125
27	4.32E-11	1.01E-09	1.27E-18	9.90E-10	3.79E-25	0.08257571
27.1	4.81E-11	1.03E-09	1.38E-18	9.71E-10	4.34E-25	0.0867733
27.2	4.76E-11	1.03E-09	1.48E-18	9.47E-10	4.27E-25	0.08948293
27.3	4.77E-11	1.12E-09	1.52E-18	9.09E-10	4.59E-25	0.09096926
27.4	4.63E-11	1.14E-09	1.61E-18	9.20E-10	4.75E-25	0.09383885
27.5	4.37E-11	1.09E-09	1.64E-18	7.75E-10	5.04E-25	0.09609353
27.6	4.76E-11	1.23E-09	1.68E-18	6.99E-10	5.85E-25	0.09797479
27.7	4.69E-11	1.26E-09	1.64E-18	5.86E-10	5.98E-25	0.09901484
27.8	4.60E-11	1.13E-09	1.56E-18	5.23E-10	6.28E-25	0.09985741
27.9	4.80E-11	1.22E-09	1.58E-18	5.11E-10	6.89E-25	0.09832143
28	5.00E-11	1.21E-09	1.67E-18	5.00E-10	7.40E-25	0.10148831
28.1	5.17E-11	1.24E-09	1.73E-18	4.93E-10	7.84E-25	0.10316502
28.2	5.17E-11	1.33E-09	1.88E-18	4.77E-10	8.58E-25	0.10328169
28.3	5.53E-11	1.31E-09	1.86E-18	4.44E-10	9.06E-25	0.1032316
28.4	4.95E-11	1.26E-09	1.77E-18	4.17E-10	9.59E-25	0.1059852
28.5	4.88E-11	1.21E-09	1.74E-18	3.93E-10	9.68E-25	0.11051506
28.6	5.53E-11	1.39E-09	1.98E-18	3.78E-10	1.35E-24	0.11043793
28.7	5.41E-11	1.49E-09	1.99E-18	3.52E-10	1.39E-24	0.11303805
28.8	5.61E-11	1.58E-09	2.08E-18	3.42E-10	1.62E-24	0.11653139
28.9	5.22E-11	1.55E-09	1.90E-18	3.48E-10	1.59E-24	0.11835673
29	5.25E-11	1.54E-09	1.82E-18	3.13E-10	1.79E-24	0.12274282
29.1	5.24E-11	1.54E-09	1.82E-18	3.11E-10	1.91E-24	0.12577325
29.2	5.02E-11	1.49E-09	1.89E-18	2.87E-10	2.06E-24	0.12830794
29.3	5.52E-11	1.70E-09	1.99E-18	2.36E-10	2.66E-24	0.13154126
29.4	5.73E-11	1.84E-09	1.98E-18	2.27E-10	3.04E-24	0.13134988
29.5	6.39E-11	1.86E-09	2.02E-18	2.20E-10	3.25E-24	0.13131002
29.6	6.99E-11	2.04E-09	2.05E-18	2.06E-10	3.63E-24	0.13141162
29.7	6.77E-11	2.09E-09	2.10E-18	1.92E-10	3.72E-24	0.13333089
29.8	7.55E-11	2.15E-09	2.43E-18	1.73E-10	4.57E-24	0.13124596
29.9	7.67E-11	2.04E-09	2.36E-18	1.63E-10	4.72E-24	0.13432002

75	3.67E-09	8.33E-08	9.80E-17	2.75E-14	2.25E-17	0.62848206
75.1	3.67E-09	8.33E-08	9.80E-17	2.75E-14	2.25E-17	0.62848206
75.2	3.67E-09	8.33E-08	9.80E-17	2.75E-14	2.25E-17	0.62848206
75.3	3.76E-09	8.62E-08	9.98E-17	2.65E-14	2.53E-17	0.62373075
75.4	3.76E-09	8.62E-08	9.98E-17	2.65E-14	2.53E-17	0.62373075
75.5	3.65E-09	8.59E-08	9.85E-17	2.54E-14	2.59E-17	0.62416158
75.6	3.65E-09	8.59E-08	9.85E-17	2.54E-14	2.59E-17	0.62416158
75.7	3.65E-09	8.59E-08	9.85E-17	2.54E-14	2.59E-17	0.62416158
75.8	3.65E-09	8.59E-08	9.85E-17	2.54E-14	2.59E-17	0.62416158
75.9	3.74E-09	8.91E-08	1.01E-16	2.57E-14	2.79E-17	0.62255298
76	3.74E-09	8.81E-08	1.01E-16	2.57E-14	2.84E-17	0.62255237
76.1	3.94E-09	9.25E-08	1.05E-16	2.62E-14	3.05E-17	0.61960711
76.2	3.94E-09	9.25E-08	1.05E-16	2.62E-14	3.05E-17	0.61960711
76.3	3.94E-09	9.25E-08	1.05E-16	2.62E-14	3.05E-17	0.61960711
76.4	3.94E-09	9.25E-08	1.05E-16	2.62E-14	3.05E-17	0.61960711
76.5	4.20E-09	1.01E-07	1.11E-16	2.51E-14	3.53E-17	0.6226242
76.6	4.19E-09	9.90E-08	1.08E-16	2.50E-14	3.49E-17	0.62383089
76.7	4.19E-09	9.90E-08	1.08E-16	2.50E-14	3.49E-17	0.62383089
76.8	4.22E-09	9.80E-08	1.06E-16	2.52E-14	3.48E-17	0.62769486
76.9	4.22E-09	9.80E-08	1.06E-16	2.52E-14	3.48E-17	0.62769486
77	4.34E-09	1.02E-07	1.08E-16	2.54E-14	3.69E-17	0.62704127
77.1	4.34E-09	1.02E-07	1.08E-16	2.54E-14	3.69E-17	0.62704127
77.2	4.83E-09	1.07E-07	1.12E-16	2.53E-14	4.13E-17	0.62900447
77.3	5.00E-09	1.04E-07	1.14E-16	2.46E-14	4.55E-17	0.62782552
77.4	5.00E-09	1.04E-07	1.14E-16	2.46E-14	4.55E-17	0.62782552
77.5	5.00E-09	1.04E-07	1.14E-16	2.46E-14	4.55E-17	0.62782552
77.6	5.00E-09	1.04E-07	1.14E-16	2.46E-14	4.55E-17	0.62782552
77.7	4.92E-09	1.04E-07	1.16E-16	2.47E-14	4.77E-17	0.62844044
77.8	5.14E-09	1.07E-07	1.19E-16	2.44E-14	5.00E-17	0.62620121
77.9	5.14E-09	1.07E-07	1.19E-16	2.44E-14	5.00E-17	0.62620121
78	5.20E-09	1.08E-07	1.20E-16	2.47E-14	5.04E-17	0.62885193
78.1	5.20E-09	1.08E-07	1.20E-16	2.47E-14	5.04E-17	0.62885193
78.2	5.15E-09	1.08E-07	1.24E-16	2.49E-14	4.93E-17	0.62659952
78.3	5.15E-09	1.08E-07	1.24E-16	2.49E-14	4.93E-17	0.62659952
78.4	5.30E-09	1.06E-07	1.22E-16	2.40E-14	4.80E-17	0.62405371
78.5	5.30E-09	1.06E-07	1.22E-16	2.40E-14	4.80E-17	0.62405371
78.6	5.30E-09	1.06E-07	1.22E-16	2.40E-14	4.80E-17	0.62405371
78.7	5.33E-09	1.06E-07	1.22E-16	2.28E-14	4.79E-17	0.62451102
78.8	5.31E-09	1.05E-07	1.23E-16	2.29E-14	4.71E-17	0.62805602
78.9	5.08E-09	1.05E-07	1.16E-16	2.17E-14	4.78E-17	0.62656193
79	5.26E-09	1.11E-07	1.18E-16	2.04E-14	5.45E-17	0.62767231
79.1	5.30E-09	1.12E-07	1.18E-16	1.98E-14	5.45E-17	0.62868818
79.2	5.30E-09	1.12E-07	1.18E-16	1.98E-14	5.45E-17	0.62868818
79.3	5.26E-09	1.12E-07	1.16E-16	1.96E-14	5.45E-17	0.63145072
79.4	5.26E-09	1.12E-07	1.16E-16	1.96E-14	5.45E-17	0.63145072
79.5	5.26E-09	1.12E-07	1.16E-16	1.96E-14	5.45E-17	0.63145072
79.6	5.26E-09	1.12E-07	1.16E-16	1.96E-14	5.45E-17	0.63145072
79.7	5.26E-09	1.13E-07	1.15E-16	1.97E-14	5.45E-17	0.63208037
79.8	5.55E-09	1.12E-07	1.18E-16	1.97E-14	5.59E-17	0.63571731
79.9	5.55E-09	1.12E-07	1.18E-16	1.97E-14	5.59E-17	0.63571731
80	5.55E-09	1.12E-07	1.18E-16	1.97E-14	5.59E-17	0.63571731
80.1	5.55E-09	1.09E-07	1.24E-16	1.99E-14	5.52E-17	0.64359477
80.2	5.61E-09	1.10E-07	1.32E-16	2.03E-14	5.66E-17	0.64130883
80.3	5.88E-09	1.11E-07	1.33E-16	1.97E-14	6.04E-17	0.64277185
80.4	5.88E-09	1.11E-07	1.33E-16	1.97E-14	6.04E-17	0.64277185
80.5	5.96E-09	1.12E-07	1.32E-16	1.90E-14	5.95E-17	0.64701155
80.6	5.96E-09	1.12E-07	1.32E-16	1.90E-14	5.95E-17	0.64701155
80.7	5.95E-09	1.12E-07	1.34E-16	1.87E-14	6.10E-17	0.64656209
80.8	5.87E-09	1.13E-07	1.35E-16	1.82E-14	6.17E-17	0.64799513
80.9	6.02E-09	1.14E-07	1.36E-16	1.83E-14	6.15E-17	0.64663213
81	6.02E-09	1.14E-07	1.36E-16	1.83E-14	6.15E-17	0.64663213
81.1	5.97E-09	1.15E-07	1.34E-16	1.77E-14	5.99E-17	0.64617156
81.2	6.24E-09	1.19E-07	1.38E-16	1.80E-14	6.17E-17	0.64572387
81.3	6.28E-09	1.18E-07	1.40E-16	1.80E-14	6.24E-17	0.64628733
81.4	6.28E-09	1.18E-07	1.40E-16	1.80E-14	6.24E-17	0.64628733
81.5	6.05E-09	1.19E-07	1.42E-16	1.83E-14	6.58E-17	0.64746712
81.6	6.05E-09	1.19E-07	1.42E-16	1.83E-14	6.58E-17	0.64746712
81.7	6.09E-09	1.19E-07	1.47E-16	1.83E-14	6.97E-17	0.64347477
81.8	6.09E-09	1.19E-07	1.47E-16	1.83E-14	6.97E-17	0.64347477
81.9	6.06E-09	1.19E-07	1.49E-16	1.77E-14	7.06E-17	0.64506761
82	6.06E-09	1.19E-07	1.49E-16	1.77E-14	7.06E-17	0.64506761
82.1	5.97E-09	1.20E-07	1.51E-16	1.78E-14	7.12E-17	0.64656506
82.2	5.89E-09	1.16E-07	1.45E-16	1.74E-14	7.31E-17	0.64652358
82.3	5.89E-09	1.16E-07	1.45E-16	1.74E-14	7.31E-17	0.64652358
82.4	5.85E-09	1.16E-07	1.39E-16	1.58E-14	7.54E-17	0.65526468
82.5	5.85E-09	1.16E-07	1.39E-16	1.58E-14	7.54E-17	0.65526468
82.6	5.67E-09	1.14E-07	1.41E-16	1.62E-14	8.05E-17	0.65736316
82.7	5.67E-09	1.14E-07	1.41E-16	1.62E-14	8.05E-17	0.65736316
82.8	5.67E-09	1.14E-07	1.41E-16	1.62E-14	8.05E-17	0.65736316
82.9	5.67E-09	1.14E-07	1.41E-16	1.62E-14	8.05E-17	0.65736316
83	5.67E-09	1.14E-07	1.41E-16	1.62E-14	8.05E-17	0.65736316
83.1	5.67E-09	1.13E-07	1.37E-16	1.45E-14	8.11E-17	0.65814053
83.2	5.61E-09	1.10E-07	1.37E-16	1.47E-14	7.74E-17	0.66255579
83.3	5.61E-09	1.10E-07	1.37E-16	1.47E-14	7.74E-17	0.66255579
83.4	5.61E-09	1.10E-07	1.37E-16	1.47E-14	7.74E-17	0.66255579
83.5	5.42E-09	1.10E-07	1.39E-16	1.42E-14	7.60E-17	0.66316581
83.6	5.42E-09	1.10E-07	1.39E-16	1.42E-14	7.60E-17	0.66316581
83.7	5.42E-09	1.10E-07	1.39E-16	1.42E-14	7.60E-17	0.66316581
83.8	5.44E-09	1.08E-07	1.38E-16	1.41E-14	7.71E-17	0.66103163
83.9	5.44E-09	1.08E-07	1.38E-16	1.41E-14	7.71E-17	0.66103163

41.9	1.88E-10	2.99E-09	4.99E-18	6.62E-12	1.40E-21	0.43118435
42	1.88E-10	2.99E-09	4.99E-18	6.62E-12	1.40E-21	0.43118435
42.1	1.96E-10	3.19E-09	5.59E-18	6.94E-12	1.62E-21	0.4359484
42.2	2.08E-10	3.14E-09	5.59E-18	6.63E-12	1.74E-21	0.43647305
42.3	2.01E-10	3.49E-09	5.79E-18	6.28E-12	1.85E-21	0.44001406
42.4	2.09E-10	3.47E-09	5.75E-18	5.85E-12	1.92E-21	0.44273396
42.5	2.07E-10	3.47E-09	5.86E-18	5.67E-12	2.03E-21	0.44075745
42.6	2.04E-10	3.49E-09	6.15E-18	5.45E-12	2.14E-21	0.44537655
42.7	2.03E-10	3.50E-09	6.24E-18	5.57E-12	2.18E-21	0.44846359
42.8	2.26E-10	3.64E-09	6.44E-18	5.65E-12	2.44E-21	0.45075203
42.9	2.25E-10	3.60E-09	6.44E-18	5.50E-12	2.40E-21	0.4535105
43	2.26E-10	3.64E-09	6.51E-18	5.30E-12	2.49E-21	0.45023279
43.1	2.19E-10	3.57E-09	6.30E-18	4.51E-12	2.42E-21	0.45509878
43.2	2.25E-10	3.56E-09	6.62E-18	4.61E-12	2.46E-21	0.45536911
43.3	2.25E-10	3.63E-09	6.76E-18	4.57E-12	2.49E-21	0.45695132
43.4	2.26E-10	3.61E-09	6.51E-18	4.11E-12	2.39E-21	0.4609417
43.5	2.26E-10	3.61E-09	6.51E-18	4.11E-12	2.39E-21	0.4609417
43.6	2.32E-10	3.63E-09	7.09E-18	3.58E-12	2.46E-21	0.47146997
43.7	2.34E-10	3.66E-09	7.25E-18	3.60E-12	2.45E-21	0.47077243
43.8	2.51E-10	3.66E-09	7.44E-18	3.44E-12	2.43E-21	0.46851351
43.9	2.46E-10	3.66E-09	7.05E-18	3.30E-12	2.63E-21	0.47395295
44	2.49E-10	3.68E-09	7.03E-18	3.41E-12	2.61E-21	0.4774135
44.1	2.44E-10	3.70E-09	6.97E-18	3.36E-12	2.65E-21	0.48101025
44.2	2.51E-10	3.65E-09	6.94E-18	3.35E-12	2.81E-21	0.47929868
44.3	2.64E-10	3.94E-09	7.16E-18	3.15E-12	2.96E-21	0.47596243
44.4	2.60E-10	3.81E-09	6.92E-18	2.95E-12	2.86E-21	0.48084868
44.5	2.50E-10	3.90E-09	7.20E-18	2.96E-12	3.01E-21	0.48256753
44.6	2.62E-10	3.87E-09	7.28E-18	2.82E-12	3.35E-21	0.47886395
44.7	2.73E-10	3.91E-09	7.65E-18	2.85E-12	3.62E-21	0.47574885
44.8	2.83E-10	4.17E-09	7.95E-18	2.76E-12	3.66E-21	0.47535274
44.9	2.83E-10	4.17E-09	7.95E-18	2.76E-12	3.66E-21	0.47535274

90	5.19E-09	9.30E-08	1.63E-16	1.04E-14	1.01E-16	0.69593929
90.1	5.19E-09	9.30E-08	1.63E-16	1.04E-14	1.01E-16	0.69593929
90.2	5.19E-09	9.30E-08	1.63E-16	1.04E-14	1.01E-16	0.69593929
90.3	5.19E-09	9.30E-08	1.63E-16	1.04E-14	1.01E-16	0.69593929
90.4	5.19E-09	9.30E-08	1.63E-16	1.04E-14	1.01E-16	0.69593929
90.5	5.28E-09	9.18E-08	1.65E-16	1.00E-14	9.71E-17	0.69619222
90.6	5.28E-09	9.18E-08	1.65E-16	1.00E-14	9.71E-17	0.69619222
90.7	5.36E-09	8.99E-08	1.65E-16	9.99E-15	9.74E-17	0.69647325
90.8	5.35E-09	8.91E-08	1.66E-16	9.92E-15	1.02E-16	0.69864331
90.9	5.35E-09	8.91E-08	1.66E-16	9.92E-15	1.02E-16	0.69864331
91	5.15E-09	8.72E-08	1.70E-16	1.02E-14	1.05E-16	0.70516905
91.1	5.15E-09	8.72E-08	1.70E-16	1.02E-14	1.05E-16	0.70516905
91.2	5.12E-09	8.69E-08	1.64E-16	1.02E-14	9.94E-17	0.70933377
91.3	5.12E-09	8.69E-08	1.64E-16	1.02E-14	9.94E-17	0.70933377
91.4	5.12E-09	8.69E-08	1.64E-16	1.02E-14	9.94E-17	0.70933377
91.5	5.12E-09	8.69E-08	1.64E-16	1.02E-14	9.94E-17	0.70933377
91.6	5.15E-09	8.52E-08	1.62E-16	9.92E-15	1.02E-16	0.70813384
91.7	5.18E-09	8.40E-08	1.58E-16	9.72E-15	1.06E-16	0.70776235
91.8	5.18E-09	8.40E-08	1.58E-16	9.72E-15	1.06E-16	0.70776235
91.9	5.19E-09	8.16E-08	1.54E-16	9.67E-15	1.08E-16	0.70754025
92	5.19E-09	8.16E-08	1.54E-16	9.67E-15	1.08E-16	0.70754025
92.1	5.24E-09	8.10E-08	1.53E-16	9.34E-15	1.07E-16	0.70018617
92.2	5.24E-09	8.10E-08	1.53E-16	9.34E-15	1.07E-16	0.70018617
92.3	5.10E-09	8.08E-08	1.62E-16	9.13E-15	1.10E-16	0.70626171
92.4	5.10E-09	8.08E-08	1.62E-16	9.13E-15	1.10E-16	0.70626171
92.5	5.10E-09	8.08E-08	1.62E-16	9.13E-15	1.10E-16	0.70626171
92.6	5.10E-09	8.08E-08	1.62E-16	9.13E-15	1.10E-16	0.70626171
92.7	5.10E-09	8.08E-08	1.62E-16	9.13E-15	1.10E-16	0.70626171
92.8	5.10E-09	8.08E-08	1.62E-16	9.13E-15	1.10E-16	0.70626171
92.9	5.10E-09	8.08E-08	1.62E-16	9.13E-15	1.10E-16	0.70626171
93	5.10E-09	8.08E-08	1.62E-16	9.13E-15	1.10E-16	0.70626171
93.1	5.10E-09	8.08E-08	1.62E-16	9.13E-15	1.10E-16	0.70626171
93.2	5.10E-09	8.08E-08	1.62E-16	9.13E-15	1.10E-16	0.70626171
93.3	5.10E-09	8.08E-08	1.62E-16	9.13E-15	1.10E-16	0.70626171
93.4	5.12E-09	7.89E-08	1.61E-16	8.78E-15	1.16E-16	0.70914069
93.5	5.12E-09	7.89E-08	1.61E-16	8.78E-15	1.16E-16	0.70914069
93.6	5.04E-09	7.96E-08	1.59E-16	8.72E-15	1.16E-16	0.70993159
93.7	5.04E-09	7.96E-08	1.59E-16	8.72E-15	1.16E-16	0.70993159
93.8	5.09E-09	7.96E-08	1.67E-16	8.64E-15	1.21E-16	0.71764901
93.9	5.09E-09	7.96E-08	1.67E-16	8.64E-15	1.21E-16	0.71764901
94	5.17E-09	7.81E-08	1.67E-16	8.36E-15	1.22E-16	0.71679591
94.1	5.17E-09	7.81E-08	1.67E-16	8.36E-15	1.22E-16	0.71679591
94.2	5.17E-09	7.81E-08	1.67E-16	8.36E-15	1.22E-16	0.71679591
94.3	5.21E-09	7.83E-08	1.68E-16	8.18E-15	1.24E-16	0.71607368
94.4	5.21E-09	7.83E-08	1.68E-16	8.18E-15	1.24E-16	0.71607368
94.5	5.21E-09	7.83E-08	1.68E-16	8.18E-15	1.24E-16	0.71607368
94.6	5.17E-09	7.83E-08	1.69E-16	8.05E-15	1.25E-16	0.71293121
94.7	5.17E-09	7.83E-08	1.69E-16	8.05E-15	1.25E-16	0.71293121
94.8	5.25E-09	7.79E-08	1.63E-16	7.68E-15	1.22E-16	0.71169383
94.9	5.38E-09	7.79E-08	1.59E-16	7.49E-15	1.20E-16	0.71515852
95	5.32E-09	7.83E-08	1.63E-16	7.49E-15	1.23E-16	0.71147221
95.1	5.32E-09	7.83E-08	1.63E-16	7.49E-15	1.23E-16	0.71147221
95.2	5.32E-09	7.83E-08	1.63E-16	7.49E-15	1.23E-16	0.71147221
95.3	5.32E-09	7.83E-08	1.63E-16	7.49E-15	1.23E-16	0.71147221
95.4	5.16E-09	7.92E-08	1.61E-16	7.38E-15	1.18E-16	0.71218934
95.5	5.16E-09	7.92E-08	1.61E-16	7.38E-15	1.18E-16	0.71218934
95.6	5.20E-09	7.82E-08	1.60E-16	7.34E-15	1.14E-16	0.7160551
95.7	5.20E-09	7.82E-08	1.60E-16	7.34E-15	1.14E-16	0.7160551
95.8	5.20E-09	7.82E-08	1.60E-16	7.34E-15	1.14E-16	0.7160551
95.9	5.20E-09	7.82E-08	1.60E-16	7.34E-15	1.14E-16	0.7160551
96	5.22E-09	7.72E-08	1.63E-16	7.07E-15	1.15E-16	0.71683838
96.1	5.28E-09	7.69E-08	1.61E-16	7.03E-15	1.17E-16	0.71556404
96.2	5.28E-09	7.69E-08	1.61E-16	7.03E-15	1.17E-16	0.71556404
96.3	5.28E-09	7.69E-08	1.61E-16	7.03E-15	1.17E-16	0.71556404
96.4	5.29E-09	7.73E-08	1.64E-16	6.99E-15	1.23E-16	0.71580683
96.5	5.20E-09	7.62E-08	1.63E-16	6.81E-15	1.28E-16	0.71642004
96.6	5.20E-09	7.62E-08	1.63E-16	6.81E-15	1.28E-16	0.71642004
96.7	5.20E-09	7.62E-08	1.63E-16	6.81E-15	1.28E-16	0.71642004
96.8	5.24E-09	7.45E-08	1.63E-16	6.64E-15	1.32E-16	0.71667195
96.9	5.24E-09	7.45E-08	1.63E-16	6.64E-15	1.32E-16	0.71667195
97	5.24E-09	7.45E-08	1.63E-16	6.64E-15	1.32E-16	0.71667195
97.1	5.24E-09	7.45E-08	1.63E-16	6.64E-15	1.32E-16	0.71667195
97.2	5.31E-09	7.42E-08	1.62E-16	6.86E-15	1.32E-16	0.71598731
97.3	5.31E-09	7.42E-08	1.62E-16	6.86E-15	1.32E-16	0.71598731
97.4	5.38E-09	7.27E-08	1.67E-16	6.67E-15	1.34E-16	0.71657544
97.5	5.38E-09	7.27E-08	1.67E-16	6.67E-15	1.34E-16	0.71657544
97.6	5.38E-09	7.27E-08	1.67E-16	6.67E-15	1.34E-16	0.71657544
97.7	5.35E-09	7.40E-08	1.68E-16	6.73E-15	1.38E-16	0.71888561
97.8	5.35E-09	7.40E-08	1.68E-16	6.73E-15	1.38E-16	0.71888561
97.9	5.35E-09	7.40E-08	1.68E-16	6.73E-15	1.38E-16	0.71888561
98	5.29E-09	7.56E-08	1.71E-16	6.79E-15	1.35E-16	0.71931058
98.1	5.29E-09	7.56E-08	1.71E-16	6.79E-15	1.35E-16	0.71931058
98.2	5.36E-09	7.46E-08	1.72E-16	6.78E-15	1.36E-16	0.71909659
98.3	5.36E-09	7.31E-08	1.71E-16	6.65E-15	1.41E-16	0.71688605
98.4	5.36E-09	7.31E-08	1.71E-16	6.65E-15	1.41E-16	0.71688605
98.5	5.36E-09	7.31E-08	1.71E-16	6.65E-15	1.41E-16	0.71688605
98.6	5.38E-09	7.32E-08	1.76E-16	6.64E-15	1.40E-16	0.71782269
98.7	5.38E-09	7.32E-08	1.76E-16	6.64E-15	1.40E-16	0.71782269
98.8	5.41E-09	7.31E-08	1.79E-16	6.45E-15	1.40E-16	0.71705447
98.9	5.41E-09	7.31E-08	1.79E-16	6.45E-15	1.40E-16	0.71705447

56.9	3.81E-10	7.65E-09	1.07E-17	1.90E-13	9.44E-20	0.57980854
57	3.78E-10	7.50E-09	1.05E-17	1.88E-13	9.72E-20	0.58038903
57.1	3.74E-10	7.55E-09	1.02E-17	1.83E-13	9.91E-20	0.58307945
57.2	3.74E-10	7.55E-09	1.02E-17	1.83E-13	9.91E-20	0.58307945
57.3	3.79E-10	7.68E-09	1.05E-17	1.79E-13	1.06E-19	0.57789586
57.4	3.76E-10	7.76E-09	1.05E-17	1.66E-13	1.13E-19	0.58233756
57.5	3.81E-10	7.95E-09	1.03E-17	1.68E-13	1.11E-19	0.58416633
57.6	3.99E-10	8.10E-09	1.07E-17	1.63E-13	1.14E-19	0.58178173
57.7	4.02E-10	8.30E-09	1.13E-17	1.65E-13	1.16E-19	0.58195689
57.8	4.14E-10	8.11E-09	1.15E-17	1.69E-13	1.19E-19	0.58453232
57.9	4.14E-10	8.11E-09	1.15E-17	1.69E-13	1.19E-19	0.58453232
58	4.19E-10	7.86E-09	1.17E-17	1.63E-13	1.20E-19	0.58597239
58.1	4.14E-10	7.84E-09	1.15E-17	1.62E-13	1.19E-19	0.58558072
58.2	4.23E-10	8.22E-09	1.16E-17	1.55E-13	1.24E-19	0.58423388
58.3	4.23E-10	8.42E-09	1.18E-17	1.45E-13	1.40E-19	0.58730137
58.4	4.48E-10	8.77E-09	1.16E-17	1.42E-13	1.52E-19	0.58689828
58.5	4.61E-10	8.68E-09	1.20E-17	1.47E-13	1.56E-19	0.58832735
58.6	4.61E-10	8.68E-09	1.20E-17	1.47E-13	1.56E-19	0.58832735
58.7	4.80E-10	8.87E-09	1.18E-17	1.46E-13	1.59E-19	0.5880406
58.8	4.81E-10	9.00E-09	1.16E-17	1.47E-13	1.62E-19	0.58824384
58.9	4.81E-10	9.00E-09	1.16E-17	1.47E-13	1.62E-19	0.58824384
59	4.97E-10	9.24E-09	1.15E-17	1.48E-13	1.65E-19	0.58579058
59.1	5.02E-10	8.94E-09	1.12E-17	1.49E-13	1.64E-19	0.59575751
59.2	5.08E-10	8.81E-09	1.11E-17	1.50E-13	1.65E-19	0.5966601
59.3	5.08E-10	8.81E-09	1.11E-17	1.50E-13	1.65E-19	0.5966601
59.4	5.08E-10	8.81E-09	1.11E-17	1.50E-13	1.65E-19	0.5966601
59.5	5.12E-10	9.07E-09	1.14E-17	1.50E-13	1.69E-19	0.59337112
59.6	5.12E-10	9.07E-09	1.14E-17	1.50E-13	1.69E-19	0.59337112
59.7	5.28E-10	9.07E-09	1.21E-17	1.49E-13	1.86E-19	0.59568184
59.8	5.40E-10	8.96E-09	1.27E-17	1.47E-13	1.88E-19	0.59619749
59.9	5.39E-10	9.09E-09	1.27E-17	1.41E-13	1.88E-19	0.59632127

105	4.71E-09	6.33E-08	1.88E-16	4.29E-15	1.94E-16	0.72062921
105.1	4.71E-09	6.33E-08	1.88E-16	4.29E-15	1.94E-16	0.72062921
105.2	4.71E-09	6.33E-08	1.88E-16	4.29E-15	1.94E-16	0.72062921
105.3	4.86E-09	6.48E-08	1.95E-16	4.36E-15	2.04E-16	0.72131067
105.4	4.86E-09	6.43E-08	1.95E-16	4.30E-15	2.09E-16	0.71908399
105.5	4.86E-09	6.43E-08	1.95E-16	4.30E-15	2.09E-16	0.71908399
105.6	4.86E-09	6.43E-08	1.95E-16	4.30E-15	2.09E-16	0.71908399
105.7	4.86E-09	6.43E-08	1.95E-16	4.30E-15	2.09E-16	0.71908399
105.8	4.86E-09	6.43E-08	1.95E-16	4.30E-15	2.09E-16	0.71908399
105.9	4.86E-09	6.54E-08	1.95E-16	4.44E-15	2.18E-16	0.71854316
106	4.77E-09	6.62E-08	1.97E-16	4.40E-15	2.13E-16	0.72060169
106.1	4.77E-09	6.62E-08	1.97E-16	4.40E-15	2.13E-16	0.72060169
106.2	4.77E-09	6.62E-08	1.97E-16	4.40E-15	2.13E-16	0.72060169
106.3	4.77E-09	6.62E-08	1.97E-16	4.40E-15	2.13E-16	0.72060169
106.4	4.77E-09	6.62E-08	1.97E-16	4.40E-15	2.13E-16	0.72060169
106.5	4.77E-09	6.62E-08	1.97E-16	4.40E-15	2.13E-16	0.72060169
106.6	4.76E-09	6.53E-08	1.98E-16	4.38E-15	2.13E-16	0.72142657
106.7	4.76E-09	6.53E-08	1.98E-16	4.38E-15	2.13E-16	0.72142657
106.8	4.76E-09	6.53E-08	1.98E-16	4.38E-15	2.13E-16	0.72142657
106.9	4.76E-09	6.53E-08	1.98E-16	4.38E-15	2.13E-16	0.72142657
107	4.75E-09	6.63E-08	2.05E-16	4.34E-15	2.15E-16	0.72341778
107.1	4.65E-09	6.62E-08	2.00E-16	4.38E-15	2.24E-16	0.72324713
107.2	4.69E-09	6.51E-08	2.05E-16	4.33E-15	2.36E-16	0.72515051
107.3	4.69E-09	6.51E-08	2.05E-16	4.33E-15	2.36E-16	0.72515051
107.4	4.69E-09	6.51E-08	2.05E-16	4.33E-15	2.36E-16	0.72515051
107.5	4.69E-09	6.51E-08	2.05E-16	4.33E-15	2.36E-16	0.72515051
107.6	4.69E-09	6.51E-08	2.05E-16	4.33E-15	2.36E-16	0.72515051
107.7	4.69E-09	6.51E-08	2.05E-16	4.33E-15	2.36E-16	0.72515051
107.8	4.69E-09	6.51E-08	2.05E-16	4.33E-15	2.36E-16	0.72515051
107.9	4.70E-09	6.56E-08	2.13E-16	4.28E-15	2.40E-16	0.72840022
108	4.70E-09	6.56E-08	2.13E-16	4.28E-15	2.40E-16	0.72840022
108.1	4.70E-09	6.56E-08	2.13E-16	4.28E-15	2.40E-16	0.72840022
108.2	4.70E-09	6.56E-08	2.13E-16	4.28E-15	2.40E-16	0.72840022
108.3	4.70E-09	6.56E-08	2.13E-16	4.28E-15	2.40E-16	0.72840022
108.4	4.66E-09	6.43E-08	2.10E-16	4.14E-15	2.44E-16	0.72650848
108.5	4.75E-09	6.29E-08	2.18E-16	4.18E-15	2.60E-16	0.72580179
108.6	4.75E-09	6.29E-08	2.18E-16	4.18E-15	2.60E-16	0.72580179
108.7	4.75E-09	6.29E-08	2.18E-16	4.18E-15	2.60E-16	0.72580179
108.8	4.75E-09	6.29E-08	2.18E-16	4.18E-15	2.60E-16	0.72580179
108.9	4.75E-09	6.29E-08	2.18E-16	4.18E-15	2.60E-16	0.72580179
109	4.83E-09	6.14E-08	2.23E-16	3.95E-15	2.77E-16	0.72296795
109.1	4.89E-09	6.14E-08	2.25E-16	3.90E-15	2.91E-16	0.72341514
109.2	4.89E-09	6.14E-08	2.25E-16	3.90E-15	2.91E-16	0.72341514
109.3	4.89E-09	6.14E-08	2.25E-16	3.90E-15	2.91E-16	0.72341514
109.4	4.89E-09	6.14E-08	2.25E-16	3.90E-15	2.91E-16	0.72341514
109.5	4.89E-09	6.14E-08	2.25E-16	3.90E-15	2.91E-16	0.72341514
109.6	4.89E-09	6.14E-08	2.25E-16	3.90E-15	2.91E-16	0.72341514
109.7	4.89E-09	6.14E-08	2.25E-16	3.90E-15	2.91E-16	0.72341514
109.8	4.89E-09	6.14E-08	2.25E-16	3.90E-15	2.91E-16	0.72341514
109.9	4.92E-09	6.06E-08	2.16E-16	3.92E-15	3.02E-16	0.72558155
110	4.92E-09	6.06E-08	2.16E-16	3.92E-15	3.02E-16	0.72558155
110.1	4.92E-09	6.06E-08	2.16E-16	3.92E-15	3.02E-16	0.72558155
110.2	4.98E-09	6.06E-08	2.20E-16	3.87E-15	3.03E-16	0.7260193
110.3	4.98E-09	6.06E-08	2.20E-16	3.87E-15	3.03E-16	0.7260193
110.4	5.00E-09	6.04E-08	2.20E-16	3.86E-15	3.02E-16	0.72849521
110.5	5.00E-09	6.04E-08	2.20E-16	3.86E-15	3.02E-16	0.72849521
110.6	5.00E-09	6.04E-08	2.20E-16	3.86E-15	3.02E-16	0.72849521
110.7	5.00E-09	6.04E-08	2.20E-16	3.86E-15	3.02E-16	0.72849521
110.8	5.00E-09	6.04E-08	2.20E-16	3.86E-15	3.02E-16	0.72849521
110.9	5.00E-09	6.04E-08	2.20E-16	3.86E-15	3.02E-16	0.72849521
111	5.00E-09	6.04E-08	2.20E-16	3.86E-15	3.02E-16	0.72849521
111.1	5.00E-09	6.04E-08	2.20E-16	3.86E-15	3.02E-16	0.72849521
111.2	5.00E-09	6.04E-08	2.20E-16	3.86E-15	3.02E-16	0.72849521
111.3	5.00E-09	6.04E-08	2.20E-16	3.86E-15	3.02E-16	0.72849521
111.4	5.00E-09	6.04E-08	2.20E-16	3.86E-15	3.02E-16	0.72849521
111.5	5.00E-09	6.04E-08	2.20E-16	3.86E-15	3.02E-16	0.72849521
111.6	4.98E-09	6.12E-08	2.24E-16	3.88E-15	3.14E-16	0.72944205
111.7	4.98E-09	6.12E-08	2.24E-16	3.88E-15	3.14E-16	0.72944205
111.8	4.98E-09	6.12E-08	2.24E-16	3.88E-15	3.14E-16	0.72944205
111.9	4.98E-09	6.12E-08	2.24E-16	3.88E-15	3.14E-16	0.72944205
112	4.92E-09	6.04E-08	2.23E-16	3.88E-15	3.23E-16	0.72834294
112.1	4.92E-09	6.04E-08	2.23E-16	3.88E-15	3.23E-16	0.72834294
112.2	4.92E-09	6.04E-08	2.23E-16	3.88E-15	3.23E-16	0.72834294
112.3	4.92E-09	6.04E-08	2.23E-16	3.88E-15	3.23E-16	0.72834294
112.4	4.92E-09	6.04E-08	2.23E-16	3.88E-15	3.23E-16	0.72834294
112.5	4.92E-09	6.04E-08	2.23E-16	3.88E-15	3.23E-16	0.72834294
112.6	4.87E-09	5.99E-08	2.32E-16	3.65E-15	3.49E-16	0.72816874
112.7	4.87E-09	5.99E-08	2.32E-16	3.65E-15	3.49E-16	0.72816874
112.8	4.87E-09	5.99E-08	2.32E-16	3.65E-15	3.49E-16	0.72816874
112.9	4.87E-09	5.99E-08	2.32E-16	3.65E-15	3.49E-16	0.72816874
113	4.87E-09	5.99E-08	2.32E-16	3.65E-15	3.49E-16	0.72816874
113.1	4.87E-09	5.99E-08	2.32E-16	3.65E-15	3.49E-16	0.72816874
113.2	4.87E-09	5.99E-08	2.32E-16	3.65E-15	3.49E-16	0.72816874
113.3	4.87E-09	5.99E-08	2.32E-16	3.65E-15	3.49E-16	0.72816874
113.4	4.87E-09	5.99E-08	2.32E-16	3.65E-15	3.49E-16	0.72816874
113.5	4.87E-09	5.99E-08	2.32E-16	3.65E-15	3.49E-16	0.72816874
113.6	4.87E-09	5.99E-08	2.32E-16	3.65E-15	3.49E-16	0.72816874
113.7	4.87E-09	5.99E-08	2.32E-16	3.65E-15	3.49E-16	0.72816874
113.8	4.87E-09	5.99E-08	2.32E-16	3.65E-15	3.49E-16	0.72816874
113.9	4.87E-09	5.99E-08	2.32E-16	3.65E-15	3.49E-16	0.72816874

69	1.71E-09	4.14E-08	6.53E-17	5.79E-14	5.12E-18	0.60091111
69.1	1.74E-09	4.21E-08	6.72E-17	5.68E-14	5.30E-18	0.60220316
69.2	1.84E-09	4.31E-08	6.81E-17	5.51E-14	5.71E-18	0.59940262
69.3	1.92E-09	4.46E-08	6.92E-17	5.52E-14	6.09E-18	0.5982019
69.4	1.94E-09	4.54E-08	6.88E-17	5.51E-14	6.27E-18	0.60036316
69.5	1.94E-09	4.54E-08	6.88E-17	5.51E-14	6.27E-18	0.60036316
69.6	1.92E-09	4.66E-08	7.00E-17	5.15E-14	6.62E-18	0.59937518
69.7	1.92E-09	4.66E-08	7.00E-17	5.15E-14	6.62E-18	0.59937518
69.8	1.92E-09	4.66E-08	7.00E-17	5.15E-14	6.62E-18	0.59937518
69.9	2.01E-09	4.79E-08	7.14E-17	5.08E-14	7.16E-18	0.60513073
70	2.04E-09	4.97E-08	6.94E-17	4.92E-14	7.50E-18	0.60434677
70.1	2.09E-09	5.09E-08	6.90E-17	4.72E-14	7.29E-18	0.60842653
70.2	2.14E-09	5.06E-08	7.00E-17	4.69E-14	7.30E-18	0.60488632
70.3	2.21E-09	5.07E-08	7.15E-17	4.64E-14	7.86E-18	0.61294562
70.4	2.14E-09	5.06E-08	7.35E-17	4.07E-14	8.47E-18	0.61470283
70.5	2.19E-09	5.20E-08	7.52E-17	4.00E-14	8.93E-18	0.61296493
70.6	2.19E-09	5.20E-08	7.52E-17	4.00E-14	8.93E-18	0.61296493
70.7	2.19E-09	5.20E-08	7.52E-17	4.00E-14	8.93E-18	0.61296493
70.8	2.21E-09	5.41E-08	7.69E-17	4.04E-14	9.43E-18	0.61448791
70.9	2.23E-09	5.49E-08	7.87E-17	3.92E-14	9.66E-18	0.61482321
71	2.33E-09	5.62E-08	7.88E-17	3.86E-14	9.92E-18	0.61685649
71.1	2.37E-09	5.64E-08	7.72E-17	3.74E-14	9.41E-18	0.61640651
71.2	2.37E-09	5.64E-08	7.72E-17	3.74E-14	9.41E-18	0.61640651
71.3	2.37E-09	5.64E-08	7.72E-17	3.74E-14	9.41E-18	0.61640651
71.4	2.39E-09	5.78E-08	7.39E-17	3.77E-14	9.80E-18	0.61989257
71.5	2.43E-09	5.92E-08	7.64E-17	3.69E-14	1.03E-17	0.62825664
71.6	2.44E-09	5.95E-08	7.64E-17	3.47E-14	1.02E-17	0.62529719
71.7	2.46E-09	5.61E-08	7.27E-17	3.53E-14	9.97E-18	0.62944233
71.8	2.67E-09	5.93E-08	7.60E-17	3.61E-14	1.15E-17	0.62457238
71.9	2.74E-09	6.15E-08	7.42E-17	3.38E-14	1.20E-17	0.62308718
72	2.74E-09	6.15E-08	7.42E-17	3.38E-14	1.20E-17	0.62308718
72.1	2.81E-09	6.36E-08	7.90E-17	3.36E-14	1.27E-17	0.62195465
72.2	2.78E-09	6.35E-08	8.12E-17	3.35E-14	1.31E-17	0.62313617
72.3	2.78E-09	6.35E-08	8.12E-17	3.35E-14	1.31E-17	0.62313617
72.4	2.76E-09	6.44E-08	8.16E-17	3.32E-14	1.35E-17	0.6247898
72.5	2.76E-09	6.44E-08	8.16E-17	3.32E-14	1.35E-17	0.6247898
72.6	2.76E-09	6.44E-08	8.16E-17	3.32E-14	1.35E-17	0.6247898
72.7	2.75E-09	6.26E-08	8.05E-17	3.24E-14	1.38E-17	0.6250321
72.8	2.75E-09	6.26E-08	8.05E-17	3.24E-14	1.38E-17	0.6250321
72.9	2.87E-09	6.45E-08	8.31E-17	3.26E-14	1.46E-17	0.62330131
73	2.87E-09	6.45E-08	8.31E-17	3.26E-14	1.46E-17	0.62330131
73.1	2.97E-09	6.33E-08	8.41E-17	3.28E-14	1.48E-17	0.6237253
73.2	2.97E-09	6.33E-08	8.41E-17	3.28E-14	1.48E-17	0.6237253
73.3	2.97E-09	6.33E-08	8.41E-17	3.28E-14	1.48E-17	0.6237253
73.4	3.04E-09	6.44E-08	8.59E-17	3.26E-14	1.57E-17	0.62343201
73.5	2.97E-09	6.57E-08	8.74E-17	3.25E-14	1.55E-17	0.62247702
73.6	3.05E-09	6.68E-08	8.81E-17	3.25E-14	1.71E-17	0.62594882
73.7	3.03E-09	6.70E-08	8.48E-17	2.97E-14	1.67E-17	0.62225207
73.8	3.03E-09	6.70E-08	8.48E-17	2.97E-14	1.67E-17	0.62225207
73.9	3.03E-09	6.70E-08	8.48E-17	2.97E-14	1.67E-17	0.62225207
74	3.02E-09	6.90E-08	8.66E-17	3.03E-14	1.71E-17	0.62226503
74.1	3.02E-09	6.90E-08	8.66E-17	3.03E-14	1.71E-17	0.62226503
74.2	3.06E-09	6.71E-08	8.44E-17	3.02E-14	1.66E-17	0.62391485
74.3	3.18E-09	7.17E-08	8.76E-17	2.76E-14	1.84E-17	0.62877563
74.4	3.18E-09	7.17E-08	8.76E-17	2.76E-14	1.84E-17	0.62877563
74.5	3.46E-09	7.79E-08	9.24E-17	2.83E-14	2.06E-17	0.62678582
74.6	3.46E-09	7.79E-08	9.24E-17	2.83E-14	2.06E-17	0.62678582
74.7	3.46E-09	7.79E-08	9.24E-17	2.83E-14	2.06E-17	0.62678582
74.8	3.62E-09	8.17E-08	9.72E-17	2.79E-14	2.21E-17	0.6258712
74.9	3.62E-09	8.17E-08	9.72E-17	2.79E-14	2.21E-17	0.6258712

84	5.53E-09	1.08E-07	1.38E-16	1.40E-14	7.56E-17	0.66402922
84.1	5.53E-09	1.08E-07	1.38E-16	1.40E-14	7.56E-17	0.66402922
84.2	5.53E-09	1.08E-07	1.38E-16	1.40E-14	7.56E-17	0.66402922
84.3	5.60E-09	1.07E-07	1.47E-16	1.41E-14	7.24E-17	0.66746464
84.4	5.55E-09	1.03E-07	1.53E-16	1.40E-14	7.51E-17	0.66759201
84.5	5.55E-09	1.03E-07	1.53E-16	1.40E-14	7.51E-17	0.66759201
84.6	5.32E-09	9.85E-08	1.47E-16	1.36E-14	7.13E-17	0.67194352
84.7	5.32E-09	9.85E-08	1.47E-16	1.36E-14	7.13E-17	0.67194352
84.8	5.33E-09	1.00E-07	1.51E-16	1.32E-14	7.15E-17	0.6786577
84.9	5.33E-09	1.00E-07	1.51E-16	1.32E-14	7.15E-17	0.6786577
85	5.33E-09	1.00E-07	1.51E-16	1.32E-14	7.15E-17	0.6786577
85.1	5.33E-09	1.00E-07	1.51E-16	1.32E-14	7.15E-17	0.6786577
85.2	5.42E-09	9.86E-08	1.56E-16	1.32E-14	7.15E-17	0.67905775
85.3	5.42E-09	9.86E-08	1.56E-16	1.32E-14	7.15E-17	0.67905775
85.4	5.42E-09	9.86E-08	1.56E-16	1.32E-14	7.15E-17	0.67905775
85.5	5.42E-09	9.86E-08	1.56E-16	1.32E-14	7.15E-17	0.67905775
85.6	5.42E-09	9.86E-08	1.56E-16	1.32E-14	7.15E-17	0.67905775
85.7	5.24E-09	9.86E-08	1.52E-16	1.28E-14	7.12E-17	0.68176127
85.8	5.26E-09	9.69E-08	1.58E-16	1.29E-14	7.59E-17	0.68124015
85.9	5.28E-09	9.52E-08	1.59E-16	1.28E-14	7.92E-17	0.67720973
86	5.34E-09	9.63E-08	1.54E-16	1.21E-14	8.11E-17	0.68575893
86.1	5.34E-09	9.63E-08	1.54E-16	1.21E-14	8.11E-17	0.68575893
86.2	5.32E-09	9.59E-08	1.53E-16	1.22E-14	8.10E-17	0.68460773
86.3	5.34E-09	9.74E-08	1.59E-16	1.24E-14	8.41E-17	0.68491627
86.4	5.34E-09	9.74E-08	1.59E-16	1.24E-14	8.41E-17	0.68491627
86.5	5.31E-09	9.54E-08	1.54E-16	1.23E-14	8.29E-17	0.68497932
86.6	5.31E-09	9.54E-08	1.54E-16	1.23E-14	8.29E-17	0.68497932
86.7	5.31E-09	9.54E-08	1.54E-16	1.23E-14	8.29E-17	0.68497932
86.8	5.31E-09	9.54E-08	1.54E-16	1.23E-14	8.29E-17	0.68497932
86.9	5.26E-09	9.61E-08	1.58E-16	1.24E-14	8.54E-17	0.68255711
87	5.26E-09	9.61E-08	1.58E-16	1.24E-14	8.54E-17	0.68255711
87.1	5.17E-09	9.60E-08	1.60E-16	1.23E-14	8.92E-17	0.68486981
87.2	5.29E-09	9.47E-08	1.63E-16	1.24E-14	8.90E-17	0.68237582
87.3	5.33E-09	9.51E-08	1.62E-16	1.22E-14	8.99E-17	0.68296872
87.4	5.33E-09	9.51E-08	1.62E-16	1.22E-14	8.99E-17	0.68296872
87.5	5.36E-09	9.42E-08	1.55E-16	1.23E-14	9.17E-17	0.67775931
87.6	5.36E-09	9.42E-08	1.55E-16	1.23E-14	9.17E-17	0.67775931
87.7	5.35E-09	9.36E-08	1.57E-16	1.23E-14	9.15E-17	0.67912978
87.8	5.35E-09	9.36E-08	1.57E-16	1.23E-14	9.15E-17	0.67912978
87.9	5.35E-09	9.36E-08	1.57E-16	1.23E-14	9.15E-17	0.67912978
88	5.41E-09	9.35E-08	1.59E-16	1.23E-14	9.14E-17	0.68203701
88.1	5.41E-09	9.35E-08	1.59E-16	1.23E-14	9.14E-17	0.68203701
88.2	5.41E-09	9.35E-08	1.59E-16	1.23E-14	9.14E-17	0.68203701
88.3	5.52E-09	9.20E-08	1.60E-16	1.17E-14	9.17E-17	0.68541599
88.4	5.52E-09	9.20E-08	1.60E-16	1.17E-14	9.17E-17	0.68541599
88.5	5.46E-09	9.22E-08	1.62E-16	1.14E-14	9.25E-17	0.68786364
88.6	5.46E-09	9.22E-08	1.62E-16	1.14E-14	9.25E-17	0.68786364
88.7	5.42E-09	9.23E-08	1.67E-16	1.12E-14	9.76E-17	0.68865539
88.8	5.42E-09	9.23E-08	1.67E-16	1.12E-14	9.76E-17	0.68865539
88.9	5.42E-09	9.23E-08	1.67E-16	1.12E-14	9.76E-17	0.68865539
89	5.33E-09	9.25E-08	1.62E-16	1.10E-14	9.92E-17	0.69230533
89.1	5.33E-09	9.25E-08	1.62E-16	1.10E-14	9.92E-17	0.69230533
89.2	5.35E-09	9.49E-08	1.68E-16	1.11E-14	9.93E-17	0.69353383
89.3	5.30E-09	9.50E-08	1.66E-16	1.11E-14	1.01E-16	0.69464403
89.4	5.34E-09	9.31E-08	1.65E-16	1.10E-14	1.02E-16	0.69603541
89.5	5.19E-09	9.30E-08	1.63E-16	1.04E-14	1.01E-16	0.69593929
89.6	5.19E-09	9.30E-08	1.63E-16	1.04E-14	1.01E-16	0.69593929
89.7	5.19E-09	9.30E-08	1.63E-16	1.04E-14	1.01E-16	0.69593929
89.8	5.19E-09	9.30E-08	1.63E-16	1.04E-14	1.01E-16	0.69593929
89.9	5.19E-09	9.30E-08	1.63E-16	1.04E-14	1.01E-16	0.69593929

99	5.41E-09	7.31E-08	1.79E-16	6.45E-15	1.40E-16	0.71705447
99.1	5.41E-09	7.31E-08	1.79E-16	6.45E-15	1.40E-16	0.71705447
99.2	5.41E-09	7.31E-08	1.79E-16	6.45E-15	1.40E-16	0.71705447
99.3	5.41E-09	7.31E-08	1.79E-16	6.45E-15	1.40E-16	0.71705447
99.4	5.41E-09	7.31E-08	1.79E-16	6.45E-15	1.40E-16	0.71705447
99.5	5.41E-09	7.31E-08	1.79E-16	6.45E-15	1.40E-16	0.71705447
99.6	5.41E-09	7.31E-08	1.79E-16	6.45E-15	1.40E-16	0.71705447
99.7	5.41E-09	7.30E-08	1.78E-16	6.47E-15	1.47E-16	0.71706598
99.8	5.52E-09	7.29E-08	1.81E-16	6.42E-15	1.45E-16	0.71741159
99.9	5.53E-09	7.30E-08	1.80E-16	6.16E-15	1.51E-16	0.72043371
100	5.32E-09	7.29E-08	1.75E-16	5.94E-15	1.53E-16	0.72035173
100.1	5.32E-09	7.29E-08	1.75E-16	5.94E-15	1.53E-16	0.72035173
100.2	5.32E-09	7.29E-08	1.75E-16	5.94E-15	1.53E-16	0.72035173
100.3	5.32E-09	7.29E-08	1.75E-16	5.94E-15	1.53E-16	0.72035173
100.4	5.31E-09	7.20E-08	1.77E-16	5.62E-15	1.59E-16	0.71918329
100.5	5.26E-09	7.38E-08	1.90E-16	5.49E-15	1.66E-16	0.71465499
100.6	5.15E-09	7.18E-08	1.78E-16	5.18E-15	1.62E-16	0.71526634
100.7	5.15E-09	7.18E-08	1.78E-16	5.18E-15	1.62E-16	0.71526634
100.8	5.15E-09	7.18E-08	1.78E-16	5.18E-15	1.62E-16	0.71526634
100.9	5.15E-09	7.18E-08	1.78E-16	5.18E-15	1.62E-16	0.71526634
101	5.15E-09	7.18E-08	1.78E-16	5.18E-15	1.62E-16	0.71526634
101.1	5.15E-09	7.18E-08	1.78E-16	5.18E-15	1.62E-16	0.71526634
101.2	5.15E-09	7.18E-08	1.78E-16	5.18E-15	1.62E-16	0.71526634
101.3	5.15E-09	7.18E-08	1.78E-16	5.18E-15	1.62E-16	0.71526634
101.4	4.99E-09	7.22E-08	1.79E-16	5.05E-15	1.63E-16	0.7159391
101.5	5.03E-09	7.22E-08	1.89E-16	5.14E-15	1.72E-16	0.71566482
101.6	5.03E-09	7.22E-08	1.89E-16	5.14E-15	1.72E-16	0.71566482
101.7	5.03E-09	7.22E-08	1.89E-16	5.14E-15	1.72E-16	0.71566482
101.8	5.03E-09	7.22E-08	1.89E-16	5.14E-15	1.72E-16	0.71566482
101.9	5.03E-09	7.22E-08	1.89E-16	5.14E-15	1.72E-16	0.71566482
102	5.03E-09	7.22E-08	1.89E-16	5.14E-15	1.72E-16	0.71566482
102.1	5.09E-09	7.04E-08	1.90E-16	5.17E-15	1.73E-16	0.71915487
102.2	5.09E-09	7.04E-08	1.90E-16	5.17E-15	1.73E-16	0.71915487
102.3	5.09E-09	7.04E-08	1.90E-16	5.17E-15	1.73E-16	0.71915487
102.4	5.09E-09	7.04E-08	1.90E-16	5.17E-15	1.73E-16	0.71915487
102.5	5.09E-09	7.04E-08	1.90E-16	5.17E-15	1.73E-16	0.71915487
102.6	5.09E-09	7.04E-08	1.90E-16	5.17E-15	1.73E-16	0.71915487
102.7	4.89E-09	6.91E-08	1.85E-16	5.04E-15	1.76E-16	0.71716973
102.8	4.89E-09	6.91E-08	1.85E-16	5.04E-15	1.76E-16	0.71716973
102.9	4.89E-09	6.91E-08	1.85E-16	5.04E-15	1.76E-16	0.71716973
103	4.89E-09	6.91E-08	1.85E-16	5.04E-15	1.76E-16	0.71716973
103.1	4.89E-09	6.91E-08	1.85E-16	5.04E-15	1.76E-16	0.71716973
103.2	4.89E-09	6.91E-08	1.85E-16	5.04E-15	1.76E-16	0.71716973
103.3	4.89E-09	6.91E-08	1.85E-16	5.04E-15	1.76E-16	0.71716973
103.4	4.89E-09	6.91E-08	1.85E-16	5.04E-15	1.76E-16	0.71716973
103.5	4.85E-09	6.97E-08	1.91E-16	5.04E-15	1.83E-16	0.71868652
103.6	4.85E-09	6.97E-08	1.91E-16	5.04E-15	1.83E-16	0.71868652
103.7	4.83E-09	6.87E-08	1.94E-16	4.83E-15	1.89E-16	0.7165832
103.8	4.83E-09	6.87E-08	1.94E-16	4.83E-15	1.89E-16	0.7165832
103.9	4.83E-09	6.87E-08	1.94E-16	4.83E-15	1.89E-16	0.7165832
104	4.83E-09	6.87E-08	1.94E-16	4.83E-15	1.89E-16	0.7165832
104.1	4.83E-09	6.87E-08	1.94E-16	4.83E-15	1.89E-16	0.7165832
104.2	4.90E-09	6.81E-08	1.95E-16	4.73E-15	1.88E-16	0.71491913
104.3	4.90E-09	6.81E-08	1.95E-16	4.73E-15	1.88E-16	0.71491913
104.4	4.95E-09	6.76E-08	1.88E-16	4.70E-15	1.83E-16	0.71819084
104.5	4.95E-09	6.76E-08	1.88E-16	4.70E-15	1.83E-16	0.71819084
104.6	4.95E-09	6.76E-08	1.88E-16	4.70E-15	1.83E-16	0.71819084
104.7	4.77E-09	6.41E-08	1.82E-16	4.37E-15	1.90E-16	0.71753565
104.8	4.76E-09	6.42E-08	1.90E-16	4.35E-15	1.92E-16	0.72179784
104.9	4.76E-09	6.42E-08	1.90E-16	4.35E-15	1.92E-16	0.72179784

114	4.87E-09	5.99E-08	2.32E-16	3.65E-15	3.49E-16	0.72816874
114.1	4.87E-09	5.99E-08	2.32E-16	3.65E-15	3.49E-16	0.72816874
114.2	4.95E-09	5.96E-08	2.40E-16	3.71E-15	3.57E-16	0.73159656
114.3	4.95E-09	5.96E-08	2.40E-16	3.71E-15	3.57E-16	0.73159656
114.4	4.95E-09	5.96E-08	2.40E-16	3.71E-15	3.57E-16	0.73159656
114.5	4.95E-09	6.02E-08	2.48E-16	3.67E-15	3.67E-16	0.73663697
114.6	5.02E-09	5.91E-08	2.48E-16	3.57E-15	3.72E-16	0.73429547
114.7	5.02E-09	5.91E-08	2.48E-16	3.57E-15	3.72E-16	0.73429547
114.8	5.02E-09	5.91E-08	2.52E-16	3.42E-15	3.60E-16	0.73476332
114.9	4.97E-09	5.87E-08	2.51E-16	3.31E-15	3.55E-16	0.73267101
115	4.97E-09	5.87E-08	2.51E-16	3.31E-15	3.55E-16	0.73267101
115.1	4.97E-09	5.87E-08	2.51E-16	3.31E-15	3.55E-16	0.73267101
115.2	4.97E-09	5.87E-08	2.51E-16	3.31E-15	3.55E-16	0.73267101
115.3	4.97E-09	5.87E-08	2.51E-16	3.31E-15	3.55E-16	0.73267101
115.4	4.97E-09	5.87E-08	2.51E-16	3.31E-15	3.55E-16	0.73267101
115.5	5.04E-09	5.80E-08	2.48E-16	3.29E-15	3.58E-16	0.73287505
115.6	5.04E-09	5.80E-08	2.48E-16	3.29E-15	3.58E-16	0.73287505
115.7	5.09E-09	5.70E-08	2.46E-16	3.12E-15	3.69E-16	0.73011122
115.8	5.22E-09	5.67E-08	2.48E-16	3.13E-15	3.62E-16	0.72858035
115.9	5.22E-09	5.67E-08	2.48E-16	3.13E-15	3.62E-16	0.72858035
116	5.22E-09	5.67E-08	2.48E-16	3.13E-15	3.62E-16	0.72858035
116.1	5.22E-09	5.67E-08	2.48E-16	3.13E-15	3.62E-16	0.72858035
116.2	5.22E-09	5.67E-08	2.48E-16	3.13E-15	3.62E-16	0.72858035
116.3	5.21E-09	5.69E-08	2.48E-16	3.02E-15	3.68E-16	0.7294761
116.4	5.14E-09	5.63E-08	2.49E-16	3.01E-15	3.75E-16	0.73065999
116.5	5.14E-09	5.63E-08	2.49E-16	3.01E-15	3.75E-16	0.73065999
116.6	5.14E-09	5.63E-08	2.49E-16	3.01E-15	3.75E-16	0.73065999
116.7	5.17E-09	5.70E-08	2.50E-16	2.96E-15	3.88E-16	0.72804456
116.8	5.17E-09	5.70E-08	2.50E-16	2.96E-15	3.88E-16	0.72804456
116.9	5.17E-09	5.70E-08	2.50E-16	2.96E-15	3.88E-16	0.72804456
117	5.17E-09	5.70E-08	2.50E-16	2.96E-15	3.88E-16	0.72804456
117.1	5.18E-09	5.72E-08	2.47E-16	2.82E-15	3.95E-16	0.7301588
117.2	5.18E-09	5.72E-08	2.47E-16	2.82E-15	3.95E-16	0.7301588
117.3	5.13E-09	5.75E-08	2.40E-16	2.81E-15	4.00E-16	0.73289011
117.4	5.17E-09	5.81E-08	2.40E-16	2.86E-15	4.05E-16	0.73238343
117.5	5.17E-09	5.81E-08	2.40E-16	2.86E-15	4.05E-16	0.73238343
117.6	5.17E-09	5.81E-08	2.40E-16	2.86E-15	4.05E-16	0.73238343
117.7	5.24E-09	5.64E-08	2.34E-16	2.98E-15	4.12E-16	0.73343828
117.8	5.24E-09	5.64E-08	2.34E-16	2.98E-15	4.12E-16	0.73343828
117.9	5.24E-09	5.64E-08	2.34E-16	2.98E-15	4.12E-16	0.73343828
118	5.24E-09	5.64E-08	2.34E-16	2.98E-15	4.12E-16	0.73343828
118.1	5.24E-09	5.64E-08	2.34E-16	2.98E-15	4.12E-16	0.73343828
118.2	5.24E-09	5.64E-08	2.34E-16	2.98E-15	4.12E-16	0.73343828
118.3	5.29E-09	5.65E-08	2.29E-16	3.00E-15	4.14E-16	0.7327884
118.4	5.57E-09	5.80E-08	2.32E-16	3.07E-15	4.25E-16	0.73382562
118.5	5.52E-09	5.76E-08	2.29E-16	3.04E-15	4.20E-16	0.73725306
118.6	5.52E-09	5.76E-08	2.29E-16	3.04E-15	4.20E-16	0.73725306
118.7	5.52E-09	5.76E-08	2.29E-16	3.04E-15	4.20E-16	0.73725306
118.8	5.52E-09	5.76E-08	2.29E-16	3.04E-15	4.20E-16	0.73725306
118.9	5.52E-09	5.76E-08	2.29E-16	3.04E-15	4.20E-16	0.73725306
119	5.52E-09	5.76E-08	2.29E-16	3.04E-15	4.20E-16	0.73725306
119.1	5.52E-09	5.76E-08	2.29E-16	3.04E-15	4.20E-16	0.73725306
119.2	5.52E-09	5.76E-08	2.29E-16	3.04E-15	4.20E-16	0.73725306
119.3	5.52E-09	5.76E-08	2.29E-16	3.04E-15	4.20E-16	0.73725306
119.4	5.52E-09	5.76E-08	2.29E-16	3.04E-15	4.20E-16	0.73725306
119.5	5.52E-09	5.76E-08	2.29E-16	3.04E-15	4.20E-16	0.73725306
119.6	5.52E-09	5.76E-08	2.29E-16	3.04E-15	4.20E-16	0.73725306
119.7	5.52E-09	5.76E-08	2.29E-16	3.04E-15	4.20E-16	0.73725306
119.8	5.52E-09	5.76E-08	2.29E-16	3.04E-15	4.20E-16	0.73725306
119.9	5.52E-09	5.76E-08	2.29E-16	3.04E-15	4.20E-16	0.73725306

120	5.43E-09	5.81E-08	2.33E-16	3.04E-15	4.38E-16	0.74062005
120.1	5.43E-09	5.81E-08	2.33E-16	3.04E-15	4.38E-16	0.74062005
120.2	5.43E-09	5.81E-08	2.33E-16	3.04E-15	4.38E-16	0.74062005
120.3	5.43E-09	5.81E-08	2.33E-16	3.04E-15	4.38E-16	0.74062005
120.4	5.42E-09	5.91E-08	2.32E-16	3.07E-15	4.20E-16	0.74477808
120.5	5.50E-09	5.90E-08	2.39E-16	3.05E-15	4.38E-16	0.74468841
120.6	5.50E-09	5.90E-08	2.39E-16	3.05E-15	4.38E-16	0.74468841
120.7	5.50E-09	5.90E-08	2.39E-16	3.05E-15	4.38E-16	0.74468841
120.8	5.47E-09	5.87E-08	2.32E-16	3.01E-15	4.24E-16	0.74442386
120.9	5.47E-09	5.87E-08	2.32E-16	3.01E-15	4.24E-16	0.74442386
121	5.47E-09	5.87E-08	2.32E-16	3.01E-15	4.24E-16	0.74442386
121.1	5.47E-09	5.87E-08	2.32E-16	3.01E-15	4.24E-16	0.74442386
121.2	5.47E-09	5.87E-08	2.32E-16	3.01E-15	4.24E-16	0.74442386
121.3	5.47E-09	5.87E-08	2.32E-16	3.01E-15	4.24E-16	0.74442386
121.4	5.47E-09	5.87E-08	2.32E-16	3.01E-15	4.24E-16	0.74442386
121.5	5.53E-09	5.78E-08	2.35E-16	3.00E-15	4.44E-16	0.74633641
121.6	5.53E-09	5.78E-08	2.35E-16	3.00E-15	4.44E-16	0.74633641
121.7	5.53E-09	5.78E-08	2.35E-16	3.00E-15	4.44E-16	0.74633641
121.8	5.46E-09	5.69E-08	2.24E-16	2.95E-15	4.32E-16	0.75020767
121.9	5.46E-09	5.69E-08	2.24E-16	2.95E-15	4.32E-16	0.75020767
122	5.46E-09	5.69E-08	2.24E-16	2.95E-15	4.32E-16	0.75020767
122.1	5.46E-09	5.69E-08	2.24E-16	2.95E-15	4.32E-16	0.75020767
122.2	5.46E-09	5.69E-08	2.24E-16	2.95E-15	4.32E-16	0.75020767
122.3	5.46E-09	5.69E-08	2.24E-16	2.95E-15	4.32E-16	0.75020767
122.4	5.46E-09	5.69E-08	2.24E-16	2.95E-15	4.32E-16	0.75020767
122.5	5.46E-09	5.69E-08	2.24E-16	2.95E-15	4.32E-16	0.75020767
122.6	5.46E-09	5.69E-08	2.24E-16	2.95E-15	4.32E-16	0.75020767
122.7	5.46E-09	5.69E-08	2.24E-16	2.95E-15	4.32E-16	0.75020767
122.8	5.45E-09	5.63E-08	2.17E-16	2.91E-15	4.26E-16	0.75063343
122.9	5.45E-09	5.63E-08	2.17E-16	2.91E-15	4.26E-16	0.75063343
123	5.45E-09	5.63E-08	2.17E-16	2.91E-15	4.26E-16	0.75063343
123.1	5.34E-09	5.52E-08	2.12E-16	2.85E-15	4.23E-16	0.75101412
123.2	5.34E-09	5.52E-08	2.12E-16	2.85E-15	4.23E-16	0.75101412
123.3	5.34E-09	5.52E-08	2.12E-16	2.85E-15	4.23E-16	0.75101412
123.4	5.34E-09	5.52E-08	2.12E-16	2.85E-15	4.23E-16	0.75101412
123.5	5.34E-09	5.52E-08	2.12E-16	2.85E-15	4.23E-16	0.75101412
123.6	5.45E-09	5.51E-08	2.17E-16	2.88E-15	4.37E-16	0.75411013
123.7	5.62E-09	5.46E-08	2.19E-16	2.87E-15	4.37E-16	0.75063394
123.8	5.67E-09	5.48E-08	2.27E-16	2.84E-15	4.58E-16	0.75052003
123.9	5.67E-09	5.48E-08	2.27E-16	2.84E-15	4.58E-16	0.75052003
124	5.67E-09	5.48E-08	2.27E-16	2.84E-15	4.58E-16	0.75052003
124.1	5.67E-09	5.48E-08	2.27E-16	2.84E-15	4.58E-16	0.75052003
124.2	5.65E-09	5.38E-08	2.30E-16	2.79E-15	4.64E-16	0.75206445
124.3	5.65E-09	5.38E-08	2.30E-16	2.79E-15	4.64E-16	0.75206445
124.4	5.65E-09	5.38E-08	2.30E-16	2.79E-15	4.64E-16	0.75206445
124.5	5.65E-09	5.38E-08	2.30E-16	2.79E-15	4.64E-16	0.75206445
124.6	5.82E-09	5.42E-08	2.38E-16	2.67E-15	4.74E-16	0.75248301
124.7	5.82E-09	5.42E-08	2.38E-16	2.67E-15	4.74E-16	0.75248301
124.8	5.83E-09	5.33E-08	2.32E-16	2.64E-15	4.56E-16	0.74820469
124.9	5.83E-09	5.33E-08	2.32E-16	2.64E-15	4.56E-16	0.74820469
125	5.83E-09	5.33E-08	2.32E-16	2.64E-15	4.56E-16	0.74820469
125.1	5.83E-09	5.33E-08	2.32E-16	2.64E-15	4.56E-16	0.74820469
125.2	5.83E-09	5.30E-08	2.28E-16	2.54E-15	4.60E-16	0.74952194
125.3	5.83E-09	5.30E-08	2.28E-16	2.54E-15	4.60E-16	0.74952194
125.4	5.83E-09	5.30E-08	2.28E-16	2.54E-15	4.60E-16	0.74952194
125.5	5.86E-09	5.34E-08	2.34E-16	2.51E-15	4.80E-16	0.74819206
125.6	5.86E-09	5.34E-08	2.34E-16	2.51E-15	4.80E-16	0.74819206
125.7	5.86E-09	5.34E-08	2.34E-16	2.51E-15	4.80E-16	0.74819206
125.8	5.86E-09	5.34E-08	2.34E-16	2.51E-15	4.80E-16	0.74819206
125.9	5.98E-09	5.57E-08	2.41E-16	2.52E-15	5.20E-16	0.74546207
126	6.10E-09	5.66E-08	2.43E-16	2.55E-15	5.37E-16	0.74694303

135	7.46E-09	6.39E-08	3.26E-16	2.40E-15	1.06E-15	0.7401049
135.1	7.46E-09	6.39E-08	3.26E-16	2.40E-15	1.06E-15	0.7401049
135.2	7.34E-09	6.27E-08	3.16E-16	2.37E-15	1.05E-15	0.742197
135.3	7.34E-09	6.27E-08	3.16E-16	2.37E-15	1.05E-15	0.742197
135.4	7.29E-09	6.34E-08	3.21E-16	2.30E-15	1.07E-15	0.74019361
135.5	7.29E-09	6.34E-08	3.21E-16	2.30E-15	1.07E-15	0.74019361
135.6	7.32E-09	6.27E-08	3.15E-16	2.33E-15	1.09E-15	0.73965968
135.7	7.35E-09	6.11E-08	2.98E-16	2.30E-15	1.05E-15	0.74344379
135.8	7.35E-09	6.11E-08	2.98E-16	2.30E-15	1.05E-15	0.74344379
135.9	7.35E-09	6.11E-08	2.98E-16	2.30E-15	1.05E-15	0.74344379
136	7.35E-09	6.11E-08	2.98E-16	2.30E-15	1.05E-15	0.74344379
136.1	7.35E-09	6.11E-08	2.98E-16	2.30E-15	1.05E-15	0.74344379
136.2	7.23E-09	6.00E-08	2.86E-16	2.29E-15	1.00E-15	0.74605718
136.3	7.23E-09	6.00E-08	2.86E-16	2.29E-15	1.00E-15	0.74605718
136.4	7.29E-09	6.02E-08	2.87E-16	2.21E-15	1.04E-15	0.74838168
136.5	7.14E-09	6.07E-08	2.89E-16	2.23E-15	1.03E-15	0.7472019
136.6	7.14E-09	6.07E-08	2.89E-16	2.23E-15	1.03E-15	0.7472019
136.7	7.14E-09	6.07E-08	2.89E-16	2.23E-15	1.03E-15	0.7472019
136.8	7.14E-09	6.07E-08	2.89E-16	2.23E-15	1.03E-15	0.7472019
136.9	7.09E-09	6.00E-08	2.84E-16	2.22E-15	1.04E-15	0.74587328
137	7.09E-09	6.00E-08	2.84E-16	2.22E-15	1.04E-15	0.74587328
137.1	7.09E-09	6.00E-08	2.84E-16	2.22E-15	1.04E-15	0.74587328
137.2	7.09E-09	6.00E-08	2.84E-16	2.22E-15	1.04E-15	0.74587328
137.3	7.09E-09	6.00E-08	2.84E-16	2.22E-15	1.04E-15	0.74587328
137.4	7.09E-09	6.00E-08	2.84E-16	2.22E-15	1.04E-15	0.74587328
137.5	7.09E-09	6.00E-08	2.84E-16	2.22E-15	1.04E-15	0.74587328
137.6	7.09E-09	6.00E-08	2.84E-16	2.22E-15	1.04E-15	0.74587328
137.7	7.25E-09	6.11E-08	2.73E-16	2.18E-15	1.07E-15	0.74441731
137.8	7.25E-09	6.11E-08	2.73E-16	2.18E-15	1.07E-15	0.74441731
137.9	7.25E-09	6.11E-08	2.73E-16	2.18E-15	1.07E-15	0.74441731
138	7.25E-09	6.11E-08	2.73E-16	2.18E-15	1.07E-15	0.74441731
138.1	7.25E-09	6.11E-08	2.73E-16	2.18E-15	1.07E-15	0.74441731
138.2	7.25E-09	6.11E-08	2.73E-16	2.18E-15	1.07E-15	0.74441731
138.3	7.25E-09	6.11E-08	2.73E-16	2.18E-15	1.07E-15	0.74441731
138.4	7.25E-09	6.11E-08	2.73E-16	2.18E-15	1.07E-15	0.74441731
138.5	7.25E-09	6.11E-08	2.73E-16	2.18E-15	1.07E-15	0.74441731
138.6	7.25E-09	6.11E-08	2.73E-16	2.18E-15	1.07E-15	0.74441731
138.7	7.25E-09	6.11E-08	2.73E-16	2.18E-15	1.07E-15	0.74441731
138.8	7.28E-09	6.04E-08	2.79E-16	2.17E-15	1.06E-15	0.74460261
138.9	7.28E-09	6.04E-08	2.79E-16	2.17E-15	1.06E-15	0.74460261
139	7.28E-09	6.04E-08	2.79E-16	2.17E-15	1.06E-15	0.74460261
139.1	7.28E-09	6.04E-08	2.79E-16	2.17E-15	1.06E-15	0.74460261
139.2	7.28E-09	6.04E-08	2.79E-16	2.17E-15	1.06E-15	0.74460261
139.3	7.28E-09	6.04E-08	2.79E-16	2.17E-15	1.06E-15	0.74460261
139.4	7.28E-09	6.04E-08	2.79E-16	2.17E-15	1.06E-15	0.74460261
139.5	7.28E-09	6.04E-08	2.79E-16	2.17E-15	1.06E-15	0.74460261
139.6	7.28E-09	6.04E-08	2.79E-16	2.17E-15	1.06E-15	0.74460261
139.7	7.28E-09	6.04E-08	2.79E-16	2.17E-15	1.06E-15	0.74460261
139.8	7.28E-09	6.04E-08	2.79E-16	2.17E-15	1.06E-15	0.74460261
139.9	7.28E-09	6.04E-08	2.79E-16	2.17E-15	1.06E-15	0.74460261
140	7.28E-09	6.04E-08	2.79E-16	2.17E-15	1.06E-15	0.74460261
140.1	7.28E-09	6.04E-08	2.79E-16	2.17E-15	1.06E-15	0.74460261
140.2	7.28E-09	6.04E-08	2.79E-16	2.17E-15	1.06E-15	0.74460261
140.3	7.28E-09	6.04E-08	2.79E-16	2.17E-15	1.06E-15	0.74460261
140.4	7.28E-09	6.04E-08	2.79E-16	2.17E-15	1.06E-15	0.74460261
140.5	7.28E-09	6.04E-08	2.79E-16	2.17E-15	1.06E-15	0.74460261
140.6	7.28E-09	6.04E-08	2.79E-16	2.17E-15	1.06E-15	0.74460261
140.7	7.35E-09	6.04E-08	2.76E-16	2.15E-15	1.09E-15	0.7435172
140.8	7.35E-09	6.04E-08	2.76E-16	2.15E-15	1.09E-15	0.7435172
140.9	7.35E-09	6.04E-08	2.76E-16	2.15E-15	1.09E-15	0.7435172
141	7.35E-09	6.04E-08	2.76E-16	2.15E-15	1.09E-15	0.7435172

150	7.18E-09	5.20E-08	2.44E-16	1.93E-15	1.15E-15	0.75528104
150.1	7.18E-09	5.20E-08	2.44E-16	1.93E-15	1.15E-15	0.75528104
150.2	7.18E-09	5.20E-08	2.44E-16	1.93E-15	1.15E-15	0.75528104
150.3	7.18E-09	5.20E-08	2.44E-16	1.93E-15	1.15E-15	0.75528104
150.4	7.18E-09	5.20E-08	2.44E-16	1.93E-15	1.15E-15	0.75528104
150.5	7.18E-09	5.20E-08	2.44E-16	1.93E-15	1.15E-15	0.75528104
150.6	7.18E-09	5.20E-08	2.44E-16	1.93E-15	1.15E-15	0.75528104
150.7	7.18E-09	5.20E-08	2.44E-16	1.93E-15	1.15E-15	0.75528104
150.8	7.30E-09	5.14E-08	2.43E-16	1.93E-15	1.17E-15	0.75163039
150.9	7.34E-09	5.07E-08	2.53E-16	1.88E-15	1.21E-15	0.75372005
151	7.34E-09	5.07E-08	2.53E-16	1.88E-15	1.21E-15	0.75372005
151.1	7.34E-09	5.07E-08	2.53E-16	1.88E-15	1.21E-15	0.75372005
151.2	7.34E-09	5.07E-08	2.53E-16	1.88E-15	1.21E-15	0.75372005
151.3	7.34E-09	5.07E-08	2.53E-16	1.88E-15	1.21E-15	0.75372005
151.4	7.53E-09	5.05E-08	2.56E-16	1.86E-15	1.21E-15	0.75023447
151.5	7.58E-09	5.02E-08	2.52E-16	1.78E-15	1.19E-15	0.75300485
151.6	7.58E-09	5.02E-08	2.52E-16	1.78E-15	1.19E-15	0.75300485
151.7	7.58E-09	5.02E-08	2.52E-16	1.78E-15	1.19E-15	0.75300485
151.8	7.58E-09	5.02E-08	2.52E-16	1.78E-15	1.19E-15	0.75300485
151.9	7.58E-09	5.02E-08	2.52E-16	1.78E-15	1.19E-15	0.75300485
152	7.58E-09	5.02E-08	2.52E-16	1.78E-15	1.19E-15	0.75300485
152.1	7.58E-09	5.02E-08	2.52E-16	1.78E-15	1.19E-15	0.75300485
152.2	7.58E-09	5.02E-08	2.52E-16	1.78E-15	1.19E-15	0.75300485
152.3	7.58E-09	5.02E-08	2.52E-16	1.78E-15	1.19E-15	0.75300485
152.4	7.58E-09	5.02E-08	2.52E-16	1.78E-15	1.19E-15	0.75300485
152.5	7.58E-09	5.02E-08	2.52E-16	1.78E-15	1.19E-15	0.75300485
152.6	7.58E-09	5.02E-08	2.52E-16	1.78E-15	1.19E-15	0.75300485
152.7	7.58E-09	5.02E-08	2.52E-16	1.78E-15	1.19E-15	0.75300485
152.8	7.58E-09	5.02E-08	2.52E-16	1.78E-15	1.19E-15	0.75300485
152.9	7.49E-09	5.06E-08	2.60E-16	1.76E-15	1.25E-15	0.75463351
153	7.49E-09	5.06E-08	2.60E-16	1.76E-15	1.25E-15	0.75463351
153.1	7.49E-09	5.06E-08	2.60E-16	1.76E-15	1.25E-15	0.75463351
153.2	7.49E-09	5.06E-08	2.60E-16	1.76E-15	1.25E-15	0.75463351
153.3	7.49E-09	5.06E-08	2.60E-16	1.76E-15	1.25E-15	0.75463351
153.4	7.49E-09	5.06E-08	2.60E-16	1.76E-15	1.25E-15	0.75463351
153.5	7.49E-09	5.06E-08	2.60E-16	1.76E-15	1.25E-15	0.75463351
153.6	7.49E-09	5.06E-08	2.60E-16	1.76E-15	1.25E-15	0.75463351
153.7	7.49E-09	5.06E-08	2.60E-16	1.76E-15	1.25E-15	0.75463351
153.8	7.49E-09	5.06E-08	2.60E-16	1.76E-15	1.25E-15	0.75463351
153.9	7.49E-09	5.06E-08	2.60E-16	1.76E-15	1.25E-15	0.75463351
154	7.49E-09	5.06E-08	2.60E-16	1.76E-15	1.25E-15	0.75463351
154.1	7.49E-09	5.06E-08	2.60E-16	1.76E-15	1.25E-15	0.75463351
154.2	7.54E-09	5.01E-08	2.56E-16	1.71E-15	1.26E-15	0.75187287
154.3	7.54E-09	5.01E-08	2.56E-16	1.71E-15	1.26E-15	0.75187287
154.4	7.54E-09	5.01E-08	2.56E-16	1.71E-15	1.26E-15	0.75187287
154.5	7.54E-09	5.01E-08	2.56E-16	1.71E-15	1.26E-15	0.75187287
154.6	7.54E-09	5.01E-08	2.56E-16	1.71E-15	1.26E-15	0.75187287
154.7	7.54E-09	5.01E-08	2.56E-16	1.71E-15	1.26E-15	0.75187287
154.8	7.54E-09	5.01E-08	2.56E-16	1.71E-15	1.26E-15	0.75187287
154.9	7.54E-09	5.01E-08	2.56E-16	1.71E-15	1.26E-15	0.75187287
155	7.54E-09	5.01E-08	2.56E-16	1.71E-15	1.26E-15	0.75187287
155.1	7.54E-09	5.01E-08	2.56E-16	1.71E-15	1.26E-15	0.75187287
155.2	7.54E-09	5.01E-08	2.56E-16	1.71E-15	1.26E-15	0.75187287
155.3	7.54E-09	5.01E-08	2.56E-16	1.71E-15	1.26E-15	0.75187287
155.4	7.52E-09	5.09E-08	2.52E-16	1.71E-15	1.26E-15	0.75026998
155.5	7.52E-09	5.09E-08	2.52E-16	1.71E-15	1.26E-15	0.75026998
155.6	7.52E-09	5.09E-08	2.52E-16	1.71E-15	1.26E-15	0.75026998
155.7	7.52E-09	5.09E-08	2.52E-16	1.71E-15	1.26E-15	0.75026998
155.8	7.52E-09	5.09E-08	2.52E-16	1.71E-15	1.26E-15	0.75026998
155.9	7.52E-09	5.09E-08	2.52E-16	1.71E-15	1.26E-15	0.75026998
156	7.52E-09	5.09E-08	2.52E-16	1.71E-15	1.26E-15	0.75026998

165	7.73E-09	5.20E-08	2.60E-16	1.67E-15	1.47E-15	0.74602856
165.1	7.90E-09	5.19E-08	2.61E-16	1.65E-15	1.47E-15	0.741349
165.2	7.90E-09	5.19E-08	2.61E-16	1.65E-15	1.47E-15	0.741349
165.3	7.90E-09	5.19E-08	2.61E-16	1.65E-15	1.47E-15	0.741349
165.4	7.90E-09	5.19E-08	2.61E-16	1.65E-15	1.47E-15	0.741349
165.5	7.90E-09	5.19E-08	2.61E-16	1.65E-15	1.47E-15	0.741349
165.6	7.90E-09	5.19E-08	2.61E-16	1.65E-15	1.47E-15	0.741349
165.7	7.90E-09	5.19E-08	2.61E-16	1.65E-15	1.47E-15	0.741349
165.8	7.90E-09	5.19E-08	2.61E-16	1.65E-15	1.47E-15	0.741349
165.9	7.90E-09	5.19E-08	2.61E-16	1.65E-15	1.47E-15	0.741349
166	7.90E-09	5.19E-08	2.61E-16	1.65E-15	1.47E-15	0.741349
166.1	7.90E-09	5.19E-08	2.61E-16	1.65E-15	1.47E-15	0.741349
166.2	7.90E-09	5.19E-08	2.61E-16	1.65E-15	1.47E-15	0.741349
166.3	7.69E-09	5.26E-08	2.59E-16	1.62E-15	1.47E-15	0.74312125
166.4	7.69E-09	5.26E-08	2.59E-16	1.62E-15	1.47E-15	0.74312125
166.5	7.69E-09	5.26E-08	2.59E-16	1.62E-15	1.47E-15	0.74312125
166.6	7.69E-09	5.26E-08	2.59E-16	1.62E-15	1.47E-15	0.74312125
166.7	7.69E-09	5.26E-08	2.59E-16	1.62E-15	1.47E-15	0.74312125
166.8	7.69E-09	5.26E-08	2.59E-16	1.62E-15	1.47E-15	0.74312125
166.9	7.69E-09	5.26E-08	2.59E-16	1.62E-15	1.47E-15	0.74312125
167	7.69E-09	5.26E-08	2.59E-16	1.62E-15	1.47E-15	0.74312125
167.1	7.69E-09	5.26E-08	2.59E-16	1.62E-15	1.47E-15	0.74312125
167.2	7.69E-09	5.26E-08	2.59E-16	1.62E-15	1.47E-15	0.74312125
167.3	7.69E-09	5.26E-08	2.59E-16	1.62E-15	1.47E-15	0.74312125
167.4	7.69E-09	5.26E-08	2.59E-16	1.62E-15	1.47E-15	0.74312125
167.5	7.69E-09	5.26E-08	2.59E-16	1.62E-15	1.47E-15	0.74312125
167.6	7.69E-09	5.26E-08	2.59E-16	1.62E-15	1.47E-15	0.74312125
167.7	7.69E-09	5.26E-08	2.59E-16	1.62E-15	1.47E-15	0.74312125
167.8	7.68E-09	5.19E-08	2.52E-16	1.54E-15	1.49E-15	0.74377902
167.9	7.68E-09	5.19E-08	2.52E-16	1.54E-15	1.49E-15	0.74377902
168	7.68E-09	5.19E-08	2.52E-16	1.54E-15	1.49E-15	0.74377902
168.1	7.68E-09	5.19E-08	2.52E-16	1.54E-15	1.49E-15	0.74377902
168.2	7.68E-09	5.19E-08	2.52E-16	1.54E-15	1.49E-15	0.74377902
168.3	7.68E-09	5.19E-08	2.52E-16	1.54E-15	1.49E-15	0.74377902
168.4	7.68E-09	5.19E-08	2.52E-16	1.54E-15	1.49E-15	0.74377902
168.5	7.68E-09	5.19E-08	2.52E-16	1.54E-15	1.49E-15	0.74377902
168.6	7.68E-09	5.19E-08	2.52E-16	1.54E-15	1.49E-15	0.74377902
168.7	7.68E-09	5.19E-08	2.52E-16	1.54E-15	1.49E-15	0.74377902
168.8	7.68E-09	5.19E-08	2.52E-16	1.54E-15	1.49E-15	0.74377902
168.9	7.59E-09	5.21E-08	2.49E-16	1.56E-15	1.44E-15	0.74631609
169	7.59E-09	5.21E-08	2.49E-16	1.56E-15	1.44E-15	0.74631609
169.1	7.59E-09	5.21E-08	2.49E-16	1.56E-15	1.44E-15	0.74631609
169.2	7.59E-09	5.21E-08	2.49E-16	1.56E-15	1.44E-15	0.74631609
169.3	7.59E-09	5.21E-08	2.49E-16	1.56E-15	1.44E-15	0.74631609
169.4	7.59E-09	5.21E-08	2.49E-16	1.56E-15	1.44E-15	0.74631609
169.5	7.59E-09	5.21E-08	2.49E-16	1.56E-15	1.44E-15	0.74631609
169.6	7.59E-09	5.21E-08	2.49E-16	1.56E-15	1.44E-15	0.74631609
169.7	7.59E-09	5.21E-08	2.49E-16	1.56E-15	1.44E-15	0.74631609
169.8	7.36E-09	5.19E-08	2.44E-16	1.49E-15	1.43E-15	0.74983256
169.9	7.36E-09	5.19E-08	2.44E-16	1.49E-15	1.43E-15	0.74983256
170	7.36E-09	5.19E-08	2.44E-16	1.49E-15	1.43E-15	0.74983256
170.1	7.36E-09	5.19E-08	2.44E-16	1.49E-15	1.43E-15	0.74983256
170.2	7.36E-09	5.19E-08	2.44E-16	1.49E-15	1.43E-15	0.74983256
170.3	7.36E-09	5.19E-08	2.44E-16	1.49E-15	1.43E-15	0.74983256
170.4	7.36E-09	5.19E-08	2.44E-16	1.49E-15	1.43E-15	0.74983256
170.5	7.36E-09	5.19E-08	2.44E-16	1.49E-15	1.43E-15	0.74983256
170.6	7.36E-09	5.19E-08	2.44E-16	1.49E-15	1.43E-15	0.74983256
170.7	7.36E-09	5.19E-08	2.44E-16	1.49E-15	1.43E-15	0.74983256
170.8	7.36E-09	5.19E-08	2.44E-16	1.49E-15	1.43E-15	0.74983256
170.9	7.38E-09	5.23E-08	2.42E-16	1.48E-15	1.41E-15	0.75086067
171	7.38E-09	5.23E-08	2.42E-16	1.48E-15	1.41E-15	0.75086067

126.1	6.10E-09	5.66E-08	2.43E-16	2.55E-15	5.37E-16	0.74694303
126.2	6.10E-09	5.66E-08	2.43E-16	2.55E-15	5.37E-16	0.74694303
126.3	6.10E-09	5.66E-08	2.43E-16	2.55E-15	5.37E-16	0.74694303
126.4	6.10E-09	5.66E-08	2.43E-16	2.55E-15	5.37E-16	0.74694303
126.5	6.10E-09	5.66E-08	2.43E-16	2.55E-15	5.37E-16	0.74694303
126.6	6.10E-09	5.66E-08	2.43E-16	2.55E-15	5.37E-16	0.74694303
126.7	6.10E-09	5.66E-08	2.43E-16	2.55E-15	5.37E-16	0.74694303
126.8	6.10E-09	5.66E-08	2.43E-16	2.55E-15	5.37E-16	0.74694303
126.9	6.10E-09	5.66E-08	2.43E-16	2.55E-15	5.37E-16	0.74694303
127	6.10E-09	5.66E-08	2.43E-16	2.55E-15	5.37E-16	0.74694303
127.1	6.10E-09	5.66E-08	2.43E-16	2.55E-15	5.37E-16	0.74694303
127.2	6.02E-09	5.76E-08	2.57E-16	2.56E-15	5.73E-16	0.75228334
127.3	6.02E-09	5.76E-08	2.57E-16	2.56E-15	5.73E-16	0.75228334
127.4	6.02E-09	5.76E-08	2.57E-16	2.56E-15	5.73E-16	0.75228334
127.5	6.02E-09	5.76E-08	2.57E-16	2.56E-15	5.73E-16	0.75228334
127.6	6.02E-09	5.76E-08	2.57E-16	2.56E-15	5.73E-16	0.75228334
127.7	6.02E-09	5.76E-08	2.57E-16	2.56E-15	5.73E-16	0.75228334
127.8	6.02E-09	5.76E-08	2.57E-16	2.56E-15	5.73E-16	0.75228334
127.9	6.02E-09	5.76E-08	2.57E-16	2.56E-15	5.73E-16	0.75228334
128	6.17E-09	5.87E-08	2.67E-16	2.58E-15	6.07E-16	0.75086121
128.1	6.21E-09	5.82E-08	2.61E-16	2.58E-15	6.04E-16	0.75241763
128.2	6.32E-09	5.87E-08	2.71E-16	2.56E-15	6.31E-16	0.75150871
128.3	6.32E-09	5.87E-08	2.71E-16	2.56E-15	6.31E-16	0.75150871
128.4	6.50E-09	5.97E-08	2.71E-16	2.58E-15	6.47E-16	0.75309729
128.5	6.50E-09	5.97E-08	2.71E-16	2.58E-15	6.47E-16	0.75309729
128.6	6.50E-09	5.97E-08	2.71E-16	2.58E-15	6.47E-16	0.75309729
128.7	6.50E-09	5.97E-08	2.71E-16	2.58E-15	6.47E-16	0.75309729
128.8	6.50E-09	5.97E-08	2.71E-16	2.58E-15	6.47E-16	0.75309729
128.9	6.50E-09	5.97E-08	2.71E-16	2.58E-15	6.47E-16	0.75309729
129	6.50E-09	5.97E-08	2.71E-16	2.58E-15	6.47E-16	0.75309729
129.1	6.50E-09	5.97E-08	2.71E-16	2.58E-15	6.47E-16	0.75309729
129.2	6.62E-09	6.03E-08	2.78E-16	2.57E-15	6.80E-16	0.75414171
129.3	6.62E-09	6.03E-08	2.78E-16	2.57E-15	6.80E-16	0.75414171
129.4	6.62E-09	6.03E-08	2.78E-16	2.57E-15	6.80E-16	0.75414171
129.5	6.62E-09	6.03E-08	2.78E-16	2.57E-15	6.80E-16	0.75414171
129.6	6.62E-09	6.03E-08	2.78E-16	2.57E-15	6.80E-16	0.75414171
129.7	6.62E-09	6.03E-08	2.78E-16	2.57E-15	6.80E-16	0.75414171
129.8	6.62E-09	6.03E-08	2.78E-16	2.57E-15	6.80E-16	0.75414171
129.9	6.76E-09	6.05E-08	2.89E-16	2.55E-15	7.15E-16	0.7545142
130	6.76E-09	6.05E-08	2.89E-16	2.55E-15	7.15E-16	0.7545142
130.1	6.76E-09	6.05E-08	2.89E-16	2.55E-15	7.15E-16	0.7545142
130.2	7.01E-09	6.17E-08	2.93E-16	2.60E-15	7.42E-16	0.75265874
130.3	7.01E-09	6.17E-08	2.93E-16	2.60E-15	7.42E-16	0.75265874
130.4	7.01E-09	6.17E-08	2.93E-16	2.60E-15	7.42E-16	0.75265874
130.5	7.01E-09	6.17E-08	2.93E-16	2.60E-15	7.42E-16	0.75265874
130.6	7.01E-09	6.17E-08	2.93E-16	2.60E-15	7.42E-16	0.75265874
130.7	7.01E-09	6.17E-08	2.93E-16	2.60E-15	7.42E-16	0.75265874
130.8	7.18E-09	6.17E-08	3.02E-16	2.60E-15	7.74E-16	0.75114446
130.9	7.18E-09	6.17E-08	3.02E-16	2.60E-15	7.74E-16	0.75114446
131	7.18E-09	6.17E-08	3.02E-16	2.60E-15	7.74E-16	0.75114446
131.1	7.18E-09	6.17E-08	3.02E-16	2.60E-15	7.74E-16	0.75114446
131.2	7.18E-09	6.17E-08	3.02E-16	2.60E-15	7.74E-16	0.75114446
131.3	7.18E-09	6.17E-08	3.02E-16	2.60E-15	7.74E-16	0.75114446
131.4	7.30E-09	6.18E-08	3.08E-16	2.54E-15	8.00E-16	0.75051919
131.5	7.30E-09	6.18E-08	3.08E-16	2.54E-15	8.00E-16	0.75051919
131.6	7.30E-09	6.18E-08	3.08E-16	2.54E-15	8.00E-16	0.75051919
131.7	7.30E-09	6.18E-08	3.08E-16	2.54E-15	8.00E-16	0.75051919
131.8	7.30E-09	6.18E-08	3.08E-16	2.54E-15	8.00E-16	0.75051919
131.9	7.30E-09	6.18E-08	3.08E-16	2.54E-15	8.00E-16	0.75051919
132	7.29E-09	6.21E-08	3.15E-16	2.54E-15	8.51E-16	0.75054375
132.1	7.29E-09	6.21E-08	3.15E-16	2.54E-15	8.51E-16	0.75054375
132.2	7.40E-09	6.25E-08	3.16E-16	2.55E-15	8.78E-16	0.74699877
132.3	7.40E-09	6.25E-08	3.16E-16	2.55E-15	8.78E-16	0.74699877
132.4	7.56E-09	6.32E-08	3.27E-16	2.54E-15	8.86E-16	0.74513719
132.5	7.56E-09	6.32E-08	3.27E-16	2.54E-15	8.86E-16	0.74513719
132.6	7.76E-09	6.17E-08	3.25E-16	2.53E-15	8.89E-16	0.74368245
132.7	7.76E-09	6.17E-08	3.25E-16	2.53E-15	8.89E-16	0.74368245
132.8	7.76E-09	6.17E-08	3.25E-16	2.53E-15	8.89E-16	0.74368245
132.9	7.76E-09	6.17E-08	3.25E-16	2.53E-15	8.89E-16	0.74368245
133	7.64E-09	6.25E-08	3.22E-16	2.50E-15	8.84E-16	0.74199533
133.1	7.64E-09	6.25E-08	3.22E-16	2.50E-15	8.84E-16	0.74199533
133.2	7.65E-09	6.33E-08	3.27E-16	2.49E-15	9.24E-16	0.74304535
133.3	7.52E-09	6.41E-08	3.27E-16	2.54E-15	9.60E-16	0.74354969
133.4	7.52E-09	6.41E-08	3.27E-16	2.54E-15	9.60E-16	0.74354969
133.5	7.52E-09	6.41E-08	3.27E-16	2.54E-15	9.60E-16	0.74354969
133.6	7.52E-09	6.41E-08	3.27E-16	2.54E-15	9.60E-16	0.74354969
133.7	7.42E-09	6.44E-08	3.22E-16	2.55E-15	9.93E-16	0.74453165
133.8	7.52E-09	6.36E-08	3.26E-16	2.44E-15	1.03E-15	0.74333136
133.9	7.52E-09	6.36E-08	3.26E-16	2.44E-15	1.03E-15	0.74333136
134	7.52E-09	6.36E-08	3.26E-16	2.44E-15	1.03E-15	0.74333136
134.1	7.52E-09	6.36E-08	3.26E-16	2.44E-15	1.03E-15	0.74333136
134.2	7.46E-09	6.39E-08	3.26E-16	2.40E-15	1.06E-15	0.7401049
134.3	7.46E-09	6.39E-08	3.26E-16	2.40E-15	1.06E-15	0.7401049
134.4	7.46E-09	6.39E-08	3.26E-16	2.40E-15	1.06E-15	0.7401049
134.5	7.46E-09	6.39E-08	3.26E-16	2.40E-15	1.06E-15	0.7401049
134.6	7.46E-09	6.39E-08	3.26E-16	2.40E-15	1.06E-15	0.7401049
134.7	7.46E-09	6.39E-08	3.26E-16	2.40E-15	1.06E-15	0.7401049
134.8	7.46E-09	6.39E-08	3.26E-16	2.40E-15	1.06E-15	0.7401049
134.9	7.46E-09	6.39E-08	3.26E-16	2.40E-15	1.06E-15	0.7401049
141.1	7.41E-09	6.19E-08	2.79E-16	2.14E-15	1.09E-15	0.74461217
141.2	7.41E-09	6.19E-08	2.79E-16	2.14E-15	1.09E-15	0.74461217
141.3	7.41E-09	6.19E-08	2.79E-16	2.14E-15	1.09E-15	0.74461217
141.4	7.41E-09	6.19E-08	2.79E-16	2.14E-15	1.09E-15	0.74461217
141.5	7.41E-09	6.19E-08	2.79E-16	2.14E-15	1.09E-15	0.74461217
141.6	7.45E-09	6.10E-08	2.77E-16	2.05E-15	1.09E-15	0.74634716
141.7	7.45E-09	6.10E-08	2.77E-16	2.05E-15	1.09E-15	0.74634716
141.8	7.45E-09	6.10E-08	2.77E-16	2.05E-15	1.09E-15	0.74634716
141.9	7.44E-09	6.10E-08	2.81E-16	2.03E-15	1.09E-15	0.75052669
142	7.44E-09	6.10E-08	2.81E-16	2.03E-15	1.09E-15	0.75052669
142.1	7.44E-09	6.10E-08	2.81E-16	2.03E-15	1.09E-15	0.75052669
142.2	7.44E-09	6.10E-08	2.81E-16	2.03E-15	1.09E-15	0.75052669
142.3	7.44E-09	6.10E-08	2.81E-16	2.03E-15	1.09E-15	0.75052669
142.4	7.44E-09	6.10E-08	2.81E-16	2.03E-15	1.09E-15	0.75052669
142.5	7.44E-09	6.10E-08	2.81E-16	2.03E-15	1.09E-15	0.75052669
142.6	7.44E-09	6.10E-08	2.81E-16	2.03E-15	1.09E-15	0.75052669
142.7	7.44E-09	6.10E-08	2.81E-16	2.03E-15	1.09E-15	0.75052669
142.8	7.44E-09	6.10E-08	2.81E-16	2.03E-15	1.09E-15	0.75052669
142.9	7.44E-09	6.10E-08	2.81E-16	2.03E-15	1.09E-15	0.75052669
143	7.44E-09	6.10E-08	2.81E-16	2.03E-15	1.09E-15	0.75052669
143.1	7.44E-09	6.10E-08	2.81E-16	2.03E-15	1.09E-15	0.75052669
143.2	7.44E-09	6.10E-08	2.81E-16	2.03E-15	1.09E-15	0.75052669
143.3	7.44E-09	6.10E-08	2.81E-16	2.03E-15	1.09E-15	0.75052669
143.4	7.44E-09	6.10E-08	2.81E-16	2.03E-15	1.09E-15	0.75052669
143.5	7.44E-09	6.10E-08	2.81E-16	2.03E-15	1.09E-15	0.75052669
143.6	7.44E-09	6.10E-08	2.81E-16	2.03E-15	1.09E-15	0.75052669
143.7	7.45E-09	5.98E-08	2.72E-16	1.94E-15	1.10E-15	0.75427347
143.8	7.45E-09	5.98E-08	2.72E-16	1.94E-15	1.10E-15	0.75427347
143.9	7.45E-09	5.98E-08	2.72E-16	1.94E-15	1.10E-15	0.75427347
144	7.45E-09	5.98E-08	2.72E-16	1.94E-15	1.10E-15	0.75427347
144.1	7.45E-09	5.98E-08	2.72E-16	1.94E-15	1.10E-15	0.75427347
144.2	7.45E-09	5.98E-08	2.72E-16	1.94E-15	1.10E-15	0.75427347
144.3	7.45E-09	5.98E-08	2.72E-16	1.94E-15	1.10E-15	0.75427347
144.4	7.45E-09	5.98E-08	2.72E-16	1.94E-15	1.10E-15	0.75427347
144.5	7.45E-09	5.98E-08	2.72E-16	1.94E-15	1.10E-15	0.75427347
144.6	7.45E-09	5.98E-08	2.72E-16	1.94E-15	1.10E-15	0.75427347
144.7	7.45E-09	5.98E-08	2.72E-16	1.94E-15	1.10E-15	0.75427347
144.8	7.45E-09	5.98E-08	2.72E-16	1.94E-15	1.10E-15	0.75427347
144.9	7.03E-09	5.83E-08	2.63E-16	1.86E-15	1.08E-15	0.75038359
145	7.03E-09	5.83E-08	2.63E-16	1.86E-15	1.08E-15	0.75038359
145.1	7.03E-09	5.83E-08	2.63E-16	1.86E-15	1.08E-15	0.75038359
145.2	7.03E-09	5.83E-08	2.63E-16	1.86E-15	1.08E-15	0.75038359
145.3	7.03E-09	5.83E-08	2.63E-16	1.86E-15	1.08E-15	0.75038359
145.4	7.26E-09	5.68E-08	2.62E-16	1.93E-15	1.07E-15	0.75446789
145.5	7.26E-09	5.68E-08	2.62E-16	1.93E-15	1.07E-15	0.75446789
145.6	7.26E-09	5.68E-08	2.62E-16	1.93E-15	1.07E-15	0.75446789
145.7	7.26E-09	5.68E-08	2.62E-16	1.93E-15	1.07E-15	0.75446789
145.8	7.26E-09	5.68E-08	2.62E-16	1.93E-15	1.07E-15	0.75446789
145.9	7.26E-09	5.68E-08	2.62E-16	1.93E-15	1.07E-15	0.75446789
146	7.36E-09	5.51E-08	2.56E-16	1.92E-15	1.08E-15	0.75496842
146.1	7.36E-09	5.51E-08	2.56E-16	1.92E-15	1.08E-15	0.75496842
146.2	7.36E-09	5.51E-08	2.56E-16	1.92E-15	1.08E-15	0.75496842
146.3	7.36E-09	5.51E-08	2.56E-16	1.92E-15	1.08E-15	0.75496842
146.4	7.36E-09	5.51E-08	2.56E-16	1.92E-15	1.08E-15	0.75496842
146.5	7.36E-09	5.51E-08	2.56E-16	1.92E-15	1.08E-15	0.75496842
146.6	7.36E-09	5.51E-08	2.56E-16	1.92E-15	1.08E-15	0.75496842
146.7	7.40E-09	5.33E-08	2.58E-16	1.93E-15	1.15E-15	0.75311437
146.8	7.40E-09	5.33E-08	2.58E-16	1.93E-15	1.15E-15	0.75311437
146.9	7.40E-09	5.33E-08	2.58E-16	1.93E-15	1.15E-15	0.75311437
147	7.40E-09	5.33E-08	2.58E-16	1.93E-15	1.15E-15	0.75311437
147.1	7.40E-09	5.33E-08	2.58E-16	1.93E-15	1.15E-15	0.75311437
147.2	7.40E-09	5.33E-08	2.58E-16	1.93E-15	1.15E-15	0.75311437
147.3	7.40E-09	5.33E-08	2.58E-16	1.93E-15	1.15E-15	0.75311437
147.4	7.40E-09	5.33E-08	2.58E-16	1.93E-15	1.15E-15	0.75311437
147.5	7.40E-09	5.33E-08	2.58E-16	1.93E-15	1.15E-15	0.75311437
147.6	7.40E-09	5.33E-08	2.58E-16	1.93E-15	1.15E-15	0.75311437
147.7	7.40E-09	5.33E-08	2.58E-16	1.93E-15	1.15E-15	0.75311437
147.8	7.40E-09	5.33E-08	2.58E-16	1.93E-15	1.15E-15	0.75311437
147.9	7.40E-09	5.33E-08	2.58E-16	1.93E-15	1.15E-15	0.75311437
148	7.40E-09	5.33E-08	2.58E-16	1.93E-15	1.15E-15	0.75311437
148.1	7.40E-09	5.33E-08	2.58E-16	1.93E-15	1.15E-15	0.75311437
148.2	7.40E-09	5.33E-08	2.58E-16	1.93E-15	1.15E-15	0.75311437
148.3	7.40E-09	5.33E-08	2.58E-16	1.93E-15	1.15E-15	0.75311437
148.4	7.40E-09	5.33E-08	2.58E-16	1.93E-15	1.15E-15	0.75311437
148.5	7.40E-09	5.33E-08	2.58E-16	1.93E-15	1.15E-15	0.75311437
148.6	7.40E-09	5.33E-08	2.58E-16	1.93E-15	1.15E-15	0.75311437
148.7	7.40E-09	5.33E-08	2.58E-16	1.93E-15	1.15E-15	0.75311437
148.8	7.40E-09	5.33E-08	2.58E-16	1.93E-15	1.15E-15	0.75311437
148.9	7.40E-09	5.33E-08	2.58E-16	1.93E-15	1.15E-15	0.75311437
149	7.40E-09	5.33E-08	2.58E-16	1.93E-15	1.15E-15	0.75311437
149.1	7.40E-09	5.33E-08	2.58E-16	1.93E-15	1.15E-15	0.75311437
149.2	7.40E-09	5.33E-08	2.58E-16	1.93E-15	1.15E-15	0.75311437
149.3	7.40E-09	5.33E-08	2.58E-16	1.93E-15	1.15E-15	0.75311437
149.4	7.36E-09	5.23E-08	2.51E-16	1.94E-15	1.12E-15	0.75708854
149.5	7.18E-09	5.20E-08	2.44E-16	1.93E-15	1.15E-15	0.75528104
149.6	7.18E-09	5.20E-08	2.44E-16	1.93E-15	1.15E-15	0.75528104
149.7	7.18E-09	5.20E-08	2.44E-16	1.93E-15	1.15E-15	0.75528104
149.8	7.18E-09	5.20E-08	2.44E-16	1.93E-15	1.15E-15	0.75528104
149.9	7.18E-09	5.20E-08	2.44E-16	1.93E-15	1.15E-15	0.75528104
156.1	7.52E-09	5.09E-08	2.52E-16	1.71E-15	1.26E-15	0.75026998
156.2	7.37E-09	5.08E-08	2.42E-16	1.68E-15	1.26E-15	0.75022321
156.3	7.37E-09	5.08E-08	2.42E-16	1.68E-15	1.26E-15	0.75022321
156.4	7.37E-09	5.08E-08	2.42E-16	1.68E-15	1.26E-15	0.75022321
156.5	7.37E-09	5.08E-08	2.42E-16	1.68E-15	1.26E-15	0.75022321
156.6	7.37E-09	5.08E-08	2.42E-16	1.68E-15	1.26E-15	0.75022321
156.7	7.37E-09	5.08E-08	2.42E-16	1.68E-15	1.26E-15	0.75022321
156.8	7.37E-09	5.08E-08	2.42E-16	1.68E-15	1.26E-15	0.75022321
156.9	7.37E-09	5.08E-08	2.42E-16	1.68E-15	1.26E-15	0.75022321
157	7.37E-09	5.08E-08	2.42E-16	1.68E-15	1.26E-15	0.75022321
157.1	7.37E-09	5.08E-08	2.42E-16	1.68E-15	1.26E-15	0.75022321
157.2	7.37E-09	5.08E-08	2.42E-16	1.68E-15	1.26E-15	0.75022321
157.3	7.37E-09	5.08E-08	2.42E-16	1.68E-15	1.26E-15	0.75022321
157.4	7.37E-09	5.08E-08	2.42E-16	1.68E-15	1.26E-15	0.75022321
157.5	7.37E-09	5.08E-08	2.42E-16	1.68E-15	1.26E-15	0.75022321
157.6	7.37E-09	5.08E-08	2.42E-16	1.68E-15	1.26E-15	0.75022321
157.7	7.37E-09	5.08E-08	2.42E-16	1.68E-15	1.26E-15	0.75022321
157.8	7.37E-09	5.08E-08	2.42E-16	1.68E-15	1.26E-15	0.75022321
157.9	7.37E-09	5.08E-08	2.42E-16	1.68E-15	1.26E-15	0.75022321
158	7.37E-09	5.08E-08	2.42E-16	1.68E-15	1.26E-15	0.75022321
158.1	7.37E-09	5.08E-08	2.42E-16	1.68E-15	1.26E-15	0.75022321
158.2	7.37E-09	5.08E-08	2.42E-16	1.68E-15	1.26E-15	0.75022321
158.3	7.37E-09	5.08E-08	2.42E-16	1.68E-15	1.26E-15	0.75022321
158.4	7.50E-09	5.03E-08	2.49E-16	1.70E-15	1.31E-15	0.75150102
158.5	7.50E-09	5.03E-08	2.49E-16	1.70E-15	1.31E-15	0.75150102
158.6	7.50E-09	5.03E-08	2.49E-16	1.70E-15	1.31E-15	0.75150102
158.7	7.50E-09	5.03E-08	2.49E-16	1.70E-15	1.31E-15	0.75150102
158.8	7.50E-09	5.03E-08	2.49E-16	1.70E-15	1.31E-15	0.75150102
158.9	7.51E-09	5.03E-08	2.45E-16	1.68E-15	1.31E-15	0.75272906
159	7.51E-09	5.03E-08	2.45E-16	1.68E-15	1.31E-15	0.75272906
159.1	7.51E-09	5.03E-08	2.45E-16	1.68E-15	1.31E-15	0.75272906
159.2	7.51E-09	5.03E-08	2.45E-16	1.68E-15	1.31E-15	0.75272906
159.3	7.51E-09	5.03E-08	2.45E-16	1.68E-15	1.31E-15	0.75272906
159.4	7.51E-09	5.03E-08	2.45E-16	1.68E-15	1.31E-15	0.75272906
159.5	7.51E-09	5.03E-08	2.45E-16	1.68E-15	1.31E-15	0.75272906
159.6	7.51E-09	5.03E-08	2.45E-16	1.68E-15	1.31E-15	0.75272906
159.7	7.74E-09	5.04E-08	2.51E-16	1.65E-15	1.32E-15	0.75042503
159.8	7.74E-09	5.04E-08	2.51E-16	1.65E-15	1.32E-15	0.75042503
159.9	7.74E-09	5.04E-08	2.51E-16	1.65E-15	1.32E-15	0.75042503
160	7.74E-09	5.04E-08	2.51E-16	1.65E-15	1.32E-15	0.75042503
160.1	7.74E-09	5.04E-08	2.51E-16	1.65E-15	1.32E-15	0.75042503
160.2	7.74E-09	5.04E-08	2.51E-16	1.65E-15	1.32E-15	0.75042503
160.3	7.74E-09	5.04E-08	2.51E-16	1.65E-15	1.32E-15	0.75042503
160.4	7.74E-09	5.04E-08	2.51E-16	1.65E-15	1.32E-15	0.75042503
160.5	7.74E-09	5.04E-08	2.51E-16	1.65E-15	1.32E-15	0.75042503
160.6	7.74E-09	5.04E-08	2.51E-16	1.65E-15	1.32E-15	0.75042503
160.7	7.74E-09	5.04E-08	2.51E-16	1.65E-15	1.32E-15	0.75042503
160.8	7.74E-09	5.04E-08	2.51E-16	1.65E-15	1.32E-15	0.75042503
160.9	7.74E-09	5.04E-08	2.51E-16	1.65E-15	1.32E-15	0.75042503
161	7.74E-09	5.04E-08	2.51E-16	1.65E-15	1.32E-15	0.75042503
161.1	7.74E-09	5.04E-08	2.51E-16	1.65E-15	1.32E-15	0.75042503
161.2	7.74E-09	5.04E-08	2.51E-16	1.65E-15	1.32E-15	0.75042503
161.3	7.74E-09	5.04E-08	2.51E-16	1.65E-15	1.32E-15	0.75042503
161.4	7.74E-09	5.04E-08	2.51E-16	1.65E-15	1.32E-15	0.75042503
161.5	7.74E-09	5.04E-08	2.51E-16	1.65E-15	1.32E-15	0.75042503
161.6	7.74E-09	5.04E-08	2.51E-16	1.65E-15	1.32E-15	0.75042503
161.7	7.74E-09	5.04E-08	2.51E-16	1.65E-15	1.32E-15	0.75042503
161.8	7.74E-09	5.04E-08	2.51E-16	1.65E-15	1.32E-15	0.75042503
161.9	7.74E-09	5.04E-08	2.51E-16	1.65E-15	1.32E-15	0.75042503
162	7.74E-09	5.04E-08	2.51E-16	1.65E-15	1.32E-15	0.75042503
162.1	7.74E-09	5.04E-08	2.51E-16	1.65E-15	1.32E-15	0.75042503
162.2	7.74E-09	5.04E-08	2.51E-16	1.65E-15	1.32E-15	0.75042503
162.3	7.74E-09	5.04E-08	2.51E-16	1.65E-15	1.32E-15	0.75042503
162.4	7.74E-09	5.04E-08	2.51E-16	1.65E-15	1.32E-15	0.75042503
162.5	7.74E-09	5.04E-08	2.51E-16	1.65E-15	1.32E-15	0.75042503
162.6	7.74E-09	5.04E-08	2.51E-16	1.65E-15	1.32E-15	0.75042503
162.7	7.74E-09	5.04E-08	2.51E-16	1.65E-15	1.32E-15	0.75042503
162.8	7.74E-09	5.04E-08	2.51E-16	1.65E-15	1.32E-15	0.75042503
162.9	7.74E-09	5.04E-08	2.51E-16	1.65E-15	1.32E-15	0.75042503
163	7.74E-09	5.04E-08	2.51E-16	1.65E-15	1.32E-15	0.75042503
163.1	7.74E-09	5.04E-08	2.51E-16	1.65E-15	1.32E-15	0.75042503
163.2	7.74E-09	5.04E-08	2.51E-16	1.65E-15	1.32E-15	0.75042503
163.3	7.88E-09	5.18E-08	2.52E-16	1.67E-15	1.39E-15	0.74810301
163.4	7.74E-09	5.16E-08	2.53E-16	1.64E-15	1.44E-15	0.74757137
163.5	7.74E-09	5.16E-08	2.53E-16	1.64E-15	1.44E-15	0.74757137
163.6	7.74E-09	5.16E-08	2.53E-16	1.64E-15	1.44E-15	0.74757137
163.7	7.74E-09	5.16E-08	2.53E-16	1.64E-15	1.44E-15	0.74757137
163.8	7.74E-09	5.16E-08	2.53E-16	1.64E-15	1.44E-15	0.74757137
163.9	7.74E-09	5.16E-08	2.53E-16	1.64E-15	1.44E-15	0.74757137
164	7.74E-09	5.16E-08	2.53E-16	1.64E-15	1.44E-15	0.74757137
164.1	7.74E-09	5.16E-08	2.53E-16	1.64E-15	1.44E-15	0.74757137
164.2	7.74E-09	5.16E-08	2.53E-16	1.64E-15	1.44E-15	0.74757137
164.3	7.74E-09	5.16E-08	2.53E-16	1.64E-15	1.44E-15	0.74757137
164.4	7.74E-09	5.16E-08	2.53E-16	1.64E-15	1.44E-15	0.74757137
164.5	7.74E-09	5.16E-08	2.53E-16	1.64E-15	1.44E-15	0.74757137
164.6	7.73E-09	5.20E-08	2.60E-16	1.67E-15	1.47E-15	0.74602856
164.7	7.73E-09	5.20E-08	2.60E-16	1.67E-15	1.47E-15	0.74602856
164.8	7.73E-09	5.20E-08	2.60E-16	1.67E-15	1.47E-15	0.74602856
164.9	7.73E-09	5.20E-08	2.60E-16	1.67E-15	1.47E-15	0.74602856
171.1	7.38E-09	5.23E-08	2.42E-16	1.48E-15	1.41E-15	0.75086067
171.2	7.38E-09	5.23E-08	2.42E-16	1.48E-15	1.41E-15	0.75086067
171.3	7.38E-09	5.23E-08	2.42E-16	1.48E-15	1.41E-15	0.75086067
171.4	7.38E-09	5.23E-08	2.42E-16	1.48E-15	1.41E-15	0.75086067
171.5	7.38E-09	5.23E-08	2.42E-16	1.48E-15	1.41E-15	0.75086067
171.6	7.12E-09	5.08E-08	2.35E-16	1.48E-15	1.38E-15	0.7524411
171.7	7.12E-09	5.08E-08	2.35E-16	1.48E-15	1.38E-15	0.7524411
171.8	7.12E-09	5.08E-08	2.35E-16	1.48E-15	1.38E-15	0.7524411
171.9	7.12E-09	5.08E-08	2.35E-16	1.48E-15	1.38E-15	0.7524411
172	7.12E-09	5.08E-08	2.35E-16	1.48E-15	1.38E-15	0.7524411
172.1	7.37E-09	5.11E-08	2.33E-16	1.50E-15	1.36E-15	0.7505485
172.2	7.37E-09	5.11E-08	2.33E-16	1.50E-15	1.36E-15	0.7505485
172.3	7.37E-09	5.11E-08	2.33E-16	1.50E-15	1.36E-15	0.7505485
172.4	7.40E-09	5.21E-08	2.35E-16	1.50E-15	1.40E-15	0.74605077
172.5	7.40E-09	5.21E-08	2.35E-16	1.50E-15	1.40E-15	0.74605077
172.6	7.40E-09	5.21E-08	2.35E-16	1.50E-15	1.40E-15	0.74605077
172.7	7.40E-09	5.21E-08	2.35E-16	1.50E-15	1.40E-15	0.74605077
172.8	7.40E-09	5.21E-08	2.35E-16	1.50E-15	1.40E-15	0.74605077
172.9	7.40E-09	5.21E-08	2.35E-16	1.50E-15	1.40E-15	0.74605077
173	7.40E-09	5.21E-08	2.35E-16	1.50E-15	1.40E-15	0.74605077
173.1	7.40E-09	5.21E-08	2.35E-16	1.50E-15	1.40E-15	0.74605077
173.2	7.40E-09	5.21E-08	2.35E-16	1.50E-15	1.40E-15	0.74605077
173.3	7.40E-09	5.21E-08	2.35E-16	1.50E-15	1.40E-15	0.74605077
173.4	7.40E-09	5.21E-08	2.35E-16	1.50E-15	1.40E-15	0.74605077
173.5	7.40E-09	5.21E-08	2.35E-16	1.50E-15	1.40E-15	0.74605077
173.6	7.40E-09	5.21E-08	2.35E-16	1.50E-15	1.40E-15	0.74605077
173.7	7.31E-09	5.10E-08	2.40E-16	1.48E-15	1.42E-15	0.74410368
173.8	7.31E-09	5.10E-08	2.40E-16	1.48E-15	1.42E-15	0.74410368
173.9	7.31E-09	5.10E-08	2.40E-16	1.48E-15	1.42E-15	0.74410368
174	7.31E-09	5.10E-08	2.40E-16	1.48E-15	1.42E-15	0.74410368
174.1	7.31E-09	5.10E-08	2.40E-16	1.48E-15	1.42E-15	0.74410368
174.2	7.31E-09	5.10E-08	2.40E-16	1.48E-15	1.42E-15	0.74410368
174.3	7.31E-09	5.10E-08	2.40E-16	1.48E-15	1.42E-15	0.74410368
174.4	7.31E-09	5.10E-08	2.40E-16	1.48E-15	1.42E-15	0.74410368
174.5	7.31E-09	5.10E-08	2.40E-16	1.48E-15	1.42E-15	0.74410368
174.6	7.31E-09	5.10E-08	2.40E-16	1.48E-15	1.42E-15	0.74410368
174.7	7.31E-09	5.10E-08	2.40E-16	1.48E-15	1.42E-15	0.74410368
174.8	7.31E-09	5.10E-08	2.40E-16	1.48E-15	1.42E-15	0.74410368
174.9	7.31E-09	5.10E-08	2.40E-16	1.48E-15	1.42E-15	0.74410368
175	7.38E-09	5.09E-08	2.35E-16	1.42E-15	1.39E-15	0.74640203
175.1	7.38E-09	5.09E-08	2.35E-16	1.42E-15	1.39E-15	0.74640203
175.2	7.14E-09	5.11E-08	2.34E-16	1.43E-15	1.43E-15	0.74787541
175.3	7.17E-09	4.97E-08	2.31E-16	1.42E-15	1.46E-15	0.74815512
175.4	7.17E-09	4.97E-08	2.31E-16	1.42E-15	1.46E-15	0.74815512
175.5	7.17E-09	4.97E-08	2.31E-16	1.42E-15	1.46E-15	0.74815512
175.6	7.17E-09	4.97E-08	2.31E-16	1.42E-15	1.46E-15	0.74815512
175.7	7.17E-09	4.97E-08	2.31E-16	1.42E-15	1.46E-15	0.74815512
175.8	7.17E-09	4.97E-08	2.31E-16	1.42E-15	1.46E-15	0.74815512
175.9	7.17E-09	4.97E-08	2.31E-16	1.42E-15	1.46E-15	0.74815512
176	7.17E-09	4.97E-08	2.31E-16	1.42E-15	1.46E-15	0.74815512
176.1	7.17E-09	4.97E-08	2.31E-16	1.42E-15	1.46E-15	0.74815512
176.2	7.17E-09	4.97E-08	2.31E-16	1.42E-15	1.46E-15	0.74815512
176.3	7.17E-09	4.97E-08	2.31E-16	1.42E-15	1.46E-15	0.74815512
176.4	7.17E-09	4.97E-08	2.31E-16	1.42E-15	1.46E-15	0.74815512
176.5	7.17E-09	4.97E-08	2.31E-16	1.42E-15	1.46E-15	0.74815512
176.6	7.17E-09	4.97E-08	2.31E-16	1.42E-15	1.46E-15	0.74815512
176.7	7.17E-09	4.97E-08	2.31E-16	1.42E-15	1.46E-15	0.74815512
176.8	7.17E-09	4.97E-08	2.31E-16	1.42E-15	1.46E-15	0.74815512
176.9	7.21E-09	4.94E-08	2.40E-16	1.42E-15	1.51E-15	0.75063475
177	7.27E-09	4.89E-08	2.37E-16	1.38E-15	1.50E-15	0.7519117
177.1	7.27E-09	4.89E-08	2.37E-16	1.38E-15	1.50E-15	0.7519117
177.2	7.27E-09	4.89E-08	2.37E-16	1.38E-15	1.50E-15	0.7519117
177.3	7.27E-09	4.89E-08	2.37E-16	1.38E-15	1.50E-15	0.7519117
177.4	7.27E-09	4.89E-08	2.37E-16	1.38E-15	1.50E-15	0.7519117
177.5	7.27E-09	4.89E-08	2.37E-16	1.38E-15	1.50E-15	0.7519117
177.6	7.27E-09	4.89E-08	2.37E-16	1.38E-15	1.50E-15	0.7519117
177.7	7.27E-09	4.89E-08	2.37E-16	1.38E-15	1.50E-15	0.7519117
177.8	7.27E-09	4.89E-08	2.37E-16	1.38E-15	1.50E-15	0.7519117
177.9	7.27E-09	4.89E-08	2.37E-16	1.38E-15	1.50E-15	0.7519117
178	7.27E-09	4.89E-08	2.37E-16	1.38E-15	1.50E-15	0.7519117
178.1	7.27E-09	4.89E-08	2.37E-16	1.38E-15	1.50E-15	0.7519117
178.2	7.27E-09	4.89E-08	2.37E-16	1.38E-15	1.50E-15	0.7519117
178.3	7.27E-09	4.89E-08	2.37E-16	1.38E-15	1.50E-15	0.7519117
178.4	7.27E-09	4.89E-08	2.37E-16	1.38E-15	1.50E-15	0.7519117
178.5	7.27E-09	4.89E-08	2.37E-16	1.38E-15	1.50E-15	0.7519117
178.6	7.20E-09	4.93E-08	2.44E-16	1.40E-15	1.53E-15	0.7520729
178.7	7.20E-09	4.93E-08	2.44E-16	1.40E-15	1.53E-15	0.7520729
178.8	7.20E-09	4.93E-08	2.44E-16	1.40E-15	1.53E-15	0.7520729
178.9	7.20E-09	4.93E-08	2.44E-16	1.40E-15	1.53E-15	0.7520729
179	7.20E-09	4.93E-08	2.44E-16	1.40E-15	1.53E-15	0.7520729
179.1	7.20E-09	4.93E-08	2.44E-16	1.40E-15	1.53E-15	0.7520729
179.2	7.20E-09	4.93E-08	2.44E-16	1.40E-15	1.53E-15	0.7520729
179.3	7.11E-09	5.05E-08	2.49E-16	1.44E-15	1.59E-15	0.75317075
179.4	7.11E-09	5.05E-08	2.49E-16	1.44E-15	1.59E-15	0.75317075
179.5	7.11E-09	5.05E-08	2.49E-16	1.44E-15	1.59E-15	0.75317075
179.6	7.40E-09	5.10E-08	2.52E-16	1.45E-15	1.61E-15	0.74838175
179.7	7.40E-09	5.10E-08	2.52E-16	1.45E-15	1.61E-15	0.74838175
179.8	7.40E-09	5.10E-08	2.52E-16	1.45E-15	1.61E-15	0.74838175
179.9	7.40E-09	5.10E-08	2.52E-16	1.45E-15	1.61E-15	0.74838175
180	7.40E-09	5.10E-08	2.52E-16	1.45E-15	1.61E-15	0.74838175
180.1	7.40E-09	5.10E-08	2.52E-16	1.45E-15	1.61E-15	0.74838175
180.2	7.40E-09	5.10E-08	2.52E-16	1.45E-15	1.61E-15	0.74838175
180.3	7.40E-09	5.10E-08	2.52E-16	1.45E-15	1.61E-15	0.74838175
180.4	7.35E-09	5.05E-08	2.49E-16	1.40E-15	1.62E-15	0.75054455
180.5	7.32E-09	5.11E-08	2.51E-16	1.38E-15	1.64E-15	0.74656101
180.6	7.32E-09	5.11E-08	2.51E-16	1.38E-15	1.64E-15	0.74656101
180.7	7.32E-09	5.11E-08	2.51E-16	1.38E-15	1.64E-15	0.74656101
180.8	7.32E-09	5.06E-08	2.43E-16	1.32E-15	1.66E-15	0.74675696
180.9	7.32E-09	5.06E-08	2.43E-16	1.32E-15	1.66E-15	0.74675696
181	7.32E-09	5.06E-08	2.43E-16	1.32E-15	1.66E-15	0.74675696
181.1	7.32E-09	5.06E-08	2.43E-16	1.32E-15	1.66E-15	0.74675696
181.2	7.32E-09	5.06E-08	2.43E-16	1.32E-15	1.66E-15	0.74675696
181.3	7.32E-09	5.06E-08	2.43E-16	1.32E-15	1.66E-15	0.74675696
181.4	7.32E-09	5.06E-08	2.43E-16	1.32E-15	1.66E-15	0.74675696
181.5	7.32E-09	5.06E-08	2.43E-16	1.32E-15	1.66E-15	0.74675696
181.6	7.32E-09	5.06E-08	2.43E-16	1.32E-15	1.66E-15	0.74675696
181.7	7.32E-09	5.06E-08	2.43E-16	1.32E-15	1.66E-15	0.74675696
181.8	7.32E-09	5.06E-08	2.43E-16	1.32E-15	1.66E-15	0.74675696
181.9	7.32E-09	5.06E-08	2.43E-16	1.32E-15	1.66E-15	0.74675696
182	7.32E-09	5.06E-08	2.43E-16	1.32E-15	1.66E-15	0.74675696
182.1	7.32E-09	5.06E-08	2.43E-16	1.32E-15	1.66E-15	0.74675696
182.2	7.32E-09	5.06E-08	2.43E-16	1.32E-15	1.66E-15	0.74675696
182.3	7.32E-09	5.06E-08	2.43E-16	1.32E-15	1.66E-15	0.74675696
182.4	7.32E-09	5.06E-08	2.43E-16	1.32E-15	1.66E-15	0.74675696
182.5	7.32E-09	5.06E-08	2.43E-16	1.32E-15	1.66E-15	0.74675696
182.6	7.32E-09	5.06E-08	2.43E-16	1.32E-15	1.66E-15	0.74675696
182.7	7.32E-09	5.06E-08	2.43E-16	1.32E-15	1.66E-15	0.74675696
182.8	7.32E-09	5.06E-08	2.43E-16	1.32E-15	1.66E-15	0.74675696
182.9	7.32E-09	5.06E-08	2.43E-16	1.32E-15	1.66E-15	0.74675696
183	7.32E-09	5.06E-08	2.43E-16	1.32E-15	1.66E-15	0.74675696
183.1	7.32E-09	5.06E-08	2.43E-16	1.32E-15	1.66E-15	0.74675696
195	9.01E-09	4.90E-08	2.56E-16	1.32E-15	1.78E-15	0.73620621
195.1	9.01E-09	4.90E-08	2.56E-16	1.32E-15	1.78E-15	0.73620621
195.2	9.01E-09	4.90E-08	2.56E-16	1.32E-15	1.78E-15	0.73620621
195.3	9.01E-09	4.90E-08	2.56E-16	1.32E-15	1.78E-15	0.73620621
195.4	9.01E-09	4.90E-08	2.56E-16	1.32E-15	1.78E-15	0.73620621
195.5	9.01E-09	4.90E-08	2.56E-16	1.32E-15	1.78E-15	0.73620621
195.6	9.01E-09	4.90E-08	2.56E-16	1.32E-15	1.78E-15	0.73620621
195.7	9.01E-09	4.90E-08	2.56E-16	1.32E-15	1.78E-15	0.73620621
195.8	9.01E-09	4.90E-08	2.56E-16	1.32E-15	1.78E-15	0.73620621
195.9	9.29E-09	4.88E-08	2.55E-16	1.29E-15	1.76E-15	0.73224175
196	9.29E-09	4.88E-08	2.55E-16	1.29E-15	1.76E-15	0.73224175
196.1	9.29E-09	4.88E-08	2.55E-16	1.29E-15	1.76E-15	0.73224175
196.2	9.29E-09	4.88E-08	2.55E-16	1.29E-15	1.76E-15	0.73224175
196.3	9.29E-09	4.88E-08	2.55E-16	1.29E-15	1.76E-15	0.73224175
196.4	9.29E-09	4.88E-08	2.55E-16	1.29E-15	1.76E-15	0.73224175
196.5	9.29E-09	4.88E-08	2.55E-16	1.29E-15	1.76E-15	0.73224175
196.6	9.29E-09	4.88E-08	2.55E-16	1.29E-15	1.76E-15	0.73224175
196.7	9.29E-09	4.88E-08	2.55E-16	1.29E-15	1.76E-15	0.73224175
196.8	9.29E-09	4.88E-08	2.55E-16	1.29E-15	1.76E-15	0.73224175
196.9	9.29E-09	4.88E-08	2.55E-16	1.29E-15	1.76E-15	0.73224175
197	9.29E-09	4.88E-08	2.55E-16	1.29E-15	1.76E-15	0.73224175
197.1	9.29E-09	4.88E-08	2.55E-16	1.29E-15	1.76E-15	0.73224175
197.2	9.29E-09	4.88E-08	2.55E-16	1.29E-15	1.76E-15	0.73224175
197.3	9.29E-09	4.88E-08	2.55E-16	1.29E-15	1.76E-15	0.73224175
197.4	9.29E-09	4.88E-08	2.55E-16	1.29E-15	1.76E-15	0.73224175
197.5	9.29E-09	4.88E-08	2.55E-16	1.29E-15	1.76E-15	0.73224175
197.6	9.29E-09	4.88E-08	2.55E-16	1.29E-15	1.76E-15	0.73224175
197.7	9.29E-09	4.88E-08	2.55E-16	1.29E-15	1.76E-15	0.73224175
197.8	9.29E-09	4.88E-08	2.55E-16	1.29E-15	1.76E-15	0.73224175
197.9	9.38E-09	4.84E-08	2.57E-16	1.30E-15	1.81E-15	0.73415867
198	9.38E-09	4.84E-08	2.57E-16	1.30E-15	1.81E-15	0.73415867
198.1	9.38E-09	4.84E-08	2.57E-16	1.30E-15	1.81E-15	0.73415867
210	9.78E-09	4.75E-08	2.59E-16	1.22E-15	1.88E-15	0.7283551
210.1	9.78E-09	4.75E-08	2.59E-16	1.22E-15	1.88E-15	0.7283551
210.2	9.78E-09	4.75E-08	2.59E-16	1.22E-15	1.88E-15	0.7283551
210.3	9.78E-09	4.75E-08	2.59E-16	1.22E-15	1.88E-15	0.7283551
210.4	9.78E-09	4.75E-08	2.59E-16	1.22E-15	1.88E-15	0.7283551
210.5	9.78E-09	4.75E-08	2.59E-16	1.22E-15	1.88E-15	0.7283551
210.6	9.78E-09	4.75E-08	2.59E-16	1.22E-15	1.88E-15	0.7283551
210.7	9.78E-09	4.75E-08	2.59E-16	1.22E-15	1.88E-15	0.7283551
210.8	9.77E-09	4.70E-08	2.58E-16	1.21E-15	1.87E-15	0.72970737
210.9	9.77E-09	4.70E-08	2.58E-16	1.21E-15	1.87E-15	0.72970737
211	9.77E-09	4.70E-08	2.58E-16	1.21E-15	1.87E-15	0.72970737
211.1	9.77E-09	4.70E-08	2.58E-16	1.21E-15	1.87E-15	0.72970737
211.2	9.77E-09	4.70E-08	2.58E-16	1.21E-15	1.87E-15	0.72970737
211.3	9.77E-09	4.70E-08	2.58E-16	1.21E-15	1.87E-15	0.72970737
211.4	9.77E-09	4.70E-08	2.58E-16	1.21E-15	1.87E-15	0.72970737
211.5	9.77E-09	4.70E-08	2.58E-16	1.21E-15	1.87E-15	0.72970737
211.6	9.77E-09	4.70E-08	2.58E-16	1.21E-15	1.87E-15	0.72970737
211.7	9.77E-09	4.70E-08	2.58E-16	1.21E-15	1.87E-15	0.72970737
211.8	9.77E-09	4.70E-08	2.58E-16	1.21E-15	1.87E-15	0.72970737
211.9	9.77E-09	4.70E-08	2.58E-16	1.21E-15	1.87E-15	0.72970737
212	1.00E-08	4.68E-08	2.54E-16	1.17E-15	1.86E-15	0.72867463
212.1	1.00E-08	4.68E-08	2.54E-16	1.17E-15	1.86E-15	0.72867463
212.2	1.00E-08	4.68E-08	2.54E-16	1.17E-15	1.86E-15	0.72867463
212.3	1.00E-08	4.68E-08	2.54E-16	1.17E-15	1.86E-15	0.72867463
212.4	1.00E-08	4.68E-08	2.54E-16	1.17E-15	1.86E-15	0.72867463
212.5	1.00E-08	4.68E-08	2.54E-16	1.17E-15	1.86E-15	0.72867463
212.6	1.00E-08	4.68E-08	2.54E-16	1.17E-15	1.86E-15	0.72867463
212.7	1.00E-08	4.68E-08	2.54E-16	1.17E-15	1.86E-15	0.72867463
212.8	1.00E-08	4.68E-08	2.54E-16	1.17E-15	1.86E-15	0.72867463
212.9	1.00E-08	4.68E-08	2.54E-16	1.17E-15	1.86E-15	0.72867463
213	1.00E-08	4.68E-08	2.54E-16	1.17E-15	1.86E-15	0.72867463
213.1	9.95E-09	4.70E-08	2.52E-16	1.13E-15	1.87E-15	0.72500555
225	9.83E-09	4.69E-08	2.46E-16	1.09E-15	1.89E-15	0.72941782
225.1	9.83E-09	4.69E-08	2.46E-16	1.09E-15	1.89E-15	0.72941782
225.2	9.83E-09	4.69E-08	2.46E-16	1.09E-15	1.89E-15	0.72941782
225.3	9.83E-09	4.69E-08	2.46E-16	1.09E-15	1.89E-15	0.72941782
225.4	9.83E-09	4.69E-08	2.46E-16	1.09E-15	1.89E-15	0.72941782
225.5	9.83E-09	4.69E-08	2.46E-16	1.09E-15	1.89E-15	0.72941782
225.6	9.83E-09	4.69E-08	2.46E-16	1.09E-15	1.89E-15	0.72941782
225.7	9.83E-09	4.69E-08	2.46E-16	1.09E-15	1.89E-15	0.72941782
225.8	9.83E-09	4.69E-08	2.46E-16	1.09E-15	1.89E-15	0.72941782
225.9	9.83E-09	4.69E-08	2.46E-16	1.09E-15	1.89E-15	0.72941782
226	9.83E-09	4.69E-08	2.46E-16	1.09E-15	1.89E-15	0.72941782
226.1	9.83E-09	4.69E-08	2.46E-16	1.09E-15	1.89E-15	0.72941782
226.2	9.83E-09	4.69E-08	2.46E-16	1.09E-15	1.89E-15	0.72941782
226.3	9.83E-09	4.69E-08	2.46E-16	1.09E-15	1.89E-15	0.72941782
226.4	9.83E-09	4.69E-08	2.46E-16	1.09E-15	1.89E-15	0.72941782
226.5	9.83E-09	4.69E-08	2.46E-16	1.09E-15	1.89E-15	0.72941782
226.6	9.83E-09	4.69E-08	2.46E-16	1.09E-15	1.89E-15	0.72941782
226.7	9.83E-09	4.69E-08	2.46E-16	1.09E-15	1.89E-15	0.72941782
226.8	9.83E-09	4.69E-08	2.46E-16	1.09E-15	1.89E-15	0.72941782
226.9	9.83E-09	4.69E-08	2.46E-16	1.09E-15	1.89E-15	0.72941782
227	9.83E-09	4.69E-08	2.46E-16	1.09E-15	1.89E-15	0.72941782
227.1	9.83E-09	4.69E-08	2.46E-16	1.09E-15	1.89E-15	0.72941782
227.2	9.83E-09	4.69E-08	2.46E-16	1.09E-15	1.89E-15	0.72941782
227.3	9.83E-09	4.69E-08	2.46E-16	1.09E-15	1.89E-15	0.72941782
227.4	9.83E-09	4.69E-08	2.46E-16	1.09E-15	1.89E-15	0.72941782
227.5	9.83E-09	4.69E-08	2.46E-16	1.09E-15	1.89E-15	0.72941782
227.6	9.83E-09	4.69E-08	2.46E-16	1.09E-15	1.89E-15	0.72941782
227.7	9.83E-09	4.69E-08	2.46E-16	1.09E-15	1.89E-15	0.72941782
227.8	9.83E-09	4.69E-08	2.46E-16	1.09E-15	1.89E-15	0.72941782
227.9	9.83E-09	4.69E-08	2.46E-16	1.09E-15	1.89E-15	0.72941782
228	9.83E-09	4.69E-08	2.46E-16	1.09E-15	1.89E-15	0.72941782
228.1	9.83E-09	4.69E-08	2.46E-16	1.09E-15	1.89E-15	0.72941782

183.2	7.32E-09	5.06E-08	2.43E-16	1.32E-15	1.66E-15	0.74675696
183.3	7.56E-09	5.07E-08	2.47E-16	1.31E-15	1.67E-15	0.7424925
183.4	7.56E-09	5.07E-08	2.47E-16	1.31E-15	1.67E-15	0.7424925
183.5	7.56E-09	5.07E-08	2.47E-16	1.31E-15	1.67E-15	0.7424925
183.6	7.56E-09	5.07E-08	2.47E-16	1.31E-15	1.67E-15	0.7424925
183.7	7.50E-09	5.07E-08	2.42E-16	1.30E-15	1.70E-15	0.74141746
183.8	7.50E-09	5.07E-08	2.42E-16	1.30E-15	1.70E-15	0.74141746
183.9	7.50E-09	5.07E-08	2.42E-16	1.30E-15	1.70E-15	0.74141746
184	7.50E-09	5.07E-08	2.42E-16	1.30E-15	1.70E-15	0.74141746
184.1	7.50E-09	5.07E-08	2.42E-16	1.30E-15	1.70E-15	0.74141746
184.2	7.50E-09	5.07E-08	2.42E-16	1.30E-15	1.70E-15	0.74141746
184.3	7.50E-09	5.07E-08	2.42E-16	1.30E-15	1.70E-15	0.74141746
184.4	7.50E-09	5.07E-08	2.42E-16	1.30E-15	1.70E-15	0.74141746
184.5	7.50E-09	5.07E-08	2.42E-16	1.30E-15	1.70E-15	0.74141746
184.6	7.50E-09	5.07E-08	2.42E-16	1.30E-15	1.70E-15	0.74141746
184.7	7.50E-09	5.07E-08	2.42E-16	1.30E-15	1.70E-15	0.74141746
184.8	7.50E-09	5.07E-08	2.42E-16	1.30E-15	1.70E-15	0.74141746
184.9	7.50E-09	5.07E-08	2.42E-16	1.30E-15	1.70E-15	0.74141746
185	7.50E-09	5.07E-08	2.42E-16	1.30E-15	1.70E-15	0.74141746
185.1	7.50E-09	5.07E-08	2.42E-16	1.30E-15	1.70E-15	0.74141746
185.2	7.78E-09	5.02E-08	2.44E-16	1.32E-15	1.68E-15	0.74113152
185.3	7.78E-09	5.02E-08	2.44E-16	1.32E-15	1.68E-15	0.74113152
185.4	7.78E-09	5.02E-08	2.44E-16	1.32E-15	1.68E-15	0.74113152
185.5	7.78E-09	5.02E-08	2.44E-16	1.32E-15	1.68E-15	0.74113152
185.6	7.78E-09	5.02E-08	2.44E-16	1.32E-15	1.68E-15	0.74113152
185.7	7.78E-09	5.02E-08	2.44E-16	1.32E-15	1.68E-15	0.74113152
185.8	7.78E-09	5.02E-08	2.44E-16	1.32E-15	1.68E-15	0.74113152
185.9	7.78E-09	5.02E-08	2.44E-16	1.32E-15	1.68E-15	0.74113152
186	7.78E-09	5.02E-08	2.44E-16	1.32E-15	1.68E-15	0.74113152
186.1	7.78E-09	5.02E-08	2.44E-16	1.32E-15	1.68E-15	0.74113152
186.2	7.78E-09	5.02E-08	2.44E-16	1.32E-15	1.68E-15	0.74113152
186.3	7.78E-09	5.02E-08	2.44E-16	1.32E-15	1.68E-15	0.74113152
186.4	7.80E-09	5.05E-08	2.55E-16	1.33E-15	1.74E-15	0.74556823
186.5	7.80E-09	5.05E-08	2.55E-16	1.33E-15	1.74E-15	0.74556823
186.6	8.08E-09	5.06E-08	2.56E-16	1.33E-15	1.76E-15	0.74324888
186.7	8.08E-09	5.06E-08	2.56E-16	1.33E-15	1.76E-15	0.74324888
186.8	8.08E-09	5.06E-08	2.56E-16	1.33E-15	1.76E-15	0.74324888
186.9	8.08E-09	5.06E-08	2.56E-16	1.33E-15	1.76E-15	0.74324888
187	8.08E-09	5.06E-08	2.56E-16	1.33E-15	1.76E-15	0.74324888
187.1	8.08E-09	5.06E-08	2.56E-16	1.33E-15	1.76E-15	0.74324888
187.2	8.08E-09	5.06E-08	2.56E-16	1.33E-15	1.76E-15	0.74324888
187.3	8.08E-09	5.06E-08	2.56E-16	1.33E-15	1.76E-15	0.74324888
187.4	8.08E-09	5.06E-08	2.56E-16	1.33E-15	1.76E-15	0.74324888
187.5	8.08E-09	5.06E-08	2.56E-16	1.33E-15	1.76E-15	0.74324888
187.6	8.08E-09	5.06E-08	2.56E-16	1.33E-15	1.76E-15	0.74324888
187.7	8.08E-09	5.06E-08	2.56E-16	1.33E-15	1.76E-15	0.74324888
187.8	8.08E-09	5.06E-08	2.56E-16	1.33E-15	1.76E-15	0.74324888
187.9	8.08E-09	5.06E-08	2.56E-16	1.33E-15	1.76E-15	0.74324888
188	8.08E-09	5.06E-08	2.56E-16	1.33E-15	1.76E-15	0.74324888
188.1	8.08E-09	5.06E-08	2.56E-16	1.33E-15	1.76E-15	0.74324888
188.2	8.06E-09	4.97E-08	2.45E-16	1.32E-15	1.74E-15	0.74343986
188.3	8.06E-09	4.97E-08	2.45E-16	1.32E-15	1.74E-15	0.74343986
188.4	8.06E-09	4.97E-08	2.45E-16	1.32E-15	1.74E-15	0.74343986
188.5	8.06E-09	4.97E-08	2.45E-16	1.32E-15	1.74E-15	0.74343986
188.6	8.05E-09	5.03E-08	2.45E-16	1.31E-15	1.77E-15	0.74140839
188.7	8.05E-09	5.03E-08	2.45E-16	1.31E-15	1.77E-15	0.74140839
188.8	8.05E-09	5.03E-08	2.45E-16	1.31E-15	1.77E-15	0.74140839
188.9	8.05E-09	5.03E-08	2.45E-16	1.31E-15	1.77E-15	0.74140839
189	8.05E-09	5.03E-08	2.45E-16	1.31E-15	1.77E-15	0.74140839
189.1	8.24E-09	4.98E-08	2.44E-16	1.31E-15	1.72E-15	0.74195736
189.2	8.24E-09	4.98E-08	2.44E-16	1.31E-15	1.72E-15	0.74195736
189.3	8.24E-09	4.98E-08	2.44E-16	1.31E-15	1.72E-15	0.74195736
189.4	8.24E-09	4.98E-08	2.44E-16	1.31E-15	1.72E-15	0.74195736
189.5	8.24E-09	4.98E-08	2.44E-16	1.31E-15	1.72E-15	0.74195736
189.6	8.24E-09	4.98E-08	2.44E-16	1.31E-15	1.72E-15	0.74195736
189.7	8.24E-09	4.98E-08	2.44E-16	1.31E-15	1.72E-15	0.74195736
189.8	8.24E-09	4.98E-08	2.44E-16	1.31E-15	1.72E-15	0.74195736
189.9	8.24E-09	4.98E-08	2.44E-16	1.31E-15	1.72E-15	0.74195736
190	8.24E-09	4.98E-08	2.44E-16	1.31E-15	1.72E-15	0.74195736
190.1	8.24E-09	4.98E-08	2.44E-16	1.31E-15	1.72E-15	0.74195736
190.2	8.24E-09	4.98E-08	2.44E-16	1.31E-15	1.72E-15	0.74195736
190.3	8.24E-09	4.98E-08	2.44E-16	1.31E-15	1.72E-15	0.74195736
190.4	8.24E-09	4.98E-08	2.44E-16	1.31E-15	1.72E-15	0.74195736
190.5	8.24E-09	4.98E-08	2.44E-16	1.31E-15	1.72E-15	0.74195736
190.6	8.24E-09	4.98E-08	2.44E-16	1.31E-15	1.72E-15	0.74195736
190.7	8.24E-09	4.98E-08	2.44E-16	1.31E-15	1.72E-15	0.74195736
190.8	8.24E-09	4.98E-08	2.44E-16	1.31E-15	1.72E-15	0.74195736
190.9	8.24E-09	4.98E-08	2.44E-16	1.31E-15	1.72E-15	0.74195736
191	8.24E-09	4.98E-08	2.44E-16	1.31E-15	1.72E-15	0.74195736
191.1	8.31E-09	5.00E-08	2.45E-16	1.33E-15	1.71E-15	0.74413001
191.2	8.31E-09	5.00E-08	2.45E-16	1.33E-15	1.71E-15	0.74413001
191.3	8.74E-09	4.97E-08	2.54E-16	1.30E-15	1.73E-15	0.73928775
191.4	8.74E-09	4.97E-08	2.54E-16	1.30E-15	1.73E-15	0.73928775
191.5	8.74E-09	4.97E-08	2.54E-16	1.30E-15	1.73E-15	0.73928775
191.6	8.74E-09	4.97E-08	2.54E-16	1.30E-15	1.73E-15	0.73928775
191.7	8.74E-09	4.97E-08	2.54E-16	1.30E-15	1.73E-15	0.73928775
191.8	8.74E-09	4.97E-08	2.54E-16	1.30E-15	1.73E-15	0.73928775
191.9	8.74E-09	4.97E-08	2.54E-16	1.30E-15	1.73E-15	0.73928775
192	8.74E-09	4.97E-08	2.54E-16	1.30E-15	1.73E-15	0.73928775
192.1	8.74E-09	4.97E-08	2.54E-16	1.30E-15	1.73E-15	0.73928775
192.2	8.74E-09	4.97E-08	2.54E-16	1.30E-15	1.73E-15	0.73928775
192.3	8.74E-09	4.97E-08	2.54E-16	1.30E-15	1.73E-15	0.73928775
192.4	8.74E-09	4.97E-08	2.54E-16	1.30E-15	1.73E-15	0.73928775
192.5	8.74E-09	4.97E-08	2.54E-16	1.30E-15	1.73E-15	0.73928775
192.6	8.74E-09	4.97E-08	2.54E-16	1.30E-15	1.73E-15	0.73928775
192.7	8.74E-09	4.97E-08	2.54E-16	1.30E-15	1.73E-15	0.73928775
192.8	8.74E-09	4.97E-08	2.54E-16	1.30E-15	1.73E-15	0.73928775
192.9	8.74E-09	4.97E-08	2.54E-16	1.30E-15	1.73E-15	0.73928775
193	8.74E-09	4.97E-08	2.54E-16	1.30E-15	1.73E-15	0.73928775
193.1	8.65E-09	4.93E-08	2.52E-16	1.29E-15	1.80E-15	0.73719415
193.2	8.65E-09	4.93E-08	2.52E-16	1.29E-15	1.80E-15	0.73719415
193.3	8.65E-09	4.93E-08	2.52E-16	1.29E-15	1.80E-15	0.73719415
193.4	8.65E-09	4.93E-08	2.52E-16	1.29E-15	1.80E-15	0.73719415
193.5	8.65E-09	4.93E-08	2.52E-16	1.29E-15	1.80E-15	0.73719415
193.6	8.65E-09	4.93E-08	2.52E-16	1.29E-15	1.80E-15	0.73719415
193.7	8.65E-09	4.93E-08	2.52E-16	1.29E-15	1.80E-15	0.73719415
193.8	8.65E-09	4.93E-08	2.52E-16	1.29E-15	1.80E-15	0.73719415
193.9	9.00E-09	4.89E-08	2.54E-16	1.30E-15	1.80E-15	0.7323489
194	9.00E-09	4.89E-08	2.54E-16	1.30E-15	1.80E-15	0.7323489
194.1	9.00E-09	4.89E-08	2.54E-16	1.30E-15	1.80E-15	0.7323489
194.2	9.00E-09	4.89E-08	2.54E-16	1.30E-15	1.80E-15	0.7323489
194.3	9.00E-09	4.89E-08	2.54E-16	1.30E-15	1.80E-15	0.7323489
194.4	9.00E-09	4.89E-08	2.54E-16	1.30E-15	1.80E-15	0.7323489
194.5	9.00E-09	4.89E-08	2.54E-16	1.30E-15	1.80E-15	0.7323489
194.6	9.00E-09	4.89E-08	2.54E-16	1.30E-15	1.80E-15	0.7323489
194.7	9.00E-09	4.89E-08	2.54E-16	1.30E-15	1.80E-15	0.7323489
194.8	9.00E-09	4.89E-08	2.54E-16	1.30E-15	1.80E-15	0.7323489
194.9	9.00E-09	4.89E-08	2.54E-16	1.30E-15	1.80E-15	0.7323489
198.2	9.38E-09	4.84E-08	2.57E-16	1.30E-15	1.81E-15	0.73415867
198.3	9.38E-09	4.84E-08	2.57E-16	1.30E-15	1.81E-15	0.73415867
198.4	9.38E-09	4.84E-08	2.57E-16	1.30E-15	1.81E-15	0.73415867
198.5	9.38E-09	4.84E-08	2.57E-16	1.30E-15	1.81E-15	0.73415867
198.6	9.38E-09	4.84E-08	2.57E-16	1.30E-15	1.81E-15	0.73415867
198.7	9.38E-09	4.84E-08	2.57E-16	1.30E-15	1.81E-15	0.73415867
198.8	9.38E-09	4.84E-08	2.57E-16	1.30E-15	1.81E-15	0.73415867
198.9	9.38E-09	4.84E-08	2.57E-16	1.30E-15	1.81E-15	0.73415867
199	9.47E-09	4.81E-08	2.52E-16	1.30E-15	1.77E-15	0.73785647
199.1	9.47E-09	4.81E-08	2.52E-16	1.30E-15	1.77E-15	0.73785647
199.2	9.47E-09	4.81E-08	2.52E-16	1.30E-15	1.77E-15	0.73785647
199.3	9.47E-09	4.81E-08	2.52E-16	1.30E-15	1.77E-15	0.73785647
199.4	9.47E-09	4.81E-08	2.52E-16	1.30E-15	1.77E-15	0.73785647
199.5	9.47E-09	4.81E-08	2.52E-16	1.30E-15	1.77E-15	0.73785647
199.6	9.47E-09	4.81E-08	2.52E-16	1.30E-15	1.77E-15	0.73785647
199.7	9.47E-09	4.81E-08	2.52E-16	1.30E-15	1.77E-15	0.73785647
199.8	9.54E-09	4.78E-08	2.57E-16	1.31E-15	1.83E-15	0.73616656
199.9	9.54E-09	4.78E-08	2.57E-16	1.31E-15	1.83E-15	0.73616656
200	9.54E-09	4.78E-08	2.57E-16	1.31E-15	1.83E-15	0.73616656
200.1	9.54E-09	4.78E-08	2.57E-16	1.31E-15	1.83E-15	0.73616656
200.2	9.54E-09	4.78E-08	2.57E-16	1.31E-15	1.83E-15	0.73616656
200.3	9.54E-09	4.78E-08	2.57E-16	1.31E-15	1.83E-15	0.73616656
200.4	9.54E-09	4.78E-08	2.57E-16	1.31E-15	1.83E-15	0.73616656
200.5	9.54E-09	4.78E-08	2.57E-16	1.31E-15	1.83E-15	0.73616656
200.6	9.54E-09	4.78E-08	2.57E-16	1.31E-15	1.83E-15	0.73616656
200.7	9.53E-09	4.79E-08	2.61E-16	1.29E-15	1.86E-15	0.73347949
200.8	9.53E-09	4.79E-08	2.61E-16	1.29E-15	1.86E-15	0.73347949
200.9	9.53E-09	4.79E-08	2.61E-16	1.29E-15	1.86E-15	0.73347949
201	9.53E-09	4.79E-08	2.61E-16	1.29E-15	1.86E-15	0.73347949
201.1	9.53E-09	4.79E-08	2.61E-16	1.29E-15	1.86E-15	0.73347949
201.2	9.53E-09	4.79E-08	2.61E-16	1.29E-15	1.86E-15	0.73347949
201.3	9.53E-09	4.79E-08	2.61E-16	1.29E-15	1.86E-15	0.73347949
201.4	9.53E-09	4.79E-08	2.61E-16	1.29E-15	1.86E-15	0.73347949
201.5	9.53E-09	4.79E-08	2.61E-16	1.29E-15	1.86E-15	0.73347949
201.6	9.53E-09	4.79E-08	2.61E-16	1.29E-15	1.86E-15	0.73347949
201.7	9.53E-09	4.79E-08	2.61E-16	1.29E-15	1.86E-15	0.73347949
201.8	9.53E-09	4.79E-08	2.61E-16	1.29E-15	1.86E-15	0.73347949
201.9	9.53E-09	4.79E-08	2.61E-16	1.29E-15	1.86E-15	0.73347949
202	9.53E-09	4.79E-08	2.61E-16	1.29E-15	1.86E-15	0.73347949
202.1	9.53E-09	4.79E-08	2.61E-16	1.29E-15	1.86E-15	0.73347949
202.2	9.53E-09	4.79E-08	2.61E-16	1.29E-15	1.86E-15	0.73347949
202.3	9.53E-09	4.79E-08	2.61E-16	1.29E-15	1.86E-15	0.73347949
202.4	9.53E-09	4.79E-08	2.61E-16	1.29E-15	1.86E-15	0.73347949
202.5	9.53E-09	4.79E-08	2.61E-16	1.29E-15	1.86E-15	0.73347949
202.6	9.53E-09	4.79E-08	2.61E-16	1.29E-15	1.86E-15	0.73347949
202.7	9.53E-09	4.79E-08	2.61E-16	1.29E-15	1.86E-15	0.73347949
202.8	9.53E-09	4.79E-08	2.61E-16	1.29E-15	1.86E-15	0.73347949
202.9	9.53E-09	4.79E-08	2.61E-16	1.29E-15	1.86E-15	0.73347949
203	9.53E-09	4.79E-08	2.61E-16	1.29E-15	1.86E-15	0.73347949
203.1	9.53E-09	4.79E-08	2.61E-16	1.29E-15	1.86E-15	0.73347949
203.2	9.53E-09	4.79E-08	2.61E-16	1.29E-15	1.86E-15	0.73347949
203.3	9.53E-09	4.79E-08	2.61E-16	1.29E-15	1.86E-15	0.73347949
203.4	9.53E-09	4.79E-08	2.61E-16	1.29E-15	1.86E-15	0.73347949
203.5	9.53E-09	4.79E-08	2.61E-16	1.29E-15	1.86E-15	0.73347949
203.6	9.53E-09	4.79E-08	2.61E-16	1.29E-15	1.86E-15	0.73347949
203.7	9.53E-09	4.79E-08	2.61E-16	1.29E-15	1.86E-15	0.73347949
203.8	9.53E-09	4.79E-08	2.61E-16	1.29E-15	1.86E-15	0.73347949
203.9	9.53E-09	4.79E-08	2.61E-16	1.29E-15	1.86E-15	0.73347949
204	9.53E-09	4.79E-08	2.61E-16	1.29E-15	1.86E-15	0.73347949
204.1	9.53E-09	4.79E-08	2.61E-16	1.29E-15	1.86E-15	0.73347949
204.2	9.53E-09	4.79E-08	2.61E-16	1.29E-15	1.86E-15	0.73347949
204.3	9.53E-09	4.79E-08	2.61E-16	1.29E-15	1.86E-15	0.73347949
204.4	9.74E-09	4.75E-08	2.58E-16	1.23E-15	1.86E-15	0.73290598
204.5	9.74E-09	4.75E-08	2.58E-16	1.23E-15	1.86E-15	0.73290598
204.6	9.74E-09	4.75E-08	2.58E-16	1.23E-15	1.86E-15	0.73290598
204.7	9.74E-09	4.75E-08	2.58E-16	1.23E-15	1.86E-15	0.73290598
204.8	9.74E-09	4.75E-08	2.58E-16	1.23E-15	1.86E-15	0.73290598
204.9	9.74E-09	4.75E-08	2.58E-16	1.23E-15	1.86E-15	0.73290598
205	9.74E-09	4.75E-08	2.58E-16	1.23E-15	1.86E-15	0.73290598
205.1	9.74E-09	4.75E-08	2.58E-16	1.23E-15	1.86E-15	0.73290598
205.2	9.74E-09	4.75E-08	2.58E-16	1.23E-15	1.86E-15	0.73290598
205.3	9.74E-09	4.75E-08	2.58E-16	1.23E-15	1.86E-15	0.73290598
205.4	9.74E-09	4.75E-08	2.58E-16	1.23E-15	1.86E-15	0.73290598
205.5	9.74E-09	4.75E-08	2.58E-16	1.23E-15	1.86E-15	0.73290598
205.6	9.74E-09	4.75E-08	2.58E-16	1.23E-15	1.86E-15	0.73290598
205.7	9.74E-09	4.75E-08	2.58E-16	1.23E-15	1.86E-15	0.73290598
205.8	9.74E-09	4.75E-08	2.58E-16	1.23E-15	1.86E-15	0.73290598
205.9	9.74E-09	4.75E-08	2.58E-16	1.23E-15	1.86E-15	0.73290598
206	9.74E-09	4.75E-08	2.58E-16	1.23E-15	1.86E-15	0.73290598
206.1	9.74E-09	4.75E-08	2.58E-16	1.23E-15	1.86E-15	0.73290598
206.2	9.74E-09	4.75E-08	2.58E-16	1.23E-15	1.86E-15	0.73290598
206.3	9.74E-09	4.75E-08	2.58E-16	1.23E-15	1.86E-15	0.73290598
206.4	9.74E-09	4.75E-08	2.58E-16	1.23E-15	1.86E-15	0.73290598
206.5	9.74E-09	4.75E-08	2.58E-16	1.23E-15	1.86E-15	0.73290598
206.6	9.74E-09	4.75E-08	2.58E-16	1.23E-15	1.86E-15	0.73290598
206.7	9.74E-09	4.75E-08	2.58E-16	1.23E-15	1.86E-15	0.73290598
206.8	9.74E-09	4.75E-08	2.58E-16	1.23E-15	1.86E-15	0.73290598
206.9	9.74E-09	4.75E-08	2.58E-16	1.23E-15	1.86E-15	0.73290598
207	9.74E-09	4.75E-08	2.58E-16	1.23E-15	1.86E-15	0.73290598
207.1	9.74E-09	4.75E-08	2.58E-16	1.23E-15	1.86E-15	0.73290598
207.2	9.74E-09	4.75E-08	2.58E-16	1.23E-15	1.86E-15	0.73290598
207.3	9.74E-09	4.75E-08	2.58E-16	1.23E-15	1.86E-15	0.73290598
207.4	9.74E-09	4.75E-08	2.58E-16	1.23E-15	1.86E-15	0.73290598
207.5	9.78E-09	4.75E-08	2.59E-16	1.22E-15	1.88E-15	0.7283551
207.6	9.78E-09	4.75E-08	2.59E-16	1.22E-15	1.88E-15	0.7283551
207.7	9.78E-09	4.75E-08	2.59E-16	1.22E-15	1.88E-15	0.7283551
207.8	9.78E-09	4.75E-08	2.59E-16	1.22E-15	1.88E-15	0.7283551
207.9	9.78E-09	4.75E-08	2.59E-16	1.22E-15	1.88E-15	0.7283551
208	9.78E-09	4.75E-08	2.59E-16	1.22E-15	1.88E-15	0.7283551
208.1	9.78E-09	4.75E-08	2.59E-16	1.22E-15	1.88E-15	0.7283551
208.2	9.78E-09	4.75E-08	2.59E-16	1.22E-15	1.88E-15	0.7283551
208.3	9.78E-09	4.75E-08	2.59E-16	1.22E-15	1.88E-15	0.7283551
208.4	9.78E-09	4.75E-08	2.59E-16	1.22E-15	1.88E-15	0.7283551
208.5	9.78E-09	4.75E-08	2.59E-16	1.22E-15	1.88E-15	0.7283551
208.6	9.78E-09	4.75E-08	2.59E-16	1.22E-15	1.88E-15	0.7283551
208.7	9.78E-09	4.75E-08	2.59E-16	1.22E-15	1.88E-15	0.7283551
208.8	9.78E-09	4.75E-08	2.59E-16	1.22E-15	1.88E-15	0.7283551
208.9	9.78E-09	4.75E-08	2.59E-16	1.22E-15	1.88E-15	0.7283551
209	9.78E-09	4.75E-08	2.59E-16	1.22E-15	1.88E-15	0.7283551
209.1	9.78E-09	4.75E-08	2.59E-16	1.22E-15	1.88E-15	0.7283551
209.2	9.78E-09	4.75E-08	2.59E-16	1.22E-15	1.88E-15	0.7283551
209.3	9.78E-09	4.75E-08	2.59E-16	1.22E-15	1.88E-15	0.7283551
209.4	9.78E-09	4.75E-08	2.59E-16	1.22E-15	1.88E-15	0.7283551
209.5	9.78E-09	4.75E-08	2.59E-16	1.22E-15	1.88E-15	0.7283551
209.6	9.78E-09	4.75E-08	2.59E-16	1.22E-15	1.88E-15	0.7283551
209.7	9.78E-09	4.75E-08	2.59E-16	1.22E-15	1.88E-15	0.7283551
209.8	9.78E-09	4.75E-08	2.59E-16	1.22E-15	1.88E-15	0.7283551
209.9	9.78E-09	4.75E-08	2.59E-16	1.22E-15	1.88E-15	0.7283551
213.2	9.95E-09	4.70E-08	2.52E-16	1.13E-15	1.87E-15	0.72500555
213.3	9.95E-09	4.70E-08	2.52E-16	1.13E-15	1.87E-15	0.72500555
213.4	9.95E-09	4.70E-08	2.52E-16	1.13E-15	1.87E-15	0.72500555
213.5	9.95E-09	4.70E-08	2.52E-16	1.13E-15	1.87E-15	0.72500555
213.6	9.95E-09	4.70E-08	2.52E-16	1.13E-15	1.87E-15	0.72500555
213.7	9.95E-09	4.70E-08	2.52E-16	1.13E-15	1.87E-15	0.72500555
213.8	9.95E-09	4.70E-08	2.52E-16	1.13E-15	1.87E-15	0.72500555
213.9	9.95E-09	4.70E-08	2.52E-16	1.13E-15	1.87E-15	0.72500555
214	9.95E-09	4.70E-08	2.52E-16	1.13E-15	1.87E-15	0.72500555
214.1	9.95E-09	4.70E-08	2.52E-16	1.13E-15	1.87E-15	0.72500555
214.2	9.95E-09	4.70E-08	2.52E-16	1.13E-15	1.87E-15	0.72500555
214.3	9.95E-09	4.70E-08	2.52E-16	1.13E-15	1.87E-15	0.72500555
214.4	9.95E-09	4.70E-08	2.52E-16	1.13E-15	1.87E-15	0.72500555
214.5	9.95E-09	4.70E-08	2.52E-16	1.13E-15	1.87E-15	0.72500555
214.6	9.95E-09	4.70E-08	2.52E-16	1.13E-15	1.87E-15	0.72500555
214.7	9.95E-09	4.70E-08	2.52E-16	1.13E-15	1.87E-15	0.72500555
214.8	9.95E-09	4.70E-08	2.52E-16	1.13E-15	1.87E-15	0.72500555
214.9	9.95E-09	4.70E-08	2.52E-16	1.13E-15	1.87E-15	0.72500555
215	9.95E-09	4.70E-08	2.52E-16	1.13E-15	1.87E-15	0.72500555
215.1	9.95E-09	4.70E-08	2.52E-16	1.13E-15	1.87E-15	0.72500555
215.2	9.95E-09	4.70E-08	2.52E-16	1.13E-15	1.87E-15	0.72500555
215.3	9.95E-09	4.70E-08	2.52E-16	1.13E-15	1.87E-15	0.72500555
215.4	9.95E-09	4.70E-08	2.52E-16	1.13E-15	1.87E-15	0.72500555
215.5	9.95E-09	4.70E-08	2.52E-16	1.13E-15	1.87E-15	0.72500555
215.6	9.95E-09	4.70E-08	2.52E-16	1.13E-15	1.87E-15	0.72500555
215.7	9.95E-09	4.70E-08	2.52E-16	1.13E-15	1.87E-15	0.72500555
215.8	9.95E-09	4.70E-08	2.52E-16	1.13E-15	1.87E-15	0.72500555
215.9	9.95E-09	4.70E-08	2.52E-16	1.13E-15	1.87E-15	0.72500555
216	9.95E-09	4.70E-08	2.52E-16	1.13E-15	1.87E-15	0.72500555
216.1	9.95E-09	4.70E-08	2.52E-16	1.13E-15	1.87E-15	0.72500555
216.2	9.95E-09	4.70E-08	2.52E-16	1.13E-15	1.87E-15	0.72500555
216.3	9.95E-09	4.70E-08	2.52E-16	1.13E-15	1.87E-15	0.72500555
216.4	9.95E-09	4.70E-08	2.52E-16	1.13E-15	1.87E-15	0.72500555
216.5	9.95E-09	4.70E-08	2.52E-16	1.13E-15	1.87E-15	0.72500555
216.6	9.95E-09	4.70E-08	2.52E-16	1.13E-15	1.87E-15	0.72500555
216.7	9.95E-09	4.70E-08	2.52E-16	1.13E-15	1.87E-15	0.72500555
216.8	9.95E-09	4.70E-08	2.52E-16	1.13E-15	1.87E-15	0.72500555
216.9	9.95E-09	4.70E-08	2.52E-16	1.13E-15	1.87E-15	0.72500555
217	9.95E-09	4.70E-08	2.52E-16	1.13E-15	1.87E-15	0.72500555
217.1	9.95E-09	4.70E-08	2.52E-16	1.13E-15	1.87E-15	0.72500555
217.2	9.95E-09	4.70E-08	2.52E-16	1.13E-15	1.87E-15	0.72500555
217.3	9.95E-09	4.70E-08	2.52E-16	1.13E-15	1.87E-15	0.72500555
217.4	9.95E-09	4.70E-08	2.52E-16	1.13E-15	1.87E-15	0.72500555
217.5	9.95E-09	4.70E-08	2.52E-16	1.13E-15	1.87E-15	0.72500555
217.6	9.95E-09	4.70E-08	2.52E-16	1.13E-15	1.87E-15	0.72500555
217.7	9.95E-09	4.70E-08	2.52E-16	1.13E-15	1.87E-15	0.72500555
217.8	9.95E-09	4.70E-08	2.52E-16	1.13E-15	1.87E-15	0.72500555
217.9	9.95E-09	4.70E-08	2.52E-16	1.13E-15	1.87E-15	0.72500555
218	9.95E-09	4.70E-08	2.52E-16	1.13E-15	1.87E-15	0.72500555
218.1	9.95E-09	4.70E-08	2.52E-16	1.13E-15	1.87E-15	0.72500555
218.2	9.95E-09	4.70E-08	2.52E-16	1.13E-15	1.87E-15	0.72500555
218.3	9.95E-09	4.70E-08	2.52E-16	1.13E-15	1.87E-15	0.72500555
218.4	9.95E-09	4.70E-08	2.52E-16	1.13E-15	1.87E-15	0.72500555
218.5	9.95E-09	4.70E-08	2.52E-16	1.13E-15	1.87E-15	0.72500555
218.6	9.95E-09	4.70E-08	2.52E-16	1.13E-15	1.87E-15	0.72500555
218.7	9.95E-09	4.70E-08	2.52E-16	1.13E-15	1.87E-15	0.72500555
218.8	9.95E-09	4.70E-08	2.52E-16	1.13E-15	1.87E-15	0.72500555
218.9	9.95E-09	4.70E-08	2.52E-16	1.13E-15	1.87E-15	0.72500555
219	9.95E-09	4.70E-08	2.52E-16	1.13E-15	1.87E-15	0.72500555
219.1	9.95E-09	4.70E-08	2.52E-16	1.13E-15	1.87E-15	0.72500555
219.2	9.95E-09	4.70E-08	2.52E-16	1.13E-15	1.87E-15	0.72500555
219.3	9.95E-09	4.70E-08	2.52E-16	1.13E-15	1.87E-15	0.72500555
219.4	9.95E-09	4.70E-08	2.52E-16	1.13E-15	1.87E-15	0.72500555
219.5	9.95E-09	4.70E-08	2.52E-16	1.13E-15	1.87E-15	0.72500555
219.6	9.95E-09	4.70E-08	2.52E-16	1.13E-15	1.87E-15	0.72500555
219.7	9.95E-09	4.70E-08	2.52E-16	1.13E-15	1.87E-15	0.72500555
219.8	9.95E-09	4.70E-08	2.52E-16	1.13E-15	1.87E-15	0.72500555
219.9	9.95E-09	4.70E-08	2.52E-16	1.13E-15	1.87E-15	0.72500555
220	9.95E-09	4.70E-08	2.52E-16	1.13E-15	1.87E-15	0.72500555
220.1	9.95E-09	4.70E-08	2.52E-16	1.13E-15	1.87E-15	0.72500555
220.2	9.95E-09	4.70E-08	2.52E-16	1.13E-15	1.87E-15	0.72500555
220.3	9.95E-09	4.70E-08	2.52E-16	1.13E-15	1.87E-15	0.72500555
220.4	9.95E-09	4.70E-08	2.52E-16	1.13E-15	1.87E-15	0.72500555
220.5	9.95E-09	4.70E-08	2.52E-16	1.13E-15	1.87E-15	0.72500555
220.6	9.95E-09	4.70E-08	2.52E-16	1.13E-15	1.87E-15	0.72500555
220.7	9.95E-09	4.70E-08	2.52E-16	1.13E-15	1.87E-15	0.72500555
220.8	9.95E-09	4.70E-08	2.52E-16	1.13E-15	1.87E-15	0.72500555
220.9	9.95E-09	4.70E-08	2.52E-16	1.13E-15	1.87E-15	0.72500555
221	9.95E-09	4.70E-08	2.52E-16	1.13E-15	1.87E-15	0.72500555
221.1	9.95E-09	4.70E-08	2.52E-16	1.13E-15	1.87E-15	0.72500555
221.2	9.95E-09	4.70E-08	2.52E-16	1.13E-15	1.87E-15	0.72500555
221.3	9.95E-09	4.70E-08	2.52E-16	1.13E-15	1.87E-15	0.72500555
221.4	9.95E-09	4.70E-08	2.52E-16	1.13E-15	1.87E-15	0.72500555
221.5	9.95E-09	4.70E-08	2.52E-16	1.13E-15	1.87E-15	0.72500555
221.6	9.95E-09	4.70E-08	2.52E-16	1.13E-15	1.87E-15	0.72500555
221.7	9.95E-09	4.70E-08	2.52E-16	1.13E-15	1.87E-15	0.72500555
221.8	9.95E-09	4.70E-08	2.52E-16	1.13E-15	1.87E-15	0.72500555
221.9	9.95E-09	4.70E-08	2.52E-16	1.13E-15	1.87E-15	0.72500555
222	9.95E-09	4.70E-08	2.52E-16	1.13E-15	1.87E-15	0.72500555
222.1	9.95E-09	4.70E-08	2.52E-16	1.13E-15	1.87E-15	0.72500555
222.2	9.95E-09	4.70E-08	2.52E-16	1.13E-15	1.87E-15	0.72500555
222.3	9.95E-09	4.70E-08	2.52E-16	1.13E-15	1.87E-15	0.72500555
222.4	9.95E-09	4.70E-08	2.52E-16	1.13E-15	1.87E-15	0.72500555
222.5	9.95E-09	4.70E-08	2.52E-16	1.13E-15	1.87E-15	0.72500555
222.6	9.95E-09	4.70E-08	2.52E-16	1.13E-15	1.87E-15	0.72500555
222.7	9.95E-09	4.70E-08	2.52E-16	1.13E-15	1.87E-15	0.72500555
222.8	9.95E-09	4.70E-08	2.52E-16	1.13E-15	1.87E-15	0.72500555
222.9	9.95E-09	4.70E-08	2.52E-16	1.13E-15	1.87E-15	0.72500555
223	9.95E-09	4.70E-08	2.52E-16	1.13E-15	1.87E-15	0.72500555
223.1	9.95E-09	4.70E-08	2.52E-16	1.13E-15	1.87E-15	0.72500555
223.2	9.95E-09	4.70E-08	2.52E-16	1.13E-15	1.87E-15	0.72500555
223.3	9.95E-09	4.70E-08	2.52E-16	1.13E-15	1.87E-15	0.72500555
223.4	9.95E-09	4.70E-08	2.52E-16	1.13E-15	1.87E-15	0.72500555
223.5	9.95E-09	4.70E-08	2.52E-16	1.13E-15	1.87E-15	0.72500555
223.6	9.83E-09	4.69E-08	2.46E-16	1.09E-15	1.89E-15	0.72941782
223.7	9.83E-09	4.69E-08	2.46E-16	1.09E-15	1.89E-15	0.72941782
223.8	9.83E-09	4.69E-08	2.46E-16	1.09E-15	1.89E-15	0.72941782
223.9	9.83E-09	4.69E-08	2.46E-16	1.09E-15	1.89E-15	0.72941782
224	9.83E-09	4.69E-08	2.46E-16	1.09E-15	1.89E-15	0.72941782
224.1	9.83E-09	4.69E-08	2.46E-16	1.09E-15	1.89E-15	0.72941782
224.2	9.83E-09	4.69E-08	2.46E-16	1.09E-15	1.89E-15	0.72941782
224.3	9.83E-09	4.69E-08	2.46E-16	1.09E-15	1.89E-15	0.72941782
224.4	9.83E-09	4.69E-08	2.46E-16	1.09E-15	1.89E-15	0.72941782
224.5	9.83E-09	4.69E-08	2.46E-16	1.09E-15	1.89E-15	0.72941782
224.6	9.83E-09	4.69E-08	2.46E-16	1.09E-15	1.89E-15	0.72941782
224.7	9.83E-09	4.69E-08	2.46E-16	1.09E-15	1.89E-15	0.72941782
224.8	9.83E-09	4.69E-08	2.46E-16	1.09E-15	1.89E-15	0.72941782
224.9	9.83E-09	4.69E-08	2.46E-16	1.09E-15	1.89E-15	0.72941782
228.2	9.83E-09	4.69E-08	2.46E-16	1.09E-15	1.89E-15	0.72941782
228.3	9.83E-09	4.69E-08	2.46E-16	1.09E-15	1.89E-15	0.72941782
228.4	9.83E-09	4.69E-08	2.46E-16	1.09E-15	1.89E-15	0.72941782
228.5	9.83E-09	4.69E-08	2.46E-16	1.09E-15	1.89E-15	0.72941782
228.6	9.83E-09	4.69E-08	2.46E-16	1.09E-15	1.89E-15	0.72941782
228.7	9.83E-09	4.69E-08	2.46E-16	1.09E-15	1.89E-15	0.72941782
228.8	9.83E-09	4.69E-08	2.46E-16	1.09E-15	1.89E-15	0.72941782
228.9	1.00E-08	4.71E-08	2.45E-16	1.09E-15	1.89E-15	0.72712618
229	1.00E-08	4.71E-08	2.45E-16	1.09E-15	1.89E-15	0.72712618
229.1	1.00E-08	4.71E-08	2.45E-16	1.09E-15	1.89E-15	0.72712618
229.2	1.00E-08	4.71E-08	2.45E-16	1.09E-15	1.89E-15	0.72712618
229.3	1.00E-08	4.71E-08	2.45E-16	1.09E-15	1.89E-15	0.72712618
229.4	1.00E-08	4.71E-08	2.45E-16	1.09E-15	1.89E-15	0.72712618
229.5	1.00E-08	4.71E-08	2.45E-16	1.09E-15	1.89E-15	0.72712618
229.6	1.00E-08	4.71E-08	2.45E-16	1.09E-15	1.89E-15	0.72712618
229.7	1.00E-08	4.71E-08	2.45E-16	1.09E-15	1.89E-15	0.72712618
229.8	1.00E-08	4.71E-08	2.45E-16	1.09E-15	1.89E-15	0.72712618
229.9	1.00E-08	4.71E-08	2.45E-16	1.09E-15	1.89E-15	0.72712618
230	1.00E-08	4.71E-08	2.45E-16	1.09E-15	1.89E-15	0.72712618
230.1	1.00E-08	4.71E-08	2.45E-16	1.09E-15	1.89E-15	0.72712618
230.2	1.00E-08	4.71E-08	2.45E-16	1.09E-15	1.89E-15	0.72712618
230.3	1.00E-08	4.71E-08	2.45E-16	1.09E-15	1.89E-15	0.72712618
230.4	1.00E-08	4.71E-08	2.45E-16	1.09E-15	1.89E-15	0.72712618
230.5	1.00E-08	4.71E-08	2.45E-16	1.09E-15	1.89E-15	0.72712618
230.6	1.00E-08	4.71E-08	2.45E-16	1.09E-15	1.89E-15	0.72712618
230.7	1.00E-08	4.71E-08	2.45E-16	1.09E-15	1.89E-15	0.72712618
230.8	1.00E-08	4.71E-08	2.45E-16	1.09E-15	1.89E-15	0.72712618
230.9	1.00E-08	4.71E-08	2.45E-16	1.09E-15	1.89E-15	0.72712618
231	1.00E-08	4.71E-08	2.45E-16	1.09E-15	1.89E-15	0.72712618
231.1	1.00E-08	4.71E-08	2.45E-16	1.09E-15	1.89E-15	0.72712618
231.2	1.00E-08	4.71E-08	2.45E-16	1.09E-15	1.89E-15	0.72712618
231.3	1.00E-08	4.71E-08	2.45E-16	1.09E-15	1.89E-15	0.72712618
231.4	1.00E-08	4.71E-08	2.45E-16	1.09E-15	1.89E-15	0.72712618
231.5	1.00E-08	4.71E-08	2.45E-16	1.09E-15	1.89E-15	0.72712618
231.6	1.00E-08	4.71E-08	2.45E-16	1.09E-15	1.89E-15	0.72712618
231.7	1.01E-08	4.57E-08	2.36E-16	1.10E-15	1.88E-15	0.72899023
231.8	1.01E-08	4.57E-08	2.36E-16	1.10E-15	1.88E-15	0.72899023
231.9	1.01E-08	4.57E-08	2.36E-16	1.10E-15	1.88E-15	0.72899023
232	1.01E-08	4.57E-08	2.36E-16	1.10E-15	1.88E-15	0.72899023
232.1	1.01E-08	4.57E-08	2.36E-16	1.10E-15	1.88E-15	0.72899023
232.2	1.01E-08	4.57E-08	2.36E-16	1.10E-15	1.88E-15	0.72899023
232.3	1.01E-08	4.57E-08	2.36E-16	1.10E-15	1.88E-15	0.72899023
232.4	1.01E-08	4.57E-08	2.36E-16	1.10E-15	1.88E-15	0.72899023
232.5	1.01E-08	4.57E-08	2.36E-16	1.10E-15	1.88E-15	0.72899023
232.6	1.01E-08	4.57E-08	2.36E-16	1.10E-15	1.88E-15	0.72899023
232.7	1.01E-08	4.57E-08	2.36E-16	1.10E-15	1.88E-15	0.72899023
232.8	1.01E-08	4.57E-08	2.36E-16	1.10E-15	1.88E-15	0.72899023
232.9	1.01E-08	4.57E-08	2.36E-16	1.10E-15	1.88E-15	0.72899023
233	1.01E-08	4.57E-08	2.36E-16	1.10E-15	1.88E-15	0.72899023
233.1	1.01E-08	4.57E-08	2.36E-16	1.10E-15	1.88E-15	0.72899023
233.2	1.01E-08	4.57E-08	2.36E-16	1.10E-15	1.88E-15	0.72899023
233.3	1.01E-08	4.57E-08	2.36E-16	1.10E-15	1.88E-15	0.72899023
233.4	1.01E-08	4.57E-08	2.36E-16	1.10E-15	1.88E-15	0.72899023
233.5	1.01E-08	4.57E-08	2.36E-16	1.10E-15	1.88E-15	0.72899023
233.6	1.01E-08	4.57E-08	2.36E-16	1.10E-15	1.88E-15	0.72899023
233.7	1.01E-08	4.57E-08	2.36E-16	1.10E-15	1.88E-15	0.72899023
233.8	1.01E-08	4.57E-08	2.36E-16	1.10E-15	1.88E-15	0.72899023
233.9	1.01E-08	4.57E-08	2.36E-16	1.10E-15	1.88E-15	0.72899023
234	1.01E-08	4.57E-08	2.36E-16	1.10E-15	1.88E-15	0.72899023
234.1	1.01E-08	4.57E-08	2.36E-16	1.10E-15	1.88E-15	0.72899023
234.2	1.01E-08	4.57E-08	2.36E-16	1.10E-15	1.88E-15	0.72899023
234.3	1.01E-08	4.57E-08	2.36E-16	1.10E-15	1.88E-15	0.72899023
234.4	1.01E-08	4.57E-08	2.36E-16	1.10E-15	1.88E-15	0.72899023
234.5	1.01E-08	4.57E-08	2.36E-16	1.10E-15	1.88E-15	0.72899023
234.6	1.01E-08	4.57E-08	2.36E-16	1.10E-15	1.88E-15	0.72899023
234.7	1.01E-08	4.57E-08	2.36E-16	1.10E-15	1.88E-15	0.72899023
234.8	1.01E-08	4.57E-08	2.36E-16	1.10E-15	1.88E-15	0.72899023
234.9	1.01E-08	4.57E-08	2.36E-16	1.10E-15	1.88E-15	0.72899023
235	1.01E-08	4.57E-08	2.36E-16	1.10E-15	1.88E-15	0.72899023
235.1	1.01E-08	4.57E-08	2.36E-16	1.10E-15	1.88E-15	0.72899023
235.2	1.01E-08	4.57E-08	2.36E-16	1.10E-15	1.88E-15	0.72899023
235.3	1.01E-08	4.57E-08	2.36E-16	1.10E-15	1.88E-15	0.72899023
235.4	1.01E-08	4.57E-08	2.36E-16	1.10E-15	1.88E-15	0.72899023
235.5	1.01E-08	4.57E-08	2.36E-16	1.10E-15	1.88E-15	0.72899023
235.6	1.01E-08	4.57E-08	2.36E-16	1.10E-15	1.88E-15	0.72899023
235.7	1.01E-08	4.57E-08	2.36E-16	1.10E-15	1.88E-15	0.72899023
235.8	1.01E-08	4.57E-08	2.36E-16	1.10E-15	1.88E-15	0.72899023
235.9	1.01E-08	4.57E-08	2.36E-16	1.10E-15	1.88E-15	0.72899023
236	1.01E-08	4.57E-08	2.36E-16	1.10E-15	1.88E-15	0.72899023
236.1	1.01E-08	4.57E-08	2.36E-16	1.10E-15	1.88E-15	0.72899023
236.2	1.01E-08	4.57E-08	2.36E-16	1.10E-15	1.88E-15	0.72899023
236.3	1.01E-08	4.57E-08	2.36E-16	1.10E-15	1.88E-15	0.72899023
236.4	1.01E-08	4.57E-08	2.36E-16	1.10E-15	1.88E-15	0.72899023
236.5	1.01E-08	4.57E-08	2.36E-16	1.10E-15	1.88E-15	0.72899023
236.6	1.01E-08	4.57E-08	2.36E-16	1.10E-15	1.88E-15	0.72899023
236.7	1.01E-08	4.57E-08	2.36E-16	1.10E-15	1.88E-15	0.72899023
236.8	1.01E-08	4.57E-08	2.36E-16	1.10E-15	1.88E-15	0.72899023
236.9	1.01E-08	4.57E-08	2.36E-16	1.10E-15	1.88E-15	0.72899023
237	1.01E-08	4.57E-08	2.36E-16	1.10E-15	1.88E-15	0.72899023
237.1	1.01E-08	4.57E-08	2.36E-16	1.10E-15	1.88E-15	0.72899023
237.2	1.01E-08	4.57E-08	2.36E-16	1.10E-15	1.88E-15	0.72899023
237.3	1.01E-08	4.57E-08	2.36E-16	1.10E-15	1.88E-15	0.72899023
237.4	1.01E-08	4.57E-08	2.36E-16	1.10E-15	1.88E-15	0.72899023
237.5	1.01E-08	4.57E-08	2.36E-16	1.10E-15	1.88E-15	0.72899023
237.6	1.01E-08	4.57E-08	2.36E-16	1.10E-15	1.88E-15	0.72899023
237.7	9.83E-09	4.44E-08	2.26E-16	1.09E-15	1.81E-15	0.73228845
237.8	9.83E-09	4.44E-08	2.26E-16	1.09E-15	1.81E-15	0.73228845
237.9	9.83E-09	4.44E-08	2.26E-16	1.09E-15	1.81E-15	0.73228845
238	9.83E-09	4.44E-08	2.26E-16	1.09E-15	1.81E-15	0.73228845
238.1	9.83E-09	4.44E-08	2.26E-16	1.09E-15	1.81E-15	0.73228845
238.2	9.83E-09	4.44E-08	2.26E-16	1.09E-15	1.81E-15	0.73228845
238.3	9.83E-09	4.44E-08	2.26E-16	1.09E-15	1.81E-15	0.73228845
238.4	9.69E-09	4.44E-08	2.22E-16	1.03E-15	1.82E-15	0.73199897
238.5	9.69E-09	4.44E-08	2.22E-16	1.03E-15	1.82E-15	0.73199897
238.6	9.69E-09	4.44E-08	2.22E-16	1.03E-15	1.82E-15	0.73199897
238.7	9.69E-09	4.44E-08	2.22E-16	1.03E-15	1.82E-15	0.73199897
238.8	9.69E-09	4.44E-08	2.22E-16	1.03E-15	1.82E-15	0.73199897
238.9	9.69E-09	4.44E-08	2.22E-16	1.03E-15	1.82E-15	0.73199897
239	9.69E-09	4.44E-08	2.22E-16	1.03E-15	1.82E-15	0.73199897
239.1	9.69E-09	4.44E-08	2.22E-16	1.03E-15	1.82E-15	0.73199897
239.2	9.69E-09	4.44E-08	2.22E-16	1.03E-15	1.82E-15	0.73199897
239.3	9.69E-09	4.44E-08	2.22E-16	1.03E-15	1.82E-15	0.73199897
239.4	9.69E-09	4.44E-08	2.22E-16	1.03E-15	1.82E-15	0.73199897
239.5	9.87E-09	4.27E-08	2.12E-16	1.04E-15	1.79E-15	0.73693407
239.6	9.87E-09	4.27E-08	2.12E-16	1.04E-15	1.79E-15	0.73693407
239.7	9.87E-09	4.27E-08	2.12E-16	1.04E-15	1.79E-15	0.73693407
239.8	9.87E-09	4.27E-08	2.12E-16	1.04E-15	1.79E-15	0.73693407
239.9	9.87E-09	4.27E-08	2.12E-16	1.04E-15	1.79E-15	0.73693407
240	9.87E-09	4.27E-08	2.12E-16	1.04E-15	1.79E-15	0.73693407

Table S2: PRCC analysis data for [G] with [G](0)=2 mM: PRCC sensitivity indices and their corresponding p-values

Sensitivity analysis data from PRCC for [G] with [G](0)=2 mM (only the parameters to which [G]'s level is sensitive to appear)

Time discretization	V_{[G]}	K_{[G]}	V_{[PYR]}	K_{[PYR]}	alpha	G_E	[G]_0
0	0	0	0	0	0	0	1
0.1	-0.0197408	-0.01806	-0.5014279	0.29793074	0.10770581	-0.0955391	0.99993154
0.2	-0.0438913	-0.0012059	-0.6885004	0.36130119	0.12362293	-0.109565	0.99985242
0.3	-0.128316	-0.0042376	-0.7844852	0.38291294	0.13535129	-0.0164923	0.99973489
0.4	-0.0999379	0.00093644	-0.8325933	0.42352556	0.1360992	-0.0494704	0.99963345
0.5	-0.0250263	-0.0445222	-0.8656837	0.45419456	0.09683606	-0.0201367	0.99954421
0.6	-0.0816433	0.0099392	-0.8808349	0.5133217	0.08295719	-0.0280668	0.99940178
0.7	-0.05249	0.01976983	-0.8931273	0.52657913	0.00347458	-0.040354	0.99926811
0.8	0.00919364	0.03627542	-0.8918282	0.53087715	0.01093118	-0.0012038	0.99904703
0.9	0.00709572	0.01764777	-0.8938778	0.53378865	-0.0152879	0.0191456	0.99875961
1	0.06302583	0.01010084	-0.9003626	0.54569157	-0.014833	0.02594125	0.9985455
1.1	0.09028209	0.02613066	-0.8982653	0.55350164	-0.0093495	0.00341091	0.99824138
1.2	0.08764284	-0.0210919	-0.8982229	0.55629138	-0.0383681	0.05617927	0.99782957
1.3	0.12751637	-0.0500375	-0.8963781	0.54838717	-0.0405819	0.08260116	0.99743506
1.4	0.14501016	-0.0535424	-0.8917682	0.53104754	-0.0745504	0.11368932	0.99682604
1.5	0.13496225	-0.076183	-0.8899335	0.54096106	-0.1027012	0.11653637	0.99634314
1.6	0.15083124	-0.1047356	-0.8902438	0.54105491	-0.1121543	0.12369188	0.99583353
1.7	0.17164762	-0.1186546	-0.8827951	0.52332676	-0.1299425	0.128619	0.99486117
1.8	0.16157173	-0.1397269	-0.8732247	0.510351	-0.1097435	0.15545163	0.99355691
1.9	0.16268148	-0.1634158	-0.8684655	0.50800663	-0.1455802	0.16923351	0.99228684
2	0.17466469	-0.1841867	-0.8569892	0.49032285	-0.1390068	0.18076228	0.99058942
2.1	0.17016197	-0.2144855	-0.8544967	0.49206381	-0.1356575	0.17954843	0.98926367
2.2	0.1731082	-0.2199506	-0.8467641	0.47290691	-0.134647	0.20566404	0.98756587
2.3	0.17586834	-0.2358266	-0.8415549	0.46862132	-0.1360685	0.21367791	0.98597113
2.4	0.19119206	-0.2583188	-0.8389715	0.47876497	-0.149867	0.22091942	0.98430053
2.5	0.19604164	-0.2718084	-0.8353606	0.46173098	-0.1487544	0.22814914	0.9821172
2.6	0.20026372	-0.2699263	-0.8268156	0.44568741	-0.1601848	0.23289302	0.979337
2.7	0.20380026	-0.2764663	-0.8206533	0.43709206	-0.1570729	0.23781476	0.97670842
2.8	0.20447879	-0.2844403	-0.810001	0.4250459	-0.1592039	0.25051312	0.97291759
2.9	0.19930853	-0.2909139	-0.7947412	0.40917013	-0.1671747	0.24336309	0.96708515
3	0.20148701	-0.2903549	-0.7856241	0.40416752	-0.1662557	0.2449293	0.96280234
3.1	0.21017612	-0.3018604	-0.7819947	0.40482718	-0.1732683	0.25468488	0.96000113
3.2	0.21931886	-0.3068977	-0.7819436	0.41423282	-0.1820523	0.25751505	0.95676537
3.3	0.22282649	-0.3134239	-0.7730336	0.40851547	-0.1916578	0.25869932	0.95171635
3.4	0.22515114	-0.3198206	-0.7679098	0.40098204	-0.1967122	0.25542237	0.94747402
3.5	0.22892451	-0.331543	-0.7678135	0.4089905	-0.2067572	0.25330804	0.94460707
3.6	0.23214815	-0.3426681	-0.7686688	0.41366827	-0.2031883	0.2509053	0.9422947
3.7	0.23120393	-0.3535616	-0.7697391	0.41728799	-0.2108352	0.25974018	0.9404795
3.8	0.23756405	-0.3639401	-0.7685964	0.40997803	-0.2080106	0.26071907	0.93744962
3.9	0.24590577	-0.3649646	-0.7692248	0.40813257	-0.2157663	0.27038754	0.9345356
4	0.24435112	-0.3695732	-0.7673018	0.41112048	-0.2196286	0.27697691	0.93208259
4.1	0.24094629	-0.3739867	-0.7666438	0.41206712	-0.2260555	0.27775804	0.92962792
4.2	0.24359468	-0.3790664	-0.7683516	0.41432811	-0.22992	0.28015563	0.92797889
4.3	0.24895759	-0.382643	-0.7675585	0.41129072	-0.2378229	0.28241391	0.92546966
4.4	0.25070738	-0.3896062	-0.7653458	0.41204835	-0.2388602	0.28350241	0.92177361
4.5	0.25543065	-0.3999763	-0.7672999	0.41935133	-0.2437968	0.28796061	0.91948432
4.6	0.25535215	-0.412046	-0.7634974	0.41806513	-0.2475548	0.28314705	0.91423566
4.7	0.25948306	-0.4101434	-0.760627	0.42144973	-0.2516671	0.2790368	0.91015696
4.8	0.26192073	-0.4133843	-0.7586166	0.42061969	-0.2574165	0.27689699	0.90685167
4.9	0.25994966	-0.4172822	-0.7576164	0.42069032	-0.2689746	0.2819983	0.90295497
5	0.25948491	-0.4216776	-0.7561688	0.42013323	-0.2724079	0.28352636	0.89886514
5.1	0.26363687	-0.4281718	-0.7556708	0.41940388	-0.2766581	0.28650443	0.89383602
5.2	0.26682792	-0.4326049	-0.75704	0.42323998	-0.2881864	0.29507837	0.88953338
5.3	0.26868904	-0.4342543	-0.7545263	0.42260036	-0.2943532	0.29412041	0.8850916
5.4	0.27617291	-0.4409675	-0.7554407	0.42213439	-0.3012699	0.29318907	0.88222139
5.5	0.27638497	-0.4411557	-0.7561648	0.42447462	-0.29846	0.28965772	0.87873487
5.6	0.27951368	-0.4416133	-0.7553334	0.42502887	-0.3012019	0.28912585	0.8735364
5.7	0.28340134	-0.4450572	-0.7528425	0.42477039	-0.304984	0.28699226	0.86941486
5.8	0.28724256	-0.4449867	-0.7523687	0.42579053	-0.3075106	0.28964156	0.86477188
5.9	0.28623816	-0.4478022	-0.7532922	0.43449814	-0.3188165	0.28804449	0.85891329
6	0.29238181	-0.448166	-0.7536497	0.4349558	-0.3270662	0.2891092	0.85524895
6.1	0.29363218	-0.4502173	-0.753485	0.43550442	-0.3312472	0.2882187	0.84988429
6.2	0.29851502	-0.450231	-0.7524383	0.43680753	-0.3360538	0.2900487	0.84506206
6.3	0.2977311	-0.4522655	-0.7529321	0.43304474	-0.3369863	0.29144933	0.84068026
6.4	0.30113267	-0.4523558	-0.7532352	0.43543622	-0.3422146	0.29366282	0.83621708
6.5	0.29730677	-0.4548026	-0.7500929	0.43185935	-0.3428046	0.29173854	0.82978573
6.6	0.2990634	-0.453405	-0.7495897	0.43406073	-0.3506132	0.28999167	0.82597366
6.7	0.30186408	-0.4562114	-0.7514258	0.43694491	-0.3587666	0.29102598	0.82398964
6.8	0.30237362	-0.4551738	-0.7503898	0.43778357	-0.3670397	0.29135304	0.81858703
6.9	0.30356019	-0.4557771	-0.7507511	0.43866183	-0.3737986	0.29378437	0.81461713
7	0.30562344	-0.4566963	-0.7527653	0.44363532	-0.3806694	0.29259558	0.81069323
7.1	0.30772848	-0.4577024	-0.75298	0.44349288	-0.3855711	0.29072046	0.80789459
7.2	0.30769374	-0.4582159	-0.754038	0.44458609	-0.3871267	0.29116779	0.80390133
7.3	0.31302407	-0.4597398	-0.7557434	0.44870308	-0.3976138	0.29358248	0.80095917
7.4	0.31194343	-0.4603547	-0.7566132	0.45103922	-0.4041653	0.29139169	0.79711703
7.5	0.31543492	-0.4603239	-0.7578333	0.45395857	-0.4078315	0.29471309	0.79427878
7.6	0.3191191	-0.4616089	-0.7596792	0.45585607	-0.4142998	0.29452396	0.78950215
7.7	0.32445093	-0.4627753	-0.7605174	0.4570766	-0.420303	0.29782717	0.78491014
7.8	0.32555275	-0.4626471	-0.7619429	0.45872027	-0.427611	0.29701492	0.77993342
7.9	0.32979904	-0.4617371	-0.7626975	0.45843872	-0.4313839	0.2996814	0.77564762
8	0.33108448	-0.4636101	-0.7622975	0.45819049	-0.4332528	0.29969009	0.76999133
8.1	0.33548553	-0.4601601	-0.7626739	0.4592416	-0.4362997	0.30258094	0.76663366
8.2	0.33530829	-0.4609766	-0.7633982	0.46241087	-0.4431664	0.30373454	0.76144083
8.3	0.33674181	-0.4617448	-0.7613035	0.46204067	-0.4475346	0.30732808	0.75731142
8.4	0.33796428	-0.4604716	-0.7602978	0.46073884	-0.4510941	0.30563599	0.75078819
8.5	0.3401781	-0.4591288	-0.7603788	0.45984157	-0.4527572	0.30563323	0.74531799
8.6	0.33855402	-0.45984	-0.7602304	0.46040259	-0.4547227	0.3068923	0.74069481
8.7	0.34132356	-0.461237	-0.7603502	0.45960738	-0.4582315	0.3091098	0.73714513
8.8	0.34187302	-0.4594103	-0.7592675	0.46157366	-0.4633391	0.31249292	0.73019255
8.9	0.34733812	-0.46341	-0.7619036	0.46518202	-0.4705065	0.31426427	0.72540003
9	0.34727627	-0.4625584	-0.760755	0.46664922	-0.4723115	0.31340154	0.71957889
9.1	0.35077078	-0.4608474	-0.7613789	0.46517415	-0.4778914	0.31503576	0.71605527
9.2	0.35104088	-0.4625107	-0.7617364	0.46534252	-0.4761782	0.31171373	0.70908719
9.3	0.35274209	-0.460963	-0.7621819	0.46697929	-0.4797578	0.31200659	0.70434421
9.4	0.35125421	-0.4614812	-0.760803	0.46742441	-0.4812353	0.31146318	0.69871461
9.5	0.35479343	-0.4600299	-0.7607212	0.46609808	-0.484996	0.31116395	0.6947232
9.6	0.3560743	-0.4608431	-0.7606135	0.46614902	-0.4910436	0.3087487	0.69168266
9.7	0.35465943	-0.4584808	-0.7594081	0.46579265	-0.4914076	0.30710727	0.68613416
9.8	0.35488745	-0.4589459	-0.7597144	0.46537142	-0.4925375	0.30698419	0.67984393
9.9	0.35782595	-0.4587855	-0.7601448	0.46480525	-0.498025	0.30645622	0.6759397
10	0.35675968	-0.4579128	-0.7604013	0.4657929	-0.5011484	0.30636318	0.67274209
10.1	0.36256892	-0.4576763	-0.7605145	0.46731935	-0.5052549	0.30776013	0.66714813
10.2	0.3645884	-0.4577788	-0.7604487	0.46673737	-0.5080529	0.30837809	0.66235677
10.3	0.36607508	-0.4561506	-0.7600047	0.46680496	-0.5115551	0.30644226	0.6575565
10.4	0.36731743	-0.4566014	-0.759284	0.46588631	-0.5169814	0.30479552	0.65287775
10.5	0.36932805	-0.4537004	-0.7583554	0.46634779	-0.5213394	0.3062005	0.64923664
10.6	0.37064664	-0.4512236	-0.7576016	0.46819932	-0.5255153	0.30526987	0.64212247
10.7	0.37382495	-0.4497871	-0.7573385	0.46661362	-0.5277649	0.30731628	0.63680776
10.8	0.37354176	-0.4507268	-0.7589125	0.46708621	-0.5295805	0.30688634	0.63279383
10.9	0.37531849	-0.4503996	-0.7588295	0.46694538	-0.5319914	0.30441785	0.62796583
11	0.37573504	-0.4508496	-0.7575653	0.46464855	-0.5325445	0.30552989	0.62389774
11.1	0.37795317	-0.450376	-0.7565215	0.46388764	-0.5344477	0.30444836	0.61785562
11.2	0.37710673	-0.4505094	-0.7546888	0.46238397	-0.5353799	0.30123631	0.61290402
11.3	0.37766699	-0.4473621	-0.7536846	0.46145977	-0.5385471	0.30201826	0.6075013
11.4	0.3798396	-0.4463257	-0.7536617	0.45831992	-0.5412586	0.30037847	0.60322705
11.5	0.38065078	-0.4439376	-0.7539038	0.45969541	-0.5420949	0.30190398	0.59770328
11.6	0.3840757	-0.4426485	-0.7534772	0.45876839	-0.5464047	0.3029592	0.59173744
11.7	0.3839054	-0.4410542	-0.7522703	0.45727534	-0.5490564	0.30341722	0.58848814
11.8	0.3852251	-0.4421716	-0.7524171	0.45811873	-0.5532468	0.30613656	0.58493134

Time discretization	V_{[G]}	K_{[G]}	V_{[PYR]}	K_{[PYR]}	alpha	G_E	[G]_0
15	0.41566111	-0.42561	-0.7327188	0.43366142	-0.6069906	0.30258781	0.43053518
15.1	0.41635952	-0.4265743	-0.7325603	0.43297568	-0.6098317	0.3041362	0.42754249
15.2	0.41573947	-0.4265782	-0.7315446	0.43251781	-0.611257	0.30261973	0.42401595
15.3	0.41643205	-0.4264753	-0.7317317	0.43270462	-0.6117017	0.30402648	0.41975952
15.4	0.41859117	-0.4262719	-0.7317582	0.43399979	-0.615292	0.30397449	0.41604492
15.5	0.4199426	-0.4253192	-0.731473	0.436397	-0.6166687	0.30510376	0.41341771
15.6	0.42057922	-0.4269407	-0.731444	0.43567185	-0.6184654	0.3034494	0.41122116
15.7	0.42074476	-0.4257386	-0.7297189	0.43457649	-0.6188648	0.30406144	0.40749184
15.8	0.42050089	-0.424877	-0.7292036	0.43580406	-0.6202894	0.30375717	0.40259218
15.9	0.42163827	-0.4251881	-0.7293331	0.43472693	-0.6218038	0.30471891	0.3996297
16	0.4226262	-0.4252701	-0.7285409	0.43424138	-0.6245904	0.30524239	0.39674245
16.1	0.4239675	-0.4272205	-0.7287709	0.43338349	-0.6261598	0.30668032	0.39441452
16.2	0.4261423	-0.4268834	-0.7286207	0.43279349	-0.6282218	0.30652857	0.38939676
16.3	0.42644984	-0.4264379	-0.7286944	0.43345319	-0.6295601	0.30681637	0.38563907
16.4	0.42671812	-0.4253086	-0.728808	0.43332187	-0.6308456	0.30641843	0.38294249
16.5	0.4276117	-0.4247869	-0.7280861	0.43366354	-0.6327313	0.30583441	0.37981621
16.6	0.42900858	-0.4230135	-0.7273456	0.43342077	-0.6337814	0.30568338	0.37675647
16.7	0.43060881	-0.4219975	-0.7267935	0.4329398	-0.635319	0.30476215	0.37410518
16.8	0.43255267	-0.4210822	-0.7266446	0.43310284	-0.6364425	0.3067319	0.37062125
16.9	0.4329261	-0.421729	-0.7262544	0.43131487	-0.6365868	0.30558409	0.36866587
17	0.43269954	-0.4220936	-0.7253448	0.43145651	-0.6365922	0.30494163	0.36309852
17.1	0.43309801	-0.4199389	-0.7241639	0.43166548	-0.6379027	0.30396723	0.35841948
17.2	0.43357798	-0.4201642	-0.7248854	0.43294972	-0.6402139	0.30557076	0.35309675
17.3	0.43429363	-0.4189985	-0.7247673	0.43331626	-0.6405483	0.30436352	0.35113867
17.4	0.43450287	-0.4183567	-0.7244335	0.43334635	-0.6416696	0.30400445	0.34881849
17.5	0.43635004	-0.418245	-0.7240789	0.43174473	-0.641934	0.30479221	0.34571451
17.6	0.43763748	-0.4188408	-0.7242308	0.43172368	-0.6441146	0.30658071	0.34175292
17.7	0.43756619	-0.4218276	-0.7245198	0.43165717	-0.645069	0.30743375	0.33847642
17.8	0.43776569	-0.4226254	-0.7244903	0.43133505	-0.6461095	0.30727964	0.33543258
17.9	0.43899552	-0.4217327	-0.723704	0.42989195	-0.6471647	0.30655533	0.33340758
18	0.43911997	-0.4220644	-0.7226035	0.4294489	-0.6488394	0.30552472	0.33220785
18.1	0.44034017	-0.419739	-0.7215352	0.4304149	-0.6493793	0.3052014	0.32899336
18.2	0.44122936	-0.419781	-0.7215422	0.43015078	-0.6508906	0.30564229	0.32756874
18.3	0.44124582	-0.4195405	-0.720881	0.42977429	-0.6508916	0.30517056	0.32494892
18.4	0.44145624	-0.4197297	-0.7204652	0.43016759	-0.6507296	0.3051873	0.32215054
18.5	0.44224406	-0.4193916	-0.7198638	0.42892902	-0.6516185	0.30330089	0.32079442
18.6	0.44161631	-0.4204827	-0.7199479	0.42853898	-0.6516539	0.30458704	0.31824219
18.7	0.44227139	-0.4182626	-0.7191694	0.42935625	-0.6519064	0.30464116	0.31442409
18.8	0.44165624	-0.4171562	-0.7185235	0.4283756	-0.6530172	0.30350781	0.30963395
18.9	0.44290758	-0.4169706	-0.7174189	0.42801458	-0.6539427	0.30132972	0.30719172
19	0.44482835	-0.4165879	-0.7166363	0.42594525	-0.6544645	0.30053414	0.3034223
19.1	0.44458929	-0.4159184	-0.7158867	0.42511943	-0.6539852	0.30024806	0.29999601
19.2	0.44459624	-0.4152913	-0.7151606	0.42614286	-0.6544539	0.30128351	0.29569821
19.3	0.44586184	-0.41558	-0.714928	0.42652494	-0.6549561	0.30184484	0.29534024
19.4	0.44802248	-0.4149276	-0.7141646	0.42562131	-0.6560296	0.30172537	0.29027045
19.5	0.44746899	-0.4150903	-0.7139892	0.42427148	-0.6554114	0.30325175	0.28767564
19.6	0.44712395	-0.4135269	-0.713021	0.42286607	-0.6554372	0.30258878	0.28584284
19.7	0.44791488	-0.4163967	-0.7133866	0.42161467	-0.6555832	0.30303781	0.28108647
19.8	0.4482289	-0.4164111	-0.7131059	0.42193033	-0.656149	0.30237775	0.27912692
19.9	0.44799803	-0.416488	-0.7123574	0.42037717	-0.6559652	0.3022144	0.2767462
20	0.4484617	-0.4171826	-0.712706	0.41955253	-0.6573918	0.30150731	0.27370841
20.1	0.44902392	-0.4172702	-0.7127454	0.42041556	-0.6585908	0.30190721	0.2722916
20.2	0.44979233	-0.4189385	-0.7125734	0.41948515	-0.6595679	0.30265439	0.26855582
20.3	0.44948276	-0.4180496	-0.711078	0.41802867	-0.6599443	0.30397978	0.26530276
20.4	0.44906888	-0.4180467	-0.710058	0.41815369	-0.6604671	0.30395695	0.26300794
20.5	0.4500065	-0.4167229	-0.7094975	0.41830367	-0.6615143	0.30461683	0.26125025
20.6	0.4503514	-0.4158834	-0.7095447	0.41874206	-0.6627191	0.30559454	0.25962921
20.7	0.45035095	-0.4147074	-0.7089585	0.41877568	-0.6632075	0.30501433	0.25699939
20.8	0.45055441	-0.4152869	-0.7086557	0.41836713	-0.6638008	0.30479152	0.25564131
20.9	0.45164736	-0.4161147	-0.7079689	0.41820007	-0.6645288	0.30452475	0.25272124
21	0.45141473	-0.4166106	-0.7081132	0.418059	-0.6651383	0.30501435	0.24924459
21.1	0.45147209	-0.4177802	-0.7081222	0.41873929	-0.6653247	0.30569922	0.24744464
21.2	0.4517512	-0.4172163	-0.7077962	0.41829097	-0.6661464	0.30543983	0.24395407
21.3	0.45211834	-0.4169826	-0.7078521	0.41801128	-0.6663246	0.30529267	0.24159205
21.4	0.45261289	-0.4166867	-0.7083004	0.41839887	-0.6666025	0.30582503	0.24031942
21.5	0.45253002	-0.4154274	-0.7078001	0.41765143	-0.666372	0.30522995	0.23911018
21.6	0.4537343	-0.4146646	-0.706886	0.4160644	-0.6671907	0.30376594	0.23757893
21.7	0.45381233	-0.4147909	-0.7060014	0.41699413	-0.6673483	0.30322007	0.23552217
21.8	0.45519586	-0.4159728	-0.7064837	0.41823055	-0.6685674	0.30363015	0.23277594
21.9	0.45525349	-0.4172675	-0.7070066	0.41948683	-0.6699972	0.30464388	0.23027588
22	0.45509733	-0.4176714	-0.7062838	0.41787729	-0.6705247	0.30479928	0.22766894
22.1	0.45536682	-0.4174581	-0.7053941	0.4168874	-0.6717084	0.30385513	0.22488932
22.2	0.45553639	-0.4186635	-0.7047409	0.41632058	-0.6723799	0.30465882	0.22034763
22.3	0.45592001	-0.417211	-0.7037215	0.41616502	-0.6730747	0.30542637	0.21845673
22.4	0.4562031	-0.4164478	-0.7033228	0.41717148	-0.674376	0.30650734	0.21687433
22.5	0.4568408	-0.4176933	-0.7037367	0.41768456	-0.6745013	0.30611172	0.21368858
22.6	0.45766408	-0.4176641	-0.7038788	0.4179565	-0.6751556	0.30636336	0.20985889
22.7	0.45795105	-0.4183103	-0.7036286	0.41862191	-0.6763285	0.30586054	0.2095359
22.8	0.45827697	-0.4189232	-0.703778	0.41853814	-0.6773916	0.30589488	0.20763946
22.9	0.46006587	-0.4194415	-0.7034012	0.41848931	-0.6793406	0.304656	0.20722502
23	0.46079012	-0.4203598	-0.7029437	0.41887555	-0.6799856	0.30647092	0.20408479
23.1	0.46100745	-0.4198207	-0.7030192	0.41919993	-0.6806895	0.30606003	0.20199066
23.2	0.46189709	-0.4194612	-0.7027137	0.41839339	-0.6817296	0.3060298	0.20200018
23.3	0.46088849	-0.4202967	-0.702056	0.41819071	-0.6826287	0.30556308	0.19901286
23.4	0.46156821	-0.4201524	-0.7015361	0.41805839	-0.6827871	0.30532428	0.19794962
23.5	0.46220018	-0.4204541	-0.7010892	0.41762396	-0.6835525	0.30433887	0.1969474
23.6	0.46296537	-0.4206907	-0.7007192	0.41795445	-0.6841229	0.30440716	0.19616937
23.7	0.46276526	-0.4197181	-0.6993623	0.41674801	-0.6842982	0.30414605	0.19390936
23.8	0.4620083	-0.4194673	-0.6986597	0.41594988	-0.6843895	0.30277766	0.19230504
23.9	0.46201514	-0.4198512	-0.6984773	0.41559409	-0.6848505	0.30411717	0.19026722
24	0.46190675	-0.4197111	-0.6979672	0.41504879	-0.6851805	0.30362775	0.18857218
24.1	0.46291123	-0.420275	-0.6986215	0.41590818	-0.6862284	0.30424848	0.18622564
24.2	0.46277846	-0.4206853	-0.6983346	0.41606588	-0.6864515	0.30448977	0.1853038
24.3	0.46258477	-0.4201181	-0.6977807	0.41565759	-0.6864983	0.30385848	0.1833509
24.4	0.46209335	-0.4201053	-0.6972603	0.4153641	-0.6863543	0.30410328	0.18189798
24.5	0.46253643	-0.4208933	-0.697209	0.41537944	-0.6869837	0.30519639	0.18014913
24.6	0.46216659	-0.4210104	-0.6969196	0.41499725	-0.6872918	0.30582474	0.17926631
24.7	0.46253893	-0.4209431	-0.6966934	0.41590835	-0.6879727	0.30607688	0.17714302
24.8	0.46357563	-0.4216371	-0.6968004	0.41648181	-0.6894069	0.30588163	0.17636228
24.9	0.46445257	-0.4217154	-0.6968413	0.41755906	-0.6902608	0.30587832	0.1757911
25	0.46504872	-0.4231438	-0.6961307	0.41772517	-0.6913016	0.30547295	0.1743832
25.1	0.46648856	-0.4244879	-0.6968656	0.41770255	-0.6926813	0.30454235	0.17128832
25.2	0.46728194	-0.4240571	-0.6964558	0.41818908	-0.6934173	0.3053264	0.17079336
25.3	0.46783972	-0.4248694	-0.6964219	0.41838814	-0.6940865	0.30441329	0.16834084
25.4	0.46804008	-0.4245213	-0.6959586	0.4186654	-0.6945011	0.30404515	0.16799202
25.5	0.46891562	-0.4246167	-0.6962839	0.41926422	-0.6951184	0.30442949	0.16601976
25.6	0.46875858	-0.4247744	-0.6964005	0.41922219	-0.6952165	0.30475406	0.16435785
25.7	0.4687884	-0.4251447	-0.6958132	0.41830045	-0.6959077	0.3046533	0.16301462
25.8	0.46934078	-0.4245416	-0.6953788	0.41939342	-0.6964675	0.3045594	0.160827
25.9	0.46927514	-0.4245506	-0.6949919	0.41892204	-0.6976757	0.30518035	0.15900739
26	0.46951365	-0.4245602	-0.694964	0.4198719	-0.6985166	0.3065829	0.15740493
26.1	0.47032258	-0.424046	-0.6946015	0.42025265	-0.6992193	0.30648857	0.15653947
26.2	0.46978316	-0.423563	-0.6944488	0.4206304	-0.6989859	0.30668012	0.15479928
26.3	0.47008543	-0.4233432	-0.6944566	0.42089662	-0.6996481	0.30681002	0.1533449
26.4	0.47048876	-0.4242191	-0.6948437	0.42045037	-0.7008816	0.30710779	0.15274083
26.5	0.47018855	-0.4240689	-0.6947643	0.42004106	-0.7009252	0.30788021	0.15139805
26.6	0.47069791	-0.4249811	-0.694694	0.42048432	-0.701543	0.30887171	0.15106921
26.7	0.47104646	-0.4255885	-0.6947258	0.42023079	-0.7020087	0.30868477	0.15009943
26.8	0.47131347	-0.4256562	-0.6947197	0.42140172	-0.7026392	0.30857929	0.14967803

Time discretization	V_{[G]}	K_{[G]}	V_{[PYR]}	K_{[PYR]}	alpha	G_E	[G]_0
30	0.48082107	-0.436883	-0.6912102	0.42634157	-0.7174375	0.31789549	0.11505362
30.1	0.48141892	-0.4374686	-0.691097	0.42557253	-0.7178146	0.31727644	0.11376043
30.2	0.48185449	-0.4389812	-0.6908225	0.42491984	-0.7178668	0.31787898	0.11287046
30.3	0.48219224	-0.438908	-0.6905941	0.42553248	-0.7181958	0.31799786	0.11215447
30.4	0.48305981	-0.439175	-0.6908607	0.42551068	-0.718972	0.31772735	0.11175399
30.5	0.48358586	-0.4393397	-0.6910186	0.42550805	-0.7194205	0.3182373	0.11117093
30.6	0.48330336	-0.4400897	-0.6902652	0.42573155	-0.7196538	0.31810132	0.10979962
30.7	0.48423056	-0.4398577	-0.6902013	0.42560079	-0.7197783	0.31846196	0.10873258
30.8	0.48399138	-0.440506	-0.6902491	0.4252938	-0.7202149	0.31903477	0.10899053
30.9	0.48430927	-0.440713	-0.69024	0.42531386	-0.7202031	0.31949402	0.10910256
31	0.48512237	-0.4402626	-0.6899065	0.42685287	-0.7211588	0.31853765	0.10743358
31.1	0.48490435	-0.4401209	-0.6893336	0.42686273	-0.7211445	0.31879455	0.10624087
31.2	0.48530563	-0.4407784	-0.6894919	0.42751275	-0.7214942	0.31808542	0.10617752
31.3	0.485963	-0.4409747	-0.6893838	0.42618875	-0.7216912	0.31833413	0.10666896
31.4	0.48664915	-0.4414995	-0.6890186	0.42573346	-0.7219791	0.31850055	0.10647671
31.5	0.48751344	-0.4410962	-0.6888639	0.42578529	-0.7221798	0.31720491	0.10398998
31.6	0.48848431	-0.4412815	-0.6890902	0.42488163	-0.7227657	0.31781876	0.10409263
31.7	0.4900753	-0.4426715	-0.6894714	0.42603286	-0.7243967	0.31954064	0.10430288
31.8	0.48945054	-0.4423923	-0.6887183	0.42614481	-0.7248161	0.31933177	0.10199261
31.9	0.48914876	-0.4417998	-0.6881706	0.42535419	-0.7247755	0.3191729	0.10047091
32	0.48914876	-0.4417998	-0.6881706	0.42535419	-0.7247755	0.3191729	0.10047091
32.1	0.48916452	-0.4422424	-0.6881778	0.42587986	-0.7249136	0.31950088	0.09862745
32.2	0.48886244	-0.4421824	-0.6882915	0.42556907	-0.7250312	0.31945614	0.09867162
32.3	0.48878483	-0.4418379	-0.6879271	0.42509376	-0.7250168	0.31945744	0.09805531
32.4	0.48822387	-0.4415569	-0.6874946	0.42403113	-0.7251264	0.31934857	0.09755142
32.5	0.48843582	-0.4407996	-0.6869626	0.42353284	-0.7252292	0.31906805	0.09693323
32.6	0.48893617	-0.4404455	-0.6868501	0.42373069	-0.7255984	0.31851431	0.09615402
32.7	0.48987587	-0.4409693	-0.6871076	0.42377414	-0.7262366	0.31899924	0.09668068
32.8	0.49068973	-0.4412439	-0.686551	0.42279172	-0.7266628	0.3189168	0.09606396
32.9	0.49105247	-0.4404329	-0.6864432	0.42189109	-0.7268653	0.31984843	0.09599557
33	0.49174558	-0.4415784	-0.6864319	0.42156375	-0.7271153	0.32006171	0.09608106
33.1	0.49145115	-0.4423344	-0.6865813	0.42179046	-0.7276707	0.32036218	0.09530439
33.2	0.49228499	-0.4422871	-0.6869166	0.42084691	-0.728653	0.32113036	0.0937764
33.3	0.49193186	-0.443058	-0.6866577	0.42132907	-0.728794	0.32165302	0.09224385
33.4	0.4919627	-0.4432666	-0.6872196	0.42183265	-0.7298349	0.32380805	0.09247746
33.5	0.49179499	-0.4442153	-0.6864843	0.42116537	-0.7303317	0.32372389	0.09198812
33.6	0.4926065	-0.4451315	-0.6864042	0.42221514	-0.731675	0.32322356	0.09236815
33.7	0.4923273	-0.4449377	-0.6860603	0.42168512	-0.731928	0.32354012	0.0914815
33.8	0.49172371	-0.4455306	-0.6857756	0.42177056	-0.73231	0.32371151	0.09059724
33.9	0.49175141	-0.445804	-0.6850472	0.4228456	-0.7332165	0.32292307	0.08801294
34	0.49272326	-0.4469967	-0.6854333	0.42296692	-0.7338175	0.32435851	0.08678074
34.1	0.49203276	-0.4472331	-0.685211	0.42402205	-0.7343828	0.32533418	0.08596033
34.2	0.4920781	-0.4483611	-0.6847934	0.42438422	-0.7349891	0.32486699	0.08690826
34.3	0.4923629	-0.4496998	-0.6846212	0.4242656	-0.7356427	0.32475134	0.08626884
34.4	0.49429173	-0.4499677	-0.6848689	0.42470393	-0.7368092	0.32410581	0.08559528
34.5	0.49403409	-0.4514483	-0.6840897	0.42471606	-0.7369772	0.32369028	0.08454975
34.6	0.49413912	-0.4517918	-0.68412	0.42325556	-0.7366569	0.32291698	0.08373396
34.7	0.4936504	-0.4517638	-0.6836777	0.42205116	-0.7371018	0.32230077	0.08467663
34.8	0.49332821	-0.4509581	-0.683131	0.42174462	-0.7373364	0.32076889	0.08396143
34.9	0.49311759	-0.451531	-0.6832961	0.42225342	-0.7375124	0.32142225	0.08285818
35	0.4932428	-0.4512074	-0.6828042	0.42235349	-0.7377051	0.32108641	0.08212365
35.1	0.49329193	-0.4514896	-0.6826324	0.42170708	-0.7382222	0.32073907	0.08186546
35.2	0.49361493	-0.4517059	-0.6824758	0.42157048	-0.7381796	0.3206338	0.08078138
35.3	0.49402619	-0.4519396	-0.6824418	0.42189824	-0.7382483	0.32129631	0.08072298
35.4	0.49461106	-0.4521575	-0.6825428	0.42196757	-0.7388878	0.32079142	0.08019525
35.5	0.49505153	-0.4529733	-0.6823126	0.42143293	-0.73908	0.32120308	0.07952247
35.6	0.49489035	-0.4529736	-0.6823361	0.42156518	-0.739185	0.32100977	0.07911252
35.7	0.49405878	-0.4531278	-0.6821823	0.42222418	-0.7392231	0.32083752	0.07763937
35.8	0.49477488	-0.4531493	-0.6816756	0.42148983	-0.7394235	0.32062173	0.07785893
35.9	0.49493126	-0.4544292	-0.6814284	0.42213437	-0.7394183	0.31989511	0.07784611
36	0.49576671	-0.4544541	-0.6811809	0.42286474	-0.7396852	0.31995557	0.07791537
36.1	0.4965059	-0.454936	-0.6812477	0.42359805	-0.7403007	0.32026226	0.07776441
36.2	0.49658573	-0.4560423	-0.6809049	0.42372668	-0.7408973	0.32108527	0.07776057
36.3	0.49717461	-0.4575554	-0.6808056	0.42381926	-0.7412349	0.32234178	0.07733155
36.4	0.49759841	-0.4581695	-0.680979	0.42418756	-0.7417148	0.32291147	0.07705193
36.5	0.49788259	-0.4588097	-0.6811678	0.42394941	-0.7421369	0.32403703	0.07714736
36.6	0.49783897	-0.4595341	-0.6812314	0.42429696	-0.7425442	0.32414094	0.07742658
36.7	0.49780303	-0.4599641	-0.681569	0.42487133	-0.7431241	0.32448099	0.07642939
36.8	0.49871902	-0.4599821	-0.6811306	0.4244561	-0.7433108	0.32423897	0.07591455
36.9	0.49969545	-0.4597749	-0.681043	0.42455922	-0.7441845	0.32387718	0.07560027
37	0.50152081	-0.4599925	-0.6809525	0.42477543	-0.7451661	0.32578459	0.07523921
37.1	0.5012907	-0.4600841	-0.6806233	0.42492243	-0.7452686	0.32585023	0.07537731
37.2	0.50045861	-0.4599875	-0.6800652	0.42415033	-0.745035	0.32549189	0.07434251
37.3	0.50052871	-0.4603255	-0.6797346	0.42466112	-0.7456468	0.32602789	0.0744519
37.4	0.50087414	-0.460357	-0.6797289	0.42481186	-0.7456321	0.32629978	0.07416256
37.5	0.50176083	-0.4618631	-0.6803302	0.42513435	-0.7467134	0.32733947	0.07512787
37.6	0.50173967	-0.4623423	-0.6804658	0.42529582	-0.7468146	0.32729712	0.07440771
37.7	0.50170707	-0.463115	-0.6803709	0.42567028	-0.7471825	0.32830709	0.0737532
37.8	0.50145611	-0.4631814	-0.6800767	0.42584982	-0.7474087	0.32869898	0.07355572
37.9	0.50144031	-0.4640862	-0.6796448	0.42653666	-0.7473624	0.32827007	0.07277682
38	0.50116882	-0.4645341	-0.6795694	0.42622976	-0.7474522	0.32840118	0.07293179
38.1	0.50098142	-0.4642297	-0.6792206	0.42613617	-0.7474656	0.32876586	0.07276351
38.2	0.50113473	-0.4645338	-0.6789605	0.42612691	-0.7475165	0.32844718	0.07339982
38.3	0.50023251	-0.4650952	-0.6783756	0.42619793	-0.7477102	0.32825434	0.07309806
38.4	0.50136081	-0.4668166	-0.6784432	0.42596297	-0.748638	0.32733484	0.07307349
38.5	0.50202817	-0.4668628	-0.6779357	0.42582633	-0.7492941	0.32765577	0.07120562
38.6	0.50235083	-0.4666589	-0.6774338	0.42481996	-0.7494728	0.32762153	0.07122059
38.7	0.50326101	-0.4676071	-0.6770594	0.42465853	-0.750298	0.32765336	0.07101719
38.8	0.50450104	-0.4680001	-0.6774699	0.42543422	-0.7509166	0.32766412	0.06980526
38.9	0.50467296	-0.4675366	-0.6776073	0.4252108	-0.751381	0.32761798	0.06963347
39	0.50441764	-0.4680004	-0.6776513	0.42507819	-0.7514276	0.32792975	0.06942271
39.1	0.50456583	-0.4690424	-0.6777858	0.42530802	-0.7517337	0.32766371	0.0682704
39.2	0.50515313	-0.4697115	-0.6775547	0.4240008	-0.7524452	0.32841511	0.06807637
39.3	0.50554941	-0.4697866	-0.6776978	0.42366137	-0.7524983	0.32860799	0.06693647
39.4	0.50586011	-0.4700845	-0.6776398	0.42343798	-0.752719	0.32899997	0.06660575
39.5	0.50622352	-0.4702847	-0.6775186	0.42363185	-0.7534528	0.32906677	0.06680421
39.6	0.50590242	-0.4702665	-0.6771528	0.42346193	-0.7533991	0.32887511	0.06653937
39.7	0.50596827	-0.469619	-0.6766075	0.42373295	-0.7535326	0.32880297	0.06469309
39.8	0.50666329	-0.4702614	-0.6765642	0.42302671	-0.7541444	0.32948246	0.06489103
39.9	0.50663238	-0.4700364	-0.6764593	0.42226365	-0.7541363	0.32944779	0.06314614
40	0.50699765	-0.4702344	-0.6765441	0.422572	-0.7545743	0.32914603	0.06255399
40.1	0.50719641	-0.4706521	-0.6766536	0.4222752	-0.7551411	0.32921186	0.06180022
40.2	0.5074225	-0.4712642	-0.6765678	0.422407	-0.7554611	0.32923498	0.06116588
40.3	0.5079313	-0.4720307	-0.676548	0.42147654	-0.7557518	0.32912769	0.06008854
40.4	0.5080282	-0.4723009	-0.6766923	0.42116139	-0.7558884	0.32915263	0.06000608
40.5	0.50902041	-0.4725972	-0.6770307	0.42110212	-0.756928	0.32925324	0.05858851
40.6	0.50954159	-0.4726304	-0.6768606	0.42104136	-0.7571905	0.32988583	0.05846639
40.7	0.51036204	-0.4725008	-0.6768388	0.42018782	-0.7571875	0.32980708	0.05841411
40.8	0.51034028	-0.472789	-0.6767629	0.41977773	-0.7572943	0.33022074	0.05826727
40.9	0.51070759	-0.4732331	-0.676796	0.41939021	-0.7573406	0.33069405	0.05754906
41	0.51097991	-0.4730066	-0.6770112	0.41959552	-0.7575593	0.3317574	0.05739644
41.1	0.51205003	-0.4747894	-0.6770842	0.41988614	-0.7583794	0.33190205	0.05766852
41.2	0.51228431	-0.4755612	-0.6764535	0.42092238	-0.7590911	0.33213966	0.05799347
41.3	0.51316719	-0.4757103	-0.6761652	0.4197209	-0.759672	0.3320606	0.0581624
41.4	0.51416225	-0.4777757	-0.6767211	0.41927966	-0.7602352	0.33228174	0.05784159
41.5	0.51424731	-0.4783904	-0.6763422	0.41871348	-0.7608276	0.33215677	0.05781132
41.6	0.51494216	-0.4789681	-0.6763124	0.41861675	-0.7609926	0.33260545	0.05754728
41.7	0.5155786	-0.4791455	-0.6759539	0.41892221	-0.7614127	0.33272228	0.05807976
41.8	0.51596967	-0.4805905	-0.6758204	0.41837727	-0.7619547	0.33313258	0.0564047

Time discretization	V_{[G]}	K_{[G]}	V_{[PYR]}	K_{[PYR]}	alpha	G_E	[G]_0
45	0.53031326	-0.4938646	-0.6750846	0.41468456	-0.7728991	0.34288439	0.04156318
45.1	0.53026134	-0.4937884	-0.6744898	0.41470205	-0.7726443	0.3435006	0.04163392
45.2	0.5304599	-0.4944445	-0.6742029	0.41394532	-0.7728098	0.34318056	0.04190042
45.3	0.53044126	-0.4946273	-0.6738457	0.41356432	-0.7728454	0.34373749	0.04047233
45.4	0.52983057	-0.4952158	-0.6734273	0.41348681	-0.7726602	0.34399692	0.0400673
45.5	0.5292918	-0.4955643	-0.6731982	0.41347146	-0.7726898	0.34338729	0.03967065
45.6	0.5300317	-0.4964989	-0.6731455	0.41217354	-0.7731328	0.34423338	0.03982716
45.7	0.53027449	-0.4971055	-0.6732076	0.41170649	-0.7733822	0.34476572	0.0393941
45.8	0.53053542	-0.4969254	-0.6730943	0.41122223	-0.7737095	0.345497	0.03887238
45.9	0.53029415	-0.4972472	-0.6726468	0.41157279	-0.7739972	0.34617462	0.03899846
46	0.53158245	-0.4982739	-0.6728612	0.41180483	-0.7749297	0.34730148	0.03842326
46.1	0.53145455	-0.4994228	-0.6729382	0.41224105	-0.7751963	0.34704292	0.03749975
46.2	0.53133624	-0.4996506	-0.672528	0.41225096	-0.7751402	0.34791148	0.03703205
46.3	0.53150726	-0.4999514	-0.6728054	0.41200339	-0.7753267	0.34838906	0.03738958
46.4	0.5321215	-0.50016	-0.6731951	0.41208112	-0.775925	0.34941451	0.03621279
46.5	0.53308887	-0.4996651	-0.6727929	0.41157579	-0.7763787	0.35064543	0.03625859
46.6	0.5339642	-0.5001187	-0.6730173	0.4115878	-0.7767456	0.35145886	0.03566186
46.7	0.53433698	-0.5002314	-0.6728802	0.41179673	-0.7768242	0.35220958	0.03618149
46.8	0.53519748	-0.5009025	-0.6733322	0.41208446	-0.7771889	0.35333221	0.03554707
46.9	0.53586866	-0.5009345	-0.6733811	0.4109427	-0.7771939	0.35427471	0.03623567
47	0.53611639	-0.5006528	-0.6730193	0.41087371	-0.7771049	0.35402514	0.03610969
47.1	0.53674767	-0.5012003	-0.6731647	0.41044515	-0.7774871	0.35412544	0.03644219
47.2	0.53719104	-0.5014113	-0.6731531	0.40995036	-0.777245	0.3549153	0.03561715
47.3	0.53734689	-0.5026034	-0.6730416	0.40962073	-0.777622	0.35516714	0.03618939
47.4	0.53702953	-0.5023418	-0.6725958	0.40927216	-0.7772474	0.35495293	0.03559422
47.5	0.53719589	-0.5024342	-0.6724141	0.40909454	-0.7773818	0.35460933	0.03557812
47.6	0.53814098	-0.5036403	-0.6724713	0.40986962	-0.7776807	0.35585503	0.03472456
47.7	0.53908672	-0.5043901	-0.6727502	0.41007896	-0.7779614	0.3559596	0.03402141
47.8	0.53925814	-0.5044603	-0.6725294	0.40959407	-0.7782063	0.35561607	0.03372664
47.9	0.53884859	-0.5045565	-0.6719699	0.40817823	-0.7781773	0.35621	0.03276475
48	0.53912868	-0.5048447	-0.6717126	0.40764185	-0.7780654	0.35658372	0.03287023
48.1	0.54066362	-0.5054171	-0.6724456	0.40748292	-0.7790879	0.35731544	0.03228875
48.2	0.54119599	-0.5060181	-0.6722943	0.40797092	-0.7794158	0.35786007	0.03199366
48.3	0.54127299	-0.5066367	-0.6724272	0.40790131	-0.7795547	0.35840941	0.03144858
48.4	0.54133177	-0.5067145	-0.6717752	0.40857701	-0.7794716	0.35866702	0.03080897
48.5	0.54223496	-0.5064037	-0.6717455	0.40799774	-0.7799981	0.3588969	0.03122723
48.6	0.54321276	-0.505922	-0.6717456	0.40745714	-0.7802936	0.36033157	0.03075373
48.7	0.54330899	-0.5058323	-0.6713677	0.40677033	-0.7808208	0.36155768	0.03259347
48.8	0.54315873	-0.5060525	-0.6712763	0.40642375	-0.7807611	0.36136366	0.03253539
48.9	0.54381155	-0.5063651	-0.6714039	0.40580604	-0.7809152	0.36148041	0.03188411
49	0.54402972	-0.5066607	-0.6713954	0.40586078	-0.7809357	0.36178352	0.03171571
49.1	0.54404406	-0.5067634	-0.671432	0.40602563	-0.7810786	0.36179546	0.03182442
49.2	0.54482684	-0.5068492	-0.670959	0.40579624	-0.7815744	0.36220764	0.03249933
49.3	0.5447865	-0.5073653	-0.6708334	0.40526818	-0.7820322	0.36278731	0.03131625
49.4	0.54568441	-0.5077833	-0.6709845	0.40529907	-0.7824185	0.36356717	0.03117115
49.5	0.54585742	-0.5078346	-0.6710829	0.40510833	-0.7825635	0.36374417	0.03106745
49.6	0.54600224	-0.5077677	-0.6706672	0.40499071	-0.7827143	0.36408353	0.030909
49.7	0.54614769	-0.5085991	-0.671235	0.40529702	-0.7831859	0.36516432	0.03068673
49.8	0.54652753	-0.5093591	-0.6712767	0.40614428	-0.783553	0.36564248	0.03015055
49.9	0.54709249	-0.5099625	-0.6713745	0.40623199	-0.7836447	0.36593216	0.02962954
50	0.54777369	-0.5103034	-0.6711009	0.40648075	-0.7837276	0.36576218	0.03053
50.1	0.54825451	-0.5099816	-0.6708335	0.40699366	-0.7839631	0.36769385	0.02990571
50.2	0.54760859	-0.5099681	-0.670516	0.40686194	-0.7839003	0.36720191	0.03024091
50.3	0.54864044	-0.5101529	-0.6706864	0.40682326	-0.7843982	0.36694545	0.0291713
50.4	0.54942465	-0.5109586	-0.6706176	0.40625321	-0.7847642	0.36809865	0.02972471
50.5	0.54963809	-0.5112864	-0.6709091	0.40675553	-0.7850033	0.36837807	0.03018804
50.6	0.54958837	-0.5108167	-0.6702993	0.4060894	-0.7847833	0.36784131	0.02997416
50.7	0.55011415	-0.5127965	-0.6708801	0.40603017	-0.7853337	0.36885168	0.02912145
50.8	0.5503867	-0.5136264	-0.6707828	0.40694832	-0.7856896	0.36976184	0.0286827
50.9	0.55111498	-0.5136421	-0.6706993	0.40647742	-0.7858502	0.36989684	0.02797521
51	0.55092122	-0.5136279	-0.6704741	0.40671807	-0.7858104	0.36917659	0.02701945
51.1	0.55130663	-0.5135539	-0.6702824	0.40601382	-0.7859538	0.36930146	0.02604188
51.2	0.55191232	-0.5155349	-0.6710996	0.40503562	-0.7868218	0.37048287	0.02588959
51.3	0.55156194	-0.5151602	-0.6706011	0.4049275	-0.7866666	0.37026134	0.02549134
51.4	0.55197738	-0.5161948	-0.6699791	0.4045526	-0.7868045	0.37225502	0.02505721
51.5	0.55244191	-0.5159536	-0.6699455	0.4040125	-0.7866997	0.37234993	0.0247171
51.6	0.55303372	-0.5170901	-0.6703391	0.4041899	-0.7873821	0.37295729	0.02480308
51.7	0.5533076	-0.5176383	-0.6702239	0.40413055	-0.7877975	0.3731061	0.02370637
51.8	0.55359156	-0.5177776	-0.6697395	0.40326601	-0.7880924	0.37340045	0.02365891
51.9	0.55352513	-0.5184882	-0.6698444	0.40367227	-0.7885142	0.37481057	0.02249605
52	0.55479564	-0.5188566	-0.6702063	0.40307381	-0.7889036	0.37477309	0.02043358
52.1	0.55504556	-0.5188439	-0.6700768	0.40238441	-0.7889575	0.37535208	0.02036359
52.2	0.55548546	-0.5201577	-0.6703764	0.40285305	-0.7896836	0.37545074	0.01998446
52.3	0.55523109	-0.5198287	-0.6697903	0.40221346	-0.7895889	0.37531092	0.01955852
52.4	0.55568149	-0.5204483	-0.6700516	0.40200534	-0.7900177	0.37564579	0.01979099
52.5	0.55577535	-0.5211871	-0.6698761	0.40149545	-0.7905391	0.37524281	0.02009584
52.6	0.55627622	-0.5207634	-0.6694938	0.40032691	-0.7906673	0.37558163	0.02135955
52.7	0.55683316	-0.5211154	-0.6693964	0.40073016	-0.791127	0.37679516	0.02019843
52.8	0.55707686	-0.5213509	-0.6694026	0.40135243	-0.7912363	0.37664401	0.01895882
52.9	0.55820691	-0.522182	-0.6695916	0.40035353	-0.7919348	0.37670146	0.01904967
53	0.55773744	-0.5213663	-0.6683581	0.39935429	-0.7919762	0.37637571	0.01942954
53.1	0.55781046	-0.520583	-0.6678555	0.39785033	-0.7917882	0.3765702	0.01849992
53.2	0.55817181	-0.5204178	-0.6678301	0.39759365	-0.7921273	0.37702586	0.01838685
53.3	0.55952353	-0.5209031	-0.6679377	0.39773852	-0.7925324	0.37720319	0.01751663
53.4	0.55974721	-0.5211003	-0.6675956	0.3974062	-0.7927489	0.37782706	0.01792428
53.5	0.56043177	-0.5217199	-0.6675547	0.39670343	-0.7933791	0.3784211	0.01733112
53.6	0.56140229	-0.5237813	-0.6684105	0.39728154	-0.7948334	0.37965572	0.01680362
53.7	0.5625386	-0.5239375	-0.6683281	0.39743914	-0.7957681	0.38072835	0.01673445
53.8	0.56325381	-0.5252686	-0.6684566	0.39760908	-0.7966093	0.3826425	0.01772919
53.9	0.56410839	-0.5254781	-0.6681449	0.39795276	-0.7968953	0.38288986	0.01727394
54	0.56431788	-0.5264735	-0.6676324	0.39926538	-0.7972306	0.38396352	0.01706743
54.1	0.56491875	-0.5260904	-0.6677444	0.39946637	-0.7974172	0.38455376	0.01547541
54.2	0.564664	-0.5261533	-0.6674897	0.39924122	-0.7973906	0.38580649	0.01436416
54.3	0.56522127	-0.5257643	-0.6675944	0.39845573	-0.7977056	0.38614129	0.01467395
54.4	0.56553317	-0.5258085	-0.6674038	0.39888396	-0.798075	0.38583339	0.01424147
54.5	0.56532372	-0.5259153	-0.6671981	0.39914795	-0.7980621	0.3861328	0.01409838
54.6	0.56530349	-0.525599	-0.6669118	0.39879738	-0.7980636	0.38605988	0.01426295
54.7	0.56526956	-0.5253997	-0.6665372	0.39904843	-0.7980472	0.38580778	0.01513617
54.8	0.56526956	-0.5253997	-0.6665372	0.39904843	-0.7980472	0.38580778	0.01513617
54.9	0.56585991	-0.5258542	-0.6665554	0.39857249	-0.7982389	0.38677109	0.0148738
55	0.56626782	-0.5258669	-0.6663222	0.39821943	-0.7982133	0.38719689	0.01465901
55.1	0.5663737	-0.5257426	-0.6660883	0.39798847	-0.7982874	0.38760179	0.01464524
55.2	0.56629482	-0.525682	-0.6660512	0.39742411	-0.7983467	0.38718122	0.014148
55.3	0.56658276	-0.5262314	-0.6659179	0.39747644	-0.7987337	0.38706454	0.01523962
55.4	0.56729293	-0.5274028	-0.6663528	0.39752979	-0.7993222	0.38852921	0.01510658
55.5	0.5675506	-0.5284534	-0.6658519	0.39760111	-0.7996733	0.38860273	0.01391413
55.6	0.5675506	-0.5284534	-0.6658519	0.39760111	-0.7996733	0.38860273	0.01391413
55.7	0.56910068	-0.5295045	-0.666447	0.39789202	-0.8002396	0.3896676	0.01308175
55.8	0.56901222	-0.5300422	-0.6663391	0.39780688	-0.8004519	0.39001711	0.01274563
55.9	0.56871747	-0.5297476	-0.6655552	0.39818701	-0.8004444	0.38976567	0.01272557
56	0.56850008	-0.5303085	-0.6652739	0.39821459	-0.8008682	0.38993353	0.01306501
56.1	0.56837435	-0.5305709	-0.6646971	0.39740381	-0.8008961	0.39115809	0.013139
56.2	0.56834708	-0.5308775	-0.6647529	0.3970703	-0.8008552	0.3914758	0.01309092
56.3	0.56808108	-0.5308514	-0.6643815	0.39761083	-0.8010659	0.39205519	0.01270761
56.4	0.56850503	-0.5307716	-0.6644314	0.39784226	-0.8013806	0.3925453	0.01190691
56.5	0.56918427	-0.5312698	-0.6649254	0.39740858	-0.8018392	0.392975	0.01180218
56.6	0.56928604	-0.5314463	-0.6650072	0.39720498	-0.8019262	0.39314207	0.01177067
56.7	0.5699688	-0.532011	-0.6652064	0.39731071	-0.8022557	0.39384733	0.01128432
56.8	0.57123745	-0.5325135	-0.6657701	0.39819745	-0.8032096	0.39489274	0.01082104

11.9	0.38564714	-0.4407783	-0.7520639	0.45768915	-0.5537022	0.3056971	0.58039466
12	0.38696483	-0.4385259	-0.7506886	0.45692816	-0.554982	0.30345413	0.57527556
12.1	0.38890095	-0.4405661	-0.7515616	0.45503182	-0.5585327	0.30530927	0.57065981
12.2	0.3897326	-0.4437246	-0.7512288	0.45514551	-0.5600595	0.30694012	0.56569095
12.3	0.3908274	-0.44146	-0.7515395	0.4528198	-0.5621845	0.30791019	0.56104397
12.4	0.39176192	-0.4417556	-0.7510071	0.45403613	-0.5637454	0.30640323	0.55536957
12.5	0.39233538	-0.4389905	-0.7494432	0.45308824	-0.5644873	0.30296584	0.5508921
12.6	0.39402892	-0.4378488	-0.7491789	0.45343822	-0.5679008	0.30301567	0.54751625
12.7	0.3957304	-0.4379159	-0.7491622	0.45372396	-0.5701459	0.30203247	0.54218672
12.8	0.39625906	-0.438392	-0.7491276	0.45541348	-0.5731857	0.30388384	0.53700766
12.9	0.3994328	-0.4402498	-0.7497733	0.45470707	-0.5751439	0.30557777	0.53499014
13	0.4011302	-0.4401173	-0.7500001	0.45479275	-0.578228	0.30613207	0.52862975
13.1	0.40085518	-0.4388639	-0.7493377	0.45404097	-0.5809873	0.30641522	0.52300566
13.2	0.40311794	-0.4363907	-0.7493022	0.45432962	-0.5821531	0.30633104	0.51940944
13.3	0.40422765	-0.4361908	-0.748021	0.45291762	-0.5836288	0.30407776	0.51602338
13.4	0.40575354	-0.4355986	-0.7469344	0.45274911	-0.5847701	0.30389081	0.51114038
13.5	0.40585159	-0.434121	-0.7443328	0.45042105	-0.5863555	0.30197687	0.50557302
13.6	0.40548481	-0.4344318	-0.7446954	0.45037864	-0.5871555	0.30126002	0.50256224
13.7	0.40674603	-0.4345514	-0.7441547	0.44864206	-0.5908072	0.3028541	0.49493432
13.8	0.40782335	-0.4336318	-0.7441194	0.44626411	-0.592149	0.30428611	0.49057895
13.9	0.40790101	-0.4344162	-0.7431371	0.44673751	-0.5946579	0.30417522	0.48474102
14	0.40833193	-0.432995	-0.7428611	0.44433287	-0.5948933	0.30391948	0.47979523
14.1	0.4088952	-0.4320199	-0.7418421	0.44202772	-0.5964943	0.30293655	0.47528555
14.2	0.40828847	-0.4299664	-0.739779	0.43950513	-0.5988096	0.30138195	0.46721275
14.3	0.41114787	-0.4303939	-0.7398652	0.44071232	-0.6010349	0.3024835	0.4630231
14.4	0.41171116	-0.4298184	-0.7385379	0.44226034	-0.6019414	0.30133042	0.45648061
14.5	0.41080776	-0.4301666	-0.7376684	0.44172864	-0.6030416	0.30249722	0.45333047
14.6	0.41145731	-0.4279799	-0.7366212	0.43989513	-0.6027621	0.30167861	0.44775966
14.7	0.4142869	-0.428449	-0.7363775	0.43908391	-0.605233	0.30324303	0.44386474
14.8	0.4149525	-0.4277376	-0.7349838	0.43657706	-0.6057382	0.30083673	0.43981227
14.9	0.41563936	-0.4273303	-0.7329717	0.43368197	-0.6065517	0.30220782	0.43515341
60	0.57642075	-0.5399879	-0.6592901	0.39423461	-0.8089384	0.41016137	0.00730741
60.1	0.57642075	-0.5399879	-0.6592901	0.39423461	-0.8089384	0.41016137	0.00730741
60.2	0.57624657	-0.5401033	-0.6588073	0.39334779	-0.8089335	0.40944661	0.00767336
60.3	0.5760382	-0.5400613	-0.6585629	0.39399873	-0.8090967	0.40983222	0.00777553
60.4	0.57627543	-0.5406437	-0.6581397	0.39318993	-0.8090333	0.41010652	0.00775674
60.5	0.57668443	-0.541067	-0.6581072	0.3927493	-0.8091567	0.4099739	0.00732346
60.6	0.57604511	-0.5404476	-0.657646	0.39184861	-0.8088516	0.4101989	0.00753103
60.7	0.57635971	-0.5413298	-0.6571214	0.39179771	-0.8088369	0.41102181	0.00783992
60.8	0.57708587	-0.5416843	-0.6570482	0.39156703	-0.8091208	0.41237383	0.00757201
60.9	0.5771221	-0.5420068	-0.657021	0.39158472	-0.8091626	0.41308679	0.00694721
61	0.57744797	-0.5424763	-0.6568127	0.3910036	-0.8091896	0.41342341	0.00777381
61.1	0.57726374	-0.5428854	-0.6563431	0.39184002	-0.8094847	0.41360249	0.00730304
61.2	0.57801488	-0.5447192	-0.6570745	0.39173194	-0.8102714	0.41508252	0.00614806
61.3	0.57845494	-0.5450568	-0.6567917	0.39204962	-0.8103345	0.41608579	0.00611678
61.4	0.57888337	-0.5450014	-0.6563634	0.39133255	-0.8105808	0.41658243	0.00627554
61.5	0.57909519	-0.5448609	-0.6559483	0.39051183	-0.8105396	0.41655527	0.00671344
61.6	0.57958415	-0.5444436	-0.6549153	0.38928277	-0.8102684	0.41680368	0.00689953
61.7	0.57992447	-0.5449638	-0.6544109	0.3883626	-0.8105936	0.41670944	0.00601156
61.8	0.57934133	-0.5441231	-0.6531862	0.387687	-0.8102509	0.41600402	0.00577162
61.9	0.57916034	-0.544515	-0.6527005	0.38704018	-0.8103667	0.41589765	0.00481961
62	0.57982962	-0.5450155	-0.6529069	0.38687125	-0.8107857	0.41630283	0.00647691
62.1	0.5801862	-0.5448702	-0.6528175	0.38719248	-0.8109089	0.41611637	0.00612768
62.2	0.58016995	-0.5450163	-0.6528907	0.38656347	-0.8109108	0.4158448	0.00640513
62.3	0.5805513	-0.5456441	-0.6527879	0.38666911	-0.8109512	0.41599108	0.00613328
62.4	0.58101606	-0.5456695	-0.6528299	0.38673618	-0.8111572	0.41616358	0.00604693
62.5	0.58196109	-0.546505	-0.6527124	0.38627309	-0.8110985	0.4162311	0.00542258
62.6	0.582269	-0.5467109	-0.6527258	0.38597747	-0.8113948	0.4162176	0.00606119
62.7	0.58270172	-0.5467376	-0.6525472	0.3871302	-0.8118685	0.41742191	0.00619552
62.8	0.58276469	-0.5468854	-0.6523283	0.38685646	-0.812066	0.41736618	0.00594257
62.9	0.58304583	-0.547245	-0.6523062	0.38686846	-0.8122535	0.41764022	0.00613193
63	0.58284731	-0.5476752	-0.6522695	0.38618015	-0.8122302	0.41802368	0.00633646
63.1	0.58271723	-0.547045	-0.6514719	0.38584527	-0.8123097	0.41735494	0.00524016
63.2	0.58266141	-0.5474763	-0.6513172	0.38592418	-0.8126015	0.41826801	0.00448804
63.3	0.58266127	-0.5480534	-0.6511725	0.38655077	-0.8127437	0.41869019	0.00346564
63.4	0.58297199	-0.548848	-0.6511542	0.38681564	-0.8130648	0.41918664	0.00290183
63.5	0.58256934	-0.5484359	-0.6505145	0.38695034	-0.8130375	0.41927628	0.00365481
63.6	0.58263456	-0.5484914	-0.6504279	0.38701231	-0.8131683	0.41950282	0.00332231
63.7	0.58295352	-0.549398	-0.6505843	0.38733652	-0.8136265	0.41953384	0.00334999
63.8	0.58262014	-0.5497268	-0.6505527	0.38755584	-0.8136089	0.41987994	0.00282664
63.9	0.58261262	-0.5498973	-0.6503045	0.38712648	-0.8135799	0.41926231	0.00187017
64	0.5831578	-0.5507056	-0.6505911	0.3868614	-0.8140543	0.41946497	0.00135241
64.1	0.58275991	-0.5509142	-0.6501316	0.38688699	-0.8139421	0.42060155	-0.0006938
64.2	0.58345368	-0.5515879	-0.6500373	0.38793013	-0.8145948	0.42166223	-6.16E-05
64.3	0.58355957	-0.5517202	-0.6499046	0.38726956	-0.8146808	0.42178962	-6.83E-05
64.4	0.58365948	-0.5520744	-0.6500413	0.38766919	-0.8145832	0.42203829	-0.0003578
64.5	0.58353272	-0.5516383	-0.6494859	0.38732541	-0.8145753	0.4224124	-0.0012454
64.6	0.58372924	-0.552043	-0.6491726	0.38824741	-0.8148778	0.4231789	-0.0024545
64.7	0.58368468	-0.5519846	-0.6490821	0.38849733	-0.8149392	0.42337952	-0.0025572
64.8	0.58370257	-0.5521877	-0.6484488	0.3883555	-0.8147705	0.42321523	-0.0022299
64.9	0.58427395	-0.552975	-0.6481986	0.38811909	-0.8151395	0.42392847	-0.0021617
65	0.58436269	-0.5530612	-0.6486623	0.38810368	-0.8154392	0.42430011	-0.0022248
65.1	0.5846913	-0.5536067	-0.6485507	0.38850602	-0.8156257	0.42475455	-0.0017097
65.2	0.58475158	-0.5538728	-0.6484599	0.3888171	-0.8155767	0.4240373	-0.0019498
65.3	0.58499597	-0.5544606	-0.6487038	0.38922447	-0.8157586	0.42423896	-0.0025819
65.4	0.58505042	-0.5550159	-0.6489447	0.38901345	-0.8159642	0.4243978	-0.0028994
65.5	0.58476351	-0.5548193	-0.6487229	0.3885578	-0.8160517	0.42498196	-0.0029408
65.6	0.58486518	-0.554859	-0.6478707	0.39016783	-0.8162106	0.42444258	-0.0036399
65.7	0.58552571	-0.5548311	-0.6472232	0.39007012	-0.8162693	0.42406092	-0.0030825
65.8	0.58577837	-0.5542359	-0.6469491	0.38967667	-0.8164638	0.42429193	-0.0015629
65.9	0.58571051	-0.5547091	-0.6468548	0.38925966	-0.8165757	0.42467162	-0.001231
66	0.58559869	-0.5550668	-0.6467343	0.38909535	-0.8165895	0.42446325	-0.0018858
66.1	0.58585584	-0.5550944	-0.6461133	0.38883524	-0.8165515	0.42441932	-0.0019239
66.2	0.58614963	-0.5555715	-0.6461908	0.38863207	-0.8165825	0.4242127	-0.0021446
66.3	0.58633906	-0.5551921	-0.6459295	0.38812607	-0.8165621	0.42515766	-0.0026445
66.4	0.58653931	-0.5551337	-0.6454724	0.38799341	-0.8165632	0.42569261	-0.0019748
66.5	0.5867214	-0.5545929	-0.6445707	0.38775709	-0.8164303	0.42501666	-0.0018943
66.6	0.58665344	-0.5550308	-0.6444412	0.38778214	-0.8167285	0.42583275	-0.0021139
66.7	0.58665344	-0.5550308	-0.6444412	0.38778214	-0.8167285	0.42583275	-0.0021139
66.8	0.58687574	-0.5548164	-0.6444933	0.38733152	-0.8168761	0.426993	-0.0023502
66.9	0.5871644	-0.5551203	-0.6447871	0.38708649	-0.8170312	0.42706131	-0.002779
67	0.58764045	-0.5556635	-0.6445124	0.38690392	-0.8172999	0.42845824	-0.0035726
67.1	0.58783552	-0.5557771	-0.6445529	0.38678007	-0.8172387	0.42874099	-0.0035212
67.2	0.58815787	-0.5558508	-0.6442957	0.38702493	-0.81751	0.4295677	-0.00294
67.3	0.58847082	-0.55681	-0.6448617	0.38713091	-0.8177887	0.42975808	-0.0031493
67.4	0.58870361	-0.5576959	-0.6447521	0.38661132	-0.818099	0.42963718	-0.0023054
67.5	0.5897886	-0.5589198	-0.6445471	0.38672298	-0.8184313	0.42964447	-0.0029998
67.6	0.58967909	-0.5592122	-0.6443271	0.38659952	-0.8184856	0.42999599	-0.003692
67.7	0.58982401	-0.5601936	-0.644386	0.38638014	-0.8186131	0.4303221	-0.0045297
67.8	0.5907866	-0.5609312	-0.64419	0.38736836	-0.8191887	0.43026958	-0.005088
67.9	0.59073717	-0.5611837	-0.6438242	0.38665436	-0.8191931	0.43075544	-0.0057782
68	0.59124795	-0.5616059	-0.6439504	0.38638632	-0.8191142	0.43036832	-0.0058453
68.1	0.5912026	-0.5617659	-0.6438716	0.38552378	-0.8194034	0.43113146	-0.005669
68.2	0.591321	-0.5619822	-0.6439733	0.38552692	-0.81955	0.43100498	-0.0053293
68.3	0.59150779	-0.5623618	-0.6438068	0.38599819	-0.8198044	0.43131666	-0.0046237
68.4	0.59178122	-0.5627748	-0.6436554	0.38592659	-0.8205102	0.43208626	-0.0039557
68.5	0.59186709	-0.5630393	-0.6436333	0.38560557	-0.8204509	0.43194412	-0.0034727
68.6	0.59170169	-0.5634951	-0.6438134	0.38496655	-0.8204557	0.43206168	-0.0032428
68.7	0.59206095	-0.5637693	-0.6438127	0.38491633	-0.8207239	0.43275487	-0.0050332
68.8	0.59222832	-0.5638648	-0.6431783	0.38468548	-0.8207856	0.43343028	-0.0046179
68.9	0.59267689	-0.564385	-0.6429724	0.38505125	-0.8210941	0.43383393	-0.0051168

26.9	0.47183275	-0.425674	-0.6945456	0.4218518	-0.7031084	0.30868341	0.14853658
27	0.47116388	-0.4260299	-0.6945496	0.42260658	-0.7036208	0.3086736	0.14769436
27.1	0.47120735	-0.4264289	-0.6945163	0.42294004	-0.7035881	0.30873345	0.14732532
27.2	0.47170016	-0.4281345	-0.6952946	0.4230228	-0.7044538	0.30861196	0.14528799
27.3	0.47219743	-0.429118	-0.6955761	0.4228723	-0.7050707	0.30857306	0.14454839
27.4	0.4727459	-0.429094	-0.6950185	0.42310988	-0.7057851	0.30920317	0.14290468
27.5	0.4735751	-0.4290097	-0.6944482	0.42307842	-0.7066162	0.30897339	0.14215963
27.6	0.47407861	-0.429946	-0.69427	0.42297548	-0.7072281	0.30931624	0.14085409
27.7	0.47472317	-0.4301212	-0.6940686	0.42359611	-0.7080909	0.3093404	0.14043255
27.8	0.47434259	-0.4304396	-0.6942916	0.42349386	-0.7081328	0.31018261	0.13936238
27.9	0.47451757	-0.4307486	-0.6940408	0.42383196	-0.7083617	0.31076785	0.13832778
28	0.47481904	-0.4300375	-0.6934292	0.42387815	-0.7083917	0.31052803	0.13724798
28.1	0.47468152	-0.4301309	-0.6933048	0.42389607	-0.7086051	0.31087317	0.1369346
28.2	0.47589274	-0.4293928	-0.6933706	0.42419362	-0.7089511	0.31094898	0.13507725
28.3	0.47765758	-0.4304321	-0.6939677	0.42489361	-0.709722	0.31031405	0.13339249
28.4	0.47859548	-0.4310251	-0.6940119	0.42452047	-0.7100572	0.31083524	0.13255591
28.5	0.47955173	-0.4320083	-0.6938877	0.42482034	-0.7103613	0.31160446	0.13041853
28.6	0.47971886	-0.4323218	-0.6938812	0.42494373	-0.7107276	0.31211338	0.12940152
28.7	0.47980418	-0.432286	-0.6937181	0.42490579	-0.710934	0.31268012	0.12899703
28.8	0.47914158	-0.433331	-0.6936338	0.42610982	-0.7115583	0.31404815	0.1288248
28.9	0.47909223	-0.4337959	-0.693745	0.42718064	-0.7128322	0.31442632	0.12657901
29	0.48064051	-0.4344827	-0.693482	0.42799043	-0.7144035	0.31383779	0.1259627
29.1	0.48079942	-0.4347435	-0.6931813	0.42778542	-0.7147979	0.31458091	0.12476916
29.2	0.48048222	-0.4343518	-0.6925056	0.42704444	-0.715043	0.31478281	0.12308748
29.3	0.48060794	-0.4344028	-0.6924935	0.42692374	-0.7159814	0.31524016	0.12261212
29.4	0.48107288	-0.4341085	-0.6921334	0.42622885	-0.7157017	0.31531772	0.1210513
29.5	0.48158397	-0.4342894	-0.6919988	0.42604678	-0.7157233	0.31604793	0.11964102
29.6	0.48171243	-0.4338412	-0.691412	0.42625833	-0.7157114	0.31615919	0.11900895
29.7	0.48219624	-0.4351773	-0.6917412	0.42709627	-0.7166648	0.3180728	0.11797475
29.8	0.48121676	-0.4356076	-0.6914554	0.42677414	-0.7164146	0.31777845	0.11609609
29.9	0.48109868	-0.4363293	-0.6913347	0.42677801	-0.7167097	0.31786294	0.11515317
75	0.59629815	-0.5762184	-0.6309189	0.37419985	-0.8260684	0.44820486	-0.0057878
75.1	0.59676931	-0.5763924	-0.6309159	0.37484987	-0.8263624	0.44865163	-0.0054375
75.2	0.5968285	-0.5762929	-0.6307649	0.3745775	-0.8265119	0.44862889	-0.0045846
75.3	0.59729731	-0.5761866	-0.6306275	0.37405349	-0.8263953	0.44878961	-0.0047512
75.4	0.59706753	-0.5756325	-0.6300901	0.37365418	-0.8261809	0.44948911	-0.0058552
75.5	0.59719263	-0.57562	-0.6298146	0.37308499	-0.8259966	0.44979513	-0.0061688
75.6	0.59781062	-0.575998	-0.6298573	0.37302488	-0.8261949	0.45049362	-0.0062873
75.7	0.59726878	-0.575445	-0.6294189	0.37234249	-0.8259033	0.44986417	-0.0064788
75.8	0.59719779	-0.575749	-0.6294889	0.37250505	-0.8260029	0.45021692	-0.0064338
75.9	0.59744502	-0.5761235	-0.6295874	0.37252374	-0.826541	0.45142638	-0.0066394
76	0.59704117	-0.5758946	-0.6293748	0.37238953	-0.8264776	0.45168198	-0.006403
76.1	0.59776031	-0.5754384	-0.6286574	0.37174878	-0.826679	0.45136657	-0.0065982
76.2	0.59777388	-0.5757985	-0.628338	0.37196691	-0.8268054	0.45229959	-0.0075113
76.3	0.59799798	-0.5754549	-0.6278558	0.37102457	-0.8270924	0.45257812	-0.0058901
76.4	0.59789235	-0.5755801	-0.6277327	0.37069943	-0.8271763	0.45268717	-0.0065383
76.5	0.59779049	-0.5758723	-0.6273623	0.37097132	-0.8270619	0.45309458	-0.0065868
76.6	0.59808183	-0.5754007	-0.6267097	0.37094485	-0.827361	0.45307006	-0.0064024
76.7	0.59806471	-0.5755002	-0.6267279	0.37058176	-0.8272532	0.45303208	-0.0067433
76.8	0.59838436	-0.575766	-0.6267131	0.37067991	-0.8272262	0.45299561	-0.0067547
76.9	0.59872958	-0.5759875	-0.6267164	0.36962429	-0.827125	0.45316538	-0.0066407
77	0.59905902	-0.5766742	-0.6264193	0.36862664	-0.8271115	0.45325361	-0.0066432
77.1	0.59838681	-0.577592	-0.6267634	0.36800413	-0.8272967	0.45344593	-0.0054506
77.2	0.59860041	-0.5772743	-0.6265731	0.36789918	-0.8273599	0.45391758	-0.0052721
77.3	0.59809488	-0.5770987	-0.6259571	0.3675786	-0.8273153	0.45469595	-0.0057058
77.4	0.59785814	-0.5771091	-0.6250968	0.36673829	-0.827318	0.45524101	-0.0069259
77.5	0.59775982	-0.5772562	-0.6252097	0.36677319	-0.827384	0.45535249	-0.0066474
77.6	0.59751243	-0.5771649	-0.6244737	0.3667568	-0.827255	0.45547115	-0.0066261
77.7	0.59739794	-0.5772607	-0.6245251	0.36595727	-0.8274017	0.45585259	-0.0066021
77.8	0.59739794	-0.5772607	-0.6245251	0.36595727	-0.8274017	0.45585259	-0.0066021
77.9	0.59769417	-0.5778223	-0.6241671	0.36605161	-0.827673	0.4560282	-0.0064819
78	0.59750092	-0.5776684	-0.6241197	0.36659788	-0.8276846	0.45611012	-0.0066877
78.1	0.59747224	-0.5781759	-0.6239864	0.36731723	-0.8280445	0.45646277	-0.0063647
78.2	0.5977893	-0.5784082	-0.6237579	0.36648927	-0.8282612	0.4573829	-0.0054471
78.3	0.59775874	-0.5786354	-0.6238885	0.36612997	-0.8282772	0.45771042	-0.0057843
78.4	0.59670284	-0.5788336	-0.6230214	0.36486334	-0.8281093	0.45786394	-0.0059556
78.5	0.59675626	-0.5790246	-0.6230212	0.36505784	-0.8281376	0.45780868	-0.0063659
78.6	0.59681505	-0.5796158	-0.6228068	0.36552505	-0.8284328	0.45798792	-0.0057179
78.7	0.59566353	-0.5789906	-0.6222122	0.364994	-0.828023	0.45909267	-0.0075719
78.8	0.59607359	-0.5793651	-0.6220864	0.36500216	-0.8282138	0.45939323	-0.0073398
78.9	0.59598318	-0.5791387	-0.6220974	0.36472784	-0.8282281	0.45952589	-0.0077116
79	0.59579006	-0.5793311	-0.6221034	0.36522563	-0.8283173	0.45976197	-0.0078618
79.1	0.59592007	-0.5789965	-0.6221197	0.36511618	-0.8282459	0.46015078	-0.0077773
79.2	0.59640158	-0.5791082	-0.6221826	0.36478462	-0.8285011	0.46070907	-0.0071971
79.3	0.59642659	-0.5795065	-0.62233	0.36464959	-0.8284613	0.46043796	-0.0066419
79.4	0.59626232	-0.5795384	-0.6224393	0.36477732	-0.8284099	0.46007404	-0.006806
79.5	0.5957559	-0.5797424	-0.6220751	0.36482609	-0.8284076	0.45991813	-0.0074068
79.6	0.59511594	-0.5795548	-0.6215148	0.3644454	-0.8282528	0.46024731	-0.0071575
79.7	0.59543735	-0.5794945	-0.6209483	0.36435973	-0.828281	0.46055677	-0.007436
79.8	0.59500949	-0.5790949	-0.620129	0.36436422	-0.8281285	0.46071768	-0.0082881
79.9	0.59500949	-0.5790949	-0.620129	0.36436422	-0.8281285	0.46071768	-0.0082881
80	0.59519124	-0.5794451	-0.6199174	0.36419479	-0.8284268	0.46111309	-0.0080115
80.1	0.59536352	-0.5793191	-0.6195059	0.36326114	-0.8283177	0.4613726	-0.0087261
80.2	0.59518855	-0.5791989	-0.6195436	0.36397079	-0.8287576	0.4618104	-0.0089459
80.3	0.59498835	-0.5793239	-0.6193064	0.36449129	-0.8288006	0.4620959	-0.0092201
80.4	0.59498835	-0.5793239	-0.6193064	0.36449129	-0.8288006	0.4620959	-0.0092201
80.5	0.59543885	-0.5791537	-0.6192516	0.3643802	-0.8291421	0.46269573	-0.0088807
80.6	0.59556423	-0.5790634	-0.6194678	0.3642925	-0.8291409	0.46243031	-0.0091207
80.7	0.59561487	-0.5790041	-0.6195643	0.36264452	-0.8289261	0.46284297	-0.0097836
80.8	0.59569217	-0.5790774	-0.6194882	0.3627974	-0.8289612	0.46242345	-0.0097754
80.9	0.59565508	-0.5789837	-0.6190919	0.36259485	-0.8290873	0.46307612	-0.0104784
81	0.59583326	-0.5791564	-0.6189299	0.36140189	-0.8291027	0.4642365	-0.0117327
81.1	0.59550356	-0.5798353	-0.6184834	0.3597926	-0.8291087	0.46510706	-0.0121031
81.2	0.5953335	-0.5802345	-0.618472	0.35915418	-0.8289679	0.46521014	-0.0131965
81.3	0.59515008	-0.5805668	-0.6180939	0.35929525	-0.8292848	0.46539553	-0.0138657
81.4	0.59536613	-0.5807715	-0.6181658	0.35885936	-0.8292702	0.46565749	-0.0137963
81.5	0.59550717	-0.581303	-0.6181307	0.35819351	-0.8294181	0.46596898	-0.0137087
81.6	0.59545989	-0.5810826	-0.6179628	0.35819795	-0.8294372	0.46626857	-0.013257
81.7	0.59548892	-0.5811424	-0.6169956	0.358013	-0.8291499	0.46694777	-0.013751
81.8	0.59507632	-0.5810114	-0.6162786	0.35757125	-0.8290936	0.46661739	-0.0146624
81.9	0.5951326	-0.5812901	-0.6160144	0.35701039	-0.8293771	0.4666969	-0.0147035
82	0.59521199	-0.5810326	-0.6160214	0.35660049	-0.829279	0.46726968	-0.0153181
82.1	0.59553468	-0.5814429	-0.6159533	0.35654119	-0.829136	0.46730504	-0.0160841
82.2	0.59498413	-0.5813543	-0.6158065	0.3557508	-0.8291002	0.46671129	-0.0157305
82.3	0.59466966	-0.5813692	-0.6158964	0.35542746	-0.8289474	0.46676756	-0.016213
82.4	0.59458063	-0.5811632	-0.6156491	0.355422	-0.8288169	0.4667837	-0.0162801
82.5	0.59384425	-0.5803221	-0.6147059	0.35583702	-0.8285313	0.46681092	-0.017225
82.6	0.59390813	-0.5804797	-0.6144832	0.35639274	-0.8286361	0.46693644	-0.0165277
82.7	0.59301518	-0.5804614	-0.6139709	0.35614664	-0.8284565	0.46729967	-0.0167014
82.8	0.59295503	-0.5810549	-0.6139004	0.35593727	-0.8285922	0.46775298	-0.016282
82.9	0.59289076	-0.5809673	-0.6133719	0.35439689	-0.8281237	0.4682345	-0.0168651
83	0.59295128	-0.5806524	-0.6127585	0.35500224	-0.8282464	0.46845914	-0.0175235
83.1	0.5928283	-0.5802647	-0.6125849	0.35493613	-0.828083	0.46820456	-0.0182874
83.2	0.59301595	-0.5804665	-0.6123191	0.35460137	-0.8281803	0.46860387	-0.018313
83.3	0.59352259	-0.5807692	-0.6123668	0.35508114	-0.8284984	0.46879328	-0.0176467
83.4	0.59391821	-0.5811017	-0.612539	0.35535916	-0.8288174	0.46957406	-0.0177086
83.5	0.59424572	-0.581052	-0.6120555	0.35454142	-0.828751	0.46981353	-0.0184645
83.6	0.59391727	-0.5807498	-0.6111492	0.35351796	-0.8283671	0.4691764	-0.0187849
83.7	0.59366702	-0.5806917	-0.6100699	0.35299465	-0.8280506	0.46920171	-0.0184843
83.8	0.59399102	-0.5801578	-0.6100138	0.35236812	-0.8280611	0.46980663	-0.0183678
83.9	0.59434852	-0.5801591	-0.608973	0.35165525	-0.8280698	0.47014911	-0.018478

41.9	0.5162966	-0.4803995	-0.6756549	0.41836534	-0.7620666	0.33296692	0.05610355
42	0.51700404	-0.4803991	-0.6755766	0.41730535	-0.7625606	0.33293466	0.05482537
42.1	0.51752011	-0.4809755	-0.6757911	0.4175234	-0.7628748	0.33338655	0.05465048
42.2	0.51790295	-0.481935	-0.6759337	0.41754094	-0.7634189	0.33347918	0.05449239
42.3	0.51872092	-0.4823874	-0.6763104	0.41689912	-0.7637792	0.33399407	0.05433182
42.4	0.51899354	-0.482252	-0.6762115	0.41656865	-0.7640063	0.33370781	0.05449183
42.5	0.51985298	-0.4830473	-0.6762937	0.41728659	-0.7647824	0.33481995	0.05330397
42.6	0.52049162	-0.4834372	-0.6762187	0.41765334	-0.7651682	0.33493612	0.05340176
42.7	0.5207252	-0.4835397	-0.6760352	0.41759117	-0.7651816	0.33444372	0.05371586
42.8	0.52134355	-0.483602	-0.6760653	0.41794474	-0.7655555	0.33466366	0.05277811
42.9	0.5220494	-0.4835607	-0.6760359	0.41728465	-0.765895	0.33479253	0.05278205
43	0.52375052	-0.4832292	-0.6764714	0.41766124	-0.7663767	0.33592459	0.05129715
43.1	0.52477451	-0.4835105	-0.6764181	0.41703345	-0.7669607	0.33676669	0.05136745
43.2	0.52486524	-0.4839105	-0.6758125	0.41581576	-0.7667952	0.3366561	0.05181193
43.3	0.5256782	-0.4851797	-0.6764937	0.41604084	-0.7675468	0.33746184	0.05223931
43.4	0.52500085	-0.4862641	-0.6763284	0.41603285	-0.7683867	0.33701713	0.05178608
43.5	0.52559554	-0.4862207	-0.6758024	0.41631506	-0.7691025	0.3375268	0.04951919
43.6	0.52559156	-0.4859431	-0.6758144	0.41620664	-0.7695271	0.3381596	0.04941519
43.7	0.5267894	-0.4860023	-0.6758034	0.41590125	-0.7700414	0.33895863	0.04917351
43.8	0.5269501	-0.4859626	-0.6758214	0.41627827	-0.7702413	0.33930454	0.04903312
43.9	0.52694566	-0.4861562	-0.6754663	0.416118	-0.7702654	0.33935007	0.04931434
44	0.52730895	-0.487414	-0.6757085	0.41558686	-0.77073	0.33960103	0.0494884
44.1	0.5266863	-0.4879597	-0.6754026	0.41640732	-0.7711792	0.33999969	0.04816765
44.2	0.52681084	-0.488948	-0.6756155	0.41635686	-0.7713603	0.34123959	0.04698138
44.3	0.52786467	-0.4897714	-0.675331	0.41611481	-0.7716726	0.34146019	0.04630536
44.4	0.52855043	-0.4917618	-0.6759463	0.41701002	-0.7722476	0.34190749	0.0454002
44.5	0.52906605	-0.4920508	-0.6758517	0.41651717	-0.7721023	0.3429706	0.04459248
44.6	0.52895433	-0.4924227	-0.675762	0.41626561	-0.7722912	0.34298231	0.04442487
44.7	0.52986253	-0.4932478	-0.6756598	0.41589337	-0.7726278	0.3437901	0.04334397
44.8	0.53031751	-0.4934937	-0.6750067	0.41518855	-0.7728636	0.34320671	0.0426127
44.9	0.53026632	-0.4940787	-0.6750508	0.41517545	-0.772942	0.34300487	0.04207913
90	0.58830211	-0.5790384	-0.5897868	0.33732125	-0.8268272	0.47940633	-0.0330281
90.1	0.58815371	-0.579291	-0.5890542	0.33674233	-0.8267219	0.47972893	-0.0336664
90.2	0.58794669	-0.5793841	-0.5892525	0.33679423	-0.8267007	0.48005822	-0.0338489
90.3	0.58788738	-0.5791206	-0.5882761	0.33742167	-0.826655	0.479997	-0.0344096
90.4	0.58765996	-0.5783124	-0.5880909	0.33762563	-0.8266874	0.48044324	-0.0353724
90.5	0.58778497	-0.5784507	-0.5879039	0.33725841	-0.826578	0.48044927	-0.0355508
90.6	0.5879626	-0.5782933	-0.5877352	0.3369394	-0.8267092	0.48071414	-0.0361687
90.7	0.58751759	-0.5787996	-0.5870823	0.33591377	-0.8265719	0.48069842	-0.0356415
90.8	0.58674555	-0.5784284	-0.5866473	0.33545469	-0.8262136	0.48088663	-0.0363729
90.9	0.58720803	-0.5787463	-0.5854958	0.33509692	-0.8259311	0.48131066	-0.0359718
91	0.58696439	-0.5792005	-0.5852658	0.33530361	-0.8260265	0.48146729	-0.0358317
91.1	0.58754811	-0.5790742	-0.5851758	0.335336	-0.8262529	0.48175572	-0.0353287
91.2	0.58784727	-0.5790208	-0.5852487	0.3347891	-0.8263657	0.48198484	-0.0357125
91.3	0.58816686	-0.578777	-0.5849652	0.3349378	-0.8264038	0.48258569	-0.0364575
91.4	0.5880368	-0.5790129	-0.5848386	0.33507686	-0.8265985	0.48258036	-0.0368971
91.5	0.5869933	-0.5782631	-0.5841305	0.33428292	-0.8260638	0.48189701	-0.037231
91.6	0.58683482	-0.5784596	-0.5835917	0.33412869	-0.8260591	0.4818692	-0.0379682
91.7	0.58637204	-0.5792763	-0.5831574	0.33427673	-0.8262613	0.48112603	-0.0378598
91.8	0.58624103	-0.5787913	-0.5822798	0.33427509	-0.8260886	0.48081547	-0.0380663
91.9	0.58645086	-0.5790654	-0.5821691	0.33334594	-0.8261194	0.48087522	-0.0377299
92	0.5868729	-0.5798247	-0.5823516	0.33346921	-0.8263796	0.4818738	-0.0380813
92.1	0.58659165	-0.5785704	-0.5809655	0.33324733	-0.8263562	0.48077958	-0.0380981
92.2	0.58674395	-0.5786461	-0.5809905	0.33318561	-0.8265007	0.48125842	-0.0383085
92.3	0.58640721	-0.5788961	-0.5803206	0.33317198	-0.8264417	0.48112075	-0.0386882
92.4	0.58690893	-0.5791012	-0.5798033	0.33282077	-0.8265526	0.48139676	-0.0395826
92.5	0.58651869	-0.5797584	-0.5798093	0.3325957	-0.8263987	0.48145578	-0.0402726
92.6	0.5859535	-0.579889	-0.5790176	0.33272133	-0.826369	0.4812573	-0.0399458
92.7	0.5860097	-0.5796508	-0.5789016	0.33268837	-0.826463	0.48166592	-0.0409956
92.8	0.58608875	-0.5797317	-0.5785708	0.33219893	-0.8266085	0.48226797	-0.0406028
92.9	0.5856424	-0.5794333	-0.578195	0.33243275	-0.8265108	0.4821274	-0.0405175
93	0.58565563	-0.5799392	-0.5783987	0.33248291	-0.8265214	0.48193353	-0.0400539
93.1	0.58565563	-0.5799392	-0.5783987	0.33248291	-0.8265214	0.48193353	-0.0400539
93.2	0.58565563	-0.5799392	-0.5783987	0.33248291	-0.8265214	0.48193353	-0.0400539
93.3	0.5856299	-0.5797764	-0.5773298	0.33211488	-0.8262532	0.48211679	-0.0406068
93.4	0.58596044	-0.5796838	-0.5776376	0.33184134	-0.8262672	0.48236081	-0.0414409
93.5	0.58596044	-0.5796838	-0.5776376	0.33184134	-0.8262672	0.48236081	-0.0414409
93.6	0.58563935	-0.5797619	-0.5773229	0.33108299	-0.8259356	0.48190311	-0.0415124
93.7	0.58636186	-0.5798449	-0.5771382	0.33083778	-0.8259501	0.48269831	-0.0418049
93.8	0.58651349	-0.5797147	-0.5765597	0.33056729	-0.825984	0.48296874	-0.0422089
93.9	0.58606159	-0.5788597	-0.5759402	0.33052701	-0.8256158	0.48259905	-0.041234
94	0.58504801	-0.577751	-0.574956	0.32949691	-0.8253063	0.48176536	-0.0420435
94.1	0.58504801	-0.577751	-0.574956	0.32949691	-0.8253063	0.48176536	-0.0420435
94.2	0.58509306	-0.5774665	-0.5740967	0.32940623	-0.8250536	0.48133646	-0.0424064
94.3	0.58491042	-0.5775514	-0.5736489	0.32935532	-0.8249631	0.48149964	-0.0418596
94.4	0.58455385	-0.5766223	-0.5730284	0.32864684	-0.8247132	0.48184431	-0.0417882
94.5	0.58400989	-0.5765123	-0.5723802	0.32815971	-0.8245615	0.48149201	-0.0431495
94.6	0.58369049	-0.5758636	-0.5716098	0.32788791	-0.8241974	0.48063771	-0.0436896
94.7	0.58426054	-0.5758853	-0.5714428	0.32752598	-0.8246023	0.48120899	-0.043443
94.8	0.58367407	-0.5762677	-0.5710142	0.32764356	-0.8244027	0.48112957	-0.0433445
94.9	0.58372905	-0.5762545	-0.5707392	0.32788571	-0.8244868	0.48091938	-0.0431888
95	0.5836534	-0.5761502	-0.5706282	0.32809596	-0.8245056	0.48111706	-0.0432935
95.1	0.58376675	-0.5761746	-0.5703385	0.32811131	-0.8245652	0.48084333	-0.0423977
95.2	0.58419775	-0.5767628	-0.5704968	0.32820477	-0.8248054	0.48154797	-0.0429025
95.3	0.58401142	-0.5768613	-0.5704844	0.32781814	-0.8247781	0.48191008	-0.0433663
95.4	0.58401142	-0.5768613	-0.5704844	0.32781814	-0.8247781	0.48191008	-0.0433663
95.5	0.58397152	-0.5770575	-0.570244	0.32822543	-0.8247513	0.48201213	-0.0437095
95.6	0.58343039	-0.5767995	-0.569698	0.32800788	-0.8246573	0.48191491	-0.043887
95.7	0.58375811	-0.577178	-0.5695254	0.32882313	-0.8247432	0.48194863	-0.0431411
95.8	0.58361097	-0.5775111	-0.5693525	0.32876663	-0.8247791	0.48213955	-0.0431324
95.9	0.58357309	-0.5779234	-0.5694928	0.32875082	-0.8248582	0.48216859	-0.0430764
96	0.58306585	-0.57794	-0.5690736	0.32857812	-0.8248275	0.48221955	-0.0433987
96.1	0.58253149	-0.578333	-0.5690612	0.32880273	-0.8248571	0.48258958	-0.0435293
96.2	0.5822135	-0.5782762	-0.5690924	0.32890377	-0.8248208	0.48351517	-0.0433691
96.3	0.58188036	-0.5780396	-0.5679662	0.32758383	-0.8247407	0.48396851	-0.0430251
96.4	0.58188036	-0.5780396	-0.5679662	0.32758383	-0.8247407	0.48396851	-0.0430251
96.5	0.5811387	-0.5779525	-0.5670886	0.32746239	-0.8245632	0.48364215	-0.0435039
96.6	0.58097629	-0.5785808	-0.5674949	0.32799486	-0.8246958	0.48410251	-0.0444638
96.7	0.58097629	-0.5785808	-0.5674949	0.32799486	-0.8246958	0.48410251	-0.0444638
96.8	0.58022584	-0.577983	-0.5669979	0.32799156	-0.8245903	0.48415339	-0.0442637
96.9	0.58003452	-0.5776276	-0.5665446	0.32815259	-0.8245381	0.48406353	-0.0445629
97	0.57990536	-0.5777131	-0.5663096	0.32754863	-0.8244988	0.48382815	-0.043992
97.1	0.57962821	-0.5774253	-0.5661654	0.32742726	-0.824402	0.48378986	-0.0441621
97.2	0.57848604	-0.577249	-0.5656935	0.32791945	-0.8239746	0.48365073	-0.0451219
97.3	0.57894244	-0.5772326	-0.5655875	0.32852644	-0.8242517	0.48364853	-0.0453703
97.4	0.57919978	-0.5770375	-0.5655044	0.327727	-0.8240763	0.48407158	-0.0456108
97.5	0.57917689	-0.5770038	-0.565767	0.32674706	-0.8238468	0.48383387	-0.0456575
97.6	0.57958626	-0.577388	-0.565104	0.32719538	-0.823973	0.48376248	-0.0458888
97.7	0.57937068	-0.5768685	-0.5647314	0.3277194	-0.823797	0.48373581	-0.0466938
97.8	0.57910352	-0.5765127	-0.5639389	0.32636378	-0.8233588	0.48373332	-0.0465077
97.9	0.57912011	-0.5764178	-0.5641615	0.32650791	-0.8234207	0.48358076	-0.0462817
98	0.57900659	-0.5759195	-0.563536	0.32630041	-0.8231559	0.48330946	-0.0468304
98.1	0.5785034	-0.5756151	-0.5629637	0.32599522	-0.8229244	0.48389518	-0.0476352
98.2	0.57762016	-0.5750801	-0.5624351	0.32540777	-0.8225053	0.48325451	-0.0481414
98.3	0.57740197	-0.5747051	-0.5621443	0.32507001	-0.8223374	0.48298539	-0.0481445
98.4	0.57708912	-0.5743958	-0.5616772	0.32498246	-0.8223519	0.48319935	-0.0483992
98.5	0.57707631	-0.574101	-0.5614018	0.3251916	-0.8226411	0.48389869	-0.0481799
98.6	0.57707772	-0.5740895	-0.5612984	0.32552358	-0.8225886	0.48373389	-0.0485582
98.7	0.57707772	-0.5740895	-0.5612984	0.32552358	-0.8225886	0.48373389	-0.0485582
98.8	0.57688691	-0.5741782	-0.5613017	0.3253422	-0.8225318	0.4836361	-0.0490453
98.9	0.57692862	-0.574098	-0.5611156	0.32529598	-0.822498	0.48391426	-0.0496877

56.9	0.57171477	-0.5331276	-0.6657204	0.39772536	-0.8035985	0.39560802	0.01070538
57	0.57161549	-0.533018	-0.6655197	0.3973956	-0.8043215	0.39573716	0.01077978
57.1	0.57259688	-0.5336566	-0.6652735	0.39704883	-0.8045286	0.39650219	0.01086036
57.2	0.57238887	-0.5340561	-0.6648952	0.39715684	-0.8046951	0.39708905	0.01053322
57.3	0.57213017	-0.5343138	-0.6647235	0.39710718	-0.8047973	0.39731721	0.01106436
57.4	0.57223352	-0.534643	-0.6645982	0.39741166	-0.8048878	0.39723248	0.01096064
57.5	0.57222286	-0.5344911	-0.664324	0.39715082	-0.8046804	0.39708795	0.01092148
57.6	0.57205688	-0.5359755	-0.6637746	0.39695193	-0.8048821	0.39806392	0.00991471
57.7	0.57189818	-0.5357478	-0.6632429	0.39692073	-0.8046289	0.39744043	0.00982697
57.8	0.57225772	-0.535539	-0.6633275	0.39748928	-0.8048832	0.39828563	0.00913649
57.9	0.57210959	-0.5364939	-0.663515	0.39787801	-0.8052197	0.39896881	0.00891176
58	0.57181354	-0.5362054	-0.6636155	0.39813416	-0.8056167	0.39969113	0.00881548
58.1	0.57198315	-0.5362682	-0.6626605	0.39724609	-0.8055712	0.40029268	0.00851035
58.2	0.5724597	-0.536356	-0.6623875	0.3972228	-0.8053551	0.40029025	0.00696382
58.3	0.57304695	-0.5371691	-0.6625428	0.39657089	-0.8055303	0.40107964	0.00642611
58.4	0.57337877	-0.5370705	-0.6627123	0.3967307	-0.8056933	0.4011948	0.00575964
58.5	0.57349909	-0.5373617	-0.6626906	0.39696914	-0.8058526	0.4021185	0.00539721
58.6	0.57427248	-0.5377716	-0.6628373	0.39684798	-0.8063393	0.4045022	0.0059609
58.7	0.57416816	-0.5381455	-0.6629656	0.39715348	-0.8067732	0.40496256	0.0068542
58.8	0.57488183	-0.5391915	-0.6622894	0.39672602	-0.8072069	0.4045776	0.00818816
58.9	0.57574103	-0.5393769	-0.6622055	0.39683786	-0.8078153	0.40622876	0.00892085
59	0.57589518	-0.5399999	-0.6618801	0.39669619	-0.8077286	0.40647788	0.00897253
59.1	0.57607365	-0.5402941	-0.6619324	0.39635602	-0.8078075	0.40682485	0.00851138
59.2	0.57620305	-0.540273	-0.6615791	0.39557142	-0.8079522	0.40739701	0.00842439
59.3	0.57641232	-0.5399654	-0.6607839	0.39501087	-0.8080715	0.40786587	0.00681549
59.4	0.57631869	-0.5398667	-0.6601957	0.39552642	-0.8084341	0.40832879	0.00752029
59.5	0.57631869	-0.5398667	-0.6601957	0.39552642	-0.8084341	0.40832879	0.00752029
59.6	0.57638003	-0.5403602	-0.6603492	0.39480099	-0.8088628	0.40833557	0.00692551
59.7	0.57661757	-0.5402036	-0.6599475	0.39470765	-0.808869	0.40900388	0.00667998
59.8	0.57675782	-0.5404121	-0.6599378	0.39423302	-0.8089916	0.40943181	0.00719243
59.9	0.57649889	-0.5399075	-0.6594851	0.39436431	-0.8090038	0.41011093	0.00705637
105	0.56876073	-0.57068	-0.5489741	0.31512526	-0.8190494	0.48017165	-0.0534636
105.1	0.5687271	-0.5708274	-0.5495755	0.314961	-0.8190858	0.48014117	-0.0532032
105.2	0.56894104	-0.5705688	-0.5495536	0.31532794	-0.8190609	0.48020489	-0.0522088
105.3	0.56894823	-0.5705222	-0.5492809	0.31537024	-0.8190273	0.48027096	-0.0519233
105.4	0.5683613	-0.5707554	-0.5488717	0.31614741	-0.8189057	0.47998352	-0.0516106
105.5	0.56778773	-0.5708161	-0.5488895	0.31590979	-0.8186634	0.47967906	-0.0519866
105.6	0.56789958	-0.5706702	-0.5492149	0.31519015	-0.8185513	0.47974458	-0.0519283
105.7	0.56786125	-0.570829	-0.5491785	0.31449945	-0.8184732	0.47949815	-0.0513931
105.8	0.56774854	-0.5708016	-0.5492634	0.31458765	-0.8185884	0.47936543	-0.0506874
105.9	0.56788053	-0.5706638	-0.5488116	0.3145023	-0.8185128	0.47959155	-0.0512466
106	0.56737665	-0.570382	-0.548335	0.31471628	-0.8184858	0.4798378	-0.0513785
106.1	0.5676707	-0.5706227	-0.5485762	0.31462266	-0.8185756	0.48024039	-0.051616
106.2	0.56742571	-0.5698013	-0.5476835	0.31426744	-0.8184623	0.48066545	-0.0513537
106.3	0.56739514	-0.5697422	-0.5481199	0.31455445	-0.8185802	0.48038766	-0.0513932
106.4	0.56709423	-0.5697708	-0.5476805	0.3143221	-0.8187942	0.48049561	-0.0504523
106.5	0.56700835	-0.5699902	-0.5477823	0.31381989	-0.8187492	0.48057035	-0.0502618
106.6	0.56639348	-0.5695177	-0.5472226	0.31351399	-0.8184911	0.48006715	-0.0504936
106.7	0.56639348	-0.5695177	-0.5472226	0.31351399	-0.8184911	0.48006715	-0.0504936
106.8	0.56659877	-0.5699697	-0.5472075	0.31364364	-0.8186637	0.47980221	-0.0499379
106.9	0.56647822	-0.5698871	-0.5471798	0.31449321	-0.8187461	0.48034619	-0.0505154
107	0.56674643	-0.5700311	-0.5471905	0.31362924	-0.818709	0.48065064	-0.050347
107.1	0.56674643	-0.5700311	-0.5471905	0.31362924	-0.818709	0.48065064	-0.050347
107.2	0.56654569	-0.5698278	-0.5472318	0.31359662	-0.8187909	0.48071027	-0.0502257
107.3	0.56609809	-0.5695275	-0.547002	0.31348212	-0.8186824	0.48089859	-0.0505165
107.4	0.56578341	-0.5690006	-0.5465639	0.31344171	-0.818715	0.48112168	-0.0508176
107.5	0.56531528	-0.5690739	-0.5459462	0.31298037	-0.8185379	0.48066306	-0.0517467
107.6	0.56531528	-0.5690739	-0.5459462	0.31298037	-0.8185379	0.48066306	-0.0517467
107.7	0.5655884	-0.5691009	-0.5461535	0.31285298	-0.8187293	0.48097002	-0.0517356
107.8	0.56540097	-0.5684282	-0.545914	0.31343638	-0.8189049	0.48130565	-0.0506242
107.9	0.56476914	-0.5687768	-0.5459538	0.3132962	-0.8191952	0.48211962	-0.0500886
108	0.56444227	-0.5691261	-0.5454082	0.31266914	-0.819385	0.48193799	-0.0498364
108.1	0.56444227	-0.5691261	-0.5454082	0.31266914	-0.819385	0.48193799	-0.0498364
108.2	0.56449201	-0.5696549	-0.5454063	0.31288091	-0.8194831	0.4823812	-0.0506296
108.3	0.56449201	-0.5696549	-0.5454063	0.31288091	-0.8194831	0.4823812	-0.0506296
108.4	0.56382609	-0.5693872	-0.5446651	0.3131111	-0.8192741	0.48206925	-0.0498
108.5	0.56286527	-0.5688757	-0.5439378	0.31310461	-0.8189282	0.48197098	-0.0499869
108.6	0.56256793	-0.568922	-0.5441525	0.31302586	-0.8189181	0.48278896	-0.0504231
108.7	0.56217422	-0.569382	-0.5437715	0.31253102	-0.8188367	0.48273937	-0.0495896
108.8	0.56163174	-0.5693864	-0.543808	0.31153328	-0.8183616	0.48266481	-0.0503543
108.9	0.5613976	-0.5692574	-0.5437474	0.31176919	-0.818438	0.48269905	-0.0505213
109	0.56118454	-0.5693443	-0.5433618	0.31144587	-0.8183193	0.48283619	-0.0511457
109.1	0.56089397	-0.5697164	-0.5432975	0.31140472	-0.8184801	0.48251961	-0.0522555
109.2	0.56058253	-0.5704559	-0.543307	0.31077335	-0.8185773	0.48342456	-0.0523424
109.3	0.56025342	-0.5701575	-0.5425364	0.31074019	-0.818388	0.48319279	-0.0521004
109.4	0.55999041	-0.5706823	-0.5424115	0.31050893	-0.8184466	0.48335467	-0.0520797
109.5	0.5599745	-0.5704762	-0.5421672	0.31025279	-0.8184149	0.48306372	-0.0519243
109.6	0.55993454	-0.5712687	-0.5421436	0.30963411	-0.8185704	0.48353842	-0.0523556
109.7	0.55968943	-0.5711465	-0.5420427	0.30924762	-0.8187523	0.48346641	-0.0518318
109.8	0.55928935	-0.5705307	-0.5416464	0.30867451	-0.8183461	0.48289651	-0.0514744
109.9	0.55943665	-0.5706676	-0.5416361	0.30875797	-0.8182771	0.48297957	-0.0513011
110	0.5593107	-0.5706844	-0.5412531	0.30836281	-0.8179166	0.48283429	-0.0511777
110.1	0.55932525	-0.5708268	-0.5406852	0.30835344	-0.8177309	0.48273747	-0.0509891
110.2	0.55922972	-0.5704817	-0.5405802	0.30788002	-0.817656	0.48275518	-0.0510264
110.3	0.55922972	-0.5704817	-0.5405802	0.30788002	-0.817656	0.48275518	-0.0510264
110.4	0.55943697	-0.5706476	-0.5402321	0.3079118	-0.8176277	0.48310786	-0.0520364
110.5	0.55916738	-0.5700532	-0.5401149	0.3075975	-0.8175614	0.4827901	-0.051863
110.6	0.5590171	-0.5700345	-0.5403326	0.30757046	-0.8176336	0.48296797	-0.0523229
110.7	0.55893194	-0.5698465	-0.5403915	0.30715855	-0.817579	0.48313724	-0.0520593
110.8	0.55876745	-0.5694005	-0.5403139	0.30621342	-0.8173717	0.48260215	-0.0520651
110.9	0.55826492	-0.5695065	-0.5402486	0.30612166	-0.8173225	0.48240322	-0.0518484
111	0.55822556	-0.5688668	-0.5393777	0.30663339	-0.8171732	0.48260925	-0.0516491
111.1	0.55719763	-0.5684962	-0.5390854	0.3054085	-0.8167455	0.48213908	-0.051369
111.2	0.55738153	-0.568525	-0.5393506	0.30528002	-0.8169493	0.48246242	-0.0519198
111.3	0.55776361	-0.568715	-0.5394725	0.30448901	-0.8168131	0.48207923	-0.0517478
111.4	0.55738804	-0.5687075	-0.5394255	0.3041146	-0.8166197	0.48159636	-0.0521344
111.5	0.5573085	-0.5685469	-0.5387678	0.30390615	-0.8165532	0.48155362	-0.0521276
111.6	0.55713591	-0.5685309	-0.5387218	0.30390679	-0.8166634	0.48152866	-0.0521774
111.7	0.557002	-0.5678103	-0.5382834	0.30368618	-0.8167787	0.48242823	-0.0526709
111.8	0.55734093	-0.5676061	-0.5374023	0.30316731	-0.8165313	0.48212206	-0.0537198
111.9	0.55777204	-0.5677129	-0.5370421	0.30301673	-0.8165298	0.48267624	-0.0544403
112	0.55756984	-0.5676392	-0.5369887	0.30263379	-0.8166426	0.48299449	-0.0548556
112.1	0.55723792	-0.5675894	-0.5372357	0.30272328	-0.8166462	0.48299761	-0.0547808
112.2	0.55696722	-0.567586	-0.5371492	0.30237887	-0.8164857	0.4828345	-0.0550543
112.3	0.55701249	-0.5674401	-0.5369301	0.3020575	-0.8164304	0.48274004	-0.0545287
112.4	0.5569381	-0.5675444	-0.537015	0.30245025	-0.8165498	0.48288335	-0.0546319
112.5	0.55658294	-0.5672555	-0.5370425	0.30260449	-0.8164793	0.48274983	-0.0553238
112.6	0.55584469	-0.5670127	-0.5361286	0.30287495	-0.8163062	0.48224695	-0.056285
112.7	0.55584469	-0.5670127	-0.5361286	0.30287495	-0.8163062	0.48224695	-0.056285
112.8	0.55572044	-0.566511	-0.5359058	0.30221993	-0.8162335	0.48298696	-0.0554969
112.9	0.55491473	-0.565533	-0.5351134	0.30154515	-0.8162756	0.4845761	-0.0555053
113	0.55484733	-0.5655233	-0.5350373	0.30127478	-0.8161464	0.48436229	-0.0552499
113.1	0.55477592	-0.5657264	-0.5350145	0.30064698	-0.8163859	0.48422913	-0.0552974
113.2	0.55446228	-0.5653285	-0.5346551	0.30075085	-0.8167308	0.48419855	-0.0544615
113.3	0.55436755	-0.5652276	-0.5346318	0.30097213	-0.8166916	0.48421182	-0.0544741
113.4	0.55418574	-0.5652963	-0.5343658	0.30155429	-0.8167486	0.48448262	-0.0549338
113.5	0.55416652	-0.5653663	-0.5342419	0.30166512	-0.8169141	0.48441991	-0.0554055
113.6	0.55388181	-0.5649845	-0.5338675	0.30210302	-0.8167983	0.48413843	-0.0554183
113.7	0.5535677	-0.5643339	-0.533153	0.30167118	-0.8165972	0.48442729	-0.0554117
113.8	0.55364158	-0.5643024	-0.5328386	0.30213008	-0.8167896	0.48514852	-0.0554329
113.9	0.55340451	-0.5639967	-0.5328594	0.30220213	-0.8166779	0.48521363	-0.055276

69	0.59316007	-0.5652423	-0.6434063	0.38474742	-0.8215496	0.4347563	-0.0043909
69.1	0.59257916	-0.5650102	-0.6421669	0.38393364	-0.8211584	0.43396189	-0.0051092
69.2	0.59257528	-0.5652571	-0.6421296	0.38353103	-0.8211946	0.4335188	-0.0054367
69.3	0.59227038	-0.5658388	-0.6422859	0.38326945	-0.8213073	0.4343923	-0.0053945
69.4	0.59249428	-0.5666016	-0.6424494	0.38291591	-0.8212425	0.43421563	-0.0058686
69.5	0.59301414	-0.5669396	-0.6419601	0.3833405	-0.8213825	0.4351959	-0.0046379
69.6	0.59309753	-0.5674068	-0.6420941	0.38346987	-0.821755	0.43587705	-0.0049071
69.7	0.59313359	-0.5674334	-0.6415281	0.38259475	-0.8220439	0.43597401	-0.0052166
69.8	0.59278909	-0.5672454	-0.64115	0.38298691	-0.8221119	0.43636404	-0.0050977
69.9	0.59299328	-0.5671217	-0.6409185	0.38277575	-0.8221047	0.43621421	-0.0053903
70	0.59440563	-0.568253	-0.6413047	0.38299595	-0.8225986	0.43699222	-0.0058934
70.1	0.59470536	-0.568487	-0.6412482	0.38291264	-0.8226525	0.43729453	-0.0055625
70.2	0.59472016	-0.5682842	-0.641203	0.38205327	-0.8225651	0.43733262	-0.0053919
70.3	0.59472122	-0.5684304	-0.6409561	0.38208872	-0.8226254	0.43791456	-0.0050634
70.4	0.59470177	-0.5690533	-0.6409891	0.3824714	-0.8229125	0.43854013	-0.0056359
70.5	0.59388778	-0.5689567	-0.640426	0.38227855	-0.8228782	0.43896848	-0.0060204
70.6	0.59359835	-0.5690543	-0.6399666	0.38213525	-0.8229417	0.43900886	-0.0057617
70.7	0.59337116	-0.569455	-0.6397501	0.38163506	-0.8228724	0.43948892	-0.0062424
70.8	0.59356185	-0.5692878	-0.6395865	0.38125643	-0.8227164	0.43945555	-0.0066678
70.9	0.59354608	-0.5693025	-0.6393584	0.38098909	-0.8226781	0.43962933	-0.0063835
71	0.59356813	-0.5696697	-0.6389078	0.38234645	-0.8230486	0.43976107	-0.0064837
71.1	0.59361307	-0.5697992	-0.6388189	0.38262687	-0.8230794	0.43993881	-0.0062405
71.2	0.59373114	-0.569979	-0.6384178	0.38281149	-0.8230685	0.44086585	-0.0054222
71.3	0.59380437	-0.5701821	-0.6384491	0.38243757	-0.823059	0.44100821	-0.0053882
71.4	0.59396719	-0.5698665	-0.6386027	0.38237414	-0.8232697	0.44216904	-0.0064775
71.5	0.59435978	-0.5700597	-0.6385319	0.38194432	-0.8235358	0.44311307	-0.0055194
71.6	0.59468049	-0.5702318	-0.6381765	0.38157174	-0.8236503	0.44418897	-0.0055318
71.7	0.59475581	-0.5696952	-0.6376344	0.38124369	-0.8234399	0.44405994	-0.0058287
71.8	0.59475412	-0.5700737	-0.637627	0.38122897	-0.8234869	0.44376124	-0.0059517
71.9	0.59480964	-0.5700731	-0.6370666	0.3809931	-0.8233765	0.44376146	-0.0057565
72	0.59475757	-0.570115	-0.636918	0.38075011	-0.8235281	0.44406098	-0.0052651
72.1	0.59505573	-0.5701968	-0.6370716	0.38020635	-0.8234605	0.44380878	-0.0056803
72.2	0.59563557	-0.5702961	-0.6368646	0.38058165	-0.823956	0.44454824	-0.0064964
72.3	0.59506929	-0.5702325	-0.636647	0.37980889	-0.8239212	0.44498731	-0.0065752
72.4	0.59568254	-0.5708596	-0.6367869	0.37916798	-0.8241592	0.44505715	-0.0065115
72.5	0.59608238	-0.5712178	-0.6361212	0.37813275	-0.8244311	0.44450907	-0.0060311
72.6	0.59601615	-0.5712981	-0.6356819	0.37862098	-0.8244373	0.44475388	-0.0063204
72.7	0.59642105	-0.5718169	-0.6358144	0.37831769	-0.824771	0.44549695	-0.0067284
72.8	0.59654219	-0.5717036	-0.6357782	0.37820659	-0.8247735	0.44547337	-0.0063804
72.9	0.59594104	-0.5718454	-0.6353759	0.37785967	-0.8248156	0.44602037	-0.006506
73	0.59559571	-0.5718707	-0.6349865	0.37710267	-0.8246865	0.44595854	-0.0057303
73.1	0.59569367	-0.5722731	-0.6349166	0.37709447	-0.8249785	0.44643873	-0.0054882
73.2	0.59560746	-0.5723118	-0.6346946	0.37725137	-0.824976	0.44672553	-0.0059065
73.3	0.59531359	-0.5722964	-0.6345472	0.3773413	-0.8249803	0.44684606	-0.005754
73.4	0.59557964	-0.5724554	-0.6344162	0.3768219	-0.824904	0.4467107	-0.0055811
73.5	0.59594423	-0.5725808	-0.6342466	0.37693107	-0.8250476	0.44716405	-0.0054918
73.6	0.59578227	-0.5722392	-0.6340227	0.37633757	-0.8250083	0.44681191	-0.0057449
73.7	0.59577581	-0.5722701	-0.6339346	0.37623927	-0.8250983	0.44649032	-0.005997
73.8	0.59633717	-0.5733626	-0.6344512	0.37621151	-0.8254902	0.44703519	-0.0064516
73.9	0.5963812	-0.5740588	-0.6341546	0.37609673	-0.8255149	0.44743012	-0.0062413
74	0.5964872	-0.5743738	-0.6339355	0.37611237	-0.8256687	0.44748115	-0.0068812
74.1	0.59620267	-0.5739873	-0.63372	0.37580389	-0.8256351	0.44736668	-0.0064235
74.2	0.59642251	-0.5743284	-0.6332532	0.37514529	-0.8256293	0.44717732	-0.006266
74.3	0.59673776	-0.5739296	-0.6325973	0.37527035	-0.825631	0.44696332	-0.0051527
74.4	0.59622909	-0.5738028	-0.6324272	0.37528184	-0.8253695	0.44709488	-0.0054344
74.5	0.59636001	-0.5741279	-0.6323765	0.37534874	-0.8254452	0.44730478	-0.0056113
74.6	0.59655254	-0.5743877	-0.6324788	0.3756606	-0.825686	0.44793327	-0.0057609
74.7	0.59658505	-0.5748339	-0.6321993	0.37481688	-0.825915	0.44837682	-0.0053219
74.8	0.59637217	-0.5755511	-0.6315835	0.37525591	-0.8258562	0.44866322	-0.0056713
74.9	0.59647617	-0.5762116	-0.6315139	0.37448551	-0.8260724	0.44876449	-0.0060265
120	0.54100253	-0.5569696	-0.5191104	0.29432144	-0.8129961	0.48519142	-0.0646203
120.1	0.54092111	-0.5569743	-0.5189448	0.29459559	-0.8131194	0.48533384	-0.0644482
120.2	0.54118066	-0.5570487	-0.5190139	0.29396738	-0.8132417	0.48586981	-0.0646168
120.3	0.54108007	-0.5571455	-0.5187467	0.29410373	-0.8131049	0.48584172	-0.0649159
120.4	0.54108007	-0.5571455	-0.5187467	0.29410373	-0.8131049	0.48584172	-0.0649159
120.5	0.54103013	-0.5568177	-0.5185659	0.2941671	-0.8129568	0.48599853	-0.0653296
120.6	0.54114274	-0.5565011	-0.5180045	0.29401554	-0.8130445	0.48630257	-0.0647169
120.7	0.54114274	-0.5565011	-0.5180045	0.29401554	-0.8130445	0.48630257	-0.0647169
120.8	0.54077665	-0.5564522	-0.5179213	0.29397106	-0.8129729	0.48638627	-0.0645695
120.9	0.54050294	-0.5563901	-0.5178232	0.29397655	-0.812867	0.48587921	-0.0647935
121	0.54072829	-0.5574343	-0.5180508	0.29396919	-0.8132215	0.48605879	-0.0644624
121.1	0.54072829	-0.5574343	-0.5180508	0.29396919	-0.8132215	0.48605879	-0.0644624
121.2	0.5397145	-0.5568347	-0.5172304	0.29388644	-0.8129285	0.48509465	-0.0644208
121.3	0.5397145	-0.5568347	-0.5172304	0.29388644	-0.8129285	0.48509465	-0.0644208
121.4	0.54005707	-0.55624	-0.5170921	0.29396616	-0.812742	0.48540351	-0.0647608
121.5	0.53994316	-0.5557926	-0.5167245	0.29349127	-0.8125856	0.48534636	-0.0644576
121.6	0.53949799	-0.5551444	-0.5158308	0.2944305	-0.8126217	0.48555864	-0.0645543
121.7	0.53970936	-0.5549536	-0.5159577	0.29454252	-0.8128681	0.48572884	-0.0650111
121.8	0.53970936	-0.5549536	-0.5159577	0.29454252	-0.8128681	0.48572884	-0.0650111
121.9	0.53936831	-0.5555448	-0.5154135	0.29449908	-0.8128715	0.48525574	-0.0652422
122	0.53927858	-0.555659	-0.5152543	0.29436679	-0.8127777	0.48504503	-0.0654793
122.1	0.53912716	-0.5558762	-0.5155876	0.29445674	-0.8127617	0.48522923	-0.0657902
122.2	0.5391392	-0.556103	-0.5157092	0.29412095	-0.812707	0.48487526	-0.0656515
122.3	0.53870215	-0.5559527	-0.515604	0.29377898	-0.8124845	0.48442143	-0.0658921
122.4	0.53870215	-0.5559527	-0.515604	0.29377898	-0.8124845	0.48442143	-0.0658921
122.5	0.53849017	-0.5558002	-0.5156177	0.2936754	-0.8124548	0.48471589	-0.065725
122.6	0.53880289	-0.5548304	-0.5156746	0.2941298	-0.8125803	0.48505965	-0.066181
122.7	0.53838786	-0.5546914	-0.515195	0.29384789	-0.8123208	0.48450932	-0.0665605
122.8	0.53842607	-0.5544649	-0.5147803	0.29447034	-0.812341	0.48484927	-0.0666511
122.9	0.53808995	-0.5543013	-0.5147005	0.29474344	-0.8122286	0.4846739	-0.0667371
123	0.53755447	-0.5544463	-0.5149772	0.29477257	-0.812133	0.484572	-0.0671134
123.1	0.53713001	-0.5540414	-0.5143523	0.29436736	-0.8119332	0.48429594	-0.0673374
123.2	0.53695037	-0.5540922	-0.5144314	0.29477132	-0.8119657	0.48419511	-0.0670912
123.3	0.53706322	-0.5540902	-0.5142399	0.29434754	-0.8118803	0.4840811	-0.0667549
123.4	0.53699318	-0.5540793	-0.5140888	0.29444243	-0.8117424	0.483882	-0.0671767
123.5	0.5362292	-0.5536996	-0.5132796	0.29401458	-0.8114167	0.48367299	-0.0672871
123.6	0.5362292	-0.5536996	-0.5132796	0.29401458	-0.8114167	0.48367299	-0.0672871
123.7	0.53603149	-0.5534167	-0.5128224	0.2938479	-0.8113587	0.48334565	-0.0670114
123.8	0.53603149	-0.5534167	-0.5128224	0.2938479	-0.8113587	0.48334565	-0.0670114
123.9	0.53603149	-0.5534167	-0.5128224	0.2938479	-0.8113587	0.48334565	-0.0670114
124	0.53595246	-0.5530928	-0.5125541	0.29365903	-0.8112292	0.48298269	-0.0668165
124.1	0.53582641	-0.5530816	-0.5125227	0.29333688	-0.8113027	0.4829853	-0.0668987
124.2	0.53550295	-0.5533801	-0.5127866	0.2934697	-0.8112371	0.4825897	-0.0674405
124.3	0.53523691	-0.5529969	-0.5123484	0.29347898	-0.811399	0.4832415	-0.0673676
124.4	0.53546633	-0.5528613	-0.5121228	0.2934522	-0.8113549	0.48307489	-0.0669451
124.5	0.53508829	-0.5525699	-0.5118922	0.29376568	-0.81124	0.48273271	-0.067177
124.6	0.53478464	-0.5522138	-0.5114918	0.2931445	-0.8110971	0.48295388	-0.0670851
124.7	0.53478464	-0.5522138	-0.5114918	0.2931445	-0.8110971	0.48295388	-0.0670851
124.8	0.53489508	-0.5520226	-0.5117162	0.29315166	-0.8112302	0.48291923	-0.0670024
124.9	0.53489508	-0.5520226	-0.5117162	0.29315166	-0.8112302	0.48291923	-0.0670024
125	0.53487039	-0.5519903	-0.5115457	0.29324972	-0.8110658	0.48289872	-0.0667928
125.1	0.53487039	-0.5519903	-0.5115457	0.29324972	-0.8110658	0.48289872	-0.0667928
125.2	0.53480172	-0.5515432	-0.5112304	0.29285838	-0.8108049	0.48271591	-0.0669864
125.3	0.53469521	-0.5516963	-0.5110929	0.29252263	-0.8106769	0.48295119	-0.0669003
125.4	0.53469521	-0.5516963	-0.5110929	0.29252263	-0.8106769	0.48295119	-0.0669003
125.5	0.53453549	-0.5513557	-0.5106016	0.29218215	-0.8106189	0.48280965	-0.0666209
125.6	0.53449312	-0.5517735	-0.5105835	0.29224234	-0.8106022	0.48309886	-0.0670015
125.7	0.53429178	-0.551633	-0.5106406	0.2922061	-0.8104623	0.48322224	-0.0672307
125.8	0.53432473	-0.5515768	-0.5105471	0.29204474	-0.8104074	0.4832409	-0.0669753
125.9	0.53414757	-0.5516081	-0.5105725	0.2920243	-0.8103885	0.48322069	-0.0668154
126	0.53424065	-0.5515415	-0.5108752	0.29155655	-0.8103061	0.48325363	-0.0669958

84	0.59385689	-0.579811	-0.6082004	0.3518373	-0.8280019	0.46925817	-0.0180894
84.1	0.59426518	-0.5802031	-0.6078195	0.35104751	-0.8282602	0.46992761	-0.0182043
84.2	0.59406147	-0.5798296	-0.6078588	0.35065134	-0.828125	0.469638	-0.0189239
84.3	0.59486396	-0.5797745	-0.6082973	0.35062168	-0.828255	0.47116242	-0.0201212
84.4	0.59493373	-0.5806949	-0.608546	0.35068647	-0.8285937	0.47147848	-0.0201675
84.5	0.59466	-0.5805936	-0.6081369	0.3503193	-0.8286643	0.471819	-0.0198371
84.6	0.59417094	-0.5805286	-0.6074226	0.3502312	-0.8284767	0.4717295	-0.020451
84.7	0.59401827	-0.5805906	-0.6073902	0.34985573	-0.8284883	0.47255663	-0.0206291
84.8	0.59368054	-0.5805045	-0.6072893	0.34972428	-0.8282265	0.47248374	-0.0211937
84.9	0.59323843	-0.5800105	-0.6070909	0.34990182	-0.8280578	0.47213791	-0.0213985
85	0.59334882	-0.5798134	-0.6071706	0.35005297	-0.8280745	0.47215647	-0.0218441
85.1	0.59286421	-0.5801675	-0.6068247	0.34847359	-0.8278779	0.47158223	-0.0223025
85.2	0.59261434	-0.5801845	-0.6068025	0.34805995	-0.8277761	0.47170936	-0.0218359
85.3	0.59223472	-0.5795267	-0.6052945	0.34697278	-0.8273602	0.47114067	-0.0220805
85.4	0.59254011	-0.579227	-0.6050664	0.34630327	-0.8273718	0.47156503	-0.0224018
85.5	0.59269803	-0.5789336	-0.6042382	0.34598325	-0.8272768	0.47134475	-0.0225023
85.6	0.59242394	-0.579001	-0.6039856	0.34489413	-0.8272873	0.47178692	-0.0219354
85.7	0.59223687	-0.5785849	-0.6037711	0.34483688	-0.8273817	0.47218116	-0.021493
85.8	0.5923041	-0.5782057	-0.6030164	0.34472255	-0.8274016	0.47086726	-0.0211341
85.9	0.59275921	-0.5787961	-0.6029988	0.34424724	-0.8279534	0.47117029	-0.0212432
86	0.59326197	-0.5788634	-0.6032262	0.3443158	-0.8281772	0.47161747	-0.0209404
86.1	0.59340366	-0.5792655	-0.6036899	0.34498561	-0.8285938	0.47242166	-0.0216602
86.2	0.59319795	-0.579511	-0.6039633	0.34395094	-0.8287065	0.4728636	-0.0220728
86.3	0.59277321	-0.5790352	-0.6033186	0.34377636	-0.8286093	0.47309184	-0.0225013
86.4	0.59291192	-0.5785543	-0.6029941	0.34425769	-0.828652	0.47373031	-0.0228836
86.5	0.59248051	-0.5787672	-0.602663	0.34412799	-0.8284577	0.47406238	-0.0227746
86.6	0.59236085	-0.5787983	-0.6028395	0.34383509	-0.8284202	0.47407423	-0.0227867
86.7	0.59259556	-0.5786723	-0.6026328	0.34414478	-0.8285374	0.47400651	-0.022989
86.8	0.59205487	-0.57798	-0.6022686	0.34417645	-0.8283464	0.47428249	-0.0246078
86.9	0.59132757	-0.5772859	-0.601101	0.34391147	-0.8278664	0.47377342	-0.0246735
87	0.59160405	-0.5778989	-0.6005674	0.34318431	-0.8281539	0.47447378	-0.0255398
87.1	0.59198545	-0.5787035	-0.6003599	0.34327462	-0.8282068	0.47535504	-0.0257521
87.2	0.59213059	-0.578696	-0.6001615	0.34308985	-0.8283962	0.47519647	-0.0265959
87.3	0.59162466	-0.5785594	-0.598576	0.34280094	-0.8278816	0.47460482	-0.027415
87.4	0.59138372	-0.5782653	-0.5979463	0.34332858	-0.8278331	0.47382624	-0.0275316
87.5	0.5908199	-0.5780161	-0.5977332	0.34311038	-0.827754	0.47398746	-0.0278243
87.6	0.59104791	-0.5774695	-0.5977669	0.34259667	-0.8275109	0.47421665	-0.0283522
87.7	0.59063903	-0.5768279	-0.5974896	0.3427853	-0.8272704	0.47414903	-0.0279496
87.8	0.5902962	-0.577345	-0.5968601	0.34256821	-0.8273327	0.47423752	-0.0279635
87.9	0.58957728	-0.5769406	-0.5961116	0.34202291	-0.8270381	0.47423939	-0.0285285
88	0.5895465	-0.5767522	-0.5948786	0.34348399	-0.8269971	0.47441361	-0.0284919
88.1	0.58970247	-0.5774311	-0.5948249	0.34371848	-0.8271143	0.47507882	-0.0285842
88.2	0.58973249	-0.5778342	-0.5949972	0.34373528	-0.827091	0.47550578	-0.0289191
88.3	0.58986511	-0.5782288	-0.5941511	0.34377661	-0.8269471	0.47550635	-0.0288351
88.4	0.58971652	-0.5782467	-0.5940119	0.34267608	-0.8268508	0.4757322	-0.0288495
88.5	0.58973476	-0.578107	-0.5937723	0.34163245	-0.8267591	0.47633273	-0.0292718
88.6	0.58922852	-0.5778297	-0.5934336	0.34250998	-0.826675	0.47668783	-0.0307045
88.7	0.58963609	-0.5776742	-0.5931362	0.34240763	-0.8268551	0.47664409	-0.0308717
88.8	0.58946511	-0.5777283	-0.5929448	0.34230907	-0.8270865	0.47760233	-0.0306198
88.9	0.58933032	-0.5782949	-0.5928993	0.3418769	-0.8270234	0.47798211	-0.0302765
89	0.58908687	-0.5782723	-0.5926373	0.34147321	-0.8269256	0.4778233	-0.0299306
89.1	0.58915399	-0.5780653	-0.5915835	0.34133067	-0.826704	0.47754231	-0.029809
89.2	0.58904757	-0.5776857	-0.5910182	0.34106915	-0.8265898	0.47699066	-0.0298101
89.3	0.58862598	-0.5780116	-0.5902987	0.34019116	-0.8264914	0.47605355	-0.0303378
89.4	0.58867563	-0.5789461	-0.5901307	0.3393328	-0.8267761	0.47733199	-0.0302017
89.5	0.5889083	-0.5791325	-0.590331	0.33858171	-0.8268116	0.47774008	-0.0321925
89.6	0.58852037	-0.5788853	-0.5899429	0.33839295	-0.82691	0.47826467	-0.0324406
89.7	0.5880163	-0.5791207	-0.5898537	0.33821291	-0.826802	0.47855809	-0.0327583
89.8	0.58812221	-0.5792538	-0.5896321	0.3379116	-0.8266547	0.47837898	-0.0326865
89.9	0.58805049	-0.5790421	-0.5894574	0.33751381	-0.8266695	0.47874568	-0.0325722
135	0.52498741	-0.5465683	-0.5021977	0.28736388	-0.807971	0.48614966	-0.0790816
135.1	0.52498097	-0.5463839	-0.501808	0.2870901	-0.8077837	0.48590175	-0.0794292
135.2	0.524559	-0.5462377	-0.5021935	0.28722281	-0.8076192	0.48576075	-0.0798975
135.3	0.52442568	-0.5465852	-0.5021765	0.28724985	-0.8077512	0.48582994	-0.0802634
135.4	0.52361237	-0.5463909	-0.5018394	0.28749307	-0.8076797	0.48609413	-0.0809484
135.5	0.52361237	-0.5463909	-0.5018394	0.28749307	-0.8076797	0.48609413	-0.0809484
135.6	0.52263001	-0.5458757	-0.5016519	0.2871398	-0.807328	0.48668668	-0.0815863
135.7	0.52253345	-0.5457858	-0.5017774	0.28666937	-0.8072634	0.48675802	-0.0817521
135.8	0.52179391	-0.5453091	-0.5011167	0.28649373	-0.8068459	0.48686343	-0.082136
135.9	0.52198146	-0.5448143	-0.5014016	0.28669059	-0.8069412	0.48682053	-0.0819316
136	0.52206005	-0.5449352	-0.5013126	0.28632839	-0.8069016	0.48702784	-0.0820626
136.1	0.52205522	-0.5449185	-0.5013644	0.2861913	-0.8069563	0.48707669	-0.0821972
136.2	0.52147334	-0.5449806	-0.5011411	0.28598273	-0.806888	0.48717254	-0.0824088
136.3	0.52134992	-0.5449048	-0.5000253	0.28595992	-0.8067859	0.48677367	-0.08212
136.4	0.52134992	-0.5449048	-0.5000253	0.28595992	-0.8067859	0.48677367	-0.08212
136.5	0.52140417	-0.5441098	-0.499708	0.28578776	-0.8064397	0.48640502	-0.0820381
136.6	0.52086775	-0.5436751	-0.4996386	0.28529347	-0.806186	0.48654606	-0.0827034
136.7	0.52086775	-0.5436751	-0.4996386	0.28529347	-0.806186	0.48654606	-0.0827034
136.8	0.52026272	-0.5434402	-0.4995282	0.28476792	-0.805873	0.48696624	-0.0833657
136.9	0.5199335	-0.5427765	-0.4994205	0.28524882	-0.8056935	0.48699985	-0.083596
137	0.5200481	-0.5425484	-0.4992064	0.2850323	-0.8057196	0.48733455	-0.0830801
137.1	0.51978926	-0.5421306	-0.4987448	0.28504248	-0.805657	0.48708685	-0.0833335
137.2	0.51902091	-0.5423001	-0.4981337	0.28460178	-0.8053623	0.48693446	-0.0835283
137.3	0.51920155	-0.5424234	-0.49791	0.28434157	-0.8056644	0.48718666	-0.0840303
137.4	0.51920155	-0.5424234	-0.49791	0.28434157	-0.8056644	0.48718666	-0.0840303
137.5	0.5184812	-0.5423414	-0.4981468	0.28370204	-0.8054641	0.48763818	-0.0847063
137.6	0.5185475	-0.5420887	-0.4978307	0.28400787	-0.805228	0.48754232	-0.0853803
137.7	0.51728895	-0.5421672	-0.4977093	0.28421244	-0.8048777	0.4880266	-0.0860011
137.8	0.51707978	-0.5421042	-0.4978066	0.28448805	-0.8048747	0.48823033	-0.0858489
137.9	0.51707978	-0.5421042	-0.4978066	0.28448805	-0.8048747	0.48823033	-0.0858489
138	0.51689752	-0.5418814	-0.4977205	0.28427262	-0.804892	0.48846402	-0.085538
138.1	0.51634752	-0.5422355	-0.4975554	0.2839639	-0.805001	0.48848591	-0.0860517
138.2	0.51630201	-0.5417101	-0.4970059	0.28338406	-0.80494	0.48840259	-0.0860878
138.3	0.51630201	-0.5417101	-0.4970059	0.28338406	-0.80494	0.48840259	-0.0860878
138.4	0.5161795	-0.5415151	-0.4969272	0.28341584	-0.8048165	0.48868545	-0.0858235
138.5	0.51689992	-0.5412001	-0.4967858	0.28367823	-0.8050002	0.48872282	-0.0851802
138.6	0.51689992	-0.5412001	-0.4967858	0.28367823	-0.8050002	0.48872282	-0.0851802
138.7	0.51705845	-0.5411714	-0.496888	0.28379537	-0.8049797	0.48848486	-0.0856746
138.8	0.51654672	-0.5410283	-0.4969773	0.28354477	-0.8049967	0.48853092	-0.0854001
138.9	0.51610291	-0.5408274	-0.4973565	0.28325709	-0.8049044	0.48832542	-0.0858581
139	0.51610291	-0.5408274	-0.4973565	0.28325709	-0.8049044	0.48832542	-0.0858581
139.1	0.5163275	-0.5409899	-0.49744	0.28295368	-0.8049669	0.48833723	-0.0856402
139.2	0.5163275	-0.5409899	-0.49744	0.28295368	-0.8049669	0.48833723	-0.0856402
139.3	0.5163275	-0.5409899	-0.49744	0.28295368	-0.8049669	0.48833723	-0.0856402
139.4	0.5161123	-0.5410737	-0.497454	0.28208144	-0.8047577	0.48874774	-0.0858603
139.5	0.5157935	-0.5412518	-0.4975134	0.28200261	-0.8047566	0.48877454	-0.0864674
139.6	0.51569478	-0.5411886	-0.4974035	0.28129773	-0.8047357	0.48925756	-0.0865065
139.7	0.51531184	-0.5411047	-0.4973241	0.28077112	-0.804577	0.48960945	-0.0863669
139.8	0.51539491	-0.5409237	-0.4975389	0.28080547	-0.8044968	0.48989665	-0.0864032
139.9	0.5156871	-0.5408054	-0.4976562	0.28056307	-0.8044194	0.48998081	-0.0865915
140	0.5156871	-0.5408054	-0.4976562	0.28056307	-0.8044194	0.48998081	-0.0865915
140.1	0.5157985	-0.5405669	-0.4975333	0.28039744	-0.8043483	0.49009861	-0.0872017
140.2	0.51515755	-0.5402328	-0.4974996	0.28095479	-0.804125	0.48973092	-0.0878829
140.3	0.5148958	-0.5406646	-0.4976114	0.28092793	-0.8041507	0.48937349	-0.08815
140.4	0.51462004	-0.5404116	-0.4975561	0.28065631	-0.8040617	0.48930556	-0.0885922
140.5	0.5140008	-0.5400122	-0.4978466	0.28120226	-0.8038894	0.48871088	-0.0891004
140.6	0.51384058	-0.5395478	-0.4974207	0.28144885	-0.8037009	0.48837298	-0.0896226
140.7	0.51384058	-0.5395478	-0.4974207	0.28144885	-0.8037009	0.48837298	-0.0896226
140.8	0.51408514	-0.5396652	-0.4975469	0.28200346	-0.8038191	0.48874379	-0.0897834
140.9	0.51406735	-0.5392815	-0.4975147	0.28174909	-0.8036359	0.48847616	-0.0898422
141	0.51357894	-0.5392988	-0.4969195	0.28095687	-0.8034179	0.48832384	-0.089522

99	0.57679556	-0.5740062	-0.5608663	0.32497291	-0.822282	0.483851	-0.0494746
99.1	0.57596082	-0.5739935	-0.5602182	0.32497418	-0.8219134	0.48387316	-0.0496575
99.2	0.5756121	-0.5738699	-0.560382	0.32544567	-0.8218976	0.48349892	-0.050528
99.3	0.5756121	-0.5738699	-0.560382	0.32544567	-0.8218976	0.48349892	-0.050528
99.4	0.57505997	-0.5734529	-0.5598357	0.32525485	-0.8217197	0.48311065	-0.0507986
99.5	0.57448289	-0.5729694	-0.559779	0.32493136	-0.8216094	0.48339391	-0.0508061
99.6	0.57334749	-0.5723674	-0.5592401	0.32433043	-0.8211356	0.48306805	-0.0512082
99.7	0.57351833	-0.5728828	-0.5594017	0.32417293	-0.8211838	0.48313993	-0.0522265
99.8	0.57313635	-0.5728016	-0.5591315	0.3234653	-0.8210909	0.48301349	-0.0525631
99.9	0.57287136	-0.5726911	-0.558397	0.32307206	-0.8208634	0.48228702	-0.0531449
100	0.57253824	-0.5730443	-0.5586761	0.32306851	-0.8208719	0.4821856	-0.0531431
100.1	0.57198955	-0.5731331	-0.558526	0.32297554	-0.8208356	0.48164647	-0.0524623
100.2	0.57219755	-0.5730565	-0.5586358	0.32224626	-0.8207433	0.48156362	-0.0528101
100.3	0.57202891	-0.5733876	-0.558878	0.32193246	-0.8208074	0.48166641	-0.0530925
100.4	0.57153405	-0.5729046	-0.5578955	0.3213155	-0.8202435	0.48113923	-0.0530632
100.5	0.57142342	-0.5731377	-0.5576686	0.32110301	-0.8201313	0.48070513	-0.0530105
100.6	0.57155518	-0.57322	-0.5576713	0.32092591	-0.82004	0.48087953	-0.0528509
100.7	0.57105365	-0.5731152	-0.5571394	0.3204619	-0.8198351	0.48083957	-0.0530097
100.8	0.57125038	-0.5735319	-0.5569988	0.31996052	-0.8198085	0.48080516	-0.0522563
100.9	0.57129238	-0.5739294	-0.5573	0.3202911	-0.8198498	0.48023423	-0.0522462
101	0.57069964	-0.5734623	-0.5567753	0.32062797	-0.8197852	0.47984594	-0.0523427
101.1	0.5707897	-0.5735012	-0.5570511	0.32008447	-0.8197008	0.47968394	-0.0525626
101.2	0.5708397	-0.57375	-0.5573839	0.31941029	-0.8196765	0.47945558	-0.0532868
101.3	0.57083377	-0.5732406	-0.5568505	0.31948899	-0.8195794	0.47935546	-0.0528249
101.4	0.57119609	-0.5737473	-0.5572446	0.31945257	-0.8197801	0.47951752	-0.0533962
101.5	0.57091066	-0.5739457	-0.5570793	0.31940641	-0.8196847	0.47944543	-0.0532543
101.6	0.57083857	-0.573675	-0.5572349	0.3185355	-0.8196838	0.47982469	-0.0532202
101.7	0.57090584	-0.5733499	-0.557791	0.31841339	-0.8199329	0.48027611	-0.053485
101.8	0.57056396	-0.5735623	-0.5572369	0.3187618	-0.8201112	0.48038313	-0.0528722
101.9	0.57028452	-0.5735332	-0.5567662	0.31843863	-0.8199417	0.4800217	-0.0535399
102	0.57012135	-0.5730545	-0.5566036	0.31933811	-0.8197875	0.47945014	-0.0533648
102.1	0.5694207	-0.5725796	-0.5556771	0.31882669	-0.8194121	0.47948301	-0.0545139
102.2	0.5693261	-0.572751	-0.5552137	0.31884556	-0.8197277	0.479836	-0.0546188
102.3	0.56893426	-0.5722761	-0.5549335	0.31879016	-0.8195013	0.47928519	-0.054507
102.4	0.56846976	-0.5720213	-0.5547972	0.31861051	-0.819322	0.47876304	-0.0540354
102.5	0.56849279	-0.5720217	-0.5541841	0.31806318	-0.8194629	0.47859058	-0.0540525
102.6	0.56849279	-0.5720217	-0.5541841	0.31806318	-0.8194629	0.47859058	-0.0540525
102.7	0.56886366	-0.571806	-0.5541595	0.31825834	-0.8194587	0.47874527	-0.0548467
102.8	0.56827673	-0.5715114	-0.5538341	0.31855019	-0.8193068	0.4787275	-0.0549872
102.9	0.56823325	-0.5714558	-0.5534832	0.31837045	-0.8192715	0.47893019	-0.0544923
103	0.56807116	-0.5707484	-0.5526173	0.31802679	-0.8188062	0.47831726	-0.0532554
103.1	0.56790276	-0.5707282	-0.5526242	0.31812516	-0.818953	0.47851632	-0.0530745
103.2	0.5677317	-0.5704656	-0.5521216	0.31834101	-0.8190368	0.4785625	-0.0535496
103.3	0.56755905	-0.5706331	-0.5520311	0.31794646	-0.818974	0.47877185	-0.0536267
103.4	0.56780381	-0.5706487	-0.5521008	0.31761885	-0.8190103	0.47878262	-0.0533919
103.5	0.56797298	-0.5706779	-0.5514132	0.31811984	-0.818936	0.47877392	-0.0539905
103.6	0.56797298	-0.5706779	-0.5514132	0.31811984	-0.818936	0.47877392	-0.0539905
103.7	0.56834723	-0.5705193	-0.5512863	0.3173979	-0.8187419	0.47842578	-0.0544586
103.8	0.56883926	-0.5706535	-0.5512702	0.317858	-0.8188896	0.47855801	-0.054194
103.9	0.56858926	-0.5704797	-0.5508108	0.31774064	-0.8189534	0.47826244	-0.0542329
104	0.56811923	-0.5705021	-0.5503569	0.31739709	-0.8188463	0.47823133	-0.0541098
104.1	0.56811923	-0.5705021	-0.5503569	0.31739709	-0.8188463	0.47823133	-0.0541098
104.2	0.56834778	-0.570504	-0.5507794	0.31685784	-0.8189723	0.4780912	-0.0542327
104.3	0.56823294	-0.5698541	-0.5506467	0.3168444	-0.8189223	0.47836542	-0.0540235
104.4	0.56836015	-0.5697096	-0.5505954	0.31672335	-0.819008	0.47851096	-0.0542807
104.5	0.56857826	-0.5699842	-0.5503366	0.31600309	-0.8190299	0.47892714	-0.0544646
104.6	0.5682417	-0.5703453	-0.5497227	0.31568259	-0.8189413	0.47906323	-0.0540446
104.7	0.56784318	-0.5704111	-0.5494521	0.3158383	-0.8188821	0.47953889	-0.053893
104.8	0.56828834	-0.5701912	-0.548644	0.31526263	-0.818901	0.47991818	-0.0530098
104.9	0.56876073	-0.57068	-0.5489741	0.31512526	-0.8190494	0.48017165	-0.0534636
150	0.50285513	-0.5325874	-0.4849697	0.27048679	-0.8010518	0.49018451	-0.092398
150.1	0.50219627	-0.5323612	-0.4844589	0.27023061	-0.8009023	0.49037444	-0.0928954
150.2	0.50214059	-0.5321698	-0.4842245	0.27071026	-0.8007417	0.49064804	-0.0930835
150.3	0.50193123	-0.5324115	-0.4843371	0.2703631	-0.8006729	0.49086181	-0.0927724
150.4	0.50215626	-0.5322257	-0.484525	0.27070801	-0.8006818	0.49105906	-0.0925825
150.5	0.50190606	-0.5323184	-0.4847952	0.27091875	-0.8008783	0.49131823	-0.0926491
150.6	0.50152384	-0.5320453	-0.4845475	0.27073335	-0.8009445	0.49166903	-0.0917672
150.7	0.50152384	-0.5320453	-0.4845475	0.27073335	-0.8009445	0.49166903	-0.0917672
150.8	0.50117317	-0.5320799	-0.4845465	0.27064011	-0.800832	0.49156435	-0.0922021
150.9	0.50133608	-0.5317599	-0.4843753	0.27099911	-0.8009129	0.49159294	-0.0919014
151	0.50117619	-0.5322331	-0.4845261	0.27097164	-0.8010947	0.49168973	-0.0918073
151.1	0.50117619	-0.5322331	-0.4845261	0.27097164	-0.8010947	0.49168973	-0.0918073
151.2	0.50098128	-0.5323827	-0.4848444	0.27118289	-0.801276	0.49147826	-0.0917086
151.3	0.50098128	-0.5323827	-0.4848444	0.27118289	-0.801276	0.49147826	-0.0917086
151.4	0.50098128	-0.5323827	-0.4848444	0.27118289	-0.801276	0.49147826	-0.0917086
151.5	0.50072128	-0.5324792	-0.4848845	0.27105364	-0.8011459	0.49141693	-0.0919397
151.6	0.50072128	-0.5324792	-0.4848845	0.27105364	-0.8011459	0.49141693	-0.0919397
151.7	0.50091951	-0.5326286	-0.4847324	0.271059	-0.8012813	0.49143036	-0.091609
151.8	0.50079959	-0.5325845	-0.4848755	0.27133913	-0.8012283	0.49130393	-0.0915444
151.9	0.50079959	-0.5325845	-0.4848755	0.27133913	-0.8012283	0.49130393	-0.0915444
152	0.50018955	-0.5323295	-0.4846048	0.27077935	-0.8009367	0.49108398	-0.0917815
152.1	0.50018955	-0.5323295	-0.4846048	0.27077935	-0.8009367	0.49108398	-0.0917815
152.2	0.50016978	-0.5322733	-0.4843717	0.27118265	-0.8010197	0.49118671	-0.0919039
152.3	0.50016978	-0.5322733	-0.4843717	0.27118265	-0.8010197	0.49118671	-0.0919039
152.4	0.50016425	-0.5322281	-0.4839928	0.27094627	-0.8010007	0.49114029	-0.0920447
152.5	0.50056418	-0.5322795	-0.4842149	0.27123172	-0.8010664	0.49105815	-0.0922037
152.6	0.50056418	-0.5322795	-0.4842149	0.27123172	-0.8010664	0.49105815	-0.0922037
152.7	0.50056418	-0.5322795	-0.4842149	0.27123172	-0.8010664	0.49105815	-0.0922037
152.8	0.50056418	-0.5322795	-0.4842149	0.27123172	-0.8010664	0.49105815	-0.0922037
152.9	0.5006063	-0.5320157	-0.4843874	0.27169413	-0.8010471	0.49104449	-0.0926168
153	0.5006063	-0.5320157	-0.4843874	0.27169413	-0.8010471	0.49104449	-0.0926168
153.1	0.49982412	-0.5316626	-0.4838967	0.27116803	-0.8007019	0.49071929	-0.0930714
153.2	0.49982412	-0.5316626	-0.4838967	0.27116803	-0.8007019	0.49071929	-0.0930714
153.3	0.49981613	-0.5312175	-0.4838204	0.27143797	-0.8006617	0.49062487	-0.0930628
153.4	0.49981613	-0.5312175	-0.4838204	0.27143797	-0.8006617	0.49062487	-0.0930628
153.5	0.49999257	-0.530988	-0.484606	0.27160753	-0.8007205	0.49058173	-0.0930602
153.6	0.49999257	-0.530988	-0.484606	0.27160753	-0.8007205	0.49058173	-0.0930602
153.7	0.49999257	-0.530988	-0.484606	0.27160753	-0.8007205	0.49058173	-0.0930602
153.8	0.49999257	-0.530988	-0.484606	0.27160753	-0.8007205	0.49058173	-0.0930602
153.9	0.49982039	-0.5308897	-0.4843861	0.27164113	-0.8006671	0.49047648	-0.0932788
154	0.49986388	-0.5310131	-0.484645	0.27212219	-0.8006174	0.49063372	-0.0936326
154.1	0.49986388	-0.5310131	-0.484645	0.27212219	-0.8006174	0.49063372	-0.0936326
154.2	0.49986388	-0.5310131	-0.484645	0.27212219	-0.8006174	0.49063372	-0.0936326
154.3	0.49986388	-0.5310131	-0.484645	0.27212219	-0.8006174	0.49063372	-0.0936326
154.4	0.49913323	-0.5311266	-0.4844917	0.27204942	-0.8003647	0.49069053	-0.0931514
154.5	0.49933703	-0.5307076	-0.4843106	0.27245497	-0.8003493	0.49056769	-0.0935835
154.6	0.4989111	-0.5304268	-0.4838763	0.27191263	-0.8001059	0.49030142	-0.0936381
154.7	0.4989111	-0.5304268	-0.4838763	0.27191263	-0.8001059	0.49030142	-0.0936381
154.8	0.4989111	-0.5304268	-0.4838763	0.27191263	-0.8001059	0.49030142	-0.0936381
154.9	0.49897051	-0.5309781	-0.4837767	0.27229029	-0.8000181	0.4900858	-0.0940431
155	0.49818913	-0.5306937	-0.4835071	0.27205713	-0.7999148	0.49034408	-0.0934641
155.1	0.49828858	-0.5309088	-0.4834209	0.27204765	-0.8000991	0.49041534	-0.093312
155.2	0.49828858	-0.5309088	-0.4834209	0.27204765	-0.8000991	0.49041534	-0.093312
155.3	0.49828858	-0.5309088	-0.4834209	0.27204765	-0.8000991	0.49041534	-0.093312
155.4	0.49828858	-0.5309088	-0.4834209	0.27204765	-0.8000991	0.49041534	-0.093312
155.5	0.49820239	-0.5305969	-0.4832745	0.27218666	-0.800208	0.49050726	-0.0932909
155.6	0.49760054	-0.5305783	-0.4830106	0.27125613	-0.7998351	0.49059858	-0.0935228
155.7	0.49682379	-0.5298431	-0.4820995	0.27002382	-0.7994738	0.49072156	-0.092704
155.8	0.49682379	-0.5298431	-0.4820995	0.27002382	-0.7994738	0.49072156	-0.092704
155.9	0.49680081	-0.5300004	-0.4819987	0.27007606	-0.7996375	0.49116907	-0.0924783
156	0.49678647	-0.5295189	-0.4817069	0.27020265	-0.7995623	0.49112257	-0.09219

114	0.55360585	-0.5640337	-0.5329543	0.30234174	-0.8165557	0.48529257	-0.055311
114.1	0.55321089	-0.5636617	-0.5326458	0.30256945	-0.8165657	0.48533259	-0.0552659
114.2	0.55303163	-0.5635558	-0.5325382	0.3029126	-0.8165613	0.4854122	-0.055386
114.3	0.55283767	-0.5631762	-0.5323371	0.30217817	-0.8164952	0.4852691	-0.0554262
114.4	0.55292522	-0.5628765	-0.5319529	0.30179874	-0.8163667	0.48491048	-0.0552928
114.5	0.55292522	-0.5628765	-0.5319529	0.30179874	-0.8163667	0.48491048	-0.0552928
114.6	0.55249942	-0.5626842	-0.5314406	0.30148721	-0.8164476	0.48513318	-0.0548837
114.7	0.55252244	-0.5624689	-0.5317486	0.30176392	-0.8165044	0.48524995	-0.055608
114.8	0.55257695	-0.5621268	-0.5314651	0.30168029	-0.816474	0.48515264	-0.0555802
114.9	0.55290146	-0.5618961	-0.5312021	0.30098833	-0.8165091	0.48600198	-0.0571804
115	0.55290146	-0.5618961	-0.5312021	0.30098833	-0.8165091	0.48600198	-0.0571804
115.1	0.55315555	-0.5617835	-0.5310743	0.30092244	-0.8165059	0.48608924	-0.0569052
115.2	0.55315555	-0.5617835	-0.5310743	0.30092244	-0.8165059	0.48608924	-0.0569052
115.3	0.55346059	-0.5618366	-0.5307329	0.30041513	-0.8165903	0.48608721	-0.0571008
115.4	0.55294281	-0.5617501	-0.5305557	0.30014258	-0.81626	0.48528461	-0.0575662
115.5	0.55267727	-0.5617056	-0.5302088	0.30025377	-0.8163717	0.48546767	-0.0566311
115.6	0.55231473	-0.5621371	-0.5300959	0.29935603	-0.8160842	0.48548355	-0.0568457
115.7	0.55104812	-0.5617737	-0.5286417	0.29945422	-0.8155163	0.48457173	-0.0568136
115.8	0.55047545	-0.5618524	-0.5282298	0.29929279	-0.8153595	0.48461953	-0.0571488
115.9	0.55015554	-0.5619114	-0.5285187	0.29930677	-0.8154553	0.48470975	-0.0572109
116	0.54986693	-0.5618209	-0.5284497	0.29927879	-0.815258	0.48423599	-0.0578905
116.1	0.54962868	-0.5611762	-0.5275156	0.29826745	-0.8150263	0.48370964	-0.0579711
116.2	0.54943063	-0.5608043	-0.5272742	0.29870232	-0.8149731	0.48366085	-0.0576506
116.3	0.54829074	-0.5607228	-0.5271935	0.29784585	-0.8148029	0.48380536	-0.057662
116.4	0.54787828	-0.5603011	-0.5265933	0.29829944	-0.8146155	0.48368686	-0.0580739
116.5	0.54759136	-0.5602627	-0.5261822	0.29810161	-0.8146513	0.48377298	-0.0578138
116.6	0.54677392	-0.5603394	-0.5256113	0.29745257	-0.8143402	0.48327026	-0.0581421
116.7	0.54677392	-0.5603394	-0.5256113	0.29745257	-0.8143402	0.48327026	-0.0581421
116.8	0.54677392	-0.5603394	-0.5256113	0.29745257	-0.8143402	0.48327026	-0.0581421
116.9	0.54680658	-0.5603731	-0.5254648	0.29718293	-0.8144105	0.48333757	-0.0577222
117	0.54674855	-0.5605828	-0.5253701	0.29742227	-0.8144707	0.48358727	-0.0579687
117.1	0.5462324	-0.5604847	-0.5251978	0.29761079	-0.8143519	0.48328717	-0.058003
117.2	0.54597697	-0.5606503	-0.5252437	0.2975777	-0.8143792	0.48330973	-0.0581792
117.3	0.54576829	-0.5603072	-0.5250142	0.29778094	-0.8143763	0.48329504	-0.0590691
117.4	0.54540379	-0.5602289	-0.5248836	0.2980265	-0.8143698	0.48329295	-0.0596186
117.5	0.54508759	-0.5601028	-0.5245042	0.29789724	-0.8143678	0.48288757	-0.0599734
117.6	0.54515497	-0.5603568	-0.523885	0.29851864	-0.8145835	0.48272636	-0.0611615
117.7	0.54485131	-0.5601864	-0.5236918	0.29905853	-0.8146084	0.4828761	-0.0611271
117.8	0.54444807	-0.5599366	-0.5235143	0.29922641	-0.8143422	0.48326552	-0.0615413
117.9	0.54469224	-0.5596788	-0.5235204	0.29920282	-0.8143394	0.48352854	-0.0614773
118	0.54488365	-0.5594319	-0.5233564	0.29942415	-0.8144976	0.48371522	-0.0620368
118.1	0.54500037	-0.5595737	-0.5230605	0.29944272	-0.8145557	0.48399275	-0.0618411
118.2	0.54435866	-0.5593033	-0.5224905	0.29945553	-0.8142621	0.4842705	-0.0620853
118.3	0.5444546	-0.5590816	-0.5222516	0.29947806	-0.8142104	0.48492261	-0.0624529
118.4	0.54433671	-0.5591196	-0.5221066	0.29912404	-0.8140892	0.48476349	-0.0627261
118.5	0.54371359	-0.5584622	-0.5215884	0.29848868	-0.8137791	0.48448466	-0.0627575
118.6	0.54364453	-0.55854	-0.5214711	0.29801729	-0.8137935	0.48408389	-0.0628915
118.7	0.54369741	-0.5586809	-0.5213733	0.29757468	-0.8137857	0.48411852	-0.0633052
118.8	0.54412721	-0.5589807	-0.5214764	0.29756182	-0.8138746	0.48466282	-0.0626471
118.9	0.54403082	-0.5580827	-0.5205464	0.29671306	-0.8135427	0.48492534	-0.0633628
119	0.54382666	-0.558042	-0.5201891	0.29604474	-0.8134566	0.48460322	-0.0634457
119.1	0.54364986	-0.5575327	-0.5199708	0.29574769	-0.8133638	0.48430606	-0.0636778
119.2	0.54300332	-0.5578346	-0.5196078	0.29545961	-0.8133569	0.48483198	-0.0635317
119.3	0.54285331	-0.5575582	-0.5191642	0.29460887	-0.8131009	0.48438448	-0.0638231
119.4	0.5424788	-0.5575285	-0.5191796	0.29453532	-0.8131725	0.485039	-0.063538
119.5	0.54254897	-0.5578489	-0.5192916	0.29373313	-0.813135	0.48485238	-0.0641319
119.6	0.54160848	-0.5576059	-0.5192105	0.29318858	-0.8130223	0.48475815	-0.0641711
119.7	0.5410097	-0.5571912	-0.5192918	0.29379921	-0.8128855	0.48491075	-0.0638849
119.8	0.54095231	-0.5570143	-0.5194148	0.29409	-0.8129315	0.48523245	-0.064301
119.9	0.54100253	-0.5569696	-0.5191104	0.29432144	-0.8129961	0.48519142	-0.0646203
165	0.49249863	-0.5257761	-0.4758513	0.26070286	-0.797002	0.49268803	-0.0884236
165.1	0.492489	-0.5257088	-0.4753008	0.26033821	-0.7970931	0.49241696	-0.0879235
165.2	0.492489	-0.5257088	-0.4753008	0.26033821	-0.7970931	0.49241696	-0.0879235
165.3	0.49236092	-0.5259719	-0.4752966	0.2599857	-0.7970337	0.49271241	-0.0880026
165.4	0.49215692	-0.5258369	-0.4749392	0.25976209	-0.7972594	0.49297395	-0.0877015
165.5	0.49215692	-0.5258369	-0.4749392	0.25976209	-0.7972594	0.49297395	-0.0877015
165.6	0.49215692	-0.5258369	-0.4749392	0.25976209	-0.7972594	0.49297395	-0.0877015
165.7	0.49215692	-0.5258369	-0.4749392	0.25976209	-0.7972594	0.49297395	-0.0877015
165.8	0.49215692	-0.5258369	-0.4749392	0.25976209	-0.7972594	0.49297395	-0.0877015
165.9	0.49233287	-0.5254489	-0.4748208	0.25936181	-0.79713	0.4926912	-0.0874554
166	0.49233287	-0.5254489	-0.4748208	0.25936181	-0.79713	0.4926912	-0.0874554
166.1	0.49258138	-0.5257554	-0.4743102	0.25909249	-0.7971462	0.49263241	-0.0871377
166.2	0.49253091	-0.5262573	-0.4742087	0.25897327	-0.7973009	0.49288374	-0.0868723
166.3	0.49253091	-0.5262573	-0.4742087	0.25897327	-0.7973009	0.49288374	-0.0868723
166.4	0.49253091	-0.5262573	-0.4742087	0.25897327	-0.7973009	0.49288374	-0.0868723
166.5	0.49253091	-0.5262573	-0.4742087	0.25897327	-0.7973009	0.49288374	-0.0868723
166.6	0.49253091	-0.5262573	-0.4742087	0.25897327	-0.7973009	0.49288374	-0.0868723
166.7	0.49203563	-0.526512	-0.4741789	0.25888342	-0.7972423	0.49270703	-0.0870731
166.8	0.49225062	-0.5263806	-0.4738083	0.25843702	-0.797153	0.49247943	-0.0870556
166.9	0.49204972	-0.526093	-0.4734172	0.25861515	-0.7970406	0.49237116	-0.0875
167	0.4921964	-0.5258646	-0.4733772	0.25837397	-0.7969639	0.49247062	-0.0879362
167.1	0.4921964	-0.5258646	-0.4733772	0.25837397	-0.7969639	0.49247062	-0.0879362
167.2	0.4921964	-0.5258646	-0.4733772	0.25837397	-0.7969639	0.49247062	-0.0879362
167.3	0.49203583	-0.5254914	-0.4729053	0.25796766	-0.7968534	0.49227022	-0.0877651
167.4	0.49177141	-0.5256477	-0.4726561	0.25815272	-0.7968389	0.49208371	-0.0879304
167.5	0.49174511	-0.525929	-0.4722424	0.25784237	-0.7968867	0.49194542	-0.0878982
167.6	0.49174511	-0.525929	-0.4722424	0.25784237	-0.7968867	0.49194542	-0.0878982
167.7	0.49120752	-0.5251902	-0.4725846	0.25687299	-0.7966077	0.4921931	-0.0887717
167.8	0.49120752	-0.5251902	-0.4725846	0.25687299	-0.7966077	0.4921931	-0.0887717
167.9	0.49120752	-0.5251902	-0.4725846	0.25687299	-0.7966077	0.4921931	-0.0887717
168	0.49107729	-0.5250902	-0.4725925	0.25715093	-0.7965953	0.49224652	-0.0887106
168.1	0.49107729	-0.5250902	-0.4725925	0.25715093	-0.7965953	0.49224652	-0.0887106
168.2	0.49066552	-0.5249803	-0.4725135	0.25699691	-0.7963933	0.49231067	-0.0885844
168.3	0.49106984	-0.5251724	-0.4725128	0.25692883	-0.7965512	0.49249995	-0.0887104
168.4	0.49051025	-0.5252045	-0.4721591	0.25681434	-0.7965458	0.49281653	-0.0887802
168.5	0.49051025	-0.5252045	-0.4721591	0.25681434	-0.7965458	0.49281653	-0.0887802
168.6	0.48994149	-0.525497	-0.4716211	0.25680632	-0.7963925	0.49294512	-0.0886614
168.7	0.4897278	-0.5257746	-0.4714459	0.25686451	-0.7965134	0.49342058	-0.0880661
168.8	0.48980665	-0.5256026	-0.4714492	0.25715259	-0.7966785	0.49332134	-0.0882269
168.9	0.48980665	-0.5256026	-0.4714492	0.25715259	-0.7966785	0.49332134	-0.0882269
169	0.48980665	-0.5256026	-0.4714492	0.25715259	-0.7966785	0.49332134	-0.0882269
169.1	0.48980665	-0.5256026	-0.4714492	0.25715259	-0.7966785	0.49332134	-0.0882269
169.2	0.48980665	-0.5256026	-0.4714492	0.25715259	-0.7966785	0.49332134	-0.0882269
169.3	0.48980665	-0.5256026	-0.4714492	0.25715259	-0.7966785	0.49332134	-0.0882269
169.4	0.48997183	-0.5256491	-0.4713197	0.25721551	-0.7967642	0.49334752	-0.0882376
169.5	0.48934487	-0.5252652	-0.4707432	0.25693567	-0.7965062	0.49309321	-0.0885431
169.6	0.48935599	-0.5252318	-0.4710553	0.25712112	-0.7965728	0.49310146	-0.0890216
169.7	0.48935599	-0.5252318	-0.4710553	0.25712112	-0.7965728	0.49310146	-0.0890216
169.8	0.48837058	-0.5245752	-0.4704103	0.2576177	-0.7962301	0.4925454	-0.0899483
169.9	0.48846867	-0.5242436	-0.4700921	0.25707571	-0.7962528	0.49240625	-0.08924
170	0.48849831	-0.5244813	-0.469751	0.25692444	-0.7963758	0.4921808	-0.0890619
170.1	0.48820073	-0.5245152	-0.469788	0.25669753	-0.7961679	0.49202984	-0.089387
170.2	0.48820073	-0.5245152	-0.469788	0.25669753	-0.7961679	0.49202984	-0.089387
170.3	0.48750291	-0.5243862	-0.469262	0.25740954	-0.7959734	0.49143013	-0.0893909
170.4	0.48750291	-0.5243862	-0.469262	0.25740954	-0.7959734	0.49143013	-0.0893909
170.5	0.48750291	-0.5243862	-0.469262	0.25740954	-0.7959734	0.49143013	-0.0893909
170.6	0.48759459	-0.5245761	-0.4692471	0.25735184	-0.7960038	0.49162365	-0.0893781
170.7	0.48759459	-0.5245761	-0.4692471	0.25735184	-0.7960038	0.49162365	-0.0893781
170.8	0.48759459	-0.5245761	-0.4692471	0.25735184	-0.7960038	0.49162365	-0.0893781
170.9	0.48759459	-0.5245761	-0.4692471	0.25735184	-0.7960038	0.49162365	-0.0893781
171	0.48759459	-0.5245761	-0.4692471	0.25735184	-0.7960038	0.49162365	-0.0893781

126.1	0.53402596	-0.5520065	-0.511067	0.29157426	-0.8103785	0.4830951	-0.0677319
126.2	0.53400268	-0.5519243	-0.5108659	0.29166176	-0.8104321	0.48327462	-0.0675938
126.3	0.53411404	-0.5521224	-0.5108624	0.29164395	-0.8103192	0.48335331	-0.0679167
126.4	0.5340831	-0.5519372	-0.5108137	0.29143079	-0.8102596	0.48325202	-0.0676158
126.5	0.53414726	-0.5520472	-0.5110605	0.29137797	-0.8104118	0.48341688	-0.0678982
126.6	0.53393955	-0.5519954	-0.5108967	0.29164356	-0.8103892	0.48340528	-0.068097
126.7	0.53355354	-0.5522914	-0.5108068	0.29154958	-0.8102999	0.48346475	-0.0682479
126.8	0.53253956	-0.5519884	-0.5104062	0.29192837	-0.810164	0.48277095	-0.0680389
126.9	0.53252525	-0.5518971	-0.5102065	0.29179673	-0.8100456	0.48239474	-0.0681009
127	0.53251044	-0.5518343	-0.5100762	0.2924311	-0.810138	0.48227445	-0.0677848
127.1	0.53251418	-0.5517874	-0.5100321	0.29224163	-0.8101311	0.48241437	-0.0676799
127.2	0.53266548	-0.551856	-0.5102952	0.29197447	-0.8099225	0.48238011	-0.0669475
127.3	0.53208758	-0.5520709	-0.5100183	0.29190824	-0.8097775	0.48260883	-0.0674549
127.4	0.53179251	-0.5523046	-0.5099159	0.29155537	-0.8096955	0.48272477	-0.0676052
127.5	0.53179257	-0.5524775	-0.5096672	0.29127327	-0.8096726	0.4830766	-0.0678183
127.6	0.53088209	-0.5523283	-0.5092331	0.29090962	-0.8095868	0.48292402	-0.0679609
127.7	0.53088209	-0.5523283	-0.5092331	0.29090962	-0.8095868	0.48292402	-0.0679609
127.8	0.53046884	-0.5520208	-0.5087381	0.29048255	-0.8093639	0.48249472	-0.0687226
127.9	0.5302334	-0.5523057	-0.5082967	0.29046015	-0.8094385	0.48241177	-0.0689594
128	0.53016762	-0.5523483	-0.5083428	0.29070909	-0.8093811	0.48257038	-0.0685412
128.1	0.53016762	-0.5523483	-0.5083428	0.29070909	-0.8093811	0.48257038	-0.0685412
128.2	0.53005054	-0.5521453	-0.5080264	0.29081448	-0.8093988	0.48289529	-0.0681815
128.3	0.52954497	-0.5518376	-0.5081166	0.29016547	-0.8091243	0.48271444	-0.0684977
128.4	0.52964221	-0.5519556	-0.5081471	0.29042844	-0.8092984	0.48287	-0.0683985
128.5	0.52987921	-0.5518479	-0.5083329	0.29034915	-0.8093986	0.48311929	-0.068775
128.6	0.52987346	-0.5517133	-0.5074986	0.29017835	-0.8093956	0.48310808	-0.0694096
128.7	0.52982235	-0.5518878	-0.507268	0.29025541	-0.8094757	0.48326804	-0.0693759
128.8	0.52930899	-0.5524786	-0.5076454	0.2904498	-0.8096165	0.48329726	-0.0703515
128.9	0.52941592	-0.5523147	-0.5076182	0.29088713	-0.8096343	0.48340094	-0.0704906
129	0.52940858	-0.5515502	-0.5066765	0.29049546	-0.809515	0.48390205	-0.0703627
129.1	0.52940858	-0.5515502	-0.5066765	0.29049546	-0.809515	0.48390205	-0.0703627
129.2	0.52994237	-0.5510092	-0.5068976	0.29024702	-0.8095497	0.48436264	-0.0710633
129.3	0.52977601	-0.5509606	-0.5073304	0.28984379	-0.809338	0.48382223	-0.0711698
129.4	0.5300423	-0.5507889	-0.5070898	0.28990382	-0.8093909	0.48404215	-0.0713322
129.5	0.53018893	-0.5505521	-0.5069184	0.28975957	-0.8093043	0.48424682	-0.0713011
129.6	0.53040673	-0.5504912	-0.5071645	0.28954847	-0.8093028	0.48409225	-0.0717442
129.7	0.53022157	-0.5509908	-0.5072943	0.29025909	-0.809593	0.48450031	-0.072558
129.8	0.53060523	-0.550937	-0.5068433	0.28993853	-0.8097697	0.48469399	-0.0725136
129.9	0.5306159	-0.5507781	-0.5069987	0.28984111	-0.8096191	0.48478404	-0.0728318
130	0.53080927	-0.5506127	-0.5071438	0.29000923	-0.8095146	0.48474951	-0.0731398
130.1	0.53072599	-0.5509095	-0.5070434	0.29009681	-0.8096265	0.48484039	-0.072989
130.2	0.53014705	-0.5506359	-0.5066673	0.28998539	-0.809362	0.48482473	-0.0729359
130.3	0.53014705	-0.5506359	-0.5066673	0.28998539	-0.809362	0.48482473	-0.0729359
130.4	0.52995788	-0.5503398	-0.5066525	0.28970436	-0.8091842	0.48470649	-0.0732327
130.5	0.52967794	-0.5507894	-0.5064858	0.28959436	-0.809184	0.48453601	-0.0734524
130.6	0.52992183	-0.5504083	-0.5057316	0.28994657	-0.8090836	0.48462331	-0.0736006
130.7	0.52945108	-0.5502083	-0.505772	0.2896954	-0.8091764	0.48470673	-0.0733253
130.8	0.52968896	-0.5502109	-0.5056263	0.28962535	-0.8092923	0.48485886	-0.0733202
130.9	0.52937866	-0.5502411	-0.5058059	0.28984189	-0.8091232	0.48460511	-0.0734876
131	0.52948569	-0.5501629	-0.5058101	0.29000239	-0.8093314	0.48471531	-0.0736572
131.1	0.52959608	-0.5502771	-0.5059925	0.29034014	-0.8096036	0.48495512	-0.0736596
131.2	0.52980245	-0.549912	-0.5058815	0.29000035	-0.8096277	0.48520079	-0.0740573
131.3	0.52960176	-0.5500855	-0.5058221	0.2896811	-0.8096426	0.48533344	-0.0738136
131.4	0.52925547	-0.5506482	-0.5061983	0.2902291	-0.8096822	0.48547638	-0.0742012
131.5	0.52908316	-0.5507862	-0.5065618	0.29045189	-0.8096197	0.48518042	-0.0745558
131.6	0.52839821	-0.5500631	-0.5061924	0.29083855	-0.8094533	0.48454176	-0.074455
131.7	0.52837781	-0.5498274	-0.5062451	0.29014199	-0.8093452	0.48448798	-0.0744317
131.8	0.52837781	-0.5498274	-0.5062451	0.29014199	-0.8093452	0.48448798	-0.0744317
131.9	0.52807875	-0.5491161	-0.5058251	0.29024538	-0.8091268	0.48423875	-0.0745115
132	0.52773381	-0.5483167	-0.5055379	0.28997039	-0.8088859	0.48449259	-0.0752216
132.1	0.52773381	-0.5483167	-0.5055379	0.28997039	-0.8088859	0.48449259	-0.0752216
132.2	0.52774564	-0.5482729	-0.5055548	0.28999704	-0.8087301	0.48442061	-0.0758502
132.3	0.52770507	-0.5481533	-0.505207	0.29020105	-0.8087969	0.48442434	-0.075526
132.4	0.52805582	-0.5478115	-0.5051105	0.28981979	-0.8087983	0.48493317	-0.0760198
132.5	0.52805582	-0.5478115	-0.5051105	0.28981979	-0.8087983	0.48493317	-0.0760198
132.6	0.52806511	-0.548006	-0.5052372	0.28950836	-0.8088021	0.48505917	-0.0762651
132.7	0.52815575	-0.5474977	-0.5048376	0.28938147	-0.8087033	0.48501812	-0.0762155
132.8	0.52815575	-0.5474977	-0.5048376	0.28938147	-0.8087033	0.48501812	-0.0762155
132.9	0.52791292	-0.5469185	-0.504188	0.28922294	-0.8084376	0.48519781	-0.0760923
133	0.52805035	-0.5468924	-0.5039132	0.28860026	-0.8084581	0.48548142	-0.0765867
133.1	0.5281665	-0.5467717	-0.5039171	0.28821739	-0.808338	0.48575622	-0.0764126
133.2	0.52800465	-0.546701	-0.5041109	0.2878506	-0.8083164	0.48558128	-0.0771059
133.3	0.52728276	-0.546218	-0.504098	0.28751757	-0.8079746	0.48555076	-0.0769095
133.4	0.52726727	-0.5462324	-0.503843	0.28718993	-0.8079644	0.48559237	-0.0765486
133.5	0.52737138	-0.5464743	-0.5035908	0.28720057	-0.8080544	0.48524923	-0.0767689
133.6	0.52730277	-0.5462619	-0.503423	0.28744564	-0.8079386	0.48537615	-0.0770775
133.7	0.5268503	-0.5464105	-0.5031191	0.28795875	-0.8081857	0.48542841	-0.0766359
133.8	0.5268503	-0.5464105	-0.5031191	0.28795875	-0.8081857	0.48542841	-0.0766359
133.9	0.52676526	-0.5468675	-0.5031194	0.28823116	-0.8081867	0.48548146	-0.0770421
134	0.52667194	-0.5471138	-0.5028805	0.2877853	-0.8082051	0.48557872	-0.0776038
134.1	0.52669456	-0.5467943	-0.5029729	0.28788875	-0.8082224	0.4857264	-0.0776211
134.2	0.52723625	-0.546363	-0.5031324	0.28812976	-0.8084129	0.48631113	-0.0774088
134.3	0.52695376	-0.546735	-0.5033342	0.28764941	-0.8083568	0.4864947	-0.0771811
134.4	0.5264666	-0.5466482	-0.5035259	0.28777651	-0.8082597	0.48612865	-0.0774157
134.5	0.52651823	-0.5465194	-0.5033652	0.28764201	-0.8082151	0.48641251	-0.0779813
134.6	0.52686086	-0.5470816	-0.5032812	0.28754471	-0.8085772	0.48643599	-0.0777527
134.7	0.5265933	-0.546733	-0.5037049	0.28764506	-0.8085588	0.48613777	-0.0785212
134.8	0.52644407	-0.546585	-0.5030302	0.28749323	-0.8084687	0.48620228	-0.0780888
134.9	0.5260807	-0.5469685	-0.5028068	0.2877564	-0.8086648	0.48607713	-0.0784963

180	0.47292593	-0.5270244	-0.4518661	0.24749028	-0.7920314	0.4913962	-0.0882405
180.1	0.4726687	-0.526843	-0.4519481	0.24745133	-0.7921518	0.49167573	-0.0880669
180.2	0.47218532	-0.5266519	-0.4515294	0.24767684	-0.7919824	0.49142483	-0.0885043
180.3	0.47178068	-0.5265449	-0.4517221	0.24776877	-0.7918411	0.49155393	-0.0888492
180.4	0.47110068	-0.5263845	-0.4516776	0.24747652	-0.7915482	0.49162851	-0.088735
180.5	0.47045456	-0.5260235	-0.4511137	0.24788153	-0.7912504	0.49140876	-0.0886601
180.6	0.47068009	-0.525999	-0.4511301	0.24764469	-0.7912019	0.49135727	-0.0886871
180.7	0.46987293	-0.5255442	-0.4498949	0.24789243	-0.7907524	0.49090549	-0.0885107
180.8	0.46987293	-0.5255442	-0.4498949	0.24789243	-0.7907524	0.49090549	-0.0885107
180.9	0.4693936	-0.5257038	-0.4491489	0.24750792	-0.7906986	0.49113833	-0.0878688
181	0.4693936	-0.5257038	-0.4491489	0.24750792	-0.7906986	0.49113833	-0.0878688
181.1	0.4693936	-0.5257038	-0.4491489	0.24750792	-0.7906986	0.49113833	-0.0878688
181.2	0.4694098	-0.5254333	-0.4489656	0.24726976	-0.7906191	0.49116375	-0.0876328
181.3	0.4694098	-0.5254333	-0.4489656	0.24726976	-0.7906191	0.49116375	-0.0876328
181.4	0.46915302	-0.5256133	-0.4487422	0.24755408	-0.79063	0.49114598	-0.0872811
181.5	0.46908255	-0.525257	-0.4480877	0.24704644	-0.7905665	0.4908478	-0.0867611
181.6	0.46871597	-0.5252912	-0.4476303	0.2463801	-0.7903591	0.49080169	-0.0866494
181.7	0.46871597	-0.5252912	-0.4476303	0.2463801	-0.7903591	0.49080169	-0.0866494
181.8	0.46871597	-0.5252912	-0.4476303	0.2463801	-0.7903591	0.49080169	-0.0866494
181.9	0.4687651	-0.5256224	-0.447013	0.24573314	-0.7900579	0.49070693	-0.0862334
182	0.46879355	-0.5261929	-0.4468617	0.24538235	-0.7901397	0.49071885	-0.0857699
182.1	0.4684887	-0.5263181	-0.4473196	0.24547015	-0.7901011	0.49075243	-0.0858512
182.2	0.46857736	-0.526696	-0.4471535	0.24572267	-0.7900203	0.49062686	-0.0855639
182.3	0.46857736	-0.526696	-0.4471535	0.24572267	-0.7900203	0.49062686	-0.0855639
182.4	0.46857736	-0.526696	-0.4471535	0.24572267	-0.7900203	0.49062686	-0.0855639
182.5	0.46850178	-0.5267973	-0.4466912	0.24560643	-0.7899168	0.49104395	-0.0856494
182.6	0.46850178	-0.5267973	-0.4466912	0.24560643	-0.7899168	0.49104395	-0.0856494
182.7	0.46850178	-0.5267973	-0.4466912	0.24560643	-0.7899168	0.49104395	-0.0856494
182.8	0.46850178	-0.5267973	-0.4466912	0.24560643	-0.7899168	0.49104395	-0.0856494
182.9	0.46849906	-0.5269105	-0.4465105	0.24568077	-0.7897902	0.49070018	-0.085426
183	0.46849906	-0.5269105	-0.4465105	0.24568077	-0.7897902	0.49070018	-0.085426
183.1	0.46807918	-0.5269877	-0.4457219	0.24572914	-0.7895025	0.49089327	-0.0855188

141.1	0.51334257	-0.5392318	-0.4970542	0.28051942	-0.8034046	0.48834128	-0.08957
141.2	0.51358509	-0.5393694	-0.4967557	0.2806521	-0.8035324	0.48865874	-0.0897093
141.3	0.51355332	-0.5388137	-0.4964615	0.2805596	-0.8033466	0.48875094	-0.0897269
141.4	0.51327117	-0.5387827	-0.4960518	0.28048071	-0.8032575	0.48825821	-0.0892346
141.5	0.51319518	-0.5389679	-0.496023	0.28048227	-0.8031961	0.48838405	-0.0889156
141.6	0.51319518	-0.5389679	-0.496023	0.28048227	-0.8031961	0.48838405	-0.0889156
141.7	0.51294719	-0.5386851	-0.4960202	0.28044585	-0.8031377	0.48831784	-0.0888542
141.8	0.51294719	-0.5386851	-0.4960202	0.28044585	-0.8031377	0.48831784	-0.0888542
141.9	0.51307221	-0.5385173	-0.4961849	0.28062128	-0.8031565	0.48854565	-0.0891149
142	0.51307221	-0.5385173	-0.4961849	0.28062128	-0.8031565	0.48854565	-0.0891149
142.1	0.51292317	-0.5385793	-0.4961602	0.28019671	-0.8031249	0.48844073	-0.0887964
142.2	0.51281534	-0.5386201	-0.4960135	0.28147544	-0.8035618	0.48869203	-0.0888692
142.3	0.51243577	-0.5385057	-0.4962787	0.28139358	-0.8034152	0.48829674	-0.0889252
142.4	0.51213319	-0.5383178	-0.4956175	0.28114296	-0.8033518	0.48849024	-0.08891
142.5	0.5113789	-0.5385392	-0.4957483	0.28020718	-0.8033169	0.48823136	-0.0888751
142.6	0.51168498	-0.5376347	-0.4951418	0.28050006	-0.8033329	0.48801591	-0.0885064
142.7	0.51168498	-0.5376347	-0.4951418	0.28050006	-0.8033329	0.48801591	-0.0885064
142.8	0.51089624	-0.5377514	-0.4949773	0.27966909	-0.8031135	0.48798107	-0.0884953
142.9	0.51092901	-0.5380801	-0.4948375	0.27936927	-0.8031002	0.48761783	-0.0885775
143	0.51092901	-0.5380801	-0.4948375	0.27936927	-0.8031002	0.48761783	-0.0885775
143.1	0.51044544	-0.5378379	-0.4946017	0.27920725	-0.8028886	0.48771554	-0.0890355
143.2	0.51008599	-0.5373255	-0.4945047	0.27848489	-0.8026974	0.48793988	-0.0895225
143.3	0.51080362	-0.537374	-0.4945057	0.27831997	-0.8030215	0.48826216	-0.0893771
143.4	0.51087366	-0.5375937	-0.4941317	0.27823216	-0.8031269	0.48817712	-0.0888659
143.5	0.5104206	-0.5376704	-0.4938701	0.27765079	-0.8031176	0.48840649	-0.088821
143.6	0.50999467	-0.5374018	-0.4936123	0.27799128	-0.8030171	0.48831965	-0.0888302
143.7	0.50999467	-0.5374018	-0.4936123	0.27799128	-0.8030171	0.48831965	-0.0888302
143.8	0.51014188	-0.5376126	-0.4935694	0.27798564	-0.8029046	0.48831536	-0.0887979
143.9	0.5110469	-0.5379423	-0.4931022	0.27824105	-0.8033363	0.4890666	-0.0881813
144	0.5117132	-0.5381108	-0.4929709	0.27818763	-0.8037194	0.48965792	-0.0884231
144.1	0.51182965	-0.5379095	-0.4928643	0.2781628	-0.8037191	0.49003568	-0.0885391
144.2	0.51133587	-0.537628	-0.4921277	0.27810499	-0.8032542	0.49003545	-0.0886732
144.3	0.51100467	-0.537196	-0.4917191	0.27753888	-0.8031991	0.49004095	-0.088567
144.4	0.51044211	-0.5369286	-0.4917444	0.27776725	-0.8031946	0.48971813	-0.0890342
144.5	0.51044211	-0.5369286	-0.4917444	0.27776725	-0.8031946	0.48971813	-0.0890342
144.6	0.50994513	-0.5368135	-0.4909963	0.2771007	-0.8030828	0.49000776	-0.0900059
144.7	0.50834919	-0.5368105	-0.4906339	0.2766223	-0.8027191	0.49032634	-0.0907596
144.8	0.50834919	-0.5368105	-0.4906339	0.2766223	-0.8027191	0.49032634	-0.0907596
144.9	0.50834919	-0.5368105	-0.4906339	0.2766223	-0.8027191	0.49032634	-0.0907596
145	0.50791365	-0.5365288	-0.4901153	0.27696001	-0.8029623	0.49049873	-0.090541
145.1	0.50755849	-0.5364063	-0.4899348	0.27616542	-0.8026716	0.49032748	-0.0905302
145.2	0.50705144	-0.5368515	-0.4901895	0.27635943	-0.8027077	0.48962909	-0.0910061
145.3	0.50705144	-0.5368515	-0.4901895	0.27635943	-0.8027077	0.48962909	-0.0910061
145.4	0.5066093	-0.536664	-0.4901924	0.2766436	-0.802648	0.48961374	-0.0913567
145.5	0.50637381	-0.5367041	-0.4903658	0.27617943	-0.8026064	0.48984082	-0.0916775
145.6	0.50595643	-0.5364845	-0.4904537	0.27582283	-0.8024774	0.48973337	-0.0920775
145.7	0.50579471	-0.536271	-0.4895996	0.27525179	-0.8023152	0.48946345	-0.0917575
145.8	0.50579471	-0.536271	-0.4895996	0.27525179	-0.8023152	0.48946345	-0.0917575
145.9	0.50579471	-0.536271	-0.4895996	0.27525179	-0.8023152	0.48946345	-0.0917575
146	0.50549953	-0.536323	-0.4894079	0.27462747	-0.8021431	0.48952338	-0.0919681
146.1	0.50498067	-0.5362145	-0.4891353	0.27430952	-0.8019093	0.48957219	-0.0923927
146.2	0.50454792	-0.5361835	-0.4890936	0.27391256	-0.8018312	0.48959183	-0.0927748
146.3	0.50435469	-0.5360114	-0.4888871	0.27385666	-0.8018323	0.48991451	-0.093142
146.4	0.50435469	-0.5360114	-0.4888871	0.27385666	-0.8018323	0.48991451	-0.093142
146.5	0.50435469	-0.5360114	-0.4888871	0.27385666	-0.8018323	0.48991451	-0.093142
146.6	0.50435469	-0.5360114	-0.4888871	0.27385666	-0.8018323	0.48991451	-0.093142
146.7	0.50452909	-0.5357621	-0.4885307	0.27359626	-0.8021015	0.48998461	-0.0928951
146.8	0.5046664	-0.535416	-0.4884454	0.27341134	-0.8018448	0.48977457	-0.0929686
146.9	0.5046664	-0.535416	-0.4884454	0.27341134	-0.8018448	0.48977457	-0.0929686
147	0.5047144	-0.5351401	-0.4881327	0.27342836	-0.8018009	0.48964961	-0.0929192
147.1	0.50437155	-0.5348414	-0.4874256	0.2727566	-0.8016023	0.48985997	-0.0927171
147.2	0.50397343	-0.5348329	-0.4873523	0.27288136	-0.8016477	0.49013159	-0.0930046
147.3	0.5039911	-0.5345074	-0.4877523	0.27223331	-0.8017548	0.49021322	-0.0932485
147.4	0.50378017	-0.5345322	-0.4876072	0.27238875	-0.8016499	0.49006837	-0.093409
147.5	0.50387188	-0.5347887	-0.4874884	0.27245725	-0.8017594	0.49062758	-0.0935724
147.6	0.50387188	-0.5347887	-0.4874884	0.27245725	-0.8017594	0.49062758	-0.0935724
147.7	0.50387188	-0.5347887	-0.4874884	0.27245725	-0.8017594	0.49062758	-0.0935724
147.8	0.50387188	-0.5347887	-0.4874884	0.27245725	-0.8017594	0.49062758	-0.0935724
147.9	0.50340848	-0.5346885	-0.4876012	0.27258683	-0.8016093	0.49013103	-0.0940214
148	0.50340848	-0.5346885	-0.4876012	0.27258683	-0.8016093	0.49013103	-0.0940214
148.1	0.50340848	-0.5346885	-0.4876012	0.27258683	-0.8016093	0.49013103	-0.0940214
148.2	0.50402055	-0.5345264	-0.4876729	0.27294421	-0.8018172	0.4900268	-0.0940219
148.3	0.50402055	-0.5345264	-0.4876729	0.27294421	-0.8018172	0.4900268	-0.0940219
148.4	0.50292943	-0.5344821	-0.4866491	0.27240996	-0.8015519	0.49009612	-0.093765
148.5	0.50293626	-0.5344481	-0.4864655	0.27299986	-0.8016307	0.49012524	-0.0937303
148.6	0.50293626	-0.5344481	-0.4864655	0.27299986	-0.8016307	0.49012524	-0.0937303
148.7	0.50293626	-0.5344481	-0.4864655	0.27299986	-0.8016307	0.49012524	-0.0937303
148.8	0.50321944	-0.5342506	-0.4863317	0.27317162	-0.8018264	0.49009959	-0.0935532
148.9	0.50362199	-0.5343297	-0.4865154	0.27318108	-0.8019655	0.49021026	-0.0932412
149	0.50353844	-0.534142	-0.4861416	0.2728625	-0.801861	0.49036357	-0.0926726
149.1	0.50353844	-0.534142	-0.4861416	0.2728625	-0.801861	0.49036357	-0.0926726
149.2	0.50353844	-0.534142	-0.4861416	0.2728625	-0.801861	0.49036357	-0.0926726
149.3	0.50290591	-0.5341557	-0.485814	0.27184078	-0.8016984	0.4904863	-0.0929627
149.4	0.50314709	-0.5338542	-0.4857112	0.27200995	-0.8017664	0.49054751	-0.0929089
149.5	0.50263472	-0.5336789	-0.4851914	0.27081868	-0.8013522	0.49013724	-0.0932191
149.6	0.50256793	-0.5332662	-0.4847407	0.27051848	-0.80109	0.4898243	-0.0932497
149.7	0.50280437	-0.5327284	-0.4849401	0.27078217	-0.8010661	0.49004186	-0.0927958
149.8	0.50265624	-0.5327381	-0.4846505	0.27038787	-0.8009758	0.49016365	-0.0928528
149.9	0.50265624	-0.5327381	-0.4846505	0.27038787	-0.8009758	0.49016365	-0.0928528

195	0.45138257	-0.5282312	-0.4222678	0.23439389	-0.7786886	0.48892905	-0.0794218
195.1	0.45138257	-0.5282312	-0.4222678	0.23439389	-0.7786886	0.48892905	-0.0794218
195.2	0.45138257	-0.5282312	-0.4222678	0.23439389	-0.7786886	0.48892905	-0.0794218
195.3	0.45138257	-0.5282312	-0.4222678	0.23439389	-0.7786886	0.48892905	-0.0794218
195.4	0.45102174	-0.528111	-0.4224589	0.23406084	-0.7785851	0.48919094	-0.0789138
195.5	0.45019974	-0.5279692	-0.4215418	0.23385517	-0.77819	0.48917943	-0.0787415
195.6	0.45019974	-0.5279692	-0.4215418	0.23385517	-0.77819	0.48917943	-0.0787415
195.7	0.45019974	-0.5279692	-0.4215418	0.23385517	-0.77819	0.48917943	-0.0787415
195.8	0.45019974	-0.5279692	-0.4215418	0.23385517	-0.77819	0.48917943	-0.0787415
195.9	0.4500077	-0.5282141	-0.4216219	0.23373252	-0.7781311	0.48892521	-0.0786791
196	0.4500077	-0.5282141	-0.4216219	0.23373252	-0.7781311	0.48892521	-0.0786791
196.1	0.44964402	-0.5283641	-0.4214958	0.23377388	-0.7782132	0.48903632	-0.078673
196.2	0.44964402	-0.5283641	-0.4214958	0.23377388	-0.7782132	0.48903632	-0.078673
196.3	0.44964402	-0.5283641	-0.4214958	0.23377388	-0.7782132	0.48903632	-0.078673
196.4	0.44964402	-0.5283641	-0.4214958	0.23377388	-0.7782132	0.48903632	-0.078673
196.5	0.44918805	-0.5288013	-0.4212122	0.23366296	-0.7782478	0.48920045	-0.078721
196.6	0.44918805	-0.5288013	-0.4212122	0.23366296	-0.7782478	0.48920045	-0.078721
196.7	0.4494281	-0.5281998	-0.4207684	0.2336327	-0.7781159	0.48924886	-0.0786926
196.8	0.4494281	-0.5281998	-0.4207684	0.2336327	-0.7781159	0.48924886	-0.0786926
196.9	0.44930882	-0.5282595	-0.42068	0.23350728	-0.7781307	0.48947866	-0.0782805
197	0.44893194	-0.5285478	-0.4203092	0.23370584	-0.7780043	0.48932636	-0.0786947
197.1	0.44883545	-0.5286613	-0.4203548	0.23342879	-0.7778806	0.48949716	-0.0791139
197.2	0.44883545	-0.5286613	-0.4203548	0.23342879	-0.7778806	0.48949716	-0.0791139
197.3	0.44857834	-0.5286484	-0.4203376	0.23348288	-0.7777044	0.48961357	-0.0791548
197.4	0.44863114	-0.5290782	-0.420307	0.23302274	-0.7777412	0.48943541	-0.0789235
197.5	0.44863114	-0.5290782	-0.420307	0.23302274	-0.7777412	0.48943541	-0.0789235
197.6	0.44869628	-0.5293512	-0.4200504	0.23288915	-0.777713	0.48933809	-0.0788409
197.7	0.44869628	-0.5293512	-0.4200504	0.23288915	-0.777713	0.48933809	-0.0788409
197.8	0.44869628	-0.5293512	-0.4200504	0.23288915	-0.777713	0.48933809	-0.0788409
197.9	0.44842846	-0.5297984	-0.4198226	0.23255388	-0.7778081	0.48935046	-0.0789123
198	0.44848386	-0.529548	-0.4197857	0.23228649	-0.77771	0.48940261	-0.079153
198.1	0.44821763	-0.5292886	-0.4194549	0.23230292	-0.7775681	0.48943957	-0.0791807

156.1	0.49678647	-0.5295189	-0.4817069	0.27020265	-0.7995623	0.49112257	-0.09219
156.2	0.49678647	-0.5295189	-0.4817069	0.27020265	-0.7995623	0.49112257	-0.09219
156.3	0.49678647	-0.5295189	-0.4817069	0.27020265	-0.7995623	0.49112257	-0.09219
156.4	0.49641869	-0.529463	-0.4821263	0.26980636	-0.7992934	0.49131676	-0.0918184
156.5	0.49651517	-0.52943	-0.4819264	0.26963224	-0.7991733	0.49143119	-0.0917164
156.6	0.49651517	-0.52943	-0.4819264	0.26963224	-0.7991733	0.49143119	-0.0917164
156.7	0.49651517	-0.52943	-0.4819264	0.26963224	-0.7991733	0.49143119	-0.0917164
156.8	0.49651517	-0.52943	-0.4819264	0.26963224	-0.7991733	0.49143119	-0.0917164
156.9	0.4964342	-0.5296407	-0.481701	0.26971727	-0.7992111	0.49130187	-0.0913094
157	0.4964342	-0.5296407	-0.481701	0.26971727	-0.7992111	0.49130187	-0.0913094
157.1	0.49681417	-0.529889	-0.4810186	0.27024127	-0.7992445	0.49081414	-0.0910698
157.2	0.4971529	-0.5294365	-0.4812947	0.27043515	-0.7993248	0.49083361	-0.0910375
157.3	0.49708052	-0.5291403	-0.4810534	0.27047731	-0.799322	0.49092597	-0.09062
157.4	0.49745665	-0.5288337	-0.4810066	0.27039043	-0.7995229	0.49133428	-0.0903909
157.5	0.49732996	-0.5286411	-0.4807272	0.27052143	-0.7993413	0.49143693	-0.0905013
157.6	0.49732996	-0.5286411	-0.4807272	0.27052143	-0.7993413	0.49143693	-0.0905013
157.7	0.49732996	-0.5286411	-0.4807272	0.27052143	-0.7993413	0.49143693	-0.0905013
157.8	0.49703077	-0.5288064	-0.4805033	0.27020397	-0.7992294	0.49163053	-0.0907989
157.9	0.49614617	-0.5281105	-0.479492	0.26929346	-0.7987598	0.49116556	-0.0910812
158	0.49614617	-0.5281105	-0.479492	0.26929346	-0.7987598	0.49116556	-0.0910812
158.1	0.49658112	-0.5282916	-0.4799824	0.26908904	-0.7989471	0.49126162	-0.0901598
158.2	0.49680369	-0.5280943	-0.4793637	0.2683007	-0.7990142	0.49126202	-0.0899596
158.3	0.49669925	-0.5281939	-0.4795892	0.26786182	-0.7988664	0.49136221	-0.0899712
158.4	0.49707267	-0.5279945	-0.4795804	0.26816295	-0.7990102	0.49157898	-0.0899392
158.5	0.49674582	-0.5277572	-0.4795967	0.26799503	-0.798926	0.49159581	-0.0897281
158.6	0.49691621	-0.5279992	-0.4796372	0.26836397	-0.7988566	0.49132707	-0.0903377
158.7	0.4970537	-0.5279461	-0.4797852	0.26793224	-0.7987333	0.49137228	-0.0904934
158.8	0.4970537	-0.5279461	-0.4797852	0.26793224	-0.7987333	0.49137228	-0.0904934
158.9	0.49715127	-0.5280464	-0.4797516	0.26810239	-0.7987983	0.4916908	-0.0899432
159	0.49710166	-0.5278366	-0.4797008	0.26820841	-0.7987415	0.49191696	-0.0895599
159.1	0.49699447	-0.5281477	-0.4797329	0.26820816	-0.7987826	0.49187428	-0.0894988
159.2	0.4964613	-0.5282499	-0.4795722	0.26782597	-0.7986763	0.49175974	-0.0893543
159.3	0.4964613	-0.5282499	-0.4795722	0.26782597	-0.7986763	0.49175974	-0.0893543
159.4	0.4964613	-0.5282499	-0.4795722	0.26782597	-0.7986763	0.49175974	-0.0893543
159.5	0.4964613	-0.5282499	-0.4795722	0.26782597	-0.7986763	0.49175974	-0.0893543
159.6	0.49655123	-0.5277649	-0.4794023	0.26749938	-0.7985643	0.4916664	-0.089351
159.7	0.49655123	-0.5277649	-0.4794023	0.26749938	-0.7985643	0.4916664	-0.089351
159.8	0.49655123	-0.5277649	-0.4794023	0.26749938	-0.7985643	0.4916664	-0.089351
159.9	0.49608681	-0.5274861	-0.4790517	0.26731279	-0.7982835	0.49138305	-0.0895697
160	0.49623277	-0.5273455	-0.4793489	0.26755535	-0.7984616	0.49157763	-0.0895497
160.1	0.49663305	-0.5271145	-0.4793781	0.26759007	-0.7984431	0.4915156	-0.089465
160.2	0.49663305	-0.5271145	-0.4793781	0.26759007	-0.7984431	0.4915156	-0.089465
160.3	0.49574811	-0.5273648	-0.4791514	0.26684666	-0.7981346	0.4917137	-0.0903331
160.4	0.49535732	-0.5273965	-0.479007	0.26688065	-0.7980544	0.49169525	-0.0904998
160.5	0.49505642	-0.5274555	-0.4787934	0.26633644	-0.7978451	0.49159305	-0.0904793
160.6	0.49526927	-0.5277996	-0.4789407	0.26603648	-0.7980127	0.49203007	-0.0900492
160.7	0.49501682	-0.5278014	-0.4790964	0.26514604	-0.7978778	0.49208936	-0.0906439
160.8	0.49501682	-0.5278014	-0.4790964	0.26514604	-0.7978778	0.49208936	-0.0906439
160.9	0.49501682	-0.5278014	-0.4790964	0.26514604	-0.7978778	0.49208936	-0.0906439
161	0.49521335	-0.5275758	-0.479158	0.26506121	-0.7978788	0.49235114	-0.0904609
161.1	0.49534102	-0.5277461	-0.4787544	0.26492576	-0.7979514	0.49206497	-0.0901057
161.2	0.49534102	-0.5277461	-0.4787544	0.26492576	-0.7979514	0.49206497	-0.0901057
161.3	0.49581273	-0.5275291	-0.4789384	0.26486522	-0.7980822	0.4922043	-0.0896287
161.4	0.49581273	-0.5275291	-0.4789384	0.26486522	-0.7980822	0.4922043	-0.0896287
161.5	0.49521257	-0.5269651	-0.4785167	0.26415073	-0.7977227	0.49179569	-0.0893267
161.6	0.49516291	-0.5269602	-0.4782233	0.26447782	-0.7977849	0.49180931	-0.0895259
161.7	0.49575614	-0.5265822	-0.4788939	0.26418004	-0.7979356	0.49180592	-0.08938
161.8	0.49541061	-0.5260549	-0.4788044	0.26380582	-0.7977058	0.49222794	-0.0893355
161.9	0.49541061	-0.5260549	-0.4788044	0.26380582	-0.7977058	0.49222794	-0.0893355
162	0.49541061	-0.5260549	-0.4788044	0.26380582	-0.7977058	0.49222794	-0.0893355
162.1	0.49535107	-0.5260614	-0.4789807	0.2637141	-0.7976584	0.49241211	-0.0888009
162.2	0.49481379	-0.5257179	-0.478778	0.26375623	-0.7975582	0.49242794	-0.0886778
162.3	0.49481379	-0.5257179	-0.478778	0.26375623	-0.7975582	0.49242794	-0.0886778
162.4	0.49490927	-0.5255906	-0.4787834	0.26370416	-0.7975274	0.4926858	-0.088708
162.5	0.49474322	-0.5260161	-0.4787	0.2634565	-0.7975132	0.49278691	-0.0883472
162.6	0.49474322	-0.5260161	-0.4787	0.2634565	-0.7975132	0.49278691	-0.0883472
162.7	0.49446314	-0.5260294	-0.4785039	0.26320034	-0.7973369	0.49285055	-0.088993
162.8	0.49446314	-0.5260294	-0.4785039	0.26320034	-0.7973369	0.49285055	-0.088993
162.9	0.49476176	-0.5259366	-0.4787819	0.26309724	-0.7974964	0.49278019	-0.0888464
163	0.49476176	-0.5259366	-0.4787819	0.26309724	-0.7974964	0.49278019	-0.0888464
163.1	0.49450332	-0.5257053	-0.4783571	0.26255769	-0.7973916	0.49283361	-0.0889378
163.2	0.49450332	-0.5257053	-0.4783571	0.26255769	-0.7973916	0.49283361	-0.0889378
163.3	0.49453867	-0.5260556	-0.4784595	0.26246995	-0.797563	0.49316563	-0.0888909
163.4	0.49453867	-0.5260556	-0.4784595	0.26246995	-0.797563	0.49316563	-0.0888909
163.5	0.49406296	-0.5258446	-0.4781185	0.26195086	-0.7972321	0.49295944	-0.0890537
163.6	0.49406296	-0.5258446	-0.4781185	0.26195086	-0.7972321	0.49295944	-0.0890537
163.7	0.49421367	-0.5257014	-0.4779647	0.26172045	-0.7972294	0.49309415	-0.0885293
163.8	0.49413755	-0.5258906	-0.4779543	0.26186369	-0.7972125	0.49307559	-0.0886767
163.9	0.49413755	-0.5258906	-0.4779543	0.26186369	-0.7972125	0.49307559	-0.0886767
164	0.49413755	-0.5258906	-0.4779543	0.26186369	-0.7972125	0.49307559	-0.0886767
164.1	0.49417372	-0.5259331	-0.4777989	0.26159354	-0.797266	0.49342919	-0.0887441
164.2	0.49399637	-0.5259393	-0.4777542	0.26180879	-0.7972697	0.49332488	-0.0891278
164.3	0.49390742	-0.5258901	-0.4775318	0.26199909	-0.7973809	0.49332864	-0.0891146
164.4	0.49390742	-0.5258901	-0.4775318	0.26199909	-0.7973809	0.49332864	-0.0891146
164.5	0.49347547	-0.5257536	-0.4775512	0.26151531	-0.7972549	0.49325056	-0.0895039
164.6	0.49301598	-0.5258852	-0.4770105	0.26142886	-0.7971525	0.49316572	-0.0892798
164.7	0.49301598	-0.5258852	-0.4770105	0.26142886	-0.7971525	0.49316572	-0.0892798
164.8	0.49301598	-0.5258852	-0.4770105	0.26142886	-0.7971525	0.49316572	-0.0892798
164.9	0.49223676	-0.5261807	-0.4764395	0.26095445	-0.7969823	0.49293639	-0.0894286

210	0.44098539	-0.5279257	-0.4073992	0.22877838	-0.7725341	0.48705169	-0.0782147
210.1	0.44098539	-0.5279257	-0.4073992	0.22877838	-0.7725341	0.48705169	-0.0782147
210.2	0.44098539	-0.5279257	-0.4073992	0.22877838	-0.7725341	0.48705169	-0.0782147
210.3	0.44098539	-0.5279257	-0.4073992	0.22877838	-0.7725341	0.48705169	-0.0782147
210.4	0.44098539	-0.5279257	-0.4073992	0.22877838	-0.7725341	0.48705169	-0.0782147
210.5	0.44098539	-0.5279257	-0.4073992	0.22877838	-0.7725341	0.48705169	-0.0782147
210.6	0.44075303	-0.5278	-0.4072455	0.22869912	-0.7725151	0.4868751	-0.0782298
210.7	0.44075303	-0.5278	-0.4072455	0.22869912	-0.7725151	0.4868751	-0.0782298
210.8	0.44075303	-0.5278	-0.4072455	0.22869912	-0.7725151	0.4868751	-0.0782298
210.9	0.44075303	-0.5278	-0.4072455	0.22869912	-0.7725151	0.4868751	-0.0782298
211	0.44055834	-0.5278774	-0.4071199	0.22880457	-0.7725398	0.48687422	-0.0784289
211.1	0.44055834	-0.5278774	-0.4071199	0.22880457	-0.7725398	0.48687422	-0.0784289
211.2	0.44055834	-0.5278774	-0.4071199	0.22880457	-0.7725398	0.48687422	-0.0784289
211.3	0.44028651	-0.52771	-0.4067738	0.22883653	-0.7723798	0.48675536	-0.078495
211.4	0.44064587	-0.5276514	-0.40662	0.22941206	-0.7725639	0.48712441	-0.0786364
211.5	0.44064587	-0.5276514	-0.40662	0.22941206	-0.7725639	0.48712441	-0.0786364
211.6	0.44064587	-0.5276514	-0.40662	0.22941206	-0.7725639	0.48712441	-0.0786364
211.7	0.44064587	-0.5276514	-0.40662	0.22941206	-0.7725639	0.48712441	-0.0786364
211.8	0.44075814	-0.5276556	-0.40625	0.22933655	-0.7723909	0.48689046	-0.0787808
211.9	0.44075814	-0.5276556	-0.40625	0.22933655	-0.7723909	0.48689046	-0.0787808
212	0.44105648	-0.5276284	-0.4063198	0.22939586	-0.77238	0.48711298	-0.0788998
212.1	0.44068595	-0.5273196	-0.4060333	0.22922775	-0.7719578	0.48660156	-0.0791857
212.2	0.44068595	-0.5273196	-0.4060333	0.22922775	-0.7719578	0.48660156	-0.0791857
212.3	0.44060331	-0.5273522	-0.4057506	0.22952133	-0.7720338	0.48642135	-0.0797108
212.4	0.44060331	-0.5273522	-0.4057506	0.22952133	-0.7720338	0.48642135	-0.0797108
212.5	0.44060331	-0.5273522	-0.4057506	0.22952133	-0.7720338	0.48642135	-0.0797108
212.6	0.44060331	-0.5273522	-0.4057506	0.22952133	-0.7720338	0.48642135	-0.0797108
212.7	0.44039547	-0.5276375	-0.405739	0.22953531	-0.7719953	0.48626029	-0.0802328
212.8	0.44051023	-0.5275542	-0.4059952	0.22940799	-0.7718325	0.48610387	-0.0800057
212.9	0.44051023	-0.5275542	-0.4059952	0.22940799	-0.7718325	0.48610387	-0.0800057
213	0.44051023	-0.5275542	-0.4059952	0.22940799	-0.7718325	0.48610387	-0.0800057
213.1	0.44051023	-0.5275542	-0.4059952	0.22940799	-0.7718325	0.48610387	-0.0800057

171.1	0.48736199	-0.5245661	-0.4695618	0.25719959	-0.796046	0.49167766	-0.0894233
171.2	0.4869444	-0.5248527	-0.4694049	0.25713091	-0.7960762	0.49155768	-0.089007
171.3	0.48695003	-0.5251935	-0.469186	0.25704326	-0.7960893	0.49167017	-0.0887771
171.4	0.48695003	-0.5251935	-0.469186	0.25704326	-0.7960893	0.49167017	-0.0887771
171.5	0.48710889	-0.5254523	-0.4689667	0.25726928	-0.7963142	0.49171101	-0.0888208
171.6	0.48710889	-0.5254523	-0.4689667	0.25726928	-0.7963142	0.49171101	-0.0888208
171.7	0.48694177	-0.5253343	-0.4685173	0.25754689	-0.7962727	0.49174693	-0.0888513
171.8	0.48632904	-0.5250895	-0.4683466	0.25715204	-0.7960976	0.49175818	-0.0890409
171.9	0.48643186	-0.5250869	-0.4685937	0.2567612	-0.796037	0.49168606	-0.0893333
172	0.48643186	-0.5250869	-0.4685937	0.2567612	-0.796037	0.49168606	-0.0893333
172.1	0.48669265	-0.5252444	-0.4684008	0.25706247	-0.7959914	0.49192593	-0.089147
172.2	0.48642773	-0.5247619	-0.4682139	0.25650631	-0.795889	0.49172259	-0.0885326
172.3	0.48642773	-0.5247619	-0.4682139	0.25650631	-0.795889	0.49172259	-0.0885326
172.4	0.48638121	-0.5246487	-0.4680989	0.25627303	-0.7957633	0.49184743	-0.0885974
172.5	0.48598639	-0.5244776	-0.4676325	0.2564591	-0.7956026	0.49176622	-0.0886702
172.6	0.48598639	-0.5244776	-0.4676325	0.2564591	-0.7956026	0.49176622	-0.0886702
172.7	0.48593749	-0.5244021	-0.4674058	0.25640152	-0.7955823	0.49175307	-0.0889261
172.8	0.48596277	-0.5240654	-0.4673619	0.25599589	-0.7956826	0.49213833	-0.0887393
172.9	0.48580825	-0.524307	-0.4668978	0.2561637	-0.7955439	0.49213701	-0.0879557
173	0.48580825	-0.524307	-0.4668978	0.2561637	-0.7955439	0.49213701	-0.0879557
173.1	0.48557359	-0.5248268	-0.4668622	0.25593694	-0.7956568	0.49233181	-0.0875439
173.2	0.48554309	-0.5249646	-0.4667533	0.25568779	-0.7958375	0.49277804	-0.0875576
173.3	0.48554309	-0.5249646	-0.4667533	0.25568779	-0.7958375	0.49277804	-0.0875576
173.4	0.48548711	-0.5254387	-0.4666882	0.25584459	-0.7959178	0.49283919	-0.0877584
173.5	0.48548711	-0.5254387	-0.4666882	0.25584459	-0.7959178	0.49283919	-0.0877584
173.6	0.48548711	-0.5254387	-0.4666882	0.25584459	-0.7959178	0.49283919	-0.0877584
173.7	0.48502554	-0.5256609	-0.46664	0.2561803	-0.7960389	0.49314134	-0.0878109
173.8	0.48502554	-0.5256609	-0.46664	0.2561803	-0.7960389	0.49314134	-0.0878109
173.9	0.48502554	-0.5256609	-0.46664	0.2561803	-0.7960389	0.49314134	-0.0878109
174	0.48450476	-0.5257564	-0.4663634	0.25610671	-0.7959727	0.49325509	-0.0877391
174.1	0.48402747	-0.5256394	-0.4663487	0.25581695	-0.7958966	0.49328545	-0.0877902
174.2	0.48402747	-0.5256394	-0.4663487	0.25581695	-0.7958966	0.49328545	-0.0877902
174.3	0.48391552	-0.5254328	-0.4658408	0.25516513	-0.7959219	0.49302915	-0.0877238
174.4	0.48386258	-0.5253474	-0.4654749	0.25569682	-0.7959781	0.49263467	-0.0881958
174.5	0.48386258	-0.5253474	-0.4654749	0.25569682	-0.7959781	0.49263467	-0.0881958
174.6	0.483429	-0.5254369	-0.4652118	0.25518026	-0.7958476	0.49280739	-0.0887759
174.7	0.48364909	-0.5255372	-0.4648764	0.25543936	-0.7959321	0.49271224	-0.0888534
174.8	0.48392238	-0.5257232	-0.4649074	0.2551668	-0.7959607	0.4930119	-0.0884591
174.9	0.48395041	-0.5255313	-0.4646083	0.25473091	-0.796037	0.49337041	-0.0887217
175	0.48370496	-0.5259836	-0.4645052	0.2545558	-0.7961329	0.49351849	-0.0882896
175.1	0.48325709	-0.5261165	-0.4639088	0.25457476	-0.7962117	0.494151	-0.0879554
175.2	0.48287507	-0.5265097	-0.4635866	0.25440609	-0.7962152	0.49386824	-0.0877282
175.3	0.48259143	-0.5269109	-0.4633053	0.25442159	-0.7962928	0.49420842	-0.0877711
175.4	0.48183316	-0.5270682	-0.4621848	0.25495245	-0.7961691	0.49383424	-0.0880325
175.5	0.48137299	-0.5270055	-0.4617946	0.25491326	-0.7959628	0.49381175	-0.087995
175.6	0.48137299	-0.5270055	-0.4617946	0.25491326	-0.7959628	0.49381175	-0.087995
175.7	0.48130671	-0.5270405	-0.4616468	0.25541404	-0.7960128	0.49382258	-0.087546
175.8	0.48130671	-0.5270405	-0.4616468	0.25541404	-0.7960128	0.49382258	-0.087546
175.9	0.4812843	-0.526933	-0.4612091	0.25548234	-0.7961779	0.4936885	-0.0869454
176	0.48056128	-0.5266203	-0.4608349	0.25545798	-0.7960733	0.49389961	-0.0867133
176.1	0.48074568	-0.5268119	-0.4603817	0.25477505	-0.7957833	0.4936075	-0.0866776
176.2	0.48054816	-0.5272429	-0.4603645	0.25470951	-0.7959099	0.49381276	-0.0863916
176.3	0.47981277	-0.5269936	-0.4599435	0.2549427	-0.7956742	0.49349014	-0.0867497
176.4	0.48029487	-0.5271009	-0.4603061	0.25499623	-0.7957972	0.49315202	-0.086526
176.5	0.4795982	-0.5273205	-0.4598407	0.25521177	-0.7954642	0.49266577	-0.0869067
176.6	0.48008266	-0.5270315	-0.4595213	0.25421513	-0.795573	0.49279721	-0.0863822
176.7	0.47997248	-0.5266222	-0.4586767	0.25380429	-0.7952923	0.49262191	-0.0859344
176.8	0.47980722	-0.5271537	-0.4579962	0.25290974	-0.7952943	0.49289847	-0.0855726
176.9	0.47923552	-0.52781	-0.457761	0.25182946	-0.79482	0.49298682	-0.0853734
177	0.47903238	-0.5280272	-0.457701	0.25165136	-0.7949714	0.49313483	-0.0853393
177.1	0.47853921	-0.5282274	-0.457492	0.25105172	-0.7948036	0.49361433	-0.0850107
177.2	0.47843212	-0.5284956	-0.4576675	0.25068581	-0.7948452	0.49376141	-0.084991
177.3	0.47824124	-0.5283754	-0.4577824	0.25020971	-0.7948005	0.49416502	-0.084962
177.4	0.47824501	-0.5279533	-0.4572701	0.24990558	-0.7946735	0.49384383	-0.08543
177.5	0.47804197	-0.5280863	-0.4572173	0.24978701	-0.7947991	0.494149	-0.0849495
177.6	0.47751593	-0.528032	-0.4570885	0.25021366	-0.7948288	0.49405444	-0.0854044
177.7	0.47751593	-0.528032	-0.4570885	0.25021366	-0.7948288	0.49405444	-0.0854044
177.8	0.4774418	-0.5281282	-0.4567903	0.24977346	-0.7948858	0.49384294	-0.0856582
177.9	0.47718382	-0.5279819	-0.4563927	0.24999721	-0.7947156	0.49349151	-0.0860497
178	0.47659881	-0.5280404	-0.4560683	0.24978972	-0.7944892	0.49342332	-0.0864581
178.1	0.47631107	-0.5277827	-0.455968	0.25011456	-0.7944397	0.49307227	-0.0868314
178.2	0.47624814	-0.5275003	-0.4558841	0.25020113	-0.7943423	0.49266935	-0.0872315
178.3	0.47617866	-0.52771	-0.4554481	0.25022009	-0.7943452	0.49238944	-0.0872952
178.4	0.47621784	-0.5279283	-0.4551811	0.25027768	-0.7943002	0.49252593	-0.087273
178.5	0.47584822	-0.5283775	-0.4548153	0.25031203	-0.7942817	0.49238229	-0.0870688
178.6	0.47575973	-0.5282567	-0.4549174	0.24999312	-0.7941229	0.49219073	-0.0873225
178.7	0.47547736	-0.5283392	-0.454306	0.24932923	-0.793925	0.4918809	-0.0870974
178.8	0.47547736	-0.5283392	-0.454306	0.24932923	-0.793925	0.4918809	-0.0870974
178.9	0.47503615	-0.5281605	-0.4540516	0.24943238	-0.7937311	0.49148391	-0.0873592
179	0.47462296	-0.5282428	-0.4538894	0.24967267	-0.7936858	0.49124993	-0.0875016
179.1	0.47450035	-0.5283638	-0.454219	0.24928011	-0.7935945	0.49125877	-0.0877419
179.2	0.47462475	-0.5284308	-0.4541579	0.24936599	-0.7935655	0.49147445	-0.0879356
179.3	0.47451343	-0.5279183	-0.4536203	0.24899883	-0.7930993	0.49142547	-0.0881112
179.4	0.47411815	-0.5277129	-0.4531367	0.24871793	-0.7928439	0.49157984	-0.088641
179.5	0.47392693	-0.5273153	-0.4525238	0.24815973	-0.7926373	0.49151645	-0.0880562
179.6	0.4733557	-0.5276113	-0.4524659	0.24802172	-0.7924844	0.49166322	-0.0879814
179.7	0.47323559	-0.5277736	-0.4524863	0.24745055	-0.7923321	0.49167235	-0.0877485
179.8	0.47305874	-0.5276338	-0.451888	0.24759905	-0.7922027	0.4917781	-0.0879964
179.9	0.47305874	-0.5276338	-0.451888	0.24759905	-0.7922027	0.4917781	-0.0879964

225	0.4354906	-0.5266423	-0.3991211	0.22402248	-0.7700353	0.48214981	-0.077956
225.1	0.4354906	-0.5266423	-0.3991211	0.22402248	-0.7700353	0.48214981	-0.077956
225.2	0.4354906	-0.5266423	-0.3991211	0.22402248	-0.7700353	0.48214981	-0.077956
225.3	0.4354906	-0.5266423	-0.3991211	0.22402248	-0.7700353	0.48214981	-0.077956
225.4	0.43540019	-0.5262793	-0.3988652	0.22354933	-0.7699034	0.48210862	-0.0780275
225.5	0.43540019	-0.5262793	-0.3988652	0.22354933	-0.7699034	0.48210862	-0.0780275
225.6	0.43540019	-0.5262793	-0.3988652	0.22354933	-0.7699034	0.48210862	-0.0780275
225.7	0.43540019	-0.5262793	-0.3988652	0.22354933	-0.7699034	0.48210862	-0.0780275
225.8	0.43540019	-0.5262793	-0.3988652	0.22354933	-0.7699034	0.48210862	-0.0780275
225.9	0.43540019	-0.5262793	-0.3988652	0.22354933	-0.7699034	0.48210862	-0.0780275
226	0.43540019	-0.5262793	-0.3988652	0.22354933	-0.7699034	0.48210862	-0.0780275
226.1	0.43540019	-0.5262793	-0.3988652	0.22354933	-0.7699034	0.48210862	-0.0780275
226.2	0.43540019	-0.5262793	-0.3988652	0.22354933	-0.7699034	0.48210862	-0.0780275
226.3	0.43540019	-0.5262793	-0.3988652	0.22354933	-0.7699034	0.48210862	-0.0780275
226.4	0.43540019	-0.5262793	-0.3988652	0.22354933	-0.7699034	0.48210862	-0.0780275
226.5	0.43496478	-0.525873	-0.3982557	0.22350033	-0.7697469	0.48187995	-0.0781108
226.6	0.43496478	-0.525873	-0.3982557	0.22350033	-0.7697469	0.48187995	-0.0781108
226.7	0.43445106	-0.5256831	-0.3982705	0.22330859	-0.7696169	0.4818787	-0.0784735
226.8	0.43438551	-0.5257394	-0.3978678	0.22296043	-0.76962	0.48180831	-0.078256
226.9	0.43438551	-0.5257394	-0.3978678	0.22296043	-0.76962	0.48180831	-0.078256
227	0.43454037	-0.5258677	-0.3978242	0.22285694	-0.7696107	0.48179188	-0.0783825
227.1	0.43454037	-0.5258677	-0.3978242	0.22285694	-0.7696107	0.48179188	-0.0783825
227.2	0.43454037	-0.5258677	-0.3978242	0.22285694	-0.7696107	0.48179188	-0.0783825
227.3	0.43454037	-0.5258677	-0.3978242	0.22285694	-0.7696107	0.48179188	-0.0783825
227.4	0.43454037	-0.5258677	-0.3978242	0.22285694	-0.7696107	0.48179188	-0.0783825
227.5	0.43454037	-0.5258677	-0.3978242	0.22285694	-0.7696107	0.48179188	-0.0783825
227.6	0.43454037	-0.5258677	-0.3978242	0.22285694	-0.7696107	0.48179188	-0.0783825
227.7	0.43454037	-0.5258677	-0.3978242	0.22285694	-0.7696107	0.48179188	-0.0783825
227.8	0.43419233	-0.5255559	-0.3977545	0.22268923	-0.7692949	0.48156305	-0.0784765
227.9	0.43419233	-0.5255559	-0.3977545	0.22268923	-0.7692949	0.48156305	-0.0784765
228	0.43419233	-0.5255559	-0.3977545	0.22268923	-0.7692949	0.48156305	-0.0784765
228.1	0.43421238	-0.5256079	-0.3977289	0.22256479	-0.7693015	0.48134074	-0.0788378

183.2	0.46807918	-0.5269877	-0.4457219	0.24572914	-0.7895025	0.49089327	-0.0855188
183.3	0.46807918	-0.5269877	-0.4457219	0.24572914	-0.7895025	0.49089327	-0.0855188
183.4	0.46807918	-0.5269877	-0.4457219	0.24572914	-0.7895025	0.49089327	-0.0855188
183.5	0.46833605	-0.5269684	-0.4453343	0.24613162	-0.7895299	0.49082337	-0.0858008
183.6	0.46831921	-0.5272233	-0.4455421	0.2462049	-0.7896002	0.49134662	-0.0861651
183.7	0.46831921	-0.5272233	-0.4455421	0.2462049	-0.7896002	0.49134662	-0.0861651
183.8	0.46837716	-0.5271469	-0.4451354	0.24630311	-0.7894004	0.49132295	-0.0863683
183.9	0.46815691	-0.527357	-0.4448604	0.2461877	-0.7893994	0.49122378	-0.0858607
184	0.46815691	-0.527357	-0.4448604	0.2461877	-0.7893994	0.49122378	-0.0858607
184.1	0.46730544	-0.5279058	-0.4448521	0.24624248	-0.7892676	0.4916701	-0.0862238
184.2	0.46755373	-0.5276895	-0.4447553	0.24620114	-0.7891374	0.49159007	-0.0864353
184.3	0.46746928	-0.5276419	-0.4444637	0.24650691	-0.7889778	0.491648	-0.086576
184.4	0.46724581	-0.5278735	-0.4438081	0.2463617	-0.788676	0.49151001	-0.0861436
184.5	0.4674752	-0.5279273	-0.4433354	0.24619351	-0.7887967	0.49165253	-0.0864687
184.6	0.46757885	-0.5284587	-0.4420217	0.24556732	-0.7885207	0.4916468	-0.0860998
184.7	0.46746881	-0.5283934	-0.4418012	0.24556791	-0.7884681	0.49167778	-0.0863056
184.8	0.46728943	-0.5282573	-0.4413932	0.24548385	-0.7881045	0.49178352	-0.0867782
184.9	0.46742435	-0.5282558	-0.4410336	0.24577155	-0.7881455	0.49168022	-0.0866231
185	0.4671811	-0.5284149	-0.440651	0.24578526	-0.787961	0.49164769	-0.0865187
185.1	0.46738673	-0.5280047	-0.440778	0.24540714	-0.7879504	0.4916155	-0.0862914
185.2	0.4669061	-0.5278879	-0.4405355	0.24506652	-0.7875972	0.49147633	-0.0860591
185.3	0.4669061	-0.5278879	-0.4405355	0.24506652	-0.7875972	0.49147633	-0.0860591
185.4	0.46577078	-0.5282235	-0.4400101	0.24410394	-0.7870874	0.49147128	-0.0869118
185.5	0.46577078	-0.5282235	-0.4400101	0.24410394	-0.7870874	0.49147128	-0.0869118
185.6	0.46513999	-0.5280851	-0.4392714	0.24399734	-0.7868651	0.49106506	-0.0869008
185.7	0.46513999	-0.5280851	-0.4392714	0.24399734	-0.7868651	0.49106506	-0.0869008
185.8	0.46498812	-0.5279808	-0.4388186	0.24375061	-0.7866293	0.49087096	-0.0866959
185.9	0.46498812	-0.5279808	-0.4388186	0.24375061	-0.7866293	0.49087096	-0.0866959
186	0.46498812	-0.5279808	-0.4388186	0.24375061	-0.7866293	0.49087096	-0.0866959
186.1	0.46463377	-0.5283413	-0.4387369	0.2439027	-0.7866824	0.4910385	-0.0864415
186.2	0.46463377	-0.5283413	-0.4387369	0.2439027	-0.7866824	0.4910385	-0.0864415
186.3	0.4643736	-0.5287039	-0.4380762	0.24380256	-0.7864464	0.49101254	-0.0859087
186.4	0.4643736	-0.5287039	-0.4380762	0.24380256	-0.7864464	0.49101254	-0.0859087
186.5	0.46391185	-0.5284222	-0.4375556	0.24361026	-0.7861745	0.49046493	-0.0861402
186.6	0.46388854	-0.5280342	-0.4376639	0.24346748	-0.7862113	0.4904808	-0.0858863
186.7	0.46335948	-0.5276966	-0.4369148	0.24338487	-0.7860744	0.49060188	-0.0854954
186.8	0.46291256	-0.5273081	-0.4371228	0.2434879	-0.786035	0.49032205	-0.0849765
186.9	0.46276451	-0.5270949	-0.4365898	0.24317003	-0.7857335	0.49009836	-0.0850248
187	0.46284996	-0.5268448	-0.4365605	0.2428347	-0.7858111	0.49032576	-0.0844067
187.1	0.46316248	-0.5269122	-0.4365468	0.24270822	-0.7857811	0.49064839	-0.0842229
187.2	0.46321464	-0.527066	-0.4363978	0.24276531	-0.7859619	0.49097678	-0.0840694
187.3	0.46321464	-0.527066	-0.4363978	0.24276531	-0.7859619	0.49097678	-0.0840694
187.4	0.46332315	-0.5272042	-0.4362597	0.24257214	-0.7860026	0.49118813	-0.083704
187.5	0.46322069	-0.5271121	-0.4357806	0.24261123	-0.7858748	0.49110758	-0.0833937
187.6	0.46269104	-0.5270295	-0.4352901	0.24233352	-0.7857682	0.4909342	-0.0834429
187.7	0.46269104	-0.5270295	-0.4352901	0.24233352	-0.7857682	0.4909342	-0.0834429
187.8	0.46245885	-0.5271436	-0.4349078	0.2424635	-0.7856656	0.49096547	-0.0834044
187.9	0.46245885	-0.5271436	-0.4349078	0.2424635	-0.7856656	0.49096547	-0.0834044
188	0.46193248	-0.5273769	-0.434658	0.24164524	-0.785395	0.49092893	-0.0830059
188.1	0.46191475	-0.5273446	-0.4346433	0.24134195	-0.7853995	0.49091773	-0.0832385
188.2	0.46164713	-0.5276546	-0.4348371	0.24100978	-0.7852536	0.49080386	-0.0835245
188.3	0.46164713	-0.5276546	-0.4348371	0.24100978	-0.7852536	0.49080386	-0.0835245
188.4	0.46164713	-0.5276546	-0.4348371	0.24100978	-0.7852536	0.49080386	-0.0835245
188.5	0.46164713	-0.5276546	-0.4348371	0.24100978	-0.7852536	0.49080386	-0.0835245
188.6	0.46119587	-0.5277302	-0.4348686	0.24104141	-0.7851049	0.49038358	-0.0834884
188.7	0.46119587	-0.5277302	-0.4348686	0.24104141	-0.7851049	0.49038358	-0.0834884
188.8	0.46104146	-0.5274707	-0.4348707	0.24070537	-0.7851724	0.49046087	-0.0832564
188.9	0.46104146	-0.5274707	-0.4348707	0.24070537	-0.7851724	0.49046087	-0.0832564
189	0.46076975	-0.5274744	-0.4345149	0.24061518	-0.7849907	0.49034515	-0.0830361
189.1	0.46039385	-0.5274612	-0.4343632	0.24069357	-0.7849981	0.48998509	-0.083359
189.2	0.46039385	-0.5274612	-0.4343632	0.24069357	-0.7849981	0.48998509	-0.083359
189.3	0.4600026	-0.5273304	-0.4334796	0.24019455	-0.7846303	0.48981737	-0.083507
189.4	0.45963934	-0.5274943	-0.4336947	0.2399051	-0.784524	0.48997471	-0.0831839
189.5	0.45943257	-0.5271842	-0.4334936	0.23967348	-0.7844481	0.49010126	-0.0830011
189.6	0.45943257	-0.5271842	-0.4334936	0.23967348	-0.7844481	0.49010126	-0.0830011
189.7	0.45925602	-0.5271616	-0.433348	0.23963712	-0.7844652	0.49012847	-0.0826839
189.8	0.45937438	-0.5277143	-0.433348	0.23973188	-0.7845303	0.49043569	-0.0830202
189.9	0.45944885	-0.5278044	-0.4331757	0.23990414	-0.784696	0.49024334	-0.0833766
190	0.45944885	-0.5278044	-0.4331757	0.23990414	-0.784696	0.49024334	-0.0833766
190.1	0.45922032	-0.5273019	-0.4326143	0.24021075	-0.7842605	0.48998387	-0.0828577
190.2	0.45833516	-0.5276092	-0.431943	0.24044265	-0.7840453	0.48941548	-0.0824841
190.3	0.45814337	-0.5270779	-0.4309497	0.24010599	-0.783942	0.4890681	-0.0822173
190.4	0.45814337	-0.5270779	-0.4309497	0.24010599	-0.783942	0.4890681	-0.0822173
190.5	0.45802256	-0.5272383	-0.4307926	0.24014776	-0.784032	0.48932887	-0.081826
190.6	0.45802256	-0.5272383	-0.4307926	0.24014776	-0.784032	0.48932887	-0.081826
190.7	0.45765638	-0.5270933	-0.4298906	0.24004071	-0.7837145	0.4896249	-0.0813213
190.8	0.45743119	-0.5270261	-0.4294312	0.23934196	-0.7834715	0.48978226	-0.0805838
190.9	0.45729333	-0.5269596	-0.4296239	0.2387088	-0.7833425	0.48983478	-0.0805361
191	0.456658	-0.5269317	-0.4294515	0.2388393	-0.7831759	0.48959063	-0.080899
191.1	0.45660118	-0.5269614	-0.4289568	0.2386012	-0.7830446	0.48960464	-0.0804007
191.2	0.45660118	-0.5269614	-0.4289568	0.2386012	-0.7830446	0.48960464	-0.0804007
191.3	0.45667147	-0.5270187	-0.4288204	0.2388655	-0.7829332	0.48955524	-0.08046
191.4	0.45667147	-0.5270187	-0.4288204	0.2388655	-0.7829332	0.48955524	-0.08046
191.5	0.45667147	-0.5270187	-0.4288204	0.2388655	-0.7829332	0.48955524	-0.08046
191.6	0.45669881	-0.5266834	-0.428746	0.23875126	-0.7829129	0.48973936	-0.0797723
191.7	0.45614748	-0.5268982	-0.4283501	0.2382121	-0.7825695	0.48994401	-0.0798245
191.8	0.45614748	-0.5268982	-0.4283501	0.2382121	-0.7825695	0.48994401	-0.0798245
191.9	0.45614748	-0.5268982	-0.4283501	0.2382121	-0.7825695	0.48994401	-0.0798245
192	0.45614748	-0.5268982	-0.4283501	0.2382121	-0.7825695	0.48994401	-0.0798245
192.1	0.45617749	-0.5270249	-0.4279327	0.23828846	-0.7824799	0.49014547	-0.0799496
192.2	0.45588483	-0.5269756	-0.4277506	0.2383168	-0.7822893	0.49008812	-0.0799646
192.3	0.45580828	-0.527218	-0.4274626	0.23802311	-0.7823612	0.49032428	-0.0795702
192.4	0.45531911	-0.527413	-0.4272659	0.23799594	-0.7822712	0.49032197	-0.0798835
192.5	0.45441881	-0.5275199	-0.4270924	0.23750648	-0.7820744	0.49051713	-0.0801149
192.6	0.45438567	-0.527777	-0.4270879	0.23732598	-0.7821104	0.49065228	-0.0799389
192.7	0.45397224	-0.5277248	-0.4272323	0.23700831	-0.7819465	0.4905764	-0.0799435
192.8	0.45397224	-0.5277248	-0.4272323	0.23700831	-0.7819465	0.4905764	-0.0799435
192.9	0.45396178	-0.5276003	-0.4274167	0.23636594	-0.7818872	0.4905316	-0.0793878
193	0.45377204	-0.5276859	-0.4270932	0.23639475	-0.7818226	0.49045345	-0.0790887
193.1	0.45382834	-0.5281721	-0.4267119	0.23644349	-0.7819141	0.49007675	-0.0788978
193.2	0.45339535	-0.5281122	-0.4265446	0.23602273	-0.7815134	0.48987067	-0.0790593
193.3	0.45320585	-0.5281388	-0.4264068	0.23575246	-0.7815439	0.48964631	-0.0790021
193.4	0.45301612	-0.5283955	-0.4261497	0.23558724	-0.7815438	0.48982021	-0.078837
193.5	0.45309565	-0.5283315	-0.4259769	0.23558501	-0.7813515	0.48985087	-0.0789872
193.6	0.45276764	-0.5281982	-0.4253224	0.23554229	-0.7808968	0.48952431	-0.0788867
193.7	0.45269148	-0.5281837	-0.424241	0.23599636	-0.7806284	0.4896997	-0.0792862
193.8	0.45271411	-0.5283996	-0.4239992	0.23548662	-0.7803653	0.48969005	-0.0791436
193.9	0.45244563	-0.5284014	-0.4244211	0.2349046	-0.7801669	0.48978742	-0.079084
194	0.45220551	-0.5282238	-0.4247013	0.23469786	-0.780051	0.48982153	-0.0789885
194.1	0.45220551	-0.5282238	-0.4247013	0.23469786	-0.780051	0.48982153	-0.0789885
194.2	0.45220551	-0.5282238	-0.4247013	0.23469786	-0.780051	0.48982153	-0.0789885
194.3	0.4521002	-0.5284023	-0.4244831	0.23479294	-0.7798752	0.48962069	-0.0793208
194.4	0.45179654	-0.5283563	-0.4239071	0.23479602	-0.7796637	0.48953839	-0.0792537
194.5	0.45201458	-0.5282015	-0.4231092	0.23536131	-0.7794767	0.48952194	-0.0797661
194.6	0.45176238	-0.5283413	-0.4231487	0.2348158	-0.7792225	0.48928242	-0.0796456
194.7	0.45149744	-0.5284573	-0.422764	0.23449222	-0.7789948	0.48910216	-0.0794187
194.8	0.45149744	-0.5284573	-0.422764	0.23449222	-0.7789948	0.48910216	-0.0794187
194.9	0.45138257	-0.5282312	-0.4222678	0.23439389	-0.7786886	0.48892905	-0.0794218
198.2	0.44821763	-0.5292886	-0.4194549	0.23230292	-0.7775681	0.48943957	-0.0791807
198.3	0.44843448	-0.5291815	-0.4194933	0.23220449	-0.7772678	0.48889208	-0.0795271
198.4	0.44819511	-0.5291751	-0.419336	0.23208253	-0.7770948	0.48863806	-0.0797614
198.5	0.44788194	-0.5289741	-0.4188622	0.23224422	-0.7769035	0.48837364	-0.0799823
198.6	0.44747422	-0.5290793	-0.4185399	0.23239507	-0.7768447	0.48854025	-0.0800269
198.7	0.44747422	-0.5290793	-0.4185399	0.23239507	-0.7768447	0.48854025	-0.0800269
198.8	0.44721943	-0.5290042	-0.4186195	0.23207775	-0.7766335	0.48825875	-0.0801089
198.9	0.44712287	-0.5290637	-0.4181694	0.23196202	-0.776529	0.48844846	-0.0805121
199	0.44657925	-0.5288225	-0.4178116	0.23199736	-0.7762853	0.48824381	-0.0804938
199.1	0.44657925	-0.5288225	-0.4178116	0.23199736	-0.7762853	0.48824381	-0.0804938
199.2	0.44657925	-0.5288225	-0.4178116	0.23199736	-0.7762853	0.48824381	-0.0804938
199.3	0.44657925	-0.5288225	-0.4178116	0.23199736	-0.7762853	0.48824381	-0.0804938
199.4	0.44657925	-0.5288225	-0.4178116	0.23199736	-0.7762853	0.48824381	-0.0804938
199.5	0.44657925	-0.5288225	-0.4178116	0.23199736	-0.7762853	0.48824381	-0.0804938
199.6	0.44663506	-0.5287054	-0.4174035	0.23125072	-0.776049	0.48812604	-0.080055
199.7	0.44589478	-0.5282761	-0.4167789	0.23128762	-0.7757569	0.48751753	-0.0805227
199.8	0.44531838	-0.5283898	-0.4160003	0.2312328	-0.7754728	0.48757489	-0.0806538
199.9	0.44531838	-0.5283898	-0.4160003	0.2312328	-0.7754728	0.48757489	-0.0806538
200	0.44531838	-0.5283898	-0.4160003	0.2312328	-0.7754728	0.48757489	-0.0806538
200.1	0.44528917	-0.5282884	-0.4154519	0.23113555	-0.7752659	0.48759988	-0.0803603
200.2	0.44528917	-0.5282884	-0.4154519	0.23113555	-0.7752659	0.48759988	-0.0803603
200.3	0.44528917	-0.5282884	-0.4154519	0.23113555	-0.7752659	0.48759988	-0.0803603
200.4	0.44477058	-0.5283645	-0.4154624	0.23062555	-0.7749707	0.48753682	-0.0805704
200.5	0.44477058	-0.5283645	-0.4154624	0.23062555	-0.7749707	0.48753682	-0.0805704
200.6	0.44477058	-0.5283645	-0.4154624	0.23062555	-0.7749707	0.48753682	-0.0805704
200.7	0.44477058	-0.5283645	-0.4154624	0.23062555	-0.7749707	0.48753682	-0.0805704
200.8	0.44477058	-0.5283645	-0.4154624	0.23062555	-0.7749707	0.48753682	-0.0805704
200.9	0.44477058	-0.5283645	-0.4154624	0.23062555	-0.7749707	0.48753682	-0.0805704
201	0.44468206	-0.5283443	-0.4152744	0.23051341	-0.7747191	0.48712319	-0.0807847
201.1	0.44438791	-0.5279973	-0.4152006	0.2308095	-0.7743082	0.48676808	-0.080906
201.2	0.44389191	-0.5279509	-0.4150463	0.23073587	-0.7741812	0.48691091	-0.0810932
201.3	0.44362642	-0.528144	-0.4151654	0.23059298	-0.7741429	0.4870164	-0.081101
201.4	0.44362642	-0.528144	-0.4151654	0.23059298	-0.7741429	0.4870164	-0.081101
201.5	0.44362642	-0.528144	-0.4151654	0.23059298	-0.7741429	0.4870164	-0.081101
201.6	0.44362642	-0.528144	-0.4151654	0.23059298	-0.7741429	0.4870164	-0.081101
201.7	0.44362642	-0.528144	-0.4151654	0.23059298	-0.7741429	0.4870164	-0.081101
201.8	0.4434012	-0.5279843	-0.4147341	0.23071324	-0.7740553	0.48715633	-0.0810677
201.9	0.4434012	-0.5279843	-0.4147341	0.23071324	-0.7740553	0.48715633	-0.0810677
202	0.4434012	-0.5279843	-0.4147341	0.23071324	-0.7740553	0.48715633	-0.0810677
202.1	0.4434012	-0.5279843	-0.4147341	0.23071324	-0.7740553	0.48715633	-0.0810677
202.2	0.44305914	-0.5279349	-0.4142066	0.23089705	-0.7736824	0.48725008	-0.081136
202.3	0.44339494	-0.5278948	-0.4140139	0.23127982	-0.7737072	0.4872565	-0.0809718
202.4	0.44304571	-0.5278425	-0.4139318	0.23148883	-0.7737404	0.48727121	-0.0810434
202.5	0.44304571	-0.5278425	-0.4139318	0.23148883	-0.7737404	0.48727121	-0.0810434
202.6	0.44274948	-0.5282075	-0.413757	0.23133783	-0.7738931	0.48731205	-0.0805129
202.7	0.44274948	-0.5282075	-0.413757	0.23133783	-0.7738931	0.48731205	-0.0805129
202.8	0.44272182	-0.5282117	-0.4133976	0.23137108	-0.7738007	0.48740428	-0.0805939
202.9	0.44272182	-0.5282117	-0.4133976	0.23137108	-0.7738007	0.48740428	-0.0805939
203	0.44258365	-0.5279831	-0.4131988	0.23126855	-0.7735355	0.48718543	-0.0804779
203.1	0.44258365	-0.5279831	-0.4131988	0.23126855	-0.7735355	0.48718543	-0.0804779
203.2	0.44258365	-0.5279831	-0.4131988	0.23126855	-0.7735355	0.48718543	-0.0804779
203.3	0.4425551	-0.527559	-0.4133382	0.23114024	-0.7733994	0.48721133	-0.0807683
203.4	0.44241756	-0.5276061	-0.413243	0.23110401	-0.7733032	0.48702407	-0.0805514
203.5	0.44265326	-0.5275992	-0.4129125	0.23077896	-0.7732204	0.48703217	-0.0801079
203.6	0.44260882	-0.5274726	-0.4125847	0.23080582	-0.7730553	0.48681266	-0.080124
203.7	0.44260882	-0.5274726	-0.4125847	0.23080582	-0.7730553	0.48681266	-0.080124
203.8	0.44260882	-0.5274726	-0.4125847	0.23080582	-0.7730553	0.48681266	-0.080124
203.9	0.44260882	-0.5274726	-0.4125847	0.23080582	-0.7730553	0.48681266	-0.080124
204	0.44260882	-0.5274726	-0.4125847	0.23080582	-0.7730553	0.48681266	-0.080124
204.1	0.44260882	-0.5274726	-0.4125847	0.23080582	-0.7730553	0.48681266	-0.080124
204.2	0.44260882	-0.5274726	-0.4125847	0.23080582	-0.7730553	0.48681266	-0.080124
204.3	0.44260882	-0.5274726	-0.4125847	0.23080582	-0.7730553	0.48681266	-0.080124
204.4	0.44260882	-0.5274726	-0.4125847	0.23080582	-0.7730553	0.48681266	-0.080124
204.5	0.44260882	-0.5274726	-0.4125847	0.23080582	-0.7730553	0.48681266	-0.080124
204.6	0.44245094	-0.5279124	-0.4124172	0.23087459	-0.773207	0.48697807	-0.0804958
204.7	0.44245094	-0.5279124	-0.4124172	0.23087459	-0.773207	0.48697807	-0.0804958
204.8	0.44245094	-0.5279124	-0.4124172	0.23087459	-0.773207	0.48697807	-0.0804958
204.9	0.44245094	-0.5279124	-0.4124172	0.23087459	-0.773207	0.48697807	-0.0804958
205	0.44258018	-0.5277612	-0.4120096	0.2308458	-0.7730749	0.4866684	-0.0802739
205.1	0.44258018	-0.5277612	-0.4120096	0.2308458	-0.7730749	0.4866684	-0.0802739
205.2	0.44258018	-0.5277612	-0.4120096	0.2308458	-0.7730749	0.4866684	-0.0802739
205.3	0.44258018	-0.5277612	-0.4120096	0.2308458	-0.7730749	0.4866684	-0.0802739
205.4	0.44258018	-0.5277612	-0.4120096	0.2308458	-0.7730749	0.4866684	-0.0802739
205.5	0.44235323	-0.5277681	-0.4121808	0.23026243	-0.7729621	0.48673995	-0.0804932
205.6	0.44235323	-0.5277681	-0.4121808	0.23026243	-0.7729621	0.48673995	-0.0804932
205.7	0.44235323	-0.5277681	-0.4121808	0.23026243	-0.7729621	0.48673995	-0.0804932
205.8	0.44235323	-0.5277681	-0.4121808	0.23026243	-0.7729621	0.48673995	-0.0804932
205.9	0.44235323	-0.5277681	-0.4121808	0.23026243	-0.7729621	0.48673995	-0.0804932
206	0.44213785	-0.527532	-0.4116858	0.2302815	-0.7727806	0.4864616	-0.080466
206.1	0.44213785	-0.527532	-0.4116858	0.2302815	-0.7727806	0.4864616	-0.080466
206.2	0.44211399	-0.5277491	-0.4116645	0.23010023	-0.7728023	0.48661087	-0.0803795
206.3	0.44211399	-0.5277491	-0.4116645	0.23010023	-0.7728023	0.48661087	-0.0803795
206.4	0.44211399	-0.5277491	-0.4116645	0.23010023	-0.7728023	0.48661087	-0.0803795
206.5	0.44224125	-0.5278301	-0.4112563	0.2302275	-0.7726784	0.48639486	-0.0801243
206.6	0.44224125	-0.5278301	-0.4112563	0.2302275	-0.7726784	0.48639486	-0.0801243
206.7	0.44234153	-0.5277638	-0.4110056	0.22997447	-0.7726872	0.48649902	-0.0803128
206.8	0.4422419	-0.5280763	-0.4108452	0.22975113	-0.7726318	0.48637063	-0.0800926
206.9	0.4422419	-0.5280763	-0.4108452	0.22975113	-0.7726318	0.48637063	-0.0800926
207	0.44202164	-0.5278385	-0.4101778	0.22964903	-0.7725541	0.48590417	-0.0795885
207.1	0.44148236	-0.5274968	-0.4094096	0.23044293	-0.7726202	0.48652686	-0.079295
207.2	0.44148236	-0.5274968	-0.4094096	0.23044293	-0.7726202	0.48652686	-0.079295
207.3	0.44148236	-0.5274968	-0.4094096	0.23044293	-0.7726202	0.48652686	-0.079295
207.4	0.44140698	-0.5275663	-0.4092789	0.2306475	-0.7724554	0.48638876	-0.0789441
207.5	0.44140698	-0.5275663	-0.4092789	0.2306475	-0.7724554	0.48638876	-0.0789441
207.6	0.44133647	-0.5278293	-0.4093502	0.23050214	-0.7725437	0.4864215	-0.0789887
207.7	0.44136159	-0.5280656	-0.4091102	0.23013875	-0.7726974	0.48665934	-0.0790441
207.8	0.4412911	-0.527998	-0.4086695	0.2298882	-0.7725158	0.48662048	-0.0789547
207.9	0.44095524	-0.5275439	-0.4089734	0.22952189	-0.7725243	0.48712166	-0.0792387
208	0.44095524	-0.5275439	-0.4089734	0.22952189	-0.7725243	0.48712166	-0.0792387
208.1	0.44078055	-0.5278161	-0.4087846	0.22976329	-0.772684	0.48723079	-0.0790016
208.2	0.44078055	-0.5278161	-0.4087846	0.22976329	-0.772684	0.48723079	-0.0790016
208.3	0.44078055	-0.5278161	-0.4087846	0.22976329	-0.772684	0.48723079	-0.0790016
208.4	0.44072897	-0.5281343	-0.4085509	0.22945134	-0.77272	0.48727922	-0.0785903
208.5	0.44050153	-0.5283665	-0.4087997	0.22910045	-0.772789	0.48736744	-0.0786251
208.6	0.44050153	-0.5283665	-0.4087997	0.22910045	-0.772789	0.48736744	-0.0786251
208.7	0.44050153	-0.5283665	-0.4087997	0.22910045	-0.772789	0.48736744	-0.0786251
208.8	0.44050153	-0.5283665	-0.4087997	0.22910045	-0.772789	0.48736744	-0.0786251
208.9	0.44012661	-0.5281427	-0.4082159	0.22895513	-0.7725279	0.48712196	-0.0783715
209	0.44012661	-0.5281427	-0.4082159	0.22895513	-0.7725279	0.48712196	-0.0783715
209.1	0.44012661	-0.5281427	-0.4082159	0.22895513	-0.7725279	0.48712196	-0.0783715
209.2	0.44012661	-0.5281427	-0.4082159	0.22895513	-0.7725279	0.48712196	-0.0783715
209.3	0.44066793	-0.5279841	-0.4081995	0.22886534	-0.7726492	0.48752156	-0.078278
209.4	0.44068819	-0.527848	-0.4081427	0.22899959	-0.7725708	0.48752612	-0.0784756
209.5	0.44068819	-0.527848	-0.4081427	0.22899959	-0.7725708	0.48752612	-0.0784756
209.6	0.44068819	-0.527848	-0.4081427	0.22899959	-0.7725708	0.48752612	-0.0784756
209.7	0.44087198	-0.5278668	-0.4078298	0.22890044	-0.7725889	0.48757685	-0.0781016
209.8	0.44098539	-0.5279257	-0.4073992	0.22877838	-0.7725341	0.48705169	-0.0782147
209.9	0.44098539	-0.5279257	-0.4073992	0.22877838	-0.7725341	0.48705169	-0.0782147
213.2	0.43970594	-0.5272782	-0.4054136	0.22970423	-0.7717518	0.48578697	-0.0793625
213.3	0.43970594	-0.5272782	-0.4054136	0.22970423	-0.7717518	0.48578697	-0.0793625
213.4	0.43948105	-0.5270775	-0.4052744	0.22968816	-0.7717097	0.4857703	-0.0789483
213.5	0.43948105	-0.5270775	-0.4052744	0.22968816	-0.7717097	0.4857703	-0.0789483
213.6	0.43948105	-0.5270775	-0.4052744	0.22968816	-0.7717097	0.4857703	-0.0789483
213.7	0.43955049	-0.5271904	-0.4056327	0.22972839	-0.7718901	0.48616173	-0.0793128
213.8	0.43955049	-0.5271904	-0.4056327	0.22972839	-0.7718901	0.48616173	-0.0793128
213.9	0.43955049	-0.5271904	-0.4056327	0.22972839	-0.7718901	0.48616173	-0.0793128
214	0.43955049	-0.5271904	-0.4056327	0.22972839	-0.7718901	0.48616173	-0.0793128
214.1	0.43955049	-0.5271904	-0.4056327	0.22972839	-0.7718901	0.48616173	-0.0793128
214.2	0.43955049	-0.5271904	-0.4056327	0.22972839	-0.7718901	0.48616173	-0.0793128
214.3	0.43955049	-0.5271904	-0.4056327	0.22972839	-0.7718901	0.48616173	-0.0793128
214.4	0.4392875	-0.5277928	-0.4053529	0.22969782	-0.7720971	0.48607768	-0.079466
214.5	0.43921259	-0.5279998	-0.4051972	0.22962268	-0.7720401	0.48593909	-0.0793645
214.6	0.43885297	-0.5280987	-0.4050734	0.22954969	-0.7720613	0.48589281	-0.0791808
214.7	0.43885297	-0.5280987	-0.4050734	0.22954969	-0.7720613	0.48589281	-0.0791808
214.8	0.43885297	-0.5280987	-0.4050734	0.22954969	-0.7720613	0.48589281	-0.0791808
214.9	0.43898683	-0.5278233	-0.4050481	0.22942903	-0.7719575	0.48560446	-0.0792015
215	0.43898683	-0.5278233	-0.4050481	0.22942903	-0.7719575	0.48560446	-0.0792015
215.1	0.43898683	-0.5278233	-0.4050481	0.22942903	-0.7719575	0.48560446	-0.0792015
215.2	0.43915522	-0.5278402	-0.4048196	0.22924765	-0.7719425	0.4855632	-0.0789655
215.3	0.43915522	-0.5278402	-0.4048196	0.22924765	-0.7719425	0.4855632	-0.0789655
215.4	0.43915522	-0.5278402	-0.4048196	0.22924765	-0.7719425	0.4855632	-0.0789655
215.5	0.43915522	-0.5278402	-0.4048196	0.22924765	-0.7719425	0.4855632	-0.0789655
215.6	0.43915522	-0.5278402	-0.4048196	0.22924765	-0.7719425	0.4855632	-0.0789655
215.7	0.43915522	-0.5278402	-0.4048196	0.22924765	-0.7719425	0.4855632	-0.0789655
215.8	0.43925488	-0.5279506	-0.4044909	0.22886139	-0.771832	0.48543482	-0.0789516
215.9	0.43925488	-0.5279506	-0.4044909	0.22886139	-0.771832	0.48543482	-0.0789516
216	0.43925488	-0.5279506	-0.4044909	0.22886139	-0.771832	0.48543482	-0.0789516
216.1	0.43925488	-0.5279506	-0.4044909	0.22886139	-0.771832	0.48543482	-0.0789516
216.2	0.43908015	-0.527558	-0.4041981	0.22822662	-0.7716534	0.48526919	-0.0785759
216.3	0.43895429	-0.5278165	-0.4037661	0.2280044	-0.771683	0.4852675	-0.078943
216.4	0.43895429	-0.5278165	-0.4037661	0.2280044	-0.771683	0.4852675	-0.078943
216.5	0.43895429	-0.5278165	-0.4037661	0.2280044	-0.771683	0.4852675	-0.078943
216.6	0.43895429	-0.5278165	-0.4037661	0.2280044	-0.771683	0.4852675	-0.078943
216.7	0.43883898	-0.5277794	-0.4033781	0.22793375	-0.7715149	0.48513423	-0.0791363
216.8	0.43883898	-0.5277794	-0.4033781	0.22793375	-0.7715149	0.48513423	-0.0791363
216.9	0.43883898	-0.5277794	-0.4033781	0.22793375	-0.7715149	0.48513423	-0.0791363
217	0.43883898	-0.5277794	-0.4033781	0.22793375	-0.7715149	0.48513423	-0.0791363
217.1	0.43843981	-0.5276467	-0.4034483	0.22763153	-0.7715485	0.48482874	-0.079153
217.2	0.43843981	-0.5276467	-0.4034483	0.22763153	-0.7715485	0.48482874	-0.079153
217.3	0.43846414	-0.5276944	-0.403503	0.22726649	-0.7713986	0.48463161	-0.079309
217.4	0.43846414	-0.5276944	-0.403503	0.22726649	-0.7713986	0.48463161	-0.079309
217.5	0.43846414	-0.5276944	-0.403503	0.22726649	-0.7713986	0.48463161	-0.079309
217.6	0.43846414	-0.5276944	-0.403503	0.22726649	-0.7713986	0.48463161	-0.079309
217.7	0.43817154	-0.5277311	-0.4032246	0.22712091	-0.7712415	0.48425445	-0.0790394
217.8	0.43817154	-0.5277311	-0.4032246	0.22712091	-0.7712415	0.48425445	-0.0790394
217.9	0.43817154	-0.5277311	-0.4032246	0.22712091	-0.7712415	0.48425445	-0.0790394
218	0.43817154	-0.5277311	-0.4032246	0.22712091	-0.7712415	0.48425445	-0.0790394
218.1	0.43810245	-0.5276605	-0.4030429	0.22700218	-0.7711911	0.48422275	-0.0787531
218.2	0.43810245	-0.5276605	-0.4030429	0.22700218	-0.7711911	0.48422275	-0.0787531
218.3	0.43810245	-0.5276605	-0.4030429	0.22700218	-0.7711911	0.48422275	-0.0787531
218.4	0.43810245	-0.5276605	-0.4030429	0.22700218	-0.7711911	0.48422275	-0.0787531
218.5	0.43810245	-0.5276605	-0.4030429	0.22700218	-0.7711911	0.48422275	-0.0787531
218.6	0.43810245	-0.5276605	-0.4030429	0.22700218	-0.7711911	0.48422275	-0.0787531
218.7	0.43810245	-0.5276605	-0.4030429	0.22700218	-0.7711911	0.48422275	-0.0787531
218.8	0.43825559	-0.527517	-0.4028408	0.22703982	-0.7713941	0.48437664	-0.0782906
218.9	0.43825559	-0.527517	-0.4028408	0.22703982	-0.7713941	0.48437664	-0.0782906
219	0.43825559	-0.527517	-0.4028408	0.22703982	-0.7713941	0.48437664	-0.0782906
219.1	0.43807749	-0.5276682	-0.4027335	0.22669341	-0.7714661	0.48447055	-0.0783202
219.2	0.43770558	-0.5272033	-0.4025245	0.22699134	-0.7713057	0.48431029	-0.078622
219.3	0.43770558	-0.5272033	-0.4025245	0.22699134	-0.7713057	0.48431029	-0.078622
219.4	0.43770558	-0.5272033	-0.4025245	0.22699134	-0.7713057	0.48431029	-0.078622
219.5	0.43770558	-0.5272033	-0.4025245	0.22699134	-0.7713057	0.48431029	-0.078622
219.6	0.43770558	-0.5272033	-0.4025245	0.22699134	-0.7713057	0.48431029	-0.078622
219.7	0.43799487	-0.5270574	-0.4024567	0.22676802	-0.7711932	0.48400636	-0.0787699
219.8	0.43799487	-0.5270574	-0.4024567	0.22676802	-0.7711932	0.48400636	-0.0787699
219.9	0.43753027	-0.5270138	-0.4023219	0.22646035	-0.7709865	0.48404004	-0.0782465
220	0.43778996	-0.5269642	-0.4021385	0.22615656	-0.7711709	0.48422962	-0.0782238
220.1	0.43778996	-0.5269642	-0.4021385	0.22615656	-0.7711709	0.48422962	-0.0782238
220.2	0.43742255	-0.5266181	-0.4015645	0.2261739	-0.770994	0.48369165	-0.0783072
220.3	0.43754861	-0.5266667	-0.4015227	0.22562354	-0.770872	0.48363369	-0.0781347
220.4	0.43754861	-0.5266667	-0.4015227	0.22562354	-0.770872	0.48363369	-0.0781347
220.5	0.43754861	-0.5266667	-0.4015227	0.22562354	-0.770872	0.48363369	-0.0781347
220.6	0.43754861	-0.5266667	-0.4015227	0.22562354	-0.770872	0.48363369	-0.0781347
220.7	0.43754861	-0.5266667	-0.4015227	0.22562354	-0.770872	0.48363369	-0.0781347
220.8	0.43754861	-0.5266667	-0.4015227	0.22562354	-0.770872	0.48363369	-0.0781347
220.9	0.43754861	-0.5266667	-0.4015227	0.22562354	-0.770872	0.48363369	-0.0781347
221	0.43720548	-0.5270366	-0.4011997	0.22546492	-0.7709498	0.48356654	-0.0784948
221.1	0.4369938	-0.5269385	-0.4009474	0.22493582	-0.7706688	0.48301873	-0.0785288
221.2	0.4369938	-0.5269385	-0.4009474	0.22493582	-0.7706688	0.48301873	-0.0785288
221.3	0.4369938	-0.5269385	-0.4009474	0.22493582	-0.7706688	0.48301873	-0.0785288
221.4	0.4369938	-0.5269385	-0.4009474	0.22493582	-0.7706688	0.48301873	-0.0785288
221.5	0.4369938	-0.5269385	-0.4009474	0.22493582	-0.7706688	0.48301873	-0.0785288
221.6	0.4369938	-0.5269385	-0.4009474	0.22493582	-0.7706688	0.48301873	-0.0785288
221.7	0.4369938	-0.5269385	-0.4009474	0.22493582	-0.7706688	0.48301873	-0.0785288
221.8	0.4369938	-0.5269385	-0.4009474	0.22493582	-0.7706688	0.48301873	-0.0785288
221.9	0.4369938	-0.5269385	-0.4009474	0.22493582	-0.7706688	0.48301873	-0.0785288
222	0.4369938	-0.5269385	-0.4009474	0.22493582	-0.7706688	0.48301873	-0.0785288
222.1	0.43651054	-0.5268294	-0.4008612	0.22457584	-0.7704957	0.48284508	-0.0783302
222.2	0.43651054	-0.5268294	-0.4008612	0.22457584	-0.7704957	0.48284508	-0.0783302
222.3	0.43651054	-0.5268294	-0.4008612	0.22457584	-0.7704957	0.48284508	-0.0783302
222.4	0.43651263	-0.5267159	-0.4002701	0.2249317	-0.7704322	0.48293659	-0.0777608
222.5	0.43634807	-0.5269207	-0.3996437	0.22489415	-0.7705298	0.48290984	-0.0773419
222.6	0.43634807	-0.5269207	-0.3996437	0.22489415	-0.7705298	0.48290984	-0.0773419
222.7	0.43634807	-0.5269207	-0.3996437	0.22489415	-0.7705298	0.48290984	-0.0773419
222.8	0.43634807	-0.5269207	-0.3996437	0.22489415	-0.7705298	0.48290984	-0.0773419
222.9	0.43634807	-0.5269207	-0.3996437	0.22489415	-0.7705298	0.48290984	-0.0773419
223	0.43634807	-0.5269207	-0.3996437	0.22489415	-0.7705298	0.48290984	-0.0773419
223.1	0.43634807	-0.5269207	-0.3996437	0.22489415	-0.7705298	0.48290984	-0.0773419
223.2	0.43623859	-0.5266542	-0.3995915	0.22500036	-0.7704676	0.48265794	-0.0773341
223.3	0.43623859	-0.5266542	-0.3995915	0.22500036	-0.7704676	0.48265794	-0.0773341
223.4	0.43623859	-0.5266542	-0.3995915	0.22500036	-0.7704676	0.48265794	-0.0773341
223.5	0.43623859	-0.5266542	-0.3995915	0.22500036	-0.7704676	0.48265794	-0.0773341
223.6	0.43585575	-0.5264763	-0.3994843	0.2243102	-0.7701372	0.48252203	-0.0771154
223.7	0.43585575	-0.5264763	-0.3994843	0.2243102	-0.7701372	0.48252203	-0.0771154
223.8	0.43551563	-0.5264037	-0.3995341	0.22420247	-0.7700678	0.48249422	-0.0774566
223.9	0.43551563	-0.5264037	-0.3995341	0.22420247	-0.7700678	0.48249422	-0.0774566
224	0.43551563	-0.5264037	-0.3995341	0.22420247	-0.7700678	0.48249422	-0.0774566
224.1	0.43551563	-0.5264037	-0.3995341	0.22420247	-0.7700678	0.48249422	-0.0774566
224.2	0.43551563	-0.5264037	-0.3995341	0.22420247	-0.7700678	0.48249422	-0.0774566
224.3	0.43551563	-0.5264037	-0.3995341	0.22420247	-0.7700678	0.48249422	-0.0774566
224.4	0.43551563	-0.5264037	-0.3995341	0.22420247	-0.7700678	0.48249422	-0.0774566
224.5	0.43551563	-0.5264037	-0.3995341	0.22420247	-0.7700678	0.48249422	-0.0774566
224.6	0.43551563	-0.5264037	-0.3995341	0.22420247	-0.7700678	0.48249422	-0.0774566
224.7	0.43544439	-0.5267796	-0.3992161	0.22401923	-0.7701062	0.48241727	-0.0778371
224.8	0.43544439	-0.5267796	-0.3992161	0.22401923	-0.7701062	0.48241727	-0.0778371
224.9	0.4354906	-0.5266423	-0.3991211	0.22402248	-0.7700353	0.48214981	-0.077956
228.2	0.43421238	-0.5256079	-0.3977289	0.22256479	-0.7693015	0.48134074	-0.0788378
228.3	0.43421238	-0.5256079	-0.3977289	0.22256479	-0.7693015	0.48134074	-0.0788378
228.4	0.43421238	-0.5256079	-0.3977289	0.22256479	-0.7693015	0.48134074	-0.0788378
228.5	0.43421238	-0.5256079	-0.3977289	0.22256479	-0.7693015	0.48134074	-0.0788378
228.6	0.43421238	-0.5256079	-0.3977289	0.22256479	-0.7693015	0.48134074	-0.0788378
228.7	0.43421238	-0.5256079	-0.3977289	0.22256479	-0.7693015	0.48134074	-0.0788378
228.8	0.43421238	-0.5256079	-0.3977289	0.22256479	-0.7693015	0.48134074	-0.0788378
228.9	0.43421238	-0.5256079	-0.3977289	0.22256479	-0.7693015	0.48134074	-0.0788378
229	0.43421238	-0.5256079	-0.3977289	0.22256479	-0.7693015	0.48134074	-0.0788378
229.1	0.43421238	-0.5256079	-0.3977289	0.22256479	-0.7693015	0.48134074	-0.0788378
229.2	0.43421238	-0.5256079	-0.3977289	0.22256479	-0.7693015	0.48134074	-0.0788378
229.3	0.43421238	-0.5256079	-0.3977289	0.22256479	-0.7693015	0.48134074	-0.0788378
229.4	0.43421238	-0.5256079	-0.3977289	0.22256479	-0.7693015	0.48134074	-0.0788378
229.5	0.43421238	-0.5256079	-0.3977289	0.22256479	-0.7693015	0.48134074	-0.0788378
229.6	0.43351982	-0.5252534	-0.3973062	0.22282106	-0.7689612	0.48092507	-0.0789434
229.7	0.43351982	-0.5252534	-0.3973062	0.22282106	-0.7689612	0.48092507	-0.0789434
229.8	0.43351982	-0.5252534	-0.3973062	0.22282106	-0.7689612	0.48092507	-0.0789434
229.9	0.43351982	-0.5252534	-0.3973062	0.22282106	-0.7689612	0.48092507	-0.0789434
230	0.43351982	-0.5252534	-0.3973062	0.22282106	-0.7689612	0.48092507	-0.0789434
230.1	0.43351982	-0.5252534	-0.3973062	0.22282106	-0.7689612	0.48092507	-0.0789434
230.2	0.43319669	-0.525361	-0.3970898	0.22277788	-0.7687805	0.48066723	-0.078749
230.3	0.43319669	-0.525361	-0.3970898	0.22277788	-0.7687805	0.48066723	-0.078749
230.4	0.43319669	-0.525361	-0.3970898	0.22277788	-0.7687805	0.48066723	-0.078749
230.5	0.43319669	-0.525361	-0.3970898	0.22277788	-0.7687805	0.48066723	-0.078749
230.6	0.43319669	-0.525361	-0.3970898	0.22277788	-0.7687805	0.48066723	-0.078749
230.7	0.43319669	-0.525361	-0.3970898	0.22277788	-0.7687805	0.48066723	-0.078749
230.8	0.43323871	-0.5251466	-0.3971671	0.22247049	-0.768823	0.48068608	-0.0788171
230.9	0.43323871	-0.5251466	-0.3971671	0.22247049	-0.768823	0.48068608	-0.0788171
231	0.43323871	-0.5251466	-0.3971671	0.22247049	-0.768823	0.48068608	-0.0788171
231.1	0.43323871	-0.5251466	-0.3971671	0.22247049	-0.768823	0.48068608	-0.0788171
231.2	0.43323871	-0.5251466	-0.3971671	0.22247049	-0.768823	0.48068608	-0.0788171
231.3	0.43323871	-0.5251466	-0.3971671	0.22247049	-0.768823	0.48068608	-0.0788171
231.4	0.43323871	-0.5251466	-0.3971671	0.22247049	-0.768823	0.48068608	-0.0788171
231.5	0.43323871	-0.5251466	-0.3971671	0.22247049	-0.768823	0.48068608	-0.0788171
231.6	0.43323871	-0.5251466	-0.3971671	0.22247049	-0.768823	0.48068608	-0.0788171
231.7	0.43323871	-0.5251466	-0.3971671	0.22247049	-0.768823	0.48068608	-0.0788171
231.8	0.43323871	-0.5251466	-0.3971671	0.22247049	-0.768823	0.48068608	-0.0788171
231.9	0.43323871	-0.5251466	-0.3971671	0.22247049	-0.768823	0.48068608	-0.0788171
232	0.43323871	-0.5251466	-0.3971671	0.22247049	-0.768823	0.48068608	-0.0788171
232.1	0.43323871	-0.5251466	-0.3971671	0.22247049	-0.768823	0.48068608	-0.0788171
232.2	0.43323871	-0.5251466	-0.3971671	0.22247049	-0.768823	0.48068608	-0.0788171
232.3	0.43323871	-0.5251466	-0.3971671	0.22247049	-0.768823	0.48068608	-0.0788171
232.4	0.43323871	-0.5251466	-0.3971671	0.22247049	-0.768823	0.48068608	-0.0788171
232.5	0.43323871	-0.5251466	-0.3971671	0.22247049	-0.768823	0.48068608	-0.0788171
232.6	0.43323871	-0.5251466	-0.3971671	0.22247049	-0.768823	0.48068608	-0.0788171
232.7	0.43323871	-0.5251466	-0.3971671	0.22247049	-0.768823	0.48068608	-0.0788171
232.8	0.43323871	-0.5251466	-0.3971671	0.22247049	-0.768823	0.48068608	-0.0788171
232.9	0.43323871	-0.5251466	-0.3971671	0.22247049	-0.768823	0.48068608	-0.0788171
233	0.43323871	-0.5251466	-0.3971671	0.22247049	-0.768823	0.48068608	-0.0788171
233.1	0.43323871	-0.5251466	-0.3971671	0.22247049	-0.768823	0.48068608	-0.0788171
233.2	0.43323871	-0.5251466	-0.3971671	0.22247049	-0.768823	0.48068608	-0.0788171
233.3	0.43323871	-0.5251466	-0.3971671	0.22247049	-0.768823	0.48068608	-0.0788171
233.4	0.43323871	-0.5251466	-0.3971671	0.22247049	-0.768823	0.48068608	-0.0788171
233.5	0.43323871	-0.5251466	-0.3971671	0.22247049	-0.768823	0.48068608	-0.0788171
233.6	0.43323871	-0.5251466	-0.3971671	0.22247049	-0.768823	0.48068608	-0.0788171
233.7	0.43306873	-0.5251518	-0.3966548	0.22237551	-0.7686713	0.48042786	-0.0785106
233.8	0.43306873	-0.5251518	-0.3966548	0.22237551	-0.7686713	0.48042786	-0.0785106
233.9	0.43306873	-0.5251518	-0.3966548	0.22237551	-0.7686713	0.48042786	-0.0785106
234	0.43306873	-0.5251518	-0.3966548	0.22237551	-0.7686713	0.48042786	-0.0785106
234.1	0.43306873	-0.5251518	-0.3966548	0.22237551	-0.7686713	0.48042786	-0.0785106
234.2	0.43306873	-0.5251518	-0.3966548	0.22237551	-0.7686713	0.48042786	-0.0785106
234.3	0.43306873	-0.5251518	-0.3966548	0.22237551	-0.7686713	0.48042786	-0.0785106
234.4	0.43306873	-0.5251518	-0.3966548	0.22237551	-0.7686713	0.48042786	-0.0785106
234.5	0.43306873	-0.5251518	-0.3966548	0.22237551	-0.7686713	0.48042786	-0.0785106
234.6	0.43306873	-0.5251518	-0.3966548	0.22237551	-0.7686713	0.48042786	-0.0785106
234.7	0.43306873	-0.5251518	-0.3966548	0.22237551	-0.7686713	0.48042786	-0.0785106
234.8	0.43286307	-0.5253823	-0.3965116	0.22173869	-0.7685352	0.4805695	-0.0782771
234.9	0.43286307	-0.5253823	-0.3965116	0.22173869	-0.7685352	0.4805695	-0.0782771
235	0.43286307	-0.5253823	-0.3965116	0.22173869	-0.7685352	0.4805695	-0.0782771
235.1	0.43286307	-0.5253823	-0.3965116	0.22173869	-0.7685352	0.4805695	-0.0782771
235.2	0.43286307	-0.5253823	-0.3965116	0.22173869	-0.7685352	0.4805695	-0.0782771
235.3	0.43286307	-0.5253823	-0.3965116	0.22173869	-0.7685352	0.4805695	-0.0782771
235.4	0.43286307	-0.5253823	-0.3965116	0.22173869	-0.7685352	0.4805695	-0.0782771
235.5	0.43286307	-0.5253823	-0.3965116	0.22173869	-0.7685352	0.4805695	-0.0782771
235.6	0.43286307	-0.5253823	-0.3965116	0.22173869	-0.7685352	0.4805695	-0.0782771
235.7	0.43286307	-0.5253823	-0.3965116	0.22173869	-0.7685352	0.4805695	-0.0782771
235.8	0.43277618	-0.5252544	-0.3960277	0.22126278	-0.7682604	0.48014268	-0.0782725
235.9	0.43277618	-0.5252544	-0.3960277	0.22126278	-0.7682604	0.48014268	-0.0782725
236	0.43277618	-0.5252544	-0.3960277	0.22126278	-0.7682604	0.48014268	-0.0782725
236.1	0.43277618	-0.5252544	-0.3960277	0.22126278	-0.7682604	0.48014268	-0.0782725
236.2	0.43277618	-0.5252544	-0.3960277	0.22126278	-0.7682604	0.48014268	-0.0782725
236.3	0.43277618	-0.5252544	-0.3960277	0.22126278	-0.7682604	0.48014268	-0.0782725
236.4	0.43277618	-0.5252544	-0.3960277	0.22126278	-0.7682604	0.48014268	-0.0782725
236.5	0.43277618	-0.5252544	-0.3960277	0.22126278	-0.7682604	0.48014268	-0.0782725
236.6	0.43277618	-0.5252544	-0.3960277	0.22126278	-0.7682604	0.48014268	-0.0782725
236.7	0.43277618	-0.5252544	-0.3960277	0.22126278	-0.7682604	0.48014268	-0.0782725
236.8	0.43277618	-0.5252544	-0.3960277	0.22126278	-0.7682604	0.48014268	-0.0782725
236.9	0.43277618	-0.5252544	-0.3960277	0.22126278	-0.7682604	0.48014268	-0.0782725
237	0.43277618	-0.5252544	-0.3960277	0.22126278	-0.7682604	0.48014268	-0.0782725
237.1	0.43277618	-0.5252544	-0.3960277	0.22126278	-0.7682604	0.48014268	-0.0782725
237.2	0.43277618	-0.5252544	-0.3960277	0.22126278	-0.7682604	0.48014268	-0.0782725
237.3	0.43248545	-0.5257286	-0.3959031	0.22102547	-0.7683525	0.47984765	-0.0787421
237.4	0.43228554	-0.525372	-0.3956538	0.22039803	-0.7683462	0.48039267	-0.0785412
237.5	0.43228554	-0.525372	-0.3956538	0.22039803	-0.7683462	0.48039267	-0.0785412
237.6	0.43196109	-0.5252492	-0.3951853	0.22040731	-0.768204	0.48015131	-0.0781092
237.7	0.43137122	-0.5254371	-0.3946553	0.21985008	-0.7681908	0.48003414	-0.0774855
237.8	0.43137122	-0.5254371	-0.3946553	0.21985008	-0.7681908	0.48003414	-0.0774855
237.9	0.43137122	-0.5254371	-0.3946553	0.21985008	-0.7681908	0.48003414	-0.0774855
238	0.43137122	-0.5254371	-0.3946553	0.21985008	-0.7681908	0.48003414	-0.0774855
238.1	0.43137122	-0.5254371	-0.3946553	0.21985008	-0.7681908	0.48003414	-0.0774855
238.2	0.43137122	-0.5254371	-0.3946553	0.21985008	-0.7681908	0.48003414	-0.0774855
238.3	0.43137122	-0.5254371	-0.3946553	0.21985008	-0.7681908	0.48003414	-0.0774855
238.4	0.43137122	-0.5254371	-0.3946553	0.21985008	-0.7681908	0.48003414	-0.0774855
238.5	0.43137122	-0.5254371	-0.3946553	0.21985008	-0.7681908	0.48003414	-0.0774855
238.6	0.43137122	-0.5254371	-0.3946553	0.21985008	-0.7681908	0.48003414	-0.0774855
238.7	0.43137122	-0.5254371	-0.3946553	0.21985008	-0.7681908	0.48003414	-0.0774855
238.8	0.43137122	-0.5254371	-0.3946553	0.21985008	-0.7681908	0.48003414	-0.0774855
238.9	0.43137122	-0.5254371	-0.3946553	0.21985008	-0.7681908	0.48003414	-0.0774855
239	0.43137122	-0.5254371	-0.3946553	0.21985008	-0.7681908	0.48003414	-0.0774855
239.1	0.43137122	-0.5254371	-0.3946553	0.21985008	-0.7681908	0.48003414	-0.0774855
239.2	0.43137122	-0.5254371	-0.3946553	0.21985008	-0.7681908	0.48003414	-0.0774855
239.3	0.43105897	-0.5258582	-0.3945553	0.2197364	-0.7682756	0.47996075	-0.0775942
239.4	0.43105897	-0.5258582	-0.3945553	0.2197364	-0.7682756	0.47996075	-0.0775942
239.5	0.43105897	-0.5258582	-0.3945553	0.2197364	-0.7682756	0.47996075	-0.0775942
239.6	0.43105897	-0.5258582	-0.3945553	0.2197364	-0.7682756	0.47996075	-0.0775942
239.7	0.43105897	-0.5258582	-0.3945553	0.2197364	-0.7682756	0.47996075	-0.0775942
239.8	0.43105897	-0.5258582	-0.3945553	0.2197364	-0.7682756	0.47996075	-0.0775942
239.9	0.43105897	-0.5258582	-0.3945553	0.2197364	-0.7682756	0.47996075	-0.0775942
240	0.43105897	-0.5258582	-0.3945553	0.2197364	-0.7682756	0.47996075	-0.0775942

Corresponding p-values for PRCC sensitivtiy indices

Time discretization	V_{[G]}	K_{[G]}	V_{[PYR]}	K_{[PYR]}	alpha	G_E	[G]_0
0	0	0	0	0	0	0	0
0.1	0.7989158	0.8157174	3.81E-12	8.35E-05	0.16336536	0.21660035	0
0.2	0.57096882	0.98758527	4.46E-25	1.39E-06	0.10930624	0.156177	1.09E-296
0.3	0.09639081	0.95639322	1.74E-36	2.78E-07	0.07932927	0.83146493	1.89E-275
0.4	0.19608402	0.99035912	1.05E-44	9.62E-09	0.07766847	0.52299609	1.06E-263
0.5	0.74671205	0.56544097	4.63E-52	5.56E-10	0.21039813	0.79497154	8.41E-256
0.6	0.29131195	0.89794809	4.08E-56	9.59E-13	0.28359027	0.71717833	6.07E-246
0.7	0.49791535	0.79862638	7.82E-60	1.93E-13	0.9642393	0.60242185	1.24E-238
0.8	0.90556632	0.63961676	2.03E-59	1.13E-13	0.88782657	0.98760664	4.60E-229
0.9	0.92704694	0.81985143	4.48E-60	7.84E-14	0.84360899	0.80485538	1.64E-219
1	0.41560794	0.89629773	3.06E-62	1.69E-14	0.84820555	0.73778367	9.70E-214
1.1	0.24307227	0.73593972	1.59E-61	5.97E-15	0.9039726	0.96489414	7.36E-207
1.2	0.25717949	0.7854764	1.65E-61	4.09E-15	0.62041228	0.46815551	3.08E-199
1.3	0.09850166	0.51823738	6.82E-61	1.18E-14	0.60037238	0.28566883	3.45E-193
1.4	0.05995882	0.48932562	2.12E-59	1.11E-13	0.3353954	0.14108346	1.79E-185
1.5	0.08020444	0.32488952	7.97E-59	3.13E-14	0.1839394	0.13133075	2.40E-180
1.6	0.05029259	0.17535757	6.38E-59	3.09E-14	0.14656585	0.10910712	1.27E-175
1.7	0.0256516	0.1244171	1.11E-56	2.88E-13	0.0922083	0.09560056	4.92E-168
1.8	0.03584751	0.07000674	5.16E-54	1.36E-12	0.15549965	0.04357479	7.43E-160
1.9	0.03457837	0.03375966	9.10E-53	1.79E-12	0.05894937	0.02783541	2.36E-153
2	0.02313021	0.01652079	5.96E-50	1.32E-11	0.07147665	0.01867894	3.60E-146
2.1	0.02697742	0.00510578	2.26E-49	1.09E-11	0.07864598	0.01950056	2.05E-141
2.2	0.02440299	0.00406066	1.21E-47	8.45E-11	0.08091925	0.0073068	4.03E-136
2.3	0.02218551	0.00202427	1.57E-46	1.31E-10	0.07773608	0.00527914	8.98E-132
2.4	0.01277198	0.00069648	5.41E-46	4.58E-11	0.05179786	0.00389691	1.01E-127
2.5	0.01063516	0.00035046	2.94E-45	2.64E-10	0.0535811	0.00285088	4.86E-123
2.6	0.00903816	0.00038654	1.38E-43	1.26E-09	0.03748877	0.00231021	7.53E-118
2.7	0.00786776	0.00027416	1.94E-42	2.82E-09	0.04140312	0.00184921	1.49E-113
2.8	0.00765926	0.00017825	1.49E-40	8.41E-09	0.03868742	0.00101967	3.74E-108
2.9	0.0093796	0.00012448	4.73E-38	3.32E-08	0.02982144	0.00143105	3.47E-101
3	0.00861696	0.00012844	1.18E-36	5.05E-08	0.03074622	0.00132976	7.93E-97
3.1	0.00609335	6.65E-05	4.05E-36	4.78E-08	0.02426934	0.00083294	3.03E-94
3.2	0.00417075	4.94E-05	4.12E-36	2.16E-08	0.01783886	0.00072476	1.76E-91
3.3	0.00359183	3.33E-05	7.77E-35	3.51E-08	0.01255158	0.00068345	1.44E-87
3.4	0.00324916	2.25E-05	3.96E-34	6.57E-08	0.01036598	0.00080341	1.37E-84
3.5	0.00275534	1.07E-05	4.09E-34	3.37E-08	0.00699459	0.00089078	1.02E-82
3.6	0.00238842	5.10E-06	3.12E-34	2.27E-08	0.00806011	0.00100061	2.83E-81
3.7	0.00249101	2.41E-06	2.23E-34	1.66E-08	0.00593203	0.00064895	3.48E-80
3.8	0.0018705	1.15E-06	3.19E-34	3.10E-08	0.00665128	0.00061798	1.93E-78
3.9	0.00126997	1.07E-06	2.62E-34	3.63E-08	0.00484128	0.00037739	7.67E-77
4	0.00136636	7.61E-07	4.80E-34	2.82E-08	0.00411646	0.00026681	1.49E-75
4.1	0.00160125	5.48E-07	5.89E-34	2.60E-08	0.00312403	0.00025591	2.61E-74
4.2	0.00141564	3.73E-07	3.45E-34	2.14E-08	0.00263693	0.00022501	1.68E-73
4.3	0.00109861	2.83E-07	4.43E-34	2.78E-08	0.00184852	0.0001991	2.64E-72
4.4	0.00101019	1.64E-07	8.82E-34	2.60E-08	0.00176279	0.00018764	1.29E-70
4.5	0.00080308	7.14E-08	4.80E-34	1.39E-08	0.00140232	0.00014679	1.30E-69
4.6	0.00080618	2.60E-08	1.56E-33	1.55E-08	0.00117456	0.00019131	2.02E-67
4.7	0.00065732	3.06E-08	3.74E-33	1.15E-08	0.00096451	0.00023897	8.23E-66
4.8	0.00058182	2.32E-08	6.85E-33	1.24E-08	0.0007283	0.00026795	1.46E-64
4.9	0.00064221	1.66E-08	9.24E-33	1.23E-08	0.00040606	0.00020365	3.79E-63
5	0.00065726	1.13E-08	1.42E-32	1.30E-08	0.00033964	0.00018739	9.98E-62
5.1	0.00053355	6.36E-09	1.65E-32	1.38E-08	0.00027138	0.00015912	4.63E-60
5.2	0.0004535	4.26E-09	1.10E-32	9.86E-09	0.00014496	9.83E-05	1.06E-58
5.3	0.0004121	3.66E-09	2.31E-32	1.04E-08	0.00010249	0.00010386	2.35E-57
5.4	0.00027847	1.97E-09	1.76E-32	1.09E-08	6.88E-05	0.00010949	1.63E-56
5.5	0.00027535	1.93E-09	1.42E-32	8.84E-09	8.10E-05	0.00013355	1.60E-55
5.6	0.00023292	1.85E-09	1.82E-32	8.42E-09	6.91E-05	0.00013757	4.26E-54
5.7	0.00018867	1.34E-09	3.78E-32	8.61E-09	5.53E-05	0.00015488	5.18E-53
5.8	0.00015275	1.35E-09	4.34E-32	7.87E-09	4.76E-05	0.00013367	7.83E-52
5.9	0.00016147	1.03E-09	3.31E-32	3.58E-09	2.39E-05	0.00014611	2.09E-50
6	0.0001146	9.96E-10	2.98E-32	3.44E-09	1.42E-05	0.0001377	1.51E-49
6.1	0.00010678	8.18E-10	3.13E-32	3.27E-09	1.09E-05	0.0001447	2.50E-48
6.2	8.08E-05	8.17E-10	4.25E-32	2.90E-09	7.94E-06	0.00013066	2.83E-47
6.3	8.45E-05	6.71E-10	3.68E-32	4.09E-09	7.46E-06	0.00012079	2.40E-46
6.4	6.94E-05	6.65E-10	3.37E-32	3.29E-09	5.26E-06	0.00010659	1.98E-45
6.5	8.66E-05	5.24E-10	8.37E-32	4.56E-09	5.06E-06	0.00011884	3.70E-44
6.6	7.82E-05	6.00E-10	9.68E-32	3.73E-09	2.96E-06	0.00013108	1.99E-43
6.7	6.65E-05	4.56E-10	5.70E-32	2.86E-09	1.67E-06	0.00012369	4.68E-43
6.8	6.45E-05	5.05E-10	7.69E-32	2.65E-09	9.18E-07	0.00012144	4.59E-42
6.9	6.02E-05	4.76E-10	6.93E-32	2.44E-09	5.56E-07	0.00010586	2.34E-41
7	5.33E-05	4.35E-10	3.86E-32	1.53E-09	3.30E-07	0.00011323	1.13E-40
7.1	4.70E-05	3.94E-10	3.63E-32	1.55E-09	2.26E-07	0.00012583	3.39E-40
7.2	4.71E-05	3.74E-10	2.66E-32	1.40E-09	2.00E-07	0.00012271	1.58E-39
7.3	3.42E-05	3.22E-10	1.61E-32	9.46E-10	8.65E-08	0.00010708	4.80E-39
7.4	3.65E-05	3.03E-10	1.25E-32	7.55E-10	5.05E-08	0.00012118	1.99E-38
7.5	2.95E-05	3.04E-10	8.66E-33	5.69E-10	3.72E-08	0.00010042	5.59E-38
7.6	2.35E-05	2.67E-10	4.98E-33	4.72E-10	2.15E-08	0.0001015	3.06E-37
7.7	1.68E-05	2.38E-10	3.87E-33	4.19E-10	1.28E-08	8.40E-05	1.50E-36
7.8	1.57E-05	2.41E-10	2.51E-33	3.56E-10	6.69E-09	8.80E-05	8.09E-36
7.9	1.19E-05	2.64E-10	1.99E-33	3.66E-10	4.76E-09	7.55E-05	3.33E-35
8	1.10E-05	2.19E-10	2.25E-33	3.75E-10	4.01E-09	7.54E-05	2.06E-34
8.1	8.24E-06	3.09E-10	2.01E-33	3.38E-10	3.04E-09	6.37E-05	5.91E-34
8.2	8.34E-06	2.85E-10	1.61E-33	2.47E-10	1.60E-09	5.96E-05	2.92E-33
8.3	7.58E-06	2.64E-10	3.05E-33	2.56E-10	1.06E-09	4.82E-05	1.01E-32
8.4	6.99E-06	2.99E-10	4.13E-33	2.91E-10	7.51E-10	5.32E-05	6.85E-32
8.5	6.03E-06	3.42E-10	4.03E-33	3.19E-10	6.39E-10	5.33E-05	3.26E-31
8.6	6.72E-06	3.19E-10	4.22E-33	3.01E-10	5.28E-10	4.94E-05	1.18E-30
8.7	5.59E-06	2.77E-10	4.07E-33	3.26E-10	3.74E-10	4.33E-05	3.11E-30
8.8	5.39E-06	3.33E-10	5.64E-33	2.68E-10	2.25E-10	3.53E-05	1.99E-29
8.9	3.71E-06	2.23E-10	2.54E-33	1.86E-10	1.08E-10	3.17E-05	6.90E-29
9	3.73E-06	2.43E-10	3.60E-33	1.61E-10	8.99E-11	3.34E-05	3.02E-28
9.1	2.93E-06	2.88E-10	2.98E-33	1.87E-10	5.02E-11	3.02E-05	7.26E-28
9.2	2.88E-06	2.44E-10	2.67E-33	1.83E-10	6.01E-11	3.70E-05	3.95E-27
9.3	2.55E-06	2.85E-10	2.33E-33	1.55E-10	4.12E-11	3.63E-05	1.22E-26
9.4	2.83E-06	2.71E-10	3.55E-33	1.48E-10	3.52E-11	3.76E-05	4.49E-26
9.5	2.21E-06	3.13E-10	3.64E-33	1.70E-10	2.35E-11	3.82E-05	1.11E-25
9.6	2.02E-06	2.88E-10	3.76E-33	1.69E-10	1.22E-11	4.42E-05	2.20E-25
9.7	2.23E-06	3.65E-10	5.40E-33	1.75E-10	1.17E-11	4.88E-05	7.49E-25
9.8	2.20E-06	3.48E-10	4.93E-33	1.83E-10	1.03E-11	4.91E-05	2.90E-24
9.9	1.79E-06	3.54E-10	4.33E-33	1.94E-10	5.60E-12	5.07E-05	6.60E-24
10	1.93E-06	3.86E-10	4.00E-33	1.75E-10	3.93E-12	5.10E-05	1.28E-23
10.1	1.27E-06	3.95E-10	3.87E-33	1.50E-10	2.46E-12	4.69E-05	4.03E-23
10.2	1.10E-06	3.91E-10	3.95E-33	1.59E-10	1.78E-12	4.52E-05	1.05E-22
10.3	9.85E-07	4.59E-10	4.51E-33	1.58E-10	1.18E-12	5.08E-05	2.71E-22
10.4	8.99E-07	4.39E-10	5.61E-33	1.74E-10	6.20E-13	5.60E-05	6.68E-22
10.5	7.75E-07	5.83E-10	7.41E-33	1.66E-10	3.67E-13	5.15E-05	1.34E-21
10.6	7.03E-07	7.42E-10	9.28E-33	1.37E-10	2.20E-13	5.44E-05	5.03E-21
10.7	5.55E-07	8.52E-10	1.00E-32	1.61E-10	1.67E-13	4.82E-05	1.33E-20
10.8	5.67E-07	7.78E-10	6.27E-33	1.54E-10	1.33E-13	4.94E-05	2.72E-20
10.9	4.96E-07	8.03E-10	6.43E-33	1.56E-10	9.84E-14	5.72E-05	6.37E-20
11	4.80E-07	7.69E-10	9.38E-33	1.97E-10	9.18E-14	5.36E-05	1.29E-19
11.1	4.06E-07	8.05E-10	1.28E-32	2.12E-10	7.21E-14	5.71E-05	3.61E-19
11.2	4.33E-07	7.95E-10	2.20E-32	2.47E-10	6.41E-14	6.90E-05	8.25E-19
11.3	4.15E-07	1.08E-09	2.95E-32	2.71E-10	4.28E-14	6.59E-05	2.00E-18
11.4	3.52E-07	1.19E-09	2.97E-32	3.71E-10	3.01E-14	7.25E-05	3.98E-18
11.5	3.30E-07	1.49E-09	2.77E-32	3.23E-10	2.70E-14	6.63E-05	9.56E-18
11.6	2.54E-07	1.68E-09	3.14E-32	3.54E-10	1.54E-14	6.23E-05	2.41E-17
11.7	2.57E-07	1.95E-09	4.46E-32	4.11E-10	1.08E-14	6.07E-05	3.97E-17
11.8	2.32E-07	1.76E-09	4.27E-32	3.78E-10	6.17E-15	5.17E-05	6.79E-17

Time discretization	V_{[G]}	K_{[G]}	V_{[PYR]}	K_{[PYR]}	alpha	G_E	[G]_0
15	1.91E-08	8.00E-09	1.02E-29	3.87E-09	2.17E-18	6.37E-05	5.14E-09
15.1	1.80E-08	7.34E-09	1.06E-29	4.12E-09	1.37E-18	5.82E-05	6.73E-09
15.2	1.90E-08	7.33E-09	1.39E-29	4.29E-09	1.08E-18	6.36E-05	9.21E-09
15.3	1.79E-08	7.40E-09	1.32E-29	4.22E-09	1.01E-18	5.86E-05	1.34E-08
15.4	1.48E-08	7.54E-09	1.32E-29	3.75E-09	5.55E-19	5.87E-05	1.85E-08
15.5	1.32E-08	8.20E-09	1.42E-29	3.01E-09	4.41E-19	5.49E-05	2.32E-08
15.6	1.25E-08	7.10E-09	1.43E-29	3.22E-09	3.26E-19	6.06E-05	2.79E-08
15.7	1.23E-08	7.90E-09	2.25E-29	3.56E-09	3.04E-19	5.84E-05	3.83E-08
15.8	1.26E-08	8.53E-09	2.57E-29	3.18E-09	2.39E-19	5.95E-05	5.76E-08
15.9	1.14E-08	8.30E-09	2.49E-29	3.51E-09	1.85E-19	5.62E-05	7.34E-08
16	1.04E-08	8.24E-09	3.06E-29	3.67E-09	1.14E-19	5.45E-05	9.29E-08
16.1	9.25E-09	6.92E-09	2.88E-29	3.97E-09	8.72E-20	5.00E-05	1.12E-07
16.2	7.62E-09	7.14E-09	3.00E-29	4.19E-09	6.09E-20	5.05E-05	1.67E-07
16.3	7.42E-09	7.43E-09	2.94E-29	3.94E-09	4.82E-20	4.96E-05	2.25E-07
16.4	7.24E-09	8.21E-09	2.85E-29	3.99E-09	3.84E-20	5.08E-05	2.77E-07
16.5	6.69E-09	8.60E-09	3.45E-29	3.87E-09	2.75E-20	5.26E-05	3.52E-07
16.6	5.90E-09	1.01E-08	4.18E-29	3.95E-09	2.28E-20	5.31E-05	4.45E-07
16.7	5.11E-09	1.10E-08	4.82E-29	4.13E-09	1.73E-20	5.61E-05	5.43E-07
16.8	4.28E-09	1.19E-08	5.01E-29	4.07E-09	1.42E-20	4.99E-05	7.05E-07
16.9	4.14E-09	1.13E-08	5.54E-29	4.79E-09	1.38E-20	5.34E-05	8.14E-07
17	4.22E-09	1.09E-08	7.00E-29	4.73E-09	1.38E-20	5.55E-05	1.22E-06
17.1	4.07E-09	1.32E-08	9.47E-29	4.64E-09	1.09E-20	5.88E-05	1.71E-06
17.2	3.90E-09	1.29E-08	7.87E-29	4.13E-09	7.14E-21	5.35E-05	2.49E-06
17.3	3.65E-09	1.43E-08	8.12E-29	3.99E-09	6.72E-21	5.74E-05	2.86E-06
17.4	3.58E-09	1.51E-08	8.84E-29	3.98E-09	5.47E-21	5.86E-05	3.35E-06
17.5	3.02E-09	1.53E-08	9.68E-29	4.61E-09	5.21E-21	5.60E-05	4.15E-06
17.6	2.68E-09	1.45E-08	9.31E-29	4.61E-09	3.48E-21	5.03E-05	5.43E-06
17.7	2.70E-09	1.12E-08	8.65E-29	4.64E-09	2.92E-21	4.78E-05	6.76E-06
17.8	2.65E-09	1.04E-08	8.71E-29	4.78E-09	2.40E-21	4.83E-05	8.27E-06
17.9	2.37E-09	1.13E-08	1.07E-28	5.45E-09	1.97E-21	5.04E-05	9.44E-06
18	2.34E-09	1.09E-08	1.41E-28	5.67E-09	1.44E-21	5.36E-05	1.02E-05
18.1	2.09E-09	1.34E-08	1.85E-28	5.20E-09	1.30E-21	5.46E-05	1.26E-05
18.2	1.92E-09	1.34E-08	1.84E-28	5.32E-09	9.77E-22	5.32E-05	1.38E-05
18.3	1.92E-09	1.37E-08	2.18E-28	5.50E-09	9.77E-22	5.47E-05	1.63E-05
18.4	1.88E-09	1.34E-08	2.42E-28	5.31E-09	1.01E-21	5.47E-05	1.94E-05
18.5	1.75E-09	1.38E-08	2.81E-28	5.94E-09	8.50E-22	6.11E-05	2.12E-05
18.6	1.85E-09	1.26E-08	2.76E-28	6.15E-09	8.45E-22	5.67E-05	2.48E-05
18.7	1.74E-09	1.53E-08	3.35E-28	5.72E-09	8.05E-22	5.65E-05	3.14E-05
18.8	1.85E-09	1.68E-08	3.94E-28	6.24E-09	6.51E-22	6.04E-05	4.19E-05
18.9	1.64E-09	1.71E-08	5.18E-28	6.45E-09	5.45E-22	6.86E-05	4.85E-05
19	1.37E-09	1.76E-08	6.29E-28	7.76E-09	4.93E-22	7.18E-05	6.07E-05
19.1	1.40E-09	1.87E-08	7.57E-28	8.35E-09	5.40E-22	7.30E-05	7.41E-05
19.2	1.40E-09	1.97E-08	9.05E-28	7.62E-09	4.94E-22	6.88E-05	9.49E-05
19.3	1.24E-09	1.92E-08	9.58E-28	7.37E-09	4.48E-22	6.66E-05	9.69E-05
19.4	1.01E-09	2.04E-08	1.16E-27	7.99E-09	3.64E-22	6.70E-05	0.00012905
19.5	1.06E-09	2.01E-08	1.21E-27	9.00E-09	4.11E-22	6.13E-05	0.00014913
19.6	1.10E-09	2.30E-08	1.53E-27	1.02E-08	4.08E-22	6.37E-05	0.00016503
19.7	1.02E-09	1.79E-08	1.40E-27	1.14E-08	3.97E-22	6.21E-05	0.00021397
19.8	9.90E-10	1.79E-08	1.50E-27	1.11E-08	3.56E-22	6.45E-05	0.00023782
19.9	1.01E-09	1.78E-08	1.80E-27	1.27E-08	3.69E-22	6.51E-05	0.00027011
20	9.68E-10	1.68E-08	1.65E-27	1.36E-08	2.80E-22	6.79E-05	0.00031723
20.1	9.17E-10	1.66E-08	1.63E-27	1.26E-08	2.21E-22	6.63E-05	0.00034171
20.2	8.52E-10	1.44E-08	1.70E-27	1.37E-08	1.83E-22	6.35E-05	0.00041494
20.3	8.78E-10	1.55E-08	2.45E-27	1.56E-08	1.70E-22	5.87E-05	0.00049026
20.4	9.13E-10	1.55E-08	3.13E-27	1.54E-08	1.53E-22	5.88E-05	0.00055079
20.5	8.34E-10	1.74E-08	3.58E-27	1.52E-08	1.24E-22	5.66E-05	0.00060174
20.6	8.07E-10	1.87E-08	3.54E-27	1.46E-08	9.80E-23	5.34E-05	0.00065255
20.7	8.07E-10	2.07E-08	4.07E-27	1.46E-08	8.89E-23	5.52E-05	0.00074345
20.8	7.91E-10	1.97E-08	4.38E-27	1.51E-08	7.90E-23	5.60E-05	0.00079483
20.9	7.12E-10	1.84E-08	5.16E-27	1.53E-08	6.83E-23	5.69E-05	0.00091653
21	7.28E-10	1.76E-08	4.99E-27	1.55E-08	6.04E-23	5.52E-05	0.00108363
21.1	7.24E-10	1.59E-08	4.98E-27	1.46E-08	5.82E-23	5.30E-05	0.00118072
21.2	7.05E-10	1.67E-08	5.38E-27	1.52E-08	4.93E-23	5.39E-05	0.00139203
21.3	6.80E-10	1.70E-08	5.31E-27	1.56E-08	4.76E-23	5.43E-05	0.00155405
21.4	6.48E-10	1.75E-08	4.77E-27	1.51E-08	4.50E-23	5.27E-05	0.00164831
21.5	6.54E-10	1.95E-08	5.37E-27	1.61E-08	4.71E-23	5.45E-05	0.00174268
21.6	5.81E-10	2.08E-08	6.68E-27	1.85E-08	4.00E-23	5.95E-05	0.00186923
21.7	5.77E-10	2.06E-08	8.24E-27	1.70E-08	3.87E-23	6.14E-05	0.00205238
21.8	5.04E-10	1.86E-08	7.35E-27	1.53E-08	3.02E-23	5.99E-05	0.00232234
21.9	5.01E-10	1.66E-08	6.49E-27	1.37E-08	2.26E-23	5.65E-05	0.00259572
22	5.09E-10	1.61E-08	7.70E-27	1.58E-08	2.03E-23	5.59E-05	0.00291155
22.1	4.96E-10	1.64E-08	9.51E-27	1.72E-08	1.59E-23	5.91E-05	0.00328622
22.2	4.87E-10	1.47E-08	1.11E-26	1.81E-08	1.38E-23	5.64E-05	0.00399282
22.3	4.69E-10	1.67E-08	1.41E-26	1.83E-08	1.20E-23	5.39E-05	0.00432532
22.4	4.57E-10	1.79E-08	1.55E-26	1.68E-08	9.15E-24	5.06E-05	0.00462245
22.5	4.29E-10	1.60E-08	1.40E-26	1.60E-08	8.92E-24	5.18E-05	0.00527682
22.6	3.95E-10	1.61E-08	1.36E-26	1.57E-08	7.78E-24	5.10E-05	0.00617237
22.7	3.84E-10	1.52E-08	1.44E-26	1.48E-08	6.09E-24	5.25E-05	0.00625378
22.8	3.72E-10	1.44E-08	1.39E-26	1.49E-08	4.87E-24	5.24E-05	0.00675133
22.9	3.12E-10	1.38E-08	1.52E-26	1.50E-08	3.23E-24	5.64E-05	0.00686464
23	2.90E-10	1.27E-08	1.69E-26	1.45E-08	2.81E-24	5.07E-05	0.00777973
23.1	2.84E-10	1.33E-08	1.66E-26	1.41E-08	2.42E-24	5.19E-05	0.00844866
23.2	2.60E-10	1.37E-08	1.78E-26	1.51E-08	1.94E-24	5.20E-05	0.00844551
23.3	2.87E-10	1.28E-08	2.08E-26	1.54E-08	1.60E-24	5.35E-05	0.00948758
23.4	2.68E-10	1.29E-08	2.34E-26	1.55E-08	1.55E-24	5.42E-05	0.00988501
23.5	2.52E-10	1.26E-08	2.60E-26	1.61E-08	1.31E-24	5.75E-05	0.01027301
23.6	2.33E-10	1.23E-08	2.83E-26	1.57E-08	1.16E-24	5.73E-05	0.01058342
23.7	2.38E-10	1.34E-08	3.87E-26	1.74E-08	1.12E-24	5.81E-05	0.01153246
23.8	2.57E-10	1.37E-08	4.55E-26	1.86E-08	1.09E-24	6.30E-05	0.01225082
23.9	2.56E-10	1.33E-08	4.75E-26	1.92E-08	9.90E-25	5.82E-05	0.01321974
24	2.59E-10	1.34E-08	5.33E-26	2.01E-08	9.21E-25	5.99E-05	0.01407618
24.1	2.34E-10	1.28E-08	4.59E-26	1.87E-08	7.33E-25	5.78E-05	0.01534169
24.2	2.38E-10	1.24E-08	4.90E-26	1.85E-08	6.99E-25	5.70E-05	0.01586537
24.3	2.42E-10	1.30E-08	5.57E-26	1.91E-08	6.92E-25	5.91E-05	0.01702646
24.4	2.54E-10	1.30E-08	6.27E-26	1.96E-08	7.14E-25	5.83E-05	0.01793757
24.5	2.43E-10	1.21E-08	6.34E-26	1.96E-08	6.22E-25	5.46E-05	0.01909014
24.6	2.53E-10	1.20E-08	6.77E-26	2.02E-08	5.81E-25	5.27E-05	0.01969594
24.7	2.43E-10	1.21E-08	7.13E-26	1.87E-08	5.01E-25	5.19E-05	0.02122151
24.8	2.19E-10	1.14E-08	6.96E-26	1.78E-08	3.65E-25	5.25E-05	0.02180757
24.9	2.01E-10	1.13E-08	6.90E-26	1.62E-08	3.02E-25	5.25E-05	0.02224512
25	1.89E-10	9.95E-09	8.10E-26	1.60E-08	2.40E-25	5.38E-05	0.02335606
25.1	1.63E-10	8.83E-09	6.86E-26	1.60E-08	1.76E-25	5.68E-05	0.02596702
25.2	1.51E-10	9.18E-09	7.53E-26	1.54E-08	1.50E-25	5.42E-05	0.02640696
25.3	1.42E-10	8.54E-09	7.59E-26	1.51E-08	1.29E-25	5.72E-05	0.02868222
25.4	1.39E-10	8.81E-09	8.43E-26	1.47E-08	1.17E-25	5.85E-05	0.02901904
25.5	1.28E-10	8.73E-09	7.83E-26	1.40E-08	1.02E-25	5.72E-05	0.03098757
25.6	1.30E-10	8.61E-09	7.62E-26	1.40E-08	9.97E-26	5.61E-05	0.03273344
25.7	1.29E-10	8.33E-09	8.71E-26	1.52E-08	8.53E-26	5.64E-05	0.03420488
25.8	1.22E-10	8.79E-09	9.61E-26	1.38E-08	7.51E-26	5.67E-05	0.03672111
25.9	1.23E-10	8.78E-09	1.05E-25	1.44E-08	5.70E-26	5.47E-05	0.03893146
26	1.20E-10	8.78E-09	1.06E-25	1.33E-08	4.70E-26	5.03E-05	0.04096975
26.1	1.10E-10	9.19E-09	1.15E-25	1.28E-08	4.00E-26	5.06E-05	0.04210738
26.2	1.17E-10	9.59E-09	1.19E-25	1.24E-08	4.22E-26	5.00E-05	0.0444751
26.3	1.13E-10	9.78E-09	1.18E-25	1.21E-08	3.63E-26	4.97E-05	0.04653842
26.4	1.08E-10	9.05E-09	1.08E-25	1.26E-08	2.73E-26	4.88E-05	0.04741856
26.5	1.12E-10	9.17E-09	1.10E-25	1.31E-08	2.70E-26	4.66E-05	0.04942479
26.6	1.06E-10	8.45E-09	1.12E-25	1.26E-08	2.34E-26	4.39E-05	0.04992672
26.7	1.02E-10	8.01E-09	1.11E-25	1.29E-08	2.10E-26	4.44E-05	0.05143168
26.8	9.97E-11	7.96E-09	1.12E-25	1.16E-08	1.81E-26	4.47E-05	0.05209726

Time discretization	V_{[G]}	K_{[G]}	V_{[PYR]}	K_{[PYR]}	alpha	G_E	[G]_0
30	3.68E-11	2.88E-09	2.45E-25	7.49E-09	5.16E-28	2.53E-05	0.13634328
30.1	3.45E-11	2.73E-09	2.51E-25	8.02E-09	4.70E-28	2.63E-05	0.14083333
30.2	3.30E-11	2.37E-09	2.67E-25	8.50E-09	4.64E-28	2.54E-05	0.14398824
30.3	3.18E-11	2.38E-09	2.81E-25	8.05E-09	4.27E-28	2.52E-05	0.14656512
30.4	2.90E-11	2.33E-09	2.65E-25	8.07E-09	3.52E-28	2.56E-05	0.14802166
30.5	2.74E-11	2.29E-09	2.56E-25	8.07E-09	3.15E-28	2.48E-05	0.15016175
30.6	2.82E-11	2.14E-09	3.02E-25	7.91E-09	2.97E-28	2.50E-05	0.15528717
30.7	2.55E-11	2.18E-09	3.06E-25	8.00E-09	2.88E-28	2.45E-05	0.15936559
30.8	2.62E-11	2.06E-09	3.03E-25	8.22E-09	2.58E-28	2.36E-05	0.15837236
30.9	2.53E-11	2.02E-09	3.04E-25	8.21E-09	2.58E-28	2.29E-05	0.15794247
31	2.32E-11	2.10E-09	3.27E-25	7.16E-09	2.03E-28	2.44E-05	0.1644383
31.1	2.38E-11	2.13E-09	3.71E-25	7.15E-09	2.04E-28	2.40E-05	0.1692012
31.2	2.27E-11	2.00E-09	3.58E-25	6.75E-09	1.87E-28	2.50E-05	0.16945702
31.3	2.12E-11	1.97E-09	3.67E-25	7.59E-09	1.78E-28	2.47E-05	0.16748005
31.4	1.97E-11	1.87E-09	3.98E-25	7.91E-09	1.65E-28	2.44E-05	0.16825138
31.5	1.79E-11	1.94E-09	4.12E-25	7.87E-09	1.57E-28	2.64E-05	0.17846791
31.6	1.61E-11	1.91E-09	3.92E-25	8.53E-09	1.35E-28	2.55E-05	0.17803734
31.7	1.35E-11	1.68E-09	3.60E-25	7.70E-09	8.92E-29	2.29E-05	0.17715781
31.8	1.45E-11	1.72E-09	4.25E-25	7.62E-09	8.02E-29	2.32E-05	0.18699963
31.9	1.50E-11	1.82E-09	4.79E-25	8.18E-09	8.10E-29	2.34E-05	0.19369717
32	1.50E-11	1.82E-09	4.79E-25	8.18E-09	8.10E-29	2.34E-05	0.19369717
32.1	1.50E-11	1.75E-09	4.79E-25	7.81E-09	7.82E-29	2.29E-05	0.20204257
32.2	1.55E-11	1.76E-09	4.67E-25	8.02E-09	7.59E-29	2.30E-05	0.2018396
32.3	1.56E-11	1.81E-09	5.06E-25	8.37E-09	7.61E-29	2.30E-05	0.20468473
32.4	1.66E-11	1.86E-09	5.56E-25	9.20E-09	7.40E-29	2.32E-05	0.20703225
32.5	1.62E-11	2.00E-09	6.25E-25	9.61E-09	7.21E-29	2.36E-05	0.20993862
32.6	1.53E-11	2.07E-09	6.40E-25	9.45E-09	6.56E-29	2.44E-05	0.21364361
32.7	1.38E-11	1.97E-09	6.05E-25	9.41E-09	5.56E-29	2.37E-05	0.21113438
32.8	1.27E-11	1.92E-09	6.84E-25	1.03E-08	4.98E-29	2.38E-05	0.21407483
32.9	1.22E-11	2.07E-09	7.00E-25	1.11E-08	4.73E-29	2.24E-05	0.2144027
33	1.13E-11	1.86E-09	7.02E-25	1.14E-08	4.43E-29	2.21E-05	0.21399289
33.1	1.16E-11	1.73E-09	6.79E-25	1.12E-08	3.84E-29	2.17E-05	0.21773644
33.2	1.06E-11	1.74E-09	6.31E-25	1.22E-08	2.97E-29	2.07E-05	0.22523699
33.3	1.10E-11	1.62E-09	6.68E-25	1.17E-08	2.87E-29	2.00E-05	0.23294181
33.4	1.10E-11	1.59E-09	5.91E-25	1.12E-08	2.18E-29	1.75E-05	0.23175553
33.5	1.12E-11	1.45E-09	6.94E-25	1.18E-08	1.92E-29	1.76E-05	0.2342453
33.6	1.02E-11	1.33E-09	7.06E-25	1.08E-08	1.34E-29	1.82E-05	0.23231007
33.7	1.06E-11	1.35E-09	7.61E-25	1.13E-08	1.26E-29	1.78E-05	0.23684274
33.8	1.13E-11	1.28E-09	8.09E-25	1.12E-08	1.14E-29	1.76E-05	0.24142449
33.9	1.13E-11	1.25E-09	9.48E-25	1.02E-08	8.93E-30	1.85E-05	0.25516802
34	1.01E-11	1.11E-09	8.72E-25	1.01E-08	7.61E-30	1.69E-05	0.26190722
34.1	1.09E-11	1.09E-09	9.15E-25	9.21E-09	6.54E-30	1.59E-05	0.26646125
34.2	1.09E-11	9.77E-10	1.00E-24	8.91E-09	5.56E-30	1.64E-05	0.26120418
34.3	1.05E-11	8.59E-10	1.04E-24	9.01E-09	4.67E-30	1.65E-05	0.26474247
34.4	8.50E-12	8.38E-10	9.86E-25	8.67E-09	3.41E-30	1.72E-05	0.26850489
34.5	8.75E-12	7.26E-10	1.17E-24	8.66E-09	3.26E-30	1.76E-05	0.27441688
34.6	8.64E-12	7.02E-10	1.16E-24	9.85E-09	3.55E-30	1.85E-05	0.27909055
34.7	9.13E-12	7.04E-10	1.28E-24	1.10E-08	3.15E-30	1.93E-05	0.27369476
34.8	9.46E-12	7.61E-10	1.43E-24	1.13E-08	2.95E-30	2.12E-05	0.27778199
34.9	9.68E-12	7.20E-10	1.38E-24	1.08E-08	2.82E-30	2.03E-05	0.28416728
35	9.55E-12	7.43E-10	1.54E-24	1.07E-08	2.67E-30	2.08E-05	0.28847271
35.1	9.50E-12	7.23E-10	1.60E-24	1.13E-08	2.32E-30	2.12E-05	0.28999633
35.2	9.16E-12	7.08E-10	1.65E-24	1.14E-08	2.35E-30	2.14E-05	0.29645238
35.3	8.75E-12	6.92E-10	1.66E-24	1.11E-08	2.31E-30	2.05E-05	0.29680289
35.4	8.20E-12	6.78E-10	1.63E-24	1.10E-08	1.94E-30	2.12E-05	0.29998239
35.5	7.81E-12	6.26E-10	1.71E-24	1.16E-08	1.84E-30	2.06E-05	0.3040683
35.6	7.95E-12	6.26E-10	1.70E-24	1.14E-08	1.79E-30	2.09E-05	0.30657589
35.7	8.72E-12	6.17E-10	1.76E-24	1.08E-08	1.77E-30	2.11E-05	0.31569858
35.8	8.05E-12	6.15E-10	1.96E-24	1.15E-08	1.67E-30	2.14E-05	0.31432788
35.9	7.91E-12	5.43E-10	2.07E-24	1.09E-08	1.67E-30	2.24E-05	0.31440778
36	7.21E-12	5.42E-10	2.18E-24	1.02E-08	1.56E-30	2.23E-05	0.31397611
36.1	6.64E-12	5.17E-10	2.15E-24	9.56E-09	1.31E-30	2.19E-05	0.31491746
36.2	6.58E-12	4.64E-10	2.31E-24	9.45E-09	1.12E-30	2.08E-05	0.31494143
36.3	6.16E-12	4.00E-10	2.36E-24	9.37E-09	1.02E-30	1.92E-05	0.31762689
36.4	5.87E-12	3.76E-10	2.28E-24	9.07E-09	8.90E-31	1.85E-05	0.3193852
36.5	5.69E-12	3.53E-10	2.19E-24	9.26E-09	7.92E-31	1.73E-05	0.31878439
36.6	5.72E-12	3.28E-10	2.16E-24	8.98E-09	7.07E-31	1.71E-05	0.31703077
36.7	5.74E-12	3.15E-10	2.01E-24	8.54E-09	6.02E-31	1.68E-05	0.3233224
36.8	5.18E-12	3.14E-10	2.20E-24	8.86E-09	5.72E-31	1.70E-05	0.32660205
36.9	4.64E-12	3.21E-10	2.25E-24	8.78E-09	4.48E-31	1.74E-05	0.32861458
37	3.77E-12	3.14E-10	2.29E-24	8.61E-09	3.40E-31	1.54E-05	0.33093648
37.1	3.87E-12	3.11E-10	2.46E-24	8.50E-09	3.30E-31	1.54E-05	0.33004715
37.2	4.25E-12	3.14E-10	2.77E-24	9.10E-09	3.53E-31	1.57E-05	0.33674833
37.3	4.22E-12	3.04E-10	2.97E-24	8.70E-09	2.97E-31	1.52E-05	0.33603588
37.4	4.06E-12	3.03E-10	2.97E-24	8.58E-09	2.98E-31	1.49E-05	0.33792246
37.5	3.67E-12	2.60E-10	2.61E-24	8.34E-09	2.20E-31	1.40E-05	0.33165461
37.6	3.68E-12	2.48E-10	2.54E-24	8.22E-09	2.14E-31	1.40E-05	0.33632357
37.7	3.69E-12	2.30E-10	2.59E-24	7.95E-09	1.92E-31	1.31E-05	0.34060309
37.8	3.80E-12	2.28E-10	2.76E-24	7.83E-09	1.80E-31	1.28E-05	0.3419011
37.9	3.80E-12	2.08E-10	3.02E-24	7.36E-09	1.83E-31	1.32E-05	0.3470511
38	3.92E-12	1.99E-10	3.07E-24	7.57E-09	1.78E-31	1.31E-05	0.34602254
38.1	4.01E-12	2.05E-10	3.31E-24	7.63E-09	1.78E-31	1.28E-05	0.34713952
38.2	3.94E-12	1.99E-10	3.50E-24	7.64E-09	1.75E-31	1.30E-05	0.34292798
38.3	4.36E-12	1.88E-10	3.96E-24	7.59E-09	1.66E-31	1.32E-05	0.34492119
38.4	3.84E-12	1.58E-10	3.90E-24	7.75E-09	1.27E-31	1.40E-05	0.34508377
38.5	3.56E-12	1.57E-10	4.34E-24	7.84E-09	1.05E-31	1.37E-05	0.35758773
38.6	3.43E-12	1.61E-10	4.83E-24	8.58E-09	1.00E-31	1.37E-05	0.35748638
38.7	3.09E-12	1.46E-10	5.22E-24	8.70E-09	7.89E-32	1.37E-05	0.35886463
38.8	2.68E-12	1.40E-10	4.79E-24	8.12E-09	6.60E-32	1.37E-05	0.36714504
38.9	2.63E-12	1.47E-10	4.65E-24	8.28E-09	5.77E-32	1.37E-05	0.36832823
39	2.71E-12	1.40E-10	4.61E-24	8.38E-09	5.70E-32	1.35E-05	0.36978308
39.1	2.66E-12	1.26E-10	4.48E-24	8.21E-09	5.21E-32	1.37E-05	0.3777996
39.2	2.49E-12	1.18E-10	4.71E-24	9.22E-09	4.24E-32	1.31E-05	0.37915981
39.3	2.38E-12	1.17E-10	4.57E-24	9.50E-09	4.18E-32	1.29E-05	0.38721102
39.4	2.29E-12	1.13E-10	4.62E-24	9.69E-09	3.92E-32	1.26E-05	0.38956617
39.5	2.20E-12	1.11E-10	4.74E-24	9.53E-09	3.16E-32	1.25E-05	0.38815183
39.6	2.28E-12	1.11E-10	5.12E-24	9.67E-09	3.21E-32	1.27E-05	0.39003991
39.7	2.26E-12	1.19E-10	5.74E-24	9.44E-09	3.09E-32	1.27E-05	0.40335509
39.8	2.09E-12	1.11E-10	5.79E-24	1.01E-08	2.58E-32	1.22E-05	0.40191477
39.9	2.10E-12	1.14E-10	5.92E-24	1.08E-08	2.59E-32	1.22E-05	0.4147165
40	2.01E-12	1.11E-10	5.82E-24	1.05E-08	2.27E-32	1.25E-05	0.41911467
40.1	1.96E-12	1.07E-10	5.69E-24	1.07E-08	1.93E-32	1.24E-05	0.4247524
40.2	1.91E-12	1.00E-10	5.79E-24	1.06E-08	1.75E-32	1.24E-05	0.42953066
40.3	1.80E-12	9.25E-11	5.81E-24	1.15E-08	1.61E-32	1.25E-05	0.43771648
40.4	1.78E-12	9.00E-11	5.64E-24	1.18E-08	1.54E-32	1.24E-05	0.43834667
40.5	1.59E-12	8.73E-11	5.25E-24	1.19E-08	1.13E-32	1.24E-05	0.44926069
40.6	1.49E-12	8.70E-11	5.45E-24	1.20E-08	1.05E-32	1.19E-05	0.45020802
40.7	1.36E-12	8.81E-11	5.47E-24	1.29E-08	1.05E-32	1.19E-05	0.45061386
40.8	1.36E-12	8.55E-11	5.56E-24	1.34E-08	1.02E-32	1.16E-05	0.45175503
40.9	1.30E-12	8.17E-11	5.52E-24	1.38E-08	1.00E-32	1.13E-05	0.45735969
41	1.26E-12	8.36E-11	5.28E-24	1.36E-08	9.40E-33	1.05E-05	0.45855564
41.1	1.11E-12	6.95E-11	5.20E-24	1.32E-08	7.36E-33	1.04E-05	0.45642476
41.2	1.08E-12	6.41E-11	5.93E-24	1.21E-08	5.94E-33	1.03E-05	0.45388709
41.3	9.76E-13	6.31E-11	6.30E-24	1.34E-08	4.99E-33	1.03E-05	0.45257095
41.4	8.68E-13	5.08E-11	5.61E-24	1.40E-08	4.21E-33	1.02E-05	0.45507225
41.5	8.59E-13	4.76E-11	6.07E-24	1.47E-08	3.52E-33	1.02E-05	0.4553086
41.6	7.91E-13	4.48E-11	6.11E-24	1.48E-08	3.35E-33	9.95E-06	0.45737362
41.7	7.33E-13	4.39E-11	6.59E-24	1.44E-08	2.95E-33	9.88E-06	0.45321453
41.8	7.00E-13	3.77E-11	6.77E-24	1.51E-08	2.50E-33	9.61E-06	0.46636925

Time discretization	V_{[G]}	K_{[G]}	V_{[PYR]}	K_{[PYR]}	alpha	G_E	[G]_0
45	1.21E-13	8.91E-12	7.90E-24	2.08E-08	8.12E-35	5.03E-06	0.59158318
45.1	1.22E-13	8.99E-12	8.94E-24	2.08E-08	8.81E-35	4.82E-06	0.59095189
45.2	1.19E-13	8.36E-12	9.49E-24	2.21E-08	8.36E-35	4.93E-06	0.58857625
45.3	1.19E-13	8.19E-12	1.02E-23	2.29E-08	8.26E-35	4.75E-06	0.60135733
45.4	1.29E-13	7.67E-12	1.11E-23	2.30E-08	8.77E-35	4.67E-06	0.60500481
45.5	1.38E-13	7.38E-12	1.17E-23	2.31E-08	8.68E-35	4.86E-06	0.60858619
45.6	1.26E-13	6.64E-12	1.18E-23	2.58E-08	7.53E-35	4.59E-06	0.60717189
45.7	1.22E-13	6.21E-12	1.17E-23	2.68E-08	6.95E-35	4.43E-06	0.61108877
45.8	1.18E-13	6.33E-12	1.19E-23	2.79E-08	6.25E-35	4.21E-06	0.61582221
45.9	1.22E-13	6.11E-12	1.31E-23	2.71E-08	5.70E-35	4.02E-06	0.61467685
46	1.04E-13	5.44E-12	1.25E-23	2.66E-08	4.21E-35	3.72E-06	0.61990961
46.1	1.05E-13	4.78E-12	1.23E-23	2.56E-08	3.86E-35	3.79E-06	0.62835108
46.2	1.07E-13	4.66E-12	1.34E-23	2.56E-08	3.93E-35	3.57E-06	0.63264475
46.3	1.05E-13	4.50E-12	1.27E-23	2.61E-08	3.70E-35	3.46E-06	0.62936139
46.4	9.68E-14	4.40E-12	1.17E-23	2.60E-08	3.04E-35	3.22E-06	0.6401953
46.5	8.57E-14	4.65E-12	1.27E-23	2.71E-08	2.62E-35	2.96E-06	0.6397722
46.6	7.67E-14	4.42E-12	1.21E-23	2.71E-08	2.33E-35	2.79E-06	0.64529376
46.7	7.32E-14	4.36E-12	1.25E-23	2.66E-08	2.27E-35	2.65E-06	0.64048451
46.8	6.56E-14	4.04E-12	1.14E-23	2.60E-08	2.01E-35	2.45E-06	0.64635822
46.9	6.02E-14	4.03E-12	1.13E-23	2.86E-08	2.01E-35	2.29E-06	0.63998395
47	5.84E-14	4.16E-12	1.21E-23	2.88E-08	2.07E-35	2.34E-06	0.64114816
47.1	5.38E-14	3.91E-12	1.18E-23	2.98E-08	1.82E-35	2.32E-06	0.63807731
47.2	5.09E-14	3.82E-12	1.18E-23	3.11E-08	1.97E-35	2.19E-06	0.64570829
47.3	4.99E-14	3.33E-12	1.21E-23	3.20E-08	1.74E-35	2.16E-06	0.64041157
47.4	5.19E-14	3.43E-12	1.32E-23	3.30E-08	1.97E-35	2.19E-06	0.64592092
47.5	5.08E-14	3.40E-12	1.37E-23	3.34E-08	1.89E-35	2.24E-06	0.64607026
47.6	4.50E-14	2.96E-12	1.36E-23	3.13E-08	1.71E-35	2.05E-06	0.65400588
47.7	3.99E-14	2.71E-12	1.28E-23	3.08E-08	1.56E-35	2.04E-06	0.66057238
47.8	3.90E-14	2.69E-12	1.34E-23	3.21E-08	1.44E-35	2.09E-06	0.66333291
47.9	4.11E-14	2.66E-12	1.51E-23	3.61E-08	1.45E-35	2.00E-06	0.6723725
48	3.97E-14	2.58E-12	1.59E-23	3.78E-08	1.51E-35	1.95E-06	0.67137892
48.1	3.25E-14	2.41E-12	1.37E-23	3.83E-08	1.07E-35	1.85E-06	0.67686336
48.2	3.04E-14	2.25E-12	1.41E-23	3.68E-08	9.62E-36	1.78E-06	0.67965313
48.3	3.01E-14	2.10E-12	1.37E-23	3.70E-08	9.18E-36	1.71E-06	0.68481771
48.4	2.98E-14	2.08E-12	1.57E-23	3.49E-08	9.44E-36	1.68E-06	0.69089668
48.5	2.65E-14	2.15E-12	1.58E-23	3.67E-08	7.92E-36	1.65E-06	0.68691925
48.6	2.34E-14	2.28E-12	1.58E-23	3.84E-08	7.18E-36	1.49E-06	0.69142262
48.7	2.31E-14	2.30E-12	1.70E-23	4.07E-08	6.02E-36	1.37E-06	0.67398718
48.8	2.35E-14	2.24E-12	1.74E-23	4.19E-08	6.14E-36	1.39E-06	0.67453496
48.9	2.16E-14	2.16E-12	1.69E-23	4.41E-08	5.83E-36	1.37E-06	0.68068992
49	2.10E-14	2.09E-12	1.70E-23	4.39E-08	5.79E-36	1.34E-06	0.68228487
49.1	2.10E-14	2.06E-12	1.68E-23	4.33E-08	5.52E-36	1.34E-06	0.68125506
49.2	1.89E-14	2.04E-12	1.85E-23	4.41E-08	4.67E-36	1.30E-06	0.67487519
49.3	1.90E-14	1.93E-12	1.90E-23	4.61E-08	4.00E-36	1.25E-06	0.68607374
49.4	1.69E-14	1.83E-12	1.84E-23	4.60E-08	3.51E-36	1.18E-06	0.68745206
49.5	1.65E-14	1.82E-12	1.81E-23	4.67E-08	3.34E-36	1.17E-06	0.68843768
49.6	1.62E-14	1.84E-12	1.97E-23	4.72E-08	3.18E-36	1.14E-06	0.68994463
49.7	1.59E-14	1.67E-12	1.75E-23	4.60E-08	2.71E-36	1.05E-06	0.69206072
49.8	1.51E-14	1.53E-12	1.74E-23	4.28E-08	2.39E-36	1.02E-06	0.69717503
49.9	1.40E-14	1.42E-12	1.70E-23	4.25E-08	2.32E-36	9.95E-07	0.70215784
50	1.28E-14	1.37E-12	1.80E-23	4.17E-08	2.25E-36	1.01E-06	0.69355424
50.1	1.20E-14	1.42E-12	1.90E-23	3.99E-08	2.08E-36	8.75E-07	0.69951499
50.2	1.31E-14	1.42E-12	2.03E-23	4.04E-08	2.12E-36	9.07E-07	0.69631218
50.3	1.14E-14	1.39E-12	1.96E-23	4.05E-08	1.79E-36	9.24E-07	0.70655075
50.4	1.03E-14	1.27E-12	1.99E-23	4.25E-08	1.58E-36	8.49E-07	0.70124668
50.5	1.00E-14	1.22E-12	1.87E-23	4.07E-08	1.46E-36	8.32E-07	0.69681704
50.6	1.01E-14	1.29E-12	2.12E-23	4.30E-08	1.57E-36	8.65E-07	0.69886059
50.7	9.40E-15	1.02E-12	1.88E-23	4.33E-08	1.30E-36	8.03E-07	0.70702926
50.8	9.06E-15	9.25E-13	1.92E-23	4.01E-08	1.15E-36	7.51E-07	0.71124552
50.9	8.22E-15	9.23E-13	1.96E-23	4.17E-08	1.09E-36	7.43E-07	0.71806263
51	8.44E-15	9.24E-13	2.05E-23	4.08E-08	1.10E-36	7.84E-07	0.72730735
51.1	8.01E-15	9.33E-13	2.13E-23	4.33E-08	1.05E-36	7.77E-07	0.73680389
51.2	7.39E-15	7.37E-13	1.80E-23	4.70E-08	7.79E-37	7.12E-07	0.73828692
51.3	7.75E-15	7.71E-13	2.00E-23	4.74E-08	8.22E-37	7.24E-07	0.74216971
51.4	7.32E-15	6.81E-13	2.27E-23	4.89E-08	7.83E-37	6.24E-07	0.74640983
51.5	6.88E-15	7.01E-13	2.28E-23	5.12E-08	8.12E-37	6.19E-07	0.74973697
51.6	6.35E-15	6.12E-13	2.11E-23	5.04E-08	6.41E-37	5.92E-07	0.74889542
51.7	6.12E-15	5.73E-13	2.16E-23	5.07E-08	5.55E-37	5.85E-07	0.75965144
51.8	5.89E-15	5.64E-13	2.38E-23	5.44E-08	5.01E-37	5.73E-07	0.76011792
51.9	5.95E-15	5.18E-13	2.33E-23	5.26E-08	4.32E-37	5.15E-07	0.77157528
52	5.01E-15	4.95E-13	2.16E-23	5.53E-08	3.77E-37	5.17E-07	0.79201678
52.1	4.84E-15	4.96E-13	2.22E-23	5.85E-08	3.70E-37	4.94E-07	0.79271304
52.2	4.56E-15	4.23E-13	2.09E-23	5.63E-08	2.87E-37	4.91E-07	0.7964875
52.3	4.72E-15	4.40E-13	2.36E-23	5.94E-08	2.97E-37	4.96E-07	0.80073358
52.4	4.44E-15	4.09E-13	2.23E-23	6.04E-08	2.55E-37	4.84E-07	0.79841538
52.5	4.38E-15	3.74E-13	2.31E-23	6.30E-08	2.12E-37	4.99E-07	0.79537814
52.6	4.09E-15	3.93E-13	2.50E-23	6.93E-08	2.03E-37	4.86E-07	0.78282095
52.7	3.80E-15	3.77E-13	2.55E-23	6.71E-08	1.72E-37	4.43E-07	0.79435671
52.8	3.67E-15	3.66E-13	2.55E-23	6.37E-08	1.66E-37	4.48E-07	0.80672165
52.9	3.14E-15	3.31E-13	2.45E-23	6.92E-08	1.29E-37	4.46E-07	0.8058138
53	3.35E-15	3.66E-13	3.15E-23	7.51E-08	1.27E-37	4.58E-07	0.80202045
53.1	3.32E-15	4.02E-13	3.49E-23	8.49E-08	1.36E-37	4.51E-07	0.81131152
53.2	3.16E-15	4.10E-13	3.51E-23	8.67E-08	1.21E-37	4.36E-07	0.81244333
53.3	2.62E-15	3.87E-13	3.43E-23	8.56E-08	1.05E-37	4.30E-07	0.8211676
53.4	2.54E-15	3.78E-13	3.68E-23	8.80E-08	9.67E-38	4.10E-07	0.81707795
53.5	2.32E-15	3.50E-13	3.71E-23	9.31E-08	7.72E-38	3.92E-07	0.82303029
53.6	2.02E-15	2.72E-13	3.12E-23	8.89E-08	4.57E-38	3.57E-07	0.82833216
53.7	1.73E-15	2.67E-13	3.17E-23	8.78E-08	3.26E-38	3.28E-07	0.829028
53.8	1.56E-15	2.27E-13	3.09E-23	8.66E-08	2.40E-38	2.83E-07	0.81903455
53.9	1.39E-15	2.21E-13	3.29E-23	8.42E-08	2.16E-38	2.78E-07	0.82360461
54	1.35E-15	1.96E-13	3.65E-23	7.56E-08	1.91E-38	2.56E-07	0.82567968
54.1	1.24E-15	2.05E-13	3.57E-23	7.44E-08	1.78E-38	2.44E-07	0.84171588
54.2	1.28E-15	2.03E-13	3.76E-23	7.58E-08	1.80E-38	2.22E-07	0.85294849
54.3	1.19E-15	2.13E-13	3.68E-23	8.08E-08	1.60E-38	2.16E-07	0.84981396
54.4	1.14E-15	2.12E-13	3.83E-23	7.80E-08	1.40E-38	2.21E-07	0.85419048
54.5	1.17E-15	2.10E-13	3.99E-23	7.64E-08	1.41E-38	2.16E-07	0.85563945
54.6	1.17E-15	2.18E-13	4.23E-23	7.86E-08	1.41E-38	2.17E-07	0.85397297
54.7	1.18E-15	2.23E-13	4.56E-23	7.70E-08	1.41E-38	2.22E-07	0.8451416
54.8	1.18E-15	2.23E-13	4.56E-23	7.70E-08	1.41E-38	2.22E-07	0.8451416
54.9	1.09E-15	2.11E-13	4.54E-23	8.00E-08	1.32E-38	2.05E-07	0.84779317
55	1.03E-15	2.11E-13	4.76E-23	8.24E-08	1.33E-38	1.99E-07	0.84996515
55.1	1.01E-15	2.14E-13	4.99E-23	8.39E-08	1.29E-38	1.93E-07	0.85010439
55.2	1.02E-15	2.16E-13	5.03E-23	8.79E-08	1.27E-38	1.99E-07	0.85513689
55.3	9.81E-16	2.02E-13	5.17E-23	8.75E-08	1.10E-38	2.01E-07	0.84409669
55.4	8.87E-16	1.74E-13	4.73E-23	8.71E-08	8.83E-39	1.79E-07	0.84544058
55.5	8.56E-16	1.53E-13	5.24E-23	8.66E-08	7.75E-39	1.78E-07	0.85750591
55.6	8.56E-16	1.53E-13	5.24E-23	8.66E-08	7.75E-39	1.78E-07	0.85750591
55.7	6.87E-16	1.34E-13	4.64E-23	8.46E-08	6.28E-39	1.64E-07	0.86594784
55.8	6.95E-16	1.26E-13	4.75E-23	8.52E-08	5.80E-39	1.59E-07	0.86936106
55.9	7.25E-16	1.30E-13	5.56E-23	8.26E-08	5.82E-39	1.62E-07	0.86956485
56	7.48E-16	1.21E-13	5.88E-23	8.24E-08	4.97E-39	1.60E-07	0.86611777
56.1	7.61E-16	1.18E-13	6.60E-23	8.80E-08	4.91E-39	1.45E-07	0.86536671
56.2	7.64E-16	1.13E-13	6.53E-23	9.04E-08	4.99E-39	1.42E-07	0.86585471
56.3	7.94E-16	1.13E-13	7.03E-23	8.65E-08	4.61E-39	1.35E-07	0.86974727
56.4	7.47E-16	1.15E-13	6.96E-23	8.49E-08	4.10E-39	1.30E-07	0.87788852
56.5	6.79E-16	1.08E-13	6.31E-23	8.80E-08	3.45E-39	1.26E-07	0.87895434
56.6	6.69E-16	1.05E-13	6.20E-23	8.94E-08	3.34E-39	1.24E-07	0.87927501
56.7	6.07E-16	9.81E-14	5.96E-23	8.87E-08	2.95E-39	1.17E-07	0.88422761
56.8	5.06E-16	9.21E-14	5.32E-23	8.25E-08	2.06E-39	1.08E-07	0.88894954

11.9	2.24E-07	2.00E-09	4.74E-32	3.94E-10	5.81E-15	5.31E-05	1.33E-16
12	2.02E-07	2.47E-09	7.05E-32	4.25E-10	4.88E-15	6.06E-05	2.83E-16
12.1	1.74E-07	2.04E-09	5.48E-32	5.12E-10	3.01E-15	5.43E-05	5.50E-16
12.2	1.63E-07	1.52E-09	6.03E-32	5.06E-10	2.44E-15	4.93E-05	1.11E-15
12.3	1.49E-07	1.88E-09	5.52E-32	6.36E-10	1.82E-15	4.65E-05	2.13E-15
12.4	1.39E-07	1.83E-09	6.43E-32	5.64E-10	1.46E-15	5.09E-05	4.63E-15
12.5	1.32E-07	2.37E-09	1.01E-31	6.19E-10	1.32E-15	6.23E-05	8.47E-15
12.6	1.16E-07	2.63E-09	1.09E-31	5.98E-10	8.14E-16	6.21E-05	1.33E-14
12.7	1.01E-07	2.61E-09	1.09E-31	5.82E-10	5.92E-16	6.58E-05	2.67E-14
12.8	9.66E-08	2.50E-09	1.10E-31	4.93E-10	3.82E-16	5.91E-05	5.21E-14
12.9	7.46E-08	2.10E-09	9.18E-32	5.29E-10	2.88E-16	5.34E-05	6.73E-14
13	6.49E-08	2.13E-09	8.60E-32	5.24E-10	1.84E-16	5.17E-05	1.50E-13
13.1	6.64E-08	2.39E-09	1.04E-31	5.64E-10	1.22E-16	5.08E-05	2.99E-13
13.2	5.51E-08	3.01E-09	1.05E-31	5.49E-10	1.03E-16	5.11E-05	4.63E-13
13.3	5.03E-08	3.07E-09	1.52E-31	6.29E-10	8.25E-17	5.84E-05	6.95E-13
13.4	4.43E-08	3.24E-09	2.06E-31	6.40E-10	6.96E-17	5.90E-05	1.24E-12
13.5	4.39E-08	3.71E-09	4.30E-31	8.02E-10	5.48E-17	6.60E-05	2.37E-12
13.6	4.53E-08	3.60E-09	3.88E-31	8.05E-10	4.86E-17	6.89E-05	3.35E-12
13.7	4.07E-08	3.57E-09	4.52E-31	9.51E-10	2.79E-17	6.27E-05	7.91E-12
13.8	3.72E-08	3.88E-09	4.56E-31	1.19E-09	2.27E-17	5.77E-05	1.28E-11
13.9	3.70E-08	3.61E-09	6.00E-31	1.14E-09	1.54E-17	5.80E-05	2.42E-11
14	3.57E-08	4.11E-09	6.48E-31	1.43E-09	1.48E-17	5.89E-05	4.10E-11
14.1	3.40E-08	4.49E-09	8.59E-31	1.78E-09	1.16E-17	6.24E-05	6.60E-11
14.2	3.58E-08	5.41E-09	1.52E-30	2.26E-09	8.03E-18	6.84E-05	1.52E-10
14.3	2.81E-08	5.21E-09	1.48E-30	2.02E-09	5.65E-18	6.41E-05	2.32E-10
14.4	2.68E-08	5.48E-09	2.13E-30	1.74E-09	4.89E-18	6.86E-05	4.44E-10
14.5	2.89E-08	5.31E-09	2.70E-30	1.83E-09	4.10E-18	6.41E-05	6.05E-10
14.6	2.74E-08	6.47E-09	3.58E-30	2.18E-09	4.29E-18	6.72E-05	1.04E-09
14.7	2.15E-08	6.20E-09	3.83E-30	2.35E-09	2.89E-18	6.13E-05	1.50E-09
14.8	2.03E-08	6.61E-09	5.57E-30	2.96E-09	2.66E-18	7.06E-05	2.19E-09
14.9	1.91E-08	6.86E-09	9.53E-30	3.86E-09	2.33E-18	6.52E-05	3.37E-09

60	2.39E-16	3.55E-14	1.93E-22	1.14E-07	2.26E-40	3.06E-08	0.92487677
60.1	2.39E-16	3.55E-14	1.93E-22	1.14E-07	2.26E-40	3.06E-08	0.92487677
60.2	2.45E-16	3.50E-14	2.12E-22	1.22E-07	2.26E-40	3.25E-08	0.92112656
60.3	2.53E-16	3.52E-14	2.22E-22	1.16E-07	2.12E-40	3.14E-08	0.92007982
60.4	2.44E-16	3.26E-14	2.42E-22	1.24E-07	2.17E-40	3.07E-08	0.92027233
60.5	2.30E-16	3.09E-14	2.43E-22	1.28E-07	2.07E-40	3.11E-08	0.92471228
60.6	2.53E-16	3.35E-14	2.66E-22	1.38E-07	2.34E-40	3.05E-08	0.92258488
60.7	2.41E-16	2.99E-14	2.95E-22	1.38E-07	2.35E-40	2.84E-08	0.91942016
60.8	2.17E-16	2.85E-14	2.99E-22	1.41E-07	2.10E-40	2.53E-08	0.92216497
60.9	2.16E-16	2.73E-14	3.00E-22	1.41E-07	2.07E-40	2.38E-08	0.92856966
61	2.06E-16	2.57E-14	3.13E-22	1.47E-07	2.05E-40	2.32E-08	0.9200974
61.1	2.12E-16	2.44E-14	3.43E-22	1.38E-07	1.82E-40	2.28E-08	0.92492155
61.2	1.89E-16	1.92E-14	2.97E-22	1.39E-07	1.34E-40	2.01E-08	0.93676827
61.3	1.78E-16	1.84E-14	3.14E-22	1.35E-07	1.30E-40	1.84E-08	0.93708931
61.4	1.67E-16	1.85E-14	3.41E-22	1.43E-07	1.18E-40	1.76E-08	0.93545999
61.5	1.62E-16	1.88E-14	3.70E-22	1.53E-07	1.20E-40	1.77E-08	0.93096722
61.6	1.50E-16	1.99E-14	4.52E-22	1.69E-07	1.34E-40	1.73E-08	0.92905861
61.7	1.43E-16	1.86E-14	4.98E-22	1.81E-07	1.18E-40	1.75E-08	0.93816931
61.8	1.56E-16	2.08E-14	6.30E-22	1.91E-07	1.35E-40	1.86E-08	0.94063257
61.9	1.60E-16	1.97E-14	6.92E-22	2.01E-07	1.29E-40	1.87E-08	0.95041131
62	1.45E-16	1.85E-14	6.65E-22	2.04E-07	1.09E-40	1.81E-08	0.93339375
62.1	1.38E-16	1.88E-14	6.76E-22	1.99E-07	1.04E-40	1.84E-08	0.93697744
62.2	1.38E-16	1.85E-14	6.67E-22	2.09E-07	1.04E-40	1.88E-08	0.93413018
62.3	1.30E-16	1.70E-14	6.80E-22	2.07E-07	1.02E-40	1.86E-08	0.93692
62.4	1.22E-16	1.69E-14	6.75E-22	2.06E-07	9.40E-41	1.83E-08	0.93780629
62.5	1.06E-16	1.52E-14	6.90E-22	2.14E-07	9.62E-41	1.82E-08	0.94421689
62.6	1.01E-16	1.48E-14	6.88E-22	2.19E-07	8.55E-41	1.82E-08	0.93765988
62.7	9.48E-17	1.47E-14	7.12E-22	2.00E-07	7.08E-41	1.64E-08	0.93628118
62.8	9.39E-17	1.44E-14	7.43E-22	2.04E-07	6.55E-41	1.65E-08	0.93887757
62.9	9.00E-17	1.38E-14	7.46E-22	2.04E-07	6.07E-41	1.61E-08	0.93693378
63	9.27E-17	1.30E-14	7.51E-22	2.15E-07	6.13E-41	1.56E-08	0.93483487
63.1	9.46E-17	1.41E-14	8.74E-22	2.21E-07	5.94E-41	1.65E-08	0.94609058
63.2	9.54E-17	1.33E-14	9.01E-22	2.20E-07	5.28E-41	1.53E-08	0.95381884
63.3	9.54E-17	1.24E-14	9.26E-22	2.09E-07	4.99E-41	1.47E-08	0.96433125
63.4	9.10E-17	1.11E-14	9.29E-22	2.05E-07	4.39E-41	1.41E-08	0.97013105
63.5	9.67E-17	1.18E-14	1.05E-21	2.03E-07	4.44E-41	1.40E-08	0.96238565
63.6	9.57E-17	1.17E-14	1.07E-21	2.02E-07	4.21E-41	1.37E-08	0.96580543
63.7	9.13E-17	1.03E-14	1.04E-21	1.97E-07	3.50E-41	1.37E-08	0.96552074
63.8	9.59E-17	9.90E-15	1.04E-21	1.93E-07	3.52E-41	1.33E-08	0.97090463
63.9	9.61E-17	9.68E-15	1.09E-21	2.00E-07	3.57E-41	1.40E-08	0.98074748
64	8.86E-17	8.69E-15	1.03E-21	2.04E-07	2.94E-41	1.37E-08	0.98607692
64.1	9.40E-17	8.45E-15	1.13E-21	2.04E-07	3.08E-41	1.24E-08	0.99285756
64.2	8.47E-17	7.72E-15	1.15E-21	1.88E-07	2.37E-41	1.13E-08	0.99936589
64.3	8.34E-17	7.58E-15	1.18E-21	1.98E-07	2.28E-41	1.12E-08	0.99929667
64.4	8.22E-17	7.23E-15	1.15E-21	1.92E-07	2.38E-41	1.10E-08	0.99631678
64.5	8.37E-17	7.67E-15	1.27E-21	1.97E-07	2.38E-41	1.06E-08	0.98717806
64.6	8.13E-17	7.26E-15	1.35E-21	1.83E-07	2.11E-41	9.92E-09	0.97473351
64.7	8.19E-17	7.32E-15	1.38E-21	1.79E-07	2.06E-41	9.74E-09	0.97367658
64.8	8.16E-17	7.12E-15	1.55E-21	1.81E-07	2.20E-41	9.89E-09	0.97704502
64.9	7.49E-17	6.40E-15	1.63E-21	1.85E-07	1.90E-41	9.28E-09	0.97774701
65	7.40E-17	6.33E-15	1.49E-21	1.85E-07	1.68E-41	8.98E-09	0.97709793
65.1	7.04E-17	5.88E-15	1.52E-21	1.79E-07	1.56E-41	8.63E-09	0.98239924
65.2	6.98E-17	5.67E-15	1.55E-21	1.75E-07	1.59E-41	9.19E-09	0.97992767
65.3	6.72E-17	5.24E-15	1.48E-21	1.69E-07	1.47E-41	9.03E-09	0.97342293
65.4	6.67E-17	4.86E-15	1.41E-21	1.72E-07	1.35E-41	8.90E-09	0.97015578
65.5	6.96E-17	4.99E-15	1.47E-21	1.79E-07	1.31E-41	8.45E-09	0.96973039
65.6	6.86E-17	4.96E-15	1.73E-21	1.57E-07	1.22E-41	8.87E-09	0.96253926
65.7	6.21E-17	4.98E-15	1.95E-21	1.58E-07	1.20E-41	9.17E-09	0.96827216
65.8	5.98E-17	5.40E-15	2.05E-21	1.63E-07	1.10E-41	8.99E-09	0.98391041
65.9	6.04E-17	5.07E-15	2.09E-21	1.69E-07	1.05E-41	8.69E-09	0.98732705
66	6.14E-17	4.83E-15	2.14E-21	1.71E-07	1.05E-41	8.85E-09	0.98058654
66.1	5.91E-17	4.81E-15	2.40E-21	1.75E-07	1.06E-41	8.89E-09	0.98019428
66.2	5.65E-17	4.51E-15	2.37E-21	1.78E-07	1.05E-41	9.05E-09	0.97792333
66.3	5.49E-17	4.75E-15	2.49E-21	1.85E-07	1.06E-41	8.32E-09	0.97277879
66.4	5.33E-17	4.78E-15	2.71E-21	1.87E-07	1.06E-41	7.94E-09	0.97967034
66.5	5.19E-17	5.15E-15	3.20E-21	1.90E-07	1.12E-41	8.43E-09	0.98049872
66.6	5.24E-17	4.85E-15	3.28E-21	1.90E-07	9.90E-42	7.84E-09	0.97823906
66.7	5.24E-17	4.85E-15	3.28E-21	1.90E-07	9.90E-42	7.84E-09	0.97823906
66.8	5.07E-17	4.99E-15	3.25E-21	1.97E-07	9.32E-42	7.07E-09	0.97580722
66.9	4.85E-17	4.79E-15	3.08E-21	2.00E-07	8.74E-42	7.02E-09	0.97139504
67	4.51E-17	4.45E-15	3.24E-21	2.03E-07	7.83E-42	6.20E-09	0.96323118
67.1	4.38E-17	4.38E-15	3.21E-21	2.05E-07	8.03E-42	6.04E-09	0.96375988
67.2	4.17E-17	4.34E-15	3.37E-21	2.01E-07	7.18E-42	5.61E-09	0.96973847
67.3	3.98E-17	3.81E-15	3.03E-21	2.00E-07	6.40E-42	5.51E-09	0.96758472
67.4	3.84E-17	3.37E-15	3.10E-21	2.08E-07	5.63E-42	5.57E-09	0.97626836
67.5	3.25E-17	2.85E-15	3.22E-21	2.06E-07	4.90E-42	5.57E-09	0.96912267
67.6	3.31E-17	2.74E-15	3.35E-21	2.08E-07	4.79E-42	5.40E-09	0.96200295
67.7	3.24E-17	2.39E-15	3.31E-21	2.12E-07	4.54E-42	5.24E-09	0.95339118
67.8	2.79E-17	2.16E-15	3.44E-21	1.96E-07	3.58E-42	5.26E-09	0.94765324
67.9	2.82E-17	2.09E-15	3.68E-21	2.07E-07	3.57E-42	5.04E-09	0.94056462
68	2.60E-17	1.97E-15	3.59E-21	2.12E-07	3.69E-42	5.22E-09	0.93987651
68.1	2.62E-17	1.92E-15	3.64E-21	2.27E-07	3.27E-42	4.87E-09	0.9416858
68.2	2.57E-17	1.87E-15	3.58E-21	2.26E-07	3.08E-42	4.93E-09	0.94517509
68.3	2.50E-17	1.77E-15	3.69E-21	2.18E-07	2.76E-42	4.79E-09	0.95242425
68.4	2.40E-17	1.67E-15	3.79E-21	2.20E-07	2.06E-42	4.46E-09	0.95929173
68.5	2.37E-17	1.61E-15	3.81E-21	2.25E-07	2.11E-42	4.52E-09	0.96425827
68.6	2.43E-17	1.51E-15	3.68E-21	2.37E-07	2.10E-42	4.47E-09	0.96662334
68.7	2.30E-17	1.46E-15	3.68E-21	2.38E-07	1.88E-42	4.20E-09	0.94821654
68.8	2.24E-17	1.44E-15	4.14E-21	2.42E-07	1.83E-42	3.95E-09	0.95248467
68.9	2.09E-17	1.34E-15	4.30E-21	2.35E-07	1.61E-42	3.81E-09	0.94735793

26.9	9.44E-11	7.95E-09	1.16E-25	1.11E-08	1.63E-26	4.44E-05	0.05393606
27	1.01E-10	7.70E-09	1.16E-25	1.04E-08	1.44E-26	4.44E-05	0.05532698
27.1	1.01E-10	7.43E-09	1.17E-25	1.01E-08	1.45E-26	4.43E-05	0.0559457
27.2	9.57E-11	6.38E-09	9.80E-26	1.01E-08	1.19E-26	4.46E-05	0.05946508
27.3	9.10E-11	5.84E-09	9.19E-26	1.02E-08	1.03E-26	4.47E-05	0.0607869
27.4	8.59E-11	5.85E-09	1.04E-25	9.98E-09	8.67E-27	4.30E-05	0.06381106
27.5	7.88E-11	5.90E-09	1.19E-25	1.00E-08	7.12E-27	4.36E-05	0.06522184
27.6	7.48E-11	5.42E-09	1.23E-25	1.01E-08	6.16E-27	4.28E-05	0.06775532
27.7	7.00E-11	5.34E-09	1.29E-25	9.56E-09	5.01E-27	4.27E-05	0.06859027
27.8	7.28E-11	5.18E-09	1.23E-25	9.65E-09	4.96E-27	4.06E-05	0.07074766
27.9	7.15E-11	5.04E-09	1.30E-25	9.36E-09	4.70E-27	3.92E-05	0.07288547
28	6.93E-11	5.38E-09	1.49E-25	9.32E-09	4.67E-27	3.97E-05	0.07517223
28.1	7.03E-11	5.33E-09	1.53E-25	9.31E-09	4.43E-27	3.89E-05	0.07584667
28.2	6.19E-11	5.70E-09	1.51E-25	9.07E-09	4.08E-27	3.87E-05	0.07994494
28.3	5.14E-11	5.19E-09	1.32E-25	8.52E-09	3.39E-27	4.03E-05	0.0838149
28.4	4.66E-11	4.92E-09	1.31E-25	8.81E-09	3.13E-27	3.90E-05	0.08579167
28.5	4.21E-11	4.50E-09	1.35E-25	8.58E-09	2.91E-27	3.72E-05	0.0910119
28.6	4.14E-11	4.37E-09	1.35E-25	8.48E-09	2.66E-27	3.61E-05	0.09358313
28.7	4.10E-11	4.38E-09	1.40E-25	8.51E-09	2.53E-27	3.49E-05	0.09462169
28.8	4.40E-11	3.99E-09	1.42E-25	7.65E-09	2.18E-27	3.21E-05	0.09506666
28.9	4.42E-11	3.82E-09	1.39E-25	6.95E-09	1.60E-27	3.14E-05	0.10102234
29	3.75E-11	3.59E-09	1.47E-25	6.46E-09	1.09E-27	3.25E-05	0.10270722
29.1	3.69E-11	3.50E-09	1.58E-25	6.58E-09	9.89E-28	3.11E-05	0.10603309
29.2	3.81E-11	3.63E-09	1.83E-25	7.03E-09	9.32E-28	3.07E-05	0.11086214
29.3	3.76E-11	3.61E-09	1.84E-25	7.11E-09	7.39E-28	2.98E-05	0.1122579
29.4	3.58E-11	3.71E-09	1.99E-25	7.57E-09	7.92E-28	2.97E-05	0.11693752
29.5	3.39E-11	3.65E-09	2.05E-25	7.69E-09	7.88E-28	2.84E-05	0.1212951
29.6	3.35E-11	3.80E-09	2.34E-25	7.55E-09	7.90E-28	2.82E-05	0.12328851
29.7	3.18E-11	3.37E-09	2.18E-25	7.00E-09	6.25E-28	2.51E-05	0.12660468
29.8	3.53E-11	3.24E-09	2.32E-25	7.21E-09	6.64E-28	2.55E-05	0.13280415
29.9	3.57E-11	3.03E-09	2.38E-25	7.20E-09	6.18E-28	2.54E-05	0.13600222

75	1.19E-17	2.46E-16	3.79E-20	5.39E-07	1.91E-43	9.92E-10	0.94046597
75.1	1.11E-17	2.40E-16	3.79E-20	5.14E-07	1.68E-43	9.50E-10	0.94406354
75.2	1.10E-17	2.44E-16	3.90E-20	5.24E-07	1.57E-43	9.53E-10	0.95282638
75.3	1.02E-17	2.48E-16	3.99E-20	5.45E-07	1.65E-43	9.38E-10	0.95111387
75.4	1.06E-17	2.68E-16	4.39E-20	5.62E-07	1.81E-43	8.77E-10	0.9397745
75.5	1.04E-17	2.69E-16	4.61E-20	5.86E-07	1.97E-43	8.52E-10	0.93655519
75.6	9.40E-18	2.54E-16	4.57E-20	5.89E-07	1.80E-43	7.96E-10	0.93533958
75.7	1.02E-17	2.76E-16	4.94E-20	6.20E-07	2.05E-43	8.46E-10	0.93337431
75.8	1.03E-17	2.64E-16	4.88E-20	6.12E-07	1.96E-43	8.18E-10	0.93383568
75.9	9.96E-18	2.50E-16	4.80E-20	6.11E-07	1.55E-43	7.27E-10	0.93172658
76	1.06E-17	2.58E-16	4.98E-20	6.18E-07	1.59E-43	7.10E-10	0.93415155
76.1	9.48E-18	2.76E-16	5.64E-20	6.48E-07	1.46E-43	7.32E-10	0.93214913
76.2	9.46E-18	2.62E-16	5.97E-20	6.37E-07	1.38E-43	6.68E-10	0.92278707
76.3	9.13E-18	2.75E-16	6.49E-20	6.84E-07	1.22E-43	6.51E-10	0.93941615
76.4	9.28E-18	2.70E-16	6.64E-20	7.00E-07	1.17E-43	6.44E-10	0.93276354
76.5	9.43E-18	2.59E-16	7.08E-20	6.86E-07	1.23E-43	6.19E-10	0.93226662
76.6	9.01E-18	2.78E-16	7.93E-20	6.88E-07	1.08E-43	6.20E-10	0.93415842
76.7	9.03E-18	2.74E-16	7.90E-20	7.07E-07	1.14E-43	6.23E-10	0.93066079
76.8	8.59E-18	2.63E-16	7.93E-20	7.01E-07	1.15E-43	6.25E-10	0.93054352
76.9	8.14E-18	2.55E-16	7.92E-20	7.59E-07	1.20E-43	6.14E-10	0.93171298
77	7.72E-18	2.31E-16	8.34E-20	8.17E-07	1.21E-43	6.09E-10	0.93168739
77.1	8.59E-18	2.02E-16	7.86E-20	8.55E-07	1.11E-43	5.98E-10	0.94392931
77.2	8.30E-18	2.11E-16	8.12E-20	8.62E-07	1.08E-43	5.71E-10	0.94576257
77.3	8.99E-18	2.17E-16	9.04E-20	8.82E-07	1.10E-43	5.29E-10	0.94130858
77.4	9.33E-18	2.16E-16	1.05E-19	9.38E-07	1.10E-43	5.02E-10	0.92878783
77.5	9.48E-18	2.12E-16	1.03E-19	9.36E-07	1.07E-43	4.96E-10	0.93164466
77.6	9.85E-18	2.15E-16	1.17E-19	9.37E-07	1.13E-43	4.91E-10	0.93186282
77.7	1.00E-17	2.12E-16	1.16E-19	9.93E-07	1.06E-43	4.73E-10	0.93210952
77.8	1.00E-17	2.12E-16	1.16E-19	9.93E-07	1.06E-43	4.73E-10	0.93210952
77.9	9.57E-18	1.95E-16	1.23E-19	9.87E-07	9.44E-44	4.64E-10	0.93334284
78	9.87E-18	1.99E-16	1.24E-19	9.48E-07	9.39E-44	4.61E-10	0.93123081
78.1	9.91E-18	1.85E-16	1.27E-19	8.99E-07	8.01E-44	4.45E-10	0.93454461
78.2	9.43E-18	1.79E-16	1.32E-19	9.55E-07	7.28E-44	4.06E-10	0.94396455
78.3	9.48E-18	1.73E-16	1.29E-19	9.81E-07	7.23E-44	3.94E-10	0.94050242
78.4	1.12E-17	1.68E-16	1.50E-19	1.08E-06	7.79E-44	3.88E-10	0.93874424
78.5	1.11E-17	1.63E-16	1.50E-19	1.06E-06	7.69E-44	3.90E-10	0.93453231
78.6	1.10E-17	1.50E-16	1.56E-19	1.03E-06	6.75E-44	3.83E-10	0.94118429
78.7	1.32E-17	1.64E-16	1.72E-19	1.07E-06	8.09E-44	3.43E-10	0.9221662
78.8	1.23E-17	1.55E-16	1.76E-19	1.07E-06	7.44E-44	3.33E-10	0.92454478
78.9	1.25E-17	1.61E-16	1.76E-19	1.09E-06	7.39E-44	3.29E-10	0.92073448
79	1.29E-17	1.56E-16	1.76E-19	1.05E-06	7.10E-44	3.21E-10	0.91919561
79.1	1.26E-17	1.64E-16	1.75E-19	1.06E-06	7.33E-44	3.09E-10	0.92006187
79.2	1.17E-17	1.61E-16	1.73E-19	1.08E-06	6.55E-44	2.92E-10	0.92600714
79.3	1.17E-17	1.52E-16	1.69E-19	1.09E-06	6.67E-44	3.00E-10	0.93170131
79.4	1.20E-17	1.51E-16	1.66E-19	1.08E-06	6.82E-44	3.11E-10	0.93001787
79.5	1.30E-17	1.47E-16	1.76E-19	1.08E-06	6.83E-44	3.16E-10	0.92385789
79.6	1.43E-17	1.51E-16	1.94E-19	1.11E-06	7.31E-44	3.06E-10	0.92641355
79.7	1.36E-17	1.52E-16	2.14E-19	1.12E-06	7.22E-44	2.97E-10	0.92355841
79.8	1.46E-17	1.62E-16	2.46E-19	1.12E-06	7.72E-44	2.92E-10	0.91483023
79.9	1.46E-17	1.62E-16	2.46E-19	1.12E-06	7.72E-44	2.92E-10	0.91483023
80	1.42E-17	1.54E-16	2.55E-19	1.13E-06	6.77E-44	2.81E-10	0.91766253
80.1	1.38E-17	1.56E-16	2.73E-19	1.21E-06	7.10E-44	2.74E-10	0.91034835
80.2	1.42E-17	1.59E-16	2.71E-19	1.15E-06	5.85E-44	2.62E-10	0.90809962
80.3	1.46E-17	1.56E-16	2.83E-19	1.11E-06	5.74E-44	2.54E-10	0.90529623
80.4	1.46E-17	1.56E-16	2.83E-19	1.11E-06	5.74E-44	2.54E-10	0.90529623
80.5	1.36E-17	1.60E-16	2.85E-19	1.11E-06	4.93E-44	2.40E-10	0.90876603
80.6	1.34E-17	1.62E-16	2.75E-19	1.12E-06	4.93E-44	2.46E-10	0.90631166
80.7	1.33E-17	1.64E-16	2.70E-19	1.26E-06	5.43E-44	2.36E-10	0.89953755
80.8	1.31E-17	1.62E-16	2.74E-19	1.25E-06	5.34E-44	2.46E-10	0.89962073
80.9	1.32E-17	1.64E-16	2.93E-19	1.27E-06	5.05E-44	2.31E-10	0.89244425
81	1.28E-17	1.60E-16	3.01E-19	1.38E-06	5.02E-44	2.05E-10	0.87966174
81.1	1.35E-17	1.45E-16	3.25E-19	1.55E-06	5.00E-44	1.88E-10	0.8758928
81.2	1.38E-17	1.37E-16	3.25E-19	1.62E-06	5.33E-44	1.86E-10	0.86478341
81.3	1.42E-17	1.30E-16	3.47E-19	1.61E-06	4.63E-44	1.82E-10	0.85799627
81.4	1.38E-17	1.26E-16	3.43E-19	1.66E-06	4.66E-44	1.78E-10	0.85869984
81.5	1.35E-17	1.17E-16	3.45E-19	1.74E-06	4.36E-44	1.72E-10	0.85958752
81.6	1.36E-17	1.21E-16	3.54E-19	1.74E-06	4.32E-44	1.67E-10	0.86416888
81.7	1.35E-17	1.19E-16	4.17E-19	1.76E-06	4.91E-44	1.56E-10	0.8591594
81.8	1.44E-17	1.22E-16	4.70E-19	1.82E-06	5.04E-44	1.61E-10	0.84993054
81.9	1.43E-17	1.17E-16	4.92E-19	1.89E-06	4.44E-44	1.60E-10	0.84951524
82	1.41E-17	1.21E-16	4.91E-19	1.95E-06	4.64E-44	1.51E-10	0.84330422
82.1	1.34E-17	1.14E-16	4.97E-19	1.96E-06	4.94E-44	1.50E-10	0.83557653
82.2	1.46E-17	1.16E-16	5.09E-19	2.07E-06	5.02E-44	1.60E-10	0.83914226
82.3	1.54E-17	1.16E-16	5.02E-19	2.12E-06	5.38E-44	1.59E-10	0.83427719
82.4	1.56E-17	1.19E-16	5.23E-19	2.12E-06	5.70E-44	1.59E-10	0.83360148
82.5	1.74E-17	1.35E-16	6.12E-19	2.06E-06	6.46E-44	1.58E-10	0.82409614
82.6	1.73E-17	1.32E-16	6.35E-19	1.98E-06	6.17E-44	1.56E-10	0.83110895
82.7	1.98E-17	1.32E-16	6.91E-19	2.01E-06	6.68E-44	1.50E-10	0.8293609
82.8	2.00E-17	1.21E-16	6.99E-19	2.04E-06	6.29E-44	1.44E-10	0.83358219
82.9	2.02E-17	1.23E-16	7.64E-19	2.28E-06	7.74E-44	1.37E-10	0.82771372
83	2.00E-17	1.28E-16	8.45E-19	2.18E-06	7.33E-44	1.34E-10	0.8210983
83.1	2.04E-17	1.36E-16	8.70E-19	2.19E-06	7.88E-44	1.37E-10	0.81343971
83.2	1.98E-17	1.32E-16	9.09E-19	2.24E-06	7.55E-44	1.32E-10	0.81318314
83.3	1.83E-17	1.26E-16	9.02E-19	2.17E-06	6.56E-44	1.29E-10	0.81986206
83.4	1.72E-17	1.20E-16	8.77E-19	2.13E-06	5.69E-44	1.19E-10	0.81924073
83.5	1.64E-17	1.21E-16	9.49E-19	2.25E-06	5.86E-44	1.16E-10	0.81166626
83.6	1.73E-17	1.27E-16	1.10E-18	2.42E-06	6.95E-44	1.24E-10	0.80846082
83.7	1.79E-17	1.28E-16	1.32E-18	2.51E-06	7.99E-44	1.24E-10	0.81146743
83.8	1.71E-17	1.26E-16	1.33E-18	2.62E-06	7.96E-44	1.16E-10	0.81263418
83.9	1.61E-17	1.38E-16	1.57E-18	2.76E-06	7.92E-44	1.12E-10	0.81153109

41.9	6.73E-13	3.85E-11	7.01E-24	1.51E-08	2.42E-33	9.72E-06	0.46875632
42	6.18E-13	3.85E-11	7.13E-24	1.66E-08	2.08E-33	9.74E-06	0.47896213
42.1	5.81E-13	3.62E-11	6.81E-24	1.63E-08	1.89E-33	9.46E-06	0.48036781
42.2	5.55E-13	3.27E-11	6.61E-24	1.62E-08	1.60E-33	9.40E-06	0.48164048
42.3	5.03E-13	3.11E-11	6.11E-24	1.72E-08	1.43E-33	9.09E-06	0.48293488
42.4	4.87E-13	3.16E-11	6.24E-24	1.77E-08	1.33E-33	9.26E-06	0.48164497
42.5	4.39E-13	2.90E-11	6.13E-24	1.66E-08	1.05E-33	8.61E-06	0.49126499
42.6	4.06E-13	2.78E-11	6.23E-24	1.61E-08	9.32E-34	8.54E-06	0.49046914
42.7	3.95E-13	2.75E-11	6.47E-24	1.62E-08	9.28E-34	8.82E-06	0.48791773
42.8	3.67E-13	2.73E-11	6.43E-24	1.57E-08	8.26E-34	8.70E-06	0.49555613
42.9	3.36E-13	2.74E-11	6.47E-24	1.66E-08	7.44E-34	8.62E-06	0.49552395
43	2.73E-13	2.84E-11	5.91E-24	1.61E-08	6.40E-34	8.00E-06	0.50774697
43.1	2.41E-13	2.76E-11	5.97E-24	1.70E-08	5.34E-34	7.57E-06	0.50716479
43.2	2.38E-13	2.64E-11	6.78E-24	1.89E-08	5.62E-34	7.63E-06	0.50349183
43.3	2.16E-13	2.31E-11	5.88E-24	1.85E-08	4.44E-34	7.23E-06	0.49997338
43.4	2.35E-13	2.05E-11	6.09E-24	1.85E-08	3.41E-34	7.45E-06	0.50370505
43.5	2.18E-13	2.06E-11	6.80E-24	1.81E-08	2.72E-34	7.20E-06	0.52258607
43.6	2.18E-13	2.12E-11	6.78E-24	1.82E-08	2.38E-34	6.90E-06	0.52346085
43.7	1.88E-13	2.11E-11	6.80E-24	1.87E-08	2.02E-34	6.55E-06	0.52549659
43.8	1.84E-13	2.12E-11	6.77E-24	1.81E-08	1.90E-34	6.40E-06	0.52668093
43.9	1.85E-13	2.07E-11	7.29E-24	1.84E-08	1.88E-34	6.38E-06	0.52430983
44	1.76E-13	1.81E-11	6.93E-24	1.92E-08	1.63E-34	6.27E-06	0.52284502
44.1	1.91E-13	1.71E-11	7.39E-24	1.79E-08	1.41E-34	6.11E-06	0.53401212
44.2	1.88E-13	1.53E-11	7.07E-24	1.80E-08	1.33E-34	5.62E-06	0.54414288
44.3	1.65E-13	1.40E-11	7.50E-24	1.84E-08	1.20E-34	5.54E-06	0.54995818
44.4	1.51E-13	1.12E-11	6.60E-24	1.70E-08	1.00E-34	5.37E-06	0.55779176
44.5	1.42E-13	1.09E-11	6.73E-24	1.77E-08	1.05E-34	5.00E-06	0.56482726
44.6	1.44E-13	1.05E-11	6.85E-24	1.81E-08	9.87E-35	5.00E-06	0.56629245
44.7	1.28E-13	9.54E-12	7.00E-24	1.87E-08	8.86E-35	4.73E-06	0.57578458
44.8	1.21E-13	9.29E-12	8.03E-24	1.99E-08	8.21E-35	4.92E-06	0.58224826
44.9	1.22E-13	8.70E-12	7.95E-24	1.99E-08	8.01E-35	4.99E-06	0.58698562

90	4.08E-17	1.63E-16	3.26E-17	7.30E-06	1.37E-43	4.28E-11	0.66989291
90.1	4.17E-17	1.57E-16	3.64E-17	7.58E-06	1.43E-43	4.13E-11	0.66389735
90.2	4.31E-17	1.55E-16	3.53E-17	7.56E-06	1.45E-43	3.99E-11	0.66218754
90.3	4.35E-17	1.61E-16	4.10E-17	7.25E-06	1.48E-43	4.02E-11	0.65694384
90.4	4.50E-17	1.81E-16	4.21E-17	7.15E-06	1.45E-43	3.83E-11	0.64797961
90.5	4.41E-17	1.78E-16	4.34E-17	7.33E-06	1.53E-43	3.83E-11	0.64632318
90.6	4.30E-17	1.82E-16	4.45E-17	7.49E-06	1.44E-43	3.72E-11	0.64060301
90.7	4.60E-17	1.69E-16	4.91E-17	8.01E-06	1.53E-43	3.73E-11	0.64548238
90.8	5.17E-17	1.78E-16	5.24E-17	8.26E-06	1.79E-43	3.65E-11	0.63871682
90.9	4.82E-17	1.70E-16	6.24E-17	8.45E-06	2.02E-43	3.49E-11	0.6424232
91	5.00E-17	1.59E-16	6.46E-17	8.34E-06	1.94E-43	3.43E-11	0.64372044
91.1	4.58E-17	1.62E-16	6.55E-17	8.32E-06	1.76E-43	3.33E-11	0.64838537
91.2	4.37E-17	1.63E-16	6.47E-17	8.63E-06	1.67E-43	3.25E-11	0.64482451
91.3	4.17E-17	1.69E-16	6.76E-17	8.54E-06	1.65E-43	3.05E-11	0.63793582
91.4	4.25E-17	1.64E-16	6.89E-17	8.46E-06	1.51E-43	3.05E-11	0.6338858
91.5	4.98E-17	1.83E-16	7.66E-17	8.92E-06	1.91E-43	3.28E-11	0.63081714
91.6	5.10E-17	1.78E-16	8.30E-17	9.01E-06	1.91E-43	3.29E-11	0.62406266
91.7	5.47E-17	1.57E-16	8.86E-17	8.92E-06	1.75E-43	3.56E-11	0.62505428
91.8	5.58E-17	1.69E-16	1.01E-16	8.92E-06	1.89E-43	3.68E-11	0.62316666
91.9	5.40E-17	1.62E-16	1.03E-16	9.48E-06	1.86E-43	3.66E-11	0.62624259
92	5.07E-17	1.45E-16	9.99E-17	9.41E-06	1.66E-43	3.29E-11	0.62302923
92.1	5.29E-17	1.75E-16	1.23E-16	9.54E-06	1.68E-43	3.70E-11	0.62287629
92.2	5.17E-17	1.73E-16	1.22E-16	9.58E-06	1.58E-43	3.51E-11	0.62095585
92.3	5.44E-17	1.66E-16	1.35E-16	9.59E-06	1.62E-43	3.56E-11	0.61749663
92.4	5.04E-17	1.62E-16	1.46E-16	9.81E-06	1.54E-43	3.46E-11	0.60938246
92.5	5.35E-17	1.47E-16	1.46E-16	9.96E-06	1.65E-43	3.44E-11	0.60315516
92.6	5.82E-17	1.44E-16	1.64E-16	9.88E-06	1.67E-43	3.51E-11	0.60610115
92.7	5.77E-17	1.49E-16	1.66E-16	9.90E-06	1.60E-43	3.36E-11	0.59666013
92.8	5.71E-17	1.47E-16	1.75E-16	1.02E-05	1.51E-43	3.15E-11	0.60018476
92.9	6.10E-17	1.54E-16	1.85E-16	1.01E-05	1.57E-43	3.20E-11	0.60095148
93	6.09E-17	1.43E-16	1.79E-16	1.00E-05	1.56E-43	3.27E-11	0.60512557
93.1	6.09E-17	1.43E-16	1.79E-16	1.00E-05	1.56E-43	3.27E-11	0.60512557
93.2	6.09E-17	1.43E-16	1.79E-16	1.00E-05	1.56E-43	3.27E-11	0.60512557
93.3	6.11E-17	1.46E-16	2.09E-16	1.03E-05	1.76E-43	3.20E-11	0.60014842
93.4	5.82E-17	1.48E-16	2.00E-16	1.05E-05	1.75E-43	3.12E-11	0.59267536
93.5	5.82E-17	1.48E-16	2.00E-16	1.05E-05	1.75E-43	3.12E-11	0.59267536
93.6	6.10E-17	1.47E-16	2.10E-16	1.10E-05	2.02E-43	3.28E-11	0.59203654
93.7	5.48E-17	1.45E-16	2.15E-16	1.12E-05	2.01E-43	3.01E-11	0.58942692
93.8	5.35E-17	1.48E-16	2.34E-16	1.14E-05	1.98E-43	2.92E-11	0.58583171
93.9	5.73E-17	1.67E-16	2.57E-16	1.14E-05	2.32E-43	3.04E-11	0.59452473
94	6.67E-17	1.97E-16	2.96E-16	1.22E-05	2.65E-43	3.33E-11	0.5873023
94.1	6.67E-17	1.97E-16	2.96E-16	1.22E-05	2.65E-43	3.33E-11	0.5873023
94.2	6.63E-17	2.05E-16	3.35E-16	1.22E-05	2.96E-43	3.48E-11	0.58407771
94.3	6.81E-17	2.03E-16	3.58E-16	1.23E-05	3.08E-43	3.42E-11	0.58893986
94.4	7.19E-17	2.32E-16	3.91E-16	1.29E-05	3.43E-43	3.30E-11	0.58957638
94.5	7.80E-17	2.36E-16	4.30E-16	1.33E-05	3.66E-43	3.43E-11	0.57749984
94.6	8.18E-17	2.59E-16	4.80E-16	1.35E-05	4.28E-43	3.75E-11	0.57274096
94.7	7.51E-17	2.59E-16	4.91E-16	1.38E-05	3.60E-43	3.53E-11	0.57491144
94.8	8.20E-17	2.45E-16	5.23E-16	1.37E-05	3.92E-43	3.56E-11	0.5757802
94.9	8.13E-17	2.45E-16	5.44E-16	1.35E-05	3.78E-43	3.64E-11	0.57715319
95	8.22E-17	2.49E-16	5.52E-16	1.33E-05	3.75E-43	3.56E-11	0.57622973
95.1	8.09E-17	2.48E-16	5.76E-16	1.33E-05	3.66E-43	3.67E-11	0.58415536
95.2	7.58E-17	2.28E-16	5.63E-16	1.32E-05	3.30E-43	3.40E-11	0.57968268
95.3	7.79E-17	2.24E-16	5.64E-16	1.36E-05	3.33E-43	3.28E-11	0.57558747
95.4	7.79E-17	2.24E-16	5.64E-16	1.36E-05	3.33E-43	3.28E-11	0.57558747
95.5	7.84E-17	2.18E-16	5.83E-16	1.32E-05	3.37E-43	3.24E-11	0.57256628
95.6	8.50E-17	2.26E-16	6.31E-16	1.34E-05	3.51E-43	3.27E-11	0.57100671
95.7	8.10E-17	2.14E-16	6.46E-16	1.27E-05	3.39E-43	3.26E-11	0.57757438
95.8	8.28E-17	2.04E-16	6.63E-16	1.28E-05	3.33E-43	3.20E-11	0.57765079
95.9	8.32E-17	1.92E-16	6.49E-16	1.28E-05	3.22E-43	3.19E-11	0.57814591
96	8.98E-17	1.92E-16	6.89E-16	1.29E-05	3.26E-43	3.17E-11	0.57530204
96.1	9.72E-17	1.81E-16	6.91E-16	1.27E-05	3.22E-43	3.05E-11	0.57415152
96.2	1.02E-16	1.82E-16	6.87E-16	1.26E-05	3.27E-43	2.76E-11	0.57556292
96.3	1.07E-16	1.89E-16	8.07E-16	1.38E-05	3.39E-43	2.63E-11	0.57859922
96.4	1.07E-16	1.89E-16	8.07E-16	1.38E-05	3.39E-43	2.63E-11	0.57859922
96.5	1.20E-16	1.91E-16	9.13E-16	1.39E-05	3.66E-43	2.72E-11	0.57437562
96.6	1.22E-16	1.74E-16	8.62E-16	1.34E-05	3.46E-43	2.59E-11	0.56595165
96.7	1.22E-16	1.74E-16	8.62E-16	1.34E-05	3.46E-43	2.59E-11	0.56595165
96.8	1.37E-16	1.90E-16	9.25E-16	1.34E-05	3.62E-43	2.58E-11	0.56770313
96.9	1.41E-16	2.01E-16	9.86E-16	1.33E-05	3.70E-43	2.60E-11	0.56508568
97	1.43E-16	1.98E-16	1.02E-15	1.38E-05	3.76E-43	2.67E-11	0.57008507
97.1	1.49E-16	2.07E-16	1.04E-15	1.39E-05	3.92E-43	2.68E-11	0.56859291
97.2	1.77E-16	2.12E-16	1.11E-15	1.35E-05	4.71E-43	2.72E-11	0.56021135
97.3	1.65E-16	2.12E-16	1.13E-15	1.30E-05	4.18E-43	2.72E-11	0.55805117
97.4	1.59E-16	2.19E-16	1.14E-15	1.36E-05	4.51E-43	2.60E-11	0.55596442
97.5	1.60E-16	2.20E-16	1.10E-15	1.45E-05	4.98E-43	2.67E-11	0.55555934
97.6	1.50E-16	2.08E-16	1.21E-15	1.41E-05	4.72E-43	2.69E-11	0.55355651
97.7	1.55E-16	2.24E-16	1.27E-15	1.36E-05	5.09E-43	2.69E-11	0.54661341
97.8	1.61E-16	2.36E-16	1.42E-15	1.49E-05	6.14E-43	2.69E-11	0.54821426
97.9	1.61E-16	2.39E-16	1.38E-15	1.47E-05	5.98E-43	2.74E-11	0.55016194
98	1.64E-16	2.57E-16	1.50E-15	1.49E-05	6.70E-43	2.82E-11	0.54543882
98.1	1.76E-16	2.69E-16	1.63E-15	1.52E-05	7.39E-43	2.65E-11	0.53854785
98.2	2.01E-16	2.91E-16	1.75E-15	1.58E-05	8.84E-43	2.84E-11	0.53423494
98.3	2.07E-16	3.07E-16	1.83E-15	1.62E-05	9.49E-43	2.92E-11	0.53420866
98.4	2.17E-16	3.21E-16	1.95E-15	1.63E-05	9.44E-43	2.85E-11	0.53204541
98.5	2.17E-16	3.35E-16	2.02E-15	1.60E-05	8.34E-43	2.65E-11	0.53390838
98.6	2.17E-16	3.36E-16	2.05E-15	1.57E-05	8.53E-43	2.69E-11	0.53069775
98.7	2.17E-16	3.36E-16	2.05E-15	1.57E-05	8.53E-43	2.69E-11	0.53069775
98.8	2.24E-16	3.31E-16	2.05E-15	1.59E-05	8.74E-43	2.72E-11	0.52657827
98.9	2.22E-16	3.35E-16	2.11E-15	1.59E-05	8.87E-43	2.64E-11	0.52117037

56.9	4.73E-16	8.52E-14	5.38E-23	8.57E-08	1.77E-39	1.02E-07	0.89012897
57	4.79E-16	8.64E-14	5.60E-23	8.81E-08	1.35E-39	1.01E-07	0.8893703
57.1	4.16E-16	7.97E-14	5.88E-23	9.06E-08	1.24E-39	9.47E-08	0.88854862
57.2	4.29E-16	7.58E-14	6.34E-23	8.98E-08	1.17E-39	9.03E-08	0.89188505
57.3	4.45E-16	7.34E-14	6.57E-23	9.01E-08	1.12E-39	8.86E-08	0.886469
57.4	4.39E-16	7.04E-14	6.73E-23	8.80E-08	1.08E-39	8.92E-08	0.88752627
57.5	4.39E-16	7.17E-14	7.11E-23	8.98E-08	1.17E-39	9.03E-08	0.8879255
57.6	4.50E-16	5.94E-14	7.94E-23	9.13E-08	1.09E-39	8.34E-08	0.89819825
57.7	4.60E-16	6.12E-14	8.83E-23	9.15E-08	1.20E-39	8.77E-08	0.8990943
57.8	4.37E-16	6.28E-14	8.68E-23	8.74E-08	1.09E-39	8.19E-08	0.90615064
57.9	4.47E-16	5.56E-14	8.36E-23	8.47E-08	9.55E-40	7.75E-08	0.90844882
58	4.66E-16	5.77E-14	8.19E-23	8.29E-08	8.20E-40	7.30E-08	0.90943367
58.1	4.55E-16	5.72E-14	9.91E-23	8.91E-08	8.34E-40	6.95E-08	0.91255579
58.2	4.25E-16	5.66E-14	1.05E-22	8.93E-08	9.06E-40	6.95E-08	0.92839935
58.3	3.90E-16	5.10E-14	1.01E-22	9.42E-08	8.47E-40	6.52E-08	0.93391491
58.4	3.72E-16	5.17E-14	9.81E-23	9.29E-08	7.96E-40	6.46E-08	0.94075554
58.5	3.66E-16	4.98E-14	9.85E-23	9.12E-08	7.49E-40	5.98E-08	0.94447742
58.6	3.27E-16	4.72E-14	9.57E-23	9.21E-08	6.21E-40	4.91E-08	0.93868935
58.7	3.32E-16	4.50E-14	9.33E-23	8.98E-08	5.25E-40	4.73E-08	0.92952355
58.8	2.99E-16	3.94E-14	1.07E-22	9.30E-08	4.43E-40	4.88E-08	0.91585386
58.9	2.64E-16	3.84E-14	1.08E-22	9.21E-08	3.50E-40	4.25E-08	0.90835582
59	2.58E-16	3.55E-14	1.16E-22	9.32E-08	3.62E-40	4.17E-08	0.90782729
59.1	2.52E-16	3.41E-14	1.15E-22	9.58E-08	3.51E-40	4.05E-08	0.91254525
59.2	2.47E-16	3.42E-14	1.23E-22	1.02E-07	3.32E-40	3.86E-08	0.91343553
59.3	2.40E-16	3.56E-14	1.44E-22	1.07E-07	3.17E-40	3.71E-08	0.92992059
59.4	2.43E-16	3.61E-14	1.61E-22	1.02E-07	2.75E-40	3.57E-08	0.92269498
59.5	2.43E-16	3.61E-14	1.61E-22	1.02E-07	2.75E-40	3.57E-08	0.92269498
59.6	2.41E-16	3.38E-14	1.57E-22	1.09E-07	2.32E-40	3.57E-08	0.92879219
59.7	2.32E-16	3.45E-14	1.69E-22	1.09E-07	2.32E-40	3.37E-08	0.93131041
59.8	2.28E-16	3.36E-14	1.70E-22	1.14E-07	2.21E-40	3.25E-08	0.92605541
59.9	2.37E-16	3.59E-14	1.86E-22	1.12E-07	2.20E-40	3.07E-08	0.92745034

105	7.21E-16	5.48E-16	1.09E-14	3.01E-05	3.79E-42	3.94E-11	0.48996631
105.1	7.24E-16	5.37E-16	1.01E-14	3.04E-05	3.73E-42	3.95E-11	0.49208607
105.2	7.02E-16	5.57E-16	1.01E-14	2.97E-05	3.77E-42	3.93E-11	0.50022454
105.3	7.02E-16	5.61E-16	1.05E-14	2.96E-05	3.83E-42	3.90E-11	0.50257344
105.4	7.63E-16	5.42E-16	1.11E-14	2.82E-05	4.02E-42	4.02E-11	0.50515411
105.5	8.27E-16	5.38E-16	1.11E-14	2.86E-05	4.45E-42	4.15E-11	0.50205234
105.6	8.14E-16	5.49E-16	1.06E-14	2.99E-05	4.66E-42	4.12E-11	0.50253266
105.7	8.19E-16	5.37E-16	1.06E-14	3.12E-05	4.82E-42	4.23E-11	0.50695208
105.8	8.32E-16	5.39E-16	1.05E-14	3.11E-05	4.59E-42	4.29E-11	0.51281119
105.9	8.17E-16	5.49E-16	1.12E-14	3.12E-05	4.74E-42	4.19E-11	0.50816576
106	8.77E-16	5.72E-16	1.19E-14	3.08E-05	4.79E-42	4.08E-11	0.50707302
106.1	8.41E-16	5.53E-16	1.15E-14	3.10E-05	4.62E-42	3.91E-11	0.50510957
106.2	8.71E-16	6.21E-16	1.30E-14	3.17E-05	4.84E-42	3.74E-11	0.50727827
106.3	8.75E-16	6.27E-16	1.23E-14	3.11E-05	4.61E-42	3.85E-11	0.50695131
106.4	9.13E-16	6.24E-16	1.30E-14	3.16E-05	4.22E-42	3.81E-11	0.514771
106.5	9.24E-16	6.05E-16	1.28E-14	3.26E-05	4.29E-42	3.78E-11	0.51636124
106.6	1.01E-15	6.47E-16	1.38E-14	3.32E-05	4.78E-42	3.99E-11	0.51442641
106.7	1.01E-15	6.47E-16	1.38E-14	3.32E-05	4.78E-42	3.99E-11	0.51442641
106.8	9.79E-16	6.07E-16	1.38E-14	3.29E-05	4.45E-42	4.10E-11	0.51907195
106.9	9.96E-16	6.14E-16	1.39E-14	3.12E-05	4.30E-42	3.87E-11	0.5142446
107	9.59E-16	6.01E-16	1.39E-14	3.29E-05	4.37E-42	3.75E-11	0.51564933
107.1	9.59E-16	6.01E-16	1.39E-14	3.29E-05	4.37E-42	3.75E-11	0.51564933
107.2	9.86E-16	6.19E-16	1.38E-14	3.30E-05	4.22E-42	3.72E-11	0.51666288
107.3	1.05E-15	6.46E-16	1.42E-14	3.32E-05	4.42E-42	3.65E-11	0.51423566
107.4	1.10E-15	6.97E-16	1.51E-14	3.33E-05	4.36E-42	3.56E-11	0.5117279
107.5	1.17E-15	6.89E-16	1.63E-14	3.43E-05	4.69E-42	3.74E-11	0.50403036
107.6	1.17E-15	6.89E-16	1.63E-14	3.43E-05	4.69E-42	3.74E-11	0.50403036
107.7	1.13E-15	6.87E-16	1.59E-14	3.45E-05	4.33E-42	3.62E-11	0.50412198
107.8	1.16E-15	7.56E-16	1.64E-14	3.33E-05	4.03E-42	3.49E-11	0.5133375
107.9	1.27E-15	7.19E-16	1.63E-14	3.36E-05	3.57E-42	3.20E-11	0.51780995
108	1.33E-15	6.84E-16	1.75E-14	3.49E-05	3.29E-42	3.27E-11	0.51992218
108.1	1.33E-15	6.84E-16	1.75E-14	3.49E-05	3.29E-42	3.27E-11	0.51992218
108.2	1.32E-15	6.35E-16	1.75E-14	3.45E-05	3.16E-42	3.11E-11	0.51329299
108.3	1.32E-15	6.35E-16	1.75E-14	3.45E-05	3.16E-42	3.11E-11	0.51329299
108.4	1.44E-15	6.59E-16	1.93E-14	3.40E-05	3.45E-42	3.22E-11	0.52022787
108.5	1.65E-15	7.09E-16	2.13E-14	3.40E-05	3.99E-42	3.25E-11	0.51866138
108.6	1.72E-15	7.04E-16	2.07E-14	3.42E-05	4.00E-42	2.98E-11	0.51501409
108.7	1.82E-15	6.60E-16	2.17E-14	3.52E-05	4.14E-42	3.00E-11	0.52199461
108.8	1.96E-15	6.59E-16	2.16E-14	3.74E-05	5.05E-42	3.02E-11	0.51558846
108.9	2.03E-15	6.72E-16	2.18E-14	3.69E-05	4.89E-42	3.01E-11	0.51419544
109	2.09E-15	6.63E-16	2.29E-14	3.76E-05	5.13E-42	2.97E-11	0.50900246
109.1	2.17E-15	6.29E-16	2.31E-14	3.77E-05	4.80E-42	3.07E-11	0.49984036
109.2	2.27E-15	5.66E-16	2.31E-14	3.92E-05	4.61E-42	2.79E-11	0.49912681
109.3	2.37E-15	5.91E-16	2.55E-14	3.92E-05	4.99E-42	2.86E-11	0.50111562
109.4	2.46E-15	5.48E-16	2.59E-14	3.98E-05	4.87E-42	2.81E-11	0.5012861
109.5	2.47E-15	5.64E-16	2.68E-14	4.04E-05	4.93E-42	2.90E-11	0.50256529
109.6	2.48E-15	5.04E-16	2.69E-14	4.19E-05	4.63E-42	2.75E-11	0.49901843
109.7	2.57E-15	5.13E-16	2.72E-14	4.29E-05	4.29E-42	2.77E-11	0.50332762
109.8	2.71E-15	5.60E-16	2.87E-14	4.44E-05	5.08E-42	2.95E-11	0.50627977
109.9	2.66E-15	5.49E-16	2.87E-14	4.42E-05	5.23E-42	2.92E-11	0.5077143
110	2.70E-15	5.48E-16	3.02E-14	4.53E-05	6.07E-42	2.97E-11	0.50873742
110.1	2.70E-15	5.37E-16	3.25E-14	4.53E-05	6.55E-42	3.00E-11	0.51030236
110.2	2.73E-15	5.64E-16	3.29E-14	4.66E-05	6.76E-42	2.99E-11	0.5099927
110.3	2.73E-15	5.64E-16	3.29E-14	4.66E-05	6.76E-42	2.99E-11	0.5099927
110.4	2.66E-15	5.51E-16	3.44E-14	4.65E-05	6.84E-42	2.88E-11	0.50164246
110.5	2.76E-15	6.00E-16	3.49E-14	4.74E-05	7.03E-42	2.98E-11	0.50307095
110.6	2.81E-15	6.01E-16	3.40E-14	4.75E-05	6.82E-42	2.93E-11	0.49928655
110.7	2.85E-15	6.17E-16	3.37E-14	4.86E-05	6.98E-42	2.87E-11	0.5014541
110.8	2.91E-15	6.58E-16	3.41E-14	5.15E-05	7.60E-42	3.04E-11	0.50140599
110.9	3.12E-15	6.48E-16	3.43E-14	5.17E-05	7.75E-42	3.11E-11	0.50319089
111	3.14E-15	7.10E-16	3.84E-14	5.02E-05	8.25E-42	3.04E-11	0.50483597
111.1	3.61E-15	7.48E-16	3.99E-14	5.40E-05	9.83E-42	3.20E-11	0.50715161
111.2	3.52E-15	7.45E-16	3.86E-14	5.44E-05	9.04E-42	3.09E-11	0.50260289
111.3	3.34E-15	7.25E-16	3.80E-14	5.70E-05	9.56E-42	3.22E-11	0.50402109
111.4	3.52E-15	7.26E-16	3.82E-14	5.83E-05	1.04E-41	3.39E-11	0.50083614
111.5	3.56E-15	7.43E-16	4.16E-14	5.90E-05	1.06E-41	3.40E-11	0.50089204
111.6	3.64E-15	7.45E-16	4.18E-14	5.90E-05	1.02E-41	3.41E-11	0.50048268
111.7	3.71E-15	8.25E-16	4.42E-14	5.97E-05	9.70E-42	3.10E-11	0.49643332
111.8	3.54E-15	8.49E-16	4.95E-14	6.16E-05	1.07E-41	3.20E-11	0.48788584
111.9	3.34E-15	8.36E-16	5.19E-14	6.21E-05	1.07E-41	3.02E-11	0.48205988
112	3.43E-15	8.45E-16	5.22E-14	6.35E-05	1.03E-41	2.92E-11	0.47871906
112.1	3.59E-15	8.51E-16	5.06E-14	6.32E-05	1.02E-41	2.92E-11	0.47932031
112.2	3.73E-15	8.51E-16	5.11E-14	6.45E-05	1.09E-41	2.97E-11	0.47712555
112.3	3.70E-15	8.69E-16	5.26E-14	6.57E-05	1.12E-41	3.00E-11	0.48134829
112.4	3.74E-15	8.56E-16	5.20E-14	6.42E-05	1.07E-41	2.95E-11	0.48051703
112.5	3.93E-15	8.92E-16	5.19E-14	6.37E-05	1.10E-41	2.99E-11	0.47496791
112.6	4.34E-15	9.23E-16	5.83E-14	6.27E-05	1.18E-41	3.16E-11	0.46731705
112.7	4.34E-15	9.23E-16	5.83E-14	6.27E-05	1.18E-41	3.16E-11	0.46731705
112.8	4.42E-15	9.91E-16	5.99E-14	6.51E-05	1.21E-41	2.92E-11	0.47358506
112.9	4.93E-15	1.14E-15	6.63E-14	6.77E-05	1.19E-41	2.46E-11	0.47351811
113	4.97E-15	1.14E-15	6.69E-14	6.88E-05	1.26E-41	2.52E-11	0.47555891
113.1	5.02E-15	1.11E-15	6.71E-14	7.14E-05	1.14E-41	2.55E-11	0.47517901
113.2	5.24E-15	1.17E-15	7.03E-14	7.09E-05	9.89E-42	2.56E-11	0.48188955
113.3	5.31E-15	1.19E-15	7.05E-14	7.00E-05	1.01E-41	2.56E-11	0.48178784
113.4	5.44E-15	1.18E-15	7.29E-14	6.77E-05	9.82E-42	2.49E-11	0.47809151
113.5	5.45E-15	1.16E-15	7.40E-14	6.73E-05	9.17E-42	2.50E-11	0.47431555
113.6	5.67E-15	1.23E-15	7.76E-14	6.56E-05	9.62E-42	2.58E-11	0.47421295
113.7	5.91E-15	1.35E-15	8.50E-14	6.72E-05	1.05E-41	2.50E-11	0.47426583
113.8	5.85E-15	1.35E-15	8.84E-14	6.55E-05	9.66E-42	2.31E-11	0.474096
113.9	6.04E-15	1.41E-15	8.82E-14	6.52E-05	1.01E-41	2.30E-11	0.47535019

69	1.94E-17	1.18E-15	3.97E-21	2.41E-07	1.33E-42	3.50E-09	0.9548174
69.1	2.12E-17	1.22E-15	4.99E-21	2.56E-07	1.56E-42	3.76E-09	0.94743576
69.2	2.12E-17	1.18E-15	5.03E-21	2.65E-07	1.54E-42	3.92E-09	0.94407201
69.3	2.22E-17	1.09E-15	4.88E-21	2.70E-07	1.47E-42	3.62E-09	0.94450525
69.4	2.15E-17	9.78E-16	4.74E-21	2.77E-07	1.51E-42	3.68E-09	0.93963699
69.5	1.98E-17	9.33E-16	5.19E-21	2.68E-07	1.42E-42	3.36E-09	0.95227878
69.6	1.96E-17	8.73E-16	5.06E-21	2.66E-07	1.22E-42	3.16E-09	0.94951194
69.7	1.95E-17	8.70E-16	5.62E-21	2.84E-07	1.08E-42	3.13E-09	0.94633247
69.8	2.05E-17	8.93E-16	6.02E-21	2.76E-07	1.04E-42	3.02E-09	0.94755373
69.9	1.99E-17	9.09E-16	6.28E-21	2.80E-07	1.05E-42	3.06E-09	0.94454825
70	1.60E-17	7.75E-16	5.85E-21	2.76E-07	8.49E-43	2.85E-09	0.93938224
70.1	1.53E-17	7.49E-16	5.91E-21	2.78E-07	8.30E-43	2.77E-09	0.94277964
70.2	1.52E-17	7.71E-16	5.96E-21	2.97E-07	8.62E-43	2.76E-09	0.94453184
70.3	1.52E-17	7.55E-16	6.24E-21	2.96E-07	8.40E-43	2.62E-09	0.94790608
70.4	1.53E-17	6.91E-16	6.20E-21	2.87E-07	7.43E-43	2.47E-09	0.94202585
70.5	1.73E-17	7.01E-16	6.87E-21	2.91E-07	7.54E-43	2.37E-09	0.93807839
70.6	1.81E-17	6.91E-16	7.47E-21	2.95E-07	7.34E-43	2.36E-09	0.94073401
70.7	1.88E-17	6.53E-16	7.77E-21	3.06E-07	7.56E-43	2.26E-09	0.93579986
70.8	1.82E-17	6.69E-16	8.01E-21	3.15E-07	8.08E-43	2.27E-09	0.93143545
70.9	1.83E-17	6.67E-16	8.35E-21	3.22E-07	8.21E-43	2.23E-09	0.93435264
71	1.82E-17	6.33E-16	9.06E-21	2.90E-07	7.01E-43	2.20E-09	0.93332381
71.1	1.81E-17	6.22E-16	9.21E-21	2.84E-07	6.92E-43	2.17E-09	0.93581955
71.2	1.78E-17	6.06E-16	9.91E-21	2.80E-07	6.95E-43	1.99E-09	0.94422046
71.3	1.76E-17	5.89E-16	9.85E-21	2.88E-07	6.98E-43	1.96E-09	0.94457002
71.4	1.71E-17	6.16E-16	9.58E-21	2.89E-07	6.38E-43	1.76E-09	0.93338808
71.5	1.61E-17	5.99E-16	9.70E-21	2.99E-07	5.69E-43	1.61E-09	0.94322268
71.6	1.53E-17	5.84E-16	1.03E-20	3.08E-07	5.42E-43	1.45E-09	0.94309549
71.7	1.51E-17	6.31E-16	1.14E-20	3.16E-07	5.93E-43	1.47E-09	0.94004686
71.8	1.52E-17	5.98E-16	1.14E-20	3.16E-07	5.81E-43	1.51E-09	0.93878416
71.9	1.50E-17	5.98E-16	1.27E-20	3.22E-07	6.09E-43	1.51E-09	0.9407881
72	1.51E-17	5.94E-16	1.30E-20	3.28E-07	5.71E-43	1.47E-09	0.94583474
72.1	1.45E-17	5.87E-16	1.26E-20	3.42E-07	5.88E-43	1.51E-09	0.94156979
72.2	1.32E-17	5.79E-16	1.31E-20	3.32E-07	4.75E-43	1.41E-09	0.93319411
72.3	1.44E-17	5.84E-16	1.36E-20	3.52E-07	4.82E-43	1.35E-09	0.93238553
72.4	1.31E-17	5.34E-16	1.33E-20	3.70E-07	4.35E-43	1.34E-09	0.93303847
72.5	1.23E-17	5.08E-16	1.50E-20	4.01E-07	3.87E-43	1.41E-09	0.93796917
72.6	1.24E-17	5.02E-16	1.62E-20	3.86E-07	3.86E-43	1.38E-09	0.9349997
72.7	1.17E-17	4.66E-16	1.59E-20	3.95E-07	3.35E-43	1.28E-09	0.93081405
72.8	1.15E-17	4.73E-16	1.60E-20	3.98E-07	3.34E-43	1.29E-09	0.93438366
72.9	1.26E-17	4.64E-16	1.72E-20	4.09E-07	3.28E-43	1.22E-09	0.93309522
73	1.33E-17	4.62E-16	1.84E-20	4.33E-07	3.47E-43	1.23E-09	0.94105732
73.1	1.31E-17	4.36E-16	1.86E-20	4.33E-07	3.06E-43	1.17E-09	0.94354247
73.2	1.33E-17	4.34E-16	1.94E-20	4.28E-07	3.06E-43	1.14E-09	0.93924758
73.3	1.39E-17	4.35E-16	1.99E-20	4.25E-07	3.06E-43	1.13E-09	0.94081348
73.4	1.33E-17	4.25E-16	2.04E-20	4.42E-07	3.16E-43	1.14E-09	0.94258917
73.5	1.26E-17	4.17E-16	2.10E-20	4.39E-07	2.97E-43	1.10E-09	0.94350548
73.6	1.29E-17	4.38E-16	2.19E-20	4.59E-07	3.02E-43	1.13E-09	0.94090709
73.7	1.29E-17	4.36E-16	2.22E-20	4.62E-07	2.90E-43	1.17E-09	0.93831883
73.8	1.18E-17	3.73E-16	2.02E-20	4.63E-07	2.45E-43	1.11E-09	0.93365381
73.9	1.18E-17	3.37E-16	2.13E-20	4.67E-07	2.42E-43	1.07E-09	0.9358113
74	1.16E-17	3.22E-16	2.22E-20	4.67E-07	2.27E-43	1.06E-09	0.92924712
74.1	1.21E-17	3.41E-16	2.31E-20	4.78E-07	2.30E-43	1.07E-09	0.93394122
74.2	1.17E-17	3.24E-16	2.51E-20	5.02E-07	2.31E-43	1.09E-09	0.93555758
74.3	1.11E-17	3.44E-16	2.82E-20	4.98E-07	2.30E-43	1.12E-09	0.94698909
74.4	1.20E-17	3.50E-16	2.90E-20	4.97E-07	2.58E-43	1.10E-09	0.94409543
74.5	1.18E-17	3.34E-16	2.93E-20	4.95E-07	2.50E-43	1.08E-09	0.94227879
74.6	1.14E-17	3.21E-16	2.88E-20	4.83E-07	2.25E-43	1.02E-09	0.94074264
74.7	1.14E-17	3.01E-16	3.02E-20	5.15E-07	2.04E-43	9.76E-10	0.945251
74.8	1.18E-17	2.72E-16	3.37E-20	4.98E-07	2.09E-43	9.49E-10	0.9416622
74.9	1.16E-17	2.47E-16	3.41E-20	5.28E-07	1.90E-43	9.40E-10	0.93801616

120	3.12E-14	3.73E-15	4.80E-13	0.00010268	4.51E-41	2.30E-11	0.40388554
120.1	3.15E-14	3.72E-15	4.90E-13	0.00010109	4.29E-41	2.27E-11	0.40514094
120.2	3.04E-14	3.69E-15	4.86E-13	0.00010477	4.09E-41	2.14E-11	0.4039112
120.3	3.08E-14	3.64E-15	5.02E-13	0.00010396	4.32E-41	2.15E-11	0.4017341
120.4	3.08E-14	3.64E-15	5.02E-13	0.00010396	4.32E-41	2.15E-11	0.4017341
120.5	3.10E-14	3.80E-15	5.13E-13	0.00010358	4.58E-41	2.11E-11	0.39873468
120.6	3.06E-14	3.97E-15	5.49E-13	0.00010448	4.42E-41	2.04E-11	0.40318139
120.7	3.06E-14	3.97E-15	5.49E-13	0.00010448	4.42E-41	2.04E-11	0.40318139
120.8	3.21E-14	4.00E-15	5.54E-13	0.00010474	4.55E-41	2.02E-11	0.40425574
120.9	3.32E-14	4.03E-15	5.61E-13	0.00010471	4.75E-41	2.14E-11	0.40262383
121	3.23E-14	3.50E-15	5.46E-13	0.00010475	4.12E-41	2.10E-11	0.40503733
121.1	3.23E-14	3.50E-15	5.46E-13	0.00010475	4.12E-41	2.10E-11	0.40503733
121.2	3.68E-14	3.79E-15	6.02E-13	0.00010525	4.63E-41	2.33E-11	0.40534153
121.3	3.68E-14	3.79E-15	6.02E-13	0.00010525	4.63E-41	2.33E-11	0.40534153
121.4	3.52E-14	4.12E-15	6.12E-13	0.00010477	4.99E-41	2.25E-11	0.40286214
121.5	3.57E-14	4.37E-15	6.40E-13	0.00010763	5.32E-41	2.26E-11	0.4050725
121.6	3.78E-14	4.78E-15	7.12E-13	0.00010204	5.24E-41	2.21E-11	0.40436649
121.7	3.68E-14	4.90E-15	7.01E-13	0.0001014	4.75E-41	2.17E-11	0.4010426
121.8	3.68E-14	4.90E-15	7.01E-13	0.0001014	4.75E-41	2.17E-11	0.4010426
121.9	3.85E-14	4.52E-15	7.48E-13	0.00010165	4.74E-41	2.29E-11	0.39936677
122	3.89E-14	4.45E-15	7.62E-13	0.00010242	4.92E-41	2.34E-11	0.39765227
122.1	3.97E-14	4.32E-15	7.32E-13	0.00010189	4.96E-41	2.29E-11	0.39541037
122.2	3.96E-14	4.19E-15	7.22E-13	0.00010386	5.07E-41	2.38E-11	0.39641009
122.3	4.19E-14	4.28E-15	7.31E-13	0.00010589	5.54E-41	2.50E-11	0.3946776
122.4	4.19E-14	4.28E-15	7.31E-13	0.00010589	5.54E-41	2.50E-11	0.3946776
122.5	4.31E-14	4.37E-15	7.30E-13	0.00010652	5.60E-41	2.42E-11	0.39588011
122.6	4.14E-14	4.98E-15	7.25E-13	0.0001038	5.33E-41	2.34E-11	0.39260344
122.7	4.36E-14	5.08E-15	7.68E-13	0.00010548	5.91E-41	2.48E-11	0.38988932
122.8	4.34E-14	5.24E-15	8.06E-13	0.00010181	5.86E-41	2.39E-11	0.38924279
122.9	4.53E-14	5.35E-15	8.14E-13	0.00010024	6.13E-41	2.44E-11	0.38862998
123	4.86E-14	5.25E-15	7.88E-13	0.00010008	6.37E-41	2.46E-11	0.38595436
123.1	5.13E-14	5.55E-15	8.48E-13	0.00010241	6.90E-41	2.54E-11	0.3843677
123.2	5.25E-14	5.51E-15	8.40E-13	0.00010008	6.81E-41	2.56E-11	0.38611221
123.3	5.17E-14	5.51E-15	8.60E-13	0.00010253	7.05E-41	2.60E-11	0.38850266
123.4	5.22E-14	5.52E-15	8.75E-13	0.00010198	7.45E-41	2.65E-11	0.38550551
123.5	5.75E-14	5.81E-15	9.63E-13	0.00010449	8.48E-41	2.71E-11	0.38472378
123.6	5.75E-14	5.81E-15	9.63E-13	0.00010449	8.48E-41	2.71E-11	0.38472378
123.7	5.90E-14	6.03E-15	1.02E-12	0.00010548	8.68E-41	2.81E-11	0.38667885
123.8	5.90E-14	6.03E-15	1.02E-12	0.00010548	8.68E-41	2.81E-11	0.38667885
123.9	5.90E-14	6.03E-15	1.02E-12	0.00010548	8.68E-41	2.81E-11	0.38667885
124	5.96E-14	6.30E-15	1.05E-12	0.00010661	9.14E-41	2.92E-11	0.38806409
124.1	6.06E-14	6.31E-15	1.05E-12	0.00010858	8.87E-41	2.92E-11	0.38747983
124.2	6.31E-14	6.06E-15	1.02E-12	0.00010777	9.11E-41	3.05E-11	0.38363805
124.3	6.53E-14	6.39E-15	1.08E-12	0.00010771	8.54E-41	2.84E-11	0.38415386
124.4	6.34E-14	6.50E-15	1.10E-12	0.00010787	8.69E-41	2.89E-11	0.38714999
124.5	6.65E-14	6.76E-15	1.13E-12	0.00010597	9.10E-41	3.00E-11	0.38550385
124.6	6.91E-14	7.10E-15	1.19E-12	0.00010977	9.63E-41	2.93E-11	0.38615579
124.7	6.91E-14	7.10E-15	1.19E-12	0.00010977	9.63E-41	2.93E-11	0.38615579
124.8	6.82E-14	7.28E-15	1.16E-12	0.00010972	9.13E-41	2.94E-11	0.38674249
124.9	6.82E-14	7.28E-15	1.16E-12	0.00010972	9.13E-41	2.94E-11	0.38674249
125	6.84E-14	7.31E-15	1.18E-12	0.00010912	9.75E-41	2.95E-11	0.38823298
125.1	6.84E-14	7.31E-15	1.18E-12	0.00010912	9.75E-41	2.95E-11	0.38823298
125.2	6.90E-14	7.76E-15	1.23E-12	0.00011156	1.08E-40	3.01E-11	0.38685659
125.3	6.99E-14	7.61E-15	1.25E-12	0.0001137	1.14E-40	2.93E-11	0.38746797
125.4	6.99E-14	7.61E-15	1.25E-12	0.0001137	1.14E-40	2.93E-11	0.38746797
125.5	7.13E-14	7.96E-15	1.32E-12	0.0001159	1.16E-40	2.98E-11	0.38945777
125.6	7.17E-14	7.53E-15	1.32E-12	0.00011551	1.17E-40	2.88E-11	0.38674932
125.7	7.36E-14	7.67E-15	1.31E-12	0.00011575	1.24E-40	2.85E-11	0.38512308
125.8	7.33E-14	7.73E-15	1.33E-12	0.0001168	1.27E-40	2.84E-11	0.38693536
125.9	7.49E-14	7.70E-15	1.32E-12	0.00011694	1.28E-40	2.85E-11	0.3880721
126	7.41E-14	7.77E-15	1.28E-12	0.00012006	1.32E-40	2.84E-11	0.38678956

84	1.74E-17	1.46E-16	1.79E-18	2.72E-06	8.17E-44	1.23E-10	0.81542319
84.1	1.63E-17	1.37E-16	1.90E-18	2.88E-06	7.29E-44	1.15E-10	0.81427203
84.2	1.69E-17	1.45E-16	1.89E-18	2.96E-06	7.73E-44	1.18E-10	0.80707069
84.3	1.49E-17	1.46E-16	1.76E-18	2.96E-06	7.30E-44	1.01E-10	0.79512555
84.4	1.47E-17	1.28E-16	1.69E-18	2.95E-06	6.29E-44	9.80E-11	0.79466441
84.5	1.54E-17	1.30E-16	1.80E-18	3.02E-06	6.09E-44	9.46E-11	0.79795547
84.6	1.66E-17	1.31E-16	2.03E-18	3.04E-06	6.62E-44	9.55E-11	0.79184393
84.7	1.70E-17	1.30E-16	2.04E-18	3.12E-06	6.59E-44	8.76E-11	0.79007246
84.8	1.79E-17	1.31E-16	2.07E-18	3.15E-06	7.40E-44	8.83E-11	0.78446616
84.9	1.92E-17	1.41E-16	2.14E-18	3.11E-06	7.97E-44	9.15E-11	0.7824345
85	1.88E-17	1.45E-16	2.11E-18	3.08E-06	7.91E-44	9.13E-11	0.778021
85.1	2.03E-17	1.38E-16	2.23E-18	3.44E-06	8.62E-44	9.69E-11	0.77348739
85.2	2.11E-17	1.38E-16	2.24E-18	3.53E-06	9.02E-44	9.57E-11	0.77810206
85.3	2.24E-17	1.52E-16	2.86E-18	3.81E-06	1.08E-43	1.01E-10	0.77568146
85.4	2.13E-17	1.59E-16	2.97E-18	3.99E-06	1.08E-43	9.71E-11	0.77250593
85.5	2.08E-17	1.66E-16	3.39E-18	4.08E-06	1.12E-43	9.93E-11	0.7715139
85.6	2.17E-17	1.64E-16	3.53E-18	4.39E-06	1.12E-43	9.49E-11	0.77711698
85.7	2.24E-17	1.74E-16	3.65E-18	4.41E-06	1.07E-43	9.11E-11	0.78149852
85.8	2.21E-17	1.84E-16	4.12E-18	4.44E-06	1.06E-43	1.04E-10	0.78505719
85.9	2.06E-17	1.69E-16	4.13E-18	4.59E-06	8.34E-44	1.01E-10	0.78397507
86	1.91E-17	1.67E-16	3.99E-18	4.57E-06	7.56E-44	9.66E-11	0.78698031
86.1	1.87E-17	1.58E-16	3.70E-18	4.36E-06	6.29E-44	8.89E-11	0.77984136
86.2	1.93E-17	1.52E-16	3.54E-18	4.68E-06	5.98E-44	8.49E-11	0.77575834
86.3	2.06E-17	1.63E-16	3.93E-18	4.74E-06	6.24E-44	8.29E-11	0.7715232
86.4	2.02E-17	1.75E-16	4.14E-18	4.58E-06	6.13E-44	7.76E-11	0.76775146
86.5	2.15E-17	1.70E-16	4.36E-18	4.62E-06	6.68E-44	7.49E-11	0.76882633
86.6	2.19E-17	1.69E-16	4.24E-18	4.72E-06	6.79E-44	7.48E-11	0.76870701
86.7	2.12E-17	1.72E-16	4.38E-18	4.62E-06	6.45E-44	7.54E-11	0.76671239
86.8	2.30E-17	1.90E-16	4.64E-18	4.61E-06	7.01E-44	7.32E-11	0.75080666
86.9	2.57E-17	2.11E-16	5.59E-18	4.69E-06	8.67E-44	7.72E-11	0.75016389
87	2.46E-17	1.93E-16	6.09E-18	4.93E-06	7.64E-44	7.18E-11	0.74169702
87.1	2.32E-17	1.71E-16	6.29E-18	4.90E-06	7.46E-44	6.55E-11	0.73962712
87.2	2.27E-17	1.71E-16	6.49E-18	4.96E-06	6.86E-44	6.66E-11	0.7314168
87.3	2.46E-17	1.75E-16	8.34E-18	5.06E-06	8.61E-44	7.08E-11	0.72347605
87.4	2.55E-17	1.83E-16	9.20E-18	4.88E-06	8.80E-44	7.68E-11	0.72234855
87.5	2.78E-17	1.89E-16	9.52E-18	4.95E-06	9.11E-44	7.55E-11	0.7195197
87.6	2.68E-17	2.05E-16	9.47E-18	5.13E-06	1.01E-43	7.37E-11	0.71442737
87.7	2.86E-17	2.25E-16	9.89E-18	5.06E-06	1.13E-43	7.43E-11	0.71831009
87.8	3.01E-17	2.09E-16	1.09E-17	5.14E-06	1.10E-43	7.36E-11	0.71817534
87.9	3.36E-17	2.22E-16	1.23E-17	5.33E-06	1.25E-43	7.36E-11	0.71272912
88	3.38E-17	2.28E-16	1.49E-17	4.83E-06	1.27E-43	7.22E-11	0.7130815
88.1	3.30E-17	2.06E-16	1.50E-17	4.75E-06	1.21E-43	6.74E-11	0.71219332
88.2	3.28E-17	1.95E-16	1.46E-17	4.75E-06	1.22E-43	6.45E-11	0.70897296
88.3	3.22E-17	1.84E-16	1.66E-17	4.74E-06	1.30E-43	6.45E-11	0.70977967
88.4	3.29E-17	1.83E-16	1.70E-17	5.10E-06	1.35E-43	6.30E-11	0.70964171
88.5	3.28E-17	1.87E-16	1.76E-17	5.47E-06	1.41E-43	5.91E-11	0.70558658
88.6	3.55E-17	1.95E-16	1.86E-17	5.16E-06	1.46E-43	5.69E-11	0.69189122
88.7	3.33E-17	1.99E-16	1.95E-17	5.19E-06	1.35E-43	5.72E-11	0.69029983
88.8	3.42E-17	1.98E-16	2.00E-17	5.23E-06	1.22E-43	5.17E-11	0.69269799
88.9	3.49E-17	1.82E-16	2.02E-17	5.38E-06	1.26E-43	4.97E-11	0.69597243
89	3.62E-17	1.82E-16	2.10E-17	5.53E-06	1.31E-43	5.05E-11	0.69927661
89.1	3.59E-17	1.88E-16	2.47E-17	5.59E-06	1.44E-43	5.21E-11	0.70043979
89.2	3.64E-17	1.99E-16	2.70E-17	5.68E-06	1.52E-43	5.52E-11	0.70042935
89.3	3.89E-17	1.90E-16	3.01E-17	6.03E-06	1.58E-43	6.09E-11	0.69538761
89.4	3.86E-17	1.65E-16	3.09E-17	6.38E-06	1.40E-43	5.32E-11	0.69668664
89.5	3.72E-17	1.61E-16	3.00E-17	6.71E-06	1.38E-43	5.10E-11	0.67777258
89.6	3.95E-17	1.67E-16	3.18E-17	6.80E-06	1.32E-43	4.82E-11	0.67542923
89.7	4.26E-17	1.61E-16	3.22E-17	6.88E-06	1.38E-43	4.68E-11	0.67243354
89.8	4.19E-17	1.58E-16	3.33E-17	7.02E-06	1.48E-43	4.77E-11	0.67310983
89.9	4.24E-17	1.63E-16	3.42E-17	7.21E-06	1.47E-43	4.58E-11	0.67418805

135	2.35E-13	1.50E-14	3.49E-12	0.00015173	3.29E-40	2.08E-11	0.30676572
135.1	2.35E-13	1.54E-14	3.65E-12	0.00015405	3.54E-40	2.13E-11	0.30463747
135.2	2.48E-13	1.57E-14	3.49E-12	0.00015292	3.78E-40	2.17E-11	0.30178608
135.3	2.52E-13	1.50E-14	3.50E-12	0.00015269	3.59E-40	2.15E-11	0.29957059
135.4	2.78E-13	1.54E-14	3.63E-12	0.00015065	3.69E-40	2.09E-11	0.29545135
135.5	2.78E-13	1.54E-14	3.63E-12	0.00015065	3.69E-40	2.09E-11	0.29545135
135.6	3.13E-13	1.65E-14	3.71E-12	0.00015362	4.23E-40	1.96E-11	0.29164955
135.7	3.17E-13	1.67E-14	3.66E-12	0.00015767	4.34E-40	1.94E-11	0.29066723
135.8	3.47E-13	1.78E-14	3.95E-12	0.00015921	5.10E-40	1.92E-11	0.28840021
135.9	3.39E-13	1.90E-14	3.82E-12	0.00015749	4.92E-40	1.93E-11	0.28960555
136	3.36E-13	1.87E-14	3.86E-12	0.00016067	4.99E-40	1.89E-11	0.28883248
136.1	3.36E-13	1.87E-14	3.84E-12	0.00016189	4.89E-40	1.88E-11	0.2880394
136.2	3.61E-13	1.85E-14	3.94E-12	0.00016376	5.02E-40	1.86E-11	0.28679619
136.3	3.66E-13	1.87E-14	4.47E-12	0.00016397	5.22E-40	1.94E-11	0.28849439
136.4	3.66E-13	1.87E-14	4.47E-12	0.00016397	5.22E-40	1.94E-11	0.28849439
136.5	3.64E-13	2.08E-14	4.63E-12	0.00016553	5.97E-40	2.02E-11	0.28897686
136.6	3.88E-13	2.20E-14	4.67E-12	0.0001701	6.58E-40	1.99E-11	0.28507076
136.7	3.88E-13	2.20E-14	4.67E-12	0.0001701	6.58E-40	1.99E-11	0.28507076
136.8	4.18E-13	2.27E-14	4.72E-12	0.00017508	7.43E-40	1.90E-11	0.28121767
136.9	4.35E-13	2.47E-14	4.78E-12	0.00017051	7.96E-40	1.89E-11	0.27988593
137	4.29E-13	2.55E-14	4.90E-12	0.00017255	7.88E-40	1.83E-11	0.28287518
137.1	4.42E-13	2.69E-14	5.16E-12	0.00017246	8.07E-40	1.88E-11	0.28140443
137.2	4.85E-13	2.63E-14	5.53E-12	0.00017668	9.04E-40	1.91E-11	0.28027738
137.3	4.75E-13	2.59E-14	5.67E-12	0.00017922	8.05E-40	1.86E-11	0.2773865
137.4	4.75E-13	2.59E-14	5.67E-12	0.00017922	8.05E-40	1.86E-11	0.2773865
137.5	5.18E-13	2.62E-14	5.52E-12	0.0001856	8.69E-40	1.77E-11	0.27352621
137.6	5.14E-13	2.71E-14	5.72E-12	0.00018252	9.52E-40	1.79E-11	0.26971322
137.7	5.98E-13	2.68E-14	5.80E-12	0.00018049	1.09E-39	1.69E-11	0.26623365
137.8	6.13E-13	2.70E-14	5.74E-12	0.00017779	1.09E-39	1.66E-11	0.26708402
137.9	6.13E-13	2.70E-14	5.74E-12	0.00017779	1.09E-39	1.66E-11	0.26708402
138	6.26E-13	2.78E-14	5.79E-12	0.0001799	1.08E-39	1.61E-11	0.26882629
138.1	6.69E-13	2.65E-14	5.90E-12	0.00018296	1.04E-39	1.61E-11	0.26595128
138.2	6.73E-13	2.84E-14	6.28E-12	0.00018885	1.06E-39	1.63E-11	0.26575013
138.3	6.73E-13	2.84E-14	6.28E-12	0.00018885	1.06E-39	1.63E-11	0.26575013
138.4	6.83E-13	2.91E-14	6.33E-12	0.00018852	1.11E-39	1.58E-11	0.26722616
138.5	6.26E-13	3.04E-14	6.43E-12	0.00018584	1.04E-39	1.57E-11	0.27084161
138.6	6.26E-13	3.04E-14	6.43E-12	0.00018584	1.04E-39	1.57E-11	0.27084161
138.7	6.14E-13	3.05E-14	6.36E-12	0.00018466	1.05E-39	1.61E-11	0.26805996
138.8	6.53E-13	3.10E-14	6.30E-12	0.0001872	1.04E-39	1.60E-11	0.26960214
138.9	6.89E-13	3.19E-14	6.03E-12	0.00019016	1.08E-39	1.64E-11	0.26703249
139	6.89E-13	3.19E-14	6.03E-12	0.00019016	1.08E-39	1.64E-11	0.26703249
139.1	6.71E-13	3.12E-14	5.98E-12	0.00019334	1.05E-39	1.64E-11	0.26825304
139.2	6.71E-13	3.12E-14	5.98E-12	0.00019334	1.05E-39	1.64E-11	0.26825304
139.3	6.71E-13	3.12E-14	5.98E-12	0.00019334	1.05E-39	1.64E-11	0.26825304
139.4	6.88E-13	3.09E-14	5.97E-12	0.00020273	1.14E-39	1.57E-11	0.26701995
139.5	7.15E-13	3.02E-14	5.93E-12	0.0002036	1.14E-39	1.56E-11	0.26364023
139.6	7.23E-13	3.04E-14	6.00E-12	0.00021154	1.15E-39	1.48E-11	0.26342341
139.7	7.57E-13	3.07E-14	6.06E-12	0.00021765	1.22E-39	1.42E-11	0.26419788
139.8	7.49E-13	3.15E-14	5.91E-12	0.00021725	1.26E-39	1.38E-11	0.2639963
139.9	7.24E-13	3.20E-14	5.83E-12	0.00022011	1.30E-39	1.37E-11	0.26295296
140	7.24E-13	3.20E-14	5.83E-12	0.00022011	1.30E-39	1.37E-11	0.26295296
140.1	7.14E-13	3.30E-14	5.92E-12	0.00022209	1.33E-39	1.35E-11	0.25959113
140.2	7.71E-13	3.44E-14	5.94E-12	0.0002155	1.45E-39	1.41E-11	0.25587352
140.3	7.95E-13	3.25E-14	5.86E-12	0.00021581	1.44E-39	1.46E-11	0.25442572
140.4	8.22E-13	3.36E-14	5.90E-12	0.00021901	1.49E-39	1.47E-11	0.2520417
140.5	8.84E-13	3.54E-14	5.71E-12	0.00021263	1.59E-39	1.57E-11	0.2493206
140.6	9.01E-13	3.76E-14	5.99E-12	0.00020981	1.71E-39	1.63E-11	0.24654553
140.7	9.01E-13	3.76E-14	5.99E-12	0.00020981	1.71E-39	1.63E-11	0.24654553
140.8	8.76E-13	3.70E-14	5.91E-12	0.00020359	1.63E-39	1.57E-11	0.24569569
140.9	8.78E-13	3.89E-14	5.93E-12	0.00020642	1.75E-39	1.61E-11	0.24538533
141	9.30E-13	3.88E-14	6.34E-12	0.00021548	1.90E-39	1.64E-11	0.24707861

99	2.27E-16	3.40E-16	2.18E-15	1.63E-05	9.72E-43	2.66E-11	0.52296132
99.1	2.56E-16	3.40E-16	2.38E-15	1.63E-05	1.14E-42	2.65E-11	0.52142346
99.2	2.69E-16	3.47E-16	2.33E-15	1.58E-05	1.14E-42	2.76E-11	0.51413945
99.3	2.69E-16	3.47E-16	2.33E-15	1.58E-05	1.14E-42	2.76E-11	0.51413945
99.4	2.92E-16	3.68E-16	2.51E-15	1.60E-05	1.23E-42	2.88E-11	0.51188596
99.5	3.17E-16	3.95E-16	2.53E-15	1.63E-05	1.29E-42	2.79E-11	0.51182328
99.6	3.74E-16	4.30E-16	2.73E-15	1.69E-05	1.58E-42	2.89E-11	0.50848378
99.7	3.65E-16	4.00E-16	2.67E-15	1.71E-05	1.55E-42	2.87E-11	0.50007855
99.8	3.85E-16	4.04E-16	2.77E-15	1.79E-05	1.61E-42	2.91E-11	0.49731634
99.9	4.00E-16	4.11E-16	3.06E-15	1.83E-05	1.77E-42	3.15E-11	0.49256109
100	4.20E-16	3.90E-16	2.95E-15	1.83E-05	1.77E-42	3.18E-11	0.49257542
100.1	4.54E-16	3.85E-16	3.01E-15	1.84E-05	1.79E-42	3.37E-11	0.49814276
100.2	4.41E-16	3.90E-16	2.97E-15	1.93E-05	1.86E-42	3.40E-11	0.49529429
100.3	4.52E-16	3.72E-16	2.87E-15	1.97E-05	1.81E-42	3.36E-11	0.49298814
100.4	4.85E-16	3.98E-16	3.28E-15	2.05E-05	2.30E-42	3.56E-11	0.49322706
100.5	4.93E-16	3.85E-16	3.39E-15	2.08E-05	2.41E-42	3.72E-11	0.49365711
100.6	4.84E-16	3.81E-16	3.38E-15	2.10E-05	2.50E-42	3.66E-11	0.49496076
100.7	5.20E-16	3.86E-16	3.64E-15	2.16E-05	2.73E-42	3.67E-11	0.49366392
100.8	5.05E-16	3.64E-16	3.71E-15	2.23E-05	2.76E-42	3.69E-11	0.49983397
100.9	5.02E-16	3.44E-16	3.56E-15	2.18E-05	2.71E-42	3.92E-11	0.49991643
101	5.47E-16	3.68E-16	3.83E-15	2.14E-05	2.79E-42	4.08E-11	0.49912406
101.1	5.40E-16	3.65E-16	3.68E-15	2.21E-05	2.89E-42	4.15E-11	0.49732085
101.2	5.36E-16	3.53E-16	3.52E-15	2.31E-05	2.92E-42	4.25E-11	0.4914049
101.3	5.36E-16	3.79E-16	3.79E-15	2.30E-05	3.04E-42	4.30E-11	0.49517386
101.4	5.09E-16	3.53E-16	3.59E-15	2.30E-05	2.79E-42	4.23E-11	0.49051457
101.5	5.30E-16	3.43E-16	3.67E-15	2.31E-05	2.91E-42	4.26E-11	0.49166984
101.6	5.36E-16	3.56E-16	3.59E-15	2.44E-05	2.91E-42	4.09E-11	0.49194719
101.7	5.31E-16	3.74E-16	3.33E-15	2.45E-05	2.62E-42	3.90E-11	0.48979263
101.8	5.57E-16	3.62E-16	3.59E-15	2.40E-05	2.43E-42	3.85E-11	0.49478727
101.9	5.80E-16	3.64E-16	3.83E-15	2.45E-05	2.61E-42	4.01E-11	0.48934648
102	5.94E-16	3.90E-16	3.92E-15	2.32E-05	2.78E-42	4.26E-11	0.49077022
102.1	6.56E-16	4.17E-16	4.44E-15	2.39E-05	3.26E-42	4.24E-11	0.48146691
102.2	6.65E-16	4.07E-16	4.73E-15	2.39E-05	2.86E-42	4.08E-11	0.48062247
102.3	7.03E-16	4.36E-16	4.91E-15	2.40E-05	3.14E-42	4.33E-11	0.48152311
102.4	7.51E-16	4.52E-16	5.01E-15	2.42E-05	3.38E-42	4.58E-11	0.48532948
102.5	7.49E-16	4.52E-16	5.44E-15	2.51E-05	3.19E-42	4.66E-11	0.4851908
102.6	7.49E-16	4.52E-16	5.44E-15	2.51E-05	3.19E-42	4.66E-11	0.4851908
102.7	7.10E-16	4.66E-16	5.46E-15	2.48E-05	3.19E-42	4.59E-11	0.47879085
102.8	7.72E-16	4.87E-16	5.70E-15	2.43E-05	3.40E-42	4.59E-11	0.47766372
102.9	7.77E-16	4.91E-16	5.98E-15	2.46E-05	3.45E-42	4.50E-11	0.48164113
103	7.95E-16	5.43E-16	6.72E-15	2.51E-05	4.19E-42	4.80E-11	0.49166014
103.1	8.14E-16	5.44E-16	6.71E-15	2.50E-05	3.95E-42	4.70E-11	0.49313531
103.2	8.34E-16	5.65E-16	7.18E-15	2.47E-05	3.81E-42	4.67E-11	0.48926708
103.3	8.55E-16	5.52E-16	7.27E-15	2.53E-05	3.91E-42	4.57E-11	0.48864141
103.4	8.25E-16	5.51E-16	7.20E-15	2.58E-05	3.85E-42	4.57E-11	0.49054938
103.5	8.06E-16	5.48E-16	7.90E-15	2.50E-05	3.97E-42	4.57E-11	0.48569298
103.6	8.06E-16	5.48E-16	7.90E-15	2.50E-05	3.97E-42	4.57E-11	0.48569298
103.7	7.64E-16	5.61E-16	8.04E-15	2.61E-05	4.31E-42	4.74E-11	0.48191239
103.8	7.13E-16	5.50E-16	8.05E-15	2.54E-05	4.05E-42	4.68E-11	0.48404776
103.9	7.38E-16	5.64E-16	8.56E-15	2.56E-05	3.94E-42	4.82E-11	0.48373321
104	7.89E-16	5.62E-16	9.10E-15	2.61E-05	4.12E-42	4.84E-11	0.48472746
104.1	7.89E-16	5.62E-16	9.10E-15	2.61E-05	4.12E-42	4.84E-11	0.48472746
104.2	7.64E-16	5.62E-16	8.60E-15	2.70E-05	3.91E-42	4.91E-11	0.48373482
104.3	7.77E-16	6.17E-16	8.75E-15	2.70E-05	4.00E-42	4.77E-11	0.48542524
104.4	7.63E-16	6.30E-16	8.82E-15	2.72E-05	3.86E-42	4.70E-11	0.48334763
104.5	7.40E-16	6.05E-16	9.13E-15	2.85E-05	3.82E-42	4.50E-11	0.48186434
104.6	7.76E-16	5.75E-16	9.90E-15	2.90E-05	3.96E-42	4.43E-11	0.48525516
104.7	8.21E-16	5.70E-16	1.03E-14	2.88E-05	4.06E-42	4.22E-11	0.48648151
104.8	7.71E-16	5.88E-16	1.14E-14	2.98E-05	4.03E-42	4.05E-11	0.49366332
104.9	7.21E-16	5.48E-16	1.09E-14	3.01E-05	3.79E-42	3.94E-11	0.48996631

150	3.24E-12	9.13E-14	2.36E-11	0.00037545	4.64E-39	1.34E-11	0.23215844
150.1	3.49E-12	9.39E-14	2.49E-11	0.00038048	4.90E-39	1.31E-11	0.22964377
150.2	3.51E-12	9.62E-14	2.56E-11	0.00037111	5.21E-39	1.27E-11	0.2286981
150.3	3.60E-12	9.33E-14	2.53E-11	0.00037787	5.34E-39	1.24E-11	0.23026403
150.4	3.51E-12	9.55E-14	2.47E-11	0.00037115	5.32E-39	1.22E-11	0.23122369
150.5	3.61E-12	9.44E-14	2.40E-11	0.00036711	4.95E-39	1.18E-11	0.23088675
150.6	3.77E-12	9.77E-14	2.47E-11	0.00037067	4.83E-39	1.14E-11	0.23537554
150.7	3.77E-12	9.77E-14	2.47E-11	0.00037067	4.83E-39	1.14E-11	0.23537554
150.8	3.92E-12	9.73E-14	2.47E-11	0.00037247	5.03E-39	1.15E-11	0.23315407
150.9	3.85E-12	1.01E-13	2.51E-11	0.00036557	4.88E-39	1.15E-11	0.23468868
151	3.92E-12	9.54E-14	2.47E-11	0.0003661	4.56E-39	1.13E-11	0.23516984
151.1	3.92E-12	9.54E-14	2.47E-11	0.0003661	4.56E-39	1.13E-11	0.23516984
151.2	4.01E-12	9.36E-14	2.39E-11	0.00036209	4.26E-39	1.16E-11	0.23567613
151.3	4.01E-12	9.36E-14	2.39E-11	0.00036209	4.26E-39	1.16E-11	0.23567613
151.4	4.01E-12	9.36E-14	2.39E-11	0.00036209	4.26E-39	1.16E-11	0.23567613
151.5	4.13E-12	9.25E-14	2.38E-11	0.00036454	4.47E-39	1.17E-11	0.23449245
151.6	4.13E-12	9.25E-14	2.38E-11	0.00036454	4.47E-39	1.17E-11	0.23449245
151.7	4.04E-12	9.08E-14	2.42E-11	0.00036443	4.25E-39	1.17E-11	0.23618708
151.8	4.09E-12	9.13E-14	2.38E-11	0.00035915	4.34E-39	1.18E-11	0.23651921
151.9	4.09E-12	9.13E-14	2.38E-11	0.00035915	4.34E-39	1.18E-11	0.23651921
152	4.38E-12	9.43E-14	2.45E-11	0.00036978	4.84E-39	1.21E-11	0.23530195
152.1	4.38E-12	9.43E-14	2.45E-11	0.00036978	4.84E-39	1.21E-11	0.23530195
152.2	4.39E-12	9.49E-14	2.52E-11	0.00036209	4.69E-39	1.20E-11	0.23467595
152.3	4.39E-12	9.49E-14	2.52E-11	0.00036209	4.69E-39	1.20E-11	0.23467595
152.4	4.40E-12	9.55E-14	2.62E-11	0.00036658	4.72E-39	1.20E-11	0.23395662
152.5	4.20E-12	9.49E-14	2.56E-11	0.00036117	4.61E-39	1.22E-11	0.23314592
152.6	4.20E-12	9.49E-14	2.56E-11	0.00036117	4.61E-39	1.22E-11	0.23314592
152.7	4.20E-12	9.49E-14	2.56E-11	0.00036117	4.61E-39	1.22E-11	0.23314592
152.8	4.20E-12	9.49E-14	2.56E-11	0.00036117	4.61E-39	1.22E-11	0.23314592
152.9	4.18E-12	9.81E-14	2.51E-11	0.00035256	4.64E-39	1.22E-11	0.23105003
153	4.18E-12	9.81E-14	2.51E-11	0.00035256	4.64E-39	1.22E-11	0.23105003
153.1	4.57E-12	1.03E-13	2.65E-11	0.00036237	5.28E-39	1.26E-11	0.22875854
153.2	4.57E-12	1.03E-13	2.65E-11	0.00036237	5.28E-39	1.26E-11	0.22875854
153.3	4.57E-12	1.08E-13	2.67E-11	0.0003573	5.36E-39	1.27E-11	0.22880192
153.4	4.57E-12	1.08E-13	2.67E-11	0.0003573	5.36E-39	1.27E-11	0.22880192
153.5	4.48E-12	1.12E-13	2.45E-11	0.00035416	5.25E-39	1.28E-11	0.22881502
153.6	4.48E-12	1.12E-13	2.45E-11	0.00035416	5.25E-39	1.28E-11	0.22881502
153.7	4.48E-12	1.12E-13	2.45E-11	0.00035416	5.25E-39	1.28E-11	0.22881502
153.8	4.48E-12	1.12E-13	2.45E-11	0.00035416	5.25E-39	1.28E-11	0.22881502
153.9	4.57E-12	1.13E-13	2.51E-11	0.00035354	5.35E-39	1.30E-11	0.22771852
154	4.55E-12	1.11E-13	2.44E-11	0.00034476	5.45E-39	1.27E-11	0.22595206
154.1	4.55E-12	1.11E-13	2.44E-11	0.00034476	5.45E-39	1.27E-11	0.22595206
154.2	4.55E-12	1.11E-13	2.44E-11	0.00034476	5.45E-39	1.27E-11	0.22595206
154.3	4.55E-12	1.11E-13	2.44E-11	0.00034476	5.45E-39	1.27E-11	0.22595206
154.4	4.94E-12	1.10E-13	2.48E-11	0.00034607	5.99E-39	1.27E-11	0.22835707
154.5	4.83E-12	1.16E-13	2.53E-11	0.0003388	6.03E-39	1.28E-11	0.2261966
154.6	5.07E-12	1.20E-13	2.65E-11	0.00034856	6.60E-39	1.32E-11	0.22592481
154.7	5.07E-12	1.20E-13	2.65E-11	0.00034856	6.60E-39	1.32E-11	0.22592481
154.8	5.07E-12	1.20E-13	2.65E-11	0.00034856	6.60E-39	1.32E-11	0.22592481
154.9	5.03E-12	1.12E-13	2.68E-11	0.00034174	6.82E-39	1.35E-11	0.22391473
155	5.50E-12	1.16E-13	2.76E-11	0.00034593	7.09E-39	1.31E-11	0.22679208
155.1	5.43E-12	1.13E-13	2.79E-11	0.0003461	6.62E-39	1.30E-11	0.22755268
155.2	5.43E-12	1.13E-13	2.79E-11	0.0003461	6.62E-39	1.30E-11	0.22755268
155.3	5.43E-12	1.13E-13	2.79E-11	0.0003461	6.62E-39	1.30E-11	0.22755268
155.4	5.43E-12	1.13E-13	2.79E-11	0.0003461	6.62E-39	1.30E-11	0.22755268
155.5	5.49E-12	1.17E-13	2.83E-11	0.0003436	6.35E-39	1.29E-11	0.22765828
155.6	5.87E-12	1.17E-13	2.91E-11	0.00036071	7.30E-39	1.28E-11	0.22649928
155.7	6.41E-12	1.29E-13	3.21E-11	0.00038459	8.35E-39	1.26E-11	0.23060904
155.8	6.41E-12	1.29E-13	3.21E-11	0.00038459	8.35E-39	1.26E-11	0.23060904
155.9	6.42E-12	1.26E-13	3.24E-11	0.00038355	7.86E-39	1.20E-11	0.23175134
156	6.43E-12	1.34E-13	3.35E-11	0.00038103	8.08E-39	1.21E-11	0.23321595

114	5.88E-15	1.40E-15	8.71E-14	6.46E-05	1.06E-41	2.28E-11	0.47507085
114.1	6.20E-15	1.48E-15	9.06E-14	6.38E-05	1.06E-41	2.27E-11	0.47543138
114.2	6.36E-15	1.50E-15	9.18E-14	6.25E-05	1.06E-41	2.25E-11	0.47447136
114.3	6.52E-15	1.58E-15	9.42E-14	6.53E-05	1.09E-41	2.28E-11	0.47414957
114.4	6.45E-15	1.65E-15	9.88E-14	6.67E-05	1.15E-41	2.37E-11	0.47521613
114.5	6.45E-15	1.65E-15	9.88E-14	6.67E-05	1.15E-41	2.37E-11	0.47521613
114.6	6.83E-15	1.69E-15	1.05E-13	6.80E-05	1.11E-41	2.32E-11	0.47849371
114.7	6.81E-15	1.75E-15	1.01E-13	6.69E-05	1.09E-41	2.29E-11	0.47269929
114.8	6.76E-15	1.83E-15	1.05E-13	6.72E-05	1.10E-41	2.31E-11	0.47292085
114.9	6.47E-15	1.89E-15	1.09E-13	7.00E-05	1.08E-41	2.11E-11	0.46025147
115	6.47E-15	1.89E-15	1.09E-13	7.00E-05	1.08E-41	2.11E-11	0.46025147
115.1	6.25E-15	1.92E-15	1.10E-13	7.02E-05	1.08E-41	2.09E-11	0.46241649
115.2	6.25E-15	1.92E-15	1.10E-13	7.02E-05	1.08E-41	2.09E-11	0.46241649
115.3	6.00E-15	1.91E-15	1.15E-13	7.23E-05	1.05E-41	2.09E-11	0.46087692
115.4	6.43E-15	1.93E-15	1.18E-13	7.35E-05	1.20E-41	2.28E-11	0.4572258
115.5	6.67E-15	1.94E-15	1.23E-13	7.30E-05	1.15E-41	2.24E-11	0.46457929
115.6	7.00E-15	1.83E-15	1.25E-13	7.69E-05	1.29E-41	2.23E-11	0.46288583
115.7	8.30E-15	1.92E-15	1.50E-13	7.65E-05	1.63E-41	2.46E-11	0.46313856
115.8	8.96E-15	1.90E-15	1.57E-13	7.72E-05	1.73E-41	2.45E-11	0.46049974
115.9	9.35E-15	1.89E-15	1.52E-13	7.71E-05	1.67E-41	2.43E-11	0.46001194
116	9.72E-15	1.91E-15	1.53E-13	7.73E-05	1.81E-41	2.55E-11	0.45469002
116.1	1.00E-14	2.09E-15	1.72E-13	8.19E-05	1.99E-41	2.70E-11	0.4540613
116.2	1.03E-14	2.20E-15	1.77E-13	7.99E-05	2.03E-41	2.72E-11	0.45656488
116.3	1.20E-14	2.22E-15	1.79E-13	8.39E-05	2.17E-41	2.67E-11	0.45647609
116.4	1.27E-14	2.36E-15	1.93E-13	8.18E-05	2.35E-41	2.71E-11	0.45325988
116.5	1.31E-14	2.37E-15	2.03E-13	8.27E-05	2.31E-41	2.68E-11	0.45528945
116.6	1.46E-14	2.34E-15	2.18E-13	8.58E-05	2.62E-41	2.83E-11	0.45272933
116.7	1.46E-14	2.34E-15	2.18E-13	8.58E-05	2.62E-41	2.83E-11	0.45272933
116.8	1.46E-14	2.34E-15	2.18E-13	8.58E-05	2.62E-41	2.83E-11	0.45272933
116.9	1.46E-14	2.33E-15	2.22E-13	8.72E-05	2.55E-41	2.81E-11	0.45600518
117	1.47E-14	2.27E-15	2.24E-13	8.60E-05	2.49E-41	2.74E-11	0.45408046
117.1	1.57E-14	2.30E-15	2.29E-13	8.51E-05	2.61E-41	2.83E-11	0.45381287
117.2	1.63E-14	2.25E-15	2.28E-13	8.52E-05	2.58E-41	2.82E-11	0.45244026
117.3	1.67E-14	2.36E-15	2.34E-13	8.42E-05	2.58E-41	2.82E-11	0.44554324
117.4	1.75E-14	2.38E-15	2.38E-13	8.31E-05	2.59E-41	2.82E-11	0.44131451
117.5	1.83E-14	2.42E-15	2.49E-13	8.37E-05	2.59E-41	2.95E-11	0.43859643
117.6	1.81E-14	2.34E-15	2.69E-13	8.07E-05	2.38E-41	3.00E-11	0.42956369
117.7	1.89E-14	2.40E-15	2.75E-13	7.83E-05	2.35E-41	2.95E-11	0.42982376
117.8	1.99E-14	2.48E-15	2.81E-13	7.75E-05	2.62E-41	2.83E-11	0.42669914
117.9	1.93E-14	2.57E-15	2.81E-13	7.76E-05	2.62E-41	2.75E-11	0.42718123
118	1.88E-14	2.66E-15	2.87E-13	7.66E-05	2.46E-41	2.70E-11	0.42297818
118.1	1.85E-14	2.61E-15	2.97E-13	7.65E-05	2.40E-41	2.62E-11	0.42444585
118.2	2.01E-14	2.71E-15	3.19E-13	7.65E-05	2.71E-41	2.54E-11	0.42261517
118.3	1.99E-14	2.79E-15	3.28E-13	7.64E-05	2.76E-41	2.37E-11	0.41986784
118.4	2.02E-14	2.77E-15	3.34E-13	7.80E-05	2.90E-41	2.41E-11	0.41783369
118.5	2.19E-14	3.04E-15	3.56E-13	8.09E-05	3.29E-41	2.49E-11	0.41759986
118.6	2.21E-14	3.00E-15	3.61E-13	8.31E-05	3.27E-41	2.59E-11	0.41660423
118.7	2.19E-14	2.95E-15	3.65E-13	8.52E-05	3.28E-41	2.59E-11	0.41354001
118.8	2.07E-14	2.83E-15	3.61E-13	8.53E-05	3.17E-41	2.44E-11	0.41842127
118.9	2.10E-14	3.20E-15	4.04E-13	8.96E-05	3.62E-41	2.37E-11	0.41311427
119	2.16E-14	3.22E-15	4.22E-13	9.31E-05	3.75E-41	2.45E-11	0.41250212
119.1	2.21E-14	3.45E-15	4.33E-13	9.47E-05	3.89E-41	2.53E-11	0.41079051
119.2	2.40E-14	3.31E-15	4.52E-13	9.62E-05	3.90E-41	2.39E-11	0.41186744
119.3	2.45E-14	3.44E-15	4.77E-13	0.00010101	4.32E-41	2.51E-11	0.40972162
119.4	2.57E-14	3.45E-15	4.76E-13	0.00010144	4.20E-41	2.34E-11	0.41182074
119.5	2.55E-14	3.30E-15	4.70E-13	0.00010617	4.27E-41	2.39E-11	0.40745486
119.6	2.88E-14	3.41E-15	4.74E-13	0.00010949	4.46E-41	2.41E-11	0.40716789
119.7	3.11E-14	3.61E-15	4.70E-13	0.00010577	4.72E-41	2.37E-11	0.40926782
119.8	3.14E-14	3.70E-15	4.63E-13	0.00010404	4.63E-41	2.29E-11	0.40621694
119.9	3.12E-14	3.73E-15	4.80E-13	0.00010268	4.51E-41	2.30E-11	0.40388554

165	1.04E-11	2.13E-13	6.22E-11	0.00061848	2.08E-38	1.02E-11	0.25294898
165.1	1.04E-11	2.15E-13	6.59E-11	0.00062986	2.01E-38	1.05E-11	0.25565327
165.2	1.04E-11	2.15E-13	6.59E-11	0.00062986	2.01E-38	1.05E-11	0.25565327
165.3	1.05E-11	2.08E-13	6.59E-11	0.00064105	2.05E-38	1.01E-11	0.25522417
165.4	1.08E-11	2.12E-13	6.84E-11	0.00064824	1.89E-38	9.84E-12	0.25685972
165.5	1.08E-11	2.12E-13	6.84E-11	0.00064824	1.89E-38	9.84E-12	0.25685972
165.6	1.08E-11	2.12E-13	6.84E-11	0.00064824	1.89E-38	9.84E-12	0.25685972
165.7	1.08E-11	2.12E-13	6.84E-11	0.00064824	1.89E-38	9.84E-12	0.25685972
165.8	1.08E-11	2.12E-13	6.84E-11	0.00064824	1.89E-38	9.84E-12	0.25685972
165.9	1.06E-11	2.22E-13	6.92E-11	0.0006613	1.98E-38	1.01E-11	0.25820264
166	1.06E-11	2.22E-13	6.92E-11	0.0006613	1.98E-38	1.01E-11	0.25820264
166.1	1.03E-11	2.14E-13	7.30E-11	0.00067023	1.97E-38	1.02E-11	0.25994262
166.2	1.03E-11	2.01E-13	7.38E-11	0.00067421	1.86E-38	9.93E-12	0.26140218
166.3	1.03E-11	2.01E-13	7.38E-11	0.00067421	1.86E-38	9.93E-12	0.26140218
166.4	1.03E-11	2.01E-13	7.38E-11	0.00067421	1.86E-38	9.93E-12	0.26140218
166.5	1.03E-11	2.01E-13	7.38E-11	0.00067421	1.86E-38	9.93E-12	0.26140218
166.6	1.03E-11	2.01E-13	7.38E-11	0.00067421	1.86E-38	9.93E-12	0.26140218
166.7	1.09E-11	1.95E-13	7.40E-11	0.00067723	1.90E-38	1.01E-11	0.26029736
166.8	1.07E-11	1.98E-13	7.69E-11	0.00069241	1.96E-38	1.04E-11	0.2603935
166.9	1.09E-11	2.05E-13	8.01E-11	0.00068631	2.05E-38	1.05E-11	0.25795867
167	1.07E-11	2.11E-13	8.05E-11	0.00069458	2.11E-38	1.04E-11	0.25558424
167.1	1.07E-11	2.11E-13	8.05E-11	0.00069458	2.11E-38	1.04E-11	0.25558424
167.2	1.07E-11	2.11E-13	8.05E-11	0.00069458	2.11E-38	1.04E-11	0.25558424
167.3	1.09E-11	2.21E-13	8.45E-11	0.00070871	2.19E-38	1.06E-11	0.25651389
167.4	1.12E-11	2.17E-13	8.67E-11	0.00070224	2.20E-38	1.09E-11	0.25561558
167.5	1.13E-11	2.09E-13	9.05E-11	0.00071312	2.17E-38	1.10E-11	0.25579037
167.6	1.13E-11	2.09E-13	9.05E-11	0.00071312	2.17E-38	1.10E-11	0.25579037
167.7	1.20E-11	2.29E-13	8.74E-11	0.0007481	2.40E-38	1.07E-11	0.25107803
167.8	1.20E-11	2.29E-13	8.74E-11	0.0007481	2.40E-38	1.07E-11	0.25107803
167.9	1.20E-11	2.29E-13	8.74E-11	0.0007481	2.40E-38	1.07E-11	0.25107803
168	1.21E-11	2.32E-13	8.73E-11	0.00073791	2.41E-38	1.07E-11	0.25140564
168.1	1.21E-11	2.32E-13	8.73E-11	0.00073791	2.41E-38	1.07E-11	0.25140564
168.2	1.27E-11	2.35E-13	8.80E-11	0.00074354	2.59E-38	1.06E-11	0.25208336
168.3	1.21E-11	2.30E-13	8.80E-11	0.00074604	2.45E-38	1.04E-11	0.25140704
168.4	1.29E-11	2.29E-13	9.13E-11	0.00075027	2.45E-38	1.00E-11	0.25103251
168.5	1.29E-11	2.29E-13	9.13E-11	0.00075027	2.45E-38	1.00E-11	0.25103251
168.6	1.37E-11	2.21E-13	9.65E-11	0.00075056	2.59E-38	9.87E-12	0.2516696
168.7	1.41E-11	2.13E-13	9.83E-11	0.00074841	2.48E-38	9.36E-12	0.25487978
168.8	1.39E-11	2.18E-13	9.83E-11	0.00073785	2.34E-38	9.46E-12	0.25401009
168.9	1.39E-11	2.18E-13	9.83E-11	0.00073785	2.34E-38	9.46E-12	0.25401009
169	1.39E-11	2.18E-13	9.83E-11	0.00073785	2.34E-38	9.46E-12	0.25401009
169.1	1.39E-11	2.18E-13	9.83E-11	0.00073785	2.34E-38	9.46E-12	0.25401009
169.2	1.39E-11	2.18E-13	9.83E-11	0.00073785	2.34E-38	9.46E-12	0.25401009
169.3	1.39E-11	2.18E-13	9.83E-11	0.00073785	2.34E-38	9.46E-12	0.25401009
169.4	1.37E-11	2.17E-13	9.96E-11	0.00073556	2.27E-38	9.44E-12	0.25395249
169.5	1.47E-11	2.27E-13	1.06E-10	0.00074579	2.49E-38	9.71E-12	0.25230547
169.6	1.46E-11	2.28E-13	1.02E-10	0.000739	2.43E-38	9.70E-12	0.24974114
169.7	1.46E-11	2.28E-13	1.02E-10	0.000739	2.43E-38	9.70E-12	0.24974114
169.8	1.63E-11	2.47E-13	1.09E-10	0.00072109	2.75E-38	1.03E-11	0.24482617
169.9	1.61E-11	2.57E-13	1.13E-10	0.00074066	2.73E-38	1.05E-11	0.24857663
170	1.61E-11	2.50E-13	1.17E-10	0.0007462	2.61E-38	1.07E-11	0.24952602
170.1	1.66E-11	2.49E-13	1.17E-10	0.0007546	2.82E-38	1.09E-11	0.24779485
170.2	1.66E-11	2.49E-13	1.17E-10	0.0007546	2.82E-38	1.09E-11	0.24779485
170.3	1.79E-11	2.53E-13	1.23E-10	0.00072855	3.02E-38	1.17E-11	0.24777409
170.4	1.79E-11	2.53E-13	1.23E-10	0.00072855	3.02E-38	1.17E-11	0.24777409
170.5	1.79E-11	2.53E-13	1.23E-10	0.00072855	3.02E-38	1.17E-11	0.24777409
170.6	1.77E-11	2.47E-13	1.23E-10	0.00073063	2.99E-38	1.14E-11	0.24784204
170.7	1.77E-11	2.47E-13	1.23E-10	0.00073063	2.99E-38	1.14E-11	0.24784204
170.8	1.77E-11	2.47E-13	1.23E-10	0.00073063	2.99E-38	1.14E-11	0.24784204
170.9	1.77E-11	2.47E-13	1.23E-10	0.00073063	2.99E-38	1.14E-11	0.24784204
171	1.77E-11	2.47E-13	1.23E-10	0.00073063	2.99E-38	1.14E-11	0.24784204

126.1	7.61E-14	7.30E-15	1.25E-12	0.00011994	1.28E-40	2.89E-11	0.38158227
126.2	7.63E-14	7.38E-15	1.28E-12	0.00011935	1.25E-40	2.83E-11	0.38255545
126.3	7.53E-14	7.18E-15	1.28E-12	0.00011947	1.31E-40	2.81E-11	0.38028172
126.4	7.56E-14	7.36E-15	1.29E-12	0.00012091	1.34E-40	2.84E-11	0.38240073
126.5	7.49E-14	7.26E-15	1.25E-12	0.00012127	1.26E-40	2.79E-11	0.38041181
126.6	7.69E-14	7.31E-15	1.28E-12	0.00011947	1.27E-40	2.79E-11	0.3790151
126.7	8.08E-14	7.02E-15	1.29E-12	0.00012011	1.32E-40	2.77E-11	0.3779571
126.8	9.18E-14	7.31E-15	1.35E-12	0.00011757	1.39E-40	2.99E-11	0.3794231
126.9	9.20E-14	7.40E-15	1.38E-12	0.00011845	1.46E-40	3.11E-11	0.37898735
127	9.21E-14	7.47E-15	1.40E-12	0.00011429	1.41E-40	3.15E-11	0.38120939
127.1	9.21E-14	7.51E-15	1.41E-12	0.00011551	1.41E-40	3.10E-11	0.38194847
127.2	9.04E-14	7.45E-15	1.37E-12	0.00011727	1.53E-40	3.12E-11	0.387133
127.3	9.72E-14	7.23E-15	1.41E-12	0.00011771	1.62E-40	3.04E-11	0.3835365
127.4	1.01E-13	7.01E-15	1.43E-12	0.00012007	1.68E-40	3.00E-11	0.382475
127.5	1.01E-13	6.85E-15	1.47E-12	0.00012199	1.69E-40	2.89E-11	0.38097373
127.6	1.13E-13	6.99E-15	1.55E-12	0.00012451	1.75E-40	2.94E-11	0.37997037
127.7	1.13E-13	6.99E-15	1.55E-12	0.00012451	1.75E-40	2.94E-11	0.37997037
127.8	1.19E-13	7.28E-15	1.64E-12	0.00012752	1.91E-40	3.08E-11	0.37464107
127.9	1.23E-13	7.01E-15	1.73E-12	0.00012768	1.85E-40	3.10E-11	0.3729936
128	1.24E-13	6.97E-15	1.72E-12	0.00012591	1.90E-40	3.05E-11	0.37590638
128.1	1.24E-13	6.97E-15	1.72E-12	0.00012591	1.90E-40	3.05E-11	0.37590638
128.2	1.25E-13	7.16E-15	1.78E-12	0.00012517	1.88E-40	2.95E-11	0.37842226
128.3	1.34E-13	7.46E-15	1.76E-12	0.00012981	2.10E-40	3.01E-11	0.37620979
128.4	1.32E-13	7.35E-15	1.76E-12	0.00012791	1.96E-40	2.96E-11	0.37690335
128.5	1.28E-13	7.45E-15	1.72E-12	0.00012848	1.88E-40	2.88E-11	0.37427595
128.6	1.28E-13	7.59E-15	1.90E-12	0.00012972	1.89E-40	2.88E-11	0.36987383
128.7	1.29E-13	7.41E-15	1.95E-12	0.00012916	1.83E-40	2.83E-11	0.37010684
128.8	1.38E-13	6.85E-15	1.86E-12	0.00012776	1.73E-40	2.82E-11	0.36339829
128.9	1.36E-13	7.00E-15	1.87E-12	0.00012466	1.72E-40	2.79E-11	0.36244838
129	1.36E-13	7.76E-15	2.09E-12	0.00012743	1.80E-40	2.65E-11	0.36332162
129.1	1.36E-13	7.76E-15	2.09E-12	0.00012743	1.80E-40	2.65E-11	0.36332162
129.2	1.27E-13	8.34E-15	2.03E-12	0.00012922	1.78E-40	2.52E-11	0.35855183
129.3	1.30E-13	8.39E-15	1.93E-12	0.00013217	1.93E-40	2.67E-11	0.35783025
129.4	1.26E-13	8.59E-15	1.99E-12	0.00013172	1.89E-40	2.61E-11	0.35673185
129.5	1.23E-13	8.87E-15	2.03E-12	0.00013279	1.96E-40	2.55E-11	0.35694166
129.6	1.20E-13	8.94E-15	1.97E-12	0.00013437	1.96E-40	2.59E-11	0.35395349
129.7	1.23E-13	8.36E-15	1.94E-12	0.00012913	1.75E-40	2.48E-11	0.34850666
129.8	1.17E-13	8.42E-15	2.05E-12	0.00013147	1.63E-40	2.43E-11	0.34880267
129.9	1.17E-13	8.60E-15	2.01E-12	0.00013219	1.73E-40	2.41E-11	0.34668593
130	1.14E-13	8.79E-15	1.98E-12	0.00013095	1.80E-40	2.42E-11	0.34464476
130.1	1.15E-13	8.45E-15	2.00E-12	0.00013031	1.72E-40	2.39E-11	0.34564332
130.2	1.24E-13	8.77E-15	2.09E-12	0.00013112	1.91E-40	2.40E-11	0.34599507
130.3	1.24E-13	8.77E-15	2.09E-12	0.00013112	1.91E-40	2.40E-11	0.34599507
130.4	1.27E-13	9.12E-15	2.09E-12	0.0001332	2.05E-40	2.43E-11	0.34403103
130.5	1.31E-13	8.59E-15	2.13E-12	0.00013402	2.05E-40	2.47E-11	0.34258158
130.6	1.27E-13	9.04E-15	2.33E-12	0.00013141	2.13E-40	2.45E-11	0.34160578
130.7	1.35E-13	9.28E-15	2.32E-12	0.00013327	2.06E-40	2.43E-11	0.34341964
130.8	1.31E-13	9.28E-15	2.35E-12	0.00013379	1.96E-40	2.39E-11	0.34345319
130.9	1.36E-13	9.24E-15	2.31E-12	0.00013218	2.10E-40	2.45E-11	0.34234956
131	1.35E-13	9.34E-15	2.31E-12	0.000131	1.93E-40	2.42E-11	0.3412336
131.1	1.33E-13	9.20E-15	2.26E-12	0.00012855	1.74E-40	2.36E-11	0.34121784
131.2	1.29E-13	9.66E-15	2.29E-12	0.00013101	1.72E-40	2.30E-11	0.33861043
131.3	1.33E-13	9.44E-15	2.30E-12	0.00013337	1.71E-40	2.27E-11	0.34020649
131.4	1.39E-13	8.75E-15	2.20E-12	0.00012935	1.68E-40	2.23E-11	0.33766998
131.5	1.42E-13	8.59E-15	2.11E-12	0.00012774	1.73E-40	2.31E-11	0.33535984
131.6	1.54E-13	9.46E-15	2.21E-12	0.000125	1.84E-40	2.47E-11	0.33601541
131.7	1.55E-13	9.77E-15	2.19E-12	0.00012998	1.92E-40	2.48E-11	0.33616749
131.8	1.55E-13	9.77E-15	2.19E-12	0.00012998	1.92E-40	2.48E-11	0.33616749
131.9	1.60E-13	1.07E-14	2.30E-12	0.00012923	2.10E-40	2.55E-11	0.335648
132	1.67E-13	1.19E-14	2.38E-12	0.00013123	2.30E-40	2.48E-11	0.33105017
132.1	1.67E-13	1.19E-14	2.38E-12	0.00013123	2.30E-40	2.48E-11	0.33105017
132.2	1.67E-13	1.20E-14	2.37E-12	0.00013104	2.45E-40	2.50E-11	0.32701358
132.3	1.68E-13	1.22E-14	2.47E-12	0.00012955	2.39E-40	2.50E-11	0.32909102
132.4	1.61E-13	1.28E-14	2.50E-12	0.00013234	2.38E-40	2.37E-11	0.32592986
132.5	1.61E-13	1.28E-14	2.50E-12	0.00013234	2.38E-40	2.37E-11	0.32592986
132.6	1.61E-13	1.24E-14	2.46E-12	0.00013467	2.38E-40	2.34E-11	0.32436652
132.7	1.59E-13	1.33E-14	2.58E-12	0.00013563	2.47E-40	2.35E-11	0.32468225
132.8	1.59E-13	1.33E-14	2.58E-12	0.00013563	2.47E-40	2.35E-11	0.32468225
132.9	1.64E-13	1.44E-14	2.78E-12	0.00013683	2.75E-40	2.30E-11	0.32546743
133	1.61E-13	1.44E-14	2.87E-12	0.00014166	2.72E-40	2.23E-11	0.32232477
133.1	1.59E-13	1.47E-14	2.87E-12	0.00014471	2.85E-40	2.17E-11	0.32342917
133.2	1.62E-13	1.48E-14	2.80E-12	0.00014769	2.88E-40	2.21E-11	0.31904524
133.3	1.77E-13	1.58E-14	2.81E-12	0.00015044	3.29E-40	2.22E-11	0.32028314
133.4	1.77E-13	1.57E-14	2.89E-12	0.0001532	3.30E-40	2.21E-11	0.32256628
133.5	1.75E-13	1.52E-14	2.98E-12	0.00015311	3.19E-40	2.29E-11	0.32117135
133.6	1.77E-13	1.57E-14	3.03E-12	0.00015104	3.34E-40	2.26E-11	0.31922417
133.7	1.87E-13	1.54E-14	3.14E-12	0.0001468	3.03E-40	2.24E-11	0.32201312
133.8	1.87E-13	1.54E-14	3.14E-12	0.0001468	3.03E-40	2.24E-11	0.32201312
133.9	1.89E-13	1.45E-14	3.14E-12	0.0001446	3.03E-40	2.23E-11	0.31944717
134	1.91E-13	1.40E-14	3.23E-12	0.00014822	3.01E-40	2.21E-11	0.31592118
134.1	1.90E-13	1.46E-14	3.19E-12	0.00014738	2.99E-40	2.17E-11	0.31581312
134.2	1.78E-13	1.55E-14	3.14E-12	0.00014542	2.77E-40	2.04E-11	0.31714213
134.3	1.84E-13	1.47E-14	3.06E-12	0.00014935	2.83E-40	2.00E-11	0.31857204
134.4	1.96E-13	1.49E-14	3.00E-12	0.0001483	2.94E-40	2.08E-11	0.31709919
134.5	1.95E-13	1.51E-14	3.05E-12	0.00014941	3.00E-40	2.02E-11	0.31356572
134.6	1.86E-13	1.41E-14	3.08E-12	0.00015022	2.60E-40	2.01E-11	0.3149905
134.7	1.93E-13	1.47E-14	2.94E-12	0.00014938	2.62E-40	2.08E-11	0.31021697
134.8	1.96E-13	1.50E-14	3.17E-12	0.00015065	2.71E-40	2.06E-11	0.31289672
134.9	2.05E-13	1.43E-14	3.25E-12	0.00014846	2.51E-40	2.09E-11	0.31037055
180	8.43E-11	1.83E-13	6.97E-10	0.00117816	1.25E-37	1.17E-11	0.25393675
180.1	8.66E-11	1.87E-13	6.92E-10	0.00118034	1.20E-37	1.14E-11	0.25487558
180.2	9.11E-11	1.91E-13	7.20E-10	0.00116776	1.27E-37	1.17E-11	0.2525142
180.3	9.50E-11	1.94E-13	7.07E-10	0.00116267	1.34E-37	1.15E-11	0.25066303
180.4	1.02E-10	1.98E-13	7.10E-10	0.00117893	1.48E-37	1.14E-11	0.25127499
180.5	1.09E-10	2.07E-13	7.50E-10	0.00115645	1.65E-37	1.17E-11	0.25167699
180.6	1.06E-10	2.07E-13	7.49E-10	0.00116955	1.68E-37	1.18E-11	0.25153195
180.7	1.16E-10	2.19E-13	8.43E-10	0.00115585	1.97E-37	1.24E-11	0.25247977
180.8	1.16E-10	2.19E-13	8.43E-10	0.00115585	1.97E-37	1.24E-11	0.25247977
180.9	1.21E-10	2.15E-13	9.06E-10	0.00117718	2.01E-37	1.20E-11	0.25595004
181	1.21E-10	2.15E-13	9.06E-10	0.00117718	2.01E-37	1.20E-11	0.25595004
181.1	1.21E-10	2.15E-13	9.06E-10	0.00117718	2.01E-37	1.20E-11	0.25595004
181.2	1.21E-10	2.22E-13	9.22E-10	0.00119056	2.06E-37	1.20E-11	0.2572341
181.3	1.21E-10	2.22E-13	9.22E-10	0.00119056	2.06E-37	1.20E-11	0.2572341
181.4	1.24E-10	2.17E-13	9.42E-10	0.0011746	2.05E-37	1.20E-11	0.25915581
181.5	1.25E-10	2.27E-13	1.00E-09	0.00120325	2.10E-37	1.24E-11	0.26201572
181.6	1.30E-10	2.26E-13	1.05E-09	0.00124182	2.26E-37	1.25E-11	0.26263268
181.7	1.30E-10	2.26E-13	1.05E-09	0.00124182	2.26E-37	1.25E-11	0.26263268
181.8	1.30E-10	2.26E-13	1.05E-09	0.00124182	2.26E-37	1.25E-11	0.26263268
181.9	1.29E-10	2.17E-13	1.11E-09	0.00128036	2.51E-37	1.26E-11	0.26493976
182	1.29E-10	2.03E-13	1.13E-09	0.00130171	2.44E-37	1.26E-11	0.26752624
182.1	1.33E-10	1.99E-13	1.08E-09	0.00129633	2.48E-37	1.26E-11	0.26707111
182.2	1.32E-10	1.90E-13	1.10E-09	0.00128099	2.55E-37	1.27E-11	0.2686812
182.3	1.32E-10	1.90E-13	1.10E-09	0.00128099	2.55E-37	1.27E-11	0.2686812
182.4	1.32E-10	1.90E-13	1.10E-09	0.00128099	2.55E-37	1.27E-11	0.2686812
182.5	1.33E-10	1.88E-13	1.15E-09	0.00128803	2.64E-37	1.22E-11	0.26820145
182.6	1.33E-10	1.88E-13	1.15E-09	0.00128803	2.64E-37	1.22E-11	0.26820145
182.7	1.33E-10	1.88E-13	1.15E-09	0.00128803	2.64E-37	1.22E-11	0.26820145
182.8	1.33E-10	1.88E-13	1.15E-09	0.00128803	2.64E-37	1.22E-11	0.26820145
182.9	1.33E-10	1.85E-13	1.17E-09	0.00128352	2.76E-37	1.26E-11	0.26945587
183	1.33E-10	1.85E-13	1.17E-09	0.00128352	2.76E-37	1.26E-11	0.26945587
183.1	1.39E-10	1.84E-13	1.26E-09	0.0012806	3.06E-37	1.24E-11	0.26893429

141.1	9.56E-13	3.92E-14	6.24E-12	0.00022063	1.91E-39	1.64E-11	0.24682403
141.2	9.29E-13	3.85E-14	6.46E-12	0.00021906	1.82E-39	1.58E-11	0.24608737
141.3	9.33E-13	4.13E-14	6.67E-12	0.00022015	1.95E-39	1.56E-11	0.24599434
141.4	9.64E-13	4.15E-14	6.98E-12	0.00022109	2.02E-39	1.65E-11	0.24860558
141.5	9.73E-13	4.05E-14	7.01E-12	0.00022107	2.07E-39	1.63E-11	0.25030765
141.6	9.73E-13	4.05E-14	7.01E-12	0.00022107	2.07E-39	1.63E-11	0.25030765
141.7	1.00E-12	4.20E-14	7.01E-12	0.00022151	2.11E-39	1.64E-11	0.25063604
141.8	1.00E-12	4.20E-14	7.01E-12	0.00022151	2.11E-39	1.64E-11	0.25063604
141.9	9.87E-13	4.29E-14	6.88E-12	0.00021942	2.10E-39	1.60E-11	0.24924308
142	9.87E-13	4.29E-14	6.88E-12	0.00021942	2.10E-39	1.60E-11	0.24924308
142.1	1.00E-12	4.26E-14	6.90E-12	0.00022451	2.12E-39	1.62E-11	0.25094594
142.2	1.02E-12	4.24E-14	7.01E-12	0.00020951	1.80E-39	1.57E-11	0.2505561
142.3	1.06E-12	4.30E-14	6.81E-12	0.00021044	1.90E-39	1.64E-11	0.25025626
142.4	1.10E-12	4.40E-14	7.33E-12	0.00021332	1.95E-39	1.61E-11	0.25033756
142.5	1.21E-12	4.28E-14	7.23E-12	0.00022438	1.97E-39	1.66E-11	0.25052441
142.6	1.16E-12	4.81E-14	7.73E-12	0.00022086	1.96E-39	1.70E-11	0.25250288
142.7	1.16E-12	4.81E-14	7.73E-12	0.00022086	1.96E-39	1.70E-11	0.25250288
142.8	1.28E-12	4.74E-14	7.87E-12	0.00023098	2.13E-39	1.70E-11	0.2525625
142.9	1.27E-12	4.54E-14	8.00E-12	0.00023474	2.14E-39	1.77E-11	0.25212034
143	1.27E-12	4.54E-14	8.00E-12	0.00023474	2.14E-39	1.77E-11	0.25212034
143.1	1.34E-12	4.68E-14	8.21E-12	0.00023679	2.32E-39	1.75E-11	0.24966674
143.2	1.40E-12	5.00E-14	8.30E-12	0.00024615	2.50E-39	1.71E-11	0.24707578
143.3	1.29E-12	4.97E-14	8.30E-12	0.00024834	2.21E-39	1.65E-11	0.24784772
143.4	1.28E-12	4.83E-14	8.65E-12	0.00024951	2.12E-39	1.67E-11	0.25057376
143.5	1.35E-12	4.78E-14	8.91E-12	0.00025739	2.13E-39	1.62E-11	0.2508141
143.6	1.42E-12	4.95E-14	9.16E-12	0.00025274	2.21E-39	1.64E-11	0.25076456
143.7	1.42E-12	4.95E-14	9.16E-12	0.00025274	2.21E-39	1.64E-11	0.25076456
143.8	1.39E-12	4.82E-14	9.21E-12	0.00025282	2.31E-39	1.64E-11	0.25093747
143.9	1.25E-12	4.62E-14	9.70E-12	0.00024939	1.96E-39	1.51E-11	0.25425645
144	1.16E-12	4.52E-14	9.84E-12	0.0002501	1.69E-39	1.42E-11	0.25295148
144.1	1.14E-12	4.64E-14	9.96E-12	0.00025043	1.69E-39	1.36E-11	0.25232717
144.2	1.21E-12	4.81E-14	1.08E-11	0.00025121	2.02E-39	1.36E-11	0.25160662
144.3	1.26E-12	5.08E-14	1.13E-11	0.00025893	2.06E-39	1.36E-11	0.25217677
144.4	1.35E-12	5.26E-14	1.13E-11	0.00025579	2.07E-39	1.41E-11	0.24967375
144.5	1.35E-12	5.26E-14	1.13E-11	0.00025579	2.07E-39	1.41E-11	0.24967375
144.6	1.43E-12	5.34E-14	1.22E-11	0.00026505	2.16E-39	1.36E-11	0.24452262
144.7	1.72E-12	5.34E-14	1.27E-11	0.00027189	2.47E-39	1.32E-11	0.24057858
144.8	1.72E-12	5.34E-14	1.27E-11	0.00027189	2.47E-39	1.32E-11	0.24057858
144.9	1.72E-12	5.34E-14	1.27E-11	0.00027189	2.47E-39	1.32E-11	0.24057858
145	1.81E-12	5.54E-14	1.35E-11	0.00026705	2.26E-39	1.29E-11	0.24171815
145.1	1.88E-12	5.62E-14	1.37E-11	0.00027858	2.52E-39	1.32E-11	0.24177446
145.2	2.00E-12	5.31E-14	1.34E-11	0.00027572	2.49E-39	1.42E-11	0.23929836
145.3	2.00E-12	5.31E-14	1.34E-11	0.00027572	2.49E-39	1.42E-11	0.23929836
145.4	2.10E-12	5.44E-14	1.34E-11	0.00027159	2.54E-39	1.42E-11	0.23748568
145.5	2.16E-12	5.41E-14	1.31E-11	0.00027837	2.58E-39	1.39E-11	0.23583533
145.6	2.27E-12	5.57E-14	1.30E-11	0.00028369	2.71E-39	1.41E-11	0.23378924
145.7	2.31E-12	5.72E-14	1.43E-11	0.00029241	2.88E-39	1.45E-11	0.23542513
145.8	2.31E-12	5.72E-14	1.43E-11	0.00029241	2.88E-39	1.45E-11	0.23542513
145.9	2.31E-12	5.72E-14	1.43E-11	0.00029241	2.88E-39	1.45E-11	0.23542513
146	2.39E-12	5.68E-14	1.46E-11	0.00030222	3.08E-39	1.44E-11	0.23434734
146.1	2.54E-12	5.76E-14	1.50E-11	0.00030734	3.36E-39	1.43E-11	0.23218553
146.2	2.67E-12	5.79E-14	1.51E-11	0.00031383	3.46E-39	1.43E-11	0.23025162
146.3	2.73E-12	5.91E-14	1.54E-11	0.00031476	3.46E-39	1.38E-11	0.22840446
146.4	2.73E-12	5.91E-14	1.54E-11	0.00031476	3.46E-39	1.38E-11	0.22840446
146.5	2.73E-12	5.91E-14	1.54E-11	0.00031476	3.46E-39	1.38E-11	0.22840446
146.6	2.73E-12	5.91E-14	1.54E-11	0.00031476	3.46E-39	1.38E-11	0.22840446
146.7	2.67E-12	6.10E-14	1.60E-11	0.0003191	3.12E-39	1.37E-11	0.22964557
146.8	2.63E-12	6.38E-14	1.62E-11	0.00032222	3.44E-39	1.40E-11	0.22927534
146.9	2.63E-12	6.38E-14	1.62E-11	0.00032222	3.44E-39	1.40E-11	0.22927534
147	2.62E-12	6.61E-14	1.67E-11	0.00032193	3.50E-39	1.42E-11	0.22952406
147.1	2.72E-12	6.86E-14	1.81E-11	0.00033349	3.77E-39	1.39E-11	0.23054312
147.2	2.85E-12	6.87E-14	1.82E-11	0.00033132	3.71E-39	1.35E-11	0.22909436
147.3	2.84E-12	7.16E-14	1.74E-11	0.00034276	3.56E-39	1.33E-11	0.2278705
147.4	2.91E-12	7.14E-14	1.77E-11	0.00033998	3.70E-39	1.35E-11	0.22706748
147.5	2.88E-12	6.91E-14	1.80E-11	0.00033876	3.55E-39	1.27E-11	0.22625213
147.6	2.88E-12	6.91E-14	1.80E-11	0.00033876	3.55E-39	1.27E-11	0.22625213
147.7	2.88E-12	6.91E-14	1.80E-11	0.00033876	3.55E-39	1.27E-11	0.22625213
147.8	2.88E-12	6.91E-14	1.80E-11	0.00033876	3.55E-39	1.27E-11	0.22625213
147.9	3.04E-12	7.00E-14	1.77E-11	0.00033647	3.76E-39	1.35E-11	0.22402238
148	3.04E-12	7.00E-14	1.77E-11	0.00033647	3.76E-39	1.35E-11	0.22402238
148.1	3.04E-12	7.00E-14	1.77E-11	0.00033647	3.76E-39	1.35E-11	0.22402238
148.2	2.83E-12	7.14E-14	1.76E-11	0.00033022	3.48E-39	1.36E-11	0.22401969
148.3	2.83E-12	7.14E-14	1.76E-11	0.00033022	3.48E-39	1.36E-11	0.22401969
148.4	3.21E-12	7.18E-14	1.97E-11	0.0003396	3.84E-39	1.35E-11	0.22529354
148.5	3.21E-12	7.21E-14	2.01E-11	0.00032926	3.73E-39	1.35E-11	0.22546625
148.6	3.21E-12	7.21E-14	2.01E-11	0.00032926	3.73E-39	1.35E-11	0.22546625
148.7	3.21E-12	7.21E-14	2.01E-11	0.00032926	3.73E-39	1.35E-11	0.22546625
148.8	3.10E-12	7.40E-14	2.04E-11	0.00032631	3.47E-39	1.35E-11	0.22634774
148.9	2.96E-12	7.32E-14	2.00E-11	0.00032614	3.29E-39	1.33E-11	0.22790704
149	2.99E-12	7.50E-14	2.08E-11	0.00033164	3.42E-39	1.31E-11	0.23076804
149.1	2.99E-12	7.50E-14	2.08E-11	0.00033164	3.42E-39	1.31E-11	0.23076804
149.2	2.99E-12	7.50E-14	2.08E-11	0.00033164	3.42E-39	1.31E-11	0.23076804
149.3	3.22E-12	7.49E-14	2.15E-11	0.00034987	3.64E-39	1.29E-11	0.22930504
149.4	3.13E-12	7.78E-14	2.18E-11	0.00034679	3.54E-39	1.29E-11	0.22957569
149.5	3.32E-12	7.95E-14	2.30E-11	0.00036902	4.14E-39	1.34E-11	0.22801777
149.6	3.34E-12	8.38E-14	2.42E-11	0.00037483	4.57E-39	1.39E-11	0.22786447
149.7	3.26E-12	8.97E-14	2.37E-11	0.00036972	4.61E-39	1.36E-11	0.23014599
149.8	3.31E-12	8.95E-14	2.44E-11	0.00037738	4.77E-39	1.34E-11	0.22985842
149.9	3.31E-12	8.95E-14	2.44E-11	0.00037738	4.77E-39	1.34E-11	0.22985842
195	7.31E-10	1.57E-13	1.07E-08	0.00215964	1.22E-35	1.53E-11	0.30468266
195.1	7.31E-10	1.57E-13	1.07E-08	0.00215964	1.22E-35	1.53E-11	0.30468266
195.2	7.31E-10	1.57E-13	1.07E-08	0.00215964	1.22E-35	1.53E-11	0.30468266
195.3	7.31E-10	1.57E-13	1.07E-08	0.00215964	1.22E-35	1.53E-11	0.30468266
195.4	7.57E-10	1.60E-13	1.06E-08	0.00219226	1.27E-35	1.49E-11	0.30779659
195.5	8.19E-10	1.63E-13	1.15E-08	0.00221262	1.44E-35	1.49E-11	0.30885716
195.6	8.19E-10	1.63E-13	1.15E-08	0.00221262	1.44E-35	1.49E-11	0.30885716
195.7	8.19E-10	1.63E-13	1.15E-08	0.00221262	1.44E-35	1.49E-11	0.30885716
195.8	8.19E-10	1.63E-13	1.15E-08	0.00221262	1.44E-35	1.49E-11	0.30885716
195.9	8.34E-10	1.58E-13	1.14E-08	0.00222485	1.47E-35	1.54E-11	0.30924153
196	8.34E-10	1.58E-13	1.14E-08	0.00222485	1.47E-35	1.54E-11	0.30924153
196.1	8.64E-10	1.55E-13	1.15E-08	0.00222072	1.43E-35	1.52E-11	0.30927918
196.2	8.64E-10	1.55E-13	1.15E-08	0.00222072	1.43E-35	1.52E-11	0.30927918
196.3	8.64E-10	1.55E-13	1.15E-08	0.00222072	1.43E-35	1.52E-11	0.30927918
196.4	8.64E-10	1.55E-13	1.15E-08	0.00222072	1.43E-35	1.52E-11	0.30927918
196.5	9.03E-10	1.47E-13	1.18E-08	0.00223181	1.42E-35	1.49E-11	0.30898344
196.6	9.03E-10	1.47E-13	1.18E-08	0.00223181	1.42E-35	1.49E-11	0.30898344
196.7	8.82E-10	1.58E-13	1.23E-08	0.00223485	1.48E-35	1.48E-11	0.30915818
196.8	8.82E-10	1.58E-13	1.23E-08	0.00223485	1.48E-35	1.48E-11	0.30915818
196.9	8.92E-10	1.57E-13	1.24E-08	0.00224747	1.47E-35	1.45E-11	0.31170653
197	9.25E-10	1.51E-13	1.28E-08	0.00222752	1.54E-35	1.47E-11	0.30914569
197.1	9.34E-10	1.49E-13	1.27E-08	0.0022554	1.60E-35	1.44E-11	0.30656741
197.2	9.34E-10	1.49E-13	1.27E-08	0.0022554	1.60E-35	1.44E-11	0.30656741
197.3	9.57E-10	1.49E-13	1.27E-08	0.00224993	1.70E-35	1.42E-11	0.30631668
197.4	9.52E-10	1.42E-13	1.28E-08	0.00229683	1.68E-35	1.45E-11	0.30773644
197.5	9.52E-10	1.42E-13	1.28E-08	0.00229683	1.68E-35	1.45E-11	0.30773644
197.6	9.46E-10	1.37E-13	1.31E-08	0.00231061	1.69E-35	1.47E-11	0.30824473
197.7	9.46E-10	1.37E-13	1.31E-08	0.00231061	1.69E-35	1.47E-11	0.30824473
197.8	9.46E-10	1.37E-13	1.31E-08	0.00231061	1.69E-35	1.47E-11	0.30824473
197.9	9.71E-10	1.29E-13	1.33E-08	0.00234552	1.64E-35	1.47E-11	0.30780577
198	9.66E-10	1.34E-13	1.34E-08	0.00237371	1.69E-35	1.46E-11	0.30632788
198.1	9.91E-10	1.38E-13	1.38E-08	0.00237197	1.77E-35	1.45E-11	0.30615771

156.1	6.43E-12	1.34E-13	3.35E-11	0.00038103	8.08E-39	1.21E-11	0.23321595
156.2	6.43E-12	1.34E-13	3.35E-11	0.00038103	8.08E-39	1.21E-11	0.23321595
156.3	6.43E-12	1.34E-13	3.35E-11	0.00038103	8.08E-39	1.21E-11	0.23321595
156.4	6.70E-12	1.35E-13	3.20E-11	0.00038895	8.93E-39	1.18E-11	0.23511304
156.5	6.63E-12	1.36E-13	3.27E-11	0.00039248	9.33E-39	1.17E-11	0.235636
156.6	6.63E-12	1.36E-13	3.27E-11	0.00039248	9.33E-39	1.17E-11	0.235636
156.7	6.63E-12	1.36E-13	3.27E-11	0.00039248	9.33E-39	1.17E-11	0.235636
156.8	6.63E-12	1.36E-13	3.27E-11	0.00039248	9.33E-39	1.17E-11	0.235636
156.9	6.69E-12	1.32E-13	3.35E-11	0.00039075	9.20E-39	1.18E-11	0.23772942
157	6.69E-12	1.32E-13	3.35E-11	0.00039075	9.20E-39	1.18E-11	0.23772942
157.1	6.41E-12	1.28E-13	3.60E-11	0.00038027	9.09E-39	1.25E-11	0.23896854
157.2	6.17E-12	1.35E-13	3.50E-11	0.00037646	8.82E-39	1.25E-11	0.2391354
157.3	6.22E-12	1.41E-13	3.59E-11	0.00037563	8.83E-39	1.23E-11	0.241306
157.4	5.97E-12	1.46E-13	3.61E-11	0.00037733	8.20E-39	1.18E-11	0.24250255
157.5	6.05E-12	1.50E-13	3.72E-11	0.00037477	8.77E-39	1.17E-11	0.24192528
157.6	6.05E-12	1.50E-13	3.72E-11	0.00037477	8.77E-39	1.17E-11	0.24192528
157.7	6.05E-12	1.50E-13	3.72E-11	0.00037477	8.77E-39	1.17E-11	0.24192528
157.8	6.26E-12	1.46E-13	3.81E-11	0.00038101	9.14E-39	1.14E-11	0.24037442
157.9	6.91E-12	1.60E-13	4.24E-11	0.00039942	1.09E-38	1.20E-11	0.23890948
158	6.91E-12	1.60E-13	4.24E-11	0.00039942	1.09E-38	1.20E-11	0.23890948
158.1	6.58E-12	1.56E-13	4.02E-11	0.00040367	1.01E-38	1.19E-11	0.24371389
158.2	6.42E-12	1.60E-13	4.29E-11	0.00042043	9.90E-39	1.19E-11	0.24476627
158.3	6.50E-12	1.58E-13	4.19E-11	0.00043005	1.05E-38	1.18E-11	0.24470562
158.4	6.23E-12	1.62E-13	4.20E-11	0.00042343	9.91E-39	1.15E-11	0.24487371
158.5	6.46E-12	1.67E-13	4.19E-11	0.00042711	1.02E-38	1.15E-11	0.24598758
158.6	6.34E-12	1.62E-13	4.17E-11	0.00041907	1.05E-38	1.18E-11	0.24278105
158.7	6.24E-12	1.63E-13	4.11E-11	0.00042849	1.10E-38	1.17E-11	0.24196668
158.8	6.24E-12	1.63E-13	4.11E-11	0.00042849	1.10E-38	1.17E-11	0.24196668
158.9	6.18E-12	1.61E-13	4.12E-11	0.00042475	1.07E-38	1.13E-11	0.24485288
159	6.21E-12	1.65E-13	4.14E-11	0.00042244	1.10E-38	1.11E-11	0.24687767
159.1	6.28E-12	1.59E-13	4.13E-11	0.00042244	1.08E-38	1.11E-11	0.24720167
159.2	6.67E-12	1.57E-13	4.20E-11	0.00043084	1.12E-38	1.12E-11	0.24796893
159.3	6.67E-12	1.57E-13	4.20E-11	0.00043084	1.12E-38	1.12E-11	0.24796893
159.4	6.67E-12	1.57E-13	4.20E-11	0.00043084	1.12E-38	1.12E-11	0.24796893
159.5	6.67E-12	1.57E-13	4.20E-11	0.00043084	1.12E-38	1.12E-11	0.24796893
159.6	6.60E-12	1.67E-13	4.28E-11	0.00043814	1.17E-38	1.14E-11	0.24798615
159.7	6.60E-12	1.67E-13	4.28E-11	0.00043814	1.17E-38	1.14E-11	0.24798615
159.8	6.60E-12	1.67E-13	4.28E-11	0.00043814	1.17E-38	1.14E-11	0.24798615
159.9	6.96E-12	1.73E-13	4.44E-11	0.00044236	1.30E-38	1.17E-11	0.24682593
160	6.84E-12	1.76E-13	4.30E-11	0.00043688	1.21E-38	1.15E-11	0.24693194
160.1	6.54E-12	1.81E-13	4.29E-11	0.0004361	1.22E-38	1.16E-11	0.24738082
160.2	6.54E-12	1.81E-13	4.29E-11	0.0004361	1.22E-38	1.16E-11	0.24738082
160.3	7.23E-12	1.75E-13	4.39E-11	0.00045307	1.37E-38	1.13E-11	0.24280494
160.4	7.55E-12	1.75E-13	4.46E-11	0.00045228	1.41E-38	1.13E-11	0.24193321
160.5	7.81E-12	1.73E-13	4.56E-11	0.00046506	1.52E-38	1.15E-11	0.24204031
160.6	7.62E-12	1.66E-13	4.49E-11	0.00047225	1.43E-38	1.09E-11	0.24429493
160.7	7.84E-12	1.66E-13	4.42E-11	0.00049419	1.51E-38	1.08E-11	0.24118142
160.8	7.84E-12	1.66E-13	4.42E-11	0.00049419	1.51E-38	1.08E-11	0.24118142
160.9	7.84E-12	1.66E-13	4.42E-11	0.00049419	1.51E-38	1.08E-11	0.24118142
161	7.67E-12	1.71E-13	4.39E-11	0.00049633	1.51E-38	1.05E-11	0.24213613
161.1	7.56E-12	1.67E-13	4.58E-11	0.00049977	1.47E-38	1.09E-11	0.24399801
161.2	7.56E-12	1.67E-13	4.58E-11	0.00049977	1.47E-38	1.09E-11	0.24399801
161.3	7.17E-12	1.72E-13	4.49E-11	0.00050131	1.40E-38	1.07E-11	0.24651326
161.4	7.17E-12	1.72E-13	4.49E-11	0.00050131	1.40E-38	1.07E-11	0.24651326
161.5	7.67E-12	1.84E-13	4.70E-11	0.00051984	1.59E-38	1.12E-11	0.24811535
161.6	7.71E-12	1.84E-13	4.84E-11	0.00051128	1.56E-38	1.12E-11	0.24705818
161.7	7.22E-12	1.93E-13	4.51E-11	0.00051907	1.47E-38	1.12E-11	0.24783192
161.8	7.50E-12	2.06E-13	4.56E-11	0.00052901	1.60E-38	1.07E-11	0.24806847
161.9	7.50E-12	2.06E-13	4.56E-11	0.00052901	1.60E-38	1.07E-11	0.24806847
162	7.50E-12	2.06E-13	4.56E-11	0.00052901	1.60E-38	1.07E-11	0.24806847
162.1	7.55E-12	2.06E-13	4.47E-11	0.00053147	1.63E-38	1.05E-11	0.25092182
162.2	8.02E-12	2.15E-13	4.57E-11	0.00053034	1.69E-38	1.04E-11	0.25158192
162.3	8.02E-12	2.15E-13	4.57E-11	0.00053034	1.69E-38	1.04E-11	0.25158192
162.4	7.93E-12	2.18E-13	4.57E-11	0.00053174	1.71E-38	1.02E-11	0.25141985
162.5	8.08E-12	2.07E-13	4.61E-11	0.00053845	1.72E-38	1.00E-11	0.25336083
162.6	8.08E-12	2.07E-13	4.61E-11	0.00053845	1.72E-38	1.00E-11	0.25336083
162.7	8.34E-12	2.07E-13	4.70E-11	0.00054547	1.84E-38	9.97E-12	0.24989398
162.8	8.34E-12	2.07E-13	4.70E-11	0.00054547	1.84E-38	9.97E-12	0.24989398
162.9	8.07E-12	2.09E-13	4.57E-11	0.00054832	1.73E-38	1.00E-11	0.2506781
163	8.07E-12	2.09E-13	4.57E-11	0.00054832	1.73E-38	1.00E-11	0.2506781
163.1	8.30E-12	2.15E-13	4.78E-11	0.00056345	1.80E-38	9.99E-12	0.25018906
163.2	8.30E-12	2.15E-13	4.78E-11	0.00056345	1.80E-38	9.99E-12	0.25018906
163.3	8.27E-12	2.06E-13	4.73E-11	0.00056595	1.69E-38	9.63E-12	0.25043974
163.4	8.27E-12	2.06E-13	4.73E-11	0.00056595	1.69E-38	9.63E-12	0.25043974
163.5	8.72E-12	2.11E-13	4.90E-11	0.00058094	1.91E-38	9.85E-12	0.24956962
163.6	8.72E-12	2.11E-13	4.90E-11	0.00058094	1.91E-38	9.85E-12	0.24956962
163.7	8.57E-12	2.15E-13	4.98E-11	0.00058771	1.91E-38	9.71E-12	0.25237977
163.8	8.65E-12	2.10E-13	4.98E-11	0.00058349	1.92E-38	9.73E-12	0.25158773
163.9	8.65E-12	2.10E-13	4.98E-11	0.00058349	1.92E-38	9.73E-12	0.25158773
164	8.65E-12	2.10E-13	4.98E-11	0.00058349	1.92E-38	9.73E-12	0.25158773
164.1	8.61E-12	2.09E-13	5.07E-11	0.00059146	1.89E-38	9.35E-12	0.25122597
164.2	8.78E-12	2.09E-13	5.09E-11	0.0005851	1.88E-38	9.46E-12	0.24917428
164.3	8.87E-12	2.10E-13	5.21E-11	0.00057953	1.81E-38	9.46E-12	0.24924503
164.4	8.87E-12	2.10E-13	5.21E-11	0.00057953	1.81E-38	9.46E-12	0.24924503
164.5	9.30E-12	2.14E-13	5.20E-11	0.00059379	1.89E-38	9.54E-12	0.24717458
164.6	9.79E-12	2.10E-13	5.51E-11	0.00059638	1.97E-38	9.63E-12	0.24836482
164.7	9.79E-12	2.10E-13	5.51E-11	0.00059638	1.97E-38	9.63E-12	0.24836482
164.8	9.79E-12	2.10E-13	5.51E-11	0.00059638	1.97E-38	9.63E-12	0.24836482
164.9	1.07E-11	2.03E-13	5.85E-11	0.00061073	2.09E-38	9.88E-12	0.24757382
210	1.96E-09	1.63E-13	3.86E-08	0.00277312	9.13E-35	1.88E-11	0.31211504
210.1	1.96E-09	1.63E-13	3.86E-08	0.00277312	9.13E-35	1.88E-11	0.31211504
210.2	1.96E-09	1.63E-13	3.86E-08	0.00277312	9.13E-35	1.88E-11	0.31211504
210.3	1.96E-09	1.63E-13	3.86E-08	0.00277312	9.13E-35	1.88E-11	0.31211504
210.4	1.96E-09	1.63E-13	3.86E-08	0.00277312	9.13E-35	1.88E-11	0.31211504
210.5	1.96E-09	1.63E-13	3.86E-08	0.00277312	9.13E-35	1.88E-11	0.31211504
210.6	2.01E-09	1.66E-13	3.91E-08	0.00278281	9.19E-35	1.92E-11	0.31202135
210.7	2.01E-09	1.66E-13	3.91E-08	0.00278281	9.19E-35	1.92E-11	0.31202135
210.8	2.01E-09	1.66E-13	3.91E-08	0.00278281	9.19E-35	1.92E-11	0.31202135
210.9	2.01E-09	1.66E-13	3.91E-08	0.00278281	9.19E-35	1.92E-11	0.31202135
211	2.04E-09	1.64E-13	3.95E-08	0.00276993	9.11E-35	1.92E-11	0.31078768
211.1	2.04E-09	1.64E-13	3.95E-08	0.00276993	9.11E-35	1.92E-11	0.31078768
211.2	2.04E-09	1.64E-13	3.95E-08	0.00276993	9.11E-35	1.92E-11	0.31078768
211.3	2.10E-09	1.68E-13	4.07E-08	0.00276603	9.59E-35	1.94E-11	0.31037861
211.4	2.03E-09	1.69E-13	4.12E-08	0.00269676	9.04E-35	1.87E-11	0.30950505
211.5	2.03E-09	1.69E-13	4.12E-08	0.00269676	9.04E-35	1.87E-11	0.30950505
211.6	2.03E-09	1.69E-13	4.12E-08	0.00269676	9.04E-35	1.87E-11	0.30950505
211.7	2.03E-09	1.69E-13	4.12E-08	0.00269676	9.04E-35	1.87E-11	0.30950505
211.8	2.01E-09	1.69E-13	4.25E-08	0.00270576	9.56E-35	1.92E-11	0.30861482
211.9	2.01E-09	1.69E-13	4.25E-08	0.00270576	9.56E-35	1.92E-11	0.30861482
212	1.95E-09	1.70E-13	4.22E-08	0.00269869	9.59E-35	1.87E-11	0.30788211
212.1	2.02E-09	1.76E-13	4.32E-08	0.00271877	1.10E-34	1.98E-11	0.30612697
212.2	2.02E-09	1.76E-13	4.32E-08	0.00271877	1.10E-34	1.98E-11	0.30612697
212.3	2.04E-09	1.75E-13	4.43E-08	0.00268378	1.07E-34	2.02E-11	0.30292105
212.4	2.04E-09	1.75E-13	4.43E-08	0.00268378	1.07E-34	2.02E-11	0.30292105
212.5	2.04E-09	1.75E-13	4.43E-08	0.00268378	1.07E-34	2.02E-11	0.30292105
212.6	2.04E-09	1.75E-13	4.43E-08	0.00268378	1.07E-34	2.02E-11	0.30292105
212.7	2.08E-09	1.69E-13	4.43E-08	0.00268213	1.09E-34	2.05E-11	0.2997552
212.8	2.05E-09	1.71E-13	4.34E-08	0.00269724	1.14E-34	2.09E-11	0.30112997
212.9	2.05E-09	1.71E-13	4.34E-08	0.00269724	1.14E-34	2.09E-11	0.30112997
213	2.05E-09	1.71E-13	4.34E-08	0.00269724	1.14E-34	2.09E-11	0.30112997
213.1	2.05E-09	1.71E-13	4.34E-08	0.00269724	1.14E-34	2.09E-11	0.30112997

171.1	1.82E-11	2.47E-13	1.19E-10	0.00073614	2.94E-38	1.14E-11	0.24760227
171.2	1.90E-11	2.39E-13	1.21E-10	0.00073864	2.91E-38	1.15E-11	0.24981928
171.3	1.90E-11	2.29E-13	1.24E-10	0.00074184	2.90E-38	1.14E-11	0.25104913
171.4	1.90E-11	2.29E-13	1.24E-10	0.00074184	2.90E-38	1.14E-11	0.25104913
171.5	1.87E-11	2.22E-13	1.27E-10	0.00073361	2.67E-38	1.13E-11	0.25081517
171.6	1.87E-11	2.22E-13	1.27E-10	0.00073361	2.67E-38	1.13E-11	0.25081517
171.7	1.91E-11	2.25E-13	1.33E-10	0.00072362	2.71E-38	1.13E-11	0.25065153
171.8	2.04E-11	2.32E-13	1.35E-10	0.00073787	2.89E-38	1.13E-11	0.24963811
171.9	2.01E-11	2.32E-13	1.32E-10	0.00075223	2.95E-38	1.13E-11	0.24808055
172	2.01E-11	2.32E-13	1.32E-10	0.00075223	2.95E-38	1.13E-11	0.24808055
172.1	1.96E-11	2.28E-13	1.34E-10	0.00074114	3.00E-38	1.10E-11	0.24907199
172.2	2.01E-11	2.41E-13	1.37E-10	0.00076174	3.12E-38	1.13E-11	0.25236184
172.3	2.01E-11	2.41E-13	1.37E-10	0.00076174	3.12E-38	1.13E-11	0.25236184
172.4	2.02E-11	2.45E-13	1.39E-10	0.00077053	3.26E-38	1.11E-11	0.25201373
172.5	2.11E-11	2.50E-13	1.45E-10	0.00076351	3.46E-38	1.12E-11	0.25162268
172.6	2.11E-11	2.50E-13	1.45E-10	0.00076351	3.46E-38	1.12E-11	0.25162268
172.7	2.12E-11	2.52E-13	1.49E-10	0.00076568	3.49E-38	1.13E-11	0.25025167
172.8	2.12E-11	2.63E-13	1.49E-10	0.00078111	3.36E-38	1.08E-11	0.25125173
172.9	2.15E-11	2.55E-13	1.57E-10	0.00077469	3.53E-38	1.08E-11	0.25547835
173	2.15E-11	2.55E-13	1.57E-10	0.00077469	3.53E-38	1.08E-11	0.25547835
173.1	2.21E-11	2.40E-13	1.57E-10	0.00078337	3.39E-38	1.06E-11	0.2577193
173.2	2.22E-11	2.36E-13	1.59E-10	0.00079301	3.18E-38	1.01E-11	0.25764442
173.3	2.22E-11	2.36E-13	1.59E-10	0.00079301	3.18E-38	1.01E-11	0.25764442
173.4	2.23E-11	2.22E-13	1.60E-10	0.00078693	3.08E-38	9.98E-12	0.25655045
173.5	2.23E-11	2.22E-13	1.60E-10	0.00078693	3.08E-38	9.98E-12	0.25655045
173.6	2.23E-11	2.22E-13	1.60E-10	0.00078693	3.08E-38	9.98E-12	0.25655045
173.7	2.34E-11	2.16E-13	1.61E-10	0.00077406	2.95E-38	9.66E-12	0.25626481
173.8	2.34E-11	2.16E-13	1.61E-10	0.00077406	2.95E-38	9.66E-12	0.25626481
173.9	2.34E-11	2.16E-13	1.61E-10	0.00077406	2.95E-38	9.66E-12	0.25626481
174	2.48E-11	2.14E-13	1.65E-10	0.00077686	3.02E-38	9.53E-12	0.25665548
174.1	2.61E-11	2.17E-13	1.66E-10	0.000788	3.11E-38	9.50E-12	0.25637732
174.2	2.61E-11	2.17E-13	1.66E-10	0.000788	3.11E-38	9.50E-12	0.25637732
174.3	2.64E-11	2.22E-13	1.74E-10	0.0008136	3.08E-38	9.78E-12	0.25673863
174.4	2.66E-11	2.25E-13	1.81E-10	0.00079266	3.02E-38	1.02E-11	0.25417844
174.5	2.66E-11	2.25E-13	1.81E-10	0.00079266	3.02E-38	1.02E-11	0.25417844
174.6	2.78E-11	2.22E-13	1.86E-10	0.000813	3.16E-38	1.00E-11	0.25105559
174.7	2.72E-11	2.20E-13	1.92E-10	0.00080274	3.07E-38	1.01E-11	0.2506402
174.8	2.64E-11	2.15E-13	1.92E-10	0.00081353	3.04E-38	9.79E-12	0.25275765
174.9	2.63E-11	2.20E-13	1.98E-10	0.00083107	2.95E-38	9.41E-12	0.25134611
175	2.70E-11	2.08E-13	2.00E-10	0.00083822	2.85E-38	9.26E-12	0.25367133
175.1	2.84E-11	2.04E-13	2.12E-10	0.00083744	2.77E-38	8.63E-12	0.2554799
175.2	2.95E-11	1.95E-13	2.19E-10	0.00084437	2.77E-38	8.91E-12	0.25671475
175.3	3.05E-11	1.85E-13	2.25E-10	0.00084373	2.69E-38	8.58E-12	0.25648119
175.4	3.30E-11	1.82E-13	2.52E-10	0.00082211	2.82E-38	8.94E-12	0.2550619
175.5	3.47E-11	1.83E-13	2.62E-10	0.00082369	3.03E-38	8.96E-12	0.25526543
175.6	3.47E-11	1.83E-13	2.62E-10	0.00082369	3.03E-38	8.96E-12	0.25526543
175.7	3.49E-11	1.82E-13	2.66E-10	0.00080374	2.98E-38	8.95E-12	0.25770741
175.8	3.49E-11	1.82E-13	2.66E-10	0.00080374	2.98E-38	8.95E-12	0.25770741
175.9	3.50E-11	1.85E-13	2.78E-10	0.00080105	2.81E-38	9.09E-12	0.26099959
176	3.78E-11	1.92E-13	2.89E-10	0.00080201	2.92E-38	8.88E-12	0.26227946
176.1	3.71E-11	1.88E-13	3.02E-10	0.00082928	3.24E-38	9.17E-12	0.26247688
176.2	3.79E-11	1.78E-13	3.02E-10	0.00083194	3.09E-38	8.96E-12	0.2640607
176.3	4.09E-11	1.83E-13	3.15E-10	0.00082251	3.37E-38	9.29E-12	0.26207881
176.4	3.89E-11	1.81E-13	3.04E-10	0.00082035	3.22E-38	9.64E-12	0.26331574
176.5	4.19E-11	1.76E-13	3.19E-10	0.00081174	3.64E-38	1.02E-11	0.26121253
176.6	3.98E-11	1.83E-13	3.29E-10	0.00085228	3.50E-38	1.00E-11	0.26411266
176.7	4.03E-11	1.92E-13	3.58E-10	0.00086952	3.87E-38	1.02E-11	0.26660606
176.8	4.10E-11	1.80E-13	3.83E-10	0.00090818	3.87E-38	9.92E-12	0.26863235
176.9	4.35E-11	1.66E-13	3.92E-10	0.00095697	4.59E-38	9.82E-12	0.26975236
177	4.45E-11	1.61E-13	3.94E-10	0.00096524	4.35E-38	9.66E-12	0.26994418
177.1	4.69E-11	1.57E-13	4.02E-10	0.00099357	4.62E-38	9.16E-12	0.27179986
177.2	4.74E-11	1.52E-13	3.95E-10	0.00101123	4.55E-38	9.01E-12	0.27191113
177.3	4.84E-11	1.55E-13	3.91E-10	0.00103465	4.63E-38	8.62E-12	0.27207553
177.4	4.83E-11	1.63E-13	4.11E-10	0.00104986	4.84E-38	8.93E-12	0.26943368
177.5	4.94E-11	1.60E-13	4.13E-10	0.00105585	4.63E-38	8.63E-12	0.27214586
177.6	5.22E-11	1.61E-13	4.18E-10	0.00103445	4.58E-38	8.73E-12	0.26957742
177.7	5.22E-11	1.61E-13	4.18E-10	0.00103445	4.58E-38	8.73E-12	0.26957742
177.8	5.26E-11	1.59E-13	4.31E-10	0.00105653	4.49E-38	8.93E-12	0.26815214
177.9	5.41E-11	1.62E-13	4.48E-10	0.00104525	4.77E-38	9.29E-12	0.26596256
178	5.75E-11	1.61E-13	4.63E-10	0.00105571	5.18E-38	9.36E-12	0.2636918
178.1	5.92E-11	1.66E-13	4.67E-10	0.00103938	5.27E-38	9.73E-12	0.26162774
178.2	5.96E-11	1.72E-13	4.71E-10	0.00103507	5.46E-38	1.02E-11	0.25942806
178.3	6.01E-11	1.68E-13	4.92E-10	0.00103413	5.45E-38	1.05E-11	0.25907871
178.4	5.98E-11	1.63E-13	5.05E-10	0.00103127	5.54E-38	1.03E-11	0.25920023
178.5	6.22E-11	1.55E-13	5.23E-10	0.00102957	5.58E-38	1.05E-11	0.26032115
178.6	6.28E-11	1.57E-13	5.18E-10	0.00104546	5.91E-38	1.07E-11	0.25892925
178.7	6.47E-11	1.55E-13	5.50E-10	0.00107925	6.34E-38	1.11E-11	0.26016381
178.8	6.47E-11	1.55E-13	5.50E-10	0.00107925	6.34E-38	1.11E-11	0.26016381
178.9	6.77E-11	1.59E-13	5.64E-10	0.00107394	6.80E-38	1.16E-11	0.25872824
179	7.07E-11	1.57E-13	5.73E-10	0.00106165	6.92E-38	1.19E-11	0.25795011
179.1	7.16E-11	1.55E-13	5.55E-10	0.00108179	7.15E-38	1.19E-11	0.25663984
179.2	7.07E-11	1.54E-13	5.58E-10	0.00107736	7.22E-38	1.16E-11	0.25558762
179.3	7.15E-11	1.64E-13	5.88E-10	0.00109644	8.53E-38	1.17E-11	0.25463574
179.4	7.45E-11	1.68E-13	6.16E-10	0.00111126	9.35E-38	1.15E-11	0.25177916
179.5	7.60E-11	1.76E-13	6.54E-10	0.00114123	1.01E-37	1.16E-11	0.25493367
179.6	8.07E-11	1.70E-13	6.58E-10	0.00114876	1.06E-37	1.14E-11	0.25533878
179.7	8.17E-11	1.67E-13	6.56E-10	0.00118039	1.12E-37	1.14E-11	0.25660433
179.8	8.32E-11	1.69E-13	6.96E-10	0.00117209	1.18E-37	1.12E-11	0.25525792
179.9	8.32E-11	1.69E-13	6.96E-10	0.00117209	1.18E-37	1.12E-11	0.25525792
225	3.27E-09	1.92E-13	7.65E-08	0.00341166	2.03E-34	3.19E-11	0.31372283
225.1	3.27E-09	1.92E-13	7.65E-08	0.00341166	2.03E-34	3.19E-11	0.31372283
225.2	3.27E-09	1.92E-13	7.65E-08	0.00341166	2.03E-34	3.19E-11	0.31372283
225.3	3.27E-09	1.92E-13	7.65E-08	0.00341166	2.03E-34	3.19E-11	0.31372283
225.4	3.30E-09	2.00E-13	7.81E-08	0.00348194	2.11E-34	3.21E-11	0.31327821
225.5	3.30E-09	2.00E-13	7.81E-08	0.00348194	2.11E-34	3.21E-11	0.31327821
225.6	3.30E-09	2.00E-13	7.81E-08	0.00348194	2.11E-34	3.21E-11	0.31327821
225.7	3.30E-09	2.00E-13	7.81E-08	0.00348194	2.11E-34	3.21E-11	0.31327821
225.8	3.30E-09	2.00E-13	7.81E-08	0.00348194	2.11E-34	3.21E-11	0.31327821
225.9	3.30E-09	2.00E-13	7.81E-08	0.00348194	2.11E-34	3.21E-11	0.31327821
226	3.30E-09	2.00E-13	7.81E-08	0.00348194	2.11E-34	3.21E-11	0.31327821
226.1	3.30E-09	2.00E-13	7.81E-08	0.00348194	2.11E-34	3.21E-11	0.31327821
226.2	3.30E-09	2.00E-13	7.81E-08	0.00348194	2.11E-34	3.21E-11	0.31327821
226.3	3.30E-09	2.00E-13	7.81E-08	0.00348194	2.11E-34	3.21E-11	0.31327821
226.4	3.30E-09	2.00E-13	7.81E-08	0.00348194	2.11E-34	3.21E-11	0.31327821
226.5	3.43E-09	2.11E-13	8.21E-08	0.00348929	2.22E-34	3.29E-11	0.3127601
226.6	3.43E-09	2.11E-13	8.21E-08	0.00348929	2.22E-34	3.29E-11	0.3127601
226.7	3.60E-09	2.16E-13	8.20E-08	0.0035182	2.31E-34	3.29E-11	0.31051183
226.8	3.62E-09	2.14E-13	8.48E-08	0.00357124	2.31E-34	3.31E-11	0.31185871
226.9	3.62E-09	2.14E-13	8.48E-08	0.00357124	2.31E-34	3.31E-11	0.31185871
227	3.57E-09	2.11E-13	8.51E-08	0.00358714	2.32E-34	3.32E-11	0.31107487
227.1	3.57E-09	2.11E-13	8.51E-08	0.00358714	2.32E-34	3.32E-11	0.31107487
227.2	3.57E-09	2.11E-13	8.51E-08	0.00358714	2.32E-34	3.32E-11	0.31107487
227.3	3.57E-09	2.11E-13	8.51E-08	0.00358714	2.32E-34	3.32E-11	0.31107487
227.4	3.57E-09	2.11E-13	8.51E-08	0.00358714	2.32E-34	3.32E-11	0.31107487
227.5	3.57E-09	2.11E-13	8.51E-08	0.00358714	2.32E-34	3.32E-11	0.31107487
227.6	3.57E-09	2.11E-13	8.51E-08	0.00358714	2.32E-34	3.32E-11	0.31107487
227.7	3.57E-09	2.11E-13	8.51E-08	0.00358714	2.32E-34	3.32E-11	0.31107487
227.8	3.68E-09	2.19E-13	8.55E-08	0.00361305	2.56E-34	3.40E-11	0.31049304
227.9	3.68E-09	2.19E-13	8.55E-08	0.00361305	2.56E-34	3.40E-11	0.31049304
228	3.68E-09	2.19E-13	8.55E-08	0.00361305	2.56E-34	3.40E-11	0.31049304
228.1	3.68E-09	2.18E-13	8.57E-08	0.00363238	2.56E-34	3.48E-11	0.30826364

183.2	1.39E-10	1.84E-13	1.26E-09	0.0012806	3.06E-37	1.24E-11	0.26893429
183.3	1.39E-10	1.84E-13	1.26E-09	0.0012806	3.06E-37	1.24E-11	0.26893429
183.4	1.39E-10	1.84E-13	1.26E-09	0.0012806	3.06E-37	1.24E-11	0.26893429
183.5	1.35E-10	1.84E-13	1.30E-09	0.0012565	3.03E-37	1.25E-11	0.26735289
183.6	1.36E-10	1.78E-13	1.28E-09	0.00125215	2.95E-37	1.18E-11	0.26531947
183.7	1.36E-10	1.78E-13	1.28E-09	0.00125215	2.95E-37	1.18E-11	0.26531947
183.8	1.35E-10	1.80E-13	1.33E-09	0.00124635	3.17E-37	1.18E-11	0.26419008
183.9	1.38E-10	1.75E-13	1.36E-09	0.00125317	3.17E-37	1.19E-11	0.26701783
184	1.38E-10	1.75E-13	1.36E-09	0.00125317	3.17E-37	1.19E-11	0.26701783
184.1	1.50E-10	1.64E-13	1.37E-09	0.00124993	3.32E-37	1.14E-11	0.26499306
184.2	1.47E-10	1.68E-13	1.38E-09	0.00125237	3.47E-37	1.15E-11	0.26381793
184.3	1.48E-10	1.69E-13	1.42E-09	0.0012344	3.67E-37	1.14E-11	0.26303873
184.4	1.51E-10	1.64E-13	1.51E-09	0.0012429	4.08E-37	1.16E-11	0.26543911
184.5	1.48E-10	1.63E-13	1.58E-09	0.00125283	3.92E-37	1.14E-11	0.26363278
184.6	1.46E-10	1.53E-13	1.78E-09	0.00129041	4.31E-37	1.14E-11	0.26568314
184.7	1.48E-10	1.54E-13	1.82E-09	0.00129037	4.39E-37	1.14E-11	0.264538
184.8	1.51E-10	1.57E-13	1.89E-09	0.0012955	4.99E-37	1.12E-11	0.26192101
184.9	1.48E-10	1.57E-13	1.96E-09	0.00127804	4.92E-37	1.13E-11	0.26277807
185	1.52E-10	1.54E-13	2.03E-09	0.00127721	5.24E-37	1.14E-11	0.26335592
185.1	1.49E-10	1.62E-13	2.00E-09	0.00130019	5.26E-37	1.14E-11	0.26461688
185.2	1.57E-10	1.64E-13	2.05E-09	0.0013212	5.95E-37	1.16E-11	0.26590995
185.3	1.57E-10	1.64E-13	2.05E-09	0.0013212	5.95E-37	1.16E-11	0.26590995
185.4	1.76E-10	1.58E-13	2.15E-09	0.00138229	7.10E-37	1.16E-11	0.26118475
185.5	1.76E-10	1.58E-13	2.15E-09	0.00138229	7.10E-37	1.16E-11	0.26118475
185.6	1.87E-10	1.60E-13	2.31E-09	0.00138921	7.67E-37	1.21E-11	0.26124525
185.7	1.87E-10	1.60E-13	2.31E-09	0.00138921	7.67E-37	1.21E-11	0.26124525
185.8	1.90E-10	1.62E-13	2.40E-09	0.00140535	8.32E-37	1.24E-11	0.26237566
185.9	1.90E-10	1.62E-13	2.40E-09	0.00140535	8.32E-37	1.24E-11	0.26237566
186	1.90E-10	1.62E-13	2.40E-09	0.00140535	8.32E-37	1.24E-11	0.26237566
186.1	1.97E-10	1.55E-13	2.42E-09	0.00139538	8.17E-37	1.22E-11	0.26378379
186.2	1.97E-10	1.55E-13	2.42E-09	0.00139538	8.17E-37	1.22E-11	0.26378379
186.3	2.02E-10	1.48E-13	2.58E-09	0.00140194	8.87E-37	1.22E-11	0.26674955
186.4	2.02E-10	1.48E-13	2.58E-09	0.00140194	8.87E-37	1.22E-11	0.26674955
186.5	2.12E-10	1.54E-13	2.70E-09	0.00141461	9.74E-37	1.30E-11	0.26545816
186.6	2.12E-10	1.61E-13	2.68E-09	0.00142409	9.61E-37	1.29E-11	0.26687467
186.7	2.24E-10	1.68E-13	2.87E-09	0.0014296	1.01E-36	1.28E-11	0.26906593
186.8	2.34E-10	1.76E-13	2.81E-09	0.00142273	1.02E-36	1.32E-11	0.27199345
186.9	2.38E-10	1.81E-13	2.96E-09	0.00144401	1.13E-36	1.35E-11	0.27171983
187	2.36E-10	1.87E-13	2.96E-09	0.00146678	1.10E-36	1.32E-11	0.27523252
187.1	2.29E-10	1.85E-13	2.97E-09	0.00147546	1.12E-36	1.27E-11	0.27628312
187.2	2.27E-10	1.82E-13	3.01E-09	0.00147154	1.05E-36	1.23E-11	0.27716247
187.3	2.27E-10	1.82E-13	3.01E-09	0.00147154	1.05E-36	1.23E-11	0.27716247
187.4	2.25E-10	1.79E-13	3.05E-09	0.00148484	1.03E-36	1.20E-11	0.27926299
187.5	2.27E-10	1.81E-13	3.18E-09	0.00148214	1.08E-36	1.21E-11	0.28105582
187.6	2.40E-10	1.83E-13	3.33E-09	0.00150143	1.12E-36	1.23E-11	0.28077103
187.7	2.40E-10	1.83E-13	3.33E-09	0.00150143	1.12E-36	1.23E-11	0.28077103
187.8	2.45E-10	1.80E-13	3.45E-09	0.00149237	1.16E-36	1.23E-11	0.28099344
187.9	2.45E-10	1.80E-13	3.45E-09	0.00149237	1.16E-36	1.23E-11	0.28099344
188	2.59E-10	1.75E-13	3.53E-09	0.00155022	1.27E-36	1.23E-11	0.28330658
188.1	2.59E-10	1.76E-13	3.54E-09	0.00157218	1.27E-36	1.23E-11	0.2819553
188.2	2.66E-10	1.69E-13	3.47E-09	0.00159655	1.34E-36	1.25E-11	0.28029888
188.3	2.66E-10	1.69E-13	3.47E-09	0.00159655	1.34E-36	1.25E-11	0.28029888
188.4	2.66E-10	1.69E-13	3.47E-09	0.00159655	1.34E-36	1.25E-11	0.28029888
188.5	2.66E-10	1.69E-13	3.47E-09	0.00159655	1.34E-36	1.25E-11	0.28029888
188.6	2.78E-10	1.67E-13	3.46E-09	0.00159422	1.41E-36	1.31E-11	0.28050804
188.7	2.78E-10	1.67E-13	3.46E-09	0.00159422	1.41E-36	1.31E-11	0.28050804
188.8	2.83E-10	1.73E-13	3.46E-09	0.00161919	1.37E-36	1.30E-11	0.28185136
188.9	2.83E-10	1.73E-13	3.46E-09	0.00161919	1.37E-36	1.30E-11	0.28185136
189	2.90E-10	1.73E-13	3.58E-09	0.00162595	1.46E-36	1.31E-11	0.283131
189.1	3.02E-10	1.73E-13	3.63E-09	0.00162007	1.46E-36	1.37E-11	0.28125685
189.2	3.02E-10	1.73E-13	3.63E-09	0.00162007	1.46E-36	1.37E-11	0.28125685
189.3	3.14E-10	1.76E-13	3.93E-09	0.00165784	1.66E-36	1.39E-11	0.28040003
189.4	3.25E-10	1.72E-13	3.86E-09	0.00168011	1.72E-36	1.37E-11	0.28227183
189.5	3.32E-10	1.79E-13	3.93E-09	0.00169812	1.76E-36	1.35E-11	0.28333486
189.6	3.32E-10	1.79E-13	3.93E-09	0.00169812	1.76E-36	1.35E-11	0.28333486
189.7	3.38E-10	1.80E-13	3.98E-09	0.00170097	1.75E-36	1.35E-11	0.28518487
189.8	3.34E-10	1.68E-13	3.98E-09	0.00169356	1.71E-36	1.30E-11	0.28322323
189.9	3.31E-10	1.66E-13	4.04E-09	0.00168018	1.62E-36	1.33E-11	0.28115456
190	3.31E-10	1.66E-13	4.04E-09	0.00168018	1.62E-36	1.33E-11	0.28115456
190.1	3.39E-10	1.77E-13	4.26E-09	0.0016566	1.88E-36	1.37E-11	0.28417014
190.2	3.70E-10	1.70E-13	4.52E-09	0.00163896	2.02E-36	1.46E-11	0.28635426
190.3	3.77E-10	1.82E-13	4.95E-09	0.00166462	2.09E-36	1.51E-11	0.28792121
190.4	3.77E-10	1.82E-13	4.95E-09	0.00166462	2.09E-36	1.51E-11	0.28792121
190.5	3.82E-10	1.78E-13	5.02E-09	0.00166142	2.03E-36	1.47E-11	0.29022942
190.6	3.82E-10	1.78E-13	5.02E-09	0.00166142	2.03E-36	1.47E-11	0.29022942
190.7	3.96E-10	1.81E-13	5.45E-09	0.00166964	2.26E-36	1.42E-11	0.29322504
190.8	4.05E-10	1.83E-13	5.68E-09	0.00172422	2.46E-36	1.40E-11	0.29763909
190.9	4.10E-10	1.84E-13	5.58E-09	0.00177507	2.57E-36	1.39E-11	0.29792637
191	4.37E-10	1.85E-13	5.67E-09	0.00176448	2.72E-36	1.43E-11	0.29574759
191.1	4.39E-10	1.84E-13	5.93E-09	0.00178385	2.84E-36	1.43E-11	0.29874178
191.2	4.39E-10	1.84E-13	5.93E-09	0.00178385	2.84E-36	1.43E-11	0.29874178
191.3	4.36E-10	1.83E-13	6.00E-09	0.00176236	2.95E-36	1.43E-11	0.2983842
191.4	4.36E-10	1.83E-13	6.00E-09	0.00176236	2.95E-36	1.43E-11	0.2983842
191.5	4.36E-10	1.83E-13	6.00E-09	0.00176236	2.95E-36	1.43E-11	0.2983842
191.6	4.35E-10	1.91E-13	6.04E-09	0.00177162	2.97E-36	1.40E-11	0.30254691
191.7	4.59E-10	1.86E-13	6.26E-09	0.00181592	3.34E-36	1.37E-11	0.30222934
191.8	4.59E-10	1.86E-13	6.26E-09	0.00181592	3.34E-36	1.37E-11	0.30222934
191.9	4.59E-10	1.86E-13	6.26E-09	0.00181592	3.34E-36	1.37E-11	0.30222934
192	4.59E-10	1.86E-13	6.26E-09	0.00181592	3.34E-36	1.37E-11	0.30222934
192.1	4.58E-10	1.83E-13	6.50E-09	0.00180958	3.44E-36	1.34E-11	0.30146978
192.2	4.71E-10	1.84E-13	6.60E-09	0.00180724	3.67E-36	1.35E-11	0.30137928
192.3	4.75E-10	1.78E-13	6.78E-09	0.00183168	3.58E-36	1.32E-11	0.30377702
192.4	4.98E-10	1.74E-13	6.90E-09	0.00183396	3.69E-36	1.32E-11	0.30187129
192.5	5.44E-10	1.72E-13	7.00E-09	0.00187542	3.95E-36	1.29E-11	0.30046841
192.6	5.46E-10	1.66E-13	7.01E-09	0.00189092	3.90E-36	1.27E-11	0.301535
192.7	5.68E-10	1.68E-13	6.92E-09	0.00191849	4.12E-36	1.28E-11	0.30150725
192.8	5.68E-10	1.68E-13	6.92E-09	0.00191849	4.12E-36	1.28E-11	0.30150725
192.9	5.69E-10	1.70E-13	6.80E-09	0.00197535	4.20E-36	1.29E-11	0.30489053
193	5.79E-10	1.68E-13	7.00E-09	0.00197277	4.30E-36	1.30E-11	0.30672215
193.1	5.76E-10	1.59E-13	7.25E-09	0.00196841	4.16E-36	1.35E-11	0.30789454
193.2	6.01E-10	1.60E-13	7.36E-09	0.00200636	4.77E-36	1.38E-11	0.30690241
193.3	6.12E-10	1.59E-13	7.45E-09	0.00203109	4.72E-36	1.42E-11	0.30725369
193.4	6.23E-10	1.54E-13	7.62E-09	0.00204634	4.72E-36	1.39E-11	0.30826879
193.5	6.19E-10	1.55E-13	7.74E-09	0.00204655	5.03E-36	1.39E-11	0.30734485
193.6	6.39E-10	1.58E-13	8.20E-09	0.00205051	5.86E-36	1.44E-11	0.30796305
193.7	6.43E-10	1.58E-13	9.03E-09	0.00200876	6.42E-36	1.41E-11	0.30551175
193.8	6.42E-10	1.54E-13	9.22E-09	0.00205568	7.01E-36	1.41E-11	0.30638532
193.9	6.59E-10	1.54E-13	8.89E-09	0.00211047	7.49E-36	1.40E-11	0.30675078
194	6.75E-10	1.57E-13	8.67E-09	0.00213025	7.78E-36	1.39E-11	0.30733707
194.1	6.75E-10	1.57E-13	8.67E-09	0.00213025	7.78E-36	1.39E-11	0.30733707
194.2	6.75E-10	1.57E-13	8.67E-09	0.00213025	7.78E-36	1.39E-11	0.30733707
194.3	6.82E-10	1.54E-13	8.84E-09	0.00212113	8.25E-36	1.42E-11	0.30530026
194.4	7.02E-10	1.55E-13	9.30E-09	0.00212084	8.86E-36	1.44E-11	0.30571053
194.5	6.87E-10	1.58E-13	9.98E-09	0.00206737	9.43E-36	1.44E-11	0.30258419
194.6	7.04E-10	1.55E-13	9.94E-09	0.00211894	1.03E-35	1.48E-11	0.30331785
194.7	7.22E-10	1.53E-13	1.03E-08	0.00215009	1.11E-35	1.51E-11	0.30470148
194.8	7.22E-10	1.53E-13	1.03E-08	0.00215009	1.11E-35	1.51E-11	0.30470148
194.9	7.31E-10	1.57E-13	1.07E-08	0.00215964	1.22E-35	1.53E-11	0.30468266
198.2	9.91E-10	1.38E-13	1.38E-08	0.00237197	1.77E-35	1.45E-11	0.30615771
198.3	9.70E-10	1.40E-13	1.37E-08	0.00238242	1.96E-35	1.54E-11	0.30404007
198.4	9.93E-10	1.40E-13	1.39E-08	0.00239542	2.07E-35	1.58E-11	0.30261276
198.5	1.02E-09	1.43E-13	1.45E-08	0.0023782	2.21E-35	1.63E-11	0.30127185
198.6	1.06E-09	1.42E-13	1.49E-08	0.00236223	2.25E-35	1.60E-11	0.30100121
198.7	1.06E-09	1.42E-13	1.49E-08	0.00236223	2.25E-35	1.60E-11	0.30100121
198.8	1.09E-09	1.43E-13	1.48E-08	0.00239593	2.41E-35	1.65E-11	0.30050491
198.9	1.10E-09	1.42E-13	1.54E-08	0.00240833	2.50E-35	1.62E-11	0.29807064
199	1.16E-09	1.46E-13	1.59E-08	0.00240454	2.70E-35	1.65E-11	0.29818116
199.1	1.16E-09	1.46E-13	1.59E-08	0.00240454	2.70E-35	1.65E-11	0.29818116
199.2	1.16E-09	1.46E-13	1.59E-08	0.00240454	2.70E-35	1.65E-11	0.29818116
199.3	1.16E-09	1.46E-13	1.59E-08	0.00240454	2.70E-35	1.65E-11	0.29818116
199.4	1.16E-09	1.46E-13	1.59E-08	0.00240454	2.70E-35	1.65E-11	0.29818116
199.5	1.16E-09	1.46E-13	1.59E-08	0.00240454	2.70E-35	1.65E-11	0.29818116
199.6	1.15E-09	1.48E-13	1.64E-08	0.00248584	2.92E-35	1.68E-11	0.30083126
199.7	1.24E-09	1.56E-13	1.74E-08	0.00248176	3.21E-35	1.79E-11	0.29800661
199.8	1.31E-09	1.54E-13	1.86E-08	0.00248782	3.53E-35	1.78E-11	0.29721814
199.9	1.31E-09	1.54E-13	1.86E-08	0.00248782	3.53E-35	1.78E-11	0.29721814
200	1.31E-09	1.54E-13	1.86E-08	0.00248782	3.53E-35	1.78E-11	0.29721814
200.1	1.31E-09	1.56E-13	1.95E-08	0.00249859	3.77E-35	1.77E-11	0.29898578
200.2	1.31E-09	1.56E-13	1.95E-08	0.00249859	3.77E-35	1.77E-11	0.29898578
200.3	1.31E-09	1.56E-13	1.95E-08	0.00249859	3.77E-35	1.77E-11	0.29898578
200.4	1.38E-09	1.55E-13	1.94E-08	0.00255581	4.15E-35	1.79E-11	0.29772005
200.5	1.38E-09	1.55E-13	1.94E-08	0.00255581	4.15E-35	1.79E-11	0.29772005
200.6	1.38E-09	1.55E-13	1.94E-08	0.00255581	4.15E-35	1.79E-11	0.29772005
200.7	1.38E-09	1.55E-13	1.94E-08	0.00255581	4.15E-35	1.79E-11	0.29772005
200.8	1.38E-09	1.55E-13	1.94E-08	0.00255581	4.15E-35	1.79E-11	0.29772005
200.9	1.38E-09	1.55E-13	1.94E-08	0.00255581	4.15E-35	1.79E-11	0.29772005
201	1.39E-09	1.55E-13	1.98E-08	0.00256855	4.51E-35	1.87E-11	0.29643261
201.1	1.43E-09	1.62E-13	1.99E-08	0.00253504	5.15E-35	1.94E-11	0.2957054
201.2	1.50E-09	1.63E-13	2.01E-08	0.00254333	5.37E-35	1.91E-11	0.29458548
201.3	1.53E-09	1.59E-13	1.99E-08	0.00255951	5.43E-35	1.89E-11	0.29453899
201.4	1.53E-09	1.59E-13	1.99E-08	0.00255951	5.43E-35	1.89E-11	0.29453899
201.5	1.53E-09	1.59E-13	1.99E-08	0.00255951	5.43E-35	1.89E-11	0.29453899
201.6	1.53E-09	1.59E-13	1.99E-08	0.00255951	5.43E-35	1.89E-11	0.29453899
201.7	1.53E-09	1.59E-13	1.99E-08	0.00255951	5.43E-35	1.89E-11	0.29453899
201.8	1.57E-09	1.62E-13	2.07E-08	0.00254589	5.59E-35	1.86E-11	0.29473778
201.9	1.57E-09	1.62E-13	2.07E-08	0.00254589	5.59E-35	1.86E-11	0.29473778
202	1.57E-09	1.62E-13	2.07E-08	0.00254589	5.59E-35	1.86E-11	0.29473778
202.1	1.57E-09	1.62E-13	2.07E-08	0.00254589	5.59E-35	1.86E-11	0.29473778
202.2	1.62E-09	1.63E-13	2.17E-08	0.00252521	6.31E-35	1.84E-11	0.29432982
202.3	1.57E-09	1.64E-13	2.20E-08	0.00248262	6.26E-35	1.84E-11	0.29531145
202.4	1.62E-09	1.65E-13	2.22E-08	0.00245964	6.19E-35	1.84E-11	0.29488336
202.5	1.62E-09	1.65E-13	2.22E-08	0.00245964	6.19E-35	1.84E-11	0.29488336
202.6	1.67E-09	1.58E-13	2.25E-08	0.00247622	5.89E-35	1.83E-11	0.29806593
202.7	1.67E-09	1.58E-13	2.25E-08	0.00247622	5.89E-35	1.83E-11	0.29806593
202.8	1.67E-09	1.58E-13	2.32E-08	0.00247256	6.07E-35	1.81E-11	0.2975785
202.9	1.67E-09	1.58E-13	2.32E-08	0.00247256	6.07E-35	1.81E-11	0.2975785
203	1.69E-09	1.62E-13	2.36E-08	0.00248387	6.61E-35	1.86E-11	0.29827646
203.1	1.69E-09	1.62E-13	2.36E-08	0.00248387	6.61E-35	1.86E-11	0.29827646
203.2	1.69E-09	1.62E-13	2.36E-08	0.00248387	6.61E-35	1.86E-11	0.29827646
203.3	1.70E-09	1.71E-13	2.33E-08	0.00249807	6.91E-35	1.85E-11	0.29653105
203.4	1.72E-09	1.70E-13	2.35E-08	0.0025021	7.13E-35	1.89E-11	0.29783412
203.5	1.68E-09	1.70E-13	2.42E-08	0.00253848	7.32E-35	1.89E-11	0.3005106
203.6	1.69E-09	1.73E-13	2.49E-08	0.00253545	7.72E-35	1.93E-11	0.3004135
203.7	1.69E-09	1.73E-13	2.49E-08	0.00253545	7.72E-35	1.93E-11	0.3004135
203.8	1.69E-09	1.73E-13	2.49E-08	0.00253545	7.72E-35	1.93E-11	0.3004135
203.9	1.69E-09	1.73E-13	2.49E-08	0.00253545	7.72E-35	1.93E-11	0.3004135
204	1.69E-09	1.73E-13	2.49E-08	0.00253545	7.72E-35	1.93E-11	0.3004135
204.1	1.69E-09	1.73E-13	2.49E-08	0.00253545	7.72E-35	1.93E-11	0.3004135
204.2	1.69E-09	1.73E-13	2.49E-08	0.00253545	7.72E-35	1.93E-11	0.3004135
204.3	1.69E-09	1.73E-13	2.49E-08	0.00253545	7.72E-35	1.93E-11	0.3004135
204.4	1.69E-09	1.73E-13	2.49E-08	0.00253545	7.72E-35	1.93E-11	0.3004135
204.5	1.69E-09	1.73E-13	2.49E-08	0.00253545	7.72E-35	1.93E-11	0.3004135
204.6	1.71E-09	1.64E-13	2.52E-08	0.00252772	7.35E-35	1.90E-11	0.29816908
204.7	1.71E-09	1.64E-13	2.52E-08	0.00252772	7.35E-35	1.90E-11	0.29816908
204.8	1.71E-09	1.64E-13	2.52E-08	0.00252772	7.35E-35	1.90E-11	0.29816908
204.9	1.71E-09	1.64E-13	2.52E-08	0.00252772	7.35E-35	1.90E-11	0.29816908
205	1.69E-09	1.67E-13	2.61E-08	0.00253096	7.67E-35	1.96E-11	0.2995068
205.1	1.69E-09	1.67E-13	2.61E-08	0.00253096	7.67E-35	1.96E-11	0.2995068
205.2	1.69E-09	1.67E-13	2.61E-08	0.00253096	7.67E-35	1.96E-11	0.2995068
205.3	1.69E-09	1.67E-13	2.61E-08	0.00253096	7.67E-35	1.96E-11	0.2995068
205.4	1.69E-09	1.67E-13	2.61E-08	0.00253096	7.67E-35	1.96E-11	0.2995068
205.5	1.73E-09	1.67E-13	2.57E-08	0.00259727	7.96E-35	1.95E-11	0.29818439
205.6	1.73E-09	1.67E-13	2.57E-08	0.00259727	7.96E-35	1.95E-11	0.29818439
205.7	1.73E-09	1.67E-13	2.57E-08	0.00259727	7.96E-35	1.95E-11	0.29818439
205.8	1.73E-09	1.67E-13	2.57E-08	0.00259727	7.96E-35	1.95E-11	0.29818439
205.9	1.73E-09	1.67E-13	2.57E-08	0.00259727	7.96E-35	1.95E-11	0.29818439
206	1.76E-09	1.72E-13	2.69E-08	0.00259508	8.43E-35	2.01E-11	0.29834821
206.1	1.76E-09	1.72E-13	2.69E-08	0.00259508	8.43E-35	2.01E-11	0.29834821
206.2	1.77E-09	1.67E-13	2.69E-08	0.00261599	8.38E-35	1.98E-11	0.29886964
206.3	1.77E-09	1.67E-13	2.69E-08	0.00261599	8.38E-35	1.98E-11	0.29886964
206.4	1.77E-09	1.67E-13	2.69E-08	0.00261599	8.38E-35	1.98E-11	0.29886964
206.5	1.75E-09	1.65E-13	2.79E-08	0.00260129	8.72E-35	2.02E-11	0.30041184
206.6	1.75E-09	1.65E-13	2.79E-08	0.00260129	8.72E-35	2.02E-11	0.30041184
206.7	1.73E-09	1.67E-13	2.85E-08	0.00263058	8.69E-35	2.00E-11	0.29927231
206.8	1.75E-09	1.60E-13	2.88E-08	0.00265668	8.85E-35	2.03E-11	0.30060351
206.9	1.75E-09	1.60E-13	2.88E-08	0.00265668	8.85E-35	2.03E-11	0.30060351
207	1.78E-09	1.65E-13	3.05E-08	0.00266869	9.07E-35	2.13E-11	0.30366594
207.1	1.88E-09	1.72E-13	3.26E-08	0.00257659	8.88E-35	1.99E-11	0.30545798
207.2	1.88E-09	1.72E-13	3.26E-08	0.00257659	8.88E-35	1.99E-11	0.30545798
207.3	1.88E-09	1.72E-13	3.26E-08	0.00257659	8.88E-35	1.99E-11	0.30545798
207.4	1.89E-09	1.71E-13	3.29E-08	0.00255332	9.36E-35	2.02E-11	0.30761025
207.5	1.89E-09	1.71E-13	3.29E-08	0.00255332	9.36E-35	2.02E-11	0.30761025
207.6	1.90E-09	1.65E-13	3.27E-08	0.00256983	9.10E-35	2.02E-11	0.30733569
207.7	1.90E-09	1.61E-13	3.34E-08	0.00261153	8.66E-35	1.96E-11	0.30699567
207.8	1.91E-09	1.62E-13	3.47E-08	0.00264064	9.18E-35	1.97E-11	0.30754498
207.9	1.97E-09	1.71E-13	3.38E-08	0.00268372	9.16E-35	1.87E-11	0.30580274
208	1.97E-09	1.71E-13	3.38E-08	0.00268372	9.16E-35	1.87E-11	0.30580274
208.1	2.00E-09	1.66E-13	3.43E-08	0.00265525	8.70E-35	1.85E-11	0.30725683
208.2	2.00E-09	1.66E-13	3.43E-08	0.00265525	8.70E-35	1.85E-11	0.30725683
208.3	2.00E-09	1.66E-13	3.43E-08	0.00265525	8.70E-35	1.85E-11	0.30725683
208.4	2.01E-09	1.59E-13	3.50E-08	0.00269209	8.60E-35	1.84E-11	0.30978963
208.5	2.06E-09	1.55E-13	3.43E-08	0.00273407	8.41E-35	1.82E-11	0.3095749
208.6	2.06E-09	1.55E-13	3.43E-08	0.00273407	8.41E-35	1.82E-11	0.3095749
208.7	2.06E-09	1.55E-13	3.43E-08	0.00273407	8.41E-35	1.82E-11	0.3095749
208.8	2.06E-09	1.55E-13	3.43E-08	0.00273407	8.41E-35	1.82E-11	0.3095749
208.9	2.13E-09	1.59E-13	3.60E-08	0.00275163	9.15E-35	1.87E-11	0.31114266
209	2.13E-09	1.59E-13	3.60E-08	0.00275163	9.15E-35	1.87E-11	0.31114266
209.1	2.13E-09	1.59E-13	3.60E-08	0.00275163	9.15E-35	1.87E-11	0.31114266
209.2	2.13E-09	1.59E-13	3.60E-08	0.00275163	9.15E-35	1.87E-11	0.31114266
209.3	2.02E-09	1.62E-13	3.61E-08	0.00276253	8.80E-35	1.79E-11	0.31172207
209.4	2.02E-09	1.65E-13	3.62E-08	0.00274624	9.02E-35	1.79E-11	0.31049848
209.5	2.02E-09	1.65E-13	3.62E-08	0.00274624	9.02E-35	1.79E-11	0.31049848
209.6	2.02E-09	1.65E-13	3.62E-08	0.00274624	9.02E-35	1.79E-11	0.31049848
209.7	1.99E-09	1.65E-13	3.72E-08	0.00275826	8.97E-35	1.78E-11	0.31281757
209.8	1.96E-09	1.63E-13	3.86E-08	0.00277312	9.13E-35	1.88E-11	0.31211504
209.9	1.96E-09	1.63E-13	3.86E-08	0.00277312	9.13E-35	1.88E-11	0.31211504
213.2	2.21E-09	1.77E-13	4.55E-08	0.00266219	1.17E-34	2.16E-11	0.30504507
213.3	2.21E-09	1.77E-13	4.55E-08	0.00266219	1.17E-34	2.16E-11	0.30504507
213.4	2.26E-09	1.82E-13	4.61E-08	0.00266408	1.19E-34	2.16E-11	0.30758397
213.5	2.26E-09	1.82E-13	4.61E-08	0.00266408	1.19E-34	2.16E-11	0.30758397
213.6	2.26E-09	1.82E-13	4.61E-08	0.00266408	1.19E-34	2.16E-11	0.30758397
213.7	2.25E-09	1.79E-13	4.47E-08	0.00265935	1.12E-34	2.07E-11	0.30534919
213.8	2.25E-09	1.79E-13	4.47E-08	0.00265935	1.12E-34	2.07E-11	0.30534919
213.9	2.25E-09	1.79E-13	4.47E-08	0.00265935	1.12E-34	2.07E-11	0.30534919
214	2.25E-09	1.79E-13	4.47E-08	0.00265935	1.12E-34	2.07E-11	0.30534919
214.1	2.25E-09	1.79E-13	4.47E-08	0.00265935	1.12E-34	2.07E-11	0.30534919
214.2	2.25E-09	1.79E-13	4.47E-08	0.00265935	1.12E-34	2.07E-11	0.30534919
214.3	2.25E-09	1.79E-13	4.47E-08	0.00265935	1.12E-34	2.07E-11	0.30534919
214.4	2.30E-09	1.66E-13	4.58E-08	0.00266295	1.05E-34	2.09E-11	0.30441261
214.5	2.32E-09	1.62E-13	4.64E-08	0.0026718	1.07E-34	2.12E-11	0.30503325
214.6	2.40E-09	1.60E-13	4.69E-08	0.00268042	1.06E-34	2.13E-11	0.30615726
214.7	2.40E-09	1.60E-13	4.69E-08	0.00268042	1.06E-34	2.13E-11	0.30615726
214.8	2.40E-09	1.60E-13	4.69E-08	0.00268042	1.06E-34	2.13E-11	0.30615726
214.9	2.37E-09	1.66E-13	4.70E-08	0.00269474	1.10E-34	2.20E-11	0.30603067
215	2.37E-09	1.66E-13	4.70E-08	0.00269474	1.10E-34	2.20E-11	0.30603067
215.1	2.37E-09	1.66E-13	4.70E-08	0.00269474	1.10E-34	2.20E-11	0.30603067
215.2	2.33E-09	1.65E-13	4.79E-08	0.00271638	1.10E-34	2.21E-11	0.30747848
215.3	2.33E-09	1.65E-13	4.79E-08	0.00271638	1.10E-34	2.21E-11	0.30747848
215.4	2.33E-09	1.65E-13	4.79E-08	0.00271638	1.10E-34	2.21E-11	0.30747848
215.5	2.33E-09	1.65E-13	4.79E-08	0.00271638	1.10E-34	2.21E-11	0.30747848
215.6	2.33E-09	1.65E-13	4.79E-08	0.00271638	1.10E-34	2.21E-11	0.30747848
215.7	2.33E-09	1.65E-13	4.79E-08	0.00271638	1.10E-34	2.21E-11	0.30747848
215.8	2.31E-09	1.63E-13	4.92E-08	0.00276301	1.14E-34	2.24E-11	0.30756362
215.9	2.31E-09	1.63E-13	4.92E-08	0.00276301	1.14E-34	2.24E-11	0.30756362
216	2.31E-09	1.63E-13	4.92E-08	0.00276301	1.14E-34	2.24E-11	0.30756362
216.1	2.31E-09	1.63E-13	4.92E-08	0.00276301	1.14E-34	2.24E-11	0.30756362
216.2	2.35E-09	1.71E-13	5.04E-08	0.0028412	1.21E-34	2.28E-11	0.30987855
216.3	2.37E-09	1.66E-13	5.22E-08	0.00286905	1.20E-34	2.28E-11	0.3076168
216.4	2.37E-09	1.66E-13	5.22E-08	0.00286905	1.20E-34	2.28E-11	0.3076168
216.5	2.37E-09	1.66E-13	5.22E-08	0.00286905	1.20E-34	2.28E-11	0.3076168
216.6	2.37E-09	1.66E-13	5.22E-08	0.00286905	1.20E-34	2.28E-11	0.3076168
216.7	2.40E-09	1.66E-13	5.39E-08	0.00287795	1.27E-34	2.32E-11	0.30643001
216.8	2.40E-09	1.66E-13	5.39E-08	0.00287795	1.27E-34	2.32E-11	0.30643001
216.9	2.40E-09	1.66E-13	5.39E-08	0.00287795	1.27E-34	2.32E-11	0.30643001
217	2.40E-09	1.66E-13	5.39E-08	0.00287795	1.27E-34	2.32E-11	0.30643001
217.1	2.49E-09	1.69E-13	5.36E-08	0.00291632	1.25E-34	2.39E-11	0.30632784
217.2	2.49E-09	1.69E-13	5.36E-08	0.00291632	1.25E-34	2.39E-11	0.30632784
217.3	2.49E-09	1.68E-13	5.34E-08	0.00296329	1.31E-34	2.45E-11	0.3053722
217.4	2.49E-09	1.68E-13	5.34E-08	0.00296329	1.31E-34	2.45E-11	0.3053722
217.5	2.49E-09	1.68E-13	5.34E-08	0.00296329	1.31E-34	2.45E-11	0.3053722
217.6	2.49E-09	1.68E-13	5.34E-08	0.00296329	1.31E-34	2.45E-11	0.3053722
217.7	2.55E-09	1.67E-13	5.46E-08	0.00298221	1.38E-34	2.55E-11	0.30702467
217.8	2.55E-09	1.67E-13	5.46E-08	0.00298221	1.38E-34	2.55E-11	0.30702467
217.9	2.55E-09	1.67E-13	5.46E-08	0.00298221	1.38E-34	2.55E-11	0.30702467
218	2.55E-09	1.67E-13	5.46E-08	0.00298221	1.38E-34	2.55E-11	0.30702467
218.1	2.57E-09	1.69E-13	5.54E-08	0.00299772	1.40E-34	2.56E-11	0.30878561
218.2	2.57E-09	1.69E-13	5.54E-08	0.00299772	1.40E-34	2.56E-11	0.30878561
218.3	2.57E-09	1.69E-13	5.54E-08	0.00299772	1.40E-34	2.56E-11	0.30878561
218.4	2.57E-09	1.69E-13	5.54E-08	0.00299772	1.40E-34	2.56E-11	0.30878561
218.5	2.57E-09	1.69E-13	5.54E-08	0.00299772	1.40E-34	2.56E-11	0.30878561
218.6	2.57E-09	1.69E-13	5.54E-08	0.00299772	1.40E-34	2.56E-11	0.30878561
218.7	2.57E-09	1.69E-13	5.54E-08	0.00299772	1.40E-34	2.56E-11	0.30878561
218.8	2.53E-09	1.72E-13	5.64E-08	0.00299279	1.32E-34	2.51E-11	0.31164427
218.9	2.53E-09	1.72E-13	5.64E-08	0.00299279	1.32E-34	2.51E-11	0.31164427
219	2.53E-09	1.72E-13	5.64E-08	0.00299279	1.32E-34	2.51E-11	0.31164427
219.1	2.58E-09	1.69E-13	5.69E-08	0.0030384	1.29E-34	2.49E-11	0.31146056
219.2	2.67E-09	1.79E-13	5.79E-08	0.00299914	1.35E-34	2.53E-11	0.30959405
219.3	2.67E-09	1.79E-13	5.79E-08	0.00299914	1.35E-34	2.53E-11	0.30959405
219.4	2.67E-09	1.79E-13	5.79E-08	0.00299914	1.35E-34	2.53E-11	0.30959405
219.5	2.67E-09	1.79E-13	5.79E-08	0.00299914	1.35E-34	2.53E-11	0.30959405
219.6	2.67E-09	1.79E-13	5.79E-08	0.00299914	1.35E-34	2.53E-11	0.30959405
219.7	2.60E-09	1.82E-13	5.82E-08	0.00302852	1.40E-34	2.62E-11	0.3086819
219.8	2.60E-09	1.82E-13	5.82E-08	0.00302852	1.40E-34	2.62E-11	0.3086819
219.9	2.71E-09	1.83E-13	5.88E-08	0.00306943	1.50E-34	2.61E-11	0.31191739
220	2.65E-09	1.84E-13	5.97E-08	0.00311032	1.41E-34	2.55E-11	0.31205851
220.1	2.65E-09	1.84E-13	5.97E-08	0.00311032	1.41E-34	2.55E-11	0.31205851
220.2	2.74E-09	1.92E-13	6.26E-08	0.00310797	1.49E-34	2.71E-11	0.31154124
220.3	2.71E-09	1.91E-13	6.29E-08	0.00318324	1.55E-34	2.72E-11	0.31261176
220.4	2.71E-09	1.91E-13	6.29E-08	0.00318324	1.55E-34	2.72E-11	0.31261176
220.5	2.71E-09	1.91E-13	6.29E-08	0.00318324	1.55E-34	2.72E-11	0.31261176
220.6	2.71E-09	1.91E-13	6.29E-08	0.00318324	1.55E-34	2.72E-11	0.31261176
220.7	2.71E-09	1.91E-13	6.29E-08	0.00318324	1.55E-34	2.72E-11	0.31261176
220.8	2.71E-09	1.91E-13	6.29E-08	0.00318324	1.55E-34	2.72E-11	0.31261176
220.9	2.71E-09	1.91E-13	6.29E-08	0.00318324	1.55E-34	2.72E-11	0.31261176
221	2.79E-09	1.82E-13	6.45E-08	0.00320524	1.52E-34	2.74E-11	0.31037992
221.1	2.85E-09	1.85E-13	6.59E-08	0.00327961	1.66E-34	2.91E-11	0.31016947
221.2	2.85E-09	1.85E-13	6.59E-08	0.00327961	1.66E-34	2.91E-11	0.31016947
221.3	2.85E-09	1.85E-13	6.59E-08	0.00327961	1.66E-34	2.91E-11	0.31016947
221.4	2.85E-09	1.85E-13	6.59E-08	0.00327961	1.66E-34	2.91E-11	0.31016947
221.5	2.85E-09	1.85E-13	6.59E-08	0.00327961	1.66E-34	2.91E-11	0.31016947
221.6	2.85E-09	1.85E-13	6.59E-08	0.00327961	1.66E-34	2.91E-11	0.31016947
221.7	2.85E-09	1.85E-13	6.59E-08	0.00327961	1.66E-34	2.91E-11	0.31016947
221.8	2.85E-09	1.85E-13	6.59E-08	0.00327961	1.66E-34	2.91E-11	0.31016947
221.9	2.85E-09	1.85E-13	6.59E-08	0.00327961	1.66E-34	2.91E-11	0.31016947
222	2.85E-09	1.85E-13	6.59E-08	0.00327961	1.66E-34	2.91E-11	0.31016947
222.1	2.98E-09	1.87E-13	6.64E-08	0.0033311	1.75E-34	2.96E-11	0.31139866
222.2	2.98E-09	1.87E-13	6.64E-08	0.0033311	1.75E-34	2.96E-11	0.31139866
222.3	2.98E-09	1.87E-13	6.64E-08	0.0033311	1.75E-34	2.96E-11	0.31139866
222.4	2.98E-09	1.90E-13	6.97E-08	0.00328019	1.79E-34	2.93E-11	0.31493977
222.5	3.02E-09	1.85E-13	7.33E-08	0.00328553	1.73E-34	2.94E-11	0.31756221
222.6	3.02E-09	1.85E-13	7.33E-08	0.00328553	1.73E-34	2.94E-11	0.31756221
222.7	3.02E-09	1.85E-13	7.33E-08	0.00328553	1.73E-34	2.94E-11	0.31756221
222.8	3.02E-09	1.85E-13	7.33E-08	0.00328553	1.73E-34	2.94E-11	0.31756221
222.9	3.02E-09	1.85E-13	7.33E-08	0.00328553	1.73E-34	2.94E-11	0.31756221
223	3.02E-09	1.85E-13	7.33E-08	0.00328553	1.73E-34	2.94E-11	0.31756221
223.1	3.02E-09	1.85E-13	7.33E-08	0.00328553	1.73E-34	2.94E-11	0.31756221
223.2	3.05E-09	1.91E-13	7.36E-08	0.00327045	1.77E-34	3.02E-11	0.31761077
223.3	3.05E-09	1.91E-13	7.36E-08	0.00327045	1.77E-34	3.02E-11	0.31761077
223.4	3.05E-09	1.91E-13	7.36E-08	0.00327045	1.77E-34	3.02E-11	0.31761077
223.5	3.05E-09	1.91E-13	7.36E-08	0.00327045	1.77E-34	3.02E-11	0.31761077
223.6	3.16E-09	1.96E-13	7.43E-08	0.00336956	1.96E-34	3.07E-11	0.31898541
223.7	3.16E-09	1.96E-13	7.43E-08	0.00336956	1.96E-34	3.07E-11	0.31898541
223.8	3.26E-09	1.97E-13	7.40E-08	0.00338527	2.01E-34	3.08E-11	0.31684261
223.9	3.26E-09	1.97E-13	7.40E-08	0.00338527	2.01E-34	3.08E-11	0.31684261
224	3.26E-09	1.97E-13	7.40E-08	0.00338527	2.01E-34	3.08E-11	0.31684261
224.1	3.26E-09	1.97E-13	7.40E-08	0.00338527	2.01E-34	3.08E-11	0.31684261
224.2	3.26E-09	1.97E-13	7.40E-08	0.00338527	2.01E-34	3.08E-11	0.31684261
224.3	3.26E-09	1.97E-13	7.40E-08	0.00338527	2.01E-34	3.08E-11	0.31684261
224.4	3.26E-09	1.97E-13	7.40E-08	0.00338527	2.01E-34	3.08E-11	0.31684261
224.5	3.26E-09	1.97E-13	7.40E-08	0.00338527	2.01E-34	3.08E-11	0.31684261
224.6	3.26E-09	1.97E-13	7.40E-08	0.00338527	2.01E-34	3.08E-11	0.31684261
224.7	3.28E-09	1.88E-13	7.59E-08	0.00341214	1.98E-34	3.10E-11	0.31446378
224.8	3.28E-09	1.88E-13	7.59E-08	0.00341214	1.98E-34	3.10E-11	0.31446378
224.9	3.27E-09	1.92E-13	7.65E-08	0.00341166	2.03E-34	3.19E-11	0.31372283
228.2	3.68E-09	2.18E-13	8.57E-08	0.00363238	2.56E-34	3.48E-11	0.30826364
228.3	3.68E-09	2.18E-13	8.57E-08	0.00363238	2.56E-34	3.48E-11	0.30826364
228.4	3.68E-09	2.18E-13	8.57E-08	0.00363238	2.56E-34	3.48E-11	0.30826364
228.5	3.68E-09	2.18E-13	8.57E-08	0.00363238	2.56E-34	3.48E-11	0.30826364
228.6	3.68E-09	2.18E-13	8.57E-08	0.00363238	2.56E-34	3.48E-11	0.30826364
228.7	3.68E-09	2.18E-13	8.57E-08	0.00363238	2.56E-34	3.48E-11	0.30826364
228.8	3.68E-09	2.18E-13	8.57E-08	0.00363238	2.56E-34	3.48E-11	0.30826364
228.9	3.68E-09	2.18E-13	8.57E-08	0.00363238	2.56E-34	3.48E-11	0.30826364
229	3.68E-09	2.18E-13	8.57E-08	0.00363238	2.56E-34	3.48E-11	0.30826364
229.1	3.68E-09	2.18E-13	8.57E-08	0.00363238	2.56E-34	3.48E-11	0.30826364
229.2	3.68E-09	2.18E-13	8.57E-08	0.00363238	2.56E-34	3.48E-11	0.30826364
229.3	3.68E-09	2.18E-13	8.57E-08	0.00363238	2.56E-34	3.48E-11	0.30826364
229.4	3.68E-09	2.18E-13	8.57E-08	0.00363238	2.56E-34	3.48E-11	0.30826364
229.5	3.68E-09	2.18E-13	8.57E-08	0.00363238	2.56E-34	3.48E-11	0.30826364
229.6	3.92E-09	2.27E-13	8.87E-08	0.00359267	2.85E-34	3.64E-11	0.30761423
229.7	3.92E-09	2.27E-13	8.87E-08	0.00359267	2.85E-34	3.64E-11	0.30761423
229.8	3.92E-09	2.27E-13	8.87E-08	0.00359267	2.85E-34	3.64E-11	0.30761423
229.9	3.92E-09	2.27E-13	8.87E-08	0.00359267	2.85E-34	3.64E-11	0.30761423
230	3.92E-09	2.27E-13	8.87E-08	0.00359267	2.85E-34	3.64E-11	0.30761423
230.1	3.92E-09	2.27E-13	8.87E-08	0.00359267	2.85E-34	3.64E-11	0.30761423
230.2	4.04E-09	2.24E-13	9.03E-08	0.00359933	3.01E-34	3.74E-11	0.30881074
230.3	4.04E-09	2.24E-13	9.03E-08	0.00359933	3.01E-34	3.74E-11	0.30881074
230.4	4.04E-09	2.24E-13	9.03E-08	0.00359933	3.01E-34	3.74E-11	0.30881074
230.5	4.04E-09	2.24E-13	9.03E-08	0.00359933	3.01E-34	3.74E-11	0.30881074
230.6	4.04E-09	2.24E-13	9.03E-08	0.00359933	3.01E-34	3.74E-11	0.30881074
230.7	4.04E-09	2.24E-13	9.03E-08	0.00359933	3.01E-34	3.74E-11	0.30881074
230.8	4.02E-09	2.30E-13	8.97E-08	0.00364709	2.97E-34	3.73E-11	0.30839121
230.9	4.02E-09	2.30E-13	8.97E-08	0.00364709	2.97E-34	3.73E-11	0.30839121
231	4.02E-09	2.30E-13	8.97E-08	0.00364709	2.97E-34	3.73E-11	0.30839121
231.1	4.02E-09	2.30E-13	8.97E-08	0.00364709	2.97E-34	3.73E-11	0.30839121
231.2	4.02E-09	2.30E-13	8.97E-08	0.00364709	2.97E-34	3.73E-11	0.30839121
231.3	4.02E-09	2.30E-13	8.97E-08	0.00364709	2.97E-34	3.73E-11	0.30839121
231.4	4.02E-09	2.30E-13	8.97E-08	0.00364709	2.97E-34	3.73E-11	0.30839121
231.5	4.02E-09	2.30E-13	8.97E-08	0.00364709	2.97E-34	3.73E-11	0.30839121
231.6	4.02E-09	2.30E-13	8.97E-08	0.00364709	2.97E-34	3.73E-11	0.30839121
231.7	4.02E-09	2.30E-13	8.97E-08	0.00364709	2.97E-34	3.73E-11	0.30839121
231.8	4.02E-09	2.30E-13	8.97E-08	0.00364709	2.97E-34	3.73E-11	0.30839121
231.9	4.02E-09	2.30E-13	8.97E-08	0.00364709	2.97E-34	3.73E-11	0.30839121
232	4.02E-09	2.30E-13	8.97E-08	0.00364709	2.97E-34	3.73E-11	0.30839121
232.1	4.02E-09	2.30E-13	8.97E-08	0.00364709	2.97E-34	3.73E-11	0.30839121
232.2	4.02E-09	2.30E-13	8.97E-08	0.00364709	2.97E-34	3.73E-11	0.30839121
232.3	4.02E-09	2.30E-13	8.97E-08	0.00364709	2.97E-34	3.73E-11	0.30839121
232.4	4.02E-09	2.30E-13	8.97E-08	0.00364709	2.97E-34	3.73E-11	0.30839121
232.5	4.02E-09	2.30E-13	8.97E-08	0.00364709	2.97E-34	3.73E-11	0.30839121
232.6	4.02E-09	2.30E-13	8.97E-08	0.00364709	2.97E-34	3.73E-11	0.30839121
232.7	4.02E-09	2.30E-13	8.97E-08	0.00364709	2.97E-34	3.73E-11	0.30839121
232.8	4.02E-09	2.30E-13	8.97E-08	0.00364709	2.97E-34	3.73E-11	0.30839121
232.9	4.02E-09	2.30E-13	8.97E-08	0.00364709	2.97E-34	3.73E-11	0.30839121
233	4.02E-09	2.30E-13	8.97E-08	0.00364709	2.97E-34	3.73E-11	0.30839121
233.1	4.02E-09	2.30E-13	8.97E-08	0.00364709	2.97E-34	3.73E-11	0.30839121
233.2	4.02E-09	2.30E-13	8.97E-08	0.00364709	2.97E-34	3.73E-11	0.30839121
233.3	4.02E-09	2.30E-13	8.97E-08	0.00364709	2.97E-34	3.73E-11	0.30839121
233.4	4.02E-09	2.30E-13	8.97E-08	0.00364709	2.97E-34	3.73E-11	0.30839121
233.5	4.02E-09	2.30E-13	8.97E-08	0.00364709	2.97E-34	3.73E-11	0.30839121
233.6	4.02E-09	2.30E-13	8.97E-08	0.00364709	2.97E-34	3.73E-11	0.30839121
233.7	4.08E-09	2.30E-13	9.35E-08	0.00366197	3.12E-34	3.84E-11	0.31028195
233.8	4.08E-09	2.30E-13	9.35E-08	0.00366197	3.12E-34	3.84E-11	0.31028195
233.9	4.08E-09	2.30E-13	9.35E-08	0.00366197	3.12E-34	3.84E-11	0.31028195
234	4.08E-09	2.30E-13	9.35E-08	0.00366197	3.12E-34	3.84E-11	0.31028195
234.1	4.08E-09	2.30E-13	9.35E-08	0.00366197	3.12E-34	3.84E-11	0.31028195
234.2	4.08E-09	2.30E-13	9.35E-08	0.00366197	3.12E-34	3.84E-11	0.31028195
234.3	4.08E-09	2.30E-13	9.35E-08	0.00366197	3.12E-34	3.84E-11	0.31028195
234.4	4.08E-09	2.30E-13	9.35E-08	0.00366197	3.12E-34	3.84E-11	0.31028195
234.5	4.08E-09	2.30E-13	9.35E-08	0.00366197	3.12E-34	3.84E-11	0.31028195
234.6	4.08E-09	2.30E-13	9.35E-08	0.00366197	3.12E-34	3.84E-11	0.31028195
234.7	4.08E-09	2.30E-13	9.35E-08	0.00366197	3.12E-34	3.84E-11	0.31028195
234.8	4.16E-09	2.24E-13	9.46E-08	0.0037631	3.26E-34	3.78E-11	0.31172797
234.9	4.16E-09	2.24E-13	9.46E-08	0.0037631	3.26E-34	3.78E-11	0.31172797
235	4.16E-09	2.24E-13	9.46E-08	0.0037631	3.26E-34	3.78E-11	0.31172797
235.1	4.16E-09	2.24E-13	9.46E-08	0.0037631	3.26E-34	3.78E-11	0.31172797
235.2	4.16E-09	2.24E-13	9.46E-08	0.0037631	3.26E-34	3.78E-11	0.31172797
235.3	4.16E-09	2.24E-13	9.46E-08	0.0037631	3.26E-34	3.78E-11	0.31172797
235.4	4.16E-09	2.24E-13	9.46E-08	0.0037631	3.26E-34	3.78E-11	0.31172797
235.5	4.16E-09	2.24E-13	9.46E-08	0.0037631	3.26E-34	3.78E-11	0.31172797
235.6	4.16E-09	2.24E-13	9.46E-08	0.0037631	3.26E-34	3.78E-11	0.31172797
235.7	4.16E-09	2.24E-13	9.46E-08	0.0037631	3.26E-34	3.78E-11	0.31172797
235.8	4.19E-09	2.27E-13	9.84E-08	0.00384032	3.55E-34	3.95E-11	0.31175621
235.9	4.19E-09	2.27E-13	9.84E-08	0.00384032	3.55E-34	3.95E-11	0.31175621
236	4.19E-09	2.27E-13	9.84E-08	0.00384032	3.55E-34	3.95E-11	0.31175621
236.1	4.19E-09	2.27E-13	9.84E-08	0.00384032	3.55E-34	3.95E-11	0.31175621
236.2	4.19E-09	2.27E-13	9.84E-08	0.00384032	3.55E-34	3.95E-11	0.31175621
236.3	4.19E-09	2.27E-13	9.84E-08	0.00384032	3.55E-34	3.95E-11	0.31175621
236.4	4.19E-09	2.27E-13	9.84E-08	0.00384032	3.55E-34	3.95E-11	0.31175621
236.5	4.19E-09	2.27E-13	9.84E-08	0.00384032	3.55E-34	3.95E-11	0.31175621
236.6	4.19E-09	2.27E-13	9.84E-08	0.00384032	3.55E-34	3.95E-11	0.31175621
236.7	4.19E-09	2.27E-13	9.84E-08	0.00384032	3.55E-34	3.95E-11	0.31175621
236.8	4.19E-09	2.27E-13	9.84E-08	0.00384032	3.55E-34	3.95E-11	0.31175621
236.9	4.19E-09	2.27E-13	9.84E-08	0.00384032	3.55E-34	3.95E-11	0.31175621
237	4.19E-09	2.27E-13	9.84E-08	0.00384032	3.55E-34	3.95E-11	0.31175621
237.1	4.19E-09	2.27E-13	9.84E-08	0.00384032	3.55E-34	3.95E-11	0.31175621
237.2	4.19E-09	2.27E-13	9.84E-08	0.00384032	3.55E-34	3.95E-11	0.31175621
237.3	4.31E-09	2.14E-13	9.94E-08	0.00387935	3.45E-34	4.08E-11	0.30885357
237.4	4.38E-09	2.24E-13	1.01E-07	0.00398429	3.46E-34	3.85E-11	0.31009334
237.5	4.38E-09	2.24E-13	1.01E-07	0.00398429	3.46E-34	3.85E-11	0.31009334
237.6	4.52E-09	2.27E-13	1.05E-07	0.00398272	3.61E-34	3.95E-11	0.31277041
237.7	4.76E-09	2.22E-13	1.10E-07	0.00407801	3.63E-34	4.00E-11	0.31666147
237.8	4.76E-09	2.22E-13	1.10E-07	0.00407801	3.63E-34	4.00E-11	0.31666147
237.9	4.76E-09	2.22E-13	1.10E-07	0.00407801	3.63E-34	4.00E-11	0.31666147
238	4.76E-09	2.22E-13	1.10E-07	0.00407801	3.63E-34	4.00E-11	0.31666147
238.1	4.76E-09	2.22E-13	1.10E-07	0.00407801	3.63E-34	4.00E-11	0.31666147
238.2	4.76E-09	2.22E-13	1.10E-07	0.00407801	3.63E-34	4.00E-11	0.31666147
238.3	4.76E-09	2.22E-13	1.10E-07	0.00407801	3.63E-34	4.00E-11	0.31666147
238.4	4.76E-09	2.22E-13	1.10E-07	0.00407801	3.63E-34	4.00E-11	0.31666147
238.5	4.76E-09	2.22E-13	1.10E-07	0.00407801	3.63E-34	4.00E-11	0.31666147
238.6	4.76E-09	2.22E-13	1.10E-07	0.00407801	3.63E-34	4.00E-11	0.31666147
238.7	4.76E-09	2.22E-13	1.10E-07	0.00407801	3.63E-34	4.00E-11	0.31666147
238.8	4.76E-09	2.22E-13	1.10E-07	0.00407801	3.63E-34	4.00E-11	0.31666147
238.9	4.76E-09	2.22E-13	1.10E-07	0.00407801	3.63E-34	4.00E-11	0.31666147
239	4.76E-09	2.22E-13	1.10E-07	0.00407801	3.63E-34	4.00E-11	0.31666147
239.1	4.76E-09	2.22E-13	1.10E-07	0.00407801	3.63E-34	4.00E-11	0.31666147
239.2	4.76E-09	2.22E-13	1.10E-07	0.00407801	3.63E-34	4.00E-11	0.31666147
239.3	4.90E-09	2.11E-13	1.11E-07	0.0040977	3.53E-34	4.03E-11	0.31598127
239.4	4.90E-09	2.11E-13	1.11E-07	0.0040977	3.53E-34	4.03E-11	0.31598127
239.5	4.90E-09	2.11E-13	1.11E-07	0.0040977	3.53E-34	4.03E-11	0.31598127
239.6	4.90E-09	2.11E-13	1.11E-07	0.0040977	3.53E-34	4.03E-11	0.31598127
239.7	4.90E-09	2.11E-13	1.11E-07	0.0040977	3.53E-34	4.03E-11	0.31598127
239.8	4.90E-09	2.11E-13	1.11E-07	0.0040977	3.53E-34	4.03E-11	0.31598127
239.9	4.90E-09	2.11E-13	1.11E-07	0.0040977	3.53E-34	4.03E-11	0.31598127
240	4.90E-09	2.11E-13	1.11E-07	0.0040977	3.53E-34	4.03E-11	0.31598127

Table S3: PRCC analysis data for [G3P] with [G](0)=0.02 mM: PRCC sensitivity indices and their corresponding p-values

Sensitivity analysis data from PRCC for [G3P] with [G](0)=0.02 mM (only the parameters to which [G3P]'s level is sensitive to appear)

Time discretization	q	V_{[G3P]}	K_{[G3P]}	K_{[PYR]}	alpha	G_E	[G]_0	[G3P]_0
0	0	0	0	0	0	0	0	1
0.1	0.94572202	0.60657894	-0.9369142	-0.2634272	-0.1078292	-0.0753571	0.91553098	0.06067706
0.2	0.94880521	0.61800701	-0.9399517	-0.2283031	-0.2185086	-0.0477768	0.91868231	0.0167694
0.3	0.94806093	0.61568058	-0.9394836	-0.2011764	-0.3079724	-0.0355369	0.9160071	-0.0077287
0.4	0.94653075	0.60625895	-0.9378213	-0.1747205	-0.3851427	-0.0171669	0.91182693	-0.0283989
0.5	0.9440213	0.59143464	-0.9356452	-0.1445172	-0.4343041	0.0083904	0.90715796	-0.0379223
0.6	0.94161851	0.58530939	-0.9331509	-0.1212962	-0.4950149	0.02334265	0.90282665	-0.0444868
0.7	0.93858852	0.58487419	-0.9311338	-0.1017002	-0.5446586	0.03343481	0.89924992	-0.050245
0.8	0.93703787	0.5800805	-0.9290644	-0.0860974	-0.583862	0.06150802	0.89573857	-0.0616065
0.9	0.9356979	0.57652667	-0.9264931	-0.0636709	-0.625133	0.08095228	0.89150782	-0.0662973
1	0.93533946	0.57344093	-0.9257671	-0.0585859	-0.6628409	0.09123383	0.89024942	-0.0747756
1.1	0.93263401	0.56384611	-0.922587	-0.0323726	-0.6856126	0.10545939	0.88543763	-0.0820843
1.2	0.93023176	0.5541968	-0.9196653	-0.0201772	-0.7031953	0.1199967	0.88245903	-0.090797
1.3	0.9284052	0.54812281	-0.9178458	-0.0077694	-0.7214639	0.13257965	0.87899152	-0.0893348
1.4	0.92577074	0.54173149	-0.915501	0.01211537	-0.7390526	0.14845537	0.87517496	-0.1040302
1.5	0.92380635	0.53546282	-0.91275	0.02296425	-0.7542261	0.16074326	0.87191217	-0.1147823
1.6	0.92068442	0.52886578	-0.9097756	0.03527928	-0.7688987	0.17621632	0.86693263	-0.123774
1.7	0.91759222	0.52930325	-0.9060264	0.04884541	-0.7814081	0.18996076	0.85901409	-0.1314735
1.8	0.913982	0.5234892	-0.9022311	0.07370548	-0.792044	0.20096401	0.85240665	-0.1253922
1.9	0.91223714	0.51949093	-0.9001246	0.09441876	-0.8044797	0.20896274	0.84740123	-0.1322385
2	0.90918349	0.51146563	-0.8959937	0.10848512	-0.8116865	0.21268572	0.84050235	-0.1404681
2.1	0.9076102	0.5063878	-0.8939324	0.12526442	-0.8221848	0.21139621	0.83810197	-0.1542203
2.2	0.90402842	0.49683937	-0.8896233	0.13253007	-0.8262491	0.22047394	0.8310244	-0.1503621
2.3	0.90099003	0.49184632	-0.8870234	0.14474971	-0.8297153	0.22367662	0.82508976	-0.1560292
2.4	0.89762004	0.49044541	-0.8834058	0.15560629	-0.8345208	0.23104076	0.81929003	-0.1568874
2.5	0.89584673	0.49080293	-0.8811984	0.17531571	-0.8403787	0.23975692	0.81498037	-0.1652908
2.6	0.89378839	0.48519162	-0.8796317	0.18841371	-0.8457917	0.24877183	0.811364	-0.1720569
2.7	0.89226634	0.48027314	-0.8775093	0.1964084	-0.8500322	0.2562674	0.80833349	-0.1701085
2.8	0.8899682	0.47454089	-0.8749927	0.20652108	-0.8541397	0.26340729	0.80493975	-0.1674997
2.9	0.8876222	0.47215225	-0.8721691	0.21389242	-0.8571675	0.26600982	0.8006819	-0.1710615
3	0.88510029	0.4650509	-0.8685298	0.21878804	-0.8602312	0.27502402	0.79400399	-0.1711271
3.1	0.88370706	0.45912137	-0.8676866	0.23150626	-0.8644135	0.28412262	0.79065091	-0.1770968
3.2	0.8804975	0.4554759	-0.8639403	0.24028953	-0.8660344	0.28832608	0.78562263	-0.179077
3.3	0.87902052	0.45630932	-0.8618499	0.25030858	-0.871076	0.29809811	0.78198049	-0.1860006
3.4	0.87712718	0.45139398	-0.8584148	0.25496632	-0.8730173	0.29705884	0.7774509	-0.1828223
3.5	0.87411275	0.44378765	-0.8538999	0.26541142	-0.8750453	0.29990535	0.77140941	-0.1867005
3.6	0.87252207	0.4414948	-0.8523441	0.27613892	-0.876816	0.30943629	0.76670543	-0.1845686
3.7	0.87007668	0.43719705	-0.848432	0.28639507	-0.8784048	0.31514104	0.76065848	-0.1906157
3.8	0.86919598	0.4352861	-0.8466687	0.29461602	-0.8816933	0.32838908	0.75444709	-0.1869294
3.9	0.86820735	0.43331448	-0.8447195	0.2981813	-0.8837358	0.33560241	0.75083121	-0.1874046
4	0.86534041	0.43240535	-0.8423133	0.29954261	-0.8856854	0.33898637	0.74822241	-0.1897403
4.1	0.86344682	0.43005444	-0.8406195	0.30343308	-0.8874953	0.34572967	0.74327379	-0.1795299
4.2	0.86093977	0.4270845	-0.8374531	0.31495311	-0.8871243	0.35376505	0.73749578	-0.1764301
4.3	0.86013826	0.42767243	-0.8359075	0.3233481	-0.8900147	0.35907316	0.73497623	-0.1725838
4.4	0.85713669	0.42393846	-0.8321405	0.32978163	-0.8902735	0.36353964	0.72950131	-0.1691379
4.5	0.85490139	0.41850045	-0.829465	0.33613977	-0.8919498	0.36748969	0.72550306	-0.1702979
4.6	0.85385523	0.41434055	-0.8283867	0.33869168	-0.8937107	0.37490036	0.72224791	-0.1672262
4.7	0.85225281	0.4098269	-0.8258208	0.34511118	-0.8954623	0.38108309	0.71771327	-0.1660656
4.8	0.85042387	0.40797287	-0.8222869	0.35079889	-0.8969204	0.38500565	0.71349181	-0.1675548
4.9	0.8482371	0.40203127	-0.8202634	0.35611963	-0.8979704	0.38879072	0.70998439	-0.1665346
5	0.84623444	0.40181233	-0.8180373	0.3604042	-0.8981305	0.39359001	0.70635253	-0.1677932
5.1	0.84455475	0.39959781	-0.814718	0.36714326	-0.9000307	0.40134949	0.70111845	-0.1688942
5.2	0.841588	0.39883962	-0.8111678	0.3713857	-0.9002907	0.40441023	0.69550141	-0.1658848
5.3	0.83935002	0.39536893	-0.8073711	0.37181648	-0.9010411	0.40794652	0.69198922	-0.1638198
5.4	0.83735955	0.39600053	-0.8039584	0.37426125	-0.9014242	0.41315626	0.68869659	-0.1610749
5.5	0.83601406	0.39297502	-0.8011107	0.3798579	-0.9020277	0.41119976	0.68603708	-0.1632165
5.6	0.83235511	0.38768745	-0.7976738	0.38000123	-0.9016845	0.41259601	0.68135429	-0.1672608
5.7	0.83063012	0.38309877	-0.7958492	0.38322933	-0.9024173	0.41677742	0.67941254	-0.1624681
5.8	0.82835835	0.38249518	-0.7925148	0.3857202	-0.9024426	0.42016884	0.6755669	-0.1590745
5.9	0.82481874	0.37588767	-0.788556	0.38622964	-0.9017958	0.42298012	0.66871323	-0.1607774
6	0.82189656	0.37241357	-0.7852963	0.38971421	-0.9021203	0.42605788	0.66387932	-0.1618622
6.1	0.82062508	0.36984837	-0.7831064	0.39174387	-0.9022237	0.42788364	0.6586824	-0.1590231
6.2	0.81893729	0.36941883	-0.7808931	0.39135452	-0.9023905	0.42953714	0.6523154	-0.156722
6.3	0.81526313	0.37018505	-0.7766252	0.39429022	-0.9019214	0.43089397	0.64462827	-0.1600257
6.4	0.81411646	0.36877348	-0.7734689	0.40053657	-0.9032027	0.43454335	0.64017415	-0.159619
6.5	0.8117076	0.36594283	-0.7700414	0.40473869	-0.9029801	0.43617425	0.63268481	-0.1573415
6.6	0.81060189	0.36533894	-0.7679254	0.40864136	-0.903684	0.44359324	0.62721256	-0.1541018
6.7	0.80803464	0.36528473	-0.7648321	0.4098085	-0.9038553	0.4451586	0.62151984	-0.1482635
6.8	0.80672506	0.36453349	-0.7625876	0.41441117	-0.9042223	0.44686201	0.61694256	-0.1491227
6.9	0.80498401	0.3637371	-0.7617431	0.41641434	-0.9046099	0.45027695	0.61191259	-0.149047
7	0.80340477	0.36346721	-0.7604149	0.42068696	-0.9058264	0.45513163	0.60799732	-0.1487921
7.1	0.80167412	0.36350953	-0.7583115	0.42458257	-0.9062367	0.45771134	0.60315926	-0.1463273
7.2	0.80019432	0.36184652	-0.7564291	0.42822371	-0.9066265	0.46004638	0.60062541	-0.1489411
7.3	0.79758727	0.35842629	-0.7528731	0.4312161	-0.9064314	0.46180192	0.5959342	-0.1488907
7.4	0.79640121	0.35918197	-0.7512431	0.43471229	-0.9069227	0.46635162	0.59124577	-0.1491271
7.5	0.79242615	0.35894911	-0.7464724	0.43701428	-0.9066139	0.46985611	0.58585322	-0.1427143
7.6	0.79077662	0.3585259	-0.7444107	0.43911363	-0.9069679	0.4720557	0.58237277	-0.1410732
7.7	0.78761747	0.35914052	-0.7421961	0.44081683	-0.9067411	0.47515413	0.575572	-0.1393366
7.8	0.78666955	0.35841395	-0.7397252	0.44593865	-0.9067957	0.47433189	0.57092607	-0.1398688
7.9	0.78376871	0.3545799	-0.7345763	0.44779638	-0.9063804	0.47463239	0.5660062	-0.1405086
8	0.78061471	0.34750799	-0.7308774	0.44423258	-0.9061341	0.47528935	0.55960951	-0.1354573
8.1	0.77941737	0.34934675	-0.7294821	0.44534507	-0.9068296	0.47889645	0.55618985	-0.1334351
8.2	0.77869004	0.34738095	-0.727866	0.44980814	-0.9071023	0.48160764	0.55238457	-0.1340046
8.3	0.77586209	0.34777632	-0.7227693	0.44977316	-0.9070836	0.48338538	0.54520654	-0.1344205
8.4	0.77413041	0.34436722	-0.7208685	0.4528636	-0.9074615	0.48917788	0.54020159	-0.1322926
8.5	0.77277231	0.34322147	-0.7184652	0.45582471	-0.9076938	0.49206829	0.53775842	-0.1329897
8.6	0.77004157	0.3404265	-0.7159764	0.45781433	-0.9074278	0.49496613	0.53186673	-0.1337822
8.7	0.76722559	0.3372125	-0.7130312	0.45819335	-0.9070099	0.49605374	0.52738584	-0.1337475
8.8	0.76562873	0.33719236	-0.7105529	0.46045767	-0.907045	0.49832029	0.52417966	-0.1315836
8.9	0.76318362	0.33747689	-0.7073124	0.46349249	-0.9070697	0.499147	0.51982747	-0.1269515
9	0.76219421	0.3375655	-0.7057063	0.46500276	-0.9072042	0.50272359	0.51668609	-0.1269529
9.1	0.75891368	0.33419126	-0.7033368	0.46674484	-0.9065796	0.50215634	0.51185143	-0.1244189
9.2	0.75896156	0.3336435	-0.7011332	0.46950476	-0.9071315	0.50351128	0.50851377	-0.1259739
9.3	0.75632669	0.3354257	-0.6978469	0.47103945	-0.9070312	0.50567862	0.50448683	-0.1232789
9.4	0.75535276	0.33472367	-0.6964875	0.47167475	-0.9068557	0.50640193	0.50187501	-0.1221684
9.5	0.75386765	0.33346511	-0.6928283	0.47209974	-0.9068245	0.51029932	0.4967342	-0.1197282
9.6	0.75135438	0.33343004	-0.6898763	0.47130295	-0.9064865	0.5111731	0.49283713	-0.1192428
9.7	0.7503207	0.33262764	-0.688924	0.4724558	-0.9068519	0.51235153	0.49094555	-0.1177991
9.8	0.7470376	0.3291868	-0.6847964	0.47409857	-0.9066404	0.51184238	0.48452368	-0.1158219
9.9	0.74470241	0.32972542	-0.6825021	0.47652122	-0.9067895	0.51650495	0.48183858	-0.1126032
10	0.74220718	0.33033114	-0.6788909	0.47658284	-0.9063938	0.51742472	0.47630047	-0.1106001
10.1	0.73938122	0.32877687	-0.6756895	0.47573173	-0.9062619	0.51849534	0.47073092	-0.1083216
10.2	0.73689717	0.32907897	-0.6730085	0.4780805	-0.90618	0.51979478	0.467547	-0.108021
10.3	0.73604124	0.32764159	-0.6711004	0.4806101	-0.9060763	0.52258943	0.46365105	-0.1063975
10.4	0.73475625	0.32787175	-0.6690601	0.48356714	-0.9059109	0.5240429	0.45698625	-0.1048022
10.5	0.73384435	0.32765041	-0.6677394	0.48343883	-0.9065539	0.52835153	0.45276493	-0.1037308
10.6	0.7334237	0.32545233	-0.6648976	0.48676983	-0.9067182	0.5320023	0.44983729	-0.1013546
10.7	0.73221882	0.32553579	-0.6636432	0.48944097	-0.9068414	0.53337497	0.44622323	-0.1005309
10.8	0.73149151	0.32354658	-0.6618105	0.48998342	-0.9068726	0.53450299	0.44083056	-0.1016097
10.9	0.72965712	0.32240213	-0.6586438	0.49042112	-0.9066719	0.53322302	0.43606852	-0.0986674
11	0.72729367	0.32307237	-0.6571383	0.49162326	-0.9064311	0.53386908	0.43256046	-0.0983414
11.1	0.72646832	0.32095487	-0.6559227	0.49387028	-0.9063777	0.53661368	0.4298667	-0.0980956
11.2	0.72372575	0.32013183	-0.6536208	0.49397993	-0.9060394	0.53831767	0.424643	-0.0933434
11.3	0.72261312	0.31846227	-0.6509954	0.49508717	-0.9061526	0.53854019	0.42222311	-0.094573
11.4	0.72080846	0.31696268	-0.648284	0.49540847	-0.9059969	0.53980382	0.41759075	-0.0935519
11.5	0.72035948	0.31691519	-0.646292	0.49722493	-0.9060962	0.54297253	0.41412702	-0.0930158
11.6	0.71851018	0.31633021	-0.6449231	0.49825097	-0.9060123	0.54380995	0.41281405	-0.0922403
11.7	0.71668799	0.3203728	-0.6431491	0.50011517	-0.9064505	0.5473871	0.40884562	-0.0892553
11.8	0.7154153	0.31690365	-0.6407627	0.50164229	-0.9059371	0.54730893	0.40467471	-0.0888479
11.9	0.7132656	0.31878274	-0.6377827	0.50588459	-0.9062425	0.54929166	0.40092061	-0.0857274
12	0.71289112	0.31594827	-0.6356998	0.50872454	-0.90615	0.55149112	0.39445712	-0.0840645
12.1	0.71110901	0.31820878	-0.6336692	0.51149678	-0.9064126	0.55434812	0.3924452	-0.0819276
12.2	0.70939627	0.31878531	-0.6305029	0.51230512	-0.9061875	0.55502708	0.38721985	-0.084409
12.3	0.70757314	0.31514407	-0.6276592	0.51391416	-0.9064706	0.55476984	0.38188656	-0.084921
12.4	0.70635645	0.31387872	-0.6250271	0.51480192	-0.9062622	0.55687744	0.37894146	-0.085643
12.5	0.70420866	0.31414605	-0.6220733	0.51641998	-0.9060404	0.55632349	0.37307433	-0.0802806
12.6	0.7021279	0.3125605	-0.6180408	0.51633315	-0.905599	0.55635629	0.37252044	-0.0798478
12.7	0.70091071	0.31360339	-0.6160875	0.51767193	-0.9057744	0.55822598	0.36934357	-0.0784524
12.8	0.69755191	0.31170642	-0.6133785	0.51957711	-0.9057826	0.55968592	0.36615897	-0.0813911
12.9	0.69506885	0.30724566	-0.6086689	0.51936515	-0.9053631	0.55879303	0.36289095	-0.0797902
13	0.69356827	0.30753975	-0.6066357	0.51845415	-0.9054291	0.55880809	0.36098119	-0.0798178
13.1	0.69112151	0.30585087	-0.6036248	0.5191759	-0.9052783	0.5603366	0.36070396	-0.0799229
13.2	0.68916104	0.30530107	-0.6022191	0.51976508	-0.9050435	0.56380032	0.35756775	-0.0779999
13.3	0.68701267	0.30445301	-0.6006656	0.52095081	-0.9049162	0.56648681	0.35264823	-0.0776997
13.4	0.68593569	0.30337757	-0.5984137	0.52312114	-0.9049772	0.56856627	0.35171432	-0.0760899
13.5	0.68610048	0.3021927	-0.5973	0.52703156	-0.9052432	0.57179822	0.34786909	-0.0732676
13.6	0.68524902	0.30235391	-0.5949136	0.52709351	-0.9053221	0.57291608	0.34557789	-0.0724189
13.7	0.68257812	0.30090564	-0.5924337	0.52733606	-0.9048594	0.57341377	0.34317173	-0.0706339
13.8	0.68090984	0.30069309	-0.5914108	0.52802849	-0.9049271	0.57421243	0.34167365	-0.0683983
13.9	0.67923177	0.30138552	-0.5902862	0.52882124	-0.90505	0.57555508	0.34107246	-0.0636523
14	0.67701432	0.30324048	-0.5884118	0.53087392	-0.9050894	0.57785078	0.3370105	-0.0619074
14.1	0.67573012	0.30245106	-0.5855824	0.53169866	-0.9051671	0.5783702	0.33406471	-0.0603452
14.2	0.67439444	0.30271189	-0.5830307	0.53251222	-0.9050584	0.57851467	0.3337024	-0.0589816
14.3	0.67261622	0.30348398	-0.5819125	0.53417812	-0.9050899	0.57958601	0.33041169	-0.0609021
14.4	0.67038142	0.30238875	-0.5803682	0.53613934	-0.9049252	0.57913289	0.32858519	-0.0590123
14.5	0.66866551	0.30305182	-0.5785399	0.5381848	-0.9049337	0.58000623	0.32844004	-0.0615652
14.6	0.66723369	0.30034114	-0.5752084	0.5379085	-0.9045647	0.58050187	0.32129295	-0.0590955
14.7	0.66416974	0.30226577	-0.5709642	0.53757797	-0.9043608	0.58189691	0.31797044	-0.0566198
14.8	0.66323185	0.30394608	-0.5703572	0.53937513	-0.904582	0.58253085	0.31770131	-0.0596095
14.9	0.66254062	0.30266578	-0.569494	0.54073232	-0.9045733	0.58368554	0.31727841	-0.0583545
15	0.66125434	0.3027134	-0.5676445	0.54381735	-0.9045832	0.58549001	0.31492243	-0.0592242
15.1	0.66124205	0.30214459	-0.5671127	0.54557344	-0.9047519	0.58710605	0.31284823	-0.0587935
15.2	0.66063733	0.30056405	-0.5667039	0.546875	-0.904807	0.58823544	0.31242902	-0.0593174
15.3	0.65895345	0.29951256	-0.5641518	0.5469389	-0.9045888	0.58834111	0.31014154	-0.0581992
15.4	0.65707277	0.29872862	-0.562156	0.54616426	-0.9044775	0.5879688	0.30771994	-0.0573273
15.5	0.65623932	0.29785054	-0.5620856	0.54834558	-0.9049464	0.58944234	0.30773294	-0.0541427
15.6	0.65480314	0.29851907	-0.5590331	0.54905192	-0.9047434	0.59145208	0.30283471	-0.0531618
15.7	0.65270053	0.29670057	-0.5561977	0.54974853	-0.904558	0.59376904	0.30118099	-0.0524935
15.8	0.64936575	0.29660542	-0.5535041	0.55134399	-0.9045335	0.59262553	0.29818815	-0.0529619
15.9	0.64578349	0.29604132	-0.5502707	0.55170313	-0.9042737	0.59260674	0.2954034	-0.0526419
16	0.64458996	0.29647328	-0.5485007	0.55301431	-0.9041586	0.59344206	0.29411324	-0.0530334
16.1	0.64245955	0.29808786	-0.5454502	0.55479755	-0.904123	0.59433915	0.29441858	-0.0520311
16.2	0.64015923	0.29949301	-0.5443408	0.55899891	-0.9045973	0.59709911	0.29127966	-0.0524015
16.3	0.6382968	0.29784066	-0.5411373	0.56095812	-0.9047564	0.59806215	0.28846839	-0.0505132
16.4	0.63756839	0.29648081	-0.5394033	0.5615251	-0.9047585	0.59867541	0.28954703	-0.0500515
16.5	0.63534021	0.29464008	-0.5361878	0.56218219	-0.9040787	0.59759121	0.28438435	-0.0479933
16.6	0.63346257	0.29243214	-0.5343895	0.56237995	-0.9039345	0.59862786	0.28268758	-0.0478148
16.7	0.63188353	0.29165404	-0.5328489	0.56269015	-0.9041195	0.59998527	0.28098273	-0.0470303
16.8	0.63108459	0.29219453	-0.531278	0.56362779	-0.9042811	0.60082007	0.28109549	-0.0474055
16.9	0.63030273	0.2914191	-0.5307258	0.564213	-0.9043733	0.60196138	0.28006807	-0.0493471
17	0.62993156	0.2922647	-0.5273237	0.56557182	-0.9042032	0.6041458	0.2750669	-0.0478522
17.1	0.62898089	0.29124566	-0.5257664	0.56461593	-0.9039121	0.60438591	0.27314797	-0.0465336
17.2	0.6282853	0.2896977	-0.5256726	0.56514129	-0.9037911	0.60576832	0.27238377	-0.0452403
17.3	0.626989	0.28849882	-0.5238619	0.56594824	-0.9036173	0.60701384	0.27090762	-0.0472286
17.4	0.62541405	0.28889584	-0.5209537	0.56626938	-0.903514	0.60728077	0.2703149	-0.046324
17.5	0.62469162	0.28969411	-0.5204717	0.56663216	-0.9034103	0.60809996	0.2700018	-0.0447656
17.6	0.62247854	0.2881292	-0.5187816	0.56561927	-0.9030746	0.60963254	0.2663145	-0.0444387
17.7	0.6208243	0.28633515	-0.5177329	0.56627824	-0.9029697	0.61089362	0.26338294	-0.0453661
17.8	0.61856799	0.28625424	-0.5158178	0.56701279	-0.9027916	0.61125901	0.26026429	-0.0472235
17.9	0.61791196	0.28602763	-0.5146072	0.56808489	-0.9027214	0.61225444	0.25923097	-0.0476254
18	0.6173099	0.28659795	-0.5127851	0.56953121	-0.9026574	0.6127483	0.25886646	-0.0460874
18.1	0.61558132	0.28733261	-0.5119182	0.57053111	-0.9025459	0.61327431	0.25607141	-0.0471279
18.2	0.61518515	0.28773303	-0.5085365	0.57010759	-0.9023151	0.61330269	0.25286427	-0.0456986
18.3	0.61415776	0.28609578	-0.5073919	0.57080359	-0.9022422	0.6144745	0.24938138	-0.043977
18.4	0.61371837	0.28543623	-0.5064117	0.57162911	-0.9021751	0.61560642	0.24727234	-0.0432424
18.5	0.61300703	0.28571852	-0.5056855	0.57300017	-0.9023148	0.61731298	0.24687172	-0.0428573
18.6	0.61110313	0.28538623	-0.5043085	0.57280057	-0.9019193	0.61778165	0.24337556	-0.0420187
18.7	0.61032572	0.28506878	-0.5022185	0.57206454	-0.901782	0.61921477	0.24120522	-0.0435481
18.8	0.60799014	0.28355773	-0.4989051	0.57184455	-0.9012663	0.61917429	0.23924973	-0.041831
18.9	0.60611106	0.2815089	-0.496572	0.57212878	-0.9010117	0.61829677	0.23711679	-0.0421806
19	0.60584711	0.28039155	-0.4952281	0.57328322	-0.9009139	0.61920436	0.2358058	-0.0414343
19.1	0.6058941	0.28022207	-0.4928505	0.57343422	-0.9009907	0.62017523	0.23258758	-0.0419348
19.2	0.6040915	0.27945628	-0.492455	0.57254592	-0.9007231	0.62062669	0.22924636	-0.0404438
19.3	0.60254116	0.28006343	-0.4906739	0.57413457	-0.9009643	0.62140182	0.22894899	-0.0416231
19.4	0.60141936	0.27910055	-0.488221	0.57389992	-0.9006008	0.62190961	0.22689717	-0.0418571
19.5	0.60076714	0.27861493	-0.4868517	0.57409208	-0.9005901	0.62256696	0.22558042	-0.0415716
19.6	0.6003916	0.27861237	-0.4859315	0.57494651	-0.9007495	0.62382558	0.22362307	-0.0421333
19.7	0.59965051	0.27580111	-0.4841227	0.57493571	-0.9005472	0.62370246	0.22249111	-0.0426878
19.8	0.59764117	0.27628435	-0.4818857	0.57589632	-0.9005543	0.62461577	0.21901669	-0.0425787
19.9	0.59527945	0.27212914	-0.4802396	0.57678457	-0.9003493	0.62545257	0.21680268	-0.0417723
20	0.59551481	0.27237956	-0.4795737	0.57807531	-0.9006601	0.62639595	0.21386134	-0.0416934
20.1	0.59306114	0.27277231	-0.4776926	0.58001539	-0.9003119	0.62721829	0.21109154	-0.042315
20.2	0.59120972	0.2718482	-0.4754622	0.58120234	-0.900434	0.62739782	0.20825993	-0.0399826
20.3	0.59027223	0.27422749	-0.4748745	0.58208791	-0.9007413	0.62935811	0.20546469	-0.0400047
20.4	0.58915414	0.27536267	-0.4739867	0.58300981	-0.9007027	0.63021274	0.20419894	-0.0386235
20.5	0.58824417	0.27784233	-0.4719732	0.58274813	-0.9006893	0.62932005	0.20123943	-0.0384152
20.6	0.58580026	0.27764139	-0.4696669	0.58344047	-0.9005219	0.62925951	0.20095901	-0.0393993
20.7	0.58374361	0.27583767	-0.4668018	0.58427644	-0.9004517	0.62938425	0.19869208	-0.0370833
20.8	0.58202625	0.2734288	-0.4649173	0.58285632	-0.8999468	0.62812831	0.19480739	-0.0365953
20.9	0.58139653	0.27362947	-0.4641877	0.58344635	-0.9002179	0.62839672	0.19317278	-0.0354002
21	0.58050489	0.27333528	-0.4607592	0.58367902	-0.9001874	0.6288802	0.19201875	-0.0344794
21.1	0.57913291	0.27380923	-0.4590535	0.58350451	-0.900017	0.62873628	0.19197333	-0.0337899
21.2	0.57849036	0.27102273	-0.457843	0.58392277	-0.89978	0.62890921	0.18990435	-0.0328086
21.3	0.57820871	0.26950389	-0.4561258	0.58496906	-0.8995864	0.6290474	0.18821524	-0.0302408
21.4	0.57706136	0.26867232	-0.4551557	0.58612874	-0.8998198	0.6295334	0.18550581	-0.0295566
21.5	0.5748756	0.26855523	-0.4531921	0.58573468	-0.8996614	0.6288569	0.18417498	-0.0294787
21.6	0.57431238	0.26918478	-0.4520379	0.58641675	-0.8999109	0.62947948	0.18401858	-0.0307019
21.7	0.57378862	0.26831964	-0.4499613	0.58632076	-0.8999359	0.62980766	0.18194625	-0.0305652
21.8	0.57294469	0.26703655	-0.4482619	0.58598691	-0.8994873	0.63019151	0.18101988	-0.0300194
21.9	0.57196447	0.26463367	-0.4454559	0.58706813	-0.8992151	0.63011242	0.17811485	-0.0286504
22	0.57031183	0.26357311	-0.4455914	0.58618786	-0.8990409	0.63015598	0.17661668	-0.0300707
22.1	0.56974042	0.26297085	-0.4431222	0.58595271	-0.8991579	0.63032151	0.17424784	-0.0299941
22.2	0.56955626	0.26339471	-0.4425887	0.58616373	-0.8991667	0.63087657	0.17351575	-0.0300157
22.3	0.56695915	0.26267264	-0.4402851	0.58710284	-0.898946	0.63058299	0.17135649	-0.0308715
22.4	0.56691555	0.26303412	-0.4400471	0.5874001	-0.8990977	0.63116234	0.17076118	-0.0298435
22.5	0.56481079	0.26291113	-0.4376107	0.58743415	-0.8987368	0.63093241	0.16827965	-0.0286303
22.6	0.56376558	0.26194632	-0.4370065	0.58775826	-0.8987348	0.6316296	0.16907191	-0.0287569
22.7	0.56354123	0.26275405	-0.435541	0.58679308	-0.8987251	0.63172781	0.16613229	-0.0293992
22.8	0.56298567	0.26207926	-0.433985	0.58680208	-0.8985837	0.63170583	0.16495305	-0.0290943
22.9	0.56230542	0.2624287	-0.4334415	0.58717916	-0.8986637	0.63316886	0.16502435	-0.0292553
23	0.56128482	0.26270473	-0.4307953	0.58783299	-0.8985032	0.63317968	0.16461229	-0.0286447
23.1	0.56099524	0.26206923	-0.429995	0.58887888	-0.8985223	0.63388443	0.16245058	-0.0265699
23.2	0.55925865	0.26197175	-0.4290935	0.58968152	-0.8983328	0.63391207	0.16066171	-0.0249333
23.3	0.55882202	0.26181402	-0.4284305	0.59035909	-0.8984023	0.63436455	0.1594885	-0.0237009
23.4	0.5586512	0.26201466	-0.4284502	0.59064537	-0.8983412	0.63468486	0.15916779	-0.02352
23.5	0.55685822	0.26262755	-0.4269583	0.59023809	-0.8981194	0.63408761	0.15630253	-0.0220022
23.6	0.55535586	0.26136978	-0.4270631	0.59105197	-0.8980252	0.63433521	0.15505238	-0.0204675
23.7	0.55433015	0.25862992	-0.4247413	0.59059363	-0.8979125	0.63532392	0.15282317	-0.0201307
23.8	0.55232648	0.25854323	-0.4216722	0.59036354	-0.8975702	0.63459068	0.15276609	-0.0201242
23.9	0.55169242	0.25647786	-0.4200595	0.59261626	-0.8977075	0.63577895	0.14961201	-0.0189826
24	0.54964641	0.25642645	-0.4207248	0.59337858	-0.8974803	0.63683686	0.14845892	-0.0183524
24.1	0.54755866	0.25597201	-0.4208385	0.59331179	-0.897334	0.63713424	0.14815888	-0.0175783
24.2	0.54675527	0.25755322	-0.4199518	0.59427343	-0.8975652	0.63853301	0.14805194	-0.0175343
24.3	0.54542021	0.25787989	-0.4188427	0.59411473	-0.8973506	0.63834175	0.14792987	-0.0178636
24.4	0.54481829	0.25611655	-0.4175923	0.59419803	-0.8971505	0.63818287	0.1475663	-0.0178566
24.5	0.54311269	0.25473137	-0.4169787	0.59389634	-0.8968453	0.63825637	0.14704536	-0.0185391
24.6	0.54226742	0.25349008	-0.4149677	0.59381514	-0.8966635	0.63885034	0.1468145	-0.0165981
24.7	0.54218853	0.25345535	-0.4135323	0.59401551	-0.8965128	0.63872588	0.14628928	-0.0190425
24.8	0.54119286	0.25239434	-0.4129423	0.59514979	-0.8967129	0.63965786	0.14604717	-0.0193556
24.9	0.54006961	0.25207055	-0.4118943	0.59446086	-0.8964877	0.63882	0.14575541	-0.0184919
25	0.53975531	0.25205028	-0.4107823	0.59577126	-0.8965959	0.63912835	0.14510877	-0.018112
25.1	0.53817252	0.25308977	-0.409646	0.59594774	-0.8967066	0.63907372	0.1440902	-0.0184595
25.2	0.5366016	0.25217157	-0.4083453	0.59554049	-0.8966248	0.63951159	0.14233891	-0.018184
25.3	0.53545497	0.25238203	-0.4061339	0.59683283	-0.8966342	0.64020758	0.14062052	-0.0164522
25.4	0.53612846	0.25272325	-0.4057787	0.59742231	-0.896792	0.64071058	0.13988021	-0.017421
25.5	0.53557147	0.25328939	-0.4047654	0.59819873	-0.8968902	0.64172224	0.13855437	-0.0174606
25.6	0.53497763	0.25181782	-0.4038731	0.59824262	-0.896574	0.64222323	0.13736955	-0.0153455
25.7	0.53358355	0.25106608	-0.4025667	0.5989194	-0.8965888	0.64253989	0.13576287	-0.0145707
25.8	0.53351744	0.25102911	-0.4019707	0.59935654	-0.8966566	0.64276021	0.13480567	-0.0148726
25.9	0.53305413	0.25140025	-0.4022124	0.59935117	-0.8966644	0.64359108	0.13398694	-0.015311
26	0.53117863	0.25122	-0.4013354	0.59960206	-0.896524	0.64357819	0.13283419	-0.0148927
26.1	0.53107186	0.25205029	-0.3995625	0.60062881	-0.8965867	0.64426407	0.1309205	-0.0156806
26.2	0.53079312	0.25170106	-0.3988515	0.60076076	-0.8966002	0.64451795	0.12991606	-0.0152966
26.3	0.52945504	0.25125303	-0.3969067	0.60187669	-0.8966081	0.64425845	0.12911577	-0.0124777
26.4	0.52788899	0.25016151	-0.3958969	0.60187478	-0.8964981	0.64447851	0.12913076	-0.0119881
26.5	0.52698766	0.24853716	-0.3938313	0.60235541	-0.8962305	0.6445678	0.12669124	-0.0110082
26.6	0.52661742	0.2487337	-0.3927606	0.60234625	-0.8961268	0.64451827	0.12512054	-0.0111014
26.7	0.52565514	0.2493304	-0.3935383	0.60301079	-0.8961767	0.64549772	0.1240338	-0.011392
26.8	0.52440169	0.24952731	-0.3916292	0.60272741	-0.8960571	0.64598653	0.12261095	-0.0119052
26.9	0.52391897	0.24933753	-0.3919301	0.60287836	-0.8960435	0.64636571	0.12285276	-0.0109991
27	0.52328479	0.24785615	-0.3908569	0.60232524	-0.8958285	0.6463469	0.12232775	-0.0109315
27.1	0.52268341	0.24836831	-0.3901439	0.60244574	-0.8958045	0.64714389	0.12136915	-0.0102422
27.2	0.52225582	0.24721549	-0.3896159	0.6026277	-0.8957452	0.64739126	0.12095774	-0.0107826
27.3	0.52093396	0.24745658	-0.3872618	0.60327288	-0.895745	0.64797324	0.11995382	-0.0117678
27.4	0.52070855	0.24737438	-0.3870053	0.60352299	-0.895747	0.64796978	0.11987617	-0.0115704
27.5	0.51862664	0.24582622	-0.3867058	0.60224803	-0.8953404	0.64722773	0.11810888	-0.0101331
27.6	0.51787719	0.24536817	-0.3851341	0.60211219	-0.8952424	0.64708555	0.11687251	-0.0102782
27.7	0.51687564	0.24349754	-0.3835352	0.60280758	-0.8951174	0.64781269	0.11517685	-0.009793
27.8	0.5151987	0.24392697	-0.3828619	0.60363336	-0.8951032	0.64802302	0.11400914	-0.0100162
27.9	0.51484961	0.24347142	-0.3821595	0.60300076	-0.8949403	0.64787025	0.1135419	-0.0100876
28	0.51398755	0.24364748	-0.3808029	0.60386871	-0.8952535	0.64831625	0.11273887	-0.0108738
28.1	0.5137852	0.24294142	-0.3804498	0.60452512	-0.8952728	0.64871273	0.11230378	-0.0099647
28.2	0.51343596	0.24317087	-0.3802141	0.60479445	-0.8952776	0.6488448	0.11187822	-0.009709
28.3	0.51325277	0.24321462	-0.3790559	0.60570503	-0.895036	0.64872616	0.10911859	-0.0090954
28.4	0.51289229	0.24284451	-0.3792379	0.60635795	-0.8952788	0.64955152	0.10867188	-0.0088628
28.5	0.51300482	0.24263624	-0.3777387	0.60693344	-0.8953461	0.64949116	0.10835954	-0.0094342
28.6	0.51214986	0.24180134	-0.3765937	0.6071185	-0.8952971	0.64961454	0.10835887	-0.0094397
28.7	0.51135931	0.24112801	-0.3762527	0.60753295	-0.8951635	0.64931994	0.10762391	-0.0088957
28.8	0.51135611	0.24140973	-0.3753602	0.60746008	-0.8950739	0.64936476	0.10658067	-0.0088865
28.9	0.50952966	0.23971725	-0.3730848	0.60708288	-0.8947687	0.64835309	0.10503278	-0.0101036
29	0.50845086	0.23942506	-0.3722923	0.60736418	-0.8949112	0.64881922	0.10462842	-0.0103127
29.1	0.5071073	0.2398241	-0.3698992	0.60660433	-0.8949273	0.65007689	0.10267596	-0.010531
29.2	0.50599336	0.23879071	-0.3677755	0.60658665	-0.8946106	0.65022755	0.10146859	-0.0104585
29.3	0.50506528	0.23874725	-0.3678237	0.60657674	-0.8945493	0.65030459	0.10187387	-0.0105535
29.4	0.50378201	0.2382304	-0.3663108	0.60850069	-0.8943169	0.6516255	0.09972881	-0.008522
29.5	0.5031615	0.23745027	-0.366382	0.60944546	-0.8943351	0.65190909	0.09874969	-0.0084024
29.6	0.50220573	0.23657275	-0.3653572	0.60914533	-0.8940135	0.65175635	0.09753049	-0.0088199
29.7	0.49994971	0.23449866	-0.3636028	0.60768775	-0.8934768	0.65109642	0.09612032	-0.0069896
29.8	0.49946173	0.23368597	-0.3632927	0.60698194	-0.8932938	0.65071888	0.0952977	-0.0068874
29.9	0.4992089	0.23311575	-0.3625047	0.60684652	-0.8931235	0.65079627	0.09507225	-0.0065016
30	0.49914864	0.23171396	-0.3619447	0.60719115	-0.893037	0.65078803	0.0955972	-0.0060181
30.1	0.49756126	0.2316857	-0.3605356	0.60716545	-0.8927754	0.65067187	0.09241989	-0.0037594
30.2	0.49662711	0.23152598	-0.3603877	0.60797472	-0.8926675	0.65066126	0.09097364	-0.0031817
30.3	0.49572514	0.23153819	-0.358942	0.60880195	-0.8927929	0.65147045	0.09054466	-0.0023089
30.4	0.4946183	0.23184323	-0.3574728	0.60971473	-0.8928505	0.65249176	0.08982056	-0.0006875
30.5	0.49339031	0.23025354	-0.3550685	0.6087121	-0.8925883	0.6518783	0.08889202	-0.0023949
30.6	0.49214579	0.228939	-0.3547083	0.60835522	-0.8922934	0.65177989	0.08864612	-0.0016389
30.7	0.49199006	0.22917269	-0.3540149	0.60866705	-0.892395	0.65185934	0.08860166	-0.0021029
30.8	0.4913986	0.22887881	-0.3533165	0.60864305	-0.8922922	0.65199523	0.08870057	-0.001989
30.9	0.49014231	0.2289439	-0.3537363	0.60920833	-0.8922781	0.65214974	0.08689041	-0.0018374
31	0.48888957	0.22949465	-0.3517777	0.60894889	-0.8922498	0.65258134	0.08636595	-0.0024177
31.1	0.48860232	0.22874613	-0.3515513	0.60865987	-0.8920608	0.65263161	0.08660175	-0.0024083
31.2	0.48751205	0.22866595	-0.350365	0.60872079	-0.8918469	0.65229771	0.08488935	-0.0026233
31.3	0.48620921	0.22877353	-0.350221	0.6087395	-0.8917934	0.65216084	0.08560082	-0.0025684
31.4	0.48595555	0.22772804	-0.348584	0.60872594	-0.8916125	0.65199851	0.08521169	-0.0029349
31.5	0.48528138	0.22715691	-0.3475108	0.60909264	-0.8914685	0.65179572	0.08672574	-0.003845
31.6	0.48476359	0.2280959	-0.3470455	0.60974243	-0.8916047	0.65192063	0.08543886	-0.0035782
31.7	0.48432857	0.22872638	-0.3467941	0.60979864	-0.8916469	0.65205657	0.0854108	-0.0040402
31.8	0.4843834	0.22834417	-0.3449113	0.60934627	-0.8913354	0.65207861	0.08459424	-0.0034779
31.9	0.48382374	0.2306532	-0.3439839	0.61021992	-0.8913464	0.65271611	0.08471485	-0.0024064
32	0.48320731	0.2303596	-0.3432759	0.61041055	-0.8912254	0.65291696	0.08578387	-0.0024888
32.1	0.48263302	0.2285511	-0.3425201	0.61054084	-0.8911871	0.65274388	0.08414834	-0.0021674
32.2	0.48160696	0.22771279	-0.3415493	0.61110208	-0.8911852	0.65258584	0.08415392	-0.0012905
32.3	0.48126634	0.22850756	-0.34012	0.61273888	-0.8912666	0.65357459	0.08321752	-0.0017195
32.4	0.48098959	0.22871426	-0.339063	0.613219	-0.8912937	0.65390708	0.08069818	-0.0003638
32.5	0.47960964	0.22781325	-0.3398678	0.61357931	-0.8912948	0.65444317	0.0797678	-0.0009867
32.6	0.47908706	0.22709742	-0.3399006	0.61394475	-0.8913386	0.65473324	0.07968806	-0.0015292
32.7	0.47890181	0.22751266	-0.3396037	0.61414873	-0.8912919	0.65482862	0.07919399	-0.0010073
32.8	0.47824095	0.22743793	-0.3388612	0.61387448	-0.8912	0.65429671	0.07785413	-0.0024935
32.9	0.47667686	0.22757317	-0.3381188	0.61298313	-0.8909193	0.65414005	0.07683204	-0.0013235
33	0.47564871	0.2279203	-0.3373018	0.61314027	-0.8910834	0.65421018	0.07612862	-0.001732
33.1	0.47584571	0.22810598	-0.334866	0.61250767	-0.8908868	0.65515215	0.07435055	-0.0010007
33.2	0.47401275	0.22893325	-0.3349221	0.61211129	-0.8910631	0.65525775	0.07330018	-0.0018178
33.3	0.47366482	0.22907205	-0.333863	0.61268174	-0.891007	0.65485628	0.07371675	-0.0022011
33.4	0.47293308	0.23015032	-0.3341559	0.61264519	-0.8910277	0.65509905	0.0734107	-0.0030526
33.5	0.4727105	0.23073327	-0.3331168	0.61301148	-0.891074	0.65544338	0.07203279	-0.0028717
33.6	0.47145404	0.2301016	-0.3319988	0.61290034	-0.8908096	0.6555546	0.07224572	-0.0027637
33.7	0.47078944	0.23084393	-0.3308804	0.61341813	-0.8908092	0.65581705	0.07178393	-0.0038901
33.8	0.47019902	0.23081298	-0.3305582	0.61308923	-0.890677	0.65556071	0.07150103	-0.0045154
33.9	0.46978217	0.22990737	-0.3283156	0.61285814	-0.8903944	0.65574585	0.07084744	-0.0059998
34	0.46876771	0.23027934	-0.3272163	0.61302111	-0.8903917	0.65585152	0.07094961	-0.0075577
34.1	0.46876771	0.23027934	-0.3272163	0.61302111	-0.8903917	0.65585152	0.07094961	-0.0075577
34.2	0.46736137	0.22986457	-0.326496	0.61363018	-0.890214	0.65595356	0.07042499	-0.0073353
34.3	0.46731699	0.22994183	-0.3258722	0.61454656	-0.8904284	0.65655806	0.07013839	-0.0083877
34.4	0.46715759	0.23046228	-0.3245879	0.61487326	-0.8904784	0.65732856	0.06892072	-0.0070394
34.5	0.46599537	0.22945348	-0.3253927	0.61620614	-0.8906977	0.65869516	0.0679614	-0.0063022
34.6	0.46585739	0.22877137	-0.3253716	0.61648476	-0.8907231	0.65894409	0.06801478	-0.0061454
34.7	0.46480388	0.2283635	-0.3254757	0.61661121	-0.8907039	0.6590166	0.06716817	-0.0071762
34.8	0.46300055	0.22606662	-0.3240616	0.61649138	-0.8905322	0.65896667	0.06568109	-0.0077216
34.9	0.46249651	0.22584163	-0.3236896	0.6162892	-0.8904217	0.65886388	0.06486281	-0.0067076
35	0.46076122	0.22472887	-0.3228704	0.615735	-0.8902634	0.65846513	0.06479328	-0.0065338
35.1	0.46004194	0.22491324	-0.3224974	0.61742539	-0.8909088	0.65984799	0.06332713	-0.0058481
35.2	0.45746912	0.22567763	-0.3210913	0.61833563	-0.890773	0.65959426	0.06220373	-0.0051328
35.3	0.45647234	0.22682379	-0.3199929	0.61861991	-0.8906467	0.66024561	0.06027788	-0.0039802
35.4	0.4559729	0.22805047	-0.3190002	0.6185021	-0.890616	0.66069915	0.05991581	-0.0038452
35.5	0.45533781	0.22845398	-0.3174701	0.61846674	-0.890523	0.66084179	0.05846761	-0.0043563
35.6	0.45528165	0.22847568	-0.3166735	0.61822031	-0.8905016	0.66048576	0.05824738	-0.0049632
35.7	0.45490993	0.22734195	-0.3147066	0.61784256	-0.89038	0.6602466	0.05713184	-0.0051358
35.8	0.45508258	0.22724403	-0.3150467	0.61804138	-0.8905817	0.66091005	0.0565013	-0.0048969
35.9	0.45572744	0.22764019	-0.3151607	0.61882422	-0.8907014	0.66146847	0.05696396	-0.0053113
36	0.45434723	0.22794386	-0.3152549	0.61929943	-0.890977	0.66188477	0.05676134	-0.0066124
36.1	0.45392153	0.22802683	-0.31501	0.61923189	-0.8908955	0.66237382	0.05604387	-0.0060364
36.2	0.45391012	0.22691197	-0.3149565	0.61915215	-0.8908392	0.66249266	0.05530215	-0.0074527
36.3	0.45333107	0.22714816	-0.3141577	0.61951972	-0.890906	0.66261758	0.05486049	-0.0076091
36.4	0.45299762	0.2272088	-0.3142683	0.61954517	-0.8907472	0.66272731	0.05356305	-0.0084074
36.5	0.45299762	0.2272088	-0.3142683	0.61954517	-0.8907472	0.66272731	0.05356305	-0.0084074
36.6	0.45156831	0.22637614	-0.3140081	0.6200207	-0.8909153	0.66360299	0.05268347	-0.0076144
36.7	0.45159357	0.22581143	-0.3132719	0.61998402	-0.8908595	0.66352561	0.05194117	-0.0078241
36.8	0.45135438	0.22540621	-0.3131458	0.62066451	-0.8909151	0.66387165	0.05057408	-0.007755
36.9	0.45167092	0.2246797	-0.311995	0.62143208	-0.8911013	0.66472354	0.04914584	-0.0069144
37	0.45145857	0.22525458	-0.3108757	0.62185956	-0.8910283	0.66398571	0.04729283	-0.0068715
37.1	0.45047694	0.22444513	-0.3104687	0.62177892	-0.8909543	0.66343649	0.04711115	-0.0064935
37.2	0.45010737	0.22514793	-0.3097282	0.62243503	-0.8910758	0.66340104	0.04685178	-0.0067701
37.3	0.44831805	0.22463818	-0.3083361	0.6218894	-0.8908309	0.66351424	0.04684945	-0.0073089
37.4	0.44688406	0.22338009	-0.3071995	0.62205078	-0.8908478	0.66342745	0.04760967	-0.0080133
37.5	0.4468797	0.22315585	-0.306491	0.62203243	-0.8907565	0.66351509	0.04616167	-0.00788
37.6	0.44648848	0.22316647	-0.3056434	0.62264229	-0.8907589	0.66406148	0.04468992	-0.0067414
37.7	0.44590603	0.2232309	-0.3045735	0.62311509	-0.8908509	0.66456387	0.04579305	-0.0079388
37.8	0.44469262	0.22206176	-0.3032134	0.62292429	-0.890712	0.6644024	0.04560151	-0.007895
37.9	0.44307205	0.22167546	-0.3028469	0.62318668	-0.8908443	0.66450984	0.04539061	-0.0076327
38	0.44243363	0.22153024	-0.300949	0.62416933	-0.8906746	0.66417042	0.04436439	-0.0066893
38.1	0.44212417	0.22112373	-0.3011467	0.62429059	-0.8907774	0.66393485	0.04412268	-0.0057965
38.2	0.44169512	0.22170169	-0.2995334	0.62448402	-0.8907806	0.66390694	0.0431607	-0.0056092
38.3	0.44068026	0.22212034	-0.3001578	0.62490492	-0.8906781	0.66407233	0.0431799	-0.0048519
38.4	0.43946728	0.22115527	-0.2984633	0.62386799	-0.890364	0.66355884	0.04356342	-0.0041006
38.5	0.43915692	0.22129907	-0.2978492	0.62391721	-0.8902444	0.66354083	0.04340537	-0.0044022
38.6	0.43916694	0.22114954	-0.2969385	0.62319985	-0.8901729	0.6630163	0.04266145	-0.0048631
38.7	0.43958857	0.22064961	-0.2949059	0.6237917	-0.8902407	0.66273406	0.04083685	-0.0052404
38.8	0.43896355	0.22095666	-0.2949304	0.62376824	-0.8902004	0.66272311	0.04144256	-0.0057039
38.9	0.43883806	0.22018355	-0.2950171	0.62404071	-0.8902262	0.66286002	0.04178102	-0.00602
39	0.43938282	0.21947972	-0.2942363	0.62399213	-0.890187	0.66315797	0.04174922	-0.0060142
39.1	0.43929174	0.219328	-0.2928263	0.62340351	-0.889992	0.66243759	0.04195169	-0.0053683
39.2	0.43923731	0.21925952	-0.2923592	0.62351493	-0.8900178	0.66234866	0.04235959	-0.0059457
39.3	0.43901083	0.21900592	-0.2921056	0.62325811	-0.8899174	0.66223012	0.04159043	-0.0062592
39.4	0.43849653	0.21825418	-0.2902377	0.623109	-0.8898623	0.66190641	0.04232226	-0.0078293
39.5	0.43828098	0.2184275	-0.2878485	0.62294234	-0.8897661	0.66137888	0.04196882	-0.008369
39.6	0.43828098	0.2184275	-0.2878485	0.62294234	-0.8897661	0.66137888	0.04196882	-0.008369
39.7	0.43803575	0.21773968	-0.2867703	0.62303414	-0.8896925	0.66129708	0.04216865	-0.0077478
39.8	0.4380268	0.21785532	-0.2866314	0.62328184	-0.889681	0.66124338	0.04234475	-0.0074791
39.9	0.4381501	0.21839709	-0.2860475	0.62335894	-0.8896575	0.66078563	0.0418852	-0.0074056
40	0.43709196	0.21876939	-0.2846004	0.62305653	-0.8894794	0.6601196	0.04121931	-0.0072277
40.1	0.43657412	0.21894727	-0.283443	0.62312017	-0.8894427	0.65982673	0.04065543	-0.0079197
40.2	0.43620745	0.21886202	-0.2831553	0.62341897	-0.8894619	0.65977667	0.04037621	-0.0081749
40.3	0.43612937	0.21851814	-0.2824639	0.62373411	-0.8894021	0.6595506	0.03889199	-0.0077201
40.4	0.43436963	0.21888747	-0.2812625	0.62263218	-0.8889198	0.65917947	0.03909522	-0.0082084
40.5	0.43409428	0.21850265	-0.2801272	0.62225977	-0.8887466	0.65869244	0.03856409	-0.0092408
40.6	0.43325171	0.21812828	-0.2799943	0.62208543	-0.8886976	0.65827456	0.03820891	-0.0092693
40.7	0.4333686	0.21870994	-0.2797071	0.62217121	-0.8887822	0.65795448	0.03781578	-0.0092192
40.8	0.43270919	0.22008036	-0.2790856	0.62286654	-0.8888106	0.65805355	0.03720696	-0.0095729
40.9	0.43250293	0.22039255	-0.278991	0.62292532	-0.8888714	0.6584229	0.03647554	-0.00942
41	0.43191537	0.22011188	-0.2788599	0.62293371	-0.8889045	0.65833609	0.03662583	-0.0097643
41.1	0.43077641	0.2219228	-0.2781224	0.62383862	-0.8891523	0.65806044	0.03471754	-0.009936
41.2	0.43001642	0.2213579	-0.2768911	0.62395537	-0.8888351	0.6576472	0.03404418	-0.008791
41.3	0.42973755	0.22050416	-0.2769417	0.62455181	-0.8887639	0.6581547	0.03337144	-0.0085832
41.4	0.42892331	0.22002778	-0.2748064	0.62484457	-0.8886948	0.65821455	0.03340727	-0.0111839
41.5	0.4274645	0.22021385	-0.2743388	0.62505182	-0.8885794	0.65843369	0.03320016	-0.0124611
41.6	0.42690812	0.21972806	-0.2737777	0.62460251	-0.8884141	0.65821172	0.03385871	-0.0123934
41.7	0.42626543	0.21931477	-0.2729729	0.62471186	-0.8884416	0.65837745	0.03423014	-0.013009
41.8	0.42654402	0.21909066	-0.2729937	0.62580366	-0.88877	0.65843409	0.03364818	-0.0123095
41.9	0.42583153	0.21955616	-0.2729126	0.62628438	-0.8887142	0.65816537	0.03294803	-0.0113717
42	0.4256789	0.21922062	-0.2727618	0.62640225	-0.8886611	0.65808521	0.03313678	-0.0110305
42.1	0.42511924	0.21885837	-0.2724309	0.62655459	-0.8885839	0.65832668	0.03290631	-0.0110644
42.2	0.42502054	0.21874122	-0.2709486	0.6268189	-0.888393	0.65809874	0.03153927	-0.0097617
42.3	0.42413549	0.21843636	-0.2694251	0.62710592	-0.8882686	0.65795634	0.0306173	-0.0086895
42.4	0.42302628	0.21870423	-0.268448	0.62727805	-0.8882736	0.65763896	0.029505	-0.0079663
42.5	0.42293388	0.21863292	-0.2677794	0.62784731	-0.8883186	0.65812118	0.02840611	-0.0077197
42.6	0.42231622	0.21868696	-0.2674988	0.62895661	-0.8886846	0.65867657	0.02504382	-0.0084894
42.7	0.42169343	0.21814557	-0.2668458	0.62902394	-0.8885323	0.65859302	0.02509389	-0.0089414
42.8	0.4211002	0.2187995	-0.2674215	0.62927123	-0.8886329	0.65851406	0.0244838	-0.0093452
42.9	0.42020199	0.21867793	-0.2665386	0.6293038	-0.888637	0.6585614	0.02397873	-0.0097027
43	0.41990494	0.2190217	-0.2660993	0.62920684	-0.8886689	0.65846903	0.02387962	-0.0092386
43.1	0.41931718	0.21841125	-0.2658199	0.62934455	-0.8886431	0.65823806	0.02431769	-0.0092276
43.2	0.41825413	0.21819817	-0.2652855	0.63013385	-0.8887093	0.65890506	0.02247639	-0.0095883
43.3	0.41768014	0.21745935	-0.2648569	0.62977175	-0.888584	0.65885716	0.02287828	-0.0098818
43.4	0.41804524	0.21762202	-0.2639181	0.63021695	-0.8886667	0.65969491	0.02309378	-0.0100585
43.5	0.41735176	0.21754693	-0.2639594	0.63007433	-0.8886498	0.65956786	0.02302399	-0.0099891
43.6	0.41663638	0.21726264	-0.2636368	0.63053363	-0.8886459	0.65947301	0.02345034	-0.0100686
43.7	0.41663638	0.21726264	-0.2636368	0.63053363	-0.8886459	0.65947301	0.02345034	-0.0100686
43.8	0.41623329	0.21772761	-0.2637107	0.63024417	-0.8886436	0.65951474	0.02378072	-0.0101264
43.9	0.41474842	0.21797668	-0.2639637	0.63026083	-0.8884709	0.65937774	0.0234997	-0.010436
44	0.41422874	0.21795458	-0.2641072	0.6307214	-0.8885028	0.65948903	0.02426791	-0.0106295
44.1	0.41319777	0.21793567	-0.2631768	0.63094553	-0.8883135	0.65947127	0.02470214	-0.0103437
44.2	0.41290902	0.21782907	-0.2624448	0.63150024	-0.8883659	0.65972222	0.02438748	-0.0100636
44.3	0.41244576	0.21842457	-0.2620002	0.63151925	-0.8883162	0.65963151	0.02367265	-0.0104948
44.4	0.41244576	0.21842457	-0.2620002	0.63151925	-0.8883162	0.65963151	0.02367265	-0.0104948
44.5	0.41249629	0.21832343	-0.2616624	0.63157876	-0.8881819	0.65932295	0.02335152	-0.0107492
44.6	0.41218862	0.21828317	-0.2611961	0.63177458	-0.8882422	0.6594982	0.02362278	-0.0107703
44.7	0.41017622	0.21835554	-0.2585581	0.63153522	-0.8881574	0.65951893	0.02080626	-0.011366
44.8	0.40936816	0.21844048	-0.2585594	0.63195913	-0.8882628	0.65942906	0.02059554	-0.0122429
44.9	0.40754963	0.21736606	-0.2574954	0.63216766	-0.8880851	0.65927006	0.02173163	-0.012324
45	0.4065659	0.21731701	-0.2574357	0.63301542	-0.8881865	0.65925655	0.0217395	-0.0129601
45.1	0.40513666	0.21729419	-0.2562789	0.63361086	-0.8883372	0.65901716	0.02177447	-0.0129979
45.2	0.40391591	0.2161917	-0.2556081	0.6333413	-0.8883475	0.65914727	0.02038444	-0.0131177
45.3	0.40284518	0.21568621	-0.2547464	0.63316256	-0.8881124	0.65908167	0.01989795	-0.013269
45.4	0.40230984	0.21504311	-0.2542264	0.63302339	-0.8880596	0.65903068	0.020494	-0.0133274
45.5	0.39977753	0.21437014	-0.2539932	0.63282238	-0.8877231	0.65849442	0.01923648	-0.0132247
45.6	0.39942781	0.2137449	-0.2536018	0.63266034	-0.8876812	0.65882955	0.01831561	-0.0134155
45.7	0.39882376	0.21488332	-0.2530245	0.63331541	-0.8876463	0.65906552	0.01741033	-0.0128802
45.8	0.39790034	0.2151977	-0.2521625	0.63289603	-0.8874708	0.65888919	0.01719953	-0.0134371
45.9	0.39765542	0.21574062	-0.2521158	0.63314764	-0.8876405	0.65905561	0.01734912	-0.0129399
46	0.3977878	0.21561478	-0.2515988	0.63312035	-0.8875821	0.65907946	0.01729577	-0.0137802
46.1	0.39746555	0.21565881	-0.250974	0.63319408	-0.8875281	0.65918586	0.01718266	-0.0142827
46.2	0.39708308	0.21559327	-0.2502318	0.63326807	-0.8875159	0.65918015	0.01607425	-0.0141137
46.3	0.39708308	0.21559327	-0.2502318	0.63326807	-0.8875159	0.65918015	0.01607425	-0.0141137
46.4	0.39672082	0.2163831	-0.248962	0.63317537	-0.8874665	0.65901502	0.0151496	-0.0149736
46.5	0.39679288	0.21681236	-0.2487323	0.63319038	-0.8874175	0.65879073	0.01459506	-0.0150804
46.6	0.39618441	0.2174842	-0.2486607	0.63311858	-0.8874123	0.65851023	0.0145379	-0.0148866
46.7	0.39630385	0.21789304	-0.2489923	0.63300899	-0.8874048	0.65850411	0.01462567	-0.0150864
46.8	0.39634705	0.21778514	-0.249027	0.63295924	-0.8873771	0.6584892	0.0142056	-0.0152592
46.9	0.39660698	0.21851173	-0.2482901	0.6330674	-0.8872878	0.65848317	0.01328949	-0.0154817
47	0.39666338	0.21811793	-0.2475898	0.6328913	-0.8872233	0.65839988	0.01269905	-0.0145743
47.1	0.39579917	0.21893024	-0.2479836	0.6337482	-0.8874037	0.65872418	0.01220021	-0.0146615
47.2	0.39443612	0.21867154	-0.2467169	0.63314802	-0.8870799	0.65846566	0.01176152	-0.0139566
47.3	0.39370605	0.21782192	-0.2467998	0.63329596	-0.887237	0.65873149	0.01101314	-0.0129986
47.4	0.39301689	0.21853815	-0.246328	0.63345532	-0.8870687	0.65917019	0.01007556	-0.0130667
47.5	0.3927656	0.21779296	-0.2459248	0.63339815	-0.8870602	0.65891415	0.010186	-0.0130036
47.6	0.39260406	0.21795119	-0.2454944	0.63348429	-0.8869694	0.65900162	0.0102121	-0.0124751
47.7	0.39266339	0.21811808	-0.2453475	0.63349047	-0.8869606	0.6592761	0.01019763	-0.0132355
47.8	0.39161069	0.21793319	-0.2445682	0.63325521	-0.8868539	0.65917947	0.00968146	-0.0132425
47.9	0.39135964	0.21777424	-0.2441055	0.6331842	-0.8867981	0.6588682	0.00950796	-0.0131876
48	0.39135964	0.21777424	-0.2441055	0.6331842	-0.8867981	0.6588682	0.00950796	-0.0131876
48.1	0.39157687	0.21762274	-0.2438998	0.63328726	-0.8868496	0.65880763	0.00924405	-0.0133642
48.2	0.39176807	0.21792949	-0.2433783	0.63410306	-0.8869838	0.6588129	0.00786805	-0.0131457
48.3	0.39199299	0.21770854	-0.243821	0.63431436	-0.8870341	0.65857564	0.00851426	-0.013334
48.4	0.39190097	0.21803885	-0.2432387	0.63481271	-0.8871316	0.65903246	0.00763305	-0.0129144
48.5	0.39106021	0.21852047	-0.2419775	0.63504312	-0.8873284	0.65930297	0.00603164	-0.0127268
48.6	0.39004425	0.21761546	-0.2426981	0.63544085	-0.8874023	0.65951228	0.00562061	-0.0131822
48.7	0.38878489	0.21734738	-0.2421759	0.63571416	-0.887308	0.65924919	0.00498763	-0.0137486
48.8	0.38786261	0.21691561	-0.2422117	0.635644	-0.8871749	0.65894274	0.00539807	-0.014055
48.9	0.38780706	0.21665717	-0.2423963	0.63562019	-0.8871313	0.65910161	0.00568465	-0.0141091
49	0.3872663	0.21654169	-0.2422193	0.63523285	-0.8871193	0.65878365	0.00604935	-0.0138385
49.1	0.38652828	0.21635723	-0.2417254	0.63516933	-0.886972	0.6589741	0.00676985	-0.0134676
49.2	0.38615734	0.21654351	-0.242027	0.6354945	-0.8870877	0.65906917	0.00638018	-0.0132164
49.3	0.38588902	0.21657606	-0.2415181	0.63545382	-0.8870795	0.65915763	0.00637016	-0.013449
49.4	0.38533785	0.21653506	-0.2415444	0.63581876	-0.8871488	0.65927602	0.00634033	-0.0144368
49.5	0.38514635	0.21608961	-0.2407835	0.63688714	-0.8873422	0.65940907	0.00566098	-0.0148139
49.6	0.38442788	0.21571083	-0.2400998	0.63722856	-0.8872938	0.65924261	0.00582324	-0.014371
49.7	0.38405778	0.21599389	-0.239037	0.63749068	-0.8873057	0.65930289	0.00617445	-0.0134936
49.8	0.38412575	0.2161192	-0.2384062	0.63765536	-0.8872388	0.65922441	0.0065008	-0.0130942
49.9	0.38412575	0.2161192	-0.2384062	0.63765536	-0.8872388	0.65922441	0.0065008	-0.0130942
50	0.38380073	0.21552481	-0.237806	0.63769758	-0.8870985	0.6593668	0.00715092	-0.0131903
50.1	0.38306353	0.21610869	-0.2371825	0.63761299	-0.8871196	0.65943594	0.00630418	-0.013572
50.2	0.38295785	0.2160949	-0.2365732	0.63767922	-0.8872056	0.65942648	0.00668596	-0.0133148
50.3	0.38295785	0.2160949	-0.2365732	0.63767922	-0.8872056	0.65942648	0.00668596	-0.0133148
50.4	0.38234999	0.21663446	-0.2367604	0.6377965	-0.8872163	0.65950944	0.00607711	-0.0136209
50.5	0.38155961	0.21617985	-0.2366973	0.63749085	-0.8871415	0.65926694	0.00653058	-0.0131127
50.6	0.38150063	0.21686544	-0.2363592	0.63810445	-0.887377	0.65971774	0.00610403	-0.0129808
50.7	0.38076918	0.21567279	-0.2350955	0.63888715	-0.8874646	0.6598204	0.00703414	-0.0124133
50.8	0.38088469	0.21514486	-0.2347259	0.63904962	-0.8874739	0.66003783	0.00728754	-0.0121801
50.9	0.38018404	0.21558996	-0.2336931	0.64004272	-0.8877693	0.66050965	0.00707166	-0.0136536
51	0.38018404	0.21558996	-0.2336931	0.64004272	-0.8877693	0.66050965	0.00707166	-0.0136536
51.1	0.38018404	0.21558996	-0.2336931	0.64004272	-0.8877693	0.66050965	0.00707166	-0.0136536
51.2	0.38013244	0.21539404	-0.2336992	0.64006782	-0.8877513	0.66035347	0.00733465	-0.0140448
51.3	0.3800477	0.21452776	-0.2332638	0.63999759	-0.8876847	0.66037106	0.00716993	-0.0141706
51.4	0.37995079	0.21445381	-0.2328871	0.64019491	-0.8877207	0.66064087	0.00653909	-0.0143391
51.5	0.3797529	0.2143551	-0.2318256	0.64021159	-0.8876762	0.66064537	0.00611136	-0.0144344
51.6	0.37989827	0.21444918	-0.2317784	0.64043446	-0.8877164	0.66058628	0.00594318	-0.0136932
51.7	0.37969139	0.21497057	-0.2321981	0.64098721	-0.8878731	0.66091611	0.0053453	-0.013995
51.8	0.37836352	0.21545657	-0.2322045	0.64187313	-0.8883545	0.66121841	0.00432399	-0.0131698
51.9	0.37792657	0.21557087	-0.2323073	0.64192003	-0.8883564	0.66139809	0.00432795	-0.0126693
52	0.37761783	0.2159782	-0.2321279	0.64161412	-0.8882521	0.66159053	0.00331671	-0.0120348
52.1	0.37731993	0.21618182	-0.2306842	0.64225056	-0.8882072	0.66205839	0.00305904	-0.0114862
52.2	0.37717289	0.21645587	-0.2303611	0.64210061	-0.8881863	0.66223111	0.00281029	-0.011237
52.3	0.37737773	0.21619592	-0.2298328	0.64225316	-0.888258	0.66256616	0.00327329	-0.0111377
52.4	0.37720007	0.21703312	-0.2294423	0.64268786	-0.88828	0.66253743	0.00164119	-0.010378
52.5	0.37723782	0.21679804	-0.2290922	0.64298875	-0.8882172	0.66292541	0.00237182	-0.010876
52.6	0.37669586	0.21728364	-0.2287979	0.6433448	-0.8882849	0.66311085	0.00169897	-0.0113447
52.7	0.37586059	0.21720744	-0.2291282	0.64376943	-0.8884523	0.66344442	0.0010781	-0.0103032
52.8	0.3749957	0.21867717	-0.2290256	0.64378852	-0.8884209	0.66391139	0.00128434	-0.0094193
52.9	0.37439294	0.2172667	-0.2283395	0.64398217	-0.8883781	0.66402488	0.00098105	-0.0093274
53	0.37439294	0.2172667	-0.2283395	0.64398217	-0.8883781	0.66402488	0.00098105	-0.0093274
53.1	0.37450641	0.217073	-0.2275555	0.6435833	-0.8882639	0.66360619	0.00116294	-0.0094682
53.2	0.37444802	0.21770148	-0.2274293	0.64390575	-0.8882702	0.6635523	0.00061644	-0.0098315
53.3	0.37424766	0.21721387	-0.2267641	0.64463849	-0.8883554	0.66371109	0.00063053	-0.0104032
53.4	0.37430656	0.2168549	-0.2261807	0.64479929	-0.888363	0.6635399	0.00066262	-0.0107983
53.5	0.37403993	0.21646403	-0.2257603	0.64473531	-0.8882517	0.66369756	0.00113951	-0.0103223
53.6	0.37403993	0.21646403	-0.2257603	0.64473531	-0.8882517	0.66369756	0.00113951	-0.0103223
53.7	0.37356485	0.2167427	-0.2265328	0.64443645	-0.8883791	0.66385328	0.00125373	-0.0100881
53.8	0.3731385	0.21662452	-0.2259179	0.64407994	-0.8881615	0.66372764	0.00148456	-0.0096792
53.9	0.37264863	0.21701853	-0.2261575	0.64396318	-0.8880783	0.66388566	0.00149269	-0.0096118
54	0.37261968	0.21724211	-0.2261038	0.64411397	-0.8881337	0.66422407	0.00146542	-0.0094486
54.1	0.37233313	0.21730711	-0.226212	0.64414003	-0.8881242	0.66417703	0.00169296	-0.0099054
54.2	0.37179187	0.21776607	-0.2259123	0.6444839	-0.8882111	0.66418763	0.00098464	-0.0097975
54.3	0.3711948	0.21706764	-0.2251802	0.64470051	-0.8881222	0.66376046	0.0019399	-0.0105695
54.4	0.3711948	0.21706764	-0.2251802	0.64470051	-0.8881222	0.66376046	0.0019399	-0.0105695
54.5	0.37182783	0.21730328	-0.2241066	0.64440518	-0.8879945	0.66378335	0.0018219	-0.010256
54.6	0.37200466	0.21748549	-0.2242253	0.64454661	-0.8880447	0.66378032	0.00162922	-0.0106218
54.7	0.37200466	0.21748549	-0.2242253	0.64454661	-0.8880447	0.66378032	0.00162922	-0.0106218
54.8	0.37200466	0.21748549	-0.2242253	0.64454661	-0.8880447	0.66378032	0.00162922	-0.0106218
54.9	0.37207311	0.21710677	-0.2233468	0.64478693	-0.8880466	0.6637065	0.00232895	-0.0106146
55	0.37182686	0.21761632	-0.2227141	0.64513896	-0.8881269	0.66381138	0.00142682	-0.0104564
55.1	0.37080236	0.21809686	-0.2216041	0.64469532	-0.8879294	0.6636879	0.00188444	-0.01024
55.2	0.37080236	0.21809686	-0.2216041	0.64469532	-0.8879294	0.6636879	0.00188444	-0.01024
55.3	0.37047277	0.21802023	-0.2211473	0.64488682	-0.8879987	0.66383248	0.00197141	-0.0105778
55.4	0.36968386	0.21705364	-0.2198433	0.64448872	-0.88775	0.66290188	0.00241378	-0.0105486
55.5	0.36968386	0.21705364	-0.2198433	0.64448872	-0.88775	0.66290188	0.00241378	-0.0105486
55.6	0.36958516	0.21660014	-0.2190373	0.64421316	-0.8876132	0.66238346	0.00299075	-0.010261
55.7	0.36928345	0.21618295	-0.2181919	0.64387644	-0.8875046	0.66205794	0.00346959	-0.010562
55.8	0.36949077	0.216128	-0.2182477	0.64426249	-0.8875612	0.66252151	0.00369738	-0.0104878
55.9	0.36936935	0.21695674	-0.2179863	0.64466203	-0.8877272	0.66249434	0.00360292	-0.0106037
56	0.36867076	0.21634709	-0.2179564	0.64433425	-0.8876997	0.66236483	0.0033428	-0.0103462
56.1	0.36851005	0.21675496	-0.2174191	0.64412449	-0.8875816	0.66249115	0.00312743	-0.0102872
56.2	0.36845808	0.21606447	-0.2172726	0.64408289	-0.8875378	0.66227287	0.00279315	-0.0099126
56.3	0.3682985	0.21582127	-0.2167805	0.6442751	-0.8875316	0.66229967	0.00252656	-0.0096601
56.4	0.36773852	0.216333	-0.2164522	0.64455969	-0.8876003	0.66266328	0.00228016	-0.0094633
56.5	0.36769376	0.21697299	-0.2167689	0.64416852	-0.887578	0.66262571	0.0022643	-0.009754
56.6	0.36769376	0.21697299	-0.2167689	0.64416852	-0.887578	0.66262571	0.0022643	-0.009754
56.7	0.36755679	0.21682264	-0.2168784	0.64395595	-0.8875434	0.66277967	0.00238935	-0.009263
56.8	0.36738766	0.21706024	-0.2172501	0.64358927	-0.8874683	0.66272446	0.00270196	-0.0103605
56.9	0.36738766	0.21706024	-0.2172501	0.64358927	-0.8874683	0.66272446	0.00270196	-0.0103605
57	0.36708595	0.21676007	-0.2168472	0.64362939	-0.8874818	0.66252929	0.00281583	-0.01086
57.1	0.36708595	0.21676007	-0.2168472	0.64362939	-0.8874818	0.66252929	0.00281583	-0.01086
57.2	0.36707649	0.21677595	-0.2170844	0.64379844	-0.8875602	0.66267626	0.00293372	-0.00959
57.3	0.36707649	0.21677595	-0.2170844	0.64379844	-0.8875602	0.66267626	0.00293372	-0.00959
57.4	0.36660663	0.21722204	-0.2174986	0.64416752	-0.8876261	0.66277262	0.00302142	-0.0097731
57.5	0.36616237	0.21680516	-0.2176962	0.64390804	-0.8876144	0.66282747	0.00270211	-0.0093229
57.6	0.36616237	0.21680516	-0.2176962	0.64390804	-0.8876144	0.66282747	0.00270211	-0.0093229
57.7	0.36600223	0.21725052	-0.2170494	0.64408361	-0.8875441	0.66272162	0.00202263	-0.0089581
57.8	0.36550677	0.21689733	-0.2168494	0.64439171	-0.8874995	0.66264648	0.00166866	-0.0092612
57.9	0.36514285	0.21640527	-0.2167345	0.64432857	-0.8874391	0.66261464	0.00157828	-0.0087444
58	0.36514285	0.21640527	-0.2167345	0.64432857	-0.8874391	0.66261464	0.00157828	-0.0087444
58.1	0.36418358	0.21684253	-0.2168397	0.64446052	-0.8875714	0.66261624	0.00152415	-0.008728
58.2	0.36334456	0.21635571	-0.2160027	0.64458213	-0.8874756	0.66239243	0.00165198	-0.0090076
58.3	0.36334456	0.21635571	-0.2160027	0.64458213	-0.8874756	0.66239243	0.00165198	-0.0090076
58.4	0.36271	0.21652972	-0.2157517	0.64479805	-0.8874608	0.66230052	0.00138967	-0.0087493
58.5	0.36271	0.21652972	-0.2157517	0.64479805	-0.8874608	0.66230052	0.00138967	-0.0087493
58.6	0.36271	0.21652972	-0.2157517	0.64479805	-0.8874608	0.66230052	0.00138967	-0.0087493
58.7	0.36245179	0.21556416	-0.2159085	0.64483496	-0.8874469	0.6623783	0.00092515	-0.0094106
58.8	0.36242036	0.21569007	-0.2155358	0.64485457	-0.8873561	0.66212132	0.00024626	-0.009445
58.9	0.36230911	0.21636875	-0.215566	0.64576538	-0.8876584	0.6625574	-0.0003571	-0.0090486
59	0.36230911	0.21636875	-0.215566	0.64576538	-0.8876584	0.6625574	-0.0003571	-0.0090486
59.1	0.36162313	0.21665096	-0.2155556	0.64585189	-0.8877128	0.66285275	-0.0003924	-0.0086323
59.2	0.36162313	0.21665096	-0.2155556	0.64585189	-0.8877128	0.66285275	-0.0003924	-0.0086323
59.3	0.36162313	0.21665096	-0.2155556	0.64585189	-0.8877128	0.66285275	-0.0003924	-0.0086323
59.4	0.3594267	0.21630676	-0.21403	0.64609139	-0.8876505	0.66245763	0.00012022	-0.0086915
59.5	0.3594267	0.21630676	-0.21403	0.64609139	-0.8876505	0.66245763	0.00012022	-0.0086915
59.6	0.3594267	0.21630676	-0.21403	0.64609139	-0.8876505	0.66245763	0.00012022	-0.0086915
59.7	0.35838	0.21686915	-0.2141998	0.646415	-0.8877223	0.66253146	-0.000184	-0.0095956
59.8	0.35786085	0.21646319	-0.2140928	0.64669458	-0.8877261	0.66240983	-0.0001006	-0.0092457
59.9	0.35786085	0.21646319	-0.2140928	0.64669458	-0.8877261	0.66240983	-0.0001006	-0.0092457

60	0.35757041	0.21659031	-0.2135208	0.64677009	-0.8876328	0.66243588	-0.0002292	-0.008843
60.1	0.35759883	0.21641936	-0.2130898	0.646766	-0.8876272	0.66247228	-0.0004734	-0.0088184
60.2	0.35657635	0.21750257	-0.2113578	0.64697402	-0.8875809	0.66220479	-0.0005594	-0.0093504
60.3	0.35657635	0.21750257	-0.2113578	0.64697402	-0.8875809	0.66220479	-0.0005594	-0.0093504
60.4	0.35615996	0.21741714	-0.2112378	0.64719276	-0.8876482	0.66227545	-0.0011027	-0.0096385
60.5	0.35572834	0.21755666	-0.2110092	0.64738879	-0.8876856	0.66241898	-0.0010488	-0.0100685
60.6	0.3556869	0.21811436	-0.2107242	0.64724154	-0.8876937	0.66239135	-0.000467	-0.0104657
60.7	0.35506923	0.21917368	-0.2093599	0.64806614	-0.8876755	0.66262414	-0.0007749	-0.0105629
60.8	0.35506923	0.21917368	-0.2093599	0.64806614	-0.8876755	0.66262414	-0.0007749	-0.0105629
60.9	0.35482867	0.21949578	-0.2094329	0.64801045	-0.887681	0.66292231	-0.0010714	-0.0106332
61	0.35456865	0.21921424	-0.2097673	0.64823259	-0.8877248	0.66285642	-0.0017346	-0.0110699
61.1	0.35392561	0.21919176	-0.2097645	0.64789726	-0.8876087	0.66301038	-0.0017053	-0.0108804
61.2	0.35335469	0.21917644	-0.2098075	0.6474702	-0.8875212	0.66266571	-0.0017601	-0.0110843
61.3	0.35335469	0.21917644	-0.2098075	0.6474702	-0.8875212	0.66266571	-0.0017601	-0.0110843
61.4	0.35300435	0.21889487	-0.209692	0.64754093	-0.8875459	0.66263732	-0.0018493	-0.0111947
61.5	0.35219209	0.21833489	-0.2089204	0.647558	-0.887575	0.66243558	-0.0013773	-0.0112562
61.6	0.35219209	0.21833489	-0.2089204	0.647558	-0.887575	0.66243558	-0.0013773	-0.0112562
61.7	0.35168125	0.21827175	-0.2086726	0.6477368	-0.8875589	0.66229103	-0.0011322	-0.0113875
61.8	0.35168125	0.21827175	-0.2086726	0.6477368	-0.8875589	0.66229103	-0.0011322	-0.0113875
61.9	0.35168125	0.21827175	-0.2086726	0.6477368	-0.8875589	0.66229103	-0.0011322	-0.0113875
62	0.35121844	0.21772126	-0.2081801	0.64761423	-0.8874808	0.66232072	-0.0004705	-0.0115145
62.1	0.35117422	0.21784243	-0.2080383	0.64756656	-0.8874245	0.66200705	-0.0012675	-0.0114693
62.2	0.35094553	0.21811061	-0.2077129	0.64736247	-0.8873755	0.6618324	-0.0007058	-0.0117417
62.3	0.3508983	0.21734379	-0.2080212	0.64772362	-0.8874331	0.66214407	-0.0005414	-0.0119127
62.4	0.35049288	0.21745535	-0.2081401	0.64774245	-0.8874112	0.66211669	-0.0008724	-0.0115463
62.5	0.34991298	0.21727019	-0.2082685	0.64805438	-0.8874297	0.66233143	-0.0010009	-0.0116082
62.6	0.34991298	0.21727019	-0.2082685	0.64805438	-0.8874297	0.66233143	-0.0010009	-0.0116082
62.7	0.34924005	0.21722971	-0.207742	0.64795068	-0.8874343	0.66228008	-0.0014473	-0.0113459
62.8	0.34936753	0.21687404	-0.2076811	0.64799354	-0.8874819	0.66230421	-0.0018772	-0.0116128
62.9	0.34938279	0.21700551	-0.2076385	0.64815712	-0.8874854	0.662279	-0.0016949	-0.0115904
63	0.34938279	0.21700551	-0.2076385	0.64815712	-0.8874854	0.662279	-0.0016949	-0.0115904
63.1	0.34861779	0.21682305	-0.2062881	0.6483731	-0.8874876	0.66183982	-0.0014261	-0.0105124
63.2	0.34850373	0.2164524	-0.2058231	0.64860122	-0.8874852	0.66185934	-0.0014467	-0.0097737
63.3	0.34817135	0.21652275	-0.2053146	0.64838076	-0.8873559	0.66207577	-0.0018873	-0.0090275
63.4	0.34805491	0.21653523	-0.2051039	0.64843184	-0.8874254	0.66176259	-0.001375	-0.0088246
63.5	0.34812767	0.2163245	-0.2047492	0.64874052	-0.8874788	0.66185539	-0.0013411	-0.0089509
63.6	0.34812767	0.2163245	-0.2047492	0.64874052	-0.8874788	0.66185539	-0.0013411	-0.0089509
63.7	0.34811487	0.21575338	-0.2050668	0.64884955	-0.8874584	0.66178088	-0.0013166	-0.0089695
63.8	0.34787674	0.21626167	-0.204986	0.64904864	-0.8874821	0.66192937	-0.0016654	-0.0092689
63.9	0.34787674	0.21626167	-0.204986	0.64904864	-0.8874821	0.66192937	-0.0016654	-0.0092689
64	0.34787674	0.21626167	-0.204986	0.64904864	-0.8874821	0.66192937	-0.0016654	-0.0092689
64.1	0.34787674	0.21626167	-0.204986	0.64904864	-0.8874821	0.66192937	-0.0016654	-0.0092689
64.2	0.3467162	0.21695503	-0.2049625	0.64921988	-0.8874736	0.66207087	-0.0014992	-0.0103482
64.3	0.3467162	0.21695503	-0.2049625	0.64921988	-0.8874736	0.66207087	-0.0014992	-0.0103482
64.4	0.34636766	0.21774754	-0.2050161	0.64885294	-0.8874127	0.66167506	-0.0011895	-0.0108541
64.5	0.34683569	0.21740876	-0.2046344	0.64925105	-0.8875223	0.66209928	-0.0020017	-0.0106648
64.6	0.34683569	0.21740876	-0.2046344	0.64925105	-0.8875223	0.66209928	-0.0020017	-0.0106648
64.7	0.34683569	0.21740876	-0.2046344	0.64925105	-0.8875223	0.66209928	-0.0020017	-0.0106648
64.8	0.34687012	0.21797604	-0.2041325	0.64946936	-0.8875535	0.6621756	-0.0021898	-0.0106597
64.9	0.34687012	0.21797604	-0.2041325	0.64946936	-0.8875535	0.6621756	-0.0021898	-0.0106597
65	0.34625338	0.21771895	-0.2045707	0.64920688	-0.8874345	0.66232507	-0.0025084	-0.0103463
65.1	0.34547658	0.21796811	-0.2035309	0.64959185	-0.8875657	0.66241641	-0.0019279	-0.0096038
65.2	0.34520261	0.21817723	-0.2026322	0.64992863	-0.8876239	0.66275413	-0.0019856	-0.0087158
65.3	0.34526795	0.21839608	-0.2027264	0.65026363	-0.8877306	0.66277199	-0.0023692	-0.0092491
65.4	0.34459975	0.21835615	-0.2027044	0.65062539	-0.8877925	0.6630484	-0.0027559	-0.0093423
65.5	0.34370677	0.21899609	-0.2019019	0.65050001	-0.8875576	0.66276179	-0.0033465	-0.0090618
65.6	0.34364017	0.21862882	-0.2014709	0.6500687	-0.8874134	0.66243846	-0.003859	-0.0091501
65.7	0.3435564	0.21915621	-0.2007059	0.65014038	-0.8873812	0.66247913	-0.0036905	-0.0090794
65.8	0.3435564	0.21915621	-0.2007059	0.65014038	-0.8873812	0.66247913	-0.0036905	-0.0090794
65.9	0.34293626	0.21924384	-0.2006658	0.65008972	-0.8873795	0.66244218	-0.0035445	-0.0093037
66	0.34293626	0.21924384	-0.2006658	0.65008972	-0.8873795	0.66244218	-0.0035445	-0.0093037
66.1	0.34293626	0.21924384	-0.2006658	0.65008972	-0.8873795	0.66244218	-0.0035445	-0.0093037
66.2	0.34322441	0.21941704	-0.200626	0.6501758	-0.8874277	0.66257806	-0.0042394	-0.0092265
66.3	0.34322441	0.21941704	-0.200626	0.6501758	-0.8874277	0.66257806	-0.0042394	-0.0092265
66.4	0.34253153	0.21974177	-0.2008302	0.64981336	-0.8872623	0.66233813	-0.0043596	-0.009181
66.5	0.34253153	0.21974177	-0.2008302	0.64981336	-0.8872623	0.66233813	-0.0043596	-0.009181
66.6	0.34206096	0.21947394	-0.2000927	0.65014155	-0.8872317	0.66248671	-0.0036607	-0.0100888
66.7	0.34160658	0.21965153	-0.1999974	0.65049252	-0.8873206	0.66258545	-0.0037621	-0.0098403
66.8	0.34178702	0.22011305	-0.1998257	0.65088607	-0.8874251	0.66323799	-0.0047019	-0.0092023
66.9	0.34178702	0.22011305	-0.1998257	0.65088607	-0.8874251	0.66323799	-0.0047019	-0.0092023
67	0.34178702	0.22011305	-0.1998257	0.65088607	-0.8874251	0.66323799	-0.0047019	-0.0092023
67.1	0.34140256	0.22079123	-0.1997206	0.6505217	-0.8873791	0.6633341	-0.0043727	-0.0091662
67.2	0.34079257	0.22137082	-0.1996359	0.65024781	-0.8872944	0.66289359	-0.0048762	-0.0094517
67.3	0.34079257	0.22137082	-0.1996359	0.65024781	-0.8872944	0.66289359	-0.0048762	-0.0094517
67.4	0.34075113	0.22134089	-0.1991596	0.65061459	-0.8873967	0.66280925	-0.0048809	-0.0090716
67.5	0.34075113	0.22134089	-0.1991596	0.65061459	-0.8873967	0.66280925	-0.0048809	-0.0090716
67.6	0.34055334	0.22075566	-0.1985534	0.65071218	-0.8874339	0.66258077	-0.0051429	-0.0091247
67.7	0.34041458	0.22085912	-0.199104	0.65085657	-0.8874903	0.66239162	-0.005371	-0.0084704
67.8	0.34014823	0.22089443	-0.1986736	0.65069521	-0.8874315	0.6624891	-0.0057523	-0.0086743
67.9	0.33998339	0.2206027	-0.1982738	0.65100151	-0.887413	0.66286263	-0.0063653	-0.0096774
68	0.33990505	0.2200067	-0.1976623	0.65127582	-0.8874573	0.66259669	-0.0063715	-0.0096689
68.1	0.33972339	0.21977362	-0.1972684	0.65142578	-0.8874922	0.66234498	-0.0065579	-0.0104376
68.2	0.33972339	0.21977362	-0.1972684	0.65142578	-0.8874922	0.66234498	-0.0065579	-0.0104376
68.3	0.33972339	0.21977362	-0.1972684	0.65142578	-0.8874922	0.66234498	-0.0065579	-0.0104376
68.4	0.33865628	0.2199647	-0.1957086	0.65181809	-0.8874448	0.66204248	-0.0063122	-0.0109313
68.5	0.33875128	0.22023418	-0.19555	0.65185402	-0.8874213	0.66198571	-0.0065829	-0.0112004
68.6	0.33875128	0.22023418	-0.19555	0.65185402	-0.8874213	0.66198571	-0.0065829	-0.0112004
68.7	0.33890064	0.21997444	-0.1949115	0.65174586	-0.8873655	0.66183659	-0.0071188	-0.0107404
68.8	0.33784422	0.22009658	-0.1931399	0.65115049	-0.8871256	0.66112059	-0.0062934	-0.0116041
68.9	0.33784422	0.22009658	-0.1931399	0.65115049	-0.8871256	0.66112059	-0.0062934	-0.0116041
69	0.3372651	0.22100029	-0.1929619	0.65091063	-0.8870194	0.66072691	-0.0069778	-0.0124443
69.1	0.33730306	0.22104109	-0.1932394	0.65078739	-0.8870289	0.66087793	-0.0066794	-0.0127983
69.2	0.33677394	0.22047157	-0.1935781	0.65051156	-0.8869342	0.66056229	-0.0073114	-0.0126051
69.3	0.33677394	0.22047157	-0.1935781	0.65051156	-0.8869342	0.66056229	-0.0073114	-0.0126051
69.4	0.33677394	0.22047157	-0.1935781	0.65051156	-0.8869342	0.66056229	-0.0073114	-0.0126051
69.5	0.33680844	0.22040645	-0.1932153	0.65036512	-0.886895	0.66054189	-0.007696	-0.0126994
69.6	0.33680844	0.22040645	-0.1932153	0.65036512	-0.886895	0.66054189	-0.007696	-0.0126994
69.7	0.33620742	0.22026148	-0.1934869	0.65013598	-0.8868667	0.66054248	-0.0074497	-0.0125651
69.8	0.33620742	0.22026148	-0.1934869	0.65013598	-0.8868667	0.66054248	-0.0074497	-0.0125651
69.9	0.3357146	0.22071614	-0.1931325	0.6502161	-0.8868393	0.66053525	-0.0074552	-0.0123476
70	0.33483517	0.22139426	-0.1929366	0.65030344	-0.8868736	0.66052339	-0.0076989	-0.0125152
70.1	0.3347688	0.22182247	-0.1927206	0.65022177	-0.8868897	0.6606251	-0.0076831	-0.013181
70.2	0.33424165	0.22172065	-0.1923082	0.64996492	-0.8867854	0.66047987	-0.0075299	-0.0125906
70.3	0.3341192	0.22221223	-0.1925924	0.64991348	-0.8867957	0.66053139	-0.0074042	-0.0127663
70.4	0.33369528	0.22281923	-0.192254	0.65007338	-0.8868322	0.66073334	-0.0071596	-0.0135111
70.5	0.3333568	0.2228688	-0.1925682	0.65009086	-0.8868025	0.66058435	-0.0077531	-0.0141385
70.6	0.3334811	0.22238203	-0.1918737	0.65037777	-0.8868547	0.6604794	-0.0079876	-0.0137691
70.7	0.33369054	0.22248975	-0.1916605	0.65069783	-0.8869423	0.66065171	-0.008014	-0.0132248
70.8	0.3329097	0.22170927	-0.1915289	0.65088908	-0.8869109	0.66030039	-0.0083331	-0.0133052
70.9	0.33247446	0.22149608	-0.1898558	0.65019785	-0.8866765	0.65916669	-0.0085308	-0.012332
71	0.33226236	0.22215615	-0.1897826	0.65036674	-0.8866751	0.65940055	-0.0087292	-0.0127655
71.1	0.33187189	0.22177569	-0.1894396	0.65048426	-0.8865724	0.65939247	-0.0094514	-0.0130136
71.2	0.33187189	0.22177569	-0.1894396	0.65048426	-0.8865724	0.65939247	-0.0094514	-0.0130136
71.3	0.33187189	0.22177569	-0.1894396	0.65048426	-0.8865724	0.65939247	-0.0094514	-0.0130136
71.4	0.33120107	0.22157163	-0.1890796	0.65005143	-0.8864317	0.65879035	-0.0092601	-0.0128521
71.5	0.33120107	0.22157163	-0.1890796	0.65005143	-0.8864317	0.65879035	-0.0092601	-0.0128521
71.6	0.33120107	0.22157163	-0.1890796	0.65005143	-0.8864317	0.65879035	-0.0092601	-0.0128521
71.7	0.33120107	0.22157163	-0.1890796	0.65005143	-0.8864317	0.65879035	-0.0092601	-0.0128521
71.8	0.33080763	0.22151965	-0.1885502	0.6499897	-0.886423	0.65873193	-0.0095056	-0.0129861
71.9	0.33040216	0.22143698	-0.1883796	0.6500361	-0.8864054	0.6585359	-0.0094238	-0.0135748
72	0.33040216	0.22143698	-0.1883796	0.6500361	-0.8864054	0.6585359	-0.0094238	-0.0135748
72.1	0.33030544	0.22138564	-0.1881546	0.6496381	-0.8862975	0.65820162	-0.0099005	-0.0134484
72.2	0.33030544	0.22138564	-0.1881546	0.6496381	-0.8862975	0.65820162	-0.0099005	-0.0134484
72.3	0.33030544	0.22138564	-0.1881546	0.6496381	-0.8862975	0.65820162	-0.0099005	-0.0134484
72.4	0.32937227	0.2206588	-0.1881853	0.64950645	-0.8862438	0.65793385	-0.008583	-0.0131349
72.5	0.32874553	0.22058184	-0.1877675	0.6492952	-0.8862354	0.65783899	-0.0084455	-0.0133308
72.6	0.32857732	0.22043314	-0.1879617	0.64944926	-0.8862706	0.65773456	-0.0081542	-0.0135169
72.7	0.32857732	0.22043314	-0.1879617	0.64944926	-0.8862706	0.65773456	-0.0081542	-0.0135169
72.8	0.32857732	0.22043314	-0.1879617	0.64944926	-0.8862706	0.65773456	-0.0081542	-0.0135169
72.9	0.32873828	0.22112339	-0.1872081	0.64968335	-0.8862345	0.65795463	-0.0080163	-0.0132503
73	0.32832362	0.22136009	-0.1874372	0.64996514	-0.8862402	0.65790827	-0.0082415	-0.0130304
73.1	0.3279775	0.22110768	-0.1874448	0.65023966	-0.886315	0.65776459	-0.0080563	-0.0131108
73.2	0.3279775	0.22110768	-0.1874448	0.65023966	-0.886315	0.65776459	-0.0080563	-0.0131108
73.3	0.32732857	0.22121616	-0.1873565	0.65003858	-0.8862312	0.65752799	-0.0075787	-0.0132111
73.4	0.32732857	0.22121616	-0.1873565	0.65003858	-0.8862312	0.65752799	-0.0075787	-0.0132111
73.5	0.3274089	0.22131855	-0.1870583	0.64970299	-0.8861554	0.65749012	-0.0075585	-0.0130007
73.6	0.3274089	0.22131855	-0.1870583	0.64970299	-0.8861554	0.65749012	-0.0075585	-0.0130007
73.7	0.32636849	0.22065341	-0.1861815	0.64955941	-0.8861685	0.65696234	-0.0076764	-0.0127296
73.8	0.3260742	0.22109941	-0.1856295	0.64967988	-0.8861191	0.65701152	-0.0077065	-0.0128693
73.9	0.32591179	0.22095784	-0.1856584	0.64957493	-0.8860332	0.65685719	-0.0078306	-0.0126418
74	0.32591179	0.22095784	-0.1856584	0.64957493	-0.8860332	0.65685719	-0.0078306	-0.0126418
74.1	0.32546912	0.22158625	-0.1851934	0.64961482	-0.8859925	0.65679322	-0.0072722	-0.0132216
74.2	0.32442032	0.22172228	-0.1845663	0.64912072	-0.8859043	0.65655597	-0.0062119	-0.013084
74.3	0.32403445	0.2216472	-0.1843268	0.64893092	-0.8858659	0.65634219	-0.006685	-0.012793
74.4	0.32403445	0.2216472	-0.1843268	0.64893092	-0.8858659	0.65634219	-0.006685	-0.012793
74.5	0.32370641	0.2211692	-0.1840139	0.64895057	-0.8858408	0.65618064	-0.0069457	-0.0122643
74.6	0.32330131	0.22118362	-0.1838299	0.64922225	-0.88577	0.65638828	-0.0072768	-0.0119743
74.7	0.32330131	0.22118362	-0.1838299	0.64922225	-0.88577	0.65638828	-0.0072768	-0.0119743
74.8	0.32330131	0.22118362	-0.1838299	0.64922225	-0.88577	0.65638828	-0.0072768	-0.0119743
74.9	0.32322673	0.22153765	-0.1834109	0.64874129	-0.8856643	0.65625525	-0.0074178	-0.0131827
120	0.28796848	0.20616968	-0.1623986	0.65037913	-0.8841628	0.64376422	-0.0088595	-0.0020771
120.1	0.28796848	0.20616968	-0.1623986	0.65037913	-0.8841628	0.64376422	-0.0088595	-0.0020771
120.2	0.28796848	0.20616968	-0.1623986	0.65037913	-0.8841628	0.64376422	-0.0088595	-0.0020771

75	0.32325748	0.22166745	-0.182775	0.64876536	-0.8856118	0.65625157	-0.0068165	-0.0134981
75.1	0.32325748	0.22166745	-0.182775	0.64876536	-0.8856118	0.65625157	-0.0068165	-0.0134981
75.2	0.32291589	0.22089954	-0.1824639	0.64899277	-0.885556	0.65637335	-0.0071614	-0.013947
75.3	0.32277477	0.22080029	-0.1820702	0.64891235	-0.8855499	0.65671493	-0.0069855	-0.0135528
75.4	0.32295837	0.22071577	-0.1822066	0.64864482	-0.8855057	0.6563685	-0.0064463	-0.0132876
75.5	0.32295837	0.22071577	-0.1822066	0.64864482	-0.8855057	0.6563685	-0.0064463	-0.0132876
75.6	0.32243724	0.22007551	-0.1819435	0.64861726	-0.8854463	0.65607154	-0.006484	-0.0132016
75.7	0.32243724	0.22007551	-0.1819435	0.64861726	-0.8854463	0.65607154	-0.006484	-0.0132016
75.8	0.32238689	0.22050631	-0.1816809	0.64814308	-0.8853386	0.65583296	-0.0063641	-0.0138083
75.9	0.32238689	0.22050631	-0.1816809	0.64814308	-0.8853386	0.65583296	-0.0063641	-0.0138083
76	0.32238689	0.22050631	-0.1816809	0.64814308	-0.8853386	0.65583296	-0.0063641	-0.0138083
76.1	0.32238689	0.22050631	-0.1816809	0.64814308	-0.8853386	0.65583296	-0.0063641	-0.0138083
76.2	0.32238689	0.22050631	-0.1816809	0.64814308	-0.8853386	0.65583296	-0.0063641	-0.0138083
76.3	0.32238689	0.22050631	-0.1816809	0.64814308	-0.8853386	0.65583296	-0.0063641	-0.0138083
76.4	0.32213513	0.22013066	-0.181569	0.6482837	-0.8852963	0.65594605	-0.0069199	-0.0140234
76.5	0.32234544	0.22001031	-0.1811841	0.64821478	-0.8852803	0.6558154	-0.0070739	-0.0137996
76.6	0.32238767	0.22049163	-0.1811425	0.64813829	-0.8852808	0.65591135	-0.0064777	-0.014532
76.7	0.32238767	0.22049163	-0.1811425	0.64813829	-0.8852808	0.65591135	-0.0064777	-0.014532
76.8	0.32148865	0.21996931	-0.1811087	0.64766904	-0.8852198	0.65552282	-0.0062563	-0.0147935
76.9	0.32124884	0.22044648	-0.1811882	0.64782487	-0.8852891	0.65559538	-0.0061981	-0.0153375
77	0.32120351	0.2205279	-0.1809936	0.64787187	-0.8853675	0.65565846	-0.0062784	-0.0149086
77.1	0.32120351	0.2205279	-0.1809936	0.64787187	-0.8853675	0.65565846	-0.0062784	-0.0149086
77.2	0.32120351	0.2205279	-0.1809936	0.64787187	-0.8853675	0.65565846	-0.0062784	-0.0149086
77.3	0.32120351	0.2205279	-0.1809936	0.64787187	-0.8853675	0.65565846	-0.0062784	-0.0149086
77.4	0.32060814	0.22093255	-0.1808199	0.64796784	-0.8853692	0.65579366	-0.0068709	-0.0156005
77.5	0.32060814	0.22093255	-0.1808199	0.64796784	-0.8853692	0.65579366	-0.0068709	-0.0156005
77.6	0.32060814	0.22093255	-0.1808199	0.64796784	-0.8853692	0.65579366	-0.0068709	-0.0156005
77.7	0.32046423	0.22058324	-0.1802751	0.64782413	-0.8853124	0.65583462	-0.00692	-0.0152139
77.8	0.32046423	0.22058324	-0.1802751	0.64782413	-0.8853124	0.65583462	-0.00692	-0.0152139
77.9	0.32046423	0.22058324	-0.1802751	0.64782413	-0.8853124	0.65583462	-0.00692	-0.0152139
78	0.32022173	0.22004373	-0.1804653	0.64752871	-0.8852216	0.65542741	-0.007556	-0.0147964
78.1	0.32022173	0.22004373	-0.1804653	0.64752871	-0.8852216	0.65542741	-0.007556	-0.0147964
78.2	0.32012765	0.2196865	-0.1806153	0.64785647	-0.8852324	0.65528534	-0.0079608	-0.0146042
78.3	0.32012765	0.2196865	-0.1806153	0.64785647	-0.8852324	0.65528534	-0.0079608	-0.0146042
78.4	0.32013351	0.21961161	-0.1800086	0.64810379	-0.8852681	0.65530457	-0.0073717	-0.0147104
78.5	0.32013351	0.21961161	-0.1800086	0.64810379	-0.8852681	0.65530457	-0.0073717	-0.0147104
78.6	0.31957803	0.21928113	-0.1797603	0.6479895	-0.8851577	0.65533751	-0.0067468	-0.0149262
78.7	0.31957803	0.21928113	-0.1797603	0.6479895	-0.8851577	0.65533751	-0.0067468	-0.0149262
78.8	0.31919305	0.21953558	-0.1799877	0.64813089	-0.8851684	0.65537327	-0.0065891	-0.0148756
78.9	0.31871541	0.21839677	-0.179833	0.64740954	-0.885	0.65501222	-0.0073774	-0.0154918
79	0.31871541	0.21839677	-0.179833	0.64740954	-0.885	0.65501222	-0.0073774	-0.0154918
79.1	0.31871541	0.21839677	-0.179833	0.64740954	-0.885	0.65501222	-0.0073774	-0.0154918
79.2	0.3180227	0.2185129	-0.1800639	0.64705341	-0.8849841	0.65478926	-0.0073564	-0.0155474
79.3	0.3180227	0.2185129	-0.1800639	0.64705341	-0.8849841	0.65478926	-0.0073564	-0.0155474
79.4	0.3180227	0.2185129	-0.1800639	0.64705341	-0.8849841	0.65478926	-0.0073564	-0.0155474
79.5	0.3180227	0.2185129	-0.1800639	0.64705341	-0.8849841	0.65478926	-0.0073564	-0.0155474
79.6	0.3180227	0.2185129	-0.1800639	0.64705341	-0.8849841	0.65478926	-0.0073564	-0.0155474
79.7	0.3180227	0.2185129	-0.1800639	0.64705341	-0.8849841	0.65478926	-0.0073564	-0.0155474
79.8	0.31744282	0.21865773	-0.1801589	0.64716556	-0.8849415	0.65487363	-0.0072661	-0.0154194
79.9	0.31732257	0.2193398	-0.1807082	0.6471306	-0.8849752	0.65475758	-0.0072637	-0.0153802
80	0.3171477	0.2189825	-0.1800706	0.64732661	-0.8849559	0.65463442	-0.0066931	-0.0153629
80.1	0.3171477	0.2189825	-0.1800706	0.64732661	-0.8849559	0.65463442	-0.0066931	-0.0153629
80.2	0.3171477	0.2189825	-0.1800706	0.64732661	-0.8849559	0.65463442	-0.0066931	-0.0153629
80.3	0.31673733	0.21914343	-0.1794586	0.64701796	-0.884678	0.6539756	-0.0073073	-0.0154468
80.4	0.31673733	0.21914343	-0.1794586	0.64701796	-0.884678	0.6539756	-0.0073073	-0.0154468
80.5	0.31673733	0.21914343	-0.1794586	0.64701796	-0.884678	0.6539756	-0.0073073	-0.0154468
80.6	0.31659455	0.21875801	-0.1789827	0.64684015	-0.8845996	0.6536501	-0.0072594	-0.0149573
80.7	0.31659455	0.21875801	-0.1789827	0.64684015	-0.8845996	0.6536501	-0.0072594	-0.0149573
80.8	0.31625188	0.21881773	-0.1784078	0.6464133	-0.8844104	0.65353276	-0.0072111	-0.0146652
80.9	0.31506167	0.21958497	-0.1793777	0.64622622	-0.8844572	0.65330106	-0.0066091	-0.0150769
81	0.31463718	0.21920836	-0.179383	0.64638059	-0.8845769	0.65315871	-0.0069422	-0.0149566
81.1	0.31465779	0.21909208	-0.1793389	0.64644731	-0.8846064	0.6531252	-0.0071298	-0.0148561
81.2	0.31465779	0.21909208	-0.1793389	0.64644731	-0.8846064	0.6531252	-0.0071298	-0.0148561
81.3	0.31465779	0.21909208	-0.1793389	0.64644731	-0.8846064	0.6531252	-0.0071298	-0.0148561
81.4	0.31465779	0.21909208	-0.1793389	0.64644731	-0.8846064	0.6531252	-0.0071298	-0.0148561
81.5	0.31465779	0.21909208	-0.1793389	0.64644731	-0.8846064	0.6531252	-0.0071298	-0.0148561
81.6	0.31465779	0.21909208	-0.1793389	0.64644731	-0.8846064	0.6531252	-0.0071298	-0.0148561
81.7	0.31465779	0.21909208	-0.1793389	0.64644731	-0.8846064	0.6531252	-0.0071298	-0.0148561
81.8	0.31429183	0.21897822	-0.1788675	0.64631909	-0.8845953	0.65312115	-0.0077035	-0.0145436
81.9	0.31467803	0.21834383	-0.1790696	0.64646181	-0.8846187	0.65306265	-0.007934	-0.0144099
82	0.31467803	0.21834383	-0.1790696	0.64646181	-0.8846187	0.65306265	-0.007934	-0.0144099
82.1	0.31467803	0.21834383	-0.1790696	0.64646181	-0.8846187	0.65306265	-0.007934	-0.0144099
82.2	0.31477603	0.21863807	-0.1787273	0.64671219	-0.8847718	0.65304341	-0.008131	-0.0143724
82.3	0.31477603	0.21863807	-0.1787273	0.64671219	-0.8847718	0.65304341	-0.008131	-0.0143724
82.4	0.31477603	0.21863807	-0.1787273	0.64671219	-0.8847718	0.65304341	-0.008131	-0.0143724
82.5	0.31446286	0.21843128	-0.1788836	0.64645854	-0.8847527	0.65309171	-0.0081124	-0.0137912
82.6	0.31446286	0.21843128	-0.1788836	0.64645854	-0.8847527	0.65309171	-0.0081124	-0.0137912
82.7	0.31386288	0.21815014	-0.1784913	0.64658578	-0.8847904	0.65272494	-0.0084325	-0.0136171
82.8	0.31386288	0.21815014	-0.1784913	0.64658578	-0.8847904	0.65272494	-0.0084325	-0.0136171
82.9	0.31386288	0.21815014	-0.1784913	0.64658578	-0.8847904	0.65272494	-0.0084325	-0.0136171
83	0.31386288	0.21815014	-0.1784913	0.64658578	-0.8847904	0.65272494	-0.0084325	-0.0136171
83.1	0.31386288	0.21815014	-0.1784913	0.64658578	-0.8847904	0.65272494	-0.0084325	-0.0136171
83.2	0.31288503	0.2176664	-0.178216	0.64625358	-0.8847584	0.65274115	-0.0082322	-0.0127105
83.3	0.31272622	0.21811576	-0.178225	0.64632979	-0.8847612	0.65264406	-0.0084028	-0.0125346
83.4	0.31250713	0.2183269	-0.1780081	0.64637076	-0.8848405	0.652404	-0.0080214	-0.0119623
83.5	0.31250713	0.2183269	-0.1780081	0.64637076	-0.8848405	0.652404	-0.0080214	-0.0119623
83.6	0.31250713	0.2183269	-0.1780081	0.64637076	-0.8848405	0.652404	-0.0080214	-0.0119623
83.7	0.31250713	0.2183269	-0.1780081	0.64637076	-0.8848405	0.652404	-0.0080214	-0.0119623
83.8	0.31250713	0.2183269	-0.1780081	0.64637076	-0.8848405	0.652404	-0.0080214	-0.0119623
83.9	0.31220323	0.2182205	-0.1773861	0.64622286	-0.8847813	0.65222795	-0.0078997	-0.0125855
84	0.31220323	0.2182205	-0.1773861	0.64622286	-0.8847813	0.65222795	-0.0078997	-0.0125855
84.1	0.31220323	0.2182205	-0.1773861	0.64622286	-0.8847813	0.65222795	-0.0078997	-0.0125855
84.2	0.31220323	0.2182205	-0.1773861	0.64622286	-0.8847813	0.65222795	-0.0078997	-0.0125855
84.3	0.31220203	0.21834104	-0.1772752	0.64635991	-0.8848025	0.65229593	-0.0081136	-0.0130504
84.4	0.31237301	0.21800254	-0.1767728	0.64661546	-0.8848044	0.65255401	-0.0083818	-0.0130284
84.5	0.31134022	0.217514	-0.1762133	0.64625263	-0.884654	0.65256033	-0.008244	-0.0124033
84.6	0.31134022	0.217514	-0.1762133	0.64625263	-0.884654	0.65256033	-0.008244	-0.0124033
84.7	0.31134022	0.217514	-0.1762133	0.64625263	-0.884654	0.65256033	-0.008244	-0.0124033
84.8	0.31115379	0.21719878	-0.1761515	0.64581816	-0.8845692	0.65228194	-0.0084506	-0.0120138
84.9	0.31042815	0.21703172	-0.1758469	0.64552005	-0.8845277	0.65180525	-0.0088448	-0.0115022
85	0.31042815	0.21703172	-0.1758469	0.64552005	-0.8845277	0.65180525	-0.0088448	-0.0115022
85.1	0.31042815	0.21703172	-0.1758469	0.64552005	-0.8845277	0.65180525	-0.0088448	-0.0115022
85.2	0.31042815	0.21703172	-0.1758469	0.64552005	-0.8845277	0.65180525	-0.0088448	-0.0115022
85.3	0.31051921	0.21647712	-0.1758198	0.64516313	-0.8843857	0.65117248	-0.0087981	-0.0111016
85.4	0.30981934	0.21645005	-0.1749515	0.64519608	-0.8843822	0.65120255	-0.0096837	-0.0109524
85.5	0.30944339	0.21660087	-0.1752779	0.64523433	-0.884457	0.65096206	-0.0098368	-0.0104923
85.6	0.30929951	0.21664335	-0.1746486	0.64517493	-0.8844231	0.65099237	-0.0098559	-0.0109693
85.7	0.30785946	0.21638467	-0.1729966	0.64475859	-0.8839137	0.6506017	-0.0100004	-0.0113106
85.8	0.30774909	0.21595741	-0.1733633	0.64491154	-0.8838927	0.65076269	-0.0102983	-0.0108854
85.9	0.30722223	0.21552233	-0.1734593	0.64521287	-0.8839422	0.65080287	-0.010326	-0.0105637
86	0.30722223	0.21552233	-0.1734593	0.64521287	-0.8839422	0.65080287	-0.010326	-0.0105637
86.1	0.30722223	0.21552233	-0.1734593	0.64521287	-0.8839422	0.65080287	-0.010326	-0.0105637
86.2	0.30722223	0.21552233	-0.1734593	0.64521287	-0.8839422	0.65080287	-0.010326	-0.0105637
86.3	0.30722223	0.21552233	-0.1734593	0.64521287	-0.8839422	0.65080287	-0.010326	-0.0105637
86.4	0.30681416	0.21557933	-0.1728981	0.64511971	-0.8839542	0.65076958	-0.0110296	-0.0110228
86.5	0.30681416	0.21557933	-0.1728981	0.64511971	-0.8839542	0.65076958	-0.0110296	-0.0110228
86.6	0.30681416	0.21557933	-0.1728981	0.64511971	-0.8839542	0.65076958	-0.0110296	-0.0110228
86.7	0.30681416	0.21557933	-0.1728981	0.64511971	-0.8839542	0.65076958	-0.0110296	-0.0110228
86.8	0.30681416	0.21557933	-0.1728981	0.64511971	-0.8839542	0.65076958	-0.0110296	-0.0110228
86.9	0.30681416	0.21557933	-0.1728981	0.64511971	-0.8839542	0.65076958	-0.0110296	-0.0110228
87	0.30694645	0.21538359	-0.1732809	0.645187	-0.8839556	0.65045557	-0.0104834	-0.0105379
87.1	0.30622302	0.21502309	-0.1728201	0.645011	-0.8839589	0.65012929	-0.010711	-0.0097805
87.2	0.30622302	0.21502309	-0.1728201	0.645011	-0.8839589	0.65012929	-0.010711	-0.0097805
87.3	0.30564929	0.21522735	-0.1726018	0.64511524	-0.8839021	0.65017615	-0.010761	-0.0093495
87.4	0.30564929	0.21522735	-0.1726018	0.64511524	-0.8839021	0.65017615	-0.010761	-0.0093495
87.5	0.30556828	0.21581475	-0.1722467	0.64530014	-0.8839998	0.65010304	-0.0114354	-0.0089928
87.6	0.30631756	0.21545597	-0.1725643	0.64536821	-0.8839958	0.65006918	-0.0115769	-0.0087509
87.7	0.30551343	0.21537213	-0.1724841	0.64536548	-0.8839876	0.64972628	-0.0116221	-0.0080082
87.8	0.30526289	0.21502547	-0.1723628	0.64535429	-0.8839748	0.64965474	-0.0118731	-0.0080622
87.9	0.30526289	0.21502547	-0.1723628	0.64535429	-0.8839748	0.64965474	-0.0118731	-0.0080622
88	0.30526289	0.21502547	-0.1723628	0.64535429	-0.8839748	0.64965474	-0.0118731	-0.0080622
88.1	0.30526289	0.21502547	-0.1723628	0.64535429	-0.8839748	0.64965474	-0.0118731	-0.0080622
88.2	0.30534271	0.21516409	-0.1722617	0.64563783	-0.8839539	0.64980231	-0.0118264	-0.0081685
88.3	0.30534271	0.21516409	-0.1722617	0.64563783	-0.8839539	0.64980231	-0.0118264	-0.0081685
88.4	0.30534271	0.21516409	-0.1722617	0.64563783	-0.8839539	0.64980231	-0.0118264	-0.0081685
88.5	0.30534271	0.21516409	-0.1722617	0.64563783	-0.8839539	0.64980231	-0.0118264	-0.0081685
88.6	0.3051004	0.21516279	-0.1716904	0.64598601	-0.8840013	0.64984953	-0.0118948	-0.0080016
88.7	0.30503397	0.21486381	-0.1722949	0.64573481	-0.8839145	0.64936335	-0.0113211	-0.0080555
88.8	0.30493423	0.21474623	-0.1716917	0.64562656	-0.8839146	0.64917035	-0.0113907	-0.0085304
88.9	0.30493423	0.21474623	-0.1716917	0.64562656	-0.8839146	0.64917035	-0.0113907	-0.0085304
89	0.30458637	0.21476987	-0.1715959	0.64562618	-0.8838645	0.64905497	-0.0115976	-0.008819
89.1	0.30458637	0.21476987	-0.1715959	0.64562618	-0.8838645	0.64905497	-0.0115976	-0.008819
89.2	0.30426424	0.21463005	-0.1711259	0.64569133	-0.8839329	0.64895459	-0.0117125	-0.008546
89.3	0.30432298	0.21411636	-0.1714906	0.6457199	-0.8838937	0.64874915	-0.0117452	-0.0078663
89.4	0.30432298	0.21411636	-0.1714906	0.6457199	-0.8838937	0.64874915	-0.0117452	-0.0078663
89.5	0.30432298	0.21411636	-0.1714906	0.6457199	-0.8838937	0.64874915	-0.0117452	-0.0078663
89.6	0.30432298	0.21411636	-0.1714906	0.6457199	-0.8838937	0.64874915	-0.0117452	-0.0078663
89.7	0.30432298	0.21411636	-0.1714906	0.6457199	-0.8838937	0.64874915	-0.0117452	-0.0078663
89.8	0.30432298	0.21411636	-0.1714906	0.6457199	-0.8838937	0.64874915	-0.0117452	-0.0078663
89.9	0.30432298	0.21411636	-0.1714906	0.6457199	-0.8838937	0.64874915	-0.0117452	-0.0078663
135	0.28315126	0.20541966	-0.1597989	0.6522119	-0.8845206	0.64454743	-0.0098941	-0.0015275
135.1	0.28315126	0.20541966	-0.1597989	0.6522119	-0.8845206	0.64454743	-0.0098941	-0.0015275
135.2	0.28315126	0.20541966	-0.1597989	0.6522119	-0.8845206	0.64454743	-0.0098941	-0.0015275

90	0.30432298	0.21411636	-0.1714906	0.6457199	-0.8838937	0.64874915	-0.0117452	-0.0078663
90.1	0.30432298	0.21411636	-0.1714906	0.6457199	-0.8838937	0.64874915	-0.0117452	-0.0078663
90.2	0.30432298	0.21411636	-0.1714906	0.6457199	-0.8838937	0.64874915	-0.0117452	-0.0078663
90.3	0.30437803	0.21425268	-0.1715581	0.6459412	-0.8840345	0.64857083	-0.0121056	-0.0075704
90.4	0.30437803	0.21425268	-0.1715581	0.6459412	-0.8840345	0.64857083	-0.0121056	-0.0075704
90.5	0.30437803	0.21425268	-0.1715581	0.6459412	-0.8840345	0.64857083	-0.0121056	-0.0075704
90.6	0.30472118	0.21458632	-0.1718884	0.64636728	-0.8841628	0.6488188	-0.0124482	-0.0075699
90.7	0.30474475	0.21463352	-0.1717741	0.64648116	-0.8841067	0.64870086	-0.0123599	-0.0074062
90.8	0.30470817	0.21415834	-0.1718191	0.64656467	-0.8841098	0.64861079	-0.0129511	-0.0066801
90.9	0.30470817	0.21415834	-0.1718191	0.64656467	-0.8841098	0.64861079	-0.0129511	-0.0066801
91	0.30470817	0.21415834	-0.1718191	0.64656467	-0.8841098	0.64861079	-0.0129511	-0.0066801
91.1	0.30470817	0.21415834	-0.1718191	0.64656467	-0.8841098	0.64861079	-0.0129511	-0.0066801
91.2	0.30479682	0.2142958	-0.1717097	0.6464404	-0.8840832	0.64858145	-0.013394	-0.0069671
91.3	0.30479682	0.2142958	-0.1717097	0.6464404	-0.8840832	0.64858145	-0.013394	-0.0069671
91.4	0.30415012	0.21402561	-0.1713946	0.64647451	-0.8840767	0.6484755	-0.013138	-0.0071178
91.5	0.30415012	0.21402561	-0.1713946	0.64647451	-0.8840767	0.6484755	-0.013138	-0.0071178
91.6	0.30381925	0.21357321	-0.1712338	0.64670609	-0.8840193	0.64834599	-0.013233	-0.0072354
91.7	0.30381925	0.21357321	-0.1712338	0.64670609	-0.8840193	0.64834599	-0.013233	-0.0072354
91.8	0.30381925	0.21357321	-0.1712338	0.64670609	-0.8840193	0.64834599	-0.013233	-0.0072354
91.9	0.30381925	0.21357321	-0.1712338	0.64670609	-0.8840193	0.64834599	-0.013233	-0.0072354
92	0.30381925	0.21357321	-0.1712338	0.64670609	-0.8840193	0.64834599	-0.013233	-0.0072354
92.1	0.30386595	0.21378959	-0.1709765	0.64655769	-0.8840204	0.64846784	-0.0128079	-0.0072604
92.2	0.3038731	0.21434658	-0.1706548	0.64644191	-0.8840371	0.64853643	-0.0128318	-0.007242
92.3	0.3038731	0.21434658	-0.1706548	0.64644191	-0.8840371	0.64853643	-0.0128318	-0.007242
92.4	0.30265704	0.21369139	-0.1697341	0.64682028	-0.8841256	0.64850432	-0.0127896	-0.0064464
92.5	0.30265704	0.21369139	-0.1697341	0.64682028	-0.8841256	0.64850432	-0.0127896	-0.0064464
92.6	0.30265704	0.21369139	-0.1697341	0.64682028	-0.8841256	0.64850432	-0.0127896	-0.0064464
92.7	0.30265704	0.21369139	-0.1697341	0.64682028	-0.8841256	0.64850432	-0.0127896	-0.0064464
92.8	0.30174038	0.21424013	-0.1701624	0.64774206	-0.8844945	0.64844411	-0.0119642	-0.0051964
92.9	0.30174038	0.21424013	-0.1701624	0.64774206	-0.8844945	0.64844411	-0.0119642	-0.0051964
93	0.302007	0.21338933	-0.1693706	0.64804104	-0.8844284	0.64842116	-0.0127781	-0.0053591
93.1	0.302007	0.21338933	-0.1693706	0.64804104	-0.8844284	0.64842116	-0.0127781	-0.0053591
93.2	0.30247256	0.21304119	-0.1692392	0.64819747	-0.8843754	0.64812902	-0.0129593	-0.0052388
93.3	0.30247256	0.21304119	-0.1692392	0.64819747	-0.8843754	0.64812902	-0.0129593	-0.0052388
93.4	0.30247256	0.21304119	-0.1692392	0.64819747	-0.8843754	0.64812902	-0.0129593	-0.0052388
93.5	0.30239095	0.21246846	-0.1693265	0.64824293	-0.8843744	0.64814899	-0.0123552	-0.0054872
93.6	0.30239095	0.21246846	-0.1693265	0.64824293	-0.8843744	0.64814899	-0.0123552	-0.0054872
93.7	0.3023752	0.21248872	-0.1698756	0.64829921	-0.8844001	0.64797128	-0.011874	-0.0055053
93.8	0.30169237	0.21179745	-0.1691088	0.64807329	-0.884283	0.64764149	-0.0129002	-0.0052629
93.9	0.30169237	0.21179745	-0.1691088	0.64807329	-0.884283	0.64764149	-0.0129002	-0.0052629
94	0.30169237	0.21179745	-0.1691088	0.64807329	-0.884283	0.64764149	-0.0129002	-0.0052629
94.1	0.30169237	0.21179745	-0.1691088	0.64807329	-0.884283	0.64764149	-0.0129002	-0.0052629
94.2	0.30169237	0.21179745	-0.1691088	0.64807329	-0.884283	0.64764149	-0.0129002	-0.0052629
94.3	0.30169237	0.21179745	-0.1691088	0.64807329	-0.884283	0.64764149	-0.0129002	-0.0052629
94.4	0.30169237	0.21179745	-0.1691088	0.64807329	-0.884283	0.64764149	-0.0129002	-0.0052629
94.5	0.30169237	0.21179745	-0.1691088	0.64807329	-0.884283	0.64764149	-0.0129002	-0.0052629
94.6	0.30169237	0.21179745	-0.1691088	0.64807329	-0.884283	0.64764149	-0.0129002	-0.0052629
94.7	0.3014285	0.21171359	-0.1688306	0.6480549	-0.8843918	0.64756281	-0.0127989	-0.0054254
94.8	0.30144341	0.21117365	-0.168521	0.64826855	-0.8844349	0.64736005	-0.0125581	-0.005048
94.9	0.30144341	0.21117365	-0.168521	0.64826855	-0.8844349	0.64736005	-0.0125581	-0.005048
95	0.30144341	0.21117365	-0.168521	0.64826855	-0.8844349	0.64736005	-0.0125581	-0.005048
95.1	0.30144341	0.21117365	-0.168521	0.64826855	-0.8844349	0.64736005	-0.0125581	-0.005048
95.2	0.30144341	0.21117365	-0.168521	0.64826855	-0.8844349	0.64736005	-0.0125581	-0.005048
95.3	0.30144341	0.21117365	-0.168521	0.64826855	-0.8844349	0.64736005	-0.0125581	-0.005048
95.4	0.30144341	0.21117365	-0.168521	0.64826855	-0.8844349	0.64736005	-0.0125581	-0.005048
95.5	0.30120525	0.21077233	-0.1687145	0.64811609	-0.8842475	0.64689125	-0.0115959	-0.0045543
95.6	0.30120525	0.21077233	-0.1687145	0.64811609	-0.8842475	0.64689125	-0.0115959	-0.0045543
95.7	0.30120525	0.21077233	-0.1687145	0.64811609	-0.8842475	0.64689125	-0.0115959	-0.0045543
95.8	0.30120525	0.21077233	-0.1687145	0.64811609	-0.8842475	0.64689125	-0.0115959	-0.0045543
95.9	0.30120525	0.21077233	-0.1687145	0.64811609	-0.8842475	0.64689125	-0.0115959	-0.0045543
96	0.30120525	0.21077233	-0.1687145	0.64811609	-0.8842475	0.64689125	-0.0115959	-0.0045543
96.1	0.30123723	0.21015995	-0.1687449	0.64819006	-0.8841994	0.6466076	-0.0109109	-0.0038361
96.2	0.30130089	0.20981913	-0.1677366	0.64811973	-0.8842654	0.64647667	-0.0107479	-0.0039933
96.3	0.30077783	0.20966419	-0.1676895	0.64838284	-0.8843477	0.64628481	-0.01064	-0.0032472
96.4	0.30072654	0.20954636	-0.1675185	0.64837347	-0.8843571	0.64678192	-0.010623	-0.0027131
96.5	0.30072654	0.20954636	-0.1675185	0.64837347	-0.8843571	0.64678192	-0.010623	-0.0027131
96.6	0.30072654	0.20954636	-0.1675185	0.64837347	-0.8843571	0.64678192	-0.010623	-0.0027131
96.7	0.30072654	0.20954636	-0.1675185	0.64837347	-0.8843571	0.64678192	-0.010623	-0.0027131
96.8	0.30084109	0.20895969	-0.1679257	0.64828326	-0.8842659	0.64632575	-0.0106673	-0.0023299
96.9	0.30084109	0.20895969	-0.1679257	0.64828326	-0.8842659	0.64632575	-0.0106673	-0.0023299
97	0.3009391	0.20899258	-0.167432	0.6484008	-0.8843627	0.64643056	-0.0102545	-0.0021626
97.1	0.3009391	0.20899258	-0.167432	0.6484008	-0.8843627	0.64643056	-0.0102545	-0.0021626
97.2	0.3009391	0.20899258	-0.167432	0.6484008	-0.8843627	0.64643056	-0.0102545	-0.0021626
97.3	0.3009391	0.20899258	-0.167432	0.6484008	-0.8843627	0.64643056	-0.0102545	-0.0021626
97.4	0.3009391	0.20899258	-0.167432	0.6484008	-0.8843627	0.64643056	-0.0102545	-0.0021626
97.5	0.30059397	0.20927608	-0.1670129	0.64850054	-0.8844345	0.64652135	-0.0100251	-0.0021186
97.6	0.30059397	0.20927608	-0.1670129	0.64850054	-0.8844345	0.64652135	-0.0100251	-0.0021186
97.7	0.30041815	0.20966122	-0.1669044	0.64841729	-0.8843987	0.64663486	-0.0105281	-0.0026407
97.8	0.30041815	0.20966122	-0.1669044	0.64841729	-0.8843987	0.64663486	-0.0105281	-0.0026407
97.9	0.30068047	0.20911067	-0.1669024	0.64837702	-0.884268	0.64611407	-0.0102421	-0.0024743
98	0.30024872	0.20913895	-0.1675044	0.64809076	-0.8842618	0.6459956	-0.0105125	-0.0019774
98.1	0.30024872	0.20913895	-0.1675044	0.64809076	-0.8842618	0.6459956	-0.0105125	-0.0019774
98.2	0.30023037	0.20900545	-0.1675464	0.64792201	-0.8842543	0.64601461	-0.0106903	-0.002
98.3	0.29942216	0.20904664	-0.1672157	0.64775877	-0.8842547	0.64585737	-0.0112661	-0.0022748
98.4	0.29942216	0.20904664	-0.1672157	0.64775877	-0.8842547	0.64585737	-0.0112661	-0.0022748
98.5	0.29942216	0.20904664	-0.1672157	0.64775877	-0.8842547	0.64585737	-0.0112661	-0.0022748
98.6	0.29942216	0.20904664	-0.1672157	0.64775877	-0.8842547	0.64585737	-0.0112661	-0.0022748
98.7	0.2992126	0.20938002	-0.1676452	0.64806816	-0.8844112	0.64592117	-0.0113929	-0.0022206
98.8	0.2992126	0.20938002	-0.1676452	0.64806816	-0.8844112	0.64592117	-0.0113929	-0.0022206
98.9	0.2991258	0.20931188	-0.1671314	0.64867431	-0.8845245	0.64580258	-0.0114846	-0.0011522
99	0.29868201	0.20910084	-0.1665101	0.64846776	-0.8843243	0.64602591	-0.0110779	-0.000478
99.1	0.29868201	0.20910084	-0.1665101	0.64846776	-0.8843243	0.64602591	-0.0110779	-0.000478
99.2	0.29868201	0.20910084	-0.1665101	0.64846776	-0.8843243	0.64602591	-0.0110779	-0.000478
99.3	0.29833693	0.20882795	-0.1661969	0.64790696	-0.8841529	0.64535665	-0.0116025	-2.43E-05
99.4	0.29833693	0.20882795	-0.1661969	0.64790696	-0.8841529	0.64535665	-0.0116025	-2.43E-05
99.5	0.29833693	0.20882795	-0.1661969	0.64790696	-0.8841529	0.64535665	-0.0116025	-2.43E-05
99.6	0.29802096	0.20881497	-0.165924	0.64814026	-0.8842052	0.64541877	-0.0110826	-0.0007387
99.7	0.29802096	0.20881497	-0.165924	0.64814026	-0.8842052	0.64541877	-0.0110826	-0.0007387
99.8	0.29802096	0.20881497	-0.165924	0.64814026	-0.8842052	0.64541877	-0.0110826	-0.0007387
99.9	0.29802096	0.20881497	-0.165924	0.64814026	-0.8842052	0.64541877	-0.0110826	-0.0007387
100	0.29818888	0.20828963	-0.1662886	0.64820966	-0.8841493	0.64502507	-0.0105993	-0.0004234
100.1	0.29818888	0.20828963	-0.1662886	0.64820966	-0.8841493	0.64502507	-0.0105993	-0.0004234
100.2	0.29818888	0.20828963	-0.1662886	0.64820966	-0.8841493	0.64502507	-0.0105993	-0.0004234
100.3	0.29818888	0.20828963	-0.1662886	0.64820966	-0.8841493	0.64502507	-0.0105993	-0.0004234
100.4	0.29818888	0.20828963	-0.1662886	0.64820966	-0.8841493	0.64502507	-0.0105993	-0.0004234
100.5	0.29838265	0.20834293	-0.1661343	0.648577	-0.8842392	0.6453397	-0.0105608	-0.000106
100.6	0.29838265	0.20834293	-0.1661343	0.648577	-0.8842392	0.6453397	-0.0105608	-0.000106
100.7	0.29862273	0.20848233	-0.1660391	0.64902415	-0.8843193	0.64528732	-0.0106911	-7.06E-06
100.8	0.29862273	0.20848233	-0.1660391	0.64902415	-0.8843193	0.64528732	-0.0106911	-7.06E-06
100.9	0.29862273	0.20848233	-0.1660391	0.64902415	-0.8843193	0.64528732	-0.0106911	-7.06E-06
101	0.29862273	0.20848233	-0.1660391	0.64902415	-0.8843193	0.64528732	-0.0106911	-7.06E-06
101.1	0.29862273	0.20848233	-0.1660391	0.64902415	-0.8843193	0.64528732	-0.0106911	-7.06E-06
101.2	0.29862273	0.20848233	-0.1660391	0.64902415	-0.8843193	0.64528732	-0.0106911	-7.06E-06
101.3	0.29862273	0.20848233	-0.1660391	0.64902415	-0.8843193	0.64528732	-0.0106911	-7.06E-06
101.4	0.29862273	0.20848233	-0.1660391	0.64902415	-0.8843193	0.64528732	-0.0106911	-7.06E-06
101.5	0.29862273	0.20848233	-0.1660391	0.64902415	-0.8843193	0.64528732	-0.0106911	-7.06E-06
101.6	0.29862273	0.20848233	-0.1660391	0.64902415	-0.8843193	0.64528732	-0.0106911	-7.06E-06
101.7	0.29862273	0.20848233	-0.1660391	0.64902415	-0.8843193	0.64528732	-0.0106911	-7.06E-06
101.8	0.29862273	0.20848233	-0.1660391	0.64902415	-0.8843193	0.64528732	-0.0106911	-7.06E-06
101.9	0.29818032	0.20873342	-0.1661147	0.64900757	-0.8842949	0.64527465	-0.0108743	-0.0001305
102	0.29818032	0.20873342	-0.1661147	0.64900757	-0.8842949	0.64527465	-0.0108743	-0.0001305
102.1	0.29818032	0.20873342	-0.1661147	0.64900757	-0.8842949	0.64527465	-0.0108743	-0.0001305
102.2	0.29808593	0.20854946	-0.1659122	0.6493315	-0.8843734	0.64536617	-0.0112016	-9.58E-05
102.3	0.29808593	0.20854946	-0.1659122	0.6493315	-0.8843734	0.64536617	-0.0112016	-9.58E-05
102.4	0.29808593	0.20854946	-0.1659122	0.6493315	-0.8843734	0.64536617	-0.0112016	-9.58E-05
102.5	0.29808593	0.20854946	-0.1659122	0.6493315	-0.8843734	0.64536617	-0.0112016	-9.58E-05
102.6	0.29808593	0.20854946	-0.1659122	0.6493315	-0.8843734	0.64536617	-0.0112016	-9.58E-05
102.7	0.29808593	0.20854946	-0.1659122	0.6493315	-0.8843734	0.64536617	-0.0112016	-9.58E-05
102.8	0.29808593	0.20854946	-0.1659122	0.6493315	-0.8843734	0.64536617	-0.0112016	-9.58E-05
102.9	0.29771567	0.20835081	-0.1653765	0.64911186	-0.884285	0.64519061	-0.0107925	-0.000207
103	0.29771567	0.20835081	-0.1653765	0.64911186	-0.884285	0.64519061	-0.0107925	-0.000207
103.1	0.2978856	0.20851858	-0.1650823	0.649327	-0.8843631	0.64550053	-0.010966	-0.0004172
103.2	0.2978856	0.20851858	-0.1650823	0.649327	-0.8843631	0.64550053	-0.010966	-0.0004172
103.3	0.29733233	0.20782324	-0.1647745	0.64994425	-0.8844608	0.64551719	-0.0105481	-0.0012336
103.4	0.29733233	0.20782324	-0.1647745	0.64994425	-0.8844608	0.64551719	-0.0105481	-0.0012336
103.5	0.29733233	0.20782324	-0.1647745	0.64994425	-0.8844608	0.64551719	-0.0105481	-0.0012336
103.6	0.29733233	0.20782324	-0.1647745	0.64994425	-0.8844608	0.64551719	-0.0105481	-0.0012336
103.7	0.29733233	0.20782324	-0.1647745	0.64994425	-0.8844608	0.64551719	-0.0105481	-0.0012336
103.8	0.29701749	0.20790277	-0.1648645	0.64980197	-0.884431	0.64516452	-0.0105758	-0.0006864
103.9	0.29701749	0.20790277	-0.1648645	0.64980197	-0.884431	0.64516452	-0.0105758	-0.0006864
104	0.29701749	0.20790277	-0.1648645	0.64980197	-0.884431	0.64516452	-0.0105758	-0.0006864
104.1	0.29701749	0.20790277	-0.1648645	0.64980197	-0.884431	0.64516452	-0.0105758	-0.0006864
104.2	0.29701749	0.20790277	-0.1648645	0.64980197	-0.884431	0.64516452	-0.0105758	-0.0006864
104.3	0.29701749	0.20790277	-0.1648645	0.64980197	-0.884431	0.64516452	-0.0105758	-0.0006864
104.4	0.29701749	0.20790277	-0.1648645	0.64980197	-0.884431	0.64516452	-0.0105758	-0.0006864
104.5	0.2966341	0.20763257	-0.1648499	0.64926972	-0.8843688	0.64551505	-0.0095277	-0.0012113
104.6	0.2966341	0.20763257	-0.1648499	0.64926972	-0.8843688	0.64551505	-0.0095277	-0.0012113
104.7	0.2966341	0.20763257	-0.1648499	0.64926972	-0.8843688	0.64551505	-0.0095277	-0.0012113
104.8	0.2966341	0.20763257	-0.1648499	0.64926972	-0.8843688	0.64551505	-0.0095277	-0.0012113
104.9	0.2966341	0.20763257	-0.1648499	0.64926972	-0.8843688	0.64551505	-0.0095277	-0.0012113
150	0.27961075	0.20565183	-0.1544966	0.65189045	-0.8837558	0.64232352	-0.0118595	0.00125961
150.1	0.27961075	0.20565183	-0.1544966	0.65189045	-0.8837558	0.64232352	-0.0118595	0.00125961
150.2	0.27961075	0.20565183	-0.1544966	0.65189045	-0.8837558	0.64232352	-0.0118595	0.00125961

105	0.2969453	0.20755807	-0.164429	0.64942901	-0.8843894	0.64538758	-0.0096421	-0.0016208
105.1	0.2969453	0.20755807	-0.164429	0.64942901	-0.8843894	0.64538758	-0.0096421	-0.0016208
105.2	0.2969453	0.20755807	-0.164429	0.64942901	-0.8843894	0.64538758	-0.0096421	-0.0016208
105.3	0.2969453	0.20755807	-0.164429	0.64942901	-0.8843894	0.64538758	-0.0096421	-0.0016208
105.4	0.2969453	0.20755807	-0.164429	0.64942901	-0.8843894	0.64538758	-0.0096421	-0.0016208
105.5	0.2969075	0.2068831	-0.1640541	0.64942164	-0.8842923	0.64536526	-0.0090421	-0.0015419
105.6	0.2969075	0.2068831	-0.1640541	0.64942164	-0.8842923	0.64536526	-0.0090421	-0.0015419
105.7	0.2969075	0.2068831	-0.1640541	0.64942164	-0.8842923	0.64536526	-0.0090421	-0.0015419
105.8	0.29650035	0.20687601	-0.1639544	0.64979444	-0.8843522	0.6455601	-0.009177	-0.0016396
105.9	0.29650035	0.20687601	-0.1639544	0.64979444	-0.8843522	0.6455601	-0.009177	-0.0016396
106	0.29650035	0.20687601	-0.1639544	0.64979444	-0.8843522	0.6455601	-0.009177	-0.0016396
106.1	0.29663121	0.20657959	-0.1641008	0.65014386	-0.8844354	0.64577463	-0.0097409	-0.0013195
106.2	0.29663121	0.20657959	-0.1641008	0.65014386	-0.8844354	0.64577463	-0.0097409	-0.0013195
106.3	0.29663121	0.20657959	-0.1641008	0.65014386	-0.8844354	0.64577463	-0.0097409	-0.0013195
106.4	0.29663121	0.20657959	-0.1641008	0.65014386	-0.8844354	0.64577463	-0.0097409	-0.0013195
106.5	0.29663121	0.20657959	-0.1641008	0.65014386	-0.8844354	0.64577463	-0.0097409	-0.0013195
106.6	0.29663121	0.20657959	-0.1641008	0.65014386	-0.8844354	0.64577463	-0.0097409	-0.0013195
106.7	0.29663121	0.20657959	-0.1641008	0.65014386	-0.8844354	0.64577463	-0.0097409	-0.0013195
106.8	0.29663121	0.20657959	-0.1641008	0.65014386	-0.8844354	0.64577463	-0.0097409	-0.0013195
106.9	0.29663121	0.20657959	-0.1641008	0.65014386	-0.8844354	0.64577463	-0.0097409	-0.0013195
107	0.29663121	0.20657959	-0.1641008	0.65014386	-0.8844354	0.64577463	-0.0097409	-0.0013195
107.1	0.29663121	0.20657959	-0.1641008	0.65014386	-0.8844354	0.64577463	-0.0097409	-0.0013195
107.2	0.29644402	0.20629548	-0.1639685	0.65033004	-0.8845103	0.64593377	-0.0097394	-0.0005784
107.3	0.29606566	0.20660852	-0.1640979	0.65011083	-0.8845076	0.64571003	-0.0096878	-0.0006023
107.4	0.29606566	0.20660852	-0.1640979	0.65011083	-0.8845076	0.64571003	-0.0096878	-0.0006023
107.5	0.29613598	0.20639403	-0.1637723	0.64982216	-0.8844474	0.64563318	-0.0096723	-0.0011104
107.6	0.29601822	0.20664954	-0.1632579	0.64954097	-0.8843598	0.64536329	-0.0095688	-0.0010638
107.7	0.29601822	0.20664954	-0.1632579	0.64954097	-0.8843598	0.64536329	-0.0095688	-0.0010638
107.8	0.29601822	0.20664954	-0.1632579	0.64954097	-0.8843598	0.64536329	-0.0095688	-0.0010638
107.9	0.29601822	0.20664954	-0.1632579	0.64954097	-0.8843598	0.64536329	-0.0095688	-0.0010638
108	0.29585151	0.20621224	-0.1629847	0.64956743	-0.8843578	0.64517683	-0.0093964	-0.0015897
108.1	0.29513565	0.20562311	-0.1630021	0.64973704	-0.8843506	0.64508146	-0.0088989	-0.0017914
108.2	0.294856	0.20522089	-0.1630438	0.64980054	-0.8843004	0.64518142	-0.0090166	-0.0022834
108.3	0.294856	0.20522089	-0.1630438	0.64980054	-0.8843004	0.64518142	-0.0090166	-0.0022834
108.4	0.294856	0.20522089	-0.1630438	0.64980054	-0.8843004	0.64518142	-0.0090166	-0.0022834
108.5	0.294856	0.20522089	-0.1630438	0.64980054	-0.8843004	0.64518142	-0.0090166	-0.0022834
108.6	0.294856	0.20522089	-0.1630438	0.64980054	-0.8843004	0.64518142	-0.0090166	-0.0022834
108.7	0.294856	0.20522089	-0.1630438	0.64980054	-0.8843004	0.64518142	-0.0090166	-0.0022834
108.8	0.29474609	0.2049472	-0.163194	0.64975513	-0.8843189	0.64522854	-0.0087514	-0.0020184
108.9	0.29474609	0.2049472	-0.163194	0.64975513	-0.8843189	0.64522854	-0.0087514	-0.0020184
109	0.29474609	0.2049472	-0.163194	0.64975513	-0.8843189	0.64522854	-0.0087514	-0.0020184
109.1	0.29387284	0.20502483	-0.1632354	0.64963712	-0.8843695	0.6450898	-0.0087349	-0.0022215
109.2	0.29387284	0.20502483	-0.1632354	0.64963712	-0.8843695	0.6450898	-0.0087349	-0.0022215
109.3	0.29387284	0.20502483	-0.1632354	0.64963712	-0.8843695	0.6450898	-0.0087349	-0.0022215
109.4	0.29329601	0.2047369	-0.1631242	0.64968152	-0.8843127	0.64508153	-0.0082681	-0.0017743
109.5	0.29329601	0.2047369	-0.1631242	0.64968152	-0.8843127	0.64508153	-0.0082681	-0.0017743
109.6	0.29360381	0.20465247	-0.1631484	0.64954088	-0.8842778	0.64501385	-0.0089824	-0.0014693
109.7	0.29360381	0.20465247	-0.1631484	0.64954088	-0.8842778	0.64501385	-0.0089824	-0.0014693
109.8	0.29330186	0.20484679	-0.1636866	0.64997729	-0.8844631	0.64511115	-0.0086647	-0.0011269
109.9	0.29279107	0.20482773	-0.1640026	0.6495188	-0.8843451	0.6445861	-0.0086665	-0.0011213
110	0.29240666	0.2048911	-0.1641801	0.64959011	-0.8842709	0.64427887	-0.0086959	-0.0005878
110.1	0.29240666	0.2048911	-0.1641801	0.64959011	-0.8842709	0.64427887	-0.0086959	-0.0005878
110.2	0.29240666	0.2048911	-0.1641801	0.64959011	-0.8842709	0.64427887	-0.0086959	-0.0005878
110.3	0.29240666	0.2048911	-0.1641801	0.64959011	-0.8842709	0.64427887	-0.0086959	-0.0005878
110.4	0.29240666	0.2048911	-0.1641801	0.64959011	-0.8842709	0.64427887	-0.0086959	-0.0005878
110.5	0.2918527	0.20514681	-0.1645427	0.64962743	-0.8842414	0.64423178	-0.0090659	-0.0006434
110.6	0.29176031	0.20575097	-0.1644284	0.64975973	-0.8842935	0.64427549	-0.0084644	-0.0005045
110.7	0.29176031	0.20575097	-0.1644284	0.64975973	-0.8842935	0.64427549	-0.0084644	-0.0005045
110.8	0.29176031	0.20575097	-0.1644284	0.64975973	-0.8842935	0.64427549	-0.0084644	-0.0005045
110.9	0.29176031	0.20575097	-0.1644284	0.64975973	-0.8842935	0.64427549	-0.0084644	-0.0005045
111	0.29176031	0.20575097	-0.1644284	0.64975973	-0.8842935	0.64427549	-0.0084644	-0.0005045
111.1	0.29157811	0.20625856	-0.1645032	0.64978469	-0.8842892	0.6442674	-0.0083926	-0.0009508
111.2	0.29157811	0.20625856	-0.1645032	0.64978469	-0.8842892	0.6442674	-0.0083926	-0.0009508
111.3	0.29157811	0.20625856	-0.1645032	0.64978469	-0.8842892	0.6442674	-0.0083926	-0.0009508
111.4	0.29157811	0.20625856	-0.1645032	0.64978469	-0.8842892	0.6442674	-0.0083926	-0.0009508
111.5	0.29064049	0.20586964	-0.1648665	0.64974995	-0.8842151	0.64412027	-0.0086737	0.00011371
111.6	0.29074657	0.20568817	-0.1648742	0.64994858	-0.8842871	0.64433107	-0.0094485	-9.33E-05
111.7	0.29074657	0.20568817	-0.1648742	0.64994858	-0.8842871	0.64433107	-0.0094485	-9.33E-05
111.8	0.29019889	0.20572326	-0.1642644	0.64988815	-0.8841914	0.64420517	-0.0095976	-4.48E-05
111.9	0.29019889	0.20572326	-0.1642644	0.64988815	-0.8841914	0.64420517	-0.0095976	-4.48E-05
112	0.2901059	0.20614227	-0.1642749	0.64973962	-0.8842405	0.64424662	-0.0096989	-0.0006216
112.1	0.2901059	0.20614227	-0.1642749	0.64973962	-0.8842405	0.64424662	-0.0096989	-0.0006216
112.2	0.2901059	0.20614227	-0.1642749	0.64973962	-0.8842405	0.64424662	-0.0096989	-0.0006216
112.3	0.2899679	0.20612239	-0.1640073	0.64983344	-0.8843719	0.64410815	-0.0098914	-0.0010452
112.4	0.2899679	0.20612239	-0.1640073	0.64983344	-0.8843719	0.64410815	-0.0098914	-0.0010452
112.5	0.2899679	0.20612239	-0.1640073	0.64983344	-0.8843719	0.64410815	-0.0098914	-0.0010452
112.6	0.28914922	0.20618713	-0.1642988	0.64951616	-0.8842891	0.64365064	-0.0091787	-0.0013443
112.7	0.28914922	0.20618713	-0.1642988	0.64951616	-0.8842891	0.64365064	-0.0091787	-0.0013443
112.8	0.28914922	0.20618713	-0.1642988	0.64951616	-0.8842891	0.64365064	-0.0091787	-0.0013443
112.9	0.28914922	0.20618713	-0.1642988	0.64951616	-0.8842891	0.64365064	-0.0091787	-0.0013443
113	0.28914922	0.20618713	-0.1642988	0.64951616	-0.8842891	0.64365064	-0.0091787	-0.0013443
113.1	0.28914922	0.20618713	-0.1642988	0.64951616	-0.8842891	0.64365064	-0.0091787	-0.0013443
113.2	0.28914922	0.20618713	-0.1642988	0.64951616	-0.8842891	0.64365064	-0.0091787	-0.0013443
113.3	0.28914922	0.20618713	-0.1642988	0.64951616	-0.8842891	0.64365064	-0.0091787	-0.0013443
113.4	0.28914922	0.20618713	-0.1642988	0.64951616	-0.8842891	0.64365064	-0.0091787	-0.0013443
113.5	0.28850965	0.20548994	-0.1640411	0.64936864	-0.8841953	0.64327255	-0.0092921	-0.0014625
113.6	0.28850965	0.20548994	-0.1640411	0.64936864	-0.8841953	0.64327255	-0.0092921	-0.0014625
113.7	0.28850965	0.20548994	-0.1640411	0.64936864	-0.8841953	0.64327255	-0.0092921	-0.0014625
113.8	0.28850965	0.20548994	-0.1640411	0.64936864	-0.8841953	0.64327255	-0.0092921	-0.0014625
113.9	0.28850965	0.20548994	-0.1640411	0.64936864	-0.8841953	0.64327255	-0.0092921	-0.0014625
114	0.28850965	0.20548994	-0.1640411	0.64936864	-0.8841953	0.64327255	-0.0092921	-0.0014625
114.1	0.28850965	0.20548994	-0.1640411	0.64936864	-0.8841953	0.64327255	-0.0092921	-0.0014625
114.2	0.28850965	0.20548994	-0.1640411	0.64936864	-0.8841953	0.64327255	-0.0092921	-0.0014625
114.3	0.28822329	0.20599459	-0.1641527	0.64918632	-0.8841599	0.64314554	-0.0086649	-0.0018638
114.4	0.28875451	0.20643389	-0.1639726	0.64932043	-0.884207	0.64347539	-0.0087494	-0.0019665
114.5	0.28875952	0.20665253	-0.1636408	0.64975544	-0.8842471	0.6438392	-0.008766	-0.0024428
114.6	0.28875952	0.20665253	-0.1636408	0.64975544	-0.8842471	0.6438392	-0.008766	-0.0024428
114.7	0.28875952	0.20665253	-0.1636408	0.64975544	-0.8842471	0.6438392	-0.008766	-0.0024428
114.8	0.28840711	0.20669767	-0.1638104	0.64966555	-0.884178	0.64357183	-0.0091015	-0.0023802
114.9	0.28840711	0.20669767	-0.1638104	0.64966555	-0.884178	0.64357183	-0.0091015	-0.0023802
115	0.28840711	0.20669767	-0.1638104	0.64966555	-0.884178	0.64357183	-0.0091015	-0.0023802
115.1	0.28840711	0.20669767	-0.1638104	0.64966555	-0.884178	0.64357183	-0.0091015	-0.0023802
115.2	0.2882421	0.20698406	-0.1636795	0.64976191	-0.884191	0.64373014	-0.009078	-0.0020471
115.3	0.2882421	0.20698406	-0.1636795	0.64976191	-0.884191	0.64373014	-0.009078	-0.0020471
115.4	0.2882421	0.20698406	-0.1636795	0.64976191	-0.884191	0.64373014	-0.009078	-0.0020471
115.5	0.2882421	0.20698406	-0.1636795	0.64976191	-0.884191	0.64373014	-0.009078	-0.0020471
115.6	0.2882421	0.20698406	-0.1636795	0.64976191	-0.884191	0.64373014	-0.009078	-0.0020471
115.7	0.2882421	0.20698406	-0.1636795	0.64976191	-0.884191	0.64373014	-0.009078	-0.0020471
115.8	0.2882421	0.20698406	-0.1636795	0.64976191	-0.884191	0.64373014	-0.009078	-0.0020471
115.9	0.2882421	0.20698406	-0.1636795	0.64976191	-0.884191	0.64373014	-0.009078	-0.0020471
116	0.2882421	0.20698406	-0.1636795	0.64976191	-0.884191	0.64373014	-0.009078	-0.0020471
116.1	0.28833276	0.20629814	-0.1633006	0.64981929	-0.8841609	0.64376614	-0.0087663	-0.0019742
116.2	0.28833276	0.20629814	-0.1633006	0.64981929	-0.8841609	0.64376614	-0.0087663	-0.0019742
116.3	0.28833276	0.20629814	-0.1633006	0.64981929	-0.8841609	0.64376614	-0.0087663	-0.0019742
116.4	0.28833276	0.20629814	-0.1633006	0.64981929	-0.8841609	0.64376614	-0.0087663	-0.0019742
116.5	0.28833276	0.20629814	-0.1633006	0.64981929	-0.8841609	0.64376614	-0.0087663	-0.0019742
116.6	0.28833276	0.20629814	-0.1633006	0.64981929	-0.8841609	0.64376614	-0.0087663	-0.0019742
116.7	0.28833276	0.20629814	-0.1633006	0.64981929	-0.8841609	0.64376614	-0.0087663	-0.0019742
116.8	0.28799205	0.20621094	-0.1633716	0.64984068	-0.8842734	0.6436211	-0.0092799	-0.0020879
116.9	0.28799205	0.20621094	-0.1633716	0.64984068	-0.8842734	0.6436211	-0.0092799	-0.0020879
117	0.28799205	0.20621094	-0.1633716	0.64984068	-0.8842734	0.6436211	-0.0092799	-0.0020879
117.1	0.28799205	0.20621094	-0.1633716	0.64984068	-0.8842734	0.6436211	-0.0092799	-0.0020879
117.2	0.28799205	0.20621094	-0.1633716	0.64984068	-0.8842734	0.6436211	-0.0092799	-0.0020879
117.3	0.28799205	0.20621094	-0.1633716	0.64984068	-0.8842734	0.6436211	-0.0092799	-0.0020879
117.4	0.28799205	0.20621094	-0.1633716	0.64984068	-0.8842734	0.6436211	-0.0092799	-0.0020879
117.5	0.28799205	0.20621094	-0.1633716	0.64984068	-0.8842734	0.6436211	-0.0092799	-0.0020879
117.6	0.28799205	0.20621094	-0.1633716	0.64984068	-0.8842734	0.6436211	-0.0092799	-0.0020879
117.7	0.28799205	0.20621094	-0.1633716	0.64984068	-0.8842734	0.6436211	-0.0092799	-0.0020879
117.8	0.28799205	0.20621094	-0.1633716	0.64984068	-0.8842734	0.6436211	-0.0092799	-0.0020879
117.9	0.28799205	0.20621094	-0.1633716	0.64984068	-0.8842734	0.6436211	-0.0092799	-0.0020879
118	0.28799205	0.20621094	-0.1633716	0.64984068	-0.8842734	0.6436211	-0.0092799	-0.0020879
118.1	0.28799205	0.20621094	-0.1633716	0.64984068	-0.8842734	0.6436211	-0.0092799	-0.0020879
118.2	0.28799205	0.20621094	-0.1633716	0.64984068	-0.8842734	0.6436211	-0.0092799	-0.0020879
118.3	0.28799205	0.20621094	-0.1633716	0.64984068	-0.8842734	0.6436211	-0.0092799	-0.0020879
118.4	0.2877876	0.20640473	-0.1629699	0.65017852	-0.884245	0.64381278	-0.0096141	-0.0018409
118.5	0.28759745	0.20619487	-0.1627213	0.64988293	-0.8840687	0.64350141	-0.0093225	-0.0018027
118.6	0.28759745	0.20619487	-0.1627213	0.64988293	-0.8840687	0.64350141	-0.0093225	-0.0018027
118.7	0.28759745	0.20619487	-0.1627213	0.64988293	-0.8840687	0.64350141	-0.0093225	-0.0018027
118.8	0.28759745	0.20619487	-0.1627213	0.64988293	-0.8840687	0.64350141	-0.0093225	-0.0018027
118.9	0.28759745	0.20619487	-0.1627213	0.64988293	-0.8840687	0.64350141	-0.0093225	-0.0018027
119	0.28759745	0.20619487	-0.1627213	0.64988293	-0.8840687	0.64350141	-0.0093225	-0.0018027
119.1	0.28759745	0.20619487	-0.1627213	0.64988293	-0.8840687	0.64350141	-0.0093225	-0.0018027
119.2	0.28759745	0.20619487	-0.1627213	0.64988293	-0.8840687	0.64350141	-0.0093225	-0.0018027
119.3	0.28759745	0.20619487	-0.1627213	0.64988293	-0.8840687	0.64350141	-0.0093225	-0.0018027
119.4	0.28759745	0.20619487	-0.1627213	0.64988293	-0.8840687	0.64350141	-0.0093225	-0.0018027
119.5	0.28759745	0.20619487	-0.1627213	0.64988293	-0.8840687	0.64350141	-0.0093225	-0.0018027
119.6	0.28759745	0.20619487	-0.1627213	0.64988293	-0.8840687	0.64350141	-0.0093225	-0.0018027
119.7	0.28781892	0.20671853	-0.1625164	0.65015702	-0.8841507	0.64389848	-0.0086938	-0.0022101
119.8	0.28781892	0.20671853	-0.1625164	0.65015702	-0.8841507	0.64389848	-0.0086938	-0.0022101
119.9	0.28781892	0.20671853	-0.1625164	0.65015702	-0.8841507	0.64389848	-0.0086938	-0.0022101
165	0.27677221	0.20767453	-0.1510888	0.65099891	-0.8830297	0.63943484	-0.0118941	0.00160475
165.1	0.27668592	0.20780768	-0.1507287	0.65097619	-0.8830267	0.6392224	-0.0123208	0.00132848
165.2	0.27668592	0.20780768	-0.1507287	0.65097619	-0.8830267	0.6392224	-0.0123208	0.00132848

120.3	0.28796848	0.20616968	-0.1623986	0.65037913	-0.8841628	0.64376422	-0.0088595	-0.0020771
120.4	0.28796848	0.20616968	-0.1623986	0.65037913	-0.8841628	0.64376422	-0.0088595	-0.0020771
120.5	0.28796848	0.20616968	-0.1623986	0.65037913	-0.8841628	0.64376422	-0.0088595	-0.0020771
120.6	0.28796848	0.20616968	-0.1623986	0.65037913	-0.8841628	0.64376422	-0.0088595	-0.0020771
120.7	0.28796848	0.20616968	-0.1623986	0.65037913	-0.8841628	0.64376422	-0.0088595	-0.0020771
120.8	0.28796848	0.20616968	-0.1623986	0.65037913	-0.8841628	0.64376422	-0.0088595	-0.0020771
120.9	0.28749209	0.2059906	-0.1621063	0.65053293	-0.8840666	0.64360392	-0.0090907	-0.0024511
121	0.28745671	0.20665447	-0.1624423	0.65079842	-0.8841544	0.64408584	-0.0091154	-0.002599
121.1	0.28745671	0.20665447	-0.1624423	0.65079842	-0.8841544	0.64408584	-0.0091154	-0.002599
121.2	0.28712084	0.20670603	-0.1620266	0.65083577	-0.8841402	0.64424613	-0.0087785	-0.0022801
121.3	0.28712084	0.20670603	-0.1620266	0.65083577	-0.8841402	0.64424613	-0.0087785	-0.0022801
121.4	0.28712084	0.20670603	-0.1620266	0.65083577	-0.8841402	0.64424613	-0.0087785	-0.0022801
121.5	0.28712084	0.20670603	-0.1620266	0.65083577	-0.8841402	0.64424613	-0.0087785	-0.0022801
121.6	0.28675207	0.20666001	-0.1620086	0.65086301	-0.8841035	0.64429799	-0.0093445	-0.0023626
121.7	0.28675207	0.20666001	-0.1620086	0.65086301	-0.8841035	0.64429799	-0.0093445	-0.0023626
121.8	0.28675207	0.20666001	-0.1620086	0.65086301	-0.8841035	0.64429799	-0.0093445	-0.0023626
121.9	0.28675207	0.20666001	-0.1620086	0.65086301	-0.8841035	0.64429799	-0.0093445	-0.0023626
122	0.28675207	0.20666001	-0.1620086	0.65086301	-0.8841035	0.64429799	-0.0093445	-0.0023626
122.1	0.28649479	0.20679998	-0.1625497	0.65085448	-0.8841092	0.64438254	-0.009998	-0.0021028
122.2	0.28649479	0.20679998	-0.1625497	0.65085448	-0.8841092	0.64438254	-0.009998	-0.0021028
122.3	0.28638662	0.20634094	-0.1626994	0.65095747	-0.884123	0.64469296	-0.0102017	-0.0023656
122.4	0.28638662	0.20634094	-0.1626994	0.65095747	-0.884123	0.64469296	-0.0102017	-0.0023656
122.5	0.28638662	0.20634094	-0.1626994	0.65095747	-0.884123	0.64469296	-0.0102017	-0.0023656
122.6	0.28638662	0.20634094	-0.1626994	0.65095747	-0.884123	0.64469296	-0.0102017	-0.0023656
122.7	0.28638662	0.20634094	-0.1626994	0.65095747	-0.884123	0.64469296	-0.0102017	-0.0023656
122.8	0.28642964	0.20609485	-0.16189	0.65096451	-0.8839653	0.64484158	-0.0107085	-0.0030329
122.9	0.28642964	0.20609485	-0.16189	0.65096451	-0.8839653	0.64484158	-0.0107085	-0.0030329
123	0.28642964	0.20609485	-0.16189	0.65096451	-0.8839653	0.64484158	-0.0107085	-0.0030329
123.1	0.28642964	0.20609485	-0.16189	0.65096451	-0.8839653	0.64484158	-0.0107085	-0.0030329
123.2	0.28642964	0.20609485	-0.16189	0.65096451	-0.8839653	0.64484158	-0.0107085	-0.0030329
123.3	0.28642964	0.20609485	-0.16189	0.65096451	-0.8839653	0.64484158	-0.0107085	-0.0030329
123.4	0.28642964	0.20609485	-0.16189	0.65096451	-0.8839653	0.64484158	-0.0107085	-0.0030329
123.5	0.28642964	0.20609485	-0.16189	0.65096451	-0.8839653	0.64484158	-0.0107085	-0.0030329
123.6	0.28642964	0.20609485	-0.16189	0.65096451	-0.8839653	0.64484158	-0.0107085	-0.0030329
123.7	0.28642964	0.20609485	-0.16189	0.65096451	-0.8839653	0.64484158	-0.0107085	-0.0030329
123.8	0.28642964	0.20609485	-0.16189	0.65096451	-0.8839653	0.64484158	-0.0107085	-0.0030329
123.9	0.28642964	0.20609485	-0.16189	0.65096451	-0.8839653	0.64484158	-0.0107085	-0.0030329
124	0.28642964	0.20609485	-0.16189	0.65096451	-0.8839653	0.64484158	-0.0107085	-0.0030329
124.1	0.28642964	0.20609485	-0.16189	0.65096451	-0.8839653	0.64484158	-0.0107085	-0.0030329
124.2	0.28642964	0.20609485	-0.16189	0.65096451	-0.8839653	0.64484158	-0.0107085	-0.0030329
124.3	0.28637869	0.20602208	-0.1616048	0.65135238	-0.8841156	0.64491621	-0.010824	-0.0026779
124.4	0.28637869	0.20602208	-0.1616048	0.65135238	-0.8841156	0.64491621	-0.010824	-0.0026779
124.5	0.28637869	0.20602208	-0.1616048	0.65135238	-0.8841156	0.64491621	-0.010824	-0.0026779
124.6	0.28631194	0.20570293	-0.1619366	0.65148534	-0.8841138	0.64492546	-0.0105823	-0.0027287
124.7	0.28631194	0.20570293	-0.1619366	0.65148534	-0.8841138	0.64492546	-0.0105823	-0.0027287
124.8	0.28631194	0.20570293	-0.1619366	0.65148534	-0.8841138	0.64492546	-0.0105823	-0.0027287
124.9	0.28631194	0.20570293	-0.1619366	0.65148534	-0.8841138	0.64492546	-0.0105823	-0.0027287
125	0.28631194	0.20570293	-0.1619366	0.65148534	-0.8841138	0.64492546	-0.0105823	-0.0027287
125.1	0.28631194	0.20570293	-0.1619366	0.65148534	-0.8841138	0.64492546	-0.0105823	-0.0027287
125.2	0.28595114	0.20583211	-0.1618965	0.65147352	-0.8841904	0.64484664	-0.0105112	-0.0029816
125.3	0.28595114	0.20583211	-0.1618965	0.65147352	-0.8841904	0.64484664	-0.0105112	-0.0029816
125.4	0.28595114	0.20583211	-0.1618965	0.65147352	-0.8841904	0.64484664	-0.0105112	-0.0029816
125.5	0.28595114	0.20583211	-0.1618965	0.65147352	-0.8841904	0.64484664	-0.0105112	-0.0029816
125.6	0.28595114	0.20583211	-0.1618965	0.65147352	-0.8841904	0.64484664	-0.0105112	-0.0029816
125.7	0.28595114	0.20583211	-0.1618965	0.65147352	-0.8841904	0.64484664	-0.0105112	-0.0029816
125.8	0.28595114	0.20583211	-0.1618965	0.65147352	-0.8841904	0.64484664	-0.0105112	-0.0029816
125.9	0.28595114	0.20583211	-0.1618965	0.65147352	-0.8841904	0.64484664	-0.0105112	-0.0029816
126	0.28595114	0.20583211	-0.1618965	0.65147352	-0.8841904	0.64484664	-0.0105112	-0.0029816
126.1	0.28595114	0.20583211	-0.1618965	0.65147352	-0.8841904	0.64484664	-0.0105112	-0.0029816
126.2	0.28595114	0.20583211	-0.1618965	0.65147352	-0.8841904	0.64484664	-0.0105112	-0.0029816
126.3	0.28587039	0.20636302	-0.1618143	0.65139853	-0.8843171	0.64466736	-0.0103228	-0.0027432
126.4	0.28587039	0.20636302	-0.1618143	0.65139853	-0.8843171	0.64466736	-0.0103228	-0.0027432
126.5	0.28587039	0.20636302	-0.1618143	0.65139853	-0.8843171	0.64466736	-0.0103228	-0.0027432
126.6	0.28559944	0.20641904	-0.1613443	0.65143554	-0.884245	0.64473483	-0.0105854	-0.0025381
126.7	0.28559944	0.20641904	-0.1613443	0.65143554	-0.884245	0.64473483	-0.0105854	-0.0025381
126.8	0.28559944	0.20641904	-0.1613443	0.65143554	-0.884245	0.64473483	-0.0105854	-0.0025381
126.9	0.28559944	0.20641904	-0.1613443	0.65143554	-0.884245	0.64473483	-0.0105854	-0.0025381
127	0.28559944	0.20641904	-0.1613443	0.65143554	-0.884245	0.64473483	-0.0105854	-0.0025381
127.1	0.28563359	0.20598339	-0.1615976	0.65190598	-0.8843547	0.64497832	-0.0107065	-0.0019315
127.2	0.28563359	0.20598339	-0.1615976	0.65190598	-0.8843547	0.64497832	-0.0107065	-0.0019315
127.3	0.28563359	0.20598339	-0.1615976	0.65190598	-0.8843547	0.64497832	-0.0107065	-0.0019315
127.4	0.28563359	0.20598339	-0.1615976	0.65190598	-0.8843547	0.64497832	-0.0107065	-0.0019315
127.5	0.28563359	0.20598339	-0.1615976	0.65190598	-0.8843547	0.64497832	-0.0107065	-0.0019315
127.6	0.28563359	0.20598339	-0.1615976	0.65190598	-0.8843547	0.64497832	-0.0107065	-0.0019315
127.7	0.28563359	0.20598339	-0.1615976	0.65190598	-0.8843547	0.64497832	-0.0107065	-0.0019315
127.8	0.28563359	0.20598339	-0.1615976	0.65190598	-0.8843547	0.64497832	-0.0107065	-0.0019315
127.9	0.28563359	0.20598339	-0.1615976	0.65190598	-0.8843547	0.64497832	-0.0107065	-0.0019315
128	0.28563359	0.20598339	-0.1615976	0.65190598	-0.8843547	0.64497832	-0.0107065	-0.0019315
128.1	0.28563359	0.20598339	-0.1615976	0.65190598	-0.8843547	0.64497832	-0.0107065	-0.0019315
128.2	0.28563359	0.20598339	-0.1615976	0.65190598	-0.8843547	0.64497832	-0.0107065	-0.0019315
128.3	0.28563359	0.20598339	-0.1615976	0.65190598	-0.8843547	0.64497832	-0.0107065	-0.0019315
128.4	0.28563359	0.20598339	-0.1615976	0.65190598	-0.8843547	0.64497832	-0.0107065	-0.0019315
128.5	0.28563359	0.20598339	-0.1615976	0.65190598	-0.8843547	0.64497832	-0.0107065	-0.0019315
128.6	0.28563359	0.20598339	-0.1615976	0.65190598	-0.8843547	0.64497832	-0.0107065	-0.0019315
128.7	0.28563359	0.20598339	-0.1615976	0.65190598	-0.8843547	0.64497832	-0.0107065	-0.0019315
128.8	0.28563359	0.20598339	-0.1615976	0.65190598	-0.8843547	0.64497832	-0.0107065	-0.0019315
128.9	0.28563359	0.20598339	-0.1615976	0.65190598	-0.8843547	0.64497832	-0.0107065	-0.0019315
129	0.28563359	0.20598339	-0.1615976	0.65190598	-0.8843547	0.64497832	-0.0107065	-0.0019315
129.1	0.28563359	0.20598339	-0.1615976	0.65190598	-0.8843547	0.64497832	-0.0107065	-0.0019315
129.2	0.28563359	0.20598339	-0.1615976	0.65190598	-0.8843547	0.64497832	-0.0107065	-0.0019315
129.3	0.28535528	0.20638207	-0.1616051	0.65209859	-0.8844419	0.64493143	-0.0105337	-0.0018682
129.4	0.28515118	0.20589681	-0.1615785	0.65192484	-0.8844156	0.64481922	-0.0102458	-0.0021848
129.5	0.28515118	0.20589681	-0.1615785	0.65192484	-0.8844156	0.64481922	-0.0102458	-0.0021848
129.6	0.28515118	0.20589681	-0.1615785	0.65192484	-0.8844156	0.64481922	-0.0102458	-0.0021848
129.7	0.28515118	0.20589681	-0.1615785	0.65192484	-0.8844156	0.64481922	-0.0102458	-0.0021848
129.8	0.28515118	0.20589681	-0.1615785	0.65192484	-0.8844156	0.64481922	-0.0102458	-0.0021848
129.9	0.28515118	0.20589681	-0.1615785	0.65192484	-0.8844156	0.64481922	-0.0102458	-0.0021848
130	0.28515118	0.20589681	-0.1615785	0.65192484	-0.8844156	0.64481922	-0.0102458	-0.0021848
130.1	0.28515118	0.20589681	-0.1615785	0.65192484	-0.8844156	0.64481922	-0.0102458	-0.0021848
130.2	0.28515118	0.20589681	-0.1615785	0.65192484	-0.8844156	0.64481922	-0.0102458	-0.0021848
130.3	0.28515118	0.20589681	-0.1615785	0.65192484	-0.8844156	0.64481922	-0.0102458	-0.0021848
130.4	0.28515118	0.20589681	-0.1615785	0.65192484	-0.8844156	0.64481922	-0.0102458	-0.0021848
130.5	0.28515118	0.20589681	-0.1615785	0.65192484	-0.8844156	0.64481922	-0.0102458	-0.0021848
130.6	0.28515118	0.20589681	-0.1615785	0.65192484	-0.8844156	0.64481922	-0.0102458	-0.0021848
130.7	0.28515118	0.20589681	-0.1615785	0.65192484	-0.8844156	0.64481922	-0.0102458	-0.0021848
130.8	0.28515118	0.20589681	-0.1615785	0.65192484	-0.8844156	0.64481922	-0.0102458	-0.0021848
130.9	0.28515118	0.20589681	-0.1615785	0.65192484	-0.8844156	0.64481922	-0.0102458	-0.0021848
131	0.28515118	0.20589681	-0.1615785	0.65192484	-0.8844156	0.64481922	-0.0102458	-0.0021848
131.1	0.28515118	0.20589681	-0.1615785	0.65192484	-0.8844156	0.64481922	-0.0102458	-0.0021848
131.2	0.28515118	0.20589681	-0.1615785	0.65192484	-0.8844156	0.64481922	-0.0102458	-0.0021848
131.3	0.28515118	0.20589681	-0.1615785	0.65192484	-0.8844156	0.64481922	-0.0102458	-0.0021848
131.4	0.28457201	0.20586343	-0.16126	0.65164598	-0.8844713	0.64456519	-0.0106633	-0.0017619
131.5	0.28457201	0.20586343	-0.16126	0.65164598	-0.8844713	0.64456519	-0.0106633	-0.0017619
131.6	0.28457201	0.20586343	-0.16126	0.65164598	-0.8844713	0.64456519	-0.0106633	-0.0017619
131.7	0.28457201	0.20586343	-0.16126	0.65164598	-0.8844713	0.64456519	-0.0106633	-0.0017619
131.8	0.28457201	0.20586343	-0.16126	0.65164598	-0.8844713	0.64456519	-0.0106633	-0.0017619
131.9	0.28457201	0.20586343	-0.16126	0.65164598	-0.8844713	0.64456519	-0.0106633	-0.0017619
132	0.28457201	0.20586343	-0.16126	0.65164598	-0.8844713	0.64456519	-0.0106633	-0.0017619
132.1	0.28457201	0.20586343	-0.16126	0.65164598	-0.8844713	0.64456519	-0.0106633	-0.0017619
132.2	0.28457201	0.20586343	-0.16126	0.65164598	-0.8844713	0.64456519	-0.0106633	-0.0017619
132.3	0.28457201	0.20586343	-0.16126	0.65164598	-0.8844713	0.64456519	-0.0106633	-0.0017619
132.4	0.28457201	0.20586343	-0.16126	0.65164598	-0.8844713	0.64456519	-0.0106633	-0.0017619
132.5	0.28433244	0.20580174	-0.1609205	0.65191942	-0.8845148	0.64463427	-0.0107034	-0.0024423
132.6	0.28433244	0.20580174	-0.1609205	0.65191942	-0.8845148	0.64463427	-0.0107034	-0.0024423
132.7	0.28433244	0.20580174	-0.1609205	0.65191942	-0.8845148	0.64463427	-0.0107034	-0.0024423
132.8	0.28433244	0.20580174	-0.1609205	0.65191942	-0.8845148	0.64463427	-0.0107034	-0.0024423
132.9	0.28433244	0.20580174	-0.1609205	0.65191942	-0.8845148	0.64463427	-0.0107034	-0.0024423
133	0.28393981	0.20608481	-0.1604088	0.65205179	-0.8844672	0.64476952	-0.0104798	-0.0028946
133.1	0.28393981	0.20608481	-0.1604088	0.65205179	-0.8844672	0.64476952	-0.0104798	-0.0028946
133.2	0.28374968	0.20556881	-0.1597855	0.65215638	-0.8844145	0.64446844	-0.0098477	-0.0030353
133.3	0.28304844	0.2054144	-0.1598346	0.65211595	-0.884379	0.6442946	-0.0097393	-0.0025988
133.4	0.28304844	0.2054144	-0.1598346	0.65211595	-0.884379	0.6442946	-0.0097393	-0.0025988
133.5	0.28304844	0.2054144	-0.1598346	0.65211595	-0.884379	0.6442946	-0.0097393	-0.0025988
133.6	0.28272289	0.20555981	-0.1597341	0.65188372	-0.8844406	0.64421147	-0.0102153	-0.0019866
133.7	0.28272289	0.20555981	-0.1597341	0.65188372	-0.8844406	0.64421147	-0.0102153	-0.0019866
133.8	0.28272289	0.20555981	-0.1597341	0.65188372	-0.8844406	0.64421147	-0.0102153	-0.0019866
133.9	0.28272289	0.20555981	-0.1597341	0.65188372	-0.8844406	0.64421147	-0.0102153	-0.0019866
134	0.28272289	0.20555981	-0.1597341	0.65188372	-0.8844406	0.64421147	-0.0102153	-0.0019866
134.1	0.28272289	0.20555981	-0.1597341	0.65188372	-0.8844406	0.64421147	-0.0102153	-0.0019866
134.2	0.28272289	0.20555981	-0.1597341	0.65188372	-0.8844406	0.64421147	-0.0102153	-0.0019866
134.3	0.28272289	0.20555981	-0.1597341	0.65188372	-0.8844406	0.64421147	-0.0102153	-0.0019866
134.4	0.28272289	0.20555981	-0.1597341	0.65188372	-0.8844406	0.64421147	-0.0102153	-0.0019866
134.5	0.28272289	0.20555981	-0.1597341	0.65188372	-0.8844406	0.64421147	-0.0102153	-0.0019866
134.6	0.28272289	0.20555981	-0.1597341	0.65188372	-0.8844406	0.64421147	-0.0102153	-0.0019866
134.7	0.28315126	0.20541966	-0.1597989	0.6522119	-0.8845206	0.64454743	-0.0098941	-0.0015275
134.8	0.28315126	0.20541966	-0.1597989	0.6522119	-0.8845206	0.64454743	-0.0098941	-0.0015275
134.9	0.28315126	0.20541966	-0.1597989	0.6522119	-0.8845206	0.64454743	-0.0098941	-0.0015275
180	0.27518902	0.20811575	-0.1486598	0.6511055	-0.8827259	0.63736938	-0.0128262	0.00120394
180.1	0.27518902	0.20811575	-0.1486598	0.6511055	-0.8827259	0.63736938	-0.0128262	0.00120394
180.2	0.27518902	0.20811575	-0.1486598	0.6511055	-0.8827259	0.63736938	-0.0128262	0.00120394
180.3	0.27518902	0.20811575	-0.1486598	0.6511055	-0.8827259	0.63736938	-0.0128262	0.00120394
180.4	0.27518902	0.20811575	-0.1486598	0.6511055	-0.8827259	0.63736938	-0.0128262	0.00120394
180.5	0.27518902	0.20811575	-0.1486598	0.6511055	-0.8827259	0.63736938	-0.0128262	0.00120394
135.3	0.28315126	0.20541966	-0.1597989	0.6522119	-0.8845206	0.64454743	-0.0098941	-0.0015275
135.4	0.28315126	0.20541966	-0.1597989	0.6522119	-0.8845206	0.64454743	-0.0098941	-0.0015275
135.5	0.28269358	0.2061887	-0.1590604	0.65267648	-0.8844875	0.64460512	-0.0102616	-0.0015599
135.6	0.28263123	0.2062869	-0.1585465	0.65258817	-0.884372	0.64457503	-0.0098785	-0.0013529
135.7	0.28263123	0.2062869	-0.1585465	0.65258817	-0.884372	0.64457503	-0.0098785	-0.0013529
135.8	0.28263123	0.2062869	-0.1585465	0.65258817	-0.884372	0.64457503	-0.0098785	-0.0013529
135.9	0.2819986	0.20610664	-0.1587047	0.65261488	-0.8843226	0.64437369	-0.0095793	-0.0007291
136	0.2819986	0.20610664	-0.1587047	0.65261488	-0.8843226	0.64437369	-0.0095793	-0.0007291
136.1	0.2819986	0.20610664	-0.1587047	0.65261488	-0.8843226	0.64437369	-0.0095793	-0.0007291
136.2	0.2819986	0.20610664	-0.1587047	0.65261488	-0.8843226	0.64437369	-0.0095793	-0.0007291
136.3	0.28197518	0.20606162	-0.1581268	0.65282304	-0.8843222	0.64445031	-0.0090202	-0.0008973
136.4	0.28197518	0.20606162	-0.1581268	0.65282304	-0.8843222	0.64445031	-0.0090202	-0.0008973
136.5	0.28197518	0.20606162	-0.1581268	0.65282304	-0.8843222	0.64445031	-0.0090202	-0.0008973
136.6	0.28197518	0.20606162	-0.1581268	0.65282304	-0.8843222	0.64445031	-0.0090202	-0.0008973
136.7	0.28197518	0.20606162	-0.1581268	0.65282304	-0.8843222	0.64445031	-0.0090202	-0.0008973
136.8	0.28197518	0.20606162	-0.1581268	0.65282304	-0.8843222	0.64445031	-0.0090202	-0.0008973
136.9	0.28197518	0.20606162	-0.1581268	0.65282304	-0.8843222	0.64445031	-0.0090202	-0.0008973
137	0.28197518	0.20606162	-0.1581268	0.65282304	-0.8843222	0.64445031	-0.0090202	-0.0008973
137.1	0.28197518	0.20606162	-0.1581268	0.65282304	-0.8843222	0.64445031	-0.0090202	-0.0008973
137.2	0.28197518	0.20606162	-0.1581268	0.65282304	-0.8843222	0.64445031	-0.0090202	-0.0008973
137.3	0.28197518	0.20606162	-0.1581268	0.65282304	-0.8843222	0.64445031	-0.0090202	-0.0008973
137.4	0.28197518	0.20606162	-0.1581268	0.65282304	-0.8843222	0.64445031	-0.0090202	-0.0008973
137.5	0.28197518	0.20606162	-0.1581268	0.65282304	-0.8843222	0.64445031	-0.0090202	-0.0008973
137.6	0.28197518	0.20606162	-0.1581268	0.65282304	-0.8843222	0.64445031	-0.0090202	-0.0008973
137.7	0.28197518	0.20606162	-0.1581268	0.65282304	-0.8843222	0.64445031	-0.0090202	-0.0008973
137.8	0.28197518	0.20606162	-0.1581268	0.65282304	-0.8843222	0.64445031	-0.0090202	-0.0008973
137.9	0.28197518	0.20606162	-0.1581268	0.65282304	-0.8843222	0.64445031	-0.0090202	-0.0008973
138	0.28197518	0.20606162	-0.1581268	0.65282304	-0.8843222	0.64445031	-0.0090202	-0.0008973
138.1	0.28197518	0.20606162	-0.1581268	0.65282304	-0.8843222	0.64445031	-0.0090202	-0.0008973
138.2	0.28197518	0.20606162	-0.1581268	0.65282304	-0.8843222	0.64445031	-0.0090202	-0.0008973
138.3	0.28197518	0.20606162	-0.1581268	0.65282304	-0.8843222	0.64445031	-0.0090202	-0.0008973
138.4	0.28197518	0.20606162	-0.1581268	0.65282304	-0.8843222	0.64445031	-0.0090202	-0.0008973
138.5	0.28197518	0.20606162	-0.1581268	0.65282304	-0.8843222	0.64445031	-0.0090202	-0.0008973
138.6	0.28197518	0.20606162	-0.1581268	0.65282304	-0.8843222	0.64445031	-0.0090202	-0.0008973
138.7	0.28197518	0.20606162	-0.1581268	0.65282304	-0.8843222	0.64445031	-0.0090202	-0.0008973
138.8	0.28197518	0.20606162	-0.1581268	0.65282304	-0.8843222	0.64445031	-0.0090202	-0.0008973
138.9	0.28231106	0.20585574	-0.1583716	0.65281242	-0.8842714	0.64445135	-0.0098446	-0.0004105
139	0.28232114	0.2055351	-0.1584849	0.65313364	-0.8843703	0.64469202	-0.0101772	-0.0003299
139.1	0.28232114	0.2055351	-0.1584849	0.65313364	-0.8843703	0.64469202	-0.0101772	-0.0003299
139.2	0.28232114	0.2055351	-0.1584849	0.65313364	-0.8843703	0.64469202	-0.0101772	-0.0003299
139.3	0.28232114	0.2055351	-0.1584849	0.65313364	-0.8843703	0.64469202	-0.0101772	-0.0003299
139.4	0.28232114	0.2055351	-0.1584849	0.65313364	-0.8843703	0.64469202	-0.0101772	-0.0003299
139.5	0.28232114	0.2055351	-0.1584849	0.65313364	-0.8843703	0.64469202	-0.0101772	-0.0003299
139.6	0.28232114	0.2055351	-0.1584849	0.65313364	-0.8843703	0.64469202	-0.0101772	-0.0003299
139.7	0.28232114	0.2055351	-0.1584849	0.65313364	-0.8843703	0.64469202	-0.0101772	-0.0003299
139.8	0.28232114	0.2055351	-0.1584849	0.65313364	-0.8843703	0.64469202	-0.0101772	-0.0003299
139.9	0.28232114	0.2055351	-0.1584849	0.65313364	-0.8843703	0.64469202	-0.0101772	-0.0003299
140	0.28232114	0.2055351	-0.1584849	0.65313364	-0.8843703	0.64469202	-0.0101772	-0.0003299
140.1	0.28232114	0.2055351	-0.1584849	0.65313364	-0.8843703	0.64469202	-0.0101772	-0.0003299
140.2	0.28232114	0.2055351	-0.1584849	0.65313364	-0.8843703	0.64469202	-0.0101772	-0.0003299
140.3	0.28232114	0.2055351	-0.1584849	0.65313364	-0.8843703	0.64469202	-0.0101772	-0.0003299
140.4	0.28232114	0.2055351	-0.1584849	0.65313364	-0.8843703	0.64469202	-0.0101772	-0.0003299
140.5	0.28232114	0.2055351	-0.1584849	0.65313364	-0.8843703	0.64469202	-0.0101772	-0.0003299
140.6	0.28232114	0.2055351	-0.1584849	0.65313364	-0.8843703	0.64469202	-0.0101772	-0.0003299
140.7	0.28232114	0.2055351	-0.1584849	0.65313364	-0.8843703	0.64469202	-0.0101772	-0.0003299
140.8	0.28232114	0.2055351	-0.1584849	0.65313364	-0.8843703	0.64469202	-0.0101772	-0.0003299
140.9	0.28232114	0.2055351	-0.1584849	0.65313364	-0.8843703	0.64469202	-0.0101772	-0.0003299
141	0.28232114	0.2055351	-0.1584849	0.65313364	-0.8843703	0.64469202	-0.0101772	-0.0003299
141.1	0.2821468	0.20566084	-0.1581114	0.65274182	-0.8842709	0.64434341	-0.0100841	-0.0003268
141.2	0.28193117	0.20612766	-0.157673	0.65238218	-0.884187	0.64394065	-0.0104388	-0.00028
141.3	0.28142328	0.20590737	-0.1569982	0.65193452	-0.8839618	0.64373928	-0.0103687	-9.42E-05
141.4	0.28105104	0.20650208	-0.1570118	0.65202514	-0.8840313	0.64344407	-0.0102243	-1.33E-05
141.5	0.28105104	0.20650208	-0.1570118	0.65202514	-0.8840313	0.64344407	-0.0102243	-1.33E-05
141.6	0.28105104	0.20650208	-0.1570118	0.65202514	-0.8840313	0.64344407	-0.0102243	-1.33E-05
141.7	0.28085521	0.20597236	-0.1572716	0.65201519	-0.8840372	0.6434915	-0.0099682	-8.52E-05
141.8	0.28085521	0.20597236	-0.1572716	0.65201519	-0.8840372	0.6434915	-0.0099682	-8.52E-05
141.9	0.28085521	0.20597236	-0.1572716	0.65201519	-0.8840372	0.6434915	-0.0099682	-8.52E-05
142	0.28085521	0.20597236	-0.1572716	0.65201519	-0.8840372	0.6434915	-0.0099682	-8.52E-05
142.1	0.28085521	0.20597236	-0.1572716	0.65201519	-0.8840372	0.6434915	-0.0099682	-8.52E-05
142.2	0.28085521	0.20597236	-0.1572716	0.65201519	-0.8840372	0.6434915	-0.0099682	-8.52E-05
142.3	0.28085521	0.20597236	-0.1572716	0.65201519	-0.8840372	0.6434915	-0.0099682	-8.52E-05
142.4	0.28085521	0.20597236	-0.1572716	0.65201519	-0.8840372	0.6434915	-0.0099682	-8.52E-05
142.5	0.28039062	0.2057801	-0.1571272	0.65207938	-0.8840356	0.64331146	-0.0098661	0.00051857
142.6	0.28039062	0.2057801	-0.1571272	0.65207938	-0.8840356	0.64331146	-0.0098661	0.00051857
142.7	0.28015983	0.20600372	-0.1568304	0.65239877	-0.8841287	0.64333034	-0.0104563	0.00118939
142.8	0.28015983	0.20600372	-0.1568304	0.65239877	-0.8841287	0.64333034	-0.0104563	0.00118939
142.9	0.28015983	0.20600372	-0.1568304	0.65239877	-0.8841287	0.64333034	-0.0104563	0.00118939
143	0.28037123	0.20620509	-0.1565295	0.65236542	-0.8840408	0.64332775	-0.0106553	0.001533
143.1	0.28037123	0.20620509	-0.1565295	0.65236542	-0.8840408	0.64332775	-0.0106553	0.001533
143.2	0.28037123	0.20620509	-0.1565295	0.65236542	-0.8840408	0.64332775	-0.0106553	0.001533
143.3	0.28037123	0.20620509	-0.1565295	0.65236542	-0.8840408	0.64332775	-0.0106553	0.001533
143.4	0.28037123	0.20620509	-0.1565295	0.65236542	-0.8840408	0.64332775	-0.0106553	0.001533
143.5	0.28037123	0.20620509	-0.1565295	0.65236542	-0.8840408	0.64332775	-0.0106553	0.001533
143.6	0.28037123	0.20620509	-0.1565295	0.65236542	-0.8840408	0.64332775	-0.0106553	0.001533
143.7	0.28037123	0.20620509	-0.1565295	0.65236542	-0.8840408	0.64332775	-0.0106553	0.001533
143.8	0.28037123	0.20620509	-0.1565295	0.65236542	-0.8840408	0.64332775	-0.0106553	0.001533
143.9	0.28037123	0.20620509	-0.1565295	0.65236542	-0.8840408	0.64332775	-0.0106553	0.001533
144	0.28037123	0.20620509	-0.1565295	0.65236542	-0.8840408	0.64332775	-0.0106553	0.001533
144.1	0.28037123	0.20620509	-0.1565295	0.65236542	-0.8840408	0.64332775	-0.0106553	0.001533
144.2	0.28047989	0.20594356	-0.1565039	0.65234393	-0.884027	0.64329238	-0.0110437	0.00171509
144.3	0.28047989	0.20594356	-0.1565039	0.65234393	-0.884027	0.64329238	-0.0110437	0.00171509
144.4	0.28047989	0.20594356	-0.1565039	0.65234393	-0.884027	0.64329238	-0.0110437	0.00171509
144.5	0.28047989	0.20594356	-0.1565039	0.65234393	-0.884027	0.64329238	-0.0110437	0.00171509
144.6	0.28047989	0.20594356	-0.1565039	0.65234393	-0.884027	0.64329238	-0.0110437	0.00171509
144.7	0.28047989	0.20594356	-0.1565039	0.65234393	-0.884027	0.64329238	-0.0110437	0.00171509
144.8	0.28047989	0.20594356	-0.1565039	0.65234393	-0.884027	0.64329238	-0.0110437	0.00171509
144.9	0.28047989	0.20594356	-0.1565039	0.65234393	-0.884027	0.64329238	-0.0110437	0.00171509
145	0.28047989	0.20594356	-0.1565039	0.65234393	-0.884027	0.64329238	-0.0110437	0.00171509
145.1	0.27971225	0.20523392	-0.156517	0.65194291	-0.8839404	0.64300706	-0.0110754	0.00136213
145.2	0.27971225	0.20523392	-0.156517	0.65194291	-0.8839404	0.64300706	-0.0110754	0.00136213
145.3	0.27971225	0.20523392	-0.156517	0.65194291	-0.8839404	0.64300706	-0.0110754	0.00136213
145.4	0.27989876	0.20494648	-0.1561069	0.65235318	-0.8839863	0.64338192	-0.0111649	0.00133352
145.5	0.27989876	0.20494648	-0.1561069	0.65235318	-0.8839863	0.64338192	-0.0111649	0.00133352
145.6	0.27989876	0.20494648	-0.1561069	0.65235318	-0.8839863	0.64338192	-0.0111649	0.00133352
145.7	0.27989876	0.20494648	-0.1561069	0.65235318	-0.8839863	0.64338192	-0.0111649	0.00133352
145.8	0.27989876	0.20494648	-0.1561069	0.65235318	-0.8839863	0.64338192	-0.0111649	0.00133352
145.9	0.27989876	0.20494648	-0.1561069	0.65235318	-0.8839863	0.64338192	-0.0111649	0.00133352
146	0.27989876	0.20494648	-0.1561069	0.65235318	-0.8839863	0.64338192	-0.0111649	0.00133352
146.1	0.27989876	0.20494648	-0.1561069	0.65235318	-0.8839863	0.64338192	-0.0111649	0.00133352
146.2	0.27989876	0.20494648	-0.1561069	0.65235318	-0.8839863	0.64338192	-0.0111649	0.00133352
146.3	0.27989876	0.20494648	-0.1561069	0.65235318	-0.8839863	0.64338192	-0.0111649	0.00133352
146.4	0.27987199	0.20491149	-0.1558373	0.65247892	-0.88398	0.6432333	-0.011457	0.00167854
146.5	0.27987199	0.20491149	-0.1558373	0.65247892	-0.88398	0.6432333	-0.011457	0.00167854
146.6	0.27987199	0.20491149	-0.1558373	0.65247892	-0.88398	0.6432333	-0.011457	0.00167854
146.7	0.27987199	0.20491149	-0.1558373	0.65247892	-0.88398	0.6432333	-0.011457	0.00167854
146.8	0.27987199	0.20491149	-0.1558373	0.65247892	-0.88398	0.6432333	-0.011457	0.00167854
146.9	0.27987199	0.20491149	-0.1558373	0.65247892	-0.88398	0.6432333	-0.011457	0.00167854
147	0.27987199	0.20491149	-0.1558373	0.65247892	-0.88398	0.6432333	-0.011457	0.00167854
147.1	0.27987199	0.20491149	-0.1558373	0.65247892	-0.88398	0.6432333	-0.011457	0.00167854
147.2	0.27987199	0.20491149	-0.1558373	0.65247892	-0.88398	0.6432333	-0.011457	0.00167854
147.3	0.27987199	0.20491149	-0.1558373	0.65247892	-0.88398	0.6432333	-0.011457	0.00167854
147.4	0.27987199	0.20491149	-0.1558373	0.65247892	-0.88398	0.6432333	-0.011457	0.00167854
147.5	0.27987199	0.20491149	-0.1558373	0.65247892	-0.88398	0.6432333	-0.011457	0.00167854
147.6	0.27987199	0.20491149	-0.1558373	0.65247892	-0.88398	0.6432333	-0.011457	0.00167854
147.7	0.27987199	0.20491149	-0.1558373	0.65247892	-0.88398	0.6432333	-0.011457	0.00167854
147.8	0.27987199	0.20491149	-0.1558373	0.65247892	-0.88398	0.6432333	-0.011457	0.00167854
147.9	0.27987199	0.20491149	-0.1558373	0.65247892	-0.88398	0.6432333	-0.011457	0.00167854
148	0.27987199	0.20491149	-0.1558373	0.65247892	-0.88398	0.6432333	-0.011457	0.00167854
148.1	0.27987199	0.20491149	-0.1558373	0.65247892	-0.88398	0.6432333	-0.011457	0.00167854
148.2	0.27987199	0.20491149	-0.1558373	0.65247892	-0.88398	0.6432333	-0.011457	0.00167854
148.3	0.27982283	0.20499177	-0.1554292	0.65245645	-0.8839628	0.64308151	-0.0119602	0.00149722
148.4	0.27982283	0.20499177	-0.1554292	0.65245645	-0.8839628	0.64308151	-0.0119602	0.00149722
148.5	0.27982283	0.20499177	-0.1554292	0.65245645	-0.8839628	0.64308151	-0.0119602	0.00149722
148.6	0.27982283	0.20499177	-0.1554292	0.65245645	-0.8839628	0.64308151	-0.0119602	0.00149722
148.7	0.27982283	0.20499177	-0.1554292	0.65245645	-0.8839628	0.64308151	-0.0119602	0.00149722
148.8	0.27982283	0.20499177	-0.1554292	0.65245645	-0.8839628	0.64308151	-0.0119602	0.00149722
148.9	0.27982283	0.20499177	-0.1554292	0.65245645	-0.8839628	0.64308151	-0.0119602	0.00149722
149	0.27982283	0.20499177	-0.1554292	0.65245645	-0.8839628	0.64308151	-0.0119602	0.00149722
149.1	0.27976474	0.20539203	-0.1549096	0.65211286	-0.8838464	0.64268884	-0.0121304	0.00123471
149.2	0.27976474	0.20539203	-0.1549096	0.65211286	-0.8838464	0.64268884	-0.0121304	0.00123471
149.3	0.27976474	0.20539203	-0.1549096	0.65211286	-0.8838464	0.64268884	-0.0121304	0.00123471
149.4	0.27976474	0.20539203	-0.1549096	0.65211286	-0.8838464	0.64268884	-0.0121304	0.00123471
149.5	0.27976474	0.20539203	-0.1549096	0.65211286	-0.8838464	0.64268884	-0.0121304	0.00123471
149.6	0.27976474	0.20539203	-0.1549096	0.65211286	-0.8838464	0.64268884	-0.0121304	0.00123471
149.7	0.27976474	0.20539203	-0.1549096	0.65211286	-0.8838464	0.64268884	-0.0121304	0.00123471
149.8	0.27976474	0.20539203	-0.1549096	0.65211286	-0.8838464	0.64268884	-0.0121304	0.00123471
149.9	0.27976474	0.20539203	-0.1549096	0.65211286	-0.8838464	0.64268884	-0.0121304	0.00123471
195	0.27365656	0.20709093	-0.1471368	0.65174161	-0.8828794	0.63593498	-0.0137332	0.00216162
195.1	0.27365656	0.20709093	-0.1471368	0.65174161	-0.8828794	0.63593498	-0.0137332	0.00216162
195.2	0.27365656	0.20709093	-0.1471368	0.65174161	-0.8828794	0.63593498	-0.0137332	0.00216162
195.3	0.27365656	0.20709093	-0.1471368	0.65174161	-0.8828794	0.63593498	-0.0137332	0.00216162
195.4	0.27365656	0.20709093	-0.1471368	0.65174161	-0.8828794	0.63593498	-0.0137332	0.00216162
195.5	0.27365656	0.20709093	-0.1471368	0.65174161	-0.8828794	0.63593498	-0.0137332	0.00216162
150.3	0.27961075	0.20565183	-0.1544966	0.65189045	-0.8837558	0.64232352	-0.0118595	0.00125961
150.4	0.27961075	0.20565183	-0.1544966	0.65189045	-0.8837558	0.64232352	-0.0118595	0.00125961
150.5	0.27961075	0.20565183	-0.1544966	0.65189045	-0.8837558	0.64232352	-0.0118595	0.00125961
150.6	0.27961075	0.20565183	-0.1544966	0.65189045	-0.8837558	0.64232352	-0.0118595	0.00125961
150.7	0.27961075	0.20565183	-0.1544966	0.65189045	-0.8837558	0.64232352	-0.0118595	0.00125961
150.8	0.27961075	0.20565183	-0.1544966	0.65189045	-0.8837558	0.64232352	-0.0118595	0.00125961
150.9	0.27961075	0.20565183	-0.1544966	0.65189045	-0.8837558	0.64232352	-0.0118595	0.00125961
151	0.27961075	0.20565183	-0.1544966	0.65189045	-0.8837558	0.64232352	-0.0118595	0.00125961
151.1	0.27961075	0.20565183	-0.1544966	0.65189045	-0.8837558	0.64232352	-0.0118595	0.00125961
151.2	0.27921022	0.20521647	-0.1541833	0.65196198	-0.8837309	0.6421974	-0.0111544	0.00113536
151.3	0.27921022	0.20521647	-0.1541833	0.65196198	-0.8837309	0.6421974	-0.0111544	0.00113536
151.4	0.27945482	0.20566706	-0.1535525	0.65217894	-0.8837278	0.64226485	-0.0112789	0.00091084
151.5	0.2791298	0.20583524	-0.1529345	0.65166401	-0.8835447	0.64153178	-0.0115044	0.00098397
151.6	0.2791298	0.20583524	-0.1529345	0.65166401	-0.8835447	0.64153178	-0.0115044	0.00098397
151.7	0.2791298	0.20583524	-0.1529345	0.65166401	-0.8835447	0.64153178	-0.0115044	0.00098397
151.8	0.2791298	0.20583524	-0.1529345	0.65166401	-0.8835447	0.64153178	-0.0115044	0.00098397
151.9	0.2791298	0.20583524	-0.1529345	0.65166401	-0.8835447	0.64153178	-0.0115044	0.00098397
152	0.2791298	0.20583524	-0.1529345	0.65166401	-0.8835447	0.64153178	-0.0115044	0.00098397
152.1	0.2791298	0.20583524	-0.1529345	0.65166401	-0.8835447	0.64153178	-0.0115044	0.00098397
152.2	0.2791298	0.20583524	-0.1529345	0.65166401	-0.8835447	0.64153178	-0.0115044	0.00098397
152.3	0.2791298	0.20583524	-0.1529345	0.65166401	-0.8835447	0.64153178	-0.0115044	0.00098397
152.4	0.2791298	0.20583524	-0.1529345	0.65166401	-0.8835447	0.64153178	-0.0115044	0.00098397
152.5	0.2791298	0.20583524	-0.1529345	0.65166401	-0.8835447	0.64153178	-0.0115044	0.00098397
152.6	0.2791298	0.20583524	-0.1529345	0.65166401	-0.8835447	0.64153178	-0.0115044	0.00098397
152.7	0.2791298	0.20583524	-0.1529345	0.65166401	-0.8835447	0.64153178	-0.0115044	0.00098397
152.8	0.2791298	0.20583524	-0.1529345	0.65166401	-0.8835447	0.64153178	-0.0115044	0.00098397
152.9	0.27918165	0.20609758	-0.152734	0.6513179	-0.8834472	0.64128584	-0.0116806	0.00076833
153	0.27918165	0.20609758	-0.152734	0.6513179	-0.8834472	0.64128584	-0.0116806	0.00076833
153.1	0.27918165	0.20609758	-0.152734	0.6513179	-0.8834472	0.64128584	-0.0116806	0.00076833
153.2	0.27918165	0.20609758	-0.152734	0.6513179	-0.8834472	0.64128584	-0.0116806	0.00076833
153.3	0.27918165	0.20609758	-0.152734	0.6513179	-0.8834472	0.64128584	-0.0116806	0.00076833
153.4	0.27918165	0.20609758	-0.152734	0.6513179	-0.8834472	0.64128584	-0.0116806	0.00076833
153.5	0.27918165	0.20609758	-0.152734	0.6513179	-0.8834472	0.64128584	-0.0116806	0.00076833
153.6	0.27887132	0.20661123	-0.1525088	0.65121231	-0.8834033	0.64086495	-0.0114115	0.00093468
153.7	0.27887132	0.20661123	-0.1525088	0.65121231	-0.8834033	0.64086495	-0.0114115	0.00093468
153.8	0.27887132	0.20661123	-0.1525088	0.65121231	-0.8834033	0.64086495	-0.0114115	0.00093468
153.9	0.27887132	0.20661123	-0.1525088	0.65121231	-0.8834033	0.64086495	-0.0114115	0.00093468
154	0.27887132	0.20661123	-0.1525088	0.65121231	-0.8834033	0.64086495	-0.0114115	0.00093468
154.1	0.27887132	0.20661123	-0.1525088	0.65121231	-0.8834033	0.64086495	-0.0114115	0.00093468
154.2	0.27887132	0.20661123	-0.1525088	0.65121231	-0.8834033	0.64086495	-0.0114115	0.00093468
154.3	0.27887132	0.20661123	-0.1525088	0.65121231	-0.8834033	0.64086495	-0.0114115	0.00093468
154.4	0.27887132	0.20661123	-0.1525088	0.65121231	-0.8834033	0.64086495	-0.0114115	0.00093468
154.5	0.27887132	0.20661123	-0.1525088	0.65121231	-0.8834033	0.64086495	-0.0114115	0.00093468
154.6	0.27887132	0.20661123	-0.1525088	0.65121231	-0.8834033	0.64086495	-0.0114115	0.00093468
154.7	0.27887132	0.20661123	-0.1525088	0.65121231	-0.8834033	0.64086495	-0.0114115	0.00093468
154.8	0.27887132	0.20661123	-0.1525088	0.65121231	-0.8834033	0.64086495	-0.0114115	0.00093468
154.9	0.27887132	0.20661123	-0.1525088	0.65121231	-0.8834033	0.64086495	-0.0114115	0.00093468
155	0.27887132	0.20661123	-0.1525088	0.65121231	-0.8834033	0.64086495	-0.0114115	0.00093468
155.1	0.27887132	0.20661123	-0.1525088	0.65121231	-0.8834033	0.64086495	-0.0114115	0.00093468
155.2	0.27887132	0.20661123	-0.1525088	0.65121231	-0.8834033	0.64086495	-0.0114115	0.00093468
155.3	0.27887132	0.20661123	-0.1525088	0.65121231	-0.8834033	0.64086495	-0.0114115	0.00093468
155.4	0.27887132	0.20661123	-0.1525088	0.65121231	-0.8834033	0.64086495	-0.0114115	0.00093468
155.5	0.27823402	0.20659712	-0.1524406	0.65133687	-0.883398	0.64075067	-0.0115161	0.00098023
155.6	0.27823402	0.20659712	-0.1524406	0.65133687	-0.883398	0.64075067	-0.0115161	0.00098023
155.7	0.27823402	0.20659712	-0.1524406	0.65133687	-0.883398	0.64075067	-0.0115161	0.00098023
155.8	0.27823402	0.20659712	-0.1524406	0.65133687	-0.883398	0.64075067	-0.0115161	0.00098023
155.9	0.27823402	0.20659712	-0.1524406	0.65133687	-0.883398	0.64075067	-0.0115161	0.00098023
156	0.27823402	0.20659712	-0.1524406	0.65133687	-0.883398	0.64075067	-0.0115161	0.00098023
156.1	0.27773506	0.20701158	-0.1524473	0.65091975	-0.8832668	0.6401598	-0.0112199	0.00060793
156.2	0.27773506	0.20701158	-0.1524473	0.65091975	-0.8832668	0.6401598	-0.0112199	0.00060793
156.3	0.27773506	0.20701158	-0.1524473	0.65091975	-0.8832668	0.6401598	-0.0112199	0.00060793
156.4	0.27773506	0.20701158	-0.1524473	0.65091975	-0.8832668	0.6401598	-0.0112199	0.00060793
156.5	0.27773506	0.20701158	-0.1524473	0.65091975	-0.8832668	0.6401598	-0.0112199	0.00060793
156.6	0.27773506	0.20701158	-0.1524473	0.65091975	-0.8832668	0.6401598	-0.0112199	0.00060793
156.7	0.27735178	0.20720712	-0.1522055	0.65077542	-0.883182	0.63985076	-0.0115252	0.00096722
156.8	0.27735178	0.20720712	-0.1522055	0.65077542	-0.883182	0.63985076	-0.0115252	0.00096722
156.9	0.27766836	0.20755637	-0.151914	0.65067203	-0.8831331	0.63971695	-0.0119382	0.00089348
157	0.27766836	0.20755637	-0.151914	0.65067203	-0.8831331	0.63971695	-0.0119382	0.00089348
157.1	0.27766836	0.20755637	-0.151914	0.65067203	-0.8831331	0.63971695	-0.0119382	0.00089348
157.2	0.27766836	0.20755637	-0.151914	0.65067203	-0.8831331	0.63971695	-0.0119382	0.00089348
157.3	0.27766836	0.20755637	-0.151914	0.65067203	-0.8831331	0.63971695	-0.0119382	0.00089348
157.4	0.27766836	0.20755637	-0.151914	0.65067203	-0.8831331	0.63971695	-0.0119382	0.00089348
157.5	0.27766836	0.20755637	-0.151914	0.65067203	-0.8831331	0.63971695	-0.0119382	0.00089348
157.6	0.27766836	0.20755637	-0.151914	0.65067203	-0.8831331	0.63971695	-0.0119382	0.00089348
157.7	0.27766836	0.20755637	-0.151914	0.65067203	-0.8831331	0.63971695	-0.0119382	0.00089348
157.8	0.27766836	0.20755637	-0.151914	0.65067203	-0.8831331	0.63971695	-0.0119382	0.00089348
157.9	0.27766836	0.20755637	-0.151914	0.65067203	-0.8831331	0.63971695	-0.0119382	0.00089348
158	0.27766836	0.20755637	-0.151914	0.65067203	-0.8831331	0.63971695	-0.0119382	0.00089348
158.1	0.27766836	0.20755637	-0.151914	0.65067203	-0.8831331	0.63971695	-0.0119382	0.00089348
158.2	0.27733887	0.20725959	-0.1514045	0.65074526	-0.8830962	0.6395769	-0.0122496	0.00159033
158.3	0.27733887	0.20725959	-0.1514045	0.65074526	-0.8830962	0.6395769	-0.0122496	0.00159033
158.4	0.27733887	0.20725959	-0.1514045	0.65074526	-0.8830962	0.6395769	-0.0122496	0.00159033
158.5	0.27733887	0.20725959	-0.1514045	0.65074526	-0.8830962	0.6395769	-0.0122496	0.00159033
158.6	0.27733887	0.20725959	-0.1514045	0.65074526	-0.8830962	0.6395769	-0.0122496	0.00159033
158.7	0.27733887	0.20725959	-0.1514045	0.65074526	-0.8830962	0.6395769	-0.0122496	0.00159033
158.8	0.27733887	0.20725959	-0.1514045	0.65074526	-0.8830962	0.6395769	-0.0122496	0.00159033
158.9	0.27733887	0.20725959	-0.1514045	0.65074526	-0.8830962	0.6395769	-0.0122496	0.00159033
159	0.27733887	0.20725959	-0.1514045	0.65074526	-0.8830962	0.6395769	-0.0122496	0.00159033
159.1	0.27733887	0.20725959	-0.1514045	0.65074526	-0.8830962	0.6395769	-0.0122496	0.00159033
159.2	0.27733887	0.20725959	-0.1514045	0.65074526	-0.8830962	0.6395769	-0.0122496	0.00159033
159.3	0.27733887	0.20725959	-0.1514045	0.65074526	-0.8830962	0.6395769	-0.0122496	0.00159033
159.4	0.27733887	0.20725959	-0.1514045	0.65074526	-0.8830962	0.6395769	-0.0122496	0.00159033
159.5	0.27733887	0.20725959	-0.1514045	0.65074526	-0.8830962	0.6395769	-0.0122496	0.00159033
159.6	0.27733887	0.20725959	-0.1514045	0.65074526	-0.8830962	0.6395769	-0.0122496	0.00159033
159.7	0.27733887	0.20725959	-0.1514045	0.65074526	-0.8830962	0.6395769	-0.0122496	0.00159033
159.8	0.27733887	0.20725959	-0.1514045	0.65074526	-0.8830962	0.6395769	-0.0122496	0.00159033
159.9	0.27708295	0.20783055	-0.1516262	0.65077029	-0.8830868	0.63959343	-0.0126033	0.00147261
160	0.27708295	0.20783055	-0.1516262	0.65077029	-0.8830868	0.63959343	-0.0126033	0.00147261
160.1	0.27708295	0.20783055	-0.1516262	0.65077029	-0.8830868	0.63959343	-0.0126033	0.00147261
160.2	0.27708295	0.20783055	-0.1516262	0.65077029	-0.8830868	0.63959343	-0.0126033	0.00147261
160.3	0.27708295	0.20783055	-0.1516262	0.65077029	-0.8830868	0.63959343	-0.0126033	0.00147261
160.4	0.27708295	0.20783055	-0.1516262	0.65077029	-0.8830868	0.63959343	-0.0126033	0.00147261
160.5	0.27708295	0.20783055	-0.1516262	0.65077029	-0.8830868	0.63959343	-0.0126033	0.00147261
160.6	0.27708295	0.20783055	-0.1516262	0.65077029	-0.8830868	0.63959343	-0.0126033	0.00147261
160.7	0.27708295	0.20783055	-0.1516262	0.65077029	-0.8830868	0.63959343	-0.0126033	0.00147261
160.8	0.27708295	0.20783055	-0.1516262	0.65077029	-0.8830868	0.63959343	-0.0126033	0.00147261
160.9	0.27708295	0.20783055	-0.1516262	0.65077029	-0.8830868	0.63959343	-0.0126033	0.00147261
161	0.27708295	0.20783055	-0.1516262	0.65077029	-0.8830868	0.63959343	-0.0126033	0.00147261
161.1	0.27708295	0.20783055	-0.1516262	0.65077029	-0.8830868	0.63959343	-0.0126033	0.00147261
161.2	0.27708295	0.20783055	-0.1516262	0.65077029	-0.8830868	0.63959343	-0.0126033	0.00147261
161.3	0.27708295	0.20783055	-0.1516262	0.65077029	-0.8830868	0.63959343	-0.0126033	0.00147261
161.4	0.27708295	0.20783055	-0.1516262	0.65077029	-0.8830868	0.63959343	-0.0126033	0.00147261
161.5	0.27708295	0.20783055	-0.1516262	0.65077029	-0.8830868	0.63959343	-0.0126033	0.00147261
161.6	0.27708295	0.20783055	-0.1516262	0.65077029	-0.8830868	0.63959343	-0.0126033	0.00147261
161.7	0.27708295	0.20783055	-0.1516262	0.65077029	-0.8830868	0.63959343	-0.0126033	0.00147261
161.8	0.27708295	0.20783055	-0.1516262	0.65077029	-0.8830868	0.63959343	-0.0126033	0.00147261
161.9	0.27708295	0.20783055	-0.1516262	0.65077029	-0.8830868	0.63959343	-0.0126033	0.00147261
162	0.27708295	0.20783055	-0.1516262	0.65077029	-0.8830868	0.63959343	-0.0126033	0.00147261
162.1	0.27708295	0.20783055	-0.1516262	0.65077029	-0.8830868	0.63959343	-0.0126033	0.00147261
162.2	0.27708295	0.20783055	-0.1516262	0.65077029	-0.8830868	0.63959343	-0.0126033	0.00147261
162.3	0.27708295	0.20783055	-0.1516262	0.65077029	-0.8830868	0.63959343	-0.0126033	0.00147261
162.4	0.27708295	0.20783055	-0.1516262	0.65077029	-0.8830868	0.63959343	-0.0126033	0.00147261
162.5	0.27708295	0.20783055	-0.1516262	0.65077029	-0.8830868	0.63959343	-0.0126033	0.00147261
162.6	0.27708295	0.20783055	-0.1516262	0.65077029	-0.8830868	0.63959343	-0.0126033	0.00147261
162.7	0.27708295	0.20783055	-0.1516262	0.65077029	-0.8830868	0.63959343	-0.0126033	0.00147261
162.8	0.27708295	0.20783055	-0.1516262	0.65077029	-0.8830868	0.63959343	-0.0126033	0.00147261
162.9	0.27708295	0.20783055	-0.1516262	0.65077029	-0.8830868	0.63959343	-0.0126033	0.00147261
163	0.27708295	0.20783055	-0.1516262	0.65077029	-0.8830868	0.63959343	-0.0126033	0.00147261
163.1	0.27708295	0.20783055	-0.1516262	0.65077029	-0.8830868	0.63959343	-0.0126033	0.00147261
163.2	0.27708295	0.20783055	-0.1516262	0.65077029	-0.8830868	0.63959343	-0.0126033	0.00147261
163.3	0.27695931	0.20779711	-0.1513581	0.65073836	-0.8830093	0.6394895	-0.012185	0.00163197
163.4	0.27695931	0.20779711	-0.1513581	0.65073836	-0.8830093	0.6394895	-0.012185	0.00163197
163.5	0.27695931	0.20779711	-0.1513581	0.65073836	-0.8830093	0.6394895	-0.012185	0.00163197
163.6	0.27705309	0.20750603	-0.1513305	0.65095268	-0.8830531	0.6397755	-0.0122122	0.00181782
163.7	0.27705309	0.20750603	-0.1513305	0.65095268	-0.8830531	0.6397755	-0.0122122	0.00181782
163.8	0.27705309	0.20750603	-0.1513305	0.65095268	-0.8830531	0.6397755	-0.0122122	0.00181782
163.9	0.27677221	0.20767453	-0.1510888	0.65099891	-0.8830297	0.63943484	-0.0118941	0.00160475
164	0.27677221	0.20767453	-0.1510888	0.65099891	-0.8830297	0.63943484	-0.0118941	0.00160475
164.1	0.27677221	0.20767453	-0.1510888	0.65099891	-0.8830297	0.63943484	-0.0118941	0.00160475
164.2	0.27677221	0.20767453	-0.1510888	0.65099891	-0.8830297	0.63943484	-0.0118941	0.00160475
164.3	0.27677221	0.20767453	-0.1510888	0.65099891	-0.8830297	0.63943484	-0.0118941	0.00160475
164.4	0.27677221	0.20767453	-0.1510888	0.65099891	-0.8830297	0.63943484	-0.0118941	0.00160475
164.5	0.27677221	0.20767453	-0.1510888	0.65099891	-0.8830297	0.63943484	-0.0118941	0.00160475
164.6	0.27677221	0.20767453	-0.1510888	0.65099891	-0.8830297	0.63943484	-0.0118941	0.00160475
164.7	0.27677221	0.20767453	-0.1510888	0.65099891	-0.8830297	0.63943484	-0.0118941	0.00160475
164.8	0.27677221	0.20767453	-0.1510888	0.65099891	-0.8830297	0.63943484	-0.0118941	0.00160475
164.9	0.27677221	0.20767453	-0.1510888	0.65099891	-0.8830297	0.63943484	-0.0118941	0.00160475
210	0.27281422	0.20624177	-0.1476273	0.65195139	-0.8828751	0.63598764	-0.0136203	0.00151955
210.1	0.27281422	0.20624177	-0.1476273	0.65195139	-0.8828751	0.63598764	-0.0136203	0.00151955
210.2	0.27281422	0.20624177	-0.1476273	0.65195139	-0.8828751	0.63598764	-0.0136203	0.00151955
210.3	0.27281422	0.20624177	-0.1476273	0.65195139	-0.8828751	0.63598764	-0.0136203	0.00151955
210.4	0.27281422	0.20624177	-0.1476273	0.65195139	-0.8828751	0.63598764	-0.0136203	0.00151955
210.5	0.27281422	0.20624177	-0.1476273	0.65195139	-0.8828751	0.63598764	-0.0136203	0.00151955
165.3	0.27669031	0.20824061	-0.1504117	0.65101222	-0.8830129	0.63915645	-0.0120251	0.00095249
165.4	0.27669031	0.20824061	-0.1504117	0.65101222	-0.8830129	0.63915645	-0.0120251	0.00095249
165.5	0.27669031	0.20824061	-0.1504117	0.65101222	-0.8830129	0.63915645	-0.0120251	0.00095249
165.6	0.27669031	0.20824061	-0.1504117	0.65101222	-0.8830129	0.63915645	-0.0120251	0.00095249
165.7	0.27669031	0.20824061	-0.1504117	0.65101222	-0.8830129	0.63915645	-0.0120251	0.00095249
165.8	0.27669031	0.20824061	-0.1504117	0.65101222	-0.8830129	0.63915645	-0.0120251	0.00095249
165.9	0.27672343	0.2086026	-0.1496767	0.650997	-0.8829159	0.63876337	-0.0124768	0.00075672
166	0.27672343	0.2086026	-0.1496767	0.650997	-0.8829159	0.63876337	-0.0124768	0.00075672
166.1	0.27672343	0.2086026	-0.1496767	0.650997	-0.8829159	0.63876337	-0.0124768	0.00075672
166.2	0.27651122	0.20851578	-0.1495704	0.65076308	-0.8828209	0.63830023	-0.0128042	0.00078451
166.3	0.27651122	0.20851578	-0.1495704	0.65076308	-0.8828209	0.63830023	-0.0128042	0.00078451
166.4	0.27651122	0.20851578	-0.1495704	0.65076308	-0.8828209	0.63830023	-0.0128042	0.00078451
166.5	0.27651122	0.20851578	-0.1495704	0.65076308	-0.8828209	0.63830023	-0.0128042	0.00078451
166.6	0.27651122	0.20851578	-0.1495704	0.65076308	-0.8828209	0.63830023	-0.0128042	0.00078451
166.7	0.27651122	0.20851578	-0.1495704	0.65076308	-0.8828209	0.63830023	-0.0128042	0.00078451
166.8	0.27651122	0.20851578	-0.1495704	0.65076308	-0.8828209	0.63830023	-0.0128042	0.00078451
166.9	0.27651122	0.20851578	-0.1495704	0.65076308	-0.8828209	0.63830023	-0.0128042	0.00078451
167	0.27651122	0.20851578	-0.1495704	0.65076308	-0.8828209	0.63830023	-0.0128042	0.00078451
167.1	0.27642129	0.20837911	-0.1495249	0.65109917	-0.8828604	0.63840126	-0.0126695	0.00089856
167.2	0.27642129	0.20837911	-0.1495249	0.65109917	-0.8828604	0.63840126	-0.0126695	0.00089856
167.3	0.27642129	0.20837911	-0.1495249	0.65109917	-0.8828604	0.63840126	-0.0126695	0.00089856
167.4	0.27642129	0.20837911	-0.1495249	0.65109917	-0.8828604	0.63840126	-0.0126695	0.00089856
167.5	0.27642129	0.20837911	-0.1495249	0.65109917	-0.8828604	0.63840126	-0.0126695	0.00089856
167.6	0.27642129	0.20837911	-0.1495249	0.65109917	-0.8828604	0.63840126	-0.0126695	0.00089856
167.7	0.27642129	0.20837911	-0.1495249	0.65109917	-0.8828604	0.63840126	-0.0126695	0.00089856
167.8	0.27642129	0.20837911	-0.1495249	0.65109917	-0.8828604	0.63840126	-0.0126695	0.00089856
167.9	0.27642129	0.20837911	-0.1495249	0.65109917	-0.8828604	0.63840126	-0.0126695	0.00089856
168	0.27642129	0.20837911	-0.1495249	0.65109917	-0.8828604	0.63840126	-0.0126695	0.00089856
168.1	0.27642129	0.20837911	-0.1495249	0.65109917	-0.8828604	0.63840126	-0.0126695	0.00089856
168.2	0.27642129	0.20837911	-0.1495249	0.65109917	-0.8828604	0.63840126	-0.0126695	0.00089856
168.3	0.27642129	0.20837911	-0.1495249	0.65109917	-0.8828604	0.63840126	-0.0126695	0.00089856
168.4	0.27642129	0.20837911	-0.1495249	0.65109917	-0.8828604	0.63840126	-0.0126695	0.00089856
168.5	0.27661191	0.20807403	-0.1497207	0.65138884	-0.882923	0.63884342	-0.0128527	0.0012255
168.6	0.27661191	0.20807403	-0.1497207	0.65138884	-0.882923	0.63884342	-0.0128527	0.0012255
168.7	0.27661191	0.20807403	-0.1497207	0.65138884	-0.882923	0.63884342	-0.0128527	0.0012255
168.8	0.27661191	0.20807403	-0.1497207	0.65138884	-0.882923	0.63884342	-0.0128527	0.0012255
168.9	0.27661191	0.20807403	-0.1497207	0.65138884	-0.882923	0.63884342	-0.0128527	0.0012255
169	0.27661191	0.20807403	-0.1497207	0.65138884	-0.882923	0.63884342	-0.0128527	0.0012255
169.1	0.27661191	0.20807403	-0.1497207	0.65138884	-0.882923	0.63884342	-0.0128527	0.0012255
169.2	0.27661191	0.20807403	-0.1497207	0.65138884	-0.882923	0.63884342	-0.0128527	0.0012255
169.3	0.27661191	0.20807403	-0.1497207	0.65138884	-0.882923	0.63884342	-0.0128527	0.0012255
169.4	0.27661191	0.20807403	-0.1497207	0.65138884	-0.882923	0.63884342	-0.0128527	0.0012255
169.5	0.27661191	0.20807403	-0.1497207	0.65138884	-0.882923	0.63884342	-0.0128527	0.0012255
169.6	0.27661191	0.20807403	-0.1497207	0.65138884	-0.882923	0.63884342	-0.0128527	0.0012255
169.7	0.27661191	0.20807403	-0.1497207	0.65138884	-0.882923	0.63884342	-0.0128527	0.0012255
169.8	0.27661191	0.20807403	-0.1497207	0.65138884	-0.882923	0.63884342	-0.0128527	0.0012255
169.9	0.27661191	0.20807403	-0.1497207	0.65138884	-0.882923	0.63884342	-0.0128527	0.0012255
170	0.27638281	0.20791435	-0.1494308	0.65150307	-0.882949	0.63885308	-0.0121342	0.00098388
170.1	0.27638281	0.20791435	-0.1494308	0.65150307	-0.882949	0.63885308	-0.0121342	0.00098388
170.2	0.27638281	0.20791435	-0.1494308	0.65150307	-0.882949	0.63885308	-0.0121342	0.00098388
170.3	0.27638281	0.20791435	-0.1494308	0.65150307	-0.882949	0.63885308	-0.0121342	0.00098388
170.4	0.27619851	0.20806759	-0.1494132	0.65162376	-0.8829441	0.63866673	-0.0119719	0.00091257
170.5	0.27619851	0.20806759	-0.1494132	0.65162376	-0.8829441	0.63866673	-0.0119719	0.00091257
170.6	0.27619851	0.20806759	-0.1494132	0.65162376	-0.8829441	0.63866673	-0.0119719	0.00091257
170.7	0.27619851	0.20806759	-0.1494132	0.65162376	-0.8829441	0.63866673	-0.0119719	0.00091257
170.8	0.27619851	0.20806759	-0.1494132	0.65162376	-0.8829441	0.63866673	-0.0119719	0.00091257
170.9	0.27619851	0.20806759	-0.1494132	0.65162376	-0.8829441	0.63866673	-0.0119719	0.00091257
171	0.27619851	0.20806759	-0.1494132	0.65162376	-0.8829441	0.63866673	-0.0119719	0.00091257
171.1	0.27619851	0.20806759	-0.1494132	0.65162376	-0.8829441	0.63866673	-0.0119719	0.00091257
171.2	0.27619851	0.20806759	-0.1494132	0.65162376	-0.8829441	0.63866673	-0.0119719	0.00091257
171.3	0.27619851	0.20806759	-0.1494132	0.65162376	-0.8829441	0.63866673	-0.0119719	0.00091257
171.4	0.27619851	0.20806759	-0.1494132	0.65162376	-0.8829441	0.63866673	-0.0119719	0.00091257
171.5	0.27619851	0.20806759	-0.1494132	0.65162376	-0.8829441	0.63866673	-0.0119719	0.00091257
171.6	0.27619851	0.20806759	-0.1494132	0.65162376	-0.8829441	0.63866673	-0.0119719	0.00091257
171.7	0.27619851	0.20806759	-0.1494132	0.65162376	-0.8829441	0.63866673	-0.0119719	0.00091257
171.8	0.27619851	0.20806759	-0.1494132	0.65162376	-0.8829441	0.63866673	-0.0119719	0.00091257
171.9	0.27619851	0.20806759	-0.1494132	0.65162376	-0.8829441	0.63866673	-0.0119719	0.00091257
172	0.27619851	0.20806759	-0.1494132	0.65162376	-0.8829441	0.63866673	-0.0119719	0.00091257
172.1	0.27590476	0.20825312	-0.1492156	0.6515872	-0.8828659	0.63819919	-0.0117174	0.00071297
172.2	0.27590476	0.20825312	-0.1492156	0.6515872	-0.8828659	0.63819919	-0.0117174	0.00071297
172.3	0.27590476	0.20825312	-0.1492156	0.6515872	-0.8828659	0.63819919	-0.0117174	0.00071297
172.4	0.27590476	0.20825312	-0.1492156	0.6515872	-0.8828659	0.63819919	-0.0117174	0.00071297
172.5	0.27590476	0.20825312	-0.1492156	0.6515872	-0.8828659	0.63819919	-0.0117174	0.00071297
172.6	0.27590476	0.20825312	-0.1492156	0.6515872	-0.8828659	0.63819919	-0.0117174	0.00071297
172.7	0.27590476	0.20825312	-0.1492156	0.6515872	-0.8828659	0.63819919	-0.0117174	0.00071297
172.8	0.27590476	0.20825312	-0.1492156	0.6515872	-0.8828659	0.63819919	-0.0117174	0.00071297
172.9	0.27590476	0.20825312	-0.1492156	0.6515872	-0.8828659	0.63819919	-0.0117174	0.00071297
173	0.27590476	0.20825312	-0.1492156	0.6515872	-0.8828659	0.63819919	-0.0117174	0.00071297
173.1	0.27590476	0.20825312	-0.1492156	0.6515872	-0.8828659	0.63819919	-0.0117174	0.00071297
173.2	0.27590476	0.20825312	-0.1492156	0.6515872	-0.8828659	0.63819919	-0.0117174	0.00071297
173.3	0.27590476	0.20825312	-0.1492156	0.6515872	-0.8828659	0.63819919	-0.0117174	0.00071297
173.4	0.27590476	0.20825312	-0.1492156	0.6515872	-0.8828659	0.63819919	-0.0117174	0.00071297
173.5	0.27590476	0.20825312	-0.1492156	0.6515872	-0.8828659	0.63819919	-0.0117174	0.00071297
173.6	0.27590476	0.20825312	-0.1492156	0.6515872	-0.8828659	0.63819919	-0.0117174	0.00071297
173.7	0.27590476	0.20825312	-0.1492156	0.6515872	-0.8828659	0.63819919	-0.0117174	0.00071297
173.8	0.27590476	0.20825312	-0.1492156	0.6515872	-0.8828659	0.63819919	-0.0117174	0.00071297
173.9	0.27590476	0.20825312	-0.1492156	0.6515872	-0.8828659	0.63819919	-0.0117174	0.00071297
174	0.27590476	0.20825312	-0.1492156	0.6515872	-0.8828659	0.63819919	-0.0117174	0.00071297
174.1	0.27590476	0.20825312	-0.1492156	0.6515872	-0.8828659	0.63819919	-0.0117174	0.00071297
174.2	0.27590476	0.20825312	-0.1492156	0.6515872	-0.8828659	0.63819919	-0.0117174	0.00071297
174.3	0.27590476	0.20825312	-0.1492156	0.6515872	-0.8828659	0.63819919	-0.0117174	0.00071297
174.4	0.27590476	0.20825312	-0.1492156	0.6515872	-0.8828659	0.63819919	-0.0117174	0.00071297
174.5	0.27590476	0.20825312	-0.1492156	0.6515872	-0.8828659	0.63819919	-0.0117174	0.00071297
174.6	0.27590476	0.20825312	-0.1492156	0.6515872	-0.8828659	0.63819919	-0.0117174	0.00071297
174.7	0.27590476	0.20825312	-0.1492156	0.6515872	-0.8828659	0.63819919	-0.0117174	0.00071297
174.8	0.27590476	0.20825312	-0.1492156	0.6515872	-0.8828659	0.63819919	-0.0117174	0.00071297
174.9	0.27590476	0.20825312	-0.1492156	0.6515872	-0.8828659	0.63819919	-0.0117174	0.00071297
175	0.27590476	0.20825312	-0.1492156	0.6515872	-0.8828659	0.63819919	-0.0117174	0.00071297
175.1	0.27590476	0.20825312	-0.1492156	0.6515872	-0.8828659	0.63819919	-0.0117174	0.00071297
175.2	0.27589539	0.20797155	-0.1486848	0.651465	-0.8828107	0.63798464	-0.0121959	0.00066036
175.3	0.27534773	0.20778915	-0.1484972	0.65133094	-0.8827406	0.63763483	-0.0123456	0.0006728
175.4	0.27534773	0.20778915	-0.1484972	0.65133094	-0.8827406	0.63763483	-0.0123456	0.0006728
175.5	0.27534773	0.20778915	-0.1484972	0.65133094	-0.8827406	0.63763483	-0.0123456	0.0006728
175.6	0.27534773	0.20778915	-0.1484972	0.65133094	-0.8827406	0.63763483	-0.0123456	0.0006728
175.7	0.27534773	0.20778915	-0.1484972	0.65133094	-0.8827406	0.63763483	-0.0123456	0.0006728
175.8	0.27534773	0.20778915	-0.1484972	0.65133094	-0.8827406	0.63763483	-0.0123456	0.0006728
175.9	0.27534773	0.20778915	-0.1484972	0.65133094	-0.8827406	0.63763483	-0.0123456	0.0006728
176	0.27534773	0.20778915	-0.1484972	0.65133094	-0.8827406	0.63763483	-0.0123456	0.0006728
176.1	0.27534773	0.20778915	-0.1484972	0.65133094	-0.8827406	0.63763483	-0.0123456	0.0006728
176.2	0.27534773	0.20778915	-0.1484972	0.65133094	-0.8827406	0.63763483	-0.0123456	0.0006728
176.3	0.27534773	0.20778915	-0.1484972	0.65133094	-0.8827406	0.63763483	-0.0123456	0.0006728
176.4	0.27534773	0.20778915	-0.1484972	0.65133094	-0.8827406	0.63763483	-0.0123456	0.0006728
176.5	0.27534773	0.20778915	-0.1484972	0.65133094	-0.8827406	0.63763483	-0.0123456	0.0006728
176.6	0.27534773	0.20778915	-0.1484972	0.65133094	-0.8827406	0.63763483	-0.0123456	0.0006728
176.7	0.27534773	0.20778915	-0.1484972	0.65133094	-0.8827406	0.63763483	-0.0123456	0.0006728
176.8	0.27534773	0.20778915	-0.1484972	0.65133094	-0.8827406	0.63763483	-0.0123456	0.0006728
176.9	0.27534773	0.20778915	-0.1484972	0.65133094	-0.8827406	0.63763483	-0.0123456	0.0006728
177	0.27534773	0.20778915	-0.1484972	0.65133094	-0.8827406	0.63763483	-0.0123456	0.0006728
177.1	0.27534773	0.20778915	-0.1484972	0.65133094	-0.8827406	0.63763483	-0.0123456	0.0006728
177.2	0.27534773	0.20778915	-0.1484972	0.65133094	-0.8827406	0.63763483	-0.0123456	0.0006728
177.3	0.27534773	0.20778915	-0.1484972	0.65133094	-0.8827406	0.63763483	-0.0123456	0.0006728
177.4	0.27534773	0.20778915	-0.1484972	0.65133094	-0.8827406	0.63763483	-0.0123456	0.0006728
177.5	0.27534773	0.20778915	-0.1484972	0.65133094	-0.8827406	0.63763483	-0.0123456	0.0006728
177.6	0.27534773	0.20778915	-0.1484972	0.65133094	-0.8827406	0.63763483	-0.0123456	0.0006728
177.7	0.27534773	0.20778915	-0.1484972	0.65133094	-0.8827406	0.63763483	-0.0123456	0.0006728
177.8	0.27534773	0.20778915	-0.1484972	0.65133094	-0.8827406	0.63763483	-0.0123456	0.0006728
177.9	0.27534773	0.20778915	-0.1484972	0.65133094	-0.8827406	0.63763483	-0.0123456	0.0006728
178	0.27544353	0.20777366	-0.1487205	0.65140475	-0.8827586	0.63778916	-0.0125012	0.00081413
178.1	0.27544353	0.20777366	-0.1487205	0.65140475	-0.8827586	0.63778916	-0.0125012	0.00081413
178.2	0.27544353	0.20777366	-0.1487205	0.65140475	-0.8827586	0.63778916	-0.0125012	0.00081413
178.3	0.27544353	0.20777366	-0.1487205	0.65140475	-0.8827586	0.63778916	-0.0125012	0.00081413
178.4	0.27544353	0.20777366	-0.1487205	0.65140475	-0.8827586	0.63778916	-0.0125012	0.00081413
178.5	0.27544353	0.20777366	-0.1487205	0.65140475	-0.8827586	0.63778916	-0.0125012	0.00081413
178.6	0.27544353	0.20777366	-0.1487205	0.65140475	-0.8827586	0.63778916	-0.0125012	0.00081413
178.7	0.27544353	0.20777366	-0.1487205	0.65140475	-0.8827586	0.63778916	-0.0125012	0.00081413
178.8	0.27513421	0.20790442	-0.1485326	0.65115699	-0.8826283	0.63724134	-0.0128161	0.00087859
178.9	0.27513421	0.20790442	-0.1485326	0.65115699	-0.8826283	0.63724134	-0.0128161	0.00087859
179	0.27513421	0.20790442	-0.1485326	0.65115699	-0.8826283	0.63724134	-0.0128161	0.00087859
179.1	0.27513421	0.20790442	-0.1485326	0.65115699	-0.8826283	0.63724134	-0.0128161	0.00087859
179.2	0.27513421	0.20790442	-0.1485326	0.65115699	-0.8826283	0.63724134	-0.0128161	0.00087859
179.3	0.27513421	0.20790442	-0.1485326	0.65115699	-0.8826283	0.63724134	-0.0128161	0.00087859
179.4	0.27518902	0.20811575	-0.1486598	0.6511055	-0.8827259	0.63736938	-0.0128262	0.00120394
179.5	0.27518902	0.20811575	-0.1486598	0.6511055	-0.8827259	0.63736938	-0.0128262	0.00120394
179.6	0.27518902	0.20811575	-0.1486598	0.6511055	-0.8827259	0.63736938	-0.0128262	0.00120394
179.7	0.27518902	0.20811575	-0.1486598	0.6511055	-0.8827259	0.63736938	-0.0128262	0.00120394
179.8	0.27518902	0.20811575	-0.1486598	0.6511055	-0.8827259	0.63736938	-0.0128262	0.00120394
179.9	0.27518902	0.20811575	-0.1486598	0.6511055	-0.8827259	0.63736938	-0.0128262	0.00120394
225	0.27130521	0.20520008	-0.146371	0.65212524	-0.8829604	0.63572271	-0.0121712	0.00179779
225.1	0.27130521	0.20520008	-0.146371	0.65212524	-0.8829604	0.63572271	-0.0121712	0.00179779
225.2	0.27130521	0.20520008	-0.146371	0.65212524	-0.8829604	0.63572271	-0.0121712	0.00179779
225.3	0.27130521	0.20520008	-0.146371	0.65212524	-0.8829604	0.63572271	-0.0121712	0.00179779
225.4	0.27130521	0.20520008	-0.146371	0.65212524	-0.8829604	0.63572271	-0.0121712	0.00179779
225.5	0.27130521	0.20520008	-0.146371	0.65212524	-0.8829604	0.63572271	-0.0121712	0.00179779

180.6	0.27518902	0.20811575	-0.1486598	0.6511055	-0.8827259	0.63736938	-0.0128262	0.00120394
180.7	0.27518902	0.20811575	-0.1486598	0.6511055	-0.8827259	0.63736938	-0.0128262	0.00120394
180.8	0.27518902	0.20811575	-0.1486598	0.6511055	-0.8827259	0.63736938	-0.0128262	0.00120394
180.9	0.27518902	0.20811575	-0.1486598	0.6511055	-0.8827259	0.63736938	-0.0128262	0.00120394
181	0.27518902	0.20811575	-0.1486598	0.6511055	-0.8827259	0.63736938	-0.0128262	0.00120394
181.1	0.27518902	0.20811575	-0.1486598	0.6511055	-0.8827259	0.63736938	-0.0128262	0.00120394
181.2	0.27518902	0.20811575	-0.1486598	0.6511055	-0.8827259	0.63736938	-0.0128262	0.00120394
181.3	0.27522218	0.20819724	-0.1482932	0.65093793	-0.8826601	0.63708048	-0.0128095	0.00120739
181.4	0.27522218	0.20819724	-0.1482932	0.65093793	-0.8826601	0.63708048	-0.0128095	0.00120739
181.5	0.27522218	0.20819724	-0.1482932	0.65093793	-0.8826601	0.63708048	-0.0128095	0.00120739
181.6	0.27522218	0.20819724	-0.1482932	0.65093793	-0.8826601	0.63708048	-0.0128095	0.00120739
181.7	0.27522218	0.20819724	-0.1482932	0.65093793	-0.8826601	0.63708048	-0.0128095	0.00120739
181.8	0.27522218	0.20819724	-0.1482932	0.65093793	-0.8826601	0.63708048	-0.0128095	0.00120739
181.9	0.27522218	0.20819724	-0.1482932	0.65093793	-0.8826601	0.63708048	-0.0128095	0.00120739
182	0.27522218	0.20819724	-0.1482932	0.65093793	-0.8826601	0.63708048	-0.0128095	0.00120739
182.1	0.27522218	0.20819724	-0.1482932	0.65093793	-0.8826601	0.63708048	-0.0128095	0.00120739
182.2	0.27522218	0.20819724	-0.1482932	0.65093793	-0.8826601	0.63708048	-0.0128095	0.00120739
182.3	0.27522218	0.20819724	-0.1482932	0.65093793	-0.8826601	0.63708048	-0.0128095	0.00120739
182.4	0.27522218	0.20819724	-0.1482932	0.65093793	-0.8826601	0.63708048	-0.0128095	0.00120739
182.5	0.2753774	0.20797874	-0.1481161	0.65102864	-0.8827491	0.63693505	-0.0127599	0.00125267
182.6	0.2753774	0.20797874	-0.1481161	0.65102864	-0.8827491	0.63693505	-0.0127599	0.00125267
182.7	0.2753774	0.20797874	-0.1481161	0.65102864	-0.8827491	0.63693505	-0.0127599	0.00125267
182.8	0.2753774	0.20797874	-0.1481161	0.65102864	-0.8827491	0.63693505	-0.0127599	0.00125267
182.9	0.2753774	0.20797874	-0.1481161	0.65102864	-0.8827491	0.63693505	-0.0127599	0.00125267
183	0.2753774	0.20797874	-0.1481161	0.65102864	-0.8827491	0.63693505	-0.0127599	0.00125267
183.1	0.2753774	0.20797874	-0.1481161	0.65102864	-0.8827491	0.63693505	-0.0127599	0.00125267
183.2	0.2753774	0.20797874	-0.1481161	0.65102864	-0.8827491	0.63693505	-0.0127599	0.00125267
183.3	0.2753774	0.20797874	-0.1481161	0.65102864	-0.8827491	0.63693505	-0.0127599	0.00125267
183.4	0.2753774	0.20797874	-0.1481161	0.65102864	-0.8827491	0.63693505	-0.0127599	0.00125267
183.5	0.2753774	0.20797874	-0.1481161	0.65102864	-0.8827491	0.63693505	-0.0127599	0.00125267
183.6	0.2753774	0.20797874	-0.1481161	0.65102864	-0.8827491	0.63693505	-0.0127599	0.00125267
183.7	0.2753774	0.20797874	-0.1481161	0.65102864	-0.8827491	0.63693505	-0.0127599	0.00125267
183.8	0.2753774	0.20797874	-0.1481161	0.65102864	-0.8827491	0.63693505	-0.0127599	0.00125267
183.9	0.2753774	0.20797874	-0.1481161	0.65102864	-0.8827491	0.63693505	-0.0127599	0.00125267
184	0.2753774	0.20797874	-0.1481161	0.65102864	-0.8827491	0.63693505	-0.0127599	0.00125267
184.1	0.2753774	0.20797874	-0.1481161	0.65102864	-0.8827491	0.63693505	-0.0127599	0.00125267
184.2	0.2753774	0.20797874	-0.1481161	0.65102864	-0.8827491	0.63693505	-0.0127599	0.00125267
184.3	0.2753774	0.20797874	-0.1481161	0.65102864	-0.8827491	0.63693505	-0.0127599	0.00125267
184.4	0.2753774	0.20797874	-0.1481161	0.65102864	-0.8827491	0.63693505	-0.0127599	0.00125267
184.5	0.2753774	0.20797874	-0.1481161	0.65102864	-0.8827491	0.63693505	-0.0127599	0.00125267
184.6	0.2753774	0.20797874	-0.1481161	0.65102864	-0.8827491	0.63693505	-0.0127599	0.00125267
184.7	0.2753774	0.20797874	-0.1481161	0.65102864	-0.8827491	0.63693505	-0.0127599	0.00125267
184.8	0.2753774	0.20797874	-0.1481161	0.65102864	-0.8827491	0.63693505	-0.0127599	0.00125267
184.9	0.2753774	0.20797874	-0.1481161	0.65102864	-0.8827491	0.63693505	-0.0127599	0.00125267
185	0.27540678	0.20790853	-0.1476994	0.65097773	-0.882666	0.63660731	-0.0135056	0.00143221
185.1	0.27540678	0.20790853	-0.1476994	0.65097773	-0.882666	0.63660731	-0.0135056	0.00143221
185.2	0.27540678	0.20790853	-0.1476994	0.65097773	-0.882666	0.63660731	-0.0135056	0.00143221
185.3	0.27540678	0.20790853	-0.1476994	0.65097773	-0.882666	0.63660731	-0.0135056	0.00143221
185.4	0.27540678	0.20790853	-0.1476994	0.65097773	-0.882666	0.63660731	-0.0135056	0.00143221
185.5	0.27540678	0.20790853	-0.1476994	0.65097773	-0.882666	0.63660731	-0.0135056	0.00143221
185.6	0.27540678	0.20790853	-0.1476994	0.65097773	-0.882666	0.63660731	-0.0135056	0.00143221
185.7	0.27540678	0.20790853	-0.1476994	0.65097773	-0.882666	0.63660731	-0.0135056	0.00143221
185.8	0.27540678	0.20790853	-0.1476994	0.65097773	-0.882666	0.63660731	-0.0135056	0.00143221
185.9	0.27540678	0.20790853	-0.1476994	0.65097773	-0.882666	0.63660731	-0.0135056	0.00143221
186	0.27540678	0.20790853	-0.1476994	0.65097773	-0.882666	0.63660731	-0.0135056	0.00143221
186.1	0.27489287	0.20790858	-0.1475949	0.65098674	-0.8826249	0.63637019	-0.0136874	0.0010901
186.2	0.27489287	0.20790858	-0.1475949	0.65098674	-0.8826249	0.63637019	-0.0136874	0.0010901
186.3	0.27489287	0.20790858	-0.1475949	0.65098674	-0.8826249	0.63637019	-0.0136874	0.0010901
186.4	0.27489287	0.20790858	-0.1475949	0.65098674	-0.8826249	0.63637019	-0.0136874	0.0010901
186.5	0.27489287	0.20790858	-0.1475949	0.65098674	-0.8826249	0.63637019	-0.0136874	0.0010901
186.6	0.27450865	0.20807745	-0.1476825	0.65113764	-0.8827592	0.63624694	-0.0137701	0.00150031
186.7	0.27450865	0.20807745	-0.1476825	0.65113764	-0.8827592	0.63624694	-0.0137701	0.00150031
186.8	0.27450865	0.20807745	-0.1476825	0.65113764	-0.8827592	0.63624694	-0.0137701	0.00150031
186.9	0.27450865	0.20807745	-0.1476825	0.65113764	-0.8827592	0.63624694	-0.0137701	0.00150031
187	0.27450865	0.20807745	-0.1476825	0.65113764	-0.8827592	0.63624694	-0.0137701	0.00150031
187.1	0.27450865	0.20807745	-0.1476825	0.65113764	-0.8827592	0.63624694	-0.0137701	0.00150031
187.2	0.27450865	0.20807745	-0.1476825	0.65113764	-0.8827592	0.63624694	-0.0137701	0.00150031
187.3	0.27450865	0.20807745	-0.1476825	0.65113764	-0.8827592	0.63624694	-0.0137701	0.00150031
187.4	0.27450865	0.20807745	-0.1476825	0.65113764	-0.8827592	0.63624694	-0.0137701	0.00150031
187.5	0.27450865	0.20807745	-0.1476825	0.65113764	-0.8827592	0.63624694	-0.0137701	0.00150031
187.6	0.27450865	0.20807745	-0.1476825	0.65113764	-0.8827592	0.63624694	-0.0137701	0.00150031
187.7	0.27450865	0.20807745	-0.1476825	0.65113764	-0.8827592	0.63624694	-0.0137701	0.00150031
187.8	0.27450865	0.20807745	-0.1476825	0.65113764	-0.8827592	0.63624694	-0.0137701	0.00150031
187.9	0.27450865	0.20807745	-0.1476825	0.65113764	-0.8827592	0.63624694	-0.0137701	0.00150031
188	0.27450865	0.20807745	-0.1476825	0.65113764	-0.8827592	0.63624694	-0.0137701	0.00150031
188.1	0.27450865	0.20807745	-0.1476825	0.65113764	-0.8827592	0.63624694	-0.0137701	0.00150031
188.2	0.27450865	0.20807745	-0.1476825	0.65113764	-0.8827592	0.63624694	-0.0137701	0.00150031
188.3	0.27450865	0.20807745	-0.1476825	0.65113764	-0.8827592	0.63624694	-0.0137701	0.00150031
188.4	0.27450865	0.20807745	-0.1476825	0.65113764	-0.8827592	0.63624694	-0.0137701	0.00150031
188.5	0.2744069	0.20775773	-0.1474395	0.65135579	-0.882803	0.63615266	-0.0135892	0.00167079
188.6	0.2744069	0.20775773	-0.1474395	0.65135579	-0.882803	0.63615266	-0.0135892	0.00167079
188.7	0.2744069	0.20775773	-0.1474395	0.65135579	-0.882803	0.63615266	-0.0135892	0.00167079
188.8	0.2744069	0.20775773	-0.1474395	0.65135579	-0.882803	0.63615266	-0.0135892	0.00167079
188.9	0.2744069	0.20775773	-0.1474395	0.65135579	-0.882803	0.63615266	-0.0135892	0.00167079
189	0.2744069	0.20775773	-0.1474395	0.65135579	-0.882803	0.63615266	-0.0135892	0.00167079
189.1	0.2744069	0.20775773	-0.1474395	0.65135579	-0.882803	0.63615266	-0.0135892	0.00167079
189.2	0.2744069	0.20775773	-0.1474395	0.65135579	-0.882803	0.63615266	-0.0135892	0.00167079
189.3	0.2744069	0.20775773	-0.1474395	0.65135579	-0.882803	0.63615266	-0.0135892	0.00167079
189.4	0.2744069	0.20775773	-0.1474395	0.65135579	-0.882803	0.63615266	-0.0135892	0.00167079
189.5	0.2744069	0.20775773	-0.1474395	0.65135579	-0.882803	0.63615266	-0.0135892	0.00167079
189.6	0.2744069	0.20775773	-0.1474395	0.65135579	-0.882803	0.63615266	-0.0135892	0.00167079
189.7	0.2744069	0.20775773	-0.1474395	0.65135579	-0.882803	0.63615266	-0.0135892	0.00167079
189.8	0.2744069	0.20775773	-0.1474395	0.65135579	-0.882803	0.63615266	-0.0135892	0.00167079
189.9	0.2744069	0.20775773	-0.1474395	0.65135579	-0.882803	0.63615266	-0.0135892	0.00167079
190	0.2744069	0.20775773	-0.1474395	0.65135579	-0.882803	0.63615266	-0.0135892	0.00167079
190.1	0.2744069	0.20775773	-0.1474395	0.65135579	-0.882803	0.63615266	-0.0135892	0.00167079
190.2	0.2744069	0.20775773	-0.1474395	0.65135579	-0.882803	0.63615266	-0.0135892	0.00167079
190.3	0.2744069	0.20775773	-0.1474395	0.65135579	-0.882803	0.63615266	-0.0135892	0.00167079
190.4	0.2744069	0.20775773	-0.1474395	0.65135579	-0.882803	0.63615266	-0.0135892	0.00167079
190.5	0.2744069	0.20775773	-0.1474395	0.65135579	-0.882803	0.63615266	-0.0135892	0.00167079
190.6	0.2744069	0.20775773	-0.1474395	0.65135579	-0.882803	0.63615266	-0.0135892	0.00167079
190.7	0.2744069	0.20775773	-0.1474395	0.65135579	-0.882803	0.63615266	-0.0135892	0.00167079
190.8	0.2744069	0.20775773	-0.1474395	0.65135579	-0.882803	0.63615266	-0.0135892	0.00167079
190.9	0.2744069	0.20775773	-0.1474395	0.65135579	-0.882803	0.63615266	-0.0135892	0.00167079
191	0.2741122	0.20743148	-0.1469228	0.65161443	-0.8828438	0.63590496	-0.0134497	0.00233203
191.1	0.2741122	0.20743148	-0.1469228	0.65161443	-0.8828438	0.63590496	-0.0134497	0.00233203
191.2	0.2741122	0.20743148	-0.1469228	0.65161443	-0.8828438	0.63590496	-0.0134497	0.00233203
191.3	0.2741122	0.20743148	-0.1469228	0.65161443	-0.8828438	0.63590496	-0.0134497	0.00233203
191.4	0.2741122	0.20743148	-0.1469228	0.65161443	-0.8828438	0.63590496	-0.0134497	0.00233203
191.5	0.2741122	0.20743148	-0.1469228	0.65161443	-0.8828438	0.63590496	-0.0134497	0.00233203
191.6	0.2741122	0.20743148	-0.1469228	0.65161443	-0.8828438	0.63590496	-0.0134497	0.00233203
191.7	0.2741122	0.20743148	-0.1469228	0.65161443	-0.8828438	0.63590496	-0.0134497	0.00233203
191.8	0.2741122	0.20743148	-0.1469228	0.65161443	-0.8828438	0.63590496	-0.0134497	0.00233203
191.9	0.2741122	0.20743148	-0.1469228	0.65161443	-0.8828438	0.63590496	-0.0134497	0.00233203
192	0.2741122	0.20743148	-0.1469228	0.65161443	-0.8828438	0.63590496	-0.0134497	0.00233203
192.1	0.2741122	0.20743148	-0.1469228	0.65161443	-0.8828438	0.63590496	-0.0134497	0.00233203
192.2	0.2741122	0.20743148	-0.1469228	0.65161443	-0.8828438	0.63590496	-0.0134497	0.00233203
192.3	0.2741122	0.20743148	-0.1469228	0.65161443	-0.8828438	0.63590496	-0.0134497	0.00233203
192.4	0.2741122	0.20743148	-0.1469228	0.65161443	-0.8828438	0.63590496	-0.0134497	0.00233203
192.5	0.2741122	0.20743148	-0.1469228	0.65161443	-0.8828438	0.63590496	-0.0134497	0.00233203
192.6	0.2741122	0.20743148	-0.1469228	0.65161443	-0.8828438	0.63590496	-0.0134497	0.00233203
192.7	0.2741122	0.20743148	-0.1469228	0.65161443	-0.8828438	0.63590496	-0.0134497	0.00233203
192.8	0.27365656	0.20709093	-0.1471368	0.65174161	-0.8828794	0.63593498	-0.0137332	0.00216162
192.9	0.27365656	0.20709093	-0.1471368	0.65174161	-0.8828794	0.63593498	-0.0137332	0.00216162
193	0.27365656	0.20709093	-0.1471368	0.65174161	-0.8828794	0.63593498	-0.0137332	0.00216162
193.1	0.27365656	0.20709093	-0.1471368	0.65174161	-0.8828794	0.63593498	-0.0137332	0.00216162
193.2	0.27365656	0.20709093	-0.1471368	0.65174161	-0.8828794	0.63593498	-0.0137332	0.00216162
193.3	0.27365656	0.20709093	-0.1471368	0.65174161	-0.8828794	0.63593498	-0.0137332	0.00216162
193.4	0.27365656	0.20709093	-0.1471368	0.65174161	-0.8828794	0.63593498	-0.0137332	0.00216162
193.5	0.27365656	0.20709093	-0.1471368	0.65174161	-0.8828794	0.63593498	-0.0137332	0.00216162
193.6	0.27365656	0.20709093	-0.1471368	0.65174161	-0.8828794	0.63593498	-0.0137332	0.00216162
193.7	0.27365656	0.20709093	-0.1471368	0.65174161	-0.8828794	0.63593498	-0.0137332	0.00216162
193.8	0.27365656	0.20709093	-0.1471368	0.65174161	-0.8828794	0.63593498	-0.0137332	0.00216162
193.9	0.27365656	0.20709093	-0.1471368	0.65174161	-0.8828794	0.63593498	-0.0137332	0.00216162
194	0.27365656	0.20709093	-0.1471368	0.65174161	-0.8828794	0.63593498	-0.0137332	0.00216162
194.1	0.27365656	0.20709093	-0.1471368	0.65174161	-0.8828794	0.63593498	-0.0137332	0.00216162
194.2	0.27365656	0.20709093	-0.1471368	0.65174161	-0.8828794	0.63593498	-0.0137332	0.00216162
194.3	0.27365656	0.20709093	-0.1471368	0.65174161	-0.8828794	0.63593498	-0.0137332	0.00216162
194.4	0.27365656	0.20709093	-0.1471368	0.65174161	-0.8828794	0.63593498	-0.0137332	0.00216162
194.5	0.27365656	0.20709093	-0.1471368	0.65174161	-0.8828794	0.63593498	-0.0137332	0.00216162
194.6	0.27365656	0.20709093	-0.1471368	0.65174161	-0.8828794	0.63593498	-0.0137332	0.00216162
194.7	0.27365656	0.20709093	-0.1471368	0.65174161	-0.8828794	0.63593498	-0.0137332	0.00216162
194.8	0.27365656	0.20709093	-0.1471368	0.65174161	-0.8828794	0.63593498	-0.0137332	0.00216162
194.9	0.27365656	0.20709093	-0.1471368	0.65174161	-0.8828794	0.63593498	-0.0137332	0.00216162
195.6	0.27365656	0.20709093	-0.1471368	0.65174161	-0.8828794	0.63593498	-0.0137332	0.00216162
195.7	0.27365656	0.20709093	-0.1471368	0.65174161	-0.8828794	0.63593498	-0.0137332	0.00216162
195.8	0.27365656	0.20709093	-0.1471368	0.65174161	-0.8828794	0.63593498	-0.0137332	0.00216162
195.9	0.27365656	0.20709093	-0.1471368	0.65174161	-0.8828794	0.63593498	-0.0137332	0.00216162
196	0.27365656	0.20709093	-0.1471368	0.65174161	-0.8828794	0.63593498	-0.0137332	0.00216162
196.1	0.27365656	0.20709093	-0.1471368	0.65174161	-0.8828794	0.63593498	-0.0137332	0.00216162
196.2	0.27365656	0.20709093	-0.1471368	0.65174161	-0.8828794	0.63593498	-0.0137332	0.00216162
196.3	0.27365656	0.20709093	-0.1471368	0.65174161	-0.8828794	0.63593498	-0.0137332	0.00216162
196.4	0.27365656	0.20709093	-0.1471368	0.65174161	-0.8828794	0.63593498	-0.0137332	0.00216162
196.5	0.27365656	0.20709093	-0.1471368	0.65174161	-0.8828794	0.63593498	-0.0137332	0.00216162
196.6	0.27365656	0.20709093	-0.1471368	0.65174161	-0.8828794	0.63593498	-0.0137332	0.00216162
196.7	0.27365656	0.20709093	-0.1471368	0.65174161	-0.8828794	0.63593498	-0.0137332	0.00216162
196.8	0.27365656	0.20709093	-0.1471368	0.65174161	-0.8828794	0.63593498	-0.0137332	0.00216162
196.9	0.27365656	0.20709093	-0.1471368	0.65174161	-0.8828794	0.63593498	-0.0137332	0.00216162
197	0.27365656	0.20709093	-0.1471368	0.65174161	-0.8828794	0.63593498	-0.0137332	0.00216162
197.1	0.27365656	0.20709093	-0.1471368	0.65174161	-0.8828794	0.63593498	-0.0137332	0.00216162
197.2	0.27365656	0.20709093	-0.1471368	0.65174161	-0.8828794	0.63593498	-0.0137332	0.00216162
197.3	0.27365656	0.20709093	-0.1471368	0.65174161	-0.8828794	0.63593498	-0.0137332	0.00216162
197.4	0.27365656	0.20709093	-0.1471368	0.65174161	-0.8828794	0.63593498	-0.0137332	0.00216162
197.5	0.27365656	0.20709093	-0.1471368	0.65174161	-0.8828794	0.63593498	-0.0137332	0.00216162
197.6	0.27365656	0.20709093	-0.1471368	0.65174161	-0.8828794	0.63593498	-0.0137332	0.00216162
197.7	0.27365656	0.20709093	-0.1471368	0.65174161	-0.8828794	0.63593498	-0.0137332	0.00216162
197.8	0.27365656	0.20709093	-0.1471368	0.65174161	-0.8828794	0.63593498	-0.0137332	0.00216162
197.9	0.27365656	0.20709093	-0.1471368	0.65174161	-0.8828794	0.63593498	-0.0137332	0.00216162
198	0.27365656	0.20709093	-0.1471368	0.65174161	-0.8828794	0.63593498	-0.0137332	0.00216162
198.1	0.27365656	0.20709093	-0.1471368	0.65174161	-0.8828794	0.63593498	-0.0137332	0.00216162
198.2	0.27365656	0.20709093	-0.1471368	0.65174161	-0.8828794	0.63593498	-0.0137332	0.00216162
198.3	0.27365656	0.20709093	-0.1471368	0.65174161	-0.8828794	0.63593498	-0.0137332	0.00216162
198.4	0.27365656	0.20709093	-0.1471368	0.65174161	-0.8828794	0.63593498	-0.0137332	0.00216162
198.5	0.27365656	0.20709093	-0.1471368	0.65174161	-0.8828794	0.63593498	-0.0137332	0.00216162
198.6	0.27365656	0.20709093	-0.1471368	0.65174161	-0.8828794	0.63593498	-0.0137332	0.00216162
198.7	0.27365656	0.20709093	-0.1471368	0.65174161	-0.8828794	0.63593498	-0.0137332	0.00216162
198.8	0.27365656	0.20709093	-0.1471368	0.65174161	-0.8828794	0.63593498	-0.0137332	0.00216162
198.9	0.27365656	0.20709093	-0.1471368	0.65174161	-0.8828794	0.63593498	-0.0137332	0.00216162
199	0.27365656	0.20709093	-0.1471368	0.65174161	-0.8828794	0.63593498	-0.0137332	0.00216162
199.1	0.27365656	0.20709093	-0.1471368	0.65174161	-0.8828794	0.63593498	-0.0137332	0.00216162
199.2	0.27365656	0.20709093	-0.1471368	0.65174161	-0.8828794	0.63593498	-0.0137332	0.00216162
199.3	0.27365656	0.20709093	-0.1471368	0.65174161	-0.8828794	0.63593498	-0.0137332	0.00216162
199.4	0.27365656	0.20709093	-0.1471368	0.65174161	-0.8828794	0.63593498	-0.0137332	0.00216162
199.5	0.27365656	0.20709093	-0.1471368	0.65174161	-0.8828794	0.63593498	-0.0137332	0.00216162
199.6	0.27365656	0.20709093	-0.1471368	0.65174161	-0.8828794	0.63593498	-0.0137332	0.00216162
199.7	0.27365656	0.20709093	-0.1471368	0.65174161	-0.8828794	0.63593498	-0.0137332	0.00216162
199.8	0.27365656	0.20709093	-0.1471368	0.65174161	-0.8828794	0.63593498	-0.0137332	0.00216162
199.9	0.27365656	0.20709093	-0.1471368	0.65174161	-0.8828794	0.63593498	-0.0137332	0.00216162
200	0.27365656	0.20709093	-0.1471368	0.65174161	-0.8828794	0.63593498	-0.0137332	0.00216162
200.1	0.27365656	0.20709093	-0.1471368	0.65174161	-0.8828794	0.63593498	-0.0137332	0.00216162
200.2	0.27365656	0.20709093	-0.1471368	0.65174161	-0.8828794	0.63593498	-0.0137332	0.00216162
200.3	0.27365656	0.20709093	-0.1471368	0.65174161	-0.8828794	0.63593498	-0.0137332	0.00216162
200.4	0.27365656	0.20709093	-0.1471368	0.65174161	-0.8828794	0.63593498	-0.0137332	0.00216162
200.5	0.27365656	0.20709093	-0.1471368	0.65174161	-0.8828794	0.63593498	-0.0137332	0.00216162
200.6	0.27365656	0.20709093	-0.1471368	0.65174161	-0.8828794	0.63593498	-0.0137332	0.00216162
200.7	0.27365656	0.20709093	-0.1471368	0.65174161	-0.8828794	0.63593498	-0.0137332	0.00216162
200.8	0.27342215	0.20692264	-0.147273	0.65170412	-0.8828615	0.63604028	-0.0142328	0.00203178
200.9	0.27342215	0.20692264	-0.147273	0.65170412	-0.8828615	0.63604028	-0.0142328	0.00203178
201	0.27342215	0.20692264	-0.147273	0.65170412	-0.8828615	0.63604028	-0.0142328	0.00203178
201.1	0.27342215	0.20692264	-0.147273	0.65170412	-0.8828615	0.63604028	-0.0142328	0.00203178
201.2	0.27342215	0.20692264	-0.147273	0.65170412	-0.8828615	0.63604028	-0.0142328	0.00203178
201.3	0.27342215	0.20692264	-0.147273	0.65170412	-0.8828615	0.63604028	-0.0142328	0.00203178
201.4	0.27342215	0.20692264	-0.147273	0.65170412	-0.8828615	0.63604028	-0.0142328	0.00203178
201.5	0.27342215	0.20692264	-0.147273	0.65170412	-0.8828615	0.63604028	-0.0142328	0.00203178
201.6	0.27342215	0.20692264	-0.147273	0.65170412	-0.8828615	0.63604028	-0.0142328	0.00203178
201.7	0.27342215	0.20692264	-0.147273	0.65170412	-0.8828615	0.63604028	-0.0142328	0.00203178
201.8	0.27342215	0.20692264	-0.147273	0.65170412	-0.8828615	0.63604028	-0.0142328	0.00203178
201.9	0.27342215	0.20692264	-0.147273	0.65170412	-0.8828615	0.63604028	-0.0142328	0.00203178
202	0.27342215	0.20692264	-0.147273	0.65170412	-0.8828615	0.63604028	-0.0142328	0.00203178
202.1	0.27342215	0.20692264	-0.147273	0.65170412	-0.8828615	0.63604028	-0.0142328	0.00203178
202.2	0.27342215	0.20692264	-0.147273	0.65170412	-0.8828615	0.63604028	-0.0142328	0.00203178
202.3	0.27342215	0.20692264	-0.147273	0.65170412	-0.8828615	0.63604028	-0.0142328	0.00203178
202.4	0.27342215	0.20692264	-0.147273	0.65170412	-0.8828615	0.63604028	-0.0142328	0.00203178
202.5	0.27342215	0.20692264	-0.147273	0.65170412	-0.8828615	0.63604028	-0.0142328	0.00203178
202.6	0.27342215	0.20692264	-0.147273	0.65170412	-0.8828615	0.63604028	-0.0142328	0.00203178
202.7	0.27342215	0.20692264	-0.147273	0.65170412	-0.8828615	0.63604028	-0.0142328	0.00203178
202.8	0.27342215	0.20692264	-0.147273	0.65170412	-0.8828615	0.63604028	-0.0142328	0.00203178
202.9	0.27342215	0.20692264	-0.147273	0.65170412	-0.8828615	0.63604028	-0.0142328	0.00203178
203	0.27342215	0.20692264	-0.147273	0.65170412	-0.8828615	0.63604028	-0.0142328	0.00203178
203.1	0.27342215	0.20692264	-0.147273	0.65170412	-0.8828615	0.63604028	-0.0142328	0.00203178
203.2	0.27342215	0.20692264	-0.147273	0.65170412	-0.8828615	0.63604028	-0.0142328	0.00203178
203.3	0.27342215	0.20692264	-0.147273	0.65170412	-0.8828615	0.63604028	-0.0142328	0.00203178
203.4	0.27342215	0.20692264	-0.147273	0.65170412	-0.8828615	0.63604028	-0.0142328	0.00203178
203.5	0.27342215	0.20692264	-0.147273	0.65170412	-0.8828615	0.63604028	-0.0142328	0.00203178
203.6	0.27342215	0.20692264	-0.147273	0.65170412	-0.8828615	0.63604028	-0.0142328	0.00203178
203.7	0.27342215	0.20692264	-0.147273	0.65170412	-0.8828615	0.63604028	-0.0142328	0.00203178
203.8	0.27342215	0.20692264	-0.147273	0.65170412	-0.8828615	0.63604028	-0.0142328	0.00203178
203.9	0.27342215	0.20692264	-0.147273	0.65170412	-0.8828615	0.63604028	-0.0142328	0.00203178
204	0.27342215	0.20692264	-0.147273	0.65170412	-0.8828615	0.63604028	-0.0142328	0.00203178
204.1	0.27342215	0.20692264	-0.147273	0.65170412	-0.8828615	0.63604028	-0.0142328	0.00203178
204.2	0.27342215	0.20692264	-0.147273	0.65170412	-0.8828615	0.63604028	-0.0142328	0.00203178
204.3	0.27342215	0.20692264	-0.147273	0.65170412	-0.8828615	0.63604028	-0.0142328	0.00203178
204.4	0.27342215	0.20692264	-0.147273	0.65170412	-0.8828615	0.63604028	-0.0142328	0.00203178
204.5	0.27342215	0.20692264	-0.147273	0.65170412	-0.8828615	0.63604028	-0.0142328	0.00203178
204.6	0.27342215	0.20692264	-0.147273	0.65170412	-0.8828615	0.63604028	-0.0142328	0.00203178
204.7	0.27342215	0.20692264	-0.147273	0.65170412	-0.8828615	0.63604028	-0.0142328	0.00203178
204.8	0.27342215	0.20692264	-0.147273	0.65170412	-0.8828615	0.63604028	-0.0142328	0.00203178
204.9	0.27342215	0.20692264	-0.147273	0.65170412	-0.8828615	0.63604028	-0.0142328	0.00203178
205	0.27342215	0.20692264	-0.147273	0.65170412	-0.8828615	0.63604028	-0.0142328	0.00203178
205.1	0.27342215	0.20692264	-0.147273	0.65170412	-0.8828615	0.63604028	-0.0142328	0.00203178
205.2	0.27342215	0.20692264	-0.147273	0.65170412	-0.8828615	0.63604028	-0.0142328	0.00203178
205.3	0.27281422	0.20624177	-0.1476273	0.65195139	-0.8828751	0.63598764	-0.0136203	0.00151955
205.4	0.27281422	0.20624177	-0.1476273	0.65195139	-0.8828751	0.63598764	-0.0136203	0.00151955
205.5	0.27281422	0.20624177	-0.1476273	0.65195139	-0.8828751	0.63598764	-0.0136203	0.00151955
205.6	0.27281422	0.20624177	-0.1476273	0.65195139	-0.8828751	0.63598764	-0.0136203	0.00151955
205.7	0.27281422	0.20624177	-0.1476273	0.65195139	-0.8828751	0.63598764	-0.0136203	0.00151955
205.8	0.27281422	0.20624177	-0.1476273	0.65195139	-0.8828751	0.63598764	-0.0136203	0.00151955
205.9	0.27281422	0.20624177	-0.1476273	0.65195139	-0.8828751	0.63598764	-0.0136203	0.00151955
206	0.27281422	0.20624177	-0.1476273	0.65195139	-0.8828751	0.63598764	-0.0136203	0.00151955
206.1	0.27281422	0.20624177	-0.1476273	0.65195139	-0.8828751	0.63598764	-0.0136203	0.00151955
206.2	0.27281422	0.20624177	-0.1476273	0.65195139	-0.8828751	0.63598764	-0.0136203	0.00151955
206.3	0.27281422	0.20624177	-0.1476273	0.65195139	-0.8828751	0.63598764	-0.0136203	0.00151955
206.4	0.27281422	0.20624177	-0.1476273	0.65195139	-0.8828751	0.63598764	-0.0136203	0.00151955
206.5	0.27281422	0.20624177	-0.1476273	0.65195139	-0.8828751	0.63598764	-0.0136203	0.00151955
206.6	0.27281422	0.20624177	-0.1476273	0.65195139	-0.8828751	0.63598764	-0.0136203	0.00151955
206.7	0.27281422	0.20624177	-0.1476273	0.65195139	-0.8828751	0.63598764	-0.0136203	0.00151955
206.8	0.27281422	0.20624177	-0.1476273	0.65195139	-0.8828751	0.63598764	-0.0136203	0.00151955
206.9	0.27281422	0.20624177	-0.1476273	0.65195139	-0.8828751	0.63598764	-0.0136203	0.00151955
207	0.27281422	0.20624177	-0.1476273	0.65195139	-0.8828751	0.63598764	-0.0136203	0.00151955
207.1	0.27281422	0.20624177	-0.1476273	0.65195139	-0.8828751	0.63598764	-0.0136203	0.00151955
207.2	0.27281422	0.20624177	-0.1476273	0.65195139	-0.8828751	0.63598764	-0.0136203	0.00151955
207.3	0.27281422	0.20624177	-0.1476273	0.65195139	-0.8828751	0.63598764	-0.0136203	0.00151955
207.4	0.27281422	0.20624177	-0.1476273	0.65195139	-0.8828751	0.63598764	-0.0136203	0.00151955
207.5	0.27281422	0.20624177	-0.1476273	0.65195139	-0.8828751	0.63598764	-0.0136203	0.00151955
207.6	0.27281422	0.20624177	-0.1476273	0.65195139	-0.8828751	0.63598764	-0.0136203	0.00151955
207.7	0.27281422	0.20624177	-0.1476273	0.65195139	-0.8828751	0.63598764	-0.0136203	0.00151955
207.8	0.27281422	0.20624177	-0.1476273	0.65195139	-0.8828751	0.63598764	-0.0136203	0.00151955
207.9	0.27281422	0.20624177	-0.1476273	0.65195139	-0.8828751	0.63598764	-0.0136203	0.00151955
208	0.27281422	0.20624177	-0.1476273	0.65195139	-0.8828751	0.63598764	-0.0136203	0.00151955
208.1	0.27281422	0.20624177	-0.1476273	0.65195139	-0.8828751	0.63598764	-0.0136203	0.00151955
208.2	0.27281422	0.20624177	-0.1476273	0.65195139	-0.8828751	0.63598764	-0.0136203	0.00151955
208.3	0.27281422	0.20624177	-0.1476273	0.65195139	-0.8828751	0.63598764	-0.0136203	0.00151955
208.4	0.27281422	0.20624177	-0.1476273	0.65195139	-0.8828751	0.63598764	-0.0136203	0.00151955
208.5	0.27281422	0.20624177	-0.1476273	0.65195139	-0.8828751	0.63598764	-0.0136203	0.00151955
208.6	0.27281422	0.20624177	-0.1476273	0.65195139	-0.8828751	0.63598764	-0.0136203	0.00151955
208.7	0.27281422	0.20624177	-0.1476273	0.65195139	-0.8828751	0.63598764	-0.0136203	0.00151955
208.8	0.27281422	0.20624177	-0.1476273	0.65195139	-0.8828751	0.63598764	-0.0136203	0.00151955
208.9	0.27281422	0.20624177	-0.1476273	0.65195139	-0.8828751	0.63598764	-0.0136203	0.00151955
209	0.27281422	0.20624177	-0.1476273	0.65195139	-0.8828751	0.63598764	-0.0136203	0.00151955
209.1	0.27281422	0.20624177	-0.1476273	0.65195139	-0.8828751	0.63598764	-0.0136203	0.00151955
209.2	0.27281422	0.20624177	-0.1476273	0.65195139	-0.8828751	0.63598764	-0.0136203	0.00151955
209.3	0.27281422	0.20624177	-0.1476273	0.65195139	-0.8828751	0.63598764	-0.0136203	0.00151955
209.4	0.27281422	0.20624177	-0.1476273	0.65195139	-0.8828751	0.63598764	-0.0136203	0.00151955
209.5	0.27281422	0.20624177	-0.1476273	0.65195139	-0.8828751	0.63598764	-0.0136203	0.00151955
209.6	0.27281422	0.20624177	-0.1476273	0.65195139	-0.8828751	0.63598764	-0.0136203	0.00151955
209.7	0.27281422	0.20624177	-0.1476273	0.65195139	-0.8828751	0.63598764	-0.0136203	0.00151955
209.8	0.27281422	0.20624177	-0.1476273	0.65195139	-0.8828751	0.63598764	-0.0136203	0.00151955
209.9	0.27281422	0.20624177	-0.1476273	0.65195139	-0.8828751	0.63598764	-0.0136203	0.00151955
210.6	0.27281422	0.20624177	-0.1476273	0.65195139	-0.8828751	0.63598764	-0.0136203	0.00151955
210.7	0.27281422	0.20624177	-0.1476273	0.65195139	-0.8828751	0.63598764	-0.0136203	0.00151955
210.8	0.27281422	0.20624177	-0.1476273	0.65195139	-0.8828751	0.63598764	-0.0136203	0.00151955
210.9	0.27281422	0.20624177	-0.1476273	0.65195139	-0.8828751	0.63598764	-0.0136203	0.00151955
211	0.27281422	0.20624177	-0.1476273	0.65195139	-0.8828751	0.63598764	-0.0136203	0.00151955
211.1	0.27281422	0.20624177	-0.1476273	0.65195139	-0.8828751	0.63598764	-0.0136203	0.00151955
211.2	0.27281422	0.20624177	-0.1476273	0.65195139	-0.8828751	0.63598764	-0.0136203	0.00151955
211.3	0.27281422	0.20624177	-0.1476273	0.65195139	-0.8828751	0.63598764	-0.0136203	0.00151955
211.4	0.27281422	0.20624177	-0.1476273	0.65195139	-0.8828751	0.63598764	-0.0136203	0.00151955
211.5	0.27281422	0.20624177	-0.1476273	0.65195139	-0.8828751	0.63598764	-0.0136203	0.00151955
211.6	0.27281422	0.20624177	-0.1476273	0.65195139	-0.8828751	0.63598764	-0.0136203	0.00151955
211.7	0.27281422	0.20624177	-0.1476273	0.65195139	-0.8828751	0.63598764	-0.0136203	0.00151955
211.8	0.27281422	0.20624177	-0.1476273	0.65195139	-0.8828751	0.63598764	-0.0136203	0.00151955
211.9	0.27281422	0.20624177	-0.1476273	0.65195139	-0.8828751	0.63598764	-0.0136203	0.00151955
212	0.27281422	0.20624177	-0.1476273	0.65195139	-0.8828751	0.63598764	-0.0136203	0.00151955
212.1	0.27281422	0.20624177	-0.1476273	0.65195139	-0.8828751	0.63598764	-0.0136203	0.00151955
212.2	0.27281422	0.20624177	-0.1476273	0.65195139	-0.8828751	0.63598764	-0.0136203	0.00151955
212.3	0.27281422	0.20624177	-0.1476273	0.65195139	-0.8828751	0.63598764	-0.0136203	0.00151955
212.4	0.27281422	0.20624177	-0.1476273	0.65195139	-0.8828751	0.63598764	-0.0136203	0.00151955
212.5	0.27281422	0.20624177	-0.1476273	0.65195139	-0.8828751	0.63598764	-0.0136203	0.00151955
212.6	0.27281422	0.20624177	-0.1476273	0.65195139	-0.8828751	0.63598764	-0.0136203	0.00151955
212.7	0.27281422	0.20624177	-0.1476273	0.65195139	-0.8828751	0.63598764	-0.0136203	0.00151955
212.8	0.27281422	0.20624177	-0.1476273	0.65195139	-0.8828751	0.63598764	-0.0136203	0.00151955
212.9	0.27281422	0.20624177	-0.1476273	0.65195139	-0.8828751	0.63598764	-0.0136203	0.00151955
213	0.27281422	0.20624177	-0.1476273	0.65195139	-0.8828751	0.63598764	-0.0136203	0.00151955
213.1	0.27281422	0.20624177	-0.1476273	0.65195139	-0.8828751	0.63598764	-0.0136203	0.00151955
213.2	0.27281422	0.20624177	-0.1476273	0.65195139	-0.8828751	0.63598764	-0.0136203	0.00151955
213.3	0.27281422	0.20624177	-0.1476273	0.65195139	-0.8828751	0.63598764	-0.0136203	0.00151955
213.4	0.27281422	0.20624177	-0.1476273	0.65195139	-0.8828751	0.63598764	-0.0136203	0.00151955
213.5	0.27281422	0.20624177	-0.1476273	0.65195139	-0.8828751	0.63598764	-0.0136203	0.00151955
213.6	0.27281422	0.20624177	-0.1476273	0.65195139	-0.8828751	0.63598764	-0.0136203	0.00151955
213.7	0.27281422	0.20624177	-0.1476273	0.65195139	-0.8828751	0.63598764	-0.0136203	0.00151955
213.8	0.27281422	0.20624177	-0.1476273	0.65195139	-0.8828751	0.63598764	-0.0136203	0.00151955
213.9	0.27281422	0.20624177	-0.1476273	0.65195139	-0.8828751	0.63598764	-0.0136203	0.00151955
214	0.27281422	0.20624177	-0.1476273	0.65195139	-0.8828751	0.63598764	-0.0136203	0.00151955
214.1	0.27281422	0.20624177	-0.1476273	0.65195139	-0.8828751	0.63598764	-0.0136203	0.00151955
214.2	0.27281422	0.20624177	-0.1476273	0.65195139	-0.8828751	0.63598764	-0.0136203	0.00151955
214.3	0.27281422	0.20624177	-0.1476273	0.65195139	-0.8828751	0.63598764	-0.0136203	0.00151955
214.4	0.27281422	0.20624177	-0.1476273	0.65195139	-0.8828751	0.63598764	-0.0136203	0.00151955
214.5	0.27281422	0.20624177	-0.1476273	0.65195139	-0.8828751	0.63598764	-0.0136203	0.00151955
214.6	0.27281422	0.20624177	-0.1476273	0.65195139	-0.8828751	0.63598764	-0.0136203	0.00151955
214.7	0.27281422	0.20624177	-0.1476273	0.65195139	-0.8828751	0.63598764	-0.0136203	0.00151955
214.8	0.27281422	0.20624177	-0.1476273	0.65195139	-0.8828751	0.63598764	-0.0136203	0.00151955
214.9	0.27281422	0.20624177	-0.1476273	0.65195139	-0.8828751	0.63598764	-0.0136203	0.00151955
215	0.27281422	0.20624177	-0.1476273	0.65195139	-0.8828751	0.63598764	-0.0136203	0.00151955
215.1	0.27281422	0.20624177	-0.1476273	0.65195139	-0.8828751	0.63598764	-0.0136203	0.00151955
215.2	0.27281422	0.20624177	-0.1476273	0.65195139	-0.8828751	0.63598764	-0.0136203	0.00151955
215.3	0.27281422	0.20624177	-0.1476273	0.65195139	-0.8828751	0.63598764	-0.0136203	0.00151955
215.4	0.27281422	0.20624177	-0.1476273	0.65195139	-0.8828751	0.63598764	-0.0136203	0.00151955
215.5	0.27281422	0.20624177	-0.1476273	0.65195139	-0.8828751	0.63598764	-0.0136203	0.00151955
215.6	0.27281422	0.20624177	-0.1476273	0.65195139	-0.8828751	0.63598764	-0.0136203	0.00151955
215.7	0.27281422	0.20624177	-0.1476273	0.65195139	-0.8828751	0.63598764	-0.0136203	0.00151955
215.8	0.27281422	0.20624177	-0.1476273	0.65195139	-0.8828751	0.63598764	-0.0136203	0.00151955
215.9	0.27281422	0.20624177	-0.1476273	0.65195139	-0.8828751	0.63598764	-0.0136203	0.00151955
216	0.27281422	0.20624177	-0.1476273	0.65195139	-0.8828751	0.63598764	-0.0136203	0.00151955
216.1	0.27281422	0.20624177	-0.1476273	0.65195139	-0.8828751	0.63598764	-0.0136203	0.00151955
216.2	0.27281422	0.20624177	-0.1476273	0.65195139	-0.8828751	0.63598764	-0.0136203	0.00151955
216.3	0.27281422	0.20624177	-0.1476273	0.65195139	-0.8828751	0.63598764	-0.0136203	0.00151955
216.4	0.27281422	0.20624177	-0.1476273	0.65195139	-0.8828751	0.63598764	-0.0136203	0.00151955
216.5	0.27281422	0.20624177	-0.1476273	0.65195139	-0.8828751	0.63598764	-0.0136203	0.00151955
216.6	0.27281422	0.20624177	-0.1476273	0.65195139	-0.8828751	0.63598764	-0.0136203	0.00151955
216.7	0.27281422	0.20624177	-0.1476273	0.65195139	-0.8828751	0.63598764	-0.0136203	0.00151955
216.8	0.27281422	0.20624177	-0.1476273	0.65195139	-0.8828751	0.63598764	-0.0136203	0.00151955
216.9	0.27281422	0.20624177	-0.1476273	0.65195139	-0.8828751	0.63598764	-0.0136203	0.00151955
217	0.27281422	0.20624177	-0.1476273	0.65195139	-0.8828751	0.63598764	-0.0136203	0.00151955
217.1	0.27281422	0.20624177	-0.1476273	0.65195139	-0.8828751	0.63598764	-0.0136203	0.00151955
217.2	0.27281422	0.20624177	-0.1476273	0.65195139	-0.8828751	0.63598764	-0.0136203	0.00151955
217.3	0.27281422	0.20624177	-0.1476273	0.65195139	-0.8828751	0.63598764	-0.0136203	0.00151955
217.4	0.27281422	0.20624177	-0.1476273	0.65195139	-0.8828751	0.63598764	-0.0136203	0.00151955
217.5	0.27281422	0.20624177	-0.1476273	0.65195139	-0.8828751	0.63598764	-0.0136203	0.00151955
217.6	0.27281422	0.20624177	-0.1476273	0.65195139	-0.8828751	0.63598764	-0.0136203	0.00151955
217.7	0.27281422	0.20624177	-0.1476273	0.65195139	-0.8828751	0.63598764	-0.0136203	0.00151955
217.8	0.27281422	0.20624177	-0.1476273	0.65195139	-0.8828751	0.63598764	-0.0136203	0.00151955
217.9	0.27281422	0.20624177	-0.1476273	0.65195139	-0.8828751	0.63598764	-0.0136203	0.00151955
218	0.27281422	0.20624177	-0.1476273	0.65195139	-0.8828751	0.63598764	-0.0136203	0.00151955
218.1	0.27281422	0.20624177	-0.1476273	0.65195139	-0.8828751	0.63598764	-0.0136203	0.00151955
218.2	0.27281422	0.20624177	-0.1476273	0.65195139	-0.8828751	0.63598764	-0.0136203	0.00151955
218.3	0.27281422	0.20624177	-0.1476273	0.65195139	-0.8828751	0.63598764	-0.0136203	0.00151955
218.4	0.27281422	0.20624177	-0.1476273	0.65195139	-0.8828751	0.63598764	-0.0136203	0.00151955
218.5	0.27281422	0.20624177	-0.1476273	0.65195139	-0.8828751	0.63598764	-0.0136203	0.00151955
218.6	0.27281422	0.20624177	-0.1476273	0.65195139	-0.8828751	0.63598764	-0.0136203	0.00151955
218.7	0.27281422	0.20624177	-0.1476273	0.65195139	-0.8828751	0.63598764	-0.0136203	0.00151955
218.8	0.27281422	0.20624177	-0.1476273	0.65195139	-0.8828751	0.63598764	-0.0136203	0.00151955
218.9	0.27281422	0.20624177	-0.1476273	0.65195139	-0.8828751	0.63598764	-0.0136203	0.00151955
219	0.27281422	0.20624177	-0.1476273	0.65195139	-0.8828751	0.63598764	-0.0136203	0.00151955
219.1	0.27281422	0.20624177	-0.1476273	0.65195139	-0.8828751	0.63598764	-0.0136203	0.00151955
219.2	0.27272144	0.20578613	-0.1472093	0.65183056	-0.8828157	0.63584657	-0.013224	0.00146258
219.3	0.27272144	0.20578613	-0.1472093	0.65183056	-0.8828157	0.63584657	-0.013224	0.00146258
219.4	0.27258691	0.20566909	-0.1467259	0.651742	-0.8827721	0.63565016	-0.0132718	0.00144905
219.5	0.27258691	0.20566909	-0.1467259	0.651742	-0.8827721	0.63565016	-0.0132718	0.00144905
219.6	0.27258691	0.20566909	-0.1467259	0.651742	-0.8827721	0.63565016	-0.0132718	0.00144905
219.7	0.27228773	0.20567775	-0.146685	0.65209691	-0.8828804	0.63555498	-0.0128265	0.00147037
219.8	0.27228773	0.20567775	-0.146685	0.65209691	-0.8828804	0.63555498	-0.0128265	0.00147037
219.9	0.27228773	0.20567775	-0.146685	0.65209691	-0.8828804	0.63555498	-0.0128265	0.00147037
220	0.27228773	0.20567775	-0.146685	0.65209691	-0.8828804	0.63555498	-0.0128265	0.00147037
220.1	0.27228773	0.20567775	-0.146685	0.65209691	-0.8828804	0.63555498	-0.0128265	0.00147037
220.2	0.27228773	0.20567775	-0.146685	0.65209691	-0.8828804	0.63555498	-0.0128265	0.00147037
220.3	0.27228773	0.20567775	-0.146685	0.65209691	-0.8828804	0.63555498	-0.0128265	0.00147037
220.4	0.27228773	0.20567775	-0.146685	0.65209691	-0.8828804	0.63555498	-0.0128265	0.00147037
220.5	0.27228773	0.20567775	-0.146685	0.65209691	-0.8828804	0.63555498	-0.0128265	0.00147037
220.6	0.27228773	0.20567775	-0.146685	0.65209691	-0.8828804	0.63555498	-0.0128265	0.00147037
220.7	0.27228773	0.20567775	-0.146685	0.65209691	-0.8828804	0.63555498	-0.0128265	0.00147037
220.8	0.27228773	0.20567775	-0.146685	0.65209691	-0.8828804	0.63555498	-0.0128265	0.00147037
220.9	0.27228773	0.20567775	-0.146685	0.65209691	-0.8828804	0.63555498	-0.0128265	0.00147037
221	0.27228773	0.20567775	-0.146685	0.65209691	-0.8828804	0.63555498	-0.0128265	0.00147037
221.1	0.27228773	0.20567775	-0.146685	0.65209691	-0.8828804	0.63555498	-0.0128265	0.00147037
221.2	0.27228773	0.20567775	-0.146685	0.65209691	-0.8828804	0.63555498	-0.0128265	0.00147037
221.3	0.27228773	0.20567775	-0.146685	0.65209691	-0.8828804	0.63555498	-0.0128265	0.00147037
221.4	0.27228773	0.20567775	-0.146685	0.65209691	-0.8828804	0.63555498	-0.0128265	0.00147037
221.5	0.27228773	0.20567775	-0.146685	0.65209691	-0.8828804	0.63555498	-0.0128265	0.00147037
221.6	0.27228773	0.20567775	-0.146685	0.65209691	-0.8828804	0.63555498	-0.0128265	0.00147037
221.7	0.27228773	0.20567775	-0.146685	0.65209691	-0.8828804	0.63555498	-0.0128265	0.00147037
221.8	0.27228773	0.20567775	-0.146685	0.65209691	-0.8828804	0.63555498	-0.0128265	0.00147037
221.9	0.27228773	0.20567775	-0.146685	0.65209691	-0.8828804	0.63555498	-0.0128265	0.00147037
222	0.27228773	0.20567775	-0.146685	0.65209691	-0.8828804	0.63555498	-0.0128265	0.00147037
222.1	0.27228773	0.20567775	-0.146685	0.65209691	-0.8828804	0.63555498	-0.0128265	0.00147037
222.2	0.27228773	0.20567775	-0.146685	0.65209691	-0.8828804	0.63555498	-0.0128265	0.00147037
222.3	0.27228773	0.20567775	-0.146685	0.65209691	-0.8828804	0.63555498	-0.0128265	0.00147037
222.4	0.27228773	0.20567775	-0.146685	0.65209691	-0.8828804	0.63555498	-0.0128265	0.00147037
222.5	0.27228773	0.20567775	-0.146685	0.65209691	-0.8828804	0.63555498	-0.0128265	0.00147037
222.6	0.27228773	0.20567775	-0.146685	0.65209691	-0.8828804	0.63555498	-0.0128265	0.00147037
222.7	0.27228773	0.20567775	-0.146685	0.65209691	-0.8828804	0.63555498	-0.0128265	0.00147037
222.8	0.27228773	0.20567775	-0.146685	0.65209691	-0.8828804	0.63555498	-0.0128265	0.00147037
222.9	0.27228773	0.20567775	-0.146685	0.65209691	-0.8828804	0.63555498	-0.0128265	0.00147037
223	0.27228773	0.20567775	-0.146685	0.65209691	-0.8828804	0.63555498	-0.0128265	0.00147037
223.1	0.27228773	0.20567775	-0.146685	0.65209691	-0.8828804	0.63555498	-0.0128265	0.00147037
223.2	0.27228773	0.20567775	-0.146685	0.65209691	-0.8828804	0.63555498	-0.0128265	0.00147037
223.3	0.27228773	0.20567775	-0.146685	0.65209691	-0.8828804	0.63555498	-0.0128265	0.00147037
223.4	0.27228773	0.20567775	-0.146685	0.65209691	-0.8828804	0.63555498	-0.0128265	0.00147037
223.5	0.27228773	0.20567775	-0.146685	0.65209691	-0.8828804	0.63555498	-0.0128265	0.00147037
223.6	0.27228773	0.20567775	-0.146685	0.65209691	-0.8828804	0.63555498	-0.0128265	0.00147037
223.7	0.27228773	0.20567775	-0.146685	0.65209691	-0.8828804	0.63555498	-0.0128265	0.00147037
223.8	0.27187639	0.20523631	-0.1464681	0.65222188	-0.8829882	0.63557399	-0.0126457	0.00162107
223.9	0.27187639	0.20523631	-0.1464681	0.65222188	-0.8829882	0.63557399	-0.0126457	0.00162107
224	0.27187639	0.20523631	-0.1464681	0.65222188	-0.8829882	0.63557399	-0.0126457	0.00162107
224.1	0.27187639	0.20523631	-0.1464681	0.65222188	-0.8829882	0.63557399	-0.0126457	0.00162107
224.2	0.27187639	0.20523631	-0.1464681	0.65222188	-0.8829882	0.63557399	-0.0126457	0.00162107
224.3	0.27187639	0.20523631	-0.1464681	0.65222188	-0.8829882	0.63557399	-0.0126457	0.00162107
224.4	0.27187639	0.20523631	-0.1464681	0.65222188	-0.8829882	0.63557399	-0.0126457	0.00162107
224.5	0.27187639	0.20523631	-0.1464681	0.65222188	-0.8829882	0.63557399	-0.0126457	0.00162107
224.6	0.27187639	0.20523631	-0.1464681	0.65222188	-0.8829882	0.63557399	-0.0126457	0.00162107
224.7	0.27130521	0.20520008	-0.146371	0.65212524	-0.8829604	0.63572271	-0.0121712	0.00179779
224.8	0.27130521	0.20520008	-0.146371	0.65212524	-0.8829604	0.63572271	-0.0121712	0.00179779
224.9	0.27130521	0.20520008	-0.146371	0.65212524	-0.8829604	0.63572271	-0.0121712	0.00179779
225.6	0.27130521	0.20520008	-0.146371	0.65212524	-0.8829604	0.63572271	-0.0121712	0.00179779
225.7	0.27130521	0.20520008	-0.146371	0.65212524	-0.8829604	0.63572271	-0.0121712	0.00179779
225.8	0.27130521	0.20520008	-0.146371	0.65212524	-0.8829604	0.63572271	-0.0121712	0.00179779
225.9	0.27130521	0.20520008	-0.146371	0.65212524	-0.8829604	0.63572271	-0.0121712	0.00179779
226	0.27130521	0.20520008	-0.146371	0.65212524	-0.8829604	0.63572271	-0.0121712	0.00179779
226.1	0.27130521	0.20520008	-0.146371	0.65212524	-0.8829604	0.63572271	-0.0121712	0.00179779
226.2	0.27130521	0.20520008	-0.146371	0.65212524	-0.8829604	0.63572271	-0.0121712	0.00179779
226.3	0.27130521	0.20520008	-0.146371	0.65212524	-0.8829604	0.63572271	-0.0121712	0.00179779
226.4	0.27130521	0.20520008	-0.146371	0.65212524	-0.8829604	0.63572271	-0.0121712	0.00179779
226.5	0.27130521	0.20520008	-0.146371	0.65212524	-0.8829604	0.63572271	-0.0121712	0.00179779
226.6	0.27130521	0.20520008	-0.146371	0.65212524	-0.8829604	0.63572271	-0.0121712	0.00179779
226.7	0.27130521	0.20520008	-0.146371	0.65212524	-0.8829604	0.63572271	-0.0121712	0.00179779
226.8	0.27130521	0.20520008	-0.146371	0.65212524	-0.8829604	0.63572271	-0.0121712	0.00179779
226.9	0.27130521	0.20520008	-0.146371	0.65212524	-0.8829604	0.63572271	-0.0121712	0.00179779
227	0.27130521	0.20520008	-0.146371	0.65212524	-0.8829604	0.63572271	-0.0121712	0.00179779
227.1	0.27130521	0.20520008	-0.146371	0.65212524	-0.8829604	0.63572271	-0.0121712	0.00179779
227.2	0.27130521	0.20520008	-0.146371	0.65212524	-0.8829604	0.63572271	-0.0121712	0.00179779
227.3	0.27130521	0.20520008	-0.146371	0.65212524	-0.8829604	0.63572271	-0.0121712	0.00179779
227.4	0.27130521	0.20520008	-0.146371	0.65212524	-0.8829604	0.63572271	-0.0121712	0.00179779
227.5	0.27130521	0.20520008	-0.146371	0.65212524	-0.8829604	0.63572271	-0.0121712	0.00179779
227.6	0.27130521	0.20520008	-0.146371	0.65212524	-0.8829604	0.63572271	-0.0121712	0.00179779
227.7	0.27130521	0.20520008	-0.146371	0.65212524	-0.8829604	0.63572271	-0.0121712	0.00179779
227.8	0.27130521	0.20520008	-0.146371	0.65212524	-0.8829604	0.63572271	-0.0121712	0.00179779
227.9	0.27130521	0.20520008	-0.146371	0.65212524	-0.8829604	0.63572271	-0.0121712	0.00179779
228	0.27130521	0.20520008	-0.146371	0.65212524	-0.8829604	0.63572271	-0.0121712	0.00179779
228.1	0.27130521	0.20520008	-0.146371	0.65212524	-0.8829604	0.63572271	-0.0121712	0.00179779
228.2	0.27130521	0.20520008	-0.146371	0.65212524	-0.8829604	0.63572271	-0.0121712	0.00179779
228.3	0.27130521	0.20520008	-0.146371	0.65212524	-0.8829604	0.63572271	-0.0121712	0.00179779
228.4	0.27130521	0.20520008	-0.146371	0.65212524	-0.8829604	0.63572271	-0.0121712	0.00179779
228.5	0.27130521	0.20520008	-0.146371	0.65212524	-0.8829604	0.63572271	-0.0121712	0.00179779
228.6	0.27130521	0.20520008	-0.146371	0.65212524	-0.8829604	0.63572271	-0.0121712	0.00179779
228.7	0.27130521	0.20520008	-0.146371	0.65212524	-0.8829604	0.63572271	-0.0121712	0.00179779
228.8	0.27149317	0.2045753	-0.1461938	0.65195836	-0.8829243	0.63592475	-0.0120927	0.00195338
228.9	0.27149317	0.2045753	-0.1461938	0.65195836	-0.8829243	0.63592475	-0.0120927	0.00195338
229	0.27149317	0.2045753	-0.1461938	0.65195836	-0.8829243	0.63592475	-0.0120927	0.00195338
229.1	0.27206052	0.20383182	-0.1456368	0.65169252	-0.8828848	0.63616623	-0.01188	0.00243775
229.2	0.27267339	0.20388095	-0.14512	0.65162959	-0.8828987	0.63609914	-0.0112461	0.00289641
229.3	0.27267339	0.20388095	-0.14512	0.65162959	-0.8828987	0.63609914	-0.0112461	0.00289641
229.4	0.27267339	0.20388095	-0.14512	0.65162959	-0.8828987	0.63609914	-0.0112461	0.00289641
229.5	0.27288833	0.20388733	-0.1451821	0.65197466	-0.8830474	0.63624047	-0.0106172	0.00326114
229.6	0.27288833	0.20388733	-0.1451821	0.65197466	-0.8830474	0.63624047	-0.0106172	0.00326114
229.7	0.27288833	0.20388733	-0.1451821	0.65197466	-0.8830474	0.63624047	-0.0106172	0.00326114
229.8	0.27288833	0.20388733	-0.1451821	0.65197466	-0.8830474	0.63624047	-0.0106172	0.00326114
229.9	0.27286939	0.20372556	-0.1453812	0.65228321	-0.8831785	0.63648812	-0.0104879	0.00349625
230	0.27286939	0.20372556	-0.1453812	0.65228321	-0.8831785	0.63648812	-0.0104879	0.00349625
230.1	0.27286939	0.20372556	-0.1453812	0.65228321	-0.8831785	0.63648812	-0.0104879	0.00349625
230.2	0.27286939	0.20372556	-0.1453812	0.65228321	-0.8831785	0.63648812	-0.0104879	0.00349625
230.3	0.27286939	0.20372556	-0.1453812	0.65228321	-0.8831785	0.63648812	-0.0104879	0.00349625
230.4	0.27286939	0.20372556	-0.1453812	0.65228321	-0.8831785	0.63648812	-0.0104879	0.00349625
230.5	0.27286939	0.20372556	-0.1453812	0.65228321	-0.8831785	0.63648812	-0.0104879	0.00349625
230.6	0.27286939	0.20372556	-0.1453812	0.65228321	-0.8831785	0.63648812	-0.0104879	0.00349625
230.7	0.27286939	0.20372556	-0.1453812	0.65228321	-0.8831785	0.63648812	-0.0104879	0.00349625
230.8	0.27286939	0.20372556	-0.1453812	0.65228321	-0.8831785	0.63648812	-0.0104879	0.00349625
230.9	0.27233631	0.20423922	-0.1455376	0.65249517	-0.8831825	0.63664904	-0.0108021	0.00291744
231	0.27233631	0.20423922	-0.1455376	0.65249517	-0.8831825	0.63664904	-0.0108021	0.00291744
231.1	0.27233631	0.20423922	-0.1455376	0.65249517	-0.8831825	0.63664904	-0.0108021	0.00291744
231.2	0.27233631	0.20423922	-0.1455376	0.65249517	-0.8831825	0.63664904	-0.0108021	0.00291744
231.3	0.27233631	0.20423922	-0.1455376	0.65249517	-0.8831825	0.63664904	-0.0108021	0.00291744
231.4	0.27233631	0.20423922	-0.1455376	0.65249517	-0.8831825	0.63664904	-0.0108021	0.00291744
231.5	0.27233631	0.20423922	-0.1455376	0.65249517	-0.8831825	0.63664904	-0.0108021	0.00291744
231.6	0.27233631	0.20423922	-0.1455376	0.65249517	-0.8831825	0.63664904	-0.0108021	0.00291744
231.7	0.27233631	0.20423922	-0.1455376	0.65249517	-0.8831825	0.63664904	-0.0108021	0.00291744
231.8	0.27233631	0.20423922	-0.1455376	0.65249517	-0.8831825	0.63664904	-0.0108021	0.00291744
231.9	0.27233631	0.20423922	-0.1455376	0.65249517	-0.8831825	0.63664904	-0.0108021	0.00291744
232	0.27233631	0.20423922	-0.1455376	0.65249517	-0.8831825	0.63664904	-0.0108021	0.00291744
232.1	0.27233631	0.20423922	-0.1455376	0.65249517	-0.8831825	0.63664904	-0.0108021	0.00291744
232.2	0.27238767	0.20362363	-0.1452171	0.65289026	-0.8832175	0.63671011	-0.0107891	0.00320161
232.3	0.27238767	0.20362363	-0.1452171	0.65289026	-0.8832175	0.63671011	-0.0107891	0.00320161
232.4	0.27238767	0.20362363	-0.1452171	0.65289026	-0.8832175	0.63671011	-0.0107891	0.00320161
232.5	0.27238767	0.20362363	-0.1452171	0.65289026	-0.8832175	0.63671011	-0.0107891	0.00320161
232.6	0.27238767	0.20362363	-0.1452171	0.65289026	-0.8832175	0.63671011	-0.0107891	0.00320161
232.7	0.27238767	0.20362363	-0.1452171	0.65289026	-0.8832175	0.63671011	-0.0107891	0.00320161
232.8	0.27238767	0.20362363	-0.1452171	0.65289026	-0.8832175	0.63671011	-0.0107891	0.00320161
232.9	0.27238767	0.20362363	-0.1452171	0.65289026	-0.8832175	0.63671011	-0.0107891	0.00320161
233	0.27238767	0.20362363	-0.1452171	0.65289026	-0.8832175	0.63671011	-0.0107891	0.00320161
233.1	0.27238767	0.20362363	-0.1452171	0.65289026	-0.8832175	0.63671011	-0.0107891	0.00320161
233.2	0.27238767	0.20362363	-0.1452171	0.65289026	-0.8832175	0.63671011	-0.0107891	0.00320161
233.3	0.27238767	0.20362363	-0.1452171	0.65289026	-0.8832175	0.63671011	-0.0107891	0.00320161
233.4	0.27238767	0.20362363	-0.1452171	0.65289026	-0.8832175	0.63671011	-0.0107891	0.00320161
233.5	0.27238767	0.20362363	-0.1452171	0.65289026	-0.8832175	0.63671011	-0.0107891	0.00320161
233.6	0.27238767	0.20362363	-0.1452171	0.65289026	-0.8832175	0.63671011	-0.0107891	0.00320161
233.7	0.27238767	0.20362363	-0.1452171	0.65289026	-0.8832175	0.63671011	-0.0107891	0.00320161
233.8	0.27238767	0.20362363	-0.1452171	0.65289026	-0.8832175	0.63671011	-0.0107891	0.00320161
233.9	0.27238767	0.20362363	-0.1452171	0.65289026	-0.8832175	0.63671011	-0.0107891	0.00320161
234	0.27238767	0.20362363	-0.1452171	0.65289026	-0.8832175	0.63671011	-0.0107891	0.00320161
234.1	0.27238767	0.20362363	-0.1452171	0.65289026	-0.8832175	0.63671011	-0.0107891	0.00320161
234.2	0.27238767	0.20362363	-0.1452171	0.65289026	-0.8832175	0.63671011	-0.0107891	0.00320161
234.3	0.27238767	0.20362363	-0.1452171	0.65289026	-0.8832175	0.63671011	-0.0107891	0.00320161
234.4	0.27238767	0.20362363	-0.1452171	0.65289026	-0.8832175	0.63671011	-0.0107891	0.00320161
234.5	0.27238767	0.20362363	-0.1452171	0.65289026	-0.8832175	0.63671011	-0.0107891	0.00320161
234.6	0.27238767	0.20362363	-0.1452171	0.65289026	-0.8832175	0.63671011	-0.0107891	0.00320161
234.7	0.27238767	0.20362363	-0.1452171	0.65289026	-0.8832175	0.63671011	-0.0107891	0.00320161
234.8	0.27238767	0.20362363	-0.1452171	0.65289026	-0.8832175	0.63671011	-0.0107891	0.00320161
234.9	0.27238767	0.20362363	-0.1452171	0.65289026	-0.8832175	0.63671011	-0.0107891	0.00320161
235	0.27238767	0.20362363	-0.1452171	0.65289026	-0.8832175	0.63671011	-0.0107891	0.00320161
235.1	0.27238767	0.20362363	-0.1452171	0.65289026	-0.8832175	0.63671011	-0.0107891	0.00320161
235.2	0.27238767	0.20362363	-0.1452171	0.65289026	-0.8832175	0.63671011	-0.0107891	0.00320161
235.3	0.27238767	0.20362363	-0.1452171	0.65289026	-0.8832175	0.63671011	-0.0107891	0.00320161
235.4	0.27238767	0.20362363	-0.1452171	0.65289026	-0.8832175	0.63671011	-0.0107891	0.00320161
235.5	0.27238767	0.20362363	-0.1452171	0.65289026	-0.8832175	0.63671011	-0.0107891	0.00320161
235.6	0.27238767	0.20362363	-0.1452171	0.65289026	-0.8832175	0.63671011	-0.0107891	0.00320161
235.7	0.27238767	0.20362363	-0.1452171	0.65289026	-0.8832175	0.63671011	-0.0107891	0.00320161
235.8	0.27238767	0.20362363	-0.1452171	0.65289026	-0.8832175	0.63671011	-0.0107891	0.00320161
235.9	0.27238767	0.20362363	-0.1452171	0.65289026	-0.8832175	0.63671011	-0.0107891	0.00320161
236	0.27238767	0.20362363	-0.1452171	0.65289026	-0.8832175	0.63671011	-0.0107891	0.00320161
236.1	0.27238767	0.20362363	-0.1452171	0.65289026	-0.8832175	0.63671011	-0.0107891	0.00320161
236.2	0.27238767	0.20362363	-0.1452171	0.65289026	-0.8832175	0.63671011	-0.0107891	0.00320161
236.3	0.27295383	0.20300567	-0.1450003	0.65327526	-0.8832929	0.63700735	-0.0105933	0.00382934
236.4	0.27295383	0.20300567	-0.1450003	0.65327526	-0.8832929	0.63700735	-0.0105933	0.00382934
236.5	0.27295383	0.20300567	-0.1450003	0.65327526	-0.8832929	0.63700735	-0.0105933	0.00382934
236.6	0.27295383	0.20300567	-0.1450003	0.65327526	-0.8832929	0.63700735	-0.0105933	0.00382934
236.7	0.27295383	0.20300567	-0.1450003	0.65327526	-0.8832929	0.63700735	-0.0105933	0.00382934
236.8	0.27295383	0.20300567	-0.1450003	0.65327526	-0.8832929	0.63700735	-0.0105933	0.00382934
236.9	0.27295383	0.20300567	-0.1450003	0.65327526	-0.8832929	0.63700735	-0.0105933	0.00382934
237	0.27295383	0.20300567	-0.1450003	0.65327526	-0.8832929	0.63700735	-0.0105933	0.00382934
237.1	0.27295383	0.20300567	-0.1450003	0.65327526	-0.8832929	0.63700735	-0.0105933	0.00382934
237.2	0.27295383	0.20300567	-0.1450003	0.65327526	-0.8832929	0.63700735	-0.0105933	0.00382934
237.3	0.27295383	0.20300567	-0.1450003	0.65327526	-0.8832929	0.63700735	-0.0105933	0.00382934
237.4	0.27295383	0.20300567	-0.1450003	0.65327526	-0.8832929	0.63700735	-0.0105933	0.00382934
237.5	0.27295383	0.20300567	-0.1450003	0.65327526	-0.8832929	0.63700735	-0.0105933	0.00382934
237.6	0.27295383	0.20300567	-0.1450003	0.65327526	-0.8832929	0.63700735	-0.0105933	0.00382934
237.7	0.27295383	0.20300567	-0.1450003	0.65327526	-0.8832929	0.63700735	-0.0105933	0.00382934
237.8	0.27295383	0.20300567	-0.1450003	0.65327526	-0.8832929	0.63700735	-0.0105933	0.00382934
237.9	0.27295383	0.20300567	-0.1450003	0.65327526	-0.8832929	0.63700735	-0.0105933	0.00382934
238	0.27295383	0.20300567	-0.1450003	0.65327526	-0.8832929	0.63700735	-0.0105933	0.00382934
238.1	0.27295383	0.20300567	-0.1450003	0.65327526	-0.8832929	0.63700735	-0.0105933	0.00382934
238.2	0.27346863	0.20300381	-0.1451041	0.65326556	-0.8833333	0.63724344	-0.0104102	0.00417295
238.3	0.27346863	0.20300381	-0.1451041	0.65326556	-0.8833333	0.63724344	-0.0104102	0.00417295
238.4	0.27346863	0.20300381	-0.1451041	0.65326556	-0.8833333	0.63724344	-0.0104102	0.00417295
238.5	0.27346863	0.20300381	-0.1451041	0.65326556	-0.8833333	0.63724344	-0.0104102	0.00417295
238.6	0.27304679	0.20240435	-0.1448032	0.65323635	-0.8832674	0.63692452	-0.0096425	0.00389193
238.7	0.27304679	0.20240435	-0.1448032	0.65323635	-0.8832674	0.63692452	-0.0096425	0.00389193
238.8	0.27304679	0.20240435	-0.1448032	0.65323635	-0.8832674	0.63692452	-0.0096425	0.00389193
238.9	0.27304679	0.20240435	-0.1448032	0.65323635	-0.8832674	0.63692452	-0.0096425	0.00389193
239	0.27313911	0.20179416	-0.1444025	0.65299677	-0.8832137	0.63697186	-0.0094087	0.0039058
239.1	0.27313911	0.20179416	-0.1444025	0.65299677	-0.8832137	0.63697186	-0.0094087	0.0039058
239.2	0.27318742	0.20152205	-0.1439347	0.65278613	-0.883174	0.63696081	-0.0096194	0.00396326
239.3	0.27318742	0.20152205	-0.1439347	0.65278613	-0.883174	0.63696081	-0.0096194	0.00396326
239.4	0.27318742	0.20152205	-0.1439347	0.65278613	-0.883174	0.63696081	-0.0096194	0.00396326
239.5	0.27318742	0.20152205	-0.1439347	0.65278613	-0.883174	0.63696081	-0.0096194	0.00396326
239.6	0.27307255	0.20137465	-0.1439097	0.65302066	-0.883287	0.63705346	-0.0093339	0.00405712
239.7	0.27307255	0.20137465	-0.1439097	0.65302066	-0.883287	0.63705346	-0.0093339	0.00405712
239.8	0.27307255	0.20137465	-0.1439097	0.65302066	-0.883287	0.63705346	-0.0093339	0.00405712
239.9	0.27307255	0.20137465	-0.1439097	0.65302066	-0.883287	0.63705346	-0.0093339	0.00405712
240	0.27307255	0.20137465	-0.1439097	0.65302066	-0.883287	0.63705346	-0.0093339	0.00405712

Corresponding p-values for PRCC sensitivtiy indices

Time discretization	q	V_{[G3P]}	K_{[G3P]}	K_{[PYR]}	alpha	G_E	[G]_0	[G3P]_0
0	0	0	0	0	0	0	0	0
0.1	1.96E-83	2.32E-18	3.85E-78	0.00053924	0.16288094	0.33017715	6.00E-68	0.43323379
0.2	1.69E-85	3.52E-19	7.11E-80	0.00283168	0.00431587	0.53733938	2.87E-69	0.8286764
0.3	5.47E-85	5.20E-19	1.33E-79	0.00872221	4.63E-05	0.64645265	3.82E-68	0.92055905
0.4	5.80E-84	2.45E-18	1.19E-78	0.02308566	2.33E-07	0.82468014	1.85E-66	0.71397734
0.5	2.40E-82	2.53E-17	1.93E-77	0.06084316	3.65E-09	0.91378344	1.12E-64	0.62448304
0.6	7.27E-81	6.42E-17	4.15E-76	0.11619353	7.84E-12	0.76322898	4.21E-63	0.56575085
0.7	4.38E-79	6.85E-17	4.56E-75	0.18827337	1.93E-14	0.66607042	7.38E-62	0.51650148
0.8	3.29E-78	1.40E-16	4.96E-74	0.26569656	7.97E-17	0.42694961	1.11E-60	0.42620855
0.9	1.80E-77	2.36E-16	8.69E-73	0.41084198	1.04E-19	0.29542838	2.56E-59	0.39177031
1	2.83E-77	3.69E-16	1.92E-72	0.4492806	9.56E-23	0.23811983	6.35E-59	0.33393356
1.1	7.73E-76	1.44E-15	5.56E-71	0.67607157	8.39E-25	0.17237709	1.86E-57	0.28870463
1.2	1.30E-74	5.43E-15	1.08E-69	0.79456848	1.59E-26	0.1201844	1.39E-56	0.24038404
1.3	1.04E-73	1.23E-14	6.50E-69	0.92014225	1.88E-28	0.08573506	1.36E-55	0.24807211
1.4	1.91E-72	2.83E-14	6.17E-68	0.87576767	1.85E-30	0.05406891	1.54E-54	0.17829911
1.5	1.55E-71	6.34E-14	7.97E-67	0.76695619	2.52E-32	0.03682046	1.15E-53	0.13727611
1.6	3.89E-70	1.45E-13	1.15E-65	0.64884404	2.90E-34	0.02191867	2.24E-52	0.10887034
1.7	8.32E-69	1.38E-13	2.94E-64	0.52826669	4.94E-36	0.01337111	1.98E-50	0.08840459
1.8	2.56E-67	2.82E-13	6.83E-63	0.34091645	1.24E-37	0.00879486	6.78E-49	0.10428663
1.9	1.27E-66	4.59E-13	3.70E-62	0.2220618	1.27E-39	0.00640058	8.81E-48	0.08655131
2	1.94E-65	1.19E-12	9.14E-61	0.16032278	7.62E-41	0.00549933	2.61E-46	0.06851944
2.1	7.62E-65	2.16E-12	4.31E-60	0.10464288	1.01E-42	0.00579775	8.17E-46	0.04528715
2.2	1.57E-63	6.39E-12	9.95E-59	0.08585331	1.76E-43	0.00397146	2.13E-44	0.05102037
2.3	1.86E-62	1.11E-11	6.21E-58	0.06042473	3.82E-44	0.00346291	2.91E-43	0.0427905
2.4	2.63E-61	1.30E-11	7.39E-57	0.04336359	4.33E-45	0.00250914	3.43E-42	0.04164691
2.5	1.02E-60	1.25E-11	3.21E-56	0.02261504	2.77E-46	0.00169161	2.02E-41	0.03174332
2.6	4.79E-60	2.30E-11	8.97E-56	0.01415867	1.97E-47	0.0011084	8.66E-41	0.0252963
2.7	1.47E-59	3.90E-11	3.52E-55	0.01048718	2.32E-48	0.00077075	2.86E-40	0.02702618
2.8	7.77E-59	7.13E-11	1.72E-54	0.007061	2.73E-49	0.00053979	1.06E-39	0.02950015
2.9	4.09E-58	9.14E-11	9.85E-54	0.00523259	5.41E-50	0.00047289	5.32E-39	0.02616788
3	2.34E-57	1.89E-10	8.76E-53	0.00426532	1.01E-50	0.00029595	6.17E-38	0.0261096
3.1	6.03E-57	3.42E-10	1.44E-52	0.00245774	9.61E-52	0.00018138	2.04E-37	0.02125582
3.2	5.10E-56	4.90E-10	1.26E-51	0.00165059	3.78E-52	0.00014384	1.18E-36	0.01982798
3.3	1.33E-55	4.52E-10	4.11E-51	0.00102974	1.91E-53	8.27E-05	4.07E-36	0.0154681
3.4	4.49E-55	7.30E-10	2.75E-50	0.00082156	5.86E-54	8.78E-05	1.84E-35	0.01735314
3.5	2.98E-54	1.51E-09	3.10E-49	0.00048756	1.67E-54	7.45E-05	1.31E-34	0.01507784
3.6	7.94E-54	1.87E-09	7.01E-49	0.00027897	5.47E-55	4.24E-05	5.78E-34	0.01629412
3.7	3.49E-53	2.79E-09	5.24E-48	0.00016008	1.98E-55	3.00E-05	3.70E-33	0.01304941
3.8	5.90E-53	3.33E-09	1.27E-47	0.00010097	2.32E-56	1.31E-05	2.36E-32	0.01495214
3.9	1.06E-52	3.99E-09	3.35E-47	8.23E-05	5.91E-57	8.18E-06	6.77E-32	0.01469397
4	5.64E-52	4.34E-09	1.09E-46	7.61E-05	1.57E-57	6.53E-06	1.43E-31	0.01348095
4.1	1.67E-51	5.37E-09	2.47E-46	6.06E-05	4.47E-58	4.15E-06	5.77E-31	0.01951335
4.2	6.83E-51	7.01E-09	1.11E-45	3.04E-05	5.79E-58	2.38E-06	2.83E-30	0.02175614
4.3	1.07E-50	6.65E-09	2.28E-45	1.80E-05	7.52E-59	1.63E-06	5.58E-30	0.02484514
4.4	5.50E-50	9.27E-09	1.28E-44	1.20E-05	6.25E-59	1.18E-06	2.38E-29	0.02792511
4.5	1.82E-49	1.49E-08	4.27E-44	7.89E-06	1.86E-59	8.88E-07	6.72E-29	0.02685376
4.6	3.17E-49	2.14E-08	6.89E-44	6.66E-06	5.08E-60	5.12E-07	1.54E-28	0.02977041
4.7	7.35E-49	3.14E-08	2.12E-43	4.32E-06	1.37E-60	3.20E-07	4.82E-28	0.03094053
4.8	1.89E-48	3.68E-08	9.70E-43	2.93E-06	4.50E-61	2.36E-07	1.36E-27	0.02944594
4.9	5.78E-48	6.03E-08	2.28E-42	2.01E-06	2.00E-61	1.75E-07	3.19E-27	0.03046308
5	1.58E-47	6.14E-08	5.77E-42	1.49E-06	1.77E-61	1.20E-07	7.58E-27	0.02921249
5.1	3.64E-47	7.36E-08	2.25E-41	9.11E-07	3.99E-62	6.38E-08	2.58E-26	0.02815478
5.2	1.55E-46	7.83E-08	9.36E-41	6.66E-07	3.24E-62	4.95E-08	9.35E-26	0.03112635
5.3	4.52E-46	1.04E-07	4.16E-40	6.45E-07	1.78E-62	3.68E-08	2.06E-25	0.03331627
5.4	1.16E-45	9.86E-08	1.55E-39	5.37E-07	1.31E-62	2.37E-08	4.27E-25	0.03642839
5.5	2.17E-45	1.26E-07	4.53E-39	3.51E-07	8.05E-63	2.80E-08	7.65E-25	0.0339802
5.6	1.17E-44	1.91E-07	1.62E-38	3.47E-07	1.06E-62	2.49E-08	2.10E-24	0.02973603
5.7	2.54E-44	2.74E-07	3.16E-38	2.71E-07	5.87E-63	1.74E-08	3.18E-24	0.03481942
5.8	6.98E-44	2.87E-07	1.05E-37	2.23E-07	5.75E-63	1.29E-08	7.14E-24	0.03884797
5.9	3.28E-43	4.75E-07	4.26E-37	2.14E-07	9.71E-63	1.01E-08	2.93E-23	0.0367799
6	1.14E-42	6.16E-07	1.32E-36	1.63E-07	7.47E-63	7.68E-09	7.77E-23	0.03551157
6.1	1.96E-42	7.46E-07	2.78E-36	1.39E-07	6.87E-63	6.53E-09	2.17E-22	0.03891189
6.2	3.97E-42	7.70E-07	5.87E-36	1.43E-07	6.00E-63	5.62E-09	7.44E-22	0.04186526
6.3	1.80E-41	7.28E-07	2.42E-35	1.13E-07	8.77E-63	4.97E-09	3.17E-21	0.03768113
6.4	2.87E-41	8.08E-07	6.76E-35	6.82E-08	3.09E-63	3.57E-09	7.20E-21	0.03817634
6.5	7.55E-41	9.94E-07	2.02E-34	4.82E-08	3.71E-63	3.07E-09	2.77E-20	0.04105228
6.6	1.17E-40	1.04E-06	3.94E-34	3.48E-08	2.08E-63	1.54E-09	7.27E-20	0.04545482
6.7	3.21E-40	1.04E-06	1.03E-33	3.15E-08	1.81E-63	1.33E-09	1.94E-19	0.05438389
6.8	5.35E-40	1.10E-06	2.06E-33	2.13E-08	1.33E-63	1.13E-09	4.21E-19	0.05298527
6.9	1.05E-39	1.17E-06	2.67E-33	1.79E-08	9.67E-64	8.13E-10	9.72E-19	0.05310729
7	1.91E-39	1.19E-06	3.99E-33	1.23E-08	3.49E-64	5.07E-10	1.85E-18	0.05351987
7.1	3.67E-39	1.19E-06	7.51E-33	8.76E-09	2.46E-64	3.94E-10	4.03E-18	0.05764771
7.2	6.39E-39	1.34E-06	1.32E-32	6.33E-09	1.77E-64	3.12E-10	6.03E-18	0.05327839
7.3	1.68E-38	1.71E-06	3.74E-32	4.83E-09	2.09E-64	2.62E-10	1.26E-17	0.05336
7.4	2.59E-38	1.62E-06	6.01E-32	3.51E-09	1.37E-64	1.66E-10	2.60E-17	0.05297821
7.5	1.09E-37	1.65E-06	2.35E-31	2.84E-09	1.79E-64	1.16E-10	5.91E-17	0.06416925
7.6	1.95E-37	1.70E-06	4.20E-31	2.34E-09	1.32E-64	9.23E-11	9.95E-17	0.06732457
7.7	5.91E-37	1.63E-06	7.79E-31	2.00E-09	1.60E-64	6.69E-11	2.71E-16	0.07080027
7.8	8.21E-37	1.71E-06	1.54E-30	1.23E-09	1.53E-64	7.29E-11	5.29E-16	0.06972004
7.9	2.22E-36	2.25E-06	6.21E-30	1.03E-09	2.18E-64	7.06E-11	1.06E-15	0.06843893
8	6.45E-36	3.67E-06	1.66E-29	1.45E-09	2.69E-64	6.59E-11	2.59E-15	0.07909209
8.1	9.61E-36	3.23E-06	2.39E-29	1.30E-09	1.49E-64	4.51E-11	4.14E-15	0.08371511
8.2	1.22E-35	3.70E-06	3.65E-29	8.50E-10	1.18E-64	3.38E-11	6.93E-15	0.0823918
8.3	3.11E-35	3.60E-06	1.35E-28	8.53E-10	1.20E-64	2.80E-11	1.80E-14	0.08143596
8.4	5.46E-35	4.55E-06	2.19E-28	6.33E-10	8.66E-65	1.49E-11	3.46E-14	0.08642149
8.5	8.46E-35	4.92E-06	3.99E-28	4.74E-10	7.09E-65	1.09E-11	4.73E-14	0.08476209
8.6	2.02E-34	5.93E-06	7.40E-28	3.90E-10	8.91E-65	7.88E-12	9.99E-14	0.08290667
8.7	4.91E-34	7.35E-06	1.52E-27	3.75E-10	1.28E-64	6.98E-12	1.75E-13	0.08298721
8.8	8.08E-34	7.36E-06	2.78E-27	3.00E-10	1.24E-64	5.41E-12	2.59E-13	0.0881359
8.9	1.72E-33	7.22E-06	6.04E-27	2.21E-10	1.21E-64	4.93E-12	4.40E-13	0.10001454
9	2.32E-33	7.18E-06	8.83E-27	1.90E-10	1.08E-64	3.29E-12	6.42E-13	0.10001084
9.1	6.27E-33	8.97E-06	1.54E-26	1.59E-10	1.84E-64	3.51E-12	1.14E-12	0.1070251
9.2	6.18E-33	9.30E-06	2.57E-26	1.20E-10	1.15E-64	3.00E-12	1.69E-12	0.10267629
9.3	1.36E-32	8.27E-06	5.48E-26	1.03E-10	1.25E-64	2.34E-12	2.68E-12	0.1103038
9.4	1.81E-32	8.66E-06	7.47E-26	9.60E-11	1.46E-64	2.15E-12	3.62E-12	0.11357299
9.5	2.80E-32	9.41E-06	1.71E-25	9.19E-11	1.49E-64	1.37E-12	6.47E-12	0.12102207
9.6	5.82E-32	9.43E-06	3.29E-25	9.98E-11	1.99E-64	1.23E-12	9.99E-12	0.12254814
9.7	7.84E-32	9.94E-06	4.06E-25	8.85E-11	1.46E-64	1.07E-12	1.23E-11	0.12717456
9.8	2.00E-31	1.24E-05	1.00E-24	7.47E-11	1.75E-64	1.14E-12	2.47E-11	0.13372826
9.9	3.87E-31	1.20E-05	1.64E-24	5.80E-11	1.54E-64	6.57E-13	3.30E-11	0.14494592
10	7.77E-31	1.15E-05	3.55E-24	5.76E-11	2.16E-64	5.88E-13	5.93E-11	0.15227946
10.1	1.69E-30	1.28E-05	6.96E-24	6.30E-11	2.41E-64	5.17E-13	1.06E-10	0.16095781
10.2	3.33E-30	1.25E-05	1.22E-23	4.92E-11	2.59E-64	4.42E-13	1.47E-10	0.16212951
10.3	4.19E-30	1.37E-05	1.80E-23	3.76E-11	2.82E-64	3.15E-13	2.18E-10	0.16857013
10.4	5.92E-30	1.35E-05	2.73E-23	2.74E-11	3.25E-64	2.64E-13	4.23E-10	0.17508187
10.5	7.56E-30	1.37E-05	3.58E-23	2.78E-11	1.88E-64	1.55E-13	6.39E-10	0.17955844
10.6	8.45E-30	1.58E-05	6.34E-23	1.94E-11	1.64E-64	9.82E-14	8.48E-10	0.18978699
10.7	1.16E-29	1.57E-05	8.15E-23	1.45E-11	1.47E-64	8.26E-14	1.20E-09	0.19342994
10.8	1.41E-29	1.78E-05	1.17E-22	1.37E-11	1.43E-64	7.16E-14	1.99E-09	0.18866895
10.9	2.29E-29	1.91E-05	2.19E-22	1.30E-11	1.70E-64	8.42E-14	3.10E-09	0.20185896
11	4.23E-29	1.83E-05	2.94E-22	1.14E-11	2.09E-64	7.76E-14	4.28E-09	0.20336043
11.1	5.24E-29	2.09E-05	3.72E-22	8.91E-12	2.19E-64	5.48E-14	5.46E-09	0.20449799
11.2	1.06E-28	2.21E-05	5.80E-22	8.80E-12	2.91E-64	4.40E-14	8.71E-09	0.22739559
11.3	1.41E-28	2.45E-05	9.57E-22	7.78E-12	2.65E-64	4.28E-14	1.08E-08	0.22130417
11.4	2.22E-28	2.68E-05	1.60E-21	7.50E-12	3.02E-64	3.64E-14	1.62E-08	0.22635448
11.5	2.49E-28	2.69E-05	2.32E-21	6.12E-12	2.78E-64	2.41E-14	2.18E-08	0.2290378
11.6	3.95E-28	2.79E-05	3.00E-21	5.46E-12	2.98E-64	2.16E-14	2.44E-08	0.23296004
11.7	6.21E-28	2.17E-05	4.17E-21	4.42E-12	2.06E-64	1.35E-14	3.42E-08	0.2484952
11.8	8.50E-28	2.69E-05	6.46E-21	3.72E-12	3.18E-64	1.36E-14	4.84E-08	0.25067015
11.9	1.44E-27	2.40E-05	1.11E-20	2.29E-12	2.45E-64	1.05E-14	6.60E-08	0.267764
12	1.58E-27	2.86E-05	1.62E-20	1.64E-12	2.65E-64	7.82E-15	1.12E-07	0.27719047
12.1	2.43E-27	2.49E-05	2.33E-20	1.19E-12	2.12E-64	5.32E-15	1.31E-07	0.28962922
12.2	3.67E-27	2.40E-05	4.08E-20	1.08E-12	2.57E-64	4.85E-15	1.98E-07	0.27521959
12.3	5.67E-27	3.00E-05	6.72E-20	8.94E-13	2.02E-64	5.03E-15	3.00E-07	0.27230734
12.4	7.57E-27	3.24E-05	1.06E-19	8.04E-13	2.41E-64	3.77E-15	3.77E-07	0.26823696
12.5	1.26E-26	3.19E-05	1.76E-19	6.63E-13	2.91E-64	4.07E-15	5.87E-07	0.29946674
12.6	2.04E-26	3.51E-05	3.50E-19	6.70E-13	4.22E-64	4.05E-15	6.12E-07	0.30208816
12.7	2.71E-26	3.30E-05	4.86E-19	5.71E-13	3.64E-64	3.14E-15	7.74E-07	0.31064235
12.8	5.86E-26	3.70E-05	7.63E-19	4.54E-13	3.62E-64	2.57E-15	9.79E-07	0.29280957
12.9	1.03E-25	4.84E-05	1.65E-18	4.66E-13	5.15E-64	2.90E-15	1.24E-06	0.30243789
13	1.45E-25	4.75E-05	2.30E-18	5.20E-13	4.87E-64	2.90E-15	1.42E-06	0.30227001
13.1	2.50E-25	5.26E-05	3.74E-18	4.76E-13	5.53E-64	2.35E-15	1.45E-06	0.30163212
13.2	3.86E-25	5.43E-05	4.68E-18	4.44E-13	6.73E-64	1.45E-15	1.82E-06	0.31344967
13.3	6.18E-25	5.71E-05	5.99E-18	3.84E-13	7.49E-64	9.94E-16	2.57E-06	0.31532182
13.4	7.82E-25	6.08E-05	8.55E-18	2.95E-13	7.11E-64	7.41E-16	2.74E-06	0.32548277
13.5	7.54E-25	6.52E-05	1.02E-17	1.83E-13	5.69E-64	4.67E-16	3.58E-06	0.34380062
13.6	9.08E-25	6.46E-05	1.48E-17	1.81E-13	5.33E-64	3.98E-16	4.19E-06	0.34943388
13.7	1.62E-24	7.03E-05	2.17E-17	1.76E-13	7.85E-64	3.70E-16	4.93E-06	0.36147067
13.8	2.31E-24	7.12E-05	2.54E-17	1.61E-13	7.42E-64	3.30E-16	5.46E-06	0.37690489
13.9	3.30E-24	6.84E-05	3.02E-17	1.46E-13	6.69E-64	2.71E-16	5.68E-06	0.41097891
14	5.27E-24	6.13E-05	4.01E-17	1.13E-13	6.48E-64	1.94E-16	7.45E-06	0.42394782
14.1	6.90E-24	6.42E-05	6.16E-17	1.02E-13	6.07E-64	1.80E-16	9.05E-06	0.43575822
14.2	9.12E-24	6.33E-05	9.02E-17	9.21E-14	6.65E-64	1.76E-16	9.26E-06	0.44621923
14.3	1.32E-23	6.05E-05	1.07E-16	7.46E-14	6.47E-64	1.50E-16	1.15E-05	0.43152696
14.4	2.09E-23	6.45E-05	1.34E-16	5.82E-14	7.43E-64	1.61E-16	1.29E-05	0.44598175
14.5	2.96E-23	6.20E-05	1.75E-16	4.48E-14	7.38E-64	1.41E-16	1.30E-05	0.42651893
14.6	3.96E-23	7.26E-05	2.85E-16	4.64E-14	1.00E-63	1.31E-16	2.05E-05	0.44533972
14.7	7.34E-23	6.49E-05	5.26E-16	4.84E-14	1.19E-63	1.07E-16	2.52E-05	0.46466849
14.8	8.85E-23	5.88E-05	5.74E-16	3.84E-14	9.89E-64	9.72E-17	2.56E-05	0.44138502
14.9	1.02E-22	6.34E-05	6.49E-16	3.23E-14	9.97E-64	8.18E-17	2.63E-05	0.45107709
15	1.31E-22	6.33E-05	8.44E-16	2.16E-14	9.88E-64	6.24E-17	3.04E-05	0.44434753
15.1	1.31E-22	6.54E-05	9.10E-16	1.72E-14	8.59E-64	4.89E-17	3.45E-05	0.44767292
15.2	1.48E-22	7.17E-05	9.64E-16	1.45E-14	8.20E-64	4.12E-17	3.54E-05	0.44363008
15.3	2.06E-22	7.62E-05	1.38E-15	1.43E-14	9.84E-64	4.06E-17	4.07E-05	0.45228418
15.4	2.97E-22	7.98E-05	1.82E-15	1.59E-14	1.08E-63	4.29E-17	4.70E-05	0.45909804
15.5	3.50E-22	8.39E-05	1.84E-15	1.19E-14	7.30E-64	3.43E-17	4.70E-05	0.48446194
15.6	4.62E-22	8.07E-05	2.81E-15	1.08E-14	8.65E-64	2.52E-17	6.28E-05	0.49242333
15.7	6.92E-22	8.96E-05	4.14E-15	9.87E-15	1.01E-63	1.77E-17	6.92E-05	0.49788682
15.8	1.30E-21	9.01E-05	5.96E-15	7.97E-15	1.03E-63	2.11E-17	8.23E-05	0.49405385
15.9	2.55E-21	9.31E-05	9.21E-15	7.60E-15	1.28E-63	2.11E-17	9.65E-05	0.49667084
16	3.19E-21	9.08E-05	1.17E-14	6.37E-15	1.41E-63	1.86E-17	0.0001039	0.4934705
16.1	4.73E-21	8.28E-05	1.74E-14	5.01E-15	1.45E-63	1.62E-17	0.00010211	0.50168584
16.2	7.21E-21	7.63E-05	2.02E-14	2.82E-15	9.77E-64	1.05E-17	0.00012194	0.4986416
16.3	1.01E-20	8.40E-05	3.06E-14	2.15E-15	8.55E-64	9.04E-18	0.0001427	0.51426321
16.4	1.16E-20	9.08E-05	3.83E-14	1.99E-15	8.54E-64	8.21E-18	0.00013438	0.51812011
16.5	1.73E-20	0.00010084	5.78E-14	1.82E-15	1.50E-63	9.73E-18	0.0001788	0.53549485
16.6	2.42E-20	0.00011428	7.27E-14	1.77E-15	1.69E-63	8.27E-18	0.00019616	0.53701535
16.7	3.20E-20	0.0001194	8.83E-14	1.69E-15	1.45E-63	6.67E-18	0.00021518	0.54372346
16.8	3.68E-20	0.00011582	1.08E-13	1.49E-15	1.27E-63	5.85E-18	0.00021387	0.54050995
16.9	4.23E-20	0.00012099	1.15E-13	1.37E-15	1.18E-63	4.88E-18	0.00022607	0.52403366
17	4.51E-20	0.00011536	1.76E-13	1.13E-15	1.36E-63	3.44E-18	0.00029528	0.53669678
17.1	5.33E-20	0.00012218	2.13E-13	1.29E-15	1.72E-63	3.31E-18	0.00032671	0.54799131
17.2	6.02E-20	0.00013325	2.16E-13	1.20E-15	1.91E-63	2.65E-18	0.00034007	0.55918148
17.3	7.55E-20	0.00014246	2.70E-13	1.07E-15	2.20E-63	2.17E-18	0.00036732	0.54202383
17.4	9.93E-20	0.00013935	3.84E-13	1.03E-15	2.39E-63	2.07E-18	0.00037882	0.54979742
17.5	1.12E-19	0.00013328	4.07E-13	9.74E-16	2.61E-63	1.82E-18	0.00038503	0.56331551
17.6	1.65E-19	0.00014542	5.00E-13	1.12E-15	3.43E-63	1.41E-18	0.00046558	0.56617168
17.7	2.18E-19	0.00016061	5.67E-13	1.02E-15	3.74E-63	1.15E-18	0.00054045	0.55808785
17.8	3.20E-19	0.00016133	7.13E-13	9.23E-16	4.33E-63	1.08E-18	0.00063219	0.54206782
17.9	3.58E-19	0.00016336	8.23E-13	7.93E-16	4.58E-63	9.19E-19	0.00066562	0.53863141
18	3.96E-19	0.0001583	1.02E-12	6.46E-16	4.83E-63	8.47E-19	0.0006778	0.55183973
18.1	5.29E-19	0.00015199	1.13E-12	5.60E-16	5.29E-63	7.76E-19	0.00077821	0.54288681
18.2	5.65E-19	0.00014866	1.68E-12	5.95E-16	6.38E-63	7.72E-19	0.00091019	0.55520316
18.3	6.70E-19	0.00016275	1.92E-12	5.39E-16	6.77E-63	6.36E-19	0.00107656	0.57021618
18.4	7.21E-19	0.00016877	2.15E-12	4.78E-16	7.14E-63	5.26E-19	0.00119042	0.57668039
18.5	8.11E-19	0.00016616	2.34E-12	3.93E-16	6.38E-63	3.95E-19	0.00121325	0.58008233
18.6	1.11E-18	0.00016923	2.74E-12	4.04E-16	8.79E-63	3.65E-19	0.00143022	0.58752347
18.7	1.26E-18	0.00017221	3.48E-12	4.49E-16	9.82E-63	2.87E-19	0.00158217	0.57398629
18.8	1.85E-18	0.00018707	5.07E-12	4.64E-16	1.49E-62	2.89E-19	0.00173154	0.58919487
18.9	2.51E-18	0.00020913	6.59E-12	4.45E-16	1.82E-62	3.35E-19	0.00190903	0.58608332
19	2.62E-18	0.00022216	7.66E-12	3.77E-16	1.97E-62	2.87E-19	0.00202618	0.59273443
19.1	2.60E-18	0.0002242	9.97E-12	3.69E-16	1.86E-62	2.44E-19	0.00234199	0.58826982
19.2	3.47E-18	0.00023364	1.04E-11	4.19E-16	2.30E-62	2.26E-19	0.00271654	0.60161414
19.3	4.45E-18	0.00022613	1.27E-11	3.33E-16	1.89E-62	1.98E-19	0.00275237	0.59104841
19.4	5.32E-18	0.00023815	1.66E-11	3.45E-16	2.53E-62	1.81E-19	0.0030115	0.58896247
19.5	5.90E-18	0.00024444	1.92E-11	3.36E-16	2.55E-62	1.62E-19	0.00318921	0.59150789
19.6	6.26E-18	0.00024447	2.13E-11	2.96E-16	2.25E-62	1.31E-19	0.0034709	0.58650405
19.7	7.04E-18	0.00028402	2.58E-11	2.97E-16	2.64E-62	1.33E-19	0.00364387	0.58158257
19.8	9.65E-18	0.00027682	3.28E-11	2.58E-16	2.63E-62	1.14E-19	0.00422435	0.58254918
19.9	1.40E-17	0.00034463	3.91E-11	2.27E-16	3.09E-62	9.86E-20	0.00463633	0.58971763
20	1.35E-17	0.00034014	4.20E-11	1.88E-16	2.42E-62	8.37E-20	0.00523931	0.59042125
20.1	1.97E-17	0.00033322	5.12E-11	1.41E-16	3.19E-62	7.26E-20	0.00587034	0.58488922
20.2	2.62E-17	0.00034973	6.48E-11	1.18E-16	2.89E-62	7.03E-20	0.00658479	0.60576866
20.3	3.02E-17	0.00030867	6.89E-11	1.04E-16	2.26E-62	4.99E-20	0.00736505	0.60556929
20.4	3.59E-17	0.0002907	7.55E-11	9.05E-17	2.34E-62	4.30E-20	0.00774465	0.61808604
20.5	4.12E-17	0.00025476	9.31E-11	9.41E-17	2.36E-62	5.03E-20	0.00870078	0.6199834
20.6	5.96E-17	0.00025751	1.18E-10	8.49E-17	2.70E-62	5.08E-20	0.00879658	0.61104133
20.7	8.11E-17	0.00028347	1.58E-10	7.49E-17	2.85E-62	4.97E-20	0.00960597	0.63217361
20.8	1.05E-16	0.00032193	1.92E-10	9.26E-17	4.26E-62	6.19E-20	0.01114674	0.63666486
20.9	1.15E-16	0.00031855	2.06E-10	8.48E-17	3.44E-62	5.91E-20	0.01185754	0.64772128
21	1.31E-16	0.00032351	2.91E-10	8.19E-17	3.52E-62	5.43E-20	0.01238306	0.65629256
21.1	1.61E-16	0.00031555	3.45E-10	8.41E-17	4.03E-62	5.57E-20	0.01240416	0.66274004
21.2	1.77E-16	0.00036512	3.88E-10	7.90E-17	4.86E-62	5.40E-20	0.01339914	0.67195972
21.3	1.84E-16	0.00039509	4.60E-10	6.75E-17	5.66E-62	5.27E-20	0.01426258	0.69631338
21.4	2.18E-16	0.00041245	5.06E-10	5.67E-17	4.71E-62	4.84E-20	0.0157493	0.70285633
21.5	3.00E-16	0.00041495	6.13E-10	6.02E-17	5.34E-62	5.45E-20	0.01652779	0.70360247
21.6	3.25E-16	0.00040167	6.86E-10	5.43E-17	4.38E-62	4.89E-20	0.01662144	0.69191584
21.7	3.51E-16	0.00042002	8.38E-10	5.51E-17	4.30E-62	4.61E-20	0.01790664	0.69321898
21.8	3.96E-16	0.00044867	9.87E-10	5.79E-17	6.12E-62	4.31E-20	0.01850849	0.69842807
21.9	4.56E-16	0.00050724	1.29E-09	4.92E-17	7.58E-62	4.37E-20	0.02051104	0.71155639
22	5.78E-16	0.00053528	1.27E-09	5.62E-17	8.70E-62	4.34E-20	0.02161509	0.69793774
22.1	6.27E-16	0.00055183	1.61E-09	5.82E-17	7.93E-62	4.21E-20	0.02346534	0.69866994
22.2	6.44E-16	0.00054013	1.69E-09	5.64E-17	7.88E-62	3.82E-20	0.02406405	0.69846336
22.3	9.30E-16	0.0005602	2.10E-09	4.90E-17	9.37E-62	4.02E-20	0.02590692	0.69030189
22.4	9.36E-16	0.00055007	2.14E-09	4.68E-17	8.32E-62	3.63E-20	0.02643579	0.7001102
22.5	1.26E-15	0.00055349	2.69E-09	4.66E-17	1.10E-61	3.78E-20	0.02874106	0.71174915
22.6	1.46E-15	0.00058107	2.84E-09	4.43E-17	1.11E-61	3.34E-20	0.02798711	0.71053173
22.7	1.50E-15	0.0005579	3.26E-09	5.13E-17	1.11E-61	3.29E-20	0.03087227	0.70436495
22.8	1.62E-15	0.0005772	3.76E-09	5.12E-17	1.24E-61	3.30E-20	0.03209882	0.70729019
22.9	1.79E-15	0.00056713	3.95E-09	4.84E-17	1.17E-61	2.54E-20	0.03202349	0.70574507
23	2.06E-15	0.00055929	5.02E-09	4.38E-17	1.32E-61	2.54E-20	0.03246085	0.71161138
23.1	2.14E-15	0.00057749	5.40E-09	3.74E-17	1.30E-61	2.24E-20	0.03483925	0.73166972
23.2	2.72E-15	0.00058033	5.85E-09	3.31E-17	1.51E-61	2.23E-20	0.03691742	0.74762127
23.3	2.89E-15	0.00058495	6.21E-09	2.98E-17	1.43E-61	2.06E-20	0.03833641	0.75970478
23.4	2.96E-15	0.00057908	6.20E-09	2.86E-17	1.50E-61	1.94E-20	0.03873221	0.76148415
23.5	3.78E-15	0.00056147	7.09E-09	3.04E-17	1.79E-61	2.16E-20	0.04242341	0.7764563
23.6	4.64E-15	0.00059815	7.02E-09	2.68E-17	1.92E-61	2.07E-20	0.04412397	0.79167903
23.7	5.33E-15	0.00068581	8.64E-09	2.88E-17	2.10E-61	1.73E-20	0.04729777	0.79503091
23.8	6.99E-15	0.00068877	1.13E-08	2.98E-17	2.73E-61	1.97E-20	0.04738148	0.79509553
23.9	7.61E-15	0.00076281	1.30E-08	2.11E-17	2.46E-61	1.60E-20	0.05220218	0.80648421
24	1.00E-14	0.00076474	1.23E-08	1.88E-17	2.93E-61	1.32E-20	0.05406309	0.81278827
24.1	1.32E-14	0.00078202	1.22E-08	1.89E-17	3.28E-61	1.25E-20	0.05455621	0.82054873
24.2	1.47E-14	0.00072339	1.32E-08	1.63E-17	2.74E-61	9.70E-21	0.05473286	0.82098982
24.3	1.75E-14	0.00071179	1.45E-08	1.67E-17	3.24E-61	1.00E-20	0.05493509	0.81768621
24.4	1.89E-14	0.00077649	1.62E-08	1.65E-17	3.77E-61	1.03E-20	0.05554104	0.81775633
24.5	2.37E-14	0.00083105	1.71E-08	1.73E-17	4.77E-61	1.02E-20	0.05641889	0.81091932
24.6	2.64E-14	0.00088292	2.03E-08	1.75E-17	5.48E-61	9.16E-21	0.05681155	0.83039998
24.7	2.67E-14	0.00088442	2.29E-08	1.70E-17	6.15E-61	9.37E-21	0.05771331	0.80588513
24.8	3.04E-14	0.00093117	2.41E-08	1.42E-17	5.28E-61	7.91E-21	0.05813293	0.80275805
24.9	3.51E-14	0.00094588	2.64E-08	1.59E-17	6.27E-61	9.21E-21	0.05864195	0.81139166
25	3.66E-14	0.00094681	2.90E-08	1.29E-17	5.77E-61	8.71E-21	0.05978319	0.81519651
25.1	4.49E-14	0.00090028	3.19E-08	1.26E-17	5.31E-61	8.79E-21	0.06161782	0.81171644
25.2	5.49E-14	0.00094126	3.56E-08	1.34E-17	5.65E-61	8.12E-21	0.06488005	0.81447483
25.3	6.35E-14	0.00093172	4.29E-08	1.10E-17	5.61E-61	7.15E-21	0.06821692	0.8318691
25.4	5.83E-14	0.00091644	4.42E-08	9.99E-18	4.97E-61	6.52E-21	0.06969696	0.82212798
25.5	6.25E-14	0.00089158	4.81E-08	8.85E-18	4.61E-61	5.42E-21	0.07241283	0.82173005
25.6	6.75E-14	0.00095751	5.18E-08	8.78E-18	5.87E-61	4.94E-21	0.07491192	0.84302689
25.7	8.05E-14	0.00099289	5.77E-08	7.90E-18	5.81E-61	4.66E-21	0.07841182	0.85085835
25.8	8.12E-14	0.00099466	6.06E-08	7.37E-18	5.51E-61	4.48E-21	0.08055885	0.84780569
25.9	8.60E-14	0.00097701	5.94E-08	7.38E-18	5.48E-61	3.84E-21	0.08243262	0.84337596
26	1.09E-13	0.00098555	6.38E-08	7.09E-18	6.10E-61	3.85E-21	0.08513	0.84760247
26.1	1.10E-13	0.00094681	7.38E-08	6.03E-18	5.82E-61	3.39E-21	0.08976372	0.83964535
26.2	1.14E-13	0.00096293	7.82E-08	5.90E-18	5.76E-61	3.23E-21	0.09227511	0.84352085
26.3	1.35E-13	0.00098398	9.16E-08	4.94E-18	5.72E-61	3.39E-21	0.09431587	0.87208311
26.4	1.64E-13	0.00103704	9.94E-08	4.94E-18	6.22E-61	3.26E-21	0.09427732	0.8770627
26.5	1.84E-13	0.00112088	1.17E-07	4.58E-18	7.63E-61	3.20E-21	0.10071786	0.88704123
26.6	1.92E-13	0.00111042	1.28E-07	4.59E-18	8.26E-61	3.23E-21	0.10504526	0.88609134
26.7	2.16E-13	0.00107919	1.20E-07	4.13E-18	7.95E-61	2.69E-21	0.10812399	0.88313085
26.8	2.52E-13	0.00106907	1.40E-07	4.32E-18	8.71E-61	2.46E-21	0.11226134	0.87790622
26.9	2.68E-13	0.00107883	1.37E-07	4.21E-18	8.80E-61	2.29E-21	0.11154962	0.88713454
27	2.89E-13	0.00115785	1.49E-07	4.60E-18	1.04E-60	2.30E-21	0.11309939	0.88782361
27.1	3.11E-13	0.00112995	1.58E-07	4.51E-18	1.05E-60	1.98E-21	0.11597245	0.89485491
27.2	3.28E-13	0.00119363	1.64E-07	4.39E-18	1.10E-60	1.89E-21	0.1172228	0.88934167
27.3	3.85E-13	0.00118005	1.98E-07	3.96E-18	1.10E-60	1.70E-21	0.1203179	0.87930465
27.4	3.96E-13	0.00118467	2.02E-07	3.80E-18	1.10E-60	1.70E-21	0.1205599	0.88131412
27.5	5.09E-13	0.00127475	2.07E-07	4.66E-18	1.50E-60	1.95E-21	0.12617075	0.89596846
27.6	5.57E-13	0.00130258	2.34E-07	4.76E-18	1.61E-60	2.00E-21	0.13021433	0.89448705
27.7	6.28E-13	0.00142209	2.64E-07	4.26E-18	1.77E-60	1.75E-21	0.13592121	0.89944168
27.8	7.67E-13	0.0013938	2.79E-07	3.73E-18	1.79E-60	1.68E-21	0.13996117	0.89716193
27.9	8.00E-13	0.00142383	2.94E-07	4.13E-18	2.03E-60	1.73E-21	0.1416031	0.89643251
28	8.86E-13	0.00141215	3.27E-07	3.60E-18	1.60E-60	1.59E-21	0.14445923	0.8884119
28.1	9.07E-13	0.0014595	3.35E-07	3.24E-18	1.58E-60	1.48E-21	0.14602489	0.89768775
28.2	9.46E-13	0.00144396	3.42E-07	3.10E-18	1.57E-60	1.44E-21	0.14756866	0.9002994
28.3	9.66E-13	0.00144101	3.73E-07	2.68E-18	1.89E-60	1.47E-21	0.15788103	0.90657117
28.4	1.01E-12	0.00146611	3.68E-07	2.41E-18	1.57E-60	1.26E-21	0.15959997	0.90894997
28.5	9.95E-13	0.00148041	4.13E-07	2.19E-18	1.49E-60	1.27E-21	0.16081016	0.9031072
28.6	1.10E-12	0.00153903	4.50E-07	2.13E-18	1.55E-60	1.24E-21	0.16081278	0.90305142
28.7	1.21E-12	0.00158784	4.62E-07	1.99E-18	1.71E-60	1.32E-21	0.1636876	0.90861317
28.8	1.21E-12	0.00156725	4.94E-07	2.01E-18	1.83E-60	1.30E-21	0.16783394	0.90870727
28.9	1.50E-12	0.00169471	5.86E-07	2.14E-18	2.30E-60	1.58E-21	0.1741293	0.89626979
29	1.70E-12	0.00171764	6.22E-07	2.05E-18	2.07E-60	1.45E-21	0.1758023	0.89413523
29.1	1.98E-12	0.00168639	7.43E-07	2.31E-18	2.05E-60	1.14E-21	0.18404777	0.89190726
29.2	2.26E-12	0.00176842	8.69E-07	2.32E-18	2.59E-60	1.11E-21	0.18928661	0.89264753
29.3	2.51E-12	0.00177195	8.66E-07	2.32E-18	2.72E-60	1.09E-21	0.18751609	0.89167824
29.4	2.91E-12	0.0018144	9.68E-07	1.70E-18	3.23E-60	8.49E-22	0.19702598	0.91243666
29.5	3.13E-12	0.00188023	9.63E-07	1.46E-18	3.19E-60	8.04E-22	0.20148127	0.9136609
29.6	3.49E-12	0.00195688	1.04E-06	1.53E-18	4.05E-60	8.28E-22	0.20713018	0.90938894
29.7	4.50E-12	0.00214947	1.18E-06	1.94E-18	6.04E-60	9.39E-22	0.21380486	0.92813488
29.8	4.76E-12	0.00222951	1.21E-06	2.18E-18	6.92E-60	1.01E-21	0.21776885	0.92918278
29.9	4.90E-12	0.00228728	1.28E-06	2.23E-18	7.84E-60	9.94E-22	0.21886433	0.93314013
30	4.93E-12	0.00243511	1.33E-06	2.10E-18	8.36E-60	9.96E-22	0.21631963	0.93810183
30.1	5.90E-12	0.00243818	1.47E-06	2.11E-18	1.01E-59	1.02E-21	0.23204745	0.96131009
30.2	6.55E-12	0.00245558	1.49E-06	1.85E-18	1.10E-59	1.02E-21	0.23946669	0.9672522
30.3	7.24E-12	0.00245425	1.65E-06	1.62E-18	1.00E-59	8.75E-22	0.24169883	0.97623197
30.4	8.20E-12	0.00242112	1.83E-06	1.39E-18	9.59E-60	7.20E-22	0.24549952	0.99292208
30.5	9.39E-12	0.00259829	2.17E-06	1.64E-18	1.16E-59	8.09E-22	0.25043376	0.97534729
30.6	1.08E-11	0.00275358	2.23E-06	1.74E-18	1.44E-59	8.25E-22	0.25175188	0.98312756
30.7	1.10E-11	0.00272538	2.34E-06	1.66E-18	1.34E-59	8.12E-22	0.25199072	0.97835206
30.8	1.17E-11	0.00276089	2.45E-06	1.66E-18	1.45E-59	7.91E-22	0.25145958	0.97952481
30.9	1.34E-11	0.00275299	2.38E-06	1.52E-18	1.46E-59	7.68E-22	0.2613025	0.98108433
31	1.54E-11	0.00268695	2.73E-06	1.58E-18	1.49E-59	7.08E-22	0.26420296	0.97511287
31.1	1.59E-11	0.00277706	2.78E-06	1.66E-18	1.71E-59	7.01E-22	0.26289618	0.97520938
31.2	1.79E-11	0.00278687	3.01E-06	1.64E-18	2.00E-59	7.47E-22	0.27248697	0.97299639
31.3	2.06E-11	0.00277371	3.04E-06	1.64E-18	2.08E-59	7.67E-22	0.26847375	0.97356185
31.4	2.12E-11	0.00290402	3.41E-06	1.64E-18	2.37E-59	7.91E-22	0.27066374	0.96979119
31.5	2.28E-11	0.00297752	3.67E-06	1.54E-18	2.64E-59	8.22E-22	0.26221082	0.96042938
31.6	2.41E-11	0.00285755	3.79E-06	1.39E-18	2.39E-59	8.03E-22	0.26938377	0.96317366
31.7	2.53E-11	0.00277948	3.86E-06	1.38E-18	2.31E-59	7.82E-22	0.26954165	0.95842275
31.8	2.51E-11	0.00282657	4.38E-06	1.48E-18	2.90E-59	7.79E-22	0.27416353	0.96420535
31.9	2.67E-11	0.00255268	4.67E-06	1.28E-18	2.88E-59	6.89E-22	0.2734775	0.97522833
32	2.85E-11	0.00258612	4.90E-06	1.24E-18	3.14E-59	6.63E-22	0.26744776	0.97438044
32.1	3.03E-11	0.00280099	5.16E-06	1.22E-18	3.23E-59	6.86E-22	0.27670988	0.97768827
32.2	3.38E-11	0.00290596	5.50E-06	1.11E-18	3.24E-59	7.07E-22	0.27667794	0.98671454
32.3	3.51E-11	0.00280635	6.06E-06	8.48E-19	3.05E-59	5.85E-22	0.28207679	0.98229859
32.4	3.61E-11	0.00278096	6.50E-06	7.83E-19	2.99E-59	5.49E-22	0.2969518	0.99625473
32.5	4.18E-11	0.0028932	6.16E-06	7.38E-19	2.99E-59	4.95E-22	0.30257414	0.989842
32.6	4.42E-11	0.00298527	6.15E-06	6.94E-19	2.90E-59	4.68E-22	0.30305927	0.9842575
32.7	4.51E-11	0.00293154	6.27E-06	6.71E-19	3.00E-59	4.59E-22	0.30607649	0.98962913
32.8	4.84E-11	0.00294115	6.59E-06	7.03E-19	3.20E-59	5.09E-22	0.31435781	0.97433287
32.9	5.70E-11	0.00292379	6.92E-06	8.14E-19	3.92E-59	5.25E-22	0.32077233	0.98637436
33	6.35E-11	0.00287965	7.31E-06	7.94E-19	3.48E-59	5.18E-22	0.32523579	0.98216959
33.1	6.22E-11	0.00285629	8.58E-06	8.81E-19	4.02E-59	4.32E-22	0.33669596	0.98969749
33.2	7.53E-11	0.00275428	8.55E-06	9.41E-19	3.54E-59	4.23E-22	0.34358534	0.98128699
33.3	7.81E-11	0.00273749	9.17E-06	8.56E-19	3.68E-59	4.57E-22	0.3408424	0.97734141
33.4	8.43E-11	0.00261019	8.99E-06	8.61E-19	3.63E-59	4.36E-22	0.34285624	0.96858013
33.5	8.62E-11	0.00254363	9.62E-06	8.11E-19	3.51E-59	4.08E-22	0.35201597	0.97044074
33.6	9.82E-11	0.00261583	1.04E-05	8.26E-19	4.25E-59	3.99E-22	0.35059056	0.9715523
33.7	1.05E-10	0.00253117	1.11E-05	7.58E-19	4.25E-59	3.80E-22	0.35368653	0.95996567
33.8	1.12E-10	0.00253465	1.14E-05	8.00E-19	4.67E-59	3.99E-22	0.35559162	0.95353796
33.9	1.17E-10	0.0026384	1.31E-05	8.31E-19	5.73E-59	3.85E-22	0.36001734	0.93829045
34	1.29E-10	0.00259533	1.41E-05	8.09E-19	5.74E-59	3.77E-22	0.35932328	0.9223117
34.1	1.29E-10	0.00259533	1.41E-05	8.09E-19	5.74E-59	3.77E-22	0.35932328	0.9223117
34.2	1.49E-10	0.0026434	1.48E-05	7.32E-19	6.52E-59	3.70E-22	0.3628961	0.92459068
34.3	1.50E-10	0.00263438	1.54E-05	6.28E-19	5.59E-59	3.29E-22	0.36485724	0.91381119
34.4	1.53E-10	0.00257438	1.67E-05	5.95E-19	5.39E-59	2.83E-22	0.37326242	0.92762474
34.5	1.72E-10	0.00269183	1.58E-05	4.76E-19	4.60E-59	2.17E-22	0.37996721	0.93518661
34.6	1.74E-10	0.00277398	1.59E-05	4.54E-19	4.52E-59	2.06E-22	0.37959219	0.93679573
34.7	1.94E-10	0.00282417	1.57E-05	4.45E-19	4.58E-59	2.03E-22	0.38556623	0.92622153
34.8	2.32E-10	0.00312251	1.72E-05	4.54E-19	5.19E-59	2.05E-22	0.3961965	0.92063249
34.9	2.44E-10	0.00315322	1.76E-05	4.70E-19	5.61E-59	2.10E-22	0.40211993	0.93102666
35	2.91E-10	0.00330912	1.86E-05	5.15E-19	6.29E-59	2.27E-22	0.40262564	0.9328104
35.1	3.12E-10	0.00328282	1.90E-05	3.88E-19	3.95E-59	1.73E-22	0.41337763	0.93984692
35.2	4.03E-10	0.00317577	2.08E-05	3.33E-19	4.36E-59	1.82E-22	0.42172895	0.94719336
35.3	4.45E-10	0.00302116	2.22E-05	3.17E-19	4.78E-59	1.60E-22	0.43627138	0.95903921
35.4	4.67E-10	0.00286325	2.37E-05	3.24E-19	4.88E-59	1.46E-22	0.4390371	0.9604282
35.5	4.97E-10	0.00281297	2.60E-05	3.26E-19	5.22E-59	1.42E-22	0.45019858	0.95517351
35.6	5.00E-10	0.00281029	2.73E-05	3.39E-19	5.30E-59	1.52E-22	0.4519097	0.94893543
35.7	5.18E-10	0.00295352	3.08E-05	3.62E-19	5.79E-59	1.60E-22	0.46063321	0.94716212
35.8	5.10E-10	0.0029662	3.02E-05	3.50E-19	5.00E-59	1.40E-22	0.46560498	0.94961712
35.9	4.78E-10	0.00291522	3.00E-05	3.07E-19	4.59E-59	1.26E-22	0.46195406	0.94536018
36	5.48E-10	0.00287668	2.98E-05	2.83E-19	3.76E-59	1.16E-22	0.46355096	0.93200371
36.1	5.71E-10	0.00286623	3.03E-05	2.86E-19	3.99E-59	1.05E-22	0.46923024	0.93791432
36.2	5.71E-10	0.00300955	3.04E-05	2.90E-19	4.16E-59	1.02E-22	0.4751412	0.92338781
36.3	6.05E-10	0.00297866	3.19E-05	2.72E-19	3.96E-59	1.00E-22	0.47868013	0.92178532
36.4	6.25E-10	0.00297077	3.17E-05	2.71E-19	4.44E-59	9.78E-23	0.48915815	0.91360929
36.5	6.25E-10	0.00297077	3.17E-05	2.71E-19	4.44E-59	9.78E-23	0.48915815	0.91360929
36.6	7.18E-10	0.00308072	3.22E-05	2.50E-19	3.93E-59	8.22E-23	0.49633051	0.92173037
36.7	7.16E-10	0.00315736	3.37E-05	2.52E-19	4.10E-59	8.34E-23	0.50242654	0.91958208
36.8	7.33E-10	0.00321341	3.39E-05	2.24E-19	3.93E-59	7.79E-23	0.51375524	0.92028993
36.9	7.10E-10	0.00331617	3.64E-05	1.97E-19	3.44E-59	6.57E-23	0.52572993	0.92890667
37	7.25E-10	0.00323462	3.89E-05	1.83E-19	3.63E-59	7.61E-23	0.54147392	0.92934661
37.1	7.97E-10	0.00334997	3.99E-05	1.86E-19	3.82E-59	8.49E-23	0.54302999	0.93322351
37.2	8.26E-10	0.00324961	4.17E-05	1.66E-19	3.50E-59	8.55E-23	0.54525541	0.93038626
37.3	9.81E-10	0.00332213	4.53E-05	1.82E-19	4.18E-59	8.36E-23	0.5452754	0.92486147
37.4	1.13E-09	0.0035074	4.85E-05	1.77E-19	4.13E-59	8.51E-23	0.53876548	0.91764485
37.5	1.13E-09	0.00354138	5.06E-05	1.78E-19	4.41E-59	8.36E-23	0.55119808	0.91900971
37.6	1.17E-09	0.00353976	5.32E-05	1.60E-19	4.40E-59	7.50E-23	0.56397627	0.93068021
37.7	1.24E-09	0.00352997	5.67E-05	1.48E-19	4.12E-59	6.78E-23	0.55438527	0.91840769
37.8	1.39E-09	0.00371148	6.14E-05	1.52E-19	4.56E-59	7.00E-23	0.55604489	0.91885552
37.9	1.62E-09	0.00377328	6.28E-05	1.46E-19	4.14E-59	6.85E-23	0.55787502	0.92154283
38	1.72E-09	0.00379675	7.01E-05	1.23E-19	4.68E-59	7.34E-23	0.56682157	0.93121536
38.1	1.77E-09	0.00386315	6.93E-05	1.21E-19	4.35E-59	7.69E-23	0.56893865	0.94037675
38.2	1.84E-09	0.00376906	7.61E-05	1.17E-19	4.34E-59	7.73E-23	0.57740133	0.94229992
38.3	2.02E-09	0.00370219	7.34E-05	1.08E-19	4.67E-59	7.48E-23	0.57723189	0.95007998
38.4	2.26E-09	0.00385796	8.10E-05	1.30E-19	5.85E-59	8.29E-23	0.57385143	0.95780144
38.5	2.33E-09	0.00383438	8.39E-05	1.29E-19	6.38E-59	8.32E-23	0.57524336	0.95470072
38.6	2.33E-09	0.0038589	8.84E-05	1.45E-19	6.71E-59	9.23E-23	0.58181628	0.94996453
38.7	2.24E-09	0.00394191	9.93E-05	1.31E-19	6.39E-59	9.77E-23	0.59808322	0.94608832
38.8	2.37E-09	0.00389074	9.92E-05	1.32E-19	6.58E-59	9.79E-23	0.59266041	0.94132789
38.9	2.40E-09	0.00402074	9.87E-05	1.26E-19	6.46E-59	9.53E-23	0.58964001	0.93808234
39	2.28E-09	0.00414247	0.00010318	1.27E-19	6.65E-59	8.98E-23	0.58992349	0.93814198
39.1	2.30E-09	0.00416914	0.00011176	1.40E-19	7.64E-59	1.04E-22	0.58811964	0.94477395
39.2	2.31E-09	0.00418123	0.00011475	1.38E-19	7.50E-59	1.05E-22	0.58449334	0.93884495
39.3	2.36E-09	0.00422627	0.0001164	1.44E-19	8.06E-59	1.08E-22	0.59134	0.93562778
39.4	2.48E-09	0.00436237	0.00012929	1.48E-19	8.39E-59	1.15E-22	0.58482477	0.9195289
39.5	2.53E-09	0.00433064	0.00014771	1.52E-19	8.98E-59	1.28E-22	0.5879672	0.91400224
39.6	2.53E-09	0.00433064	0.00014771	1.52E-19	8.98E-59	1.28E-22	0.5879672	0.91400224
39.7	2.59E-09	0.00445777	0.0001568	1.50E-19	9.47E-59	1.30E-22	0.58618956	0.92036417
39.8	2.59E-09	0.00443616	0.000158	1.43E-19	9.54E-59	1.31E-22	0.58462508	0.92311675
39.9	2.56E-09	0.00433619	0.00016318	1.42E-19	9.71E-59	1.44E-22	0.58871173	0.92387041
40	2.82E-09	0.00426868	0.00017669	1.49E-19	1.10E-58	1.64E-22	0.5946565	0.92569351
40.1	2.96E-09	0.00423675	0.00018824	1.47E-19	1.13E-58	1.74E-22	0.59971174	0.91860298
40.2	3.06E-09	0.00425203	0.00019122	1.40E-19	1.12E-58	1.75E-22	0.60222205	0.91598939
40.3	3.08E-09	0.00431414	0.00019856	1.33E-19	1.16E-58	1.83E-22	0.615644	0.9206472
40.4	3.63E-09	0.00424746	0.00021194	1.60E-19	1.64E-58	1.97E-22	0.61379844	0.91564654
40.5	3.72E-09	0.00431695	0.00022535	1.71E-19	1.85E-58	2.17E-22	0.61862663	0.90508422
40.6	4.02E-09	0.00438554	0.00022697	1.76E-19	1.92E-58	2.35E-22	0.62186458	0.90479239
40.7	3.97E-09	0.00427939	0.00023051	1.73E-19	1.81E-58	2.50E-22	0.62545693	0.90530468
40.8	4.22E-09	0.00403838	0.00023834	1.54E-19	1.77E-58	2.46E-22	0.63103757	0.90169026
40.9	4.30E-09	0.00398521	0.00023956	1.52E-19	1.70E-58	2.29E-22	0.63776966	0.90325216
41	4.53E-09	0.00403298	0.00024125	1.52E-19	1.66E-58	2.32E-22	0.63638388	0.89973423
41.1	5.03E-09	0.00373361	0.00025098	1.30E-19	1.39E-58	2.45E-22	0.65407132	0.90352257
41.2	5.39E-09	0.00382477	0.00026803	1.28E-19	1.74E-58	2.66E-22	0.66035934	0.90968444
41.3	5.52E-09	0.00396636	0.00026731	1.15E-19	1.83E-58	2.41E-22	0.66666545	0.91180998
41.4	5.94E-09	0.0040474	0.00029938	1.10E-19	1.92E-58	2.38E-22	0.66632904	0.88525117
41.5	6.78E-09	0.00401557	0.00030686	1.06E-19	2.09E-58	2.28E-22	0.66827476	0.87225262
41.6	7.12E-09	0.00409914	0.00031607	1.14E-19	2.34E-58	2.38E-22	0.66209553	0.87294059
41.7	7.54E-09	0.00417147	0.00032973	1.12E-19	2.30E-58	2.31E-22	0.65862042	0.86668673
41.8	7.36E-09	0.00421117	0.00032937	9.28E-20	1.82E-58	2.28E-22	0.66406854	0.87379373
41.9	7.84E-09	0.00412909	0.00033077	8.54E-20	1.90E-58	2.40E-22	0.6706464	0.88333729
42	7.95E-09	0.00418811	0.0003334	8.37E-20	1.97E-58	2.44E-22	0.6688706	0.88681453
42.1	8.35E-09	0.00425268	0.00033923	8.15E-20	2.08E-58	2.33E-22	0.6710392	0.88646892
42.2	8.43E-09	0.00427375	0.00036653	7.78E-20	2.38E-58	2.44E-22	0.68395749	0.8997612
42.3	9.11E-09	0.00432903	0.00039671	7.40E-20	2.60E-58	2.50E-22	0.69272223	0.91072205
42.4	1.01E-08	0.00428043	0.00041725	7.18E-20	2.59E-58	2.66E-22	0.7033508	0.91812591
42.5	1.01E-08	0.00429332	0.00043188	6.50E-20	2.51E-58	2.42E-22	0.71390795	0.92065192
42.6	1.07E-08	0.00428354	0.00043815	5.36E-20	1.94E-58	2.17E-22	0.74654071	0.9127706
42.7	1.13E-08	0.00438235	0.00045308	5.29E-20	2.16E-58	2.21E-22	0.74605121	0.90814535
42.8	1.19E-08	0.00426326	0.0004399	5.07E-20	2.01E-58	2.25E-22	0.75202186	0.90401705
42.9	1.29E-08	0.00428517	0.00046027	5.04E-20	2.00E-58	2.22E-22	0.75697589	0.90036376
43	1.32E-08	0.00422346	0.00047073	5.13E-20	1.96E-58	2.27E-22	0.75794913	0.90510613
43.1	1.39E-08	0.00433361	0.0004775	5.00E-20	1.99E-58	2.37E-22	0.75365	0.90521913
43.2	1.53E-08	0.00437266	0.0004907	4.36E-20	1.90E-58	2.08E-22	0.77176952	0.90153274
43.3	1.60E-08	0.00451053	0.00050152	4.64E-20	2.08E-58	2.10E-22	0.76780376	0.8985342
43.4	1.55E-08	0.00447984	0.00052601	4.29E-20	1.96E-58	1.78E-22	0.7656797	0.89672968
43.5	1.65E-08	0.00449399	0.00052491	4.40E-20	1.99E-58	1.83E-22	0.76636734	0.89743811
43.6	1.76E-08	0.00454789	0.00053356	4.06E-20	1.99E-58	1.86E-22	0.76216916	0.89662639
43.7	1.76E-08	0.00454789	0.00053356	4.06E-20	1.99E-58	1.86E-22	0.76216916	0.89662639
43.8	1.82E-08	0.00446003	0.00053156	4.27E-20	1.99E-58	1.85E-22	0.75892075	0.89603676
43.9	2.07E-08	0.00441359	0.00052479	4.26E-20	2.25E-58	1.90E-22	0.76168362	0.89287683
44	2.16E-08	0.00441769	0.00052099	3.93E-20	2.20E-58	1.85E-22	0.75413824	0.89090268
44.1	2.36E-08	0.00442121	0.00054611	3.77E-20	2.52E-58	1.86E-22	0.74988341	0.89381861
44.2	2.42E-08	0.00444106	0.00056667	3.42E-20	2.43E-58	1.77E-22	0.7529658	0.89667752
44.3	2.52E-08	0.00433118	0.0005795	3.41E-20	2.51E-58	1.80E-22	0.75998292	0.89227688
44.4	2.52E-08	0.00433118	0.0005795	3.41E-20	2.51E-58	1.80E-22	0.75998292	0.89227688
44.5	2.51E-08	0.00434967	0.00058942	3.37E-20	2.76E-58	1.92E-22	0.76314162	0.88968195
44.6	2.57E-08	0.00435705	0.00060338	3.26E-20	2.65E-58	1.85E-22	0.76047313	0.88946704
44.7	3.05E-08	0.00434379	0.00068826	3.40E-20	2.81E-58	1.84E-22	0.78831218	0.8833955
44.8	3.27E-08	0.00432828	0.00068822	3.16E-20	2.61E-58	1.88E-22	0.79040626	0.87447058
44.9	3.81E-08	0.00452821	0.00072546	3.04E-20	2.96E-58	1.94E-22	0.77913408	0.87364609
45	4.14E-08	0.00453754	0.00072761	2.62E-20	2.75E-58	1.94E-22	0.77905616	0.86718242
45.1	4.66E-08	0.00454188	0.00077031	2.35E-20	2.48E-58	2.03E-22	0.77870998	0.86679897
45.2	5.16E-08	0.0047562	0.00079612	2.47E-20	2.46E-58	1.98E-22	0.79250563	0.86558264
45.3	5.64E-08	0.00485746	0.00083044	2.55E-20	2.90E-58	2.01E-22	0.79734939	0.86404785
45.4	5.89E-08	0.00498908	0.00085181	2.61E-20	3.01E-58	2.03E-22	0.79141591	0.86345496
45.5	7.25E-08	0.00513023	0.00086155	2.71E-20	3.81E-58	2.25E-22	0.80394779	0.86449706
45.6	7.46E-08	0.00526457	0.00087813	2.79E-20	3.92E-58	2.11E-22	0.81315671	0.86256129
45.7	7.84E-08	0.00502228	0.00090313	2.48E-20	4.02E-58	2.02E-22	0.82223486	0.86799375
45.8	8.45E-08	0.00495715	0.00094168	2.67E-20	4.55E-58	2.09E-22	0.82435213	0.8623419
45.9	8.62E-08	0.00484647	0.00094381	2.55E-20	4.04E-58	2.02E-22	0.82284951	0.86738806
46	8.53E-08	0.00487192	0.00096769	2.57E-20	4.21E-58	2.01E-22	0.82338532	0.85886323
46.1	8.76E-08	0.004863	0.0009973	2.53E-20	4.37E-58	1.97E-22	0.82452166	0.85377328
46.2	9.03E-08	0.00487629	0.00103355	2.50E-20	4.41E-58	1.97E-22	0.83567575	0.85548448
46.3	9.03E-08	0.00487629	0.00103355	2.50E-20	4.41E-58	1.97E-22	0.83567575	0.85548448
46.4	9.30E-08	0.00471835	0.00109837	2.54E-20	4.56E-58	2.04E-22	0.84500594	0.84678393
46.5	9.25E-08	0.00463445	0.00111049	2.54E-20	4.72E-58	2.13E-22	0.85061199	0.8457055
46.6	9.71E-08	0.00450583	0.0011143	2.57E-20	4.74E-58	2.25E-22	0.85119022	0.84766335
46.7	9.62E-08	0.00442914	0.00109679	2.62E-20	4.76E-58	2.25E-22	0.85030237	0.84564431
46.8	9.59E-08	0.00444926	0.00109497	2.64E-20	4.85E-58	2.26E-22	0.8545536	0.84389864
46.9	9.39E-08	0.0043153	0.00113416	2.59E-20	5.17E-58	2.26E-22	0.86383952	0.84165191
47	9.35E-08	0.00438745	0.0011726	2.67E-20	5.40E-58	2.30E-22	0.86983426	0.85082214
47.1	1.00E-07	0.0042398	0.00115085	2.30E-20	4.77E-58	2.15E-22	0.87490484	0.84994014
47.2	1.12E-07	0.00428633	0.00122219	2.55E-20	5.97E-58	2.27E-22	0.87936819	0.85707574
47.3	1.19E-07	0.00444239	0.00121739	2.49E-20	5.35E-58	2.15E-22	0.88699114	0.86679185
47.4	1.25E-07	0.0043105	0.00124488	2.42E-20	6.02E-58	1.97E-22	0.89655581	0.86610086
47.5	1.28E-07	0.0044478	0.00126883	2.44E-20	6.06E-58	2.08E-22	0.89542835	0.86674109
47.6	1.30E-07	0.00441832	0.00129485	2.41E-20	6.45E-58	2.04E-22	0.89516189	0.87210999
47.7	1.29E-07	0.00438742	0.00130385	2.40E-20	6.49E-58	1.93E-22	0.89530958	0.86438724
47.8	1.40E-07	0.00442167	0.00135251	2.51E-20	6.99E-58	1.97E-22	0.9005807	0.86431678
47.9	1.43E-07	0.0044513	0.00138219	2.54E-20	7.27E-58	2.09E-22	0.90235344	0.86487351
48	1.43E-07	0.0044513	0.00138219	2.54E-20	7.27E-58	2.09E-22	0.90235344	0.86487351
48.1	1.41E-07	0.00447971	0.00139557	2.49E-20	7.01E-58	2.12E-22	0.90505095	0.86308193
48.2	1.38E-07	0.00442235	0.00143004	2.15E-20	6.39E-58	2.12E-22	0.91913209	0.86529848
48.3	1.36E-07	0.0044636	0.00140073	2.07E-20	6.17E-58	2.22E-22	0.91251569	0.86338809
48.4	1.37E-07	0.00440207	0.00143939	1.90E-20	5.76E-58	2.03E-22	0.92153953	0.86764649
48.5	1.46E-07	0.00431371	0.00152649	1.82E-20	5.02E-58	1.92E-22	0.93796325	0.86955281
48.6	1.59E-07	0.00448108	0.00147615	1.70E-20	4.77E-58	1.85E-22	0.94218314	0.86492781
48.7	1.75E-07	0.00453176	0.00151248	1.61E-20	5.09E-58	1.94E-22	0.94868485	0.85918369
48.8	1.89E-07	0.00461448	0.00150996	1.63E-20	5.59E-58	2.06E-22	0.94446862	0.85607887
48.9	1.89E-07	0.00466463	0.00149705	1.64E-20	5.76E-58	2.00E-22	0.94152553	0.85553094
49	1.98E-07	0.0046872	0.00150943	1.76E-20	5.81E-58	2.13E-22	0.93778141	0.85827256
49.1	2.09E-07	0.00472345	0.00154447	1.78E-20	6.44E-58	2.05E-22	0.93038866	0.86203277
49.2	2.16E-07	0.00468684	0.00152298	1.68E-20	5.94E-58	2.01E-22	0.93438623	0.86458066
49.3	2.20E-07	0.00468047	0.00155939	1.69E-20	5.97E-58	1.98E-22	0.93448901	0.8622213
49.4	2.30E-07	0.0046885	0.00155749	1.58E-20	5.69E-58	1.93E-22	0.93479511	0.85221374
49.5	2.33E-07	0.00477649	0.00161335	1.31E-20	4.97E-58	1.88E-22	0.94176858	0.84839912
49.6	2.47E-07	0.00485249	0.0016651	1.23E-20	5.15E-58	1.95E-22	0.94010255	0.85287911
49.7	2.54E-07	0.0047956	0.00174854	1.17E-20	5.10E-58	1.92E-22	0.93649745	0.86176917
49.8	2.53E-07	0.0047706	0.00179985	1.14E-20	5.35E-58	1.95E-22	0.93314862	0.86582129
49.9	2.53E-07	0.0047706	0.00179985	1.14E-20	5.35E-58	1.95E-22	0.93314862	0.86582129
50	2.59E-07	0.0048902	0.00184995	1.13E-20	5.90E-58	1.90E-22	0.92648101	0.86484621
50.1	2.74E-07	0.0047727	0.00190333	1.15E-20	5.81E-58	1.87E-22	0.93516612	0.86097383
50.2	2.77E-07	0.00477544	0.00195684	1.13E-20	5.47E-58	1.88E-22	0.93124906	0.86358261
50.3	2.77E-07	0.00477544	0.00195684	1.13E-20	5.47E-58	1.88E-22	0.93124906	0.86358261
50.4	2.90E-07	0.00466906	0.00194025	1.11E-20	5.43E-58	1.85E-22	0.93749649	0.86047764
50.5	3.08E-07	0.00475855	0.00194583	1.17E-20	5.72E-58	1.94E-22	0.93284303	0.86563336
50.6	3.09E-07	0.00462417	0.00197596	1.05E-20	4.85E-58	1.77E-22	0.93722017	0.86697231
50.7	3.27E-07	0.00486018	0.00209235	9.10E-21	4.57E-58	1.74E-22	0.92767833	0.87273787
50.8	3.24E-07	0.00496805	0.00212756	8.83E-21	4.54E-58	1.66E-22	0.92508046	0.8751091
50.9	3.42E-07	0.00487696	0.0022288	7.37E-21	3.69E-58	1.52E-22	0.92729362	0.86014636
51	3.42E-07	0.00487696	0.0022288	7.37E-21	3.69E-58	1.52E-22	0.92729362	0.86014636
51.1	3.42E-07	0.00487696	0.0022288	7.37E-21	3.69E-58	1.52E-22	0.92729362	0.86014636
51.2	3.44E-07	0.00491687	0.00222819	7.34E-21	3.74E-58	1.56E-22	0.9245976	0.85618218
51.3	3.46E-07	0.00509686	0.00227215	7.43E-21	3.92E-58	1.56E-22	0.92628605	0.85490793
51.4	3.49E-07	0.00511249	0.00231082	7.17E-21	3.82E-58	1.48E-22	0.93275578	0.85320222
51.5	3.54E-07	0.00513343	0.00242302	7.15E-21	3.94E-58	1.48E-22	0.93714496	0.85223714
51.6	3.50E-07	0.00511347	0.00242813	6.86E-21	3.83E-58	1.49E-22	0.93887129	0.85974489
51.7	3.56E-07	0.00500413	0.0023831	6.20E-21	3.43E-58	1.40E-22	0.94501056	0.8566863
51.8	3.94E-07	0.0049041	0.00238242	5.27E-21	2.45E-58	1.32E-22	0.95550515	0.86505447
51.9	4.07E-07	0.00488084	0.00237151	5.22E-21	2.44E-58	1.27E-22	0.95546444	0.87013675
52	4.17E-07	0.00479873	0.00239058	5.53E-21	2.63E-58	1.23E-22	0.96586312	0.87658744
52.1	4.26E-07	0.00475816	0.00254918	4.92E-21	2.71E-58	1.12E-22	0.96851366	0.88217176
52.2	4.31E-07	0.00470403	0.00258594	5.05E-21	2.75E-58	1.08E-22	0.97107293	0.88470959
52.3	4.24E-07	0.00475536	0.00264711	4.91E-21	2.62E-58	1.01E-22	0.96630975	0.88572138
52.4	4.30E-07	0.00459183	0.00269317	4.54E-21	2.58E-58	1.02E-22	0.9831043	0.89346905
52.5	4.29E-07	0.00463723	0.00273507	4.29E-21	2.69E-58	9.40E-23	0.97558463	0.88838917
52.6	4.47E-07	0.00454389	0.00277074	4.02E-21	2.57E-58	9.06E-23	0.98250957	0.88361229
52.7	4.76E-07	0.00455842	0.00273073	3.71E-21	2.28E-58	8.48E-23	0.98890079	0.89423164
52.8	5.08E-07	0.00428531	0.0027431	3.70E-21	2.33E-58	7.72E-23	0.98677762	0.90325909
52.9	5.31E-07	0.00454712	0.00282715	3.57E-21	2.41E-58	7.55E-23	0.98989985	0.90419925
53	5.31E-07	0.00454712	0.00282715	3.57E-21	2.41E-58	7.55E-23	0.98989985	0.90419925
53.1	5.27E-07	0.00458417	0.00292605	3.84E-21	2.61E-58	8.21E-23	0.98802737	0.90275937
53.2	5.29E-07	0.00446492	0.00294226	3.62E-21	2.60E-58	8.30E-23	0.99365349	0.89904844
53.3	5.37E-07	0.00455719	0.00302904	3.16E-21	2.44E-58	8.04E-23	0.99350845	0.89321192
53.4	5.35E-07	0.00462621	0.00310705	3.07E-21	2.43E-58	8.32E-23	0.99317806	0.88918127
53.5	5.46E-07	0.00470243	0.00316439	3.11E-21	2.63E-58	8.06E-23	0.98826856	0.89403764
53.6	5.46E-07	0.00470243	0.00316439	3.11E-21	2.63E-58	8.06E-23	0.98826856	0.89403764
53.7	5.66E-07	0.00464798	0.00305975	3.28E-21	2.40E-58	7.81E-23	0.98709275	0.89642795
53.8	5.84E-07	0.004671	0.00314278	3.51E-21	2.80E-58	8.01E-23	0.98471663	0.90060391
53.9	6.06E-07	0.00459464	0.00311019	3.58E-21	2.97E-58	7.76E-23	0.98463291	0.90129274
54	6.07E-07	0.0045518	0.00311747	3.48E-21	2.86E-58	7.26E-23	0.98491359	0.9029602
54.1	6.20E-07	0.00453942	0.00310282	3.47E-21	2.88E-58	7.33E-23	0.98257152	0.89829339
54.2	6.46E-07	0.00445283	0.00314355	3.25E-21	2.71E-58	7.31E-23	0.98986286	0.89939563
54.3	6.75E-07	0.0045852	0.00324507	3.13E-21	2.88E-58	7.96E-23	0.98002976	0.89151521
54.4	6.75E-07	0.0045852	0.00324507	3.13E-21	2.88E-58	7.96E-23	0.98002976	0.89151521
54.5	6.44E-07	0.00454015	0.00339931	3.30E-21	3.15E-58	7.92E-23	0.98124435	0.8947134
54.6	6.36E-07	0.00450559	0.00338192	3.22E-21	3.04E-58	7.93E-23	0.98322756	0.8909819
54.7	6.36E-07	0.00450559	0.00338192	3.22E-21	3.04E-58	7.93E-23	0.98322756	0.8909819
54.8	6.36E-07	0.00450559	0.00338192	3.22E-21	3.04E-58	7.93E-23	0.98322756	0.8909819
54.9	6.32E-07	0.00457769	0.00351242	3.08E-21	3.04E-58	8.05E-23	0.97602582	0.89105485
55	6.44E-07	0.00448092	0.0036092	2.88E-21	2.87E-58	7.88E-23	0.98531101	0.8926691
55.1	6.95E-07	0.00439134	0.0037848	3.13E-21	3.30E-58	8.08E-23	0.9806006	0.89487746
55.2	6.95E-07	0.00439134	0.0037848	3.13E-21	3.30E-58	8.08E-23	0.9806006	0.89487746
55.3	7.12E-07	0.00440552	0.00385926	3.02E-21	3.14E-58	7.85E-23	0.97970548	0.89143036
55.4	7.55E-07	0.00458789	0.00407917	3.25E-21	3.74E-58	9.45E-23	0.97515289	0.89172782
55.5	7.55E-07	0.00458789	0.00407917	3.25E-21	3.74E-58	9.45E-23	0.97515289	0.89172782
55.6	7.61E-07	0.00467576	0.00422068	3.42E-21	4.12E-58	1.05E-22	0.96921623	0.89466323
55.7	7.78E-07	0.00475793	0.00437382	3.64E-21	4.44E-58	1.12E-22	0.9642906	0.89159161
55.8	7.66E-07	0.00476885	0.00436356	3.39E-21	4.27E-58	1.02E-22	0.96194787	0.89234886
55.9	7.73E-07	0.00460654	0.00441181	3.15E-21	3.80E-58	1.02E-22	0.96291932	0.89116651
56	8.14E-07	0.00472545	0.00441736	3.35E-21	3.87E-58	1.05E-22	0.96559476	0.89379331
56.1	8.24E-07	0.0046456	0.00451816	3.48E-21	4.21E-58	1.03E-22	0.96781019	0.8943957
56.2	8.27E-07	0.0047815	0.00454599	3.51E-21	4.34E-58	1.07E-22	0.97124924	0.89821935
56.3	8.37E-07	0.00483022	0.00464063	3.38E-21	4.36E-58	1.06E-22	0.97399234	0.90079842
56.4	8.72E-07	0.00472823	0.00470476	3.21E-21	4.15E-58	9.91E-23	0.97652796	0.90280944
56.5	8.75E-07	0.00460341	0.0046429	3.45E-21	4.22E-58	9.98E-23	0.97669115	0.89983978
56.6	8.75E-07	0.00460341	0.0046429	3.45E-21	4.22E-58	9.98E-23	0.97669115	0.89983978
56.7	8.83E-07	0.00463246	0.00462167	3.59E-21	4.32E-58	9.68E-23	0.9754043	0.90485678
56.8	8.95E-07	0.00458662	0.00455028	3.84E-21	4.56E-58	9.79E-23	0.97218753	0.89364744
56.9	8.95E-07	0.00458662	0.00455028	3.84E-21	4.56E-58	9.79E-23	0.97218753	0.89364744
57	9.15E-07	0.0046446	0.00462769	3.81E-21	4.51E-58	1.02E-22	0.97101587	0.88855234
57.1	9.15E-07	0.0046446	0.00462769	3.81E-21	4.51E-58	1.02E-22	0.97101587	0.88855234
57.2	9.15E-07	0.00464152	0.00458199	3.69E-21	4.27E-58	9.88E-23	0.96980296	0.9015154
57.3	9.15E-07	0.00464152	0.00458199	3.69E-21	4.27E-58	9.88E-23	0.96980296	0.9015154
57.4	9.47E-07	0.00455564	0.00450311	3.45E-21	4.08E-58	9.69E-23	0.96890076	0.89964482
57.5	9.79E-07	0.00463585	0.00446591	3.62E-21	4.11E-58	9.59E-23	0.97218594	0.90424481
57.6	9.79E-07	0.00463585	0.00446591	3.62E-21	4.11E-58	9.59E-23	0.97218594	0.90424481
57.7	9.90E-07	0.0045502	0.0045887	3.50E-21	4.32E-58	9.79E-23	0.97917827	0.90797525
57.8	1.03E-06	0.00461801	0.00462728	3.31E-21	4.46E-58	9.94E-23	0.98282164	0.90487574
57.9	1.05E-06	0.00471399	0.00464957	3.35E-21	4.65E-58	1.00E-22	0.98375187	0.91016076
58	1.05E-06	0.00471399	0.00464957	3.35E-21	4.65E-58	1.00E-22	0.98375187	0.91016076
58.1	1.13E-06	0.00462861	0.00462916	3.27E-21	4.24E-58	1.00E-22	0.98430904	0.91032908
58.2	1.20E-06	0.00472375	0.00479384	3.20E-21	4.53E-58	1.05E-22	0.98299328	0.90746867
58.3	1.20E-06	0.00472375	0.00479384	3.20E-21	4.53E-58	1.05E-22	0.98299328	0.90746867
58.4	1.26E-06	0.00468954	0.00484423	3.07E-21	4.58E-58	1.06E-22	0.98569335	0.91011044
58.5	1.26E-06	0.00468954	0.00484423	3.07E-21	4.58E-58	1.06E-22	0.98569335	0.91011044
58.6	1.26E-06	0.00468954	0.00484423	3.07E-21	4.58E-58	1.06E-22	0.98569335	0.91011044
58.7	1.28E-06	0.0048822	0.0048127	3.05E-21	4.62E-58	1.05E-22	0.99047536	0.90334801
58.8	1.28E-06	0.00485668	0.00488796	3.04E-21	4.93E-58	1.10E-22	0.9974646	0.9029966
58.9	1.29E-06	0.00472118	0.00488183	2.56E-21	3.99E-58	1.01E-22	0.99632355	0.90704957
59	1.29E-06	0.00472118	0.00488183	2.56E-21	3.99E-58	1.01E-22	0.99632355	0.90704957
59.1	1.36E-06	0.00466584	0.00488394	2.52E-21	3.84E-58	9.54E-23	0.99595971	0.91130765
59.2	1.36E-06	0.00466584	0.00488394	2.52E-21	3.84E-58	9.54E-23	0.99595971	0.91130765
59.3	1.36E-06	0.00466584	0.00488394	2.52E-21	3.84E-58	9.54E-23	0.99595971	0.91130765
59.4	1.59E-06	0.00473341	0.00520292	2.41E-21	4.01E-58	1.03E-22	0.99876223	0.91070186
59.5	1.59E-06	0.00473341	0.00520292	2.41E-21	4.01E-58	1.03E-22	0.99876223	0.91070186
59.6	1.59E-06	0.00473341	0.00520292	2.41E-21	4.01E-58	1.03E-22	0.99876223	0.91070186
59.7	1.72E-06	0.00462346	0.00516651	2.27E-21	3.81E-58	1.02E-22	0.99810587	0.90145757
59.8	1.78E-06	0.0047026	0.00518944	2.16E-21	3.80E-58	1.04E-22	0.99896396	0.90503412
59.9	1.78E-06	0.0047026	0.00518944	2.16E-21	3.80E-58	1.04E-22	0.99896396	0.90503412

60	1.82E-06	0.00467768	0.00531346	2.12E-21	4.06E-58	1.04E-22	0.99764019	0.90915237
60.1	1.81E-06	0.00471121	0.00540868	2.13E-21	4.08E-58	1.03E-22	0.99512595	0.90940406
60.2	1.95E-06	0.00450236	0.00580686	2.05E-21	4.21E-58	1.09E-22	0.9942406	0.90396415
60.3	1.95E-06	0.00450236	0.00580686	2.05E-21	4.21E-58	1.09E-22	0.9942406	0.90396415
60.4	2.01E-06	0.00451852	0.00583539	1.96E-21	4.02E-58	1.07E-22	0.98864734	0.90101948
60.5	2.07E-06	0.00449215	0.00589011	1.89E-21	3.91E-58	1.04E-22	0.98920219	0.89662783
60.6	2.08E-06	0.00438811	0.00595893	1.95E-21	3.89E-58	1.05E-22	0.99519225	0.89257427
60.7	2.17E-06	0.00419643	0.00629854	1.67E-21	3.94E-58	9.98E-23	0.9920225	0.89158232
60.8	2.17E-06	0.00419643	0.00629854	1.67E-21	3.94E-58	9.98E-23	0.9920225	0.89158232
60.9	2.21E-06	0.00413966	0.00627993	1.68E-21	3.93E-58	9.41E-23	0.9889693	0.89086529
61	2.25E-06	0.00418924	0.00619535	1.61E-21	3.81E-58	9.53E-23	0.9821428	0.88641265
61.1	2.35E-06	0.00419322	0.00619607	1.72E-21	4.13E-58	9.24E-23	0.98244406	0.88834422
61.2	2.45E-06	0.00419594	0.00618527	1.86E-21	4.39E-58	9.90E-23	0.98188063	0.88626609
61.3	2.45E-06	0.00419594	0.00618527	1.86E-21	4.39E-58	9.90E-23	0.98188063	0.88626609
61.4	2.51E-06	0.00424613	0.0062143	1.84E-21	4.31E-58	9.96E-23	0.9809618	0.8851409
61.5	2.65E-06	0.00434757	0.00641154	1.83E-21	4.23E-58	1.04E-22	0.9858211	0.88451365
61.6	2.65E-06	0.00434757	0.00641154	1.83E-21	4.23E-58	1.04E-22	0.9858211	0.88451365
61.7	2.75E-06	0.00435914	0.00647605	1.77E-21	4.28E-58	1.07E-22	0.98834401	0.8831768
61.8	2.75E-06	0.00435914	0.00647605	1.77E-21	4.28E-58	1.07E-22	0.98834401	0.8831768
61.9	2.75E-06	0.00435914	0.00647605	1.77E-21	4.28E-58	1.07E-22	0.98834401	0.8831768
62	2.84E-06	0.00446122	0.00660602	1.81E-21	4.52E-58	1.06E-22	0.99515583	0.88188292
62.1	2.85E-06	0.00443857	0.00664384	1.83E-21	4.70E-58	1.13E-22	0.98695137	0.882343
62.2	2.90E-06	0.0043888	0.00673143	1.90E-21	4.86E-58	1.17E-22	0.99273382	0.87957019
62.3	2.91E-06	0.00453244	0.00664844	1.78E-21	4.67E-58	1.10E-22	0.99442584	0.87782992
62.4	2.99E-06	0.00451129	0.00661665	1.77E-21	4.74E-58	1.10E-22	0.99101793	0.8815591
62.5	3.11E-06	0.00454645	0.00658253	1.67E-21	4.68E-58	1.06E-22	0.98969545	0.88092934
62.6	3.11E-06	0.00454645	0.00658253	1.67E-21	4.68E-58	1.06E-22	0.98969545	0.88092934
62.7	3.26E-06	0.00455417	0.00672354	1.70E-21	4.66E-58	1.07E-22	0.98509998	0.88360023
62.8	3.23E-06	0.00462251	0.00674004	1.69E-21	4.51E-58	1.06E-22	0.98067557	0.88088262
62.9	3.23E-06	0.00459714	0.00675159	1.64E-21	4.50E-58	1.07E-22	0.98255124	0.88111024
63	3.23E-06	0.00459714	0.00675159	1.64E-21	4.50E-58	1.07E-22	0.98255124	0.88111024
63.1	3.40E-06	0.00463238	0.00712709	1.57E-21	4.49E-58	1.17E-22	0.98531821	0.89209749
63.2	3.43E-06	0.00470472	0.00726061	1.51E-21	4.50E-58	1.16E-22	0.98510649	0.89963794
63.3	3.51E-06	0.00469091	0.00740918	1.57E-21	4.93E-58	1.11E-22	0.98057164	0.90726543
63.4	3.54E-06	0.00468846	0.00747153	1.56E-21	4.69E-58	1.18E-22	0.98584425	0.90934033
63.5	3.52E-06	0.00472991	0.00757755	1.47E-21	4.52E-58	1.16E-22	0.98619322	0.9080483
63.6	3.52E-06	0.00472991	0.00757755	1.47E-21	4.52E-58	1.16E-22	0.98619322	0.9080483
63.7	3.52E-06	0.00484389	0.00748255	1.44E-21	4.59E-58	1.18E-22	0.98644582	0.90785824
63.8	3.58E-06	0.00474233	0.00750663	1.38E-21	4.51E-58	1.15E-22	0.98285549	0.90479725
63.9	3.58E-06	0.00474233	0.00750663	1.38E-21	4.51E-58	1.15E-22	0.98285549	0.90479725
64	3.58E-06	0.00474233	0.00750663	1.38E-21	4.51E-58	1.15E-22	0.98285549	0.90479725
64.1	3.58E-06	0.00474233	0.00750663	1.38E-21	4.51E-58	1.15E-22	0.98285549	0.90479725
64.2	3.88E-06	0.00460687	0.00751362	1.34E-21	4.54E-58	1.11E-22	0.98456587	0.89377263
64.3	3.88E-06	0.00460687	0.00751362	1.34E-21	4.54E-58	1.11E-22	0.98456587	0.89377263
64.4	3.97E-06	0.0044563	0.00749765	1.44E-21	4.74E-58	1.21E-22	0.98775385	0.88861256
64.5	3.84E-06	0.00452011	0.00761215	1.33E-21	4.39E-58	1.11E-22	0.97939401	0.89054287
64.6	3.84E-06	0.00452011	0.00761215	1.33E-21	4.39E-58	1.11E-22	0.97939401	0.89054287
64.7	3.84E-06	0.00452011	0.00761215	1.33E-21	4.39E-58	1.11E-22	0.97939401	0.89054287
64.8	3.84E-06	0.00441371	0.00776505	1.28E-21	4.29E-58	1.09E-22	0.97745766	0.8905947
64.9	3.84E-06	0.00441371	0.00776505	1.28E-21	4.29E-58	1.09E-22	0.97745766	0.8905947
65	4.00E-06	0.00446165	0.0076314	1.34E-21	4.66E-58	1.06E-22	0.97417949	0.89379186
65.1	4.22E-06	0.00441518	0.00795192	1.25E-21	4.26E-58	1.04E-22	0.98015324	0.90137462
65.2	4.30E-06	0.00437652	0.0082385	1.17E-21	4.09E-58	9.73E-23	0.97955968	0.91045347
65.3	4.28E-06	0.00433638	0.00820804	1.10E-21	3.79E-58	9.69E-23	0.97561122	0.90499888
65.4	4.48E-06	0.00434368	0.00821517	1.03E-21	3.63E-58	9.18E-23	0.97163248	0.90404671
65.5	4.76E-06	0.00422803	0.00847813	1.05E-21	4.28E-58	9.71E-23	0.96555686	0.9069147
65.6	4.78E-06	0.00429406	0.00862241	1.14E-21	4.73E-58	1.04E-22	0.9602854	0.90601194
65.7	4.81E-06	0.00419953	0.00888385	1.13E-21	4.84E-58	1.03E-22	0.96201815	0.90673394
65.8	4.81E-06	0.00419953	0.00888385	1.13E-21	4.84E-58	1.03E-22	0.96201815	0.90673394
65.9	5.01E-06	0.004184	0.00889776	1.14E-21	4.85E-58	1.04E-22	0.9635204	0.90444094
66	5.01E-06	0.004184	0.00889776	1.14E-21	4.85E-58	1.04E-22	0.9635204	0.90444094
66.1	5.01E-06	0.004184	0.00889776	1.14E-21	4.85E-58	1.04E-22	0.9635204	0.90444094
66.2	4.92E-06	0.00415347	0.00891156	1.12E-21	4.69E-58	1.01E-22	0.95637513	0.90523007
66.3	4.92E-06	0.00415347	0.00891156	1.12E-21	4.69E-58	1.01E-22	0.95637513	0.90523007
66.4	5.15E-06	0.00409677	0.0088409	1.20E-21	5.26E-58	1.06E-22	0.95513901	0.90569569
66.5	5.15E-06	0.00409677	0.0088409	1.20E-21	5.26E-58	1.06E-22	0.95513901	0.90569569
66.6	5.32E-06	0.00414348	0.00909847	1.13E-21	5.37E-58	1.03E-22	0.96232521	0.896421
66.7	5.48E-06	0.00411245	0.00913222	1.05E-21	5.05E-58	1.01E-22	0.96128215	0.89895847
66.8	5.42E-06	0.00403278	0.00919335	9.78E-22	4.69E-58	8.84E-23	0.95162046	0.90547821
66.9	5.42E-06	0.00403278	0.00919335	9.78E-22	4.69E-58	8.84E-23	0.95162046	0.90547821
67	5.42E-06	0.00403278	0.00919335	9.78E-22	4.69E-58	8.84E-23	0.95162046	0.90547821
67.1	5.56E-06	0.00391823	0.00923095	1.05E-21	4.85E-58	8.67E-23	0.95500433	0.90584674
67.2	5.79E-06	0.00382267	0.00926132	1.10E-21	5.14E-58	9.46E-23	0.94982998	0.90292837
67.3	5.79E-06	0.00382267	0.00926132	1.10E-21	5.14E-58	9.46E-23	0.94982998	0.90292837
67.4	5.81E-06	0.00382755	0.00943384	1.03E-21	4.79E-58	9.62E-23	0.94978121	0.90681406
67.5	5.81E-06	0.00382755	0.00943384	1.03E-21	4.79E-58	9.62E-23	0.94978121	0.90681406
67.6	5.88E-06	0.00392417	0.00965757	1.01E-21	4.67E-58	1.01E-22	0.94708922	0.90627155
67.7	5.94E-06	0.00390693	0.00945419	9.83E-22	4.49E-58	1.05E-22	0.94474687	0.91296478
67.8	6.05E-06	0.00390106	0.00961285	1.01E-21	4.67E-58	1.03E-22	0.9408306	0.9108778
67.9	6.11E-06	0.00394978	0.0097623	9.56E-22	4.73E-58	9.52E-23	0.93453909	0.90062217
68	6.15E-06	0.00405102	0.00999489	9.08E-22	4.59E-58	1.00E-22	0.9344753	0.90070913
68.1	6.22E-06	0.00409124	0.01014729	8.82E-22	4.48E-58	1.06E-22	0.93256292	0.89286095
68.2	6.22E-06	0.00409124	0.01014729	8.82E-22	4.48E-58	1.06E-22	0.93256292	0.89286095
68.3	6.22E-06	0.00409124	0.01014729	8.82E-22	4.48E-58	1.06E-22	0.93256292	0.89286095
68.4	6.68E-06	0.00405824	0.01077113	8.19E-22	4.63E-58	1.12E-22	0.93508344	0.88782581
68.5	6.64E-06	0.00401211	0.01083638	8.13E-22	4.71E-58	1.13E-22	0.93230669	0.88508248
68.6	6.64E-06	0.00401211	0.01083638	8.13E-22	4.71E-58	1.13E-22	0.93230669	0.88508248
68.7	6.57E-06	0.00405656	0.01110276	8.30E-22	4.89E-58	1.17E-22	0.92681006	0.88977207
68.8	7.05E-06	0.0040356	0.01187224	9.30E-22	5.79E-58	1.34E-22	0.93527665	0.88097062
68.9	7.05E-06	0.0040356	0.01187224	9.30E-22	5.79E-58	1.34E-22	0.93527665	0.88097062
69	7.33E-06	0.00388352	0.01195206	9.73E-22	6.23E-58	1.45E-22	0.92825588	0.87242272
69.1	7.31E-06	0.00387677	0.0118278	9.96E-22	6.19E-58	1.41E-22	0.93131675	0.86882566
69.2	7.57E-06	0.00397186	0.01167767	1.05E-21	6.61E-58	1.50E-22	0.92483603	0.87078878
69.3	7.57E-06	0.00397186	0.01167767	1.05E-21	6.61E-58	1.50E-22	0.92483603	0.87078878
69.4	7.57E-06	0.00397186	0.01167767	1.05E-21	6.61E-58	1.50E-22	0.92483603	0.87078878
69.5	7.55E-06	0.00398286	0.01183858	1.08E-21	6.79E-58	1.51E-22	0.92089428	0.86983079
69.6	7.55E-06	0.00398286	0.01183858	1.08E-21	6.79E-58	1.51E-22	0.92089428	0.86983079
69.7	7.86E-06	0.00400746	0.01171793	1.13E-21	6.93E-58	1.51E-22	0.9234189	0.87119537
69.8	7.86E-06	0.00400746	0.01171793	1.13E-21	6.93E-58	1.51E-22	0.9234189	0.87119537
69.9	8.12E-06	0.00393077	0.01187557	1.11E-21	7.06E-58	1.51E-22	0.9233623	0.8734057
70	8.60E-06	0.00381885	0.01196349	1.09E-21	6.89E-58	1.51E-22	0.92086536	0.87170243
70.1	8.64E-06	0.00374966	0.01206109	1.11E-21	6.82E-58	1.48E-22	0.92102631	0.8649403
70.2	8.94E-06	0.003766	0.01224936	1.16E-21	7.33E-58	1.53E-22	0.92259685	0.87093609
70.3	9.01E-06	0.00368766	0.01211932	1.18E-21	7.28E-58	1.51E-22	0.92388448	0.86915102
70.4	9.27E-06	0.00359295	0.0122743	1.14E-21	7.10E-58	1.45E-22	0.92639226	0.86159161
70.5	9.47E-06	0.00358531	0.01213035	1.14E-21	7.24E-58	1.50E-22	0.9203096	0.85523302
70.6	9.40E-06	0.00366094	0.01245053	1.08E-21	6.99E-58	1.53E-22	0.9179076	0.85897564
70.7	9.27E-06	0.00364408	0.0125503	1.01E-21	6.57E-58	1.48E-22	0.91763707	0.86449576
70.8	9.76E-06	0.00376784	0.01261226	9.77E-22	6.72E-58	1.58E-22	0.91436985	0.86367995
70.9	1.00E-05	0.00380229	0.01342328	1.11E-21	7.91E-58	1.98E-22	0.91234664	0.87356469
71	1.02E-05	0.00369652	0.0134598	1.08E-21	7.91E-58	1.89E-22	0.91031608	0.86915971
71.1	1.04E-05	0.00375716	0.013632	1.06E-21	8.50E-58	1.89E-22	0.90293142	0.8666397
71.2	1.04E-05	0.00375716	0.013632	1.06E-21	8.50E-58	1.89E-22	0.90293142	0.8666397
71.3	1.04E-05	0.00375716	0.013632	1.06E-21	8.50E-58	1.89E-22	0.90293142	0.8666397
71.4	1.09E-05	0.00379005	0.01381483	1.15E-21	9.37E-58	2.13E-22	0.90488668	0.86827935
71.5	1.09E-05	0.00379005	0.01381483	1.15E-21	9.37E-58	2.13E-22	0.90488668	0.86827935
71.6	1.09E-05	0.00379005	0.01381483	1.15E-21	9.37E-58	2.13E-22	0.90488668	0.86827935
71.7	1.09E-05	0.00379005	0.01381483	1.15E-21	9.37E-58	2.13E-22	0.90488668	0.86827935
71.8	1.12E-05	0.00379847	0.01408757	1.16E-21	9.42E-58	2.15E-22	0.90237772	0.86691872
71.9	1.15E-05	0.00381189	0.01417647	1.15E-21	9.54E-58	2.24E-22	0.90321314	0.86094554
72	1.15E-05	0.00381189	0.01417647	1.15E-21	9.54E-58	2.24E-22	0.90321314	0.86094554
72.1	1.16E-05	0.00382025	0.01429448	1.24E-21	1.03E-57	2.39E-22	0.8983434	0.8622278
72.2	1.16E-05	0.00382025	0.01429448	1.24E-21	1.03E-57	2.39E-22	0.8983434	0.8622278
72.3	1.16E-05	0.00382025	0.01429448	1.24E-21	1.03E-57	2.39E-22	0.8983434	0.8622278
72.4	1.23E-05	0.00394037	0.01427832	1.27E-21	1.07E-57	2.51E-22	0.91181232	0.86540801
72.5	1.28E-05	0.00395329	0.01449946	1.32E-21	1.07E-57	2.56E-22	0.91321906	0.86342021
72.6	1.29E-05	0.00397835	0.01439628	1.28E-21	1.05E-57	2.61E-22	0.91620162	0.8615326
72.7	1.29E-05	0.00397835	0.01439628	1.28E-21	1.05E-57	2.61E-22	0.91620162	0.8615326
72.8	1.29E-05	0.00397835	0.01439628	1.28E-21	1.05E-57	2.61E-22	0.91620162	0.8615326
72.9	1.28E-05	0.0038632	0.01480023	1.23E-21	1.07E-57	2.50E-22	0.91761319	0.86423751
73	1.31E-05	0.00382442	0.01467638	1.16E-21	1.07E-57	2.53E-22	0.91530765	0.8664692
73.1	1.34E-05	0.00386579	0.01467231	1.11E-21	1.02E-57	2.60E-22	0.91720392	0.86565339
73.2	1.34E-05	0.00386579	0.01467231	1.11E-21	1.02E-57	2.60E-22	0.91720392	0.86565339
73.3	1.40E-05	0.00384796	0.01471991	1.15E-21	1.08E-57	2.72E-22	0.92209674	0.86463512
73.4	1.40E-05	0.00384796	0.01471991	1.15E-21	1.08E-57	2.72E-22	0.92209674	0.86463512
73.5	1.39E-05	0.0038312	0.01488169	1.22E-21	1.13E-57	2.74E-22	0.92230363	0.86677071
73.6	1.39E-05	0.0038312	0.01488169	1.22E-21	1.13E-57	2.74E-22	0.92230363	0.86677071
73.7	1.49E-05	0.00394127	0.01536642	1.26E-21	1.12E-57	3.04E-22	0.92109536	0.86952425
73.8	1.52E-05	0.00386715	0.01567861	1.23E-21	1.16E-57	3.01E-22	0.92078666	0.8681052
73.9	1.53E-05	0.00389054	0.01566212	1.25E-21	1.23E-57	3.10E-22	0.91951584	0.87041603
74	1.53E-05	0.00389054	0.01566212	1.25E-21	1.23E-57	3.10E-22	0.91951584	0.87041603
74.1	1.58E-05	0.00378768	0.01592914	1.24E-21	1.27E-57	3.14E-22	0.92523802	0.8645288
74.2	1.68E-05	0.00376574	0.01629549	1.37E-21	1.35E-57	3.29E-22	0.93611326	0.86592451
74.3	1.73E-05	0.00377784	0.0164373	1.42E-21	1.38E-57	3.43E-22	0.93125883	0.86887958
74.4	1.73E-05	0.00377784	0.0164373	1.42E-21	1.38E-57	3.43E-22	0.93125883	0.86887958
74.5	1.76E-05	0.00385567	0.01662425	1.41E-21	1.41E-57	3.54E-22	0.92858542	0.87425314
74.6	1.81E-05	0.0038533	0.01673501	1.34E-21	1.48E-57	3.40E-22	0.92519078	0.87720259
74.7	1.81E-05	0.0038533	0.01673501	1.34E-21	1.48E-57	3.40E-22	0.92519078	0.87720259
74.8	1.81E-05	0.0038533	0.01673501	1.34E-21	1.48E-57	3.40E-22	0.92519078	0.87720259
74.9	1.82E-05	0.00379555	0.01698972	1.47E-21	1.59E-57	3.49E-22	0.92374503	0.86492363
120	0.00014673	0.00716088	0.03489817	1.08E-21	4.43E-57	3.72E-21	0.90898311	0.97861792
120.1	0.00014673	0.00716088	0.03489817	1.08E-21	4.43E-57	3.72E-21	0.90898311	0.97861792
75	1.81E-05	0.00377457	0.01738267	1.46E-21	1.65E-57	3.49E-22	0.92991002	0.86172346
75.1	1.81E-05	0.00377457	0.01738267	1.46E-21	1.65E-57	3.49E-22	0.92991002	0.86172346
75.2	1.85E-05	0.00390021	0.01757777	1.40E-21	1.71E-57	3.41E-22	0.92637314	0.85717271
75.3	1.87E-05	0.00391672	0.01782741	1.42E-21	1.72E-57	3.19E-22	0.92817695	0.86116866
75.4	1.85E-05	0.00393083	0.0177406	1.49E-21	1.77E-57	3.41E-22	0.93370793	0.86385877
75.5	1.85E-05	0.00393083	0.0177406	1.49E-21	1.77E-57	3.41E-22	0.93370793	0.86385877
75.6	1.91E-05	0.00403921	0.0179084	1.50E-21	1.85E-57	3.61E-22	0.93332144	0.86473132
75.7	1.91E-05	0.00403921	0.0179084	1.50E-21	1.85E-57	3.61E-22	0.93332144	0.86473132
75.8	1.91E-05	0.003966	0.0180773	1.64E-21	1.99E-57	3.78E-22	0.93455133	0.85857837
75.9	1.91E-05	0.003966	0.0180773	1.64E-21	1.99E-57	3.78E-22	0.93455133	0.85857837
76	1.91E-05	0.003966	0.0180773	1.64E-21	1.99E-57	3.78E-22	0.93455133	0.85857837
76.1	1.91E-05	0.003966	0.0180773	1.64E-21	1.99E-57	3.78E-22	0.93455133	0.85857837
76.2	1.91E-05	0.003966	0.0180773	1.64E-21	1.99E-57	3.78E-22	0.93455133	0.85857837
76.3	1.91E-05	0.003966	0.0180773	1.64E-21	1.99E-57	3.78E-22	0.93455133	0.85857837
76.4	1.95E-05	0.00402977	0.01814966	1.60E-21	2.05E-57	3.70E-22	0.92884976	0.85639868
76.5	1.92E-05	0.0040504	0.01840056	1.62E-21	2.07E-57	3.80E-22	0.92727092	0.8586662
76.6	1.91E-05	0.00396847	0.01842782	1.64E-21	2.07E-57	3.73E-22	0.93338574	0.85125041
76.7	1.91E-05	0.00396847	0.01842782	1.64E-21	2.07E-57	3.73E-22	0.93338574	0.85125041
76.8	2.03E-05	0.00405745	0.01845003	1.80E-21	2.16E-57	4.02E-22	0.93565753	0.84860519
76.9	2.06E-05	0.00397609	0.01839787	1.74E-21	2.06E-57	3.96E-22	0.93625487	0.84310775
77	2.06E-05	0.00396236	0.01852584	1.73E-21	1.95E-57	3.91E-22	0.93543107	0.8474411
77.1	2.06E-05	0.00396236	0.01852584	1.73E-21	1.95E-57	3.91E-22	0.93543107	0.8474411
77.2	2.06E-05	0.00396236	0.01852584	1.73E-21	1.95E-57	3.91E-22	0.93543107	0.8474411
77.3	2.06E-05	0.00396236	0.01852584	1.73E-21	1.95E-57	3.91E-22	0.93543107	0.8474411
77.4	2.14E-05	0.00389474	0.0186407	1.70E-21	1.95E-57	3.81E-22	0.92935253	0.84045389
77.5	2.14E-05	0.00389474	0.0186407	1.70E-21	1.95E-57	3.81E-22	0.92935253	0.84045389
77.6	2.14E-05	0.00389474	0.0186407	1.70E-21	1.95E-57	3.81E-22	0.92935253	0.84045389
77.7	2.16E-05	0.00395305	0.01900503	1.74E-21	2.02E-57	3.78E-22	0.9288485	0.84435604
77.8	2.16E-05	0.00395305	0.01900503	1.74E-21	2.02E-57	3.78E-22	0.9288485	0.84435604
77.9	2.16E-05	0.00395305	0.01900503	1.74E-21	2.02E-57	3.78E-22	0.9288485	0.84435604
78	2.19E-05	0.00404466	0.01887714	1.84E-21	2.15E-57	4.09E-22	0.92232869	0.84857546
78.1	2.19E-05	0.00404466	0.01887714	1.84E-21	2.15E-57	4.09E-22	0.92232869	0.84857546
78.2	2.21E-05	0.00410637	0.01877683	1.73E-21	2.14E-57	4.21E-22	0.9181824	0.85051951
78.3	2.21E-05	0.00410637	0.01877683	1.73E-21	2.14E-57	4.21E-22	0.9181824	0.85051951
78.4	2.20E-05	0.00411941	0.01918548	1.65E-21	2.09E-57	4.19E-22	0.92421824	0.84944575
78.5	2.20E-05	0.00411941	0.01918548	1.65E-21	2.09E-57	4.19E-22	0.92421824	0.84944575
78.6	2.28E-05	0.00417741	0.01935494	1.69E-21	2.25E-57	4.16E-22	0.93062532	0.84726374
78.7	2.28E-05	0.00417741	0.01935494	1.69E-21	2.25E-57	4.16E-22	0.93062532	0.84726374
78.8	2.34E-05	0.00413269	0.01919972	1.65E-21	2.23E-57	4.14E-22	0.93224306	0.84777464
78.9	2.41E-05	0.00433625	0.01930521	1.88E-21	2.51E-57	4.43E-22	0.92415918	0.8415501
79	2.41E-05	0.00433625	0.01930521	1.88E-21	2.51E-57	4.43E-22	0.92415918	0.8415501
79.1	2.41E-05	0.00433625	0.01930521	1.88E-21	2.51E-57	4.43E-22	0.92415918	0.8415501
79.2	2.51E-05	0.00431509	0.0191479	2.02E-21	2.53E-57	4.63E-22	0.92437476	0.84098924
79.3	2.51E-05	0.00431509	0.0191479	2.02E-21	2.53E-57	4.63E-22	0.92437476	0.84098924
79.4	2.51E-05	0.00431509	0.0191479	2.02E-21	2.53E-57	4.63E-22	0.92437476	0.84098924
79.5	2.51E-05	0.00431509	0.0191479	2.02E-21	2.53E-57	4.63E-22	0.92437476	0.84098924
79.6	2.51E-05	0.00431509	0.0191479	2.02E-21	2.53E-57	4.63E-22	0.92437476	0.84098924
79.7	2.51E-05	0.00431509	0.0191479	2.02E-21	2.53E-57	4.63E-22	0.92437476	0.84098924
79.8	2.61E-05	0.00428883	0.01908349	1.97E-21	2.61E-57	4.55E-22	0.92529986	0.84228173
79.9	2.63E-05	0.00416706	0.01871491	1.99E-21	2.55E-57	4.66E-22	0.92532503	0.8426773
80	2.65E-05	0.00423045	0.01914338	1.91E-21	2.58E-57	4.77E-22	0.93117607	0.84285148
80.1	2.65E-05	0.00423045	0.01914338	1.91E-21	2.58E-57	4.77E-22	0.93117607	0.84285148
80.2	2.65E-05	0.00423045	0.01914338	1.91E-21	2.58E-57	4.77E-22	0.93117607	0.84285148
80.3	2.72E-05	0.0042018	0.0195626	2.03E-21	3.12E-57	5.41E-22	0.92487823	0.84200507
80.4	2.72E-05	0.0042018	0.0195626	2.03E-21	3.12E-57	5.41E-22	0.92487823	0.84200507
80.5	2.72E-05	0.0042018	0.0195626	2.03E-21	3.12E-57	5.41E-22	0.92487823	0.84200507
80.6	2.75E-05	0.00427073	0.01989406	2.10E-21	3.29E-57	5.76E-22	0.92536919	0.84694902
80.7	2.75E-05	0.00427073	0.01989406	2.10E-21	3.29E-57	5.76E-22	0.92536919	0.84694902
80.8	2.80E-05	0.00425998	0.02030093	2.27E-21	3.74E-57	5.90E-22	0.92586369	0.84990238
80.9	3.02E-05	0.00412406	0.01961858	2.35E-21	3.63E-57	6.16E-22	0.93203708	0.84574054
81	3.10E-05	0.00419028	0.0196149	2.29E-21	3.34E-57	6.33E-22	0.92862066	0.8469566
81.1	3.09E-05	0.00421092	0.01964548	2.26E-21	3.28E-57	6.38E-22	0.92669745	0.84797194
81.2	3.09E-05	0.00421092	0.01964548	2.26E-21	3.28E-57	6.38E-22	0.92669745	0.84797194
81.3	3.09E-05	0.00421092	0.01964548	2.26E-21	3.28E-57	6.38E-22	0.92669745	0.84797194
81.4	3.09E-05	0.00421092	0.01964548	2.26E-21	3.28E-57	6.38E-22	0.92669745	0.84797194
81.5	3.09E-05	0.00421092	0.01964548	2.26E-21	3.28E-57	6.38E-22	0.92669745	0.84797194
81.6	3.09E-05	0.00421092	0.01964548	2.26E-21	3.28E-57	6.38E-22	0.92669745	0.84797194
81.7	3.09E-05	0.00421092	0.01964548	2.26E-21	3.28E-57	6.38E-22	0.92669745	0.84797194
81.8	3.16E-05	0.00423122	0.01997505	2.31E-21	3.30E-57	6.38E-22	0.92081816	0.85113229
81.9	3.09E-05	0.00434593	0.0198332	2.25E-21	3.25E-57	6.45E-22	0.91845615	0.85248557
82	3.09E-05	0.00434593	0.0198332	2.25E-21	3.25E-57	6.45E-22	0.91845615	0.85248557
82.1	3.09E-05	0.00434593	0.0198332	2.25E-21	3.25E-57	6.45E-22	0.91845615	0.85248557
82.2	3.07E-05	0.00429238	0.02007393	2.15E-21	2.93E-57	6.48E-22	0.91643875	0.85286466
82.3	3.07E-05	0.00429238	0.02007393	2.15E-21	2.93E-57	6.48E-22	0.91643875	0.85286466
82.4	3.07E-05	0.00429238	0.02007393	2.15E-21	2.93E-57	6.48E-22	0.91643875	0.85286466
82.5	3.13E-05	0.00432995	0.01996371	2.25E-21	2.97E-57	6.42E-22	0.91662967	0.85875139
82.6	3.13E-05	0.00432995	0.01996371	2.25E-21	2.97E-57	6.42E-22	0.91662967	0.85875139
82.7	3.25E-05	0.00438151	0.02024144	2.20E-21	2.89E-57	6.88E-22	0.91335216	0.86051622
82.8	3.25E-05	0.00438151	0.02024144	2.20E-21	2.89E-57	6.88E-22	0.91335216	0.86051622
82.9	3.25E-05	0.00438151	0.02024144	2.20E-21	2.89E-57	6.88E-22	0.91335216	0.86051622
83	3.25E-05	0.00438151	0.02024144	2.20E-21	2.89E-57	6.88E-22	0.91335216	0.86051622
83.1	3.25E-05	0.00438151	0.02024144	2.20E-21	2.89E-57	6.88E-22	0.91335216	0.86051622
83.2	3.45E-05	0.00447151	0.02043831	2.34E-21	2.96E-57	6.86E-22	0.91540305	0.86971789
83.3	3.48E-05	0.00438785	0.02043183	2.31E-21	2.95E-57	6.99E-22	0.91365691	0.87150476
83.4	3.53E-05	0.00434903	0.02058803	2.29E-21	2.79E-57	7.32E-22	0.91756099	0.87732485
83.5	3.53E-05	0.00434903	0.02058803	2.29E-21	2.79E-57	7.32E-22	0.91756099	0.87732485
83.6	3.53E-05	0.00434903	0.02058803	2.29E-21	2.79E-57	7.32E-22	0.91756099	0.87732485
83.7	3.53E-05	0.00434903	0.02058803	2.29E-21	2.79E-57	7.32E-22	0.91756099	0.87732485
83.8	3.53E-05	0.00434903	0.02058803	2.29E-21	2.79E-57	7.32E-22	0.91756099	0.87732485
83.9	3.59E-05	0.00436855	0.02104181	2.35E-21	2.91E-57	7.57E-22	0.91880751	0.87098794
84	3.59E-05	0.00436855	0.02104181	2.35E-21	2.91E-57	7.57E-22	0.91880751	0.87098794
84.1	3.59E-05	0.00436855	0.02104181	2.35E-21	2.91E-57	7.57E-22	0.91880751	0.87098794
84.2	3.59E-05	0.00436855	0.02104181	2.35E-21	2.91E-57	7.57E-22	0.91880751	0.87098794
84.3	3.59E-05	0.00434644	0.02112363	2.29E-21	2.87E-57	7.47E-22	0.91661778	0.86626574
84.4	3.55E-05	0.00440879	0.02149771	2.19E-21	2.86E-57	7.11E-22	0.91387192	0.86648892
84.5	3.78E-05	0.0045002	0.02192095	2.34E-21	3.17E-57	7.10E-22	0.91528221	0.87284027
84.6	3.78E-05	0.0045002	0.02192095	2.34E-21	3.17E-57	7.10E-22	0.91528221	0.87284027
84.7	3.78E-05	0.0045002	0.02192095	2.34E-21	3.17E-57	7.10E-22	0.91528221	0.87284027
84.8	3.83E-05	0.00456008	0.02196818	2.54E-21	3.36E-57	7.49E-22	0.91316737	0.87680142
84.9	4.00E-05	0.0045921	0.02220202	2.68E-21	3.46E-57	8.21E-22	0.90913407	0.88200841
85	4.00E-05	0.0045921	0.02220202	2.68E-21	3.46E-57	8.21E-22	0.90913407	0.88200841
85.1	4.00E-05	0.0045921	0.02220202	2.68E-21	3.46E-57	8.21E-22	0.90913407	0.88200841
85.2	4.00E-05	0.0045921	0.02220202	2.68E-21	3.46E-57	8.21E-22	0.90913407	0.88200841
85.3	3.98E-05	0.00469986	0.02222294	2.87E-21	3.81E-57	9.26E-22	0.90961111	0.88609
85.4	4.15E-05	0.00470518	0.022902	2.85E-21	3.82E-57	9.20E-22	0.90055807	0.88761016
85.5	4.24E-05	0.00467562	0.0226447	2.83E-21	3.63E-57	9.64E-22	0.89899379	0.89230277
85.6	4.28E-05	0.00466733	0.0231431	2.86E-21	3.71E-57	9.58E-22	0.89879856	0.88743759
85.7	4.66E-05	0.00471804	0.02449655	3.09E-21	5.24E-57	1.03E-21	0.8973236	0.88396018
85.8	4.70E-05	0.00480289	0.02419038	3.01E-21	5.32E-57	1.00E-21	0.89428187	0.88829296
85.9	4.85E-05	0.0048907	0.02411079	2.84E-21	5.14E-57	9.93E-22	0.89399915	0.89157428
86	4.85E-05	0.0048907	0.02411079	2.84E-21	5.14E-57	9.93E-22	0.89399915	0.89157428
86.1	4.85E-05	0.0048907	0.02411079	2.84E-21	5.14E-57	9.93E-22	0.89399915	0.89157428
86.2	4.85E-05	0.0048907	0.02411079	2.84E-21	5.14E-57	9.93E-22	0.89399915	0.89157428
86.3	4.85E-05	0.0048907	0.02411079	2.84E-21	5.14E-57	9.93E-22	0.89399915	0.89157428
86.4	4.96E-05	0.00487912	0.02457936	2.89E-21	5.10E-57	9.99E-22	0.88682383	0.88689283
86.5	4.96E-05	0.00487912	0.02457936	2.89E-21	5.10E-57	9.99E-22	0.88682383	0.88689283
86.6	4.96E-05	0.00487912	0.02457936	2.89E-21	5.10E-57	9.99E-22	0.88682383	0.88689283
86.7	4.96E-05	0.00487912	0.02457936	2.89E-21	5.10E-57	9.99E-22	0.88682383	0.88689283
86.8	4.96E-05	0.00487912	0.02457936	2.89E-21	5.10E-57	9.99E-22	0.88682383	0.88689283
86.9	4.96E-05	0.00487912	0.02457936	2.89E-21	5.10E-57	9.99E-22	0.88682383	0.88689283
87	4.93E-05	0.004919	0.02425888	2.86E-21	5.10E-57	1.06E-21	0.89239289	0.89183693
87.1	5.14E-05	0.00499323	0.02464506	2.95E-21	5.09E-57	1.13E-21	0.89007122	0.8995693
87.2	5.14E-05	0.00499323	0.02464506	2.95E-21	5.09E-57	1.13E-21	0.89007122	0.8995693
87.3	5.32E-05	0.00495105	0.02482989	2.89E-21	5.28E-57	1.12E-21	0.88956138	0.90397294
87.4	5.32E-05	0.00495105	0.02482989	2.89E-21	5.28E-57	1.12E-21	0.88956138	0.90397294
87.5	5.35E-05	0.00483153	0.02513302	2.80E-21	4.95E-57	1.13E-21	0.88268911	0.90762021
87.6	5.11E-05	0.00490422	0.02486169	2.76E-21	4.96E-57	1.14E-21	0.88124771	0.91009483
87.7	5.36E-05	0.00492135	0.02493002	2.76E-21	4.99E-57	1.22E-21	0.88078724	0.91769695
87.8	5.44E-05	0.00499274	0.02503355	2.77E-21	5.03E-57	1.23E-21	0.87823306	0.91714385
87.9	5.44E-05	0.00499274	0.02503355	2.77E-21	5.03E-57	1.23E-21	0.87823306	0.91714385
88	5.44E-05	0.00499274	0.02503355	2.77E-21	5.03E-57	1.23E-21	0.87823306	0.91714385
88.1	5.44E-05	0.00499274	0.02503355	2.77E-21	5.03E-57	1.23E-21	0.87823306	0.91714385
88.2	5.42E-05	0.00496408	0.02512011	2.63E-21	5.10E-57	1.20E-21	0.87870757	0.91605511
88.3	5.42E-05	0.00496408	0.02512011	2.63E-21	5.10E-57	1.20E-21	0.87870757	0.91605511
88.4	5.42E-05	0.00496408	0.02512011	2.63E-21	5.10E-57	1.20E-21	0.87870757	0.91605511
88.5	5.42E-05	0.00496408	0.02512011	2.63E-21	5.10E-57	1.20E-21	0.87870757	0.91605511
88.6	5.50E-05	0.00496435	0.02561423	2.46E-21	4.94E-57	1.19E-21	0.87801153	0.91776453
88.7	5.52E-05	0.00502634	0.02509168	2.58E-21	5.24E-57	1.30E-21	0.88385263	0.91721247
88.8	5.55E-05	0.00505092	0.02561312	2.63E-21	5.24E-57	1.35E-21	0.88314354	0.91235033
88.9	5.55E-05	0.00505092	0.02561312	2.63E-21	5.24E-57	1.35E-21	0.88314354	0.91235033
89	5.67E-05	0.00504597	0.0256968	2.63E-21	5.42E-57	1.38E-21	0.88103705	0.90939768
89.1	5.67E-05	0.00504597	0.0256968	2.63E-21	5.42E-57	1.38E-21	0.88103705	0.90939768
89.2	5.77E-05	0.0050753	0.0261107	2.60E-21	5.18E-57	1.41E-21	0.87986691	0.91219095
89.3	5.75E-05	0.00518438	0.025789	2.59E-21	5.31E-57	1.47E-21	0.87953442	0.91915019
89.4	5.75E-05	0.00518438	0.025789	2.59E-21	5.31E-57	1.47E-21	0.87953442	0.91915019
89.5	5.75E-05	0.00518438	0.025789	2.59E-21	5.31E-57	1.47E-21	0.87953442	0.91915019
89.6	5.75E-05	0.00518438	0.025789	2.59E-21	5.31E-57	1.47E-21	0.87953442	0.91915019
89.7	5.75E-05	0.00518438	0.025789	2.59E-21	5.31E-57	1.47E-21	0.87953442	0.91915019
89.8	5.75E-05	0.00518438	0.025789	2.59E-21	5.31E-57	1.47E-21	0.87953442	0.91915019
89.9	5.75E-05	0.00518438	0.025789	2.59E-21	5.31E-57	1.47E-21	0.87953442	0.91915019
135	0.00019127	0.00737827	0.03795653	7.59E-22	3.47E-57	3.22E-21	0.89840858	0.9842747
135.1	0.00019127	0.00737827	0.03795653	7.59E-22	3.47E-57	3.22E-21	0.89840858	0.9842747
90	5.75E-05	0.00518438	0.025789	2.59E-21	5.31E-57	1.47E-21	0.87953442	0.91915019
90.1	5.75E-05	0.00518438	0.025789	2.59E-21	5.31E-57	1.47E-21	0.87953442	0.91915019
90.2	5.75E-05	0.00518438	0.025789	2.59E-21	5.31E-57	1.47E-21	0.87953442	0.91915019
90.3	5.74E-05	0.00515523	0.02572984	2.48E-21	4.83E-57	1.52E-21	0.87586682	0.92218103
90.4	5.74E-05	0.00515523	0.02572984	2.48E-21	4.83E-57	1.52E-21	0.87586682	0.92218103
90.5	5.74E-05	0.00515523	0.02572984	2.48E-21	4.83E-57	1.52E-21	0.87586682	0.92218103
90.6	5.62E-05	0.0050845	0.02544207	2.29E-21	4.43E-57	1.45E-21	0.87238385	0.92218645
90.7	5.61E-05	0.00507457	0.02554134	2.24E-21	4.60E-57	1.48E-21	0.87328096	0.92386447
90.8	5.62E-05	0.00517539	0.02550221	2.21E-21	4.59E-57	1.50E-21	0.86727399	0.93130933
90.9	5.62E-05	0.00517539	0.02550221	2.21E-21	4.59E-57	1.50E-21	0.86727399	0.93130933
91	5.62E-05	0.00517539	0.02550221	2.21E-21	4.59E-57	1.50E-21	0.86727399	0.93130933
91.1	5.62E-05	0.00517539	0.02550221	2.21E-21	4.59E-57	1.50E-21	0.86727399	0.93130933
91.2	5.60E-05	0.00514604	0.02559747	2.26E-21	4.68E-57	1.51E-21	0.86277931	0.92836534
91.3	5.60E-05	0.00514604	0.02559747	2.26E-21	4.68E-57	1.51E-21	0.86277931	0.92836534
91.4	5.81E-05	0.00520387	0.02587338	2.25E-21	4.70E-57	1.54E-21	0.86537733	0.92682039
91.5	5.81E-05	0.00520387	0.02587338	2.25E-21	4.70E-57	1.54E-21	0.86537733	0.92682039
91.6	5.93E-05	0.005302	0.02601514	2.15E-21	4.88E-57	1.58E-21	0.86441271	0.92561488
91.7	5.93E-05	0.005302	0.02601514	2.15E-21	4.88E-57	1.58E-21	0.86441271	0.92561488
91.8	5.93E-05	0.005302	0.02601514	2.15E-21	4.88E-57	1.58E-21	0.86441271	0.92561488
91.9	5.93E-05	0.005302	0.02601514	2.15E-21	4.88E-57	1.58E-21	0.86441271	0.92561488
92	5.93E-05	0.005302	0.02601514	2.15E-21	4.88E-57	1.58E-21	0.86441271	0.92561488
92.1	5.91E-05	0.00525486	0.02624348	2.21E-21	4.88E-57	1.54E-21	0.86872864	0.92535836
92.2	5.91E-05	0.00513524	0.02653129	2.26E-21	4.82E-57	1.52E-21	0.86848623	0.92554699
92.3	5.91E-05	0.00513524	0.02653129	2.26E-21	4.82E-57	1.52E-21	0.86848623	0.92554699
92.4	6.35E-05	0.00527621	0.02737	2.10E-21	4.54E-57	1.53E-21	0.86891393	0.93370693
92.5	6.35E-05	0.00527621	0.02737	2.10E-21	4.54E-57	1.53E-21	0.86891393	0.93370693
92.6	6.35E-05	0.00527621	0.02737	2.10E-21	4.54E-57	1.53E-21	0.86891393	0.93370693
92.7	6.35E-05	0.00527621	0.02737	2.10E-21	4.54E-57	1.53E-21	0.86891393	0.93370693
92.8	6.70E-05	0.00515791	0.02697708	1.77E-21	3.54E-57	1.55E-21	0.87730505	0.94654046
92.9	6.70E-05	0.00515791	0.02697708	1.77E-21	3.54E-57	1.55E-21	0.87730505	0.94654046
93	6.59E-05	0.00534236	0.02770732	1.67E-21	3.70E-57	1.56E-21	0.86903145	0.94486848
93.1	6.59E-05	0.00534236	0.02770732	1.67E-21	3.70E-57	1.56E-21	0.86903145	0.94486848
93.2	6.42E-05	0.00541952	0.02783011	1.63E-21	3.83E-57	1.65E-21	0.86719129	0.94610412
93.3	6.42E-05	0.00541952	0.02783011	1.63E-21	3.83E-57	1.65E-21	0.86719129	0.94610412
93.4	6.42E-05	0.00541952	0.02783011	1.63E-21	3.83E-57	1.65E-21	0.86719129	0.94610412
93.5	6.45E-05	0.00554862	0.02774842	1.61E-21	3.84E-57	1.64E-21	0.87332926	0.94355283
93.6	6.45E-05	0.00554862	0.02774842	1.61E-21	3.84E-57	1.64E-21	0.87332926	0.94355283
93.7	6.45E-05	0.00554401	0.0272396	1.59E-21	3.77E-57	1.70E-21	0.87822329	0.94336715
93.8	6.71E-05	0.00570338	0.02795245	1.66E-21	4.08E-57	1.80E-21	0.86779124	0.9458566
93.9	6.71E-05	0.00570338	0.02795245	1.66E-21	4.08E-57	1.80E-21	0.86779124	0.9458566
94	6.71E-05	0.00570338	0.02795245	1.66E-21	4.08E-57	1.80E-21	0.86779124	0.9458566
94.1	6.71E-05	0.00570338	0.02795245	1.66E-21	4.08E-57	1.80E-21	0.86779124	0.9458566
94.2	6.71E-05	0.00570338	0.02795245	1.66E-21	4.08E-57	1.80E-21	0.86779124	0.9458566
94.3	6.71E-05	0.00570338	0.02795245	1.66E-21	4.08E-57	1.80E-21	0.86779124	0.9458566
94.4	6.71E-05	0.00570338	0.02795245	1.66E-21	4.08E-57	1.80E-21	0.86779124	0.9458566
94.5	6.71E-05	0.00570338	0.02795245	1.66E-21	4.08E-57	1.80E-21	0.86779124	0.9458566
94.6	6.71E-05	0.00570338	0.02795245	1.66E-21	4.08E-57	1.80E-21	0.86779124	0.9458566
94.7	6.82E-05	0.00572299	0.02821496	1.67E-21	3.79E-57	1.83E-21	0.86882039	0.94418807
94.8	6.81E-05	0.0058507	0.02850957	1.60E-21	3.68E-57	1.90E-21	0.87126608	0.94806411
94.9	6.81E-05	0.0058507	0.02850957	1.60E-21	3.68E-57	1.90E-21	0.87126608	0.94806411
95	6.81E-05	0.0058507	0.02850957	1.60E-21	3.68E-57	1.90E-21	0.87126608	0.94806411
95.1	6.81E-05	0.0058507	0.02850957	1.60E-21	3.68E-57	1.90E-21	0.87126608	0.94806411
95.2	6.81E-05	0.0058507	0.02850957	1.60E-21	3.68E-57	1.90E-21	0.87126608	0.94806411
95.3	6.81E-05	0.0058507	0.02850957	1.60E-21	3.68E-57	1.90E-21	0.87126608	0.94806411
95.4	6.81E-05	0.0058507	0.02850957	1.60E-21	3.68E-57	1.90E-21	0.87126608	0.94806411
95.5	6.91E-05	0.00594726	0.02832509	1.65E-21	4.18E-57	2.08E-21	0.8810545	0.95313798
95.6	6.91E-05	0.00594726	0.02832509	1.65E-21	4.18E-57	2.08E-21	0.8810545	0.95313798
95.7	6.91E-05	0.00594726	0.02832509	1.65E-21	4.18E-57	2.08E-21	0.8810545	0.95313798
95.8	6.91E-05	0.00594726	0.02832509	1.65E-21	4.18E-57	2.08E-21	0.8810545	0.95313798
95.9	6.91E-05	0.00594726	0.02832509	1.65E-21	4.18E-57	2.08E-21	0.8810545	0.95313798
96	6.91E-05	0.00594726	0.02832509	1.65E-21	4.18E-57	2.08E-21	0.8810545	0.95313798
96.1	6.90E-05	0.00609735	0.02829622	1.63E-21	4.32E-57	2.19E-21	0.88803339	0.96052147
96.2	6.87E-05	0.00618234	0.02926782	1.65E-21	4.13E-57	2.24E-21	0.88969491	0.95890479
96.3	7.08E-05	0.00622133	0.02931389	1.57E-21	3.91E-57	2.33E-21	0.89079579	0.9665786
96.4	7.10E-05	0.00625112	0.02948165	1.57E-21	3.88E-57	2.12E-21	0.89096955	0.97207288
96.5	7.10E-05	0.00625112	0.02948165	1.57E-21	3.88E-57	2.12E-21	0.89096955	0.97207288
96.6	7.10E-05	0.00625112	0.02948165	1.57E-21	3.88E-57	2.12E-21	0.89096955	0.97207288
96.7	7.10E-05	0.00625112	0.02948165	1.57E-21	3.88E-57	2.12E-21	0.89096955	0.97207288
96.8	7.06E-05	0.00640137	0.02908342	1.60E-21	4.13E-57	2.31E-21	0.89051774	0.97601576
96.9	7.06E-05	0.00640137	0.02908342	1.60E-21	4.13E-57	2.31E-21	0.89051774	0.97601576
97	7.02E-05	0.00639287	0.02956682	1.56E-21	3.87E-57	2.26E-21	0.89472914	0.97773754
97.1	7.02E-05	0.00639287	0.02956682	1.56E-21	3.87E-57	2.26E-21	0.89472914	0.97773754
97.2	7.02E-05	0.00639287	0.02956682	1.56E-21	3.87E-57	2.26E-21	0.89472914	0.97773754
97.3	7.02E-05	0.00639287	0.02956682	1.56E-21	3.87E-57	2.26E-21	0.89472914	0.97773754
97.4	7.02E-05	0.00639287	0.02956682	1.56E-21	3.87E-57	2.26E-21	0.89472914	0.97773754
97.5	7.16E-05	0.00631995	0.02998252	1.54E-21	3.68E-57	2.23E-21	0.89707145	0.97819102
97.6	7.16E-05	0.00631995	0.02998252	1.54E-21	3.68E-57	2.23E-21	0.89707145	0.97819102
97.7	7.23E-05	0.00622208	0.03009095	1.56E-21	3.77E-57	2.18E-21	0.89193754	0.9728179
97.8	7.23E-05	0.00622208	0.03009095	1.56E-21	3.77E-57	2.18E-21	0.89193754	0.9728179
97.9	7.12E-05	0.0063624	0.03009302	1.57E-21	4.12E-57	2.40E-21	0.89485618	0.9745305
98	7.30E-05	0.00635513	0.02949553	1.66E-21	4.14E-57	2.46E-21	0.89209689	0.97964351
98.1	7.30E-05	0.00635513	0.02949553	1.66E-21	4.14E-57	2.46E-21	0.89209689	0.97964351
98.2	7.31E-05	0.00638954	0.02945428	1.71E-21	4.16E-57	2.45E-21	0.89028251	0.97941147
98.3	7.66E-05	0.0063789	0.02978081	1.77E-21	4.16E-57	2.52E-21	0.88441298	0.97658275
98.4	7.66E-05	0.0063789	0.02978081	1.77E-21	4.16E-57	2.52E-21	0.88441298	0.97658275
98.5	7.66E-05	0.0063789	0.02978081	1.77E-21	4.16E-57	2.52E-21	0.88441298	0.97658275
98.6	7.66E-05	0.0063789	0.02978081	1.77E-21	4.16E-57	2.52E-21	0.88441298	0.97658275
98.7	7.76E-05	0.0062934	0.02935728	1.67E-21	3.74E-57	2.49E-21	0.88312202	0.97714099
98.8	7.76E-05	0.0062934	0.02935728	1.67E-21	3.74E-57	2.49E-21	0.88312202	0.97714099
98.9	7.80E-05	0.00631079	0.02986448	1.49E-21	3.47E-57	2.55E-21	0.88218797	0.9881375
99	8.00E-05	0.00636493	0.03048786	1.54E-21	3.97E-57	2.44E-21	0.88633066	0.99507878
99.1	8.00E-05	0.00636493	0.03048786	1.54E-21	3.97E-57	2.44E-21	0.88633066	0.99507878
99.2	8.00E-05	0.00636493	0.03048786	1.54E-21	3.97E-57	2.44E-21	0.88633066	0.99507878
99.3	8.16E-05	0.00643555	0.03080619	1.72E-21	4.46E-57	2.77E-21	0.88098722	0.9997496
99.4	8.16E-05	0.00643555	0.03080619	1.72E-21	4.46E-57	2.77E-21	0.88098722	0.9997496
99.5	8.16E-05	0.00643555	0.03080619	1.72E-21	4.46E-57	2.77E-21	0.88098722	0.9997496
99.6	8.31E-05	0.00643893	0.03108595	1.64E-21	4.30E-57	2.73E-21	0.88628308	0.99239477
99.7	8.31E-05	0.00643893	0.03108595	1.64E-21	4.30E-57	2.73E-21	0.88628308	0.99239477
99.8	8.31E-05	0.00643893	0.03108595	1.64E-21	4.30E-57	2.73E-21	0.88628308	0.99239477
99.9	8.31E-05	0.00643893	0.03108595	1.64E-21	4.30E-57	2.73E-21	0.88628308	0.99239477
100	8.23E-05	0.00657691	0.03071276	1.62E-21	4.47E-57	2.94E-21	0.89121077	0.99564075
100.1	8.23E-05	0.00657691	0.03071276	1.62E-21	4.47E-57	2.94E-21	0.89121077	0.99564075
100.2	8.23E-05	0.00657691	0.03071276	1.62E-21	4.47E-57	2.94E-21	0.89121077	0.99564075
100.3	8.23E-05	0.00657691	0.03071276	1.62E-21	4.47E-57	2.94E-21	0.89121077	0.99564075
100.4	8.23E-05	0.00657691	0.03071276	1.62E-21	4.47E-57	2.94E-21	0.89121077	0.99564075
100.5	8.14E-05	0.00656279	0.03087022	1.51E-21	4.21E-57	2.78E-21	0.89160349	0.99890909
100.6	8.14E-05	0.00656279	0.03087022	1.51E-21	4.21E-57	2.78E-21	0.89160349	0.99890909
100.7	8.03E-05	0.00652599	0.03096777	1.39E-21	3.98E-57	2.80E-21	0.89027496	0.99992733
100.8	8.03E-05	0.00652599	0.03096777	1.39E-21	3.98E-57	2.80E-21	0.89027496	0.99992733
100.9	8.03E-05	0.00652599	0.03096777	1.39E-21	3.98E-57	2.80E-21	0.89027496	0.99992733
101	8.03E-05	0.00652599	0.03096777	1.39E-21	3.98E-57	2.80E-21	0.89027496	0.99992733
101.1	8.03E-05	0.00652599	0.03096777	1.39E-21	3.98E-57	2.80E-21	0.89027496	0.99992733
101.2	8.03E-05	0.00652599	0.03096777	1.39E-21	3.98E-57	2.80E-21	0.89027496	0.99992733
101.3	8.03E-05	0.00652599	0.03096777	1.39E-21	3.98E-57	2.80E-21	0.89027496	0.99992733
101.4	8.03E-05	0.00652599	0.03096777	1.39E-21	3.98E-57	2.80E-21	0.89027496	0.99992733
101.5	8.03E-05	0.00652599	0.03096777	1.39E-21	3.98E-57	2.80E-21	0.89027496	0.99992733
101.6	8.03E-05	0.00652599	0.03096777	1.39E-21	3.98E-57	2.80E-21	0.89027496	0.99992733
101.7	8.03E-05	0.00652599	0.03096777	1.39E-21	3.98E-57	2.80E-21	0.89027496	0.99992733
101.8	8.03E-05	0.00652599	0.03096777	1.39E-21	3.98E-57	2.80E-21	0.89027496	0.99992733
101.9	8.23E-05	0.00646018	0.03089026	1.40E-21	4.05E-57	2.81E-21	0.88840624	0.9986564
102	8.23E-05	0.00646018	0.03089026	1.40E-21	4.05E-57	2.81E-21	0.88840624	0.9986564
102.1	8.23E-05	0.00646018	0.03089026	1.40E-21	4.05E-57	2.81E-21	0.88840624	0.9986564
102.2	8.28E-05	0.00650834	0.03109814	1.31E-21	3.84E-57	2.76E-21	0.88507024	0.99901417
102.3	8.28E-05	0.00650834	0.03109814	1.31E-21	3.84E-57	2.76E-21	0.88507024	0.99901417
102.4	8.28E-05	0.00650834	0.03109814	1.31E-21	3.84E-57	2.76E-21	0.88507024	0.99901417
102.5	8.28E-05	0.00650834	0.03109814	1.31E-21	3.84E-57	2.76E-21	0.88507024	0.99901417
102.6	8.28E-05	0.00650834	0.03109814	1.31E-21	3.84E-57	2.76E-21	0.88507024	0.99901417
102.7	8.28E-05	0.00650834	0.03109814	1.31E-21	3.84E-57	2.76E-21	0.88507024	0.99901417
102.8	8.28E-05	0.00650834	0.03109814	1.31E-21	3.84E-57	2.76E-21	0.88507024	0.99901417
102.9	8.46E-05	0.00656071	0.03165372	1.37E-21	4.08E-57	2.85E-21	0.88924101	0.99786839
103	8.46E-05	0.00656071	0.03165372	1.37E-21	4.08E-57	2.85E-21	0.88924101	0.99786839
103.1	8.37E-05	0.00651646	0.03196234	1.31E-21	3.87E-57	2.69E-21	0.88747121	0.99570486
103.2	8.37E-05	0.00651646	0.03196234	1.31E-21	3.87E-57	2.69E-21	0.88747121	0.99570486
103.3	8.64E-05	0.00670161	0.03228809	1.17E-21	3.62E-57	2.69E-21	0.89173305	0.98729974
103.4	8.64E-05	0.00670161	0.03228809	1.17E-21	3.62E-57	2.69E-21	0.89173305	0.98729974
103.5	8.64E-05	0.00670161	0.03228809	1.17E-21	3.62E-57	2.69E-21	0.89173305	0.98729974
103.6	8.64E-05	0.00670161	0.03228809	1.17E-21	3.62E-57	2.69E-21	0.89173305	0.98729974
103.7	8.64E-05	0.00670161	0.03228809	1.17E-21	3.62E-57	2.69E-21	0.89173305	0.98729974
103.8	8.80E-05	0.0066802	0.03219253	1.20E-21	3.69E-57	2.87E-21	0.89145082	0.99293314
103.9	8.80E-05	0.0066802	0.03219253	1.20E-21	3.69E-57	2.87E-21	0.89145082	0.99293314
104	8.80E-05	0.0066802	0.03219253	1.20E-21	3.69E-57	2.87E-21	0.89145082	0.99293314
104.1	8.80E-05	0.0066802	0.03219253	1.20E-21	3.69E-57	2.87E-21	0.89145082	0.99293314
104.2	8.80E-05	0.0066802	0.03219253	1.20E-21	3.69E-57	2.87E-21	0.89145082	0.99293314
104.3	8.80E-05	0.0066802	0.03219253	1.20E-21	3.69E-57	2.87E-21	0.89145082	0.99293314
104.4	8.80E-05	0.0066802	0.03219253	1.20E-21	3.69E-57	2.87E-21	0.89145082	0.99293314
104.5	9.00E-05	0.0067532	0.03220804	1.33E-21	3.85E-57	2.69E-21	0.90215175	0.98752921
104.6	9.00E-05	0.0067532	0.03220804	1.33E-21	3.85E-57	2.69E-21	0.90215175	0.98752921
104.7	9.00E-05	0.0067532	0.03220804	1.33E-21	3.85E-57	2.69E-21	0.90215175	0.98752921
104.8	9.00E-05	0.0067532	0.03220804	1.33E-21	3.85E-57	2.69E-21	0.90215175	0.98752921
104.9	9.00E-05	0.0067532	0.03220804	1.33E-21	3.85E-57	2.69E-21	0.90215175	0.98752921
150	0.00023171	0.00731036	0.04489807	8.07E-22	5.83E-57	4.85E-21	0.87837115	0.9870322
150.1	0.00023171	0.00731036	0.04489807	8.07E-22	5.83E-57	4.85E-21	0.87837115	0.9870322
105	8.84E-05	0.00677345	0.03265698	1.29E-21	3.80E-57	2.75E-21	0.90098238	0.98331435
105.1	8.84E-05	0.00677345	0.03265698	1.29E-21	3.80E-57	2.75E-21	0.90098238	0.98331435
105.2	8.84E-05	0.00677345	0.03265698	1.29E-21	3.80E-57	2.75E-21	0.90098238	0.98331435
105.3	8.84E-05	0.00677345	0.03265698	1.29E-21	3.80E-57	2.75E-21	0.90098238	0.98331435
105.4	8.84E-05	0.00677345	0.03265698	1.29E-21	3.80E-57	2.75E-21	0.90098238	0.98331435
105.5	8.86E-05	0.0069594	0.03306138	1.29E-21	4.06E-57	2.76E-21	0.90711607	0.98412597
105.6	8.86E-05	0.0069594	0.03306138	1.29E-21	4.06E-57	2.76E-21	0.90711607	0.98412597
105.7	8.86E-05	0.0069594	0.03306138	1.29E-21	4.06E-57	2.76E-21	0.90711607	0.98412597
105.8	9.07E-05	0.00696138	0.03316964	1.20E-21	3.90E-57	2.66E-21	0.90573659	0.98312097
105.9	9.07E-05	0.00696138	0.03316964	1.20E-21	3.90E-57	2.66E-21	0.90573659	0.98312097
106	9.07E-05	0.00696138	0.03316964	1.20E-21	3.90E-57	2.66E-21	0.90573659	0.98312097
106.1	9.00E-05	0.00704449	0.03301082	1.13E-21	3.68E-57	2.56E-21	0.89997336	0.98641569
106.2	9.00E-05	0.00704449	0.03301082	1.13E-21	3.68E-57	2.56E-21	0.89997336	0.98641569
106.3	9.00E-05	0.00704449	0.03301082	1.13E-21	3.68E-57	2.56E-21	0.89997336	0.98641569
106.4	9.00E-05	0.00704449	0.03301082	1.13E-21	3.68E-57	2.56E-21	0.89997336	0.98641569
106.5	9.00E-05	0.00704449	0.03301082	1.13E-21	3.68E-57	2.56E-21	0.89997336	0.98641569
106.6	9.00E-05	0.00704449	0.03301082	1.13E-21	3.68E-57	2.56E-21	0.89997336	0.98641569
106.7	9.00E-05	0.00704449	0.03301082	1.13E-21	3.68E-57	2.56E-21	0.89997336	0.98641569
106.8	9.00E-05	0.00704449	0.03301082	1.13E-21	3.68E-57	2.56E-21	0.89997336	0.98641569
106.9	9.00E-05	0.00704449	0.03301082	1.13E-21	3.68E-57	2.56E-21	0.89997336	0.98641569
107	9.00E-05	0.00704449	0.03301082	1.13E-21	3.68E-57	2.56E-21	0.89997336	0.98641569
107.1	9.00E-05	0.00704449	0.03301082	1.13E-21	3.68E-57	2.56E-21	0.89997336	0.98641569
107.2	9.10E-05	0.00712498	0.03315435	1.09E-21	3.50E-57	2.48E-21	0.89998856	0.9940454
107.3	9.30E-05	0.00703634	0.03301389	1.13E-21	3.51E-57	2.59E-21	0.90051566	0.99379892
107.4	9.30E-05	0.00703634	0.03301389	1.13E-21	3.51E-57	2.59E-21	0.90051566	0.99379892
107.5	9.26E-05	0.00709697	0.03336819	1.20E-21	3.65E-57	2.63E-21	0.90067432	0.98856788
107.6	9.32E-05	0.0070248	0.03393431	1.26E-21	3.88E-57	2.76E-21	0.90173213	0.98904781
107.7	9.32E-05	0.0070248	0.03393431	1.26E-21	3.88E-57	2.76E-21	0.90173213	0.98904781
107.8	9.32E-05	0.0070248	0.03393431	1.26E-21	3.88E-57	2.76E-21	0.90173213	0.98904781
107.9	9.32E-05	0.0070248	0.03393431	1.26E-21	3.88E-57	2.76E-21	0.90173213	0.98904781
108	9.41E-05	0.00714872	0.03423824	1.26E-21	3.88E-57	2.86E-21	0.90349375	0.98363413
108.1	9.80E-05	0.00731873	0.03421884	1.22E-21	3.90E-57	2.91E-21	0.90857992	0.98155826
108.2	9.96E-05	0.00743686	0.03417229	1.20E-21	4.04E-57	2.86E-21	0.90737647	0.9764949
108.3	9.96E-05	0.00743686	0.03417229	1.20E-21	4.04E-57	2.86E-21	0.90737647	0.9764949
108.4	9.96E-05	0.00743686	0.03417229	1.20E-21	4.04E-57	2.86E-21	0.90737647	0.9764949
108.5	9.96E-05	0.00743686	0.03417229	1.20E-21	4.04E-57	2.86E-21	0.90737647	0.9764949
108.6	9.96E-05	0.00743686	0.03417229	1.20E-21	4.04E-57	2.86E-21	0.90737647	0.9764949
108.7	9.96E-05	0.00743686	0.03417229	1.20E-21	4.04E-57	2.86E-21	0.90737647	0.9764949
108.8	0.00010023	0.0075182	0.03400521	1.21E-21	3.98E-57	2.83E-21	0.91008886	0.9792221
108.9	0.00010023	0.0075182	0.03400521	1.21E-21	3.98E-57	2.83E-21	0.91008886	0.9792221
109	0.00010023	0.0075182	0.03400521	1.21E-21	3.98E-57	2.83E-21	0.91008886	0.9792221
109.1	0.00010533	0.00749505	0.03395926	1.24E-21	3.85E-57	2.91E-21	0.9102576	0.97713128
109.2	0.00010533	0.00749505	0.03395926	1.24E-21	3.85E-57	2.91E-21	0.9102576	0.97713128
109.3	0.00010533	0.00749505	0.03395926	1.24E-21	3.85E-57	2.91E-21	0.9102576	0.97713128
109.4	0.00010883	0.00758125	0.03408276	1.23E-21	4.00E-57	2.91E-21	0.91503549	0.98173473
109.5	0.00010883	0.00758125	0.03408276	1.23E-21	4.00E-57	2.91E-21	0.91503549	0.98173473
109.6	0.00010695	0.00760669	0.03405586	1.26E-21	4.10E-57	2.95E-21	0.9077259	0.98487361
109.7	0.00010695	0.00760669	0.03405586	1.26E-21	4.10E-57	2.95E-21	0.9077259	0.98487361
109.8	0.00010879	0.00754824	0.03346189	1.16E-21	3.61E-57	2.90E-21	0.91097593	0.98839835
109.9	0.00011199	0.00755396	0.03311732	1.27E-21	3.91E-57	3.19E-21	0.91095791	0.9884562
110	0.00011444	0.00753498	0.03292504	1.25E-21	4.12E-57	3.38E-21	0.91065736	0.99394863
110.1	0.00011444	0.00753498	0.03292504	1.25E-21	4.12E-57	3.38E-21	0.91065736	0.99394863
110.2	0.00011444	0.00753498	0.03292504	1.25E-21	4.12E-57	3.38E-21	0.91065736	0.99394863
110.3	0.00011444	0.00753498	0.03292504	1.25E-21	4.12E-57	3.38E-21	0.91065736	0.99394863
110.4	0.00011444	0.00753498	0.03292504	1.25E-21	4.12E-57	3.38E-21	0.91065736	0.99394863
110.5	0.00011807	0.0074588	0.03253524	1.24E-21	4.20E-57	3.41E-21	0.9068726	0.99337551
110.6	0.00011869	0.00728153	0.03265764	1.21E-21	4.05E-57	3.38E-21	0.91302618	0.99480639
110.7	0.00011869	0.00728153	0.03265764	1.21E-21	4.05E-57	3.38E-21	0.91302618	0.99480639
110.8	0.00011869	0.00728153	0.03265764	1.21E-21	4.05E-57	3.38E-21	0.91302618	0.99480639
110.9	0.00011869	0.00728153	0.03265764	1.21E-21	4.05E-57	3.38E-21	0.91302618	0.99480639
111	0.00011869	0.00728153	0.03265764	1.21E-21	4.05E-57	3.38E-21	0.91302618	0.99480639
111.1	0.00011991	0.0071355	0.03257754	1.20E-21	4.07E-57	3.39E-21	0.91376102	0.99021135
111.2	0.00011991	0.0071355	0.03257754	1.20E-21	4.07E-57	3.39E-21	0.91376102	0.99021135
111.3	0.00011991	0.0071355	0.03257754	1.20E-21	4.07E-57	3.39E-21	0.91376102	0.99021135
111.4	0.00011991	0.0071355	0.03257754	1.20E-21	4.07E-57	3.39E-21	0.91376102	0.99021135
111.5	0.0001264	0.00724715	0.0321904	1.21E-21	4.28E-57	3.48E-21	0.91088369	0.99882933
111.6	0.00012565	0.00729978	0.03218232	1.17E-21	4.07E-57	3.35E-21	0.90296135	0.99903975
111.7	0.00012565	0.00729978	0.03218232	1.17E-21	4.07E-57	3.35E-21	0.90296135	0.99903975
111.8	0.00012957	0.00728957	0.03283408	1.18E-21	4.34E-57	3.43E-21	0.901438	0.99953918
111.9	0.00012957	0.00728957	0.03283408	1.18E-21	4.34E-57	3.43E-21	0.901438	0.99953918
112	0.00013024	0.00716872	0.03282274	1.22E-21	4.20E-57	3.40E-21	0.90040216	0.99360009
112.1	0.00013024	0.00716872	0.03282274	1.22E-21	4.20E-57	3.40E-21	0.90040216	0.99360009
112.2	0.00013024	0.00716872	0.03282274	1.22E-21	4.20E-57	3.40E-21	0.90040216	0.99360009
112.3	0.00013125	0.00717442	0.03311222	1.19E-21	3.84E-57	3.49E-21	0.8984362	0.98923919
112.4	0.00013125	0.00717442	0.03311222	1.19E-21	3.84E-57	3.49E-21	0.8984362	0.98923919
112.5	0.00013125	0.00717442	0.03311222	1.19E-21	3.84E-57	3.49E-21	0.8984362	0.98923919
112.6	0.00013739	0.00715589	0.03279698	1.27E-21	4.07E-57	3.80E-21	0.90571875	0.98616055
112.7	0.00013739	0.00715589	0.03279698	1.27E-21	4.07E-57	3.80E-21	0.90571875	0.98616055
112.8	0.00013739	0.00715589	0.03279698	1.27E-21	4.07E-57	3.80E-21	0.90571875	0.98616055
112.9	0.00013739	0.00715589	0.03279698	1.27E-21	4.07E-57	3.80E-21	0.90571875	0.98616055
113	0.00013739	0.00715589	0.03279698	1.27E-21	4.07E-57	3.80E-21	0.90571875	0.98616055
113.1	0.00013739	0.00715589	0.03279698	1.27E-21	4.07E-57	3.80E-21	0.90571875	0.98616055
113.2	0.00013739	0.00715589	0.03279698	1.27E-21	4.07E-57	3.80E-21	0.90571875	0.98616055
113.3	0.00013739	0.00715589	0.03279698	1.27E-21	4.07E-57	3.80E-21	0.90571875	0.98616055
113.4	0.00013739	0.00715589	0.03279698	1.27E-21	4.07E-57	3.80E-21	0.90571875	0.98616055
113.5	0.00014238	0.00735765	0.03307547	1.30E-21	4.33E-57	4.07E-21	0.90455952	0.98494346
113.6	0.00014238	0.00735765	0.03307547	1.30E-21	4.33E-57	4.07E-21	0.90455952	0.98494346
113.7	0.00014238	0.00735765	0.03307547	1.30E-21	4.33E-57	4.07E-21	0.90455952	0.98494346
113.8	0.00014238	0.00735765	0.03307547	1.30E-21	4.33E-57	4.07E-21	0.90455952	0.98494346
113.9	0.00014238	0.00735765	0.03307547	1.30E-21	4.33E-57	4.07E-21	0.90455952	0.98494346
114	0.00014238	0.00735765	0.03307547	1.30E-21	4.33E-57	4.07E-21	0.90455952	0.98494346
114.1	0.00014238	0.00735765	0.03307547	1.30E-21	4.33E-57	4.07E-21	0.90455952	0.98494346
114.2	0.00014238	0.00735765	0.03307547	1.30E-21	4.33E-57	4.07E-21	0.90455952	0.98494346
114.3	0.00014466	0.00721111	0.03295464	1.35E-21	4.44E-57	4.17E-21	0.91097399	0.9808135
114.4	0.00014045	0.00708567	0.03314983	1.32E-21	4.30E-57	3.92E-21	0.91011014	0.97975574
114.5	0.00014041	0.00702396	0.03351215	1.21E-21	4.18E-57	3.67E-21	0.90993972	0.97485399
114.6	0.00014041	0.00702396	0.03351215	1.21E-21	4.18E-57	3.67E-21	0.90993972	0.97485399
114.7	0.00014041	0.00702396	0.03351215	1.21E-21	4.18E-57	3.67E-21	0.90993972	0.97485399
114.8	0.00014319	0.00701128	0.03332654	1.23E-21	4.38E-57	3.85E-21	0.90650815	0.97549844
114.9	0.00014319	0.00701128	0.03332654	1.23E-21	4.38E-57	3.85E-21	0.90650815	0.97549844
115	0.00014319	0.00701128	0.03332654	1.23E-21	4.38E-57	3.85E-21	0.90650815	0.97549844
115.1	0.00014319	0.00701128	0.03332654	1.23E-21	4.38E-57	3.85E-21	0.90650815	0.97549844
115.2	0.00014451	0.0069313	0.03346969	1.21E-21	4.35E-57	3.74E-21	0.9067482	0.97892602
115.3	0.00014451	0.0069313	0.03346969	1.21E-21	4.35E-57	3.74E-21	0.9067482	0.97892602
115.4	0.00014451	0.0069313	0.03346969	1.21E-21	4.35E-57	3.74E-21	0.9067482	0.97892602
115.5	0.00014451	0.0069313	0.03346969	1.21E-21	4.35E-57	3.74E-21	0.9067482	0.97892602
115.6	0.00014451	0.0069313	0.03346969	1.21E-21	4.35E-57	3.74E-21	0.9067482	0.97892602
115.7	0.00014451	0.0069313	0.03346969	1.21E-21	4.35E-57	3.74E-21	0.9067482	0.97892602
115.8	0.00014451	0.0069313	0.03346969	1.21E-21	4.35E-57	3.74E-21	0.9067482	0.97892602
115.9	0.00014451	0.0069313	0.03346969	1.21E-21	4.35E-57	3.74E-21	0.9067482	0.97892602
116	0.00014451	0.0069313	0.03346969	1.21E-21	4.35E-57	3.74E-21	0.9067482	0.97892602
116.1	0.00014379	0.00712422	0.03388697	1.20E-21	4.44E-57	3.72E-21	0.90993651	0.97967656
116.2	0.00014379	0.00712422	0.03388697	1.20E-21	4.44E-57	3.72E-21	0.90993651	0.97967656
116.3	0.00014379	0.00712422	0.03388697	1.20E-21	4.44E-57	3.72E-21	0.90993651	0.97967656
116.4	0.00014379	0.00712422	0.03388697	1.20E-21	4.44E-57	3.72E-21	0.90993651	0.97967656
116.5	0.00014379	0.00712422	0.03388697	1.20E-21	4.44E-57	3.72E-21	0.90993651	0.97967656
116.6	0.00014379	0.00712422	0.03388697	1.20E-21	4.44E-57	3.72E-21	0.90993651	0.97967656
116.7	0.00014379	0.00712422	0.03388697	1.20E-21	4.44E-57	3.72E-21	0.90993651	0.97967656
116.8	0.00014653	0.00714909	0.03380848	1.19E-21	4.11E-57	3.82E-21	0.90468428	0.97850619
116.9	0.00014653	0.00714909	0.03380848	1.19E-21	4.11E-57	3.82E-21	0.90468428	0.97850619
117	0.00014653	0.00714909	0.03380848	1.19E-21	4.11E-57	3.82E-21	0.90468428	0.97850619
117.1	0.00014653	0.00714909	0.03380848	1.19E-21	4.11E-57	3.82E-21	0.90468428	0.97850619
117.2	0.00014653	0.00714909	0.03380848	1.19E-21	4.11E-57	3.82E-21	0.90468428	0.97850619
117.3	0.00014653	0.00714909	0.03380848	1.19E-21	4.11E-57	3.82E-21	0.90468428	0.97850619
117.4	0.00014653	0.00714909	0.03380848	1.19E-21	4.11E-57	3.82E-21	0.90468428	0.97850619
117.5	0.00014653	0.00714909	0.03380848	1.19E-21	4.11E-57	3.82E-21	0.90468428	0.97850619
117.6	0.00014653	0.00714909	0.03380848	1.19E-21	4.11E-57	3.82E-21	0.90468428	0.97850619
117.7	0.00014653	0.00714909	0.03380848	1.19E-21	4.11E-57	3.82E-21	0.90468428	0.97850619
117.8	0.00014653	0.00714909	0.03380848	1.19E-21	4.11E-57	3.82E-21	0.90468428	0.97850619
117.9	0.00014653	0.00714909	0.03380848	1.19E-21	4.11E-57	3.82E-21	0.90468428	0.97850619
118	0.00014653	0.00714909	0.03380848	1.19E-21	4.11E-57	3.82E-21	0.90468428	0.97850619
118.1	0.00014653	0.00714909	0.03380848	1.19E-21	4.11E-57	3.82E-21	0.90468428	0.97850619
118.2	0.00014653	0.00714909	0.03380848	1.19E-21	4.11E-57	3.82E-21	0.90468428	0.97850619
118.3	0.00014653	0.00714909	0.03380848	1.19E-21	4.11E-57	3.82E-21	0.90468428	0.97850619
118.4	0.00014821	0.00709394	0.03425485	1.12E-21	4.19E-57	3.68E-21	0.90126917	0.98104883
118.5	0.00014978	0.00715368	0.03453357	1.18E-21	4.72E-57	3.90E-21	0.90424857	0.98144159
118.6	0.00014978	0.00715368	0.03453357	1.18E-21	4.72E-57	3.90E-21	0.90424857	0.98144159
118.7	0.00014978	0.00715368	0.03453357	1.18E-21	4.72E-57	3.90E-21	0.90424857	0.98144159
118.8	0.00014978	0.00715368	0.03453357	1.18E-21	4.72E-57	3.90E-21	0.90424857	0.98144159
118.9	0.00014978	0.00715368	0.03453357	1.18E-21	4.72E-57	3.90E-21	0.90424857	0.98144159
119	0.00014978	0.00715368	0.03453357	1.18E-21	4.72E-57	3.90E-21	0.90424857	0.98144159
119.1	0.00014978	0.00715368	0.03453357	1.18E-21	4.72E-57	3.90E-21	0.90424857	0.98144159
119.2	0.00014978	0.00715368	0.03453357	1.18E-21	4.72E-57	3.90E-21	0.90424857	0.98144159
119.3	0.00014978	0.00715368	0.03453357	1.18E-21	4.72E-57	3.90E-21	0.90424857	0.98144159
119.4	0.00014978	0.00715368	0.03453357	1.18E-21	4.72E-57	3.90E-21	0.90424857	0.98144159
119.5	0.00014978	0.00715368	0.03453357	1.18E-21	4.72E-57	3.90E-21	0.90424857	0.98144159
119.6	0.00014978	0.00715368	0.03453357	1.18E-21	4.72E-57	3.90E-21	0.90424857	0.98144159
119.7	0.00014795	0.00700543	0.0347647	1.12E-21	4.47E-57	3.63E-21	0.9106784	0.97724925
119.8	0.00014795	0.00700543	0.0347647	1.12E-21	4.47E-57	3.63E-21	0.9106784	0.97724925
119.9	0.00014795	0.00700543	0.0347647	1.12E-21	4.47E-57	3.63E-21	0.9106784	0.97724925
165	0.00026973	0.00674182	0.04989671	9.57E-22	9.51E-57	8.23E-21	0.87801888	0.98347944
165.1	0.00027097	0.00670581	0.05045093	9.61E-22	9.53E-57	8.56E-21	0.87367881	0.98632324

120.2	0.00014673	0.00716088	0.03489817	1.08E-21	4.43E-57	3.72E-21	0.90898311	0.97861792
120.3	0.00014673	0.00716088	0.03489817	1.08E-21	4.43E-57	3.72E-21	0.90898311	0.97861792
120.4	0.00014673	0.00716088	0.03489817	1.08E-21	4.43E-57	3.72E-21	0.90898311	0.97861792
120.5	0.00014673	0.00716088	0.03489817	1.08E-21	4.43E-57	3.72E-21	0.90898311	0.97861792
120.6	0.00014673	0.00716088	0.03489817	1.08E-21	4.43E-57	3.72E-21	0.90898311	0.97861792
120.7	0.00014673	0.00716088	0.03489817	1.08E-21	4.43E-57	3.72E-21	0.90898311	0.97861792
120.8	0.00014673	0.00716088	0.03489817	1.08E-21	4.43E-57	3.72E-21	0.90898311	0.97861792
120.9	0.00015065	0.00721226	0.03523135	1.05E-21	4.73E-57	3.83E-21	0.90661844	0.97476843
121	0.00015095	0.00702341	0.03484859	9.94E-22	4.46E-57	3.50E-21	0.90636674	0.97324691
121.1	0.00015095	0.00702341	0.03484859	9.94E-22	4.46E-57	3.50E-21	0.90636674	0.97324691
121.2	0.00015378	0.00700893	0.03532268	9.87E-22	4.50E-57	3.40E-21	0.90981164	0.97652841
121.3	0.00015378	0.00700893	0.03532268	9.87E-22	4.50E-57	3.40E-21	0.90981164	0.97652841
121.4	0.00015378	0.00700893	0.03532268	9.87E-22	4.50E-57	3.40E-21	0.90981164	0.97652841
121.5	0.00015378	0.00700893	0.03532268	9.87E-22	4.50E-57	3.40E-21	0.90981164	0.97652841
121.6	0.00015695	0.00702186	0.03534327	9.82E-22	4.61E-57	3.37E-21	0.90402421	0.97567909
121.7	0.00015695	0.00702186	0.03534327	9.82E-22	4.61E-57	3.37E-21	0.90402421	0.97567909
121.8	0.00015695	0.00702186	0.03534327	9.82E-22	4.61E-57	3.37E-21	0.90402421	0.97567909
121.9	0.00015695	0.00702186	0.03534327	9.82E-22	4.61E-57	3.37E-21	0.90402421	0.97567909
122	0.00015695	0.00702186	0.03534327	9.82E-22	4.61E-57	3.37E-21	0.90402421	0.97567909
122.1	0.0001592	0.00698262	0.03472707	9.83E-22	4.59E-57	3.32E-21	0.89734768	0.97835287
122.2	0.0001592	0.00698262	0.03472707	9.83E-22	4.59E-57	3.32E-21	0.89734768	0.97835287
122.3	0.00016015	0.00711205	0.03455817	9.64E-22	4.55E-57	3.13E-21	0.89526813	0.97564856
122.4	0.00016015	0.00711205	0.03455817	9.64E-22	4.55E-57	3.13E-21	0.89526813	0.97564856
122.5	0.00016015	0.00711205	0.03455817	9.64E-22	4.55E-57	3.13E-21	0.89526813	0.97564856
122.6	0.00016015	0.00711205	0.03455817	9.64E-22	4.55E-57	3.13E-21	0.89526813	0.97564856
122.7	0.00016015	0.00711205	0.03455817	9.64E-22	4.55E-57	3.13E-21	0.89526813	0.97564856
122.8	0.00015977	0.00718231	0.03547962	9.63E-22	5.06E-57	3.04E-21	0.890097	0.96878241
122.9	0.00015977	0.00718231	0.03547962	9.63E-22	5.06E-57	3.04E-21	0.890097	0.96878241
123	0.00015977	0.00718231	0.03547962	9.63E-22	5.06E-57	3.04E-21	0.890097	0.96878241
123.1	0.00015977	0.00718231	0.03547962	9.63E-22	5.06E-57	3.04E-21	0.890097	0.96878241
123.2	0.00015977	0.00718231	0.03547962	9.63E-22	5.06E-57	3.04E-21	0.890097	0.96878241
123.3	0.00015977	0.00718231	0.03547962	9.63E-22	5.06E-57	3.04E-21	0.890097	0.96878241
123.4	0.00015977	0.00718231	0.03547962	9.63E-22	5.06E-57	3.04E-21	0.890097	0.96878241
123.5	0.00015977	0.00718231	0.03547962	9.63E-22	5.06E-57	3.04E-21	0.890097	0.96878241
123.6	0.00015977	0.00718231	0.03547962	9.63E-22	5.06E-57	3.04E-21	0.890097	0.96878241
123.7	0.00015977	0.00718231	0.03547962	9.63E-22	5.06E-57	3.04E-21	0.890097	0.96878241
123.8	0.00015977	0.00718231	0.03547962	9.63E-22	5.06E-57	3.04E-21	0.890097	0.96878241
123.9	0.00015977	0.00718231	0.03547962	9.63E-22	5.06E-57	3.04E-21	0.890097	0.96878241
124	0.00015977	0.00718231	0.03547962	9.63E-22	5.06E-57	3.04E-21	0.890097	0.96878241
124.1	0.00015977	0.00718231	0.03547962	9.63E-22	5.06E-57	3.04E-21	0.890097	0.96878241
124.2	0.00015977	0.00718231	0.03547962	9.63E-22	5.06E-57	3.04E-21	0.890097	0.96878241
124.3	0.00016022	0.0072032	0.03580916	8.95E-22	4.57E-57	3.00E-21	0.88891908	0.97243462
124.4	0.00016022	0.0072032	0.03580916	8.95E-22	4.57E-57	3.00E-21	0.88891908	0.97243462
124.5	0.00016022	0.0072032	0.03580916	8.95E-22	4.57E-57	3.00E-21	0.88891908	0.97243462
124.6	0.00016082	0.00729549	0.035426	8.72E-22	4.58E-57	3.00E-21	0.89138479	0.97191255
124.7	0.00016082	0.00729549	0.035426	8.72E-22	4.58E-57	3.00E-21	0.89138479	0.97191255
124.8	0.00016082	0.00729549	0.035426	8.72E-22	4.58E-57	3.00E-21	0.89138479	0.97191255
124.9	0.00016082	0.00729549	0.035426	8.72E-22	4.58E-57	3.00E-21	0.89138479	0.97191255
125	0.00016082	0.00729549	0.035426	8.72E-22	4.58E-57	3.00E-21	0.89138479	0.97191255
125.1	0.00016082	0.00729549	0.035426	8.72E-22	4.58E-57	3.00E-21	0.89138479	0.97191255
125.2	0.00016405	0.00725801	0.03547214	8.74E-22	4.35E-57	3.04E-21	0.89210963	0.96930988
125.3	0.00016405	0.00725801	0.03547214	8.74E-22	4.35E-57	3.04E-21	0.89210963	0.96930988
125.4	0.00016405	0.00725801	0.03547214	8.74E-22	4.35E-57	3.04E-21	0.89210963	0.96930988
125.5	0.00016405	0.00725801	0.03547214	8.74E-22	4.35E-57	3.04E-21	0.89210963	0.96930988
125.6	0.00016405	0.00725801	0.03547214	8.74E-22	4.35E-57	3.04E-21	0.89210963	0.96930988
125.7	0.00016405	0.00725801	0.03547214	8.74E-22	4.35E-57	3.04E-21	0.89210963	0.96930988
125.8	0.00016405	0.00725801	0.03547214	8.74E-22	4.35E-57	3.04E-21	0.89210963	0.96930988
125.9	0.00016405	0.00725801	0.03547214	8.74E-22	4.35E-57	3.04E-21	0.89210963	0.96930988
126	0.00016405	0.00725801	0.03547214	8.74E-22	4.35E-57	3.04E-21	0.89210963	0.96930988
126.1	0.00016405	0.00725801	0.03547214	8.74E-22	4.35E-57	3.04E-21	0.89210963	0.96930988
126.2	0.00016405	0.00725801	0.03547214	8.74E-22	4.35E-57	3.04E-21	0.89210963	0.96930988
126.3	0.00016478	0.00710577	0.0355668	8.87E-22	3.99E-57	3.14E-21	0.89403243	0.97176323
126.4	0.00016478	0.00710577	0.0355668	8.87E-22	3.99E-57	3.14E-21	0.89403243	0.97176323
126.5	0.00016478	0.00710577	0.0355668	8.87E-22	3.99E-57	3.14E-21	0.89403243	0.97176323
126.6	0.00016726	0.00708988	0.0361124	8.80E-22	4.19E-57	3.11E-21	0.89135256	0.97387329
126.7	0.00016726	0.00708988	0.0361124	8.80E-22	4.19E-57	3.11E-21	0.89135256	0.97387329
126.8	0.00016726	0.00708988	0.0361124	8.80E-22	4.19E-57	3.11E-21	0.89135256	0.97387329
126.9	0.00016726	0.00708988	0.0361124	8.80E-22	4.19E-57	3.11E-21	0.89135256	0.97387329
127	0.00016726	0.00708988	0.0361124	8.80E-22	4.19E-57	3.11E-21	0.89135256	0.97387329
127.1	0.00016694	0.00721434	0.03581751	8.05E-22	3.89E-57	2.97E-21	0.89011784	0.98011621
127.2	0.00016694	0.00721434	0.03581751	8.05E-22	3.89E-57	2.97E-21	0.89011784	0.98011621
127.3	0.00016694	0.00721434	0.03581751	8.05E-22	3.89E-57	2.97E-21	0.89011784	0.98011621
127.4	0.00016694	0.00721434	0.03581751	8.05E-22	3.89E-57	2.97E-21	0.89011784	0.98011621
127.5	0.00016694	0.00721434	0.03581751	8.05E-22	3.89E-57	2.97E-21	0.89011784	0.98011621
127.6	0.00016694	0.00721434	0.03581751	8.05E-22	3.89E-57	2.97E-21	0.89011784	0.98011621
127.7	0.00016694	0.00721434	0.03581751	8.05E-22	3.89E-57	2.97E-21	0.89011784	0.98011621
127.8	0.00016694	0.00721434	0.03581751	8.05E-22	3.89E-57	2.97E-21	0.89011784	0.98011621
127.9	0.00016694	0.00721434	0.03581751	8.05E-22	3.89E-57	2.97E-21	0.89011784	0.98011621
128	0.00016694	0.00721434	0.03581751	8.05E-22	3.89E-57	2.97E-21	0.89011784	0.98011621
128.1	0.00016694	0.00721434	0.03581751	8.05E-22	3.89E-57	2.97E-21	0.89011784	0.98011621
128.2	0.00016694	0.00721434	0.03581751	8.05E-22	3.89E-57	2.97E-21	0.89011784	0.98011621
128.3	0.00016694	0.00721434	0.03581751	8.05E-22	3.89E-57	2.97E-21	0.89011784	0.98011621
128.4	0.00016694	0.00721434	0.03581751	8.05E-22	3.89E-57	2.97E-21	0.89011784	0.98011621
128.5	0.00016694	0.00721434	0.03581751	8.05E-22	3.89E-57	2.97E-21	0.89011784	0.98011621
128.6	0.00016694	0.00721434	0.03581751	8.05E-22	3.89E-57	2.97E-21	0.89011784	0.98011621
128.7	0.00016694	0.00721434	0.03581751	8.05E-22	3.89E-57	2.97E-21	0.89011784	0.98011621
128.8	0.00016694	0.00721434	0.03581751	8.05E-22	3.89E-57	2.97E-21	0.89011784	0.98011621
128.9	0.00016694	0.00721434	0.03581751	8.05E-22	3.89E-57	2.97E-21	0.89011784	0.98011621
129	0.00016694	0.00721434	0.03581751	8.05E-22	3.89E-57	2.97E-21	0.89011784	0.98011621
129.1	0.00016694	0.00721434	0.03581751	8.05E-22	3.89E-57	2.97E-21	0.89011784	0.98011621
129.2	0.00016694	0.00721434	0.03581751	8.05E-22	3.89E-57	2.97E-21	0.89011784	0.98011621
129.3	0.00016952	0.00710036	0.03580883	7.76E-22	3.67E-57	2.99E-21	0.89188064	0.98076731
129.4	0.00017143	0.0072393	0.03583965	8.02E-22	3.73E-57	3.06E-21	0.89481772	0.97750982
129.5	0.00017143	0.0072393	0.03583965	8.02E-22	3.73E-57	3.06E-21	0.89481772	0.97750982
129.6	0.00017143	0.0072393	0.03583965	8.02E-22	3.73E-57	3.06E-21	0.89481772	0.97750982
129.7	0.00017143	0.0072393	0.03583965	8.02E-22	3.73E-57	3.06E-21	0.89481772	0.97750982
129.8	0.00017143	0.0072393	0.03583965	8.02E-22	3.73E-57	3.06E-21	0.89481772	0.97750982
129.9	0.00017143	0.0072393	0.03583965	8.02E-22	3.73E-57	3.06E-21	0.89481772	0.97750982
130	0.00017143	0.0072393	0.03583965	8.02E-22	3.73E-57	3.06E-21	0.89481772	0.97750982
130.1	0.00017143	0.0072393	0.03583965	8.02E-22	3.73E-57	3.06E-21	0.89481772	0.97750982
130.2	0.00017143	0.0072393	0.03583965	8.02E-22	3.73E-57	3.06E-21	0.89481772	0.97750982
130.3	0.00017143	0.0072393	0.03583965	8.02E-22	3.73E-57	3.06E-21	0.89481772	0.97750982
130.4	0.00017143	0.0072393	0.03583965	8.02E-22	3.73E-57	3.06E-21	0.89481772	0.97750982
130.5	0.00017143	0.0072393	0.03583965	8.02E-22	3.73E-57	3.06E-21	0.89481772	0.97750982
130.6	0.00017143	0.0072393	0.03583965	8.02E-22	3.73E-57	3.06E-21	0.89481772	0.97750982
130.7	0.00017143	0.0072393	0.03583965	8.02E-22	3.73E-57	3.06E-21	0.89481772	0.97750982
130.8	0.00017143	0.0072393	0.03583965	8.02E-22	3.73E-57	3.06E-21	0.89481772	0.97750982
130.9	0.00017143	0.0072393	0.03583965	8.02E-22	3.73E-57	3.06E-21	0.89481772	0.97750982
131	0.00017143	0.0072393	0.03583965	8.02E-22	3.73E-57	3.06E-21	0.89481772	0.97750982
131.1	0.00017143	0.0072393	0.03583965	8.02E-22	3.73E-57	3.06E-21	0.89481772	0.97750982
131.2	0.00017143	0.0072393	0.03583965	8.02E-22	3.73E-57	3.06E-21	0.89481772	0.97750982
131.3	0.00017143	0.0072393	0.03583965	8.02E-22	3.73E-57	3.06E-21	0.89481772	0.97750982
131.4	0.00017697	0.00724895	0.03621099	8.46E-22	3.59E-57	3.21E-21	0.89055797	0.98186152
131.5	0.00017697	0.00724895	0.03621099	8.46E-22	3.59E-57	3.21E-21	0.89055797	0.98186152
131.6	0.00017697	0.00724895	0.03621099	8.46E-22	3.59E-57	3.21E-21	0.89055797	0.98186152
131.7	0.00017697	0.00724895	0.03621099	8.46E-22	3.59E-57	3.21E-21	0.89055797	0.98186152
131.8	0.00017697	0.00724895	0.03621099	8.46E-22	3.59E-57	3.21E-21	0.89055797	0.98186152
131.9	0.00017697	0.00724895	0.03621099	8.46E-22	3.59E-57	3.21E-21	0.89055797	0.98186152
132	0.00017697	0.00724895	0.03621099	8.46E-22	3.59E-57	3.21E-21	0.89055797	0.98186152
132.1	0.00017697	0.00724895	0.03621099	8.46E-22	3.59E-57	3.21E-21	0.89055797	0.98186152
132.2	0.00017697	0.00724895	0.03621099	8.46E-22	3.59E-57	3.21E-21	0.89055797	0.98186152
132.3	0.00017697	0.00724895	0.03621099	8.46E-22	3.59E-57	3.21E-21	0.89055797	0.98186152
132.4	0.00017697	0.00724895	0.03621099	8.46E-22	3.59E-57	3.21E-21	0.89055797	0.98186152
132.5	0.00017931	0.0072668	0.03661042	8.03E-22	3.49E-57	3.16E-21	0.89014908	0.9748589
132.6	0.00017931	0.0072668	0.03661042	8.03E-22	3.49E-57	3.16E-21	0.89014908	0.9748589
132.7	0.00017931	0.0072668	0.03661042	8.03E-22	3.49E-57	3.16E-21	0.89014908	0.9748589
132.8	0.00017931	0.0072668	0.03661042	8.03E-22	3.49E-57	3.16E-21	0.89014908	0.9748589
132.9	0.00017931	0.0072668	0.03661042	8.03E-22	3.49E-57	3.16E-21	0.89014908	0.9748589
133	0.0001832	0.00718519	0.03721948	7.83E-22	3.60E-57	3.09E-21	0.89242961	0.97020561
133.1	0.0001832	0.00718519	0.03721948	7.83E-22	3.60E-57	3.09E-21	0.89242961	0.97020561
133.2	0.00018512	0.00733458	0.03797286	7.67E-22	3.73E-57	3.26E-21	0.89888303	0.96875776
133.3	0.00019234	0.00737981	0.0379131	7.73E-22	3.83E-57	3.37E-21	0.89998942	0.97324855
133.4	0.00019234	0.00737981	0.0379131	7.73E-22	3.83E-57	3.37E-21	0.89998942	0.97324855
133.5	0.00019234	0.00737981	0.0379131	7.73E-22	3.83E-57	3.37E-21	0.89998942	0.97324855
133.6	0.00019578	0.00733721	0.03803561	8.08E-22	3.67E-57	3.42E-21	0.89512947	0.97954947
133.7	0.00019578	0.00733721	0.03803561	8.08E-22	3.67E-57	3.42E-21	0.89512947	0.97954947
133.8	0.00019578	0.00733721	0.03803561	8.08E-22	3.67E-57	3.42E-21	0.89512947	0.97954947
133.9	0.00019578	0.00733721	0.03803561	8.08E-22	3.67E-57	3.42E-21	0.89512947	0.97954947
134	0.00019578	0.00733721	0.03803561	8.08E-22	3.67E-57	3.42E-21	0.89512947	0.97954947
134.1	0.00019578	0.00733721	0.03803561	8.08E-22	3.67E-57	3.42E-21	0.89512947	0.97954947
134.2	0.00019578	0.00733721	0.03803561	8.08E-22	3.67E-57	3.42E-21	0.89512947	0.97954947
134.3	0.00019578	0.00733721	0.03803561	8.08E-22	3.67E-57	3.42E-21	0.89512947	0.97954947
134.4	0.00019578	0.00733721	0.03803561	8.08E-22	3.67E-57	3.42E-21	0.89512947	0.97954947
134.5	0.00019578	0.00733721	0.03803561	8.08E-22	3.67E-57	3.42E-21	0.89512947	0.97954947
134.6	0.00019578	0.00733721	0.03803561	8.08E-22	3.67E-57	3.42E-21	0.89512947	0.97954947
134.7	0.00019127	0.00737827	0.03795653	7.59E-22	3.47E-57	3.22E-21	0.89840858	0.9842747
134.8	0.00019127	0.00737827	0.03795653	7.59E-22	3.47E-57	3.22E-21	0.89840858	0.9842747
134.9	0.00019127	0.00737827	0.03795653	7.59E-22	3.47E-57	3.22E-21	0.89840858	0.9842747

180	0.00029338	0.00662316	0.053735	9.38E-22	1.17E-56	1.20E-20	0.86854304	0.98760534
180.1	0.00029338	0.00662316	0.053735	9.38E-22	1.17E-56	1.20E-20	0.86854304	0.98760534
180.2	0.00029338	0.00662316	0.053735	9.38E-22	1.17E-56	1.20E-20	0.86854304	0.98760534
180.3	0.00029338	0.00662316	0.053735	9.38E-22	1.17E-56	1.20E-20	0.86854304	0.98760534
180.4	0.00029338	0.00662316	0.053735	9.38E-22	1.17E-56	1.20E-20	0.86854304	0.98760534

135.2	0.00019127	0.00737827	0.03795653	7.59E-22	3.47E-57	3.22E-21	0.89840858	0.9842747
135.3	0.00019127	0.00737827	0.03795653	7.59E-22	3.47E-57	3.22E-21	0.89840858	0.9842747
135.4	0.00019127	0.00737827	0.03795653	7.59E-22	3.47E-57	3.22E-21	0.89840858	0.9842747
135.5	0.0001961	0.00715544	0.03886551	6.95E-22	3.55E-57	3.18E-21	0.89465696	0.98394066
135.6	0.00019676	0.00712742	0.03950876	7.07E-22	3.84E-57	3.20E-21	0.89856804	0.98607222
135.7	0.00019676	0.00712742	0.03950876	7.07E-22	3.84E-57	3.20E-21	0.89856804	0.98607222
135.8	0.00019676	0.00712742	0.03950876	7.07E-22	3.84E-57	3.20E-21	0.89856804	0.98607222
135.9	0.00020365	0.00717893	0.03930977	7.03E-22	3.97E-57	3.32E-21	0.90162398	0.99249403
136	0.00020365	0.00717893	0.03930977	7.03E-22	3.97E-57	3.32E-21	0.90162398	0.99249403
136.1	0.00020365	0.00717893	0.03930977	7.03E-22	3.97E-57	3.32E-21	0.90162398	0.99249403
136.2	0.00020365	0.00717893	0.03930977	7.03E-22	3.97E-57	3.32E-21	0.90162398	0.99249403
136.3	0.00020391	0.00719185	0.04004077	6.76E-22	3.98E-57	3.27E-21	0.90734017	0.99076158
136.4	0.00020391	0.00719185	0.04004077	6.76E-22	3.98E-57	3.27E-21	0.90734017	0.99076158
136.5	0.00020391	0.00719185	0.04004077	6.76E-22	3.98E-57	3.27E-21	0.90734017	0.99076158
136.6	0.00020391	0.00719185	0.04004077	6.76E-22	3.98E-57	3.27E-21	0.90734017	0.99076158
136.7	0.00020391	0.00719185	0.04004077	6.76E-22	3.98E-57	3.27E-21	0.90734017	0.99076158
136.8	0.00020391	0.00719185	0.04004077	6.76E-22	3.98E-57	3.27E-21	0.90734017	0.99076158
136.9	0.00020391	0.00719185	0.04004077	6.76E-22	3.98E-57	3.27E-21	0.90734017	0.99076158
137	0.00020391	0.00719185	0.04004077	6.76E-22	3.98E-57	3.27E-21	0.90734017	0.99076158
137.1	0.00020391	0.00719185	0.04004077	6.76E-22	3.98E-57	3.27E-21	0.90734017	0.99076158
137.2	0.00020391	0.00719185	0.04004077	6.76E-22	3.98E-57	3.27E-21	0.90734017	0.99076158
137.3	0.00020391	0.00719185	0.04004077	6.76E-22	3.98E-57	3.27E-21	0.90734017	0.99076158
137.4	0.00020391	0.00719185	0.04004077	6.76E-22	3.98E-57	3.27E-21	0.90734017	0.99076158
137.5	0.00020391	0.00719185	0.04004077	6.76E-22	3.98E-57	3.27E-21	0.90734017	0.99076158
137.6	0.00020391	0.00719185	0.04004077	6.76E-22	3.98E-57	3.27E-21	0.90734017	0.99076158
137.7	0.00020391	0.00719185	0.04004077	6.76E-22	3.98E-57	3.27E-21	0.90734017	0.99076158
137.8	0.00020391	0.00719185	0.04004077	6.76E-22	3.98E-57	3.27E-21	0.90734017	0.99076158
137.9	0.00020391	0.00719185	0.04004077	6.76E-22	3.98E-57	3.27E-21	0.90734017	0.99076158
138	0.00020391	0.00719185	0.04004077	6.76E-22	3.98E-57	3.27E-21	0.90734017	0.99076158
138.1	0.00020391	0.00719185	0.04004077	6.76E-22	3.98E-57	3.27E-21	0.90734017	0.99076158
138.2	0.00020391	0.00719185	0.04004077	6.76E-22	3.98E-57	3.27E-21	0.90734017	0.99076158
138.3	0.00020391	0.00719185	0.04004077	6.76E-22	3.98E-57	3.27E-21	0.90734017	0.99076158
138.4	0.00020391	0.00719185	0.04004077	6.76E-22	3.98E-57	3.27E-21	0.90734017	0.99076158
138.5	0.00020391	0.00719185	0.04004077	6.76E-22	3.98E-57	3.27E-21	0.90734017	0.99076158
138.6	0.00020391	0.00719185	0.04004077	6.76E-22	3.98E-57	3.27E-21	0.90734017	0.99076158
138.7	0.00020391	0.00719185	0.04004077	6.76E-22	3.98E-57	3.27E-21	0.90734017	0.99076158
138.8	0.00020391	0.00719185	0.04004077	6.76E-22	3.98E-57	3.27E-21	0.90734017	0.99076158
138.9	0.00020022	0.00725117	0.03972973	6.77E-22	4.12E-57	3.27E-21	0.89891409	0.99577363
139	0.00020011	0.00734443	0.03958641	6.36E-22	3.85E-57	3.13E-21	0.89551795	0.99660327
139.1	0.00020011	0.00734443	0.03958641	6.36E-22	3.85E-57	3.13E-21	0.89551795	0.99660327
139.2	0.00020011	0.00734443	0.03958641	6.36E-22	3.85E-57	3.13E-21	0.89551795	0.99660327
139.3	0.00020011	0.00734443	0.03958641	6.36E-22	3.85E-57	3.13E-21	0.89551795	0.99660327
139.4	0.00020011	0.00734443	0.03958641	6.36E-22	3.85E-57	3.13E-21	0.89551795	0.99660327
139.5	0.00020011	0.00734443	0.03958641	6.36E-22	3.85E-57	3.13E-21	0.89551795	0.99660327
139.6	0.00020011	0.00734443	0.03958641	6.36E-22	3.85E-57	3.13E-21	0.89551795	0.99660327
139.7	0.00020011	0.00734443	0.03958641	6.36E-22	3.85E-57	3.13E-21	0.89551795	0.99660327
139.8	0.00020011	0.00734443	0.03958641	6.36E-22	3.85E-57	3.13E-21	0.89551795	0.99660327
139.9	0.00020011	0.00734443	0.03958641	6.36E-22	3.85E-57	3.13E-21	0.89551795	0.99660327
140	0.00020011	0.00734443	0.03958641	6.36E-22	3.85E-57	3.13E-21	0.89551795	0.99660327
140.1	0.00020011	0.00734443	0.03958641	6.36E-22	3.85E-57	3.13E-21	0.89551795	0.99660327
140.2	0.00020011	0.00734443	0.03958641	6.36E-22	3.85E-57	3.13E-21	0.89551795	0.99660327
140.3	0.00020011	0.00734443	0.03958641	6.36E-22	3.85E-57	3.13E-21	0.89551795	0.99660327
140.4	0.00020011	0.00734443	0.03958641	6.36E-22	3.85E-57	3.13E-21	0.89551795	0.99660327
140.5	0.00020011	0.00734443	0.03958641	6.36E-22	3.85E-57	3.13E-21	0.89551795	0.99660327
140.6	0.00020011	0.00734443	0.03958641	6.36E-22	3.85E-57	3.13E-21	0.89551795	0.99660327
140.7	0.00020011	0.00734443	0.03958641	6.36E-22	3.85E-57	3.13E-21	0.89551795	0.99660327
140.8	0.00020011	0.00734443	0.03958641	6.36E-22	3.85E-57	3.13E-21	0.89551795	0.99660327
140.9	0.00020011	0.00734443	0.03958641	6.36E-22	3.85E-57	3.13E-21	0.89551795	0.99660327
141	0.00020011	0.00734443	0.03958641	6.36E-22	3.85E-57	3.13E-21	0.89551795	0.99660327
141.1	0.00020202	0.00730773	0.04006034	6.86E-22	4.12E-57	3.34E-21	0.89646878	0.99663547
141.2	0.0002044	0.00717291	0.04062268	7.35E-22	4.36E-57	3.60E-21	0.89284829	0.99711748
141.3	0.0002101	0.00723625	0.04150115	8.01E-22	5.08E-57	3.74E-21	0.89356335	0.99903064
141.4	0.00021438	0.00706637	0.04148334	7.87E-22	4.84E-57	3.95E-21	0.89503696	0.99986307
141.5	0.00021438	0.00706637	0.04148334	7.87E-22	4.84E-57	3.95E-21	0.89503696	0.99986307
141.6	0.00021438	0.00706637	0.04148334	7.87E-22	4.84E-57	3.95E-21	0.89503696	0.99986307
141.7	0.00021666	0.00721751	0.04114334	7.88E-22	4.82E-57	3.91E-21	0.89765191	0.99912233
141.8	0.00021666	0.00721751	0.04114334	7.88E-22	4.82E-57	3.91E-21	0.89765191	0.99912233
141.9	0.00021666	0.00721751	0.04114334	7.88E-22	4.82E-57	3.91E-21	0.89765191	0.99912233
142	0.00021666	0.00721751	0.04114334	7.88E-22	4.82E-57	3.91E-21	0.89765191	0.99912233
142.1	0.00021666	0.00721751	0.04114334	7.88E-22	4.82E-57	3.91E-21	0.89765191	0.99912233
142.2	0.00021666	0.00721751	0.04114334	7.88E-22	4.82E-57	3.91E-21	0.89765191	0.99912233
142.3	0.00021666	0.00721751	0.04114334	7.88E-22	4.82E-57	3.91E-21	0.89765191	0.99912233
142.4	0.00021666	0.00721751	0.04114334	7.88E-22	4.82E-57	3.91E-21	0.89765191	0.99912233
142.5	0.00022217	0.00727308	0.04133198	7.79E-22	4.83E-57	4.04E-21	0.89869514	0.99466113
142.6	0.00022217	0.00727308	0.04133198	7.79E-22	4.83E-57	4.04E-21	0.89869514	0.99466113
142.7	0.00022496	0.00720849	0.04172209	7.33E-22	4.53E-57	4.03E-21	0.89266968	0.98775513
142.8	0.00022496	0.00720849	0.04172209	7.33E-22	4.53E-57	4.03E-21	0.89266968	0.98775513
142.9	0.00022496	0.00720849	0.04172209	7.33E-22	4.53E-57	4.03E-21	0.89266968	0.98775513
143	0.0002224	0.00715076	0.04212066	7.37E-22	4.81E-57	4.03E-21	0.89063936	0.984218
143.1	0.0002224	0.00715076	0.04212066	7.37E-22	4.81E-57	4.03E-21	0.89063936	0.984218
143.2	0.0002224	0.00715076	0.04212066	7.37E-22	4.81E-57	4.03E-21	0.89063936	0.984218
143.3	0.0002224	0.00715076	0.04212066	7.37E-22	4.81E-57	4.03E-21	0.89063936	0.984218
143.4	0.0002224	0.00715076	0.04212066	7.37E-22	4.81E-57	4.03E-21	0.89063936	0.984218
143.5	0.0002224	0.00715076	0.04212066	7.37E-22	4.81E-57	4.03E-21	0.89063936	0.984218
143.6	0.0002224	0.00715076	0.04212066	7.37E-22	4.81E-57	4.03E-21	0.89063936	0.984218
143.7	0.0002224	0.00715076	0.04212066	7.37E-22	4.81E-57	4.03E-21	0.89063936	0.984218
143.8	0.0002224	0.00715076	0.04212066	7.37E-22	4.81E-57	4.03E-21	0.89063936	0.984218
143.9	0.0002224	0.00715076	0.04212066	7.37E-22	4.81E-57	4.03E-21	0.89063936	0.984218
144	0.0002224	0.00715076	0.04212066	7.37E-22	4.81E-57	4.03E-21	0.89063936	0.984218
144.1	0.0002224	0.00715076	0.04212066	7.37E-22	4.81E-57	4.03E-21	0.89063936	0.984218
144.2	0.0002211	0.00722581	0.04215474	7.40E-22	4.86E-57	4.06E-21	0.88667916	0.98234372
144.3	0.0002211	0.00722581	0.04215474	7.40E-22	4.86E-57	4.06E-21	0.88667916	0.98234372
144.4	0.0002211	0.00722581	0.04215474	7.40E-22	4.86E-57	4.06E-21	0.88667916	0.98234372
144.5	0.0002211	0.00722581	0.04215474	7.40E-22	4.86E-57	4.06E-21	0.88667916	0.98234372
144.6	0.0002211	0.00722581	0.04215474	7.40E-22	4.86E-57	4.06E-21	0.88667916	0.98234372
144.7	0.0002211	0.00722581	0.04215474	7.40E-22	4.86E-57	4.06E-21	0.88667916	0.98234372
144.8	0.0002211	0.00722581	0.04215474	7.40E-22	4.86E-57	4.06E-21	0.88667916	0.98234372
144.9	0.0002211	0.00722581	0.04215474	7.40E-22	4.86E-57	4.06E-21	0.88667916	0.98234372
145	0.0002211	0.00722581	0.04215474	7.40E-22	4.86E-57	4.06E-21	0.88667916	0.98234372
145.1	0.00023045	0.007433	0.04213729	7.99E-22	5.15E-57	4.28E-21	0.88635654	0.98597689
145.2	0.00023045	0.007433	0.04213729	7.99E-22	5.15E-57	4.28E-21	0.88635654	0.98597689
145.3	0.00023045	0.007433	0.04213729	7.99E-22	5.15E-57	4.28E-21	0.88635654	0.98597689
145.4	0.00022814	0.00751842	0.04268587	7.39E-22	4.99E-57	3.99E-21	0.88544402	0.98627137
145.5	0.00022814	0.00751842	0.04268587	7.39E-22	4.99E-57	3.99E-21	0.88544402	0.98627137
145.6	0.00022814	0.00751842	0.04268587	7.39E-22	4.99E-57	3.99E-21	0.88544402	0.98627137
145.7	0.00022814	0.00751842	0.04268587	7.39E-22	4.99E-57	3.99E-21	0.88544402	0.98627137
145.8	0.00022814	0.00751842	0.04268587	7.39E-22	4.99E-57	3.99E-21	0.88544402	0.98627137
145.9	0.00022814	0.00751842	0.04268587	7.39E-22	4.99E-57	3.99E-21	0.88544402	0.98627137
146	0.00022814	0.00751842	0.04268587	7.39E-22	4.99E-57	3.99E-21	0.88544402	0.98627137
146.1	0.00022814	0.00751842	0.04268587	7.39E-22	4.99E-57	3.99E-21	0.88544402	0.98627137
146.2	0.00022814	0.00751842	0.04268587	7.39E-22	4.99E-57	3.99E-21	0.88544402	0.98627137
146.3	0.00022814	0.00751842	0.04268587	7.39E-22	4.99E-57	3.99E-21	0.88544402	0.98627137
146.4	0.00022847	0.00752888	0.04304977	7.22E-22	5.01E-57	4.10E-21	0.88246913	0.98271989
146.5	0.00022847	0.00752888	0.04304977	7.22E-22	5.01E-57	4.10E-21	0.88246913	0.98271989
146.6	0.00022847	0.00752888	0.04304977	7.22E-22	5.01E-57	4.10E-21	0.88246913	0.98271989
146.7	0.00022847	0.00752888	0.04304977	7.22E-22	5.01E-57	4.10E-21	0.88246913	0.98271989
146.8	0.00022847	0.00752888	0.04304977	7.22E-22	5.01E-57	4.10E-21	0.88246913	0.98271989
146.9	0.00022847	0.00752888	0.04304977	7.22E-22	5.01E-57	4.10E-21	0.88246913	0.98271989
147	0.00022847	0.00752888	0.04304977	7.22E-22	5.01E-57	4.10E-21	0.88246913	0.98271989
147.1	0.00022847	0.00752888	0.04304977	7.22E-22	5.01E-57	4.10E-21	0.88246913	0.98271989
147.2	0.00022847	0.00752888	0.04304977	7.22E-22	5.01E-57	4.10E-21	0.88246913	0.98271989
147.3	0.00022847	0.00752888	0.04304977	7.22E-22	5.01E-57	4.10E-21	0.88246913	0.98271989
147.4	0.00022847	0.00752888	0.04304977	7.22E-22	5.01E-57	4.10E-21	0.88246913	0.98271989
147.5	0.00022847	0.00752888	0.04304977	7.22E-22	5.01E-57	4.10E-21	0.88246913	0.98271989
147.6	0.00022847	0.00752888	0.04304977	7.22E-22	5.01E-57	4.10E-21	0.88246913	0.98271989
147.7	0.00022847	0.00752888	0.04304977	7.22E-22	5.01E-57	4.10E-21	0.88246913	0.98271989
147.8	0.00022847	0.00752888	0.04304977	7.22E-22	5.01E-57	4.10E-21	0.88246913	0.98271989
147.9	0.00022847	0.00752888	0.04304977	7.22E-22	5.01E-57	4.10E-21	0.88246913	0.98271989
148	0.00022847	0.00752888	0.04304977	7.22E-22	5.01E-57	4.10E-21	0.88246913	0.98271989
148.1	0.00022847	0.00752888	0.04304977	7.22E-22	5.01E-57	4.10E-21	0.88246913	0.98271989
148.2	0.00022847	0.00752888	0.04304977	7.22E-22	5.01E-57	4.10E-21	0.88246913	0.98271989
148.3	0.00022908	0.0075049	0.04360555	7.25E-22	5.07E-57	4.22E-21	0.87734577	0.98458627
148.4	0.00022908	0.0075049	0.04360555	7.25E-22	5.07E-57	4.22E-21	0.87734577	0.98458627
148.5	0.00022908	0.0075049	0.04360555	7.25E-22	5.07E-57	4.22E-21	0.87734577	0.98458627
148.6	0.00022908	0.0075049	0.04360555	7.25E-22	5.07E-57	4.22E-21	0.87734577	0.98458627
148.7	0.00022908	0.0075049	0.04360555	7.25E-22	5.07E-57	4.22E-21	0.87734577	0.98458627
148.8	0.00022908	0.0075049	0.04360555	7.25E-22	5.07E-57	4.22E-21	0.87734577	0.98458627
148.9	0.00022908	0.0075049	0.04360555	7.25E-22	5.07E-57	4.22E-21	0.87734577	0.98458627
149	0.00022908	0.0075049	0.04360555	7.25E-22	5.07E-57	4.22E-21	0.87734577	0.98458627
149.1	0.0002298	0.00738639	0.04432183	7.74E-22	5.49E-57	4.54E-21	0.87561488	0.98728858
149.2	0.0002298	0.00738639	0.04432183	7.74E-22	5.49E-57	4.54E-21	0.87561488	0.98728858
149.3	0.0002298	0.00738639	0.04432183	7.74E-22	5.49E-57	4.54E-21	0.87561488	0.98728858
149.4	0.0002298	0.00738639	0.04432183	7.74E-22	5.49E-57	4.54E-21	0.87561488	0.98728858
149.5	0.0002298	0.00738639	0.04432183	7.74E-22	5.49E-57	4.54E-21	0.87561488	0.98728858
149.6	0.0002298	0.00738639	0.04432183	7.74E-22	5.49E-57	4.54E-21	0.87561488	0.98728858
149.7	0.0002298	0.00738639	0.04432183	7.74E-22	5.49E-57	4.54E-21	0.87561488	0.98728858
149.8	0.0002298	0.00738639	0.04432183	7.74E-22	5.49E-57	4.54E-21	0.87561488	0.98728858
149.9	0.0002298	0.00738639	0.04432183	7.74E-22	5.49E-57	4.54E-21	0.87561488	0.98728858

195	0.00031809	0.00690167	0.05626402	8.31E-22	1.05E-56	1.55E-20	0.85933914	0.97774782
195.1	0.00031809	0.00690167	0.05626402	8.31E-22	1.05E-56	1.55E-20	0.85933914	0.97774782
195.2	0.00031809	0.00690167	0.05626402	8.31E-22	1.05E-56	1.55E-20	0.85933914	0.97774782
195.3	0.00031809	0.00690167	0.05626402	8.31E-22	1.05E-56	1.55E-20	0.85933914	0.97774782
195.4	0.00031809	0.00690167	0.05626402	8.31E-22	1.05E-56	1.55E-20	0.85933914	0.97774782

150.2	0.00023171	0.00731036	0.04489807	8.07E-22	5.83E-57	4.85E-21	0.87837115	0.9870322
150.3	0.00023171	0.00731036	0.04489807	8.07E-22	5.83E-57	4.85E-21	0.87837115	0.9870322
150.4	0.00023171	0.00731036	0.04489807	8.07E-22	5.83E-57	4.85E-21	0.87837115	0.9870322
150.5	0.00023171	0.00731036	0.04489807	8.07E-22	5.83E-57	4.85E-21	0.87837115	0.9870322
150.6	0.00023171	0.00731036	0.04489807	8.07E-22	5.83E-57	4.85E-21	0.87837115	0.9870322
150.7	0.00023171	0.00731036	0.04489807	8.07E-22	5.83E-57	4.85E-21	0.87837115	0.9870322
150.8	0.00023171	0.00731036	0.04489807	8.07E-22	5.83E-57	4.85E-21	0.87837115	0.9870322
150.9	0.00023171	0.00731036	0.04489807	8.07E-22	5.83E-57	4.85E-21	0.87837115	0.9870322
151	0.00023171	0.00731036	0.04489807	8.07E-22	5.83E-57	4.85E-21	0.87837115	0.9870322
151.1	0.00023171	0.00731036	0.04489807	8.07E-22	5.83E-57	4.85E-21	0.87837115	0.9870322
151.2	0.00023675	0.00743817	0.04533951	7.96E-22	5.93E-57	4.97E-21	0.88555098	0.98831133
151.3	0.00023675	0.00743817	0.04533951	7.96E-22	5.93E-57	4.97E-21	0.88555098	0.98831133
151.4	0.00023366	0.00730592	0.04623913	7.64E-22	5.94E-57	4.90E-21	0.88428248	0.99062261
151.5	0.00023778	0.0072571	0.04713492	8.43E-22	6.73E-57	5.61E-21	0.88198599	0.98986974
151.6	0.00023778	0.0072571	0.04713492	8.43E-22	6.73E-57	5.61E-21	0.88198599	0.98986974
151.7	0.00023778	0.0072571	0.04713492	8.43E-22	6.73E-57	5.61E-21	0.88198599	0.98986974
151.8	0.00023778	0.0072571	0.04713492	8.43E-22	6.73E-57	5.61E-21	0.88198599	0.98986974
151.9	0.00023778	0.0072571	0.04713492	8.43E-22	6.73E-57	5.61E-21	0.88198599	0.98986974
152	0.00023778	0.0072571	0.04713492	8.43E-22	6.73E-57	5.61E-21	0.88198599	0.98986974
152.1	0.00023778	0.0072571	0.04713492	8.43E-22	6.73E-57	5.61E-21	0.88198599	0.98986974
152.2	0.00023778	0.0072571	0.04713492	8.43E-22	6.73E-57	5.61E-21	0.88198599	0.98986974
152.3	0.00023778	0.0072571	0.04713492	8.43E-22	6.73E-57	5.61E-21	0.88198599	0.98986974
152.4	0.00023778	0.0072571	0.04713492	8.43E-22	6.73E-57	5.61E-21	0.88198599	0.98986974
152.5	0.00023778	0.0072571	0.04713492	8.43E-22	6.73E-57	5.61E-21	0.88198599	0.98986974
152.6	0.00023778	0.0072571	0.04713492	8.43E-22	6.73E-57	5.61E-21	0.88198599	0.98986974
152.7	0.00023778	0.0072571	0.04713492	8.43E-22	6.73E-57	5.61E-21	0.88198599	0.98986974
152.8	0.00023778	0.0072571	0.04713492	8.43E-22	6.73E-57	5.61E-21	0.88198599	0.98986974
152.9	0.00023712	0.00718153	0.04742864	9.00E-22	7.18E-57	5.87E-21	0.88019204	0.99208974
153	0.00023712	0.00718153	0.04742864	9.00E-22	7.18E-57	5.87E-21	0.88019204	0.99208974
153.1	0.00023712	0.00718153	0.04742864	9.00E-22	7.18E-57	5.87E-21	0.88019204	0.99208974
153.2	0.00023712	0.00718153	0.04742864	9.00E-22	7.18E-57	5.87E-21	0.88019204	0.99208974
153.3	0.00023712	0.00718153	0.04742864	9.00E-22	7.18E-57	5.87E-21	0.88019204	0.99208974
153.4	0.00023712	0.00718153	0.04742864	9.00E-22	7.18E-57	5.87E-21	0.88019204	0.99208974
153.5	0.00023712	0.00718153	0.04742864	9.00E-22	7.18E-57	5.87E-21	0.88019204	0.99208974
153.6	0.0002411	0.00703558	0.04776038	9.19E-22	7.40E-57	6.34E-21	0.88293192	0.9903772
153.7	0.0002411	0.00703558	0.04776038	9.19E-22	7.40E-57	6.34E-21	0.88293192	0.9903772
153.8	0.0002411	0.00703558	0.04776038	9.19E-22	7.40E-57	6.34E-21	0.88293192	0.9903772
153.9	0.0002411	0.00703558	0.04776038	9.19E-22	7.40E-57	6.34E-21	0.88293192	0.9903772
154	0.0002411	0.00703558	0.04776038	9.19E-22	7.40E-57	6.34E-21	0.88293192	0.9903772
154.1	0.0002411	0.00703558	0.04776038	9.19E-22	7.40E-57	6.34E-21	0.88293192	0.9903772
154.2	0.0002411	0.00703558	0.04776038	9.19E-22	7.40E-57	6.34E-21	0.88293192	0.9903772
154.3	0.0002411	0.00703558	0.04776038	9.19E-22	7.40E-57	6.34E-21	0.88293192	0.9903772
154.4	0.0002411	0.00703558	0.04776038	9.19E-22	7.40E-57	6.34E-21	0.88293192	0.9903772
154.5	0.0002411	0.00703558	0.04776038	9.19E-22	7.40E-57	6.34E-21	0.88293192	0.9903772
154.6	0.0002411	0.00703558	0.04776038	9.19E-22	7.40E-57	6.34E-21	0.88293192	0.9903772
154.7	0.0002411	0.00703558	0.04776038	9.19E-22	7.40E-57	6.34E-21	0.88293192	0.9903772
154.8	0.0002411	0.00703558	0.04776038	9.19E-22	7.40E-57	6.34E-21	0.88293192	0.9903772
154.9	0.0002411	0.00703558	0.04776038	9.19E-22	7.40E-57	6.34E-21	0.88293192	0.9903772
155	0.0002411	0.00703558	0.04776038	9.19E-22	7.40E-57	6.34E-21	0.88293192	0.9903772
155.1	0.0002411	0.00703558	0.04776038	9.19E-22	7.40E-57	6.34E-21	0.88293192	0.9903772
155.2	0.0002411	0.00703558	0.04776038	9.19E-22	7.40E-57	6.34E-21	0.88293192	0.9903772
155.3	0.0002411	0.00703558	0.04776038	9.19E-22	7.40E-57	6.34E-21	0.88293192	0.9903772
155.4	0.0002411	0.00703558	0.04776038	9.19E-22	7.40E-57	6.34E-21	0.88293192	0.9903772
155.5	0.00024948	0.00703955	0.04786117	8.97E-22	7.42E-57	6.48E-21	0.88186667	0.98990831
155.6	0.00024948	0.00703955	0.04786117	8.97E-22	7.42E-57	6.48E-21	0.88186667	0.98990831
155.7	0.00024948	0.00703955	0.04786117	8.97E-22	7.42E-57	6.48E-21	0.88186667	0.98990831
155.8	0.00024948	0.00703955	0.04786117	8.97E-22	7.42E-57	6.48E-21	0.88186667	0.98990831
155.9	0.00024948	0.00703955	0.04786117	8.97E-22	7.42E-57	6.48E-21	0.88186667	0.98990831
156	0.00024948	0.00703955	0.04786117	8.97E-22	7.42E-57	6.48E-21	0.88186667	0.98990831
156.1	0.00025623	0.00692366	0.04785124	9.71E-22	8.11E-57	7.21E-21	0.8848838	0.99374108
156.2	0.00025623	0.00692366	0.04785124	9.71E-22	8.11E-57	7.21E-21	0.8848838	0.99374108
156.3	0.00025623	0.00692366	0.04785124	9.71E-22	8.11E-57	7.21E-21	0.8848838	0.99374108
156.4	0.00025623	0.00692366	0.04785124	9.71E-22	8.11E-57	7.21E-21	0.8848838	0.99374108
156.5	0.00025623	0.00692366	0.04785124	9.71E-22	8.11E-57	7.21E-21	0.8848838	0.99374108
156.6	0.00025623	0.00692366	0.04785124	9.71E-22	8.11E-57	7.21E-21	0.8848838	0.99374108
156.7	0.00026153	0.00686958	0.04821006	9.98E-22	8.58E-57	7.63E-21	0.88177398	0.99004219
156.8	0.00026153	0.00686958	0.04821006	9.98E-22	8.58E-57	7.63E-21	0.88177398	0.99004219
156.9	0.00025714	0.00677391	0.04864579	1.02E-21	8.87E-57	7.82E-21	0.87757025	0.99080133
157	0.00025714	0.00677391	0.04864579	1.02E-21	8.87E-57	7.82E-21	0.87757025	0.99080133
157.1	0.00025714	0.00677391	0.04864579	1.02E-21	8.87E-57	7.82E-21	0.87757025	0.99080133
157.2	0.00025714	0.00677391	0.04864579	1.02E-21	8.87E-57	7.82E-21	0.87757025	0.99080133
157.3	0.00025714	0.00677391	0.04864579	1.02E-21	8.87E-57	7.82E-21	0.87757025	0.99080133
157.4	0.00025714	0.00677391	0.04864579	1.02E-21	8.87E-57	7.82E-21	0.87757025	0.99080133
157.5	0.00025714	0.00677391	0.04864579	1.02E-21	8.87E-57	7.82E-21	0.87757025	0.99080133
157.6	0.00025714	0.00677391	0.04864579	1.02E-21	8.87E-57	7.82E-21	0.87757025	0.99080133
157.7	0.00025714	0.00677391	0.04864579	1.02E-21	8.87E-57	7.82E-21	0.87757025	0.99080133
157.8	0.00025714	0.00677391	0.04864579	1.02E-21	8.87E-57	7.82E-21	0.87757025	0.99080133
157.9	0.00025714	0.00677391	0.04864579	1.02E-21	8.87E-57	7.82E-21	0.87757025	0.99080133
158	0.00025714	0.00677391	0.04864579	1.02E-21	8.87E-57	7.82E-21	0.87757025	0.99080133
158.1	0.00025714	0.00677391	0.04864579	1.02E-21	8.87E-57	7.82E-21	0.87757025	0.99080133
158.2	0.00026171	0.00685513	0.04941505	1.00E-21	9.09E-57	8.02E-21	0.87440228	0.98362787
158.3	0.00026171	0.00685513	0.04941505	1.00E-21	9.09E-57	8.02E-21	0.87440228	0.98362787
158.4	0.00026171	0.00685513	0.04941505	1.00E-21	9.09E-57	8.02E-21	0.87440228	0.98362787
158.5	0.00026171	0.00685513	0.04941505	1.00E-21	9.09E-57	8.02E-21	0.87440228	0.98362787
158.6	0.00026171	0.00685513	0.04941505	1.00E-21	9.09E-57	8.02E-21	0.87440228	0.98362787
158.7	0.00026171	0.00685513	0.04941505	1.00E-21	9.09E-57	8.02E-21	0.87440228	0.98362787
158.8	0.00026171	0.00685513	0.04941505	1.00E-21	9.09E-57	8.02E-21	0.87440228	0.98362787
158.9	0.00026171	0.00685513	0.04941505	1.00E-21	9.09E-57	8.02E-21	0.87440228	0.98362787
159	0.00026171	0.00685513	0.04941505	1.00E-21	9.09E-57	8.02E-21	0.87440228	0.98362787
159.1	0.00026171	0.00685513	0.04941505	1.00E-21	9.09E-57	8.02E-21	0.87440228	0.98362787
159.2	0.00026171	0.00685513	0.04941505	1.00E-21	9.09E-57	8.02E-21	0.87440228	0.98362787
159.3	0.00026171	0.00685513	0.04941505	1.00E-21	9.09E-57	8.02E-21	0.87440228	0.98362787
159.4	0.00026171	0.00685513	0.04941505	1.00E-21	9.09E-57	8.02E-21	0.87440228	0.98362787
159.5	0.00026171	0.00685513	0.04941505	1.00E-21	9.09E-57	8.02E-21	0.87440228	0.98362787
159.6	0.00026171	0.00685513	0.04941505	1.00E-21	9.09E-57	8.02E-21	0.87440228	0.98362787
159.7	0.00026171	0.00685513	0.04941505	1.00E-21	9.09E-57	8.02E-21	0.87440228	0.98362787
159.8	0.00026171	0.00685513	0.04941505	1.00E-21	9.09E-57	8.02E-21	0.87440228	0.98362787
159.9	0.0002653	0.00669964	0.04907897	9.99E-22	9.15E-57	8.00E-21	0.87080715	0.9848396
160	0.0002653	0.00669964	0.04907897	9.99E-22	9.15E-57	8.00E-21	0.87080715	0.9848396
160.1	0.0002653	0.00669964	0.04907897	9.99E-22	9.15E-57	8.00E-21	0.87080715	0.9848396
160.2	0.0002653	0.00669964	0.04907897	9.99E-22	9.15E-57	8.00E-21	0.87080715	0.9848396
160.3	0.0002653	0.00669964	0.04907897	9.99E-22	9.15E-57	8.00E-21	0.87080715	0.9848396
160.4	0.0002653	0.00669964	0.04907897	9.99E-22	9.15E-57	8.00E-21	0.87080715	0.9848396
160.5	0.0002653	0.00669964	0.04907897	9.99E-22	9.15E-57	8.00E-21	0.87080715	0.9848396
160.6	0.0002653	0.00669964	0.04907897	9.99E-22	9.15E-57	8.00E-21	0.87080715	0.9848396
160.7	0.0002653	0.00669964	0.04907897	9.99E-22	9.15E-57	8.00E-21	0.87080715	0.9848396
160.8	0.0002653	0.00669964	0.04907897	9.99E-22	9.15E-57	8.00E-21	0.87080715	0.9848396
160.9	0.0002653	0.00669964	0.04907897	9.99E-22	9.15E-57	8.00E-21	0.87080715	0.9848396
161	0.0002653	0.00669964	0.04907897	9.99E-22	9.15E-57	8.00E-21	0.87080715	0.9848396
161.1	0.0002653	0.00669964	0.04907897	9.99E-22	9.15E-57	8.00E-21	0.87080715	0.9848396
161.2	0.0002653	0.00669964	0.04907897	9.99E-22	9.15E-57	8.00E-21	0.87080715	0.9848396
161.3	0.0002653	0.00669964	0.04907897	9.99E-22	9.15E-57	8.00E-21	0.87080715	0.9848396
161.4	0.0002653	0.00669964	0.04907897	9.99E-22	9.15E-57	8.00E-21	0.87080715	0.9848396
161.5	0.0002653	0.00669964	0.04907897	9.99E-22	9.15E-57	8.00E-21	0.87080715	0.9848396
161.6	0.0002653	0.00669964	0.04907897	9.99E-22	9.15E-57	8.00E-21	0.87080715	0.9848396
161.7	0.0002653	0.00669964	0.04907897	9.99E-22	9.15E-57	8.00E-21	0.87080715	0.9848396
161.8	0.0002653	0.00669964	0.04907897	9.99E-22	9.15E-57	8.00E-21	0.87080715	0.9848396
161.9	0.0002653	0.00669964	0.04907897	9.99E-22	9.15E-57	8.00E-21	0.87080715	0.9848396
162	0.0002653	0.00669964	0.04907897	9.99E-22	9.15E-57	8.00E-21	0.87080715	0.9848396
162.1	0.0002653	0.00669964	0.04907897	9.99E-22	9.15E-57	8.00E-21	0.87080715	0.9848396
162.2	0.0002653	0.00669964	0.04907897	9.99E-22	9.15E-57	8.00E-21	0.87080715	0.9848396
162.3	0.0002653	0.00669964	0.04907897	9.99E-22	9.15E-57	8.00E-21	0.87080715	0.9848396
162.4	0.0002653	0.00669964	0.04907897	9.99E-22	9.15E-57	8.00E-21	0.87080715	0.9848396
162.5	0.0002653	0.00669964	0.04907897	9.99E-22	9.15E-57	8.00E-21	0.87080715	0.9848396
162.6	0.0002653	0.00669964	0.04907897	9.99E-22	9.15E-57	8.00E-21	0.87080715	0.9848396
162.7	0.0002653	0.00669964	0.04907897	9.99E-22	9.15E-57	8.00E-21	0.87080715	0.9848396
162.8	0.0002653	0.00669964	0.04907897	9.99E-22	9.15E-57	8.00E-21	0.87080715	0.9848396
162.9	0.0002653	0.00669964	0.04907897	9.99E-22	9.15E-57	8.00E-21	0.87080715	0.9848396
163	0.0002653	0.00669964	0.04907897	9.99E-22	9.15E-57	8.00E-21	0.87080715	0.9848396
163.1	0.0002653	0.00669964	0.04907897	9.99E-22	9.15E-57	8.00E-21	0.87080715	0.9848396
163.2	0.0002653	0.00669964	0.04907897	9.99E-22	9.15E-57	8.00E-21	0.87080715	0.9848396
163.3	0.00026706	0.00670866	0.04948561	1.01E-21	9.64E-57	8.15E-21	0.87505979	0.9831993
163.4	0.00026706	0.00670866	0.04948561	1.01E-21	9.64E-57	8.15E-21	0.87505979	0.9831993
163.5	0.00026706	0.00670866	0.04948561	1.01E-21	9.64E-57	8.15E-21	0.87505979	0.9831993
163.6	0.00026573	0.00678763	0.04952763	9.65E-22	9.36E-57	7.74E-21	0.87478268	0.98128627
163.7	0.00026573	0.00678763	0.04952763	9.65E-22	9.36E-57	7.74E-21	0.87478268	0.98128627
163.8	0.00026573	0.00678763	0.04952763	9.65E-22	9.36E-57	7.74E-21	0.87478268	0.98128627
163.9	0.00026973	0.00674182	0.04989671	9.57E-22	9.51E-57	8.23E-21	0.87801888	0.98347944
164	0.00026973	0.00674182	0.04989671	9.57E-22	9.51E-57	8.23E-21	0.87801888	0.98347944
164.1	0.00026973	0.00674182	0.04989671	9.57E-22	9.51E-57	8.23E-21	0.87801888	0.98347944
164.2	0.00026973	0.00674182	0.04989671	9.57E-22	9.51E-57	8.23E-21	0.87801888	0.98347944
164.3	0.00026973	0.00674182	0.04989671	9.57E-22	9.51E-57	8.23E-21	0.87801888	0.98347944
164.4	0.00026973	0.00674182	0.04989671	9.57E-22	9.51E-57	8.23E-21	0.87801888	0.98347944
164.5	0.00026973	0.00674182	0.04989671	9.57E-22	9.51E-57	8.23E-21	0.87801888	0.98347944
164.6	0.00026973	0.00674182	0.04989671	9.57E-22	9.51E-57	8.23E-21	0.87801888	0.98347944
164.7	0.00026973	0.00674182	0.04989671	9.57E-22	9.51E-57	8.23E-21	0.87801888	0.98347944
164.8	0.00026973	0.00674182	0.04989671	9.57E-22	9.51E-57	8.23E-21	0.87801888	0.98347944
164.9	0.00026973	0.00674182	0.04989671	9.57E-22	9.51E-57	8.23E-21	0.87801888	0.98347944

210	0.00033248	0.00714029	0.055439	7.98E-22	1.05E-56	1.54E-20	0.86048427	0.98435647
210.1	0.00033248	0.00714029	0.055439	7.98E-22	1.05E-56	1.54E-20	0.86048427	0.98435647
210.2	0.00033248	0.00714029	0.055439	7.98E-22	1.05E-56	1.54E-20	0.86048427	0.98435647
210.3	0.00033248	0.00714029	0.055439	7.98E-22	1.05E-56	1.54E-20	0.86048427	0.98435647
210.4	0.00033248	0.00714029	0.055439	7.98E-22	1.05E-56	1.54E-20	0.86048427	0.98435647

165.2	0.00027097	0.00670581	0.05045093	9.61E-22	9.53E-57	8.56E-21	0.87367881	0.98632324
165.3	0.00027091	0.00658992	0.05094311	9.54E-22	9.62E-57	8.66E-21	0.87668634	0.99019383
165.4	0.00027091	0.00658992	0.05094311	9.54E-22	9.62E-57	8.66E-21	0.87668634	0.99019383
165.5	0.00027091	0.00658992	0.05094311	9.54E-22	9.62E-57	8.66E-21	0.87668634	0.99019383
165.6	0.00027091	0.00658992	0.05094311	9.54E-22	9.62E-57	8.66E-21	0.87668634	0.99019383
165.7	0.00027091	0.00658992	0.05094311	9.54E-22	9.62E-57	8.66E-21	0.87668634	0.99019383
165.8	0.00027091	0.00658992	0.05094311	9.54E-22	9.62E-57	8.66E-21	0.87668634	0.99019383
165.9	0.00027043	0.00649439	0.05209935	9.57E-22	1.03E-56	9.30E-21	0.87209236	0.9922093
166	0.00027043	0.00649439	0.05209935	9.57E-22	1.03E-56	9.30E-21	0.87209236	0.9922093
166.1	0.00027043	0.00649439	0.05209935	9.57E-22	1.03E-56	9.30E-21	0.87209236	0.9922093
166.2	0.00027351	0.00651719	0.05226838	1.00E-21	1.09E-56	1.01E-20	0.86876583	0.99192322
166.3	0.00027351	0.00651719	0.05226838	1.00E-21	1.09E-56	1.01E-20	0.86876583	0.99192322
166.4	0.00027351	0.00651719	0.05226838	1.00E-21	1.09E-56	1.01E-20	0.86876583	0.99192322
166.5	0.00027351	0.00651719	0.05226838	1.00E-21	1.09E-56	1.01E-20	0.86876583	0.99192322
166.6	0.00027351	0.00651719	0.05226838	1.00E-21	1.09E-56	1.01E-20	0.86876583	0.99192322
166.7	0.00027351	0.00651719	0.05226838	1.00E-21	1.09E-56	1.01E-20	0.86876583	0.99192322
166.8	0.00027351	0.00651719	0.05226838	1.00E-21	1.09E-56	1.01E-20	0.86876583	0.99192322
166.9	0.00027351	0.00651719	0.05226838	1.00E-21	1.09E-56	1.01E-20	0.86876583	0.99192322
167	0.00027351	0.00651719	0.05226838	1.00E-21	1.09E-56	1.01E-20	0.86876583	0.99192322
167.1	0.00027482	0.00655323	0.05234083	9.39E-22	1.07E-56	9.94E-21	0.87013428	0.99074908
167.2	0.00027482	0.00655323	0.05234083	9.39E-22	1.07E-56	9.94E-21	0.87013428	0.99074908
167.3	0.00027482	0.00655323	0.05234083	9.39E-22	1.07E-56	9.94E-21	0.87013428	0.99074908
167.4	0.00027482	0.00655323	0.05234083	9.39E-22	1.07E-56	9.94E-21	0.87013428	0.99074908
167.5	0.00027482	0.00655323	0.05234083	9.39E-22	1.07E-56	9.94E-21	0.87013428	0.99074908
167.6	0.00027482	0.00655323	0.05234083	9.39E-22	1.07E-56	9.94E-21	0.87013428	0.99074908
167.7	0.00027482	0.00655323	0.05234083	9.39E-22	1.07E-56	9.94E-21	0.87013428	0.99074908
167.8	0.00027482	0.00655323	0.05234083	9.39E-22	1.07E-56	9.94E-21	0.87013428	0.99074908
167.9	0.00027482	0.00655323	0.05234083	9.39E-22	1.07E-56	9.94E-21	0.87013428	0.99074908
168	0.00027482	0.00655323	0.05234083	9.39E-22	1.07E-56	9.94E-21	0.87013428	0.99074908
168.1	0.00027482	0.00655323	0.05234083	9.39E-22	1.07E-56	9.94E-21	0.87013428	0.99074908
168.2	0.00027482	0.00655323	0.05234083	9.39E-22	1.07E-56	9.94E-21	0.87013428	0.99074908
168.3	0.00027482	0.00655323	0.05234083	9.39E-22	1.07E-56	9.94E-21	0.87013428	0.99074908
168.4	0.00027482	0.00655323	0.05234083	9.39E-22	1.07E-56	9.94E-21	0.87013428	0.99074908
168.5	0.00027204	0.0066343	0.05202949	8.88E-22	1.02E-56	9.17E-21	0.86827304	0.98738336
168.6	0.00027204	0.0066343	0.05202949	8.88E-22	1.02E-56	9.17E-21	0.86827304	0.98738336
168.7	0.00027204	0.0066343	0.05202949	8.88E-22	1.02E-56	9.17E-21	0.86827304	0.98738336
168.8	0.00027204	0.0066343	0.05202949	8.88E-22	1.02E-56	9.17E-21	0.86827304	0.98738336
168.9	0.00027204	0.0066343	0.05202949	8.88E-22	1.02E-56	9.17E-21	0.86827304	0.98738336
169	0.00027204	0.0066343	0.05202949	8.88E-22	1.02E-56	9.17E-21	0.86827304	0.98738336
169.1	0.00027204	0.0066343	0.05202949	8.88E-22	1.02E-56	9.17E-21	0.86827304	0.98738336
169.2	0.00027204	0.0066343	0.05202949	8.88E-22	1.02E-56	9.17E-21	0.86827304	0.98738336
169.3	0.00027204	0.0066343	0.05202949	8.88E-22	1.02E-56	9.17E-21	0.86827304	0.98738336
169.4	0.00027204	0.0066343	0.05202949	8.88E-22	1.02E-56	9.17E-21	0.86827304	0.98738336
169.5	0.00027204	0.0066343	0.05202949	8.88E-22	1.02E-56	9.17E-21	0.86827304	0.98738336
169.6	0.00027204	0.0066343	0.05202949	8.88E-22	1.02E-56	9.17E-21	0.86827304	0.98738336
169.7	0.00027204	0.0066343	0.05202949	8.88E-22	1.02E-56	9.17E-21	0.86827304	0.98738336
169.8	0.00027204	0.0066343	0.05202949	8.88E-22	1.02E-56	9.17E-21	0.86827304	0.98738336
169.9	0.00027204	0.0066343	0.05202949	8.88E-22	1.02E-56	9.17E-21	0.86827304	0.98738336
170	0.00027538	0.00667709	0.05249101	8.69E-22	1.00E-56	9.15E-21	0.87557659	0.98987073
170.1	0.00027538	0.00667709	0.05249101	8.69E-22	1.00E-56	9.15E-21	0.87557659	0.98987073
170.2	0.00027538	0.00667709	0.05249101	8.69E-22	1.00E-56	9.15E-21	0.87557659	0.98987073
170.3	0.00027538	0.00667709	0.05249101	8.69E-22	1.00E-56	9.15E-21	0.87557659	0.98987073
170.4	0.00027809	0.00663602	0.05251912	8.49E-22	1.01E-56	9.47E-21	0.87722679	0.99060486
170.5	0.00027809	0.00663602	0.05251912	8.49E-22	1.01E-56	9.47E-21	0.87722679	0.99060486
170.6	0.00027809	0.00663602	0.05251912	8.49E-22	1.01E-56	9.47E-21	0.87722679	0.99060486
170.7	0.00027809	0.00663602	0.05251912	8.49E-22	1.01E-56	9.47E-21	0.87722679	0.99060486
170.8	0.00027809	0.00663602	0.05251912	8.49E-22	1.01E-56	9.47E-21	0.87722679	0.99060486
170.9	0.00027809	0.00663602	0.05251912	8.49E-22	1.01E-56	9.47E-21	0.87722679	0.99060486
171	0.00027809	0.00663602	0.05251912	8.49E-22	1.01E-56	9.47E-21	0.87722679	0.99060486
171.1	0.00027809	0.00663602	0.05251912	8.49E-22	1.01E-56	9.47E-21	0.87722679	0.99060486
171.2	0.00027809	0.00663602	0.05251912	8.49E-22	1.01E-56	9.47E-21	0.87722679	0.99060486
171.3	0.00027809	0.00663602	0.05251912	8.49E-22	1.01E-56	9.47E-21	0.87722679	0.99060486
171.4	0.00027809	0.00663602	0.05251912	8.49E-22	1.01E-56	9.47E-21	0.87722679	0.99060486
171.5	0.00027809	0.00663602	0.05251912	8.49E-22	1.01E-56	9.47E-21	0.87722679	0.99060486
171.6	0.00027809	0.00663602	0.05251912	8.49E-22	1.01E-56	9.47E-21	0.87722679	0.99060486
171.7	0.00027809	0.00663602	0.05251912	8.49E-22	1.01E-56	9.47E-21	0.87722679	0.99060486
171.8	0.00027809	0.00663602	0.05251912	8.49E-22	1.01E-56	9.47E-21	0.87722679	0.99060486
171.9	0.00027809	0.00663602	0.05251912	8.49E-22	1.01E-56	9.47E-21	0.87722679	0.99060486
172	0.00027809	0.00663602	0.05251912	8.49E-22	1.01E-56	9.47E-21	0.87722679	0.99060486
172.1	0.00028246	0.0065866	0.05283587	8.55E-22	1.06E-56	1.03E-20	0.87981736	0.99265967
172.2	0.00028246	0.0065866	0.05283587	8.55E-22	1.06E-56	1.03E-20	0.87981736	0.99265967
172.3	0.00028246	0.0065866	0.05283587	8.55E-22	1.06E-56	1.03E-20	0.87981736	0.99265967
172.4	0.00028246	0.0065866	0.05283587	8.55E-22	1.06E-56	1.03E-20	0.87981736	0.99265967
172.5	0.00028246	0.0065866	0.05283587	8.55E-22	1.06E-56	1.03E-20	0.87981736	0.99265967
172.6	0.00028246	0.0065866	0.05283587	8.55E-22	1.06E-56	1.03E-20	0.87981736	0.99265967
172.7	0.00028246	0.0065866	0.05283587	8.55E-22	1.06E-56	1.03E-20	0.87981736	0.99265967
172.8	0.00028246	0.0065866	0.05283587	8.55E-22	1.06E-56	1.03E-20	0.87981736	0.99265967
172.9	0.00028246	0.0065866	0.05283587	8.55E-22	1.06E-56	1.03E-20	0.87981736	0.99265967
173	0.00028246	0.0065866	0.05283587	8.55E-22	1.06E-56	1.03E-20	0.87981736	0.99265967
173.1	0.00028246	0.0065866	0.05283587	8.55E-22	1.06E-56	1.03E-20	0.87981736	0.99265967
173.2	0.00028246	0.0065866	0.05283587	8.55E-22	1.06E-56	1.03E-20	0.87981736	0.99265967
173.3	0.00028246	0.0065866	0.05283587	8.55E-22	1.06E-56	1.03E-20	0.87981736	0.99265967
173.4	0.00028246	0.0065866	0.05283587	8.55E-22	1.06E-56	1.03E-20	0.87981736	0.99265967
173.5	0.00028246	0.0065866	0.05283587	8.55E-22	1.06E-56	1.03E-20	0.87981736	0.99265967
173.6	0.00028246	0.0065866	0.05283587	8.55E-22	1.06E-56	1.03E-20	0.87981736	0.99265967
173.7	0.00028246	0.0065866	0.05283587	8.55E-22	1.06E-56	1.03E-20	0.87981736	0.99265967
173.8	0.00028246	0.0065866	0.05283587	8.55E-22	1.06E-56	1.03E-20	0.87981736	0.99265967
173.9	0.00028246	0.0065866	0.05283587	8.55E-22	1.06E-56	1.03E-20	0.87981736	0.99265967
174	0.00028246	0.0065866	0.05283587	8.55E-22	1.06E-56	1.03E-20	0.87981736	0.99265967
174.1	0.00028246	0.0065866	0.05283587	8.55E-22	1.06E-56	1.03E-20	0.87981736	0.99265967
174.2	0.00028246	0.0065866	0.05283587	8.55E-22	1.06E-56	1.03E-20	0.87981736	0.99265967
174.3	0.00028246	0.0065866	0.05283587	8.55E-22	1.06E-56	1.03E-20	0.87981736	0.99265967
174.4	0.00028246	0.0065866	0.05283587	8.55E-22	1.06E-56	1.03E-20	0.87981736	0.99265967
174.5	0.00028246	0.0065866	0.05283587	8.55E-22	1.06E-56	1.03E-20	0.87981736	0.99265967
174.6	0.00028246	0.0065866	0.05283587	8.55E-22	1.06E-56	1.03E-20	0.87981736	0.99265967
174.7	0.00028246	0.0065866	0.05283587	8.55E-22	1.06E-56	1.03E-20	0.87981736	0.99265967
174.8	0.00028246	0.0065866	0.05283587	8.55E-22	1.06E-56	1.03E-20	0.87981736	0.99265967
174.9	0.00028246	0.0065866	0.05283587	8.55E-22	1.06E-56	1.03E-20	0.87981736	0.99265967
175	0.00028246	0.0065866	0.05283587	8.55E-22	1.06E-56	1.03E-20	0.87981736	0.99265967
175.1	0.00028246	0.0065866	0.05283587	8.55E-22	1.06E-56	1.03E-20	0.87981736	0.99265967
175.2	0.0002826	0.00666173	0.05369427	8.76E-22	1.10E-56	1.07E-20	0.87494892	0.99320133
175.3	0.00029093	0.00671081	0.05400043	8.98E-22	1.15E-56	1.14E-20	0.87342647	0.99307325
175.4	0.00029093	0.00671081	0.05400043	8.98E-22	1.15E-56	1.14E-20	0.87342647	0.99307325
175.5	0.00029093	0.00671081	0.05400043	8.98E-22	1.15E-56	1.14E-20	0.87342647	0.99307325
175.6	0.00029093	0.00671081	0.05400043	8.98E-22	1.15E-56	1.14E-20	0.87342647	0.99307325
175.7	0.00029093	0.00671081	0.05400043	8.98E-22	1.15E-56	1.14E-20	0.87342647	0.99307325
175.8	0.00029093	0.00671081	0.05400043	8.98E-22	1.15E-56	1.14E-20	0.87342647	0.99307325
175.9	0.00029093	0.00671081	0.05400043	8.98E-22	1.15E-56	1.14E-20	0.87342647	0.99307325
176	0.00029093	0.00671081	0.05400043	8.98E-22	1.15E-56	1.14E-20	0.87342647	0.99307325
176.1	0.00029093	0.00671081	0.05400043	8.98E-22	1.15E-56	1.14E-20	0.87342647	0.99307325
176.2	0.00029093	0.00671081	0.05400043	8.98E-22	1.15E-56	1.14E-20	0.87342647	0.99307325
176.3	0.00029093	0.00671081	0.05400043	8.98E-22	1.15E-56	1.14E-20	0.87342647	0.99307325
176.4	0.00029093	0.00671081	0.05400043	8.98E-22	1.15E-56	1.14E-20	0.87342647	0.99307325
176.5	0.00029093	0.00671081	0.05400043	8.98E-22	1.15E-56	1.14E-20	0.87342647	0.99307325
176.6	0.00029093	0.00671081	0.05400043	8.98E-22	1.15E-56	1.14E-20	0.87342647	0.99307325
176.7	0.00029093	0.00671081	0.05400043	8.98E-22	1.15E-56	1.14E-20	0.87342647	0.99307325
176.8	0.00029093	0.00671081	0.05400043	8.98E-22	1.15E-56	1.14E-20	0.87342647	0.99307325
176.9	0.00029093	0.00671081	0.05400043	8.98E-22	1.15E-56	1.14E-20	0.87342647	0.99307325
177	0.00029093	0.00671081	0.05400043	8.98E-22	1.15E-56	1.14E-20	0.87342647	0.99307325
177.1	0.00029093	0.00671081	0.05400043	8.98E-22	1.15E-56	1.14E-20	0.87342647	0.99307325
177.2	0.00029093	0.00671081	0.05400043	8.98E-22	1.15E-56	1.14E-20	0.87342647	0.99307325
177.3	0.00029093	0.00671081	0.05400043	8.98E-22	1.15E-56	1.14E-20	0.87342647	0.99307325
177.4	0.00029093	0.00671081	0.05400043	8.98E-22	1.15E-56	1.14E-20	0.87342647	0.99307325
177.5	0.00029093	0.00671081	0.05400043	8.98E-22	1.15E-56	1.14E-20	0.87342647	0.99307325
177.6	0.00029093	0.00671081	0.05400043	8.98E-22	1.15E-56	1.14E-20	0.87342647	0.99307325
177.7	0.00029093	0.00671081	0.05400043	8.98E-22	1.15E-56	1.14E-20	0.87342647	0.99307325
177.8	0.00029093	0.00671081	0.05400043	8.98E-22	1.15E-56	1.14E-20	0.87342647	0.99307325
177.9	0.00029093	0.00671081	0.05400043	8.98E-22	1.15E-56	1.14E-20	0.87342647	0.99307325
178	0.00028945	0.00671499	0.05363627	8.86E-22	1.14E-56	1.11E-20	0.87184519	0.99161827
178.1	0.00028945	0.00671499	0.05363627	8.86E-22	1.14E-56	1.11E-20	0.87184519	0.99161827
178.2	0.00028945	0.00671499	0.05363627	8.86E-22	1.14E-56	1.11E-20	0.87184519	0.99161827
178.3	0.00028945	0.00671499	0.05363627	8.86E-22	1.14E-56	1.11E-20	0.87184519	0.99161827
178.4	0.00028945	0.00671499	0.05363627	8.86E-22	1.14E-56	1.11E-20	0.87184519	0.99161827
178.5	0.00028945	0.00671499	0.05363627	8.86E-22	1.14E-56	1.11E-20	0.87184519	0.99161827
178.6	0.00028945	0.00671499	0.05363627	8.86E-22	1.14E-56	1.11E-20	0.87184519	0.99161827
178.7	0.00028945	0.00671499	0.05363627	8.86E-22	1.14E-56	1.11E-20	0.87184519	0.99161827
178.8	0.00029423	0.00667976	0.05394263	9.28E-22	1.24E-56	1.23E-20	0.8686451	0.99095468
178.9	0.00029423	0.00667976	0.05394263	9.28E-22	1.24E-56	1.23E-20	0.8686451	0.99095468
179	0.00029423	0.00667976	0.05394263	9.28E-22	1.24E-56	1.23E-20	0.8686451	0.99095468
179.1	0.00029423	0.00667976	0.05394263	9.28E-22	1.24E-56	1.23E-20	0.8686451	0.99095468
179.2	0.00029423	0.00667976	0.05394263	9.28E-22	1.24E-56	1.23E-20	0.8686451	0.99095468
179.3	0.00029423	0.00667976	0.05394263	9.28E-22	1.24E-56	1.23E-20	0.8686451	0.99095468
179.4	0.00029338	0.00662316	0.053735	9.38E-22	1.17E-56	1.20E-20	0.86854304	0.98760534
179.5	0.00029338	0.00662316	0.053735	9.38E-22	1.17E-56	1.20E-20	0.86854304	0.98760534
179.6	0.00029338	0.00662316	0.053735	9.38E-22	1.17E-56	1.20E-20	0.86854304	0.98760534
179.7	0.00029338	0.00662316	0.053735	9.38E-22	1.17E-56	1.20E-20	0.86854304	0.98760534
179.8	0.00029338	0.00662316	0.053735	9.38E-22	1.17E-56	1.20E-20	0.86854304	0.98760534
179.9	0.00029338	0.00662316	0.053735	9.38E-22	1.17E-56	1.20E-20	0.86854304	0.98760534

225	0.00035979	0.00744301	0.05757221	7.72E-22	9.96E-57	1.61E-20	0.87520008	0.98149248
225.1	0.00035979	0.00744301	0.05757221	7.72E-22	9.96E-57	1.61E-20	0.87520008	0.98149248
225.2	0.00035979	0.00744301	0.05757221	7.72E-22	9.96E-57	1.61E-20	0.87520008	0.98149248
225.3	0.00035979	0.00744301	0.05757221	7.72E-22	9.96E-57	1.61E-20	0.87520008	0.98149248
225.4	0.00035979	0.00744301	0.05757221	7.72E-22	9.96E-57	1.61E-20	0.87520008	0.98149248

180.5	0.00029338	0.00662316	0.053735	9.38E-22	1.17E-56	1.20E-20	0.86854304	0.98760534
180.6	0.00029338	0.00662316	0.053735	9.38E-22	1.17E-56	1.20E-20	0.86854304	0.98760534
180.7	0.00029338	0.00662316	0.053735	9.38E-22	1.17E-56	1.20E-20	0.86854304	0.98760534
180.8	0.00029338	0.00662316	0.053735	9.38E-22	1.17E-56	1.20E-20	0.86854304	0.98760534
180.9	0.00029338	0.00662316	0.053735	9.38E-22	1.17E-56	1.20E-20	0.86854304	0.98760534
181	0.00029338	0.00662316	0.053735	9.38E-22	1.17E-56	1.20E-20	0.86854304	0.98760534
181.1	0.00029338	0.00662316	0.053735	9.38E-22	1.17E-56	1.20E-20	0.86854304	0.98760534
181.2	0.00029338	0.00662316	0.053735	9.38E-22	1.17E-56	1.20E-20	0.86854304	0.98760534
181.3	0.00029287	0.00660145	0.05433497	9.68E-22	1.22E-56	1.26E-20	0.86871232	0.9875698
181.4	0.00029287	0.00660145	0.05433497	9.68E-22	1.22E-56	1.26E-20	0.86871232	0.9875698
181.5	0.00029287	0.00660145	0.05433497	9.68E-22	1.22E-56	1.26E-20	0.86871232	0.9875698
181.6	0.00029287	0.00660145	0.05433497	9.68E-22	1.22E-56	1.26E-20	0.86871232	0.9875698
181.7	0.00029287	0.00660145	0.05433497	9.68E-22	1.22E-56	1.26E-20	0.86871232	0.9875698
181.8	0.00029287	0.00660145	0.05433497	9.68E-22	1.22E-56	1.26E-20	0.86871232	0.9875698
181.9	0.00029287	0.00660145	0.05433497	9.68E-22	1.22E-56	1.26E-20	0.86871232	0.9875698
182	0.00029287	0.00660145	0.05433497	9.68E-22	1.22E-56	1.26E-20	0.86871232	0.9875698
182.1	0.00029287	0.00660145	0.05433497	9.68E-22	1.22E-56	1.26E-20	0.86871232	0.9875698
182.2	0.00029287	0.00660145	0.05433497	9.68E-22	1.22E-56	1.26E-20	0.86871232	0.9875698
182.3	0.00029287	0.00660145	0.05433497	9.68E-22	1.22E-56	1.26E-20	0.86871232	0.9875698
182.4	0.00029287	0.00660145	0.05433497	9.68E-22	1.22E-56	1.26E-20	0.86871232	0.9875698
182.5	0.00029047	0.0066598	0.0546268	9.51E-22	1.15E-56	1.30E-20	0.86921561	0.98710363
182.6	0.00029047	0.0066598	0.0546268	9.51E-22	1.15E-56	1.30E-20	0.86921561	0.98710363
182.7	0.00029047	0.0066598	0.0546268	9.51E-22	1.15E-56	1.30E-20	0.86921561	0.98710363
182.8	0.00029047	0.0066598	0.0546268	9.51E-22	1.15E-56	1.30E-20	0.86921561	0.98710363
182.9	0.00029047	0.0066598	0.0546268	9.51E-22	1.15E-56	1.30E-20	0.86921561	0.98710363
183	0.00029047	0.0066598	0.0546268	9.51E-22	1.15E-56	1.30E-20	0.86921561	0.98710363
183.1	0.00029047	0.0066598	0.0546268	9.51E-22	1.15E-56	1.30E-20	0.86921561	0.98710363
183.2	0.00029047	0.0066598	0.0546268	9.51E-22	1.15E-56	1.30E-20	0.86921561	0.98710363
183.3	0.00029047	0.0066598	0.0546268	9.51E-22	1.15E-56	1.30E-20	0.86921561	0.98710363
183.4	0.00029047	0.0066598	0.0546268	9.51E-22	1.15E-56	1.30E-20	0.86921561	0.98710363
183.5	0.00029047	0.0066598	0.0546268	9.51E-22	1.15E-56	1.30E-20	0.86921561	0.98710363
183.6	0.00029047	0.0066598	0.0546268	9.51E-22	1.15E-56	1.30E-20	0.86921561	0.98710363
183.7	0.00029047	0.0066598	0.0546268	9.51E-22	1.15E-56	1.30E-20	0.86921561	0.98710363
183.8	0.00029047	0.0066598	0.0546268	9.51E-22	1.15E-56	1.30E-20	0.86921561	0.98710363
183.9	0.00029047	0.0066598	0.0546268	9.51E-22	1.15E-56	1.30E-20	0.86921561	0.98710363
184	0.00029047	0.0066598	0.0546268	9.51E-22	1.15E-56	1.30E-20	0.86921561	0.98710363
184.1	0.00029047	0.0066598	0.0546268	9.51E-22	1.15E-56	1.30E-20	0.86921561	0.98710363
184.2	0.00029047	0.0066598	0.0546268	9.51E-22	1.15E-56	1.30E-20	0.86921561	0.98710363
184.3	0.00029047	0.0066598	0.0546268	9.51E-22	1.15E-56	1.30E-20	0.86921561	0.98710363
184.4	0.00029047	0.0066598	0.0546268	9.51E-22	1.15E-56	1.30E-20	0.86921561	0.98710363
184.5	0.00029047	0.0066598	0.0546268	9.51E-22	1.15E-56	1.30E-20	0.86921561	0.98710363
184.6	0.00029047	0.0066598	0.0546268	9.51E-22	1.15E-56	1.30E-20	0.86921561	0.98710363
184.7	0.00029047	0.0066598	0.0546268	9.51E-22	1.15E-56	1.30E-20	0.86921561	0.98710363
184.8	0.00029047	0.0066598	0.0546268	9.51E-22	1.15E-56	1.30E-20	0.86921561	0.98710363
184.9	0.00029047	0.0066598	0.0546268	9.51E-22	1.15E-56	1.30E-20	0.86921561	0.98710363
185	0.00029002	0.00667865	0.05531852	9.61E-22	1.21E-56	1.37E-20	0.86164732	0.98525549
185.1	0.00029002	0.00667865	0.05531852	9.61E-22	1.21E-56	1.37E-20	0.86164732	0.98525549
185.2	0.00029002	0.00667865	0.05531852	9.61E-22	1.21E-56	1.37E-20	0.86164732	0.98525549
185.3	0.00029002	0.00667865	0.05531852	9.61E-22	1.21E-56	1.37E-20	0.86164732	0.98525549
185.4	0.00029002	0.00667865	0.05531852	9.61E-22	1.21E-56	1.37E-20	0.86164732	0.98525549
185.5	0.00029002	0.00667865	0.05531852	9.61E-22	1.21E-56	1.37E-20	0.86164732	0.98525549
185.6	0.00029002	0.00667865	0.05531852	9.61E-22	1.21E-56	1.37E-20	0.86164732	0.98525549
185.7	0.00029002	0.00667865	0.05531852	9.61E-22	1.21E-56	1.37E-20	0.86164732	0.98525549
185.8	0.00029002	0.00667865	0.05531852	9.61E-22	1.21E-56	1.37E-20	0.86164732	0.98525549
185.9	0.00029002	0.00667865	0.05531852	9.61E-22	1.21E-56	1.37E-20	0.86164732	0.98525549
186	0.00029002	0.00667865	0.05531852	9.61E-22	1.21E-56	1.37E-20	0.86164732	0.98525549
186.1	0.00029801	0.00667864	0.05549313	9.59E-22	1.25E-56	1.43E-20	0.85980424	0.98877718
186.2	0.00029801	0.00667864	0.05549313	9.59E-22	1.25E-56	1.43E-20	0.85980424	0.98877718
186.3	0.00029801	0.00667864	0.05549313	9.59E-22	1.25E-56	1.43E-20	0.85980424	0.98877718
186.4	0.00029801	0.00667864	0.05549313	9.59E-22	1.25E-56	1.43E-20	0.85980424	0.98877718
186.5	0.00029801	0.00667864	0.05549313	9.59E-22	1.25E-56	1.43E-20	0.85980424	0.98877718
186.6	0.00030412	0.00663339	0.05534676	9.32E-22	1.14E-56	1.47E-20	0.8589657	0.98455448
186.7	0.00030412	0.00663339	0.05534676	9.32E-22	1.14E-56	1.47E-20	0.8589657	0.98455448
186.8	0.00030412	0.00663339	0.05534676	9.32E-22	1.14E-56	1.47E-20	0.8589657	0.98455448
186.9	0.00030412	0.00663339	0.05534676	9.32E-22	1.14E-56	1.47E-20	0.8589657	0.98455448
187	0.00030412	0.00663339	0.05534676	9.32E-22	1.14E-56	1.47E-20	0.8589657	0.98455448
187.1	0.00030412	0.00663339	0.05534676	9.32E-22	1.14E-56	1.47E-20	0.8589657	0.98455448
187.2	0.00030412	0.00663339	0.05534676	9.32E-22	1.14E-56	1.47E-20	0.8589657	0.98455448
187.3	0.00030412	0.00663339	0.05534676	9.32E-22	1.14E-56	1.47E-20	0.8589657	0.98455448
187.4	0.00030412	0.00663339	0.05534676	9.32E-22	1.14E-56	1.47E-20	0.8589657	0.98455448
187.5	0.00030412	0.00663339	0.05534676	9.32E-22	1.14E-56	1.47E-20	0.8589657	0.98455448
187.6	0.00030412	0.00663339	0.05534676	9.32E-22	1.14E-56	1.47E-20	0.8589657	0.98455448
187.7	0.00030412	0.00663339	0.05534676	9.32E-22	1.14E-56	1.47E-20	0.8589657	0.98455448
187.8	0.00030412	0.00663339	0.05534676	9.32E-22	1.14E-56	1.47E-20	0.8589657	0.98455448
187.9	0.00030412	0.00663339	0.05534676	9.32E-22	1.14E-56	1.47E-20	0.8589657	0.98455448
188	0.00030412	0.00663339	0.05534676	9.32E-22	1.14E-56	1.47E-20	0.8589657	0.98455448
188.1	0.00030412	0.00663339	0.05534676	9.32E-22	1.14E-56	1.47E-20	0.8589657	0.98455448
188.2	0.00030412	0.00663339	0.05534676	9.32E-22	1.14E-56	1.47E-20	0.8589657	0.98455448
188.3	0.00030412	0.00663339	0.05534676	9.32E-22	1.14E-56	1.47E-20	0.8589657	0.98455448
188.4	0.00030412	0.00663339	0.05534676	9.32E-22	1.14E-56	1.47E-20	0.8589657	0.98455448
188.5	0.00030576	0.0067193	0.05575363	8.94E-22	1.11E-56	1.49E-20	0.86079918	0.98279969
188.6	0.00030576	0.0067193	0.05575363	8.94E-22	1.11E-56	1.49E-20	0.86079918	0.98279969
188.7	0.00030576	0.0067193	0.05575363	8.94E-22	1.11E-56	1.49E-20	0.86079918	0.98279969
188.8	0.00030576	0.0067193	0.05575363	8.94E-22	1.11E-56	1.49E-20	0.86079918	0.98279969
188.9	0.00030576	0.0067193	0.05575363	8.94E-22	1.11E-56	1.49E-20	0.86079918	0.98279969
189	0.00030576	0.0067193	0.05575363	8.94E-22	1.11E-56	1.49E-20	0.86079918	0.98279969
189.1	0.00030576	0.0067193	0.05575363	8.94E-22	1.11E-56	1.49E-20	0.86079918	0.98279969
189.2	0.00030576	0.0067193	0.05575363	8.94E-22	1.11E-56	1.49E-20	0.86079918	0.98279969
189.3	0.00030576	0.0067193	0.05575363	8.94E-22	1.11E-56	1.49E-20	0.86079918	0.98279969
189.4	0.00030576	0.0067193	0.05575363	8.94E-22	1.11E-56	1.49E-20	0.86079918	0.98279969
189.5	0.00030576	0.0067193	0.05575363	8.94E-22	1.11E-56	1.49E-20	0.86079918	0.98279969
189.6	0.00030576	0.0067193	0.05575363	8.94E-22	1.11E-56	1.49E-20	0.86079918	0.98279969
189.7	0.00030576	0.0067193	0.05575363	8.94E-22	1.11E-56	1.49E-20	0.86079918	0.98279969
189.8	0.00030576	0.0067193	0.05575363	8.94E-22	1.11E-56	1.49E-20	0.86079918	0.98279969
189.9	0.00030576	0.0067193	0.05575363	8.94E-22	1.11E-56	1.49E-20	0.86079918	0.98279969
190	0.00030576	0.0067193	0.05575363	8.94E-22	1.11E-56	1.49E-20	0.86079918	0.98279969
190.1	0.00030576	0.0067193	0.05575363	8.94E-22	1.11E-56	1.49E-20	0.86079918	0.98279969
190.2	0.00030576	0.0067193	0.05575363	8.94E-22	1.11E-56	1.49E-20	0.86079918	0.98279969
190.3	0.00030576	0.0067193	0.05575363	8.94E-22	1.11E-56	1.49E-20	0.86079918	0.98279969
190.4	0.00030576	0.0067193	0.05575363	8.94E-22	1.11E-56	1.49E-20	0.86079918	0.98279969
190.5	0.00030576	0.0067193	0.05575363	8.94E-22	1.11E-56	1.49E-20	0.86079918	0.98279969
190.6	0.00030576	0.0067193	0.05575363	8.94E-22	1.11E-56	1.49E-20	0.86079918	0.98279969
190.7	0.00030576	0.0067193	0.05575363	8.94E-22	1.11E-56	1.49E-20	0.86079918	0.98279969
190.8	0.00030576	0.0067193	0.05575363	8.94E-22	1.11E-56	1.49E-20	0.86079918	0.98279969
190.9	0.00030576	0.0067193	0.05575363	8.94E-22	1.11E-56	1.49E-20	0.86079918	0.98279969
191	0.00031055	0.00680798	0.05662705	8.51E-22	1.08E-56	1.56E-20	0.86221451	0.9759941
191.1	0.00031055	0.00680798	0.05662705	8.51E-22	1.08E-56	1.56E-20	0.86221451	0.9759941
191.2	0.00031055	0.00680798	0.05662705	8.51E-22	1.08E-56	1.56E-20	0.86221451	0.9759941
191.3	0.00031055	0.00680798	0.05662705	8.51E-22	1.08E-56	1.56E-20	0.86221451	0.9759941
191.4	0.00031055	0.00680798	0.05662705	8.51E-22	1.08E-56	1.56E-20	0.86221451	0.9759941
191.5	0.00031055	0.00680798	0.05662705	8.51E-22	1.08E-56	1.56E-20	0.86221451	0.9759941
191.6	0.00031055	0.00680798	0.05662705	8.51E-22	1.08E-56	1.56E-20	0.86221451	0.9759941
191.7	0.00031055	0.00680798	0.05662705	8.51E-22	1.08E-56	1.56E-20	0.86221451	0.9759941
191.8	0.00031055	0.00680798	0.05662705	8.51E-22	1.08E-56	1.56E-20	0.86221451	0.9759941
191.9	0.00031055	0.00680798	0.05662705	8.51E-22	1.08E-56	1.56E-20	0.86221451	0.9759941
192	0.00031055	0.00680798	0.05662705	8.51E-22	1.08E-56	1.56E-20	0.86221451	0.9759941
192.1	0.00031055	0.00680798	0.05662705	8.51E-22	1.08E-56	1.56E-20	0.86221451	0.9759941
192.2	0.00031055	0.00680798	0.05662705	8.51E-22	1.08E-56	1.56E-20	0.86221451	0.9759941
192.3	0.00031055	0.00680798	0.05662705	8.51E-22	1.08E-56	1.56E-20	0.86221451	0.9759941
192.4	0.00031055	0.00680798	0.05662705	8.51E-22	1.08E-56	1.56E-20	0.86221451	0.9759941
192.5	0.00031055	0.00680798	0.05662705	8.51E-22	1.08E-56	1.56E-20	0.86221451	0.9759941
192.6	0.00031055	0.00680798	0.05662705	8.51E-22	1.08E-56	1.56E-20	0.86221451	0.9759941
192.7	0.00031055	0.00680798	0.05662705	8.51E-22	1.08E-56	1.56E-20	0.86221451	0.9759941
192.8	0.00031809	0.00690167	0.05626402	8.31E-22	1.05E-56	1.55E-20	0.85933914	0.97774782
192.9	0.00031809	0.00690167	0.05626402	8.31E-22	1.05E-56	1.55E-20	0.85933914	0.97774782
193	0.00031809	0.00690167	0.05626402	8.31E-22	1.05E-56	1.55E-20	0.85933914	0.97774782
193.1	0.00031809	0.00690167	0.05626402	8.31E-22	1.05E-56	1.55E-20	0.85933914	0.97774782
193.2	0.00031809	0.00690167	0.05626402	8.31E-22	1.05E-56	1.55E-20	0.85933914	0.97774782
193.3	0.00031809	0.00690167	0.05626402	8.31E-22	1.05E-56	1.55E-20	0.85933914	0.97774782
193.4	0.00031809	0.00690167	0.05626402	8.31E-22	1.05E-56	1.55E-20	0.85933914	0.97774782
193.5	0.00031809	0.00690167	0.05626402	8.31E-22	1.05E-56	1.55E-20	0.85933914	0.97774782
193.6	0.00031809	0.00690167	0.05626402	8.31E-22	1.05E-56	1.55E-20	0.85933914	0.97774782
193.7	0.00031809	0.00690167	0.05626402	8.31E-22	1.05E-56	1.55E-20	0.85933914	0.97774782
193.8	0.00031809	0.00690167	0.05626402	8.31E-22	1.05E-56	1.55E-20	0.85933914	0.97774782
193.9	0.00031809	0.00690167	0.05626402	8.31E-22	1.05E-56	1.55E-20	0.85933914	0.97774782
194	0.00031809	0.00690167	0.05626402	8.31E-22	1.05E-56	1.55E-20	0.85933914	0.97774782
194.1	0.00031809	0.00690167	0.05626402	8.31E-22	1.05E-56	1.55E-20	0.85933914	0.97774782
194.2	0.00031809	0.00690167	0.05626402	8.31E-22	1.05E-56	1.55E-20	0.85933914	0.97774782
194.3	0.00031809	0.00690167	0.05626402	8.31E-22	1.05E-56	1.55E-20	0.85933914	0.97774782
194.4	0.00031809	0.00690167	0.05626402	8.31E-22	1.05E-56	1.55E-20	0.85933914	0.97774782
194.5	0.00031809	0.00690167	0.05626402	8.31E-22	1.05E-56	1.55E-20	0.85933914	0.97774782
194.6	0.00031809	0.00690167	0.05626402	8.31E-22	1.05E-56	1.55E-20	0.85933914	0.97774782
194.7	0.00031809	0.00690167	0.05626402	8.31E-22	1.05E-56	1.55E-20	0.85933914	0.97774782
194.8	0.00031809	0.00690167	0.05626402	8.31E-22	1.05E-56	1.55E-20	0.85933914	0.97774782
194.9	0.00031809	0.00690167	0.05626402	8.31E-22	1.05E-56	1.55E-20	0.85933914	0.97774782
195.5	0.00031809	0.00690167	0.05626402	8.31E-22	1.05E-56	1.55E-20	0.85933914	0.97774782
195.6	0.00031809	0.00690167	0.05626402	8.31E-22	1.05E-56	1.55E-20	0.85933914	0.97774782
195.7	0.00031809	0.00690167	0.05626402	8.31E-22	1.05E-56	1.55E-20	0.85933914	0.97774782
195.8	0.00031809	0.00690167	0.05626402	8.31E-22	1.05E-56	1.55E-20	0.85933914	0.97774782
195.9	0.00031809	0.00690167	0.05626402	8.31E-22	1.05E-56	1.55E-20	0.85933914	0.97774782
196	0.00031809	0.00690167	0.05626402	8.31E-22	1.05E-56	1.55E-20	0.85933914	0.97774782
196.1	0.00031809	0.00690167	0.05626402	8.31E-22	1.05E-56	1.55E-20	0.85933914	0.97774782
196.2	0.00031809	0.00690167	0.05626402	8.31E-22	1.05E-56	1.55E-20	0.85933914	0.97774782
196.3	0.00031809	0.00690167	0.05626402	8.31E-22	1.05E-56	1.55E-20	0.85933914	0.97774782
196.4	0.00031809	0.00690167	0.05626402	8.31E-22	1.05E-56	1.55E-20	0.85933914	0.97774782
196.5	0.00031809	0.00690167	0.05626402	8.31E-22	1.05E-56	1.55E-20	0.85933914	0.97774782
196.6	0.00031809	0.00690167	0.05626402	8.31E-22	1.05E-56	1.55E-20	0.85933914	0.97774782
196.7	0.00031809	0.00690167	0.05626402	8.31E-22	1.05E-56	1.55E-20	0.85933914	0.97774782
196.8	0.00031809	0.00690167	0.05626402	8.31E-22	1.05E-56	1.55E-20	0.85933914	0.97774782
196.9	0.00031809	0.00690167	0.05626402	8.31E-22	1.05E-56	1.55E-20	0.85933914	0.97774782
197	0.00031809	0.00690167	0.05626402	8.31E-22	1.05E-56	1.55E-20	0.85933914	0.97774782
197.1	0.00031809	0.00690167	0.05626402	8.31E-22	1.05E-56	1.55E-20	0.85933914	0.97774782
197.2	0.00031809	0.00690167	0.05626402	8.31E-22	1.05E-56	1.55E-20	0.85933914	0.97774782
197.3	0.00031809	0.00690167	0.05626402	8.31E-22	1.05E-56	1.55E-20	0.85933914	0.97774782
197.4	0.00031809	0.00690167	0.05626402	8.31E-22	1.05E-56	1.55E-20	0.85933914	0.97774782
197.5	0.00031809	0.00690167	0.05626402	8.31E-22	1.05E-56	1.55E-20	0.85933914	0.97774782
197.6	0.00031809	0.00690167	0.05626402	8.31E-22	1.05E-56	1.55E-20	0.85933914	0.97774782
197.7	0.00031809	0.00690167	0.05626402	8.31E-22	1.05E-56	1.55E-20	0.85933914	0.97774782
197.8	0.00031809	0.00690167	0.05626402	8.31E-22	1.05E-56	1.55E-20	0.85933914	0.97774782
197.9	0.00031809	0.00690167	0.05626402	8.31E-22	1.05E-56	1.55E-20	0.85933914	0.97774782
198	0.00031809	0.00690167	0.05626402	8.31E-22	1.05E-56	1.55E-20	0.85933914	0.97774782
198.1	0.00031809	0.00690167	0.05626402	8.31E-22	1.05E-56	1.55E-20	0.85933914	0.97774782
198.2	0.00031809	0.00690167	0.05626402	8.31E-22	1.05E-56	1.55E-20	0.85933914	0.97774782
198.3	0.00031809	0.00690167	0.05626402	8.31E-22	1.05E-56	1.55E-20	0.85933914	0.97774782
198.4	0.00031809	0.00690167	0.05626402	8.31E-22	1.05E-56	1.55E-20	0.85933914	0.97774782
198.5	0.00031809	0.00690167	0.05626402	8.31E-22	1.05E-56	1.55E-20	0.85933914	0.97774782
198.6	0.00031809	0.00690167	0.05626402	8.31E-22	1.05E-56	1.55E-20	0.85933914	0.97774782
198.7	0.00031809	0.00690167	0.05626402	8.31E-22	1.05E-56	1.55E-20	0.85933914	0.97774782
198.8	0.00031809	0.00690167	0.05626402	8.31E-22	1.05E-56	1.55E-20	0.85933914	0.97774782
198.9	0.00031809	0.00690167	0.05626402	8.31E-22	1.05E-56	1.55E-20	0.85933914	0.97774782
199	0.00031809	0.00690167	0.05626402	8.31E-22	1.05E-56	1.55E-20	0.85933914	0.97774782
199.1	0.00031809	0.00690167	0.05626402	8.31E-22	1.05E-56	1.55E-20	0.85933914	0.97774782
199.2	0.00031809	0.00690167	0.05626402	8.31E-22	1.05E-56	1.55E-20	0.85933914	0.97774782
199.3	0.00031809	0.00690167	0.05626402	8.31E-22	1.05E-56	1.55E-20	0.85933914	0.97774782
199.4	0.00031809	0.00690167	0.05626402	8.31E-22	1.05E-56	1.55E-20	0.85933914	0.97774782
199.5	0.00031809	0.00690167	0.05626402	8.31E-22	1.05E-56	1.55E-20	0.85933914	0.97774782
199.6	0.00031809	0.00690167	0.05626402	8.31E-22	1.05E-56	1.55E-20	0.85933914	0.97774782
199.7	0.00031809	0.00690167	0.05626402	8.31E-22	1.05E-56	1.55E-20	0.85933914	0.97774782
199.8	0.00031809	0.00690167	0.05626402	8.31E-22	1.05E-56	1.55E-20	0.85933914	0.97774782
199.9	0.00031809	0.00690167	0.05626402	8.31E-22	1.05E-56	1.55E-20	0.85933914	0.97774782
200	0.00031809	0.00690167	0.05626402	8.31E-22	1.05E-56	1.55E-20	0.85933914	0.97774782
200.1	0.00031809	0.00690167	0.05626402	8.31E-22	1.05E-56	1.55E-20	0.85933914	0.97774782
200.2	0.00031809	0.00690167	0.05626402	8.31E-22	1.05E-56	1.55E-20	0.85933914	0.97774782
200.3	0.00031809	0.00690167	0.05626402	8.31E-22	1.05E-56	1.55E-20	0.85933914	0.97774782
200.4	0.00031809	0.00690167	0.05626402	8.31E-22	1.05E-56	1.55E-20	0.85933914	0.97774782
200.5	0.00031809	0.00690167	0.05626402	8.31E-22	1.05E-56	1.55E-20	0.85933914	0.97774782
200.6	0.00031809	0.00690167	0.05626402	8.31E-22	1.05E-56	1.55E-20	0.85933914	0.97774782
200.7	0.00031809	0.00690167	0.05626402	8.31E-22	1.05E-56	1.55E-20	0.85933914	0.97774782
200.8	0.00032204	0.00694839	0.05603393	8.37E-22	1.06E-56	1.52E-20	0.85427775	0.97908414
200.9	0.00032204	0.00694839	0.05603393	8.37E-22	1.06E-56	1.52E-20	0.85427775	0.97908414
201	0.00032204	0.00694839	0.05603393	8.37E-22	1.06E-56	1.52E-20	0.85427775	0.97908414
201.1	0.00032204	0.00694839	0.05603393	8.37E-22	1.06E-56	1.52E-20	0.85427775	0.97908414
201.2	0.00032204	0.00694839	0.05603393	8.37E-22	1.06E-56	1.52E-20	0.85427775	0.97908414
201.3	0.00032204	0.00694839	0.05603393	8.37E-22	1.06E-56	1.52E-20	0.85427775	0.97908414
201.4	0.00032204	0.00694839	0.05603393	8.37E-22	1.06E-56	1.52E-20	0.85427775	0.97908414
201.5	0.00032204	0.00694839	0.05603393	8.37E-22	1.06E-56	1.52E-20	0.85427775	0.97908414
201.6	0.00032204	0.00694839	0.05603393	8.37E-22	1.06E-56	1.52E-20	0.85427775	0.97908414
201.7	0.00032204	0.00694839	0.05603393	8.37E-22	1.06E-56	1.52E-20	0.85427775	0.97908414
201.8	0.00032204	0.00694839	0.05603393	8.37E-22	1.06E-56	1.52E-20	0.85427775	0.97908414
201.9	0.00032204	0.00694839	0.05603393	8.37E-22	1.06E-56	1.52E-20	0.85427775	0.97908414
202	0.00032204	0.00694839	0.05603393	8.37E-22	1.06E-56	1.52E-20	0.85427775	0.97908414
202.1	0.00032204	0.00694839	0.05603393	8.37E-22	1.06E-56	1.52E-20	0.85427775	0.97908414
202.2	0.00032204	0.00694839	0.05603393	8.37E-22	1.06E-56	1.52E-20	0.85427775	0.97908414
202.3	0.00032204	0.00694839	0.05603393	8.37E-22	1.06E-56	1.52E-20	0.85427775	0.97908414
202.4	0.00032204	0.00694839	0.05603393	8.37E-22	1.06E-56	1.52E-20	0.85427775	0.97908414
202.5	0.00032204	0.00694839	0.05603393	8.37E-22	1.06E-56	1.52E-20	0.85427775	0.97908414
202.6	0.00032204	0.00694839	0.05603393	8.37E-22	1.06E-56	1.52E-20	0.85427775	0.97908414
202.7	0.00032204	0.00694839	0.05603393	8.37E-22	1.06E-56	1.52E-20	0.85427775	0.97908414
202.8	0.00032204	0.00694839	0.05603393	8.37E-22	1.06E-56	1.52E-20	0.85427775	0.97908414
202.9	0.00032204	0.00694839	0.05603393	8.37E-22	1.06E-56	1.52E-20	0.85427775	0.97908414
203	0.00032204	0.00694839	0.05603393	8.37E-22	1.06E-56	1.52E-20	0.85427775	0.97908414
203.1	0.00032204	0.00694839	0.05603393	8.37E-22	1.06E-56	1.52E-20	0.85427775	0.97908414
203.2	0.00032204	0.00694839	0.05603393	8.37E-22	1.06E-56	1.52E-20	0.85427775	0.97908414
203.3	0.00032204	0.00694839	0.05603393	8.37E-22	1.06E-56	1.52E-20	0.85427775	0.97908414
203.4	0.00032204	0.00694839	0.05603393	8.37E-22	1.06E-56	1.52E-20	0.85427775	0.97908414
203.5	0.00032204	0.00694839	0.05603393	8.37E-22	1.06E-56	1.52E-20	0.85427775	0.97908414
203.6	0.00032204	0.00694839	0.05603393	8.37E-22	1.06E-56	1.52E-20	0.85427775	0.97908414
203.7	0.00032204	0.00694839	0.05603393	8.37E-22	1.06E-56	1.52E-20	0.85427775	0.97908414
203.8	0.00032204	0.00694839	0.05603393	8.37E-22	1.06E-56	1.52E-20	0.85427775	0.97908414
203.9	0.00032204	0.00694839	0.05603393	8.37E-22	1.06E-56	1.52E-20	0.85427775	0.97908414
204	0.00032204	0.00694839	0.05603393	8.37E-22	1.06E-56	1.52E-20	0.85427775	0.97908414
204.1	0.00032204	0.00694839	0.05603393	8.37E-22	1.06E-56	1.52E-20	0.85427775	0.97908414
204.2	0.00032204	0.00694839	0.05603393	8.37E-22	1.06E-56	1.52E-20	0.85427775	0.97908414
204.3	0.00032204	0.00694839	0.05603393	8.37E-22	1.06E-56	1.52E-20	0.85427775	0.97908414
204.4	0.00032204	0.00694839	0.05603393	8.37E-22	1.06E-56	1.52E-20	0.85427775	0.97908414
204.5	0.00032204	0.00694839	0.05603393	8.37E-22	1.06E-56	1.52E-20	0.85427775	0.97908414
204.6	0.00032204	0.00694839	0.05603393	8.37E-22	1.06E-56	1.52E-20	0.85427775	0.97908414
204.7	0.00032204	0.00694839	0.05603393	8.37E-22	1.06E-56	1.52E-20	0.85427775	0.97908414
204.8	0.00032204	0.00694839	0.05603393	8.37E-22	1.06E-56	1.52E-20	0.85427775	0.97908414
204.9	0.00032204	0.00694839	0.05603393	8.37E-22	1.06E-56	1.52E-20	0.85427775	0.97908414
205	0.00032204	0.00694839	0.05603393	8.37E-22	1.06E-56	1.52E-20	0.85427775	0.97908414
205.1	0.00032204	0.00694839	0.05603393	8.37E-22	1.06E-56	1.52E-20	0.85427775	0.97908414
205.2	0.00032204	0.00694839	0.05603393	8.37E-22	1.06E-56	1.52E-20	0.85427775	0.97908414
205.3	0.00033248	0.00714029	0.055439	7.98E-22	1.05E-56	1.54E-20	0.86048427	0.98435647
205.4	0.00033248	0.00714029	0.055439	7.98E-22	1.05E-56	1.54E-20	0.86048427	0.98435647
205.5	0.00033248	0.00714029	0.055439	7.98E-22	1.05E-56	1.54E-20	0.86048427	0.98435647
205.6	0.00033248	0.00714029	0.055439	7.98E-22	1.05E-56	1.54E-20	0.86048427	0.98435647
205.7	0.00033248	0.00714029	0.055439	7.98E-22	1.05E-56	1.54E-20	0.86048427	0.98435647
205.8	0.00033248	0.00714029	0.055439	7.98E-22	1.05E-56	1.54E-20	0.86048427	0.98435647
205.9	0.00033248	0.00714029	0.055439	7.98E-22	1.05E-56	1.54E-20	0.86048427	0.98435647
206	0.00033248	0.00714029	0.055439	7.98E-22	1.05E-56	1.54E-20	0.86048427	0.98435647
206.1	0.00033248	0.00714029	0.055439	7.98E-22	1.05E-56	1.54E-20	0.86048427	0.98435647
206.2	0.00033248	0.00714029	0.055439	7.98E-22	1.05E-56	1.54E-20	0.86048427	0.98435647
206.3	0.00033248	0.00714029	0.055439	7.98E-22	1.05E-56	1.54E-20	0.86048427	0.98435647
206.4	0.00033248	0.00714029	0.055439	7.98E-22	1.05E-56	1.54E-20	0.86048427	0.98435647
206.5	0.00033248	0.00714029	0.055439	7.98E-22	1.05E-56	1.54E-20	0.86048427	0.98435647
206.6	0.00033248	0.00714029	0.055439	7.98E-22	1.05E-56	1.54E-20	0.86048427	0.98435647
206.7	0.00033248	0.00714029	0.055439	7.98E-22	1.05E-56	1.54E-20	0.86048427	0.98435647
206.8	0.00033248	0.00714029	0.055439	7.98E-22	1.05E-56	1.54E-20	0.86048427	0.98435647
206.9	0.00033248	0.00714029	0.055439	7.98E-22	1.05E-56	1.54E-20	0.86048427	0.98435647
207	0.00033248	0.00714029	0.055439	7.98E-22	1.05E-56	1.54E-20	0.86048427	0.98435647
207.1	0.00033248	0.00714029	0.055439	7.98E-22	1.05E-56	1.54E-20	0.86048427	0.98435647
207.2	0.00033248	0.00714029	0.055439	7.98E-22	1.05E-56	1.54E-20	0.86048427	0.98435647
207.3	0.00033248	0.00714029	0.055439	7.98E-22	1.05E-56	1.54E-20	0.86048427	0.98435647
207.4	0.00033248	0.00714029	0.055439	7.98E-22	1.05E-56	1.54E-20	0.86048427	0.98435647
207.5	0.00033248	0.00714029	0.055439	7.98E-22	1.05E-56	1.54E-20	0.86048427	0.98435647
207.6	0.00033248	0.00714029	0.055439	7.98E-22	1.05E-56	1.54E-20	0.86048427	0.98435647
207.7	0.00033248	0.00714029	0.055439	7.98E-22	1.05E-56	1.54E-20	0.86048427	0.98435647
207.8	0.00033248	0.00714029	0.055439	7.98E-22	1.05E-56	1.54E-20	0.86048427	0.98435647
207.9	0.00033248	0.00714029	0.055439	7.98E-22	1.05E-56	1.54E-20	0.86048427	0.98435647
208	0.00033248	0.00714029	0.055439	7.98E-22	1.05E-56	1.54E-20	0.86048427	0.98435647
208.1	0.00033248	0.00714029	0.055439	7.98E-22	1.05E-56	1.54E-20	0.86048427	0.98435647
208.2	0.00033248	0.00714029	0.055439	7.98E-22	1.05E-56	1.54E-20	0.86048427	0.98435647
208.3	0.00033248	0.00714029	0.055439	7.98E-22	1.05E-56	1.54E-20	0.86048427	0.98435647
208.4	0.00033248	0.00714029	0.055439	7.98E-22	1.05E-56	1.54E-20	0.86048427	0.98435647
208.5	0.00033248	0.00714029	0.055439	7.98E-22	1.05E-56	1.54E-20	0.86048427	0.98435647
208.6	0.00033248	0.00714029	0.055439	7.98E-22	1.05E-56	1.54E-20	0.86048427	0.98435647
208.7	0.00033248	0.00714029	0.055439	7.98E-22	1.05E-56	1.54E-20	0.86048427	0.98435647
208.8	0.00033248	0.00714029	0.055439	7.98E-22	1.05E-56	1.54E-20	0.86048427	0.98435647
208.9	0.00033248	0.00714029	0.055439	7.98E-22	1.05E-56	1.54E-20	0.86048427	0.98435647
209	0.00033248	0.00714029	0.055439	7.98E-22	1.05E-56	1.54E-20	0.86048427	0.98435647
209.1	0.00033248	0.00714029	0.055439	7.98E-22	1.05E-56	1.54E-20	0.86048427	0.98435647
209.2	0.00033248	0.00714029	0.055439	7.98E-22	1.05E-56	1.54E-20	0.86048427	0.98435647
209.3	0.00033248	0.00714029	0.055439	7.98E-22	1.05E-56	1.54E-20	0.86048427	0.98435647
209.4	0.00033248	0.00714029	0.055439	7.98E-22	1.05E-56	1.54E-20	0.86048427	0.98435647
209.5	0.00033248	0.00714029	0.055439	7.98E-22	1.05E-56	1.54E-20	0.86048427	0.98435647
209.6	0.00033248	0.00714029	0.055439	7.98E-22	1.05E-56	1.54E-20	0.86048427	0.98435647
209.7	0.00033248	0.00714029	0.055439	7.98E-22	1.05E-56	1.54E-20	0.86048427	0.98435647
209.8	0.00033248	0.00714029	0.055439	7.98E-22	1.05E-56	1.54E-20	0.86048427	0.98435647
209.9	0.00033248	0.00714029	0.055439	7.98E-22	1.05E-56	1.54E-20	0.86048427	0.98435647
210.5	0.00033248	0.00714029	0.055439	7.98E-22	1.05E-56	1.54E-20	0.86048427	0.98435647
210.6	0.00033248	0.00714029	0.055439	7.98E-22	1.05E-56	1.54E-20	0.86048427	0.98435647
210.7	0.00033248	0.00714029	0.055439	7.98E-22	1.05E-56	1.54E-20	0.86048427	0.98435647
210.8	0.00033248	0.00714029	0.055439	7.98E-22	1.05E-56	1.54E-20	0.86048427	0.98435647
210.9	0.00033248	0.00714029	0.055439	7.98E-22	1.05E-56	1.54E-20	0.86048427	0.98435647
211	0.00033248	0.00714029	0.055439	7.98E-22	1.05E-56	1.54E-20	0.86048427	0.98435647
211.1	0.00033248	0.00714029	0.055439	7.98E-22	1.05E-56	1.54E-20	0.86048427	0.98435647
211.2	0.00033248	0.00714029	0.055439	7.98E-22	1.05E-56	1.54E-20	0.86048427	0.98435647
211.3	0.00033248	0.00714029	0.055439	7.98E-22	1.05E-56	1.54E-20	0.86048427	0.98435647
211.4	0.00033248	0.00714029	0.055439	7.98E-22	1.05E-56	1.54E-20	0.86048427	0.98435647
211.5	0.00033248	0.00714029	0.055439	7.98E-22	1.05E-56	1.54E-20	0.86048427	0.98435647
211.6	0.00033248	0.00714029	0.055439	7.98E-22	1.05E-56	1.54E-20	0.86048427	0.98435647
211.7	0.00033248	0.00714029	0.055439	7.98E-22	1.05E-56	1.54E-20	0.86048427	0.98435647
211.8	0.00033248	0.00714029	0.055439	7.98E-22	1.05E-56	1.54E-20	0.86048427	0.98435647
211.9	0.00033248	0.00714029	0.055439	7.98E-22	1.05E-56	1.54E-20	0.86048427	0.98435647
212	0.00033248	0.00714029	0.055439	7.98E-22	1.05E-56	1.54E-20	0.86048427	0.98435647
212.1	0.00033248	0.00714029	0.055439	7.98E-22	1.05E-56	1.54E-20	0.86048427	0.98435647
212.2	0.00033248	0.00714029	0.055439	7.98E-22	1.05E-56	1.54E-20	0.86048427	0.98435647
212.3	0.00033248	0.00714029	0.055439	7.98E-22	1.05E-56	1.54E-20	0.86048427	0.98435647
212.4	0.00033248	0.00714029	0.055439	7.98E-22	1.05E-56	1.54E-20	0.86048427	0.98435647
212.5	0.00033248	0.00714029	0.055439	7.98E-22	1.05E-56	1.54E-20	0.86048427	0.98435647
212.6	0.00033248	0.00714029	0.055439	7.98E-22	1.05E-56	1.54E-20	0.86048427	0.98435647
212.7	0.00033248	0.00714029	0.055439	7.98E-22	1.05E-56	1.54E-20	0.86048427	0.98435647
212.8	0.00033248	0.00714029	0.055439	7.98E-22	1.05E-56	1.54E-20	0.86048427	0.98435647
212.9	0.00033248	0.00714029	0.055439	7.98E-22	1.05E-56	1.54E-20	0.86048427	0.98435647
213	0.00033248	0.00714029	0.055439	7.98E-22	1.05E-56	1.54E-20	0.86048427	0.98435647
213.1	0.00033248	0.00714029	0.055439	7.98E-22	1.05E-56	1.54E-20	0.86048427	0.98435647
213.2	0.00033248	0.00714029	0.055439	7.98E-22	1.05E-56	1.54E-20	0.86048427	0.98435647
213.3	0.00033248	0.00714029	0.055439	7.98E-22	1.05E-56	1.54E-20	0.86048427	0.98435647
213.4	0.00033248	0.00714029	0.055439	7.98E-22	1.05E-56	1.54E-20	0.86048427	0.98435647
213.5	0.00033248	0.00714029	0.055439	7.98E-22	1.05E-56	1.54E-20	0.86048427	0.98435647
213.6	0.00033248	0.00714029	0.055439	7.98E-22	1.05E-56	1.54E-20	0.86048427	0.98435647
213.7	0.00033248	0.00714029	0.055439	7.98E-22	1.05E-56	1.54E-20	0.86048427	0.98435647
213.8	0.00033248	0.00714029	0.055439	7.98E-22	1.05E-56	1.54E-20	0.86048427	0.98435647
213.9	0.00033248	0.00714029	0.055439	7.98E-22	1.05E-56	1.54E-20	0.86048427	0.98435647
214	0.00033248	0.00714029	0.055439	7.98E-22	1.05E-56	1.54E-20	0.86048427	0.98435647
214.1	0.00033248	0.00714029	0.055439	7.98E-22	1.05E-56	1.54E-20	0.86048427	0.98435647
214.2	0.00033248	0.00714029	0.055439	7.98E-22	1.05E-56	1.54E-20	0.86048427	0.98435647
214.3	0.00033248	0.00714029	0.055439	7.98E-22	1.05E-56	1.54E-20	0.86048427	0.98435647
214.4	0.00033248	0.00714029	0.055439	7.98E-22	1.05E-56	1.54E-20	0.86048427	0.98435647
214.5	0.00033248	0.00714029	0.055439	7.98E-22	1.05E-56	1.54E-20	0.86048427	0.98435647
214.6	0.00033248	0.00714029	0.055439	7.98E-22	1.05E-56	1.54E-20	0.86048427	0.98435647
214.7	0.00033248	0.00714029	0.055439	7.98E-22	1.05E-56	1.54E-20	0.86048427	0.98435647
214.8	0.00033248	0.00714029	0.055439	7.98E-22	1.05E-56	1.54E-20	0.86048427	0.98435647
214.9	0.00033248	0.00714029	0.055439	7.98E-22	1.05E-56	1.54E-20	0.86048427	0.98435647
215	0.00033248	0.00714029	0.055439	7.98E-22	1.05E-56	1.54E-20	0.86048427	0.98435647
215.1	0.00033248	0.00714029	0.055439	7.98E-22	1.05E-56	1.54E-20	0.86048427	0.98435647
215.2	0.00033248	0.00714029	0.055439	7.98E-22	1.05E-56	1.54E-20	0.86048427	0.98435647
215.3	0.00033248	0.00714029	0.055439	7.98E-22	1.05E-56	1.54E-20	0.86048427	0.98435647
215.4	0.00033248	0.00714029	0.055439	7.98E-22	1.05E-56	1.54E-20	0.86048427	0.98435647
215.5	0.00033248	0.00714029	0.055439	7.98E-22	1.05E-56	1.54E-20	0.86048427	0.98435647
215.6	0.00033248	0.00714029	0.055439	7.98E-22	1.05E-56	1.54E-20	0.86048427	0.98435647
215.7	0.00033248	0.00714029	0.055439	7.98E-22	1.05E-56	1.54E-20	0.86048427	0.98435647
215.8	0.00033248	0.00714029	0.055439	7.98E-22	1.05E-56	1.54E-20	0.86048427	0.98435647
215.9	0.00033248	0.00714029	0.055439	7.98E-22	1.05E-56	1.54E-20	0.86048427	0.98435647
216	0.00033248	0.00714029	0.055439	7.98E-22	1.05E-56	1.54E-20	0.86048427	0.98435647
216.1	0.00033248	0.00714029	0.055439	7.98E-22	1.05E-56	1.54E-20	0.86048427	0.98435647
216.2	0.00033248	0.00714029	0.055439	7.98E-22	1.05E-56	1.54E-20	0.86048427	0.98435647
216.3	0.00033248	0.00714029	0.055439	7.98E-22	1.05E-56	1.54E-20	0.86048427	0.98435647
216.4	0.00033248	0.00714029	0.055439	7.98E-22	1.05E-56	1.54E-20	0.86048427	0.98435647
216.5	0.00033248	0.00714029	0.055439	7.98E-22	1.05E-56	1.54E-20	0.86048427	0.98435647
216.6	0.00033248	0.00714029	0.055439	7.98E-22	1.05E-56	1.54E-20	0.86048427	0.98435647
216.7	0.00033248	0.00714029	0.055439	7.98E-22	1.05E-56	1.54E-20	0.86048427	0.98435647
216.8	0.00033248	0.00714029	0.055439	7.98E-22	1.05E-56	1.54E-20	0.86048427	0.98435647
216.9	0.00033248	0.00714029	0.055439	7.98E-22	1.05E-56	1.54E-20	0.86048427	0.98435647
217	0.00033248	0.00714029	0.055439	7.98E-22	1.05E-56	1.54E-20	0.86048427	0.98435647
217.1	0.00033248	0.00714029	0.055439	7.98E-22	1.05E-56	1.54E-20	0.86048427	0.98435647
217.2	0.00033248	0.00714029	0.055439	7.98E-22	1.05E-56	1.54E-20	0.86048427	0.98435647
217.3	0.00033248	0.00714029	0.055439	7.98E-22	1.05E-56	1.54E-20	0.86048427	0.98435647
217.4	0.00033248	0.00714029	0.055439	7.98E-22	1.05E-56	1.54E-20	0.86048427	0.98435647
217.5	0.00033248	0.00714029	0.055439	7.98E-22	1.05E-56	1.54E-20	0.86048427	0.98435647
217.6	0.00033248	0.00714029	0.055439	7.98E-22	1.05E-56	1.54E-20	0.86048427	0.98435647
217.7	0.00033248	0.00714029	0.055439	7.98E-22	1.05E-56	1.54E-20	0.86048427	0.98435647
217.8	0.00033248	0.00714029	0.055439	7.98E-22	1.05E-56	1.54E-20	0.86048427	0.98435647
217.9	0.00033248	0.00714029	0.055439	7.98E-22	1.05E-56	1.54E-20	0.86048427	0.98435647
218	0.00033248	0.00714029	0.055439	7.98E-22	1.05E-56	1.54E-20	0.86048427	0.98435647
218.1	0.00033248	0.00714029	0.055439	7.98E-22	1.05E-56	1.54E-20	0.86048427	0.98435647
218.2	0.00033248	0.00714029	0.055439	7.98E-22	1.05E-56	1.54E-20	0.86048427	0.98435647
218.3	0.00033248	0.00714029	0.055439	7.98E-22	1.05E-56	1.54E-20	0.86048427	0.98435647
218.4	0.00033248	0.00714029	0.055439	7.98E-22	1.05E-56	1.54E-20	0.86048427	0.98435647
218.5	0.00033248	0.00714029	0.055439	7.98E-22	1.05E-56	1.54E-20	0.86048427	0.98435647
218.6	0.00033248	0.00714029	0.055439	7.98E-22	1.05E-56	1.54E-20	0.86048427	0.98435647
218.7	0.00033248	0.00714029	0.055439	7.98E-22	1.05E-56	1.54E-20	0.86048427	0.98435647
218.8	0.00033248	0.00714029	0.055439	7.98E-22	1.05E-56	1.54E-20	0.86048427	0.98435647
218.9	0.00033248	0.00714029	0.055439	7.98E-22	1.05E-56	1.54E-20	0.86048427	0.98435647
219	0.00033248	0.00714029	0.055439	7.98E-22	1.05E-56	1.54E-20	0.86048427	0.98435647
219.1	0.00033248	0.00714029	0.055439	7.98E-22	1.05E-56	1.54E-20	0.86048427	0.98435647
219.2	0.00033411	0.00727133	0.0561414	8.17E-22	1.10E-56	1.58E-20	0.86450424	0.98494288
219.3	0.00033411	0.00727133	0.0561414	8.17E-22	1.10E-56	1.58E-20	0.86450424	0.98494288
219.4	0.00033647	0.00730533	0.0569628	8.31E-22	1.13E-56	1.63E-20	0.8640185	0.98508216
219.5	0.00033647	0.00730533	0.0569628	8.31E-22	1.13E-56	1.63E-20	0.8640185	0.98508216
219.6	0.00033647	0.00730533	0.0569628	8.31E-22	1.13E-56	1.63E-20	0.8640185	0.98508216
219.7	0.00034178	0.00730281	0.05703278	7.76E-22	1.05E-56	1.66E-20	0.86853996	0.98486271
219.8	0.00034178	0.00730281	0.05703278	7.76E-22	1.05E-56	1.66E-20	0.86853996	0.98486271
219.9	0.00034178	0.00730281	0.05703278	7.76E-22	1.05E-56	1.66E-20	0.86853996	0.98486271
220	0.00034178	0.00730281	0.05703278	7.76E-22	1.05E-56	1.66E-20	0.86853996	0.98486271
220.1	0.00034178	0.00730281	0.05703278	7.76E-22	1.05E-56	1.66E-20	0.86853996	0.98486271
220.2	0.00034178	0.00730281	0.05703278	7.76E-22	1.05E-56	1.66E-20	0.86853996	0.98486271
220.3	0.00034178	0.00730281	0.05703278	7.76E-22	1.05E-56	1.66E-20	0.86853996	0.98486271
220.4	0.00034178	0.00730281	0.05703278	7.76E-22	1.05E-56	1.66E-20	0.86853996	0.98486271
220.5	0.00034178	0.00730281	0.05703278	7.76E-22	1.05E-56	1.66E-20	0.86853996	0.98486271
220.6	0.00034178	0.00730281	0.05703278	7.76E-22	1.05E-56	1.66E-20	0.86853996	0.98486271
220.7	0.00034178	0.00730281	0.05703278	7.76E-22	1.05E-56	1.66E-20	0.86853996	0.98486271
220.8	0.00034178	0.00730281	0.05703278	7.76E-22	1.05E-56	1.66E-20	0.86853996	0.98486271
220.9	0.00034178	0.00730281	0.05703278	7.76E-22	1.05E-56	1.66E-20	0.86853996	0.98486271
221	0.00034178	0.00730281	0.05703278	7.76E-22	1.05E-56	1.66E-20	0.86853996	0.98486271
221.1	0.00034178	0.00730281	0.05703278	7.76E-22	1.05E-56	1.66E-20	0.86853996	0.98486271
221.2	0.00034178	0.00730281	0.05703278	7.76E-22	1.05E-56	1.66E-20	0.86853996	0.98486271
221.3	0.00034178	0.00730281	0.05703278	7.76E-22	1.05E-56	1.66E-20	0.86853996	0.98486271
221.4	0.00034178	0.00730281	0.05703278	7.76E-22	1.05E-56	1.66E-20	0.86853996	0.98486271
221.5	0.00034178	0.00730281	0.05703278	7.76E-22	1.05E-56	1.66E-20	0.86853996	0.98486271
221.6	0.00034178	0.00730281	0.05703278	7.76E-22	1.05E-56	1.66E-20	0.86853996	0.98486271
221.7	0.00034178	0.00730281	0.05703278	7.76E-22	1.05E-56	1.66E-20	0.86853996	0.98486271
221.8	0.00034178	0.00730281	0.05703278	7.76E-22	1.05E-56	1.66E-20	0.86853996	0.98486271
221.9	0.00034178	0.00730281	0.05703278	7.76E-22	1.05E-56	1.66E-20	0.86853996	0.98486271
222	0.00034178	0.00730281	0.05703278	7.76E-22	1.05E-56	1.66E-20	0.86853996	0.98486271
222.1	0.00034178	0.00730281	0.05703278	7.76E-22	1.05E-56	1.66E-20	0.86853996	0.98486271
222.2	0.00034178	0.00730281	0.05703278	7.76E-22	1.05E-56	1.66E-20	0.86853996	0.98486271
222.3	0.00034178	0.00730281	0.05703278	7.76E-22	1.05E-56	1.66E-20	0.86853996	0.98486271
222.4	0.00034178	0.00730281	0.05703278	7.76E-22	1.05E-56	1.66E-20	0.86853996	0.98486271
222.5	0.00034178	0.00730281	0.05703278	7.76E-22	1.05E-56	1.66E-20	0.86853996	0.98486271
222.6	0.00034178	0.00730281	0.05703278	7.76E-22	1.05E-56	1.66E-20	0.86853996	0.98486271
222.7	0.00034178	0.00730281	0.05703278	7.76E-22	1.05E-56	1.66E-20	0.86853996	0.98486271
222.8	0.00034178	0.00730281	0.05703278	7.76E-22	1.05E-56	1.66E-20	0.86853996	0.98486271
222.9	0.00034178	0.00730281	0.05703278	7.76E-22	1.05E-56	1.66E-20	0.86853996	0.98486271
223	0.00034178	0.00730281	0.05703278	7.76E-22	1.05E-56	1.66E-20	0.86853996	0.98486271
223.1	0.00034178	0.00730281	0.05703278	7.76E-22	1.05E-56	1.66E-20	0.86853996	0.98486271
223.2	0.00034178	0.00730281	0.05703278	7.76E-22	1.05E-56	1.66E-20	0.86853996	0.98486271
223.3	0.00034178	0.00730281	0.05703278	7.76E-22	1.05E-56	1.66E-20	0.86853996	0.98486271
223.4	0.00034178	0.00730281	0.05703278	7.76E-22	1.05E-56	1.66E-20	0.86853996	0.98486271
223.5	0.00034178	0.00730281	0.05703278	7.76E-22	1.05E-56	1.66E-20	0.86853996	0.98486271
223.6	0.00034178	0.00730281	0.05703278	7.76E-22	1.05E-56	1.66E-20	0.86853996	0.98486271
223.7	0.00034178	0.00730281	0.05703278	7.76E-22	1.05E-56	1.66E-20	0.86853996	0.98486271
223.8	0.00034922	0.0074323	0.05740495	7.58E-22	9.78E-57	1.66E-20	0.87037583	0.98331145
223.9	0.00034922	0.0074323	0.05740495	7.58E-22	9.78E-57	1.66E-20	0.87037583	0.98331145
224	0.00034922	0.0074323	0.05740495	7.58E-22	9.78E-57	1.66E-20	0.87037583	0.98331145
224.1	0.00034922	0.0074323	0.05740495	7.58E-22	9.78E-57	1.66E-20	0.87037583	0.98331145
224.2	0.00034922	0.0074323	0.05740495	7.58E-22	9.78E-57	1.66E-20	0.87037583	0.98331145
224.3	0.00034922	0.0074323	0.05740495	7.58E-22	9.78E-57	1.66E-20	0.87037583	0.98331145
224.4	0.00034922	0.0074323	0.05740495	7.58E-22	9.78E-57	1.66E-20	0.87037583	0.98331145
224.5	0.00034922	0.0074323	0.05740495	7.58E-22	9.78E-57	1.66E-20	0.87037583	0.98331145
224.6	0.00034922	0.0074323	0.05740495	7.58E-22	9.78E-57	1.66E-20	0.87037583	0.98331145
224.7	0.00035979	0.00744301	0.05757221	7.72E-22	9.96E-57	1.61E-20	0.87520008	0.98149248
224.8	0.00035979	0.00744301	0.05757221	7.72E-22	9.96E-57	1.61E-20	0.87520008	0.98149248
224.9	0.00035979	0.00744301	0.05757221	7.72E-22	9.96E-57	1.61E-20	0.87520008	0.98149248
225.5	0.00035979	0.00744301	0.05757221	7.72E-22	9.96E-57	1.61E-20	0.87520008	0.98149248
225.6	0.00035979	0.00744301	0.05757221	7.72E-22	9.96E-57	1.61E-20	0.87520008	0.98149248
225.7	0.00035979	0.00744301	0.05757221	7.72E-22	9.96E-57	1.61E-20	0.87520008	0.98149248
225.8	0.00035979	0.00744301	0.05757221	7.72E-22	9.96E-57	1.61E-20	0.87520008	0.98149248
225.9	0.00035979	0.00744301	0.05757221	7.72E-22	9.96E-57	1.61E-20	0.87520008	0.98149248
226	0.00035979	0.00744301	0.05757221	7.72E-22	9.96E-57	1.61E-20	0.87520008	0.98149248
226.1	0.00035979	0.00744301	0.05757221	7.72E-22	9.96E-57	1.61E-20	0.87520008	0.98149248
226.2	0.00035979	0.00744301	0.05757221	7.72E-22	9.96E-57	1.61E-20	0.87520008	0.98149248
226.3	0.00035979	0.00744301	0.05757221	7.72E-22	9.96E-57	1.61E-20	0.87520008	0.98149248
226.4	0.00035979	0.00744301	0.05757221	7.72E-22	9.96E-57	1.61E-20	0.87520008	0.98149248
226.5	0.00035979	0.00744301	0.05757221	7.72E-22	9.96E-57	1.61E-20	0.87520008	0.98149248
226.6	0.00035979	0.00744301	0.05757221	7.72E-22	9.96E-57	1.61E-20	0.87520008	0.98149248
226.7	0.00035979	0.00744301	0.05757221	7.72E-22	9.96E-57	1.61E-20	0.87520008	0.98149248
226.8	0.00035979	0.00744301	0.05757221	7.72E-22	9.96E-57	1.61E-20	0.87520008	0.98149248
226.9	0.00035979	0.00744301	0.05757221	7.72E-22	9.96E-57	1.61E-20	0.87520008	0.98149248
227	0.00035979	0.00744301	0.05757221	7.72E-22	9.96E-57	1.61E-20	0.87520008	0.98149248
227.1	0.00035979	0.00744301	0.05757221	7.72E-22	9.96E-57	1.61E-20	0.87520008	0.98149248
227.2	0.00035979	0.00744301	0.05757221	7.72E-22	9.96E-57	1.61E-20	0.87520008	0.98149248
227.3	0.00035979	0.00744301	0.05757221	7.72E-22	9.96E-57	1.61E-20	0.87520008	0.98149248
227.4	0.00035979	0.00744301	0.05757221	7.72E-22	9.96E-57	1.61E-20	0.87520008	0.98149248
227.5	0.00035979	0.00744301	0.05757221	7.72E-22	9.96E-57	1.61E-20	0.87520008	0.98149248
227.6	0.00035979	0.00744301	0.05757221	7.72E-22	9.96E-57	1.61E-20	0.87520008	0.98149248
227.7	0.00035979	0.00744301	0.05757221	7.72E-22	9.96E-57	1.61E-20	0.87520008	0.98149248
227.8	0.00035979	0.00744301	0.05757221	7.72E-22	9.96E-57	1.61E-20	0.87520008	0.98149248
227.9	0.00035979	0.00744301	0.05757221	7.72E-22	9.96E-57	1.61E-20	0.87520008	0.98149248
228	0.00035979	0.00744301	0.05757221	7.72E-22	9.96E-57	1.61E-20	0.87520008	0.98149248
228.1	0.00035979	0.00744301	0.05757221	7.72E-22	9.96E-57	1.61E-20	0.87520008	0.98149248
228.2	0.00035979	0.00744301	0.05757221	7.72E-22	9.96E-57	1.61E-20	0.87520008	0.98149248
228.3	0.00035979	0.00744301	0.05757221	7.72E-22	9.96E-57	1.61E-20	0.87520008	0.98149248
228.4	0.00035979	0.00744301	0.05757221	7.72E-22	9.96E-57	1.61E-20	0.87520008	0.98149248
228.5	0.00035979	0.00744301	0.05757221	7.72E-22	9.96E-57	1.61E-20	0.87520008	0.98149248
228.6	0.00035979	0.00744301	0.05757221	7.72E-22	9.96E-57	1.61E-20	0.87520008	0.98149248
228.7	0.00035979	0.00744301	0.05757221	7.72E-22	9.96E-57	1.61E-20	0.87520008	0.98149248
228.8	0.00035628	0.00763001	0.05787853	7.97E-22	1.02E-56	1.55E-20	0.87599855	0.97989106
228.9	0.00035628	0.00763001	0.05787853	7.97E-22	1.02E-56	1.55E-20	0.87599855	0.97989106
229	0.00035628	0.00763001	0.05787853	7.97E-22	1.02E-56	1.55E-20	0.87599855	0.97989106
229.1	0.00034587	0.00785795	0.05884999	8.38E-22	1.05E-56	1.49E-20	0.87816189	0.97490619
229.2	0.00033495	0.00784271	0.0597633	8.49E-22	1.04E-56	1.51E-20	0.88461659	0.97018683
229.3	0.00033495	0.00784271	0.0597633	8.49E-22	1.04E-56	1.51E-20	0.88461659	0.97018683
229.4	0.00033495	0.00784271	0.0597633	8.49E-22	1.04E-56	1.51E-20	0.88461659	0.97018683
229.5	0.00033119	0.00784073	0.05965288	7.94E-22	9.40E-57	1.47E-20	0.89102863	0.96643473
229.6	0.00033119	0.00784073	0.05965288	7.94E-22	9.40E-57	1.47E-20	0.89102863	0.96643473
229.7	0.00033119	0.00784073	0.05965288	7.94E-22	9.40E-57	1.47E-20	0.89102863	0.96643473
229.8	0.00033119	0.00784073	0.05965288	7.94E-22	9.40E-57	1.47E-20	0.89102863	0.96643473
229.9	0.00033152	0.00789102	0.05930015	7.49E-22	8.61E-57	1.40E-20	0.89234781	0.96401644
230	0.00033152	0.00789102	0.05930015	7.49E-22	8.61E-57	1.40E-20	0.89234781	0.96401644
230.1	0.00033152	0.00789102	0.05930015	7.49E-22	8.61E-57	1.40E-20	0.89234781	0.96401644
230.2	0.00033152	0.00789102	0.05930015	7.49E-22	8.61E-57	1.40E-20	0.89234781	0.96401644
230.3	0.00033152	0.00789102	0.05930015	7.49E-22	8.61E-57	1.40E-20	0.89234781	0.96401644
230.4	0.00033152	0.00789102	0.05930015	7.49E-22	8.61E-57	1.40E-20	0.89234781	0.96401644
230.5	0.00033152	0.00789102	0.05930015	7.49E-22	8.61E-57	1.40E-20	0.89234781	0.96401644
230.6	0.00033152	0.00789102	0.05930015	7.49E-22	8.61E-57	1.40E-20	0.89234781	0.96401644
230.7	0.00033152	0.00789102	0.05930015	7.49E-22	8.61E-57	1.40E-20	0.89234781	0.96401644
230.8	0.00033152	0.00789102	0.05930015	7.49E-22	8.61E-57	1.40E-20	0.89234781	0.96401644
230.9	0.00034092	0.00773231	0.05902435	7.19E-22	8.58E-57	1.36E-20	0.8891428	0.96997049
231	0.00034092	0.00773231	0.05902435	7.19E-22	8.58E-57	1.36E-20	0.8891428	0.96997049
231.1	0.00034092	0.00773231	0.05902435	7.19E-22	8.58E-57	1.36E-20	0.8891428	0.96997049
231.2	0.00034092	0.00773231	0.05902435	7.19E-22	8.58E-57	1.36E-20	0.8891428	0.96997049
231.3	0.00034092	0.00773231	0.05902435	7.19E-22	8.58E-57	1.36E-20	0.8891428	0.96997049
231.4	0.00034092	0.00773231	0.05902435	7.19E-22	8.58E-57	1.36E-20	0.8891428	0.96997049
231.5	0.00034092	0.00773231	0.05902435	7.19E-22	8.58E-57	1.36E-20	0.8891428	0.96997049
231.6	0.00034092	0.00773231	0.05902435	7.19E-22	8.58E-57	1.36E-20	0.8891428	0.96997049
231.7	0.00034092	0.00773231	0.05902435	7.19E-22	8.58E-57	1.36E-20	0.8891428	0.96997049
231.8	0.00034092	0.00773231	0.05902435	7.19E-22	8.58E-57	1.36E-20	0.8891428	0.96997049
231.9	0.00034092	0.00773231	0.05902435	7.19E-22	8.58E-57	1.36E-20	0.8891428	0.96997049
232	0.00034092	0.00773231	0.05902435	7.19E-22	8.58E-57	1.36E-20	0.8891428	0.96997049
232.1	0.00034092	0.00773231	0.05902435	7.19E-22	8.58E-57	1.36E-20	0.8891428	0.96997049
232.2	0.00034	0.00792286	0.05959066	6.67E-22	8.38E-57	1.35E-20	0.88927478	0.96704706
232.3	0.00034	0.00792286	0.05959066	6.67E-22	8.38E-57	1.35E-20	0.88927478	0.96704706
232.4	0.00034	0.00792286	0.05959066	6.67E-22	8.38E-57	1.35E-20	0.88927478	0.96704706
232.5	0.00034	0.00792286	0.05959066	6.67E-22	8.38E-57	1.35E-20	0.88927478	0.96704706
232.6	0.00034	0.00792286	0.05959066	6.67E-22	8.38E-57	1.35E-20	0.88927478	0.96704706
232.7	0.00034	0.00792286	0.05959066	6.67E-22	8.38E-57	1.35E-20	0.88927478	0.96704706
232.8	0.00034	0.00792286	0.05959066	6.67E-22	8.38E-57	1.35E-20	0.88927478	0.96704706
232.9	0.00034	0.00792286	0.05959066	6.67E-22	8.38E-57	1.35E-20	0.88927478	0.96704706
233	0.00034	0.00792286	0.05959066	6.67E-22	8.38E-57	1.35E-20	0.88927478	0.96704706
233.1	0.00034	0.00792286	0.05959066	6.67E-22	8.38E-57	1.35E-20	0.88927478	0.96704706
233.2	0.00034	0.00792286	0.05959066	6.67E-22	8.38E-57	1.35E-20	0.88927478	0.96704706
233.3	0.00034	0.00792286	0.05959066	6.67E-22	8.38E-57	1.35E-20	0.88927478	0.96704706
233.4	0.00034	0.00792286	0.05959066	6.67E-22	8.38E-57	1.35E-20	0.88927478	0.96704706
233.5	0.00034	0.00792286	0.05959066	6.67E-22	8.38E-57	1.35E-20	0.88927478	0.96704706
233.6	0.00034	0.00792286	0.05959066	6.67E-22	8.38E-57	1.35E-20	0.88927478	0.96704706
233.7	0.00034	0.00792286	0.05959066	6.67E-22	8.38E-57	1.35E-20	0.88927478	0.96704706
233.8	0.00034	0.00792286	0.05959066	6.67E-22	8.38E-57	1.35E-20	0.88927478	0.96704706
233.9	0.00034	0.00792286	0.05959066	6.67E-22	8.38E-57	1.35E-20	0.88927478	0.96704706
234	0.00034	0.00792286	0.05959066	6.67E-22	8.38E-57	1.35E-20	0.88927478	0.96704706
234.1	0.00034	0.00792286	0.05959066	6.67E-22	8.38E-57	1.35E-20	0.88927478	0.96704706
234.2	0.00034	0.00792286	0.05959066	6.67E-22	8.38E-57	1.35E-20	0.88927478	0.96704706
234.3	0.00034	0.00792286	0.05959066	6.67E-22	8.38E-57	1.35E-20	0.88927478	0.96704706
234.4	0.00034	0.00792286	0.05959066	6.67E-22	8.38E-57	1.35E-20	0.88927478	0.96704706
234.5	0.00034	0.00792286	0.05959066	6.67E-22	8.38E-57	1.35E-20	0.88927478	0.96704706
234.6	0.00034	0.00792286	0.05959066	6.67E-22	8.38E-57	1.35E-20	0.88927478	0.96704706
234.7	0.00034	0.00792286	0.05959066	6.67E-22	8.38E-57	1.35E-20	0.88927478	0.96704706
234.8	0.00034	0.00792286	0.05959066	6.67E-22	8.38E-57	1.35E-20	0.88927478	0.96704706
234.9	0.00034	0.00792286	0.05959066	6.67E-22	8.38E-57	1.35E-20	0.88927478	0.96704706
235	0.00034	0.00792286	0.05959066	6.67E-22	8.38E-57	1.35E-20	0.88927478	0.96704706
235.1	0.00034	0.00792286	0.05959066	6.67E-22	8.38E-57	1.35E-20	0.88927478	0.96704706
235.2	0.00034	0.00792286	0.05959066	6.67E-22	8.38E-57	1.35E-20	0.88927478	0.96704706
235.3	0.00034	0.00792286	0.05959066	6.67E-22	8.38E-57	1.35E-20	0.88927478	0.96704706
235.4	0.00034	0.00792286	0.05959066	6.67E-22	8.38E-57	1.35E-20	0.88927478	0.96704706
235.5	0.00034	0.00792286	0.05959066	6.67E-22	8.38E-57	1.35E-20	0.88927478	0.96704706
235.6	0.00034	0.00792286	0.05959066	6.67E-22	8.38E-57	1.35E-20	0.88927478	0.96704706
235.7	0.00034	0.00792286	0.05959066	6.67E-22	8.38E-57	1.35E-20	0.88927478	0.96704706
235.8	0.00034	0.00792286	0.05959066	6.67E-22	8.38E-57	1.35E-20	0.88927478	0.96704706
235.9	0.00034	0.00792286	0.05959066	6.67E-22	8.38E-57	1.35E-20	0.88927478	0.96704706
236	0.00034	0.00792286	0.05959066	6.67E-22	8.38E-57	1.35E-20	0.88927478	0.96704706
236.1	0.00034	0.00792286	0.05959066	6.67E-22	8.38E-57	1.35E-20	0.88927478	0.96704706
236.2	0.00034	0.00792286	0.05959066	6.67E-22	8.38E-57	1.35E-20	0.88927478	0.96704706
236.3	0.00033006	0.00811832	0.05997645	6.19E-22	7.97E-57	1.28E-20	0.89127255	0.96059081
236.4	0.00033006	0.00811832	0.05997645	6.19E-22	7.97E-57	1.28E-20	0.89127255	0.96059081
236.5	0.00033006	0.00811832	0.05997645	6.19E-22	7.97E-57	1.28E-20	0.89127255	0.96059081
236.6	0.00033006	0.00811832	0.05997645	6.19E-22	7.97E-57	1.28E-20	0.89127255	0.96059081
236.7	0.00033006	0.00811832	0.05997645	6.19E-22	7.97E-57	1.28E-20	0.89127255	0.96059081
236.8	0.00033006	0.00811832	0.05997645	6.19E-22	7.97E-57	1.28E-20	0.89127255	0.96059081
236.9	0.00033006	0.00811832	0.05997645	6.19E-22	7.97E-57	1.28E-20	0.89127255	0.96059081
237	0.00033006	0.00811832	0.05997645	6.19E-22	7.97E-57	1.28E-20	0.89127255	0.96059081
237.1	0.00033006	0.00811832	0.05997645	6.19E-22	7.97E-57	1.28E-20	0.89127255	0.96059081
237.2	0.00033006	0.00811832	0.05997645	6.19E-22	7.97E-57	1.28E-20	0.89127255	0.96059081
237.3	0.00033006	0.00811832	0.05997645	6.19E-22	7.97E-57	1.28E-20	0.89127255	0.96059081
237.4	0.00033006	0.00811832	0.05997645	6.19E-22	7.97E-57	1.28E-20	0.89127255	0.96059081
237.5	0.00033006	0.00811832	0.05997645	6.19E-22	7.97E-57	1.28E-20	0.89127255	0.96059081
237.6	0.00033006	0.00811832	0.05997645	6.19E-22	7.97E-57	1.28E-20	0.89127255	0.96059081
237.7	0.00033006	0.00811832	0.05997645	6.19E-22	7.97E-57	1.28E-20	0.89127255	0.96059081
237.8	0.00033006	0.00811832	0.05997645	6.19E-22	7.97E-57	1.28E-20	0.89127255	0.96059081
237.9	0.00033006	0.00811832	0.05997645	6.19E-22	7.97E-57	1.28E-20	0.89127255	0.96059081
238	0.00033006	0.00811832	0.05997645	6.19E-22	7.97E-57	1.28E-20	0.89127255	0.96059081
238.1	0.00033006	0.00811832	0.05997645	6.19E-22	7.97E-57	1.28E-20	0.89127255	0.96059081
238.2	0.00032125	0.00811892	0.05979154	6.21E-22	7.75E-57	1.23E-20	0.89313975	0.95705788
238.3	0.00032125	0.00811892	0.05979154	6.21E-22	7.75E-57	1.23E-20	0.89313975	0.95705788
238.4	0.00032125	0.00811892	0.05979154	6.21E-22	7.75E-57	1.23E-20	0.89313975	0.95705788
238.5	0.00032125	0.00811892	0.05979154	6.21E-22	7.75E-57	1.23E-20	0.89313975	0.95705788
238.6	0.00032845	0.00831261	0.06032872	6.24E-22	8.11E-57	1.30E-20	0.90097854	0.95994726
238.7	0.00032845	0.00831261	0.06032872	6.24E-22	8.11E-57	1.30E-20	0.90097854	0.95994726
238.8	0.00032845	0.00831261	0.06032872	6.24E-22	8.11E-57	1.30E-20	0.90097854	0.95994726
238.9	0.00032845	0.00831261	0.06032872	6.24E-22	8.11E-57	1.30E-20	0.90097854	0.95994726
239	0.00032686	0.00851398	0.06105056	6.53E-22	8.40E-57	1.29E-20	0.90336826	0.95980459
239.1	0.00032686	0.00851398	0.06105056	6.53E-22	8.40E-57	1.29E-20	0.90336826	0.95980459
239.2	0.00032604	0.00860516	0.06190202	6.80E-22	8.63E-57	1.29E-20	0.90121481	0.95921386
239.3	0.00032604	0.00860516	0.06190202	6.80E-22	8.63E-57	1.29E-20	0.90121481	0.95921386
239.4	0.00032604	0.00860516	0.06190202	6.80E-22	8.63E-57	1.29E-20	0.90121481	0.95921386
239.5	0.00032604	0.00860516	0.06190202	6.80E-22	8.63E-57	1.29E-20	0.90121481	0.95921386
239.6	0.00032801	0.00865491	0.06194777	6.50E-22	8.00E-57	1.27E-20	0.9041325	0.95824874
239.7	0.00032801	0.00865491	0.06194777	6.50E-22	8.00E-57	1.27E-20	0.9041325	0.95824874
239.8	0.00032801	0.00865491	0.06194777	6.50E-22	8.00E-57	1.27E-20	0.9041325	0.95824874
239.9	0.00032801	0.00865491	0.06194777	6.50E-22	8.00E-57	1.27E-20	0.9041325	0.95824874
240	0.00032801	0.00865491	0.06194777	6.50E-22	8.00E-57	1.27E-20	0.9041325	0.95824874

Table S4: PRCC analysis data for [G3P] with [G](0)=2 mM: PRCC sensitivity indices and their corresponding p-values

Sensitivity analysis data from PRCC for [G3P] with [G](0)=2 mM (only the parameters to which [G3P]'s level is sensitive to appear)

Time discretization	V_{[G]}	K_{[G]}	q	V_{[G3P]}	alpha	G_E	[G3P]_0
0	0	0	0	0	0	0	1
0.1	-0.03935	-0.0342423	0.99307604	0.92088906	-0.2715117	-0.0276403	-0.0601311
0.2	-0.0142666	-0.0302665	0.99209469	0.91125183	-0.4820689	-0.0287217	-0.057692
0.3	-0.0239625	-0.0065273	0.99152323	0.90657462	-0.6036038	-0.0253111	-0.0796973
0.4	-0.0247772	0.00761714	0.99058952	0.89897791	-0.6912376	0.0149231	-0.1041006
0.5	-0.0139984	0.02535236	0.98954573	0.88840986	-0.7536164	0.03175941	-0.1106922
0.6	-0.0012115	0.03387278	0.98872069	0.87992204	-0.7907888	0.0410549	-0.1292986
0.7	-0.0137289	0.01982871	0.98826305	0.87444581	-0.8255769	0.03331667	-0.1254147
0.8	-0.0200314	0.02552155	0.98742666	0.8647775	-0.8526002	0.03679293	-0.127445
0.9	-0.0388344	0.02818305	0.9863773	0.85564572	-0.8637758	0.02203446	-0.1195435
1	-0.0501218	0.018172	0.98575334	0.84924997	-0.8795496	0.02157752	-0.1122998
1.1	-0.0729193	0.03210895	0.98542018	0.84584764	-0.8902003	0.01959525	-0.1188226
1.2	-0.0642057	0.06107998	0.98474475	0.83927333	-0.8977431	0.0016605	-0.1230209
1.3	-0.0717903	0.06996257	0.98403209	0.8332231	-0.9052378	0.00375784	-0.128252
1.4	-0.0634607	0.0662108	0.98271357	0.82600572	-0.9086488	0.00674749	-0.1229397
1.5	-0.0714768	0.06846692	0.98153749	0.81365356	-0.9132763	-0.0018564	-0.1293199
1.6	-0.0733358	0.0786451	0.97999048	0.79979031	-0.9155555	0.00752219	-0.1247412
1.7	-0.0674259	0.07319431	0.97922215	0.79553662	-0.9201723	0.0155198	-0.1174994
1.8	-0.0740282	0.07278694	0.97823699	0.78769179	-0.9220711	0.0124131	-0.117177
1.9	-0.066889	0.07649198	0.9776422	0.78425536	-0.9273566	0.02218145	-0.106788
2	-0.0650992	0.0631396	0.97672903	0.77917344	-0.928936	0.02452413	-0.1100755
2.1	-0.0611043	0.06227023	0.97550941	0.770948	-0.930447	0.02581741	-0.1055162
2.2	-0.0588467	0.04863743	0.97428966	0.76496288	-0.9328226	0.03634063	-0.1081137
2.3	-0.0569771	0.04832922	0.97305543	0.7588764	-0.934353	0.03341758	-0.1071483
2.4	-0.0584209	0.04980332	0.97225851	0.75460126	-0.93617	0.03969714	-0.1060473
2.5	-0.0582465	0.05697512	0.97119622	0.74758525	-0.9374746	0.03833535	-0.0948306
2.6	-0.0605124	0.06190041	0.97064664	0.74626412	-0.9397525	0.03314593	-0.1009076
2.7	-0.0591338	0.05910193	0.96994409	0.74374285	-0.94152	0.03768705	-0.0978929
2.8	-0.0528121	0.06022753	0.96845938	0.73544209	-0.9419772	0.03933324	-0.102325
2.9	-0.033734	0.06711362	0.96751489	0.73041346	-0.9436009	0.04102911	-0.1005042
3	-0.034645	0.07186236	0.96657862	0.72476737	-0.9443511	0.04145188	-0.0911972
3.1	-0.0374975	0.06886578	0.96528663	0.71743031	-0.9449482	0.03526451	-0.0831987
3.2	-0.0344088	0.06985109	0.96432808	0.7115837	-0.9457307	0.02976031	-0.0829626
3.3	-0.0231182	0.0726296	0.96341898	0.70542778	-0.946753	0.03070786	-0.0925258
3.4	-0.0153666	0.07300047	0.96298546	0.70222017	-0.9486034	0.02597543	-0.0914741
3.5	-0.0098769	0.06480652	0.96222573	0.69559044	-0.9492817	0.02700677	-0.0881408
3.6	-0.0025622	0.05863775	0.96127821	0.69133174	-0.9501983	0.02863497	-0.0871379
3.7	0.00182941	0.06431749	0.96031662	0.68975223	-0.9504042	0.03002627	-0.0879322
3.8	0.00829356	0.07289975	0.95942553	0.6850688	-0.9516765	0.03765089	-0.0913441
3.9	0.00404075	0.07071916	0.95926365	0.68521579	-0.9528917	0.03315821	-0.0963437
4	0.01159493	0.06897901	0.9588395	0.68198626	-0.9536586	0.03685544	-0.094805
4.1	0.01307757	0.06808852	0.95819141	0.67706736	-0.954331	0.03871407	-0.0854248
4.2	0.01529599	0.06154023	0.95754844	0.67586967	-0.9547838	0.03595568	-0.0839169
4.3	0.01074707	0.06357467	0.95687759	0.67396021	-0.9553824	0.02769978	-0.0857672
4.4	0.01164911	0.05554403	0.95578575	0.66949553	-0.9553081	0.03008647	-0.0861893
4.5	0.00968014	0.06193252	0.95543975	0.66825374	-0.9560044	0.02327049	-0.0874688
4.6	0.00762898	0.06170824	0.95511477	0.66375821	-0.9567725	0.02336713	-0.084378
4.7	0.00840266	0.05972774	0.95506467	0.66442552	-0.9574752	0.0268874	-0.0910306
4.8	0.0028012	0.05504509	0.95426193	0.66107275	-0.9575325	0.02592118	-0.0937661
4.9	0.00370464	0.05791027	0.95373589	0.659135	-0.9579212	0.02139854	-0.0982894
5	-0.0002139	0.05774361	0.95348669	0.65665788	-0.9586404	0.02118109	-0.0998651
5.1	9.38E-05	0.0566287	0.95279979	0.65341147	-0.9588579	0.01814516	-0.0998666
5.2	-0.000499	0.0595166	0.95231455	0.64886252	-0.9592978	0.02251175	-0.094486
5.3	0.00548365	0.05515514	0.95153429	0.64355941	-0.9595372	0.02590022	-0.0927076
5.4	0.00675117	0.0579289	0.95106	0.64261339	-0.9600012	0.0248011	-0.0918464
5.5	0.015783	0.05376121	0.950127	0.63870275	-0.9601325	0.01857704	-0.0933814
5.6	0.01256545	0.0573511	0.94914391	0.63596175	-0.9602622	0.01573421	-0.0954717
5.7	0.01902251	0.05471105	0.94810327	0.63357276	-0.9599838	0.01727893	-0.0942
5.8	0.02177605	0.05962774	0.94777989	0.63102613	-0.9602585	0.0186762	-0.0947868
5.9	0.02172157	0.05854805	0.94757685	0.63083656	-0.9607127	0.02160198	-0.0950849
6	0.02423985	0.05965011	0.94713309	0.62995904	-0.9609924	0.01894313	-0.0935024
6.1	0.02644843	0.05201638	0.94603101	0.62384046	-0.961134	0.02728129	-0.0917788
6.2	0.02771271	0.05109819	0.94576677	0.62287823	-0.9613034	0.02647592	-0.0914131
6.3	0.03039661	0.05029239	0.94538106	0.62155136	-0.961553	0.02330463	-0.0900971
6.4	0.02895074	0.04612867	0.94501878	0.62137015	-0.9617488	0.02208049	-0.0864477
6.5	0.0294775	0.047791	0.94407399	0.62191741	-0.9614452	0.0198979	-0.088115
6.6	0.03174594	0.04668988	0.94344194	0.620035	-0.9614002	0.01996552	-0.0851502
6.7	0.03391732	0.04823307	0.94281171	0.61701734	-0.9613405	0.01531135	-0.083816
6.8	0.03611359	0.04492947	0.94284073	0.61569924	-0.9618315	0.01532056	-0.082628
6.9	0.03581103	0.0453463	0.94252175	0.61202622	-0.9619356	0.01194367	-0.0806603
7	0.03815742	0.04403897	0.94212194	0.60833899	-0.9621033	0.01263874	-0.0814196
7.1	0.03466042	0.04233693	0.94206659	0.60511657	-0.9623969	0.01478758	-0.0789288
7.2	0.03433027	0.04066121	0.94174421	0.60311893	-0.9625901	0.00771831	-0.0754791
7.3	0.03767735	0.04144114	0.94109935	0.59928526	-0.9627389	0.0102148	-0.0730896
7.4	0.03730002	0.03978016	0.94097652	0.59939342	-0.9628957	0.01016275	-0.076088
7.5	0.03691371	0.03522642	0.94031247	0.59586497	-0.9628012	0.00512156	-0.0710984
7.6	0.03633988	0.03436442	0.93981172	0.5946003	-0.9627148	0.00429299	-0.0695326
7.7	0.03845464	0.0324429	0.93916463	0.59194323	-0.9627055	0.00156149	-0.0717892
7.8	0.04057546	0.03367954	0.93811215	0.5886879	-0.9623974	0.00026042	-0.0687525
7.9	0.03901026	0.03227682	0.9377935	0.58640511	-0.9625252	-0.0029664	-0.0669378
8	0.03813153	0.03398379	0.93766694	0.58617157	-0.9627096	-0.0019305	-0.0649076
8.1	0.03568759	0.0331535	0.93747006	0.58538661	-0.9627236	-0.0012684	-0.0651952
8.2	0.03734334	0.03204525	0.93686915	0.58426802	-0.9628403	-1.20E-05	-0.0648975
8.3	0.03866767	0.03253179	0.93537739	0.57922934	-0.9622938	0.0002121	-0.0651717
8.4	0.03943868	0.03233724	0.93481088	0.57796811	-0.9621728	-0.0003645	-0.0613546
8.5	0.04008055	0.03573515	0.93445986	0.57773959	-0.9621259	0.00075172	-0.0621163
8.6	0.04084161	0.03496841	0.93427085	0.57688508	-0.9622884	0.00316279	-0.061868
8.7	0.04156089	0.03337052	0.9339734	0.57385123	-0.9623402	0.00385774	-0.0575301
8.8	0.04119299	0.03336959	0.93312705	0.57105554	-0.9621503	0.00418662	-0.0554317
8.9	0.0391278	0.03643933	0.93274664	0.57176065	-0.9622509	-0.0006772	-0.0565468
9	0.04154766	0.03433295	0.9322402	0.56994636	-0.9621087	-0.0015707	-0.0552135
9.1	0.04147138	0.03386205	0.93187974	0.56834139	-0.9620196	-0.001302	-0.0536767
9.2	0.03992612	0.03244946	0.93152659	0.56513761	-0.9619185	-0.0023113	-0.053449
9.3	0.03937299	0.03224112	0.93080023	0.56305042	-0.9617782	-0.0029756	-0.0505069
9.4	0.03766841	0.03157208	0.9303332	0.56011566	-0.9616759	-0.0047063	-0.0511528
9.5	0.03549319	0.03033907	0.92990436	0.55817141	-0.9616053	-0.0077808	-0.0523137
9.6	0.0354505	0.02965759	0.92953188	0.55900462	-0.9616027	-0.0113937	-0.0532878
9.7	0.03857969	0.02985427	0.92940551	0.55917987	-0.9618355	-0.0115903	-0.0517049
9.8	0.03877667	0.03207738	0.9293479	0.55778639	-0.9619369	-0.0128141	-0.0537049
9.9	0.04087845	0.0309605	0.92879846	0.55754754	-0.9617632	-0.0114799	-0.0505555
10	0.04101044	0.02950327	0.92831092	0.55661121	-0.9616195	-0.0107737	-0.0502864
10.1	0.04053937	0.02808277	0.92736319	0.55513599	-0.9613619	-0.007192	-0.0484454
10.2	0.04148622	0.02480276	0.9266532	0.55272155	-0.9612526	-0.0075113	-0.0454354
10.3	0.04320515	0.02314199	0.92626893	0.55147661	-0.9612672	-0.0069109	-0.0443171
10.4	0.04354244	0.02328369	0.92560274	0.54987762	-0.9611378	-0.0069233	-0.0454795
10.5	0.04474324	0.02378042	0.92547339	0.54951867	-0.9612467	-0.009264	-0.0459561
10.6	0.04490427	0.02212521	0.92509742	0.54856273	-0.9611552	-0.0134215	-0.0430726
10.7	0.04461239	0.02286412	0.92378981	0.54365777	-0.9607667	-0.0139769	-0.0462172
10.8	0.04357297	0.02110791	0.92325472	0.54277925	-0.9606687	-0.0111091	-0.0464954
10.9	0.04499884	0.0208375	0.92308811	0.54361044	-0.9607875	-0.0096247	-0.0465787
11	0.04892699	0.02298997	0.9227481	0.54376978	-0.9608694	-0.0090451	-0.0461455
11.1	0.04830643	0.02347391	0.92196696	0.54197905	-0.9606199	-0.0108151	-0.0453103
11.2	0.04961693	0.02311766	0.92197431	0.54022836	-0.9607367	-0.0099979	-0.0461191
11.3	0.05237228	0.02391172	0.92208271	0.54063288	-0.9609601	-0.0104778	-0.0441909
11.4	0.05029956	0.02151734	0.92161826	0.53747694	-0.9608424	-0.0114255	-0.0437524
11.5	0.04937503	0.02196145	0.92142791	0.53523351	-0.9607641	-0.011643	-0.0441647
11.6	0.05030086	0.01980763	0.92127825	0.53454198	-0.9607804	-0.0129693	-0.043789
11.7	0.05049265	0.02165894	0.92102528	0.53372085	-0.9608615	-0.0110662	-0.0426607
11.8	0.04945665	0.02183509	0.9206708	0.53295728	-0.9607333	-0.0090144	-0.0426714
15	0.04791054	0.01077056	0.91435845	0.5177103	-0.9595381	-0.0146742	-0.045928
15.1	0.04857363	0.00992159	0.91406082	0.51602958	-0.9594478	-0.0146618	-0.0450761
15.2	0.04963746	0.0104226	0.91406183	0.51570039	-0.9595156	-0.0146947	-0.0450189
15.3	0.04997713	0.00929892	0.91382643	0.51459172	-0.9594854	-0.0149022	-0.0433916
15.4	0.05088687	0.00777525	0.91356147	0.51354511	-0.9593448	-0.0162784	-0.0434169
15.5	0.05088687	0.00777525	0.91356147	0.51354511	-0.9593448	-0.0162784	-0.0434169
15.6	0.05125	0.0073092	0.91318772	0.51309486	-0.9592359	-0.0165129	-0.0447878
15.7	0.05162467	0.00628969	0.91280606	0.51193535	-0.9591808	-0.0170433	-0.0439167
15.8	0.05174969	0.00477876	0.91268655	0.51095643	-0.9590892	-0.0172708	-0.0435434
15.9	0.05000201	0.00413195	0.91240882	0.51021698	-0.9589758	-0.0181217	-0.0439559
16	0.05028866	0.00334502	0.91186327	0.50789877	-0.958778	-0.0171613	-0.0429724
16.1	0.05028866	0.00334502	0.91186327	0.50789877	-0.958778	-0.0171613	-0.0429724
16.2	0.05028848	0.00333636	0.91173663	0.50698917	-0.9587326	-0.0163412	-0.0423622
16.3	0.04979182	0.00367033	0.91168576	0.50691144	-0.9587599	-0.0156717	-0.0427417
16.4	0.0494855	0.00322227	0.91168143	0.50722552	-0.9588439	-0.0165047	-0.042167
16.5	0.0494855	0.00322227	0.91168143	0.50722552	-0.9588439	-0.0165047	-0.042167
16.6	0.05095668	0.00241603	0.91148588	0.50666896	-0.9587532	-0.0166109	-0.0425333
16.7	0.05322518	0.00386861	0.91075734	0.50564851	-0.9585154	-0.0172043	-0.0419679
16.8	0.05382054	0.00380006	0.91066761	0.50613023	-0.958561	-0.0168131	-0.041999
16.9	0.05371245	0.00415175	0.91053298	0.50624833	-0.9585105	-0.017158	-0.0417096
17	0.05397765	0.00278439	0.91002184	0.50440342	-0.9583632	-0.0175823	-0.0402482
17.1	0.05570176	0.00174534	0.90985141	0.50456185	-0.9584161	-0.0165483	-0.0395676
17.2	0.05882929	0.00171107	0.90961888	0.50431106	-0.9584763	-0.0156542	-0.0389842
17.3	0.05882929	0.00171107	0.90961888	0.50431106	-0.9584763	-0.0156542	-0.0389842
17.4	0.05982128	0.00085689	0.9096297	0.50340369	-0.9584876	-0.0154479	-0.038712
17.5	0.05911848	-0.000421	0.90924849	0.50228419	-0.9583439	-0.0163102	-0.0384333
17.6	0.05911848	-0.000421	0.90924849	0.50228419	-0.9583439	-0.0163102	-0.0384333
17.7	0.05764189	-0.0009312	0.90893854	0.50021636	-0.9581957	-0.015853	-0.0370342
17.8	0.05759743	-0.0016795	0.90888178	0.50007847	-0.9581877	-0.0159479	-0.0360174
17.9	0.05784784	-0.0009727	0.90870328	0.49985076	-0.9581753	-0.0167076	-0.0366918
18	0.0578858	0.0006492	0.90867118	0.4986373	-0.9581529	-0.0159267	-0.0356807
18.1	0.05880345	0.00142488	0.90803584	0.49659704	-0.9579836	-0.0166799	-0.0351762
18.2	0.05880345	0.00142488	0.90803584	0.49659704	-0.9579836	-0.0166799	-0.0351762
18.3	0.05835123	0.00174335	0.90748949	0.4944447	-0.9578133	-0.0145147	-0.0347971
18.4	0.05709796	0.00079741	0.90750404	0.4940502	-0.9578295	-0.0155855	-0.0335676
18.5	0.05931736	0.0013902	0.90688475	0.4921328	-0.957583	-0.0142587	-0.0323108
18.6	0.05927576	0.00093735	0.9067852	0.49160098	-0.9575243	-0.0151429	-0.0319774
18.7	0.05892426	-0.0003826	0.90640545	0.49156956	-0.9573354	-0.0161926	-0.0309586
18.8	0.05762075	3.32E-05	0.90640536	0.49223227	-0.9574191	-0.0169888	-0.0303589
18.9	0.05802001	0.00024643	0.90631284	0.49200071	-0.95737	-0.0152983	-0.0299814
19	0.05802001	0.00024643	0.90631284	0.49200071	-0.95737	-0.0152983	-0.0299814
19.1	0.05830621	-0.0008178	0.90628335	0.49238398	-0.9573645	-0.0149363	-0.0306493
19.2	0.05835558	-0.0008458	0.90614464	0.49207892	-0.9573072	-0.0162919	-0.0313295
19.3	0.05848902	-0.0019471	0.90610347	0.49125116	-0.9573011	-0.0164275	-0.0316091
19.4	0.0584717	-0.0025403	0.90603307	0.49093963	-0.9573945	-0.0176197	-0.0298154
19.5	0.05896822	-0.0031035	0.90570825	0.48979688	-0.9573049	-0.0180403	-0.0298708
19.6	0.0583311	-0.003416	0.90530926	0.48949014	-0.9571777	-0.0186179	-0.030214
19.7	0.05856128	-0.0036398	0.90484018	0.48723287	-0.9570085	-0.0176838	-0.0302618
19.8	0.05837453	-0.0050576	0.90459766	0.4862175	-0.956917	-0.0190491	-0.0298488
19.9	0.05814083	-0.0058271	0.90442462	0.48558461	-0.9567958	-0.0192184	-0.0298281
20	0.05691111	-0.0054349	0.90380006	0.48540771	-0.9565606	-0.0174174	-0.0305508
20.1	0.05657582	-0.0045707	0.9036013	0.48460556	-0.9565864	-0.0178029	-0.030059
20.2	0.05657582	-0.0045707	0.9036013	0.48460556	-0.9565864	-0.0178029	-0.030059
20.3	0.05657582	-0.0045707	0.9036013	0.48460556	-0.9565864	-0.0178029	-0.030059
20.4	0.05496409	-0.0066214	0.90350306	0.48304334	-0.9564922	-0.0160931	-0.0286754
20.5	0.05496409	-0.0066214	0.90350306	0.48304334	-0.9564922	-0.0160931	-0.0286754
20.6	0.05496409	-0.0066214	0.90350306	0.48304334	-0.9564922	-0.0160931	-0.0286754
20.7	0.05460189	-0.0069463	0.9032767	0.48176443	-0.9564553	-0.0172881	-0.0286193
20.8	0.05460189	-0.0069463	0.9032767	0.48176443	-0.9564553	-0.0172881	-0.0286193
20.9	0.05716522	-0.0076275	0.90262916	0.48051018	-0.9562569	-0.0181043	-0.0303584
21	0.05789755	-0.0087676	0.90234963	0.47985625	-0.9561691	-0.0173512	-0.0290773
21.1	0.05807265	-0.0097213	0.90198315	0.47801233	-0.9559023	-0.0194932	-0.0280223
21.2	0.05748995	-0.0088619	0.9016452	0.47709923	-0.9557774	-0.0206496	-0.0268712
21.3	0.05821526	-0.0071308	0.90135045	0.47730759	-0.9557445	-0.0201999	-0.0283632
21.4	0.05762816	-0.0081812	0.90148993	0.47861683	-0.9558854	-0.0196443	-0.0279554
21.5	0.05734663	-0.0075742	0.90118755	0.47801693	-0.9557267	-0.020985	-0.0264065
21.6	0.05773454	-0.0076074	0.90115865	0.47784973	-0.9557474	-0.0203253	-0.0264278
21.7	0.05675542	-0.0070685	0.9012091	0.47779198	-0.9558247	-0.0197293	-0.0249751
21.8	0.05606498	-0.0089831	0.90091611	0.47613279	-0.9556871	-0.020267	-0.0241298
21.9	0.05747127	-0.0083128	0.90076374	0.47597599	-0.9556861	-0.0202395	-0.0244486
22	0.05732569	-0.0083505	0.90045825	0.47458582	-0.9555682	-0.0203612	-0.0238817
22.1	0.05732569	-0.0083505	0.90045825	0.47458582	-0.9555682	-0.0203612	-0.0238817
22.2	0.05689616	-0.0089435	0.89999469	0.4739999	-0.9554127	-0.0196622	-0.0225217
22.3	0.05689616	-0.0089435	0.89999469	0.4739999	-0.9554127	-0.0196622	-0.0225217
22.4	0.05662144	-0.0093797	0.89990454	0.47339594	-0.9554041	-0.0191143	-0.0229272
22.5	0.05636789	-0.0092469	0.89963387	0.47255313	-0.955341	-0.0198812	-0.023932
22.6	0.05532976	-0.0098681	0.89910211	0.47087968	-0.9551525	-0.019581	-0.0228003
22.7	0.05694961	-0.0089168	0.89916084	0.47122731	-0.9552163	-0.0193801	-0.0240408
22.8	0.05684314	-0.008369	0.89907197	0.47057548	-0.95518	-0.0194434	-0.0239743
22.9	0.05684314	-0.008369	0.89907197	0.47057548	-0.95518	-0.0194434	-0.0239743
23	0.0571097	-0.0082907	0.89901569	0.47026753	-0.9552231	-0.0184606	-0.0245356
23.1	0.05790124	-0.0072776	0.89887583	0.46993674	-0.9551926	-0.0197727	-0.023056
23.2	0.05790124	-0.0072776	0.89887583	0.46993674	-0.9551926	-0.0197727	-0.023056
23.3	0.05827475	-0.0083529	0.89857202	0.46939946	-0.955107	-0.0188159	-0.0234684
23.4	0.05816358	-0.0089118	0.89846585	0.46849622	-0.95502	-0.0194967	-0.0226925
23.5	0.05815555	-0.009597	0.89843499	0.46905068	-0.9550216	-0.0193865	-0.0236571
23.6	0.05881651	-0.0096064	0.89844423	0.46851399	-0.9549861	-0.0198419	-0.0240421
23.7	0.0590809	-0.0091474	0.89832557	0.46810768	-0.9549753	-0.0194543	-0.0249565
23.8	0.05953372	-0.0095107	0.89803474	0.46729607	-0.9548959	-0.0193487	-0.0251416
23.9	0.05910549	-0.0098616	0.8976503	0.46563814	-0.9546689	-0.0215841	-0.0239437
24	0.05848099	-0.0101045	0.8976833	0.46579477	-0.9546889	-0.0201409	-0.0238554
24.1	0.05882038	-0.0112335	0.89767144	0.46594678	-0.9546606	-0.020598	-0.0233979
24.2	0.06107803	-0.0108331	0.89758089	0.46547961	-0.9545543	-0.0211226	-0.0230233
24.3	0.06050039	-0.0119566	0.89741605	0.46542042	-0.9545153	-0.0203794	-0.0232078
24.4	0.06050039	-0.0119566	0.89741605	0.46542042	-0.9545153	-0.0203794	-0.0232078
24.5	0.0604657	-0.012208	0.89730236	0.46515986	-0.9544756	-0.0197518	-0.0227458
24.6	0.05988955	-0.0130339	0.89697905	0.46482118	-0.9543579	-0.019657	-0.0219494
24.7	0.05939251	-0.0140881	0.89689718	0.46422228	-0.9543296	-0.0195621	-0.0218159
24.8	0.06053214	-0.0151094	0.89671185	0.46376069	-0.9542279	-0.0211252	-0.0203893
24.9	0.05981432	-0.0162828	0.89673584	0.46389906	-0.9542532	-0.0207128	-0.020709
25	0.05977524	-0.0190267	0.89608067	0.46182808	-0.9538964	-0.0225907	-0.018818
25.1	0.05961602	-0.0192905	0.89580532	0.4620161	-0.9538062	-0.0225305	-0.0178227
25.2	0.05969837	-0.0197288	0.89529498	0.46033868	-0.9536209	-0.0215305	-0.0172142
25.3	0.06021682	-0.0207935	0.89486558	0.45848483	-0.9535053	-0.0235209	-0.0188774
25.4	0.05945309	-0.0199754	0.89477904	0.45874867	-0.9534913	-0.0229443	-0.019062
25.5	0.06104723	-0.0199617	0.89459067	0.45895037	-0.9534591	-0.0231701	-0.0185461
25.6	0.06054779	-0.0208569	0.89431042	0.45828763	-0.9533179	-0.0229049	-0.0174487
25.7	0.06054779	-0.0208569	0.89431042	0.45828763	-0.9533179	-0.0229049	-0.0174487
25.8	0.06193379	-0.0205432	0.89409025	0.45789269	-0.9532522	-0.0234925	-0.0161057
25.9	0.06193379	-0.0205432	0.89409025	0.45789269	-0.9532522	-0.0234925	-0.0161057
26	0.06201221	-0.02081	0.89407045	0.457613	-0.9532364	-0.0240725	-0.0162372
26.1	0.0617668	-0.0204786	0.89376735	0.4573597	-0.9531428	-0.0241684	-0.0160321
26.2	0.062935	-0.019525	0.89356993	0.45789151	-0.9530752	-0.0237446	-0.0167394
26.3	0.06344934	-0.0200075	0.89367056	0.4573706	-0.9531252	-0.023856	-0.0154904
26.4	0.06333306	-0.0204436	0.89364268	0.45751957	-0.9531137	-0.0243501	-0.0153617
26.5	0.06336313	-0.0211019	0.89330449	0.45612304	-0.9529721	-0.0240125	-0.0149925
26.6	0.06336313	-0.0211019	0.89330449	0.45612304	-0.9529721	-0.0240125	-0.0149925
26.7	0.06292886	-0.0203485	0.89338135	0.45540317	-0.9530482	-0.0235551	-0.0153967
26.8	0.06324905	-0.0210911	0.89320692	0.45449454	-0.9529941	-0.0230791	-0.0154934
30	0.07233191	-0.0238958	0.88648749	0.43551157	-0.9501899	-0.0182356	-0.009004
30.1	0.0719663	-0.0244634	0.88640493	0.43505859	-0.9502011	-0.0183235	-0.0080094
30.2	0.07191526	-0.0254124	0.88629832	0.43438508	-0.9501245	-0.0185833	-0.0065138
30.3	0.07295704	-0.0249948	0.88628684	0.43371876	-0.9501038	-0.0173199	-0.0071126
30.4	0.07286007	-0.0256063	0.88603193	0.43394063	-0.9500313	-0.0167901	-0.0066954
30.5	0.07357059	-0.0256644	0.8857705	0.43345714	-0.9498631	-0.0163486	-0.0068616
30.6	0.07459811	-0.0244474	0.88555954	0.43284031	-0.9497422	-0.0166965	-0.0082885
30.7	0.07459811	-0.0244474	0.88555954	0.43284031	-0.9497422	-0.0166965	-0.0082885
30.8	0.07562044	-0.0242015	0.8854228	0.4331576	-0.9496978	-0.0158865	-0.0094323
30.9	0.07630081	-0.023821	0.88533046	0.4325109	-0.9497354	-0.015624	-0.0109223
31	0.0751989	-0.0235929	0.88515718	0.43197941	-0.9496994	-0.0149581	-0.0118871
31.1	0.07489845	-0.0242995	0.88486201	0.43268714	-0.9495065	-0.0171999	-0.0113756
31.2	0.07489845	-0.0242995	0.88486201	0.43268714	-0.9495065	-0.0171999	-0.0113756
31.3	0.07348775	-0.0253585	0.88424897	0.43198448	-0.9492524	-0.017158	-0.012137
31.4	0.07267872	-0.0264362	0.88403421	0.43139053	-0.9491048	-0.0167613	-0.0117834
31.5	0.07254649	-0.024731	0.88382032	0.42920228	-0.9488893	-0.018127	-0.013023
31.6	0.07425254	-0.0247604	0.88355902	0.42929459	-0.9488823	-0.0150256	-0.0144659
31.7	0.07448335	-0.0243362	0.88352107	0.42847056	-0.9487948	-0.0146061	-0.0146841
31.8	0.07580505	-0.0282619	0.8831427	0.42761732	-0.9485682	-0.0142599	-0.0123813
31.9	0.07584097	-0.0287994	0.88300502	0.42685738	-0.9484914	-0.014672	-0.0121334
32	0.07525502	-0.0297947	0.88267933	0.42630049	-0.9482864	-0.0155091	-0.0123888
32.1	0.07579702	-0.0312609	0.88250721	0.42532706	-0.948235	-0.0137661	-0.0111802
32.2	0.07747257	-0.0321264	0.88230122	0.42454161	-0.9480859	-0.0128829	-0.0109184
32.3	0.07858676	-0.0322184	0.88229411	0.42511352	-0.9481425	-0.0131989	-0.0106612
32.4	0.07904399	-0.0326346	0.88219546	0.42502775	-0.9481048	-0.0118154	-0.011642
32.5	0.07848822	-0.0323686	0.88191768	0.42469928	-0.9480852	-0.01054	-0.0118664
32.6	0.0786725	-0.0323816	0.88164972	0.42437795	-0.9480115	-0.0102114	-0.0108636
32.7	0.07863871	-0.0334963	0.88147898	0.4237079	-0.9478779	-0.0096983	-0.0105109
32.8	0.0788269	-0.0330606	0.88129719	0.42144695	-0.9478516	-0.0104636	-0.0099912
32.9	0.0796327	-0.0342866	0.88113253	0.42190723	-0.9477885	-0.0093833	-0.0088842
33	0.08080591	-0.0342055	0.88116836	0.42204045	-0.9478801	-0.0103343	-0.0091509
33.1	0.08062877	-0.0371072	0.88064385	0.41924333	-0.9475826	-0.0094877	-0.0091273
33.2	0.08148943	-0.0371817	0.88008348	0.41762512	-0.9473182	-0.0092663	-0.009962
33.3	0.08207635	-0.0368098	0.8799553	0.41755439	-0.9472904	-0.0089364	-0.0099795
33.4	0.0826615	-0.0367509	0.8799559	0.41783724	-0.9472837	-0.0081599	-0.0100884
33.5	0.0826615	-0.0367509	0.8799559	0.41783724	-0.9472837	-0.0081599	-0.0100884
33.6	0.08259999	-0.0371334	0.87924811	0.41470126	-0.9468424	-0.0089858	-0.0101319
33.7	0.08269861	-0.0389819	0.87913316	0.41407425	-0.9468446	-0.0070601	-0.011464
33.8	0.08325473	-0.0413321	0.87909343	0.41262818	-0.9467702	-0.0069111	-0.0124317
33.9	0.08336338	-0.0409812	0.87889317	0.41281927	-0.9467211	-0.0063703	-0.0126174
34	0.08293217	-0.0415021	0.87889696	0.41168386	-0.9467467	-0.0067842	-0.0119962
34.1	0.08228965	-0.0413327	0.87882843	0.41157541	-0.9467593	-0.007071	-0.0122817
34.2	0.08338702	-0.0410196	0.87886748	0.41098837	-0.9467766	-0.0062098	-0.0131857
34.3	0.08339795	-0.0425541	0.87877541	0.41053853	-0.9468632	-0.004848	-0.0131575
34.4	0.08251484	-0.0439192	0.87850633	0.4098584	-0.9467343	-0.0055931	-0.0114728
34.5	0.08244237	-0.0442582	0.87847922	0.40949109	-0.9467357	-0.0050273	-0.0113833
34.6	0.08228541	-0.0445621	0.87786963	0.4081362	-0.9465207	-0.0062989	-0.010483
34.7	0.08491235	-0.0455202	0.87751274	0.4065645	-0.9463972	-0.0068793	-0.0116513
34.8	0.08416901	-0.0469063	0.87730647	0.40591197	-0.9463784	-0.0068809	-0.0122842
34.9	0.08685812	-0.0483436	0.87716358	0.40483893	-0.9464237	-0.0046777	-0.0137792
35	0.08759159	-0.0488269	0.87722971	0.40530176	-0.9465532	-0.0047003	-0.0140915
35.1	0.08753945	-0.0501912	0.87691758	0.40450615	-0.9464271	-0.0041038	-0.0136899
35.2	0.08755035	-0.0508269	0.87649235	0.40309543	-0.946194	-0.0043452	-0.0138787
35.3	0.08758226	-0.0512077	0.87625806	0.40275626	-0.9460808	-0.0044332	-0.0138508
35.4	0.08870958	-0.0510033	0.87622835	0.40255304	-0.9461541	-0.0043661	-0.0137871
35.5	0.08906168	-0.0524142	0.87588078	0.40136858	-0.9460221	-0.0037779	-0.0137392
35.6	0.0883222	-0.0522598	0.87579979	0.40181003	-0.9460071	-0.0044166	-0.0133591
35.7	0.08851646	-0.0522306	0.87551861	0.40037826	-0.9458298	-0.0055177	-0.013111
35.8	0.08849262	-0.0513449	0.87571141	0.40045042	-0.9458242	-0.0040687	-0.0125197
35.9	0.09023205	-0.0512777	0.87553369	0.39952542	-0.9457435	-0.0037102	-0.0129393
36	0.09041027	-0.0512248	0.87505098	0.39846155	-0.9455537	-0.0005896	-0.0142861
36.1	0.09222333	-0.0529601	0.87522234	0.3960848	-0.9457282	0.00191365	-0.0129967
36.2	0.09224558	-0.0534929	0.87495884	0.39536324	-0.9456766	0.002305	-0.0118925
36.3	0.09168694	-0.0552313	0.87462553	0.393195	-0.945469	0.00111833	-0.0103194
36.4	0.09120119	-0.05428	0.87449506	0.39271918	-0.9454112	0.00058902	-0.0104204
36.5	0.0915688	-0.055026	0.87407338	0.39131962	-0.9452166	0.0023984	-0.0100177
36.6	0.09170862	-0.0547807	0.8740724	0.39124688	-0.9452524	0.0029973	-0.0104466
36.7	0.09267163	-0.0559746	0.87387624	0.38963609	-0.9451166	0.00486336	-0.0111066
36.8	0.09364858	-0.0566002	0.87295853	0.38666618	-0.9446643	0.00251317	-0.0094674
36.9	0.09355482	-0.0564471	0.87300353	0.38625968	-0.9446492	0.00211251	-0.0095651
37	0.09430035	-0.0567106	0.873203	0.38630713	-0.944809	0.00171003	-0.0102335
37.1	0.09390447	-0.058038	0.87304567	0.38656711	-0.9447727	0.00093257	-0.0095801
37.2	0.09387579	-0.0589528	0.87273275	0.38628234	-0.9447363	0.00250947	-0.008425
37.3	0.09373796	-0.0605851	0.87209362	0.3846358	-0.9443613	0.00239109	-0.0058606
37.4	0.09383304	-0.0610334	0.87180096	0.38271648	-0.9442601	0.00173956	-0.0055457
37.5	0.09289286	-0.0629337	0.8715252	0.3813832	-0.944081	0.00215783	-0.004981
37.6	0.09298724	-0.0654733	0.87041397	0.37728879	-0.9434348	0.00176991	-0.0055581
37.7	0.09392431	-0.0702096	0.86952651	0.37407748	-0.9431141	-0.0004925	-0.00465
37.8	0.09151722	-0.0728409	0.86913621	0.37315571	-0.942889	0.0007904	-0.0038303
37.9	0.09229781	-0.0743389	0.86860668	0.37103194	-0.942588	0.00096738	-0.0033804
38	0.09177707	-0.0763595	0.86836836	0.36954616	-0.9424692	0.0023311	-0.0029131
38.1	0.09327264	-0.0773592	0.86807049	0.36882646	-0.942353	0.00443872	-0.002106
38.2	0.09366098	-0.078003	0.86779576	0.36896116	-0.9423591	0.00346243	-0.0016544
38.3	0.0939232	-0.0790063	0.86704672	0.36627406	-0.9419659	0.00264941	-0.0012362
38.4	0.09445509	-0.081369	0.86641085	0.36473436	-0.9416996	0.00278482	0.00013866
38.5	0.09369999	-0.0806605	0.86588981	0.36335392	-0.9414913	0.00383439	-0.0006738
38.6	0.09347183	-0.0821721	0.86484843	0.36103389	-0.9411047	0.00489497	-0.0002319
38.7	0.0935844	-0.0831053	0.8644099	0.36090527	-0.9409192	0.00478728	0.00020934
38.8	0.09268361	-0.0853389	0.86383989	0.35825851	-0.9406923	0.00512066	0.00112012
38.9	0.09062662	-0.0862237	0.86314524	0.35724963	-0.9406544	0.0068234	0.00126782
39	0.09045891	-0.0874332	0.86268914	0.35495639	-0.9405251	0.00794826	0.000415
39.1	0.09139614	-0.0883457	0.86202759	0.35343865	-0.9403676	0.01018138	-0.0012919
39.2	0.09157867	-0.0905909	0.86165775	0.35167966	-0.9401407	0.00941023	0.00017223
39.3	0.09241217	-0.0905427	0.86142807	0.35100064	-0.9400878	0.00925081	0.00078434
39.4	0.09430923	-0.0926173	0.86071618	0.34937842	-0.9398916	0.00917573	0.00166891
39.5	0.09408488	-0.0956365	0.85980388	0.34680544	-0.9394603	0.01104108	0.00335129
39.6	0.09392083	-0.0984493	0.85908511	0.34559917	-0.9391535	0.01210747	0.00362242
39.7	0.09532566	-0.1002581	0.85831509	0.34346642	-0.9389464	0.01451684	0.0061002
39.8	0.09702549	-0.1009132	0.85739516	0.34253242	-0.9386246	0.01449276	0.00812743
39.9	0.09821918	-0.101777	0.85693222	0.34109386	-0.9385366	0.01488907	0.00708291
40	0.09849884	-0.1022873	0.85546372	0.33824989	-0.9379063	0.0135247	0.00698684
40.1	0.09914449	-0.1015958	0.85503266	0.33738058	-0.9377975	0.01505378	0.00712796
40.2	0.09956659	-0.1025017	0.85463922	0.33755603	-0.9378505	0.0148211	0.00789289
40.3	0.09990546	-0.104785	0.85408578	0.33655475	-0.9375471	0.01503927	0.00917409
40.4	0.10098641	-0.1063635	0.85381071	0.33490628	-0.93753	0.01576899	0.0101644
40.5	0.10076071	-0.1069359	0.85322034	0.33441408	-0.9373371	0.01625023	0.00971757
40.6	0.10155951	-0.1065895	0.85273482	0.3340291	-0.9371186	0.0158847	0.00979485
40.7	0.10107967	-0.1079883	0.85216733	0.33240019	-0.9369761	0.01666551	0.01133424
40.8	0.10107967	-0.1079883	0.85216733	0.33240019	-0.9369761	0.01666551	0.01133424
40.9	0.10135738	-0.1113839	0.85095435	0.32848266	-0.9365896	0.01545204	0.01104912
41	0.10285047	-0.112228	0.84991697	0.32762244	-0.936046	0.01779647	0.01031755
41.1	0.10312258	-0.1134716	0.84909761	0.32610361	-0.935724	0.0201606	0.01018356
41.2	0.10577855	-0.1143196	0.84855992	0.32336629	-0.9356151	0.02041051	0.01067121
41.3	0.10674295	-0.1151016	0.84711402	0.32225588	-0.9351251	0.0231289	0.01147314
41.4	0.1046464	-0.1174151	0.84461892	0.3179312	-0.9340012	0.02266002	0.0128983
41.5	0.10398395	-0.1191525	0.84456689	0.31707281	-0.9338502	0.02361671	0.01437008
41.6	0.10356581	-0.1217857	0.84369893	0.31644252	-0.9335997	0.0235459	0.01515553
41.7	0.10568786	-0.121905	0.84287999	0.31394206	-0.93325	0.02129185	0.01546387
41.8	0.10766978	-0.1222578	0.84256064	0.31176445	-0.9333363	0.01963449	0.01575797
45	0.13117918	-0.1597634	0.81837534	0.27751508	-0.9261271	0.04662563	0.02164752
45.1	0.13328136	-0.1612164	0.81769123	0.27776971	-0.9259317	0.0468297	0.02364867
45.2	0.13405511	-0.1620067	0.81714099	0.27798396	-0.9257859	0.04687275	0.02569884
45.3	0.13408091	-0.16156	0.81599506	0.27632167	-0.9252682	0.04612117	0.02569905
45.4	0.13588941	-0.1618844	0.81545116	0.27609464	-0.9251177	0.04624749	0.0276058
45.5	0.13673761	-0.1626986	0.81501271	0.27516098	-0.9250591	0.04718892	0.0265518
45.6	0.13893532	-0.1635019	0.81370818	0.27143377	-0.9247894	0.04403223	0.02727408
45.7	0.13990222	-0.1645436	0.81323881	0.27118445	-0.924641	0.04318849	0.02890719
45.8	0.14012852	-0.1648981	0.81281537	0.27094523	-0.9245278	0.04285513	0.02992375
45.9	0.14085757	-0.165098	0.81195741	0.27255684	-0.9242731	0.0406672	0.0321221
46	0.14149594	-0.1651107	0.81102727	0.27078764	-0.9239322	0.03968637	0.03198513
46.1	0.1418899	-0.1655418	0.81055617	0.26960058	-0.9236886	0.04049042	0.03374659
46.2	0.14259659	-0.1652716	0.80988145	0.26930894	-0.9234908	0.03939247	0.03405025
46.3	0.14489813	-0.1644506	0.80910816	0.26907284	-0.923349	0.0400411	0.03509848
46.4	0.14542213	-0.163886	0.80747409	0.26789191	-0.922936	0.04080721	0.03524212
46.5	0.14569613	-0.1645078	0.80696719	0.26649012	-0.9228463	0.04300423	0.03299821
46.6	0.14558043	-0.164957	0.80668948	0.26485225	-0.9228126	0.04411697	0.03203354
46.7	0.14738477	-0.1657333	0.80636098	0.26415985	-0.9227619	0.04473515	0.0319229
46.8	0.14765316	-0.1651991	0.80595036	0.2644161	-0.9225907	0.04500952	0.03392946
46.9	0.14894422	-0.1658258	0.80436754	0.26406104	-0.9222854	0.04813936	0.03358455
47	0.15016849	-0.1660109	0.80417622	0.26356921	-0.9223059	0.04751495	0.03393267
47.1	0.1504772	-0.1668628	0.80380001	0.26429467	-0.9222573	0.04805951	0.03448523
47.2	0.15144724	-0.1679646	0.80291948	0.26213574	-0.9220655	0.04649695	0.03548605
47.3	0.15195848	-0.1679653	0.80214732	0.26192582	-0.9218239	0.04550889	0.03657393
47.4	0.15193074	-0.1676992	0.80121103	0.26057521	-0.9215136	0.04582338	0.03755316
47.5	0.15387834	-0.1687697	0.800998	0.25994806	-0.9216844	0.04699719	0.03698527
47.6	0.15217191	-0.1719548	0.79983821	0.2577269	-0.9212971	0.04602108	0.03892026
47.7	0.15361145	-0.1732747	0.79878614	0.25539735	-0.9211667	0.04723572	0.04021848
47.8	0.15502353	-0.1735946	0.79799136	0.25546651	-0.9209602	0.04572205	0.03983889
47.9	0.15564164	-0.1753061	0.79664189	0.25623801	-0.9205933	0.04507102	0.03859775
48	0.15669251	-0.1756101	0.79626892	0.25460082	-0.9205716	0.04679803	0.03932467
48.1	0.15881236	-0.1764308	0.79544696	0.25364448	-0.9205407	0.0459061	0.03698799
48.2	0.15980721	-0.1763575	0.79483783	0.25314094	-0.920421	0.04546368	0.03803965
48.3	0.16143845	-0.1772165	0.79359815	0.25457534	-0.9202942	0.04595001	0.03846175
48.4	0.16198424	-0.1792883	0.79209911	0.25265302	-0.9197419	0.04435007	0.03806436
48.5	0.16275926	-0.179265	0.79111538	0.25252007	-0.9193756	0.0445867	0.03876041
48.6	0.16437033	-0.1803928	0.78860353	0.25256747	-0.9185691	0.04370443	0.04154272
48.7	0.16476562	-0.1804902	0.78804732	0.2508285	-0.9185021	0.04405656	0.04046133
48.8	0.16525886	-0.1825823	0.78716675	0.24975901	-0.918238	0.04396506	0.04127069
48.9	0.16540105	-0.1841902	0.78609956	0.25000022	-0.9180151	0.04208578	0.0413669
49	0.1663972	-0.1839387	0.78554873	0.25082364	-0.9180394	0.04186919	0.04217546
49.1	0.16516267	-0.1840742	0.78473809	0.24900237	-0.9178417	0.04260618	0.04414396
49.2	0.16648789	-0.1843291	0.78407477	0.2482629	-0.917795	0.04335708	0.0448316
49.3	0.16870366	-0.1838022	0.7827742	0.24761906	-0.9173113	0.04329864	0.04380668
49.4	0.16894365	-0.1845506	0.78170338	0.24657466	-0.9172541	0.04337463	0.04373597
49.5	0.16964924	-0.1841445	0.78070206	0.24557443	-0.9170571	0.04543523	0.04314946
49.6	0.1716422	-0.184238	0.78011583	0.24448445	-0.9169796	0.04479429	0.04116366
49.7	0.17271822	-0.1842516	0.77859989	0.24243996	-0.9164005	0.04542848	0.04033927
49.8	0.17404383	-0.1839972	0.77839523	0.24233712	-0.9164622	0.04552842	0.04023729
49.9	0.17428418	-0.1839821	0.77856727	0.24232336	-0.916433	0.04592773	0.0404869
50	0.17633226	-0.1863124	0.7773794	0.24019879	-0.9163551	0.04601622	0.03880786
50.1	0.17984939	-0.1860725	0.77560956	0.23924203	-0.9160405	0.04592729	0.0407012
50.2	0.18129181	-0.1859372	0.77441706	0.23650512	-0.9158121	0.04670264	0.04061441
50.3	0.18249408	-0.185889	0.77330374	0.23559495	-0.9154985	0.04773252	0.04132267
50.4	0.1827022	-0.1866353	0.77268681	0.23457432	-0.9153239	0.04915745	0.04140808
50.5	0.18288134	-0.1853707	0.77160011	0.23366217	-0.9149881	0.04949661	0.04202463
50.6	0.18490398	-0.1855723	0.77097202	0.23293036	-0.9149766	0.05041501	0.0425581
50.7	0.18386765	-0.1871375	0.7701253	0.23134876	-0.9145011	0.05174853	0.04315337
50.8	0.18375234	-0.1869481	0.76992456	0.2296747	-0.9145542	0.0501648	0.04222968
50.9	0.18677619	-0.1873616	0.76856018	0.22949221	-0.9144907	0.04910599	0.04247906
51	0.18742724	-0.1877858	0.76707855	0.22807228	-0.9140122	0.04820931	0.04125575
51.1	0.18922441	-0.1919172	0.7658449	0.22597805	-0.9139551	0.04969012	0.04230839
51.2	0.19044635	-0.1918557	0.76516923	0.22695291	-0.9137759	0.05026392	0.04405371
51.3	0.19144027	-0.1930704	0.7634942	0.22610889	-0.9134272	0.05005167	0.04583503
51.4	0.19160926	-0.1933955	0.76258227	0.22493514	-0.912989	0.04900618	0.04718755
51.5	0.1933446	-0.1934063	0.76218179	0.22487022	-0.9129117	0.04874386	0.04719618
51.6	0.19340013	-0.1928706	0.76053444	0.22351297	-0.9126535	0.04771464	0.04891949
51.7	0.19475189	-0.1917116	0.75914856	0.22267007	-0.9122209	0.04843184	0.04841438
51.8	0.19525103	-0.1915375	0.75783271	0.22066499	-0.9119312	0.04830391	0.04720793
51.9	0.19703849	-0.1917623	0.75732812	0.2193923	-0.9120595	0.04828768	0.04900976
52	0.19851382	-0.1921648	0.75637774	0.21960184	-0.9122691	0.04718628	0.04986055
52.1	0.19985631	-0.1929623	0.75495537	0.22025109	-0.9119784	0.04629125	0.05011939
52.2	0.19953891	-0.1941104	0.75323421	0.21988055	-0.9114587	0.04514215	0.05240334
52.3	0.20143765	-0.1937439	0.75256314	0.21972639	-0.9114581	0.04532587	0.05169295
52.4	0.20230649	-0.194486	0.75212652	0.21927992	-0.9113924	0.04554402	0.05258427
52.5	0.2021537	-0.1935699	0.75084723	0.2177549	-0.9112565	0.04385186	0.05251588
52.6	0.20350485	-0.1933524	0.74950536	0.21462964	-0.9109039	0.04318594	0.05124951
52.7	0.20528671	-0.1928081	0.74865415	0.21414634	-0.9107713	0.04418914	0.04987302
52.8	0.20801847	-0.1914567	0.74641758	0.2112624	-0.9102575	0.04188231	0.04995892
52.9	0.20835435	-0.1924453	0.74570296	0.21145102	-0.9100665	0.04202499	0.0499545
53	0.21031822	-0.1934214	0.74495176	0.21013302	-0.9099589	0.04097867	0.05037104
53.1	0.20978127	-0.193326	0.74365356	0.2083872	-0.9096484	0.04123167	0.05144123
53.2	0.21203352	-0.1940032	0.7429104	0.20630817	-0.9095731	0.04073938	0.0519944
53.3	0.21166529	-0.1948984	0.74211509	0.20499184	-0.9093642	0.04104898	0.05276077
53.4	0.21461285	-0.1951329	0.74024035	0.20213631	-0.909243	0.04328469	0.05250214
53.5	0.2174061	-0.1967239	0.73876532	0.19805384	-0.9090778	0.04421274	0.05216835
53.6	0.21889685	-0.1978292	0.7375544	0.19658144	-0.9092498	0.04256974	0.05181599
53.7	0.22011327	-0.20067	0.73574727	0.19479084	-0.9090825	0.04278076	0.0509133
53.8	0.22121151	-0.2010678	0.73551902	0.19444719	-0.9091368	0.04356887	0.05100816
53.9	0.22332281	-0.2038254	0.73317838	0.19006872	-0.909185	0.04339736	0.05300483
54	0.22648546	-0.2046145	0.7323692	0.18928851	-0.9091626	0.04352421	0.05513949
54.1	0.22644987	-0.2080666	0.73123007	0.18803301	-0.9091226	0.04314169	0.05507504
54.2	0.22890371	-0.2080661	0.72938313	0.18659501	-0.9087732	0.04498954	0.05597273
54.3	0.23066591	-0.2095574	0.72828737	0.18587886	-0.9088802	0.04466876	0.05611163
54.4	0.23171923	-0.2108766	0.72704349	0.18471212	-0.9086964	0.04332212	0.05658837
54.5	0.23167969	-0.2128215	0.72580463	0.18468743	-0.9085668	0.043945	0.05757502
54.6	0.23370078	-0.2123071	0.72434896	0.18313072	-0.9083939	0.04484255	0.05648701
54.7	0.23471633	-0.2148728	0.72309361	0.18537885	-0.9085018	0.04410282	0.05739507
54.8	0.23571331	-0.2150057	0.72289669	0.18394654	-0.9085707	0.04428164	0.0575698
54.9	0.23794079	-0.2141115	0.72046599	0.18331903	-0.9081428	0.04503935	0.05760633
55	0.23826612	-0.2147398	0.71874284	0.18198189	-0.9079517	0.04463223	0.05755993
55.1	0.2394332	-0.2136058	0.71676071	0.1834197	-0.9072941	0.04543805	0.05604746
55.2	0.23965095	-0.2166893	0.7148244	0.18194884	-0.9070171	0.04561851	0.05614504
55.3	0.24002591	-0.2163563	0.71382443	0.18057155	-0.9067657	0.04505829	0.05571862
55.4	0.23979094	-0.2166496	0.71235694	0.17924285	-0.9063138	0.04548549	0.05469443
55.5	0.24109091	-0.2189701	0.71101569	0.17914708	-0.9061828	0.04581024	0.05402433
55.6	0.24163687	-0.2198116	0.70993764	0.17822958	-0.906025	0.04612938	0.05460249
55.7	0.24259405	-0.2203266	0.70879518	0.17720507	-0.9059938	0.04683438	0.05361655
55.8	0.24416142	-0.2205012	0.70839197	0.17627426	-0.905928	0.04748416	0.05375533
55.9	0.2440813	-0.2227262	0.7065697	0.17498544	-0.9057115	0.04651141	0.05347717
56	0.24416973	-0.2231325	0.70608573	0.17372386	-0.9056276	0.04676597	0.05338016
56.1	0.24541876	-0.2233472	0.70571685	0.17274421	-0.9054893	0.0471529	0.0520635
56.2	0.24562888	-0.2238582	0.70269405	0.1708901	-0.9049192	0.04851436	0.0524671
56.3	0.24666941	-0.2254174	0.70185149	0.17179181	-0.9050067	0.04852391	0.05362361
56.4	0.24766861	-0.2270378	0.70062202	0.17058193	-0.9049656	0.05044869	0.05248219
56.5	0.24913093	-0.2268892	0.7000049	0.16978823	-0.9050015	0.05007155	0.05243677
56.6	0.24906941	-0.2273315	0.69871381	0.17074664	-0.9048348	0.05076389	0.05174168
56.7	0.25057768	-0.2282331	0.69716553	0.17114916	-0.9046494	0.05200811	0.05168524
56.8	0.25097468	-0.2291173	0.69639326	0.17220877	-0.9045704	0.05323194	0.05218573

11.9	0.04893969	0.02202063	0.92025977	0.53173056	-0.9606899	-0.009371	-0.0425064
12	0.04978318	0.02134442	0.92023798	0.5307984	-0.9606881	-0.0093282	-0.0413049
12.1	0.04987053	0.02088511	0.91999223	0.53073657	-0.9606151	-0.010094	-0.043326
12.2	0.04939245	0.02180497	0.91977922	0.52923486	-0.9605946	-0.0116387	-0.0428126
12.3	0.04889462	0.02089196	0.91931226	0.52790309	-0.9605148	-0.011084	-0.0431688
12.4	0.05016929	0.02107311	0.91902495	0.52742182	-0.960407	-0.009495	-0.0432571
12.5	0.05108571	0.0209879	0.9188507	0.5270942	-0.9603635	-0.0089315	-0.0430045
12.6	0.05051862	0.01918294	0.91855415	0.52553637	-0.960315	-0.0094548	-0.0435887
12.7	0.04855446	0.01813373	0.9181369	0.52531236	-0.9602133	-0.0086081	-0.0443911
12.8	0.04877944	0.01881171	0.91822161	0.52626332	-0.9602959	-0.010048	-0.0438227
12.9	0.04727443	0.0149593	0.91775326	0.52494424	-0.9601969	-0.0098358	-0.0443934
13	0.0474412	0.01570117	0.91747309	0.52425903	-0.9601117	-0.009379	-0.0445083
13.1	0.04689986	0.01626852	0.9172617	0.52345519	-0.9601071	-0.0107334	-0.0453008
13.2	0.0483633	0.01564936	0.91709103	0.52373649	-0.9600855	-0.0120038	-0.044786
13.3	0.0483633	0.01564936	0.91709103	0.52373649	-0.9600855	-0.0120038	-0.044786
13.4	0.04955669	0.01413178	0.91687522	0.52328108	-0.9600052	-0.0120229	-0.0444594
13.5	0.05045341	0.01472435	0.91678749	0.5241017	-0.9600507	-0.0134313	-0.0442224
13.6	0.05051519	0.01401013	0.91644255	0.52399268	-0.9599182	-0.0127793	-0.043443
13.7	0.05051519	0.01401013	0.91644255	0.52399268	-0.9599182	-0.0127793	-0.043443
13.8	0.05035081	0.01481947	0.91568715	0.5247398	-0.9597166	-0.0103618	-0.0440499
13.9	0.05035081	0.01481947	0.91568715	0.5247398	-0.9597166	-0.0103618	-0.0440499
14	0.05099193	0.01351246	0.91557911	0.52468587	-0.9597336	-0.0108538	-0.0439981
14.1	0.04778063	0.01214662	0.91533927	0.52332666	-0.9596566	-0.0111763	-0.0461793
14.2	0.04817085	0.01290847	0.9151647	0.52237029	-0.9596236	-0.0122979	-0.0477142
14.3	0.0474305	0.01274464	0.91496694	0.5214764	-0.9595501	-0.0118379	-0.0470624
14.4	0.04741464	0.01200351	0.91499212	0.52165882	-0.9595342	-0.0116713	-0.0466073
14.5	0.04722751	0.01155364	0.91488233	0.52132041	-0.9595416	-0.0125694	-0.0465334
14.6	0.04736112	0.01078837	0.91497402	0.52120778	-0.9595886	-0.0139054	-0.0459037
14.7	0.04859084	0.01124646	0.91488944	0.52094539	-0.9595734	-0.013122	-0.0456877
14.8	0.04774695	0.01215329	0.91459775	0.51904346	-0.959577	-0.0150481	-0.0457808
14.9	0.04878792	0.01143447	0.91448421	0.51857526	-0.9595695	-0.0140766	-0.046714

60	0.27741237	-0.2347254	0.66148028	0.14588056	-0.9008362	0.07883254	0.04298498
60.1	0.27804098	-0.2352998	0.66033049	0.14494002	-0.9006764	0.0781747	0.04337285
60.2	0.27772926	-0.2359042	0.65939864	0.14399479	-0.9005811	0.07919286	0.04264168
60.3	0.27909047	-0.2358601	0.65809312	0.14263961	-0.9003803	0.08117798	0.04151794
60.4	0.27986061	-0.2361955	0.65706036	0.14414919	-0.9003512	0.08185738	0.04323458
60.5	0.27961739	-0.235779	0.65658048	0.14364406	-0.90041	0.08282094	0.04216563
60.6	0.27981102	-0.2353041	0.6551889	0.14331753	-0.9001323	0.0839478	0.0423962
60.7	0.2786711	-0.2359616	0.65344181	0.14263378	-0.899809	0.08482289	0.04367854
60.8	0.28079825	-0.2367381	0.65216185	0.14164475	-0.8996059	0.08597337	0.04268989
60.9	0.28169341	-0.2374961	0.65117257	0.14098524	-0.8994876	0.08645867	0.04366902
61	0.28234729	-0.2383475	0.65107505	0.14013532	-0.8994735	0.0864735	0.04334825
61.1	0.28284463	-0.2398346	0.65042251	0.13867924	-0.899207	0.08606795	0.04424804
61.2	0.28360409	-0.240994	0.64886528	0.13914324	-0.8993837	0.08676384	0.04434495
61.3	0.28363651	-0.2410651	0.64857506	0.13931162	-0.8994167	0.08698487	0.04378865
61.4	0.285381	-0.2419961	0.64697066	0.13779546	-0.8991288	0.08897193	0.04318771
61.5	0.28500386	-0.2410206	0.64502857	0.13701011	-0.8988692	0.08976768	0.04320825
61.6	0.28560006	-0.2409885	0.6432969	0.13675025	-0.8985423	0.09059423	0.04424603
61.7	0.28600741	-0.2408425	0.64179894	0.13743242	-0.8983891	0.09201564	0.04394628
61.8	0.28632235	-0.2408204	0.64060224	0.13724598	-0.8981823	0.09283129	0.04329244
61.9	0.28693582	-0.2415132	0.63945796	0.13768655	-0.8982294	0.09398788	0.04365964
62	0.28864023	-0.2401955	0.63772052	0.13558251	-0.8980286	0.0949622	0.04154687
62.1	0.2886274	-0.2412546	0.63690142	0.13514879	-0.8979751	0.09491758	0.04103921
62.2	0.28873592	-0.2422991	0.63642165	0.13537995	-0.8980341	0.09503615	0.04182529
62.3	0.28937465	-0.2422781	0.6356125	0.13532263	-0.8979502	0.09383337	0.04126177
62.4	0.28991492	-0.2433384	0.63457204	0.13529709	-0.8977028	0.09479462	0.04338176
62.5	0.28941959	-0.243378	0.63354216	0.13361073	-0.8975967	0.0955941	0.04305789
62.6	0.28849652	-0.2434213	0.63199683	0.13294276	-0.8974352	0.09571732	0.04383482
62.7	0.28933202	-0.2451263	0.6305473	0.13505308	-0.897283	0.09819747	0.04446424
62.8	0.29164661	-0.2449859	0.62896239	0.13406803	-0.897377	0.09917844	0.04482904
62.9	0.29245822	-0.2464233	0.62780641	0.13381	-0.8974739	0.09879755	0.0444307
63	0.29302827	-0.24801	0.6268061	0.13343727	-0.89721	0.10013165	0.0440351
63.1	0.29451463	-0.2497514	0.6264024	0.13348767	-0.8971991	0.09936033	0.04453887
63.2	0.29607558	-0.2497262	0.62561978	0.13335954	-0.8971873	0.10080272	0.04405339
63.3	0.29620755	-0.2505261	0.62477285	0.13142605	-0.8970189	0.10193021	0.04360203
63.4	0.29744128	-0.2501081	0.62369439	0.13174046	-0.8971214	0.10343288	0.04267027
63.5	0.29691154	-0.2508353	0.62235919	0.12989554	-0.8966664	0.10373335	0.0409411
63.6	0.29784138	-0.2515214	0.62013587	0.1302423	-0.8964078	0.10495386	0.04008326
63.7	0.29883971	-0.2512784	0.61829644	0.12815538	-0.896199	0.10635374	0.0397244
63.8	0.29940602	-0.251581	0.61774224	0.12866708	-0.8961325	0.10722085	0.03975134
63.9	0.29980028	-0.2525752	0.61673197	0.12746262	-0.895982	0.10551275	0.0376003
64	0.29983022	-0.2531235	0.61638063	0.12730276	-0.8960153	0.10646768	0.03683939
64.1	0.299901	-0.2552091	0.61472188	0.12696605	-0.895668	0.10752995	0.03617728
64.2	0.29996324	-0.2552483	0.61445341	0.12635814	-0.8957419	0.10812869	0.03567292
64.3	0.301463	-0.2560414	0.61467621	0.12545778	-0.8956511	0.10787759	0.03422613
64.4	0.30186942	-0.2563174	0.61377453	0.12422616	-0.8955362	0.10822263	0.03376895
64.5	0.30314107	-0.2574358	0.61358098	0.12305116	-0.8955518	0.10868107	0.03441189
64.6	0.30360009	-0.2588547	0.61269043	0.12163059	-0.8953824	0.10802063	0.03389616
64.7	0.30371646	-0.2599215	0.61113181	0.1208984	-0.8953579	0.10872567	0.03329196
64.8	0.30574573	-0.2614257	0.60968268	0.12015191	-0.8951481	0.11004239	0.03283455
64.9	0.30612359	-0.261969	0.60876611	0.11950497	-0.8949912	0.11046323	0.03193486
65	0.30739762	-0.2621478	0.60789308	0.11859383	-0.8948799	0.11083298	0.03120846
65.1	0.30700652	-0.2623989	0.60672346	0.11884054	-0.8948202	0.11212133	0.03072762
65.2	0.30805512	-0.2633678	0.6058939	0.11849419	-0.8947498	0.11241496	0.03059554
65.3	0.3095973	-0.2629474	0.6054805	0.11890878	-0.8948496	0.11423954	0.02928566
65.4	0.31025352	-0.264107	0.60385413	0.11895916	-0.8947359	0.11519671	0.02858167
65.5	0.31131119	-0.2639168	0.60236373	0.11778704	-0.8943914	0.11630092	0.02784416
65.6	0.31166177	-0.2642003	0.60161487	0.1176067	-0.8941363	0.11644325	0.02683242
65.7	0.31177817	-0.2656833	0.6004541	0.11734618	-0.8938768	0.11790555	0.02530312
65.8	0.31287198	-0.2669015	0.60029789	0.11532487	-0.8937478	0.1205476	0.02426253
65.9	0.31244711	-0.2683884	0.59933614	0.11553583	-0.8937194	0.11974912	0.02435439
66	0.31291046	-0.2678956	0.59832504	0.11481077	-0.893664	0.12065291	0.02404915
66.1	0.3127347	-0.2687833	0.59810645	0.11446963	-0.8937443	0.12060026	0.02367515
66.2	0.31412184	-0.2692659	0.59753435	0.11448599	-0.8937401	0.12198711	0.02373378
66.3	0.31519679	-0.2675746	0.59671931	0.11536047	-0.8939279	0.12298768	0.02325237
66.4	0.31508771	-0.2675502	0.59624135	0.11447121	-0.8938402	0.12345093	0.02280881
66.5	0.31606126	-0.268645	0.59571815	0.11472139	-0.8938558	0.12303617	0.02199769
66.6	0.31653713	-0.2691499	0.59505754	0.11384009	-0.8937867	0.1229962	0.02209243
66.7	0.31796866	-0.2691912	0.59483297	0.11311727	-0.8938188	0.12354564	0.0226208
66.8	0.31806041	-0.2695455	0.59455993	0.11277764	-0.8936337	0.12355578	0.02292739
66.9	0.31814348	-0.2699346	0.59387415	0.11223376	-0.8935115	0.12335357	0.02340702
67	0.31848382	-0.2695808	0.59341048	0.11211833	-0.8934723	0.12354871	0.02293189
67.1	0.31910519	-0.2686749	0.59198555	0.11262201	-0.8932533	0.12386976	0.02322292
67.2	0.31889758	-0.2690947	0.59032719	0.11180594	-0.8927777	0.12281673	0.02237373
67.3	0.31900547	-0.2691193	0.58915173	0.11123204	-0.8925516	0.12356653	0.02174889
67.4	0.31898165	-0.2686966	0.58698714	0.11025458	-0.8919759	0.12365947	0.02089398
67.5	0.31950681	-0.2691557	0.58672025	0.10950725	-0.8918146	0.12363797	0.01990771
67.6	0.31907543	-0.2684965	0.58593208	0.1080248	-0.8916475	0.12392558	0.01991659
67.7	0.32040964	-0.2691895	0.58529185	0.10863502	-0.8915571	0.12464942	0.01953548
67.8	0.32007059	-0.269239	0.58480859	0.1079223	-0.8913875	0.12501601	0.01920988
67.9	0.32190042	-0.2694254	0.58405429	0.10787496	-0.891519	0.12592993	0.01828702
68	0.32387105	-0.2716101	0.58408323	0.10701223	-0.891622	0.12596324	0.01772427
68.1	0.32329	-0.2718554	0.58303331	0.10635743	-0.8914784	0.12729955	0.01843132
68.2	0.32342766	-0.2722937	0.58179751	0.10604409	-0.8913226	0.12840189	0.01772414
68.3	0.32418569	-0.27271	0.58094323	0.10618659	-0.891139	0.12869261	0.01725199
68.4	0.32586282	-0.2734527	0.58027219	0.10481431	-0.8914163	0.1298534	0.01715377
68.5	0.32489116	-0.2724927	0.57882133	0.105238	-0.8912446	0.13192476	0.0159568
68.6	0.32550463	-0.2719269	0.57726982	0.10478524	-0.8911013	0.13230232	0.01381556
68.7	0.3253588	-0.2717247	0.57647322	0.10473235	-0.8910588	0.1327353	0.01322041
68.8	0.32548821	-0.2720297	0.57564307	0.10419777	-0.8909331	0.13333453	0.01331995
68.9	0.32632098	-0.2719294	0.57491833	0.10386395	-0.8909328	0.13388906	0.01236001

26.9	0.06320216	-0.0222412	0.89278347	0.45460489	-0.9528088	-0.0227168	-0.0153491
27	0.06400132	-0.0228803	0.89250592	0.45235835	-0.9526453	-0.020319	-0.0140667
27.1	0.06463274	-0.0230411	0.89242546	0.45128238	-0.952525	-0.0196925	-0.015239
27.2	0.06664623	-0.0214121	0.8920684	0.45036924	-0.9524181	-0.0191989	-0.0125815
27.3	0.06696624	-0.0222433	0.89177998	0.44938501	-0.9523594	-0.0179638	-0.0117931
27.4	0.06774598	-0.0218508	0.89129996	0.44922467	-0.9521159	-0.0190398	-0.0090315
27.5	0.06728931	-0.0223298	0.89099658	0.44866864	-0.9519495	-0.0192864	-0.0078304
27.6	0.06708817	-0.0212895	0.89088807	0.4486572	-0.951932	-0.0188578	-0.0073707
27.7	0.06846671	-0.0206835	0.89077575	0.44860736	-0.9519393	-0.0185037	-0.0084698
27.8	0.07021761	-0.0201291	0.89011367	0.44723064	-0.9517814	-0.0174343	-0.0078604
27.9	0.07074407	-0.0200841	0.88996281	0.44661259	-0.9517962	-0.0174511	-0.0082501
28	0.07065722	-0.0196622	0.88995482	0.44662183	-0.9518805	-0.01702	-0.0071608
28.1	0.07027323	-0.0192419	0.88998963	0.44606731	-0.9519027	-0.017762	-0.0072366
28.2	0.07042593	-0.0193494	0.89005648	0.44702761	-0.9519049	-0.0167188	-0.0071438
28.3	0.07019777	-0.0198535	0.88991723	0.44747249	-0.9518439	-0.017064	-0.0077702
28.4	0.0697813	-0.0197589	0.88943346	0.44562291	-0.9515462	-0.0183464	-0.0081678
28.5	0.07082824	-0.0204502	0.88927613	0.4438523	-0.9514698	-0.0160816	-0.0094746
28.6	0.07098821	-0.0214707	0.88917337	0.44353092	-0.9514646	-0.0154359	-0.010098
28.7	0.07016763	-0.0215958	0.8889091	0.44285807	-0.9513563	-0.0167845	-0.0081044
28.8	0.06941325	-0.023644	0.88857628	0.44218633	-0.9511774	-0.0163491	-0.0073627
28.9	0.0695413	-0.0236719	0.88840996	0.44204749	-0.9510853	-0.0169665	-0.0074924
29	0.06920326	-0.0248655	0.88827468	0.44100305	-0.9509766	-0.0179359	-0.0073052
29.1	0.07005041	-0.0238639	0.88814652	0.44094807	-0.9509192	-0.0181281	-0.0070401
29.2	0.07015265	-0.0233008	0.88826984	0.44134771	-0.9510046	-0.0183263	-0.0081775
29.3	0.07082397	-0.0230212	0.88799612	0.44101202	-0.9508767	-0.0191048	-0.008897
29.4	0.06931684	-0.0235149	0.8875608	0.43931442	-0.9506743	-0.0196692	-0.0081415
29.5	0.06931684	-0.0235149	0.8875608	0.43931442	-0.9506743	-0.0196692	-0.0081415
29.6	0.07013014	-0.0244683	0.8875007	0.43901714	-0.9506422	-0.0185201	-0.0089912
29.7	0.07011216	-0.02442	0.8873934	0.43861257	-0.9505277	-0.0191328	-0.0085293
29.8	0.07135368	-0.0246053	0.88707535	0.43721468	-0.9504544	-0.0183653	-0.0096021
29.9	0.07233191	-0.0238958	0.88648749	0.43551157	-0.9501899	-0.0182356	-0.009004

75	0.34881191	-0.3032355	0.52664289	0.08284468	-0.8885895	0.16142602	-0.0149653
75.1	0.34825607	-0.3036576	0.52558381	0.08190547	-0.8882459	0.16103799	-0.0160528
75.2	0.3479601	-0.3039489	0.52490301	0.0815024	-0.8882781	0.16167367	-0.0169915
75.3	0.34728434	-0.303358	0.52308821	0.08061069	-0.8878516	0.16202572	-0.017723
75.4	0.34908961	-0.3048938	0.52248624	0.0796468	-0.8876847	0.16390304	-0.0189648
75.5	0.3514583	-0.3068668	0.52138951	0.07849456	-0.8877034	0.16473589	-0.0190287
75.6	0.35108012	-0.3074663	0.52083977	0.07827083	-0.8875519	0.16520681	-0.0199682
75.7	0.35154225	-0.3078367	0.52122671	0.07848086	-0.887626	0.16637688	-0.0191453
75.8	0.35190802	-0.3077093	0.52107136	0.07848421	-0.8875829	0.16624395	-0.0193565
75.9	0.35260525	-0.30789	0.52098443	0.07856975	-0.8874616	0.16651158	-0.0195625
76	0.3522614	-0.3102585	0.52003117	0.07869489	-0.8877195	0.16858413	-0.0206413
76.1	0.35218467	-0.3119315	0.51888382	0.0785483	-0.88797	0.16992352	-0.0206971
76.2	0.35152801	-0.3113236	0.51746372	0.07807289	-0.8879929	0.17111246	-0.0199487
76.3	0.35229988	-0.3106823	0.516327	0.07727829	-0.8879015	0.17289816	-0.0206263
76.4	0.35255344	-0.3098436	0.51533395	0.07676507	-0.8876657	0.17289286	-0.0203332
76.5	0.35239106	-0.3092757	0.51503524	0.07612452	-0.8875645	0.17370683	-0.0211861
76.6	0.35297017	-0.3107239	0.51457027	0.07629041	-0.8875089	0.17453974	-0.02157
76.7	0.35442718	-0.31249	0.51339764	0.07619849	-0.8874908	0.1749895	-0.0222666
76.8	0.3549855	-0.3129515	0.51296492	0.07570126	-0.8873039	0.17699259	-0.0224874
76.9	0.35540148	-0.3127109	0.51161399	0.07573247	-0.8872314	0.17733059	-0.0235477
77	0.35555761	-0.3129561	0.51080277	0.07500085	-0.8870327	0.17791369	-0.0229194
77.1	0.35673851	-0.3132349	0.50981751	0.07438917	-0.8868979	0.17765118	-0.0228588
77.2	0.35603279	-0.3134198	0.50992212	0.0730987	-0.8869204	0.17829985	-0.0235865
77.3	0.35700088	-0.3133812	0.51014273	0.07160089	-0.8867727	0.17938673	-0.0246931
77.4	0.35637505	-0.3148001	0.50920441	0.07242613	-0.8865678	0.18003777	-0.025625
77.5	0.35580808	-0.314998	0.50914414	0.07247313	-0.8865128	0.17998854	-0.0254596
77.6	0.35497498	-0.315312	0.50908257	0.07221602	-0.886431	0.17971759	-0.0255782
77.7	0.35491512	-0.3140802	0.50858544	0.07185034	-0.886527	0.18062088	-0.0261359
77.8	0.35460075	-0.3159157	0.50666933	0.07189868	-0.8864414	0.18079194	-0.02736
77.9	0.35471286	-0.3162636	0.50534429	0.07034146	-0.8863621	0.18194457	-0.0280281
78	0.35495544	-0.3165843	0.50494583	0.06934056	-0.8862873	0.18195337	-0.0281986
78.1	0.35572453	-0.3178206	0.504287	0.06795992	-0.8864334	0.18280317	-0.0293204
78.2	0.35676061	-0.3187655	0.50375975	0.06822534	-0.8864368	0.18326662	-0.0294632
78.3	0.35646423	-0.3192962	0.50287542	0.06742325	-0.8862848	0.18378475	-0.0298168
78.4	0.35747217	-0.3189424	0.50212086	0.06732654	-0.8858671	0.18422082	-0.0291884
78.5	0.35738105	-0.3195548	0.5019245	0.06689786	-0.8858694	0.18487641	-0.0297676
78.6	0.3574156	-0.3207224	0.5008442	0.06655528	-0.8859907	0.1857311	-0.0304014
78.7	0.35810793	-0.3203498	0.500083	0.06596722	-0.8860255	0.1861544	-0.0289271
78.8	0.35882584	-0.3212423	0.50001381	0.06613813	-0.8861481	0.18588546	-0.028479
78.9	0.36012847	-0.3218692	0.49896802	0.06601601	-0.8864099	0.18647625	-0.0278498
79	0.35993576	-0.321971	0.49820083	0.06462476	-0.8860577	0.18626994	-0.0285372
79.1	0.36052873	-0.3220191	0.49785824	0.06444466	-0.8858012	0.18540714	-0.0289016
79.2	0.36097302	-0.3221492	0.49768036	0.06505026	-0.885696	0.18549133	-0.028566
79.3	0.36044773	-0.3223789	0.49663014	0.06487312	-0.8855056	0.18531169	-0.0288104
79.4	0.3610624	-0.3218045	0.49648022	0.06532463	-0.8853599	0.18522805	-0.029238
79.5	0.36135312	-0.3218058	0.49649262	0.06621581	-0.8853897	0.1852647	-0.0287941
79.6	0.36122906	-0.3225587	0.49646508	0.06608677	-0.885423	0.18582085	-0.0289547
79.7	0.36165634	-0.3218612	0.49494212	0.06646174	-0.8852311	0.18610024	-0.0294695
79.8	0.36196749	-0.3227933	0.4939665	0.06602772	-0.8849068	0.18833758	-0.0302483
79.9	0.36157838	-0.3232224	0.49309463	0.06460262	-0.8847384	0.18844603	-0.0300846
80	0.36222093	-0.3236823	0.49235903	0.06377285	-0.8846049	0.18834705	-0.0295569
80.1	0.36266145	-0.3243197	0.4915379	0.06410578	-0.8846532	0.18894563	-0.0300472
80.2	0.36376722	-0.3233575	0.49081786	0.06310482	-0.8846298	0.18962113	-0.0312516
80.3	0.36443798	-0.3228664	0.49026735	0.06178418	-0.884845	0.19114871	-0.0330017
80.4	0.36511913	-0.3232562	0.48963082	0.06196866	-0.8849598	0.19240547	-0.0341136
80.5	0.36501754	-0.3224883	0.48943192	0.06159183	-0.8849076	0.19303289	-0.0340253
80.6	0.36477519	-0.3221623	0.48878015	0.06118083	-0.8850312	0.19351596	-0.0349409
80.7	0.3650877	-0.3240017	0.48811013	0.06085228	-0.8850656	0.19383968	-0.0367871
80.8	0.36481887	-0.3239889	0.48750292	0.06024116	-0.884989	0.19362377	-0.0371554
80.9	0.36439783	-0.3240753	0.48761907	0.06000887	-0.885022	0.19407763	-0.0374518
81	0.36546111	-0.3252547	0.48668603	0.05992833	-0.8850274	0.19546102	-0.0382255
81.1	0.36599776	-0.3254374	0.48638018	0.05852105	-0.885077	0.19588038	-0.037957
81.2	0.3657064	-0.3258546	0.48587811	0.05873028	-0.8850671	0.19555053	-0.0381179
81.3	0.36685454	-0.3267352	0.48503106	0.05865035	-0.8851785	0.19543564	-0.03826
81.4	0.36636505	-0.3269013	0.4845928	0.0573041	-0.8850393	0.19591689	-0.037437
81.5	0.36664477	-0.3269685	0.48463992	0.05758849	-0.8850427	0.19554598	-0.0369932
81.6	0.36677121	-0.3274726	0.48360127	0.05723431	-0.8849591	0.19591195	-0.0387397
81.7	0.3668997	-0.3290017	0.48300783	0.05829947	-0.8849817	0.19734738	-0.0389576
81.8	0.36649589	-0.328453	0.48299545	0.0579838	-0.884923	0.19825212	-0.0400211
81.9	0.36639999	-0.3284014	0.48246246	0.05757542	-0.8849393	0.19823273	-0.0398391
82	0.36626749	-0.32871	0.48193302	0.05778043	-0.8848016	0.19822968	-0.0403492
82.1	0.36642347	-0.3286537	0.48153233	0.05784998	-0.8847404	0.19823661	-0.0401795
82.2	0.36556234	-0.3281461	0.4808607	0.05821268	-0.8845874	0.19835927	-0.0409681
82.3	0.36565006	-0.3295216	0.4813317	0.05681002	-0.8845176	0.19778256	-0.0412558
82.4	0.36648121	-0.328647	0.48040738	0.05697833	-0.8841741	0.19758178	-0.0413535
82.5	0.36701367	-0.3294233	0.47980019	0.05780336	-0.8843077	0.19728993	-0.041219
82.6	0.36660937	-0.3293699	0.4791724	0.05874805	-0.8841539	0.19735082	-0.0413498
82.7	0.36636266	-0.3296809	0.47915788	0.05806987	-0.8840641	0.1975274	-0.0415598
82.8	0.36712964	-0.3303618	0.47902262	0.05925593	-0.8841352	0.19873877	-0.0410165
82.9	0.36845213	-0.3317777	0.47757219	0.06008965	-0.8838915	0.20041535	-0.0419074
83	0.36866481	-0.3326797	0.47724678	0.05986455	-0.8837865	0.20057686	-0.041611
83.1	0.36815068	-0.3332824	0.47738231	0.05994507	-0.8837132	0.2012172	-0.0407602
83.2	0.36864831	-0.3349818	0.47793989	0.05959746	-0.883815	0.20182551	-0.0416514
83.3	0.36971654	-0.3350188	0.47716475	0.05955184	-0.883898	0.20219224	-0.0422123
83.4	0.36930865	-0.3346655	0.47621562	0.0598371	-0.8836555	0.20217572	-0.0428276
83.5	0.37027306	-0.3352917	0.47566315	0.05995312	-0.8837526	0.20351288	-0.0415248
83.6	0.37101546	-0.3370192	0.47544528	0.05982316	-0.8837616	0.20495713	-0.042093
83.7	0.37066011	-0.3369565	0.47558481	0.05991285	-0.8837635	0.20526501	-0.0422313
83.8	0.37087269	-0.3369733	0.47523081	0.06001578	-0.8839257	0.20583158	-0.0423461
83.9	0.37031165	-0.3383001	0.47581745	0.05895931	-0.8839283	0.20667203	-0.0417011

41.9	0.10799905	-0.1240891	0.84116677	0.31008273	-0.9328084	0.02325632	0.01505248
42	0.10980432	-0.1245802	0.84083504	0.31057063	-0.9327257	0.02420962	0.01434866
42.1	0.11120746	-0.1257657	0.84053888	0.3103331	-0.9325865	0.02449378	0.0148477
42.2	0.11041081	-0.1262533	0.83998868	0.30890716	-0.932397	0.02501881	0.01486378
42.3	0.11197482	-0.1264815	0.83923172	0.30736774	-0.9321223	0.02769695	0.01609117
42.4	0.11298215	-0.1275668	0.83862763	0.30649383	-0.9318333	0.02773543	0.01630053
42.5	0.11434121	-0.1289013	0.83816238	0.30599664	-0.9317578	0.02920045	0.01600032
42.6	0.11488561	-0.1323523	0.83785732	0.30354072	-0.931869	0.03012959	0.01589543
42.7	0.11548112	-0.1324017	0.83792166	0.30351996	-0.9318827	0.03175755	0.01713154
42.8	0.11442874	-0.1320512	0.83761794	0.30320446	-0.9317317	0.03205457	0.01742738
42.9	0.11594921	-0.1341545	0.83685537	0.30262742	-0.9317213	0.03417514	0.01588728
43	0.11515746	-0.1333139	0.83497833	0.30008154	-0.9309436	0.03637205	0.01809727
43.1	0.1169767	-0.1348323	0.83477442	0.29909134	-0.9309946	0.03574979	0.01908483
43.2	0.11695149	-0.1372122	0.83355984	0.29685137	-0.9306212	0.03727146	0.02036188
43.3	0.11702413	-0.138198	0.83303946	0.29677261	-0.9303972	0.03659474	0.02064653
43.4	0.11917473	-0.1393025	0.83201529	0.29618568	-0.9299472	0.03776235	0.02043269
43.5	0.11993705	-0.140933	0.831642	0.29589245	-0.9298047	0.0395347	0.01977267
43.6	0.11925348	-0.1410519	0.83073948	0.29352411	-0.9295743	0.03939797	0.02057205
43.7	0.11919036	-0.1444162	0.82907751	0.29183797	-0.9289709	0.04018897	0.02115192
43.8	0.11897987	-0.1465248	0.82798404	0.29028265	-0.928679	0.04037855	0.02391312
43.9	0.12105192	-0.1471586	0.82717455	0.28954869	-0.9284881	0.04224181	0.02219913
44	0.12111869	-0.1490324	0.82675976	0.28897001	-0.928412	0.04197277	0.02206613
44.1	0.12555955	-0.1505068	0.8252703	0.28471135	-0.9282563	0.04256452	0.02190156
44.2	0.12510538	-0.1522547	0.82414738	0.28367768	-0.9278171	0.04176376	0.0229814
44.3	0.125956	-0.1535918	0.82344151	0.28231175	-0.9278169	0.04443078	0.02271704
44.4	0.12645501	-0.1539739	0.82288337	0.2820535	-0.9277271	0.04510217	0.02191459
44.5	0.12802295	-0.1539669	0.82253385	0.28272403	-0.9276584	0.0453098	0.02150997
44.6	0.12853585	-0.1551179	0.82170028	0.28067613	-0.9274759	0.04660484	0.02025112
44.7	0.12821474	-0.1558328	0.82128281	0.27949605	-0.9274397	0.04761349	0.01936278
44.8	0.13099644	-0.1559105	0.82028676	0.28018331	-0.9270324	0.0468993	0.01982954
44.9	0.13037361	-0.1573951	0.81933352	0.27874793	-0.9264086	0.04638901	0.02033512

90	0.38783262	-0.3575745	0.43975658	0.0499301	-0.8824348	0.23783316	-0.0373442
90.1	0.38773995	-0.3582926	0.43997317	0.05027939	-0.8824146	0.23833814	-0.0369299
90.2	0.38831084	-0.358452	0.43863407	0.05027125	-0.8823787	0.24013283	-0.0374658
90.3	0.38797537	-0.3588687	0.43777061	0.05063845	-0.8822054	0.23980762	-0.0369917
90.4	0.38807118	-0.3577341	0.43739111	0.05070582	-0.8821931	0.23954156	-0.0366627
90.5	0.38790663	-0.3586276	0.43587639	0.05063513	-0.8820342	0.24040688	-0.0377112
90.6	0.38794015	-0.3590142	0.43424002	0.04988494	-0.8818884	0.23999133	-0.0377436
90.7	0.38836337	-0.3593972	0.43391995	0.04978918	-0.8818819	0.23998104	-0.0376977
90.8	0.38827852	-0.3602333	0.4339814	0.05026277	-0.8818246	0.24017762	-0.0377783
90.9	0.38860274	-0.3608943	0.43296788	0.04981936	-0.8816122	0.2407792	-0.0373246
91	0.38843744	-0.3611572	0.43352946	0.04876605	-0.8816581	0.24088834	-0.0369316
91.1	0.38965391	-0.3608124	0.43218236	0.04828301	-0.881515	0.24195834	-0.0375287
91.2	0.38968306	-0.3609404	0.43214749	0.04831284	-0.8815838	0.24213034	-0.0370174
91.3	0.38980503	-0.3616164	0.43140761	0.04803839	-0.8815094	0.24248005	-0.0375412
91.4	0.39002768	-0.3617937	0.43074698	0.04743051	-0.8814723	0.24071414	-0.0376442
91.5	0.3896781	-0.3609367	0.42994058	0.0477611	-0.881146	0.24100565	-0.0364024
91.6	0.38926816	-0.3629653	0.42854387	0.04736224	-0.8811454	0.2412451	-0.0365013
91.7	0.38975328	-0.3633794	0.42705962	0.04730627	-0.8807891	0.2407609	-0.0374252
91.8	0.38978246	-0.3640405	0.42614889	0.0480101	-0.8809081	0.2415143	-0.0376738
91.9	0.39035047	-0.3642278	0.42572192	0.04807647	-0.8808477	0.24111863	-0.0372815
92	0.39098459	-0.3635859	0.42556177	0.04754797	-0.8809124	0.24275488	-0.0369666
92.1	0.3910649	-0.3632399	0.42467845	0.04733705	-0.880549	0.24291274	-0.0371335
92.2	0.39158899	-0.3638673	0.42384337	0.04843443	-0.8804969	0.24338663	-0.0364756
92.3	0.39195959	-0.3640052	0.42304281	0.04965264	-0.8807648	0.24353512	-0.0359584
92.4	0.39222399	-0.3646867	0.4219447	0.05115374	-0.8807732	0.24335659	-0.0360862
92.5	0.39249797	-0.3646417	0.42157446	0.05164514	-0.8806577	0.2434031	-0.0357991
92.6	0.39154635	-0.3656232	0.42007339	0.05236043	-0.8805005	0.24367138	-0.0354613
92.7	0.39266712	-0.3665092	0.41935852	0.05303705	-0.8806364	0.24457907	-0.0349502
92.8	0.39319391	-0.3669368	0.41916608	0.05262573	-0.8806123	0.24518729	-0.0354687
92.9	0.39440003	-0.3668005	0.41895593	0.05193047	-0.8807476	0.24564898	-0.0345344
93	0.3943507	-0.3674225	0.41775299	0.05183313	-0.8806204	0.24636501	-0.0335168
93.1	0.39420545	-0.3674395	0.41675472	0.05090576	-0.8804798	0.2470741	-0.033643
93.2	0.3944236	-0.3680874	0.41590937	0.05119076	-0.8804636	0.24720467	-0.0338298
93.3	0.39461334	-0.3680339	0.41592785	0.05166729	-0.8803759	0.24780765	-0.0338553
93.4	0.39512819	-0.3679456	0.4159163	0.05179765	-0.8802728	0.24783371	-0.0332821
93.5	0.39614071	-0.3683435	0.41549249	0.0522588	-0.880319	0.24840749	-0.0344331
93.6	0.39626736	-0.3682933	0.4152379	0.05215007	-0.8803492	0.24836189	-0.0344172
93.7	0.39670368	-0.3675538	0.41371758	0.05218524	-0.8802863	0.24903482	-0.0339786
93.8	0.39633849	-0.3676751	0.41303327	0.05178084	-0.880224	0.24890532	-0.0340638
93.9	0.39554115	-0.3670721	0.41248376	0.05163831	-0.8799421	0.24946679	-0.0338244
94	0.39634813	-0.3673266	0.41116907	0.05197461	-0.8799013	0.24982472	-0.0338448
94.1	0.39669442	-0.3671145	0.41034538	0.05131102	-0.8795877	0.24983186	-0.0333498
94.2	0.39697133	-0.3668196	0.40943893	0.05109623	-0.8794861	0.25024759	-0.0335166
94.3	0.39725637	-0.3664356	0.40798695	0.05128892	-0.8795546	0.25142066	-0.0327997
94.4	0.39746532	-0.3671642	0.40768355	0.05096778	-0.8795576	0.2517005	-0.0326514
94.5	0.39843375	-0.3674134	0.40770418	0.05085756	-0.8797146	0.25153045	-0.0320979
94.6	0.39855107	-0.3687354	0.40726309	0.05217149	-0.8793839	0.25228931	-0.0310693
94.7	0.39886702	-0.3685429	0.40713305	0.05200356	-0.8793537	0.25275075	-0.0310521
94.8	0.39843432	-0.3678741	0.4070864	0.05234769	-0.8796117	0.25359332	-0.0310649
94.9	0.39857475	-0.3679657	0.40693003	0.052175	-0.8797486	0.25408963	-0.0311389
95	0.39872063	-0.3687468	0.40714267	0.05262833	-0.8797918	0.25468805	-0.0319844
95.1	0.39949848	-0.3686585	0.40604323	0.05235309	-0.8796029	0.25491889	-0.0311675
95.2	0.39949848	-0.3686585	0.40604323	0.05235309	-0.8796029	0.25491889	-0.0311675
95.3	0.39935882	-0.3693362	0.40631673	0.05277214	-0.8798836	0.25580955	-0.0310557
95.4	0.39929671	-0.3688161	0.40490276	0.05259382	-0.879628	0.25617277	-0.0312495
95.5	0.40003632	-0.3689632	0.40468057	0.05203768	-0.8794566	0.25641552	-0.0301761
95.6	0.40025199	-0.3695404	0.40444503	0.05296307	-0.8793636	0.25719536	-0.0304334
95.7	0.40034213	-0.369685	0.40459373	0.05283841	-0.8794863	0.25765486	-0.0303699
95.8	0.40054284	-0.3705498	0.40427001	0.05281772	-0.8794245	0.25849297	-0.0299802
95.9	0.40082667	-0.3704732	0.40303768	0.05291955	-0.879433	0.25954244	-0.0281968
96	0.40073173	-0.3703044	0.40262999	0.05256923	-0.8793108	0.25954406	-0.0287551
96.1	0.4007718	-0.3703097	0.4032085	0.05227388	-0.8793741	0.26003492	-0.0286997
96.2	0.40050893	-0.3705701	0.4032569	0.05162717	-0.8793379	0.26014966	-0.0290659
96.3	0.40041075	-0.3702872	0.40338007	0.05157355	-0.8793617	0.26008422	-0.0287717
96.4	0.40076933	-0.3702837	0.40172577	0.05233786	-0.8788682	0.26024846	-0.0288317
96.5	0.4004224	-0.369494	0.40049946	0.05201798	-0.8787325	0.26017742	-0.0283758
96.6	0.3998843	-0.3694734	0.40018045	0.05264342	-0.8787678	0.26004098	-0.0283033
96.7	0.40054685	-0.3700096	0.40002014	0.05253642	-0.8787835	0.26132668	-0.0278422
96.8	0.40082985	-0.3714814	0.39831962	0.05211164	-0.8788482	0.26287641	-0.0274933
96.9	0.40157106	-0.3724313	0.39741112	0.05258568	-0.8793562	0.26412466	-0.0278505
97	0.40171633	-0.3725634	0.39676539	0.05208252	-0.8792528	0.26387559	-0.0281415
97.1	0.40199137	-0.3730181	0.39640728	0.05247861	-0.8794065	0.26470777	-0.0278807
97.2	0.40228403	-0.3729146	0.3962011	0.05233296	-0.8793612	0.2641834	-0.02722
97.3	0.40225921	-0.3729141	0.3959659	0.05285325	-0.8793612	0.26427942	-0.0267074
97.4	0.40230609	-0.372948	0.39624991	0.05172946	-0.8793189	0.26576992	-0.0265731
97.5	0.40256222	-0.3737003	0.39592841	0.05258784	-0.8790167	0.26536826	-0.0260558
97.6	0.4020182	-0.3725897	0.39404842	0.05195015	-0.8785256	0.26519698	-0.0261993
97.7	0.40215451	-0.3734898	0.39394999	0.05159922	-0.8783699	0.26512167	-0.0260145
97.8	0.40234332	-0.3736537	0.39414701	0.05137004	-0.8784968	0.26569902	-0.0258836
97.9	0.40225952	-0.3731956	0.39309719	0.05127735	-0.878572	0.26600642	-0.0255683
98	0.40171193	-0.3734261	0.39231496	0.05077633	-0.8785523	0.26646226	-0.0258293
98.1	0.40176859	-0.3735159	0.3921387	0.05097743	-0.8786178	0.26657838	-0.0260269
98.2	0.40261909	-0.3735021	0.39130261	0.05149947	-0.8787916	0.26647159	-0.0267955
98.3	0.40320419	-0.373552	0.39030621	0.05147831	-0.8790087	0.26781673	-0.027217
98.4	0.40274644	-0.3742373	0.38875886	0.05131261	-0.8789213	0.26783051	-0.027907
98.5	0.40334681	-0.3741494	0.38868757	0.05081614	-0.8788424	0.26839197	-0.0289666
98.6	0.40409489	-0.373782	0.3875287	0.05165326	-0.8791569	0.26948908	-0.029609
98.7	0.40328531	-0.3729732	0.38560396	0.0520697	-0.8789887	0.27052041	-0.0306369
98.8	0.40365528	-0.3740864	0.38490061	0.05143255	-0.8791539	0.27079738	-0.0306656
98.9	0.40479727	-0.3737037	0.38461774	0.05099579	-0.879257	0.27129525	-0.030671

56.9	0.25210834	-0.2279591	0.69521842	0.170496	-0.9043495	0.05295441	0.05242986
57	0.25474707	-0.2286327	0.69385239	0.1700424	-0.9043363	0.05485266	0.05347209
57.1	0.25587612	-0.2292705	0.69299292	0.16866834	-0.9042747	0.05660119	0.05250766
57.2	0.25528053	-0.2293227	0.69261581	0.16841042	-0.9043722	0.05747757	0.05227733
57.3	0.2557989	-0.2286297	0.69171639	0.16759536	-0.9043289	0.05740266	0.05197496
57.4	0.25649395	-0.2291257	0.69129687	0.16694376	-0.9043457	0.05703499	0.05058162
57.5	0.25653317	-0.2294159	0.69066179	0.16476443	-0.9042622	0.05671161	0.05162835
57.6	0.25835981	-0.2287179	0.68885503	0.16392092	-0.9041904	0.05899712	0.052045
57.7	0.26096257	-0.2291613	0.68825755	0.16371517	-0.9043739	0.0603547	0.05102495
57.8	0.2605766	-0.230312	0.68687681	0.16319151	-0.9043186	0.06204008	0.05072065
57.9	0.26226016	-0.2299034	0.68610845	0.16329958	-0.9043642	0.06348847	0.05074872
58	0.2618007	-0.2304916	0.68392524	0.16160875	-0.9038978	0.06523453	0.05143608
58.1	0.26232422	-0.2299451	0.68398272	0.16176669	-0.9038598	0.06631242	0.05128848
58.2	0.26234837	-0.2302882	0.68292568	0.16064838	-0.9036163	0.0671741	0.05058024
58.3	0.2621047	-0.2300736	0.68211945	0.16148603	-0.9034625	0.06763913	0.05022336
58.4	0.26124137	-0.2300575	0.68095813	0.1615274	-0.9031117	0.06821283	0.05030067
58.5	0.26314095	-0.2304763	0.68004258	0.16232357	-0.9029402	0.06907157	0.05053643
58.6	0.26330772	-0.2296644	0.67943929	0.16129946	-0.9028691	0.06944448	0.04967684
58.7	0.26490276	-0.23101	0.67848349	0.159397	-0.9026312	0.06934103	0.04720367
58.8	0.26483328	-0.2308875	0.67796956	0.15916596	-0.9024932	0.06942322	0.04699542
58.9	0.26544288	-0.231245	0.67490235	0.15600065	-0.9019737	0.07103674	0.04881403
59	0.26580225	-0.233303	0.67299318	0.15366182	-0.9014409	0.0715576	0.04901838
59.1	0.26749559	-0.2313349	0.6711622	0.15341374	-0.9014589	0.07312992	0.0481687
59.2	0.26950378	-0.2313662	0.66942995	0.15029675	-0.9013025	0.07524809	0.04594163
59.3	0.26862269	-0.2312877	0.66821136	0.14958748	-0.9010398	0.07484643	0.04706256
59.4	0.26975592	-0.2315518	0.66702808	0.14927962	-0.9010999	0.07548488	0.04549533
59.5	0.27034161	-0.2307947	0.66619197	0.14803913	-0.9010018	0.07552619	0.04469424
59.6	0.27175124	-0.2306081	0.66589054	0.14742262	-0.90107	0.07667769	0.04419338
59.7	0.27254319	-0.2329766	0.66414576	0.1463684	-0.9010595	0.07674672	0.04371075
59.8	0.27323734	-0.2324309	0.66258053	0.1460188	-0.9007984	0.0768513	0.04508558
59.9	0.27614815	-0.2326415	0.66181189	0.14560881	-0.900896	0.07851429	0.04330093

105	0.40430339	-0.3745974	0.35679575	0.05508986	-0.8770127	0.28533934	-0.0325332
105.1	0.40457852	-0.3749507	0.35637418	0.05527512	-0.8770368	0.28573039	-0.0320453
105.2	0.40476991	-0.3743858	0.35566044	0.05564265	-0.8769268	0.28626583	-0.0319699
105.3	0.40476991	-0.3743858	0.35566044	0.05564265	-0.8769268	0.28626583	-0.0319699
105.4	0.40570806	-0.3748271	0.35486113	0.05686979	-0.8767804	0.28620614	-0.0310641
105.5	0.40580021	-0.3752939	0.35458084	0.05674878	-0.8769889	0.28704448	-0.0308934
105.6	0.40615125	-0.3758122	0.35397393	0.05747445	-0.8771521	0.2871766	-0.0316711
105.7	0.40661416	-0.3772286	0.35393027	0.05817408	-0.8774215	0.28817415	-0.0322589
105.8	0.40652164	-0.3774655	0.35433605	0.05721414	-0.8774678	0.28903186	-0.0321634
105.9	0.40707226	-0.3768385	0.35322384	0.05670871	-0.8770753	0.28876657	-0.0329359
106	0.40638843	-0.376171	0.3536119	0.05615965	-0.8770764	0.28899299	-0.0328285
106.1	0.40607219	-0.3758321	0.35235421	0.05658013	-0.8768741	0.28863516	-0.0334025
106.2	0.40682229	-0.3758307	0.35116445	0.05713232	-0.8767447	0.28813504	-0.0337553
106.3	0.40720065	-0.3769855	0.3509592	0.05647068	-0.8767573	0.28949033	-0.0329481
106.4	0.40714563	-0.3772193	0.35104619	0.0573567	-0.8767804	0.29022321	-0.0335852
106.5	0.40719853	-0.3783937	0.34948771	0.05860618	-0.8768355	0.2909028	-0.0342249
106.6	0.40707779	-0.3786394	0.34956965	0.05889041	-0.876898	0.29103224	-0.0344418
106.7	0.40678213	-0.3788556	0.34925447	0.05862701	-0.8769366	0.29118159	-0.0341296
106.8	0.40649771	-0.3793044	0.34870755	0.05820319	-0.8771068	0.29097884	-0.0332435
106.9	0.40677379	-0.3789587	0.3490389	0.05844215	-0.8771812	0.2912576	-0.0337148
107	0.40640318	-0.3787746	0.34802716	0.05774696	-0.8772399	0.29255261	-0.0329641
107.1	0.40643898	-0.3789704	0.34810737	0.05766929	-0.8773348	0.29292376	-0.0319184
107.2	0.40653713	-0.3791332	0.34836679	0.05768001	-0.8773363	0.29320496	-0.0320937
107.3	0.40653713	-0.3791332	0.34836679	0.05768001	-0.8773363	0.29320496	-0.0320937
107.4	0.40705872	-0.3788233	0.34676721	0.05815574	-0.8774355	0.29343781	-0.0322417
107.5	0.406926	-0.3793792	0.34653038	0.05846148	-0.8772997	0.29359806	-0.0318366
107.6	0.40700324	-0.3795549	0.34698468	0.05842488	-0.877397	0.29377381	-0.0312804
107.7	0.40707038	-0.3794885	0.34700071	0.05858921	-0.8775679	0.2942353	-0.0319189
107.8	0.40780627	-0.3787741	0.34619308	0.0580219	-0.8776334	0.29400646	-0.0322508
107.9	0.40769271	-0.3782191	0.34527695	0.05803541	-0.8774841	0.29394247	-0.0316523
108	0.40754092	-0.3782438	0.34511324	0.05741524	-0.8775023	0.29414382	-0.0315725
108.1	0.40797437	-0.3782273	0.34475563	0.05832117	-0.8776092	0.29499421	-0.0314253
108.2	0.40720529	-0.378633	0.34523172	0.05758242	-0.8775764	0.29492559	-0.0307428
108.3	0.40771386	-0.379659	0.34432336	0.05754443	-0.8776695	0.29519775	-0.0303823
108.4	0.40730982	-0.3798803	0.34439169	0.05762918	-0.877736	0.29583539	-0.0305128
108.5	0.40726616	-0.379708	0.34443202	0.05796128	-0.87786	0.29595441	-0.0302525
108.6	0.40731608	-0.3795299	0.3436509	0.05779755	-0.8777601	0.29598507	-0.0305741
108.7	0.40696992	-0.3795151	0.34426486	0.05901359	-0.8778466	0.29660103	-0.0302143
108.8	0.4071879	-0.3799482	0.34384562	0.05888865	-0.8778326	0.29632658	-0.0298742
108.9	0.40723105	-0.3798062	0.34396053	0.0583819	-0.8778663	0.2961558	-0.0296083
109	0.40726903	-0.3803356	0.34323613	0.05847827	-0.877865	0.29607369	-0.0301929
109.1	0.4075033	-0.3794265	0.34359043	0.05763964	-0.8780095	0.29693136	-0.0291325
109.2	0.40689059	-0.3798347	0.34362251	0.0561821	-0.8780842	0.29693717	-0.0287195
109.3	0.40622855	-0.3800538	0.34327512	0.05751029	-0.877926	0.29715873	-0.029162
109.4	0.40609564	-0.3803235	0.34319669	0.05782546	-0.8779064	0.29706666	-0.0289207
109.5	0.40664909	-0.3797213	0.34324747	0.05701909	-0.8780827	0.2970071	-0.028682
109.6	0.40666183	-0.3797525	0.34324584	0.05714287	-0.8781371	0.29700813	-0.0283342
109.7	0.40659242	-0.3800488	0.34310039	0.05746839	-0.8782009	0.29691435	-0.0280832
109.8	0.40603959	-0.3788935	0.34221813	0.05810956	-0.8776666	0.29812755	-0.0272971
109.9	0.40587811	-0.3787315	0.34216488	0.05801778	-0.8777627	0.29881978	-0.0271624
110	0.40577699	-0.3791453	0.34234185	0.05815159	-0.8779103	0.29994022	-0.0272568
110.1	0.40599239	-0.3787706	0.34199019	0.05844918	-0.8778034	0.30057674	-0.0270104
110.2	0.40577931	-0.3783022	0.34138431	0.05837048	-0.8778062	0.30090547	-0.0267349
110.3	0.4044031	-0.3781513	0.34128978	0.057922	-0.8776711	0.30170394	-0.0268536
110.4	0.40402696	-0.3783486	0.34137354	0.05881185	-0.8775071	0.30153689	-0.0276378
110.5	0.40400419	-0.378854	0.34046306	0.05789591	-0.8776392	0.30290304	-0.0272138
110.6	0.40388915	-0.3789225	0.33964936	0.05783773	-0.8775217	0.30263082	-0.0272095
110.7	0.40375749	-0.3786116	0.33896768	0.05744879	-0.8772698	0.30287359	-0.0278421
110.8	0.40393257	-0.37905	0.33840764	0.05803788	-0.8773069	0.30317834	-0.0275332
110.9	0.4036354	-0.379115	0.33752936	0.0584886	-0.8771445	0.30401798	-0.027241
111	0.40395026	-0.3792809	0.33692774	0.05856421	-0.8771767	0.30404396	-0.0275127
111.1	0.40343954	-0.3790381	0.33636066	0.05823267	-0.8773242	0.30510522	-0.0269661
111.2	0.40355581	-0.3790603	0.33611695	0.0587116	-0.8776165	0.30646025	-0.025943
111.3	0.40358576	-0.3789641	0.33561563	0.05925811	-0.8775349	0.30684054	-0.0256149
111.4	0.40420121	-0.3792812	0.33480687	0.0591666	-0.8775563	0.30668809	-0.025592
111.5	0.4040512	-0.3793683	0.33470993	0.05930755	-0.8775948	0.3070926	-0.0250033
111.6	0.40338746	-0.3785623	0.33443189	0.05854746	-0.8776389	0.30742071	-0.0247463
111.7	0.40330702	-0.3784026	0.33413692	0.05878198	-0.8776198	0.30762006	-0.0242173
111.8	0.40344471	-0.3779806	0.33410788	0.05912464	-0.8776968	0.30799828	-0.024852
111.9	0.40347056	-0.378051	0.33400039	0.05863426	-0.8777061	0.3080919	-0.0249737
112	0.40349634	-0.3791071	0.3334283	0.05847314	-0.8778197	0.30882016	-0.0249505
112.1	0.40321575	-0.3791665	0.33378752	0.05828868	-0.8778387	0.30851705	-0.0247659
112.2	0.40345576	-0.3786191	0.33283869	0.05800365	-0.8779364	0.30831343	-0.0250545
112.3	0.40345576	-0.3786191	0.33283869	0.05800365	-0.8779364	0.30831343	-0.0250545
112.4	0.40293326	-0.3787176	0.33259513	0.05803681	-0.8777961	0.30844699	-0.025224
112.5	0.40231504	-0.3785087	0.33218709	0.05838025	-0.8777505	0.30866889	-0.0250154
112.6	0.40283509	-0.3788815	0.33073216	0.05902376	-0.877624	0.30833097	-0.0252363
112.7	0.4017082	-0.3791865	0.33063001	0.0596451	-0.8773419	0.30880421	-0.0251856
112.8	0.40099146	-0.3782569	0.33052195	0.0610019	-0.8773886	0.31030938	-0.0257805
112.9	0.4012797	-0.3773757	0.33051503	0.06107048	-0.8773865	0.31054029	-0.0244858
113	0.40039235	-0.3782415	0.32988857	0.06066458	-0.8772631	0.31051074	-0.0246203
113.1	0.40046236	-0.378835	0.32977766	0.06054132	-0.8772152	0.31113104	-0.0239138
113.2	0.40022568	-0.3788698	0.32956896	0.06011099	-0.8771523	0.31151078	-0.0239381
113.3	0.40015499	-0.3791929	0.32910733	0.05984004	-0.8771441	0.31136409	-0.0236128
113.4	0.3998755	-0.379522	0.32920615	0.05979213	-0.8770753	0.31110661	-0.0235887
113.5	0.3992193	-0.379878	0.32903474	0.05990226	-0.8775037	0.3121835	-0.0232556
113.6	0.39861727	-0.3793631	0.32898471	0.05964202	-0.8775875	0.31257324	-0.0225022
113.7	0.39927923	-0.379401	0.32956281	0.05938927	-0.8776557	0.31292844	-0.0225395
113.8	0.39894753	-0.3792942	0.32994397	0.05836824	-0.8777653	0.31320504	-0.0228096
113.9	0.3988269	-0.3788763	0.3301195	0.05860391	-0.8777286	0.31382385	-0.02339

69	0.32680674	-0.2727063	0.5746564	0.10401762	-0.8910681	0.13488042	0.01275722
69.1	0.32682857	-0.2737083	0.57409311	0.1036846	-0.8911458	0.13623189	0.01316166
69.2	0.32771353	-0.2736675	0.57348302	0.10367808	-0.8912203	0.13693792	0.01289104
69.3	0.32930827	-0.2740395	0.5730689	0.10385217	-0.8914009	0.1368518	0.01334612
69.4	0.32969854	-0.2740195	0.57281705	0.10410328	-0.8912354	0.13662199	0.01298533
69.5	0.33121239	-0.2754765	0.57041641	0.1035214	-0.8909257	0.13603402	0.01247503
69.6	0.33173831	-0.2763313	0.57010387	0.10312579	-0.8908475	0.1362486	0.01239373
69.7	0.33148676	-0.2761738	0.5696215	0.1030176	-0.8907492	0.13658856	0.01309164
69.8	0.3323509	-0.2769	0.56914699	0.10213788	-0.8909016	0.13791947	0.01199934
69.9	0.33314856	-0.2784922	0.56830286	0.10182276	-0.8907161	0.13836995	0.01169188
70	0.33293646	-0.2789352	0.56874064	0.10191053	-0.8907333	0.13941898	0.01186817
70.1	0.33275386	-0.2782024	0.56787397	0.10138489	-0.8906637	0.13990305	0.01087858
70.2	0.33319804	-0.2778836	0.56808544	0.10107775	-0.8908298	0.14004341	0.00995759
70.3	0.33375644	-0.2786576	0.56791278	0.10110247	-0.8908741	0.14066803	0.01000052
70.4	0.33279281	-0.279016	0.56730465	0.09989915	-0.8908859	0.14072867	0.00900271
70.5	0.33330867	-0.280573	0.56713384	0.0990732	-0.8909811	0.14095935	0.00898566
70.6	0.3342514	-0.2822113	0.56578441	0.09767148	-0.8910222	0.14311132	0.00956718
70.7	0.33530499	-0.2826159	0.56514801	0.09744518	-0.8910583	0.14260109	0.01005909
70.8	0.33542032	-0.2820966	0.56523336	0.09670126	-0.8909494	0.14338894	0.00926424
70.9	0.33540917	-0.2827407	0.56457855	0.09682772	-0.8909956	0.14337056	0.00794007
71	0.33530528	-0.2830806	0.56427637	0.09656346	-0.8912079	0.14407988	0.00797151
71.1	0.3355039	-0.2831984	0.56353321	0.0970378	-0.8914395	0.14406205	0.00829406
71.2	0.3354916	-0.2830571	0.56342504	0.09713478	-0.891472	0.14425269	0.00762177
71.3	0.33536803	-0.2834084	0.56294031	0.09705153	-0.8913911	0.1441895	0.00730268
71.4	0.33682723	-0.2839045	0.56206381	0.09740485	-0.8914597	0.14438684	0.00623048
71.5	0.33622258	-0.2842414	0.56084307	0.0975142	-0.8915854	0.14676352	0.00657864
71.6	0.33670501	-0.2843017	0.56069989	0.09741861	-0.8916573	0.14728653	0.00628972
71.7	0.33678646	-0.2838352	0.56041976	0.09805917	-0.8916842	0.14767714	0.00642485
71.8	0.3371221	-0.2829464	0.55949242	0.09800001	-0.8918295	0.14988067	0.00544742
71.9	0.33671284	-0.2828087	0.55951726	0.09862098	-0.8917578	0.15018585	0.00563288
72	0.33710785	-0.2832584	0.55854488	0.09794057	-0.8918784	0.15013658	0.00597035
72.1	0.33663531	-0.283587	0.55609522	0.10013224	-0.891859	0.15168248	0.00640584
72.2	0.33677521	-0.2841134	0.55521259	0.09910294	-0.8918142	0.15191519	0.00634439
72.3	0.33758714	-0.2842051	0.55394781	0.09839336	-0.8918577	0.15406075	0.00537781
72.4	0.33731422	-0.2843356	0.55315164	0.09761919	-0.8916543	0.15498786	0.00447063
72.5	0.33739146	-0.2860368	0.55286172	0.09827893	-0.8916742	0.15603229	0.0043331
72.6	0.33750867	-0.2871753	0.5524004	0.09761885	-0.891756	0.15592875	0.00469857
72.7	0.3387088	-0.2871839	0.55028467	0.09718874	-0.8915825	0.15633361	0.00385774
72.8	0.33923211	-0.2887288	0.54920524	0.09625112	-0.8914691	0.15691103	0.00209084
72.9	0.33983977	-0.2886752	0.54877441	0.09414821	-0.8914265	0.15594723	0.00138974
73	0.3393924	-0.2890064	0.54794045	0.09333261	-0.8913865	0.15707121	0.00085703
73.1	0.33959455	-0.2886564	0.5475958	0.09364924	-0.8912975	0.15728353	0.00047155
73.2	0.33939047	-0.2892339	0.54708162	0.09312075	-0.8911438	0.15753696	0.00017373
73.3	0.33991272	-0.2904397	0.54565501	0.09365819	-0.8911089	0.15766849	8.37E-05
73.4	0.34080751	-0.2903731	0.54463408	0.09326905	-0.8909629	0.15857556	-0.0006643
73.5	0.3420847	-0.2908982	0.54415036	0.09285497	-0.8908794	0.15905245	-0.0010773
73.6	0.34362693	-0.2916042	0.54288789	0.09243005	-0.8908139	0.15996922	-0.000953
73.7	0.34247186	-0.2930447	0.54202333	0.09151255	-0.8905691	0.16092749	-0.0025635
73.8	0.34233976	-0.2928157	0.54034459	0.09051749	-0.8903285	0.16097153	-0.0026078
73.9	0.34323567	-0.2933642	0.53932024	0.09091464	-0.8903551	0.16013015	-0.0032534
74	0.34336916	-0.294073	0.53830761	0.09046063	-0.8901984	0.15914222	-0.0035855
74.1	0.34357311	-0.2953424	0.53746178	0.09037103	-0.8901761	0.1591609	-0.0042652
74.2	0.34349215	-0.2964379	0.53677736	0.0892869	-0.8900428	0.15859829	-0.0045268
74.3	0.34433156	-0.2974499	0.53528597	0.08766014	-0.8898245	0.15758621	-0.005845
74.4	0.34672951	-0.2977917	0.5325809	0.08596936	-0.889586	0.15970651	-0.0077706
74.5	0.34671321	-0.2987379	0.53144607	0.0855078	-0.8895742	0.16056596	-0.0092685
74.6	0.34781434	-0.2988597	0.53064482	0.08569315	-0.8893221	0.16063113	-0.0108577
74.7	0.34791637	-0.2998273	0.52896148	0.08452243	-0.8886203	0.15955737	-0.0121784
74.8	0.34829839	-0.300946	0.52829404	0.08426714	-0.8886807	0.15976154	-0.013683
74.9	0.34823434	-0.3023268	0.52790595	0.08313135	-0.8886224	0.16052888	-0.0145695

120	0.39526367	-0.3787299	0.32294005	0.06679512	-0.8792034	0.34051991	-0.0184705
120.1	0.39533978	-0.3787531	0.32338181	0.06678242	-0.8793221	0.34124542	-0.0169596
120.2	0.39558004	-0.3792604	0.32368627	0.06723645	-0.8796666	0.34134197	-0.0168627
120.3	0.3951509	-0.3798015	0.32372564	0.06716397	-0.8798105	0.34180547	-0.0166853
120.4	0.39459808	-0.3796849	0.32398454	0.06655269	-0.8799214	0.34194797	-0.0165062
120.5	0.39439471	-0.3799313	0.32389776	0.06621721	-0.8799246	0.34182769	-0.0166581
120.6	0.39429156	-0.3801559	0.32371294	0.06637251	-0.8798593	0.34191671	-0.0168078
120.7	0.39402279	-0.3795454	0.32368835	0.06581276	-0.8800311	0.3428249	-0.0161153
120.8	0.39362932	-0.3796892	0.32373372	0.06651576	-0.8800379	0.34327027	-0.0160706
120.9	0.39375528	-0.3797837	0.32380466	0.06716915	-0.879982	0.34362822	-0.016462
121	0.39359997	-0.3798425	0.32393667	0.06729603	-0.8801284	0.34368248	-0.0166967
121.1	0.39227437	-0.3809475	0.32271648	0.06810298	-0.8800273	0.34498849	-0.016986
121.2	0.39266245	-0.3809268	0.32327149	0.0691793	-0.8804603	0.34593449	-0.0172912
121.3	0.39263654	-0.3805334	0.32320803	0.06954947	-0.8804557	0.34604415	-0.0175074
121.4	0.39268647	-0.3803862	0.32296131	0.07005985	-0.8805051	0.34691757	-0.017378
121.5	0.39330903	-0.3810107	0.32323461	0.06982597	-0.8806591	0.34696665	-0.0167869
121.6	0.39330921	-0.3810107	0.32323461	0.06982597	-0.8806572	0.34696665	-0.0167869
121.7	0.39357277	-0.3804126	0.32357374	0.06986956	-0.8809715	0.34773385	-0.0163815
121.8	0.39296924	-0.3811864	0.32321708	0.07107285	-0.880741	0.34810014	-0.0171322
121.9	0.39278018	-0.3807727	0.32295925	0.07129157	-0.8807324	0.34807124	-0.0175862
122	0.39268302	-0.3808082	0.32343725	0.0718351	-0.8808402	0.34917284	-0.0165605
122.1	0.39237951	-0.3802466	0.32336177	0.07148249	-0.8809971	0.34978701	-0.0167603
122.2	0.39101811	-0.3798487	0.32232513	0.07087204	-0.8808735	0.3499713	-0.0163191
122.3	0.39048354	-0.3800494	0.32228914	0.07184384	-0.8808655	0.35048121	-0.0166855
122.4	0.39013316	-0.3802094	0.32245912	0.07234782	-0.8807862	0.35044415	-0.0165015
122.5	0.39000092	-0.3802366	0.32248635	0.07227172	-0.880715	0.35033228	-0.0161349
122.6	0.38982697	-0.3804198	0.32193878	0.07268683	-0.8807267	0.35071012	-0.0160955
122.7	0.38913785	-0.3811085	0.32150201	0.07312376	-0.8806204	0.35099944	-0.0161433
122.8	0.38957186	-0.3800138	0.32139109	0.07306584	-0.8805859	0.35159923	-0.0161587
122.9	0.38947645	-0.3791616	0.32196753	0.07270746	-0.8807356	0.35226692	-0.0158345
123	0.38965038	-0.3789169	0.32215308	0.07326299	-0.8808567	0.35261988	-0.0153119
123.1	0.3890871	-0.3784225	0.32139984	0.07308285	-0.8808534	0.35278984	-0.0151114
123.2	0.38879667	-0.3787976	0.32129145	0.07263376	-0.8808251	0.3526971	-0.0146784
123.3	0.38856179	-0.378429	0.32041757	0.07313221	-0.8807798	0.35286783	-0.0144243
123.4	0.38813359	-0.3779571	0.3197078	0.07332487	-0.8805873	0.35269724	-0.0141607
123.5	0.38734448	-0.3779285	0.32006287	0.07376345	-0.8806569	0.35291888	-0.0138292
123.6	0.38734448	-0.3779285	0.32006287	0.07376345	-0.8806569	0.35291888	-0.0138292
123.7	0.38700321	-0.3773337	0.32040169	0.07418508	-0.8805035	0.35314591	-0.0135868
123.8	0.38660821	-0.3773168	0.32013443	0.07401704	-0.88041	0.35343902	-0.0132649
123.9	0.38590314	-0.3773318	0.3202441	0.07392081	-0.8803338	0.35355286	-0.0134059
124	0.38579744	-0.3770423	0.32038435	0.07358276	-0.8804793	0.35402447	-0.0134964
124.1	0.38545033	-0.3767175	0.32048797	0.07326016	-0.88051	0.35428447	-0.0136364
124.2	0.38504806	-0.3760741	0.32009345	0.07343415	-0.8805258	0.35464019	-0.0135113
124.3	0.38468272	-0.3766008	0.32019129	0.07324753	-0.8804087	0.3553596	-0.0127186
124.4	0.38417047	-0.3765862	0.31962276	0.07316161	-0.8803125	0.3560413	-0.0120613
124.5	0.3840365	-0.3772771	0.31959232	0.07247866	-0.8804063	0.35699294	-0.01145
124.6	0.38416407	-0.3769439	0.31922311	0.07160935	-0.8805307	0.35788841	-0.0102169
124.7	0.38356148	-0.3765223	0.31912772	0.07102619	-0.8808414	0.35790316	-0.0102553
124.8	0.38306237	-0.3761395	0.31933009	0.07100322	-0.8808887	0.3578413	-0.0100449
124.9	0.38247588	-0.3755532	0.32017859	0.07057918	-0.8806326	0.35838429	-0.010183
125	0.38247588	-0.3755532	0.32017859	0.07057918	-0.8806326	0.35838429	-0.010183
125.1	0.38275942	-0.3755681	0.32046643	0.07098774	-0.8807536	0.35835999	-0.010039
125.2	0.38261768	-0.3756689	0.32019403	0.0716777	-0.8807279	0.35877823	-0.0099759
125.3	0.38238704	-0.3759287	0.3193873	0.07177985	-0.8807497	0.35915411	-0.0095819
125.4	0.38214168	-0.3765194	0.31884995	0.0719587	-0.8808132	0.35979195	-0.0090221
125.5	0.38214168	-0.3765194	0.31884995	0.0719587	-0.8808132	0.35979195	-0.0090221
125.6	0.3806235	-0.3773802	0.31903343	0.07208908	-0.8808152	0.36078285	-0.00886
125.7	0.38047868	-0.3769754	0.31803124	0.07242336	-0.8807334	0.3610075	-0.0092587
125.8	0.38047868	-0.3769754	0.31803124	0.07242336	-0.8807334	0.3610075	-0.0092587
125.9	0.37985842	-0.3767808	0.31818134	0.07173082	-0.8807362	0.36140999	-0.0089114
126	0.37982684	-0.3765619	0.31854942	0.07184412	-0.8807427	0.36173056	-0.0088123

84	0.37062621	-0.3379742	0.47556103	0.05845693	-0.8838676	0.20707801	-0.0424927
84.1	0.37062621	-0.3379742	0.47556103	0.05845693	-0.8838676	0.20707801	-0.0424927
84.2	0.37104254	-0.3382062	0.47553265	0.05886272	-0.883917	0.20764031	-0.0426151
84.3	0.37073032	-0.3387664	0.47460317	0.05795022	-0.88378	0.20852772	-0.0432503
84.4	0.3709732	-0.3390174	0.47416833	0.05886363	-0.8834679	0.20913715	-0.0433217
84.5	0.37029138	-0.3393909	0.47381406	0.05792751	-0.8830654	0.20976898	-0.0437035
84.6	0.37264896	-0.3399261	0.47240223	0.05810145	-0.8829083	0.21026102	-0.0440278
84.7	0.37325126	-0.3404691	0.47163172	0.05867597	-0.8828148	0.21177654	-0.0446051
84.8	0.37338568	-0.3409089	0.47148713	0.05883152	-0.8828946	0.21269135	-0.0452019
84.9	0.37357949	-0.3416816	0.47036236	0.05926867	-0.8830287	0.21314354	-0.0457894
85	0.37321753	-0.341615	0.46867537	0.0586232	-0.8828497	0.21444876	-0.0450066
85.1	0.37466279	-0.3409826	0.46850971	0.05640057	-0.8830634	0.21612053	-0.046517
85.2	0.37466604	-0.3420428	0.4676162	0.05597648	-0.8833485	0.21680158	-0.04674
85.3	0.3747611	-0.341929	0.46783158	0.05593546	-0.8835174	0.21711383	-0.0465471
85.4	0.37579313	-0.3415377	0.46669038	0.0550645	-0.8835323	0.21784131	-0.0458258
85.5	0.37620038	-0.341177	0.4654077	0.054803	-0.8833337	0.21783554	-0.0451356
85.6	0.37635137	-0.341974	0.4650368	0.05614795	-0.8832405	0.21883464	-0.0449168
85.7	0.37601923	-0.3424	0.46522636	0.05599678	-0.8832681	0.2184067	-0.0439407
85.8	0.37634727	-0.3425683	0.46497727	0.0567316	-0.8833015	0.2204649	-0.0416343
85.9	0.37655881	-0.3429852	0.46506751	0.05628412	-0.8833599	0.22048339	-0.0416495
86	0.37560391	-0.3437252	0.4645304	0.05653535	-0.8829957	0.22146136	-0.0405428
86.1	0.37704892	-0.3424006	0.46348846	0.05579988	-0.8829659	0.22149036	-0.0412051
86.2	0.37721779	-0.3427825	0.46262636	0.05575215	-0.8829126	0.22172213	-0.0416086
86.3	0.3777114	-0.3427299	0.46296905	0.05532712	-0.8829927	0.22227812	-0.0410215
86.4	0.37799639	-0.3433622	0.46255769	0.05528635	-0.882897	0.22267609	-0.0417495
86.5	0.37795445	-0.3446579	0.46283811	0.05374891	-0.8830524	0.22384432	-0.0420496
86.6	0.37867974	-0.345585	0.46184774	0.05398964	-0.8830894	0.22498334	-0.0417746
86.7	0.37849794	-0.3457376	0.46099344	0.05341794	-0.8829563	0.22523206	-0.0422682
86.8	0.37847657	-0.3461609	0.45968634	0.05249207	-0.8830247	0.22554743	-0.0420692
86.9	0.37856088	-0.346332	0.45944516	0.05298553	-0.8827216	0.22668853	-0.0425352
87	0.37909133	-0.3463812	0.45914874	0.05299334	-0.8827066	0.22652016	-0.0421763
87.1	0.37952212	-0.3463617	0.45908352	0.05351829	-0.882611	0.22717366	-0.0415278
87.2	0.37947223	-0.3464123	0.45948893	0.05326953	-0.8827148	0.22785686	-0.0416711
87.3	0.37940173	-0.3465171	0.45949291	0.05265358	-0.8828275	0.22850893	-0.0406351
87.4	0.38020448	-0.3468415	0.45724148	0.05146891	-0.8827706	0.22971492	-0.0407076
87.5	0.38034156	-0.347565	0.45688626	0.05155557	-0.8825181	0.22939383	-0.0403944
87.6	0.38116087	-0.3477821	0.45623845	0.05114807	-0.8826572	0.22969128	-0.0413473
87.7	0.38233066	-0.3476217	0.45577897	0.05193131	-0.882668	0.2298961	-0.0424477
87.8	0.38218451	-0.3476784	0.45532001	0.05273429	-0.882667	0.2306175	-0.0419991
87.9	0.38218796	-0.3475247	0.45550011	0.05220697	-0.8827353	0.23008508	-0.0414697
88	0.38275754	-0.3483083	0.45526793	0.0519607	-0.8827326	0.23017854	-0.0401647
88.1	0.38303965	-0.3495843	0.45385705	0.05134584	-0.8827498	0.23174694	-0.0408017
88.2	0.38287245	-0.3500298	0.45302562	0.0510983	-0.8825527	0.23229114	-0.0408502
88.3	0.38319848	-0.3503773	0.45251106	0.05123995	-0.8826218	0.2316933	-0.0411789
88.4	0.38286593	-0.3519206	0.45224044	0.04989493	-0.8826341	0.2315502	-0.0417987
88.5	0.38277597	-0.3524877	0.45158836	0.05074987	-0.8827166	0.23227013	-0.0414021
88.6	0.38376944	-0.3532343	0.45043293	0.04979865	-0.8827217	0.23293514	-0.0409946
88.7	0.38393426	-0.352803	0.45005399	0.04991484	-0.8826981	0.23357681	-0.0412588
88.8	0.38386852	-0.3530998	0.44952942	0.04911417	-0.8825289	0.23361829	-0.0405333
88.9	0.38425807	-0.3533077	0.44935145	0.04862678	-0.8827406	0.23317672	-0.040924
89	0.38484362	-0.3533189	0.44869327	0.04863288	-0.8826643	0.23232259	-0.0411389
89.1	0.38508138	-0.3539341	0.44823397	0.04843962	-0.882707	0.23275214	-0.0415365
89.2	0.385538	-0.3533148	0.44544958	0.04820437	-0.8823639	0.2329577	-0.0416673
89.3	0.38586447	-0.3524233	0.4454484	0.04876632	-0.8825531	0.23318942	-0.0395847
89.4	0.38644248	-0.3530438	0.44438762	0.04957528	-0.8825717	0.23384523	-0.0393158
89.5	0.38675225	-0.3543096	0.44303679	0.04892297	-0.8826411	0.23546374	-0.0394959
89.6	0.38546509	-0.3554022	0.44322498	0.04938512	-0.8828591	0.23624333	-0.0386963
89.7	0.38544917	-0.35633	0.44182667	0.04940622	-0.8828512	0.2356816	-0.0383189
89.8	0.38674764	-0.3565242	0.4413491	0.0509079	-0.8828912	0.23650587	-0.0374201
89.9	0.3872399	-0.3565052	0.44092827	0.05094115	-0.8829724	0.23675392	-0.0378489

135	0.3697393	-0.3737889	0.31698843	0.0694859	-0.8811714	0.3860223	0.00167126
135.1	0.36910002	-0.3743339	0.3182741	0.06997666	-0.881407	0.38651913	0.00214243
135.2	0.36910002	-0.3743339	0.3182741	0.06997666	-0.881407	0.38651913	0.00214243
135.3	0.36911307	-0.3734651	0.31836498	0.06906082	-0.8814134	0.38706655	0.00302391
135.4	0.36885574	-0.3736905	0.31810063	0.06957678	-0.8813059	0.38665149	0.00310821
135.5	0.36824963	-0.3731667	0.3180971	0.06923148	-0.8812252	0.38682977	0.00284122
135.6	0.36889876	-0.3732071	0.31780217	0.06937358	-0.8813359	0.38728889	0.00320348
135.7	0.36854488	-0.374158	0.31805215	0.06901437	-0.8814172	0.38692705	0.00373681
135.8	0.36854234	-0.3745692	0.3183383	0.06934348	-0.8814993	0.38705887	0.00348891
135.9	0.36756418	-0.3756924	0.31889738	0.06897634	-0.881651	0.38771326	0.00198706
136	0.36776756	-0.3759371	0.3189322	0.06834998	-0.8818748	0.38787304	0.00208263
136.1	0.36776756	-0.3759371	0.3189322	0.06834998	-0.8818748	0.38787304	0.00208263
136.2	0.367839	-0.3757869	0.32003868	0.06879419	-0.881731	0.38892252	0.00196021
136.3	0.36731031	-0.3754537	0.31959581	0.06939039	-0.8816432	0.38895626	0.00270833
136.4	0.36741661	-0.3750787	0.31848017	0.07025092	-0.8816256	0.38934049	0.00242364
136.5	0.36725879	-0.3747873	0.31834175	0.06959331	-0.8814491	0.38872355	0.00242843
136.6	0.36739048	-0.3751447	0.31869253	0.07045496	-0.8815792	0.38986444	0.00161384
136.7	0.36732855	-0.3755173	0.3185327	0.0702445	-0.8818198	0.39039368	0.00125252
136.8	0.36732855	-0.3755173	0.3185327	0.0702445	-0.8818198	0.39039368	0.00125252
136.9	0.36732855	-0.3755173	0.3185327	0.0702445	-0.8818198	0.39039368	0.00125252
137	0.36732855	-0.3755173	0.3185327	0.0702445	-0.8818198	0.39039368	0.00125252
137.1	0.36732855	-0.3755173	0.3185327	0.0702445	-0.8818198	0.39039368	0.00125252
137.2	0.36710516	-0.3755227	0.31897594	0.07037617	-0.8816437	0.39056337	0.00163345
137.3	0.36696871	-0.3757887	0.31937868	0.07028599	-0.8817698	0.39085909	0.00173344
137.4	0.36680896	-0.3750199	0.32010225	0.07070039	-0.8817824	0.39141551	0.00216642
137.5	0.36708215	-0.3751658	0.32093511	0.07013709	-0.8819915	0.39199552	0.0028928
137.6	0.36715328	-0.3746316	0.32056365	0.06969616	-0.8819133	0.39206923	0.00270094
137.7	0.36741811	-0.3751219	0.32039362	0.07005698	-0.8820246	0.39160779	0.0029906
137.8	0.36758054	-0.3754717	0.32034003	0.07047436	-0.8821136	0.39201741	0.00242216
137.9	0.36741257	-0.3759134	0.32128531	0.07103262	-0.8823528	0.39241297	0.00261376
138	0.36626138	-0.3759406	0.32123695	0.07143129	-0.8823683	0.39273391	0.00254195
138.1	0.36594015	-0.376425	0.32157164	0.07043059	-0.8826396	0.39328965	0.00231754
138.2	0.36621545	-0.3757002	0.32136598	0.07001539	-0.882466	0.39317509	0.0028268
138.3	0.36659321	-0.3754316	0.32135961	0.06974555	-0.882617	0.3939924	0.00318317
138.4	0.3664677	-0.3756574	0.32224982	0.07024466	-0.882792	0.39444316	0.00364501
138.5	0.36671615	-0.3753938	0.32271444	0.0700892	-0.8829778	0.3945799	0.00353459
138.6	0.366541	-0.3750754	0.32266569	0.07007532	-0.882966	0.39477092	0.00340188
138.7	0.366541	-0.3750754	0.32266569	0.07007532	-0.882966	0.39477092	0.00340188
138.8	0.366541	-0.3750754	0.32266569	0.07007532	-0.882966	0.39477092	0.00340188
138.9	0.36599391	-0.3751397	0.32320895	0.06986479	-0.882971	0.39469517	0.00404018
139	0.36585142	-0.3753526	0.32347704	0.06991216	-0.8830292	0.39481341	0.00372964
139.1	0.36525014	-0.3756449	0.32422551	0.06912362	-0.8831228	0.39478044	0.00386504
139.2	0.36442468	-0.3757593	0.32424824	0.06920567	-0.8830008	0.39528492	0.00359601
139.3	0.36442493	-0.3754599	0.32413747	0.06916784	-0.8830464	0.39537852	0.00429611
139.4	0.36430477	-0.3750685	0.32393103	0.06953578	-0.8829307	0.39561766	0.00380934
139.5	0.36430417	-0.3750685	0.32393103	0.06929441	-0.8827858	0.39587186	0.00380934
139.6	0.36435166	-0.3745589	0.32356141	0.06955721	-0.8827782	0.39597435	0.00405211
139.7	0.36481932	-0.3732112	0.32422098	0.06904374	-0.8828211	0.39624252	0.00465359
139.8	0.36381385	-0.373354	0.32410901	0.06785491	-0.8828202	0.39634914	0.00502966
139.9	0.3635764	-0.3734227	0.32459379	0.06798977	-0.8828728	0.39655609	0.00479309
140	0.36287858	-0.3753439	0.32453733	0.06746786	-0.8829129	0.39665555	0.00438091
140.1	0.36287046	-0.374056	0.32460215	0.06698886	-0.882992	0.39677461	0.00417231
140.2	0.3628596	-0.3750214	0.32422199	0.06815885	-0.8828778	0.3968971	0.00390324
140.3	0.36281156	-0.3749841	0.3239937	0.06783603	-0.8828769	0.39702931	0.00464877
140.4	0.3627292	-0.375138	0.32449814	0.06766679	-0.882771	0.39751907	0.00432394
140.5	0.3627292	-0.375138	0.32449814	0.06766679	-0.882771	0.39751907	0.00432394
140.6	0.36277758	-0.3751553	0.32419192	0.06765892	-0.8827187	0.39769648	0.00391701
140.7	0.36295796	-0.3750242	0.32465158	0.06787974	-0.8827444	0.39778395	0.00399805
140.8	0.36372296	-0.3747669	0.32458734	0.06783056	-0.8827857	0.39841045	0.00406617
140.9	0.36405644	-0.3737467	0.32431341	0.06855647	-0.882886	0.39890834	0.00445105
141	0.36425937	-0.3734692	0.32449146	0.06844166	-0.8829065	0.39898417	0.00483598

99	0.40504044	-0.3739762	0.38447565	0.05115068	-0.879174	0.27185105	-0.0306962
99.1	0.40545339	-0.3738378	0.38398285	0.05170897	-0.8792402	0.2730858	-0.0305168
99.2	0.40569802	-0.3741541	0.38285529	0.05126114	-0.8791484	0.27284406	-0.0307229
99.3	0.40575785	-0.3741408	0.38306495	0.0507712	-0.8791792	0.27274208	-0.0303304
99.4	0.40566925	-0.3744175	0.38285498	0.05047735	-0.8792133	0.27335789	-0.0303235
99.5	0.40562061	-0.3748105	0.38196636	0.05021983	-0.8790394	0.27305053	-0.0303571
99.6	0.406011	-0.3755453	0.38200294	0.04992052	-0.8791061	0.27335541	-0.0306054
99.7	0.4057852	-0.375486	0.38186049	0.04961993	-0.8790561	0.27311256	-0.0301585
99.8	0.40567302	-0.375745	0.38091202	0.04951789	-0.8788664	0.27344545	-0.0301392
99.9	0.40516449	-0.3750761	0.37977473	0.04911201	-0.8786657	0.27372232	-0.0299745
100	0.40487052	-0.3756878	0.37933475	0.04813905	-0.8786197	0.2747714	-0.0294113
100.1	0.4050002	-0.3756221	0.3790322	0.04801781	-0.8786344	0.27512125	-0.0294316
100.2	0.40485612	-0.3751386	0.37886449	0.04894625	-0.8786102	0.27617064	-0.0298212
100.3	0.40456094	-0.3751242	0.37842975	0.04888193	-0.8786159	0.27604853	-0.0299284
100.4	0.40462045	-0.3762172	0.37818228	0.04839529	-0.878609	0.27602533	-0.0320372
100.5	0.40446359	-0.3756188	0.3767687	0.04887407	-0.8782466	0.27551955	-0.0316599
100.6	0.40444778	-0.3756425	0.37610564	0.0491895	-0.8782216	0.27571534	-0.0319556
100.7	0.4048199	-0.3754291	0.37573802	0.04917189	-0.8780908	0.27544378	-0.0319808
100.8	0.40490477	-0.3758208	0.37526296	0.04953685	-0.8781094	0.27560625	-0.0321461
100.9	0.4057336	-0.3760628	0.37389905	0.04885833	-0.8781632	0.27538235	-0.0326381
101	0.40582076	-0.3757992	0.37370853	0.04932297	-0.8781561	0.27544221	-0.0326839
101.1	0.40546214	-0.3761385	0.37243391	0.04997707	-0.8776999	0.2757405	-0.0332724
101.2	0.40537007	-0.3755299	0.37108864	0.04973276	-0.8774483	0.27549783	-0.0329458
101.3	0.40524978	-0.3749589	0.37043252	0.0491339	-0.8772513	0.27544719	-0.0331812
101.4	0.40569604	-0.3748617	0.3702743	0.04926971	-0.8773241	0.27527091	-0.0329364
101.5	0.40563404	-0.3745788	0.36962355	0.05018428	-0.8775194	0.27628	-0.0321852
101.6	0.40537421	-0.3750349	0.36966045	0.05078583	-0.8776369	0.27661048	-0.0308305
101.7	0.40552677	-0.3746838	0.36883793	0.05081099	-0.8776303	0.27631153	-0.0308192
101.8	0.40543187	-0.3750688	0.36867398	0.05134615	-0.8775955	0.276087	-0.0313394
101.9	0.40533409	-0.3745767	0.36815578	0.05133167	-0.8774888	0.2765379	-0.0314853
102	0.40547927	-0.3747181	0.36822777	0.05144157	-0.8775542	0.2768343	-0.0315464
102.1	0.40560757	-0.3742289	0.36821001	0.05123204	-0.8775987	0.27718203	-0.0317351
102.2	0.40486174	-0.3736632	0.36774678	0.05103544	-0.8773357	0.27662447	-0.0322861
102.3	0.40470055	-0.3738189	0.3678821	0.0512752	-0.8773589	0.27731821	-0.0322187
102.4	0.40509382	-0.373194	0.3670014	0.05203992	-0.877367	0.2772867	-0.0320165
102.5	0.40555453	-0.3737903	0.36645501	0.05232997	-0.8775452	0.27811121	-0.0320681
102.6	0.40561805	-0.3731587	0.36651114	0.05292613	-0.8775238	0.27904698	-0.0315132
102.7	0.40546478	-0.3739587	0.36511607	0.05334294	-0.8775299	0.27910048	-0.0307079
102.8	0.40558454	-0.3740215	0.36484798	0.0536516	-0.8774137	0.27917709	-0.0304774
102.9	0.40559368	-0.3736151	0.36427184	0.05364022	-0.8772911	0.27952153	-0.0296036
103	0.40541643	-0.3729892	0.36360084	0.05360375	-0.8773017	0.28019092	-0.0305
103.1	0.40490074	-0.3729951	0.36351068	0.054097	-0.877207	0.28056372	-0.030614
103.2	0.40488288	-0.3734691	0.36264714	0.05512259	-0.8771394	0.27993581	-0.0312579
103.3	0.40477028	-0.373406	0.36262932	0.05462613	-0.8770952	0.28058807	-0.0310644
103.4	0.40513482	-0.3737023	0.36207718	0.05474278	-0.8771759	0.28074206	-0.030498
103.5	0.40548006	-0.3736594	0.36079269	0.05463044	-0.8773181	0.28157273	-0.0302324
103.6	0.40548196	-0.3733606	0.36058162	0.05511972	-0.8772024	0.28184562	-0.0299468
103.7	0.40452265	-0.3737818	0.36124086	0.05528659	-0.8771669	0.2816995	-0.0306955
103.8	0.40476993	-0.3732965	0.36093413	0.055351	-0.8772645	0.28240074	-0.0312541
103.9	0.40516196	-0.3731293	0.36060002	0.05568495	-0.8772658	0.28295557	-0.0318183
104	0.40456467	-0.3732798	0.36005813	0.05547641	-0.8769894	0.28295169	-0.0318313
104.1	0.40437985	-0.3731617	0.35991472	0.05528017	-0.8770143	0.28314837	-0.032032
104.2	0.40395117	-0.3728268	0.36030164	0.05452347	-0.8770423	0.28328482	-0.0313342
104.3	0.40448743	-0.3735741	0.35929076	0.05579188	-0.8773017	0.2838089	-0.0308728
104.4	0.40467796	-0.3732632	0.35895216	0.05557274	-0.8772761	0.28465984	-0.0304453
104.5	0.40365294	-0.3736171	0.3585789	0.05480792	-0.8770121	0.28407927	-0.0307071
104.6	0.40337921	-0.3739097	0.35831077	0.05456203	-0.8769333	0.28413622	-0.0305649
104.7	0.40366699	-0.3743811	0.35817731	0.05457675	-0.8770252	0.28435822	-0.0310433
104.8	0.40423364	-0.3746241	0.35851826	0.05429114	-0.8770836	0.28475704	-0.0323417
104.9	0.404338	-0.3747301	0.35688209	0.05467955	-0.8769663	0.28510072	-0.0327034

150	0.35588029	-0.3684072	0.34669031	0.06749243	-0.8854763	0.41964518	0.0161283
150.1	0.35498284	-0.368363	0.34646086	0.067967	-0.8854578	0.41973602	0.01665847
150.2	0.35561752	-0.3677274	0.34672733	0.0673539	-0.8855546	0.42003183	0.01674703
150.3	0.35584752	-0.3679616	0.34641601	0.06711886	-0.8856389	0.42026962	0.01697163
150.4	0.35566521	-0.3677052	0.34610663	0.06798067	-0.8855548	0.42059797	0.01764112
150.5	0.35588381	-0.3676478	0.34572898	0.0678815	-0.8854771	0.42129244	0.0176378
150.6	0.35577855	-0.3672016	0.34734746	0.06686497	-0.8855316	0.4223254	0.0181846
150.7	0.35520794	-0.3669342	0.34745483	0.06675318	-0.8853625	0.42212359	0.01872904
150.8	0.35525008	-0.3664388	0.34891301	0.06705102	-0.8854651	0.42331291	0.01980785
150.9	0.35548911	-0.3661808	0.34880733	0.06691356	-0.8853924	0.42327608	0.01967557
151	0.35497896	-0.3668065	0.34974057	0.06674008	-0.8855881	0.42431107	0.0199127
151.1	0.35451116	-0.3662459	0.3493168	0.06612864	-0.8853237	0.4245551	0.0199885
151.2	0.35674235	-0.3649037	0.34899528	0.06592585	-0.8853147	0.42470026	0.02034606
151.3	0.35633816	-0.3652942	0.34911587	0.06504101	-0.8852571	0.42470492	0.02062872
151.4	0.35584096	-0.3654078	0.34973447	0.06539685	-0.8852576	0.42500585	0.02001643
151.5	0.35545714	-0.3644359	0.34945021	0.06489822	-0.8850539	0.42518799	0.02007889
151.6	0.3557225	-0.3647976	0.3502036	0.06577267	-0.8852006	0.42566521	0.02015218
151.7	0.35556974	-0.3642148	0.35072453	0.06453682	-0.8853005	0.42608446	0.01951856
151.8	0.35524229	-0.3647303	0.35140045	0.06481577	-0.8855615	0.42669699	0.01938831
151.9	0.35496471	-0.3640799	0.35133602	0.06412593	-0.8853022	0.42670919	0.02031879
152	0.35419994	-0.3643777	0.35195626	0.06559129	-0.8853845	0.42771762	0.02156852
152.1	0.35419994	-0.3643777	0.35195626	0.06559129	-0.8853845	0.42771762	0.02156852
152.2	0.35350895	-0.3647107	0.35237409	0.064828	-0.8855126	0.4277471	0.02212535
152.3	0.35350895	-0.3647107	0.35237409	0.064828	-0.8855126	0.4277471	0.02212535
152.4	0.35321947	-0.3646408	0.3523864	0.0652453	-0.8855157	0.42825141	0.02151683
152.5	0.35419402	-0.364485	0.35283387	0.06567083	-0.8855812	0.42893339	0.02137036
152.6	0.35419402	-0.364485	0.35283387	0.06567083	-0.8855812	0.42893339	0.02137036
152.7	0.35421754	-0.3648342	0.35326018	0.06610698	-0.8859525	0.42882611	0.02172286
152.8	0.35407011	-0.3649002	0.35396661	0.06644462	-0.8860129	0.42898885	0.02193333
152.9	0.35382706	-0.365482	0.35440299	0.06618613	-0.8860743	0.42966909	0.02247053
153	0.35363783	-0.3655973	0.35422334	0.06604437	-0.8860847	0.4302349	0.0226463
153.1	0.35363783	-0.3655973	0.35422334	0.06604437	-0.8860847	0.4302349	0.0226463
153.2	0.35298922	-0.3655408	0.35421863	0.06576701	-0.8860882	0.43078347	0.02197676
153.3	0.35229621	-0.3659299	0.35366159	0.06596747	-0.8860136	0.43027858	0.02220512
153.4	0.3530139	-0.3656705	0.35374307	0.06559147	-0.8861627	0.43017926	0.02237482
153.5	0.3530139	-0.3656705	0.35374307	0.06559147	-0.8861627	0.43017926	0.02237482
153.6	0.35276707	-0.3654627	0.35382568	0.0654656	-0.8861661	0.43017458	0.02249121
153.7	0.35276707	-0.3654627	0.35382568	0.0654656	-0.8861661	0.43017458	0.02249121
153.8	0.35355585	-0.3648598	0.35365411	0.06477688	-0.8862743	0.43106348	0.02214832
153.9	0.35388292	-0.3643718	0.35418864	0.06443314	-0.8862259	0.43119907	0.02223653
154	0.35384443	-0.36479	0.35462201	0.06419418	-0.8861572	0.43091189	0.02284352
154.1	0.35384443	-0.36479	0.35462201	0.06419418	-0.8861572	0.43091189	0.02284352
154.2	0.35375968	-0.3638837	0.35438122	0.0636299	-0.8858946	0.43141753	0.02323431
154.3	0.35375968	-0.3638837	0.35438122	0.0636299	-0.8858946	0.43141753	0.02323431
154.4	0.35375968	-0.3638837	0.35438122	0.0636299	-0.8858946	0.43141753	0.02323431
154.5	0.35380454	-0.3637458	0.35440689	0.06351208	-0.8858501	0.43144695	0.02385067
154.6	0.35386349	-0.3646959	0.35500689	0.06387124	-0.8855245	0.43180983	0.02347563
154.7	0.35310408	-0.3644338	0.35493847	0.06263213	-0.885704	0.43183811	0.02420785
154.8	0.35310408	-0.3644338	0.35493847	0.06263213	-0.885704	0.43183811	0.02420785
154.9	0.35331885	-0.3641469	0.35561934	0.0623111	-0.8858106	0.43224698	0.02426839
155	0.35331885	-0.3641469	0.35561934	0.0623111	-0.8858106	0.43224698	0.02426839
155.1	0.35301941	-0.3637204	0.3551734	0.06289283	-0.8856953	0.43255259	0.02483561
155.2	0.3533231	-0.3635199	0.35553204	0.06168449	-0.8857552	0.43268237	0.02439668
155.3	0.35296057	-0.3633893	0.35551209	0.06218248	-0.8857321	0.43256097	0.02519693
155.4	0.35283198	-0.3636746	0.355399	0.06213326	-0.8856979	0.43292836	0.0244721
155.5	0.35192154	-0.3634028	0.35469415	0.06247544	-0.8856178	0.43406525	0.02466672
155.6	0.35166837	-0.3646397	0.35463631	0.06244847	-0.8855245	0.4334848	0.02515122
155.7	0.35234537	-0.3636823	0.35465493	0.06247275	-0.8855355	0.43426564	0.02576193
155.8	0.35235512	-0.3634954	0.35468478	0.06285701	-0.8856505	0.43427059	0.0258557
155.9	0.35224668	-0.3631688	0.35495167	0.06316001	-0.885694	0.4347766	0.02562263
156	0.35195382	-0.3628243	0.35517153	0.06380429	-0.8854389	0.43498951	0.02547829

114	0.39894256	-0.3784958	0.32953802	0.0592314	-0.8777218	0.31425099	-0.0230472
114.1	0.39794352	-0.3781587	0.32910419	0.06001912	-0.877738	0.31535354	-0.0223125
114.2	0.39817159	-0.3791962	0.32950704	0.06064341	-0.8778935	0.31623946	-0.0219358
114.3	0.39808741	-0.3795689	0.32971441	0.06098103	-0.8778198	0.31630435	-0.0213395
114.4	0.39771673	-0.3795298	0.32979578	0.06100866	-0.8775932	0.31622455	-0.0207441
114.5	0.39769832	-0.3795613	0.32957671	0.06081738	-0.8775433	0.31623391	-0.0211602
114.6	0.39769316	-0.3784196	0.32970563	0.06109284	-0.8775749	0.31703325	-0.0213086
114.7	0.39769316	-0.3784196	0.32970563	0.06109284	-0.8775749	0.31703325	-0.0213086
114.8	0.3980418	-0.3785986	0.32951342	0.06087074	-0.8777338	0.3172522	-0.0213094
114.9	0.39815183	-0.3786959	0.3290969	0.06160785	-0.8777069	0.31692464	-0.0217436
115	0.39778471	-0.37838	0.32726896	0.06197741	-0.8776515	0.31761303	-0.0203961
115.1	0.39796862	-0.3782252	0.32658755	0.06219305	-0.8774839	0.31717656	-0.0197751
115.2	0.39849471	-0.378461	0.3261873	0.06230073	-0.8777063	0.31759981	-0.0194231
115.3	0.3986741	-0.3782668	0.32607996	0.06245622	-0.877747	0.31809146	-0.0199986
115.4	0.39813195	-0.3782185	0.32565968	0.06243814	-0.8777884	0.31833349	-0.0197137
115.5	0.39788818	-0.3782388	0.32568557	0.06225641	-0.8777058	0.31859868	-0.0196834
115.6	0.39859344	-0.3783406	0.32551918	0.06215046	-0.8777647	0.31910398	-0.018871
115.7	0.39851253	-0.3782506	0.32553414	0.06344747	-0.877945	0.31971353	-0.0181729
115.8	0.39842105	-0.3780529	0.32513436	0.06381143	-0.8780119	0.31953979	-0.0185167
115.9	0.39828407	-0.3779735	0.32452063	0.06312971	-0.8782528	0.3197334	-0.0188872
116	0.3981509	-0.3779982	0.32496785	0.06311968	-0.8783285	0.31985098	-0.0190585
116.1	0.39771264	-0.3784298	0.32473911	0.06363873	-0.8784065	0.32039021	-0.0186988
116.2	0.39768538	-0.3781998	0.32457113	0.06350558	-0.8785359	0.32093481	-0.019127
116.3	0.39710429	-0.3786035	0.32386915	0.06378438	-0.878414	0.32077169	-0.0191305
116.4	0.39710429	-0.3786035	0.32386915	0.06378438	-0.878414	0.32077169	-0.0191305
116.5	0.39686404	-0.3780278	0.32383167	0.06391872	-0.8784023	0.32110431	-0.0187959
116.6	0.396004	-0.3789979	0.32379335	0.06425936	-0.8782116	0.32166048	-0.0178979
116.7	0.39623485	-0.3792529	0.32417823	0.0638885	-0.8781471	0.32268195	-0.0173134
116.8	0.39641015	-0.3790454	0.32391757	0.06303209	-0.8782143	0.32274922	-0.0174254
116.9	0.39597176	-0.3794134	0.32384787	0.06127953	-0.8783853	0.32314053	-0.0174468
117	0.3959403	-0.3793429	0.32376982	0.061265	-0.8784907	0.32338159	-0.0172415
117.1	0.39530201	-0.3798242	0.32441885	0.06225283	-0.8784731	0.32455233	-0.016782
117.2	0.39516302	-0.3798254	0.3244243	0.06212397	-0.878514	0.32512183	-0.0166461
117.3	0.39486108	-0.379763	0.32467557	0.06276065	-0.8783747	0.32531818	-0.0166211
117.4	0.39458934	-0.3797686	0.325223	0.06275139	-0.8785158	0.3257797	-0.0161502
117.5	0.39493089	-0.3801662	0.32521628	0.06329247	-0.8786324	0.32584952	-0.0155947
117.6	0.39512541	-0.3805226	0.32553331	0.06356767	-0.8786087	0.32679773	-0.0149246
117.7	0.3946425	-0.3808174	0.32578622	0.06338971	-0.8786301	0.32742926	-0.0149177
117.8	0.39434812	-0.3807812	0.32542547	0.06297515	-0.8785379	0.32864056	-0.015014
117.9	0.394577	-0.3809451	0.32627586	0.06155385	-0.8785958	0.32982233	-0.0154915
118	0.39411731	-0.3803344	0.32680964	0.06177261	-0.8784859	0.33018402	-0.0155108
118.1	0.39411731	-0.3803344	0.32680964	0.06177261	-0.8784859	0.33018402	-0.0155108
118.2	0.3944964	-0.3810462	0.32650686	0.06241452	-0.8784792	0.33179851	-0.0172482
118.3	0.39427952	-0.381532	0.32641052	0.06223761	-0.8785447	0.3322033	-0.017553
118.4	0.39388355	-0.3820072	0.32600063	0.06284713	-0.8785274	0.33223011	-0.0180383
118.5	0.39448409	-0.381989	0.32541069	0.062644	-0.8785847	0.33227963	-0.0180253
118.6	0.39439503	-0.381842	0.32537733	0.06282631	-0.87853	0.33224946	-0.0177607
118.7	0.39438941	-0.3815679	0.32576111	0.06344109	-0.878593	0.33357491	-0.0180847
118.8	0.39551354	-0.3812582	0.32655463	0.06431483	-0.8789436	0.33387836	-0.0178526
118.9	0.39532895	-0.3811216	0.32708569	0.06426023	-0.8791149	0.33439352	-0.0177586
119	0.39489988	-0.3810932	0.32660713	0.06468308	-0.8790019	0.3341453	-0.0171618
119.1	0.39472425	-0.3810156	0.32614135	0.06491312	-0.8789207	0.3349125	-0.0178054
119.2	0.39438544	-0.3795611	0.32551013	0.06635822	-0.878733	0.336791	-0.0178713
119.3	0.3948146	-0.3795515	0.32471717	0.06632064	-0.8788486	0.33716584	-0.0182462
119.4	0.39516861	-0.3792888	0.32462999	0.06704374	-0.8789654	0.33829672	-0.0177676
119.5	0.39570874	-0.3790838	0.32476273	0.06688761	-0.8790192	0.33892448	-0.017613
119.6	0.39572312	-0.3797197	0.32432063	0.0669017	-0.8790891	0.33890766	-0.0176812
119.7	0.39568854	-0.3792599	0.3233402	0.06760925	-0.879331	0.33985002	-0.0184569
119.8	0.39598776	-0.3795419	0.32300236	0.06780018	-0.8792917	0.34009613	-0.0185291
119.9	0.39529843	-0.3789237	0.32247471	0.06725552	-0.8791394	0.34027383	-0.0184811

165	0.3416781	-0.3620116	0.36102788	0.06450503	-0.8854781	0.44704971	0.02735345
165.1	0.34149786	-0.3622497	0.36075281	0.06453297	-0.8854251	0.44713952	0.02728685
165.2	0.34087941	-0.3619785	0.36068456	0.06399647	-0.8853112	0.44722583	0.02764355
165.3	0.34126101	-0.3617615	0.36067065	0.06343148	-0.8853504	0.44765485	0.02747312
165.4	0.34121262	-0.3616343	0.36074362	0.06352286	-0.8853132	0.44782104	0.02803804
165.5	0.34134697	-0.3616529	0.3608765	0.06315123	-0.8853046	0.44778874	0.02826172
165.6	0.34075502	-0.3617727	0.36133837	0.06354177	-0.885336	0.44824421	0.02868563
165.7	0.3406463	-0.3615968	0.36135298	0.06292631	-0.8853319	0.44834919	0.02878415
165.8	0.33984631	-0.3613466	0.36071958	0.06235922	-0.8852495	0.44841319	0.02929246
165.9	0.33989891	-0.3616656	0.36151185	0.06290193	-0.8855815	0.44864736	0.02909728
166	0.33977627	-0.3612352	0.36182226	0.06272013	-0.8856502	0.44904002	0.02899934
166.1	0.33946941	-0.3599083	0.36168204	0.06209247	-0.8854223	0.44866805	0.02912451
166.2	0.33811338	-0.3609649	0.36278889	0.06276928	-0.885467	0.44856793	0.0287405
166.3	0.3377595	-0.3614009	0.36230069	0.06214004	-0.8854392	0.44850715	0.02832191
166.4	0.3387211	-0.3619488	0.36268988	0.06231414	-0.8855217	0.44847577	0.02817533
166.5	0.33824571	-0.3631857	0.36279244	0.06169427	-0.8854838	0.44881345	0.02800066
166.6	0.3378825	-0.3628485	0.36313605	0.06127645	-0.8856239	0.44915457	0.02789203
166.7	0.3378825	-0.3628485	0.36313605	0.06127645	-0.8856239	0.44915457	0.02789203
166.8	0.3378825	-0.3628485	0.36313605	0.06127645	-0.8856239	0.44915457	0.02789203
166.9	0.3378825	-0.3628485	0.36313605	0.06127645	-0.8856239	0.44915457	0.02789203
167	0.33694213	-0.3624382	0.36366393	0.06242289	-0.8857475	0.44968505	0.02762952
167.1	0.33791036	-0.3625981	0.36362342	0.06266238	-0.8857227	0.4498706	0.02731333
167.2	0.33734402	-0.3633204	0.36404341	0.06230814	-0.8856119	0.45000948	0.02689604
167.3	0.33708267	-0.3632314	0.36468864	0.06335825	-0.8855906	0.45026115	0.02667143
167.4	0.3365318	-0.3632788	0.36447794	0.06350224	-0.8855795	0.45061901	0.02672219
167.5	0.33571721	-0.3637155	0.36476747	0.06349771	-0.8855894	0.45085879	0.02743433
167.6	0.33485857	-0.3636185	0.36399799	0.06377726	-0.8855899	0.45118499	0.02800026
167.7	0.33404133	-0.3633382	0.36382677	0.06381989	-0.8853576	0.4516729	0.02752309
167.8	0.33404133	-0.3633382	0.36382677	0.06381989	-0.8853576	0.4516729	0.02752309
167.9	0.33386347	-0.3632881	0.36431298	0.06426821	-0.8855547	0.45208722	0.02731691
168	0.33342425	-0.3630396	0.36462189	0.06422041	-0.8855918	0.45220052	0.02729311
168.1	0.33342425	-0.3630396	0.36462189	0.06422041	-0.8855918	0.45220052	0.02729311
168.2	0.33326941	-0.3630812	0.36516271	0.06486846	-0.8858073	0.45255753	0.02644768
168.3	0.3326941	-0.3630812	0.36516271	0.06486846	-0.8858073	0.45255753	0.02644768
168.4	0.33225514	-0.3637568	0.36558265	0.06258468	-0.8858242	0.4524157	0.02675142
168.5	0.33203787	-0.3629715	0.36557819	0.06585293	-0.88591	0.45291121	0.02746747
168.6	0.33177874	-0.3632469	0.36542774	0.06612331	-0.8858769	0.4528255	0.02781828
168.7	0.33171622	-0.3633366	0.36516014	0.06661589	-0.8858391	0.45306094	0.02810874
168.8	0.33199632	-0.3629622	0.36610018	0.06716562	-0.8859162	0.45316762	0.02794463
168.9	0.33186431	-0.362949	0.36596615	0.06678287	-0.8859204	0.45274997	0.02734152
169	0.33190114	-0.3623635	0.36562778	0.06708706	-0.8859975	0.45345866	0.02692532
169.1	0.33190114	-0.3623635	0.36562778	0.06708706	-0.8859975	0.45345866	0.02692532
169.2	0.33190114	-0.3623635	0.36562778	0.06708706	-0.8859975	0.45345866	0.02692532
169.3	0.33237975	-0.3619138	0.36552382	0.06769903	-0.8859848	0.45381222	0.02709038
169.4	0.33193571	-0.3623908	0.36562048	0.06847719	-0.8859848	0.45366393	0.02699297
169.5	0.33124891	-0.362626	0.36563402	0.06879981	-0.8857974	0.45430328	0.02689175
169.6	0.33124891	-0.362626	0.36563402	0.06879981	-0.8857974	0.45430328	0.02689175
169.7	0.33060748	-0.3625836	0.36564498	0.06909968	-0.8856507	0.4543954	0.02680304
169.8	0.33075942	-0.3622511	0.36536418	0.06886562	-0.8855622	0.45428085	0.02657102
169.9	0.33083105	-0.3623787	0.36677695	0.06952873	-0.8858124	0.45461379	0.0255557
170	0.33083105	-0.3623787	0.36677695	0.06952873	-0.8858124	0.45461379	0.0255557
170.1	0.33144977	-0.3629491	0.3675855	0.06829556	-0.8859748	0.45460079	0.02543679
170.2	0.33144977	-0.3629491	0.3675855	0.06829556	-0.8859748	0.45460079	0.02543679
170.3	0.33103894	-0.3633342	0.36744661	0.06895861	-0.885921	0.45477176	0.02543575
170.4	0.330896	-0.364166	0.36714918	0.06893563	-0.8859294	0.45451396	0.02551966
170.5	0.33086662	-0.3648128	0.3669699	0.0687127	-0.8859623	0.45501102	0.02529468
170.6	0.33086662	-0.3648128	0.3669699	0.0687127	-0.8859623	0.45501102	0.02529468
170.7	0.33086662	-0.3648128	0.3669699	0.0687127	-0.8859623	0.45501102	0.02529468
170.8	0.33040133	-0.3647532	0.36688902	0.06846068	-0.8859773	0.45498751	0.02563173
170.9	0.33052254	-0.3646851	0.36621134	0.06845478	-0.8858823	0.45516837	0.02566299
171	0.33079634	-0.3641244	0.36791867	0.06874405	-0.8860727	0.45606574	0.02560789

126.1	0.37984477	-0.3763425	0.31813374	0.07191238	-0.8807089	0.3622057	-0.0088538
126.2	0.380069	-0.3763406	0.31837599	0.0718889	-0.8807642	0.36250832	-0.0091639
126.3	0.37988422	-0.3758714	0.31784173	0.07091288	-0.8805988	0.36287846	-0.008884
126.4	0.37946729	-0.3752395	0.31761434	0.0702355	-0.880587	0.36300542	-0.0095529
126.5	0.37995796	-0.3750191	0.31803785	0.0701831	-0.8806174	0.36373108	-0.0093687
126.6	0.37968963	-0.3749965	0.31773737	0.07026674	-0.8806395	0.36392988	-0.0090164
126.7	0.37891493	-0.3742268	0.3173571	0.06979556	-0.8806741	0.36408882	-0.0097969
126.8	0.37860598	-0.3741181	0.3175492	0.06967927	-0.8805794	0.36455177	-0.0093869
126.9	0.37886233	-0.3736266	0.31748286	0.06869137	-0.8807644	0.3649953	-0.010051
127	0.37899805	-0.3734487	0.3170368	0.06889389	-0.8806754	0.36528917	-0.0093016
127.1	0.37930693	-0.373404	0.31702784	0.0687428	-0.8806604	0.3658974	-0.0085123
127.2	0.37914309	-0.3730877	0.31674894	0.06849464	-0.8806663	0.36582338	-0.0090106
127.3	0.37885757	-0.3736898	0.31597926	0.06856058	-0.8807777	0.36701572	-0.0087332
127.4	0.37903675	-0.3738865	0.31697863	0.06941361	-0.8808363	0.36762804	-0.0082873
127.5	0.37822256	-0.3743336	0.31731379	0.06989567	-0.8807757	0.36786026	-0.008232
127.6	0.37857812	-0.374153	0.31711747	0.06984124	-0.880925	0.3678011	-0.0079514
127.7	0.37905575	-0.3738592	0.31694782	0.07058086	-0.8810331	0.36844542	-0.0076138
127.8	0.37905575	-0.3738592	0.31694782	0.07058086	-0.8810331	0.36844542	-0.0076138
127.9	0.37909462	-0.3737712	0.31653948	0.07097073	-0.8810167	0.36861855	-0.0072702
128	0.37909462	-0.3737712	0.31653948	0.07097073	-0.8810167	0.36861855	-0.0072702
128.1	0.37897078	-0.3731365	0.3163522	0.0703065	-0.8809283	0.36957027	-0.005982
128.2	0.37883302	-0.3730234	0.31655704	0.07017532	-0.8809342	0.3697751	-0.0062822
128.3	0.37830702	-0.3727746	0.31591442	0.06971896	-0.8807499	0.36935345	-0.0061039
128.4	0.37802008	-0.3725188	0.31623714	0.06941566	-0.8807984	0.36960366	-0.0066523
128.5	0.37781596	-0.372414	0.31634971	0.06924919	-0.8808704	0.36964317	-0.0065008
128.6	0.37733046	-0.3722971	0.31617681	0.06867587	-0.8808224	0.36970221	-0.0064949
128.7	0.37727541	-0.372416	0.31628024	0.06850659	-0.8808246	0.36996965	-0.0071512
128.8	0.37727541	-0.372416	0.31628024	0.06850659	-0.8808246	0.36996965	-0.0071512
128.9	0.37727541	-0.372416	0.31628024	0.06850659	-0.8808246	0.36996965	-0.0071512
129	0.37762444	-0.3723965	0.3163109	0.06824373	-0.8808224	0.37057378	-0.0067592
129.1	0.37762444	-0.3723965	0.3163109	0.06824373	-0.8808224	0.37057378	-0.0067592
129.2	0.37749821	-0.372733	0.31657836	0.06799137	-0.8808266	0.37080969	-0.0066916
129.3	0.3769465	-0.3726669	0.31590883	0.06791883	-0.8809004	0.37126833	-0.0069352
129.4	0.37692839	-0.3720525	0.31651443	0.0690716	-0.8809287	0.37271231	-0.0055174
129.5	0.37648731	-0.3719449	0.31722324	0.06851394	-0.8809164	0.37188983	-0.005479
129.6	0.37648731	-0.3719449	0.31722324	0.06851394	-0.8809164	0.37188983	-0.005479
129.7	0.37637376	-0.3716611	0.31704453	0.06902175	-0.8808547	0.37209258	-0.0049259
129.8	0.37557961	-0.3713952	0.31700302	0.06860087	-0.8806795	0.37232248	-0.005039
129.9	0.37529867	-0.3715222	0.31821868	0.06893844	-0.880905	0.37369819	-0.00411
130	0.37534698	-0.3713463	0.31783967	0.06976404	-0.8810172	0.37428484	-0.0035883
130.1	0.37510684	-0.371188	0.3184509	0.06932523	-0.8810591	0.37462097	-0.0031871
130.2	0.37489083	-0.3714659	0.31796829	0.06936376	-0.8809769	0.37488242	-0.0033057
130.3	0.37509437	-0.3707097	0.31774563	0.069932	-0.8810117	0.37644755	-0.0028947
130.4	0.37547505	-0.3703438	0.31839825	0.06987631	-0.8809411	0.3767744	-0.0028774
130.5	0.37552305	-0.3703827	0.31863445	0.07008764	-0.8810664	0.37680446	-0.002263
130.6	0.37552305	-0.3703827	0.31863445	0.07008764	-0.8810664	0.37680446	-0.002263
130.7	0.37507504	-0.3705867	0.31858847	0.07052693	-0.8811705	0.37755865	-0.0015144
130.8	0.37523355	-0.3707473	0.31780745	0.07046849	-0.8811981	0.37816864	-0.001415
130.9	0.37540956	-0.3703912	0.31781773	0.07047667	-0.8813468	0.37883412	-0.0018006
131	0.37486963	-0.3713085	0.31794288	0.07021293	-0.8813855	0.37945516	-0.0019249
131.1	0.37511075	-0.3716066	0.31780748	0.06979301	-0.8813669	0.37988101	-0.0015514
131.2	0.37511075	-0.3716066	0.31780748	0.06979301	-0.8813669	0.37988101	-0.0015514
131.3	0.37518016	-0.3715293	0.31764425	0.07041627	-0.8810844	0.3801061	-0.0010047
131.4	0.37397406	-0.3719911	0.3174912	0.071376	-0.8809831	0.38070103	0.0001226
131.5	0.37426191	-0.3716834	0.31844312	0.07204929	-0.881024	0.38109044	0.00042231
131.6	0.37422817	-0.371578	0.31793666	0.07224282	-0.8809428	0.38152112	-6.24E-05
131.7	0.37359102	-0.3711234	0.31786019	0.07130029	-0.8807562	0.38129723	-2.31E-06
131.8	0.37295815	-0.3713859	0.31729587	0.07098568	-0.8809193	0.38120907	0.00040221
131.9	0.37312067	-0.3714023	0.3166175	0.07118438	-0.8808274	0.38116654	3.66E-05
132	0.37312067	-0.3714023	0.3166175	0.07118438	-0.8808274	0.38116654	3.66E-05
132.1	0.37312067	-0.3714023	0.3166175	0.07118438	-0.8808274	0.38116654	3.66E-05
132.2	0.3731939	-0.3714416	0.31676216	0.07122991	-0.8808973	0.38141088	9.24E-05
132.3	0.3730058	-0.3717382	0.3167434	0.07159919	-0.8809278	0.3814722	0.00019366
132.4	0.3722721	-0.3716159	0.31698045	0.07195311	-0.8808415	0.38138632	0.00057938
132.5	0.37188753	-0.371768	0.31713936	0.07230348	-0.8809589	0.38190853	0.00088967
132.6	0.37149251	-0.371906	0.31669416	0.07231744	-0.8808842	0.38219415	0.00019135
132.7	0.37107429	-0.3714716	0.31641327	0.07225424	-0.880814	0.38216939	9.47E-05
132.8	0.37012995	-0.3725304	0.31661142	0.0722808	-0.8808129	0.38240566	0.00081646
132.9	0.37012995	-0.3725304	0.31661142	0.0722808	-0.8808129	0.38240566	0.00081646
133	0.37017272	-0.3727861	0.31646488	0.07248364	-0.8808263	0.38241304	0.00109046
133.1	0.37006063	-0.3728288	0.31662108	0.07217461	-0.880885	0.38244283	0.00088541
133.2	0.36988802	-0.3725631	0.31616136	0.07209902	-0.8807921	0.38243341	0.0009771
133.3	0.36988802	-0.3725631	0.31616136	0.07209902	-0.8807921	0.38243341	0.0009771
133.4	0.36966232	-0.3728294	0.31688356	0.07231327	-0.8809223	0.3829536	0.00147232
133.5	0.3701528	-0.3723622	0.31682782	0.07189266	-0.8810164	0.38293637	0.00210836
133.6	0.36987282	-0.3726534	0.31657043	0.07230626	-0.8810273	0.38302226	0.00153395
133.7	0.36990018	-0.3731037	0.31642319	0.07145257	-0.8811351	0.38347032	0.00229211
133.8	0.36978389	-0.3734019	0.31656712	0.07152283	-0.8810997	0.3842985	0.00251174
133.9	0.36978389	-0.3734019	0.31656712	0.07152283	-0.8810997	0.3842985	0.00251174
134	0.36994305	-0.3741582	0.31693782	0.0711055	-0.8810964	0.38443364	0.00196092
134.1	0.37015958	-0.3744389	0.31700321	0.07066793	-0.8811595	0.38432893	0.00144485
134.2	0.37015958	-0.3744389	0.31700321	0.07066793	-0.8811595	0.38432893	0.00144485
134.3	0.36985281	-0.3743017	0.31678004	0.07096597	-0.8811688	0.3843423	0.0012335
134.4	0.36998713	-0.3738002	0.31688155	0.0705143	-0.8809887	0.38481986	0.00134549
134.5	0.36944684	-0.3741949	0.31690476	0.07070305	-0.8809819	0.38489472	0.00135312
134.6	0.36956654	-0.3742526	0.31748317	0.07089258	-0.8811134	0.38506498	0.00123601
134.7	0.36949649	-0.3738606	0.31709467	0.07061832	-0.8811981	0.38531404	0.00141637
134.8	0.36942595	-0.3740306	0.31709321	0.06998284	-0.8811708	0.38568144	0.00166376
134.9	0.36942595	-0.3740306	0.31709321	0.06998284	-0.8811708	0.38568144	0.00166376

180	0.31903665	-0.3741793	0.37544982	0.06373582	-0.8865058	0.46742812	0.01485104
180.1	0.31903665	-0.3741793	0.37544982	0.06373582	-0.8865058	0.46742812	0.01485104
180.2	0.3186153	-0.374055	0.37584719	0.06358597	-0.8864863	0.46783869	0.01440883
180.3	0.3186153	-0.374055	0.37584719	0.06358597	-0.8864863	0.46783869	0.01440883
180.4	0.31742001	-0.3748708	0.37596647	0.0640626	-0.8864588	0.46788291	0.01362306
180.5	0.31762402	-0.3751389	0.37600563	0.06386285	-0.8864768	0.46825043	0.01321907
180.6	0.31735802	-0.3754525	0.37605292	0.06369991	-0.8864504	0.46899301	0.01262181
180.7	0.31638182	-0.3749235	0.37735548	0.06302303	-0.8865772	0.4698974	0.01156842
180.8	0.31638182	-0.3749235	0.37735548	0.06302303	-0.8865772	0.4698974	0.01156842
180.9	0.31658711	-0.3753856	0.37768462	0.0624017	-0.8864737	0.46968044	0.01096431
181	0.31542168	-0.3751065	0.37689474	0.06212972	-0.8862117	0.46928834	0.01069718
181.1	0.31570635	-0.3749605	0.37672214	0.06218467	-0.8861989	0.46981082	0.01106452
181.2	0.3145677	-0.3762158	0.37678796	0.06191085	-0.8859904	0.47005853	0.00963727
181.3	0.31449011	-0.3766964	0.37628103	0.06198932	-0.8858348	0.47004205	0.00900923
181.4	0.31445578	-0.3771698	0.37632275	0.06158003	-0.8859601	0.47047055	0.00904188
181.5	0.31457107	-0.3780416	0.3764221	0.06013739	-0.8861095	0.47086591	0.00877892
181.6	0.31400023	-0.3787666	0.37692011	0.06051848	-0.8863594	0.47109773	0.0091752
181.7	0.31389504	-0.3793024	0.37696764	0.05978229	-0.8863748	0.47108385	0.0086774
181.8	0.31389504	-0.3793024	0.37696764	0.05978229	-0.8863748	0.47108385	0.0086774
181.9	0.31389504	-0.3793024	0.37696764	0.05978229	-0.8863748	0.47108385	0.0086774
182	0.31348463	-0.3800573	0.37719508	0.05983427	-0.8864964	0.47137972	0.00874617
182.1	0.31363463	-0.3802469	0.37725281	0.05983285	-0.8864331	0.47136175	0.00827761
182.2	0.31357443	-0.3802756	0.37737062	0.06007785	-0.8865268	0.4713436	0.00863637
182.3	0.31233804	-0.3805841	0.37849712	0.06006012	-0.8866604	0.47212043	0.00860415
182.4	0.31200228	-0.3806817	0.37884175	0.06084824	-0.8865861	0.47212707	0.00853943
182.5	0.31163565	-0.3813611	0.37923972	0.06053382	-0.8869457	0.47281809	0.00787952
182.6	0.31076725	-0.3821629	0.37982428	0.06032699	-0.8869967	0.47328628	0.00725914
182.7	0.31067312	-0.3821404	0.37941008	0.06077874	-0.8869196	0.47326381	0.00691321
182.8	0.31022582	-0.3821762	0.37989451	0.06105059	-0.8870045	0.47362522	0.00666243
182.9	0.3103124	-0.3822673	0.38062251	0.06093977	-0.8870895	0.47399697	0.00691432
183	0.310862	-0.3818225	0.38131831	0.06100535	-0.8873081	0.47447816	0.00641934
183.1	0.31127957	-0.3822538	0.38154963	0.06105014	-0.8874397	0.47500021	0.0065915

141.1	0.36398552	-0.3735682	0.32444875	0.06841405	-0.8828099	0.39910058	0.00466007
141.2	0.3641677	-0.373907	0.32369143	0.06961873	-0.8827497	0.39953983	0.00473721
141.3	0.36426069	-0.3729002	0.32443574	0.07011206	-0.883011	0.40070538	0.00385714
141.4	0.36437183	-0.372895	0.32517249	0.07025982	-0.8832535	0.40098016	0.00431957
141.5	0.36381631	-0.3734743	0.32603166	0.07095322	-0.8833932	0.40124158	0.00457406
141.6	0.36381631	-0.3734743	0.32603166	0.07095322	-0.8833932	0.40124158	0.00457406
141.7	0.36381631	-0.3734743	0.32603166	0.07095322	-0.8833932	0.40124158	0.00457406
141.8	0.3636494	-0.3733377	0.32665945	0.07106359	-0.883571	0.40180443	0.00465239
141.9	0.36330346	-0.3733238	0.32687482	0.07097785	-0.883587	0.4021833	0.00488462
142	0.36330346	-0.3733238	0.32687482	0.07097785	-0.883587	0.4021833	0.00488462
142.1	0.36317391	-0.3733785	0.32715689	0.07111327	-0.8836937	0.40216495	0.00484054
142.2	0.36317391	-0.3733785	0.32715689	0.07111327	-0.8836937	0.40216495	0.00484054
142.3	0.36302203	-0.3735329	0.32811488	0.07172922	-0.8840767	0.40251849	0.00413118
142.4	0.36281923	-0.3736499	0.32803779	0.07187705	-0.8841324	0.40313523	0.00319906
142.5	0.36269505	-0.373626	0.32889168	0.0712081	-0.8843378	0.40372748	0.00342138
142.6	0.36209444	-0.3736659	0.32919777	0.07129719	-0.8842547	0.40389787	0.00369133
142.7	0.36209444	-0.3736659	0.32919777	0.07129719	-0.8842547	0.40389787	0.00369133
142.8	0.3620397	-0.3736126	0.32914355	0.07180677	-0.8843161	0.40387558	0.00373257
142.9	0.3620397	-0.3736126	0.32914355	0.07180677	-0.8843161	0.40387558	0.00373257
143	0.36233432	-0.3737209	0.3288768	0.07207427	-0.8843041	0.40396464	0.00370445
143.1	0.36242237	-0.3734096	0.32915605	0.07150001	-0.8844787	0.40414728	0.00394115
143.2	0.36239651	-0.373119	0.32901587	0.07068162	-0.8844121	0.40429624	0.00414699
143.3	0.36214467	-0.3725933	0.32910675	0.07072354	-0.8844009	0.40432595	0.00405238
143.4	0.36214467	-0.3725933	0.32910675	0.07072354	-0.8844009	0.40432595	0.00405238
143.5	0.36210539	-0.372315	0.32979167	0.07006493	-0.8844845	0.40463364	0.00452668
143.6	0.36165744	-0.3719121	0.33044593	0.06984769	-0.8845462	0.40506037	0.00483379
143.7	0.36149152	-0.3718867	0.33077477	0.06990994	-0.8846047	0.4054691	0.00528461
143.8	0.36149152	-0.3718867	0.33077477	0.06990994	-0.8846047	0.4054691	0.00528461
143.9	0.36150768	-0.3714043	0.3309723	0.06946257	-0.8845719	0.40549966	0.00540234
144	0.36138168	-0.3721493	0.33208347	0.06928707	-0.8847433	0.40609199	0.00523196
144.1	0.36161803	-0.372158	0.33244073	0.06924648	-0.8848699	0.40650677	0.00470468
144.2	0.36115623	-0.371829	0.33209849	0.06904168	-0.8848209	0.40646508	0.00518204
144.3	0.36168201	-0.3717801	0.3328966	0.06916152	-0.8850321	0.40682309	0.00517482
144.4	0.36201795	-0.3724315	0.3331217	0.06968925	-0.8852092	0.40684672	0.00591756
144.5	0.36168225	-0.3722189	0.33349944	0.06955382	-0.8852323	0.40686436	0.00623809
144.6	0.36079165	-0.3721635	0.33366031	0.07055541	-0.8849912	0.40708987	0.0064836
144.7	0.36079165	-0.3721635	0.33366031	0.07055541	-0.8849912	0.40708987	0.0064836
144.8	0.36054173	-0.371772	0.3340167	0.07013103	-0.8849967	0.40747654	0.00654678
144.9	0.36078002	-0.3713601	0.33408038	0.06975656	-0.8850401	0.40775033	0.00626972
145	0.36119318	-0.3710983	0.3346212	0.06875597	-0.8851753	0.40773992	0.00606074
145.1	0.36115615	-0.3716212	0.33481511	0.06826856	-0.8853484	0.40760814	0.00604039
145.2	0.36116311	-0.37159	0.33424725	0.06944461	-0.8851973	0.40785336	0.00617367
145.3	0.36095176	-0.3714535	0.3345281	0.06956236	-0.8850491	0.40840255	0.00588138
145.4	0.36122014	-0.3713931	0.33501828	0.06962495	-0.885232	0.40855725	0.00594698
145.5	0.36150543	-0.3716107	0.33488959	0.07041828	-0.8851756	0.40868931	0.00585213
145.6	0.36206356	-0.3712467	0.33494644	0.07020053	-0.8852706	0.40908611	0.00575605
145.7	0.36168299	-0.3709707	0.3353643	0.07054502	-0.8853181	0.40956381	0.00619005
145.8	0.36164115	-0.3712192	0.33606558	0.07087115	-0.8853337	0.4100909	0.00585981
145.9	0.36164115	-0.3712192	0.33606558	0.07087115	-0.8853337	0.4100909	0.00585981
146	0.36204182	-0.3711049	0.33627254	0.07079856	-0.885411	0.41026243	0.00596944
146.1	0.36156608	-0.3710374	0.33706508	0.07091552	-0.8854237	0.41091128	0.0054867
146.2	0.3616896	-0.3708643	0.33733668	0.07083817	-0.8855549	0.41095212	0.00509395
146.3	0.3616896	-0.3708643	0.33733668	0.07083817	-0.8855549	0.41095212	0.00509395
146.4	0.36176565	-0.3708836	0.3375286	0.07057425	-0.8855836	0.41099618	0.00543942
146.5	0.36210031	-0.3704644	0.33810354	0.07015911	-0.8855726	0.41129306	0.00574454
146.6	0.36210031	-0.3704644	0.33810354	0.07015911	-0.8855726	0.41129306	0.00574454
146.7	0.36195223	-0.3708348	0.33777462	0.07022172	-0.8855909	0.41158127	0.00620012
146.8	0.36186523	-0.37029	0.33817697	0.0697229	-0.8857136	0.41201231	0.00672621
146.9	0.36193092	-0.3703143	0.33879455	0.06954488	-0.8857675	0.4122367	0.00731728
147	0.36193092	-0.3703143	0.33879455	0.06954488	-0.8857675	0.4122367	0.00731728
147.1	0.36237333	-0.370132	0.33863906	0.06917372	-0.8857155	0.41252655	0.00751171
147.2	0.36178602	-0.3694222	0.339598	0.0695437	-0.8857745	0.41308809	0.00855122
147.3	0.36058605	-0.3687859	0.33917141	0.06928408	-0.8853658	0.4132374	0.009132
147.4	0.36046316	-0.3694915	0.33988751	0.06982282	-0.8854782	0.41329282	0.00943931
147.5	0.35996669	-0.3698238	0.33981329	0.07003845	-0.8854336	0.41341994	0.00997962
147.6	0.35996669	-0.3698238	0.33981329	0.07003845	-0.8854336	0.41341994	0.00997962
147.7	0.35996669	-0.3698238	0.33981329	0.07003845	-0.8854336	0.41341994	0.00997962
147.8	0.36026395	-0.3688334	0.340643	0.0689231	-0.8853007	0.41402313	0.01146274
147.9	0.35964121	-0.368562	0.34122035	0.06874547	-0.885403	0.41390192	0.01210294
148	0.35927702	-0.3683492	0.34141855	0.06869851	-0.8852496	0.41403636	0.01224132
148.1	0.35902567	-0.3682599	0.34187968	0.06831185	-0.8852197	0.4143258	0.01159883
148.2	0.35839036	-0.3686434	0.34237562	0.06856112	-0.8852163	0.41423654	0.01191435
148.3	0.35853048	-0.3683775	0.34241864	0.06831889	-0.8852236	0.41435269	0.01236065
148.4	0.3588461	-0.368091	0.34337514	0.06827231	-0.8852881	0.41485928	0.01329404
148.5	0.35842487	-0.3681855	0.3438716	0.06902995	-0.8853734	0.4150605	0.01332655
148.6	0.35842487	-0.3681855	0.3438716	0.06902995	-0.8853734	0.4150605	0.01332655
148.7	0.35842487	-0.3681855	0.3438716	0.06902995	-0.8853734	0.4150605	0.01332655
148.8	0.35784975	-0.3677041	0.34460442	0.06890761	-0.8852993	0.41557902	0.01310907
148.9	0.35758269	-0.3674685	0.34419225	0.06889656	-0.885282	0.41542777	0.01312873
149	0.35812214	-0.3673421	0.34436748	0.06847392	-0.8852476	0.41530504	0.01299486
149.1	0.35869824	-0.3672979	0.34425442	0.06842245	-0.8854323	0.41543131	0.0128933
149.2	0.35857491	-0.3673963	0.34343171	0.06924498	-0.8854376	0.41601921	0.01278408
149.3	0.35857491	-0.3673963	0.34343171	0.06924498	-0.8854376	0.41601921	0.01278408
149.4	0.35723897	-0.367201	0.34433381	0.06959193	-0.8850843	0.41701901	0.0131838
149.5	0.35659104	-0.3676428	0.34540715	0.06901234	-0.8851255	0.41747865	0.01383387
149.6	0.35572139	-0.3672519	0.34595966	0.06800521	-0.885152	0.41876311	0.01440706
149.7	0.35595675	-0.3684615	0.34617387	0.06831593	-0.8854007	0.41921625	0.01493066
149.8	0.35595675	-0.3684615	0.34617387	0.06831593	-0.8854007	0.41921625	0.01493066
149.9	0.35588029	-0.3684072	0.34669031	0.06749243	-0.8854763	0.41964518	0.0161283

195	0.29080204	-0.3995856	0.38542421	0.0542112	-0.8882446	0.48405911	-0.0090259
195.1	0.29080204	-0.3995856	0.38542421	0.0542112	-0.8882446	0.48405911	-0.0090259
195.2	0.29075253	-0.399834	0.38533013	0.05476414	-0.888253	0.48413709	-0.0090724
195.3	0.29084474	-0.3993918	0.38521187	0.0546759	-0.8883161	0.48427507	-0.0087412
195.4	0.29084474	-0.3993918	0.38521187	0.0546759	-0.8883161	0.48427507	-0.0087412
195.5	0.29053706	-0.3995388	0.38467359	0.05502437	-0.8881792	0.48420739	-0.0088435
195.6	0.29053706	-0.3995388	0.38467359	0.05502437	-0.8881792	0.48420739	-0.0088435
195.7	0.29020049	-0.3995721	0.38469561	0.0547933	-0.8880904	0.48454981	-0.0091605
195.8	0.29020049	-0.3995721	0.38469561	0.0547933	-0.8880904	0.48454981	-0.0091605
195.9	0.28986814	-0.3998735	0.38436309	0.05451819	-0.8881013	0.48438583	-0.0088074
196	0.28974994	-0.3997717	0.38483733	0.05472887	-0.8882897	0.48506443	-0.0091089
196.1	0.28974994	-0.3997717	0.38483733	0.05472887	-0.8882897	0.48506443	-0.0091089
196.2	0.28984987	-0.3993036	0.38429223	0.05445143	-0.8881147	0.48513749	-0.0093985
196.3	0.28895062	-0.3998943	0.38447653	0.05478322	-0.8882333	0.48542703	-0.0091904
196.4	0.2884452	-0.3999543	0.384749	0.05517423	-0.8882012	0.48545008	-0.0092223
196.5	0.2883705	-0.4004702	0.3849154	0.05545968	-0.8883186	0.485609	-0.0098874
196.6	0.28806767	-0.4007011	0.38496524	0.05538753	-0.8882713	0.4856151	-0.0100463
196.7	0.28862393	-0.4009287	0.38486695	0.0556726	-0.8882627	0.4856376	-0.0097973
196.8	0.28847639	-0.4006101	0.38480243	0.05548735	-0.8882256	0.48582773	-0.0092868
196.9	0.28847639	-0.4006101	0.38480243	0.05548735	-0.8882256	0.48582773	-0.0092868
197	0.28847639	-0.4006101	0.38480243	0.05548735	-0.8882256	0.48582773	-0.0092868
197.1	0.28820268	-0.4002589	0.38455678	0.05523691	-0.8882297	0.48567434	-0.0087835
197.2	0.28820268	-0.4002589	0.38455678	0.05523691	-0.8882297	0.48567434	-0.0087835
197.3	0.28820268	-0.4002589	0.38455678	0.05523691	-0.8882297	0.48567434	-0.0087835
197.4	0.28820268	-0.4002589	0.38455678	0.05523691	-0.8882297	0.48567434	-0.0087835
197.5	0.28820268	-0.4002589	0.38455678	0.05523691	-0.8882297	0.48567434	-0.0087835
197.6	0.28821749	-0.399895	0.38418453	0.05501185	-0.8882797	0.48556962	-0.0088505
197.7	0.28821749	-0.399895	0.38418453	0.05501185	-0.8882797	0.48556962	-0.0088505
197.8	0.28821749	-0.399895	0.38418453	0.05501185	-0.8882797	0.48556962	-0.0088505
197.9	0.28782301	-0.4003238	0.38427618	0.05518248	-0.8883733	0.4857977	-0.0089513
198	0.28782301	-0.4003238	0.38427618	0.05518248	-0.8883733	0.4857977	-0.0089513
198.1	0.28772667	-0.4007303	0.38414726	0.05476643	-0.8883616	0.48555843	-0.0090047

156.1	0.35195382	-0.3628243	0.35517153	0.06380429	-0.8854389	0.43498951	0.02547829
156.2	0.35207541	-0.3628819	0.35556083	0.06364985	-0.8853847	0.43504127	0.02549404
156.3	0.35185962	-0.3633563	0.35601111	0.06325174	-0.8853654	0.43550024	0.02576216
156.4	0.3516056	-0.3635881	0.35613538	0.06281433	-0.8853719	0.43550482	0.02583946
156.5	0.35145272	-0.3633873	0.35618724	0.06266232	-0.8853013	0.43550314	0.02653786
156.6	0.35169956	-0.3631236	0.35617391	0.06242769	-0.8852298	0.43566864	0.02671454
156.7	0.35169956	-0.3631236	0.35617391	0.06242769	-0.8852298	0.43566864	0.02671454
156.8	0.35169956	-0.3631236	0.35617391	0.06242769	-0.8852298	0.43566864	0.02671454
156.9	0.35169956	-0.3631236	0.35617391	0.06242769	-0.8852298	0.43566864	0.02671454
157	0.35171695	-0.36218	0.3570813	0.06299145	-0.8853575	0.4364304	0.02629864
157.1	0.35171695	-0.36218	0.3570813	0.06299145	-0.8853575	0.4364304	0.02629864
157.2	0.35141392	-0.3625513	0.35733524	0.06310888	-0.8854347	0.43659286	0.02658992
157.3	0.35162577	-0.3627261	0.35820464	0.06258402	-0.8854244	0.43678908	0.02635553
157.4	0.35110471	-0.3634251	0.35831183	0.06257503	-0.8854812	0.43703132	0.02650679
157.5	0.35110471	-0.3634251	0.35831183	0.06257503	-0.8854812	0.43703132	0.02650679
157.6	0.3506706	-0.3635095	0.35836972	0.06275158	-0.8855861	0.43767669	0.02644171
157.7	0.3505403	-0.3634765	0.35777022	0.06336953	-0.8855472	0.4373811	0.02579237
157.8	0.35058826	-0.364416	0.35827362	0.06320423	-0.8857291	0.43765288	0.02662091
157.9	0.35068311	-0.3642357	0.35887528	0.06383295	-0.8858704	0.43816156	0.02675411
158	0.35033145	-0.3641339	0.35973409	0.06415454	-0.8859988	0.43945552	0.02724016
158.1	0.35033145	-0.3641339	0.35973409	0.06415454	-0.8859988	0.43945552	0.02724016
158.2	0.35058075	-0.3647025	0.36009739	0.0645686	-0.8861097	0.43987729	0.02801343
158.3	0.35012237	-0.3650343	0.35974114	0.06442081	-0.8861572	0.43962633	0.02834557
158.4	0.35036654	-0.3646028	0.35943202	0.06447722	-0.8860102	0.43961288	0.02874143
158.5	0.35029778	-0.36467	0.35954932	0.06473964	-0.8860357	0.43973684	0.02863619
158.6	0.35029778	-0.36467	0.35954932	0.06473964	-0.8860357	0.43973684	0.02863619
158.7	0.34968813	-0.3648911	0.35953759	0.06436657	-0.8860232	0.43969308	0.02838196
158.8	0.34913899	-0.3649317	0.35934627	0.06467893	-0.8858226	0.43925938	0.0278505
158.9	0.3484886	-0.3649118	0.35951264	0.06418308	-0.8856624	0.43968338	0.02767835
159	0.34844622	-0.3649368	0.35988075	0.0640967	-0.8857466	0.43985635	0.02767749
159.1	0.34844622	-0.3649368	0.35988075	0.0640967	-0.8857466	0.43985635	0.02767749
159.2	0.34844622	-0.3649368	0.35988075	0.0640967	-0.8857466	0.43985635	0.02767749
159.3	0.34778885	-0.3658189	0.35960155	0.06429083	-0.8857238	0.43981909	0.02843447
159.4	0.34762778	-0.3657945	0.35960946	0.06459197	-0.8858421	0.44008008	0.02887914
159.5	0.34644434	-0.3654555	0.3595688	0.06432678	-0.8857847	0.44014685	0.02931007
159.6	0.34599116	-0.365413	0.35913764	0.06352339	-0.88562	0.4400616	0.02903536
159.7	0.34586671	-0.3651311	0.35871587	0.0632641	-0.8855821	0.44003135	0.02887453
159.8	0.34623161	-0.3644223	0.35833782	0.06284499	-0.8855618	0.44025799	0.02863225
159.9	0.34504468	-0.3644438	0.35926623	0.06236719	-0.8854838	0.44014666	0.02842845
160	0.34419208	-0.3645456	0.3588557	0.06156372	-0.8853092	0.44024375	0.02862192
160.1	0.34362829	-0.3644494	0.35799053	0.06185347	-0.8852408	0.44052483	0.02938396
160.2	0.34336309	-0.3641017	0.35776818	0.06215666	-0.8852371	0.44132229	0.02895149
160.3	0.34336927	-0.3640418	0.3577821	0.06236379	-0.885198	0.441367	0.02815697
160.4	0.34336927	-0.3640418	0.3577821	0.06236379	-0.885198	0.441367	0.02815697
160.5	0.34336192	-0.3636987	0.35807389	0.06189415	-0.8852993	0.44155123	0.02818295
160.6	0.34332293	-0.3627938	0.35741029	0.06236093	-0.885241	0.44123604	0.02846593
160.7	0.34345208	-0.3624952	0.35756442	0.0627266	-0.8852271	0.44171814	0.02790712
160.8	0.34336826	-0.3625819	0.35767714	0.06239505	-0.8851985	0.44176938	0.02801827
160.9	0.34336826	-0.3625819	0.35767714	0.06239505	-0.8851985	0.44176938	0.02801827
161	0.34311373	-0.3626742	0.35785876	0.06230672	-0.885163	0.44166838	0.02742935
161.1	0.34311373	-0.3626742	0.35785876	0.06230672	-0.885163	0.44166838	0.02742935
161.2	0.34357821	-0.3619731	0.3576973	0.06176813	-0.8850505	0.44154877	0.02750624
161.3	0.34357821	-0.3619731	0.3576973	0.06176813	-0.8850505	0.44154877	0.02750624
161.4	0.34377888	-0.3610501	0.35738667	0.06349898	-0.8852418	0.44263301	0.0273359
161.5	0.34326487	-0.360964	0.35878164	0.06308439	-0.8852034	0.44252769	0.02721417
161.6	0.34321609	-0.3609784	0.35841959	0.06332692	-0.8851347	0.44269347	0.02764968
161.7	0.34321609	-0.3609784	0.35841959	0.06332692	-0.8851347	0.44269347	0.02764968
161.8	0.34329292	-0.3613764	0.35826359	0.06350878	-0.8850648	0.44318423	0.02721102
161.9	0.34329292	-0.3613764	0.35826359	0.06350878	-0.8850648	0.44318423	0.02721102
162	0.34301413	-0.3610103	0.3591175	0.06329002	-0.8850927	0.4437403	0.02702868
162.1	0.34305955	-0.3605355	0.35914385	0.06344429	-0.8851441	0.44385171	0.0275179
162.2	0.34293665	-0.360247	0.35884226	0.06326302	-0.884942	0.4437497	0.02788023
162.3	0.34296684	-0.3605176	0.3590261	0.06359466	-0.8851676	0.4444645	0.02725966
162.4	0.3428363	-0.3603937	0.35908534	0.06339916	-0.8851045	0.44452473	0.02720096
162.5	0.3428363	-0.3603937	0.35908534	0.06339916	-0.8851045	0.44452473	0.02720096
162.6	0.3428363	-0.3603937	0.35908534	0.06339916	-0.8851045	0.44452473	0.02720096
162.7	0.34291179	-0.3603278	0.35947777	0.0631634	-0.8851313	0.44472658	0.02728227
162.8	0.34291179	-0.3603278	0.35947777	0.0631634	-0.8851313	0.44472658	0.02728227
162.9	0.34291179	-0.3603278	0.35947777	0.0631634	-0.8851313	0.44472658	0.02728227
163	0.34225718	-0.3601951	0.3596222	0.06327978	-0.8850868	0.44491605	0.02720855
163.1	0.34190009	-0.360057	0.35969625	0.06268394	-0.8849717	0.44505161	0.02682593
163.2	0.34190009	-0.360057	0.35969625	0.06268394	-0.8849717	0.44505161	0.02682593
163.3	0.34190009	-0.360057	0.35969625	0.06268394	-0.8849717	0.44505161	0.02682593
163.4	0.34190009	-0.360057	0.35969625	0.06268394	-0.8849717	0.44505161	0.02682593
163.5	0.34191531	-0.36032	0.36045501	0.06267379	-0.88509	0.44543272	0.02715485
163.6	0.34191531	-0.36032	0.36045501	0.06267379	-0.88509	0.44543272	0.02715485
163.7	0.34212423	-0.3606023	0.36042219	0.061934	-0.8851449	0.44511034	0.02696037
163.8	0.34207186	-0.3605675	0.36032324	0.06227532	-0.8850849	0.4453526	0.02664751
163.9	0.34156322	-0.3607379	0.36022926	0.06255203	-0.8851978	0.4457135	0.02672965
164	0.34119121	-0.3607636	0.36020785	0.06269915	-0.8851901	0.44576948	0.02719148
164.1	0.34119121	-0.3607636	0.36020785	0.06269915	-0.8851901	0.44576948	0.02719148
164.2	0.34144194	-0.3613787	0.36052277	0.06280571	-0.8852845	0.44588754	0.0276498
164.3	0.34099221	-0.3614217	0.35986252	0.06322438	-0.885139	0.44605404	0.0275419
164.4	0.341236	-0.3611874	0.35964052	0.06386927	-0.8852356	0.44597698	0.02771727
164.5	0.341236	-0.3611874	0.35964052	0.06386927	-0.8852356	0.44597698	0.02771727
164.6	0.341236	-0.3611874	0.35964052	0.06386927	-0.8852356	0.44597698	0.02771727
164.7	0.34153271	-0.361667	0.36052798	0.06409507	-0.8854065	0.44673736	0.02760652
164.8	0.34153271	-0.361667	0.36052798	0.06409507	-0.8854065	0.44673736	0.02760652
164.9	0.3416781	-0.3620116	0.36102788	0.06450503	-0.8854781	0.44704971	0.02735345

210	0.26998067	-0.4024992	0.38647697	0.05526881	-0.887599	0.48606123	-0.0180328
210.1	0.26998067	-0.4024992	0.38647697	0.05526881	-0.887599	0.48606123	-0.0180328
210.2	0.26998067	-0.4024992	0.38647697	0.05526881	-0.887599	0.48606123	-0.0180328
210.3	0.26998067	-0.4024992	0.38647697	0.05526881	-0.887599	0.48606123	-0.0180328
210.4	0.26948034	-0.4026216	0.38665601	0.05528156	-0.8876253	0.48601238	-0.0177146
210.5	0.26948034	-0.4026216	0.38665601	0.05528156	-0.8876253	0.48601238	-0.0177146
210.6	0.26948034	-0.4026216	0.38665601	0.05528156	-0.8876253	0.48601238	-0.0177146
210.7	0.26948034	-0.4026216	0.38665601	0.05528156	-0.8876253	0.48601238	-0.0177146
210.8	0.26948034	-0.4026216	0.38665601	0.05528156	-0.8876253	0.48601238	-0.0177146
210.9	0.26933549	-0.4031881	0.38721474	0.05515544	-0.8877593	0.48607815	-0.0179812
211	0.26933549	-0.4031881	0.38721474	0.05515544	-0.8877593	0.48607815	-0.0179812
211.1	0.26933549	-0.4031881	0.38721474	0.05515544	-0.8877593	0.48607815	-0.0179812
211.2	0.26933549	-0.4031881	0.38721474	0.05515544	-0.8877593	0.48607815	-0.0179812
211.3	0.26920228	-0.4032031	0.38747798	0.05465677	-0.8877746	0.48605611	-0.018333
211.4	0.26912017	-0.4037052	0.38809076	0.05550713	-0.8878662	0.48615206	-0.0182816
211.5	0.26912017	-0.4037052	0.38809076	0.05550713	-0.8878662	0.48615206	-0.0182816
211.6	0.26900707	-0.403533	0.38865468	0.0556857	-0.8879543	0.48651725	-0.0184175
211.7	0.26872492	-0.4033391	0.38878908	0.05525356	-0.8878629	0.48662001	-0.0184244
211.8	0.26872492	-0.4033391	0.38878908	0.05525356	-0.8878629	0.48662001	-0.0184244
211.9	0.26872492	-0.4033391	0.38878908	0.05525356	-0.8878629	0.48662001	-0.0184244
212	0.26872492	-0.4033391	0.38878908	0.05525356	-0.8878629	0.48662001	-0.0184244
212.1	0.26872492	-0.4033391	0.38878908	0.05525356	-0.8878629	0.48662001	-0.0184244
212.2	0.26872492	-0.4033391	0.38878908	0.05525356	-0.8878629	0.48662001	-0.0184244
212.3	0.26872492	-0.4033391	0.38878908	0.05525356	-0.8878629	0.48662001	-0.0184244
212.4	0.26872492	-0.4033391	0.38878908	0.05525356	-0.8878629	0.48662001	-0.0184244
212.5	0.26882566	-0.4034966	0.38888929	0.05534981	-0.8879872	0.48699882	-0.0187558
212.6	0.26882566	-0.4034966	0.38888929	0.05534981	-0.8879872	0.48699882	-0.0187558
212.7	0.26859944	-0.4034017	0.3884749	0.05525633	-0.8878924	0.48668316	-0.0184041
212.8	0.26815919	-0.4043117	0.38947223	0.05532278	-0.8879721	0.48695748	-0.0190483
212.9	0.26815919	-0.4043117	0.38947223	0.05532278	-0.8879721	0.48695748	-0.0190483
213	0.26815919	-0.4043117	0.38947223	0.05532278	-0.8879721	0.48695748	-0.0190483
213.1	0.26815919	-0.4043117	0.38947223	0.05532278	-0.8879721	0.48695748	-0.0190483

171.1	0.33118992	-0.364314	0.36779627	0.06866108	-0.8861362	0.45599353	0.02493083
171.2	0.33039937	-0.3643562	0.36841048	0.06871857	-0.8860352	0.45603569	0.02423177
171.3	0.33031294	-0.3642276	0.36828849	0.06903592	-0.8861586	0.45637975	0.02414911
171.4	0.33036315	-0.3643343	0.36816464	0.06856532	-0.8859648	0.4559778	0.02367686
171.5	0.33008445	-0.3647068	0.36805015	0.06842981	-0.8859601	0.45603876	0.0235956
171.6	0.33007982	-0.3644751	0.3683485	0.06840111	-0.8860519	0.45621852	0.02379496
171.7	0.32969344	-0.363661	0.36852687	0.0685896	-0.8858186	0.45684683	0.02424607
171.8	0.32942003	-0.3641172	0.36819461	0.06862338	-0.8858076	0.4564515	0.02482031
171.9	0.32967241	-0.3636232	0.36796061	0.06864539	-0.8858868	0.45658293	0.02520059
172	0.32967241	-0.3636232	0.36796061	0.06864539	-0.8858868	0.45658293	0.02520059
172.1	0.32925108	-0.3634807	0.36781018	0.06870403	-0.885813	0.45692881	0.02463478
172.2	0.32888466	-0.3635233	0.36840862	0.06907702	-0.8859088	0.45709491	0.02499929
172.3	0.32889103	-0.3635195	0.36850339	0.06897953	-0.8859111	0.45729644	0.02531043
172.4	0.32889103	-0.3635195	0.36850339	0.06897953	-0.8859111	0.45729644	0.02531043
172.5	0.32840219	-0.3634629	0.36876283	0.06891983	-0.8858839	0.45731821	0.02510336
172.6	0.32814337	-0.3632486	0.36878934	0.06848407	-0.8858744	0.45750423	0.02514179
172.7	0.32814337	-0.3632486	0.36878934	0.06848407	-0.8858744	0.45750423	0.02514179
172.8	0.32803309	-0.3631375	0.36908155	0.06836877	-0.8858904	0.4573939	0.02560631
172.9	0.32790087	-0.3633159	0.36923835	0.06820887	-0.8857904	0.45735789	0.02484179
173	0.32783997	-0.3626124	0.36946129	0.06772466	-0.8858691	0.45750637	0.02488881
173.1	0.32763455	-0.3620147	0.36940761	0.06666493	-0.8859217	0.45788356	0.02461317
173.2	0.32692079	-0.3621656	0.37042355	0.06745451	-0.8858801	0.45814339	0.02470668
173.3	0.32678016	-0.3618305	0.37051355	0.06728728	-0.8859265	0.45821775	0.02444509
173.4	0.32678016	-0.3618305	0.37051355	0.06728728	-0.8859265	0.45821775	0.02444509
173.5	0.32649867	-0.3617811	0.37021049	0.06740897	-0.8858123	0.45794231	0.0248236
173.6	0.3269395	-0.3620218	0.37029491	0.06741175	-0.8858624	0.45812223	0.0246143
173.7	0.32675995	-0.3615627	0.36988478	0.06712522	-0.8858122	0.45860975	0.02527629
173.8	0.32620432	-0.36212	0.37058457	0.0671715	-0.8859747	0.45919659	0.02493114
173.9	0.32706768	-0.3617166	0.37010483	0.06741718	-0.8860351	0.45937317	0.02507129
174	0.32706768	-0.3617166	0.37010483	0.06741718	-0.8860351	0.45937317	0.02507129
174.1	0.32646724	-0.3618272	0.37061746	0.06811204	-0.88606	0.45943011	0.02456093
174.2	0.32646724	-0.3618272	0.37061746	0.06811204	-0.88606	0.45943011	0.02456093
174.3	0.32640287	-0.3618755	0.37070161	0.06795524	-0.8860886	0.4597242	0.02483797
174.4	0.32640287	-0.3618755	0.37070161	0.06795524	-0.8860886	0.4597242	0.02483797
174.5	0.32553554	-0.3618173	0.37116102	0.06783916	-0.8860485	0.45995144	0.02460037
174.6	0.32543015	-0.3616341	0.37153383	0.06746249	-0.8860157	0.46020611	0.02496895
174.7	0.32538145	-0.3618113	0.37170539	0.06759075	-0.8859419	0.46027656	0.02482926
174.8	0.32538145	-0.3618113	0.37170539	0.06759075	-0.8859419	0.46027656	0.02482926
174.9	0.32497595	-0.3622261	0.37138158	0.06754016	-0.8859393	0.46048141	0.02501502
175	0.3246788	-0.3628273	0.37164225	0.06718322	-0.8860507	0.46167392	0.02416015
175.1	0.32406473	-0.3626027	0.37196741	0.06712728	-0.8859771	0.46195289	0.02361287
175.2	0.32429019	-0.3629349	0.3715668	0.06757293	-0.8859264	0.46235781	0.02325558
175.3	0.32415361	-0.3628862	0.37123173	0.0677866	-0.8859085	0.46266622	0.02344431
175.4	0.3238039	-0.3634908	0.3715717	0.06763084	-0.8860048	0.46282605	0.02331278
175.5	0.32317478	-0.363519	0.37159645	0.06712839	-0.8858522	0.46280813	0.02296547
175.6	0.32317478	-0.363519	0.37159645	0.06712839	-0.8858522	0.46280813	0.02296547
175.7	0.32309715	-0.3640849	0.3720332	0.06749424	-0.8859359	0.46305008	0.02329667
175.8	0.32309715	-0.3640849	0.3720332	0.06749424	-0.8859359	0.46305008	0.02329667
175.9	0.32309715	-0.3640849	0.3720332	0.06749424	-0.8859359	0.46305008	0.02329667
176	0.32205585	-0.3641757	0.37229646	0.06761039	-0.8859744	0.46328417	0.02229026
176.1	0.32205585	-0.3641757	0.37229646	0.06761039	-0.8859744	0.46328417	0.02229026
176.2	0.32205199	-0.3640102	0.37217913	0.06822952	-0.88591	0.46329991	0.0224805
176.3	0.32205056	-0.3638057	0.37213771	0.06788951	-0.8858386	0.46400344	0.02234162
176.4	0.32177673	-0.3635981	0.37231206	0.0680222	-0.885832	0.4638692	0.02280616
176.5	0.32206241	-0.3638194	0.3722167	0.06755135	-0.8859287	0.46391195	0.02281621
176.6	0.32206241	-0.3638194	0.3722167	0.06755135	-0.8859287	0.46391195	0.02281621
176.7	0.32259218	-0.3640465	0.37269225	0.06685091	-0.8860336	0.46413448	0.0227885
176.8	0.32208109	-0.3639862	0.37290215	0.06643597	-0.886053	0.46407108	0.02291376
176.9	0.3221338	-0.3643365	0.37372874	0.06644535	-0.886236	0.46454508	0.02272281
177	0.32216284	-0.364576	0.37394402	0.06661526	-0.886184	0.46464472	0.02248492
177.1	0.32111864	-0.3660149	0.37345975	0.0667096	-0.8861797	0.46481513	0.02252984
177.2	0.32111864	-0.3660149	0.37345975	0.0667096	-0.8861797	0.46481513	0.02252984
177.3	0.3214148	-0.3663727	0.37377225	0.0660441	-0.8861481	0.46452142	0.02240112
177.4	0.32088388	-0.3667387	0.3738363	0.06587509	-0.8862197	0.46471552	0.02171751
177.5	0.32088388	-0.3667387	0.3738363	0.06587509	-0.8862197	0.46471552	0.02171751
177.6	0.32088388	-0.3667387	0.3738363	0.06587509	-0.8862197	0.46471552	0.02171751
177.7	0.32100014	-0.3665673	0.37376476	0.06552129	-0.8861524	0.46491049	0.02110069
177.8	0.32159594	-0.3667054	0.37359764	0.06575312	-0.8861311	0.46474112	0.0204816
177.9	0.3211342	-0.3670182	0.37300719	0.06577543	-0.8860469	0.46507471	0.02022495
178	0.32139069	-0.3668523	0.37287361	0.06583583	-0.8860135	0.46512706	0.01991516
178.1	0.32141884	-0.3672286	0.37312422	0.06503483	-0.8859369	0.464851	0.01947139
178.2	0.32066738	-0.3686625	0.37402634	0.06600372	-0.8862222	0.46524356	0.01883899
178.3	0.3213606	-0.3683091	0.3743584	0.06585977	-0.8862963	0.46562874	0.01893788
178.4	0.32153098	-0.3688797	0.37431981	0.0653401	-0.8861255	0.46515743	0.01826979
178.5	0.3218061	-0.3694735	0.37442357	0.06467648	-0.8861227	0.46525387	0.01776651
178.6	0.32124831	-0.3703902	0.37454247	0.06403475	-0.8862648	0.46555664	0.01734089
178.7	0.32121045	-0.3706231	0.37486363	0.06405497	-0.8863225	0.46564922	0.01703633
178.8	0.32121045	-0.3706231	0.37486363	0.06405497	-0.8863225	0.46564922	0.01703633
178.9	0.32158038	-0.3703114	0.3746116	0.06433181	-0.8864394	0.46570524	0.01727697
179	0.32130873	-0.3712817	0.37452209	0.06388851	-0.8864244	0.46620422	0.01709489
179.1	0.32163133	-0.3714907	0.37431406	0.06416324	-0.8864232	0.46638197	0.01710633
179.2	0.3210016	-0.3718612	0.37456254	0.0640728	-0.8864383	0.46687253	0.01647215
179.3	0.3210016	-0.3718612	0.37456254	0.0640728	-0.8864383	0.46687253	0.01647215
179.4	0.32044977	-0.3719653	0.3744401	0.06441261	-0.8863368	0.4672778	0.01638893
179.5	0.32044977	-0.3719653	0.3744401	0.06441261	-0.8863368	0.4672778	0.01638893
179.6	0.32035876	-0.3721752	0.3743835	0.06389713	-0.8862632	0.46730887	0.0161443
179.7	0.32000909	-0.3725659	0.37422039	0.06431767	-0.8863802	0.46693534	0.01574862
179.8	0.32004238	-0.3725324	0.37434597	0.063942	-0.8863979	0.46706884	0.01548088
179.9	0.31959176	-0.3746343	0.37515557	0.06307366	-0.8866142	0.46723921	0.01532237

225	0.26150459	-0.403226	0.39506672	0.05805105	-0.8886035	0.48886549	-0.0258202
225.1	0.26150459	-0.403226	0.39506672	0.05805105	-0.8886035	0.48886549	-0.0258202
225.2	0.26150459	-0.403226	0.39506672	0.05805105	-0.8886035	0.48886549	-0.0258202
225.3	0.26150459	-0.403226	0.39506672	0.05805105	-0.8886035	0.48886549	-0.0258202
225.4	0.26150459	-0.403226	0.39506672	0.05805105	-0.8886035	0.48886549	-0.0258202
225.5	0.26150459	-0.403226	0.39506672	0.05805105	-0.8886035	0.48886549	-0.0258202
225.6	0.26150459	-0.403226	0.39506672	0.05805105	-0.8886035	0.48886549	-0.0258202
225.7	0.26150459	-0.403226	0.39506672	0.05805105	-0.8886035	0.48886549	-0.0258202
225.8	0.2611603	-0.4035046	0.39499236	0.05805218	-0.888457	0.48873535	-0.0260083
225.9	0.2611603	-0.4035046	0.39499236	0.05805218	-0.888457	0.48873535	-0.0260083
226	0.2611603	-0.4035046	0.39499236	0.05805218	-0.888457	0.48873535	-0.0260083
226.1	0.2611603	-0.4035046	0.39499236	0.05805218	-0.888457	0.48873535	-0.0260083
226.2	0.26050775	-0.4034612	0.39505392	0.05830469	-0.888381	0.48865411	-0.0265044
226.3	0.26050775	-0.4034612	0.39505392	0.05830469	-0.888381	0.48865411	-0.0265044
226.4	0.26070885	-0.4033527	0.39577484	0.05848723	-0.8884851	0.48883759	-0.0265866
226.5	0.26070885	-0.4033527	0.39577484	0.05848723	-0.8884851	0.48883759	-0.0265866
226.6	0.26060507	-0.4033029	0.39599307	0.05896104	-0.8886116	0.48890817	-0.0267132
226.7	0.26060507	-0.4033029	0.39599307	0.05896104	-0.8886116	0.48890817	-0.0267132
226.8	0.25983975	-0.4033371	0.39591094	0.05896367	-0.8885542	0.48882092	-0.0262361
226.9	0.25983975	-0.4033371	0.39591094	0.05896367	-0.8885542	0.48882092	-0.0262361
227	0.25983975	-0.4033371	0.39591094	0.05896367	-0.8885542	0.48882092	-0.0262361
227.1	0.25983975	-0.4033371	0.39591094	0.05896367	-0.8885542	0.48882092	-0.0262361
227.2	0.25983975	-0.4033371	0.39591094	0.05896367	-0.8885542	0.48882092	-0.0262361
227.3	0.25983975	-0.4033371	0.39591094	0.05896367	-0.8885542	0.48882092	-0.0262361
227.4	0.25983975	-0.4033371	0.39591094	0.05896367	-0.8885542	0.48882092	-0.0262361
227.5	0.25983975	-0.4033371	0.39591094	0.05896367	-0.8885542	0.48882092	-0.0262361
227.6	0.25983975	-0.4033371	0.39591094	0.05896367	-0.8885542	0.48882092	-0.0262361
227.7	0.25983975	-0.4033371	0.39591094	0.05896367	-0.8885542	0.48882092	-0.0262361
227.8	0.25983975	-0.4033371	0.39591094	0.05896367	-0.8885542	0.48882092	-0.0262361
227.9	0.26005146	-0.4030387	0.39643951	0.05934094	-0.8885915	0.48889812	-0.0263743
228	0.26005146	-0.4030387	0.39643951	0.05934094	-0.8885915	0.48889812	-0.0263743
228.1	0.26009469	-0.4027785	0.39634097	0.05914425	-0.8885357	0.48920804	-0.0267629

183.2	0.31143973	-0.3826306	0.38155793	0.06046402	-0.8873726	0.4748718	0.00657156
183.3	0.31171636	-0.3819126	0.38100367	0.06000395	-0.8873785	0.47506608	0.00666323
183.4	0.31141215	-0.3816849	0.38032612	0.06009064	-0.8871966	0.47490915	0.00677086
183.5	0.31141215	-0.3816849	0.38032612	0.06009064	-0.8871966	0.47490915	0.00677086
183.6	0.31098932	-0.38238	0.38067235	0.06025079	-0.8871811	0.47510806	0.00565819
183.7	0.31100708	-0.3822061	0.38046939	0.06044844	-0.8871078	0.47512451	0.00620273
183.8	0.31106345	-0.3822187	0.38082317	0.06017379	-0.88715	0.47553159	0.0054917
183.9	0.31053579	-0.3818076	0.38074601	0.05958643	-0.887073	0.47566892	0.00547967
184	0.31053579	-0.3818076	0.38074601	0.05958643	-0.887073	0.47566892	0.00547967
184.1	0.31058144	-0.3818428	0.38039447	0.05908425	-0.8870606	0.4755054	0.00552886
184.2	0.31057285	-0.3818562	0.38066871	0.05953143	-0.887105	0.47570032	0.00568061
184.3	0.31047927	-0.3825016	0.38139236	0.05945114	-0.887261	0.4762037	0.00578025
184.4	0.31047927	-0.3825016	0.38139236	0.05945114	-0.887261	0.4762037	0.00578025
184.5	0.31056971	-0.3826913	0.38125254	0.05951982	-0.8871816	0.47614503	0.00607993
184.6	0.31105252	-0.3839439	0.38184638	0.05889357	-0.8874322	0.47754871	0.00488326
184.7	0.31105252	-0.3839439	0.38184638	0.05889357	-0.8874322	0.47754871	0.00488326
184.8	0.31107512	-0.3848108	0.38189279	0.05936978	-0.8874602	0.47728526	0.00472109
184.9	0.31055841	-0.3851572	0.38189514	0.05962341	-0.8875467	0.47730643	0.00482538
185	0.31060243	-0.3859528	0.3822899	0.05954972	-0.8876787	0.4777186	0.00461492
185.1	0.31047361	-0.3856546	0.38199594	0.0597418	-0.8878055	0.47794748	0.00472862
185.2	0.31047361	-0.3856546	0.38199594	0.0597418	-0.8878055	0.47794748	0.00472862
185.3	0.31049307	-0.3854655	0.38206902	0.0601747	-0.8878669	0.47820965	0.00458408
185.4	0.31025778	-0.3852678	0.38184064	0.06078732	-0.8878163	0.47825516	0.00442138
185.5	0.30939469	-0.3864085	0.38230949	0.06016092	-0.8878478	0.47819515	0.00440588
185.6	0.30942435	-0.3868991	0.38210957	0.05946841	-0.887928	0.47924913	0.00291217
185.7	0.3093757	-0.3870498	0.38218112	0.05992754	-0.8880411	0.47952998	0.00216899
185.8	0.30818348	-0.3873099	0.38226331	0.06034965	-0.8880467	0.48012791	0.00050275
185.9	0.30744318	-0.3880116	0.38247796	0.05978753	-0.88809	0.4805871	-0.0006195
186	0.30744318	-0.3880116	0.38247796	0.05978753	-0.88809	0.4805871	-0.0006195
186.1	0.30744318	-0.3880116	0.38247796	0.05978753	-0.88809	0.4805871	-0.0006195
186.2	0.30713774	-0.3880225	0.38246467	0.05985548	-0.8880459	0.48049059	-0.0005406
186.3	0.30728577	-0.3878534	0.38279641	0.06011603	-0.8881011	0.48044729	-0.0004768
186.4	0.30728577	-0.3878534	0.38279641	0.06011603	-0.8881011	0.48044729	-0.0004768
186.5	0.30728577	-0.3878534	0.38279641	0.06011603	-0.8881011	0.48044729	-0.0004768
186.6	0.30672872	-0.3879875	0.38307021	0.05999834	-0.8881767	0.48068339	-0.0007493
186.7	0.30609211	-0.3881396	0.38292288	0.05996904	-0.8882201	0.48044088	-0.0005284
186.8	0.30554294	-0.3880084	0.38235039	0.05993349	-0.8881247	0.48020645	-0.0002778
186.9	0.30553253	-0.3883772	0.38253003	0.06010014	-0.888194	0.48046972	-0.0003022
187	0.30524459	-0.3883593	0.38303327	0.06071768	-0.8883088	0.48053204	2.29E-06
187.1	0.30503112	-0.3881615	0.38279053	0.06072336	-0.88818	0.48025754	-0.0002381
187.2	0.30503112	-0.3881615	0.38279053	0.06072336	-0.88818	0.48025754	-0.0002381
187.3	0.30503112	-0.3881615	0.38279053	0.06072336	-0.88818	0.48025754	-0.0002381
187.4	0.30503112	-0.3881615	0.38279053	0.06072336	-0.88818	0.48025754	-0.0002381
187.5	0.30493734	-0.3878159	0.38309003	0.06005156	-0.8882513	0.4802601	-0.0001041
187.6	0.30505748	-0.3877942	0.38267487	0.05982057	-0.8882811	0.48044978	0.00015191
187.7	0.30508887	-0.3879339	0.38258803	0.06006109	-0.8881808	0.48039337	-1.76E-05
187.8	0.30508887	-0.3879339	0.38258803	0.06006109	-0.8881808	0.48039337	-1.76E-05
187.9	0.30461873	-0.3883431	0.38355749	0.06101771	-0.8882935	0.48077646	0.00018865
188	0.30476957	-0.388446	0.38321997	0.06085946	-0.8882227	0.48066504	0.00026633
188.1	0.30461343	-0.3885841	0.38343033	0.06027699	-0.8882224	0.48088478	-0.0002428
188.2	0.30392686	-0.3888007	0.38287739	0.0605138	-0.8881002	0.48039191	-0.0002
188.3	0.30353238	-0.3891252	0.38196273	0.06053405	-0.8878946	0.48034321	-0.0016053
188.4	0.30405962	-0.3898234	0.38194509	0.060342	-0.8877963	0.48087873	-0.0021959
188.5	0.30405962	-0.3898234	0.38194509	0.060342	-0.8877963	0.48087873	-0.0021959
188.6	0.30405962	-0.3898234	0.38194509	0.060342	-0.8877963	0.48087873	-0.0021959
188.7	0.30433275	-0.3897823	0.38168353	0.06033119	-0.8877553	0.48121826	-0.0023278
188.8	0.30433275	-0.3897823	0.38168353	0.06033119	-0.8877553	0.48121826	-0.0023278
188.9	0.30321739	-0.3901839	0.38066038	0.06062656	-0.8875128	0.48093699	-0.0024474
189	0.30331695	-0.3904797	0.38020694	0.06015621	-0.887484	0.48067517	-0.0023078
189.1	0.30277006	-0.3909482	0.38036295	0.05969882	-0.8875991	0.48059193	-0.0027322
189.2	0.30277006	-0.3909482	0.38036295	0.05969882	-0.8875991	0.48059193	-0.0027322
189.3	0.30277006	-0.3909482	0.38036295	0.05969882	-0.8875991	0.48059193	-0.0027322
189.4	0.30262008	-0.3912397	0.38081052	0.05920637	-0.8876968	0.48049339	-0.0029906
189.5	0.30245618	-0.3906133	0.38083251	0.05904991	-0.8875323	0.48036266	-0.0032994
189.6	0.30213603	-0.3905012	0.38136451	0.05921872	-0.8875568	0.48069261	-0.0035823
189.7	0.30213603	-0.3905012	0.38136451	0.05921872	-0.8875568	0.48069261	-0.0035823
189.8	0.30107152	-0.3911693	0.3809857	0.05840727	-0.8874864	0.48103936	-0.0032765
189.9	0.30078885	-0.3914524	0.38070732	0.0579141	-0.8874326	0.48116391	-0.0035276
190	0.30078885	-0.3914524	0.38070732	0.0579141	-0.8874326	0.48116391	-0.0035276
190.1	0.30080246	-0.3917682	0.38082765	0.05795588	-0.8874067	0.48109619	-0.0042461
190.2	0.30091335	-0.3918381	0.38157583	0.05697834	-0.8874521	0.48166241	-0.0043714
190.3	0.30042869	-0.3920288	0.38176249	0.05723308	-0.8875076	0.4816162	-0.0045158
190.4	0.30042869	-0.3920288	0.38176249	0.05723308	-0.8875076	0.4816162	-0.0045158
190.5	0.30042869	-0.3920288	0.38176249	0.05723308	-0.8875076	0.4816162	-0.0045158
190.6	0.29968845	-0.3927273	0.38159443	0.05719207	-0.8874088	0.48159263	-0.0046417
190.7	0.29968845	-0.3927273	0.38159443	0.05719207	-0.8874088	0.48159263	-0.0046417
190.8	0.29886212	-0.3923514	0.38200331	0.05708447	-0.8874992	0.48183327	-0.0053181
190.9	0.29868052	-0.3926821	0.38217275	0.05674092	-0.8876085	0.48207882	-0.0058714
191	0.2983767	-0.3932716	0.38236776	0.05636471	-0.8876118	0.48219288	-0.0062246
191.1	0.2983767	-0.3932716	0.38236776	0.05636471	-0.8876118	0.48219288	-0.0062246
191.2	0.29850954	-0.3929725	0.38217132	0.05652017	-0.8875793	0.48228376	-0.0060623
191.3	0.29850954	-0.3929725	0.38217132	0.05652017	-0.8875793	0.48228376	-0.0060623
191.4	0.29779389	-0.393191	0.38224332	0.05625881	-0.8875747	0.48249003	-0.0063397
191.5	0.29785129	-0.393322	0.38185786	0.0561376	-0.8874715	0.48278216	-0.0062294
191.6	0.29780547	-0.393943	0.38172278	0.0563893	-0.8875324	0.48288656	-0.0063076
191.7	0.29784963	-0.3940995	0.3820371	0.05664555	-0.8876231	0.48313953	-0.0064272
191.8	0.29781128	-0.3938451	0.38217705	0.05643685	-0.8876124	0.48313095	-0.0067105
191.9	0.29758787	-0.3943489	0.38181955	0.05705621	-0.8875959	0.48328377	-0.0069566
192	0.29754705	-0.3940052	0.3819049	0.05691317	-0.8875497	0.4831821	-0.0071774
192.1	0.29739283	-0.395097	0.38230866	0.05724564	-0.8877184	0.48331368	-0.0070999
192.2	0.29739283	-0.395097	0.38230866	0.05724564	-0.8877184	0.48331368	-0.0070999
192.3	0.29739283	-0.395097	0.38230866	0.05724564	-0.8877184	0.48331368	-0.0070999
192.4	0.29739283	-0.395097	0.38230866	0.05724564	-0.8877184	0.48331368	-0.0070999
192.5	0.29665994	-0.3949774	0.38330266	0.05692529	-0.8877656	0.4835712	-0.0066664
192.6	0.29647055	-0.3955926	0.38324999	0.05617589	-0.8877342	0.48356557	-0.0069586
192.7	0.29647055	-0.3955926	0.38324999	0.05617589	-0.8877342	0.48356557	-0.0069586
192.8	0.29613745	-0.3957538	0.38297339	0.05647019	-0.8877296	0.48316384	-0.0068496
192.9	0.29577089	-0.395845	0.3833613	0.05690749	-0.8876582	0.48335337	-0.0073495
193	0.2958218	-0.3962426	0.383637	0.05704881	-0.8878229	0.48379344	-0.0080869
193.1	0.29561205	-0.3963928	0.38371894	0.05658486	-0.8878923	0.48405112	-0.0081109
193.2	0.29533219	-0.3966858	0.38417269	0.05624033	-0.8880383	0.48413101	-0.0082501
193.3	0.29533219	-0.3966858	0.38417269	0.05624033	-0.8880383	0.48413101	-0.0082501
193.4	0.29560324	-0.3964683	0.38395555	0.05571084	-0.8879326	0.48406826	-0.0087935
193.5	0.29364784	-0.3984697	0.3837389	0.05458846	-0.8880713	0.48403182	-0.0083518
193.6	0.29349566	-0.3987349	0.38395808	0.05445862	-0.8881126	0.48398551	-0.009071
193.7	0.29334037	-0.3991138	0.38445118	0.0543084	-0.8882216	0.48406701	-0.0094104
193.8	0.29334037	-0.3991138	0.38445118	0.0543084	-0.8882216	0.48406701	-0.0094104
193.9	0.29334037	-0.3991138	0.38445118	0.0543084	-0.8882216	0.48406701	-0.0094104
194	0.29334037	-0.3991138	0.38445118	0.0543084	-0.8882216	0.48406701	-0.0094104
194.1	0.29303954	-0.3990594	0.38460513	0.0539934	-0.8882253	0.48421057	-0.0096201
194.2	0.29300401	-0.3993148	0.38501006	0.05412804	-0.8882587	0.48418835	-0.009498
194.3	0.29300401	-0.3993148	0.38501006	0.05412804	-0.8882587	0.48418835	-0.009498
194.4	0.29247048	-0.3990856	0.38489356	0.05401335	-0.888244	0.48408264	-0.0095038
194.5	0.29227661	-0.39962	0.38535104	0.05502331	-0.8882198	0.48442982	-0.0090904
194.6	0.29227661	-0.39962	0.38535104	0.05502331	-0.8882198	0.48442982	-0.0090904
194.7	0.29175995	-0.3994814	0.38526518	0.05466321	-0.8882711	0.48425318	-0.0091178
194.8	0.29154565	-0.3995808	0.38586576	0.05511456	-0.8882803	0.48449942	-0.0092272
194.9	0.29154565	-0.3995808	0.38586576	0.05511456	-0.8882803	0.48449942	-0.0092272

198.2	0.28772667	-0.4007303	0.38414726	0.05476643	-0.8883616	0.48555843	-0.0090047
198.3	0.28772667	-0.4007303	0.38414726	0.05476643	-0.8883616	0.48555843	-0.0090047
198.4	0.28763438	-0.4009279	0.38418846	0.05500908	-0.8885021	0.48578	-0.0089466
198.5	0.28763438	-0.4009279	0.38418846	0.05500908	-0.8885021	0.48578	-0.0089466
198.6	0.28763438	-0.4009279	0.38418846	0.05500908	-0.8885021	0.48578	-0.0089466
198.7	0.28763438	-0.4009279	0.38418846	0.05500908	-0.8885021	0.48578	-0.0089466
198.8	0.28763438	-0.4009279	0.38418846	0.05500908	-0.8885021	0.48578	-0.0089466
198.9	0.28763438	-0.4009279	0.38418846	0.05500908	-0.8885021	0.48578	-0.0089466
199	0.28763438	-0.4009279	0.38418846	0.05500908	-0.8885021	0.48578	-0.0089466
199.1	0.28736386	-0.4010869	0.38373371	0.05484562	-0.8884225	0.48567956	-0.0087457
199.2	0.28698031	-0.4004101	0.38361799	0.05453125	-0.8883236	0.48554954	-0.009035
199.3	0.28698031	-0.4004101	0.38361799	0.05453125	-0.8883236	0.48554954	-0.009035
199.4	0.28709337	-0.4004903	0.38427428	0.05418645	-0.8883488	0.48574842	-0.0093517
199.5	0.2866003	-0.4008402	0.38431443	0.05440107	-0.8882678	0.48592002	-0.0094047
199.6	0.2866003	-0.4008402	0.38431443	0.05440107	-0.8882678	0.48592002	-0.0094047
199.7	0.28584793	-0.400939	0.38450244	0.05436186	-0.8882926	0.48595656	-0.0103264
199.8	0.28569449	-0.401126	0.38450202	0.05432056	-0.8882813	0.4858892	-0.0100983
199.9	0.28534497	-0.4010827	0.38513063	0.05439467	-0.8884995	0.48636113	-0.009932
200	0.28534497	-0.4010827	0.38513063	0.05439467	-0.8884995	0.48636113	-0.009932
200.1	0.28534497	-0.4010827	0.38513063	0.05439467	-0.8884995	0.48636113	-0.009932
200.2	0.28538291	-0.4012025	0.38479267	0.05495687	-0.8884855	0.4864758	-0.0092377
200.3	0.28538291	-0.4012025	0.38479267	0.05495687	-0.8884855	0.4864758	-0.0092377
200.4	0.28538291	-0.4012025	0.38479267	0.05495687	-0.8884855	0.4864758	-0.0092377
200.5	0.28532677	-0.4007892	0.38427108	0.05493433	-0.8883352	0.48644897	-0.009349
200.6	0.28482033	-0.4014943	0.3846652	0.05502864	-0.8883774	0.48632523	-0.0092413
200.7	0.2845426	-0.4014851	0.38464922	0.05511551	-0.8883439	0.48617843	-0.0094139
200.8	0.2845426	-0.4014851	0.38464922	0.05511551	-0.8883439	0.48617843	-0.0094139
200.9	0.28456311	-0.4014717	0.38492937	0.0550966	-0.8883804	0.48613912	-0.0102649
201	0.28418656	-0.4014541	0.38503751	0.05511107	-0.8883873	0.48640121	-0.0101955
201.1	0.28355325	-0.4013424	0.38582536	0.05496224	-0.8885541	0.48640496	-0.0100812
201.2	0.28352798	-0.4013672	0.38530371	0.05490186	-0.8883423	0.4861941	-0.010178
201.3	0.28325668	-0.4015034	0.38517649	0.05490884	-0.8883033	0.48615921	-0.0105433
201.4	0.28235302	-0.4010448	0.38575906	0.05507228	-0.8883182	0.48643495	-0.0103351
201.5	0.28222419	-0.401041	0.38595223	0.0551709	-0.8883628	0.48634087	-0.0101156
201.6	0.28201969	-0.4012697	0.38638478	0.05493229	-0.888437	0.48646314	-0.0103008
201.7	0.28193467	-0.4007636	0.3860944	0.05460776	-0.888319	0.48634122	-0.010392
201.8	0.28193467	-0.4007636	0.3860944	0.05460776	-0.888319	0.48634122	-0.010392
201.9	0.28151547	-0.4007202	0.38595877	0.05464595	-0.8883223	0.48602403	-0.0100947
202	0.28151547	-0.4007202	0.38595877	0.05464595	-0.8883223	0.48602403	-0.0100947
202.1	0.28151547	-0.4007202	0.38595877	0.05464595	-0.8883223	0.48602403	-0.0100947
202.2	0.28166496	-0.4009917	0.38576179	0.05433302	-0.8881778	0.48567363	-0.0104458
202.3	0.28169252	-0.4016624	0.38640003	0.05497364	-0.8884625	0.48613809	-0.0104346
202.4	0.28136827	-0.4020585	0.38703011	0.05501068	-0.8886197	0.48642708	-0.0102543
202.5	0.28091532	-0.4023549	0.38717776	0.0554035	-0.8886913	0.48647529	-0.0109416
202.6	0.28091532	-0.4023549	0.38717776	0.0554035	-0.8886913	0.48647529	-0.0109416
202.7	0.28091532	-0.4023549	0.38717776	0.0554035	-0.8886913	0.48647529	-0.0109416
202.8	0.28028658	-0.4028476	0.3875763	0.05581877	-0.8887035	0.48662662	-0.0106491
202.9	0.27991219	-0.4031486	0.38781809	0.0557	-0.8887405	0.48665673	-0.0108968
203	0.27989236	-0.4033116	0.38792736	0.05627051	-0.8887424	0.48694813	-0.0110224
203.1	0.27915756	-0.403099	0.38775229	0.05592234	-0.8884843	0.48672625	-0.0118545
203.2	0.27915756	-0.403099	0.38775229	0.05592234	-0.8884843	0.48672625	-0.0118545
203.3	0.27915756	-0.403099	0.38775229	0.05592234	-0.8884843	0.48672625	-0.0118545
203.4	0.2789902	-0.4032515	0.38788574	0.05601752	-0.8886043	0.48686656	-0.0121207
203.5	0.2789902	-0.4032515	0.38788574	0.05601752	-0.8886043	0.48686656	-0.0121207
203.6	0.2789902	-0.4032515	0.38788574	0.05601752	-0.8886043	0.48686656	-0.0121207
203.7	0.27907874	-0.4031454	0.38747537	0.05566314	-0.8886381	0.48652313	-0.0117906
203.8	0.27827005	-0.4022812	0.38793771	0.05579251	-0.8886616	0.48653088	-0.0129356
203.9	0.27827005	-0.4022812	0.38793771	0.05579251	-0.8886616	0.48653088	-0.0129356
204	0.27760909	-0.4022543	0.38753723	0.05542021	-0.8885717	0.4862242	-0.0127875
204.1	0.27760909	-0.4022543	0.38753723	0.05542021	-0.8885717	0.4862242	-0.0127875
204.2	0.27743442	-0.4025658	0.38748476	0.05485852	-0.8884675	0.48637273	-0.0134029
204.3	0.27743442	-0.4025658	0.38748476	0.05485852	-0.8884675	0.48637273	-0.0134029
204.4	0.27743442	-0.4025658	0.38748476	0.05485852	-0.8884675	0.48637273	-0.0134029
204.5	0.27751529	-0.4027425	0.38705804	0.05489355	-0.888377	0.48653605	-0.0138259
204.6	0.27751529	-0.4027425	0.38705804	0.05489355	-0.888377	0.48653605	-0.0138259
204.7	0.27751529	-0.4027425	0.38705804	0.05489355	-0.888377	0.48653605	-0.0138259
204.8	0.27751529	-0.4027425	0.38705804	0.05489355	-0.888377	0.48653605	-0.0138259
204.9	0.27736122	-0.4024698	0.38780642	0.05534138	-0.8885331	0.48699804	-0.0140777
205	0.27736122	-0.4024698	0.38780642	0.05534138	-0.8885331	0.48699804	-0.0140777
205.1	0.27678962	-0.4025971	0.38758106	0.05548044	-0.8886002	0.4869402	-0.0145284
205.2	0.27602683	-0.40319	0.38732543	0.05623452	-0.8885512	0.48709262	-0.0147129
205.3	0.27602683	-0.40319	0.38732543	0.05623452	-0.8885512	0.48709262	-0.0147129
205.4	0.27602683	-0.40319	0.38732543	0.05623452	-0.8885512	0.48709262	-0.0147129
205.5	0.27602683	-0.40319	0.38732543	0.05623452	-0.8885512	0.48709262	-0.0147129
205.6	0.27602683	-0.40319	0.38732543	0.05623452	-0.8885512	0.48709262	-0.0147129
205.7	0.27511304	-0.4029131	0.38669872	0.05634446	-0.8884894	0.48654907	-0.0149772
205.8	0.27436423	-0.4029938	0.38671076	0.05632648	-0.8883666	0.48658793	-0.0151583
205.9	0.27436423	-0.4029938	0.38671076	0.05632648	-0.8883666	0.48658793	-0.0151583
206	0.27436423	-0.4029938	0.38671076	0.05632648	-0.8883666	0.48658793	-0.0151583
206.1	0.27436423	-0.4029938	0.38671076	0.05632648	-0.8883666	0.48658793	-0.0151583
206.2	0.27436423	-0.4029938	0.38671076	0.05632648	-0.8883666	0.48658793	-0.0151583
206.3	0.27436423	-0.4029938	0.38671076	0.05632648	-0.8883666	0.48658793	-0.0151583
206.4	0.2738816	-0.402942	0.38668279	0.05533645	-0.8883833	0.48637222	-0.0156215
206.5	0.2738816	-0.402942	0.38668279	0.05533645	-0.8883833	0.48637222	-0.0156215
206.6	0.2735662	-0.4027687	0.38690665	0.05513026	-0.8883726	0.48622535	-0.0155299
206.7	0.2735662	-0.4027687	0.38690665	0.05513026	-0.8883726	0.48622535	-0.0155299
206.8	0.2735662	-0.4027687	0.38690665	0.05513026	-0.8883726	0.48622535	-0.0155299
206.9	0.2735662	-0.4027687	0.38690665	0.05513026	-0.8883726	0.48622535	-0.0155299
207	0.2735662	-0.4027687	0.38690665	0.05513026	-0.8883726	0.48622535	-0.0155299
207.1	0.27295406	-0.4030585	0.3872004	0.05517403	-0.8882687	0.48627027	-0.0160059
207.2	0.27295477	-0.4030177	0.38763791	0.05518841	-0.8882496	0.48617957	-0.0165492
207.3	0.27295477	-0.4030177	0.38763791	0.05518841	-0.8882496	0.48617957	-0.0165492
207.4	0.27256083	-0.4026199	0.38707181	0.05478698	-0.8881186	0.48593687	-0.0165901
207.5	0.27278657	-0.4026093	0.38697347	0.05460743	-0.8881592	0.48614872	-0.0172831
207.6	0.27278657	-0.4026093	0.38697347	0.05460743	-0.8881592	0.48614872	-0.0172831
207.7	0.2724714	-0.4025275	0.38725368	0.05433528	-0.8881849	0.48609695	-0.0172556
207.8	0.2724714	-0.4025275	0.38725368	0.05433528	-0.8881849	0.48609695	-0.0172556
207.9	0.2724714	-0.4025275	0.38725368	0.05433528	-0.8881849	0.48609695	-0.0172556
208	0.27220924	-0.4023524	0.38705156	0.05424755	-0.8880944	0.48609123	-0.0171157
208.1	0.27220924	-0.4023524	0.38705156	0.05424755	-0.8880944	0.48609123	-0.0171157
208.2	0.27169021	-0.4027007	0.38740151	0.05440217	-0.8880393	0.48622202	-0.0171919
208.3	0.27171435	-0.402855	0.38761575	0.05475897	-0.8881242	0.48627151	-0.0176331
208.4	0.27098688	-0.4032473	0.38709717	0.05473177	-0.8879969	0.48605783	-0.017882
208.5	0.27098688	-0.4032473	0.38709717	0.05473177	-0.8879969	0.48605783	-0.017882
208.6	0.27098688	-0.4032473	0.38709717	0.05473177	-0.8879969	0.48605783	-0.017882
208.7	0.27082166	-0.4031894	0.3869322	0.05449717	-0.8878195	0.48580684	-0.0182989
208.8	0.27082166	-0.4031894	0.3869322	0.05449717	-0.8878195	0.48580684	-0.0182989
208.9	0.27032569	-0.4033724	0.38743536	0.05487949	-0.8879371	0.48588958	-0.0182869
209	0.27032569	-0.4033724	0.38743536	0.05487949	-0.8879371	0.48588958	-0.0182869
209.1	0.27032569	-0.4033724	0.38743536	0.05487949	-0.8879371	0.48588958	-0.0182869
209.2	0.27032569	-0.4033724	0.38743536	0.05487949	-0.8879371	0.48588958	-0.0182869
209.3	0.27032569	-0.4033724	0.38743536	0.05487949	-0.8879371	0.48588958	-0.0182869
209.4	0.27032569	-0.4033724	0.38743536	0.05487949	-0.8879371	0.48588958	-0.0182869
209.5	0.27032569	-0.4033724	0.38743536	0.05487949	-0.8879371	0.48588958	-0.0182869
209.6	0.27032569	-0.4033724	0.38743536	0.05487949	-0.8879371	0.48588958	-0.0182869
209.7	0.27084168	-0.4031412	0.38712232	0.05492441	-0.8878809	0.48612156	-0.0180186
209.8	0.2702358	-0.4029489	0.38717316	0.05507691	-0.8878085	0.48634726	-0.0175371
209.9	0.2702358	-0.4029489	0.38717316	0.05507691	-0.8878085	0.48634726	-0.0175371

213.2	0.26815919	-0.4043117	0.38947223	0.05532278	-0.8879721	0.48695748	-0.0190483
213.3	0.26786234	-0.4043828	0.38961565	0.05556582	-0.8880353	0.48708023	-0.0189244
213.4	0.26755694	-0.4039215	0.38995153	0.05561654	-0.8880718	0.48707255	-0.0186338
213.5	0.26755694	-0.4039215	0.38995153	0.05561654	-0.8880718	0.48707255	-0.0186338
213.6	0.26720588	-0.4040266	0.39006292	0.05576711	-0.8880071	0.48690762	-0.019376
213.7	0.26720588	-0.4040266	0.39006292	0.05576711	-0.8880071	0.48690762	-0.019376
213.8	0.26720588	-0.4040266	0.39006292	0.05576711	-0.8880071	0.48690762	-0.019376
213.9	0.26720588	-0.4040266	0.39006292	0.05576711	-0.8880071	0.48690762	-0.019376
214	0.26686385	-0.404292	0.3903559	0.05589624	-0.8881387	0.4869349	-0.0199699
214.1	0.26687458	-0.4041842	0.39082651	0.05582894	-0.8881232	0.48703272	-0.0198591
214.2	0.26687458	-0.4041842	0.39082651	0.05582894	-0.8881232	0.48703272	-0.0198591
214.3	0.26687458	-0.4041842	0.39082651	0.05582894	-0.8881232	0.48703272	-0.0198591
214.4	0.26687458	-0.4041842	0.39082651	0.05582894	-0.8881232	0.48703272	-0.0198591
214.5	0.26687458	-0.4041842	0.39082651	0.05582894	-0.8881232	0.48703272	-0.0198591
214.6	0.26687458	-0.4041842	0.39082651	0.05582894	-0.8881232	0.48703272	-0.0198591
214.7	0.26687458	-0.4041842	0.39082651	0.05582894	-0.8881232	0.48703272	-0.0198591
214.8	0.26651837	-0.4039912	0.39072261	0.05638564	-0.8880434	0.48708793	-0.0205646
214.9	0.26651837	-0.4039912	0.39072261	0.05638564	-0.8880434	0.48708793	-0.0205646
215	0.26568036	-0.4047897	0.39152609	0.05647187	-0.8880172	0.48718238	-0.021276
215.1	0.26550837	-0.4047027	0.39220016	0.05648466	-0.8881132	0.48738456	-0.0213811
215.2	0.26550837	-0.4047027	0.39220016	0.05648466	-0.8881132	0.48738456	-0.0213811
215.3	0.26550837	-0.4047027	0.39220016	0.05648466	-0.8881132	0.48738456	-0.0213811
215.4	0.26550837	-0.4047027	0.39220016	0.05648466	-0.8881132	0.48738456	-0.0213811
215.5	0.26550837	-0.4047027	0.39220016	0.05648466	-0.8881132	0.48738456	-0.0213811
215.6	0.26550837	-0.4047027	0.39220016	0.05648466	-0.8881132	0.48738456	-0.0213811
215.7	0.26550837	-0.4047027	0.39220016	0.05648466	-0.8881132	0.48738456	-0.0213811
215.8	0.26550837	-0.4047027	0.39220016	0.05648466	-0.8881132	0.48738456	-0.0213811
215.9	0.26550837	-0.4047027	0.39220016	0.05648466	-0.8881132	0.48738456	-0.0213811
216	0.26555553	-0.4046254	0.39259141	0.05602567	-0.8881658	0.48735982	-0.0213705
216.1	0.26555553	-0.4046254	0.39259141	0.05602567	-0.8881658	0.48735982	-0.0213705
216.2	0.26555553	-0.4046254	0.39259141	0.05602567	-0.8881658	0.48735982	-0.0213705
216.3	0.26439828	-0.4042242	0.39206487	0.05560881	-0.8880372	0.48675064	-0.0207498
216.4	0.26398923	-0.4044052	0.39203506	0.05589536	-0.887971	0.48668763	-0.0208211
216.5	0.26398923	-0.4044052	0.39203506	0.05589536	-0.887971	0.48668763	-0.0208211
216.6	0.26398923	-0.4044052	0.39203506	0.05589536	-0.887971	0.48668763	-0.0208211
216.7	0.26398923	-0.4044052	0.39203506	0.05589536	-0.887971	0.48668763	-0.0208211
216.8	0.26398923	-0.4044052	0.39203506	0.05589536	-0.887971	0.48668763	-0.0208211
216.9	0.26398923	-0.4044052	0.39203506	0.05589536	-0.887971	0.48668763	-0.0208211
217	0.26398923	-0.4044052	0.39203506	0.05589536	-0.887971	0.48668763	-0.0208211
217.1	0.26398923	-0.4044052	0.39203506	0.05589536	-0.887971	0.48668763	-0.0208211
217.2	0.26398923	-0.4044052	0.39203506	0.05589536	-0.887971	0.48668763	-0.0208211
217.3	0.26398923	-0.4044052	0.39203506	0.05589536	-0.887971	0.48668763	-0.0208211
217.4	0.26400349	-0.4039053	0.39203743	0.05528017	-0.887961	0.48687348	-0.0213931
217.5	0.26400349	-0.4039053	0.39203743	0.05528017	-0.887961	0.48687348	-0.0213931
217.6	0.26400349	-0.4039053	0.39203743	0.05528017	-0.887961	0.48687348	-0.0213931
217.7	0.26400349	-0.4039053	0.39203743	0.05528017	-0.887961	0.48687348	-0.0213931
217.8	0.26400349	-0.4039053	0.39203743	0.05528017	-0.887961	0.48687348	-0.0213931
217.9	0.26400349	-0.4039053	0.39203743	0.05528017	-0.887961	0.48687348	-0.0213931
218	0.26400349	-0.4039053	0.39203743	0.05528017	-0.887961	0.48687348	-0.0213931
218.1	0.26406887	-0.403629	0.39214263	0.05541484	-0.8881481	0.48743914	-0.0217431
218.2	0.26406887	-0.403629	0.39214263	0.05541484	-0.8881481	0.48743914	-0.0217431
218.3	0.26406887	-0.403629	0.39214263	0.05541484	-0.8881481	0.48743914	-0.0217431
218.4	0.26406887	-0.403629	0.39214263	0.05541484	-0.8881481	0.48743914	-0.0217431
218.5	0.26406887	-0.403629	0.39214263	0.05541484	-0.8881481	0.48743914	-0.0217431
218.6	0.26410648	-0.4035448	0.39163803	0.05505076	-0.888054	0.48751776	-0.0220278
218.7	0.26410648	-0.4035448	0.39163803	0.05505076	-0.888054	0.48751776	-0.0220278
218.8	0.26410648	-0.4035448	0.39163803	0.05505076	-0.888054	0.48751776	-0.0220278
218.9	0.26410648	-0.4035448	0.39163803	0.05505076	-0.888054	0.48751776	-0.0220278
219	0.26403203	-0.4038407	0.39176466	0.05551903	-0.8880632	0.48752568	-0.0216494
219.1	0.26403203	-0.4038407	0.39176466	0.05551903	-0.8880632	0.48752568	-0.0216494
219.2	0.26409006	-0.4037331	0.39156457	0.05590157	-0.8879707	0.48779043	-0.0214097
219.3	0.26409006	-0.4037331	0.39156457	0.05590157	-0.8879707	0.48779043	-0.0214097
219.4	0.2639835	-0.4037888	0.39202446	0.05521674	-0.8879492	0.48775777	-0.0216726
219.5	0.2639835	-0.4037888	0.39202446	0.05521674	-0.8879492	0.48775777	-0.0216726
219.6	0.2639835	-0.4037888	0.39202446	0.05521674	-0.8879492	0.48775777	-0.0216726
219.7	0.2639835	-0.4037888	0.39202446	0.05521674	-0.8879492	0.48775777	-0.0216726
219.8	0.2639835	-0.4037888	0.39202446	0.05521674	-0.8879492	0.48775777	-0.0216726
219.9	0.2639835	-0.4037888	0.39202446	0.05521674	-0.8879492	0.48775777	-0.0216726
220	0.26383438	-0.4034076	0.39214781	0.0550128	-0.8879988	0.48769248	-0.0219871
220.1	0.26383438	-0.4034076	0.39214781	0.0550128	-0.8879988	0.48769248	-0.0219871
220.2	0.26383438	-0.4034076	0.39214781	0.0550128	-0.8879988	0.48769248	-0.0219871
220.3	0.26383438	-0.4034076	0.39214781	0.0550128	-0.8879988	0.48769248	-0.0219871
220.4	0.26383438	-0.4034076	0.39214781	0.0550128	-0.8879988	0.48769248	-0.0219871
220.5	0.26383438	-0.4034076	0.39214781	0.0550128	-0.8879988	0.48769248	-0.0219871
220.6	0.26383438	-0.4034076	0.39214781	0.0550128	-0.8879988	0.48769248	-0.0219871
220.7	0.26384032	-0.403792	0.39271188	0.05492807	-0.8881456	0.48782998	-0.0224876
220.8	0.26371031	-0.4036931	0.39288133	0.05441557	-0.8880811	0.48807191	-0.0235066
220.9	0.26371031	-0.4036931	0.39288133	0.05441557	-0.8880811	0.48807191	-0.0235066
221	0.26371031	-0.4036931	0.39288133	0.05441557	-0.8880811	0.48807191	-0.0235066
221.1	0.26371031	-0.4036931	0.39288133	0.05441557	-0.8880811	0.48807191	-0.0235066
221.2	0.26349685	-0.4034583	0.39311176	0.05462701	-0.8881683	0.48820826	-0.023478
221.3	0.26349685	-0.4034583	0.39311176	0.05462701	-0.8881683	0.48820826	-0.023478
221.4	0.26360697	-0.4035662	0.39365437	0.05507224	-0.8883562	0.48830985	-0.0235788
221.5	0.26360697	-0.4035662	0.39365437	0.05507224	-0.8883562	0.48830985	-0.0235788
221.6	0.26360697	-0.4035662	0.39365437	0.05507224	-0.8883562	0.48830985	-0.0235788
221.7	0.26367282	-0.4032612	0.3939721	0.05543646	-0.8883148	0.48833251	-0.0241038
221.8	0.26367282	-0.4032612	0.3939721	0.05543646	-0.8883148	0.48833251	-0.0241038
221.9	0.26320902	-0.4028672	0.39406655	0.05552543	-0.8882638	0.48818307	-0.0244365
222	0.26320902	-0.4028672	0.39406655	0.05552543	-0.8882638	0.48818307	-0.0244365
222.1	0.262848	-0.4026094	0.39425949	0.0556562	-0.8882576	0.48814048	-0.0240227
222.2	0.262848	-0.4026094	0.39425949	0.0556562	-0.8882576	0.48814048	-0.0240227
222.3	0.26236379	-0.4031629	0.39474893	0.05594195	-0.8883384	0.48834975	-0.0241793
222.4	0.26251648	-0.4030113	0.39519425	0.05619241	-0.8884708	0.48876239	-0.0246695
222.5	0.2619672	-0.4031523	0.39471178	0.05629143	-0.888339	0.48884169	-0.0248603
222.6	0.26121433	-0.4031669	0.39468211	0.05677969	-0.8883195	0.48876016	-0.025006
222.7	0.26121433	-0.4031669	0.39468211	0.05677969	-0.8883195	0.48876016	-0.025006
222.8	0.26121433	-0.4031669	0.39468211	0.05677969	-0.8883195	0.48876016	-0.025006
222.9	0.26121433	-0.4031669	0.39468211	0.05677969	-0.8883195	0.48876016	-0.025006
223	0.26121433	-0.4031669	0.39468211	0.05677969	-0.8883195	0.48876016	-0.025006
223.1	0.26121433	-0.4031669	0.39468211	0.05677969	-0.8883195	0.48876016	-0.025006
223.2	0.26121433	-0.4031669	0.39468211	0.05677969	-0.8883195	0.48876016	-0.025006
223.3	0.2616693	-0.403018	0.39466506	0.05722254	-0.8883871	0.48893751	-0.0252219
223.4	0.2616693	-0.403018	0.39466506	0.05722254	-0.8883871	0.48893751	-0.0252219
223.5	0.2616693	-0.403018	0.39466506	0.05722254	-0.8883871	0.48893751	-0.0252219
223.6	0.2616693	-0.403018	0.39466506	0.05722254	-0.8883871	0.48893751	-0.0252219
223.7	0.2616693	-0.403018	0.39466506	0.05722254	-0.8883871	0.48893751	-0.0252219
223.8	0.2616693	-0.403018	0.39466506	0.05722254	-0.8883871	0.48893751	-0.0252219
223.9	0.2616693	-0.403018	0.39466506	0.05722254	-0.8883871	0.48893751	-0.0252219
224	0.2616693	-0.403018	0.39466506	0.05722254	-0.8883871	0.48893751	-0.0252219
224.1	0.2616693	-0.403018	0.39466506	0.05722254	-0.8883871	0.48893751	-0.0252219
224.2	0.2616693	-0.403018	0.39466506	0.05722254	-0.8883871	0.48893751	-0.0252219
224.3	0.26206483	-0.402976	0.3948484	0.05728285	-0.8885467	0.48885006	-0.0256798
224.4	0.26206483	-0.402976	0.3948484	0.05728285	-0.8885467	0.48885006	-0.0256798
224.5	0.26206483	-0.402976	0.3948484	0.05728285	-0.8885467	0.48885006	-0.0256798
224.6	0.26206483	-0.402976	0.3948484	0.05728285	-0.8885467	0.48885006	-0.0256798
224.7	0.26206483	-0.402976	0.3948484	0.05728285	-0.8885467	0.48885006	-0.0256798
224.8	0.26206483	-0.402976	0.3948484	0.05728285	-0.8885467	0.48885006	-0.0256798
224.9	0.26157401	-0.4031434	0.39477127	0.05765217	-0.8884801	0.48891942	-0.0253329

228.2	0.26009469	-0.4027785	0.39634097	0.05914425	-0.8885357	0.48920804	-0.0267629
228.3	0.25974293	-0.4029618	0.39661273	0.05902856	-0.8886302	0.48921712	-0.0268924
228.4	0.25974293	-0.4029618	0.39661273	0.05902856	-0.8886302	0.48921712	-0.0268924
228.5	0.25978083	-0.4024545	0.39696831	0.05922022	-0.888684	0.48978916	-0.0274126
228.6	0.25931447	-0.4023967	0.39716963	0.06003635	-0.88878	0.48983047	-0.0273924
228.7	0.25931447	-0.4023967	0.39716963	0.06003635	-0.88878	0.48983047	-0.0273924
228.8	0.25966341	-0.4021405	0.39661003	0.06003084	-0.8886603	0.48990618	-0.0275054
228.9	0.25966341	-0.4021405	0.39661003	0.06003084	-0.8886603	0.48990618	-0.0275054
229	0.25966341	-0.4021405	0.39661003	0.06003084	-0.8886603	0.48990618	-0.0275054
229.1	0.25966633	-0.4019332	0.39713399	0.06036218	-0.8887482	0.48992678	-0.0277703
229.2	0.25997109	-0.4019369	0.39667227	0.06055331	-0.8886838	0.4896921	-0.0274945
229.3	0.26033876	-0.4015311	0.39615682	0.06015904	-0.8885624	0.48931047	-0.0278302
229.4	0.26033876	-0.4015311	0.39615682	0.06015904	-0.8885624	0.48931047	-0.0278302
229.5	0.26010667	-0.4017626	0.39682145	0.05975185	-0.8886283	0.48945336	-0.0277347
229.6	0.26003456	-0.4023016	0.39734135	0.05988934	-0.8887206	0.48961987	-0.0283736
229.7	0.26003456	-0.4023016	0.39734135	0.05988934	-0.8887206	0.48961987	-0.0283736
229.8	0.26003456	-0.4023016	0.39734135	0.05988934	-0.8887206	0.48961987	-0.0283736
229.9	0.26003456	-0.4023016	0.39734135	0.05988934	-0.8887206	0.48961987	-0.0283736
230	0.26023724	-0.4020381	0.39773637	0.05947234	-0.8887857	0.48966589	-0.0285959
230.1	0.25971443	-0.402147	0.3981027	0.06004535	-0.8887648	0.48970303	-0.0288325
230.2	0.25971443	-0.402147	0.3981027	0.06004535	-0.8887648	0.48970303	-0.0288325
230.3	0.25963499	-0.4018078	0.39830316	0.06036126	-0.8887609	0.48955716	-0.0288885
230.4	0.25963499	-0.4018078	0.39830316	0.06036126	-0.8887609	0.48955716	-0.0288885
230.5	0.25963499	-0.4018078	0.39830316	0.06036126	-0.8887609	0.48955716	-0.0288885
230.6	0.25963499	-0.4018078	0.39830316	0.06036126	-0.8887609	0.48955716	-0.0288885
230.7	0.25963499	-0.4018078	0.39830316	0.06036126	-0.8887609	0.48955716	-0.0288885
230.8	0.25963499	-0.4018078	0.39830316	0.06036126	-0.8887609	0.48955716	-0.0288885
230.9	0.25963499	-0.4018078	0.39830316	0.06036126	-0.8887609	0.48955716	-0.0288885
231	0.25963499	-0.4018078	0.39830316	0.06036126	-0.8887609	0.48955716	-0.0288885
231.1	0.25990048	-0.4014625	0.39858283	0.06075113	-0.8888303	0.4895207	-0.0288338
231.2	0.25990048	-0.4014625	0.39858283	0.06075113	-0.8888303	0.4895207	-0.0288338
231.3	0.25980558	-0.4015836	0.39938799	0.0611634	-0.888927	0.48976878	-0.0286423
231.4	0.26001096	-0.4017507	0.39932117	0.06168077	-0.8889271	0.48988821	-0.0280795
231.5	0.26001096	-0.4017507	0.39932117	0.06168077	-0.8889271	0.48988821	-0.0280795
231.6	0.25953833	-0.4019987	0.39978607	0.06187542	-0.8889401	0.48990115	-0.0284335
231.7	0.25953833	-0.4019987	0.39978607	0.06187542	-0.8889401	0.48990115	-0.0284335
231.8	0.25953833	-0.4019987	0.39978607	0.06187542	-0.8889401	0.48990115	-0.0284335
231.9	0.25937669	-0.40177	0.39970136	0.0619518	-0.8888675	0.489754	-0.0285656
232	0.25884573	-0.4019478	0.40013276	0.06242417	-0.8889162	0.48998906	-0.0283622
232.1	0.25884573	-0.4019478	0.40013276	0.06242417	-0.8889162	0.48998906	-0.0283622
232.2	0.25884573	-0.4019478	0.40013276	0.06242417	-0.8889162	0.48998906	-0.0283622
232.3	0.25893405	-0.4017542	0.40020085	0.06295346	-0.8889932	0.48997055	-0.0281226
232.4	0.25893405	-0.4017542	0.40020085	0.06295346	-0.8889932	0.48997055	-0.0281226
232.5	0.25893405	-0.4017542	0.40020085	0.06295346	-0.8889932	0.48997055	-0.0281226
232.6	0.25878638	-0.4016617	0.4002139	0.06246374	-0.8889623	0.48987008	-0.0282502
232.7	0.25831997	-0.4019037	0.40150464	0.0634476	-0.8891793	0.49023498	-0.0291568
232.8	0.25801585	-0.4015056	0.40135874	0.0641664	-0.8891672	0.49047994	-0.0299151
232.9	0.25801585	-0.4015056	0.40135874	0.0641664	-0.8891672	0.49047994	-0.0299151
233	0.25801585	-0.4015056	0.40135874	0.0641664	-0.8891672	0.49047994	-0.0299151
233.1	0.25801585	-0.4015056	0.40135874	0.0641664	-0.8891672	0.49047994	-0.0299151
233.2	0.25786098	-0.4010352	0.401455	0.06371306	-0.8891782	0.49039205	-0.0297594
233.3	0.25786098	-0.4010352	0.401455	0.06371306	-0.8891782	0.49039205	-0.0297594
233.4	0.25786098	-0.4010352	0.401455	0.06371306	-0.8891782	0.49039205	-0.0297594
233.5	0.25786098	-0.4010352	0.401455	0.06371306	-0.8891782	0.49039205	-0.0297594
233.6	0.25786098	-0.4010352	0.401455	0.06371306	-0.8891782	0.49039205	-0.0297594
233.7	0.25719791	-0.4012163	0.40210045	0.06378655	-0.8892028	0.49041283	-0.0294602
233.8	0.25719791	-0.4012163	0.40210045	0.06378655	-0.8892028	0.49041283	-0.0294602
233.9	0.2569445	-0.4014642	0.40216778	0.06394593	-0.8892074	0.49041172	-0.0299811
234	0.2569445	-0.4014642	0.40216778	0.06394593	-0.8892074	0.49041172	-0.0299811
234.1	0.25707025	-0.4013506	0.40251166	0.06374661	-0.8892163	0.49048149	-0.030336
234.2	0.25707025	-0.4013506	0.40251166	0.06374661	-0.8892163	0.49048149	-0.030336
234.3	0.25701797	-0.4013594	0.4026951	0.06410396	-0.8893193	0.49046866	-0.0302726
234.4	0.25701797	-0.4013594	0.4026951	0.06410396	-0.8893193	0.49046866	-0.0302726
234.5	0.25701797	-0.4013594	0.4026951	0.06410396	-0.8893193	0.49046866	-0.0302726
234.6	0.25701797	-0.4013594	0.4026951	0.06410396	-0.8893193	0.49046866	-0.0302726
234.7	0.25701797	-0.4013594	0.4026951	0.06410396	-0.8893193	0.49046866	-0.0302726
234.8	0.25701797	-0.4013594	0.4026951	0.06410396	-0.8893193	0.49046866	-0.0302726
234.9	0.25701797	-0.4013594	0.4026951	0.06410396	-0.8893193	0.49046866	-0.0302726
235	0.2562854	-0.4017716	0.40293213	0.06467842	-0.889331	0.49051373	-0.0300939
235.1	0.25624753	-0.4018599	0.40343938	0.06504806	-0.8894267	0.49044022	-0.0298104
235.2	0.25624753	-0.4018599	0.40343938	0.06504806	-0.8894267	0.49044022	-0.0298104
235.3	0.25624753	-0.4018599	0.40343938	0.06504806	-0.8894267	0.49044022	-0.0298104
235.4	0.25624753	-0.4018599	0.40343938	0.06504806	-0.8894267	0.49044022	-0.0298104
235.5	0.25624753	-0.4018599	0.40343938	0.06504806	-0.8894267	0.49044022	-0.0298104
235.6	0.25624753	-0.4018599	0.40343938	0.06504806	-0.8894267	0.49044022	-0.0298104
235.7	0.25588949	-0.4015777	0.40377979	0.06513915	-0.889523	0.49059446	-0.0302205
235.8	0.25588949	-0.4015777	0.40377979	0.06513915	-0.889523	0.49059446	-0.0302205
235.9	0.25580132	-0.4011754	0.40448126	0.06517793	-0.8896735	0.49070795	-0.0307734
236	0.25580132	-0.4011754	0.40448126	0.06517793	-0.8896735	0.49070795	-0.0307734
236.1	0.25581205	-0.4011056	0.40470341	0.06520007	-0.8897177	0.49071209	-0.0308718
236.2	0.25581733	-0.4011234	0.40497911	0.0651562	-0.8897955	0.49080678	-0.0310363
236.3	0.25585032	-0.4008836	0.40533799	0.06572233	-0.8898256	0.49097563	-0.0311034
236.4	0.25585032	-0.4008836	0.40533799	0.06572233	-0.8898256	0.49097563	-0.0311034
236.5	0.25579771	-0.4004768	0.40522804	0.06554117	-0.8897472	0.49087732	-0.0313375
236.6	0.25579771	-0.4004768	0.40522804	0.06554117	-0.8897472	0.49087732	-0.0313375
236.7	0.25579771	-0.4004768	0.40522804	0.06554117	-0.8897472	0.49087732	-0.0313375
236.8	0.25579771	-0.4004768	0.40522804	0.06554117	-0.8897472	0.49087732	-0.0313375
236.9	0.25579771	-0.4004768	0.40522804	0.06554117	-0.8897472	0.49087732	-0.0313375
237	0.25579771	-0.4004768	0.40522804	0.06554117	-0.8897472	0.49087732	-0.0313375
237.1	0.25579771	-0.4004768	0.40522804	0.06554117	-0.8897472	0.49087732	-0.0313375
237.2	0.25579771	-0.4004768	0.40522804	0.06554117	-0.8897472	0.49087732	-0.0313375
237.3	0.25553128	-0.4002762	0.40590505	0.06517635	-0.8898793	0.49121698	-0.0312626
237.4	0.25553128	-0.4002762	0.40590505	0.06517635	-0.8898793	0.49121698	-0.0312626
237.5	0.25553128	-0.4002762	0.40590505	0.06517635	-0.8898793	0.49121698	-0.0312626
237.6	0.25585799	-0.4003728	0.4058998	0.06540513	-0.8899294	0.49135875	-0.0314536
237.7	0.25585799	-0.4003728	0.4058998	0.06540513	-0.8899294	0.49135875	-0.0314536
237.8	0.25582738	-0.4003972	0.40612947	0.06579006	-0.8899741	0.49156773	-0.0319978
237.9	0.25582738	-0.4003972	0.40612947	0.06579006	-0.8899741	0.49156773	-0.0319978
238	0.25582738	-0.4003972	0.40612947	0.06579006	-0.8899741	0.49156773	-0.0319978
238.1	0.25582738	-0.4003972	0.40612947	0.06579006	-0.8899741	0.49156773	-0.0319978
238.2	0.25582738	-0.4003972	0.40612947	0.06579006	-0.8899741	0.49156773	-0.0319978
238.3	0.25582738	-0.4003972	0.40612947	0.06579006	-0.8899741	0.49156773	-0.0319978
238.4	0.25582738	-0.4003972	0.40612947	0.06579006	-0.8899741	0.49156773	-0.0319978
238.5	0.25582738	-0.4003972	0.40612947	0.06579006	-0.8899741	0.49156773	-0.0319978
238.6	0.25582738	-0.4003972	0.40612947	0.06579006	-0.8899741	0.49156773	-0.0319978
238.7	0.25582738	-0.4003972	0.40612947	0.06579006	-0.8899741	0.49156773	-0.0319978
238.8	0.25602826	-0.3997399	0.40595024	0.06544853	-0.8898904	0.49146868	-0.0317091
238.9	0.25602826	-0.3997399	0.40595024	0.06544853	-0.8898904	0.49146868	-0.0317091
239	0.25607913	-0.3998998	0.40654977	0.065548	-0.8899612	0.49166542	-0.0314761
239.1	0.25607913	-0.3998998	0.40654977	0.065548	-0.8899612	0.49166542	-0.0314761
239.2	0.25607913	-0.3998998	0.40654977	0.065548	-0.8899612	0.49166542	-0.0314761
239.3	0.25607913	-0.3998998	0.40654977	0.065548	-0.8899612	0.49166542	-0.0314761
239.4	0.25607913	-0.3998998	0.40654977	0.065548	-0.8899612	0.49166542	-0.0314761
239.5	0.25607913	-0.3998998	0.40654977	0.065548	-0.8899612	0.49166542	-0.0314761
239.6	0.25619263	-0.3998687	0.40700568	0.06608174	-0.8900103	0.49179543	-0.0318834
239.7	0.25619263	-0.3998687	0.40700568	0.06608174	-0.8900103	0.49179543	-0.0318834
239.8	0.25619263	-0.3998687	0.40700568	0.06608174	-0.8900103	0.49179543	-0.0318834
239.9	0.25619263	-0.3998687	0.40700568	0.06608174	-0.8900103	0.49179543	-0.0318834
240	0.25644736	-0.3998381	0.40767008	0.0666309	-0.8901348	0.49197528	-0.0319029

Corresponding p-values for PRCC sensitivtiy indices

Time discretization	V_{[G]}	K_{[G]}	q	V_{[G3P]}	alpha	G_E	[G3P]_0
0	0	0	0	0	0	0	0
0.1	0.6114885	0.65850719	2.97E-157	3.17E-70	0.00035593	0.72129702	0.4373911
0.2	0.85393588	0.6960679	1.83E-152	3.10E-66	3.22E-11	0.7108706	0.45624093
0.3	0.75713512	0.93287639	6.06E-150	1.85E-64	3.75E-18	0.74392869	0.30300329
0.4	0.74914847	0.92170255	3.59E-146	9.14E-62	2.43E-25	0.84729475	0.17800396
0.5	0.85665238	0.74352632	2.25E-142	2.35E-58	3.01E-32	0.68187081	0.15193629
0.6	0.98752773	0.6619638	1.23E-139	7.42E-56	1.94E-37	0.59612844	0.09384644
0.7	0.85938321	0.79803943	3.35E-138	2.42E-54	2.36E-43	0.66717993	0.10422399
0.8	0.79601994	0.74187492	1.02E-135	7.80E-52	6.13E-49	0.63484463	0.09869189
0.9	0.61616743	0.71605763	7.85E-133	1.23E-49	1.38E-51	0.77613709	0.12160114
1	0.5175316	0.81459518	3.22E-131	3.46E-48	9.46E-56	0.78066056	0.14603916
1.1	0.34610526	0.67856265	2.19E-130	1.92E-47	6.58E-59	0.80036721	0.12388088
1.2	0.40691485	0.43018011	9.34E-129	4.69E-46	2.39E-61	0.98290559	0.11105677
1.3	0.35364365	0.36606359	4.11E-127	7.85E-45	5.72E-64	0.96132608	0.09655844
1.4	0.41239153	0.39238992	2.93E-124	1.96E-43	3.10E-65	0.93061798	0.1112947
1.5	0.35575501	0.37642498	6.81E-122	3.46E-41	4.92E-67	0.98088967	0.09379193
1.6	0.34335002	0.30945152	5.29E-119	7.42E-39	5.86E-68	0.92267548	0.106112
1.7	0.38374156	0.34428462	1.19E-117	3.54E-38	6.52E-70	0.84126785	0.12815163
1.8	0.33880083	0.34698388	5.47E-116	5.76E-37	9.47E-71	0.8727402	0.1292092
1.9	0.38754882	0.32292512	5.07E-115	1.88E-36	3.36E-73	0.77468361	0.1670037
2	0.40040314	0.41476496	1.38E-113	1.04E-35	5.73E-74	0.75162668	0.15424556
2.1	0.4299961	0.4212319	9.36E-112	1.52E-34	1.02E-74	0.73899011	0.17214466
2.2	0.44726155	0.53002637	5.15E-110	9.93E-34	6.16E-76	0.63901468	0.16176762
2.3	0.46185022	0.53263963	2.46E-108	6.34E-33	9.60E-77	0.66623218	0.16556842
2.4	0.45056123	0.52019997	2.71E-107	2.26E-32	9.94E-78	0.60834671	0.16998375
2.5	0.45191638	0.46186617	5.98E-106	1.72E-31	1.87E-78	0.62071112	0.22004282
2.6	0.43448524	0.42400053	2.83E-105	2.50E-31	9.30E-80	0.66878456	0.1917573
2.7	0.44504459	0.44529029	1.98E-104	5.07E-31	8.33E-81	0.62663511	0.20543923
2.8	0.49527789	0.43665544	1.04E-102	4.93E-30	4.41E-81	0.6116401	0.18555944
2.9	0.66326369	0.38595306	1.18E-101	1.87E-29	4.41E-82	0.59635949	0.1935486
3	0.65474715	0.3531595	1.22E-100	8.12E-29	1.49E-82	0.5925772	0.23830925
3.1	0.62837194	0.3736444	2.74E-99	5.17E-28	6.20E-83	0.64898126	0.28218592
3.2	0.65695117	0.36682973	2.56E-98	2.17E-27	1.94E-83	0.70090602	0.2835589
3.3	0.76543876	0.34803	2.02E-97	9.43E-27	4.13E-84	0.69185947	0.23151061
3.4	0.84281474	0.34556737	5.29E-97	2.00E-26	2.33E-85	0.73745085	0.23688096
3.5	0.89858467	0.4025293	2.80E-96	9.16E-26	7.91E-86	0.72743023	0.25447566
3.6	0.97362555	0.44887905	2.13E-95	2.38E-25	1.79E-86	0.71170464	0.25994152
3.7	0.98116702	0.40609652	1.59E-94	3.38E-25	1.28E-86	0.69836246	0.25560566
3.8	0.9147747	0.34623507	9.76E-94	9.44E-25	1.54E-87	0.62696627	0.23755056
3.9	0.95841709	0.36088999	1.35E-93	9.14E-25	1.93E-88	0.66866915	0.21273737
4	0.8810641	0.37285735	3.15E-93	1.84E-24	5.06E-89	0.63426923	0.22016778
4.1	0.86599024	0.37907452	1.13E-92	5.21E-24	1.54E-89	0.61726166	0.26946315
4.2	0.84352732	0.42670708	3.94E-92	6.70E-24	6.82E-90	0.64257258	0.27803789
4.3	0.88970379	0.41155061	1.42E-91	9.98E-24	2.30E-90	0.7207226	0.26754113
4.4	0.8805125	0.47320917	1.09E-90	2.50E-23	2.63E-90	0.69778722	0.26518508
4.5	0.90059423	0.42375966	2.07E-90	3.22E-23	7.30E-91	0.76393931	0.25812934
4.6	0.92158126	0.42544334	3.74E-90	7.96E-23	1.73E-91	0.76298799	0.27539636
4.7	0.91365793	0.44047764	4.10E-90	6.97E-23	4.54E-92	0.72858769	0.23917113
4.8	0.97116639	0.4771992	1.74E-89	1.36E-22	4.06E-92	0.73797915	0.2252883
4.9	0.96187317	0.45453611	4.42E-89	1.99E-22	1.92E-92	0.78243433	0.20360048
5	0.99779815	0.45583766	6.85E-89	3.22E-22	4.68E-93	0.78459096	0.19641147
5.1	0.99903427	0.46459807	2.26E-88	6.03E-22	3.04E-93	0.81486409	0.19640494
5.2	0.99486285	0.442098	5.21E-88	1.43E-21	1.26E-93	0.7714203	0.22173134
5.3	0.94358966	0.47631755	1.96E-87	3.86E-21	7.79E-94	0.73818334	0.23059091
5.4	0.93058028	0.4543907	4.33E-87	4.60E-21	3.03E-94	0.74891473	0.23496978
5.5	0.83861229	0.48754988	2.01E-86	9.41E-21	2.32E-94	0.8105397	0.22720519
5.6	0.87119174	0.45891129	9.86E-86	1.54E-20	1.77E-94	0.8391044	0.2169259
5.7	0.80608522	0.47988076	5.12E-85	2.37E-20	3.14E-94	0.82355449	0.22313953
5.8	0.77869428	0.44124464	8.49E-85	3.72E-20	1.79E-94	0.80954762	0.2202569
5.9	0.77923375	0.44957441	1.16E-84	3.85E-20	6.98E-95	0.78041824	0.21880257
6	0.75441339	0.44107297	2.31E-84	4.49E-20	3.89E-95	0.80687853	0.22660134
6.1	0.7328495	0.50180706	1.24E-83	1.30E-19	2.89E-95	0.72477067	0.23531606
6.2	0.72059765	0.50939661	1.84E-83	1.54E-19	2.02E-95	0.73258237	0.23719491
6.3	0.69482633	0.51610584	3.27E-83	1.93E-19	1.19E-95	0.76360319	0.24404315
6.4	0.70866867	0.55148305	5.59E-83	1.99E-19	7.82E-96	0.7756819	0.26374952
6.5	0.70361426	0.53721839	2.23E-82	1.81E-19	1.49E-95	0.79734994	0.25461512
6.6	0.68199847	0.54664671	5.55E-82	2.50E-19	1.64E-95	0.79667619	0.27101115
6.7	0.6615467	0.53345614	1.36E-81	4.16E-19	1.87E-95	0.84337228	0.27861796
6.8	0.64111209	0.56188679	1.31E-81	5.18E-19	6.55E-96	0.84327927	0.28551161
6.9	0.64391165	0.55825996	2.05E-81	9.54E-19	5.24E-96	0.87751444	0.29717909
7	0.62233456	0.56967271	3.60E-81	1.75E-18	3.65E-96	0.87044702	0.29264032
7.1	0.65460377	0.58469449	3.89E-81	2.94E-18	1.93E-96	0.84866491	0.3077042
7.2	0.65768478	0.59965982	6.10E-81	4.05E-18	1.26E-96	0.92066601	0.32939233
7.3	0.62672395	0.59267308	1.49E-80	7.45E-18	9.12E-97	0.89513433	0.3449771
7.4	0.63018315	0.6075965	1.76E-80	7.33E-18	6.46E-97	0.8956657	0.32549501
7.5	0.63373309	0.6493352	4.36E-80	1.27E-17	7.95E-97	0.94730889	0.3583138
7.6	0.63902153	0.65736584	8.58E-80	1.55E-17	9.62E-97	0.95582383	0.36902404
7.7	0.61962363	0.67540775	2.04E-79	2.34E-17	9.82E-97	0.98392473	0.35365121
7.8	0.60043022	0.66377446	8.18E-79	3.85E-17	1.93E-96	0.99731885	0.37443266
7.9	0.6145697	0.67697603	1.24E-78	5.44E-17	1.46E-96	0.96946653	0.38720128
8	0.62257091	0.6609245	1.46E-78	5.63E-17	9.73E-97	0.98012701	0.40179408
8.1	0.64505534	0.66871343	1.88E-78	6.34E-17	9.43E-97	0.98694126	0.39970783
8.2	0.62978563	0.67916511	4.08E-78	7.50E-17	7.30E-97	0.99987658	0.4018674
8.3	0.61768377	0.67456899	2.70E-77	1.59E-16	2.41E-96	0.99781637	0.39987789
8.4	0.61068502	0.67640538	5.45E-77	1.91E-16	3.14E-96	0.99624766	0.42810556
8.5	0.60488531	0.64461458	8.42E-77	1.97E-16	3.47E-96	0.99226077	0.42238266
8.6	0.59804046	0.6517347	1.06E-76	2.24E-16	2.44E-96	0.96744641	0.42424398
8.7	0.59160369	0.66667411	1.53E-76	3.47E-16	2.18E-96	0.96029881	0.45750797
8.8	0.59489204	0.66668288	4.27E-76	5.19E-16	3.29E-96	0.95691736	0.47410588
8.9	0.61350284	0.63810372	6.75E-76	4.70E-16	2.65E-96	0.99302837	0.46524548
9	0.59172175	0.65765976	1.24E-75	6.09E-16	3.61E-96	0.98383041	0.47585041
9.1	0.59240299	0.66206428	1.90E-75	7.65E-16	4.37E-96	0.98659544	0.48823545
9.2	0.60627844	0.67534582	2.88E-75	1.20E-15	5.43E-96	0.97620787	0.49008479
9.3	0.61127998	0.6773134	6.74E-75	1.61E-15	7.34E-96	0.96937225	0.51431538
9.4	0.62680578	0.68364635	1.16E-74	2.42E-15	9.14E-96	0.95157544	0.50894367
9.5	0.64685809	0.69537535	1.90E-74	3.16E-15	1.06E-95	0.92002621	0.49936259
9.6	0.64725417	0.70188923	2.91E-74	2.82E-15	1.07E-95	0.88311359	0.49139637
9.7	0.6184846	0.70000704	3.36E-74	2.75E-15	6.49E-96	0.88111142	0.50437532
9.8	0.61669227	0.67886125	3.59E-74	3.33E-15	5.22E-96	0.86866516	0.48800643
9.9	0.59771004	0.68945471	6.70E-74	3.44E-15	7.58E-96	0.88223536	0.51390985
10	0.59652678	0.70336735	1.16E-73	3.91E-15	1.03E-95	0.88943226	0.51615582
10.1	0.60075456	0.71702474	3.34E-73	4.78E-15	1.78E-95	0.92606025	0.53165418
10.2	0.59227044	0.74889851	7.29E-73	6.63E-15	2.25E-95	0.92278758	0.557486
10.3	0.57700897	0.76520472	1.11E-72	7.83E-15	2.18E-95	0.92894197	0.56723568
10.4	0.57403611	0.76380933	2.29E-72	9.70E-15	2.86E-95	0.92881501	0.55710367
10.5	0.5635109	0.75892367	2.63E-72	1.02E-14	2.28E-95	0.90484734	0.55297432
10.6	0.56210643	0.77523965	3.94E-72	1.16E-14	2.76E-95	0.86249978	0.57817926
10.7	0.56465325	0.76794339	1.58E-71	2.21E-14	6.24E-95	0.85687042	0.55071903
10.8	0.57376734	0.78531709	2.77E-71	2.47E-14	7.65E-95	0.88601334	0.54832007
10.9	0.56128247	0.78800185	3.30E-71	2.22E-14	5.97E-95	0.90116048	0.54760313
11	0.52757716	0.76670264	4.70E-71	2.17E-14	5.03E-95	0.90708506	0.55133783
11.1	0.53283312	0.76193728	1.05E-70	2.74E-14	8.47E-95	0.88901034	0.55857275
11.2	0.52176458	0.7654444	1.05E-70	3.44E-14	6.64E-95	0.89734866	0.55156596
11.3	0.49888139	0.75763386	9.36E-71	3.27E-14	4.16E-95	0.89245009	0.56834068
11.4	0.51604592	0.78125686	1.51E-70	4.90E-14	5.32E-95	0.88278952	0.57218888
11.5	0.52379886	0.77685941	1.83E-70	6.53E-14	6.27E-95	0.88057488	0.56856996
11.6	0.51603508	0.79824958	2.13E-70	7.13E-14	6.06E-95	0.86708907	0.57186747
11.7	0.51443419	0.77985403	2.76E-70	7.91E-14	5.11E-95	0.88645016	0.58182257
11.8	0.52311202	0.77810991	3.95E-70	8.71E-14	6.69E-95	0.90739938	0.58172834

Time discretization	V_{[G]}	K_{[G]}	q	V_{[G3P]}	alpha	G_E	[G3P]_0
15	0.53619979	0.88946425	1.80E-67	5.68E-13	7.78E-94	0.84981122	0.55321735
15.1	0.53056679	0.89812793	2.38E-67	6.95E-13	9.33E-94	0.84993733	0.56061007
15.2	0.52159215	0.89301363	2.38E-67	7.23E-13	8.14E-94	0.8496037	0.56110738
15.3	0.51874301	0.90448996	2.96E-67	8.25E-13	8.65E-94	0.84750654	0.57536511
15.4	0.51115172	0.92008264	3.78E-67	9.34E-13	1.15E-93	0.83361902	0.575142
15.5	0.51115172	0.92008264	3.78E-67	9.34E-13	1.15E-93	0.83361902	0.575142
15.6	0.50813769	0.92485839	5.34E-67	9.85E-13	1.43E-93	0.8312579	0.56312177
15.7	0.50503761	0.9353148	7.57E-67	1.13E-12	1.60E-93	0.82592242	0.57074542
15.8	0.50400533	0.95083112	8.45E-67	1.27E-12	1.92E-93	0.82363643	0.57402778
15.9	0.51853468	0.95747937	1.09E-66	1.38E-12	2.41E-93	0.81509928	0.57040206
16	0.51613701	0.96557184	1.79E-66	1.81E-12	3.56E-93	0.8247365	0.57906427
16.1	0.51613701	0.96557184	1.79E-66	1.81E-12	3.56E-93	0.8247365	0.57906427
16.2	0.51613847	0.96566101	2.00E-66	2.01E-12	3.90E-93	0.83298663	0.58446996
16.3	0.52029646	0.96222609	2.10E-66	2.03E-12	3.70E-93	0.83973476	0.58110567
16.4	0.52286939	0.96683456	2.10E-66	1.96E-12	3.13E-93	0.83133982	0.58620409
16.5	0.52286939	0.96683456	2.10E-66	1.96E-12	3.13E-93	0.83133982	0.58620409
16.6	0.51057154	0.97512971	2.51E-66	2.09E-12	3.74E-93	0.83027076	0.58295229
16.7	0.49190664	0.96018708	4.82E-66	2.35E-12	5.99E-93	0.82430442	0.58797515
16.8	0.48706896	0.960892	5.23E-66	2.22E-12	5.48E-93	0.82823726	0.58769825
16.9	0.4879454	0.95727586	5.89E-66	2.19E-12	6.05E-93	0.8247696	0.59027656
17	0.4857966	0.97133934	9.28E-66	2.71E-12	8.08E-93	0.8205089	0.60337426
17.1	0.47195158	0.98203229	1.08E-65	2.66E-12	7.29E-93	0.83090161	0.60951792
17.2	0.44739622	0.9823851	1.32E-65	2.74E-12	6.47E-93	0.83991118	0.61480678
17.3	0.44739622	0.9823851	1.32E-65	2.74E-12	6.47E-93	0.83991118	0.61480678
17.4	0.43976078	0.9911781	1.31E-65	3.04E-12	6.33E-93	0.84199318	0.61728003
17.5	0.44516261	0.99566597	1.83E-65	3.45E-12	8.40E-93	0.83329846	0.61981823
17.6	0.44516261	0.99566597	1.83E-65	3.45E-12	8.40E-93	0.83329846	0.61981823
17.7	0.45663306	0.99041323	2.41E-65	4.37E-12	1.12E-92	0.83790661	0.63262504
17.8	0.45698097	0.98271003	2.53E-65	4.44E-12	1.14E-92	0.83694923	0.64200117
17.9	0.45502341	0.98998573	2.96E-65	4.56E-12	1.17E-92	0.82929779	0.63577592
18	0.45472706	0.99331624	3.04E-65	5.22E-12	1.22E-92	0.83716331	0.64511909
18.1	0.4475961	0.98533096	5.28E-65	6.57E-12	1.70E-92	0.82957668	0.64980155
18.2	0.4475961	0.98533096	5.28E-65	6.57E-12	1.70E-92	0.82957668	0.64980155
18.3	0.45110239	0.98205284	8.45E-65	8.36E-12	2.36E-92	0.85142475	0.65332962
18.4	0.46089958	0.9917904	8.35E-65	8.73E-12	2.29E-92	0.84060505	0.66482408
18.5	0.44363019	0.98568788	1.42E-64	1.08E-11	3.69E-92	0.85401584	0.67665541
18.6	0.44395046	0.99034971	1.55E-64	1.14E-11	4.13E-92	0.84507334	0.67980687
18.7	0.446662	0.99606114	2.14E-64	1.15E-11	5.93E-92	0.83448353	0.68947286
18.8	0.45679852	0.99965783	2.14E-64	1.07E-11	5.05E-92	0.82646959	0.6951861
18.9	0.45368018	0.99746283	2.31E-64	1.10E-11	5.55E-92	0.8435045	0.69879178
19	0.45368018	0.99746283	2.31E-64	1.10E-11	5.55E-92	0.8435045	0.69879178
19.1	0.45145225	0.99158043	2.37E-64	1.05E-11	5.61E-92	0.84716179	0.69241724
19.2	0.45106856	0.99129188	2.66E-64	1.09E-11	6.26E-92	0.83348257	0.685948
19.3	0.4500324	0.9799555	2.76E-64	1.19E-11	6.34E-92	0.83211752	0.68329571
19.4	0.45016676	0.9738506	2.93E-64	1.23E-11	5.30E-92	0.82013277	0.70037908
19.5	0.44632241	0.96805659	3.85E-64	1.40E-11	6.29E-92	0.8159152	0.69984936
19.6	0.45125881	0.96484186	5.39E-64	1.44E-11	8.02E-92	0.81013037	0.69656875
19.7	0.44947183	0.96253977	7.98E-64	1.85E-11	1.11E-91	0.81948971	0.6961125
19.8	0.4509213	0.94796568	9.76E-64	2.06E-11	1.32E-91	0.80581948	0.70005972
19.9	0.45273893	0.94006276	1.13E-63	2.21E-11	1.66E-91	0.80412802	0.70025707
20	0.46237029	0.94409058	1.89E-63	2.25E-11	2.58E-91	0.82216425	0.69335622
20.1	0.46501586	0.95296881	2.23E-63	2.45E-11	2.46E-91	0.81829504	0.69805014
20.2	0.46501586	0.95296881	2.23E-63	2.45E-11	2.46E-91	0.81829504	0.69805014
20.3	0.46501586	0.95296881	2.23E-63	2.45E-11	2.46E-91	0.81829504	0.69805014
20.4	0.47784872	0.93191109	2.42E-63	2.90E-11	2.94E-91	0.83548583	0.71131576
20.5	0.47784872	0.93191109	2.42E-63	2.90E-11	2.94E-91	0.83548583	0.71131576
20.6	0.47784872	0.93191109	2.42E-63	2.90E-11	2.94E-91	0.83548583	0.71131576
20.7	0.48075881	0.92857929	2.91E-63	3.33E-11	3.15E-91	0.82346219	0.71185587
20.8	0.48075881	0.92857929	2.91E-63	3.33E-11	3.15E-91	0.82346219	0.71185587
20.9	0.46037078	0.92159637	4.94E-63	3.80E-11	4.56E-91	0.81527401	0.69519119
21	0.45463535	0.90992331	6.20E-63	4.08E-11	5.37E-91	0.82282873	0.70745285
21.1	0.45326996	0.90017371	8.35E-63	4.95E-11	8.82E-91	0.80138543	0.7176081
21.2	0.45782267	0.90895866	1.10E-62	5.45E-11	1.11E-90	0.78986882	0.72874448
21.3	0.45215957	0.92668761	1.39E-62	5.34E-11	1.18E-90	0.79434224	0.71432082
21.4	0.45674052	0.91592473	1.24E-62	4.65E-11	9.10E-91	0.79987767	0.71825361
21.5	0.45894634	0.92214278	1.58E-62	4.95E-11	1.22E-90	0.78653664	0.7332569
21.6	0.45590853	0.921802	1.62E-62	5.04E-11	1.17E-90	0.79309415	0.73304953
21.7	0.46359767	0.92732631	1.56E-62	5.07E-11	1.02E-90	0.79903085	0.74721297
21.8	0.46906255	0.90771889	1.97E-62	6.04E-11	1.31E-90	0.7936744	0.75549295
21.9	0.45796907	0.91457796	2.22E-62	6.14E-11	1.31E-90	0.79394748	0.75236702
22	0.45911061	0.91419178	2.84E-62	7.10E-11	1.63E-90	0.79273668	0.75792842
22.1	0.45911061	0.91419178	2.84E-62	7.10E-11	1.63E-90	0.79273668	0.75792842
22.2	0.46248805	0.90812439	4.10E-62	7.54E-11	2.17E-90	0.79969969	0.77132231
22.3	0.46248805	0.90812439	4.10E-62	7.54E-11	2.17E-90	0.79969969	0.77132231
22.4	0.46465537	0.9036643	4.40E-62	8.03E-11	2.21E-90	0.80516795	0.76732173
22.5	0.46666066	0.90502216	5.45E-62	8.77E-11	2.48E-90	0.79751653	0.75743491
22.6	0.47492045	0.89867434	8.29E-62	1.04E-10	3.49E-90	0.80050919	0.76857273
22.7	0.46206703	0.90839733	7.91E-62	1.01E-10	3.11E-90	0.80251433	0.7563661
22.8	0.46290591	0.91400241	8.49E-62	1.08E-10	3.32E-90	0.8018817	0.75701886
22.9	0.46290591	0.91400241	8.49E-62	1.08E-10	3.32E-90	0.8018817	0.75701886
23	0.46080731	0.91480452	8.87E-62	1.11E-10	3.07E-90	0.81170495	0.7515146
23.1	0.45460654	0.92518215	9.90E-62	1.15E-10	3.25E-90	0.79859731	0.76605222
23.2	0.45460654	0.92518215	9.90E-62	1.15E-10	3.25E-90	0.79859731	0.76605222
23.3	0.45169683	0.91416735	1.25E-61	1.21E-10	3.80E-90	0.80815068	0.76199173
23.4	0.4525618	0.9084488	1.36E-61	1.33E-10	4.45E-90	0.80135012	0.76963626
23.5	0.45262431	0.90144355	1.40E-61	1.26E-10	4.43E-90	0.8024504	0.76013529
23.6	0.44749502	0.90134765	1.39E-61	1.33E-10	4.73E-90	0.79790828	0.75635372
23.7	0.4454525	0.90603867	1.52E-61	1.38E-10	4.82E-90	0.80177386	0.7473945
23.8	0.44196653	0.90232558	1.91E-61	1.50E-10	5.57E-90	0.80282734	0.74558478
23.9	0.44526282	0.89874041	2.57E-61	1.78E-10	8.38E-90	0.78059584	0.75732023
24	0.45009466	0.8962608	2.50E-61	1.75E-10	8.09E-90	0.79492903	0.75818744
24.1	0.4474651	0.88474582	2.53E-61	1.73E-10	8.51E-90	0.79038176	0.76268486
24.2	0.43019485	0.88882629	2.71E-61	1.81E-10	1.03E-89	0.78517088	0.76637379
24.3	0.43457672	0.87738327	3.08E-61	1.82E-10	1.11E-89	0.79255627	0.76455665
24.4	0.43457672	0.87738327	3.08E-61	1.82E-10	1.11E-89	0.79255627	0.76455665
24.5	0.43484063	0.87482565	3.36E-61	1.87E-10	1.19E-89	0.79880576	0.76911005
24.6	0.43923805	0.86643374	4.31E-61	1.93E-10	1.47E-89	0.79975115	0.7769783
24.7	0.44305195	0.85574361	4.58E-61	2.05E-10	1.54E-89	0.80069747	0.77829953
24.8	0.43433515	0.84541162	5.28E-61	2.15E-10	1.85E-89	0.78514535	0.79245769
24.9	0.43981416	0.83357407	5.19E-61	2.12E-10	1.77E-89	0.7892412	0.78927871
25	0.44011357	0.80604321	8.55E-61	2.61E-10	3.33E-89	0.7706412	0.8081296
25.1	0.44133461	0.80340814	1.05E-60	2.56E-10	3.90E-89	0.77123476	0.81809673
25.2	0.44070283	0.79903581	1.55E-60	3.03E-10	5.41E-89	0.78112686	0.82420462
25.3	0.43673713	0.78843844	2.14E-60	3.65E-10	6.63E-89	0.76147495	0.80753525
25.4	0.44258613	0.79657787	2.29E-60	3.55E-10	6.80E-89	0.76715264	0.80569039
25.5	0.43042781	0.79671431	2.63E-60	3.48E-10	7.19E-89	0.76492749	0.81084977
25.6	0.4342162	0.78780891	3.25E-60	3.72E-10	9.21E-89	0.7675413	0.82184988
25.7	0.4342162	0.78780891	3.25E-60	3.72E-10	9.21E-89	0.7675413	0.82184988
25.8	0.42375019	0.79092688	3.83E-60	3.87E-10	1.03E-88	0.76175449	0.83535859
25.9	0.42375019	0.79092688	3.83E-60	3.87E-10	1.03E-88	0.76175449	0.83535859
26	0.42316244	0.78827539	3.89E-60	3.97E-10	1.06E-88	0.75605497	0.83403424
26.1	0.42500335	0.79156862	4.87E-60	4.07E-10	1.25E-88	0.75511462	0.8361008
26.2	0.41628164	0.8010681	5.64E-60	3.87E-10	1.40E-88	0.7592754	0.82897781
26.3	0.41247506	0.79625806	5.23E-60	4.07E-10	1.29E-88	0.75818104	0.8415642
26.4	0.41333383	0.79191721	5.34E-60	4.01E-10	1.31E-88	0.75333262	0.84286359
26.5	0.41311162	0.78537685	6.86E-60	4.60E-10	1.68E-88	0.75664466	0.84659335
26.6	0.41311162	0.78537685	6.86E-60	4.60E-10	1.68E-88	0.75664466	0.84659335
26.7	0.41632721	0.79286275	6.48E-60	4.94E-10	1.47E-88	0.76113852	0.84251065
26.8	0.41395496	0.78548361	7.37E-60	5.40E-10	1.62E-88	0.76582423	0.84153383

Time discretization	V_{[G]}	K_{[G]}	q	V_{[G3P]}	alpha	G_E	[G3P]_0
30	0.35001464	0.75779021	9.01E-58	3.26E-09	1.82E-86	0.81395786	0.90750547
30.1	0.35246183	0.752222	9.54E-58	3.40E-09	1.78E-86	0.8130778	0.9176845
30.2	0.35280431	0.74294041	1.03E-57	3.62E-09	2.02E-86	0.81047669	0.93301555
30.3	0.34585519	0.7470199	1.04E-57	3.85E-09	2.09E-86	0.82314262	0.92687341
30.4	0.34649831	0.74104778	1.23E-57	3.77E-09	2.35E-86	0.8284686	0.93115223
30.5	0.34180321	0.74048204	1.48E-57	3.94E-09	3.09E-86	0.83291151	0.92944777
30.6	0.3350851	0.75237829	1.71E-57	4.17E-09	3.76E-86	0.82940955	0.91482678
30.7	0.3350851	0.75237829	1.71E-57	4.17E-09	3.76E-86	0.82940955	0.91482678
30.8	0.3284852	0.75478997	1.88E-57	4.05E-09	4.04E-86	0.83756876	0.90312641
30.9	0.32413951	0.75852511	2.00E-57	4.30E-09	3.81E-86	0.84021615	0.88791745
31	0.33119633	0.76076712	2.25E-57	4.51E-09	4.03E-86	0.84694092	0.87809013
31.1	0.33313746	0.75382878	2.75E-57	4.23E-09	5.51E-86	0.8243483	0.88329743
31.2	0.33313746	0.75382878	2.75E-57	4.23E-09	5.51E-86	0.8243483	0.88329743
31.3	0.34234857	0.74346585	4.18E-57	4.51E-09	8.29E-86	0.82476934	0.87554764
31.4	0.34770314	0.73296833	4.83E-57	4.76E-09	1.05E-85	0.82875788	0.87914571
31.5	0.34858335	0.74960094	5.58E-57	5.80E-09	1.48E-85	0.81504596	0.8665438
31.6	0.33733504	0.74931302	6.66E-57	5.75E-09	1.50E-85	0.84625898	0.85191848
31.7	0.33583118	0.75346862	6.83E-57	6.19E-09	1.72E-85	0.8505008	0.84971158
31.8	0.32730235	0.71529786	8.81E-57	6.68E-09	2.46E-85	0.85400391	0.87306396
31.9	0.32707248	0.7101229	9.67E-57	7.15E-09	2.78E-85	0.84983388	0.8755842
32	0.33083458	0.70057669	1.20E-56	7.52E-09	3.84E-85	0.84137559	0.87298727
32.1	0.32735372	0.68659898	1.35E-56	8.20E-09	4.17E-85	0.85900578	0.88528845
32.2	0.31674258	0.67839779	1.55E-56	8.79E-09	5.26E-85	0.86796636	0.88795647
32.3	0.30981165	0.67752809	1.55E-56	8.36E-09	4.82E-85	0.86475831	0.89057908
32.4	0.30699636	0.67359911	1.66E-56	8.42E-09	5.11E-85	0.87881938	0.88058521
32.5	0.3104206	0.67610894	2.00E-56	8.67E-09	5.27E-85	0.89181628	0.87830078
32.6	0.30928247	0.67598631	2.39E-56	8.92E-09	5.92E-85	0.89516874	0.88851589
32.7	0.30949094	0.66549319	2.67E-56	9.47E-09	7.29E-85	0.9004083	0.89211288
32.8	0.30833093	0.66958711	3.01E-56	1.16E-08	7.59E-85	0.89259506	0.89741703
32.9	0.30339638	0.65809268	3.36E-56	1.11E-08	8.38E-85	0.90362756	0.90873075
33	0.29630529	0.65885108	3.28E-56	1.10E-08	7.26E-85	0.89391506	0.9060031
33.1	0.29736883	0.63195382	4.63E-56	1.40E-08	1.15E-84	0.90256089	0.90624488
33.2	0.29222502	0.63126984	6.68E-56	1.61E-08	1.74E-84	0.90482372	0.89771525
33.3	0.28875143	0.63468895	7.26E-56	1.62E-08	1.81E-84	0.90819641	0.89753662
33.4	0.28531587	0.63523121	7.26E-56	1.58E-08	1.83E-84	0.91614297	0.89642494
33.5	0.28531587	0.63523121	7.26E-56	1.58E-08	1.83E-84	0.91614297	0.89642494
33.6	0.28567573	0.63171297	1.15E-55	2.08E-08	3.61E-84	0.90769134	0.89598053
33.7	0.28509893	0.61482739	1.24E-55	2.19E-08	3.60E-84	0.92741256	0.88239761
33.8	0.28186093	0.59364809	1.27E-55	2.48E-08	4.03E-84	0.92894034	0.87255074
33.9	0.28123123	0.59678884	1.45E-55	2.44E-08	4.34E-84	0.93448732	0.87066374
34	0.28373597	0.59212879	1.44E-55	2.69E-08	4.18E-84	0.93024096	0.87698025
34.1	0.28749588	0.59364259	1.51E-55	2.71E-08	4.10E-84	0.92730028	0.87407624
34.2	0.28109432	0.59644429	1.47E-55	2.85E-08	3.99E-84	0.93613511	0.86489293
34.3	0.28103102	0.58276808	1.56E-55	2.96E-08	3.49E-84	0.95011909	0.86517936
34.4	0.28617434	0.57072353	1.86E-55	3.14E-08	4.25E-84	0.94246552	0.88230777
34.5	0.2865992	0.56775104	1.89E-55	3.23E-08	4.25E-84	0.9482773	0.88321892
34.6	0.28752082	0.56509247	2.80E-55	3.63E-08	5.89E-84	0.93521985	0.89239717
34.7	0.27235662	0.55675054	3.51E-55	4.14E-08	7.10E-84	0.92926656	0.88049027
34.8	0.2765915	0.54478707	4.01E-55	4.37E-08	7.31E-84	0.92924926	0.87405087
34.9	0.26148048	0.53251759	4.39E-55	4.78E-08	6.82E-84	0.95186991	0.85887305
35	0.25745893	0.52842329	4.21E-55	4.60E-08	5.61E-84	0.95163694	0.85570915
35.1	0.25774335	0.5169518	5.13E-55	4.91E-08	6.79E-84	0.95776914	0.85977832
35.2	0.25768391	0.51165073	6.72E-55	5.52E-08	9.66E-84	0.95528716	0.85786533
35.3	0.25750982	0.50848836	7.79E-55	5.68E-08	1.15E-83	0.95438247	0.85814732
35.4	0.25141125	0.51018429	7.94E-55	5.77E-08	1.03E-83	0.95507189	0.85879317
35.5	0.24952711	0.49853737	9.88E-55	6.37E-08	1.25E-83	0.96112007	0.85927911
35.6	0.25349551	0.49980479	1.04E-54	6.14E-08	1.28E-83	0.95455354	0.86313317
35.7	0.25244883	0.50004518	1.24E-54	6.90E-08	1.67E-83	0.94324001	0.86565095
35.8	0.25257715	0.50735147	1.10E-54	6.86E-08	1.69E-83	0.95812967	0.87165717
35.9	0.24333464	0.50790773	1.23E-54	7.40E-08	1.90E-83	0.96181569	0.86739445
36	0.24240111	0.50834641	1.66E-54	8.08E-08	2.53E-83	0.99392967	0.85373901
36.1	0.23304618	0.49406908	1.49E-54	9.79E-08	1.95E-83	0.98029997	0.8668108
36.2	0.23293303	0.48972802	1.76E-54	1.04E-07	2.10E-83	0.97627228	0.87803509
36.3	0.23578701	0.4757078	2.17E-54	1.24E-07	2.87E-83	0.98848665	0.89406668
36.4	0.23828849	0.48335304	2.35E-54	1.28E-07	3.12E-83	0.99393582	0.89303569
36.5	0.23639368	0.47735226	3.06E-54	1.43E-07	4.17E-83	0.97531113	0.89714629
36.6	0.2356758	0.47932091	3.06E-54	1.44E-07	3.95E-83	0.96914885	0.8927687
36.7	0.23077277	0.46978042	3.45E-54	1.64E-07	4.84E-83	0.94996177	0.88603904
36.8	0.22587263	0.46482335	6.08E-54	2.07E-07	9.41E-83	0.97413016	0.90276758
36.9	0.22633969	0.46603338	5.91E-54	2.14E-07	9.63E-83	0.97825325	0.90176911
37	0.22264471	0.46395126	5.23E-54	2.13E-07	7.61E-83	0.9823958	0.8949435
37.1	0.2246014	0.45353964	5.76E-54	2.09E-07	8.03E-83	0.99039891	0.90161613
37.2	0.22474362	0.44644179	6.98E-54	2.14E-07	8.47E-83	0.97416819	0.9134298
37.3	0.22542801	0.43393221	1.03E-53	2.43E-07	1.47E-82	0.97538641	0.93971883
37.4	0.22495574	0.43053242	1.23E-53	2.82E-07	1.70E-82	0.98209183	0.94295191
37.5	0.22965664	0.4162916	1.46E-53	3.12E-07	2.21E-82	0.97778689	0.9487526
37.6	0.2291816	0.39769554	2.85E-53	4.27E-07	5.60E-82	0.98177938	0.94282462
37.7	0.22450304	0.36436966	4.85E-53	5.44E-07	8.86E-82	0.99492952	0.9521545
37.8	0.23665892	0.34662534	6.12E-53	5.83E-07	1.22E-81	0.99186261	0.96058136
37.9	0.23266744	0.33677201	8.37E-53	6.83E-07	1.87E-81	0.99004057	0.96520763
38	0.23532491	0.32376633	9.64E-53	7.63E-07	2.21E-81	0.97600368	0.97001524
38.1	0.22774947	0.31745332	1.15E-52	8.05E-07	2.60E-81	0.95432588	0.97831991
38.2	0.22581089	0.31343029	1.35E-52	7.97E-07	2.58E-81	0.96436423	0.98296853
38.3	0.22450853	0.30722762	2.10E-52	9.71E-07	4.48E-81	0.97272824	0.98727314
38.4	0.22188317	0.29294142	3.04E-52	1.09E-06	6.49E-81	0.9713349	0.99857242
38.5	0.2256168	0.29717806	4.11E-52	1.20E-06	8.67E-81	0.96053893	0.99306303
38.6	0.22675366	0.28818765	7.49E-52	1.42E-06	1.48E-80	0.94963698	0.99761265
38.7	0.22619223	0.28272836	9.63E-52	1.43E-06	1.91E-80	0.95074349	0.99784477
38.8	0.23071224	0.26994622	1.33E-51	1.73E-06	2.60E-80	0.94731818	0.98846817
38.9	0.24127128	0.26499354	1.98E-51	1.86E-06	2.74E-80	0.92983942	0.98694767
39	0.24214678	0.25832355	2.56E-51	2.19E-06	3.27E-80	0.91831055	0.99572738
39.1	0.23728234	0.25336889	3.72E-51	2.43E-06	4.05E-80	0.89547546	0.98670027
39.2	0.23634297	0.24145771	4.57E-51	2.75E-06	5.51E-80	0.90335224	0.99822679
39.3	0.23208664	0.24170898	5.20E-51	2.88E-06	5.92E-80	0.90498177	0.99192494
39.4	0.22260096	0.23104731	7.73E-51	3.23E-06	7.71E-80	0.90574935	0.98281899
39.5	0.22370819	0.21613009	1.28E-50	3.85E-06	1.38E-79	0.88670632	0.96550739
39.6	0.2245203	0.20286282	1.90E-50	4.18E-06	2.07E-79	0.87584803	0.96271874
39.7	0.21763327	0.19464756	2.90E-50	4.84E-06	2.73E-79	0.85140337	0.93725952
39.8	0.20950304	0.19173288	4.78E-50	5.15E-06	4.17E-79	0.85164695	0.91647568
39.9	0.20392542	0.18793809	6.14E-50	5.67E-06	4.68E-79	0.84763884	0.92717827
40	0.20263433	0.18572244	1.35E-49	6.86E-06	1.07E-78	0.86145355	0.92816332
40.1	0.19967612	0.18872975	1.70E-49	7.27E-06	1.23E-78	0.8459741	0.92671636
40.2	0.19775916	0.18479724	2.10E-49	7.19E-06	1.15E-78	0.8483259	0.91887761
40.3	0.19622986	0.17515327	2.81E-49	7.68E-06	1.71E-78	0.8461207	0.90576613
40.4	0.191409	0.16870681	3.25E-49	8.56E-06	1.74E-78	0.83875351	0.89564885
40.5	0.19240835	0.16641343	4.43E-49	8.84E-06	2.24E-78	0.83390265	0.90021175
40.6	0.18888836	0.16779835	5.71E-49	9.07E-06	2.96E-78	0.83758665	0.89942244
40.7	0.19099716	0.16225753	7.68E-49	1.01E-05	3.56E-78	0.82972157	0.88371907
40.8	0.19099716	0.16225753	7.68E-49	1.01E-05	3.56E-78	0.82972157	0.88371907
40.9	0.1897746	0.14937754	1.44E-48	1.30E-05	5.84E-78	0.84195178	0.88662434
41	0.18329943	0.14629873	2.46E-48	1.37E-05	1.16E-77	0.81835964	0.89408562
41.1	0.18213704	0.14185138	3.73E-48	1.51E-05	1.75E-77	0.79473335	0.89545327
41.2	0.17107475	0.13887825	4.91E-48	1.80E-05	2.00E-77	0.79224629	0.89047752
41.3	0.16718389	0.13617879	1.02E-47	1.93E-05	3.69E-77	0.76533372	0.88230426
41.4	0.17572766	0.12842757	3.53E-47	2.53E-05	1.48E-76	0.76995671	0.86781045
41.5	0.17849321	0.12283348	3.62E-47	2.67E-05	1.78E-76	0.76053287	0.85288853
41.6	0.1802553	0.1147172	5.54E-47	2.77E-05	2.41E-76	0.76122913	0.84494603
41.7	0.17144406	0.11435964	8.27E-47	3.23E-05	3.68E-76	0.78349225	0.84183233
41.8	0.16350707	0.11330711	9.66E-47	3.69E-05	3.32E-76	0.79997578	0.8388647

Time discretization	V_{[G]}	K_{[G]}	q	V_{[G3P]}	alpha	G_E	[G3P]_0
45	0.08912584	0.03799987	5.02E-42	0.00025926	1.30E-72	0.54719937	0.77996706
45.1	0.08407536	0.03626207	6.66E-42	0.00025575	1.60E-72	0.54544505	0.76021859
45.2	0.08227528	0.0353454	8.36E-42	0.00025284	1.88E-72	0.54507526	0.74014589
45.3	0.08221578	0.03586118	1.34E-41	0.00027628	3.28E-72	0.55154786	0.74014375
45.4	0.07813145	0.03548605	1.67E-41	0.00027963	3.86E-72	0.55045736	0.721631
45.5	0.07627311	0.03455909	2.00E-41	0.00029382	4.11E-72	0.54236363	0.73184515
45.6	0.07162385	0.03366472	3.39E-41	0.00035738	5.48E-72	0.56973185	0.72484049
45.7	0.06965259	0.03253421	4.09E-41	0.00036206	6.42E-72	0.57715602	0.70908708
45.8	0.06919764	0.03215696	4.85E-41	0.0003666	7.24E-72	0.58010169	0.69934248
45.9	0.06774845	0.03194583	6.84E-41	0.000337	9.49E-72	0.599606	0.67843835
46	0.06649992	0.03193249	9.90E-41	0.00036962	1.36E-71	0.6084441	0.67973389
46.1	0.06573882	0.03148135	1.19E-40	0.00039312	1.76E-71	0.60119468	0.66314594
46.2	0.06439138	0.03176344	1.56E-40	0.0003991	2.17E-71	0.61110358	0.66030256
46.3	0.06015887	0.03263384	2.11E-40	0.000404	2.51E-71	0.60524103	0.65052466
46.4	0.05922789	0.03324404	4.00E-40	0.00042938	3.87E-71	0.59834913	0.64918934
46.5	0.05874582	0.03257259	4.87E-40	0.00046142	4.25E-71	0.57878333	0.67017418
46.6	0.05894898	0.03209463	5.42E-40	0.00050164	4.40E-71	0.56898868	0.67927583
46.7	0.05584565	0.03128271	6.15E-40	0.0005196	4.64E-71	0.5635815	0.68032274
46.8	0.05539577	0.03183958	7.21E-40	0.00051288	5.54E-71	0.56118946	0.66143304
46.9	0.05327331	0.03118712	1.32E-39	0.00052221	7.59E-71	0.53425257	0.66466529
47	0.05132328	0.0309967	1.42E-39	0.00053538	7.43E-71	0.53957448	0.66140298
47.1	0.05084102	0.03013261	1.64E-39	0.00051605	7.82E-71	0.53493171	0.65623791
47.2	0.04935008	0.02904567	2.29E-39	0.00057556	9.52E-71	0.54830701	0.64692429
47.3	0.04857904	0.02904494	3.07E-39	0.00058167	1.22E-70	0.55684829	0.6368623
47.4	0.04862062	0.02930438	4.37E-39	0.00062244	1.68E-70	0.55412271	0.62786154
47.5	0.04577254	0.02827271	4.73E-39	0.00064226	1.41E-70	0.54400722	0.63307488
47.6	0.04826013	0.02538454	7.29E-39	0.0007172	2.09E-70	0.55241261	0.61538708
47.7	0.04615439	0.02426402	1.08E-38	0.00080439	2.39E-70	0.54196288	0.6036422
47.8	0.04416387	0.02399896	1.44E-38	0.00080167	2.95E-70	0.55500024	0.60706597
47.9	0.04331543	0.02262258	2.37E-38	0.00077186	4.27E-70	0.56065393	0.61832023
48	0.04190431	0.02238532	2.71E-38	0.00083637	4.36E-70	0.54571713	0.61171775
48.1	0.03917488	0.02175559	3.66E-38	0.00087631	4.50E-70	0.55340685	0.63304979
48.2	0.03794646	0.02181121	4.56E-38	0.00089804	5.08E-70	0.55724065	0.6234101
48.3	0.03600255	0.02116705	7.14E-38	0.00083742	5.77E-70	0.55302713	0.61955892
48.4	0.03537124	0.01968066	1.22E-37	0.00091956	1.00E-69	0.56694691	0.62318444
48.5	0.03449087	0.01969683	1.73E-37	0.00092551	1.44E-69	0.56487773	0.61684011
48.6	0.03272003	0.01892583	4.19E-37	0.00092339	3.21E-69	0.57261086	0.59176586
48.7	0.03229752	0.01886045	5.09E-37	0.00100431	3.42E-69	0.56951841	0.60145627
48.8	0.03177683	0.01750329	6.91E-37	0.00105726	4.44E-69	0.57032124	0.59419683
48.9	0.03162805	0.01651869	9.99E-37	0.0010451	5.51E-69	0.58692642	0.5933366
49	0.03060229	0.01666946	1.21E-36	0.00100455	5.39E-69	0.58885437	0.586129
49.1	0.0318778	0.01658805	1.60E-36	0.00109626	6.53E-69	0.58230607	0.56875215
49.2	0.03051033	0.01643594	2.00E-36	0.00113564	6.83E-69	0.57566895	0.56274003
49.3	0.02833543	0.01675175	3.11E-36	0.00117097	1.09E-68	0.57618423	0.57171209
49.4	0.02810801	0.01630474	4.47E-36	0.00123044	1.15E-68	0.57551425	0.57233355
49.5	0.0274484	0.01654602	6.26E-36	0.00128998	1.40E-68	0.55748758	0.57750058
49.6	0.02565634	0.01649019	7.62E-36	0.00135784	1.51E-68	0.56306541	0.59515457
49.7	0.02473116	0.0164821	1.26E-35	0.00149401	2.62E-68	0.55754622	0.60255455
49.8	0.02363088	0.0166343	1.35E-35	0.00150118	2.47E-68	0.55667884	0.60347273
49.9	0.02343596	0.01664333	1.28E-35	0.00150214	2.54E-68	0.55321975	0.60122632
50	0.02183039	0.01529322	1.89E-35	0.00165751	2.74E-68	0.55245468	0.61640868
50.1	0.01929399	0.01542764	3.37E-35	0.00173215	3.70E-68	0.55322358	0.59930067
50.2	0.01833003	0.01550391	4.97E-35	0.0019629	4.60E-68	0.54653694	0.60008026
50.3	0.01755876	0.01553114	7.13E-35	0.00204563	6.19E-68	0.53771712	0.59373203
50.4	0.01742816	0.01511385	8.69E-35	0.00214215	7.30E-68	0.52563199	0.59296853
50.5	0.01731641	0.01582684	1.23E-34	0.00223189	1.00E-67	0.52277597	0.58747043
50.6	0.0160973	0.01571128	1.50E-34	0.00230635	1.01E-67	0.51508193	0.58273224
50.7	0.01671226	0.01483859	1.97E-34	0.00247502	1.58E-67	0.50401492	0.57746604
50.8	0.01678193	0.01494188	2.10E-34	0.00266567	1.50E-67	0.51717227	0.58564716
50.9	0.01503618	0.01471715	3.23E-34	0.00268723	1.60E-67	0.52606601	0.58343309
51	0.01468177	0.01448971	5.14E-34	0.00286052	2.49E-67	0.53365801	0.59433047
51.1	0.01374105	0.01243027	7.55E-34	0.00313457	2.63E-67	0.5211499	0.58494789
51.2	0.01313195	0.01245893	9.31E-34	0.00300418	3.10E-67	0.51634374	0.56954344
51.3	0.0126541	0.01190337	1.56E-33	0.00311678	4.28E-67	0.51811885	0.55402185
51.4	0.01257439	0.0117584	2.06E-33	0.0032797	6.40E-67	0.52690839	0.5423754
51.5	0.011781	0.01175362	2.33E-33	0.00328894	6.87E-67	0.52912548	0.54230144
51.6	0.01175635	0.01199321	3.85E-33	0.0034874	8.70E-67	0.53786964	0.52764054
51.7	0.01117025	0.01252632	5.84E-33	0.00361602	1.29E-66	0.53176884	0.53191692
51.8	0.01096042	0.01260819	8.66E-33	0.00393933	1.68E-66	0.53285447	0.54220085
51.9	0.0102372	0.01250261	1.01E-32	0.00415782	1.49E-66	0.5329923	0.52687813
52	0.00967232	0.01231546	1.34E-32	0.00412111	1.24E-66	0.54238629	0.51972004
52.1	0.00918242	0.01195192	2.03E-32	0.00400923	1.61E-66	0.55007985	0.51755216
52.2	0.00929622	0.01144511	3.37E-32	0.00407274	2.57E-66	0.56003485	0.49862644
52.3	0.00863362	0.01160481	4.10E-32	0.00409943	2.57E-66	0.55843748	0.50447366
52.4	0.00834462	0.01128346	4.65E-32	0.00417763	2.73E-66	0.55654348	0.49714293
52.5	0.00839481	0.0116813	6.74E-32	0.00445492	3.09E-66	0.57131511	0.49770343
52.6	0.0079601	0.01177754	9.91E-32	0.00507539	4.23E-66	0.57717856	0.50814169
52.7	0.00741741	0.01202146	1.27E-31	0.00517796	4.76E-66	0.56835617	0.51961546
52.8	0.00664916	0.01264631	2.39E-31	0.00582953	7.53E-66	0.58873754	0.51889555
52.9	0.00655977	0.01218651	2.92E-31	0.00578478	8.92E-66	0.58746729	0.51893263
53	0.00605824	0.01174691	3.61E-31	0.00610403	9.81E-66	0.59681155	0.51544901
53.1	0.00619185	0.01178927	5.19E-31	0.00655109	1.29E-65	0.59454598	0.50655416
53.2	0.0056485	0.0114916	6.39E-31	0.00712137	1.38E-65	0.59895791	0.5019881
53.3	0.00573431	0.0111083	7.97E-31	0.00750488	1.66E-65	0.59618147	0.49569795
53.4	0.00507892	0.01100974	1.34E-30	0.00840054	1.84E-65	0.57630729	0.49781608
53.5	0.00452062	0.01036133	2.00E-30	0.00984541	2.13E-65	0.56814941	0.50055666
53.6	0.00424578	0.00993093	2.78E-30	0.010418	1.83E-65	0.58262907	0.5034584
53.7	0.00403275	0.00889628	4.54E-30	0.01115375	2.12E-65	0.58075979	0.51093203
53.8	0.00384872	0.00875929	4.82E-30	0.01130007	2.02E-65	0.57380342	0.51014395
53.9	0.00351605	0.00785995	9.02E-30	0.01331761	1.94E-65	0.57531395	0.4937036
54	0.00306608	0.00761815	1.12E-29	0.0137085	1.98E-65	0.57419656	0.47644292
54.1	0.00307084	0.00663628	1.51E-29	0.01435858	2.05E-65	0.57756916	0.47695924
54.2	0.00275787	0.00663642	2.46E-29	0.01513613	2.78E-65	0.56136341	0.46979536
54.3	0.00255124	0.00624834	3.27E-29	0.0155369	2.53E-65	0.564161	0.4686922
54.4	0.00243454	0.00592204	4.52E-29	0.01620964	2.97E-65	0.57597718	0.46491671
54.5	0.00243883	0.00546871	6.22E-29	0.01622415	3.33E-65	0.57049733	0.45715644
54.6	0.00222803	0.00558548	9.03E-29	0.01716189	3.87E-65	0.56264455	0.46571796
54.7	0.00212847	0.00502447	1.24E-28	0.01582216	3.52E-65	0.5691128	0.45856638
54.8	0.00203469	0.00499685	1.31E-28	0.01666474	3.32E-65	0.56754595	0.4571973
54.9	0.00183859	0.00518542	2.42E-28	0.01704601	4.81E-65	0.56092968	0.45691136
55	0.00181143	0.00505227	3.73E-28	0.01788382	5.68E-65	0.56448005	0.45727458
55.1	0.001717	0.00529487	6.10E-28	0.01698433	1.00E-64	0.55746312	0.46920166
55.2	0.00169988	0.00465837	9.83E-28	0.01790497	1.27E-64	0.55589756	0.46842708
55.3	0.00167078	0.00472363	1.26E-27	0.01880602	1.57E-64	0.5607647	0.47181725
55.4	0.00168896	0.00466611	1.80E-27	0.01971227	2.31E-64	0.55705139	0.48001441
55.5	0.00159057	0.00423268	2.48E-27	0.01977902	2.58E-64	0.5542365	0.4854189
55.6	0.00155082	0.00408466	3.22E-27	0.02042854	2.95E-64	0.55147692	0.48075398
55.7	0.00148333	0.0039964	4.24E-27	0.02117552	3.03E-64	0.54540482	0.48872368
55.8	0.00137857	0.00396686	4.67E-27	0.02187451	3.20E-64	0.53983758	0.48759755
55.9	0.00138376	0.00360732	7.20E-27	0.02287514	3.84E-64	0.54818243	0.48985597
56	0.00137804	0.00354493	8.07E-27	0.02389254	4.12E-64	0.54599256	0.49064492
56.1	0.00129947	0.00351237	8.81E-27	0.02470917	4.63E-64	0.54267225	0.50141919
56.2	0.00128667	0.00343591	1.79E-26	0.02632047	7.47E-64	0.53106908	0.49810336
56.3	0.00122494	0.00321185	2.18E-26	0.02552594	6.94E-64	0.53098817	0.48866631
56.4	0.00116822	0.00299306	2.90E-26	0.02659683	7.18E-64	0.51480091	0.49797967
56.5	0.00108954	0.00301255	3.34E-26	0.02732004	6.97E-64	0.5179525	0.49835215
56.6	0.00109275	0.00295487	4.50E-26	0.02644881	8.01E-64	0.51217449	0.5040714
56.7	0.00101651	0.0028404	6.41E-26	0.02609007	9.35E-64	0.50187521	0.50453737
56.8	0.00099727	0.00273204	7.64E-26	0.02516556	9.99E-64	0.49185156	0.50041382

11.9	0.52746989	0.7762739	5.97E-70	1.02E-13	7.32E-95	0.903753	0.58319061
12	0.52036893	0.78297092	6.11E-70	1.14E-13	7.35E-95	0.90419055	0.59389126
12.1	0.51963633	0.78752898	7.81E-70	1.15E-13	8.55E-95	0.89636717	0.5759433
12.2	0.52365224	0.77840806	9.67E-70	1.39E-13	8.92E-95	0.88061853	0.58047844
12.3	0.52785074	0.78746097	1.54E-69	1.64E-13	1.05E-94	0.88626906	0.57733011
12.4	0.51713475	0.78566238	2.04E-69	1.74E-13	1.32E-94	0.90248593	0.57655107
12.5	0.50950015	0.78650824	2.43E-69	1.81E-13	1.44E-94	0.9082466	0.57878066
12.6	0.51421765	0.80448243	3.25E-69	2.20E-13	1.59E-94	0.90289628	0.57362899
12.7	0.5307292	0.81497867	4.90E-69	2.26E-13	1.96E-94	0.91155553	0.56658827
12.8	0.5288245	0.80819235	4.51E-69	2.01E-13	1.66E-94	0.89683753	0.57157099
12.9	0.54163145	0.84692887	7.12E-69	2.36E-13	2.03E-94	0.8990044	0.56656806
13	0.54020477	0.83943769	9.34E-69	2.57E-13	2.42E-94	0.90367111	0.56556321
13.1	0.54484248	0.83371842	1.15E-68	2.83E-13	2.44E-94	0.88984319	0.55865565
13.2	0.53235034	0.83996039	1.35E-68	2.74E-13	2.55E-94	0.8769026	0.56313804
13.3	0.53235034	0.83996039	1.35E-68	2.74E-13	2.55E-94	0.8769026	0.56313804
13.4	0.52227083	0.85530122	1.66E-68	2.90E-13	3.01E-94	0.87670854	0.56599013
13.5	0.51476153	0.84930428	1.81E-68	2.62E-13	2.74E-94	0.86240111	0.56806497
13.6	0.51424623	0.8565333	2.52E-68	2.65E-13	3.59E-94	0.86901928	0.57491212
13.7	0.51424623	0.8565333	2.52E-68	2.65E-13	3.59E-94	0.86901928	0.57491212
13.8	0.51561792	0.84834248	5.18E-68	2.42E-13	5.42E-94	0.89363361	0.5695767
13.9	0.51561792	0.84834248	5.18E-68	2.42E-13	5.42E-94	0.89363361	0.5695767
14	0.51027871	0.86157767	5.73E-68	2.44E-13	5.23E-94	0.88861579	0.57003132
14.1	0.53730681	0.87544979	7.19E-68	2.88E-13	6.12E-94	0.88532863	0.55104559
14.2	0.53398485	0.86770714	8.48E-68	3.24E-13	6.54E-94	0.87391181	0.53787302
14.3	0.54029629	0.86937114	1.02E-67	3.61E-13	7.59E-94	0.8785908	0.54344765
14.4	0.54043188	0.8769056	9.97E-68	3.53E-13	7.84E-94	0.88028623	0.54735688
14.5	0.54203319	0.88148458	1.11E-67	3.68E-13	7.72E-94	0.87115148	0.54799328
14.6	0.54088959	0.88928267	1.01E-67	3.73E-13	7.02E-94	0.85759481	0.5534279
14.7	0.53042099	0.8846134	1.10E-67	3.85E-13	7.24E-94	0.86553887	0.55529795
14.8	0.53759403	0.87538204	1.44E-67	4.84E-13	7.19E-94	0.84603111	0.55449141
14.9	0.52875281	0.88269816	1.60E-67	5.12E-13	7.30E-94	0.85586045	0.54643939
60	0.00026068	0.0021276	1.25E-22	0.05842317	2.10E-62	0.30829626	0.57895351
60.1	0.00025207	0.00207312	1.57E-22	0.060084	2.38E-62	0.31236323	0.57552996
60.2	0.00025631	0.00201717	1.89E-22	0.06179202	2.57E-62	0.30608338	0.58199144
60.3	0.00023828	0.0020212	2.44E-22	0.06431009	3.02E-62	0.29407957	0.59198718
60.4	0.00022861	0.00199069	2.98E-22	0.06151034	3.09E-62	0.29004416	0.57674933
60.5	0.00023163	0.00202865	3.27E-22	0.06243582	2.95E-62	0.28438452	0.58621641
60.6	0.00022922	0.00207272	4.29E-22	0.06304014	3.68E-62	0.27786026	0.58416834
60.7	0.00024371	0.00201193	6.00E-22	0.06432111	4.75E-62	0.27286397	0.5728385
60.8	0.00021733	0.00194222	7.67E-22	0.06621159	5.58E-62	0.26638842	0.58156443
60.9	0.00020705	0.0018763	9.26E-22	0.06749724	6.12E-62	0.26368863	0.57292223
61	0.00019983	0.0018047	9.43E-22	0.06918401	6.19E-62	0.26360642	0.57574678
61.1	0.00019449	0.00168557	1.07E-21	0.07215344	7.63E-62	0.26586081	0.5678402
61.2	0.0001866	0.00159772	1.43E-21	0.07119616	6.64E-62	0.26200051	0.56699168
61.3	0.00018627	0.00159247	1.51E-21	0.07085135	6.47E-62	0.26078244	0.5718705
61.4	0.00016928	0.00152517	2.05E-21	0.07400565	8.12E-62	0.25000645	0.5771629
61.5	0.00017282	0.00159575	2.94E-21	0.07568371	9.95E-62	0.24577869	0.57698166
61.6	0.00016725	0.00159812	4.05E-21	0.07624569	1.28E-61	0.2414402	0.5678578
61.7	0.00016354	0.00160895	5.34E-21	0.07477757	1.45E-61	0.23410478	0.57048605
61.8	0.00016072	0.0016106	6.65E-21	0.07517651	1.70E-61	0.22996688	0.5762389
61.9	0.00015537	0.00155975	8.20E-21	0.07423654	1.64E-61	0.22418813	0.57300472
62	0.00014135	0.00165776	1.12E-20	0.07881279	1.92E-61	0.2194005	0.59172882
62.1	0.00014145	0.00157855	1.30E-20	0.07978383	2.00E-61	0.21961815	0.59626904
62.2	0.0001406	0.00150383	1.42E-20	0.07926511	1.91E-61	0.21904011	0.5892455
62.3	0.00013568	0.0015053	1.64E-20	0.07939349	2.04E-61	0.22495412	0.59427669
62.4	0.00013164	0.00143271	1.98E-20	0.07945073	2.47E-61	0.22021874	0.57545146
62.5	0.00013534	0.00143005	2.38E-20	0.08330528	2.68E-61	0.21633458	0.57830923
62.6	0.00014248	0.00142716	3.13E-20	0.08487297	3.03E-61	0.21574035	0.57146481
62.7	0.000136	0.00131749	4.05E-20	0.07999941	3.41E-61	0.20402591	0.56594811
62.8	0.00011945	0.00132622	5.35E-20	0.08224549	3.17E-61	0.19952143	0.56276234
62.9	0.00011411	0.00123929	6.55E-20	0.08284212	2.94E-61	0.20126181	0.56624147
63	0.00011049	0.0011494	7.80E-20	0.08371013	3.61E-61	0.19521384	0.56970665
63.1	0.00010156	0.00105765	8.36E-20	0.08359233	3.64E-61	0.19869421	0.5652957
63.2	9.29E-05	0.00105893	9.58E-20	0.08389207	3.67E-61	0.19222207	0.56954625
63.3	9.22E-05	0.00101903	1.11E-19	0.08852048	4.18E-61	0.18727092	0.57351159
63.4	8.59E-05	0.00103971	1.34E-19	0.08775425	3.86E-61	0.18081814	0.58173817
63.5	8.86E-05	0.00100399	1.68E-19	0.092327	5.47E-61	0.17954775	0.59714825
63.6	8.39E-05	0.00097132	2.45E-19	0.09145337	6.67E-61	0.1744549	0.60486085
63.7	7.93E-05	0.00098277	3.35E-19	0.09681201	7.82E-61	0.16874612	0.60810038
63.8	7.67E-05	0.00096853	3.68E-19	0.09547561	8.22E-61	0.16528041	0.60785692
63.9	7.50E-05	0.00092304	4.36E-19	0.09864483	9.22E-61	0.17215884	0.62742966
64	7.48E-05	0.0008988	4.62E-19	0.09907164	8.99E-61	0.16828766	0.63441701
64.1	7.45E-05	0.00081185	6.10E-19	0.09997542	1.17E-60	0.16405786	0.64052348
64.2	7.43E-05	0.00081029	6.38E-19	0.10162362	1.11E-60	0.16170906	0.64519124
64.3	6.81E-05	0.00077936	6.15E-19	0.10410403	1.19E-60	0.16269103	0.65865787
64.4	6.65E-05	0.00076885	7.14E-19	0.10757392	1.29E-60	0.1613428	0.66293639
64.5	6.17E-05	0.0007276	7.38E-19	0.11096829	1.28E-60	0.15956445	0.65692253
64.6	6.00E-05	0.0006782	8.55E-19	0.1151833	1.45E-60	0.16213108	0.66174487
64.7	5.96E-05	0.00064311	1.11E-18	0.11740401	1.48E-60	0.15939226	0.667412
64.8	5.29E-05	0.00059647	1.40E-18	0.11970222	1.73E-60	0.15437034	0.67171495
64.9	5.17E-05	0.00058041	1.63E-18	0.12172203	1.95E-60	0.15279068	0.68020956
65	4.80E-05	0.00057521	1.88E-18	0.12461142	2.12E-60	0.15141285	0.68709749
65.1	4.91E-05	0.00056798	2.27E-18	0.12382389	2.22E-60	0.14668524	0.69167124
65.2	4.61E-05	0.00054087	2.60E-18	0.1249306	2.34E-60	0.14562359	0.69292959
65.3	4.20E-05	0.00055248	2.77E-18	0.12360672	2.17E-60	0.13915688	0.70545352
65.4	4.04E-05	0.00052099	3.60E-18	0.1234466	2.36E-60	0.13585325	0.71221758
65.5	3.79E-05	0.00052604	4.57E-18	0.12721389	3.06E-60	0.1321171	0.71932785
65.6	3.71E-05	0.00051853	5.15E-18	0.12780129	3.70E-60	0.13164133	0.72912107
65.7	3.69E-05	0.00048084	6.20E-18	0.12865355	4.49E-60	0.12682901	0.74400711
65.8	3.45E-05	0.00045179	6.35E-18	0.13541554	4.94E-60	0.11847968	0.75419096
65.9	3.54E-05	0.00041854	7.39E-18	0.13469735	5.04E-60	0.12095671	0.75329019
66	3.44E-05	0.0004293	8.67E-18	0.13717802	5.26E-60	0.11815598	0.75628453
66.1	3.48E-05	0.0004101	8.97E-18	0.13835718	4.95E-60	0.11831771	0.75995832
66.2	3.20E-05	0.00039999	9.82E-18	0.13830045	4.97E-60	0.11411391	0.75938208
66.3	2.99E-05	0.00043645	1.12E-17	0.13529411	4.32E-60	0.11115402	0.76411767
66.4	3.01E-05	0.000437	1.20E-17	0.13835171	4.61E-60	0.10980414	0.76848885
66.5	2.84E-05	0.00041303	1.30E-17	0.13748621	4.56E-60	0.11101212	0.77650086
66.6	2.76E-05	0.0004024	1.45E-17	0.14055354	4.80E-60	0.11112909	0.77556379
66.7	2.52E-05	0.00040154	1.50E-17	0.14310798	4.69E-60	0.10952975	0.7703438
66.8	2.51E-05	0.00039424	1.56E-17	0.14432033	5.38E-60	0.10950042	0.7673195
66.9	2.50E-05	0.00038637	1.74E-17	0.14627806	5.89E-60	0.11008677	0.7625954
67	2.44E-05	0.00039352	1.87E-17	0.14669613	6.06E-60	0.10952087	0.76727513
67.1	2.35E-05	0.0004124	2.32E-17	0.1448785	7.13E-60	0.10859479	0.76440773
67.2	2.38E-05	0.00040355	3.00E-17	0.14783209	1.01E-59	0.11165543	0.77278341
67.3	2.37E-05	0.00040303	3.59E-17	0.14993636	1.19E-59	0.10946932	0.77896323
67.4	2.37E-05	0.00041193	4.98E-17	0.15357232	1.82E-59	0.10920067	0.78744091
67.5	2.29E-05	0.00040228	5.19E-17	0.15639678	2.05E-59	0.10926277	0.79725218
67.6	2.36E-05	0.00041621	5.84E-17	0.16211477	2.31E-59	0.10843439	0.79716366
67.7	2.17E-05	0.00040158	6.43E-17	0.15974244	2.47E-59	0.10637136	0.80096345
67.8	2.21E-05	0.00040055	6.92E-17	0.16251585	2.79E-59	0.10533839	0.80421341
67.9	1.97E-05	0.0003967	7.74E-17	0.16270134	2.54E-59	0.10279742	0.81344306
68	1.74E-05	0.00035411	7.71E-17	0.16610934	2.36E-59	0.10270575	0.81908396
68.1	1.81E-05	0.0003496	9.02E-17	0.16873129	2.62E-59	0.09908025	0.81199816
68.2	1.79E-05	0.00034168	1.08E-16	0.16999679	2.93E-59	0.09616637	0.81908519
68.3	1.71E-05	0.00033431	1.23E-16	0.16942038	3.35E-59	0.09540933	0.82382514
68.4	1.54E-05	0.00032152	1.36E-16	0.17503174	2.74E-59	0.09243363	0.8248119
68.5	1.63E-05	0.00033814	1.68E-16	0.17328478	3.10E-59	0.08730757	0.83685972
68.6	1.57E-05	0.0003483	2.11E-16	0.17515209	3.44E-59	0.08639818	0.85850477
68.7	1.59E-05	0.000352	2.37E-16	0.17537118	3.55E-59	0.08536466	0.86454048
68.8	1.57E-05	0.00034643	2.68E-16	0.1775971	3.88E-59	0.08395068	0.8635304
68.9	1.49E-05	0.00034825	2.98E-16	0.17899759	3.88E-59	0.08265896	0.87327997

26.9	0.41430183	0.77409267	1.01E-59	5.34E-10	2.23E-88	0.76939692	0.8429911
27	0.40841271	0.76778337	1.24E-59	6.65E-10	2.96E-88	0.79315674	0.85596071
27.1	0.4037948	0.76619902	1.31E-59	7.38E-10	3.63E-88	0.79939762	0.84410317
27.2	0.38927743	0.78229975	1.70E-59	8.06E-10	4.37E-88	0.80432323	0.87102902
27.3	0.3869995	0.77407252	2.10E-59	8.86E-10	4.83E-88	0.81668146	0.87904651
27.4	0.38148284	0.77795412	2.98E-59	9.00E-10	7.31E-88	0.80591216	0.90722379
27.5	0.38470795	0.77321707	3.71E-59	9.49E-10	9.70E-88	0.80344938	0.91951818
27.6	0.38613368	0.78351572	4.01E-59	9.50E-10	1.00E-87	0.8077319	0.9242281
27.7	0.37642645	0.78953179	4.35E-59	9.54E-10	9.87E-88	0.8112734	0.91297102
27.8	0.36431453	0.79504721	7.00E-59	1.09E-09	1.29E-87	0.82199417	0.91921054
27.9	0.36072045	0.79549503	7.80E-59	1.16E-09	1.26E-87	0.8218251	0.91521991
28	0.36131181	0.79969918	7.85E-59	1.15E-09	1.09E-87	0.82615678	0.92637965
28.1	0.36393374	0.80389368	7.65E-59	1.22E-09	1.05E-87	0.81870585	0.92560277
28.2	0.36288969	0.80282054	7.30E-59	1.11E-09	1.05E-87	0.8291857	0.92655383
28.3	0.3644504	0.79779228	8.06E-59	1.06E-09	1.16E-87	0.82571368	0.9201348
28.4	0.36730987	0.79873566	1.14E-58	1.27E-09	1.92E-87	0.812848	0.91606193
28.5	0.36014792	0.79185174	1.27E-58	1.50E-09	2.18E-87	0.83560203	0.90269463
28.6	0.35906126	0.78171903	1.37E-58	1.55E-09	2.20E-87	0.84211419	0.89632625
28.7	0.3646569	0.78047945	1.65E-58	1.65E-09	2.64E-87	0.82852406	0.91671136
28.8	0.36984844	0.76026407	2.09E-58	1.76E-09	3.56E-87	0.83290687	0.92431002
28.9	0.36896402	0.75999056	2.35E-58	1.78E-09	4.15E-87	0.82669418	0.9229806
29	0.37130161	0.74828418	2.59E-58	1.96E-09	4.97E-87	0.8169619	0.92489892
29.1	0.36546061	0.75810336	2.83E-58	1.97E-09	5.47E-87	0.81503536	0.92761679
29.2	0.36475952	0.76364119	2.60E-58	1.90E-09	4.74E-87	0.81305015	0.9159628
29.3	0.36017692	0.76639483	3.15E-58	1.96E-09	5.87E-87	0.80526267	0.90860018
29.4	0.37051518	0.76153457	4.27E-58	2.30E-09	8.20E-87	0.79962997	0.91633196
29.5	0.37051518	0.76153457	4.27E-58	2.30E-09	8.20E-87	0.79962997	0.91633196
29.6	0.36491379	0.75217358	4.45E-58	2.36E-09	8.64E-87	0.81110941	0.90763643
29.7	0.3650371	0.75264685	4.80E-58	2.45E-09	1.04E-86	0.804983	0.91236204
29.8	0.35658638	0.75083158	5.99E-58	2.79E-09	1.18E-86	0.81265884	0.90139144
29.9	0.35001464	0.75779021	9.01E-58	3.26E-09	1.82E-86	0.81395786	0.90750547
75	3.36E-06	6.13E-05	1.92E-13	0.28424601	2.07E-58	0.03601705	0.84686873
75.1	3.49E-06	5.98E-05	2.18E-13	0.28975991	2.64E-58	0.0364718	0.83589192
75.2	3.56E-06	5.88E-05	2.37E-13	0.29214798	2.58E-58	0.03572932	0.82644252
75.3	3.73E-06	6.09E-05	2.96E-13	0.29747754	3.48E-58	0.03532364	0.81909662
75.4	3.29E-06	5.56E-05	3.19E-13	0.30331047	3.92E-58	0.03322553	0.80666148
75.5	2.79E-06	4.95E-05	3.65E-13	0.31038143	3.86E-58	0.03232914	0.80602318
75.6	2.87E-06	4.78E-05	3.90E-13	0.31176678	4.30E-58	0.03183143	0.79664953
75.7	2.78E-06	4.67E-05	3.72E-13	0.3104661	4.08E-58	0.03062293	0.80485809
75.8	2.71E-06	4.71E-05	3.79E-13	0.31044538	4.20E-58	0.03075822	0.80274967
75.9	2.58E-06	4.66E-05	3.83E-13	0.30991672	4.58E-58	0.03048635	0.80069422
76	2.64E-06	4.04E-05	4.30E-13	0.30914435	3.82E-58	0.02844928	0.78995128
76.1	2.66E-06	3.65E-05	4.94E-13	0.31004925	3.21E-58	0.02719559	0.78939663
76.2	2.78E-06	3.79E-05	5.85E-13	0.31299581	3.15E-58	0.02612261	0.79684376
76.3	2.63E-06	3.94E-05	6.71E-13	0.31796132	3.36E-58	0.02457927	0.79010021
76.4	2.59E-06	4.14E-05	7.55E-13	0.32119554	3.97E-58	0.02458374	0.79301569
76.5	2.62E-06	4.29E-05	7.82E-13	0.32526188	4.26E-58	0.02390653	0.78454111
76.6	2.51E-06	3.93E-05	8.27E-13	0.32420561	4.43E-58	0.02323023	0.78073493
76.7	2.27E-06	3.53E-05	9.50E-13	0.32479065	4.48E-58	0.02287193	0.7738418
76.8	2.18E-06	3.43E-05	1.00E-12	0.32796698	5.11E-58	0.02133336	0.77166043
76.9	2.12E-06	3.48E-05	1.17E-12	0.32776702	5.37E-58	0.02108276	0.76121144
77	2.10E-06	3.43E-05	1.29E-12	0.33247508	6.17E-58	0.02065639	0.76739802
77.1	1.93E-06	3.37E-05	1.45E-12	0.3364443	6.78E-58	0.0208474	0.76799613
77.2	2.03E-06	3.34E-05	1.43E-12	0.34491695	6.67E-58	0.02037815	0.76083009
77.3	1.89E-06	3.34E-05	1.39E-12	0.35491839	7.39E-58	0.01961234	0.74997151
77.4	1.98E-06	3.07E-05	1.55E-12	0.34938569	8.52E-58	0.01916566	0.7408658
77.5	2.06E-06	3.03E-05	1.57E-12	0.34907223	8.86E-58	0.01919912	0.74247899
77.6	2.18E-06	2.97E-05	1.58E-12	0.35078918	9.37E-58	0.01938422	0.74132218
77.7	2.19E-06	3.20E-05	1.67E-12	0.35324025	8.77E-58	0.01877308	0.73588904
77.8	2.24E-06	2.86E-05	2.09E-12	0.35291561	9.30E-58	0.01865925	0.72400851
77.9	2.23E-06	2.80E-05	2.43E-12	0.36346702	9.83E-58	0.01790771	0.71755207
78	2.19E-06	2.75E-05	2.55E-12	0.37035111	1.04E-57	0.01790207	0.71590765
78.1	2.07E-06	2.55E-05	2.75E-12	0.37997759	9.36E-58	0.0173651	0.7051201
78.2	1.93E-06	2.40E-05	2.92E-12	0.37811522	9.33E-58	0.01707819	0.70375161
78.3	1.97E-06	2.32E-05	3.23E-12	0.38376034	1.04E-57	0.01676232	0.70036513
78.4	1.83E-06	2.37E-05	3.52E-12	0.38444441	1.38E-57	0.01650043	0.70638669
78.5	1.84E-06	2.29E-05	3.60E-12	0.38748555	1.38E-57	0.0161134	0.70083581
78.6	1.84E-06	2.13E-05	4.07E-12	0.38992634	1.27E-57	0.01562073	0.69478038
78.7	1.75E-06	2.18E-05	4.44E-12	0.39413758	1.24E-57	0.01538162	0.70889556
78.8	1.66E-06	2.06E-05	4.47E-12	0.39291082	1.14E-57	0.01553316	0.71320562
78.9	1.51E-06	1.98E-05	5.03E-12	0.39378719	9.51E-58	0.01520196	0.71927358
79	1.54E-06	1.97E-05	5.49E-12	0.40385296	1.21E-57	0.01531691	0.71264582
79.1	1.47E-06	1.96E-05	5.70E-12	0.40516713	1.45E-57	0.0158059	0.70914116
79.2	1.43E-06	1.94E-05	5.82E-12	0.4007584	1.56E-57	0.0157576	0.71236826
79.3	1.48E-06	1.92E-05	6.55E-12	0.40204498	1.77E-57	0.01586083	0.71001708
79.4	1.42E-06	1.99E-05	6.66E-12	0.39877044	1.96E-57	0.01590909	0.7059107
79.5	1.39E-06	1.99E-05	6.65E-12	0.39235402	1.92E-57	0.01588792	0.710174
79.6	1.40E-06	1.89E-05	6.67E-12	0.39327928	1.88E-57	0.01556976	0.70863056
79.7	1.36E-06	1.98E-05	7.91E-12	0.39059434	2.14E-57	0.01541204	0.70369063
79.8	1.33E-06	1.87E-05	8.81E-12	0.39370308	2.67E-57	0.01419845	0.69624184
79.9	1.36E-06	1.82E-05	9.71E-12	0.40401442	3.00E-57	0.01414181	0.69780519
80	1.30E-06	1.76E-05	1.05E-11	0.41009126	3.28E-57	0.0141935	0.70285311
80.1	1.26E-06	1.69E-05	1.15E-11	0.40764663	3.17E-57	0.01388342	0.69816282
80.2	1.17E-06	1.80E-05	1.25E-11	0.41502256	3.23E-57	0.01354064	0.68668783
80.3	1.11E-06	1.86E-05	1.33E-11	0.42487283	2.79E-57	0.01279267	0.67014149
80.4	1.06E-06	1.81E-05	1.42E-11	0.42348876	2.58E-57	0.01220472	0.65970956
80.5	1.06E-06	1.90E-05	1.45E-11	0.42631871	2.67E-57	0.01192018	0.66053555
80.6	1.08E-06	1.94E-05	1.56E-11	0.42941768	2.45E-57	0.01170509	0.65199061
80.7	1.06E-06	1.73E-05	1.68E-11	0.43190433	2.40E-57	0.01156287	0.63489857
80.8	1.08E-06	1.73E-05	1.79E-11	0.43655145	2.53E-57	0.01165756	0.63151107
80.9	1.11E-06	1.72E-05	1.77E-11	0.43832528	2.47E-57	0.0114593	0.62879078
81	1.03E-06	1.60E-05	1.96E-11	0.43894129	2.46E-57	0.01087318	0.62171272
81.1	9.90E-07	1.58E-05	2.03E-11	0.44978389	2.38E-57	0.0107008	0.6241654
81.2	1.01E-06	1.54E-05	2.14E-11	0.44816233	2.39E-57	0.01083619	0.62269554
81.3	9.30E-07	1.45E-05	2.34E-11	0.44878145	2.22E-57	0.0108837	0.62139799
81.4	9.64E-07	1.44E-05	2.46E-11	0.45928003	2.44E-57	0.01068591	0.62892616
81.5	9.45E-07	1.43E-05	2.44E-11	0.45705101	2.43E-57	0.01083806	0.6330019
81.6	9.36E-07	1.39E-05	2.73E-11	0.45982804	2.58E-57	0.01068792	0.61702843
81.7	9.27E-07	1.26E-05	2.91E-11	0.45150466	2.54E-57	0.01011658	0.61504826
81.8	9.55E-07	1.30E-05	2.92E-11	0.4539625	2.64E-57	0.00977047	0.60542144
81.9	9.62E-07	1.31E-05	3.09E-11	0.4571533	2.61E-57	0.00977777	0.60706396
82	9.71E-07	1.28E-05	3.27E-11	0.45554993	2.87E-57	0.00977892	0.60246493
82.1	9.60E-07	1.29E-05	3.41E-11	0.45500674	2.99E-57	0.00977631	0.60399366
82.2	1.02E-06	1.33E-05	3.66E-11	0.45217967	3.32E-57	0.00973018	0.59690643
82.3	1.02E-06	1.22E-05	3.48E-11	0.46316703	3.48E-57	0.00994877	0.59433043
82.4	9.56E-07	1.29E-05	3.84E-11	0.46184094	4.40E-57	0.01002588	0.5934567
82.5	9.19E-07	1.22E-05	4.10E-11	0.45537083	4.02E-57	0.01013892	0.59465888
82.6	9.47E-07	1.23E-05	4.38E-11	0.44802478	4.46E-57	0.01011524	0.59348912
82.7	9.64E-07	1.20E-05	4.39E-11	0.45329164	4.74E-57	0.01004686	0.59161339
82.8	9.12E-07	1.15E-05	4.45E-11	0.44410321	4.51E-57	0.00958866	0.5964727
82.9	8.27E-07	1.05E-05	5.19E-11	0.43770796	5.32E-57	0.00898498	0.58851416
83	8.14E-07	9.90E-06	5.37E-11	0.43942944	5.71E-57	0.00892864	0.59115601
83.1	8.46E-07	9.52E-06	5.29E-11	0.43881322	6.00E-57	0.00870834	0.59877111
83.2	8.15E-07	8.52E-06	4.99E-11	0.44147708	5.60E-57	0.00850353	0.59079612
83.3	7.53E-07	8.50E-06	5.42E-11	0.44182733	5.30E-57	0.00838213	0.58580175
83.4	7.76E-07	8.70E-06	5.98E-11	0.43963963	6.24E-57	0.00838756	0.58034539
83.5	7.23E-07	8.35E-06	6.34E-11	0.43875165	5.85E-57	0.00795758	0.59192576
83.6	6.84E-07	7.45E-06	6.49E-11	0.43974639	5.81E-57	0.00751524	0.58686219
83.7	7.02E-07	7.48E-06	6.39E-11	0.43905977	5.80E-57	0.00742382	0.58563234
83.8	6.91E-07	7.47E-06	6.63E-11	0.43827249	5.20E-57	0.00725816	0.58461319
83.9	7.21E-07	6.84E-06	6.24E-11	0.4463912	5.19E-57	0.00701848	0.59035252

41.9	0.16221546	0.10796556	1.90E-46	4.08E-05	6.27E-76	0.76407877	0.84598719
42	0.15526939	0.10656718	2.22E-46	3.96E-05	6.92E-76	0.75470995	0.85310531
42.1	0.15002699	0.10325051	2.56E-46	4.02E-05	8.18E-76	0.75192405	0.84805705
42.2	0.15298676	0.10190989	3.34E-46	4.38E-05	1.03E-75	0.74678518	0.84789447
42.3	0.14721714	0.10128757	4.78E-46	4.80E-05	1.42E-75	0.72074994	0.83550531
42.4	0.14358937	0.09836758	6.37E-46	5.06E-05	2.00E-75	0.72037809	0.83339591
42.5	0.13880307	0.09486889	7.94E-46	5.21E-05	2.19E-75	0.70627108	0.83642102
42.6	0.13692035	0.08627834	9.17E-46	6.03E-05	1.92E-75	0.69737528	0.83747844
42.7	0.13488331	0.0861601	8.89E-46	6.03E-05	1.89E-75	0.68188843	0.8250353
42.8	0.13849906	0.08700206	1.03E-45	6.15E-05	2.26E-75	0.67907695	0.82206362
42.9	0.13329847	0.08204639	1.47E-45	6.36E-05	2.29E-75	0.65913453	0.83756058
43	0.13598756	0.083999	3.51E-45	7.37E-05	5.70E-75	0.63872462	0.81534397
43.1	0.12986976	0.0804985	3.85E-45	7.81E-05	5.37E-75	0.64447891	0.80546251
43.2	0.12995307	0.07524909	6.73E-45	8.89E-05	8.30E-75	0.63044534	0.79273012
43.3	0.12971316	0.0731572	8.54E-45	8.93E-05	1.08E-74	0.63667048	0.7898994
43.4	0.12276322	0.07087001	1.36E-44	9.23E-05	1.81E-74	0.62594585	0.79202562
43.5	0.12037013	0.06759989	1.61E-44	9.39E-05	2.13E-74	0.60981587	0.79859806
43.6	0.12251434	0.0673663	2.42E-44	0.00010743	2.77E-74	0.61105373	0.79063974
43.7	0.12271381	0.06102576	5.07E-44	0.00011817	5.51E-74	0.60390811	0.78488035
43.8	0.12338083	0.05730751	8.23E-44	0.00012896	7.67E-74	0.60220101	0.75762009
43.9	0.11693564	0.05622718	1.17E-43	0.00013436	9.50E-74	0.58553936	0.77450884
44	0.11673238	0.05313081	1.41E-43	0.00013877	1.04E-73	0.58793198	0.77582395
44.1	0.1038213	0.05079501	2.70E-43	0.00017562	1.23E-73	0.58267528	0.77745195
44.2	0.10508774	0.04813697	4.38E-43	0.00018585	2.02E-73	0.58979388	0.76678711
44.3	0.10272565	0.04618263	5.93E-43	0.00020021	2.02E-73	0.5662407	0.76939409
44.4	0.10135955	0.04563649	7.52E-43	0.00020304	2.23E-73	0.56038279	0.77732304
44.5	0.09716027	0.04564641	8.73E-43	0.00019577	2.40E-73	0.55857711	0.78132992
44.6	0.09581695	0.04403338	1.24E-42	0.00021877	2.95E-73	0.5473782	0.79383222
44.7	0.09665621	0.04305587	1.48E-42	0.00023314	3.07E-73	0.53873289	0.80268684
44.8	0.08957608	0.04295073	2.26E-42	0.00022467	4.81E-73	0.54484732	0.7980312
44.9	0.09112427	0.04098259	3.37E-42	0.0002427	9.53E-73	0.54923691	0.79299637
90	1.89E-07	1.82E-06	2.20E-09	0.51913709	1.42E-56	0.00184765	0.62977755
90.1	1.90E-07	1.73E-06	2.16E-09	0.51621447	1.43E-56	0.00180547	0.63358417
90.2	1.82E-07	1.71E-06	2.45E-09	0.51628248	1.47E-56	0.00166256	0.62866272
90.3	1.87E-07	1.66E-06	2.65E-09	0.51321893	1.65E-56	0.00168767	0.63301584
90.4	1.86E-07	1.80E-06	2.75E-09	0.51265789	1.66E-56	0.00170846	0.63604433
90.5	1.88E-07	1.69E-06	3.16E-09	0.51324658	1.85E-56	0.00164167	0.62641428
90.6	1.87E-07	1.64E-06	3.67E-09	0.5195155	2.04E-56	0.00167344	0.62611768
90.7	1.81E-07	1.60E-06	3.78E-09	0.52031853	2.04E-56	0.00167424	0.62653806
90.8	1.83E-07	1.50E-06	3.76E-09	0.51635329	2.12E-56	0.00165913	0.62579979
90.9	1.78E-07	1.43E-06	4.12E-09	0.52006543	2.45E-56	0.00161367	0.62995715
91	1.80E-07	1.41E-06	3.91E-09	0.52893777	2.37E-56	0.00160555	0.63356827
91.1	1.64E-07	1.44E-06	4.43E-09	0.53303192	2.61E-56	0.00152785	0.6280858
91.2	1.63E-07	1.43E-06	4.44E-09	0.53277865	2.49E-56	0.00151568	0.632779
91.3	1.62E-07	1.36E-06	4.75E-09	0.53511134	2.62E-56	0.00149122	0.62797157
91.4	1.59E-07	1.34E-06	5.04E-09	0.5402962	2.68E-56	0.00161853	0.62702771
91.5	1.63E-07	1.43E-06	5.42E-09	0.53747341	3.33E-56	0.00159686	0.63844425
91.6	1.69E-07	1.23E-06	6.15E-09	0.54088006	3.33E-56	0.00157925	0.63753165
91.7	1.62E-07	1.20E-06	7.03E-09	0.5413589	4.21E-56	0.00161504	0.62903473
91.8	1.62E-07	1.14E-06	7.62E-09	0.53535209	3.89E-56	0.00155967	0.62675627
91.9	1.55E-07	1.13E-06	7.92E-09	0.53478738	4.05E-56	0.00158853	0.63035327
92	1.47E-07	1.18E-06	8.03E-09	0.53929241	3.88E-56	0.00147225	0.63324616
92.1	1.46E-07	1.21E-06	8.69E-09	0.54109556	4.93E-56	0.00146146	0.63171221
92.2	1.40E-07	1.16E-06	9.35E-09	0.53174682	5.10E-56	0.00142948	0.6377692
92.3	1.36E-07	1.15E-06	1.00E-08	0.52146463	4.28E-56	0.00141959	0.64254758
92.4	1.34E-07	1.09E-06	1.11E-08	0.50893575	4.25E-56	0.00143149	0.64136526
92.5	1.31E-07	1.09E-06	1.14E-08	0.50486847	4.59E-56	0.00142838	0.64402233
92.6	1.41E-07	1.02E-06	1.30E-08	0.49897858	5.09E-56	0.00141057	0.64715357
92.7	1.29E-07	9.54E-07	1.39E-08	0.49344066	4.65E-56	0.00135182	0.65190417
92.8	1.24E-07	9.25E-07	1.41E-08	0.49680329	4.73E-56	0.00131372	0.64708516
92.9	1.12E-07	9.34E-07	1.44E-08	0.50251463	4.32E-56	0.00128545	0.65577915
93	1.13E-07	8.92E-07	1.59E-08	0.50331702	4.70E-56	0.00124271	0.6653005
93.1	1.14E-07	8.91E-07	1.74E-08	0.51099465	5.16E-56	0.00120167	0.66411671
93.2	1.12E-07	8.50E-07	1.87E-08	0.50862872	5.21E-56	0.00119425	0.66236649
93.3	1.10E-07	8.53E-07	1.87E-08	0.50468558	5.52E-56	0.00116052	0.66212705
93.4	1.06E-07	8.59E-07	1.87E-08	0.50360965	5.90E-56	0.00115908	0.66750439
93.5	9.75E-08	8.34E-07	1.94E-08	0.49981329	5.73E-56	0.00112784	0.65672453
93.6	9.65E-08	8.37E-07	1.98E-08	0.50070703	5.62E-56	0.00113029	0.6568734
93.7	9.31E-08	8.84E-07	2.26E-08	0.50041785	5.85E-56	0.00109456	0.66097267
93.8	9.59E-08	8.76E-07	2.39E-08	0.50374835	6.10E-56	0.00110135	0.66017571
93.9	1.02E-07	9.16E-07	2.51E-08	0.50492487	7.33E-56	0.00107217	0.66241727
94	9.59E-08	8.99E-07	2.81E-08	0.50215107	7.52E-56	0.00105394	0.66222607
94.1	9.32E-08	9.13E-07	3.01E-08	0.50763209	9.23E-56	0.00105358	0.66686869
94.2	9.11E-08	9.33E-07	3.25E-08	0.50941284	9.86E-56	0.00103276	0.66530302
94.3	8.91E-08	9.59E-07	3.67E-08	0.50781519	9.43E-56	0.00097605	0.6720428
94.4	8.76E-08	9.09E-07	3.77E-08	0.51047932	9.41E-56	0.00096295	0.6734406
94.5	8.09E-08	8.93E-07	3.76E-08	0.51139536	8.50E-56	0.00097089	0.67866677
94.6	8.02E-08	8.10E-07	3.90E-08	0.50053085	1.05E-55	0.00093591	0.68841969
94.7	7.81E-08	8.22E-07	3.94E-08	0.50191263	1.07E-55	0.00091521	0.68858349
94.8	8.09E-08	8.63E-07	3.96E-08	0.49908317	9.09E-56	0.0008785	0.68846217
94.9	8.00E-08	8.57E-07	4.01E-08	0.50050201	8.31E-56	0.00085751	0.68775888
95	7.91E-08	8.09E-07	3.94E-08	0.49678199	8.08E-56	0.00083282	0.67974102
95.1	7.42E-08	8.15E-07	4.32E-08	0.49903891	9.14E-56	0.00082347	0.68748684
95.2	7.42E-08	8.15E-07	4.32E-08	0.49903891	9.14E-56	0.00082347	0.68748684
95.3	7.51E-08	7.75E-07	4.22E-08	0.49560498	7.61E-56	0.00078829	0.68854955
95.4	7.54E-08	8.05E-07	4.75E-08	0.4970647	8.99E-56	0.00077434	0.68670744
95.5	7.10E-08	7.97E-07	4.84E-08	0.50163173	1.00E-55	0.00076515	0.69693104
95.6	6.98E-08	7.63E-07	4.94E-08	0.49404456	1.07E-55	0.00073629	0.69447529
95.7	6.93E-08	7.55E-07	4.88E-08	0.49506307	9.86E-56	0.00071976	0.69508148
95.8	6.81E-08	7.08E-07	5.01E-08	0.49523222	1.03E-55	0.00069049	0.69880292
95.9	6.66E-08	7.12E-07	5.55E-08	0.49439999	1.02E-55	0.00065538	0.71592492
96	6.71E-08	7.21E-07	5.74E-08	0.49726614	1.10E-55	0.00065533	0.71054915
96.1	6.69E-08	7.21E-07	5.47E-08	0.49968936	1.06E-55	0.00063948	0.71108178
96.2	6.83E-08	7.07E-07	5.45E-08	0.5050169	1.09E-55	0.00063582	0.70756305
96.3	6.89E-08	7.22E-07	5.39E-08	0.50545993	1.07E-55	0.00063791	0.71038984
96.4	6.69E-08	7.22E-07	6.18E-08	0.4991639	1.47E-55	0.00063269	0.70981231
96.5	6.88E-08	7.66E-07	6.84E-08	0.50179392	1.61E-55	0.00063494	0.71420012
96.6	7.19E-08	7.67E-07	7.02E-08	0.49665841	1.57E-55	0.00063928	0.71489833
96.7	6.81E-08	7.37E-07	7.11E-08	0.49753506	1.55E-55	0.00059944	0.71934641
96.8	6.65E-08	6.61E-07	8.17E-08	0.50102313	1.49E-55	0.00055447	0.72271847
96.9	6.26E-08	6.16E-07	8.80E-08	0.49713139	1.07E-55	0.00052053	0.71926689
97	6.19E-08	6.10E-07	9.27E-08	0.50126271	1.15E-55	0.00052714	0.71645821
97.1	6.05E-08	5.89E-07	9.54E-08	0.49800898	1.04E-55	0.00050534	0.71897496
97.2	5.90E-08	5.94E-07	9.70E-08	0.4992041	1.07E-55	0.00051898	0.72536413
97.3	5.92E-08	5.94E-07	9.89E-08	0.49494175	1.07E-55	0.00051645	0.73033446
97.4	5.89E-08	5.92E-07	9.66E-08	0.50417231	1.10E-55	0.00047872	0.73163839
97.5	5.77E-08	5.60E-07	9.92E-08	0.49711368	1.34E-55	0.00048863	0.73666852
97.6	6.03E-08	6.08E-07	1.15E-07	0.50235255	1.83E-55	0.00049291	0.73527195
97.7	5.97E-08	5.69E-07	1.16E-07	0.5052478	2.03E-55	0.00049481	0.73707082
97.8	5.87E-08	5.62E-07	1.14E-07	0.50714335	1.87E-55	0.00048046	0.73834524
97.9	5.92E-08	5.81E-07	1.25E-07	0.50791103	1.78E-55	0.00047297	0.74141858
98	6.19E-08	5.72E-07	1.33E-07	0.51207101	1.80E-55	0.00046207	0.73887467
98.1	6.16E-08	5.68E-07	1.34E-07	0.51039918	1.73E-55	0.00045934	0.73695001
98.2	5.74E-08	5.68E-07	1.44E-07	0.50607236	1.55E-55	0.00046185	0.72947898
98.3	5.47E-08	5.66E-07	1.56E-07	0.50624742	1.34E-55	0.00043105	0.72539317
98.4	5.68E-08	5.38E-07	1.76E-07	0.50761896	1.42E-55	0.00043074	0.71872069
98.5	5.41E-08	5.41E-07	1.77E-07	0.5117398	1.50E-55	0.00041846	0.70851603
98.6	5.08E-08	5.56E-07	1.94E-07	0.50480145	1.22E-55	0.0003954	0.70235458
98.7	5.43E-08	5.91E-07	2.25E-07	0.50136818	1.36E-55	0.00037479	0.69253514
98.8	5.27E-08	5.44E-07	2.38E-07	0.506626	1.22E-55	0.00036943	0.69226163
98.9	4.79E-08	5.60E-07	2.43E-07	0.51024663	1.14E-55	0.00035997	0.6922109

56.9	0.00094415	0.00287475	9.97E-26	0.02667433	1.20E-63	0.49411528	0.49840881
57	0.00083042	0.00279095	1.36E-25	0.02708663	1.21E-63	0.47874301	0.48989728
57.1	0.00078572	0.00271365	1.64E-25	0.02836903	1.28E-63	0.46481541	0.49777077
57.2	0.00080901	0.00270741	1.79E-25	0.02861543	1.18E-63	0.45791971	0.49966098
57.3	0.0007887	0.00279133	2.19E-25	0.02940615	1.22E-63	0.45850688	0.5021482
57.4	0.0007622	0.00273103	2.40E-25	0.03005161	1.20E-63	0.46139495	0.51369243
57.5	0.00076073	0.0026963	2.77E-25	0.03229878	1.29E-63	0.46394341	0.50500715
57.6	0.00069507	0.00278051	4.12E-25	0.03320607	1.37E-63	0.44609919	0.50157151
57.7	0.00061049	0.00272675	4.70E-25	0.03343062	1.18E-63	0.43568592	0.51000452
57.8	0.0006224	0.00259157	6.37E-25	0.03400796	1.23E-63	0.42295365	0.51253441
57.9	0.00057196	0.00263886	7.53E-25	0.03388812	1.19E-63	0.41218632	0.51230079
58	0.00058534	0.00257103	1.21E-24	0.03580455	1.74E-63	0.39942262	0.5065968
58.1	0.00057012	0.00263399	1.19E-24	0.03562175	1.80E-63	0.39166224	0.50781884
58.2	0.00056943	0.0025943	1.50E-24	0.0369333	2.20E-63	0.38552417	0.51370395
58.3	0.00057646	0.00261907	1.78E-24	0.03594714	2.50E-63	0.38223596	0.51668271
58.4	0.00060201	0.00262094	2.29E-24	0.03589902	3.33E-63	0.37820289	0.51603662
58.5	0.00054711	0.00257278	2.78E-24	0.03498346	3.83E-63	0.37221476	0.51406911
58.6	0.00054251	0.00266688	3.16E-24	0.03616484	4.06E-63	0.36963261	0.52126146
58.7	0.00050035	0.00251257	3.87E-24	0.03844899	4.93E-63	0.37034785	0.5422373
58.8	0.00050212	0.00252628	4.31E-24	0.03873448	5.52E-63	0.36977951	0.54402238
58.9	0.00048677	0.00248646	8.20E-24	0.04282894	8.41E-63	0.35873203	0.528532
59	0.00047793	0.00226816	1.22E-23	0.04608212	1.29E-62	0.3552101	0.52680536
59.1	0.00043822	0.00247654	1.78E-23	0.04643897	1.27E-62	0.34471042	0.53400316
59.2	0.0003951	0.0024731	2.54E-23	0.05112246	1.44E-62	0.33087926	0.5530996
59.3	0.00041351	0.00248175	3.25E-23	0.05224122	1.78E-62	0.3334743	0.54344656
59.4	0.00038997	0.00245276	4.13E-23	0.05273308	1.70E-62	0.32935548	0.55696597
59.5	0.00037829	0.00253671	4.89E-23	0.05475406	1.84E-62	0.32909013	0.56393853
59.6	0.00035151	0.00255779	5.19E-23	0.05578203	1.74E-62	0.3217483	0.568319
59.7	0.00033724	0.00230158	7.37E-23	0.05757672	1.76E-62	0.32131154	0.57255529
59.8	0.00032518	0.00235845	1.01E-22	0.05818227	2.16E-62	0.32065064	0.56052713
59.9	0.00027883	0.00233636	1.17E-22	0.0588991	2.00E-62	0.31025944	0.57616402
105	4.99E-08	5.23E-07	1.92E-06	0.47684049	4.83E-55	0.00016967	0.6745556
105.1	4.88E-08	5.10E-07	1.98E-06	0.47535743	4.76E-55	0.00016606	0.67916481
105.2	4.80E-08	5.32E-07	2.08E-06	0.47242263	5.10E-55	0.00016123	0.67987782
105.3	4.80E-08	5.32E-07	2.08E-06	0.47242263	5.10E-55	0.00016123	0.67987782
105.4	4.44E-08	5.14E-07	2.20E-06	0.4626959	5.60E-55	0.00016176	0.6884699
105.5	4.41E-08	4.97E-07	2.25E-06	0.46365006	4.90E-55	0.00015444	0.6900934
105.6	4.28E-08	4.78E-07	2.34E-06	0.45794413	4.42E-55	0.00015331	0.68270716
105.7	4.12E-08	4.29E-07	2.35E-06	0.45248004	3.72E-55	0.00014506	0.67714535
105.8	4.15E-08	4.21E-07	2.28E-06	0.45998643	3.62E-55	0.0001383	0.67804756
105.9	3.96E-08	4.42E-07	2.47E-06	0.46396633	4.64E-55	0.00014036	0.67076038
106	4.20E-08	4.65E-07	2.40E-06	0.46831114	4.64E-55	0.0001386	0.67177237
106.1	4.31E-08	4.77E-07	2.63E-06	0.46498179	5.28E-55	0.00014139	0.66637364
106.2	4.05E-08	4.77E-07	2.85E-06	0.46062944	5.73E-55	0.00014537	0.66306466
106.3	3.92E-08	4.37E-07	2.89E-06	0.46584715	5.68E-55	0.0001348	0.67064602
106.4	3.94E-08	4.29E-07	2.88E-06	0.45886733	5.60E-55	0.00012939	0.66465882
106.5	3.92E-08	3.93E-07	3.20E-06	0.44912374	5.41E-55	0.00012455	0.65866939
106.6	3.96E-08	3.85E-07	3.19E-06	0.44692359	5.20E-55	0.00012365	0.65664317
106.7	4.06E-08	3.79E-07	3.26E-06	0.44896225	5.07E-55	0.00012262	0.65956063
106.8	4.16E-08	3.66E-07	3.38E-06	0.45225354	4.55E-55	0.00012402	0.66786697
106.9	4.07E-08	3.76E-07	3.30E-06	0.45039617	4.34E-55	0.0001221	0.66344376
107	4.19E-08	3.81E-07	3.54E-06	0.45581148	4.18E-55	0.0001135	0.67049556
107.1	4.18E-08	3.76E-07	3.52E-06	0.45641877	3.94E-55	0.00011115	0.68036507
107.2	4.15E-08	3.71E-07	3.46E-06	0.45633493	3.93E-55	0.00010939	0.67870644
107.3	4.15E-08	3.71E-07	3.46E-06	0.45633493	3.93E-55	0.00010939	0.67870644
107.4	3.97E-08	3.80E-07	3.86E-06	0.45262286	3.69E-55	0.00010796	0.67730791
107.5	4.01E-08	3.64E-07	3.93E-06	0.45024611	4.02E-55	0.00010698	0.68113961
107.6	3.99E-08	3.59E-07	3.81E-06	0.4505303	3.78E-55	0.00010592	0.68641401
107.7	3.97E-08	3.61E-07	3.80E-06	0.44925525	3.39E-55	0.00010318	0.68036026
107.8	3.73E-08	3.81E-07	4.02E-06	0.45366544	3.25E-55	0.00010453	0.67722145
107.9	3.76E-08	3.98E-07	4.28E-06	0.45356017	3.58E-55	0.00010491	0.68288567
108	3.81E-08	3.97E-07	4.32E-06	0.45840821	3.54E-55	0.00010372	0.68364266
108.1	3.68E-08	3.98E-07	4.43E-06	0.45133595	3.30E-55	9.88E-05	0.68503858
108.2	3.92E-08	3.86E-07	4.29E-06	0.45709849	3.37E-55	9.92E-05	0.69152709
108.3	3.76E-08	3.56E-07	4.56E-06	0.45739594	3.18E-55	9.77E-05	0.69496274
108.4	3.89E-08	3.50E-07	4.54E-06	0.45673257	3.05E-55	9.42E-05	0.69371854
108.5	3.90E-08	3.55E-07	4.53E-06	0.45413814	2.81E-55	9.36E-05	0.69620176
108.6	3.88E-08	3.60E-07	4.78E-06	0.45541617	3.00E-55	9.34E-05	0.69313393
108.7	4.00E-08	3.60E-07	4.58E-06	0.44597204	2.84E-55	9.02E-05	0.69656667
108.8	3.93E-08	3.49E-07	4.71E-06	0.44693719	2.86E-55	9.16E-05	0.6998168
108.9	3.91E-08	3.52E-07	4.68E-06	0.45086406	2.80E-55	9.25E-05	0.70236095
109	3.90E-08	3.38E-07	4.91E-06	0.45011578	2.80E-55	9.29E-05	0.69677066
109.1	3.82E-08	3.63E-07	4.80E-06	0.4566507	2.56E-55	8.85E-05	0.70692346
109.2	4.03E-08	3.52E-07	4.79E-06	0.46813307	2.44E-55	8.84E-05	0.71089185
109.3	4.25E-08	3.46E-07	4.90E-06	0.45766332	2.70E-55	8.73E-05	0.70664015
109.4	4.30E-08	3.39E-07	4.92E-06	0.45519819	2.73E-55	8.78E-05	0.70895726
109.5	4.11E-08	3.55E-07	4.91E-06	0.46152004	2.44E-55	8.81E-05	0.71125242
109.6	4.10E-08	3.54E-07	4.91E-06	0.4605465	2.36E-55	8.81E-05	0.71460075
109.7	4.13E-08	3.46E-07	4.96E-06	0.45799163	2.26E-55	8.85E-05	0.7170203
109.8	4.32E-08	3.78E-07	5.26E-06	0.45298243	3.18E-55	8.26E-05	0.72461772
109.9	4.38E-08	3.83E-07	5.28E-06	0.45369754	2.99E-55	7.93E-05	0.72592231
110	4.42E-08	3.71E-07	5.22E-06	0.4526551	2.72E-55	7.44E-05	0.72500753
110.1	4.34E-08	3.81E-07	5.34E-06	0.4503416	2.92E-55	7.17E-05	0.72739483
110.2	4.42E-08	3.95E-07	5.57E-06	0.45095283	2.91E-55	7.03E-05	0.73006766
110.3	4.95E-08	4.00E-07	5.60E-06	0.45444456	3.17E-55	6.71E-05	0.72891556
110.4	5.11E-08	3.94E-07	5.57E-06	0.44753107	3.53E-55	6.78E-05	0.72132202
110.5	5.12E-08	3.79E-07	5.92E-06	0.45464815	3.24E-55	6.26E-05	0.72542398
110.6	5.17E-08	3.77E-07	6.25E-06	0.45510239	3.49E-55	6.36E-05	0.72546538
110.7	5.23E-08	3.86E-07	6.54E-06	0.4581452	4.10E-55	6.27E-05	0.71934724
110.8	5.15E-08	3.73E-07	6.79E-06	0.45354093	4.01E-55	6.16E-05	0.7223329
110.9	5.28E-08	3.72E-07	7.20E-06	0.45003562	4.44E-55	5.86E-05	0.72516107
111	5.14E-08	3.67E-07	7.49E-06	0.4494491	4.35E-55	5.85E-05	0.72253158
111.1	5.37E-08	3.74E-07	7.78E-06	0.45202415	3.96E-55	5.49E-05	0.72782491
111.2	5.31E-08	3.73E-07	7.90E-06	0.44830697	3.29E-55	5.07E-05	0.73776712
111.3	5.30E-08	3.76E-07	8.17E-06	0.44408643	3.46E-55	4.96E-05	0.74096442
111.4	5.04E-08	3.67E-07	8.62E-06	0.4447916	3.42E-55	5.00E-05	0.74118758
111.5	5.10E-08	3.64E-07	8.67E-06	0.44370572	3.33E-55	4.88E-05	0.74693691
111.6	5.39E-08	3.88E-07	8.83E-06	0.44957904	3.24E-55	4.79E-05	0.74945069
111.7	5.42E-08	3.92E-07	9.00E-06	0.44776222	3.28E-55	4.73E-05	0.75463459
111.8	5.36E-08	4.05E-07	9.02E-06	0.44511512	3.12E-55	4.63E-05	0.74841694
111.9	5.35E-08	4.03E-07	9.08E-06	0.44890606	3.10E-55	4.60E-05	0.74722583
112	5.34E-08	3.72E-07	9.43E-06	0.45015558	2.89E-55	4.40E-05	0.74745283
112.1	5.47E-08	3.70E-07	9.21E-06	0.45158857	2.85E-55	4.49E-05	0.74925967
112.2	5.36E-08	3.86E-07	9.80E-06	0.45380777	2.68E-55	4.54E-05	0.746436
112.3	5.36E-08	3.86E-07	9.80E-06	0.45380777	2.68E-55	4.54E-05	0.746436
112.4	5.60E-08	3.83E-07	9.96E-06	0.45354924	2.93E-55	4.50E-05	0.74477981
112.5	5.89E-08	3.89E-07	1.02E-05	0.45087688	3.02E-55	4.44E-05	0.74681808
112.6	5.64E-08	3.78E-07	1.12E-05	0.44589352	3.27E-55	4.54E-05	0.74466009
112.7	6.19E-08	3.70E-07	1.13E-05	0.44111145	3.92E-55	4.41E-05	0.74515518
112.8	6.57E-08	3.97E-07	1.14E-05	0.43077089	3.80E-55	4.03E-05	0.73935014
112.9	6.41E-08	4.24E-07	1.14E-05	0.43025193	3.81E-55	3.97E-05	0.75200262
113	6.90E-08	3.97E-07	1.19E-05	0.43332864	4.12E-55	3.98E-05	0.75068508
113.1	6.86E-08	3.80E-07	1.20E-05	0.43426537	4.25E-55	3.83E-05	0.75761318
113.2	6.99E-08	3.79E-07	1.21E-05	0.43754494	4.42E-55	3.75E-05	0.75737478
113.3	7.03E-08	3.69E-07	1.25E-05	0.43961713	4.44E-55	3.78E-05	0.76057102
113.4	7.20E-08	3.60E-07	1.24E-05	0.43998409	4.64E-55	3.84E-05	0.76080815
113.5	7.59E-08	3.51E-07	1.25E-05	0.43914081	3.53E-55	3.60E-05	0.76408544
113.6	7.97E-08	3.65E-07	1.26E-05	0.4411351	3.35E-55	3.51E-05	0.77151486
113.7	7.55E-08	3.64E-07	1.21E-05	0.44307685	3.21E-55	3.44E-05	0.77114671
113.8	7.76E-08	3.67E-07	1.18E-05	0.45097017	2.99E-55	3.38E-05	0.7684811
113.9	7.84E-08	3.78E-07	1.17E-05	0.44914132	3.06E-55	3.25E-05	0.7627629

69	1.45E-05	0.00033437	3.09E-16	0.1783519	3.52E-59	0.08038949	0.86924337
69.1	1.44E-05	0.00031723	3.35E-16	0.1797534	3.33E-59	0.07737675	0.86513666
69.2	1.37E-05	0.00031791	3.66E-16	0.17978092	3.15E-59	0.07583948	0.86788419
69.3	1.23E-05	0.00031174	3.89E-16	0.1790472	2.77E-59	0.07602567	0.86326488
69.4	1.20E-05	0.00031207	4.03E-16	0.17799269	3.12E-59	0.07652429	0.86692667
69.5	1.09E-05	0.00028895	5.69E-16	0.18044319	3.90E-59	0.07781207	0.87211072
69.6	1.05E-05	0.00027613	5.95E-16	0.18212339	4.13E-59	0.07734008	0.87293712
69.7	1.07E-05	0.00027846	6.38E-16	0.18258484	4.44E-59	0.07659704	0.86584746
69.8	1.01E-05	0.0002679	6.82E-16	0.18636922	3.97E-59	0.07374345	0.876948
69.9	9.60E-06	0.00024605	7.69E-16	0.18773869	4.54E-59	0.07279731	0.88007703
70	9.74E-06	0.00024028	7.23E-16	0.18735651	4.49E-59	0.07063218	0.87828271
70.1	9.86E-06	0.0002499	8.17E-16	0.18965381	4.72E-59	0.06965091	0.8883628
70.2	9.57E-06	0.0002542	7.93E-16	0.1910056	4.18E-59	0.06936846	0.89776031
70.3	9.23E-06	0.00024388	8.13E-16	0.19089655	4.05E-59	0.06812281	0.89732195
70.4	9.83E-06	0.00023924	8.86E-16	0.19625825	4.02E-59	0.06800287	0.90751865
70.5	9.50E-06	0.00021999	9.08E-16	0.20000122	3.75E-59	0.06754812	0.90769301
70.6	8.94E-06	0.00020131	1.10E-15	0.20647116	3.64E-59	0.06342425	0.90174836
70.7	8.34E-06	0.00019693	1.20E-15	0.20752965	3.55E-59	0.06438287	0.89672394
70.8	8.28E-06	0.00020257	1.19E-15	0.21103673	3.84E-59	0.06290757	0.9048445
70.9	8.28E-06	0.00019559	1.30E-15	0.2104376	3.71E-59	0.06294167	0.91839438
71	8.34E-06	0.000192	1.36E-15	0.21169103	3.18E-59	0.06163665	0.91807237
71.1	8.23E-06	0.00019077	1.50E-15	0.20944494	2.69E-59	0.06166918	0.91476959
71.2	8.24E-06	0.00019225	1.53E-15	0.20898785	2.63E-59	0.06132209	0.92165516
71.3	8.30E-06	0.0001886	1.63E-15	0.20938021	2.79E-59	0.06143697	0.92492528
71.4	7.54E-06	0.00018356	1.85E-15	0.20771869	2.65E-59	0.06107881	0.93592238
71.5	7.85E-06	0.0001802	2.19E-15	0.20720638	2.42E-59	0.05689858	0.93235008
71.6	7.60E-06	0.00017961	2.23E-15	0.20765419	2.30E-59	0.05601107	0.93531449
71.7	7.56E-06	0.00018425	2.32E-15	0.20466679	2.25E-59	0.05535572	0.93392787
71.8	7.40E-06	0.00019341	2.64E-15	0.20494141	2.03E-59	0.05177632	0.94396171
71.9	7.60E-06	0.00019487	2.63E-15	0.20207228	2.14E-59	0.05129606	0.9420572
72	7.40E-06	0.00019015	3.00E-15	0.20521756	1.96E-59	0.05137334	0.93859234
72.1	7.64E-06	0.00018677	4.20E-15	0.19521122	1.98E-59	0.04899404	0.93412294
72.2	7.57E-06	0.00018147	4.73E-15	0.19986556	2.05E-59	0.04864394	0.93475344
72.3	7.17E-06	0.00018056	5.62E-15	0.20312059	1.99E-59	0.04551307	0.94467667
72.4	7.30E-06	0.00017928	6.25E-15	0.20671539	2.30E-59	0.04421325	0.95399783
72.5	7.26E-06	0.00016328	6.50E-15	0.2036491	2.27E-59	0.04278629	0.95541149
72.6	7.21E-06	0.00015332	6.92E-15	0.20671699	2.14E-59	0.042926	0.95165511
72.7	6.66E-06	0.00015325	9.19E-15	0.20873385	2.43E-59	0.04238185	0.96029884
72.8	6.43E-06	0.00014065	1.06E-14	0.21317938	2.63E-59	0.04161578	0.97847631
72.9	6.17E-06	0.00014107	1.12E-14	0.22339522	2.72E-59	0.04290103	0.98569262
73	6.36E-06	0.00013849	1.26E-14	0.22744931	2.80E-59	0.04140533	0.99117664
73.1	6.27E-06	0.00014122	1.31E-14	0.22586932	2.98E-59	0.04112776	0.99514515
73.2	6.36E-06	0.00013675	1.41E-14	0.22851087	3.33E-59	0.04079849	0.99821135
73.3	6.14E-06	0.00012783	1.70E-14	0.2258248	3.42E-59	0.04062848	0.9991382
73.4	5.78E-06	0.00012831	1.94E-14	0.22776744	3.80E-59	0.03947214	0.99316096
73.5	5.31E-06	0.00012459	2.07E-14	0.22984752	4.04E-59	0.0388754	0.98890938
73.6	4.78E-06	0.00011974	2.44E-14	0.23199592	4.23E-59	0.03774952	0.99018884
73.7	5.17E-06	0.00011039	2.73E-14	0.23668292	5.05E-59	0.0366022	0.97361269
73.8	5.22E-06	0.00011183	3.39E-14	0.24184071	6.00E-59	0.03655018	0.97315686
73.9	4.91E-06	0.00010841	3.87E-14	0.2397728	5.89E-59	0.03755476	0.96651383
74	4.87E-06	0.00010414	4.41E-14	0.24213779	6.59E-59	0.03876392	0.96309877
74.1	4.80E-06	9.69E-05	4.91E-14	0.24260643	6.70E-59	0.03874075	0.95610951
74.2	4.83E-06	9.10E-05	5.36E-14	0.24832703	7.37E-59	0.03944353	0.9534207
74.3	4.56E-06	8.59E-05	6.49E-14	0.25708527	8.61E-59	0.04073477	0.93987958
74.4	3.87E-06	8.42E-05	9.13E-14	0.26641085	1.02E-58	0.0380693	0.92013057
74.5	3.88E-06	7.97E-05	1.05E-13	0.2689962	1.03E-58	0.03703155	0.904801
74.6	3.59E-06	7.92E-05	1.16E-13	0.26795593	1.23E-58	0.03695384	0.88857574
74.7	3.57E-06	7.48E-05	1.44E-13	0.27457253	2.03E-58	0.03825187	0.8751267
74.8	3.48E-06	7.01E-05	1.56E-13	0.27602991	1.94E-58	0.03800213	0.85984867
74.9	3.49E-06	6.47E-05	1.64E-13	0.28257715	2.02E-58	0.03707582	0.85087067
120	1.05E-07	3.83E-07	1.85E-05	0.38821659	1.18E-55	5.90E-06	0.81160621
120.1	1.04E-07	3.82E-07	1.80E-05	0.38830696	1.10E-55	5.62E-06	0.82676312
120.2	1.02E-07	3.67E-07	1.76E-05	0.38508229	8.77E-56	5.58E-06	0.82773816
120.3	1.06E-07	3.53E-07	1.76E-05	0.38559597	7.98E-56	5.41E-06	0.82952272
120.4	1.10E-07	3.56E-07	1.73E-05	0.38994479	7.43E-56	5.36E-06	0.83132463
120.5	1.12E-07	3.49E-07	1.74E-05	0.39234403	7.41E-56	5.40E-06	0.82979626
120.6	1.13E-07	3.43E-07	1.76E-05	0.39123229	7.73E-56	5.37E-06	0.82828973
120.7	1.16E-07	3.60E-07	1.76E-05	0.39524824	6.91E-56	5.05E-06	0.83526257
120.8	1.19E-07	3.56E-07	1.76E-05	0.39020846	6.88E-56	4.90E-06	0.83571283
120.9	1.18E-07	3.53E-07	1.75E-05	0.38555926	7.14E-56	4.78E-06	0.83176974
121	1.20E-07	3.51E-07	1.74E-05	0.38466033	6.49E-56	4.77E-06	0.82940768
121.1	1.33E-07	3.23E-07	1.88E-05	0.37897307	6.08E-56	4.36E-06	0.82649792
121.2	1.29E-07	3.23E-07	1.81E-05	0.37146765	5.22E-56	4.09E-06	0.82343173
121.3	1.29E-07	3.33E-07	1.82E-05	0.36890765	5.24E-56	4.06E-06	0.82126014
121.4	1.29E-07	3.37E-07	1.85E-05	0.36539583	5.07E-56	3.82E-06	0.82255935
121.5	1.22E-07	3.21E-07	1.82E-05	0.36700256	4.59E-56	3.81E-06	0.82850011
121.6	1.22E-07	3.21E-07	1.82E-05	0.36700256	4.59E-56	3.81E-06	0.82850011
121.7	1.20E-07	3.36E-07	1.78E-05	0.36670271	3.73E-56	3.61E-06	0.83258004
121.8	1.26E-07	3.17E-07	1.82E-05	0.35848712	4.34E-56	3.52E-06	0.82502899
121.9	1.28E-07	3.27E-07	1.85E-05	0.3570062	4.37E-56	3.53E-06	0.82046966
122	1.29E-07	3.26E-07	1.79E-05	0.35334264	4.07E-56	3.27E-06	0.8307781
122.1	1.32E-07	3.41E-07	1.80E-05	0.35571669	3.67E-56	3.14E-06	0.82876812
122.2	1.47E-07	3.51E-07	1.92E-05	0.35985019	3.98E-56	3.10E-06	0.83320863
122.3	1.53E-07	3.46E-07	1.93E-05	0.35614628	3.78E-56	2.99E-06	0.84010086
122.4	1.58E-07	3.42E-07	1.91E-05	0.3499084	4.22E-56	3.00E-06	0.83137276
122.5	1.59E-07	3.41E-07	1.90E-05	0.35041678	4.42E-56	3.02E-06	0.83506507
122.6	1.62E-07	3.35E-07	1.97E-05	0.34764922	4.38E-56	2.94E-06	0.83546162
122.7	1.71E-07	3.19E-07	2.02E-05	0.34475113	4.70E-56	2.88E-06	0.83498011
122.8	1.65E-07	3.47E-07	2.04E-05	0.34184678	4.59E-56	2.77E-06	0.83482468
122.9	1.66E-07	3.70E-07	1.97E-05	0.34751204	4.36E-56	2.64E-06	0.83809306
123	1.64E-07	3.77E-07	1.94E-05	0.34383089	4.03E-56	2.58E-06	0.84336695
123.1	1.71E-07	3.92E-07	2.04E-05	0.34502183	4.03E-56	2.55E-06	0.84539139
123.2	1.75E-07	3.81E-07	2.08E-05	0.34382843	3.90E-56	2.51E-06	0.84976903
123.3	1.79E-07	3.92E-07	2.17E-05	0.34469523	4.23E-56	2.53E-06	0.85233961
123.4	1.85E-07	4.20E-07	2.26E-05	0.34342237	4.80E-56	2.56E-06	0.85500806
123.5	1.96E-07	4.07E-07	2.21E-05	0.34053578	4.59E-56	2.52E-06	0.8583667
123.6	1.96E-07	4.07E-07	2.21E-05	0.34053578	4.59E-56	2.52E-06	0.8583667
123.7	2.02E-07	4.24E-07	2.17E-05	0.33777535	5.08E-56	2.48E-06	0.86082382
123.8	2.08E-07	4.26E-07	2.20E-05	0.33887381	5.40E-56	2.43E-06	0.86408887
123.9	2.20E-07	4.26E-07	2.19E-05	0.33950389	5.67E-56	2.41E-06	0.86265834
124	2.22E-07	4.35E-07	2.17E-05	0.34172314	5.16E-56	2.34E-06	0.86174085
124.1	2.28E-07	4.46E-07	2.16E-05	0.34384958	5.05E-56	2.29E-06	0.86032081
124.2	2.35E-07	4.68E-07	2.21E-05	0.34270165	5.00E-56	2.24E-06	0.86158918
124.3	2.42E-07	4.41E-07	2.20E-05	0.34393299	5.40E-56	2.13E-06	0.86963579
124.4	2.52E-07	4.50E-07	2.28E-05	0.34450083	5.75E-56	2.03E-06	0.87631786
124.5	2.54E-07	4.27E-07	2.28E-05	0.34903539	5.41E-56	1.89E-06	0.88254028
124.6	2.52E-07	4.38E-07	2.33E-05	0.35486142	4.99E-56	1.78E-06	0.89511324
124.7	2.64E-07	4.53E-07	2.35E-05	0.35880359	5.15E-56	1.78E-06	0.89472088
124.8	2.74E-07	4.66E-07	2.32E-05	0.35895937	5.13E-56	1.78E-06	0.89686909
124.9	2.87E-07	4.87E-07	2.20E-05	0.36184374	4.66E-56	1.72E-06	0.89545869
125	2.87E-07	4.87E-07	2.20E-05	0.36184374	4.66E-56	1.72E-06	0.89545869
125.1	2.81E-07	4.86E-07	2.16E-05	0.35906443	4.31E-56	1.72E-06	0.8969288
125.2	2.84E-07	4.83E-07	2.20E-05	0.35440115	4.38E-56	1.67E-06	0.89757365
125.3	2.89E-07	4.73E-07	2.31E-05	0.35371398	4.32E-56	1.62E-06	0.90159808
125.4	2.95E-07	4.53E-07	2.39E-05	0.35251282	4.14E-56	1.55E-06	0.90732026
125.5	2.95E-07	4.53E-07	2.39E-05	0.35251282	4.14E-56	1.55E-06	0.90732026
125.6	3.31E-07	4.24E-07	2.36E-05	0.35163882	4.14E-56	1.45E-06	0.90897824
125.7	3.35E-07	4.37E-07	2.51E-05	0.34940423	4.37E-56	1.42E-06	0.90490131
125.8	3.35E-07	4.37E-07	2.51E-05	0.34940423	4.37E-56	1.42E-06	0.90490131
125.9	3.51E-07	4.44E-07	2.49E-05	0.35404367	4.36E-56	1.38E-06	0.90845256
126	3.52E-07	4.51E-07	2.43E-05	0.35328205	4.34E-56	1.35E-06	0.90946579

84	7.04E-07	6.99E-06	6.41E-11	0.45028145	5.41E-57	0.00690525	0.58331249
84.1	7.04E-07	6.99E-06	6.41E-11	0.45028145	5.41E-57	0.00690525	0.58331249
84.2	6.83E-07	6.88E-06	6.43E-11	0.44713769	5.23E-57	0.0067511	0.58222686
84.3	6.99E-07	6.63E-06	7.08E-11	0.45422443	5.74E-57	0.00651405	0.57661071
84.4	6.86E-07	6.52E-06	7.41E-11	0.44713063	7.08E-57	0.00635559	0.57598089
84.5	7.22E-07	6.36E-06	7.69E-11	0.45440158	9.28E-57	0.00619494	0.57261924
84.6	6.06E-07	6.14E-06	8.90E-11	0.45304557	1.03E-56	0.00607235	0.56977094
84.7	5.79E-07	5.92E-06	9.64E-11	0.44858297	1.10E-56	0.00570826	0.56471705
84.8	5.73E-07	5.75E-06	9.79E-11	0.44737892	1.04E-56	0.00549805	0.55951469
84.9	5.65E-07	5.45E-06	1.10E-10	0.4440051	9.52E-57	0.00539673	0.55441678
85	5.81E-07	5.48E-06	1.31E-10	0.44899184	1.07E-56	0.00511356	0.56121479
85.1	5.21E-07	5.72E-06	1.33E-10	0.46640194	9.30E-57	0.00477034	0.54813417
85.2	5.21E-07	5.32E-06	1.46E-10	0.46976561	7.68E-57	0.00463654	0.54621583
85.3	5.17E-07	5.36E-06	1.42E-10	0.47009161	6.85E-57	0.00457634	0.5478753
85.4	4.78E-07	5.51E-06	1.60E-10	0.47704365	6.78E-57	0.00443877	0.55410147
85.5	4.64E-07	5.64E-06	1.82E-10	0.47914183	7.75E-57	0.00443985	0.56009166
85.6	4.58E-07	5.35E-06	1.89E-10	0.468404	8.25E-57	0.00425694	0.56199696
85.7	4.70E-07	5.20E-06	1.86E-10	0.46960426	8.10E-57	0.00433444	0.57053502
85.8	4.59E-07	5.14E-06	1.90E-10	0.46378562	7.92E-57	0.00397298	0.59094859
85.9	4.51E-07	5.00E-06	1.89E-10	0.46732428	7.62E-57	0.00396986	0.59081255
86	4.85E-07	4.75E-06	1.99E-10	0.46533578	9.73E-57	0.00380793	0.60072354
86.1	4.35E-07	5.20E-06	2.21E-10	0.47117015	9.92E-57	0.00380322	0.59478399
86.2	4.29E-07	5.06E-06	2.41E-10	0.47155021	1.03E-56	0.00376577	0.59117752
86.3	4.14E-07	5.08E-06	2.33E-10	0.47494156	9.75E-57	0.00367727	0.59642804
86.4	4.05E-07	4.87E-06	2.43E-10	0.47526756	1.04E-56	0.00361509	0.58992105
86.5	4.06E-07	4.46E-06	2.36E-10	0.48764968	9.37E-57	0.00343797	0.58724828
86.6	3.84E-07	4.19E-06	2.61E-10	0.48569953	9.14E-57	0.00327287	0.58969747
86.7	3.90E-07	4.14E-06	2.84E-10	0.49033754	9.99E-57	0.00323778	0.58530515
86.8	3.90E-07	4.03E-06	3.24E-10	0.49789865	9.54E-57	0.00319378	0.58707387
86.9	3.88E-07	3.98E-06	3.31E-10	0.49386119	1.17E-56	0.00303904	0.58293552
87	3.72E-07	3.97E-06	3.41E-10	0.49379741	1.18E-56	0.00306144	0.58612136
87.1	3.60E-07	3.97E-06	3.44E-10	0.48952177	1.26E-56	0.00297534	0.59189915
87.2	3.62E-07	3.96E-06	3.30E-10	0.49154544	1.17E-56	0.00288767	0.5906206
87.3	3.64E-07	3.93E-06	3.30E-10	0.49657521	1.09E-56	0.00280618	0.59989448
87.4	3.42E-07	3.84E-06	4.12E-10	0.50632519	1.13E-56	0.00266094	0.59924335
87.5	3.38E-07	3.66E-06	4.27E-10	0.50560854	1.34E-56	0.00269893	0.60205862
87.6	3.18E-07	3.60E-06	4.55E-10	0.50898279	1.22E-56	0.00266372	0.59351141
87.7	2.90E-07	3.64E-06	4.76E-10	0.50250773	1.21E-56	0.00263971	0.58371115
87.8	2.94E-07	3.63E-06	4.98E-10	0.49591459	1.21E-56	0.00255672	0.58769809
87.9	2.93E-07	3.67E-06	4.89E-10	0.50023921	1.16E-56	0.00261774	0.59241757
88	2.81E-07	3.47E-06	5.00E-10	0.50226561	1.16E-56	0.00260693	0.60412715
88.1	2.75E-07	3.18E-06	5.74E-10	0.50734375	1.15E-56	0.00243153	0.59839877
88.2	2.78E-07	3.09E-06	6.23E-10	0.5093957	1.31E-56	0.00237322	0.59796335
88.3	2.71E-07	3.01E-06	6.55E-10	0.50822091	1.25E-56	0.00243735	0.59501827
88.4	2.79E-07	2.71E-06	6.72E-10	0.5194318	1.24E-56	0.00245293	0.58948274
88.5	2.80E-07	2.60E-06	7.16E-10	0.51229115	1.17E-56	0.00237545	0.59302187
88.6	2.60E-07	2.47E-06	8.01E-10	0.52023909	1.17E-56	0.00230586	0.59666914
88.7	2.56E-07	2.54E-06	8.31E-10	0.51926494	1.19E-56	0.00224046	0.5943033
88.8	2.58E-07	2.49E-06	8.74E-10	0.52599699	1.33E-56	0.0022363	0.60080913
88.9	2.50E-07	2.46E-06	8.89E-10	0.53011661	1.15E-56	0.00228104	0.59730148
89	2.39E-07	2.45E-06	9.47E-10	0.53006495	1.21E-56	0.00236989	0.59537657
89.1	2.35E-07	2.35E-06	9.89E-10	0.53170278	1.18E-56	0.00232482	0.5918213
89.2	2.26E-07	2.45E-06	1.29E-09	0.53370001	1.48E-56	0.00230353	0.59065373
89.3	2.21E-07	2.61E-06	1.29E-09	0.52893548	1.31E-56	0.00227974	0.6093637
89.4	2.11E-07	2.50E-06	1.43E-09	0.52211462	1.29E-56	0.00221361	0.6117982
89.5	2.06E-07	2.29E-06	1.62E-09	0.52761121	1.23E-56	0.00205781	0.61016702
89.6	2.28E-07	2.12E-06	1.59E-09	0.52371389	1.07E-56	0.00198638	0.61742309
89.7	2.28E-07	1.99E-06	1.82E-09	0.52353634	1.07E-56	0.00203762	0.6208611
89.8	2.06E-07	1.96E-06	1.90E-09	0.51097688	1.04E-56	0.00196284	0.62908154
89.9	1.98E-07	1.96E-06	1.98E-09	0.51070055	9.88E-57	0.00194083	0.62515358
135	7.52E-07	5.56E-07	2.68E-05	0.36934651	3.27E-56	2.18E-07	0.98279488
135.1	7.89E-07	5.34E-07	2.48E-05	0.36596685	2.80E-56	2.10E-07	0.97794537
135.2	7.89E-07	5.34E-07	2.48E-05	0.36596685	2.80E-56	2.10E-07	0.97794537
135.3	7.88E-07	5.70E-07	2.46E-05	0.37228933	2.79E-56	2.01E-07	0.96887515
135.4	8.03E-07	5.60E-07	2.50E-05	0.36871922	2.99E-56	2.07E-07	0.96800783
135.5	8.40E-07	5.83E-07	2.50E-05	0.37110613	3.16E-56	2.05E-07	0.97075469
135.6	8.00E-07	5.81E-07	2.55E-05	0.37012267	2.94E-56	1.97E-07	0.96702784
135.7	8.22E-07	5.41E-07	2.51E-05	0.3726118	2.78E-56	2.03E-07	0.96154233
135.8	8.22E-07	5.24E-07	2.47E-05	0.37033088	2.63E-56	2.01E-07	0.96409187
135.9	8.83E-07	4.82E-07	2.38E-05	0.37287592	2.38E-56	1.91E-07	0.97954445
136	8.70E-07	4.73E-07	2.38E-05	0.37724257	2.68E-56	1.88E-07	0.97856083
136.1	8.70E-07	4.73E-07	2.38E-05	0.37724257	2.68E-56	1.88E-07	0.97856083
136.2	8.65E-07	4.78E-07	2.22E-05	0.37414257	2.26E-56	1.74E-07	0.97982076
136.3	9.00E-07	4.91E-07	2.28E-05	0.37000647	2.40E-56	1.73E-07	0.97212192
136.4	8.93E-07	5.05E-07	2.44E-05	0.36408646	2.42E-56	1.68E-07	0.9750514
136.5	9.03E-07	5.16E-07	2.46E-05	0.36612718	2.48E-56	1.66E-07	0.97897558
136.6	8.94E-07	5.02E-07	2.47E-05	0.36269143	2.50E-56	1.61E-07	0.9833859
136.7	8.98E-07	4.88E-07	2.44E-05	0.36413044	2.40E-56	1.54E-07	0.98710517
136.8	8.98E-07	4.88E-07	2.44E-05	0.36413044	2.40E-56	1.54E-07	0.98710517
136.9	8.98E-07	4.88E-07	2.44E-05	0.36413044	2.40E-56	1.54E-07	0.98710517
137	8.98E-07	4.88E-07	2.44E-05	0.36413044	2.40E-56	1.54E-07	0.98710517
137.1	8.98E-07	4.88E-07	2.44E-05	0.36413044	2.40E-56	1.54E-07	0.98710517
137.2	9.13E-07	4.88E-07	2.37E-05	0.36322973	2.39E-56	1.52E-07	0.98318399
137.3	9.20E-07	4.78E-07	2.31E-05	0.36384642	2.20E-56	1.49E-07	0.98019959
137.4	9.33E-07	4.89E-07	2.21E-05	0.36101782	2.18E-56	1.42E-07	0.97771663
137.5	9.15E-07	5.01E-07	2.10E-05	0.36486615	1.90E-56	1.36E-07	0.97022398
137.6	9.10E-07	5.22E-07	2.15E-05	0.3678962	1.99E-56	1.35E-07	0.97210538
137.7	8.93E-07	5.03E-07	2.17E-05	0.36541551	1.86E-56	1.40E-07	0.96921781
137.8	8.82E-07	4.80E-07	2.10E-05	0.36255898	1.75E-56	1.36E-07	0.96990133
137.9	8.93E-07	4.74E-07	2.05E-05	0.35875999	1.50E-56	1.32E-07	0.97309501
138	9.72E-07	4.73E-07	2.06E-05	0.35606222	1.48E-56	1.28E-07	0.97383394
138.1	9.95E-07	4.56E-07	2.02E-05	0.36285788	1.23E-56	1.23E-07	0.97614329
138.2	9.75E-07	4.82E-07	2.04E-05	0.36570092	1.39E-56	1.24E-07	0.97090306
138.3	9.48E-07	4.91E-07	2.04E-05	0.36755602	1.25E-56	1.16E-07	0.96723675
138.4	9.57E-07	4.86E-07	1.93E-05	0.36262521	1.11E-56	1.12E-07	0.96248643
138.5	9.40E-07	4.93E-07	1.88E-05	0.3651945	9.85E-57	1.11E-07	0.96362209
138.6	9.52E-07	5.05E-07	1.88E-05	0.36528968	9.92E-57	1.09E-07	0.96498702
138.7	9.52E-07	5.05E-07	1.88E-05	0.36528968	9.92E-57	1.09E-07	0.96498702
138.8	9.52E-07	5.05E-07	1.88E-05	0.36528968	9.92E-57	1.09E-07	0.96498702
138.9	9.91E-07	5.02E-07	1.82E-05	0.36673556	9.89E-57	1.10E-07	0.95842297
139	1.00E-06	4.94E-07	1.79E-05	0.36640993	9.51E-57	1.09E-07	0.96161608
139.1	1.05E-06	4.84E-07	1.70E-05	0.3718537	8.93E-57	1.09E-07	0.96022374
139.2	1.11E-06	4.79E-07	1.70E-05	0.37128494	9.69E-57	1.04E-07	0.96299036
139.3	1.11E-06	4.90E-07	1.71E-05	0.37154709	9.47E-57	1.04E-07	0.95579178
139.4	1.12E-06	5.05E-07	1.74E-05	0.37067039	1.05E-56	9.96E-08	0.96079652
139.5	1.12E-06	5.05E-07	1.74E-05	0.37067039	1.05E-56	9.96E-08	0.96079652
139.6	1.12E-06	5.25E-07	1.78E-05	0.36885423	1.12E-56	9.88E-08	0.95830028
139.7	1.08E-06	5.81E-07	1.71E-05	0.37240787	1.09E-56	9.67E-08	0.95211745
139.8	1.16E-06	5.75E-07	1.72E-05	0.380716	1.09E-56	9.59E-08	0.94825309
139.9	1.18E-06	5.72E-07	1.67E-05	0.37976785	1.06E-56	9.43E-08	0.9506838
140	1.24E-06	5.71E-07	1.67E-05	0.38344505	1.03E-56	9.35E-08	0.95492004
140.1	1.24E-06	5.45E-07	1.66E-05	0.3868388	9.75E-57	9.26E-08	0.95706446
140.2	1.24E-06	5.07E-07	1.71E-05	0.37858122	1.05E-56	9.17E-08	0.95983097
140.3	1.25E-06	5.08E-07	1.73E-05	0.38084883	1.20E-56	9.07E-08	0.95216696
140.4	1.26E-06	5.03E-07	1.68E-05	0.38204091	1.13E-56	8.72E-08	0.95550565
140.5	1.26E-06	5.03E-07	1.68E-05	0.38204091	1.13E-56	8.72E-08	0.95550565
140.6	1.25E-06	5.02E-07	1.71E-05	0.38209638	1.17E-56	8.59E-08	0.95968936
140.7	1.24E-06	5.07E-07	1.66E-05	0.38054128	1.15E-56	8.53E-08	0.95885605
140.8	1.17E-06	5.17E-07	1.67E-05	0.38088734	1.12E-56	8.11E-08	0.95815572
140.9	1.14E-06	5.58E-07	1.70E-05	0.37579962	1.05E-56	7.79E-08	0.95419913
141	1.12E-06	5.70E-07	1.68E-05	0.37660149	1.03E-56	7.74E-08	0.95024304

99	4.70E-08	5.48E-07	2.46E-07	0.50896113	1.21E-55	0.00034968	0.69197045
99.1	4.54E-08	5.54E-07	2.55E-07	0.50434141	1.16E-55	0.00032778	0.69367966
99.2	4.45E-08	5.41E-07	2.79E-07	0.50804535	1.23E-55	0.00033196	0.69171576
99.3	4.43E-08	5.42E-07	2.74E-07	0.51211368	1.20E-55	0.00033374	0.69545838
99.4	4.46E-08	5.31E-07	2.79E-07	0.51456177	1.18E-55	0.00032313	0.695524
99.5	4.48E-08	5.15E-07	2.99E-07	0.51671222	1.32E-55	0.00032839	0.69520299
99.6	4.33E-08	4.87E-07	2.98E-07	0.51921731	1.26E-55	0.00032317	0.692836
99.7	4.42E-08	4.89E-07	3.01E-07	0.52173945	1.30E-55	0.00032732	0.6970992
99.8	4.46E-08	4.80E-07	3.24E-07	0.52259699	1.47E-55	0.00032164	0.69728307
99.9	4.65E-08	5.05E-07	3.53E-07	0.52601526	1.68E-55	0.00031699	0.69885732
100	4.76E-08	4.82E-07	3.65E-07	0.53425518	1.73E-55	0.00029993	0.70424907
100.1	4.71E-08	4.84E-07	3.74E-07	0.53528647	1.71E-55	0.00029444	0.70405461
100.2	4.77E-08	5.02E-07	3.79E-07	0.5274145	1.74E-55	0.0002785	0.70032373
100.3	4.89E-08	5.03E-07	3.92E-07	0.52795798	1.73E-55	0.00028031	0.69929838
100.4	4.87E-08	4.63E-07	3.99E-07	0.53207885	1.74E-55	0.00028066	0.67924111
100.5	4.93E-08	4.85E-07	4.44E-07	0.52802442	2.20E-55	0.00028829	0.68281356
100.6	4.94E-08	4.84E-07	4.67E-07	0.52536175	2.23E-55	0.00028531	0.68001374
100.7	4.79E-08	4.92E-07	4.80E-07	0.52551024	2.43E-55	0.00028945	0.6797745
100.8	4.75E-08	4.77E-07	4.98E-07	0.52243764	2.40E-55	0.00028697	0.67821124
100.9	4.43E-08	4.69E-07	5.52E-07	0.5281574	2.32E-55	0.00029039	0.67356614
101	4.40E-08	4.78E-07	5.60E-07	0.52423716	2.33E-55	0.00028947	0.67313416
101.1	4.54E-08	4.66E-07	6.16E-07	0.51874356	3.12E-55	0.00028493	0.66759615
101.2	4.57E-08	4.88E-07	6.80E-07	0.52079198	3.66E-55	0.00028862	0.67066755
101.3	4.62E-08	5.09E-07	7.14E-07	0.52583059	4.15E-55	0.0002894	0.6684532
101.4	4.45E-08	5.13E-07	7.23E-07	0.5246858	3.96E-55	0.00029211	0.6707557
101.5	4.47E-08	5.24E-07	7.59E-07	0.51700941	3.50E-55	0.00027689	0.67784169
101.6	4.57E-08	5.06E-07	7.57E-07	0.51199198	3.25E-55	0.00027206	0.69069212
101.7	4.51E-08	5.20E-07	8.04E-07	0.51178265	3.26E-55	0.00027642	0.69079966
101.8	4.55E-08	5.05E-07	8.14E-07	0.50734116	3.33E-55	0.00027974	0.68585381
101.9	4.58E-08	5.24E-07	8.45E-07	0.50746109	3.57E-55	0.00027312	0.68446921
102	4.53E-08	5.19E-07	8.41E-07	0.50655135	3.42E-55	0.00026884	0.68388966
102.1	4.48E-08	5.38E-07	8.42E-07	0.50828653	3.33E-55	0.00026391	0.68210101
102.2	4.77E-08	5.61E-07	8.71E-07	0.50991743	3.93E-55	0.00027186	0.67688799
102.3	4.83E-08	5.55E-07	8.63E-07	0.50792887	3.88E-55	0.000262	0.67752527
102.4	4.68E-08	5.82E-07	9.20E-07	0.50161331	3.86E-55	0.00026244	0.67943713
102.5	4.50E-08	5.56E-07	9.58E-07	0.49922873	3.44E-55	0.00025113	0.67894886
102.6	4.48E-08	5.83E-07	9.54E-07	0.49434624	3.49E-55	0.00023884	0.68420428
102.7	4.53E-08	5.49E-07	1.06E-06	0.49094778	3.47E-55	0.00023815	0.69185873
102.8	4.49E-08	5.47E-07	1.08E-06	0.48843913	3.74E-55	0.00023718	0.69405624
102.9	4.49E-08	5.63E-07	1.12E-06	0.48853151	4.05E-55	0.00023282	0.702406
103	4.55E-08	5.91E-07	1.18E-06	0.4888276	4.02E-55	0.00022458	0.6938399
103.1	4.75E-08	5.90E-07	1.19E-06	0.48483121	4.27E-55	0.0002201	0.69275394
103.2	4.76E-08	5.70E-07	1.26E-06	0.4765783	4.46E-55	0.00022769	0.68662795
103.3	4.80E-08	5.72E-07	1.27E-06	0.48056378	4.58E-55	0.00021982	0.68846624
103.4	4.66E-08	5.60E-07	1.32E-06	0.47962568	4.35E-55	0.00021799	0.69385957
103.5	4.53E-08	5.62E-07	1.44E-06	0.48052903	3.98E-55	0.00020841	0.69639305
103.6	4.53E-08	5.74E-07	1.47E-06	0.47660125	4.28E-55	0.00020535	0.69912177
103.7	4.90E-08	5.56E-07	1.40E-06	0.47526565	4.38E-55	0.00020698	0.69197712
103.8	4.80E-08	5.77E-07	1.43E-06	0.47475067	4.12E-55	0.00019925	0.6866643
103.9	4.65E-08	5.84E-07	1.46E-06	0.47208554	4.11E-55	0.00019332	0.68131322
104	4.89E-08	5.78E-07	1.52E-06	0.47374883	4.90E-55	0.00019336	0.68118975
104.1	4.96E-08	5.83E-07	1.54E-06	0.47531701	4.83E-55	0.0001913	0.67929049
104.2	5.14E-08	5.98E-07	1.50E-06	0.48139008	4.74E-55	0.00018988	0.68590324
104.3	4.92E-08	5.65E-07	1.61E-06	0.47123388	4.02E-55	0.00018452	0.69028876
104.4	4.84E-08	5.79E-07	1.65E-06	0.47298014	4.09E-55	0.00017612	0.69436191
104.5	5.27E-08	5.63E-07	1.69E-06	0.47910228	4.83E-55	0.00018181	0.69186656
104.6	5.39E-08	5.51E-07	1.72E-06	0.48107966	5.08E-55	0.00018124	0.69322169
104.7	5.27E-08	5.32E-07	1.74E-06	0.48096116	4.79E-55	0.00017905	0.68866746
104.8	5.02E-08	5.22E-07	1.70E-06	0.48326309	4.62E-55	0.00017518	0.67636292
104.9	4.98E-08	5.18E-07	1.91E-06	0.48013407	4.98E-55	0.00017191	0.67295073
150	2.05E-06	8.30E-07	3.88E-06	0.38327145	1.81E-57	1.35E-08	0.8351311
150.1	2.18E-06	8.33E-07	3.94E-06	0.37992788	1.83E-57	1.34E-08	0.82979245
150.2	2.09E-06	8.72E-07	3.87E-06	0.3842508	1.71E-57	1.31E-08	0.82890142
150.3	2.05E-06	8.58E-07	3.96E-06	0.38591589	1.62E-57	1.28E-08	0.82664263
150.4	2.08E-06	8.74E-07	4.04E-06	0.37983178	1.71E-57	1.24E-08	0.81991815
150.5	2.05E-06	8.78E-07	4.15E-06	0.38052895	1.81E-57	1.17E-08	0.81995151
150.6	2.06E-06	9.07E-07	3.71E-06	0.38771949	1.74E-57	1.07E-08	0.81446895
150.7	2.15E-06	9.25E-07	3.68E-06	0.38851523	1.96E-57	1.09E-08	0.80901904
150.8	2.14E-06	9.59E-07	3.33E-06	0.38639734	1.82E-57	9.80E-09	0.79824738
150.9	2.11E-06	9.77E-07	3.36E-06	0.3873739	1.92E-57	9.83E-09	0.79956615
151	2.18E-06	9.34E-07	3.15E-06	0.38860855	2.06E-57	8.97E-09	0.79720248
151.1	2.08E-06	9.73E-07	3.24E-06	0.38871138	2.01E-57	8.78E-09	0.79747846
151.2	1.93E-06	1.07E-06	3.31E-06	0.3944349	2.02E-57	8.67E-09	0.79288752
151.3	1.98E-06	1.04E-06	3.29E-06	0.40082551	2.10E-57	8.64E-09	0.79168173
151.4	2.06E-06	1.03E-06	3.15E-06	0.39824815	2.10E-57	8.44E-09	0.79616902
151.5	2.04E-06	1.11E-06	3.21E-06	0.40186249	2.42E-57	8.30E-09	0.79554691
151.6	2.07E-06	1.08E-06	3.05E-06	0.39553681	2.19E-57	7.96E-09	0.79481716
151.7	2.09E-06	1.13E-06	2.94E-06	0.40449433	1.97E-57	7.66E-09	0.80113222
151.8	2.14E-06	1.09E-06	2.80E-06	0.40246202	1.65E-57	7.26E-09	0.80243197
151.9	2.23E-06	1.14E-06	2.86E-06	0.40409793	2.04E-57	6.93E-09	0.79446156
152	2.31E-06	1.11E-06	2.70E-06	0.39684399	1.93E-57	6.62E-09	0.78074967
152.1	2.31E-06	1.11E-06	2.70E-06	0.39684399	1.93E-57	6.62E-09	0.78074967
152.2	2.42E-06	1.09E-06	2.62E-06	0.40237305	1.76E-57	6.61E-09	0.77523827
152.3	2.42E-06	1.09E-06	2.62E-06	0.40237305	1.76E-57	6.61E-09	0.77523827
152.4	2.47E-06	1.09E-06	2.62E-06	0.39934463	1.74E-57	6.31E-09	0.78126187
152.5	2.31E-06	1.11E-06	2.54E-06	0.3962704	1.43E-57	5.94E-09	0.7827137
152.6	2.31E-06	1.11E-06	2.54E-06	0.3962704	1.43E-57	5.94E-09	0.7827137
152.7	2.30E-06	1.08E-06	2.46E-06	0.39313424	1.30E-57	6.00E-09	0.77922101
152.8	2.34E-06	1.07E-06	2.41E-06	0.39071672	1.25E-57	5.70E-09	0.77318079
152.9	2.37E-06	1.03E-06	2.27E-06	0.39256671	1.20E-57	5.41E-09	0.77182735
153	2.40E-06	1.02E-06	2.30E-06	0.39359075	1.19E-57	5.28E-09	0.77009211
153.1	2.40E-06	1.02E-06	2.30E-06	0.39359075	1.19E-57	5.28E-09	0.77009211
153.2	2.51E-06	1.02E-06	2.30E-06	0.39557757	1.19E-57	5.02E-09	0.77650122
153.3	2.64E-06	9.95E-07	2.40E-06	0.3941358	1.25E-57	5.26E-09	0.77444959
153.4	2.51E-06	1.01E-06	2.38E-06	0.3968427	1.13E-57	5.31E-09	0.7727727
153.5	2.51E-06	1.01E-06	2.38E-06	0.3968427	1.13E-57	5.31E-09	0.7727727
153.6	2.55E-06	1.03E-06	2.37E-06	0.39775128	1.13E-57	5.31E-09	0.77162308
153.7	2.55E-06	1.03E-06	2.37E-06	0.39775128	1.13E-57	5.31E-09	0.77162308
153.8	2.41E-06	1.08E-06	2.40E-06	0.40274498	1.04E-57	4.90E-09	0.77501117
153.9	2.36E-06	1.12E-06	2.21E-06	0.40598207	1.08E-57	4.84E-09	0.77413912
154	2.37E-06	1.08E-06	2.24E-06	0.40699897	1.13E-57	4.97E-09	0.76814649
154.1	2.37E-06	1.08E-06	2.24E-06	0.40699897	1.13E-57	4.97E-09	0.76814649
154.2	2.38E-06	1.16E-06	2.28E-06	0.41114357	1.36E-57	4.74E-09	0.76429558
154.3	2.38E-06	1.16E-06	2.28E-06	0.41114357	1.36E-57	4.74E-09	0.76429558
154.4	2.38E-06	1.16E-06	2.28E-06	0.41114357	1.36E-57	4.74E-09	0.76429558
154.5	2.37E-06	1.17E-06	2.27E-06	0.41201214	1.40E-57	4.69E-09	0.75823347
154.6	2.36E-06	1.09E-06	2.18E-06	0.40936789	1.31E-57	4.58E-09	0.76192037
154.7	2.49E-06	1.11E-06	2.19E-06	0.41853275	1.55E-57	4.57E-09	0.75472732
154.8	2.49E-06	1.11E-06	2.19E-06	0.41853275	1.55E-57	4.57E-09	0.75472732
154.9	2.45E-06	1.13E-06	2.09E-06	0.42092651	1.44E-57	4.40E-09	0.75413351
155	2.45E-06	1.13E-06	2.09E-06	0.42092651	1.44E-57	4.40E-09	0.75413351
155.1	2.51E-06	1.17E-06	2.15E-06	0.41659466	1.56E-57	4.28E-09	0.74857704
155.2	2.45E-06	1.19E-06	2.10E-06	0.42562181	1.49E-57	4.23E-09	0.75287568
155.3	2.52E-06	1.20E-06	2.16E-06	0.42188785	1.52E-57	4.24E-09	0.74504436
155.4	2.54E-06	1.17E-06	2.12E-06	0.42225608	1.55E-57	4.14E-09	0.75213651
155.5	2.71E-06	1.12E-06	2.23E-06	0.41970015	1.64E-57	4.05E-09	0.75023019
155.6	2.75E-06	1.09E-06	2.24E-06	0.41990129	1.75E-57	3.93E-09	0.74549102
155.7	2.63E-06	1.17E-06	2.23E-06	0.41972015	1.74E-57	3.66E-09	0.73953083
155.8	2.62E-06	1.19E-06	2.20E-06	0.4168606	1.61E-57	3.48E-09	0.73861706
155.9	2.64E-06	1.22E-06	2.19E-06	0.41461385	1.56E-57	3.49E-09	0.74088899
156	2.70E-06	1.25E-06	2.15E-06	0.40986005	1.86E-57	3.42E-09	0.74229712

114	7.76E-08	3.90E-07	1.21E-05	0.44429218	3.07E-55	3.17E-05	0.76613907
114.1	8.42E-08	4.00E-07	1.25E-05	0.43824691	3.04E-55	2.96E-05	0.77338809
114.2	8.27E-08	3.69E-07	1.22E-05	0.43348941	2.75E-55	2.81E-05	0.77711296
114.3	8.33E-08	3.59E-07	1.20E-05	0.43092887	2.89E-55	2.80E-05	0.78301924
114.4	8.58E-08	3.60E-07	1.19E-05	0.43071974	3.34E-55	2.81E-05	0.78892929
114.5	8.59E-08	3.59E-07	1.21E-05	0.43216893	3.45E-55	2.81E-05	0.78479837
114.6	8.60E-08	3.92E-07	1.20E-05	0.4300828	3.38E-55	2.67E-05	0.78332635
114.7	8.60E-08	3.92E-07	1.20E-05	0.4300828	3.38E-55	2.67E-05	0.78332635
114.8	8.36E-08	3.87E-07	1.22E-05	0.43176441	3.05E-55	2.64E-05	0.78331829
114.9	8.28E-08	3.84E-07	1.25E-05	0.42619816	3.10E-55	2.69E-05	0.77901606
115	8.53E-08	3.93E-07	1.40E-05	0.42342321	3.22E-55	2.58E-05	0.7923897
115.1	8.41E-08	3.98E-07	1.47E-05	0.4218088	3.58E-55	2.65E-05	0.79857367
115.2	8.05E-08	3.91E-07	1.51E-05	0.42100397	3.10E-55	2.58E-05	0.80208513
115.3	7.94E-08	3.96E-07	1.52E-05	0.41984344	3.02E-55	2.50E-05	0.79634681
115.4	8.29E-08	3.98E-07	1.56E-05	0.41997831	2.95E-55	2.47E-05	0.79918559
115.5	8.46E-08	3.97E-07	1.55E-05	0.42133512	3.11E-55	2.43E-05	0.79948778
115.6	7.99E-08	3.94E-07	1.57E-05	0.42212735	2.99E-55	2.35E-05	0.80759986
115.7	8.04E-08	3.97E-07	1.57E-05	0.41248888	2.66E-55	2.26E-05	0.81458586
115.8	8.10E-08	4.03E-07	1.61E-05	0.40980757	2.55E-55	2.29E-05	0.81114363
115.9	8.19E-08	4.05E-07	1.67E-05	0.41483816	2.19E-55	2.26E-05	0.8074371
116	8.28E-08	4.05E-07	1.63E-05	0.41491248	2.08E-55	2.24E-05	0.80572579
116.1	8.58E-08	3.92E-07	1.65E-05	0.41107852	1.98E-55	2.17E-05	0.80932131
116.2	8.60E-08	3.98E-07	1.67E-05	0.41206008	1.82E-55	2.10E-05	0.80504084
116.3	9.02E-08	3.86E-07	1.74E-05	0.4100065	1.97E-55	2.12E-05	0.80500667
116.4	9.02E-08	3.86E-07	1.74E-05	0.4100065	1.97E-55	2.12E-05	0.80500667
116.5	9.19E-08	4.04E-07	1.75E-05	0.40901908	1.99E-55	2.08E-05	0.80835054
116.6	9.86E-08	3.75E-07	1.75E-05	0.40652179	2.25E-55	2.00E-05	0.8173423
116.7	9.67E-08	3.68E-07	1.71E-05	0.4092411	2.34E-55	1.88E-05	0.8232081
116.8	9.54E-08	3.74E-07	1.74E-05	0.41556153	2.24E-55	1.87E-05	0.82208366
116.9	9.88E-08	3.63E-07	1.75E-05	0.4286723	2.01E-55	1.83E-05	0.82186845
117	9.91E-08	3.65E-07	1.75E-05	0.42878202	1.88E-55	1.80E-05	0.82393014
117.1	1.04E-07	3.52E-07	1.68E-05	0.42136189	1.90E-55	1.67E-05	0.82854988
117.2	1.05E-07	3.52E-07	1.68E-05	0.4223256	1.85E-55	1.61E-05	0.82991656
117.3	1.08E-07	3.54E-07	1.66E-05	0.41757666	2.02E-55	1.59E-05	0.83016804
117.4	1.10E-07	3.53E-07	1.60E-05	0.41764548	1.85E-55	1.54E-05	0.83491079
117.5	1.07E-07	3.43E-07	1.60E-05	0.41363385	1.71E-55	1.54E-05	0.84051227
117.6	1.06E-07	3.34E-07	1.57E-05	0.41160218	1.74E-55	1.45E-05	0.84727921
117.7	1.10E-07	3.26E-07	1.54E-05	0.41291529	1.72E-55	1.39E-05	0.84734974
117.8	1.13E-07	3.27E-07	1.58E-05	0.41598377	1.82E-55	1.29E-05	0.84637589
117.9	1.11E-07	3.23E-07	1.50E-05	0.42660451	1.75E-55	1.19E-05	0.8415532
118	1.15E-07	3.38E-07	1.45E-05	0.42495973	1.88E-55	1.16E-05	0.8413591
118.1	1.15E-07	3.38E-07	1.45E-05	0.42495973	1.88E-55	1.16E-05	0.8413591
118.2	1.11E-07	3.20E-07	1.47E-05	0.42015452	1.89E-55	1.05E-05	0.82386301
118.3	1.13E-07	3.09E-07	1.48E-05	0.42147562	1.81E-55	1.02E-05	0.82080263
118.4	1.17E-07	2.98E-07	1.52E-05	0.416934	1.83E-55	1.02E-05	0.81593507
118.5	1.11E-07	2.98E-07	1.58E-05	0.41844438	1.77E-55	1.02E-05	0.8160648
118.6	1.12E-07	3.01E-07	1.58E-05	0.41708865	1.83E-55	1.02E-05	0.81871845
118.7	1.12E-07	3.08E-07	1.55E-05	0.41253593	1.76E-55	9.34E-06	0.8154704
118.8	1.03E-07	3.15E-07	1.47E-05	0.40611598	1.40E-55	9.16E-06	0.81779704
118.9	1.04E-07	3.19E-07	1.42E-05	0.40651545	1.25E-55	8.85E-06	0.81873986
119	1.08E-07	3.19E-07	1.47E-05	0.403428	1.35E-55	9.00E-06	0.82473105
119.1	1.09E-07	3.21E-07	1.51E-05	0.40175426	1.42E-55	8.56E-06	0.81827057
119.2	1.12E-07	3.59E-07	1.57E-05	0.3913345	1.61E-55	7.56E-06	0.81760944
119.3	1.08E-07	3.59E-07	1.65E-05	0.3916034	1.49E-55	7.37E-06	0.81385214
119.4	1.05E-07	3.67E-07	1.66E-05	0.38644904	1.38E-55	6.84E-06	0.81864912
119.5	1.01E-07	3.72E-07	1.65E-05	0.38755845	1.33E-55	6.56E-06	0.8202006
119.6	1.01E-07	3.55E-07	1.69E-05	0.38745828	1.28E-55	6.57E-06	0.81951584
119.7	1.01E-07	3.68E-07	1.80E-05	0.38244675	1.09E-55	6.17E-06	0.8117423
119.8	9.87E-08	3.60E-07	1.84E-05	0.38110115	1.12E-55	6.07E-06	0.81101895
119.9	1.04E-07	3.77E-07	1.90E-05	0.38494721	1.23E-55	6.00E-06	0.81150014
165	5.46E-06	1.32E-06	1.42E-06	0.40472631	1.81E-57	1.11E-09	0.72407207
165.1	5.52E-06	1.30E-06	1.45E-06	0.4045224	1.87E-57	1.10E-09	0.7247168
165.2	5.76E-06	1.33E-06	1.46E-06	0.40844831	2.03E-57	1.09E-09	0.72126608
165.3	5.61E-06	1.35E-06	1.46E-06	0.4126069	1.97E-57	1.05E-09	0.72291413
165.4	5.63E-06	1.36E-06	1.45E-06	0.41193259	2.02E-57	1.03E-09	0.7174563
165.5	5.58E-06	1.36E-06	1.44E-06	0.41467887	2.04E-57	1.03E-09	0.71529916
165.6	5.81E-06	1.35E-06	1.39E-06	0.41179318	1.99E-57	9.88E-10	0.71121725
165.7	5.85E-06	1.36E-06	1.39E-06	0.41634612	2.00E-57	9.78E-10	0.71026977
165.8	6.17E-06	1.39E-06	1.45E-06	0.42056726	2.11E-57	9.72E-10	0.70538828
165.9	6.15E-06	1.36E-06	1.37E-06	0.41652711	1.68E-57	9.51E-10	0.70726126
166	6.20E-06	1.40E-06	1.34E-06	0.41787792	1.61E-57	9.16E-10	0.70820182
166.1	6.35E-06	1.54E-06	1.35E-06	0.42256138	1.88E-57	9.49E-10	0.71282085
166.2	6.92E-06	1.43E-06	1.25E-06	0.41751248	1.82E-57	9.58E-10	0.71068956
166.3	7.09E-06	1.38E-06	1.30E-06	0.42220529	1.86E-57	9.64E-10	0.71471911
166.4	6.65E-06	1.33E-06	1.26E-06	0.42090388	1.75E-57	9.67E-10	0.71613202
166.5	6.99E-06	1.22E-06	1.25E-06	0.42554828	1.80E-57	9.34E-10	0.71781757
166.6	7.03E-06	1.25E-06	1.22E-06	0.42869556	1.63E-57	9.06E-10	0.71886557
166.7	7.03E-06	1.25E-06	1.22E-06	0.42869556	1.63E-57	9.06E-10	0.71886557
166.8	7.03E-06	1.25E-06	1.22E-06	0.42869556	1.63E-57	9.06E-10	0.71886557
166.9	7.03E-06	1.25E-06	1.22E-06	0.42869556	1.63E-57	9.06E-10	0.71886557
167	7.48E-06	1.28E-06	1.17E-06	0.42009208	1.78E-57	8.61E-10	0.72140167
167.1	7.02E-06	1.27E-06	1.18E-06	0.41830759	1.53E-57	8.45E-10	0.72446045
167.2	7.29E-06	1.20E-06	1.14E-06	0.41381374	1.65E-57	8.33E-10	0.72860948
167.3	7.42E-06	1.20E-06	1.09E-06	0.41314769	1.67E-57	8.14E-10	0.73026727
167.4	7.69E-06	1.16E-06	1.11E-06	0.41208471	1.69E-57	7.87E-10	0.73019075
167.5	8.11E-06	1.17E-06	1.08E-06	0.41211816	1.67E-57	7.69E-10	0.72328945
167.6	8.59E-06	1.18E-06	1.15E-06	0.41008826	1.92E-57	7.45E-10	0.71782089
167.7	9.06E-06	1.20E-06	1.16E-06	0.40974534	1.96E-57	7.10E-10	0.72243075
167.8	9.06E-06	1.20E-06	1.16E-06	0.40974534	1.96E-57	7.10E-10	0.72243075
167.9	9.17E-06	1.21E-06	1.12E-06	0.40645703	1.71E-57	6.82E-10	0.72425587
168	9.43E-06	1.23E-06	1.09E-06	0.40680689	1.67E-57	6.75E-10	0.72465626
168.1	9.43E-06	1.23E-06	1.09E-06	0.40680689	1.67E-57	6.75E-10	0.72465626
168.2	9.89E-06	1.17E-06	1.05E-06	0.40207884	1.44E-57	6.52E-10	0.73285678
168.3	9.89E-06	1.17E-06	1.05E-06	0.40207884	1.44E-57	6.52E-10	0.73285678
168.4	1.02E-05	1.17E-06	1.02E-06	0.39905949	1.42E-57	6.59E-10	0.72990704
168.5	1.03E-05	1.23E-06	1.02E-06	0.39495919	1.34E-57	6.30E-10	0.72296874
168.6	1.05E-05	1.21E-06	1.03E-06	0.39301713	1.37E-57	6.35E-10	0.71957779
168.7	1.05E-05	1.20E-06	1.05E-06	0.38949381	1.41E-57	6.21E-10	0.71677425
168.8	1.04E-05	1.24E-06	9.79E-07	0.38558431	1.34E-57	6.14E-10	0.71835779
168.9	1.04E-05	1.24E-06	1.01E-06	0.38830377	1.33E-57	6.40E-10	0.72418758
169	1.04E-05	1.29E-06	1.02E-06	0.38614155	1.26E-57	5.97E-10	0.72821996
169.1	1.04E-05	1.29E-06	1.02E-06	0.38614155	1.26E-57	5.97E-10	0.72821996
169.2	1.04E-05	1.29E-06	1.02E-06	0.38614155	1.26E-57	5.97E-10	0.72821996
169.3	1.01E-05	1.33E-06	1.03E-06	0.38181363	1.40E-57	5.77E-10	0.7266199
169.4	1.04E-05	1.29E-06	1.03E-06	0.37628334	1.45E-57	5.54E-10	0.72756398
169.5	1.09E-05	1.27E-06	1.02E-06	0.37410347	1.45E-57	5.50E-10	0.72854556
169.6	1.09E-05	1.27E-06	1.02E-06	0.37410347	1.45E-57	5.50E-10	0.72854556
169.7	1.13E-05	1.27E-06	1.03E-06	0.37201969	1.61E-57	5.45E-10	0.72940611
169.8	1.12E-05	1.30E-06	1.04E-06	0.37364554	1.64E-57	5.51E-10	0.73165855
169.9	1.12E-05	1.29E-06	9.36E-07	0.36905075	1.44E-57	5.34E-10	0.74154184
170	1.12E-05	1.29E-06	9.36E-07	0.36905075	1.44E-57	5.34E-10	0.74154184
170.1	1.07E-05	1.24E-06	8.82E-07	0.37762347	1.28E-57	5.34E-10	0.74270212
170.2	1.07E-05	1.24E-06	8.82E-07	0.37762347	1.28E-57	5.34E-10	0.74270212
170.3	1.10E-05	1.20E-06	8.91E-07	0.37299907	1.33E-57	5.25E-10	0.74271223
170.4	1.11E-05	1.13E-06	9.10E-07	0.37315878	1.32E-57	5.39E-10	0.74189341
170.5	1.11E-05	1.08E-06	9.22E-07	0.37471006	1.30E-57	5.13E-10	0.74408952
170.6	1.11E-05	1.08E-06	9.22E-07	0.37471006	1.30E-57	5.13E-10	0.74408952
170.7	1.11E-05	1.08E-06	9.22E-07	0.37471006	1.30E-57	5.13E-10	0.74408952
170.8	1.13E-05	1.08E-06	9.28E-07	0.37646854	1.28E-57	5.14E-10	0.74080021
170.9	1.14E-05	1.09E-06	9.75E-07	0.37650977	1.37E-57	5.05E-10	0.74049541
171	1.12E-05	1.14E-06	8.60E-07	0.37449168	1.20E-57	4.63E-10	0.74103267

126.1	3.51E-07	4.59E-07	2.50E-05	0.35282363	4.44E-56	1.30E-06	0.9090414
126.2	3.45E-07	4.59E-07	2.46E-05	0.35298131	4.28E-56	1.28E-06	0.90587064
126.3	3.50E-07	4.75E-07	2.54E-05	0.35957272	4.77E-56	1.24E-06	0.9087331
126.4	3.62E-07	4.99E-07	2.58E-05	0.36419202	4.81E-56	1.23E-06	0.90189384
126.5	3.48E-07	5.07E-07	2.51E-05	0.36455086	4.71E-56	1.17E-06	0.90377636
126.6	3.56E-07	5.08E-07	2.56E-05	0.36397821	4.64E-56	1.15E-06	0.90737857
126.7	3.77E-07	5.38E-07	2.62E-05	0.36721174	4.54E-56	1.14E-06	0.89940116
126.8	3.86E-07	5.43E-07	2.59E-05	0.36801258	4.83E-56	1.10E-06	0.90359029
126.9	3.79E-07	5.63E-07	2.60E-05	0.37485872	4.28E-56	1.07E-06	0.89680658
127	3.75E-07	5.71E-07	2.67E-05	0.37344891	4.53E-56	1.04E-06	0.90446268
127.1	3.66E-07	5.72E-07	2.67E-05	0.37450037	4.58E-56	9.98E-07	0.91253605
127.2	3.71E-07	5.86E-07	2.72E-05	0.3762313	4.56E-56	1.00E-06	0.90743787
127.3	3.79E-07	5.60E-07	2.85E-05	0.37577088	4.24E-56	9.19E-07	0.91027592
127.4	3.74E-07	5.52E-07	2.68E-05	0.36984597	4.08E-56	8.79E-07	0.91483914
127.5	3.98E-07	5.34E-07	2.63E-05	0.36652326	4.25E-56	8.64E-07	0.91540467
127.6	3.87E-07	5.41E-07	2.66E-05	0.36689751	3.85E-56	8.68E-07	0.91827881
127.7	3.73E-07	5.53E-07	2.69E-05	0.36183228	3.58E-56	8.28E-07	0.92173707
127.8	3.73E-07	5.53E-07	2.69E-05	0.36183228	3.58E-56	8.28E-07	0.92173707
127.9	3.72E-07	5.57E-07	2.76E-05	0.3591799	3.62E-56	8.17E-07	0.92525783
128	3.72E-07	5.57E-07	2.76E-05	0.3591799	3.62E-56	8.17E-07	0.92525783
128.1	3.76E-07	5.84E-07	2.79E-05	0.36370612	3.84E-56	7.62E-07	0.93847272
128.2	3.80E-07	5.89E-07	2.75E-05	0.3646042	3.83E-56	7.50E-07	0.93539156
128.3	3.95E-07	6.00E-07	2.86E-05	0.36773909	4.32E-56	7.74E-07	0.93722153
128.4	4.04E-07	6.12E-07	2.81E-05	0.36983174	4.18E-56	7.60E-07	0.93159448
128.5	4.10E-07	6.16E-07	2.79E-05	0.37098345	3.99E-56	7.57E-07	0.93314853
128.6	4.26E-07	6.22E-07	2.82E-05	0.37496675	4.12E-56	7.54E-07	0.93320955
128.7	4.27E-07	6.16E-07	2.80E-05	0.37614783	4.11E-56	7.39E-07	0.92647848
128.8	4.27E-07	6.16E-07	2.80E-05	0.37614783	4.11E-56	7.39E-07	0.92647848
128.9	4.27E-07	6.16E-07	2.80E-05	0.37614783	4.11E-56	7.39E-07	0.92647848
129	4.16E-07	6.17E-07	2.79E-05	0.37798637	4.12E-56	7.07E-07	0.93049806
129.1	4.16E-07	6.17E-07	2.79E-05	0.37798637	4.12E-56	7.07E-07	0.93049806
129.2	4.20E-07	6.02E-07	2.75E-05	0.37975667	4.11E-56	6.95E-07	0.93119124
129.3	4.38E-07	6.05E-07	2.86E-05	0.38026644	3.91E-56	6.71E-07	0.92869305
129.4	4.39E-07	6.33E-07	2.76E-05	0.37221452	3.84E-56	6.03E-07	0.9432434
129.5	4.54E-07	6.38E-07	2.64E-05	0.37609655	3.87E-56	6.41E-07	0.94363767
129.6	4.54E-07	6.38E-07	2.64E-05	0.37609655	3.87E-56	6.41E-07	0.94363767
129.7	4.58E-07	6.52E-07	2.67E-05	0.37256055	4.03E-56	6.31E-07	0.94931939
129.8	4.86E-07	6.65E-07	2.68E-05	0.37548979	4.52E-56	6.21E-07	0.94815679
129.9	4.96E-07	6.59E-07	2.48E-05	0.37313925	3.90E-56	5.60E-07	0.95770479
130	4.95E-07	6.68E-07	2.54E-05	0.3674287	3.62E-56	5.36E-07	0.96306996
130.1	5.04E-07	6.75E-07	2.45E-05	0.37045714	3.52E-56	5.22E-07	0.96719671
130.2	5.12E-07	6.62E-07	2.52E-05	0.37019058	3.72E-56	5.12E-07	0.96597673
130.3	5.04E-07	7.00E-07	2.56E-05	0.36627357	3.64E-56	4.55E-07	0.97020428
130.4	4.90E-07	7.19E-07	2.46E-05	0.36665636	3.81E-56	4.44E-07	0.9703828
130.5	4.88E-07	7.17E-07	2.42E-05	0.36520524	3.51E-56	4.43E-07	0.97670444
130.6	4.88E-07	7.17E-07	2.42E-05	0.36520524	3.51E-56	4.43E-07	0.97670444
130.7	5.05E-07	7.06E-07	2.43E-05	0.36220017	3.27E-56	4.18E-07	0.98440906
130.8	4.99E-07	6.98E-07	2.55E-05	0.36259902	3.21E-56	3.99E-07	0.98543217
130.9	4.92E-07	7.17E-07	2.55E-05	0.3625432	2.91E-56	3.80E-07	0.98146344
131	5.13E-07	6.69E-07	2.53E-05	0.36434655	2.84E-56	3.62E-07	0.98018446
131.1	5.04E-07	6.55E-07	2.55E-05	0.36722933	2.88E-56	3.50E-07	0.98402821
131.2	5.04E-07	6.55E-07	2.55E-05	0.36722933	2.88E-56	3.50E-07	0.98402821
131.3	5.01E-07	6.59E-07	2.57E-05	0.3629557	3.47E-56	3.44E-07	0.98965674
131.4	5.48E-07	6.36E-07	2.60E-05	0.35643562	3.70E-56	3.29E-07	0.99873774
131.5	5.37E-07	6.51E-07	2.45E-05	0.3519054	3.61E-56	3.19E-07	0.99565216
131.6	5.38E-07	6.56E-07	2.53E-05	0.35060996	3.80E-56	3.09E-07	0.99935748
131.7	5.64E-07	6.79E-07	2.54E-05	0.35694726	4.30E-56	3.14E-07	0.99997618
131.8	5.92E-07	6.66E-07	2.63E-05	0.3590784	3.86E-56	3.16E-07	0.9958591
131.9	5.85E-07	6.65E-07	2.74E-05	0.35773152	4.10E-56	3.18E-07	0.99962308
132	5.85E-07	6.65E-07	2.74E-05	0.35773152	4.10E-56	3.18E-07	0.99962308
132.1	5.85E-07	6.65E-07	2.74E-05	0.35773152	4.10E-56	3.18E-07	0.99962308
132.2	5.82E-07	6.63E-07	2.72E-05	0.35742332	3.92E-56	3.12E-07	0.99904911
132.3	5.90E-07	6.48E-07	2.72E-05	0.35492983	3.84E-56	3.10E-07	0.99800622
132.4	6.23E-07	6.54E-07	2.68E-05	0.35255035	4.07E-56	3.12E-07	0.99403499
132.5	6.41E-07	6.47E-07	2.66E-05	0.35020458	3.76E-56	3.00E-07	0.99084056
132.6	6.60E-07	6.40E-07	2.73E-05	0.35011132	3.95E-56	2.93E-07	0.99802999
132.7	6.81E-07	6.61E-07	2.78E-05	0.35053362	4.14E-56	2.94E-07	0.99902496
132.8	7.31E-07	6.11E-07	2.74E-05	0.35035613	4.14E-56	2.89E-07	0.99159424
132.9	7.31E-07	6.11E-07	2.74E-05	0.35035613	4.14E-56	2.89E-07	0.99159424
133	7.28E-07	6.00E-07	2.77E-05	0.3490022	4.11E-56	2.88E-07	0.9887735
133.1	7.34E-07	5.98E-07	2.74E-05	0.35106623	3.95E-56	2.88E-07	0.99088445
133.2	7.44E-07	6.10E-07	2.82E-05	0.35157222	4.20E-56	2.88E-07	0.98994048
133.3	7.44E-07	6.10E-07	2.82E-05	0.35157222	4.20E-56	2.88E-07	0.98994048
133.4	7.56E-07	5.98E-07	2.70E-05	0.35013915	3.86E-56	2.77E-07	0.98484261
133.5	7.29E-07	6.19E-07	2.71E-05	0.35295601	3.62E-56	2.77E-07	0.97829598
133.6	7.45E-07	6.06E-07	2.75E-05	0.35018601	3.60E-56	2.75E-07	0.98420818
133.7	7.43E-07	5.85E-07	2.78E-05	0.35591854	3.35E-56	2.66E-07	0.97640497
133.8	7.50E-07	5.73E-07	2.75E-05	0.35544455	3.43E-56	2.49E-07	0.97414479
133.9	7.50E-07	5.73E-07	2.75E-05	0.35544455	3.43E-56	2.49E-07	0.97414479
134	7.41E-07	5.41E-07	2.69E-05	0.35826587	3.44E-56	2.47E-07	0.97981344
134.1	7.29E-07	5.30E-07	2.68E-05	0.36123886	3.30E-56	2.49E-07	0.98512538
134.2	7.29E-07	5.30E-07	2.68E-05	0.36123886	3.30E-56	2.49E-07	0.98512538
134.3	7.46E-07	5.35E-07	2.71E-05	0.35921218	3.28E-56	2.48E-07	0.98730097
134.4	7.38E-07	5.56E-07	2.70E-05	0.36228636	3.69E-56	2.39E-07	0.98614819
134.5	7.69E-07	5.39E-07	2.69E-05	0.36099972	3.71E-56	2.38E-07	0.98606959
134.6	7.62E-07	5.37E-07	2.60E-05	0.35971062	3.40E-56	2.35E-07	0.98727516
134.7	7.66E-07	5.53E-07	2.66E-05	0.3615769	3.21E-56	2.30E-07	0.98541853
134.8	7.70E-07	5.46E-07	2.66E-05	0.36592439	3.27E-56	2.24E-07	0.98287201
134.9	7.70E-07	5.46E-07	2.66E-05	0.36592439	3.27E-56	2.24E-07	0.98287201
180	2.36E-05	5.40E-07	4.91E-07	0.41036375	8.90E-58	1.48E-10	0.84802322
180.1	2.36E-05	5.40E-07	4.91E-07	0.41036375	8.90E-58	1.48E-10	0.84802322
180.2	2.42E-05	5.45E-07	4.76E-07	0.41146732	9.02E-58	1.42E-10	0.85249636
180.3	2.42E-05	5.45E-07	4.76E-07	0.41146732	9.02E-58	1.42E-10	0.85249636
180.4	2.61E-05	5.13E-07	4.72E-07	0.40796316	9.19E-58	1.42E-10	0.86045614
180.5	2.58E-05	5.02E-07	4.71E-07	0.40942956	9.08E-58	1.36E-10	0.86455402
180.6	2.62E-05	4.91E-07	4.69E-07	0.41062802	9.25E-58	1.27E-10	0.87061903
180.7	2.78E-05	5.11E-07	4.25E-07	0.41562866	8.47E-58	1.15E-10	0.88133408
180.8	2.78E-05	5.11E-07	4.25E-07	0.41562866	8.47E-58	1.15E-10	0.88133408
180.9	2.75E-05	4.93E-07	4.14E-07	0.42025017	9.10E-58	1.18E-10	0.88748882
181	2.95E-05	5.04E-07	4.40E-07	0.42228257	1.09E-57	1.23E-10	0.8902126
181.1	2.90E-05	5.09E-07	4.46E-07	0.42187144	1.10E-57	1.16E-10	0.88646739
181.2	3.11E-05	4.63E-07	4.44E-07	0.42392222	1.27E-57	1.13E-10	0.90103214
181.3	3.12E-05	4.47E-07	4.61E-07	0.42333389	1.41E-57	1.14E-10	0.90745199
181.4	3.13E-05	4.31E-07	4.59E-07	0.42640753	1.30E-57	1.09E-10	0.907118
181.5	3.11E-05	4.03E-07	4.56E-07	0.43734334	1.17E-57	1.04E-10	0.90980769
181.6	3.22E-05	3.82E-07	4.39E-07	0.4344391	9.85E-58	1.02E-10	0.90575485
181.7	3.24E-05	3.66E-07	4.38E-07	0.44005949	9.74E-58	1.02E-10	0.91084634
181.8	3.24E-05	3.66E-07	4.38E-07	0.44005949	9.74E-58	1.02E-10	0.91084634
181.9	3.24E-05	3.66E-07	4.38E-07	0.44005949	9.74E-58	1.02E-10	0.91084634
182	3.32E-05	3.46E-07	4.30E-07	0.43966135	8.96E-58	9.90E-11	0.9101427
182.1	3.29E-05	3.41E-07	4.28E-07	0.43967219	9.36E-58	9.92E-11	0.914938
182.2	3.30E-05	3.40E-07	4.24E-07	0.43779813	8.77E-58	9.93E-11	0.91126612
182.3	3.56E-05	3.32E-07	3.90E-07	0.43793357	7.99E-58	9.17E-11	0.91159585
182.4	3.64E-05	3.30E-07	3.79E-07	0.43193494	8.42E-58	9.16E-11	0.91225811
182.5	3.72E-05	3.13E-07	3.68E-07	0.43432241	6.56E-58	8.53E-11	0.91901458
182.6	3.92E-05	2.94E-07	3.52E-07	0.43589707	6.33E-58	8.12E-11	0.92537161
182.7	3.94E-05	2.95E-07	3.63E-07	0.432462	6.68E-58	8.14E-11	0.92891837
182.8	4.05E-05	2.94E-07	3.50E-07	0.43040245	6.29E-58	7.84E-11	0.93149044
182.9	4.03E-05	2.92E-07	3.31E-07	0.43124136	5.93E-58	7.55E-11	0.92890698
183	3.90E-05	3.02E-07	3.14E-07	0.43074477	5.09E-58	7.18E-11	0.93398442
183.1	3.80E-05	2.92E-07	3.08E-07	0.43040586	4.65E-58	6.80E-11	0.93221806
141.1	1.15E-06	5.65E-07	1.68E-05	0.37679446	1.10E-56	7.67E-08	0.95205082
141.2	1.13E-06	5.51E-07	1.76E-05	0.36842985	1.15E-56	7.40E-08	0.95125807
141.3	1.12E-06	5.94E-07	1.68E-05	0.36503774	9.63E-57	6.72E-08	0.96030495
141.4	1.12E-06	5.95E-07	1.61E-05	0.36402551	8.18E-57	6.57E-08	0.95555065
141.5	1.16E-06	5.69E-07	1.52E-05	0.35929877	7.45E-57	6.43E-08	0.95293474
141.6	1.16E-06	5.69E-07	1.52E-05	0.35929877	7.45E-57	6.43E-08	0.95293474
141.7	1.16E-06	5.69E-07	1.52E-05	0.35929877	7.45E-57	6.43E-08	0.95293474
141.8	1.18E-06	5.75E-07	1.46E-05	0.3585499	6.61E-57	6.14E-08	0.95212971
141.9	1.20E-06	5.76E-07	1.44E-05	0.3591316	6.54E-57	5.95E-08	0.9497433
142	1.20E-06	5.76E-07	1.44E-05	0.3591316	6.54E-57	5.95E-08	0.9497433
142.1	1.22E-06	5.74E-07	1.41E-05	0.35821319	6.08E-57	5.96E-08	0.95019622
142.2	1.22E-06	5.74E-07	1.41E-05	0.35821319	6.08E-57	5.96E-08	0.95019622
142.3	1.23E-06	5.67E-07	1.33E-05	0.35405448	4.70E-57	5.79E-08	0.95748727
142.4	1.25E-06	5.62E-07	1.34E-05	0.35306089	4.52E-57	5.50E-08	0.96707332
142.5	1.26E-06	5.63E-07	1.27E-05	0.35757093	3.93E-57	5.24E-08	0.96478642
142.6	1.32E-06	5.61E-07	1.24E-05	0.35696822	4.16E-57	5.17E-08	0.9620101
142.7	1.32E-06	5.61E-07	1.24E-05	0.35696822	4.16E-57	5.17E-08	0.9620101
142.8	1.32E-06	5.64E-07	1.25E-05	0.35353302	3.99E-57	5.18E-08	0.96158603
142.9	1.32E-06	5.64E-07	1.25E-05	0.35353302	3.99E-57	5.18E-08	0.96158603
143	1.29E-06	5.59E-07	1.27E-05	0.35173803	4.02E-57	5.14E-08	0.96187521
143.1	1.28E-06	5.72E-07	1.24E-05	0.35559849	3.58E-57	5.06E-08	0.95944112
143.2	1.29E-06	5.85E-07	1.26E-05	0.36114566	3.74E-57	5.00E-08	0.95732481
143.3	1.31E-06	6.08E-07	1.25E-05	0.36086023	3.77E-57	4.99E-08	0.95829745
143.4	1.31E-06	6.08E-07	1.25E-05	0.36086023	3.77E-57	4.99E-08	0.95829745
143.5	1.31E-06	6.21E-07	1.19E-05	0.36536102	3.56E-57	4.86E-08	0.95342176
143.6	1.36E-06	6.40E-07	1.14E-05	0.36685311	3.41E-57	4.69E-08	0.95026564
143.7	1.37E-06	6.41E-07	1.12E-05	0.36642517	3.28E-57	4.53E-08	0.94563394
143.8	1.37E-06	6.41E-07	1.12E-05	0.36642517	3.28E-57	4.53E-08	0.94563394
143.9	1.37E-06	6.65E-07	1.11E-05	0.36950763	3.36E-57	4.52E-08	0.9444247
144	1.38E-06	6.29E-07	1.03E-05	0.37072119	2.99E-57	4.30E-08	0.9461748
144.1	1.36E-06	6.28E-07	1.01E-05	0.37100223	2.74E-57	4.16E-08	0.95159233
144.2	1.41E-06	6.44E-07	1.03E-05	0.37242219	2.83E-57	4.17E-08	0.94668758
144.3	1.35E-06	6.46E-07	9.76E-06	0.37159089	2.45E-57	4.05E-08	0.94676171
144.4	1.32E-06	6.16E-07	9.62E-06	0.36794377	2.17E-57	4.04E-08	0.93913428
144.5	1.35E-06	6.26E-07	9.39E-06	0.36887765	2.14E-57	4.03E-08	0.93584433
144.6	1.44E-06	6.28E-07	9.29E-06	0.3620059	2.52E-57	3.96E-08	0.93332509
144.7	1.44E-06	6.28E-07	9.29E-06	0.3620059	2.52E-57	3.96E-08	0.93332509
144.8	1.47E-06	6.47E-07	9.07E-06	0.36490772	2.51E-57	3.83E-08	0.93267687
144.9	1.45E-06	6.67E-07	9.04E-06	0.3674802	2.44E-57	3.75E-08	0.93551971
145	1.40E-06	6.80E-07	8.72E-06	0.3744087	2.22E-57	3.75E-08	0.93766451
145.1	1.41E-06	6.54E-07	8.61E-06	0.3778125	1.97E-57	3.79E-08	0.93787336
145.2	1.41E-06	6.56E-07	8.94E-06	0.36963175	2.19E-57	3.71E-08	0.93650541
145.3	1.43E-06	6.62E-07	8.78E-06	0.36881871	2.42E-57	3.55E-08	0.93950569
145.4	1.40E-06	6.65E-07	8.50E-06	0.36838697	2.14E-57	3.50E-08	0.93883224
145.5	1.37E-06	6.55E-07	8.57E-06	0.36294197	2.22E-57	3.46E-08	0.93980598
145.6	1.32E-06	6.73E-07	8.54E-06	0.36443151	2.08E-57	3.35E-08	0.94079247
145.7	1.35E-06	6.86E-07	8.31E-06	0.36207673	2.02E-57	3.22E-08	0.93633734
145.8	1.36E-06	6.74E-07	7.93E-06	0.35985621	1.99E-57	3.07E-08	0.93972713
145.9	1.36E-06	6.74E-07	7.93E-06	0.35985621	1.99E-57	3.07E-08	0.93972713
146	1.32E-06	6.80E-07	7.82E-06	0.36034976	1.89E-57	3.03E-08	0.93860169
146.1	1.37E-06	6.83E-07	7.42E-06	0.35955479	1.88E-57	2.87E-08	0.94355828
146.2	1.35E-06	6.92E-07	7.29E-06	0.36008038	1.71E-57	2.86E-08	0.94759253
146.3	1.35E-06	6.92E-07	7.29E-06	0.36008038	1.71E-57	2.86E-08	0.94759253
146.4	1.35E-06	6.91E-07	7.20E-06	0.36187741	1.68E-57	2.85E-08	0.94404392
146.5	1.31E-06	7.13E-07	6.93E-06	0.36471523	1.69E-57	2.78E-08	0.94091064
146.6	1.31E-06	7.13E-07	6.93E-06	0.36471523	1.69E-57	2.78E-08	0.94091064
146.7	1.33E-06	6.93E-07	7.08E-06	0.36428639	1.67E-57	2.71E-08	0.93623397
146.8	1.34E-06	7.22E-07	6.90E-06	0.36771196	1.54E-57	2.61E-08	0.9308363
146.9	1.33E-06	7.21E-07	6.62E-06	0.36893929	1.48E-57	2.56E-08	0.92477564
147	1.33E-06	7.21E-07	6.62E-06	0.36893929	1.48E-57	2.56E-08	0.92477564
147.1	1.29E-06	7.31E-07	6.69E-06	0.37150634	1.54E-57	2.50E-08	0.92278294
147.2	1.34E-06	7.70E-07	6.27E-06	0.36894747	1.47E-57	2.38E-08	0.91213751
147.3	1.47E-06	8.07E-07	6.45E-06	0.3707419	1.95E-57	2.35E-08	0.9061965
147.4	1.48E-06	7.66E-07	6.15E-06	0.36702417	1.81E-57	2.34E-08	0.90305503
147.5	1.53E-06	7.47E-07	6.18E-06	0.36554266	1.86E-57	2.32E-08	0.8975354
147.6	1.53E-06	7.47E-07	6.18E-06	0.36554266	1.86E-57	2.32E-08	0.8975354
147.7	1.53E-06	7.47E-07	6.18E-06	0.36554266	1.86E-57	2.32E-08	0.8975354
147.8	1.50E-06	8.04E-07	5.85E-06	0.37324587	2.04E-57	2.20E-08	0.88241025
147.9	1.57E-06	8.20E-07	5.63E-06	0.37448177	1.90E-57	2.22E-08	0.87589415
148	1.61E-06	8.33E-07	5.55E-06	0.37480897	2.11E-57	2.20E-08	0.87448675
148.1	1.64E-06	8.39E-07	5.38E-06	0.3775094	2.16E-57	2.14E-08	0.8810244
148.2	1.72E-06	8.16E-07	5.21E-06	0.37576711	2.16E-57	2.16E-08	0.87781283
148.3	1.70E-06	8.32E-07	5.19E-06	0.37746013	2.15E-57	2.14E-08	0.87327338
148.4	1.66E-06	8.49E-07	4.87E-06	0.3777862	2.06E-57	2.05E-08	0.86379332
148.5	1.71E-06	8.44E-07	4.70E-06	0.37250362	1.94E-57	2.01E-08	0.86346348
148.6	1.71E-06	8.44E-07	4.70E-06	0.37250362	1.94E-57	2.01E-08	0.86346348
148.7	1.71E-06	8.44E-07	4.70E-06	0.37250362	1.94E-57	2.01E-08	0.86346348
148.8	1.78E-06	8.74E-07	4.48E-06	0.37335357	2.04E-57	1.92E-08	0.86567047
148.9	1.82E-06	8.89E-07	4.60E-06	0.37343037	2.07E-57	1.95E-08	0.86547091
149	1.75E-06	8.98E-07	4.55E-06	0.37637606	2.12E-57	1.97E-08	0.86682991
149.1	1.68E-06	9.00E-07	4.58E-06	0.37673577	1.86E-57	1.95E-08	0.86786124
149.2	1.69E-06	8.94E-07	4.85E-06	0.3710126	1.86E-57	1.85E-08	0.86897046
149.3	1.69E-06	8.94E-07	4.85E-06	0.3710126	1.86E-57	1.85E-08	0.86897046
149.4	1.86E-06	9.07E-07	4.56E-06	0.36861468	2.37E-57	1.70E-08	0.86491198
149.5	1.95E-06	8.78E-07	4.24E-06	0.37262588	2.30E-57	1.63E-08	0.85831918
149.6	2.07E-06	9.04E-07	4.08E-06	0.37965941	2.26E-57	1.46E-08	0.85251425
149.7	2.04E-06	8.27E-07	4.02E-06	0.37748082	1.91E-57	1.40E-08	0.84721839
149.8	2.04E-06	8.27E-07	4.02E-06	0.37748082	1.91E-57	1.40E-08	0.84721839
149.9	2.05E-06	8.30E-07	3.88E-06	0.38327145	1.81E-57	1.35E-08	0.8351311
195	0.00012526	7.37E-08	2.28E-07	0.48390844	2.64E-58	2.60E-11	0.90728153
195.1	0.00012526	7.37E-08	2.28E-07	0.48390844	2.64E-58	2.60E-11	0.90728153
195.2	0.00012561	7.22E-08	2.30E-07	0.47945401	2.63E-58	2.58E-11	0.90680635
195.3	0.00012496	7.49E-08	2.32E-07	0.48016338	2.51E-58	2.54E-11	0.9101931
195.4	0.00012496	7.49E-08	2.32E-07	0.48016338	2.51E-58	2.54E-11	0.9101931
195.5	0.00012714	7.40E-08	2.42E-07	0.47736533	2.77E-58	2.56E-11	0.9091469
195.6	0.00012714	7.40E-08	2.42E-07	0.47736533	2.77E-58	2.56E-11	0.9091469
195.7	0.00012956	7.38E-08	2.42E-07	0.47921973	2.95E-58	2.47E-11	0.90590534
195.8	0.00012956	7.38E-08	2.42E-07	0.47921973	2.95E-58	2.47E-11	0.90590534
195.9	0.00013199	7.20E-08	2.48E-07	0.48143266	2.92E-58	2.51E-11	0.9095167
196	0.00013286	7.26E-08	2.39E-07	0.47973746	2.56E-58	2.33E-11	0.90643307
196.1	0.00013286	7.26E-08	2.39E-07	0.47973746	2.56E-58	2.33E-11	0.90643307
196.2	0.00013212	7.54E-08	2.49E-07	0.4819705	2.90E-58	2.32E-11	0.9034718
196.3	0.00013892	7.18E-08	2.46E-07	0.47930069	2.66E-58	2.25E-11	0.90559979
196.4	0.00014289	7.15E-08	2.41E-07	0.47616475	2.72E-58	2.24E-11	0.90527295
196.5	0.00014348	6.85E-08	2.38E-07	0.47388241	2.51E-58	2.20E-11	0.89847699
196.6	0.00014592	6.72E-08	2.37E-07	0.47445878	2.59E-58	2.20E-11	0.89685467
196.7	0.00014147	6.60E-08	2.38E-07	0.47218395	2.61E-58	2.19E-11	0.89939703
196.8	0.00014264	6.77E-08	2.40E-07	0.4736615	2.68E-58	2.15E-11	0.90461356
196.9	0.00014264	6.77E-08	2.40E-07	0.4736615	2.68E-58	2.15E-11	0.90461356
197	0.00014264	6.77E-08	2.40E-07	0.4736615	2.68E-58	2.15E-11	0.90461356
197.1	0.00014483	6.97E-08	2.44E-07	0.47566306	2.67E-58	2.19E-11	0.90976035
197.2	0.00014483	6.97E-08	2.44E-07	0.47566306	2.67E-58	2.19E-11	0.90976035
197.3	0.00014483	6.97E-08	2.44E-07	0.47566306	2.67E-58	2.19E-11	0.90976035
197.4	0.00014483	6.97E-08	2.44E-07	0.47566306	2.67E-58	2.19E-11	0.90976035
197.5	0.00014483	6.97E-08	2.44E-07	0.47566306	2.67E-58	2.19E-11	0.90976035
197.6	0.00014471	7.18E-08	2.51E-07	0.47746572	2.58E-58	2.21E-11	0.90907514
197.7	0.00014471	7.18E-08	2.51E-07	0.47746572	2.58E-58	2.21E-11	0.90907514
197.8	0.00014471	7.18E-08	2.51E-07	0.47746572	2.58E-58	2.21E-11	0.90907514
197.9	0.00014791	6.94E-08	2.50E-07	0.4760987	2.41E-58	2.16E-11	0.90804403
198	0.00014791	6.94E-08	2.50E-07	0.4760987	2.41E-58	2.16E-11	0.90804403
198.1	0.00014871	6.71E-08	2.52E-07	0.47943565	2.43E-58	2.21E-11	0.90749781
156.1	2.70E-06	1.25E-06	2.15E-06	0.40986005	1.86E-57	3.42E-09	0.74229712
156.2	2.68E-06	1.24E-06	2.10E-06	0.41099665	1.93E-57	3.41E-09	0.74214338
156.3	2.72E-06	1.20E-06	2.03E-06	0.41393507	1.95E-57	3.27E-09	0.73952861
156.4	2.77E-06	1.18E-06	2.01E-06	0.41717766	1.94E-57	3.27E-09	0.73877527
156.5	2.80E-06	1.20E-06	2.01E-06	0.41830806	2.04E-57	3.27E-09	0.73198063
156.6	2.75E-06	1.22E-06	2.01E-06	0.42005622	2.14E-57	3.22E-09	0.73026498
156.7	2.75E-06	1.22E-06	2.01E-06	0.42005622	2.14E-57	3.22E-09	0.73026498
156.8	2.75E-06	1.22E-06	2.01E-06	0.42005622	2.14E-57	3.22E-09	0.73026498
156.9	2.75E-06	1.22E-06	2.01E-06	0.42005622	2.14E-57	3.22E-09	0.73026498
157	2.74E-06	1.31E-06	1.88E-06	0.41586288	1.96E-57	3.00E-09	0.73430567
157.1	2.74E-06	1.31E-06	1.88E-06	0.41586288	1.96E-57	3.00E-09	0.73430567
157.2	2.80E-06	1.27E-06	1.85E-06	0.4149925	1.86E-57	2.96E-09	0.73147496
157.3	2.76E-06	1.26E-06	1.74E-06	0.41889099	1.87E-57	2.90E-09	0.73375247
157.4	2.86E-06	1.19E-06	1.72E-06	0.41895793	1.80E-57	2.84E-09	0.73228242
157.5	2.86E-06	1.19E-06	1.72E-06	0.41895793	1.80E-57	2.84E-09	0.73228242
157.6	2.95E-06	1.19E-06	1.72E-06	0.41764405	1.68E-57	2.67E-09	0.73291484
157.7	2.98E-06	1.19E-06	1.79E-06	0.41306434	1.72E-57	2.75E-09	0.73923421
157.8	2.97E-06	1.11E-06	1.73E-06	0.41428655	1.52E-57	2.68E-09	0.73117402
157.9	2.95E-06	1.13E-06	1.66E-06	0.40964931	1.38E-57	2.56E-09	0.7298809
158	3.02E-06	1.13E-06	1.56E-06	0.40728932	1.26E-57	2.27E-09	0.72516896
158.1	3.02E-06	1.13E-06	1.56E-06	0.40728932	1.26E-57	2.27E-09	0.72516896
158.2	2.97E-06	1.09E-06	1.52E-06	0.40426252	1.17E-57	2.18E-09	0.71769373
158.3	3.07E-06	1.06E-06	1.56E-06	0.40534128	1.13E-57	2.23E-09	0.71449108
158.4	3.01E-06	1.10E-06	1.59E-06	0.40492933	1.25E-57	2.23E-09	0.71068058
158.5	3.03E-06	1.09E-06	1.58E-06	0.40301607	1.23E-57	2.21E-09	0.71169292
158.6	3.03E-06	1.09E-06	1.58E-06	0.40301607	1.23E-57	2.21E-09	0.71169292
158.7	3.16E-06	1.07E-06	1.58E-06	0.40573764	1.24E-57	2.22E-09	0.71414053
158.8	3.28E-06	1.07E-06	1.60E-06	0.40345824	1.43E-57	2.31E-09	0.71926663
158.9	3.43E-06	1.07E-06	1.58E-06	0.40708021	1.59E-57	2.22E-09	0.7209297
159	3.44E-06	1.07E-06	1.54E-06	0.40771319	1.50E-57	2.18E-09	0.72093804
159.1	3.44E-06	1.07E-06	1.54E-06	0.40771319	1.50E-57	2.18E-09	0.72093804
159.2	3.44E-06	1.07E-06	1.54E-06	0.40771319	1.50E-57	2.18E-09	0.72093804
159.3	3.60E-06	1.00E-06	1.57E-06	0.40629154	1.53E-57	2.19E-09	0.71363471
159.4	3.64E-06	1.01E-06	1.57E-06	0.40409207	1.41E-57	2.14E-09	0.70935668
159.5	3.95E-06	1.03E-06	1.58E-06	0.40602857	1.46E-57	2.13E-09	0.70521944
159.6	4.07E-06	1.03E-06	1.63E-06	0.41192871	1.64E-57	2.14E-09	0.70785589
159.7	4.11E-06	1.06E-06	1.68E-06	0.41384361	1.68E-57	2.15E-09	0.70940093
159.8	4.01E-06	1.11E-06	1.72E-06	0.41694993	1.71E-57	2.10E-09	0.71173087
159.9	4.34E-06	1.11E-06	1.61E-06	0.42050773	1.80E-57	2.13E-09	0.7136927
160	4.60E-06	1.10E-06	1.66E-06	0.42653021	2.03E-57	2.11E-09	0.71183028
160.1	4.78E-06	1.11E-06	1.76E-06	0.42435265	2.13E-57	2.05E-09	0.70451091
160.2	4.87E-06	1.14E-06	1.79E-06	0.42208098	2.13E-57	1.90E-09	0.70866147
160.3	4.87E-06	1.14E-06	1.79E-06	0.42053312	2.19E-57	1.90E-09	0.71630906
160.4	4.87E-06	1.14E-06	1.79E-06	0.42053312	2.19E-57	1.90E-09	0.71630906
160.5	4.87E-06	1.17E-06	1.75E-06	0.42404747	2.04E-57	1.86E-09	0.71605858
160.6	4.88E-06	1.25E-06	1.84E-06	0.4205545	2.13E-57	1.92E-09	0.71333177
160.7	4.84E-06	1.28E-06	1.82E-06	0.41782981	2.15E-57	1.83E-09	0.71871987
160.8	4.87E-06	1.27E-06	1.80E-06	0.4202998	2.19E-57	1.83E-09	0.71764709
160.9	4.87E-06	1.27E-06	1.80E-06	0.4202998	2.19E-57	1.83E-09	0.71764709
161	4.95E-06	1.26E-06	1.78E-06	0.42095923	2.24E-57	1.84E-09	0.72333758
161.1	4.95E-06	1.26E-06	1.78E-06	0.42095923	2.24E-57	1.84E-09	0.72333758
161.2	4.80E-06	1.33E-06	1.80E-06	0.42499339	2.42E-57	1.86E-09	0.72259377
161.3	4.80E-06	1.33E-06	1.80E-06	0.42499339	2.42E-57	1.86E-09	0.72259377
161.4	4.73E-06	1.42E-06	1.84E-06	0.41210877	2.12E-57	1.68E-09	0.724242
161.5	4.90E-06	1.43E-06	1.67E-06	0.4151739	2.18E-57	1.70E-09	0.72542065
161.6	4.92E-06	1.43E-06	1.71E-06	0.41337921	2.29E-57	1.67E-09	0.72120681
161.7	4.92E-06	1.43E-06	1.71E-06	0.41337921	2.29E-57	1.67E-09	0.72120681
161.8	4.89E-06	1.38E-06	1.73E-06	0.41203649	2.40E-57	1.60E-09	0.72545115
161.9	4.89E-06	1.38E-06	1.73E-06	0.41203649	2.40E-57	1.60E-09	0.72545115
162	4.99E-06	1.42E-06	1.63E-06	0.41365198	2.35E-57	1.52E-09	0.72721782
162.1	4.97E-06	1.47E-06	1.63E-06	0.41251235	2.27E-57	1.50E-09	0.722481
162.2	5.01E-06	1.50E-06	1.66E-06	0.4138516	2.61E-57	1.52E-09	0.71897955
162.3	5.00E-06	1.47E-06	1.64E-06	0.41140326	2.24E-57	1.42E-09	0.72498009
162.4	5.05E-06	1.49E-06	1.63E-06	0.41284549	2.33E-57	1.41E-09	0.72554858
162.5	5.05E-06	1.49E-06	1.63E-06	0.41284549	2.33E-57	1.41E-09	0.72554858
162.6	5.05E-06	1.49E-06	1.63E-06	0.41284549	2.33E-57	1.41E-09	0.72554858
162.7	5.02E-06	1.49E-06	1.59E-06	0.41458873	2.29E-57	1.38E-09	0.72476113
162.8	5.02E-06	1.49E-06	1.59E-06	0.41458873	2.29E-57	1.38E-09	0.72476113
162.9	5.02E-06	1.49E-06	1.59E-06	0.41458873	2.29E-57	1.38E-09	0.72476113
163	5.25E-06	1.51E-06	1.57E-06	0.41372767	2.36E-57	1.36E-09	0.72547505
163.1	5.38E-06	1.52E-06	1.56E-06	0.41814719	2.56E-57	1.34E-09	0.729184
163.2	5.38E-06	1.52E-06	1.56E-06	0.41814719	2.56E-57	1.34E-09	0.729184
163.3	5.38E-06	1.52E-06	1.56E-06	0.41814719	2.56E-57	1.34E-09	0.729184
163.4	5.38E-06	1.52E-06	1.56E-06	0.41814719	2.56E-57	1.34E-09	0.729184
163.5	5.37E-06	1.49E-06	1.48E-06	0.41822268	2.36E-57	1.29E-09	0.72599525
163.6	5.37E-06	1.49E-06	1.48E-06	0.41822268	2.36E-57	1.29E-09	0.72599525
163.7	5.29E-06	1.46E-06	1.48E-06	0.42374861	2.27E-57	1.33E-09	0.72788012
163.8	5.31E-06	1.47E-06	1.49E-06	0.42119383	2.37E-57	1.30E-09	0.73091571
163.9	5.50E-06	1.45E-06	1.50E-06	0.4191293	2.19E-57	1.26E-09	0.73011834
164	5.64E-06	1.45E-06	1.51E-06	0.41803401	2.20E-57	1.25E-09	0.72564039
164.1	5.64E-06	1.45E-06	1.51E-06	0.41803401	2.20E-57	1.25E-09	0.72564039
164.2	5.54E-06	1.38E-06	1.47E-06	0.41724175	2.06E-57	1.24E-09	0.72120566
164.3	5.71E-06	1.38E-06	1.54E-06	0.41413741	2.28E-57	1.22E-09	0.72224888
164.4	5.62E-06	1.40E-06	1.57E-06	0.40938242	2.13E-57	1.23E-09	0.7205536
164.5	5.62E-06	1.40E-06	1.57E-06	0.40938242	2.13E-57	1.23E-09	0.7205536
164.6	5.62E-06	1.40E-06	1.57E-06	0.40938242	2.13E-57	1.23E-09	0.7205536
164.7	5.51E-06	1.36E-06	1.47E-06	0.40772511	1.90E-57	1.14E-09	0.72162408
164.8	5.51E-06	1.36E-06	1.47E-06	0.40772511	1.90E-57	1.14E-09	0.72162408
164.9	5.46E-06	1.32E-06	1.42E-06	0.40472631	1.81E-57	1.11E-09	0.72407207
210	0.00038545	5.80E-08	2.10E-07	0.47540785	4.16E-58	2.10E-11	0.81599019
210.1	0.00038545	5.80E-08	2.10E-07	0.47540785	4.16E-58	2.10E-11	0.81599019
210.2	0.00038545	5.80E-08	2.10E-07	0.47540785	4.16E-58	2.10E-11	0.81599019
210.3	0.00038545	5.80E-08	2.10E-07	0.47540785	4.16E-58	2.10E-11	0.81599019
210.4	0.00039558	5.74E-08	2.07E-07	0.47530592	4.08E-58	2.11E-11	0.81918075
210.5	0.00039558	5.74E-08	2.07E-07	0.47530592	4.08E-58	2.11E-11	0.81918075
210.6	0.00039558	5.74E-08	2.07E-07	0.47530592	4.08E-58	2.11E-11	0.81918075
210.7	0.00039558	5.74E-08	2.07E-07	0.47530592	4.08E-58	2.11E-11	0.81918075
210.8	0.00039558	5.74E-08	2.07E-07	0.47530592	4.08E-58	2.11E-11	0.81918075
210.9	0.00039855	5.48E-08	1.98E-07	0.47631519	3.72E-58	2.09E-11	0.81650746
211	0.00039855	5.48E-08	1.98E-07	0.47631519	3.72E-58	2.09E-11	0.81650746
211.1	0.00039855	5.48E-08	1.98E-07	0.47631519	3.72E-58	2.09E-11	0.81650746
211.2	0.00039855	5.48E-08	1.98E-07	0.47631519	3.72E-58	2.09E-11	0.81650746
211.3	0.00040131	5.47E-08	1.94E-07	0.48031722	3.68E-58	2.10E-11	0.81298239
211.4	0.00040302	5.25E-08	1.85E-07	0.47350364	3.45E-58	2.08E-11	0.81349697
211.5	0.00040302	5.25E-08	1.85E-07	0.47350364	3.45E-58	2.08E-11	0.81349697
211.6	0.00040538	5.32E-08	1.77E-07	0.47207955	3.24E-58	2.00E-11	0.81213678
211.7	0.00041133	5.41E-08	1.75E-07	0.47552984	3.46E-58	1.97E-11	0.81206752
211.8	0.00041133	5.41E-08	1.75E-07	0.47552984	3.46E-58	1.97E-11	0.81206752
211.9	0.00041133	5.41E-08	1.75E-07	0.47552984	3.46E-58	1.97E-11	0.81206752
212	0.00041133	5.41E-08	1.75E-07	0.47552984	3.46E-58	1.97E-11	0.81206752
212.1	0.00041133	5.41E-08	1.75E-07	0.47552984	3.46E-58	1.97E-11	0.81206752
212.2	0.00041133	5.41E-08	1.75E-07	0.47552984	3.46E-58	1.97E-11	0.81206752
212.3	0.00041133	5.41E-08	1.75E-07	0.47552984	3.46E-58	1.97E-11	0.81206752
212.4	0.00041133	5.41E-08	1.75E-07	0.47552984	3.46E-58	1.97E-11	0.81206752
212.5	0.0004092	5.34E-08	1.74E-07	0.47476016	3.17E-58	1.89E-11	0.80875134
212.6	0.0004092	5.34E-08	1.74E-07	0.47476016	3.17E-58	1.89E-11	0.80875134
212.7	0.00041401	5.38E-08	1.80E-07	0.47550774	3.38E-58	1.96E-11	0.81227082
212.8	0.00042351	4.99E-08	1.66E-07	0.47497625	3.20E-58	1.90E-11	0.80582765
212.9	0.00042351	4.99E-08	1.66E-07	0.47497625	3.20E-58	1.90E-11	0.80582765
213	0.00042351	4.99E-08	1.66E-07	0.47497625	3.20E-58	1.90E-11	0.80582765
213.1	0.00042351	4.99E-08	1.66E-07	0.47497625	3.20E-58	1.90E-11	0.80582765
171.1	1.09E-05	1.12E-06	8.68E-07	0.37506984	1.15E-57	4.66E-10	0.74764553
171.2	1.15E-05	1.12E-06	8.30E-07	0.37466916	1.23E-57	4.64E-10	0.75449268
171.3	1.15E-05	1.13E-06	8.37E-07	0.37246214	1.13E-57	4.49E-10	0.75530354
171.4	1.15E-05	1.12E-06	8.45E-07	0.37573784	1.29E-57	4.67E-10	0.75994153
171.5	1.17E-05	1.09E-06	8.52E-07	0.37668432	1.30E-57	4.64E-10	0.76074037
171.6	1.17E-05	1.11E-06	8.33E-07	0.37688498	1.22E-57	4.56E-10	0.75878086
171.7	1.20E-05	1.17E-06	8.23E-07	0.37556842	1.43E-57	4.29E-10	0.75435242
171.8	1.22E-05	1.14E-06	8.43E-07	0.37533273	1.44E-57	4.46E-10	0.74872679
171.9	1.20E-05	1.18E-06	8.58E-07	0.37517927	1.36E-57	4.40E-10	0.74500861
172	1.20E-05	1.18E-06	8.58E-07	0.37517927	1.36E-57	4.40E-10	0.74500861
172.1	1.24E-05	1.19E-06	8.67E-07	0.37477051	1.44E-57	4.25E-10	0.75054296
172.2	1.27E-05	1.19E-06	8.30E-07	0.37217691	1.34E-57	4.18E-10	0.74697607
172.3	1.27E-05	1.19E-06	8.24E-07	0.37285374	1.34E-57	4.10E-10	0.74393569
172.4	1.27E-05	1.19E-06	8.24E-07	0.37285374	1.34E-57	4.10E-10	0.74393569
172.5	1.31E-05	1.19E-06	8.08E-07	0.37326856	1.37E-57	4.09E-10	0.74595869
172.6	1.33E-05	1.21E-06	8.07E-07	0.37630513	1.38E-57	4.02E-10	0.74558313
172.7	1.33E-05	1.21E-06	8.07E-07	0.37630513	1.38E-57	4.02E-10	0.74558313
172.8	1.34E-05	1.22E-06	7.90E-07	0.37711115	1.36E-57	4.06E-10	0.74104815
172.9	1.35E-05	1.20E-06	7.81E-07	0.37823063	1.46E-57	4.07E-10	0.74851663
173	1.35E-05	1.27E-06	7.68E-07	0.38163304	1.38E-57	4.02E-10	0.74805656
173.1	1.37E-05	1.32E-06	7.71E-07	0.38914409	1.33E-57	3.87E-10	0.75075453
173.2	1.44E-05	1.31E-06	7.15E-07	0.38353935	1.37E-57	3.77E-10	0.74983897
173.3	1.45E-05	1.34E-06	7.10E-07	0.38472231	1.33E-57	3.74E-10	0.75240115
173.4	1.45E-05	1.34E-06	7.10E-07	0.38472231	1.33E-57	3.74E-10	0.75240115
173.5	1.48E-05	1.35E-06	7.26E-07	0.38386127	1.44E-57	3.85E-10	0.74869456
173.6	1.43E-05	1.32E-06	7.22E-07	0.38384164	1.39E-57	3.78E-10	0.75074349
173.7	1.45E-05	1.37E-06	7.44E-07	0.38587079	1.44E-57	3.60E-10	0.74426908
173.8	1.50E-05	1.31E-06	7.06E-07	0.38554258	1.28E-57	3.40E-10	0.74764258
173.9	1.42E-05	1.35E-06	7.32E-07	0.38380323	1.23E-57	3.34E-10	0.74627219
174	1.42E-05	1.35E-06	7.32E-07	0.38380323	1.23E-57	3.34E-10	0.74627219
174.1	1.48E-05	1.34E-06	7.05E-07	0.37890956	1.21E-57	3.32E-10	0.75126617
174.2	1.48E-05	1.34E-06	7.05E-07	0.37890956	1.21E-57	3.32E-10	0.75126617
174.3	1.48E-05	1.34E-06	7.00E-07	0.38001052	1.19E-57	3.22E-10	0.74855394
174.4	1.48E-05	1.34E-06	7.00E-07	0.38001052	1.19E-57	3.22E-10	0.74855394
174.5	1.57E-05	1.34E-06	6.77E-07	0.38082678	1.22E-57	3.15E-10	0.75087992
174.6	1.58E-05	1.36E-06	6.58E-07	0.38348298	1.25E-57	3.07E-10	0.74727276
174.7	1.58E-05	1.34E-06	6.50E-07	0.38257722	1.31E-57	3.05E-10	0.74863916
174.8	1.58E-05	1.34E-06	6.50E-07	0.38257722	1.31E-57	3.05E-10	0.74863916
174.9	1.63E-05	1.30E-06	6.66E-07	0.38293434	1.32E-57	2.99E-10	0.74682224
175	1.66E-05	1.25E-06	6.53E-07	0.3854595	1.22E-57	2.65E-10	0.75519521
175.1	1.72E-05	1.27E-06	6.37E-07	0.38585619	1.28E-57	2.58E-10	0.76057064
175.2	1.70E-05	1.24E-06	6.57E-07	0.38270299	1.33E-57	2.48E-10	0.76408608
175.3	1.71E-05	1.24E-06	6.73E-07	0.38119678	1.34E-57	2.40E-10	0.7622285
175.4	1.75E-05	1.19E-06	6.56E-07	0.38229438	1.26E-57	2.36E-10	0.76352294
175.5	1.82E-05	1.19E-06	6.55E-07	0.38584829	1.40E-57	2.37E-10	0.7669441
175.6	1.82E-05	1.19E-06	6.55E-07	0.38584829	1.40E-57	2.37E-10	0.7669441
175.7	1.83E-05	1.14E-06	6.34E-07	0.38325861	1.32E-57	2.31E-10	0.76368156
175.8	1.83E-05	1.14E-06	6.34E-07	0.38325861	1.32E-57	2.31E-10	0.76368156
175.9	1.83E-05	1.14E-06	6.34E-07	0.38325861	1.32E-57	2.31E-10	0.76368156
176	1.95E-05	1.13E-06	6.22E-07	0.38243867	1.28E-57	2.26E-10	0.77360815
176.1	1.95E-05	1.13E-06	6.22E-07	0.38243867	1.28E-57	2.26E-10	0.77360815
176.2	1.96E-05	1.14E-06	6.27E-07	0.37808594	1.34E-57	2.25E-10	0.77172889
176.3	1.96E-05	1.16E-06	6.29E-07	0.38047258	1.41E-57	2.10E-10	0.77310069
176.4	1.99E-05	1.18E-06	6.21E-07	0.3795401	1.42E-57	2.13E-10	0.76851497
176.5	1.95E-05	1.16E-06	6.26E-07	0.38285533	1.33E-57	2.12E-10	0.76841587
176.6	1.95E-05	1.16E-06	6.26E-07	0.38285533	1.33E-57	2.12E-10	0.76841587
176.7	1.89E-05	1.14E-06	6.04E-07	0.38781953	1.23E-57	2.07E-10	0.76868912
176.8	1.95E-05	1.15E-06	5.94E-07	0.39077854	1.22E-57	2.09E-10	0.76745387
176.9	1.95E-05	1.12E-06	5.59E-07	0.39071148	1.07E-57	1.99E-10	0.76933716
177	1.94E-05	1.10E-06	5.50E-07	0.3894983	1.11E-57	1.97E-10	0.77168521
177.1	2.07E-05	9.89E-07	5.70E-07	0.38882568	1.11E-57	1.94E-10	0.77124174
177.2	2.07E-05	9.89E-07	5.70E-07	0.38882568	1.11E-57	1.94E-10	0.77124174
177.3	2.04E-05	9.64E-07	5.57E-07	0.39358548	1.14E-57	1.99E-10	0.77251289
177.4	2.10E-05	9.38E-07	5.54E-07	0.39479983	1.08E-57	1.95E-10	0.77927392
177.5	2.10E-05	9.38E-07	5.54E-07	0.39479983	1.08E-57	1.95E-10	0.77927392
177.6	2.10E-05	9.38E-07	5.54E-07	0.39479983	1.08E-57	1.95E-10	0.77927392
177.7	2.09E-05	9.50E-07	5.57E-07	0.39734911	1.14E-57	1.92E-10	0.78538869
177.8	2.01E-05	9.40E-07	5.64E-07	0.39567756	1.15E-57	1.95E-10	0.79153915
177.9	2.07E-05	9.19E-07	5.90E-07	0.39551692	1.22E-57	1.89E-10	0.79409276
178	2.04E-05	9.30E-07	5.96E-07	0.39508222	1.25E-57	1.88E-10	0.79717792
178.1	2.03E-05	9.05E-07	5.85E-07	0.40087038	1.32E-57	1.93E-10	0.80160287
178.2	2.13E-05	8.14E-07	5.46E-07	0.39387539	1.08E-57	1.85E-10	0.8079196
178.3	2.04E-05	8.36E-07	5.33E-07	0.39491003	1.03E-57	1.78E-10	0.80693099
178.4	2.02E-05	8.01E-07	5.34E-07	0.39865847	1.16E-57	1.87E-10	0.81361561
178.5	1.99E-05	7.67E-07	5.30E-07	0.40347607	1.16E-57	1.85E-10	0.81866017
178.6	2.06E-05	7.17E-07	5.26E-07	0.40816742	1.05E-57	1.80E-10	0.82293217
178.7	2.06E-05	7.04E-07	5.13E-07	0.40801917	1.01E-57	1.78E-10	0.82599222
178.8	2.06E-05	7.04E-07	5.13E-07	0.40801917	1.01E-57	1.78E-10	0.82599222
178.9	2.01E-05	7.21E-07	5.23E-07	0.40599183	9.32E-58	1.77E-10	0.82357415
179	2.05E-05	6.71E-07	5.26E-07	0.40924101	9.41E-58	1.68E-10	0.82540363
179.1	2.01E-05	6.60E-07	5.35E-07	0.40722557	9.42E-58	1.65E-10	0.82528867
179.2	2.09E-05	6.42E-07	5.25E-07	0.40788837	9.32E-58	1.57E-10	0.83166773
179.3	2.09E-05	6.42E-07	5.25E-07	0.40788837	9.32E-58	1.57E-10	0.83166773
179.4	2.16E-05	6.37E-07	5.30E-07	0.40540121	1.00E-57	1.51E-10	0.8325057
179.5	2.16E-05	6.37E-07	5.30E-07	0.40540121	1.00E-57	1.51E-10	0.8325057
179.6	2.17E-05	6.28E-07	5.32E-07	0.40917773	1.05E-57	1.50E-10	0.83496987
179.7	2.22E-05	6.10E-07	5.38E-07	0.40609521	9.71E-58	1.56E-10	0.83895907
179.8	2.22E-05	6.11E-07	5.33E-07	0.40884816	9.59E-58	1.54E-10	0.84166068
179.9	2.28E-05	5.22E-07	5.02E-07	0.41525342	8.25E-58	1.51E-10	0.84326095
225	0.00059411	5.46E-08	1.06E-07	0.45343824	2.05E-58	1.55E-11	0.73896334
225.1	0.00059411	5.46E-08	1.06E-07	0.45343824	2.05E-58	1.55E-11	0.73896334
225.2	0.00059411	5.46E-08	1.06E-07	0.45343824	2.05E-58	1.55E-11	0.73896334
225.3	0.00059411	5.46E-08	1.06E-07	0.45343824	2.05E-58	1.55E-11	0.73896334
225.4	0.00059411	5.46E-08	1.06E-07	0.45343824	2.05E-58	1.55E-11	0.73896334
225.5	0.00059411	5.46E-08	1.06E-07	0.45343824	2.05E-58	1.55E-11	0.73896334
225.6	0.00059411	5.46E-08	1.06E-07	0.45343824	2.05E-58	1.55E-11	0.73896334
225.7	0.00059411	5.46E-08	1.06E-07	0.45343824	2.05E-58	1.55E-11	0.73896334
225.8	0.00060447	5.34E-08	1.07E-07	0.45342948	2.28E-58	1.57E-11	0.73713078
225.9	0.00060447	5.34E-08	1.07E-07	0.45342948	2.28E-58	1.57E-11	0.73713078
226	0.00060447	5.34E-08	1.07E-07	0.45342948	2.28E-58	1.57E-11	0.73713078
226.1	0.00060447	5.34E-08	1.07E-07	0.45342948	2.28E-58	1.57E-11	0.73713078
226.2	0.00062455	5.36E-08	1.06E-07	0.4514641	2.40E-58	1.58E-11	0.73230533
226.3	0.00062455	5.36E-08	1.06E-07	0.4514641	2.40E-58	1.58E-11	0.73230533
226.4	0.00061829	5.40E-08	1.00E-07	0.45004625	2.23E-58	1.55E-11	0.7315072
226.5	0.00061829	5.40E-08	1.00E-07	0.45004625	2.23E-58	1.55E-11	0.7315072
226.6	0.00062151	5.43E-08	9.87E-08	0.44637786	2.04E-58	1.54E-11	0.73027784
226.7	0.00062151	5.43E-08	9.87E-08	0.44637786	2.04E-58	1.54E-11	0.73027784
226.8	0.00064574	5.41E-08	9.93E-08	0.44635755	2.12E-58	1.55E-11	0.73491382
226.9	0.00064574	5.41E-08	9.93E-08	0.44635755	2.12E-58	1.55E-11	0.73491382
227	0.00064574	5.41E-08	9.93E-08	0.44635755	2.12E-58	1.55E-11	0.73491382
227.1	0.00064574	5.41E-08	9.93E-08	0.44635755	2.12E-58	1.55E-11	0.73491382
227.2	0.00064574	5.41E-08	9.93E-08	0.44635755	2.12E-58	1.55E-11	0.73491382
227.3	0.00064574	5.41E-08	9.93E-08	0.44635755	2.12E-58	1.55E-11	0.73491382
227.4	0.00064574	5.41E-08	9.93E-08	0.44635755	2.12E-58	1.55E-11	0.73491382
227.5	0.00064574	5.41E-08	9.93E-08	0.44635755	2.12E-58	1.55E-11	0.73491382
227.6	0.00064574	5.41E-08	9.93E-08	0.44635755	2.12E-58	1.55E-11	0.73491382
227.7	0.00064574	5.41E-08	9.93E-08	0.44635755	2.12E-58	1.55E-11	0.73491382
227.8	0.00064574	5.41E-08	9.93E-08	0.44635755	2.12E-58	1.55E-11	0.73491382
227.9	0.00063895	5.55E-08	9.52E-08	0.44344869	2.07E-58	1.54E-11	0.73356982
228	0.00063895	5.55E-08	9.52E-08	0.44344869	2.07E-58	1.54E-11	0.73356982
228.1	0.00063757	5.67E-08	9.59E-08	0.44496386	2.15E-58	1.49E-11	0.72979517

183.2	3.76E-05	2.84E-07	3.08E-07	0.43485346	4.87E-58	6.89E-11	0.93242264
183.3	3.70E-05	3.00E-07	3.22E-07	0.43836292	4.85E-58	6.75E-11	0.93148225
183.4	3.77E-05	3.05E-07	3.39E-07	0.43770043	5.51E-58	6.86E-11	0.93037832
183.5	3.77E-05	3.05E-07	3.39E-07	0.43770043	5.51E-58	6.86E-11	0.93037832
183.6	3.87E-05	2.89E-07	3.30E-07	0.43647803	5.57E-58	6.72E-11	0.9417973
183.7	3.86E-05	2.93E-07	3.35E-07	0.43497206	5.86E-58	6.71E-11	0.93620718
183.8	3.85E-05	2.93E-07	3.26E-07	0.43706549	5.69E-58	6.43E-11	0.94350698
183.9	3.97E-05	3.02E-07	3.28E-07	0.44156178	6.00E-58	6.34E-11	0.94363051
184	3.97E-05	3.02E-07	3.28E-07	0.44156178	6.00E-58	6.34E-11	0.94363051
184.1	3.96E-05	3.01E-07	3.37E-07	0.4454267	6.05E-58	6.45E-11	0.94312535
184.2	3.96E-05	3.01E-07	3.30E-07	0.44198412	5.87E-58	6.32E-11	0.94156708
184.3	3.99E-05	2.86E-07	3.12E-07	0.4426011	5.26E-58	5.99E-11	0.94054396
184.4	3.99E-05	2.86E-07	3.12E-07	0.4426011	5.26E-58	5.99E-11	0.94054396
184.5	3.96E-05	2.82E-07	3.15E-07	0.44207331	5.56E-58	6.03E-11	0.93746755
184.6	3.85E-05	2.56E-07	3.01E-07	0.44689922	4.67E-58	5.20E-11	0.94975726
184.7	3.85E-05	2.56E-07	3.01E-07	0.44689922	4.67E-58	5.20E-11	0.94975726
184.8	3.85E-05	2.39E-07	3.00E-07	0.44322678	4.58E-58	5.35E-11	0.95142367
184.9	3.97E-05	2.33E-07	3.00E-07	0.44127792	4.31E-58	5.34E-11	0.950352
185	3.96E-05	2.19E-07	2.91E-07	0.44184362	3.93E-58	5.11E-11	0.95251486
185.1	3.99E-05	2.24E-07	2.98E-07	0.44036985	3.60E-58	4.99E-11	0.95134637
185.2	3.99E-05	2.24E-07	2.98E-07	0.44036985	3.60E-58	4.99E-11	0.95134637
185.3	3.98E-05	2.28E-07	2.96E-07	0.43705854	3.45E-58	4.85E-11	0.95283183
185.4	4.04E-05	2.31E-07	3.01E-07	0.43239696	3.57E-58	4.83E-11	0.95450406
185.5	4.26E-05	2.11E-07	2.91E-07	0.43716372	3.49E-58	4.86E-11	0.95466342
185.6	4.25E-05	2.03E-07	2.95E-07	0.44246836	3.30E-58	4.35E-11	0.97002473
185.7	4.26E-05	2.01E-07	2.94E-07	0.43894737	3.05E-58	4.22E-11	0.97767198
185.8	4.58E-05	1.97E-07	2.92E-07	0.43572441	3.04E-58	3.96E-11	0.99482401
185.9	4.78E-05	1.86E-07	2.87E-07	0.44001935	2.95E-58	3.77E-11	0.99362191
186	4.78E-05	1.86E-07	2.87E-07	0.44001935	2.95E-58	3.77E-11	0.99362191
186.1	4.78E-05	1.86E-07	2.87E-07	0.44001935	2.95E-58	3.77E-11	0.99362191
186.2	4.87E-05	1.86E-07	2.87E-07	0.43949889	3.04E-58	3.81E-11	0.9944345
186.3	4.83E-05	1.89E-07	2.80E-07	0.43750649	2.92E-58	3.83E-11	0.99509148
186.4	4.83E-05	1.89E-07	2.80E-07	0.43750649	2.92E-58	3.83E-11	0.99509148
186.5	4.83E-05	1.89E-07	2.80E-07	0.43750649	2.92E-58	3.83E-11	0.99509148
186.6	4.99E-05	1.87E-07	2.74E-07	0.4384058	2.77E-58	3.73E-11	0.99228533
186.7	5.18E-05	1.85E-07	2.77E-07	0.43862987	2.69E-58	3.83E-11	0.9945599
186.8	5.35E-05	1.86E-07	2.90E-07	0.4389018	2.88E-58	3.93E-11	0.99714022
186.9	5.36E-05	1.81E-07	2.86E-07	0.43762788	2.74E-58	3.82E-11	0.99688827
187	5.45E-05	1.81E-07	2.75E-07	0.43292537	2.53E-58	3.79E-11	0.99997647
187.1	5.52E-05	1.84E-07	2.80E-07	0.43288232	2.77E-58	3.91E-11	0.99754852
187.2	5.52E-05	1.84E-07	2.80E-07	0.43288232	2.77E-58	3.91E-11	0.99754852
187.3	5.52E-05	1.84E-07	2.80E-07	0.43288232	2.77E-58	3.91E-11	0.99754852
187.4	5.52E-05	1.84E-07	2.80E-07	0.43288232	2.77E-58	3.91E-11	0.99754852
187.5	5.55E-05	1.89E-07	2.74E-07	0.43799901	2.63E-58	3.91E-11	0.99892773
187.6	5.51E-05	1.90E-07	2.83E-07	0.43976622	2.58E-58	3.83E-11	0.99843604
187.7	5.50E-05	1.88E-07	2.85E-07	0.43792616	2.76E-58	3.85E-11	0.99981844
187.8	5.50E-05	1.88E-07	2.85E-07	0.43792616	2.76E-58	3.85E-11	0.99981844
187.9	5.65E-05	1.82E-07	2.64E-07	0.43065126	2.55E-58	3.70E-11	0.99805772
188	5.60E-05	1.80E-07	2.71E-07	0.43184985	2.68E-58	3.74E-11	0.99725797
188.1	5.66E-05	1.78E-07	2.67E-07	0.43627816	2.68E-58	3.65E-11	0.9975005
188.2	5.89E-05	1.75E-07	2.78E-07	0.43447471	2.92E-58	3.85E-11	0.99794128
188.3	6.03E-05	1.71E-07	2.99E-07	0.43432063	3.38E-58	3.87E-11	0.98347396
188.4	5.84E-05	1.62E-07	2.99E-07	0.43578269	3.62E-58	3.66E-11	0.97739518
188.5	5.84E-05	1.62E-07	2.99E-07	0.43578269	3.62E-58	3.66E-11	0.97739518
188.6	5.84E-05	1.62E-07	2.99E-07	0.43578269	3.62E-58	3.66E-11	0.97739518
188.7	5.75E-05	1.62E-07	3.05E-07	0.43586506	3.73E-58	3.53E-11	0.97603805
188.8	5.75E-05	1.62E-07	3.05E-07	0.43586506	3.73E-58	3.53E-11	0.97603805
188.9	6.14E-05	1.57E-07	3.30E-07	0.43361746	4.42E-58	3.63E-11	0.97480736
189	6.11E-05	1.53E-07	3.42E-07	0.4371997	4.51E-58	3.74E-11	0.97624311
189.1	6.30E-05	1.48E-07	3.38E-07	0.44069937	4.16E-58	3.77E-11	0.97187639
189.2	6.30E-05	1.48E-07	3.38E-07	0.44069937	4.16E-58	3.77E-11	0.97187639
189.3	6.30E-05	1.48E-07	3.38E-07	0.44069937	4.16E-58	3.77E-11	0.97187639
189.4	6.36E-05	1.44E-07	3.26E-07	0.44448502	3.88E-58	3.81E-11	0.96921791
189.5	6.42E-05	1.52E-07	3.26E-07	0.44569166	4.36E-58	3.86E-11	0.96604127
189.6	6.54E-05	1.53E-07	3.13E-07	0.44438985	4.28E-58	3.73E-11	0.96313167
189.7	6.54E-05	1.53E-07	3.13E-07	0.44438985	4.28E-58	3.73E-11	0.96313167
189.8	6.96E-05	1.45E-07	3.22E-07	0.45066705	4.50E-58	3.59E-11	0.9662763
189.9	7.08E-05	1.42E-07	3.29E-07	0.45450622	4.67E-58	3.55E-11	0.96369373
190	7.08E-05	1.42E-07	3.29E-07	0.45450622	4.67E-58	3.55E-11	0.96369373
190.1	7.07E-05	1.38E-07	3.26E-07	0.45418023	4.76E-58	3.57E-11	0.95630606
190.2	7.03E-05	1.38E-07	3.08E-07	0.46184083	4.61E-58	3.36E-11	0.95501756
190.3	7.23E-05	1.36E-07	3.03E-07	0.45983767	4.43E-58	3.38E-11	0.95353373
190.4	7.23E-05	1.36E-07	3.03E-07	0.45983767	4.43E-58	3.38E-11	0.95353373
190.5	7.23E-05	1.36E-07	3.03E-07	0.45983767	4.43E-58	3.38E-11	0.95353373
190.6	7.55E-05	1.28E-07	3.07E-07	0.46015984	4.75E-58	3.39E-11	0.95223984
190.7	7.55E-05	1.28E-07	3.07E-07	0.46015984	4.75E-58	3.39E-11	0.95223984
190.8	7.91E-05	1.32E-07	2.98E-07	0.4610057	4.46E-58	3.30E-11	0.94528988
190.9	8.00E-05	1.29E-07	2.94E-07	0.46371214	4.13E-58	3.22E-11	0.93960779
191	8.14E-05	1.23E-07	2.89E-07	0.46668586	4.12E-58	3.18E-11	0.93598285
191.1	8.14E-05	1.23E-07	2.89E-07	0.46668586	4.12E-58	3.18E-11	0.93598285
191.2	8.08E-05	1.26E-07	2.94E-07	0.46545574	4.21E-58	3.15E-11	0.93764878
191.3	8.08E-05	1.26E-07	2.94E-07	0.46545574	4.21E-58	3.15E-11	0.93764878
191.4	8.42E-05	1.24E-07	2.92E-07	0.46752484	4.23E-58	3.08E-11	0.93480121
191.5	8.39E-05	1.22E-07	3.01E-07	0.4684861	4.54E-58	2.98E-11	0.93593371
191.6	8.41E-05	1.16E-07	3.04E-07	0.46649119	4.36E-58	2.95E-11	0.93513053
191.7	8.39E-05	1.15E-07	2.97E-07	0.46446501	4.09E-58	2.87E-11	0.93390333
191.8	8.41E-05	1.17E-07	2.94E-07	0.4661148	4.12E-58	2.87E-11	0.93099737
191.9	8.52E-05	1.13E-07	3.02E-07	0.461228	4.17E-58	2.83E-11	0.92847389
192	8.54E-05	1.16E-07	3.00E-07	0.46235404	4.30E-58	2.86E-11	0.9262096
192.1	8.61E-05	1.06E-07	2.91E-07	0.459739	3.82E-58	2.82E-11	0.92700369
192.2	8.61E-05	1.06E-07	2.91E-07	0.459739	3.82E-58	2.82E-11	0.92700369
192.3	8.61E-05	1.06E-07	2.91E-07	0.459739	3.82E-58	2.82E-11	0.92700369
192.4	8.61E-05	1.06E-07	2.91E-07	0.459739	3.82E-58	2.82E-11	0.92700369
192.5	8.98E-05	1.07E-07	2.69E-07	0.46225858	3.70E-58	2.74E-11	0.93144962
192.6	9.08E-05	1.02E-07	2.70E-07	0.46818231	3.78E-58	2.74E-11	0.92845256
192.7	9.08E-05	1.02E-07	2.70E-07	0.46818231	3.78E-58	2.74E-11	0.92845256
192.8	9.26E-05	1.01E-07	2.76E-07	0.46585107	3.79E-58	2.86E-11	0.92957083
192.9	9.45E-05	9.98E-08	2.68E-07	0.46239881	3.99E-58	2.81E-11	0.92444527
193	9.43E-05	9.67E-08	2.62E-07	0.4612862	3.55E-58	2.68E-11	0.91689065
193.1	9.54E-05	9.55E-08	2.61E-07	0.46494442	3.38E-58	2.60E-11	0.91664499
193.2	9.69E-05	9.33E-08	2.52E-07	0.46767136	3.06E-58	2.58E-11	0.91521976
193.3	9.69E-05	9.33E-08	2.52E-07	0.46767136	3.06E-58	2.58E-11	0.91521976
193.4	9.54E-05	9.49E-08	2.56E-07	0.4718792	3.29E-58	2.60E-11	0.90965816
193.5	0.00010668	8.07E-08	2.60E-07	0.48086692	2.98E-58	2.61E-11	0.91417822
193.6	0.00010761	7.90E-08	2.56E-07	0.48191254	2.90E-58	2.62E-11	0.90682026
193.7	0.00010856	7.66E-08	2.46E-07	0.48312384	2.69E-58	2.60E-11	0.90335032
193.8	0.00010856	7.66E-08	2.46E-07	0.48312384	2.69E-58	2.60E-11	0.90335032
193.9	0.00010856	7.66E-08	2.46E-07	0.48312384	2.69E-58	2.60E-11	0.90335032
194	0.00010856	7.66E-08	2.46E-07	0.48312384	2.69E-58	2.60E-11	0.90335032
194.1	0.00011042	7.69E-08	2.43E-07	0.48566912	2.68E-58	2.56E-11	0.90120769
194.2	0.00011064	7.53E-08	2.36E-07	0.48458028	2.62E-58	2.57E-11	0.90245566
194.3	0.00011064	7.53E-08	2.36E-07	0.48458028	2.62E-58	2.57E-11	0.90245566
194.4	0.00011403	7.67E-08	2.38E-07	0.48550776	2.64E-58	2.60E-11	0.90239547
194.5	0.00011529	7.35E-08	2.30E-07	0.47737379	2.69E-58	2.50E-11	0.90662197
194.6	0.00011529	7.35E-08	2.30E-07	0.47737379	2.69E-58	2.50E-11	0.90662197
194.7	0.00011869	7.43E-08	2.31E-07	0.48026542	2.59E-58	2.55E-11	0.90634172
194.8	0.00012013	7.37E-08	2.21E-07	0.47664257	2.58E-58	2.48E-11	0.90522316
194.9	0.00012013	7.37E-08	2.21E-07	0.47664257	2.58E-58	2.48E-11	0.90522316

198.2	0.00014871	6.71E-08	2.52E-07	0.47943565	2.43E-58	2.21E-11	0.90749781
198.3	0.00014871	6.71E-08	2.52E-07	0.47943565	2.43E-58	2.21E-11	0.90749781
198.4	0.00014947	6.60E-08	2.51E-07	0.47748795	2.20E-58	2.16E-11	0.90809292
198.5	0.00014947	6.60E-08	2.51E-07	0.47748795	2.20E-58	2.16E-11	0.90809292
198.6	0.00014947	6.60E-08	2.51E-07	0.47748795	2.20E-58	2.16E-11	0.90809292
198.7	0.00014947	6.60E-08	2.51E-07	0.47748795	2.20E-58	2.16E-11	0.90809292
198.8	0.00014947	6.60E-08	2.51E-07	0.47748795	2.20E-58	2.16E-11	0.90809292
198.9	0.00014947	6.60E-08	2.51E-07	0.47748795	2.20E-58	2.16E-11	0.90809292
199	0.00014947	6.60E-08	2.51E-07	0.47748795	2.20E-58	2.16E-11	0.90809292
199.1	0.00015173	6.51E-08	2.60E-07	0.47879949	2.33E-58	2.18E-11	0.91014719
199.2	0.00015499	6.89E-08	2.63E-07	0.48132746	2.50E-58	2.22E-11	0.90718797
199.3	0.00015499	6.89E-08	2.63E-07	0.48132746	2.50E-58	2.22E-11	0.90718797
199.4	0.00015402	6.84E-08	2.50E-07	0.48410838	2.46E-58	2.17E-11	0.90395029
199.5	0.00015828	6.65E-08	2.49E-07	0.48237635	2.60E-58	2.13E-11	0.90340924
199.6	0.00015828	6.65E-08	2.49E-07	0.48237635	2.60E-58	2.13E-11	0.90340924
199.7	0.00016498	6.59E-08	2.45E-07	0.48269257	2.55E-58	2.12E-11	0.893995
199.8	0.00016638	6.49E-08	2.45E-07	0.48302573	2.57E-58	2.14E-11	0.89632343
199.9	0.00016961	6.52E-08	2.34E-07	0.48242801	2.21E-58	2.03E-11	0.89802133
200	0.00016961	6.52E-08	2.34E-07	0.48242801	2.21E-58	2.03E-11	0.89802133
200.1	0.00016961	6.52E-08	2.34E-07	0.48242801	2.21E-58	2.03E-11	0.89802133
200.2	0.00016926	6.45E-08	2.40E-07	0.47790664	2.23E-58	2.00E-11	0.90511533
200.3	0.00016926	6.45E-08	2.40E-07	0.47790664	2.23E-58	2.00E-11	0.90511533
200.4	0.00016926	6.45E-08	2.40E-07	0.47790664	2.23E-58	2.00E-11	0.90511533
200.5	0.00016978	6.68E-08	2.50E-07	0.47808747	2.48E-58	2.01E-11	0.90397763
200.6	0.00017457	6.30E-08	2.42E-07	0.47733114	2.41E-58	2.04E-11	0.90507896
200.7	0.00017725	6.30E-08	2.43E-07	0.47663497	2.46E-58	2.07E-11	0.90331496
200.8	0.00017725	6.30E-08	2.43E-07	0.47663497	2.46E-58	2.07E-11	0.90331496
200.9	0.00017706	6.31E-08	2.37E-07	0.47678646	2.40E-58	2.08E-11	0.89462294
201	0.00018075	6.32E-08	2.35E-07	0.47667058	2.39E-58	2.02E-11	0.89533116
201.1	0.00018712	6.38E-08	2.21E-07	0.47786355	2.12E-58	2.02E-11	0.89649861
201.2	0.00018737	6.37E-08	2.30E-07	0.47834804	2.47E-58	2.07E-11	0.89550976
201.3	0.00019017	6.30E-08	2.33E-07	0.47829197	2.54E-58	2.07E-11	0.89178188
201.4	0.00019976	6.54E-08	2.22E-07	0.47698136	2.51E-58	2.01E-11	0.89390604
201.5	0.00020117	6.54E-08	2.19E-07	0.47619144	2.43E-58	2.03E-11	0.89614734
201.6	0.00020342	6.42E-08	2.12E-07	0.4781038	2.31E-58	2.01E-11	0.89425664
201.7	0.00020436	6.69E-08	2.17E-07	0.48071158	2.51E-58	2.03E-11	0.89332557
201.8	0.00020436	6.69E-08	2.17E-07	0.48071158	2.51E-58	2.03E-11	0.89332557
201.9	0.00020906	6.71E-08	2.19E-07	0.48040426	2.50E-58	2.10E-11	0.89636029
202	0.00020906	6.71E-08	2.19E-07	0.48040426	2.50E-58	2.10E-11	0.89636029
202.1	0.00020906	6.71E-08	2.19E-07	0.48040426	2.50E-58	2.10E-11	0.89636029
202.2	0.00020737	6.57E-08	2.22E-07	0.48292522	2.77E-58	2.19E-11	0.89277689
202.3	0.00020706	6.21E-08	2.12E-07	0.47777214	2.27E-58	2.08E-11	0.89289142
202.4	0.00021073	6.01E-08	2.01E-07	0.47747512	2.03E-58	2.01E-11	0.89473072
202.5	0.00021596	5.87E-08	1.99E-07	0.47433112	1.93E-58	2.00E-11	0.88772053
202.6	0.00021596	5.87E-08	1.99E-07	0.47433112	1.93E-58	2.00E-11	0.88772053
202.7	0.00021596	5.87E-08	1.99E-07	0.47433112	1.93E-58	2.00E-11	0.88772053
202.8	0.00022342	5.63E-08	1.93E-07	0.47101986	1.91E-58	1.97E-11	0.89070329
202.9	0.00022798	5.50E-08	1.89E-07	0.47196561	1.86E-58	1.97E-11	0.88817669
203	0.00022822	5.42E-08	1.88E-07	0.46743209	1.86E-58	1.90E-11	0.88689632
203.1	0.00023742	5.52E-08	1.90E-07	0.47019594	2.23E-58	1.95E-11	0.87842143
203.2	0.00023742	5.52E-08	1.90E-07	0.47019594	2.23E-58	1.95E-11	0.87842143
203.3	0.00023742	5.52E-08	1.90E-07	0.47019594	2.23E-58	1.95E-11	0.87842143
203.4	0.00023957	5.45E-08	1.88E-07	0.46943947	2.05E-58	1.92E-11	0.87571387
203.5	0.00023957	5.45E-08	1.88E-07	0.46943947	2.05E-58	1.92E-11	0.87571387
203.6	0.00023957	5.45E-08	1.88E-07	0.46943947	2.05E-58	1.92E-11	0.87571387
203.7	0.00023843	5.50E-08	1.94E-07	0.47225932	2.00E-58	1.99E-11	0.87907234
203.8	0.000249	5.90E-08	1.88E-07	0.47122882	1.97E-58	1.99E-11	0.86743162
203.9	0.000249	5.90E-08	1.88E-07	0.47122882	1.97E-58	1.99E-11	0.86743162
204	0.00025796	5.92E-08	1.94E-07	0.47419769	2.10E-58	2.06E-11	0.86893536
204.1	0.00025796	5.92E-08	1.94E-07	0.47419769	2.10E-58	2.06E-11	0.86893536
204.2	0.00026038	5.77E-08	1.94E-07	0.47869596	2.26E-58	2.03E-11	0.86268903
204.3	0.00026038	5.77E-08	1.94E-07	0.47869596	2.26E-58	2.03E-11	0.86268903
204.4	0.00026038	5.77E-08	1.94E-07	0.47869596	2.26E-58	2.03E-11	0.86268903
204.5	0.00025925	5.68E-08	2.01E-07	0.47841471	2.41E-58	1.99E-11	0.85840002
204.6	0.00025925	5.68E-08	2.01E-07	0.47841471	2.41E-58	1.99E-11	0.85840002
204.7	0.00025925	5.68E-08	2.01E-07	0.47841471	2.41E-58	1.99E-11	0.85840002
204.8	0.00025925	5.68E-08	2.01E-07	0.47841471	2.41E-58	1.99E-11	0.85840002
204.9	0.0002614	5.81E-08	1.89E-07	0.47482758	2.16E-58	1.89E-11	0.85584901
205	0.0002614	5.81E-08	1.89E-07	0.47482758	2.16E-58	1.89E-11	0.85584901
205.1	0.00026948	5.75E-08	1.93E-07	0.4737167	2.06E-58	1.91E-11	0.85128667
205.2	0.00028064	5.48E-08	1.97E-07	0.46771737	2.13E-58	1.87E-11	0.84941986
205.3	0.00028064	5.48E-08	1.97E-07	0.46771737	2.13E-58	1.87E-11	0.84941986
205.4	0.00028064	5.48E-08	1.97E-07	0.46771737	2.13E-58	1.87E-11	0.84941986
205.5	0.00028064	5.48E-08	1.97E-07	0.46771737	2.13E-58	1.87E-11	0.84941986
205.6	0.00028064	5.48E-08	1.97E-07	0.46771737	2.13E-58	1.87E-11	0.84941986
205.7	0.00029456	5.60E-08	2.07E-07	0.46684621	2.22E-58	1.99E-11	0.84674833
205.8	0.00030645	5.57E-08	2.06E-07	0.46698861	2.42E-58	1.98E-11	0.84491833
205.9	0.00030645	5.57E-08	2.06E-07	0.46698861	2.42E-58	1.98E-11	0.84491833
206	0.00030645	5.57E-08	2.06E-07	0.46698861	2.42E-58	1.98E-11	0.84491833
206.1	0.00030645	5.57E-08	2.06E-07	0.46698861	2.42E-58	1.98E-11	0.84491833
206.2	0.00030645	5.57E-08	2.06E-07	0.46698861	2.42E-58	1.98E-11	0.84491833
206.3	0.00030645	5.57E-08	2.06E-07	0.46698861	2.42E-58	1.98E-11	0.84491833
206.4	0.00031435	5.59E-08	2.07E-07	0.47486695	2.40E-58	2.03E-11	0.8402418
206.5	0.00031435	5.59E-08	2.07E-07	0.47486695	2.40E-58	2.03E-11	0.8402418
206.6	0.00031961	5.67E-08	2.03E-07	0.47651684	2.41E-58	2.06E-11	0.84116601
206.7	0.00031961	5.67E-08	2.03E-07	0.47651684	2.41E-58	2.06E-11	0.84116601
206.8	0.00031961	5.67E-08	2.03E-07	0.47651684	2.41E-58	2.06E-11	0.84116601
206.9	0.00031961	5.67E-08	2.03E-07	0.47651684	2.41E-58	2.06E-11	0.84116601
207	0.00031961	5.67E-08	2.03E-07	0.47651684	2.41E-58	2.06E-11	0.84116601
207.1	0.00033005	5.54E-08	1.99E-07	0.47616632	2.60E-58	2.05E-11	0.83636435
207.2	0.00033004	5.56E-08	1.92E-07	0.47605124	2.63E-58	2.07E-11	0.8308925
207.3	0.00033004	5.56E-08	1.92E-07	0.47605124	2.63E-58	2.07E-11	0.8308925
207.4	0.00033693	5.74E-08	2.01E-07	0.47927049	2.89E-58	2.12E-11	0.83048081
207.5	0.00033297	5.75E-08	2.02E-07	0.48071422	2.81E-58	2.08E-11	0.82351309
207.6	0.00033297	5.75E-08	2.02E-07	0.48071422	2.81E-58	2.08E-11	0.82351309
207.7	0.00033851	5.79E-08	1.98E-07	0.48290698	2.76E-58	2.09E-11	0.8237892
207.8	0.00033851	5.79E-08	1.98E-07	0.48290698	2.76E-58	2.09E-11	0.8237892
207.9	0.00033851	5.79E-08	1.98E-07	0.48290698	2.76E-58	2.09E-11	0.8237892
208	0.00034319	5.87E-08	2.01E-07	0.48361499	2.94E-58	2.09E-11	0.82519482
208.1	0.00034319	5.87E-08	2.01E-07	0.48361499	2.94E-58	2.09E-11	0.82519482
208.2	0.00035263	5.70E-08	1.96E-07	0.48236754	3.05E-58	2.06E-11	0.82442901
208.3	0.00035219	5.63E-08	1.92E-07	0.47949558	2.88E-58	2.05E-11	0.81999884
208.4	0.00036581	5.45E-08	2.00E-07	0.47971419	3.15E-58	2.10E-11	0.81750226
208.5	0.00036581	5.45E-08	2.00E-07	0.47971419	3.15E-58	2.10E-11	0.81750226
208.6	0.00036581	5.45E-08	2.00E-07	0.47971419	3.15E-58	2.10E-11	0.81750226
208.7	0.00036897	5.48E-08	2.03E-07	0.48160195	3.56E-58	2.15E-11	0.81332372
208.8	0.00036897	5.48E-08	2.03E-07	0.48160195	3.56E-58	2.15E-11	0.81332372
208.9	0.00037861	5.40E-08	1.95E-07	0.47852754	3.28E-58	2.14E-11	0.81344403
209	0.00037861	5.40E-08	1.95E-07	0.47852754	3.28E-58	2.14E-11	0.81344403
209.1	0.00037861	5.40E-08	1.95E-07	0.47852754	3.28E-58	2.14E-11	0.81344403
209.2	0.00037861	5.40E-08	1.95E-07	0.47852754	3.28E-58	2.14E-11	0.81344403
209.3	0.00037861	5.40E-08	1.95E-07	0.47852754	3.28E-58	2.14E-11	0.81344403
209.4	0.00037861	5.40E-08	1.95E-07	0.47852754	3.28E-58	2.14E-11	0.81344403
209.5	0.00037861	5.40E-08	1.95E-07	0.47852754	3.28E-58	2.14E-11	0.81344403
209.6	0.00037861	5.40E-08	1.95E-07	0.47852754	3.28E-58	2.14E-11	0.81344403
209.7	0.00036858	5.50E-08	2.00E-07	0.47816705	3.41E-58	2.08E-11	0.81613254
209.8	0.00038038	5.59E-08	1.99E-07	0.47694424	3.59E-58	2.03E-11	0.82096255
209.9	0.00038038	5.59E-08	1.99E-07	0.47694424	3.59E-58	2.03E-11	0.82096255

213.2	0.00042351	4.99E-08	1.66E-07	0.47497625	3.20E-58	1.90E-11	0.80582765
213.3	0.00043004	4.96E-08	1.64E-07	0.47303532	3.06E-58	1.88E-11	0.80706547
213.4	0.00043684	5.16E-08	1.60E-07	0.47263078	2.98E-58	1.88E-11	0.80997157
213.5	0.00043684	5.16E-08	1.60E-07	0.47263078	2.98E-58	1.88E-11	0.80997157
213.6	0.00044479	5.11E-08	1.59E-07	0.47143108	3.12E-58	1.91E-11	0.80255499
213.7	0.00044479	5.11E-08	1.59E-07	0.47143108	3.12E-58	1.91E-11	0.80255499
213.8	0.00044479	5.11E-08	1.59E-07	0.47143108	3.12E-58	1.91E-11	0.80255499
213.9	0.00044479	5.11E-08	1.59E-07	0.47143108	3.12E-58	1.91E-11	0.80255499
214	0.00045267	5.00E-08	1.55E-07	0.47040348	2.85E-58	1.91E-11	0.79663226
214.1	0.00045242	5.04E-08	1.49E-07	0.47093893	2.88E-58	1.89E-11	0.79773698
214.2	0.00045242	5.04E-08	1.49E-07	0.47093893	2.88E-58	1.89E-11	0.79773698
214.3	0.00045242	5.04E-08	1.49E-07	0.47093893	2.88E-58	1.89E-11	0.79773698
214.4	0.00045242	5.04E-08	1.49E-07	0.47093893	2.88E-58	1.89E-11	0.79773698
214.5	0.00045242	5.04E-08	1.49E-07	0.47093893	2.88E-58	1.89E-11	0.79773698
214.6	0.00045242	5.04E-08	1.49E-07	0.47093893	2.88E-58	1.89E-11	0.79773698
214.7	0.00045242	5.04E-08	1.49E-07	0.47093893	2.88E-58	1.89E-11	0.79773698
214.8	0.00046075	5.13E-08	1.50E-07	0.46652016	3.04E-58	1.88E-11	0.79071339
214.9	0.00046075	5.13E-08	1.50E-07	0.46652016	3.04E-58	1.88E-11	0.79071339
215	0.00048091	4.80E-08	1.41E-07	0.46583772	3.10E-58	1.86E-11	0.78364933
215.1	0.00048515	4.83E-08	1.34E-07	0.46573655	2.90E-58	1.82E-11	0.78260751
215.2	0.00048515	4.83E-08	1.34E-07	0.46573655	2.90E-58	1.82E-11	0.78260751
215.3	0.00048515	4.83E-08	1.34E-07	0.46573655	2.90E-58	1.82E-11	0.78260751
215.4	0.00048515	4.83E-08	1.34E-07	0.46573655	2.90E-58	1.82E-11	0.78260751
215.5	0.00048515	4.83E-08	1.34E-07	0.46573655	2.90E-58	1.82E-11	0.78260751
215.6	0.00048515	4.83E-08	1.34E-07	0.46573655	2.90E-58	1.82E-11	0.78260751
215.7	0.00048515	4.83E-08	1.34E-07	0.46573655	2.90E-58	1.82E-11	0.78260751
215.8	0.00048515	4.83E-08	1.34E-07	0.46573655	2.90E-58	1.82E-11	0.78260751
215.9	0.00048515	4.83E-08	1.34E-07	0.46573655	2.90E-58	1.82E-11	0.78260751
216	0.00048399	4.86E-08	1.30E-07	0.46937474	2.79E-58	1.82E-11	0.78271265
216.1	0.00048399	4.86E-08	1.30E-07	0.46937474	2.79E-58	1.82E-11	0.78271265
216.2	0.00048399	4.86E-08	1.30E-07	0.46937474	2.79E-58	1.82E-11	0.78271265
216.3	0.00051335	5.03E-08	1.35E-07	0.47269242	3.06E-58	1.95E-11	0.7888727
216.4	0.00052411	4.95E-08	1.36E-07	0.4704105	3.20E-58	1.96E-11	0.78816519
216.5	0.00052411	4.95E-08	1.36E-07	0.4704105	3.20E-58	1.96E-11	0.78816519
216.6	0.00052411	4.95E-08	1.36E-07	0.4704105	3.20E-58	1.96E-11	0.78816519
216.7	0.00052411	4.95E-08	1.36E-07	0.4704105	3.20E-58	1.96E-11	0.78816519
216.8	0.00052411	4.95E-08	1.36E-07	0.4704105	3.20E-58	1.96E-11	0.78816519
216.9	0.00052411	4.95E-08	1.36E-07	0.4704105	3.20E-58	1.96E-11	0.78816519
217	0.00052411	4.95E-08	1.36E-07	0.4704105	3.20E-58	1.96E-11	0.78816519
217.1	0.00052411	4.95E-08	1.36E-07	0.4704105	3.20E-58	1.96E-11	0.78816519
217.2	0.00052411	4.95E-08	1.36E-07	0.4704105	3.20E-58	1.96E-11	0.78816519
217.3	0.00052411	4.95E-08	1.36E-07	0.4704105	3.20E-58	1.96E-11	0.78816519
217.4	0.00052373	5.16E-08	1.36E-07	0.47531703	3.23E-58	1.92E-11	0.78248856
217.5	0.00052373	5.16E-08	1.36E-07	0.47531703	3.23E-58	1.92E-11	0.78248856
217.6	0.00052373	5.16E-08	1.36E-07	0.47531703	3.23E-58	1.92E-11	0.78248856
217.7	0.00052373	5.16E-08	1.36E-07	0.47531703	3.23E-58	1.92E-11	0.78248856
217.8	0.00052373	5.16E-08	1.36E-07	0.47531703	3.23E-58	1.92E-11	0.78248856
217.9	0.00052373	5.16E-08	1.36E-07	0.47531703	3.23E-58	1.92E-11	0.78248856
218	0.00052373	5.16E-08	1.36E-07	0.47531703	3.23E-58	1.92E-11	0.78248856
218.1	0.000522	5.28E-08	1.34E-07	0.47424052	2.83E-58	1.81E-11	0.77902064
218.2	0.000522	5.28E-08	1.34E-07	0.47424052	2.83E-58	1.81E-11	0.77902064
218.3	0.000522	5.28E-08	1.34E-07	0.47424052	2.83E-58	1.81E-11	0.77902064
218.4	0.000522	5.28E-08	1.34E-07	0.47424052	2.83E-58	1.81E-11	0.77902064
218.5	0.000522	5.28E-08	1.34E-07	0.47424052	2.83E-58	1.81E-11	0.77902064
218.6	0.00052101	5.32E-08	1.40E-07	0.4771538	3.02E-58	1.79E-11	0.77620271
218.7	0.00052101	5.32E-08	1.40E-07	0.4771538	3.02E-58	1.79E-11	0.77620271
218.8	0.00052101	5.32E-08	1.40E-07	0.4771538	3.02E-58	1.79E-11	0.77620271
218.9	0.00052101	5.32E-08	1.40E-07	0.4771538	3.02E-58	1.79E-11	0.77620271
219	0.00052298	5.19E-08	1.39E-07	0.47340862	3.00E-58	1.79E-11	0.77994832
219.1	0.00052298	5.19E-08	1.39E-07	0.47340862	3.00E-58	1.79E-11	0.77994832
219.2	0.00052144	5.24E-08	1.41E-07	0.47036112	3.20E-58	1.74E-11	0.78232395
219.3	0.00052144	5.24E-08	1.41E-07	0.47036112	3.20E-58	1.74E-11	0.78232395
219.4	0.00052427	5.21E-08	1.36E-07	0.4758245	3.25E-58	1.74E-11	0.77971871
219.5	0.00052427	5.21E-08	1.36E-07	0.4758245	3.25E-58	1.74E-11	0.77971871
219.6	0.00052427	5.21E-08	1.36E-07	0.4758245	3.25E-58	1.74E-11	0.77971871
219.7	0.00052427	5.21E-08	1.36E-07	0.4758245	3.25E-58	1.74E-11	0.77971871
219.8	0.00052427	5.21E-08	1.36E-07	0.4758245	3.25E-58	1.74E-11	0.77971871
219.9	0.00052427	5.21E-08	1.36E-07	0.4758245	3.25E-58	1.74E-11	0.77971871
220	0.00052824	5.38E-08	1.34E-07	0.4774581	3.14E-58	1.76E-11	0.77660534
220.1	0.00052824	5.38E-08	1.34E-07	0.4774581	3.14E-58	1.76E-11	0.77660534
220.2	0.00052824	5.38E-08	1.34E-07	0.4774581	3.14E-58	1.76E-11	0.77660534
220.3	0.00052824	5.38E-08	1.34E-07	0.4774581	3.14E-58	1.76E-11	0.77660534
220.4	0.00052824	5.38E-08	1.34E-07	0.4774581	3.14E-58	1.76E-11	0.77660534
220.5	0.00052824	5.38E-08	1.34E-07	0.4774581	3.14E-58	1.76E-11	0.77660534
220.6	0.00052824	5.38E-08	1.34E-07	0.4774581	3.14E-58	1.76E-11	0.77660534
220.7	0.00052808	5.21E-08	1.28E-07	0.47813766	2.83E-58	1.73E-11	0.77165865
220.8	0.00053157	5.25E-08	1.27E-07	0.48225948	2.96E-58	1.69E-11	0.76161559
220.9	0.00053157	5.25E-08	1.27E-07	0.48225948	2.96E-58	1.69E-11	0.76161559
221	0.00053157	5.25E-08	1.27E-07	0.48225948	2.96E-58	1.69E-11	0.76161559
221.1	0.00053157	5.25E-08	1.27E-07	0.48225948	2.96E-58	1.69E-11	0.76161559
221.2	0.00053735	5.36E-08	1.24E-07	0.48055664	2.79E-58	1.66E-11	0.76189678
221.3	0.00053735	5.36E-08	1.24E-07	0.48055664	2.79E-58	1.66E-11	0.76189678
221.4	0.00053436	5.31E-08	1.19E-07	0.47698161	2.44E-58	1.64E-11	0.76090597
221.5	0.00053436	5.31E-08	1.19E-07	0.47698161	2.44E-58	1.64E-11	0.76090597
221.6	0.00053436	5.31E-08	1.19E-07	0.47698161	2.44E-58	1.64E-11	0.76090597
221.7	0.00053258	5.45E-08	1.16E-07	0.47406786	2.51E-58	1.64E-11	0.75574812
221.8	0.00053258	5.45E-08	1.16E-07	0.47406786	2.51E-58	1.64E-11	0.75574812
221.9	0.00054523	5.63E-08	1.15E-07	0.47335755	2.61E-58	1.66E-11	0.75248516
222	0.00054523	5.63E-08	1.15E-07	0.47335755	2.61E-58	1.66E-11	0.75248516
222.1	0.00055526	5.75E-08	1.13E-07	0.4723146	2.62E-58	1.67E-11	0.75654391
222.2	0.00055526	5.75E-08	1.13E-07	0.4723146	2.62E-58	1.67E-11	0.75654391
222.3	0.00056899	5.49E-08	1.09E-07	0.47004004	2.47E-58	1.63E-11	0.75500758
222.4	0.00056462	5.56E-08	1.05E-07	0.46805128	2.25E-58	1.56E-11	0.7502031
222.5	0.00058046	5.49E-08	1.09E-07	0.46726635	2.47E-58	1.55E-11	0.74833541
222.6	0.00060283	5.49E-08	1.10E-07	0.46340622	2.51E-58	1.56E-11	0.74691092
222.7	0.00060283	5.49E-08	1.10E-07	0.46340622	2.51E-58	1.56E-11	0.74691092
222.8	0.00060283	5.49E-08	1.10E-07	0.46340622	2.51E-58	1.56E-11	0.74691092
222.9	0.00060283	5.49E-08	1.10E-07	0.46340622	2.51E-58	1.56E-11	0.74691092
223	0.00060283	5.49E-08	1.10E-07	0.46340622	2.51E-58	1.56E-11	0.74691092
223.1	0.00060283	5.49E-08	1.10E-07	0.46340622	2.51E-58	1.56E-11	0.74691092
223.2	0.00060283	5.49E-08	1.10E-07	0.46340622	2.51E-58	1.56E-11	0.74691092
223.3	0.00058922	5.56E-08	1.10E-07	0.45992042	2.39E-58	1.53E-11	0.7448003
223.4	0.00058922	5.56E-08	1.10E-07	0.45992042	2.39E-58	1.53E-11	0.7448003
223.5	0.00058922	5.56E-08	1.10E-07	0.45992042	2.39E-58	1.53E-11	0.7448003
223.6	0.00058922	5.56E-08	1.10E-07	0.45992042	2.39E-58	1.53E-11	0.7448003
223.7	0.00058922	5.56E-08	1.10E-07	0.45992042	2.39E-58	1.53E-11	0.7448003
223.8	0.00058922	5.56E-08	1.10E-07	0.45992042	2.39E-58	1.53E-11	0.7448003
223.9	0.00058922	5.56E-08	1.10E-07	0.45992042	2.39E-58	1.53E-11	0.7448003
224	0.00058922	5.56E-08	1.10E-07	0.45992042	2.39E-58	1.53E-11	0.7448003
224.1	0.00058922	5.56E-08	1.10E-07	0.45992042	2.39E-58	1.53E-11	0.7448003
224.2	0.00058922	5.56E-08	1.10E-07	0.45992042	2.39E-58	1.53E-11	0.7448003
224.3	0.00057762	5.58E-08	1.08E-07	0.45944687	2.14E-58	1.55E-11	0.74033129
224.4	0.00057762	5.58E-08	1.08E-07	0.45944687	2.14E-58	1.55E-11	0.74033129
224.5	0.00057762	5.58E-08	1.08E-07	0.45944687	2.14E-58	1.55E-11	0.74033129
224.6	0.00057762	5.58E-08	1.08E-07	0.45944687	2.14E-58	1.55E-11	0.74033129
224.7	0.00057762	5.58E-08	1.08E-07	0.45944687	2.14E-58	1.55E-11	0.74033129
224.8	0.00057762	5.58E-08	1.08E-07	0.45944687	2.14E-58	1.55E-11	0.74033129
224.9	0.00059205	5.50E-08	1.09E-07	0.45655271	2.24E-58	1.54E-11	0.74371675

228.2	0.00063757	5.67E-08	9.59E-08	0.44496386	2.15E-58	1.49E-11	0.72979517
228.3	0.00064886	5.58E-08	9.38E-08	0.44585641	2.01E-58	1.49E-11	0.72853905
228.4	0.00064886	5.58E-08	9.38E-08	0.44585641	2.01E-58	1.49E-11	0.72853905
228.5	0.00064764	5.82E-08	9.12E-08	0.44437834	1.94E-58	1.40E-11	0.72349991
228.6	0.00066286	5.85E-08	8.97E-08	0.43811527	1.81E-58	1.39E-11	0.72369557
228.7	0.00066286	5.85E-08	8.97E-08	0.43811527	1.81E-58	1.39E-11	0.72369557
228.8	0.00065144	5.97E-08	9.39E-08	0.43815738	1.97E-58	1.38E-11	0.72260185
228.9	0.00065144	5.97E-08	9.39E-08	0.43815738	1.97E-58	1.38E-11	0.72260185
229	0.00065144	5.97E-08	9.39E-08	0.43815738	1.97E-58	1.38E-11	0.72260185
229.1	0.00065135	6.08E-08	9.00E-08	0.43562894	1.85E-58	1.38E-11	0.7200411
229.2	0.00064152	6.07E-08	9.34E-08	0.43417422	1.94E-58	1.41E-11	0.72270697
229.3	0.00062985	6.28E-08	9.74E-08	0.4371781	2.11E-58	1.47E-11	0.71946276
229.4	0.00062985	6.28E-08	9.74E-08	0.4371781	2.11E-58	1.47E-11	0.71946276
229.5	0.00063719	6.16E-08	9.23E-08	0.44029283	2.02E-58	1.45E-11	0.72038489
229.6	0.00063949	5.89E-08	8.85E-08	0.4392397	1.89E-58	1.42E-11	0.71422072
229.7	0.00063949	5.89E-08	8.85E-08	0.4392397	1.89E-58	1.42E-11	0.71422072
229.8	0.00063949	5.89E-08	8.85E-08	0.4392397	1.89E-58	1.42E-11	0.71422072
229.9	0.00063949	5.89E-08	8.85E-08	0.4392397	1.89E-58	1.42E-11	0.71422072
230	0.00063305	6.02E-08	8.57E-08	0.44243815	1.80E-58	1.42E-11	0.71208023
230.1	0.00064979	5.97E-08	8.31E-08	0.43804643	1.83E-58	1.41E-11	0.70980491
230.2	0.00064979	5.97E-08	8.31E-08	0.43804643	1.83E-58	1.41E-11	0.70980491
230.3	0.00065236	6.14E-08	8.18E-08	0.4356359	1.84E-58	1.43E-11	0.70926665
230.4	0.00065236	6.14E-08	8.18E-08	0.4356359	1.84E-58	1.43E-11	0.70926665
230.5	0.00065236	6.14E-08	8.18E-08	0.4356359	1.84E-58	1.43E-11	0.70926665
230.6	0.00065236	6.14E-08	8.18E-08	0.4356359	1.84E-58	1.43E-11	0.70926665
230.7	0.00065236	6.14E-08	8.18E-08	0.4356359	1.84E-58	1.43E-11	0.70926665
230.8	0.00065236	6.14E-08	8.18E-08	0.4356359	1.84E-58	1.43E-11	0.70926665
230.9	0.00065236	6.14E-08	8.18E-08	0.4356359	1.84E-58	1.43E-11	0.70926665
231	0.00065236	6.14E-08	8.18E-08	0.4356359	1.84E-58	1.43E-11	0.70926665
231.1	0.00064378	6.32E-08	8.00E-08	0.43267147	1.75E-58	1.44E-11	0.70979279
231.2	0.00064378	6.32E-08	8.00E-08	0.43267147	1.75E-58	1.44E-11	0.70979279
231.3	0.00064684	6.25E-08	7.49E-08	0.42954938	1.63E-58	1.40E-11	0.71163391
231.4	0.00064024	6.17E-08	7.53E-08	0.42564981	1.63E-58	1.38E-11	0.71705583
231.5	0.00064024	6.17E-08	7.53E-08	0.42564981	1.63E-58	1.38E-11	0.71705583
231.6	0.00065551	6.04E-08	7.25E-08	0.42418795	1.62E-58	1.38E-11	0.71364422
231.7	0.00065551	6.04E-08	7.25E-08	0.42418795	1.62E-58	1.38E-11	0.71364422
231.8	0.00065551	6.04E-08	7.25E-08	0.42418795	1.62E-58	1.38E-11	0.71364422
231.9	0.00066081	6.16E-08	7.30E-08	0.42361512	1.70E-58	1.40E-11	0.71237184
232	0.0006785	6.07E-08	7.05E-08	0.42008253	1.64E-58	1.37E-11	0.71433137
232.1	0.0006785	6.07E-08	7.05E-08	0.42008253	1.64E-58	1.37E-11	0.71433137
232.2	0.0006785	6.07E-08	7.05E-08	0.42008253	1.64E-58	1.37E-11	0.71433137
232.3	0.00067553	6.17E-08	7.01E-08	0.41614468	1.56E-58	1.37E-11	0.71664011
232.4	0.00067553	6.17E-08	7.01E-08	0.41614468	1.56E-58	1.37E-11	0.71664011
232.5	0.00067553	6.17E-08	7.01E-08	0.41614468	1.56E-58	1.37E-11	0.71664011
232.6	0.0006805	6.21E-08	7.00E-08	0.4197874	1.59E-58	1.38E-11	0.71541056
232.7	0.00069644	6.09E-08	6.29E-08	0.41248786	1.36E-58	1.33E-11	0.70669003
232.8	0.00070702	6.29E-08	6.37E-08	0.40720238	1.38E-58	1.29E-11	0.69942568
232.9	0.00070702	6.29E-08	6.37E-08	0.40720238	1.38E-58	1.29E-11	0.69942568
233	0.00070702	6.29E-08	6.37E-08	0.40720238	1.38E-58	1.29E-11	0.69942568
233.1	0.00070702	6.29E-08	6.37E-08	0.40720238	1.38E-58	1.29E-11	0.69942568
233.2	0.00071246	6.54E-08	6.32E-08	0.41053126	1.37E-58	1.31E-11	0.70091456
233.3	0.00071246	6.54E-08	6.32E-08	0.41053126	1.37E-58	1.31E-11	0.70091456
233.4	0.00071246	6.54E-08	6.32E-08	0.41053126	1.37E-58	1.31E-11	0.70091456
233.5	0.00071246	6.54E-08	6.32E-08	0.41053126	1.37E-58	1.31E-11	0.70091456
233.6	0.00071246	6.54E-08	6.32E-08	0.41053126	1.37E-58	1.31E-11	0.70091456
233.7	0.0007362	6.45E-08	5.99E-08	0.40999052	1.34E-58	1.30E-11	0.70377989
233.8	0.0007362	6.45E-08	5.99E-08	0.40999052	1.34E-58	1.30E-11	0.70377989
233.9	0.00074547	6.32E-08	5.96E-08	0.40881929	1.34E-58	1.30E-11	0.69879388
234	0.00074547	6.32E-08	5.96E-08	0.40881929	1.34E-58	1.30E-11	0.69879388
234.1	0.00074086	6.38E-08	5.79E-08	0.41028433	1.33E-58	1.29E-11	0.69540446
234.2	0.00074086	6.38E-08	5.79E-08	0.41028433	1.33E-58	1.29E-11	0.69540446
234.3	0.00074277	6.37E-08	5.71E-08	0.40765996	1.24E-58	1.30E-11	0.69600926
234.4	0.00074277	6.37E-08	5.71E-08	0.40765996	1.24E-58	1.30E-11	0.69600926
234.5	0.00074277	6.37E-08	5.71E-08	0.40765996	1.24E-58	1.30E-11	0.69600926
234.6	0.00074277	6.37E-08	5.71E-08	0.40765996	1.24E-58	1.30E-11	0.69600926
234.7	0.00074277	6.37E-08	5.71E-08	0.40765996	1.24E-58	1.30E-11	0.69600926
234.8	0.00074277	6.37E-08	5.71E-08	0.40765996	1.24E-58	1.30E-11	0.69600926
234.9	0.00074277	6.37E-08	5.71E-08	0.40765996	1.24E-58	1.30E-11	0.69600926
235	0.00077007	6.16E-08	5.60E-08	0.40346196	1.22E-58	1.29E-11	0.69771604
235.1	0.0007715	6.11E-08	5.37E-08	0.40077436	1.14E-58	1.30E-11	0.70042704
235.2	0.0007715	6.11E-08	5.37E-08	0.40077436	1.14E-58	1.30E-11	0.70042704
235.3	0.0007715	6.11E-08	5.37E-08	0.40077436	1.14E-58	1.30E-11	0.70042704
235.4	0.0007715	6.11E-08	5.37E-08	0.40077436	1.14E-58	1.30E-11	0.70042704
235.5	0.0007715	6.11E-08	5.37E-08	0.40077436	1.14E-58	1.30E-11	0.70042704
235.6	0.0007715	6.11E-08	5.37E-08	0.40077436	1.14E-58	1.30E-11	0.70042704
235.7	0.0007852	6.26E-08	5.22E-08	0.40011369	1.07E-58	1.28E-11	0.69650698
235.8	0.0007852	6.26E-08	5.22E-08	0.40011369	1.07E-58	1.28E-11	0.69650698
235.9	0.00078861	6.47E-08	4.92E-08	0.3998326	9.60E-59	1.26E-11	0.69123506
236	0.00078861	6.47E-08	4.92E-08	0.3998326	9.60E-59	1.26E-11	0.69123506
236.1	0.00078819	6.50E-08	4.83E-08	0.39967221	9.30E-59	1.26E-11	0.69029885
236.2	0.00078799	6.50E-08	4.72E-08	0.39999008	8.80E-59	1.25E-11	0.6887337
236.3	0.00078671	6.62E-08	4.58E-08	0.39589936	8.61E-59	1.23E-11	0.68809567
236.4	0.00078671	6.62E-08	4.58E-08	0.39589936	8.61E-59	1.23E-11	0.68809567
236.5	0.00078875	6.85E-08	4.63E-08	0.39720564	9.10E-59	1.24E-11	0.68587183
236.6	0.00078875	6.85E-08	4.63E-08	0.39720564	9.10E-59	1.24E-11	0.68587183
236.7	0.00078875	6.85E-08	4.63E-08	0.39720564	9.10E-59	1.24E-11	0.68587183
236.8	0.00078875	6.85E-08	4.63E-08	0.39720564	9.10E-59	1.24E-11	0.68587183
236.9	0.00078875	6.85E-08	4.63E-08	0.39720564	9.10E-59	1.24E-11	0.68587183
237	0.00078875	6.85E-08	4.63E-08	0.39720564	9.10E-59	1.24E-11	0.68587183
237.1	0.00078875	6.85E-08	4.63E-08	0.39720564	9.10E-59	1.24E-11	0.68587183
237.2	0.00078875	6.85E-08	4.63E-08	0.39720564	9.10E-59	1.24E-11	0.68587183
237.3	0.00079913	6.96E-08	4.37E-08	0.39984407	8.28E-59	1.19E-11	0.68658285
237.4	0.00079913	6.96E-08	4.37E-08	0.39984407	8.28E-59	1.19E-11	0.68658285
237.5	0.00079913	6.96E-08	4.37E-08	0.39984407	8.28E-59	1.19E-11	0.68658285
237.6	0.00078642	6.91E-08	4.37E-08	0.39818828	7.99E-59	1.18E-11	0.68476986
237.7	0.00078642	6.91E-08	4.37E-08	0.39818828	7.99E-59	1.18E-11	0.68476986
237.8	0.0007876	6.89E-08	4.29E-08	0.39541159	7.74E-59	1.15E-11	0.67961355
237.9	0.0007876	6.89E-08	4.29E-08	0.39541159	7.74E-59	1.15E-11	0.67961355
238	0.0007876	6.89E-08	4.29E-08	0.39541159	7.74E-59	1.15E-11	0.67961355
238.1	0.0007876	6.89E-08	4.29E-08	0.39541159	7.74E-59	1.15E-11	0.67961355
238.2	0.0007876	6.89E-08	4.29E-08	0.39541159	7.74E-59	1.15E-11	0.67961355
238.3	0.0007876	6.89E-08	4.29E-08	0.39541159	7.74E-59	1.15E-11	0.67961355
238.4	0.0007876	6.89E-08	4.29E-08	0.39541159	7.74E-59	1.15E-11	0.67961355
238.5	0.0007876	6.89E-08	4.29E-08	0.39541159	7.74E-59	1.15E-11	0.67961355
238.6	0.0007876	6.89E-08	4.29E-08	0.39541159	7.74E-59	1.15E-11	0.67961355
238.7	0.0007876	6.89E-08	4.29E-08	0.39541159	7.74E-59	1.15E-11	0.67961355
238.8	0.00077986	7.28E-08	4.35E-08	0.39787465	8.22E-59	1.16E-11	0.68234785
238.9	0.00077986	7.28E-08	4.35E-08	0.39787465	8.22E-59	1.16E-11	0.68234785
239	0.00077792	7.18E-08	4.14E-08	0.39715631	7.81E-59	1.14E-11	0.68455618
239.1	0.00077792	7.18E-08	4.14E-08	0.39715631	7.81E-59	1.14E-11	0.68455618
239.2	0.00077792	7.18E-08	4.14E-08	0.39715631	7.81E-59	1.14E-11	0.68455618
239.3	0.00077792	7.18E-08	4.14E-08	0.39715631	7.81E-59	1.14E-11	0.68455618
239.4	0.00077792	7.18E-08	4.14E-08	0.39715631	7.81E-59	1.14E-11	0.68455618
239.5	0.00077792	7.18E-08	4.14E-08	0.39715631	7.81E-59	1.14E-11	0.68455618
239.6	0.00077359	7.20E-08	3.99E-08	0.39331538	7.54E-59	1.12E-11	0.68069678
239.7	0.00077359	7.20E-08	3.99E-08	0.39331538	7.54E-59	1.12E-11	0.68069678
239.8	0.00077359	7.20E-08	3.99E-08	0.39331538	7.54E-59	1.12E-11	0.68069678
239.9	0.00077359	7.20E-08	3.99E-08	0.39331538	7.54E-59	1.12E-11	0.68069678
240	0.00076395	7.22E-08	3.77E-08	0.38938674	6.90E-59	1.10E-11	0.6805119

Table S5: PRCC analysis data for [PYR] with [G](0)=0.02 mM: PRCC sensitivity indices and their corresponding p-values

Sensitivity analysis data from PRCC for [PYR] with [G](0)=0.02 mM (only the parameters to which [PYR]'s level is sensitive to appear)

Time discretization	V_{[PYR]}	K_{[PYR]}	alpha	K_{[ACoA]}	G_E	[G]_0	[PYR]_0
0	0	0	0	0	0	0	1
0.1	0.81217261	-0.9332108	-0.0119196	-0.0093057	0.0054733	0.64420847	0.02818229
0.2	0.81214899	-0.9341645	-0.0163341	0.01601896	0.00748876	0.64838144	0.01907735
0.3	0.8120948	-0.9344795	-0.0214832	0.04632315	0.00757408	0.65030598	0.01156335
0.4	0.81174876	-0.9346577	-0.0276789	0.07163834	0.00850727	0.65157982	0.00569687
0.5	0.81108069	-0.9350825	-0.0344209	0.09629927	0.01173051	0.65445242	0.00169198
0.6	0.80999877	-0.9353126	-0.0422744	0.12680608	0.0140188	0.65476947	-0.0058232
0.7	0.80910062	-0.935212	-0.0399032	0.15368723	0.02442781	0.65536724	-0.0052466
0.8	0.8077058	-0.9349716	-0.0429959	0.17792357	0.02632781	0.65408504	-0.0091611
0.9	0.80620407	-0.9345964	-0.0413371	0.19874731	0.02989418	0.65162499	-0.0056097
1	0.80388378	-0.9342833	-0.0464706	0.21859661	0.03398082	0.65115206	-0.0064569
1.1	0.80241559	-0.9339539	-0.0469461	0.23930862	0.03898307	0.65082939	-0.0046074
1.2	0.80108366	-0.9334066	-0.0455058	0.25619948	0.04244616	0.65162607	-0.0094715
1.3	0.80038067	-0.9338449	-0.0435092	0.27418352	0.04832757	0.65135202	-0.0089437
1.4	0.80015593	-0.9337633	-0.0369742	0.29272565	0.05107671	0.64708997	-0.0178809
1.5	0.798401	-0.933219	-0.0391407	0.30109755	0.05104693	0.64763098	-0.0186324
1.6	0.7973138	-0.9327947	-0.0396987	0.31189023	0.05039238	0.64536271	-0.0250833
1.7	0.79645504	-0.9324329	-0.0379941	0.32402239	0.05109829	0.64487601	-0.027537
1.8	0.79425702	-0.9318345	-0.0380644	0.33462135	0.05735227	0.64147089	-0.0265126
1.9	0.79310593	-0.931594	-0.0368712	0.34394337	0.06227126	0.6410965	-0.0283468
2	0.79084701	-0.9306923	-0.0408231	0.35262223	0.0655586	0.63896204	-0.0238907
2.1	0.79043861	-0.9307586	-0.0422792	0.36436333	0.06894938	0.63756844	-0.0258497
2.2	0.79038298	-0.9309259	-0.0369351	0.37239541	0.0679184	0.63536465	-0.0212726
2.3	0.7901284	-0.9308829	-0.0365396	0.38033966	0.07291165	0.63578972	-0.0242659
2.4	0.78860916	-0.9307317	-0.0332418	0.39113933	0.07484663	0.63438379	-0.0239763
2.5	0.78824382	-0.9306074	-0.0306115	0.39525902	0.07697524	0.63176505	-0.0214396
2.6	0.78746668	-0.9306211	-0.0333039	0.40572701	0.08446472	0.62892742	-0.0234119
2.7	0.78723371	-0.9306621	-0.0346167	0.41402987	0.08705776	0.62833285	-0.0214628
2.8	0.786423	-0.9303576	-0.0293006	0.41984627	0.08867381	0.62531978	-0.019878
2.9	0.78541039	-0.9299098	-0.0306294	0.42331953	0.08994875	0.62356198	-0.0178947
3	0.78494283	-0.9295972	-0.0308459	0.43042326	0.09424844	0.62188393	-0.017379
3.1	0.78454749	-0.9296197	-0.0313061	0.43489167	0.09843974	0.62069981	-0.0169671
3.2	0.78277167	-0.9291559	-0.031576	0.44061738	0.1009801	0.6188248	-0.0194917
3.3	0.78180304	-0.9288991	-0.0286408	0.44675124	0.10488966	0.61504424	-0.0215969
3.4	0.78170264	-0.9288591	-0.0312487	0.45307743	0.10555916	0.61424083	-0.0237067
3.5	0.78014149	-0.9282216	-0.0299543	0.45651636	0.10889396	0.61058002	-0.0180608
3.6	0.77964719	-0.9284366	-0.0291901	0.46072072	0.11280925	0.60912054	-0.0146088
3.7	0.77985622	-0.9286526	-0.0288469	0.46725424	0.11853873	0.60800064	-0.0146772
3.8	0.78087155	-0.9286197	-0.0240059	0.47176263	0.12239189	0.60677243	-0.01751
3.9	0.78105324	-0.9290324	-0.0257102	0.47737387	0.12363926	0.60734688	-0.0181879
4	0.7814648	-0.9290887	-0.0236977	0.47835553	0.12537742	0.60647471	-0.0143727
4.1	0.78156389	-0.9290943	-0.0233263	0.48189083	0.12726004	0.60574659	-0.0170814
4.2	0.78162516	-0.9290545	-0.0238759	0.48595395	0.13092382	0.60370024	-0.0157196
4.3	0.7811896	-0.9288679	-0.0245704	0.49203793	0.13610629	0.6019594	-0.0144738
4.4	0.7808474	-0.9286653	-0.0259633	0.49383942	0.13890656	0.60148255	-0.0141461
4.5	0.78044798	-0.9282331	-0.024414	0.49564456	0.14220433	0.60041378	-0.0120477
4.6	0.78067353	-0.927968	-0.0234905	0.49859866	0.14788647	0.59910716	-0.0092314
4.7	0.77932139	-0.9278071	-0.0244634	0.50040875	0.15152374	0.59655365	-0.0094972
4.8	0.77908992	-0.9276466	-0.0276326	0.50149042	0.15371029	0.59482608	-0.0092801
4.9	0.77863617	-0.9275009	-0.0259236	0.50221845	0.15463623	0.59377026	-0.0106172
5	0.78048577	-0.9283041	-0.0265882	0.50937585	0.16067068	0.5950743	-0.0107416
5.1	0.77975561	-0.9277719	-0.0266853	0.51299386	0.16294219	0.59303434	-0.0100061
5.2	0.7790081	-0.9276816	-0.0295426	0.51588675	0.16735182	0.59088131	-0.0092382
5.3	0.7794293	-0.9280298	-0.0300626	0.51809249	0.17129032	0.58942172	-0.0107591
5.4	0.77995988	-0.9279117	-0.0292541	0.51997574	0.17193192	0.58826372	-0.0104978
5.5	0.78062861	-0.9284972	-0.0292629	0.52413428	0.17927683	0.58699605	-0.0055362
5.6	0.78108906	-0.9284525	-0.0301337	0.52728815	0.18608278	0.58530255	-0.0066673
5.7	0.78110102	-0.9284262	-0.0291749	0.52932706	0.1904147	0.58488314	-0.0086339
5.8	0.78059116	-0.9283611	-0.0293912	0.53144928	0.19425408	0.58312048	-0.0091175
5.9	0.77886373	-0.9278053	-0.0278754	0.53077485	0.19861896	0.57996852	-0.0089912
6	0.77906177	-0.9279893	-0.0283576	0.53326583	0.19978753	0.5791838	-0.0072667
6.1	0.77930865	-0.9279527	-0.0276072	0.5360937	0.20430548	0.57805382	-0.008684
6.2	0.77931777	-0.9278921	-0.0318341	0.53673145	0.20793198	0.57704994	-0.0082903
6.3	0.78005911	-0.9282588	-0.0312225	0.53906779	0.21247862	0.57706307	-0.0089232
6.4	0.78041266	-0.9281534	-0.030946	0.54013196	0.21449876	0.5767243	-0.0090818
6.5	0.78074146	-0.9279343	-0.0315163	0.54003498	0.21662608	0.57461735	-0.0098869
6.6	0.7809922	-0.9277193	-0.0339194	0.54163408	0.21895617	0.57370339	-0.0115808
6.7	0.78050027	-0.9276609	-0.0357055	0.54219183	0.22067178	0.57283393	-0.0115008
6.8	0.78069122	-0.9273845	-0.0360017	0.54226151	0.2248358	0.57058427	-0.0133874
6.9	0.78075876	-0.9274143	-0.0389982	0.54247701	0.22781472	0.57164324	-0.0153555
7	0.78034023	-0.9272031	-0.0405317	0.54301093	0.22966452	0.57066386	-0.0158212
7.1	0.78063412	-0.9272466	-0.0421041	0.54400924	0.23265095	0.56850976	-0.0147599
7.2	0.781214	-0.9276428	-0.0408548	0.54518409	0.23854378	0.56773736	-0.0140215
7.3	0.78093851	-0.9273118	-0.0410367	0.54590586	0.24049138	0.56654401	-0.0131684
7.4	0.78012544	-0.9270884	-0.0439745	0.54623596	0.24450179	0.56428233	-0.0165606
7.5	0.78008594	-0.9270748	-0.046299	0.54485425	0.24818977	0.56223415	-0.0128488
7.6	0.78013516	-0.9268931	-0.0470276	0.54378139	0.25086313	0.56136073	-0.0137444
7.7	0.78101602	-0.927098	-0.0472299	0.54433292	0.25436922	0.56077621	-0.0135278
7.8	0.78122949	-0.9270735	-0.0509925	0.54628119	0.25893273	0.55952054	-0.0126567
7.9	0.78070655	-0.9269253	-0.050969	0.5463684	0.26055579	0.55767335	-0.0142035
8	0.7808596	-0.9268665	-0.0509634	0.54583978	0.26383445	0.55668829	-0.0164835
8.1	0.78107059	-0.926939	-0.0537909	0.545879	0.26751306	0.55533815	-0.0157528
8.2	0.78053848	-0.9267996	-0.0534851	0.54728632	0.27014999	0.55615647	-0.0162856
8.3	0.78135601	-0.9270363	-0.0551789	0.54979574	0.27486009	0.55478447	-0.016418
8.4	0.78071362	-0.9269031	-0.0549932	0.5493357	0.27815719	0.55226904	-0.0173361
8.5	0.78103968	-0.9271605	-0.054848	0.55167814	0.28248839	0.54948273	-0.0131957
8.6	0.78177573	-0.927125	-0.0573814	0.55295357	0.28847541	0.54915108	-0.0154801
8.7	0.78095775	-0.9269207	-0.0581932	0.55327889	0.2908313	0.54690452	-0.0163786
8.8	0.78084348	-0.9264472	-0.0602861	0.55123816	0.2922452	0.54414079	-0.014472
8.9	0.77998558	-0.9261769	-0.0623533	0.54909023	0.29238182	0.54292987	-0.012895
9	0.77996738	-0.9262148	-0.0650294	0.54897904	0.29321263	0.54114569	-0.0129955
9.1	0.78007331	-0.9258339	-0.0637975	0.54823324	0.29690409	0.53898346	-0.0112229
9.2	0.77973162	-0.9258063	-0.0658043	0.54961254	0.30239567	0.53688665	-0.0123087
9.3	0.77955595	-0.9255512	-0.0677557	0.55076917	0.30605094	0.53555192	-0.0143663
9.4	0.77930764	-0.9255941	-0.0689872	0.54931866	0.31097649	0.53393312	-0.0155862
9.5	0.7784916	-0.9252858	-0.0712354	0.54724017	0.3138879	0.53332868	-0.0154209
9.6	0.77809033	-0.9250535	-0.0724643	0.54725851	0.3164068	0.53125571	-0.0157703
9.7	0.77694359	-0.9243858	-0.0723088	0.54594146	0.31963325	0.52667329	-0.0163585
9.8	0.77639361	-0.924086	-0.0719791	0.54759771	0.32357732	0.52397058	-0.015802
9.9	0.77580229	-0.9237846	-0.0753046	0.54740016	0.32558187	0.52293302	-0.0143946
10	0.77593496	-0.9236832	-0.0770709	0.54520186	0.32746068	0.52180402	-0.0132665
10.1	0.77572274	-0.9235085	-0.0765586	0.54615617	0.32914023	0.52015439	-0.0142846
10.2	0.77437481	-0.92264	-0.0771475	0.54332122	0.32935626	0.51663405	-0.013786
10.3	0.77412246	-0.9223173	-0.0794002	0.54277206	0.33209997	0.51490336	-0.0133737
10.4	0.77343417	-0.9218736	-0.081494	0.54322406	0.33629559	0.51250656	-0.0150356
10.5	0.77221429	-0.9213052	-0.081181	0.54111159	0.33561743	0.50971206	-0.010774
10.6	0.77038056	-0.9207819	-0.0863612	0.53903736	0.3399563	0.50654669	-0.0103708
10.7	0.7703245	-0.9206596	-0.0856151	0.53862453	0.34199117	0.50484421	-0.0113538
10.8	0.76733297	-0.9195262	-0.0877967	0.53550189	0.34233006	0.49917762	-0.0103564
10.9	0.76484399	-0.9186796	-0.0883826	0.5320421	0.34639036	0.49355585	-0.0069723
11	0.76149142	-0.9172104	-0.0911296	0.5253847	0.34512105	0.4883961	-0.0061863
11.1	0.76103033	-0.9169828	-0.0938903	0.52382734	0.3469044	0.48743929	-0.0058932
11.2	0.75596415	-0.9151602	-0.0951688	0.51610589	0.34600742	0.48098018	-0.0050815
11.3	0.75226938	-0.9138373	-0.1027411	0.50932921	0.34714242	0.47689219	-0.0065587
11.4	0.7486112	-0.9125393	-0.1028108	0.50454734	0.34642179	0.47304039	-0.0060588
11.5	0.74639203	-0.9115922	-0.1020867	0.50122544	0.34812346	0.46965602	-0.003141
11.6	0.74337908	-0.9106905	-0.1042062	0.49845853	0.34943586	0.46368755	-0.0009401
11.7	0.74311771	-0.9103748	-0.1049329	0.49780162	0.35102442	0.46243162	-0.0016929
11.8	0.73834049	-0.9085776	-0.1078585	0.49325441	0.35037033	0.45437233	-0.0010043

Time discretization	V_{[PYR]}	K_{[PYR]}	alpha	K_{[ACoA]}	G_E	[G]_0	[PYR]_0
15	0.70043045	-0.8885508	-0.1420202	0.45892771	0.38950118	0.3808439	-0.0004674
15.1	0.70059293	-0.8883131	-0.1439981	0.45798131	0.39004478	0.38058113	0.00019915
15.2	0.70069093	-0.8879735	-0.1476011	0.45763237	0.39199113	0.37847601	0.00223318
15.3	0.69944357	-0.8872431	-0.1497055	0.45710799	0.39263258	0.37603501	6.97E-05
15.4	0.69805655	-0.8867643	-0.151718	0.45868416	0.39633645	0.37158791	-0.001596
15.5	0.697299	-0.8862335	-0.1552201	0.45735215	0.39742755	0.37078327	-0.0015368
15.6	0.69687001	-0.8858308	-0.154337	0.45578834	0.39893251	0.36846329	-0.0014637
15.7	0.69564499	-0.8854685	-0.1571452	0.45605868	0.39931035	0.36710773	-0.002672
15.8	0.69470031	-0.8851727	-0.159037	0.45528138	0.39886666	0.36613702	-0.0022007
15.9	0.69381083	-0.8851206	-0.1615407	0.45497148	0.40273418	0.36414344	-0.0022542
16	0.69397449	-0.8851066	-0.1640005	0.45451315	0.40402956	0.36366037	-0.0031994
16.1	0.69315502	-0.8847858	-0.1656988	0.45399497	0.40364279	0.36359356	-0.0045618
16.2	0.69284577	-0.8842876	-0.1683058	0.45405786	0.40463498	0.3632125	-0.0054329
16.3	0.69312595	-0.8842565	-0.1684187	0.45417953	0.40604782	0.36203319	-0.0056729
16.4	0.69168672	-0.8836706	-0.1695714	0.4547386	0.40779075	0.35985436	-0.0052207
16.5	0.69066312	-0.883108	-0.1697381	0.4553795	0.40795844	0.35904124	-0.0042232
16.6	0.68979192	-0.8828619	-0.1733429	0.4561732	0.40969825	0.35622108	-0.0053284
16.7	0.68986108	-0.8823605	-0.1744183	0.45442911	0.41056922	0.35547589	-0.0059621
16.8	0.68888998	-0.8820252	-0.1747345	0.45412885	0.41106378	0.35381958	-0.0050942
16.9	0.68884564	-0.8817795	-0.1749204	0.45481013	0.41277851	0.35193473	-0.006474
17	0.68789978	-0.8814499	-0.1773094	0.45329218	0.41320278	0.35115459	-0.0056695
17.1	0.68777485	-0.8811171	-0.1772944	0.45350619	0.41319925	0.35110043	-0.0049808
17.2	0.68714124	-0.8803577	-0.1770784	0.45225378	0.41397148	0.35122176	-0.0051819
17.3	0.6866488	-0.8801576	-0.1780738	0.45179998	0.41495824	0.35059921	-0.0035521
17.4	0.68592805	-0.8796536	-0.1803228	0.45154084	0.41704948	0.35074364	-0.0027883
17.5	0.68562911	-0.8793112	-0.1838549	0.4513143	0.41731616	0.35063934	-0.0026557
17.6	0.68522031	-0.8791368	-0.1859662	0.45050376	0.41972506	0.35127396	-0.003785
17.7	0.68432927	-0.8783429	-0.187918	0.45141445	0.42097887	0.35078137	-0.0031539
17.8	0.68408504	-0.8780207	-0.1899047	0.45024261	0.42001934	0.3508059	-0.0023034
17.9	0.68331935	-0.8772934	-0.1938134	0.45059575	0.42178565	0.35013543	-0.0038926
18	0.6821109	-0.8768734	-0.1970461	0.4492765	0.42340916	0.34920863	-0.0035634
18.1	0.68081953	-0.8760671	-0.1990672	0.44701839	0.42186566	0.34832021	-0.0025625
18.2	0.67872996	-0.8752209	-0.2005775	0.44696965	0.42169145	0.34596134	-0.0025618
18.3	0.67680293	-0.8742307	-0.2035784	0.44531203	0.42195451	0.34430571	-0.0012945
18.4	0.67649604	-0.8734343	-0.2055777	0.4437621	0.42157419	0.34447214	-0.0004911
18.5	0.67636296	-0.8732852	-0.2075038	0.44313742	0.42275453	0.34497555	-0.0024681
18.6	0.67477348	-0.8726849	-0.2089447	0.44143043	0.42331614	0.34354433	-0.0011016
18.7	0.67269224	-0.871708	-0.2117767	0.4397417	0.42181676	0.34217158	-0.0011071
18.8	0.67150982	-0.8709345	-0.2139464	0.43834809	0.42229788	0.33988619	-0.000907
18.9	0.67034111	-0.8701971	-0.2180599	0.43563601	0.42357696	0.33772731	-0.0009621
19	0.66886465	-0.8686567	-0.2208497	0.43405509	0.42310377	0.33652263	0.00238401
19.1	0.66835668	-0.8682182	-0.2221112	0.43391514	0.42501549	0.33821969	0.00479844
19.2	0.66694937	-0.8673433	-0.2235215	0.432008	0.42507782	0.33645338	0.00580239
19.3	0.66302504	-0.8653873	-0.2268088	0.42942518	0.4241163	0.3345537	0.00775579
19.4	0.6601562	-0.863678	-0.2292166	0.42671249	0.42376509	0.33253408	0.00974312
19.5	0.65808904	-0.8630004	-0.2301271	0.42702169	0.4243447	0.32897945	0.01268953
19.6	0.6555673	-0.8612859	-0.2315637	0.4246209	0.42342508	0.32725463	0.0135077
19.7	0.65368987	-0.8600359	-0.231173	0.4235273	0.42376696	0.32487558	0.01445647
19.8	0.65233258	-0.8589282	-0.2334238	0.42258189	0.42331689	0.32430595	0.01481743
19.9	0.65001314	-0.8576685	-0.232854	0.4223077	0.42323658	0.32204467	0.01459287
20	0.64686166	-0.8557083	-0.2355482	0.41819583	0.42311897	0.32071746	0.01578224
20.1	0.64599551	-0.8547621	-0.2372747	0.41651896	0.4232513	0.31960786	0.01546403
20.2	0.64533864	-0.8538526	-0.2390718	0.4151917	0.42392976	0.31945562	0.01526923
20.3	0.64307933	-0.8523107	-0.240251	0.41398534	0.42470776	0.31806942	0.01544312
20.4	0.64262925	-0.8518218	-0.2400022	0.41530724	0.42559766	0.31817032	0.01627276
20.5	0.64068289	-0.8504739	-0.2425206	0.41232059	0.42412074	0.31758531	0.01512433
20.6	0.63889053	-0.8496272	-0.2437809	0.41070728	0.42412214	0.31593693	0.0165971
20.7	0.6377941	-0.8485414	-0.2444157	0.40892403	0.42432209	0.3133435	0.01658279
20.8	0.63601179	-0.8469911	-0.2467485	0.40682982	0.42325378	0.31126414	0.01840047
20.9	0.63362909	-0.8458058	-0.2478636	0.40605787	0.4239141	0.31015236	0.01892834
21	0.63217579	-0.844689	-0.2499418	0.40591306	0.42434636	0.30904183	0.01930925
21.1	0.6290073	-0.8421689	-0.2515237	0.40198147	0.42315247	0.30615448	0.02203121
21.2	0.6283628	-0.8417486	-0.2520433	0.40167528	0.42338555	0.3055722	0.02257696
21.3	0.62697985	-0.8414899	-0.2544126	0.39934901	0.4248303	0.30433933	0.02372697
21.4	0.62528833	-0.8404427	-0.2567363	0.39919654	0.42369218	0.30326038	0.02417518
21.5	0.62110759	-0.8380701	-0.2577724	0.39601443	0.42244945	0.3001609	0.02499303
21.6	0.62007255	-0.8378489	-0.2593055	0.39527205	0.42390996	0.30056823	0.02402824
21.7	0.61939381	-0.8373482	-0.260143	0.39536577	0.42535764	0.30018064	0.0234615
21.8	0.61940682	-0.8368645	-0.2601157	0.39524218	0.42641448	0.29908747	0.02341797
21.9	0.6180607	-0.8357281	-0.2610025	0.39421437	0.42613397	0.29851037	0.02389894
22	0.61685952	-0.835187	-0.2638676	0.39438514	0.42704343	0.29797502	0.02387495
22.1	0.61664393	-0.8348901	-0.2638172	0.39522322	0.4281954	0.29741955	0.02267213
22.2	0.61576317	-0.8341809	-0.2639795	0.3942202	0.42732482	0.29760237	0.02385045
22.3	0.61580356	-0.8336295	-0.2641065	0.39460814	0.42795459	0.29656856	0.02416596
22.4	0.61539223	-0.8332565	-0.2642099	0.39495694	0.4301328	0.2955129	0.02381679
22.5	0.61453204	-0.8324753	-0.2645983	0.39405712	0.42974494	0.29542727	0.02240939
22.6	0.61364907	-0.8316384	-0.264897	0.39307727	0.43071072	0.29424082	0.02295235
22.7	0.61358144	-0.831812	-0.2661553	0.39205833	0.43105654	0.29421962	0.02372773
22.8	0.6119962	-0.8310007	-0.2680488	0.39245688	0.43105628	0.29304119	0.02167384
22.9	0.61164412	-0.8304214	-0.2702836	0.39175741	0.43194867	0.29260425	0.0198004
23	0.61116243	-0.8299533	-0.271668	0.39193603	0.43248376	0.29252259	0.01895293
23.1	0.61117786	-0.829828	-0.2722854	0.39181085	0.4342751	0.29245609	0.01744519
23.2	0.61080527	-0.8291128	-0.272708	0.39158142	0.43398033	0.29242268	0.01716822
23.3	0.61024017	-0.8286416	-0.2729262	0.39143687	0.43431884	0.29146396	0.01785556
23.4	0.61013018	-0.8286694	-0.2735787	0.3920873	0.4355988	0.29057202	0.01777238
23.5	0.61015499	-0.8284886	-0.2747102	0.39220318	0.43574745	0.29068696	0.01769437
23.6	0.60952662	-0.8284739	-0.2759176	0.39223784	0.4361916	0.29108307	0.01807819
23.7	0.60946596	-0.8281204	-0.276378	0.39326783	0.4361626	0.29056112	0.01686001
23.8	0.608265	-0.8274376	-0.276814	0.39225084	0.43800677	0.28983033	0.01639963
23.9	0.6082242	-0.8274194	-0.2776616	0.39266979	0.43818195	0.28985806	0.01790141
24	0.60840571	-0.8274698	-0.2782531	0.39307892	0.43942959	0.28908815	0.01801681
24.1	0.60772859	-0.8275467	-0.2803167	0.39291878	0.44047064	0.28829575	0.01895431
24.2	0.60822139	-0.8275569	-0.2811225	0.39218918	0.44066255	0.28923149	0.01819291
24.3	0.60756441	-0.8268692	-0.2824147	0.39221298	0.44261803	0.28839882	0.01529918
24.4	0.60669318	-0.8263523	-0.2836896	0.3915544	0.44369902	0.28774383	0.01611759
24.5	0.60664866	-0.8261537	-0.284682	0.39113126	0.44392828	0.28773589	0.0158315
24.6	0.60685335	-0.8258816	-0.2852837	0.3903568	0.44486527	0.28765897	0.01579838
24.7	0.60612102	-0.8256147	-0.2860755	0.39034792	0.44644667	0.28700299	0.01561432
24.8	0.60604416	-0.8255987	-0.2867852	0.39035998	0.4467453	0.28706931	0.01568252
24.9	0.60523951	-0.8251241	-0.2869628	0.39008119	0.44732764	0.28601522	0.01577217
25	0.60521274	-0.8249116	-0.2884034	0.38942754	0.44733552	0.28533584	0.01474775
25.1	0.60374018	-0.8244888	-0.2896475	0.38977998	0.44772224	0.28594036	0.01389289
25.2	0.60322569	-0.8242457	-0.2898531	0.38830551	0.44783574	0.28671819	0.01435157
25.3	0.60315223	-0.8243222	-0.2911286	0.38821984	0.44848552	0.28642935	0.01506324
25.4	0.60223046	-0.8240397	-0.294956	0.38843499	0.44839828	0.28683819	0.01607542
25.5	0.60233538	-0.8238344	-0.2946766	0.38768409	0.44861189	0.28626304	0.01536848
25.6	0.60248347	-0.8239789	-0.2965279	0.3876611	0.44940044	0.2864816	0.01687688
25.7	0.60159026	-0.8234546	-0.2978986	0.38755824	0.45123545	0.28547145	0.01658282
25.8	0.60105845	-0.8231079	-0.2989427	0.38799601	0.45199815	0.28540284	0.01691617
25.9	0.60004422	-0.8217764	-0.2986345	0.38691996	0.45215216	0.28445112	0.01475147
26	0.59971715	-0.8209982	-0.3003645	0.38645004	0.45177714	0.28542795	0.01447465
26.1	0.59982746	-0.8206799	-0.3014863	0.38716313	0.45319503	0.28523811	0.01435914
26.2	0.59983522	-0.8204183	-0.303827	0.38785126	0.45399826	0.28489349	0.01473761
26.3	0.60040546	-0.8200754	-0.305071	0.38767142	0.45523109	0.28331685	0.01526109
26.4	0.60009239	-0.8199021	-0.3060691	0.38709856	0.4558472	0.28240007	0.01624009
26.5	0.59938937	-0.8195513	-0.3074377	0.38634846	0.45563476	0.28196075	0.0168369
26.6	0.59929529	-0.8195264	-0.3095238	0.38666863	0.45663862	0.28246827	0.01693207
26.7	0.59879546	-0.8195297	-0.3114537	0.38734873	0.45764053	0.28252376	0.01839865
26.8	0.59890432	-0.8195436	-0.3117111	0.38828053	0.45869092	0.28179018	0.01867486

Time discretization	V_{[PYR]}	K_{[PYR]}	alpha	K_{[ACoA]}	G_E	[G]_0	[PYR]_0
30	0.59014869	-0.8111711	-0.3321629	0.3822505	0.47246825	0.27047361	0.02582722
30.1	0.59050906	-0.8110565	-0.3337387	0.38282853	0.47292514	0.27086709	0.02534631
30.2	0.59056696	-0.8111398	-0.3338044	0.38338546	0.4734417	0.27070872	0.02446079
30.3	0.59056651	-0.8109759	-0.3352905	0.38403024	0.4749137	0.27082619	0.02309211
30.4	0.59051419	-0.8107388	-0.336892	0.38301565	0.47443098	0.27094476	0.02421263
30.5	0.5901334	-0.8105184	-0.3374271	0.38302899	0.47405133	0.27125847	0.02482221
30.6	0.58877545	-0.810114	-0.3382582	0.38330218	0.4733481	0.27098982	0.02501599
30.7	0.58816328	-0.8098011	-0.3410348	0.38283701	0.47287814	0.27031515	0.02454105
30.8	0.58777985	-0.8096433	-0.3421973	0.38234553	0.47250939	0.27062135	0.02496977
30.9	0.58786783	-0.8096436	-0.3433286	0.38269049	0.47224128	0.27057063	0.02491345
31	0.58760896	-0.8095199	-0.3443589	0.38258544	0.47216863	0.27000503	0.02544214
31.1	0.58734917	-0.809376	-0.3454337	0.38270975	0.47192354	0.26930299	0.02585068
31.2	0.58762917	-0.8097001	-0.3461359	0.38208576	0.47240315	0.27071268	0.02591303
31.3	0.58748561	-0.8094311	-0.3468868	0.38202187	0.47207948	0.2702272	0.02591344
31.4	0.58738518	-0.8094775	-0.347072	0.38195552	0.47231336	0.27003068	0.02662712
31.5	0.58657672	-0.8091826	-0.3473395	0.38255639	0.47204941	0.26906446	0.02652368
31.6	0.58657409	-0.8089531	-0.3472756	0.38177275	0.47217251	0.26839475	0.02651439
31.7	0.58631252	-0.8090925	-0.3480928	0.38159124	0.47253895	0.26807399	0.02745259
31.8	0.58633167	-0.8092602	-0.3484498	0.38175359	0.47350231	0.26790727	0.0264786
31.9	0.5860227	-0.8087834	-0.3488008	0.38158935	0.47338418	0.26765983	0.02651993
32	0.58558623	-0.8083739	-0.3495415	0.37987161	0.47280943	0.26738473	0.02623696
32.1	0.58528291	-0.8082619	-0.3509857	0.37947843	0.47259298	0.26779425	0.0262848
32.2	0.58450021	-0.8079991	-0.3510227	0.37923344	0.47187707	0.2680985	0.02644632
32.3	0.58376581	-0.8077096	-0.3517714	0.37860235	0.47301722	0.2682946	0.02547128
32.4	0.58280206	-0.8075068	-0.353065	0.37730224	0.4722355	0.26864239	0.02689448
32.5	0.58321048	-0.8070197	-0.3542076	0.37676229	0.47240691	0.26891695	0.02632844
32.6	0.58249528	-0.8063972	-0.3555794	0.37626354	0.47161841	0.26842586	0.02614646
32.7	0.58183461	-0.8059358	-0.3569285	0.37587912	0.47130488	0.2686278	0.02763554
32.8	0.58159745	-0.8057713	-0.3574145	0.37508969	0.47161183	0.2681524	0.02830633
32.9	0.58149216	-0.8055299	-0.3579594	0.37492214	0.47183133	0.26841527	0.02839181
33	0.58089881	-0.8055774	-0.3592967	0.3756003	0.47241112	0.26775293	0.02920176
33.1	0.58073144	-0.8054945	-0.3613489	0.37608088	0.47393257	0.26640482	0.02877278
33.2	0.58010698	-0.8052891	-0.3632381	0.3757392	0.47398999	0.26588273	0.02977767
33.3	0.57974989	-0.8049993	-0.3640964	0.37640129	0.47420116	0.26632511	0.02953158
33.4	0.57879649	-0.8047645	-0.3672008	0.37774439	0.47417982	0.26587513	0.03048859
33.5	0.57875746	-0.8048186	-0.3682023	0.37771835	0.47502765	0.26571608	0.02962935
33.6	0.5777003	-0.8042227	-0.3698863	0.37692079	0.47435862	0.26503293	0.03064651
33.7	0.57713165	-0.8040449	-0.3712827	0.37584159	0.47373422	0.26407567	0.03097684
33.8	0.57596192	-0.8034615	-0.3724945	0.37533474	0.47278681	0.26459809	0.03235521
33.9	0.57606026	-0.8034112	-0.373183	0.37566339	0.47334032	0.26463494	0.03265366
34	0.57432083	-0.8025857	-0.3736078	0.37463095	0.47321204	0.26480063	0.03307961
34.1	0.57372421	-0.8019947	-0.3750342	0.37467155	0.47230833	0.26527448	0.03408371
34.2	0.57390846	-0.8021507	-0.3753585	0.37451038	0.47317219	0.26558617	0.03397209
34.3	0.5740015	-0.801899	-0.3766173	0.37434709	0.47279198	0.26631207	0.03419274
34.4	0.57359061	-0.8012319	-0.3771087	0.37348743	0.47270414	0.26623486	0.03263062
34.5	0.57292056	-0.8008373	-0.3780765	0.37314358	0.47284584	0.26587855	0.03175326
34.6	0.57268216	-0.8004432	-0.3784836	0.37349106	0.47300044	0.26550342	0.03111837
34.7	0.57235738	-0.800615	-0.3802694	0.37240403	0.47248283	0.26719869	0.03154141
34.8	0.57279901	-0.8005425	-0.3804014	0.37317425	0.47331491	0.26717089	0.03162649
34.9	0.5715042	-0.7999495	-0.3810796	0.37304318	0.47313267	0.2660868	0.03282077
35	0.57040188	-0.7991448	-0.3821006	0.37186072	0.47199272	0.26546559	0.03267406
35.1	0.56912927	-0.7980592	-0.3844411	0.37057744	0.47115978	0.26413931	0.03353832
35.2	0.56865669	-0.7976845	-0.3870142	0.36919397	0.47066514	0.26429554	0.03466576
35.3	0.56799517	-0.7970249	-0.3882354	0.36846434	0.4704445	0.26353906	0.03561872
35.4	0.56732579	-0.7964801	-0.389245	0.36793404	0.47012279	0.26300235	0.03628725
35.5	0.56725052	-0.7962647	-0.3899279	0.36738304	0.47085007	0.26272949	0.03623354
35.6	0.56740603	-0.7960652	-0.3919634	0.36686629	0.47174816	0.26304252	0.03675867
35.7	0.56757109	-0.7958924	-0.3935408	0.36637959	0.4714102	0.26331854	0.03717543
35.8	0.56687212	-0.7955343	-0.3941612	0.36607357	0.47095214	0.26213335	0.03753145
35.9	0.5658729	-0.7953314	-0.3956929	0.36595999	0.47059206	0.26091152	0.03831876
36	0.56550298	-0.7948599	-0.3967748	0.36583558	0.46968656	0.26093895	0.038985
36.1	0.56529306	-0.7940908	-0.3973117	0.36600023	0.4682991	0.26126844	0.03864775
36.2	0.56429375	-0.7936446	-0.3980941	0.3658579	0.46821677	0.26142753	0.03843485
36.3	0.5630582	-0.7929889	-0.3999595	0.36632017	0.46786355	0.26174067	0.0388075
36.4	0.56261363	-0.7929403	-0.4014683	0.36533976	0.4674113	0.26225645	0.03901842
36.5	0.56269727	-0.7929541	-0.4049429	0.36508613	0.4672751	0.26413728	0.03987436
36.6	0.56118372	-0.7923324	-0.4055724	0.36373141	0.46672794	0.26321562	0.04089499
36.7	0.56082258	-0.7919125	-0.4084295	0.36382795	0.46692695	0.26216895	0.03988675
36.8	0.56017009	-0.791442	-0.4086984	0.36332371	0.46614525	0.26178265	0.04038787
36.9	0.55812658	-0.7903612	-0.4097899	0.3612019	0.46558654	0.26100506	0.04115734
37	0.55863129	-0.7903374	-0.4104765	0.36129147	0.4655734	0.261335	0.04129577
37.1	0.55827155	-0.7898191	-0.4107989	0.36005176	0.46479877	0.26160773	0.04145412
37.2	0.5570007	-0.7894943	-0.411371	0.35953454	0.46523046	0.26163559	0.04139304
37.3	0.55702708	-0.7891997	-0.4114533	0.35913932	0.46534826	0.26183677	0.04124028
37.4	0.55686536	-0.788872	-0.4122502	0.35847654	0.46568166	0.26083472	0.04072315
37.5	0.55649533	-0.7885451	-0.4138737	0.35822708	0.46565773	0.26160916	0.04080725
37.6	0.55691386	-0.7884388	-0.4141878	0.35819956	0.4660864	0.26157441	0.04037563
37.7	0.55600343	-0.7878342	-0.4159512	0.35717957	0.46592547	0.26171431	0.04068209
37.8	0.55543309	-0.7873033	-0.417661	0.356624	0.46497453	0.26107542	0.04031144
37.9	0.55475629	-0.7869551	-0.4181033	0.35687593	0.46511876	0.26115713	0.04040201
38	0.55507947	-0.7866235	-0.4188426	0.35812382	0.46702012	0.26063324	0.04033472
38.1	0.55505259	-0.7862704	-0.4193939	0.35755523	0.46700731	0.26084004	0.04124009
38.2	0.55570895	-0.7859543	-0.4212283	0.35722156	0.46740619	0.26023424	0.04105342
38.3	0.5558757	-0.784941	-0.4228127	0.35629859	0.46673806	0.25985238	0.04070255
38.4	0.55509291	-0.7846167	-0.4235436	0.35603915	0.46619408	0.26006006	0.04076
38.5	0.55407979	-0.7835523	-0.4239073	0.35535488	0.46504843	0.258974	0.04087994
38.6	0.55405354	-0.783203	-0.4246344	0.35570071	0.46493802	0.25905883	0.04027826
38.7	0.5535583	-0.7823659	-0.4261008	0.35406617	0.46413358	0.25840353	0.04027259
38.8	0.55371952	-0.7821204	-0.4270354	0.3532092	0.46378314	0.25810881	0.04027591
38.9	0.5535996	-0.7819556	-0.4271829	0.35303884	0.4637509	0.25824547	0.04054079
39	0.55370273	-0.7817848	-0.4277368	0.35305262	0.4636364	0.25812414	0.04123085
39.1	0.55269693	-0.7811167	-0.4288928	0.35214382	0.46329979	0.25748581	0.04216347
39.2	0.55192949	-0.779861	-0.4297378	0.35111748	0.46192541	0.25661734	0.04264667
39.3	0.55049345	-0.7788622	-0.4302142	0.35118137	0.46058174	0.25593506	0.04314947
39.4	0.55023114	-0.7786162	-0.430988	0.35082912	0.45991012	0.25552375	0.04285914
39.5	0.55040904	-0.7784671	-0.4317677	0.3508322	0.4596038	0.25512022	0.04292308
39.6	0.55036046	-0.7780294	-0.432306	0.35048311	0.45929513	0.25485758	0.04321584
39.7	0.54981976	-0.7778478	-0.4329124	0.34995897	0.45993858	0.25423266	0.043663
39.8	0.54887744	-0.7773315	-0.4337205	0.34974708	0.46009666	0.25366902	0.04379045
39.9	0.54899417	-0.7771095	-0.4337427	0.34920593	0.45996078	0.2534557	0.0436559
40	0.54922904	-0.7771316	-0.4344867	0.34967791	0.46069455	0.25352726	0.04310747
40.1	0.54863959	-0.7768067	-0.4356067	0.34822605	0.45981473	0.25468954	0.043013
40.2	0.54841808	-0.7765731	-0.436165	0.34778888	0.46017958	0.25449249	0.0426545
40.3	0.54767825	-0.7759984	-0.4360746	0.34734402	0.46060615	0.25396632	0.04177364
40.4	0.54710728	-0.775668	-0.4365286	0.3468984	0.46003643	0.25357842	0.0416207
40.5	0.54569114	-0.774786	-0.4371302	0.34639979	0.4591793	0.25303075	0.04124516
40.6	0.54479883	-0.7744793	-0.4379554	0.34686162	0.45893471	0.25191971	0.04152403
40.7	0.54427964	-0.7740717	-0.4390942	0.34591344	0.45868851	0.25235346	0.04142937
40.8	0.54365519	-0.7734017	-0.4397513	0.3448313	0.45739835	0.25272181	0.04275803
40.9	0.54360645	-0.7730757	-0.4402052	0.34399151	0.45709893	0.25267442	0.04245595
41	0.54286617	-0.7725932	-0.4403845	0.34384281	0.45662249	0.25237739	0.043368
41.1	0.54261039	-0.7722606	-0.4406724	0.34325325	0.45718002	0.25198828	0.04272403
41.2	0.54262633	-0.7720259	-0.440881	0.34298131	0.4568092	0.25235867	0.04272462
41.3	0.54267447	-0.771961	-0.4416016	0.34248518	0.45685966	0.25201831	0.04149509
41.4	0.54309172	-0.7716975	-0.4423917	0.34225119	0.45669433	0.25156724	0.04123471
41.5	0.5423321	-0.7710616	-0.4432206	0.34075613	0.4553792	0.25144823	0.04045354
41.6	0.5411412	-0.7704426	-0.4433365	0.34067485	0.45507304	0.25154342	0.04125244
41.7	0.54122718	-0.770075	-0.4441024	0.34123711	0.45500814	0.25096131	0.04026718
41.8	0.54115751	-0.7695364	-0.4444813	0.34050255	0.45497175	0.25083029	0.04056922

Time discretization	V_{[PYR]}	K_{[PYR]}	alpha	K_{[ACoA]}	G_E	[G]_0	[PYR]_0
45	0.53410227	-0.7597176	-0.4573419	0.33140397	0.44960823	0.24063921	0.03375285
45.1	0.53363759	-0.7593455	-0.4572683	0.33043984	0.44969301	0.24006927	0.03372716
45.2	0.53417194	-0.7592612	-0.4582716	0.33077802	0.44986871	0.24084393	0.0330777
45.3	0.53380032	-0.7589493	-0.458089	0.33043615	0.44975065	0.24061787	0.03281024
45.4	0.53354039	-0.7586943	-0.4581343	0.33043726	0.44996357	0.23975396	0.0330525
45.5	0.533197	-0.7583487	-0.4581791	0.33042204	0.44956924	0.24007712	0.03259993
45.6	0.53275778	-0.7582808	-0.4586796	0.33140846	0.4497089	0.23960273	0.03260251
45.7	0.53275778	-0.7582808	-0.4586796	0.33140846	0.4497089	0.23960273	0.03260251
45.8	0.5321347	-0.7579492	-0.4588919	0.33035952	0.44956451	0.23907882	0.03243371
45.9	0.53208757	-0.7580727	-0.4587809	0.33039371	0.44957498	0.23911393	0.03300509
46	0.53136958	-0.7577289	-0.4585216	0.32980347	0.44951734	0.23955755	0.03308525
46.1	0.53079815	-0.7575297	-0.458375	0.32974807	0.44972743	0.23892497	0.03220282
46.2	0.53012028	-0.7574742	-0.4601147	0.32969064	0.45058672	0.23929052	0.03160251
46.3	0.53051482	-0.7571322	-0.4603805	0.32915081	0.45009375	0.23904254	0.03108358
46.4	0.53083562	-0.7568035	-0.4615934	0.32883132	0.44974936	0.23957921	0.03065852
46.5	0.53009239	-0.7560754	-0.4614621	0.32884933	0.44996204	0.23836884	0.02991145
46.6	0.52972717	-0.7557879	-0.4617982	0.32869445	0.44981876	0.23833234	0.02918644
46.7	0.52935152	-0.7554892	-0.4619116	0.32946121	0.45028006	0.23779991	0.0289989
46.8	0.52912659	-0.7551039	-0.4618284	0.32880203	0.45098369	0.23698197	0.02824679
46.9	0.52878271	-0.7549428	-0.4616273	0.32890838	0.45105812	0.23656243	0.02803651
47	0.52879289	-0.7546274	-0.4621367	0.32877525	0.45065609	0.2366915	0.02768705
47.1	0.52886356	-0.7543013	-0.4624568	0.32812921	0.44990163	0.23684372	0.02741719
47.2	0.52820923	-0.7540624	-0.4627033	0.32839719	0.44950914	0.2365439	0.02784584
47.3	0.52827338	-0.7537089	-0.4630372	0.32755693	0.44959865	0.23625575	0.02770531
47.4	0.52848668	-0.7538627	-0.4631209	0.32736273	0.45005956	0.23646427	0.02743054
47.5	0.52824676	-0.7538088	-0.4632267	0.32729305	0.45024313	0.23622794	0.02744442
47.6	0.52849694	-0.7536521	-0.4640727	0.32661487	0.450028	0.23657704	0.02633947
47.7	0.52883795	-0.753491	-0.4650589	0.32646641	0.45077361	0.2358567	0.02578176
47.8	0.52848859	-0.7529739	-0.4650984	0.32636518	0.45078115	0.23559499	0.02529193
47.9	0.52835357	-0.752882	-0.4652061	0.3263463	0.4507657	0.23502342	0.02537603
48	0.52811605	-0.7526299	-0.4658947	0.32631793	0.45093775	0.23355669	0.02415795
48.1	0.52801652	-0.7523919	-0.4666383	0.32577118	0.45023689	0.23379146	0.02410538
48.2	0.52756744	-0.7519716	-0.4666615	0.32535555	0.45024714	0.23392401	0.02392515
48.3	0.52763831	-0.7517976	-0.4668795	0.32540187	0.45090633	0.2343276	0.02406735
48.4	0.52732135	-0.7514508	-0.4669877	0.32518133	0.45067721	0.23407544	0.02407305
48.5	0.52702866	-0.7514957	-0.4676462	0.32419906	0.45138094	0.23333999	0.02412753
48.6	0.52664979	-0.7513405	-0.4679261	0.32425455	0.45136669	0.23291432	0.0246222
48.7	0.52593415	-0.7509132	-0.468354	0.3243575	0.45159493	0.23216466	0.02443522
48.8	0.52463731	-0.7505288	-0.4687317	0.32425691	0.45103162	0.23142776	0.02462322
48.9	0.52452443	-0.7504412	-0.468622	0.32390421	0.45086063	0.23113891	0.02475289
49	0.52436373	-0.750192	-0.4686455	0.32381897	0.45055442	0.23105878	0.02470029
49.1	0.52465238	-0.7502695	-0.4690077	0.32413911	0.45083098	0.23121052	0.02437789
49.2	0.52443936	-0.7502564	-0.4692955	0.32403075	0.45087635	0.23088176	0.02442063
49.3	0.52413167	-0.7498825	-0.4698554	0.32330433	0.45050047	0.23072298	0.02430766
49.4	0.52400937	-0.7497973	-0.4702342	0.32276296	0.45077028	0.23056671	0.02401753
49.5	0.52412276	-0.7492819	-0.4701668	0.3221041	0.45034966	0.23031915	0.0237282
49.6	0.5243578	-0.7490299	-0.4699951	0.3218744	0.45007267	0.22995641	0.02319109
49.7	0.52436186	-0.7489434	-0.4703088	0.32193698	0.45007335	0.23005213	0.02275194
49.8	0.52408867	-0.7487495	-0.4705104	0.32258824	0.44995318	0.22997916	0.02263201
49.9	0.52361617	-0.748332	-0.4704361	0.32213131	0.44974934	0.22921559	0.02227952
50	0.52316207	-0.7478576	-0.4705745	0.32267449	0.45034556	0.22766981	0.02129003
50.1	0.52294183	-0.7477286	-0.4708395	0.32243327	0.45040268	0.22748092	0.0216919
50.2	0.52286403	-0.747257	-0.4712828	0.32214464	0.45055319	0.2273958	0.02071866
50.3	0.52277561	-0.7469343	-0.4711729	0.32153528	0.45064752	0.22757451	0.02053301
50.4	0.52215146	-0.7467198	-0.4722709	0.32040803	0.45017767	0.22814322	0.0200608
50.5	0.52173285	-0.746519	-0.4722507	0.32013766	0.45025536	0.22779847	0.01960768
50.6	0.52148911	-0.7462982	-0.4729593	0.32048035	0.45060127	0.22798886	0.01960639
50.7	0.52125459	-0.7462216	-0.4729378	0.32033064	0.45046663	0.22770264	0.01965558
50.8	0.52052522	-0.7455514	-0.473087	0.32048693	0.45049316	0.22706425	0.01907797
50.9	0.52014174	-0.7453526	-0.4728828	0.32029716	0.4506188	0.22664847	0.01883006
51	0.51934398	-0.7446669	-0.4727344	0.31915965	0.45017017	0.22615755	0.01867837
51.1	0.51934398	-0.7446669	-0.4727344	0.31915965	0.45017017	0.22615755	0.01867837
51.2	0.51942692	-0.7445457	-0.4729557	0.31919118	0.45044018	0.22656029	0.01789014
51.3	0.5194316	-0.7445748	-0.4733241	0.31946706	0.45064428	0.22646141	0.01770083
51.4	0.51926474	-0.7443982	-0.473419	0.31971341	0.45079084	0.22607978	0.01694763
51.5	0.51915114	-0.7441961	-0.4738876	0.31916276	0.45101385	0.2266369	0.01687237
51.6	0.51861037	-0.7436756	-0.4741432	0.31888665	0.45087715	0.22572218	0.01611499
51.7	0.51815366	-0.7434602	-0.4744554	0.31865026	0.45033667	0.22585683	0.01563758
51.8	0.51733719	-0.7431143	-0.4747174	0.31839638	0.45047408	0.22502948	0.01552279
51.9	0.51718518	-0.7429861	-0.4748337	0.31829903	0.45047405	0.2245849	0.0152359
52	0.51631676	-0.7425522	-0.4744289	0.31747591	0.45072545	0.22333486	0.01566398
52.1	0.51593829	-0.7425892	-0.4750601	0.31683025	0.45055622	0.22251411	0.0159109
52.2	0.51563494	-0.742021	-0.4750819	0.31638316	0.45020056	0.22183519	0.0162122
52.3	0.51481721	-0.7415253	-0.4751463	0.31630983	0.45014763	0.22132912	0.01535639
52.4	0.515099	-0.7413514	-0.4749674	0.31633561	0.45037513	0.22112491	0.01481516
52.5	0.51510381	-0.741213	-0.4749801	0.31655178	0.45021242	0.22063251	0.01498344
52.6	0.51460636	-0.741109	-0.4757614	0.31626831	0.4504267	0.22091412	0.0148674
52.7	0.5144027	-0.7410226	-0.4764642	0.31614857	0.45022224	0.22093271	0.01503093
52.8	0.5144027	-0.7410226	-0.4764642	0.31614857	0.45022224	0.22093271	0.01503093
52.9	0.51419635	-0.7409706	-0.4768082	0.31609139	0.45019057	0.2206247	0.01449867
53	0.51406647	-0.7408436	-0.4770447	0.31643204	0.45050985	0.22044943	0.01478521
53.1	0.51351128	-0.7402639	-0.477004	0.316173	0.45098614	0.22033521	0.01517584
53.2	0.51303991	-0.7403925	-0.4772011	0.31583921	0.45105636	0.21924281	0.01565605
53.3	0.51283356	-0.7403841	-0.4771281	0.31557121	0.45085855	0.21868167	0.01568489
53.4	0.5126587	-0.7403552	-0.4770685	0.31527234	0.45057671	0.21878997	0.01566551
53.5	0.51192162	-0.7401211	-0.4772682	0.3156099	0.4508548	0.21811293	0.01545884
53.6	0.51126962	-0.7396172	-0.4771659	0.31523142	0.45077938	0.2179316	0.01530909
53.7	0.51107151	-0.7393574	-0.4770631	0.31533058	0.45074303	0.21812644	0.01519519
53.8	0.51025268	-0.7390456	-0.4771741	0.31505986	0.45014619	0.2178001	0.01583314
53.9	0.50974019	-0.7387738	-0.4771666	0.31490935	0.44986368	0.21705175	0.01517275
54	0.50943055	-0.7381354	-0.4769514	0.31479835	0.44986961	0.21720146	0.01454291
54.1	0.50890948	-0.737962	-0.477521	0.3146923	0.45009957	0.21711166	0.01483535
54.2	0.50835873	-0.7375798	-0.4772787	0.31403254	0.44991708	0.21711646	0.01501014
54.3	0.5075675	-0.7371403	-0.4772654	0.31377016	0.44964955	0.21697539	0.01460196
54.4	0.50754071	-0.7368937	-0.4782012	0.31400824	0.44965836	0.21669328	0.01403113
54.5	0.50732687	-0.7367379	-0.4783594	0.31401381	0.44936244	0.21697685	0.0139951
54.6	0.50704229	-0.7364886	-0.4787692	0.31341658	0.44892497	0.21652402	0.01480953
54.7	0.5068354	-0.73628	-0.4786652	0.31278985	0.44918005	0.21629744	0.01500719
54.8	0.50667966	-0.7362513	-0.4790658	0.31264818	0.44899191	0.21621544	0.01478792
54.9	0.50606717	-0.7358327	-0.4787678	0.31210776	0.4498463	0.21523579	0.01538729
55	0.50597508	-0.735946	-0.4788802	0.31204422	0.44987378	0.21578512	0.01536863
55.1	0.50584347	-0.7357688	-0.4788019	0.31245987	0.45002277	0.21462259	0.01509017
55.2	0.50460834	-0.7349015	-0.4789023	0.3124582	0.44967835	0.21327047	0.01469466
55.3	0.5042142	-0.7346022	-0.4792558	0.31294278	0.45009245	0.21213802	0.0155013
55.4	0.5042142	-0.7346022	-0.4792558	0.31294278	0.45009245	0.21213802	0.0155013
55.5	0.5042142	-0.7346022	-0.4792558	0.31294278	0.45009245	0.21213802	0.0155013
55.6	0.50418238	-0.7346757	-0.479384	0.3129785	0.45061555	0.2117531	0.01504718
55.7	0.50418238	-0.7346757	-0.479384	0.3129785	0.45061555	0.2117531	0.01504718
55.8	0.50374718	-0.7345833	-0.4804524	0.31317878	0.45043653	0.21155444	0.01448965
55.9	0.50340825	-0.7340243	-0.4799658	0.31314608	0.45085906	0.2114972	0.01393612
56	0.50334252	-0.7340001	-0.4801387	0.31301021	0.45090691	0.2114371	0.01420633
56.1	0.50284917	-0.7338529	-0.4810958	0.31288213	0.45023015	0.21139811	0.01460614
56.2	0.50248888	-0.7337219	-0.4813648	0.31255966	0.45030655	0.2118333	0.01432983
56.3	0.50182081	-0.7332942	-0.4815458	0.31240634	0.45042666	0.21125882	0.01404633
56.4	0.50194039	-0.7332508	-0.4814327	0.31281989	0.45041314	0.21104373	0.01365075
56.5	0.50095	-0.7325031	-0.481917	0.31295311	0.45114982	0.21093	0.01368028
56.6	0.50032881	-0.7318427	-0.4817881	0.31271293	0.45094195	0.2104126	0.01308963
56.7	0.49990171	-0.7314089	-0.4817896	0.31253752	0.45104105	0.21024751	0.01287545
56.8	0.49944383	-0.7311835	-0.4826187	0.3124576	0.45175927	0.21059232	0.01305199

11.9	0.73593407	-0.9073507	-0.1080449	0.48996708	0.35206987	0.4506762	-0.0014919
12	0.73435782	-0.9065373	-0.1085327	0.48763536	0.35358289	0.4478875	-0.0002369
12.1	0.73237888	-0.905357	-0.1074982	0.48534772	0.35240456	0.44411021	-0.0001849
12.2	0.72956046	-0.9044712	-0.1096354	0.48369106	0.35363852	0.44068694	0.00053677
12.3	0.72604317	-0.902927	-0.109414	0.47934248	0.35281865	0.43623623	0.00270703
12.4	0.72406389	-0.9018782	-0.1105985	0.4776988	0.35210022	0.43449278	0.00498352
12.5	0.72289604	-0.9005743	-0.1131028	0.4738002	0.35270075	0.42866004	0.00551435
12.6	0.71795388	-0.898212	-0.1147499	0.46789837	0.35159342	0.42192583	0.00774023
12.7	0.71495839	-0.8965754	-0.1166867	0.46549555	0.35174488	0.41711655	0.00900889
12.8	0.7150476	-0.896188	-0.1170258	0.46424084	0.35248649	0.41627964	0.01019889
12.9	0.71210825	-0.8943993	-0.1161881	0.45930526	0.35284335	0.41235131	0.00987509
13	0.71044173	-0.8936782	-0.1167283	0.4560441	0.35317193	0.41011699	0.00923423
13.1	0.70980523	-0.8934853	-0.1186164	0.45554114	0.35490223	0.40755173	0.00873169
13.2	0.70918462	-0.893138	-0.1198089	0.45446148	0.35756126	0.4059195	0.00913793
13.3	0.70706479	-0.8919866	-0.1236676	0.45182557	0.35882588	0.40330689	0.00678648
13.4	0.7066889	-0.8915235	-0.1248879	0.45253743	0.3599639	0.40251423	0.00556812
13.5	0.70615217	-0.8915034	-0.1249485	0.45297183	0.36205724	0.4017823	0.0085535
13.6	0.70619748	-0.8914062	-0.1265285	0.4538056	0.36323541	0.39970465	0.00641708
13.7	0.70500748	-0.8908301	-0.1265949	0.45321388	0.36581638	0.3981895	0.00713224
13.8	0.705369	-0.8906155	-0.128403	0.4536753	0.36938601	0.39691785	0.00772817
13.9	0.70477336	-0.8906003	-0.1292052	0.45198378	0.37070988	0.3946126	0.00966776
14	0.70403631	-0.890187	-0.1288499	0.45206307	0.37299689	0.39214108	0.0088704
14.1	0.70417895	-0.8904539	-0.1287286	0.45376411	0.3754046	0.39140397	0.0080359
14.2	0.70421105	-0.8903253	-0.1302279	0.45533362	0.37728137	0.38966391	0.00538645
14.3	0.70350424	-0.8901001	-0.133837	0.45617114	0.37936109	0.39042429	0.00345138
14.4	0.70351485	-0.8898752	-0.1353112	0.45590608	0.38093667	0.38979512	0.0025055
14.5	0.70123619	-0.8892258	-0.1353492	0.45533422	0.38345609	0.38822155	0.00155743
14.6	0.69978452	-0.8886644	-0.1369755	0.45600447	0.38441319	0.38623209	0.00165622
14.7	0.69959488	-0.888429	-0.1383107	0.45687299	0.38637386	0.38345873	0.00172429
14.8	0.69999496	-0.8885523	-0.1392444	0.45723259	0.3878845	0.3830748	0.00015455
14.9	0.70021565	-0.888617	-0.1405996	0.45777073	0.388477	0.38116678	-0.0009761

60	0.48133663	-0.7188095	-0.4888545	0.29967754	0.44504485	0.20327707	0.00736753
60.1	0.48087184	-0.7185825	-0.4892087	0.29917258	0.44494548	0.20301674	0.00681747
60.2	0.48087184	-0.7185825	-0.4892087	0.29917258	0.44494548	0.20301674	0.00681747
60.3	0.48028612	-0.7179192	-0.4894621	0.29956078	0.44490632	0.20301331	0.00588402
60.4	0.47970339	-0.7173445	-0.4895651	0.29913973	0.44422814	0.2029591	0.00531786
60.5	0.47982397	-0.7173918	-0.4900163	0.2992676	0.44498479	0.20219267	0.00535841
60.6	0.47853566	-0.7164341	-0.4897717	0.29866783	0.44430166	0.20135561	0.00511833
60.7	0.47778983	-0.7158682	-0.489361	0.29811071	0.4442905	0.20093052	0.00508315
60.8	0.47742498	-0.7157071	-0.4894006	0.29777633	0.44445332	0.20107235	0.00478825
60.9	0.47738295	-0.7156085	-0.4893677	0.29740502	0.4443309	0.20074029	0.0045603
61	0.47710121	-0.7146343	-0.4898698	0.29692966	0.44360478	0.2008836	0.00372863
61.1	0.47610976	-0.7140869	-0.4900152	0.29760611	0.44386733	0.20116604	0.0044869
61.2	0.47517596	-0.7134038	-0.4897234	0.29726103	0.44390514	0.20077095	0.00504553
61.3	0.4750687	-0.7128052	-0.4896385	0.2973118	0.44354332	0.20127686	0.00482675
61.4	0.47462353	-0.7123325	-0.4894957	0.29714741	0.44343093	0.20153121	0.00429469
61.5	0.47248213	-0.7103837	-0.4893442	0.29593855	0.44270232	0.19860057	0.00281155
61.6	0.47167839	-0.7096065	-0.4894474	0.29551036	0.44217815	0.19802467	0.00249607
61.7	0.47209968	-0.7092381	-0.4895078	0.29623479	0.44243949	0.19679344	0.00225557
61.8	0.47152951	-0.7086474	-0.4893923	0.29620477	0.44210752	0.19642419	0.00224885
61.9	0.47178137	-0.7084386	-0.4894798	0.2962429	0.44236776	0.19576599	0.00241453
62	0.47058037	-0.7073486	-0.4898824	0.2946172	0.44081449	0.19625116	0.00308655
62.1	0.47046526	-0.706932	-0.4902992	0.29432975	0.4413896	0.19494005	0.00300346
62.2	0.46992417	-0.7061455	-0.4902956	0.29356014	0.44121573	0.19445377	0.00304785
62.3	0.46986299	-0.7059431	-0.4900253	0.2933843	0.44138332	0.19441809	0.00339817
62.4	0.46968617	-0.7058384	-0.4904281	0.29331372	0.44130172	0.19464221	0.00303161
62.5	0.46909699	-0.7058717	-0.4904004	0.29312166	0.44107515	0.19497361	0.00351147
62.6	0.4690575	-0.7055813	-0.4906355	0.29306762	0.44086656	0.19512754	0.00281452
62.7	0.4689776	-0.7052477	-0.4905008	0.29299273	0.44076943	0.19545854	0.00290999
62.8	0.4689776	-0.7052477	-0.4905008	0.29299273	0.44076943	0.19545854	0.00290999
62.9	0.46717406	-0.7036281	-0.4902464	0.29233506	0.44080606	0.19528122	0.00151583
63	0.46598355	-0.7031651	-0.4898348	0.29181247	0.44045223	0.19450656	0.00163965
63.1	0.46519816	-0.7027602	-0.4900135	0.29132217	0.44026595	0.19377309	0.00178389
63.2	0.46467926	-0.7024371	-0.4899568	0.29113515	0.44022221	0.19360385	0.00158544
63.3	0.46413952	-0.7019924	-0.4900619	0.29085963	0.44021812	0.19362671	0.00133101
63.4	0.46386956	-0.7014672	-0.4906713	0.2902824	0.43989022	0.19396127	0.00069099
63.5	0.46338987	-0.7011784	-0.4912633	0.28979549	0.43993377	0.19346174	0.00084734
63.6	0.46266388	-0.7004149	-0.4908585	0.28946378	0.44026159	0.19319982	0.00049391
63.7	0.46231674	-0.700293	-0.4911852	0.28993409	0.44024378	0.19345112	0.00075461
63.8	0.4608176	-0.6991285	-0.4912236	0.28950659	0.43954334	0.19289633	-0.0001041
63.9	0.46031976	-0.6983488	-0.4911561	0.28926797	0.43925175	0.19277688	-0.000196
64	0.46027642	-0.6978904	-0.4918592	0.2890365	0.43912413	0.1925604	-0.0008672
64.1	0.45951826	-0.6972759	-0.492115	0.28871201	0.43894458	0.19232873	-0.0013552
64.2	0.45907664	-0.6966784	-0.4923593	0.2881627	0.43862811	0.19251961	-0.0021963
64.3	0.45879844	-0.6960385	-0.4925581	0.28775224	0.43852704	0.19203544	-0.0035292
64.4	0.4577107	-0.6957415	-0.4926199	0.28738187	0.43836389	0.19143433	-0.003946
64.5	0.45757774	-0.695594	-0.4929257	0.28721509	0.43851166	0.19105766	-0.0042685
64.6	0.45747533	-0.6957906	-0.4932232	0.28690302	0.4384114	0.19086889	-0.0045248
64.7	0.45691998	-0.6952879	-0.4931493	0.28647008	0.43812618	0.19051956	-0.0045779
64.8	0.45556841	-0.6947664	-0.4935718	0.28611949	0.43774505	0.18999318	-0.0048718
64.9	0.45556432	-0.694501	-0.4941011	0.2862405	0.43820778	0.18982655	-0.0053782
65	0.45519622	-0.6943514	-0.4945123	0.28588357	0.43776391	0.19007779	-0.0053247
65.1	0.45480991	-0.6938695	-0.4946322	0.28536675	0.43771979	0.1902752	-0.0056811
65.2	0.45450287	-0.6935294	-0.4943485	0.28494802	0.43769202	0.1903777	-0.0054923
65.3	0.45419446	-0.6931056	-0.4944922	0.28464693	0.4373426	0.19075332	-0.0063104
65.4	0.45345517	-0.6922455	-0.4946508	0.28415492	0.43708153	0.19020293	-0.00689
65.5	0.4530173	-0.6919277	-0.4942044	0.28398244	0.43745657	0.19078075	-0.0058878
65.6	0.45280262	-0.6914308	-0.4944409	0.28318768	0.43646037	0.19086575	-0.0057683
65.7	0.45251692	-0.6913044	-0.4944901	0.28342397	0.4364965	0.19047577	-0.0059289
65.8	0.45221088	-0.69103	-0.4944341	0.28357134	0.43640031	0.19038691	-0.006154
65.9	0.45172693	-0.6902311	-0.4941216	0.28340187	0.43605692	0.19049751	-0.0067556
66	0.45138202	-0.6895689	-0.4937553	0.28288311	0.43596192	0.19087248	-0.0066764
66.1	0.45079967	-0.6891818	-0.4938217	0.28287634	0.43581634	0.19056585	-0.0064974
66.2	0.45079967	-0.6891818	-0.4938217	0.28287634	0.43581634	0.19056585	-0.0064974
66.3	0.45052632	-0.689168	-0.4942804	0.28288095	0.43595754	0.19060463	-0.0071688
66.4	0.4494326	-0.6877114	-0.4943246	0.28172169	0.43559643	0.19040094	-0.0073655
66.5	0.44921614	-0.6872747	-0.4942332	0.28171509	0.43544022	0.19060522	-0.0075759
66.6	0.44869903	-0.6865448	-0.4938399	0.28155278	0.43511921	0.19051121	-0.0074952
66.7	0.44813622	-0.6860173	-0.4939161	0.28137022	0.4351491	0.19001706	-0.0080653
66.8	0.44751727	-0.685759	-0.4939182	0.28088448	0.43498464	0.189343	-0.0085275
66.9	0.44704531	-0.6854464	-0.4936411	0.28038464	0.43475354	0.18898831	-0.0084305
67	0.44676727	-0.6847767	-0.49378	0.28021216	0.43433864	0.18815355	-0.0085669
67.1	0.44660882	-0.6846996	-0.4939962	0.28038561	0.43427959	0.18835839	-0.0087117
67.2	0.44658848	-0.6845298	-0.4941464	0.28039476	0.43413548	0.18830847	-0.0088803
67.3	0.44570547	-0.6839706	-0.4941228	0.28093262	0.43395615	0.18752172	-0.0096217
67.4	0.44578342	-0.6834062	-0.4944184	0.28049292	0.43375601	0.18693687	-0.0090884
67.5	0.44496733	-0.6829062	-0.4952386	0.27994523	0.4332792	0.18766724	-0.0100885
67.6	0.44487568	-0.6829836	-0.4955704	0.28015054	0.43333256	0.18769906	-0.0104678
67.7	0.4451132	-0.6826408	-0.4953839	0.28013575	0.43351817	0.18738191	-0.011287
67.8	0.44516428	-0.682371	-0.495886	0.28018337	0.43333135	0.18783742	-0.0119014
67.9	0.44409712	-0.6821112	-0.4966368	0.27912673	0.43310655	0.18757674	-0.0122085
68	0.44341788	-0.6817827	-0.4973965	0.27847685	0.43266538	0.18810289	-0.0123607
68.1	0.44313419	-0.6815716	-0.4973431	0.27854378	0.43268313	0.18815549	-0.0123978
68.2	0.44271419	-0.6810974	-0.497127	0.27845785	0.43222077	0.18768095	-0.0128835
68.3	0.44271419	-0.6810974	-0.497127	0.27845785	0.43222077	0.18768095	-0.0128835
68.4	0.44195744	-0.6802905	-0.4977475	0.27812671	0.43166605	0.18722494	-0.0133503
68.5	0.44134697	-0.6794542	-0.4973601	0.27780071	0.43140163	0.18651109	-0.014067
68.6	0.44123378	-0.6784696	-0.4974454	0.27792216	0.43100564	0.18604552	-0.0149677
68.7	0.44064779	-0.6779929	-0.4983832	0.27731204	0.43064491	0.18571351	-0.0156882
68.8	0.43980635	-0.6775497	-0.4981656	0.27656015	0.43024868	0.18553491	-0.0151274
68.9	0.43958375	-0.6774219	-0.4987508	0.27571571	0.42920247	0.18582029	-0.0156456

26.9	0.59795643	-0.8183942	-0.3126367	0.38680602	0.45876183	0.28128243	0.0184578
27	0.59718134	-0.8180327	-0.3140934	0.38661211	0.45963517	0.2800142	0.01838874
27.1	0.59689824	-0.8174006	-0.3153193	0.38664842	0.45965233	0.28010443	0.01861
27.2	0.59691158	-0.8170654	-0.3161864	0.38608184	0.46032517	0.27944975	0.01905988
27.3	0.59684312	-0.8169289	-0.3164328	0.38619661	0.4613049	0.27865115	0.0189223
27.4	0.59571652	-0.8165562	-0.3166641	0.38583878	0.46230169	0.27795844	0.01915063
27.5	0.59558022	-0.8164463	-0.3172119	0.38566987	0.46175145	0.277347	0.0199916
27.6	0.595415	-0.8162079	-0.3178502	0.38482193	0.46239313	0.27718187	0.0200133
27.7	0.59519665	-0.816048	-0.3179127	0.38401974	0.46255281	0.27708899	0.01988264
27.8	0.59438423	-0.8157815	-0.3179418	0.38324772	0.46191659	0.27694953	0.01971494
27.9	0.59428836	-0.8154803	-0.3181243	0.38311762	0.46271376	0.27568955	0.019771
28	0.59396301	-0.8151677	-0.3187256	0.38255488	0.46330666	0.27542266	0.02145313
28.1	0.59296813	-0.8148444	-0.3197738	0.38193431	0.46364372	0.27572187	0.02235984
28.2	0.59262974	-0.814929	-0.3211097	0.38145582	0.46356694	0.27609037	0.02186638
28.3	0.59294505	-0.8147246	-0.3221438	0.38102259	0.46390961	0.27653461	0.02144807
28.4	0.59261087	-0.8145416	-0.3241635	0.38167231	0.46412841	0.27627246	0.02261335
28.5	0.59230917	-0.8143919	-0.3237885	0.38191646	0.46499408	0.27551141	0.02277803
28.6	0.59194185	-0.8144261	-0.3240952	0.38181181	0.46569507	0.27513127	0.02321723
28.7	0.59299651	-0.8141848	-0.3251346	0.38120313	0.46769554	0.27498479	0.02300297
28.8	0.59335278	-0.8139262	-0.3262239	0.3805118	0.46868947	0.27354833	0.02234089
28.9	0.59322699	-0.8136464	-0.3269968	0.38125272	0.46910201	0.27324917	0.02263368
29	0.59338612	-0.8128987	-0.3278238	0.38164101	0.46910453	0.27432684	0.02178216
29.1	0.59281561	-0.8127145	-0.3278663	0.381397	0.47073872	0.27369049	0.0220998
29.2	0.59251969	-0.8125538	-0.3290922	0.38190964	0.47164389	0.27348738	0.02286976
29.3	0.59240492	-0.8125125	-0.3295759	0.38212848	0.4715959	0.27278487	0.02363509
29.4	0.59238998	-0.8122371	-0.329712	0.38213729	0.47183926	0.27246562	0.0239496
29.5	0.59235541	-0.8120786	-0.3300307	0.38245157	0.47181806	0.27276078	0.02388483
29.6	0.59214948	-0.8118922	-0.3298248	0.38223712	0.4718154	0.2721522	0.02350126
29.7	0.59138893	-0.8116348	-0.3298936	0.3829062	0.47223733	0.27093793	0.02397463
29.8	0.59097217	-0.8114312	-0.3303832	0.38232892	0.47268177	0.27078752	0.02407999
29.9	0.59032773	-0.8113463	-0.3323677	0.38246921	0.4725977	0.27075767	0.0256378

75	0.41217042	-0.6517797	-0.5085441	0.25391559	0.40939571	0.17268092	-0.0246992
75.1	0.41129733	-0.6512163	-0.5088326	0.25299802	0.40915333	0.17251013	-0.0249709
75.2	0.41146047	-0.6509428	-0.5089949	0.25300369	0.40921962	0.17234847	-0.0253808
75.3	0.41117053	-0.6507683	-0.5090642	0.25293578	0.40886954	0.17198215	-0.025287
75.4	0.41043763	-0.650056	-0.5087308	0.25229985	0.40835573	0.17152302	-0.0255875
75.5	0.41039968	-0.6497578	-0.5087897	0.25219724	0.40760757	0.17203838	-0.0259574
75.6	0.41024882	-0.6496794	-0.5088917	0.25224778	0.40740626	0.17169392	-0.0255606
75.7	0.41006868	-0.649672	-0.5087769	0.25191924	0.40704752	0.17125736	-0.0252248
75.8	0.41008534	-0.6493226	-0.5087927	0.25165824	0.40689568	0.17130422	-0.0255235
75.9	0.40979495	-0.6493457	-0.5088672	0.25142593	0.40701238	0.17116913	-0.0253918
76	0.40950409	-0.6492591	-0.5089878	0.25174476	0.406902	0.17129016	-0.0255296
76.1	0.40873344	-0.6486901	-0.5092724	0.25125084	0.40620682	0.17095108	-0.0256444
76.2	0.4080209	-0.6483496	-0.5090715	0.25031902	0.40559709	0.17033232	-0.0252103
76.3	0.40767405	-0.648063	-0.5089519	0.25026448	0.40556908	0.17040108	-0.025432
76.4	0.40728363	-0.6473793	-0.5088387	0.25031098	0.40535357	0.17031154	-0.025458
76.5	0.40704039	-0.6474265	-0.5089345	0.2502268	0.40523883	0.17049785	-0.0256321
76.6	0.40704039	-0.6474265	-0.5089345	0.2502268	0.40523883	0.17049785	-0.0256321
76.7	0.40684529	-0.647433	-0.5092029	0.25002024	0.40487106	0.17100574	-0.0252854
76.8	0.40635957	-0.6469431	-0.5097025	0.24984636	0.40452125	0.17069758	-0.0254221
76.9	0.4059022	-0.646855	-0.5099838	0.25006483	0.40464963	0.17071808	-0.025108
77	0.4059877	-0.6468931	-0.5100643	0.24947796	0.40472771	0.17104943	-0.0255073
77.1	0.40587851	-0.6467726	-0.5102078	0.24928577	0.40484934	0.17090138	-0.0254526
77.2	0.40487616	-0.6465301	-0.5109934	0.2484615	0.40392349	0.17033711	-0.025757
77.3	0.40487616	-0.6465301	-0.5109934	0.2484615	0.40392349	0.17033711	-0.025757
77.4	0.40461275	-0.6463032	-0.5112583	0.24852469	0.40357198	0.17051973	-0.0259982
77.5	0.40460733	-0.646237	-0.511931	0.24891266	0.40347463	0.17074964	-0.0265191
77.6	0.40433408	-0.6460804	-0.5116269	0.2488846	0.4031153	0.17082332	-0.0265839
77.7	0.40380968	-0.6452401	-0.5113601	0.24851542	0.40262802	0.17052335	-0.0266688
77.8	0.40318688	-0.6450231	-0.5114442	0.24873126	0.40274365	0.17050333	-0.0263141
77.9	0.40257631	-0.6447229	-0.5119218	0.24891507	0.40259925	0.17065214	-0.0269827
78	0.40273613	-0.6448899	-0.5125292	0.24837578	0.40249875	0.1704395	-0.0272086
78.1	0.40237551	-0.6445589	-0.5126298	0.24810515	0.40219385	0.17039831	-0.0270467
78.2	0.40279953	-0.6442799	-0.5131794	0.24862137	0.40230466	0.17081735	-0.0262036
78.3	0.4022745	-0.6433319	-0.5131006	0.24814959	0.40163387	0.17018687	-0.0264434
78.4	0.40227545	-0.643258	-0.5132963	0.24796282	0.40132026	0.17026491	-0.026472
78.5	0.40228808	-0.6429998	-0.51344	0.24734243	0.4007967	0.16961957	-0.0262753
78.6	0.40214654	-0.6428394	-0.513756	0.24728475	0.40018601	0.1690826	-0.0263494
78.7	0.40169106	-0.6427599	-0.5142664	0.24744703	0.40035119	0.16843413	-0.0260714
78.8	0.40171578	-0.6426724	-0.5144018	0.24735776	0.40023999	0.16866157	-0.0257174
78.9	0.40097904	-0.6421324	-0.5147223	0.24747051	0.39998284	0.16888538	-0.0261969
79	0.4009658	-0.64161	-0.5145401	0.24678973	0.39963009	0.16806887	-0.0265916
79.1	0.40017287	-0.6413828	-0.5154051	0.24647424	0.39928479	0.16734232	-0.0265851
79.2	0.40018495	-0.641078	-0.5151617	0.24580242	0.39924451	0.16787666	-0.0269393
79.3	0.40018495	-0.641078	-0.5151617	0.24580242	0.39924451	0.16787666	-0.0269393
79.4	0.40008408	-0.6408843	-0.5152918	0.24577242	0.3991069	0.16755362	-0.0266027
79.5	0.39919347	-0.6405068	-0.5153826	0.24574341	0.39865977	0.16693245	-0.0267079
79.6	0.39916182	-0.6404569	-0.5161503	0.2463627	0.39905405	0.16652158	-0.0262184
79.7	0.39918972	-0.6399903	-0.5161893	0.24610516	0.39870627	0.16650681	-0.0264486
79.8	0.39910047	-0.6397183	-0.516293	0.24617678	0.39807628	0.16707663	-0.0267323
79.9	0.39907415	-0.6393879	-0.5166748	0.2468489	0.39763477	0.16779617	-0.0273941
80	0.3995757	-0.6387718	-0.5167626	0.24678114	0.39668019	0.16843174	-0.02833
80.1	0.39961291	-0.638454	-0.5169804	0.24662422	0.3965223	0.16818394	-0.0285759
80.2	0.39961291	-0.638454	-0.5169804	0.24662422	0.3965223	0.16818394	-0.0285759
80.3	0.39965659	-0.6382573	-0.5172274	0.24578644	0.39646281	0.16807088	-0.0289819
80.4	0.39965659	-0.6382573	-0.5172274	0.24578644	0.39646281	0.16807088	-0.0289819
80.5	0.39917309	-0.6382194	-0.517534	0.24557314	0.39698176	0.16802079	-0.0289167
80.6	0.39916833	-0.6379019	-0.5176869	0.24546838	0.39753307	0.16745086	-0.0286517
80.7	0.39862838	-0.6378492	-0.518325	0.24491263	0.39680401	0.16725174	-0.028613
80.8	0.39875199	-0.6383778	-0.5190161	0.2445862	0.39710853	0.16821666	-0.0287624
80.9	0.39875199	-0.6383778	-0.5190161	0.2445862	0.39710853	0.16821666	-0.0287624
81	0.39853079	-0.6382068	-0.5191525	0.24441554	0.39665274	0.16849215	-0.0287148
81.1	0.39800396	-0.6377673	-0.5194482	0.24382109	0.39607327	0.16867636	-0.0284119
81.2	0.39800396	-0.6377673	-0.5194482	0.24382109	0.39607327	0.16867636	-0.0284119
81.3	0.39764416	-0.6374015	-0.5194732	0.2436358	0.39556848	0.16874812	-0.0291252
81.4	0.39715055	-0.6371426	-0.5196393	0.24285725	0.39495493	0.16888133	-0.0291619
81.5	0.39703656	-0.6370199	-0.5195946	0.24298118	0.39536626	0.16871208	-0.0293635
81.6	0.39702935	-0.6366261	-0.5198135	0.24222968	0.39493379	0.16885574	-0.0296884
81.7	0.39702473	-0.6364657	-0.5199583	0.24142835	0.39450335	0.16894729	-0.0293816
81.8	0.39630769	-0.6359887	-0.5199565	0.24126392	0.39392944	0.16904867	-0.0291644
81.9	0.39606145	-0.6358284	-0.5201056	0.24130342	0.39370809	0.16910556	-0.0293447
82	0.39597101	-0.6356092	-0.5200314	0.24112566	0.39339693	0.16938299	-0.0297805
82.1	0.39544909	-0.6352781	-0.5200555	0.24061562	0.39258853	0.16900723	-0.0299704
82.2	0.39544909	-0.6352781	-0.5200555	0.24061562	0.39258853	0.16900723	-0.0299704
82.3	0.39456812	-0.6341522	-0.5204671	0.24027866	0.39111411	0.16852188	-0.0301556
82.4	0.39371002	-0.6334934	-0.5207431	0.23923554	0.38964158	0.16792159	-0.0304404
82.5	0.39357034	-0.6330773	-0.5210843	0.2389617	0.3891938	0.16805541	-0.0309354
82.6	0.39317096	-0.6328375	-0.5213115	0.23850736	0.38853071	0.16797031	-0.0308069
82.7	0.39323978	-0.6327236	-0.5213442	0.23849758	0.38862072	0.1682915	-0.0310342
82.8	0.39327846	-0.632726	-0.521862	0.23813243	0.38891631	0.16846077	-0.0312018
82.9	0.39267894	-0.6326072	-0.5221354	0.23784251	0.38846307	0.16848473	-0.0314845
83	0.39198603	-0.632059	-0.5219455	0.23675924	0.38782621	0.16925303	-0.031235
83.1	0.39179148	-0.6317891	-0.521937	0.23687084	0.3877817	0.16944433	-0.0316578
83.2	0.39153523	-0.6316816	-0.5219254	0.23715311	0.38755124	0.16946338	-0.0317637
83.3	0.39140671	-0.6314173	-0.5222777	0.2366963	0.38730221	0.16943158	-0.0317508
83.4	0.39140974	-0.6314058	-0.5223365	0.23673454	0.38701783	0.16962204	-0.0316479
83.5	0.39094945	-0.6311819	-0.5229032	0.23630692	0.38691898	0.1698024	-0.0320121
83.6	0.38951514	-0.6303538	-0.523055	0.2355175	0.38614592	0.1687881	-0.0318967
83.7	0.38932867	-0.6303426	-0.5232658	0.23552089	0.3859682	0.16906557	-0.0317586
83.8	0.38881544	-0.6297172	-0.5233606	0.23555051	0.38539698	0.16885676	-0.0316349
83.9	0.38909321	-0.6295147	-0.5236246	0.2356589	0.38579379	0.16848479	-0.0318049

41.9	0.5408166	-0.7693707	-0.4448679	0.34038684	0.45471041	0.2505286	0.0406403
42	0.54048294	-0.7688621	-0.4452897	0.34017041	0.45498699	0.25005383	0.04036228
42.1	0.53962011	-0.7686674	-0.4456422	0.34045422	0.45509447	0.24951581	0.03975252
42.2	0.53968354	-0.7681137	-0.4460664	0.34038467	0.45426108	0.24992452	0.03984814
42.3	0.5398507	-0.7681688	-0.4464491	0.33989453	0.45410131	0.25006642	0.03916518
42.4	0.5395667	-0.7676556	-0.4467736	0.3395816	0.45406711	0.24994188	0.03833846
42.5	0.53968519	-0.76754	-0.4475408	0.33975407	0.45425332	0.25000078	0.0384241
42.6	0.53905998	-0.766844	-0.4481415	0.33925522	0.45254609	0.25056009	0.03938786
42.7	0.53909191	-0.7669684	-0.4486131	0.33951467	0.45261299	0.25015641	0.03917639
42.8	0.5390557	-0.7662449	-0.4485891	0.33933148	0.45270697	0.24955092	0.03841687
42.9	0.53882532	-0.7661311	-0.4489642	0.33896715	0.45216399	0.24992682	0.03842263
43	0.53848377	-0.7655231	-0.4487892	0.33836486	0.45173781	0.24968052	0.03826359
43.1	0.53812114	-0.7652231	-0.4490648	0.33867499	0.45172162	0.24942379	0.03794185
43.2	0.53819655	-0.7649675	-0.4497574	0.33842798	0.45156877	0.2489322	0.03830411
43.3	0.53848298	-0.7643978	-0.4497276	0.33785167	0.45103959	0.24867507	0.03733564
43.4	0.53794678	-0.7642909	-0.4505409	0.33792469	0.45115202	0.24788563	0.03786175
43.5	0.53832917	-0.7641648	-0.4510165	0.33776527	0.45110579	0.24782235	0.03731551
43.6	0.53891835	-0.7641457	-0.4526228	0.33639604	0.45057742	0.24886952	0.03695668
43.7	0.5386909	-0.7637641	-0.4528731	0.33615747	0.45173555	0.24800605	0.0364824
43.8	0.53840097	-0.7636348	-0.4531238	0.3356761	0.45155552	0.24754767	0.03573184
43.9	0.53788076	-0.7633498	-0.452681	0.33456229	0.45188903	0.24760131	0.03572102
44	0.53698557	-0.7631174	-0.4532745	0.33499632	0.4523711	0.24640068	0.03593713
44.1	0.53590203	-0.7626828	-0.4539804	0.33436644	0.45177073	0.24632026	0.03531802
44.2	0.53609251	-0.7623889	-0.4544327	0.33424017	0.45137569	0.24614619	0.03487691
44.3	0.53612711	-0.7619549	-0.455293	0.33285027	0.45051751	0.2453375	0.034619
44.4	0.5353085	-0.761281	-0.4562914	0.33266986	0.4501565	0.2446498	0.03446309
44.5	0.53545763	-0.7609033	-0.4566694	0.33215069	0.44985926	0.24420849	0.03359786
44.6	0.53503455	-0.7606254	-0.4566991	0.33177627	0.4500173	0.24400552	0.03365752
44.7	0.53478157	-0.760297	-0.4567652	0.33209798	0.44936554	0.24304797	0.0339014
44.8	0.5349302	-0.7603752	-0.4574838	0.33179541	0.44965219	0.24207459	0.03410642
44.9	0.53460489	-0.7601554	-0.4576068	0.33134359	0.44969507	0.24152881	0.03451624

90	0.37090398	-0.6109593	-0.5335321	0.22605946	0.36702417	0.16956657	-0.0396097
90.1	0.37055138	-0.6106997	-0.5340854	0.22629505	0.36705673	0.16997969	-0.0399439
90.2	0.37001001	-0.6104215	-0.5341009	0.226257	0.36678399	0.16972167	-0.039825
90.3	0.36943191	-0.6101204	-0.5345401	0.22639116	0.36584949	0.16986739	-0.0397645
90.4	0.36915668	-0.6089968	-0.5346597	0.22620647	0.36514333	0.16977	-0.0399047
90.5	0.36896763	-0.6089164	-0.5347827	0.2262439	0.36491477	0.16999595	-0.040141
90.6	0.36865242	-0.6087822	-0.534882	0.22626652	0.36468478	0.17007063	-0.0400709
90.7	0.36839244	-0.6086443	-0.5350901	0.22613125	0.36445921	0.17065121	-0.0399467
90.8	0.36826489	-0.6084673	-0.5352474	0.22602556	0.36444202	0.17023581	-0.0396635
90.9	0.36732293	-0.6078514	-0.535385	0.2260455	0.36413078	0.16987544	-0.0390901
91	0.36732293	-0.6078514	-0.535385	0.2260455	0.36413078	0.16987544	-0.0390901
91.1	0.36732293	-0.6078514	-0.535385	0.2260455	0.36413078	0.16987544	-0.0390901
91.2	0.36646592	-0.6073987	-0.5354931	0.22607454	0.36370078	0.17080693	-0.0395782
91.3	0.3664441	-0.6073465	-0.5356703	0.22584858	0.36397083	0.17065316	-0.0397731
91.4	0.3664441	-0.6073465	-0.5356703	0.22584858	0.36397083	0.17065316	-0.0397731
91.5	0.36618877	-0.6071939	-0.535911	0.22587752	0.36401869	0.17072079	-0.0399462
91.6	0.3658979	-0.6068584	-0.5361385	0.22535467	0.36399717	0.17103601	-0.0399575
91.7	0.36530835	-0.6064276	-0.5367466	0.22562775	0.36388813	0.17047164	-0.0403641
91.8	0.36530835	-0.6064276	-0.5367466	0.22562775	0.36388813	0.17047164	-0.0403641
91.9	0.36518144	-0.6062154	-0.536859	0.22587826	0.364101	0.17035963	-0.0407744
92	0.36471407	-0.605979	-0.5371994	0.22634166	0.36438956	0.17097049	-0.0406384
92.1	0.36421006	-0.6056624	-0.5370859	0.22622337	0.36504393	0.17021226	-0.04121
92.2	0.36336876	-0.6049849	-0.5375366	0.22698785	0.36458437	0.16994744	-0.0405604
92.3	0.36318782	-0.6048728	-0.5378793	0.2266503	0.3642104	0.1696355	-0.0405632
92.4	0.36318782	-0.6048728	-0.5378793	0.2266503	0.3642104	0.1696355	-0.0405632
92.5	0.36308724	-0.6047378	-0.5380351	0.22671754	0.36431637	0.16974852	-0.0409812
92.6	0.36260783	-0.6044084	-0.5382857	0.22655737	0.36417274	0.16972067	-0.0406331
92.7	0.36228003	-0.6042906	-0.538753	0.22679676	0.36384191	0.16987472	-0.0409703
92.8	0.36228003	-0.6042906	-0.538753	0.22679676	0.36384191	0.16987472	-0.0409703
92.9	0.36197641	-0.6041512	-0.5392879	0.22668606	0.36319284	0.17006399	-0.0405487
93	0.36177793	-0.6040376	-0.5391627	0.22657332	0.3630296	0.17020963	-0.0407557
93.1	0.36113072	-0.6034632	-0.5392396	0.22635886	0.36241351	0.17018997	-0.0400808
93.2	0.36056992	-0.6032362	-0.5394482	0.22570827	0.36244674	0.16986013	-0.0403037
93.3	0.36056992	-0.6032362	-0.5394482	0.22570827	0.36244674	0.16986013	-0.0403037
93.4	0.36062116	-0.6030304	-0.5394815	0.22560361	0.36249174	0.16966437	-0.0400933
93.5	0.36054979	-0.6029263	-0.5394858	0.22552543	0.36264189	0.16976124	-0.0402042
93.6	0.35986758	-0.6023405	-0.5393412	0.22531	0.36213968	0.16977598	-0.0403036
93.7	0.35986758	-0.6023405	-0.5393412	0.22531	0.36213968	0.16977598	-0.0403036
93.8	0.35993889	-0.6024962	-0.5394011	0.22517907	0.36213736	0.16960303	-0.0401137
93.9	0.36028875	-0.602362	-0.5398497	0.22524595	0.3623994	0.16963548	-0.0402299
94	0.35986926	-0.6019581	-0.5401276	0.22486827	0.36198076	0.1697607	-0.039384
94.1	0.35969008	-0.6014901	-0.5404232	0.22547109	0.3616192	0.16973553	-0.0394546
94.2	0.35964008	-0.6012574	-0.5403342	0.22552225	0.36188695	0.169711	-0.0391751
94.3	0.35964008	-0.6012574	-0.5403342	0.22552225	0.36188695	0.169711	-0.0391751
94.4	0.35956067	-0.6011734	-0.5403126	0.22581418	0.361722	0.1699769	-0.0393978
94.5	0.35885929	-0.6008449	-0.540543	0.22613933	0.3615551	0.17000424	-0.0396194
94.6	0.35806182	-0.6005235	-0.5408266	0.22567212	0.36175561	0.16980099	-0.0397427
94.7	0.35806182	-0.6005235	-0.5408266	0.22567212	0.36175561	0.16980099	-0.0397427
94.8	0.35789841	-0.600309	-0.540794	0.22580546	0.36164451	0.17001187	-0.0398646
94.9	0.35771413	-0.5999329	-0.5407292	0.2256591	0.36147462	0.17015392	-0.0399232
95	0.35718762	-0.5991996	-0.5410021	0.22605967	0.36104321	0.16993213	-0.0404106
95.1	0.35695489	-0.5984209	-0.5411276	0.22627301	0.36058258	0.17005759	-0.0407548
95.2	0.35648934	-0.5978388	-0.5413572	0.22616446	0.36029823	0.16915936	-0.0406943
95.3	0.35633491	-0.5975724	-0.5418805	0.22609134	0.3602889	0.16866046	-0.041003
95.4	0.35633491	-0.5975724	-0.5418805	0.22609134	0.3602889	0.16866046	-0.041003
95.5	0.3561284	-0.5974454	-0.5421137	0.22605228	0.36044663	0.16865436	-0.041137
95.6	0.3557586	-0.5968752	-0.542348	0.2263566	0.36022557	0.16794125	-0.0410154
95.7	0.35565581	-0.5968122	-0.5426578	0.22626795	0.36031446	0.16751096	-0.0412386
95.8	0.35554777	-0.5963867	-0.542742	0.22648328	0.36028398	0.16780465	-0.0417819
95.9	0.35554777	-0.5963867	-0.542742	0.22648328	0.36028398	0.16780465	-0.0417819
96	0.35539567	-0.5961647	-0.5428065	0.22664684	0.36037687	0.16762853	-0.0418098
96.1	0.35479348	-0.5955826	-0.5428906	0.22585925	0.36041884	0.16738401	-0.0408019
96.2	0.35461939	-0.5952939	-0.5429597	0.22612634	0.3605792	0.16797504	-0.0407591
96.3	0.35443805	-0.5951079	-0.5432034	0.22607233	0.36039611	0.16788392	-0.0407148
96.4	0.35417377	-0.5949052	-0.5431045	0.22600462	0.36037964	0.16754952	-0.0407027
96.5	0.35438168	-0.5948767	-0.5431511	0.22600991	0.36026618	0.1678847	-0.0407434
96.6	0.35438168	-0.5948767	-0.5431511	0.22600991	0.36026618	0.1678847	-0.0407434
96.7	0.35386474	-0.5944594	-0.5437591	0.22568331	0.36049638	0.16776339	-0.041209
96.8	0.35386474	-0.5944594	-0.5437591	0.22568331	0.36049638	0.16776339	-0.041209
96.9	0.35386474	-0.5944594	-0.5437591	0.22568331	0.36049638	0.16776339	-0.041209
97	0.35386474	-0.5944594	-0.5437591	0.22568331	0.36049638	0.16776339	-0.041209
97.1	0.35357262	-0.5941979	-0.5441988	0.22510369	0.36060411	0.16745431	-0.0406483
97.2	0.35315599	-0.5938693	-0.5442641	0.22509928	0.36032177	0.16729758	-0.0408285
97.3	0.35287051	-0.5935417	-0.5443012	0.22537803	0.36023267	0.1670768	-0.0411757
97.4	0.35274544	-0.5930234	-0.5442635	0.2255746	0.3598728	0.16712216	-0.0418481
97.5	0.35258001	-0.592862	-0.5444816	0.22574409	0.35961862	0.16725968	-0.0420255
97.6	0.35201305	-0.5925224	-0.5447629	0.22532257	0.35987825	0.16749769	-0.0422335
97.7	0.351612	-0.5922423	-0.5448463	0.22534462	0.35967255	0.16767946	-0.0424038
97.8	0.35133622	-0.5921262	-0.5448879	0.22542851	0.35965014	0.16776048	-0.0424489
97.9	0.35133622	-0.5921262	-0.5448879	0.22542851	0.35965014	0.16776048	-0.0424489
98	0.35137401	-0.5919433	-0.545082	0.22558216	0.35952397	0.16795462	-0.0424984
98.1	0.35124974	-0.5917684	-0.5452332	0.22536662	0.35959112	0.16798932	-0.0424044
98.2	0.35124974	-0.5917684	-0.5452332	0.22536662	0.35959112	0.16798932	-0.0424044
98.3	0.35030943	-0.5910384	-0.5452505	0.22501807	0.35898278	0.16754904	-0.0426159
98.4	0.35018188	-0.5908882	-0.5454954	0.22458678	0.35869246	0.16700026	-0.0427633
98.5	0.35002543	-0.5906412	-0.5455331	0.22444534	0.35857629	0.16646763	-0.0429271
98.6	0.35002543	-0.5906412	-0.5455331	0.22444534	0.35857629	0.16646763	-0.0429271
98.7	0.34982002	-0.5900684	-0.5461204	0.22439067	0.3583167	0.1668681	-0.0434953
98.8	0.35040459	-0.5902006	-0.5468547	0.22389022	0.35815547	0.16688089	-0.0437553
98.9	0.35034121	-0.5899304	-0.5469749	0.22396289	0.35812592	0.16706137	-0.0432936

56.9	0.49963325	-0.7311798	-0.4830183	0.31291433	0.45192044	0.21036208	0.01310488
57	0.49865564	-0.7308383	-0.4836169	0.31243517	0.45162694	0.21096989	0.0127784
57.1	0.49874411	-0.7306405	-0.4836825	0.31206159	0.45127817	0.21114184	0.01236193
57.2	0.49832715	-0.7306892	-0.4842141	0.31162402	0.45118453	0.21076914	0.01266246
57.3	0.49749834	-0.7305105	-0.4845111	0.31094859	0.45104525	0.21028172	0.01296281
57.4	0.49732923	-0.7304612	-0.484697	0.31100452	0.45091894	0.20994224	0.0135419
57.5	0.49699549	-0.730332	-0.4846865	0.31116116	0.45100703	0.20976939	0.0130326
57.6	0.49614049	-0.7300462	-0.4847584	0.31042071	0.45076909	0.20970488	0.01259612
57.7	0.49582654	-0.7297478	-0.4853593	0.30978167	0.45060193	0.20933809	0.01207659
57.8	0.49538444	-0.7290939	-0.485153	0.30918339	0.45055559	0.20848674	0.01190358
57.9	0.49383571	-0.7279147	-0.4851526	0.30902467	0.44994537	0.20762478	0.01115461
58	0.49367763	-0.727718	-0.4852641	0.30906012	0.44993813	0.20732667	0.01099248
58.1	0.49276886	-0.7270281	-0.4857561	0.30903874	0.44886385	0.20748726	0.01074745
58.2	0.49240437	-0.726704	-0.4860442	0.30906611	0.4488785	0.20733556	0.00998013
58.3	0.49211802	-0.7265776	-0.4859477	0.30902483	0.4487166	0.20706211	0.009709
58.4	0.49179438	-0.7260596	-0.4863787	0.30807586	0.44790466	0.20765455	0.00903785
58.5	0.49128967	-0.7258903	-0.4863846	0.30789292	0.44799335	0.20693153	0.00931094
58.6	0.49004305	-0.7251153	-0.4869479	0.30727259	0.4474474	0.20483154	0.00851434
58.7	0.48991548	-0.7246553	-0.4873665	0.30716852	0.44752288	0.20464461	0.00871696
58.8	0.48915991	-0.7236158	-0.4871342	0.30621831	0.44697073	0.20456175	0.00857806
58.9	0.48936124	-0.7230507	-0.4867076	0.30573439	0.44679368	0.20544124	0.00886332
59	0.48859582	-0.7226423	-0.4867123	0.30522939	0.44620881	0.2057061	0.00920088
59.1	0.48720493	-0.7219128	-0.4867316	0.3039625	0.44614843	0.20469327	0.00918625
59.2	0.48669118	-0.7214973	-0.4866307	0.30369522	0.44608707	0.20530721	0.00868301
59.3	0.48648044	-0.7215123	-0.4866101	0.30352021	0.44586387	0.20481616	0.00824147
59.4	0.4850999	-0.7207659	-0.4871799	0.30207438	0.44484086	0.20473146	0.007697
59.5	0.4848022	-0.7204574	-0.4870916	0.30160319	0.44529536	0.20416801	0.00784767
59.6	0.48430069	-0.7198454	-0.4876407	0.30134236	0.44474802	0.20518412	0.00728636
59.7	0.48335521	-0.7197143	-0.4884405	0.30123885	0.44478912	0.2043163	0.00712335
59.8	0.48261219	-0.7190976	-0.4883464	0.30114778	0.44476399	0.2032824	0.00683181
59.9	0.48206166	-0.7188256	-0.4885132	0.30054777	0.44488625	0.20351918	0.00682757

105	0.34507544	-0.5831829	-0.5505607	0.21868384	0.35353082	0.16530698	-0.0419376
105.1	0.34507544	-0.5831829	-0.5505607	0.21868384	0.35353082	0.16530698	-0.0419376
105.2	0.34507544	-0.5831829	-0.5505607	0.21868384	0.35353082	0.16530698	-0.0419376
105.3	0.34506816	-0.5833625	-0.5506471	0.21859822	0.35337274	0.16566794	-0.0417274
105.4	0.34503172	-0.5831468	-0.5505592	0.21841721	0.35339285	0.16580801	-0.0416041
105.5	0.34504903	-0.5830995	-0.5508787	0.21869467	0.3535644	0.16596739	-0.0412633
105.6	0.34504903	-0.5830995	-0.5508787	0.21869467	0.3535644	0.16596739	-0.0412633
105.7	0.34504903	-0.5830995	-0.5508787	0.21869467	0.3535644	0.16596739	-0.0412633
105.8	0.34518939	-0.5829385	-0.5509676	0.21866282	0.35356056	0.16593986	-0.0410256
105.9	0.34518939	-0.5829385	-0.5509676	0.21866282	0.35356056	0.16593986	-0.0410256
106	0.34513676	-0.5826686	-0.5510677	0.21878518	0.35336351	0.16605358	-0.040709
106.1	0.34534297	-0.5828589	-0.5512271	0.21885937	0.35309957	0.16620276	-0.0406051
106.2	0.34534297	-0.5828589	-0.5512271	0.21885937	0.35309957	0.16620276	-0.0406051
106.3	0.34534297	-0.5828589	-0.5512271	0.21885937	0.35309957	0.16620276	-0.0406051
106.4	0.34534297	-0.5828589	-0.5512271	0.21885937	0.35309957	0.16620276	-0.0406051
106.5	0.34549027	-0.5828207	-0.5512572	0.21860279	0.352745	0.16638908	-0.0405975
106.6	0.34576526	-0.582763	-0.5512765	0.21823939	0.35273475	0.16645953	-0.0405527
106.7	0.34576526	-0.582763	-0.5512765	0.21823939	0.35273475	0.16645953	-0.0405527
106.8	0.34575387	-0.5824238	-0.5512209	0.21797193	0.35239615	0.1665147	-0.0406877
106.9	0.34575387	-0.5824238	-0.5512209	0.21797193	0.35239615	0.1665147	-0.0406877
107	0.34575387	-0.5824238	-0.5512209	0.21797193	0.35239615	0.1665147	-0.0406877
107.1	0.34575387	-0.5824238	-0.5512209	0.21797193	0.35239615	0.1665147	-0.0406877
107.2	0.34546183	-0.5821832	-0.5511949	0.21784919	0.35248782	0.16654643	-0.0401806
107.3	0.34514032	-0.5821673	-0.551253	0.21781061	0.35281562	0.16682504	-0.0401342
107.4	0.34514032	-0.5821673	-0.551253	0.21781061	0.35281562	0.16682504	-0.0401342
107.5	0.34514032	-0.5821673	-0.551253	0.21781061	0.35281562	0.16682504	-0.0401342
107.6	0.3453606	-0.5822202	-0.551503	0.2176751	0.35260794	0.16688871	-0.040187
107.7	0.34514082	-0.5820446	-0.551525	0.21760823	0.35214044	0.16710851	-0.0402305
107.8	0.34533112	-0.5817263	-0.5512693	0.21739718	0.35216912	0.16718663	-0.0404752
107.9	0.34533112	-0.5817263	-0.5512693	0.21739718	0.35216912	0.16718663	-0.0404752
108	0.34524608	-0.5816265	-0.5511636	0.21706737	0.35213177	0.16690166	-0.0406025
108.1	0.34515022	-0.5814012	-0.5510674	0.21693719	0.35209803	0.16708981	-0.0402599
108.2	0.34515022	-0.5814012	-0.5510674	0.21693719	0.35209803	0.16708981	-0.0402599
108.3	0.34515022	-0.5814012	-0.5510674	0.21693719	0.35209803	0.16708981	-0.0402599
108.4	0.34511746	-0.5810862	-0.5511168	0.21657728	0.3520122	0.16742554	-0.0401371
108.5	0.34489141	-0.5808777	-0.5510892	0.21654213	0.35194118	0.16736589	-0.0399218
108.6	0.34478683	-0.5805421	-0.5510561	0.21644151	0.35184324	0.16723761	-0.03961
108.7	0.34496913	-0.5803751	-0.5510615	0.21601214	0.35157817	0.16719698	-0.0393202
108.8	0.34496913	-0.5803751	-0.5510615	0.21601214	0.35157817	0.16719698	-0.0393202
108.9	0.34496913	-0.5803751	-0.5510615	0.21601214	0.35157817	0.16719698	-0.0393202
109	0.34496913	-0.5803751	-0.5510615	0.21601214	0.35157817	0.16719698	-0.0393202
109.1	0.3447861	-0.5800238	-0.5510732	0.21608117	0.35146383	0.16732522	-0.0391375
109.2	0.3448803	-0.5798583	-0.5513933	0.2164377	0.35149503	0.16722247	-0.0392042
109.3	0.34480359	-0.5796356	-0.5514583	0.21626802	0.35129694	0.1669928	-0.0387253
109.4	0.34480359	-0.5796356	-0.5514583	0.21626802	0.35129694	0.1669928	-0.0387253
109.5	0.34469318	-0.5794855	-0.5516804	0.21620999	0.35130286	0.16681211	-0.0387041
109.6	0.34474997	-0.5796144	-0.5516878	0.21587498	0.35160642	0.16691141	-0.0383776
109.7	0.34474997	-0.5796144	-0.5516878	0.21587498	0.35160642	0.16691141	-0.0383776
109.8	0.34474997	-0.5796144	-0.5516878	0.21587498	0.35160642	0.16691141	-0.0383776
109.9	0.34474997	-0.5796144	-0.5516878	0.21587498	0.35160642	0.16691141	-0.0383776
110	0.34482122	-0.5797434	-0.5520322	0.21587098	0.35163231	0.1669474	-0.0384781
110.1	0.3449141	-0.5798447	-0.552118	0.21560772	0.35164626	0.16675885	-0.0384533
110.2	0.34510117	-0.5797244	-0.5522729	0.21530319	0.35142874	0.16682091	-0.0385835
110.3	0.34497828	-0.5795205	-0.5521794	0.21525972	0.35137631	0.1667672	-0.0383286
110.4	0.34494569	-0.5792796	-0.5522378	0.21528823	0.35148739	0.16655322	-0.0377769
110.5	0.34494569	-0.5792796	-0.5522378	0.21528823	0.35148739	0.16655322	-0.0377769
110.6	0.34494569	-0.5792796	-0.5522378	0.21528823	0.35148739	0.16655322	-0.0377769
110.7	0.34494569	-0.5792796	-0.5522378	0.21528823	0.35148739	0.16655322	-0.0377769
110.8	0.34494569	-0.5792796	-0.5522378	0.21528823	0.35148739	0.16655322	-0.0377769
110.9	0.34494569	-0.5792796	-0.5522378	0.21528823	0.35148739	0.16655322	-0.0377769
111	0.34468045	-0.5791088	-0.5522097	0.21523656	0.3516167	0.16620328	-0.037668
111.1	0.34483751	-0.5792199	-0.5522803	0.21520587	0.35191777	0.16631143	-0.0377279
111.2	0.34483751	-0.5792199	-0.5522803	0.21520587	0.35191777	0.16631143	-0.0377279
111.3	0.34477647	-0.5791083	-0.5524082	0.21491731	0.35168103	0.16627264	-0.0374625
111.4	0.34497744	-0.5788521	-0.5522202	0.21471394	0.35149511	0.16630288	-0.0378364
111.5	0.3447058	-0.5785596	-0.55204	0.21467866	0.35139574	0.1662156	-0.0375834
111.6	0.3447058	-0.5785596	-0.55204	0.21467866	0.35139574	0.1662156	-0.0375834
111.7	0.34465936	-0.5783019	-0.5521197	0.21465226	0.35127846	0.16573429	-0.0374508
111.8	0.34473725	-0.5783876	-0.5521387	0.21436076	0.35144146	0.1654705	-0.0372308
111.9	0.34473725	-0.5783876	-0.5521387	0.21436076	0.35144146	0.1654705	-0.0372308
112	0.34487784	-0.5783905	-0.5523612	0.21442545	0.35137961	0.16588291	-0.0371341
112.1	0.34487784	-0.5783905	-0.5523612	0.21442545	0.35137961	0.16588291	-0.0371341
112.2	0.34487784	-0.5783905	-0.5523612	0.21442545	0.35137961	0.16588291	-0.0371341
112.3	0.34487784	-0.5783905	-0.5523612	0.21442545	0.35137961	0.16588291	-0.0371341
112.4	0.34487784	-0.5783905	-0.5523612	0.21442545	0.35137961	0.16588291	-0.0371341
112.5	0.34487784	-0.5783905	-0.5523612	0.21442545	0.35137961	0.16588291	-0.0371341
112.6	0.34477025	-0.5782034	-0.552541	0.21411314	0.35127998	0.16577543	-0.0366292
112.7	0.34477025	-0.5782034	-0.552541	0.21411314	0.35127998	0.16577543	-0.0366292
112.8	0.34477025	-0.5782034	-0.552541	0.21411314	0.35127998	0.16577543	-0.0366292
112.9	0.34477025	-0.5782034	-0.552541	0.21411314	0.35127998	0.16577543	-0.0366292
113	0.34477025	-0.5782034	-0.552541	0.21411314	0.35127998	0.16577543	-0.0366292
113.1	0.34469043	-0.578095	-0.5527552	0.21385558	0.35101023	0.16601613	-0.0364016
113.2	0.34469043	-0.578095	-0.5527552	0.21385558	0.35101023	0.16601613	-0.0364016
113.3	0.34469043	-0.578095	-0.5527552	0.21385558	0.35101023	0.16601613	-0.0364016
113.4	0.34487095	-0.5781934	-0.5530699	0.21354387	0.35105836	0.16597405	-0.0362141
113.5	0.34462605	-0.5782728	-0.5533919	0.2137065	0.35117593	0.16587785	-0.0359334
113.6	0.34432117	-0.5780432	-0.5534919	0.21304956	0.35108804	0.16581116	-0.035464
113.7	0.34432117	-0.5780432	-0.5534919	0.21304956	0.35108804	0.16581116	-0.035464
113.8	0.34432117	-0.5780432	-0.5534919	0.21304956	0.35108804	0.16581116	-0.035464
113.9	0.34433129	-0.5781054	-0.5535579	0.21305577	0.35087252	0.1657627	-0.0355924

69	0.43872913	-0.6766693	-0.4988578	0.27521077	0.42862566	0.186288	-0.0161677
69.1	0.43872913	-0.6766693	-0.4988578	0.27521077	0.42862566	0.186288	-0.0161677
69.2	0.43767741	-0.675592	-0.4991768	0.27459162	0.42780508	0.18644195	-0.0163064
69.3	0.43728928	-0.6753962	-0.4998692	0.27394052	0.42759171	0.1869491	-0.0163322
69.4	0.43634821	-0.6749507	-0.5003275	0.27373804	0.42687059	0.18663585	-0.0158629
69.5	0.4356389	-0.6742581	-0.4999444	0.27308674	0.42690422	0.18673249	-0.0166142
69.6	0.43522379	-0.6740297	-0.5001749	0.27270015	0.42697795	0.18614165	-0.0165234
69.7	0.43415246	-0.6736894	-0.5001118	0.27233332	0.42656363	0.18558102	-0.0159564
69.8	0.43363724	-0.6734898	-0.5008028	0.27140521	0.42544036	0.18515091	-0.0162231
69.9	0.43336974	-0.67293	-0.5008973	0.27114997	0.42525229	0.18529398	-0.0165855
70	0.43286978	-0.6723638	-0.5006467	0.27099827	0.42476216	0.18521811	-0.0172732
70.1	0.43225743	-0.6718128	-0.5011228	0.27057879	0.42416943	0.1842589	-0.0173786
70.2	0.43224333	-0.6718159	-0.5019393	0.26996307	0.42388437	0.18371549	-0.017258
70.3	0.43146795	-0.6709181	-0.5024345	0.26903759	0.42336431	0.18272065	-0.0174727
70.4	0.43024078	-0.6703183	-0.5029712	0.2683011	0.42264699	0.18213168	-0.0179308
70.5	0.42990065	-0.6697768	-0.5033385	0.26795253	0.42266627	0.18143162	-0.0183937
70.6	0.42940279	-0.6691182	-0.5030422	0.26771631	0.42258561	0.1816562	-0.0186913
70.7	0.42940279	-0.6691182	-0.5030422	0.26771631	0.42258561	0.1816562	-0.0186913
70.8	0.42908989	-0.6688345	-0.5029664	0.26663362	0.42183564	0.18163377	-0.0185203
70.9	0.42833885	-0.6682534	-0.5028106	0.26635232	0.42180229	0.18161892	-0.0189975
71	0.42814116	-0.6682313	-0.5028765	0.26635382	0.42191655	0.1814449	-0.0188173
71.1	0.42761078	-0.6680022	-0.5037002	0.26567625	0.42183304	0.1813398	-0.0190584
71.2	0.42659176	-0.666966	-0.5039761	0.26448307	0.42119625	0.18166841	-0.0202247
71.3	0.4262251	-0.6668206	-0.5038528	0.26431958	0.42110424	0.18135169	-0.0202884
71.4	0.42561235	-0.666339	-0.5042465	0.26364485	0.42035872	0.18136253	-0.0202111
71.5	0.42471815	-0.6654354	-0.5043239	0.26343478	0.42005066	0.18093866	-0.0192836
71.6	0.42455147	-0.6653615	-0.5047152	0.26274023	0.41963294	0.18112826	-0.0199045
71.7	0.42373993	-0.6645678	-0.5046692	0.26211509	0.41923863	0.18031667	-0.020797
71.8	0.42374501	-0.6642569	-0.5046505	0.2618545	0.4189454	0.18066229	-0.0211063
71.9	0.4237639	-0.6640681	-0.5044994	0.26183804	0.41897372	0.18027303	-0.0210119
72	0.42319671	-0.6636713	-0.5046927	0.2619514	0.4185036	0.18028908	-0.0215781
72.1	0.42295676	-0.663575	-0.5047376	0.26194856	0.4184638	0.18023972	-0.0216885
72.2	0.42263878	-0.66271	-0.5043708	0.26146923	0.41807622	0.18046604	-0.021538
72.3	0.42157349	-0.6618923	-0.5041999	0.26034088	0.41755807	0.17978635	-0.0215862
72.4	0.42157349	-0.6618923	-0.5041999	0.26034088	0.41755807	0.17978635	-0.0215862
72.5	0.42126333	-0.6618302	-0.5041841	0.26012995	0.41758095	0.17990738	-0.021194
72.6	0.42078689	-0.6613889	-0.5043165	0.25989826	0.41697606	0.17969784	-0.0220855
72.7	0.42055798	-0.6607465	-0.5047625	0.25991118	0.41659448	0.1796453	-0.0223317
72.8	0.42043544	-0.6602832	-0.5045572	0.25997654	0.41659809	0.17965013	-0.0220394
72.9	0.41979271	-0.6597712	-0.5045711	0.25960359	0.41663678	0.17971697	-0.0220164
73	0.41964643	-0.6594414	-0.5043964	0.25933898	0.4164251	0.17971076	-0.0222021
73.1	0.41964643	-0.6594414	-0.5043964	0.25933898	0.4164251	0.17971076	-0.0222021
73.2	0.41847144	-0.658344	-0.5044546	0.25864562	0.41567622	0.17828179	-0.0222621
73.3	0.41737573	-0.6572328	-0.5043418	0.25842224	0.41488211	0.17738737	-0.0223747
73.4	0.41675168	-0.6565934	-0.5040807	0.2583285	0.4143688	0.17638477	-0.0233114
73.5	0.41624496	-0.6563457	-0.5039196	0.25823577	0.41429963	0.17633273	-0.0228413
73.6	0.41589805	-0.655957	-0.5043214	0.25793641	0.41408176	0.1753595	-0.0221246
73.7	0.41539936	-0.6558338	-0.5044262	0.25802857	0.41390517	0.17496116	-0.021728
73.8	0.41539936	-0.6558338	-0.5044262	0.25802857	0.41390517	0.17496116	-0.021728
73.9	0.4149976	-0.6554902	-0.5046373	0.25800081	0.41351033	0.17536289	-0.0212475
74	0.41463972	-0.6551586	-0.5052603	0.25742639	0.41275427	0.1742137	-0.0211339
74.1	0.41441735	-0.6550874	-0.5054316	0.25738222	0.41276645	0.17383158	-0.0208208
74.2	0.41448718	-0.6548768	-0.505943	0.25697524	0.41244168	0.17362111	-0.0214151
74.3	0.4135365	-0.6544096	-0.5063793	0.25586922	0.41227225	0.17368596	-0.021777
74.4	0.41365381	-0.6540584	-0.5070949	0.25511128	0.41187842	0.1738703	-0.0228471
74.5	0.41361606	-0.6541131	-0.5073563	0.25511804	0.41165858	0.17361369	-0.0230191
74.6	0.41325108	-0.6540211	-0.5076412	0.25467336	0.41090392	0.17336204	-0.0229314
74.7	0.4118997	-0.6530464	-0.5077922	0.2545022	0.41015119	0.17279958	-0.0235517
74.8	0.41173132	-0.6523669	-0.5079048	0.25405939	0.40925125	0.17275376	-0.0243764
74.9	0.41217042	-0.6517797	-0.5085441	0.25391559	0.40939571	0.17268092	-0.0246992

120	0.34541204	-0.5741403	-0.5549379	0.21032486	0.34756178	0.16532754	-0.0345592
120.1	0.34537632	-0.5741133	-0.5552195	0.21033795	0.34769442	0.16539583	-0.0343368
120.2	0.34514313	-0.5739191	-0.5553843	0.2100453	0.3474777	0.16526287	-0.0341973
120.3	0.3455422	-0.5740162	-0.5555907	0.20996066	0.34785505	0.16507227	-0.033869
120.4	0.34547088	-0.5739382	-0.5556733	0.21011123	0.34790398	0.16477072	-0.0338628
120.5	0.34564446	-0.5741167	-0.555935	0.21025599	0.34815678	0.16489602	-0.0343104
120.6	0.34571451	-0.5736894	-0.5557426	0.21013797	0.34812294	0.1648627	-0.0344774
120.7	0.34571451	-0.5736894	-0.5557426	0.21013797	0.34812294	0.1648627	-0.0344774
120.8	0.34571451	-0.5736894	-0.5557426	0.21013797	0.34812294	0.1648627	-0.0344774
120.9	0.34566311	-0.5736971	-0.5559788	0.20980495	0.34782341	0.16490714	-0.0345279
121	0.34580311	-0.5737323	-0.556084	0.21001192	0.34756639	0.16522888	-0.0346111
121.1	0.34580311	-0.5737323	-0.556084	0.21001192	0.34756639	0.16522888	-0.0346111
121.2	0.34580311	-0.5737323	-0.556084	0.21001192	0.34756639	0.16522888	-0.0346111
121.3	0.3453239	-0.5733617	-0.5560408	0.20979269	0.34763784	0.16525138	-0.0341846
121.4	0.3453239	-0.5733617	-0.5560408	0.20979269	0.34763784	0.16525138	-0.0341846
121.5	0.3453239	-0.5733617	-0.5560408	0.20979269	0.34763784	0.16525138	-0.0341846
121.6	0.3453239	-0.5733617	-0.5560408	0.20979269	0.34763784	0.16525138	-0.0341846
121.7	0.3453239	-0.5733617	-0.5560408	0.20979269	0.34763784	0.16525138	-0.0341846
121.8	0.34538555	-0.5734788	-0.5561583	0.20937325	0.3476471	0.16506885	-0.034356
121.9	0.34538555	-0.5734788	-0.5561583	0.20937325	0.3476471	0.16506885	-0.034356
122	0.34538555	-0.5734788	-0.5561583	0.20937325	0.3476471	0.16506885	-0.034356
122.1	0.3454685	-0.5736473	-0.5562579	0.20932027	0.34775949	0.16499048	-0.0341229
122.2	0.3454685	-0.5736473	-0.5562579	0.20932027	0.34775949	0.16499048	-0.0341229
122.3	0.3454685	-0.5736473	-0.5562579	0.20932027	0.34775949	0.16499048	-0.0341229
122.4	0.34572429	-0.5734813	-0.5563473	0.2085752	0.34750235	0.16531515	-0.0342938
122.5	0.34572429	-0.5734813	-0.5563473	0.2085752	0.34750235	0.16531515	-0.0342938
122.6	0.34572429	-0.5734813	-0.5563473	0.2085752	0.34750235	0.16531515	-0.0342938
122.7	0.34549919	-0.5731041	-0.5565424	0.20828965	0.34724816	0.16560839	-0.0340108
122.8	0.34561468	-0.5730406	-0.5568479	0.20822659	0.34724415	0.16547725	-0.0337691
122.9	0.34561468	-0.5730406	-0.5568479	0.20822659	0.34724415	0.16547725	-0.0337691
123	0.3452544	-0.5728122	-0.5567555	0.20808409	0.34700287	0.16543083	-0.0332961
123.1	0.34511944	-0.5727968	-0.5569615	0.20780768	0.34660716	0.16572935	-0.0331007
123.2	0.34511944	-0.5727968	-0.5569615	0.20780768	0.34660716	0.16572935	-0.0331007
123.3	0.34511944	-0.5727968	-0.5569615	0.20780768	0.34660716	0.16572935	-0.0331007
123.4	0.34511944	-0.5727968	-0.5569615	0.20780768	0.34660716	0.16572935	-0.0331007
123.5	0.34511944	-0.5727968	-0.5569615	0.20780768	0.34660716	0.16572935	-0.0331007
123.6	0.34492954	-0.5729978	-0.5570473	0.2077558	0.34679207	0.16592482	-0.0327423
123.7	0.34516603	-0.5729848	-0.5572558	0.20747988	0.34655296	0.16604539	-0.0327444
123.8	0.34516603	-0.5729848	-0.5572558	0.20747988	0.34655296	0.16604539	-0.0327444
123.9	0.34516603	-0.5729848	-0.5572558	0.20747988	0.34655296	0.16604539	-0.0327444
124	0.34516603	-0.5729848	-0.5572558	0.20747988	0.34655296	0.16604539	-0.0327444
124.1	0.34516603	-0.5729848	-0.5572558	0.20747988	0.34655296	0.16604539	-0.0327444
124.2	0.34532594	-0.5731764	-0.5574042	0.20768589	0.34680132	0.16633699	-0.0327895
124.3	0.34571916	-0.5730531	-0.5574636	0.20756196	0.34658521	0.16635851	-0.0330923
124.4	0.34571916	-0.5730531	-0.5574636	0.20756196	0.34658521	0.16635851	-0.0330923
124.5	0.34578027	-0.5730119	-0.5577326	0.20723592	0.34654242	0.16613658	-0.0329694
124.6	0.34578027	-0.5730119	-0.5577326	0.20723592	0.34654242	0.16613658	-0.0329694
124.7	0.34578027	-0.5730119	-0.5577326	0.20723592	0.34654242	0.16613658	-0.0329694
124.8	0.34608451	-0.5728906	-0.5576829	0.20674773	0.34638207	0.16601028	-0.0331266
124.9	0.34608451	-0.5728906	-0.5576829	0.20674773	0.34638207	0.16601028	-0.0331266
125	0.34608451	-0.5728906	-0.5576829	0.20674773	0.34638207	0.16601028	-0.0331266
125.1	0.34618037	-0.5728757	-0.5576707	0.20640851	0.34620593	0.16571965	-0.0332261
125.2	0.34618037	-0.5728757	-0.5576707	0.20640851	0.34620593	0.16571965	-0.0332261
125.3	0.34592146	-0.5722236	-0.5575179	0.20609008	0.3458929	0.16580488	-0.032359
125.4	0.34592146	-0.5722236	-0.5575179	0.20609008	0.3458929	0.16580488	-0.032359
125.5	0.34592146	-0.5722236	-0.5575179	0.20609008	0.3458929	0.16580488	-0.032359
125.6	0.34600073	-0.5722497	-0.5575842	0.206106	0.34605642	0.16592646	-0.0325972
125.7	0.34600073	-0.5722497	-0.5575842	0.206106	0.34605642	0.16592646	-0.0325972
125.8	0.34600073	-0.5722497	-0.5575842	0.206106	0.34605642	0.16592646	-0.0325972
125.9	0.34623173	-0.5721496	-0.5577022	0.20558012	0.34582479	0.1661196	-0.0325679
126	0.34623173	-0.5721496	-0.5577022	0.20558012	0.34582479	0.1661196	-0.0325679

84	0.3890857	-0.6293177	-0.5237009	0.23542683	0.38576863	0.16860906	-0.0322592
84.1	0.38872643	-0.628977	-0.52394	0.23489144	0.38495932	0.1687027	-0.0327313
84.2	0.38872202	-0.6288187	-0.5239402	0.23529471	0.38499169	0.16852959	-0.0327143
84.3	0.38841939	-0.6282641	-0.5246281	0.2352169	0.38497284	0.16824147	-0.0331361
84.4	0.38840373	-0.6281455	-0.5245953	0.23504333	0.38463946	0.16850258	-0.0332504
84.5	0.38813179	-0.6277838	-0.5248709	0.23514351	0.38402699	0.16853345	-0.0334512
84.6	0.38795082	-0.6277034	-0.5253525	0.23492017	0.38405914	0.16864085	-0.0330326
84.7	0.38780784	-0.6275204	-0.525536	0.23486387	0.38387079	0.16854029	-0.0327242
84.8	0.3869942	-0.6267486	-0.5254443	0.23366288	0.38275754	0.16814665	-0.0329504
84.9	0.38697291	-0.6267146	-0.5258235	0.2338391	0.38298375	0.16809005	-0.0334675
85	0.38704955	-0.6266246	-0.5259895	0.23381326	0.38289586	0.16800041	-0.0333759
85.1	0.38641408	-0.6264147	-0.5258879	0.2336891	0.38238397	0.16747697	-0.0333266
85.2	0.38654576	-0.6258842	-0.5260517	0.23371652	0.3817655	0.16762406	-0.0337646
85.3	0.38612633	-0.6256133	-0.5264089	0.23317168	0.3815145	0.16720005	-0.033596
85.4	0.3855843	-0.6253879	-0.5265987	0.23285877	0.3811023	0.16778333	-0.0340057
85.5	0.38477436	-0.6248797	-0.5264825	0.23247606	0.38091111	0.16837935	-0.0340024
85.6	0.38462435	-0.6247043	-0.5264978	0.23270899	0.38076366	0.16863638	-0.0342755
85.7	0.38424548	-0.6242286	-0.5264261	0.23220228	0.38017152	0.16883647	-0.0349557
85.8	0.38404706	-0.6239698	-0.5263824	0.23213522	0.37989269	0.16880577	-0.0352381
85.9	0.38367718	-0.6231466	-0.526452	0.23187384	0.37927914	0.16892145	-0.0352343
86	0.38280119	-0.6220821	-0.5269029	0.23107961	0.37808492	0.16883618	-0.0353372
86.1	0.38212076	-0.6216778	-0.5274689	0.23120152	0.37795394	0.16870757	-0.0359324
86.2	0.38217861	-0.6215644	-0.5276771	0.23146772	0.37828855	0.16862035	-0.0359022
86.3	0.38146354	-0.6203559	-0.5276415	0.23190098	0.37776857	0.16836444	-0.0364727
86.4	0.3813008	-0.6202083	-0.5275729	0.2314903	0.37716611	0.16808582	-0.0364501
86.5	0.38131318	-0.6200052	-0.5274811	0.23144449	0.37704278	0.16807026	-0.0367382
86.6	0.38119584	-0.6198511	-0.5277824	0.23173972	0.37681271	0.16841063	-0.0369054
86.7	0.38113294	-0.6198661	-0.5277375	0.23146707	0.37674203	0.16847541	-0.036944
86.8	0.38104587	-0.6196198	-0.527909	0.23111473	0.3764296	0.16839194	-0.0369252
86.9	0.38114292	-0.6194118	-0.5279251	0.23143602	0.37624759	0.16852507	-0.0373625
87	0.38078459	-0.6191975	-0.5284642	0.23166784	0.37617253	0.16811554	-0.0371329
87.1	0.38011602	-0.6187368	-0.5286369	0.23089452	0.37589537	0.1679234	-0.0370924
87.2	0.38011602	-0.6187368	-0.5286369	0.23089452	0.37589537	0.1679234	-0.0370924
87.3	0.37937391	-0.6180576	-0.5291489	0.22972023	0.37495376	0.16669965	-0.0357399
87.4	0.37931395	-0.6178727	-0.5292324	0.22963596	0.37475849	0.16701055	-0.0358356
87.5	0.3788243	-0.6174171	-0.5292895	0.22939936	0.37432483	0.16702218	-0.0360163
87.6	0.3785416	-0.6165203	-0.5292938	0.22909893	0.37381615	0.16609526	-0.0368496
87.7	0.37803203	-0.6162023	-0.5295505	0.22870384	0.37327564	0.16639965	-0.0369818
87.8	0.37756064	-0.616047	-0.5300923	0.22884321	0.37335781	0.16643658	-0.0371136
87.9	0.37702339	-0.6158089	-0.5305362	0.2287667	0.37297989	0.16695282	-0.0374904
88	0.37644093	-0.6155721	-0.5304909	0.22868254	0.37262127	0.16662602	-0.0373683
88.1	0.37635803	-0.6154525	-0.5311951	0.22820649	0.37234028	0.16714933	-0.037422
88.2	0.37635803	-0.6154525	-0.5311951	0.22820649	0.37234028	0.16714933	-0.037422
88.3	0.37596994	-0.6148327	-0.5308492	0.22732152	0.37169588	0.16741704	-0.0379844
88.4	0.37531288	-0.614759	-0.5311428	0.22748226	0.37111041	0.16795602	-0.0377518
88.5	0.37531288	-0.614759	-0.5311428	0.22748226	0.37111041	0.16795602	-0.0377518
88.6	0.37537663	-0.6145818	-0.5312219	0.22748459	0.370935	0.16811632	-0.0374146
88.7	0.37554628	-0.6142748	-0.5312728	0.22720995	0.37065498	0.16838028	-0.0377153
88.8	0.3749922	-0.6139745	-0.5317875	0.22754666	0.37054102	0.16845159	-0.0383683
88.9	0.37441285	-0.6135497	-0.532022	0.22660835	0.36972991	0.16848979	-0.0382464
89	0.37395844	-0.6134639	-0.5322973	0.22648933	0.37012415	0.16839152	-0.0379293
89.1	0.37399496	-0.6132647	-0.5326056	0.2268057	0.36965934	0.16859287	-0.0381433
89.2	0.37298123	-0.6124808	-0.5324373	0.22638695	0.36851032	0.16875343	-0.0382698
89.3	0.37238867	-0.6123054	-0.5323982	0.22637241	0.3683568	0.16870619	-0.0383715
89.4	0.37238867	-0.6123054	-0.5323982	0.22637241	0.3683568	0.16870619	-0.0383715
89.5	0.37201181	-0.612034	-0.532666	0.22640044	0.36826367	0.16859804	-0.038536
89.6	0.37165282	-0.6115384	-0.5329807	0.22615641	0.36765953	0.16890417	-0.0389756
89.7	0.37113576	-0.6112883	-0.5332114	0.2266173	0.36739121	0.16921103	-0.0395183
89.8	0.37096184	-0.6112371	-0.5332818	0.22677039	0.36705725	0.16930465	-0.0397549
89.9	0.37082628	-0.6110311	-0.5332237	0.2267976	0.36682936	0.16937664	-0.0394993

135	0.34772713	-0.5700837	-0.5620884	0.19624985	0.33947577	0.16810029	-0.0327184
135.1	0.34771661	-0.5699802	-0.5621119	0.19663969	0.33996773	0.16817736	-0.0331025
135.2	0.34771661	-0.5699802	-0.5621119	0.19663969	0.33996773	0.16817736	-0.0331025
135.3	0.34774472	-0.5699442	-0.5621306	0.1965548	0.34013394	0.1684048	-0.0333317
135.4	0.34763412	-0.5696153	-0.5624439	0.19629488	0.33976127	0.16797095	-0.0329149
135.5	0.34763412	-0.5696153	-0.5624439	0.19629488	0.33976127	0.16797095	-0.0329149
135.6	0.34786625	-0.5694925	-0.5624133	0.19646647	0.33966732	0.16808583	-0.0330831
135.7	0.34819647	-0.5692326	-0.5625601	0.19609464	0.33970656	0.16837917	-0.0334457
135.8	0.34819647	-0.5692326	-0.5625601	0.19609464	0.33970656	0.16837917	-0.0334457
135.9	0.34819872	-0.569256	-0.5626737	0.19606158	0.33953691	0.16854138	-0.033633
136	0.34819872	-0.569256	-0.5626737	0.19606158	0.33953691	0.16854138	-0.033633
136.1	0.34818022	-0.5692078	-0.5627221	0.19615653	0.33951758	0.16842533	-0.0336599
136.2	0.34818022	-0.5692078	-0.5627221	0.19615653	0.33951758	0.16842533	-0.0336599
136.3	0.34820381	-0.5691754	-0.56285	0.19566617	0.33941321	0.16857165	-0.033623
136.4	0.34820381	-0.5691754	-0.56285	0.19566617	0.33941321	0.16857165	-0.033623
136.5	0.34820381	-0.5691754	-0.56285	0.19566617	0.33941321	0.16857165	-0.033623
136.6	0.34820381	-0.5691754	-0.56285	0.19566617	0.33941321	0.16857165	-0.033623
136.7	0.34842028	-0.5692688	-0.5632298	0.19549425	0.33909812	0.16835335	-0.0337438
136.8	0.34842028	-0.5692688	-0.5632298	0.19549425	0.33909812	0.16835335	-0.0337438
136.9	0.34842028	-0.5692688	-0.5632298	0.19549425	0.33909812	0.16835335	-0.0337438
137	0.34841324	-0.5688986	-0.563184	0.19525242	0.3387105	0.16811509	-0.0336004
137.1	0.34841324	-0.5688986	-0.563184	0.19525242	0.3387105	0.16811509	-0.0336004
137.2	0.3484326	-0.5687089	-0.5632157	0.19536039	0.33864902	0.16816377	-0.0338031
137.3	0.3484326	-0.5687089	-0.5632157	0.19536039	0.33864902	0.16816377	-0.0338031
137.4	0.3484326	-0.5687089	-0.5632157	0.19536039	0.33864902	0.16816377	-0.0338031
137.5	0.34860642	-0.5687468	-0.5632904	0.19503529	0.33839972	0.16829133	-0.0337631
137.6	0.34874914	-0.5684317	-0.5633206	0.1950801	0.33831482	0.16827368	-0.0338703
137.7	0.34868318	-0.5682824	-0.5632681	0.19457174	0.33820443	0.16821131	-0.0336691
137.8	0.34864577	-0.5680138	-0.56325	0.19398255	0.33773861	0.16806697	-0.0337255
137.9	0.34864577	-0.5680138	-0.56325	0.19398255	0.33773861	0.16806697	-0.0337255
138	0.34864577	-0.5680138	-0.56325	0.19398255	0.33773861	0.16806697	-0.0337255
138.1	0.34864577	-0.5680138	-0.56325	0.19398255	0.33773861	0.16806697	-0.0337255
138.2	0.34864577	-0.5680138	-0.56325	0.19398255	0.33773861	0.16806697	-0.0337255
138.3	0.34864577	-0.5680138	-0.56325	0.19398255	0.33773861	0.16806697	-0.0337255
138.4	0.34864577	-0.5680138	-0.56325	0.19398255	0.33773861	0.16806697	-0.0337255
138.5	0.34864577	-0.5680138	-0.56325	0.19398255	0.33773861	0.16806697	-0.0337255
138.6	0.3489951	-0.5679377	-0.5634254	0.19375988	0.33760757	0.16794539	-0.0338251
138.7	0.34936358	-0.5675116	-0.5635551	0.19358805	0.33714678	0.1682439	-0.0339608
138.8	0.34963022	-0.5673233	-0.5637648	0.19348271	0.33714108	0.16826843	-0.0340015
138.9	0.34963022	-0.5673233	-0.5637648	0.19348271	0.33714108	0.16826843	-0.0340015
139	0.34963022	-0.5673233	-0.5637648	0.19348271	0.33714108	0.16826843	-0.0340015
139.1	0.34963022	-0.5673233	-0.5637648	0.19348271	0.33714108	0.16826843	-0.0340015
139.2	0.34963022	-0.5673233	-0.5637648	0.19348271	0.33714108	0.16826843	-0.0340015
139.3	0.34955346	-0.5671691	-0.5638293	0.19317271	0.33710967	0.16820589	-0.033835
139.4	0.34955346	-0.5671691	-0.5638293	0.19317271	0.33710967	0.16820589	-0.033835
139.5	0.34955346	-0.5671691	-0.5638293	0.19317271	0.33710967	0.16820589	-0.033835
139.6	0.34955346	-0.5671691	-0.5638293	0.19317271	0.33710967	0.16820589	-0.033835
139.7	0.34955346	-0.5671691	-0.5638293	0.19317271	0.33710967	0.16820589	-0.033835
139.8	0.34955346	-0.5671691	-0.5638293	0.19317271	0.33710967	0.16820589	-0.033835
139.9	0.34963636	-0.5671198	-0.5639638	0.19249204	0.33664502	0.16825654	-0.0339389
140	0.34973143	-0.5667489	-0.564082	0.19198522	0.33622815	0.16788227	-0.0340889
140.1	0.35014624	-0.5665358	-0.5643726	0.19134791	0.3357603	0.1679658	-0.0340693
140.2	0.35014099	-0.5663354	-0.5644976	0.19090242	0.33538541	0.16779558	-0.0341444
140.3	0.35033483	-0.5662559	-0.564915	0.19054977	0.33519534	0.16721898	-0.033933
140.4	0.35043933	-0.5661974	-0.5652209	0.1903046	0.33507849	0.16694707	-0.034248
140.5	0.35043933	-0.5661974	-0.5652209	0.1903046	0.33507849	0.16694707	-0.034248
140.6	0.35043933	-0.5661974	-0.5652209	0.1903046	0.33507849	0.16694707	-0.034248
140.7	0.3503839	-0.5662364	-0.5656922	0.19046031	0.33539101	0.16737726	-0.034528
140.8	0.3503839	-0.5662364	-0.5656922	0.19046031	0.33539101	0.16737726	-0.034528
140.9	0.35071374	-0.5656321	-0.5659042	0.19028913	0.33509968	0.16715929	-0.0346344
141	0.35071374	-0.5656321	-0.5659042	0.19028913	0.33509968	0.16715929	-0.0346344

99	0.3500202	-0.5897356	-0.5470789	0.22369462	0.35808543	0.16697827	-0.0432422
99.1	0.34971448	-0.5895441	-0.5473097	0.22366117	0.35778741	0.16686267	-0.0435653
99.2	0.34980638	-0.5894938	-0.5472745	0.22376529	0.35800326	0.16702183	-0.0439642
99.3	0.34992397	-0.5893959	-0.5474889	0.22362816	0.35804177	0.16695094	-0.0437924
99.4	0.34992397	-0.5893959	-0.5474889	0.22362816	0.35804177	0.16695094	-0.0437924
99.5	0.34992397	-0.5893959	-0.5474889	0.22362816	0.35804177	0.16695094	-0.0437924
99.6	0.34883922	-0.5884787	-0.5475071	0.22295526	0.35760678	0.16630612	-0.0431719
99.7	0.34840389	-0.5882498	-0.547474	0.22306809	0.35728124	0.16627742	-0.0431557
99.8	0.34808104	-0.5879757	-0.5476188	0.22274713	0.35717071	0.16596058	-0.0426202
99.9	0.34808104	-0.5879757	-0.5476188	0.22274713	0.35717071	0.16596058	-0.0426202
100	0.3482031	-0.5880085	-0.5476302	0.2227056	0.35700943	0.16616322	-0.0426334
100.1	0.34801766	-0.5876042	-0.5480412	0.22226786	0.35647549	0.16630824	-0.0432173
100.2	0.34817384	-0.5874358	-0.54819	0.22199718	0.35648693	0.16629451	-0.0432668
100.3	0.34817384	-0.5874358	-0.54819	0.22199718	0.35648693	0.16629451	-0.0432668
100.4	0.34815678	-0.587061	-0.5481832	0.22190416	0.35659986	0.16660311	-0.0433621
100.5	0.34815678	-0.587061	-0.5481832	0.22190416	0.35659986	0.16660311	-0.0433621
100.6	0.34808992	-0.58651	-0.5482382	0.22212486	0.3570555	0.16662972	-0.0441982
100.7	0.34787466	-0.5864657	-0.5487214	0.22167622	0.35697759	0.16632362	-0.0442201
100.8	0.34792224	-0.5865013	-0.5490336	0.22162426	0.35674454	0.16610825	-0.0444441
100.9	0.34792224	-0.5865013	-0.5490336	0.22162426	0.35674454	0.16610825	-0.0444441
101	0.34792224	-0.5865013	-0.5490336	0.22162426	0.35674454	0.16610825	-0.0444441
101.1	0.34790421	-0.5867393	-0.5494561	0.22127424	0.3569856	0.1658909	-0.0443095
101.2	0.34707584	-0.5858451	-0.5494186	0.22121479	0.35649783	0.16581904	-0.0442289
101.3	0.34697983	-0.5855996	-0.5494815	0.2212087	0.35628678	0.16617646	-0.0440133
101.4	0.34662406	-0.5854561	-0.5494683	0.22103107	0.35612571	0.16588325	-0.0437761
101.5	0.34662406	-0.5854561	-0.5494683	0.22103107	0.35612571	0.16588325	-0.0437761
101.6	0.34662406	-0.5854561	-0.5494683	0.22103107	0.35612571	0.16588325	-0.0437761
101.7	0.34662985	-0.5854618	-0.5497703	0.22105214	0.35594135	0.16575785	-0.0435603
101.8	0.34614891	-0.5849947	-0.5499265	0.22090171	0.35586957	0.16571391	-0.0437427
101.9	0.34578473	-0.5846871	-0.5498455	0.22099526	0.3556507	0.16565841	-0.0438053
102	0.34578473	-0.5846871	-0.5498455	0.22099526	0.3556507	0.16565841	-0.0438053
102.1	0.34578473	-0.5846871	-0.5498455	0.22099526	0.3556507	0.16565841	-0.0438053
102.2	0.34573261	-0.5845846	-0.5501128	0.22085133	0.35544762	0.16548066	-0.0438546
102.3	0.345948	-0.584381	-0.55001	0.22046643	0.35520806	0.16555123	-0.0438427
102.4	0.34587715	-0.5843918	-0.5501721	0.22077997	0.35551134	0.16549595	-0.0439947
102.5	0.34587715	-0.5843918	-0.5501721	0.22077997	0.35551134	0.16549595	-0.0439947
102.6	0.34593663	-0.5841236	-0.5504689	0.22046011	0.35508777	0.16527476	-0.0438183
102.7	0.34593663	-0.5841236	-0.5504689	0.22046011	0.35508777	0.16527476	-0.0438183
102.8	0.34593663	-0.5841236	-0.5504689	0.22046011	0.35508777	0.16527476	-0.0438183
102.9	0.34569447	-0.5836509	-0.5504793	0.2200744	0.35493921	0.16487094	-0.0434243
103	0.34593944	-0.5837837	-0.5505322	0.22016441	0.35508964	0.16465374	-0.0438636
103.1	0.34590109	-0.5837296	-0.5505169	0.220026	0.35496491	0.16487815	-0.0434832
103.2	0.34590109	-0.5837296	-0.5505169	0.220026	0.35496491	0.16487815	-0.0434832
103.3	0.34590109	-0.5837296	-0.5505169	0.220026	0.35496491	0.16487815	-0.0434832
103.4	0.34590109	-0.5837296	-0.5505169	0.220026	0.35496491	0.16487815	-0.0434832
103.5	0.345859	-0.5837279	-0.5506417	0.22026152	0.35512281	0.16531675	-0.0432816
103.6	0.34586485	-0.5839456	-0.550703	0.22016964	0.35503609	0.16520496	-0.0430103
103.7	0.34557927	-0.5837228	-0.5506413	0.21997215	0.3549806	0.16488489	-0.0428525
103.8	0.34557927	-0.5837228	-0.5506413	0.21997215	0.3549806	0.16488489	-0.0428525
103.9	0.34533271	-0.5836984	-0.5505802	0.21982875	0.35496949	0.16477607	-0.0424319
104	0.34545998	-0.583666	-0.5505175	0.21960354	0.35467936	0.16496061	-0.0427208
104.1	0.34517721	-0.5834262	-0.5503687	0.21927931	0.35468845	0.16499213	-0.0421771
104.2	0.34534201	-0.5834038	-0.5504001	0.21913593	0.35433443	0.16530793	-0.0422116
104.3	0.34508451	-0.5830409	-0.5502837	0.21872543	0.3539854	0.16525468	-0.0419261
104.4	0.34511442	-0.583165	-0.5504398	0.21821256	0.35392975	0.16504274	-0.0417533
104.5	0.34520335	-0.5831638	-0.55055	0.21857722	0.35387479	0.16519119	-0.0419005
104.6	0.34507544	-0.5831829	-0.5505607	0.21868384	0.35353082	0.16530698	-0.0419376
104.7	0.34507544	-0.5831829	-0.5505607	0.21868384	0.35353082	0.16530698	-0.0419376
104.8	0.34507544	-0.5831829	-0.5505607	0.21868384	0.35353082	0.16530698	-0.0419376
104.9	0.34507544	-0.5831829	-0.5505607	0.21868384	0.35353082	0.16530698	-0.0419376

150	0.35166635	-0.561298	-0.5694872	0.18327496	0.33092054	0.16575713	-0.035319
150.1	0.35176759	-0.5606606	-0.5693749	0.18346973	0.33065205	0.16545543	-0.0352754
150.2	0.35176759	-0.5606606	-0.5693749	0.18346973	0.33065205	0.16545543	-0.0352754
150.3	0.35176759	-0.5606606	-0.5693749	0.18346973	0.33065205	0.16545543	-0.0352754
150.4	0.35176759	-0.5606606	-0.5693749	0.18346973	0.33065205	0.16545543	-0.0352754
150.5	0.35176759	-0.5606606	-0.5693749	0.18346973	0.33065205	0.16545543	-0.0352754
150.6	0.35176759	-0.5606606	-0.5693749	0.18346973	0.33065205	0.16545543	-0.0352754
150.7	0.35176759	-0.5606606	-0.5693749	0.18346973	0.33065205	0.16545543	-0.0352754
150.8	0.35176759	-0.5606606	-0.5693749	0.18346973	0.33065205	0.16545543	-0.0352754
150.9	0.35176759	-0.5606606	-0.5693749	0.18346973	0.33065205	0.16545543	-0.0352754
151	0.35176759	-0.5606606	-0.5693749	0.18346973	0.33065205	0.16545543	-0.0352754
151.1	0.35178637	-0.5604467	-0.569297	0.1836126	0.33076295	0.16534333	-0.0352909
151.2	0.35178637	-0.5604467	-0.569297	0.1836126	0.33076295	0.16534333	-0.0352909
151.3	0.35178637	-0.5604467	-0.569297	0.1836126	0.33076295	0.16534333	-0.0352909
151.4	0.35178637	-0.5604467	-0.569297	0.1836126	0.33076295	0.16534333	-0.0352909
151.5	0.35178637	-0.5604467	-0.569297	0.1836126	0.33076295	0.16534333	-0.0352909
151.6	0.35178637	-0.5604467	-0.569297	0.1836126	0.33076295	0.16534333	-0.0352909
151.7	0.35192538	-0.5602778	-0.5694269	0.18392715	0.33105063	0.16544199	-0.0353017
151.8	0.35192538	-0.5602778	-0.5694269	0.18392715	0.33105063	0.16544199	-0.0353017
151.9	0.35192538	-0.5602778	-0.5694269	0.18392715	0.33105063	0.16544199	-0.0353017
152	0.35192538	-0.5602778	-0.5694269	0.18392715	0.33105063	0.16544199	-0.0353017
152.1	0.35192538	-0.5602778	-0.5694269	0.18392715	0.33105063	0.16544199	-0.0353017
152.2	0.35192538	-0.5602778	-0.5694269	0.18392715	0.33105063	0.16544199	-0.0353017
152.3	0.35192538	-0.5602778	-0.5694269	0.18392715	0.33105063	0.16544199	-0.0353017
152.4	0.35192538	-0.5602778	-0.5694269	0.18392715	0.33105063	0.16544199	-0.0353017
152.5	0.35192538	-0.5602778	-0.5694269	0.18392715	0.33105063	0.16544199	-0.0353017
152.6	0.35192538	-0.5602778	-0.5694269	0.18392715	0.33105063	0.16544199	-0.0353017
152.7	0.35192538	-0.5602778	-0.5694269	0.18392715	0.33105063	0.16544199	-0.0353017
152.8	0.35192538	-0.5602778	-0.5694269	0.18392715	0.33105063	0.16544199	-0.0353017
152.9	0.35192538	-0.5602778	-0.5694269	0.18392715	0.33105063	0.16544199	-0.0353017
153	0.35192538	-0.5602778	-0.5694269	0.18392715	0.33105063	0.16544199	-0.0353017
153.1	0.35192538	-0.5602778	-0.5694269	0.18392715	0.33105063	0.16544199	-0.0353017
153.2	0.35192538	-0.5602778	-0.5694269	0.18392715	0.33105063	0.16544199	-0.0353017
153.3	0.35192538	-0.5602778	-0.5694269	0.18392715	0.33105063	0.16544199	-0.0353017
153.4	0.35192538	-0.5602778	-0.5694269	0.18392715	0.33105063	0.16544199	-0.0353017
153.5	0.35177724	-0.5602729	-0.5694352	0.18380023	0.33108382	0.16580612	-0.0349964
153.6	0.35177724	-0.5602729	-0.5694352	0.18380023	0.33108382	0.16580612	-0.0349964
153.7	0.35177724	-0.5602729	-0.5694352	0.18380023	0.33108382	0.16580612	-0.0349964
153.8	0.35177724	-0.5602729	-0.5694352	0.18380023	0.33108382	0.16580612	-0.0349964
153.9	0.35177724	-0.5602729	-0.5694352	0.18380023	0.33108382	0.16580612	-0.0349964
154	0.35177724	-0.5602729	-0.5694352	0.18380023	0.33108382	0.16580612	-0.0349964
154.1	0.35177724	-0.5602729	-0.5694352	0.18380023	0.33108382	0.16580612	-0.0349964
154.2	0.35177724	-0.5602729	-0.5694352	0.18380023	0.33108382	0.16580612	-0.0349964
154.3	0.35177724	-0.5602729	-0.5694352	0.18380023	0.33108382	0.16580612	-0.0349964
154.4	0.35177724	-0.5602729	-0.5694352	0.18380023	0.33108382	0.16580612	-0.0349964
154.5	0.35177724	-0.5602729	-0.5694352	0.18380023	0.33108382	0.16580612	-0.0349964
154.6	0.35177724	-0.5602729	-0.5694352	0.18380023	0.33108382	0.16580612	-0.0349964
154.7	0.35177724	-0.5602729	-0.5694352	0.18380023	0.33108382	0.16580612	-0.0349964
154.8	0.35177724	-0.5602729	-0.5694352	0.18380023	0.33108382	0.16580612	-0.0349964
154.9	0.35177724	-0.5602729	-0.5694352	0.18380023	0.33108382	0.16580612	-0.0349964
155	0.35177724	-0.5602729	-0.5694352	0.18380023	0.33108382	0.16580612	-0.0349964
155.1	0.35177724	-0.5602729	-0.5694352	0.18380023	0.33108382	0.16580612	-0.0349964
155.2	0.35177724	-0.5602729	-0.5694352	0.18380023	0.33108382	0.16580612	-0.0349964
155.3	0.35177724	-0.5602729	-0.5694352	0.18380023	0.33108382	0.16580612	-0.0349964
155.4	0.35177724	-0.5602729	-0.5694352	0.18380023	0.33108382	0.16580612	-0.0349964
155.5	0.35180604	-0.5600229	-0.5692794	0.18381682	0.33101812	0.1656961	-0.0352719
155.6	0.35180604	-0.5600229	-0.5692794	0.18381682	0.33101812	0.1656961	-0.0352719
155.7	0.35180604	-0.5600229	-0.5692794	0.18381682	0.33101812	0.1656961	-0.0352719
155.8	0.35180604	-0.5600229	-0.5692794	0.18381682	0.33101812	0.1656961	-0.0352719
155.9	0.35188478	-0.559874	-0.5693752	0.18399349	0.33124769	0.16546074	-0.0352224
156	0.35188478	-0.559874	-0.5693752	0.18399349	0.33124769	0.16546074	-0.0352224

114	0.34433129	-0.5781054	-0.5535579	0.21305577	0.35087252	0.1657627	-0.0355924
114.1	0.34433129	-0.5781054	-0.5535579	0.21305577	0.35087252	0.1657627	-0.0355924
114.2	0.34433129	-0.5781054	-0.5535579	0.21305577	0.35087252	0.1657627	-0.0355924
114.3	0.34433129	-0.5781054	-0.5535579	0.21305577	0.35087252	0.1657627	-0.0355924
114.4	0.34433129	-0.5781054	-0.5535579	0.21305577	0.35087252	0.1657627	-0.0355924
114.5	0.34413742	-0.5781545	-0.5536458	0.21327127	0.3509474	0.16552859	-0.0356824
114.6	0.34413742	-0.5781545	-0.5536458	0.21327127	0.3509474	0.16552859	-0.0356824
114.7	0.34413742	-0.5781545	-0.5536458	0.21327127	0.3509474	0.16552859	-0.0356824
114.8	0.34413742	-0.5781545	-0.5536458	0.21327127	0.3509474	0.16552859	-0.0356824
114.9	0.34413742	-0.5781545	-0.5536458	0.21327127	0.3509474	0.16552859	-0.0356824
115	0.34388567	-0.5779513	-0.5538759	0.21259661	0.35050686	0.16538706	-0.0356356
115.1	0.34384813	-0.5778867	-0.5538826	0.21306998	0.35083167	0.1652364	-0.0357914
115.2	0.34391935	-0.5777005	-0.5538384	0.21286841	0.35041985	0.16551295	-0.0358751
115.3	0.34370652	-0.5773666	-0.5538139	0.21288809	0.35041309	0.16527601	-0.0357585
115.4	0.34373834	-0.5773483	-0.553842	0.21272131	0.35014518	0.16518396	-0.0357368
115.5	0.34357714	-0.5770141	-0.5537991	0.21261432	0.34994891	0.16490131	-0.0356599
115.6	0.34357714	-0.5770141	-0.5537991	0.21261432	0.34994891	0.16490131	-0.0356599
115.7	0.34363296	-0.5769501	-0.5537994	0.21249304	0.34965582	0.1649397	-0.0358717
115.8	0.34363296	-0.5769501	-0.5537994	0.21249304	0.34965582	0.1649397	-0.0358717
115.9	0.34353859	-0.5767852	-0.5539517	0.21240173	0.34948206	0.16505728	-0.0355863
116	0.34361891	-0.5764202	-0.5538235	0.2122883	0.34923104	0.16497491	-0.0358808
116.1	0.34361891	-0.5764202	-0.5538235	0.2122883	0.34923104	0.16497491	-0.0358808
116.2	0.34362885	-0.5765734	-0.5538096	0.21201227	0.34901419	0.16484563	-0.0358857
116.3	0.34362885	-0.5765734	-0.5538096	0.21201227	0.34901419	0.16484563	-0.0358857
116.4	0.34362885	-0.5765734	-0.5538096	0.21201227	0.34901419	0.16484563	-0.0358857
116.5	0.34330363	-0.5762926	-0.5538329	0.21177842	0.34889377	0.16500887	-0.0355953
116.6	0.34330363	-0.5762926	-0.5538329	0.21177842	0.34889377	0.16500887	-0.0355953
116.7	0.34330363	-0.5762926	-0.5538329	0.21177842	0.34889377	0.16500887	-0.0355953
116.8	0.3433826	-0.5761609	-0.5540072	0.21174013	0.34889821	0.16520257	-0.0358889
116.9	0.34363986	-0.5760615	-0.5540564	0.2118797	0.34926056	0.16493088	-0.0361499
117	0.34363986	-0.5760615	-0.5540564	0.2118797	0.34926056	0.16493088	-0.0361499
117.1	0.34381051	-0.5757793	-0.5539144	0.21184424	0.34910076	0.16459871	-0.0362849
117.2	0.34392091	-0.5755942	-0.5540466	0.21173821	0.34905694	0.1647734	-0.0358734
117.3	0.34392091	-0.5755942	-0.5540466	0.21173821	0.34905694	0.1647734	-0.0358734
117.4	0.34421769	-0.5754856	-0.5541171	0.21195294	0.34901641	0.16508541	-0.036077
117.5	0.34427897	-0.5752375	-0.5540078	0.21174609	0.3488211	0.16541	-0.0360325
117.6	0.34456117	-0.5751553	-0.5540479	0.21169513	0.34881312	0.16513187	-0.0358932
117.7	0.34456117	-0.5751553	-0.5540479	0.21169513	0.34881312	0.16513187	-0.0358932
117.8	0.34494687	-0.5750158	-0.5541976	0.21180556	0.34881998	0.16521313	-0.0360738
117.9	0.34463873	-0.5746274	-0.5542145	0.21131974	0.34860853	0.16493173	-0.0356666
118	0.344494	-0.5744787	-0.5542014	0.21105606	0.34832645	0.16496326	-0.0352138
118.1	0.344494	-0.5744787	-0.5542014	0.21105606	0.34832645	0.16496326	-0.0352138
118.2	0.344494	-0.5744787	-0.5542014	0.21105606	0.34832645	0.16496326	-0.0352138
118.3	0.344494	-0.5744787	-0.5542014	0.21105606	0.34832645	0.16496326	-0.0352138
118.4	0.344494	-0.5744787	-0.5542014	0.21105606	0.34832645	0.16496326	-0.0352138
118.5	0.344494	-0.5744787	-0.5542014	0.21105606	0.34832645	0.16496326	-0.0352138
118.6	0.344494	-0.5744787	-0.5542014	0.21105606	0.34832645	0.16496326	-0.0352138
118.7	0.34468738	-0.5745641	-0.5544886	0.21072245	0.34809925	0.16491712	-0.0350906
118.8	0.34468738	-0.5745641	-0.5544886	0.21072245	0.34809925	0.16491712	-0.0350906
118.9	0.34468738	-0.5745641	-0.5544886	0.21072245	0.34809925	0.16491712	-0.0350906
119	0.3452183	-0.5742364	-0.554793	0.21076014	0.34791596	0.16509583	-0.0350001
119.1	0.3452183	-0.5742364	-0.554793	0.21076014	0.34791596	0.16509583	-0.0350001
119.2	0.34536508	-0.5739773	-0.5548589	0.21052021	0.34784377	0.16532605	-0.0348083
119.3	0.34536508	-0.5739773	-0.5548589	0.21052021	0.34784377	0.16532605	-0.0348083
119.4	0.34536508	-0.5739773	-0.5548589	0.21052021	0.34784377	0.16532605	-0.0348083
119.5	0.34541204	-0.5741403	-0.5549379	0.21032486	0.34756178	0.16532754	-0.0345592
119.6	0.34541204	-0.5741403	-0.5549379	0.21032486	0.34756178	0.16532754	-0.0345592
119.7	0.34541204	-0.5741403	-0.5549379	0.21032486	0.34756178	0.16532754	-0.0345592
119.8	0.34541204	-0.5741403	-0.5549379	0.21032486	0.34756178	0.16532754	-0.0345592
119.9	0.34541204	-0.5741403	-0.5549379	0.21032486	0.34756178	0.16532754	-0.0345592

165	0.35371219	-0.5587979	-0.5708717	0.18324913	0.3313022	0.1668074	-0.0364213
165.1	0.35371219	-0.5587979	-0.5708717	0.18324913	0.3313022	0.1668074	-0.0364213
165.2	0.35371219	-0.5587979	-0.5708717	0.18324913	0.3313022	0.1668074	-0.0364213
165.3	0.35382964	-0.5587719	-0.5708271	0.18282858	0.33094961	0.16658467	-0.0362388
165.4	0.35382964	-0.5587719	-0.5708271	0.18282858	0.33094961	0.16658467	-0.0362388
165.5	0.3538402	-0.5587584	-0.5708774	0.18276495	0.33128464	0.16674401	-0.036331
165.6	0.3538402	-0.5587584	-0.5708774	0.18276495	0.33128464	0.16674401	-0.036331
165.7	0.3538402	-0.5587584	-0.5708774	0.18276495	0.33128464	0.16674401	-0.036331
165.8	0.3538402	-0.5587584	-0.5708774	0.18276495	0.33128464	0.16674401	-0.036331
165.9	0.3538402	-0.5587584	-0.5708774	0.18276495	0.33128464	0.16674401	-0.036331
166	0.35400223	-0.5586388	-0.5707876	0.18255508	0.33138063	0.16649952	-0.0364575
166.1	0.35400223	-0.5586388	-0.5707876	0.18255508	0.33138063	0.16649952	-0.0364575
166.2	0.35400223	-0.5586388	-0.5707876	0.18255508	0.33138063	0.16649952	-0.0364575
166.3	0.35400223	-0.5586388	-0.5707876	0.18255508	0.33138063	0.16649952	-0.0364575
166.4	0.35400223	-0.5586388	-0.5707876	0.18255508	0.33138063	0.16649952	-0.0364575
166.5	0.35400223	-0.5586388	-0.5707876	0.18255508	0.33138063	0.16649952	-0.0364575
166.6	0.35400223	-0.5586388	-0.5707876	0.18255508	0.33138063	0.16649952	-0.0364575
166.7	0.35400223	-0.5586388	-0.5707876	0.18255508	0.33138063	0.16649952	-0.0364575
166.8	0.35400223	-0.5586388	-0.5707876	0.18255508	0.33138063	0.16649952	-0.0364575
166.9	0.35400223	-0.5586388	-0.5707876	0.18255508	0.33138063	0.16649952	-0.0364575
167	0.35400223	-0.5586388	-0.5707876	0.18255508	0.33138063	0.16649952	-0.0364575
167.1	0.35400223	-0.5586388	-0.5707876	0.18255508	0.33138063	0.16649952	-0.0364575
167.2	0.35400223	-0.5586388	-0.5707876	0.18255508	0.33138063	0.16649952	-0.0364575
167.3	0.35400223	-0.5586388	-0.5707876	0.18255508	0.33138063	0.16649952	-0.0364575
167.4	0.35400223	-0.5586388	-0.5707876	0.18255508	0.33138063	0.16649952	-0.0364575
167.5	0.35400223	-0.5586388	-0.5707876	0.18255508	0.33138063	0.16649952	-0.0364575
167.6	0.35400223	-0.5586388	-0.5707876	0.18255508	0.33138063	0.16649952	-0.0364575
167.7	0.35400223	-0.5586388	-0.5707876	0.18255508	0.33138063	0.16649952	-0.0364575
167.8	0.35400223	-0.5586388	-0.5707876	0.18255508	0.33138063	0.16649952	-0.0364575
167.9	0.35400223	-0.5586388	-0.5707876	0.18255508	0.33138063	0.16649952	-0.0364575
168	0.35400223	-0.5586388	-0.5707876	0.18255508	0.33138063	0.16649952	-0.0364575
168.1	0.35400223	-0.5586388	-0.5707876	0.18255508	0.33138063	0.16649952	-0.0364575
168.2	0.35400223	-0.5586388	-0.5707876	0.18255508	0.33138063	0.16649952	-0.0364575
168.3	0.35402762	-0.5584552	-0.5709968	0.18229888	0.33144454	0.1669116	-0.0366846
168.4	0.35402762	-0.5584552	-0.5709968	0.18229888	0.33144454	0.1669116	-0.0366846
168.5	0.35402762	-0.5584552	-0.5709968	0.18229888	0.33144454	0.1669116	-0.0366846
168.6	0.35402762	-0.5584552	-0.5709968	0.18229888	0.33144454	0.1669116	-0.0366846
168.7	0.35402762	-0.5584552	-0.5709968	0.18229888	0.33144454	0.1669116	-0.0366846
168.8	0.35398786	-0.5584975	-0.5711702	0.18252062	0.33148494	0.16726715	-0.0368934
168.9	0.35398786	-0.5584975	-0.5711702	0.18252062	0.33148494	0.16726715	-0.0368934
169	0.35398786	-0.5584975	-0.5711702	0.18252062	0.33148494	0.16726715	-0.0368934
169.1	0.3541903	-0.5586097	-0.571468	0.18277348	0.33160622	0.16706269	-0.0371435
169.2	0.354213	-0.5585334	-0.5714748	0.18252578	0.33143972	0.16726958	-0.0370808
169.3	0.354213	-0.5585334	-0.5714748	0.18252578	0.33143972	0.16726958	-0.0370808
169.4	0.354213	-0.5585334	-0.5714748	0.18252578	0.33143972	0.16726958	-0.0370808
169.5	0.354213	-0.5585334	-0.5714748	0.18252578	0.33143972	0.16726958	-0.0370808
169.6	0.354213	-0.5585334	-0.5714748	0.18252578	0.33143972	0.16726958	-0.0370808
169.7	0.354213	-0.5585334	-0.5714748	0.18252578	0.33143972	0.16726958	-0.0370808
169.8	0.354213	-0.5585334	-0.5714748	0.18252578	0.33143972	0.16726958	-0.0370808
169.9	0.354213	-0.5585334	-0.5714748	0.18252578	0.33143972	0.16726958	-0.0370808
170	0.354213	-0.5585334	-0.5714748	0.18252578	0.33143972	0.16726958	-0.0370808
170.1	0.354213	-0.5585334	-0.5714748	0.18252578	0.33143972	0.16726958	-0.0370808
170.2	0.354213	-0.5585334	-0.5714748	0.18252578	0.33143972	0.16726958	-0.0370808
170.3	0.354213	-0.5585334	-0.5714748	0.18252578	0.33143972	0.16726958	-0.0370808
170.4	0.354213	-0.5585334	-0.5714748	0.18252578	0.33143972	0.16726958	-0.0370808
170.5	0.354213	-0.5585334	-0.5714748	0.18252578	0.33143972	0.16726958	-0.0370808
170.6	0.35428493	-0.5586152	-0.5714931	0.1825007	0.33146633	0.167433	-0.0366101
170.7	0.35428493	-0.5586152	-0.5714931	0.1825007	0.33146633	0.167433	-0.0366101
170.8	0.35428493	-0.5586152	-0.5714931	0.1825007	0.33146633	0.167433	-0.0366101
170.9	0.35428895	-0.5584181	-0.5715286	0.18277068	0.33157234	0.16729361	-0.0367831
171	0.35428895	-0.5584181	-0.5715286	0.18277068	0.33157234	0.16729361	-0.0367831

126.1	0.34623173	-0.5721496	-0.5577022	0.20558012	0.34582479	0.1661196	-0.0325679
126.2	0.34623173	-0.5721496	-0.5577022	0.20558012	0.34582479	0.1661196	-0.0325679
126.3	0.34619747	-0.5720307	-0.5575948	0.20543954	0.34581274	0.16609182	-0.0326155
126.4	0.34619747	-0.5720307	-0.5575948	0.20543954	0.34581274	0.16609182	-0.0326155
126.5	0.34622008	-0.5721074	-0.5578729	0.2052541	0.34550758	0.1662637	-0.0328191
126.6	0.34622008	-0.5721074	-0.5578729	0.2052541	0.34550758	0.1662637	-0.0328191
126.7	0.34616801	-0.5722225	-0.5581016	0.20548318	0.34531166	0.16625817	-0.0329159
126.8	0.34616801	-0.5722225	-0.5581016	0.20548318	0.34531166	0.16625817	-0.0329159
126.9	0.34587651	-0.571912	-0.5579611	0.20532062	0.34517551	0.1661839	-0.0325218
127	0.34589825	-0.572029	-0.5583024	0.20485411	0.34506311	0.16629821	-0.0324133
127.1	0.34589825	-0.572029	-0.5583024	0.20485411	0.34506311	0.16629821	-0.0324133
127.2	0.34589825	-0.572029	-0.5583024	0.20485411	0.34506311	0.16629821	-0.0324133
127.3	0.34589825	-0.572029	-0.5583024	0.20485411	0.34506311	0.16629821	-0.0324133
127.4	0.34580358	-0.5717956	-0.5583394	0.2044135	0.34502825	0.16651655	-0.032119
127.5	0.34580358	-0.5717956	-0.5583394	0.2044135	0.34502825	0.16651655	-0.032119
127.6	0.34580358	-0.5717956	-0.5583394	0.2044135	0.34502825	0.16651655	-0.032119
127.7	0.34580358	-0.5717956	-0.5583394	0.2044135	0.34502825	0.16651655	-0.032119
127.8	0.34592825	-0.571907	-0.5584578	0.20425736	0.34494643	0.16655125	-0.0319073
127.9	0.34592825	-0.571907	-0.5584578	0.20425736	0.34494643	0.16655125	-0.0319073
128	0.34589989	-0.5720112	-0.5587472	0.20372986	0.34462805	0.16674339	-0.0316521
128.1	0.34589989	-0.5720112	-0.5587472	0.20372986	0.34462805	0.16674339	-0.0316521
128.2	0.34601849	-0.5719378	-0.5587804	0.20385987	0.34452609	0.16702346	-0.0319894
128.3	0.34601849	-0.5719378	-0.5587804	0.20385987	0.34452609	0.16702346	-0.0319894
128.4	0.34607543	-0.5718314	-0.5587801	0.20377944	0.34386535	0.16737231	-0.0316677
128.5	0.34610489	-0.5716864	-0.5587518	0.20332744	0.3436816	0.16702839	-0.0320517
128.6	0.34610489	-0.5716864	-0.5587518	0.20332744	0.3436816	0.16702839	-0.0320517
128.7	0.34610489	-0.5716864	-0.5587518	0.20332744	0.3436816	0.16702839	-0.0320517
128.8	0.34610489	-0.5716864	-0.5587518	0.20332744	0.3436816	0.16702839	-0.0320517
128.9	0.34616133	-0.5717179	-0.5589202	0.20321354	0.34354995	0.16709684	-0.0323607
129	0.34616133	-0.5717179	-0.5589202	0.20321354	0.34354995	0.16709684	-0.0323607
129.1	0.34616133	-0.5717179	-0.5589202	0.20321354	0.34354995	0.16709684	-0.0323607
129.2	0.34616133	-0.5717179	-0.5589202	0.20321354	0.34354995	0.16709684	-0.0323607
129.3	0.34644969	-0.5716291	-0.5590943	0.20303502	0.34325918	0.16716407	-0.0323694
129.4	0.34644969	-0.5716291	-0.5590943	0.20303502	0.34325918	0.16716407	-0.0323694
129.5	0.34644969	-0.5716291	-0.5590943	0.20303502	0.34325918	0.16716407	-0.0323694
129.6	0.34644969	-0.5716291	-0.5590943	0.20303502	0.34325918	0.16716407	-0.0323694
129.7	0.34644969	-0.5716291	-0.5590943	0.20303502	0.34325918	0.16716407	-0.0323694
129.8	0.34644969	-0.5716291	-0.5590943	0.20303502	0.34325918	0.16716407	-0.0323694
129.9	0.34644969	-0.5716291	-0.5590943	0.20303502	0.34325918	0.16716407	-0.0323694
130	0.34653975	-0.5715149	-0.5591418	0.20288376	0.34291856	0.16714619	-0.0324576
130.1	0.34653975	-0.5715149	-0.5591418	0.20288376	0.34291856	0.16714619	-0.0324576
130.2	0.34653975	-0.5715149	-0.5591418	0.20288376	0.34291856	0.16714619	-0.0324576
130.3	0.34670527	-0.5716422	-0.5592182	0.20290752	0.34303955	0.16742015	-0.0327782
130.4	0.34670527	-0.5716422	-0.5592182	0.20290752	0.34303955	0.16742015	-0.0327782
130.5	0.34679559	-0.57157	-0.5592751	0.20212934	0.34269966	0.16742764	-0.0329694
130.6	0.34679559	-0.57157	-0.5592751	0.20212934	0.34269966	0.16742764	-0.0329694
130.7	0.34679559	-0.57157	-0.5592751	0.20212934	0.34269966	0.16742764	-0.0329694
130.8	0.3469788	-0.5715521	-0.5594107	0.20187084	0.34251882	0.16743846	-0.0331147
130.9	0.34692426	-0.5715453	-0.5596487	0.2017586	0.34238805	0.16737146	-0.0331355
131	0.34692426	-0.5715453	-0.5596487	0.2017586	0.34238805	0.16737146	-0.0331355
131.1	0.34692426	-0.5715453	-0.5596487	0.2017586	0.34238805	0.16737146	-0.0331355
131.2	0.34692426	-0.5715453	-0.5596487	0.2017586	0.34238805	0.16737146	-0.0331355
131.3	0.34699058	-0.5714501	-0.5599253	0.20170969	0.3421061	0.16723496	-0.0330085
131.4	0.34684119	-0.5712751	-0.5598401	0.20170535	0.34176976	0.16714063	-0.0328192
131.5	0.34659421	-0.571087	-0.559893	0.20123036	0.34146673	0.16678083	-0.0325824
131.6	0.34659421	-0.571087	-0.559893	0.20123036	0.34146673	0.16678083	-0.0325824
131.7	0.34665441	-0.5710438	-0.5603129	0.20047476	0.3407422	0.16669055	-0.0324315
131.8	0.34665441	-0.5710438	-0.5603129	0.20047476	0.3407422	0.16669055	-0.0324315
131.9	0.34665441	-0.5710438	-0.5603129	0.20047476	0.3407422	0.16669055	-0.0324315
132	0.34692562	-0.5709255	-0.5604418	0.20018775	0.34067018	0.16640769	-0.0325622
132.1	0.34692562	-0.5709255	-0.5604418	0.20018775	0.34067018	0.16640769	-0.0325622
132.2	0.34667159	-0.5708621	-0.5604635	0.20021177	0.34072943	0.16646627	-0.0326468
132.3	0.34667159	-0.5708621	-0.5604635	0.20021177	0.34072943	0.16646627	-0.0326468
132.4	0.34686603	-0.5708332	-0.5606943	0.19934157	0.34012902	0.1663318	-0.0321804
132.5	0.34686603	-0.5708332	-0.5606943	0.19934157	0.34012902	0.1663318	-0.0321804
132.6	0.34686603	-0.5708332	-0.5606943	0.19934157	0.34012902	0.1663318	-0.0321804
132.7	0.34686603	-0.5708332	-0.5606943	0.19934157	0.34012902	0.1663318	-0.0321804
132.8	0.34696107	-0.5708429	-0.5608407	0.19893179	0.33985789	0.16644115	-0.0322892
132.9	0.34696107	-0.5708429	-0.5608407	0.19893179	0.33985789	0.16644115	-0.0322892
133	0.34696107	-0.5708429	-0.5608407	0.19893179	0.33985789	0.16644115	-0.0322892
133.1	0.34696107	-0.5708429	-0.5608407	0.19893179	0.33985789	0.16644115	-0.0322892
133.2	0.34716253	-0.5709775	-0.5610074	0.19903412	0.33991133	0.16684475	-0.0323557
133.3	0.34716253	-0.5709775	-0.5610074	0.19903412	0.33991133	0.16684475	-0.0323557
133.4	0.34717732	-0.5709596	-0.5610865	0.19860771	0.33947458	0.16683206	-0.0322264
133.5	0.34730801	-0.5704842	-0.5611862	0.19754505	0.3392599	0.16735796	-0.0323763
133.6	0.34730801	-0.5704842	-0.5611862	0.19754505	0.3392599	0.16735796	-0.0323763
133.7	0.34730801	-0.5704842	-0.5611862	0.19754505	0.3392599	0.16735796	-0.0323763
133.8	0.34730801	-0.5704842	-0.5611862	0.19754505	0.3392599	0.16735796	-0.0323763
133.9	0.34730801	-0.5704842	-0.5611862	0.19754505	0.3392599	0.16735796	-0.0323763
134	0.34733674	-0.5704134	-0.5614497	0.19712365	0.3392662	0.1673135	-0.0324225
134.1	0.34747342	-0.5703298	-0.561581	0.19693606	0.33921117	0.16779705	-0.032294
134.2	0.34747342	-0.5703298	-0.561581	0.19693606	0.33921117	0.16779705	-0.032294
134.3	0.34747342	-0.5703298	-0.561581	0.19693606	0.33921117	0.16779705	-0.032294
134.4	0.34747342	-0.5703298	-0.561581	0.19693606	0.33921117	0.16779705	-0.032294
134.5	0.3475741	-0.5702679	-0.5618797	0.19636228	0.33962674	0.16801463	-0.0328374
134.6	0.3475741	-0.5702679	-0.5618797	0.19636228	0.33962674	0.16801463	-0.0328374
134.7	0.3475741	-0.5702679	-0.5618797	0.19636228	0.33962674	0.16801463	-0.0328374
134.8	0.3475741	-0.5702679	-0.5618797	0.19636228	0.33962674	0.16801463	-0.0328374
134.9	0.3475741	-0.5702679	-0.5618797	0.19636228	0.33962674	0.16801463	-0.0328374

141.1	0.35071374	-0.5656321	-0.5659042	0.19028913	0.33509968	0.16715929	-0.0346344
141.2	0.35071374	-0.5656321	-0.5659042	0.19028913	0.33509968	0.16715929	-0.0346344
141.3	0.35071374	-0.5656321	-0.5659042	0.19028913	0.33509968	0.16715929	-0.0346344
141.4	0.35061474	-0.5657274	-0.5661001	0.19013859	0.33513131	0.16730779	-0.0346749
141.5	0.35074344	-0.5655841	-0.5661894	0.18985215	0.33518078	0.16702117	-0.0345794
141.6	0.35074344	-0.5655841	-0.5661894	0.18985215	0.33518078	0.16702117	-0.0345794
141.7	0.35068148	-0.5653773	-0.5660973	0.1896352	0.33495791	0.16683877	-0.0346645
141.8	0.35068148	-0.5653773	-0.5660973	0.1896352	0.33495791	0.16683877	-0.0346645
141.9	0.35068148	-0.5653773	-0.5660973	0.1896352	0.33495791	0.16683877	-0.0346645
142	0.35079072	-0.5652847	-0.5663646	0.18913614	0.33473751	0.16664059	-0.0344992
142.1	0.35079072	-0.5652847	-0.5663646	0.18913614	0.33473751	0.16664059	-0.0344992
142.2	0.35079072	-0.5652847	-0.5663646	0.18913614	0.33473751	0.16664059	-0.0344992
142.3	0.35079072	-0.5652847	-0.5663646	0.18913614	0.33473751	0.16664059	-0.0344992
142.4	0.35078041	-0.56517	-0.5666013	0.18854961	0.33466457	0.16685022	-0.0346466
142.5	0.35078041	-0.56517	-0.5666013	0.18854961	0.33466457	0.16685022	-0.0346466
142.6	0.35109735	-0.5649176	-0.5667597	0.18880122	0.33454738	0.16685471	-0.0345122
142.7	0.35109735	-0.5649176	-0.5667597	0.18880122	0.33454738	0.16685471	-0.0345122
142.8	0.35109735	-0.5649176	-0.5667597	0.18880122	0.33454738	0.16685471	-0.0345122
142.9	0.35109735	-0.5649176	-0.5667597	0.18880122	0.33454738	0.16685471	-0.0345122
143	0.35097679	-0.5646806	-0.5668602	0.18835803	0.33423157	0.16659296	-0.0345687
143.1	0.35097679	-0.5646806	-0.5668602	0.18835803	0.33423157	0.16659296	-0.0345687
143.2	0.3509776	-0.5644346	-0.5669249	0.18819488	0.33410375	0.16626414	-0.0347885
143.3	0.35103007	-0.5641917	-0.5669726	0.18790727	0.33378716	0.16589064	-0.0350479
143.4	0.35103007	-0.5641917	-0.5669726	0.18790727	0.33378716	0.16589064	-0.0350479
143.5	0.35114732	-0.5641153	-0.5672078	0.187627	0.33348608	0.16591325	-0.0350996
143.6	0.35114732	-0.5641153	-0.5672078	0.187627	0.33348608	0.16591325	-0.0350996
143.7	0.35114732	-0.5641153	-0.5672078	0.187627	0.33348608	0.16591325	-0.0350996
143.8	0.35103775	-0.5639254	-0.567319	0.18720904	0.33324417	0.16598226	-0.0351463
143.9	0.35117722	-0.5638001	-0.5675974	0.18729455	0.33326396	0.16591372	-0.035541
144	0.35150162	-0.5637091	-0.5678201	0.18700117	0.33309849	0.16599591	-0.0354675
144.1	0.35150162	-0.5637091	-0.5678201	0.18700117	0.33309849	0.16599591	-0.0354675
144.2	0.35150162	-0.5637091	-0.5678201	0.18700117	0.33309849	0.16599591	-0.0354675
144.3	0.35150162	-0.5637091	-0.5678201	0.18700117	0.33309849	0.16599591	-0.0354675
144.4	0.3513973	-0.5630559	-0.567615	0.18572747	0.33204619	0.1659513	-0.0354177
144.5	0.3513973	-0.5630559	-0.567615	0.18572747	0.33204619	0.1659513	-0.0354177
144.6	0.3513973	-0.5630559	-0.567615	0.18572747	0.33204619	0.1659513	-0.0354177
144.7	0.3513973	-0.5630559	-0.567615	0.18572747	0.33204619	0.1659513	-0.0354177
144.8	0.3513973	-0.5630559	-0.567615	0.18572747	0.33204619	0.1659513	-0.0354177
144.9	0.35175313	-0.5628855	-0.5677682	0.1852982	0.33184912	0.16608661	-0.0355619
145	0.35175313	-0.5628855	-0.5677682	0.1852982	0.33184912	0.16608661	-0.0355619
145.1	0.35175313	-0.5628855	-0.5677682	0.1852982	0.33184912	0.16608661	-0.0355619
145.2	0.35175313	-0.5628855	-0.5677682	0.1852982	0.33184912	0.16608661	-0.0355619
145.3	0.35181032	-0.5629449	-0.5678978	0.18516065	0.33202442	0.16588475	-0.0358958
145.4	0.35170122	-0.5629054	-0.5682019	0.18477948	0.33183907	0.16566328	-0.0358489
145.5	0.35170122	-0.5629054	-0.5682019	0.18477948	0.33183907	0.16566328	-0.0358489
145.6	0.35170122	-0.5629054	-0.5682019	0.18477948	0.33183907	0.16566328	-0.0358489
145.7	0.35170122	-0.5629054	-0.5682019	0.18477948	0.33183907	0.16566328	-0.0358489
145.8	0.35148368	-0.5626552	-0.568203	0.18437547	0.3316263	0.16561825	-0.0358209
145.9	0.35148368	-0.5626552	-0.568203	0.18437547	0.3316263	0.16561825	-0.0358209
146	0.3516976	-0.5625652	-0.5684328	0.18469788	0.33171657	0.16546938	-0.0356091
146.1	0.3516976	-0.5625652	-0.5684328	0.18469788	0.33171657	0.16546938	-0.0356091
146.2	0.35156069	-0.5629137	-0.5688293	0.18468604	0.33206108	0.16577872	-0.0350003
146.3	0.35148916	-0.5626376	-0.56899	0.18431914	0.33169726	0.16582973	-0.0351556
146.4	0.35148916	-0.5626376	-0.56899	0.18431914	0.33169726	0.16582973	-0.0351556
146.5	0.35148916	-0.5626376	-0.56899	0.18431914	0.33169726	0.16582973	-0.0351556
146.6	0.3516484	-0.5626127	-0.5690846	0.18462019	0.33204033	0.16623319	-0.0354706
146.7	0.3516484	-0.5626127	-0.5690846	0.18462019	0.33204033	0.16623319	-0.0354706
146.8	0.3516484	-0.5626127	-0.5690846	0.18462019	0.33204033	0.16623319	-0.0354706
146.9	0.35173458	-0.5625532	-0.5692509	0.18464882	0.33243307	0.16619224	-0.0358442
147	0.35158403	-0.5624123	-0.5692428	0.18432169	0.33206496	0.1660503	-0.0357074
147.1	0.35158403	-0.5624123	-0.5692428	0.18432169	0.33206496	0.1660503	-0.0357074
147.2	0.35158403	-0.5624123	-0.5692428	0.18432169	0.33206496	0.1660503	-0.0357074
147.3	0.35174352	-0.5619356	-0.5692637	0.18363388	0.33150314	0.16594614	-0.0355123
147.4	0.35174352	-0.5619356	-0.5692637	0.18363388	0.33150314	0.16594614	-0.0355123
147.5	0.35174352	-0.5619356	-0.5692637	0.18363388	0.33150314	0.16594614	-0.0355123
147.6	0.35174352	-0.5619356	-0.5692637	0.18363388	0.33150314	0.16594614	-0.0355123
147.7	0.35174352	-0.5619356	-0.5692637	0.18363388	0.33150314	0.16594614	-0.0355123
147.8	0.35164436	-0.5616761	-0.5693128	0.18337429	0.33104148	0.16613528	-0.0355942
147.9	0.35164436	-0.5616761	-0.5693128	0.18337429	0.33104148	0.16613528	-0.0355942
148	0.35156486	-0.5614552	-0.5692625	0.18302815	0.33078249	0.16596776	-0.0355356
148.1	0.35156486	-0.5614552	-0.5692625	0.18302815	0.33078249	0.16596776	-0.0355356
148.2	0.35156486	-0.5614552	-0.5692625	0.18302815	0.33078249	0.16596776	-0.0355356
148.3	0.35156486	-0.5614552	-0.5692625	0.18302815	0.33078249	0.16596776	-0.0355356
148.4	0.35156486	-0.5614552	-0.5692625	0.18302815	0.33078249	0.16596776	-0.0355356
148.5	0.35156486	-0.5614552	-0.5692625	0.18302815	0.33078249	0.16596776	-0.0355356
148.6	0.35156486	-0.5614552	-0.5692625	0.18302815	0.33078249	0.16596776	-0.0355356
148.7	0.35156486	-0.5614552	-0.5692625	0.18302815	0.33078249	0.16596776	-0.0355356
148.8	0.35156486	-0.5614552	-0.5692625	0.18302815	0.33078249	0.16596776	-0.0355356
148.9	0.35156486	-0.5614552	-0.5692625	0.18302815	0.33078249	0.16596776	-0.0355356
149	0.35156486	-0.5614552	-0.5692625	0.18302815	0.33078249	0.16596776	-0.0355356
149.1	0.35156486	-0.5614552	-0.5692625	0.18302815	0.33078249	0.16596776	-0.0355356
149.2	0.35156486	-0.5614552	-0.5692625	0.18302815	0.33078249	0.16596776	-0.0355356
149.3	0.35156486	-0.5614552	-0.5692625	0.18302815	0.33078249	0.16596776	-0.0355356
149.4	0.35156486	-0.5614552	-0.5692625	0.18302815	0.33078249	0.16596776	-0.0355356
149.5	0.35156486	-0.5614552	-0.5692625	0.18302815	0.33078249	0.16596776	-0.0355356
149.6	0.3516852	-0.5612684	-0.5692309	0.18312848	0.33073991	0.16602212	-0.035698
149.7	0.3516852	-0.5612684	-0.5692309	0.18312848	0.33073991	0.16602212	-0.035698
149.8	0.3516852	-0.5612684	-0.5692309	0.18312848	0.33073991	0.16602212	-0.035698
149.9	0.35166635	-0.561298	-0.5694872	0.18327496	0.33092054	0.16575713	-0.035319

156.1	0.35188478	-0.559874	-0.5693752	0.18399349	0.33124769	0.16546074	-0.0352224
156.2	0.35188478	-0.559874	-0.5693752	0.18399349	0.33124769	0.16546074	-0.0352224
156.3	0.35188478	-0.559874	-0.5693752	0.18399349	0.33124769	0.16546074	-0.0352224
156.4	0.35188478	-0.559874	-0.5693752	0.18399349	0.33124769	0.16546074	-0.0352224
156.5	0.35188478	-0.559874	-0.5693752	0.18399349	0.33124769	0.16546074	-0.0352224
156.6	0.35167264	-0.5597301	-0.5694387	0.18441455	0.33152593	0.16529206	-0.0351151
156.7	0.35167264	-0.5597301	-0.5694387	0.18441455	0.33152593	0.16529206	-0.0351151
156.8	0.35167264	-0.5597301	-0.5694387	0.18441455	0.33152593	0.16529206	-0.0351151
156.9	0.35172943	-0.5597015	-0.5695327	0.18476779	0.33164113	0.16554272	-0.0354184
157	0.35181601	-0.5599105	-0.5699131	0.18438254	0.33136585	0.16592741	-0.0354006
157.1	0.35181601	-0.5599105	-0.5699131	0.18438254	0.33136585	0.16592741	-0.0354006
157.2	0.35181601	-0.5599105	-0.5699131	0.18438254	0.33136585	0.16592741	-0.0354006
157.3	0.35181601	-0.5599105	-0.5699131	0.18438254	0.33136585	0.16592741	-0.0354006
157.4	0.35181601	-0.5599105	-0.5699131	0.18438254	0.33136585	0.16592741	-0.0354006
157.5	0.35181601	-0.5599105	-0.5699131	0.18438254	0.33136585	0.16592741	-0.0354006
157.6	0.35181601	-0.5599105	-0.5699131	0.18438254	0.33136585	0.16592741	-0.0354006
157.7	0.35182787	-0.5599295	-0.5699226	0.18444031	0.33118624	0.1660943	-0.035661
157.8	0.35182787	-0.5599295	-0.5699226	0.18444031	0.33118624	0.1660943	-0.035661
157.9	0.35182787	-0.5599295	-0.5699226	0.18444031	0.33118624	0.1660943	-0.035661
158	0.35199032	-0.5597166	-0.5699847	0.18448425	0.33137186	0.16601103	-0.0356456
158.1	0.35199032	-0.5597166	-0.5699847	0.18448425	0.33137186	0.16601103	-0.0356456
158.2	0.35199032	-0.5597166	-0.5699847	0.18448425	0.33137186	0.16601103	-0.0356456
158.3	0.35213376	-0.5593776	-0.5699083	0.1845653	0.33146158	0.16583407	-0.0355663
158.4	0.35213376	-0.5593776	-0.5699083	0.1845653	0.33146158	0.16583407	-0.0355663
158.5	0.35213376	-0.5593776	-0.5699083	0.1845653	0.33146158	0.16583407	-0.0355663
158.6	0.35213376	-0.5593776	-0.5699083	0.1845653	0.33146158	0.16583407	-0.0355663
158.7	0.35213376	-0.5593776	-0.5699083	0.1845653	0.33146158	0.16583407	-0.0355663
158.8	0.35241197	-0.5593488	-0.5700443	0.1845259	0.33149114	0.16590043	-0.0354782
158.9	0.35259421	-0.5591822	-0.5699464	0.18453183	0.33163631	0.16601382	-0.0356446
159	0.35259421	-0.5591822	-0.5699464	0.18453183	0.33163631	0.16601382	-0.0356446
159.1	0.35259421	-0.5591822	-0.5699464	0.18453183	0.33163631	0.16601382	-0.0356446
159.2	0.35259421	-0.5591822	-0.5699464	0.18453183	0.33163631	0.16601382	-0.0356446
159.3	0.3527611	-0.5591445	-0.5700487	0.18457216	0.33213705	0.16602527	-0.0360586
159.4	0.3527611	-0.5591445	-0.5700487	0.18457216	0.33213705	0.16602527	-0.0360586
159.5	0.3527611	-0.5591445	-0.5700487	0.18457216	0.33213705	0.16602527	-0.0360586
159.6	0.3527611	-0.5591445	-0.5700487	0.18457216	0.33213705	0.16602527	-0.0360586
159.7	0.3527611	-0.5591445	-0.5700487	0.18457216	0.33213705	0.16602527	-0.0360586
159.8	0.3527611	-0.5591445	-0.5700487	0.18457216	0.33213705	0.16602527	-0.0360586
159.9	0.35252423	-0.5590957	-0.5701323	0.18490808	0.33201094	0.16597182	-0.036164
160	0.35252423	-0.5590957	-0.5701323	0.18490808	0.33201094	0.16597182	-0.036164
160.1	0.35252423	-0.5590957	-0.5701323	0.18490808	0.33201094	0.16597182	-0.036164
160.2	0.3524689	-0.5592795	-0.5704705	0.18497829	0.33244465	0.16622123	-0.0362527
160.3	0.3524689	-0.5592795	-0.5704705	0.18497829	0.33244465	0.16622123	-0.0362527
160.4	0.3524689	-0.5592795	-0.5704705	0.18497829	0.33244465	0.16622123	-0.0362527
160.5	0.35274604	-0.5594468	-0.5705717	0.18475934	0.33233927	0.16628811	-0.0361158
160.6	0.35274604	-0.5594468	-0.5705717	0.18475934	0.33233927	0.16628811	-0.0361158
160.7	0.35274604	-0.5594468	-0.5705717	0.18475934	0.33233927	0.16628811	-0.0361158
160.8	0.35274604	-0.5594468	-0.5705717	0.18475934	0.33233927	0.16628811	-0.0361158
160.9	0.35274604	-0.5594468	-0.5705717	0.18475934	0.33233927	0.16628811	-0.0361158
161	0.35274604	-0.5594468	-0.5705717	0.18475934	0.33233927	0.16628811	-0.0361158
161.1	0.35274604	-0.5594468	-0.5705717	0.18475934	0.33233927	0.16628811	-0.0361158
161.2	0.35274604	-0.5594468	-0.5705717	0.18475934	0.33233927	0.16628811	-0.0361158
161.3	0.35303044	-0.5595532	-0.5706051	0.1843621	0.33210987	0.16633926	-0.0362529
161.4	0.3533028	-0.5596403	-0.5706289	0.18390696	0.33205998	0.1662234	-0.0361294
161.5	0.35327517	-0.5593651	-0.5705114	0.18402234	0.33197427	0.16624443	-0.0362937
161.6	0.35327517	-0.5593651	-0.5705114	0.18402234	0.33197427	0.16624443	-0.0362937
161.7	0.35327517	-0.5593651	-0.5705114	0.18402234	0.33197427	0.16624443	-0.0362937
161.8	0.35327517	-0.5593651	-0.5705114	0.18402234	0.33197427	0.16624443	-0.0362937
161.9	0.35327517	-0.5593651	-0.5705114	0.18402234	0.33197427	0.16624443	-0.0362937
162	0.35359132	-0.5594721	-0.5706285	0.18370014	0.33167186	0.16642251	-0.0361779
162.1	0.35359132	-0.5594721	-0.5706285	0.18370014	0.33167186	0.16642251	-0.0361779
162.2	0.35359132	-0.5594721	-0.5706285	0.18370014	0.33167186	0.16642251	-0.0361779
162.3	0.35359132	-0.5594721	-0.5706285	0.18370014	0.33167186	0.16642251	-0.0361779
162.4	0.35359132	-0.5594721	-0.5706285	0.18370014	0.33167186	0.16642251	-0.0361779
162.5	0.35359132	-0.5594721	-0.5706285	0.18370014	0.33167186	0.16642251	-0.0361779
162.6	0.35363535	-0.5593783	-0.5706542	0.18354599	0.33184515	0.1660753	-0.0364603
162.7	0.35363535	-0.5593783	-0.5706542	0.18354599	0.33184515	0.1660753	-0.0364603
162.8	0.35363535	-0.5593783	-0.5706542	0.18354599	0.33184515	0.1660753	-0.0364603
162.9	0.35363535	-0.5593783	-0.5706542	0.18354599	0.33184515	0.1660753	-0.0364603
163	0.35337283	-0.5593433	-0.5708073	0.18366365	0.33167033	0.16652823	-0.0361137
163.1	0.35337283	-0.5593433	-0.5708073	0.18366365	0.33167033	0.16652823	-0.0361137
163.2	0.35340139	-0.5592053	-0.5706327	0.18356147	0.33187855	0.16646763	-0.0363005
163.3	0.35354667	-0.559046	-0.5708733	0.1833937	0.33179612	0.16647367	-0.0362158
163.4	0.35354667	-0.559046	-0.5708733	0.1833937	0.33179612	0.16647367	-0.0362158
163.5	0.35354667	-0.559046	-0.5708733	0.1833937	0.33179612	0.16647367	-0.0362158
163.6	0.35354667	-0.559046	-0.5708733	0.1833937	0.33179612	0.16647367	-0.0362158
163.7	0.35354667	-0.559046	-0.5708733	0.1833937	0.33179612	0.16647367	-0.0362158
163.8	0.35325929	-0.5589022	-0.5707703	0.183435	0.33154751	0.16659823	-0.0361677
163.9	0.35325929	-0.5589022	-0.5707703	0.183435	0.33154751	0.16659823	-0.0361677
164	0.35325929	-0.5589022	-0.5707703	0.183435	0.33154751	0.16659823	-0.0361677
164.1	0.35325929	-0.5589022	-0.5707703	0.183435	0.33154751	0.16659823	-0.0361677
164.2	0.35325929	-0.5589022	-0.5707703	0.183435	0.33154751	0.16659823	-0.0361677
164.3	0.35325929	-0.5589022	-0.5707703	0.183435	0.33154751	0.16659823	-0.0361677
164.4	0.35325929	-0.5589022	-0.5707703	0.183435	0.33154751	0.16659823	-0.0361677
164.5	0.353537	-0.5588517	-0.5708215	0.1833157	0.33153872	0.16677898	-0.0363002
164.6	0.353537	-0.5588517	-0.5708215	0.1833157	0.33153872	0.16677898	-0.0363002
164.7	0.353537	-0.5588517	-0.5708215	0.1833157	0.33153872	0.16677898	-0.0363002
164.8	0.35371219	-0.5587979	-0.5708717	0.18324913	0.3313022	0.1668074	-0.0364213
164.9	0.35371219	-0.5587979	-0.5708717	0.18324913	0.3313022	0.1668074	-0.0364213

171.1	0.35428895	-0.5584181	-0.5715286	0.18277068	0.33157234	0.16729361	-0.0367831
171.2	0.35428895	-0.5584181	-0.5715286	0.18277068	0.33157234	0.16729361	-0.0367831
171.3	0.35428895	-0.5584181	-0.5715286	0.18277068	0.33157234	0.16729361	-0.0367831
171.4	0.35428895	-0.5584181	-0.5715286	0.18277068	0.33157234	0.16729361	-0.0367831
171.5	0.35464535	-0.5582958	-0.5714432	0.1823999	0.33145266	0.16719381	-0.0370039
171.6	0.35464535	-0.5582958	-0.5714432	0.1823999	0.33145266	0.16719381	-0.0370039
171.7	0.35464535	-0.5582958	-0.5714432	0.1823999	0.33145266	0.16719381	-0.0370039
171.8	0.35464535	-0.5582958	-0.5714432	0.1823999	0.33145266	0.16719381	-0.0370039
171.9	0.35464535	-0.5582958	-0.5714432	0.1823999	0.33145266	0.16719381	-0.0370039
172	0.35464535	-0.5582958	-0.5714432	0.1823999	0.33145266	0.16719381	-0.0370039
172.1	0.35464535	-0.5582958	-0.5714432	0.1823999	0.33145266	0.16719381	-0.0370039
172.2	0.35483976	-0.5582933	-0.5714476	0.18223911	0.33123703	0.16733859	-0.0370983
172.3	0.35483976	-0.5582933	-0.5714476	0.18223911	0.33123703	0.16733859	-0.0370983
172.4	0.35483976	-0.5582933	-0.5714476	0.18223911	0.33123703	0.16733859	-0.0370983
172.5	0.35483976	-0.5582933	-0.5714476	0.18223911	0.33123703	0.16733859	-0.0370983
172.6	0.35483976	-0.5582933	-0.5714476	0.18223911	0.33123703	0.16733859	-0.0370983
172.7	0.35503473	-0.558219	-0.5716343	0.18239882	0.33112237	0.16769614	-0.0370093
172.8	0.35503473	-0.558219	-0.5716343	0.18239882	0.33112237	0.16769614	-0.0370093
172.9	0.35511387	-0.5582403	-0.5715851	0.18219582	0.33102529	0.16784373	-0.0368124
173	0.35511387	-0.5582403	-0.5715851	0.18219582	0.33102529	0.16784373	-0.0368124
173.1	0.35511387	-0.5582403	-0.5715851	0.18219582	0.33102529	0.16784373	-0.0368124
173.2	0.35511387	-0.5582403	-0.5715851	0.18219582	0.33102529	0.16784373	-0.0368124
173.3	0.35511387	-0.5582403	-0.5715851	0.18219582	0.33102529	0.16784373	-0.0368124
173.4	0.35511387	-0.5582403	-0.5715851	0.18219582	0.33102529	0.16784373	-0.0368124
173.5	0.35511387	-0.5582403	-0.5715851	0.18219582	0.33102529	0.16784373	-0.0368124
173.6	0.35511387	-0.5582403	-0.5715851	0.18219582	0.33102529	0.16784373	-0.0368124
173.7	0.35511387	-0.5582403	-0.5715851	0.18219582	0.33102529	0.16784373	-0.0368124
173.8	0.35511387	-0.5582403	-0.5715851	0.18219582	0.33102529	0.16784373	-0.0368124
173.9	0.35511387	-0.5582403	-0.5715851	0.18219582	0.33102529	0.16784373	-0.0368124
174	0.35511387	-0.5582403	-0.5715851	0.18219582	0.33102529	0.16784373	-0.0368124
174.1	0.35520645	-0.5581394	-0.5717004	0.1821056	0.33087867	0.16780042	-0.037209
174.2	0.35520645	-0.5581394	-0.5717004	0.1821056	0.33087867	0.16780042	-0.037209
174.3	0.35520645	-0.5581394	-0.5717004	0.1821056	0.33087867	0.16780042	-0.037209
174.4	0.35520645	-0.5581394	-0.5717004	0.1821056	0.33087867	0.16780042	-0.037209
174.5	0.35520645	-0.5581394	-0.5717004	0.1821056	0.33087867	0.16780042	-0.037209
174.6	0.35520645	-0.5581394	-0.5717004	0.1821056	0.33087867	0.16780042	-0.037209
174.7	0.35512332	-0.5578344	-0.5717762	0.18199477	0.33070139	0.1677432	-0.0373452
174.8	0.35512332	-0.5578344	-0.5717762	0.18199477	0.33070139	0.1677432	-0.0373452
174.9	0.35512332	-0.5578344	-0.5717762	0.18199477	0.33070139	0.1677432	-0.0373452
175	0.35519039	-0.5578363	-0.5717174	0.18173383	0.33078389	0.16772388	-0.0368879
175.1	0.35519039	-0.5578363	-0.5717174	0.18173383	0.33078389	0.16772388	-0.0368879
175.2	0.35519039	-0.5578363	-0.5717174	0.18173383	0.33078389	0.16772388	-0.0368879
175.3	0.35519039	-0.5578363	-0.5717174	0.18173383	0.33078389	0.16772388	-0.0368879
175.4	0.35519039	-0.5578363	-0.5717174	0.18173383	0.33078389	0.16772388	-0.0368879
175.5	0.35519039	-0.5578363	-0.5717174	0.18173383	0.33078389	0.16772388	-0.0368879
175.6	0.35519039	-0.5578363	-0.5717174	0.18173383	0.33078389	0.16772388	-0.0368879
175.7	0.35530133	-0.5578577	-0.5717515	0.18160561	0.33061337	0.16799823	-0.0364384
175.8	0.35530133	-0.5578577	-0.5717515	0.18160561	0.33061337	0.16799823	-0.0364384
175.9	0.35530133	-0.5578577	-0.5717515	0.18160561	0.33061337	0.16799823	-0.0364384
176	0.35545978	-0.5579154	-0.5717494	0.18170769	0.33080254	0.16813858	-0.0365274
176.1	0.35549617	-0.5576005	-0.571789	0.18187683	0.33081278	0.16801401	-0.0368265
176.2	0.35549617	-0.5576005	-0.571789	0.18187683	0.33081278	0.16801401	-0.0368265
176.3	0.35549617	-0.5576005	-0.571789	0.18187683	0.33081278	0.16801401	-0.0368265
176.4	0.35549617	-0.5576005	-0.571789	0.18187683	0.33081278	0.16801401	-0.0368265
176.5	0.35549617	-0.5576005	-0.571789	0.18187683	0.33081278	0.16801401	-0.0368265
176.6	0.35549617	-0.5576005	-0.571789	0.18187683	0.33081278	0.16801401	-0.0368265
176.7	0.35549617	-0.5576005	-0.571789	0.18187683	0.33081278	0.16801401	-0.0368265
176.8	0.35549617	-0.5576005	-0.571789	0.18187683	0.33081278	0.16801401	-0.0368265
176.9	0.35558509	-0.5572911	-0.5718135	0.18192277	0.33063585	0.16784554	-0.0371651
177	0.35550719	-0.5571599	-0.5716881	0.18184291	0.33096643	0.16779734	-0.0370935
177.1	0.35550719	-0.5571599	-0.5716881	0.18184291	0.33096643	0.16779734	-0.0370935
177.2	0.35550719	-0.5571599	-0.5716881	0.18184291	0.33096643	0.16779734	-0.0370935
177.3	0.35550719	-0.5571599	-0.5716881	0.18184291	0.33096643	0.16779734	-0.0370935
177.4	0.35550719	-0.5571599	-0.5716881	0.18184291	0.33096643	0.16779734	-0.0370935
177.5	0.35573866	-0.5571107	-0.5717429	0.18147119	0.33061734	0.16794196	-0.0370268
177.6	0.35573866	-0.5571107	-0.5717429	0.18147119	0.33061734	0.16794196	-0.0370268
177.7	0.35573866	-0.5571107	-0.5717429	0.18147119	0.33061734	0.16794196	-0.0370268
177.8	0.35573866	-0.5571107	-0.5717429	0.18147119	0.33061734	0.16794196	-0.0370268
177.9	0.35568975	-0.5568608	-0.5716521	0.18131284	0.3305935	0.16781302	-0.0372114
178	0.35568975	-0.5568608	-0.5716521	0.18131284	0.3305935	0.16781302	-0.0372114
178.1	0.35568975	-0.5568608	-0.5716521	0.18131284	0.3305935	0.16781302	-0.0372114
178.2	0.35576072	-0.5567802	-0.5716108	0.1812934	0.33070259	0.16778753	-0.0372894
178.3	0.35576072	-0.5567802	-0.5716108	0.1812934	0.33070259	0.16778753	-0.0372894
178.4	0.35576072	-0.5567802	-0.5716108	0.1812934	0.33070259	0.16778753	-0.0372894
178.5	0.35576072	-0.5567802	-0.5716108	0.1812934	0.33070259	0.16778753	-0.0372894
178.6	0.35576072	-0.5567802	-0.5716108	0.1812934	0.33070259	0.16778753	-0.0372894
178.7	0.35591316	-0.5566354	-0.571819	0.18134395	0.33053863	0.16801328	-0.0374211
178.8	0.35591316	-0.5566354	-0.571819	0.18134395	0.33053863	0.16801328	-0.0374211
178.9	0.35591316	-0.5566354	-0.571819	0.18134395	0.33053863	0.16801328	-0.0374211
179	0.35591316	-0.5566354	-0.571819	0.18134395	0.33053863	0.16801328	-0.0374211
179.1	0.35591316	-0.5566354	-0.571819	0.18134395	0.33053863	0.16801328	-0.0374211
179.2	0.35591316	-0.5566354	-0.571819	0.18134395	0.33053863	0.16801328	-0.0374211
179.3	0.35591316	-0.5566354	-0.571819	0.18134395	0.33053863	0.16801328	-0.0374211
179.4	0.35591316	-0.5566354	-0.571819	0.18134395	0.33053863	0.16801328	-0.0374211
179.5	0.35591316	-0.5566354	-0.571819	0.18134395	0.33053863	0.16801328	-0.0374211
179.6	0.35591316	-0.5566354	-0.571819	0.18134395	0.33053863	0.16801328	-0.0374211
179.7	0.35591316	-0.5566354	-0.571819	0.18134395	0.33053863	0.16801328	-0.0374211
179.8	0.35591316	-0.5566354	-0.571819	0.18134395	0.33053863	0.16801328	-0.0374211
179.9	0.35591316	-0.5566354	-0.571819	0.18134395	0.33053863	0.16801328	-0.0374211

180	0.35591316	-0.5566354	-0.571819	0.18134395	0.33053863	0.16801328	-0.0374211
180.1	0.35591316	-0.5566354	-0.571819	0.18134395	0.33053863	0.16801328	-0.0374211
180.2	0.35591316	-0.5566354	-0.571819	0.18134395	0.33053863	0.16801328	-0.0374211
180.3	0.35591316	-0.5566354	-0.571819	0.18134395	0.33053863	0.16801328	-0.0374211
180.4	0.35591316	-0.5566354	-0.571819	0.18134395	0.33053863	0.16801328	-0.0374211
180.5	0.35591316	-0.5566354	-0.571819	0.18134395	0.33053863	0.16801328	-0.0374211
180.6	0.35591316	-0.5566354	-0.571819	0.18134395	0.33053863	0.16801328	-0.0374211
180.7	0.35591316	-0.5566354	-0.571819	0.18134395	0.33053863	0.16801328	-0.0374211
180.8	0.35591316	-0.5566354	-0.571819	0.18134395	0.33053863	0.16801328	-0.0374211
180.9	0.35591316	-0.5566354	-0.571819	0.18134395	0.33053863	0.16801328	-0.0374211
181	0.35615035	-0.5564022	-0.5717901	0.18106564	0.33035443	0.16780189	-0.0375629
181.1	0.35615035	-0.5564022	-0.5717901	0.18106564	0.33035443	0.16780189	-0.0375629
181.2	0.35607789	-0.5561447	-0.5718765	0.18098011	0.33025119	0.16807392	-0.0376893
181.3	0.35607789	-0.5561447	-0.5718765	0.18098011	0.33025119	0.16807392	-0.0376893
181.4	0.35607789	-0.5561447	-0.5718765	0.18098011	0.33025119	0.16807392	-0.0376893
181.5	0.35607789	-0.5561447	-0.5718765	0.18098011	0.33025119	0.16807392	-0.0376893
181.6	0.35607789	-0.5561447	-0.5718765	0.18098011	0.33025119	0.16807392	-0.0376893
181.7	0.35607789	-0.5561447	-0.5718765	0.18098011	0.33025119	0.16807392	-0.0376893
181.8	0.35607789	-0.5561447	-0.5718765	0.18098011	0.33025119	0.16807392	-0.0376893
181.9	0.35607497	-0.5560421	-0.5719374	0.18116766	0.33008522	0.16834546	-0.037679
182	0.35607497	-0.5560421	-0.5719374	0.18116766	0.33008522	0.16834546	-0.037679
182.1	0.35607497	-0.5560421	-0.5719374	0.18116766	0.33008522	0.16834546	-0.037679
182.2	0.35607497	-0.5560421	-0.5719374	0.18116766	0.33008522	0.16834546	-0.037679
182.3	0.35607497	-0.5560421	-0.5719374	0.18116766	0.33008522	0.16834546	-0.037679
182.4	0.35607497	-0.5560421	-0.5719374	0.18116766	0.33008522	0.16834546	-0.037679
182.5	0.35607497	-0.5560421	-0.5719374	0.18116766	0.33008522	0.16834546	-0.037679
182.6	0.35607497	-0.5560421	-0.5719374	0.18116766	0.33008522	0.16834546	-0.037679
182.7	0.35607497	-0.5560421	-0.5719374	0.18116766	0.33008522	0.16834546	-0.037679
182.8	0.35607497	-0.5560421	-0.5719374	0.18116766	0.33008522	0.16834546	-0.037679
182.9	0.35607497	-0.5560421	-0.5719374	0.18116766	0.33008522	0.16834546	-0.037679
183	0.35607497	-0.5560421	-0.5719374	0.18116766	0.33008522	0.16834546	-0.037679
183.1	0.35607497	-0.5560421	-0.5719374	0.18116766	0.33008522	0.16834546	-0.037679

195	0.3579165	-0.5527676	-0.5734761	0.18280735	0.33142487	0.16954778	-0.0400587
195.1	0.3579165	-0.5527676	-0.5734761	0.18280735	0.33142487	0.16954778	-0.0400587
195.2	0.3579165	-0.5527676	-0.5734761	0.18280735	0.33142487	0.16954778	-0.0400587
195.3	0.3579165	-0.5527676	-0.5734761	0.18280735	0.33142487	0.16954778	-0.0400587
195.4	0.3579165	-0.5527676	-0.5734761	0.18280735	0.33142487	0.16954778	-0.0400587
195.5	0.3579165	-0.5527676	-0.5734761	0.18280735	0.33142487	0.16954778	-0.0400587
195.6	0.3579165	-0.5527676	-0.5734761	0.18280735	0.33142487	0.16954778	-0.0400587
195.7	0.3579165	-0.5527676	-0.5734761	0.18280735	0.33142487	0.16954778	-0.0400587
195.8	0.3579165	-0.5527676	-0.5734761	0.18280735	0.33142487	0.16954778	-0.0400587
195.9	0.3579165	-0.5527676	-0.5734761	0.18280735	0.33142487	0.16954778	-0.0400587
196	0.3578089	-0.5523456	-0.5733743	0.18293329	0.33161148	0.16957091	-0.0403215
196.1	0.3578089	-0.5523456	-0.5733743	0.18293329	0.33161148	0.16957091	-0.0403215
196.2	0.35797697	-0.5522126	-0.5734472	0.18320662	0.3320438	0.16928179	-0.0402402
196.3	0.35797697	-0.5522126	-0.5734472	0.18320662	0.3320438	0.16928179	-0.0402402
196.4	0.35797697	-0.5522126	-0.5734472	0.18320662	0.3320438	0.16928179	-0.0402402
196.5	0.35797697	-0.5522126	-0.5734472	0.18320662	0.3320438	0.16928179	-0.0402402
196.6	0.35797697	-0.5522126	-0.5734472	0.18320662	0.3320438	0.16928179	-0.0402402
196.7	0.35797697	-0.5522126	-0.5734472	0.18320662	0.3320438	0.16928179	-0.0402402
196.8	0.35797697	-0.5522126	-0.5734472	0.18320662	0.3320438	0.16928179	-0.0402402
196.9	0.35797697	-0.5522126	-0.5734472	0.18320662	0.3320438	0.16928179	-0.0402402
197	0.35797697	-0.5522126	-0.5734472	0.18320662	0.3320438	0.16928179	-0.0402402
197.1	0.35797697	-0.5522126	-0.5734472	0.18320662	0.3320438	0.16928179	-0.0402402
197.2	0.35797697	-0.5522126	-0.5734472	0.18320662	0.3320438	0.16928179	-0.0402402
197.3	0.35797697	-0.5522126	-0.5734472	0.18320662	0.3320438	0.16928179	-0.0402402
197.4	0.35797697	-0.5522126	-0.5734472	0.18320662	0.3320438	0.16928179	-0.0402402
197.5	0.35797697	-0.5522126	-0.5734472	0.18320662	0.3320438	0.16928179	-0.0402402
197.6	0.35797697	-0.5522126	-0.5734472	0.18320662	0.3320438	0.16928179	-0.0402402
197.7	0.35797697	-0.5522126	-0.5734472	0.18320662	0.3320438	0.16928179	-0.0402402
197.8	0.35797697	-0.5522126	-0.5734472	0.18320662	0.3320438	0.16928179	-0.0402402
197.9	0.35797697	-0.5522126	-0.5734472	0.18320662	0.3320438	0.16928179	-0.0402402
198	0.35797697	-0.5522126	-0.5734472	0.18320662	0.3320438	0.16928179	-0.0402402
198.1	0.35797697	-0.5522126	-0.5734472	0.18320662	0.3320438	0.16928179	-0.0402402

210	0.35824209	-0.5490276	-0.5750957	0.1834328	0.33384772	0.1693004	-0.0422192
210.1	0.35824209	-0.5490276	-0.5750957	0.1834328	0.33384772	0.1693004	-0.0422192
210.2	0.35824209	-0.5490276	-0.5750957	0.1834328	0.33384772	0.1693004	-0.0422192
210.3	0.35824209	-0.5490276	-0.5750957	0.1834328	0.33384772	0.1693004	-0.0422192
210.4	0.35824209	-0.5490276	-0.5750957	0.1834328	0.33384772	0.1693004	-0.0422192
210.5	0.35824209	-0.5490276	-0.5750957	0.1834328	0.33384772	0.1693004	-0.0422192
210.6	0.35824209	-0.5490276	-0.5750957	0.1834328	0.33384772	0.1693004	-0.0422192
210.7	0.35824209	-0.5490276	-0.5750957	0.1834328	0.33384772	0.1693004	-0.0422192
210.8	0.35824209	-0.5490276	-0.5750957	0.1834328	0.33384772	0.1693004	-0.0422192
210.9	0.35824209	-0.5490276	-0.5750957	0.1834328	0.33384772	0.1693004	-0.0422192
211	0.35824209	-0.5490276	-0.5750957	0.1834328	0.33384772	0.1693004	-0.0422192
211.1	0.35858857	-0.548878	-0.5751805	0.18343453	0.33409535	0.16945207	-0.0424934
211.2	0.35846729	-0.5488449	-0.5753156	0.18334964	0.33400122	0.16970326	-0.0422055
211.3	0.35846729	-0.5488449	-0.5753156	0.18334964	0.33400122	0.16970326	-0.0422055
211.4	0.35846729	-0.5488449	-0.5753156	0.18334964	0.33400122	0.16970326	-0.0422055
211.5	0.35846729	-0.5488449	-0.5753156	0.18334964	0.33400122	0.16970326	-0.0422055
211.6	0.35846729	-0.5488449	-0.5753156	0.18334964	0.33400122	0.16970326	-0.0422055
211.7	0.35846729	-0.5488449	-0.5753156	0.18334964	0.33400122	0.16970326	-0.0422055
211.8	0.35846729	-0.5488449	-0.5753156	0.18334964	0.33400122	0.16970326	-0.0422055
211.9	0.35846729	-0.5488449	-0.5753156	0.18334964	0.33400122	0.16970326	-0.0422055
212	0.35846729	-0.5488449	-0.5753156	0.18334964	0.33400122	0.16970326	-0.0422055
212.1	0.35846729	-0.5488449	-0.5753156	0.18334964	0.33400122	0.16970326	-0.0422055
212.2	0.35846729	-0.5488449	-0.5753156	0.18334964	0.33400122	0.16970326	-0.0422055
212.3	0.35846729	-0.5488449	-0.5753156	0.18334964	0.33400122	0.16970326	-0.0422055
212.4	0.35843408	-0.5487557	-0.5754834	0.18329536	0.33409658	0.16949092	-0.04253
212.5	0.35843408	-0.5487557	-0.5754834	0.18329536	0.33409658	0.16949092	-0.04253
212.6	0.35843408	-0.5487557	-0.5754834	0.18329536	0.33409658	0.16949092	-0.04253
212.7	0.35843408	-0.5487557	-0.5754834	0.18329536	0.33409658	0.16949092	-0.04253
212.8	0.35843408	-0.5487557	-0.5754834	0.18329536	0.33409658	0.16949092	-0.04253
212.9	0.35843408	-0.5487557	-0.5754834	0.18329536	0.33409658	0.16949092	-0.04253
213	0.35856477	-0.5487916	-0.575601	0.18318717	0.33394511	0.16968528	-0.0421586
213.1	0.35856477	-0.5487916	-0.575601	0.18318717	0.33394511	0.16968528	-0.0421586

225	0.35783519	-0.5477135	-0.5768199	0.1819586	0.33545968	0.17281825	-0.0426929
225.1	0.35771661	-0.5478274	-0.5769154	0.18179425	0.33565958	0.172957	-0.0429941
225.2	0.35773953	-0.5479398	-0.5771384	0.18173808	0.33600059	0.17326416	-0.043128
225.3	0.35773953	-0.5479398	-0.5771384	0.18173808	0.33600059	0.17326416	-0.043128
225.4	0.35773953	-0.5479398	-0.5771384	0.18173808	0.33600059	0.17326416	-0.043128
225.5	0.35773953	-0.5479398	-0.5771384	0.18173808	0.33600059	0.17326416	-0.043128
225.6	0.35765738	-0.5479737	-0.5771253	0.18123172	0.33660991	0.17410181	-0.0435533
225.7	0.35736621	-0.5480616	-0.5772539	0.18118199	0.3367891	0.17453488	-0.0437768
225.8	0.35736621	-0.5480616	-0.5772539	0.18118199	0.3367891	0.17453488	-0.0437768
225.9	0.35736621	-0.5480616	-0.5772539	0.18118199	0.3367891	0.17453488	-0.0437768
226	0.35724879	-0.548103	-0.5772463	0.1809421	0.33700892	0.1748489	-0.0438709
226.1	0.35724879	-0.548103	-0.5772463	0.1809421	0.33700892	0.1748489	-0.0438709
226.2	0.35724879	-0.548103	-0.5772463	0.1809421	0.33700892	0.1748489	-0.0438709
226.3	0.35724879	-0.548103	-0.5772463	0.1809421	0.33700892	0.1748489	-0.0438709
226.4	0.35724879	-0.548103	-0.5772463	0.1809421	0.33700892	0.1748489	-0.0438709
226.5	0.35724879	-0.548103	-0.5772463	0.1809421	0.33700892	0.1748489	-0.0438709
226.6	0.35724879	-0.548103	-0.5772463	0.1809421	0.33700892	0.1748489	-0.0438709
226.7	0.35724879	-0.548103	-0.5772463	0.1809421	0.33700892	0.1748489	-0.0438709
226.8	0.35720865	-0.5480804	-0.5774463	0.18086884	0.33737321	0.1752668	-0.0441103
226.9	0.35720865	-0.5480804	-0.5774463	0.18086884	0.33737321	0.1752668	-0.0441103
227	0.35706767	-0.548215	-0.5776783	0.18075561	0.33770758	0.17567252	-0.0442107
227.1	0.35706767	-0.548215	-0.5776783	0.18075561	0.33770758	0.17567252	-0.0442107
227.2	0.35706767	-0.548215	-0.5776783	0.18075561	0.33770758	0.17567252	-0.0442107
227.3	0.35711524	-0.5481504	-0.5778306	0.18070501	0.33786367	0.17588525	-0.044665
227.4	0.35707181	-0.5481911	-0.5778712	0.18074561	0.33816684	0.17614202	-0.0447229
227.5	0.35720401	-0.5483542	-0.5780887	0.18054491	0.33836502	0.17633132	-0.0451579
227.6	0.35719659	-0.5484613	-0.5784147	0.18045709	0.33874614	0.17698097	-0.0456446
227.7	0.35719659	-0.5484613	-0.5784147	0.18045709	0.33874614	0.17698097	-0.0456446
227.8	0.3571685	-0.5485458	-0.5785723	0.18020563	0.33873978	0.17700221	-0.0459923
227.9	0.3571685	-0.5485458	-0.5785723	0.18020563	0.33873978	0.17700221	-0.0459923
228	0.3571685	-0.5485458	-0.5785723	0.18020563	0.33873978	0.17700221	-0.0459923
228.1	0.35718798	-0.5483903	-0.578576	0.17990107	0.33898626	0.1774247	-0.0462896

183.2	0.35607497	-0.5560421	-0.5719374	0.18116766	0.33008522	0.16834546	-0.037679
183.3	0.3564083	-0.5559818	-0.5720355	0.18137078	0.33039253	0.16823793	-0.0379077
183.4	0.3564083	-0.5559818	-0.5720355	0.18137078	0.33039253	0.16823793	-0.0379077
183.5	0.3564083	-0.5559818	-0.5720355	0.18137078	0.33039253	0.16823793	-0.0379077
183.6	0.35676891	-0.5558244	-0.572235	0.18140932	0.3303665	0.16852451	-0.0379151
183.7	0.35676891	-0.5558244	-0.572235	0.18140932	0.3303665	0.16852451	-0.0379151
183.8	0.35676891	-0.5558244	-0.572235	0.18140932	0.3303665	0.16852451	-0.0379151
183.9	0.35690987	-0.5556508	-0.572337	0.1817735	0.3304797	0.16855455	-0.0380662
184	0.35690987	-0.5556508	-0.572337	0.1817735	0.3304797	0.16855455	-0.0380662
184.1	0.35690987	-0.5556508	-0.572337	0.1817735	0.3304797	0.16855455	-0.0380662
184.2	0.35690987	-0.5556508	-0.572337	0.1817735	0.3304797	0.16855455	-0.0380662
184.3	0.35690987	-0.5556508	-0.572337	0.1817735	0.3304797	0.16855455	-0.0380662
184.4	0.35690987	-0.5556508	-0.572337	0.1817735	0.3304797	0.16855455	-0.0380662
184.5	0.35690987	-0.5556508	-0.572337	0.1817735	0.3304797	0.16855455	-0.0380662
184.6	0.35690987	-0.5556508	-0.572337	0.1817735	0.3304797	0.16855455	-0.0380662
184.7	0.35690987	-0.5556508	-0.572337	0.1817735	0.3304797	0.16855455	-0.0380662
184.8	0.35702791	-0.5556834	-0.5724184	0.18192807	0.33021267	0.16866757	-0.0382467
184.9	0.35702791	-0.5556834	-0.5724184	0.18192807	0.33021267	0.16866757	-0.0382467
185	0.35702791	-0.5556834	-0.5724184	0.18192807	0.33021267	0.16866757	-0.0382467
185.1	0.35702791	-0.5556834	-0.5724184	0.18192807	0.33021267	0.16866757	-0.0382467
185.2	0.35702791	-0.5556834	-0.5724184	0.18192807	0.33021267	0.16866757	-0.0382467
185.3	0.35702791	-0.5556834	-0.5724184	0.18192807	0.33021267	0.16866757	-0.0382467
185.4	0.35702791	-0.5556834	-0.5724184	0.18192807	0.33021267	0.16866757	-0.0382467
185.5	0.35702791	-0.5556834	-0.5724184	0.18192807	0.33021267	0.16866757	-0.0382467
185.6	0.35702791	-0.5556834	-0.5724184	0.18192807	0.33021267	0.16866757	-0.0382467
185.7	0.35702791	-0.5556834	-0.5724184	0.18192807	0.33021267	0.16866757	-0.0382467
185.8	0.35702791	-0.5556834	-0.5724184	0.18192807	0.33021267	0.16866757	-0.0382467
185.9	0.35702791	-0.5556834	-0.5724184	0.18192807	0.33021267	0.16866757	-0.0382467
186	0.35702791	-0.5556834	-0.5724184	0.18192807	0.33021267	0.16866757	-0.0382467
186.1	0.35702791	-0.5556834	-0.5724184	0.18192807	0.33021267	0.16866757	-0.0382467
186.2	0.35695847	-0.5553532	-0.5723049	0.1817693	0.33018127	0.16868794	-0.0384476
186.3	0.35713462	-0.5550936	-0.5723253	0.18172574	0.33003543	0.16910616	-0.0382113
186.4	0.35713462	-0.5550936	-0.5723253	0.18172574	0.33003543	0.16910616	-0.0382113
186.5	0.35714839	-0.5549722	-0.5723851	0.181986	0.32967747	0.16952392	-0.0380064
186.6	0.35714839	-0.5549722	-0.5723851	0.181986	0.32967747	0.16952392	-0.0380064
186.7	0.35714839	-0.5549722	-0.5723851	0.181986	0.32967747	0.16952392	-0.0380064
186.8	0.35714839	-0.5549722	-0.5723851	0.181986	0.32967747	0.16952392	-0.0380064
186.9	0.35714839	-0.5549722	-0.5723851	0.181986	0.32967747	0.16952392	-0.0380064
187	0.35714839	-0.5549722	-0.5723851	0.181986	0.32967747	0.16952392	-0.0380064
187.1	0.35714839	-0.5549722	-0.5723851	0.181986	0.32967747	0.16952392	-0.0380064
187.2	0.35714839	-0.5549722	-0.5723851	0.181986	0.32967747	0.16952392	-0.0380064
187.3	0.35714839	-0.5549722	-0.5723851	0.181986	0.32967747	0.16952392	-0.0380064
187.4	0.35714839	-0.5549722	-0.5723851	0.181986	0.32967747	0.16952392	-0.0380064
187.5	0.35707649	-0.5547791	-0.5724388	0.18211983	0.33004547	0.16991001	-0.0381348
187.6	0.35707649	-0.5547791	-0.5724388	0.18211983	0.33004547	0.16991001	-0.0381348
187.7	0.35707649	-0.5547791	-0.5724388	0.18211983	0.33004547	0.16991001	-0.0381348
187.8	0.35707649	-0.5547791	-0.5724388	0.18211983	0.33004547	0.16991001	-0.0381348
187.9	0.35707649	-0.5547791	-0.5724388	0.18211983	0.33004547	0.16991001	-0.0381348
188	0.35707649	-0.5547791	-0.5724388	0.18211983	0.33004547	0.16991001	-0.0381348
188.1	0.35707649	-0.5547791	-0.5724388	0.18211983	0.33004547	0.16991001	-0.0381348
188.2	0.35707649	-0.5547791	-0.5724388	0.18211983	0.33004547	0.16991001	-0.0381348
188.3	0.35707649	-0.5547791	-0.5724388	0.18211983	0.33004547	0.16991001	-0.0381348
188.4	0.35707649	-0.5547791	-0.5724388	0.18211983	0.33004547	0.16991001	-0.0381348
188.5	0.35707649	-0.5547791	-0.5724388	0.18211983	0.33004547	0.16991001	-0.0381348
188.6	0.35734393	-0.5545656	-0.5725407	0.18204839	0.33021425	0.16977606	-0.0379902
188.7	0.35734393	-0.5545656	-0.5725407	0.18204839	0.33021425	0.16977606	-0.0379902
188.8	0.35734393	-0.5545656	-0.5725407	0.18204839	0.33021425	0.16977606	-0.0379902
188.9	0.35734393	-0.5545656	-0.5725407	0.18204839	0.33021425	0.16977606	-0.0379902
189	0.35734393	-0.5545656	-0.5725407	0.18204839	0.33021425	0.16977606	-0.0379902
189.1	0.35734393	-0.5545656	-0.5725407	0.18204839	0.33021425	0.16977606	-0.0379902
189.2	0.35734393	-0.5545656	-0.5725407	0.18204839	0.33021425	0.16977606	-0.0379902
189.3	0.35734393	-0.5545656	-0.5725407	0.18204839	0.33021425	0.16977606	-0.0379902
189.4	0.35734393	-0.5545656	-0.5725407	0.18204839	0.33021425	0.16977606	-0.0379902
189.5	0.35734393	-0.5545656	-0.5725407	0.18204839	0.33021425	0.16977606	-0.0379902
189.6	0.35734393	-0.5545656	-0.5725407	0.18204839	0.33021425	0.16977606	-0.0379902
189.7	0.35734393	-0.5545656	-0.5725407	0.18204839	0.33021425	0.16977606	-0.0379902
189.8	0.35734393	-0.5545656	-0.5725407	0.18204839	0.33021425	0.16977606	-0.0379902
189.9	0.35734393	-0.5545656	-0.5725407	0.18204839	0.33021425	0.16977606	-0.0379902
190	0.35734393	-0.5545656	-0.5725407	0.18204839	0.33021425	0.16977606	-0.0379902
190.1	0.35718864	-0.5544665	-0.5726903	0.18208448	0.3305516	0.16964857	-0.0382766
190.2	0.35757964	-0.5542271	-0.5728201	0.18198695	0.33049347	0.16998769	-0.0385012
190.3	0.35757964	-0.5542271	-0.5728201	0.18198695	0.33049347	0.16998769	-0.0385012
190.4	0.35757964	-0.5542271	-0.5728201	0.18198695	0.33049347	0.16998769	-0.0385012
190.5	0.35759212	-0.5540074	-0.5726739	0.18190698	0.33019764	0.17006131	-0.0388118
190.6	0.35759212	-0.5540074	-0.5726739	0.18190698	0.33019764	0.17006131	-0.0388118
190.7	0.35759212	-0.5540074	-0.5726739	0.18190698	0.33019764	0.17006131	-0.0388118
190.8	0.35759212	-0.5540074	-0.5726739	0.18190698	0.33019764	0.17006131	-0.0388118
190.9	0.35759212	-0.5540074	-0.5726739	0.18190698	0.33019764	0.17006131	-0.0388118
191	0.35769331	-0.5538852	-0.5725948	0.18201862	0.33047987	0.17016659	-0.0389373
191.1	0.35769331	-0.5538852	-0.5725948	0.18201862	0.33047987	0.17016659	-0.0389373
191.2	0.35769331	-0.5538852	-0.5725948	0.18201862	0.33047987	0.17016659	-0.0389373
191.3	0.35769331	-0.5538852	-0.5725948	0.18201862	0.33047987	0.17016659	-0.0389373
191.4	0.35766831	-0.5537813	-0.572581	0.18222602	0.33072437	0.17002767	-0.0391886
191.5	0.35766831	-0.5537813	-0.572581	0.18222602	0.33072437	0.17002767	-0.0391886
191.6	0.35768031	-0.5534403	-0.5724424	0.18214587	0.33072823	0.16969783	-0.0388286
191.7	0.35757668	-0.5534357	-0.5724397	0.18219795	0.33049918	0.16954482	-0.0388169
191.8	0.35757668	-0.5534357	-0.5724397	0.18219795	0.33049918	0.16954482	-0.0388169
191.9	0.35757668	-0.5534357	-0.5724397	0.18219795	0.33049918	0.16954482	-0.0388169
192	0.35780491	-0.5533755	-0.5727024	0.18222584	0.33058634	0.16964668	-0.0389167
192.1	0.35775918	-0.5534863	-0.572763	0.18228894	0.33071783	0.16961853	-0.0391522
192.2	0.35775918	-0.5534863	-0.572763	0.18228894	0.33071783	0.16961853	-0.0391522
192.3	0.35764754	-0.5534968	-0.5727814	0.18217041	0.33077142	0.16976936	-0.0389804
192.4	0.35764754	-0.5534968	-0.5727814	0.18217041	0.33077142	0.16976936	-0.0389804
192.5	0.35781067	-0.5533406	-0.5726836	0.18216033	0.33093965	0.16986132	-0.0391425
192.6	0.35781067	-0.5533406	-0.5726836	0.18216033	0.33093965	0.16986132	-0.0391425
192.7	0.35781067	-0.5533406	-0.5726836	0.18216033	0.33093965	0.16986132	-0.0391425
192.8	0.35782748	-0.5530878	-0.5727063	0.18247487	0.33114169	0.16990814	-0.0394258
192.9	0.35782748	-0.5530878	-0.5727063	0.18247487	0.33114169	0.16990814	-0.0394258
193	0.35782748	-0.5530878	-0.5727063	0.18247487	0.33114169	0.16990814	-0.0394258
193.1	0.35782748	-0.5530878	-0.5727063	0.18247487	0.33114169	0.16990814	-0.0394258
193.2	0.35784166	-0.5529115	-0.5729239	0.18232342	0.33093438	0.16988498	-0.0397013
193.3	0.35784166	-0.5529115	-0.5729239	0.18232342	0.33093438	0.16988498	-0.0397013
193.4	0.35784166	-0.5529115	-0.5729239	0.18232342	0.33093438	0.16988498	-0.0397013
193.5	0.35796998	-0.5528016	-0.5730068	0.1823984	0.33105681	0.16958214	-0.0396925
193.6	0.35796998	-0.5528016	-0.5730068	0.1823984	0.33105681	0.16958214	-0.0396925
193.7	0.35796998	-0.5528016	-0.5730068	0.1823984	0.33105681	0.16958214	-0.0396925
193.8	0.35796998	-0.5528016	-0.5730068	0.1823984	0.33105681	0.16958214	-0.0396925
193.9	0.35806503	-0.5527643	-0.573237	0.18258255	0.33138269	0.169327	-0.0401362
194	0.35806503	-0.5527643	-0.573237	0.18258255	0.33138269	0.169327	-0.0401362
194.1	0.35806503	-0.5527643	-0.573237	0.18258255	0.33138269	0.169327	-0.0401362
194.2	0.35806503	-0.5527643	-0.573237	0.18258255	0.33138269	0.169327	-0.0401362
194.3	0.3579165	-0.5527676	-0.5734761	0.18280735	0.33142487	0.16954778	-0.0400587
194.4	0.3579165	-0.5527676	-0.5734761	0.18280735	0.33142487	0.16954778	-0.0400587
194.5	0.3579165	-0.5527676	-0.5734761	0.18280735	0.33142487	0.16954778	-0.0400587
194.6	0.3579165	-0.5527676	-0.5734761	0.18280735	0.33142487	0.16954778	-0.0400587
194.7	0.3579165	-0.5527676	-0.5734761	0.18280735	0.33142487	0.16954778	-0.0400587
194.8	0.3579165	-0.5527676	-0.5734761	0.18280735	0.33142487	0.16954778	-0.0400587
194.9	0.3579165	-0.5527676	-0.5734761	0.18280735	0.33142487	0.16954778	-0.0400587
198.2	0.35797697	-0.5522126	-0.5734472	0.18320662	0.3320438	0.16928179	-0.0402402
198.3	0.35797697	-0.5522126	-0.5734472	0.18320662	0.3320438	0.16928179	-0.0402402
198.4	0.35797697	-0.5522126	-0.5734472	0.18320662	0.3320438	0.16928179	-0.0402402
198.5	0.35797697	-0.5522126	-0.5734472	0.18320662	0.3320438	0.16928179	-0.0402402
198.6	0.35795351	-0.5521282	-0.5737313	0.18349628	0.33193789	0.16943652	-0.040017
198.7	0.35795351	-0.5521282	-0.5737313	0.18349628	0.33193789	0.16943652	-0.040017
198.8	0.35795351	-0.5521282	-0.5737313	0.18349628	0.33193789	0.16943652	-0.040017
198.9	0.35795351	-0.5521282	-0.5737313	0.18349628	0.33193789	0.16943652	-0.040017
199	0.35807102	-0.5520031	-0.5737484	0.18363917	0.33242923	0.16916605	-0.0400514
199.1	0.35803733	-0.5516573	-0.5738855	0.18360518	0.33220559	0.16942064	-0.0402859
199.2	0.35803733	-0.5516573	-0.5738855	0.18360518	0.33220559	0.16942064	-0.0402859
199.3	0.35803733	-0.5516573	-0.5738855	0.18360518	0.33220559	0.16942064	-0.0402859
199.4	0.35803733	-0.5516573	-0.5738855	0.18360518	0.33220559	0.16942064	-0.0402859
199.5	0.35803733	-0.5516573	-0.5738855	0.18360518	0.33220559	0.16942064	-0.0402859
199.6	0.35803733	-0.5516573	-0.5738855	0.18360518	0.33220559	0.16942064	-0.0402859
199.7	0.35803733	-0.5516573	-0.5738855	0.18360518	0.33220559	0.16942064	-0.0402859
199.8	0.35803733	-0.5516573	-0.5738855	0.18360518	0.33220559	0.16942064	-0.0402859
199.9	0.35803733	-0.5516573	-0.5738855	0.18360518	0.33220559	0.16942064	-0.0402859
200	0.35803733	-0.5516573	-0.5738855	0.18360518	0.33220559	0.16942064	-0.0402859
200.1	0.35803733	-0.5516573	-0.5738855	0.18360518	0.33220559	0.16942064	-0.0402859
200.2	0.35803733	-0.5516573	-0.5738855	0.18360518	0.33220559	0.16942064	-0.0402859
200.3	0.35803733	-0.5516573	-0.5738855	0.18360518	0.33220559	0.16942064	-0.0402859
200.4	0.35770109	-0.5514793	-0.5739482	0.18347058	0.33207974	0.16935174	-0.0399512
200.5	0.35770109	-0.5514793	-0.5739482	0.18347058	0.33207974	0.16935174	-0.0399512
200.6	0.35770109	-0.5514793	-0.5739482	0.18347058	0.33207974	0.16935174	-0.0399512
200.7	0.35770109	-0.5514793	-0.5739482	0.18347058	0.33207974	0.16935174	-0.0399512
200.8	0.35762601	-0.5512903	-0.5737833	0.18368355	0.33229745	0.16918327	-0.0401636
200.9	0.35762601	-0.5512903	-0.5737833	0.18368355	0.33229745	0.16918327	-0.0401636
201	0.35772554	-0.5512186	-0.573976	0.18361416	0.33251967	0.16900146	-0.0401286
201.1	0.35772554	-0.5512186	-0.573976	0.18361416	0.33251967	0.16900146	-0.0401286
201.2	0.35772554	-0.5512186	-0.573976	0.18361416	0.33251967	0.16900146	-0.0401286
201.3	0.35772554	-0.5512186	-0.573976	0.18361416	0.33251967	0.16900146	-0.0401286
201.4	0.35772554	-0.5512186	-0.573976	0.18361416	0.33251967	0.16900146	-0.0401286
201.5	0.35772554	-0.5512186	-0.573976	0.18361416	0.33251967	0.16900146	-0.0401286
201.6	0.35772554	-0.5512186	-0.573976	0.18361416	0.33251967	0.16900146	-0.0401286
201.7	0.35772554	-0.5512186	-0.573976	0.18361416	0.33251967	0.16900146	-0.0401286
201.8	0.35772554	-0.5512186	-0.573976	0.18361416	0.33251967	0.16900146	-0.0401286
201.9	0.35772554	-0.5512186	-0.573976	0.18361416	0.33251967	0.16900146	-0.0401286
202	0.35791994	-0.5512736	-0.5739557	0.1833366	0.33256399	0.16876039	-0.0402921
202.1	0.35791994	-0.5512736	-0.5739557	0.1833366	0.33256399	0.16876039	-0.0402921
202.2	0.35791994	-0.5512736	-0.5739557	0.1833366	0.33256399	0.16876039	-0.0402921
202.3	0.35791994	-0.5512736	-0.5739557	0.1833366	0.33256399	0.16876039	-0.0402921
202.4	0.35791994	-0.5512736	-0.5739557	0.1833366	0.33256399	0.16876039	-0.0402921
202.5	0.35791994	-0.5512736	-0.5739557	0.1833366	0.33256399	0.16876039	-0.0402921
202.6	0.35787725	-0.5512922	-0.574221	0.18320152	0.33243804	0.16902145	-0.0402451
202.7	0.35780235	-0.551262	-0.5743964	0.18325718	0.33219403	0.16925202	-0.0399477
202.8	0.3577808	-0.551163	-0.5745558	0.1830983	0.33256081	0.16947483	-0.0402242
202.9	0.3577808	-0.551163	-0.5745558	0.1830983	0.33256081	0.16947483	-0.0402242
203	0.3577808	-0.551163	-0.5745558	0.1830983	0.33256081	0.16947483	-0.0402242
203.1	0.3577808	-0.551163	-0.5745558	0.1830983	0.33256081	0.16947483	-0.0402242
203.2	0.3577808	-0.551163	-0.5745558	0.1830983	0.33256081	0.16947483	-0.0402242
203.3	0.3577808	-0.551163	-0.5745558	0.1830983	0.33256081	0.16947483	-0.0402242
203.4	0.3577808	-0.551163	-0.5745558	0.1830983	0.33256081	0.16947483	-0.0402242
203.5	0.3577808	-0.551163	-0.5745558	0.1830983	0.33256081	0.16947483	-0.0402242
203.6	0.3577808	-0.551163	-0.5745558	0.1830983	0.33256081	0.16947483	-0.0402242
203.7	0.3577808	-0.551163	-0.5745558	0.1830983	0.33256081	0.16947483	-0.0402242
203.8	0.3577808	-0.551163	-0.5745558	0.1830983	0.33256081	0.16947483	-0.0402242
203.9	0.3577808	-0.551163	-0.5745558	0.1830983	0.33256081	0.16947483	-0.0402242
204	0.3577808	-0.551163	-0.5745558	0.1830983	0.33256081	0.16947483	-0.0402242
204.1	0.35800753	-0.5505412	-0.5745265	0.18307283	0.3322734	0.16977523	-0.0401878
204.2	0.35800753	-0.5505412	-0.5745265	0.18307283	0.3322734	0.16977523	-0.0401878
204.3	0.35800753	-0.5505412	-0.5745265	0.18307283	0.3322734	0.16977523	-0.0401878
204.4	0.35800753	-0.5505412	-0.5745265	0.18307283	0.3322734	0.16977523	-0.0401878
204.5	0.35800753	-0.5505412	-0.5745265	0.18307283	0.3322734	0.16977523	-0.0401878
204.6	0.35800753	-0.5505412	-0.5745265	0.18307283	0.3322734	0.16977523	-0.0401878
204.7	0.35800753	-0.5505412	-0.5745265	0.18307283	0.3322734	0.16977523	-0.0401878
204.8	0.35800753	-0.5505412	-0.5745265	0.18307283	0.3322734	0.16977523	-0.0401878
204.9	0.35800753	-0.5505412	-0.5745265	0.18307283	0.3322734	0.16977523	-0.0401878
205	0.35800753	-0.5505412	-0.5745265	0.18307283	0.3322734	0.16977523	-0.0401878
205.1	0.35788083	-0.5503244	-0.5745576	0.18306124	0.33240245	0.16953078	-0.0403733
205.2	0.35788083	-0.5503244	-0.5745576	0.18306124	0.33240245	0.16953078	-0.0403733
205.3	0.35788083	-0.5503244	-0.5745576	0.18306124	0.33240245	0.16953078	-0.0403733
205.4	0.35788083	-0.5503244	-0.5745576	0.18306124	0.33240245	0.16953078	-0.0403733
205.5	0.35788083	-0.5503244	-0.5745576	0.18306124	0.33240245	0.16953078	-0.0403733
205.6	0.3578726	-0.5500969	-0.5745518	0.18332894	0.3327179	0.16921867	-0.0407213
205.7	0.3578726	-0.5500969	-0.5745518	0.18332894	0.3327179	0.16921867	-0.0407213
205.8	0.3578726	-0.5500969	-0.5745518	0.18332894	0.3327179	0.16921867	-0.0407213
205.9	0.35791828	-0.5498779	-0.5745331	0.1834744	0.33284801	0.16886351	-0.0411088
206	0.35791828	-0.5498779	-0.5745331	0.1834744	0.33284801	0.16886351	-0.0411088
206.1	0.35791828	-0.5498779	-0.5745331	0.1834744	0.33284801	0.16886351	-0.0411088
206.2	0.35791828	-0.5498779	-0.5745331	0.1834744	0.33284801	0.16886351	-0.0411088
206.3	0.35791828	-0.5498779	-0.5745331	0.1834744	0.33284801	0.16886351	-0.0411088
206.4	0.35791828	-0.5498779	-0.5745331	0.1834744	0.33284801	0.16886351	-0.0411088
206.5	0.35791828	-0.5498779	-0.5745331	0.1834744	0.33284801	0.16886351	-0.0411088
206.6	0.35820219	-0.5497129	-0.5748354	0.18367449	0.3330948	0.16891017	-0.0413229
206.7	0.35820219	-0.5497129	-0.5748354	0.18367449	0.3330948	0.16891017	-0.0413229
206.8	0.35815801	-0.5494202	-0.5747614	0.18365938	0.33297328	0.16894549	-0.0413434
206.9	0.35815801	-0.5494202	-0.5747614	0.18365938	0.33297328	0.16894549	-0.0413434
207	0.35815801	-0.5494202	-0.5747614	0.18365938	0.33297328	0.16894549	-0.0413434
207.1	0.35815622	-0.5494597	-0.5748422	0.18352897	0.33287083	0.16908052	-0.0415034
207.2	0.35815622	-0.5494597	-0.5748422	0.18352897	0.33287083	0.16908052	-0.0415034
207.3	0.35815622	-0.5494597	-0.5748422	0.18352897	0.33287083	0.16908052	-0.0415034
207.4	0.35815622	-0.5494597	-0.5748422	0.18352897	0.33287083	0.16908052	-0.0415034
207.5	0.35803909	-0.5492908	-0.5748825	0.18354193	0.33307252	0.16916465	-0.0416788
207.6	0.35803909	-0.5492908	-0.5748825	0.18354193	0.33307252	0.16916465	-0.0416788
207.7	0.35803909	-0.5492908	-0.5748825	0.18354193	0.33307252	0.16916465	-0.0416788
207.8	0.35803909	-0.5492908	-0.5748825	0.18354193	0.33307252	0.16916465	-0.0416788
207.9	0.35803909	-0.5492908	-0.5748825	0.18354193	0.33307252	0.16916465	-0.0416788
208	0.35803909	-0.5492908	-0.5748825	0.18354193	0.33307252	0.16916465	-0.0416788
208.1	0.35803909	-0.5492908	-0.5748825	0.18354193	0.33307252	0.16916465	-0.0416788
208.2	0.35803909	-0.5492908	-0.5748825	0.18354193	0.33307252	0.16916465	-0.0416788
208.3	0.35803909	-0.5492908	-0.5748825	0.18354193	0.33307252	0.16916465	-0.0416788
208.4	0.35835526	-0.5492229	-0.5750367	0.18367976	0.33335245	0.16926386	-0.0417363
208.5	0.35835526	-0.5492229	-0.5750367	0.18367976	0.33335245	0.16926386	-0.0417363
208.6	0.35835526	-0.5492229	-0.5750367	0.18367976	0.33335245	0.16926386	-0.0417363
208.7	0.35835526	-0.5492229	-0.5750367	0.18367976	0.33335245	0.16926386	-0.0417363
208.8	0.35835526	-0.5492229	-0.5750367	0.18367976	0.33335245	0.16926386	-0.0417363
208.9	0.35835526	-0.5492229	-0.5750367	0.18367976	0.33335245	0.16926386	-0.0417363
209	0.35835526	-0.5492229	-0.5750367	0.18367976	0.33335245	0.16926386	-0.0417363
209.1	0.35835526	-0.5492229	-0.5750367	0.18367976	0.33335245	0.16926386	-0.0417363
209.2	0.35835526	-0.5492229	-0.5750367	0.18367976	0.33335245	0.16926386	-0.0417363
209.3	0.35835526	-0.5492229	-0.5750367	0.18367976	0.33335245	0.16926386	-0.0417363
209.4	0.35829961	-0.5493034	-0.575204	0.18351414	0.3335408	0.16951541	-0.0419557
209.5	0.35824209	-0.5490276	-0.5750957	0.1834328	0.33384772	0.1693004	-0.0422192
209.6	0.35824209	-0.5490276	-0.5750957	0.1834328	0.33384772	0.1693004	-0.0422192
209.7	0.35824209	-0.5490276	-0.5750957	0.1834328	0.33384772	0.1693004	-0.0422192
209.8	0.35824209	-0.5490276	-0.5750957	0.1834328	0.33384772	0.1693004	-0.0422192
209.9	0.35824209	-0.5490276	-0.5750957	0.1834328	0.33384772	0.1693004	-0.0422192
213.2	0.35856477	-0.5487916	-0.575601	0.18318717	0.33394511	0.16968528	-0.0421586
213.3	0.35856477	-0.5487916	-0.575601	0.18318717	0.33394511	0.16968528	-0.0421586
213.4	0.35856477	-0.5487916	-0.575601	0.18318717	0.33394511	0.16968528	-0.0421586
213.5	0.35856477	-0.5487916	-0.575601	0.18318717	0.33394511	0.16968528	-0.0421586
213.6	0.35856477	-0.5487916	-0.575601	0.18318717	0.33394511	0.16968528	-0.0421586
213.7	0.35833775	-0.5485587	-0.5755303	0.1832126	0.33417297	0.1696541	-0.0422588
213.8	0.35833775	-0.5485587	-0.5755303	0.1832126	0.33417297	0.1696541	-0.0422588
213.9	0.35833775	-0.5485587	-0.5755303	0.1832126	0.33417297	0.1696541	-0.0422588
214	0.35833775	-0.5485587	-0.5755303	0.1832126	0.33417297	0.1696541	-0.0422588
214.1	0.35833775	-0.5485587	-0.5755303	0.1832126	0.33417297	0.1696541	-0.0422588
214.2	0.35833775	-0.5485587	-0.5755303	0.1832126	0.33417297	0.1696541	-0.0422588
214.3	0.35833775	-0.5485587	-0.5755303	0.1832126	0.33417297	0.1696541	-0.0422588
214.4	0.358199	-0.5488243	-0.5758251	0.18305724	0.33427568	0.16983535	-0.0423063
214.5	0.358199	-0.5488243	-0.5758251	0.18305724	0.33427568	0.16983535	-0.0423063
214.6	0.358199	-0.5488243	-0.5758251	0.18305724	0.33427568	0.16983535	-0.0423063
214.7	0.358199	-0.5488243	-0.5758251	0.18305724	0.33427568	0.16983535	-0.0423063
214.8	0.358199	-0.5488243	-0.5758251	0.18305724	0.33427568	0.16983535	-0.0423063
214.9	0.35808206	-0.5486111	-0.5757897	0.18306055	0.33404346	0.16989492	-0.0422482
215	0.35808206	-0.5486111	-0.5757897	0.18306055	0.33404346	0.16989492	-0.0422482
215.1	0.35800216	-0.5483537	-0.5758788	0.18312441	0.33412312	0.16996077	-0.0425314
215.2	0.35800216	-0.5483537	-0.5758788	0.18312441	0.33412312	0.16996077	-0.0425314
215.3	0.35800216	-0.5483537	-0.5758788	0.18312441	0.33412312	0.16996077	-0.0425314
215.4	0.35800216	-0.5483537	-0.5758788	0.18312441	0.33412312	0.16996077	-0.0425314
215.5	0.35800216	-0.5483537	-0.5758788	0.18312441	0.33412312	0.16996077	-0.0425314
215.6	0.35800216	-0.5483537	-0.5758788	0.18312441	0.33412312	0.16996077	-0.0425314
215.7	0.35800216	-0.5483537	-0.5758788	0.18312441	0.33412312	0.16996077	-0.0425314
215.8	0.35800216	-0.5483537	-0.5758788	0.18312441	0.33412312	0.16996077	-0.0425314
215.9	0.35800216	-0.5483537	-0.5758788	0.18312441	0.33412312	0.16996077	-0.0425314
216	0.35800216	-0.5483537	-0.5758788	0.18312441	0.33412312	0.16996077	-0.0425314
216.1	0.35800216	-0.5483537	-0.5758788	0.18312441	0.33412312	0.16996077	-0.0425314
216.2	0.35800216	-0.5483537	-0.5758788	0.18312441	0.33412312	0.16996077	-0.0425314
216.3	0.35815681	-0.5481487	-0.5757172	0.18295056	0.33413925	0.16995921	-0.0424178
216.4	0.35815681	-0.5481487	-0.5757172	0.18295056	0.33413925	0.16995921	-0.0424178
216.5	0.35815681	-0.5481487	-0.5757172	0.18295056	0.33413925	0.16995921	-0.0424178
216.6	0.35815681	-0.5481487	-0.5757172	0.18295056	0.33413925	0.16995921	-0.0424178
216.7	0.35815681	-0.5481487	-0.5757172	0.18295056	0.33413925	0.16995921	-0.0424178
216.8	0.35815681	-0.5481487	-0.5757172	0.18295056	0.33413925	0.16995921	-0.0424178
216.9	0.35815681	-0.5481487	-0.5757172	0.18295056	0.33413925	0.16995921	-0.0424178
217	0.35815681	-0.5481487	-0.5757172	0.18295056	0.33413925	0.16995921	-0.0424178
217.1	0.35815681	-0.5481487	-0.5757172	0.18295056	0.33413925	0.16995921	-0.0424178
217.2	0.35815681	-0.5481487	-0.5757172	0.18295056	0.33413925	0.16995921	-0.0424178
217.3	0.35815681	-0.5481487	-0.5757172	0.18295056	0.33413925	0.16995921	-0.0424178
217.4	0.35816378	-0.548181	-0.5758785	0.18288208	0.33404995	0.17035961	-0.0424287
217.5	0.35816378	-0.548181	-0.5758785	0.18288208	0.33404995	0.17035961	-0.0424287
217.6	0.35816378	-0.548181	-0.5758785	0.18288208	0.33404995	0.17035961	-0.0424287
217.7	0.35829625	-0.5477358	-0.5757531	0.18305129	0.33436702	0.17028187	-0.0424896
217.8	0.35829625	-0.5477358	-0.5757531	0.18305129	0.33436702	0.17028187	-0.0424896
217.9	0.35829625	-0.5477358	-0.5757531	0.18305129	0.33436702	0.17028187	-0.0424896
218	0.35829625	-0.5477358	-0.5757531	0.18305129	0.33436702	0.17028187	-0.0424896
218.1	0.35829625	-0.5477358	-0.5757531	0.18305129	0.33436702	0.17028187	-0.0424896
218.2	0.35829625	-0.5477358	-0.5757531	0.18305129	0.33436702	0.17028187	-0.0424896
218.3	0.35829625	-0.5477358	-0.5757531	0.18305129	0.33436702	0.17028187	-0.0424896
218.4	0.35829625	-0.5477358	-0.5757531	0.18305129	0.33436702	0.17028187	-0.0424896
218.5	0.35829625	-0.5477358	-0.5757531	0.18305129	0.33436702	0.17028187	-0.0424896
218.6	0.35829625	-0.5477358	-0.5757531	0.18305129	0.33436702	0.17028187	-0.0424896
218.7	0.35829625	-0.5477358	-0.5757531	0.18305129	0.33436702	0.17028187	-0.0424896
218.8	0.35829625	-0.5477358	-0.5757531	0.18305129	0.33436702	0.17028187	-0.0424896
218.9	0.35829625	-0.5477358	-0.5757531	0.18305129	0.33436702	0.17028187	-0.0424896
219	0.35829625	-0.5477358	-0.5757531	0.18305129	0.33436702	0.17028187	-0.0424896
219.1	0.35829625	-0.5477358	-0.5757531	0.18305129	0.33436702	0.17028187	-0.0424896
219.2	0.3582051	-0.5475321	-0.5756312	0.18313355	0.33454746	0.17012812	-0.0425589
219.3	0.35815866	-0.5477274	-0.5759205	0.18294044	0.33478798	0.17065727	-0.043056
219.4	0.35815866	-0.5477274	-0.5759205	0.18294044	0.33478798	0.17065727	-0.043056
219.5	0.35815866	-0.5477274	-0.5759205	0.18294044	0.33478798	0.17065727	-0.043056
219.6	0.35815866	-0.5477274	-0.5759205	0.18294044	0.33478798	0.17065727	-0.043056
219.7	0.35815866	-0.5477274	-0.5759205	0.18294044	0.33478798	0.17065727	-0.043056
219.8	0.35815866	-0.5477274	-0.5759205	0.18294044	0.33478798	0.17065727	-0.043056
219.9	0.35815866	-0.5477274	-0.5759205	0.18294044	0.33478798	0.17065727	-0.043056
220	0.35813617	-0.5477042	-0.5759094	0.1825954	0.33497899	0.17088094	-0.0429926
220.1	0.35813617	-0.5477042	-0.5759094	0.1825954	0.33497899	0.17088094	-0.0429926
220.2	0.35813617	-0.5477042	-0.5759094	0.1825954	0.33497899	0.17088094	-0.0429926
220.3	0.35813617	-0.5477042	-0.5759094	0.1825954	0.33497899	0.17088094	-0.0429926
220.4	0.35813617	-0.5477042	-0.5759094	0.1825954	0.33497899	0.17088094	-0.0429926
220.5	0.35813617	-0.5477042	-0.5759094	0.1825954	0.33497899	0.17088094	-0.0429926
220.6	0.35813617	-0.5477042	-0.5759094	0.1825954	0.33497899	0.17088094	-0.0429926
220.7	0.35813617	-0.5477042	-0.5759094	0.1825954	0.33497899	0.17088094	-0.0429926
220.8	0.35813617	-0.5477042	-0.5759094	0.1825954	0.33497899	0.17088094	-0.0429926
220.9	0.35813617	-0.5477042	-0.5759094	0.1825954	0.33497899	0.17088094	-0.0429926
221	0.35813617	-0.5477042	-0.5759094	0.1825954	0.33497899	0.17088094	-0.0429926
221.1	0.35813617	-0.5477042	-0.5759094	0.1825954	0.33497899	0.17088094	-0.0429926
221.2	0.35813617	-0.5477042	-0.5759094	0.1825954	0.33497899	0.17088094	-0.0429926
221.3	0.35813617	-0.5477042	-0.5759094	0.1825954	0.33497899	0.17088094	-0.0429926
221.4	0.35813617	-0.5477042	-0.5759094	0.1825954	0.33497899	0.17088094	-0.0429926
221.5	0.35813617	-0.5477042	-0.5759094	0.1825954	0.33497899	0.17088094	-0.0429926
221.6	0.3580445	-0.54778	-0.5761283	0.18241179	0.33518539	0.17136932	-0.0429726
221.7	0.3580445	-0.54778	-0.5761283	0.18241179	0.33518539	0.17136932	-0.0429726
221.8	0.3580445	-0.54778	-0.5761283	0.18241179	0.33518539	0.17136932	-0.0429726
221.9	0.3580445	-0.54778	-0.5761283	0.18241179	0.33518539	0.17136932	-0.0429726
222	0.3580445	-0.54778	-0.5761283	0.18241179	0.33518539	0.17136932	-0.0429726
222.1	0.3580445	-0.54778	-0.5761283	0.18241179	0.33518539	0.17136932	-0.0429726
222.2	0.3580445	-0.54778	-0.5761283	0.18241179	0.33518539	0.17136932	-0.0429726
222.3	0.3580445	-0.54778	-0.5761283	0.18241179	0.33518539	0.17136932	-0.0429726
222.4	0.3580445	-0.54778	-0.5761283	0.18241179	0.33518539	0.17136932	-0.0429726
222.5	0.35821901	-0.5478968	-0.5764843	0.18212866	0.33519075	0.1713566	-0.0427262
222.6	0.35821901	-0.5478968	-0.5764843	0.18212866	0.33519075	0.1713566	-0.0427262
222.7	0.35821901	-0.5478968	-0.5764843	0.18212866	0.33519075	0.1713566	-0.0427262
222.8	0.35821901	-0.5478968	-0.5764843	0.18212866	0.33519075	0.1713566	-0.0427262
222.9	0.35821901	-0.5478968	-0.5764843	0.18212866	0.33519075	0.1713566	-0.0427262
223	0.35821901	-0.5478968	-0.5764843	0.18212866	0.33519075	0.1713566	-0.0427262
223.1	0.35821901	-0.5478968	-0.5764843	0.18212866	0.33519075	0.1713566	-0.0427262
223.2	0.35821901	-0.5478968	-0.5764843	0.18212866	0.33519075	0.1713566	-0.0427262
223.3	0.35821901	-0.5478968	-0.5764843	0.18212866	0.33519075	0.1713566	-0.0427262
223.4	0.35821901	-0.5478968	-0.5764843	0.18212866	0.33519075	0.1713566	-0.0427262
223.5	0.35821901	-0.5478968	-0.5764843	0.18212866	0.33519075	0.1713566	-0.0427262
223.6	0.35821901	-0.5478968	-0.5764843	0.18212866	0.33519075	0.1713566	-0.0427262
223.7	0.35821901	-0.5478968	-0.5764843	0.18212866	0.33519075	0.1713566	-0.0427262
223.8	0.35797161	-0.5478633	-0.5765982	0.18182699	0.33531894	0.1719625	-0.0425364
223.9	0.35797161	-0.5478633	-0.5765982	0.18182699	0.33531894	0.1719625	-0.0425364
224	0.35797161	-0.5478633	-0.5765982	0.18182699	0.33531894	0.1719625	-0.0425364
224.1	0.35797161	-0.5478633	-0.5765982	0.18182699	0.33531894	0.1719625	-0.0425364
224.2	0.35797161	-0.5478633	-0.5765982	0.18182699	0.33531894	0.1719625	-0.0425364
224.3	0.35788519	-0.5477538	-0.5767123	0.18213154	0.33555295	0.17231611	-0.0426917
224.4	0.35788519	-0.5477538	-0.5767123	0.18213154	0.33555295	0.17231611	-0.0426917
224.5	0.35788519	-0.5477538	-0.5767123	0.18213154	0.33555295	0.17231611	-0.0426917
224.6	0.35783519	-0.5477135	-0.5768199	0.1819586	0.33545968	0.17281825	-0.0426929
224.7	0.35783519	-0.5477135	-0.5768199	0.1819586	0.33545968	0.17281825	-0.0426929
224.8	0.35783519	-0.5477135	-0.5768199	0.1819586	0.33545968	0.17281825	-0.0426929
224.9	0.35783519	-0.5477135	-0.5768199	0.1819586	0.33545968	0.17281825	-0.0426929
228.2	0.35718798	-0.5483903	-0.578576	0.17990107	0.33898626	0.1774247	-0.0462896
228.3	0.35712009	-0.5482279	-0.5785949	0.17962746	0.33919312	0.17790885	-0.0463018
228.4	0.35712009	-0.5482279	-0.5785949	0.17962746	0.33919312	0.17790885	-0.0463018
228.5	0.35712009	-0.5482279	-0.5785949	0.17962746	0.33919312	0.17790885	-0.0463018
228.6	0.35712009	-0.5482279	-0.5785949	0.17962746	0.33919312	0.17790885	-0.0463018
228.7	0.35712009	-0.5482279	-0.5785949	0.17962746	0.33919312	0.17790885	-0.0463018
228.8	0.35714385	-0.5483145	-0.5788347	0.17946702	0.33933604	0.17837498	-0.0464966
228.9	0.35714385	-0.5483145	-0.5788347	0.17946702	0.33933604	0.17837498	-0.0464966
229	0.35714385	-0.5483145	-0.5788347	0.17946702	0.33933604	0.17837498	-0.0464966
229.1	0.35714385	-0.5483145	-0.5788347	0.17946702	0.33933604	0.17837498	-0.0464966
229.2	0.35714385	-0.5483145	-0.5788347	0.17946702	0.33933604	0.17837498	-0.0464966
229.3	0.35714385	-0.5483145	-0.5788347	0.17946702	0.33933604	0.17837498	-0.0464966
229.4	0.35714385	-0.5483145	-0.5788347	0.17946702	0.33933604	0.17837498	-0.0464966
229.5	0.35714385	-0.5483145	-0.5788347	0.17946702	0.33933604	0.17837498	-0.0464966
229.6	0.35714385	-0.5483145	-0.5788347	0.17946702	0.33933604	0.17837498	-0.0464966
229.7	0.35708503	-0.5482549	-0.5791081	0.17938177	0.33952233	0.17862381	-0.0464478
229.8	0.35699151	-0.5481839	-0.5790808	0.17921249	0.33945636	0.17873802	-0.0464953
229.9	0.35705107	-0.5481018	-0.5791524	0.17882284	0.33951507	0.17908045	-0.0468585
230	0.35705107	-0.5481018	-0.5791524	0.17882284	0.33951507	0.17908045	-0.0468585
230.1	0.35698405	-0.547975	-0.5792308	0.17867374	0.33947674	0.17942077	-0.0469312
230.2	0.35698405	-0.547975	-0.5792308	0.17867374	0.33947674	0.17942077	-0.0469312
230.3	0.35701613	-0.5479078	-0.5793962	0.17852271	0.33942684	0.1795045	-0.0470502
230.4	0.35679436	-0.5479128	-0.5795535	0.17849377	0.33958201	0.17993218	-0.0471925
230.5	0.35675794	-0.5480216	-0.5797682	0.17831115	0.33982308	0.18012138	-0.047274
230.6	0.35675794	-0.5480216	-0.5797682	0.17831115	0.33982308	0.18012138	-0.047274
230.7	0.35669366	-0.5479214	-0.5797723	0.17799528	0.33990139	0.18051981	-0.047388
230.8	0.35669366	-0.5479214	-0.5797723	0.17799528	0.33990139	0.18051981	-0.047388
230.9	0.35661536	-0.5479245	-0.5797552	0.17796194	0.34014936	0.18076628	-0.0477422
231	0.35661536	-0.5479245	-0.5797552	0.17796194	0.34014936	0.18076628	-0.0477422
231.1	0.35661536	-0.5479245	-0.5797552	0.17796194	0.34014936	0.18076628	-0.0477422
231.2	0.35661536	-0.5479245	-0.5797552	0.17796194	0.34014936	0.18076628	-0.0477422
231.3	0.35661536	-0.5479245	-0.5797552	0.17796194	0.34014936	0.18076628	-0.0477422
231.4	0.35661536	-0.5479245	-0.5797552	0.17796194	0.34014936	0.18076628	-0.0477422
231.5	0.35645195	-0.5478017	-0.5796725	0.1780648	0.34021864	0.18063532	-0.047732
231.6	0.35629785	-0.5478051	-0.5797636	0.17777574	0.34021178	0.18085753	-0.0480295
231.7	0.35623404	-0.5478155	-0.5799761	0.17765514	0.34022355	0.18122449	-0.0483997
231.8	0.35571597	-0.5477776	-0.5801723	0.17753646	0.34072162	0.18205833	-0.0487423
231.9	0.35571597	-0.5477776	-0.5801723	0.17753646	0.34072162	0.18205833	-0.0487423
232	0.35557091	-0.5477062	-0.5801855	0.1773438	0.3410008	0.1821678	-0.0489441
232.1	0.35557091	-0.5477062	-0.5801855	0.1773438	0.3410008	0.1821678	-0.0489441
232.2	0.35557091	-0.5477062	-0.5801855	0.1773438	0.3410008	0.1821678	-0.0489441
232.3	0.35557091	-0.5477062	-0.5801855	0.1773438	0.3410008	0.1821678	-0.0489441
232.4	0.35557091	-0.5477062	-0.5801855	0.1773438	0.3410008	0.1821678	-0.0489441
232.5	0.35563211	-0.5476049	-0.5802607	0.1773792	0.34134916	0.18259981	-0.0493062
232.6	0.35563211	-0.5476049	-0.5802607	0.1773792	0.34134916	0.18259981	-0.0493062
232.7	0.35519295	-0.5475648	-0.5805296	0.17724227	0.34151366	0.1830278	-0.0496427
232.8	0.35519295	-0.5475648	-0.5805296	0.17724227	0.34151366	0.1830278	-0.0496427
232.9	0.35519295	-0.5475648	-0.5805296	0.17724227	0.34151366	0.1830278	-0.0496427
233	0.35518548	-0.5474498	-0.5805979	0.17692605	0.34166599	0.1834857	-0.0501013
233.1	0.35511027	-0.5474343	-0.5806351	0.17670413	0.34175211	0.18381476	-0.0505909
233.2	0.35511027	-0.5474343	-0.5806351	0.17670413	0.34175211	0.18381476	-0.0505909
233.3	0.35500763	-0.5475299	-0.5809031	0.17626343	0.34184366	0.18432765	-0.0507872
233.4	0.35465165	-0.5476136	-0.5810541	0.17596586	0.34207016	0.18486156	-0.0515389
233.5	0.35447568	-0.5475354	-0.5810114	0.17588002	0.34230135	0.18503304	-0.0517728
233.6	0.35447568	-0.5475354	-0.5810114	0.17588002	0.34230135	0.18503304	-0.0517728
233.7	0.35419472	-0.5475789	-0.5812262	0.17570484	0.34264207	0.18530315	-0.0519277
233.8	0.35407833	-0.5476836	-0.5815668	0.17526008	0.34311558	0.18607036	-0.0524178
233.9	0.35407833	-0.5476836	-0.5815668	0.17526008	0.34311558	0.18607036	-0.0524178
234	0.35407833	-0.5476836	-0.5815668	0.17526008	0.34311558	0.18607036	-0.0524178
234.1	0.35338053	-0.5477454	-0.5818174	0.17470541	0.34354203	0.18672247	-0.0530536
234.2	0.35313002	-0.5476848	-0.581785	0.17432481	0.34372419	0.18685132	-0.0532048
234.3	0.35313036	-0.5477457	-0.5817976	0.17386331	0.34381243	0.18694046	-0.0534362
234.4	0.35313036	-0.5477457	-0.5817976	0.17386331	0.34381243	0.18694046	-0.0534362
234.5	0.35313036	-0.5477457	-0.5817976	0.17386331	0.34381243	0.18694046	-0.0534362
234.6	0.35313036	-0.5477457	-0.5817976	0.17386331	0.34381243	0.18694046	-0.0534362
234.7	0.35313036	-0.5477457	-0.5817976	0.17386331	0.34381243	0.18694046	-0.0534362
234.8	0.35313036	-0.5477457	-0.5817976	0.17386331	0.34381243	0.18694046	-0.0534362
234.9	0.35313036	-0.5477457	-0.5817976	0.17386331	0.34381243	0.18694046	-0.0534362
235	0.35255601	-0.5477043	-0.5818639	0.17369359	0.34395754	0.1875237	-0.0539801
235.1	0.35255601	-0.5477043	-0.5818639	0.17369359	0.34395754	0.1875237	-0.0539801
235.2	0.35255601	-0.5477043	-0.5818639	0.17369359	0.34395754	0.1875237	-0.0539801
235.3	0.35232224	-0.5477374	-0.5819189	0.17332635	0.34383238	0.18773865	-0.054175
235.4	0.35189392	-0.5477793	-0.5825718	0.17214519	0.34467008	0.18856128	-0.055079
235.5	0.35122842	-0.5475967	-0.5832676	0.17156894	0.34516825	0.18949364	-0.0561966
235.6	0.35103082	-0.547537	-0.5834814	0.17144237	0.34551417	0.18971375	-0.0564826
235.7	0.35098085	-0.547512	-0.5836274	0.17115472	0.34555062	0.18983887	-0.0567762
235.8	0.35098085	-0.547512	-0.5836274	0.17115472	0.34555062	0.18983887	-0.0567762
235.9	0.35069953	-0.5475788	-0.5836572	0.17069142	0.34573735	0.18990799	-0.0567777
236	0.35069953	-0.5475788	-0.5836572	0.17069142	0.34573735	0.18990799	-0.0567777
236.1	0.35053313	-0.5474701	-0.5835705	0.17049105	0.34562493	0.19036572	-0.0569422
236.2	0.35044785	-0.5474695	-0.5836975	0.17041682	0.34566795	0.19070986	-0.0571688
236.3	0.35016895	-0.5472891	-0.5836828	0.17044682	0.34577621	0.19100254	-0.0574032
236.4	0.35012661	-0.5471572	-0.5835837	0.17013672	0.34600618	0.19134533	-0.0575304
236.5	0.35012661	-0.5471572	-0.5835837	0.17013672	0.34600618	0.19134533	-0.0575304
236.6	0.34975639	-0.5469729	-0.5835885	0.16990586	0.34601327	0.19158283	-0.0574391
236.7	0.34975639	-0.5469729	-0.5835885	0.16990586	0.34601327	0.19158283	-0.0574391
236.8	0.34963098	-0.547012	-0.5837792	0.16964264	0.34630885	0.19182655	-0.0574312
236.9	0.34963098	-0.547012	-0.5837792	0.16964264	0.34630885	0.19182655	-0.0574312
237	0.34940551	-0.5470753	-0.5838711	0.16927607	0.34630457	0.19213257	-0.0578376
237.1	0.34912079	-0.5469852	-0.583776	0.16916792	0.34627492	0.19231316	-0.0579857
237.2	0.34912079	-0.5469852	-0.583776	0.16916792	0.34627492	0.19231316	-0.0579857
237.3	0.34912079	-0.5469852	-0.583776	0.16916792	0.34627492	0.19231316	-0.0579857
237.4	0.34909894	-0.5468151	-0.5838473	0.16916015	0.34662005	0.19267611	-0.05795
237.5	0.34909894	-0.5468151	-0.5838473	0.16916015	0.34662005	0.19267611	-0.05795
237.6	0.34901716	-0.5466983	-0.5840438	0.1687065	0.346893	0.1931534	-0.0579871
237.7	0.3493418	-0.5467406	-0.5840568	0.16863912	0.34680541	0.19340864	-0.0582068
237.8	0.3493418	-0.5467406	-0.5840568	0.16863912	0.34680541	0.19340864	-0.0582068
237.9	0.34898107	-0.5466845	-0.5841644	0.16836231	0.34675025	0.19372771	-0.0584288
238	0.34898107	-0.5466845	-0.5841644	0.16836231	0.34675025	0.19372771	-0.0584288
238.1	0.34872791	-0.5467397	-0.5841556	0.16799575	0.34699878	0.19382057	-0.0587693
238.2	0.3484736	-0.5466538	-0.5841523	0.16794738	0.34722434	0.19424202	-0.0588412
238.3	0.34770813	-0.5463296	-0.5842249	0.16746179	0.34768092	0.19509964	-0.0592308
238.4	0.34763587	-0.5462507	-0.5843138	0.16723957	0.34768863	0.19549803	-0.0594082
238.5	0.34772512	-0.5460653	-0.584268	0.16718084	0.34772508	0.19579914	-0.059882
238.6	0.34748419	-0.5459666	-0.5844181	0.16691483	0.34793404	0.19586222	-0.0599336
238.7	0.34748419	-0.5459666	-0.5844181	0.16691483	0.34793404	0.19586222	-0.0599336
238.8	0.34748419	-0.5459666	-0.5844181	0.16691483	0.34793404	0.19586222	-0.0599336
238.9	0.3474069	-0.545618	-0.5842684	0.16601713	0.34804013	0.19647977	-0.0604018
239	0.34746872	-0.5456639	-0.5845051	0.16588927	0.34781468	0.19730463	-0.0607466
239.1	0.34718746	-0.5457275	-0.5847256	0.16547252	0.3479534	0.19770115	-0.0608166
239.2	0.34718746	-0.5457275	-0.5847256	0.16547252	0.3479534	0.19770115	-0.0608166
239.3	0.34718746	-0.5457275	-0.5847256	0.16547252	0.3479534	0.19770115	-0.0608166
239.4	0.34649817	-0.5457062	-0.5847869	0.16459451	0.34799546	0.19852126	-0.0607021
239.5	0.34580143	-0.5455134	-0.5846818	0.16415714	0.34795727	0.19893817	-0.0608891
239.6	0.34586212	-0.5455051	-0.5847348	0.1639765	0.34816053	0.19937186	-0.0613286
239.7	0.34585035	-0.5453575	-0.5848016	0.1638605	0.34812596	0.19972058	-0.0615126
239.8	0.34585035	-0.5453575	-0.5848016	0.1638605	0.34812596	0.19972058	-0.0615126
239.9	0.34563901	-0.5451829	-0.5847461	0.1637472	0.34809399	0.19998217	-0.061773
240	0.34544387	-0.5450547	-0.5846905	0.1635136	0.34807449	0.20040307	-0.0622086

Corresponding p-values for PRCC sensitivtiy indices

Time discretization	V_{[PYR]}	K_{[PYR]}	alpha	K_{[ACoA]}	G_E	[G]_0	[PYR]_0
0	0	0	0	0	0	0	0
0.1	6.27E-41	3.86E-76	0.87775939	0.90442082	0.94369592	3.42E-21	0.71606492
0.2	6.33E-41	1.21E-76	0.83305771	0.8362331	0.92301812	1.57E-21	0.80553726
0.3	6.47E-41	8.21E-77	0.78159539	0.54980475	0.9221438	1.09E-21	0.88138569
0.4	7.43E-41	6.59E-77	0.72092445	0.35466612	0.9125873	8.57E-22	0.9414001
0.5	9.69E-41	3.89E-77	0.65683887	0.21294946	0.87968381	4.94E-22	0.98258155
0.6	1.49E-40	2.92E-77	0.58524943	0.10040709	0.85644549	4.65E-22	0.94010303
0.7	2.12E-40	3.31E-77	0.60648493	0.04604571	0.7525705	4.14E-22	0.94602453
0.8	3.65E-40	4.47E-77	0.57885734	0.02064923	0.73402205	5.30E-22	0.90589879
0.9	6.54E-40	7.11E-77	0.59360269	0.00958549	0.69962531	8.49E-22	0.94229526
1	1.59E-39	1.05E-76	0.54853425	0.00429989	0.66095222	9.29E-22	0.93359874
1.1	2.78E-39	1.57E-76	0.54444542	0.00172686	0.61481656	9.88E-22	0.95259222
1.2	4.58E-39	3.05E-76	0.55687521	0.00077333	0.583725	8.49E-22	0.90272577
1.3	5.96E-39	1.79E-76	0.57432835	0.00030939	0.53265357	8.95E-22	0.90812173
1.4	6.48E-39	1.98E-76	0.63317662	0.0001124	0.50957487	2.00E-21	0.81751343
1.5	1.24E-38	3.82E-76	0.6133854	6.95E-05	0.50982202	1.81E-21	0.8099855
1.6	1.85E-38	6.37E-76	0.6083323	3.66E-05	0.51527086	2.76E-21	0.74615516
1.7	2.54E-38	9.83E-76	0.62382616	1.73E-05	0.50939571	3.03E-21	0.72229655
1.8	5.63E-38	2.00E-75	0.62318408	8.72E-06	0.45890206	5.67E-21	0.73222636
1.9	8.51E-38	2.66E-75	0.63412405	4.68E-06	0.42122417	6.08E-21	0.7144792
2	1.90E-37	7.64E-75	0.59820634	2.58E-06	0.39707985	8.97E-21	0.75784056
2.1	2.20E-37	7.07E-75	0.58520689	1.12E-06	0.37306324	1.16E-20	0.73867527
2.2	2.24E-37	5.82E-75	0.63353644	6.17E-07	0.38026947	1.72E-20	0.78368303
2.3	2.45E-37	6.12E-75	0.63717888	3.38E-07	0.34615615	1.59E-20	0.7541583
2.4	4.18E-37	7.30E-75	0.66788343	1.46E-07	0.33347296	2.05E-20	0.75699937
2.5	4.75E-37	8.43E-75	0.69277717	1.05E-07	0.31986852	3.27E-20	0.78202731
2.6	6.23E-37	8.30E-75	0.66729956	4.44E-08	0.27490153	5.38E-20	0.76254707
2.7	6.75E-37	7.91E-75	0.65501125	2.20E-08	0.26038159	5.97E-20	0.78179714
2.8	8.94E-37	1.13E-74	0.70530991	1.33E-08	0.25160319	1.01E-19	0.79754797
2.9	1.27E-36	1.89E-74	0.69260681	9.80E-09	0.24482364	1.37E-19	0.81737486
3	1.49E-36	2.70E-74	0.69054473	5.19E-09	0.22290059	1.82E-19	0.82254918
3.1	1.70E-36	2.63E-74	0.68617017	3.46E-09	0.20290668	2.23E-19	0.82668815
3.2	3.12E-36	4.47E-74	0.68360932	2.03E-09	0.19143686	3.06E-19	0.8013999
3.3	4.32E-36	5.98E-74	0.71164859	1.14E-09	0.17472011	5.78E-19	0.78046834
3.4	4.47E-36	6.25E-74	0.68671504	6.20E-10	0.17196916	6.61E-19	0.75964828
3.5	7.55E-36	1.28E-73	0.69905049	4.43E-10	0.15874367	1.21E-18	0.8157091
3.6	8.91E-36	1.01E-73	0.70637002	2.92E-10	0.14420718	1.54E-18	0.85047338
3.7	8.31E-36	7.90E-74	0.7096663	1.51E-10	0.12478782	1.84E-18	0.84978072
3.8	5.91E-36	8.20E-74	0.75670881	9.51E-11	0.11290917	2.25E-18	0.82123428
3.9	5.56E-36	5.14E-74	0.74003481	5.30E-11	0.10925904	2.05E-18	0.81443584
4	4.85E-36	4.82E-74	0.75973645	4.78E-11	0.10432772	2.36E-18	0.85286246
4.1	4.69E-36	4.79E-74	0.76339006	3.28E-11	0.09918597	2.66E-18	0.82553952
4.2	4.59E-36	5.01E-74	0.75798537	2.12E-11	0.08975551	3.69E-18	0.83925137
4.3	5.32E-36	6.19E-74	0.75117313	1.09E-11	0.07765284	4.88E-18	0.85183916
4.4	5.96E-36	7.78E-74	0.73756885	8.94E-12	0.07168315	5.26E-18	0.85515604
4.5	6.82E-36	1.27E-73	0.75270627	7.31E-12	0.06513648	6.24E-18	0.8764564
4.6	6.32E-36	1.70E-73	0.76177415	5.25E-12	0.05500713	7.67E-18	0.90518067
4.7	9.93E-36	2.04E-73	0.75222143	4.28E-12	0.04923406	1.14E-17	0.90246363
4.8	1.07E-35	2.44E-73	0.72137204	3.78E-12	0.04601267	1.50E-17	0.90468242
4.9	1.25E-35	2.86E-73	0.73795569	3.48E-12	0.04470255	1.76E-17	0.89102799
5	6.73E-36	1.17E-73	0.73149153	1.52E-12	0.03690675	1.44E-17	0.88976
5.1	8.59E-36	2.12E-73	0.73054846	9.96E-13	0.03428579	1.98E-17	0.89726541
5.2	1.10E-35	2.34E-73	0.70299038	7.07E-13	0.029646	2.75E-17	0.905111
5.3	9.58E-36	1.59E-73	0.69801547	5.43E-13	0.02596525	3.44E-17	0.88958141
5.4	8.02E-36	1.81E-73	0.70575585	4.33E-13	0.02540436	4.11E-17	0.89224682
5.5	6.42E-36	9.41E-74	0.70567205	2.61E-13	0.01968862	4.98E-17	0.94305003
5.6	5.50E-36	9.89E-74	0.6973363	1.77E-13	0.01542185	6.42E-17	0.93144024
5.7	5.48E-36	1.02E-73	0.70651618	1.37E-13	0.01314743	6.84E-17	0.9112911
5.8	6.50E-36	1.10E-73	0.70444103	1.05E-13	0.01138303	8.90E-17	0.90634515
5.9	1.16E-35	2.04E-73	0.71902592	1.15E-13	0.00963314	1.42E-16	0.90763629
6	1.08E-35	1.66E-73	0.71437513	8.38E-14	0.00920698	1.60E-16	0.92529377
6.1	9.97E-36	1.73E-73	0.72161789	5.85E-14	0.00771205	1.88E-16	0.91077862
6.2	9.94E-36	1.85E-73	0.68116338	5.40E-14	0.00667235	2.18E-16	0.91480812
6.3	7.76E-36	1.23E-73	0.68696431	4.00E-14	0.00554631	2.18E-16	0.90833199
6.4	6.90E-36	1.38E-73	0.6895922	3.49E-14	0.00510298	2.29E-16	0.90670952
6.5	6.18E-36	1.77E-73	0.68417537	3.53E-14	0.0046707	3.11E-16	0.89848202
6.6	5.68E-36	2.25E-73	0.66152764	2.87E-14	0.00423516	3.55E-16	0.88120765
6.7	6.70E-36	2.40E-73	0.64488912	2.67E-14	0.00393819	4.02E-16	0.88202255
6.8	6.28E-36	3.26E-73	0.64214684	2.65E-14	0.00329384	5.56E-16	0.86284602
6.9	6.14E-36	3.15E-73	0.61467921	2.57E-14	0.00289301	4.78E-16	0.84292685
7	7.07E-36	3.98E-73	0.60082372	2.40E-14	0.00266687	5.49E-16	0.83822744
7.1	6.40E-36	3.80E-73	0.58676339	2.11E-14	0.00233537	7.47E-16	0.84894524
7.2	5.27E-36	2.45E-73	0.59792239	1.81E-14	0.00178855	8.33E-16	0.85641771
7.3	5.78E-36	3.53E-73	0.5962914	1.64E-14	0.00163528	9.86E-16	0.86506834
7.4	7.59E-36	4.52E-73	0.57023802	1.57E-14	0.00135674	1.36E-15	0.83077731
7.5	7.69E-36	4.59E-73	0.55001292	1.89E-14	0.0011396	1.80E-15	0.86831346
7.6	7.57E-36	5.61E-73	0.5437468	2.17E-14	0.00100264	2.04E-15	0.85922648
7.7	5.63E-36	4.47E-73	0.54201272	2.02E-14	0.00084589	2.21E-15	0.86142198
7.8	5.24E-36	4.60E-73	0.51027364	1.56E-14	0.00067557	2.63E-15	0.87026498
7.9	6.25E-36	5.41E-73	0.51046912	1.55E-14	0.00062305	3.38E-15	0.85457482
8	5.94E-36	5.77E-73	0.51051559	1.66E-14	0.00052824	3.87E-15	0.83155317
8.1	5.53E-36	5.33E-73	0.48730882	1.65E-14	0.00043783	4.65E-15	0.83891701
8.2	6.61E-36	6.21E-73	0.48979165	1.37E-14	0.00038208	4.16E-15	0.83354609
8.3	5.03E-36	4.79E-73	0.47612756	9.81E-15	0.00029853	5.02E-15	0.83221249
8.4	6.24E-36	5.54E-73	0.47761535	1.04E-14	0.00025051	7.04E-15	0.82298047
8.5	5.59E-36	4.17E-73	0.47878078	7.63E-15	0.0001983	1.02E-14	0.86479165
8.6	4.36E-36	4.34E-73	0.45867327	6.42E-15	0.00014265	1.07E-14	0.84166863
8.7	5.75E-36	5.44E-73	0.45233143	6.15E-15	0.00012505	1.44E-14	0.83260994
8.8	5.97E-36	9.14E-73	0.43620879	8.09E-15	0.00011549	2.07E-14	0.8518567
8.9	7.96E-36	1.23E-72	0.42061154	1.08E-14	0.0001146	2.43E-14	0.86784403
9	8.00E-36	1.18E-72	0.4009097	1.09E-14	0.00010935	3.06E-14	0.86682373
9.1	7.73E-36	1.78E-72	0.40990981	1.21E-14	8.86E-05	4.04E-14	0.88485319
9.2	8.66E-36	1.84E-72	0.39530894	1.01E-14	6.44E-05	5.29E-14	0.87380157
9.3	9.18E-36	2.42E-72	0.3814146	8.61E-15	5.20E-05	6.27E-14	0.85292702
9.4	9.97E-36	2.31E-72	0.37280025	1.05E-14	3.87E-05	7.70E-14	0.84059765
9.5	1.31E-35	3.22E-72	0.35738649	1.38E-14	3.24E-05	8.31E-14	0.84226565
9.6	1.49E-35	4.13E-72	0.34913133	1.37E-14	2.78E-05	1.08E-13	0.83874083
9.7	2.18E-35	8.42E-72	0.35016873	1.63E-14	2.27E-05	1.91E-13	0.83281218
9.8	2.61E-35	1.16E-71	0.35237628	1.31E-14	1.78E-05	2.66E-13	0.83842078
9.9	3.17E-35	1.59E-71	0.33051495	1.35E-14	1.56E-05	3.02E-13	0.85264035
10	3.03E-35	1.77E-71	0.31926556	1.80E-14	1.39E-05	3.47E-13	0.864073
10.1	3.25E-35	2.13E-71	0.32250277	1.59E-14	1.25E-05	4.23E-13	0.8537543
10.2	5.04E-35	5.26E-71	0.31878353	2.30E-14	1.23E-05	6.46E-13	0.85880466
10.3	5.47E-35	7.35E-71	0.30481459	2.48E-14	1.03E-05	7.95E-13	0.86298532
10.4	6.83E-35	1.16E-70	0.29219788	2.33E-14	7.81E-06	1.06E-12	0.8461577
10.5	1.01E-34	2.07E-70	0.29406173	3.07E-14	8.17E-06	1.47E-12	0.88942926
10.6	1.82E-34	3.53E-70	0.26422917	4.01E-14	6.12E-06	2.12E-12	0.89354234
10.7	1.85E-34	3.99E-70	0.26839377	4.23E-14	5.34E-06	2.58E-12	0.8835199
10.8	4.75E-34	1.24E-69	0.25634184	6.31E-14	5.22E-06	4.92E-12	0.89368908
10.9	1.03E-33	2.88E-69	0.2531695	9.77E-14	3.96E-06	9.22E-12	0.92831233
11	2.88E-33	1.20E-68	0.23865864	2.24E-13	4.32E-06	1.63E-11	0.9363757
11.1	3.31E-33	1.50E-68	0.2246714	2.71E-13	3.83E-06	1.81E-11	0.93938458
11.2	1.51E-32	8.51E-68	0.21839479	6.89E-13	4.07E-06	3.62E-11	0.94772024
11.3	4.46E-32	2.93E-67	0.18376818	1.53E-12	3.76E-06	5.57E-11	0.93255465
11.4	1.28E-31	9.66E-67	0.18346937	2.67E-12	3.95E-06	8.33E-11	0.93768473
11.5	2.41E-31	2.28E-66	0.18659122	3.90E-12	3.52E-06	1.18E-10	0.96767033
11.6	5.61E-31	5.12E-66	0.1775618	5.33E-12	3.21E-06	2.17E-10	0.9903215
11.7	6.03E-31	6.78E-66	0.17454141	5.74E-12	2.88E-06	2.46E-10	0.98257215
11.8	2.25E-30	3.30E-65	0.1627658	9.54E-12	3.01E-06	5.46E-10	0.98966012

Time discretization	V_{[PYR]}	K_{[PYR]}	alpha	K_{[ACoA]}	G_E	[G]_0	[PYR]_0
15	3.03E-26	2.13E-58	0.06548871	3.49E-10	1.66E-07	3.25E-07	0.99518821
15.1	2.92E-26	2.52E-58	0.06178602	3.83E-10	1.59E-07	3.32E-07	0.9979497
15.2	2.85E-26	3.20E-58	0.05548273	3.97E-10	1.36E-07	3.90E-07	0.97701141
15.3	3.80E-26	5.33E-58	0.05205366	4.18E-10	1.29E-07	4.70E-07	0.99928285
15.4	5.23E-26	7.44E-58	0.0489405	3.57E-10	9.60E-08	6.56E-07	0.98356989
15.5	6.21E-26	1.07E-57	0.04389258	4.08E-10	8.78E-08	6.96E-07	0.98417886
15.6	6.85E-26	1.42E-57	0.04512253	4.76E-10	7.77E-08	8.26E-07	0.9849309
15.7	9.05E-26	1.82E-57	0.04130846	4.63E-10	7.54E-08	9.13E-07	0.9724961
15.8	1.12E-25	2.23E-57	0.03889459	5.00E-10	7.81E-08	9.80E-07	0.97734578
15.9	1.37E-25	2.31E-57	0.03588352	5.15E-10	5.69E-08	1.13E-06	0.97679529
16	1.32E-25	2.33E-57	0.03311952	5.39E-10	5.11E-08	1.17E-06	0.96706933
16.1	1.59E-25	2.90E-57	0.03131846	5.67E-10	5.28E-08	1.18E-06	0.9530603
16.2	1.70E-25	4.07E-57	0.02871592	5.63E-10	4.86E-08	1.21E-06	0.94411059
16.3	1.60E-25	4.16E-57	0.02860746	5.57E-10	4.32E-08	1.32E-06	0.94164609
16.4	2.20E-25	6.18E-57	0.02752048	5.27E-10	3.73E-08	1.54E-06	0.94629032
16.5	2.77E-25	9.02E-57	0.02736625	4.95E-10	3.68E-08	1.64E-06	0.95654082
16.6	3.35E-25	1.06E-56	0.02420729	4.58E-10	3.18E-08	2.00E-06	0.94518462
16.7	3.30E-25	1.49E-56	0.02332779	5.43E-10	2.95E-08	2.11E-06	0.9386771
16.8	4.09E-25	1.86E-56	0.02307448	5.59E-10	2.83E-08	2.37E-06	0.94759039
16.9	4.13E-25	2.19E-56	0.02292663	5.23E-10	2.45E-08	2.70E-06	0.93342315
17	5.09E-25	2.72E-56	0.02109841	6.07E-10	2.36E-08	2.85E-06	0.94168127
17.1	5.23E-25	3.39E-56	0.02110946	5.94E-10	2.36E-08	2.86E-06	0.94875496
17.2	6.01E-25	5.58E-56	0.02126948	6.71E-10	2.21E-08	2.84E-06	0.94668946
17.3	6.69E-25	6.37E-56	0.02054063	7.02E-10	2.03E-08	2.97E-06	0.96344207
17.4	7.83E-25	8.84E-56	0.01897286	7.19E-10	1.70E-08	2.94E-06	0.9712992
17.5	8.36E-25	1.10E-55	0.01671995	7.35E-10	1.66E-08	2.96E-06	0.97266347
17.6	9.13E-25	1.24E-55	0.01548753	7.95E-10	1.34E-08	2.83E-06	0.96104706
17.7	1.11E-24	2.06E-55	0.01441948	7.28E-10	1.20E-08	2.93E-06	0.96753796
17.8	1.17E-24	2.54E-55	0.01339895	8.16E-10	1.31E-08	2.92E-06	0.97628875
17.9	1.38E-24	4.04E-55	0.01157438	7.88E-10	1.12E-08	3.06E-06	0.95994029
18	1.79E-24	5.28E-55	0.0102342	8.95E-10	9.72E-09	3.27E-06	0.96332552
18.1	2.36E-24	8.79E-55	0.00946765	1.11E-09	1.11E-08	3.47E-06	0.97362258
18.2	3.67E-24	1.50E-54	0.00892841	1.12E-09	1.13E-08	4.08E-06	0.97362985
18.3	5.51E-24	2.77E-54	0.00793703	1.31E-09	1.10E-08	4.57E-06	0.98667256
18.4	5.88E-24	4.54E-54	0.00733199	1.51E-09	1.14E-08	4.52E-06	0.99494395
18.5	6.04E-24	4.97E-54	0.00678825	1.61E-09	1.03E-08	4.37E-06	0.97459398
18.6	8.43E-24	7.19E-54	0.00640524	1.88E-09	9.80E-09	4.81E-06	0.98865919
18.7	1.30E-23	1.30E-53	0.00570823	2.21E-09	1.12E-08	5.28E-06	0.98860238
18.8	1.66E-23	2.08E-53	0.00522093	2.51E-09	1.07E-08	6.15E-06	0.99066252
18.9	2.10E-23	3.25E-53	0.00439817	3.23E-09	9.58E-09	7.10E-06	0.99009521
19	2.85E-23	8.13E-53	0.0039085	3.73E-09	9.98E-09	7.69E-06	0.97545926
19.1	3.15E-23	1.05E-52	0.00370364	3.78E-09	8.43E-09	6.88E-06	0.95062882
19.2	4.20E-23	1.76E-52	0.00348612	4.50E-09	8.38E-09	7.73E-06	0.94031665
19.3	9.22E-23	5.49E-52	0.00302314	5.68E-09	9.13E-09	8.76E-06	0.92028204
19.4	1.63E-22	1.46E-51	0.0027201	7.25E-09	9.42E-09	1.00E-05	0.89995075
19.5	2.44E-22	2.15E-51	0.00261287	7.05E-09	8.95E-09	1.26E-05	0.86993103
19.6	3.98E-22	5.63E-51	0.00245146	8.73E-09	9.70E-09	1.41E-05	0.86162591
19.7	5.72E-22	1.13E-50	0.00249444	9.62E-09	9.42E-09	1.64E-05	0.85201417
19.8	7.42E-22	2.07E-50	0.0022559	1.05E-08	9.80E-09	1.70E-05	0.84836306
19.9	1.15E-21	4.12E-50	0.00231425	1.07E-08	9.87E-09	1.96E-05	0.85063412
20	2.09E-21	1.19E-49	0.00204996	1.53E-08	9.97E-09	2.13E-05	0.83861991
20.1	2.46E-21	1.96E-49	0.00189535	1.77E-08	9.85E-09	2.28E-05	0.84183071
20.2	2.78E-21	3.18E-49	0.00174575	1.99E-08	9.28E-09	2.30E-05	0.84379762
20.3	4.22E-21	7.13E-49	0.00165352	2.21E-08	8.66E-09	2.51E-05	0.84204184
20.4	4.59E-21	9.20E-49	0.00167261	1.97E-08	8.00E-09	2.49E-05	0.83367571
20.5	6.56E-21	1.85E-48	0.00148841	2.54E-08	9.13E-09	2.58E-05	0.84526126
20.6	9.09E-21	2.85E-48	0.00140336	2.92E-08	9.12E-09	2.86E-05	0.83041002
20.7	1.11E-20	4.95E-48	0.00136223	3.39E-08	8.96E-09	3.35E-05	0.83055407
20.8	1.53E-20	1.08E-47	0.00122036	4.05E-08	9.85E-09	3.80E-05	0.81230705
20.9	2.34E-20	1.96E-47	0.00115743	4.32E-08	9.29E-09	4.07E-05	0.8070263
21	3.04E-20	3.41E-47	0.00104803	4.37E-08	8.94E-09	4.35E-05	0.80322123
21.1	5.31E-20	1.17E-46	0.00097121	6.05E-08	9.94E-09	5.16E-05	0.77616924
21.2	5.94E-20	1.43E-46	0.00094713	6.21E-08	9.74E-09	5.34E-05	0.77077653
21.3	7.57E-20	1.62E-46	0.0008441	7.51E-08	8.57E-09	5.75E-05	0.75944892
21.4	1.01E-19	2.68E-46	0.00075316	7.61E-08	9.48E-09	6.13E-05	0.7550478
21.5	2.08E-19	8.29E-46	0.00071559	9.85E-08	1.06E-08	7.34E-05	0.74703729
21.6	2.48E-19	9.20E-46	0.00066316	1.05E-07	9.30E-09	7.17E-05	0.75648975
21.7	2.78E-19	1.16E-45	0.00063603	1.04E-07	8.18E-09	7.33E-05	0.76205943
21.8	2.78E-19	1.46E-45	0.0006369	1.05E-07	7.44E-09	7.81E-05	0.76248772
21.9	3.49E-19	2.48E-45	0.00060926	1.14E-07	7.63E-09	8.08E-05	0.75775934
22	4.27E-19	3.19E-45	0.00052735	1.12E-07	7.04E-09	8.33E-05	0.75799499
22.1	4.42E-19	3.65E-45	0.0005287	1.05E-07	6.35E-09	8.60E-05	0.76983723
22.2	5.13E-19	5.06E-45	0.00052437	1.14E-07	6.86E-09	8.51E-05	0.75823565
22.3	5.09E-19	6.52E-45	0.00052101	1.10E-07	6.48E-09	9.03E-05	0.75513822
22.4	5.46E-19	7.73E-45	0.00051828	1.07E-07	5.33E-09	9.59E-05	0.75856631
22.5	6.30E-19	1.10E-44	0.00050816	1.15E-07	5.52E-09	9.64E-05	0.77243116
22.6	7.29E-19	1.61E-44	0.0005005	1.25E-07	5.06E-09	0.00010315	0.76707349
22.7	7.38E-19	1.49E-44	0.00046939	1.35E-07	4.90E-09	0.00010328	0.7594415
22.8	9.59E-19	2.15E-44	0.00042593	1.31E-07	4.90E-09	0.00011041	0.77970642
22.9	1.02E-18	2.79E-44	0.00037943	1.39E-07	4.52E-09	0.00011317	0.79832165
23	1.10E-18	3.43E-44	0.00035304	1.37E-07	4.31E-09	0.0001137	0.80678053
23.1	1.10E-18	3.63E-44	0.00034183	1.38E-07	3.66E-09	0.00011412	0.82188481
23.2	1.17E-18	4.99E-44	0.00033434	1.41E-07	3.76E-09	0.00011434	0.82466677
23.3	1.28E-18	6.16E-44	0.00033054	1.42E-07	3.64E-09	0.00012069	0.81776708
23.4	1.30E-18	6.08E-44	0.0003194	1.35E-07	3.24E-09	0.00012689	0.81860133
23.5	1.30E-18	6.59E-44	0.0003009	1.34E-07	3.19E-09	0.00012607	0.81938389
23.6	1.44E-18	6.63E-44	0.00028227	1.33E-07	3.07E-09	0.0001233	0.81553518
23.7	1.45E-18	7.75E-44	0.00027545	1.23E-07	3.07E-09	0.00012696	0.82776505
23.8	1.77E-18	1.05E-43	0.00026913	1.33E-07	2.59E-09	0.00013227	0.83239791
23.9	1.78E-18	1.06E-43	0.00025724	1.29E-07	2.55E-09	0.00013206	0.8173073
24	1.73E-18	1.03E-43	0.00024923	1.25E-07	2.27E-09	0.00013786	0.81615038
24.1	1.93E-18	9.98E-44	0.00022306	1.26E-07	2.06E-09	0.00014408	0.8067667
24.2	1.78E-18	9.93E-44	0.00021355	1.34E-07	2.03E-09	0.00013677	0.81438572
24.3	1.98E-18	1.34E-43	0.0001991	1.34E-07	1.69E-09	0.00014326	0.84349515
24.4	2.28E-18	1.68E-43	0.00018573	1.41E-07	1.52E-09	0.00014857	0.83523902
24.5	2.30E-18	1.84E-43	0.0001759	1.46E-07	1.49E-09	0.00014863	0.83812314
24.6	2.22E-18	2.07E-43	0.00017019	1.55E-07	1.36E-09	0.00014927	0.83845711
24.7	2.50E-18	2.32E-43	0.00016293	1.55E-07	1.17E-09	0.00015479	0.84031391
24.8	2.53E-18	2.34E-43	0.00015667	1.55E-07	1.14E-09	0.00015422	0.83962578
24.9	2.88E-18	2.87E-43	0.00015514	1.58E-07	1.08E-09	0.00016347	0.83872152
25	2.90E-18	3.15E-43	0.00014322	1.67E-07	1.08E-09	0.0001697	0.84906767
25.1	3.67E-18	3.78E-43	0.00013363	1.62E-07	1.04E-09	0.00016415	0.85772111
25.2	3.99E-18	4.20E-43	0.0001321	1.82E-07	1.03E-09	0.00015725	0.85307585
25.3	4.03E-18	4.06E-43	0.00012298	1.83E-07	9.66E-10	0.00015978	0.84587845
25.4	4.67E-18	4.58E-43	9.90E-05	1.80E-07	9.74E-10	0.00015621	0.83566401
25.5	4.59E-18	5.01E-43	0.00010063	1.91E-07	9.54E-10	0.00016125	0.84279535
25.6	4.49E-18	4.71E-43	9.05E-05	1.92E-07	8.84E-10	0.00015932	0.82759535
25.7	5.17E-18	5.89E-43	8.37E-05	1.93E-07	7.41E-10	0.00016844	0.83055372
25.8	5.63E-18	6.84E-43	7.88E-05	1.87E-07	6.88E-10	0.00016908	0.82720025
25.9	6.61E-18	1.20E-42	8.02E-05	2.03E-07	6.78E-10	0.00017815	0.84903005
26	6.96E-18	1.67E-42	7.25E-05	2.11E-07	7.03E-10	0.00016884	0.85183028
26.1	6.84E-18	1.91E-42	6.80E-05	1.99E-07	6.13E-10	0.00017061	0.85299923
26.2	6.83E-18	2.14E-42	5.92E-05	1.89E-07	5.67E-10	0.00017387	0.84917015
26.3	6.24E-18	2.47E-42	5.51E-05	1.91E-07	5.02E-10	0.00018955	0.84387979
26.4	6.56E-18	2.65E-42	5.19E-05	2.00E-07	4.73E-10	0.00019925	0.83400478
26.5	7.33E-18	3.07E-42	4.78E-05	2.12E-07	4.83E-10	0.00020407	0.82799741
26.6	7.44E-18	3.11E-42	4.22E-05	2.07E-07	4.37E-10	0.00019852	0.8270404
26.7	8.05E-18	3.10E-42	3.76E-05	1.96E-07	3.96E-10	0.00019792	0.8123252
26.8	7.92E-18	3.08E-42	3.70E-05	1.83E-07	3.57E-10	0.00020597	0.80956102

Time discretization	V_{[PYR]}	K_{[PYR]}	alpha	K_{[ACoA]}	G_E	[G]_0	[PYR]_0
30	3.08E-17	9.35E-41	1.02E-05	2.92E-07	8.84E-11	0.00037571	0.73889457
30.1	2.92E-17	9.79E-41	9.24E-06	2.79E-07	8.43E-11	0.00036809	0.74358533
30.2	2.89E-17	9.47E-41	9.20E-06	2.68E-07	7.99E-11	0.00037114	0.75224733
30.3	2.89E-17	1.01E-40	8.35E-06	2.54E-07	6.86E-11	0.00036888	0.76569618
30.4	2.91E-17	1.11E-40	7.51E-06	2.75E-07	7.21E-11	0.00036661	0.75468039
30.5	3.09E-17	1.21E-40	7.25E-06	2.75E-07	7.50E-11	0.00036066	0.74870818
30.6	3.80E-17	1.42E-40	6.86E-06	2.69E-07	8.07E-11	0.00036575	0.74681275
30.7	4.17E-17	1.61E-40	5.70E-06	2.79E-07	8.48E-11	0.00037881	0.75146097
30.8	4.42E-17	1.71E-40	5.27E-06	2.90E-07	8.81E-11	0.00037283	0.74726474
30.9	4.36E-17	1.71E-40	4.88E-06	2.82E-07	9.05E-11	0.00037382	0.74781552
31	4.53E-17	1.80E-40	4.55E-06	2.85E-07	9.12E-11	0.00038496	0.74264989
31.1	4.72E-17	1.90E-40	4.23E-06	2.82E-07	9.36E-11	0.00039922	0.73866598
31.2	4.52E-17	1.67E-40	4.03E-06	2.96E-07	8.90E-11	0.00037106	0.73805855
31.3	4.62E-17	1.86E-40	3.83E-06	2.97E-07	9.21E-11	0.00038055	0.73805457
31.4	4.69E-17	1.83E-40	3.78E-06	2.99E-07	8.99E-11	0.00038445	0.73111369
31.5	5.30E-17	2.05E-40	3.71E-06	2.85E-07	9.24E-11	0.00040418	0.73211835
31.6	5.30E-17	2.24E-40	3.73E-06	3.03E-07	9.12E-11	0.0004184	0.73220858
31.7	5.52E-17	2.12E-40	3.53E-06	3.07E-07	8.78E-11	0.00042538	0.72311271
31.8	5.50E-17	1.99E-40	3.44E-06	3.03E-07	7.94E-11	0.00042904	0.73255631
31.9	5.76E-17	2.40E-40	3.36E-06	3.07E-07	8.04E-11	0.00043454	0.73215476
32	6.15E-17	2.82E-40	3.19E-06	3.51E-07	8.54E-11	0.00044073	0.73490552
32.1	6.44E-17	2.94E-40	2.89E-06	3.61E-07	8.73E-11	0.00043154	0.73444023
32.2	7.24E-17	3.26E-40	2.88E-06	3.68E-07	9.40E-11	0.00042484	0.73287003
32.3	8.09E-17	3.65E-40	2.73E-06	3.86E-07	8.35E-11	0.00042057	0.74236545
32.4	9.34E-17	3.95E-40	2.50E-06	4.27E-07	9.06E-11	0.00041309	0.72851901
32.5	8.79E-17	4.77E-40	2.31E-06	4.44E-07	8.90E-11	0.00040727	0.7340159
32.6	9.77E-17	6.07E-40	2.09E-06	4.62E-07	9.66E-11	0.00041773	0.73578597
32.7	1.08E-16	7.25E-40	1.90E-06	4.75E-07	9.97E-11	0.0004134	0.72134348
32.8	1.12E-16	7.72E-40	1.84E-06	5.04E-07	9.66E-11	0.00042366	0.71486926
32.9	1.13E-16	8.48E-40	1.77E-06	5.11E-07	9.45E-11	0.00041796	0.71404561
33	1.24E-16	8.32E-40	1.61E-06	4.85E-07	8.90E-11	0.00043246	0.70625842
33.1	1.27E-16	8.59E-40	1.39E-06	4.68E-07	7.60E-11	0.00046343	0.71037909
33.2	1.39E-16	9.30E-40	1.21E-06	4.80E-07	7.55E-11	0.00047597	0.70073988
33.3	1.47E-16	1.04E-39	1.14E-06	4.57E-07	7.39E-11	0.00046533	0.70309612
33.4	1.69E-16	1.14E-39	9.07E-07	4.13E-07	7.40E-11	0.00047616	0.69394908
33.5	1.70E-16	1.11E-39	8.43E-07	4.13E-07	6.78E-11	0.00048004	0.7021596
33.6	1.98E-16	1.40E-39	7.44E-07	4.39E-07	7.27E-11	0.00049705	0.69244385
33.7	2.16E-16	1.50E-39	6.71E-07	4.77E-07	7.75E-11	0.00052182	0.68929926
33.8	2.56E-16	1.87E-39	6.13E-07	4.95E-07	8.56E-11	0.00050816	0.67623565
33.9	2.52E-16	1.90E-39	5.82E-07	4.83E-07	8.08E-11	0.00050721	0.67341959
34	3.25E-16	2.60E-39	5.64E-07	5.22E-07	8.19E-11	0.00050296	0.66940836
34.1	3.54E-16	3.25E-39	5.06E-07	5.20E-07	8.99E-11	0.00049097	0.65998959
34.2	3.45E-16	3.07E-39	4.94E-07	5.27E-07	8.22E-11	0.00048323	0.66103399
34.3	3.40E-16	3.37E-39	4.49E-07	5.33E-07	8.55E-11	0.00046564	0.65896998
34.4	3.61E-16	4.33E-39	4.33E-07	5.69E-07	8.63E-11	0.00046748	0.67363686
34.5	3.97E-16	5.02E-39	4.02E-07	5.84E-07	8.50E-11	0.00047607	0.68192906
34.6	4.11E-16	5.82E-39	3.90E-07	5.69E-07	8.37E-11	0.00048527	0.68795362
34.7	4.31E-16	5.46E-39	3.40E-07	6.17E-07	8.83E-11	0.00044496	0.68393712
34.8	4.04E-16	5.61E-39	3.37E-07	5.82E-07	8.10E-11	0.00044559	0.68313047
34.9	4.87E-16	7.00E-39	3.20E-07	5.88E-07	8.25E-11	0.00047103	0.67184479
35	5.70E-16	9.43E-39	2.95E-07	6.42E-07	9.29E-11	0.00048621	0.67322722
35.1	6.84E-16	1.41E-38	2.46E-07	7.07E-07	1.01E-10	0.00052014	0.66509892
35.2	7.31E-16	1.62E-38	2.02E-07	7.83E-07	1.07E-10	0.00051603	0.65455393
35.3	8.03E-16	2.06E-38	1.83E-07	8.26E-07	1.09E-10	0.0005362	0.64569376
35.4	8.83E-16	2.51E-38	1.69E-07	8.59E-07	1.13E-10	0.00055095	0.63950749
35.5	8.93E-16	2.72E-38	1.60E-07	8.95E-07	1.05E-10	0.00055859	0.6400036
35.6	8.73E-16	2.92E-38	1.36E-07	9.29E-07	9.53E-11	0.00054983	0.63516016
35.7	8.53E-16	3.11E-38	1.20E-07	9.63E-07	9.87E-11	0.00054222	0.63132716
35.8	9.42E-16	3.55E-38	1.14E-07	9.85E-07	1.03E-10	0.00057563	0.62806053
35.9	1.08E-15	3.82E-38	1.01E-07	9.93E-07	1.07E-10	0.00061205	0.62086235
36	1.14E-15	4.53E-38	9.26E-08	1.00E-06	1.18E-10	0.00061121	0.61479903
36.1	1.18E-15	5.98E-38	8.87E-08	9.90E-07	1.36E-10	0.0006012	0.61786511
36.2	1.35E-15	7.02E-38	8.32E-08	1.00E-06	1.37E-10	0.00059642	0.61980397
36.3	1.61E-15	8.88E-38	7.15E-08	9.67E-07	1.42E-10	0.00058711	0.61641189
36.4	1.71E-15	9.03E-38	6.31E-08	1.04E-06	1.49E-10	0.00057207	0.61449559
36.5	1.69E-15	8.99E-38	4.74E-08	1.06E-06	1.51E-10	0.00052019	0.60674573
36.6	2.09E-15	1.12E-37	4.49E-08	1.17E-06	1.59E-10	0.00054505	0.59756167
36.7	2.19E-15	1.30E-37	3.54E-08	1.16E-06	1.56E-10	0.0005746	0.60663386
36.8	2.40E-15	1.54E-37	3.46E-08	1.20E-06	1.69E-10	0.00058587	0.60211715
36.9	3.18E-15	2.26E-37	3.15E-08	1.40E-06	1.79E-10	0.00060919	0.59521118
37	2.97E-15	2.28E-37	2.98E-08	1.39E-06	1.79E-10	0.00059919	0.59397255
37.1	3.12E-15	2.74E-37	2.90E-08	1.52E-06	1.94E-10	0.00059104	0.59255716
37.2	3.71E-15	3.07E-37	2.76E-08	1.58E-06	1.86E-10	0.00059022	0.59310292
37.3	3.70E-15	3.40E-37	2.74E-08	1.63E-06	1.83E-10	0.00058428	0.59446889
37.4	3.78E-15	3.81E-37	2.56E-08	1.70E-06	1.77E-10	0.00061441	0.59910358
37.5	3.97E-15	4.28E-37	2.23E-08	1.74E-06	1.78E-10	0.000591	0.59834872
37.6	3.75E-15	4.44E-37	2.17E-08	1.74E-06	1.70E-10	0.00059203	0.60222729
37.7	4.25E-15	5.48E-37	1.86E-08	1.87E-06	1.73E-10	0.00058789	0.59947228
37.8	4.59E-15	6.59E-37	1.61E-08	1.94E-06	1.90E-10	0.00060704	0.60280507
37.9	5.03E-15	7.43E-37	1.55E-08	1.91E-06	1.88E-10	0.00060456	0.60198988
38	4.82E-15	8.34E-37	1.45E-08	1.75E-06	1.55E-10	0.00062064	0.60259545
38.1	4.84E-15	9.42E-37	1.38E-08	1.82E-06	1.55E-10	0.00061425	0.59447056
38.2	4.42E-15	1.05E-36	1.18E-08	1.86E-06	1.49E-10	0.00063314	0.59614171
38.3	4.32E-15	1.49E-36	1.02E-08	1.99E-06	1.59E-10	0.00064533	0.59928859
38.4	4.81E-15	1.66E-36	9.60E-09	2.03E-06	1.68E-10	0.00063868	0.59877277
38.5	5.52E-15	2.39E-36	9.30E-09	2.13E-06	1.89E-10	0.00067419	0.59769669
38.6	5.54E-15	2.69E-36	8.72E-09	2.08E-06	1.91E-10	0.00067135	0.60310381
38.7	5.92E-15	3.58E-36	7.65E-09	2.33E-06	2.07E-10	0.00069356	0.60315481
38.8	5.79E-15	3.88E-36	7.04E-09	2.47E-06	2.15E-10	0.00070377	0.603125
38.9	5.89E-15	4.11E-36	6.95E-09	2.50E-06	2.15E-10	0.00069902	0.60074184
39	5.81E-15	4.35E-36	6.61E-09	2.50E-06	2.18E-10	0.00070323	0.59455324
39.1	6.65E-15	5.45E-36	5.96E-09	2.66E-06	2.25E-10	0.00072581	0.58623555
39.2	7.37E-15	8.29E-36	5.52E-09	2.86E-06	2.59E-10	0.00075758	0.58194722
39.3	8.94E-15	1.16E-35	5.29E-09	2.85E-06	2.96E-10	0.00078344	0.57750044
39.4	9.25E-15	1.25E-35	4.93E-09	2.92E-06	3.16E-10	0.00079942	0.58006625
39.5	9.04E-15	1.32E-35	4.60E-09	2.92E-06	3.26E-10	0.00081539	0.5795007
39.6	9.10E-15	1.52E-35	4.38E-09	2.99E-06	3.36E-10	0.00082594	0.57691464
39.7	9.78E-15	1.62E-35	4.14E-09	3.10E-06	3.16E-10	0.00085155	0.57297516
39.8	1.11E-14	1.92E-35	3.85E-09	3.15E-06	3.11E-10	0.00087527	0.57185468
39.9	1.09E-14	2.06E-35	3.84E-09	3.27E-06	3.15E-10	0.0008844	0.57303767
40	1.06E-14	2.05E-35	3.59E-09	3.16E-06	2.93E-10	0.00088133	0.57787128
40.1	1.14E-14	2.28E-35	3.24E-09	3.49E-06	3.19E-10	0.00083275	0.57870587
40.2	1.18E-14	2.46E-35	3.07E-09	3.60E-06	3.08E-10	0.00084081	0.58187792
40.3	1.30E-14	2.97E-35	3.10E-09	3.71E-06	2.95E-10	0.00086268	0.58970586
40.4	1.40E-14	3.31E-35	2.97E-09	3.83E-06	3.12E-10	0.00087914	0.59106983
40.5	1.69E-14	4.41E-35	2.81E-09	3.96E-06	3.40E-10	0.00090286	0.59442521
40.6	1.90E-14	4.87E-35	2.61E-09	3.84E-06	3.49E-10	0.0009528	0.59193279
40.7	2.03E-14	5.56E-35	2.34E-09	4.09E-06	3.57E-10	0.00093301	0.59277829
40.8	2.21E-14	6.90E-35	2.20E-09	4.41E-06	4.06E-10	0.0009165	0.58096106
40.9	2.22E-14	7.67E-35	2.11E-09	4.67E-06	4.18E-10	0.00091861	0.58363815
41	2.45E-14	8.96E-35	2.08E-09	4.71E-06	4.38E-10	0.00093193	0.57557268
41.1	2.53E-14	9.97E-35	2.02E-09	4.91E-06	4.15E-10	0.00094965	0.58126204
41.2	2.52E-14	1.07E-34	1.98E-09	5.00E-06	4.30E-10	0.00093278	0.58125683
41.3	2.51E-14	1.10E-34	1.85E-09	5.17E-06	4.28E-10	0.00094827	0.5921912
41.4	2.37E-14	1.19E-34	1.72E-09	5.25E-06	4.35E-10	0.00096917	0.59451878
41.5	2.62E-14	1.46E-34	1.59E-09	5.80E-06	4.95E-10	0.00097475	0.60152637
41.6	3.06E-14	1.78E-34	1.58E-09	5.84E-06	5.10E-10	0.00097029	0.59436012
41.7	3.03E-14	2.00E-34	1.47E-09	5.62E-06	5.13E-10	0.00099791	0.60320355
41.8	3.05E-14	2.37E-34	1.41E-09	5.90E-06	5.15E-10	0.00100423	0.60048626

Time discretization	V_{[PYR]}	K_{[PYR]}	alpha	K_{[ACoA]}	G_E	[G]_0	[PYR]_0
45	7.54E-14	4.92E-33	4.08E-10	1.08E-05	8.67E-10	0.00162415	0.6630873
45.1	7.99E-14	5.51E-33	4.11E-10	1.15E-05	8.60E-10	0.00166744	0.66332811
45.2	7.47E-14	5.65E-33	3.72E-10	1.12E-05	8.46E-10	0.00160885	0.6694263
45.3	7.83E-14	6.20E-33	3.79E-10	1.15E-05	8.55E-10	0.00162575	0.67194398
45.4	8.09E-14	6.70E-33	3.77E-10	1.15E-05	8.38E-10	0.00169184	0.66966331
45.5	8.45E-14	7.43E-33	3.76E-10	1.15E-05	8.70E-10	0.00166684	0.6739262
45.6	8.93E-14	7.58E-33	3.58E-10	1.08E-05	8.59E-10	0.00170366	0.67390187
45.7	8.93E-14	7.58E-33	3.58E-10	1.08E-05	8.59E-10	0.00170366	0.67390187
45.8	9.66E-14	8.37E-33	3.50E-10	1.15E-05	8.71E-10	0.00174519	0.67549452
45.9	9.72E-14	8.06E-33	3.54E-10	1.15E-05	8.70E-10	0.00174238	0.67010938
46	1.06E-13	8.94E-33	3.63E-10	1.19E-05	8.75E-10	0.00170721	0.66935528
46.1	1.14E-13	9.48E-33	3.69E-10	1.20E-05	8.57E-10	0.00175756	0.67767528
46.2	1.24E-13	9.64E-33	3.10E-10	1.20E-05	7.89E-10	0.0017283	0.68335781
46.3	1.18E-13	1.07E-32	3.02E-10	1.24E-05	8.27E-10	0.0017481	0.68828432
46.4	1.14E-13	1.18E-32	2.68E-10	1.27E-05	8.55E-10	0.00170551	0.69232943
46.5	1.25E-13	1.46E-32	2.71E-10	1.27E-05	8.38E-10	0.00180294	0.69946016
46.6	1.31E-13	1.59E-32	2.62E-10	1.28E-05	8.50E-10	0.00180595	0.70640546
46.7	1.37E-13	1.74E-32	2.59E-10	1.22E-05	8.13E-10	0.00185046	0.70820599
46.8	1.41E-13	1.95E-32	2.61E-10	1.27E-05	7.59E-10	0.00192079	0.71544311
46.9	1.47E-13	2.04E-32	2.67E-10	1.26E-05	7.54E-10	0.0019578	0.717471
47	1.47E-13	2.24E-32	2.53E-10	1.28E-05	7.84E-10	0.00194634	0.72084567
47.1	1.45E-13	2.46E-32	2.45E-10	1.33E-05	8.43E-10	0.00193291	0.72345529
47.2	1.58E-13	2.64E-32	2.39E-10	1.31E-05	8.75E-10	0.00195945	0.71931162
47.3	1.57E-13	2.93E-32	2.31E-10	1.38E-05	8.68E-10	0.00198526	0.72066915
47.4	1.52E-13	2.80E-32	2.30E-10	1.40E-05	8.30E-10	0.00196655	0.7233261
47.5	1.57E-13	2.85E-32	2.27E-10	1.40E-05	8.16E-10	0.00198777	0.72319182
47.6	1.52E-13	2.98E-32	2.09E-10	1.46E-05	8.33E-10	0.0019565	0.73390868
47.7	1.46E-13	3.13E-32	1.89E-10	1.48E-05	7.75E-10	0.00202152	0.73933757
47.8	1.52E-13	3.63E-32	1.88E-10	1.49E-05	7.74E-10	0.00204562	0.74411635
47.9	1.55E-13	3.73E-32	1.86E-10	1.49E-05	7.75E-10	0.00209918	0.74329517
48	1.60E-13	4.02E-32	1.73E-10	1.49E-05	7.63E-10	0.00224249	0.75521681
48.1	1.62E-13	4.31E-32	1.61E-10	1.55E-05	8.16E-10	0.00221897	0.75573266
48.2	1.71E-13	4.87E-32	1.60E-10	1.59E-05	8.15E-10	0.00220579	0.75750196
48.3	1.69E-13	5.12E-32	1.57E-10	1.58E-05	7.65E-10	0.00216609	0.75610592
48.4	1.76E-13	5.66E-32	1.55E-10	1.60E-05	7.82E-10	0.00219082	0.75604995
48.5	1.83E-13	5.59E-32	1.45E-10	1.71E-05	7.31E-10	0.0022644	0.75551528
48.6	1.91E-13	5.84E-32	1.41E-10	1.70E-05	7.32E-10	0.00230801	0.75066606
48.7	2.09E-13	6.61E-32	1.35E-10	1.69E-05	7.16E-10	0.00238666	0.75249795
48.8	2.45E-13	7.39E-32	1.30E-10	1.70E-05	7.56E-10	0.00246634	0.7506561
48.9	2.49E-13	7.58E-32	1.31E-10	1.74E-05	7.68E-10	0.00249822	0.74938662
49	2.54E-13	8.14E-32	1.31E-10	1.75E-05	7.91E-10	0.00250713	0.74990151
49.1	2.45E-13	7.96E-32	1.26E-10	1.71E-05	7.71E-10	0.00249028	0.7530598
49.2	2.51E-13	7.99E-32	1.23E-10	1.73E-05	7.67E-10	0.00252692	0.75264087
49.3	2.61E-13	8.90E-32	1.16E-10	1.81E-05	7.96E-10	0.00254479	0.75374839
49.4	2.65E-13	9.12E-32	1.11E-10	1.87E-05	7.75E-10	0.00256249	0.75659489
49.5	2.61E-13	1.06E-31	1.12E-10	1.95E-05	8.07E-10	0.00259076	0.75943689
49.6	2.54E-13	1.14E-31	1.14E-10	1.98E-05	8.29E-10	0.00263268	0.7647212
49.7	2.54E-13	1.16E-31	1.11E-10	1.97E-05	8.29E-10	0.00262156	0.76904978
49.8	2.62E-13	1.23E-31	1.08E-10	1.89E-05	8.39E-10	0.00263003	0.7702331
49.9	2.78E-13	1.39E-31	1.09E-10	1.95E-05	8.55E-10	0.00272023	0.77371434
50	2.94E-13	1.59E-31	1.08E-10	1.88E-05	8.08E-10	0.00291144	0.78351026
50.1	3.02E-13	1.65E-31	1.05E-10	1.91E-05	8.03E-10	0.00293562	0.77952755
50.2	3.05E-13	1.88E-31	1.00E-10	1.94E-05	7.92E-10	0.00294657	0.78918249
50.3	3.08E-13	2.06E-31	1.01E-10	2.02E-05	7.84E-10	0.00292361	0.79102799
50.4	3.32E-13	2.19E-31	9.03E-11	2.17E-05	8.21E-10	0.00285162	0.79572714
50.5	3.50E-13	2.32E-31	9.04E-11	2.20E-05	8.15E-10	0.00289507	0.80024321
50.6	3.60E-13	2.47E-31	8.40E-11	2.16E-05	7.88E-10	0.002871	0.80025611
50.7	3.71E-13	2.53E-31	8.42E-11	2.18E-05	7.98E-10	0.00290725	0.79976549
50.8	4.05E-13	3.05E-31	8.29E-11	2.16E-05	7.96E-10	0.0029896	0.80553103
50.9	4.24E-13	3.23E-31	8.47E-11	2.18E-05	7.87E-10	0.00304436	0.80800888
51	4.67E-13	3.91E-31	8.60E-11	2.34E-05	8.21E-10	0.00311018	0.80952591
51.1	4.67E-13	3.91E-31	8.60E-11	2.34E-05	8.21E-10	0.00311018	0.80952591
51.2	4.62E-13	4.05E-31	8.41E-11	2.34E-05	8.00E-10	0.00305609	0.81742034
51.3	4.62E-13	4.01E-31	8.09E-11	2.30E-05	7.85E-10	0.00306929	0.81931905
51.4	4.71E-13	4.22E-31	8.01E-11	2.26E-05	7.74E-10	0.00312073	0.82688392
51.5	4.78E-13	4.46E-31	7.63E-11	2.34E-05	7.57E-10	0.0030459	0.82764076
51.6	5.10E-13	5.16E-31	7.43E-11	2.38E-05	7.67E-10	0.00316963	0.83526527
51.7	5.39E-13	5.48E-31	7.19E-11	2.42E-05	8.08E-10	0.00315113	0.84007921
51.8	5.94E-13	6.04E-31	7.00E-11	2.46E-05	7.98E-10	0.00326633	0.84123766
51.9	6.05E-13	6.26E-31	6.92E-11	2.47E-05	7.98E-10	0.00332979	0.84413425
52	6.71E-13	7.06E-31	7.21E-11	2.60E-05	7.78E-10	0.00351423	0.83981291
52.1	7.02E-13	6.99E-31	6.75E-11	2.71E-05	7.91E-10	0.00364028	0.83732243
52.2	7.28E-13	8.18E-31	6.74E-11	2.78E-05	8.19E-10	0.00374762	0.83428572
52.3	8.03E-13	9.38E-31	6.69E-11	2.79E-05	8.23E-10	0.00382947	0.84291745
52.4	7.76E-13	9.84E-31	6.82E-11	2.79E-05	8.05E-10	0.00386295	0.84838596
52.5	7.76E-13	1.02E-30	6.81E-11	2.75E-05	8.18E-10	0.00394478	0.84668494
52.6	8.23E-13	1.05E-30	6.28E-11	2.80E-05	8.01E-10	0.00389779	0.84785784
52.7	8.43E-13	1.08E-30	5.83E-11	2.82E-05	8.17E-10	0.00389471	0.84620502
52.8	8.43E-13	1.08E-30	5.83E-11	2.82E-05	8.17E-10	0.00389471	0.84620502
52.9	8.64E-13	1.09E-30	5.62E-11	2.83E-05	8.20E-10	0.00394609	0.85158714
53	8.78E-13	1.13E-30	5.49E-11	2.77E-05	7.95E-10	0.0039756	0.84868888
53.1	9.37E-13	1.33E-30	5.51E-11	2.82E-05	7.59E-10	0.00399493	0.84474092
53.2	9.91E-13	1.28E-30	5.40E-11	2.88E-05	7.54E-10	0.00418418	0.83989284
53.3	1.02E-12	1.29E-30	5.44E-11	2.92E-05	7.69E-10	0.0042845	0.83960192
53.4	1.04E-12	1.30E-30	5.47E-11	2.98E-05	7.90E-10	0.00426497	0.83979746
53.5	1.13E-12	1.38E-30	5.36E-11	2.92E-05	7.69E-10	0.00438837	0.84188314
53.6	1.22E-12	1.59E-30	5.42E-11	2.99E-05	7.74E-10	0.00442196	0.84339505
53.7	1.25E-12	1.70E-30	5.47E-11	2.97E-05	7.77E-10	0.00438588	0.84454547
53.8	1.38E-12	1.85E-30	5.41E-11	3.02E-05	8.23E-10	0.00444647	0.83810659
53.9	1.46E-12	2.00E-30	5.42E-11	3.05E-05	8.46E-10	0.00458825	0.84477208
54	1.51E-12	2.38E-30	5.54E-11	3.07E-05	8.45E-10	0.00455956	0.85113955
54.1	1.61E-12	2.49E-30	5.22E-11	3.09E-05	8.27E-10	0.00457675	0.84818184
54.2	1.72E-12	2.76E-30	5.35E-11	3.21E-05	8.42E-10	0.00457583	0.84641506
54.3	1.88E-12	3.11E-30	5.36E-11	3.27E-05	8.64E-10	0.00460294	0.85054216
54.4	1.89E-12	3.33E-30	4.86E-11	3.22E-05	8.63E-10	0.00465759	0.85632063
54.5	1.93E-12	3.47E-30	4.78E-11	3.22E-05	8.88E-10	0.00460266	0.85668554
54.6	2.00E-12	3.72E-30	4.57E-11	3.34E-05	9.26E-10	0.00469066	0.84844296
54.7	2.05E-12	3.93E-30	4.62E-11	3.47E-05	9.03E-10	0.00473526	0.84644487
54.8	2.08E-12	3.96E-30	4.43E-11	3.50E-05	9.20E-10	0.00475149	0.84866142
54.9	2.24E-12	4.43E-30	4.57E-11	3.61E-05	8.47E-10	0.00494932	0.84260548
55	2.26E-12	4.30E-30	4.52E-11	3.63E-05	8.45E-10	0.00483749	0.84279382
55.1	2.30E-12	4.51E-30	4.56E-11	3.54E-05	8.33E-10	0.00507687	0.84560636
55.2	2.65E-12	5.69E-30	4.51E-11	3.54E-05	8.61E-10	0.00536859	0.84960452
55.3	2.77E-12	6.17E-30	4.34E-11	3.43E-05	8.27E-10	0.00562436	0.84145449
55.4	2.77E-12	6.17E-30	4.34E-11	3.43E-05	8.27E-10	0.00562436	0.84145449
55.5	2.77E-12	6.17E-30	4.34E-11	3.43E-05	8.27E-10	0.00562436	0.84145449
55.6	2.78E-12	6.05E-30	4.29E-11	3.43E-05	7.87E-10	0.00571374	0.84604083
55.7	2.78E-12	6.05E-30	4.29E-11	3.43E-05	7.87E-10	0.00571374	0.84604083
55.8	2.92E-12	6.20E-30	3.83E-11	3.38E-05	8.00E-10	0.00576037	0.85167845
55.9	3.04E-12	7.20E-30	4.03E-11	3.39E-05	7.69E-10	0.00577387	0.85728311
56	3.06E-12	7.25E-30	3.96E-11	3.42E-05	7.65E-10	0.00578807	0.8545463
56.1	3.24E-12	7.54E-30	3.57E-11	3.45E-05	8.17E-10	0.0057973	0.85049985
56.2	3.38E-12	7.81E-30	3.47E-11	3.51E-05	8.11E-10	0.00569502	0.85329589
56.3	3.64E-12	8.75E-30	3.41E-11	3.55E-05	8.01E-10	0.00583039	0.85616663
56.4	3.59E-12	8.85E-30	3.45E-11	3.46E-05	8.02E-10	0.0058818	0.86017542
56.5	4.02E-12	1.08E-29	3.27E-11	3.43E-05	7.47E-10	0.00590916	0.859876
56.6	4.32E-12	1.29E-29	3.32E-11	3.48E-05	7.62E-10	0.00603502	0.86586785
56.7	4.53E-12	1.44E-29	3.32E-11	3.52E-05	7.55E-10	0.00607569	0.86804247
56.8	4.77E-12	1.53E-29	3.04E-11	3.54E-05	7.04E-10	0.00599103	0.86624994

11.9	4.31E-30	9.53E-65	0.16203619	1.37E-11	2.68E-06	7.82E-10	0.98464082
12	6.59E-30	1.91E-64	0.16013833	1.77E-11	2.41E-06	1.02E-09	0.99756131
12.1	1.12E-29	5.18E-64	0.16418317	2.26E-11	2.62E-06	1.46E-09	0.99809585
12.2	2.34E-29	1.08E-63	0.15590951	2.71E-11	2.40E-06	2.02E-09	0.9944737
12.3	5.85E-29	3.87E-63	0.15675188	4.30E-11	2.54E-06	3.05E-09	0.97213539
12.4	9.72E-29	9.09E-63	0.15228534	5.12E-11	2.67E-06	3.58E-09	0.94872708
12.5	1.31E-28	2.59E-62	0.14315961	7.70E-11	2.56E-06	6.09E-09	0.94327435
12.6	4.54E-28	1.66E-61	0.13738792	1.41E-10	2.77E-06	1.11E-08	0.92044141
12.7	9.51E-28	5.87E-61	0.13083058	1.81E-10	2.74E-06	1.69E-08	0.90745542
12.8	9.31E-28	7.88E-61	0.12970777	2.05E-10	2.60E-06	1.81E-08	0.89529679
12.9	1.91E-27	3.04E-60	0.13249521	3.36E-10	2.54E-06	2.54E-08	0.89860289
13	2.85E-27	5.20E-60	0.13069224	4.64E-10	2.48E-06	3.07E-08	0.90515128
13.1	3.33E-27	6.00E-60	0.12453915	4.87E-10	2.20E-06	3.81E-08	0.91029088
13.2	3.86E-27	7.76E-60	0.12076987	5.42E-10	1.82E-06	4.37E-08	0.90613593
13.3	6.40E-27	1.81E-59	0.10917713	7.00E-10	1.66E-06	5.42E-08	0.93021802
13.4	7.00E-27	2.53E-59	0.10569836	6.53E-10	1.53E-06	5.79E-08	0.9427222
13.5	7.95E-27	2.57E-59	0.10552788	6.26E-10	1.32E-06	6.15E-08	0.91211412
13.6	7.86E-27	2.76E-59	0.10115947	5.77E-10	1.21E-06	7.30E-08	0.93400755
13.7	1.04E-26	4.18E-59	0.10097918	6.12E-10	1.00E-06	8.26E-08	0.92667249
13.8	9.56E-27	4.88E-59	0.09616349	5.85E-10	7.72E-07	9.15E-08	0.92056501
13.9	1.10E-26	4.94E-59	0.09408609	6.89E-10	7.00E-07	1.10E-07	0.90072068
14	1.31E-26	6.65E-59	0.09500168	6.84E-10	5.90E-07	1.34E-07	0.90887183
14.1	1.27E-26	5.49E-59	0.09531594	5.80E-10	4.93E-07	1.43E-07	0.91741295
14.2	1.26E-26	6.02E-59	0.0914895	4.97E-10	4.27E-07	1.64E-07	0.9445879
14.3	1.48E-26	7.07E-59	0.0827795	4.58E-10	3.65E-07	1.54E-07	0.96447786
14.4	1.48E-26	8.31E-59	0.0794191	4.70E-10	3.23E-07	1.62E-07	0.97420902
14.5	2.51E-26	1.32E-58	0.07933393	4.97E-10	2.66E-07	1.83E-07	0.98396653
14.6	3.51E-26	1.97E-58	0.07575842	4.66E-10	2.47E-07	2.14E-07	0.9829497
14.7	3.67E-26	2.32E-58	0.07292122	4.27E-10	2.12E-07	2.66E-07	0.982249
14.8	3.35E-26	2.13E-58	0.07098885	4.13E-10	1.88E-07	2.74E-07	0.99840886
14.9	3.18E-26	2.03E-58	0.06825843	3.91E-10	1.80E-07	3.18E-07	0.98995055

26.9	9.19E-18	4.98E-42	3.50E-05	2.05E-07	3.55E-10	0.00021171	0.81173313
27	1.04E-17	5.78E-42	3.20E-05	2.08E-07	3.25E-10	0.00022673	0.81242445
27.1	1.08E-17	7.51E-42	2.97E-05	2.07E-07	3.25E-10	0.00022563	0.81020985
27.2	1.08E-17	8.62E-42	2.82E-05	2.17E-07	3.04E-10	0.00023372	0.80571177
27.3	1.09E-17	9.12E-42	2.77E-05	2.15E-07	2.75E-10	0.00024397	0.80708667
27.4	1.30E-17	1.06E-41	2.73E-05	2.21E-07	2.49E-10	0.00025319	0.80480518
27.5	1.33E-17	1.11E-41	2.64E-05	2.24E-07	2.63E-10	0.00026159	0.79641638
27.6	1.37E-17	1.23E-41	2.54E-05	2.39E-07	2.47E-10	0.00026391	0.79620023
27.7	1.41E-17	1.31E-41	2.53E-05	2.55E-07	2.43E-10	0.00026522	0.79750198
27.8	1.60E-17	1.46E-41	2.53E-05	2.70E-07	2.59E-10	0.0002672	0.79917358
27.9	1.63E-17	1.65E-41	2.50E-05	2.73E-07	2.39E-10	0.0002857	0.79861468
28	1.71E-17	1.87E-41	2.41E-05	2.85E-07	2.25E-10	0.00028977	0.78189321
28.1	2.00E-17	2.14E-41	2.25E-05	2.99E-07	2.18E-10	0.00028522	0.77292065
28.2	2.10E-17	2.07E-41	2.07E-05	3.11E-07	2.19E-10	0.00027969	0.7778002
28.3	2.00E-17	2.24E-41	1.94E-05	3.21E-07	2.12E-10	0.00027316	0.78194336
28.4	2.11E-17	2.42E-41	1.71E-05	3.05E-07	2.07E-10	0.000277	0.7704173
28.5	2.21E-17	2.57E-41	1.75E-05	3.00E-07	1.90E-10	0.00028841	0.7687924
28.6	2.34E-17	2.53E-41	1.72E-05	3.02E-07	1.77E-10	0.00029428	0.76446376
28.7	1.99E-17	2.79E-41	1.61E-05	3.17E-07	1.44E-10	0.00029657	0.76657448
28.8	1.88E-17	3.10E-41	1.50E-05	3.34E-07	1.30E-10	0.00031991	0.77310793
28.9	1.92E-17	3.47E-41	1.43E-05	3.15E-07	1.25E-10	0.00032498	0.7702167
29	1.87E-17	4.69E-41	1.36E-05	3.06E-07	1.25E-10	0.00030706	0.77863386
29.1	2.05E-17	5.05E-41	1.35E-05	3.12E-07	1.06E-10	0.00031753	0.7754909
29.2	2.14E-17	5.39E-41	1.25E-05	3.00E-07	9.63E-11	0.00032094	0.76788772
29.3	2.18E-17	5.48E-41	1.21E-05	2.95E-07	9.68E-11	0.000333	0.76035217
29.4	2.18E-17	6.11E-41	1.20E-05	2.95E-07	9.44E-11	0.00033862	0.75726188
29.5	2.20E-17	6.51E-41	1.18E-05	2.88E-07	9.46E-11	0.00033342	0.757898
29.6	2.27E-17	7.02E-41	1.19E-05	2.92E-07	9.46E-11	0.00034422	0.7616682
29.7	2.55E-17	7.78E-41	1.19E-05	2.78E-07	9.06E-11	0.00036674	0.75701613
29.8	2.72E-17	8.43E-41	1.15E-05	2.90E-07	8.65E-11	0.00036962	0.75598184
29.9	3.00E-17	8.72E-41	1.01E-05	2.87E-07	8.73E-11	0.0003702	0.74074102

41.9	3.19E-14	2.50E-34	1.36E-09	5.95E-06	5.29E-10	0.00101891	0.5998476
42	3.33E-14	2.94E-34	1.31E-09	6.04E-06	5.14E-10	0.00104242	0.60234743
42.1	3.72E-14	3.12E-34	1.27E-09	5.92E-06	5.09E-10	0.00106966	0.60784619
42.2	3.69E-14	3.72E-34	1.22E-09	5.95E-06	5.52E-10	0.00104891	0.60698243
42.3	3.62E-14	3.65E-34	1.17E-09	6.15E-06	5.61E-10	0.00104179	0.61316373
42.4	3.75E-14	4.29E-34	1.14E-09	6.28E-06	5.63E-10	0.00104803	0.62068268
42.5	3.69E-14	4.45E-34	1.06E-09	6.21E-06	5.53E-10	0.00104508	0.61990202
42.6	4.00E-14	5.53E-34	9.98E-10	6.42E-06	6.53E-10	0.00101737	0.61114532
42.7	3.99E-14	5.32E-34	9.54E-10	6.31E-06	6.48E-10	0.0010373	0.61306205
42.8	4.01E-14	6.67E-34	9.56E-10	6.39E-06	6.43E-10	0.00106786	0.61996783
42.9	4.13E-14	6.91E-34	9.22E-10	6.54E-06	6.77E-10	0.00104879	0.61991542
43	4.31E-14	8.35E-34	9.38E-10	6.81E-06	7.06E-10	0.00106125	0.62136562
43.1	4.52E-14	9.16E-34	9.13E-10	6.67E-06	7.07E-10	0.00107438	0.62430396
43.2	4.47E-14	9.91E-34	8.55E-10	6.78E-06	7.18E-10	0.00109994	0.62099603
43.3	4.31E-14	1.18E-33	8.57E-10	7.05E-06	7.55E-10	0.00111353	0.6298563
43.4	4.62E-14	1.22E-33	7.92E-10	7.01E-06	7.47E-10	0.00115622	0.6250364
43.5	4.40E-14	1.27E-33	7.57E-10	7.09E-06	7.50E-10	0.00115971	0.63004099
43.6	4.08E-14	1.28E-33	6.48E-10	7.76E-06	7.90E-10	0.00110324	0.63333783
43.7	4.20E-14	1.44E-33	6.32E-10	7.88E-06	7.06E-10	0.00114962	0.63770633
43.8	4.36E-14	1.49E-33	6.17E-10	8.14E-06	7.18E-10	0.00117495	0.64464526
43.9	4.66E-14	1.63E-33	6.44E-10	8.76E-06	6.96E-10	0.00117196	0.64474553
44	5.22E-14	1.75E-33	6.08E-10	8.51E-06	6.64E-10	0.00124061	0.64274429
44.1	6.00E-14	2.00E-33	5.68E-10	8.87E-06	7.04E-10	0.00124534	0.64848419
44.2	5.85E-14	2.19E-33	5.43E-10	8.94E-06	7.31E-10	0.00125563	0.65258652
44.3	5.83E-14	2.50E-33	4.99E-10	9.79E-06	7.94E-10	0.00130446	0.65499
44.4	6.47E-14	3.07E-33	4.53E-10	9.91E-06	8.22E-10	0.00134734	0.65644455
44.5	6.35E-14	3.44E-33	4.36E-10	1.03E-05	8.46E-10	0.00137553	0.66454043
44.6	6.70E-14	3.74E-33	4.35E-10	1.05E-05	8.34E-10	0.00138868	0.66398088
44.7	6.92E-14	4.13E-33	4.32E-10	1.03E-05	8.87E-10	0.00145227	0.66169577
44.8	6.79E-14	4.04E-33	4.02E-10	1.05E-05	8.63E-10	0.00151962	0.65977715
44.9	7.07E-14	4.31E-33	3.98E-10	1.08E-05	8.60E-10	0.00155862	0.65594859

56.9	4.67E-12	1.53E-29	2.91E-11	3.44E-05	6.93E-10	0.00604744	0.86571301
57	5.21E-12	1.68E-29	2.73E-11	3.54E-05	7.13E-10	0.00589955	0.86902821
57.1	5.16E-12	1.77E-29	2.71E-11	3.62E-05	7.38E-10	0.0058583	0.87326042
57.2	5.41E-12	1.74E-29	2.56E-11	3.72E-05	7.45E-10	0.00594804	0.87020597
57.3	5.94E-12	1.83E-29	2.48E-11	3.88E-05	7.55E-10	0.00606724	0.86715535
57.4	6.05E-12	1.85E-29	2.43E-11	3.86E-05	7.64E-10	0.00615152	0.86127914
57.5	6.28E-12	1.92E-29	2.43E-11	3.83E-05	7.58E-10	0.00619484	0.86644681
57.6	6.92E-12	2.06E-29	2.41E-11	4.00E-05	7.75E-10	0.00621107	0.87088006
57.7	7.16E-12	2.23E-29	2.26E-11	4.16E-05	7.88E-10	0.0063041	0.87616219
57.8	7.52E-12	2.65E-29	2.31E-11	4.31E-05	7.91E-10	0.00652483	0.87792235
57.9	8.94E-12	3.60E-29	2.31E-11	4.35E-05	8.39E-10	0.00675531	0.88554933
58	9.10E-12	3.79E-29	2.28E-11	4.34E-05	8.40E-10	0.0068367	0.88720171
58.1	1.01E-11	4.53E-29	2.17E-11	4.35E-05	9.31E-10	0.00679275	0.88969991
58.2	1.05E-11	4.93E-29	2.10E-11	4.34E-05	9.30E-10	0.00683426	0.89753015
58.3	1.08E-11	5.09E-29	2.12E-11	4.35E-05	9.45E-10	0.00690965	0.90029935
58.4	1.12E-11	5.82E-29	2.03E-11	4.61E-05	1.02E-09	0.00674723	0.90715931
58.5	1.18E-11	6.08E-29	2.02E-11	4.66E-05	1.01E-09	0.00694591	0.90436712
58.6	1.36E-11	7.42E-29	1.90E-11	4.83E-05	1.07E-09	0.00755282	0.91251491
58.7	1.38E-11	8.35E-29	1.82E-11	4.86E-05	1.06E-09	0.00760906	0.9104416
58.8	1.50E-11	1.09E-28	1.87E-11	5.14E-05	1.12E-09	0.00763411	0.91186278
58.9	1.46E-11	1.26E-28	1.95E-11	5.29E-05	1.14E-09	0.00737193	0.90894428
59	1.59E-11	1.40E-28	1.95E-11	5.45E-05	1.20E-09	0.00729456	0.9054923
59.1	1.85E-11	1.68E-28	1.95E-11	5.88E-05	1.21E-09	0.00759438	0.90564188
59.2	1.96E-11	1.87E-28	1.97E-11	5.97E-05	1.21E-09	0.00741136	0.91078894
59.3	2.00E-11	1.86E-28	1.98E-11	6.03E-05	1.24E-09	0.00755743	0.91530804
59.4	2.33E-11	2.24E-28	1.86E-11	6.57E-05	1.37E-09	0.00758288	0.92088437
59.5	2.40E-11	2.42E-28	1.87E-11	6.75E-05	1.31E-09	0.00775414	0.91934083
59.6	2.54E-11	2.83E-28	1.77E-11	6.85E-05	1.38E-09	0.00744774	0.92509255
59.7	2.81E-11	2.92E-28	1.62E-11	6.89E-05	1.37E-09	0.00770874	0.92676366
59.8	3.04E-11	3.41E-28	1.64E-11	6.93E-05	1.38E-09	0.00803027	0.92975315
59.9	3.22E-11	3.65E-28	1.61E-11	7.18E-05	1.36E-09	0.0079556	0.92979661

60	3.48E-11	3.66E-28	1.55E-11	7.55E-05	1.34E-09	0.00803196	0.92426051
60.1	3.66E-11	3.88E-28	1.49E-11	7.77E-05	1.35E-09	0.00811478	0.92990025
60.2	3.66E-11	3.88E-28	1.49E-11	7.77E-05	1.35E-09	0.00811478	0.92990025
60.3	3.89E-11	4.57E-28	1.45E-11	7.60E-05	1.36E-09	0.00811588	0.93947859
60.4	4.14E-11	5.28E-28	1.43E-11	7.79E-05	1.45E-09	0.00813322	0.94529247
60.5	4.09E-11	5.22E-28	1.36E-11	7.73E-05	1.35E-09	0.00838199	0.94487594
60.6	4.69E-11	6.61E-28	1.40E-11	8.00E-05	1.44E-09	0.00866136	0.94734209
60.7	5.07E-11	7.60E-28	1.46E-11	8.27E-05	1.44E-09	0.00880636	0.9477035
60.8	5.27E-11	7.91E-28	1.46E-11	8.43E-05	1.42E-09	0.00875775	0.95073359
60.9	5.29E-11	8.11E-28	1.46E-11	8.61E-05	1.43E-09	0.00887195	0.95307618
61	5.45E-11	1.03E-27	1.38E-11	8.85E-05	1.54E-09	0.0088225	0.96162652
61.1	6.05E-11	1.18E-27	1.36E-11	8.51E-05	1.50E-09	0.00872576	0.95383058
61.2	6.67E-11	1.39E-27	1.41E-11	8.68E-05	1.49E-09	0.00886135	0.94809
61.3	6.75E-11	1.61E-27	1.42E-11	8.65E-05	1.55E-09	0.00868806	0.95033788
61.4	7.07E-11	1.81E-27	1.44E-11	8.74E-05	1.56E-09	0.00860208	0.95580632
61.5	8.83E-11	2.89E-27	1.47E-11	9.36E-05	1.67E-09	0.00963998	0.97105996
61.6	9.60E-11	3.49E-27	1.45E-11	9.60E-05	1.76E-09	0.00985648	0.97430604
61.7	9.19E-11	3.81E-27	1.44E-11	9.21E-05	1.71E-09	0.01033379	0.97678098
61.8	9.75E-11	4.39E-27	1.46E-11	9.22E-05	1.77E-09	0.01048085	0.97685015
61.9	9.49E-11	4.61E-27	1.44E-11	9.20E-05	1.73E-09	0.01074758	0.97514515
62	1.07E-10	5.98E-27	1.38E-11	0.00010097	2.00E-09	0.0105504	0.96823074
62.1	1.09E-10	6.61E-27	1.32E-11	0.00010263	1.89E-09	0.01109074	0.96908554
62.2	1.15E-10	7.96E-27	1.32E-11	0.00010721	1.92E-09	0.01129725	0.9686288
62.3	1.16E-10	8.35E-27	1.36E-11	0.00010829	1.89E-09	0.01131253	0.96502524
62.4	1.18E-10	8.56E-27	1.30E-11	0.00010872	1.91E-09	0.01121683	0.96879591
62.5	1.25E-10	8.49E-27	1.31E-11	0.00010991	1.95E-09	0.01107661	0.96385989
62.6	1.26E-10	9.09E-27	1.27E-11	0.00011025	1.99E-09	0.01101201	0.97102937
62.7	1.27E-10	9.84E-27	1.29E-11	0.00011072	2.01E-09	0.01087421	0.97004713
62.8	1.27E-10	9.84E-27	1.29E-11	0.00011072	2.01E-09	0.01087421	0.97004713
62.9	1.52E-10	1.44E-26	1.33E-11	0.00011491	2.00E-09	0.01094784	0.9843947
63	1.72E-10	1.60E-26	1.39E-11	0.00011834	2.07E-09	0.01127467	0.98312024
63.1	1.86E-10	1.76E-26	1.36E-11	0.00012165	2.10E-09	0.011592	0.98163552
63.2	1.96E-10	1.90E-26	1.37E-11	0.00012294	2.11E-09	0.01166633	0.98367815
63.3	2.07E-10	2.11E-26	1.36E-11	0.00012486	2.11E-09	0.01165626	0.98629718
63.4	2.13E-10	2.38E-26	1.27E-11	0.00012896	2.18E-09	0.01150985	0.99288595
63.5	2.23E-10	2.55E-26	1.19E-11	0.00013252	2.17E-09	0.01172906	0.99127637
63.6	2.40E-10	3.04E-26	1.24E-11	0.000135	2.10E-09	0.01184547	0.99491496
63.7	2.49E-10	3.13E-26	1.20E-11	0.0001315	2.11E-09	0.01173377	0.99223101
63.8	2.89E-10	4.09E-26	1.19E-11	0.00013468	2.25E-09	0.01198162	0.9989281
63.9	3.04E-10	4.89E-26	1.20E-11	0.00013649	2.31E-09	0.01203557	0.99798243
64	3.05E-10	5.43E-26	1.11E-11	0.00013826	2.34E-09	0.01213392	0.99107204
64.1	3.29E-10	6.25E-26	1.08E-11	0.00014078	2.38E-09	0.01223994	0.98604827
64.2	3.44E-10	7.16E-26	1.05E-11	0.00014515	2.45E-09	0.01215252	0.97739046
64.3	3.53E-10	8.28E-26	1.03E-11	0.0001485	2.47E-09	0.01237532	0.96367795
64.4	3.94E-10	8.85E-26	1.02E-11	0.00015158	2.51E-09	0.01265691	0.95939131
64.5	3.99E-10	9.15E-26	9.89E-12	0.00015298	2.47E-09	0.01283621	0.95607524
64.6	4.03E-10	8.75E-26	9.57E-12	0.00015565	2.50E-09	0.01292691	0.95344094
64.7	4.25E-10	9.81E-26	9.65E-12	0.00015942	2.56E-09	0.01309623	0.95289481
64.8	4.86E-10	1.10E-25	9.21E-12	0.00016253	2.66E-09	0.01335503	0.94987548
64.9	4.86E-10	1.17E-25	8.68E-12	0.00016145	2.55E-09	0.01343788	0.94467219
65	5.04E-10	1.21E-25	8.29E-12	0.00016466	2.65E-09	0.01331313	0.945222
65.1	5.23E-10	1.35E-25	8.18E-12	0.00016941	2.66E-09	0.01321582	0.94156235
65.2	5.39E-10	1.46E-25	8.44E-12	0.00017336	2.67E-09	0.01316554	0.94350058
65.3	5.56E-10	1.60E-25	8.31E-12	0.00017624	2.76E-09	0.01298271	0.93510215
65.4	5.97E-10	1.94E-25	8.17E-12	0.00018106	2.82E-09	0.01325137	0.92915623
65.5	6.23E-10	2.09E-25	8.58E-12	0.00018278	2.73E-09	0.01296945	0.93943997
65.6	6.37E-10	2.33E-25	8.36E-12	0.00019089	2.99E-09	0.01292842	0.94066697
65.7	6.54E-10	2.40E-25	8.31E-12	0.00018844	2.98E-09	0.01311759	0.93901788
65.8	6.74E-10	2.55E-25	8.37E-12	0.00018693	3.01E-09	0.01316103	0.93670722
65.9	7.07E-10	3.04E-25	8.66E-12	0.00018867	3.10E-09	0.01310698	0.9305353
66	7.31E-10	3.52E-25	9.02E-12	0.00019408	3.13E-09	0.01292518	0.93134711
66.1	7.73E-10	3.84E-25	8.95E-12	0.00019415	3.17E-09	0.01307368	0.93318313
66.2	7.73E-10	3.84E-25	8.95E-12	0.00019415	3.17E-09	0.01307368	0.93318313
66.3	7.94E-10	3.85E-25	8.51E-12	0.00019411	3.13E-09	0.01305481	0.92629785
66.4	8.82E-10	5.30E-25	8.47E-12	0.00020673	3.24E-09	0.01315416	0.92428169
66.5	9.00E-10	5.84E-25	8.55E-12	0.00020681	3.29E-09	0.01305453	0.92212484
66.6	9.46E-10	6.85E-25	8.94E-12	0.00020863	3.38E-09	0.0131003	0.92295201
66.7	9.99E-10	7.68E-25	8.86E-12	0.00021071	3.38E-09	0.01334319	0.91711184
66.8	1.06E-09	8.12E-25	8.86E-12	0.00021632	3.43E-09	0.01368087	0.91237995
66.9	1.11E-09	8.70E-25	9.14E-12	0.00022224	3.50E-09	0.01386154	0.91337324
67	1.14E-09	1.01E-24	9.00E-12	0.00022432	3.64E-09	0.01429502	0.91197714
67.1	1.16E-09	1.02E-24	8.78E-12	0.00022223	3.66E-09	0.01418757	0.91049496
67.2	1.16E-09	1.06E-24	8.64E-12	0.00022212	3.70E-09	0.01421369	0.90877101
67.3	1.26E-09	1.20E-24	8.66E-12	0.00021576	3.76E-09	0.01463095	0.90119174
67.4	1.25E-09	1.35E-24	8.38E-12	0.00022095	3.83E-09	0.01494803	0.90664259
67.5	1.35E-09	1.51E-24	7.65E-12	0.00022757	4.01E-09	0.01455297	0.89642319
67.6	1.36E-09	1.48E-24	7.37E-12	0.00022507	3.99E-09	0.01453597	0.89255287
67.7	1.33E-09	1.59E-24	7.53E-12	0.00022525	3.92E-09	0.01470621	0.88419992
67.8	1.33E-09	1.69E-24	7.11E-12	0.00022467	3.99E-09	0.01446225	0.8779448
67.9	1.47E-09	1.79E-24	6.54E-12	0.00023782	4.07E-09	0.01460143	0.8748207
68	1.56E-09	1.92E-24	6.01E-12	0.00024626	4.24E-09	0.0143217	0.87327303
68.1	1.61E-09	2.01E-24	6.04E-12	0.00024538	4.23E-09	0.014294	0.87289529
68.2	1.67E-09	2.22E-24	6.19E-12	0.00024651	4.41E-09	0.01454565	0.86796063
68.3	1.67E-09	2.22E-24	6.19E-12	0.00024651	4.41E-09	0.01454565	0.86796063
68.4	1.79E-09	2.64E-24	5.78E-12	0.00025092	4.64E-09	0.01479111	0.86322235
68.5	1.90E-09	3.15E-24	6.03E-12	0.00025533	4.75E-09	0.01518263	0.85595691
68.6	1.92E-09	3.88E-24	5.97E-12	0.00025368	4.92E-09	0.01544282	0.84684392
68.7	2.03E-09	4.29E-24	5.38E-12	0.00026208	5.09E-09	0.01563073	0.83956865
68.8	2.19E-09	4.71E-24	5.51E-12	0.00027279	5.27E-09	0.01573264	0.8452303
68.9	2.24E-09	4.84E-24	5.16E-12	0.00028531	5.80E-09	0.01557008	0.83999786

75	2.58E-08	8.25E-22	1.68E-12	0.00086482	3.26E-08	0.02476275	0.74991255
75.1	2.78E-08	9.18E-22	1.62E-12	0.0009043	3.33E-08	0.02490781	0.74725395
75.2	2.74E-08	9.67E-22	1.59E-12	0.00090405	3.31E-08	0.02504579	0.74324861
75.3	2.81E-08	1.00E-21	1.58E-12	0.00090703	3.41E-08	0.02536089	0.74416453
75.4	2.99E-08	1.14E-21	1.64E-12	0.00093544	3.56E-08	0.02576061	0.74123199
75.5	3.00E-08	1.21E-21	1.63E-12	0.0009401	3.79E-08	0.0253123	0.73762646
75.6	3.03E-08	1.23E-21	1.61E-12	0.0009378	3.86E-08	0.02561119	0.74149368
75.7	3.08E-08	1.23E-21	1.63E-12	0.00095283	3.97E-08	0.02599435	0.74477215
75.8	3.08E-08	1.31E-21	1.63E-12	0.00096492	4.02E-08	0.02595299	0.74185598
75.9	3.15E-08	1.31E-21	1.62E-12	0.0009758	3.98E-08	0.02607238	0.74314117
76	3.23E-08	1.33E-21	1.59E-12	0.0009609	4.02E-08	0.02596539	0.74179645
76.1	3.45E-08	1.48E-21	1.54E-12	0.00098408	4.26E-08	0.02626608	0.74067681
76.2	3.66E-08	1.58E-21	1.58E-12	0.00102923	4.49E-08	0.02682248	0.74491348
76.3	3.77E-08	1.67E-21	1.60E-12	0.00103193	4.50E-08	0.02676015	0.7427492
76.4	3.90E-08	1.90E-21	1.62E-12	0.00102962	4.58E-08	0.02684134	0.74249481
76.5	3.98E-08	1.88E-21	1.60E-12	0.0010338	4.62E-08	0.02667266	0.74079621
76.6	3.98E-08	1.88E-21	1.60E-12	0.0010338	4.62E-08	0.02667266	0.74079621
76.7	4.04E-08	1.88E-21	1.55E-12	0.0010441	4.76E-08	0.02621742	0.7441803
76.8	4.21E-08	2.06E-21	1.47E-12	0.00105285	4.91E-08	0.02649283	0.74284506
76.9	4.37E-08	2.09E-21	1.42E-12	0.00104187	4.85E-08	0.02647443	0.74591302
77	4.34E-08	2.08E-21	1.41E-12	0.0010716	4.82E-08	0.02617856	0.74201438
77.1	4.38E-08	2.12E-21	1.38E-12	0.0010815	4.77E-08	0.02631041	0.74254741
77.2	4.76E-08	2.22E-21	1.26E-12	0.00112494	5.15E-08	0.02681813	0.73957844
77.3	4.76E-08	2.22E-21	1.26E-12	0.00112494	5.15E-08	0.02681813	0.73957844
77.4	4.87E-08	2.32E-21	1.22E-12	0.00112155	5.31E-08	0.0266529	0.73722932
77.5	4.87E-08	2.35E-21	1.13E-12	0.00110097	5.35E-08	0.02644613	0.73216309
77.6	4.98E-08	2.42E-21	1.17E-12	0.00110245	5.51E-08	0.02638015	0.73153323
77.7	5.20E-08	2.83E-21	1.21E-12	0.00112205	5.74E-08	0.02664964	0.73070935
77.8	5.48E-08	2.94E-21	1.20E-12	0.00111055	5.68E-08	0.02666771	0.7341551
77.9	5.76E-08	3.11E-21	1.13E-12	0.00110084	5.75E-08	0.02653365	0.7276634
78	5.69E-08	3.02E-21	1.05E-12	0.00112954	5.80E-08	0.02672538	0.72547474
78.1	5.86E-08	3.21E-21	1.04E-12	0.0011442	5.95E-08	0.02676267	0.72704314
78.2	5.66E-08	3.38E-21	9.75E-13	0.00111639	5.89E-08	0.02638549	0.73523004
78.3	5.91E-08	4.03E-21	9.84E-13	0.00114179	6.23E-08	0.02695473	0.73289851
78.4	5.91E-08	4.08E-21	9.61E-13	0.00115198	6.39E-08	0.0268837	0.73262043
78.5	5.90E-08	4.28E-21	9.45E-13	0.00118646	6.67E-08	0.02747587	0.73453225
78.6	5.97E-08	4.41E-21	9.10E-13	0.00118972	7.01E-08	0.02797705	0.7338124
78.7	6.20E-08	4.48E-21	8.57E-13	0.00118059	6.92E-08	0.02859271	0.73651688
78.8	6.19E-08	4.55E-21	8.43E-13	0.0011856	6.98E-08	0.02837548	0.73996473
78.9	6.57E-08	5.02E-21	8.12E-13	0.00117927	7.13E-08	0.02816309	0.73529561
79	6.58E-08	5.53E-21	8.30E-13	0.00121798	7.34E-08	0.02894454	0.73145892
79.1	7.02E-08	5.77E-21	7.49E-13	0.00123631	7.55E-08	0.02965539	0.73152152
79.2	7.02E-08	6.10E-21	7.71E-13	0.00127618	7.58E-08	0.02913117	0.7280847
79.3	7.02E-08	6.10E-21	7.71E-13	0.00127618	7.58E-08	0.02913117	0.7280847
79.4	7.07E-08	6.32E-21	7.59E-13	0.00127799	7.66E-08	0.02944714	0.73135042
79.5	7.61E-08	6.77E-21	7.51E-13	0.00127974	7.95E-08	0.03006292	0.73032902
79.6	7.63E-08	6.83E-21	6.85E-13	0.00124285	7.69E-08	0.03047622	0.73508627
79.7	7.61E-08	7.44E-21	6.82E-13	0.00125807	7.92E-08	0.03049117	0.73284747
79.8	7.67E-08	7.82E-21	6.73E-13	0.00125382	8.33E-08	0.02991902	0.73009291
79.9	7.68E-08	8.30E-21	6.43E-13	0.00121457	8.64E-08	0.02920963	0.72367893
80	7.37E-08	9.29E-21	6.37E-13	0.00121847	9.33E-08	0.028595	0.71464073
80.1	7.35E-08	9.84E-21	6.20E-13	0.00122756	9.45E-08	0.02883331	0.71227297
80.2	7.35E-08	9.84E-21	6.20E-13	0.00122756	9.45E-08	0.02883331	0.71227297
80.3	7.33E-08	1.02E-20	6.02E-13	0.00127714	9.50E-08	0.0289426	0.70836925
80.4	7.33E-08	1.02E-20	6.02E-13	0.00127714	9.50E-08	0.0289426	0.70836925
80.5	7.62E-08	1.03E-20	5.80E-13	0.00129005	9.11E-08	0.02899114	0.70899585
80.6	7.62E-08	1.09E-20	5.70E-13	0.00129644	8.71E-08	0.02954826	0.71154387
80.7	7.97E-08	1.10E-20	5.28E-13	0.0013308	9.24E-08	0.02974504	0.71191655
80.8	7.89E-08	9.98E-21	4.86E-13	0.00135137	9.01E-08	0.02880174	0.71047925
80.9	7.89E-08	9.98E-21	4.86E-13	0.00135137	9.01E-08	0.02880174	0.71047925
81	8.03E-08	1.03E-20	4.78E-13	0.00136224	9.35E-08	0.02853716	0.71093655
81.1	8.38E-08	1.11E-20	4.61E-13	0.00140073	9.80E-08	0.0283614	0.7138519
81.2	8.38E-08	1.11E-20	4.61E-13	0.00140073	9.80E-08	0.0283614	0.7138519
81.3	8.63E-08	1.19E-20	4.60E-13	0.00141292	1.02E-07	0.02829318	0.70699338
81.4	8.98E-08	1.25E-20	4.51E-13	0.00146524	1.07E-07	0.02816692	0.70664117
81.5	9.07E-08	1.28E-20	4.53E-13	0.0014568	1.04E-07	0.02832742	0.70470661
81.6	9.07E-08	1.37E-20	4.41E-13	0.0015087	1.07E-07	0.02819113	0.70159453
81.7	9.08E-08	1.41E-20	4.34E-13	0.00156589	1.11E-07	0.02810458	0.7045336
81.8	9.62E-08	1.54E-20	4.34E-13	0.00157787	1.16E-07	0.02800898	0.70661747
81.9	9.81E-08	1.58E-20	4.26E-13	0.00157499	1.19E-07	0.02795547	0.70488711
82	9.88E-08	1.64E-20	4.30E-13	0.00158801	1.22E-07	0.02769573	0.70071293
82.1	1.03E-07	1.75E-20	4.28E-13	0.00162592	1.30E-07	0.02804803	0.69889676
82.2	1.03E-07	1.75E-20	4.28E-13	0.00162592	1.30E-07	0.02804803	0.69889676
82.3	1.11E-07	2.14E-20	4.08E-13	0.00165142	1.46E-07	0.02850872	0.69712645
82.4	1.19E-07	2.40E-20	3.94E-13	0.00173267	1.64E-07	0.02908745	0.69440877
82.5	1.20E-07	2.59E-20	3.78E-13	0.0017546	1.70E-07	0.02895758	0.68969354
82.6	1.24E-07	2.70E-20	3.68E-13	0.00179154	1.79E-07	0.02904011	0.69091652
82.7	1.23E-07	2.75E-20	3.67E-13	0.00179234	1.78E-07	0.02872966	0.68875335
82.8	1.23E-07	2.75E-20	3.44E-13	0.00182255	1.74E-07	0.02856719	0.68716034
82.9	1.29E-07	2.81E-20	3.33E-13	0.00184686	1.80E-07	0.02854426	0.68447699
83	1.36E-07	3.10E-20	3.41E-13	0.00194036	1.89E-07	0.02781715	0.6868454
83.1	1.38E-07	3.25E-20	3.41E-13	0.00193053	1.90E-07	0.02763858	0.68283319
83.2	1.41E-07	3.31E-20	3.42E-13	0.00190588	1.93E-07	0.02762086	0.68182986
83.3	1.43E-07	3.47E-20	3.27E-13	0.00194592	1.97E-07	0.02765045	0.68195235
83.4	1.42E-07	3.48E-20	3.25E-13	0.00194254	2.02E-07	0.02747358	0.68292739
83.5	1.48E-07	3.62E-20	3.03E-13	0.00198065	2.03E-07	0.02730698	0.6794787
83.6	1.66E-07	4.19E-20	2.98E-13	0.00205281	2.16E-07	0.02825523	0.68057057
83.7	1.68E-07	4.20E-20	2.90E-13	0.00205249	2.19E-07	0.02799308	0.68187855
83.8	1.75E-07	4.69E-20	2.87E-13	0.00204974	2.29E-07	0.02819017	0.68305079
83.9	1.71E-07	4.86E-20	2.78E-13	0.00203971	2.22E-07	0.0285442	0.68144033

90	6.90E-07	1.14E-18	8.10E-14	0.00312349	9.19E-07	0.02752499	0.60913756
90.1	7.08E-07	1.19E-18	7.55E-14	0.00309162	9.17E-07	0.02714406	0.60611759
90.2	7.37E-07	1.24E-18	7.54E-14	0.00309675	9.35E-07	0.02738145	0.6071916
90.3	7.69E-07	1.31E-18	7.13E-14	0.0030787	1.00E-06	0.02724717	0.60773774
90.4	7.85E-07	1.57E-18	7.02E-14	0.00310357	1.05E-06	0.02733685	0.60647221
90.5	7.96E-07	1.59E-18	6.91E-14	0.00309851	1.07E-06	0.02712916	0.60434049
90.6	8.15E-07	1.62E-18	6.83E-14	0.00309546	1.09E-06	0.02706081	0.60497265
90.7	8.31E-07	1.66E-18	6.65E-14	0.00311374	1.11E-06	0.02653448	0.60609229
90.8	8.39E-07	1.71E-18	6.52E-14	0.0031281	1.11E-06	0.02691016	0.60865112
90.9	8.99E-07	1.89E-18	6.40E-14	0.00312538	1.13E-06	0.02723976	0.61384524
91	8.99E-07	1.89E-18	6.40E-14	0.00312538	1.13E-06	0.02723976	0.61384524
91.1	8.99E-07	1.89E-18	6.40E-14	0.00312538	1.13E-06	0.02723976	0.61384524
91.2	9.57E-07	2.03E-18	6.32E-14	0.00312144	1.17E-06	0.02639482	0.609422
91.3	9.59E-07	2.05E-18	6.18E-14	0.00315226	1.15E-06	0.02653274	0.60766072
91.4	9.59E-07	2.05E-18	6.18E-14	0.00315226	1.15E-06	0.02653274	0.60766072
91.5	9.77E-07	2.10E-18	5.99E-14	0.0031483	1.14E-06	0.026472	0.60609689
91.6	9.98E-07	2.22E-18	5.82E-14	0.00322061	1.15E-06	0.02619049	0.60599542
91.7	1.04E-06	2.38E-18	5.38E-14	0.00318266	1.15E-06	0.02669633	0.60233109
91.8	1.04E-06	2.38E-18	5.38E-14	0.00318266	1.15E-06	0.02669633	0.60233109
91.9	1.05E-06	2.46E-18	5.31E-14	0.0031482	1.14E-06	0.02679771	0.59864328
92	1.09E-06	2.56E-18	5.08E-14	0.00308535	1.11E-06	0.02624879	0.59986459
92.1	1.13E-06	2.69E-18	5.16E-14	0.00310128	1.06E-06	0.0269316	0.59473943
92.2	1.20E-06	3.01E-18	4.87E-14	0.00299959	1.10E-06	0.02717364	0.60056527
92.3	1.22E-06	3.06E-18	4.66E-14	0.00304412	1.13E-06	0.02746112	0.60054034
92.4	1.22E-06	3.06E-18	4.66E-14	0.00304412	1.13E-06	0.02746112	0.60054034
92.5	1.22E-06	3.13E-18	4.57E-14	0.0030352	1.12E-06	0.02735666	0.59678904
92.6	1.27E-06	3.30E-18	4.42E-14	0.00305648	1.13E-06	0.02738237	0.59991188
92.7	1.30E-06	3.36E-18	4.16E-14	0.00302473	1.16E-06	0.02724042	0.5968866
92.8	1.30E-06	3.36E-18	4.16E-14	0.00302473	1.16E-06	0.02724042	0.5968866
92.9	1.33E-06	3.44E-18	3.89E-14	0.00303937	1.21E-06	0.02706689	0.60067029
93	1.35E-06	3.50E-18	3.95E-14	0.00305435	1.23E-06	0.02693399	0.59881136
93.1	1.41E-06	3.84E-18	3.91E-14	0.00308304	1.29E-06	0.0269519	0.60488337
93.2	1.47E-06	3.98E-18	3.81E-14	0.00317154	1.28E-06	0.02725384	0.60287448
93.3	1.47E-06	3.98E-18	3.81E-14	0.00317154	1.28E-06	0.02725384	0.60287448
93.4	1.46E-06	4.11E-18	3.79E-14	0.003186	1.28E-06	0.0274344	0.60477009
93.5	1.47E-06	4.18E-18	3.79E-14	0.00319683	1.26E-06	0.02734493	0.60377091
93.6	1.54E-06	4.59E-18	3.86E-14	0.00322685	1.31E-06	0.02733133	0.60287574
93.7	1.54E-06	4.59E-18	3.86E-14	0.00322685	1.31E-06	0.02733133	0.60287574
93.8	1.54E-06	4.48E-18	3.83E-14	0.00324523	1.31E-06	0.02749119	0.60458622
93.9	1.50E-06	4.58E-18	3.62E-14	0.00323583	1.29E-06	0.02746113	0.60353931
94	1.54E-06	4.88E-18	3.49E-14	0.00328922	1.33E-06	0.02734542	0.61118018
94.1	1.56E-06	5.26E-18	3.36E-14	0.00320438	1.36E-06	0.02736865	0.61054066
94.2	1.57E-06	5.45E-18	3.40E-14	0.00319727	1.33E-06	0.0273913	0.61307383
94.3	1.57E-06	5.45E-18	3.40E-14	0.00319727	1.33E-06	0.0273913	0.61307383
94.4	1.58E-06	5.53E-18	3.41E-14	0.00315698	1.35E-06	0.02714662	0.61105506
94.5	1.66E-06	5.82E-18	3.31E-14	0.00311265	1.37E-06	0.02712156	0.60904922
94.6	1.76E-06	6.13E-18	3.19E-14	0.00317653	1.35E-06	0.02730828	0.6079348
94.7	1.76E-06	6.13E-18	3.19E-14	0.00317653	1.35E-06	0.02730828	0.6079348
94.8	1.78E-06	6.34E-18	3.20E-14	0.00315818	1.36E-06	0.02711458	0.60683351
94.9	1.80E-06	6.73E-18	3.23E-14	0.00317833	1.38E-06	0.02698476	0.60630451
95	1.87E-06	7.56E-18	3.12E-14	0.00312346	1.42E-06	0.02718768	0.60191221
95.1	1.90E-06	8.54E-18	3.06E-14	0.00309459	1.47E-06	0.02707274	0.59881913
95.2	1.96E-06	9.36E-18	2.98E-14	0.00310925	1.50E-06	0.02790493	0.59936294
95.3	1.98E-06	9.76E-18	2.78E-14	0.00311916	1.50E-06	0.02837654	0.59659354
95.4	1.98E-06	9.76E-18	2.78E-14	0.00311916	1.50E-06	0.02837654	0.59659354
95.5	2.01E-06	9.96E-18	2.70E-14	0.00312446	1.48E-06	0.02838234	0.59539367
95.6	2.07E-06	1.09E-17	2.62E-14	0.00308334	1.50E-06	0.02906835	0.59648209
95.7	2.08E-06	1.10E-17	2.51E-14	0.00309527	1.49E-06	0.02948908	0.59448376
95.8	2.10E-06	1.17E-17	2.49E-14	0.00306637	1.50E-06	0.02920136	0.58963209
95.9	2.10E-06	1.17E-17	2.49E-14	0.00306637	1.50E-06	0.02920136	0.58963209
96	2.12E-06	1.22E-17	2.46E-14	0.00304458	1.49E-06	0.02937361	0.58938312
96.1	2.21E-06	1.33E-17	2.44E-14	0.0031508	1.48E-06	0.0296142	0.59839639
96.2	2.24E-06	1.39E-17	2.42E-14	0.00311441	1.47E-06	0.02903552	0.59878099
96.3	2.27E-06	1.43E-17	2.34E-14	0.00312174	1.49E-06	0.0291241	0.5991789
96.4	2.31E-06	1.48E-17	2.37E-14	0.00313095	1.49E-06	0.02945117	0.5992868
96.5	2.28E-06	1.49E-17	2.36E-14	0.00313023	1.50E-06	0.02912334	0.59892222
96.6	2.28E-06	1.49E-17	2.36E-14	0.00313023	1.50E-06	0.02912334	0.59892222
96.7	2.36E-06	1.59E-17	2.18E-14	0.00317499	1.48E-06	0.02924163	0.59474834
96.8	2.36E-06	1.59E-17	2.18E-14	0.00317499	1.48E-06	0.02924163	0.59474834
96.9	2.36E-06	1.59E-17	2.18E-14	0.00317499	1.48E-06	0.02924163	0.59474834
97	2.36E-06	1.59E-17	2.18E-14	0.00317499	1.48E-06	0.02924163	0.59474834
97.1	2.41E-06	1.65E-17	2.05E-14	0.00325585	1.46E-06	0.02954486	0.59977619
97.2	2.48E-06	1.74E-17	2.04E-14	0.00325647	1.49E-06	0.02969964	0.59815852
97.3	2.53E-06	1.83E-17	2.03E-14	0.00321735	1.50E-06	0.02991885	0.59504673
97.4	2.55E-06	1.98E-17	2.04E-14	0.00319001	1.54E-06	0.0298737	0.58904209
97.5	2.58E-06	2.03E-17	1.98E-14	0.00316661	1.57E-06	0.02973717	0.58746286
97.6	2.69E-06	2.14E-17	1.91E-14	0.00322509	1.54E-06	0.02950214	0.58561295
97.7	2.76E-06	2.23E-17	1.89E-14	0.00322201	1.56E-06	0.02932371	0.58410077
97.8	2.82E-06	2.27E-17	1.88E-14	0.00321031	1.57E-06	0.02924448	0.58370068
97.9	2.82E-06	2.27E-17	1.88E-14	0.00321031	1.57E-06	0.02924448	0.58370068
98	2.81E-06	2.34E-17	1.83E-14	0.00318896	1.58E-06	0.02905536	0.58326156
98.1	2.83E-06	2.40E-17	1.79E-14	0.00321894	1.57E-06	0.02902166	0.58409545
98.2	2.83E-06	2.40E-17	1.79E-14	0.00321894	1.57E-06	0.02902166	0.58409545
98.3	3.03E-06	2.69E-17	1.79E-14	0.00326795	1.64E-06	0.02945163	0.58222004
98.4	3.05E-06	2.75E-17	1.73E-14	0.00332952	1.68E-06	0.02999516	0.58091472
98.5	3.09E-06	2.86E-17	1.72E-14	0.00334994	1.69E-06	0.03053085	0.57946519
98.6	3.09E-06	2.86E-17	1.72E-14	0.00334994	1.69E-06	0.03053085	0.57946519
98.7	3.13E-06	3.12E-17	1.60E-14	0.00335786	1.72E-06	0.03012733	0.5744508
98.8	3.01E-06	3.06E-17	1.45E-14	0.00343118	1.74E-06	0.03011452	0.57216346
98.9	3.02E-06	3.19E-17	1.43E-14	0.00342044	1.75E-06	0.02993422	0.5762283

105	4.34E-06	8.82E-17	8.86E-15	0.00428411	2.42E-06	0.03172642	0.58824498
105.1	4.34E-06	8.82E-17	8.86E-15	0.00428411	2.42E-06	0.03172642	0.58824498
105.2	4.34E-06	8.82E-17	8.86E-15	0.00428411	2.42E-06	0.03172642	0.58824498
105.3	4.34E-06	8.59E-17	8.75E-15	0.0042996	2.44E-06	0.0313504	0.59011845
105.4	4.35E-06	8.87E-17	8.86E-15	0.00433252	2.44E-06	0.03120551	0.59121789
105.5	4.34E-06	8.93E-17	8.49E-15	0.00428215	2.41E-06	0.03104135	0.59426321
105.6	4.34E-06	8.93E-17	8.49E-15	0.00428215	2.41E-06	0.03104135	0.59426321
105.7	4.34E-06	8.93E-17	8.49E-15	0.00428215	2.41E-06	0.03104135	0.59426321
105.8	4.30E-06	9.15E-17	8.39E-15	0.00428791	2.41E-06	0.03106966	0.59639129
105.9	4.30E-06	9.15E-17	8.39E-15	0.00428791	2.41E-06	0.03106966	0.59639129
106	4.32E-06	9.53E-17	8.28E-15	0.00426583	2.45E-06	0.03095288	0.59923101
106.1	4.26E-06	9.26E-17	8.10E-15	0.0042525	2.49E-06	0.03080026	0.6001637
106.2	4.26E-06	9.26E-17	8.10E-15	0.0042525	2.49E-06	0.03080026	0.6001637
106.3	4.26E-06	9.26E-17	8.10E-15	0.0042525	2.49E-06	0.03080026	0.6001637
106.4	4.26E-06	9.26E-17	8.10E-15	0.0042525	2.49E-06	0.03080026	0.6001637
106.5	4.21E-06	9.31E-17	8.07E-15	0.00429877	2.55E-06	0.03061053	0.60023241
106.6	4.14E-06	9.39E-17	8.05E-15	0.00436508	2.56E-06	0.03053906	0.60063489
106.7	4.14E-06	9.39E-17	8.05E-15	0.00436508	2.56E-06	0.03053906	0.60063489
106.8	4.14E-06	9.88E-17	8.11E-15	0.00441447	2.62E-06	0.03048318	0.59942188
106.9	4.14E-06	9.88E-17	8.11E-15	0.00441447	2.62E-06	0.03048318	0.59942188
107	4.14E-06	9.88E-17	8.11E-15	0.00441447	2.62E-06	0.03048318	0.59942188
107.1	4.14E-06	9.88E-17	8.11E-15	0.00441447	2.62E-06	0.03048318	0.59942188
107.2	4.22E-06	1.02E-16	8.14E-15	0.00443731	2.60E-06	0.03045109	0.60398345
107.3	4.32E-06	1.03E-16	8.07E-15	0.0044445	2.54E-06	0.0301705	0.60440128
107.4	4.32E-06	1.03E-16	8.07E-15	0.0044445	2.54E-06	0.0301705	0.60440128
107.5	4.32E-06	1.03E-16	8.07E-15	0.0044445	2.54E-06	0.0301705	0.60440128
107.6	4.25E-06	1.02E-16	7.81E-15	0.00446987	2.58E-06	0.03010669	0.60392557
107.7	4.32E-06	1.05E-16	7.78E-15	0.00448244	2.66E-06	0.02988729	0.60353403
107.8	4.26E-06	1.10E-16	8.05E-15	0.00452231	2.66E-06	0.02980963	0.60133183
107.9	4.26E-06	1.10E-16	8.05E-15	0.00452231	2.66E-06	0.02980963	0.60133183
108	4.29E-06	1.11E-16	8.17E-15	0.00458525	2.67E-06	0.03009373	0.60018752
108.1	4.31E-06	1.15E-16	8.28E-15	0.00461031	2.67E-06	0.0299059	0.60326916
108.2	4.31E-06	1.15E-16	8.28E-15	0.00461031	2.67E-06	0.0299059	0.60326916
108.3	4.31E-06	1.15E-16	8.28E-15	0.00461031	2.67E-06	0.0299059	0.60326916
108.4	4.32E-06	1.20E-16	8.22E-15	0.00468023	2.69E-06	0.02957322	0.60437557
108.5	4.39E-06	1.24E-16	8.25E-15	0.00468711	2.70E-06	0.0296321	0.60631718
108.6	4.42E-06	1.31E-16	8.29E-15	0.00470686	2.72E-06	0.02975905	0.60913449
108.7	4.37E-06	1.34E-16	8.28E-15	0.00479195	2.77E-06	0.02979936	0.61175786
108.8	4.37E-06	1.34E-16	8.28E-15	0.00479195	2.77E-06	0.02979936	0.61175786
108.9	4.37E-06	1.34E-16	8.28E-15	0.00479195	2.77E-06	0.02979936	0.61175786
109	4.37E-06	1.34E-16	8.28E-15	0.00479195	2.77E-06	0.02979936	0.61175786
109.1	4.42E-06	1.41E-16	8.27E-15	0.00477818	2.79E-06	0.02967229	0.61341476
109.2	4.39E-06	1.44E-16	7.92E-15	0.0047076	2.79E-06	0.02977406	0.61280944
109.3	4.42E-06	1.49E-16	7.85E-15	0.00474108	2.83E-06	0.03000262	0.6171596
109.4	4.42E-06	1.49E-16	7.85E-15	0.00474108	2.83E-06	0.03000262	0.6171596
109.5	4.45E-06	1.53E-16	7.62E-15	0.00475257	2.82E-06	0.03018348	0.61735274
109.6	4.43E-06	1.50E-16	7.62E-15	0.00481942	2.77E-06	0.03008397	0.62032612
109.7	4.43E-06	1.50E-16	7.62E-15	0.00481942	2.77E-06	0.03008397	0.62032612
109.8	4.43E-06	1.50E-16	7.62E-15	0.00481942	2.77E-06	0.03008397	0.62032612
109.9	4.43E-06	1.50E-16	7.62E-15	0.00481942	2.77E-06	0.03008397	0.62032612
110	4.41E-06	1.47E-16	7.27E-15	0.00482023	2.76E-06	0.03004798	0.61940993
110.1	4.38E-06	1.45E-16	7.19E-15	0.00487336	2.76E-06	0.03023697	0.61963599
110.2	4.33E-06	1.47E-16	7.04E-15	0.00493547	2.80E-06	0.03017465	0.6184496
110.3	4.36E-06	1.52E-16	7.13E-15	0.0049444	2.81E-06	0.03022857	0.62077231
110.4	4.37E-06	1.57E-16	7.07E-15	0.00493854	2.79E-06	0.03044423	0.62581292
110.5	4.37E-06	1.57E-16	7.07E-15	0.00493854	2.79E-06	0.03044423	0.62581292
110.6	4.37E-06	1.57E-16	7.07E-15	0.00493854	2.79E-06	0.03044423	0.62581292
110.7	4.37E-06	1.57E-16	7.07E-15	0.00493854	2.79E-06	0.03044423	0.62581292
110.8	4.37E-06	1.57E-16	7.07E-15	0.00493854	2.79E-06	0.03044423	0.62581292
110.9	4.37E-06	1.57E-16	7.07E-15	0.00493854	2.79E-06	0.03044423	0.62581292
111	4.45E-06	1.61E-16	7.10E-15	0.00494916	2.76E-06	0.03079972	0.62680941
111.1	4.41E-06	1.59E-16	7.03E-15	0.00495547	2.71E-06	0.03068948	0.6262612
111.2	4.41E-06	1.59E-16	7.03E-15	0.00495547	2.71E-06	0.03068948	0.6262612
111.3	4.42E-06	1.61E-16	6.91E-15	0.0050152	2.75E-06	0.03072899	0.62869295
111.4	4.36E-06	1.68E-16	7.09E-15	0.00505768	2.79E-06	0.03069818	0.62526855
111.5	4.45E-06	1.75E-16	7.26E-15	0.00506509	2.81E-06	0.03078715	0.62758481
111.6	4.45E-06	1.75E-16	7.26E-15	0.00506509	2.81E-06	0.03078715	0.62758481
111.7	4.46E-06	1.82E-16	7.19E-15	0.00507063	2.83E-06	0.0312817	0.62880027
111.8	4.44E-06	1.79E-16	7.17E-15	0.00513222	2.80E-06	0.0315556	0.63081893
111.9	4.44E-06	1.79E-16	7.17E-15	0.00513222	2.80E-06	0.0315556	0.63081893
112	4.39E-06	1.79E-16	6.96E-15	0.0051185	2.81E-06	0.03112828	0.63170664
112.1	4.39E-06	1.79E-16	6.96E-15	0.0051185	2.81E-06	0.03112828	0.63170664
112.2	4.39E-06	1.79E-16	6.96E-15	0.0051185	2.81E-06	0.03112828	0.63170664
112.3	4.39E-06	1.79E-16	6.96E-15	0.0051185	2.81E-06	0.03112828	0.63170664
112.4	4.39E-06	1.79E-16	6.96E-15	0.0051185	2.81E-06	0.03112828	0.63170664
112.5	4.39E-06	1.79E-16	6.96E-15	0.0051185	2.81E-06	0.03112828	0.63170664
112.6	4.43E-06	1.84E-16	6.79E-15	0.00518507	2.83E-06	0.03123917	0.6363533
112.7	4.43E-06	1.84E-16	6.79E-15	0.00518507	2.83E-06	0.03123917	0.6363533
112.8	4.43E-06	1.84E-16	6.79E-15	0.00518507	2.83E-06	0.03123917	0.6363533
112.9	4.43E-06	1.84E-16	6.79E-15	0.00518507	2.83E-06	0.03123917	0.6363533
113	4.43E-06	1.84E-16	6.79E-15	0.00518507	2.83E-06	0.03123917	0.6363533
113.1	4.45E-06	1.87E-16	6.60E-15	0.00524056	2.88E-06	0.03099129	0.63845218
113.2	4.45E-06	1.87E-16	6.60E-15	0.00524056	2.88E-06	0.03099129	0.63845218
113.3	4.45E-06	1.87E-16	6.60E-15	0.00524056	2.88E-06	0.03099129	0.63845218
113.4	4.40E-06	1.85E-16	6.32E-15	0.00530842	2.87E-06	0.0310345	0.64018357
113.5	4.47E-06	1.82E-16	6.05E-15	0.00527292	2.85E-06	0.03113349	0.64277903
113.6	4.56E-06	1.89E-16	5.97E-15	0.00541765	2.87E-06	0.03120227	0.64712845
113.7	4.56E-06	1.89E-16	5.97E-15	0.00541765	2.87E-06	0.03120227	0.64712845
113.8	4.56E-06	1.89E-16	5.97E-15	0.00541765	2.87E-06	0.03120227	0.64712845
113.9	4.56E-06	1.87E-16	5.92E-15	0.00541626	2.91E-06	0.03125233	0.64593764

69	2.42E-09	5.67E-24	5.10E-12	0.00029304	6.10E-09	0.01530682	0.83473396
69.1	2.42E-09	5.67E-24	5.10E-12	0.00029304	6.10E-09	0.01530682	0.83473396
69.2	2.67E-09	7.10E-24	4.92E-12	0.00030279	6.57E-09	0.01522102	0.83333659
69.3	2.77E-09	7.40E-24	4.55E-12	0.00031337	6.70E-09	0.01494134	0.83307715
69.4	3.02E-09	8.12E-24	4.32E-12	0.00031673	7.14E-09	0.01511355	0.83780663
69.5	3.23E-09	9.38E-24	4.51E-12	0.00032776	7.12E-09	0.01506024	0.83023792
69.6	3.35E-09	9.84E-24	4.39E-12	0.00033448	7.08E-09	0.01538878	0.83115187
69.7	3.70E-09	1.06E-23	4.42E-12	0.00034097	7.34E-09	0.01570628	0.83686368
69.8	3.88E-09	1.10E-23	4.09E-12	0.00035791	8.12E-09	0.01595371	0.83417599
69.9	3.97E-09	1.24E-23	4.05E-12	0.00036271	8.25E-09	0.01587103	0.83052724
70	4.16E-09	1.39E-23	4.16E-12	0.00036559	8.62E-09	0.01591483	0.82361164
70.1	4.40E-09	1.56E-23	3.94E-12	0.00037366	9.09E-09	0.01647773	0.82255332
70.2	4.40E-09	1.55E-23	3.59E-12	0.0003858	9.32E-09	0.01680425	0.82376482
70.3	4.72E-09	1.87E-23	3.40E-12	0.00040474	9.76E-09	0.01741662	0.82160898
70.4	5.28E-09	2.11E-23	3.19E-12	0.00042043	1.04E-08	0.01778822	0.81701304
70.5	5.44E-09	2.36E-23	3.06E-12	0.00042804	1.04E-08	0.01823886	0.81237473
70.6	5.69E-09	2.70E-23	3.17E-12	0.00043328	1.05E-08	0.01809322	0.80939702
70.7	5.69E-09	2.70E-23	3.17E-12	0.00043328	1.05E-08	0.01809322	0.80939702
70.8	5.86E-09	2.86E-23	3.20E-12	0.00045804	1.12E-08	0.01810773	0.81110761
70.9	6.26E-09	3.22E-23	3.25E-12	0.00046468	1.12E-08	0.01811733	0.80633537
71	6.38E-09	3.24E-23	3.23E-12	0.00046465	1.11E-08	0.01823022	0.80813604
71.1	6.69E-09	3.39E-23	2.94E-12	0.00048101	1.12E-08	0.01829869	0.80572635
71.2	7.32E-09	4.18E-23	2.85E-12	0.00051114	1.18E-08	0.01808534	0.79409523
71.3	7.57E-09	4.31E-23	2.89E-12	0.0005154	1.19E-08	0.01829093	0.7934611
71.4	7.99E-09	4.75E-23	2.76E-12	0.00053334	1.27E-08	0.01828386	0.79423039
71.5	8.65E-09	5.69E-23	2.74E-12	0.00053904	1.31E-08	0.01856209	0.80347769
71.6	8.78E-09	5.78E-23	2.62E-12	0.00055829	1.35E-08	0.01843719	0.79728446
71.7	9.44E-09	6.77E-23	2.63E-12	0.00057616	1.40E-08	0.01897701	0.78840373
71.8	9.43E-09	7.21E-23	2.63E-12	0.00058376	1.44E-08	0.01874547	0.78533308
71.9	9.42E-09	7.49E-23	2.68E-12	0.00058424	1.43E-08	0.01900642	0.78627048
72	9.90E-09	8.10E-23	2.62E-12	0.00058092	1.49E-08	0.0189956	0.78065513
72.1	1.01E-08	8.26E-23	2.61E-12	0.000581	1.50E-08	0.0190289	0.77956135
72.2	1.04E-08	9.81E-23	2.72E-12	0.00059517	1.55E-08	0.01887664	0.78105249
72.3	1.14E-08	1.15E-22	2.77E-12	0.00062978	1.62E-08	0.0193371	0.78057465
72.4	1.14E-08	1.15E-22	2.77E-12	0.00062978	1.62E-08	0.0193371	0.78057465
72.5	1.17E-08	1.17E-22	2.78E-12	0.00063645	1.62E-08	0.01925441	0.78446302
72.6	1.22E-08	1.28E-22	2.74E-12	0.00064385	1.71E-08	0.01939777	0.77563248
72.7	1.25E-08	1.45E-22	2.60E-12	0.00064344	1.76E-08	0.01943387	0.773199
72.8	1.26E-08	1.59E-22	2.66E-12	0.00064134	1.76E-08	0.01943054	0.7760885
72.9	1.34E-08	1.75E-22	2.66E-12	0.00065339	1.76E-08	0.01938464	0.77631566
73	1.35E-08	1.87E-22	2.71E-12	0.00066206	1.79E-08	0.01938891	0.77447991
73.1	1.35E-08	1.87E-22	2.71E-12	0.00066206	1.79E-08	0.01938891	0.77447991
73.2	1.50E-08	2.32E-22	2.69E-12	0.00068528	1.91E-08	0.02039109	0.77388601
73.3	1.65E-08	2.88E-22	2.73E-12	0.00069292	2.04E-08	0.02104091	0.77277408
73.4	1.74E-08	3.26E-22	2.81E-12	0.00069615	2.14E-08	0.0217905	0.76353657
73.5	1.82E-08	3.43E-22	2.87E-12	0.00069935	2.15E-08	0.02183002	0.76816789
73.6	1.87E-08	3.69E-22	2.74E-12	0.0007098	2.19E-08	0.02258074	0.77524541
73.7	1.95E-08	3.78E-22	2.70E-12	0.00070657	2.22E-08	0.02289436	0.77917021
73.8	1.95E-08	3.78E-22	2.70E-12	0.00070657	2.22E-08	0.02289436	0.77917021
73.9	2.02E-08	4.04E-22	2.64E-12	0.00070754	2.30E-08	0.02257809	0.78393168
74	2.09E-08	4.31E-22	2.46E-12	0.00072794	2.45E-08	0.02349298	0.78505959
74.1	2.13E-08	4.37E-22	2.41E-12	0.00072953	2.45E-08	0.02380417	0.78816733
74.2	2.11E-08	4.55E-22	2.27E-12	0.00074434	2.52E-08	0.02397709	0.78227054
74.3	2.29E-08	4.98E-22	2.16E-12	0.00078598	2.55E-08	0.02392369	0.77868492
74.4	2.27E-08	5.33E-22	1.99E-12	0.00081575	2.64E-08	0.02377248	0.76811098
74.5	2.28E-08	5.27E-22	1.93E-12	0.00081548	2.69E-08	0.0239832	0.76641584
74.6	2.35E-08	5.37E-22	1.86E-12	0.00083341	2.87E-08	0.02419141	0.76727965
74.7	2.64E-08	6.47E-22	1.83E-12	0.00084041	3.06E-08	0.02466238	0.761172
74.8	2.68E-08	7.37E-22	1.81E-12	0.00085878	3.30E-08	0.0247011	0.75307433
74.9	2.58E-08	8.25E-22	1.68E-12	0.00086482	3.26E-08	0.02476275	0.74991255

84	1.71E-07	5.03E-20	2.75E-13	0.00206125	2.22E-07	0.0284255	0.67714265
84.1	1.76E-07	5.34E-20	2.67E-13	0.00211172	2.37E-07	0.02833635	0.6726875
84.2	1.76E-07	5.49E-20	2.67E-13	0.0020736	2.36E-07	0.02850136	0.67284788
84.3	1.81E-07	6.05E-20	2.45E-13	0.00208091	2.36E-07	0.02877783	0.66887716
84.4	1.81E-07	6.17E-20	2.46E-13	0.00209729	2.43E-07	0.02852718	0.66780264
84.5	1.85E-07	6.58E-20	2.38E-13	0.00208782	2.55E-07	0.02849767	0.66591651
84.6	1.87E-07	6.67E-20	2.25E-13	0.00210899	2.54E-07	0.0283952	0.66985073
84.7	1.89E-07	6.89E-20	2.20E-13	0.00211435	2.58E-07	0.02849114	0.67275444
84.8	2.02E-07	7.88E-20	2.22E-13	0.00223182	2.81E-07	0.02886932	0.67062389
84.9	2.02E-07	7.92E-20	2.12E-13	0.00221422	2.76E-07	0.02892405	0.66576392
85	2.01E-07	8.05E-20	2.08E-13	0.0022168	2.78E-07	0.0290109	0.66662354
85.1	2.11E-07	8.35E-20	2.10E-13	0.0022292	2.89E-07	0.02952254	0.6670865
85.2	2.09E-07	9.15E-20	2.06E-13	0.00222645	3.03E-07	0.02937799	0.66297717
85.3	2.16E-07	9.59E-20	1.97E-13	0.00228155	3.09E-07	0.02979631	0.66455812
85.4	2.25E-07	9.97E-20	1.93E-13	0.00231375	3.19E-07	0.02922216	0.6607196
85.5	2.40E-07	1.09E-19	1.95E-13	0.0023537	3.24E-07	0.02864524	0.66075057
85.6	2.43E-07	1.12E-19	1.95E-13	0.00232931	3.28E-07	0.02839946	0.65819656
85.7	2.50E-07	1.22E-19	1.97E-13	0.00238266	3.43E-07	0.02820939	0.65185313
85.8	2.54E-07	1.27E-19	1.98E-13	0.0023898	3.50E-07	0.02823848	0.64922678
85.9	2.62E-07	1.47E-19	1.96E-13	0.00241782	3.67E-07	0.02812898	0.64926166
86	2.80E-07	1.76E-19	1.86E-13	0.00250481	4.02E-07	0.02820966	0.64830645
86.1	2.95E-07	1.89E-19	1.73E-13	0.00249128	4.06E-07	0.02833171	0.64278827
86.2	2.94E-07	1.92E-19	1.69E-13	0.00246195	3.96E-07	0.02841474	0.64306802
86.3	3.10E-07	2.36E-19	1.69E-13	0.0024149	4.12E-07	0.02865956	0.63779612
86.4	3.14E-07	2.42E-19	1.71E-13	0.00245948	4.31E-07	0.02892814	0.63800433
86.5	3.14E-07	2.51E-19	1.73E-13	0.0024645	4.35E-07	0.0289432	0.63534878
86.6	3.17E-07	2.58E-19	1.66E-13	0.00243232	4.43E-07	0.02861523	0.63380954
86.7	3.18E-07	2.57E-19	1.67E-13	0.00246203	4.45E-07	0.02855317	0.63345483
86.8	3.20E-07	2.68E-19	1.64E-13	0.00250091	4.56E-07	0.02863316	0.63362749
86.9	3.18E-07	2.78E-19	1.63E-13	0.00246543	4.62E-07	0.02850568	0.62960937
87	3.27E-07	2.88E-19	1.53E-13	0.00244012	4.65E-07	0.02889938	0.63171741
87.1	3.44E-07	3.11E-19	1.50E-13	0.00252549	4.75E-07	0.0290857	0.63209008
87.2	3.44E-07	3.11E-19	1.50E-13	0.00252549	4.75E-07	0.0290857	0.63209008
87.3	3.64E-07	3.49E-19	1.40E-13	0.00266031	5.10E-07	0.03029651	0.64457026
87.4	3.66E-07	3.60E-19	1.39E-13	0.00267023	5.17E-07	0.0299849	0.64368389
87.5	3.80E-07	3.89E-19	1.38E-13	0.00269827	5.34E-07	0.0299733	0.64201165
87.6	3.88E-07	4.52E-19	1.38E-13	0.00273425	5.55E-07	0.03091018	0.63432308
87.7	4.04E-07	4.76E-19	1.34E-13	0.00278223	5.78E-07	0.03059981	0.63310636
87.8	4.18E-07	4.89E-19	1.25E-13	0.00276522	5.74E-07	0.03056233	0.63189562
87.9	4.36E-07	5.09E-19	1.18E-13	0.00277455	5.91E-07	0.03004255	0.62843648
88	4.55E-07	5.29E-19	1.19E-13	0.00278484	6.07E-07	0.03037071	0.62955685
88.1	4.58E-07	5.40E-19	1.09E-13	0.00284371	6.20E-07	0.02984669	0.6290641
88.2	4.58E-07	5.40E-19	1.09E-13	0.00284371	6.20E-07	0.02984669	0.6290641
88.3	4.72E-07	5.99E-19	1.14E-13	0.00295616	6.50E-07	0.0295816	0.62391544
88.4	4.96E-07	6.06E-19	1.09E-13	0.00293545	6.79E-07	0.029054	0.62604239
88.5	4.96E-07	6.06E-19	1.09E-13	0.00293545	6.79E-07	0.029054	0.62604239
88.6	4.94E-07	6.25E-19	1.08E-13	0.00293515	6.88E-07	0.02889863	0.62913181
88.7	4.87E-07	6.57E-19	1.08E-13	0.00297062	7.03E-07	0.02864435	0.62637643
88.8	5.08E-07	6.91E-19	1.01E-13	0.00292718	7.09E-07	0.02857598	0.62041041
88.9	5.31E-07	7.41E-19	9.80E-14	0.00304969	7.53E-07	0.02853942	0.62152268
89	5.49E-07	7.52E-19	9.47E-14	0.00306556	7.31E-07	0.02863356	0.62441873
89.1	5.48E-07	7.77E-19	9.10E-14	0.00302355	7.57E-07	0.02844094	0.62246354
89.2	5.91E-07	8.85E-19	9.30E-14	0.00307927	8.24E-07	0.02828813	0.62130873
89.3	6.18E-07	9.11E-19	9.35E-14	0.00308122	8.33E-07	0.02833302	0.6203818
89.4	6.18E-07	9.11E-19	9.35E-14	0.00308122	8.33E-07	0.02833302	0.6203818
89.5	6.35E-07	9.53E-19	9.04E-14	0.00307746	8.39E-07	0.02843601	0.61888259
89.6	6.52E-07	1.03E-18	8.68E-14	0.00311034	8.77E-07	0.02814532	0.61488482
89.7	6.78E-07	1.08E-18	8.44E-14	0.0030485	8.94E-07	0.02785648	0.60996471
89.8	6.87E-07	1.09E-18	8.36E-14	0.00302821	9.17E-07	0.02776886	0.60782494
89.9	6.94E-07	1.12E-18	8.42E-14	0.00302462	9.32E-07	0.02770165	0.6101363

99	3.09E-06	3.28E-17	1.41E-14	0.00346022	1.75E-06	0.03001712	0.57668249
99.1	3.15E-06	3.38E-17	1.36E-14	0.00346521	1.79E-06	0.03013278	0.57383524
99.2	3.13E-06	3.40E-17	1.37E-14	0.0034497	1.76E-06	0.02997364	0.57032844
99.3	3.11E-06	3.46E-17	1.33E-14	0.00347014	1.76E-06	0.03004444	0.57183744
99.4	3.11E-06	3.46E-17	1.33E-14	0.00347014	1.76E-06	0.03004444	0.57183744
99.5	3.11E-06	3.46E-17	1.33E-14	0.00347014	1.76E-06	0.03004444	0.57183744
99.6	3.35E-06	3.97E-17	1.33E-14	0.00357203	1.81E-06	0.03069488	0.57730225
99.7	3.45E-06	4.11E-17	1.34E-14	0.00355476	1.86E-06	0.03072411	0.5774457
99.8	3.53E-06	4.29E-17	1.31E-14	0.00360409	1.87E-06	0.03104835	0.58218202
99.9	3.53E-06	4.29E-17	1.31E-14	0.00360409	1.87E-06	0.03104835	0.58218202
100	3.50E-06	4.27E-17	1.31E-14	0.00361051	1.89E-06	0.03084064	0.58206516
100.1	3.54E-06	4.54E-17	1.24E-14	0.00367889	1.96E-06	0.03069272	0.57690169
100.2	3.51E-06	4.65E-17	1.21E-14	0.00372175	1.96E-06	0.03070671	0.57646507
100.3	3.51E-06	4.65E-17	1.21E-14	0.00372175	1.96E-06	0.03070671	0.57646507
100.4	3.51E-06	4.93E-17	1.22E-14	0.00373659	1.95E-06	0.03039383	0.57562436
100.5	3.51E-06	4.93E-17	1.22E-14	0.00373659	1.95E-06	0.03039383	0.57562436
100.6	3.53E-06	5.35E-17	1.21E-14	0.00370148	1.89E-06	0.03036698	0.56827662
100.7	3.58E-06	5.39E-17	1.13E-14	0.00377316	1.90E-06	0.03067707	0.56808466
100.8	3.57E-06	5.36E-17	1.09E-14	0.00378154	1.93E-06	0.03089688	0.56612441
100.9	3.57E-06	5.36E-17	1.09E-14	0.00378154	1.93E-06	0.03089688	0.56612441
101	3.57E-06	5.36E-17	1.09E-14	0.00378154	1.93E-06	0.03089688	0.56612441
101.1	3.57E-06	5.17E-17	1.03E-14	0.00383844	1.90E-06	0.03112004	0.56730169
101.2	3.78E-06	5.92E-17	1.03E-14	0.00384819	1.96E-06	0.03119414	0.56800744
101.3	3.81E-06	6.14E-17	1.02E-14	0.00384919	1.99E-06	0.03082711	0.56989776
101.4	3.90E-06	6.28E-17	1.02E-14	0.00387843	2.01E-06	0.03112793	0.57198062
101.5	3.90E-06	6.28E-17	1.02E-14	0.00387843	2.01E-06	0.03112793	0.57198062
101.6	3.90E-06	6.28E-17	1.02E-14	0.00387843	2.01E-06	0.03112793	0.57198062
101.7	3.90E-06	6.27E-17	9.84E-15	0.00387495	2.04E-06	0.03125733	0.57387898
101.8	4.03E-06	6.73E-17	9.64E-15	0.00389985	2.05E-06	0.0313028	0.57227398
101.9	4.13E-06	7.04E-17	9.74E-15	0.00388435	2.08E-06	0.03136028	0.57172376
102	4.13E-06	7.04E-17	9.74E-15	0.00388435	2.08E-06	0.03136028	0.57172376
102.1	4.13E-06	7.04E-17	9.74E-15	0.00388435	2.08E-06	0.03136028	0.57172376
102.2	4.15E-06	7.15E-17	9.40E-15	0.00390823	2.11E-06	0.03154502	0.57129106
102.3	4.09E-06	7.37E-17	9.53E-15	0.00397272	2.15E-06	0.03147156	0.57139585
102.4	4.10E-06	7.36E-17	9.33E-15	0.00392011	2.10E-06	0.03152909	0.57006121
102.5	4.10E-06	7.36E-17	9.33E-15	0.00392011	2.10E-06	0.03152909	0.57006121
102.6	4.09E-06	7.66E-17	8.97E-15	0.00397379	2.17E-06	0.03176016	0.57161034
102.7	4.09E-06	7.66E-17	8.97E-15	0.00397379	2.17E-06	0.03176016	0.57161034
102.8	4.09E-06	7.66E-17	8.97E-15	0.00397379	2.17E-06	0.03176016	0.57161034
102.9	4.16E-06	8.23E-17	8.95E-15	0.0040394	2.19E-06	0.03218573	0.57507642
103	4.09E-06	8.06E-17	8.89E-15	0.00402401	2.17E-06	0.03241663	0.57121195
103.1	4.10E-06	8.13E-17	8.91E-15	0.0040477	2.19E-06	0.0321781	0.57455758
103.2	4.10E-06	8.13E-17	8.91E-15	0.0040477	2.19E-06	0.0321781	0.57455758
103.3	4.10E-06	8.13E-17	8.91E-15	0.0040477	2.19E-06	0.0321781	0.57455758
103.4	4.10E-06	8.13E-17	8.91E-15	0.0040477	2.19E-06	0.0321781	0.57455758
103.5	4.11E-06	8.13E-17	8.76E-15	0.00400745	2.16E-06	0.03171619	0.57633474
103.6	4.11E-06	7.87E-17	8.69E-15	0.00402311	2.18E-06	0.03183338	0.5787299
103.7	4.19E-06	8.14E-17	8.76E-15	0.00405696	2.18E-06	0.03217095	0.58012491
103.8	4.19E-06	8.14E-17	8.76E-15	0.00405696	2.18E-06	0.03217095	0.58012491
103.9	4.26E-06	8.17E-17	8.83E-15	0.00408169	2.19E-06	0.03228642	0.5838517
104	4.22E-06	8.21E-17	8.91E-15	0.00412082	2.23E-06	0.03209082	0.58129077
104.1	4.31E-06	8.51E-17	9.09E-15	0.00417773	2.23E-06	0.03205751	0.5861147
104.2	4.26E-06	8.54E-17	9.05E-15	0.00420313	2.29E-06	0.03172541	0.58580812
104.3	4.33E-06	9.01E-17	9.19E-15	0.0042766	2.34E-06	0.03178121	0.58834774
104.4	4.32E-06	8.85E-17	9.00E-15	0.00437001	2.35E-06	0.03200409	0.58988699
104.5	4.30E-06	8.85E-17	8.87E-15	0.00430341	2.36E-06	0.03184784	0.58857537
104.6	4.34E-06	8.82E-17	8.86E-15	0.00428411	2.42E-06	0.03172642	0.58824498
104.7	4.34E-06	8.82E-17	8.86E-15	0.00428411	2.42E-06	0.03172642	0.58824498
104.8	4.34E-06	8.82E-17	8.86E-15	0.00428411	2.42E-06	0.03172642	0.58824498
104.9	4.34E-06	8.82E-17	8.86E-15	0.00428411	2.42E-06	0.03172642	0.58824498

114	4.56E-06	1.87E-16	5.92E-15	0.00541626	2.91E-06	0.03125233	0.64593764
114.1	4.56E-06	1.87E-16	5.92E-15	0.00541626	2.91E-06	0.03125233	0.64593764
114.2	4.56E-06	1.87E-16	5.92E-15	0.00541626	2.91E-06	0.03125233	0.64593764
114.3	4.56E-06	1.87E-16	5.92E-15	0.00541626	2.91E-06	0.03125233	0.64593764
114.4	4.56E-06	1.87E-16	5.92E-15	0.00541626	2.91E-06	0.03125233	0.64593764
114.5	4.62E-06	1.86E-16	5.85E-15	0.00536841	2.90E-06	0.03149511	0.64510303
114.6	4.62E-06	1.86E-16	5.85E-15	0.00536841	2.90E-06	0.03149511	0.64510303
114.7	4.62E-06	1.86E-16	5.85E-15	0.00536841	2.90E-06	0.03149511	0.64510303
114.8	4.62E-06	1.86E-16	5.85E-15	0.00536841	2.90E-06	0.03149511	0.64510303
114.9	4.62E-06	1.86E-16	5.85E-15	0.00536841	2.90E-06	0.03149511	0.64510303
115	4.70E-06	1.91E-16	5.67E-15	0.0055195	2.99E-06	0.03164267	0.64553732
115.1	4.71E-06	1.93E-16	5.67E-15	0.0054131	2.92E-06	0.03180038	0.64409391
115.2	4.69E-06	1.98E-16	5.70E-15	0.00545818	3.00E-06	0.03151139	0.64331813
115.3	4.76E-06	2.08E-16	5.72E-15	0.00545376	3.00E-06	0.03175885	0.64439841
115.4	4.75E-06	2.09E-16	5.70E-15	0.00549129	3.06E-06	0.03185544	0.64459958
115.5	4.80E-06	2.19E-16	5.73E-15	0.00551548	3.10E-06	0.03215356	0.64531158
115.6	4.80E-06	2.19E-16	5.73E-15	0.00551548	3.10E-06	0.03215356	0.64531158
115.7	4.78E-06	2.21E-16	5.73E-15	0.00554303	3.17E-06	0.03211293	0.64335008
115.8	4.78E-06	2.21E-16	5.73E-15	0.00554303	3.17E-06	0.03211293	0.64335008
115.9	4.81E-06	2.27E-16	5.61E-15	0.00556384	3.20E-06	0.03198876	0.64599447
116	4.79E-06	2.39E-16	5.71E-15	0.0055898	3.26E-06	0.0320757	0.64326575
116.1	4.79E-06	2.39E-16	5.71E-15	0.0055898	3.26E-06	0.0320757	0.64326575
116.2	4.78E-06	2.34E-16	5.72E-15	0.00565342	3.31E-06	0.03221257	0.64322015
116.3	4.78E-06	2.34E-16	5.72E-15	0.00565342	3.31E-06	0.03221257	0.64322015
116.4	4.78E-06	2.34E-16	5.72E-15	0.00565342	3.31E-06	0.03221257	0.64322015
116.5	4.89E-06	2.44E-16	5.70E-15	0.00570783	3.34E-06	0.03203983	0.64591116
116.6	4.89E-06	2.44E-16	5.70E-15	0.00570783	3.34E-06	0.03203983	0.64591116
116.7	4.89E-06	2.44E-16	5.70E-15	0.00570783	3.34E-06	0.03203983	0.64591116
116.8	4.86E-06	2.48E-16	5.57E-15	0.00571678	3.34E-06	0.03183589	0.64319077
116.9	4.78E-06	2.52E-16	5.53E-15	0.0056842	3.25E-06	0.03212226	0.64077666
117	4.78E-06	2.52E-16	5.53E-15	0.0056842	3.25E-06	0.03212226	0.64077666
117.1	4.72E-06	2.63E-16	5.64E-15	0.00569247	3.29E-06	0.03247536	0.63952946
117.2	4.69E-06	2.70E-16	5.54E-15	0.00571723	3.30E-06	0.03228925	0.64333428
117.3	4.69E-06	2.70E-16	5.54E-15	0.00571723	3.30E-06	0.03228925	0.64333428
117.4	4.60E-06	2.74E-16	5.49E-15	0.00566718	3.31E-06	0.03195911	0.64145054
117.5	4.58E-06	2.84E-16	5.57E-15	0.00571538	3.35E-06	0.03161871	0.64186169
117.6	4.49E-06	2.88E-16	5.54E-15	0.00572731	3.36E-06	0.0319102	0.64315094
117.7	4.49E-06	2.88E-16	5.54E-15	0.00572731	3.36E-06	0.0319102	0.64315094
117.8	4.37E-06	2.94E-16	5.43E-15	0.00570149	3.35E-06	0.0318248	0.64147957
117.9	4.47E-06	3.11E-16	5.42E-15	0.0058159	3.40E-06	0.03212136	0.64524955
118	4.51E-06	3.17E-16	5.43E-15	0.00587885	3.47E-06	0.03208802	0.64945294
118.1	4.51E-06	3.17E-16	5.43E-15	0.00587885	3.47E-06	0.03208802	0.64945294
118.2	4.51E-06	3.17E-16	5.43E-15	0.00587885	3.47E-06	0.03208802	0.64945294
118.3	4.51E-06	3.17E-16	5.43E-15	0.00587885	3.47E-06	0.03208802	0.64945294
118.4	4.51E-06	3.17E-16	5.43E-15	0.00587885	3.47E-06	0.03208802	0.64945294
118.5	4.51E-06	3.17E-16	5.43E-15	0.00587885	3.47E-06	0.03208802	0.64945294
118.6	4.51E-06	3.17E-16	5.43E-15	0.00587885	3.47E-06	0.03208802	0.64945294
118.7	4.45E-06	3.13E-16	5.22E-15	0.00595936	3.53E-06	0.03213683	0.65059787
118.8	4.45E-06	3.13E-16	5.22E-15	0.00595936	3.53E-06	0.03213683	0.65059787
118.9	4.45E-06	3.13E-16	5.22E-15	0.00595936	3.53E-06	0.03213683	0.65059787
119	4.29E-06	3.29E-16	5.01E-15	0.00595022	3.57E-06	0.03194814	0.6514402
119.1	4.29E-06	3.29E-16	5.01E-15	0.00595022	3.57E-06	0.03194814	0.6514402
119.2	4.25E-06	3.41E-16	4.96E-15	0.00600865	3.59E-06	0.03170645	0.65322548
119.3	4.25E-06	3.41E-16	4.96E-15	0.00600865	3.59E-06	0.03170645	0.65322548
119.4	4.25E-06	3.41E-16	4.96E-15	0.00600865	3.59E-06	0.03170645	0.65322548
119.5	4.24E-06	3.33E-16	4.91E-15	0.00605661	3.66E-06	0.03170489	0.65554765
119.6	4.24E-06	3.33E-16	4.91E-15	0.00605661	3.66E-06	0.03170489	0.65554765
119.7	4.24E-06	3.33E-16	4.91E-15	0.00605661	3.66E-06	0.03170489	0.65554765
119.8	4.24E-06	3.33E-16	4.91E-15	0.00605661	3.66E-06	0.03170489	0.65554765
119.9	4.24E-06	3.33E-16	4.91E-15	0.00605661	3.66E-06	0.03170489	0.65554765

120	4.24E-06	3.33E-16	4.91E-15	0.00605661	3.66E-06	0.03170489	0.65554765
120.1	4.25E-06	3.35E-16	4.73E-15	0.00605338	3.62E-06	0.0316335	0.65762391
120.2	4.32E-06	3.44E-16	4.62E-15	0.00612583	3.68E-06	0.03177262	0.65892735
120.3	4.20E-06	3.39E-16	4.50E-15	0.00614692	3.58E-06	0.03197296	0.66199877
120.4	4.22E-06	3.43E-16	4.45E-15	0.00610944	3.57E-06	0.0322921	0.66205765
120.5	4.17E-06	3.34E-16	4.29E-15	0.00607359	3.51E-06	0.03215917	0.65787013
120.6	4.15E-06	3.56E-16	4.40E-15	0.0061028	3.52E-06	0.03219447	0.65631128
120.7	4.15E-06	3.56E-16	4.40E-15	0.0061028	3.52E-06	0.03219447	0.65631128
120.8	4.15E-06	3.56E-16	4.40E-15	0.0061028	3.52E-06	0.03219447	0.65631128
120.9	4.17E-06	3.55E-16	4.26E-15	0.0061859	3.59E-06	0.03214739	0.65583978
121	4.13E-06	3.53E-16	4.20E-15	0.00613414	3.66E-06	0.03180827	0.65506358
121.1	4.13E-06	3.53E-16	4.20E-15	0.00613414	3.66E-06	0.03180827	0.65506358
121.2	4.13E-06	3.53E-16	4.20E-15	0.00613414	3.66E-06	0.03180827	0.65506358
121.3	4.26E-06	3.73E-16	4.23E-15	0.00618898	3.64E-06	0.03178467	0.65904603
121.4	4.26E-06	3.73E-16	4.23E-15	0.00618898	3.64E-06	0.03178467	0.65904603
121.5	4.26E-06	3.73E-16	4.23E-15	0.00618898	3.64E-06	0.03178467	0.65904603
121.6	4.26E-06	3.73E-16	4.23E-15	0.00618898	3.64E-06	0.03178467	0.65904603
121.7	4.26E-06	3.73E-16	4.23E-15	0.00618898	3.64E-06	0.03178467	0.65904603
121.8	4.24E-06	3.67E-16	4.16E-15	0.00629513	3.64E-06	0.03197657	0.65744467
121.9	4.24E-06	3.67E-16	4.16E-15	0.00629513	3.64E-06	0.03197657	0.65744467
122	4.24E-06	3.67E-16	4.16E-15	0.00629513	3.64E-06	0.03197657	0.65744467
122.1	4.22E-06	3.58E-16	4.11E-15	0.00630865	3.61E-06	0.03205925	0.65962319
122.2	4.22E-06	3.58E-16	4.11E-15	0.00630865	3.61E-06	0.03205925	0.65962319
122.3	4.22E-06	3.58E-16	4.11E-15	0.00630865	3.61E-06	0.03205925	0.65962319
122.4	4.15E-06	3.67E-16	4.06E-15	0.00650158	3.67E-06	0.03171786	0.65802563
122.5	4.15E-06	3.67E-16	4.06E-15	0.00650158	3.67E-06	0.03171786	0.65802563
122.6	4.15E-06	3.67E-16	4.06E-15	0.00650158	3.67E-06	0.03171786	0.65802563
122.7	4.21E-06	3.87E-16	3.95E-15	0.00657691	3.74E-06	0.03141217	0.66067136
122.8	4.18E-06	3.91E-16	3.79E-15	0.00659365	3.74E-06	0.03154857	0.66293545
122.9	4.18E-06	3.91E-16	3.79E-15	0.00659365	3.74E-06	0.03154857	0.66293545
123	4.28E-06	4.04E-16	3.84E-15	0.00663161	3.80E-06	0.03159697	0.66737297
123.1	4.32E-06	4.05E-16	3.73E-15	0.00670581	3.91E-06	0.03128681	0.66920996
123.2	4.32E-06	4.05E-16	3.73E-15	0.00670581	3.91E-06	0.03128681	0.66920996
123.3	4.32E-06	4.05E-16	3.73E-15	0.00670581	3.91E-06	0.03128681	0.66920996
123.4	4.32E-06	4.05E-16	3.73E-15	0.00670581	3.91E-06	0.03128681	0.66920996
123.5	4.32E-06	4.05E-16	3.73E-15	0.00670581	3.91E-06	0.03128681	0.66920996
123.6	4.38E-06	3.93E-16	3.69E-15	0.00671982	3.86E-06	0.03108512	0.67258392
123.7	4.31E-06	3.94E-16	3.58E-15	0.00679476	3.92E-06	0.03096128	0.67256426
123.8	4.31E-06	3.94E-16	3.58E-15	0.00679476	3.92E-06	0.03096128	0.67256426
123.9	4.31E-06	3.94E-16	3.58E-15	0.00679476	3.92E-06	0.03096128	0.67256426
124	4.31E-06	3.94E-16	3.58E-15	0.00679476	3.92E-06	0.03096128	0.67256426
124.1	4.31E-06	3.94E-16	3.58E-15	0.00679476	3.92E-06	0.03096128	0.67256426
124.2	4.26E-06	3.83E-16	3.51E-15	0.00673874	3.85E-06	0.03066348	0.67213896
124.3	4.15E-06	3.90E-16	3.48E-15	0.00677239	3.91E-06	0.0306416	0.66928857
124.4	4.15E-06	3.90E-16	3.48E-15	0.00677239	3.91E-06	0.0306416	0.66928857
124.5	4.13E-06	3.92E-16	3.36E-15	0.00686164	3.92E-06	0.03086789	0.67044571
124.6	4.13E-06	3.92E-16	3.36E-15	0.00686164	3.92E-06	0.03086789	0.67044571
124.7	4.13E-06	3.92E-16	3.36E-15	0.00686164	3.92E-06	0.03086789	0.67044571
124.8	4.05E-06	3.99E-16	3.38E-15	0.00699724	3.97E-06	0.0309973	0.66896623
124.9	4.05E-06	3.99E-16	3.38E-15	0.00699724	3.97E-06	0.0309973	0.66896623
125	4.05E-06	3.99E-16	3.38E-15	0.00699724	3.97E-06	0.0309973	0.66896623
125.1	4.02E-06	4.00E-16	3.38E-15	0.00709286	4.01E-06	0.03129685	0.66803117
125.2	4.02E-06	4.00E-16	3.38E-15	0.00709286	4.01E-06	0.03129685	0.66803117
125.3	4.09E-06	4.39E-16	3.46E-15	0.00718368	4.10E-06	0.03120875	0.67619954
125.4	4.09E-06	4.39E-16	3.46E-15	0.00718368	4.10E-06	0.03120875	0.67619954
125.5	4.09E-06	4.39E-16	3.46E-15	0.00718368	4.10E-06	0.03120875	0.67619954
125.6	4.07E-06	4.38E-16	3.42E-15	0.00717911	4.05E-06	0.03108344	0.67395181
125.7	4.07E-06	4.38E-16	3.42E-15	0.00717911	4.05E-06	0.03108344	0.67395181
125.8	4.07E-06	4.38E-16	3.42E-15	0.00717911	4.05E-06	0.03108344	0.67395181
125.9	4.01E-06	4.44E-16	3.37E-15	0.00733127	4.12E-06	0.03088525	0.67422868
126	4.01E-06	4.44E-16	3.37E-15	0.00733127	4.12E-06	0.03088525	0.67422868

135	3.62E-06	5.97E-16	1.84E-15	0.01055093	6.32E-06	0.02891413	0.67280896
135.1	3.62E-06	6.06E-16	1.83E-15	0.0103948	6.12E-06	0.02883966	0.66919336
135.2	3.62E-06	6.06E-16	1.83E-15	0.0103948	6.12E-06	0.02883966	0.66919336
135.3	3.61E-06	6.09E-16	1.83E-15	0.01042862	6.05E-06	0.02862082	0.66703886
135.4	3.64E-06	6.38E-16	1.75E-15	0.01053279	6.20E-06	0.0290395	0.67095796
135.5	3.64E-06	6.38E-16	1.75E-15	0.01053279	6.20E-06	0.0290395	0.67095796
135.6	3.58E-06	6.49E-16	1.76E-15	0.01046392	6.24E-06	0.02892812	0.66937567
135.7	3.50E-06	6.74E-16	1.72E-15	0.01061366	6.23E-06	0.02864541	0.66596841
135.8	3.50E-06	6.74E-16	1.72E-15	0.01061366	6.23E-06	0.02864541	0.66596841
135.9	3.50E-06	6.72E-16	1.70E-15	0.01062707	6.30E-06	0.0284901	0.6642107
136	3.50E-06	6.72E-16	1.70E-15	0.01062707	6.30E-06	0.0284901	0.6642107
136.1	3.51E-06	6.76E-16	1.69E-15	0.01058861	6.31E-06	0.02860114	0.66395825
136.2	3.51E-06	6.76E-16	1.69E-15	0.01058861	6.31E-06	0.02860114	0.66395825
136.3	3.50E-06	6.79E-16	1.66E-15	0.01078855	6.35E-06	0.02846119	0.66430467
136.4	3.50E-06	6.79E-16	1.66E-15	0.01078855	6.35E-06	0.02846119	0.66430467
136.5	3.50E-06	6.79E-16	1.66E-15	0.01078855	6.35E-06	0.02846119	0.66430467
136.6	3.50E-06	6.79E-16	1.66E-15	0.01078855	6.35E-06	0.02846119	0.66430467
136.7	3.45E-06	6.70E-16	1.57E-15	0.01085944	6.49E-06	0.02867021	0.66317185
136.8	3.45E-06	6.70E-16	1.57E-15	0.01085944	6.49E-06	0.02867021	0.66317185
136.9	3.45E-06	6.70E-16	1.57E-15	0.01085944	6.49E-06	0.02867021	0.66317185
137	3.45E-06	7.07E-16	1.58E-15	0.01095984	6.66E-06	0.02889982	0.66451615
137.1	3.45E-06	7.07E-16	1.58E-15	0.01095984	6.66E-06	0.02889982	0.66451615
137.2	3.45E-06	7.26E-16	1.57E-15	0.01091491	6.68E-06	0.02885278	0.66261602
137.3	3.45E-06	7.26E-16	1.57E-15	0.01091491	6.68E-06	0.02885278	0.66261602
137.4	3.45E-06	7.26E-16	1.57E-15	0.01091491	6.68E-06	0.02885278	0.66261602
137.5	3.40E-06	7.22E-16	1.56E-15	0.01105068	6.79E-06	0.02872982	0.66299114
137.6	3.37E-06	7.55E-16	1.55E-15	0.01103188	6.83E-06	0.02874681	0.66198659
137.7	3.39E-06	7.71E-16	1.56E-15	0.01124684	6.88E-06	0.02880691	0.66387213
137.8	3.39E-06	8.01E-16	1.57E-15	0.01150059	7.10E-06	0.02894638	0.66334348
137.9	3.39E-06	8.01E-16	1.57E-15	0.01150059	7.10E-06	0.02894638	0.66334348
138	3.39E-06	8.01E-16	1.57E-15	0.01150059	7.10E-06	0.02894638	0.66334348
138.1	3.39E-06	8.01E-16	1.57E-15	0.01150059	7.10E-06	0.02894638	0.66334348
138.2	3.39E-06	8.01E-16	1.57E-15	0.01150059	7.10E-06	0.02894638	0.66334348
138.3	3.39E-06	8.01E-16	1.57E-15	0.01150059	7.10E-06	0.02894638	0.66334348
138.4	3.39E-06	8.01E-16	1.57E-15	0.01150059	7.10E-06	0.02894638	0.66334348
138.5	3.39E-06	8.01E-16	1.57E-15	0.01150059	7.10E-06	0.02894638	0.66334348
138.6	3.31E-06	8.10E-16	1.53E-15	0.01159779	7.16E-06	0.02906432	0.66241003
138.7	3.23E-06	8.60E-16	1.50E-15	0.01167329	7.38E-06	0.02877549	0.66113926
138.8	3.17E-06	8.84E-16	1.46E-15	0.01171979	7.39E-06	0.02875186	0.66075863
138.9	3.17E-06	8.84E-16	1.46E-15	0.01171979	7.39E-06	0.02875186	0.66075863
139	3.17E-06	8.84E-16	1.46E-15	0.01171979	7.39E-06	0.02875186	0.66075863
139.1	3.17E-06	8.84E-16	1.46E-15	0.01171979	7.39E-06	0.02875186	0.66075863
139.2	3.17E-06	8.84E-16	1.46E-15	0.01171979	7.39E-06	0.02875186	0.66075863
139.3	3.19E-06	9.03E-16	1.44E-15	0.01185757	7.40E-06	0.02881213	0.6623175
139.4	3.19E-06	9.03E-16	1.44E-15	0.01185757	7.40E-06	0.02881213	0.6623175
139.5	3.19E-06	9.03E-16	1.44E-15	0.01185757	7.40E-06	0.02881213	0.6623175
139.6	3.19E-06	9.03E-16	1.44E-15	0.01185757	7.40E-06	0.02881213	0.6623175
139.7	3.19E-06	9.03E-16	1.44E-15	0.01185757	7.40E-06	0.02881213	0.6623175
139.8	3.19E-06	9.03E-16	1.44E-15	0.01185757	7.40E-06	0.02881213	0.6623175
139.9	3.17E-06	9.09E-16	1.42E-15	0.01216511	7.63E-06	0.02876332	0.66134496
140	3.15E-06	9.58E-16	1.39E-15	0.01239864	7.85E-06	0.02912571	0.65994133
140.1	3.06E-06	9.88E-16	1.34E-15	0.01269786	8.09E-06	0.0290445	0.66012476
140.2	3.06E-06	1.02E-15	1.32E-15	0.01291076	8.29E-06	0.0292102	0.65942183
140.3	3.02E-06	1.03E-15	1.24E-15	0.01308151	8.40E-06	0.02977752	0.66139951
140.4	3.00E-06	1.04E-15	1.19E-15	0.01320138	8.46E-06	0.0300483	0.65845389
140.5	3.00E-06	1.04E-15	1.19E-15	0.01320138	8.46E-06	0.0300483	0.65845389
140.6	3.00E-06	1.04E-15	1.19E-15	0.01320138	8.46E-06	0.0300483	0.65845389
140.7	3.01E-06	1.03E-15	1.11E-15	0.01312514	8.29E-06	0.02962087	0.65583895
140.8	3.01E-06	1.03E-15	1.11E-15	0.01312514	8.29E-06	0.02962087	0.65583895
140.9	2.94E-06	1.12E-15	1.08E-15	0.01320897	8.45E-06	0.02983679	0.65484636
141	2.94E-06	1.12E-15	1.08E-15	0.01320897	8.45E-06	0.02983679	0.65484636

150	2.75E-06	2.05E-15	6.50E-16	0.01707306	1.11E-05	0.03125808	0.64847549
150.1	2.73E-06	2.24E-15	6.60E-16	0.01695375	1.13E-05	0.03157131	0.64887989
150.2	2.73E-06	2.24E-15	6.60E-16	0.01695375	1.13E-05	0.03157131	0.64887989
150.3	2.73E-06	2.24E-15	6.60E-16	0.01695375	1.13E-05	0.03157131	0.64887989
150.4	2.73E-06	2.24E-15	6.60E-16	0.01695375	1.13E-05	0.03157131	0.64887989
150.5	2.73E-06	2.24E-15	6.60E-16	0.01695375	1.13E-05	0.03157131	0.64887989
150.6	2.73E-06	2.24E-15	6.60E-16	0.01695375	1.13E-05	0.03157131	0.64887989
150.7	2.73E-06	2.24E-15	6.60E-16	0.01695375	1.13E-05	0.03157131	0.64887989
150.8	2.73E-06	2.24E-15	6.60E-16	0.01695375	1.13E-05	0.03157131	0.64887989
150.9	2.73E-06	2.24E-15	6.60E-16	0.01695375	1.13E-05	0.03157131	0.64887989
151	2.73E-06	2.24E-15	6.60E-16	0.01695375	1.13E-05	0.03157131	0.64887989
151.1	2.73E-06	2.31E-15	6.68E-16	0.0168667	1.12E-05	0.03168837	0.64873573
151.2	2.73E-06	2.31E-15	6.68E-16	0.0168667	1.12E-05	0.03168837	0.64873573
151.3	2.73E-06	2.31E-15	6.68E-16	0.0168667	1.12E-05	0.03168837	0.64873573
151.4	2.73E-06	2.31E-15	6.68E-16	0.0168667	1.12E-05	0.03168837	0.64873573
151.5	2.73E-06	2.31E-15	6.68E-16	0.0168667	1.12E-05	0.03168837	0.64873573
151.6	2.73E-06	2.31E-15	6.68E-16	0.0168667	1.12E-05	0.03168837	0.64873573
151.7	2.70E-06	2.37E-15	6.56E-16	0.0166764	1.10E-05	0.03158533	0.64863609
151.8	2.70E-06	2.37E-15	6.56E-16	0.0166764	1.10E-05	0.03158533	0.64863609
151.9	2.70E-06	2.37E-15	6.56E-16	0.0166764	1.10E-05	0.03158533	0.64863609
152	2.70E-06	2.37E-15	6.56E-16	0.0166764	1.10E-05	0.03158533	0.64863609
152.1	2.70E-06	2.37E-15	6.56E-16	0.0166764	1.10E-05	0.03158533	0.64863609
152.2	2.70E-06	2.37E-15	6.56E-16	0.0166764	1.10E-05	0.03158533	0.64863609
152.3	2.70E-06	2.37E-15	6.56E-16	0.0166764	1.10E-05	0.03158533	0.64863609
152.4	2.70E-06	2.37E-15	6.56E-16	0.0166764	1.10E-05	0.03158533	0.64863609
152.5	2.70E-06	2.37E-15	6.56E-16	0.0166764	1.10E-05	0.03158533	0.64863609
152.6	2.70E-06	2.37E-15	6.56E-16	0.0166764	1.10E-05	0.03158533	0.64863609
152.7	2.70E-06	2.37E-15	6.56E-16	0.0166764	1.10E-05	0.03158533	0.64863609
152.8	2.70E-06	2.37E-15	6.56E-16	0.0166764	1.10E-05	0.03158533	0.64863609
152.9	2.70E-06	2.37E-15	6.56E-16	0.0166764	1.10E-05	0.03158533	0.64863609
153	2.70E-06	2.37E-15	6.56E-16	0.0166764	1.10E-05	0.03158533	0.64863609
153.1	2.70E-06	2.37E-15	6.56E-16	0.0166764	1.10E-05	0.03158533	0.64863609
153.2	2.70E-06	2.37E-15	6.56E-16	0.0166764	1.10E-05	0.03158533	0.64863609
153.3	2.70E-06	2.37E-15	6.56E-16	0.0166764	1.10E-05	0.03158533	0.64863609
153.4	2.70E-06	2.37E-15	6.56E-16	0.0166764	1.10E-05	0.03158533	0.64863609
153.5	2.73E-06	2.37E-15	6.55E-16	0.01675296	1.10E-05	0.03120747	0.65147439
153.6	2.73E-06	2.37E-15	6.55E-16	0.01675296	1.10E-05	0.03120747	0.65147439
153.7	2.73E-06	2.37E-15	6.55E-16	0.01675296	1.10E-05	0.03120747	0.65147439
153.8	2.73E-06	2.37E-15	6.55E-16	0.01675296	1.10E-05	0.03120747	0.65147439
153.9	2.73E-06	2.37E-15	6.55E-16	0.01675296	1.10E-05	0.03120747	0.65147439
154	2.73E-06	2.37E-15	6.55E-16	0.01675296	1.10E-05	0.03120747	0.65147439
154.1	2.73E-06	2.37E-15	6.55E-16	0.01675296	1.10E-05	0.03120747	0.65147439
154.2	2.73E-06	2.37E-15	6.55E-16	0.01675296	1.10E-05	0.03120747	0.65147439
154.3	2.73E-06	2.37E-15	6.55E-16	0.01675296	1.10E-05	0.03120747	0.65147439
154.4	2.73E-06	2.37E-15	6.55E-16	0.01675296	1.10E-05	0.03120747	0.65147439
154.5	2.73E-06	2.37E-15	6.55E-16	0.01675296	1.10E-05	0.03120747	0.65147439
154.6	2.73E-06	2.37E-15	6.55E-16	0.01675296	1.10E-05	0.03120747	0.65147439
154.7	2.73E-06	2.37E-15	6.55E-16	0.01675296	1.10E-05	0.03120747	0.65147439
154.8	2.73E-06	2.37E-15	6.55E-16	0.01675296	1.10E-05	0.03120747	0.65147439
154.9	2.73E-06	2.37E-15	6.55E-16	0.01675296	1.10E-05	0.03120747	0.65147439
155	2.73E-06	2.37E-15	6.55E-16	0.01675296	1.10E-05	0.03120747	0.65147439
155.1	2.73E-06	2.37E-15	6.55E-16	0.01675296	1.10E-05	0.03120747	0.65147439
155.2	2.73E-06	2.37E-15	6.55E-16	0.01675296	1.10E-05	0.03120747	0.65147439
155.3	2.73E-06	2.37E-15	6.55E-16	0.01675296	1.10E-05	0.03120747	0.65147439
155.4	2.73E-06	2.37E-15	6.55E-16	0.01675296	1.10E-05	0.03120747	0.65147439
155.5	2.73E-06	2.45E-15	6.69E-16	0.01674294	1.10E-05	0.03132123	0.64891278
155.6	2.73E-06	2.45E-15	6.69E-16	0.01674294	1.10E-05	0.03132123	0.64891278
155.7	2.73E-06	2.45E-15	6.69E-16	0.01674294	1.10E-05	0.03132123	0.64891278
155.8	2.73E-06	2.45E-15	6.69E-16	0.01674294	1.10E-05	0.03132123	0.64891278
155.9	2.71E-06	2.50E-15	6.60E-16	0.01663651	1.09E-05	0.03156578	0.64937256
156	2.71E-06	2.50E-15	6.60E-16	0.01663651	1.09E-05	0.03156578	0.64937256

165	2.39E-06	2.90E-15	5.33E-16	0.01708894	1.08E-05	0.0301882	0.63827018
165.1	2.39E-06	2.90E-15	5.33E-16	0.01708894	1.08E-05	0.0301882	0.63827018
165.2	2.39E-06	2.90E-15	5.33E-16	0.01708894	1.08E-05	0.0301882	0.63827018
165.3	2.37E-06	2.91E-15	5.37E-16	0.01734926	1.11E-05	0.03041244	0.63995487
165.4	2.37E-06	2.91E-15	5.37E-16	0.01734926	1.11E-05	0.03041244	0.63995487
165.5	2.37E-06	2.92E-15	5.33E-16	0.01738895	1.08E-05	0.03025188	0.63910316
165.6	2.37E-06	2.92E-15	5.33E-16	0.01738895	1.08E-05	0.03025188	0.63910316
165.7	2.37E-06	2.92E-15	5.33E-16	0.01738895	1.08E-05	0.03025188	0.63910316
165.8	2.37E-06	2.92E-15	5.33E-16	0.01738895	1.08E-05	0.03025188	0.63910316
165.9	2.37E-06	2.92E-15	5.33E-16	0.01738895	1.08E-05	0.03025188	0.63910316
166	2.34E-06	2.96E-15	5.40E-16	0.01752039	1.08E-05	0.03049855	0.6379359
166.1	2.34E-06	2.96E-15	5.40E-16	0.01752039	1.08E-05	0.03049855	0.6379359
166.2	2.34E-06	2.96E-15	5.40E-16	0.01752039	1.08E-05	0.03049855	0.6379359
166.3	2.34E-06	2.96E-15	5.40E-16	0.01752039	1.08E-05	0.03049855	0.6379359
166.4	2.34E-06	2.96E-15	5.40E-16	0.01752039	1.08E-05	0.03049855	0.6379359
166.5	2.34E-06	2.96E-15	5.40E-16	0.01752039	1.08E-05	0.03049855	0.6379359
166.6	2.34E-06	2.96E-15	5.40E-16	0.01752039	1.08E-05	0.03049855	0.6379359
166.7	2.34E-06	2.96E-15	5.40E-16	0.01752039	1.08E-05	0.03049855	0.6379359
166.8	2.34E-06	2.96E-15	5.40E-16	0.01752039	1.08E-05	0.03049855	0.6379359
166.9	2.34E-06	2.96E-15	5.40E-16	0.01752039	1.08E-05	0.03049855	0.6379359
167	2.34E-06	2.96E-15	5.40E-16	0.01752039	1.08E-05	0.03049855	0.6379359
167.1	2.34E-06	2.96E-15	5.40E-16	0.01752039	1.08E-05	0.03049855	0.6379359
167.2	2.34E-06	2.96E-15	5.40E-16	0.01752039	1.08E-05	0.03049855	0.6379359
167.3	2.34E-06	2.96E-15	5.40E-16	0.01752039	1.08E-05	0.03049855	0.6379359
167.4	2.34E-06	2.96E-15	5.40E-16	0.01752039	1.08E-05	0.03049855	0.6379359
167.5	2.34E-06	2.96E-15	5.40E-16	0.01752039	1.08E-05	0.03049855	0.6379359
167.6	2.34E-06	2.96E-15	5.40E-16	0.01752039	1.08E-05	0.03049855	0.6379359
167.7	2.34E-06	2.96E-15	5.40E-16	0.01752039	1.08E-05	0.03049855	0.6379359
167.8	2.34E-06	2.96E-15	5.40E-16	0.01752039	1.08E-05	0.03049855	0.6379359
167.9	2.34E-06	2.96E-15	5.40E-16	0.01752039	1.08E-05	0.03049855	0.6379359
168	2.34E-06	2.96E-15	5.40E-16	0.01752039	1.08E-05	0.03049855	0.6379359
168.1	2.34E-06	2.96E-15	5.40E-16	0.01752039	1.08E-05	0.03049855	0.6379359
168.2	2.34E-06	2.96E-15	5.40E-16	0.01752039	1.08E-05	0.03049855	0.6379359
168.3	2.33E-06	3.04E-15	5.24E-16	0.01768204	1.07E-05	0.03008377	0.63584268
168.4	2.33E-06	3.04E-15	5.24E-16	0.01768204	1.07E-05	0.03008377	0.63584268
168.5	2.33E-06	3.04E-15	5.24E-16	0.01768204	1.07E-05	0.03008377	0.63584268
168.6	2.33E-06	3.04E-15	5.24E-16	0.01768204	1.07E-05	0.03008377	0.63584268
168.7	2.33E-06	3.04E-15	5.24E-16	0.01768204	1.07E-05	0.03008377	0.63584268
168.8	2.34E-06	3.02E-15	5.11E-16	0.01754206	1.07E-05	0.02972978	0.63391984
168.9	2.34E-06	3.02E-15	5.11E-16	0.01754206	1.07E-05	0.02972978	0.63391984
169	2.34E-06	3.02E-15	5.11E-16	0.01754206	1.07E-05	0.02972978	0.63391984
169.1	2.31E-06	2.98E-15	4.90E-16	0.01738362	1.06E-05	0.02993291	0.63162028
169.2	2.30E-06	3.01E-15	4.89E-16	0.01753882	1.07E-05	0.02972736	0.63219628
169.3	2.30E-06	3.01E-15	4.89E-16	0.01753882	1.07E-05	0.02972736	0.63219628
169.4	2.30E-06	3.01E-15	4.89E-16	0.01753882	1.07E-05	0.02972736	0.63219628
169.5	2.30E-06	3.01E-15	4.89E-16	0.01753882	1.07E-05	0.02972736	0.63219628
169.6	2.30E-06	3.01E-15	4.89E-16	0.01753882	1.07E-05	0.02972736	0.63219628
169.7	2.30E-06	3.01E-15	4.89E-16	0.01753882	1.07E-05	0.02972736	0.63219628
169.8	2.30E-06	3.01E-15	4.89E-16	0.01753882	1.07E-05	0.02972736	0.63219628
169.9	2.30E-06	3.01E-15	4.89E-16	0.01753882	1.07E-05	0.02972736	0.63219628
170	2.30E-06	3.01E-15	4.89E-16	0.01753882	1.07E-05	0.02972736	0.63219628
170.1	2.30E-06	3.01E-15	4.89E-16	0.01753882	1.07E-05	0.02972736	0.63219628
170.2	2.30E-06	3.01E-15	4.89E-16	0.01753882	1.07E-05	0.02972736	0.63219628
170.3	2.30E-06	3.01E-15	4.89E-16	0.01753882	1.07E-05	0.02972736	0.63219628
170.4	2.30E-06	3.01E-15	4.89E-16	0.01753882	1.07E-05	0.02972736	0.63219628
170.5	2.30E-06	3.01E-15	4.89E-16	0.01753882	1.07E-05	0.02972736	0.63219628
170.6	2.29E-06	2.97E-15	4.88E-16	0.0175546	1.07E-05	0.02956586	0.63652887
170.7	2.29E-06	2.97E-15	4.88E-16	0.0175546	1.07E-05	0.02956586	0.63652887
170.8	2.29E-06	2.97E-15	4.88E-16	0.0175546	1.07E-05	0.02956586	0.63652887
170.9	2.29E-06	3.06E-15	4.85E-16	0.01738537	1.06E-05	0.02970357	0.63493547
171	2.29E-06	3.06E-15	4.85E-16	0.01738537	1.06E-05	0.02970357	0.63493547

126.1	4.01E-06	4.44E-16	3.37E-15	0.00733127	4.12E-06	0.03088525	0.67422868
126.2	4.01E-06	4.44E-16	3.37E-15	0.00733127	4.12E-06	0.03088525	0.67422868
126.3	4.02E-06	4.52E-16	3.42E-15	0.00737243	4.12E-06	0.0309137	0.67377986
126.4	4.02E-06	4.52E-16	3.42E-15	0.00737243	4.12E-06	0.0309137	0.67377986
126.5	4.01E-06	4.47E-16	3.29E-15	0.00742704	4.21E-06	0.0307381	0.67186072
126.6	4.01E-06	4.47E-16	3.29E-15	0.00742704	4.21E-06	0.0307381	0.67186072
126.7	4.02E-06	4.39E-16	3.19E-15	0.00735964	4.27E-06	0.03074373	0.67094898
126.8	4.02E-06	4.39E-16	3.19E-15	0.00735964	4.27E-06	0.03074373	0.67094898
126.9	4.11E-06	4.59E-16	3.25E-15	0.00740741	4.31E-06	0.03081951	0.67466349
127	4.10E-06	4.52E-16	3.10E-15	0.00754605	4.34E-06	0.03070294	0.67568747
127.1	4.10E-06	4.52E-16	3.10E-15	0.00754605	4.34E-06	0.03070294	0.67568747
127.2	4.10E-06	4.52E-16	3.10E-15	0.00754605	4.34E-06	0.03070294	0.67568747
127.3	4.10E-06	4.52E-16	3.10E-15	0.00754605	4.34E-06	0.03070294	0.67568747
127.4	4.13E-06	4.67E-16	3.09E-15	0.00767911	4.35E-06	0.03048131	0.67846806
127.5	4.13E-06	4.67E-16	3.09E-15	0.00767911	4.35E-06	0.03048131	0.67846806
127.6	4.13E-06	4.67E-16	3.09E-15	0.00767911	4.35E-06	0.03048131	0.67846806
127.7	4.13E-06	4.67E-16	3.09E-15	0.00767911	4.35E-06	0.03048131	0.67846806
127.8	4.09E-06	4.60E-16	3.04E-15	0.00772676	4.37E-06	0.03044622	0.68047052
127.9	4.09E-06	4.60E-16	3.04E-15	0.00772676	4.37E-06	0.03044622	0.68047052
128	4.10E-06	4.53E-16	2.92E-15	0.00788968	4.47E-06	0.0302525	0.6828873
128.1	4.10E-06	4.53E-16	2.92E-15	0.00788968	4.47E-06	0.0302525	0.6828873
128.2	4.07E-06	4.58E-16	2.91E-15	0.00784925	4.50E-06	0.02997202	0.67969318
128.3	4.07E-06	4.58E-16	2.91E-15	0.00784925	4.50E-06	0.02997202	0.67969318
128.4	4.05E-06	4.65E-16	2.91E-15	0.00787424	4.71E-06	0.02962575	0.68273972
128.5	4.04E-06	4.75E-16	2.92E-15	0.00801602	4.77E-06	0.0299671	0.67910427
128.6	4.04E-06	4.75E-16	2.92E-15	0.00801602	4.77E-06	0.0299671	0.67910427
128.7	4.04E-06	4.75E-16	2.92E-15	0.00801602	4.77E-06	0.0299671	0.67910427
128.8	4.04E-06	4.75E-16	2.92E-15	0.00801602	4.77E-06	0.0299671	0.67910427
128.9	4.03E-06	4.72E-16	2.85E-15	0.0080521	4.81E-06	0.0298989	0.67618376
129	4.03E-06	4.72E-16	2.85E-15	0.0080521	4.81E-06	0.0298989	0.67618376
129.1	4.03E-06	4.72E-16	2.85E-15	0.0080521	4.81E-06	0.0298989	0.67618376
129.2	4.03E-06	4.72E-16	2.85E-15	0.0080521	4.81E-06	0.0298989	0.67618376
129.3	3.95E-06	4.78E-16	2.78E-15	0.00810894	4.90E-06	0.02983204	0.67610201
129.4	3.95E-06	4.78E-16	2.78E-15	0.00810894	4.90E-06	0.02983204	0.67610201
129.5	3.95E-06	4.78E-16	2.78E-15	0.00810894	4.90E-06	0.02983204	0.67610201
129.6	3.95E-06	4.78E-16	2.78E-15	0.00810894	4.90E-06	0.02983204	0.67610201
129.7	3.95E-06	4.78E-16	2.78E-15	0.00810894	4.90E-06	0.02983204	0.67610201
129.8	3.95E-06	4.78E-16	2.78E-15	0.00810894	4.90E-06	0.02983204	0.67610201
129.9	3.95E-06	4.78E-16	2.78E-15	0.00810894	4.90E-06	0.02983204	0.67610201
130	3.92E-06	4.86E-16	2.77E-15	0.00815738	5.02E-06	0.02984981	0.67526882
130.1	3.92E-06	4.86E-16	2.77E-15	0.00815738	5.02E-06	0.02984981	0.67526882
130.2	3.92E-06	4.86E-16	2.77E-15	0.00815738	5.02E-06	0.02984981	0.67526882
130.3	3.88E-06	4.78E-16	2.74E-15	0.00814976	4.98E-06	0.02957853	0.67224535
130.4	3.88E-06	4.78E-16	2.74E-15	0.00814976	4.98E-06	0.02957853	0.67224535
130.5	3.86E-06	4.83E-16	2.72E-15	0.00840284	5.09E-06	0.02957115	0.67044526
130.6	3.86E-06	4.83E-16	2.72E-15	0.00840284	5.09E-06	0.02957115	0.67044526
130.7	3.86E-06	4.83E-16	2.72E-15	0.00840284	5.09E-06	0.02957115	0.67044526
130.8	3.81E-06	4.84E-16	2.67E-15	0.00848844	5.16E-06	0.02956048	0.66907861
130.9	3.82E-06	4.84E-16	2.58E-15	0.00852585	5.20E-06	0.02962659	0.66888226
131	3.82E-06	4.84E-16	2.58E-15	0.00852585	5.20E-06	0.02962659	0.66888226
131.1	3.82E-06	4.84E-16	2.58E-15	0.00852585	5.20E-06	0.02962659	0.66888226
131.2	3.82E-06	4.84E-16	2.58E-15	0.00852585	5.20E-06	0.02962659	0.66888226
131.3	3.80E-06	4.91E-16	2.48E-15	0.00854219	5.30E-06	0.02976167	0.6700769
131.4	3.84E-06	5.03E-16	2.51E-15	0.00854364	5.42E-06	0.02985534	0.67185982
131.5	3.91E-06	5.17E-16	2.49E-15	0.00870386	5.53E-06	0.03021488	0.67409112
131.6	3.91E-06	5.17E-16	2.49E-15	0.00870386	5.53E-06	0.03021488	0.67409112
131.7	3.89E-06	5.20E-16	2.35E-15	0.00896422	5.81E-06	0.03030567	0.67551534
131.8	3.89E-06	5.20E-16	2.35E-15	0.00896422	5.81E-06	0.03030567	0.67551534
131.9	3.89E-06	5.20E-16	2.35E-15	0.00896422	5.81E-06	0.03030567	0.67551534
132	3.82E-06	5.29E-16	2.31E-15	0.00906491	5.84E-06	0.03059164	0.67428231
132.1	3.82E-06	5.29E-16	2.31E-15	0.00906491	5.84E-06	0.03059164	0.67428231
132.2	3.89E-06	5.34E-16	2.31E-15	0.00905644	5.82E-06	0.03053223	0.67348434
132.3	3.89E-06	5.34E-16	2.31E-15	0.00905644	5.82E-06	0.03053223	0.67348434
132.4	3.84E-06	5.36E-16	2.23E-15	0.0093676	6.05E-06	0.03066876	0.67788692
132.5	3.84E-06	5.36E-16	2.23E-15	0.0093676	6.05E-06	0.03066876	0.67788692
132.6	3.84E-06	5.36E-16	2.23E-15	0.0093676	6.05E-06	0.03066876	0.67788692
132.7	3.84E-06	5.36E-16	2.23E-15	0.0093676	6.05E-06	0.03066876	0.67788692
132.8	3.81E-06	5.36E-16	2.19E-15	0.00951738	6.16E-06	0.03055769	0.67685894
132.9	3.81E-06	5.36E-16	2.19E-15	0.00951738	6.16E-06	0.03055769	0.67685894
133	3.81E-06	5.36E-16	2.19E-15	0.00951738	6.16E-06	0.03055769	0.67685894
133.1	3.81E-06	5.36E-16	2.19E-15	0.00951738	6.16E-06	0.03055769	0.67685894
133.2	3.76E-06	5.25E-16	2.14E-15	0.00947978	6.14E-06	0.03015073	0.6762314
133.3	3.76E-06	5.25E-16	2.14E-15	0.00947978	6.14E-06	0.03015073	0.6762314
133.4	3.76E-06	5.27E-16	2.11E-15	0.00963733	6.32E-06	0.03016346	0.6774522
133.5	3.72E-06	5.64E-16	2.09E-15	0.01004005	6.42E-06	0.02963993	0.67603672
133.6	3.72E-06	5.64E-16	2.09E-15	0.01004005	6.42E-06	0.02963993	0.67603672
133.7	3.72E-06	5.64E-16	2.09E-15	0.01004005	6.42E-06	0.02963993	0.67603672
133.8	3.72E-06	5.64E-16	2.09E-15	0.01004005	6.42E-06	0.02963993	0.67603672
133.9	3.72E-06	5.64E-16	2.09E-15	0.01004005	6.42E-06	0.02963993	0.67603672
134	3.71E-06	5.69E-16	2.01E-15	0.01020382	6.41E-06	0.02968389	0.67560057
134.1	3.68E-06	5.76E-16	1.97E-15	0.01027747	6.44E-06	0.02920877	0.6768139
134.2	3.68E-06	5.76E-16	1.97E-15	0.01027747	6.44E-06	0.02920877	0.6768139
134.3	3.68E-06	5.76E-16	1.97E-15	0.01027747	6.44E-06	0.02920877	0.6768139
134.4	3.68E-06	5.76E-16	1.97E-15	0.01027747	6.44E-06	0.02920877	0.6768139
134.5	3.65E-06	5.81E-16	1.89E-15	0.01050569	6.26E-06	0.02899711	0.67168818
134.6	3.65E-06	5.81E-16	1.89E-15	0.01050569	6.26E-06	0.02899711	0.67168818
134.7	3.65E-06	5.81E-16	1.89E-15	0.01050569	6.26E-06	0.02899711	0.67168818
134.8	3.65E-06	5.81E-16	1.89E-15	0.01050569	6.26E-06	0.02899711	0.67168818
134.9	3.65E-06	5.81E-16	1.89E-15	0.01050569	6.26E-06	0.02899711	0.67168818

180	2.04E-06	3.90E-15	4.66E-16	0.01829598	1.14E-05	0.02899842	0.62907239
180.1	2.04E-06	3.90E-15	4.66E-16	0.01829598	1.14E-05	0.02899842	0.62907239
180.2	2.04E-06	3.90E-15	4.66E-16	0.01829598	1.14E-05	0.02899842	0.62907239
180.3	2.04E-06	3.90E-15	4.66E-16	0.01829598	1.14E-05	0.02899842	0.62907239
180.4	2.04E-06	3.90E-15	4.66E-16	0.01829598	1.14E-05	0.02899842	0.62907239
180.5	2.04E-06	3.90E-15	4.66E-16	0.01829598	1.14E-05	0.02899842	0.62907239
180.6	2.04E-06	3.90E-15	4.66E-16	0.01829598	1.14E-05	0.02899842	0.62907239
180.7	2.04E-06	3.90E-15	4.66E-16	0.01829598	1.14E-05	0.02899842	0.62907239
180.8	2.04E-06	3.90E-15	4.66E-16	0.01829598	1.14E-05	0.02899842	0.62907239
180.9	2.04E-06	3.90E-15	4.66E-16	0.01829598	1.14E-05	0.02899842	0.62907239
181	2.01E-06	4.03E-15	4.68E-16	0.01847836	1.15E-05	0.02920405	0.62777234
181.1	2.01E-06	4.03E-15	4.68E-16	0.01847836	1.15E-05	0.02920405	0.62777234
181.2	2.02E-06	4.17E-15	4.62E-16	0.01853472	1.16E-05	0.02893965	0.62661427
181.3	2.02E-06	4.17E-15	4.62E-16	0.01853472	1.16E-05	0.02893965	0.62661427
181.4	2.02E-06	4.17E-15	4.62E-16	0.01853472	1.16E-05	0.02893965	0.62661427
181.5	2.02E-06	4.17E-15	4.62E-16	0.01853472	1.16E-05	0.02893965	0.62661427
181.6	2.02E-06	4.17E-15	4.62E-16	0.01853472	1.16E-05	0.02893965	0.62661427
181.7	2.02E-06	4.17E-15	4.62E-16	0.01853472	1.16E-05	0.02893965	0.62661427
181.8	2.02E-06	4.17E-15	4.62E-16	0.01853472	1.16E-05	0.02893965	0.62661427
181.9	2.02E-06	4.23E-15	4.58E-16	0.01841132	1.17E-05	0.02867778	0.62670915
182	2.02E-06	4.23E-15	4.58E-16	0.01841132	1.17E-05	0.02867778	0.62670915
182.1	2.02E-06	4.23E-15	4.58E-16	0.01841132	1.17E-05	0.02867778	0.62670915
182.2	2.02E-06	4.23E-15	4.58E-16	0.01841132	1.17E-05	0.02867778	0.62670915
182.3	2.02E-06	4.23E-15	4.58E-16	0.01841132	1.17E-05	0.02867778	0.62670915
182.4	2.02E-06	4.23E-15	4.58E-16	0.01841132	1.17E-05	0.02867778	0.62670915
182.5	2.02E-06	4.23E-15	4.58E-16	0.01841132	1.17E-05	0.02867778	0.62670915
182.6	2.02E-06	4.23E-15	4.58E-16	0.01841132	1.17E-05	0.02867778	0.62670915
182.7	2.02E-06	4.23E-15	4.58E-16	0.01841132	1.17E-05	0.02867778	0.62670915
182.8	2.02E-06	4.23E-15	4.58E-16	0.01841132	1.17E-05	0.02867778	0.62670915
182.9	2.02E-06	4.23E-15	4.58E-16	0.01841132	1.17E-05	0.02867778	0.62670915
183	2.02E-06	4.23E-15	4.58E-16	0.01841132	1.17E-05	0.02867778	0.62670915
183.1	2.02E-06	4.23E-15	4.58E-16	0.01841132	1.17E-05	0.02867778	0.62670915

141.1	2.94E-06	1.12E-15	1.08E-15	0.01320897	8.45E-06	0.02983679	0.65484636
141.2	2.94E-06	1.12E-15	1.08E-15	0.01320897	8.45E-06	0.02983679	0.65484636
141.3	2.94E-06	1.12E-15	1.08E-15	0.01320897	8.45E-06	0.02983679	0.65484636
141.4	2.96E-06	1.11E-15	1.05E-15	0.01328309	8.43E-06	0.02968954	0.65446869
141.5	2.94E-06	1.13E-15	1.04E-15	0.01342512	8.41E-06	0.0299743	0.65535908
141.6	2.94E-06	1.13E-15	1.04E-15	0.01342512	8.41E-06	0.0299743	0.65535908
141.7	2.95E-06	1.16E-15	1.05E-15	0.01353358	8.53E-06	0.03015674	0.6545658
141.8	2.95E-06	1.16E-15	1.05E-15	0.01353358	8.53E-06	0.03015674	0.6545658
141.9	2.95E-06	1.16E-15	1.05E-15	0.01353358	8.53E-06	0.03015674	0.6545658
142	2.93E-06	1.18E-15	1.01E-15	0.01378599	8.66E-06	0.03035601	0.65610798
142.1	2.93E-06	1.18E-15	1.01E-15	0.01378599	8.66E-06	0.03035601	0.65610798
142.2	2.93E-06	1.18E-15	1.01E-15	0.01378599	8.66E-06	0.03035601	0.65610798
142.3	2.93E-06	1.18E-15	1.01E-15	0.01378599	8.66E-06	0.03035601	0.65610798
142.4	2.93E-06	1.20E-15	9.78E-16	0.0140879	8.70E-06	0.03014525	0.6547328
142.5	2.93E-06	1.20E-15	9.78E-16	0.0140879	8.70E-06	0.03014525	0.6547328
142.6	2.87E-06	1.24E-15	9.57E-16	0.01395769	8.76E-06	0.03014075	0.65598639
142.7	2.87E-06	1.24E-15	9.57E-16	0.01395769	8.76E-06	0.03014075	0.65598639
142.8	2.87E-06	1.24E-15	9.57E-16	0.01395769	8.76E-06	0.03014075	0.65598639
142.9	2.87E-06	1.24E-15	9.57E-16	0.01395769	8.76E-06	0.03014075	0.65598639
143	2.89E-06	1.28E-15	9.43E-16	0.01418776	8.95E-06	0.03040408	0.65545951
143.1	2.89E-06	1.28E-15	9.43E-16	0.01418776	8.95E-06	0.03040408	0.65545951
143.2	2.89E-06	1.33E-15	9.35E-16	0.01427329	9.02E-06	0.03073764	0.65341011
143.3	2.88E-06	1.37E-15	9.28E-16	0.01442515	9.21E-06	0.03112031	0.65099464
143.4	2.88E-06	1.37E-15	9.28E-16	0.01442515	9.21E-06	0.03112031	0.65099464
143.5	2.86E-06	1.39E-15	8.98E-16	0.0145745	9.40E-06	0.03109703	0.65051456
143.6	2.86E-06	1.39E-15	8.98E-16	0.0145745	9.40E-06	0.03109703	0.65051456
143.7	2.86E-06	1.39E-15	8.98E-16	0.0145745	9.40E-06	0.03109703	0.65051456
143.8	2.88E-06	1.42E-15	8.84E-16	0.01479973	9.54E-06	0.03102607	0.65007984
143.9	2.85E-06	1.45E-15	8.50E-16	0.01475341	9.53E-06	0.03109655	0.64641458
144	2.79E-06	1.47E-15	8.24E-16	0.01491288	9.64E-06	0.03101205	0.64709674
144.1	2.79E-06	1.47E-15	8.24E-16	0.01491288	9.64E-06	0.03101205	0.64709674
144.2	2.79E-06	1.47E-15	8.24E-16	0.01491288	9.64E-06	0.03101205	0.64709674
144.3	2.79E-06	1.47E-15	8.24E-16	0.01491288	9.64E-06	0.03101205	0.64709674
144.4	2.81E-06	1.61E-15	8.48E-16	0.01562279	1.03E-05	0.03105789	0.64755865
144.5	2.81E-06	1.61E-15	8.48E-16	0.01562279	1.03E-05	0.03105789	0.64755865
144.6	2.81E-06	1.61E-15	8.48E-16	0.01562279	1.03E-05	0.03105789	0.64755865
144.7	2.81E-06	1.61E-15	8.48E-16	0.01562279	1.03E-05	0.03105789	0.64755865
144.8	2.81E-06	1.61E-15	8.48E-16	0.01562279	1.03E-05	0.03105789	0.64755865
144.9	2.74E-06	1.65E-15	8.30E-16	0.0158686	1.05E-05	0.03091904	0.64622032
145	2.74E-06	1.65E-15	8.30E-16	0.0158686	1.05E-05	0.03091904	0.64622032
145.1	2.74E-06	1.65E-15	8.30E-16	0.0158686	1.05E-05	0.03091904	0.64622032
145.2	2.74E-06	1.65E-15	8.30E-16	0.0158686	1.05E-05	0.03091904	0.64622032
145.3	2.73E-06	1.63E-15	8.15E-16	0.01594807	1.03E-05	0.03112638	0.64312723
145.4	2.75E-06	1.64E-15	7.80E-16	0.01617012	1.05E-05	0.03135524	0.6435612
145.5	2.75E-06	1.64E-15	7.80E-16	0.01617012	1.05E-05	0.03135524	0.6435612
145.6	2.75E-06	1.64E-15	7.80E-16	0.01617012	1.05E-05	0.03135524	0.6435612
145.7	2.75E-06	1.64E-15	7.80E-16	0.01617012	1.05E-05	0.03135524	0.6435612
145.8	2.79E-06	1.70E-15	7.80E-16	0.01640841	1.06E-05	0.03140194	0.64382025
145.9	2.79E-06	1.70E-15	7.80E-16	0.01640841	1.06E-05	0.03140194	0.64382025
146	2.75E-06	1.72E-15	7.55E-16	0.016218	1.05E-05	0.03155677	0.64578296
146.1	2.75E-06	1.72E-15	7.55E-16	0.016218	1.05E-05	0.03155677	0.64578296
146.2	2.77E-06	1.64E-15	7.14E-16	0.01622496	1.03E-05	0.03123576	0.65143804
146.3	2.79E-06	1.71E-15	6.98E-16	0.01644188	1.06E-05	0.0311831	0.64999391
146.4	2.79E-06	1.71E-15	6.98E-16	0.01644188	1.06E-05	0.0311831	0.64999391
146.5	2.79E-06	1.71E-15	6.98E-16	0.01644188	1.06E-05	0.0311831	0.64999391
146.6	2.76E-06	1.71E-15	6.88E-16	0.01626371	1.03E-05	0.03076921	0.64706749
146.7	2.76E-06	1.71E-15	6.88E-16	0.01626371	1.03E-05	0.03076921	0.64706749
146.8	2.76E-06	1.71E-15	6.88E-16	0.01626371	1.03E-05	0.03076921	0.64706749
146.9	2.74E-06	1.73E-15	6.72E-16	0.01624685	1.01E-05	0.030811	0.64360491
147	2.77E-06	1.76E-15	6.73E-16	0.01644036	1.03E-05	0.03095624	0.64487201
147.1	2.77E-06	1.76E-15	6.73E-16	0.01644036	1.03E-05	0.03095624	0.64487201
147.2	2.77E-06	1.76E-15	6.73E-16	0.01644036	1.03E-05	0.03095624	0.64487201
147.3	2.74E-06	1.88E-15	6.71E-16	0.01685377	1.07E-05	0.0310632	0.64668114
147.4	2.74E-06	1.88E-15	6.71E-16	0.01685377	1.07E-05	0.0310632	0.64668114
147.5	2.74E-06	1.88E-15	6.71E-16	0.01685377	1.07E-05	0.0310632	0.64668114
147.6	2.74E-06	1.88E-15	6.71E-16	0.01685377	1.07E-05	0.0310632	0.64668114
147.7	2.74E-06	1.88E-15	6.71E-16	0.01685377	1.07E-05	0.0310632	0.64668114
147.8	2.76E-06	1.95E-15	6.66E-16	0.01701213	1.10E-05	0.03086922	0.64592136
147.9	2.76E-06	1.95E-15	6.66E-16	0.01701213	1.10E-05	0.03086922	0.64592136
148	2.77E-06	2.01E-15	6.71E-16	0.0172253	1.12E-05	0.03104097	0.64646414
148.1	2.77E-06	2.01E-15	6.71E-16	0.0172253	1.12E-05	0.03104097	0.64646414
148.2	2.77E-06	2.01E-15	6.71E-16	0.0172253	1.12E-05	0.03104097	0.64646414
148.3	2.77E-06	2.01E-15	6.71E-16	0.0172253	1.12E-05	0.03104097	0.64646414
148.4	2.77E-06	2.01E-15	6.71E-16	0.0172253	1.12E-05	0.03104097	0.64646414
148.5	2.77E-06	2.01E-15	6.71E-16	0.0172253	1.12E-05	0.03104097	0.64646414
148.6	2.77E-06	2.01E-15	6.71E-16	0.0172253	1.12E-05	0.03104097	0.64646414
148.7	2.77E-06	2.01E-15	6.71E-16	0.0172253	1.12E-05	0.03104097	0.64646414
148.8	2.77E-06	2.01E-15	6.71E-16	0.0172253	1.12E-05	0.03104097	0.64646414
148.9	2.77E-06	2.01E-15	6.71E-16	0.0172253	1.12E-05	0.03104097	0.64646414
149	2.77E-06	2.01E-15	6.71E-16	0.0172253	1.12E-05	0.03104097	0.64646414
149.1	2.77E-06	2.01E-15	6.71E-16	0.0172253	1.12E-05	0.03104097	0.64646414
149.2	2.77E-06	2.01E-15	6.71E-16	0.0172253	1.12E-05	0.03104097	0.64646414
149.3	2.77E-06	2.01E-15	6.71E-16	0.0172253	1.12E-05	0.03104097	0.64646414
149.4	2.77E-06	2.01E-15	6.71E-16	0.0172253	1.12E-05	0.03104097	0.64646414
149.5	2.77E-06	2.01E-15	6.71E-16	0.0172253	1.12E-05	0.03104097	0.64646414
149.6	2.75E-06	2.06E-15	6.74E-16	0.01716327	1.12E-05	0.03098515	0.64495855
149.7	2.75E-06	2.06E-15	6.74E-16	0.01716327	1.12E-05	0.03098515	0.64495855
149.8	2.75E-06	2.06E-15	6.74E-16	0.01716327	1.12E-05	0.03098515	0.64495855
149.9	2.75E-06	2.05E-15	6.50E-16	0.01707306	1.11E-05	0.03125808	0.64847549

195	1.77E-06	6.59E-15	3.67E-16	0.01736249	1.07E-05	0.02754242	0.60508238
195.1	1.77E-06	6.59E-15	3.67E-16	0.01736249	1.07E-05	0.02754242	0.60508238
195.2	1.77E-06	6.59E-15	3.67E-16	0.01736249	1.07E-05	0.02754242	0.60508238
195.3	1.77E-06	6.59E-15	3.67E-16	0.01736249	1.07E-05	0.02754242	0.60508238
195.4	1.77E-06	6.59E-15	3.67E-16	0.01736249	1.07E-05	0.02754242	0.60508238
195.5	1.77E-06	6.59E-15	3.67E-16	0.01736249	1.07E-05	0.02754242	0.60508238
195.6	1.77E-06	6.59E-15	3.67E-16	0.01736249	1.07E-05	0.02754242	0.60508238
195.7	1.77E-06	6.59E-15	3.67E-16	0.01736249	1.07E-05	0.02754242	0.60508238
195.8	1.77E-06	6.59E-15	3.67E-16	0.01736249	1.07E-05	0.02754242	0.60508238
195.9	1.77E-06	6.59E-15	3.67E-16	0.01736249	1.07E-05	0.02754242	0.60508238
196	1.79E-06	6.97E-15	3.72E-16	0.01728413	1.06E-05	0.02752096	0.6027143
196.1	1.79E-06	6.97E-15	3.72E-16	0.01728413	1.06E-05	0.02752096	0.6027143
196.2	1.77E-06	7.10E-15	3.68E-16	0.0171151	1.03E-05	0.02779024	0.60344685
196.3	1.77E-06	7.10E-15	3.68E-16	0.0171151	1.03E-05	0.02779024	0.60344685
196.4	1.77E-06	7.10E-15	3.68E-16	0.0171151	1.03E-05	0.02779024	0.60344685
196.5	1.77E-06	7.10E-15	3.68E-16	0.0171151	1.03E-05	0.02779024	0.60344685
196.6	1.77E-06	7.10E-15	3.68E-16	0.0171151	1.03E-05	0.02779024	0.60344685
196.7	1.77E-06	7.10E-15	3.68E-16	0.0171151	1.03E-05	0.02779024	0.60344685
196.8	1.77E-06	7.10E-15	3.68E-16	0.0171151	1.03E-05	0.02779024	0.60344685
196.9	1.77E-06	7.10E-15	3.68E-16	0.0171151	1.03E-05	0.02779024	0.60344685
197	1.77E-06	7.10E-15	3.68E-16	0.0171151	1.03E-05	0.02779024	0.60344685
197.1	1.77E-06	7.10E-15	3.68E-16	0.0171151	1.03E-05	0.02779024	0.60344685
197.2	1.77E-06	7.10E-15	3.68E-16	0.0171151	1.03E-05	0.02779024	0.60344685
197.3	1.77E-06	7.10E-15	3.68E-16	0.0171151	1.03E-05	0.02779024	0.60344685
197.4	1.77E-06	7.10E-15	3.68E-16	0.0171151	1.03E-05	0.02779024	0.60344685
197.5	1.77E-06	7.10E-15	3.68E-16	0.0171151	1.03E-05	0.02779024	0.60344685
197.6	1.77E-06	7.10E-15	3.68E-16	0.0171151	1.03E-05	0.02779024	0.60344685
197.7	1.77E-06	7.10E-15	3.68E-16	0.0171151	1.03E-05	0.02779024	0.60344685
197.8	1.77E-06	7.10E-15	3.68E-16	0.0171151	1.03E-05	0.02779024	0.60344685
197.9	1.77E-06	7.10E-15	3.68E-16	0.0171151	1.03E-05	0.02779024	0.60344685
198	1.77E-06	7.10E-15	3.68E-16	0.0171151	1.03E-05	0.02779024	0.60344685
198.1	1.77E-06	7.10E-15	3.68E-16	0.0171151	1.03E-05	0.02779024	0.60344685

156.1	2.71E-06	2.50E-15	6.60E-16	0.01663651	1.09E-05	0.03156578	0.64937256
156.2	2.71E-06	2.50E-15	6.60E-16	0.01663651	1.09E-05	0.03156578	0.64937256
156.3	2.71E-06	2.50E-15	6.60E-16	0.01663651	1.09E-05	0.03156578	0.64937256
156.4	2.71E-06	2.50E-15	6.60E-16	0.01663651	1.09E-05	0.03156578	0.64937256
156.5	2.71E-06	2.50E-15	6.60E-16	0.01663651	1.09E-05	0.03156578	0.64937256
156.6	2.75E-06	2.55E-15	6.54E-16	0.01638523	1.07E-05	0.03174204	0.6503699
156.7	2.75E-06	2.55E-15	6.54E-16	0.01638523	1.07E-05	0.03174204	0.6503699
156.8	2.75E-06	2.55E-15	6.54E-16	0.01638523	1.07E-05	0.03174204	0.6503699
156.9	2.74E-06	2.56E-15	6.46E-16	0.01617697	1.06E-05	0.03148042	0.64755201
157	2.73E-06	2.49E-15	6.12E-16	0.01640421	1.08E-05	0.03108246	0.64771742
157.1	2.73E-06	2.49E-15	6.12E-16	0.01640421	1.08E-05	0.03108246	0.64771742
157.2	2.73E-06	2.49E-15	6.12E-16	0.01640421	1.08E-05	0.03108246	0.64771742
157.3	2.73E-06	2.49E-15	6.12E-16	0.01640421	1.08E-05	0.03108246	0.64771742
157.4	2.73E-06	2.49E-15	6.12E-16	0.01640421	1.08E-05	0.03108246	0.64771742
157.5	2.73E-06	2.49E-15	6.12E-16	0.01640421	1.08E-05	0.03108246	0.64771742
157.6	2.73E-06	2.49E-15	6.12E-16	0.01640421	1.08E-05	0.03108246	0.64771742
157.7	2.72E-06	2.48E-15	6.11E-16	0.01636996	1.09E-05	0.03091116	0.64530221
157.8	2.72E-06	2.48E-15	6.11E-16	0.01636996	1.09E-05	0.03091116	0.64530221
157.9	2.72E-06	2.48E-15	6.11E-16	0.01636996	1.09E-05	0.03091116	0.64530221
158	2.69E-06	2.56E-15	6.05E-16	0.01634395	1.08E-05	0.03099653	0.6454444
158.1	2.69E-06	2.56E-15	6.05E-16	0.01634395	1.08E-05	0.03099653	0.6454444
158.2	2.69E-06	2.56E-15	6.05E-16	0.01634395	1.08E-05	0.03099653	0.6454444
158.3	2.67E-06	2.68E-15	6.12E-16	0.01629607	1.07E-05	0.03117863	0.64617971
158.4	2.67E-06	2.68E-15	6.12E-16	0.01629607	1.07E-05	0.03117863	0.64617971
158.5	2.67E-06	2.68E-15	6.12E-16	0.01629607	1.07E-05	0.03117863	0.64617971
158.6	2.67E-06	2.68E-15	6.12E-16	0.01629607	1.07E-05	0.03117863	0.64617971
158.7	2.67E-06	2.68E-15	6.12E-16	0.01629607	1.07E-05	0.03117863	0.64617971
158.8	2.61E-06	2.69E-15	6.00E-16	0.01631933	1.07E-05	0.03111024	0.64699692
158.9	2.58E-06	2.75E-15	6.09E-16	0.01631583	1.06E-05	0.03099366	0.64545426
159	2.58E-06	2.75E-15	6.09E-16	0.01631583	1.06E-05	0.03099366	0.64545426
159.1	2.58E-06	2.75E-15	6.09E-16	0.01631583	1.06E-05	0.03099366	0.64545426
159.2	2.58E-06	2.75E-15	6.09E-16	0.01631583	1.06E-05	0.03099366	0.64545426
159.3	2.55E-06	2.76E-15	6.00E-16	0.01629202	1.03E-05	0.03098192	0.64162063
159.4	2.55E-06	2.76E-15	6.00E-16	0.01629202	1.03E-05	0.03098192	0.64162063
159.5	2.55E-06	2.76E-15	6.00E-16	0.01629202	1.03E-05	0.03098192	0.64162063
159.6	2.55E-06	2.76E-15	6.00E-16	0.01629202	1.03E-05	0.03098192	0.64162063
159.7	2.55E-06	2.76E-15	6.00E-16	0.01629202	1.03E-05	0.03098192	0.64162063
159.8	2.55E-06	2.76E-15	6.00E-16	0.01629202	1.03E-05	0.03098192	0.64162063
159.9	2.59E-06	2.78E-15	5.93E-16	0.01609491	1.03E-05	0.0310368	0.6406459
160	2.59E-06	2.78E-15	5.93E-16	0.01609491	1.03E-05	0.0310368	0.6406459
160.1	2.59E-06	2.78E-15	5.93E-16	0.01609491	1.03E-05	0.0310368	0.6406459
160.2	2.60E-06	2.71E-15	5.65E-16	0.01605397	1.01E-05	0.03078141	0.63982685
160.3	2.60E-06	2.71E-15	5.65E-16	0.01605397	1.01E-05	0.03078141	0.63982685
160.4	2.60E-06	2.71E-15	5.65E-16	0.01605397	1.01E-05	0.03078141	0.63982685
160.5	2.55E-06	2.65E-15	5.57E-16	0.01618193	1.01E-05	0.03071322	0.64109166
160.6	2.55E-06	2.65E-15	5.57E-16	0.01618193	1.01E-05	0.03071322	0.64109166
160.7	2.55E-06	2.65E-15	5.57E-16	0.01618193	1.01E-05	0.03071322	0.64109166
160.8	2.55E-06	2.65E-15	5.57E-16	0.01618193	1.01E-05	0.03071322	0.64109166
160.9	2.55E-06	2.65E-15	5.57E-16	0.01618193	1.01E-05	0.03071322	0.64109166
161	2.55E-06	2.65E-15	5.57E-16	0.01618193	1.01E-05	0.03071322	0.64109166
161.1	2.55E-06	2.65E-15	5.57E-16	0.01618193	1.01E-05	0.03071322	0.64109166
161.2	2.55E-06	2.65E-15	5.57E-16	0.01618193	1.01E-05	0.03071322	0.64109166
161.3	2.50E-06	2.61E-15	5.54E-16	0.01641635	1.03E-05	0.03066117	0.63982493
161.4	2.46E-06	2.58E-15	5.52E-16	0.01668856	1.03E-05	0.03077919	0.64096571
161.5	2.46E-06	2.68E-15	5.62E-16	0.01661919	1.04E-05	0.03075774	0.63944785
161.6	2.46E-06	2.68E-15	5.62E-16	0.01661919	1.04E-05	0.03075774	0.63944785
161.7	2.46E-06	2.68E-15	5.62E-16	0.01661919	1.04E-05	0.03075774	0.63944785
161.8	2.46E-06	2.68E-15	5.62E-16	0.01661919	1.04E-05	0.03075774	0.63944785
161.9	2.46E-06	2.68E-15	5.62E-16	0.01661919	1.04E-05	0.03075774	0.63944785
162	2.41E-06	2.64E-15	5.52E-16	0.01681355	1.06E-05	0.0305766	0.64051737
162.1	2.41E-06	2.64E-15	5.52E-16	0.01681355	1.06E-05	0.0305766	0.64051737
162.2	2.41E-06	2.64E-15	5.52E-16	0.01681355	1.06E-05	0.0305766	0.64051737
162.3	2.41E-06	2.64E-15	5.52E-16	0.01681355	1.06E-05	0.0305766	0.64051737
162.4	2.41E-06	2.64E-15	5.52E-16	0.01681355	1.06E-05	0.0305766	0.64051737
162.5	2.41E-06	2.64E-15	5.52E-16	0.01681355	1.06E-05	0.0305766	0.64051737
162.6	2.40E-06	2.68E-15	5.50E-16	0.01690724	1.05E-05	0.03093062	0.63790993
162.7	2.40E-06	2.68E-15	5.50E-16	0.01690724	1.05E-05	0.03093062	0.63790993
162.8	2.40E-06	2.68E-15	5.50E-16	0.01690724	1.05E-05	0.03093062	0.63790993
162.9	2.40E-06	2.68E-15	5.50E-16	0.01690724	1.05E-05	0.03093062	0.63790993
163	2.44E-06	2.69E-15	5.38E-16	0.01683569	1.06E-05	0.0304695	0.64111137
163.1	2.44E-06	2.69E-15	5.38E-16	0.01683569	1.06E-05	0.0304695	0.64111137
163.2	2.44E-06	2.74E-15	5.52E-16	0.01689781	1.04E-05	0.03053085	0.63938482
163.3	2.41E-06	2.80E-15	5.33E-16	0.01700025	1.05E-05	0.03052473	0.64016744
163.4	2.41E-06	2.80E-15	5.33E-16	0.01700025	1.05E-05	0.03052473	0.64016744
163.5	2.41E-06	2.80E-15	5.33E-16	0.01700025	1.05E-05	0.03052473	0.64016744
163.6	2.41E-06	2.80E-15	5.33E-16	0.01700025	1.05E-05	0.03052473	0.64016744
163.7	2.41E-06	2.80E-15	5.33E-16	0.01700025	1.05E-05	0.03052473	0.64016744
163.8	2.46E-06	2.86E-15	5.41E-16	0.01697498	1.07E-05	0.03039876	0.64061186
163.9	2.46E-06	2.86E-15	5.41E-16	0.01697498	1.07E-05	0.03039876	0.64061186
164	2.46E-06	2.86E-15	5.41E-16	0.01697498	1.07E-05	0.03039876	0.64061186
164.1	2.46E-06	2.86E-15	5.41E-16	0.01697498	1.07E-05	0.03039876	0.64061186
164.2	2.46E-06	2.86E-15	5.41E-16	0.01697498	1.07E-05	0.03039876	0.64061186
164.3	2.46E-06	2.86E-15	5.41E-16	0.01697498	1.07E-05	0.03039876	0.64061186
164.4	2.46E-06	2.86E-15	5.41E-16	0.01697498	1.07E-05	0.03039876	0.64061186
164.5	2.42E-06	2.88E-15	5.37E-16	0.01704805	1.07E-05	0.03021674	0.63938827
164.6	2.42E-06	2.88E-15	5.37E-16	0.01704805	1.07E-05	0.03021674	0.63938827
164.7	2.42E-06	2.88E-15	5.37E-16	0.01704805	1.07E-05	0.03021674	0.63938827
164.8	2.39E-06	2.90E-15	5.33E-16	0.01708894	1.08E-05	0.0301882	0.63827018
164.9	2.39E-06	2.90E-15	5.33E-16	0.01708894	1.08E-05	0.0301882	0.63827018

210	1.73E-06	1.09E-14	2.90E-16	0.01697632	9.18E-06	0.02777284	0.58574037
210.1	1.73E-06	1.09E-14	2.90E-16	0.01697632	9.18E-06	0.02777284	0.58574037
210.2	1.73E-06	1.09E-14	2.90E-16	0.01697632	9.18E-06	0.02777284	0.58574037
210.3	1.73E-06	1.09E-14	2.90E-16	0.01697632	9.18E-06	0.02777284	0.58574037
210.4	1.73E-06	1.09E-14	2.90E-16	0.01697632	9.18E-06	0.02777284	0.58574037
210.5	1.73E-06	1.09E-14	2.90E-16	0.01697632	9.18E-06	0.02777284	0.58574037
210.6	1.73E-06	1.09E-14	2.90E-16	0.01697632	9.18E-06	0.02777284	0.58574037
210.7	1.73E-06	1.09E-14	2.90E-16	0.01697632	9.18E-06	0.02777284	0.58574037
210.8	1.73E-06	1.09E-14	2.90E-16	0.01697632	9.18E-06	0.02777284	0.58574037
210.9	1.73E-06	1.09E-14	2.90E-16	0.01697632	9.18E-06	0.02777284	0.58574037
211	1.73E-06	1.09E-14	2.90E-16	0.01697632	9.18E-06	0.02777284	0.58574037
211.1	1.69E-06	1.11E-14	2.87E-16	0.01697526	9.03E-06	0.02763138	0.58330605
211.2	1.71E-06	1.11E-14	2.81E-16	0.01702724	9.08E-06	0.02739845	0.58586172
211.3	1.71E-06	1.11E-14	2.81E-16	0.01702724	9.08E-06	0.02739845	0.58586172
211.4	1.71E-06	1.11E-14	2.81E-16	0.01702724	9.08E-06	0.02739845	0.58586172
211.5	1.71E-06	1.11E-14	2.81E-16	0.01702724	9.08E-06	0.02739845	0.58586172
211.6	1.71E-06	1.11E-14	2.81E-16	0.01702724	9.08E-06	0.02739845	0.58586172
211.7	1.71E-06	1.11E-14	2.81E-16	0.01702724	9.08E-06	0.02739845	0.58586172
211.8	1.71E-06	1.11E-14	2.81E-16	0.01702724	9.08E-06	0.02739845	0.58586172
211.9	1.71E-06	1.11E-14	2.81E-16	0.01702724	9.08E-06	0.02739845	0.58586172
212	1.71E-06	1.11E-14	2.81E-16	0.01702724	9.08E-06	0.02739845	0.58586172
212.1	1.71E-06	1.11E-14	2.81E-16	0.01702724	9.08E-06	0.02739845	0.58586172
212.2	1.71E-06	1.11E-14	2.81E-16	0.01702724	9.08E-06	0.02739845	0.58586172
212.3	1.71E-06	1.11E-14	2.81E-16	0.01702724	9.08E-06	0.02739845	0.58586172
212.4	1.71E-06	1.13E-14	2.74E-16	0.01706054	9.03E-06	0.02759524	0.58298111
212.5	1.71E-06	1.13E-14	2.74E-16	0.01706054	9.03E-06	0.02759524	0.58298111
212.6	1.71E-06	1.13E-14	2.74E-16	0.01706054	9.03E-06	0.02759524	0.58298111
212.7	1.71E-06	1.13E-14	2.74E-16	0.01706054	9.03E-06	0.02759524	0.58298111
212.8	1.71E-06	1.13E-14	2.74E-16	0.01706054	9.03E-06	0.02759524	0.58298111
212.9	1.71E-06	1.13E-14	2.74E-16	0.01706054	9.03E-06	0.02759524	0.58298111
213	1.69E-06	1.12E-14	2.70E-16	0.01712708	9.12E-06	0.02741507	0.5862786
213.1	1.69E-06	1.12E-14	2.70E-16	0.01712708	9.12E-06	0.02741507	0.5862786

171.1	2.29E-06	3.06E-15	4.85E-16	0.01738537	1.06E-05	0.02970357	0.63493547
171.2	2.29E-06	3.06E-15	4.85E-16	0.01738537	1.06E-05	0.02970357	0.63493547
171.3	2.29E-06	3.06E-15	4.85E-16	0.01738537	1.06E-05	0.02970357	0.63493547
171.4	2.29E-06	3.06E-15	4.85E-16	0.01738537	1.06E-05	0.02970357	0.63493547
171.5	2.24E-06	3.11E-15	4.91E-16	0.01761815	1.07E-05	0.0298025	0.63290332
171.6	2.24E-06	3.11E-15	4.91E-16	0.01761815	1.07E-05	0.0298025	0.63290332
171.7	2.24E-06	3.11E-15	4.91E-16	0.01761815	1.07E-05	0.0298025	0.63290332
171.8	2.24E-06	3.11E-15	4.91E-16	0.01761815	1.07E-05	0.0298025	0.63290332
171.9	2.24E-06	3.11E-15	4.91E-16	0.01761815	1.07E-05	0.0298025	0.63290332
172	2.24E-06	3.11E-15	4.91E-16	0.01761815	1.07E-05	0.0298025	0.63290332
172.1	2.24E-06	3.11E-15	4.91E-16	0.01761815	1.07E-05	0.0298025	0.63290332
172.2	2.21E-06	3.11E-15	4.91E-16	0.01771993	1.09E-05	0.02965908	0.63203581
172.3	2.21E-06	3.11E-15	4.91E-16	0.01771993	1.09E-05	0.02965908	0.63203581
172.4	2.21E-06	3.11E-15	4.91E-16	0.01771993	1.09E-05	0.02965908	0.63203581
172.5	2.21E-06	3.11E-15	4.91E-16	0.01771993	1.09E-05	0.02965908	0.63203581
172.6	2.21E-06	3.11E-15	4.91E-16	0.01771993	1.09E-05	0.02965908	0.63203581
172.7	2.18E-06	3.14E-15	4.78E-16	0.01761883	1.10E-05	0.02930738	0.63285368
172.8	2.18E-06	3.14E-15	4.78E-16	0.01761883	1.10E-05	0.02930738	0.63285368
172.9	2.16E-06	3.13E-15	4.82E-16	0.01774742	1.10E-05	0.02916325	0.63466511
173	2.16E-06	3.13E-15	4.82E-16	0.01774742	1.10E-05	0.02916325	0.63466511
173.1	2.16E-06	3.13E-15	4.82E-16	0.01774742	1.10E-05	0.02916325	0.63466511
173.2	2.16E-06	3.13E-15	4.82E-16	0.01774742	1.10E-05	0.02916325	0.63466511
173.3	2.16E-06	3.13E-15	4.82E-16	0.01774742	1.10E-05	0.02916325	0.63466511
173.4	2.16E-06	3.13E-15	4.82E-16	0.01774742	1.10E-05	0.02916325	0.63466511
173.5	2.16E-06	3.13E-15	4.82E-16	0.01774742	1.10E-05	0.02916325	0.63466511
173.6	2.16E-06	3.13E-15	4.82E-16	0.01774742	1.10E-05	0.02916325	0.63466511
173.7	2.16E-06	3.13E-15	4.82E-16	0.01774742	1.10E-05	0.02916325	0.63466511
173.8	2.16E-06	3.13E-15	4.82E-16	0.01774742	1.10E-05	0.02916325	0.63466511
173.9	2.16E-06	3.13E-15	4.82E-16	0.01774742	1.10E-05	0.02916325	0.63466511
174	2.16E-06	3.13E-15	4.82E-16	0.01774742	1.10E-05	0.02916325	0.63466511
174.1	2.15E-06	3.17E-15	4.74E-16	0.01780484	1.11E-05	0.02920548	0.63101887
174.2	2.15E-06	3.17E-15	4.74E-16	0.01780484	1.11E-05	0.02920548	0.63101887
174.3	2.15E-06	3.17E-15	4.74E-16	0.01780484	1.11E-05	0.02920548	0.63101887
174.4	2.15E-06	3.17E-15	4.74E-16	0.01780484	1.11E-05	0.02920548	0.63101887
174.5	2.15E-06	3.17E-15	4.74E-16	0.01780484	1.11E-05	0.02920548	0.63101887
174.6	2.15E-06	3.17E-15	4.74E-16	0.01780484	1.11E-05	0.02920548	0.63101887
174.7	2.16E-06	3.31E-15	4.68E-16	0.01787558	1.13E-05	0.02926135	0.62976858
174.8	2.16E-06	3.31E-15	4.68E-16	0.01787558	1.13E-05	0.02926135	0.62976858
174.9	2.16E-06	3.31E-15	4.68E-16	0.01787558	1.13E-05	0.02926135	0.62976858
175	2.15E-06	3.31E-15	4.72E-16	0.01804312	1.12E-05	0.02928025	0.63397037
175.1	2.15E-06	3.31E-15	4.72E-16	0.01804312	1.12E-05	0.02928025	0.63397037
175.2	2.15E-06	3.31E-15	4.72E-16	0.01804312	1.12E-05	0.02928025	0.63397037
175.3	2.15E-06	3.31E-15	4.72E-16	0.01804312	1.12E-05	0.02928025	0.63397037
175.4	2.15E-06	3.31E-15	4.72E-16	0.01804312	1.12E-05	0.02928025	0.63397037
175.5	2.15E-06	3.31E-15	4.72E-16	0.01804312	1.12E-05	0.02928025	0.63397037
175.6	2.15E-06	3.31E-15	4.72E-16	0.01804312	1.12E-05	0.02928025	0.63397037
175.7	2.13E-06	3.30E-15	4.70E-16	0.01812594	1.13E-05	0.02901302	0.63811261
175.8	2.13E-06	3.30E-15	4.70E-16	0.01812594	1.13E-05	0.02901302	0.63811261
175.9	2.13E-06	3.30E-15	4.70E-16	0.01812594	1.13E-05	0.02901302	0.63811261
176	2.11E-06	3.27E-15	4.70E-16	0.01805998	1.12E-05	0.02887711	0.63729102
176.1	2.11E-06	3.42E-15	4.68E-16	0.01795114	1.12E-05	0.0289977	0.63453571
176.2	2.11E-06	3.42E-15	4.68E-16	0.01795114	1.12E-05	0.0289977	0.63453571
176.3	2.11E-06	3.42E-15	4.68E-16	0.01795114	1.12E-05	0.0289977	0.63453571
176.4	2.11E-06	3.42E-15	4.68E-16	0.01795114	1.12E-05	0.0289977	0.63453571
176.5	2.11E-06	3.42E-15	4.68E-16	0.01795114	1.12E-05	0.0289977	0.63453571
176.6	2.11E-06	3.42E-15	4.68E-16	0.01795114	1.12E-05	0.0289977	0.63453571
176.7	2.11E-06	3.42E-15	4.68E-16	0.01795114	1.12E-05	0.0289977	0.63453571
176.8	2.11E-06	3.42E-15	4.68E-16	0.01795114	1.12E-05	0.0289977	0.63453571
176.9	2.09E-06	3.57E-15	4.66E-16	0.01792168	1.13E-05	0.02916148	0.6314218
177	2.10E-06	3.63E-15	4.74E-16	0.01797292	1.11E-05	0.02920848	0.63208001
177.1	2.10E-06	3.63E-15	4.74E-16	0.01797292	1.11E-05	0.02920848	0.63208001
177.2	2.10E-06	3.63E-15	4.74E-16	0.01797292	1.11E-05	0.02920848	0.63208001
177.3	2.10E-06	3.63E-15	4.74E-16	0.01797292	1.11E-05	0.02920848	0.63208001
177.4	2.10E-06	3.63E-15	4.74E-16	0.01797292	1.11E-05	0.02920848	0.63208001
177.5	2.07E-06	3.65E-15	4.71E-16	0.01821312	1.13E-05	0.02906766	0.63269296
177.6	2.07E-06	3.65E-15	4.71E-16	0.01821312	1.13E-05	0.02906766	0.63269296
177.7	2.07E-06	3.65E-15	4.71E-16	0.01821312	1.13E-05	0.02906766	0.63269296
177.8	2.07E-06	3.65E-15	4.71E-16	0.01821312	1.13E-05	0.02906766	0.63269296
177.9	2.08E-06	3.78E-15	4.77E-16	0.01831629	1.13E-05	0.02919319	0.63099685
178	2.08E-06	3.78E-15	4.77E-16	0.01831629	1.13E-05	0.02919319	0.63099685
178.1	2.08E-06	3.78E-15	4.77E-16	0.01831629	1.13E-05	0.02919319	0.63099685
178.2	2.07E-06	3.82E-15	4.80E-16	0.01832899	1.13E-05	0.02921806	0.6302803
178.3	2.07E-06	3.82E-15	4.80E-16	0.01832899	1.13E-05	0.02921806	0.6302803
178.4	2.07E-06	3.82E-15	4.80E-16	0.01832899	1.13E-05	0.02921806	0.6302803
178.5	2.07E-06	3.82E-15	4.80E-16	0.01832899	1.13E-05	0.02921806	0.6302803
178.6	2.07E-06	3.82E-15	4.80E-16	0.01832899	1.13E-05	0.02921806	0.6302803
178.7	2.04E-06	3.90E-15	4.66E-16	0.01829598	1.14E-05	0.02899842	0.62907239
178.8	2.04E-06	3.90E-15	4.66E-16	0.01829598	1.14E-05	0.02899842	0.62907239
178.9	2.04E-06	3.90E-15	4.66E-16	0.01829598	1.14E-05	0.02899842	0.62907239
179	2.04E-06	3.90E-15	4.66E-16	0.01829598	1.14E-05	0.02899842	0.62907239
179.1	2.04E-06	3.90E-15	4.66E-16	0.01829598	1.14E-05	0.02899842	0.62907239
179.2	2.04E-06	3.90E-15	4.66E-16	0.01829598	1.14E-05	0.02899842	0.62907239
179.3	2.04E-06	3.90E-15	4.66E-16	0.01829598	1.14E-05	0.02899842	0.62907239
179.4	2.04E-06	3.90E-15	4.66E-16	0.01829598	1.14E-05	0.02899842	0.62907239
179.5	2.04E-06	3.90E-15	4.66E-16	0.01829598	1.14E-05	0.02899842	0.62907239
179.6	2.04E-06	3.90E-15	4.66E-16	0.01829598	1.14E-05	0.02899842	0.62907239
179.7	2.04E-06	3.90E-15	4.66E-16	0.01829598	1.14E-05	0.02899842	0.62907239
179.8	2.04E-06	3.90E-15	4.66E-16	0.01829598	1.14E-05	0.02899842	0.62907239
179.9	2.04E-06	3.90E-15	4.66E-16	0.01829598	1.14E-05	0.02899842	0.62907239

225	1.78E-06	1.29E-14	2.26E-16	0.01789873	8.25E-06	0.02464663	0.58153807
225.1	1.80E-06	1.27E-14	2.23E-16	0.01800421	8.15E-06	0.02452978	0.5788728
225.2	1.80E-06	1.26E-14	2.15E-16	0.01804038	7.96E-06	0.02427281	0.57768962
225.3	1.80E-06	1.26E-14	2.15E-16	0.01804038	7.96E-06	0.02427281	0.57768962
225.4	1.80E-06	1.26E-14	2.15E-16	0.01804038	7.96E-06	0.02427281	0.57768962
225.5	1.80E-06	1.26E-14	2.15E-16	0.01804038	7.96E-06	0.02427281	0.57768962
225.6	1.81E-06	1.25E-14	2.16E-16	0.01836934	7.65E-06	0.02358373	0.57394051
225.7	1.84E-06	1.24E-14	2.12E-16	0.01840193	7.56E-06	0.02323413	0.5719744
225.8	1.84E-06	1.24E-14	2.12E-16	0.01840193	7.56E-06	0.02323413	0.5719744
225.9	1.84E-06	1.24E-14	2.12E-16	0.01840193	7.56E-06	0.02323413	0.5719744
226	1.86E-06	1.23E-14	2.12E-16	0.01855982	7.45E-06	0.02298342	0.57114794
226.1	1.86E-06	1.23E-14	2.12E-16	0.01855982	7.45E-06	0.02298342	0.57114794
226.2	1.86E-06	1.23E-14	2.12E-16	0.01855982	7.45E-06	0.02298342	0.57114794
226.3	1.86E-06	1.23E-14	2.12E-16	0.01855982	7.45E-06	0.02298342	0.57114794
226.4	1.86E-06	1.23E-14	2.12E-16	0.01855982	7.45E-06	0.02298342	0.57114794
226.5	1.86E-06	1.23E-14	2.12E-16	0.01855982	7.45E-06	0.02298342	0.57114794
226.6	1.86E-06	1.23E-14	2.12E-16	0.01855982	7.45E-06	0.02298342	0.57114794
226.7	1.86E-06	1.23E-14	2.12E-16	0.01855982	7.45E-06	0.02298342	0.57114794
226.8	1.87E-06	1.23E-14	2.06E-16	0.01860828	7.27E-06	0.0226534	0.56904712
226.9	1.87E-06	1.23E-14	2.06E-16	0.01860828	7.27E-06	0.0226534	0.56904712
227	1.88E-06	1.21E-14	1.99E-16	0.01868338	7.11E-06	0.0223369	0.5681675
227.1	1.88E-06	1.21E-14	1.99E-16	0.01868338	7.11E-06	0.0223369	0.5681675
227.2	1.88E-06	1.21E-14	1.99E-16	0.01868338	7.11E-06	0.0223369	0.5681675
227.3	1.88E-06	1.22E-14	1.95E-16	0.01871702	7.04E-06	0.02217248	0.56419395
227.4	1.88E-06	1.21E-14	1.94E-16	0.01869002	6.90E-06	0.02197542	0.56368812
227.5	1.87E-06	1.19E-14	1.87E-16	0.01882383	6.81E-06	0.0218311	0.5598976
227.6	1.87E-06	1.17E-14	1.79E-16	0.01888264	6.64E-06	0.02134202	0.55567112
227.7	1.87E-06	1.17E-14	1.79E-16	0.01888264	6.64E-06	0.02134202	0.55567112
227.8	1.87E-06	1.16E-14	1.75E-16	0.01905192	6.64E-06	0.02132619	0.5526611
227.9	1.87E-06	1.16E-14	1.75E-16	0.01905192	6.64E-06	0.02132619	0.5526611
228	1.87E-06	1.16E-14	1.75E-16	0.01905192	6.64E-06	0.02132619	0.5526611
228.1	1.87E-06	1.18E-14	1.75E-16	0.01925871	6.53E-06	0.02101343	0.550094

183.2	2.02E-06	4.23E-15	4.58E-16	0.01841132	1.17E-05	0.02867778	0.62670915
183.3	1.97E-06	4.26E-15	4.51E-16	0.01827848	1.15E-05	0.02878124	0.62461581
183.4	1.97E-06	4.26E-15	4.51E-16	0.01827848	1.15E-05	0.02878124	0.62461581
183.5	1.97E-06	4.26E-15	4.51E-16	0.01827848	1.15E-05	0.02878124	0.62461581
183.6	1.92E-06	4.35E-15	4.39E-16	0.01825338	1.15E-05	0.02850622	0.62454847
183.7	1.92E-06	4.35E-15	4.39E-16	0.01825338	1.15E-05	0.02850622	0.62454847
183.8	1.92E-06	4.35E-15	4.39E-16	0.01825338	1.15E-05	0.02850622	0.62454847
183.9	1.91E-06	4.46E-15	4.32E-16	0.01801756	1.14E-05	0.02847751	0.62316714
184	1.91E-06	4.46E-15	4.32E-16	0.01801756	1.14E-05	0.02847751	0.62316714
184.1	1.91E-06	4.46E-15	4.32E-16	0.01801756	1.14E-05	0.02847751	0.62316714
184.2	1.91E-06	4.46E-15	4.32E-16	0.01801756	1.14E-05	0.02847751	0.62316714
184.3	1.91E-06	4.46E-15	4.32E-16	0.01801756	1.14E-05	0.02847751	0.62316714
184.4	1.91E-06	4.46E-15	4.32E-16	0.01801756	1.14E-05	0.02847751	0.62316714
184.5	1.91E-06	4.46E-15	4.32E-16	0.01801756	1.14E-05	0.02847751	0.62316714
184.6	1.91E-06	4.46E-15	4.32E-16	0.01801756	1.14E-05	0.02847751	0.62316714
184.7	1.91E-06	4.46E-15	4.32E-16	0.01801756	1.14E-05	0.02847751	0.62316714
184.8	1.89E-06	4.44E-15	4.27E-16	0.01791828	1.16E-05	0.02836976	0.62151999
184.9	1.89E-06	4.44E-15	4.27E-16	0.01791828	1.16E-05	0.02836976	0.62151999
185	1.89E-06	4.44E-15	4.27E-16	0.01791828	1.16E-05	0.02836976	0.62151999
185.1	1.89E-06	4.44E-15	4.27E-16	0.01791828	1.16E-05	0.02836976	0.62151999
185.2	1.89E-06	4.44E-15	4.27E-16	0.01791828	1.16E-05	0.02836976	0.62151999
185.3	1.89E-06	4.44E-15	4.27E-16	0.01791828	1.16E-05	0.02836976	0.62151999
185.4	1.89E-06	4.44E-15	4.27E-16	0.01791828	1.16E-05	0.02836976	0.62151999
185.5	1.89E-06	4.44E-15	4.27E-16	0.01791828	1.16E-05	0.02836976	0.62151999
185.6	1.89E-06	4.44E-15	4.27E-16	0.01791828	1.16E-05	0.02836976	0.62151999
185.7	1.89E-06	4.44E-15	4.27E-16	0.01791828	1.16E-05	0.02836976	0.62151999
185.8	1.89E-06	4.44E-15	4.27E-16	0.01791828	1.16E-05	0.02836976	0.62151999
185.9	1.89E-06	4.44E-15	4.27E-16	0.01791828	1.16E-05	0.02836976	0.62151999
186	1.89E-06	4.44E-15	4.27E-16	0.01791828	1.16E-05	0.02836976	0.62151999
186.1	1.89E-06	4.44E-15	4.27E-16	0.01791828	1.16E-05	0.02836976	0.62151999
186.2	1.90E-06	4.64E-15	4.34E-16	0.01802027	1.16E-05	0.02835038	0.61968769
186.3	1.88E-06	4.81E-15	4.33E-16	0.01804833	1.18E-05	0.02795491	0.62184241
186.4	1.88E-06	4.81E-15	4.33E-16	0.01804833	1.18E-05	0.02795491	0.62184241
186.5	1.87E-06	4.89E-15	4.29E-16	0.0178812	1.20E-05	0.02756458	0.62371429
186.6	1.87E-06	4.89E-15	4.29E-16	0.0178812	1.20E-05	0.02756458	0.62371429
186.7	1.87E-06	4.89E-15	4.29E-16	0.0178812	1.20E-05	0.02756458	0.62371429
186.8	1.87E-06	4.89E-15	4.29E-16	0.0178812	1.20E-05	0.02756458	0.62371429
186.9	1.87E-06	4.89E-15	4.29E-16	0.0178812	1.20E-05	0.02756458	0.62371429
187	1.87E-06	4.89E-15	4.29E-16	0.0178812	1.20E-05	0.02756458	0.62371429
187.1	1.87E-06	4.89E-15	4.29E-16	0.0178812	1.20E-05	0.02756458	0.62371429
187.2	1.87E-06	4.89E-15	4.29E-16	0.0178812	1.20E-05	0.02756458	0.62371429
187.3	1.87E-06	4.89E-15	4.29E-16	0.0178812	1.20E-05	0.02756458	0.62371429
187.4	1.87E-06	4.89E-15	4.29E-16	0.0178812	1.20E-05	0.02756458	0.62371429
187.5	1.88E-06	5.02E-15	4.26E-16	0.01779577	1.17E-05	0.02720799	0.62254108
187.6	1.88E-06	5.02E-15	4.26E-16	0.01779577	1.17E-05	0.02720799	0.62254108
187.7	1.88E-06	5.02E-15	4.26E-16	0.01779577	1.17E-05	0.02720799	0.62254108
187.8	1.88E-06	5.02E-15	4.26E-16	0.01779577	1.17E-05	0.02720799	0.62254108
187.9	1.88E-06	5.02E-15	4.26E-16	0.01779577	1.17E-05	0.02720799	0.62254108
188	1.88E-06	5.02E-15	4.26E-16	0.01779577	1.17E-05	0.02720799	0.62254108
188.1	1.88E-06	5.02E-15	4.26E-16	0.01779577	1.17E-05	0.02720799	0.62254108
188.2	1.88E-06	5.02E-15	4.26E-16	0.01779577	1.17E-05	0.02720799	0.62254108
188.3	1.88E-06	5.02E-15	4.26E-16	0.01779577	1.17E-05	0.02720799	0.62254108
188.4	1.88E-06	5.02E-15	4.26E-16	0.01779577	1.17E-05	0.02720799	0.62254108
188.5	1.88E-06	5.02E-15	4.26E-16	0.01779577	1.17E-05	0.02720799	0.62254108
188.6	1.85E-06	5.17E-15	4.20E-16	0.01784133	1.16E-05	0.02733126	0.62386223
188.7	1.85E-06	5.17E-15	4.20E-16	0.01784133	1.16E-05	0.02733126	0.62386223
188.8	1.85E-06	5.17E-15	4.20E-16	0.01784133	1.16E-05	0.02733126	0.62386223
188.9	1.85E-06	5.17E-15	4.20E-16	0.01784133	1.16E-05	0.02733126	0.62386223
189	1.85E-06	5.17E-15	4.20E-16	0.01784133	1.16E-05	0.02733126	0.62386223
189.1	1.85E-06	5.17E-15	4.20E-16	0.01784133	1.16E-05	0.02733126	0.62386223
189.2	1.85E-06	5.17E-15	4.20E-16	0.01784133	1.16E-05	0.02733126	0.62386223
189.3	1.85E-06	5.17E-15	4.20E-16	0.01784133	1.16E-05	0.02733126	0.62386223
189.4	1.85E-06	5.17E-15	4.20E-16	0.01784133	1.16E-05	0.02733126	0.62386223
189.5	1.85E-06	5.17E-15	4.20E-16	0.01784133	1.16E-05	0.02733126	0.62386223
189.6	1.85E-06	5.17E-15	4.20E-16	0.01784133	1.16E-05	0.02733126	0.62386223
189.7	1.85E-06	5.17E-15	4.20E-16	0.01784133	1.16E-05	0.02733126	0.62386223
189.8	1.85E-06	5.17E-15	4.20E-16	0.01784133	1.16E-05	0.02733126	0.62386223
189.9	1.85E-06	5.17E-15	4.20E-16	0.01784133	1.16E-05	0.02733126	0.62386223
190	1.85E-06	5.17E-15	4.20E-16	0.01784133	1.16E-05	0.02733126	0.62386223
190.1	1.87E-06	5.24E-15	4.11E-16	0.0178183	1.14E-05	0.02744902	0.62124648
190.2	1.82E-06	5.41E-15	4.03E-16	0.01788059	1.14E-05	0.02713673	0.61919921
190.3	1.82E-06	5.41E-15	4.03E-16	0.01788059	1.14E-05	0.02713673	0.61919921
190.4	1.82E-06	5.41E-15	4.03E-16	0.01788059	1.14E-05	0.02713673	0.61919921
190.5	1.82E-06	5.57E-15	4.12E-16	0.0179318	1.16E-05	0.02706934	0.61637254
190.6	1.82E-06	5.57E-15	4.12E-16	0.0179318	1.16E-05	0.02706934	0.61637254
190.7	1.82E-06	5.57E-15	4.12E-16	0.0179318	1.16E-05	0.02706934	0.61637254
190.8	1.82E-06	5.57E-15	4.12E-16	0.0179318	1.16E-05	0.02706934	0.61637254
190.9	1.82E-06	5.57E-15	4.12E-16	0.0179318	1.16E-05	0.02706934	0.61637254
191	1.80E-06	5.66E-15	4.16E-16	0.01786034	1.14E-05	0.02697321	0.61523206
191.1	1.80E-06	5.66E-15	4.16E-16	0.01786034	1.14E-05	0.02697321	0.61523206
191.2	1.80E-06	5.66E-15	4.16E-16	0.01786034	1.14E-05	0.02697321	0.61523206
191.3	1.80E-06	5.66E-15	4.16E-16	0.01786034	1.14E-05	0.02697321	0.61523206
191.4	1.81E-06	5.74E-15	4.17E-16	0.01772824	1.12E-05	0.02710011	0.61295137
191.5	1.81E-06	5.74E-15	4.17E-16	0.01772824	1.12E-05	0.02710011	0.61295137
191.6	1.80E-06	6.02E-15	4.26E-16	0.01777919	1.12E-05	0.02740347	0.6162204
191.7	1.82E-06	6.02E-15	4.26E-16	0.01774607	1.14E-05	0.02754517	0.61632688
191.8	1.82E-06	6.02E-15	4.26E-16	0.01774607	1.14E-05	0.02754517	0.61632688
191.9	1.82E-06	6.02E-15	4.26E-16	0.01774607	1.14E-05	0.02754517	0.61632688
192	1.79E-06	6.07E-15	4.10E-16	0.01772836	1.13E-05	0.02745076	0.61541973
192.1	1.79E-06	5.98E-15	4.07E-16	0.01768833	1.12E-05	0.02747683	0.61328124
192.2	1.79E-06	5.98E-15	4.07E-16	0.01768833	1.12E-05	0.02747683	0.61328124
192.3	1.81E-06	5.97E-15	4.05E-16	0.01776358	1.12E-05	0.02733743	0.61484045
192.4	1.81E-06	5.97E-15	4.05E-16	0.01776358	1.12E-05	0.02733743	0.61484045
192.5	1.79E-06	6.10E-15	4.11E-16	0.01776999	1.11E-05	0.02725274	0.61336927
192.6	1.79E-06	6.10E-15	4.11E-16	0.01776999	1.11E-05	0.02725274	0.61336927
192.7	1.79E-06	6.10E-15	4.11E-16	0.01776999	1.11E-05	0.02725274	0.61336927
192.8	1.79E-06	6.31E-15	4.10E-16	0.01757087	1.09E-05	0.02720971	0.61080173
192.9	1.79E-06	6.31E-15	4.10E-16	0.01757087	1.09E-05	0.02720971	0.61080173
193	1.79E-06	6.31E-15	4.10E-16	0.01757087	1.09E-05	0.02720971	0.61080173
193.1	1.79E-06	6.31E-15	4.10E-16	0.01757087	1.09E-05	0.02720971	0.61080173
193.2	1.78E-06	6.46E-15	3.97E-16	0.0176665	1.11E-05	0.02723099	0.6083091
193.3	1.78E-06	6.46E-15	3.97E-16	0.0176665	1.11E-05	0.02723099	0.6083091
193.4	1.78E-06	6.46E-15	3.97E-16	0.0176665	1.11E-05	0.02723099	0.6083091
193.5	1.77E-06	6.56E-15	3.92E-16	0.01761909	1.10E-05	0.02751055	0.6083889
193.6	1.77E-06	6.56E-15	3.92E-16	0.01761909	1.10E-05	0.02751055	0.6083889
193.7	1.77E-06	6.56E-15	3.92E-16	0.01761909	1.10E-05	0.02751055	0.6083889
193.8	1.77E-06	6.56E-15	3.92E-16	0.01761909	1.10E-05	0.02751055	0.6083889
193.9	1.76E-06	6.59E-15	3.80E-16	0.01750314	1.08E-05	0.02774798	0.60438324
194	1.76E-06	6.59E-15	3.80E-16	0.01750314	1.08E-05	0.02774798	0.60438324
194.1	1.76E-06	6.59E-15	3.80E-16	0.01750314	1.08E-05	0.02774798	0.60438324
194.2	1.76E-06	6.59E-15	3.80E-16	0.01750314	1.08E-05	0.02774798	0.60438324
194.3	1.77E-06	6.59E-15	3.67E-16	0.01736249	1.07E-05	0.02754242	0.60508238
194.4	1.77E-06	6.59E-15	3.67E-16	0.01736249	1.07E-05	0.02754242	0.60508238
194.5	1.77E-06	6.59E-15	3.67E-16	0.01736249	1.07E-05	0.02754242	0.60508238
194.6	1.77E-06	6.59E-15	3.67E-16	0.01736249	1.07E-05	0.02754242	0.60508238
194.7	1.77E-06	6.59E-15	3.67E-16	0.01736249	1.07E-05	0.02754242	0.60508238
194.8	1.77E-06	6.59E-15	3.67E-16	0.01736249	1.07E-05	0.02754242	0.60508238
194.9	1.77E-06	6.59E-15	3.67E-16	0.01736249	1.07E-05	0.02754242	0.60508238

198.2	1.77E-06	7.10E-15	3.68E-16	0.0171151	1.03E-05	0.02779024	0.60344685
198.3	1.77E-06	7.10E-15	3.68E-16	0.0171151	1.03E-05	0.02779024	0.60344685
198.4	1.77E-06	7.10E-15	3.68E-16	0.0171151	1.03E-05	0.02779024	0.60344685
198.5	1.77E-06	7.10E-15	3.68E-16	0.0171151	1.03E-05	0.02779024	0.60344685
198.6	1.77E-06	7.18E-15	3.54E-16	0.01693755	1.04E-05	0.02764585	0.60545826
198.7	1.77E-06	7.18E-15	3.54E-16	0.01693755	1.04E-05	0.02764585	0.60545826
198.8	1.77E-06	7.18E-15	3.54E-16	0.01693755	1.04E-05	0.02764585	0.60545826
198.9	1.77E-06	7.18E-15	3.54E-16	0.01693755	1.04E-05	0.02764585	0.60545826
199	1.75E-06	7.30E-15	3.53E-16	0.01685055	1.01E-05	0.02789866	0.6051479
199.1	1.76E-06	7.65E-15	3.46E-16	0.01687121	1.02E-05	0.02766064	0.60303472
199.2	1.76E-06	7.65E-15	3.46E-16	0.01687121	1.02E-05	0.02766064	0.60303472
199.3	1.76E-06	7.65E-15	3.46E-16	0.01687121	1.02E-05	0.02766064	0.60303472
199.4	1.76E-06	7.65E-15	3.46E-16	0.01687121	1.02E-05	0.02766064	0.60303472
199.5	1.76E-06	7.65E-15	3.46E-16	0.01687121	1.02E-05	0.02766064	0.60303472
199.6	1.76E-06	7.65E-15	3.46E-16	0.01687121	1.02E-05	0.02766064	0.60303472
199.7	1.76E-06	7.65E-15	3.46E-16	0.01687121	1.02E-05	0.02766064	0.60303472
199.8	1.76E-06	7.65E-15	3.46E-16	0.01687121	1.02E-05	0.02766064	0.60303472
199.9	1.76E-06	7.65E-15	3.46E-16	0.01687121	1.02E-05	0.02766064	0.60303472
200	1.76E-06	7.65E-15	3.46E-16	0.01687121	1.02E-05	0.02766064	0.60303472
200.1	1.76E-06	7.65E-15	3.46E-16	0.01687121	1.02E-05	0.02766064	0.60303472
200.2	1.76E-06	7.65E-15	3.46E-16	0.01687121	1.02E-05	0.02766064	0.60303472
200.3	1.76E-06	7.65E-15	3.46E-16	0.01687121	1.02E-05	0.02766064	0.60303472
200.4	1.80E-06	7.83E-15	3.43E-16	0.01695324	1.03E-05	0.02772488	0.60605188
200.5	1.80E-06	7.83E-15	3.43E-16	0.01695324	1.03E-05	0.02772488	0.60605188
200.6	1.80E-06	7.83E-15	3.43E-16	0.01695324	1.03E-05	0.02772488	0.60605188
200.7	1.80E-06	7.83E-15	3.43E-16	0.01695324	1.03E-05	0.02772488	0.60605188
200.8	1.81E-06	8.03E-15	3.51E-16	0.01682361	1.02E-05	0.0278825	0.60413682
200.9	1.81E-06	8.03E-15	3.51E-16	0.01682361	1.02E-05	0.0278825	0.60413682
201	1.80E-06	8.11E-15	3.41E-16	0.01686575	1.00E-05	0.02805347	0.60445234
201.1	1.80E-06	8.11E-15	3.41E-16	0.01686575	1.00E-05	0.02805347	0.60445234
201.2	1.80E-06	8.11E-15	3.41E-16	0.01686575	1.00E-05	0.02805347	0.60445234
201.3	1.80E-06	8.11E-15	3.41E-16	0.01686575	1.00E-05	0.02805347	0.60445234
201.4	1.80E-06	8.11E-15	3.41E-16	0.01686575	1.00E-05	0.02805347	0.60445234
201.5	1.80E-06	8.11E-15	3.41E-16	0.01686575	1.00E-05	0.02805347	0.60445234
201.6	1.80E-06	8.11E-15	3.41E-16	0.01686575	1.00E-05	0.02805347	0.60445234
201.7	1.80E-06	8.11E-15	3.41E-16	0.01686575	1.00E-05	0.02805347	0.60445234
201.8	1.80E-06	8.11E-15	3.41E-16	0.01686575	1.00E-05	0.02805347	0.60445234
201.9	1.80E-06	8.11E-15	3.41E-16	0.01686575	1.00E-05	0.02805347	0.60445234
202	1.77E-06	8.05E-15	3.42E-16	0.01703523	9.98E-06	0.02828153	0.60297887
202.1	1.77E-06	8.05E-15	3.42E-16	0.01703523	9.98E-06	0.02828153	0.60297887
202.2	1.77E-06	8.05E-15	3.42E-16	0.01703523	9.98E-06	0.02828153	0.60297887
202.3	1.77E-06	8.05E-15	3.42E-16	0.01703523	9.98E-06	0.02828153	0.60297887
202.4	1.77E-06	8.05E-15	3.42E-16	0.01703523	9.98E-06	0.02828153	0.60297887
202.5	1.77E-06	8.05E-15	3.42E-16	0.01703523	9.98E-06	0.02828153	0.60297887
202.6	1.78E-06	8.03E-15	3.29E-16	0.01711824	1.01E-05	0.02803463	0.60340205
202.7	1.79E-06	8.06E-15	3.21E-16	0.017084	1.02E-05	0.02781809	0.6060836
202.8	1.79E-06	8.17E-15	3.14E-16	0.01718191	9.98E-06	0.0276102	0.60359031
202.9	1.79E-06	8.17E-15	3.14E-16	0.01718191	9.98E-06	0.0276102	0.60359031
203	1.79E-06	8.17E-15	3.14E-16	0.01718191	9.98E-06	0.0276102	0.60359031
203.1	1.79E-06	8.17E-15	3.14E-16	0.01718191	9.98E-06	0.0276102	0.60359031
203.2	1.79E-06	8.17E-15	3.14E-16	0.01718191	9.98E-06	0.0276102	0.60359031
203.3	1.79E-06	8.17E-15	3.14E-16	0.01718191	9.98E-06	0.0276102	0.60359031
203.4	1.79E-06	8.17E-15	3.14E-16	0.01718191	9.98E-06	0.0276102	0.60359031
203.5	1.79E-06	8.17E-15	3.14E-16	0.01718191	9.98E-06	0.0276102	0.60359031
203.6	1.79E-06	8.17E-15	3.14E-16	0.01718191	9.98E-06	0.0276102	0.60359031
203.7	1.79E-06	8.17E-15	3.14E-16	0.01718191	9.98E-06	0.0276102	0.60359031
203.8	1.79E-06	8.17E-15	3.14E-16	0.01718191	9.98E-06	0.0276102	0.60359031
203.9	1.79E-06	8.17E-15	3.14E-16	0.01718191	9.98E-06	0.0276102	0.60359031
204	1.79E-06	8.17E-15	3.14E-16	0.01718191	9.98E-06	0.0276102	0.60359031
204.1	1.76E-06	8.88E-15	3.15E-16	0.01719766	1.02E-05	0.02733202	0.60391847
204.2	1.76E-06	8.88E-15	3.15E-16	0.01719766	1.02E-05	0.02733202	0.60391847
204.3	1.76E-06	8.88E-15	3.15E-16	0.01719766	1.02E-05	0.02733202	0.60391847
204.4	1.76E-06	8.88E-15	3.15E-16	0.01719766	1.02E-05	0.02733202	0.60391847
204.5	1.76E-06	8.88E-15	3.15E-16	0.01719766	1.02E-05	0.02733202	0.60391847
204.6	1.76E-06	8.88E-15	3.15E-16	0.01719766	1.02E-05	0.02733202	0.60391847
204.7	1.76E-06	8.88E-15	3.15E-16	0.01719766	1.02E-05	0.02733202	0.60391847
204.8	1.76E-06	8.88E-15	3.15E-16	0.01719766	1.02E-05	0.02733202	0.60391847
204.9	1.76E-06	8.88E-15	3.15E-16	0.01719766	1.02E-05	0.02733202	0.60391847
205	1.76E-06	8.88E-15	3.15E-16	0.01719766	1.02E-05	0.02733202	0.60391847
205.1	1.78E-06	9.14E-15	3.14E-16	0.01720482	1.01E-05	0.02755821	0.60224823
205.2	1.78E-06	9.14E-15	3.14E-16	0.01720482	1.01E-05	0.02755821	0.60224823
205.3	1.78E-06	9.14E-15	3.14E-16	0.01720482	1.01E-05	0.02755821	0.60224823
205.4	1.78E-06	9.14E-15	3.14E-16	0.01720482	1.01E-05	0.02755821	0.60224823
205.5	1.78E-06	9.14E-15	3.14E-16	0.01720482	1.01E-05	0.02755821	0.60224823
205.6	1.78E-06	9.42E-15	3.14E-16	0.01703992	9.88E-06	0.02784932	0.59911999
205.7	1.78E-06	9.42E-15	3.14E-16	0.01703992	9.88E-06	0.02784932	0.59911999
205.8	1.78E-06	9.42E-15	3.14E-16	0.01703992	9.88E-06	0.02784932	0.59911999
205.9	1.77E-06	9.70E-15	3.15E-16	0.01695091	9.80E-06	0.02818377	0.59564591
206	1.77E-06	9.70E-15	3.15E-16	0.01695091	9.80E-06	0.02818377	0.59564591
206.1	1.77E-06	9.70E-15	3.15E-16	0.01695091	9.80E-06	0.02818377	0.59564591
206.2	1.77E-06	9.70E-15	3.15E-16	0.01695091	9.80E-06	0.02818377	0.59564591
206.3	1.77E-06	9.70E-15	3.15E-16	0.01695091	9.80E-06	0.02818377	0.59564591
206.4	1.77E-06	9.70E-15	3.15E-16	0.01695091	9.80E-06	0.02818377	0.59564591
206.5	1.77E-06	9.70E-15	3.15E-16	0.01695091	9.80E-06	0.02818377	0.59564591
206.6	1.74E-06	9.92E-15	3.01E-16	0.01682911	9.64E-06	0.02813965	0.59372996
206.7	1.74E-06	9.92E-15	3.01E-16	0.01682911	9.64E-06	0.02813965	0.59372996
206.8	1.74E-06	1.03E-14	3.05E-16	0.01683828	9.72E-06	0.02810628	0.59354686
206.9	1.74E-06	1.03E-14	3.05E-16	0.01683828	9.72E-06	0.02810628	0.59354686
207	1.74E-06	1.03E-14	3.05E-16	0.01683828	9.72E-06	0.02810628	0.59354686
207.1	1.74E-06	1.03E-14	3.01E-16	0.01691761	9.78E-06	0.02797902	0.59211682
207.2	1.74E-06	1.03E-14	3.01E-16	0.01691761	9.78E-06	0.02797902	0.59211682
207.3	1.74E-06	1.03E-14	3.01E-16	0.01691761	9.78E-06	0.02797902	0.59211682
207.4	1.74E-06	1.03E-14	3.01E-16	0.01691761	9.78E-06	0.02797902	0.59211682
207.5	1.76E-06	1.05E-14	2.99E-16	0.01690972	9.65E-06	0.02789997	0.59055112
207.6	1.76E-06	1.05E-14	2.99E-16	0.01690972	9.65E-06	0.02789997	0.59055112
207.7	1.76E-06	1.05E-14	2.99E-16	0.01690972	9.65E-06	0.02789997	0.59055112
207.8	1.76E-06	1.05E-14	2.99E-16	0.01690972	9.65E-06	0.02789997	0.59055112
207.9	1.76E-06	1.05E-14	2.99E-16	0.01690972	9.65E-06	0.02789997	0.59055112
208	1.76E-06	1.05E-14	2.99E-16	0.01690972	9.65E-06	0.02789997	0.59055112
208.1	1.76E-06	1.05E-14	2.99E-16	0.01690972	9.65E-06	0.02789997	0.59055112
208.2	1.76E-06	1.05E-14	2.99E-16	0.01690972	9.65E-06	0.02789997	0.59055112
208.3	1.76E-06	1.05E-14	2.99E-16	0.01690972	9.65E-06	0.02789997	0.59055112
208.4	1.72E-06	1.06E-14	2.93E-16	0.01682591	9.48E-06	0.027807	0.59003868
208.5	1.72E-06	1.06E-14	2.93E-16	0.01682591	9.48E-06	0.027807	0.59003868
208.6	1.72E-06	1.06E-14	2.93E-16	0.01682591	9.48E-06	0.027807	0.59003868
208.7	1.72E-06	1.06E-14	2.93E-16	0.01682591	9.48E-06	0.027807	0.59003868
208.8	1.72E-06	1.06E-14	2.93E-16	0.01682591	9.48E-06	0.027807	0.59003868
208.9	1.72E-06	1.06E-14	2.93E-16	0.01682591	9.48E-06	0.027807	0.59003868
209	1.72E-06	1.06E-14	2.93E-16	0.01682591	9.48E-06	0.027807	0.59003868
209.1	1.72E-06	1.06E-14	2.93E-16	0.01682591	9.48E-06	0.027807	0.59003868
209.2	1.72E-06	1.06E-14	2.93E-16	0.01682591	9.48E-06	0.027807	0.59003868
209.3	1.72E-06	1.06E-14	2.93E-16	0.01682591	9.48E-06	0.027807	0.59003868
209.4	1.73E-06	1.05E-14	2.84E-16	0.01692665	9.36E-06	0.02757248	0.58808368
209.5	1.73E-06	1.09E-14	2.90E-16	0.01697632	9.18E-06	0.02777284	0.58574037
209.6	1.73E-06	1.09E-14	2.90E-16	0.01697632	9.18E-06	0.02777284	0.58574037
209.7	1.73E-06	1.09E-14	2.90E-16	0.01697632	9.18E-06	0.02777284	0.58574037
209.8	1.73E-06	1.09E-14	2.90E-16	0.01697632	9.18E-06	0.02777284	0.58574037
209.9	1.73E-06	1.09E-14	2.90E-16	0.01697632	9.18E-06	0.02777284	0.58574037

213.2	1.69E-06	1.12E-14	2.70E-16	0.01712708	9.12E-06	0.02741507	0.5862786
213.3	1.69E-06	1.12E-14	2.70E-16	0.01712708	9.12E-06	0.02741507	0.5862786
213.4	1.69E-06	1.12E-14	2.70E-16	0.01712708	9.12E-06	0.02741507	0.5862786
213.5	1.69E-06	1.12E-14	2.70E-16	0.01712708	9.12E-06	0.02741507	0.5862786
213.6	1.69E-06	1.12E-14	2.70E-16	0.01712708	9.12E-06	0.02741507	0.5862786
213.7	1.72E-06	1.16E-14	2.72E-16	0.01711142	8.98E-06	0.0274439	0.58538853
213.8	1.72E-06	1.16E-14	2.72E-16	0.01711142	8.98E-06	0.0274439	0.58538853
213.9	1.72E-06	1.16E-14	2.72E-16	0.01711142	8.98E-06	0.0274439	0.58538853
214	1.72E-06	1.16E-14	2.72E-16	0.01711142	8.98E-06	0.0274439	0.58538853
214.1	1.72E-06	1.16E-14	2.72E-16	0.01711142	8.98E-06	0.0274439	0.58538853
214.2	1.72E-06	1.16E-14	2.72E-16	0.01711142	8.98E-06	0.0274439	0.58538853
214.3	1.72E-06	1.16E-14	2.72E-16	0.01711142	8.98E-06	0.0274439	0.58538853
214.4	1.74E-06	1.12E-14	2.61E-16	0.0172073	8.92E-06	0.02727663	0.5849665
214.5	1.74E-06	1.12E-14	2.61E-16	0.0172073	8.92E-06	0.02727663	0.5849665
214.6	1.74E-06	1.12E-14	2.61E-16	0.0172073	8.92E-06	0.02727663	0.5849665
214.7	1.74E-06	1.12E-14	2.61E-16	0.0172073	8.92E-06	0.02727663	0.5849665
214.8	1.74E-06	1.12E-14	2.61E-16	0.0172073	8.92E-06	0.02727663	0.5849665
214.9	1.75E-06	1.15E-14	2.62E-16	0.01720525	9.06E-06	0.02722186	0.58548263
215	1.75E-06	1.15E-14	2.62E-16	0.01720525	9.06E-06	0.02722186	0.58548263
215.1	1.76E-06	1.19E-14	2.59E-16	0.01716579	9.01E-06	0.02716141	0.58296865
215.2	1.76E-06	1.19E-14	2.59E-16	0.01716579	9.01E-06	0.02716141	0.58296865
215.3	1.76E-06	1.19E-14	2.59E-16	0.01716579	9.01E-06	0.02716141	0.58296865
215.4	1.76E-06	1.19E-14	2.59E-16	0.01716579	9.01E-06	0.02716141	0.58296865
215.5	1.76E-06	1.19E-14	2.59E-16	0.01716579	9.01E-06	0.02716141	0.58296865
215.6	1.76E-06	1.19E-14	2.59E-16	0.01716579	9.01E-06	0.02716141	0.58296865
215.7	1.76E-06	1.19E-14	2.59E-16	0.01716579	9.01E-06	0.02716141	0.58296865
215.8	1.76E-06	1.19E-14	2.59E-16	0.01716579	9.01E-06	0.02716141	0.58296865
215.9	1.76E-06	1.19E-14	2.59E-16	0.01716579	9.01E-06	0.02716141	0.58296865
216	1.76E-06	1.19E-14	2.59E-16	0.01716579	9.01E-06	0.02716141	0.58296865
216.1	1.76E-06	1.19E-14	2.59E-16	0.01716579	9.01E-06	0.02716141	0.58296865
216.2	1.76E-06	1.19E-14	2.59E-16	0.01716579	9.01E-06	0.02716141	0.58296865
216.3	1.74E-06	1.22E-14	2.65E-16	0.0172734	9.00E-06	0.02716284	0.58397647
216.4	1.74E-06	1.22E-14	2.65E-16	0.0172734	9.00E-06	0.02716284	0.58397647
216.5	1.74E-06	1.22E-14	2.65E-16	0.0172734	9.00E-06	0.02716284	0.58397647
216.6	1.74E-06	1.22E-14	2.65E-16	0.0172734	9.00E-06	0.02716284	0.58397647
216.7	1.74E-06	1.22E-14	2.65E-16	0.0172734	9.00E-06	0.02716284	0.58397647
216.8	1.74E-06	1.22E-14	2.65E-16	0.0172734	9.00E-06	0.02716284	0.58397647
216.9	1.74E-06	1.22E-14	2.65E-16	0.0172734	9.00E-06	0.02716284	0.58397647
217	1.74E-06	1.22E-14	2.65E-16	0.0172734	9.00E-06	0.02716284	0.58397647
217.1	1.74E-06	1.22E-14	2.65E-16	0.0172734	9.00E-06	0.02716284	0.58397647
217.2	1.74E-06	1.22E-14	2.65E-16	0.0172734	9.00E-06	0.02716284	0.58397647
217.3	1.74E-06	1.22E-14	2.65E-16	0.0172734	9.00E-06	0.02716284	0.58397647
217.4	1.74E-06	1.22E-14	2.59E-16	0.01731595	9.05E-06	0.02679773	0.58387972
217.5	1.74E-06	1.22E-14	2.59E-16	0.01731595	9.05E-06	0.02679773	0.58387972
217.6	1.74E-06	1.22E-14	2.59E-16	0.01731595	9.05E-06	0.02679773	0.58387972
217.7	1.73E-06	1.29E-14	2.64E-16	0.01721098	8.87E-06	0.02686829	0.58333929
217.8	1.73E-06	1.29E-14	2.64E-16	0.01721098	8.87E-06	0.02686829	0.58333929
217.9	1.73E-06	1.29E-14	2.64E-16	0.01721098	8.87E-06	0.02686829	0.58333929
218	1.73E-06	1.29E-14	2.64E-16	0.01721098	8.87E-06	0.02686829	0.58333929
218.1	1.73E-06	1.29E-14	2.64E-16	0.01721098	8.87E-06	0.02686829	0.58333929
218.2	1.73E-06	1.29E-14	2.64E-16	0.01721098	8.87E-06	0.02686829	0.58333929
218.3	1.73E-06	1.29E-14	2.64E-16	0.01721098	8.87E-06	0.02686829	0.58333929
218.4	1.73E-06	1.29E-14	2.64E-16	0.01721098	8.87E-06	0.02686829	0.58333929
218.5	1.73E-06	1.29E-14	2.64E-16	0.01721098	8.87E-06	0.02686829	0.58333929
218.6	1.73E-06	1.29E-14	2.64E-16	0.01721098	8.87E-06	0.02686829	0.58333929
218.7	1.73E-06	1.29E-14	2.64E-16	0.01721098	8.87E-06	0.02686829	0.58333929
218.8	1.73E-06	1.29E-14	2.64E-16	0.01721098	8.87E-06	0.02686829	0.58333929
218.9	1.73E-06	1.29E-14	2.64E-16	0.01721098	8.87E-06	0.02686829	0.58333929
219	1.73E-06	1.29E-14	2.64E-16	0.01721098	8.87E-06	0.02686829	0.58333929
219.1	1.73E-06	1.29E-14	2.64E-16	0.01721098	8.87E-06	0.02686829	0.58333929
219.2	1.74E-06	1.32E-14	2.68E-16	0.01716015	8.76E-06	0.0270083	0.58272512
219.3	1.74E-06	1.29E-14	2.57E-16	0.01727968	8.63E-06	0.02652904	0.57832633
219.4	1.74E-06	1.29E-14	2.57E-16	0.01727968	8.63E-06	0.02652904	0.57832633
219.5	1.74E-06	1.29E-14	2.57E-16	0.01727968	8.63E-06	0.02652904	0.57832633
219.6	1.74E-06	1.29E-14	2.57E-16	0.01727968	8.63E-06	0.02652904	0.57832633
219.7	1.74E-06	1.29E-14	2.57E-16	0.01727968	8.63E-06	0.02652904	0.57832633
219.8	1.74E-06	1.29E-14	2.57E-16	0.01727968	8.63E-06	0.02652904	0.57832633
219.9	1.74E-06	1.29E-14	2.57E-16	0.01727968	8.63E-06	0.02652904	0.57832633
220	1.75E-06	1.30E-14	2.58E-16	0.01749508	8.52E-06	0.02632865	0.57888623
220.1	1.75E-06	1.30E-14	2.58E-16	0.01749508	8.52E-06	0.02632865	0.57888623
220.2	1.75E-06	1.30E-14	2.58E-16	0.01749508	8.52E-06	0.02632865	0.57888623
220.3	1.75E-06	1.30E-14	2.58E-16	0.01749508	8.52E-06	0.02632865	0.57888623
220.4	1.75E-06	1.30E-14	2.58E-16	0.01749508	8.52E-06	0.02632865	0.57888623
220.5	1.75E-06	1.30E-14	2.58E-16	0.01749508	8.52E-06	0.02632865	0.57888623
220.6	1.75E-06	1.30E-14	2.58E-16	0.01749508	8.52E-06	0.02632865	0.57888623
220.7	1.75E-06	1.30E-14	2.58E-16	0.01749508	8.52E-06	0.02632865	0.57888623
220.8	1.75E-06	1.30E-14	2.58E-16	0.01749508	8.52E-06	0.02632865	0.57888623
220.9	1.75E-06	1.30E-14	2.58E-16	0.01749508	8.52E-06	0.02632865	0.57888623
221	1.75E-06	1.30E-14	2.58E-16	0.01749508	8.52E-06	0.02632865	0.57888623
221.1	1.75E-06	1.30E-14	2.58E-16	0.01749508	8.52E-06	0.02632865	0.57888623
221.2	1.75E-06	1.30E-14	2.58E-16	0.01749508	8.52E-06	0.02632865	0.57888623
221.3	1.75E-06	1.30E-14	2.58E-16	0.01749508	8.52E-06	0.02632865	0.57888623
221.4	1.75E-06	1.30E-14	2.58E-16	0.01749508	8.52E-06	0.02632865	0.57888623
221.5	1.75E-06	1.30E-14	2.58E-16	0.01749508	8.52E-06	0.02632865	0.57888623
221.6	1.76E-06	1.28E-14	2.50E-16	0.01761064	8.40E-06	0.02589562	0.57906304
221.7	1.76E-06	1.28E-14	2.50E-16	0.01761064	8.40E-06	0.02589562	0.57906304
221.8	1.76E-06	1.28E-14	2.50E-16	0.01761064	8.40E-06	0.02589562	0.57906304
221.9	1.76E-06	1.28E-14	2.50E-16	0.01761064	8.40E-06	0.02589562	0.57906304
222	1.76E-06	1.28E-14	2.50E-16	0.01761064	8.40E-06	0.02589562	0.57906304
222.1	1.76E-06	1.28E-14	2.50E-16	0.01761064	8.40E-06	0.02589562	0.57906304
222.2	1.76E-06	1.28E-14	2.50E-16	0.01761064	8.40E-06	0.02589562	0.57906304
222.3	1.76E-06	1.28E-14	2.50E-16	0.01761064	8.40E-06	0.02589562	0.57906304
222.4	1.76E-06	1.28E-14	2.50E-16	0.01761064	8.40E-06	0.02589562	0.57906304
222.5	1.74E-06	1.26E-14	2.37E-16	0.01779015	8.40E-06	0.02590682	0.58124324
222.6	1.74E-06	1.26E-14	2.37E-16	0.01779015	8.40E-06	0.02590682	0.58124324
222.7	1.74E-06	1.26E-14	2.37E-16	0.01779015	8.40E-06	0.02590682	0.58124324
222.8	1.74E-06	1.26E-14	2.37E-16	0.01779015	8.40E-06	0.02590682	0.58124324
222.9	1.74E-06	1.26E-14	2.37E-16	0.01779015	8.40E-06	0.02590682	0.58124324
223	1.74E-06	1.26E-14	2.37E-16	0.01779015	8.40E-06	0.02590682	0.58124324
223.1	1.74E-06	1.26E-14	2.37E-16	0.01779015	8.40E-06	0.02590682	0.58124324
223.2	1.74E-06	1.26E-14	2.37E-16	0.01779015	8.40E-06	0.02590682	0.58124324
223.3	1.74E-06	1.26E-14	2.37E-16	0.01779015	8.40E-06	0.02590682	0.58124324
223.4	1.74E-06	1.26E-14	2.37E-16	0.01779015	8.40E-06	0.02590682	0.58124324
223.5	1.74E-06	1.26E-14	2.37E-16	0.01779015	8.40E-06	0.02590682	0.58124324
223.6	1.74E-06	1.26E-14	2.37E-16	0.01779015	8.40E-06	0.02590682	0.58124324
223.7	1.74E-06	1.26E-14	2.37E-16	0.01779015	8.40E-06	0.02590682	0.58124324
223.8	1.77E-06	1.27E-14	2.33E-16	0.01798315	8.33E-06	0.02537788	0.58292452
223.9	1.77E-06	1.27E-14	2.33E-16	0.01798315	8.33E-06	0.02537788	0.58292452
224	1.77E-06	1.27E-14	2.33E-16	0.01798315	8.33E-06	0.02537788	0.58292452
224.1	1.77E-06	1.27E-14	2.33E-16	0.01798315	8.33E-06	0.02537788	0.58292452
224.2	1.77E-06	1.27E-14	2.33E-16	0.01798315	8.33E-06	0.02537788	0.58292452
224.3	1.78E-06	1.29E-14	2.29E-16	0.01778831	8.20E-06	0.02507349	0.581548
224.4	1.78E-06	1.29E-14	2.29E-16	0.01778831	8.20E-06	0.02507349	0.581548
224.5	1.78E-06	1.29E-14	2.29E-16	0.01778831	8.20E-06	0.02507349	0.581548
224.6	1.78E-06	1.29E-14	2.26E-16	0.01789873	8.25E-06	0.02464663	0.58153807
224.7	1.78E-06	1.29E-14	2.26E-16	0.01789873	8.25E-06	0.02464663	0.58153807
224.8	1.78E-06	1.29E-14	2.26E-16	0.01789873	8.25E-06	0.02464663	0.58153807
224.9	1.78E-06	1.29E-14	2.26E-16	0.01789873	8.25E-06	0.02464663	0.58153807

228.2	1.87E-06	1.18E-14	1.75E-16	0.01925871	6.53E-06	0.02101343	0.550094
228.3	1.88E-06	1.21E-14	1.74E-16	0.01944613	6.44E-06	0.0206599	0.54998916
228.4	1.88E-06	1.21E-14	1.74E-16	0.01944613	6.44E-06	0.0206599	0.54998916
228.5	1.88E-06	1.21E-14	1.74E-16	0.01944613	6.44E-06	0.0206599	0.54998916
228.6	1.88E-06	1.21E-14	1.74E-16	0.01944613	6.44E-06	0.0206599	0.54998916
228.7	1.88E-06	1.21E-14	1.74E-16	0.01944613	6.44E-06	0.0206599	0.54998916
228.8	1.87E-06	1.19E-14	1.68E-16	0.01955677	6.38E-06	0.0203244	0.54831003
228.9	1.87E-06	1.19E-14	1.68E-16	0.01955677	6.38E-06	0.0203244	0.54831003
229	1.87E-06	1.19E-14	1.68E-16	0.01955677	6.38E-06	0.0203244	0.54831003
229.1	1.87E-06	1.19E-14	1.68E-16	0.01955677	6.38E-06	0.0203244	0.54831003
229.2	1.87E-06	1.19E-14	1.68E-16	0.01955677	6.38E-06	0.0203244	0.54831003
229.3	1.87E-06	1.19E-14	1.68E-16	0.01955677	6.38E-06	0.0203244	0.54831003
229.4	1.87E-06	1.19E-14	1.68E-16	0.01955677	6.38E-06	0.0203244	0.54831003
229.5	1.87E-06	1.19E-14	1.68E-16	0.01955677	6.38E-06	0.0203244	0.54831003
229.6	1.87E-06	1.19E-14	1.68E-16	0.01955677	6.38E-06	0.0203244	0.54831003
229.7	1.88E-06	1.20E-14	1.61E-16	0.01961578	6.30E-06	0.02014723	0.54872997
229.8	1.89E-06	1.22E-14	1.62E-16	0.01973341	6.33E-06	0.02006636	0.5483213
229.9	1.89E-06	1.23E-14	1.60E-16	0.02000649	6.31E-06	0.01982558	0.54519742
230	1.89E-06	1.23E-14	1.60E-16	0.02000649	6.31E-06	0.01982558	0.54519742
230.1	1.90E-06	1.25E-14	1.58E-16	0.02011184	6.32E-06	0.01958876	0.54457321
230.2	1.90E-06	1.25E-14	1.58E-16	0.02011184	6.32E-06	0.01958876	0.54457321
230.3	1.89E-06	1.26E-14	1.55E-16	0.02021905	6.34E-06	0.01953088	0.5435524
230.4	1.92E-06	1.26E-14	1.51E-16	0.02023965	6.28E-06	0.0192375	0.54233268
230.5	1.93E-06	1.24E-14	1.46E-16	0.02037006	6.18E-06	0.01910893	0.5416354
230.6	1.93E-06	1.24E-14	1.46E-16	0.02037006	6.18E-06	0.01910893	0.5416354
230.7	1.93E-06	1.26E-14	1.46E-16	0.02059733	6.15E-06	0.01884062	0.54065994
230.8	1.93E-06	1.26E-14	1.46E-16	0.02059733	6.15E-06	0.01884062	0.54065994
230.9	1.95E-06	1.26E-14	1.47E-16	0.02062145	6.05E-06	0.01867629	0.53763453
231	1.95E-06	1.26E-14	1.47E-16	0.02062145	6.05E-06	0.01867629	0.53763453
231.1	1.95E-06	1.26E-14	1.47E-16	0.02062145	6.05E-06	0.01867629	0.53763453
231.2	1.95E-06	1.26E-14	1.47E-16	0.02062145	6.05E-06	0.01867629	0.53763453
231.3	1.95E-06	1.26E-14	1.47E-16	0.02062145	6.05E-06	0.01867629	0.53763453
231.4	1.95E-06	1.26E-14	1.47E-16	0.02062145	6.05E-06	0.01867629	0.53763453
231.5	1.97E-06	1.28E-14	1.49E-16	0.02054713	6.02E-06	0.01876345	0.53772134
231.6	1.99E-06	1.28E-14	1.47E-16	0.02075658	6.02E-06	0.01861576	0.53518667
231.7	2.00E-06	1.28E-14	1.42E-16	0.02084451	6.02E-06	0.01837407	0.53204127
231.8	2.07E-06	1.28E-14	1.38E-16	0.02093136	5.82E-06	0.01783498	0.52913872
231.9	2.07E-06	1.28E-14	1.38E-16	0.02093136	5.82E-06	0.01783498	0.52913872
232	2.09E-06	1.29E-14	1.38E-16	0.02107301	5.71E-06	0.01776524	0.52743225
232.1	2.09E-06	1.29E-14	1.38E-16	0.02107301	5.71E-06	0.01776524	0.52743225
232.2	2.09E-06	1.29E-14	1.38E-16	0.02107301	5.71E-06	0.01776524	0.52743225
232.3	2.09E-06	1.29E-14	1.38E-16	0.02107301	5.71E-06	0.01776524	0.52743225
232.4	2.09E-06	1.29E-14	1.38E-16	0.02107301	5.71E-06	0.01776524	0.52743225
232.5	2.09E-06	1.31E-14	1.36E-16	0.02104692	5.58E-06	0.01749231	0.52437853
232.6	2.09E-06	1.31E-14	1.36E-16	0.02104692	5.58E-06	0.01749231	0.52437853
232.7	2.15E-06	1.32E-14	1.31E-16	0.02114799	5.52E-06	0.01722552	0.5215479
232.8	2.15E-06	1.32E-14	1.31E-16	0.02114799	5.52E-06	0.01722552	0.5215479
232.9	2.15E-06	1.32E-14	1.31E-16	0.02114799	5.52E-06	0.01722552	0.5215479
233	2.15E-06	1.34E-14	1.30E-16	0.021383	5.46E-06	0.016944	0.51770377
233.1	2.16E-06	1.34E-14	1.29E-16	0.02154927	5.43E-06	0.01674418	0.51361524
233.2	2.16E-06	1.34E-14	1.29E-16	0.02154927	5.43E-06	0.01674418	0.51361524
233.3	2.18E-06	1.33E-14	1.24E-16	0.02188276	5.40E-06	0.01643681	0.51198061
233.4	2.23E-06	1.31E-14	1.21E-16	0.02211045	5.31E-06	0.01612208	0.50574617
233.5	2.26E-06	1.32E-14	1.22E-16	0.02217651	5.23E-06	0.01602211	0.50381433
233.6	2.26E-06	1.32E-14	1.22E-16	0.02217651	5.23E-06	0.01602211	0.50381433
233.7	2.31E-06	1.32E-14	1.18E-16	0.02231186	5.11E-06	0.01586575	0.5025375
233.8	2.33E-06	1.30E-14	1.12E-16	0.02265867	4.95E-06	0.01542883	0.49850813
233.9	2.33E-06	1.30E-14	1.12E-16	0.02265867	4.95E-06	0.01542883	0.49850813
234	2.33E-06	1.30E-14	1.12E-16	0.02265867	4.95E-06	0.01542883	0.49850813
234.1	2.44E-06	1.29E-14	1.08E-16	0.02309769	4.81E-06	0.01506576	0.49330576
234.2	2.49E-06	1.30E-14	1.09E-16	0.02340316	4.75E-06	0.01499491	0.49207296
234.3	2.49E-06	1.29E-14	1.08E-16	0.0237782	4.72E-06	0.01494607	0.49018876
234.4	2.49E-06	1.29E-14	1.08E-16	0.0237782	4.72E-06	0.01494607	0.49018876
234.5	2.49E-06	1.29E-14	1.08E-16	0.0237782	4.72E-06	0.01494607	0.49018876
234.6	2.49E-06	1.29E-14	1.08E-16	0.0237782	4.72E-06	0.01494607	0.49018876
234.7	2.49E-06	1.29E-14	1.08E-16	0.0237782	4.72E-06	0.01494607	0.49018876
234.8	2.49E-06	1.29E-14	1.08E-16	0.0237782	4.72E-06	0.01494607	0.49018876
234.9	2.49E-06	1.29E-14	1.08E-16	0.0237782	4.72E-06	0.01494607	0.49018876
235	2.59E-06	1.30E-14	1.07E-16	0.02391742	4.68E-06	0.01462989	0.48577705
235.1	2.59E-06	1.30E-14	1.07E-16	0.02391742	4.68E-06	0.01462989	0.48577705
235.2	2.59E-06	1.30E-14	1.07E-16	0.02391742	4.68E-06	0.01462989	0.48577705
235.3	2.63E-06	1.29E-14	1.06E-16	0.02422106	4.72E-06	0.01451485	0.48420059
235.4	2.71E-06	1.28E-14	9.66E-17	0.02522023	4.46E-06	0.01408184	0.47692739
235.5	2.84E-06	1.31E-14	8.71E-17	0.02572039	4.31E-06	0.01360477	0.46801843
235.6	2.88E-06	1.32E-14	8.44E-17	0.02583137	4.21E-06	0.01349423	0.46575264
235.7	2.89E-06	1.33E-14	8.26E-17	0.02608514	4.20E-06	0.01343174	0.46343401
235.8	2.89E-06	1.33E-14	8.26E-17	0.02608514	4.20E-06	0.01343174	0.46343401
235.9	2.95E-06	1.32E-14	8.22E-17	0.02649836	4.14E-06	0.01339733	0.46342169
236	2.95E-06	1.32E-14	8.22E-17	0.02649836	4.14E-06	0.01339733	0.46342169
236.1	2.98E-06	1.34E-14	8.33E-17	0.0266788	4.18E-06	0.01317141	0.46212574
236.2	3.00E-06	1.34E-14	8.17E-17	0.02674591	4.16E-06	0.01300375	0.4603428
236.3	3.06E-06	1.37E-14	8.19E-17	0.02671876	4.13E-06	0.01286264	0.45850284
236.4	3.06E-06	1.39E-14	8.31E-17	0.02700046	4.07E-06	0.01269908	0.45750615
236.5	3.06E-06	1.39E-14	8.31E-17	0.02700046	4.07E-06	0.01269908	0.45750615
236.6	3.14E-06	1.43E-14	8.30E-17	0.02721181	4.07E-06	0.01258683	0.45822119
236.7	3.14E-06	1.43E-14	8.30E-17	0.02721181	4.07E-06	0.01258683	0.45822119
236.8	3.17E-06	1.42E-14	8.07E-17	0.02745451	3.99E-06	0.01247254	0.45828307
236.9	3.17E-06	1.42E-14	8.07E-17	0.02745451	3.99E-06	0.01247254	0.45828307
237	3.22E-06	1.41E-14	7.96E-17	0.02779558	3.99E-06	0.01233034	0.455103
237.1	3.29E-06	1.42E-14	8.07E-17	0.0278969	3.99E-06	0.01224709	0.45394752
237.2	3.29E-06	1.42E-14	8.07E-17	0.0278969	3.99E-06	0.01224709	0.45394752
237.3	3.29E-06	1.42E-14	8.07E-17	0.0278969	3.99E-06	0.01224709	0.45394752
237.4	3.29E-06	1.46E-14	7.99E-17	0.0279042	3.90E-06	0.01208126	0.45422609
237.5	3.29E-06	1.46E-14	7.99E-17	0.0279042	3.90E-06	0.01208126	0.45422609
237.6	3.31E-06	1.48E-14	7.76E-17	0.02833273	3.83E-06	0.0118662	0.45393639
237.7	3.24E-06	1.47E-14	7.74E-17	0.02839686	3.85E-06	0.01175258	0.45222513
237.8	3.24E-06	1.47E-14	7.74E-17	0.02839686	3.85E-06	0.01175258	0.45222513
237.9	3.32E-06	1.48E-14	7.62E-17	0.0286616	3.87E-06	0.01161189	0.45050013
238	3.32E-06	1.48E-14	7.62E-17	0.0286616	3.87E-06	0.01161189	0.45050013
238.1	3.38E-06	1.47E-14	7.63E-17	0.02901542	3.80E-06	0.01157123	0.44786034
238.2	3.44E-06	1.49E-14	7.63E-17	0.02906239	3.74E-06	0.01138823	0.44730407
238.3	3.62E-06	1.55E-14	7.55E-17	0.02953749	3.63E-06	0.01102369	0.44429703
238.4	3.64E-06	1.57E-14	7.45E-17	0.0297571	3.63E-06	0.01085787	0.44293155
238.5	3.62E-06	1.61E-14	7.50E-17	0.02981538	3.62E-06	0.01073401	0.4392961
238.6	3.68E-06	1.63E-14	7.33E-17	0.03008055	3.57E-06	0.01070822	0.43890134
238.7	3.68E-06	1.63E-14	7.33E-17	0.03008055	3.57E-06	0.01070822	0.43890134
238.8	3.68E-06	1.63E-14	7.33E-17	0.03008055	3.57E-06	0.01070822	0.43890134
238.9	3.70E-06	1.71E-14	7.50E-17	0.03099026	3.54E-06	0.0104586	0.43532693
239	3.68E-06	1.70E-14	7.24E-17	0.03112173	3.59E-06	0.0101332	0.43270624
239.1	3.75E-06	1.68E-14	7.00E-17	0.03155349	3.56E-06	0.00997997	0.43217477
239.2	3.75E-06	1.68E-14	7.00E-17	0.03155349	3.56E-06	0.00997997	0.43217477
239.3	3.75E-06	1.68E-14	7.00E-17	0.03155349	3.56E-06	0.00997997	0.43217477
239.4	3.93E-06	1.69E-14	6.94E-17	0.03247984	3.55E-06	0.00966955	0.43304354
239.5	4.13E-06	1.73E-14	7.05E-17	0.03294984	3.56E-06	0.00951503	0.43162503
239.6	4.11E-06	1.73E-14	6.99E-17	0.03314562	3.51E-06	0.00935662	0.42830219
239.7	4.11E-06	1.76E-14	6.92E-17	0.03327188	3.52E-06	0.00923094	0.42691493
239.8	4.11E-06	1.76E-14	6.92E-17	0.03327188	3.52E-06	0.00923094	0.42691493
239.9	4.17E-06	1.81E-14	6.98E-17	0.0333655	3.54E-06	0.00913764	0.42495654
240	4.23E-06	1.84E-14	7.04E-17	0.03365187	3.53E-06	0.00898927	0.42169258

Table S6: PRCC analysis data for [PYR] with [G](0)=2 mM: PRCC sensitivity indices and their corresponding p-values

Sensitivity analysis data from PRCC for [PYR] with [G](0)=2 mM (only the parameters to which [PYR]'s level is sensitive to appear)

Time discretization	V_{[G]}	K_{[G]}	V_{[PYR]}	K_{[PYR]}	alpha	K_{[LACT]}	G_E	[G]_0	[PYR]_0
0	0	0	0	0	0	0	0	0	1
0.1	0.1167174	-0.1109664	0.97236094	-0.8066506	-0.0329099	-0.0264146	0.01015484	0.61698294	0.02529193
0.2	0.10345407	-0.1085613	0.9716563	-0.8032101	-0.0318787	0.01168823	0.00538029	0.60937407	0.01339639
0.3	0.09426976	-0.111253	0.97132535	-0.8002251	-0.0262222	0.03760387	0.00383321	0.60776805	0.00811069
0.4	0.09286874	-0.1245886	0.97090855	-0.7984237	-0.015936	0.06809119	0.00483599	0.60500445	0.01012798
0.5	0.08728501	-0.1276531	0.97042068	-0.7974242	-0.0151395	0.09111804	0.00216661	0.60220579	0.01259455
0.6	0.08075772	-0.1295814	0.96980969	-0.7941166	-0.0103804	0.11594085	0.00318792	0.60160269	0.00903313
0.7	0.07005204	-0.1306477	0.96885192	-0.7895461	-0.004463	0.1368928	0.00453076	0.59885699	0.008144
0.8	0.06550113	-0.1349049	0.96782169	-0.7856747	-0.0021671	0.1530404	0.0019303	0.59339666	0.00516866
0.9	0.0618057	-0.1319214	0.96702182	-0.7817364	-0.0004135	0.17265446	0.00514389	0.5890216	0.00377763
1	0.06014997	-0.1307052	0.96603773	-0.7780363	-0.0007514	0.1903119	-7.44E-05	0.58515142	0.00359533
1.1	0.05910092	-0.1306099	0.96539183	-0.7759967	0.00229445	0.20467562	0.00535316	0.58283845	0.01005105
1.2	0.05398079	-0.1321324	0.96487405	-0.7731463	0.01009228	0.22272654	-0.0008264	0.58361119	0.01093138
1.3	0.05167922	-0.1346979	0.96384621	-0.7682521	0.01320916	0.2411527	0.00374447	0.57967801	0.00908196
1.4	0.05496833	-0.1345666	0.96295196	-0.7650089	0.00992086	0.25234561	0.00775959	0.57834147	0.00852973
1.5	0.05772099	-0.1322715	0.96231885	-0.7629108	0.01216955	0.2619038	0.00728428	0.57708638	0.00432659
1.6	0.05926149	-0.1354556	0.96142438	-0.7586885	0.01199988	0.27096735	0.00805967	0.57213058	0.00300319
1.7	0.05534984	-0.1352709	0.96095237	-0.7578554	0.01240614	0.28555562	0.00613227	0.57007556	-0.0006623
1.8	0.05427079	-0.1391861	0.96068375	-0.7572301	0.01167381	0.29525294	0.01069048	0.570703	-0.0004343
1.9	0.04776268	-0.1402006	0.96002406	-0.7536403	0.01290342	0.30840996	0.01155451	0.57036133	0.00111822
2	0.04970518	-0.137729	0.95930277	-0.7511925	0.01312587	0.31601895	0.01487602	0.57149307	0.00331976
2.1	0.05199728	-0.1354745	0.95873404	-0.7493738	0.01508752	0.32063185	0.01739905	0.56914011	0.00573826
2.2	0.0498131	-0.1362504	0.95846379	-0.7488022	0.01515625	0.32717207	0.02222983	0.5688529	0.00357476
2.3	0.0467771	-0.1416876	0.95821376	-0.7477383	0.01867253	0.33578115	0.02104766	0.57012459	0.0048548
2.4	0.04785582	-0.1445079	0.95752886	-0.7460346	0.02258402	0.34440885	0.02340138	0.56752646	0.00074827
2.5	0.04450891	-0.1474155	0.95651423	-0.7426034	0.02057403	0.35100807	0.02640418	0.56470273	0.00046604
2.6	0.04341131	-0.1511806	0.95616397	-0.7421812	0.02341733	0.35677836	0.02460378	0.56530018	-0.0011439
2.7	0.04324049	-0.1569013	0.95559033	-0.7402394	0.01980073	0.36137961	0.02424506	0.56420832	0.00246819
2.8	0.04344898	-0.1586122	0.95508664	-0.7397717	0.02122284	0.36235723	0.0257736	0.56338536	-0.0005699
2.9	0.04162611	-0.1603312	0.95471664	-0.7387371	0.02309329	0.36997424	0.02653709	0.56400746	0.0039419
3	0.04563507	-0.1627519	0.95419243	-0.7369878	0.02247552	0.37289012	0.02655957	0.56260736	0.00275281
3.1	0.04502134	-0.1672176	0.95335949	-0.7343943	0.02684728	0.38226695	0.02596437	0.56119417	0.00043153
3.2	0.04154098	-0.169379	0.95275921	-0.7309314	0.02635975	0.38989388	0.02463938	0.56067261	0.00361905
3.3	0.04331725	-0.1694196	0.95187661	-0.7287228	0.02259392	0.39474484	0.02490483	0.55815936	0.00486239
3.4	0.04083697	-0.1714548	0.95114535	-0.7257729	0.02242467	0.39952601	0.02706049	0.55839035	0.00647898
3.5	0.0421561	-0.1745783	0.95038523	-0.7238315	0.01802514	0.40234632	0.02734507	0.55556998	0.00740874
3.6	0.04386322	-0.1769191	0.94982153	-0.7228116	0.01783411	0.40802448	0.02699387	0.55573429	0.00891658
3.7	0.04211718	-0.1788185	0.94892345	-0.7196528	0.01675694	0.41311472	0.02675868	0.55264859	0.01016135
3.8	0.04295337	-0.1785459	0.94818471	-0.7166763	0.01461267	0.41662044	0.02855308	0.5503248	0.01487652
3.9	0.04201493	-0.1802336	0.94778167	-0.7150173	0.01634594	0.42044183	0.02764924	0.54987051	0.01616129
4	0.04430049	-0.1810894	0.94699122	-0.7119778	0.01320585	0.42441238	0.0272016	0.54938148	0.01616306
4.1	0.04612046	-0.1819426	0.94618477	-0.7092695	0.01231984	0.42682466	0.02907885	0.54793212	0.01676709
4.2	0.04302628	-0.184905	0.945465	-0.7063881	0.01530846	0.43070479	0.02885358	0.54682408	0.01917028
4.3	0.04323431	-0.1864279	0.94485535	-0.7041431	0.01359436	0.43265326	0.02941519	0.54533651	0.0224624
4.4	0.04678948	-0.1906327	0.94429053	-0.7032308	0.01451324	0.43992442	0.02943979	0.5419497	0.02315865
4.5	0.0494907	-0.1930459	0.94358662	-0.7015131	0.01698177	0.44385408	0.03060353	0.54206241	0.02450837
4.6	0.05227943	-0.1956915	0.9434475	-0.700678	0.01676955	0.44846884	0.03218592	0.54376285	0.02560489
4.7	0.05184728	-0.1967262	0.94258613	-0.6968227	0.01418046	0.45384525	0.03587475	0.54237268	0.02649606
4.8	0.05357027	-0.1983981	0.94224478	-0.6961741	0.01416166	0.45785729	0.03657377	0.54116811	0.02663382
4.9	0.05491299	-0.2004148	0.94171436	-0.6934601	0.01241116	0.46248236	0.0340227	0.54148586	0.03144561
5	0.05552915	-0.207103	0.94091761	-0.6909054	0.01205156	0.46693172	0.04005941	0.54064702	0.03513423
5.1	0.05437638	-0.2054981	0.9401648	-0.6881771	0.00886654	0.46999225	0.04383172	0.54090205	0.03510248
5.2	0.05858223	-0.2039393	0.93972806	-0.6878521	0.00481582	0.47198748	0.0492918	0.54192942	0.03519259
5.3	0.05725965	-0.2050105	0.93873005	-0.6836216	0.00578195	0.47126327	0.05181461	0.53860719	0.03609295
5.4	0.05944552	-0.2075665	0.93786984	-0.6820983	0.00709074	0.47343375	0.05309444	0.53633609	0.03583258
5.5	0.05848733	-0.2094489	0.93743693	-0.6820119	0.01128598	0.47635073	0.0558754	0.5347781	0.03164286
5.6	0.05640494	-0.2118203	0.93692415	-0.6804185	0.00868004	0.47956456	0.05995175	0.53290424	0.03054867
5.7	0.05914808	-0.2103261	0.93574713	-0.6776491	0.00827245	0.48110976	0.06118141	0.5310253	0.03213875
5.8	0.05852635	-0.2139948	0.93518528	-0.6761867	0.00690612	0.48706929	0.06058389	0.52869769	0.03378388
5.9	0.05823694	-0.2183403	0.93451988	-0.6742572	0.00726932	0.48931092	0.05775719	0.52796714	0.03311896
6	0.06016914	-0.2210552	0.93438007	-0.6732755	0.00511917	0.49283326	0.05733965	0.52780707	0.03494799
6.1	0.06198414	-0.2207368	0.93349099	-0.6699772	0.00331906	0.49433525	0.0597839	0.52508945	0.03458731
6.2	0.06322513	-0.2212384	0.93303439	-0.6688512	0.00398413	0.49743148	0.06445125	0.52496445	0.03450871
6.3	0.06622559	-0.2226189	0.93252906	-0.6670881	0.00428464	0.50223034	0.06763785	0.52323568	0.03267458
6.4	0.06858066	-0.2257478	0.93176464	-0.6639143	0.00243185	0.50643702	0.06933958	0.52379336	0.03915762
6.5	0.0711551	-0.2256091	0.93185453	-0.6640146	0.00364101	0.50994706	0.07018385	0.52443914	0.03899493
6.6	0.07357102	-0.227236	0.93143129	-0.663348	0.00190483	0.51188618	0.06899337	0.52363375	0.03928115
6.7	0.07371054	-0.2276052	0.93083586	-0.6610618	-0.0019805	0.51522231	0.06978387	0.52160415	0.03824118
6.8	0.07861369	-0.230968	0.93061205	-0.6608612	-0.0056945	0.51790967	0.07278838	0.52199108	0.03841049
6.9	0.08141352	-0.2325781	0.92956063	-0.6580943	-0.0055991	0.51986912	0.07463373	0.52128064	0.04031316
7	0.07925264	-0.236488	0.92886084	-0.6563016	-0.0080426	0.52061509	0.07446948	0.51979633	0.04089097
7.1	0.07959658	-0.2390834	0.92804439	-0.6547987	-0.008101	0.52234153	0.07607756	0.51963015	0.04346206
7.2	0.08282777	-0.2382004	0.9270338	-0.6527715	-0.0133883	0.52438481	0.07868358	0.51819813	0.04806067
7.3	0.08217225	-0.2399176	0.92643652	-0.6498815	-0.0149649	0.52717811	0.07873475	0.51821804	0.04483521
7.4	0.08333567	-0.2415032	0.92562782	-0.6482771	-0.0162629	0.52920245	0.0796989	0.51655804	0.03795093
7.5	0.08355895	-0.2434453	0.92507228	-0.6458083	-0.0195905	0.5321594	0.08344715	0.51516219	0.0377985
7.6	0.08311536	-0.2455964	0.92369263	-0.6432401	-0.0212605	0.53183108	0.08404851	0.51265348	0.04223971
7.7	0.07961699	-0.2502491	0.92257473	-0.6392063	-0.0233295	0.53419107	0.08731305	0.51141388	0.04064363
7.8	0.08141769	-0.2527544	0.92186587	-0.6380773	-0.0242176	0.53472349	0.08953503	0.51085106	0.04028046
7.9	0.08074059	-0.2558506	0.92121393	-0.6377408	-0.0281435	0.53708532	0.08956368	0.50882263	0.04050696
8	0.08261455	-0.2607616	0.92026729	-0.6361889	-0.0313743	0.53677917	0.08772204	0.50955289	0.04217344
8.1	0.08387539	-0.2610473	0.91916977	-0.6341543	-0.0322431	0.53553073	0.08760264	0.50850778	0.0430845
8.2	0.08466671	-0.2600546	0.91838558	-0.6319412	-0.0349012	0.53740066	0.08760269	0.50840323	0.04718156
8.3	0.0853249	-0.2608421	0.91739608	-0.6295764	-0.0366632	0.53807148	0.0890995	0.50572545	0.0477071
8.4	0.08683057	-0.2611235	0.91713695	-0.6291862	-0.0381957	0.53963084	0.08983219	0.50632698	0.04990687
8.5	0.08906531	-0.2639666	0.91674347	-0.6276773	-0.0395646	0.54163722	0.08987754	0.50461287	0.04942014
8.6	0.08796084	-0.2630266	0.91598227	-0.6254253	-0.0400571	0.54229412	0.08991163	0.50481463	0.04755783
8.7	0.08973432	-0.2629216	0.91539638	-0.6223859	-0.0450765	0.54575063	0.0929626	0.50375401	0.04802998
8.8	0.09022824	-0.261901	0.9142274	-0.6202242	-0.0451378	0.54590639	0.09341456	0.50116441	0.04908429
8.9	0.09215578	-0.2648872	0.91406519	-0.6193032	-0.0477402	0.55082093	0.09827044	0.49966168	0.04963002
9	0.09319089	-0.2664477	0.91294194	-0.6170149	-0.0498552	0.55206613	0.09744647	0.49701715	0.04962855
9.1	0.09481826	-0.2707169	0.91249446	-0.6156657	-0.0525858	0.55365518	0.09817737	0.49686061	0.04772266
9.2	0.09709168	-0.2717068	0.91165293	-0.6134322	-0.0543422	0.55522424	0.10040287	0.49813712	0.04805745
9.3	0.09906498	-0.2720134	0.91141274	-0.6131742	-0.05606	0.55647565	0.1023281	0.49835965	0.05192795
9.4	0.10331717	-0.2722061	0.91104147	-0.6130073	-0.0599727	0.5585439	0.10456053	0.49879367	0.05155032
9.5	0.10452839	-0.2738116	0.91028695	-0.6116967	-0.0634181	0.5577318	0.10530872	0.4977736	0.05167552
9.6	0.10642984	-0.2768808	0.90944906	-0.609065	-0.0662544	0.559534	0.10607587	0.49347191	0.05107548
9.7	0.10779416	-0.2782031	0.90898579	-0.6083651	-0.0676513	0.56097618	0.10606578	0.49355447	0.05433298
9.8	0.10816252	-0.2790337	0.90840978	-0.6074148	-0.0695038	0.56149227	0.10697876	0.49241023	0.05200357
9.9	0.10907118	-0.2830049	0.90723987	-0.6051734	-0.0690725	0.56168482	0.10704863	0.49223613	0.0538369
10	0.10912503	-0.2861211	0.90622888	-0.6017057	-0.0703478	0.56307359	0.10982854	0.4920614	0.05418175
10.1	0.1087267	-0.2860451	0.90570531	-0.5999504	-0.0684215	0.56638179	0.11240135	0.49066982	0.05597961
10.2	0.10949419	-0.287445	0.90544785	-0.5985013	-0.0712896	0.56890279	0.11293827	0.49006963	0.05663979
10.3	0.1082029	-0.2884909	0.90443941	-0.5960177	-0.0728377	0.57063332	0.11342362	0.48893956	0.05750083
10.4	0.10901847	-0.2899798	0.90381954	-0.5942851	-0.0736077	0.57089048	0.11529556	0.48943138	0.0579562
10.5	0.10971473	-0.290566	0.90281132	-0.5925402	-0.0758738	0.57225744	0.11571592	0.48931588	0.05840826
10.6	0.11071278	-0.2909031	0.90247339	-0.5925092	-0.0776203	0.57342016	0.11544735	0.48966636	0.05883903
10.7	0.11450213	-0.2916596	0.9016619	-0.5913832	-0.0796201	0.57551823	0.117382	0.49091854	0.06202953
10.8	0.11328548	-0.294477	0.90102471	-0.5890048	-0.0806793	0.57814323	0.11903589	0.48840704	0.06309618
10.9	0.11445331	-0.2959083	0.89981636	-0.5856933	-0.0818923	0.57868939	0.12193695	0.48655606	0.06181074
11	0.116709	-0.2973626	0.8993005	-0.5847987	-0.083768	0.57976156	0.12594189	0.48587808	0.06103242
11.1	0.11847433	-0.2968053	0.89810467	-0.5823494	-0.0874915	0.57948287	0.12614889	0.48470181	0.06076395
11.2	0.11873372	-0.2966694	0.89717529	-0.5803111	-0.0905935	0.58175575	0.12464332	0.48150295	0.06287679
11.3	0.11924154	-0.2979412	0.89596532	-0.577033	-0.0926361	0.58310401	0.12466675	0.48039545	0.06412877
11.4	0.11972714	-0.2999598	0.89553348	-0.5759646	-0.0890118	0.58420041	0.12496152	0.47863098	0.06216033
11.5	0.11934535	-0.2987848	0.89471203	-0.5739631	-0.0918536	0.58419124	0.12635774	0.4781281	0.06228512
11.6	0.12234766	-0.2983331	0.89352884	-0.5727509	-0.0937272	0.58422989	0.12792988	0.47770856	0.0640538
11.7	0.12412795	-0.2984601	0.89298977	-0.5714394	-0.0958781	0.58512814	0.13033287	0.4765201	0.0660576
11.8	0.12750075	-0.2986074	0.89211716	-0.5698191	-0.0974246	0.58583249	0.13303769	0.47617012	0.06625375
11.9	0.13103062	-0.2995891	0.89093093	-0.5682234	-0.1003072	0.58778017	0.13236235	0.47656043	0.06917742
12	0.13073726	-0.3000444	0.89014799	-0.5661811	-0.1022532	0.58771138	0.13280069	0.47585249	0.07015008
12.1	0.13101779	-0.299578	0.88891813	-0.5638597	-0.103794	0.58820553	0.13401679	0.47405802	0.06876174
12.2	0.13225483	-0.3000233	0.88807515	-0.5612672	-0.1043601	0.5877782	0.13213475	0.47333289	0.06620378
12.3	0.13292521	-0.3016548	0.88735318	-0.5604045	-0.1034388	0.58829375	0.1339542	0.47130752	0.06394241
12.4	0.13490988	-0.301418	0.88643908	-0.5586837	-0.10378	0.58841074	0.13587761	0.46863292	0.06026609
12.5	0.13627956	-0.3021537	0.88567939	-0.5567433	-0.1060006	0.58736712	0.13352183	0.46876616	0.05901994
12.6	0.13483008	-0.3042935	0.88526052	-0.5549862	-0.1067845	0.58880867	0.13476606	0.46737664	0.0602344
12.7	0.13600405	-0.3070703	0.88407104	-0.5548741	-0.1079677	0.5901703	0.136268	0.46551053	0.05988844
12.8	0.13578964	-0.308331	0.88323057	-0.5538309	-0.1084108	0.59126016	0.13831395	0.46459875	0.06004432
12.9	0.13874377	-0.3076375	0.88246096	-0.5519999	-0.1142685	0.592217	0.13767301	0.46483632	0.06071972
13	0.14142565	-0.312226	0.8809659	-0.5490049	-0.1141753	0.59257488	0.13831929	0.46401953	0.05955559
13.1	0.14309986	-0.3119462	0.8798563	-0.5466899	-0.1135721	0.59231693	0.13774225	0.46387843	0.05959048
13.2	0.14411794	-0.3126819	0.87885175	-0.5432029	-0.1123699	0.59149238	0.13856518	0.46432084	0.06047474
13.3	0.14251358	-0.3154827	0.87789507	-0.5409682	-0.1111782	0.59268803	0.14023743	0.46176661	0.06114231
13.4	0.14181903	-0.3172642	0.87694612	-0.5393738	-0.1108664	0.59429761	0.14065658	0.46000553	0.06036205
13.5	0.14268388	-0.3168236	0.87617239	-0.5380926	-0.1118091	0.594005	0.14242354	0.45974907	0.05938601
13.6	0.14347641	-0.3166935	0.87500802	-0.5364558	-0.1120727	0.59504364	0.14563655	0.45802441	0.05904481
13.7	0.14430025	-0.316489	0.87422146	-0.5361724	-0.1141506	0.5952935	0.14640849	0.45886525	0.05929977
13.8	0.14505418	-0.3152562	0.87365616	-0.5353054	-0.1154035	0.59567337	0.14873204	0.45879112	0.05916526
13.9	0.14848628	-0.3152343	0.87305497	-0.5342225	-0.1177508	0.59555718	0.14838573	0.45875083	0.05938663
14	0.14900869	-0.3155932	0.87255009	-0.5318351	-0.119406	0.59660847	0.14879271	0.45811232	0.06011347
14.1	0.15039363	-0.3149694	0.87152946	-0.5280218	-0.1232592	0.59653751	0.15013872	0.45472013	0.05853654
14.2	0.15061944	-0.31662	0.87044166	-0.5254464	-0.1253415	0.59723834	0.15283972	0.45354642	0.05722201
14.3	0.1513799	-0.3158765	0.86964343	-0.523699	-0.1256562	0.59672119	0.15390593	0.45288818	0.05734221
14.4	0.15066822	-0.3178091	0.86874494	-0.5213992	-0.124152	0.59822871	0.14818135	0.45149287	0.05782794
14.5	0.15156453	-0.3175611	0.86799927	-0.5201725	-0.1271589	0.59781128	0.14837856	0.45071047	0.05688764
14.6	[illegible]	[illegible]	[illegible]	[illegible]	[illegible]	[illegible]	[illegible]	[illegible]	[illegible]
14.7	[illegible]	[illegible]	[illegible]	[illegible]	[illegible]	[illegible]	[illegible]	[illegible]	[illegible]
14.8	[illegible]	[illegible]	[illegible]	[illegible]	[illegible]	[illegible]	[illegible]	[illegible]	[illegible]
14.9	[illegible]	[illegible]	[illegible]	[illegible]	[illegible]	[illegible]	[illegible]	[illegible]	[illegible]
15	0.15115158	-0.3193373	0.86276058	-0.5084334	-0.1311343	0.60262726	0.15165241	0.44552484	0.05510432
15.1	0.15414411	-0.3209445	0.86163182	-0.5071216	-0.1341053	0.60397268	0.15367252	0.4447322	0.05680441
15.2	0.15658151	-0.319789	0.86049321	-0.5052676	-0.1354934	0.60505671	0.15470048	0.44283492	0.05789414
15.3	0.15799024	-0.3220844	0.8590635	-0.5021647	-0.1375869	0.60697969	0.15585365	0.44162228	0.059373
15.4	0.15551341	-0.3239748	0.85751249	-0.4995894	-0.1400886	0.60785805	0.15636955	0.44075896	0.05917369
15.5	0.15645584	-0.3254178	0.85619371	-0.4959411	-0.1441175	0.60787466	0.15855194	0.43920974	0.0615693
15.6	0.15555747	-0.3283175	0.85501153	-0.4939221	-0.1441341	0.6097345	0.15930914	0.4379454	0.0608322
15.7	0.15587456	-0.3304548	0.85401888	-0.491395	-0.1472516	0.610398	0.15819413	0.43656489	0.06157641
15.8	0.15646865	-0.3298862	0.85301245	-0.4906076	-0.1492995	0.61095654	0.15965199	0.43467334	0.05894806
15.9	0.15686624	-0.3298412	0.85150757	-0.4862474	-0.1533124	0.61056607	0.15704049	0.4328049	0.05952676
16	0.15700017	-0.3300514	0.85120496	-0.4844442	-0.1541115	0.61132231	0.15682779	0.43334185	0.06082611
16.1	0.1585238	-0.3290766	0.84964618	-0.4799586	-0.1556948	0.61238108	0.15802407	0.43089541	0.06224292
16.2	0.15769565	-0.3311967	0.84867041	-0.4766983	-0.1578412	0.61325166	0.15794447	0.42921105	0.06010905
16.3	0.15896326	-0.3320132	0.84750292	-0.4745857	-0.1602639	0.6135835	0.15975001	0.42752755	0.06165754
16.4	0.15851349	-0.3342072	0.84645769	-0.472541	-0.1615033	0.61587461	0.1630953	0.42929525	0.06164564
16.5	0.15843835	-0.334394	0.84566294	-0.4723774	-0.1638637	0.61629202	0.16312341	0.42793583	0.06113341
16.6	0.15959035	-0.3329877	0.84429297	-0.4702656	-0.1654716	0.61592876	0.16319749	0.42671727	0.06096639
16.7	0.15935679	-0.3336332	0.84267662	-0.4677966	-0.1673204	0.61616973	0.16471535	0.4256634	0.06450391
16.8	0.15850598	-0.3341541	0.84139519	-0.4666403	-0.168485	0.61659602	0.16644782	0.42626111	0.06456555
16.9	0.15835417	-0.3357286	0.8407778	-0.4675202	-0.1704751	0.61697275	0.1658188	0.4267326	0.06464138
17	0.15859187	-0.3368122	0.83951338	-0.4668017	-0.1736648	0.61614844	0.16584198	0.42534851	0.0642687
17.1	0.15897967	-0.3382406	0.83833003	-0.464629	-0.1734062	0.61635617	0.16420986	0.42472636	0.06510893
17.2	0.16057045	-0.3391949	0.83766355	-0.4635552	-0.1753277	0.61674692	0.16664519	0.42383563	0.06408216
17.3	0.1606464	-0.3405096	0.83688356	-0.4630513	-0.1759453	0.61769663	0.1677718	0.42349497	0.06300112
17.4	0.16111207	-0.3411463	0.83559726	-0.460559	-0.1773239	0.61757769	0.16822532	0.42128626	0.06285468
17.5	0.16237171	-0.3419745	0.83540113	-0.4606666	-0.1786981	0.61748304	0.16784434	0.4215303	0.06294315
17.6	0.16462373	-0.3429088	0.83491436	-0.4603735	-0.1795573	0.61730479	0.16924927	0.42088817	0.06219416
17.7	0.16704961	-0.3428706	0.83358051	-0.4593671	-0.1805694	0.61649533	0.16982012	0.42099553	0.06219515
17.8	0.16620986	-0.3439363	0.83311812	-0.4576585	-0.182868	0.61827013	0.16956764	0.42161073	0.06144381
17.9	0.16668362	-0.3449086	0.83221876	-0.4564459	-0.1832137	0.61834508	0.16971217	0.42034285	0.06053529
18	0.17011544	-0.3440845	0.83082302	-0.4534612	-0.184745	0.61860335	0.17121315	0.42062469	0.06221046
18.1	0.17146761	-0.3461103	0.83005275	-0.4529408	-0.1857988	0.61894545	0.17144612	0.41971626	0.06043199
18.2	0.17282179	-0.348098	0.82916735	-0.450773	-0.1872755	0.61923046	0.17162021	0.41775186	0.0622908
18.3	0.17297257	-0.3481979	0.82760401	-0.4475999	-0.1902991	0.6188277	0.17041211	0.41807409	0.06021511
18.4	0.17436273	-0.3477472	0.82679896	-0.4460351	-0.1926202	0.61821642	0.17054525	0.41764493	0.06215079
18.5	0.17362939	-0.3475716	0.82577064	-0.443341	-0.1941008	0.61825133	0.17096722	0.41592577	0.06108603
18.6	0.17231373	-0.348712	0.82480601	-0.4408462	-0.1940582	0.61890839	0.17102872	0.41597511	0.05997753
18.7	0.17337867	-0.3489283	0.82356642	-0.4392309	-0.1937286	0.61926216	0.1703158	0.41395386	0.05741224
18.8	0.17235812	-0.3494997	0.82221394	-0.437059	-0.194838	0.61941563	0.17013234	0.41284991	0.060034
18.9	0.17457964	-0.3498091	0.82083513	-0.435334	-0.1971919	0.61876806	0.16903257	0.41251025	0.06114434
19	0.17535177	-0.3499181	0.8196283	-0.433454	-0.1976675	0.61904376	0.16988649	0.4129376	0.06198184
19.1	0.17585139	-0.3503146	0.81873215	-0.4327697	-0.2023192	0.6199462	0.17161663	0.41115527	0.06181939
19.2	0.17782044	-0.3505916	0.81736928	-0.4297733	-0.2050871	0.62106591	0.17499312	0.41091516	0.06231694
19.3	0.17747512	-0.350319	0.81595944	-0.4274128	-0.2057891	0.6209754	0.17209852	0.40873515	0.06033276
19.4	0.17752321	-0.3502975	0.81435856	-0.4262722	-0.2068286	0.62081899	0.17281078	0.40684888	0.06186194
19.5	0.18032389	-0.3492807	0.813206	-0.425188	-0.2105064	0.62201008	0.17254466	0.40442529	0.06086666
19.6	0.17977488	-0.351837	0.81160979	-0.4214462	-0.2115351	0.62266043	0.17247627	0.40196138	0.0596559
19.7	0.17945134	-0.352783	0.81086937	-0.4208752	-0.2119626	0.62225423	0.17412121	0.40050237	0.05989858
19.8	0.18022343	-0.3538751	0.80943526	-0.4185662	-0.2137502	0.62249337	0.17335502	0.40061492	0.05973094
19.9	0.18221296	-0.3515021	0.80804295	-0.4153443	-0.2149153	0.62170432	0.17344902	0.40037076	0.05910346
20	0.18100957	-0.3513674	0.80683302	-0.4130426	-0.2159501	0.62131051	0.17366105	0.39975471	0.05873063
20.1	0.18097476	-0.3510678	0.80564583	-0.4113133	-0.2166851	0.62140061	0.17486765	0.39881175	0.05948859
20.2	0.18116533	-0.3501638	0.80404824	-0.4094662	-0.2158432	0.62265061	0.17758532	0.39911622	0.06203388
20.3	0.18471617	-0.350363	0.80314772	-0.4092977	-0.2156998	0.62197393	0.17935665	0.39821455	0.06219716
20.4	0.18446641	-0.3505612	0.80186782	-0.4081964	-0.2152536	0.62130207	0.17791707	0.39695536	0.06260035
20.5	0.18421289	-0.3507182	0.80007811	-0.4067432	-0.2165261	0.6206249	0.17748754	0.39587354	0.06262848
20.6	0.18361957	-0.3512343	0.79881456	-0.4034873	-0.2175906	0.62041213	0.17736035	0.39523392	0.06157215
20.7	0.182998	-0.3516519	0.79760682	-0.4021206	-0.2188883	0.6198351	0.17671906	0.39217558	0.05988226
20.8	0.18485178	-0.3519809	0.7956943	-0.3994966	-0.2196362	0.61835381	0.17864008	0.38921243	0.05842565
20.9	0.18389229	-0.3515421	0.79463456	-0.3975464	-0.2206656	0.61845665	0.17819674	0.38969834	0.0590222
21	0.18349465	-0.354013	0.79328913	-0.3956661	-0.2219734	0.61987535	0.17790121	0.38887464	0.05842999
21.1	0.18274755	-0.3542436	0.79252353	-0.3939524	-0.2220981	0.61989084	0.17676186	0.38868583	0.0582408
21.2	0.18373153	-0.3542697	0.79148928	-0.3919477	-0.2226647	0.62119852	0.17518921	0.38748844	0.05814038
21.3	0.18581694	-0.3545852	0.79042639	-0.3909453	-0.2237991	0.62147022	0.17403251	0.38799142	0.05865792
21.4	0.18518779	-0.3555614	0.78934622	-0.3892811	-0.2241149	0.62177026	0.17313502	0.38514844	0.05801419
21.5	0.1836161	-0.3559509	0.78803829	-0.3881665	-0.2269074	0.62106723	0.17210301	0.38360396	0.05794163
21.6	0.18297661	-0.3555213	0.78604358	-0.3862549	-0.2290078	0.62041047	0.17273243	0.38072965	0.05851832
21.7	0.18298256	-0.3568744	0.78438465	-0.3839755	-0.2306735	0.62140613	0.17232492	0.37985306	0.05941048
21.8	0.1849293	-0.3561497	0.78377467	-0.3816756	-0.2339297	0.62246966	0.17200504	0.37961116	0.06000763
21.9	0.1842066	-0.3561313	0.78283389	-0.3808887	-0.2349116	0.62353864	0.17221925	0.38032052	0.0589137
22	0.18371132	-0.3559631	0.78136888	-0.3828398	-0.2365051	0.62404328	0.17137728	0.3784947	0.05818953
22.1	0.1863791	-0.3554661	0.78015495	-0.3820439	-0.2377442	0.62363078	0.17171416	0.37853959	0.05790053
22.2	0.18884851	-0.3557571	0.77965501	-0.3823643	-0.23886	0.62376649	0.17401082	0.37838296	0.05677272
22.3	0.19139732	-0.3554532	0.77843819	-0.3819411	-0.239497	0.62414026	0.17605368	0.37725093	0.0557694
22.4	0.19161365	-0.3558939	0.77708494	-0.3811802	-0.2412127	0.62455977	0.1772874	0.37652249	0.0554749
22.5	0.1930754	-0.3563143	0.77591286	-0.3788436	-0.2423636	0.62514107	0.17871916	0.37637112	0.05516214
22.6	0.19507897	-0.355531	0.77380578	-0.3763269	-0.2462325	0.62310816	0.18005053	0.37544938	0.05626131
22.7	0.19527381	-0.3559662	0.77305558	-0.3759637	-0.2470644	0.62303072	0.17960619	0.37336045	0.05582269
22.8	0.19516326	-0.3557068	0.77186177	-0.3739848	-0.2480985	0.62388231	0.18171672	0.37204075	0.0558122
22.9	0.19329252	-0.3562491	0.76947656	-0.3741803	-0.2448749	0.62407609	0.18152931	0.36920583	0.05495367
23	0.19469828	-0.3560865	0.76780955	-0.3717836	-0.2470972	0.6237641	0.18145729	0.36752084	0.0532803
23.1	0.19493404	-0.3588168	0.76753869	-0.3709462	-0.2480964	0.62468734	0.18234299	0.36646456	0.05299647
23.2	0.19523068	-0.359342	0.76698543	-0.3710328	-0.2498732	0.62548936	0.18375725	0.36567609	0.0532599
23.3	0.19412838	-0.3581131	0.76536211	-0.3691713	-0.2501304	0.62631844	0.18365231	0.36434988	0.0547614
23.4	0.1939454	-0.3597428	0.76511738	-0.3685216	-0.2502817	0.62738607	0.18387721	0.36357521	0.0557253
23.5	0.19434355	-0.3597739	0.76323683	-0.3683601	-0.2530518	0.62693834	0.18314463	0.36186535	0.05600597
23.6	0.19514125	-0.3592301	0.76190918	-0.3661966	-0.2526177	0.62657463	0.18307326	0.35983805	0.05446889
23.7	0.19431845	-0.360212	0.76134338	-0.364767	-0.2529575	0.62718817	0.18324867	0.35782806	0.05312856
23.8	0.19459555	-0.3599507	0.75983863	-0.3637475	-0.2530819	0.62752373	0.18438088	0.35735644	0.05515802
23.9	0.19441592	-0.361118	0.75929864	-0.3628597	-0.2550749	0.62836003	0.18354632	0.35874779	0.05586008
24	0.19332837	-0.3616959	0.75711842	-0.3594647	-0.2571962	0.62869524	0.1842298	0.35656869	0.0570751
24.1	0.19504761	-0.3609145	0.75506436	-0.3578147	-0.2607134	0.62864263	0.18558776	0.35519787	0.05714639
24.2	0.19469623	-0.358913	0.75370458	-0.3563107	-0.2604713	0.62918649	0.18455297	0.35564229	0.05790311
24.3	0.19452543	-0.3600851	0.75305951	-0.3554243	-0.2630475	0.6291042	0.1858441	0.35391026	0.05762525
24.4	0.19625872	-0.3590728	0.75225738	-0.3538451	-0.2649278	0.62915049	0.18412239	0.35194858	0.05698572
24.5	0.19624081	-0.3596008	0.75100452	-0.3524443	-0.2648158	0.62893196	0.18362054	0.35122581	0.05729709
24.6	0.19499005	-0.3577178	0.74919402	-0.3510224	-0.2656518	0.62848684	0.18261128	0.34945061	0.05851015
24.7	0.19591546	-0.3579852	0.74784801	-0.3507331	-0.2656992	0.62793767	0.18109344	0.34781673	0.05711051
24.8	0.19757678	-0.3571078	0.74662633	-0.3490115	-0.2664334	0.62808526	0.18053537	0.34546909	0.05599971
24.9	0.19500724	-0.357346	0.74485108	-0.3461182	-0.2668696	0.62835679	0.18006608	0.34214271	0.05689985
25	0.19532054	-0.3567912	0.74255504	-0.3448717	-0.2651055	0.62745225	0.17965521	0.33859866	0.05504374
25.1	0.19536993	-0.3562171	0.74161224	-0.3446192	-0.266148	0.62701081	0.17895007	0.33814679	0.05420407
25.2	0.19604762	-0.3559434	0.7406938	-0.3443643	-0.2668514	0.62711466	0.18178202	0.33633089	0.05410034
25.3	0.19601616	-0.3560243	0.73952608	-0.3426455	-0.2678665	0.62748587	0.18107789	0.33596409	0.05500718
25.4	0.19864932	-0.3547747	0.73802947	-0.3402166	-0.2695973	0.62783513	0.1836726	0.33562748	0.05579776
25.5	0.19842743	-0.3558902	0.73587585	-0.3407223	-0.2695571	0.62726742	0.18180234	0.33347232	0.05433186
25.6	0.20060758	-0.3552119	0.73386294	-0.3392402	-0.2718299	0.62705922	0.18055462	0.3344957	0.05604229
25.7	0.19857895	-0.3564689	0.73162931	-0.3369554	-0.2733928	0.62659226	0.18026642	0.33251156	0.05678333
25.8	0.1968669	-0.3567087	0.73028906	-0.3340071	-0.2733349	0.62691586	0.18003973	0.33063263	0.0558784
25.9	0.19772215	-0.3571782	0.72911262	-0.3340234	-0.2751071	0.62673072	0.17948955	0.33099634	0.05650802
26	0.19688806	-0.3573206	0.72801133	-0.3334928	-0.2754465	0.62632806	0.18034214	0.32912532	0.05398615
26.1	0.19624377	-0.3572748	0.72702164	-0.3337912	-0.2762898	0.62573986	0.18127206	0.32788363	0.05398871
26.2	0.19688678	-0.3575017	0.72578409	-0.3322843	-0.2778696	0.62553034	0.18089766	0.32758296	0.05451751
26.3	0.19739372	-0.3573121	0.72537826	-0.3318028	-0.2788195	0.62550832	0.18243231	0.32735725	0.0550476
26.4	0.19683489	-0.3570092	0.72340528	-0.3297406	-0.2789288	0.62528895	0.18346201	0.32574355	0.05473382
26.5	0.19751527	-0.3565774	0.72242445	-0.3285587	-0.2813971	0.6243797	0.1831129	0.32483274	0.05509808
26.6	0.19795111	-0.3573292	0.72078044	-0.3274038	-0.2830319	0.62504113	0.18362141	0.32350611	0.05595085
26.7	0.19848074	-0.3591454	0.71881125	-0.3264091	-0.2845896	0.62459105	0.18397673	0.32140299	0.05638799
26.8	0.19991834	-0.3600675	0.71773482	-0.3256006	-0.2865018	0.62472898	0.18408254	0.31948876	0.05652634
26.9	0.19986432	-0.3590866	0.71663637	-0.3233685	-0.2876867	0.62419462	0.18410994	0.31848162	0.05566597
27	0.19931726	-0.3614651	0.71577498	-0.3213155	-0.2893947	0.62476902	0.18314175	0.31767602	0.05517946
27.1	0.19976016	-0.3621431	0.71355087	-0.3219104	-0.2897882	0.62519537	0.182241	0.31787311	0.05484094
27.2	0.19974614	-0.3614	0.71295081	-0.3215724	-0.2914639	0.62557333	0.18336397	0.31706933	0.05501111
27.3	0.20102409	-0.3627519	0.71222384	-0.3212091	-0.2927997	0.62576186	0.18341474	0.31695392	0.05386566
27.4	0.20124583	-0.3630155	0.71246	-0.3204745	-0.2949101	0.62562531	0.18366523	0.31691563	0.05270746
27.5	0.20053139	-0.3630761	0.71159102	-0.3195616	-0.2949387	0.62556557	0.18355098	0.31653537	0.05335448
27.6	0.20096226	-0.3640977	0.71024449	-0.3168766	-0.2972694	0.6262736	0.18469811	0.31661966	0.05401822
27.7	0.20011171	-0.3628216	0.70891473	-0.3162754	-0.2974152	0.6268595	0.18514431	0.31553392	0.05311314
27.8	0.20103633	-0.3632083	0.70756416	-0.3148347	-0.3000994	0.62662735	0.185381	0.31379027	0.05463012
27.9	0.20111118	-0.3622072	0.70585807	-0.3141481	-0.3015831	0.62666849	0.18624614	0.31318393	0.05423768
28	0.20103506	-0.3629373	0.70454152	-0.3138091	-0.3015546	0.62707492	0.18667932	0.3119132	0.05455187
28.1	0.19975322	-0.3636225	0.70251577	-0.3113249	-0.3035586	0.62713725	0.18493231	0.30863159	0.05443012
28.2	0.20017807	-0.3621431	0.70087803	-0.3098821	-0.3034357	0.62702825	0.18386754	0.30679346	0.05362315
28.3	0.20175146	-0.3623757	0.69998526	-0.309008	-0.3055647	0.6268896	0.18519725	0.30557038	0.05385343
28.4	0.2025773	-0.3625671	0.69925097	-0.3073244	-0.3062573	0.62722193	0.18611819	0.30447344	0.05436904
28.5	0.20254443	-0.3628422	0.69766571	-0.3055739	-0.3065622	0.62701265	0.18601119	0.30300072	0.05439825
28.6	0.20403282	-0.3617188	0.69610271	-0.3048496	-0.3074747	0.6268344	0.18466499	0.30249514	0.05502838
28.7	0.20438957	-0.3622255	0.69535262	-0.3040976	-0.3090994	0.62660531	0.1850317	0.30151572	0.0542309
28.8	0.20515268	-0.3619749	0.69432031	-0.3038071	-0.3097907	0.62580718	0.18443299	0.30010151	0.05448714
28.9	0.20572868	-0.3617919	0.6931814	-0.3024529	-0.3102546	0.62662753	0.18339654	0.29912273	0.05542463
29	0.20545293	-0.3625982	0.69202255	-0.3012586	-0.311207	0.62658653	0.1846149	0.29972897	0.05463284
29.1	0.20538976	-0.3625326	0.69046379	-0.3008194	-0.311864	0.62708923	0.18504157	0.30049167	0.05553706
29.2	0.20659047	-0.3608813	0.68765945	-0.2991294	-0.3109816	0.6267337	0.18698286	0.29892374	0.05636582
29.3	0.20746913	-0.3620275	0.68603956	-0.297598	-0.3123122	0.62617608	0.18619964	0.29790576	0.05613854
29.4	0.20783255	-0.3616153	0.68487687	-0.2961673	-0.3132258	0.62565694	0.18792393	0.29751868	0.05609777
29.5	0.20864385	-0.3621322	0.68268432	-0.2945833	-0.3150826	0.62572999	0.18873804	0.29551123	0.0536634
29.6	[illegible]	[illegible]	[illegible]	[illegible]	[illegible]	[illegible]	[illegible]	[illegible]	[illegible]
29.7	[illegible]	[illegible]	[illegible]	[illegible]	[illegible]	[illegible]	[illegible]	[illegible]	[illegible]
29.8	[illegible]	[illegible]	[illegible]	[illegible]	[illegible]	[illegible]	[illegible]	[illegible]	[illegible]
29.9	[illegible]	[illegible]	[illegible]	[illegible]	[illegible]	[illegible]	[illegible]	[illegible]	[illegible]
30	0.20987746	-0.3636156	0.67518855	-0.2906391	-0.318782	0.62391224	0.19355825	0.2897695	0.05209906
30.1	0.20931648	-0.3642243	0.67373535	-0.2905605	-0.3194674	0.62405417	0.19380889	0.28907811	0.05167628
30.2	0.20849616	-0.3646314	0.67187578	-0.2906628	-0.3198818	0.62316996	0.19237011	0.28928159	0.05222314
30.3	0.20834193	-0.3639244	0.67060675	-0.289168	-0.3219857	0.62380098	0.19336877	0.28930739	0.05307703
30.4	0.20878458	-0.364146	0.66986226	-0.2867248	-0.3222537	0.6240421	0.19285322	0.28914392	0.05286728
30.5	0.20802691	-0.3639496	0.66839266	-0.2840511	-0.3250481	0.62423469	0.19445076	0.28603025	0.05389308
30.6	0.20966257	-0.3638177	0.66698409	-0.283461	-0.3265981	0.62325121	0.19710064	0.28465114	0.05355945
30.7	0.20959541	-0.3633472	0.666535	-0.2831587	-0.3272717	0.62365287	0.19644698	0.28510407	0.05223947
30.8	0.21029154	-0.3639262	0.6649023	-0.2821787	-0.3285356	0.62344257	0.19624246	0.2837598	0.05151788
30.9	0.20968742	-0.3647793	0.66272615	-0.2810519	-0.3295063	0.623022	0.19608036	0.28261852	0.0518664
31	0.21102315	-0.3646854	0.66222365	-0.2814044	-0.3312791	0.6234386	0.19686895	0.28219243	0.05289189
31.1	0.20975774	-0.3653042	0.66146285	-0.2797428	-0.3323569	0.62379605	0.1980333	0.28080161	0.05201337
31.2	0.20994268	-0.3652313	0.66064543	-0.2788242	-0.3331133	0.62448501	0.19815395	0.27988515	0.05061145
31.3	0.21053076	-0.365947	0.65907242	-0.2788318	-0.3328339	0.62506507	0.19714435	0.27841379	0.05091902
31.4	0.21060874	-0.3661927	0.65813054	-0.2770739	-0.3335932	0.62551267	0.19650308	0.27836095	0.05069735
31.5	0.21042826	-0.3657188	0.65719505	-0.2766011	-0.3335693	0.62579308	0.19747112	0.27735614	0.04999115
31.6	0.20976116	-0.365263	0.65592767	-0.2744774	-0.3343124	0.6264479	0.19665071	0.27546743	0.0499988
31.7	0.20946714	-0.3662983	0.65469432	-0.2730731	-0.33544	0.62714044	0.19723668	0.27419474	0.05073936
31.8	0.21064925	-0.3662806	0.65397915	-0.2721683	-0.3361036	0.62727419	0.19718958	0.27372406	0.04974873
31.9	0.2121759	-0.367022	0.65327298	-0.27183	-0.3364513	0.62853569	0.19687363	0.27335991	0.05009565
32	0.21273031	-0.3664187	0.65188801	-0.2702727	-0.3371438	0.62840834	0.1973906	0.27182554	0.04814228
32.1	0.2128865	-0.3666054	0.65116184	-0.2688043	-0.3390504	0.62882496	0.19718883	0.27074096	0.04698655
32.2	0.21380399	-0.3670226	0.64950928	-0.2683675	-0.3390744	0.62842257	0.19762308	0.27078852	0.04757516
32.3	0.21374734	-0.3679623	0.64898208	-0.2674077	-0.3396279	0.62859956	0.19804577	0.26994481	0.04749422
32.4	0.2126993	-0.3664787	0.64682468	-0.2652518	-0.3399127	0.62874925	0.19810705	0.2671405	0.04745593
32.5	0.21324571	-0.3664409	0.64534215	-0.2640499	-0.3418972	0.62842417	0.19620541	0.26660432	0.04652092
32.6	0.21347541	-0.3647223	0.64414663	-0.2615923	-0.3435624	0.62833122	0.1954601	0.26516291	0.04755211
32.7	0.21427604	-0.3649406	0.64313174	-0.2603481	-0.3451396	0.62866039	0.19528498	0.26361821	0.04817294
32.8	0.21509265	-0.3650213	0.64170914	-0.2589257	-0.3452084	0.62925858	0.19593991	0.2630282	0.04781041
32.9	0.21485247	-0.3665776	0.64058339	-0.2573399	-0.3463223	0.62886491	0.19494409	0.26334342	0.04774547
33	0.21484314	-0.3672834	0.63895504	-0.2560985	-0.346964	0.62883479	0.19443514	0.26307431	0.0478052
33.1	0.21565143	-0.3686405	0.63725301	-0.2555343	-0.3480541	0.62845166	0.19375162	0.26312442	0.04782775
33.2	0.21562846	-0.3684155	0.6352424	-0.2544444	-0.34888	0.62791797	0.19576662	0.26222423	0.04691907
33.3	0.21482557	-0.3685663	0.63434609	-0.2530951	-0.3491939	0.62736825	0.19556778	0.26155144	0.04691575
33.4	0.21546989	-0.3695195	0.63332526	-0.2521222	-0.3504454	0.62722375	0.19580031	0.25969186	0.0471623
33.5	0.2149581	-0.3696876	0.63268532	-0.251032	-0.3512545	0.62717006	0.19698832	0.25892469	0.04689694
33.6	0.21667742	-0.3699376	0.63138773	-0.2500287	-0.352335	0.62720552	0.19792091	0.25884836	0.04757111
33.7	0.21770003	-0.3701606	0.62939949	-0.2484443	-0.3531544	0.6271701	0.19836533	0.25920516	0.04681451
33.8	0.21752272	-0.370041	0.62712548	-0.2484995	-0.3534543	0.62683158	0.19782962	0.25834944	0.04745167
33.9	0.21839777	-0.3709982	0.62585644	-0.248652	-0.3544183	0.62679273	0.19748136	0.25843464	0.04667428
34	0.2187077	-0.3721508	0.62437975	-0.2468461	-0.3551482	0.62693765	0.198076	0.25757104	0.04629884
34.1	0.21826452	-0.3724462	0.62344102	-0.2467126	-0.3564781	0.62699669	0.19826894	0.2564178	0.04602366
34.2	0.21869221	-0.3724849	0.62268608	-0.2459984	-0.3583109	0.62677673	0.19772849	0.25534898	0.04507598
34.3	0.21802429	-0.3737616	0.62133047	-0.2458664	-0.3582064	0.62653717	0.19674419	0.25532369	0.04550098
34.4	0.21902758	-0.3744819	0.61978944	-0.2437246	-0.3607247	0.62705928	0.19767636	0.25559449	0.04596046
34.5	0.21951883	-0.3746062	0.61739522	-0.2426513	-0.3604844	0.62712979	0.19757183	0.25330987	0.04511369
34.6	0.21955783	-0.3741491	0.61574836	-0.241533	-0.3610424	0.62649598	0.19583492	0.25287437	0.04488646
34.7	0.21975666	-0.3747115	0.61447876	-0.2403983	-0.3621804	0.62637631	0.19559904	0.25202814	0.04535665
34.8	0.21982723	-0.3750224	0.61368506	-0.2386303	-0.3640707	0.62593607	0.19711349	0.25113415	0.04377949
34.9	0.21861401	-0.3756246	0.61162789	-0.2371631	-0.3651267	0.62558857	0.19768329	0.24938217	0.04343371
35	0.21920035	-0.375896	0.61085778	-0.236815	-0.3651494	0.62538104	0.19795943	0.24886849	0.04309563
35.1	0.22059964	-0.3754388	0.60888505	-0.2359976	-0.3671107	0.62557052	0.19829496	0.24725204	0.04213873
35.2	0.22168679	-0.3753328	0.6073119	-0.2341581	-0.3694361	0.62613544	0.19784416	0.24585018	0.04295471
35.3	0.22084384	-0.3757701	0.60595072	-0.2338765	-0.3716801	0.62655566	0.19733701	0.24491821	0.04293519
35.4	0.22086919	-0.3766172	0.60497392	-0.2330908	-0.3725574	0.62706439	0.1957249	0.24522072	0.04346955
35.5	0.22091446	-0.3771181	0.60348736	-0.2318583	-0.372885	0.62724561	0.19444992	0.24425281	0.04345079
35.6	0.22201402	-0.3770429	0.60205929	-0.2297733	-0.3736058	0.62737986	0.19356519	0.24221763	0.04441056
35.7	0.22257391	-0.3770612	0.60084582	-0.2283685	-0.374682	0.62722834	0.19427115	0.24180988	0.04407093
35.8	0.22347709	-0.3771342	0.60017536	-0.2282485	-0.3761901	0.62760804	0.19493103	0.24191403	0.0441724
35.9	0.22402684	-0.377049	0.59889559	-0.2265244	-0.3780793	0.62803115	0.19622343	0.24152044	0.04395269
36	0.22587308	-0.3754812	0.59823519	-0.2269295	-0.3801198	0.62866616	0.19638379	0.24183524	0.04404864
36.1	0.22580677	-0.3745432	0.5977927	-0.225829	-0.3803573	0.62813678	0.195955	0.24215808	0.04365533
36.2	0.22583295	-0.3741351	0.59637314	-0.2260026	-0.3790697	0.62807731	0.19515213	0.2416777	0.04332341
36.3	0.22729144	-0.3743793	0.59473297	-0.2251461	-0.3805452	0.62788066	0.19504483	0.24073623	0.04375985
36.4	0.22691528	-0.3750205	0.59406717	-0.2244027	-0.3806447	0.62774514	0.19615903	0.2398193	0.04389603
36.5	0.22618855	-0.3757548	0.5917979	-0.2234012	-0.380922	0.62781497	0.19731567	0.2378771	0.04339257
36.6	0.2253073	-0.3755958	0.5906052	-0.2221938	-0.3816148	0.62749544	0.19697	0.23719047	0.04304145
36.7	0.22552196	-0.3758523	0.58941168	-0.2216615	-0.3817541	0.62711047	0.19709591	0.23638676	0.04251574
36.8	0.22574647	-0.3760006	0.58898959	-0.2210672	-0.3819548	0.62710816	0.19669676	0.23626894	0.04294468
36.9	0.22783472	-0.376561	0.58676084	-0.2197572	-0.3839458	0.62727334	0.19744698	0.23606631	0.04340398
37	0.22745041	-0.3768817	0.58599482	-0.2201431	-0.3847171	0.62682722	0.19733811	0.23546618	0.04199923
37.1	0.22865906	-0.3773385	0.58447826	-0.2190962	-0.385128	0.62688079	0.19636079	0.23510097	0.04234758
37.2	0.22881055	-0.3779562	0.58257494	-0.2179171	-0.3855925	0.62607722	0.19535918	0.23427833	0.04227583
37.3	0.2296223	-0.3784319	0.58145319	-0.2165227	-0.3873788	0.62673281	0.19543188	0.23179862	0.04242878
37.4	0.2295466	-0.3782248	0.58043229	-0.2162771	-0.3884144	0.62683111	0.19599994	0.23209536	0.0425836
37.5	0.23001272	-0.3794833	0.57972485	-0.2149543	-0.3891777	0.62717964	0.19788055	0.23218843	0.04262546
37.6	0.22974339	-0.3810412	0.57855109	-0.2134078	-0.3905293	0.62619407	0.19686722	0.23153504	0.04221482
37.7	0.23059948	-0.3818491	0.5779427	-0.213022	-0.3914384	0.62625936	0.19775986	0.23190141	0.0417768
37.8	0.23045453	-0.3814706	0.57714174	-0.2124752	-0.392347	0.62654537	0.19753823	0.23184711	0.04214288
37.9	0.23017457	-0.3828307	0.57609868	-0.2112151	-0.3941474	0.6269747	0.19755529	0.23241394	0.04277679
38	0.23010375	-0.3827366	0.57501164	-0.209471	-0.3941184	0.62715038	0.19743745	0.2308068	0.04188356
38.1	0.2309012	-0.3837184	0.57373933	-0.2091898	-0.3950372	0.62728027	0.19803848	0.2313332	0.04241974
38.2	0.2322098	-0.384392	0.57320718	-0.2091212	-0.3955267	0.62803667	0.19806599	0.23126721	0.04269585
38.3	0.23242072	-0.3843793	0.57222587	-0.2074732	-0.3963542	0.62841044	0.19925159	0.23063955	0.04352524
38.4	0.23391571	-0.3843634	0.57052741	-0.2076375	-0.3979168	0.62870823	0.20051849	0.23075605	0.04362329
38.5	0.2330806	-0.3852613	0.56915751	-0.207396	-0.3990438	0.62899303	0.20060899	0.22994262	0.04306354
38.6	0.23322533	-0.3856988	0.56872434	-0.2061688	-0.399817	0.6301224	0.20054461	0.22892021	0.04399374
38.7	0.23352965	-0.3859593	0.56653759	-0.204465	-0.4010745	0.62989582	0.19899004	0.22687142	0.04344396
38.8	0.23358885	-0.3847068	0.56531892	-0.2051946	-0.4027315	0.62904437	0.19828024	0.22643566	0.04290805
38.9	0.23301531	-0.3855138	0.5636268	-0.2052164	-0.4030362	0.62832823	0.19755283	0.22520999	0.04191728
39	0.23507524	-0.3850919	0.56126576	-0.203408	-0.404429	0.6284523	0.19708308	0.22455774	0.04371513
39.1	0.23660182	-0.3849281	0.55961677	-0.2006053	-0.4063614	0.62766049	0.19727756	0.22468033	0.04288465
39.2	0.23659815	-0.3849709	0.55852585	-0.1992379	-0.4075355	0.62699725	0.19684475	0.22366481	0.04252061
39.3	0.2376791	-0.3842904	0.55799299	-0.1986564	-0.4078894	0.62676232	0.19687582	0.22345589	0.04263843
39.4	0.23826206	-0.3835128	0.55614587	-0.198404	-0.409139	0.62755165	0.19855412	0.22426096	0.04286902
39.5	0.23997909	-0.3841185	0.55477357	-0.1979106	-0.4106751	0.62815636	0.19991524	0.22446144	0.04398782
39.6	0.2402212	-0.3839425	0.55255952	-0.1968644	-0.4120698	0.62832387	0.19964947	0.22270474	0.04358891
39.7	0.24008687	-0.3840171	0.55181443	-0.196015	-0.412454	0.62854157	0.19864677	0.22309005	0.04347741
39.8	0.2408826	-0.3841995	0.55024789	-0.195142	-0.4133369	0.62860957	0.1988138	0.22291362	0.04381122
39.9	0.24126123	-0.3850637	0.55006672	-0.194684	-0.4137173	0.62868613	0.19854355	0.22266974	0.04433011
40	0.24123201	-0.3854676	0.54764065	-0.19364	-0.4137401	0.62882807	0.19757358	0.22175574	0.04475363
40.1	0.24044021	-0.3856547	0.5454004	-0.1939767	-0.4142326	0.62914654	0.19619488	0.22073704	0.04434123
40.2	0.24132441	-0.3862452	0.54445203	-0.1946783	-0.4160919	0.62908764	0.19647295	0.22157919	0.04387685
40.3	0.24214443	-0.3856404	0.54313673	-0.1943735	-0.4169805	0.62893019	0.19623391	0.22167724	0.04456708
40.4	0.24233595	-0.3869436	0.54184738	-0.1934017	-0.4178485	0.62944514	0.19578599	0.2209594	0.04430472
40.5	0.24289828	-0.3863119	0.54068021	-0.1930877	-0.417499	0.62937174	0.19501461	0.21996639	0.04414619
40.6	0.2425309	-0.3862988	0.53974971	-0.1918948	-0.4185569	0.62987687	0.19396193	0.22036522	0.04408571
40.7	0.24264382	-0.3879687	0.53872756	-0.1911946	-0.4206316	0.63055373	0.19472334	0.21970708	0.04291514
40.8	0.24386264	-0.3884032	0.53722056	-0.1893332	-0.4212247	0.63021748	0.19480639	0.21783292	0.04238655
40.9	0.24476556	-0.3885442	0.53598827	-0.1898601	-0.4218618	0.63032051	0.19475342	0.21784902	0.04166962
41	0.24546956	-0.3905476	0.53413192	-0.1887589	-0.4224654	0.63046889	0.19547458	0.21696936	0.04158985
41.1	0.24531441	-0.3907755	0.53359357	-0.1881061	-0.4231453	0.63053884	0.19486262	0.2160207	0.04126758
41.2	0.24725116	-0.3913681	0.53245172	-0.1866441	-0.4247191	0.63027417	0.19484106	0.21646768	0.0418765
41.3	0.24769754	-0.3923732	0.5306121	-0.1862148	-0.4257945	0.63091786	0.19522319	0.2164777	0.04255475
41.4	0.24864559	-0.3920159	0.52941113	-0.1844477	-0.4272688	0.63039272	0.19529829	0.21497635	0.04257563
41.5	0.24779615	-0.3910404	0.52855076	-0.1839379	-0.4289243	0.63018579	0.19603052	0.21561789	0.04277242
41.6	0.24925321	-0.3912537	0.52724373	-0.1834837	-0.4300913	0.63004731	0.19647654	0.21579972	0.04246394
41.7	0.24992249	-0.3908809	0.52679012	-0.1823742	-0.4316708	0.63088708	0.19664246	0.21676751	0.04293121
41.8	0.25076017	-0.3906073	0.52530974	-0.1817638	-0.4339814	0.63176293	0.19949505	0.21762558	0.04330272
41.9	0.25055723	-0.3910146	0.52492733	-0.1808321	-0.4347948	0.63180355	0.19887269	0.21734894	0.04218085
42	0.25081832	-0.3904787	0.52465115	-0.1803883	-0.4349582	0.63184399	0.19854097	0.21731449	0.04262059
42.1	0.25130292	-0.3912095	0.52311684	-0.1804621	-0.4354712	0.63185532	0.19862384	0.21796196	0.0429585
42.2	0.25181653	-0.3920355	0.52213031	-0.1792272	-0.4370412	0.63241237	0.19943453	0.21705697	0.0434866
42.3	0.2514559	-0.392277	0.52004777	-0.1779968	-0.4376893	0.63201363	0.19906698	0.21738473	0.04353715
42.4	0.25172259	-0.3924862	0.51874444	-0.1776444	-0.4385126	0.63258768	0.19915255	0.21547512	0.04247273
42.5	0.25133432	-0.3932463	0.51627647	-0.1751778	-0.439345	0.63235338	0.19963024	0.21461896	0.04178193
42.6	0.25249357	-0.3919769	0.51504268	-0.1735792	-0.441042	0.6321056	0.20034752	0.21450487	0.04160025
42.7	0.25206939	-0.3932304	0.51371251	-0.1740756	-0.4418849	0.63180387	0.20192534	0.21325321	0.04115796
42.8	0.25207596	-0.3937644	0.51279142	-0.1738861	-0.442853	0.63198013	0.2030795	0.21392996	0.04160194
42.9	0.25269992	-0.3935404	0.51149398	-0.1737966	-0.444594	0.63248587	0.2020256	0.21354383	0.04246595
43	0.25240827	-0.3945409	0.51006037	-0.1737425	-0.4451628	0.63203837	0.20091839	0.21332694	0.04202875
43.1	0.25283086	-0.3961769	0.50885414	-0.1732414	-0.4466195	0.63267408	0.19993883	0.21312921	0.04149834
43.2	0.25328732	-0.3974056	0.50686817	-0.1725657	-0.4464513	0.63184317	0.19976048	0.21186732	0.04172622
43.3	0.2529838	-0.3981683	0.50569491	-0.1717522	-0.4470978	0.63192735	0.20056843	0.21187543	0.04227133
43.4	0.25255798	-0.3980016	0.50492664	-0.1709704	-0.4474607	0.63168459	0.20113038	0.21011775	0.04252406
43.5	0.25298541	-0.3977696	0.50377159	-0.1704833	-0.4484984	0.63194306	0.20151863	0.20955839	0.04336357
43.6	0.25332315	-0.3974626	0.50363184	-0.1702744	-0.4489763	0.63184299	0.20151752	0.20968529	0.04341382
43.7	0.2535604	-0.3982268	0.50334262	-0.1699726	-0.4497668	0.63279783	0.20135098	0.20963036	0.04275032
43.8	0.25453224	-0.3982431	0.50218431	-0.1698526	-0.4507872	0.63254813	0.20145369	0.20956831	0.04223151
43.9	0.25555945	-0.3992262	0.49966156	-0.1683549	-0.4523142	0.63226432	0.2023963	0.20974696	0.04204683
44	0.25646768	-0.398613	0.49858201	-0.168119	-0.4534624	0.63195694	0.20293697	0.21007013	0.04156551
44.1	0.25747981	-0.3986543	0.49734851	-0.1677856	-0.4543066	0.63155713	0.2028952	0.21003396	0.04169898
44.2	0.25895081	-0.3986128	0.49585719	-0.1675094	-0.456012	0.63177652	0.20141364	0.21096635	0.04256679
44.3	0.25814295	-0.3985504	0.49471144	-0.165784	-0.4569501	0.63183533	0.20194857	0.21025998	0.04245642
44.4	0.2588295	-0.3985583	0.49486449	-0.1652286	-0.457816	0.63206159	0.20243074	0.21080651	0.0421941
44.5	0.25884856	-0.3987794	0.49335566	-0.1642528	-0.458115	0.63182237	0.20242972	0.20998141	0.04218889
44.6	[illegible]	[illegible]	[illegible]	[illegible]	[illegible]	[illegible]	[illegible]	[illegible]	[illegible]
44.7	[illegible]	[illegible]	[illegible]	[illegible]	[illegible]	[illegible]	[illegible]	[illegible]	[illegible]
44.8	[illegible]	[illegible]	[illegible]	[illegible]	[illegible]	[illegible]	[illegible]	[illegible]	[illegible]
44.9	[illegible]	[illegible]	[illegible]	[illegible]	[illegible]	[illegible]	[illegible]	[illegible]	[illegible]
45	0.25905107	-0.3996884	0.48720808	-0.1587327	-0.4605478	0.63292447	0.20398342	0.20930429	0.0445472
45.1	0.25954847	-0.3998429	0.48634474	-0.1577232	-0.4614639	0.63284474	0.20446057	0.20832786	0.04387814
45.2	0.26006344	-0.400258	0.48502094	-0.1574137	-0.462595	0.63283029	0.20455252	0.20863412	0.04369374
45.3	0.26115347	-0.4007307	0.48391637	-0.1564702	-0.4637078	0.63368089	0.2049396	0.20913114	0.04482761
45.4	0.26230849	-0.4036	0.48108282	-0.1561935	-0.4662537	0.63341951	0.20552851	0.20802418	0.04563074
45.5	0.26265753	-0.404968	0.47991316	-0.1555418	-0.4673259	0.63283604	0.2062057	0.2087495	0.04541472
45.6	0.26294062	-0.4050339	0.47896657	-0.1542822	-0.4678462	0.63231474	0.2059121	0.20922854	0.04501094
45.7	0.26240276	-0.4058955	0.47788919	-0.1529356	-0.4696255	0.63316125	0.20660389	0.20984451	0.04560954
45.8	0.2627095	-0.4058052	0.47649318	-0.1527488	-0.4708474	0.63306771	0.20763825	0.20913731	0.04622203
45.9	0.26431739	-0.405847	0.4747203	-0.1521966	-0.4712193	0.63313172	0.2074129	0.20649663	0.04656219
46	0.26554252	-0.4059948	0.47348317	-0.1515231	-0.4719434	0.63307717	0.20723689	0.20731556	0.04650584
46.1	0.26701053	-0.4070325	0.47271531	-0.1515958	-0.4738742	0.63416954	0.20804033	0.20761964	0.04680769
46.2	0.26731427	-0.4074985	0.47211291	-0.1518599	-0.4739128	0.63375603	0.20728041	0.20760273	0.04650806
46.3	0.2684336	-0.4077278	0.47119295	-0.1502928	-0.4755365	0.63391678	0.2084631	0.20784929	0.04627337
46.4	0.26955318	-0.4081251	0.46924539	-0.1508049	-0.4760193	0.63404365	0.20867253	0.20817557	0.04634108
46.5	0.26931564	-0.4088775	0.46814307	-0.1493112	-0.4774469	0.63399594	0.20729118	0.20617224	0.04651111
46.6	0.27026932	-0.408943	0.46631801	-0.1487217	-0.4773088	0.63348602	0.20792792	0.20405835	0.04505229
46.7	0.27064542	-0.4086408	0.46553579	-0.1490446	-0.4772145	0.63318942	0.20789628	0.20378421	0.04486988
46.8	0.27165056	-0.4080409	0.46373637	-0.1477878	-0.4784968	0.63273896	0.20838683	0.20340008	0.04514993
46.9	0.27242359	-0.408188	0.4619464	-0.1456525	-0.4796663	0.63335655	0.20839172	0.20294013	0.04474803
47	0.27456907	-0.4104446	0.46000528	-0.1449633	-0.4815843	0.63333533	0.20801698	0.20286445	0.04570823
47.1	0.27461515	-0.4108539	0.45794529	-0.1447049	-0.4816472	0.6329864	0.20976324	0.20308048	0.04585419
47.2	0.27440864	-0.4103555	0.45736191	-0.1437395	-0.4823793	0.63295932	0.21048962	0.2022717	0.04572439
47.3	0.27501249	-0.4098674	0.45614386	-0.1429094	-0.4829711	0.63172128	0.21143796	0.20236413	0.04449784
47.4	0.27599148	-0.4091675	0.45458279	-0.1428285	-0.4838051	0.6312698	0.21153897	0.20104523	0.04444513
47.5	0.27629694	-0.4101898	0.45305105	-0.1420481	-0.4847585	0.63229877	0.21130478	0.20025216	0.04387099
47.6	0.27623282	-0.4110872	0.45141859	-0.1416962	-0.4850659	0.63159121	0.21096418	0.19973475	0.04325601
47.7	0.27668784	-0.4109949	0.44947057	-0.1410505	-0.4861725	0.63158506	0.21189742	0.20014471	0.0434656
47.8	0.27626124	-0.4119316	0.44869711	-0.1403243	-0.4876048	0.63178492	0.21237015	0.19955673	0.04330193
47.9	0.2761564	-0.4121751	0.44855155	-0.1399361	-0.4885209	0.63177059	0.21193579	0.19861558	0.04266736
48	0.2766907	-0.4123332	0.44731413	-0.1391757	-0.4888887	0.63152986	0.21143892	0.1976419	0.04284472
48.1	0.27589776	-0.4127042	0.44623364	-0.1383953	-0.4898865	0.6311834	0.21228584	0.19688694	0.0422501
48.2	0.27663011	-0.4138589	0.44454203	-0.1383743	-0.4912163	0.63068095	0.21345043	0.19725684	0.04323336
48.3	0.27880205	-0.4125	0.44287254	-0.1373605	-0.4929578	0.63088625	0.21372957	0.19819606	0.04373192
48.4	0.27941319	-0.4119172	0.44131201	-0.1363855	-0.4927381	0.63053112	0.21380724	0.19834162	0.04459369
48.5	0.28066188	-0.4119568	0.43929366	-0.1367437	-0.4934335	0.63061993	0.21381055	0.19827876	0.04536526
48.6	0.28031995	-0.4127839	0.43816342	-0.1355978	-0.494427	0.63058159	0.21503416	0.19731876	0.0444682
48.7	0.28118347	-0.4134293	0.43487822	-0.1338565	-0.4958823	0.63045119	0.21597425	0.19787784	0.04537398
48.8	0.28161467	-0.4136615	0.43380514	-0.1332249	-0.4965733	0.63027419	0.21523689	0.19807358	0.04581635
48.9	0.28117587	-0.4140104	0.43173706	-0.1318708	-0.4971399	0.63012621	0.21380031	0.19806761	0.04560801
49	0.28153074	-0.4162963	0.43067277	-0.1316136	-0.4982133	0.62987737	0.21493467	0.1983514	0.04630312
49.1	0.28194389	-0.4163886	0.43004003	-0.1305787	-0.4991957	0.62979331	0.21505061	0.19844778	0.04631321
49.2	0.2828196	-0.4168031	0.42925827	-0.1289222	-0.5011069	0.63062097	0.21501515	0.19858695	0.04617618
49.3	0.28370969	-0.4168721	0.42872	-0.1283042	-0.5021619	0.63058562	0.2144448	0.198765	0.04634967
49.4	0.28328523	-0.4175628	0.426887	-0.1277586	-0.502569	0.63003342	0.21445803	0.19871785	0.04785655
49.5	0.28351623	-0.4183037	0.42506747	-0.1274632	-0.5033995	0.62967637	0.21500534	0.197536	0.04780008
49.6	0.28448809	-0.4178922	0.42401575	-0.127217	-0.5039611	0.6295111	0.21480028	0.19779037	0.04667708
49.7	0.2846049	-0.4167862	0.42241477	-0.1260597	-0.5040136	0.62893242	0.21496523	0.19776416	0.04703767
49.8	0.28489224	-0.4180218	0.42173719	-0.1265482	-0.5048101	0.62874248	0.2150244	0.19815973	0.04669048
49.9	0.28496484	-0.4182039	0.42148803	-0.1261346	-0.5055778	0.62849174	0.21515953	0.19852014	0.04654254
50	0.28429177	-0.419203	0.42122752	-0.1259536	-0.5068278	0.62911001	0.21456449	0.1983967	0.04651624
50.1	0.28428945	-0.4196352	0.41956234	-0.1259295	-0.5078991	0.62915538	0.21410555	0.19854107	0.04658674
50.2	0.28572476	-0.4195982	0.41821724	-0.1246743	-0.5077208	0.62885458	0.213651	0.19773758	0.04723626
50.3	0.28534135	-0.4197389	0.41652557	-0.1241286	-0.5091131	0.62870382	0.21297094	0.19713996	0.04762001
50.4	0.28633783	-0.4209714	0.41516844	-0.1222454	-0.5107639	0.62859401	0.21229944	0.19634629	0.04668785
50.5	0.28716067	-0.4210015	0.41395952	-0.1228664	-0.5113465	0.62846429	0.2112814	0.19667863	0.04587174
50.6	0.28704951	-0.4211854	0.41359831	-0.12177	-0.5120229	0.62880999	0.21123214	0.19627289	0.04547381
50.7	0.28873858	-0.4219168	0.41143723	-0.1220552	-0.5135481	0.6285	0.2113626	0.19577951	0.04531863
50.8	0.28940143	-0.4223267	0.410975	-0.1221957	-0.514022	0.62796941	0.21134248	0.19518899	0.04478064
50.9	0.29101754	-0.4217196	0.41029916	-0.1210938	-0.5150475	0.62816958	0.21194884	0.19532837	0.04556864
51	0.29172997	-0.4222715	0.40807011	-0.1185305	-0.5166494	0.62801588	0.21213134	0.19579363	0.0462376
51.1	0.29136518	-0.4223221	0.40708538	-0.1179799	-0.5175623	0.62840377	0.21371458	0.19653535	0.04703689
51.2	0.29160773	-0.4222622	0.40678876	-0.1173875	-0.5180244	0.62849595	0.21346835	0.19558701	0.04706675
51.3	0.29205897	-0.4223522	0.40564212	-0.1166812	-0.5184586	0.62838304	0.21365692	0.19517479	0.04736357
51.4	0.29302414	-0.4223394	0.40405837	-0.1155226	-0.5179966	0.62751132	0.21285147	0.19577166	0.04736547
51.5	0.29429743	-0.4225667	0.40327474	-0.1156751	-0.5194467	0.62692356	0.21160593	0.1961328	0.04844963
51.6	0.29511285	-0.4229773	0.40277493	-0.1147684	-0.5202828	0.62708156	0.21156324	0.19721452	0.048566
51.7	0.29676912	-0.4241507	0.40210131	-0.1157649	-0.5219516	0.62690982	0.21088534	0.19749867	0.04765895
51.8	0.29712927	-0.4228248	0.40050247	-0.1135916	-0.522457	0.62577878	0.20914828	0.19571373	0.04701994
51.9	0.29780912	-0.4231404	0.39891897	-0.1124813	-0.5243396	0.62602048	0.20908905	0.19456244	0.04849586
52	0.29700889	-0.4225224	0.39763179	-0.1115704	-0.5242204	0.62597997	0.20850694	0.19493774	0.04728244
52.1	0.2975323	-0.4234448	0.39749325	-0.1117912	-0.5250772	0.62619379	0.20908116	0.19480817	0.04802604
52.2	0.29826268	-0.4241352	0.39695974	-0.1113785	-0.5257548	0.62609823	0.20850444	0.1948814	0.04868992
52.3	0.29857263	-0.4240971	0.39594623	-0.110063	-0.5264445	0.62596194	0.20826283	0.19508988	0.04857468
52.4	0.29830571	-0.4239896	0.39392498	-0.1101104	-0.5271871	0.62528786	0.20950445	0.19454662	0.04781544
52.5	0.29916501	-0.4247556	0.39106415	-0.1092177	-0.5279861	0.62457779	0.21024268	0.19429075	0.04823879
52.6	0.29948204	-0.425299	0.39084552	-0.1075932	-0.5285019	0.62477772	0.20971208	0.1935886	0.04784384
52.7	0.29957613	-0.4256427	0.3904958	-0.1078633	-0.5290323	0.62482261	0.20975498	0.19217935	0.04758549
52.8	0.30111278	-0.4251066	0.38793113	-0.1045124	-0.5301901	0.62446824	0.210434	0.19024896	0.04856426
52.9	0.30206031	-0.4238614	0.38590007	-0.1046509	-0.5317817	0.62363769	0.21020368	0.19164404	0.04902125
53	0.30221272	-0.4242467	0.38553762	-0.10444	-0.532387	0.6234707	0.21020977	0.19083795	0.04886446
53.1	0.30213653	-0.4252182	0.38318001	-0.1039001	-0.5335344	0.62348643	0.21054392	0.19062036	0.04796802
53.2	0.30147606	-0.4258852	0.38143425	-0.1040632	-0.5341372	0.62343401	0.21113589	0.19123225	0.04792749
53.3	0.30268918	-0.4256482	0.3806993	-0.1034164	-0.5345435	0.62311759	0.21091233	0.1913354	0.048311
53.4	0.30305609	-0.4259685	0.3801797	-0.1031831	-0.5359272	0.62326319	0.21041721	0.19011534	0.04746572
53.5	0.30327447	-0.425739	0.37852719	-0.1016823	-0.5372186	0.62257615	0.20990974	0.18898764	0.04529777
53.6	0.30335271	-0.4252403	0.37795579	-0.1023818	-0.5380045	0.62196538	0.20899028	0.18953788	0.04466289
53.7	0.30411056	-0.425707	0.37738223	-0.1023846	-0.5382459	0.62210765	0.20998613	0.18965714	0.04349997
53.8	0.30473017	-0.4257538	0.37594102	-0.1011319	-0.5393989	0.62126387	0.21039419	0.18939813	0.04355834
53.9	0.30534816	-0.4255823	0.37596235	-0.0996742	-0.5402599	0.62127834	0.21109098	0.1891355	0.04405933
54	0.30740406	-0.4265532	0.374993	-0.098893	-0.5425858	0.62157822	0.21120865	0.18888761	0.04410879
54.1	0.30860106	-0.4259374	0.37312993	-0.0989023	-0.543346	0.62041811	0.21186105	0.18925294	0.04338793
54.2	0.3086073	-0.4258679	0.3728018	-0.0987333	-0.5434346	0.62038103	0.21199525	0.18905862	0.04326339
54.3	0.30937166	-0.4261752	0.37143715	-0.0978384	-0.5445455	0.62026724	0.21211727	0.18933324	0.04357241
54.4	0.30978959	-0.425847	0.3712095	-0.0977537	-0.5449073	0.62010436	0.21315903	0.18933993	0.04299342
54.5	0.31047289	-0.4260797	0.37065256	-0.0966817	-0.5455698	0.62041494	0.21292302	0.18817475	0.04269487
54.6	0.31095654	-0.4268013	0.36960631	-0.0979932	-0.5467235	0.6200504	0.21367594	0.18889677	0.04366005
54.7	0.31175799	-0.4271999	0.36856925	-0.0971386	-0.5481517	0.62047543	0.21429631	0.18948002	0.04472624
54.8	0.31345439	-0.4282707	0.36645551	-0.0956945	-0.5491566	0.62037607	0.2148669	0.19012521	0.04450031
54.9	0.31304326	-0.4283281	0.36529945	-0.095236	-0.5490834	0.62015395	0.21478448	0.1898335	0.04380009
55	0.31348469	-0.4283689	0.36362896	-0.0942631	-0.5495528	0.61944743	0.21678607	0.18860982	0.04330376
55.1	0.31297462	-0.4281577	0.3609154	-0.0935677	-0.5495525	0.61822366	0.21698263	0.1872225	0.04223813
55.2	0.31457916	-0.4299197	0.35961132	-0.0922139	-0.5520139	0.61838977	0.21761272	0.18871918	0.04296314
55.3	0.31508684	-0.4294646	0.35864808	-0.0912427	-0.5530317	0.61827433	0.21730651	0.18703228	0.04337856
55.4	0.31556368	-0.4294622	0.35642694	-0.0904754	-0.5535136	0.61732019	0.21776211	0.1864388	0.04211598
55.5	0.3160054	-0.4290526	0.35543927	-0.0916009	-0.5544728	0.61657523	0.21788842	0.18695695	0.0423903
55.6	0.3161447	-0.4292149	0.35335198	-0.0893661	-0.5553287	0.61580509	0.21631599	0.18573741	0.04249473
55.7	0.31704547	-0.4298505	0.35205336	-0.0885329	-0.556853	0.61641029	0.2174952	0.18566942	0.04318919
55.8	0.31797759	-0.4296601	0.35089099	-0.0874694	-0.5582569	0.61627279	0.21848675	0.18627233	0.04337794
55.9	0.31840022	-0.4298095	0.34974463	-0.0853183	-0.559084	0.61665027	0.21774881	0.1843114	0.04417276
56	0.31946124	-0.4297623	0.34823727	-0.0844636	-0.5595069	0.61616575	0.21726842	0.1838159	0.0440418
56.1	0.32119088	-0.4299772	0.34755587	-0.0828693	-0.5606266	0.61578412	0.2184622	0.18349973	0.04378739
56.2	0.32257417	-0.4304982	0.34664332	-0.0812266	-0.5621347	0.61550046	0.21859848	0.18383206	0.04304488
56.3	0.32291465	-0.4303557	0.34575507	-0.0809113	-0.5628238	0.61472825	0.21878068	0.1830169	0.04269569
56.4	0.32327884	-0.4309361	0.34534115	-0.0806556	-0.563684	0.61465393	0.21916807	0.18336292	0.04240561
56.5	0.32520216	-0.4320541	0.3431443	-0.0787438	-0.5643037	0.61423582	0.21785063	0.1825346	0.0416551
56.6	0.32521901	-0.4332307	0.34255291	-0.0780465	-0.5654115	0.61412485	0.21947566	0.18254352	0.04123466
56.7	0.32707882	-0.4342598	0.3398887	-0.0782369	-0.5676747	0.61339041	0.21973873	0.18296878	0.04119622
56.8	0.32804063	-0.434159	0.33801235	-0.0780317	-0.5681889	0.6134155	0.2197723	0.18237281	0.04106672
56.9	0.32897278	-0.433574	0.33740201	-0.0776491	-0.5685718	0.61280956	0.21950473	0.18155891	0.04175016
57	0.32923801	-0.4337409	0.33721048	-0.0776415	-0.5692282	0.6124453	0.21945184	0.18135887	0.04139932
57.1	0.32963597	-0.4333512	0.33578462	-0.0762207	-0.5696433	0.61194382	0.21862626	0.18172899	0.04247311
57.2	0.33026843	-0.4332253	0.33392761	-0.0755304	-0.5708938	0.61166891	0.2184314	0.18238867	0.04236339
57.3	0.33037108	-0.4333824	0.332595	-0.07502	-0.5719615	0.61077161	0.2191404	0.18253635	0.04249866
57.4	0.33128459	-0.4347841	0.33094304	-0.0751036	-0.5737334	0.61098548	0.2197974	0.18348166	0.04164599
57.5	0.33074766	-0.4351091	0.33069826	-0.0751102	-0.573909	0.61034353	0.2197214	0.18295994	0.04147059
57.6	0.33087888	-0.4359784	0.33012381	-0.076038	-0.575005	0.61078448	0.22053796	0.18371963	0.04259066
57.7	0.33101897	-0.435821	0.32879546	-0.0748623	-0.5753308	0.61087042	0.22088422	0.18285161	0.04325202
57.8	0.33188878	-0.4361854	0.32794476	-0.0745069	-0.5765887	0.61100886	0.22217029	0.18232103	0.04236628
57.9	0.33281386	-0.4358001	0.32681258	-0.0737563	-0.5779716	0.61063868	0.22182626	0.18295282	0.04173766
58	0.333025	-0.4351948	0.32645772	-0.0720523	-0.5787211	0.61080818	0.22174961	0.18345801	0.04187276
58.1	0.33398591	-0.4344566	0.32592102	-0.0712988	-0.5796544	0.60998407	0.22268827	0.18311337	0.04181008
58.2	0.3339292	-0.435696	0.32392462	-0.0693144	-0.5809854	0.61035906	0.22377276	0.1820616	0.04157465
58.3	0.33406551	-0.4354	0.32380063	-0.0688747	-0.5809093	0.60935417	0.2248225	0.18235151	0.04139477
58.4	0.33495312	-0.4351024	0.32265422	-0.0677679	-0.5818855	0.60878698	0.22433506	0.18276075	0.04202493
58.5	0.33544008	-0.4356605	0.32062382	-0.0673713	-0.5827289	0.60912335	0.22478202	0.18251078	0.04306232
58.6	0.33556076	-0.4360085	0.3198353	-0.0674762	-0.5834982	0.60896329	0.22496611	0.18180182	0.04335665
58.7	0.33628833	-0.4357338	0.31736758	-0.0677584	-0.5839843	0.60856069	0.22640979	0.18213621	0.04414605
58.8	0.33668112	-0.4360177	0.31638241	-0.0664796	-0.5848859	0.60837944	0.22721323	0.18143694	0.04341168
58.9	0.33645346	-0.4374595	0.31567256	-0.065661	-0.5857423	0.60831207	0.22741867	0.18175944	0.04330119
59	0.33657331	-0.436986	0.31511411	-0.0658858	-0.5863608	0.60825975	0.22814273	0.18137945	0.04319663
59.1	0.33729487	-0.4361692	0.31321123	-0.0649024	-0.5870617	0.6076709	0.22866701	0.18136094	0.04386613
59.2	0.33740676	-0.4365758	0.31303481	-0.0653574	-0.5877351	0.608018	0.22869078	0.18125212	0.04387506
59.3	0.33651501	-0.4369237	0.3112943	-0.063745	-0.5887119	0.60716233	0.22987187	0.18060744	0.04395911
59.4	0.33616657	-0.4371731	0.30926895	-0.063109	-0.5881928	0.60660244	0.22967585	0.18011326	0.04328769
59.5	0.33729709	-0.4368762	0.30696185	-0.0625168	-0.5891255	0.60656455	0.22852435	0.17949246	0.04345567
59.6	[illegible]	[illegible]	[illegible]	[illegible]	[illegible]	[illegible]	[illegible]	[illegible]	[illegible]
59.7	[illegible]	[illegible]	[illegible]	[illegible]	[illegible]	[illegible]	[illegible]	[illegible]	[illegible]
59.8	[illegible]	[illegible]	[illegible]	[illegible]	[illegible]	[illegible]	[illegible]	[illegible]	[illegible]
59.9	[illegible]	[illegible]	[illegible]	[illegible]	[illegible]	[illegible]	[illegible]	[illegible]	[illegible]

60	0.33817882	-0.4378511	0.29978534	-0.0604832	-0.5925698	0.60448595	0.22960831	0.17673892	0.04291807
60.1	0.33819293	-0.4389261	0.29763415	-0.0608159	-0.5924367	0.60378756	0.22947829	0.17699355	0.04205752
60.2	0.33885285	-0.4392335	0.29746061	-0.0612528	-0.592759	0.60377125	0.2302795	0.17615632	0.04262856
60.3	0.3402656	-0.4412369	0.29571073	-0.0612085	-0.594358	0.60328733	0.23107573	0.17614919	0.04268495
60.4	0.34025631	-0.441631	0.2943135	-0.0604587	-0.5954476	0.60233043	0.23095208	0.17603702	0.0417833
60.5	0.34076739	-0.4411972	0.29266467	-0.0587221	-0.5961058	0.60181211	0.23237499	0.17623281	0.04267596
60.6	0.34147612	-0.4417841	0.29138354	-0.0584866	-0.5963324	0.60141269	0.23223922	0.17490962	0.04354972
60.7	0.34151041	-0.4429943	0.28893059	-0.0564366	-0.5977138	0.60186397	0.23140054	0.17523605	0.04360778
60.8	0.34055447	-0.443578	0.28731957	-0.0565315	-0.5985776	0.60089914	0.23104447	0.17354389	0.04445916
60.9	0.34147186	-0.4438299	0.28613795	-0.0569888	-0.5987004	0.60075666	0.23149678	0.17331267	0.04518658
61	0.34091181	-0.4442879	0.28439779	-0.0561021	-0.5989007	0.60064537	0.23148586	0.17474995	0.04501246
61.1	0.34172826	-0.4446654	0.28220151	-0.0569932	-0.5995088	0.60021159	0.23218377	0.17401519	0.04510308
61.2	0.34237099	-0.4441929	0.28040296	-0.0569872	-0.5997154	0.59966422	0.23261809	0.17337381	0.04488024
61.3	0.34214052	-0.4444042	0.27913793	-0.0567772	-0.6007456	0.59947616	0.23243695	0.17399103	0.04617103
61.4	0.34242219	-0.4448435	0.2782081	-0.0557775	-0.6014504	0.59917023	0.23159077	0.17307406	0.04563101
61.5	0.34261967	-0.4458367	0.27695553	-0.054597	-0.6014902	0.59854321	0.23166594	0.17326082	0.04515659
61.6	0.34290474	-0.4453857	0.27627596	-0.0550409	-0.601311	0.59795489	0.23089168	0.17267798	0.04477127
61.7	0.34378101	-0.4452149	0.27411129	-0.0541828	-0.6022921	0.59764738	0.23141178	0.17343997	0.04659913
61.8	0.34424033	-0.4448272	0.27246557	-0.0535249	-0.6022228	0.59623785	0.23226032	0.1741046	0.04583587
61.9	0.34443073	-0.4444603	0.2710822	-0.0524596	-0.6028283	0.59580314	0.23197966	0.17423697	0.0467545
62	0.34477409	-0.4444699	0.26877257	-0.0518968	-0.6027362	0.59447435	0.23307714	0.17433052	0.04634512
62.1	0.34541316	-0.4449998	0.26849613	-0.0522301	-0.6030057	0.59423407	0.2321249	0.1741868	0.04635568
62.2	0.34526268	-0.4450484	0.26626222	-0.0520903	-0.603365	0.59446459	0.23316768	0.17373205	0.04628791
62.3	0.34511699	-0.4441284	0.26589949	-0.0516011	-0.6035482	0.59389554	0.23313335	0.173121	0.04575445
62.4	0.34489962	-0.4442903	0.26552174	-0.0508791	-0.6037004	0.59371118	0.23287972	0.17169044	0.04532746
62.5	0.34512057	-0.4439442	0.26429102	-0.0503055	-0.6040735	0.59360272	0.23244278	0.17123406	0.04459007
62.6	0.34497402	-0.4446061	0.26243898	-0.0492734	-0.6043789	0.59373925	0.23314931	0.17118998	0.04499416
62.7	0.34503671	-0.4452784	0.26125945	-0.0487228	-0.6048792	0.59345202	0.23414088	0.17031623	0.04558659
62.8	0.34605227	-0.4448428	0.26099868	-0.049174	-0.6056943	0.59344782	0.23512785	0.17085082	0.04539909
62.9	0.34593378	-0.4454886	0.25975234	-0.0483121	-0.6060092	0.59271957	0.23488615	0.17044134	0.04509223
63	0.34661806	-0.4456506	0.25920623	-0.0482448	-0.6061663	0.59220422	0.23532976	0.17096534	0.04591405
63.1	0.34718887	-0.4468923	0.25800108	-0.0474853	-0.6073895	0.59239274	0.23600975	0.17164114	0.04615451
63.2	0.34696806	-0.4475755	0.25691423	-0.0473678	-0.6075789	0.59243317	0.237117	0.17093754	0.04599317
63.3	0.34758611	-0.4477313	0.25646526	-0.0475704	-0.6086725	0.59248274	0.23669296	0.17157737	0.04632022
63.4	0.34869677	-0.4486418	0.25471245	-0.047441	-0.6094701	0.59199041	0.23706598	0.17056783	0.04662889
63.5	0.34897358	-0.4488611	0.25287209	-0.0451769	-0.6111381	0.5918722	0.23689766	0.16853016	0.04700134
63.6	0.3490931	-0.4498421	0.25194398	-0.0451899	-0.6121284	0.59179785	0.23789543	0.16847827	0.04652105
63.7	0.35051805	-0.450645	0.25038503	-0.0455601	-0.6128989	0.5912001	0.23875991	0.16923795	0.04804143
63.8	0.35240955	-0.4504953	0.24865477	-0.0461657	-0.6134978	0.59062145	0.23888962	0.16910139	0.04853612
63.9	0.35339674	-0.4516922	0.24675424	-0.046707	-0.6142621	0.5901447	0.23916731	0.1686146	0.04796971
64	0.35347001	-0.451464	0.24584465	-0.0463927	-0.6142033	0.58927576	0.23800231	0.16846822	0.04706526
64.1	0.3546124	-0.4519299	0.24507543	-0.0466066	-0.6151307	0.58883454	0.23892095	0.16801705	0.04750253
64.2	0.35650973	-0.4519834	0.24295181	-0.0454454	-0.6157781	0.58867769	0.24079047	0.16679852	0.04755294
64.3	0.35624513	-0.4531571	0.24239251	-0.0447103	-0.6162136	0.58915535	0.24188176	0.16572622	0.04704504
64.4	0.35675008	-0.4534481	0.24160154	-0.0441323	-0.6168072	0.58924852	0.24148955	0.16602134	0.04784048
64.5	0.35880628	-0.4544212	0.23788041	-0.0439345	-0.6173798	0.58776888	0.24210404	0.16499013	0.04840979
64.6	0.35949559	-0.4547691	0.23542312	-0.043431	-0.61797	0.58828923	0.24284028	0.16428927	0.04925101
64.7	0.36081019	-0.4561693	0.23451293	-0.0430035	-0.618956	0.58865429	0.24281141	0.16503093	0.0494921
64.8	0.36247375	-0.4564169	0.2333652	-0.0427803	-0.6196138	0.58839472	0.24274628	0.16425135	0.0491628
64.9	0.36297406	-0.4564873	0.2326192	-0.0425215	-0.6206609	0.58749016	0.2431394	0.16401235	0.0492243
65	0.36345639	-0.4568753	0.23200092	-0.0419533	-0.6216183	0.58717586	0.24474574	0.16287065	0.05037644
65.1	0.36376772	-0.4577215	0.23051404	-0.0415407	-0.6228368	0.58676315	0.24507106	0.16267486	0.05092791
65.2	0.3649728	-0.4575236	0.2285265	-0.0424209	-0.6237665	0.5861999	0.2459117	0.16211898	0.05130127
65.3	0.36457606	-0.4584579	0.22870961	-0.0421731	-0.6242506	0.58596402	0.24636546	0.16130132	0.05046641
65.4	0.36441702	-0.4590017	0.22706617	-0.0422218	-0.6248853	0.58459799	0.24665473	0.16038792	0.05142748
65.5	0.36446134	-0.45884	0.2253648	-0.0408952	-0.6247703	0.58402197	0.24743816	0.15895592	0.05126436
65.6	0.36529228	-0.4593147	0.2244249	-0.0405126	-0.6258527	0.58391279	0.24792933	0.1585041	0.05215495
65.7	0.3657553	-0.4600436	0.22165922	-0.0386107	-0.6267844	0.58371881	0.24956433	0.15649919	0.05278356
65.8	0.36669175	-0.459599	0.21931523	-0.0377597	-0.627178	0.58313713	0.2501925	0.15629308	0.05461139
65.9	0.36749209	-0.4603019	0.21815933	-0.0380095	-0.6278018	0.58356049	0.25042821	0.15623461	0.05497247
66	0.36840797	-0.4609812	0.21754495	-0.0384329	-0.6288725	0.58275704	0.24997261	0.1556886	0.05429523
66.1	0.36740682	-0.4621815	0.21616643	-0.0383793	-0.6292255	0.5814583	0.25104884	0.15364613	0.05335946
66.2	0.36756712	-0.4625897	0.2136858	-0.0382725	-0.6303925	0.58061029	0.25107547	0.15244868	0.05467411
66.3	0.36736387	-0.4631843	0.21268261	-0.0372299	-0.6305077	0.58040146	0.25130684	0.15157304	0.05446484
66.4	0.36720933	-0.4638237	0.21250368	-0.0366469	-0.6305428	0.57988334	0.25208015	0.15107606	0.05338855
66.5	0.36767701	-0.4637615	0.21122343	-0.0370332	-0.6306749	0.57894929	0.25368718	0.15037938	0.05362877
66.6	0.36840887	-0.4638863	0.20996499	-0.0349616	-0.6317739	0.57894086	0.25522062	0.14798232	0.05299461
66.7	0.3696389	-0.463592	0.20823295	-0.0343913	-0.6317264	0.57796156	0.25593246	0.14758297	0.05268312
66.8	0.36904188	-0.4630747	0.20754883	-0.0346019	-0.6314311	0.57731826	0.2564016	0.14658034	0.05302586
66.9	0.37026801	-0.4618879	0.20617881	-0.0339588	-0.6321674	0.57712338	0.25618394	0.14627306	0.05423075
67	0.37082695	-0.4627069	0.20609885	-0.0337068	-0.6326985	0.576998	0.25524673	0.14533137	0.05510957
67.1	0.37172587	-0.4628769	0.20600269	-0.0332291	-0.633254	0.57712696	0.25534485	0.14548031	0.05597088
67.2	0.37164981	-0.4633342	0.20448068	-0.0329223	-0.6345211	0.57634769	0.25530588	0.14509406	0.05670655
67.3	0.37214301	-0.4636273	0.20429648	-0.0326825	-0.6349832	0.57602581	0.25681027	0.14443178	0.05759561
67.4	0.37341401	-0.4636366	0.20356317	-0.0323873	-0.6355208	0.57624551	0.25782587	0.1435893	0.05804534
67.5	0.37383105	-0.4651789	0.20148201	-0.0326775	-0.6366304	0.57574589	0.25972409	0.14284625	0.05815197
67.6	0.37446681	-0.4660009	0.20027093	-0.0320913	-0.6377092	0.5755504	0.26011166	0.14171216	0.05843663
67.7	0.375099	-0.4664464	0.19966603	-0.0299098	-0.6384913	0.57574212	0.26087665	0.14072964	0.05991931
67.8	0.37635292	-0.4670557	0.19893253	-0.0296865	-0.6389855	0.57591735	0.26145402	0.14148988	0.06112729
67.9	0.37745591	-0.4676706	0.19934828	-0.0294796	-0.6401774	0.57629333	0.26206358	0.14200984	0.06061422
68	0.37816075	-0.4669727	0.19886125	-0.0295812	-0.6405791	0.57557952	0.26366281	0.14248932	0.06183111
68.1	0.37859486	-0.4675022	0.19847668	-0.0293684	-0.6411385	0.57517476	0.26423696	0.14181746	0.06238856
68.2	0.379003	-0.4678121	0.19839584	-0.0302417	-0.6417048	0.57427412	0.26397942	0.14143107	0.06184432
68.3	0.37988509	-0.4684356	0.19714911	-0.0290741	-0.6420104	0.57388794	0.26369886	0.13975674	0.06153029
68.4	0.38035709	-0.4693826	0.19618714	-0.027936	-0.6430722	0.57358002	0.26424613	0.13952256	0.06097171
68.5	0.38119138	-0.4697396	0.19455477	-0.0280943	-0.6436439	0.57309075	0.26430179	0.13910833	0.06092696
68.6	0.38095551	-0.4704687	0.19313748	-0.0281009	-0.6437668	0.57332128	0.2645671	0.13848939	0.06134335
68.7	0.38071799	-0.4700599	0.19196612	-0.0289502	-0.6439227	0.57289709	0.2649814	0.13904906	0.0608708
68.8	0.38108404	-0.470818	0.19043809	-0.027776	-0.6441391	0.57248976	0.26479855	0.1384118	0.06049314
68.9	0.38099752	-0.4717854	0.19009616	-0.0275277	-0.6451922	0.57204654	0.26563302	0.13832201	0.0592166
69	0.3804483	-0.4721949	0.19006438	-0.0271371	-0.6462772	0.57219158	0.26663903	0.13826382	0.0601079
69.1	0.38159415	-0.4716973	0.19011706	-0.0266139	-0.6471262	0.57191088	0.26779047	0.13848716	0.06116767
69.2	0.3821627	-0.4713801	0.18895537	-0.0261294	-0.6478776	0.57197395	0.26874158	0.13792934	0.06087894
69.3	0.38242509	-0.4710799	0.18769081	-0.0258001	-0.6482663	0.57161844	0.26976402	0.13789026	0.06136317
69.4	0.38231594	-0.4708129	0.18714412	-0.0257296	-0.6488379	0.57150702	0.27089162	0.1375756	0.06154933
69.5	0.3825325	-0.4705524	0.18728316	-0.0248454	-0.6492332	0.57126038	0.27194641	0.13613829	0.06208068
69.6	0.3823477	-0.47173	0.18635052	-0.0232085	-0.6501422	0.57127376	0.27259434	0.13544943	0.06178225
69.7	0.38306527	-0.4713025	0.18453345	-0.0226165	-0.6506896	0.57106568	0.27326748	0.13439998	0.06196937
69.8	0.38337235	-0.4713009	0.18320849	-0.0217378	-0.6513031	0.57108669	0.27336978	0.13363253	0.0622317
69.9	0.38277954	-0.4714908	0.1828716	-0.0210671	-0.6514111	0.57070748	0.27371771	0.13315126	0.06228179
70	0.38373552	-0.4722282	0.18235651	-0.0206751	-0.6524048	0.57097773	0.27326601	0.13387768	0.06262628
70.1	0.38443813	-0.4718761	0.18110233	-0.0202214	-0.6527947	0.56959339	0.27285993	0.13374454	0.06257078
70.2	0.38489185	-0.4717575	0.18099869	-0.019786	-0.6533005	0.56959226	0.27312974	0.13380602	0.06300916
70.3	0.38577042	-0.472812	0.18117992	-0.0203474	-0.6548218	0.56977417	0.2740239	0.13468763	0.06359545
70.4	0.38584436	-0.473338	0.17998681	-0.019192	-0.6550751	0.56951516	0.27429228	0.1333446	0.06355631
70.5	0.38574877	-0.4737535	0.17875602	-0.0185508	-0.655719	0.56885991	0.27373322	0.1319712	0.06302797
70.6	0.38637302	-0.4738144	0.17736356	-0.019486	-0.6567051	0.56922173	0.27470732	0.13181474	0.0633061
70.7	0.3866791	-0.4739442	0.17679901	-0.0193987	-0.6572009	0.56904865	0.27605992	0.13146535	0.06349092
70.8	0.38618265	-0.4743644	0.17595827	-0.0194065	-0.6575208	0.56901182	0.27629668	0.13036041	0.06264859
70.9	0.38588878	-0.4746912	0.17561456	-0.0182911	-0.6580463	0.56891002	0.27658411	0.13007837	0.06259645
71	0.38542015	-0.4747886	0.17466866	-0.0173716	-0.6580703	0.56821651	0.27615208	0.1295313	0.06260385
71.1	0.38538048	-0.4744709	0.17337138	-0.0169365	-0.6584865	0.56778736	0.27596957	0.12772738	0.06262994
71.2	0.38536014	-0.4748961	0.17350367	-0.0170241	-0.6587762	0.56729456	0.27664217	0.12785221	0.06245709
71.3	0.38528891	-0.474569	0.17250415	-0.0159114	-0.6592436	0.56665909	0.27685985	0.12720868	0.06258154
71.4	0.3857519	-0.4745189	0.17194159	-0.0159379	-0.6596833	0.56614416	0.27668685	0.12717725	0.06166866
71.5	0.38625233	-0.4746876	0.17184617	-0.0154837	-0.6601475	0.56584603	0.27751948	0.12744169	0.06185524
71.6	0.38593174	-0.474964	0.17085966	-0.0144765	-0.6603179	0.56520858	0.27707245	0.12693711	0.06149267
71.7	0.38672837	-0.4749491	0.16968361	-0.0148779	-0.6605329	0.5645017	0.27810388	0.1274511	0.06172869
71.8	0.38627977	-0.4747066	0.16849288	-0.0146794	-0.6603639	0.56407001	0.2777784	0.12697229	0.0614177
71.9	0.38618893	-0.4750641	0.16806484	-0.0144332	-0.6611553	0.56400932	0.27864823	0.1266382	0.06040415
72	0.38618977	-0.4751044	0.16668432	-0.0148132	-0.6619202	0.56307687	0.27953597	0.12711495	0.06012672
72.1	0.38732096	-0.4743122	0.1670637	-0.014398	-0.662168	0.56267607	0.2797704	0.12740832	0.06051753
72.2	0.38722869	-0.4739656	0.1667178	-0.0138115	-0.6629358	0.56290635	0.28060953	0.12714865	0.06100623
72.3	0.3877805	-0.4734168	0.16657605	-0.0142671	-0.6637005	0.56239979	0.28041294	0.1259948	0.06055557
72.4	0.38747074	-0.4740893	0.16626435	-0.0145936	-0.6640715	0.56241114	0.28111405	0.1267293	0.06036294
72.5	0.38775835	-0.4745982	0.16623316	-0.0144598	-0.6651078	0.56196527	0.28220031	0.12668939	0.06082938
72.6	0.3877511	-0.4748844	0.16612087	-0.0141182	-0.6656468	0.56139297	0.28285341	0.12618955	0.06007649
72.7	0.38745074	-0.4751204	0.16471931	-0.0144096	-0.6657848	0.55984774	0.28279377	0.12437292	0.05906319
72.8	0.38795898	-0.4753799	0.16437901	-0.0139238	-0.6666195	0.56008821	0.28395944	0.12457122	0.05934651
72.9	0.3890276	-0.4750644	0.16359241	-0.0143491	-0.667217	0.55973716	0.28481918	0.12351154	0.05960194
73	0.38875272	-0.474267	0.16235774	-0.0138908	-0.6670613	0.55911193	0.28497706	0.12244177	0.05960391
73.1	0.38899822	-0.4741269	0.16167111	-0.0134303	-0.6672211	0.55904411	0.28508141	0.12208188	0.05970518
73.2	0.3888856	-0.474527	0.16048671	-0.0126145	-0.6684654	0.55906865	0.285834	0.12144528	0.05967905
73.3	0.38805676	-0.4740638	0.15967843	-0.0126076	-0.6679204	0.55818742	0.28662231	0.12072157	0.05945362
73.4	0.38833001	-0.4748427	0.15836893	-0.0121743	-0.6685429	0.55767532	0.28682066	0.1200245	0.0588232
73.5	0.38864042	-0.4750096	0.15826422	-0.0122566	-0.6689002	0.55792083	0.28664061	0.12041938	0.05876675
73.6	0.38827751	-0.4748599	0.15766773	-0.0119254	-0.6690651	0.55680231	0.2870864	0.11979136	0.0581635
73.7	0.38841941	-0.4747615	0.15705304	-0.0113269	-0.6693772	0.55645864	0.28833272	0.11844404	0.05802822
73.8	0.38818079	-0.4750067	0.15541319	-0.0103435	-0.669765	0.55550249	0.28831877	0.11862706	0.05852964
73.9	0.38908552	-0.4747999	0.15566921	-0.0093649	-0.6701964	0.55521019	0.28702405	0.11820507	0.05768587
74	0.38814884	-0.4744319	0.15514737	-0.008774	-0.6705388	0.55509737	0.28717702	0.11702772	0.05724197
74.1	0.38945868	-0.4747433	0.15457297	-0.0080381	-0.6714785	0.55491436	0.28840108	0.11688336	0.0565353
74.2	0.38985961	-0.4750092	0.15422421	-0.0084931	-0.671947	0.55372179	0.28931241	0.11602163	0.05586348
74.3	0.39079104	-0.4751942	0.15358994	-0.0082253	-0.6724874	0.55285323	0.28949971	0.11474324	0.05562295
74.4	0.39169991	-0.4757524	0.15286752	-0.0086014	-0.673423	0.55232782	0.29000287	0.11447313	0.05465827
74.5	0.3926114	-0.4754863	0.15212597	-0.008566	-0.6736643	0.55137222	0.29026748	0.1123068	0.05315967
74.6	0.3924585	-0.4761101	0.15150576	-0.0083611	-0.6740535	0.55028529	0.29056382	0.11116009	0.05166273
74.7	0.39251415	-0.4757029	0.15171409	-0.0079798	-0.6742361	0.54984742	0.2912949	0.11150943	0.0521005
74.8	0.39207377	-0.4758719	0.15069924	-0.0073651	-0.6745412	0.54963148	0.29151797	0.11051735	0.05283625
74.9	0.39211898	-0.4758642	0.15016073	-0.0068254	-0.6748602	0.54936351	0.29070464	0.10988029	0.05303704
120	0.50132194	-0.5206709	-0.0160489	-0.0059798	-0.7739141	0.29497768	0.41322068	-0.1102917	0.05621746
120.1	0.50198861	-0.5209016	-0.0155674	-0.0054703	-0.7741521	0.29496015	0.41337654	-0.1104971	0.05720247
120.2	0.50117676	-0.5208645	-0.0150827	-0.0052459	-0.7741388	0.29543212	0.41354332	-0.1107331	0.05734654

75	0.39233616	-0.4764741	0.14867555	-0.0071403	-0.6754096	0.54825441	0.29092701	0.11007987	0.05334343
75.1	0.39306985	-0.4760359	0.14825	-0.0073004	-0.6752608	0.54749478	0.29126458	0.11015865	0.05419712
75.2	0.39390372	-0.4763135	0.14552415	-0.0066292	-0.6758932	0.54740888	0.29275405	0.10885651	0.05375397
75.3	0.39386724	-0.4763545	0.14458509	-0.0052806	-0.6762042	0.54725628	0.29298924	0.10839994	0.05347522
75.4	0.39389371	-0.4763169	0.14438115	-0.0054108	-0.6764712	0.54726702	0.29303996	0.1083818	0.05341893
75.5	0.39357216	-0.4772387	0.14362159	-0.004793	-0.6768044	0.54699068	0.29365854	0.10813389	0.05299296
75.6	0.39376415	-0.4772616	0.1438405	-0.0050497	-0.6769229	0.54709878	0.29420868	0.10813464	0.053331
75.7	0.39313773	-0.4774175	0.1440201	-0.0049106	-0.6772278	0.54644647	0.29425702	0.10805745	0.05249845
75.8	0.39299103	-0.4774618	0.14283268	-0.0051255	-0.6775777	0.54607301	0.29406416	0.10783286	0.05222189
75.9	0.39371745	-0.4783546	0.14219892	-0.0053912	-0.6783446	0.54562713	0.29377529	0.10756271	0.05175498
76	0.39373639	-0.4788812	0.14191588	-0.0058444	-0.6786849	0.54569054	0.29454075	0.10706785	0.05234092
76.1	0.39365808	-0.4784274	0.14159385	-0.0061363	-0.6788692	0.5452249	0.29437194	0.10657747	0.05254269
76.2	0.39394982	-0.4789334	0.14128992	-0.0058229	-0.6788813	0.54544807	0.29378614	0.10561974	0.05200238
76.3	0.39459023	-0.4788104	0.14029503	-0.005135	-0.67949	0.54500542	0.29468113	0.10487083	0.05230874
76.4	0.39508542	-0.4791975	0.13943	-0.004792	-0.6800926	0.5449975	0.29499032	0.10491511	0.05256008
76.5	0.39539986	-0.4789788	0.13876225	-0.0029116	-0.6804507	0.54497206	0.29676254	0.10383248	0.05189577
76.6	0.39553455	-0.4795755	0.13799489	-0.003859	-0.6804405	0.54399089	0.29739303	0.10339006	0.0505422
76.7	0.39641388	-0.4792836	0.13703984	-0.0031053	-0.6813829	0.5431854	0.2980293	0.10350601	0.04982492
76.8	0.39657107	-0.479204	0.13619593	-0.0025432	-0.6816863	0.54254382	0.29870606	0.10330803	0.04955395
76.9	0.39660704	-0.4799414	0.13636389	-0.0016703	-0.6821467	0.54253414	0.29926011	0.10281057	0.04952155
77	0.39681069	-0.4798399	0.13596117	-0.00195	-0.6821648	0.54204035	0.29934505	0.10219179	0.0481468
77.1	0.39573661	-0.4804786	0.13556648	-0.0024233	-0.6821542	0.54110964	0.29912156	0.10109398	0.04761684
77.2	0.39621062	-0.4810019	0.13433077	-0.0017323	-0.6826644	0.5409428	0.30022187	0.09939514	0.04793485
77.3	0.39643948	-0.4813016	0.13315872	-0.0018175	-0.6837129	0.54073532	0.30045467	0.09961433	0.04789769
77.4	0.3967137	-0.481624	0.13262313	-0.0015819	-0.6840295	0.54078099	0.30021555	0.09884973	0.04792432
77.5	0.39702553	-0.4817668	0.13149474	-0.0006395	-0.6844778	0.54076515	0.30072888	0.09869515	0.04786489
77.6	0.39789323	-0.481236	0.13105647	0.00134433	-0.6852668	0.5392261	0.30109918	0.09836581	0.04852009
77.7	0.39747876	-0.4814833	0.13083849	0.00120934	-0.6853074	0.53913869	0.30121816	0.09821636	0.0484446
77.8	0.39819909	-0.4819711	0.13082638	0.00103186	-0.6855208	0.53918168	0.30129632	0.09792675	0.04778903
77.9	0.39863514	-0.4817492	0.12960111	0.00112839	-0.6863712	0.53917834	0.30201986	0.09783706	0.04693843
78	0.39916231	-0.4827171	0.13050351	0.00108799	-0.6868611	0.53929423	0.30263898	0.09682797	0.0451165
78.1	0.39932429	-0.4824735	0.12978639	0.00207919	-0.6868836	0.53898747	0.30295076	0.09518939	0.04458559
78.2	0.40024783	-0.4824741	0.12938747	0.00247773	-0.6872862	0.53914954	0.30379515	0.09518634	0.04439401
78.3	0.40044312	-0.4827059	0.12729775	0.0030383	-0.6882101	0.53831704	0.30405612	0.09444982	0.04368938
78.4	0.40123998	-0.4820266	0.12650263	0.00306213	-0.6886323	0.53779233	0.30482182	0.09542866	0.04455513
78.5	0.40172327	-0.4826491	0.12567181	0.00291802	-0.6888976	0.53783645	0.30564463	0.09531	0.04494195
78.6	0.40247604	-0.4823166	0.12494268	0.0025183	-0.6895661	0.53689312	0.30657446	0.09565027	0.04442097
78.7	0.40208202	-0.4826139	0.12337235	0.00309123	-0.6895007	0.53594348	0.30690629	0.09525885	0.04458638
78.8	0.40270711	-0.4826744	0.12216713	0.00382328	-0.6901522	0.535201	0.30676712	0.09600634	0.04388426
78.9	0.403058	-0.4836421	0.12044959	0.00443637	-0.6912412	0.53455536	0.30754039	0.09464796	0.04284852
79	0.40365935	-0.4842807	0.11957663	0.00389634	-0.6923131	0.53383479	0.30727255	0.09484573	0.04230188
79.1	0.40415157	-0.4844623	0.119432	0.0042025	-0.6926029	0.53393316	0.30746068	0.09429914	0.04188801
79.2	0.40537811	-0.4851532	0.1188897	0.00457803	-0.6928506	0.53338542	0.3075606	0.09347766	0.04111306
79.3	0.40579921	-0.4853645	0.11728262	0.00455851	-0.693521	0.5314066	0.30841512	0.09299012	0.04001998
79.4	0.40638684	-0.4852103	0.11739695	0.00498065	-0.6942831	0.53144728	0.30813088	0.09308323	0.0398803
79.5	0.40695076	-0.4857952	0.11647583	0.00500988	-0.694718	0.53134785	0.30908861	0.09261522	0.04013844
79.6	0.40841018	-0.4866901	0.11563533	0.00598818	-0.6955662	0.53032307	0.30961652	0.09092082	0.04047366
79.7	0.40821086	-0.4865227	0.11536604	0.00608277	-0.6957833	0.53050352	0.30994783	0.09069923	0.04089756
79.8	0.41079613	-0.4876615	0.11477706	0.00565098	-0.6974473	0.53136862	0.31118579	0.09059325	0.04058389
79.9	0.41191589	-0.4870624	0.11487377	0.0048856	-0.6979246	0.53069054	0.31183814	0.09071294	0.03999951
80	0.41207177	-0.4879002	0.11426725	0.00432937	-0.6981068	0.530568	0.31171496	0.09060315	0.03929575
80.1	0.41307664	-0.4877758	0.11268589	0.00323839	-0.6985672	0.52981294	0.31126116	0.08978397	0.03877585
80.2	0.41380667	-0.4887542	0.11203853	0.00248175	-0.6992055	0.52952551	0.31173424	0.08925715	0.03677374
80.3	0.41408439	-0.488844	0.11113218	0.00224626	-0.6997859	0.52932644	0.31217435	0.09002522	0.03624065
80.4	0.41368814	-0.4888637	0.11101043	0.00278985	-0.6996806	0.52897574	0.31176543	0.08930987	0.03578708
80.5	0.41481399	-0.4893932	0.10931033	0.00263293	-0.7006537	0.52898969	0.31355418	0.08921007	0.03556195
80.6	0.41574174	-0.4890714	0.10848611	0.00288642	-0.7019239	0.52842536	0.31450611	0.08891418	0.03620659
80.7	0.4160431	-0.4903738	0.10729784	0.00204709	-0.7022059	0.52779522	0.3147701	0.08833359	0.03735578
80.8	0.41604094	-0.4907627	0.10678973	0.0017717	-0.7025197	0.52794948	0.31507354	0.08787541	0.03717676
80.9	0.41674479	-0.4907066	0.10555831	0.00143911	-0.7022375	0.52714061	0.31534905	0.08631794	0.03668627
81	0.41663913	-0.4911626	0.10375515	0.00127597	-0.702819	0.52633593	0.31692003	0.08576163	0.03633724
81.1	0.41798827	-0.4918394	0.10209432	0.0018251	-0.7033909	0.5254755	0.31710032	0.08352803	0.03686219
81.2	0.41961194	-0.4923019	0.10077016	0.00281948	-0.7042897	0.52429374	0.31808809	0.08118462	0.03689021
81.3	0.42038539	-0.4925358	0.09997087	0.00349047	-0.7045454	0.52468094	0.3189256	0.08026256	0.03726643
81.4	0.42152608	-0.4925968	0.09876665	0.00368003	-0.7052757	0.52444191	0.31877185	0.07931412	0.03766812
81.5	0.42253962	-0.49348	0.09754559	0.00426955	-0.7058383	0.52374642	0.31968238	0.07803431	0.03737822
81.6	0.42445542	-0.4932167	0.09772232	0.0051874	-0.7069359	0.52315391	0.31850719	0.07707597	0.03676884
81.7	0.42518642	-0.4945862	0.09621086	0.00545393	-0.7082308	0.52340344	0.32018942	0.077148	0.03682331
81.8	0.42696952	-0.4945752	0.09473167	0.00541579	-0.709431	0.52253441	0.32133596	0.07806162	0.03607131
81.9	0.4274185	-0.4954932	0.0946353	0.00538153	-0.7100433	0.52269295	0.32120606	0.07755507	0.03578458
82	0.42841446	-0.4958179	0.09447845	0.00577317	-0.7104967	0.52235632	0.32166851	0.07790798	0.03491425
82.1	0.42806452	-0.4953557	0.09365259	0.00525773	-0.7105773	0.52207135	0.32187085	0.07767631	0.03490324
82.2	0.42798351	-0.4956558	0.09348843	0.00553153	-0.710536	0.52166399	0.32162771	0.07666725	0.03463926
82.3	0.42820514	-0.4964586	0.09208168	0.00565921	-0.7106344	0.52159297	0.32207852	0.07601327	0.03467395
82.4	0.42850272	-0.4967976	0.09106157	0.00621187	-0.7109381	0.52059072	0.32187769	0.07492057	0.03368673
82.5	0.42882996	-0.4967486	0.08917022	0.0069077	-0.7113313	0.52052664	0.32276924	0.07409794	0.03498954
82.6	0.42979703	-0.4970967	0.08618301	0.00814306	-0.7126011	0.5192875	0.32350884	0.0740185	0.0342924
82.7	0.42995727	-0.4973121	0.08614219	0.00786301	-0.7140497	0.51944503	0.32442965	0.0727238	0.0354898
82.8	0.43027614	-0.4975796	0.08493921	0.00894265	-0.7143104	0.51852296	0.32456537	0.07194418	0.03484562
82.9	0.43135095	-0.4977223	0.08495845	0.01007616	-0.7151075	0.51874929	0.32585146	0.07198265	0.03533734
83	0.43290263	-0.4991028	0.08324824	0.00940907	-0.7164379	0.51845597	0.32639384	0.07182288	0.03484274
83.1	0.43286596	-0.5000498	0.08239863	0.01001913	-0.7171819	0.51826679	0.32706785	0.07065016	0.03564712
83.2	0.43298904	-0.500701	0.08039296	0.01015343	-0.7177502	0.51792946	0.32873976	0.06988057	0.03669398
83.3	0.43334691	-0.5003345	0.08048161	0.00947409	-0.7175323	0.51653267	0.3300685	0.06969627	0.03636081
83.4	0.43386445	-0.5017277	0.07988908	0.00943987	-0.7182115	0.51625481	0.33111575	0.06916702	0.03633889
83.5	0.43393708	-0.5013701	0.0798507	0.01042094	-0.7181001	0.51619528	0.33143789	0.06834791	0.03701952
83.6	0.43376705	-0.5010763	0.07952557	0.01134484	-0.7183329	0.51549968	0.33104048	0.06764213	0.036679
83.7	0.43433813	-0.5014115	0.0796464	0.01138507	-0.7186434	0.51536214	0.33139629	0.06748401	0.03681215
83.8	0.43578963	-0.5019839	0.07936288	0.01201113	-0.7198312	0.51472969	0.33248394	0.06680226	0.0370568
83.9	0.43620824	-0.5019423	0.07744443	0.01224593	-0.7199284	0.513394	0.33352267	0.06485457	0.03733969
84	0.43581365	-0.5019548	0.07615742	0.01190368	-0.7197129	0.51209092	0.33458326	0.06337257	0.03765507
84.1	0.43790834	-0.5021179	0.0759189	0.01050673	-0.7203493	0.51075952	0.33506083	0.06280281	0.03687848
84.2	0.43877399	-0.5018339	0.07394481	0.01114795	-0.7206727	0.5088361	0.33598134	0.06189025	0.0381487
84.3	0.43833571	-0.5012547	0.0714758	0.01170961	-0.7208408	0.50687316	0.33613854	0.05927267	0.03864219
84.4	0.43784417	-0.5020056	0.07051662	0.01086619	-0.7213358	0.50609316	0.33634893	0.05740175	0.03761533
84.5	0.43835561	-0.5023785	0.07035198	0.01047758	-0.7216803	0.50611944	0.33679578	0.05698421	0.03795112
84.6	0.43829206	-0.5024981	0.06953365	0.00901537	-0.7217701	0.50526515	0.337464	0.05586843	0.03698422
84.7	0.43853688	-0.5036701	0.06874753	0.00872681	-0.7220715	0.50539598	0.33756607	0.0542114	0.03711416
84.8	0.43932023	-0.5040742	0.06799791	0.00828836	-0.722762	0.5047441	0.33906322	0.05351438	0.03788954
84.9	0.43888327	-0.5041126	0.06796425	0.0097077	-0.7235948	0.50342292	0.34042392	0.05254407	0.03930462
85	0.43968719	-0.5043279	0.06740377	0.0104971	-0.7240571	0.5032032	0.34143	0.05141933	0.03971042
85.1	0.44012266	-0.5032487	0.06673916	0.01022249	-0.7244262	0.50247455	0.34107346	0.0522598	0.03921531
85.2	0.43943498	-0.5037647	0.06728694	0.01071513	-0.7246712	0.50126015	0.34192963	0.05139848	0.03861156
85.3	0.43963435	-0.503702	0.06656204	0.01097085	-0.724694	0.50144252	0.34179327	0.05077649	0.03929531
85.4	0.43936938	-0.5034893	0.06521717	0.00962154	-0.7248076	0.500168	0.3419858	0.04891174	0.03855958
85.5	0.4390662	-0.5030841	0.06452864	0.01039108	-0.7257572	0.49940964	0.34218647	0.0481984	0.03856286
85.6	0.44012082	-0.5055651	0.06337856	0.01156371	-0.7273423	0.49841678	0.34320999	0.04677875	0.03874533
85.7	0.44128053	-0.5059219	0.06335589	0.0118691	-0.7276859	0.49690176	0.34413295	0.04589477	0.03891423
85.8	0.44204827	-0.5069251	0.06352969	0.01297979	-0.728381	0.49690873	0.34456425	0.04538947	0.03906935
85.9	0.44088983	-0.5069434	0.0624639	0.01306435	-0.7284353	0.49545647	0.34605684	0.0444575	0.03954297
86	0.44057193	-0.5070592	0.06251932	0.01308946	-0.7282378	0.49492476	0.34626221	0.04386029	0.03944522
86.1	0.44043603	-0.5062517	0.06184112	0.01332633	-0.7276204	0.49329544	0.34559402	0.04208506	0.03918254
86.2	0.43976829	-0.5059333	0.06067216	0.01464803	-0.7276208	0.49165983	0.34543488	0.04029347	0.03891323
86.3	0.43990854	-0.5061331	0.05990087	0.01539236	-0.7279781	0.4913971	0.34539951	0.04060438	0.03917857
86.4	0.43944461	-0.5057616	0.05937645	0.01512848	-0.7276827	0.4905932	0.34569671	0.03996638	0.03813048
86.5	0.43904358	-0.50483	0.05735743	0.01612467	-0.7276079	0.48933411	0.3468877	0.04034603	0.0383844
86.6	0.43873868	-0.5049565	0.05639549	0.01607822	-0.7279945	0.48829174	0.34791907	0.04009699	0.03804651
86.7	0.43916844	-0.50536	0.05562575	0.01634153	-0.7284453	0.48804412	0.34867626	0.03964093	0.03787394
86.8	0.44011271	-0.5054387	0.05411234	0.01719388	-0.7294743	0.48711108	0.34795724	0.03968254	0.03801735
86.9	0.43903417	-0.5052448	0.05327275	0.01700703	-0.729285	0.48530495	0.34730108	0.03913987	0.03723022
87	0.43932902	-0.5060435	0.05386909	0.01716392	-0.72934	0.48474672	0.3471365	0.03903353	0.03703831
87.1	0.43861489	-0.5066853	0.05403421	0.01744112	-0.7296739	0.48366919	0.34710053	0.03815825	0.03697468
87.2	0.43755228	-0.5066756	0.05246393	0.01846681	-0.7298553	0.48222309	0.34679818	0.03743269	0.03699933
87.3	0.43792037	-0.5064578	0.05220262	0.01804302	-0.7300165	0.48127502	0.34755743	0.0372654	0.03643177
87.4	0.43837614	-0.5060532	0.05249002	0.01826968	-0.7304889	0.48032108	0.34792299	0.03756971	0.03637801
87.5	0.4392503	-0.5067744	0.05184217	0.0186485	-0.7311905	0.47913387	0.34839255	0.03813108	0.03612261
87.6	0.43989449	-0.5076401	0.05130439	0.01830341	-0.7318687	0.47945447	0.34858482	0.03835856	0.03687825
87.7	0.43984111	-0.5078794	0.05122203	0.01886669	-0.7323156	0.48003218	0.34792781	0.03845076	0.03666603
87.8	0.44041726	-0.508361	0.05148211	0.01972031	-0.7329441	0.47956998	0.34907112	0.03899325	0.03700313
87.9	0.44050234	-0.508188	0.05186857	0.01978496	-0.7332849	0.47950598	0.34944067	0.03922927	0.03691351
88	0.44195782	-0.5087493	0.0520291	0.019262	-0.7343387	0.47899848	0.34991516	0.03805113	0.03720887
88.1	0.44142394	-0.5094674	0.05114225	0.01911501	-0.7347635	0.47863423	0.35082784	0.03774187	0.03693091
88.2	0.44134771	-0.5106892	0.05048832	0.01988658	-0.7347769	0.47769102	0.3504216	0.03609119	0.03543065
88.3	0.44286871	-0.5106543	0.04968935	0.0195251	-0.7352344	0.47689669	0.35014448	0.03562975	0.03569416
88.4	0.44406946	-0.5109231	0.04931164	0.01941823	-0.7359946	0.47669669	0.3505147	0.03587124	0.03571266
88.5	0.44440694	-0.5105784	0.04918424	0.01919166	-0.7368241	0.47571603	0.35110832	0.03667215	0.03652683
88.6	0.44348805	-0.511313	0.04791759	0.01909741	-0.7377481	0.47477184	0.35290225	0.03735051	0.03647021
88.7	0.44349321	-0.511745	0.04720224	0.01941714	-0.7385669	0.4750654	0.35225809	0.03539091	0.03544506
88.8	0.44277788	-0.512236	0.04626483	0.01990433	-0.7388195	0.47458337	0.35279723	0.03531544	0.03525298
88.9	0.44251324	-0.5122017	0.04567766	0.02009571	-0.738739	0.47441604	0.35267883	0.03535367	0.03550526
89	0.44249765	-0.5121835	0.04503104	0.0203369	-0.7391362	0.47238195	0.3523186	0.0351313	0.03551949
89.1	0.44377499	-0.5130682	0.045228	0.0190087	-0.7402323	0.47132999	0.35357154	0.03427664	0.03464964
89.2	0.44305482	-0.5122427	0.04548393	0.01968299	-0.7401704	0.47046192	0.35372909	0.03309632	0.03437943
89.3	0.44331878	-0.5127429	0.04336545	0.02105986	-0.7409286	0.47059975	0.35430444	0.03082306	0.03412083
89.4	0.44311979	-0.5130959	0.04269323	0.02101784	-0.7409733	0.47032566	0.35517768	0.02872308	0.03470074
89.5	0.44365505	-0.513199	0.04216248	0.02136609	-0.7412511	0.46991023	0.35569134	0.02841116	0.03503913
89.6	0.44379573	-0.5127407	0.04160606	0.02149963	-0.7413593	0.46879569	0.3559431	0.02892477	0.03517472
89.7	0.44269322	-0.5139143	0.03913405	0.02288093	-0.7418354	0.46902017	0.35652594	0.02831492	0.03530397
89.8	0.44322076	-0.5140848	0.03769628	0.0228809	-0.7421954	0.46823055	0.35774876	0.02786453	0.03461924
89.9	0.44369746	-0.5143398	0.03844878	0.02271305	-0.7423008	0.4679373	0.35776447	0.02821072	0.03394532
135	0.48298179	-0.5069117	-0.0045764	-0.0105854	-0.7744518	0.26928637	0.429869	-0.1235346	0.05352694
135.1	0.4834629	-0.5069514	-0.0040776	-0.0102393	-0.7744931	0.26896314	0.43049512	-0.1235184	0.05357326
135.2	0.48305102	-0.5066757	-0.0044327	-0.0098827	-0.7743736	0.2684802	0.43020192	-0.1236864	0.05351735

90	0.44404194	-0.5142311	0.03782032	0.022352	-0.7425736	0.46712291	0.35797874	0.02833708	0.0342979
90.1	0.44411813	-0.5139748	0.03741342	0.02223336	-0.7425869	0.46669409	0.35842524	0.02759011	0.03420744
90.2	0.44415171	-0.5142952	0.03615031	0.02134991	-0.7428278	0.46581697	0.35849585	0.02713824	0.03427914
90.3	0.44435487	-0.5139143	0.03561999	0.02078246	-0.7424114	0.46471965	0.35786818	0.02652413	0.0345389
90.4	0.44433244	-0.5139984	0.03604459	0.02047187	-0.7426541	0.46433431	0.35828817	0.02671237	0.03421933
90.5	0.44457427	-0.5137939	0.0360118	0.02045804	-0.7428882	0.46406757	0.35846343	0.02604984	0.03439404
90.6	0.44441533	-0.5137594	0.0363628	0.02064848	-0.7432679	0.46378162	0.35892037	0.02566224	0.03472743
90.7	0.44402176	-0.5134921	0.03529158	0.02105577	-0.7432787	0.46223881	0.35871444	0.02635521	0.03432447
90.8	0.44432622	-0.5132546	0.03392099	0.02046397	-0.7432062	0.4603538	0.35895134	0.02594053	0.03400439
90.9	0.4438413	-0.5135061	0.03378491	0.0204316	-0.7436092	0.45957573	0.35942802	0.02572725	0.03476764
91	0.44492098	-0.5150048	0.03429628	0.02060729	-0.7443611	0.45840267	0.35961886	0.0257232	0.03567677
91.1	0.44443798	-0.5145788	0.03425666	0.02112643	-0.7439343	0.45767704	0.35896803	0.02471942	0.03484598
91.2	0.44492917	-0.514683	0.03434112	0.02205134	-0.744699	0.45707001	0.36032006	0.02436362	0.03473139
91.3	0.44608293	-0.515159	0.03469337	0.02221724	-0.745397	0.45696505	0.36061404	0.02410455	0.03485959
91.4	0.44577427	-0.5149161	0.03390706	0.02205599	-0.7454046	0.4567081	0.36087656	0.02388499	0.03444696
91.5	0.44603115	-0.5148926	0.03358619	0.02100039	-0.745605	0.45587918	0.36025269	0.02430202	0.03429078
91.6	0.44735097	-0.5148366	0.0313957	0.021929	-0.7456621	0.45481467	0.36138397	0.02412164	0.03543588
91.7	0.44746716	-0.5144058	0.03109158	0.02249458	-0.7455566	0.4543104	0.36085913	0.02399186	0.03527607
91.8	0.44766759	-0.5148266	0.03053164	0.02264197	-0.7456023	0.45344961	0.36183073	0.02413647	0.03498256
91.9	0.44731714	-0.5148045	0.03098243	0.02337704	-0.7455779	0.45237417	0.36281991	0.023925	0.0363452
92	0.44652613	-0.5150488	0.03003447	0.02542242	-0.7455162	0.45168976	0.36219137	0.02331973	0.03670228
92.1	0.44673177	-0.514837	0.02964123	0.025139	-0.7457416	0.45135432	0.36222977	0.02301188	0.0367604
92.2	0.4477417	-0.5158938	0.02943922	0.02647059	-0.7466752	0.4516011	0.3631773	0.02208074	0.03795737
92.3	0.44769081	-0.5156654	0.02956437	0.02588454	-0.7469698	0.45050654	0.36356138	0.02069118	0.03727684
92.4	0.44791871	-0.515271	0.02873104	0.02577939	-0.747282	0.45072399	0.36437329	0.02108955	0.03820589
92.5	0.44762502	-0.5146377	0.02830033	0.02572159	-0.7468615	0.44941726	0.36462274	0.02025424	0.03737385
92.6	0.44794532	-0.516318	0.02928646	0.02720069	-0.7476904	0.44928179	0.36441656	0.01968433	0.03751544
92.7	0.44818017	-0.516234	0.02895403	0.02725098	-0.7476942	0.44878922	0.36433855	0.01943329	0.03744839
92.8	0.44825546	-0.5160821	0.02833357	0.02736362	-0.7477416	0.44843599	0.3642063	0.02056894	0.03736932
92.9	0.44847529	-0.5162413	0.0272452	0.02817953	-0.7473687	0.44728682	0.36428691	0.01991969	0.03743352
93	0.44863907	-0.5158915	0.02663566	0.02862352	-0.7473674	0.44688026	0.36501736	0.0194561	0.0376163
93.1	0.44906939	-0.5153984	0.02701232	0.02872121	-0.7475512	0.44656102	0.36485689	0.01917563	0.03745816
93.2	0.44906989	-0.5151361	0.02643442	0.02919876	-0.7477911	0.44527347	0.36572737	0.01929269	0.03678221
93.3	0.44896896	-0.5159468	0.0264084	0.03002205	-0.7479748	0.44459129	0.36544371	0.01899854	0.03666303
93.4	0.44913903	-0.5165532	0.02537118	0.03042553	-0.7484597	0.44415136	0.36662445	0.01838729	0.0366119
93.5	0.44968792	-0.5166786	0.02460858	0.03067918	-0.7484524	0.44435004	0.36675042	0.017958	0.03748193
93.6	0.45023462	-0.5161822	0.02445514	0.03132469	-0.7488421	0.44374092	0.36796359	0.01574683	0.03747045
93.7	0.45006284	-0.516241	0.02428476	0.03121156	-0.7487697	0.44292752	0.36804071	0.01476036	0.03633891
93.8	0.45120695	-0.5166213	0.02266429	0.03087021	-0.749503	0.44209159	0.36858943	0.01420296	0.03613671
93.9	0.45090645	-0.5166269	0.02237889	0.03096614	-0.7495906	0.44047905	0.36802926	0.01386732	0.03658522
94	0.4516222	-0.5163012	0.02180597	0.03143533	-0.7495936	0.44029593	0.36849741	0.01255402	0.03618111
94.1	0.45224452	-0.5162753	0.02081398	0.03203499	-0.7499034	0.43923149	0.36865623	0.01202898	0.03662607
94.2	0.45312731	-0.5162695	0.02201547	0.03275728	-0.7505276	0.43970178	0.36845103	0.01170257	0.03601593
94.3	0.45431188	-0.5160638	0.02199172	0.03368643	-0.7509681	0.4369117	0.36887038	0.01230013	0.03486636
94.4	0.45390368	-0.5160918	0.02015413	0.03368892	-0.7504856	0.43585622	0.36946414	0.01182586	0.03432637
94.5	0.45538462	-0.5154419	0.01875328	0.03359199	-0.7504395	0.43536852	0.36952867	0.01170017	0.03495061
94.6	0.45623966	-0.5159395	0.01851049	0.0332978	-0.7508663	0.43522069	0.37025373	0.01149862	0.03535099
94.7	0.45629759	-0.5161569	0.01800357	0.03392102	-0.7507022	0.43235181	0.37023764	0.01002172	0.0348406
94.8	0.45695423	-0.5160619	0.0179262	0.03372846	-0.7512609	0.4315698	0.370137	0.00889714	0.03479437
94.9	0.45704856	-0.5167146	0.0179274	0.03480185	-0.7515138	0.43202419	0.37096108	0.00781168	0.03512484
95	0.45737192	-0.517103	0.01687593	0.03557698	-0.7520385	0.43205304	0.3709961	0.00876218	0.03570982
95.1	0.45835391	-0.5161539	0.01580122	0.03555808	-0.7523779	0.43101743	0.37163061	0.00830598	0.03631247
95.2	0.45799225	-0.5161228	0.01575513	0.03568844	-0.7524738	0.4304089	0.37177733	0.00807984	0.03625557
95.3	0.4588368	-0.515264	0.01566179	0.03614414	-0.7523609	0.42988569	0.37173215	0.00702322	0.03605051
95.4	0.458658	-0.5151674	0.01558836	0.03543521	-0.7521296	0.42994139	0.37190686	0.00608717	0.03581003
95.5	0.45862931	-0.5142337	0.01529225	0.03646548	-0.7525348	0.43021672	0.37252015	0.00658786	0.03678427
95.6	0.45927769	-0.5149005	0.01468573	0.03627088	-0.7527711	0.4302475	0.37317421	0.00544023	0.03610734
95.7	0.46004435	-0.5160027	0.01341472	0.03728352	-0.753021	0.42959179	0.37327819	0.00528996	0.03586423
95.8	0.46116646	-0.517665	0.01323425	0.03785137	-0.7540546	0.42929754	0.37422977	0.00312291	0.03592085
95.9	0.46178789	-0.5179211	0.01386818	0.03833714	-0.7543863	0.42927594	0.37555802	0.00250098	0.0353047
96	0.46215549	-0.5178416	0.01357777	0.03886446	-0.7547182	0.42931581	0.37576611	0.00244328	0.03552809
96.1	0.46287193	-0.5187074	0.01264717	0.03823636	-0.7552385	0.42879023	0.376319	0.00224067	0.03655765
96.2	0.46391053	-0.5192596	0.01215816	0.0380513	-0.7557724	0.42879524	0.37725894	0.00121636	0.03764644
96.3	0.46463648	-0.5199347	0.01211795	0.03732713	-0.7557712	0.42843033	0.37778677	0.00023506	0.03750543
96.4	0.4658089	-0.5211991	0.01189529	0.03788278	-0.7566697	0.428943	0.37854282	3.54E-06	0.03872635
96.5	0.46561278	-0.5212728	0.01152414	0.03820749	-0.7567393	0.42881927	0.3783435	-7.99E-05	0.03935087
96.6	0.4657647	-0.5213199	0.01143792	0.03881293	-0.7569934	0.42782776	0.3787333	-0.0006682	0.03859679
96.7	0.46551267	-0.5201527	0.01093944	0.0389287	-0.7570829	0.42646044	0.37877152	-0.0014868	0.03825743
96.8	0.46523134	-0.5203154	0.01009386	0.03886956	-0.7570507	0.42588205	0.37897311	-0.0021843	0.03821313
96.9	0.46514899	-0.5204349	0.00919306	0.03947828	-0.7572506	0.42642728	0.37993683	-0.003819	0.03910849
97	0.46614001	-0.5205839	0.00717016	0.04014966	-0.7573574	0.4250204	0.37921358	-0.0048162	0.03939064
97.1	0.46726125	-0.5208585	0.00654446	0.04117784	-0.7585324	0.42415216	0.38059478	-0.0054385	0.0403472
97.2	0.4674169	-0.5202198	0.00580015	0.04172698	-0.7585042	0.42402841	0.38024535	-0.0062907	0.04169621
97.3	0.46758455	-0.5210945	0.00494578	0.04191254	-0.7588808	0.42250288	0.38095238	-0.0058769	0.04184314
97.4	0.46760931	-0.5203073	0.00509738	0.04182775	-0.7590296	0.42207087	0.38127099	-0.0055134	0.04271356
97.5	0.46692642	-0.5212605	0.00598009	0.04228233	-0.7589101	0.42148748	0.38219195	-0.0068979	0.0419541
97.6	0.46738804	-0.5211019	0.00590345	0.04274714	-0.7588566	0.42095087	0.38220291	-0.0073761	0.041998
97.7	0.46784747	-0.5216505	0.00530253	0.04392188	-0.7589072	0.41984104	0.38179253	-0.0081918	0.04210104
97.8	0.46798343	-0.5215251	0.00415646	0.04469049	-0.7592886	0.4188197	0.38256729	-0.0081145	0.04325614
97.9	0.46810415	-0.5207885	0.00426563	0.04474616	-0.7590253	0.4180097	0.38327293	-0.0087739	0.04399017
98	0.46862466	-0.5204733	0.00394501	0.04430966	-0.7594586	0.41822081	0.38371843	-0.0089337	0.04449545
98.1	0.46902261	-0.5203173	0.00381041	0.0448493	-0.7594006	0.4174603	0.38370978	-0.0099735	0.04506826
98.2	0.46899721	-0.5204987	0.0032528	0.04540908	-0.759676	0.41659139	0.3839044	-0.0096969	0.04519986
98.3	0.47012102	-0.5214632	0.00288746	0.0458412	-0.7598778	0.41709869	0.38528174	-0.0103176	0.04688713
98.4	0.47038339	-0.5216863	0.00314456	0.04548046	-0.7598893	0.41669318	0.38570285	-0.0105209	0.04776044
98.5	0.47125546	-0.5220835	0.00270248	0.04583426	-0.7606234	0.41615502	0.38551652	-0.0102142	0.04804637
98.6	0.47188598	-0.5218172	0.00181932	0.04490685	-0.7610708	0.414007	0.3864514	-0.010228	0.04749046
98.7	0.47323084	-0.5209822	0.00148339	0.04584224	-0.7616281	0.41312299	0.38686546	-0.0113205	0.04873583
98.8	0.47373083	-0.5213442	0.00196836	0.04571854	-0.7624578	0.41231471	0.38803564	-0.0117981	0.04939361
98.9	0.47454765	-0.5221007	0.00227319	0.04483473	-0.7623988	0.41038161	0.38958012	-0.0130756	0.0486868
99	0.47556466	-0.5227667	0.00112461	0.04441139	-0.762731	0.41029042	0.3900948	-0.0137324	0.05032784
99.1	0.47646776	-0.5228027	0.00020923	0.04424065	-0.7626587	0.40840489	0.38945667	-0.015036	0.04949021
99.2	0.47794581	-0.5226664	0.00042847	0.0450217	-0.7635697	0.40719516	0.38891311	-0.0153704	0.04980488
99.3	0.47747934	-0.52251	-0.0004595	0.04445941	-0.7634239	0.40571377	0.38882431	-0.0164698	0.04923147
99.4	0.47788849	-0.5229621	-0.0011267	0.04452638	-0.7635086	0.40494798	0.38872686	-0.0177476	0.04947862
99.5	0.47825422	-0.5231824	-0.0003573	0.04497409	-0.7638719	0.40336285	0.3893206	-0.0183781	0.04991958
99.6	0.47837763	-0.5239281	-0.0001093	0.04489289	-0.7639339	0.40299798	0.38984809	-0.0181866	0.0498328
99.7	0.47920441	-0.5241997	-6.02E-05	0.04480575	-0.7641538	0.40261311	0.38976862	-0.0189914	0.04998428
99.8	0.47949271	-0.5250474	-0.0007299	0.04459488	-0.7647904	0.40286995	0.39003677	-0.0198572	0.05058662
99.9	0.47872296	-0.5254709	-0.0011131	0.04482674	-0.7647777	0.40260548	0.3901572	-0.0212521	0.05090661
100	0.48011347	-0.5255739	-0.0017054	0.04334348	-0.7657953	0.40091602	0.3914291	-0.0234014	0.05124541
100.1	0.48109806	-0.5252604	-0.0014617	0.0432549	-0.7660695	0.40043228	0.39189321	-0.0241659	0.05215656
100.2	0.48040766	-0.5252775	-0.0014474	0.0437374	-0.7659004	0.39941957	0.39223171	-0.0244456	0.05178974
100.3	0.48128606	-0.5254446	-0.0016837	0.04363114	-0.7659239	0.39818611	0.39228754	-0.0248507	0.05134317
100.4	0.48086928	-0.5247602	-0.0025218	0.04305074	-0.7656803	0.39630485	0.39329985	-0.0250563	0.05282678
100.5	0.48158751	-0.5241296	-0.003187	0.04179203	-0.7660351	0.39476068	0.3933458	-0.0273639	0.05281159
100.6	0.48322171	-0.5249554	-0.0024262	0.0403397	-0.7663087	0.39255516	0.39386653	-0.0282397	0.05197843
100.7	0.48337041	-0.5247901	-0.0023757	0.04004799	-0.7668406	0.39111682	0.39375116	-0.0277142	0.05149047
100.8	0.48414793	-0.5251217	-0.0018787	0.04040979	-0.7671483	0.39050755	0.39422506	-0.0282662	0.05137306
100.9	0.48471147	-0.5247141	-0.0034451	0.04026892	-0.7666145	0.38789202	0.39265089	-0.0307512	0.05079624
101	0.48546968	-0.5249675	-0.0034168	0.04046442	-0.7668069	0.38726736	0.39360516	-0.0314748	0.05141327
101.1	0.485584	-0.525276	-0.0032913	0.04046867	-0.7666263	0.38628797	0.3940498	-0.0323052	0.05123354
101.2	0.48508143	-0.5245242	-0.0031738	0.04085554	-0.7664471	0.38524879	0.39407059	-0.03304	0.05009836
101.3	0.48518677	-0.5245396	-0.0060172	0.04213764	-0.7664881	0.3836399	0.39334465	-0.0339932	0.0507641
101.4	0.48583521	-0.5251906	-0.0047356	0.04166598	-0.7665674	0.38262062	0.3942278	-0.0347565	0.05027592
101.5	0.48626919	-0.5242114	-0.0051451	0.04236805	-0.7667047	0.38122544	0.39383307	-0.0364649	0.05044498
101.6	0.48650461	-0.5243415	-0.0045655	0.04222377	-0.7668159	0.38043263	0.39432265	-0.036344	0.05013552
101.7	0.48726919	-0.5248787	-0.0046732	0.04161632	-0.7670895	0.37975651	0.39477019	-0.0371996	0.05009933
101.8	0.48880212	-0.5245697	-0.0045186	0.04105529	-0.7679407	0.37812876	0.39494329	-0.0377394	0.05074681
101.9	0.48833779	-0.5252399	-0.005391	0.0402876	-0.7680209	0.37745944	0.39597784	-0.0396312	0.05200128
102	0.4883394	-0.5250995	-0.0055342	0.04044687	-0.7682538	0.37703146	0.39595406	-0.0405406	0.05206686
102.1	0.48821005	-0.5242924	-0.005845	0.03890892	-0.7681935	0.37497284	0.39730044	-0.0416333	0.05187962
102.2	0.49015671	-0.5237138	-0.0048611	0.03781872	-0.7682657	0.37288317	0.39787016	-0.0430767	0.05321689
102.3	0.49144541	-0.5234009	-0.0041266	0.03845863	-0.7684576	0.37188709	0.39923121	-0.0448796	0.05468698
102.4	0.49213033	-0.5232836	-0.0037947	0.0394869	-0.7685052	0.37145789	0.39874435	-0.0461995	0.05427886
102.5	0.49262667	-0.5236661	-0.0041187	0.03782313	-0.7682642	0.36997579	0.3988832	-0.048011	0.05335174
102.6	0.49179281	-0.5242285	-0.0041947	0.03747515	-0.7683626	0.36901235	0.39920532	-0.0492445	0.05320331
102.7	0.49235478	-0.5245158	-0.0046426	0.03747714	-0.7680946	0.36756868	0.39929629	-0.0499251	0.05416263
102.8	0.49183471	-0.5238552	-0.0044016	0.03726761	-0.7675934	0.36712189	0.39944757	-0.0522032	0.05416129
102.9	0.49303556	-0.524061	-0.0049418	0.03645484	-0.7683059	0.36544188	0.39976818	-0.0534126	0.05457196
103	0.49380733	-0.5243308	-0.0061634	0.03700632	-0.7683686	0.36540169	0.3996078	-0.0538096	0.05496344
103.1	0.49423139	-0.5244129	-0.006796	0.03624264	-0.7681654	0.36427462	0.3997838	-0.0548048	0.05401035
103.2	0.49446211	-0.523949	-0.0074949	0.03554973	-0.7680864	0.36401958	0.39990344	-0.0556442	0.05468219
103.3	0.49452826	-0.524963	-0.0090035	0.03546252	-0.7680674	0.36365647	0.39987514	-0.0554663	0.05333574
103.4	0.49427978	-0.5253945	-0.0086491	0.03436868	-0.7679953	0.36282489	0.39974051	-0.0564508	0.05281675
103.5	0.49480114	-0.5254885	-0.0086906	0.03510337	-0.7682546	0.36209981	0.39951826	-0.0577678	0.0522243
103.6	0.49513253	-0.5258006	-0.0089806	0.03384801	-0.768494	0.36061679	0.40001491	-0.058474	0.05162688
103.7	0.49484291	-0.5257666	-0.0087117	0.0325808	-0.768025	0.36033296	0.40026267	-0.0592895	0.05100206
103.8	0.49455442	-0.5250661	-0.0071526	0.03251145	-0.767704	0.35910235	0.39947772	-0.0603271	0.05033416
103.9	0.49530009	-0.5241244	-0.0092572	0.03074209	-0.7671566	0.35710993	0.39963824	-0.0613508	0.04957548
104	0.49561823	-0.5239585	-0.008697	0.03050871	-0.7672761	0.35704663	0.39984691	-0.0605613	0.05092506
104.1	0.49612718	-0.524244	-0.0084032	0.03017574	-0.7675547	0.35607484	0.40035918	-0.0610173	0.05143933
104.2	0.49604177	-0.5244584	-0.0081526	0.02950664	-0.7676657	0.35594252	0.40041347	-0.061116	0.05141096
104.3	0.49578969	-0.523687	-0.0090195	0.02940081	-0.7677034	0.35402327	0.39890513	-0.0622236	0.05120869
104.4	0.49699295	-0.5231707	-0.0083531	0.02724206	-0.7674907	0.35187802	0.39938833	-0.0648571	0.0498778
104.5	0.49724534	-0.5226916	-0.0081105	0.02636278	-0.7673312	0.34993034	0.39953306	-0.0650274	0.04963326
104.6	0.49799937	-0.5226515	-0.0084844	0.02523486	-0.7671837	0.34960564	0.40014525	-0.0657062	0.04961415
104.7	0.49761507	-0.5224615	-0.0092142	0.02477492	-0.7671037	0.34957741	0.40031605	-0.0659142	0.05073102
104.8	0.49817436	-0.5222716	-0.008984	0.02355908	-0.7669873	0.34813323	0.40054256	-0.0668666	0.0511914
104.9	0.49788986	-0.5215412	-0.0082269	0.02260034	-0.7667605	0.34751649	0.39937677	-0.0671405	0.05115214
150	0.45470254	-0.4985063	0.00383944	-0.0304696	-0.7646805	0.26687065	0.44394954	-0.145936	0.05868655
150.1	0.45470254	-0.4985063	0.00383944	-0.0304696	-0.7646805	0.26687065	0.44394954	-0.145936	0.05868655
150.2	0.45470254	-0.4985063	0.00383944	-0.0304696	-0.7646805	0.26687065	0.44394954	-0.145936	0.05868655

105	0.49830955	-0.5218169	-0.008222	0.02241604	-0.7667996	0.34705214	0.39995704	-0.0677502	0.05077971
105.1	0.49793326	-0.5213535	-0.0083012	0.02259514	-0.766656	0.34595775	0.39996089	-0.0669974	0.05059941
105.2	0.49779287	-0.5211844	-0.0079193	0.02295954	-0.7664605	0.34685889	0.40071993	-0.0673081	0.05181763
105.3	0.49778543	-0.5216485	-0.0075977	0.02290268	-0.7664735	0.34711248	0.40056674	-0.0682762	0.05154776
105.4	0.49800341	-0.5217357	-0.0078632	0.02219746	-0.7666037	0.34613254	0.40055494	-0.0680976	0.05214238
105.5	0.4980442	-0.5219584	-0.0069618	0.0211704	-0.7663641	0.34584293	0.40086274	-0.0683269	0.0514902
105.6	0.49827738	-0.5221347	-0.0064243	0.02115417	-0.7664361	0.34529147	0.40083233	-0.0684201	0.05068847
105.7	0.49757796	-0.5216399	-0.006149	0.02081549	-0.7661475	0.34499791	0.40091736	-0.0687938	0.05030857
105.8	0.4975391	-0.5214491	-0.0059704	0.02069796	-0.7659333	0.3443141	0.40053706	-0.0689696	0.04965537
105.9	0.4970433	-0.5217548	-0.0054864	0.0202571	-0.7657849	0.34469401	0.40086289	-0.0688367	0.05003204
106	0.4977953	-0.5215595	-0.0058519	0.01968614	-0.7657977	0.34405568	0.40084444	-0.0693604	0.04949289
106.1	0.49745726	-0.521742	-0.0063132	0.01934553	-0.7658966	0.34356261	0.40086574	-0.0697174	0.04888312
106.2	0.4984415	-0.5216321	-0.0064506	0.01829002	-0.7659769	0.34325969	0.40063539	-0.0697075	0.04837334
106.3	0.49837187	-0.5217501	-0.0063114	0.01893412	-0.7662857	0.34289786	0.40081788	-0.0700961	0.0492439
106.4	0.49832037	-0.5219382	-0.0069035	0.01845152	-0.766354	0.34192054	0.39926487	-0.0710578	0.0494657
106.5	0.49837442	-0.5222175	-0.0072867	0.0183842	-0.7664313	0.3416269	0.39912712	-0.0711664	0.04936526
106.6	0.49918962	-0.5229051	-0.0066903	0.01699364	-0.7670647	0.34058624	0.3994657	-0.0735219	0.04903524
106.7	0.49987881	-0.5227965	-0.006882	0.01585152	-0.7672416	0.33936199	0.40003541	-0.0748627	0.04854952
106.8	0.49973361	-0.5231608	-0.0071776	0.01500375	-0.7668408	0.33911317	0.3997769	-0.0756723	0.04861153
106.9	0.49952317	-0.5229266	-0.0071614	0.01429383	-0.7666373	0.33949755	0.40037081	-0.0754522	0.04895503
107	0.49981126	-0.5230154	-0.00702	0.01353615	-0.7665988	0.33897823	0.40078	-0.075576	0.04912033
107.1	0.499555	-0.5228023	-0.0066019	0.01359611	-0.7666146	0.33913839	0.40093062	-0.076079	0.04954634
107.2	0.49953756	-0.5227528	-0.0059113	0.01260739	-0.7667992	0.3386512	0.40166275	-0.0760093	0.05057566
107.3	0.49937377	-0.522549	-0.0065203	0.01335239	-0.7668208	0.33733564	0.40148185	-0.075493	0.05028801
107.4	0.49913968	-0.5221082	-0.0062156	0.0133361	-0.7667901	0.33743731	0.40177244	-0.0757121	0.05074972
107.5	0.49889431	-0.5220431	-0.0061542	0.01350061	-0.7668683	0.33754791	0.40167601	-0.0757842	0.0508752
107.6	0.49849732	-0.5218122	-0.0060606	0.0126157	-0.7665829	0.33670575	0.40126823	-0.0764935	0.05043304
107.7	0.49867344	-0.5212423	-0.0055975	0.01307412	-0.7667142	0.33640451	0.40125477	-0.0758627	0.05081883
107.8	0.49976833	-0.5216714	-0.0061199	0.01201647	-0.7668269	0.33571092	0.40166364	-0.0768845	0.05120016
107.9	0.50035082	-0.5220343	-0.0060703	0.01173039	-0.7668995	0.33559984	0.4017887	-0.0777892	0.05189962
108	0.50067459	-0.5214811	-0.0070218	0.01147344	-0.7666445	0.33436571	0.40146171	-0.0782479	0.05179112
108.1	0.50004302	-0.5215738	-0.0077975	0.01120687	-0.7664768	0.33359652	0.40204331	-0.0789449	0.05234124
108.2	0.49999148	-0.5217202	-0.0087004	0.01133751	-0.7666425	0.33294109	0.40270047	-0.0790298	0.05192023
108.3	0.49992702	-0.5216586	-0.0088455	0.01128873	-0.7666841	0.33287858	0.40263086	-0.0791484	0.05194897
108.4	0.49926477	-0.5213474	-0.0088552	0.01069068	-0.7668023	0.33299681	0.40294156	-0.0791745	0.05221835
108.5	0.49944533	-0.5211824	-0.0085376	0.01131991	-0.7669087	0.33196294	0.40321655	-0.0802306	0.0515912
108.6	0.4985057	-0.5211462	-0.0087263	0.01070601	-0.7665228	0.3311012	0.40302533	-0.0810862	0.05120295
108.7	0.4985057	-0.5211462	-0.0087263	0.01070601	-0.7665228	0.3311012	0.40302533	-0.0810862	0.05120295
108.8	0.49815042	-0.5214501	-0.008384	0.01040134	-0.7666475	0.33027862	0.40327135	-0.0821485	0.05059643
108.9	0.49845991	-0.5215131	-0.00815	0.01056334	-0.7664524	0.32980531	0.40362852	-0.082364	0.0497858
109	0.49809857	-0.5215663	-0.0081092	0.01029855	-0.7664972	0.33003409	0.40386008	-0.0821255	0.04948005
109.1	0.4981842	-0.5208803	-0.0085944	0.00995313	-0.7664336	0.32915723	0.40392798	-0.0825358	0.04975046
109.2	0.49822261	-0.5207175	-0.008007	0.00993506	-0.7664687	0.32753193	0.40354366	-0.0819854	0.05084165
109.3	0.49836223	-0.5207363	-0.0095048	0.00916454	-0.7665912	0.3265327	0.40336152	-0.0823127	0.05114619
109.4	0.49816276	-0.5208171	-0.0103245	0.00897531	-0.7665587	0.32601688	0.40301833	-0.0827894	0.05136474
109.5	0.4985128	-0.5210167	-0.0103551	0.00920056	-0.7667764	0.32591772	0.4029764	-0.0830391	0.0514514
109.6	0.49845568	-0.5215995	-0.0108781	0.0094715	-0.7669432	0.32581837	0.40265057	-0.0829413	0.05172179
109.7	0.49859282	-0.5217831	-0.0109984	0.00952441	-0.7670379	0.32562761	0.40275946	-0.0829438	0.05167458
109.8	0.49886524	-0.5214335	-0.0107479	0.00903306	-0.7671784	0.32447778	0.40308209	-0.0828743	0.05086718
109.9	0.49896319	-0.5214415	-0.0111047	0.00936014	-0.7672607	0.32456629	0.40294275	-0.0830226	0.05073247
110	0.49932749	-0.5214633	-0.0116632	0.00924575	-0.7671794	0.3239212	0.40285776	-0.0834109	0.05077496
110.1	0.49915361	-0.522451	-0.0120329	0.00887585	-0.7674081	0.32354323	0.40256559	-0.0836179	0.05066107
110.2	0.49968447	-0.5226636	-0.0122619	0.00932337	-0.7678001	0.32362865	0.40248598	-0.0832732	0.0501322
110.3	0.49905209	-0.5225933	-0.01311	0.00876403	-0.7676341	0.32371808	0.40228563	-0.0845105	0.050505
110.4	0.4990823	-0.5229137	-0.0133004	0.00913084	-0.7678134	0.32396492	0.40227394	-0.0849977	0.05095796
110.5	0.49954759	-0.5227217	-0.013425	0.00892194	-0.7680661	0.32321422	0.40245056	-0.0846947	0.05048474
110.6	0.49959339	-0.5227644	-0.01352	0.00854193	-0.7679263	0.32314063	0.40236466	-0.0847329	0.05052313
110.7	0.49963946	-0.5225692	-0.0140037	0.00780952	-0.7677645	0.32376211	0.4032415	-0.0865148	0.05158292
110.8	0.49980929	-0.5224107	-0.0139066	0.00690146	-0.7677764	0.3231975	0.4035725	-0.0874828	0.05224624
110.9	0.49923344	-0.5228245	-0.0135501	0.0064576	-0.7678846	0.32277935	0.40342589	-0.0881468	0.05176731
111	0.49903749	-0.5225286	-0.0130031	0.00612387	-0.7677835	0.32230119	0.40325621	-0.0881829	0.05223111
111.1	0.49986657	-0.5228937	-0.0130168	0.00634059	-0.7684552	0.32229753	0.40329123	-0.0891341	0.05231361
111.2	0.49956755	-0.5231327	-0.0135758	0.00581183	-0.7685155	0.32192	0.40354134	-0.0906304	0.05274883
111.3	0.50054362	-0.5233963	-0.012776	0.00474313	-0.7686733	0.3218319	0.404227	-0.0899542	0.05279544
111.4	0.49993535	-0.5227908	-0.0122771	0.00507515	-0.7687028	0.3223657	0.40453967	-0.0895927	0.05329501
111.5	0.50029645	-0.5228628	-0.0114886	0.00514805	-0.7692912	0.32249463	0.40537809	-0.0897037	0.05333848
111.6	0.49969716	-0.5224323	-0.0114229	0.00541444	-0.7693979	0.32284063	0.40601139	-0.0899706	0.05434262
111.7	0.49967586	-0.5224039	-0.0114054	0.00514008	-0.7692923	0.32301606	0.40622112	-0.090054	0.05415445
111.8	0.49955249	-0.523385	-0.0114776	0.00410778	-0.7697045	0.32286961	0.40658004	-0.0898853	0.05382557
111.9	0.49951977	-0.5228048	-0.0116965	0.00394475	-0.7696629	0.32296772	0.40675695	-0.089826	0.0550196
112	0.49941126	-0.5221591	-0.0117965	0.0035241	-0.7693641	0.32253563	0.40673719	-0.0902635	0.05491929
112.1	0.49943102	-0.5217905	-0.011453	0.00253342	-0.768941	0.32201091	0.40640905	-0.0908419	0.05473652
112.2	0.49992577	-0.5221845	-0.0120975	0.00267773	-0.7691245	0.32173688	0.40706824	-0.091839	0.05450293
112.3	0.50019218	-0.5224469	-0.0120604	0.00190287	-0.7692104	0.32149807	0.40705046	-0.0920809	0.0545916
112.4	0.50013174	-0.522471	-0.0121336	0.0013928	-0.7692389	0.32135026	0.40642016	-0.0916771	0.05425168
112.5	0.4999444	-0.5222741	-0.0118121	0.00124552	-0.7695372	0.32045862	0.40694069	-0.0925051	0.05353057
112.6	0.4998582	-0.5222429	-0.0120905	0.00090733	-0.7695972	0.3201887	0.40653334	-0.0929987	0.05375538
112.7	0.49980974	-0.5216999	-0.0118649	0.00104683	-0.7696147	0.32005961	0.40608956	-0.0929978	0.05388227
112.8	0.49931364	-0.5216381	-0.0118246	0.00021796	-0.7692333	0.31905144	0.40575698	-0.0939057	0.05433184
112.9	0.49950072	-0.5219698	-0.0117108	0.00059747	-0.7693782	0.31862222	0.4051989	-0.0938173	0.05402266
113	0.50001125	-0.5219382	-0.0117603	0.0005127	-0.7692857	0.31825214	0.40475028	-0.0934636	0.05421005
113.1	0.50040655	-0.5222305	-0.0120884	0.00024756	-0.7695387	0.31787024	0.40469016	-0.0934771	0.05435819
113.2	0.49996149	-0.5217932	-0.0121769	0.00043458	-0.7694629	0.31666712	0.40467905	-0.0939649	0.05432215
113.3	0.50051258	-0.5221885	-0.0124856	3.37E-05	-0.7697993	0.31675731	0.40488312	-0.0941545	0.05439634
113.4	0.50096293	-0.5219774	-0.0133178	-0.0003783	-0.769897	0.31586182	0.40534608	-0.0952373	0.05528992
113.5	0.50061053	-0.5219606	-0.0135018	-0.0008767	-0.769843	0.31585561	0.40553382	-0.0951764	0.05531734
113.6	0.50069821	-0.5217277	-0.0135565	-0.0010872	-0.7700497	0.3153429	0.40643474	-0.0950282	0.05556218
113.7	0.50073527	-0.5216395	-0.0147736	-0.0013346	-0.7698502	0.31417111	0.406497	-0.0959583	0.05569452
113.8	0.50172515	-0.5213817	-0.0153001	-0.0013025	-0.7700865	0.3138885	0.4066811	-0.0951676	0.05638896
113.9	0.50169703	-0.5210502	-0.0154846	-0.0021307	-0.7703519	0.31294979	0.40750337	-0.0951102	0.05590162
114	0.50148792	-0.5206096	-0.0151013	-0.0013312	-0.7701698	0.31170669	0.4079417	-0.096455	0.05530635
114.1	0.50232522	-0.5209893	-0.0153946	-0.0012071	-0.7705086	0.3110775	0.4070541	-0.0962357	0.05535846
114.2	0.50197353	-0.5200467	-0.0146594	-0.0001002	-0.7705486	0.31175995	0.40766144	-0.0964104	0.05590402
114.3	0.50214635	-0.5199752	-0.014765	0.00060191	-0.7708306	0.31207916	0.4080454	-0.0966937	0.05625557
114.4	0.5024252	-0.5199614	-0.0150182	0.00077639	-0.7711544	0.31066561	0.40879071	-0.0972733	0.0550773
114.5	0.50213764	-0.5196217	-0.0149055	0.00088392	-0.7710635	0.31032424	0.40905818	-0.0971136	0.05542815
114.6	0.50197841	-0.5200616	-0.015427	0.00060707	-0.7711383	0.31072879	0.40899338	-0.0974975	0.0554621
114.7	0.50241613	-0.5200827	-0.0154633	0.00071471	-0.7712776	0.31076324	0.40893779	-0.0982526	0.05489302
114.8	0.50268687	-0.5197908	-0.0159477	0.00039709	-0.7711551	0.31004591	0.40872196	-0.098887	0.0551525
114.9	0.50241026	-0.5194655	-0.0156539	0.00014685	-0.7711254	0.30985118	0.40858528	-0.0992096	0.05514007
115	0.50232957	-0.5191749	-0.0151795	0.00032905	-0.7709774	0.3092592	0.40801192	-0.0996517	0.05484562
115.1	0.50254174	-0.5193621	-0.0152883	0.00041951	-0.7710834	0.30856135	0.40791253	-0.099411	0.05475512
115.2	0.50223986	-0.5193875	-0.0155209	0.00092967	-0.7716761	0.30821507	0.40807364	-0.1000082	0.05435457
115.3	0.50249862	-0.5195909	-0.0155267	0.0003574	-0.7718553	0.30784917	0.40825567	-0.1003975	0.0544061
115.4	0.50206515	-0.519076	-0.0151567	0.00022845	-0.7716638	0.30762213	0.40827383	-0.1002191	0.05439851
115.5	0.50232274	-0.5195538	-0.0153788	0.00011434	-0.7717457	0.3076121	0.40823675	-0.1003874	0.05459539
115.6	0.50196786	-0.5192325	-0.0150453	0.00020955	-0.7715944	0.30760699	0.40798996	-0.1003952	0.05468914
115.7	0.50200632	-0.5193065	-0.0156938	-0.0002855	-0.7715699	0.3072385	0.40823073	-0.1013728	0.05483542
115.8	0.50194314	-0.5188083	-0.0161026	-0.000603	-0.7713742	0.30679158	0.40760093	-0.1015869	0.05472262
115.9	0.50193092	-0.5187882	-0.0156417	-0.0012657	-0.77134	0.30676763	0.40799509	-0.101029	0.05513593
116	0.50165709	-0.5191899	-0.0152109	-0.0015199	-0.7714551	0.30636812	0.40814183	-0.1010259	0.05478805
116.1	0.50114014	-0.5195337	-0.0158354	-0.0013335	-0.7714361	0.30622492	0.40833432	-0.1017944	0.05444726
116.2	0.50105694	-0.519796	-0.015588	-0.00098	-0.7714176	0.30649199	0.40843645	-0.1020266	0.0545771
116.3	0.50083908	-0.5195603	-0.0153653	-0.0007258	-0.7712952	0.30685177	0.4083305	-0.1019806	0.05467626
116.4	0.50047342	-0.5192588	-0.0162604	-0.0006897	-0.7714527	0.30648308	0.40853184	-0.1018647	0.05492775
116.5	0.50031594	-0.519155	-0.0164294	-0.0012708	-0.7716394	0.30633561	0.40907369	-0.101265	0.05524792
116.6	0.50031594	-0.519155	-0.0164294	-0.0012708	-0.7716394	0.30633561	0.40907369	-0.101265	0.05524792
116.7	0.50050078	-0.518991	-0.0158581	-0.0015563	-0.7719251	0.30581439	0.40903631	-0.1007652	0.05509179
116.8	0.50047834	-0.5183064	-0.0159633	-0.0016595	-0.7717535	0.30484127	0.40878114	-0.1013516	0.05528985
116.9	0.50099081	-0.5186541	-0.0153966	-0.0017722	-0.7718206	0.30473359	0.40888605	-0.1014426	0.0550974
117	0.50066402	-0.5182269	-0.0149024	-0.0018666	-0.771767	0.30462164	0.40900333	-0.1017704	0.05493446
117.1	0.50079496	-0.5186711	-0.0153258	-0.0024009	-0.771831	0.30408278	0.40943992	-0.1023558	0.05466852
117.2	0.50034269	-0.5186382	-0.0141185	-0.0022166	-0.7718771	0.30421174	0.41015664	-0.1030237	0.05473687
117.3	0.49984983	-0.5188304	-0.0140694	-0.0028663	-0.7716798	0.30327479	0.40984352	-0.1030301	0.05429069
117.4	0.50076869	-0.5191286	-0.0142118	-0.0032642	-0.7719147	0.30236045	0.40965823	-0.1023713	0.05390907
117.5	0.50086887	-0.5191274	-0.0142525	-0.0032509	-0.7720223	0.3022483	0.40984807	-0.102667	0.05426134
117.6	0.50116302	-0.519679	-0.014786	-0.003402	-0.7721586	0.30219236	0.41012342	-0.1029189	0.05431264
117.7	0.50074951	-0.5193996	-0.0156223	-0.0039052	-0.7718352	0.30205211	0.4102781	-0.1034878	0.05444239
117.8	0.50099732	-0.5198429	-0.0162611	-0.0052679	-0.771587	0.30199762	0.41032475	-0.1041232	0.05469534
117.9	0.50090492	-0.5199258	-0.0161848	-0.0048264	-0.7717368	0.30156533	0.4102646	-0.1038874	0.05485975
118	0.50077815	-0.5200159	-0.0161653	-0.0048244	-0.7718288	0.30111495	0.41021757	-0.1048822	0.0545452
118.1	0.50006735	-0.5201526	-0.0158944	-0.0051279	-0.7714666	0.30101305	0.41022031	-0.1049219	0.05464648
118.2	0.50017931	-0.5203955	-0.016092	-0.0054883	-0.7716536	0.30053238	0.41043347	-0.1053652	0.05463555
118.3	0.50032521	-0.520326	-0.0161953	-0.0054264	-0.7718957	0.30047887	0.41044355	-0.1053198	0.05495957
118.4	0.50091264	-0.5205463	-0.0159172	-0.0062439	-0.7721421	0.29978516	0.41028715	-0.1058497	0.05415188
118.5	0.5011604	-0.5206297	-0.0159467	-0.0061966	-0.7724613	0.29924148	0.41094666	-0.1063624	0.0536979
118.6	0.50154764	-0.5209017	-0.0159979	-0.0061052	-0.7726115	0.29938804	0.41087389	-0.1061518	0.05440684
118.7	0.50153358	-0.5204053	-0.016348	-0.0055132	-0.7727015	0.29913052	0.41104013	-0.1061573	0.05517029
118.8	0.50096299	-0.5202379	-0.0161313	-0.0058199	-0.7724756	0.29916929	0.41090991	-0.1066127	0.05482434
118.9	0.5008881	-0.520846	-0.0155755	-0.0065579	-0.7726478	0.29836309	0.41148195	-0.1069066	0.05494963
119	0.5009633	-0.5208121	-0.0150531	-0.0064751	-0.7727017	0.29799153	0.41187612	-0.1071271	0.05515477
119.1	0.50098202	-0.5207673	-0.0150672	-0.0061784	-0.7727371	0.29779244	0.41205314	-0.1077505	0.05520197
119.2	0.50054115	-0.5202512	-0.0153321	-0.0062061	-0.7727121	0.29682029	0.41257253	-0.1079176	0.05504437
119.3	0.50125947	-0.520925	-0.015659	-0.0056398	-0.7733157	0.29700716	0.41353018	-0.1083429	0.05547143
119.4	0.50145769	-0.5206987	-0.0158499	-0.0055706	-0.7735284	0.29525508	0.41379392	-0.1083802	0.05490325
119.5	0.50147049	-0.5203056	-0.0161887	-0.0058743	-0.773389	0.29486219	0.41339388	-0.1088318	0.05533098
119.6	0.5014128	-0.5202625	-0.0165215	-0.0062382	-0.7732832	0.29514912	0.41339836	-0.1095852	0.05532653
119.7	0.50097136	-0.5204563	-0.015581	-0.0058683	-0.773753	0.29527209	0.41299585	-0.1098814	0.0550944
119.8	0.50055319	-0.520459	-0.0155639	-0.005535	-0.7736727	0.29543597	0.41313649	-0.1099534	0.05526214
119.9	0.50095402	-0.5204744	-0.015648	-0.0063492	-0.7735774	0.29493424	0.41309962	-0.1098848	0.05560251
165	0.44552999	-0.5023354	0.02662475	-0.0464787	-0.7632893	0.27193139	0.45328166	-0.1437427	0.05362224
165.1	0.44472466	-0.5020853	0.02615761	-0.0472518	-0.7629086	0.27163861	0.45322825	-0.1433189	0.05337703
165.2	0.44481239	-0.5022214	0.02622127	-0.0476541	-0.763091	0.27201103	0.45358287	-0.1428742	0.05312701

120.3	0.5011216	-0.5203767	-0.0147536	-0.0055916	-0.7740122	0.29480123	0.41356612	-0.1112045	0.05686987
120.4	0.50131926	-0.5204153	-0.0148266	-0.0059441	-0.7740618	0.29429916	0.41322521	-0.1116901	0.05637885
120.5	0.50144657	-0.5202086	-0.0147345	-0.0059566	-0.7743841	0.29467846	0.41367115	-0.1124413	0.05724899
120.6	0.50213203	-0.5206195	-0.0148023	-0.0071565	-0.7744118	0.29493469	0.41378722	-0.1120139	0.05799363
120.7	0.50230878	-0.5204767	-0.0150371	-0.0083556	-0.7745225	0.2938314	0.41364222	-0.1119627	0.05814125
120.8	0.50234661	-0.5207137	-0.0150815	-0.0086481	-0.7746118	0.29372453	0.41345254	-0.1116713	0.05811409
120.9	0.50144715	-0.5209523	-0.0144832	-0.0087856	-0.7746367	0.29399313	0.41300218	-0.1115192	0.05807707
121	0.50159364	-0.5210127	-0.0146111	-0.008482	-0.7749062	0.2937736	0.41321142	-0.1117946	0.05847455
121.1	0.50206357	-0.5206448	-0.0143014	-0.0076982	-0.7750499	0.29332039	0.41318326	-0.1117357	0.05872426
121.2	0.50218684	-0.5202939	-0.0136317	-0.0075964	-0.7754156	0.29364353	0.41400696	-0.1119816	0.05953791
121.3	0.50226231	-0.5206029	-0.0142443	-0.0088217	-0.7755052	0.29331658	0.41364623	-0.1126665	0.06018989
121.4	0.50218603	-0.5208679	-0.0145799	-0.0089179	-0.775415	0.29344691	0.41367789	-0.1127897	0.06008708
121.5	0.5025302	-0.5211334	-0.0152936	-0.0084393	-0.7757756	0.29347847	0.41343055	-0.1125336	0.05981656
121.6	0.50183666	-0.5213803	-0.015089	-0.0078097	-0.7759999	0.29323559	0.41384939	-0.1121849	0.05946193
121.7	0.50072143	-0.5213805	-0.0139848	-0.007378	-0.7757542	0.29425903	0.41402124	-0.1116836	0.05968556
121.8	0.50084944	-0.5213061	-0.0140533	-0.0078694	-0.7757921	0.29394146	0.4145738	-0.111727	0.05969248
121.9	0.50099462	-0.5214991	-0.0142833	-0.0076615	-0.7758168	0.29378569	0.41453558	-0.11183	0.05960567
122	0.50038365	-0.5216327	-0.0138668	-0.0066211	-0.7759892	0.29442365	0.41535461	-0.1125619	0.05994169
122.1	0.50038365	-0.5216327	-0.0138668	-0.0066211	-0.7759892	0.29442365	0.41535461	-0.1125619	0.05994169
122.2	0.50041233	-0.5215919	-0.0139144	-0.0063444	-0.7761452	0.29463853	0.41546693	-0.1123703	0.06017062
122.3	0.49965828	-0.5219841	-0.0139446	-0.0066539	-0.7757369	0.29428336	0.41520059	-0.1129155	0.05961584
122.4	0.49929629	-0.5215424	-0.014837	-0.0066438	-0.7756927	0.29356227	0.41549791	-0.1130397	0.05918487
122.5	0.49919067	-0.5218348	-0.0150338	-0.0064997	-0.7757055	0.29349991	0.41544635	-0.1126883	0.05959912
122.6	0.49882331	-0.5216439	-0.0150493	-0.0066028	-0.775432	0.29295619	0.41540317	-0.1131673	0.05891574
122.7	0.49897101	-0.5215157	-0.0149037	-0.0063006	-0.7752847	0.29290547	0.41586317	-0.1132432	0.0589173
122.8	0.49871953	-0.521402	-0.0145991	-0.006714	-0.7751551	0.29295327	0.41575897	-0.1133273	0.05853689
122.9	0.49825394	-0.5213118	-0.0146871	-0.0066799	-0.7751887	0.29240162	0.41561528	-0.112916	0.05899164
123	0.4985467	-0.5214223	-0.0146057	-0.0063006	-0.7753816	0.29208647	0.41557774	-0.1127701	0.05929108
123.1	0.49838005	-0.5216329	-0.0141772	-0.0063672	-0.7754064	0.29180172	0.41583415	-0.1124284	0.05899493
123.2	0.49814018	-0.5219727	-0.0143792	-0.0064854	-0.7754842	0.29146915	0.41568632	-0.1125579	0.05911134
123.3	0.49839603	-0.5224457	-0.0143626	-0.006633	-0.7756345	0.29127917	0.41593382	-0.1129815	0.05936139
123.4	0.4984313	-0.5222805	-0.0140806	-0.0060187	-0.7756913	0.29105365	0.4163386	-0.1129686	0.05957593
123.5	0.4980559	-0.5222482	-0.0140015	-0.0063913	-0.7758944	0.29065894	0.41673603	-0.1125827	0.05977888
123.6	0.49766156	-0.5221523	-0.0132117	-0.0066956	-0.7759329	0.29085039	0.41698308	-0.1124114	0.05958191
123.7	0.49668003	-0.521914	-0.0124499	-0.0063248	-0.7758338	0.29130071	0.41634403	-0.1126659	0.06023379
123.8	0.49665556	-0.521843	-0.0119852	-0.0063522	-0.7759966	0.29129414	0.41631331	-0.1125904	0.06019465
123.9	0.4962187	-0.5216927	-0.0119342	-0.0062033	-0.7760336	0.29074119	0.41641976	-0.1122478	0.06040985
124	0.49602984	-0.5215151	-0.011564	-0.00631	-0.7760896	0.29069156	0.41632456	-0.1120801	0.06063735
124.1	0.49602984	-0.5215151	-0.011564	-0.00631	-0.7760896	0.29069156	0.41632456	-0.1120801	0.06063735
124.2	0.49621228	-0.5220906	-0.0117717	-0.0065268	-0.776471	0.29078736	0.41651545	-0.1127024	0.06114076
124.3	0.49588401	-0.5219324	-0.0122187	-0.0071376	-0.7763998	0.29015967	0.41674636	-0.1124328	0.0609327
124.4	0.49569566	-0.5218247	-0.0128765	-0.006881	-0.7764385	0.28970901	0.41685821	-0.1124805	0.06136179
124.5	0.49539889	-0.5208455	-0.012382	-0.0072825	-0.7761141	0.28887132	0.41643443	-0.113007	0.06099932
124.6	0.49494218	-0.5208415	-0.0125506	-0.0071922	-0.7761197	0.28849587	0.41633871	-0.1130759	0.06104401
124.7	0.49494218	-0.5208415	-0.0125506	-0.0071922	-0.7761197	0.28849587	0.41633871	-0.1130759	0.06104401
124.8	0.49466143	-0.5206132	-0.0124805	-0.0074787	-0.7762162	0.28779496	0.41664806	-0.1132812	0.06097864
124.9	0.49453425	-0.5204667	-0.0119162	-0.0082181	-0.7760499	0.28710564	0.41669584	-0.1129077	0.060789
125	0.49421675	-0.5206981	-0.0123464	-0.0085811	-0.7760973	0.28704155	0.41702424	-0.1123574	0.06142122
125.1	0.49410065	-0.5204292	-0.0119574	-0.0080652	-0.7761738	0.287123	0.41707727	-0.1125915	0.06163634
125.2	0.49399389	-0.5204881	-0.0126087	-0.007752	-0.7763178	0.28699309	0.41730037	-0.1130474	0.06114598
125.3	0.49399389	-0.5204881	-0.0126087	-0.007752	-0.7763178	0.28699309	0.41730037	-0.1130474	0.06114598
125.4	0.49409167	-0.5203859	-0.0126029	-0.0074538	-0.7762983	0.28563915	0.41711732	-0.1130659	0.06111771
125.5	0.49367324	-0.5200762	-0.0121691	-0.0070762	-0.7760475	0.28591755	0.41684769	-0.1132826	0.06124143
125.6	0.49373528	-0.5197059	-0.0125891	-0.0067852	-0.7760228	0.28546631	0.4170514	-0.1130423	0.06118536
125.7	0.49373528	-0.5197059	-0.0125891	-0.0067852	-0.7760228	0.28546631	0.4170514	-0.1130423	0.06118536
125.8	0.49303867	-0.519557	-0.0122814	-0.0061881	-0.7760712	0.28504758	0.41627202	-0.1131351	0.06069001
125.9	0.49362076	-0.5192336	-0.0119766	-0.0056001	-0.7761655	0.28410851	0.41669201	-0.1128997	0.06002941
126	0.49320855	-0.5190042	-0.0114787	-0.0050153	-0.7761723	0.28364187	0.41685493	-0.1130281	0.05983156
126.1	0.49316554	-0.5186849	-0.0118604	-0.0054931	-0.7762902	0.28285213	0.41744948	-0.1126752	0.06051172
126.2	0.49278738	-0.5189244	-0.0110875	-0.0057267	-0.7763019	0.28227301	0.41778002	-0.1124175	0.0608609
126.3	0.49258506	-0.5187282	-0.0109049	-0.0055882	-0.7762368	0.28156173	0.4172578	-0.1133503	0.06089702
126.4	0.49224624	-0.5190873	-0.0092497	-0.0055002	-0.7763791	0.28180561	0.41814984	-0.1136699	0.06037439
126.5	0.49156523	-0.5182846	-0.0094961	-0.0052409	-0.7762213	0.28155667	0.41803913	-0.1138559	0.06000014
126.6	0.49156523	-0.5182846	-0.0094961	-0.0052409	-0.7762213	0.28155667	0.41803913	-0.1138559	0.06000014
126.7	0.4910784	-0.5184523	-0.009315	-0.0056388	-0.7761936	0.28112003	0.41834999	-0.114295	0.05995556
126.8	0.49028283	-0.5181601	-0.0090634	-0.0053725	-0.7761565	0.28132613	0.41814632	-0.1144712	0.05985906
126.9	0.49015282	-0.5171119	-0.0091813	-0.0055166	-0.776112	0.28077467	0.41889238	-0.1139491	0.06001016
127	0.49029118	-0.5170685	-0.0085876	-0.0056585	-0.7764041	0.28094171	0.41931795	-0.1142475	0.05927323
127.1	0.49040978	-0.5171298	-0.0090159	-0.0054583	-0.7765188	0.28095235	0.41961153	-0.1150777	0.05964923
127.2	0.48934942	-0.5162815	-0.0087191	-0.005607	-0.7758229	0.28036322	0.41933192	-0.115094	0.06003889
127.3	0.48976469	-0.5157903	-0.0090746	-0.0053569	-0.7760326	0.28046181	0.41931057	-0.1153908	0.06045586
127.4	0.48932076	-0.5157888	-0.0082794	-0.0052064	-0.7760138	0.28075226	0.41976438	-0.1153381	0.06019263
127.5	0.48957043	-0.5152601	-0.0077496	-0.005647	-0.7761506	0.28073523	0.42020697	-0.1151351	0.06024894
127.6	0.48957043	-0.5152601	-0.0077496	-0.005647	-0.7761506	0.28073523	0.42020697	-0.1151351	0.06024894
127.7	0.4895928	-0.5152288	-0.0081826	-0.0052208	-0.7761903	0.28051052	0.42035001	-0.1152543	0.06024798
127.8	0.4895928	-0.5152288	-0.0081826	-0.0052208	-0.7761903	0.28051052	0.42035001	-0.1152543	0.06024798
127.9	0.48925215	-0.5151455	-0.0075887	-0.0051591	-0.7761212	0.28058758	0.42065563	-0.1151987	0.06011314
128	0.48935137	-0.5148133	-0.0072676	-0.0046288	-0.7762661	0.28015518	0.42087004	-0.1146744	0.05968977
128.1	0.48930991	-0.5144544	-0.0075227	-0.0046259	-0.7762072	0.2799547	0.42102303	-0.1148613	0.05936623
128.2	0.48929414	-0.5139773	-0.0078571	-0.0043026	-0.7760736	0.27936959	0.42083838	-0.1149925	0.05921645
128.3	0.48899322	-0.5138123	-0.0078907	-0.0043119	-0.7759516	0.27884065	0.42073998	-0.1153518	0.05848661
128.4	0.48922887	-0.513673	-0.0075635	-0.0041208	-0.7759511	0.27860052	0.4209373	-0.1161465	0.05844477
128.5	0.48962282	-0.5137735	-0.0071909	-0.0034774	-0.775971	0.27861078	0.42103163	-0.1166021	0.05828879
128.6	0.488938	-0.5136977	-0.0061964	-0.003388	-0.7755978	0.27898421	0.42151542	-0.1168956	0.05807082
128.7	0.48915287	-0.5140105	-0.0058878	-0.004015	-0.7757642	0.27940144	0.42151618	-0.1168049	0.05881634
128.8	0.48830283	-0.5144616	-0.0059769	-0.0042353	-0.7755433	0.27887148	0.42181901	-0.1168709	0.05841679
128.9	0.48830283	-0.5144616	-0.0059769	-0.0042353	-0.7755433	0.27887148	0.42181901	-0.1168709	0.05841679
129	0.48867951	-0.5144245	-0.0061399	-0.0047219	-0.7757131	0.27980562	0.42257287	-0.1173247	0.05877708
129.1	0.48814729	-0.5143212	-0.0059775	-0.0050333	-0.7758273	0.27952665	0.42333957	-0.1175749	0.05760235
129.2	0.48852193	-0.5143179	-0.0063654	-0.0046045	-0.7759843	0.27959767	0.42347677	-0.1177492	0.05767934
129.3	0.48843123	-0.5143116	-0.0069029	-0.0045811	-0.7759969	0.27951019	0.42315311	-0.1177841	0.05783591
129.4	0.48824794	-0.5141291	-0.0069755	-0.0050036	-0.7757312	0.27848869	0.42311136	-0.1179158	0.05729909
129.5	0.4876473	-0.5139635	-0.0061536	-0.005619	-0.7755548	0.27861997	0.42325811	-0.1184214	0.05714112
129.6	0.4876473	-0.5139635	-0.0061536	-0.005619	-0.7755548	0.27861997	0.42325811	-0.1184214	0.05714112
129.7	0.4873055	-0.5137092	-0.0061025	-0.0055309	-0.7753554	0.27871849	0.42327823	-0.1183444	0.05753848
129.8	0.4873055	-0.5137092	-0.0061025	-0.0055309	-0.7753554	0.27871849	0.42327823	-0.1183444	0.05753848
129.9	0.48650418	-0.5134551	-0.0056629	-0.0050728	-0.7753163	0.27803599	0.42369986	-0.1192554	0.05675443
130	0.48611793	-0.513264	-0.0061486	-0.0056135	-0.7750851	0.2772343	0.42336143	-0.1191937	0.05655656
130.1	0.48617888	-0.5128555	-0.0060629	-0.0058287	-0.7751966	0.27635615	0.42351047	-0.1195323	0.05621913
130.2	0.4865513	-0.5126719	-0.0055188	-0.0059457	-0.7752885	0.2758053	0.4236962	-0.1188963	0.0561117
130.3	0.48615732	-0.5123985	-0.0063858	-0.0065351	-0.7753745	0.27624909	0.42398262	-0.119254	0.05627
130.4	0.4857045	-0.5121778	-0.0064942	-0.0061656	-0.7754842	0.27552164	0.42508401	-0.1190697	0.05618748
130.5	0.48573093	-0.5119279	-0.0067476	-0.006927	-0.7753306	0.27514966	0.42540375	-0.119171	0.0562945
130.6	0.4854101	-0.5122288	-0.0062512	-0.0075799	-0.7753544	0.27514974	0.4255469	-0.1197476	0.05605532
130.7	0.48468457	-0.5122705	-0.0064353	-0.0073266	-0.7752852	0.27456488	0.42586799	-0.1199798	0.05479781
130.8	0.48519121	-0.5127018	-0.0060974	-0.0077075	-0.7755242	0.27470815	0.42629412	-0.1205869	0.0547201
130.9	0.48540663	-0.5124163	-0.0060866	-0.0077411	-0.775496	0.27442541	0.42652336	-0.1205237	0.0547908
131	0.4850154	-0.512193	-0.0060974	-0.0079345	-0.7754394	0.27434825	0.42671864	-0.1199158	0.05529257
131.1	0.48465088	-0.5124663	-0.0055346	-0.0081323	-0.7755969	0.27450588	0.42697893	-0.1204377	0.0550922
131.2	0.48439244	-0.5115303	-0.0051262	-0.0080063	-0.7757701	0.27384403	0.42731728	-0.1197363	0.05519942
131.3	0.48485226	-0.5108595	-0.005709	-0.0075309	-0.7756363	0.27313951	0.42749448	-0.1201745	0.05505651
131.4	0.48399283	-0.5106948	-0.0060936	-0.0070878	-0.7754745	0.27333814	0.42739823	-0.1197726	0.05478039
131.5	0.48433803	-0.5104489	-0.005065	-0.0074436	-0.775395	0.27279945	0.42720726	-0.1201519	0.05466117
131.6	0.48529388	-0.5099621	-0.0044403	-0.0071417	-0.775845	0.27212663	0.42730222	-0.119857	0.05477295
131.7	0.48480979	-0.509791	-0.004706	-0.0081263	-0.7756443	0.27228951	0.42779897	-0.1198119	0.05536863
131.8	0.48480625	-0.5097802	-0.0044898	-0.0083348	-0.775404	0.27259525	0.42766234	-0.1197214	0.05578375
131.9	0.48464561	-0.5100689	-0.0042211	-0.0086485	-0.7753885	0.27312976	0.42798673	-0.1200341	0.0556246
132	0.48460833	-0.5092089	-0.003411	-0.0085554	-0.7753207	0.27282422	0.42833243	-0.1205626	0.05560434
132.1	0.48369642	-0.5087799	-0.0033652	-0.0084866	-0.7748516	0.27281344	0.42853076	-0.1211425	0.05571806
132.2	0.48382869	-0.5087388	-0.0036363	-0.0086879	-0.7747727	0.27232219	0.42846496	-0.1215307	0.05539947
132.3	0.48382869	-0.5087388	-0.0036363	-0.0086879	-0.7747727	0.27232219	0.42846496	-0.1215307	0.05539947
132.4	0.48382869	-0.5087388	-0.0036363	-0.0086879	-0.7747727	0.27232219	0.42846496	-0.1215307	0.05539947
132.5	0.48382869	-0.5087388	-0.0036363	-0.0086879	-0.7747727	0.27232219	0.42846496	-0.1215307	0.05539947
132.6	0.48382869	-0.5087388	-0.0036363	-0.0086879	-0.7747727	0.27232219	0.42846496	-0.1215307	0.05539947
132.7	0.48323097	-0.5085419	-0.0037274	-0.0086056	-0.7745889	0.27245129	0.42805622	-0.1219561	0.05528754
132.8	0.48323097	-0.5085419	-0.0037274	-0.0086056	-0.7745889	0.27245129	0.42805622	-0.1219561	0.05528754
132.9	0.48333729	-0.5085389	-0.0035952	-0.0086041	-0.774817	0.27223898	0.42804351	-0.1220529	0.05544235
133	0.48333729	-0.5085389	-0.0035952	-0.0086041	-0.774817	0.27223898	0.42804351	-0.1220529	0.05544235
133.1	0.48340787	-0.5083294	-0.003938	-0.0088795	-0.7748099	0.27197162	0.42828007	-0.121937	0.05590715
133.2	0.48358807	-0.5077643	-0.0046219	-0.0088768	-0.7748503	0.27214841	0.42840956	-0.1218526	0.05656841
133.3	0.48385157	-0.5075366	-0.0044602	-0.0090976	-0.774843	0.27186111	0.42862111	-0.1222549	0.05646861
133.4	0.48363897	-0.5073584	-0.004241	-0.0089035	-0.774815	0.27136822	0.42859096	-0.1219439	0.05622357
133.5	0.48419115	-0.5072182	-0.0044644	-0.0092159	-0.7749052	0.27166729	0.42895103	-0.1217962	0.05640557
133.6	0.48419115	-0.5072182	-0.0044644	-0.0092159	-0.7749052	0.27166729	0.42895103	-0.1217962	0.05640557
133.7	0.48422593	-0.5067088	-0.0041662	-0.009003	-0.7748537	0.27076266	0.42878713	-0.121778	0.05550288
133.8	0.48395776	-0.5068602	-0.0041216	-0.0087101	-0.7748584	0.27085201	0.42864251	-0.1221294	0.05518701
133.9	0.48414029	-0.506671	-0.0045478	-0.0088612	-0.7746888	0.27079634	0.42855801	-0.1222085	0.05509669
134	0.48414029	-0.506671	-0.0045478	-0.0088612	-0.7746888	0.27079634	0.42855801	-0.1222085	0.05509669
134.1	0.48414029	-0.506671	-0.0045478	-0.0088612	-0.7746888	0.27079634	0.42855801	-0.1222085	0.05509669
134.2	0.48381818	-0.5065726	-0.004654	-0.0089339	-0.774503	0.27106129	0.42826179	-0.1228312	0.05462158
134.3	0.4836928	-0.5066487	-0.0044116	-0.0090099	-0.7745372	0.2712029	0.42849561	-0.1229405	0.0545345
134.4	0.48322249	-0.5067253	-0.0050885	-0.0085013	-0.7745521	0.26950702	0.42886318	-0.123196	0.05425151
134.5	0.48305894	-0.5071023	-0.0048721	-0.0090968	-0.7746309	0.26977028	0.42938867	-0.1237762	0.05389193
134.6	0.48305894	-0.5071023	-0.0048721	-0.0090968	-0.7746309	0.26977028	0.42938867	-0.1237762	0.05389193
134.7	0.48314876	-0.507006	-0.0047547	-0.0095597	-0.7745774	0.26993297	0.42988967	-0.1242996	0.05356035
134.8	0.48314876	-0.507006	-0.0047547	-0.0095597	-0.7745774	0.26993297	0.42988967	-0.1242996	0.05356035
134.9	0.48358896	-0.5065057	-0.0049504	-0.0095317	-0.7744604	0.26933373	0.42927581	-0.1243878	0.05357403
180	0.42962001	-0.5096267	0.06529053	-0.0591621	-0.7584053	0.26811706	0.45455525	-0.1375016	0.03638596
180.1	0.42955136	-0.5097304	0.0656407	-0.0590895	-0.7584075	0.26789152	0.45486747	-0.1377609	0.0365801
180.2	0.42947215	-0.5104105	0.06643218	-0.0587629	-0.7582538	0.26841278	0.4549714	-0.1373711	0.03678765
180.3	0.42969414	-0.5104008	0.06768438	-0.0589276	-0.7580573	0.26823174	0.45536409	-0.13742	0.03657764
180.4	0.42969414	-0.5104008	0.06768438	-0.0589276	-0.7580573	0.26823174	0.45536409	-0.13742	0.03657764
180.5	0.42963903	-0.5104606	0.06751045	-0.059403	-0.7581523	0.268103	0.45542889	-0.1374206	0.03685244

135.3	0.48305102	-0.5066757	-0.0044327	-0.0098827	-0.7743736	0.2684802	0.43020192	-0.1236864	0.05351735
135.4	0.48316517	-0.5064777	-0.0046436	-0.0094302	-0.7743381	0.26853663	0.43053267	-0.1243714	0.05384087
135.5	0.48292851	-0.5063197	-0.0044748	-0.0090864	-0.7745699	0.26816417	0.43049154	-0.1245846	0.05419202
135.6	0.4828916	-0.5063861	-0.0046681	-0.0095386	-0.7745463	0.26758331	0.43076845	-0.1243669	0.05399785
135.7	0.48290716	-0.5068378	-0.0046672	-0.0101076	-0.7742426	0.26662208	0.43156056	-0.1253182	0.05370602
135.8	0.48275878	-0.506435	-0.0044686	-0.0101678	-0.7740241	0.26663937	0.4313336	-0.1252129	0.0537374
135.9	0.48203702	-0.5061749	-0.0049998	-0.0097981	-0.7736607	0.26633322	0.43121455	-0.1263095	0.05373721
136	0.48209769	-0.5062568	-0.0048612	-0.0096246	-0.7738278	0.26627075	0.4313317	-0.1262326	0.05399587
136.1	0.48047997	-0.5062972	-0.0042276	-0.0097343	-0.7734469	0.26542372	0.43098861	-0.1274137	0.05354666
136.2	0.48057913	-0.5063609	-0.0038234	-0.0097458	-0.77348	0.2657683	0.431285	-0.1277191	0.05444391
136.3	0.48055484	-0.5065947	-0.0035954	-0.0097268	-0.7735878	0.26559225	0.4312287	-0.127921	0.05431578
136.4	0.48063808	-0.5064334	-0.0037634	-0.0102947	-0.7735526	0.26558629	0.43218898	-0.1278751	0.05467724
136.5	0.48108643	-0.506257	-0.0037498	-0.0105563	-0.7737453	0.26508269	0.43264697	-0.1284936	0.05556454
136.6	0.48073573	-0.5061129	-0.0026738	-0.0110102	-0.7736005	0.26500416	0.43348007	-0.1291928	0.05566687
136.7	0.48073573	-0.5061129	-0.0026738	-0.0110102	-0.7736005	0.26500416	0.43348007	-0.1291928	0.05566687
136.8	0.48085565	-0.5059778	-0.0015124	-0.0111141	-0.7737284	0.26441416	0.43395405	-0.1287775	0.05553734
136.9	0.48000895	-0.5053568	-0.0021015	-0.0118859	-0.7730906	0.26324328	0.43382437	-0.1293998	0.05606071
137	0.47966041	-0.5046932	-0.0017259	-0.0121454	-0.7733944	0.26295119	0.43429867	-0.1287438	0.0574761
137.1	0.47910483	-0.5047691	-0.0017429	-0.0124574	-0.773314	0.26316212	0.43465313	-0.1292175	0.05752843
137.2	0.47928174	-0.5052439	-0.0018414	-0.0120714	-0.7733489	0.26315766	0.43485423	-0.1292403	0.05774874
137.3	0.47904248	-0.5051576	-0.0013042	-0.0124656	-0.7734449	0.26321356	0.43467124	-0.129203	0.05727074
137.4	0.47915384	-0.5049544	-0.0010168	-0.0123729	-0.7734537	0.26324329	0.43461512	-0.1291687	0.05739417
137.5	0.47927162	-0.5048002	-0.000915	-0.0125488	-0.7734641	0.2631975	0.43475913	-0.1289731	0.05748698
137.6	0.47927162	-0.5048002	-0.000915	-0.0125488	-0.7734641	0.2631975	0.43475913	-0.1289731	0.05748698
137.7	0.47907762	-0.5046323	-0.0014401	-0.0131866	-0.7731755	0.26279981	0.43536852	-0.1288799	0.05723029
137.8	0.47907762	-0.5046323	-0.0014401	-0.0131866	-0.7731755	0.26279981	0.43536852	-0.1288799	0.05723029
137.9	0.47873331	-0.5047186	-0.0008955	-0.0128093	-0.7731095	0.26306327	0.43541893	-0.1285832	0.05708949
138	0.479105	-0.5043828	-0.0008284	-0.0130999	-0.7734291	0.26315391	0.43588805	-0.1279868	0.05756855
138.1	0.4789167	-0.5044751	0.00026463	-0.0124565	-0.7736902	0.26312525	0.43625781	-0.1283106	0.05784532
138.2	0.47900025	-0.5043438	0.00054889	-0.0122761	-0.7737529	0.26368715	0.4365169	-0.1284912	0.05845518
138.3	0.47910101	-0.5045527	0.00082324	-0.0116224	-0.7739344	0.26397822	0.4370672	-0.1288957	0.0585025
138.4	0.4789498	-0.5051503	0.00061545	-0.0120257	-0.773962	0.26369422	0.43709076	-0.1296625	0.05853518
138.5	0.4789498	-0.5051503	0.00061545	-0.0120257	-0.773962	0.26369422	0.43709076	-0.1296625	0.05853518
138.6	0.47802997	-0.5053123	0.00022225	-0.012176	-0.7737649	0.26430373	0.43764599	-0.1301199	0.05914976
138.7	0.47774749	-0.5051271	-0.0003607	-0.0122039	-0.7733223	0.264951	0.43773571	-0.1311331	0.06019127
138.8	0.47712863	-0.5054448	-5.35E-05	-0.0136255	-0.7731986	0.26471076	0.43800168	-0.1326852	0.05989385
138.9	0.47703834	-0.5050379	-9.88E-05	-0.0134428	-0.7729813	0.26469484	0.43775422	-0.1330289	0.06010545
139	0.47668411	-0.5046516	8.66E-05	-0.0137631	-0.7727582	0.26452897	0.43803266	-0.1330494	0.06001982
139.1	0.47639608	-0.504412	-0.0004081	-0.014167	-0.7725085	0.26429221	0.43824425	-0.1330076	0.0601601
139.2	0.47560389	-0.5041573	0.00013495	-0.0144827	-0.7721253	0.26403237	0.43872501	-0.1339108	0.06028973
139.3	0.47509046	-0.5042505	0.00013905	-0.0148931	-0.7718788	0.26429644	0.43885639	-0.1338904	0.06014244
139.4	0.47463192	-0.5035232	5.36E-05	-0.0149572	-0.7714899	0.26396452	0.43867695	-0.1342266	0.06074119
139.5	0.47463192	-0.5035232	5.36E-05	-0.0149572	-0.7714899	0.26396452	0.43867695	-0.1342266	0.06074119
139.6	0.47496341	-0.5033531	0.00050824	-0.0144655	-0.7717314	0.26428471	0.43863158	-0.1342475	0.06103524
139.7	0.47449267	-0.5035199	0.00061266	-0.0147119	-0.7716901	0.26430597	0.43883727	-0.1337538	0.06083889
139.8	0.47477735	-0.5032822	0.00026453	-0.0143953	-0.7713827	0.26384325	0.43870982	-0.1340039	0.06133965
139.9	0.47475418	-0.5031769	2.79E-05	-0.0145483	-0.7713494	0.26365627	0.43862158	-0.1341749	0.0615759
140	0.47475418	-0.5031769	2.79E-05	-0.0145483	-0.7713494	0.26365627	0.43862158	-0.1341749	0.0615759
140.1	0.47548128	-0.5033988	0.00045629	-0.0152244	-0.7716937	0.26320415	0.43902565	-0.1340555	0.06146231
140.2	0.47512277	-0.5034909	0.00010734	-0.015284	-0.7717078	0.26330252	0.43873806	-0.1343303	0.06150686
140.3	0.47427969	-0.5039247	-0.0004262	-0.0165632	-0.7713132	0.2630726	0.43902074	-0.1355168	0.06071085
140.4	0.47415737	-0.5035889	0.00014999	-0.0172355	-0.7714971	0.26285467	0.43933224	-0.1356634	0.06086579
140.5	0.47337144	-0.5038762	0.00042515	-0.0175822	-0.7711771	0.26341128	0.43964083	-0.1363472	0.06050228
140.6	0.47348245	-0.5040731	0.000141	-0.0186978	-0.7712225	0.26335958	0.4401342	-0.1367112	0.06016503
140.7	0.47320396	-0.5041488	-0.0002503	-0.0192284	-0.7710651	0.26355754	0.44021271	-0.1370744	0.06018159
140.8	0.47268565	-0.5041831	-0.0005682	-0.0189616	-0.7710904	0.2636795	0.44018272	-0.1374664	0.06007152
140.9	0.47276585	-0.5039926	-0.0005095	-0.0185248	-0.771088	0.26343389	0.44025716	-0.1375814	0.06020487
141	0.47299652	-0.503478	-0.0008035	-0.0186627	-0.7709524	0.26298842	0.44040706	-0.1377351	0.06064686
141.1	0.47234681	-0.502932	-0.0006468	-0.0188075	-0.7706747	0.26291995	0.44029318	-0.1374611	0.06096759
141.2	0.47240325	-0.5032954	-0.0001566	-0.0191857	-0.7708502	0.26310483	0.44064573	-0.1371514	0.06082863
141.3	0.4723695	-0.5028504	-0.0004481	-0.0191991	-0.7706144	0.26285387	0.44048141	-0.1371229	0.06118436
141.4	0.4719616	-0.5026019	9.85E-05	-0.018723	-0.7705773	0.2632383	0.44081135	-0.1373095	0.06114676
141.5	0.47154941	-0.5028102	0.00019861	-0.0190586	-0.7705526	0.2633974	0.44080387	-0.1372041	0.06086636
141.6	0.47169828	-0.5027606	0.00058454	-0.019152	-0.770607	0.26358698	0.44122809	-0.1374742	0.06092619
141.7	0.47174629	-0.5026484	0.0009807	-0.0191237	-0.7705367	0.26389504	0.44135899	-0.1372375	0.06083545
141.8	0.4715845	-0.5025814	0.00090672	-0.0190804	-0.7704699	0.26440267	0.44147855	-0.1377383	0.06023004
141.9	0.47114785	-0.5028742	0.00087161	-0.0191348	-0.7704436	0.26478856	0.4413442	-0.1379293	0.06038187
142	0.47114785	-0.5028742	0.00087161	-0.0191348	-0.7704436	0.26478856	0.4413442	-0.1379293	0.06038187
142.1	0.4703778	-0.5031563	0.00048309	-0.019362	-0.7703723	0.26514019	0.44104727	-0.1380489	0.06064215
142.2	0.47032012	-0.5031533	0.00070348	-0.0199273	-0.7703836	0.26536236	0.44087115	-0.1378823	0.06069494
142.3	0.47032012	-0.5031533	0.00070348	-0.0199273	-0.7703836	0.26536236	0.44087115	-0.1378823	0.06069494
142.4	0.47024017	-0.5033754	0.0009103	-0.0203256	-0.7703666	0.26533927	0.44095569	-0.137741	0.06016888
142.5	0.47032201	-0.503282	0.00070044	-0.0201216	-0.7703286	0.26576368	0.44116719	-0.1375758	0.06056168
142.6	0.47032201	-0.503282	0.00070044	-0.0201216	-0.7703286	0.26576368	0.44116719	-0.1375758	0.06056168
142.7	0.46956937	-0.5033571	0.00068735	-0.019614	-0.7702551	0.26613194	0.44116787	-0.1378431	0.06034701
142.8	0.46920113	-0.5034884	0.00062699	-0.0202671	-0.7701244	0.26648184	0.44087396	-0.1385528	0.06019613
142.9	0.4690081	-0.5036482	0.00076395	-0.0207513	-0.7699821	0.26677268	0.44087531	-0.1385549	0.05990812
143	0.46899133	-0.5039226	0.00113616	-0.0205939	-0.7701772	0.2669525	0.44071176	-0.138386	0.06034354
143.1	0.46840169	-0.5039231	0.0006933	-0.0198736	-0.7702505	0.26700347	0.44023436	-0.1385427	0.06014232
143.2	0.46805167	-0.5038193	0.00049816	-0.0198382	-0.7700453	0.26734573	0.43990298	-0.1386825	0.06015493
143.3	0.46784956	-0.5037953	0.00021949	-0.020032	-0.7701848	0.26737594	0.44016346	-0.1396324	0.06015999
143.4	0.46780534	-0.503977	-8.96E-05	-0.0202127	-0.7701848	0.26718781	0.44001638	-0.1399446	0.06035204
143.5	0.46734712	-0.5041452	-2.36E-05	-0.0201512	-0.7701648	0.26766731	0.44008237	-0.1399844	0.06022995
143.6	0.46767178	-0.503761	-0.0004044	-0.0202996	-0.7701534	0.26802844	0.44019178	-0.1399666	0.06029801
143.7	0.46736253	-0.5035822	-0.000262	-0.0202476	-0.7702209	0.26765196	0.44022018	-0.1398499	0.06004365
143.8	0.46751889	-0.5031293	-0.0005065	-0.0209393	-0.770276	0.26823513	0.44085195	-0.1394673	0.06051888
143.9	0.4671239	-0.5033611	0.00010312	-0.0209734	-0.7702671	0.26798508	0.44101644	-0.1397415	0.06024335
144	0.46669475	-0.5029094	0.00023255	-0.02046	-0.7699778	0.26822063	0.44121259	-0.1398088	0.06071164
144.1	0.46669475	-0.5029094	0.00023255	-0.02046	-0.7699778	0.26822063	0.44121259	-0.1398088	0.06071164
144.2	0.46656422	-0.5033665	0.00025961	-0.0198373	-0.7700889	0.26865252	0.4412472	-0.1400395	0.06088927
144.3	0.46642866	-0.5027684	0.00018971	-0.0193946	-0.7697824	0.26811157	0.44113883	-0.1412339	0.06049699
144.4	0.46623447	-0.5030187	0.00015805	-0.0193246	-0.7697994	0.26871545	0.44118218	-0.1415254	0.06056944
144.5	0.46554239	-0.5029065	0.00017634	-0.0194703	-0.769524	0.26873153	0.44097848	-0.1415822	0.06061541
144.6	0.46549125	-0.503036	0.00105647	-0.0196317	-0.7692558	0.26788824	0.44080604	-0.1410567	0.05990839
144.7	0.46500598	-0.5027422	0.00109931	-0.0201001	-0.7690123	0.26798565	0.44045894	-0.141545	0.06026071
144.8	0.46421028	-0.502487	0.00120592	-0.0203089	-0.7687883	0.26811611	0.44041264	-0.1419931	0.06012268
144.9	0.46314777	-0.5023227	0.00139392	-0.0211542	-0.7682776	0.26854146	0.44063051	-0.1427292	0.05974813
145	0.46291577	-0.5023207	0.00107395	-0.0213653	-0.7682219	0.26847641	0.44073853	-0.1427744	0.05933465
145.1	0.46329031	-0.5018634	0.00096043	-0.0215907	-0.7680941	0.26819221	0.44069522	-0.1431886	0.05950288
145.2	0.46340969	-0.5018625	0.00148569	-0.0221188	-0.7682359	0.26792031	0.44057762	-0.1438218	0.0598152
145.3	0.46290528	-0.5021793	0.00197589	-0.0221624	-0.7680655	0.26857898	0.44086265	-0.14406	0.05976297
145.4	0.46262823	-0.5019465	0.00215639	-0.0220837	-0.7678724	0.26918495	0.44100848	-0.1439789	0.0600152
145.5	0.46283096	-0.5018703	0.00260362	-0.0220452	-0.7679882	0.26909433	0.44115452	-0.1436808	0.06021771
145.6	0.46306752	-0.501826	0.00235386	-0.0214405	-0.7681722	0.26935505	0.44162712	-0.1439244	0.06074291
145.7	0.46297495	-0.5013813	0.00246029	-0.0213381	-0.7680252	0.26909691	0.44140636	-0.1439821	0.06083288
145.8	0.46274144	-0.5014648	0.00179519	-0.0213234	-0.7678671	0.26882939	0.44160342	-0.1438746	0.06128837
145.9	0.46253798	-0.5011754	0.00207634	-0.0214061	-0.7678099	0.26869789	0.44150729	-0.1443013	0.06134838
146	0.46253798	-0.5011754	0.00207634	-0.0214061	-0.7678099	0.26869789	0.44150729	-0.1443013	0.06134838
146.1	0.46256339	-0.5008575	0.00191302	-0.0216049	-0.7680553	0.26871299	0.44162985	-0.1443048	0.06108389
146.2	0.46256339	-0.5008575	0.00191302	-0.0216049	-0.7680553	0.26871299	0.44162985	-0.1443048	0.06108389
146.3	0.46256339	-0.5008575	0.00191302	-0.0216049	-0.7680553	0.26871299	0.44162985	-0.1443048	0.06108389
146.4	0.46213182	-0.501265	0.00157976	-0.0221541	-0.7680498	0.26856942	0.44155727	-0.1449357	0.0610553
146.5	0.4615628	-0.5015436	0.00155441	-0.0233056	-0.7678659	0.26871133	0.44211461	-0.144516	0.06065313
146.6	0.46136048	-0.5014039	0.00233634	-0.0232098	-0.7677771	0.26920982	0.44196924	-0.1444887	0.06101959
146.7	0.46132044	-0.5014172	0.00231931	-0.0238419	-0.767541	0.26912045	0.44216219	-0.1443645	0.0611071
146.8	0.46123702	-0.5014459	0.00277593	-0.0246401	-0.7675524	0.26922503	0.44207238	-0.1446807	0.06037144
146.9	0.46099637	-0.500846	0.00283099	-0.0245114	-0.7674016	0.26952326	0.4422443	-0.1446071	0.06037874
147	0.460844	-0.5009182	0.00266625	-0.0250102	-0.7674157	0.26946257	0.44210937	-0.1443561	0.06014555
147.1	0.46072362	-0.5010703	0.00217169	-0.0250632	-0.7674199	0.26976287	0.4421069	-0.1446708	0.05977002
147.2	0.46034654	-0.5013479	0.00160594	-0.0254298	-0.7671371	0.26905497	0.44237416	-0.1448076	0.05976206
147.3	0.46053254	-0.501077	0.00207642	-0.0255311	-0.7670046	0.26880825	0.4428362	-0.1442303	0.05984677
147.4	0.46013616	-0.5009723	0.00159032	-0.025171	-0.766734	0.26884685	0.44260134	-0.1448653	0.06001537
147.5	0.45981648	-0.5005075	0.00195908	-0.0256193	-0.7668284	0.26884755	0.44330964	-0.1443869	0.05988041
147.6	0.45957517	-0.5003556	0.00198962	-0.0253955	-0.7666604	0.2683559	0.4431613	-0.1443821	0.05945273
147.7	0.45957517	-0.5003556	0.00198962	-0.0253955	-0.7666604	0.2683559	0.4431613	-0.1443821	0.05945273
147.8	0.45938198	-0.5006712	0.00143461	-0.0262459	-0.7666071	0.26805472	0.44315633	-0.1445061	0.0591946
147.9	0.45962611	-0.5005761	0.00158292	-0.0257389	-0.7666936	0.26882662	0.44341373	-0.1445641	0.05966117
148	0.45955536	-0.50044	0.00175625	-0.0254908	-0.7665379	0.26868391	0.44357437	-0.1446589	0.05931073
148.1	0.45879181	-0.5008843	0.00247191	-0.0256953	-0.7665146	0.26932981	0.44341842	-0.1444408	0.05907758
148.2	0.45852704	-0.5008934	0.00284099	-0.0257151	-0.7664524	0.26897913	0.44346061	-0.1446064	0.05913459
148.3	0.45879648	-0.5008724	0.00314522	-0.0254683	-0.7665986	0.26883122	0.44363166	-0.1450203	0.05923201
148.4	0.45874052	-0.5007946	0.00344977	-0.0262219	-0.766542	0.26791087	0.44401599	-0.1445096	0.05890544
148.5	0.45957629	-0.5004133	0.00337926	-0.0260949	-0.7669052	0.26865521	0.44388847	-0.1444709	0.05882945
148.6	0.45957629	-0.5004133	0.00337926	-0.0260949	-0.7669052	0.26865521	0.44388847	-0.1444709	0.05882945
148.7	0.45957629	-0.5004133	0.00337926	-0.0260949	-0.7669052	0.26865521	0.44388847	-0.1444709	0.05882945
148.8	0.45913349	-0.5003869	0.0033135	-0.0269932	-0.7666262	0.26890531	0.44427495	-0.1450163	0.05847371
148.9	0.45919822	-0.499903	0.00304216	-0.0273391	-0.7665916	0.26876703	0.44414091	-0.1444726	0.0587596
149	0.45876726	-0.4993353	0.00302342	-0.0273035	-0.7662281	0.26913941	0.44401836	-0.1444349	0.05881815
149.1	0.45876726	-0.4993353	0.00302342	-0.0273035	-0.7662281	0.26913941	0.44401836	-0.1444349	0.05881815
149.2	0.45866616	-0.4993592	0.00288598	-0.0273632	-0.7661982	0.26905331	0.44402982	-0.1447133	0.0588707
149.3	0.45695787	-0.4995238	0.00233593	-0.0288201	-0.7654753	0.26823954	0.44407776	-0.145194	0.05860997
149.4	0.4565518	-0.4991946	0.00244965	-0.0289864	-0.7651404	0.26813034	0.44385232	-0.1451284	0.05864087
149.5	0.45683619	-0.4988035	0.00259142	-0.0285925	-0.764986	0.26824658	0.44384153	-0.1450833	0.05883207
149.6	0.45630858	-0.4989985	0.00247117	-0.02879	-0.764763	0.26814491	0.44400973	-0.1449322	0.0587543
149.7	0.45543222	-0.498971	0.00320697	-0.0293614	-0.7648691	0.26826818	0.44364159	-0.1450452	0.05849001
149.8	0.45514921	-0.4986574	0.00363382	-0.029496	-0.7647216	0.26736301	0.44351431	-0.1458608	0.05847311
149.9	0.4546696	-0.4984251	0.00398777	-0.0300706	-0.7647755	0.26699489	0.44379384	-0.1454642	0.05872686
195	0.41966428	-0.5145049	0.09844826	-0.06891	-0.7511118	0.27428533	0.44589018	-0.1327768	0.02879623
195.1	0.41975409	-0.514595	0.09844192	-0.0689279	-0.7508284	0.27433805	0.44592753	-0.1326858	0.02910057
195.2	0.41932562	-0.5146868	0.09823798	-0.0688061	-0.7506826	0.27432209	0.44552657	-0.1326431	0.02942092
195.3	0.41932562	-0.5146868	0.09823798	-0.0688061	-0.7506826	0.27432209	0.44552657	-0.1326431	0.02942092
195.4	0.41887496	-0.5144297	0.09860683	-0.0689316	-0.7505041	0.27414957	0.4454055	-0.1326056	0.02922378
195.5	0.41885917	-0.5142663	0.09893021	-0.0687292	-0.7503074	0.2742058	0.44498856	-0.1325904	0.02940261

150.3	0.45484741	-0.4983667	0.00358831	-0.0305333	-0.7644278	0.26683089	0.44356134	-0.1457651	0.05839004
150.4	0.45484741	-0.4983667	0.00358831	-0.0305333	-0.7644278	0.26683089	0.44356134	-0.1457651	0.05839004
150.5	0.45469599	-0.4982652	0.00310891	-0.0302845	-0.7641901	0.266929	0.44354128	-0.1462003	0.05840592
150.6	0.45469599	-0.4982652	0.00310891	-0.0302845	-0.7641901	0.266929	0.44354128	-0.1462003	0.05840592
150.7	0.45469599	-0.4982652	0.00310891	-0.0302845	-0.7641901	0.266929	0.44354128	-0.1462003	0.05840592
150.8	0.45511942	-0.4983905	0.00335065	-0.030407	-0.7644213	0.26736513	0.44419218	-0.1467319	0.05839338
150.9	0.45550622	-0.4982126	0.00337577	-0.0301679	-0.7644259	0.26743979	0.44411904	-0.1462736	0.05826357
151	0.45535475	-0.497951	0.00387324	-0.0307252	-0.7642473	0.26711498	0.44379692	-0.1461229	0.0579864
151.1	0.45520136	-0.4982611	0.00362409	-0.0307306	-0.764164	0.2668133	0.44341529	-0.1460473	0.05841843
151.2	0.45521146	-0.4983191	0.00390436	-0.030628	-0.764382	0.26672572	0.44339813	-0.1457478	0.05847404
151.3	0.45537792	-0.4981741	0.00419948	-0.0307526	-0.7644893	0.26654363	0.44364252	-0.1458729	0.05870933
151.4	0.45553631	-0.4979732	0.00452814	-0.030906	-0.7645235	0.26661061	0.44388243	-0.1455778	0.05885302
151.5	0.45487902	-0.4982445	0.004918	-0.0315932	-0.7644277	0.26639904	0.44371818	-0.1449734	0.05870646
151.6	0.45496117	-0.4985721	0.00531016	-0.0317223	-0.7645391	0.26655409	0.44392772	-0.1449961	0.05844855
151.7	0.45482522	-0.4984248	0.00570228	-0.0318022	-0.764687	0.26643854	0.44422477	-0.1444623	0.0584413
151.8	0.45461024	-0.4987282	0.00653059	-0.0324116	-0.7649337	0.26685187	0.44475612	-0.1440679	0.05857968
151.9	0.45409989	-0.4988488	0.00584581	-0.0335704	-0.7648845	0.26647409	0.44531309	-0.1437739	0.05920247
152	0.45397121	-0.4986023	0.00596543	-0.0340365	-0.7648894	0.26658247	0.44552888	-0.1438322	0.05901945
152.1	0.45397121	-0.4986023	0.00596543	-0.0340365	-0.7648894	0.26658247	0.44552888	-0.1438322	0.05901945
152.2	0.45399475	-0.4981965	0.00597779	-0.0337107	-0.7648711	0.2669307	0.44546643	-0.1438254	0.05916476
152.3	0.45376244	-0.4978691	0.00622287	-0.033199	-0.7649446	0.26720506	0.44577753	-0.143175	0.05972212
152.4	0.45376244	-0.4978691	0.00622287	-0.033199	-0.7649446	0.26720506	0.44577753	-0.143175	0.05972212
152.5	0.45376244	-0.4978691	0.00622287	-0.033199	-0.7649446	0.26720506	0.44577753	-0.143175	0.05972212
152.6	0.45376244	-0.4978691	0.00622287	-0.033199	-0.7649446	0.26720506	0.44577753	-0.143175	0.05972212
152.7	0.4539658	-0.4980862	0.00669941	-0.0333736	-0.7650538	0.2674605	0.44578421	-0.1435222	0.05935688
152.8	0.45389358	-0.4975755	0.00695187	-0.0340424	-0.7649343	0.26736664	0.44599204	-0.1434579	0.05930022
152.9	0.45410952	-0.4977115	0.00702264	-0.0343585	-0.7650766	0.26783037	0.44609167	-0.143479	0.05944882
153	0.45324883	-0.497728	0.00695657	-0.0346448	-0.764987	0.2675953	0.44626994	-0.1430891	0.05944341
153.1	0.45319891	-0.497827	0.00731909	-0.0343173	-0.7650302	0.26785769	0.44660045	-0.14323	0.05914447
153.2	0.45287667	-0.4974099	0.00733779	-0.0344219	-0.764867	0.26774019	0.44636363	-0.1428024	0.05921617
153.3	0.45287667	-0.4974099	0.00733779	-0.0344219	-0.764867	0.26774019	0.44636363	-0.1428024	0.05921617
153.4	0.45289141	-0.4980695	0.00681811	-0.0340927	-0.7650608	0.26775409	0.44668605	-0.1434006	0.05934518
153.5	0.45289141	-0.4980695	0.00681811	-0.0340927	-0.7650608	0.26775409	0.44668605	-0.1434006	0.05934518
153.6	0.45293723	-0.4981129	0.00684122	-0.0337222	-0.7651726	0.26803422	0.44726554	-0.1434936	0.05964966
153.7	0.45324792	-0.498109	0.00695595	-0.0335851	-0.7650512	0.26829984	0.44730652	-0.1440485	0.05914231
153.8	0.45285583	-0.4985292	0.00737456	-0.0339328	-0.7651137	0.26872917	0.44777111	-0.1444935	0.05886607
153.9	0.4523066	-0.4983591	0.00781183	-0.0340674	-0.7649953	0.26883346	0.44819713	-0.1444149	0.0585445
154	0.4523066	-0.4983591	0.00781183	-0.0340674	-0.7649953	0.26883346	0.44819713	-0.1444149	0.0585445
154.1	0.45218609	-0.4985575	0.00800183	-0.0340574	-0.7649432	0.26950427	0.44836581	-0.1450058	0.05860237
154.2	0.45218609	-0.4985575	0.00800183	-0.0340574	-0.7649432	0.26950427	0.44836581	-0.1450058	0.05860237
154.3	0.45217849	-0.4986353	0.00818482	-0.0342145	-0.7649756	0.26962029	0.44824021	-0.1451566	0.05870972
154.4	0.45217849	-0.4986353	0.00818482	-0.0342145	-0.7649756	0.26962029	0.44824021	-0.1451566	0.05870972
154.5	0.45217849	-0.4986353	0.00818482	-0.0342145	-0.7649756	0.26962029	0.44824021	-0.1451566	0.05870972
154.6	0.45217722	-0.4991127	0.00852146	-0.0345474	-0.7651115	0.27019583	0.44843086	-0.1450468	0.05885681
154.7	0.45198708	-0.4988651	0.00837109	-0.0348246	-0.7650813	0.27057612	0.44859151	-0.1446695	0.05933349
154.8	0.45179309	-0.4988809	0.00828571	-0.0352745	-0.7649343	0.27015351	0.44882204	-0.1442589	0.05950505
154.9	0.45179309	-0.4988809	0.00828571	-0.0352745	-0.7649343	0.27015351	0.44882204	-0.1442589	0.05950505
155	0.45134885	-0.4991242	0.0087371	-0.036214	-0.7646421	0.27013673	0.44837771	-0.1447845	0.05883721
155.1	0.45134885	-0.4991242	0.0087371	-0.036214	-0.7646421	0.27013673	0.44837771	-0.1447845	0.05883721
155.2	0.45090809	-0.4984824	0.0091005	-0.0360431	-0.7643267	0.27016033	0.44839551	-0.1443934	0.05854479
155.3	0.45106939	-0.4984335	0.00886583	-0.0355751	-0.7643774	0.27014503	0.4488298	-0.144422	0.05907395
155.4	0.45101057	-0.4979945	0.00957503	-0.0354423	-0.7642746	0.26951297	0.44868046	-0.1445966	0.05904064
155.5	0.45069055	-0.4979736	0.00918676	-0.0355388	-0.7642209	0.26960405	0.44848597	-0.1449899	0.05920204
155.6	0.45063921	-0.497865	0.00959812	-0.0357127	-0.7639863	0.26940586	0.44836728	-0.145034	0.05905482
155.7	0.45079447	-0.497974	0.00977386	-0.035492	-0.7639846	0.26936969	0.44841087	-0.144696	0.05902547
155.8	0.45079447	-0.497974	0.00977386	-0.035492	-0.7639846	0.26936969	0.44841087	-0.144696	0.05902547
155.9	0.45087571	-0.4980051	0.01002315	-0.0357689	-0.7641026	0.26974155	0.44875761	-0.1444467	0.05925217
156	0.45058232	-0.4978321	0.01015364	-0.0367933	-0.764005	0.26986626	0.44861221	-0.1446965	0.05933808
156.1	0.45025299	-0.4980888	0.01091297	-0.0370901	-0.7641477	0.26985933	0.4488138	-0.1444995	0.05868041
156.2	0.45024891	-0.4976505	0.01092765	-0.0371181	-0.7640693	0.26993648	0.44856097	-0.1444942	0.0586106
156.3	0.45048651	-0.4976609	0.0112455	-0.0371626	-0.764168	0.26998016	0.4485535	-0.1442996	0.05865916
156.4	0.45048651	-0.4976609	0.0112455	-0.0371626	-0.764168	0.26998016	0.4485535	-0.1442996	0.05865916
156.5	0.45047801	-0.4975166	0.01133097	-0.0375858	-0.7640617	0.26987265	0.44859189	-0.1440775	0.05862215
156.6	0.45047801	-0.4975166	0.01133097	-0.0375858	-0.7640617	0.26987265	0.44859189	-0.1440775	0.05862215
156.7	0.45008327	-0.4972065	0.01136443	-0.0376857	-0.7639543	0.26984315	0.4487115	-0.1438223	0.05890572
156.8	0.44981207	-0.4971579	0.01167762	-0.0376714	-0.7638569	0.27007438	0.44879001	-0.1443072	0.05890775
156.9	0.44961708	-0.4969476	0.01175821	-0.0382048	-0.7639012	0.2705351	0.448636	-0.1451408	0.05961815
157	0.44941035	-0.4975062	0.01229676	-0.03856	-0.7640782	0.27089145	0.44887972	-0.145006	0.05957503
157.1	0.44907279	-0.4972316	0.01221457	-0.0387831	-0.763978	0.27088553	0.4487092	-0.1453036	0.05926813
157.2	0.44907279	-0.4972316	0.01221457	-0.0387831	-0.763978	0.27088553	0.4487092	-0.1453036	0.05926813
157.3	0.44897863	-0.4974171	0.01207851	-0.0393058	-0.763832	0.27061355	0.44874504	-0.1450861	0.0590265
157.4	0.44839567	-0.497171	0.01197352	-0.0392214	-0.7635799	0.27037529	0.44878756	-0.1450544	0.05924509
157.5	0.44780513	-0.4972834	0.01155991	-0.0391734	-0.7634388	0.27076081	0.44866031	-0.1453606	0.05916005
157.6	0.44780513	-0.4972834	0.01155991	-0.0391734	-0.7634388	0.27076081	0.44866031	-0.1453606	0.05916005
157.7	0.44780513	-0.4972834	0.01155991	-0.0391734	-0.7634388	0.27076081	0.44866031	-0.1453606	0.05916005
157.8	0.44766355	-0.4981712	0.01252973	-0.039888	-0.763858	0.27134064	0.44948882	-0.1449273	0.05866111
157.9	0.44746609	-0.4983949	0.01292817	-0.0399987	-0.7639215	0.27127252	0.44974313	-0.1449699	0.05816202
158	0.4474944	-0.4983636	0.01313367	-0.0395901	-0.7638953	0.27119227	0.44994774	-0.1450363	0.05872804
158.1	0.44732271	-0.4988128	0.013369	-0.0397812	-0.7640551	0.27141881	0.45017143	-0.1448141	0.05868177
158.2	0.44696576	-0.4991383	0.01447721	-0.0396254	-0.7641905	0.27184971	0.4502848	-0.1440583	0.05860213
158.3	0.44674362	-0.4991132	0.01438383	-0.039627	-0.763956	0.27133437	0.45031033	-0.1435725	0.05854499
158.4	0.44693568	-0.4987834	0.01510553	-0.0401913	-0.7639449	0.27126706	0.45017225	-0.1425786	0.05797875
158.5	0.44693568	-0.4987834	0.01510553	-0.0401913	-0.7639449	0.27126706	0.45017225	-0.1425786	0.05797875
158.6	0.44660078	-0.4990006	0.01521656	-0.0405824	-0.7639788	0.27073625	0.45035257	-0.1421552	0.05774293
158.7	0.44660078	-0.4990006	0.01521656	-0.0405824	-0.7639788	0.27073625	0.45035257	-0.1421552	0.05774293
158.8	0.44667882	-0.4988614	0.01545012	-0.0412179	-0.7638746	0.2716777	0.45011967	-0.1416153	0.05808596
158.9	0.44691972	-0.4988084	0.01579451	-0.0410531	-0.7638043	0.27116111	0.45012497	-0.1414837	0.05801315
159	0.44687868	-0.4987262	0.0158944	-0.0408988	-0.7637664	0.27100434	0.45033968	-0.1415436	0.05765189
159.1	0.44684531	-0.4987414	0.01708365	-0.0414873	-0.7635331	0.27090054	0.45071009	-0.1413183	0.05743431
159.2	0.44699032	-0.4985303	0.01717124	-0.0414448	-0.763546	0.27096167	0.45063663	-0.1408127	0.05757559
159.3	0.44695016	-0.4989516	0.01801544	-0.0415478	-0.7637867	0.27120125	0.45126997	-0.1406994	0.05774691
159.4	0.44730384	-0.4985936	0.01762164	-0.041428	-0.763881	0.27137399	0.45118616	-0.1406038	0.05799917
159.5	0.44730384	-0.4985936	0.01762164	-0.041428	-0.763881	0.27137399	0.45118616	-0.1406038	0.05799917
159.6	0.4461452	-0.4988972	0.01739652	-0.0421526	-0.7635464	0.27149898	0.45130126	-0.1408852	0.05731344
159.7	0.44624088	-0.4990465	0.01731404	-0.0417721	-0.7634869	0.27162376	0.45150053	-0.1410784	0.05744327
159.8	0.44624088	-0.4990465	0.01731404	-0.0417721	-0.7634869	0.27162376	0.45150053	-0.1410784	0.05744327
159.9	0.44637723	-0.4992773	0.01713142	-0.0415457	-0.763465	0.2719049	0.45148477	-0.1412115	0.05793456
160	0.44637723	-0.4992773	0.01713142	-0.0415457	-0.763465	0.2719049	0.45148477	-0.1412115	0.05793456
160.1	0.44637723	-0.4992773	0.01713142	-0.0415457	-0.763465	0.2719049	0.45148477	-0.1412115	0.05793456
160.2	0.44630541	-0.499173	0.01684601	-0.041786	-0.7633029	0.27225154	0.45131134	-0.1414298	0.05765798
160.3	0.44647045	-0.49897	0.01736629	-0.0420171	-0.7632658	0.27185808	0.45110963	-0.1414966	0.05740015
160.4	0.44647045	-0.49897	0.01736629	-0.0420171	-0.7632658	0.27185808	0.45110963	-0.1414966	0.05740015
160.5	0.44633845	-0.4993273	0.01759746	-0.0423799	-0.7634848	0.27193136	0.45120536	-0.1414419	0.05724916
160.6	0.44599352	-0.4995966	0.01713837	-0.0425079	-0.7634674	0.27202424	0.45095908	-0.1419801	0.05736713
160.7	0.4456511	-0.4998102	0.01711051	-0.0426709	-0.763329	0.27158477	0.45105678	-0.1421575	0.05726449
160.8	0.44539953	-0.4996318	0.01758658	-0.0422243	-0.7631796	0.27173916	0.4511093	-0.1424283	0.05715965
160.9	0.44535489	-0.5001135	0.01788437	-0.0421845	-0.763284	0.271941	0.45118109	-0.1426423	0.05680857
161	0.44576365	-0.5004507	0.0187448	-0.0427412	-0.7634836	0.27163576	0.45150696	-0.1431176	0.05625854
161.1	0.44599731	-0.5006198	0.01950682	-0.0430574	-0.7635583	0.27219019	0.45170162	-0.1433299	0.05626022
161.2	0.44618776	-0.5007998	0.02047949	-0.0435011	-0.7637592	0.2719963	0.4520418	-0.1433227	0.05647399
161.3	0.44592386	-0.5011066	0.02015549	-0.0438003	-0.7636004	0.27189193	0.4519257	-0.1435762	0.05607557
161.4	0.44676999	-0.5010783	0.01946185	-0.0439303	-0.763744	0.27243099	0.4517988	-0.1435627	0.05653744
161.5	0.44713175	-0.5010447	0.01933817	-0.0440864	-0.7637841	0.27297924	0.45175908	-0.1433654	0.05692156
161.6	0.44722669	-0.5012209	0.01952742	-0.0441249	-0.7637823	0.27270163	0.45138948	-0.1435556	0.0569156
161.7	0.44752	-0.5007611	0.01969273	-0.0446515	-0.7637352	0.27283877	0.45155233	-0.143354	0.05682897
161.8	0.4472695	-0.501362	0.01974514	-0.0447725	-0.7636768	0.27291698	0.45122008	-0.1432055	0.05684764
161.9	0.44659942	-0.5014867	0.01959395	-0.0445386	-0.7634822	0.27344198	0.45107418	-0.1432506	0.05695299
162	0.44659942	-0.5014867	0.01959395	-0.0445386	-0.7634822	0.27344198	0.45107418	-0.1432506	0.05695299
162.1	0.44659942	-0.5014867	0.01959395	-0.0445386	-0.7634822	0.27344198	0.45107418	-0.1432506	0.05695299
162.2	0.446365	-0.5012032	0.02064252	-0.0441112	-0.7632613	0.27334977	0.45096475	-0.1431437	0.05633607
162.3	0.446365	-0.5012032	0.02064252	-0.0441112	-0.7632613	0.27334977	0.45096475	-0.1431437	0.05633607
162.4	0.446365	-0.5012032	0.02064252	-0.0441112	-0.7632613	0.27334977	0.45096475	-0.1431437	0.05633607
162.5	0.4460123	-0.5011723	0.02041234	-0.0443667	-0.7630806	0.27275763	0.45053722	-0.1437929	0.05622008
162.6	0.44575588	-0.5012931	0.02066145	-0.0442784	-0.7631363	0.27238092	0.45073079	-0.143859	0.05581509
162.7	0.44555086	-0.5013673	0.02048505	-0.0440857	-0.7630608	0.27253941	0.4506249	-0.1444426	0.05580292
162.8	0.44463164	-0.5011894	0.02070754	-0.0447221	-0.7627332	0.27184873	0.45060401	-0.1444376	0.0553106
162.9	0.44463164	-0.5011894	0.02070754	-0.0447221	-0.7627332	0.27184873	0.45060401	-0.1444376	0.0553106
163	0.44469823	-0.500978	0.02050609	-0.0445828	-0.762867	0.27191729	0.45071986	-0.144415	0.05524222
163.1	0.44485159	-0.501102	0.02089481	-0.0443764	-0.7627727	0.27195164	0.45054099	-0.14417	0.05522258
163.2	0.44524671	-0.5009822	0.02080195	-0.0441915	-0.7627895	0.27129176	0.45047719	-0.1442296	0.05502182
163.3	0.44524671	-0.5009822	0.02080195	-0.0441915	-0.7627895	0.27129176	0.45047719	-0.1442296	0.05502182
163.4	0.44507883	-0.5013606	0.02132958	-0.0444189	-0.7627924	0.2711745	0.45056742	-0.1440962	0.05487756
163.5	0.44507883	-0.5013606	0.02132958	-0.0444189	-0.7627924	0.2711745	0.45056742	-0.1440962	0.05487756
163.6	0.44541533	-0.5014314	0.02191585	-0.0440591	-0.7628978	0.27156982	0.45058046	-0.144399	0.05455142
163.7	0.44541533	-0.5014314	0.02191585	-0.0440591	-0.7628978	0.27156982	0.45058046	-0.144399	0.05455142
163.8	0.44559477	-0.5016097	0.02229221	-0.0441024	-0.7628459	0.27177271	0.45053781	-0.1439136	0.05508782
163.9	0.44559477	-0.5016097	0.02229221	-0.0441024	-0.7628459	0.27177271	0.45053781	-0.1439136	0.05508782
164	0.44557205	-0.501691	0.02306599	-0.044155	-0.7629372	0.27183101	0.45058955	-0.1443617	0.05510974
164.1	0.44629081	-0.5014362	0.02358211	-0.0447494	-0.7630946	0.27274809	0.45129608	-0.1443092	0.05553935
164.2	0.44620092	-0.5018778	0.02477012	-0.0453457	-0.7631748	0.27191287	0.45180001	-0.1442656	0.05446836
164.3	0.44585107	-0.5015696	0.02531506	-0.0450524	-0.7631052	0.27221449	0.45233274	-0.144238	0.05444934
164.4	0.44575576	-0.5018395	0.02622175	-0.0455023	-0.7630259	0.27224643	0.4524725	-0.1435988	0.05406418
164.5	0.44557053	-0.5018375	0.026615	-0.045407	-0.763165	0.27170471	0.45266481	-0.1433672	0.0539964
164.6	0.44557053	-0.5018375	0.026615	-0.045407	-0.763165	0.27170471	0.45266481	-0.1433672	0.0539964
164.7	0.44553504	-0.5022083	0.02660266	-0.0461282	-0.7632688	0.27161343	0.45289262	-0.1434855	0.05361661
164.8	0.44520778	-0.5023568	0.02650459	-0.0462199	-0.7631428	0.27142867	0.45306415	-0.1435887	0.05350364
164.9	0.44520778	-0.5023568	0.02650459	-0.0462199	-0.7631428	0.27142867	0.45306415	-0.1435887	0.05350364
210	0.41098696	-0.5122501	0.11167069	-0.0754194	-0.7452506	0.27456728	0.43919681	-0.1309973	0.02458244
210.1	0.41098696	-0.5122501	0.11167069	-0.0754194	-0.7452506	0.27456728	0.43919681	-0.1309973	0.02458244
210.2	0.41098696	-0.5122501	0.11167069	-0.0754194	-0.7452506	0.27456728	0.43919681	-0.1309973	0.02458244
210.3	0.41065371	-0.5121926	0.11197696	-0.0755181	-0.7451375	0.27454735	0.43891097	-0.1313801	0.02464821
210.4	0.41014704	-0.5121043	0.11187899	-0.0754677	-0.7448497	0.27465992	0.43857114	-0.131485	0.02449166
210.5	0.41014704	-0.5121043	0.11187899	-0.0754677	-0.7448497	0.27465992	0.43857114	-0.131485	0.02449166

165.3	0.44494428	-0.501898	0.02631693	-0.0476698	-0.7630358	0.27217621	0.45360876	-0.142562	0.05300519
165.4	0.44439788	-0.5021076	0.02703308	-0.0479731	-0.7627274	0.27238119	0.45396492	-0.1428713	0.05245067
165.5	0.44485602	-0.5019003	0.02748848	-0.0484165	-0.7627398	0.27318727	0.45322143	-0.1429791	0.05255487
165.6	0.44479487	-0.5022792	0.02742861	-0.0488103	-0.7630327	0.27358591	0.45341299	-0.1435902	0.05210956
165.7	0.44458337	-0.5022042	0.02751279	-0.0487475	-0.7629095	0.2734774	0.45343627	-0.1432389	0.05213403
165.8	0.44415657	-0.5025991	0.02794447	-0.0488216	-0.762923	0.27398335	0.45367103	-0.1429594	0.05204243
165.9	0.44419655	-0.5029431	0.02835236	-0.0489375	-0.7630557	0.27412209	0.45394543	-0.1432197	0.05183196
166	0.44419655	-0.5029431	0.02835236	-0.0489375	-0.7630557	0.27412209	0.45394543	-0.1432197	0.05183196
166.1	0.44426268	-0.503263	0.02884856	-0.0494645	-0.7632402	0.27424473	0.45402199	-0.1430647	0.05128394
166.2	0.44433771	-0.5035768	0.02809058	-0.0501502	-0.7631294	0.27395815	0.45439468	-0.143161	0.05144598
166.3	0.44433771	-0.5035768	0.02809058	-0.0501502	-0.7631294	0.27395815	0.45439468	-0.143161	0.05144598
166.4	0.44378027	-0.5028492	0.02812006	-0.0493493	-0.7630935	0.27367185	0.45406241	-0.1433205	0.05160987
166.5	0.44378027	-0.5028492	0.02812006	-0.0493493	-0.7630935	0.27367185	0.45406241	-0.1433205	0.05160987
166.6	0.44345761	-0.5027323	0.02772597	-0.049339	-0.7630112	0.27351187	0.45409576	-0.143319	0.05154448
166.7	0.44291164	-0.5028461	0.0288779	-0.0502978	-0.7627112	0.27333052	0.45339727	-0.1435139	0.05097419
166.8	0.44291164	-0.5028461	0.0288779	-0.0502978	-0.7627112	0.27333052	0.45339727	-0.1435139	0.05097419
166.9	0.44322743	-0.5027577	0.02886005	-0.0503174	-0.7628112	0.27329543	0.45412948	-0.1436865	0.05137999
167	0.44333007	-0.5023794	0.02892589	-0.049651	-0.7628365	0.27339291	0.45434602	-0.1439062	0.051701
167.1	0.44325847	-0.502682	0.02881869	-0.0500999	-0.7629135	0.27363601	0.45452713	-0.1439182	0.05174813
167.2	0.44356762	-0.5028578	0.0291244	-0.0503937	-0.763077	0.27377811	0.45465191	-0.1437425	0.0516064
167.3	0.44356762	-0.5028578	0.0291244	-0.0503937	-0.763077	0.27377811	0.45465191	-0.1437425	0.0516064
167.4	0.4433501	-0.5032912	0.02951157	-0.050655	-0.7632899	0.27398423	0.45475091	-0.1432892	0.05135506
167.5	0.44308552	-0.5029793	0.02947495	-0.0505077	-0.7634616	0.2733839	0.45497342	-0.1431014	0.05112739
167.6	0.44308552	-0.5029793	0.02947495	-0.0505077	-0.7634616	0.2733839	0.45497342	-0.1431014	0.05112739
167.7	0.44347934	-0.5029687	0.0297586	-0.0505028	-0.7634638	0.2736451	0.45520853	-0.1428203	0.05130042
167.8	0.44339828	-0.5026227	0.02961782	-0.0503764	-0.7633546	0.27378381	0.45516063	-0.1426903	0.05147293
167.9	0.44277956	-0.502339	0.02950135	-0.0506376	-0.7630378	0.27327009	0.45500624	-0.1426607	0.05148194
168	0.44277956	-0.502339	0.02950135	-0.0506376	-0.7630378	0.27327009	0.45500624	-0.1426607	0.05148194
168.1	0.44232755	-0.501988	0.02932499	-0.0507507	-0.762904	0.27280966	0.45470362	-0.1427584	0.05117306
168.2	0.44232755	-0.501988	0.02932499	-0.0507507	-0.762904	0.27280966	0.45470362	-0.1427584	0.05117306
168.3	0.44226491	-0.502643	0.03004158	-0.0508389	-0.7630166	0.2727927	0.45489472	-0.1428782	0.05044737
168.4	0.44231952	-0.5024507	0.03097682	-0.0504716	-0.7630172	0.27280502	0.4549148	-0.1429263	0.05027056
168.5	0.44231952	-0.5024507	0.03097682	-0.0504716	-0.7630172	0.27280502	0.4549148	-0.1429263	0.05027056
168.6	0.44225452	-0.5023644	0.03130462	-0.0504694	-0.7628959	0.27251194	0.45504037	-0.1433542	0.04973473
168.7	0.44225452	-0.5023644	0.03130462	-0.0504694	-0.7628959	0.27251194	0.45504037	-0.1433542	0.04973473
168.8	0.44226517	-0.502745	0.03172618	-0.0508747	-0.7629837	0.27284466	0.45514235	-0.1430057	0.04947544
168.9	0.44226517	-0.502745	0.03172618	-0.0508747	-0.7629837	0.27284466	0.45514235	-0.1430057	0.04947544
169	0.44199525	-0.5031324	0.03208135	-0.0507418	-0.7631961	0.27336462	0.45530761	-0.1426941	0.04938002
169.1	0.44145506	-0.5032435	0.03258242	-0.0502688	-0.7630711	0.27312731	0.45498465	-0.1427543	0.04928308
169.2	0.44145506	-0.5032435	0.03258242	-0.0502688	-0.7630711	0.27312731	0.45498465	-0.1427543	0.04928308
169.3	0.44144788	-0.5033725	0.03302108	-0.0505594	-0.7630479	0.27279823	0.45505259	-0.1430068	0.04901698
169.4	0.44101771	-0.5036499	0.03370152	-0.0506168	-0.7629121	0.27250349	0.45490561	-0.1425607	0.04858735
169.5	0.44056524	-0.5040366	0.03451292	-0.0510181	-0.7629049	0.27237012	0.45494754	-0.1422001	0.04809797
169.6	0.44052596	-0.5041	0.03468472	-0.0512877	-0.7628793	0.2719606	0.45497312	-0.1422719	0.04796469
169.7	0.44052596	-0.5041	0.03468472	-0.0512877	-0.7628793	0.2719606	0.45497312	-0.1422719	0.04796469
169.8	0.44052596	-0.5041	0.03468472	-0.0512877	-0.7628793	0.2719606	0.45497312	-0.1422719	0.04796469
169.9	0.44052596	-0.5041	0.03468472	-0.0512877	-0.7628793	0.2719606	0.45497312	-0.1422719	0.04796469
170	0.43950454	-0.504277	0.03575223	-0.0520313	-0.7629265	0.27211899	0.45519003	-0.1417467	0.04693724
170.1	0.43934173	-0.5047787	0.03598947	-0.0522126	-0.7630017	0.2726207	0.45514057	-0.1418704	0.0465194
170.2	0.43982871	-0.5045914	0.03604154	-0.0525457	-0.7631371	0.27262056	0.45522766	-0.141899	0.04638034
170.3	0.43964874	-0.5049819	0.03619756	-0.0526364	-0.763068	0.27249028	0.45529713	-0.1419708	0.04610658
170.4	0.4389552	-0.5056665	0.03748951	-0.052815	-0.7631237	0.27305673	0.45551827	-0.1413923	0.04622414
170.5	0.43940985	-0.5054745	0.0375166	-0.0533339	-0.7632786	0.2731744	0.4560025	-0.141525	0.04600659
170.6	0.43918003	-0.505798	0.03817932	-0.0535812	-0.7632304	0.27359523	0.45603327	-0.1419342	0.04566663
170.7	0.43893116	-0.5056614	0.03822006	-0.0535634	-0.7631198	0.27358818	0.4557208	-0.1417237	0.04512379
170.8	0.43925311	-0.506024	0.03892066	-0.0539965	-0.7632212	0.27345769	0.45614702	-0.1413512	0.04484899
170.9	0.43925311	-0.506024	0.03892066	-0.0539965	-0.7632212	0.27345769	0.45614702	-0.1413512	0.04484899
171	0.43918988	-0.5063962	0.03983926	-0.054074	-0.7629414	0.27394349	0.45615779	-0.1413795	0.04511623
171.1	0.43918988	-0.5063962	0.03983926	-0.054074	-0.7629414	0.27394349	0.45615779	-0.1413795	0.04511623
171.2	0.43910399	-0.5064961	0.0402894	-0.0540638	-0.762864	0.27397137	0.45598771	-0.1410909	0.04537398
171.3	0.43910399	-0.5064961	0.0402894	-0.0540638	-0.762864	0.27397137	0.45598771	-0.1410909	0.04537398
171.4	0.43893841	-0.5064148	0.04035804	-0.0547135	-0.7627285	0.27358723	0.45598543	-0.1410026	0.04557061
171.5	0.43876774	-0.5066728	0.04072759	-0.0548422	-0.7627704	0.27331989	0.45593433	-0.1405519	0.04522042
171.6	0.43887154	-0.5068705	0.0412956	-0.0547266	-0.7630061	0.27392757	0.45600189	-0.1404198	0.0452003
171.7	0.43887154	-0.5068705	0.0412956	-0.0547266	-0.7630061	0.27392757	0.45600189	-0.1404198	0.0452003
171.8	0.43886696	-0.5071555	0.04144148	-0.054998	-0.763068	0.27400828	0.45616254	-0.1402835	0.04513587
171.9	0.43864586	-0.507268	0.04211018	-0.0544848	-0.763036	0.27402173	0.45597821	-0.14012	0.0446129
172	0.43864586	-0.507268	0.04211018	-0.0544848	-0.763036	0.27402173	0.45597821	-0.14012	0.0446129
172.1	0.43864586	-0.507268	0.04211018	-0.0544848	-0.763036	0.27402173	0.45597821	-0.14012	0.0446129
172.2	0.43894209	-0.5072972	0.04260352	-0.05463	-0.7629647	0.27404867	0.4560847	-0.1402393	0.04437486
172.3	0.43873259	-0.5073189	0.04287279	-0.0543716	-0.7629717	0.27392842	0.45632156	-0.1404978	0.0440517
172.4	0.43879308	-0.5075237	0.04327563	-0.0546446	-0.7628879	0.27378808	0.45627303	-0.1401518	0.04369415
172.5	0.43867458	-0.5070627	0.0438567	-0.0548225	-0.7628141	0.27362059	0.45602245	-0.1403368	0.0435793
172.6	0.43844936	-0.5072725	0.04419377	-0.0545557	-0.7628533	0.27366631	0.45603508	-0.1400272	0.04338557
172.7	0.43834085	-0.5073595	0.04377061	-0.0549428	-0.7626792	0.27313873	0.45612807	-0.1400232	0.04334168
172.8	0.43834085	-0.5073595	0.04377061	-0.0549428	-0.7626792	0.27313873	0.45612807	-0.1400232	0.04334168
172.9	0.4376561	-0.5080427	0.04454398	-0.0553206	-0.7627646	0.27190133	0.45648056	-0.1401783	0.04242317
173	0.43715043	-0.5082726	0.04458853	-0.0551789	-0.7626897	0.27237472	0.45598557	-0.140668	0.04279462
173.1	0.43733029	-0.5085214	0.04484727	-0.0549158	-0.7626937	0.27242158	0.45619475	-0.1408539	0.04275214
173.2	0.43641977	-0.5084791	0.04528776	-0.0544841	-0.7624596	0.27137964	0.45564403	-0.1410204	0.04175372
173.3	0.43621943	-0.5089061	0.04544714	-0.0544688	-0.7623083	0.27183427	0.45530952	-0.1409393	0.04151035
173.4	0.43621943	-0.5089061	0.04544714	-0.0544688	-0.7623083	0.27183427	0.45530952	-0.1409393	0.04151035
173.5	0.43589727	-0.5095171	0.0460461	-0.054585	-0.7622955	0.27201783	0.45538274	-0.140894	0.04161084
173.6	0.43570474	-0.5094458	0.04605257	-0.0547762	-0.7619472	0.27141911	0.45527112	-0.1408263	0.04137653
173.7	0.43556537	-0.5101021	0.047101	-0.055152	-0.7617689	0.27227032	0.45577638	-0.1412075	0.04178699
173.8	0.43578962	-0.5097712	0.04702272	-0.0557964	-0.7619373	0.27225316	0.45578302	-0.141098	0.04205888
173.9	0.43587939	-0.5096894	0.04772685	-0.0556693	-0.761727	0.27156988	0.45596669	-0.1409878	0.04159214
174	0.43587939	-0.5096894	0.04772685	-0.0556693	-0.761727	0.27156988	0.45596669	-0.1409878	0.04159214
174.1	0.4359601	-0.5099591	0.04828288	-0.0561576	-0.7616074	0.27159504	0.45605688	-0.1408587	0.04111049
174.2	0.43583078	-0.5106469	0.0488325	-0.0565216	-0.7618692	0.27195821	0.45639341	-0.1403193	0.0409291
174.3	0.43574929	-0.5106161	0.049482	-0.0561436	-0.761621	0.27174387	0.4560717	-0.1402594	0.04095634
174.4	0.43583882	-0.5106963	0.05034863	-0.0558742	-0.7617672	0.27224467	0.45612462	-0.1397034	0.04089244
174.5	0.43561146	-0.5104801	0.05042729	-0.0560414	-0.7618235	0.27196952	0.45620663	-0.1395373	0.04123816
174.6	0.43538778	-0.5104973	0.0511048	-0.0563744	-0.7619592	0.2722856	0.4566641	-0.1386927	0.04126398
174.7	0.43520063	-0.5106055	0.05116242	-0.0566715	-0.761959	0.27234973	0.45661526	-0.1383783	0.04134484
174.8	0.43495284	-0.5107764	0.05142666	-0.0564417	-0.761963	0.27223759	0.45645194	-0.1385299	0.04110352
174.9	0.43486578	-0.5112125	0.05160676	-0.05688	-0.7618443	0.27197751	0.45635801	-0.1382464	0.04111497
175	0.43480517	-0.5115432	0.05214551	-0.0569281	-0.7620723	0.27237571	0.45654739	-0.137866	0.04080983
175.1	0.43493437	-0.5117751	0.05270843	-0.0568702	-0.7620659	0.27273441	0.45685397	-0.1380228	0.04066568
175.2	0.43493437	-0.5117751	0.05270843	-0.0568702	-0.7620659	0.27273441	0.45685397	-0.1380228	0.04066568
175.3	0.4347597	-0.5112218	0.05243663	-0.0561364	-0.7619682	0.27288939	0.45680366	-0.1384782	0.04085468
175.4	0.43422682	-0.5112019	0.05292158	-0.055782	-0.7617697	0.27287175	0.45689102	-0.1388275	0.04082158
175.5	0.43428425	-0.5117716	0.05351477	-0.0560451	-0.7619457	0.27239363	0.45708179	-0.1385258	0.04028387
175.6	0.43420887	-0.511996	0.05389074	-0.0560002	-0.7619699	0.27219938	0.45710309	-0.1381249	0.04007378
175.7	0.43415506	-0.5116366	0.05406775	-0.0562865	-0.7619253	0.27195201	0.45719189	-0.1375528	0.04035562
175.8	0.43417367	-0.5115245	0.05447653	-0.0564566	-0.7616553	0.27178491	0.45688066	-0.1372275	0.04052277
175.9	0.43377285	-0.5112684	0.05445335	-0.0567205	-0.761574	0.2717898	0.45670248	-0.137691	0.04058972
176	0.43398094	-0.5115916	0.05526589	-0.0568866	-0.7616288	0.27180104	0.45673384	-0.1375468	0.04041824
176.1	0.43397439	-0.5115391	0.05538602	-0.057247	-0.7618559	0.27230546	0.45716925	-0.1377073	0.0405117
176.2	0.43393324	-0.5117159	0.0557902	-0.0569754	-0.7619157	0.27189394	0.45719989	-0.1375393	0.04026394
176.3	0.43393324	-0.5117159	0.0557902	-0.0569754	-0.7619157	0.27189394	0.45719989	-0.1375393	0.04026394
176.4	0.43361492	-0.5115526	0.05604252	-0.0569718	-0.7618629	0.27183714	0.4570491	-0.1375256	0.04027117
176.5	0.43402944	-0.5119745	0.05734356	-0.0575195	-0.7618489	0.27197442	0.45746533	-0.1380026	0.03986074
176.6	0.43402944	-0.5119745	0.05734356	-0.0575195	-0.7618489	0.27197442	0.45746533	-0.1380026	0.03986074
176.7	0.43402944	-0.5119745	0.05734356	-0.0575195	-0.7618489	0.27197442	0.45746533	-0.1380026	0.03986074
176.8	0.4339398	-0.5116352	0.05748652	-0.0574705	-0.7617739	0.27182564	0.4574803	-0.1376957	0.04009787
176.9	0.43398884	-0.5114544	0.05745562	-0.0573331	-0.7615967	0.27134676	0.45732401	-0.1377005	0.04000396
177	0.43398884	-0.5114544	0.05745562	-0.0573331	-0.7615967	0.27134676	0.45732401	-0.1377005	0.04000396
177.1	0.43347885	-0.5111413	0.05730503	-0.0578511	-0.7612818	0.27111251	0.45701446	-0.1370651	0.03966858
177.2	0.43374908	-0.5108948	0.05695182	-0.0574802	-0.7613094	0.2710548	0.45678141	-0.1370482	0.03976518
177.3	0.43389109	-0.511179	0.05769149	-0.0573994	-0.7613736	0.27116807	0.45645262	-0.1372309	0.03993525
177.4	0.43389109	-0.511179	0.05769149	-0.0573994	-0.7613736	0.27116807	0.45645262	-0.1372309	0.03993525
177.5	0.43389109	-0.511179	0.05769149	-0.0573994	-0.7613736	0.27116807	0.45645262	-0.1372309	0.03993525
177.6	0.43399198	-0.5113446	0.05766895	-0.057337	-0.761359	0.27101493	0.45660449	-0.1369921	0.03991527
177.7	0.43376605	-0.5113397	0.05795487	-0.0574832	-0.7610129	0.2704239	0.4566132	-0.1370847	0.03982615
177.8	0.43378484	-0.511288	0.05821458	-0.0575897	-0.7608086	0.27067024	0.45632084	-0.1370265	0.04000054
177.9	0.43378484	-0.511288	0.05821458	-0.0575897	-0.7608086	0.27067024	0.45632084	-0.1370265	0.04000054
178	0.43374008	-0.5113608	0.05844962	-0.0576527	-0.7607998	0.27052109	0.45630084	-0.1371652	0.03983254
178.1	0.43334856	-0.5112604	0.05921301	-0.0574862	-0.7606703	0.27037971	0.45630107	-0.1373501	0.03913992
178.2	0.43330273	-0.5114993	0.05934793	-0.0575305	-0.7606373	0.2705416	0.45619068	-0.1372561	0.03944451
178.3	0.43312589	-0.5113148	0.05958957	-0.0577446	-0.7604258	0.27027038	0.45584885	-0.137115	0.03943576
178.4	0.43312589	-0.5113148	0.05958957	-0.0577446	-0.7604258	0.27027038	0.45584885	-0.137115	0.03943576
178.5	0.43293268	-0.5111049	0.05961682	-0.0574178	-0.7604693	0.27021256	0.4557827	-0.137064	0.03994266
178.6	0.43272761	-0.5110609	0.05994019	-0.0573059	-0.7604579	0.27010499	0.45594731	-0.1371495	0.04027897
178.7	0.43216745	-0.5108394	0.06011789	-0.0576301	-0.7601724	0.26956331	0.45531521	-0.1373888	0.03932084
178.8	0.43166575	-0.5108534	0.05999135	-0.0581854	-0.7603039	0.26976073	0.45556533	-0.1371935	0.03936508
178.9	0.43166575	-0.5108534	0.05999135	-0.0581854	-0.7603039	0.26976073	0.45556533	-0.1371935	0.03936508
179	0.4316771	-0.5107622	0.06081235	-0.0586884	-0.7601561	0.26916675	0.45553172	-0.1373291	0.03914532
179.1	0.43174243	-0.5107629	0.06135115	-0.0587043	-0.7600259	0.26913869	0.45566035	-0.1370667	0.03902944
179.2	0.43190835	-0.5110665	0.06180783	-0.0587991	-0.7600521	0.26964994	0.45574443	-0.1371602	0.03925155
179.3	0.43139201	-0.5101216	0.06246497	-0.059144	-0.7597188	0.26917182	0.45520645	-0.1377473	0.03816956
179.4	0.43139201	-0.5101216	0.06246497	-0.059144	-0.7597188	0.26917182	0.45520645	-0.1377473	0.03816956
179.5	0.43117041	-0.5101965	0.062948	-0.0591028	-0.7598514	0.2694768	0.45556057	-0.1377176	0.03818086
179.6	0.4309642	-0.5101252	0.06337504	-0.0591499	-0.7597683	0.26905507	0.45530256	-0.1374987	0.03790081
179.7	0.43102965	-0.5101152	0.0639549	-0.0592107	-0.7595599	0.26892868	0.45484143	-0.137294	0.03702362
179.8	0.42931035	-0.509708	0.06477965	-0.0591671	-0.7586992	0.26812261	0.45453643	-0.1371729	0.0368911
179.9	0.42941957	-0.5097972	0.06495787	-0.0592182	-0.7586666	0.26775555	0.45442653	-0.1369672	0.03660324
225	0.40324533	-0.5086263	0.12257245	-0.0865968	-0.7415903	0.27158609	0.43316618	-0.1243972	0.02379572
225.1	0.40324533	-0.5086263	0.12257245	-0.0865968	-0.7415903	0.27158609	0.43316618	-0.1243972	0.02379572
225.2	0.40324533	-0.5086263	0.12257245	-0.0865968	-0.7415903	0.27158609	0.43316618	-0.1243972	0.02379572
225.3	0.40324533	-0.5086263	0.12257245	-0.0865968	-0.7415903	0.27158609	0.43316618	-0.1243972	0.02379572
225.4	0.40324533	-0.5086263	0.12257245	-0.0865968	-0.7415903	0.27158609	0.43316618	-0.1243972	0.02379572
225.5	0.40324533	-0.5086263	0.12257245	-0.0865968	-0.7415903	0.27158609	0.43316618	-0.1243972	0.02379572

180.6	0.42974643	-0.5101885	0.06748141	-0.0588202	-0.7582681	0.26804734	0.45565064	-0.1370763	0.03702317
180.7	0.43009885	-0.5101838	0.0680649	-0.0588269	-0.7582547	0.26780924	0.45592217	-0.1369456	0.03681176
180.8	0.43009885	-0.5101838	0.0680649	-0.0588269	-0.7582547	0.26780924	0.45592217	-0.1369456	0.03681176
180.9	0.42990445	-0.5100606	0.06830179	-0.058657	-0.7580968	0.26783183	0.45581356	-0.1370005	0.03690105
181	0.42969299	-0.5099467	0.06892669	-0.0588746	-0.7580542	0.26758758	0.45567378	-0.1365844	0.03666711
181.1	0.42950727	-0.5100795	0.06905986	-0.0592253	-0.7581901	0.26746294	0.45574416	-0.1365869	0.03621115
181.2	0.42976244	-0.5102023	0.06947863	-0.0595926	-0.7582354	0.26742863	0.45573495	-0.1368354	0.03618429
181.3	0.43009963	-0.5100091	0.06935051	-0.059973	-0.7581277	0.2675614	0.45577934	-0.1372004	0.03629205
181.4	0.4300332	-0.5100235	0.06969872	-0.0603549	-0.7580643	0.26805439	0.45601619	-0.1368133	0.03660168
181.5	0.42968428	-0.5101351	0.07009744	-0.0600637	-0.7580186	0.26796602	0.45585617	-0.1368827	0.03603077
181.6	0.42955528	-0.5100773	0.07019812	-0.0599735	-0.7579122	0.26807631	0.45569042	-0.1365682	0.0355505
181.7	0.42955528	-0.5100773	0.07019812	-0.0599735	-0.7579122	0.26807631	0.45569042	-0.1365682	0.0355505
181.8	0.42935142	-0.5100948	0.07050421	-0.0601105	-0.7579916	0.26791173	0.45609976	-0.1366889	0.03536983
181.9	0.42927131	-0.5101598	0.07090453	-0.0606309	-0.7580996	0.26787038	0.45611603	-0.1363757	0.03542309
182	0.42927131	-0.5101598	0.07090453	-0.0606309	-0.7580996	0.26787038	0.45611603	-0.1363757	0.03542309
182.1	0.42927131	-0.5101598	0.07090453	-0.0606309	-0.7580996	0.26787038	0.45611603	-0.1363757	0.03542309
182.2	0.42927131	-0.5101598	0.07090453	-0.0606309	-0.7580996	0.26787038	0.45611603	-0.1363757	0.03542309
182.3	0.42942129	-0.5101411	0.07212495	-0.0608796	-0.7580767	0.26774844	0.45578626	-0.1363225	0.03425711
182.4	0.42933524	-0.5102739	0.07209178	-0.0609598	-0.7579979	0.26754167	0.45596881	-0.1360457	0.03418055
182.5	0.42944678	-0.5103744	0.07250526	-0.0610601	-0.7578586	0.26765738	0.4559594	-0.1358911	0.03391078
182.6	0.42978613	-0.5101405	0.07257638	-0.0611945	-0.7578528	0.2678155	0.45596196	-0.1353449	0.03404645
182.7	0.42960242	-0.5102409	0.072927	-0.0607568	-0.7578343	0.26811346	0.4560084	-0.1352241	0.0339386
182.8	0.42960242	-0.5102409	0.072927	-0.0607568	-0.7578343	0.26811346	0.4560084	-0.1352241	0.0339386
182.9	0.42895906	-0.5103742	0.07326678	-0.0610588	-0.7577168	0.26755605	0.45598714	-0.1348784	0.03401221
183	0.42880894	-0.5104973	0.07378781	-0.0607974	-0.7575809	0.26749939	0.45603416	-0.13512	0.03388237
183.1	0.42880894	-0.5104973	0.07378781	-0.0607974	-0.7575809	0.26749939	0.45603416	-0.13512	0.03388237
183.2	0.42880894	-0.5104973	0.07378781	-0.0607974	-0.7575809	0.26749939	0.45603416	-0.13512	0.03388237
183.3	0.42822241	-0.5106104	0.07473595	-0.0606157	-0.7574544	0.2674992	0.45573441	-0.1348396	0.03337376
183.4	0.42822241	-0.5106104	0.07473595	-0.0606157	-0.7574544	0.2674992	0.45573441	-0.1348396	0.03337376
183.5	0.42800007	-0.5105702	0.07509629	-0.0605024	-0.7573942	0.26731684	0.45559762	-0.1348673	0.0330427
183.6	0.42787218	-0.5106357	0.07554935	-0.0601708	-0.7572764	0.26752145	0.45572414	-0.1350362	0.03290301
183.7	0.42780755	-0.5108549	0.07577691	-0.0600462	-0.7571227	0.26780571	0.45556241	-0.1353558	0.03247617
183.8	0.42780755	-0.5108549	0.07577691	-0.0600462	-0.7571227	0.26780571	0.45556241	-0.1353558	0.03247617
183.9	0.42771109	-0.5110776	0.07623401	-0.0599811	-0.7568708	0.26831434	0.45475492	-0.1353554	0.03259626
184	0.42771109	-0.5110776	0.07623401	-0.0599811	-0.7568708	0.26831434	0.45475492	-0.1353554	0.03259626
184.1	0.42771109	-0.5110776	0.07623401	-0.0599811	-0.7568708	0.26831434	0.45475492	-0.1353554	0.03259626
184.2	0.42761059	-0.5112461	0.076331	-0.0600741	-0.7564788	0.26849768	0.45443997	-0.1355686	0.03191679
184.3	0.4276035	-0.5113909	0.07645808	-0.0602885	-0.7565039	0.26850239	0.45419871	-0.1356445	0.03211517
184.4	0.4276035	-0.5113909	0.07645808	-0.0602885	-0.7565039	0.26850239	0.45419871	-0.1356445	0.03211517
184.5	0.42745404	-0.5114757	0.07594204	-0.060642	-0.756374	0.26791493	0.45417966	-0.1358467	0.03200133
184.6	0.42754476	-0.5114733	0.07607256	-0.0606042	-0.7562	0.26777174	0.45422058	-0.1359043	0.03192946
184.7	0.42776525	-0.5109551	0.07623314	-0.0604667	-0.756011	0.26770399	0.45395609	-0.1359266	0.03194683
184.8	0.42776525	-0.5109551	0.07623314	-0.0604667	-0.756011	0.26770399	0.45395609	-0.1359266	0.03194683
184.9	0.42776525	-0.5109551	0.07623314	-0.0604667	-0.756011	0.26770399	0.45395609	-0.1359266	0.03194683
185	0.42776744	-0.5110115	0.07665603	-0.0608847	-0.75599	0.26794477	0.45384516	-0.1358181	0.03195139
185.1	0.42781178	-0.511512	0.07754716	-0.0613909	-0.7560809	0.26848654	0.45375318	-0.1356268	0.03229695
185.2	0.42781178	-0.511512	0.07754716	-0.0613909	-0.7560809	0.26848654	0.45375318	-0.1356268	0.03229695
185.3	0.42802413	-0.511853	0.07823244	-0.0616792	-0.7560047	0.26851877	0.45363585	-0.1353551	0.03220566
185.4	0.42809094	-0.5116553	0.07976688	-0.061907	-0.75603	0.26885803	0.45376715	-0.1343179	0.03197061
185.5	0.42809094	-0.5116553	0.07976688	-0.061907	-0.75603	0.26885803	0.45376715	-0.1343179	0.03197061
185.6	0.42839994	-0.5117268	0.08010577	-0.0619472	-0.756045	0.26842327	0.45355506	-0.1341692	0.03222136
185.7	0.42839994	-0.5117268	0.08010577	-0.0619472	-0.756045	0.26842327	0.45355506	-0.1341692	0.03222136
185.8	0.42787673	-0.5117337	0.08049008	-0.0622563	-0.7556999	0.26837164	0.45373899	-0.13449	0.03173869
185.9	0.42787673	-0.5117337	0.08049008	-0.0622563	-0.7556999	0.26837164	0.45373899	-0.13449	0.03173869
186	0.42807834	-0.5120298	0.08073259	-0.0623457	-0.7558439	0.26873976	0.45362284	-0.1347338	0.0315286
186.1	0.42815828	-0.5120234	0.081143	-0.062457	-0.7556994	0.26846105	0.45373987	-0.1346979	0.0313857
186.2	0.42785093	-0.5124999	0.08126687	-0.0626068	-0.7558928	0.26907964	0.45396912	-0.1346536	0.03135004
186.3	0.427886	-0.5122758	0.08156307	-0.0622798	-0.7557434	0.26922585	0.45398999	-0.1344219	0.03139786
186.4	0.42760658	-0.5119716	0.08156914	-0.0617927	-0.7556877	0.26962765	0.45412873	-0.1341921	0.03156213
186.5	0.42708318	-0.511848	0.08174348	-0.0615034	-0.7552067	0.26932819	0.45363372	-0.1340825	0.03171156
186.6	0.42723546	-0.5119993	0.08242706	-0.0609663	-0.7551222	0.26979096	0.45370232	-0.1339684	0.03120831
186.7	0.42698702	-0.5117844	0.08243273	-0.0610838	-0.7549442	0.26945714	0.45346703	-0.1335604	0.0311611
186.8	0.42681012	-0.5119864	0.08280479	-0.0612157	-0.7547373	0.26884483	0.45311514	-0.1334696	0.03037076
186.9	0.42684662	-0.5123191	0.08282341	-0.0615021	-0.7547416	0.2688555	0.45292139	-0.1338189	0.03064411
187	0.42684596	-0.5123829	0.08323785	-0.0610604	-0.7547397	0.2686947	0.45298137	-0.133839	0.03048277
187.1	0.42658462	-0.5131043	0.08403177	-0.0613422	-0.7546351	0.26938021	0.45309105	-0.1335356	0.03082096
187.2	0.42675321	-0.5132294	0.08432267	-0.0613042	-0.7545655	0.26944642	0.45302244	-0.1339259	0.03096407
187.3	0.42675321	-0.5132294	0.08432267	-0.0613042	-0.7545655	0.26944642	0.45302244	-0.1339259	0.03096407
187.4	0.42633373	-0.5126673	0.08436162	-0.0608398	-0.7544689	0.26932037	0.452385	-0.1340665	0.03082472
187.5	0.4261144	-0.5125496	0.08447773	-0.061003	-0.7544445	0.26946863	0.45227485	-0.1337277	0.03060487
187.6	0.4261144	-0.5125496	0.08447773	-0.061003	-0.7544445	0.26946863	0.45227485	-0.1337277	0.03060487
187.7	0.4261144	-0.5125496	0.08447773	-0.061003	-0.7544445	0.26946863	0.45227485	-0.1337277	0.03060487
187.8	0.4261144	-0.5125496	0.08447773	-0.061003	-0.7544445	0.26946863	0.45227485	-0.1337277	0.03060487
187.9	0.42635764	-0.5124783	0.08487992	-0.0614789	-0.7543946	0.26987126	0.45241429	-0.1336654	0.03050446
188	0.42619537	-0.5125493	0.08544262	-0.0621514	-0.7542918	0.26951004	0.452575	-0.1333198	0.03067585
188.1	0.42619537	-0.5125493	0.08544262	-0.0621514	-0.7542918	0.26951004	0.452575	-0.1333198	0.03067585
188.2	0.42619537	-0.5125493	0.08544262	-0.0621514	-0.7542918	0.26951004	0.452575	-0.1333198	0.03067585
188.3	0.42577409	-0.5128317	0.08590057	-0.062408	-0.7543553	0.26937917	0.45233748	-0.1328763	0.03093355
188.4	0.42577409	-0.5128317	0.08590057	-0.062408	-0.7543553	0.26937917	0.45233748	-0.1328763	0.03093355
188.5	0.42577409	-0.5128317	0.08590057	-0.062408	-0.7543553	0.26937917	0.45233748	-0.1328763	0.03093355
188.6	0.42577409	-0.5128317	0.08590057	-0.062408	-0.7543553	0.26937917	0.45233748	-0.1328763	0.03093355
188.7	0.42586622	-0.5131039	0.08609033	-0.0622196	-0.7542632	0.26951799	0.45207876	-0.1332253	0.03093285
188.8	0.42556221	-0.5129251	0.08659077	-0.0621538	-0.7540533	0.26927969	0.45190857	-0.1331216	0.03034963
188.9	0.42556221	-0.5129251	0.08659077	-0.0621538	-0.7540533	0.26927969	0.45190857	-0.1331216	0.03034963
189	0.42562051	-0.5130101	0.08636509	-0.0624456	-0.7539281	0.2694522	0.45174849	-0.1332641	0.03075317
189.1	0.42572027	-0.5132466	0.08614821	-0.0624527	-0.7538384	0.26917901	0.45153851	-0.1334245	0.03091895
189.2	0.42564184	-0.5134784	0.08642447	-0.0625622	-0.753869	0.26932504	0.45131205	-0.1331054	0.03102183
189.3	0.42551837	-0.5132695	0.08687973	-0.0622123	-0.7537668	0.26940724	0.45154822	-0.1332709	0.0310284
189.4	0.42551837	-0.5132695	0.08687973	-0.0622123	-0.7537668	0.26940724	0.45154822	-0.1332709	0.0310284
189.5	0.42546652	-0.5135007	0.08684054	-0.0624582	-0.7536449	0.26994154	0.45122139	-0.1333081	0.03106963
189.6	0.42533461	-0.5130147	0.08730349	-0.062299	-0.7537852	0.27028883	0.45116647	-0.1331027	0.0315171
189.7	0.42510629	-0.5135141	0.08768024	-0.0626321	-0.753883	0.27054057	0.45107895	-0.1331389	0.0313871
189.8	0.42489446	-0.5136461	0.08829656	-0.0630687	-0.7539038	0.27055982	0.45101858	-0.1330048	0.03120087
189.9	0.42489446	-0.5136461	0.08829656	-0.0630687	-0.7539038	0.27055982	0.45101858	-0.1330048	0.03120087
190	0.42468798	-0.5140544	0.08846796	-0.0632221	-0.7540442	0.27070718	0.45078784	-0.1333195	0.0313081
190.1	0.4245984	-0.5138999	0.08857406	-0.063262	-0.7539422	0.27036019	0.45073667	-0.1335164	0.03084868
190.2	0.4245984	-0.5138999	0.08857406	-0.063262	-0.7539422	0.27036019	0.45073667	-0.1335164	0.03084868
190.3	0.4243464	-0.5138015	0.0885546	-0.0631883	-0.7539459	0.27055954	0.45066821	-0.1337181	0.03073744
190.4	0.4243464	-0.5138015	0.0885546	-0.0631883	-0.7539459	0.27055954	0.45066821	-0.1337181	0.03073744
190.5	0.4243644	-0.5135806	0.08892409	-0.0633967	-0.7538165	0.2702958	0.45056382	-0.1336005	0.03078696
190.6	0.42424149	-0.5133903	0.08874038	-0.063837	-0.7538809	0.26993841	0.45081323	-0.1337974	0.03096004
190.7	0.42444808	-0.5136104	0.08962685	-0.0646296	-0.7539215	0.27058482	0.45082924	-0.1340889	0.0310572
190.8	0.42426397	-0.5140305	0.09010433	-0.064931	-0.7539286	0.27075803	0.45095074	-0.1339698	0.03099722
190.9	0.42448351	-0.5137415	0.09025609	-0.0653284	-0.7539039	0.27087809	0.45073698	-0.1339901	0.03100341
191	0.4246817	-0.5138525	0.09047623	-0.0651842	-0.7539825	0.27144874	0.45034294	-0.1342461	0.03057698
191.1	0.4246817	-0.5138525	0.09047623	-0.0651842	-0.7539825	0.27144874	0.45034294	-0.1342461	0.03057698
191.2	0.4246379	-0.5139278	0.0918865	-0.0658341	-0.7538309	0.27174927	0.4500445	-0.1342468	0.03021659
191.3	0.4246379	-0.5139278	0.0918865	-0.0658341	-0.7538309	0.27174927	0.4500445	-0.1342468	0.03021659
191.4	0.42449345	-0.514101	0.09212789	-0.0655492	-0.7538486	0.2721995	0.44987063	-0.134065	0.03047496
191.5	0.42449345	-0.514101	0.09212789	-0.0655492	-0.7538486	0.2721995	0.44987063	-0.134065	0.03047496
191.6	0.42449345	-0.514101	0.09212789	-0.0655492	-0.7538486	0.2721995	0.44987063	-0.134065	0.03047496
191.7	0.42417357	-0.5137557	0.09270238	-0.0665947	-0.7540564	0.27203335	0.44993749	-0.1328478	0.03024351
191.8	0.42366737	-0.5130212	0.0937979	-0.0668017	-0.7536311	0.2717438	0.4495803	-0.1329059	0.02983562
191.9	0.42313813	-0.513008	0.09384474	-0.066673	-0.7534096	0.27170681	0.44930196	-0.1327624	0.03025612
192	0.42303987	-0.5127521	0.09426859	-0.0670031	-0.7532434	0.27168304	0.44904714	-0.1325802	0.02994245
192.1	0.42309548	-0.5128628	0.09444197	-0.0667944	-0.7531628	0.27202516	0.44893393	-0.1325034	0.03012269
192.2	0.42311517	-0.5125608	0.09515551	-0.066796	-0.7529041	0.27200234	0.44917106	-0.1327177	0.02994472
192.3	0.42292028	-0.5127096	0.09530174	-0.0671995	-0.7529174	0.27203943	0.4490056	-0.1324506	0.02961975
192.4	0.42292028	-0.5127096	0.09530174	-0.0671995	-0.7529174	0.27203943	0.4490056	-0.1324506	0.02961975
192.5	0.42267953	-0.5127755	0.0956353	-0.0673531	-0.7528136	0.27230768	0.44845986	-0.1329525	0.03002871
192.6	0.4222934	-0.5128637	0.0959571	-0.0674215	-0.7527767	0.27259216	0.4485522	-0.1328266	0.02986195
192.7	0.4222934	-0.5128637	0.0959571	-0.0674215	-0.7527767	0.27259216	0.4485522	-0.1328266	0.02986195
192.8	0.42227013	-0.5128658	0.09645931	-0.0675712	-0.7526811	0.27236435	0.4483891	-0.132586	0.02947208
192.9	0.42227013	-0.5128658	0.09645931	-0.0675712	-0.7526811	0.27236435	0.4483891	-0.132586	0.02947208
193	0.42209301	-0.5131908	0.09647027	-0.0678081	-0.7525202	0.27182816	0.44778765	-0.1329745	0.03000555
193.1	0.42209301	-0.5131908	0.09647027	-0.0678081	-0.7525202	0.27182816	0.44778765	-0.1329745	0.03000555
193.2	0.42192146	-0.5133864	0.09706622	-0.0679991	-0.7523103	0.27209249	0.447441	-0.1332452	0.03047061
193.3	0.42117216	-0.5137688	0.09703929	-0.068324	-0.7522906	0.27241391	0.44728506	-0.1335249	0.02974719
193.4	0.42120718	-0.5138808	0.09676086	-0.0684385	-0.7523391	0.27246656	0.44718668	-0.1333629	0.02997277
193.5	0.42118926	-0.5141769	0.09687892	-0.0684657	-0.7522251	0.27290556	0.4468092	-0.1336224	0.02991387
193.6	0.42118926	-0.5141769	0.09687892	-0.0684657	-0.7522251	0.27290556	0.4468092	-0.1336224	0.02991387
193.7	0.4206451	-0.5143554	0.0975237	-0.0684189	-0.752129	0.273226	0.44687213	-0.1338564	0.02962845
193.8	0.4206451	-0.5143554	0.0975237	-0.0684189	-0.752129	0.273226	0.44687213	-0.1338564	0.02962845
193.9	0.42036989	-0.5144097	0.09717572	-0.0684074	-0.7520016	0.27366255	0.44654405	-0.133663	0.02944618
194	0.42014686	-0.5142794	0.09743631	-0.0682874	-0.7517964	0.27380541	0.44638752	-0.1336184	0.02920327
194.1	0.42014686	-0.5142794	0.09743631	-0.0682874	-0.7517964	0.27380541	0.44638752	-0.1336184	0.02920327
194.2	0.42014686	-0.5142794	0.09743631	-0.0682874	-0.7517964	0.27380541	0.44638752	-0.1336184	0.02920327
194.3	0.42014686	-0.5142794	0.09743631	-0.0682874	-0.7517964	0.27380541	0.44638752	-0.1336184	0.02920327
194.4	0.42009403	-0.514529	0.09804444	-0.0688094	-0.7516496	0.27426353	0.44627875	-0.1335653	0.02898476
194.5	0.42009403	-0.514529	0.09804444	-0.0688094	-0.7516496	0.27426353	0.44627875	-0.1335653	0.02898476
194.6	0.42009403	-0.514529	0.09804444	-0.0688094	-0.7516496	0.27426353	0.44627875	-0.1335653	0.02898476
194.7	0.42009403	-0.514529	0.09804444	-0.0688094	-0.7516496	0.27426353	0.44627875	-0.1335653	0.02898476
194.8	0.42009403	-0.514529	0.09804444	-0.0688094	-0.7516496	0.27426353	0.44627875	-0.1335653	0.02898476
194.9	0.42020801	-0.5144581	0.09833722	-0.0687612	-0.7515259	0.2743827	0.44643071	-0.133168	0.02903808
195.6	0.41885917	-0.5142663	0.09893021	-0.0687292	-0.7503074	0.2742058	0.44498856	-0.1325904	0.02940261
195.7	0.41885917	-0.5142663	0.09893021	-0.0687292	-0.7503074	0.2742058	0.44498856	-0.1325904	0.02940261
195.8	0.41789245	-0.5144786	0.09848125	-0.0687886	-0.7501988	0.27362539	0.44486886	-0.1329175	0.0292276
195.9	0.41789245	-0.5144786	0.09848125	-0.0687886	-0.7501988	0.27362539	0.44486886	-0.1329175	0.0292276
196	0.41797021	-0.5146477	0.09867656	-0.0686885	-0.7502814	0.27364506	0.44486815	-0.1331482	0.02943195
196.1	0.41770499	-0.5149529	0.09882928	-0.0689509	-0.7502019	0.27395572	0.44459263	-0.1331601	0.02928358
196.2	0.41770499	-0.5149529	0.09882928	-0.0689509	-0.7502019	0.27395572	0.44459263	-0.1331601	0.02928358
196.3	0.41780792	-0.5146685	0.0987931	-0.0686399	-0.7499617	0.27373292	0.44447525	-0.1333547	0.02928173
196.4	0.41780792	-0.5146685	0.0987931	-0.0686399	-0.7499617	0.27373292	0.44447525	-0.1333547	0.02928173
196.5	0.41780792	-0.5146685	0.0987931	-0.0686399	-0.7499617	0.27373292	0.44447525	-0.1333547	0.02928173
196.6	0.41780792	-0.5146685	0.0987931	-0.0686399	-0.7499617	0.27373292	0.44447525	-0.1333547	0.02928173
196.7	0.41790168	-0.5147408	0.0987237	-0.0687214	-0.7498731	0.273685	0.44418625	-0.1335561	0.02950277
196.8	0.41790168	-0.5147408	0.0987237	-0.0687214	-0.7498731	0.273685	0.44418625	-0.1335561	0.02950277
196.9	0.41790258	-0.5149005	0.09921553	-0.0690876	-0.7500729	0.27398171	0.44437134	-0.1339889	0.02947455
197	0.41746558	-0.5144328	0.09895343	-0.0689023	-0.7496981	0.2739524	0.44422232	-0.1337101	0.02987362
197.1	0.41746558	-0.5144328	0.09895343	-0.0689023	-0.7496981	0.2739524	0.44422232	-0.1337101	0.02987362
197.2	0.41768821	-0.5141551	0.09870178	-0.06876	-0.7496348	0.27419362	0.44410605	-0.1337299	0.0301751
197.3	0.41768821	-0.5141551	0.09870178	-0.06876	-0.7496348	0.27419362	0.44410605	-0.1337299	0.0301751
197.4	0.41768821	-0.5141551	0.09870178	-0.06876	-0.7496348	0.27419362	0.44410605	-0.1337299	0.0301751
197.5	0.41703938	-0.5138144	0.09898761	-0.0684516	-0.7493982	0.27392008	0.44358544	-0.1341408	0.02969486
197.6	0.41716858	-0.5137288	0.0995847	-0.0690421	-0.749482	0.27361916	0.44360589	-0.1342598	0.0291788
197.7	0.41741135	-0.513719	0.10002884	-0.0687758	-0.749526	0.27415234	0.44333458	-0.1342602	0.02951188
197.8	0.41760034	-0.5136022	0.10043241	-0.0692704	-0.7495818	0.27406229	0.44343225	-0.1342371	0.02906475
197.9	0.41760034	-0.5136022	0.10043241	-0.0692704	-0.7495818	0.27406229	0.44343225	-0.1342371	0.02906475
198	0.41760034	-0.5136022	0.10043241	-0.0692704	-0.7495818	0.27406229	0.44343225	-0.1342371	0.02906475
198.1	0.41760034	-0.5136022	0.10043241	-0.0692704	-0.7495818	0.27406229	0.44343225	-0.1342371	0.02906475
198.2	0.41769112	-0.5138426	0.10081021	-0.069567	-0.7496435	0.27413377	0.44352786	-0.1342375	0.02928193
198.3	0.41783954	-0.5138976	0.10054864	-0.0698979	-0.7494635	0.27413005	0.44294077	-0.1343628	0.02926303
198.4	0.41783954	-0.5138976	0.10054864	-0.0698979	-0.7494635	0.27413005	0.44294077	-0.1343628	0.02926303
198.5	0.41776348	-0.5138587	0.10067489	-0.0699357	-0.7494744	0.27429358	0.44279672	-0.1342061	0.02977804
198.6	0.41776348	-0.5138587	0.10067489	-0.0699357	-0.7494744	0.27429358	0.44279672	-0.1342061	0.02977804
198.7	0.4178208	-0.5137457	0.10061782	-0.0699675	-0.749402	0.27428626	0.44288291	-0.1343299	0.02990001
198.8	0.41780507	-0.5138475	0.10101544	-0.0701427	-0.7492805	0.27506779	0.44262072	-0.134426	0.03008229
198.9	0.41780507	-0.5138475	0.10101544	-0.0701427	-0.7492805	0.27506779	0.44262072	-0.134426	0.03008229
199	0.41767593	-0.5139994	0.10130979	-0.0701534	-0.749376	0.27465968	0.44287803	-0.1340729	0.0297309
199.1	0.4172456	-0.5138616	0.10173901	-0.069661	-0.7491968	0.27428677	0.44260681	-0.1339427	0.02960528
199.2	0.41671092	-0.5136051	0.1012511	-0.0701654	-0.7490067	0.27415996	0.44253352	-0.1336047	0.02987696
199.3	0.41691102	-0.5132953	0.10080167	-0.0698429	-0.7488269	0.27426874	0.44263001	-0.1332134	0.03083739
199.4	0.41691102	-0.5132953	0.10080167	-0.0698429	-0.7488269	0.27426874	0.44263001	-0.1332134	0.03083739
199.5	0.41675821	-0.513293	0.10155707	-0.0699835	-0.7487476	0.273949	0.44301683	-0.1330062	0.03037536
199.6	0.41683545	-0.5134127	0.10196713	-0.0698778	-0.7487821	0.27427437	0.44286774	-0.1327636	0.03042935
199.7	0.41662128	-0.5132231	0.10206951	-0.0698555	-0.7487452	0.27430526	0.44286225	-0.1323695	0.03034113
199.8	0.41662128	-0.5132231	0.10206951	-0.0698555	-0.7487452	0.27430526	0.44286225	-0.1323695	0.03034113
199.9	0.4166493	-0.513105	0.10175166	-0.0701566	-0.7486546	0.27461789	0.44268111	-0.1320685	0.02998775
200	0.41650864	-0.5134294	0.10216107	-0.070477	-0.7486049	0.27494771	0.44298246	-0.132892	0.02942917
200.1	0.41683163	-0.5134909	0.10190449	-0.0705734	-0.748431	0.2757894	0.44232742	-0.1330644	0.02964534
200.2	0.41683163	-0.5134909	0.10190449	-0.0705734	-0.748431	0.2757894	0.44232742	-0.1330644	0.02964534
200.3	0.41681754	-0.5133688	0.10203488	-0.070865	-0.748524	0.27584388	0.44253079	-0.1327543	0.02972621
200.4	0.41681754	-0.5133688	0.10203488	-0.070865	-0.748524	0.27584388	0.44253079	-0.1327543	0.02972621
200.5	0.41658711	-0.5135461	0.10235434	-0.0710156	-0.7482629	0.27619719	0.44205766	-0.1326275	0.02915997
200.6	0.41662562	-0.5136807	0.10265602	-0.0707289	-0.7482972	0.27619043	0.44189552	-0.1325863	0.02936293
200.7	0.41662562	-0.5136807	0.10265602	-0.0707289	-0.7482972	0.27619043	0.44189552	-0.1325863	0.02936293
200.8	0.41662562	-0.5136807	0.10265602	-0.0707289	-0.7482972	0.27619043	0.44189552	-0.1325863	0.02936293
200.9	0.41692907	-0.5136096	0.10292722	-0.0703528	-0.7482956	0.27600942	0.44186988	-0.1324257	0.0293791
201	0.41692907	-0.5136096	0.10292722	-0.0703528	-0.7482956	0.27600942	0.44186988	-0.1324257	0.0293791
201.1	0.41674401	-0.5135978	0.10281871	-0.0703515	-0.7483764	0.27589119	0.44204084	-0.1324463	0.02973127
201.2	0.41717959	-0.5133522	0.10242049	-0.0701718	-0.7484926	0.27585197	0.44214307	-0.1324297	0.02995372
201.3	0.41702762	-0.5131398	0.10251509	-0.0696241	-0.7483266	0.27567217	0.4419425	-0.1325136	0.03004215
201.4	0.41702762	-0.5131398	0.10251509	-0.0696241	-0.7483266	0.27567217	0.4419425	-0.1325136	0.03004215
201.5	0.41702762	-0.5131398	0.10251509	-0.0696241	-0.7483266	0.27567217	0.4419425	-0.1325136	0.03004215
201.6	0.41702762	-0.5131398	0.10251509	-0.0696241	-0.7483266	0.27567217	0.4419425	-0.1325136	0.03004215
201.7	0.41683077	-0.5129678	0.10262759	-0.0697357	-0.7481314	0.27566878	0.44180096	-0.1324367	0.02964257
201.8	0.41694715	-0.5131281	0.10289579	-0.0694112	-0.7479989	0.27556494	0.441662	-0.1327992	0.02943341
201.9	0.41698415	-0.5131176	0.10296849	-0.0691858	-0.7477815	0.2754398	0.44152223	-0.1329311	0.02902069
202	0.41698415	-0.5131176	0.10296849	-0.0691858	-0.7477815	0.2754398	0.44152223	-0.1329311	0.02902069
202.1	0.4162122	-0.5133517	0.10316896	-0.0695233	-0.7474853	0.2750465	0.44161142	-0.1324912	0.02897909
202.2	0.4162122	-0.5133517	0.10316896	-0.0695233	-0.7474853	0.2750465	0.44161142	-0.1324912	0.02897909
202.3	0.4161588	-0.5134405	0.10321766	-0.0692507	-0.7473264	0.27503938	0.44149529	-0.132157	0.02895173
202.4	0.41592057	-0.513626	0.10306725	-0.069346	-0.7472156	0.27520949	0.44118657	-0.1320773	0.02880068
202.5	0.41592057	-0.513626	0.10306725	-0.069346	-0.7472156	0.27520949	0.44118657	-0.1320773	0.02880068
202.6	0.41592057	-0.513626	0.10306725	-0.069346	-0.7472156	0.27520949	0.44118657	-0.1320773	0.02880068
202.7	0.41592057	-0.513626	0.10306725	-0.069346	-0.7472156	0.27520949	0.44118657	-0.1320773	0.02880068
202.8	0.41592057	-0.513626	0.10306725	-0.069346	-0.7472156	0.27520949	0.44118657	-0.1320773	0.02880068
202.9	0.41592057	-0.513626	0.10306725	-0.069346	-0.7472156	0.27520949	0.44118657	-0.1320773	0.02880068
203	0.41592057	-0.513626	0.10306725	-0.069346	-0.7472156	0.27520949	0.44118657	-0.1320773	0.02880068
203.1	0.41592057	-0.513626	0.10306725	-0.069346	-0.7472156	0.27520949	0.44118657	-0.1320773	0.02880068
203.2	0.41568483	-0.5135229	0.10351291	-0.0695423	-0.7471661	0.27559707	0.44113649	-0.1319045	0.02917055
203.3	0.41568483	-0.5135229	0.10351291	-0.0695423	-0.7471661	0.27559707	0.44113649	-0.1319045	0.02917055
203.4	0.41526854	-0.5132318	0.103787	-0.0694818	-0.7469691	0.275404	0.44077055	-0.1319913	0.02899504
203.5	0.41526854	-0.5132318	0.103787	-0.0694818	-0.7469691	0.275404	0.44077055	-0.1319913	0.02899504
203.6	0.41522987	-0.5131275	0.10430398	-0.069974	-0.7469686	0.27521868	0.44069378	-0.132244	0.02869282
203.7	0.41522987	-0.5131275	0.10430398	-0.069974	-0.7469686	0.27521868	0.44069378	-0.132244	0.02869282
203.8	0.41522987	-0.5131275	0.10430398	-0.069974	-0.7469686	0.27521868	0.44069378	-0.132244	0.02869282
203.9	0.41521409	-0.5129959	0.10489676	-0.0703624	-0.7469109	0.27507605	0.44063788	-0.1317996	0.02845551
204	0.41521409	-0.5129959	0.10489676	-0.0703624	-0.7469109	0.27507605	0.44063788	-0.1317996	0.02845551
204.1	0.41521409	-0.5129959	0.10489676	-0.0703624	-0.7469109	0.27507605	0.44063788	-0.1317996	0.02845551
204.2	0.41533735	-0.5131529	0.10520659	-0.0706337	-0.7469723	0.27494417	0.44086757	-0.131484	0.02823635
204.3	0.41533735	-0.5131529	0.10520659	-0.0706337	-0.7469723	0.27494417	0.44086757	-0.131484	0.02823635
204.4	0.41502474	-0.5128794	0.10550503	-0.0710063	-0.7470772	0.27498227	0.4408284	-0.131032	0.02832344
204.5	0.41502474	-0.5128794	0.10550503	-0.0710063	-0.7470772	0.27498227	0.4408284	-0.131032	0.02832344
204.6	0.41488139	-0.5129946	0.10582382	-0.0710596	-0.7469865	0.27474951	0.44098508	-0.1311027	0.02781994
204.7	0.41488139	-0.5129946	0.10582382	-0.0710596	-0.7469865	0.27474951	0.44098508	-0.1311027	0.02781994
204.8	0.41488139	-0.5129946	0.10582382	-0.0710596	-0.7469865	0.27474951	0.44098508	-0.1311027	0.02781994
204.9	0.41488139	-0.5129946	0.10582382	-0.0710596	-0.7469865	0.27474951	0.44098508	-0.1311027	0.02781994
205	0.41475029	-0.5129326	0.10612763	-0.0714958	-0.7470532	0.2744884	0.4407756	-0.1310521	0.02752963
205.1	0.41475029	-0.5129326	0.10612763	-0.0714958	-0.7470532	0.2744884	0.4407756	-0.1310521	0.02752963
205.2	0.41449273	-0.5126473	0.10662125	-0.0712571	-0.7467782	0.27449803	0.44064034	-0.1311364	0.02704056
205.3	0.41449273	-0.5126473	0.10662125	-0.0712571	-0.7467782	0.27449803	0.44064034	-0.1311364	0.02704056
205.4	0.41449273	-0.5126473	0.10662125	-0.0712571	-0.7467782	0.27449803	0.44064034	-0.1311364	0.02704056
205.5	0.41449273	-0.5126473	0.10662125	-0.0712571	-0.7467782	0.27449803	0.44064034	-0.1311364	0.02704056
205.6	0.41418887	-0.512379	0.10640816	-0.0712633	-0.7464776	0.27460085	0.44045874	-0.1312057	0.0269957
205.7	0.41418887	-0.512379	0.10640816	-0.0712633	-0.7464776	0.27460085	0.44045874	-0.1312057	0.0269957
205.8	0.41382882	-0.51233	0.10663277	-0.0715896	-0.7463734	0.27462202	0.44039987	-0.1311909	0.02658058
205.9	0.41347587	-0.5124346	0.10715966	-0.0718473	-0.7463266	0.27433007	0.44034644	-0.1312873	0.0262101
206	0.41347587	-0.5124346	0.10715966	-0.0718473	-0.7463266	0.27433007	0.44034644	-0.1312873	0.0262101
206.1	0.41347587	-0.5124346	0.10715966	-0.0718473	-0.7463266	0.27433007	0.44034644	-0.1312873	0.0262101
206.2	0.41347587	-0.5124346	0.10715966	-0.0718473	-0.7463266	0.27433007	0.44034644	-0.1312873	0.0262101
206.3	0.41339231	-0.512563	0.10736	-0.0723554	-0.7463756	0.27407861	0.44018914	-0.131508	0.02599421
206.4	0.41339231	-0.512563	0.10736	-0.0723554	-0.7463756	0.27407861	0.44018914	-0.131508	0.02599421
206.5	0.41331788	-0.5128691	0.10781789	-0.072699	-0.7461627	0.27473565	0.44002524	-0.1314549	0.02557739
206.6	0.41290355	-0.5128556	0.10829762	-0.072845	-0.7460237	0.27490958	0.43997148	-0.1311886	0.02535593
206.7	0.41287095	-0.5126428	0.10909539	-0.0728334	-0.7459295	0.27494081	0.43995802	-0.1315523	0.0253813
206.8	0.41287095	-0.5126428	0.10909539	-0.0728334	-0.7459295	0.27494081	0.43995802	-0.1315523	0.0253813
206.9	0.41287095	-0.5126428	0.10909539	-0.0728334	-0.7459295	0.27494081	0.43995802	-0.1315523	0.0253813
207	0.41287095	-0.5126428	0.10909539	-0.0728334	-0.7459295	0.27494081	0.43995802	-0.1315523	0.0253813
207.1	0.41272471	-0.5122827	0.10950016	-0.0726479	-0.7458044	0.27531421	0.43998289	-0.1316957	0.02561192
207.2	0.41272471	-0.5122827	0.10950016	-0.0726479	-0.7458044	0.27531421	0.43998289	-0.1316957	0.02561192
207.3	0.41272471	-0.5122827	0.10950016	-0.0726479	-0.7458044	0.27531421	0.43998289	-0.1316957	0.02561192
207.4	0.41272471	-0.5122827	0.10950016	-0.0726479	-0.7458044	0.27531421	0.43998289	-0.1316957	0.02561192
207.5	0.41272471	-0.5122827	0.10950016	-0.0726479	-0.7458044	0.27531421	0.43998289	-0.1316957	0.02561192
207.6	0.41272471	-0.5122827	0.10950016	-0.0726479	-0.7458044	0.27531421	0.43998289	-0.1316957	0.02561192
207.7	0.41272471	-0.5122827	0.10950016	-0.0726479	-0.7458044	0.27531421	0.43998289	-0.1316957	0.02561192
207.8	0.41272471	-0.5122827	0.10950016	-0.0726479	-0.7458044	0.27531421	0.43998289	-0.1316957	0.02561192
207.9	0.41272471	-0.5122827	0.10950016	-0.0726479	-0.7458044	0.27531421	0.43998289	-0.1316957	0.02561192
208	0.41299772	-0.5121491	0.11014551	-0.0730293	-0.7458698	0.27496986	0.44007088	-0.1313812	0.02527881
208.1	0.41286619	-0.5121933	0.11028419	-0.0731687	-0.745917	0.27517295	0.440145	-0.1312851	0.02543028
208.2	0.41286619	-0.5121933	0.11028419	-0.0731687	-0.745917	0.27517295	0.440145	-0.1312851	0.02543028
208.3	0.41257273	-0.5123329	0.11028357	-0.0736589	-0.7458283	0.27509523	0.44047542	-0.1314165	0.02562344
208.4	0.41209422	-0.512007	0.11079689	-0.0739409	-0.7457357	0.27496349	0.44027026	-0.1311196	0.02582326
208.5	0.41150931	-0.5121547	0.1106232	-0.0739235	-0.7455003	0.27497189	0.43995284	-0.1312027	0.02540367
208.6	0.41150931	-0.5121547	0.1106232	-0.0739235	-0.7455003	0.27497189	0.43995284	-0.1312027	0.02540367
208.7	0.41150931	-0.5121547	0.1106232	-0.0739235	-0.7455003	0.27497189	0.43995284	-0.1312027	0.02540367
208.8	0.41150931	-0.5121547	0.1106232	-0.0739235	-0.7455003	0.27497189	0.43995284	-0.1312027	0.02540367
208.9	0.41150931	-0.5121547	0.1106232	-0.0739235	-0.7455003	0.27497189	0.43995284	-0.1312027	0.02540367
209	0.41151927	-0.512407	0.11112045	-0.0741965	-0.74555	0.27485043	0.44000583	-0.131406	0.02543603
209.1	0.41151927	-0.512407	0.11112045	-0.0741965	-0.74555	0.27485043	0.44000583	-0.131406	0.02543603
209.2	0.41151927	-0.512407	0.11112045	-0.0741965	-0.74555	0.27485043	0.44000583	-0.131406	0.02543603
209.3	0.41151927	-0.512407	0.11112045	-0.0741965	-0.74555	0.27485043	0.44000583	-0.131406	0.02543603
209.4	0.41151927	-0.512407	0.11112045	-0.0741965	-0.74555	0.27485043	0.44000583	-0.131406	0.02543603
209.5	0.41151927	-0.512407	0.11112045	-0.0741965	-0.74555	0.27485043	0.44000583	-0.131406	0.02543603
209.6	0.41141167	-0.5122084	0.11106675	-0.074891	-0.7453996	0.27456401	0.43961279	-0.1315564	0.0252595
209.7	0.41141167	-0.5122084	0.11106675	-0.074891	-0.7453996	0.27456401	0.43961279	-0.1315564	0.0252595
209.8	0.41119305	-0.5122276	0.11122122	-0.0750873	-0.7451808	0.27469045	0.43939347	-0.1314384	0.02496625
209.9	0.41098696	-0.5122501	0.11167069	-0.0754194	-0.7452506	0.27456728	0.43919681	-0.1309973	0.02458244
210.6	0.41014704	-0.5121043	0.11187899	-0.0754677	-0.7448497	0.27465992	0.43857114	-0.131485	0.02449166
210.7	0.41014704	-0.5121043	0.11187899	-0.0754677	-0.7448497	0.27465992	0.43857114	-0.131485	0.02449166
210.8	0.41014704	-0.5121043	0.11187899	-0.0754677	-0.7448497	0.27465992	0.43857114	-0.131485	0.02449166
210.9	0.41021787	-0.5119669	0.11212914	-0.0758181	-0.7447643	0.27455621	0.43825676	-0.1313122	0.02490358
211	0.40997232	-0.511948	0.11218986	-0.0760123	-0.744786	0.27455208	0.43826915	-0.1312718	0.02517467
211.1	0.40981172	-0.5118754	0.11195734	-0.0763827	-0.7446733	0.27399064	0.43849196	-0.1308321	0.02528901
211.2	0.40981172	-0.5118754	0.11195734	-0.0763827	-0.7446733	0.27399064	0.43849196	-0.1308321	0.02528901
211.3	0.40981172	-0.5118754	0.11195734	-0.0763827	-0.7446733	0.27399064	0.43849196	-0.1308321	0.02528901
211.4	0.40896808	-0.5116507	0.11172867	-0.0765233	-0.7442573	0.27323455	0.43833288	-0.1307982	0.0250007
211.5	0.40896808	-0.5116507	0.11172867	-0.0765233	-0.7442573	0.27323455	0.43833288	-0.1307982	0.0250007
211.6	0.40914821	-0.5114064	0.11161502	-0.0765894	-0.7442317	0.27308711	0.43856538	-0.1306218	0.0254351
211.7	0.40937813	-0.511454	0.11161023	-0.0768364	-0.7442713	0.27316645	0.4387175	-0.1302989	0.02573496
211.8	0.40937813	-0.511454	0.11161023	-0.0768364	-0.7442713	0.27316645	0.4387175	-0.1302989	0.02573496
211.9	0.40937813	-0.511454	0.11161023	-0.0768364	-0.7442713	0.27316645	0.4387175	-0.1302989	0.02573496
212	0.40933243	-0.5113965	0.11210256	-0.0771645	-0.7443111	0.27309147	0.43869442	-0.1302087	0.02533063
212.1	0.40894479	-0.5111934	0.11218075	-0.077305	-0.7439313	0.27285017	0.43819408	-0.1300759	0.02533237
212.2	0.40894479	-0.5111934	0.11218075	-0.077305	-0.7439313	0.27285017	0.43819408	-0.1300759	0.02533237
212.3	0.40894479	-0.5111934	0.11218075	-0.077305	-0.7439313	0.27285017	0.43819408	-0.1300759	0.02533237
212.4	0.40899806	-0.5112817	0.11175095	-0.0771309	-0.7441179	0.27293757	0.43846708	-0.1305111	0.02519744
212.5	0.40865989	-0.5110984	0.11148973	-0.0774214	-0.7437718	0.27264495	0.43840249	-0.1302266	0.02534762
212.6	0.40865989	-0.5110984	0.11148973	-0.0774214	-0.7437718	0.27264495	0.43840249	-0.1302266	0.02534762
212.7	0.4086933	-0.5110688	0.11196232	-0.0778902	-0.7437078	0.27264632	0.43821033	-0.1298911	0.02496045
212.8	0.4086933	-0.5110688	0.11196232	-0.0778902	-0.7437078	0.27264632	0.43821033	-0.1298911	0.02496045
212.9	0.4084216	-0.5110343	0.11237648	-0.0778347	-0.7437653	0.27282257	0.438068	-0.1295866	0.02473071
213	0.4084216	-0.5110343	0.11237648	-0.0778347	-0.7437653	0.27282257	0.438068	-0.1295866	0.02473071
213.1	0.40820653	-0.5106903	0.11235349	-0.0779603	-0.7436613	0.27290019	0.43785148	-0.1294259	0.0250208
213.2	0.40820653	-0.5106903	0.11235349	-0.0779603	-0.7436613	0.27290019	0.43785148	-0.1294259	0.0250208
213.3	0.40820653	-0.5106903	0.11235349	-0.0779603	-0.7436613	0.27290019	0.43785148	-0.1294259	0.0250208
213.4	0.40820653	-0.5106903	0.11235349	-0.0779603	-0.7436613	0.27290019	0.43785148	-0.1294259	0.0250208
213.5	0.40820653	-0.5106903	0.11235349	-0.0779603	-0.7436613	0.27290019	0.43785148	-0.1294259	0.0250208
213.6	0.40786416	-0.5102287	0.11261075	-0.0782074	-0.7436154	0.2726901	0.43758523	-0.1289739	0.02532758
213.7	0.40762785	-0.510215	0.11318453	-0.0783215	-0.7435498	0.27251928	0.43744439	-0.1290252	0.0250774
213.8	0.40762785	-0.510215	0.11318453	-0.0783215	-0.7435498	0.27251928	0.43744439	-0.1290252	0.0250774
213.9	0.40762785	-0.510215	0.11318453	-0.0783215	-0.7435498	0.27251928	0.43744439	-0.1290252	0.0250774
214	0.40765014	-0.5099079	0.11339431	-0.0785112	-0.7435113	0.2728385	0.43762669	-0.1286906	0.02518658
214.1	0.40741889	-0.5095629	0.11330761	-0.0784805	-0.7436275	0.27247521	0.43779335	-0.1282641	0.02533662
214.2	0.40741889	-0.5095629	0.11330761	-0.0784805	-0.7436275	0.27247521	0.43779335	-0.1282641	0.02533662
214.3	0.40741889	-0.5095629	0.11330761	-0.0784805	-0.7436275	0.27247521	0.43779335	-0.1282641	0.02533662
214.4	0.40741889	-0.5095629	0.11330761	-0.0784805	-0.7436275	0.27247521	0.43779335	-0.1282641	0.02533662
214.5	0.40714024	-0.5094761	0.11345944	-0.0783499	-0.7434514	0.27261596	0.4371869	-0.1281509	0.02530651
214.6	0.40680493	-0.5095027	0.11378827	-0.0784152	-0.7436396	0.27198967	0.43744149	-0.1276168	0.02544877
214.7	0.40657378	-0.5095137	0.11370847	-0.0783051	-0.7434655	0.27226918	0.43758988	-0.127652	0.0253245
214.8	0.40654245	-0.5095777	0.11406958	-0.0783434	-0.7434109	0.27214017	0.43736376	-0.1278699	0.02484149
214.9	0.40654245	-0.5095777	0.11406958	-0.0783434	-0.7434109	0.27214017	0.43736376	-0.1278699	0.02484149
215	0.40654245	-0.5095777	0.11406958	-0.0783434	-0.7434109	0.27214017	0.43736376	-0.1278699	0.02484149
215.1	0.40639534	-0.5098896	0.11424412	-0.078479	-0.7433996	0.27218329	0.43746514	-0.1276663	0.02477947
215.2	0.40639534	-0.5098896	0.11424412	-0.078479	-0.7433996	0.27218329	0.43746514	-0.1276663	0.02477947
215.3	0.40639534	-0.5098896	0.11424412	-0.078479	-0.7433996	0.27218329	0.43746514	-0.1276663	0.02477947
215.4	0.40670318	-0.5101658	0.11427733	-0.078887	-0.7435201	0.27243121	0.43769213	-0.1276362	0.02531565
215.5	0.40649461	-0.5101018	0.11478689	-0.0789337	-0.7434789	0.27251895	0.43763701	-0.1276184	0.02505624
215.6	0.40649461	-0.5101018	0.11478689	-0.0789337	-0.7434789	0.27251895	0.43763701	-0.1276184	0.02505624
215.7	0.406654	-0.5104989	0.11521163	-0.078939	-0.7436063	0.27267121	0.43768269	-0.127635	0.02481596
215.8	0.406654	-0.5104989	0.11521163	-0.078939	-0.7436063	0.27267121	0.43768269	-0.127635	0.02481596
215.9	0.40690725	-0.5105041	0.11522915	-0.0791985	-0.7435711	0.27294418	0.43765533	-0.1276712	0.02494579
216	0.40690725	-0.5105041	0.11522915	-0.0791985	-0.7435711	0.27294418	0.43765533	-0.1276712	0.02494579
216.1	0.40690725	-0.5105041	0.11522915	-0.0791985	-0.7435711	0.27294418	0.43765533	-0.1276712	0.02494579
216.2	0.40680607	-0.5104415	0.11570133	-0.0797633	-0.7433955	0.27313358	0.43720377	-0.1277585	0.02429849
216.3	0.40680607	-0.5104415	0.11570133	-0.0797633	-0.7433955	0.27313358	0.43720377	-0.1277585	0.02429849
216.4	0.40680607	-0.5104415	0.11570133	-0.0797633	-0.7433955	0.27313358	0.43720377	-0.1277585	0.02429849
216.5	0.40680607	-0.5104415	0.11570133	-0.0797633	-0.7433955	0.27313358	0.43720377	-0.1277585	0.02429849
216.6	0.40680607	-0.5104415	0.11570133	-0.0797633	-0.7433955	0.27313358	0.43720377	-0.1277585	0.02429849
216.7	0.40680607	-0.5104415	0.11570133	-0.0797633	-0.7433955	0.27313358	0.43720377	-0.1277585	0.02429849
216.8	0.40680607	-0.5104415	0.11570133	-0.0797633	-0.7433955	0.27313358	0.43720377	-0.1277585	0.02429849
216.9	0.40680607	-0.5104415	0.11570133	-0.0797633	-0.7433955	0.27313358	0.43720377	-0.1277585	0.02429849
217	0.40669929	-0.5106149	0.11597091	-0.0797799	-0.7431725	0.2735485	0.43672925	-0.1273333	0.02481628
217.1	0.40652204	-0.5102077	0.11634515	-0.0799272	-0.7429738	0.27364576	0.43655325	-0.1274777	0.02484024
217.2	0.40652204	-0.5102077	0.11634515	-0.0799272	-0.7429738	0.27364576	0.43655325	-0.1274777	0.02484024
217.3	0.40652204	-0.5102077	0.11634515	-0.0799272	-0.7429738	0.27364576	0.43655325	-0.1274777	0.02484024
217.4	0.40630918	-0.5101623	0.11649058	-0.0803432	-0.7428014	0.27360093	0.43636689	-0.127337	0.02441128
217.5	0.40630918	-0.5101623	0.11649058	-0.0803432	-0.7428014	0.27360093	0.43636689	-0.127337	0.02441128
217.6	0.40630918	-0.5101623	0.11649058	-0.0803432	-0.7428014	0.27360093	0.43636689	-0.127337	0.02441128
217.7	0.40608728	-0.510329	0.1167114	-0.0801664	-0.7426872	0.27357036	0.43652343	-0.1274835	0.02433149
217.8	0.40614496	-0.5103817	0.11712359	-0.0804181	-0.7426164	0.2737959	0.43649348	-0.1276297	0.02476671
217.9	0.40614496	-0.5103817	0.11712359	-0.0804181	-0.7426164	0.2737959	0.43649348	-0.1276297	0.02476671
218	0.40571397	-0.5104623	0.11675586	-0.0806494	-0.7424285	0.27340941	0.43658696	-0.1273023	0.02503689
218.1	0.40569911	-0.5104337	0.11675205	-0.0816372	-0.7424313	0.27314555	0.43627623	-0.1267682	0.02510377
218.2	0.40569911	-0.5104337	0.11675205	-0.0816372	-0.7424313	0.27314555	0.43627623	-0.1267682	0.02510377
218.3	0.40570002	-0.510424	0.11717042	-0.0818531	-0.7424141	0.27312449	0.43601954	-0.1268018	0.02512898
218.4	0.40570002	-0.510424	0.11717042	-0.0818531	-0.7424141	0.27312449	0.43601954	-0.1268018	0.02512898
218.5	0.40570002	-0.510424	0.11717042	-0.0818531	-0.7424141	0.27312449	0.43601954	-0.1268018	0.02512898
218.6	0.40570002	-0.510424	0.11717042	-0.0818531	-0.7424141	0.27312449	0.43601954	-0.1268018	0.02512898
218.7	0.4055082	-0.5109617	0.11757115	-0.0821628	-0.7426645	0.27343279	0.43612838	-0.126516	0.02483927
218.8	0.40563442	-0.5111606	0.11783765	-0.0822036	-0.7426518	0.27346946	0.43607831	-0.1268364	0.02493902
218.9	0.40542248	-0.5105593	0.11870547	-0.0822774	-0.7423965	0.27307247	0.43536725	-0.1264399	0.0244232
219	0.40529921	-0.510514	0.11895141	-0.082118	-0.7424745	0.27263169	0.43542494	-0.126389	0.02445189
219.1	0.40512208	-0.5103864	0.11909925	-0.0821263	-0.7424881	0.27284821	0.43559657	-0.1261536	0.02467544
219.2	0.40512208	-0.5103864	0.11909925	-0.0821263	-0.7424881	0.27284821	0.43559657	-0.1261536	0.02467544
219.3	0.40486323	-0.5104374	0.11937191	-0.0821751	-0.7425404	0.27271022	0.43561701	-0.1257314	0.02472698
219.4	0.40486323	-0.5104374	0.11937191	-0.0821751	-0.7425404	0.27271022	0.43561701	-0.1257314	0.02472698
219.5	0.40486323	-0.5104374	0.11937191	-0.0821751	-0.7425404	0.27271022	0.43561701	-0.1257314	0.02472698
219.6	0.40481735	-0.5104026	0.11942874	-0.0821299	-0.742495	0.27294885	0.43526035	-0.1258569	0.02478135
219.7	0.40481735	-0.5104026	0.11942874	-0.0821299	-0.742495	0.27294885	0.43526035	-0.1258569	0.02478135
219.8	0.40478674	-0.5101769	0.11890396	-0.0824836	-0.7422829	0.27279166	0.43522514	-0.1257696	0.02524277
219.9	0.40478674	-0.5101769	0.11890396	-0.0824836	-0.7422829	0.27279166	0.43522514	-0.1257696	0.02524277
220	0.40478674	-0.5101769	0.11890396	-0.0824836	-0.7422829	0.27279166	0.43522514	-0.1257696	0.02524277
220.1	0.40483581	-0.5101483	0.11913212	-0.0823631	-0.7421897	0.27266021	0.43510088	-0.1257975	0.02510577
220.2	0.40483581	-0.5101483	0.11913212	-0.0823631	-0.7421897	0.27266021	0.43510088	-0.1257975	0.02510577
220.3	0.40483581	-0.5101483	0.11913212	-0.0823631	-0.7421897	0.27266021	0.43510088	-0.1257975	0.02510577
220.4	0.40483581	-0.5101483	0.11913212	-0.0823631	-0.7421897	0.27266021	0.43510088	-0.1257975	0.02510577
220.5	0.40480657	-0.5100168	0.11923867	-0.0826635	-0.7421406	0.27234923	0.43484626	-0.1258727	0.02484248
220.6	0.40456389	-0.5099703	0.11930795	-0.0825343	-0.7422306	0.27210371	0.43471602	-0.125722	0.02512617
220.7	0.40456389	-0.5099703	0.11930795	-0.0825343	-0.7422306	0.27210371	0.43471602	-0.125722	0.02512617
220.8	0.40456389	-0.5099703	0.11930795	-0.0825343	-0.7422306	0.27210371	0.43471602	-0.125722	0.02512617
220.9	0.40456389	-0.5099703	0.11930795	-0.0825343	-0.7422306	0.27210371	0.43471602	-0.125722	0.02512617
221	0.40456389	-0.5099703	0.11930795	-0.0825343	-0.7422306	0.27210371	0.43471602	-0.125722	0.02512617
221.1	0.40438361	-0.5101433	0.11970974	-0.0825751	-0.7422198	0.27207549	0.43461633	-0.1256087	0.02539124
221.2	0.40438361	-0.5101433	0.11970974	-0.0825751	-0.7422198	0.27207549	0.43461633	-0.1256087	0.02539124
221.3	0.40438361	-0.5101433	0.11970974	-0.0825751	-0.7422198	0.27207549	0.43461633	-0.1256087	0.02539124
221.4	0.40438361	-0.5101433	0.11970974	-0.0825751	-0.7422198	0.27207549	0.43461633	-0.1256087	0.02539124
221.5	0.40438361	-0.5101433	0.11970974	-0.0825751	-0.7422198	0.27207549	0.43461633	-0.1256087	0.02539124
221.6	0.40438361	-0.5101433	0.11970974	-0.0825751	-0.7422198	0.27207549	0.43461633	-0.1256087	0.02539124
221.7	0.40443012	-0.5103321	0.11960855	-0.0826473	-0.7422006	0.27208417	0.43466736	-0.1258777	0.02546464
221.8	0.40443012	-0.5103321	0.11960855	-0.0826473	-0.7422006	0.27208417	0.43466736	-0.1258777	0.02546464
221.9	0.40399753	-0.5105265	0.11986158	-0.0830932	-0.7422603	0.27185078	0.43435652	-0.1261111	0.02529784
222	0.40399753	-0.5105265	0.11986158	-0.0830932	-0.7422603	0.27185078	0.43435652	-0.1261111	0.02529784
222.1	0.40399753	-0.5105265	0.11986158	-0.0830932	-0.7422603	0.27185078	0.43435652	-0.1261111	0.02529784
222.2	0.40371353	-0.5102953	0.11931894	-0.0831872	-0.7420833	0.2714213	0.43434896	-0.1259877	0.0256292
222.3	0.40371353	-0.5102953	0.11931894	-0.0831872	-0.7420833	0.2714213	0.43434896	-0.1259877	0.0256292
222.4	0.40368247	-0.5100064	0.11970305	-0.0832685	-0.7422343	0.27132455	0.43463885	-0.1259552	0.02578026
222.5	0.40368247	-0.5100064	0.11970305	-0.0832685	-0.7422343	0.27132455	0.43463885	-0.1259552	0.02578026
222.6	0.40391079	-0.5097609	0.12055468	-0.0838157	-0.7422702	0.27118786	0.4342493	-0.1257036	0.02521442
222.7	0.40391061	-0.5099512	0.12018114	-0.084441	-0.742143	0.27053075	0.43434504	-0.1257046	0.02495585
222.8	0.40391061	-0.5099512	0.12018114	-0.084441	-0.742143	0.27053075	0.43434504	-0.1257046	0.02495585
222.9	0.40330757	-0.5096864	0.12033804	-0.083983	-0.741854	0.27063928	0.43401786	-0.1257952	0.02499281
223	0.4031007	-0.5094381	0.12063667	-0.0842408	-0.7419007	0.27053323	0.43390176	-0.1255138	0.02501896
223.1	0.40316697	-0.5096302	0.12097783	-0.084089	-0.7418682	0.27050935	0.43378992	-0.1254409	0.02501447
223.2	0.40316697	-0.5096302	0.12097783	-0.084089	-0.7418682	0.27050935	0.43378992	-0.1254409	0.02501447
223.3	0.40312965	-0.5092844	0.12132613	-0.0841352	-0.741799	0.2705413	0.43363352	-0.1250335	0.02494031
223.4	0.40304123	-0.5093278	0.12148961	-0.084414	-0.7417865	0.27080444	0.43361642	-0.1254516	0.02468401
223.5	0.40311574	-0.5094136	0.12162674	-0.0848262	-0.7417666	0.27079305	0.43341896	-0.1257111	0.02421121
223.6	0.40290208	-0.5092291	0.12210165	-0.0848727	-0.7417612	0.27079404	0.43341768	-0.1255562	0.02386277
223.7	0.40290208	-0.5092291	0.12210165	-0.0848727	-0.7417612	0.27079404	0.43341768	-0.1255562	0.02386277
223.8	0.40290208	-0.5092291	0.12210165	-0.0848727	-0.7417612	0.27079404	0.43341768	-0.1255562	0.02386277
223.9	0.40290208	-0.5092291	0.12210165	-0.0848727	-0.7417612	0.27079404	0.43341768	-0.1255562	0.02386277
224	0.40273662	-0.5089244	0.12218967	-0.0852184	-0.7415977	0.27052291	0.43306846	-0.1252599	0.02381759
224.1	0.40273662	-0.5089244	0.12218967	-0.0852184	-0.7415977	0.27052291	0.43306846	-0.1252599	0.02381759
224.2	0.40280745	-0.5087065	0.12200772	-0.0854927	-0.7415508	0.27067242	0.43318521	-0.1249984	0.02419258
224.3	0.40280745	-0.5087065	0.12200772	-0.0854927	-0.7415508	0.27067242	0.43318521	-0.1249984	0.02419258
224.4	0.40280745	-0.5087065	0.12200772	-0.0854927	-0.7415508	0.27067242	0.43318521	-0.1249984	0.02419258
224.5	0.40276156	-0.5086247	0.12258991	-0.0856135	-0.7416292	0.27073269	0.43307138	-0.1246341	0.02376451
224.6	0.40276156	-0.5086247	0.12258991	-0.0856135	-0.7416292	0.27073269	0.43307138	-0.1246341	0.02376451
224.7	0.40289143	-0.5086731	0.12253345	-0.0859282	-0.7415753	0.27092512	0.43294576	-0.1247517	0.02336002
224.8	0.40278363	-0.5087021	0.1224663	-0.0861386	-0.7415566	0.27122309	0.43283166	-0.1246663	0.02330331
224.9	0.40278363	-0.5087021	0.1224663	-0.0861386	-0.7415566	0.27122309	0.43283166	-0.1246663	0.02330331
225.6	0.4031887	-0.5085251	0.12327318	-0.0866361	-0.7416574	0.27148159	0.43284358	-0.1241141	0.02320482
225.7	0.4031887	-0.5085251	0.12327318	-0.0866361	-0.7416574	0.27148159	0.43284358	-0.1241141	0.02320482
225.8	0.4031887	-0.5085251	0.12327318	-0.0866361	-0.7416574	0.27148159	0.43284358	-0.1241141	0.02320482
225.9	0.40293234	-0.5084716	0.12363506	-0.086515	-0.7415669	0.27128631	0.43267083	-0.1237099	0.02323378
226	0.40293234	-0.5084716	0.12363506	-0.086515	-0.7415669	0.27128631	0.43267083	-0.1237099	0.02323378
226.1	0.40293234	-0.5084716	0.12363506	-0.086515	-0.7415669	0.27128631	0.43267083	-0.1237099	0.02323378
226.2	0.40293234	-0.5084716	0.12363506	-0.086515	-0.7415669	0.27128631	0.43267083	-0.1237099	0.02323378
226.3	0.40293234	-0.5084716	0.12363506	-0.086515	-0.7415669	0.27128631	0.43267083	-0.1237099	0.02323378
226.4	0.40293234	-0.5084716	0.12363506	-0.086515	-0.7415669	0.27128631	0.43267083	-0.1237099	0.02323378
226.5	0.40293234	-0.5084716	0.12363506	-0.086515	-0.7415669	0.27128631	0.43267083	-0.1237099	0.02323378
226.6	0.40293234	-0.5084716	0.12363506	-0.086515	-0.7415669	0.27128631	0.43267083	-0.1237099	0.02323378
226.7	0.40293234	-0.5084716	0.12363506	-0.086515	-0.7415669	0.27128631	0.43267083	-0.1237099	0.02323378
226.8	0.40293234	-0.5084716	0.12363506	-0.086515	-0.7415669	0.27128631	0.43267083	-0.1237099	0.02323378
226.9	0.40293234	-0.5084716	0.12363506	-0.086515	-0.7415669	0.27128631	0.43267083	-0.1237099	0.02323378
227	0.40293234	-0.5084716	0.12363506	-0.086515	-0.7415669	0.27128631	0.43267083	-0.1237099	0.02323378
227.1	0.40296752	-0.5088159	0.1238552	-0.086757	-0.7415799	0.2717335	0.43260003	-0.123897	0.02372899
227.2	0.40296752	-0.5088159	0.1238552	-0.086757	-0.7415799	0.2717335	0.43260003	-0.123897	0.02372899
227.3	0.40315865	-0.5090203	0.1241652	-0.0866982	-0.7418163	0.27176925	0.43268197	-0.12354	0.02374408
227.4	0.40328129	-0.5090647	0.1239197	-0.0868567	-0.7417375	0.2722089	0.43262335	-0.1235906	0.02371541
227.5	0.40300059	-0.5091163	0.12409187	-0.0865692	-0.7415319	0.27264252	0.43278303	-0.1233783	0.02378371
227.6	0.40300059	-0.5091163	0.12409187	-0.0865692	-0.7415319	0.27264252	0.43278303	-0.1233783	0.02378371
227.7	0.40335595	-0.5088116	0.12465685	-0.0868625	-0.7413712	0.2727778	0.4323375	-0.1230982	0.0239292
227.8	0.40335595	-0.5088116	0.12465685	-0.0868625	-0.7413712	0.2727778	0.4323375	-0.1230982	0.0239292
227.9	0.40318824	-0.5087657	0.12479574	-0.0868856	-0.7412099	0.27307388	0.43197362	-0.1230015	0.02405195
228	0.40318824	-0.5087657	0.12479574	-0.0868856	-0.7412099	0.27307388	0.43197362	-0.1230015	0.02405195
228.1	0.40318824	-0.5087657	0.12479574	-0.0868856	-0.7412099	0.27307388	0.43197362	-0.1230015	0.02405195
228.2	0.40318824	-0.5087657	0.12479574	-0.0868856	-0.7412099	0.27307388	0.43197362	-0.1230015	0.02405195
228.3	0.40346895	-0.5086136	0.1251307	-0.086976	-0.7411101	0.27322617	0.43203184	-0.1230832	0.02411006
228.4	0.40346895	-0.5086136	0.1251307	-0.086976	-0.7411101	0.27322617	0.43203184	-0.1230832	0.02411006
228.5	0.40346895	-0.5086136	0.1251307	-0.086976	-0.7411101	0.27322617	0.43203184	-0.1230832	0.02411006
228.6	0.40346895	-0.5086136	0.1251307	-0.086976	-0.7411101	0.27322617	0.43203184	-0.1230832	0.02411006
228.7	0.40320789	-0.5086808	0.12526178	-0.0867441	-0.7411828	0.27326596	0.43194328	-0.1229716	0.02419855
228.8	0.40277452	-0.5084701	0.12522792	-0.0866599	-0.740982	0.27332056	0.4317748	-0.1225864	0.024116
228.9	0.40277452	-0.5084701	0.12522792	-0.0866599	-0.740982	0.27332056	0.4317748	-0.1225864	0.024116
229	0.40277452	-0.5084701	0.12522792	-0.0866599	-0.740982	0.27332056	0.4317748	-0.1225864	0.024116
229.1	0.40277452	-0.5084701	0.12522792	-0.0866599	-0.740982	0.27332056	0.4317748	-0.1225864	0.024116
229.2	0.40277452	-0.5084701	0.12522792	-0.0866599	-0.740982	0.27332056	0.4317748	-0.1225864	0.024116
229.3	0.40277452	-0.5084701	0.12522792	-0.0866599	-0.740982	0.27332056	0.4317748	-0.1225864	0.024116
229.4	0.40277452	-0.5084701	0.12522792	-0.0866599	-0.740982	0.27332056	0.4317748	-0.1225864	0.024116
229.5	0.40277452	-0.5084701	0.12522792	-0.0866599	-0.740982	0.27332056	0.4317748	-0.1225864	0.024116
229.6	0.40277452	-0.5084701	0.12522792	-0.0866599	-0.740982	0.27332056	0.4317748	-0.1225864	0.024116
229.7	0.40240824	-0.5083692	0.12528725	-0.0866543	-0.740878	0.27357632	0.4317087	-0.1225662	0.02407734
229.8	0.40257037	-0.5085187	0.1250858	-0.0865317	-0.7410413	0.27380008	0.43182935	-0.1230989	0.02413777
229.9	0.40252698	-0.5085183	0.12457595	-0.0861258	-0.7410138	0.27388473	0.43201503	-0.1229309	0.02404467
230	0.40252698	-0.5085183	0.12457595	-0.0861258	-0.7410138	0.27388473	0.43201503	-0.1229309	0.02404467
230.1	0.40231564	-0.5086741	0.12453304	-0.0864117	-0.7408951	0.27384674	0.4323126	-0.1227895	0.02423176
230.2	0.40231564	-0.5086741	0.12453304	-0.0864117	-0.7408951	0.27384674	0.4323126	-0.1227895	0.02423176
230.3	0.40231564	-0.5086741	0.12453304	-0.0864117	-0.7408951	0.27384674	0.4323126	-0.1227895	0.02423176
230.4	0.40231564	-0.5086741	0.12453304	-0.0864117	-0.7408951	0.27384674	0.4323126	-0.1227895	0.02423176
230.5	0.40231564	-0.5086741	0.12453304	-0.0864117	-0.7408951	0.27384674	0.4323126	-0.1227895	0.02423176
230.6	0.40231564	-0.5086741	0.12453304	-0.0864117	-0.7408951	0.27384674	0.4323126	-0.1227895	0.02423176
230.7	0.40231564	-0.5086741	0.12453304	-0.0864117	-0.7408951	0.27384674	0.4323126	-0.1227895	0.02423176
230.8	0.40231564	-0.5086741	0.12453304	-0.0864117	-0.7408951	0.27384674	0.4323126	-0.1227895	0.02423176
230.9	0.40231564	-0.5086741	0.12453304	-0.0864117	-0.7408951	0.27384674	0.4323126	-0.1227895	0.02423176
231	0.40231564	-0.5086741	0.12453304	-0.0864117	-0.7408951	0.27384674	0.4323126	-0.1227895	0.02423176
231.1	0.40231564	-0.5086741	0.12453304	-0.0864117	-0.7408951	0.27384674	0.4323126	-0.1227895	0.02423176
231.2	0.40228915	-0.508495	0.12500188	-0.0865012	-0.7407962	0.27393322	0.43232034	-0.1224384	0.02445489
231.3	0.40228915	-0.508495	0.12500188	-0.0865012	-0.7407962	0.27393322	0.43232034	-0.1224384	0.02445489
231.4	0.40228915	-0.508495	0.12500188	-0.0865012	-0.7407962	0.27393322	0.43232034	-0.1224384	0.02445489
231.5	0.40246177	-0.508469	0.12515495	-0.0862532	-0.7406911	0.27398765	0.43248932	-0.1227445	0.02440095
231.6	0.40246177	-0.508469	0.12515495	-0.0862532	-0.7406911	0.27398765	0.43248932	-0.1227445	0.02440095
231.7	0.40246177	-0.508469	0.12515495	-0.0862532	-0.7406911	0.27398765	0.43248932	-0.1227445	0.02440095
231.8	0.40246177	-0.508469	0.12515495	-0.0862532	-0.7406911	0.27398765	0.43248932	-0.1227445	0.02440095
231.9	0.40246177	-0.508469	0.12515495	-0.0862532	-0.7406911	0.27398765	0.43248932	-0.1227445	0.02440095
232	0.40246177	-0.508469	0.12515495	-0.0862532	-0.7406911	0.27398765	0.43248932	-0.1227445	0.02440095
232.1	0.40183185	-0.5081604	0.12528122	-0.0861249	-0.740331	0.27382242	0.43196492	-0.1228396	0.02423398
232.2	0.40183185	-0.5081604	0.12528122	-0.0861249	-0.740331	0.27382242	0.43196492	-0.1228396	0.02423398
232.3	0.40183185	-0.5081604	0.12528122	-0.0861249	-0.740331	0.27382242	0.43196492	-0.1228396	0.02423398
232.4	0.40183185	-0.5081604	0.12528122	-0.0861249	-0.740331	0.27382242	0.43196492	-0.1228396	0.02423398
232.5	0.40183185	-0.5081604	0.12528122	-0.0861249	-0.740331	0.27382242	0.43196492	-0.1228396	0.02423398
232.6	0.40183185	-0.5081604	0.12528122	-0.0861249	-0.740331	0.27382242	0.43196492	-0.1228396	0.02423398
232.7	0.40183185	-0.5081604	0.12528122	-0.0861249	-0.740331	0.27382242	0.43196492	-0.1228396	0.02423398
232.8	0.40183185	-0.5081604	0.12528122	-0.0861249	-0.740331	0.27382242	0.43196492	-0.1228396	0.02423398
232.9	0.40183185	-0.5081604	0.12528122	-0.0861249	-0.740331	0.27382242	0.43196492	-0.1228396	0.02423398
233	0.40183185	-0.5081604	0.12528122	-0.0861249	-0.740331	0.27382242	0.43196492	-0.1228396	0.02423398
233.1	0.40183185	-0.5081604	0.12528122	-0.0861249	-0.740331	0.27382242	0.43196492	-0.1228396	0.02423398
233.2	0.40150257	-0.5088059	0.1259165	-0.0863184	-0.7405932	0.2739371	0.43200942	-0.1233627	0.02413749
233.3	0.4016339	-0.5087548	0.12623397	-0.0860419	-0.7406693	0.27370051	0.43216159	-0.122922	0.02407857
233.4	0.40162694	-0.5090827	0.12676475	-0.0865127	-0.7407383	0.27398569	0.43210326	-0.1231513	0.02357501
233.5	0.40162694	-0.5090827	0.12676475	-0.0865127	-0.7407383	0.27398569	0.43210326	-0.1231513	0.02357501
233.6	0.40162694	-0.5090827	0.12676475	-0.0865127	-0.7407383	0.27398569	0.43210326	-0.1231513	0.02357501
233.7	0.40162694	-0.5090827	0.12676475	-0.0865127	-0.7407383	0.27398569	0.43210326	-0.1231513	0.02357501
233.8	0.40162694	-0.5090827	0.12676475	-0.0865127	-0.7407383	0.27398569	0.43210326	-0.1231513	0.02357501
233.9	0.4014263	-0.5087503	0.12726895	-0.0865371	-0.7407175	0.27395467	0.43216134	-0.1231324	0.02346039
234	0.4014263	-0.5087503	0.12726895	-0.0865371	-0.7407175	0.27395467	0.43216134	-0.1231324	0.02346039
234.1	0.4014263	-0.5087503	0.12726895	-0.0865371	-0.7407175	0.27395467	0.43216134	-0.1231324	0.02346039
234.2	0.4014263	-0.5087503	0.12726895	-0.0865371	-0.7407175	0.27395467	0.43216134	-0.1231324	0.02346039
234.3	0.40124776	-0.5086443	0.12705433	-0.086912	-0.7406021	0.27404018	0.43220498	-0.1227766	0.02351451
234.4	0.40124776	-0.5086443	0.12705433	-0.086912	-0.7406021	0.27404018	0.43220498	-0.1227766	0.02351451
234.5	0.40124776	-0.5086443	0.12705433	-0.086912	-0.7406021	0.27404018	0.43220498	-0.1227766	0.02351451
234.6	0.40147774	-0.5085186	0.1271622	-0.0871224	-0.7405937	0.27432189	0.43238537	-0.1228293	0.02370889
234.7	0.40147774	-0.5085186	0.1271622	-0.0871224	-0.7405937	0.27432189	0.43238537	-0.1228293	0.02370889
234.8	0.40147774	-0.5085186	0.1271622	-0.0871224	-0.7405937	0.27432189	0.43238537	-0.1228293	0.02370889
234.9	0.40147774	-0.5085186	0.1271622	-0.0871224	-0.7405937	0.27432189	0.43238537	-0.1228293	0.02370889
235	0.40147774	-0.5085186	0.1271622	-0.0871224	-0.7405937	0.27432189	0.43238537	-0.1228293	0.02370889
235.1	0.40147774	-0.5085186	0.1271622	-0.0871224	-0.7405937	0.27432189	0.43238537	-0.1228293	0.02370889
235.2	0.40147774	-0.5085186	0.1271622	-0.0871224	-0.7405937	0.27432189	0.43238537	-0.1228293	0.02370889
235.3	0.40169882	-0.5083594	0.12757061	-0.08702	-0.7404737	0.27441307	0.43238168	-0.1225189	0.02366324
235.4	0.40169882	-0.5083594	0.12757061	-0.08702	-0.7404737	0.27441307	0.43238168	-0.1225189	0.02366324
235.5	0.40169882	-0.5083594	0.12757061	-0.08702	-0.7404737	0.27441307	0.43238168	-0.1225189	0.02366324
235.6	0.40169882	-0.5083594	0.12757061	-0.08702	-0.7404737	0.27441307	0.43238168	-0.1225189	0.02366324
235.7	0.40169882	-0.5083594	0.12757061	-0.08702	-0.7404737	0.27441307	0.43238168	-0.1225189	0.02366324
235.8	0.40169882	-0.5083594	0.12757061	-0.08702	-0.7404737	0.27441307	0.43238168	-0.1225189	0.02366324
235.9	0.40173683	-0.5082054	0.12761306	-0.0872524	-0.7403866	0.2745559	0.43235516	-0.1225793	0.0233059
236	0.40173683	-0.5082054	0.12761306	-0.0872524	-0.7403866	0.2745559	0.43235516	-0.1225793	0.0233059
236.1	0.40173683	-0.5082054	0.12761306	-0.0872524	-0.7403866	0.2745559	0.43235516	-0.1225793	0.0233059
236.2	0.40173683	-0.5082054	0.12761306	-0.0872524	-0.7403866	0.2745559	0.43235516	-0.1225793	0.0233059
236.3	0.40173683	-0.5082054	0.12761306	-0.0872524	-0.7403866	0.2745559	0.43235516	-0.1225793	0.0233059
236.4	0.40177562	-0.5085439	0.12810566	-0.0876755	-0.740266	0.27492198	0.43217198	-0.1224384	0.02320295
236.5	0.40177562	-0.5085439	0.12810566	-0.0876755	-0.740266	0.27492198	0.43217198	-0.1224384	0.02320295
236.6	0.40177562	-0.5085439	0.12810566	-0.0876755	-0.740266	0.27492198	0.43217198	-0.1224384	0.02320295
236.7	0.40177562	-0.5085439	0.12810566	-0.0876755	-0.740266	0.27492198	0.43217198	-0.1224384	0.02320295
236.8	0.40177562	-0.5085439	0.12810566	-0.0876755	-0.740266	0.27492198	0.43217198	-0.1224384	0.02320295
236.9	0.40156887	-0.5087413	0.12848075	-0.0879416	-0.740404	0.2749875	0.43202259	-0.1228055	0.02287018
237	0.40156887	-0.5087413	0.12848075	-0.0879416	-0.740404	0.2749875	0.43202259	-0.1228055	0.02287018
237.1	0.40145392	-0.5083378	0.12853583	-0.087935	-0.7403401	0.2748613	0.43211394	-0.1227677	0.02279494
237.2	0.40145392	-0.5083378	0.12853583	-0.087935	-0.7403401	0.2748613	0.43211394	-0.1227677	0.02279494
237.3	0.40145392	-0.5083378	0.12853583	-0.087935	-0.7403401	0.2748613	0.43211394	-0.1227677	0.02279494
237.4	0.40145392	-0.5083378	0.12853583	-0.087935	-0.7403401	0.2748613	0.43211394	-0.1227677	0.02279494
237.5	0.40145392	-0.5083378	0.12853583	-0.087935	-0.7403401	0.2748613	0.43211394	-0.1227677	0.02279494
237.6	0.40127455	-0.5080202	0.12900067	-0.088023	-0.7402032	0.27421903	0.4316937	-0.122415	0.0219858
237.7	0.40127455	-0.5080202	0.12900067	-0.088023	-0.7402032	0.27421903	0.4316937	-0.122415	0.0219858
237.8	0.40127455	-0.5080202	0.12900067	-0.088023	-0.7402032	0.27421903	0.4316937	-0.122415	0.0219858
237.9	0.40123464	-0.5079936	0.12872557	-0.0878315	-0.7399979	0.27450928	0.43160436	-0.122435	0.02253208
238	0.40125136	-0.5084131	0.12942273	-0.088383	-0.7399877	0.27530592	0.43163069	-0.1225486	0.02329397
238.1	0.40125136	-0.5084131	0.12942273	-0.088383	-0.7399877	0.27530592	0.43163069	-0.1225486	0.02329397
238.2	0.40095152	-0.5085285	0.12957429	-0.0888001	-0.7400378	0.27552511	0.43160883	-0.1222809	0.02290164
238.3	0.40095152	-0.5085285	0.12957429	-0.0888001	-0.7400378	0.27552511	0.43160883	-0.1222809	0.02290164
238.4	0.40077953	-0.508637	0.1300225	-0.0892116	-0.7399476	0.27544564	0.4312726	-0.1224989	0.02269156
238.5	0.40077953	-0.508637	0.1300225	-0.0892116	-0.7399476	0.27544564	0.4312726	-0.1224989	0.02269156
238.6	0.40077953	-0.508637	0.1300225	-0.0892116	-0.7399476	0.27544564	0.4312726	-0.1224989	0.02269156
238.7	0.40077953	-0.508637	0.1300225	-0.0892116	-0.7399476	0.27544564	0.4312726	-0.1224989	0.02269156
238.8	0.40077953	-0.508637	0.1300225	-0.0892116	-0.7399476	0.27544564	0.4312726	-0.1224989	0.02269156
238.9	0.40077953	-0.508637	0.1300225	-0.0892116	-0.7399476	0.27544564	0.4312726	-0.1224989	0.02269156
239	0.40077953	-0.508637	0.1300225	-0.0892116	-0.7399476	0.27544564	0.4312726	-0.1224989	0.02269156
239.1	0.40077953	-0.508637	0.1300225	-0.0892116	-0.7399476	0.27544564	0.4312726	-0.1224989	0.02269156
239.2	0.40077953	-0.508637	0.1300225	-0.0892116	-0.7399476	0.27544564	0.4312726	-0.1224989	0.02269156
239.3	0.40073184	-0.5083019	0.13044214	-0.0897766	-0.7399073	0.27508402	0.43099717	-0.1222065	0.02262035
239.4	0.40073184	-0.5083019	0.13044214	-0.0897766	-0.7399073	0.27508402	0.43099717	-0.1222065	0.02262035
239.5	0.40073184	-0.5083019	0.13044214	-0.0897766	-0.7399073	0.27508402	0.43099717	-0.1222065	0.02262035
239.6	0.40073184	-0.5083019	0.13044214	-0.0897766	-0.7399073	0.27508402	0.43099717	-0.1222065	0.02262035
239.7	0.40073184	-0.5083019	0.13044214	-0.0897766	-0.7399073	0.27508402	0.43099717	-0.1222065	0.02262035
239.8	0.40081457	-0.5079993	0.13065451	-0.0895596	-0.7399814	0.27507211	0.43135368	-0.1221006	0.02341887
239.9	0.40081457	-0.5079993	0.13065451	-0.0895596	-0.7399814	0.27507211	0.43135368	-0.1221006	0.02341887
240	0.40081457	-0.5079993	0.13065451	-0.0895596	-0.7399814	0.27507211	0.43135368	-0.1221006	0.02341887

Corresponding p-values for PRCC sensitivtiy indices

Time discretization	V_{[G]}	K_{[G]}	V_{[PYR]}	K_{[PYR]}	alpha	K_{[LACT]}	G_E	[G]_0	[PYR]_0
0	0	0	0	0	0	0	0	0	0
0.1	0.13072857	0.15091802	2.00E-107	5.50E-40	0.67100535	0.73317859	0.89574646	4.18E-19	0.74411643
0.2	0.18072833	0.16002778	1.59E-106	2.05E-39	0.68074083	0.88011426	0.94465125	1.47E-18	0.86275495
0.3	0.22279545	0.14985913	4.13E-106	6.31E-39	0.7350493	0.62739694	0.96055108	1.92E-18	0.91664703
0.4	0.22977814	0.10654352	1.35E-105	1.23E-38	0.83706958	0.37905584	0.95024298	3.00E-18	0.89602058
0.5	0.25913466	0.0981382	5.32E-105	1.78E-38	0.84510844	0.23871855	0.97769649	4.69E-18	0.87089605
0.6	0.29659438	0.09312411	2.86E-104	5.92E-38	0.89344428	0.13332664	0.96718793	5.16E-18	0.90720756
0.7	0.36544944	0.09044024	3.73E-103	3.01E-37	0.95407677	0.07593698	0.95337979	7.97E-18	0.916306
0.8	0.39749469	0.08033404	5.41E-102	1.16E-36	0.97769189	0.04698036	0.98012856	1.87E-17	0.94682508
0.9	0.42471121	0.08731564	4.07E-101	4.42E-36	0.99574245	0.02478517	0.94707951	3.66E-17	0.96112261
1	0.43724736	0.09029744	4.55E-100	1.52E-35	0.99226457	0.0131978	0.99923391	6.57E-17	0.96299735
1.1	0.44529807	0.09053445	2.14E-99	2.97E-35	0.97638084	0.0075997	0.9449299	9.29E-17	0.89680597
1.2	0.48577117	0.08680645	7.22E-99	7.50E-35	0.8963851	0.00360727	0.99149164	8.28E-17	0.88782455
1.3	0.50458706	0.08080347	7.69E-98	3.56E-34	0.86465459	0.00158602	0.96146362	1.48E-16	0.9067082
1.4	0.47781469	0.08110238	5.70E-97	9.79E-34	0.8981354	0.00093337	0.92024305	1.81E-16	0.91235744
1.5	0.45601447	0.0864722	2.29E-96	1.87E-33	0.87521663	0.00058231	0.92511381	2.17E-16	0.95547848
1.6	0.44406038	0.07909602	1.56E-95	6.71E-33	0.87694252	0.00036618	0.91716956	4.45E-16	0.96908831
1.7	0.47475997	0.07950937	4.23E-95	8.61E-33	0.87281092	0.00016766	0.93693038	5.98E-16	0.99318137
1.8	0.48342733	0.07110823	7.41E-95	1.04E-32	0.88026105	9.74E-05	0.89028092	5.46E-16	0.99552902
1.9	0.53745988	0.06905326	2.89E-94	2.99E-32	0.86775837	4.51E-05	0.88147565	5.74E-16	0.98848777
2	0.5210235	0.07414649	1.25E-93	6.10E-32	0.86549995	2.85E-05	0.84777074	4.88E-16	0.96583167
2.1	0.50196437	0.07905378	3.89E-93	1.03E-31	0.84563317	2.14E-05	0.82234812	6.83E-16	0.94097509
2.2	0.52011789	0.07733603	6.63E-93	1.21E-31	0.84493877	1.41E-05	0.77420539	7.11E-16	0.96320893
2.3	0.54589696	0.06612881	1.08E-92	1.64E-31	0.80958429	8.08E-06	0.78591501	5.93E-16	0.95004974
2.4	0.53666592	0.06086001	4.09E-92	2.66E-31	0.77070685	4.54E-06	0.76265094	8.59E-16	0.99229631
2.5	0.56555752	0.05579399	2.82E-91	6.96E-31	0.79062012	2.88E-06	0.73327959	1.28E-15	0.99520188
2.6	0.57519099	0.04975616	5.43E-91	7.82E-31	0.76249393	1.92E-06	0.75084648	1.18E-15	0.98822351
2.7	0.57669717	0.04162861	1.57E-90	1.34E-30	0.79831831	1.38E-06	0.75436225	1.37E-15	0.97459296
2.8	0.57485916	0.039426	3.94E-90	1.52E-30	0.78417673	1.29E-06	0.73941708	1.54E-15	0.99413222
2.9	0.59102158	0.03731264	7.69E-90	2.02E-30	0.76568448	7.39E-07	0.7319881	1.41E-15	0.95943344
3	0.55575398	0.03449913	1.97E-89	3.25E-30	0.77177804	5.95E-07	0.73176969	1.71E-15	0.97166431
3.1	0.56108644	0.02977887	8.56E-89	6.52E-30	0.72897688	2.92E-07	0.73755853	2.08E-15	0.99555718
3.2	0.59178139	0.02769944	2.43E-88	1.64E-29	0.73371151	1.61E-07	0.75049786	2.24E-15	0.96275339
3.3	0.57602014	0.02766165	1.10E-87	2.92E-29	0.77060908	1.09E-07	0.74789988	3.17E-15	0.9499717
3.4	0.59808213	0.02582043	3.75E-87	6.27E-29	0.77228022	7.40E-08	0.72690954	3.07E-15	0.93337245
3.5	0.58630114	0.02319935	1.32E-86	1.03E-28	0.8160669	5.87E-08	0.72415323	4.51E-15	0.92383813
3.6	0.57121538	0.02138822	3.31E-86	1.34E-28	0.81798217	3.66E-08	0.72755532	4.41E-15	0.90839953
3.7	0.58664716	0.02000957	1.40E-85	2.97E-28	0.82880177	2.38E-08	0.72983653	6.69E-15	0.89567995
3.8	0.5792329	0.02020258	4.51E-85	6.23E-28	0.85043385	1.76E-08	0.71249275	9.14E-15	0.84776563
3.9	0.58755679	0.01903301	8.47E-85	9.37E-28	0.8329386	1.26E-08	0.72121103	9.71E-15	0.83479867
4	0.5673809	0.01846271	2.87E-84	1.97E-27	0.86468824	8.89E-09	0.72554233	1.04E-14	0.83478083
4.1	0.55155401	0.01790897	9.80E-84	3.78E-27	0.87368836	7.17E-09	0.70743825	1.26E-14	0.82869961
4.2	0.57858853	0.01609669	2.88E-83	7.52E-27	0.84340141	5.06E-09	0.70960237	1.45E-14	0.80460887
4.3	0.57675171	0.01522881	7.11E-83	1.28E-26	0.86074715	4.24E-09	0.70421152	1.77E-14	0.77190758
4.4	0.54579053	0.01304116	1.63E-82	1.58E-26	0.8514398	2.17E-09	0.70397571	2.75E-14	0.76504066
4.5	0.52282562	0.01191434	4.50E-82	2.36E-26	0.82654067	1.50E-09	0.69285339	2.71E-14	0.75178109
4.6	0.49964376	0.01077816	5.50E-82	2.86E-26	0.82867491	9.67E-10	0.67783499	2.18E-14	0.74106199
4.7	0.50320035	0.01036045	1.87E-81	6.93E-26	0.85480817	5.75E-10	0.64332163	2.61E-14	0.7323867
4.8	0.48909947	0.0097156	3.03E-81	8.03E-26	0.85499858	3.88E-10	0.63686381	3.05E-14	0.73104863
4.9	0.47825868	0.00898517	6.36E-81	1.48E-25	0.87275991	2.45E-10	0.66056032	2.93E-14	0.68484589
5	0.47332788	0.00689832	1.91E-80	2.62E-25	0.87641677	1.56E-10	0.60507589	3.26E-14	0.65019219
5.1	0.48257549	0.00735526	5.33E-80	4.79E-25	0.90891135	1.14E-10	0.57149211	3.16E-14	0.65048741
5.2	0.44930938	0.00782462	9.61E-80	5.14E-25	0.95045024	9.29E-11	0.52449968	2.76E-14	0.64964968
5.3	0.459629	0.00749932	3.63E-79	1.29E-24	0.9405265	1.00E-10	0.50346976	4.24E-14	0.64130291
5.4	0.44264434	0.00677116	1.12E-78	1.79E-24	0.92709803	8.00E-11	0.49297245	5.67E-14	0.64371213
5.5	0.45004545	0.00627586	1.97E-78	1.83E-24	0.88421073	5.90E-11	0.47056924	6.92E-14	0.68297523
5.6	0.46636739	0.00569805	3.80E-78	2.57E-24	0.91081928	4.20E-11	0.4387621	8.77E-14	0.69337625
5.7	0.44493438	0.00605629	1.70E-77	4.61E-24	0.91499085	3.57E-11	0.42941333	1.11E-13	0.67828093
5.8	0.44974274	0.00521049	3.43E-77	6.27E-24	0.92899105	1.88E-11	0.43394168	1.48E-13	0.66279649
5.9	0.45199096	0.00434658	7.82E-77	9.38E-24	0.92526718	1.47E-11	0.4557315	1.63E-13	0.66903821
6	0.437101	0.00387445	9.28E-77	1.15E-23	0.94733345	9.99E-12	0.45900109	1.66E-13	0.65192478
6.1	0.42337273	0.00392732	2.75E-76	2.27E-23	0.96583891	8.46E-12	0.44004716	2.32E-13	0.6552855
6.2	0.41413186	0.00384431	4.78E-76	2.85E-23	0.95899925	5.98E-12	0.40511894	2.36E-13	0.65601879
6.3	0.39228401	0.00362397	8.76E-76	4.08E-23	0.95590971	3.48E-12	0.38224497	2.91E-13	0.6732224
6.4	0.37563078	0.00316611	2.17E-75	7.72E-23	0.97496693	2.14E-12	0.37035783	2.72E-13	0.61323234
6.5	0.35792978	0.00318523	1.95E-75	7.57E-23	0.96252755	1.43E-12	0.36454573	2.51E-13	0.61470892
6.6	0.3418004	0.00296724	3.22E-75	8.64E-23	0.9803907	1.14E-12	0.37275762	2.77E-13	0.61211215
6.7	0.34088319	0.00291969	6.46E-75	1.36E-22	0.97961188	7.65E-13	0.36729219	3.55E-13	0.62157011
6.8	0.30964537	0.00251726	8.39E-75	1.42E-22	0.94142446	5.55E-13	0.34697428	3.39E-13	0.62002605
6.9	0.29267629	0.00234299	2.82E-74	2.44E-22	0.94240382	4.38E-13	0.33485375	3.69E-13	0.60278956
7	0.30571725	0.00196443	6.24E-74	3.46E-22	0.9173442	4.00E-13	0.33592148	4.42E-13	0.59759773
7.1	0.30361643	0.00174482	1.56E-73	4.62E-22	0.91674631	3.25E-13	0.32556135	4.51E-13	0.57474387
7.2	0.28434469	0.00181689	4.80E-73	6.82E-22	0.86283744	2.53E-13	0.30921414	5.36E-13	0.53492177
7.3	0.28818651	0.00167914	9.25E-73	1.18E-21	0.84687213	1.79E-13	0.30889862	5.35E-13	0.56270856
7.4	0.28139175	0.00156047	2.23E-72	1.60E-21	0.83377477	1.39E-13	0.30299329	6.52E-13	0.62422092
7.5	0.28010014	0.00142556	4.05E-72	2.54E-21	0.80041413	9.63E-14	0.28074632	7.71E-13	0.62561498
7.6	0.28267008	0.00128864	1.75E-71	4.10E-21	0.78380331	1.00E-13	0.27728218	1.04E-12	0.58555802
7.7	0.30349206	0.00103269	5.63E-71	8.58E-21	0.76335877	7.45E-14	0.2589811	1.20E-12	0.59981768
7.8	0.2926515	0.00091505	1.17E-70	1.05E-20	0.75463209	6.97E-14	0.24700958	1.28E-12	0.60308397
7.9	0.29669718	0.0007867	2.28E-70	1.12E-20	0.71643939	5.16E-14	0.24685777	1.63E-12	0.60104601
8	0.28559048	0.00061666	5.93E-70	1.48E-20	0.68552245	5.36E-14	0.25674816	1.49E-12	0.58614698
8.1	0.27827645	0.0006079	1.77E-69	2.13E-20	0.67729503	6.29E-14	0.25739863	1.69E-12	0.57807421
8.2	0.27375114	0.00063885	3.84E-69	3.17E-20	0.65236075	4.95E-14	0.25739836	1.71E-12	0.54242673
8.3	0.27002539	0.00061418	1.01E-68	4.80E-20	0.63603924	4.54E-14	0.24932529	2.33E-12	0.53793396
8.4	0.26163232	0.00060558	1.29E-68	5.15E-20	0.62198513	3.72E-14	0.24543813	2.17E-12	0.51933175
8.5	0.24950772	0.00052471	1.89E-68	6.70E-20	0.60954578	2.87E-14	0.24519895	2.65E-12	0.52341918
8.6	0.25545055	0.00055028	3.91E-68	9.91E-20	0.6050965	2.63E-14	0.24501925	2.59E-12	0.53920815
8.7	0.24595495	0.0005532	6.81E-68	1.67E-19	0.56060664	1.68E-14	0.22930555	2.92E-12	0.53518296
8.8	0.24335461	0.0005824	2.04E-67	2.42E-19	0.5600724	1.64E-14	0.22703964	3.92E-12	0.52624911
8.9	0.23339011	0.00050075	2.37E-67	2.83E-19	0.53765138	8.55E-15	0.20368832	4.65E-12	0.52165465
9	0.22815906	0.00046242	6.69E-67	4.16E-19	0.51976449	7.24E-15	0.20752362	6.27E-12	0.521667
9.1	0.22010316	0.00037098	1.01E-66	5.21E-19	0.49713003	5.84E-15	0.20411896	6.38E-12	0.53780121
9.2	0.20919089	0.00035232	2.16E-66	7.56E-19	0.48285153	4.72E-15	0.19400062	5.53E-12	0.53494919
9.3	0.20003872	0.00034673	2.68E-66	7.89E-19	0.46910237	3.98E-15	0.1855461	5.39E-12	0.50253541
9.4	0.18130915	0.00034325	3.74E-66	8.11E-19	0.43860159	3.00E-15	0.17608434	5.13E-12	0.50565194
9.5	0.17621801	0.00031551	7.33E-66	1.01E-18	0.41270562	3.36E-15	0.17299444	5.76E-12	0.50461756
9.6	0.16843982	0.00026818	1.54E-65	1.55E-18	0.39207739	2.62E-15	0.16986811	9.31E-12	0.50958501
9.7	0.16301825	0.00024989	2.31E-65	1.74E-18	0.38214992	2.15E-15	0.16990897	9.22E-12	0.48292552
9.8	0.16157707	0.00023901	3.82E-65	2.03E-18	0.36922305	2.00E-15	0.16624262	1.05E-11	0.50191254
9.9	0.15806278	0.0001928	1.05E-64	2.92E-18	0.37220823	1.95E-15	0.16596448	1.07E-11	0.48693638
10	0.15785632	0.00016252	2.48E-64	5.08E-18	0.36342349	1.60E-15	0.15517773	1.09E-11	0.4841463
10.1	0.15938826	0.0001632	3.86E-64	6.71E-18	0.37674244	1.01E-15	0.14567268	1.27E-11	0.46974075
10.2	0.15644649	0.00015105	4.80E-64	8.43E-18	0.35701935	7.06E-16	0.14374597	1.35E-11	0.46451047
10.3	0.16141966	0.00014253	1.11E-63	1.24E-17	0.3466468	5.52E-16	0.14202106	1.53E-11	0.45773746
10.4	0.15826507	0.00013117	1.86E-63	1.63E-17	0.34155943	5.32E-16	0.13551553	1.45E-11	0.45417778
10.5	0.15560875	0.00012693	4.26E-63	2.13E-17	0.32686279	4.37E-16	0.13408654	1.47E-11	0.45065935
10.6	0.15185972	0.00012455	5.61E-63	2.14E-17	0.31581807	3.70E-16	0.1349982	1.42E-11	0.44732089
10.7	0.13824453	0.00011937	1.08E-62	2.55E-17	0.30347332	2.73E-16	0.12853611	1.23E-11	0.42303264
10.8	0.14251038	0.00010178	1.81E-62	3.67E-17	0.29706492	1.86E-16	0.12320304	1.62E-11	0.41508657
10.9	0.13841379	9.38E-05	4.72E-62	6.06E-17	0.28983751	1.72E-16	0.1142639	1.99E-11	0.42467341
11	0.13075648	8.63E-05	7.09E-62	6.93E-17	0.27889451	1.47E-16	0.10276451	2.14E-11	0.43053989
11.1	0.12499429	8.91E-05	1.81E-61	9.99E-17	0.25800538	1.53E-16	0.10219589	2.43E-11	0.43257421
11.2	0.12416438	8.98E-05	3.70E-61	1.35E-16	0.24144379	1.09E-16	0.10638862	3.42E-11	0.41671371
11.3	0.12255204	8.35E-05	9.34E-61	2.19E-16	0.23095253	8.93E-17	0.10632237	3.85E-11	0.40747815
11.4	0.12102546	7.43E-05	1.30E-60	2.56E-16	0.24979339	7.58E-17	0.10549142	4.64E-11	0.42205355
11.5	0.12222445	7.95E-05	2.40E-60	3.42E-16	0.23493311	7.59E-17	0.10162472	4.89E-11	0.42112062
11.6	0.11304031	8.16E-05	5.81E-60	4.07E-16	0.22548143	7.54E-17	0.09740563	5.12E-11	0.40802773
11.7	0.10785448	8.10E-05	8.66E-60	4.92E-16	0.21496666	6.59E-17	0.09122626	5.80E-11	0.39348856
11.8	0.09854325	8.03E-05	1.64E-59	6.20E-16	0.20762597	5.93E-17	0.08464875	6.01E-11	0.39208227
11.9	0.08949173	7.59E-05	3.89E-59	7.78E-16	0.194428	4.42E-17	0.08625431	5.77E-11	0.37148069
12	0.09021776	7.39E-05	6.83E-59	1.04E-15	0.18586998	4.46E-17	0.08520944	6.22E-11	0.36477717
12.1	0.08952337	7.59E-05	1.64E-58	1.44E-15	0.17929209	4.14E-17	0.08236369	7.50E-11	0.37436846
12.2	0.08651216	7.40E-05	2.98E-58	2.06E-15	0.17691897	4.42E-17	0.08680086	8.08E-11	0.39244027
12.3	0.08491449	6.73E-05	4.94E-58	2.32E-15	0.18079314	4.09E-17	0.08250826	9.97E-11	0.40884514
12.4	0.08032283	6.82E-05	9.32E-58	2.95E-15	0.1793513	4.01E-17	0.07815757	1.31E-10	0.43636133
12.5	0.07727219	6.54E-05	1.57E-57	3.84E-15	0.17017305	4.70E-17	0.08351258	1.29E-10	0.44592298
12.6	0.08050351	5.76E-05	2.10E-57	4.88E-15	0.16701764	3.78E-17	0.08064871	1.49E-10	0.43660301
12.7	0.07787818	4.89E-05	4.71E-57	4.95E-15	0.16233804	3.07E-17	0.07729752	1.80E-10	0.43924657
12.8	0.07835244	4.54E-05	8.31E-57	5.71E-15	0.16061096	2.60E-17	0.07291438	1.98E-10	0.43805434
12.9	0.07201968	4.73E-05	1.39E-56	7.30E-15	0.13905618	2.24E-17	0.07426527	1.93E-10	0.43290995
13	0.06663645	3.59E-05	3.75E-56	1.09E-14	0.13938069	2.12E-17	0.07290323	2.10E-10	0.44179851
13.1	0.06344565	3.65E-05	7.75E-56	1.48E-14	0.14149658	2.21E-17	0.07411838	2.13E-10	0.44153065
13.2	0.06156726	3.49E-05	1.49E-55	2.34E-14	0.14578625	2.51E-17	0.07239034	2.03E-10	0.43477188
13.3	0.06454848	2.94E-05	2.75E-55	3.13E-14	0.15013488	2.09E-17	0.06897957	2.63E-10	0.4297088
13.4	0.0658752	2.63E-05	5.04E-55	3.84E-14	0.15128891	1.63E-17	0.06814549	3.13E-10	0.43562994
13.5	0.06422652	2.71E-05	8.22E-55	4.53E-14	0.14782048	1.70E-17	0.06471923	3.22E-10	0.44310193
13.6	0.06274551	2.73E-05	1.71E-54	5.59E-14	0.14686151	1.45E-17	0.05885037	3.82E-10	0.44573098
13.7	0.06123576	2.76E-05	2.79E-54	5.79E-14	0.13946681	1.39E-17	0.05750761	3.51E-10	0.44376566
13.8	0.05988036	2.98E-05	3.96E-54	6.47E-14	0.13514764	1.31E-17	0.05361743	3.54E-10	0.4448019
13.9	0.05401832	2.99E-05	5.73E-54	7.42E-14	0.12733187	1.34E-17	0.05418304	3.55E-10	0.44309715
14	[illegible]	[illegible]	[illegible]	[illegible]	[illegible]	[illegible]	[illegible]	[illegible]	[illegible]
14.1	[illegible]	[illegible]	[illegible]	[illegible]	[illegible]	[illegible]	[illegible]	[illegible]	[illegible]
14.2	[illegible]	[illegible]	[illegible]	[illegible]	[illegible]	[illegible]	[illegible]	[illegible]	[illegible]
14.3	[illegible]	[illegible]	[illegible]	[illegible]	[illegible]	[illegible]	[illegible]	[illegible]	[illegible]
14.4	[illegible]	[illegible]	[illegible]	[illegible]	[illegible]	[illegible]	[illegible]	[illegible]	[illegible]
14.5	[illegible]	[illegible]	[illegible]	[illegible]	[illegible]	[illegible]	[illegible]	[illegible]	[illegible]
14.6	[illegible]	[illegible]	[illegible]	[illegible]	[illegible]	[illegible]	[illegible]	[illegible]	[illegible]
14.7	[illegible]	[illegible]	[illegible]	[illegible]	[illegible]	[illegible]	[illegible]	[illegible]	[illegible]
14.8	[illegible]	[illegible]	[illegible]	[illegible]	[illegible]	[illegible]	[illegible]	[illegible]	[illegible]
14.9	[illegible]	[illegible]	[illegible]	[illegible]	[illegible]	[illegible]	[illegible]	[illegible]	[illegible]

Time discretization	V_{[G]}	K_{[G]}	V_{[PYR]}	K_{[PYR]}	alpha	K_{[LACT]}	G_E	[G]_0	[PYR]_0
15	0.0498006	2.32E-05	2.46E-51	1.70E-12	0.08923621	4.39E-18	0.04903943	1.28E-09	0.47672462
15.1	0.04539492	2.10E-05	4.64E-51	1.98E-12	0.08215967	3.54E-18	0.04606679	1.38E-09	0.46321129
15.2	0.04205152	2.25E-05	8.75E-51	2.45E-12	0.07901161	2.97E-18	0.04461281	1.65E-09	0.454662
15.3	0.0402151	1.95E-05	1.93E-50	3.50E-12	0.07444825	2.18E-18	0.04302758	1.85E-09	0.44320203
15.4	0.04349031	1.73E-05	4.49E-50	4.69E-12	0.06927766	1.89E-18	0.04233382	2.01E-09	0.44473688
15.5	0.0422187	1.58E-05	9.14E-50	7.07E-12	0.06156813	1.88E-18	0.0395019	2.32E-09	0.42648821
15.6	0.04343016	1.31E-05	1.72E-49	8.85E-12	0.06153786	1.39E-18	0.03855734	2.61E-09	0.43205655
15.7	0.04299928	1.14E-05	2.91E-49	1.17E-11	0.05607006	1.25E-18	0.03995497	2.96E-09	0.42643475
15.8	0.04220163	1.19E-05	4.94E-49	1.28E-11	0.05270127	1.14E-18	0.03813595	3.53E-09	0.44647812
15.9	0.04167478	1.19E-05	1.08E-48	2.05E-11	0.04658546	1.21E-18	0.04144561	4.18E-09	0.44202001
16	0.04149855	1.17E-05	1.27E-48	2.50E-11	0.04544106	1.07E-18	0.04172549	3.98E-09	0.4321027
16.1	0.03953738	1.25E-05	2.82E-48	4.03E-11	0.04324307	9.00E-19	0.04017184	4.97E-09	0.42143595
16.2	0.04059344	1.09E-05	4.64E-48	5.69E-11	0.04040621	7.79E-19	0.04027369	5.79E-09	0.43755976
16.3	0.03898642	1.03E-05	8.38E-48	7.10E-11	0.03739347	7.37E-19	0.0380162	6.74E-09	0.42582448
16.4	0.03955038	8.96E-06	1.41E-47	8.78E-11	0.035927	5.03E-19	0.03411495	5.75E-09	0.42591393
16.5	0.03964526	8.85E-06	2.10E-47	8.93E-11	0.03326842	4.69E-19	0.03408366	6.49E-09	0.4297761
16.6	0.03821144	9.71E-06	4.14E-47	1.11E-10	0.03155446	4.99E-19	0.03400133	7.24E-09	0.4310397
16.7	0.03849855	9.31E-06	9.13E-47	1.43E-10	0.02967708	4.79E-19	0.032351	7.96E-09	0.40473449
16.8	0.03955986	8.99E-06	1.70E-46	1.61E-10	0.02854402	4.46E-19	0.03055093	7.54E-09	0.40428471
16.9	0.03975179	8.11E-06	2.29E-46	1.47E-10	0.02669318	4.19E-19	0.03119438	7.23E-09	0.40373184
17	0.03945162	7.55E-06	4.18E-46	1.58E-10	0.02394111	4.81E-19	0.03117047	8.18E-09	0.40645345
17.1	0.03896597	6.87E-06	7.33E-46	1.97E-10	0.02415474	4.64E-19	0.03289288	8.65E-09	0.40033277
17.2	0.03702619	6.44E-06	1.00E-45	2.20E-10	0.02260561	4.35E-19	0.03035138	9.36E-09	0.40781974
17.3	0.03693566	5.90E-06	1.45E-45	2.31E-10	0.02212628	3.71E-19	0.02923341	9.65E-09	0.41579113
17.4	0.03638461	5.65E-06	2.63E-45	2.97E-10	0.02108765	3.78E-19	0.02879339	1.17E-08	0.41687793
17.5	0.03492875	5.35E-06	2.88E-45	2.93E-10	0.0200946	3.84E-19	0.02916265	1.15E-08	0.41622114
17.6	0.03244864	5.02E-06	3.61E-45	3.02E-10	0.01949443	3.96E-19	0.02782067	1.21E-08	0.42180048
17.7	0.02994594	5.03E-06	6.67E-45	3.34E-10	0.01880748	4.54E-19	0.02729066	1.20E-08	0.42179311
17.8	0.03079301	4.68E-06	8.24E-45	3.96E-10	0.01732474	3.37E-19	0.02752399	1.14E-08	0.42743327
17.9	0.03031265	4.38E-06	1.24E-44	4.46E-10	0.01711077	3.32E-19	0.02739022	1.27E-08	0.43431124
18	0.02701987	4.64E-06	2.33E-44	5.97E-10	0.01619032	3.18E-19	0.02603343	1.24E-08	0.4216786
18.1	0.02580921	4.04E-06	3.29E-44	6.28E-10	0.01558226	3.00E-19	0.02582808	1.34E-08	0.43509726
18.2	0.02464364	3.53E-06	4.87E-44	7.75E-10	0.01476374	2.86E-19	0.02567554	1.59E-08	0.42107817
18.3	0.0245167	3.50E-06	9.73E-44	1.05E-09	0.01320408	3.06E-19	0.02675017	1.55E-08	0.4367502
18.4	0.02337257	3.61E-06	1.39E-43	1.22E-09	0.01210669	3.40E-19	0.02662989	1.61E-08	0.42212486
18.5	0.02397026	3.66E-06	2.17E-43	1.57E-09	0.01144927	3.38E-19	0.0262517	1.87E-08	0.43013434
18.6	0.02507553	3.38E-06	3.29E-43	1.99E-09	0.01146774	3.02E-19	0.02619698	1.86E-08	0.43856497
18.7	0.0241776	3.33E-06	5.62E-43	2.31E-09	0.01161148	2.85E-19	0.02683748	2.21E-08	0.45843171
18.8	0.02503754	3.20E-06	1.00E-42	2.83E-09	0.01113378	2.77E-19	0.02700445	2.43E-08	0.43813324
18.9	0.02319825	3.13E-06	1.79E-42	3.32E-09	0.01017712	3.09E-19	0.02802415	2.50E-08	0.42969346
19	0.02258679	3.11E-06	2.98E-42	3.94E-09	0.00999288	2.95E-19	0.02722961	2.41E-08	0.42339
19.1	0.02219858	3.03E-06	4.33E-42	4.20E-09	0.00834047	2.53E-19	0.02567867	2.81E-08	0.42460844
19.2	0.02072407	2.97E-06	7.61E-42	5.51E-09	0.00747654	2.10E-19	0.02286906	2.87E-08	0.42088292
19.3	0.02097637	3.02E-06	1.36E-41	6.81E-09	0.00727047	2.13E-19	0.02526042	3.45E-08	0.43585311
19.4	0.02094108	3.03E-06	2.60E-41	7.54E-09	0.00697462	2.19E-19	0.02465293	4.04E-08	0.42428909
19.5	0.01897215	3.25E-06	4.15E-41	8.30E-09	0.00601203	1.78E-19	0.02487843	4.94E-08	0.43179531
19.6	0.01934495	2.72E-06	7.85E-41	1.16E-08	0.00576493	1.60E-19	0.02493665	6.06E-08	0.44102859
19.7	0.01956761	2.55E-06	1.05E-40	1.21E-08	0.00566493	1.71E-19	0.02356798	6.83E-08	0.43916899
19.8	0.0190399	2.36E-06	1.86E-40	1.49E-08	0.00526341	1.64E-19	0.02419724	6.77E-08	0.4404531
19.9	0.01773654	2.79E-06	3.20E-40	1.96E-08	0.00501563	1.88E-19	0.02411927	6.91E-08	0.44527844
20	0.01851529	2.81E-06	5.13E-40	2.39E-08	0.00480437	2.01E-19	0.02394419	7.27E-08	0.44815969
20.1	0.01853825	2.87E-06	8.11E-40	2.77E-08	0.00465918	1.98E-19	0.02296853	7.85E-08	0.44231321
20.2	0.01841285	3.06E-06	1.49E-39	3.24E-08	0.00482582	1.60E-19	0.02089557	7.66E-08	0.42300005
20.3	0.01620726	3.01E-06	2.10E-39	3.29E-08	0.00485471	1.79E-19	0.01963319	8.24E-08	0.42177807
20.4	0.0163545	2.97E-06	3.41E-39	3.61E-08	0.00494566	2.01E-19	0.02065394	9.13E-08	0.41876939
20.5	0.01650516	2.94E-06	6.67E-39	4.08E-08	0.00469025	2.26E-19	0.02096725	9.96E-08	0.41855991
20.6	0.01686246	2.84E-06	1.07E-38	5.34E-08	0.00448576	2.34E-19	0.02106081	1.05E-07	0.42646682
20.7	0.01724398	2.76E-06	1.66E-38	5.98E-08	0.00424732	2.58E-19	0.02153804	1.34E-07	0.4392939
20.8	0.0161278	2.69E-06	3.35E-38	7.42E-08	0.00411512	3.32E-19	0.0201357	1.70E-07	0.45052428
20.9	0.0166974	2.78E-06	4.91E-38	8.70E-08	0.00393924	3.26E-19	0.02045212	1.63E-07	0.44590556
21	0.01693854	2.34E-06	7.97E-38	1.01E-07	0.00372553	2.57E-19	0.02066544	1.74E-07	0.45049058
21.1	0.01739981	2.30E-06	1.05E-37	1.16E-07	0.00370572	2.56E-19	0.0215059	1.77E-07	0.45196093
21.2	0.01679453	2.30E-06	1.52E-37	1.36E-07	0.00361685	2.05E-19	0.02271436	1.94E-07	0.45274245
21.3	0.01557198	2.25E-06	2.21E-37	1.48E-07	0.00344469	1.96E-19	0.0236401	1.87E-07	0.44872275
21.4	0.01593237	2.10E-06	3.23E-37	1.69E-07	0.00339809	1.86E-19	0.02438056	2.33E-07	0.45372556
21.5	0.01686457	2.04E-06	5.10E-37	1.84E-07	0.00301015	2.09E-19	0.02525656	2.63E-07	0.45429144
21.6	0.01725724	2.10E-06	1.02E-36	2.14E-07	0.00274525	2.34E-19	0.02471914	3.28E-07	0.44980504
21.7	0.01725355	1.91E-06	1.80E-36	2.56E-07	0.00255038	1.98E-19	0.02506595	3.51E-07	0.4429137
21.8	0.01608253	2.01E-06	2.22E-36	3.05E-07	0.00220522	1.65E-19	0.0253411	3.58E-07	0.43833477
21.9	0.01650891	2.01E-06	3.05E-36	3.24E-07	0.00210981	1.37E-19	0.02515656	3.39E-07	0.4467436
22	0.01680678	2.04E-06	5.00E-36	2.79E-07	0.00196291	1.26E-19	0.02588861	3.90E-07	0.45235979
22.1	0.015256	2.11E-06	7.52E-36	2.97E-07	0.00185517	1.35E-19	0.02559355	3.88E-07	0.4546121
22.2	0.01393333	2.07E-06	8.88E-36	2.90E-07	0.0017628	1.32E-19	0.02365777	3.93E-07	0.46346123
22.3	0.01267443	2.11E-06	1.33E-35	2.99E-07	0.00171197	1.24E-19	0.02204304	4.28E-07	0.47141286
22.4	0.01257233	2.05E-06	2.08E-35	3.17E-07	0.00158162	1.15E-19	0.02111463	4.53E-07	0.4737609
22.5	0.01190112	1.99E-06	3.05E-35	3.79E-07	0.00149933	1.04E-19	0.0200797	4.58E-07	0.47626156
22.6	0.01103235	2.10E-06	6.06E-35	4.59E-07	0.00125052	1.48E-19	0.01915699	4.91E-07	0.46750502
22.7	0.01095093	2.04E-06	7.72E-35	4.72E-07	0.00120222	1.50E-19	0.01946077	5.74E-07	0.47098861
22.8	0.01099706	2.07E-06	1.13E-34	5.48E-07	0.00114457	1.29E-19	0.01805416	6.34E-07	0.47107209
22.9	0.01180415	2.00E-06	2.42E-34	5.40E-07	0.00133316	1.25E-19	0.01817538	7.82E-07	0.47793227
23	0.01119299	2.02E-06	4.09E-34	6.46E-07	0.00120035	1.32E-19	0.01822216	8.86E-07	0.49145769
23.1	0.01109327	1.66E-06	4.45E-34	6.88E-07	0.00114468	1.13E-19	0.01765412	9.57E-07	0.49377186
23.2	0.0109689	1.60E-06	5.29E-34	6.83E-07	0.00105149	9.80E-20	0.01677896	1.01E-06	0.49162385
23.3	0.01143732	1.75E-06	8.77E-34	7.84E-07	0.00103859	8.49E-20	0.01684257	1.12E-06	0.47947606
23.4	0.01151675	1.56E-06	9.46E-34	8.23E-07	0.00103107	7.05E-20	0.01670649	1.18E-06	0.47176402
23.5	0.01134453	1.55E-06	1.69E-33	8.33E-07	0.00090194	7.62E-20	0.01715331	1.34E-06	0.46953128
23.6	0.01100626	1.62E-06	2.53E-33	9.76E-07	0.00092114	8.12E-20	0.01719739	1.55E-06	0.48182981
23.7	0.01135531	1.51E-06	3.01E-33	1.08E-06	0.00090608	7.30E-20	0.01708923	1.79E-06	0.49269418
23.8	0.01123669	1.53E-06	4.75E-33	1.17E-06	0.00090062	6.88E-20	0.0164052	1.85E-06	0.47629456
23.9	0.01131346	1.41E-06	5.58E-33	1.24E-06	0.0008172	5.95E-20	0.01690704	1.67E-06	0.47069114
24	0.01178821	1.35E-06	1.07E-32	1.59E-06	0.00073627	5.61E-20	0.01649507	1.95E-06	0.46107939
24.1	0.01104551	1.43E-06	1.97E-32	1.79E-06	0.00061815	5.66E-20	0.01570242	2.15E-06	0.46051882
24.2	0.01119386	1.65E-06	2.94E-32	1.99E-06	0.00062569	5.14E-20	0.01630334	2.08E-06	0.45459201
24.3	0.01126661	1.52E-06	3.55E-32	2.12E-06	0.00054969	5.22E-20	0.01555658	2.35E-06	0.45676332
24.4	0.01054735	1.63E-06	4.48E-32	2.36E-06	0.00049971	5.18E-20	0.01655923	2.70E-06	0.46178275
24.5	0.01055458	1.57E-06	6.44E-32	2.61E-06	0.00050257	5.38E-20	0.01686187	2.84E-06	0.45933511
24.6	0.01106969	1.80E-06	1.08E-31	2.88E-06	0.00048162	5.82E-20	0.01748511	3.21E-06	0.4498684
24.7	0.01068649	1.77E-06	1.59E-31	2.94E-06	0.00048045	6.40E-20	0.01846007	3.59E-06	0.46080093
24.8	0.01002781	1.88E-06	2.25E-31	3.31E-06	0.00046276	6.24E-20	0.01883021	4.22E-06	0.46958097
24.9	0.01106246	1.85E-06	3.71E-31	4.04E-06	0.00045253	5.95E-20	0.01914643	5.29E-06	0.46245901
25	0.01093147	1.92E-06	7.05E-31	4.40E-06	0.00049522	6.97E-20	0.01942705	6.70E-06	0.47721003
25.1	0.01091095	2.00E-06	9.16E-31	4.47E-06	0.00046956	7.53E-20	0.01991696	6.91E-06	0.48396608
25.2	0.01063273	2.04E-06	1.18E-30	4.55E-06	0.00045296	7.39E-20	0.01801208	7.79E-06	0.48480421
25.3	0.01064551	2.03E-06	1.63E-30	5.11E-06	0.00042994	6.93E-20	0.0184703	7.98E-06	0.47750314
25.4	0.00962185	2.22E-06	2.45E-30	6.02E-06	0.00039319	6.52E-20	0.01683026	8.16E-06	0.47118705
25.5	0.00970462	2.05E-06	4.38E-30	5.82E-06	0.00039401	7.20E-20	0.017999	9.40E-06	0.48293457
25.6	0.00891796	2.15E-06	7.52E-30	6.42E-06	0.00035007	7.46E-20	0.01881734	8.79E-06	0.46924275
25.7	0.00964803	1.97E-06	1.36E-29	7.48E-06	0.00032254	8.09E-20	0.01901088	1.00E-05	0.46337757
25.8	0.01030475	1.93E-06	1.94E-29	9.08E-06	0.00032352	7.65E-20	0.01916432	1.13E-05	0.47054537
25.9	0.00997192	1.87E-06	2.64E-29	9.07E-06	0.00029466	7.90E-20	0.0195412	1.10E-05	0.46555183
26	0.0102964	1.85E-06	3.51E-29	9.39E-06	0.00028941	8.47E-20	0.01895986	1.25E-05	0.48572779
26.1	0.01055338	1.86E-06	4.54E-29	9.21E-06	0.00027674	9.38E-20	0.01834294	1.35E-05	0.48570713
26.2	0.0102969	1.83E-06	6.25E-29	1.02E-05	0.00025439	9.73E-20	0.0185892	1.38E-05	0.48143814
26.3	0.01009859	1.85E-06	6.94E-29	1.05E-05	0.00024177	9.77E-20	0.01759769	1.40E-05	0.4771791
26.4	0.01031739	1.89E-06	1.15E-28	1.20E-05	0.00024036	1.01E-19	0.01695847	1.55E-05	0.47969768
26.5	0.01005155	1.95E-06	1.48E-28	1.29E-05	0.0002104	1.19E-19	0.01717289	1.64E-05	0.47677463
26.6	0.00988444	1.85E-06	2.24E-28	1.39E-05	0.00019251	1.06E-19	0.01686134	1.78E-05	0.46996927
26.7	0.00968468	1.63E-06	3.66E-28	1.48E-05	0.0001768	1.14E-19	0.01664658	2.04E-05	0.46650156
26.8	0.00916033	1.52E-06	4.79E-28	1.56E-05	0.00015914	1.12E-19	0.01658309	2.30E-05	0.46540696
26.9	0.00917957	1.63E-06	6.29E-28	1.80E-05	0.00014904	1.23E-19	0.01656668	2.44E-05	0.47223676
27	0.00937643	1.38E-06	7.78E-28	2.05E-05	0.00013553	1.11E-19	0.01715509	2.57E-05	0.47612288
27.1	0.00921677	1.31E-06	1.34E-27	1.97E-05	0.00013258	1.03E-19	0.01771873	2.54E-05	0.47883706
27.2	0.00922178	1.38E-06	1.55E-27	2.02E-05	0.00012069	9.66E-20	0.01701845	2.67E-05	0.47747165
27.3	0.00877426	1.25E-06	1.85E-27	2.06E-05	0.00011193	9.35E-20	0.01698736	2.69E-05	0.48670337
27.4	0.0086986	1.23E-06	1.75E-27	2.16E-05	9.93E-05	9.57E-20	0.01683473	2.69E-05	0.49613418
27.5	0.00894447	1.22E-06	2.16E-27	2.28E-05	9.91E-05	9.67E-20	0.0169042	2.76E-05	0.49085386
27.6	0.00879546	1.14E-06	2.99E-27	2.70E-05	8.68E-05	8.55E-20	0.01621787	2.74E-05	0.4854683
27.7	0.00909175	1.25E-06	4.12E-27	2.80E-05	8.60E-05	7.73E-20	0.01595754	2.93E-05	0.49281991
27.8	0.00877007	1.21E-06	5.68E-27	3.06E-05	7.37E-05	8.04E-20	0.01582093	3.26E-05	0.48053168
27.9	0.00874448	1.30E-06	8.52E-27	3.19E-05	6.76E-05	7.99E-20	0.01533022	3.38E-05	0.48369465
28	0.00877051	1.24E-06	1.16E-26	3.26E-05	6.77E-05	7.44E-20	0.01508955	3.66E-05	0.48116145
28.1	0.00921925	1.18E-06	1.87E-26	3.79E-05	6.02E-05	7.36E-20	0.01608077	4.45E-05	0.4821422
28.2	0.00906832	1.31E-06	2.73E-26	4.13E-05	6.06E-05	7.50E-20	0.01671232	4.97E-05	0.4886701
28.3	0.00852823	1.29E-06	3.36E-26	4.36E-05	5.35E-05	7.69E-20	0.01592689	5.35E-05	0.48680245
28.4	0.00825631	1.27E-06	3.97E-26	4.82E-05	5.13E-05	7.25E-20	0.01540195	5.70E-05	0.48263468
28.5	0.00826699	1.25E-06	5.71E-26	5.34E-05	5.04E-05	7.52E-20	0.01546215	6.22E-05	0.48239912
28.6	0.00779574	1.35E-06	8.16E-26	5.58E-05	4.77E-05	7.76E-20	0.01623734	6.41E-05	0.47733319
28.7	0.00768639	1.30E-06	9.67E-26	5.83E-05	4.33E-05	8.08E-20	0.01602289	6.78E-05	0.48374938
28.8	0.00745706	1.33E-06	1.22E-25	5.93E-05	4.16E-05	9.28E-20	0.01637429	7.37E-05	0.48168274
28.9	[illegible]	[illegible]	[illegible]	[illegible]	[illegible]	[illegible]	[illegible]	[illegible]	[illegible]
29	[illegible]	[illegible]	[illegible]	[illegible]	[illegible]	[illegible]	[illegible]	[illegible]	[illegible]
29.1	[illegible]	[illegible]	[illegible]	[illegible]	[illegible]	[illegible]	[illegible]	[illegible]	[illegible]
29.2	[illegible]	[illegible]	[illegible]	[illegible]	[illegible]	[illegible]	[illegible]	[illegible]	[illegible]
29.3	[illegible]	[illegible]	[illegible]	[illegible]	[illegible]	[illegible]	[illegible]	[illegible]	[illegible]
29.4	[illegible]	[illegible]	[illegible]	[illegible]	[illegible]	[illegible]	[illegible]	[illegible]	[illegible]
29.5	[illegible]	[illegible]	[illegible]	[illegible]	[illegible]	[illegible]	[illegible]	[illegible]	[illegible]
29.6	[illegible]	[illegible]	[illegible]	[illegible]	[illegible]	[illegible]	[illegible]	[illegible]	[illegible]
29.7	[illegible]	[illegible]	[illegible]	[illegible]	[illegible]	[illegible]	[illegible]	[illegible]	[illegible]
29.8	[illegible]	[illegible]	[illegible]	[illegible]	[illegible]	[illegible]	[illegible]	[illegible]	[illegible]
29.9	[illegible]	[illegible]	[illegible]	[illegible]	[illegible]	[illegible]	[illegible]	[illegible]	[illegible]

Time discretization	V_{[G]}	K_{[G]}	V_{[PYR]}	K_{[PYR]}	alpha	K_{[LACT]}	G_E	[G]_0	[PYR]_0
30	0.00616772	1.18E-06	7.73E-24	0.00012641	2.40E-05	1.29E-19	0.01168643	0.00013272	0.50112656
30.1	0.00630962	1.13E-06	1.05E-23	0.00012697	2.30E-05	1.26E-19	0.01157633	0.00013794	0.5046113
30.2	0.00652235	1.09E-06	1.54E-23	0.00012624	2.24E-05	1.46E-19	0.01222094	0.00013638	0.50010628
30.3	0.00656306	1.15E-06	1.99E-23	0.00013725	1.96E-05	1.31E-19	0.01177027	0.00013619	0.49311445
30.4	0.00644684	1.13E-06	2.32E-23	0.00015719	1.93E-05	1.26E-19	0.01200107	0.00013743	0.49482713
30.5	0.0066469	1.15E-06	3.13E-23	0.00018209	1.62E-05	1.22E-19	0.01129854	0.00016333	0.48648123
30.6	0.00622174	1.16E-06	4.17E-23	0.00018806	1.47E-05	1.44E-19	0.01021283	0.0001762	0.48918733
30.7	0.00622387	1.20E-06	4.56E-23	0.00019119	1.40E-05	1.35E-19	0.01047172	0.00017188	0.4999721
30.8	0.00606482	1.15E-06	6.34E-23	0.00020167	1.30E-05	1.39E-19	0.01055391	0.00018502	0.50592015
30.9	0.00621547	1.08E-06	9.78E-23	0.00021437	1.22E-05	1.50E-19	0.01061945	0.0001969	0.50304271
31	0.00588674	1.09E-06	1.08E-22	0.00021032	1.08E-05	1.40E-19	0.01030394	0.00020152	0.49462597
31.1	0.00619776	1.04E-06	1.26E-22	0.00023007	1.01E-05	1.31E-19	0.0098532	0.00021729	0.5018319
31.2	0.00615141	1.05E-06	1.48E-22	0.00024171	9.63E-06	1.17E-19	0.00980751	0.00022831	0.51344388
31.3	0.00600607	9.94E-07	2.01E-22	0.00024161	9.80E-06	1.05E-19	0.01019572	0.00024709	0.51088449
31.4	0.00598703	9.76E-07	2.42E-22	0.00026543	9.33E-06	9.76E-20	0.01044928	0.00024779	0.5127284
31.5	0.00603118	1.01E-06	2.90E-22	0.0002722	9.34E-06	9.30E-20	0.01006861	0.00026147	0.51862562
31.6	0.0061969	1.05E-06	3.71E-22	0.00030463	8.90E-06	8.30E-20	0.01039042	0.00028909	0.51856154
31.7	0.00627123	9.69E-07	4.71E-22	0.000328	8.26E-06	7.36E-20	0.01015966	0.0003092	0.51237863
31.8	0.00597716	9.70E-07	5.41E-22	0.00034393	7.91E-06	7.19E-20	0.01017804	0.00031696	0.52065798
31.9	0.00561563	9.19E-07	6.20E-22	0.00035007	7.73E-06	5.77E-20	0.01030209	0.00032309	0.51775083
32	0.00548926	9.60E-07	8.08E-22	0.00037965	7.39E-06	5.90E-20	0.0100998	0.00035015	0.53422771
32.1	0.00545412	9.47E-07	9.28E-22	0.00040965	6.51E-06	5.48E-20	0.01017833	0.00037052	0.54409855
32.2	0.00525174	9.19E-07	1.27E-21	0.00041899	6.50E-06	5.88E-20	0.01000998	0.0003696	0.53906013
32.3	0.00526403	8.58E-07	1.40E-21	0.00044021	6.26E-06	5.70E-20	0.00984847	0.00038617	0.53975159
32.4	0.00549626	9.56E-07	2.10E-21	0.00049154	6.14E-06	5.55E-20	0.00982525	0.00044629	0.54007885
32.5	0.00537407	9.59E-07	2.77E-21	0.0005225	5.38E-06	5.88E-20	0.01056886	0.00045873	0.54810056
32.6	0.00532343	1.09E-06	3.46E-21	0.0005915	4.80E-06	5.98E-20	0.01087356	0.00049377	0.53925704
32.7	0.00515025	1.07E-06	4.18E-21	0.00062955	4.32E-06	5.64E-20	0.01094627	0.00053406	0.53396712
32.8	0.00497883	1.06E-06	5.43E-21	0.00067581	4.30E-06	5.08E-20	0.01067653	0.00055023	0.53705296
32.9	0.00502871	9.49E-07	6.68E-21	0.00073106	3.98E-06	5.44E-20	0.01108903	0.00054154	0.53760664
33	0.00503066	9.01E-07	8.99E-21	0.00077718	3.81E-06	5.47E-20	0.01130523	0.00054895	0.53709736
33.1	0.0048645	8.16E-07	1.22E-20	0.00079901	3.54E-06	5.85E-20	0.01160141	0.00054756	0.53690516
33.2	0.00486915	8.29E-07	1.76E-20	0.00084279	3.34E-06	6.42E-20	0.01074733	0.000573	0.54467758
33.3	0.00503432	8.20E-07	2.06E-20	0.00090004	3.27E-06	7.07E-20	0.01082907	0.00059272	0.54470609
33.4	0.00490138	7.64E-07	2.48E-20	0.00094352	3.00E-06	7.25E-20	0.01073353	0.00065052	0.54259169
33.5	0.00500672	7.55E-07	2.77E-20	0.00099452	2.83E-06	7.32E-20	0.01025691	0.00067584	0.54486761
33.6	0.00466068	7.41E-07	3.49E-20	0.00104368	2.63E-06	7.27E-20	0.00989594	0.00067841	0.53909475
33.7	0.0044652	7.29E-07	4.96E-20	0.00112586	2.48E-06	7.32E-20	0.0097279	0.00066648	0.54557553
33.8	0.00449855	7.36E-07	7.38E-20	0.0011229	2.43E-06	7.76E-20	0.00993077	0.00069542	0.54011524
33.9	0.00433607	6.85E-07	9.20E-20	0.00111476	2.27E-06	7.82E-20	0.01006465	0.00069249	0.54678088
34	0.0042798	6.29E-07	1.19E-19	0.00121473	2.16E-06	7.62E-20	0.00983701	0.00072275	0.55001437
34.1	0.00436047	6.15E-07	1.40E-19	0.00122244	1.96E-06	7.54E-20	0.00976413	0.00076507	0.55239035
34.2	0.0042826	6.13E-07	1.59E-19	0.00126443	1.72E-06	7.84E-20	0.00996949	0.0008063	0.56061071
34.3	0.00440476	5.57E-07	2.00E-19	0.00127233	1.74E-06	8.17E-20	0.0103533	0.0008073	0.55691692
34.4	0.00422241	5.28E-07	2.60E-19	0.00140706	1.45E-06	7.46E-20	0.00998949	0.00079665	0.55293675
34.5	0.00413562	5.23E-07	3.90E-19	0.00147938	1.48E-06	7.37E-20	0.01002972	0.0008907	0.56028254
34.6	0.0041288	5.41E-07	5.14E-19	0.00155831	1.42E-06	8.23E-20	0.01071937	0.00090974	0.56226168
34.7	0.00409418	5.19E-07	6.35E-19	0.00164232	1.31E-06	8.40E-20	0.01081618	0.00094782	0.55817
34.8	0.00408196	5.07E-07	7.25E-19	0.00178147	1.14E-06	9.07E-20	0.01020779	0.00098963	0.57195101
34.9	0.00429674	4.84E-07	1.02E-18	0.00190501	1.06E-06	9.63E-20	0.00998683	0.00107652	0.5749937
35	0.0041917	4.75E-07	1.16E-18	0.00193544	1.05E-06	9.99E-20	0.00988127	0.00110329	0.57797585
35.1	0.00395029	4.91E-07	1.60E-18	0.00200865	9.13E-07	9.66E-20	0.00975434	0.00119157	0.58645558
35.2	0.00377146	4.95E-07	2.06E-18	0.00218268	7.69E-07	8.76E-20	0.00992522	0.00127331	0.57922103
35.3	0.00390947	4.79E-07	2.57E-18	0.0022105	6.51E-07	8.15E-20	0.01012061	0.00133045	0.57939364
35.4	0.00390526	4.49E-07	3.01E-18	0.00228983	6.10E-07	7.46E-20	0.01076443	0.00131165	0.57467797
35.5	0.00389774	4.33E-07	3.82E-18	0.0024195	5.95E-07	7.22E-20	0.0112989	0.00137268	0.57484318
35.6	0.00371907	4.35E-07	4.80E-18	0.00265408	5.64E-07	7.06E-20	0.01168337	0.00150954	0.56641758
35.7	0.00363096	4.34E-07	5.82E-18	0.00282356	5.20E-07	7.25E-20	0.01137567	0.00153842	0.56939245
35.8	0.00349279	4.32E-07	6.48E-18	0.00283848	4.64E-07	6.78E-20	0.01109453	0.001531	0.56850286
35.9	0.00341102	4.35E-07	7.93E-18	0.00306088	4.02E-07	6.30E-20	0.01056158	0.00155922	0.57042979
36	0.00314891	4.90E-07	8.79E-18	0.00300725	3.44E-07	5.64E-20	0.01049705	0.00153661	0.56958789
36.1	0.003158	5.26E-07	9.43E-18	0.00315494	3.38E-07	6.18E-20	0.01067038	0.00151373	0.57304264
36.2	0.00315441	5.42E-07	1.18E-17	0.00313123	3.73E-07	6.25E-20	0.01100172	0.00154789	0.57596586
36.3	0.00296006	5.32E-07	1.52E-17	0.00324987	3.33E-07	6.47E-20	0.01104667	0.00161688	0.57212364
36.4	0.00300912	5.07E-07	1.69E-17	0.00335612	3.31E-07	6.62E-20	0.0105876	0.00168676	0.57092719
36.5	0.00310599	4.80E-07	2.39E-17	0.00350422	3.24E-07	6.54E-20	0.0101289	0.00184395	0.57535621
36.6	0.00322723	4.85E-07	2.87E-17	0.00369058	3.07E-07	6.92E-20	0.01026411	0.00190263	0.57845447
36.7	0.00319731	4.76E-07	3.45E-17	0.00377553	3.03E-07	7.40E-20	0.01021468	0.00197349	0.58310784
36.8	0.00316629	4.71E-07	3.68E-17	0.00387247	2.99E-07	7.40E-20	0.01037212	0.00198407	0.57930969
36.9	0.00289047	4.51E-07	5.16E-17	0.00409409	2.56E-07	7.19E-20	0.01007795	0.0020024	0.57525559
37	0.00293954	4.40E-07	5.79E-17	0.00402765	2.41E-07	7.77E-20	0.01012018	0.00205758	0.5876965
37.1	0.00278772	4.25E-07	7.27E-17	0.00421019	2.34E-07	7.70E-20	0.01050629	0.00209184	0.58459996
37.2	0.0027692	4.06E-07	9.66E-17	0.00442465	2.25E-07	8.85E-20	0.01091541	0.0021709	0.58523712
37.3	0.00267184	3.91E-07	1.14E-16	0.00469093	1.96E-07	7.90E-20	0.01088525	0.00242594	0.58387918
37.4	0.00268079	3.98E-07	1.33E-16	0.00473927	1.81E-07	7.76E-20	0.0106521	0.00239405	0.58250616
37.5	0.00262613	3.61E-07	1.47E-16	0.00500751	1.70E-07	7.31E-20	0.00991133	0.00238413	0.58213515
37.6	0.00265759	3.21E-07	1.75E-16	0.00533829	1.53E-07	8.67E-20	0.01030462	0.00245459	0.58577922
37.7	0.00255877	3.01E-07	1.92E-16	0.00542379	1.42E-07	8.58E-20	0.00995746	0.00241485	0.58967765
37.8	0.00257526	3.10E-07	2.15E-16	0.00554709	1.32E-07	8.16E-20	0.01004268	0.0024207	0.58641868
37.9	0.00260739	2.79E-07	2.51E-16	0.00584081	1.14E-07	7.57E-20	0.0100361	0.00236024	0.58079494
38	0.00261558	2.81E-07	2.94E-16	0.00627024	1.15E-07	7.34E-20	0.01008164	0.00253534	0.58872639
38.1	0.00252474	2.61E-07	3.53E-16	0.00634206	1.07E-07	7.18E-20	0.00985124	0.00247673	0.58395946
38.2	0.00238186	2.47E-07	3.81E-16	0.00635968	1.02E-07	6.29E-20	0.00984081	0.00248401	0.58151164
38.3	0.00235953	2.48E-07	4.39E-16	0.00679657	9.58E-08	5.89E-20	0.00940031	0.00255422	0.57418747
38.4	0.00220661	2.48E-07	5.60E-16	0.00675186	8.44E-08	5.59E-20	0.00894896	0.00254106	0.57332453
38.5	0.00229088	2.31E-07	6.81E-16	0.00681769	7.70E-08	5.32E-20	0.00891747	0.00263429	0.57825927
38.6	0.00227607	2.23E-07	7.24E-16	0.00716114	7.23E-08	4.36E-20	0.00893986	0.00275586	0.57006953
38.7	0.00224521	2.19E-07	9.87E-16	0.00766345	6.52E-08	4.54E-20	0.00949596	0.00301489	0.57490335
38.8	0.00223925	2.41E-07	1.17E-15	0.00744464	5.69E-08	5.27E-20	0.00975988	0.00307274	0.57963361
38.9	0.00229759	2.27E-07	1.49E-15	0.00743818	5.55E-08	5.98E-20	0.01003705	0.00324088	0.58842607
39	0.00209427	2.34E-07	2.06E-15	0.00797798	4.94E-08	5.85E-20	0.01021971	0.0033337	0.57251678
39.1	0.0019543	2.37E-07	2.59E-15	0.00891876	4.21E-08	6.72E-20	0.01014373	0.00331608	0.57984057
39.2	0.00195462	2.37E-07	3.01E-15	0.00940531	3.81E-08	7.54E-20	0.0103135	0.00346467	0.58306461
39.3	0.0018607	2.49E-07	3.24E-15	0.00961921	3.70E-08	7.86E-20	0.01030123	0.00349597	0.58202031
39.4	0.00181177	2.65E-07	4.17E-15	0.00971339	3.33E-08	6.85E-20	0.00965729	0.00337673	0.5799788
39.5	0.00167439	2.53E-07	5.02E-15	0.00989987	2.93E-08	6.16E-20	0.00916143	0.00334761	0.57012151
39.6	0.0016558	2.56E-07	6.77E-15	0.01030572	2.60E-08	5.98E-20	0.00925644	0.00361065	0.57362703
39.7	0.00166609	2.55E-07	7.49E-15	0.01064596	2.52E-08	5.76E-20	0.0096228	0.00355141	0.57460868
39.8	0.00160597	2.51E-07	9.23E-15	0.01100595	2.33E-08	5.69E-20	0.0095609	0.00357842	0.57167218
39.9	0.00157807	2.35E-07	9.46E-15	0.01119905	2.26E-08	5.62E-20	0.00966123	0.00361607	0.56712157
40	0.00158021	2.28E-07	1.31E-14	0.01165044	2.25E-08	5.48E-20	0.01002904	0.00376036	0.56342016
40.1	0.00163915	2.24E-07	1.76E-14	0.01150312	2.16E-08	5.18E-20	0.01057311	0.00392728	0.56702426
40.2	0.00157346	2.14E-07	1.99E-14	0.01120146	1.84E-08	5.23E-20	0.01046133	0.00378883	0.57109565
40.3	0.00151469	2.24E-07	2.36E-14	0.01133167	1.71E-08	5.38E-20	0.01055736	0.00377299	0.56504918
40.4	0.00150126	2.03E-07	2.79E-14	0.01175566	1.58E-08	4.92E-20	0.01073939	0.00389029	0.5673439
40.5	0.00146244	2.13E-07	3.25E-14	0.01189562	1.63E-08	4.98E-20	0.01105937	0.00405795	0.56873254
40.6	0.0014877	2.13E-07	3.66E-14	0.01244069	1.49E-08	4.56E-20	0.01150956	0.00398984	0.56926278
40.7	0.00147989	1.87E-07	4.18E-14	0.01277079	1.24E-08	4.04E-20	0.01118235	0.00410279	0.57957089
40.8	0.001398	1.81E-07	5.07E-14	0.01368582	1.18E-08	4.29E-20	0.01114717	0.0044034	0.584254
40.9	0.00134003	1.79E-07	5.93E-14	0.01342116	1.11E-08	4.21E-20	0.0111696	0.00443734	0.59063337
41	0.00129637	1.53E-07	7.51E-14	0.01397953	1.06E-08	4.11E-20	0.01086757	0.00460411	0.59134512
41.1	0.00130588	1.50E-07	8.04E-14	0.01432003	9.95E-09	4.06E-20	0.01112339	0.00479024	0.59422467
41.2	0.00119162	1.43E-07	9.28E-14	0.015109	8.65E-09	4.25E-20	0.0111325	0.00470171	0.58878926
41.3	0.00116661	1.32E-07	1.17E-13	0.01534778	7.86E-09	3.79E-20	0.01097203	0.00469975	0.58276189
41.4	0.0011151	1.36E-07	1.36E-13	0.01636558	6.89E-09	4.16E-20	0.01094073	0.00500293	0.58257681
41.5	0.00116116	1.47E-07	1.51E-13	0.01666991	5.94E-09	4.32E-20	0.01063967	0.00487129	0.5808336
41.6	0.00108319	1.44E-07	1.78E-13	0.01694521	5.35E-09	4.42E-20	0.01045989	0.00483456	0.5835673
41.7	0.00104901	1.49E-07	1.88E-13	0.01763436	4.64E-09	3.81E-20	0.0103937	0.00464316	0.57942885
41.8	0.00100762	1.52E-07	2.26E-13	0.01802381	3.76E-09	3.27E-20	0.00931205	0.00447918	0.57614829
41.9	0.00101751	1.47E-07	2.37E-13	0.01863264	3.49E-09	3.24E-20	0.00953915	0.00453146	0.58608113
42	0.00100481	1.53E-07	2.45E-13	0.01892884	3.43E-09	3.22E-20	0.00966219	0.00453801	0.58217833
42.1	0.00098161	1.45E-07	2.95E-13	0.0188793	3.28E-09	3.21E-20	0.00963132	0.00441632	0.57918751
42.2	0.00095757	1.36E-07	3.33E-13	0.01972318	2.84E-09	2.91E-20	0.00933392	0.00458725	0.57452778
42.3	0.00097439	1.33E-07	4.29E-13	0.02059621	2.67E-09	3.12E-20	0.00946773	0.00452467	0.57408268
42.4	0.00096193	1.31E-07	5.02E-13	0.02085233	2.47E-09	2.82E-20	0.00943643	0.00490032	0.58348925
42.5	0.00098013	1.23E-07	6.75E-13	0.02272331	2.29E-09	2.94E-20	0.00926335	0.00507763	0.58963191
42.6	0.0009267	1.36E-07	7.82E-13	0.02401165	1.95E-09	3.07E-20	0.00900873	0.00510169	0.59125236
42.7	0.00094593	1.23E-07	9.15E-13	0.02360501	1.81E-09	3.24E-20	0.00847033	0.00537241	0.59520556
42.8	0.00094563	1.18E-07	1.02E-12	0.02375954	1.65E-09	3.14E-20	0.00809475	0.00522448	0.59123727
42.9	0.00091747	1.20E-07	1.19E-12	0.02383286	1.40E-09	2.87E-20	0.0084371	0.00530843	0.58354944
43	0.00093054	1.11E-07	1.41E-12	0.02387726	1.33E-09	3.11E-20	0.00881053	0.00535611	0.58743379
43.1	0.00091166	9.72E-08	1.62E-12	0.02429176	1.15E-09	2.78E-20	0.00915304	0.00539991	0.59216213
43.2	0.00089167	8.80E-08	2.04E-12	0.02486054	1.17E-09	3.22E-20	0.00921665	0.00568709	0.5901286
43.3	0.00090492	8.27E-08	2.34E-12	0.02556045	1.10E-09	3.17E-20	0.00893157	0.0056852	0.58527713
43.4	0.00092381	8.38E-08	2.55E-12	0.0262489	1.07E-09	3.31E-20	0.00873792	0.00610782	0.58303403
43.5	0.00090485	8.54E-08	2.91E-12	0.02668584	9.65E-10	3.16E-20	0.00860631	0.00624807	0.57561175
43.6	0.00089012	8.76E-08	2.96E-12	0.02687507	9.21E-10	3.22E-20	0.00860668	0.00621601	0.57516895
43.7	0.00087991	8.23E-08	3.06E-12	0.02715052	8.54E-10	2.72E-20	0.00866293	0.00622987	0.58102927
43.8	0.00083918	8.22E-08	3.49E-12	0.02726075	7.74E-10	2.84E-20	0.0086282	0.00624556	0.58563091
43.9	[illegible]	[illegible]	[illegible]	[illegible]	[illegible]	[illegible]	[illegible]	[illegible]	[illegible]
44	[illegible]	[illegible]	[illegible]	[illegible]	[illegible]	[illegible]	[illegible]	[illegible]	[illegible]
44.1	[illegible]	[illegible]	[illegible]	[illegible]	[illegible]	[illegible]	[illegible]	[illegible]	[illegible]
44.2	[illegible]	[illegible]	[illegible]	[illegible]	[illegible]	[illegible]	[illegible]	[illegible]	[illegible]
44.3	[illegible]	[illegible]	[illegible]	[illegible]	[illegible]	[illegible]	[illegible]	[illegible]	[illegible]
44.4	[illegible]	[illegible]	[illegible]	[illegible]	[illegible]	[illegible]	[illegible]	[illegible]	[illegible]
44.5	[illegible]	[illegible]	[illegible]	[illegible]	[illegible]	[illegible]	[illegible]	[illegible]	[illegible]
44.6	[illegible]	[illegible]	[illegible]	[illegible]	[illegible]	[illegible]	[illegible]	[illegible]	[illegible]
44.7	[illegible]	[illegible]	[illegible]	[illegible]	[illegible]	[illegible]	[illegible]	[illegible]	[illegible]
44.8	[illegible]	[illegible]	[illegible]	[illegible]	[illegible]	[illegible]	[illegible]	[illegible]	[illegible]
44.9	[illegible]	[illegible]	[illegible]	[illegible]	[illegible]	[illegible]	[illegible]	[illegible]	[illegible]

Time discretization	V_{[G]}	K_{[G]}	V_{[PYR]}	K_{[PYR]}	alpha	K_{[LACT]}	G_E	[G]_0	[PYR]_0
45	0.00067161	7.31E-08	1.85E-11	0.03927464	2.97E-10	2.66E-20	0.00781099	0.00631273	0.5652229
45.1	0.00065518	7.21E-08	2.03E-11	0.04055791	2.71E-10	2.70E-20	0.00766479	0.00656678	0.57108435
45.2	0.00063857	6.97E-08	2.35E-11	0.04095833	2.42E-10	2.70E-20	0.0076369	0.00648614	0.57270486
45.3	0.00060467	6.71E-08	2.64E-11	0.04219962	2.16E-10	2.32E-20	0.00752047	0.00635714	0.56277485
45.4	0.00057057	5.29E-08	3.58E-11	0.04256945	1.67E-10	2.43E-20	0.00734636	0.00664763	0.55579148
45.5	0.00056062	4.73E-08	4.05E-11	0.04345148	1.50E-10	2.70E-20	0.00715058	0.00645598	0.55766573
45.6	0.00055267	4.70E-08	4.48E-11	0.04519971	1.42E-10	2.96E-20	0.00723489	0.00633212	0.56117706
45.7	0.00056787	4.37E-08	5.02E-11	0.04713325	1.19E-10	2.55E-20	0.00703765	0.00617598	0.55597529
45.8	0.00055916	4.41E-08	5.81E-11	0.04740683	1.05E-10	2.59E-20	0.00675166	0.00635555	0.5506771
45.9	0.00051546	4.39E-08	7.00E-11	0.04822329	1.01E-10	2.56E-20	0.00681306	0.00706791	0.54774528
46	0.00048431	4.34E-08	7.96E-11	0.04923506	9.34E-11	2.59E-20	0.00686138	0.00683975	0.54823046
46.1	0.00044927	3.98E-08	8.62E-11	0.04912496	7.64E-11	2.13E-20	0.00664331	0.00675671	0.54563413
46.2	0.00044232	3.83E-08	9.17E-11	0.04872691	7.61E-11	2.29E-20	0.0068494	0.0067613	0.54821128
46.3	0.00041756	3.75E-08	1.01E-10	0.05112859	6.43E-11	2.23E-20	0.00653106	0.00669459	0.55023411
46.4	0.00039409	3.63E-08	1.23E-10	0.05033327	6.11E-11	2.18E-20	0.00647608	0.00660722	0.54965009
46.5	0.00039896	3.41E-08	1.38E-10	0.0526825	5.26E-11	2.20E-20	0.00684644	0.00716015	0.54818508
46.6	0.00037972	3.39E-08	1.66E-10	0.0536342	5.34E-11	2.41E-20	0.00667344	0.00778787	0.56081699
46.7	0.00037236	3.48E-08	1.80E-10	0.0531111	5.39E-11	2.54E-20	0.00668195	0.00787276	0.56240623
46.8	0.00035336	3.66E-08	2.16E-10	0.05527114	4.71E-11	2.75E-20	0.00655118	0.00799308	0.5599672
46.9	0.00033936	3.61E-08	2.58E-10	0.05882243	4.16E-11	2.46E-20	0.00654989	0.0081393	0.56346907
47	0.00030316	2.98E-08	3.13E-10	0.06004244	3.39E-11	2.47E-20	0.00664956	0.00816358	0.55511993
47.1	0.00030242	2.88E-08	3.85E-10	0.06050517	3.37E-11	2.63E-20	0.00619638	0.00809443	0.55385604
47.2	0.00030573	3.01E-08	4.07E-10	0.06226009	3.12E-11	2.64E-20	0.00601614	0.00835603	0.55497991
47.3	0.00029613	3.13E-08	4.59E-10	0.0638022	2.92E-11	3.29E-20	0.00578787	0.00832576	0.56565431
47.4	0.00028116	3.32E-08	5.35E-10	0.06395416	2.67E-11	3.56E-20	0.00576402	0.00876702	0.56611519
47.5	0.00027664	3.05E-08	6.21E-10	0.06543522	2.41E-11	2.97E-20	0.00581945	0.00904222	0.57114712
47.6	0.00027758	2.83E-08	7.28E-10	0.06611217	2.33E-11	3.37E-20	0.00590092	0.00922586	0.57656022
47.7	0.00027095	2.85E-08	8.79E-10	0.06736913	2.07E-11	3.37E-20	0.00568008	0.00908009	0.57471273
47.8	0.00027716	2.63E-08	9.46E-10	0.06880605	1.77E-11	3.25E-20	0.00557106	0.0092898	0.57615523
47.9	0.00027871	2.58E-08	9.60E-10	0.06958434	1.60E-11	3.26E-20	0.00567116	0.0096344	0.581764
48	0.00027091	2.54E-08	1.08E-09	0.07112964	1.54E-11	3.40E-20	0.00578764	0.01000274	0.58019379
48.1	0.00028257	2.46E-08	1.20E-09	0.07274434	1.38E-11	3.62E-20	0.00559036	0.01029684	0.58546567
48.2	0.00027178	2.23E-08	1.41E-09	0.07278825	1.19E-11	3.96E-20	0.00532891	0.0101518	0.57676008
48.3	0.000242	2.51E-08	1.65E-09	0.07493136	9.85E-12	3.81E-20	0.0052679	0.0097916	0.57236911
48.4	0.00023418	2.63E-08	1.91E-09	0.07704011	1.01E-11	4.06E-20	0.00525103	0.0097368	0.56481668
48.5	0.00021894	2.62E-08	2.30E-09	0.07625984	9.35E-12	4.00E-20	0.00525031	0.00976044	0.5580952
48.6	0.00022302	2.45E-08	2.56E-09	0.07877881	8.37E-12	4.03E-20	0.00499094	0.0101277	0.56591351
48.7	0.00021285	2.31E-08	3.46E-09	0.08273433	7.12E-12	4.12E-20	0.00479952	0.00991236	0.55801952
48.8	0.00020794	2.27E-08	3.82E-09	0.084208	6.59E-12	4.25E-20	0.00494909	0.00983793	0.55418362
48.9	0.00021294	2.20E-08	4.61E-09	0.08743808	6.18E-12	4.36E-20	0.00525253	0.00984019	0.55598854
49	0.00020888	1.81E-08	5.08E-09	0.08806286	5.48E-12	4.56E-20	0.00501159	0.00973313	0.54997749
49.1	0.00020425	1.79E-08	5.37E-09	0.09061206	4.91E-12	4.62E-20	0.00498753	0.00969701	0.54989048
49.2	0.00019475	1.73E-08	5.77E-09	0.09481478	3.95E-12	4.00E-20	0.00499488	0.00964506	0.5510728
49.3	0.00018552	1.72E-08	6.05E-09	0.09642174	3.50E-12	4.02E-20	0.0051144	0.00957894	0.5495761
49.4	0.00018987	1.62E-08	7.13E-09	0.09785851	3.34E-12	4.43E-20	0.0051116	0.00959641	0.53665975
49.5	0.00018749	1.52E-08	8.39E-09	0.09864322	3.04E-12	4.72E-20	0.00499691	0.01004354	0.53714106
49.6	0.00017778	1.58E-08	9.21E-09	0.09930129	2.85E-12	4.86E-20	0.00503961	0.00994578	0.54675675
49.7	0.00017665	1.73E-08	1.06E-08	0.10244051	2.83E-12	5.38E-20	0.00500524	0.00995582	0.54366004
49.8	0.00017389	1.56E-08	1.13E-08	0.10110602	2.59E-12	5.56E-20	0.00499296	0.00980533	0.54664153
49.9	0.0001732	1.53E-08	1.15E-08	0.10223509	2.37E-12	5.81E-20	0.00496502	0.00966996	0.54791445
50	0.00017971	1.41E-08	1.18E-08	0.1027324	2.05E-12	5.21E-20	0.00508911	0.00971614	0.54814085
50.1	0.00017973	1.35E-08	1.36E-08	0.1027986	1.82E-12	5.17E-20	0.0051867	0.00966215	0.547534
50.2	0.00016611	1.36E-08	1.53E-08	0.10630109	1.85E-12	5.45E-20	0.00528501	0.009966	0.54195819
50.3	0.00016965	1.34E-08	1.77E-08	0.10785265	1.57E-12	5.60E-20	0.00543521	0.01019743	0.53867722
50.4	0.00016059	1.20E-08	1.99E-08	0.11334402	1.30E-12	5.71E-20	0.00558725	0.01051211	0.54666417
50.5	0.00015345	1.20E-08	2.21E-08	0.11150964	1.21E-12	5.84E-20	0.00582501	0.01037932	0.55370421
50.6	0.00015439	1.18E-08	2.28E-08	0.11476421	1.12E-12	5.50E-20	0.00583674	0.01054164	0.5571527
50.7	0.00014057	1.11E-08	2.74E-08	0.11391049	9.33E-13	5.80E-20	0.00580572	0.01074204	0.55850036
50.8	0.00013547	1.07E-08	2.85E-08	0.11349195	8.82E-13	6.37E-20	0.0058105	0.01098631	0.5631845
50.9	0.00012375	1.13E-08	3.02E-08	0.11680815	7.81E-13	6.15E-20	0.00566813	0.01092822	0.55633001
51	0.00011889	1.07E-08	3.65E-08	0.12481414	6.45E-13	6.32E-20	0.0056259	0.01073626	0.55054272
51.1	0.00012136	1.07E-08	3.96E-08	0.12658794	5.78E-13	5.90E-20	0.00527116	0.01043639	0.54366669
51.2	0.00011971	1.08E-08	4.06E-08	0.12851824	5.47E-13	5.81E-20	0.00532498	0.01082114	0.54341063
51.3	0.00011671	1.07E-08	4.47E-08	0.1308488	5.19E-13	5.92E-20	0.00528372	0.01099224	0.54086861
51.4	0.00011052	1.07E-08	5.10E-08	0.1347422	5.49E-13	6.90E-20	0.00546198	0.01074526	0.54085236
51.5	0.00010282	1.05E-08	5.44E-08	0.13422483	4.61E-13	7.64E-20	0.00574825	0.01059821	0.53161791
51.6	9.82E-05	1.01E-08	5.67E-08	0.13732408	4.17E-13	7.43E-20	0.0057583	0.0101683	0.53063144
51.7	8.93E-05	9.10E-09	5.99E-08	0.13392077	3.40E-13	7.66E-20	0.00591993	0.01005796	0.53834482
51.8	8.75E-05	1.02E-08	6.83E-08	0.14142762	3.20E-13	9.32E-20	0.00635273	0.01076902	0.54381208
51.9	8.41E-05	9.95E-09	7.78E-08	0.14538447	2.54E-13	8.94E-20	0.00636797	0.01125081	0.53122593
52	8.81E-05	1.05E-08	8.64E-08	0.14869282	2.58E-13	9.00E-20	0.00651952	0.01109171	0.54156285
52.1	8.55E-05	9.69E-09	8.74E-08	0.14788593	2.32E-13	8.67E-20	0.00637	0.01114641	0.53521644
52.2	8.19E-05	9.11E-09	9.12E-08	0.14939722	2.14E-13	8.82E-20	0.00652018	0.01111547	0.52958199
52.3	8.05E-05	9.14E-09	9.90E-08	0.15429253	1.96E-13	9.03E-20	0.00658402	0.01102778	0.53055794
52.4	8.17E-05	9.23E-09	1.17E-07	0.1541144	1.79E-13	1.01E-19	0.00626175	0.01125756	0.53701009
52.5	7.78E-05	8.63E-09	1.46E-07	0.1575015	1.62E-13	1.15E-19	0.00607688	0.01136724	0.53340753
52.6	7.64E-05	8.22E-09	1.49E-07	0.16380852	1.52E-13	1.11E-19	0.00620926	0.01167305	0.53676806
52.7	7.59E-05	7.97E-09	1.53E-07	0.1627471	1.42E-13	1.10E-19	0.00619846	0.01230873	0.53897197
52.8	6.95E-05	8.36E-09	1.88E-07	0.17628469	1.23E-13	1.17E-19	0.00602977	0.01322871	0.53064619
52.9	6.57E-05	9.34E-09	2.20E-07	0.17570899	1.01E-13	1.35E-19	0.00608653	0.01255804	0.52678116
53	6.51E-05	9.02E-09	2.26E-07	0.1765859	9.36E-14	1.39E-19	0.00608502	0.01294183	0.5281056
53.1	6.54E-05	8.28E-09	2.72E-07	0.17884543	8.10E-14	1.38E-19	0.00600285	0.01304717	0.53571028
53.2	6.80E-05	7.80E-09	3.11E-07	0.17816059	7.50E-14	1.40E-19	0.00585972	0.01275283	0.53605543
53.3	6.33E-05	7.97E-09	3.29E-07	0.18088803	7.13E-14	1.48E-19	0.00591341	0.01270379	0.53279428
53.4	6.20E-05	7.74E-09	3.43E-07	0.18187915	5.98E-14	1.44E-19	0.00603389	0.01329457	0.53999518
53.5	6.12E-05	7.90E-09	3.89E-07	0.18835158	5.07E-14	1.62E-19	0.00615965	0.01386189	0.55868163
53.6	6.09E-05	8.26E-09	4.06E-07	0.18531436	4.58E-14	1.80E-19	0.00639346	0.01358248	0.56421222
53.7	5.83E-05	7.93E-09	4.24E-07	0.1853021	4.44E-14	1.75E-19	0.00614057	0.01352258	0.57440999
53.8	5.62E-05	7.89E-09	4.73E-07	0.19076664	3.83E-14	2.03E-19	0.00603955	0.01365297	0.57389608
53.9	5.42E-05	8.01E-09	4.72E-07	0.19727265	3.43E-14	2.02E-19	0.00587047	0.01378632	0.56949411
54	4.79E-05	7.35E-09	5.08E-07	0.20082467	2.54E-14	1.92E-19	0.00584234	0.01391322	0.56906039
54.1	4.46E-05	7.77E-09	5.84E-07	0.20078229	2.30E-14	2.34E-19	0.00568855	0.01372655	0.57539707
54.2	4.46E-05	7.81E-09	5.99E-07	0.20155625	2.27E-14	2.35E-19	0.00565736	0.01382557	0.57649512
54.3	4.26E-05	7.60E-09	6.63E-07	0.20569293	1.96E-14	2.40E-19	0.00562914	0.01368582	0.57377229
54.4	4.16E-05	7.83E-09	6.74E-07	0.20608762	1.87E-14	2.47E-19	0.00539329	0.01368243	0.57887889
54.5	3.99E-05	7.67E-09	7.03E-07	0.21112967	1.72E-14	2.34E-19	0.00544593	0.01428386	0.58152025
54.6	3.87E-05	7.19E-09	7.60E-07	0.20497326	1.47E-14	2.49E-19	0.00527957	0.01390851	0.57300118
54.7	3.69E-05	6.94E-09	8.20E-07	0.20896989	1.22E-14	2.32E-19	0.00514593	0.01361163	0.56365925
54.8	3.33E-05	6.30E-09	9.58E-07	0.21585019	1.07E-14	2.36E-19	0.0050257	0.0132897	0.5656327
54.9	3.41E-05	6.27E-09	1.04E-06	0.21806804	1.08E-14	2.45E-19	0.00504283	0.01343442	0.57176997
55	3.32E-05	6.25E-09	1.18E-06	0.22282831	1.01E-14	2.76E-19	0.00463955	0.01405664	0.57613911
55.1	3.43E-05	6.37E-09	1.43E-06	0.22627558	1.01E-14	3.39E-19	0.00460155	0.01479243	0.58557205
55.2	3.11E-05	5.43E-09	1.57E-06	0.23309408	7.29E-15	3.30E-19	0.00448159	0.01400003	0.5791465
55.3	3.01E-05	5.66E-09	1.68E-06	0.23807419	6.36E-15	3.34E-19	0.00453953	0.0148959	0.57547962
55.4	2.93E-05	5.66E-09	1.97E-06	0.24206057	5.96E-15	3.95E-19	0.00445357	0.01522277	0.58665787
55.5	2.85E-05	5.87E-09	2.11E-06	0.23622891	5.23E-15	4.48E-19	0.00443	0.01493705	0.58422072
55.6	2.82E-05	5.79E-09	2.45E-06	0.247906	4.66E-15	5.09E-19	0.00473159	0.01561714	0.58329416
55.7	2.67E-05	5.47E-09	2.68E-06	0.25236042	3.79E-15	4.60E-19	0.00450375	0.01565583	0.57714982
55.8	2.52E-05	5.56E-09	2.91E-06	0.25812572	3.12E-15	4.71E-19	0.00431985	0.01531558	0.57548512
55.9	2.46E-05	5.49E-09	3.15E-06	0.27006262	2.79E-15	4.42E-19	0.00445606	0.01644648	0.56849967
56	2.30E-05	5.51E-09	3.49E-06	0.27490818	2.63E-15	4.79E-19	0.00454679	0.0167435	0.56964791
56.1	2.06E-05	5.40E-09	3.66E-06	0.28410248	2.25E-15	5.11E-19	0.00432432	0.01693545	0.5718816
56.2	1.89E-05	5.16E-09	3.90E-06	0.29378955	1.83E-15	5.36E-19	0.00429955	0.01673373	0.57842416
56.3	1.85E-05	5.22E-09	4.14E-06	0.29567373	1.66E-15	6.10E-19	0.00426664	0.01723227	0.58151306
56.4	1.81E-05	4.96E-09	4.26E-06	0.29720758	1.47E-15	6.17E-19	0.00419742	0.01701909	0.5840848
56.5	1.60E-05	4.48E-09	4.94E-06	0.30884259	1.35E-15	6.61E-19	0.00443704	0.01753327	0.59076295
56.6	1.60E-05	4.02E-09	5.14E-06	0.31316014	1.16E-15	6.74E-19	0.00414323	0.01752766	0.59451914
56.7	1.42E-05	3.66E-09	6.15E-06	0.3119774	8.41E-16	7.61E-19	0.00409729	0.0172621	0.59486314
56.8	1.34E-05	3.70E-09	6.97E-06	0.31325173	7.82E-16	7.58E-19	0.00409147	0.01763526	0.59602264
56.9	1.26E-05	3.90E-09	7.26E-06	0.31563803	7.40E-16	8.38E-19	0.00413809	0.01815619	0.58991516
57	1.24E-05	3.84E-09	7.35E-06	0.31568546	6.74E-16	8.90E-19	0.00414736	0.01828625	0.59304684
57.1	1.21E-05	3.98E-09	8.08E-06	0.32464951	6.36E-16	9.67E-19	0.00429452	0.01804624	0.58348589
57.2	1.16E-05	4.02E-09	9.13E-06	0.32906326	5.32E-16	1.01E-18	0.00432993	0.01762524	0.58445953
57.3	1.15E-05	3.97E-09	9.96E-06	0.33235116	4.56E-16	1.17E-18	0.00420233	0.01753217	0.58325926
57.4	1.08E-05	3.49E-09	1.11E-05	0.33181117	3.53E-16	1.13E-18	0.00408712	0.01694647	0.59084416
57.5	1.12E-05	3.39E-09	1.13E-05	0.33176891	3.45E-16	1.26E-18	0.0041003	0.01726758	0.59240998
57.6	1.11E-05	3.13E-09	1.17E-05	0.32581399	2.94E-16	1.17E-18	0.00396067	0.01680174	0.58244361
57.7	1.10E-05	3.17E-09	1.27E-05	0.33337178	2.80E-16	1.15E-18	0.00390342	0.01733491	0.57659544
57.8	1.04E-05	3.07E-09	1.35E-05	0.33567785	2.33E-16	1.13E-18	0.00369429	0.01766801	0.58443393
57.9	9.82E-06	3.18E-09	1.45E-05	0.34058242	1.91E-16	1.20E-18	0.00374905	0.017272	0.59002663
58	9.68E-06	3.36E-09	1.48E-05	0.35188552	1.71E-16	1.17E-18	0.00376135	0.01696091	0.58882262
58.1	9.09E-06	3.60E-09	1.53E-05	0.35695762	1.49E-16	1.33E-18	0.0036132	0.0171726	0.58938103
58.2	9.13E-06	3.21E-09	1.74E-05	0.37053234	1.22E-16	1.26E-18	0.00344859	0.01783289	0.59148087
58.3	9.05E-06	3.30E-09	1.75E-05	0.37358205	1.24E-16	1.48E-18	0.00329574	0.01764873	0.59308748
58.4	8.53E-06	3.39E-09	1.88E-05	0.38132809	1.07E-16	1.62E-18	0.00336594	0.01739157	0.58746779
58.5	8.26E-06	3.22E-09	2.14E-05	0.38412757	9.44E-17	1.54E-18	0.00330152	0.01754825	0.57827004
58.6	8.20E-06	3.12E-09	2.25E-05	0.38338617	8.42E-17	1.58E-18	0.00327531	0.01799934	0.5756728
58.7	7.81E-06	3.20E-09	2.62E-05	0.38139548	7.83E-17	1.68E-18	0.0030762	0.01778534	0.56873383
58.8	7.61E-06	3.12E-09	2.78E-05	0.39046682	6.84E-17	1.73E-18	0.0029702	0.0182354	0.57518779
58.9	7.73E-06	2.73E-09	2.91E-05	0.39634126	6.01E-17	1.75E-18	0.00294363	0.01802662	0.57616175
59	7.67E-06	2.85E-09	3.01E-05	0.39472301	5.48E-17	1.77E-18	0.00285169	0.01827283	0.57708416
59.1	7.31E-06	3.07E-09	3.38E-05	0.40183187	4.93E-17	1.95E-18	0.00278674	0.0182849	0.57118986
59.2	7.26E-06	2.96E-09	3.41E-05	0.40580484	4.45E-17	1.84E-18	0.00278383	0.01835598	0.57111138
59.3	7.70E-06	2.87E-09	3.79E-05	0.41029633	3.84E-17	2.11E-18	0.00264254	0.01878205	0.57037348
59.4	7.88E-06	2.80E-09	4.29E-05	0.41499163	4.15E-17	2.31E-18	0.00266553	0.01911443	0.57628078
59.5	7.31E-06	2.88E-09	4.92E-05	0.41939186	3.60E-17	2.33E-18	0.00280426	0.01953919	0.57480021
59.6	7.72E-06	2.77E-09	5.36E-05	0.4212739	2.87E-17	2.16E-18	0.00278843	0.01994574	0.57039496
59.7	7.15E-06	3.04E-09	5.84E-05	0.42663856	2.77E-17	2.63E-18	0.00275831	0.01988861	0.57841606
59.8	7.48E-06	3.00E-09	6.41E-05	0.43843157	2.64E-17	3.23E-18	0.00269407	0.02007619	0.58093827
59.9	7.25E-06	2.89E-09	6.77E-05	0.4337667	2.57E-17	3.61E-18	0.00272109	0.02066841	0.58030197

60	6.89E-06	2.63E-09	7.50E-05	0.43470779	2.12E-17	3.26E-18	0.0026735	0.02152313	0.57954501
60.1	6.89E-06	2.38E-09	8.50E-05	0.4321799	2.17E-17	3.64E-18	0.00268889	0.02133265	0.58717779
60.2	6.59E-06	2.31E-09	8.58E-05	0.42887397	2.06E-17	3.65E-18	0.00259531	0.02196448	0.58210773
60.3	6.00E-06	1.92E-09	9.49E-05	0.4292085	1.61E-17	3.95E-18	0.00250525	0.02196993	0.58160813
60.4	6.00E-06	1.85E-09	0.00010273	0.43489428	1.36E-17	4.60E-18	0.00251904	0.02205581	0.58961974
60.5	5.80E-06	1.93E-09	0.00011279	0.44822582	1.23E-17	4.99E-18	0.00236435	0.0219061	0.58168777
60.6	5.53E-06	1.82E-09	0.00012123	0.45005133	1.18E-17	5.32E-18	0.00237873	0.02293522	0.57397201
60.7	5.52E-06	1.63E-09	0.00013908	0.46611695	9.55E-18	4.95E-18	0.00246933	0.02267754	0.57346104
60.8	5.88E-06	1.54E-09	0.0001521	0.46536627	8.33E-18	5.77E-18	0.00250873	0.02404079	0.56599253
60.9	5.53E-06	1.50E-09	0.00016237	0.46175817	8.17E-18	5.91E-18	0.00245877	0.02423243	0.55964836
61	5.74E-06	1.44E-09	0.00017867	0.46876794	7.92E-18	6.01E-18	0.00245997	0.02306216	0.56116378
61.1	5.44E-06	1.39E-09	0.00020142	0.46172372	7.20E-18	6.44E-18	0.00238462	0.0236542	0.56037482
61.2	5.21E-06	1.45E-09	0.00022202	0.46177079	6.96E-18	7.02E-18	0.0023388	0.02418163	0.56231595
61.3	5.29E-06	1.42E-09	0.00023768	0.46342619	5.92E-18	7.23E-18	0.00235781	0.02367389	0.55111728
61.4	5.19E-06	1.37E-09	0.00024983	0.47134831	5.29E-18	7.59E-18	0.00244851	0.02443157	0.5557892
61.5	5.12E-06	1.24E-09	0.00026711	0.48079822	5.26E-18	8.38E-18	0.00244032	0.02427559	0.55990919
61.6	5.02E-06	1.30E-09	0.00027695	0.47723293	5.41E-18	9.19E-18	0.00252581	0.02476524	0.56326624
61.7	4.73E-06	1.32E-09	0.00031057	0.48413803	4.63E-18	9.65E-18	0.00246809	0.02412676	0.54742734
61.8	4.59E-06	1.37E-09	0.00033862	0.48946789	4.68E-18	1.20E-17	0.00237649	0.02358147	0.55401457
61.9	4.53E-06	1.42E-09	0.00036399	0.49816456	4.25E-18	1.29E-17	0.00240644	0.02347414	0.54609117
62	4.43E-06	1.42E-09	0.00041032	0.50279187	4.31E-18	1.58E-17	0.00229124	0.02339855	0.54961533
62.1	4.24E-06	1.35E-09	0.00041622	0.5000487	4.13E-18	1.64E-17	0.0023909	0.02351477	0.54952429
62.2	4.28E-06	1.34E-09	0.00046683	0.50119878	3.90E-18	1.58E-17	0.00228196	0.02388581	0.55010863
62.3	4.32E-06	1.46E-09	0.00047556	0.50523257	3.79E-18	1.73E-17	0.00228547	0.02439229	0.55471955
62.4	4.39E-06	1.44E-09	0.00048482	0.51121661	3.69E-18	1.78E-17	0.00231158	0.02561423	0.55842364
62.5	4.32E-06	1.49E-09	0.00051615	0.51599641	3.48E-18	1.81E-17	0.0023572	0.02601493	0.56484826
62.6	4.37E-06	1.40E-09	0.00056683	0.52465463	3.31E-18	1.77E-17	0.00228384	0.02605393	0.56132316
62.7	4.35E-06	1.31E-09	0.00060147	0.52930349	3.06E-18	1.85E-17	0.00218438	0.02683709	0.55617431
62.8	4.06E-06	1.37E-09	0.00060938	0.52549253	2.68E-18	1.86E-17	0.0020893	0.02635556	0.55780141
62.9	4.09E-06	1.29E-09	0.00064856	0.53278514	2.55E-18	2.08E-17	0.00211223	0.02672372	0.56046932
63	3.90E-06	1.27E-09	0.00066644	0.53335625	2.48E-18	2.25E-17	0.00207032	0.02625338	0.55333815
63.1	3.75E-06	1.12E-09	0.00070753	0.53982756	2.04E-18	2.18E-17	0.00200754	0.02565726	0.55125994
63.2	3.81E-06	1.05E-09	0.00074658	0.54083284	1.98E-18	2.17E-17	0.00190901	0.02627816	0.55265386
63.3	3.65E-06	1.04E-09	0.00076328	0.53910039	1.65E-18	2.15E-17	0.00194621	0.02571301	0.54982998
63.4	3.38E-06	9.51E-10	0.00083182	0.54020661	1.45E-18	2.32E-17	0.00191346	0.02660953	0.54717125
63.5	3.32E-06	9.32E-10	0.00090984	0.55973251	1.10E-18	2.37E-17	0.00192818	0.02850082	0.5439716
63.6	3.29E-06	8.48E-10	0.00095169	0.55961988	9.38E-19	2.39E-17	0.0018424	0.02855043	0.54809944
63.7	2.98E-06	7.85E-10	0.00102597	0.55640404	8.26E-19	2.62E-17	0.00177092	0.02783126	0.53508553
63.8	2.61E-06	7.96E-10	0.00111461	0.55116314	7.48E-19	2.87E-17	0.00176041	0.02795939	0.53088465
63.9	2.44E-06	7.09E-10	0.00122003	0.54649972	6.59E-19	3.08E-17	0.00173811	0.02842022	0.53569592
64	2.43E-06	7.25E-10	0.00127364	0.54920504	6.65E-19	3.52E-17	0.00183343	0.02856006	0.54342342
64.1	2.24E-06	6.93E-10	0.00132065	0.54736303	5.70E-19	3.76E-17	0.00175788	0.02899476	0.53968058
64.2	1.96E-06	6.89E-10	0.0014588	0.55739927	5.12E-19	3.86E-17	0.00161283	0.03019711	0.53924988
64.3	2.00E-06	6.15E-10	0.00149731	0.56379843	4.76E-19	3.58E-17	0.00153329	0.03129005	0.54359684
64.4	1.93E-06	5.98E-10	0.00155337	0.56885443	4.31E-19	3.53E-17	0.00156146	0.03098595	0.53679666
64.5	1.66E-06	5.44E-10	0.00184367	0.57058952	3.91E-19	4.43E-17	0.00151754	0.03205963	0.53195583
64.6	1.58E-06	5.25E-10	0.00206159	0.57501725	3.54E-19	4.09E-17	0.0014664	0.03280724	0.52484332
64.7	1.44E-06	4.58E-10	0.00214808	0.57878979	3.00E-19	3.87E-17	0.00146838	0.03201655	0.52281389
64.8	1.28E-06	4.47E-10	0.00226184	0.58076412	2.68E-19	4.02E-17	0.00147284	0.03284812	0.52558686
64.9	1.23E-06	4.44E-10	0.00233868	0.58305702	2.24E-19	4.62E-17	0.00144608	0.0331067	0.52506841
65	1.19E-06	4.27E-10	0.00240416	0.5881056	1.91E-19	4.84E-17	0.00134128	0.03436587	0.51540389
65.1	1.17E-06	3.93E-10	0.00256848	0.59178386	1.55E-19	5.15E-17	0.00132092	0.03458583	0.51081055
65.2	1.07E-06	4.01E-10	0.00280402	0.58394917	1.32E-19	5.61E-17	0.00126961	0.03521683	0.50771288
65.3	1.10E-06	3.66E-10	0.00278153	0.58615014	1.21E-19	5.81E-17	0.00124268	0.03616267	0.51465307
65.4	1.11E-06	3.46E-10	0.00298935	0.58571694	1.09E-19	7.14E-17	0.00122579	0.03724454	0.5066679
65.5	1.11E-06	3.52E-10	0.00321919	0.59755952	1.11E-19	7.78E-17	0.00118108	0.03899557	0.50801864
65.6	1.04E-06	3.36E-10	0.0033529	0.60099491	9.20E-20	7.91E-17	0.00115382	0.03956223	0.5006669
65.7	1.01E-06	3.12E-10	0.0037759	0.61820222	7.83E-20	8.14E-17	0.00106717	0.04216097	0.49551158
65.8	9.41E-07	3.26E-10	0.00417139	0.62597051	7.31E-20	8.88E-17	0.0010355	0.04243606	0.48068236
65.9	8.88E-07	3.04E-10	0.00437981	0.62368523	6.56E-20	8.34E-17	0.00102384	0.04251437	0.47778149
66	8.30E-07	2.84E-10	0.00449436	0.61982201	5.44E-20	9.40E-17	0.00104649	0.04325154	0.48323014
66.1	8.93E-07	2.52E-10	0.00476122	0.62031003	5.11E-20	1.14E-16	0.00099371	0.04610463	0.4908133
66.2	8.83E-07	2.42E-10	0.00527742	0.6212843	4.16E-20	1.29E-16	0.00099244	0.04784918	0.48017777
66.3	8.96E-07	2.28E-10	0.00550003	0.63082725	4.08E-20	1.33E-16	0.00098143	0.04915941	0.48186237
66.4	9.06E-07	2.14E-10	0.0055406	0.63618937	4.05E-20	1.44E-16	0.00094544	0.04991622	0.49057663
66.5	8.76E-07	2.15E-10	0.00583882	0.63263413	3.96E-20	1.65E-16	0.00087449	0.05099342	0.48862441
66.6	8.30E-07	2.13E-10	0.00614584	0.65179826	3.26E-20	1.65E-16	0.00081139	0.05484812	0.49378707
66.7	7.58E-07	2.19E-10	0.00659195	0.6571149	3.29E-20	1.91E-16	0.00078354	0.05551314	0.49633343
66.8	7.92E-07	2.31E-10	0.00677597	0.65514952	3.46E-20	2.10E-16	0.00076568	0.05721213	0.49353193
66.9	7.23E-07	2.60E-10	0.00715827	0.66115817	3.04E-20	2.16E-16	0.00077392	0.05774134	0.48375058
67	6.94E-07	2.39E-10	0.00718116	0.66351846	2.77E-20	2.20E-16	0.00081035	0.0593883	0.47668257
67.1	6.49E-07	2.35E-10	0.00720878	0.66800255	2.51E-20	2.16E-16	0.00080647	0.05912526	0.4698101
67.2	6.53E-07	2.25E-10	0.00765869	0.67088905	2.00E-20	2.42E-16	0.00080801	0.05980936	0.46398334
67.3	6.29E-07	2.18E-10	0.0077148	0.67314737	1.84E-20	2.53E-16	0.00075042	0.06099749	0.45699525
67.4	5.72E-07	2.18E-10	0.0079418	0.67593245	1.67E-20	2.45E-16	0.0007137	0.06253683	0.45348278
67.5	5.54E-07	1.87E-10	0.00861865	0.67319526	1.37E-20	2.64E-16	0.00064947	0.06392079	0.45265219
67.6	5.29E-07	1.72E-10	0.00903562	0.67872981	1.13E-20	2.72E-16	0.00063703	0.0660813	0.45043903
67.7	5.04E-07	1.64E-10	0.0092505	0.69947558	9.78E-21	2.64E-16	0.00061312	0.06800095	0.43901033
67.8	4.58E-07	1.54E-10	0.00951711	0.70161223	8.94E-21	2.57E-16	0.00059562	0.06651167	0.42982236
67.9	4.22E-07	1.45E-10	0.00936517	0.70359429	7.19E-21	2.44E-16	0.00057765	0.06550852	0.4337112
68	4.00E-07	1.55E-10	0.00954337	0.70262117	6.68E-21	2.70E-16	0.00053285	0.06459445	0.42452049
68.1	3.87E-07	1.47E-10	0.0096862	0.70466025	6.03E-21	2.87E-16	0.00051757	0.06587823	0.42034826
68.2	3.75E-07	1.43E-10	0.00971646	0.69630431	5.44E-21	3.27E-16	0.00052437	0.06662593	0.4244213
68.3	3.50E-07	1.34E-10	0.01019386	0.70748368	5.14E-21	3.46E-16	0.00053188	0.06994633	0.42678189
68.4	3.38E-07	1.22E-10	0.01057624	0.71844131	4.23E-21	3.61E-16	0.00051733	0.07042129	0.43099946
68.5	3.17E-07	1.17E-10	0.01125408	0.71691308	3.80E-21	3.88E-16	0.00051587	0.07126783	0.43133837
68.6	3.23E-07	1.09E-10	0.01187332	0.71684947	3.72E-21	3.75E-16	0.00050896	0.07254811	0.42819069
68.7	3.29E-07	1.13E-10	0.01240751	0.70867403	3.61E-21	3.99E-16	0.00049835	0.07138964	0.43176391
68.8	3.20E-07	1.05E-10	0.01313599	0.71998649	3.47E-21	4.23E-16	0.00050301	0.07270993	0.43463186
68.9	3.22E-07	9.49E-11	0.01330405	0.72238587	2.85E-21	4.51E-16	0.00048208	0.07289753	0.4444062
69	3.36E-07	9.10E-11	0.01331976	0.72616735	2.33E-21	4.41E-16	0.00045791	0.07301933	0.43756856
69.1	3.07E-07	9.58E-11	0.01329372	0.73124223	1.99E-21	4.60E-16	0.00043163	0.07255276	0.42951716
69.2	2.94E-07	9.90E-11	0.01387843	0.73595154	1.73E-21	4.55E-16	0.00041098	0.07372261	0.43170225
69.3	2.88E-07	1.02E-10	0.01454038	0.73915848	1.60E-21	4.79E-16	0.0003898	0.07380515	0.42804121
69.4	2.91E-07	1.05E-10	0.01483499	0.73984578	1.44E-21	4.87E-16	0.00036762	0.07447235	0.42663857
69.5	2.86E-07	1.08E-10	0.01475957	0.74848141	1.34E-21	5.04E-16	0.00034794	0.07758244	0.42264957
69.6	2.90E-07	9.55E-11	0.01527192	0.76454991	1.13E-21	5.03E-16	0.00033634	0.07910972	0.42488732
69.7	2.74E-07	9.98E-11	0.01631487	0.77038598	1.01E-21	5.19E-16	0.00032467	0.081483	0.42348345
69.8	2.68E-07	9.98E-11	0.01711395	0.77907268	9.03E-22	5.17E-16	0.00032293	0.08325452	0.42151982
69.9	2.80E-07	9.78E-11	0.01732248	0.78572199	8.85E-22	5.46E-16	0.00031707	0.08438112	0.42114552
70	2.60E-07	9.07E-11	0.01764556	0.78961522	7.32E-22	5.25E-16	0.00032469	0.08268529	0.41857629
70.1	2.47E-07	9.40E-11	0.01845423	0.79412779	6.79E-22	6.40E-16	0.00033169	0.08299405	0.41898962
70.2	2.38E-07	9.52E-11	0.01852247	0.79846548	6.16E-22	6.40E-16	0.00032702	0.08285136	0.41573149
70.3	2.22E-07	8.53E-11	0.01840328	0.79287437	4.60E-22	6.24E-16	0.000312	0.08082687	0.41139748
70.4	2.21E-07	8.08E-11	0.0192003	0.8043915	4.38E-22	6.47E-16	0.00030762	0.08392707	0.41168592
70.5	2.23E-07	7.74E-11	0.02005364	0.81080244	3.87E-22	7.11E-16	0.00031681	0.08719531	0.41559209
70.6	2.12E-07	7.69E-11	0.02105845	0.8014566	3.19E-22	6.75E-16	0.00030095	0.08757399	0.41353312
70.7	2.07E-07	7.59E-11	0.02147804	0.80232855	2.90E-22	6.92E-16	0.00028014	0.0884244	0.41216821
70.8	2.15E-07	7.26E-11	0.02211628	0.80225078	2.73E-22	6.95E-16	0.00027664	0.0911573	0.41841021
70.9	2.20E-07	7.02E-11	0.02238188	0.81340256	2.46E-22	7.06E-16	0.00027245	0.09186555	0.41879841
71	2.28E-07	6.95E-11	0.02312704	0.82262335	2.45E-22	7.79E-16	0.00027878	0.09325184	0.41874329
71.1	2.29E-07	7.18E-11	0.02418365	0.82699608	2.26E-22	8.27E-16	0.00028149	0.09794116	0.41854904
71.2	2.29E-07	6.87E-11	0.02407403	0.82611561	2.13E-22	8.87E-16	0.00027161	0.09761074	0.419837
71.3	2.31E-07	7.11E-11	0.0249129	0.8373172	1.95E-22	9.70E-16	0.00026848	0.09932353	0.41890944
71.4	2.23E-07	7.15E-11	0.02539598	0.83705006	1.79E-22	1.04E-15	0.00027096	0.0994078	0.42574087
71.5	2.14E-07	7.02E-11	0.02547871	0.84163261	1.63E-22	1.09E-15	0.0002592	0.09870064	0.42433936
71.6	2.19E-07	6.82E-11	0.02634767	0.85181162	1.58E-22	1.19E-15	0.00026545	0.10005342	0.42706518
71.7	2.06E-07	6.83E-11	0.02741661	0.84775217	1.51E-22	1.31E-15	0.00025122	0.09867554	0.42528961
71.8	2.14E-07	7.01E-11	0.02853646	0.8497593	1.56E-22	1.40E-15	0.00025564	0.09995862	0.4276301
71.9	2.15E-07	6.75E-11	0.02894845	0.85224955	1.34E-22	1.41E-15	0.00024401	0.10086167	0.43530926
72	2.15E-07	6.72E-11	0.03031195	0.84840555	1.15E-22	1.60E-15	0.00023264	0.09957496	0.43742481
72.1	1.97E-07	7.30E-11	0.0299319	0.85260639	1.09E-22	1.70E-15	0.00022973	0.09878965	0.43444629
72.2	1.98E-07	7.57E-11	0.03027825	0.85854607	9.38E-23	1.64E-15	0.00021956	0.09948451	0.43073814
72.3	1.90E-07	8.01E-11	0.03042116	0.85393122	8.06E-23	1.76E-15	0.0002219	0.10261892	0.43415701
72.4	1.95E-07	7.47E-11	0.03073743	0.85062644	7.48E-23	1.76E-15	0.00021365	0.1006148	0.43562309
72.5	1.90E-07	7.09E-11	0.03076923	0.8519807	6.08E-23	1.87E-15	0.00020143	0.10072289	0.43207794
72.6	1.90E-07	6.88E-11	0.03088395	0.8554384	5.46E-23	2.03E-15	0.0001944	0.1020845	0.43780848
72.7	1.95E-07	6.71E-11	0.03234678	0.85248886	5.31E-23	2.51E-15	0.00019503	0.10715575	0.44558918
72.8	1.87E-07	6.53E-11	0.0327107	0.85740772	4.48E-23	2.43E-15	0.00018301	0.10659275	0.44340584
72.9	1.72E-07	6.75E-11	0.03356522	0.8531013	3.97E-23	2.55E-15	0.00017459	0.10962847	0.44144265
73	1.76E-07	7.34E-11	0.03494462	0.85774187	4.10E-23	2.78E-15	0.00017308	0.1127614	0.44142757
73.1	1.72E-07	7.44E-11	0.03573228	0.86241085	3.97E-23	2.80E-15	0.00017209	0.11383096	0.44065058
73.2	1.74E-07	7.14E-11	0.03712623	0.87069293	3.09E-23	2.79E-15	0.00016511	0.11574221	0.44085097
73.3	1.86E-07	7.49E-11	0.03810362	0.87076377	3.45E-23	3.15E-15	0.00015808	0.11794527	0.442582
73.4	1.82E-07	6.91E-11	0.0397331	0.87516861	3.04E-23	3.38E-15	0.00015636	0.12009792	0.44744331
73.5	1.77E-07	6.79E-11	0.03986589	0.8743311	2.82E-23	3.27E-15	0.00015792	0.11887476	0.44788006
73.6	1.83E-07	6.90E-11	0.04062947	0.87770053	2.73E-23	3.81E-15	0.00015408	0.12082468	0.45256244
73.7	1.81E-07	6.97E-11	0.04142915	0.88379428	2.56E-23	3.99E-15	0.00014379	0.12509145	0.45361617
73.8	1.84E-07	6.79E-11	0.04362741	0.8938213	2.37E-23	4.55E-15	0.0001439	0.12450511	0.44971719
73.9	1.71E-07	6.94E-11	0.04327791	0.90381559	2.17E-23	4.73E-15	0.00015461	0.12586026	0.45628907
74	1.84E-07	7.21E-11	0.04399278	0.90985814	2.02E-23	4.81E-15	0.00015331	0.12970132	0.45976786
74.1	1.66E-07	6.98E-11	0.04479104	0.91739009	1.67E-23	4.93E-15	0.00014324	0.13017844	0.46533614
74.2	1.61E-07	6.79E-11	0.04528161	0.9127319	1.51E-23	5.79E-15	0.00013615	0.13305456	0.47066406
74.3	1.50E-07	6.66E-11	0.04618529	0.91547377	1.35E-23	6.51E-15	0.00013473	0.13741084	0.4725797
74.4	1.39E-07	6.28E-11	0.04723282	0.91162444	1.12E-23	6.99E-15	0.000131	0.13834504	0.48030519
74.5	1.29E-07	6.46E-11	0.04832863	0.91198658	1.06E-23	7.94E-15	0.00012907	0.14601398	0.49424054
74.6	1.31E-07	6.05E-11	0.04926131	0.91408314	9.79E-24	9.19E-15	0.00012694	0.15020176	0.50472324
74.7	1.30E-07	6.31E-11	0.04894637	0.91798701	9.42E-24	9.74E-15	0.00012184	0.14891647	0.50111476
74.8	1.35E-07	6.20E-11	0.05049649	0.92428538	8.84E-24	1.00E-14	0.00012032	0.15258841	0.49508071
74.9	1.35E-07	6.21E-11	0.05133545	0.9298189	8.27E-24	1.04E-14	0.00012595	0.15498208	0.49344074
120	3.85E-12	3.98E-13	0.83593149	0.93849583	5.85E-35	9.89E-05	2.36E-08	0.1534329	0.46785263
120.1	3.57E-12	3.87E-13	0.84078717	0.94372647	5.42E-35	9.90E-05	2.33E-08	0.15266396	0.46007813
120.2	3.92E-12	3.88E-13	0.84568195	0.94603172	5.44E-35	9.64E-05	2.29E-08	0.15178426	0.45894705

75	1.32E-07	5.82E-11	0.05370936	0.92658977	7.38E-24	1.20E-14	0.00012438	0.15422917	0.4909438
75.1	1.25E-07	6.10E-11	0.05440606	0.9249491	7.61E-24	1.33E-14	0.00012205	0.15393273	0.48402215
75.2	1.17E-07	5.92E-11	0.05904803	0.93183118	6.67E-24	1.35E-14	0.00011222	0.15888782	0.4876086
75.3	1.17E-07	5.90E-11	0.06072075	0.94567526	6.25E-24	1.37E-14	0.00011074	0.16065326	0.48987184
75.4	1.17E-07	5.92E-11	0.06108911	0.94433786	5.91E-24	1.37E-14	0.00011042	0.16072371	0.49032953
75.5	1.20E-07	5.37E-11	0.06247725	0.95068457	5.51E-24	1.42E-14	0.00010662	0.16168876	0.49380056
75.6	1.18E-07	5.36E-11	0.06207457	0.94804685	5.37E-24	1.40E-14	0.00010334	0.16168584	0.49104498
75.7	1.24E-07	5.27E-11	0.06174578	0.94947607	5.04E-24	1.53E-14	0.00010306	0.1619872	0.49784629
75.8	1.26E-07	5.25E-11	0.0639463	0.9472687	4.68E-24	1.61E-14	0.00010419	0.1628664	0.50011657
75.9	1.18E-07	4.78E-11	0.06514681	0.94453901	3.98E-24	1.70E-14	0.00010591	0.1639287	0.50396169
76	1.18E-07	4.52E-11	0.06568888	0.93988571	3.71E-24	1.69E-14	0.00010141	0.16588803	0.49913879
76.1	1.19E-07	4.74E-11	0.06631008	0.93688891	3.56E-24	1.80E-14	0.00010239	0.16784679	0.49748363
76.2	1.16E-07	4.49E-11	0.06690078	0.94010587	3.56E-24	1.74E-14	0.00010585	0.17172186	0.50192233
76.3	1.10E-07	4.55E-11	0.06886446	0.94717077	3.13E-24	1.85E-14	0.0001006	0.17479793	0.49940303
76.4	1.06E-07	4.37E-11	0.07060974	0.95069503	2.75E-24	1.85E-14	9.88E-05	0.17461495	0.49734114
76.5	1.03E-07	4.47E-11	0.07198142	0.97003017	2.55E-24	1.86E-14	8.93E-05	0.17913005	0.50280062
76.6	1.02E-07	4.20E-11	0.07358434	0.9602862	2.55E-24	2.11E-14	8.61E-05	0.18099975	0.51402098
76.7	9.54E-08	4.33E-11	0.07561963	0.96803755	2.09E-24	2.35E-14	8.30E-05	0.18050835	0.52001878
76.8	9.42E-08	4.37E-11	0.07745573	0.97382067	1.96E-24	2.55E-14	7.99E-05	0.18134796	0.52229388
76.9	9.39E-08	4.04E-11	0.07708744	0.9828052	1.77E-24	2.55E-14	7.73E-05	0.18347035	0.5225662
77	9.23E-08	4.08E-11	0.07797285	0.97992626	1.77E-24	2.72E-14	7.70E-05	0.18613566	0.53418929
77.1	1.01E-07	3.82E-11	0.07884852	0.97505493	1.77E-24	3.07E-14	7.80E-05	0.19093399	0.53870423
77.2	9.69E-08	3.61E-11	0.08164151	0.98216618	1.59E-24	3.14E-14	7.32E-05	0.19853617	0.53599274
77.3	9.52E-08	3.50E-11	0.08436356	0.98142628	1.27E-24	3.22E-14	7.22E-05	0.1975432	0.53630925
77.4	9.31E-08	3.38E-11	0.08563146	0.98371512	1.18E-24	3.21E-14	7.32E-05	0.20102274	0.53608239
77.5	9.07E-08	3.33E-11	0.08835262	0.99341637	1.07E-24	3.21E-14	7.10E-05	0.20173156	0.53658866
77.6	8.46E-08	3.52E-11	0.08942797	0.98616011	9.04E-25	3.92E-14	6.95E-05	0.20324773	0.53102051
77.7	8.75E-08	3.43E-11	0.08996671	0.98754968	8.96E-25	3.96E-14	6.90E-05	0.20393848	0.53166062
77.8	8.25E-08	3.25E-11	0.08999671	0.98937673	8.56E-25	3.94E-14	6.87E-05	0.20528184	0.53723518
77.9	7.96E-08	3.33E-11	0.09307401	0.98838304	7.11E-25	3.94E-14	6.59E-05	0.20569915	0.5445114
78	7.63E-08	3.00E-11	0.09079963	0.98879899	6.39E-25	3.88E-14	6.35E-05	0.21043639	0.56025809
78.1	7.53E-08	3.08E-11	0.09260338	0.97859616	6.36E-25	4.04E-14	6.24E-05	0.21829465	0.56488738
78.2	6.98E-08	3.08E-11	0.09361906	0.97449482	5.82E-25	3.96E-14	5.94E-05	0.21830947	0.56656244
78.3	6.87E-08	3.01E-11	0.09908505	0.96872703	4.75E-25	4.40E-14	5.85E-05	0.22190909	0.57274322
78.4	6.43E-08	3.24E-11	0.10122996	0.96848193	4.33E-25	4.71E-14	5.59E-05	0.21713433	0.56515358
78.5	6.18E-08	3.03E-11	0.10351013	0.96996447	4.09E-25	4.68E-14	5.32E-05	0.21770922	0.56177806
78.6	5.81E-08	3.14E-11	0.10554438	0.97407735	3.53E-25	5.28E-14	5.04E-05	0.21606358	0.5663265
78.7	6.00E-08	3.04E-11	0.11003221	0.96818252	3.58E-25	5.97E-14	4.94E-05	0.21795738	0.56488049
78.8	5.70E-08	3.02E-11	0.1135769	0.96065318	3.10E-25	6.56E-14	4.98E-05	0.21435102	0.57103057
78.9	5.54E-08	2.72E-11	0.1187816	0.95434995	2.43E-25	7.12E-14	4.75E-05	0.2209366	0.58016014
79	5.27E-08	2.54E-11	0.12149702	0.95990192	1.91E-25	7.80E-14	4.83E-05	0.21996892	0.58500575
79.1	5.06E-08	2.49E-11	0.12195151	0.95675412	1.79E-25	7.70E-14	4.78E-05	0.22265067	0.58868672
79.2	4.57E-08	2.31E-11	0.12366742	0.95289399	1.70E-25	8.25E-14	4.75E-05	0.22752456	0.59560754
79.3	4.41E-08	2.26E-11	0.12886216	0.95309455	1.46E-25	1.06E-13	4.51E-05	0.22916711	0.6054315
79.4	4.20E-08	2.30E-11	0.12848714	0.94875658	1.23E-25	1.05E-13	4.59E-05	0.22869923	0.60669212
79.5	4.01E-08	2.16E-11	0.1315326	0.94845625	1.12E-25	1.07E-13	4.33E-05	0.23105799	0.60436344
79.6	3.54E-08	1.96E-11	0.1343596	0.93840929	9.21E-26	1.21E-13	4.20E-05	0.23974075	0.6013454
79.7	3.60E-08	1.99E-11	0.13527515	0.93743842	8.77E-26	1.19E-13	4.12E-05	0.24089289	0.59753866
79.8	2.90E-08	1.76E-11	0.1372942	0.94187126	6.01E-26	1.06E-13	3.82E-05	0.24144528	0.60035448
79.9	2.63E-08	1.88E-11	0.13696111	0.94973325	5.39E-26	1.16E-13	3.67E-05	0.24082148	0.60561617
80	2.60E-08	1.72E-11	0.13906039	0.95544982	5.17E-26	1.18E-13	3.70E-05	0.24139366	0.61197981
80.1	2.39E-08	1.74E-11	0.14464921	0.9666687	4.65E-26	1.29E-13	3.80E-05	0.24569267	0.61669971
80.2	2.24E-08	1.56E-11	0.14698567	0.9744534	4.02E-26	1.34E-13	3.70E-05	0.24848536	0.63502134
80.3	2.19E-08	1.55E-11	0.15030481	0.97687679	3.51E-26	1.37E-13	3.60E-05	0.24442112	0.63993794
80.4	2.26E-08	1.55E-11	0.15075494	0.97128324	3.60E-26	1.43E-13	3.69E-05	0.24820486	0.64413355
80.5	2.06E-08	1.46E-11	0.15714747	0.97289779	2.88E-26	1.43E-13	3.31E-05	0.24873598	0.64622017
80.6	1.90E-08	1.51E-11	0.16031895	0.97028964	2.14E-26	1.54E-13	3.12E-05	0.25031523	0.64025258
80.7	1.85E-08	1.31E-11	0.16497525	0.97892653	2.01E-26	1.66E-13	3.07E-05	0.25343407	0.62967147
80.8	1.85E-08	1.26E-11	0.16699683	0.98176103	1.86E-26	1.63E-13	3.02E-05	0.25591422	0.63131493
80.9	1.74E-08	1.26E-11	0.17197265	0.98518447	1.99E-26	1.80E-13	2.96E-05	0.2644696	0.63582699
81	1.76E-08	1.20E-11	0.17945584	0.98686382	1.74E-26	1.99E-13	2.69E-05	0.26757228	0.63904591
81.1	1.56E-08	1.11E-11	0.18655811	0.98121135	1.52E-26	2.21E-13	2.66E-05	0.28027875	0.63420712
81.2	1.36E-08	1.06E-11	0.19236645	0.97097831	1.23E-26	2.56E-13	2.50E-05	0.29403991	0.6339493
81.3	1.27E-08	1.03E-11	0.19593564	0.96407581	1.16E-26	2.44E-13	2.38E-05	0.29957556	0.63049153
81.4	1.15E-08	1.03E-11	0.20140346	0.96212632	9.77E-27	2.51E-13	2.40E-05	0.30534106	0.62680847
81.5	1.05E-08	9.30E-12	0.20705953	0.95606485	8.56E-27	2.74E-13	2.27E-05	0.31323573	0.62946556
81.6	8.86E-09	9.58E-12	0.20623388	0.94663253	6.60E-27	2.94E-13	2.44E-05	0.31923377	0.63506651
81.7	8.30E-09	8.22E-12	0.21337175	0.94389491	4.85E-27	2.85E-13	2.20E-05	0.31878035	0.63456501
81.8	7.08E-09	8.23E-12	0.22052663	0.94428654	3.64E-27	3.17E-13	2.05E-05	0.31306589	0.64150301
81.9	6.80E-09	7.43E-12	0.22099865	0.94463847	3.14E-27	3.11E-13	2.06E-05	0.31622594	0.64415664
82	6.22E-09	7.17E-12	0.22176837	0.94061667	2.82E-27	3.24E-13	2.00E-05	0.31402216	0.65223891
82.1	6.42E-09	7.55E-12	0.22585268	0.94591006	2.76E-27	3.36E-13	1.98E-05	0.31546771	0.6523414
82.2	6.47E-09	7.30E-12	0.22667082	0.94309795	2.79E-27	3.53E-13	2.01E-05	0.32181439	0.65480105
82.3	6.34E-09	6.67E-12	0.23376779	0.94178673	2.72E-27	3.56E-13	1.95E-05	0.3259715	0.65447759
82.4	6.17E-09	6.42E-12	0.2390109	0.93611336	2.53E-27	4.02E-13	1.98E-05	0.33299429	0.66370703
82.5	5.99E-09	6.46E-12	0.24894825	0.92897484	2.30E-27	4.05E-13	1.87E-05	0.33834472	0.65153811
82.6	5.49E-09	6.21E-12	0.26521989	0.91631564	1.69E-27	4.70E-13	1.78E-05	0.33886427	0.65803863
82.7	5.41E-09	6.06E-12	0.2654471	0.91918366	1.19E-27	4.61E-13	1.68E-05	0.3474034	0.64688954
82.8	5.26E-09	5.89E-12	0.27220441	0.90813285	1.11E-27	5.15E-13	1.67E-05	0.35261022	0.65287799
82.9	4.77E-09	5.79E-12	0.27209543	0.89654967	9.17E-28	5.02E-13	1.54E-05	0.3523522	0.64830474
83	4.15E-09	4.96E-12	0.28189859	0.90336411	6.61E-28	5.20E-13	1.49E-05	0.35342473	0.65290473
83.1	4.16E-09	4.45E-12	0.28685386	0.89713197	5.49E-28	5.32E-13	1.42E-05	0.36135992	0.64543043
83.2	4.11E-09	4.14E-12	0.29878603	0.89576063	4.77E-28	5.54E-13	1.28E-05	0.36662704	0.635756
83.3	3.98E-09	4.31E-12	0.29825432	0.90269957	5.04E-28	6.54E-13	1.17E-05	0.3678954	0.63882838
83.4	3.80E-09	3.68E-12	0.30183729	0.90304927	4.25E-28	6.76E-13	1.10E-05	0.37155279	0.63903074
83.5	3.77E-09	3.83E-12	0.30207031	0.89303058	4.37E-28	6.81E-13	1.07E-05	0.37725708	0.63275993
83.6	3.83E-09	3.96E-12	0.30404939	0.88361112	4.13E-28	7.40E-13	1.10E-05	0.38221478	0.63589396
83.7	3.64E-09	3.82E-12	0.30331291	0.88320136	3.82E-28	7.52E-13	1.08E-05	0.38333091	0.6346677
83.8	3.18E-09	3.58E-12	0.30504628	0.87682807	2.84E-28	8.11E-13	1.00E-05	0.38816573	0.63241723
83.9	3.06E-09	3.59E-12	0.31691893	0.87443985	2.77E-28	9.50E-13	9.37E-06	0.40217988	0.62981909
84	3.18E-09	3.59E-12	0.32505227	0.87792138	2.92E-28	1.11E-12	8.74E-06	0.41304194	0.62692796
84.1	2.62E-09	3.52E-12	0.32657425	0.89215528	2.49E-28	1.30E-12	8.47E-06	0.41726328	0.63405718
84.2	2.41E-09	3.64E-12	0.33934665	0.88561714	2.30E-28	1.62E-12	7.97E-06	0.42407674	0.62241414
84.3	2.52E-09	3.88E-12	0.35576183	0.87989657	2.20E-28	2.04E-12	7.89E-06	0.44397429	0.61791566
84.4	2.63E-09	3.57E-12	0.36227053	0.88848918	1.94E-28	2.23E-12	7.78E-06	0.458514	0.62729199
84.5	2.51E-09	3.42E-12	0.36339511	0.89245273	1.78E-28	2.22E-12	7.56E-06	0.46179459	0.62421916
84.6	2.53E-09	3.37E-12	0.36901685	0.90738918	1.74E-28	2.45E-12	7.23E-06	0.47062473	0.63308451
84.7	2.47E-09	2.95E-12	0.37446745	0.91034081	1.61E-28	2.42E-12	7.18E-06	0.48390688	0.63188998
84.8	2.30E-09	2.81E-12	0.37971069	0.91482799	1.35E-28	2.61E-12	6.50E-06	0.48955356	0.62478224
84.9	2.39E-09	2.80E-12	0.37994717	0.90031261	1.10E-28	3.03E-12	5.94E-06	0.49747232	0.61189944
85	2.22E-09	2.73E-12	0.38389803	0.89225355	9.73E-29	3.11E-12	5.55E-06	0.50673538	0.6082267
85.1	2.13E-09	3.09E-12	0.38861508	0.89505584	8.86E-29	3.38E-12	5.68E-06	0.49980502	0.61270908
85.2	2.27E-09	2.92E-12	0.38472472	0.89002953	8.32E-29	3.88E-12	5.36E-06	0.50690791	0.6181945
85.3	2.23E-09	2.94E-12	0.38987806	0.88742218	8.27E-29	3.80E-12	5.41E-06	0.51206965	0.61198385
85.4	2.28E-09	3.01E-12	0.39954829	0.90119287	8.03E-29	4.39E-12	5.34E-06	0.52770603	0.61866772
85.5	2.35E-09	3.15E-12	0.40455403	0.89333524	6.29E-29	4.79E-12	5.27E-06	0.5337507	0.6186379
85.6	2.13E-09	2.37E-12	0.41299766	0.88138196	4.18E-29	5.36E-12	4.92E-06	0.54588277	0.61697723
85.7	1.91E-09	2.28E-12	0.41316516	0.87827322	3.82E-29	6.35E-12	4.62E-06	0.55350488	0.61544193
85.8	1.78E-09	2.03E-12	0.4118822	0.86698293	3.19E-29	6.35E-12	4.49E-06	0.55788496	0.61403328
85.9	1.98E-09	2.02E-12	0.41978622	0.86612449	3.15E-29	7.46E-12	4.05E-06	0.56600706	0.60974109
86	2.04E-09	2.00E-12	0.41937305	0.86586959	3.31E-29	7.92E-12	4.00E-06	0.57124111	0.61062587
86.1	2.07E-09	2.19E-12	0.42444537	0.86346572	3.89E-29	9.49E-12	4.18E-06	0.58693282	0.61300628
86.2	2.20E-09	2.27E-12	0.43327106	0.85007613	3.89E-29	1.14E-11	4.23E-06	0.60296686	0.61545097
86.3	2.17E-09	2.22E-12	0.43915145	0.84255424	3.54E-29	1.17E-11	4.24E-06	0.60017033	0.61304226
86.4	2.27E-09	2.32E-12	0.44317547	0.84521937	3.83E-29	1.28E-11	4.16E-06	0.60591508	0.62258049
86.5	2.36E-09	2.58E-12	0.45886159	0.83516772	3.90E-29	1.47E-11	3.83E-06	0.6024937	0.62026383
86.6	2.42E-09	2.54E-12	0.46644216	0.83563574	3.53E-29	1.65E-11	3.57E-06	0.60473708	0.6233475
86.7	2.33E-09	2.43E-12	0.47255736	0.83298305	3.14E-29	1.69E-11	3.39E-06	0.60885496	0.62492491
86.8	2.13E-09	2.41E-12	0.48470719	0.82440889	2.40E-29	1.87E-11	3.56E-06	0.60847874	0.62361392
86.9	2.36E-09	2.46E-12	0.49151918	0.82628675	2.52E-29	2.27E-11	3.72E-06	0.61339332	0.63082397
87	2.29E-09	2.24E-12	0.48667561	0.82470992	2.48E-29	2.42E-11	3.77E-06	0.61435845	0.63258713
87.1	2.45E-09	2.08E-12	0.48533899	0.82192571	2.26E-29	2.71E-11	3.78E-06	0.62232698	0.63317221
87.2	2.70E-09	2.09E-12	0.49812937	0.81164288	2.17E-29	3.17E-11	3.85E-06	0.62896594	0.63294555
87.3	2.61E-09	2.14E-12	0.50027498	0.81588766	2.08E-29	3.51E-11	3.66E-06	0.63050092	0.63817343
87.4	2.51E-09	2.24E-12	0.49791546	0.81361676	1.84E-29	3.88E-11	3.57E-06	0.62770988	0.63866957
87.5	2.31E-09	2.06E-12	0.50324245	0.80982471	1.53E-29	4.40E-11	3.45E-06	0.62257505	0.64102871
87.6	2.18E-09	1.87E-12	0.50768703	0.8132789	1.28E-29	4.25E-11	3.41E-06	0.62049941	0.63405936
87.7	2.19E-09	1.81E-12	0.50836952	0.80764257	1.13E-29	4.00E-11	3.57E-06	0.61965907	0.63601346
87.8	2.07E-09	1.72E-12	0.50621595	0.79912002	9.60E-30	4.20E-11	3.30E-06	0.61472417	0.63291057
87.9	2.06E-09	1.75E-12	0.50302483	0.7984755	8.77E-30	4.23E-11	3.21E-06	0.61258251	0.63373486
88	1.79E-09	1.64E-12	0.50170235	0.80369298	6.62E-30	4.46E-11	3.11E-06	0.62330524	0.63101999
88.1	1.89E-09	1.51E-12	0.50903102	0.80516102	5.91E-30	4.64E-11	2.92E-06	0.62613326	0.63357484
88.2	1.90E-09	1.31E-12	0.51447029	0.79746271	5.89E-30	5.12E-11	3.00E-06	0.6413192	0.64743848
88.3	1.65E-09	1.31E-12	0.52115642	0.80106697	5.21E-30	5.57E-11	3.06E-06	0.64559146	0.64499438
88.4	1.47E-09	1.27E-12	0.52433261	0.80213335	4.24E-30	5.69E-11	2.98E-06	0.64335421	0.64482295
88.5	1.42E-09	1.32E-12	0.52540611	0.80439538	3.39E-30	6.31E-11	2.86E-06	0.63595706	0.6372966
88.6	1.55E-09	1.21E-12	0.53613971	0.80533686	2.64E-30	6.96E-11	2.53E-06	0.62971981	0.63781882
88.7	1.55E-09	1.15E-12	0.54224961	0.80214427	2.11E-30	6.75E-11	2.64E-06	0.64780732	0.64730472
88.8	1.66E-09	1.09E-12	0.55030773	0.79728581	1.97E-30	7.10E-11	2.55E-06	0.64850821	0.64908844
88.9	1.70E-09	1.09E-12	0.5553848	0.7953794	2.02E-30	7.22E-11	2.57E-06	0.64815311	0.64674606
89	1.71E-09	1.10E-12	0.56100199	0.79297861	1.81E-30	8.92E-11	2.63E-06	0.65021948	0.64661407
89.1	1.51E-09	9.88E-13	0.55928816	0.80622321	1.34E-30	9.95E-11	2.41E-06	0.65818586	0.65470426
89.2	1.62E-09	1.09E-12	0.55706489	0.79949217	1.36E-30	1.09E-10	2.38E-06	0.66925117	0.65722565
89.3	1.58E-09	1.03E-12	0.57559518	0.78579392	1.11E-30	1.07E-10	2.29E-06	0.69076259	0.65964237
89.4	1.61E-09	9.84E-13	0.58153481	0.78621104	1.09E-30	1.10E-10	2.15E-06	0.7108571	0.65422788
89.5	1.53E-09	9.73E-13	0.58624441	0.78275605	1.01E-30	1.15E-10	2.08E-06	0.71385924	0.65107668
89.6	1.51E-09	1.03E-12	0.59120051	0.78143234	9.82E-31	1.29E-10	2.04E-06	0.7089182	0.64981578
89.7	1.67E-09	8.94E-13	0.61344613	0.76777757	8.61E-31	1.26E-10	1.96E-06	0.71478648	0.64861468
89.8	1.59E-09	8.76E-13	0.62655762	0.76777793	7.79E-31	1.37E-10	1.80E-06	0.71913112	0.65498774
89.9	1.52E-09	8.50E-13	0.61967707	0.76943347	7.57E-31	1.41E-10	1.79E-06	0.71579078	0.66128452
135	2.92E-11	2.03E-12	0.95291072	0.89135278	4.92E-35	0.00039957	5.46E-09	0.1095618	0.48945145
135.1	2.77E-11	2.02E-12	0.95803845	0.89488395	4.85E-35	0.0004063	5.16E-09	0.10960874	0.48907519
135.2	2.90E-11	2.09E-12	0.95438808	0.89852516	5.04E-35	0.00041656	5.30E-09	0.10912299	0.4895294

90	1.47E-09	8.61E-13	0.62541533	0.77299814	7.02E-31	1.53E-10	1.77E-06	0.71457292	0.6579872
90.1	1.46E-09	8.87E-13	0.62914266	0.77417048	6.99E-31	1.60E-10	1.71E-06	0.72178275	0.65883253
90.2	1.46E-09	8.54E-13	0.64077273	0.78291653	6.54E-31	1.75E-10	1.70E-06	0.72615613	0.65816246
90.3	1.43E-09	8.94E-13	0.64568198	0.78854862	7.34E-31	1.95E-10	1.78E-06	0.73211396	0.65573713
90.4	1.43E-09	8.85E-13	0.64175012	0.79163599	6.86E-31	2.03E-10	1.73E-06	0.73028606	0.65872144
90.5	1.40E-09	9.06E-13	0.64205338	0.79177353	6.43E-31	2.09E-10	1.71E-06	0.73672641	0.65708926
90.6	1.42E-09	9.10E-13	0.63880997	0.78987998	5.78E-31	2.15E-10	1.65E-06	0.74050267	0.65397914
90.7	1.48E-09	9.39E-13	0.64872983	0.78583451	5.77E-31	2.51E-10	1.68E-06	0.73375565	0.65773894
90.8	1.44E-09	9.66E-13	0.66151235	0.79171459	5.88E-31	3.03E-10	1.65E-06	0.73779074	0.66073169
90.9	1.50E-09	9.38E-13	0.66278686	0.79203654	5.26E-31	3.27E-10	1.59E-06	0.73986892	0.65360436
91	1.36E-09	7.85E-13	0.65800238	0.79028941	4.26E-31	3.68E-10	1.57E-06	0.73990836	0.64515556
91.1	1.42E-09	8.26E-13	0.65837259	0.78513325	4.80E-31	3.95E-10	1.65E-06	0.74971422	0.65287458
91.2	1.36E-09	8.16E-13	0.65758349	0.7759702	3.88E-31	4.19E-10	1.49E-06	0.7531997	0.65394221
91.3	1.21E-09	7.71E-13	0.65429661	0.7743298	3.19E-31	4.24E-10	1.46E-06	0.75574081	0.65274786
91.4	1.25E-09	7.93E-13	0.66164274	0.77592417	3.18E-31	4.34E-10	1.44E-06	0.75789638	0.65659515
91.5	1.22E-09	7.96E-13	0.66464991	0.78638425	3.00E-31	4.71E-10	1.50E-06	0.75380372	0.65805376
91.6	1.08E-09	8.01E-13	0.68531961	0.77718048	2.96E-31	5.23E-10	1.38E-06	0.75557305	0.6473899
91.7	1.06E-09	8.43E-13	0.68820829	0.77158982	3.05E-31	5.50E-10	1.44E-06	0.75684694	0.6488739
91.8	1.04E-09	8.02E-13	0.69353867	0.77013484	3.01E-31	5.98E-10	1.34E-06	0.75542762	0.65160301
91.9	1.08E-09	8.04E-13	0.68924609	0.76289052	3.03E-31	6.64E-10	1.25E-06	0.75750348	0.63897247
92	1.16E-09	7.81E-13	0.69828414	0.74284235	3.08E-31	7.09E-10	1.31E-06	0.7634546	0.63567948
92.1	1.14E-09	8.01E-13	0.70204584	0.7456104	2.89E-31	7.33E-10	1.30E-06	0.76648674	0.63514417
92.2	1.04E-09	7.06E-13	0.70398111	0.73263419	2.22E-31	7.15E-10	1.22E-06	0.77567945	0.62416209
92.3	1.04E-09	7.26E-13	0.70278194	0.7383361	2.04E-31	7.95E-10	1.18E-06	0.78945562	0.63039592
92.4	1.02E-09	7.61E-13	0.71078048	0.73936062	1.87E-31	7.79E-10	1.11E-06	0.78549926	0.62189217
92.5	1.05E-09	8.20E-13	0.71492703	0.73992401	2.11E-31	8.83E-10	1.09E-06	0.79380119	0.62950571
92.6	1.02E-09	6.71E-13	0.70544586	0.72555122	1.67E-31	8.95E-10	1.11E-06	0.79947878	0.62820731
92.7	9.94E-10	6.78E-13	0.70863706	0.72506411	1.66E-31	9.38E-10	1.12E-06	0.80198307	0.628822
92.8	9.87E-10	6.91E-13	0.71460677	0.72397366	1.64E-31	9.70E-10	1.13E-06	0.79067066	0.62954723
92.9	9.67E-10	6.78E-13	0.72512007	0.71609153	1.82E-31	1.08E-09	1.12E-06	0.79713281	0.62895835
93	9.52E-10	7.06E-13	0.73103074	0.7118148	1.83E-31	1.13E-09	1.06E-06	0.80175546	0.62728311
93.1	9.13E-10	7.49E-13	0.7273764	0.71087503	1.73E-31	1.16E-09	1.08E-06	0.80455546	0.62873245
93.2	9.13E-10	7.73E-13	0.73298564	0.70628721	1.62E-31	1.31E-09	1.01E-06	0.80338649	0.63494336
93.3	9.22E-10	7.02E-13	0.73323854	0.69840277	1.54E-31	1.40E-09	1.03E-06	0.80632467	0.63604115
93.4	9.07E-10	6.53E-13	0.74334251	0.69455049	1.34E-31	1.46E-09	9.46E-07	0.81243896	0.63651233
93.5	8.60E-10	6.43E-13	0.75079946	0.69213264	1.34E-31	1.43E-09	9.37E-07	0.81673995	0.62851444
93.6	8.16E-10	6.82E-13	0.75230274	0.68599366	1.20E-31	1.52E-09	8.57E-07	0.83897705	0.62861972
93.7	8.30E-10	6.78E-13	0.7539729	0.68706804	1.22E-31	1.64E-09	8.53E-07	0.84894012	0.63903055
93.8	7.43E-10	6.47E-13	0.76991459	0.69031374	9.92E-32	1.77E-09	8.19E-07	0.85458042	0.64089841
93.9	7.65E-10	6.47E-13	0.7727325	0.68940103	9.68E-32	2.06E-09	8.53E-07	0.85798023	0.63675827
94	7.14E-10	6.73E-13	0.77839814	0.68494344	9.67E-32	2.10E-09	8.24E-07	0.87130795	0.64048801
94.1	6.72E-10	6.75E-13	0.78823549	0.67926215	8.84E-32	2.31E-09	8.15E-07	0.87664645	0.63638169
94.2	6.17E-10	6.75E-13	0.776325	0.67244291	7.39E-32	2.22E-09	8.27E-07	0.87996823	0.64201518
94.3	5.50E-10	6.92E-13	0.77655989	0.66370991	6.51E-32	2.87E-09	8.02E-07	0.87388871	0.6526848
94.4	5.72E-10	6.90E-13	0.79479771	0.66368657	7.48E-32	3.16E-09	7.68E-07	0.87871327	0.6577212
94.5	4.95E-10	7.45E-13	0.80877663	0.66459548	7.58E-32	3.31E-09	7.64E-07	0.87999262	0.65190037
94.6	4.55E-10	7.02E-13	0.8112057	0.66735714	6.70E-32	3.35E-09	7.24E-07	0.88204485	0.64817802
94.7	4.52E-10	6.84E-13	0.81628311	0.66151208	7.03E-32	4.36E-09	7.25E-07	0.89710547	0.65292467
94.8	4.24E-10	6.92E-13	0.81705875	0.66331593	5.98E-32	4.68E-09	7.30E-07	0.90859836	0.65335538
94.9	4.20E-10	6.40E-13	0.81704672	0.65328562	5.56E-32	4.49E-09	6.87E-07	0.91970944	0.65027953
95	4.07E-10	6.11E-13	0.82760495	0.64608082	4.77E-32	4.48E-09	6.85E-07	0.90997899	0.64484934
95.1	3.69E-10	6.85E-13	0.83842845	0.64625607	4.32E-32	4.92E-09	6.54E-07	0.91464756	0.6392746
95.2	3.83E-10	6.87E-13	0.83889333	0.64504743	4.20E-32	5.20E-09	6.46E-07	0.91696297	0.6398001
95.3	3.52E-10	7.61E-13	0.83983496	0.64082976	4.35E-32	5.45E-09	6.49E-07	0.9277903	0.64169534
95.4	3.58E-10	7.70E-13	0.84057588	0.64739615	4.65E-32	5.42E-09	6.40E-07	0.93739319	0.64392092
95.5	3.59E-10	8.60E-13	0.84356507	0.63786245	4.13E-32	5.29E-09	6.12E-07	0.93225546	0.63492438
95.6	3.37E-10	7.95E-13	0.84969483	0.63965875	3.86E-32	5.27E-09	5.82E-07	0.94403554	0.64116989
95.7	3.12E-10	6.97E-13	0.86256904	0.63033462	3.59E-32	5.60E-09	5.78E-07	0.94557903	0.64341906
95.8	2.79E-10	5.71E-13	0.86440005	0.62513132	2.65E-32	5.75E-09	5.38E-07	0.96785662	0.64289493
95.9	2.62E-10	5.54E-13	0.85797151	0.62069475	2.40E-32	5.76E-09	4.87E-07	0.97425556	0.64860793
96	2.53E-10	5.59E-13	0.86091541	0.61589415	2.18E-32	5.74E-09	4.79E-07	0.97484934	0.64653428
96.1	2.35E-10	5.04E-13	0.87036139	0.62161411	1.87E-32	6.02E-09	4.60E-07	0.97693436	0.63701238
96.2	2.12E-10	4.72E-13	0.87533245	0.62330369	1.60E-32	6.01E-09	4.28E-07	0.98747745	0.62700703
96.3	1.97E-10	4.35E-13	0.87574147	0.62993434	1.60E-32	6.21E-09	4.11E-07	0.99757992	0.62829908
96.4	1.75E-10	3.73E-13	0.87800676	0.62484405	1.23E-32	5.93E-09	3.88E-07	0.99996357	0.61714989
96.5	1.79E-10	3.70E-13	0.88178491	0.6218775	1.20E-32	6.00E-09	3.94E-07	0.99917706	0.61148042
96.6	1.76E-10	3.68E-13	0.88266303	0.61636256	1.11E-32	6.56E-09	3.83E-07	0.99312091	0.6183289
96.7	1.80E-10	4.23E-13	0.88774241	0.61531041	1.08E-32	7.41E-09	3.81E-07	0.98469405	0.6214218
96.8	1.86E-10	4.15E-13	0.89636894	0.61584782	1.09E-32	7.80E-09	3.76E-07	0.97751489	0.62182604
96.9	1.87E-10	4.09E-13	0.90557225	0.61032656	1.03E-32	7.43E-09	3.49E-07	0.96069767	0.61367801
97	1.69E-10	4.02E-13	0.92628375	0.60426232	9.98E-33	8.43E-09	3.69E-07	0.95044678	0.61112009
97.1	1.51E-10	3.89E-13	0.93270066	0.59502767	7.03E-33	9.10E-09	3.32E-07	0.94405326	0.60248315
97.2	1.49E-10	4.20E-13	0.94033969	0.59012179	7.09E-33	9.20E-09	3.41E-07	0.93530424	0.59039622
97.3	1.46E-10	3.78E-13	0.94911492	0.58846824	6.33E-33	1.05E-08	3.23E-07	0.93955194	0.58908647
97.4	1.46E-10	4.16E-13	0.94755735	0.58922355	6.05E-33	1.09E-08	3.15E-07	0.94328364	0.58135473
97.5	1.56E-10	3.70E-13	0.9384924	0.58517935	6.28E-33	1.15E-08	2.93E-07	0.92907537	0.58809819
97.6	1.49E-10	3.77E-13	0.93927914	0.58105746	6.38E-33	1.21E-08	2.93E-07	0.92417282	0.58770748
97.7	1.42E-10	3.53E-13	0.94544988	0.57070028	6.28E-33	1.33E-08	3.03E-07	0.91581684	0.58679071
97.8	1.40E-10	3.59E-13	0.95722742	0.56397126	5.60E-33	1.45E-08	2.85E-07	0.91660844	0.57655913
97.9	1.39E-10	3.92E-13	0.95610513	0.56348538	6.06E-33	1.56E-08	2.70E-07	0.9098592	0.57010089
98	1.31E-10	4.07E-13	0.95940149	0.56730059	5.32E-33	1.53E-08	2.61E-07	0.90822405	0.56567526
98.1	1.26E-10	4.15E-13	0.96078548	0.5625857	5.42E-33	1.64E-08	2.61E-07	0.90158614	0.56067794
98.2	1.26E-10	4.06E-13	0.96652053	0.55771463	4.98E-33	1.76E-08	2.57E-07	0.90042269	0.55953286
98.3	1.13E-10	3.61E-13	0.97027892	0.55396846	4.69E-33	1.69E-08	2.31E-07	0.89408501	0.54495182
98.4	1.10E-10	3.52E-13	0.9676339	0.55709503	4.67E-33	1.75E-08	2.23E-07	0.89201098	0.53747901
98.5	1.00E-10	3.35E-13	0.97218216	0.55402851	3.74E-33	1.83E-08	2.27E-07	0.89514093	0.53504346
98.6	9.39E-11	3.46E-13	0.98127089	0.562084	3.27E-33	2.20E-08	2.11E-07	0.89499959	0.53978376
98.7	8.17E-11	3.83E-13	0.98472866	0.55395945	2.76E-33	2.38E-08	2.04E-07	0.88385939	0.52919345
98.8	7.76E-11	3.67E-13	0.97973688	0.55503062	2.14E-33	2.55E-08	1.86E-07	0.87899573	0.52364246
98.9	7.12E-11	3.34E-13	0.97659962	0.56271276	2.18E-33	3.00E-08	1.65E-07	0.86601068	0.52960844
99	6.41E-11	3.08E-13	0.98842198	0.56641032	1.97E-33	3.02E-08	1.58E-07	0.85934792	0.51580972
99.1	5.83E-11	3.07E-13	0.99784587	0.56790488	2.02E-33	3.55E-08	1.66E-07	0.84615329	0.52282975
99.2	4.99E-11	3.12E-13	0.99558875	0.56108333	1.52E-33	3.92E-08	1.74E-07	0.84277614	0.5201869
99.3	5.24E-11	3.18E-13	0.9952696	0.5659903	1.59E-33	4.44E-08	1.75E-07	0.83169123	0.525008
99.4	5.02E-11	3.01E-13	0.98840062	0.56540486	1.55E-33	4.73E-08	1.76E-07	0.81884976	0.52292722
99.5	4.83E-11	2.93E-13	0.99632189	0.561498	1.39E-33	5.40E-08	1.68E-07	0.81253105	0.51922518
99.6	4.77E-11	2.67E-13	0.99887474	0.56220562	1.36E-33	5.57E-08	1.61E-07	0.81444942	0.51995269
99.7	4.37E-11	2.59E-13	0.99937989	0.5629655	1.27E-33	5.75E-08	1.62E-07	0.80639571	0.51868317
99.8	4.24E-11	2.33E-13	0.99248575	0.56480629	1.05E-33	5.62E-08	1.59E-07	0.79775533	0.51365076
99.9	4.60E-11	2.21E-13	0.98854083	0.56278239	1.05E-33	5.75E-08	1.57E-07	0.78388642	0.51098758
100	3.97E-11	2.19E-13	0.98244315	0.57578891	7.67E-34	6.61E-08	1.42E-07	0.76265074	0.50817571
100.1	3.57E-11	2.27E-13	0.98495194	0.57657006	7.04E-34	6.87E-08	1.37E-07	0.75513899	0.50065362
100.2	3.84E-11	2.27E-13	0.98509943	0.57232095	7.42E-34	7.47E-08	1.33E-07	0.7523964	0.50367486
100.3	3.50E-11	2.22E-13	0.98266649	0.57325545	7.37E-34	8.26E-08	1.33E-07	0.74842902	0.50736581
100.4	3.66E-11	2.42E-13	0.97404082	0.57837238	7.95E-34	9.62E-08	1.23E-07	0.74641824	0.49515818
100.5	3.39E-11	2.61E-13	0.96719776	0.58954192	7.12E-34	1.09E-07	1.22E-07	0.72397095	0.49528237
100.6	2.85E-11	2.36E-13	0.97502499	0.60255064	6.54E-34	1.30E-07	1.17E-07	0.71551157	0.50211961
100.7	2.80E-11	2.41E-13	0.97554429	0.60517893	5.54E-34	1.46E-07	1.18E-07	0.72058361	0.50614686
100.8	2.58E-11	2.31E-13	0.98065956	0.60191989	5.03E-34	1.53E-07	1.14E-07	0.71525564	0.50711835
100.9	2.43E-11	2.43E-13	0.96454219	0.6031879	5.94E-34	1.88E-07	1.29E-07	0.69144674	0.51190533
101	2.23E-11	2.35E-13	0.964834	0.60142847	5.60E-34	1.98E-07	1.20E-07	0.68456898	0.5067855
101.1	2.21E-11	2.27E-13	0.96612435	0.60139028	5.92E-34	2.13E-07	1.15E-07	0.67670798	0.50827407
101.2	2.33E-11	2.49E-13	0.9673328	0.59791555	6.26E-34	2.31E-07	1.15E-07	0.66978079	0.51772809
101.3	2.30E-11	2.48E-13	0.93811109	0.58646525	6.18E-34	2.62E-07	1.22E-07	0.66083627	0.51217278
101.4	2.15E-11	2.29E-13	0.95127471	0.59066584	6.03E-34	2.84E-07	1.14E-07	0.65370834	0.5162434
101.5	2.05E-11	2.58E-13	0.94706727	0.58441816	5.78E-34	3.16E-07	1.17E-07	0.63786734	0.51483181
101.6	2.00E-11	2.54E-13	0.9530228	0.5856996	5.58E-34	3.36E-07	1.13E-07	0.63898372	0.51741725
101.7	1.84E-11	2.38E-13	0.95191596	0.59110895	5.12E-34	3.54E-07	1.09E-07	0.63110494	0.51772
101.8	1.56E-11	2.47E-13	0.95350458	0.59612501	3.93E-34	4.01E-07	1.07E-07	0.62615522	0.51231667
101.9	1.64E-11	2.28E-13	0.944541	0.60301973	3.83E-34	4.22E-07	9.88E-08	0.60894317	0.5019314
102	1.64E-11	2.32E-13	0.94307006	0.60158632	3.56E-34	4.35E-07	9.90E-08	0.60074342	0.50139154
102.1	1.66E-11	2.56E-13	0.93987929	0.61549017	3.63E-34	5.09E-07	8.87E-08	0.5909574	0.50293372
102.2	1.34E-11	2.75E-13	0.94998474	0.62543003	3.54E-34	5.95E-07	8.47E-08	0.57814329	0.49197417
102.3	1.16E-11	2.85E-13	0.95753413	0.61958728	3.34E-34	6.41E-07	7.58E-08	0.56232111	0.48007429
102.4	1.08E-11	2.89E-13	0.96094743	0.61024855	3.29E-34	6.62E-07	7.89E-08	0.55087173	0.48336222
102.5	1.02E-11	2.76E-13	0.957616	0.62538966	3.55E-34	7.39E-07	7.80E-08	0.53534463	0.49087614
102.6	1.12E-11	2.58E-13	0.95683434	0.62857663	3.44E-34	7.94E-07	7.60E-08	0.52489832	0.49208488
102.7	1.05E-11	2.49E-13	0.95222987	0.62855838	3.74E-34	8.83E-07	7.54E-08	0.51917869	0.48430083
102.8	1.12E-11	2.70E-13	0.9547071	0.63048065	4.38E-34	9.12E-07	7.45E-08	0.50027054	0.48431167
102.9	9.77E-12	2.63E-13	0.94915543	0.63796058	3.50E-34	1.03E-06	7.26E-08	0.49038109	0.4809997
103	8.97E-12	2.55E-13	0.93661092	0.63288128	3.43E-34	1.03E-06	7.35E-08	0.48715792	0.47785397
103.1	8.56E-12	2.52E-13	0.93012085	0.63991956	3.66E-34	1.12E-06	7.25E-08	0.47912713	0.48553202
103.2	8.34E-12	2.67E-13	0.92295536	0.64633348	3.75E-34	1.14E-06	7.18E-08	0.47241054	0.48011278
103.3	8.28E-12	2.36E-13	0.90751098	0.64714261	3.77E-34	1.17E-06	7.20E-08	0.47382991	0.49100639
103.4	8.51E-12	2.23E-13	0.91113548	0.65732605	3.86E-34	1.25E-06	7.27E-08	0.46600463	0.49524014
103.5	8.03E-12	2.21E-13	0.91071093	0.65047915	3.56E-34	1.31E-06	7.41E-08	0.45564855	0.50009676
103.6	7.74E-12	2.13E-13	0.90774462	0.66219576	3.30E-34	1.46E-06	7.11E-08	0.45014879	0.5050193
103.7	7.99E-12	2.13E-13	0.91049557	0.67410664	3.82E-34	1.49E-06	6.97E-08	0.44384501	0.51019459
103.8	8.25E-12	2.33E-13	0.90584139	0.6747609	4.23E-34	1.63E-06	7.43E-08	0.43589637	0.51575691
103.9	7.60E-12	2.61E-13	0.90491656	0.69153343	5.02E-34	1.88E-06	7.34E-08	0.42813482	0.52211294
104	7.33E-12	2.66E-13	0.91064592	0.69375727	4.83E-34	1.89E-06	7.21E-08	0.43411361	0.51083428
104.1	6.93E-12	2.57E-13	0.91365193	0.69693453	4.43E-34	2.02E-06	6.92E-08	0.43065407	0.50656991
104.2	6.99E-12	2.51E-13	0.91621778	0.70333502	4.28E-34	2.04E-06	6.88E-08	0.42990755	0.50680463
104.3	7.19E-12	2.75E-13	0.9073474	0.7043493	4.23E-34	2.34E-06	7.79E-08	0.42158049	0.50848009
104.4	6.29E-12	2.93E-13	0.91416493	0.72515048	4.52E-34	2.71E-06	7.49E-08	0.40216158	0.5195754
104.5	6.11E-12	3.11E-13	0.91664882	0.73368205	4.75E-34	3.11E-06	7.40E-08	0.40092459	0.52162744
104.6	5.61E-12	3.13E-13	0.91282126	0.7446738	4.98E-34	3.18E-06	7.04E-08	0.39601585	0.52178795
104.7	5.86E-12	3.20E-13	0.9053564	0.74917092	5.10E-34	3.18E-06	6.94E-08	0.39451829	0.51244806
104.8	5.50E-12	3.27E-13	0.90771013	0.76109952	5.29E-34	3.52E-06	6.81E-08	0.38770755	0.50862338
104.9	5.68E-12	3.58E-13	0.91545701	0.7705457	5.68E-34	3.67E-06	7.49E-08	0.3857627	0.50894896
150	5.29E-10	5.30E-12	0.96048702	0.69413024	1.08E-33	0.00045251	1.49E-09	0.0583264	0.448501
150.1	5.29E-10	5.30E-12	0.96048702	0.69413024	1.08E-33	0.00045251	1.49E-09	0.0583264	0.448501
150.2	5.29E-10	5.30E-12	0.96048702	0.69413024	1.08E-33	0.00045251	1.49E-09	0.0583264	0.448501

105	5.42E-12	3.46E-13	0.91550752	0.77236554	5.61E-34	3.79E-06	7.15E-08	0.38145294	0.51204287
105.1	5.66E-12	3.66E-13	0.91469604	0.77059707	5.87E-34	4.08E-06	7.14E-08	0.38677779	0.51354421
105.2	5.75E-12	3.74E-13	0.91860713	0.7670026	6.24E-34	3.84E-06	6.71E-08	0.38457496	0.50344488
105.3	5.75E-12	3.53E-13	0.92190148	0.76756317	6.21E-34	3.77E-06	6.80E-08	0.37775897	0.50567311
105.4	5.61E-12	3.50E-13	0.9191819	0.77452539	5.96E-34	4.03E-06	6.81E-08	0.37901111	0.50077029
105.5	5.59E-12	3.40E-13	0.92842031	0.78469703	6.43E-34	4.11E-06	6.64E-08	0.37740437	0.5061491
105.6	5.44E-12	3.33E-13	0.93393376	0.78485802	6.28E-34	4.27E-06	6.65E-08	0.37675206	0.51280235
105.7	5.89E-12	3.54E-13	0.93675856	0.78822044	6.87E-34	4.36E-06	6.61E-08	0.37414504	0.51597066
105.8	5.91E-12	3.62E-13	0.93859209	0.78938825	7.35E-34	4.57E-06	6.82E-08	0.37292303	0.52144177
105.9	6.25E-12	3.49E-13	0.94356105	0.7937728	7.69E-34	4.45E-06	6.64E-08	0.37384667	0.51828323
106	5.74E-12	3.57E-13	0.9398086	0.79946079	7.66E-34	4.65E-06	6.65E-08	0.37021356	0.52280721
106.1	5.97E-12	3.49E-13	0.9350735	0.80285898	7.43E-34	4.80E-06	6.63E-08	0.36775005	0.52794789
106.2	5.34E-12	3.54E-13	0.93366358	0.813413	7.25E-34	4.90E-06	6.76E-08	0.36781839	0.5322651
106.3	5.38E-12	3.49E-13	0.93509208	0.80696853	6.59E-34	5.03E-06	6.66E-08	0.36514702	0.52490324
106.4	5.41E-12	3.41E-13	0.92901826	0.81179598	6.45E-34	5.37E-06	7.56E-08	0.35858938	0.52303592
106.5	5.38E-12	3.30E-13	0.92508949	0.81246994	6.29E-34	5.48E-06	7.65E-08	0.35785337	0.52388113
106.6	4.91E-12	3.03E-13	0.93120467	0.82642133	5.16E-34	5.87E-06	7.44E-08	0.34212388	0.52666308
106.7	4.54E-12	3.07E-13	0.92923834	0.83792125	4.89E-34	6.37E-06	7.10E-08	0.33336864	0.53077111
106.8	4.62E-12	2.94E-13	0.92620762	0.84647962	5.54E-34	6.48E-06	7.25E-08	0.32815275	0.53024572
106.9	4.73E-12	3.02E-13	0.92637366	0.85366033	5.90E-34	6.31E-06	6.91E-08	0.3295652	0.52734035
107	4.58E-12	2.99E-13	0.92782285	0.86133741	5.97E-34	6.54E-06	6.68E-08	0.32877059	0.52594507
107.1	4.71E-12	3.07E-13	0.93211162	0.86072935	5.94E-34	6.47E-06	6.60E-08	0.32555243	0.52235784
107.2	4.72E-12	3.09E-13	0.93919821	0.87076552	5.61E-34	6.68E-06	6.21E-08	0.32599672	0.51374211
107.3	4.81E-12	3.17E-13	0.93294832	0.86320128	5.57E-34	7.29E-06	6.31E-08	0.32930362	0.51614238
107.4	4.94E-12	3.34E-13	0.93607469	0.86336656	5.63E-34	7.24E-06	6.16E-08	0.32789735	0.5122924
107.5	5.08E-12	3.37E-13	0.93670525	0.86169785	5.49E-34	7.19E-06	6.21E-08	0.327436	0.51124869
107.6	5.31E-12	3.46E-13	0.93766565	0.87068108	6.00E-34	7.60E-06	6.42E-08	0.32291535	0.51493151
107.7	5.20E-12	3.71E-13	0.9424208	0.86602529	5.76E-34	7.76E-06	6.43E-08	0.32693335	0.51171745
107.8	4.60E-12	3.52E-13	0.93705718	0.87677373	5.56E-34	8.12E-06	6.21E-08	0.32044095	0.50855078
107.9	4.30E-12	3.37E-13	0.93756589	0.87968506	5.44E-34	8.18E-06	6.15E-08	0.31476272	0.5027689
108	4.15E-12	3.60E-13	0.92780535	0.88230125	5.89E-34	8.87E-06	6.32E-08	0.31190894	0.5036635
108.1	4.46E-12	3.56E-13	0.91985449	0.88501673	6.20E-34	9.33E-06	6.02E-08	0.30760532	0.49913613
108.2	4.48E-12	3.50E-13	0.91061146	0.88368581	5.89E-34	9.74E-06	5.70E-08	0.30708369	0.502599
108.3	4.52E-12	3.53E-13	0.90912687	0.88418276	5.82E-34	9.78E-06	5.74E-08	0.30635601	0.50236225
108.4	4.87E-12	3.66E-13	0.90902752	0.89027891	5.61E-34	9.70E-06	5.59E-08	0.30619572	0.50014566
108.5	4.77E-12	3.74E-13	0.91227636	0.88386509	5.42E-34	1.04E-05	5.47E-08	0.29976858	0.50531408
108.6	5.30E-12	3.75E-13	0.91034576	0.89012251	6.12E-34	1.10E-05	5.55E-08	0.29462748	0.50852762
108.7	5.30E-12	3.75E-13	0.91034576	0.89012251	6.12E-34	1.10E-05	5.55E-08	0.29462748	0.50852762
108.8	5.52E-12	3.62E-13	0.91384929	0.89323059	5.88E-34	1.16E-05	5.44E-08	0.28832618	0.51356896
108.9	5.33E-12	3.59E-13	0.91624471	0.89157777	6.25E-34	1.19E-05	5.28E-08	0.287059	0.52034689
109	5.55E-12	3.57E-13	0.91666188	0.89427958	6.17E-34	1.18E-05	5.18E-08	0.28846198	0.52291524
109.1	5.50E-12	3.88E-13	0.91169582	0.89780588	6.29E-34	1.24E-05	5.15E-08	0.28605163	0.52064346
109.2	5.47E-12	3.95E-13	0.91770864	0.89799044	6.22E-34	1.38E-05	5.32E-08	0.28928815	0.51152766
109.3	5.39E-12	3.95E-13	0.90238612	0.90586385	5.99E-34	1.47E-05	5.40E-08	0.28736034	0.50899834
109.4	5.51E-12	3.91E-13	0.89401428	0.90779885	6.05E-34	1.52E-05	5.56E-08	0.28456853	0.50718725
109.5	5.30E-12	3.81E-13	0.89370293	0.9054955	5.65E-34	1.53E-05	5.58E-08	0.28311329	0.50647001
109.6	5.33E-12	3.55E-13	0.88836783	0.90272609	5.36E-34	1.54E-05	5.73E-08	0.28368258	0.50423559
109.7	5.25E-12	3.48E-13	0.88714132	0.90218537	5.21E-34	1.56E-05	5.68E-08	0.28366847	0.50462535
109.8	5.09E-12	3.63E-13	0.8896951	0.90720822	4.98E-34	1.68E-05	5.53E-08	0.28407346	0.51131534
109.9	5.04E-12	3.62E-13	0.88605774	0.90386422	4.86E-34	1.67E-05	5.59E-08	0.28320974	0.51243597
110	4.83E-12	3.61E-13	0.88036946	0.90503348	4.98E-34	1.74E-05	5.63E-08	0.2809563	0.51208237
110.1	4.93E-12	3.20E-13	0.87660704	0.90881612	4.64E-34	1.78E-05	5.77E-08	0.27975972	0.51303046
110.2	4.64E-12	3.12E-13	0.87427779	0.90424	4.10E-34	1.77E-05	5.81E-08	0.28175398	0.51744499
110.3	4.99E-12	3.15E-13	0.86566134	0.90996004	4.32E-34	1.76E-05	5.90E-08	0.27464022	0.51433123
110.4	4.97E-12	3.03E-13	0.86372835	0.90620842	4.09E-34	1.73E-05	5.91E-08	0.27187312	0.51056092
110.5	4.71E-12	3.10E-13	0.86246517	0.90834471	3.77E-34	1.82E-05	5.82E-08	0.27359205	0.51450013
110.6	4.69E-12	3.08E-13	0.8615015	0.91223258	3.94E-34	1.83E-05	5.86E-08	0.27337515	0.51418003
110.7	4.67E-12	3.16E-13	0.85659834	0.91973163	4.15E-34	1.76E-05	5.45E-08	0.26337769	0.50538255
110.8	4.58E-12	3.22E-13	0.857582	0.92903891	4.13E-34	1.82E-05	5.31E-08	0.25805273	0.49991642
110.9	4.88E-12	3.06E-13	0.86119574	0.93359181	4.00E-34	1.87E-05	5.37E-08	0.25444317	0.50385998
111	4.99E-12	3.17E-13	0.86674649	0.93701655	4.12E-34	1.92E-05	5.45E-08	0.254248	0.50004078
111.1	4.55E-12	3.04E-13	0.86660725	0.93479249	3.34E-34	1.93E-05	5.43E-08	0.24914081	0.49936303
111.2	4.70E-12	2.95E-13	0.86093492	0.94021975	3.28E-34	1.97E-05	5.32E-08	0.24125171	0.49579566
111.3	4.21E-12	2.85E-13	0.86905269	0.95119724	3.12E-34	1.98E-05	5.03E-08	0.24479519	0.49541439
111.4	4.51E-12	3.07E-13	0.87412277	0.94778569	3.09E-34	1.92E-05	4.90E-08	0.24670409	0.49133792
111.5	4.33E-12	3.05E-13	0.88214641	0.94703673	2.57E-34	1.90E-05	4.57E-08	0.24611699	0.49098405
111.6	4.64E-12	3.21E-13	0.88281633	0.94430045	2.48E-34	1.86E-05	4.33E-08	0.24470847	0.48284775
111.7	4.65E-12	3.22E-13	0.88299388	0.94711862	2.56E-34	1.84E-05	4.26E-08	0.24426951	0.48436693
111.8	4.71E-12	2.86E-13	0.88225858	0.95772793	2.25E-34	1.86E-05	4.13E-08	0.24515809	0.48702818
111.9	4.73E-12	3.07E-13	0.88003019	0.95940409	2.28E-34	1.85E-05	4.07E-08	0.24547084	0.47740358
112	4.79E-12	3.32E-13	0.87901222	0.96372993	2.51E-34	1.90E-05	4.08E-08	0.24316961	0.47820818
112.1	4.78E-12	3.47E-13	0.88250984	0.97392169	2.87E-34	1.96E-05	4.19E-08	0.24015076	0.47967603
112.2	4.52E-12	3.31E-13	0.87594912	0.9724368	2.70E-34	1.99E-05	3.97E-08	0.23500779	0.48155557
112.3	4.38E-12	3.21E-13	0.87632696	0.9804109	2.63E-34	2.02E-05	3.97E-08	0.23377177	0.48084157
112.4	4.41E-12	3.20E-13	0.8755819	0.98566113	2.61E-34	2.04E-05	4.19E-08	0.23583739	0.48358165
112.5	4.51E-12	3.27E-13	0.87885362	0.98717731	2.37E-34	2.16E-05	4.01E-08	0.23161564	0.489422
112.6	4.55E-12	3.29E-13	0.8760209	0.99065876	2.33E-34	2.20E-05	4.15E-08	0.22912411	0.48759718
112.7	4.58E-12	3.51E-13	0.87831606	0.98922267	2.32E-34	2.22E-05	4.30E-08	0.22912869	0.48656881
112.8	4.84E-12	3.54E-13	0.87872576	0.99775602	2.61E-34	2.36E-05	4.43E-08	0.22459518	0.48293474
112.9	4.74E-12	3.40E-13	0.87988468	0.99384876	2.50E-34	2.42E-05	4.64E-08	0.22503406	0.48543244
113	4.47E-12	3.41E-13	0.87938042	0.99472155	2.57E-34	2.48E-05	4.81E-08	0.22679464	0.48391772
113.1	4.28E-12	3.29E-13	0.87604168	0.99745128	2.37E-34	2.54E-05	4.84E-08	0.22672752	0.48272217
113.2	4.50E-12	3.47E-13	0.8751418	0.99552582	2.43E-34	2.73E-05	4.84E-08	0.22430204	0.48301285
113.3	4.23E-12	3.31E-13	0.87200303	0.99965295	2.18E-34	2.72E-05	4.76E-08	0.22336415	0.48241454
113.4	4.02E-12	3.39E-13	0.86355225	0.99610568	2.12E-34	2.87E-05	4.58E-08	0.21806212	0.47523899
113.5	4.18E-12	3.40E-13	0.86168615	0.99097413	2.15E-34	2.87E-05	4.51E-08	0.21835788	0.47501978
113.6	4.14E-12	3.50E-13	0.86113118	0.98880728	2.02E-34	2.97E-05	4.18E-08	0.21907906	0.47306439
113.7	4.12E-12	3.54E-13	0.84880589	0.98626017	2.15E-34	3.19E-05	4.16E-08	0.21458168	0.47200929
113.8	3.68E-12	3.65E-13	0.84348578	0.98659091	1.99E-34	3.24E-05	4.10E-08	0.21840058	0.46649386
113.9	3.69E-12	3.80E-13	0.84162305	0.97806616	1.83E-34	3.43E-05	3.82E-08	0.21867958	0.4703607
114	3.78E-12	4.01E-13	0.84549412	0.98629558	1.94E-34	3.70E-05	3.69E-08	0.21220691	0.47510764
114.1	3.44E-12	3.83E-13	0.84253176	0.98757326	1.74E-34	3.85E-05	3.97E-08	0.21325297	0.47469107
114.2	3.58E-12	4.29E-13	0.84996111	0.99896799	1.72E-34	3.69E-05	3.77E-08	0.21241964	0.47034165
114.3	3.51E-12	4.33E-13	0.84889335	0.99380312	1.57E-34	3.62E-05	3.65E-08	0.21107238	0.46755055
114.4	3.40E-12	4.33E-13	0.84633381	0.99200675	1.42E-34	3.94E-05	3.43E-08	0.20833603	0.47694109
114.5	3.51E-12	4.52E-13	0.84747296	0.99089976	1.46E-34	4.02E-05	3.36E-08	0.20908766	0.47413425
114.6	3.58E-12	4.28E-13	0.8422043	0.99374993	1.43E-34	3.93E-05	3.37E-08	0.2072848	0.47386309
114.7	3.40E-12	4.27E-13	0.84183764	0.99264175	1.37E-34	3.92E-05	3.39E-08	0.20377069	0.47841898
114.8	3.30E-12	4.42E-13	0.83695105	0.99591172	1.42E-34	4.09E-05	3.45E-08	0.2008523	0.47633871
114.9	3.41E-12	4.60E-13	0.83991492	0.99848812	1.43E-34	4.14E-05	3.49E-08	0.19937939	0.47643823
115	3.44E-12	4.77E-13	0.84470358	0.99661226	1.50E-34	4.29E-05	3.66E-08	0.19737432	0.47879952
115.1	3.35E-12	4.66E-13	0.84360493	0.995681	1.45E-34	4.47E-05	3.69E-08	0.19846441	0.47952651
115.2	3.47E-12	4.64E-13	0.84125672	0.9904288	1.20E-34	4.57E-05	3.65E-08	0.19576804	0.48275133
115.3	3.37E-12	4.53E-13	0.84119828	0.99632037	1.14E-34	4.67E-05	3.59E-08	0.19402445	0.4823358
115.4	3.54E-12	4.82E-13	0.84493463	0.99764796	1.21E-34	4.73E-05	3.58E-08	0.19482216	0.48239704
115.5	3.44E-12	4.55E-13	0.84269081	0.9988228	1.18E-34	4.73E-05	3.60E-08	0.19406948	0.48081112
115.6	3.58E-12	4.73E-13	0.8460595	0.99784253	1.23E-34	4.74E-05	3.67E-08	0.19403506	0.4800569
115.7	3.57E-12	4.69E-13	0.83951165	0.99706082	1.24E-34	4.84E-05	3.60E-08	0.18970677	0.47888139
115.8	3.59E-12	4.98E-13	0.8353898	0.99379223	1.32E-34	4.97E-05	3.79E-08	0.18876832	0.47978775
115.9	3.60E-12	4.99E-13	0.84003747	0.98696961	1.34E-34	4.98E-05	3.67E-08	0.19122062	0.47647146
116	3.71E-12	4.76E-13	0.84438716	0.98435272	1.29E-34	5.10E-05	3.62E-08	0.19123458	0.47926191
116.1	3.94E-12	4.56E-13	0.8380833	0.98627188	1.30E-34	5.14E-05	3.57E-08	0.1878623	0.48200406
116.2	3.97E-12	4.42E-13	0.8405799	0.98991061	1.31E-34	5.06E-05	3.54E-08	0.18685207	0.48095834
116.3	4.07E-12	4.55E-13	0.84282741	0.99252776	1.36E-34	4.95E-05	3.57E-08	0.18705185	0.48016051
116.4	4.24E-12	4.72E-13	0.83380033	0.99289954	1.29E-34	5.06E-05	3.51E-08	0.18755611	0.47814028
116.5	4.32E-12	4.78E-13	0.83209796	0.98691707	1.22E-34	5.11E-05	3.35E-08	0.19018085	0.475575
116.6	4.32E-12	4.78E-13	0.83209796	0.98691707	1.22E-34	5.11E-05	3.35E-08	0.19018085	0.475575
116.7	4.23E-12	4.87E-13	0.83785449	0.98397801	1.11E-34	5.27E-05	3.36E-08	0.19238832	0.476825
116.8	4.24E-12	5.29E-13	0.83679398	0.98291584	1.17E-34	5.58E-05	3.43E-08	0.18979985	0.47523961
116.9	4.00E-12	5.07E-13	0.84251135	0.98175618	1.15E-34	5.62E-05	3.40E-08	0.18940074	0.47678002
117	4.15E-12	5.34E-13	0.84750421	0.98078372	1.17E-34	5.65E-05	3.37E-08	0.18796674	0.4780864
117.1	4.09E-12	5.06E-13	0.84322637	0.97528553	1.14E-34	5.84E-05	3.25E-08	0.18542662	0.48022273
117.2	4.31E-12	5.08E-13	0.85543527	0.97718205	1.13E-34	5.79E-05	3.06E-08	0.18255872	0.47967316
117.3	4.56E-12	4.97E-13	0.85593291	0.97049702	1.20E-34	6.12E-05	3.14E-08	0.18253142	0.48326677
117.4	4.11E-12	4.79E-13	0.85449047	0.96640291	1.11E-34	6.46E-05	3.19E-08	0.18535941	0.48635179
117.5	4.06E-12	4.79E-13	0.85407905	0.96654005	1.08E-34	6.50E-05	3.14E-08	0.18408618	0.48350365
117.6	3.93E-12	4.48E-13	0.84868061	0.96498626	1.03E-34	6.52E-05	3.07E-08	0.18300653	0.48308963
117.7	4.11E-12	4.64E-13	0.84023349	0.9598105	1.14E-34	6.57E-05	3.03E-08	0.18058555	0.48204334
117.8	4.00E-12	4.40E-13	0.83379267	0.94580559	1.24E-34	6.60E-05	3.01E-08	0.17790942	0.48000706
117.9	4.04E-12	4.35E-13	0.83456176	0.95034168	1.18E-34	6.76E-05	3.03E-08	0.17889903	0.47868608
118	4.10E-12	4.31E-13	0.83475877	0.9503616	1.14E-34	6.94E-05	3.04E-08	0.17475094	0.48121515
118.1	4.44E-12	4.23E-13	0.83748873	0.94724332	1.29E-34	6.99E-05	3.04E-08	0.17458707	0.48040005
118.2	4.39E-12	4.11E-13	0.83549653	0.94354172	1.21E-34	7.18E-05	2.99E-08	0.17276301	0.48048795
118.3	4.32E-12	4.15E-13	0.83445641	0.9441779	1.12E-34	7.21E-05	2.98E-08	0.17294882	0.47788493
118.4	4.04E-12	4.04E-13	0.83725843	0.93578499	1.04E-34	7.50E-05	3.02E-08	0.17078522	0.4843877
118.5	3.93E-12	4.00E-13	0.8369612	0.93627061	9.35E-35	7.74E-05	2.86E-08	0.1687113	0.48806337
118.6	3.76E-12	3.87E-13	0.83644493	0.93720863	8.91E-35	7.68E-05	2.88E-08	0.16956111	0.48232984
118.7	3.76E-12	4.11E-13	0.8329183	0.94328575	8.65E-35	7.79E-05	2.84E-08	0.16953871	0.47619626
118.8	4.02E-12	4.19E-13	0.83510068	0.94013689	9.30E-35	7.78E-05	2.87E-08	0.16770552	0.4789704
118.9	4.05E-12	3.89E-13	0.84070593	0.93256246	8.80E-35	8.15E-05	2.73E-08	0.16653029	0.47796471
119	4.02E-12	3.91E-13	0.84598059	0.93341236	8.65E-35	8.32E-05	2.64E-08	0.16565242	0.47632054
119.1	4.01E-12	3.93E-13	0.84583887	0.93645708	8.55E-35	8.42E-05	2.60E-08	0.16183983	0.47594269
119.2	4.21E-12	4.18E-13	0.84316283	0.93617283	8.62E-35	8.90E-05	2.49E-08	0.16253411	0.47720502
119.3	3.88E-12	3.86E-13	0.83986326	0.94198599	7.10E-35	8.81E-05	2.29E-08	0.16087473	0.47378858
119.4	3.80E-12	3.96E-13	0.83793752	0.94269652	6.63E-35	9.74E-05	2.24E-08	0.16072993	0.4783369
119.5	3.79E-12	4.16E-13	0.83452281	0.9395782	6.93E-35	9.96E-05	2.32E-08	0.15898312	0.47491073
119.6	3.82E-12	4.18E-13	0.83117123	0.93584294	7.17E-35	9.80E-05	2.32E-08	0.15610023	0.4749463
119.7	4.01E-12	4.08E-13	0.84064998	0.93964028	6.16E-35	9.73E-05	2.40E-08	0.15497805	0.47680412
119.8	4.21E-12	4.08E-13	0.84082263	0.943062	6.33E-35	9.64E-05	2.37E-08	0.15470591	0.47546122
119.9	4.02E-12	4.07E-13	0.83997458	0.93470435	6.52E-35	9.92E-05	2.38E-08	0.15496501	0.47274266
165	1.28E-09	3.43E-12	0.73113673	0.54846386	1.66E-33	0.00034821	6.08E-10	0.06225422	0.48867747
165.1	1.38E-09	3.53E-12	0.73567748	0.54182521	1.87E-33	0.00035358	6.11E-10	0.06303757	0.49067038
165.2	1.37E-09	3.48E-12	0.73505816	0.53838608	1.77E-33	0.00034677	5.90E-10	0.06386822	0.49270681

120.3	3.94E-12	4.12E-13	0.84900884	0.94248134	5.67E-35	9.99E-05	2.29E-08	0.15003774	0.46269521
120.4	3.86E-12	4.10E-13	0.84827027	0.93886202	5.58E-35	0.00010281	2.36E-08	0.14825517	0.46657388
120.5	3.80E-12	4.21E-13	0.84920158	0.93873335	5.02E-35	0.00010062	2.27E-08	0.14552856	0.45971274
120.6	3.52E-12	4.00E-13	0.8485165	0.92642375	4.98E-35	9.92E-05	2.24E-08	0.1470752	0.45388589
120.7	3.45E-12	4.07E-13	0.84614272	0.91414005	4.80E-35	0.00010558	2.27E-08	0.14726127	0.45273563
120.8	3.43E-12	3.96E-13	0.84569438	0.91114639	4.67E-35	0.00010622	2.31E-08	0.14832385	0.45294715
120.9	3.80E-12	3.84E-13	0.8517436	0.90973983	4.63E-35	0.00010461	2.40E-08	0.14888053	0.45323552
121	3.74E-12	3.82E-13	0.85044999	0.91284627	4.24E-35	0.00010592	2.36E-08	0.14787336	0.45014465
121.1	3.54E-12	3.99E-13	0.85358351	0.92087247	4.05E-35	0.00010868	2.36E-08	0.14808838	0.44820895
121.2	3.49E-12	4.16E-13	0.8603683	0.92191467	3.59E-35	0.00010671	2.20E-08	0.14719236	0.44193435
121.3	3.46E-12	4.01E-13	0.85416226	0.90936972	3.49E-35	0.0001087	2.27E-08	0.14471892	0.43694264
121.4	3.49E-12	3.88E-13	0.8507654	0.90838554	3.59E-35	0.0001079	2.27E-08	0.14427703	0.43772757
121.5	3.36E-12	3.76E-13	0.84355102	0.91328293	3.19E-35	0.00010771	2.31E-08	0.14519621	0.43979699
121.6	3.64E-12	3.65E-13	0.84561836	0.91973024	2.97E-35	0.0001092	2.23E-08	0.14645486	0.44251816
121.7	4.13E-12	3.65E-13	0.85678989	0.92415296	3.22E-35	0.00010304	2.20E-08	0.14827876	0.44080106
121.8	4.07E-12	3.68E-13	0.85609653	0.91911795	3.18E-35	0.00010492	2.10E-08	0.14812037	0.44074801
121.9	4.00E-12	3.60E-13	0.85376693	0.92124853	3.15E-35	0.00010585	2.11E-08	0.14774425	0.44141407
122	4.29E-12	3.54E-13	0.85798598	0.93191414	2.98E-35	0.00010208	1.96E-08	0.14509439	0.4388391
122.1	4.29E-12	3.54E-13	0.85798598	0.93191414	2.98E-35	0.00010208	1.96E-08	0.14509439	0.4388391
122.2	4.27E-12	3.56E-13	0.8575037	0.93475385	2.83E-35	0.00010084	1.94E-08	0.14578468	0.43708969
122.3	4.66E-12	3.39E-13	0.85719742	0.93157771	3.24E-35	0.0001029	1.99E-08	0.1438274	0.44133595
122.4	4.85E-12	3.58E-13	0.84816497	0.93168171	3.28E-35	0.0001072	1.94E-08	0.14338423	0.44465074
122.5	4.91E-12	3.45E-13	0.84617639	0.93315992	3.27E-35	0.00010758	1.95E-08	0.14464059	0.4414643
122.6	5.12E-12	3.53E-13	0.84601967	0.9321017	3.57E-35	0.00011094	1.95E-08	0.14293019	0.4467278
122.7	5.03E-12	3.59E-13	0.84749123	0.93520249	3.75E-35	0.00011126	1.88E-08	0.14266049	0.44671575
122.8	5.18E-12	3.64E-13	0.85057074	0.93096098	3.91E-35	0.00011096	1.89E-08	0.14236219	0.44966096
122.9	5.46E-12	3.68E-13	0.84968129	0.93131135	3.87E-35	0.00011448	1.92E-08	0.14382554	0.44614149
123	5.28E-12	3.63E-13	0.85050428	0.93520249	3.63E-35	0.00011653	1.92E-08	0.1443473	0.44383254
123.1	5.38E-12	3.54E-13	0.8548409	0.93451969	3.60E-35	0.00011841	1.88E-08	0.14557499	0.44611611
123.2	5.53E-12	3.40E-13	0.85279602	0.93330705	3.51E-35	0.00012065	1.91E-08	0.14510881	0.44521767
123.3	5.37E-12	3.21E-13	0.85296448	0.93179265	3.35E-35	0.00012195	1.87E-08	0.14359164	0.44329135
123.4	5.35E-12	3.27E-13	0.85581912	0.93809621	3.28E-35	0.0001235	1.80E-08	0.14363789	0.44164235
123.5	5.58E-12	3.28E-13	0.85662117	0.93427219	3.07E-35	0.00012627	1.74E-08	0.14501985	0.44008567
123.6	5.83E-12	3.32E-13	0.86462903	0.93115016	3.03E-35	0.00012492	1.70E-08	0.14563637	0.44159646
123.7	6.51E-12	3.42E-13	0.87236566	0.93495433	3.13E-35	0.0001218	1.80E-08	0.14472079	0.43660769
123.8	6.53E-12	3.45E-13	0.87709175	0.93467302	2.97E-35	0.00012185	1.81E-08	0.14499204	0.43690629
123.9	6.86E-12	3.51E-13	0.87761032	0.93620151	2.94E-35	0.00012569	1.79E-08	0.1462274	0.43526583
124	7.00E-12	3.59E-13	0.88137908	0.93510634	2.88E-35	0.00012604	1.80E-08	0.14683495	0.43353545
124.1	7.00E-12	3.59E-13	0.88137908	0.93510634	2.88E-35	0.00012604	1.80E-08	0.14683495	0.43353545
124.2	6.86E-12	3.35E-13	0.87926495	0.93288208	2.54E-35	0.00012536	1.78E-08	0.1445901	0.42972048
124.3	7.12E-12	3.41E-13	0.87471712	0.92661784	2.60E-35	0.00012985	1.74E-08	0.14555935	0.43129492
124.4	7.27E-12	3.46E-13	0.86803143	0.92924879	2.57E-35	0.00013317	1.72E-08	0.1453875	0.42805164
124.5	7.51E-12	3.89E-13	0.87305649	0.92513245	2.86E-35	0.00013954	1.79E-08	0.1435006	0.43079044
124.6	7.91E-12	3.90E-13	0.87134276	0.92605801	2.85E-35	0.00014249	1.80E-08	0.14325512	0.4304522
124.7	7.91E-12	3.90E-13	0.87134276	0.92605801	2.85E-35	0.00014249	1.80E-08	0.14325512	0.4304522
124.8	8.16E-12	4.01E-13	0.87205531	0.92312106	2.77E-35	0.00014815	1.75E-08	0.14252545	0.43094699
124.9	8.27E-12	4.08E-13	0.87779414	0.91554732	2.92E-35	0.00015391	1.75E-08	0.14385503	0.43238421
125	8.57E-12	3.96E-13	0.87341786	0.91183124	2.88E-35	0.00015446	1.70E-08	0.14583108	0.42760358
125.1	8.68E-12	4.10E-13	0.87737437	0.9171126	2.80E-35	0.00015377	1.69E-08	0.14498794	0.42598385
125.2	8.78E-12	4.07E-13	0.8707527	0.92032127	2.68E-35	0.00015488	1.66E-08	0.14335673	0.42968104
125.3	8.78E-12	4.07E-13	0.8707527	0.92032127	2.68E-35	0.00015488	1.66E-08	0.14335673	0.42968104
125.4	8.69E-12	4.12E-13	0.87081129	0.92337657	2.69E-35	0.00016689	1.69E-08	0.14329102	0.42989477
125.5	9.10E-12	4.27E-13	0.87522124	0.92724707	2.92E-35	0.00016435	1.72E-08	0.14252073	0.42895993
125.6	9.04E-12	4.47E-13	0.87095099	0.93023162	2.95E-35	0.00016849	1.69E-08	0.14337484	0.42938348
125.7	9.04E-12	4.47E-13	0.87095099	0.93023162	2.95E-35	0.00016849	1.69E-08	0.14337484	0.42938348
125.8	9.77E-12	4.55E-13	0.87407886	0.9363574	2.90E-35	0.00017241	1.81E-08	0.14304443	0.43313545
125.9	9.16E-12	4.73E-13	0.87717957	0.94239427	2.81E-35	0.00018152	1.75E-08	0.14388388	0.4381683
126	9.58E-12	4.86E-13	0.88224782	0.94840083	2.81E-35	0.00018621	1.72E-08	0.14342556	0.4396821
126.1	9.63E-12	5.05E-13	0.87836195	0.94349232	2.70E-35	0.00019441	1.64E-08	0.14468765	0.43449051
126.2	1.00E-11	4.91E-13	0.88623302	0.9410933	2.69E-35	0.00020063	1.59E-08	0.14561427	0.43183899
126.3	1.03E-11	5.03E-13	0.88809468	0.94251575	2.75E-35	0.00020853	1.66E-08	0.14228067	0.43156517
126.4	1.07E-11	4.82E-13	0.90499351	0.94342002	2.62E-35	0.00020579	1.54E-08	0.14115198	0.43553593
126.5	1.15E-11	5.30E-13	0.9024748	0.94608334	2.76E-35	0.00020859	1.56E-08	0.14049797	0.43839203
126.6	1.15E-11	5.30E-13	0.9024748	0.94608334	2.76E-35	0.00020859	1.56E-08	0.14049797	0.43839203
126.7	1.21E-11	5.20E-13	0.90432609	0.94199616	2.79E-35	0.00021358	1.51E-08	0.13896376	0.43873296
126.8	1.32E-11	5.38E-13	0.90689776	0.94473171	2.82E-35	0.00021121	1.54E-08	0.13835164	0.43947151
126.9	1.34E-11	6.11E-13	0.90569268	0.94325101	2.86E-35	0.00021761	1.44E-08	0.14017142	0.43831542
127	1.32E-11	6.14E-13	0.91176492	0.94179368	2.60E-35	0.00021565	1.39E-08	0.13912905	0.44396997
127.1	1.30E-11	6.09E-13	0.90738411	0.94385049	2.50E-35	0.00021553	1.36E-08	0.1362606	0.44107977
127.2	1.47E-11	6.74E-13	0.91041997	0.94232331	3.15E-35	0.0002225	1.39E-08	0.136205	0.43809584
127.3	1.40E-11	7.15E-13	0.90678303	0.94489117	2.94E-35	0.00022132	1.39E-08	0.13519075	0.43491559
127.4	1.47E-11	7.15E-13	0.91491945	0.94643694	2.96E-35	0.00021787	1.34E-08	0.13537045	0.43692172
127.5	1.43E-11	7.62E-13	0.92034578	0.94191188	2.83E-35	0.00021807	1.29E-08	0.13606393	0.43649207
127.6	1.43E-11	7.62E-13	0.92034578	0.94191188	2.83E-35	0.00021807	1.29E-08	0.13606393	0.43649207
127.7	1.43E-11	7.64E-13	0.91591065	0.94628892	2.79E-35	0.00022074	1.27E-08	0.1356564	0.43649942
127.8	1.43E-11	7.64E-13	0.91591065	0.94628892	2.79E-35	0.00022074	1.27E-08	0.1356564	0.43649942
127.9	1.48E-11	7.72E-13	0.92199441	0.94692355	2.85E-35	0.00021982	1.24E-08	0.13584652	0.43752854
128	1.47E-11	8.03E-13	0.92528529	0.95237169	2.72E-35	0.00022501	1.22E-08	0.13764831	0.44076879
128.1	1.47E-11	8.38E-13	0.92267035	0.95240224	2.77E-35	0.00022746	1.20E-08	0.13700397	0.44325415
128.2	1.47E-11	8.87E-13	0.91924446	0.95572487	2.90E-35	0.00023473	1.22E-08	0.1365531	0.44440734
128.3	1.52E-11	9.04E-13	0.9188996	0.9556293	3.02E-35	0.0002415	1.23E-08	0.13532377	0.45005108
128.4	1.49E-11	9.19E-13	0.92225212	0.95759386	3.02E-35	0.00024463	1.21E-08	0.13263466	0.45037584
128.5	1.42E-11	9.09E-13	0.92607081	0.96420992	3.00E-35	0.0002445	1.20E-08	0.13111201	0.45158769
128.6	1.53E-11	9.17E-13	0.93627202	0.96512983	3.39E-35	0.00023965	1.15E-08	0.13013807	0.45328424
128.7	1.50E-11	8.83E-13	0.93944026	0.95868221	3.21E-35	0.00023433	1.15E-08	0.13043823	0.44749634
128.8	1.64E-11	8.37E-13	0.93852539	0.95641651	3.45E-35	0.0002411	1.12E-08	0.1302197	0.45059307
128.9	1.64E-11	8.37E-13	0.93852539	0.95641651	3.45E-35	0.0002411	1.12E-08	0.1302197	0.45059307
129	1.58E-11	8.41E-13	0.93685161	0.95141566	3.26E-35	0.00022929	1.05E-08	0.12872393	0.4478001
129.1	1.67E-11	8.51E-13	0.93851948	0.9482152	3.14E-35	0.00023276	9.78E-09	0.12790521	0.45694248
129.2	1.60E-11	8.52E-13	0.93453784	0.95262192	2.98E-35	0.00023187	9.66E-09	0.127337	0.45634019
129.3	1.62E-11	8.52E-13	0.92902374	0.95286217	2.97E-35	0.00023297	9.94E-09	0.12722338	0.45511661
129.4	1.65E-11	8.71E-13	0.92827968	0.9485208	3.24E-35	0.0002461	9.98E-09	0.12679583	0.45931942
129.5	1.76E-11	8.88E-13	0.93671131	0.94219957	3.43E-35	0.00024438	9.85E-09	0.12516421	0.46056025
129.6	1.76E-11	8.88E-13	0.93671131	0.94219957	3.43E-35	0.00024438	9.85E-09	0.12516421	0.46056025
129.7	1.83E-11	9.16E-13	0.93723558	0.94310447	3.66E-35	0.00024309	9.83E-09	0.12541144	0.45744254
129.8	1.83E-11	9.16E-13	0.93723558	0.94310447	3.66E-35	0.00024309	9.83E-09	0.12541144	0.45744254
129.9	2.00E-11	9.44E-13	0.94174839	0.94780947	3.71E-35	0.00025214	9.47E-09	0.12250841	0.46360555
130	2.08E-11	9.65E-13	0.93676283	0.94225618	4.00E-35	0.00026317	9.76E-09	0.12270324	0.46516807
130.1	2.07E-11	1.01E-12	0.93764205	0.9400468	3.86E-35	0.00027577	9.63E-09	0.12163626	0.46783941
130.2	1.99E-11	1.03E-12	0.94322891	0.93884554	3.74E-35	0.00028396	9.47E-09	0.12364648	0.46869168
130.3	2.07E-11	1.07E-12	0.93432809	0.93279633	3.64E-35	0.00027734	9.24E-09	0.12251279	0.46743615
130.4	2.18E-11	1.10E-12	0.93321642	0.9365884	3.51E-35	0.00028826	8.38E-09	0.12309591	0.46809038
130.5	2.17E-11	1.13E-12	0.93061673	0.92877712	3.69E-35	0.00029399	8.14E-09	0.12277495	0.46724201
130.6	2.25E-11	1.09E-12	0.93571027	0.92208458	3.67E-35	0.00029399	8.04E-09	0.12096154	0.46913929
130.7	2.43E-11	1.09E-12	0.93382082	0.92468029	3.75E-35	0.00030322	7.81E-09	0.12023697	0.4791835
130.8	2.30E-11	1.03E-12	0.93728813	0.92077688	3.47E-35	0.00030094	7.52E-09	0.11835868	0.47980802
130.9	2.25E-11	1.07E-12	0.93739898	0.92043253	3.50E-35	0.00030546	7.37E-09	0.11855329	0.47923978
131	2.35E-11	1.10E-12	0.93728828	0.91845103	3.57E-35	0.00030671	7.24E-09	0.12043624	0.47521783
131.1	2.44E-11	1.06E-12	0.94306645	0.91642573	3.39E-35	0.00030417	7.08E-09	0.11881838	0.47682173
131.2	2.51E-11	1.18E-12	0.94726093	0.91771607	3.20E-35	0.00031497	6.86E-09	0.12099668	0.47596313
131.3	2.39E-11	1.28E-12	0.94127541	0.92258618	3.34E-35	0.00032686	6.76E-09	0.11963216	0.47710767
131.4	2.62E-11	1.31E-12	0.93732742	0.92712851	3.52E-35	0.00032346	6.82E-09	0.12088327	0.47932348
131.5	2.52E-11	1.34E-12	0.94789034	0.92348104	3.62E-35	0.00033274	6.93E-09	0.11970222	0.48028187
131.6	2.28E-11	1.42E-12	0.95430934	0.9265757	3.12E-35	0.00034468	6.87E-09	0.12061966	0.47938322
131.7	2.40E-11	1.45E-12	0.95157879	0.91648769	3.33E-35	0.00034175	6.58E-09	0.12076062	0.47460975
131.8	2.40E-11	1.45E-12	0.95380113	0.91435238	3.61E-35	0.00033632	6.66E-09	0.12104346	0.47129857
131.9	2.44E-11	1.41E-12	0.95656275	0.91114154	3.62E-35	0.00032702	6.47E-09	0.120068	0.47256652
132	2.45E-11	1.55E-12	0.96489337	0.9120947	3.71E-35	0.00033231	6.27E-09	0.11843351	0.47272811
132.1	2.71E-11	1.63E-12	0.96536394	0.91279894	4.32E-35	0.0003325	6.16E-09	0.1166601	0.4718217
132.2	2.67E-11	1.64E-12	0.96257566	0.91073926	4.43E-35	0.00034117	6.19E-09	0.11548434	0.47436334
132.3	2.67E-11	1.64E-12	0.96257566	0.91073926	4.43E-35	0.00034117	6.19E-09	0.11548434	0.47436334
132.4	2.67E-11	1.64E-12	0.96257566	0.91073926	4.43E-35	0.00034117	6.19E-09	0.11548434	0.47436334
132.5	2.67E-11	1.64E-12	0.96257566	0.91073926	4.43E-35	0.00034117	6.19E-09	0.11548434	0.47436334
132.6	2.67E-11	1.64E-12	0.96257566	0.91073926	4.43E-35	0.00034117	6.19E-09	0.11548434	0.47436334
132.7	2.84E-11	1.68E-12	0.96163868	0.9115812	4.70E-35	0.00033887	6.43E-09	0.11420673	0.47525807
132.8	2.84E-11	1.68E-12	0.96163868	0.9115812	4.70E-35	0.00033887	6.43E-09	0.11420673	0.47525807
132.9	2.81E-11	1.68E-12	0.96299861	0.91159623	4.37E-35	0.00034266	6.43E-09	0.11391736	0.47402081
133	2.81E-11	1.68E-12	0.96299861	0.91159623	4.37E-35	0.00034266	6.43E-09	0.11391736	0.47402081
133.1	2.79E-11	1.72E-12	0.95947387	0.90877904	4.38E-35	0.00034748	6.30E-09	0.11426389	0.4703167
133.2	2.74E-11	1.84E-12	0.9524436	0.90880602	4.32E-35	0.00034429	6.22E-09	0.11451634	0.46507438
133.3	2.66E-11	1.89E-12	0.95410488	0.90654877	4.33E-35	0.0003495	6.11E-09	0.11331586	0.46586352
133.4	2.72E-11	1.93E-12	0.95635824	0.90853282	4.37E-35	0.00035861	6.12E-09	0.11424299	0.46780423
133.5	2.57E-11	1.96E-12	0.95406217	0.90533848	4.24E-35	0.00035305	5.93E-09	0.11468564	0.46636238
133.6	2.57E-11	1.96E-12	0.95406217	0.90533848	4.24E-35	0.00035305	5.93E-09	0.11468564	0.46636238
133.7	2.56E-11	2.08E-12	0.95712756	0.90751571	4.31E-35	0.0003701	6.02E-09	0.11474033	0.47353752
133.8	2.63E-11	2.04E-12	0.95758619	0.910512	4.31E-35	0.00036838	6.10E-09	0.11368925	0.47606242
133.9	2.58E-11	2.09E-12	0.95320423	0.90896599	4.55E-35	0.00036945	6.14E-09	0.11345369	0.47678576
134	2.58E-11	2.09E-12	0.95320423	0.90896599	4.55E-35	0.00036945	6.14E-09	0.11345369	0.47678576
134.1	2.58E-11	2.09E-12	0.95320423	0.90896599	4.55E-35	0.00036945	6.14E-09	0.11345369	0.47678576
134.2	2.67E-11	2.11E-12	0.95211287	0.90822252	4.83E-35	0.00036439	6.31E-09	0.11161291	0.48060032
134.3	2.71E-11	2.09E-12	0.95460442	0.90744501	4.78E-35	0.00036171	6.18E-09	0.11129236	0.48130131
134.4	2.85E-11	2.07E-12	0.94764903	0.91264859	4.76E-35	0.00039503	5.98E-09	0.11054539	0.48358295
134.5	2.90E-11	1.99E-12	0.94987155	0.90655679	4.64E-35	0.00038968	5.70E-09	0.10886412	0.48649058
134.6	2.90E-11	1.99E-12	0.94987155	0.90655679	4.64E-35	0.00038968	5.70E-09	0.10886412	0.48649058
134.7	2.87E-11	2.01E-12	0.95107848	0.90182493	4.72E-35	0.0003864	5.45E-09	0.10736461	0.48918001
134.8	2.87E-11	2.01E-12	0.95107848	0.90182493	4.72E-35	0.0003864	5.45E-09	0.10736461	0.48918001
134.9	2.74E-11	2.13E-12	0.94906776	0.90211131	4.90E-35	0.00039859	5.76E-09	0.10711339	0.48906891
180	5.58E-09	1.48E-12	0.39901713	0.44482658	7.30E-33	0.00042443	5.37E-10	0.07463006	0.63859621
180.1	5.62E-09	1.46E-12	0.3964876	0.4453858	7.30E-33	0.00042939	5.20E-10	0.07407875	0.63680544
180.2	5.66E-09	1.35E-12	0.39080561	0.44790986	7.64E-33	0.00041801	5.15E-10	0.07490856	0.63489325
180.3	5.54E-09	1.35E-12	0.3819169	0.44663637	8.10E-33	0.00042193	4.96E-10	0.07480413	0.63682807
180.4	5.54E-09	1.35E-12	0.3819169	0.44663637	8.10E-33	0.00042193	4.96E-10	0.07480413	0.63682807
180.5	5.57E-09	1.34E-12	0.38314412	0.44297097	7.88E-33	0.00042474	4.93E-10	0.07480286	0.63429681

135.3	2.90E-11	2.09E-12	0.95438808	0.89852516	5.04E-35	0.00041656	5.30E-09	0.10912299	0.4895294
135.4	2.86E-11	2.13E-12	0.95222005	0.90314826	5.10E-35	0.00041535	5.14E-09	0.1071602	0.48690425
135.5	2.94E-11	2.17E-12	0.95395504	0.90666267	4.73E-35	0.0004234	5.16E-09	0.1065548	0.48406339
135.6	2.95E-11	2.16E-12	0.95196777	0.90204048	4.77E-35	0.00043625	5.03E-09	0.10717298	0.48563316
135.7	2.94E-11	2.05E-12	0.95197729	0.89622877	5.26E-35	0.00045831	4.68E-09	0.10449287	0.48799756
135.8	2.99E-11	2.14E-12	0.95401882	0.89561447	5.65E-35	0.0004579	4.78E-09	0.1047869	0.487743
135.9	3.23E-11	2.21E-12	0.94855977	0.89938877	6.35E-35	0.00046514	4.83E-09	0.10175637	0.48774456
136	3.21E-11	2.19E-12	0.94998428	0.9011618	6.02E-35	0.00046662	4.78E-09	0.10196653	0.48564914
136.1	3.81E-11	2.18E-12	0.95649565	0.90004045	6.80E-35	0.00048725	4.93E-09	0.09877525	0.48929129
136.2	3.77E-11	2.16E-12	0.96065208	0.89992345	6.73E-35	0.00047876	4.80E-09	0.09796311	0.4820311
136.3	3.78E-11	2.11E-12	0.96299662	0.90011747	6.50E-35	0.00048308	4.83E-09	0.09742903	0.48306426
136.4	3.75E-11	2.15E-12	0.96126913	0.8943185	6.58E-35	0.00048323	4.42E-09	0.09755023	0.48015261
136.5	3.58E-11	2.19E-12	0.96140864	0.89164965	6.18E-35	0.00049579	4.24E-09	0.09592691	0.47304553
136.6	3.71E-11	2.23E-12	0.9724769	0.8870209	6.48E-35	0.00049778	3.93E-09	0.09411798	0.47222959
136.7	3.71E-11	2.23E-12	0.9724769	0.8870209	6.48E-35	0.00049778	3.93E-09	0.09411798	0.47222959
136.8	3.67E-11	2.26E-12	0.98443003	0.88596256	6.21E-35	0.00051293	3.77E-09	0.09518921	0.47326255
136.9	4.01E-11	2.43E-12	0.97836671	0.87810229	7.63E-35	0.00054428	3.81E-09	0.09358754	0.46909648
137	4.16E-11	2.62E-12	0.98223253	0.87546232	6.92E-35	0.00055238	3.65E-09	0.09527644	0.45793116
137.1	4.41E-11	2.60E-12	0.9820571	0.87229015	7.10E-35	0.00054652	3.53E-09	0.09405438	0.4575212
137.2	4.33E-11	2.46E-12	0.9810435	0.87621518	7.02E-35	0.00054664	3.47E-09	0.0939961	0.45579758
137.3	4.44E-11	2.49E-12	0.98657304	0.87220685	6.81E-35	0.0005451	3.53E-09	0.09409173	0.45954195
137.4	4.39E-11	2.54E-12	0.98953163	0.87314918	6.79E-35	0.00054428	3.54E-09	0.09417969	0.45857347
137.5	4.34E-11	2.59E-12	0.99057992	0.87136062	6.77E-35	0.00054554	3.50E-09	0.09468351	0.45784592
137.6	4.34E-11	2.59E-12	0.99057992	0.87136062	6.77E-35	0.00054554	3.50E-09	0.09468351	0.45784592
137.7	4.43E-11	2.64E-12	0.98517469	0.8648834	7.43E-35	0.00055661	3.31E-09	0.09492424	0.45985955
137.8	4.43E-11	2.64E-12	0.98517469	0.8648834	7.43E-35	0.00055661	3.31E-09	0.09492424	0.45985955
137.9	4.59E-11	2.61E-12	0.99078076	0.8687142	7.59E-35	0.00054926	3.29E-09	0.09569355	0.46096622
138	4.41E-11	2.72E-12	0.99147101	0.86576332	6.84E-35	0.00054675	3.15E-09	0.09725553	0.45720707
138.1	4.50E-11	2.69E-12	0.99727548	0.87229944	6.29E-35	0.00054754	3.05E-09	0.09640509	0.45504307
138.2	4.46E-11	2.73E-12	0.99434895	0.87413312	6.16E-35	0.0005322	2.98E-09	0.09593327	0.45029499
138.3	4.42E-11	2.66E-12	0.9915245	0.88078395	5.81E-35	0.00052441	2.83E-09	0.09488324	0.4499278
138.4	4.49E-11	2.49E-12	0.99366372	0.87668012	5.76E-35	0.00053201	2.82E-09	0.09291779	0.44967428
138.5	4.49E-11	2.49E-12	0.99366372	0.87668012	5.76E-35	0.00053201	2.82E-09	0.09291779	0.44967428
138.6	4.94E-11	2.44E-12	0.99771178	0.87515078	6.14E-35	0.00051582	2.68E-09	0.09176089	0.44492139
138.7	5.09E-11	2.49E-12	0.99628608	0.87486762	7.08E-35	0.00049912	2.66E-09	0.08923912	0.43693209
138.8	5.44E-11	2.40E-12	0.99944956	0.86043169	7.37E-35	0.00050526	2.59E-09	0.08548379	0.4392052
138.9	5.49E-11	2.52E-12	0.99898261	0.86228445	7.91E-35	0.00050567	2.65E-09	0.08466943	0.43758725
139	5.70E-11	2.63E-12	0.99910852	0.85903651	8.50E-35	0.00050995	2.59E-09	0.08462101	0.43824159
139.1	5.87E-11	2.71E-12	0.99579872	0.85494466	9.21E-35	0.00051612	2.54E-09	0.08471969	0.43716997
139.2	6.38E-11	2.79E-12	0.99861064	0.8517489	1.04E-34	0.00052297	2.43E-09	0.08260872	0.43618108
139.3	6.73E-11	2.76E-12	0.99856843	0.84759776	1.13E-34	0.00051601	2.40E-09	0.08265586	0.43730484
139.4	7.06E-11	3.00E-12	0.99944768	0.84695042	1.28E-34	0.00052477	2.44E-09	0.08188049	0.43274693
139.5	7.06E-11	3.00E-12	0.99944768	0.84695042	1.28E-34	0.00052477	2.44E-09	0.08188049	0.43274693
139.6	6.82E-11	3.06E-12	0.99476749	0.85192316	1.18E-34	0.00051632	2.45E-09	0.08183254	0.43051857
139.7	7.17E-11	3.00E-12	0.99369241	0.84943013	1.20E-34	0.00051576	2.40E-09	0.08297245	0.43200583
139.8	6.96E-11	3.08E-12	0.99727648	0.85263315	1.32E-34	0.00052801	2.43E-09	0.0823934	0.42821867
139.9	6.97E-11	3.12E-12	0.99971226	0.85108517	1.33E-34	0.00053303	2.45E-09	0.08199944	0.42643861
140	6.97E-11	3.12E-12	0.99971226	0.85108517	1.33E-34	0.00053303	2.45E-09	0.08199944	0.42643861
140.1	6.46E-11	3.04E-12	0.99530233	0.84424989	1.20E-34	0.00054536	2.36E-09	0.08227448	0.42729392
140.2	6.71E-11	3.01E-12	0.99889486	0.84364892	1.19E-34	0.00054266	2.42E-09	0.08164265	0.42695835
140.3	7.33E-11	2.86E-12	0.99561241	0.83075095	1.35E-34	0.000549	2.36E-09	0.07895926	0.43297723
140.4	7.42E-11	2.98E-12	0.99845579	0.82399079	1.27E-34	0.00055507	2.29E-09	0.07863267	0.43180186
140.5	8.05E-11	2.88E-12	0.99562288	0.82050923	1.41E-34	0.00053968	2.23E-09	0.077124	0.43456235
140.6	7.96E-11	2.82E-12	0.99854831	0.80933172	1.39E-34	0.00054109	2.13E-09	0.0763305	0.43713237
140.7	8.19E-11	2.79E-12	0.99742336	0.80402831	1.46E-34	0.0005357	2.11E-09	0.07554526	0.43700597
140.8	8.65E-11	2.78E-12	0.99415039	0.80669417	1.45E-34	0.0005324	2.12E-09	0.0747051	0.4378465
140.9	8.57E-11	2.84E-12	0.99475457	0.81106255	1.45E-34	0.00053906	2.10E-09	0.07446004	0.43682828
141	8.37E-11	3.01E-12	0.99172808	0.80968283	1.51E-34	0.00055134	2.07E-09	0.0741335	0.43346323
141.1	8.96E-11	3.21E-12	0.99334123	0.8082349	1.65E-34	0.00055325	2.10E-09	0.07471636	0.43103064
141.2	8.90E-11	3.08E-12	0.99838805	0.80445514	1.56E-34	0.00054811	2.03E-09	0.0753796	0.43208361
141.3	8.93E-11	3.24E-12	0.99538624	0.80432115	1.69E-34	0.0005551	2.06E-09	0.07544075	0.42939102
141.4	9.32E-11	3.33E-12	0.99898567	0.80907916	1.71E-34	0.00054442	2.00E-09	0.07504048	0.42967513
141.5	9.73E-11	3.25E-12	0.99795524	0.80572437	1.72E-34	0.00054006	2.00E-09	0.07526643	0.43179758
141.6	9.58E-11	3.27E-12	0.99398194	0.80479198	1.69E-34	0.0005349	1.92E-09	0.07468847	0.43134419
141.7	9.53E-11	3.31E-12	0.98990348	0.80507452	1.73E-34	0.00052662	1.90E-09	0.07519475	0.43203188
141.8	9.69E-11	3.34E-12	0.99066506	0.80550679	1.77E-34	0.00051323	1.88E-09	0.0741267	0.43663625
141.9	1.01E-10	3.23E-12	0.99102655	0.80496343	1.78E-34	0.00050327	1.90E-09	0.07372259	0.43547895
142	1.01E-10	3.23E-12	0.99102655	0.80496343	1.78E-34	0.00050327	1.90E-09	0.07372259	0.43547895
142.1	1.10E-10	3.13E-12	0.99502636	0.8026949	1.82E-34	0.00049434	1.95E-09	0.07347055	0.43349895
142.2	1.10E-10	3.13E-12	0.99275742	0.79705729	1.81E-34	0.00048878	1.99E-09	0.07382202	0.43309805
142.3	1.10E-10	3.13E-12	0.99275742	0.79705729	1.81E-34	0.00048878	1.99E-09	0.07382202	0.43309805
142.4	1.11E-10	3.05E-12	0.99062819	0.793091	1.82E-34	0.00048935	1.97E-09	0.07412097	0.43710298
142.5	1.10E-10	3.08E-12	0.99278875	0.79512158	1.85E-34	0.00047887	1.93E-09	0.07447187	0.43411056
142.6	1.10E-10	3.08E-12	0.99278875	0.79512158	1.85E-34	0.00047887	1.93E-09	0.07447187	0.43411056
142.7	1.19E-10	3.06E-12	0.9929235	0.80018013	1.89E-34	0.00046995	1.93E-09	0.07390473	0.43574445
142.8	1.24E-10	3.01E-12	0.99354486	0.79367301	1.97E-34	0.00046161	1.99E-09	0.07241617	0.43689503
142.9	1.26E-10	2.96E-12	0.99213488	0.78885836	2.06E-34	0.00045479	1.99E-09	0.07241179	0.43909596
143	1.27E-10	2.86E-12	0.98830301	0.79042297	1.94E-34	0.00045061	2.02E-09	0.07276382	0.4357709
143.1	1.34E-10	2.86E-12	0.99286219	0.79759188	1.89E-34	0.00044944	2.11E-09	0.0724371	0.4373057
143.2	1.39E-10	2.90E-12	0.99487127	0.79794457	2.02E-34	0.00044161	2.17E-09	0.07214677	0.43720948
143.3	1.42E-10	2.91E-12	0.99774023	0.79601441	1.93E-34	0.00044093	2.12E-09	0.07019828	0.43717086
143.4	1.43E-10	2.85E-12	0.99907783	0.79421426	1.93E-34	0.00044521	2.15E-09	0.06956727	0.43570617
143.5	1.50E-10	2.79E-12	0.99975672	0.79482702	1.95E-34	0.00043437	2.14E-09	0.06948707	0.43663694
143.6	1.45E-10	2.92E-12	0.99583669	0.79335023	1.95E-34	0.00042637	2.12E-09	0.06952287	0.43611792
143.7	1.49E-10	2.98E-12	0.99730278	0.79386744	1.91E-34	0.00043471	2.11E-09	0.06975802	0.43805946
143.8	1.47E-10	3.14E-12	0.9947849	0.7869911	1.88E-34	0.00042186	1.99E-09	0.07053378	0.43443602
143.9	1.53E-10	3.05E-12	0.99893829	0.78665206	1.88E-34	0.00042733	1.96E-09	0.06997709	0.43653474
144	1.60E-10	3.22E-12	0.99760573	0.79175413	2.06E-34	0.00042217	1.92E-09	0.06984108	0.43297121
144.1	1.60E-10	3.22E-12	0.99760573	0.79175413	2.06E-34	0.00042217	1.92E-09	0.06984108	0.43297121
144.2	1.62E-10	3.05E-12	0.99732716	0.79795375	1.99E-34	0.00041287	1.92E-09	0.06937622	0.4316239
144.3	1.64E-10	3.27E-12	0.99804686	0.80236935	2.20E-34	0.00042455	1.94E-09	0.06701017	0.43460255
144.4	1.68E-10	3.18E-12	0.99837278	0.80306829	2.18E-34	0.00041153	1.93E-09	0.06644278	0.4340516
144.5	1.80E-10	3.22E-12	0.99818447	0.80161333	2.38E-34	0.00041119	1.97E-09	0.06633268	0.43370216
144.6	1.81E-10	3.17E-12	0.98912344	0.80000383	2.59E-34	0.00042946	2.00E-09	0.06735694	0.43909388
144.7	1.90E-10	3.28E-12	0.98868238	0.79533584	2.80E-34	0.00042731	2.06E-09	0.06640477	0.43640237
144.8	2.06E-10	3.38E-12	0.98758488	0.79325771	3.01E-34	0.00042445	2.07E-09	0.06554054	0.43745571
144.9	2.29E-10	3.44E-12	0.98564968	0.78485789	3.53E-34	0.00041525	2.03E-09	0.06414111	0.44032135
145	2.34E-10	3.44E-12	0.98894351	0.78276348	3.59E-34	0.00041664	2.01E-09	0.0640559	0.44349716
145.1	2.26E-10	3.62E-12	0.99011215	0.78053029	3.74E-34	0.00042279	2.02E-09	0.06328002	0.44220345
145.2	2.23E-10	3.62E-12	0.98470502	0.77530317	3.58E-34	0.00042875	2.04E-09	0.06210887	0.4398074
145.3	2.35E-10	3.50E-12	0.9796594	0.77487177	3.77E-34	0.00041444	1.99E-09	0.06167299	0.44020756
145.4	2.41E-10	3.59E-12	0.97780169	0.77565058	4.01E-34	0.00040167	1.96E-09	0.06182107	0.43827691
145.5	2.36E-10	3.62E-12	0.97319936	0.77603138	3.87E-34	0.00040356	1.93E-09	0.06236818	0.4367303
145.6	2.31E-10	3.64E-12	0.97576944	0.78201818	3.65E-34	0.00039815	1.85E-09	0.06192071	0.43273388
145.7	2.33E-10	3.83E-12	0.97467423	0.78303391	3.82E-34	0.0004035	1.89E-09	0.06181526	0.43205136
145.8	2.38E-10	3.79E-12	0.98151921	0.78317958	4.02E-34	0.00040912	1.85E-09	0.06201201	0.4286056
145.9	2.43E-10	3.92E-12	0.97862553	0.78235962	4.09E-34	0.00041191	1.87E-09	0.06123384	0.42815277
146	2.43E-10	3.92E-12	0.97862553	0.78235962	4.09E-34	0.00041191	1.87E-09	0.06123384	0.42815277
146.1	2.43E-10	4.06E-12	0.9803064	0.78038882	3.79E-34	0.00041159	1.85E-09	0.06122741	0.4301505
146.2	2.43E-10	4.06E-12	0.9803064	0.78038882	3.79E-34	0.00041159	1.85E-09	0.06122741	0.4301505
146.3	2.43E-10	4.06E-12	0.9803064	0.78038882	3.79E-34	0.00041159	1.85E-09	0.06122741	0.4301505
146.4	2.53E-10	3.88E-12	0.98373671	0.77495443	3.79E-34	0.00041465	1.86E-09	0.06009169	0.43036675
146.5	2.68E-10	3.76E-12	0.9839976	0.76359361	4.02E-34	0.00041162	1.77E-09	0.06084538	0.43341559
146.6	2.74E-10	3.82E-12	0.97594974	0.76453716	4.13E-34	0.00040115	1.79E-09	0.06089471	0.43063699
146.7	2.75E-10	3.81E-12	0.97612505	0.75831946	4.45E-34	0.00040301	1.76E-09	0.06111924	0.42997497
146.8	2.77E-10	3.80E-12	0.97142647	0.75049123	4.43E-34	0.00040084	1.77E-09	0.06054868	0.43555839
146.9	2.84E-10	4.07E-12	0.97085991	0.75175129	4.65E-34	0.0003947	1.75E-09	0.06068101	0.43550275
147	2.88E-10	4.04E-12	0.97255491	0.74686957	4.63E-34	0.00039594	1.77E-09	0.06113442	0.43728105
147.1	2.92E-10	3.97E-12	0.97764421	0.74635092	4.62E-34	0.00038983	1.77E-09	0.0605664	0.44015352
147.2	3.03E-10	3.84E-12	0.98346721	0.74277074	5.05E-34	0.00040438	1.72E-09	0.06032091	0.44021457
147.3	2.97E-10	3.96E-12	0.97862476	0.74178145	5.26E-34	0.00040957	1.65E-09	0.06136274	0.43956559
147.4	3.09E-10	4.01E-12	0.98362794	0.74529801	5.73E-34	0.00040875	1.69E-09	0.06021762	0.43827565
147.5	3.19E-10	4.23E-12	0.97983239	0.74092184	5.56E-34	0.00040874	1.58E-09	0.06107871	0.43930808
147.6	3.27E-10	4.30E-12	0.97951811	0.74310538	5.86E-34	0.00041924	1.60E-09	0.06108746	0.44258889
147.7	3.27E-10	4.30E-12	0.97951811	0.74310538	5.86E-34	0.00041924	1.60E-09	0.06108746	0.44258889
147.8	3.33E-10	4.15E-12	0.98523078	0.73481813	5.96E-34	0.0004258	1.60E-09	0.06086313	0.44457572
147.9	3.26E-10	4.20E-12	0.98370419	0.73975568	5.80E-34	0.00040918	1.56E-09	0.06075862	0.44098816
148	3.28E-10	4.26E-12	0.98192006	0.74217471	6.09E-34	0.0004122	1.54E-09	0.06058796	0.44368124
148.1	3.54E-10	4.05E-12	0.97455473	0.74018075	6.13E-34	0.00039867	1.56E-09	0.06098114	0.4454781
148.2	3.63E-10	4.05E-12	0.97075708	0.73998689	6.25E-34	0.00040597	1.56E-09	0.06068239	0.44503835
148.3	3.53E-10	4.06E-12	0.96762713	0.74239473	5.97E-34	0.00040908	1.53E-09	0.0599408	0.44428748
148.4	3.55E-10	4.09E-12	0.96449448	0.73505197	6.08E-34	0.00042896	1.48E-09	0.06085695	0.44680745
148.5	3.27E-10	4.27E-12	0.96521968	0.73628805	5.43E-34	0.00041282	1.50E-09	0.06092669	0.44739494
148.6	3.27E-10	4.27E-12	0.96521968	0.73628805	5.43E-34	0.00041282	1.50E-09	0.06092669	0.44739494
148.7	3.27E-10	4.27E-12	0.96521968	0.73628805	5.43E-34	0.00041282	1.50E-09	0.06092669	0.44739494
148.8	3.42E-10	4.29E-12	0.96589608	0.72756192	5.92E-34	0.00040752	1.44E-09	0.05994784	0.45015119
148.9	3.40E-10	4.53E-12	0.96868731	0.72421115	5.99E-34	0.00041044	1.46E-09	0.06092367	0.44793537
149	3.54E-10	4.83E-12	0.96888012	0.72455601	6.70E-34	0.00040262	1.48E-09	0.0609919	0.44748239
149.1	3.54E-10	4.83E-12	0.96888012	0.72455601	6.70E-34	0.00040262	1.48E-09	0.0609919	0.44748239
149.2	3.58E-10	4.82E-12	0.97029416	0.72397785	6.77E-34	0.00040441	1.48E-09	0.06049015	0.44707595
149.3	4.24E-10	4.73E-12	0.97595404	0.70992451	8.47E-34	0.00042176	1.47E-09	0.05963171	0.44909438
149.4	4.41E-10	4.91E-12	0.97478371	0.70832614	9.40E-34	0.00042414	1.50E-09	0.05974822	0.4488549
149.5	4.29E-10	5.13E-12	0.97332488	0.71211342	9.86E-34	0.00042161	1.50E-09	0.05982844	0.44737474
149.6	4.52E-10	5.02E-12	0.97456228	0.71021328	1.06E-33	0.00042382	1.48E-09	0.06009803	0.44797646
149.7	4.92E-10	5.03E-12	0.96699195	0.70472739	1.02E-33	0.00042114	1.53E-09	0.05989641	0.45002472
149.8	5.06E-10	5.21E-12	0.96260151	0.70343659	1.07E-33	0.00044122	1.55E-09	0.05845762	0.45015587
149.9	5.31E-10	5.35E-12	0.95896177	0.69793875	1.05E-33	0.00044963	1.51E-09	0.0591536	0.44818881
195	1.35E-08	8.33E-13	0.20286739	0.37333716	6.24E-32	0.00030773	1.24E-09	0.08526603	0.71015369
195.1	1.34E-08	8.24E-13	0.20289664	0.37321222	6.77E-32	0.00030687	1.23E-09	0.08548239	0.70722973
195.2	1.39E-08	8.15E-13	0.20383846	0.37405938	7.07E-32	0.00030713	1.28E-09	0.08558385	0.70415659
195.3	1.39E-08	8.15E-13	0.20383846	0.37405938	7.07E-32	0.00030713	1.28E-09	0.08558385	0.70415659
195.4	1.45E-08	8.41E-13	0.20213733	0.37318705	7.44E-32	0.00030994	1.30E-09	0.08567331	0.70604718
195.5	1.45E-08	8.57E-13	0.2006544	0.37459506	7.87E-32	0.00030902	1.35E-09	0.08570949	0.70433207

150.3	5.21E-10	5.39E-12	0.96306958	0.6935227	1.17E-33	0.00045343	1.54E-09	0.05862496	0.45080086
150.4	5.21E-10	5.39E-12	0.96306958	0.6935227	1.17E-33	0.00045343	1.54E-09	0.05862496	0.45080086
150.5	5.29E-10	5.45E-12	0.9680007	0.69589588	1.26E-33	0.00045116	1.55E-09	0.0578672	0.45067747
150.6	5.29E-10	5.45E-12	0.9680007	0.69589588	1.26E-33	0.00045116	1.55E-09	0.0578672	0.45067747
150.7	5.29E-10	5.45E-12	0.9680007	0.69589588	1.26E-33	0.00045116	1.55E-09	0.0578672	0.45067747
150.8	5.08E-10	5.37E-12	0.96551394	0.69472766	1.17E-33	0.00044117	1.45E-09	0.05695267	0.45077486
150.9	4.89E-10	5.48E-12	0.96525557	0.69700902	1.17E-33	0.00043948	1.46E-09	0.05774034	0.4517838
151	4.96E-10	5.64E-12	0.96013947	0.69169471	1.24E-33	0.00044687	1.51E-09	0.05800147	0.45394224
151.1	5.04E-10	5.45E-12	0.96270161	0.6916432	1.27E-33	0.00045384	1.56E-09	0.05813264	0.45058038
151.2	5.03E-10	5.42E-12	0.9598194	0.69262038	1.19E-33	0.00045588	1.57E-09	0.05865526	0.45014863
151.3	4.95E-10	5.50E-12	0.95678512	0.69143334	1.15E-33	0.00046015	1.53E-09	0.05843655	0.44832459
151.4	4.87E-10	5.63E-12	0.95340669	0.6899735	1.14E-33	0.00045858	1.50E-09	0.05895361	0.44721272
151.5	5.20E-10	5.46E-12	0.94940028	0.68344574	1.17E-33	0.00046357	1.52E-09	0.06002441	0.44834682
151.6	5.16E-10	5.26E-12	0.94537156	0.68222287	1.13E-33	0.00045991	1.49E-09	0.0599839	0.45034647
151.7	5.23E-10	5.35E-12	0.94134452	0.68146588	1.08E-33	0.00046264	1.45E-09	0.06094229	0.45040279
151.8	5.34E-10	5.17E-12	0.93284294	0.6757028	1.00E-33	0.00045294	1.38E-09	0.06165849	0.44932913
151.9	5.61E-10	5.10E-12	0.93987086	0.66479841	1.02E-33	0.00046179	1.31E-09	0.06219689	0.44451508
152	5.68E-10	5.25E-12	0.93864287	0.66043096	1.02E-33	0.00045924	1.28E-09	0.06208974	0.4459268
152.1	5.68E-10	5.25E-12	0.93864287	0.66043096	1.02E-33	0.00045924	1.28E-09	0.06208974	0.4459268
152.2	5.67E-10	5.49E-12	0.93851604	0.66348266	1.02E-33	0.00045112	1.29E-09	0.06210221	0.44480572
152.3	5.80E-10	5.70E-12	0.93600045	0.66828585	9.98E-34	0.00044481	1.25E-09	0.06330537	0.44052068
152.4	5.80E-10	5.70E-12	0.93600045	0.66828585	9.98E-34	0.00044481	1.25E-09	0.06330537	0.44052068
152.5	5.80E-10	5.70E-12	0.93600045	0.66828585	9.98E-34	0.00044481	1.25E-09	0.06330537	0.44052068
152.6	5.80E-10	5.70E-12	0.93600045	0.66828585	9.98E-34	0.00044481	1.25E-09	0.06330537	0.44052068
152.7	5.68E-10	5.56E-12	0.93111113	0.66664541	9.65E-34	0.00043901	1.25E-09	0.06266075	0.44332608
152.8	5.72E-10	5.89E-12	0.92852194	0.66037628	1.00E-33	0.00044114	1.23E-09	0.06277986	0.44376217
152.9	5.60E-10	5.80E-12	0.92779622	0.657421	9.58E-34	0.00043074	1.21E-09	0.06274069	0.44261892
153	6.10E-10	5.79E-12	0.92847372	0.65474956	1.01E-33	0.00043598	1.19E-09	0.06346582	0.44266051
153.1	6.12E-10	5.72E-12	0.92475705	0.65780633	9.72E-34	0.00043014	1.16E-09	0.06320302	0.44496218
153.2	6.32E-10	6.00E-12	0.92456538	0.65682926	1.02E-33	0.00043275	1.18E-09	0.06400324	0.44440952
153.3	6.32E-10	6.00E-12	0.92456538	0.65682926	1.02E-33	0.00043275	1.18E-09	0.06400324	0.44440952
153.4	6.31E-10	5.57E-12	0.92989365	0.6599059	9.63E-34	0.00043244	1.15E-09	0.06288591	0.44341606
153.5	6.31E-10	5.57E-12	0.92989365	0.6599059	9.63E-34	0.00043244	1.15E-09	0.06288591	0.44341606
153.6	6.28E-10	5.54E-12	0.92965662	0.66337484	9.30E-34	0.00042625	1.09E-09	0.06271374	0.44107649
153.7	6.10E-10	5.56E-12	0.92848011	0.6646599	9.66E-34	0.00042045	1.08E-09	0.06169384	0.44497882
153.8	6.33E-10	5.29E-12	0.92418852	0.661402	9.47E-34	0.00041124	1.03E-09	0.06088589	0.44711177
153.9	6.68E-10	5.39E-12	0.91970793	0.66014233	9.83E-34	0.00040903	9.93E-10	0.06102795	0.44960199
154	6.68E-10	5.39E-12	0.91970793	0.66014233	9.83E-34	0.00040903	9.93E-10	0.06102795	0.44960199
154.1	6.76E-10	5.27E-12	0.91776188	0.66023557	9.99E-34	0.00039509	9.77E-10	0.05996669	0.44915327
154.2	6.76E-10	5.27E-12	0.91776188	0.66023557	9.99E-34	0.00039509	9.77E-10	0.05996669	0.44915327
154.3	6.76E-10	5.23E-12	0.91588808	0.65876649	9.89E-34	0.00039272	9.89E-10	0.05969824	0.44832153
154.4	6.76E-10	5.23E-12	0.91588808	0.65876649	9.89E-34	0.00039272	9.89E-10	0.05969824	0.44832153
154.5	6.76E-10	5.23E-12	0.91588808	0.65876649	9.89E-34	0.00039272	9.89E-10	0.05969824	0.44832153
154.6	6.76E-10	4.95E-12	0.91244206	0.655658	9.48E-34	0.00038117	9.71E-10	0.05989349	0.44718337
154.7	6.89E-10	5.09E-12	0.91398109	0.6530738	9.57E-34	0.00037371	9.56E-10	0.06056886	0.44350607
154.8	7.02E-10	5.08E-12	0.91485514	0.64888884	1.00E-33	0.00038201	9.35E-10	0.06131077	0.44218675
154.9	7.02E-10	5.08E-12	0.91485514	0.64888884	1.00E-33	0.00038201	9.35E-10	0.06131077	0.44218675
155	7.33E-10	4.95E-12	0.91023556	0.64018441	1.10E-33	0.00038234	9.76E-10	0.06036233	0.44733499
155.1	7.33E-10	4.95E-12	0.91023556	0.64018441	1.10E-33	0.00038234	9.76E-10	0.06036233	0.44733499
155.2	7.65E-10	5.32E-12	0.90651861	0.64176422	1.21E-33	0.00038187	9.74E-10	0.06106699	0.44959972
155.3	7.53E-10	5.35E-12	0.90891861	0.64609832	1.19E-33	0.00038217	9.34E-10	0.06101519	0.4455061
155.4	7.57E-10	5.62E-12	0.90166807	0.6473303	1.23E-33	0.00039491	9.48E-10	0.06069995	0.44576321
155.5	7.81E-10	5.63E-12	0.90563659	0.64643451	1.25E-33	0.00039305	9.66E-10	0.05999504	0.44451839
155.6	7.85E-10	5.70E-12	0.90143222	0.64482264	1.34E-33	0.0003971	9.77E-10	0.05991624	0.44565375
155.7	7.73E-10	5.63E-12	0.89963684	0.6468695	1.34E-33	0.00039785	9.73E-10	0.06052123	0.4458803
155.8	7.73E-10	5.63E-12	0.89963684	0.6468695	1.34E-33	0.00039785	9.73E-10	0.06052123	0.4458803
155.9	7.67E-10	5.61E-12	0.89709093	0.64430171	1.29E-33	0.00039026	9.41E-10	0.06097052	0.4441322
156	7.89E-10	5.72E-12	0.89575862	0.63484121	1.33E-33	0.00038774	9.54E-10	0.06052031	0.44347071
156.1	8.15E-10	5.56E-12	0.8880122	0.63211096	1.28E-33	0.00038788	9.36E-10	0.06087512	0.44854858
156.2	8.15E-10	5.84E-12	0.8878626	0.63185353	1.31E-33	0.00038633	9.59E-10	0.06088475	0.44908945
156.3	7.97E-10	5.83E-12	0.88462316	0.63144504	1.27E-33	0.00038546	9.59E-10	0.06123688	0.44871315
156.4	7.97E-10	5.83E-12	0.88462316	0.63144504	1.27E-33	0.00038546	9.59E-10	0.06123688	0.44871315
156.5	7.97E-10	5.93E-12	0.88375242	0.62756267	1.31E-33	0.00038761	9.56E-10	0.06164102	0.44899993
156.6	7.97E-10	5.93E-12	0.88375242	0.62756267	1.31E-33	0.00038761	9.56E-10	0.06164102	0.44899993
156.7	8.28E-10	6.14E-12	0.88341158	0.62664727	1.35E-33	0.00038821	9.45E-10	0.06210795	0.44680524
156.8	8.50E-10	6.17E-12	0.88022227	0.62677842	1.40E-33	0.00038358	9.38E-10	0.06122311	0.44678957
156.9	8.66E-10	6.32E-12	0.87940185	0.62190189	1.38E-33	0.00037451	9.52E-10	0.0597263	0.44131826
157	8.84E-10	5.93E-12	0.87392304	0.61866434	1.30E-33	0.00036763	9.30E-10	0.05996617	0.44164926
157.1	9.13E-10	6.12E-12	0.87475875	0.61663421	1.35E-33	0.00036774	9.45E-10	0.05943736	0.44400924
157.2	9.13E-10	6.12E-12	0.87475875	0.61663421	1.35E-33	0.00036774	9.45E-10	0.05943736	0.44400924
157.3	9.21E-10	5.99E-12	0.87614266	0.61188901	1.41E-33	0.00037298	9.42E-10	0.05982362	0.44587238
157.4	9.74E-10	6.16E-12	0.87721075	0.61265426	1.52E-33	0.00037763	9.38E-10	0.05987992	0.44418675
157.5	1.03E-09	6.08E-12	0.88142067	0.61308898	1.59E-33	0.00037014	9.50E-10	0.05933657	0.44484203
157.6	1.03E-09	6.08E-12	0.88142067	0.61308898	1.59E-33	0.00037014	9.50E-10	0.05933657	0.44484203
157.7	1.03E-09	6.08E-12	0.88142067	0.61308898	1.59E-33	0.00037014	9.50E-10	0.05933657	0.44484203
157.8	1.04E-09	5.51E-12	0.87654175	0.60662271	1.40E-33	0.00035912	8.77E-10	0.0601067	0.44869803
157.9	1.06E-09	5.37E-12	0.86750707	0.60562312	1.37E-33	0.0003604	8.56E-10	0.06003616	0.45257398
158	1.06E-09	5.39E-12	0.86542084	0.60931456	1.38E-33	0.00036191	8.39E-10	0.05991225	0.44817969
158.1	1.08E-09	5.12E-12	0.86303279	0.60758701	1.31E-33	0.00035766	8.21E-10	0.06030919	0.44853805
158.2	1.12E-09	4.94E-12	0.85180438	0.60899552	1.26E-33	0.0003497	8.12E-10	0.0616476	0.44915509
158.3	1.14E-09	4.95E-12	0.85274934	0.60898087	1.35E-33	0.00035924	8.10E-10	0.06256793	0.44959814
158.4	1.12E-09	5.14E-12	0.8454512	0.60388666	1.36E-33	0.0003605	8.21E-10	0.06442546	0.45400188
158.5	1.12E-09	5.14E-12	0.8454512	0.60388666	1.36E-33	0.0003605	8.21E-10	0.06442546	0.45400188
158.6	1.16E-09	5.01E-12	0.84432952	0.60036797	1.34E-33	0.00037061	8.07E-10	0.06523028	0.455843
158.7	1.16E-09	5.01E-12	0.84432952	0.60036797	1.34E-33	0.00037061	8.07E-10	0.06523028	0.455843
158.8	1.15E-09	5.09E-12	0.8419711	0.59466952	1.39E-33	0.00035286	8.25E-10	0.06626859	0.45316629
158.9	1.12E-09	5.12E-12	0.83849613	0.59614437	1.42E-33	0.0003625	8.25E-10	0.06652366	0.45373368
159	1.13E-09	5.17E-12	0.83748884	0.59752792	1.44E-33	0.00036547	8.08E-10	0.06640743	0.45655488
159.1	1.13E-09	5.16E-12	0.82551664	0.59226104	1.54E-33	0.00036745	7.80E-10	0.06684553	0.45825871
159.2	1.11E-09	5.29E-12	0.8246364	0.59264069	1.56E-33	0.00036629	7.85E-10	0.06783686	0.45715196
159.3	1.12E-09	5.04E-12	0.81616408	0.59172074	1.43E-33	0.00036174	7.39E-10	0.06806076	0.45581189
159.4	1.08E-09	5.25E-12	0.82011362	0.59279038	1.39E-33	0.0003585	7.45E-10	0.06825014	0.45384265
159.5	1.08E-09	5.25E-12	0.82011362	0.59279038	1.39E-33	0.0003585	7.45E-10	0.06825014	0.45384265
159.6	1.21E-09	5.07E-12	0.82237354	0.58633227	1.54E-33	0.00035617	7.36E-10	0.06769395	0.4592068
159.7	1.20E-09	4.99E-12	0.82320182	0.58972	1.56E-33	0.00035386	7.22E-10	0.06731441	0.45818852
159.8	1.20E-09	4.99E-12	0.82320182	0.58972	1.56E-33	0.00035386	7.22E-10	0.06731441	0.45818852
159.9	1.18E-09	4.86E-12	0.8250365	0.591739	1.57E-33	0.0003487	7.23E-10	0.06705394	0.45434658
160	1.18E-09	4.86E-12	0.8250365	0.591739	1.57E-33	0.0003487	7.23E-10	0.06705394	0.45434658
160.1	1.18E-09	4.86E-12	0.8250365	0.591739	1.57E-33	0.0003487	7.23E-10	0.06705394	0.45434658
160.2	1.19E-09	4.92E-12	0.82790578	0.58959536	1.66E-33	0.00034243	7.36E-10	0.06662848	0.45650722
160.3	1.17E-09	5.03E-12	0.82267709	0.58753732	1.67E-33	0.00034955	7.50E-10	0.06649864	0.45852657
160.4	1.17E-09	5.03E-12	0.82267709	0.58753732	1.67E-33	0.00034955	7.50E-10	0.06649864	0.45852657
160.5	1.19E-09	4.83E-12	0.82035633	0.58431303	1.57E-33	0.00034822	7.43E-10	0.06660483	0.45971138
160.6	1.23E-09	4.69E-12	0.82496666	0.58317701	1.57E-33	0.00034653	7.61E-10	0.06556556	0.45878552
160.7	1.27E-09	4.58E-12	0.8252467	0.58173275	1.64E-33	0.00035458	7.54E-10	0.065226	0.45959102
160.8	1.30E-09	4.67E-12	0.82046554	0.58569532	1.72E-33	0.00035173	7.50E-10	0.06471014	0.46041463
160.9	1.30E-09	4.42E-12	0.81747811	0.58604906	1.67E-33	0.00034804	7.45E-10	0.06430499	0.46317847
161	1.25E-09	4.26E-12	0.80886141	0.58111032	1.57E-33	0.00035363	7.22E-10	0.06341256	0.46752695
161.1	1.22E-09	4.18E-12	0.80124933	0.57831371	1.53E-33	0.00034353	7.08E-10	0.0630171	0.46751368
161.2	1.20E-09	4.09E-12	0.79156019	0.57439981	1.44E-33	0.00034703	6.85E-10	0.06303061	0.46582098
161.3	1.23E-09	3.95E-12	0.79478424	0.5717677	1.51E-33	0.00034893	6.93E-10	0.06256095	0.4689785
161.4	1.14E-09	3.96E-12	0.80169806	0.57062595	1.45E-33	0.00033923	7.02E-10	0.06258599	0.4653192
161.5	1.10E-09	3.98E-12	0.80293249	0.56925635	1.43E-33	0.00032962	7.04E-10	0.06295127	0.46228794
161.6	1.09E-09	3.90E-12	0.80104387	0.56891933	1.43E-33	0.00033445	7.30E-10	0.06259907	0.46233494
161.7	1.06E-09	4.11E-12	0.79939507	0.56431197	1.45E-33	0.00033206	7.19E-10	0.06297248	0.46301766
161.8	1.08E-09	3.84E-12	0.79887248	0.56325557	1.48E-33	0.0003307	7.42E-10	0.06324867	0.46287043
161.9	1.16E-09	3.78E-12	0.80038011	0.56529831	1.57E-33	0.0003217	7.53E-10	0.06316464	0.46204039
162	1.16E-09	3.78E-12	0.80038011	0.56529831	1.57E-33	0.0003217	7.53E-10	0.06316464	0.46204039
162.1	1.16E-09	3.78E-12	0.80038011	0.56529831	1.57E-33	0.0003217	7.53E-10	0.06316464	0.46204039
162.2	1.18E-09	3.91E-12	0.78993928	0.56903933	1.68E-33	0.00032327	7.61E-10	0.06336374	0.46691264
162.3	1.18E-09	3.91E-12	0.78993928	0.56903933	1.68E-33	0.00032327	7.61E-10	0.06336374	0.46691264
162.4	1.18E-09	3.91E-12	0.78993928	0.56903933	1.68E-33	0.00032327	7.61E-10	0.06336374	0.46691264
162.5	1.22E-09	3.92E-12	0.79222804	0.5668011	1.77E-33	0.00033347	7.93E-10	0.06216188	0.46783187
162.6	1.25E-09	3.87E-12	0.78975109	0.5675746	1.74E-33	0.00034012	7.78E-10	0.06204055	0.4710491
162.7	1.28E-09	3.84E-12	0.79150491	0.56926274	1.78E-33	0.00033731	7.86E-10	0.06097795	0.471146
162.8	1.39E-09	3.91E-12	0.78929307	0.56369494	1.97E-33	0.00034972	7.88E-10	0.06098697	0.47507363
162.9	1.39E-09	3.91E-12	0.78929307	0.56369494	1.97E-33	0.00034972	7.88E-10	0.06098697	0.47507363
163	1.39E-09	4.01E-12	0.79129569	0.56491222	1.89E-33	0.00034847	7.79E-10	0.06102782	0.47562061
163.1	1.37E-09	3.95E-12	0.78843261	0.56671685	1.95E-33	0.00034785	7.92E-10	0.06147247	0.47577977
163.2	1.32E-09	4.01E-12	0.78835503	0.56833536	1.94E-33	0.00036004	7.97E-10	0.06136399	0.47738579
163.3	1.32E-09	4.01E-12	0.78835503	0.56833536	1.94E-33	0.00036004	7.97E-10	0.06136399	0.47738579
163.4	1.34E-09	3.84E-12	0.7831181	0.5663444	1.94E-33	0.00036225	7.90E-10	0.06160689	0.47854307
163.5	1.34E-09	3.84E-12	0.7831181	0.5663444	1.94E-33	0.00036225	7.90E-10	0.06160689	0.47854307
163.6	1.29E-09	3.81E-12	0.77731057	0.56949606	1.87E-33	0.00035485	7.89E-10	0.06105684	0.48116504
163.7	1.29E-09	3.81E-12	0.77731057	0.56949606	1.87E-33	0.00035485	7.89E-10	0.06105684	0.48116504
163.8	1.27E-09	3.73E-12	0.77358892	0.56911639	1.90E-33	0.00035111	7.93E-10	0.0619405	0.4768568
163.9	1.27E-09	3.73E-12	0.77358892	0.56911639	1.90E-33	0.00035111	7.93E-10	0.0619405	0.4768568
164	1.28E-09	3.70E-12	0.76595347	0.56865535	1.85E-33	0.00035005	7.89E-10	0.06112433	0.47668116
164.1	1.19E-09	3.81E-12	0.76087311	0.56345699	1.76E-33	0.00033364	7.37E-10	0.06121959	0.47324648
164.2	1.20E-09	3.62E-12	0.74921797	0.55826532	1.72E-33	0.00034855	7.02E-10	0.06129872	0.48183401
164.3	1.24E-09	3.75E-12	0.74389049	0.56081579	1.76E-33	0.0003431	6.66E-10	0.06134872	0.48198729
164.4	1.25E-09	3.63E-12	0.73505352	0.55690513	1.80E-33	0.00034252	6.57E-10	0.06251924	0.48509656
164.5	1.28E-09	3.64E-12	0.73123143	0.55773277	1.73E-33	0.00035236	6.45E-10	0.062948	0.48564482
164.6	1.28E-09	3.64E-12	0.73123143	0.55773277	1.73E-33	0.00035236	6.45E-10	0.062948	0.48564482
164.7	1.28E-09	3.48E-12	0.73135123	0.55148705	1.67E-33	0.00035405	6.31E-10	0.06272874	0.48872313
164.8	1.32E-09	3.43E-12	0.73230381	0.55069559	1.74E-33	0.00035748	6.21E-10	0.06253802	0.48964082
164.9	1.32E-09	3.43E-12	0.73230381	0.55069559	1.74E-33	0.00035748	6.21E-10	0.06253802	0.48964082
210	2.85E-08	1.09E-12	0.14832597	0.32977624	3.32E-31	0.00030318	2.32E-09	0.08957394	0.75105547
210.1	2.85E-08	1.09E-12	0.14832597	0.32977624	3.32E-31	0.00030318	2.32E-09	0.08957394	0.75105547
210.2	2.85E-08	1.09E-12	0.14832597	0.32977624	3.32E-31	0.00030318	2.32E-09	0.08957394	0.75105547
210.3	2.93E-08	1.10E-12	0.14720937	0.32914191	3.43E-31	0.0003035	2.38E-09	0.08863281	0.75041144
210.4	3.06E-08	1.11E-12	0.14756587	0.32946606	3.72E-31	0.0003017	2.46E-09	0.08837646	0.75194484
210.5	3.06E-08	1.11E-12	0.14756587	0.32946606	3.72E-31	0.0003017	2.46E-09	0.08837646	0.75194484

165.3	1.35E-09	3.61E-12	0.73412776	0.53825261	1.80E-33	0.00034378	5.89E-10	0.06445673	0.49370068
165.4	1.43E-09	3.53E-12	0.72717524	0.53566669	1.97E-33	0.00034012	5.68E-10	0.06387375	0.49823812
165.5	1.36E-09	3.61E-12	0.72276557	0.53189909	1.97E-33	0.00032604	6.11E-10	0.06367159	0.49738381
165.6	1.37E-09	3.46E-12	0.72334473	0.5285637	1.80E-33	0.00031928	6.00E-10	0.06253519	0.50104018
165.7	1.40E-09	3.49E-12	0.72253038	0.52909504	1.87E-33	0.0003211	5.99E-10	0.06318641	0.50083894
165.8	1.46E-09	3.33E-12	0.71835931	0.52846795	1.86E-33	0.00031267	5.85E-10	0.06370838	0.50159259
165.9	1.45E-09	3.20E-12	0.71442568	0.52748823	1.79E-33	0.00031039	5.70E-10	0.06322212	0.50332666
166	1.45E-09	3.20E-12	0.71442568	0.52748823	1.79E-33	0.00031039	5.70E-10	0.06322212	0.50332666
166.1	1.44E-09	3.09E-12	0.70965056	0.52304587	1.69E-33	0.00030839	5.65E-10	0.06351131	0.5078564
166.2	1.43E-09	2.98E-12	0.71694937	0.51729442	1.75E-33	0.00031308	5.45E-10	0.06333147	0.50651489
166.3	1.43E-09	2.98E-12	0.71694937	0.51729442	1.75E-33	0.00031308	5.45E-10	0.06333147	0.50651489
166.4	1.51E-09	3.24E-12	0.71666505	0.52401524	1.76E-33	0.00031784	5.63E-10	0.06303471	0.50515985
166.5	1.51E-09	3.24E-12	0.71666505	0.52401524	1.76E-33	0.00031784	5.63E-10	0.06303471	0.50515985
166.6	1.56E-09	3.28E-12	0.72046956	0.52410227	1.81E-33	0.00032052	5.61E-10	0.06303735	0.50570023
166.7	1.64E-09	3.24E-12	0.7093686	0.51606066	1.98E-33	0.00032359	6.01E-10	0.06267608	0.51042609
166.8	1.64E-09	3.24E-12	0.7093686	0.51606066	1.98E-33	0.00032359	6.01E-10	0.06267608	0.51042609
166.9	1.59E-09	3.27E-12	0.70954012	0.51589674	1.92E-33	0.00032419	5.59E-10	0.0623576	0.50706101
167	1.58E-09	3.42E-12	0.70890737	0.5214783	1.91E-33	0.00032253	5.48E-10	0.06195403	0.50440722
167.1	1.59E-09	3.30E-12	0.70993773	0.51771548	1.86E-33	0.00031844	5.38E-10	0.06193218	0.5040182
167.2	1.54E-09	3.24E-12	0.70700092	0.51525998	1.77E-33	0.00031606	5.32E-10	0.06225454	0.50518855
167.3	1.54E-09	3.24E-12	0.70700092	0.51525998	1.77E-33	0.00031606	5.32E-10	0.06225454	0.50518855
167.4	1.57E-09	3.08E-12	0.70328781	0.513081	1.66E-33	0.00031265	5.26E-10	0.0630928	0.5072674
167.5	1.61E-09	3.19E-12	0.70363869	0.51430848	1.58E-33	0.00032269	5.15E-10	0.06344268	0.50915432
167.6	1.61E-09	3.19E-12	0.70363869	0.51430848	1.58E-33	0.00032269	5.15E-10	0.06344268	0.50915432
167.7	1.55E-09	3.19E-12	0.70092233	0.51434927	1.58E-33	0.00031828	5.03E-10	0.06396949	0.5077199
167.8	1.57E-09	3.32E-12	0.70227007	0.5154041	1.63E-33	0.00031597	5.06E-10	0.06421446	0.50629187
167.9	1.66E-09	3.43E-12	0.70338578	0.51322619	1.80E-33	0.00032462	5.13E-10	0.06427032	0.50621738
168	1.66E-09	3.43E-12	0.70338578	0.51322619	1.80E-33	0.00032462	5.13E-10	0.06427032	0.50621738
168.1	1.73E-09	3.57E-12	0.70507633	0.512284	1.87E-33	0.00033256	5.29E-10	0.06408615	0.50877548
168.2	1.73E-09	3.57E-12	0.70507633	0.512284	1.87E-33	0.00033256	5.29E-10	0.06408615	0.50877548
168.3	1.74E-09	3.32E-12	0.69821619	0.51155057	1.81E-33	0.00033286	5.19E-10	0.06386081	0.51481193
168.4	1.73E-09	3.39E-12	0.68929944	0.51460988	1.81E-33	0.00033264	5.18E-10	0.06377056	0.51628822
168.5	1.73E-09	3.39E-12	0.68929944	0.51460988	1.81E-33	0.00033264	5.18E-10	0.06377056	0.51628822
168.6	1.74E-09	3.42E-12	0.68618423	0.51462839	1.87E-33	0.00033779	5.12E-10	0.06297197	0.52077547
168.7	1.74E-09	3.42E-12	0.68618423	0.51462839	1.87E-33	0.00033779	5.12E-10	0.06297197	0.52077547
168.8	1.74E-09	3.28E-12	0.68218565	0.51125247	1.83E-33	0.00033195	5.07E-10	0.06362181	0.52295402
168.9	1.74E-09	3.28E-12	0.68218565	0.51125247	1.83E-33	0.00033195	5.07E-10	0.06362181	0.52295402
169	1.79E-09	3.14E-12	0.67882369	0.51235807	1.71E-33	0.00032301	4.98E-10	0.06420722	0.52375687
169.1	1.88E-09	3.10E-12	0.67409134	0.51630312	1.78E-33	0.00032707	5.15E-10	0.06409378	0.52457313
169.2	1.88E-09	3.10E-12	0.67409134	0.51630312	1.78E-33	0.00032707	5.15E-10	0.06409378	0.52457313
169.3	1.88E-09	3.05E-12	0.66995891	0.51387757	1.79E-33	0.00033276	5.11E-10	0.06361967	0.52681722
169.4	1.96E-09	2.96E-12	0.66356844	0.51339921	1.87E-33	0.00033794	5.19E-10	0.06445936	0.5304506
169.5	2.04E-09	2.83E-12	0.65597958	0.5100616	1.87E-33	0.00034031	5.16E-10	0.06514452	0.5346045
169.6	2.05E-09	2.81E-12	0.65437717	0.50782494	1.88E-33	0.00034768	5.15E-10	0.06500766	0.53573868
169.7	2.05E-09	2.81E-12	0.65437717	0.50782494	1.88E-33	0.00034768	5.15E-10	0.06500766	0.53573868
169.8	2.05E-09	2.81E-12	0.65437717	0.50782494	1.88E-33	0.00034768	5.15E-10	0.06500766	0.53573868
169.9	2.05E-09	2.81E-12	0.65437717	0.50782494	1.88E-33	0.00034768	5.15E-10	0.06500766	0.53573868
170	2.26E-09	2.75E-12	0.64445638	0.50168464	1.86E-33	0.00034482	5.04E-10	0.06601468	0.5445216
170.1	2.29E-09	2.60E-12	0.64226001	0.50019302	1.82E-33	0.00033588	5.07E-10	0.06577641	0.54811363
170.2	2.19E-09	2.65E-12	0.64177833	0.4974589	1.74E-33	0.00033588	5.02E-10	0.06572127	0.5493117
170.3	2.23E-09	2.54E-12	0.64033605	0.49671626	1.78E-33	0.00033818	4.99E-10	0.06558349	0.55167385
170.4	2.37E-09	2.34E-12	0.62844496	0.49525423	1.75E-33	0.00032828	4.88E-10	0.06670123	0.55065883
170.5	2.28E-09	2.40E-12	0.62819666	0.49102127	1.67E-33	0.00032626	4.66E-10	0.06644343	0.55253789
170.6	2.33E-09	2.31E-12	0.62213461	0.48901104	1.69E-33	0.00031912	4.64E-10	0.06565366	0.55548044
170.7	2.38E-09	2.35E-12	0.62176278	0.48915545	1.75E-33	0.00031924	4.79E-10	0.06605894	0.5601946
170.8	2.31E-09	2.25E-12	0.61538349	0.48564414	1.70E-33	0.00032144	4.59E-10	0.06678135	0.5625884
170.9	2.31E-09	2.25E-12	0.61538349	0.48564414	1.70E-33	0.00032144	4.59E-10	0.06678135	0.5625884
171	2.32E-09	2.15E-12	0.60706268	0.485017	1.85E-33	0.00031332	4.59E-10	0.06672631	0.56026042
171.1	2.32E-09	2.15E-12	0.60706268	0.485017	1.85E-33	0.00031332	4.59E-10	0.06672631	0.56026042
171.2	2.34E-09	2.13E-12	0.60300352	0.48509967	1.89E-33	0.00031286	4.66E-10	0.06728992	0.55801954
171.3	2.34E-09	2.13E-12	0.60300352	0.48509967	1.89E-33	0.00031286	4.66E-10	0.06728992	0.55801954
171.4	2.38E-09	2.15E-12	0.60238559	0.47986067	1.97E-33	0.00031925	4.66E-10	0.06746307	0.5563129
171.5	2.42E-09	2.09E-12	0.59906374	0.4788272	1.95E-33	0.00032377	4.69E-10	0.06835295	0.55935404
171.6	2.39E-09	2.04E-12	0.59397442	0.47975587	1.81E-33	0.00031359	4.66E-10	0.06861562	0.55952903
171.7	2.39E-09	2.04E-12	0.59397442	0.47975587	1.81E-33	0.00031359	4.66E-10	0.06861562	0.55952903
171.8	2.39E-09	1.97E-12	0.59267003	0.4775769	1.78E-33	0.00031226	4.58E-10	0.06888755	0.56008946
171.9	2.44E-09	1.95E-12	0.58670941	0.48170174	1.80E-33	0.00031203	4.67E-10	0.06921479	0.5646488
172	2.44E-09	1.95E-12	0.58670941	0.48170174	1.80E-33	0.00031203	4.67E-10	0.06921479	0.5646488
172.1	2.44E-09	1.95E-12	0.58670941	0.48170174	1.80E-33	0.00031203	4.67E-10	0.06921479	0.5646488
172.2	2.38E-09	1.94E-12	0.5823296	0.48053266	1.84E-33	0.00031159	4.62E-10	0.06897577	0.56672996
172.3	2.42E-09	1.89E-12	0.57994545	0.48261418	1.83E-33	0.00031357	4.51E-10	0.06846039	0.56956109
172.4	2.41E-09	1.89E-12	0.57638717	0.48041529	1.88E-33	0.0003159	4.53E-10	0.06915104	0.57270127
172.5	2.44E-09	1.99E-12	0.57127268	0.47898496	1.92E-33	0.0003187	4.65E-10	0.06878108	0.5737116
172.6	2.49E-09	1.95E-12	0.56831563	0.48113086	1.90E-33	0.00031793	4.64E-10	0.06940104	0.5754179
172.7	2.51E-09	1.93E-12	0.57202902	0.47801964	2.00E-33	0.00032687	4.60E-10	0.06940912	0.57580471
172.8	2.51E-09	1.93E-12	0.57202902	0.47801964	2.00E-33	0.00032687	4.60E-10	0.06940912	0.57580471
172.9	2.68E-09	1.78E-12	0.56525101	0.47499358	1.95E-33	0.00034876	4.44E-10	0.06909783	0.58392902
173	2.81E-09	1.73E-12	0.56486169	0.47612733	2.00E-33	0.00034023	4.66E-10	0.06812281	0.58063713
173.1	2.76E-09	1.68E-12	0.56260335	0.47823612	1.99E-33	0.0003394	4.57E-10	0.06775571	0.58101313
173.2	3.00E-09	1.69E-12	0.55876865	0.48170742	2.14E-33	0.00035839	4.82E-10	0.06742819	0.5898834
173.3	3.06E-09	1.61E-12	0.55738419	0.48183013	2.24E-33	0.00034999	4.98E-10	0.06758748	0.59205488
173.4	3.06E-09	1.61E-12	0.55738419	0.48183013	2.24E-33	0.00034999	4.98E-10	0.06758748	0.59205488
173.5	3.15E-09	1.50E-12	0.55219644	0.48089439	2.25E-33	0.00034664	4.95E-10	0.06767673	0.59115785
173.6	3.21E-09	1.51E-12	0.55214046	0.47935686	2.50E-33	0.00035766	5.00E-10	0.06781018	0.5932505
173.7	3.25E-09	1.40E-12	0.54311699	0.47634243	2.64E-33	0.0003421	4.76E-10	0.06706163	0.5895868
173.8	3.18E-09	1.46E-12	0.54378825	0.4711975	2.51E-33	0.0003424	4.76E-10	0.06727608	0.58716569
173.9	3.16E-09	1.47E-12	0.53776549	0.47221018	2.68E-33	0.00035485	4.67E-10	0.06749226	0.59132475
174	3.16E-09	1.47E-12	0.53776549	0.47221018	2.68E-33	0.00035485	4.67E-10	0.06749226	0.59132475
174.1	3.13E-09	1.42E-12	0.53303306	0.46832746	2.78E-33	0.00035439	4.63E-10	0.0677462	0.59563056
174.2	3.17E-09	1.31E-12	0.52837582	0.46544465	2.57E-33	0.00034773	4.48E-10	0.06881605	0.59725587
174.3	3.19E-09	1.32E-12	0.5228988	0.46843862	2.77E-33	0.00035164	4.62E-10	0.06893563	0.59701169
174.4	3.17E-09	1.31E-12	0.51563612	0.47057879	2.65E-33	0.00034256	4.60E-10	0.0700542	0.59758453
174.5	3.23E-09	1.34E-12	0.51497946	0.46925004	2.60E-33	0.00034752	4.56E-10	0.07039133	0.59448786
174.6	3.30E-09	1.34E-12	0.50934174	0.46660895	2.50E-33	0.00034177	4.36E-10	0.07212558	0.59425688
174.7	3.36E-09	1.32E-12	0.50886377	0.46426029	2.50E-33	0.00034068	4.38E-10	0.07277986	0.59353376
174.8	3.44E-09	1.29E-12	0.50667471	0.46607666	2.49E-33	0.00034268	4.46E-10	0.07246378	0.59569305
174.9	3.46E-09	1.23E-12	0.50518556	0.46261523	2.58E-33	0.00034738	4.50E-10	0.07305577	0.59559047
175	3.48E-09	1.18E-12	0.50074451	0.46223635	2.41E-33	0.00034021	4.41E-10	0.07385636	0.59832563
175.1	3.44E-09	1.15E-12	0.49612622	0.46269292	2.42E-33	0.00033388	4.28E-10	0.07352562	0.59961964
175.2	3.44E-09	1.15E-12	0.49612622	0.46269292	2.42E-33	0.00033388	4.28E-10	0.07352562	0.59961964
175.3	3.50E-09	1.23E-12	0.4983533	0.46849552	2.49E-33	0.00033118	4.30E-10	0.07257142	0.59792322
175.4	3.67E-09	1.23E-12	0.49438344	0.47131291	2.64E-33	0.00033148	4.27E-10	0.07184645	0.59822018
175.5	3.65E-09	1.15E-12	0.48955038	0.46922059	2.51E-33	0.00033989	4.19E-10	0.07247232	0.60305332
175.6	3.68E-09	1.12E-12	0.48650026	0.46957682	2.49E-33	0.00034337	4.18E-10	0.07331074	0.6049463
175.7	3.70E-09	1.17E-12	0.48506773	0.46730548	2.52E-33	0.00034784	4.14E-10	0.0745208	0.60240736
175.8	3.69E-09	1.18E-12	0.4817682	0.46595894	2.74E-33	0.00035089	4.27E-10	0.0752161	0.60090381
175.9	3.83E-09	1.22E-12	0.481955	0.46387318	2.81E-33	0.0003508	4.35E-10	0.07422713	0.60030208
176	3.76E-09	1.18E-12	0.47543124	0.46256356	2.76E-33	0.00035059	4.33E-10	0.07453375	0.60184389
176.1	3.76E-09	1.18E-12	0.47447081	0.45972827	2.58E-33	0.00034147	4.15E-10	0.07419258	0.60100333
176.2	3.77E-09	1.16E-12	0.47124726	0.46186387	2.53E-33	0.0003489	4.14E-10	0.0745497	0.60323274
176.3	3.77E-09	1.16E-12	0.47124726	0.46186387	2.53E-33	0.0003489	4.14E-10	0.0745497	0.60323274
176.4	3.88E-09	1.18E-12	0.46924091	0.46189212	2.57E-33	0.00034993	4.20E-10	0.07457875	0.60316768
176.5	3.74E-09	1.12E-12	0.45897044	0.45759096	2.58E-33	0.00034743	4.03E-10	0.07356807	0.6068687
176.6	3.74E-09	1.12E-12	0.45897044	0.45759096	2.58E-33	0.00034743	4.03E-10	0.07356807	0.6068687
176.7	3.74E-09	1.12E-12	0.45897044	0.45759096	2.58E-33	0.00034743	4.03E-10	0.07356807	0.6068687
176.8	3.77E-09	1.17E-12	0.45784958	0.45797487	2.64E-33	0.00035014	4.03E-10	0.07421721	0.60472914
176.9	3.75E-09	1.19E-12	0.4580917	0.45905244	2.79E-33	0.00035901	4.09E-10	0.07420695	0.60557609
177	3.75E-09	1.19E-12	0.4580917	0.45905244	2.79E-33	0.00035901	4.09E-10	0.07420695	0.60557609
177.1	3.93E-09	1.24E-12	0.45927277	0.45499822	3.07E-33	0.00036342	4.22E-10	0.07556525	0.60860494
177.2	3.84E-09	1.28E-12	0.46204964	0.45789903	3.04E-33	0.00036451	4.31E-10	0.07560153	0.60773182
177.3	3.79E-09	1.23E-12	0.4562451	0.4585322	2.98E-33	0.00036237	4.45E-10	0.07520888	0.60619604
177.4	3.79E-09	1.23E-12	0.4562451	0.4585322	2.98E-33	0.00036237	4.45E-10	0.07520888	0.60619604
177.5	3.79E-09	1.23E-12	0.4562451	0.4585322	2.98E-33	0.00036237	4.45E-10	0.07520888	0.60619604
177.6	3.75E-09	1.21E-12	0.45487807	0.45904793	3.00E-33	0.00036527	4.39E-10	0.07572244	0.60637637
177.7	3.83E-09	1.21E-12	0.45418817	0.45787568	3.33E-33	0.00037668	4.39E-10	0.07552295	0.60718108
177.8	3.82E-09	1.22E-12	0.45216487	0.45704182	3.54E-33	0.00037188	4.51E-10	0.07564836	0.6056069
177.9	3.82E-09	1.22E-12	0.45216487	0.45704182	3.54E-33	0.00037188	4.51E-10	0.07564836	0.6056069
178	3.84E-09	1.21E-12	0.45033818	0.45654818	3.55E-33	0.00037478	4.52E-10	0.07534989	0.60712336
178.1	3.98E-09	1.22E-12	0.44443387	0.45785205	3.69E-33	0.00037754	4.52E-10	0.07495348	0.61339284
178.2	4.00E-09	1.19E-12	0.44339491	0.45750466	3.73E-33	0.00037438	4.57E-10	0.07515484	0.61063231
178.3	4.06E-09	1.21E-12	0.44153766	0.45582977	3.98E-33	0.00037969	4.73E-10	0.07545782	0.61071149
178.4	4.06E-09	1.21E-12	0.44153766	0.45582977	3.98E-33	0.00037969	4.73E-10	0.07545782	0.61071149
178.5	4.13E-09	1.24E-12	0.44132848	0.45838807	3.92E-33	0.00038084	4.76E-10	0.07556768	0.60612919
178.6	4.21E-09	1.25E-12	0.43885057	0.45926568	3.94E-33	0.00038297	4.68E-10	0.07538372	0.60309739
178.7	4.43E-09	1.28E-12	0.43749227	0.45672526	4.29E-33	0.00039388	4.98E-10	0.07487073	0.61175244
178.8	4.64E-09	1.28E-12	0.43845926	0.45239161	4.12E-33	0.00038987	4.86E-10	0.07528903	0.61135164
178.9	4.64E-09	1.28E-12	0.43845926	0.45239161	4.12E-33	0.00038987	4.86E-10	0.07528903	0.61135164
179	4.63E-09	1.30E-12	0.43220704	0.44848676	4.31E-33	0.00040205	4.88E-10	0.07499843	0.61334388
179.1	4.61E-09	1.30E-12	0.42813192	0.44836336	4.49E-33	0.00040263	4.82E-10	0.07556173	0.61439552
179.2	4.54E-09	1.25E-12	0.4246952	0.44762943	4.45E-33	0.00039212	4.78E-10	0.07536055	0.61238053
179.3	4.76E-09	1.40E-12	0.41977819	0.44496575	4.92E-33	0.00040194	5.03E-10	0.07410761	0.62222377
179.4	4.76E-09	1.40E-12	0.41977819	0.44496575	4.92E-33	0.00040194	5.03E-10	0.07410761	0.62222377
179.5	4.85E-09	1.38E-12	0.4161852	0.4452836	4.73E-33	0.00039565	4.86E-10	0.07417055	0.62212058
179.6	4.94E-09	1.40E-12	0.4130237	0.44492038	4.85E-33	0.00040438	4.99E-10	0.07463617	0.6246792
179.7	4.91E-09	1.40E-12	0.40875342	0.44445136	5.16E-33	0.00040703	5.22E-10	0.07507365	0.63272226
179.8	5.74E-09	1.47E-12	0.40272482	0.44478749	6.69E-33	0.00042431	5.38E-10	0.07533333	0.6339411
179.9	5.68E-09	1.45E-12	0.40142913	0.44439407	6.75E-33	0.0004324	5.43E-10	0.07577625	0.63659214
225	5.45E-08	1.66E-12	0.11237498	0.2629235	9.21E-31	0.00035455	4.05E-09	0.10708682	0.75877332
225.1	5.45E-08	1.66E-12	0.11237498	0.2629235	9.21E-31	0.00035455	4.05E-09	0.10708682	0.75877332
225.2	5.45E-08	1.66E-12	0.11237498	0.2629235	9.21E-31	0.00035455	4.05E-09	0.10708682	0.75877332
225.3	5.45E-08	1.66E-12	0.11237498	0.2629235	9.21E-31	0.00035455	4.05E-09	0.10708682	0.75877332
225.4	5.45E-08	1.66E-12	0.11237498	0.2629235	9.21E-31	0.00035455	4.05E-09	0.10708682	0.75877332
225.5	5.45E-08	1.66E-12	0.11237498	0.2629235	9.21E-31	0.00035455	4.05E-09	0.10708682	0.75877332

180.6	5.52E-09	1.39E-12	0.38334926	0.44746632	7.61E-33	0.00042596	4.82E-10	0.07554106	0.63272636
180.7	5.35E-09	1.39E-12	0.37924031	0.4474146	7.64E-33	0.00043121	4.69E-10	0.07582281	0.63467131
180.8	5.35E-09	1.39E-12	0.37924031	0.4474146	7.64E-33	0.00043121	4.69E-10	0.07582281	0.63467131
180.9	5.44E-09	1.41E-12	0.37757985	0.44872977	8.01E-33	0.00043071	4.74E-10	0.07570439	0.63384951
181	5.55E-09	1.43E-12	0.37322091	0.44704605	8.11E-33	0.00043616	4.81E-10	0.07660602	0.63600355
181.1	5.64E-09	1.40E-12	0.37229599	0.44433898	7.79E-33	0.00043896	4.78E-10	0.07660071	0.64021047
181.2	5.51E-09	1.38E-12	0.36939669	0.44151458	7.68E-33	0.00043973	4.78E-10	0.07606115	0.64045865
181.3	5.35E-09	1.42E-12	0.37028227	0.43859987	7.93E-33	0.00043674	4.76E-10	0.07527429	0.63946323
181.4	5.38E-09	1.41E-12	0.36787854	0.43568444	8.09E-33	0.0004258	4.65E-10	0.07610896	0.63660652
181.5	5.55E-09	1.39E-12	0.36513799	0.43790624	8.20E-33	0.00042775	4.72E-10	0.07595873	0.64187791
181.6	5.61E-09	1.40E-12	0.36444802	0.43859557	8.46E-33	0.00042532	4.80E-10	0.07664145	0.6463264
181.7	5.61E-09	1.40E-12	0.36444802	0.43859557	8.46E-33	0.00042532	4.80E-10	0.07664145	0.6463264
181.8	5.72E-09	1.40E-12	0.36235522	0.43754833	8.26E-33	0.00042894	4.61E-10	0.07637881	0.64800303
181.9	5.76E-09	1.39E-12	0.35962942	0.43358431	8.00E-33	0.00042986	4.60E-10	0.07706155	0.64750861
182	5.76E-09	1.39E-12	0.35962942	0.43358431	8.00E-33	0.00042986	4.60E-10	0.07706155	0.64750861
182.1	5.76E-09	1.39E-12	0.35962942	0.43358431	8.00E-33	0.00042986	4.60E-10	0.07706155	0.64750861
182.2	5.76E-09	1.39E-12	0.35962942	0.43358431	8.00E-33	0.00042986	4.60E-10	0.07706155	0.64750861
182.3	5.68E-09	1.39E-12	0.35139858	0.43169729	8.06E-33	0.00043256	4.76E-10	0.07717811	0.6583683
182.4	5.73E-09	1.37E-12	0.35162073	0.43108976	8.25E-33	0.00043719	4.67E-10	0.07778627	0.65908393
182.5	5.67E-09	1.36E-12	0.34885809	0.43033079	8.60E-33	0.00043459	4.68E-10	0.07812779	0.66160794
182.6	5.50E-09	1.39E-12	0.34838423	0.42931407	8.61E-33	0.00043107	4.67E-10	0.07934354	0.6603381
182.7	5.59E-09	1.38E-12	0.34605435	0.43262854	8.66E-33	0.00042451	4.65E-10	0.07961451	0.66134743
182.8	5.59E-09	1.38E-12	0.34605435	0.43262854	8.66E-33	0.00042451	4.65E-10	0.07961451	0.66134743
182.9	5.92E-09	1.36E-12	0.34380581	0.43034035	8.97E-33	0.00043686	4.66E-10	0.08039398	0.66065847
183	6.00E-09	1.34E-12	0.34037593	0.43232046	9.34E-33	0.00043814	4.64E-10	0.07984871	0.66187396
183.1	6.00E-09	1.34E-12	0.34037593	0.43232046	9.34E-33	0.00043814	4.64E-10	0.07984871	0.66187396
183.2	6.00E-09	1.34E-12	0.34037593	0.43232046	9.34E-33	0.00043814	4.64E-10	0.07984871	0.66187396
183.3	6.33E-09	1.32E-12	0.33419034	0.43369972	9.70E-33	0.00043814	4.78E-10	0.08048184	0.66664365
183.4	6.33E-09	1.32E-12	0.33419034	0.43369972	9.70E-33	0.00043814	4.78E-10	0.08048184	0.66664365
183.5	6.46E-09	1.33E-12	0.33185845	0.43456121	9.88E-33	0.00044227	4.85E-10	0.08041919	0.66975552
183.6	6.53E-09	1.32E-12	0.32894142	0.43708844	1.02E-32	0.00043764	4.79E-10	0.0800375	0.67107026
183.7	6.57E-09	1.28E-12	0.32748247	0.43803975	1.07E-32	0.00043129	4.86E-10	0.07931918	0.67509381
183.8	6.57E-09	1.28E-12	0.32748247	0.43803975	1.07E-32	0.00043129	4.86E-10	0.07931918	0.67509381
183.9	6.63E-09	1.25E-12	0.3245645	0.43853749	1.15E-32	0.00042014	5.26E-10	0.07931998	0.67396083
184	6.63E-09	1.25E-12	0.3245645	0.43853749	1.15E-32	0.00042014	5.26E-10	0.07931998	0.67396083
184.1	6.63E-09	1.25E-12	0.3245645	0.43853749	1.15E-32	0.00042014	5.26E-10	0.07931998	0.67396083
184.2	6.69E-09	1.22E-12	0.32394749	0.43782692	1.30E-32	0.00041619	5.43E-10	0.0788439	0.68038063
184.3	6.69E-09	1.20E-12	0.32314028	0.4361908	1.29E-32	0.00041608	5.56E-10	0.07867475	0.67850382
184.4	6.69E-09	1.20E-12	0.32314028	0.4361908	1.29E-32	0.00041608	5.56E-10	0.07867475	0.67850382
184.5	6.78E-09	1.19E-12	0.32642642	0.43350027	1.34E-32	0.00042887	5.57E-10	0.07822598	0.67958055
184.6	6.73E-09	1.19E-12	0.32559319	0.43378722	1.41E-32	0.00043204	5.54E-10	0.07809844	0.68026066
184.7	6.59E-09	1.27E-12	0.32457004	0.43483313	1.49E-32	0.00043355	5.69E-10	0.07804927	0.68009627
184.8	6.59E-09	1.27E-12	0.32457004	0.43483313	1.49E-32	0.00043355	5.69E-10	0.07804927	0.68009627
184.9	6.59E-09	1.27E-12	0.32457004	0.43483313	1.49E-32	0.00043355	5.69E-10	0.07804927	0.68009627
185	6.59E-09	1.26E-12	0.32188544	0.43165861	1.50E-32	0.00042821	5.75E-10	0.07828934	0.68005308
185.1	6.57E-09	1.19E-12	0.31627546	0.42783247	1.46E-32	0.00041642	5.80E-10	0.07871419	0.6767859
185.2	6.57E-09	1.19E-12	0.31627546	0.42783247	1.46E-32	0.00041642	5.80E-10	0.07871419	0.6767859
185.3	6.44E-09	1.14E-12	0.31200488	0.42566195	1.49E-32	0.00041573	5.87E-10	0.07932073	0.67764842
185.4	6.41E-09	1.17E-12	0.30257971	0.42395103	1.48E-32	0.00040852	5.80E-10	0.08167105	0.67987124
185.5	6.41E-09	1.17E-12	0.30257971	0.42395103	1.48E-32	0.00040852	5.80E-10	0.08167105	0.67987124
185.6	6.23E-09	1.16E-12	0.3005237	0.42364926	1.47E-32	0.00041779	5.92E-10	0.08201247	0.67750013
185.7	6.23E-09	1.16E-12	0.3005237	0.42364926	1.47E-32	0.00041779	5.92E-10	0.08201247	0.67750013
185.8	6.53E-09	1.16E-12	0.2982033	0.42133586	1.63E-32	0.0004189	5.81E-10	0.08127711	0.68206718
185.9	6.53E-09	1.16E-12	0.2982033	0.42133586	1.63E-32	0.0004189	5.81E-10	0.08127711	0.68206718
186	6.41E-09	1.12E-12	0.29674519	0.42066854	1.56E-32	0.00041102	5.88E-10	0.08072192	0.68405866
186.1	6.37E-09	1.12E-12	0.29428835	0.41983778	1.63E-32	0.00041697	5.81E-10	0.08080359	0.68541445
186.2	6.54E-09	1.06E-12	0.29354945	0.41872128	1.54E-32	0.00040386	5.68E-10	0.08090417	0.685753
186.3	6.52E-09	1.08E-12	0.29178765	0.42116038	1.61E-32	0.00040082	5.67E-10	0.08143276	0.68529905
186.4	6.69E-09	1.12E-12	0.29175167	0.42480897	1.64E-32	0.00039257	5.59E-10	0.08195991	0.68374063
186.5	7.01E-09	1.14E-12	0.29071805	0.42698408	1.89E-32	0.0003987	5.87E-10	0.08221204	0.68232423
186.6	6.92E-09	1.12E-12	0.28668904	0.43104071	1.94E-32	0.00038926	5.83E-10	0.08247552	0.68709899
186.7	7.07E-09	1.15E-12	0.28665575	0.43015105	2.04E-32	0.00039605	5.97E-10	0.08342249	0.68754751
186.8	7.18E-09	1.12E-12	0.28447874	0.42915405	2.17E-32	0.00040879	6.18E-10	0.08363456	0.69507298
186.9	7.16E-09	1.08E-12	0.2843701	0.42699431	2.17E-32	0.00040857	6.29E-10	0.08282144	0.69246673
187	7.16E-09	1.07E-12	0.28195881	0.43032823	2.17E-32	0.00041198	6.26E-10	0.08277495	0.69400459
187.1	7.33E-09	9.83E-13	0.27737817	0.42819915	2.23E-32	0.00039763	6.19E-10	0.08348039	0.6907825
187.2	7.22E-09	9.69E-13	0.27571246	0.42848648	2.28E-32	0.00039627	6.23E-10	0.08257364	0.68942072
187.3	7.22E-09	9.69E-13	0.27571246	0.42848648	2.28E-32	0.00039627	6.23E-10	0.08257364	0.68942072
187.4	7.50E-09	1.04E-12	0.27548993	0.43199906	2.35E-32	0.00039886	6.63E-10	0.08224908	0.69074677
187.5	7.64E-09	1.05E-12	0.2748273	0.43076258	2.36E-32	0.00039582	6.70E-10	0.08303326	0.69284064
187.6	7.64E-09	1.05E-12	0.2748273	0.43076258	2.36E-32	0.00039582	6.70E-10	0.08303326	0.69284064
187.7	7.64E-09	1.05E-12	0.2748273	0.43076258	2.36E-32	0.00039582	6.70E-10	0.08303326	0.69284064
187.8	7.64E-09	1.05E-12	0.2748273	0.43076258	2.36E-32	0.00039582	6.70E-10	0.08303326	0.69284064
187.9	7.48E-09	1.06E-12	0.27254046	0.42716861	2.40E-32	0.00038764	6.61E-10	0.08317797	0.69379776
188	7.59E-09	1.05E-12	0.26936266	0.42212045	2.47E-32	0.00039497	6.51E-10	0.08398521	0.69216438
188.1	7.59E-09	1.05E-12	0.26936266	0.42212045	2.47E-32	0.00039497	6.51E-10	0.08398521	0.69216438
188.2	7.59E-09	1.05E-12	0.26936266	0.42212045	2.47E-32	0.00039497	6.51E-10	0.08398521	0.69216438
188.3	7.88E-09	1.02E-12	0.26679506	0.4202032	2.43E-32	0.00039765	6.66E-10	0.08503013	0.68971113
188.4	7.88E-09	1.02E-12	0.26679506	0.4202032	2.43E-32	0.00039765	6.66E-10	0.08503013	0.68971113
188.5	7.88E-09	1.02E-12	0.26679506	0.4202032	2.43E-32	0.00039765	6.66E-10	0.08503013	0.68971113
188.6	7.88E-09	1.02E-12	0.26679506	0.4202032	2.43E-32	0.00039765	6.66E-10	0.08503013	0.68971113
188.7	7.81E-09	9.84E-13	0.26573607	0.42161017	2.49E-32	0.0003948	6.83E-10	0.08420701	0.6897178
188.8	8.03E-09	1.00E-12	0.26295693	0.42210266	2.65E-32	0.00039971	6.94E-10	0.08445104	0.69527453
188.9	8.03E-09	1.00E-12	0.26295693	0.42210266	2.65E-32	0.00039971	6.94E-10	0.08445104	0.69527453
189	7.99E-09	9.94E-13	0.26420775	0.41992242	2.75E-32	0.00039615	7.05E-10	0.08411591	0.69142796
189.1	7.92E-09	9.67E-13	0.26541363	0.41986998	2.82E-32	0.00040179	7.20E-10	0.0837401	0.68985
189.2	7.97E-09	9.41E-13	0.26387825	0.41905336	2.80E-32	0.00039877	7.36E-10	0.08448911	0.68887139
189.3	8.06E-09	9.64E-13	0.26136137	0.42166472	2.88E-32	0.00039708	7.19E-10	0.08409989	0.6888089
189.4	8.06E-09	9.64E-13	0.26136137	0.42166472	2.88E-32	0.00039708	7.19E-10	0.08409989	0.6888089
189.5	8.10E-09	9.38E-13	0.26157736	0.41982882	2.99E-32	0.00038623	7.42E-10	0.08401267	0.68841696
189.6	8.19E-09	9.94E-13	0.25903346	0.42101714	2.87E-32	0.00037933	7.46E-10	0.08449554	0.68416768
189.7	8.36E-09	9.37E-13	0.25697572	0.41853317	2.79E-32	0.0003744	7.52E-10	0.08441033	0.68540115
189.8	8.52E-09	9.22E-13	0.25363389	0.41529022	2.77E-32	0.00037403	7.57E-10	0.08472648	0.68716961
189.9	8.52E-09	9.22E-13	0.25363389	0.41529022	2.77E-32	0.00037403	7.57E-10	0.08472648	0.68716961
190	8.68E-09	8.79E-13	0.25270986	0.41415449	2.66E-32	0.00037117	7.74E-10	0.0839858	0.6861512
190.1	8.75E-09	8.95E-13	0.25213905	0.4138588	2.74E-32	0.00037793	7.78E-10	0.0835252	0.69051872
190.2	8.75E-09	8.95E-13	0.25213905	0.4138588	2.74E-32	0.00037793	7.78E-10	0.0835252	0.69051872
190.3	8.94E-09	9.06E-13	0.25224369	0.41440427	2.74E-32	0.00037403	7.83E-10	0.08305538	0.69157773
190.4	8.94E-09	9.06E-13	0.25224369	0.41440427	2.74E-32	0.00037403	7.83E-10	0.08305538	0.69157773
190.5	8.93E-09	9.30E-13	0.25026222	0.41286349	2.84E-32	0.00037919	7.91E-10	0.08332916	0.69110625
190.6	9.03E-09	9.51E-13	0.25124606	0.40961956	2.79E-32	0.00038629	7.72E-10	0.08287126	0.68945905
190.7	8.86E-09	9.26E-13	0.24652324	0.40381747	2.76E-32	0.00037354	7.71E-10	0.08219739	0.68853512
190.8	9.01E-09	8.81E-13	0.24400512	0.40162437	2.75E-32	0.00037019	7.62E-10	0.08247223	0.68910548
190.9	8.84E-09	9.12E-13	0.24320855	0.39874347	2.77E-32	0.00036788	7.78E-10	0.08242537	0.68904659
191	8.68E-09	9.00E-13	0.24205626	0.39978721	2.71E-32	0.0003571	8.08E-10	0.0818358	0.69310643
191.1	8.68E-09	9.00E-13	0.24205626	0.39978721	2.71E-32	0.0003571	8.08E-10	0.0818358	0.69310643
191.2	8.72E-09	8.92E-13	0.23476469	0.39509441	2.83E-32	0.00035154	8.31E-10	0.08183414	0.69654445
191.3	8.72E-09	8.92E-13	0.23476469	0.39509441	2.83E-32	0.00035154	8.31E-10	0.08183414	0.69654445
191.4	8.83E-09	8.74E-13	0.23353226	0.39714771	2.81E-32	0.00034337	8.45E-10	0.08225239	0.69407908
191.5	8.83E-09	8.74E-13	0.23353226	0.39714771	2.81E-32	0.00034337	8.45E-10	0.08225239	0.69407908
191.6	8.83E-09	8.74E-13	0.23353226	0.39714771	2.81E-32	0.00034337	8.45E-10	0.08225239	0.69407908
191.7	9.08E-09	9.11E-13	0.2306174	0.38964534	2.65E-32	0.00034636	8.40E-10	0.08509779	0.69628741
191.8	9.50E-09	9.93E-13	0.22513022	0.38816976	3.00E-32	0.00035164	8.69E-10	0.08496006	0.70018544
191.9	9.95E-09	9.95E-13	0.22489765	0.38908627	3.20E-32	0.00035232	8.93E-10	0.0853003	0.69616703
192	1.00E-08	1.03E-12	0.22280123	0.38673749	3.36E-32	0.00035276	9.15E-10	0.08573376	0.69916373
192.1	9.99E-09	1.01E-12	0.22194766	0.38822165	3.44E-32	0.00034651	9.25E-10	0.08591704	0.69744122
192.2	9.97E-09	1.05E-12	0.21845933	0.38821034	3.71E-32	0.00034693	9.04E-10	0.08540644	0.69914206
192.3	1.01E-08	1.03E-12	0.21774926	0.38534415	3.70E-32	0.00034625	9.19E-10	0.08604323	0.70225157
192.4	1.01E-08	1.03E-12	0.21774926	0.38534415	3.70E-32	0.00034625	9.19E-10	0.08604323	0.70225157
192.5	1.04E-08	1.02E-12	0.21613578	0.38425664	3.81E-32	0.00034143	9.68E-10	0.08484996	0.69833915
192.6	1.07E-08	1.01E-12	0.21458726	0.38377277	3.85E-32	0.00033638	9.60E-10	0.08514812	0.69993356
192.7	1.07E-08	1.01E-12	0.21458726	0.38377277	3.85E-32	0.00033638	9.60E-10	0.08514812	0.69993356
192.8	1.07E-08	1.01E-12	0.21218649	0.38271541	3.96E-32	0.00034042	9.75E-10	0.08571995	0.7036662
192.9	1.07E-08	1.01E-12	0.21218649	0.38271541	3.96E-32	0.00034042	9.75E-10	0.08571995	0.7036662
193	1.09E-08	9.73E-13	0.21213429	0.38104558	4.15E-32	0.0003501	1.03E-09	0.08479804	0.69856049
193.1	1.09E-08	9.73E-13	0.21213429	0.38104558	4.15E-32	0.0003501	1.03E-09	0.08479804	0.69856049
193.2	1.11E-08	9.51E-13	0.20931091	0.37970226	4.41E-32	0.00034529	1.07E-09	0.08416032	0.69412056
193.3	1.18E-08	9.09E-13	0.20943793	0.37742448	4.44E-32	0.00033953	1.08E-09	0.08350541	0.70103152
193.4	1.18E-08	8.97E-13	0.21075421	0.37662364	4.37E-32	0.0003386	1.09E-09	0.08388422	0.69887388
193.5	1.18E-08	8.66E-13	0.21019538	0.37643358	4.52E-32	0.0003309	1.13E-09	0.0832781	0.69943695
193.6	1.18E-08	8.66E-13	0.21019538	0.37643358	4.52E-32	0.0003309	1.13E-09	0.0832781	0.69943695
193.7	1.24E-08	8.48E-13	0.20716195	0.37676054	4.65E-32	0.00032538	1.13E-09	0.08273455	0.70216822
193.8	1.24E-08	8.48E-13	0.20716195	0.37676054	4.65E-32	0.00032538	1.13E-09	0.08273455	0.70216822
193.9	1.27E-08	8.43E-13	0.20879512	0.37684083	4.82E-32	0.00031799	1.16E-09	0.08318357	0.70391447
194	1.29E-08	8.56E-13	0.20757122	0.37768077	5.12E-32	0.00031561	1.18E-09	0.08328739	0.70624399
194.1	1.29E-08	8.56E-13	0.20757122	0.37768077	5.12E-32	0.00031561	1.18E-09	0.08328739	0.70624399
194.2	1.29E-08	8.56E-13	0.20757122	0.37768077	5.12E-32	0.00031561	1.18E-09	0.08328739	0.70624399
194.3	1.29E-08	8.56E-13	0.20757122	0.37768077	5.12E-32	0.00031561	1.18E-09	0.08328739	0.70624399
194.4	1.30E-08	8.31E-13	0.20473514	0.37403635	5.34E-32	0.00030808	1.19E-09	0.08341109	0.70834184
194.5	1.30E-08	8.31E-13	0.20473514	0.37403635	5.34E-32	0.00030808	1.19E-09	0.08341109	0.70834184
194.6	1.30E-08	8.31E-13	0.20473514	0.37403635	5.34E-32	0.00030808	1.19E-09	0.08341109	0.70834184
194.7	1.30E-08	8.31E-13	0.20473514	0.37403635	5.34E-32	0.00030808	1.19E-09	0.08341109	0.70834184
194.8	1.30E-08	8.31E-13	0.20473514	0.37403635	5.34E-32	0.00030808	1.19E-09	0.08341109	0.70834184
194.9	1.29E-08	8.38E-13	0.20337977	0.37437219	5.54E-32	0.00030615	1.18E-09	0.08434179	0.70782969

195.6	1.45E-08	8.57E-13	0.2006544	0.37459506	7.87E-32	0.00030902	1.35E-09	0.08570949	0.70433207
195.7	1.45E-08	8.57E-13	0.2006544	0.37459506	7.87E-32	0.00030902	1.35E-09	0.08570949	0.70433207
195.8	1.58E-08	8.36E-13	0.20271537	0.37418166	8.12E-32	0.00031861	1.36E-09	0.08493282	0.7060105
195.9	1.58E-08	8.36E-13	0.20271537	0.37418166	8.12E-32	0.00031861	1.36E-09	0.08493282	0.7060105
196	1.57E-08	8.19E-13	0.20181691	0.37487901	7.93E-32	0.00031829	1.36E-09	0.08438824	0.70405085
196.1	1.60E-08	7.90E-13	0.20111642	0.37305263	8.12E-32	0.00031312	1.40E-09	0.08436028	0.70547348
196.2	1.60E-08	7.90E-13	0.20111642	0.37305263	8.12E-32	0.00031312	1.40E-09	0.08436028	0.70547348
196.3	1.59E-08	8.17E-13	0.20128221	0.37521729	8.70E-32	0.00031682	1.41E-09	0.08390335	0.70549119
196.4	1.59E-08	8.17E-13	0.20128221	0.37521729	8.70E-32	0.00031682	1.41E-09	0.08390335	0.70549119
196.5	1.59E-08	8.17E-13	0.20128221	0.37521729	8.70E-32	0.00031682	1.41E-09	0.08390335	0.70549119
196.6	1.59E-08	8.17E-13	0.20128221	0.37521729	8.70E-32	0.00031682	1.41E-09	0.08390335	0.70549119
196.7	1.57E-08	8.10E-13	0.20160051	0.37464946	8.92E-32	0.00031762	1.45E-09	0.08343259	0.70337213
196.8	1.57E-08	8.10E-13	0.20160051	0.37464946	8.92E-32	0.00031762	1.45E-09	0.08343259	0.70337213
196.9	1.57E-08	7.95E-13	0.19935254	0.37210362	8.42E-32	0.00031269	1.43E-09	0.08242799	0.70364253
197	1.64E-08	8.40E-13	0.20054824	0.37339046	9.38E-32	0.00031318	1.45E-09	0.08307404	0.69982197
197.1	1.64E-08	8.40E-13	0.20054824	0.37339046	9.38E-32	0.00031318	1.45E-09	0.08307404	0.69982197
197.2	1.60E-08	8.68E-13	0.20170112	0.37438036	9.55E-32	0.00030922	1.47E-09	0.08302802	0.69694056
197.3	1.60E-08	8.68E-13	0.20170112	0.37438036	9.55E-32	0.00030922	1.47E-09	0.08302802	0.69694056
197.4	1.60E-08	8.68E-13	0.20170112	0.37438036	9.55E-32	0.00030922	1.47E-09	0.08302802	0.69694056
197.5	1.70E-08	9.04E-13	0.20039204	0.37653212	1.02E-31	0.00031371	1.54E-09	0.08207779	0.70153239
197.6	1.68E-08	9.13E-13	0.1976772	0.37241896	9.98E-32	0.00031872	1.54E-09	0.08180428	0.70647883
197.7	1.64E-08	9.14E-13	0.19567519	0.37427092	9.86E-32	0.00030989	1.58E-09	0.08180335	0.70328483
197.8	1.62E-08	9.27E-13	0.19386884	0.37083633	9.70E-32	0.00031137	1.56E-09	0.08185645	0.7075736
197.9	1.62E-08	9.27E-13	0.19386884	0.37083633	9.70E-32	0.00031137	1.56E-09	0.08185645	0.7075736
198	1.62E-08	9.27E-13	0.19386884	0.37083633	9.70E-32	0.00031137	1.56E-09	0.08185645	0.7075736
198.1	1.62E-08	9.27E-13	0.19386884	0.37083633	9.70E-32	0.00031137	1.56E-09	0.08185645	0.7075736
198.2	1.60E-08	9.01E-13	0.19218887	0.36878698	9.53E-32	0.0003102	1.55E-09	0.08185552	0.70548928
198.3	1.58E-08	8.95E-13	0.19335086	0.36650813	1.00E-31	0.00031026	1.64E-09	0.08156814	0.70567061
198.4	1.58E-08	8.95E-13	0.19335086	0.36650813	1.00E-31	0.00031026	1.64E-09	0.08156814	0.70567061
198.5	1.59E-08	8.99E-13	0.19278938	0.36624787	1.00E-31	0.00030759	1.66E-09	0.08192772	0.70073628
198.6	1.59E-08	8.99E-13	0.19278938	0.36624787	1.00E-31	0.00030759	1.66E-09	0.08192772	0.70073628
198.7	1.59E-08	9.12E-13	0.19304305	0.36602987	1.02E-31	0.00030771	1.64E-09	0.0816436	0.69956958
198.8	1.59E-08	9.01E-13	0.1912807	0.36482769	1.06E-31	0.00029527	1.69E-09	0.08142357	0.69782718
198.9	1.59E-08	9.01E-13	0.1912807	0.36482769	1.06E-31	0.00029527	1.69E-09	0.08142357	0.69782718
199	1.61E-08	8.85E-13	0.18998368	0.36475442	1.03E-31	0.00030171	1.65E-09	0.08223432	0.7011874
199.1	1.67E-08	8.99E-13	0.18810387	0.36813869	1.08E-31	0.00030771	1.69E-09	0.08253476	0.70239011
199.2	1.75E-08	9.27E-13	0.19024178	0.36467228	1.14E-31	0.00030977	1.70E-09	0.08331924	0.69978998
199.3	1.72E-08	9.62E-13	0.19222674	0.36688619	1.20E-31	0.000308	1.68E-09	0.08423491	0.69062614
199.4	1.72E-08	9.62E-13	0.19222674	0.36688619	1.20E-31	0.000308	1.68E-09	0.08423491	0.69062614
199.5	1.74E-08	9.62E-13	0.18889902	0.36591995	1.23E-31	0.00031323	1.62E-09	0.08472311	0.69502904
199.6	1.73E-08	9.48E-13	0.18711038	0.36664582	1.22E-31	0.00030791	1.65E-09	0.08529736	0.69451403
199.7	1.76E-08	9.70E-13	0.18666573	0.36679955	1.23E-31	0.00030741	1.65E-09	0.08623707	0.69535564
199.8	1.76E-08	9.70E-13	0.18666573	0.36679955	1.23E-31	0.00030741	1.65E-09	0.08623707	0.69535564
199.9	1.75E-08	9.83E-13	0.1880487	0.36473227	1.26E-31	0.00030237	1.68E-09	0.08696037	0.69873068
200	1.78E-08	9.46E-13	0.18626874	0.36254115	1.28E-31	0.00029715	1.63E-09	0.08499318	0.70407748
200.1	1.73E-08	9.40E-13	0.18738282	0.36188345	1.35E-31	0.0002842	1.73E-09	0.08458564	0.70200654
200.2	1.73E-08	9.40E-13	0.18738282	0.36188345	1.35E-31	0.0002842	1.73E-09	0.08458564	0.70200654
200.3	1.73E-08	9.53E-13	0.18681606	0.35989808	1.31E-31	0.00028338	1.70E-09	0.08531964	0.70123237
200.4	1.73E-08	9.53E-13	0.18681606	0.35989808	1.31E-31	0.00028338	1.70E-09	0.08531964	0.70123237
200.5	1.76E-08	9.33E-13	0.18543276	0.35887527	1.41E-31	0.00027811	1.78E-09	0.08562106	0.70665951
200.6	1.76E-08	9.19E-13	0.1841334	0.36082369	1.40E-31	0.00027821	1.80E-09	0.08571914	0.70471251
200.7	1.76E-08	9.19E-13	0.1841334	0.36082369	1.40E-31	0.00027821	1.80E-09	0.08571914	0.70471251
200.8	1.76E-08	9.19E-13	0.1841334	0.36082369	1.40E-31	0.00027821	1.80E-09	0.08571914	0.70471251
200.9	1.71E-08	9.26E-13	0.18297103	0.3633894	1.40E-31	0.0002809	1.81E-09	0.08610259	0.70455748
201	1.71E-08	9.26E-13	0.18297103	0.3633894	1.40E-31	0.0002809	1.81E-09	0.08610259	0.70455748
201.1	1.74E-08	9.28E-13	0.18343545	0.36339812	1.37E-31	0.00028266	1.78E-09	0.08605344	0.70118391
201.2	1.68E-08	9.55E-13	0.18514728	0.36462833	1.32E-31	0.00028325	1.76E-09	0.08609298	0.69905596
201.3	1.70E-08	9.79E-13	0.18473957	0.36839268	1.39E-31	0.00028597	1.80E-09	0.08589269	0.69821071
201.4	1.70E-08	9.79E-13	0.18473957	0.36839268	1.39E-31	0.00028597	1.80E-09	0.08589269	0.69821071
201.5	1.70E-08	9.79E-13	0.18473957	0.36839268	1.39E-31	0.00028597	1.80E-09	0.08589269	0.69821071
201.6	1.70E-08	9.79E-13	0.18473957	0.36839268	1.39E-31	0.00028597	1.80E-09	0.08589269	0.69821071
201.7	1.73E-08	9.99E-13	0.18425555	0.36762363	1.47E-31	0.00028602	1.82E-09	0.08607629	0.70203303
201.8	1.71E-08	9.81E-13	0.18310546	0.3698628	1.53E-31	0.0002876	1.84E-09	0.08521297	0.70403681
201.9	1.70E-08	9.82E-13	0.18279462	0.37142253	1.62E-31	0.00028951	1.87E-09	0.08490051	0.70799669
202	1.70E-08	9.82E-13	0.18279462	0.37142253	1.62E-31	0.00028951	1.87E-09	0.08490051	0.70799669
202.1	1.82E-08	9.55E-13	0.18193949	0.3690884	1.77E-31	0.0002956	1.85E-09	0.085946	0.70839627
202.2	1.82E-08	9.55E-13	0.18193949	0.3690884	1.77E-31	0.0002956	1.85E-09	0.085946	0.70839627
202.3	1.83E-08	9.45E-13	0.18173218	0.37097332	1.85E-31	0.00029571	1.87E-09	0.08674719	0.70865912
202.4	1.87E-08	9.25E-13	0.18237296	0.37031342	1.91E-31	0.00029306	1.93E-09	0.08693932	0.71011085
202.5	1.87E-08	9.25E-13	0.18237296	0.37031342	1.91E-31	0.00029306	1.93E-09	0.08693932	0.71011085
202.6	1.87E-08	9.25E-13	0.18237296	0.37031342	1.91E-31	0.00029306	1.93E-09	0.08693932	0.71011085
202.7	1.87E-08	9.25E-13	0.18237296	0.37031342	1.91E-31	0.00029306	1.93E-09	0.08693932	0.71011085
202.8	1.87E-08	9.25E-13	0.18237296	0.37031342	1.91E-31	0.00029306	1.93E-09	0.08693932	0.71011085
202.9	1.87E-08	9.25E-13	0.18237296	0.37031342	1.91E-31	0.00029306	1.93E-09	0.08693932	0.71011085
203	1.87E-08	9.25E-13	0.18237296	0.37031342	1.91E-31	0.00029306	1.93E-09	0.08693932	0.71011085
203.1	1.87E-08	9.25E-13	0.18237296	0.37031342	1.91E-31	0.00029306	1.93E-09	0.08693932	0.71011085
203.2	1.91E-08	9.36E-13	0.18047914	0.36895737	1.93E-31	0.00028711	1.94E-09	0.08735651	0.70655797
203.3	1.91E-08	9.36E-13	0.18047914	0.36895737	1.93E-31	0.00028711	1.94E-09	0.08735651	0.70655797
203.4	1.98E-08	9.69E-13	0.17932161	0.36937506	2.04E-31	0.00029006	2.00E-09	0.08714681	0.70824312
203.5	1.98E-08	9.69E-13	0.17932161	0.36937506	2.04E-31	0.00029006	2.00E-09	0.08714681	0.70824312
203.6	1.98E-08	9.81E-13	0.17715321	0.36598511	2.04E-31	0.00029292	2.02E-09	0.08653825	0.71114811
203.7	1.98E-08	9.81E-13	0.17715321	0.36598511	2.04E-31	0.00029292	2.02E-09	0.08653825	0.71114811
203.8	1.98E-08	9.81E-13	0.17715321	0.36598511	2.04E-31	0.00029292	2.02E-09	0.08653825	0.71114811
203.9	1.99E-08	9.96E-13	0.17469077	0.3633242	2.08E-31	0.00029514	2.03E-09	0.08761063	0.71343213
204	1.99E-08	9.96E-13	0.17469077	0.3633242	2.08E-31	0.00029514	2.03E-09	0.08761063	0.71343213
204.1	1.99E-08	9.96E-13	0.17469077	0.3633242	2.08E-31	0.00029514	2.03E-09	0.08761063	0.71343213
204.2	1.97E-08	9.78E-13	0.17341383	0.3614723	2.04E-31	0.00029721	1.99E-09	0.08837883	0.71554374
204.3	1.97E-08	9.78E-13	0.17341383	0.3614723	2.04E-31	0.00029721	1.99E-09	0.08837883	0.71554374
204.4	2.02E-08	1.01E-12	0.17219039	0.35893868	1.98E-31	0.00029661	1.99E-09	0.08948824	0.71470433
204.5	2.02E-08	1.01E-12	0.17219039	0.35893868	1.98E-31	0.00029661	1.99E-09	0.08948824	0.71470433
204.6	2.04E-08	9.96E-13	0.17089063	0.35857674	2.03E-31	0.00030028	1.96E-09	0.08931407	0.7195617
204.7	2.04E-08	9.96E-13	0.17089063	0.35857674	2.03E-31	0.00030028	1.96E-09	0.08931407	0.7195617
204.8	2.04E-08	9.96E-13	0.17089063	0.35857674	2.03E-31	0.00030028	1.96E-09	0.08931407	0.7195617
204.9	2.04E-08	9.96E-13	0.17089063	0.35857674	2.03E-31	0.00030028	1.96E-09	0.08931407	0.7195617
205	2.07E-08	1.00E-12	0.16965871	0.35562687	2.00E-31	0.00030445	2.00E-09	0.08943871	0.72236753
205.1	2.07E-08	1.00E-12	0.16965871	0.35562687	2.00E-31	0.00030445	2.00E-09	0.08943871	0.72236753
205.2	2.11E-08	1.04E-12	0.1676712	0.35723964	2.16E-31	0.00030429	2.03E-09	0.08923103	0.72710269
205.3	2.11E-08	1.04E-12	0.1676712	0.35723964	2.16E-31	0.00030429	2.03E-09	0.08923103	0.72710269
205.4	2.11E-08	1.04E-12	0.1676712	0.35723964	2.16E-31	0.00030429	2.03E-09	0.08923103	0.72710269
205.5	2.11E-08	1.04E-12	0.1676712	0.35723964	2.16E-31	0.00030429	2.03E-09	0.08923103	0.72710269
205.6	2.17E-08	1.07E-12	0.16852707	0.35719717	2.35E-31	0.00030265	2.06E-09	0.08906069	0.72753755
205.7	2.17E-08	1.07E-12	0.16852707	0.35719717	2.35E-31	0.00030265	2.06E-09	0.08906069	0.72753755
205.8	2.24E-08	1.08E-12	0.16762504	0.35499466	2.42E-31	0.00030231	2.08E-09	0.08909697	0.7315657
205.9	2.31E-08	1.06E-12	0.16552321	0.35326078	2.45E-31	0.000307	2.09E-09	0.08886037	0.73516679
206	2.31E-08	1.06E-12	0.16552321	0.35326078	2.45E-31	0.000307	2.09E-09	0.08886037	0.73516679
206.1	2.31E-08	1.06E-12	0.16552321	0.35326078	2.45E-31	0.000307	2.09E-09	0.08886037	0.73516679
206.2	2.31E-08	1.06E-12	0.16552321	0.35326078	2.45E-31	0.000307	2.09E-09	0.08886037	0.73516679
206.3	2.32E-08	1.05E-12	0.16472921	0.34985752	2.42E-31	0.0003111	2.12E-09	0.08832024	0.73726802
206.4	2.32E-08	1.05E-12	0.16472921	0.34985752	2.42E-31	0.0003111	2.12E-09	0.08832024	0.73726802
206.5	2.34E-08	1.01E-12	0.16292514	0.34756807	2.57E-31	0.0003005	2.15E-09	0.08844987	0.74133023
206.6	2.42E-08	1.01E-12	0.16105091	0.34659854	2.67E-31	0.00029775	2.16E-09	0.08910261	0.74349142
206.7	2.43E-08	1.04E-12	0.15796995	0.34667514	2.74E-31	0.00029726	2.16E-09	0.08821211	0.74324375
206.8	2.43E-08	1.04E-12	0.15796995	0.34667514	2.74E-31	0.00029726	2.16E-09	0.08821211	0.74324375
206.9	2.43E-08	1.04E-12	0.15796995	0.34667514	2.74E-31	0.00029726	2.16E-09	0.08821211	0.74324375
207	2.43E-08	1.04E-12	0.15796995	0.34667514	2.74E-31	0.00029726	2.16E-09	0.08821211	0.74324375
207.1	2.46E-08	1.08E-12	0.15642374	0.34790834	2.84E-31	0.00029144	2.16E-09	0.0878631	0.74099339
207.2	2.46E-08	1.08E-12	0.15642374	0.34790834	2.84E-31	0.00029144	2.16E-09	0.0878631	0.74099339
207.3	2.46E-08	1.08E-12	0.15642374	0.34790834	2.84E-31	0.00029144	2.16E-09	0.0878631	0.74099339
207.4	2.46E-08	1.08E-12	0.15642374	0.34790834	2.84E-31	0.00029144	2.16E-09	0.0878631	0.74099339
207.5	2.46E-08	1.08E-12	0.15642374	0.34790834	2.84E-31	0.00029144	2.16E-09	0.0878631	0.74099339
207.6	2.46E-08	1.08E-12	0.15642374	0.34790834	2.84E-31	0.00029144	2.16E-09	0.0878631	0.74099339
207.7	2.46E-08	1.08E-12	0.15642374	0.34790834	2.84E-31	0.00029144	2.16E-09	0.0878631	0.74099339
207.8	2.46E-08	1.08E-12	0.15642374	0.34790834	2.84E-31	0.00029144	2.16E-09	0.0878631	0.74099339
207.9	2.46E-08	1.08E-12	0.15642374	0.34790834	2.84E-31	0.00029144	2.16E-09	0.0878631	0.74099339
208	2.40E-08	1.10E-12	0.15398211	0.34537606	2.79E-31	0.0002968	2.14E-09	0.08863015	0.74424449
208.1	2.43E-08	1.10E-12	0.15346122	0.3444541	2.75E-31	0.00029363	2.13E-09	0.08886564	0.74276568
208.2	2.43E-08	1.10E-12	0.15346122	0.3444541	2.75E-31	0.00029363	2.13E-09	0.08886564	0.74276568
208.3	2.49E-08	1.08E-12	0.15346354	0.34122221	2.82E-31	0.00029484	2.06E-09	0.08854378	0.7408811
208.4	2.59E-08	1.12E-12	0.15154691	0.33937249	2.90E-31	0.0002969	2.10E-09	0.08927252	0.73893313
208.5	2.73E-08	1.10E-12	0.1521934	0.33948656	3.09E-31	0.00029677	2.16E-09	0.08906809	0.74302534
208.6	2.73E-08	1.10E-12	0.1521934	0.33948656	3.09E-31	0.00029677	2.16E-09	0.08906809	0.74302534
208.7	2.73E-08	1.10E-12	0.1521934	0.33948656	3.09E-31	0.00029677	2.16E-09	0.08906809	0.74302534
208.8	2.73E-08	1.10E-12	0.1521934	0.33948656	3.09E-31	0.00029677	2.16E-09	0.08906809	0.74302534
208.9	2.73E-08	1.10E-12	0.1521934	0.33948656	3.09E-31	0.00029677	2.16E-09	0.08906809	0.74302534
209	2.72E-08	1.07E-12	0.15034815	0.33770098	3.05E-31	0.00029868	2.15E-09	0.08856945	0.74270954
209.1	2.72E-08	1.07E-12	0.15034815	0.33770098	3.05E-31	0.00029868	2.15E-09	0.08856945	0.74270954
209.2	2.72E-08	1.07E-12	0.15034815	0.33770098	3.05E-31	0.00029868	2.15E-09	0.08856945	0.74270954
209.3	2.72E-08	1.07E-12	0.15034815	0.33770098	3.05E-31	0.00029868	2.15E-09	0.08856945	0.74270954
209.4	2.72E-08	1.07E-12	0.15034815	0.33770098	3.05E-31	0.00029868	2.15E-09	0.08856945	0.74270954
209.5	2.72E-08	1.07E-12	0.15034815	0.33770098	3.05E-31	0.00029868	2.15E-09	0.08856945	0.74270954
209.6	2.75E-08	1.09E-12	0.15054658	0.33318568	3.18E-31	0.00030324	2.23E-09	0.08820213	0.74443308
209.7	2.75E-08	1.09E-12	0.15054658	0.33318568	3.18E-31	0.00030324	2.23E-09	0.08820213	0.74443308
209.8	2.80E-08	1.09E-12	0.14997623	0.33191632	3.39E-31	0.00030122	2.28E-09	0.08849035	0.74729918
209.9	2.85E-08	1.09E-12	0.14832597	0.32977624	3.32E-31	0.00030318	2.32E-09	0.08957394	0.75105547

210.6	3.06E-08	1.11E-12	0.14756587	0.32946606	3.72E-31	0.0003017	2.46E-09	0.08837646	0.75194484
210.7	3.06E-08	1.11E-12	0.14756587	0.32946606	3.72E-31	0.0003017	2.46E-09	0.08837646	0.75194484
210.8	3.06E-08	1.11E-12	0.14756587	0.32946606	3.72E-31	0.0003017	2.46E-09	0.08837646	0.75194484
210.9	3.04E-08	1.12E-12	0.14665693	0.32721908	3.81E-31	0.00030336	2.53E-09	0.08879916	0.74791213
211	3.11E-08	1.13E-12	0.14643692	0.3259775	3.78E-31	0.00030343	2.53E-09	0.08889828	0.74526183
211.1	3.15E-08	1.14E-12	0.14728073	0.3236191	3.90E-31	0.00031255	2.48E-09	0.08998244	0.74414494
211.2	3.15E-08	1.14E-12	0.14728073	0.3236191	3.90E-31	0.00031255	2.48E-09	0.08998244	0.74414494
211.3	3.15E-08	1.14E-12	0.14728073	0.3236191	3.90E-31	0.00031255	2.48E-09	0.08998244	0.74414494
211.4	3.38E-08	1.17E-12	0.14811409	0.32272652	4.39E-31	0.00032523	2.52E-09	0.09006647	0.74696225
211.5	3.38E-08	1.17E-12	0.14811409	0.32272652	4.39E-31	0.00032523	2.52E-09	0.09006647	0.74696225
211.6	3.33E-08	1.20E-12	0.1485296	0.32230765	4.42E-31	0.00032776	2.46E-09	0.09050468	0.74271855
211.7	3.27E-08	1.19E-12	0.14854716	0.32074473	4.37E-31	0.00032639	2.43E-09	0.09131149	0.73979369
211.8	3.27E-08	1.19E-12	0.14854716	0.32074473	4.37E-31	0.00032639	2.43E-09	0.09131149	0.73979369
211.9	3.27E-08	1.19E-12	0.14854716	0.32074473	4.37E-31	0.00032639	2.43E-09	0.09131149	0.73979369
212	3.28E-08	1.20E-12	0.14675329	0.31867647	4.32E-31	0.00032768	2.43E-09	0.09153771	0.7437385
212.1	3.39E-08	1.23E-12	0.14646992	0.31779372	4.81E-31	0.00033186	2.55E-09	0.09187166	0.74372149
212.2	3.39E-08	1.23E-12	0.14646992	0.31779372	4.81E-31	0.00033186	2.55E-09	0.09187166	0.74372149
212.3	3.39E-08	1.23E-12	0.14646992	0.31779372	4.81E-31	0.00033186	2.55E-09	0.09187166	0.74372149
212.4	3.37E-08	1.22E-12	0.14803273	0.31888768	4.56E-31	0.00033034	2.48E-09	0.09078057	0.74503936
212.5	3.47E-08	1.25E-12	0.14898873	0.317063	5.03E-31	0.00033545	2.50E-09	0.09149279	0.74357252
212.6	3.47E-08	1.25E-12	0.14898873	0.317063	5.03E-31	0.00033545	2.50E-09	0.09149279	0.74357252
212.7	3.46E-08	1.25E-12	0.1472626	0.31413285	5.12E-31	0.00033542	2.54E-09	0.09233832	0.74735587
212.8	3.46E-08	1.25E-12	0.1472626	0.31413285	5.12E-31	0.00033542	2.54E-09	0.09233832	0.74735587
212.9	3.54E-08	1.26E-12	0.14576239	0.31447884	5.03E-31	0.00033234	2.58E-09	0.09311107	0.74960367
213	3.54E-08	1.26E-12	0.14576239	0.31447884	5.03E-31	0.00033234	2.58E-09	0.09311107	0.74960367
213.1	3.60E-08	1.31E-12	0.14584538	0.3136961	5.18E-31	0.00033099	2.63E-09	0.09352071	0.74676575
213.2	3.60E-08	1.31E-12	0.14584538	0.3136961	5.18E-31	0.00033099	2.63E-09	0.09352071	0.74676575
213.3	3.60E-08	1.31E-12	0.14584538	0.3136961	5.18E-31	0.00033099	2.63E-09	0.09352071	0.74676575
213.4	3.60E-08	1.31E-12	0.14584538	0.3136961	5.18E-31	0.00033099	2.63E-09	0.09352071	0.74676575
213.5	3.60E-08	1.31E-12	0.14584538	0.3136961	5.18E-31	0.00033099	2.63E-09	0.09352071	0.74676575
213.6	3.71E-08	1.38E-12	0.14491895	0.31216046	5.25E-31	0.00033466	2.70E-09	0.09468132	0.74376823
213.7	3.78E-08	1.38E-12	0.14286879	0.31145297	5.35E-31	0.00033766	2.73E-09	0.09454895	0.74621247
213.8	3.78E-08	1.38E-12	0.14286879	0.31145297	5.35E-31	0.00033766	2.73E-09	0.09454895	0.74621247
213.9	3.78E-08	1.38E-12	0.14286879	0.31145297	5.35E-31	0.00033766	2.73E-09	0.09454895	0.74621247
214	3.78E-08	1.43E-12	0.14212479	0.31027851	5.40E-31	0.00033206	2.69E-09	0.09541453	0.74514549
214.1	3.85E-08	1.49E-12	0.14243192	0.31046817	5.23E-31	0.00033845	2.64E-09	0.09652689	0.74367997
214.2	3.85E-08	1.49E-12	0.14243192	0.31046817	5.23E-31	0.00033845	2.64E-09	0.09652689	0.74367997
214.3	3.85E-08	1.49E-12	0.14243192	0.31046817	5.23E-31	0.00033845	2.64E-09	0.09652689	0.74367997
214.4	3.85E-08	1.49E-12	0.14243192	0.31046817	5.23E-31	0.00033845	2.64E-09	0.09652689	0.74367997
214.5	3.94E-08	1.51E-12	0.14189439	0.31127656	5.50E-31	0.00033596	2.80E-09	0.09682373	0.74397401
214.6	4.05E-08	1.50E-12	0.1407355	0.31087264	5.21E-31	0.00034716	2.73E-09	0.09823465	0.74258515
214.7	4.13E-08	1.50E-12	0.14101607	0.31155407	5.47E-31	0.00034212	2.70E-09	0.09814108	0.7437983
214.8	4.14E-08	1.49E-12	0.13974984	0.31131668	5.56E-31	0.00034443	2.75E-09	0.0975641	0.74851949
214.9	4.14E-08	1.49E-12	0.13974984	0.31131668	5.56E-31	0.00034443	2.75E-09	0.0975641	0.74851949
215	4.14E-08	1.49E-12	0.13974984	0.31131668	5.56E-31	0.00034443	2.75E-09	0.0975641	0.74851949
215.1	4.20E-08	1.44E-12	0.1391409	0.31047758	5.58E-31	0.00034366	2.73E-09	0.09810327	0.74912644
215.2	4.20E-08	1.44E-12	0.1391409	0.31047758	5.58E-31	0.00034366	2.73E-09	0.09810327	0.74912644
215.3	4.20E-08	1.44E-12	0.1391409	0.31047758	5.58E-31	0.00034366	2.73E-09	0.09810327	0.74912644
215.4	4.09E-08	1.39E-12	0.1390253	0.30796121	5.39E-31	0.00033923	2.67E-09	0.09818306	0.74388476
215.5	4.16E-08	1.40E-12	0.1372603	0.30767378	5.45E-31	0.00033767	2.68E-09	0.09823028	0.74641924
215.6	4.16E-08	1.40E-12	0.1372603	0.30767378	5.45E-31	0.00033767	2.68E-09	0.09823028	0.74641924
215.7	4.11E-08	1.34E-12	0.13580225	0.30764107	5.26E-31	0.00033499	2.67E-09	0.09818615	0.74876934
215.8	4.11E-08	1.34E-12	0.13580225	0.30764107	5.26E-31	0.00033499	2.67E-09	0.09818615	0.74876934
215.9	4.02E-08	1.34E-12	0.13574236	0.3060488	5.32E-31	0.00033023	2.68E-09	0.09809016	0.74749928
216	4.02E-08	1.34E-12	0.13574236	0.3060488	5.32E-31	0.00033023	2.68E-09	0.09809016	0.74749928
216.1	4.02E-08	1.34E-12	0.13574236	0.3060488	5.32E-31	0.00033023	2.68E-09	0.09809016	0.74749928
216.2	4.05E-08	1.35E-12	0.13413593	0.30260127	5.58E-31	0.00032696	2.79E-09	0.09785878	0.75383832
216.3	4.05E-08	1.35E-12	0.13413593	0.30260127	5.58E-31	0.00032696	2.79E-09	0.09785878	0.75383832
216.4	4.05E-08	1.35E-12	0.13413593	0.30260127	5.58E-31	0.00032696	2.79E-09	0.09785878	0.75383832
216.5	4.05E-08	1.35E-12	0.13413593	0.30260127	5.58E-31	0.00032696	2.79E-09	0.09785878	0.75383832
216.6	4.05E-08	1.35E-12	0.13413593	0.30260127	5.58E-31	0.00032696	2.79E-09	0.09785878	0.75383832
216.7	4.05E-08	1.35E-12	0.13413593	0.30260127	5.58E-31	0.00032696	2.79E-09	0.09785878	0.75383832
216.8	4.05E-08	1.35E-12	0.13413593	0.30260127	5.58E-31	0.00032696	2.79E-09	0.09785878	0.75383832
216.9	4.05E-08	1.35E-12	0.13413593	0.30260127	5.58E-31	0.00032696	2.79E-09	0.09785878	0.75383832
217	4.09E-08	1.32E-12	0.13322536	0.30250075	5.94E-31	0.00031991	2.92E-09	0.09899	0.74876618
217.1	4.15E-08	1.38E-12	0.13196912	0.301606	6.28E-31	0.00031827	2.97E-09	0.09860464	0.74853181
217.2	4.15E-08	1.38E-12	0.13196912	0.301606	6.28E-31	0.00031827	2.97E-09	0.09860464	0.74853181
217.3	4.15E-08	1.38E-12	0.13196912	0.301606	6.28E-31	0.00031827	2.97E-09	0.09860464	0.74853181
217.4	4.23E-08	1.39E-12	0.13148341	0.29908897	6.59E-31	0.00031902	3.02E-09	0.09898007	0.75273256
217.5	4.23E-08	1.39E-12	0.13148341	0.29908897	6.59E-31	0.00031902	3.02E-09	0.09898007	0.75273256
217.6	4.23E-08	1.39E-12	0.13148341	0.29908897	6.59E-31	0.00031902	3.02E-09	0.09898007	0.75273256
217.7	4.31E-08	1.36E-12	0.1307485	0.30015689	6.80E-31	0.00031954	2.97E-09	0.09858929	0.7535147
217.8	4.28E-08	1.35E-12	0.12938522	0.29863702	6.93E-31	0.00031577	2.98E-09	0.09820025	0.74925136
217.9	4.28E-08	1.35E-12	0.12938522	0.29863702	6.93E-31	0.00031577	2.98E-09	0.09820025	0.74925136
218	4.44E-08	1.34E-12	0.13060091	0.29724476	7.31E-31	0.00032225	2.96E-09	0.09907296	0.7466085
218.1	4.45E-08	1.35E-12	0.13061358	0.2913478	7.30E-31	0.00032675	3.04E-09	0.1005094	0.74595473
218.2	4.45E-08	1.35E-12	0.13061358	0.2913478	7.30E-31	0.00032675	3.04E-09	0.1005094	0.74595473
218.3	4.45E-08	1.35E-12	0.12923103	0.29006931	7.33E-31	0.00032711	3.12E-09	0.1004187	0.74570828
218.4	4.45E-08	1.35E-12	0.12923103	0.29006931	7.33E-31	0.00032711	3.12E-09	0.1004187	0.74570828
218.5	4.45E-08	1.35E-12	0.12923103	0.29006931	7.33E-31	0.00032711	3.12E-09	0.1004187	0.74570828
218.6	4.45E-08	1.35E-12	0.12923103	0.29006931	7.33E-31	0.00032711	3.12E-09	0.1004187	0.74570828
218.7	4.52E-08	1.27E-12	0.12791733	0.2882424	6.84E-31	0.00032186	3.08E-09	0.10119348	0.74854126
218.8	4.47E-08	1.24E-12	0.1270494	0.28800175	6.87E-31	0.00032124	3.10E-09	0.10032521	0.74756542
218.9	4.55E-08	1.33E-12	0.12425457	0.28756781	7.37E-31	0.00032801	3.31E-09	0.10140076	0.75261569
219	4.60E-08	1.33E-12	0.12347121	0.28850622	7.21E-31	0.00033568	3.29E-09	0.10153937	0.7523345
219.1	4.67E-08	1.35E-12	0.12300219	0.28845709	7.18E-31	0.00033189	3.24E-09	0.10218298	0.75014474
219.2	4.67E-08	1.35E-12	0.12300219	0.28845709	7.18E-31	0.00033189	3.24E-09	0.10218298	0.75014474
219.3	4.77E-08	1.35E-12	0.12214074	0.28816978	7.08E-31	0.0003343	3.23E-09	0.10334518	0.74964016
219.4	4.77E-08	1.35E-12	0.12214074	0.28816978	7.08E-31	0.0003343	3.23E-09	0.10334518	0.74964016
219.5	4.77E-08	1.35E-12	0.12214074	0.28816978	7.08E-31	0.0003343	3.23E-09	0.10334518	0.74964016
219.6	4.79E-08	1.35E-12	0.12196179	0.28843594	7.17E-31	0.00033014	3.34E-09	0.10299864	0.74910805
219.7	4.79E-08	1.35E-12	0.12196179	0.28843594	7.17E-31	0.00033014	3.34E-09	0.10299864	0.74910805
219.8	4.80E-08	1.39E-12	0.12362207	0.28635742	7.61E-31	0.00033288	3.35E-09	0.10323974	0.74459651
219.9	4.80E-08	1.39E-12	0.12362207	0.28635742	7.61E-31	0.00033288	3.35E-09	0.10323974	0.74459651
220	4.80E-08	1.39E-12	0.12362207	0.28635742	7.61E-31	0.00033288	3.35E-09	0.10323974	0.74459651
220.1	4.78E-08	1.39E-12	0.12289808	0.28706448	7.81E-31	0.00033518	3.39E-09	0.10316265	0.74593517
220.2	4.78E-08	1.39E-12	0.12289808	0.28706448	7.81E-31	0.00033518	3.39E-09	0.10316265	0.74593517
220.3	4.78E-08	1.39E-12	0.12289808	0.28706448	7.81E-31	0.00033518	3.39E-09	0.10316265	0.74593517
220.4	4.78E-08	1.39E-12	0.12289808	0.28706448	7.81E-31	0.00033518	3.39E-09	0.10316265	0.74593517
220.5	4.79E-08	1.41E-12	0.12256109	0.28530392	7.91E-31	0.00034069	3.47E-09	0.10295507	0.74850985
220.6	4.89E-08	1.42E-12	0.1223424	0.28606025	7.72E-31	0.00034509	3.51E-09	0.10337126	0.74573577
220.7	4.89E-08	1.42E-12	0.1223424	0.28606025	7.72E-31	0.00034509	3.51E-09	0.10337126	0.74573577
220.8	4.89E-08	1.42E-12	0.1223424	0.28606025	7.72E-31	0.00034509	3.51E-09	0.10337126	0.74573577
220.9	4.89E-08	1.42E-12	0.1223424	0.28606025	7.72E-31	0.00034509	3.51E-09	0.10337126	0.74573577
221	4.89E-08	1.42E-12	0.1223424	0.28606025	7.72E-31	0.00034509	3.51E-09	0.10337126	0.74573577
221.1	4.96E-08	1.39E-12	0.1210799	0.28582156	7.74E-31	0.0003456	3.54E-09	0.10368492	0.74314667
221.2	4.96E-08	1.39E-12	0.1210799	0.28582156	7.74E-31	0.0003456	3.54E-09	0.10368492	0.74314667
221.3	4.96E-08	1.39E-12	0.1210799	0.28582156	7.74E-31	0.0003456	3.54E-09	0.10368492	0.74314667
221.4	4.96E-08	1.39E-12	0.1210799	0.28582156	7.74E-31	0.0003456	3.54E-09	0.10368492	0.74314667
221.5	4.96E-08	1.39E-12	0.1210799	0.28582156	7.74E-31	0.0003456	3.54E-09	0.10368492	0.74314667
221.6	4.96E-08	1.39E-12	0.1210799	0.28582156	7.74E-31	0.0003456	3.54E-09	0.10368492	0.74314667
221.7	4.94E-08	1.36E-12	0.1213969	0.28539869	7.78E-31	0.00034544	3.53E-09	0.10294136	0.74243025
221.8	4.94E-08	1.36E-12	0.1213969	0.28539869	7.78E-31	0.00034544	3.53E-09	0.10294136	0.74243025
221.9	5.12E-08	1.33E-12	0.12060542	0.28279907	7.65E-31	0.00034968	3.63E-09	0.1022996	0.74405871
222	5.12E-08	1.33E-12	0.12060542	0.28279907	7.65E-31	0.00034968	3.63E-09	0.1022996	0.74405871
222.1	5.12E-08	1.33E-12	0.12060542	0.28279907	7.65E-31	0.00034968	3.63E-09	0.1022996	0.74405871
222.2	5.25E-08	1.37E-12	0.12230772	0.28225291	8.04E-31	0.00035761	3.63E-09	0.10263855	0.74082485
222.3	5.25E-08	1.37E-12	0.12230772	0.28225291	8.04E-31	0.00035761	3.63E-09	0.10263855	0.74082485
222.4	5.26E-08	1.42E-12	0.12110082	0.28178125	7.71E-31	0.00035942	3.54E-09	0.10272786	0.73935216
222.5	5.26E-08	1.42E-12	0.12110082	0.28178125	7.71E-31	0.00035942	3.54E-09	0.10272786	0.73935216
222.6	5.16E-08	1.46E-12	0.1184579	0.27862011	7.63E-31	0.00036199	3.67E-09	0.10342218	0.74487351
222.7	5.16E-08	1.42E-12	0.11961158	0.2750366	7.91E-31	0.00037459	3.63E-09	0.10341949	0.74740086
222.8	5.16E-08	1.42E-12	0.11961158	0.2750366	7.91E-31	0.00037459	3.63E-09	0.10341949	0.74740086
222.9	5.42E-08	1.47E-12	0.11912593	0.27765807	8.57E-31	0.00037248	3.74E-09	0.10316903	0.74703942
223	5.52E-08	1.51E-12	0.11820584	0.27618033	8.46E-31	0.00037454	3.78E-09	0.10394838	0.74678376
223.1	5.49E-08	1.48E-12	0.11716149	0.27705018	8.53E-31	0.00037501	3.82E-09	0.10415097	0.74682769
223.2	5.49E-08	1.48E-12	0.11716149	0.27705018	8.53E-31	0.00037501	3.82E-09	0.10415097	0.74682769
223.3	5.51E-08	1.54E-12	0.11610271	0.27678516	8.70E-31	0.00037439	3.88E-09	0.10528932	0.74755286
223.4	5.55E-08	1.53E-12	0.11560832	0.27519109	8.73E-31	0.0003693	3.88E-09	0.10412125	0.75006086
223.5	5.51E-08	1.52E-12	0.1151949	0.272845	8.78E-31	0.00036952	3.95E-09	0.1034013	0.75469432
223.6	5.61E-08	1.55E-12	0.113772	0.27258113	8.79E-31	0.0003695	3.95E-09	0.10383071	0.75811463
223.7	5.61E-08	1.55E-12	0.113772	0.27258113	8.79E-31	0.0003695	3.95E-09	0.10383071	0.75811463
223.8	5.61E-08	1.55E-12	0.113772	0.27258113	8.79E-31	0.0003695	3.95E-09	0.10383071	0.75811463
223.9	5.61E-08	1.55E-12	0.113772	0.27258113	8.79E-31	0.0003695	3.95E-09	0.10383071	0.75811463
224	5.69E-08	1.61E-12	0.11350978	0.27062561	9.20E-31	0.00037474	4.08E-09	0.10465561	0.75855844
224.1	5.69E-08	1.61E-12	0.11350978	0.27062561	9.20E-31	0.00037474	4.08E-09	0.10465561	0.75855844
224.2	5.65E-08	1.65E-12	0.11405233	0.26908097	9.32E-31	0.00037184	4.04E-09	0.10538779	0.75487708
224.3	5.65E-08	1.65E-12	0.11405233	0.26908097	9.32E-31	0.00037184	4.04E-09	0.10538779	0.75487708
224.4	5.65E-08	1.65E-12	0.11405233	0.26908097	9.32E-31	0.00037184	4.04E-09	0.10538779	0.75487708
224.5	5.68E-08	1.66E-12	0.11232345	0.2684028	9.12E-31	0.00037068	4.08E-09	0.10641481	0.75908006
224.6	5.68E-08	1.66E-12	0.11232345	0.2684028	9.12E-31	0.00037068	4.08E-09	0.10641481	0.75908006
224.7	5.61E-08	1.65E-12	0.1124902	0.26664089	9.25E-31	0.00036698	4.13E-09	0.10608224	0.76305797
224.8	5.66E-08	1.65E-12	0.11268879	0.26546703	9.30E-31	0.00036133	4.17E-09	0.10632362	0.76361622
224.9	5.66E-08	1.65E-12	0.11268879	0.26546703	9.30E-31	0.00036133	4.17E-09	0.10632362	0.76361622

225.6	5.48E-08	1.68E-12	0.11032056	0.26270614	9.05E-31	0.00035649	4.17E-09	0.10789399	0.76458593
225.7	5.48E-08	1.68E-12	0.11032056	0.26270614	9.05E-31	0.00035649	4.17E-09	0.10789399	0.76458593
225.8	5.48E-08	1.68E-12	0.11032056	0.26270614	9.05E-31	0.00035649	4.17E-09	0.10789399	0.76458593
225.9	5.60E-08	1.69E-12	0.10927118	0.26337628	9.27E-31	0.00036014	4.23E-09	0.1090551	0.76430072
226	5.60E-08	1.69E-12	0.10927118	0.26337628	9.27E-31	0.00036014	4.23E-09	0.1090551	0.76430072
226.1	5.60E-08	1.69E-12	0.10927118	0.26337628	9.27E-31	0.00036014	4.23E-09	0.1090551	0.76430072
226.2	5.60E-08	1.69E-12	0.10927118	0.26337628	9.27E-31	0.00036014	4.23E-09	0.1090551	0.76430072
226.3	5.60E-08	1.69E-12	0.10927118	0.26337628	9.27E-31	0.00036014	4.23E-09	0.1090551	0.76430072
226.4	5.60E-08	1.69E-12	0.10927118	0.26337628	9.27E-31	0.00036014	4.23E-09	0.1090551	0.76430072
226.5	5.60E-08	1.69E-12	0.10927118	0.26337628	9.27E-31	0.00036014	4.23E-09	0.1090551	0.76430072
226.6	5.60E-08	1.69E-12	0.10927118	0.26337628	9.27E-31	0.00036014	4.23E-09	0.1090551	0.76430072
226.7	5.60E-08	1.69E-12	0.10927118	0.26337628	9.27E-31	0.00036014	4.23E-09	0.1090551	0.76430072
226.8	5.60E-08	1.69E-12	0.10927118	0.26337628	9.27E-31	0.00036014	4.23E-09	0.1090551	0.76430072
226.9	5.60E-08	1.69E-12	0.10927118	0.26337628	9.27E-31	0.00036014	4.23E-09	0.1090551	0.76430072
227	5.60E-08	1.69E-12	0.10927118	0.26337628	9.27E-31	0.00036014	4.23E-09	0.1090551	0.76430072
227.1	5.58E-08	1.63E-12	0.10863665	0.26203825	9.24E-31	0.00035183	4.26E-09	0.10851663	0.75942909
227.2	5.58E-08	1.63E-12	0.10863665	0.26203825	9.24E-31	0.00035183	4.26E-09	0.10851663	0.75942909
227.3	5.49E-08	1.59E-12	0.107748	0.26236313	8.66E-31	0.00035118	4.23E-09	0.10954601	0.7592808
227.4	5.44E-08	1.58E-12	0.10845129	0.26148815	8.85E-31	0.0003432	4.25E-09	0.10939957	0.75956255
227.5	5.56E-08	1.57E-12	0.10795771	0.26307636	9.36E-31	0.00033549	4.19E-09	0.11001493	0.75889135
227.6	5.56E-08	1.57E-12	0.10795771	0.26307636	9.36E-31	0.00033549	4.19E-09	0.11001493	0.75889135
227.7	5.40E-08	1.63E-12	0.10635037	0.26145658	9.79E-31	0.00033312	4.36E-09	0.11083094	0.75746215
227.8	5.40E-08	1.63E-12	0.10635037	0.26145658	9.79E-31	0.00033312	4.36E-09	0.11083094	0.75746215
227.9	5.48E-08	1.64E-12	0.1059581	0.26132904	1.02E-30	0.00032798	4.51E-09	0.11111347	0.75625701
228	5.48E-08	1.64E-12	0.1059581	0.26132904	1.02E-30	0.00032798	4.51E-09	0.11111347	0.75625701
228.1	5.48E-08	1.64E-12	0.1059581	0.26132904	1.02E-30	0.00032798	4.51E-09	0.11111347	0.75625701
228.2	5.48E-08	1.64E-12	0.1059581	0.26132904	1.02E-30	0.00032798	4.51E-09	0.11111347	0.75625701
228.3	5.35E-08	1.67E-12	0.10501682	0.26083117	1.05E-30	0.00032537	4.49E-09	0.11087477	0.75568677
228.4	5.35E-08	1.67E-12	0.10501682	0.26083117	1.05E-30	0.00032537	4.49E-09	0.11087477	0.75568677
228.5	5.35E-08	1.67E-12	0.10501682	0.26083117	1.05E-30	0.00032537	4.49E-09	0.11087477	0.75568677
228.6	5.35E-08	1.67E-12	0.10501682	0.26083117	1.05E-30	0.00032537	4.49E-09	0.11087477	0.75568677
228.7	5.47E-08	1.65E-12	0.10465025	0.2621095	1.03E-30	0.00032469	4.52E-09	0.11120103	0.75481851
228.8	5.67E-08	1.69E-12	0.10474486	0.26257435	1.09E-30	0.00032376	4.59E-09	0.11233367	0.7556284
228.9	5.67E-08	1.69E-12	0.10474486	0.26257435	1.09E-30	0.00032376	4.59E-09	0.11233367	0.7556284
229	5.67E-08	1.69E-12	0.10474486	0.26257435	1.09E-30	0.00032376	4.59E-09	0.11233367	0.7556284
229.1	5.67E-08	1.69E-12	0.10474486	0.26257435	1.09E-30	0.00032376	4.59E-09	0.11233367	0.7556284
229.2	5.67E-08	1.69E-12	0.10474486	0.26257435	1.09E-30	0.00032376	4.59E-09	0.11233367	0.7556284
229.3	5.67E-08	1.69E-12	0.10474486	0.26257435	1.09E-30	0.00032376	4.59E-09	0.11233367	0.7556284
229.4	5.67E-08	1.69E-12	0.10474486	0.26257435	1.09E-30	0.00032376	4.59E-09	0.11233367	0.7556284
229.5	5.67E-08	1.69E-12	0.10474486	0.26257435	1.09E-30	0.00032376	4.59E-09	0.11233367	0.7556284
229.6	5.67E-08	1.69E-12	0.10474486	0.26257435	1.09E-30	0.00032376	4.59E-09	0.11233367	0.7556284
229.7	5.84E-08	1.71E-12	0.10457915	0.26260557	1.12E-30	0.00031944	4.62E-09	0.11239342	0.75600781
229.8	5.77E-08	1.68E-12	0.1051426	0.2632839	1.07E-30	0.0003157	4.57E-09	0.11082883	0.75541482
229.9	5.79E-08	1.68E-12	0.10657935	0.26553816	1.08E-30	0.00031429	4.49E-09	0.11132025	0.75632847
230	5.79E-08	1.68E-12	0.10657935	0.26553816	1.08E-30	0.00031429	4.49E-09	0.11132025	0.75632847
230.1	5.89E-08	1.65E-12	0.10670096	0.26394916	1.12E-30	0.00031492	4.37E-09	0.11173551	0.75449274
230.2	5.89E-08	1.65E-12	0.10670096	0.26394916	1.12E-30	0.00031492	4.37E-09	0.11173551	0.75449274
230.3	5.89E-08	1.65E-12	0.10670096	0.26394916	1.12E-30	0.00031492	4.37E-09	0.11173551	0.75449274
230.4	5.89E-08	1.65E-12	0.10670096	0.26394916	1.12E-30	0.00031492	4.37E-09	0.11173551	0.75449274
230.5	5.89E-08	1.65E-12	0.10670096	0.26394916	1.12E-30	0.00031492	4.37E-09	0.11173551	0.75449274
230.6	5.89E-08	1.65E-12	0.10670096	0.26394916	1.12E-30	0.00031492	4.37E-09	0.11173551	0.75449274
230.7	5.89E-08	1.65E-12	0.10670096	0.26394916	1.12E-30	0.00031492	4.37E-09	0.11173551	0.75449274
230.8	5.89E-08	1.65E-12	0.10670096	0.26394916	1.12E-30	0.00031492	4.37E-09	0.11173551	0.75449274
230.9	5.89E-08	1.65E-12	0.10670096	0.26394916	1.12E-30	0.00031492	4.37E-09	0.11173551	0.75449274
231	5.89E-08	1.65E-12	0.10670096	0.26394916	1.12E-30	0.00031492	4.37E-09	0.11173551	0.75449274
231.1	5.89E-08	1.65E-12	0.10670096	0.26394916	1.12E-30	0.00031492	4.37E-09	0.11173551	0.75449274
231.2	5.90E-08	1.69E-12	0.10537805	0.26345284	1.15E-30	0.00031349	4.37E-09	0.11277133	0.75230512
231.3	5.90E-08	1.69E-12	0.10537805	0.26345284	1.15E-30	0.00031349	4.37E-09	0.11277133	0.75230512
231.4	5.90E-08	1.69E-12	0.10537805	0.26345284	1.15E-30	0.00031349	4.37E-09	0.11277133	0.75230512
231.5	5.82E-08	1.69E-12	0.10494892	0.26482947	1.18E-30	0.0003126	4.30E-09	0.11186771	0.75283384
231.6	5.82E-08	1.69E-12	0.10494892	0.26482947	1.18E-30	0.0003126	4.30E-09	0.11186771	0.75283384
231.7	5.82E-08	1.69E-12	0.10494892	0.26482947	1.18E-30	0.0003126	4.30E-09	0.11186771	0.75283384
231.8	5.82E-08	1.69E-12	0.10494892	0.26482947	1.18E-30	0.0003126	4.30E-09	0.11186771	0.75283384
231.9	5.82E-08	1.69E-12	0.10494892	0.26482947	1.18E-30	0.0003126	4.30E-09	0.11186771	0.75283384
232	5.82E-08	1.69E-12	0.10494892	0.26482947	1.18E-30	0.0003126	4.30E-09	0.11186771	0.75283384
232.1	6.13E-08	1.76E-12	0.10459599	0.26554317	1.30E-30	0.00031533	4.51E-09	0.11158819	0.75447097
232.2	6.13E-08	1.76E-12	0.10459599	0.26554317	1.30E-30	0.00031533	4.51E-09	0.11158819	0.75447097
232.3	6.13E-08	1.76E-12	0.10459599	0.26554317	1.30E-30	0.00031533	4.51E-09	0.11158819	0.75447097
232.4	6.13E-08	1.76E-12	0.10459599	0.26554317	1.30E-30	0.00031533	4.51E-09	0.11158819	0.75447097
232.5	6.13E-08	1.76E-12	0.10459599	0.26554317	1.30E-30	0.00031533	4.51E-09	0.11158819	0.75447097
232.6	6.13E-08	1.76E-12	0.10459599	0.26554317	1.30E-30	0.00031533	4.51E-09	0.11158819	0.75447097
232.7	6.13E-08	1.76E-12	0.10459599	0.26554317	1.30E-30	0.00031533	4.51E-09	0.11158819	0.75447097
232.8	6.13E-08	1.76E-12	0.10459599	0.26554317	1.30E-30	0.00031533	4.51E-09	0.11158819	0.75447097
232.9	6.13E-08	1.76E-12	0.10459599	0.26554317	1.30E-30	0.00031533	4.51E-09	0.11158819	0.75447097
233	6.13E-08	1.76E-12	0.10459599	0.26554317	1.30E-30	0.00031533	4.51E-09	0.11158819	0.75447097
233.1	6.13E-08	1.76E-12	0.10459599	0.26554317	1.30E-30	0.00031533	4.51E-09	0.11158819	0.75447097
233.2	6.30E-08	1.63E-12	0.1028344	0.26446703	1.21E-30	0.00031343	4.50E-09	0.11006017	0.75541753
233.3	6.23E-08	1.64E-12	0.10196292	0.26600621	1.19E-30	0.00031736	4.43E-09	0.11134661	0.75599579
233.4	6.23E-08	1.58E-12	0.10051885	0.26338928	1.17E-30	0.00031263	4.46E-09	0.11067574	0.76094288
233.5	6.23E-08	1.58E-12	0.10051885	0.26338928	1.17E-30	0.00031263	4.46E-09	0.11067574	0.76094288
233.6	6.23E-08	1.58E-12	0.10051885	0.26338928	1.17E-30	0.00031263	4.46E-09	0.11067574	0.76094288
233.7	6.23E-08	1.58E-12	0.10051885	0.26338928	1.17E-30	0.00031263	4.46E-09	0.11067574	0.76094288
233.8	6.23E-08	1.58E-12	0.10051885	0.26338928	1.17E-30	0.00031263	4.46E-09	0.11067574	0.76094288
233.9	6.34E-08	1.64E-12	0.09916211	0.26325394	1.17E-30	0.00031314	4.43E-09	0.11073081	0.76207029
234	6.34E-08	1.64E-12	0.09916211	0.26325394	1.17E-30	0.00031314	4.43E-09	0.11073081	0.76207029
234.1	6.34E-08	1.64E-12	0.09916211	0.26325394	1.17E-30	0.00031314	4.43E-09	0.11073081	0.76207029
234.2	6.34E-08	1.64E-12	0.09916211	0.26325394	1.17E-30	0.00031314	4.43E-09	0.11073081	0.76207029
234.3	6.43E-08	1.66E-12	0.09973784	0.26118363	1.21E-30	0.00031173	4.42E-09	0.11177329	0.76153791
234.4	6.43E-08	1.66E-12	0.09973784	0.26118363	1.21E-30	0.00031173	4.42E-09	0.11177329	0.76153791
234.5	6.43E-08	1.66E-12	0.09973784	0.26118363	1.21E-30	0.00031173	4.42E-09	0.11177329	0.76153791
234.6	6.31E-08	1.68E-12	0.09944815	0.26002626	1.21E-30	0.00030714	4.35E-09	0.11161838	0.7596267
234.7	6.31E-08	1.68E-12	0.09944815	0.26002626	1.21E-30	0.00030714	4.35E-09	0.11161838	0.7596267
234.8	6.31E-08	1.68E-12	0.09944815	0.26002626	1.21E-30	0.00030714	4.35E-09	0.11161838	0.7596267
234.9	6.31E-08	1.68E-12	0.09944815	0.26002626	1.21E-30	0.00030714	4.35E-09	0.11161838	0.7596267
235	6.31E-08	1.68E-12	0.09944815	0.26002626	1.21E-30	0.00030714	4.35E-09	0.11161838	0.7596267
235.1	6.31E-08	1.68E-12	0.09944815	0.26002626	1.21E-30	0.00030714	4.35E-09	0.11161838	0.7596267
235.2	6.31E-08	1.68E-12	0.09944815	0.26002626	1.21E-30	0.00030714	4.35E-09	0.11161838	0.7596267
235.3	6.19E-08	1.72E-12	0.09835734	0.26058903	1.25E-30	0.00030566	4.35E-09	0.11253329	0.76007538
235.4	6.19E-08	1.72E-12	0.09835734	0.26058903	1.25E-30	0.00030566	4.35E-09	0.11253329	0.76007538
235.5	6.19E-08	1.72E-12	0.09835734	0.26058903	1.25E-30	0.00030566	4.35E-09	0.11253329	0.76007538
235.6	6.19E-08	1.72E-12	0.09835734	0.26058903	1.25E-30	0.00030566	4.35E-09	0.11253329	0.76007538
235.7	6.19E-08	1.72E-12	0.09835734	0.26058903	1.25E-30	0.00030566	4.35E-09	0.11253329	0.76007538
235.8	6.19E-08	1.72E-12	0.09835734	0.26058903	1.25E-30	0.00030566	4.35E-09	0.11253329	0.76007538
235.9	6.18E-08	1.75E-12	0.0982445	0.25931326	1.28E-30	0.00030337	4.36E-09	0.11235466	0.76359067
236	6.18E-08	1.75E-12	0.0982445	0.25931326	1.28E-30	0.00030337	4.36E-09	0.11235466	0.76359067
236.1	6.18E-08	1.75E-12	0.0982445	0.25931326	1.28E-30	0.00030337	4.36E-09	0.11235466	0.76359067
236.2	6.18E-08	1.75E-12	0.0982445	0.25931326	1.28E-30	0.00030337	4.36E-09	0.11235466	0.76359067
236.3	6.18E-08	1.75E-12	0.0982445	0.25931326	1.28E-30	0.00030337	4.36E-09	0.11235466	0.76359067
236.4	6.16E-08	1.68E-12	0.09694264	0.2570018	1.33E-30	0.00029755	4.43E-09	0.11277134	0.76460431
236.5	6.16E-08	1.68E-12	0.09694264	0.2570018	1.33E-30	0.00029755	4.43E-09	0.11277134	0.76460431
236.6	6.16E-08	1.68E-12	0.09694264	0.2570018	1.33E-30	0.00029755	4.43E-09	0.11277134	0.76460431
236.7	6.16E-08	1.68E-12	0.09694264	0.2570018	1.33E-30	0.00029755	4.43E-09	0.11277134	0.76460431
236.8	6.16E-08	1.68E-12	0.09694264	0.2570018	1.33E-30	0.00029755	4.43E-09	0.11277134	0.76460431
236.9	6.26E-08	1.64E-12	0.09596055	0.25555499	1.28E-30	0.00029653	4.49E-09	0.11168843	0.76788356
237	6.26E-08	1.64E-12	0.09596055	0.25555499	1.28E-30	0.00029653	4.49E-09	0.11168843	0.76788356
237.1	6.32E-08	1.72E-12	0.09581698	0.25559095	1.30E-30	0.00029851	4.45E-09	0.11179971	0.7686256
237.2	6.32E-08	1.72E-12	0.09581698	0.25559095	1.30E-30	0.00029851	4.45E-09	0.11179971	0.7686256
237.3	6.32E-08	1.72E-12	0.09581698	0.25559095	1.30E-30	0.00029851	4.45E-09	0.11179971	0.7686256
237.4	6.32E-08	1.72E-12	0.09581698	0.25559095	1.30E-30	0.00029851	4.45E-09	0.11179971	0.7686256
237.5	6.32E-08	1.72E-12	0.09581698	0.25559095	1.30E-30	0.00029851	4.45E-09	0.11179971	0.7686256
237.6	6.42E-08	1.78E-12	0.0946123	0.25511326	1.35E-30	0.00030881	4.63E-09	0.11284062	0.77661852
237.7	6.42E-08	1.78E-12	0.0946123	0.25511326	1.35E-30	0.00030881	4.63E-09	0.11284062	0.77661852
237.8	6.42E-08	1.78E-12	0.0946123	0.25511326	1.35E-30	0.00030881	4.63E-09	0.11284062	0.77661852
237.9	6.44E-08	1.79E-12	0.0953238	0.25615271	1.43E-30	0.00030411	4.66E-09	0.11278148	0.77121957
238	6.43E-08	1.70E-12	0.09352892	0.25316735	1.43E-30	0.00029157	4.65E-09	0.11244541	0.76370818
238.1	6.43E-08	1.70E-12	0.09352892	0.25316735	1.43E-30	0.00029157	4.65E-09	0.11244541	0.76370818
238.2	6.59E-08	1.68E-12	0.09314229	0.25092575	1.41E-30	0.00028821	4.66E-09	0.11323859	0.76757337
238.3	6.59E-08	1.68E-12	0.09314229	0.25092575	1.41E-30	0.00028821	4.66E-09	0.11323859	0.76757337
238.4	6.68E-08	1.66E-12	0.09200638	0.24872788	1.45E-30	0.00028942	4.81E-09	0.11259245	0.76964549
238.5	6.68E-08	1.66E-12	0.09200638	0.24872788	1.45E-30	0.00028942	4.81E-09	0.11259245	0.76964549
238.6	6.68E-08	1.66E-12	0.09200638	0.24872788	1.45E-30	0.00028942	4.81E-09	0.11259245	0.76964549
238.7	6.68E-08	1.66E-12	0.09200638	0.24872788	1.45E-30	0.00028942	4.81E-09	0.11259245	0.76964549
238.8	6.68E-08	1.66E-12	0.09200638	0.24872788	1.45E-30	0.00028942	4.81E-09	0.11259245	0.76964549
238.9	6.68E-08	1.66E-12	0.09200638	0.24872788	1.45E-30	0.00028942	4.81E-09	0.11259245	0.76964549
239	6.68E-08	1.66E-12	0.09200638	0.24872788	1.45E-30	0.00028942	4.81E-09	0.11259245	0.76964549
239.1	6.68E-08	1.66E-12	0.09200638	0.24872788	1.45E-30	0.00028942	4.81E-09	0.11259245	0.76964549
239.2	6.68E-08	1.66E-12	0.09200638	0.24872788	1.45E-30	0.00028942	4.81E-09	0.11259245	0.76964549
239.3	6.71E-08	1.73E-12	0.09095287	0.24573174	1.46E-30	0.00029502	4.93E-09	0.11345962	0.77034827
239.4	6.71E-08	1.73E-12	0.09095287	0.24573174	1.46E-30	0.00029502	4.93E-09	0.11345962	0.77034827
239.5	6.71E-08	1.73E-12	0.09095287	0.24573174	1.46E-30	0.00029502	4.93E-09	0.11345962	0.77034827
239.6	6.71E-08	1.73E-12	0.09095287	0.24573174	1.46E-30	0.00029502	4.93E-09	0.11345962	0.77034827
239.7	6.71E-08	1.73E-12	0.09095287	0.24573174	1.46E-30	0.00029502	4.93E-09	0.11345962	0.77034827
239.8	6.66E-08	1.79E-12	0.09042339	0.24687958	1.44E-30	0.0002952	4.77E-09	0.11377514	0.76247887
239.9	6.66E-08	1.79E-12	0.09042339	0.24687958	1.44E-30	0.0002952	4.77E-09	0.11377514	0.76247887
240	6.66E-08	1.79E-12	0.09042339	0.24687958	1.44E-30	0.0002952	4.77E-09	0.11377514	0.76247887

Table S7: PRCC analysis data for [LACT] with [G](0)=0.02 mM: PRCC sensitivity indices and their corresponding p-values

Sensitivity analysis data from PRCC for [LACT] with [G](0)=0.02 mM (only the parameters to which [LACT]'s level is sensitive to appear)

Time discretization	alpha	[LACT]_0
0	0	1
0.1	0	1
0.2	0	1
0.3	0	1
0.4	0	1
0.5	0.03396159	0.99999855
0.6	0.03396159	0.99999855
0.7	0.03396159	0.99999855
0.8	0.03396159	0.99999855
0.9	0.03396159	0.99999855
1	0.03396159	0.99999855
1.1	0.03396159	0.99999855
1.2	0.03396159	0.99999855
1.3	0.03396159	0.99999855
1.4	0.03396159	0.99999855
1.5	0.03396159	0.99999855
1.6	0.03396159	0.99999855
1.7	0.03396159	0.99999855
1.8	0.03396159	0.99999855
1.9	0.03396159	0.99999855
2	0.03396159	0.99999855
2.1	0.03396159	0.99999855
2.2	0.03396159	0.99999855
2.3	0.03396159	0.99999855
2.4	0.03396159	0.99999855
2.5	0.03396159	0.99999855
2.6	0.03396159	0.99999855
2.7	0.03396159	0.99999855
2.8	0.03396159	0.99999855
2.9	0.03396159	0.99999855
3	0.03396159	0.99999855
3.1	0.03396159	0.99999855
3.2	0.03396159	0.99999855
3.3	0.03396159	0.99999855
3.4	0.03396159	0.99999855
3.5	0.03396159	0.99999855
3.6	0.03396159	0.99999855
3.7	0.03396159	0.99999855
3.8	0.03396159	0.99999855
3.9	0.03396159	0.99999855
4	0.03396159	0.99999855
4.1	0.03396159	0.99999855
4.2	0.03396159	0.99999855
4.3	0.03396159	0.99999855
4.4	0.03396159	0.99999855
4.5	0.03396159	0.99999855
4.6	0.03396159	0.99999855
4.7	0.03396159	0.99999855
4.8	0.03396159	0.99999855
4.9	0.05551429	0.999997
5	0.05551429	0.999997
5.1	0.05551429	0.999997
5.2	0.05551429	0.999997
5.3	0.05551429	0.999997
5.4	0.05551429	0.999997
5.5	0.05551429	0.999997
5.6	0.05551429	0.999997
5.7	0.05551429	0.999997
5.8	0.05551429	0.999997
5.9	0.05551429	0.999997
6	0.05551429	0.999997
6.1	0.05551429	0.999997
6.2	0.05551429	0.999997
6.3	0.05551429	0.999997
6.4	0.05551429	0.999997
6.5	0.05551429	0.999997
6.6	0.05551429	0.999997
6.7	0.05551429	0.999997
6.8	0.05551429	0.999997
6.9	0.05551429	0.999997
7	0.05551429	0.999997
7.1	0.05551429	0.999997
7.2	0.05551429	0.999997
7.3	0.05551429	0.999997
7.4	0.05551429	0.999997
7.5	0.01577905	0.9999955
7.6	0.01577905	0.9999955
7.7	0.01577905	0.9999955
7.8	0.01577905	0.9999955
7.9	0.01577905	0.9999955
8	0.01577905	0.9999955
8.1	0.01577905	0.9999955
8.2	0.01577905	0.9999955
8.3	0.01577905	0.9999955
8.4	0.01577905	0.9999955
8.5	0.01577905	0.9999955
8.6	0.01577905	0.9999955
8.7	0.01577905	0.9999955
8.8	0.01577905	0.9999955
8.9	0.01577905	0.9999955
9	0.01577905	0.9999955
9.1	0.01577905	0.9999955
9.2	0.01577905	0.9999955
9.3	0.01577905	0.9999955
9.4	0.01577905	0.9999955
9.5	0.01577905	0.9999955
9.6	0.01577905	0.9999955
9.7	0.01577905	0.9999955
9.8	0.01577905	0.9999955
9.9	0.01577905	0.9999955
10	0.01577905	0.9999955
10.1	0.01577905	0.9999955
10.2	0.01577905	0.9999955
10.3	0.01577905	0.9999955
10.4	0.01577905	0.9999955
10.5	0.01577905	0.9999955
10.6	0.01577905	0.9999955
10.7	0.01577905	0.9999955
10.8	0.01577905	0.9999955
10.9	0.01577905	0.9999955
11	0.01577905	0.9999955
11.1	0.01577905	0.9999955
11.2	0.01577905	0.9999955
11.3	0.01577905	0.9999955
11.4	0.01577905	0.9999955
11.5	0.01577905	0.9999955
11.6	0.01577905	0.9999955
11.7	0.01577905	0.9999955

Time discretization	alpha	[LACT]_0
15	0.01577905	0.9999955
15.1	0.01577905	0.9999955
15.2	0.01577905	0.9999955
15.3	0.01577905	0.9999955
15.4	0.01577905	0.9999955
15.5	0.01577905	0.9999955
15.6	0.01577905	0.9999955
15.7	0.01577905	0.9999955
15.8	0.01577905	0.9999955
15.9	0.01577905	0.9999955
16	0.01577905	0.9999955
16.1	0.01577905	0.9999955
16.2	0.01577905	0.9999955
16.3	0.01577905	0.9999955
16.4	0.01577905	0.9999955
16.5	0.01577905	0.9999955
16.6	-0.0079442	0.99999399
16.7	-0.0079442	0.99999399
16.8	-0.0079442	0.99999399
16.9	-0.0079442	0.99999399
17	-0.0079442	0.99999399
17.1	-0.0017259	0.99999264
17.2	-0.0017259	0.99999264
17.3	-0.0017259	0.99999264
17.4	-0.0017259	0.99999264
17.5	-0.0017259	0.99999264
17.6	-0.0017259	0.99999264
17.7	-0.0017259	0.99999264
17.8	-0.0017259	0.99999264
17.9	-0.0017259	0.99999264
18	-0.0433299	0.99999015
18.1	-0.0433299	0.99999015
18.2	-0.0433299	0.99999015
18.3	-0.0433299	0.99999015
18.4	-0.0433299	0.99999015
18.5	-0.0441256	0.99998599
18.6	-0.0441256	0.99998599
18.7	-0.0441256	0.99998599
18.8	-0.0441256	0.99998599
18.9	-0.0441256	0.99998599
19	-0.0441256	0.99998599
19.1	-0.0441256	0.99998599
19.2	-0.0441256	0.99998599
19.3	-0.0441256	0.99998599
19.4	-0.0399819	0.99998087
19.5	-0.0399819	0.99998087
19.6	-0.0399819	0.99998087
19.7	-0.0399819	0.99998087
19.8	-0.0399819	0.99998087
19.9	-0.0454076	0.99997395
20	-0.0454076	0.99997395
20.1	-0.0681923	0.9999663
20.2	-0.0681923	0.9999663
20.3	-0.0681923	0.9999663
20.4	-0.0681923	0.9999663
20.5	-0.0681923	0.9999663
20.6	-0.0681923	0.9999663
20.7	-0.0681923	0.9999663
20.8	-0.0681923	0.9999663
20.9	-0.0681923	0.9999663
21	-0.0681923	0.9999663
21.1	-0.0681923	0.9999663
21.2	-0.0674727	0.99994509
21.3	-0.0674727	0.99994509
21.4	-0.0674727	0.99994509
21.5	-0.0674727	0.99994509
21.6	-0.0674727	0.99994509
21.7	-0.0674727	0.99994509
21.8	-0.0696436	0.99993303
21.9	-0.0696436	0.99993303
22	-0.0696436	0.99993303
22.1	-0.0616364	0.9999198
22.2	-0.0616364	0.9999198
22.3	-0.0616364	0.9999198
22.4	-0.0560587	0.99990443
22.5	-0.0560587	0.99990443
22.6	-0.0560587	0.99990443
22.7	-0.0560587	0.99990443
22.8	-0.0560587	0.99990443
22.9	-0.0560587	0.99990443
23	-0.0560587	0.99990443
23.1	-0.0568329	0.99988777
23.2	-0.0568329	0.99988777
23.3	-0.0744494	0.99988707
23.4	-0.0568401	0.99988458
23.5	-0.0568401	0.99988458
23.6	-0.055569	0.99986731
23.7	-0.055569	0.99986731
23.8	-0.0483279	0.99984939
23.9	-0.0483279	0.99984939
24	-0.0483279	0.99984939
24.1	-0.0483279	0.99984939
24.2	-0.0483279	0.99984939
24.3	-0.0450711	0.99982926
24.4	-0.0450711	0.99982926
24.5	-0.0467267	0.99980835
24.6	-0.0467267	0.99980835
24.7	-0.0467267	0.99980835
24.8	-0.0467267	0.99980835
24.9	-0.0467267	0.99980835
25	-0.0467267	0.99980835
25.1	-0.0556457	0.99978399
25.2	-0.0600088	0.99976181
25.3	-0.0600088	0.99976181
25.4	-0.0648415	0.99973855
25.5	-0.0747546	0.99973839
25.6	-0.0747546	0.99973839
25.7	-0.0747546	0.99973839
25.8	-0.0766648	0.99971139
25.9	-0.0766648	0.99971139
26	-0.0766648	0.99971139
26.1	-0.0779952	0.99968144
26.2	-0.0779952	0.99968144
26.3	-0.0779952	0.99968144
26.4	-0.0744241	0.99965119
26.5	-0.0744241	0.99965119
26.6	-0.0744241	0.99965119
26.7	-0.0744241	0.99965119

Time discretization	alpha	[LACT]_0
30	-0.1309393	0.99919182
30.1	-0.1309393	0.99919182
30.2	-0.1309393	0.99919182
30.3	-0.1318738	0.99919299
30.4	-0.1320204	0.99915144
30.5	-0.140396	0.99909172
30.6	-0.1474338	0.99907503
30.7	-0.1474338	0.99907503
30.8	-0.1474338	0.99907503
30.9	-0.1508444	0.99905752
31	-0.1481927	0.99899073
31.1	-0.1469856	0.99894217
31.2	-0.148972	0.99894003
31.3	-0.1519855	0.99892332
31.4	-0.1519855	0.99892332
31.5	-0.1544147	0.99885093
31.6	-0.1544147	0.99885093
31.7	-0.1574121	0.99883003
31.8	-0.1529968	0.99877904
31.9	-0.1512725	0.99875011
32	-0.1529635	0.99869737
32.1	-0.156736	0.99867418
32.2	-0.156736	0.99867418
32.3	-0.156736	0.99867418
32.4	-0.1582676	0.99859535
32.5	-0.158427	0.99853785
32.6	-0.161886	0.99851122
32.7	-0.161886	0.99851122
32.8	-0.163947	0.99842078
32.9	-0.163947	0.99842078
33	-0.1659675	0.99839753
33.1	-0.167923	0.99839197
33.2	-0.1672479	0.99835918
33.3	-0.1686579	0.99825728
33.4	-0.1697451	0.99820307
33.5	-0.1700108	0.99817082
33.6	-0.1700108	0.99817082
33.7	-0.1700108	0.99817082
33.8	-0.1688335	0.99813709
33.9	-0.1699912	0.99806886
34	-0.1705347	0.99797591
34.1	-0.1741373	0.99793463
34.2	-0.1741373	0.99793463
34.3	-0.1729121	0.99783471
34.4	-0.1725586	0.99782728
34.5	-0.1749612	0.99779018
34.6	-0.1743861	0.99772675
34.7	-0.1739407	0.99767814
34.8	-0.1778126	0.99762377
34.9	-0.177194	0.99758371
35	-0.1771623	0.9975476
35.1	-0.1767466	0.99750262
35.2	-0.1767466	0.99750262
35.3	-0.1767466	0.99750262
35.4	-0.1794783	0.99738651
35.5	-0.1794783	0.99738651
35.6	-0.1760323	0.99726357
35.7	-0.1792851	0.99715083
35.8	-0.1810301	0.99710372
35.9	-0.1810301	0.99710372
36	-0.18148	0.99703318
36.1	-0.1824996	0.99698537
36.2	-0.1823258	0.99692642
36.3	-0.1814025	0.99679383
36.4	-0.1836546	0.99679254
36.5	-0.1836284	0.9967328
36.6	-0.1833082	0.9966694
36.7	-0.1825427	0.9966126
36.8	-0.1825427	0.9966126
36.9	-0.1813529	0.9965491
37	-0.1793375	0.99647358
37.1	-0.17664	0.99633707
37.2	-0.1768707	0.99627588
37.3	-0.1771374	0.9961317
37.4	-0.1780916	0.99612394
37.5	-0.1805474	0.99606502
37.6	-0.1807124	0.99600179
37.7	-0.1807124	0.99600179
37.8	-0.1791993	0.99586249
37.9	-0.1791993	0.99586249
38	-0.1817381	0.9958517
38.1	-0.182646	0.9957839
38.2	-0.1816971	0.99556972
38.3	-0.1832278	0.99555733
38.4	-0.1851654	0.99555583
38.5	-0.1882356	0.99546581
38.6	-0.1880493	0.99532624
38.7	-0.1903271	0.99532309
38.8	-0.1926044	0.9953048
38.9	-0.1938307	0.99507673
39	-0.1939193	0.99507576
39.1	-0.192952	0.99490815
39.2	-0.1949983	0.99492431
39.3	-0.1966542	0.99484295
39.4	-0.1962297	0.99469037
39.5	-0.1962297	0.99469037
39.6	-0.1949993	0.99461253
39.7	-0.1939197	0.9945384
39.8	-0.1915253	0.99437381
39.9	-0.1934148	0.9942797
40	-0.1925545	0.99420084
40.1	-0.1917878	0.99410264
40.2	-0.1930298	0.9940214
40.3	-0.1920668	0.99394899
40.4	-0.1934379	0.99379683
40.5	-0.1929317	0.99370592
40.6	-0.1941783	0.99368989
40.7	-0.1946771	0.99361517
40.8	-0.1957995	0.99359417
40.9	-0.1975494	0.99340792
41	-0.2005307	0.99340236
41.1	-0.2005307	0.99340236
41.2	-0.2033252	0.993379
41.3	-0.2048002	0.9932825
41.4	-0.2057302	0.99326941
41.5	-0.2045767	0.99317044
41.6	-0.2055677	0.99317352
41.7	-0.2067057	0.99306196

Time discretization	alpha	[LACT]_0
45	-0.2220871	0.99169523
45.1	-0.221327	0.99165686
45.2	-0.2227972	0.99161873
45.3	-0.2235541	0.99162683
45.4	-0.2235541	0.99162683
45.5	-0.2242362	0.99150805
45.6	-0.2260782	0.99145968
45.7	-0.2274464	0.99140695
45.8	-0.228651	0.99139472
45.9	-0.2294326	0.99115579
46	-0.2323476	0.99102151
46.1	-0.2323476	0.99102151
46.2	-0.2321103	0.99100799
46.3	-0.2308816	0.99089242
46.4	-0.2315202	0.99087486
46.5	-0.232896	0.99082574
46.6	-0.2348299	0.99066448
46.7	-0.2348299	0.99066448
46.8	-0.237958	0.99067217
46.9	-0.2380599	0.99053513
47	-0.2385955	0.99048613
47.1	-0.2393935	0.99030011
47.2	-0.2405997	0.99029991
47.3	-0.2405997	0.99029991
47.4	-0.241101	0.99025686
47.5	-0.2394429	0.99013839
47.6	-0.2393388	0.99002323
47.7	-0.2423939	0.98995614
47.8	-0.2425636	0.98982648
47.9	-0.2427504	0.98975858
48	-0.2401648	0.98951923
48.1	-0.2401648	0.98951923
48.2	-0.2407903	0.98949005
48.3	-0.2407903	0.98949005
48.4	-0.2416791	0.98943928
48.5	-0.2421366	0.98919859
48.6	-0.2421366	0.98919859
48.7	-0.2438257	0.98913226
48.8	-0.2434444	0.98911861
48.9	-0.2442629	0.98906012
49	-0.2475818	0.98899509
49.1	-0.2473391	0.98899609
49.2	-0.2477265	0.98893513
49.3	-0.2468943	0.98879041
49.4	-0.2473182	0.98874791
49.5	-0.2473182	0.98874791
49.6	-0.2473298	0.9887294
49.7	-0.2473298	0.9887294
49.8	-0.2478132	0.98867597
49.9	-0.2453307	0.9884825
50	-0.2467002	0.9884802
50.1	-0.2470723	0.98839
50.2	-0.2475662	0.98839145
50.3	-0.2475662	0.98839145
50.4	-0.2481512	0.98832206
50.5	-0.247291	0.98826299
50.6	-0.2504366	0.98827913
50.7	-0.2514733	0.9882346
50.8	-0.2519424	0.98820797
50.9	-0.2523148	0.98813137
51	-0.2530193	0.98811399
51.1	-0.2529171	0.98803155
51.2	-0.2540422	0.98803304
51.3	-0.2540422	0.98803304
51.4	-0.2556105	0.98794951
51.5	-0.2556105	0.98794951
51.6	-0.2557484	0.98786202
51.7	-0.2557484	0.98786202
51.8	-0.2557484	0.98786202
51.9	-0.2540144	0.9876596
52	-0.2535307	0.98765185
52.1	-0.2525776	0.98757793
52.2	-0.2533667	0.98747502
52.3	-0.252863	0.98745555
52.4	-0.252863	0.98745555
52.5	-0.2560894	0.98737297
52.6	-0.2570593	0.98735142
52.7	-0.2570593	0.98735142
52.8	-0.256692	0.98725613
52.9	-0.2593861	0.98721085
53	-0.2607332	0.98717868
53.1	-0.2613633	0.98709318
53.2	-0.2629525	0.98707337
53.3	-0.2629525	0.98707337
53.4	-0.2642859	0.98701029
53.5	-0.2654207	0.98689839
53.6	-0.2649615	0.98681187
53.7	-0.2640761	0.98678089
53.8	-0.2654777	0.98665724
53.9	-0.2656772	0.986622
54	-0.2656772	0.986622
54.1	-0.264759	0.98649588
54.2	-0.2640521	0.98640648
54.3	-0.2652271	0.98636419
54.4	-0.2653069	0.98636735
54.5	-0.2660664	0.98636986
54.6	-0.2691121	0.98618534
54.7	-0.2703007	0.98616485
54.8	-0.2703041	0.98605693
54.9	-0.2699768	0.98595746
55	-0.2699768	0.98595746
55.1	-0.2691335	0.98583216
55.2	-0.2709756	0.98578855
55.3	-0.2709756	0.98578855
55.4	-0.2722224	0.9856099
55.5	-0.273618	0.98549452
55.6	-0.2748261	0.98544795
55.7	-0.2748261	0.98544795
55.8	-0.2748011	0.98528569
55.9	-0.2748011	0.98528569
56	-0.2760825	0.98527117
56.1	-0.2760825	0.98527117
56.2	-0.2770293	0.98517448
56.3	-0.2761727	0.98513119
56.4	-0.2823606	0.98504718
56.5	-0.2823606	0.98504718
56.6	-0.2823606	0.98504718
56.7	-0.2837434	0.98502144

11.8	0.01577905	0.9999955	26.8	-0.0752688	0.99961935	41.8	-0.2079259	0.99303068	56.8	-0.2851838	0.98491199
11.9	0.01577905	0.9999955	26.9	-0.0752688	0.99961935	41.9	-0.2079259	0.99303068	56.9	-0.2848763	0.98484375
12	0.01577905	0.9999955	27	-0.0752688	0.99961935	42	-0.2081232	0.99303322	57	-0.2848763	0.98484375
12.1	0.01577905	0.9999955	27.1	-0.0752688	0.99961935	42.1	-0.2071725	0.99292845	57.1	-0.2845696	0.98462613
12.2	0.01577905	0.9999955	27.2	-0.0752688	0.99961935	42.2	-0.2057584	0.99282995	57.2	-0.2827654	0.98449168
12.3	0.01577905	0.9999955	27.3	-0.0829201	0.99961801	42.3	-0.2059414	0.9927958	57.3	-0.2833363	0.98444771
12.4	0.01577905	0.9999955	27.4	-0.0849844	0.99958615	42.4	-0.2087916	0.99277689	57.4	-0.2832189	0.98428911
12.5	0.01577905	0.9999955	27.5	-0.0947895	0.9995838	42.5	-0.2112993	0.99279275	57.5	-0.2840354	0.98425942
12.6	0.01577905	0.9999955	27.6	-0.1030386	0.99955161	42.6	-0.2112993	0.99279275	57.6	-0.2856457	0.98423738
12.7	0.01577905	0.9999955	27.7	-0.1030386	0.99955161	42.7	-0.2096475	0.9926942	57.7	-0.285267	0.98409284
12.8	0.01577905	0.9999955	27.8	-0.107144	0.99954925	42.8	-0.2104578	0.99269111	57.8	-0.2845177	0.98406779
12.9	0.01577905	0.9999955	27.9	-0.107144	0.99954925	42.9	-0.2116426	0.99269053	57.9	-0.2868903	0.98395785
13	0.01577905	0.9999955	28	-0.1155005	0.99950877	43	-0.2123509	0.99257083	58	-0.2867462	0.98386197
13.1	0.01577905	0.9999955	28.1	-0.118394	0.99950406	43.1	-0.2129707	0.99245364	58.1	-0.2887402	0.98387795
13.2	0.01577905	0.9999955	28.2	-0.1181089	0.99947022	43.2	-0.2123513	0.99241934	58.2	-0.290802	0.98383091
13.3	0.01577905	0.9999955	28.3	-0.1226298	0.99946457	43.3	-0.2126962	0.99241263	58.3	-0.290683	0.98370122
13.4	0.01577905	0.9999955	28.4	-0.1226298	0.99946457	43.4	-0.2111078	0.99230495	58.4	-0.2906197	0.98346795
13.5	0.01577905	0.9999955	28.5	-0.1239019	0.99942599	43.5	-0.2111078	0.99230495	58.5	-0.2906197	0.98346795
13.6	0.01577905	0.9999955	28.6	-0.1239019	0.99942599	43.6	-0.213362	0.99229697	58.6	-0.2900861	0.98331767
13.7	0.01577905	0.9999955	28.7	-0.12387	0.99941552	43.7	-0.2130411	0.99217084	58.7	-0.2902592	0.98318249
13.8	0.01577905	0.9999955	28.8	-0.12387	0.99941552	43.8	-0.2129159	0.99205141	58.8	-0.2913606	0.98320259
13.9	0.01577905	0.9999955	28.9	-0.12387	0.99941552	43.9	-0.2129159	0.99205141	58.9	-0.2917741	0.98317901
14	0.01577905	0.9999955	29	-0.1214683	0.99937455	44	-0.2149222	0.99205458	59	-0.292587	0.98314601
14.1	0.01577905	0.9999955	29.1	-0.1200419	0.99932866	44.1	-0.2161922	0.99202546	59.1	-0.292653	0.9829422
14.2	0.01577905	0.9999955	29.2	-0.1276461	0.99931914	44.2	-0.2193248	0.99188519	59.2	-0.292653	0.9829422
14.3	0.01577905	0.9999955	29.3	-0.1276461	0.99931914	44.3	-0.2199436	0.99188773	59.3	-0.2948425	0.98291295
14.4	0.01577905	0.9999955	29.4	-0.1276461	0.99931914	44.4	-0.2204344	0.99185473	59.4	-0.2946434	0.98285236
14.5	0.01577905	0.9999955	29.5	-0.1276461	0.99931914	44.5	-0.2204344	0.99185473	59.5	-0.2942292	0.98280009
14.6	0.01577905	0.9999955	29.6	-0.1276461	0.99931914	44.6	-0.2204344	0.99185473	59.6	-0.2956243	0.98280462
14.7	0.01577905	0.9999955	29.7	-0.1309862	0.99926589	44.7	-0.2187584	0.99182059	59.7	-0.2950589	0.98263346
14.8	0.01577905	0.9999955	29.8	-0.136099	0.99925215	44.8	-0.2187832	0.99170979	59.8	-0.2966677	0.98261671
14.9	0.01577905	0.9999955	29.9	-0.131141	0.99920648	44.9	-0.2203963	0.99170835	59.9	-0.2968603	0.98251627

60	-0.2968603	0.98251627	75	-0.3747312	0.96441523	90	-0.3873753	0.91188172	105	-0.4168153	0.89766747
60.1	-0.2968603	0.98251627	75.1	-0.375815	0.96408322	90.1	-0.3873753	0.91188172	105.1	-0.4168153	0.89766747
60.2	-0.2978097	0.98251413	75.2	-0.3744719	0.9637615	90.2	-0.3877809	0.91161212	105.2	-0.4169937	0.89767201
60.3	-0.2978097	0.98251413	75.3	-0.3756244	0.96350473	90.3	-0.3874221	0.91127582	105.3	-0.4172578	0.89759666
60.4	-0.2974613	0.98242572	75.4	-0.3770644	0.963215	90.4	-0.3877864	0.91125305	105.4	-0.4175473	0.89759061
60.5	-0.3001387	0.98240453	75.5	-0.37748	0.96290615	90.5	-0.3875173	0.91101223	105.5	-0.4176174	0.89744357
60.6	-0.3016767	0.98237168	75.6	-0.3795831	0.96252712	90.6	-0.3879945	0.91083201	105.6	-0.4176174	0.89744357
60.7	-0.3030129	0.98234609	75.7	-0.3807104	0.96228854	90.7	-0.388172	0.91060209	105.7	-0.4182226	0.89743006
60.8	-0.3038018	0.98225569	75.8	-0.3804171	0.96217989	90.8	-0.3878376	0.9104103	105.8	-0.4176193	0.8971314
60.9	-0.3036142	0.98220211	75.9	-0.3811543	0.96176827	90.9	-0.3874481	0.90991331	105.9	-0.4176193	0.8971314
61	-0.304798	0.98216175	76	-0.3822053	0.961635	91	-0.3874481	0.90991331	106	-0.4176193	0.8971314
61.1	-0.30546	0.98213564	76.1	-0.3830089	0.96150123	91.1	-0.3870589	0.90958211	106.1	-0.4181103	0.89709222
61.2	-0.3061224	0.98200852	76.2	-0.3835018	0.96132473	91.2	-0.3873105	0.90901378	106.2	-0.4181103	0.89709222
61.3	-0.3061224	0.98200852	76.3	-0.383129	0.96109189	91.3	-0.3875182	0.90897707	106.3	-0.4180335	0.89704335
61.4	-0.3068387	0.98201461	76.4	-0.3823422	0.96067765	91.4	-0.3875182	0.90897707	106.4	-0.4179218	0.89698881
61.5	-0.3069875	0.98197118	76.5	-0.382769	0.9604211	91.5	-0.3879021	0.90851397	106.5	-0.4183426	0.89698079
61.6	-0.3081039	0.98196166	76.6	-0.3834372	0.96012704	91.6	-0.3879021	0.90851397	106.6	-0.4183887	0.89700113
61.7	-0.3088275	0.98192553	76.7	-0.3828196	0.95975973	91.7	-0.3884439	0.90805041	106.7	-0.4185522	0.89691519
61.8	-0.3103501	0.98185868	76.8	-0.3835678	0.95964601	91.8	-0.3883483	0.90789206	106.8	-0.4185522	0.89691519
61.9	-0.3102244	0.9817746	76.9	-0.382986	0.95952658	91.9	-0.3883283	0.90766003	106.9	-0.4185347	0.8968797
62	-0.3136627	0.98171504	77	-0.3834765	0.95913709	92	-0.3884534	0.90763282	107	-0.4192908	0.89689452
62.1	-0.3125659	0.98164204	77.1	-0.3837673	0.95904005	92.1	-0.3887041	0.90748173	107.1	-0.4193433	0.89689149
62.2	-0.3121897	0.98158587	77.2	-0.3859074	0.95886587	92.2	-0.3894723	0.90722537	107.2	-0.419487	0.89677054
62.3	-0.3117869	0.9815344	77.3	-0.3868006	0.95845043	92.3	-0.3897413	0.90718146	107.3	-0.4198662	0.89667161
62.4	-0.3117869	0.9815344	77.4	-0.3869254	0.95831355	92.4	-0.3899223	0.90710157	107.4	-0.4200138	0.89664366
62.5	-0.3113515	0.98143746	77.5	-0.3858353	0.95782136	92.5	-0.3901015	0.90703752	107.5	-0.4194095	0.89658117
62.6	-0.3121014	0.98137678	77.6	-0.3858163	0.95772657	92.6	-0.3901015	0.90703752	107.6	-0.4194095	0.89658117
62.7	-0.3137756	0.98132174	77.7	-0.3863106	0.95719859	92.7	-0.3903659	0.90687972	107.7	-0.4194095	0.89658117
62.8	-0.3143563	0.98123035	77.8	-0.3858244	0.95663279	92.8	-0.3901565	0.90670731	107.8	-0.4201732	0.8965236
62.9	-0.3138237	0.98112712	77.9	-0.386814	0.9565501	92.9	-0.3901868	0.90672255	107.9	-0.4200301	0.89639767
63	-0.3154527	0.98113195	78	-0.3861888	0.95604637	93	-0.3902079	0.90666328	108	-0.420486	0.89643453
63.1	-0.3162166	0.98108917	78.1	-0.3866645	0.95568755	93.1	-0.3907945	0.90669526	108.1	-0.4207098	0.89642833
63.2	-0.3170842	0.98103838	78.2	-0.3852077	0.9554751	93.2	-0.3909086	0.90666636	108.2	-0.4206938	0.89617095
63.3	-0.3177457	0.98098155	78.3	-0.3855219	0.95463195	93.3	-0.3916688	0.9064493	108.3	-0.4206938	0.89617095
63.4	-0.3179404	0.98088899	78.4	-0.3859219	0.95449969	93.4	-0.392427	0.90642807	108.4	-0.4210337	0.89605854
63.5	-0.3176631	0.98082454	78.5	-0.3867616	0.95436416	93.5	-0.392536	0.90638167	108.5	-0.4210337	0.89605854
63.6	-0.3215472	0.9807396	78.6	-0.38541	0.95356975	93.6	-0.3928604	0.9060113	108.6	-0.4208301	0.89599667
63.7	-0.3215472	0.9807396	78.7	-0.3852183	0.95335498	93.7	-0.3931635	0.90597932	108.7	-0.4207761	0.89595816
63.8	-0.3215472	0.9807396	78.8	-0.3860815	0.95305621	93.8	-0.3931635	0.90597932	108.8	-0.4211889	0.89592516
63.9	-0.3216162	0.9806259	78.9	-0.3859151	0.95284124	93.9	-0.3934592	0.90579532	108.9	-0.4211345	0.89575421
64	-0.3236726	0.98073209	79	-0.3862595	0.95236665	94	-0.3935013	0.90574345	109	-0.4215864	0.89576507
64.1	-0.3248471	0.98066214	79.1	-0.387044	0.95185258	94.1	-0.3941353	0.90572228	109.1	-0.4215864	0.89576507
64.2	-0.3250739	0.980652	79.2	-0.3867024	0.95149614	94.2	-0.3948232	0.90554538	109.2	-0.4215864	0.89576507
64.3	-0.3252542	0.98059541	79.3	-0.386619	0.95121786	94.3	-0.3949715	0.90551185	109.3	-0.4218935	0.89567333
64.4	-0.3273203	0.98052443	79.4	-0.3867823	0.95089333	94.4	-0.3957044	0.90532416	109.4	-0.4222761	0.89567355
64.5	-0.3280482	0.98041692	79.5	-0.3864175	0.95060257	94.5	-0.3955348	0.90513373	109.5	-0.4222761	0.89567355
64.6	-0.3269303	0.98033632	79.6	-0.3865356	0.95017211	94.6	-0.3959779	0.90496197	109.6	-0.4222761	0.89567355
64.7	-0.3284466	0.98029	79.7	-0.3872679	0.94982458	94.7	-0.396007	0.90475376	109.7	-0.4226604	0.89567351
64.8	-0.3265471	0.98008061	79.8	-0.3862013	0.94923813	94.8	-0.3962523	0.90481536	109.8	-0.4230958	0.8956586
64.9	-0.326792	0.9800572	79.9	-0.3855102	0.94884636	94.9	-0.3966366	0.90462995	109.9	-0.4230958	0.8956586
65	-0.3272857	0.97992622	80	-0.385553	0.94838402	95	-0.3965045	0.90457244	110	-0.4230958	0.8956586
65.1	-0.3281179	0.97984584	80.1	-0.3848431	0.94785261	95.1	-0.396714	0.90438841	110.1	-0.4232196	0.89561047
65.2	-0.329923	0.97980858	80.2	-0.3849984	0.94761672	95.2	-0.3971573	0.90431086	110.2	-0.4232196	0.89561047
65.3	-0.3303128	0.97961675	80.3	-0.3857418	0.94724887	95.3	-0.3969016	0.90409771	110.3	-0.4232196	0.89561047
65.4	-0.330357	0.97959391	80.4	-0.3849042	0.94677829	95.4	-0.3967732	0.90380771	110.4	-0.4234392	0.89550156
65.5	-0.3313292	0.97949835	80.5	-0.3859713	0.94637015	95.5	-0.3973084	0.90380584	110.5	-0.4239039	0.89552724
65.6	-0.331541	0.97944266	80.6	-0.386014	0.946069	95.6	-0.3976208	0.90349326	110.6	-0.4239039	0.89552724
65.7	-0.3312068	0.97937461	80.7	-0.3869883	0.94567513	95.7	-0.3981755	0.90346275	110.7	-0.4242163	0.89546743
65.8	-0.332043	0.97920666	80.8	-0.3870679	0.94518496	95.8	-0.3988467	0.90326398	110.8	-0.4242262	0.89544729
65.9	-0.3328976	0.97910354	80.9	-0.3865201	0.94486958	95.9	-0.3990482	0.90303197	110.9	-0.4254072	0.89541259
66	-0.3353533	0.97899708	81	-0.3861943	0.94416038	96	-0.3990482	0.90303197	111	-0.4254072	0.89541259
66.1	-0.3347663	0.9788132	81.1	-0.3863025	0.94377783	96.1	-0.3994089	0.9030519	111.1	-0.4255371	0.89535086
66.2	-0.3337829	0.97859188	81.2	-0.3859722	0.94331327	96.2	-0.4001382	0.90308683	111.2	-0.4254751	0.89528708
66.3	-0.3346262	0.9785143	81.3	-0.385896	0.94288553	96.3	-0.4005938	0.90270603	111.3	-0.4249423	0.89521693
66.4	-0.3349428	0.97847137	81.4	-0.3858161	0.94250719	96.4	-0.4005938	0.90270603	111.4	-0.4249817	0.89514003
66.5	-0.336377	0.97837211	81.5	-0.3860784	0.94214272	96.5	-0.4006509	0.90232743	111.5	-0.4249817	0.89514003
66.6	-0.336377	0.97837211	81.6	-0.3856947	0.94162103	96.6	-0.4011205	0.90230003	111.6	-0.4253	0.8949991
66.7	-0.3387327	0.97819444	81.7	-0.3848712	0.94093601	96.7	-0.401269	0.90229884	111.7	-0.4253	0.8949991
66.8	-0.3383411	0.97812727	81.8	-0.3834484	0.94013429	96.8	-0.4019391	0.90225205	111.8	-0.425375	0.89493383
66.9	-0.3391136	0.97798109	81.9	-0.3853103	0.93994061	96.9	-0.4018229	0.90215631	111.9	-0.425264	0.89487857
67	-0.3398252	0.9778938	82	-0.3849554	0.93929592	97	-0.4018229	0.90215631	112	-0.4253622	0.89480593
67.1	-0.3409103	0.97783889	82.1	-0.384796	0.9389815	97.1	-0.4018436	0.90195676	112.1	-0.4253622	0.89480593
67.2	-0.3407286	0.9777784	82.2	-0.3848607	0.93869741	97.2	-0.4016899	0.9017608	112.2	-0.4259814	0.89470411
67.3	-0.3437644	0.97763074	82.3	-0.3848754	0.93828462	97.3	-0.4016899	0.9017608	112.3	-0.4259873	0.89465799
67.4	-0.3439433	0.97755629	82.4	-0.385225	0.93782458	97.4	-0.4020124	0.90167635	112.4	-0.4260908	0.89448941
67.5	-0.3451304	0.97745056	82.5	-0.384928	0.93757839	97.5	-0.4022038	0.9015465	112.5	-0.4260908	0.89448941
67.6	-0.3459177	0.97742757	82.6	-0.3843736	0.93729001	97.6	-0.4022436	0.90128986	112.6	-0.4260908	0.89448941
67.7	-0.3472636	0.97718194	82.7	-0.3846399	0.93672317	97.7	-0.4022227	0.90119983	112.7	-0.4260908	0.89448941
67.8	-0.3480433	0.97707358	82.8	-0.3853105	0.93580908	97.8	-0.4022227	0.90119983	112.8	-0.4259707	0.89444243
67.9	-0.3493081	0.97695267	82.9	-0.3856773	0.93559031	97.9	-0.402785	0.9011586	112.9	-0.4261315	0.89437117
68	-0.3505662	0.97688633	83	-0.3858937	0.93528733	98	-0.4025984	0.90103267	113	-0.4265005	0.89434753
68.1	-0.3504454	0.97677367	83.1	-0.3857606	0.93440923	98.1	-0.4025984	0.90103267	113.1	-0.4265005	0.89434753
68.2	-0.3520075	0.97671374	83.2	-0.3861333	0.93399058	98.2	-0.4025984	0.90103267	113.2	-0.4265005	0.89434753
68.3	-0.3522361	0.97660352	83.3	-0.3849408	0.93324766	98.3	-0.403647	0.90081713	113.3	-0.4269025	0.8943265
68.4	-0.3538876	0.97656124	83.4	-0.3839787	0.93261603	98.4	-0.4042237	0.90086597	113.4	-0.4272947	0.89432795
68.5	-0.3553199	0.9765133	83.5	-0.3839853	0.93215722	98.5	-0.4043569	0.90076881	113.5	-0.4272947	0.89432795
68.6	-0.3574397	0.97622968	83.6	-0.3843902	0.93147325	98.6	-0.4043569	0.90076881	113.6	-0.4273445	0.89428516
68.7	-0.3575067	0.97602804	83.7	-0.3826742	0.9306692	98.7	-0.4047593	0.9007745	113.7	-0.4274035	0.89427153
68.8	-0.359925	0.97594318	83.8	-0.3827041	0.93033859	98.8	-0.4047454	0.90062427	113.8	-0.4274035	0.89427153

68.9	-0.3606664	0.97592261
69	-0.3631498	0.97559001
69.1	-0.363508	0.97542444
69.2	-0.3636473	0.97536339
69.3	-0.363528	0.97496295
69.4	-0.3636434	0.97477202
69.5	-0.3636434	0.97477202
69.6	-0.3639269	0.9746674
69.7	-0.3660222	0.97443436
69.8	-0.367357	0.97427548
69.9	-0.3676413	0.97419983
70	-0.3677244	0.97400988
70.1	-0.3652681	0.97358281
70.2	-0.3652681	0.97358281
70.3	-0.3657507	0.9734609
70.4	-0.3670315	0.97339158
70.5	-0.3670367	0.97320639
70.6	-0.367381	0.97295364
70.7	-0.3682472	0.9728022
70.8	-0.3676441	0.97270556
70.9	-0.3685227	0.97261377
71	-0.3692897	0.97241613
71.1	-0.3686982	0.97222094
71.2	-0.3690688	0.97206353
71.3	-0.3689291	0.97192221
71.4	-0.3684661	0.97141161
71.5	-0.369316	0.97128843
71.6	-0.3704281	0.97117354
71.7	-0.3704746	0.97109019
71.8	-0.37061	0.97102471
71.9	-0.37061	0.97102471
72	-0.3706683	0.97088221
72.1	-0.3714992	0.97077989
72.2	-0.3722588	0.97044592
72.3	-0.3725687	0.97007566
72.4	-0.373224	0.97001399
72.5	-0.3709937	0.96963115
72.6	-0.3714631	0.96949824
72.7	-0.3730118	0.96925159
72.8	-0.3728935	0.96909052
72.9	-0.3735929	0.96893807
73	-0.3738149	0.96869138
73.1	-0.3723367	0.96847573
73.2	-0.3723207	0.96813105
73.3	-0.3714814	0.96793197
73.4	-0.371954	0.96772308
73.5	-0.3722222	0.96749351
73.6	-0.372543	0.96733121
73.7	-0.3725795	0.967053
73.8	-0.3730146	0.96688919
73.9	-0.3739474	0.96672694
74	-0.3740597	0.96659982
74.1	-0.3735122	0.96633717
74.2	-0.3749304	0.9660839
74.3	-0.373374	0.96562305
74.4	-0.3739599	0.96554645
74.5	-0.3737131	0.96532736
74.6	-0.3755234	0.96503601
74.7	-0.3755804	0.96481057
74.8	-0.3753062	0.96463871
74.9	-0.3753062	0.96463871
120	-0.43886	0.89219484
120.1	-0.4390105	0.89217307
120.2	-0.4390105	0.89217307
120.3	-0.4390105	0.89217307
120.4	-0.4393589	0.89218694
120.5	-0.4395158	0.892102
120.6	-0.4395158	0.892102
120.7	-0.4398712	0.89213954
120.8	-0.4397269	0.89203015
120.9	-0.4401667	0.8919283
121	-0.4405204	0.89187621
121.1	-0.4405204	0.89187621
121.2	-0.4408082	0.89175649
121.3	-0.4408082	0.89175649
121.4	-0.4409791	0.89166184
121.5	-0.4409791	0.89166184
121.6	-0.4410917	0.89163746
121.7	-0.4410917	0.89163746
121.8	-0.4410917	0.89163746
121.9	-0.4416566	0.89156233
122	-0.4416566	0.89156233
122.1	-0.4419317	0.89148206
122.2	-0.4419317	0.89148206
122.3	-0.441847	0.89126879
122.4	-0.4421387	0.89128766
122.5	-0.4425935	0.89116672
122.6	-0.4425935	0.89116672
122.7	-0.4425935	0.89116672
122.8	-0.4426507	0.89098301
122.9	-0.4426507	0.89098301
123	-0.4426507	0.89098301
123.1	-0.4434302	0.890877
123.2	-0.4434302	0.890877
123.3	-0.4439365	0.89084236
123.4	-0.4439064	0.89077884
123.5	-0.443931	0.89069984
123.6	-0.4439917	0.89061201
123.7	-0.4443684	0.89058214
123.8	-0.4443684	0.89058214
123.9	-0.4443684	0.89058214
124	-0.4451479	0.89055915
124.1	-0.4451299	0.890461
124.2	-0.4452643	0.8903974
124.3	-0.4457827	0.8903825
124.4	-0.446232	0.89036821
124.5	-0.4468421	0.89038649
124.6	-0.4463597	0.89021654
124.7	-0.4461599	0.89008076
124.8	-0.4459295	0.88996626
124.9	-0.4463672	0.88992575
125	-0.4463672	0.88992575
125.1	-0.446395	0.8898499
125.2	-0.4469637	0.88973374
125.3	-0.4471468	0.88966805
125.4	-0.4471468	0.88966805
125.5	-0.4472091	0.88961588
125.6	-0.4475079	0.88957285
125.7	-0.4473713	0.88943799
125.8	-0.4478089	0.88946464
125.9	-0.4481114	0.88952735
83.9	-0.383178	0.92953531
84	-0.3829524	0.92894782
84.1	-0.3833135	0.92872477
84.2	-0.3822198	0.92798753
84.3	-0.3822783	0.92755103
84.4	-0.3819563	0.92728677
84.5	-0.3813766	0.92678413
84.6	-0.3825489	0.92637328
84.7	-0.3826227	0.92624142
84.8	-0.3829797	0.92614828
84.9	-0.3829797	0.92614828
85	-0.3841507	0.92560343
85.1	-0.3846099	0.9250357
85.2	-0.3849113	0.92458708
85.3	-0.384667	0.92445635
85.4	-0.3851187	0.92407469
85.5	-0.3856962	0.92398333
85.6	-0.3860894	0.92347344
85.7	-0.3862168	0.92328374
85.8	-0.3867043	0.92301291
85.9	-0.3860748	0.9226377
86	-0.3856713	0.922438
86.1	-0.3853362	0.92168896
86.2	-0.3856547	0.92160849
86.3	-0.3849612	0.92086485
86.4	-0.3860602	0.92078927
86.5	-0.384901	0.92038933
86.6	-0.3850112	0.91985334
86.7	-0.3845282	0.91955863
86.8	-0.3843481	0.91933776
86.9	-0.3849753	0.91906446
87	-0.3845956	0.91879953
87.1	-0.3841041	0.91823261
87.2	-0.3840574	0.91799237
87.3	-0.3835244	0.91761448
87.4	-0.3839057	0.91759012
87.5	-0.3840181	0.91732716
87.6	-0.3833116	0.91686965
87.7	-0.3834917	0.91671753
87.8	-0.383053	0.91621914
87.9	-0.383053	0.91621914
88	-0.3820328	0.91580146
88.1	-0.3814902	0.91544707
88.2	-0.3817439	0.91516397
88.3	-0.3815192	0.9148378
88.4	-0.3816665	0.91484848
88.5	-0.3826688	0.91467834
88.6	-0.3824103	0.91449738
88.7	-0.3825103	0.91421445
88.8	-0.382535	0.91421656
88.9	-0.3827345	0.91407732
89	-0.3838647	0.91393209
89.1	-0.3842522	0.91383378
89.2	-0.3842522	0.91383378
89.3	-0.3848003	0.91355776
89.4	-0.38513	0.91331878
89.5	-0.3856316	0.91321281
89.6	-0.38606	0.91274329
89.7	-0.3861749	0.91259618
89.8	-0.385789	0.91224439
89.9	-0.3865522	0.91219868
135	-0.4664837	0.88392191
135.1	-0.4665659	0.88389223
135.2	-0.4675154	0.883541
135.3	-0.467476	0.8834552
135.4	-0.467476	0.8834552
135.5	-0.4673753	0.88330461
135.6	-0.4675482	0.88319656
135.7	-0.4675482	0.88319656
135.8	-0.4676199	0.88303742
135.9	-0.4677151	0.88288779
136	-0.4677151	0.88288779
136.1	-0.468318	0.88289594
136.2	-0.4686545	0.88280794
136.3	-0.4694269	0.88269879
136.4	-0.4697024	0.88258437
136.5	-0.4698198	0.88256254
136.6	-0.4701032	0.88247988
136.7	-0.4704584	0.88248657
136.8	-0.4706335	0.88222585
136.9	-0.4706335	0.88222585
137	-0.470701	0.88203744
137.1	-0.4707662	0.88173341
137.2	-0.4710668	0.88164016
137.3	-0.4710095	0.8815115
137.4	-0.4711752	0.88131403
137.5	-0.4711752	0.88131403
137.6	-0.4714616	0.88114378
137.7	-0.4711853	0.88116221
137.8	-0.4712753	0.88114279
137.9	-0.4709874	0.88109071
138	-0.470701	0.88110837
138.1	-0.4709999	0.88098532
138.2	-0.4709443	0.88083019
138.3	-0.4707609	0.88064842
138.4	-0.4708128	0.88063592
138.5	-0.4710206	0.88049612
138.6	-0.4711904	0.88033269
138.7	-0.4711107	0.8801809
138.8	-0.4713745	0.880179
138.9	-0.4715946	0.87995706
139	-0.4715569	0.87982359
139.1	-0.4717734	0.87957048
139.2	-0.4716048	0.87943815
139.3	-0.4718952	0.87920951
139.4	-0.4718952	0.87920951
139.5	-0.471918	0.87905476
139.6	-0.4726422	0.87881132
139.7	-0.4726422	0.87881132
139.8	-0.4729596	0.8788576
139.9	-0.4729596	0.8788576
140	-0.4730517	0.87870181
140.1	-0.4734845	0.87853362
140.2	-0.4738981	0.87839042
140.3	-0.4738981	0.87839042
140.4	-0.4738981	0.87839042
140.5	-0.4738613	0.87821824
140.6	-0.4740309	0.87801332
140.7	-0.4740534	0.87809008
140.8	-0.4740534	0.87809008
140.9	-0.4742609	0.87795777
98.9	-0.4048933	0.90063619
99	-0.4048933	0.90063619
99.1	-0.4050443	0.90053174
99.2	-0.4051124	0.90046738
99.3	-0.4055857	0.90047587
99.4	-0.4058038	0.90030191
99.5	-0.4058038	0.90030191
99.6	-0.4058038	0.90030191
99.7	-0.4072393	0.90031091
99.8	-0.4074881	0.90024443
99.9	-0.4074881	0.90024443
100	-0.4072634	0.90020983
100.1	-0.4072617	0.90016985
100.2	-0.4072617	0.90016985
100.3	-0.4073841	0.90016621
100.4	-0.4082418	0.90014762
100.5	-0.4082418	0.90014762
100.6	-0.4086578	0.90011069
100.7	-0.4086578	0.90011069
100.8	-0.4086578	0.90011069
100.9	-0.4086578	0.90011069
101	-0.4088862	0.9001147
101.1	-0.4096409	0.90021155
101.2	-0.4096409	0.90021155
101.3	-0.4100895	0.90013389
101.4	-0.4100895	0.90013389
101.5	-0.410376	0.90013956
101.6	-0.4108759	0.90010248
101.7	-0.4115008	0.8999556
101.8	-0.4115008	0.8999556
101.9	-0.4114997	0.89994181
102	-0.4114997	0.89994181
102.1	-0.4119779	0.89963176
102.2	-0.4120491	0.89957946
102.3	-0.4116136	0.89913857
102.4	-0.4120559	0.89915637
102.5	-0.4125577	0.8990404
102.6	-0.4125577	0.8990404
102.7	-0.4125577	0.8990404
102.8	-0.4129726	0.8989781
102.9	-0.4129726	0.8989781
103	-0.412774	0.89890582
103.1	-0.4130135	0.89887344
103.2	-0.4129097	0.89877431
103.3	-0.4133627	0.89881473
103.4	-0.4130933	0.89877111
103.5	-0.4132036	0.89865513
103.6	-0.4132036	0.89865513
103.7	-0.4135608	0.89856624
103.8	-0.4131853	0.89808909
103.9	-0.4130927	0.89796907
104	-0.4130927	0.89796907
104.1	-0.4140935	0.89788007
104.2	-0.4142761	0.89782629
104.3	-0.4147547	0.89782853
104.4	-0.4147547	0.89782853
104.5	-0.4147547	0.89782853
104.6	-0.416036	0.89778991
104.7	-0.4162961	0.89771488
104.8	-0.4162961	0.89771488
104.9	-0.4162961	0.89771488
150	-0.4788402	0.86840898
150.1	-0.4788402	0.86840898
150.2	-0.4788652	0.86823387
150.3	-0.4788652	0.86823387
150.4	-0.4789908	0.8683939
150.5	-0.4790335	0.86815041
150.6	-0.4792555	0.86810503
150.7	-0.4795603	0.86808927
150.8	-0.4795603	0.86808927
150.9	-0.4795603	0.86808927
151	-0.4798972	0.86816788
151.1	-0.4797336	0.86806418
151.2	-0.4796493	0.86795707
151.3	-0.4796493	0.86795707
151.4	-0.4796493	0.86795707
151.5	-0.4800204	0.86797384
151.6	-0.479902	0.86769678
151.7	-0.4801366	0.86767105
151.8	-0.4801366	0.86767105
151.9	-0.4801366	0.86767105
152	-0.4801366	0.86767105
152.1	-0.4801366	0.86767105
152.2	-0.4801366	0.86767105
152.3	-0.4803688	0.86755849
152.4	-0.4810504	0.86728777
152.5	-0.4812922	0.86737625
152.6	-0.4812922	0.86737625
152.7	-0.4812922	0.86737625
152.8	-0.4812922	0.86737625
152.9	-0.4812221	0.86732037
153	-0.4812221	0.86732037
153.1	-0.4814076	0.8670783
153.2	-0.4815187	0.86694845
153.3	-0.4815187	0.86694845
153.4	-0.4815187	0.86694845
153.5	-0.4815187	0.86694845
153.6	-0.4815602	0.86679364
153.7	-0.4815437	0.86669099
153.8	-0.4815136	0.86661395
153.9	-0.48152	0.86651922
154	-0.4812846	0.86631297
154.1	-0.4812846	0.86631297
154.2	-0.4812846	0.86631297
154.3	-0.481195	0.86611645
154.4	-0.481195	0.86611645
154.5	-0.4811925	0.86614552
154.6	-0.4815177	0.86612461
154.7	-0.4815177	0.86612461
154.8	-0.4814021	0.86597096
154.9	-0.4810694	0.86571698
155	-0.480851	0.86549877
155.1	-0.4811793	0.86549
155.2	-0.4811793	0.86549
155.3	-0.4811793	0.86549
155.4	-0.4811793	0.86549
155.5	-0.4811793	0.86549
155.6	-0.4809721	0.86527876
155.7	-0.4809721	0.86527876
155.8	-0.4809721	0.86527876
155.9	-0.4805884	0.86501906
113.9	-0.4275672	0.89419434
114	-0.4280136	0.89419768
114.1	-0.4285107	0.89416767
114.2	-0.4285773	0.8941655
114.3	-0.4284413	0.89406155
114.4	-0.4284413	0.89406155
114.5	-0.4289223	0.89410782
114.6	-0.4291426	0.89398225
114.7	-0.4289542	0.89376762
114.8	-0.4293224	0.89375641
114.9	-0.4296584	0.89373163
115	-0.4302665	0.89368037
115.1	-0.4302798	0.89363864
115.2	-0.4306353	0.89361628
115.3	-0.4308935	0.89358745
115.4	-0.4308935	0.89358745
115.5	-0.4309884	0.89343947
115.6	-0.4309884	0.89343947
115.7	-0.4309884	0.89343947
115.8	-0.4309476	0.89339723
115.9	-0.4314115	0.89341475
116	-0.4317235	0.89336014
116.1	-0.4317235	0.89336014
116.2	-0.4317379	0.89343803
116.3	-0.4326806	0.89333891
116.4	-0.4326806	0.89333891
116.5	-0.4330143	0.89332255
116.6	-0.4337456	0.89331764
116.7	-0.4337456	0.89331764
116.8	-0.4337456	0.89331764
116.9	-0.4337456	0.89331764
117	-0.4339139	0.89326517
117.1	-0.4341249	0.89340307
117.2	-0.4344485	0.89327124
117.3	-0.4343426	0.89323599
117.4	-0.4343426	0.89323599
117.5	-0.4347183	0.89316484
117.6	-0.4348652	0.89315522
117.7	-0.4348652	0.89315522
117.8	-0.4348821	0.89302519
117.9	-0.4348821	0.89302519
118	-0.4354733	0.89281364
118.1	-0.4354733	0.89281364
118.2	-0.4354733	0.89281364
118.3	-0.435747	0.89273617
118.4	-0.435747	0.89273617
118.5	-0.4359544	0.89263972
118.6	-0.4363791	0.8925919
118.7	-0.4363791	0.8925919
118.8	-0.4363791	0.8925919
118.9	-0.4368552	0.89256674
119	-0.437077	0.89259982
119.1	-0.4372644	0.8925566
119.2	-0.4374173	0.89244497
119.3	-0.4374173	0.89244497
119.4	-0.4379553	0.89241632
119.5	-0.4378889	0.89235054
119.6	-0.4378108	0.89223029
119.7	-0.4380438	0.89221994
119.8	-0.4380438	0.89221994
119.9	-0.43886	0.89219484
165	-0.4833746	0.86092884
165.1	-0.4839716	0.86098897
165.2	-0.4839716	0.86098897
165.3	-0.4839716	0.86098897
165.4	-0.4839716	0.86098897
165.5	-0.4839908	0.8606551
165.6	-0.4839908	0.8606551
165.7	-0.4839908	0.8606551
165.8	-0.483951	0.86044902
165.9	-0.483951	0.86044902
166	-0.4838679	0.86020163
166.1	-0.4838679	0.86020163
166.2	-0.4838679	0.86020163
166.3	-0.4838868	0.86028028
166.4	-0.4838104	0.86015451
166.5	-0.4841867	0.86021237
166.6	-0.4845179	0.86026233
166.7	-0.4843589	0.86014718
166.8	-0.4843589	0.86014718
166.9	-0.4843246	0.85992378
167	-0.4843246	0.85992378
167.1	-0.4841986	0.85970802
167.2	-0.4841302	0.85958707
167.3	-0.4847134	0.85961722
167.4	-0.4847134	0.85961722
167.5	-0.4847134	0.85961722
167.6	-0.4847773	0.85957995
167.7	-0.484536	0.85940993
167.8	-0.484952	0.85932963
167.9	-0.484952	0.85932963
168	-0.4854034	0.85933566
168.1	-0.4858373	0.8593642
168.2	-0.4858373	0.8593642
168.3	-0.4858373	0.8593642
168.4	-0.4858373	0.8593642
168.5	-0.4858373	0.8593642
168.6	-0.48561	0.85908785
168.7	-0.48561	0.85908785
168.8	-0.4861042	0.85915189
168.9	-0.4861042	0.85915189
169	-0.4861042	0.85915189
169.1	-0.4865732	0.85917859
169.2	-0.4865732	0.85917859
169.3	-0.4865732	0.85917859
169.4	-0.4863611	0.85905378
169.5	-0.4862893	0.85907754
169.6	-0.4865972	0.85893189
169.7	-0.486824	0.8589183
169.8	-0.486824	0.8589183
169.9	-0.4869025	0.858881
170	-0.4873541	0.85891007
170.1	-0.4872623	0.85887695
170.2	-0.4876976	0.85888971
170.3	-0.4876976	0.85888971
170.4	-0.4876504	0.85867763
170.5	-0.4876504	0.85867763
170.6	-0.4879082	0.85868403
170.7	-0.4879082	0.85868403
170.8	-0.4879165	0.85856837
170.9	-0.4879165	0.85856837

126	-0.4481114	0.88952735
126.1	-0.4481114	0.88952735
126.2	-0.4483625	0.88958494
126.3	-0.4484133	0.88957626
126.4	-0.4484133	0.88957626
126.5	-0.4478526	0.88936392
126.6	-0.4481806	0.88929264
126.7	-0.4485715	0.88926586
126.8	-0.4485715	0.88926586
126.9	-0.4485899	0.88916924
127	-0.4490271	0.88922462
127.1	-0.4498824	0.88906502
127.2	-0.4504596	0.88912928
127.3	-0.4504596	0.88912928
127.4	-0.450561	0.88904956
127.5	-0.4510771	0.88901403
127.6	-0.4510771	0.88901403
127.7	-0.4509958	0.88890481
127.8	-0.4509958	0.88890481
127.9	-0.4513884	0.88890279
128	-0.4516207	0.88886104
128.1	-0.4516207	0.88886104
128.2	-0.4516207	0.88886104
128.3	-0.4524005	0.88866776
128.4	-0.4529393	0.88850878
128.5	-0.4529259	0.88829408
128.6	-0.4531212	0.88819779
128.7	-0.4532071	0.88801627
128.8	-0.4538537	0.88797944
128.9	-0.4538537	0.88797944
129	-0.4539306	0.88788476
129.1	-0.4539306	0.88788476
129.2	-0.4539423	0.88777167
129.3	-0.4540339	0.88770552
129.4	-0.4543273	0.88771249
129.5	-0.454779	0.88759158
129.6	-0.455615	0.88739018
129.7	-0.4557335	0.88730771
129.8	-0.4555884	0.88722792
129.9	-0.4557847	0.88722162
130	-0.4561046	0.8872334
130.1	-0.4565739	0.88719172
130.2	-0.4568128	0.88710743
130.3	-0.4574353	0.88697279
130.4	-0.4576925	0.88689658
130.5	-0.4577484	0.88671092
130.6	-0.4576957	0.8865875
130.7	-0.4580328	0.88634535
130.8	-0.4580328	0.88634535
130.9	-0.4580573	0.88622966
131	-0.4582968	0.88623465
131.1	-0.458266	0.8860589
131.2	-0.458266	0.8860589
131.3	-0.4585231	0.88603315
131.4	-0.4587325	0.88607298
131.5	-0.4587325	0.88607298
131.6	-0.4598604	0.88581951
131.7	-0.4600166	0.88575003
131.8	-0.4602801	0.88551099
131.9	-0.4602801	0.88551099
132	-0.4604645	0.88529939
132.1	-0.4604645	0.88529939
132.2	-0.4612184	0.88526279
132.3	-0.4615988	0.8851825
132.4	-0.4617643	0.88498212
132.5	-0.4622036	0.8848893
132.6	-0.4628646	0.88511879
132.7	-0.46322	0.88497562
132.8	-0.46322	0.88497562
132.9	-0.4635	0.88491702
133	-0.4635009	0.88489869
133.1	-0.4635009	0.88489869
133.2	-0.4641653	0.88473058
133.3	-0.4644483	0.88466863
133.4	-0.4648167	0.88453255
133.5	-0.4650112	0.88450962
133.6	-0.4650112	0.88450962
133.7	-0.4652742	0.8843969
133.8	-0.4653483	0.88429003
133.9	-0.4653483	0.88429003
134	-0.4655155	0.88445994
134.1	-0.4655155	0.88445994
134.2	-0.4652783	0.8843084
134.3	-0.4654883	0.88406788
134.4	-0.4659322	0.88404548
134.5	-0.4660228	0.88412434
134.6	-0.4663897	0.88392903
134.7	-0.4663897	0.88392903
134.8	-0.4663897	0.88392903
134.9	-0.4664837	0.88392191

141	-0.4743233	0.87766547
141.1	-0.474261	0.87746471
141.2	-0.4749664	0.87749945
141.3	-0.4747036	0.87727741
141.4	-0.4750252	0.87722492
141.5	-0.4751699	0.87681004
141.6	-0.4752823	0.8766741
141.7	-0.4754316	0.87645458
141.8	-0.4758051	0.87648839
141.9	-0.4755136	0.87629583
142	-0.4755136	0.87629583
142.1	-0.4756548	0.87598055
142.2	-0.475787	0.87594225
142.3	-0.475787	0.87594225
142.4	-0.475787	0.87594225
142.5	-0.4759395	0.8756722
142.6	-0.475846	0.8755043
142.7	-0.4758999	0.87508944
142.8	-0.476241	0.87506307
142.9	-0.4760631	0.87483506
143	-0.4760144	0.87468037
143.1	-0.4761941	0.87462184
143.2	-0.4763746	0.87444283
143.3	-0.4765687	0.87411493
143.4	-0.4765687	0.87411493
143.5	-0.476552	0.8740753
143.6	-0.4764312	0.87381508
143.7	-0.476529	0.87378569
143.8	-0.4766667	0.87359261
143.9	-0.4765046	0.87336096
144	-0.4765881	0.87305318
144.1	-0.4765881	0.87305318
144.2	-0.4765881	0.87305318
144.3	-0.4768668	0.87253567
144.4	-0.4768668	0.87253567
144.5	-0.4770174	0.87246817
144.6	-0.4771308	0.87239208
144.7	-0.4771308	0.87239208
144.8	-0.477148	0.87203147
144.9	-0.477148	0.87203147
145	-0.4770758	0.8719627
145.1	-0.4772737	0.87192561
145.2	-0.4772737	0.87192561
145.3	-0.4773065	0.87173822
145.4	-0.4773065	0.87173822
145.5	-0.4780187	0.87170824
145.6	-0.4780187	0.87170824
145.7	-0.4778306	0.87160191
145.8	-0.4781558	0.87156601
145.9	-0.4781558	0.87156601
146	-0.4779731	0.87149597
146.1	-0.4778072	0.87129941
146.2	-0.4778072	0.87129941
146.3	-0.4776675	0.87111139
146.4	-0.4776675	0.87111139
146.5	-0.477656	0.87106452
146.6	-0.477656	0.87106452
146.7	-0.4776203	0.87104298
146.8	-0.4779894	0.87099343
146.9	-0.477758	0.87073302
147	-0.477758	0.87073302
147.1	-0.4776259	0.87063418
147.2	-0.4778597	0.87055085
147.3	-0.4781431	0.87037522
147.4	-0.4781431	0.87037522
147.5	-0.4782744	0.87032527
147.6	-0.4779003	0.8700547
147.7	-0.4778871	0.86995833
147.8	-0.4778871	0.86995833
147.9	-0.4778871	0.86995833
148	-0.4778871	0.86995833
148.1	-0.4786699	0.8699372
148.2	-0.4787301	0.86986433
148.3	-0.4787301	0.86986433
148.4	-0.4786999	0.86959424
148.5	-0.4786999	0.86959424
148.6	-0.4787949	0.8695371
148.7	-0.4787949	0.8695371
148.8	-0.4788664	0.86936963
148.9	-0.478962	0.8694278
149	-0.478962	0.8694278
149.1	-0.4787265	0.86923109
149.2	-0.4786642	0.86917028
149.3	-0.4786642	0.86917028
149.4	-0.4789843	0.86909938
149.5	-0.4792826	0.86904044
149.6	-0.4789254	0.86881591
149.7	-0.4788567	0.86872928
149.8	-0.4788402	0.86840898
149.9	-0.4788402	0.86840898

156	-0.4802871	0.8648463
156.1	-0.4802871	0.8648463
156.2	-0.480021	0.86483144
156.3	-0.480021	0.86483144
156.4	-0.4798278	0.86474516
156.5	-0.4798278	0.86474516
156.6	-0.4797735	0.86472101
156.7	-0.4797735	0.86472101
156.8	-0.4796762	0.86461863
156.9	-0.480181	0.8647116
157	-0.4804652	0.8646762
157.1	-0.4802185	0.86455837
157.2	-0.4798673	0.86434399
157.3	-0.4797898	0.86428061
157.4	-0.4795618	0.86404055
157.5	-0.4794876	0.86396245
157.6	-0.4794876	0.86396245
157.7	-0.4796992	0.86388738
157.8	-0.4796992	0.86388738
157.9	-0.4796992	0.86388738
158	-0.4795597	0.86369139
158.1	-0.4794703	0.86358652
158.2	-0.4792983	0.86351544
158.3	-0.4792983	0.86351544
158.4	-0.4792983	0.86351544
158.5	-0.4792983	0.86351544
158.6	-0.4792807	0.86326238
158.7	-0.4790574	0.86300934
158.8	-0.4792651	0.86298686
158.9	-0.4790561	0.8628535
159	-0.4794821	0.86290548
159.1	-0.4794821	0.86290548
159.2	-0.4794821	0.86290548
159.3	-0.4793883	0.8628357
159.4	-0.4793883	0.8628357
159.5	-0.4793883	0.8628357
159.6	-0.4797681	0.86291166
159.7	-0.4799363	0.86283081
159.8	-0.4799363	0.86283081
159.9	-0.4799363	0.86283081
160	-0.4799363	0.86283081
160.1	-0.4799363	0.86283081
160.2	-0.4798265	0.86272942
160.3	-0.4798098	0.86251874
160.4	-0.4796357	0.86233316
160.5	-0.4797198	0.86225221
160.6	-0.4800818	0.86232479
160.7	-0.4800818	0.86232479
160.8	-0.4800818	0.86232479
160.9	-0.4802564	0.86250779
161	-0.4801205	0.86242021
161.1	-0.4801205	0.86242021
161.2	-0.4805594	0.86246755
161.3	-0.4806412	0.86245555
161.4	-0.481227	0.86242276
161.5	-0.481227	0.86242276
161.6	-0.4810959	0.86238198
161.7	-0.4810296	0.86230279
161.8	-0.4810615	0.86208085
161.9	-0.4810615	0.86208085
162	-0.4810615	0.86208085
162.1	-0.4810615	0.86208085
162.2	-0.4810615	0.86208085
162.3	-0.4815315	0.86167342
162.4	-0.4815315	0.86167342
162.5	-0.4822102	0.86177983
162.6	-0.4822102	0.86177983
162.7	-0.4822102	0.86177983
162.8	-0.4821692	0.86181232
162.9	-0.4821112	0.86148014
163	-0.4821112	0.86148014
163.1	-0.4826117	0.86154949
163.2	-0.4826117	0.86154949
163.3	-0.4826117	0.86154949
163.4	-0.4831413	0.86162533
163.5	-0.4831501	0.86141121
163.6	-0.4831501	0.86141121
163.7	-0.4831501	0.86141121
163.8	-0.4830256	0.86128752
163.9	-0.4829081	0.86110211
164	-0.4832905	0.86090308
164.1	-0.4832905	0.86090308
164.2	-0.4832905	0.86090308
164.3	-0.4832905	0.86090308
164.4	-0.4832905	0.86090308
164.5	-0.483499	0.86094765
164.6	-0.4836553	0.86091542
164.7	-0.483552	0.86095537
164.8	-0.483552	0.86095537
164.9	-0.483552	0.86095537

171	-0.4882626	0.85859669
171.1	-0.4882626	0.85859669
171.2	-0.4880964	0.85845892
171.3	-0.4883671	0.85843486
171.4	-0.4882735	0.85826854
171.5	-0.4887605	0.85830894
171.6	-0.4887605	0.85830894
171.7	-0.4887605	0.85830894
171.8	-0.4886923	0.8582535
171.9	-0.4890225	0.85823941
172	-0.4892025	0.85832314
172.1	-0.4893979	0.85818463
172.2	-0.4893979	0.85818463
172.3	-0.4896634	0.8581747
172.4	-0.4896634	0.8581747
172.5	-0.4896634	0.8581747
172.6	-0.4896634	0.8581747
172.7	-0.4896634	0.8581747
172.8	-0.4896388	0.85824728
172.9	-0.4900141	0.85826406
173	-0.4901548	0.85822394
173.1	-0.490555	0.85822918
173.2	-0.4906475	0.8582259
173.3	-0.4910305	0.8582015
173.4	-0.4911375	0.85807923
173.5	-0.4911375	0.85807923
173.6	-0.4921138	0.85811045
173.7	-0.4921138	0.85811045
173.8	-0.4921138	0.85811045
173.9	-0.4922088	0.85822126
174	-0.4922088	0.85822126
174.1	-0.4926211	0.85816421
174.2	-0.4927185	0.85808259
174.3	-0.4929396	0.85803745
174.4	-0.4931472	0.85806262
174.5	-0.4933691	0.85806912
174.6	-0.4933691	0.85806912
174.7	-0.4930288	0.85786115
174.8	-0.4930288	0.85786115
174.9	-0.4930288	0.85786115
175	-0.4930288	0.85786115
175.1	-0.4933876	0.85786277
175.2	-0.4933876	0.85786277
175.3	-0.493941	0.8577254
175.4	-0.4940353	0.85765626
175.5	-0.4940353	0.85765626
175.6	-0.4940353	0.85765626
175.7	-0.4940353	0.85765626
175.8	-0.4941809	0.85759478
175.9	-0.4946576	0.85741412
176	-0.4946082	0.85726605
176.1	-0.4946082	0.85726605
176.2	-0.4946082	0.85726605
176.3	-0.4948552	0.85723561
176.4	-0.4948552	0.85723561
176.5	-0.4950629	0.85718141
176.6	-0.4950305	0.85709301
176.7	-0.4948676	0.85684818
176.8	-0.4948676	0.85684818
176.9	-0.4949412	0.85676748
177	-0.4950767	0.85669527
177.1	-0.4950767	0.85669527
177.2	-0.4950767	0.85669527
177.3	-0.4952377	0.85670698
177.4	-0.4952377	0.85670698
177.5	-0.4953655	0.85657958
177.6	-0.4953655	0.85657958
177.7	-0.4952817	0.85652389
177.8	-0.495465	0.8564623
177.9	-0.495465	0.8564623
178	-0.4964556	0.85632495
178.1	-0.4964556	0.85632495
178.2	-0.4967254	0.85627896
178.3	-0.4970294	0.85614875
178.4	-0.497248	0.8561001
178.5	-0.497248	0.8561001
178.6	-0.4975995	0.8560773
178.7	-0.4977886	0.85600672
178.8	-0.4977886	0.85600672
178.9	-0.4977886	0.85600672
179	-0.4977886	0.85600672
179.1	-0.4980149	0.85590735
179.2	-0.4982902	0.85584689
179.3	-0.4983309	0.85562612
179.4	-0.4985351	0.85555967
179.5	-0.4985351	0.85555967
179.6	-0.498863	0.855501
179.7	-0.498863	0.855501
179.8	-0.4989627	0.8555792
179.9	-0.4993409	0.85546397

180	-0.499434	0.85532377
180.1	-0.499434	0.85532377
180.2	-0.4997474	0.85528911
180.3	-0.4997474	0.85528911
180.4	-0.4994894	0.85511582
180.5	-0.4999043	0.8548102
180.6	-0.5000838	0.85480355
180.7	-0.5000838	0.85480355
180.8	-0.5003618	0.85464155
180.9	-0.5003618	0.85464155
181	-0.5003618	0.85464155
181.1	-0.5003618	0.85464155
181.2	-0.5003618	0.85464155
181.3	-0.5007574	0.85458072
181.4	-0.5007574	0.85458072
181.5	-0.5009556	0.85452284
181.6	-0.5012867	0.85427962
181.7	-0.5012867	0.85427962
181.8	-0.5012867	0.85427962
181.9	-0.5012996	0.85414654
182	-0.5021874	0.85406272
182.1	-0.5021874	0.85406272
182.2	-0.5021874	0.85406272
182.3	-0.5021495	0.8539129
182.4	-0.5023717	0.85382711
182.5	-0.5025285	0.85371926
182.6	-0.5027155	0.8536167
182.7	-0.5029472	0.85352668
182.8	-0.5029472	0.85352668
182.9	-0.5028253	0.85326757
183	-0.5032401	0.853151

195	-0.5064555	0.84566155
195.1	-0.5062164	0.84559189
195.2	-0.5061058	0.84553746
195.3	-0.5061058	0.84553746
195.4	-0.5061058	0.84553746
195.5	-0.5061058	0.84553746
195.6	-0.5059967	0.84544445
195.7	-0.5059967	0.84544445
195.8	-0.5059967	0.84544445
195.9	-0.5059967	0.84544445
196	-0.5059967	0.84544445
196.1	-0.5059967	0.84544445
196.2	-0.5059967	0.84544445
196.3	-0.5059967	0.84544445
196.4	-0.5059967	0.84544445
196.5	-0.5059967	0.84544445
196.6	-0.5059967	0.84544445
196.7	-0.5059967	0.84544445
196.8	-0.5062832	0.84564253
196.9	-0.5061576	0.84565095
197	-0.5061576	0.84565095
197.1	-0.5063881	0.84561368
197.2	-0.5063881	0.84561368
197.3	-0.5064556	0.84558517
197.4	-0.5064556	0.84558517
197.5	-0.5064556	0.84558517
197.6	-0.5066622	0.84579612
197.7	-0.5066622	0.84579612
197.8	-0.5066622	0.84579612
197.9	-0.5066622	0.84579612
198	-0.5070332	0.84585057

210	-0.5084875	0.84635396
210.1	-0.5084875	0.84635396
210.2	-0.5084875	0.84635396
210.3	-0.5084875	0.84635396
210.4	-0.5084875	0.84635396
210.5	-0.5084875	0.84635396
210.6	-0.5084875	0.84635396
210.7	-0.5084875	0.84635396
210.8	-0.5084875	0.84635396
210.9	-0.5084875	0.84635396
211	-0.5084875	0.84635396
211.1	-0.5084875	0.84635396
211.2	-0.5084875	0.84635396
211.3	-0.5084875	0.84635396
211.4	-0.5084875	0.84635396
211.5	-0.5082776	0.84631367
211.6	-0.5082776	0.84631367
211.7	-0.5083448	0.8462617
211.8	-0.5077533	0.84604384
211.9	-0.5078661	0.84610448
212	-0.5078661	0.84610448
212.1	-0.5078661	0.84610448
212.2	-0.5078661	0.84610448
212.3	-0.5078661	0.84610448
212.4	-0.5078661	0.84610448
212.5	-0.5078661	0.84610448
212.6	-0.5078661	0.84610448
212.7	-0.5078661	0.84610448
212.8	-0.5078661	0.84610448
212.9	-0.5078661	0.84610448
213	-0.5081026	0.84602055

225	-0.5088101	0.84490441
225.1	-0.5088101	0.84490441
225.2	-0.5088101	0.84490441
225.3	-0.5088101	0.84490441
225.4	-0.5088101	0.84490441
225.5	-0.5088101	0.84490441
225.6	-0.5088101	0.84490441
225.7	-0.5086561	0.84484185
225.8	-0.5086561	0.84484185
225.9	-0.5086561	0.84484185
226	-0.5086561	0.84484185
226.1	-0.5086561	0.84484185
226.2	-0.5086561	0.84484185
226.3	-0.5086561	0.84484185
226.4	-0.5086561	0.84484185
226.5	-0.5086561	0.84484185
226.6	-0.5086561	0.84484185
226.7	-0.5086561	0.84484185
226.8	-0.5086561	0.84484185
226.9	-0.5086561	0.84484185
227	-0.5086526	0.84475796
227.1	-0.5088935	0.84495428
227.2	-0.5088935	0.84495428
227.3	-0.5088935	0.84495428
227.4	-0.5088935	0.84495428
227.5	-0.5088935	0.84495428
227.6	-0.5088935	0.84495428
227.7	-0.5088935	0.84495428
227.8	-0.5088935	0.84495428
227.9	-0.5088935	0.84495428
228	-0.5088543	0.84496032

183.1	-0.5032401	0.853151
183.2	-0.5035525	0.85308388
183.3	-0.5038128	0.85294933
183.4	-0.5038128	0.85294933
183.5	-0.5038128	0.85294933
183.6	-0.5039762	0.8526617
183.7	-0.5038388	0.85252447
183.8	-0.5037781	0.85236252
183.9	-0.5037781	0.85236252
184	-0.503404	0.85208355
184.1	-0.5035962	0.85225422
184.2	-0.5037652	0.85236454
184.3	-0.5036205	0.85220064
184.4	-0.503915	0.85208862
184.5	-0.5039189	0.85193936
184.6	-0.5038043	0.85175793
184.7	-0.5038043	0.85175793
184.8	-0.504056	0.85163811
184.9	-0.504048	0.85149562
185	-0.504048	0.85149562
185.1	-0.5040238	0.85137299
185.2	-0.5040512	0.85124957
185.3	-0.5040512	0.85124957
185.4	-0.5040512	0.85124957
185.5	-0.5046931	0.85117489
185.6	-0.5046654	0.85100877
185.7	-0.5050041	0.85093736
185.8	-0.5049481	0.850698
185.9	-0.5049481	0.850698
186	-0.505093	0.8505553
186.1	-0.505093	0.8505553
186.2	-0.505117	0.85039225
186.3	-0.505117	0.85039225
186.4	-0.5051551	0.850237
186.5	-0.505009	0.85001967
186.6	-0.505009	0.85001967
186.7	-0.5046546	0.84966281
186.8	-0.5046228	0.84953287
186.9	-0.5046174	0.84933459
187	-0.5046174	0.84933459
187.1	-0.5047506	0.84914557
187.2	-0.5046907	0.84896877
187.3	-0.5046961	0.84876063
187.4	-0.5047912	0.8486057
187.5	-0.5047912	0.8486057
187.6	-0.5047912	0.8486057
187.7	-0.504992	0.84844583
187.8	-0.504992	0.84844583
187.9	-0.5051313	0.84854575
188	-0.5046494	0.8482566
188.1	-0.504904	0.84808846
188.2	-0.504904	0.84808846
188.3	-0.5050738	0.84768795
188.4	-0.5050738	0.84768795
188.5	-0.5049599	0.84745677
188.6	-0.5051843	0.84729266
188.7	-0.5051843	0.84729266
188.8	-0.5054119	0.84713923
188.9	-0.5054835	0.84715019
189	-0.5054351	0.84682517
189.1	-0.5054351	0.84682517
189.2	-0.5056342	0.84665127
189.3	-0.5053392	0.846213
189.4	-0.5053392	0.846213
189.5	-0.5053392	0.846213
189.6	-0.505634	0.84586421
189.7	-0.505634	0.84586421
189.8	-0.5055859	0.84585711
189.9	-0.5055859	0.84585711
190	-0.5055903	0.8456306
190.1	-0.5055903	0.8456306
190.2	-0.5055903	0.8456306
190.3	-0.5055903	0.8456306
190.4	-0.5055903	0.8456306
190.5	-0.5055903	0.8456306
190.6	-0.5055903	0.8456306
190.7	-0.5055903	0.8456306
190.8	-0.5055903	0.8456306
190.9	-0.5055903	0.8456306
191	-0.5055903	0.8456306
191.1	-0.505644	0.84559123
191.2	-0.505644	0.84559123
191.3	-0.5058083	0.84578094
191.4	-0.5058083	0.84578094
191.5	-0.5058083	0.84578094
191.6	-0.5058083	0.84578094
191.7	-0.5058083	0.84578094
191.8	-0.5059235	0.84581599
191.9	-0.5057137	0.84565414
192	-0.5058054	0.84583204
192.1	-0.506203	0.84586062
192.2	-0.506203	0.84586062
192.3	-0.506203	0.84586062
192.4	-0.506203	0.84586062
192.5	-0.506203	0.84586062
192.6	-0.506203	0.84586062
192.7	-0.506203	0.84586062
192.8	-0.506203	0.84586062
192.9	-0.506203	0.84586062
193	-0.506203	0.84586062
193.1	-0.506203	0.84586062
193.2	-0.506203	0.84586062
193.3	-0.506203	0.84586062
193.4	-0.506203	0.84586062
193.5	-0.506203	0.84586062
193.6	-0.506203	0.84586062
193.7	-0.506203	0.84586062
193.8	-0.506203	0.84586062
193.9	-0.506203	0.84586062
194	-0.506203	0.84586062
194.1	-0.506203	0.84586062
194.2	-0.5062976	0.84564827
194.3	-0.50637	0.84567675
194.4	-0.50637	0.84567675
194.5	-0.50637	0.84567675
194.6	-0.50637	0.84567675
194.7	-0.50637	0.84567675
194.8	-0.50637	0.84567675
194.9	-0.5064555	0.84566155

198.1	-0.5070332	0.84585057
198.2	-0.5072022	0.84604238
198.3	-0.5072022	0.84604238
198.4	-0.5071365	0.84601723
198.5	-0.5071365	0.84601723
198.6	-0.5071365	0.84601723
198.7	-0.5071365	0.84601723
198.8	-0.507095	0.84611103
198.9	-0.5072951	0.84614865
199	-0.5073609	0.84617894
199.1	-0.5073609	0.84617894
199.2	-0.5074835	0.84614818
199.3	-0.5074854	0.84613188
199.4	-0.5073621	0.84607507
199.5	-0.5072804	0.84592449
199.6	-0.5072804	0.84592449
199.7	-0.5072804	0.84592449
199.8	-0.507331	0.84585652
199.9	-0.507331	0.84585652
200	-0.507331	0.84585652
200.1	-0.5072784	0.84575965
200.2	-0.5072784	0.84575965
200.3	-0.5072784	0.84575965
200.4	-0.5072784	0.84575965
200.5	-0.5072784	0.84575965
200.6	-0.5072784	0.84575965
200.7	-0.5072676	0.8458011
200.8	-0.5073355	0.84593272
200.9	-0.5073355	0.84593272
201	-0.5073355	0.84593272
201.1	-0.5073355	0.84593272
201.2	-0.5074647	0.84613578
201.3	-0.5074647	0.84613578
201.4	-0.5074647	0.84613578
201.5	-0.5074647	0.84613578
201.6	-0.5074549	0.84595711
201.7	-0.5074549	0.84595711
201.8	-0.5074844	0.84601115
201.9	-0.5074844	0.84601115
202	-0.5074844	0.84601115
202.1	-0.5074844	0.84601115
202.2	-0.5076678	0.84598877
202.3	-0.50781	0.8461275
202.4	-0.50781	0.8461275
202.5	-0.50781	0.8461275
202.6	-0.50781	0.8461275
202.7	-0.50781	0.8461275
202.8	-0.50781	0.8461275
202.9	-0.50781	0.8461275
203	-0.50781	0.8461275
203.1	-0.50781	0.8461275
203.2	-0.50781	0.8461275
203.3	-0.5079647	0.8461336
203.4	-0.5079647	0.8461336
203.5	-0.5082113	0.84637106
203.6	-0.5082113	0.84637106
203.7	-0.5081562	0.84637163
203.8	-0.5081562	0.84637163
203.9	-0.5081562	0.84637163
204	-0.5081562	0.84637163
204.1	-0.5081562	0.84637163
204.2	-0.5081562	0.84637163
204.3	-0.5081562	0.84637163
204.4	-0.5081562	0.84637163
204.5	-0.5081562	0.84637163
204.6	-0.5081562	0.84637163
204.7	-0.5081562	0.84637163
204.8	-0.5081562	0.84637163
204.9	-0.5080806	0.84634107
205	-0.5080806	0.84634107
205.1	-0.5080806	0.84634107
205.2	-0.5080806	0.84634107
205.3	-0.5080806	0.84634107
205.4	-0.5080806	0.84634107
205.5	-0.5080806	0.84634107
205.6	-0.5080806	0.84634107
205.7	-0.5080806	0.84634107
205.8	-0.5080806	0.84634107
205.9	-0.5080806	0.84634107
206	-0.5080806	0.84634107
206.1	-0.5080806	0.84634107
206.2	-0.5080806	0.84634107
206.3	-0.5080806	0.84634107
206.4	-0.5080806	0.84634107
206.5	-0.5080806	0.84634107
206.6	-0.5080806	0.84634107
206.7	-0.5080806	0.84634107
206.8	-0.5080806	0.84634107
206.9	-0.5080806	0.84634107
207	-0.5080806	0.84634107
207.1	-0.5082619	0.84650619
207.2	-0.5083791	0.84654064
207.3	-0.5086449	0.84657266
207.4	-0.5086449	0.84657266
207.5	-0.5086449	0.84657266
207.6	-0.5084324	0.84651347
207.7	-0.5084324	0.84651347
207.8	-0.5084324	0.84651347
207.9	-0.5084324	0.84651347
208	-0.5084324	0.84651347
208.1	-0.5084324	0.84651347
208.2	-0.5084324	0.84651347
208.3	-0.5084324	0.84651347
208.4	-0.5084324	0.84651347
208.5	-0.5086912	0.84651446
208.6	-0.5086912	0.84651446
208.7	-0.5086912	0.84651446
208.8	-0.5086912	0.84651446
208.9	-0.5086912	0.84651446
209	-0.5085202	0.84645778
209.1	-0.5085202	0.84645778
209.2	-0.5085202	0.84645778
209.3	-0.5084875	0.84635396
209.4	-0.5084875	0.84635396
209.5	-0.5084875	0.84635396
209.6	-0.5084875	0.84635396
209.7	-0.5084875	0.84635396
209.8	-0.5084875	0.84635396
209.9	-0.5084875	0.84635396

213.1	-0.508139	0.8460262
213.2	-0.508139	0.8460262
213.3	-0.508139	0.8460262
213.4	-0.508139	0.8460262
213.5	-0.5081136	0.84593306
213.6	-0.508188	0.84598674
213.7	-0.5081633	0.84593484
213.8	-0.5081633	0.84593484
213.9	-0.5081633	0.84593484
214	-0.5081633	0.84593484
214.1	-0.5081616	0.84593284
214.2	-0.5081616	0.84593284
214.3	-0.5079798	0.84576889
214.4	-0.5079798	0.84576889
214.5	-0.5079831	0.84580954
214.6	-0.5079831	0.84580954
214.7	-0.5079831	0.84580954
214.8	-0.5079831	0.84580954
214.9	-0.5079831	0.84580954
215	-0.5079831	0.84580954
215.1	-0.5079831	0.84580954
215.2	-0.5079831	0.84580954
215.3	-0.5079831	0.84580954
215.4	-0.5080584	0.8458419
215.5	-0.5080584	0.8458419
215.6	-0.5083714	0.84583522
215.7	-0.5083958	0.84588694
215.8	-0.5083958	0.84588694
215.9	-0.5083958	0.84588694
216	-0.5083958	0.84588694
216.1	-0.5083958	0.84588694
216.2	-0.5085218	0.8457721
216.3	-0.5085218	0.8457721
216.4	-0.5085218	0.8457721
216.5	-0.5085218	0.8457721
216.6	-0.5085218	0.8457721
216.7	-0.5085218	0.8457721
216.8	-0.5080537	0.84561163
216.9	-0.5080537	0.84561163
217	-0.5080537	0.84561163
217.1	-0.5080537	0.84561163
217.2	-0.5080537	0.84561163
217.3	-0.5081197	0.84568444
217.4	-0.5082925	0.84563052
217.5	-0.5082925	0.84563052
217.6	-0.5082925	0.84563052
217.7	-0.5082925	0.84563052
217.8	-0.5084074	0.84560519
217.9	-0.508317	0.84561465
218	-0.508317	0.84561465
218.1	-0.508317	0.84561465
218.2	-0.508317	0.84561465
218.3	-0.508317	0.84561465
218.4	-0.508317	0.84561465
218.5	-0.508317	0.84561465
218.6	-0.508317	0.84561465
218.7	-0.508317	0.84561465
218.8	-0.508317	0.84561465
218.9	-0.508317	0.84561465
219	-0.508317	0.84561465
219.1	-0.507948	0.84540869
219.2	-0.507948	0.84540869
219.3	-0.507948	0.84540869
219.4	-0.50807	0.84549893
219.5	-0.50807	0.84549893
219.6	-0.50807	0.84549893
219.7	-0.50807	0.84549893
219.8	-0.50807	0.84549893
219.9	-0.5088538	0.84550227
220	-0.5088453	0.84538535
220.1	-0.5088453	0.84538535
220.2	-0.5088453	0.84538535
220.3	-0.5088453	0.84538535
220.4	-0.5088453	0.84538535
220.5	-0.5088453	0.84538535
220.6	-0.5088453	0.84538535
220.7	-0.5088453	0.84538535
220.8	-0.5089471	0.84546629
220.9	-0.5088405	0.84533251
221	-0.5088405	0.84533251
221.1	-0.5088405	0.84533251
221.2	-0.5088405	0.84533251
221.3	-0.5087826	0.84532884
221.4	-0.5087826	0.84532884
221.5	-0.5087826	0.84532884
221.6	-0.5087826	0.84532884
221.7	-0.5086112	0.84521789
221.8	-0.5086112	0.84521789
221.9	-0.5086112	0.84521789
222	-0.5086112	0.84521789
222.1	-0.5088228	0.84529831
222.2	-0.5088228	0.84529831
222.3	-0.5088228	0.84529831
222.4	-0.5088228	0.84529831
222.5	-0.5088228	0.84529831
222.6	-0.5091065	0.84529692
222.7	-0.5091065	0.84529692
222.8	-0.5089226	0.84529471
222.9	-0.5089226	0.84529471
223	-0.5089226	0.84529471
223.1	-0.5089226	0.84529471
223.2	-0.5089226	0.84529471
223.3	-0.5089762	0.84530051
223.4	-0.5088457	0.84511514
223.5	-0.5086739	0.84490847
223.6	-0.5086739	0.84490847
223.7	-0.5086225	0.84494644
223.8	-0.5086225	0.84494644
223.9	-0.5086225	0.84494644
224	-0.5085732	0.84489296
224.1	-0.5085732	0.84489296
224.2	-0.5085732	0.84489296
224.3	-0.5088587	0.84490124
224.4	-0.5088587	0.84490124
224.5	-0.5088587	0.84490124
224.6	-0.5088587	0.84490124
224.7	-0.5088587	0.84490124
224.8	-0.5087412	0.84491507
224.9	-0.5087412	0.84491507

228.1	-0.5083544	0.84477703
228.2	-0.5083544	0.84477703
228.3	-0.5082583	0.84474306
228.4	-0.5084571	0.84491773
228.5	-0.5084571	0.84491773
228.6	-0.5084571	0.84491773
228.7	-0.5084571	0.84491773
228.8	-0.5084571	0.84491773
228.9	-0.5084571	0.84491773
229	-0.5084571	0.84491773
229.1	-0.5084571	0.84491773
229.2	-0.5084571	0.84491773
229.3	-0.5084571	0.84491773
229.4	-0.508589	0.84485265
229.5	-0.508589	0.84485265
229.6	-0.508589	0.84485265
229.7	-0.508589	0.84485265
229.8	-0.508589	0.84485265
229.9	-0.508589	0.84485265
230	-0.508589	0.84485265
230.1	-0.508589	0.84485265
230.2	-0.508589	0.84485265
230.3	-0.508589	0.84485265
230.4	-0.508589	0.84485265
230.5	-0.508589	0.84485265
230.6	-0.508589	0.84485265
230.7	-0.508589	0.84485265
230.8	-0.5088478	0.8448413
230.9	-0.5088478	0.8448413
231	-0.5088478	0.8448413
231.1	-0.5088478	0.8448413
231.2	-0.5088478	0.8448413
231.3	-0.5088478	0.8448413
231.4	-0.5088478	0.8448413
231.5	-0.5087607	0.84479083
231.6	-0.5086973	0.8446678
231.7	-0.508788	0.84474276
231.8	-0.508788	0.84474276
231.9	-0.508788	0.84474276
232	-0.5088439	0.84475348
232.1	-0.5088439	0.84475348
232.2	-0.5088439	0.84475348
232.3	-0.5088439	0.84475348
232.4	-0.5089864	0.84474581
232.5	-0.5089864	0.84474581
232.6	-0.5089864	0.84474581
232.7	-0.5089864	0.84474581
232.8	-0.5089864	0.84474581
232.9	-0.5089864	0.84474581
233	-0.5089864	0.84474581
233.1	-0.5089864	0.84474581
233.2	-0.5089864	0.84474581
233.3	-0.5089864	0.84474581
233.4	-0.5089864	0.84474581
233.5	-0.5089864	0.84474581
233.6	-0.5089864	0.84474581
233.7	-0.5089864	0.84474581
233.8	-0.509349	0.84480142
233.9	-0.509349	0.84480142
234	-0.509349	0.84480142
234.1	-0.5091842	0.84462219
234.2	-0.5091842	0.84462219
234.3	-0.5091842	0.84462219
234.4	-0.5094162	0.8446048
234.5	-0.5094162	0.8446048
234.6	-0.5094162	0.8446048
234.7	-0.5094162	0.8446048
234.8	-0.5096465	0.84458427
234.9	-0.5096465	0.84458427
235	-0.5096465	0.84458427
235.1	-0.5096465	0.84458427
235.2	-0.5096465	0.84458427
235.3	-0.5096465	0.84458427
235.4	-0.5096465	0.84458427
235.5	-0.5096465	0.84458427
235.6	-0.5096465	0.84458427
235.7	-0.5097582	0.84456146
235.8	-0.5097582	0.84456146
235.9	-0.5097582	0.84456146
236	-0.5099363	0.8445552
236.1	-0.5099363	0.8445552
236.2	-0.5099363	0.8445552
236.3	-0.5099363	0.8445552
236.4	-0.5099363	0.8445552
236.5	-0.5099363	0.8445552
236.6	-0.5099363	0.8445552
236.7	-0.5099363	0.8445552
236.8	-0.5099363	0.8445552
236.9	-0.5099363	0.8445552
237	-0.5102205	0.84463843
237.1	-0.5102205	0.84463843
237.2	-0.5102205	0.84463843
237.3	-0.5102205	0.84463843
237.4	-0.5103798	0.84466846
237.5	-0.5103798	0.84466846
237.6	-0.5103798	0.84466846
237.7	-0.5103798	0.84466846
237.8	-0.5102918	0.84469446
237.9	-0.5102918	0.84469446
238	-0.5102918	0.84469446
238.1	-0.5102918	0.84469446
238.2	-0.5102918	0.84469446
238.3	-0.5102093	0.8446527
238.4	-0.5102093	0.8446527
238.5	-0.5102472	0.84467782
238.6	-0.5102472	0.84467782
238.7	-0.5100995	0.84464714
238.8	-0.5100995	0.84464714
238.9	-0.5100995	0.84464714
239	-0.5100995	0.84464714
239.1	-0.5100995	0.84464714
239.2	-0.5100995	0.84464714
239.3	-0.5100995	0.84464714
239.4	-0.5100995	0.84464714
239.5	-0.5102087	0.84466969
239.6	-0.5102087	0.84466969
239.7	-0.5102087	0.84466969
239.8	-0.509866	0.84464755
239.9	-0.509866	0.84464755
240	-0.5100268	0.84468224

Corresponding p-values for PRCC sensitivtiy indices

Time discretization	alpha	[LACT]_0	Time discretization	alpha	[LACT]_0	Time discretization	alpha	[LACT]_0	Time discretization	alpha	[LACT]_0
0	0	0	15	0.8386521	0	30	0.08971732	4.88E-235	45	0.00370747	1.10E-150
0.1	0	0	15.1	0.8386521	0	30.1	0.08971732	4.88E-235	45.1	0.00382982	1.62E-150
0.2	0	0	15.2	0.8386521	0	30.2	0.08971732	4.88E-235	45.2	0.00359635	2.36E-150
0.3	0	0	15.3	0.8386521	0	30.3	0.08743097	4.33E-235	45.3	0.00348123	2.18E-150
0.4	0	0	15.4	0.8386521	0	30.4	0.08707645	2.86E-233	45.4	0.00348123	2.18E-150
0.5	0.66113227	0	15.5	0.8386521	0	30.5	0.068663	8.34E-231	45.5	0.00338034	7.04E-150
0.6	0.66113227	0	15.6	0.8386521	0	30.6	0.05576323	3.81E-230	45.6	0.00312095	1.13E-149
0.7	0.66113227	0	15.7	0.8386521	0	30.7	0.05576323	3.81E-230	45.7	0.00294005	1.88E-149
0.8	0.66113227	0	15.8	0.8386521	0	30.8	0.05576323	3.81E-230	45.8	0.0027887	2.12E-149
0.9	0.66113227	0	15.9	0.8386521	0	30.9	0.05027226	1.82E-229	45.9	0.00269431	2.07E-148
1	0.66113227	0	16	0.8386521	0	31	0.05450039	5.53E-227	46	0.00236724	7.23E-148
1.1	0.66113227	0	16.1	0.8386521	0	31.1	0.05652024	2.79E-225	46.1	0.00236724	7.23E-148
1.2	0.66113227	0	16.2	0.8386521	0	31.2	0.05322837	3.31E-225	46.2	0.00239246	8.19E-148
1.3	0.66113227	0	16.3	0.8386521	0	31.3	0.04853862	1.22E-224	46.3	0.00252694	2.37E-147
1.4	0.66113227	0	16.4	0.8386521	0	31.4	0.04853862	1.22E-224	46.4	0.00245621	2.78E-147
1.5	0.66113227	0	16.5	0.8386521	0	31.5	0.04501309	2.78E-222	46.5	0.00230989	4.34E-147
1.6	0.66113227	0	16.6	0.91835194	0	31.6	0.04501309	2.78E-222	46.6	0.0021176	1.85E-146
1.7	0.66113227	0	16.7	0.91835194	0	31.7	0.04096038	1.25E-221	46.7	0.0021176	1.85E-146
1.8	0.66113227	0	16.8	0.91835194	0	31.8	0.04704389	4.40E-220	46.8	0.00183714	1.73E-146
1.9	0.66113227	0	16.9	0.91835194	0	31.9	0.04961592	3.11E-219	46.9	0.0018286	5.80E-146
2	0.66113227	0	17	0.91835194	0	32	0.04709257	9.78E-218	47	0.00178431	8.91E-146
2.1	0.66113227	0	17.1	0.98223293	0	32.1	0.04184677	4.26E-217	47.1	0.00172014	4.45E-145
2.2	0.66113227	0	17.2	0.98223293	0	32.2	0.04184677	4.26E-217	47.2	0.00162711	4.46E-145
2.3	0.66113227	0	17.3	0.98223293	0	32.3	0.04184677	4.26E-217	47.3	0.00162711	4.46E-145
2.4	0.66113227	0	17.4	0.98223293	0	32.4	0.03986155	5.28E-215	47.4	0.00158983	6.45E-145
2.5	0.66113227	0	17.5	0.98223293	0	32.5	0.03965965	1.50E-213	47.5	0.00171623	1.76E-144
2.6	0.66113227	0	17.6	0.98223293	0	32.6	0.03548413	6.78E-213	47.6	0.00172447	4.62E-144
2.7	0.66113227	0	17.7	0.98223293	0	32.7	0.03548413	6.78E-213	47.7	0.00149721	8.06E-144
2.8	0.66113227	0	17.8	0.98223293	0	32.8	0.03317775	9.29E-211	47.8	0.00148543	2.34E-143
2.9	0.66113227	0	17.9	0.98223293	0	32.9	0.03317775	9.29E-211	47.9	0.00147256	4.07E-143
3	0.66113227	0	18	0.57590848	0	33	0.03104126	3.14E-210	48	0.00166011	2.77E-142
3.1	0.66113227	0	18.1	0.57590848	0	33.1	0.02908611	4.20E-210	48.1	0.00166011	2.77E-142
3.2	0.66113227	0	18.2	0.57590848	0	33.2	0.02974887	2.26E-209	48.2	0.00161284	3.49E-142
3.3	0.66113227	0	18.3	0.57590848	0	33.3	0.02837895	3.45E-207	48.3	0.00161284	3.49E-142
3.4	0.66113227	0	18.4	0.57590848	0	33.4	0.02735983	4.44E-206	48.4	0.00154778	5.21E-142
3.5	0.66113227	0	18.5	0.56891349	0	33.5	0.0271156	1.96E-205	48.5	0.00151524	3.39E-141
3.6	0.66113227	0	18.6	0.56891349	0	33.6	0.0271156	1.96E-205	48.6	0.00151524	3.39E-141
3.7	0.66113227	0	18.7	0.56891349	0	33.7	0.0271156	1.96E-205	48.7	0.00140043	5.63E-141
3.8	0.66113227	0	18.8	0.56891349	0	33.8	0.02821221	8.99E-205	48.8	0.00142562	6.25E-141
3.9	0.66113227	0	18.9	0.56891349	0	33.9	0.0271335	1.81E-203	48.9	0.00137203	9.76E-141
4	0.66113227	0	19	0.56891349	0	34	0.0266394	9.13E-202	49	0.00117305	1.60E-140
4.1	0.66113227	0	19.1	0.56891349	0	34.1	0.02355493	4.92E-201	49.1	0.00118665	1.58E-140
4.2	0.66113227	0	19.2	0.56891349	0	34.2	0.02355493	4.92E-201	49.2	0.00116501	2.51E-140
4.3	0.66113227	0	19.3	0.56891349	0	34.3	0.0245675	2.53E-199	49.3	0.00121196	7.37E-140
4.4	0.66113227	0	19.4	0.6057749	0	34.4	0.02486658	3.37E-199	49.4	0.00118783	1.01E-139
4.5	0.66113227	0	19.5	0.6057749	0	34.5	0.02289437	1.38E-198	49.5	0.00118783	1.01E-139
4.6	0.66113227	0	19.6	0.6057749	0	34.6	0.02335373	1.46E-197	49.6	0.00118717	1.16E-139
4.7	0.66113227	0	19.7	0.6057749	0	34.7	0.02371497	8.55E-197	49.7	0.00118717	1.16E-139
4.8	0.66113227	0	19.8	0.6057749	0	34.8	0.02072974	5.90E-196	49.8	0.00116022	1.71E-139
4.9	0.47344649	0	19.9	0.55772782	0	34.9	0.02118372	2.38E-195	49.9	0.00130488	7.00E-139
5	0.47344649	0	20	0.55772782	0	35	0.02120724	8.19E-195	50	0.00122315	7.11E-139
5.1	0.47344649	0	20.1	0.37834702	0	35.1	0.02151738	3.73E-194	50.1	0.00120177	1.36E-138
5.2	0.47344649	0	20.2	0.37834702	0	35.2	0.02151738	3.73E-194	50.2	0.00117392	1.35E-138
5.3	0.47344649	0	20.3	0.37834702	0	35.3	0.02151738	3.73E-194	50.3	0.00117392	1.35E-138
5.4	0.47344649	0	20.4	0.37834702	0	35.4	0.01954895	1.65E-192	50.4	0.0011417	2.21E-138
5.5	0.47344649	0	20.5	0.37834702	0	35.5	0.01954895	1.65E-192	50.5	0.00118937	3.35E-138
5.6	0.47344649	0	20.6	0.37834702	0	35.6	0.02205941	7.63E-191	50.6	0.00102343	2.99E-138
5.7	0.47344649	0	20.7	0.37834702	0	35.7	0.01968285	2.21E-189	50.7	0.00097358	4.10E-138
5.8	0.47344649	0	20.8	0.37834702	0	35.8	0.01850176	8.68E-189	50.8	0.00095176	4.95E-138
5.9	0.47344649	0	20.9	0.37834702	0	35.9	0.01850176	8.68E-189	50.9	0.00093476	8.47E-138
6	0.47344649	0	21	0.37834702	0	36	0.01820742	6.45E-188	51	0.00090336	9.56E-138
6.1	0.47344649	0	21.1	0.37834702	0	36.1	0.01755528	2.45E-187	51.1	0.00090786	1.70E-137
6.2	0.47344649	0	21.2	0.38341063	0	36.2	0.01766501	1.23E-186	51.2	0.00085949	1.68E-137
6.3	0.47344649	0	21.3	0.38341063	0	36.3	0.01825783	4.16E-185	51.3	0.00085949	1.68E-137
6.4	0.47344649	0	21.4	0.38341063	0	36.4	0.01684117	4.30E-185	51.4	0.00079603	2.99E-137
6.5	0.47344649	0	21.5	0.38341063	0	36.5	0.01685711	2.00E-184	51.5	0.00079603	2.99E-137
6.6	0.47344649	0	21.6	0.38341063	0	36.6	0.01705266	9.94E-184	51.6	0.00079066	5.45E-137
6.7	0.47344649	0	21.7	0.38341063	0	36.7	0.01752815	4.07E-183	51.7	0.00079066	5.45E-137
6.8	0.47344649	0	21.8	0.36825823	0	36.8	0.01752815	4.07E-183	51.8	0.00079066	5.45E-137
6.9	0.47344649	0	21.9	0.36825823	0	36.9	0.01829011	1.91E-182	51.9	0.00086066	2.15E-136
7	0.47344649	0	22	0.36825823	0	37	0.01964651	1.16E-181	52	0.00088118	2.27E-136
7.1	0.47344649	0	22.1	0.42598351	8.33716000000000e-319	37.1	0.02159751	2.76E-180	52.1	0.00092293	3.72E-136
7.2	0.47344649	0	22.2	0.42598351	8.33716000000000e-319	37.2	0.02142435	1.10E-179	52.2	0.00088824	7.38E-136
7.3	0.47344649	0	22.3	0.42598351	8.33716000000000e-319	37.3	0.02122565	2.60E-178	52.3	0.00091024	8.39E-136
7.4	0.47344649	0	22.4	0.46911225	1.91107297463000e-312	37.4	0.02052779	3.08E-178	52.4	0.00091024	8.39E-136
7.5	0.8386521	0	22.5	0.46911225	1.91107297463000e-312	37.5	0.01882219	1.08E-177	52.5	0.00077753	1.45E-135
7.6	0.8386521	0	22.6	0.46911225	1.91107297463000e-312	37.6	0.0187121	4.08E-177	52.6	0.00074125	1.67E-135
7.7	0.8386521	0	22.7	0.46911225	1.91107297463000e-312	37.7	0.0187121	4.08E-177	52.7	0.00074125	1.67E-135
7.8	0.8386521	0	22.8	0.46911225	1.91107297463000e-312	37.8	0.01974259	7.08E-176	52.8	0.0007548	3.11E-135
7.9	0.8386521	0	22.9	0.46911225	1.91107297463000e-312	37.9	0.01974259	7.08E-176	52.9	0.0006605	4.17E-135
8	0.8386521	0	23	0.46911225	1.91107297463000e-312	38	0.01804038	8.80E-176	53	0.00061754	5.14E-135
8.1	0.8386521	0	23.1	0.46298667	1.28E-306	38.1	0.01746332	3.40E-175	53.1	0.00059834	8.92E-135
8.2	0.8386521	0	23.2	0.46298667	1.28E-306	38.2	0.01806683	2.11E-173	53.2	0.00055234	1.01E-134
8.3	0.8386521	0	23.3	0.33605222	2.15E-306	38.3	0.01710208	2.66E-173	53.3	0.00055234	1.01E-134
8.4	0.8386521	0	23.4	0.46292967	1.34E-305	38.4	0.01594531	2.74E-173	53.4	0.00051629	1.52E-134
8.5	0.8386521	0	23.5	0.46292967	1.34E-305	38.5	0.01425192	1.46E-172	53.5	0.00048733	3.09E-134
8.6	0.8386521	0	23.6	0.47301011	1.52E-300	38.6	0.01434998	1.82E-171	53.6	0.00049886	5.33E-134
8.7	0.8386521	0	23.7	0.47301011	1.52E-300	38.7	0.01319032	1.93E-171	53.7	0.00052181	6.48E-134
8.8	0.8386521	0	23.8	0.53265101	5.94E-296	38.8	0.01211387	2.67E-171	53.8	0.00048591	1.40E-133
8.9	0.8386521	0	23.9	0.53265101	5.94E-296	38.9	0.01156679	1.39E-169	53.9	0.00048099	1.75E-133
9	0.8386521	0	24	0.53265101	5.94E-296	39	0.01152811	1.41E-169	54	0.00048099	1.75E-133
9.1	0.8386521	0	24.1	0.53265101	5.94E-296	39.1	0.01195653	2.29E-168	54.1	0.00050402	3.80E-133
9.2	0.8386521	0	24.2	0.53265101	5.94E-296	39.2	0.01106621	1.76E-168	54.2	0.00052244	6.57E-133
9.3	0.8386521	0	24.3	0.56065302	2.11E-291	39.3	0.01038904	6.61E-168	54.3	0.00049216	8.50E-133
9.4	0.8386521	0	24.4	0.56065302	2.11E-291	39.4	0.01055906	7.49E-167	54.4	0.00049016	8.34E-133
9.5	0.8386521	0	24.5	0.54632974	3.25E-287	39.5	0.01055906	7.49E-167	54.5	0.00047152	8.22E-133
9.6	0.8386521	0	24.6	0.54632974	3.25E-287	39.6	0.01106579	2.52E-166	54.6	0.00040319	2.51E-132
9.7	0.8386521	0	24.7	0.54632974	3.25E-287	39.7	0.01152795	7.86E-166	54.7	0.0003791	2.83E-132
9.8	0.8386521	0	24.8	0.54632974	3.25E-287	39.8	0.01261395	9.31E-165	54.8	0.00037903	5.40E-132
9.9	0.8386521	0	24.9	0.54632974	3.25E-287	39.9	0.01174983	3.71E-164	54.9	0.00038553	9.73E-132
10	0.8386521	0	25	0.54632974	3.25E-287	40	0.01213659	1.16E-163	55	0.00038553	9.73E-132
10.1	0.8386521	0	25.1	0.472398	7.10E-283	40.1	0.01249064	4.69E-163	55.1	0.00040274	2.03E-131
10.2	0.8386521	0	25.2	0.43832566	2.48E-279	40.2	0.01192156	1.47E-162	55.2	0.00036602	2.62E-131
10.3	0.8386521	0	25.3	0.43832566	2.48E-279	40.3	0.0123608	3.99E-162	55.3	0.00036602	2.62E-131
10.4	0.8386521	0	25.4	0.40227522	5.93E-276	40.4	0.01173962	3.16E-161	55.4	0.00034296	7.39E-131
10.5	0.8386521	0	25.5	0.33406954	6.25E-276	40.5	0.01196569	1.06E-160	55.5	0.00031874	1.43E-130
10.6	0.8386521	0	25.6	0.33406954	6.25E-276	40.6	0.01141572	1.31E-160	55.6	0.00029907	1.87E-130
10.7	0.8386521	0	25.7	0.33406954	6.25E-276	40.7	0.01120199	3.49E-160	55.7	0.00029907	1.87E-130
10.8	0.8386521	0	25.8	0.32182989	2.27E-272	40.8	0.01073387	4.59E-160	55.8	0.00029946	4.69E-130
10.9	0.8386521	0	25.9	0.32182989	2.27E-272	40.9	0.01003838	4.98E-159	55.9	0.00029946	4.69E-130
11	0.8386521	0	26	0.32182989	2.27E-272	41	0.0089447	5.34E-159	56	0.00027981	5.09E-130
11.1	0.8386521	0	26.1	0.3134792	8.65E-269	41.1	0.0089447	5.34E-159	56.1	0.00027981	5.09E-130
11.2	0.8386521	0	26.2	0.3134792	8.65E-269	41.2	0.00801672	7.17E-159	56.2	0.00026606	8.75E-130
11.3	0.8386521	0	26.3	0.3134792	8.65E-269	41.3	0.00756222	2.39E-158	56.3	0.00027847	1.11E-129
11.4	0.8386521	0	26.4	0.33621682	1.68E-265	41.4	0.00728757	2.81E-158	56.4	0.00019968	1.78E-129
11.5	0.8386521	0	26.5	0.33621682	1.68E-265	41.5	0.0076296	9.48E-158	56.5	0.00019968	1.78E-129
11.6	0.8386521	0	26.6	0.33621682	1.68E-265	41.6	0.0073349	9.13E-158	56.6	0.00019968	1.78E-129
11.7	0.8386521	0	26.7	0.33621682	1.68E-265	41.7	0.00700902	3.52E-157	56.7	0.00018518	2.05E-129
11.8	0.8386521	0	26.8	0.33074602	2.47E-262	41.8	0.00667398	5.11E-157	56.8	0.00017112	3.75E-129

11.9	0.8386521	0	26.9	0.33074602	2.47E-262	41.9	0.00667398	5.11E-157	56.9	0.00017404	5.45E-129
12	0.8386521	0	27	0.33074602	2.47E-262	42	0.00662117	4.96E-157	57	0.00017404	5.45E-129
12.1	0.8386521	0	27.1	0.33074602	2.47E-262	42.1	0.00687914	1.72E-156	57.1	0.00017699	1.77E-128
12.2	0.8386521	0	27.2	0.33074602	2.47E-262	42.2	0.00727937	5.43E-156	57.2	0.00019533	3.65E-128
12.3	0.8386521	0	27.3	0.28380657	3.32E-262	42.3	0.00722642	8.06E-156	57.3	0.00018934	4.62E-128
12.4	0.8386521	0	27.4	0.27194875	2.66E-259	42.4	0.00644501	1.00E-155	57.4	0.00019056	1.07E-127
12.5	0.8386521	0	27.5	0.22024368	4.27E-259	42.5	0.00582076	8.35E-156	57.5	0.00018225	1.25E-127
12.6	0.8386521	0	27.6	0.18249516	2.15E-256	42.6	0.00582076	8.35E-156	57.6	0.00016683	1.41E-127
12.7	0.8386521	0	27.7	0.18249516	2.15E-256	42.7	0.00622553	2.59E-155	57.7	0.00017034	2.99E-127
12.8	0.8386521	0	27.8	0.16558547	3.32E-256	42.8	0.00602393	2.68E-155	57.8	0.0001775	3.41E-127
12.9	0.8386521	0	27.9	0.16558547	3.32E-256	42.9	0.00573963	2.70E-155	57.9	0.00015576	6.03E-127
13	0.8386521	0	28	0.13481747	4.36E-253	43	0.00557546	1.04E-154	58	0.000157	9.88E-127
13.1	0.8386521	0	28.1	0.12525214	9.66E-253	43.1	0.00543526	3.83E-154	58.1	0.00014056	9.10E-127
13.2	0.8386521	0	28.2	0.12617082	2.39E-250	43.2	0.00557538	5.58E-154	58.2	0.00012526	1.16E-126
13.3	0.8386521	0	28.3	0.11220587	5.80E-250	43.3	0.00549696	6.01E-154	58.3	0.0001261	2.24E-126
13.4	0.8386521	0	28.4	0.11220587	5.80E-250	43.4	0.00586645	1.94E-153	58.4	0.00012655	7.28E-126
13.5	0.8386521	0	28.5	0.10850232	1.93E-247	43.5	0.00586645	1.94E-153	58.5	0.00012655	7.28E-126
13.6	0.8386521	0	28.6	0.10850232	1.93E-247	43.6	0.00534837	2.12E-153	58.6	0.00013039	1.54E-125
13.7	0.8386521	0	28.7	0.10859419	8.73E-247	43.7	0.00541953	8.17E-153	58.7	0.00012913	3.01E-125
13.8	0.8386521	0	28.8	0.10859419	8.73E-247	43.8	0.00544752	2.88E-152	58.8	0.00012139	2.72E-125
13.9	0.8386521	0	28.9	0.10859419	8.73E-247	43.9	0.00544752	2.88E-152	58.9	0.0001186	3.06E-125
14	0.8386521	0	29	0.11567278	2.49E-244	44	0.00501418	2.78E-152	59	0.00011328	3.60E-125
14.1	0.8386521	0	29.1	0.1200439	9.20E-242	44.1	0.00475609	3.78E-152	59.1	0.00011286	9.73E-125
14.2	0.8386521	0	29.2	0.09815682	2.98E-241	44.2	0.0041697	1.61E-151	59.2	0.00011286	9.73E-125
14.3	0.8386521	0	29.3	0.09815682	2.98E-241	44.3	0.00406188	1.57E-151	59.3	9.97E-05	1.12E-124
14.4	0.8386521	0	29.4	0.09815682	2.98E-241	44.4	0.00397814	2.20E-151	59.4	0.00010082	1.50E-124
14.5	0.8386521	0	29.5	0.09815682	2.98E-241	44.5	0.00397814	2.20E-151	59.5	0.00010322	1.93E-124
14.6	0.8386521	0	29.6	0.09815682	2.98E-241	44.6	0.00397814	2.20E-151	59.6	9.53E-05	1.89E-124
14.7	0.8386521	0	29.7	0.08960128	1.60E-238	44.7	0.00427065	3.11E-151	59.7	9.85E-05	4.30E-124
14.8	0.8386521	0	29.8	0.07766891	7.52E-238	44.8	0.00426619	9.53E-151	59.8	8.98E-05	4.65E-124
14.9	0.8386521	0	29.9	0.08921983	1.06E-235	44.9	0.00398459	9.67E-151	59.9	8.88E-05	7.50E-124

60	8.88E-05	7.50E-124	75	5.18E-07	2.09E-98	90	1.96E-07	1.76E-66	105	1.73E-08	2.53E-61
60.1	8.88E-05	7.50E-124	75.1	4.77E-07	4.49E-98	90.1	1.96E-07	1.76E-66	105.1	1.73E-08	2.53E-61
60.2	8.41E-05	7.58E-124	75.2	5.28E-07	9.32E-98	90.2	1.90E-07	2.24E-66	105.2	1.70E-08	2.52E-61
60.3	8.41E-05	7.58E-124	75.3	4.84E-07	1.66E-97	90.3	1.95E-07	3.03E-66	105.3	1.66E-08	2.68E-61
60.4	8.58E-05	1.15E-123	75.4	4.34E-07	3.18E-97	90.4	1.90E-07	3.09E-66	105.4	1.62E-08	2.69E-61
60.5	7.35E-05	1.27E-123	75.5	4.21E-07	6.31E-97	90.5	1.94E-07	3.84E-66	105.5	1.61E-08	3.01E-61
60.6	6.72E-05	1.48E-123	75.6	3.59E-07	1.45E-96	90.6	1.87E-07	4.51E-66	105.6	1.61E-08	3.01E-61
60.7	6.22E-05	1.67E-123	75.7	3.29E-07	2.44E-96	90.7	1.84E-07	5.54E-66	105.7	1.53E-08	3.04E-61
60.8	5.93E-05	2.55E-123	75.8	3.36E-07	3.09E-96	90.8	1.89E-07	6.57E-66	105.8	1.61E-08	3.83E-61
60.9	6.00E-05	3.28E-123	75.9	3.18E-07	7.50E-96	90.9	1.95E-07	1.02E-65	105.9	1.61E-08	3.83E-61
61	5.60E-05	3.95E-123	76	2.93E-07	9.98E-96	91	1.95E-07	1.02E-65	106	1.61E-08	3.83E-61
61.1	5.38E-05	4.46E-123	76.1	2.75E-07	1.33E-95	91.1	2.01E-07	1.37E-65	106.1	1.55E-08	3.95E-61
61.2	5.17E-05	8.02E-123	76.2	2.65E-07	1.93E-95	91.2	1.97E-07	2.25E-65	106.2	1.55E-08	3.95E-61
61.3	5.17E-05	8.02E-123	76.3	2.73E-07	3.15E-95	91.3	1.94E-07	2.33E-65	106.3	1.56E-08	4.10E-61
61.4	4.96E-05	7.80E-123	76.4	2.90E-07	7.51E-95	91.4	1.94E-07	2.33E-65	106.4	1.57E-08	4.27E-61
61.5	4.91E-05	9.52E-123	76.5	2.81E-07	1.28E-94	91.5	1.88E-07	3.49E-65	106.5	1.52E-08	4.30E-61
61.6	4.60E-05	9.95E-123	76.6	2.66E-07	2.34E-94	91.6	1.88E-07	3.49E-65	106.6	1.51E-08	4.23E-61
61.7	4.40E-05	1.17E-122	76.7	2.80E-07	4.96E-94	91.7	1.80E-07	5.21E-65	106.7	1.49E-08	4.52E-61
61.8	4.02E-05	1.59E-122	76.8	2.64E-07	6.25E-94	91.8	1.82E-07	5.98E-65	106.8	1.49E-08	4.52E-61
61.9	4.05E-05	2.34E-122	76.9	2.76E-07	7.96E-94	91.9	1.82E-07	7.30E-65	106.9	1.49E-08	4.65E-61
62	3.29E-05	3.06E-122	77	2.66E-07	1.74E-93	92	1.80E-07	7.47E-65	107	1.40E-08	4.59E-61
62.1	3.51E-05	4.25E-122	77.1	2.60E-07	2.12E-93	92.1	1.77E-07	8.51E-65	107.1	1.39E-08	4.60E-61
62.2	3.59E-05	5.48E-122	77.2	2.20E-07	2.99E-93	92.2	1.66E-07	1.06E-64	107.2	1.37E-08	5.05E-61
62.3	3.68E-05	6.90E-122	77.3	2.05E-07	6.81E-93	92.3	1.63E-07	1.10E-64	107.3	1.33E-08	5.45E-61
62.4	3.68E-05	6.90E-122	77.4	2.03E-07	8.91E-93	92.4	1.60E-07	1.18E-64	107.4	1.31E-08	5.57E-61
62.5	3.78E-05	1.06E-121	77.5	2.21E-07	2.33E-92	92.5	1.58E-07	1.25E-64	107.5	1.38E-08	5.84E-61
62.6	3.61E-05	1.39E-121	77.6	2.21E-07	2.80E-92	92.6	1.58E-07	1.25E-64	107.6	1.38E-08	5.84E-61
62.7	3.26E-05	1.78E-121	77.7	2.13E-07	7.71E-92	92.7	1.55E-07	1.43E-64	107.7	1.38E-08	5.84E-61
62.8	3.15E-05	2.67E-121	77.8	2.21E-07	2.25E-91	92.8	1.57E-07	1.65E-64	107.8	1.29E-08	6.10E-61
62.9	3.25E-05	4.20E-121	77.9	2.05E-07	2.63E-91	92.9	1.57E-07	1.63E-64	107.9	1.31E-08	6.72E-61
63	2.95E-05	4.11E-121	78	2.15E-07	6.75E-91	93	1.57E-07	1.71E-64	108	1.26E-08	6.53E-61
63.1	2.81E-05	4.95E-121	78.1	2.07E-07	1.31E-90	93.1	1.50E-07	1.67E-64	108.1	1.23E-08	6.56E-61
63.2	2.66E-05	6.18E-121	78.2	2.32E-07	1.94E-90	93.2	1.48E-07	1.71E-64	108.2	1.23E-08	7.99E-61
63.3	2.56E-05	7.92E-121	78.3	2.27E-07	8.96E-90	93.3	1.40E-07	2.06E-64	108.3	1.23E-08	7.99E-61
63.4	2.53E-05	1.18E-120	78.4	2.20E-07	1.14E-89	93.4	1.31E-07	2.09E-64	108.4	1.20E-08	8.70E-61
63.5	2.57E-05	1.56E-120	78.5	2.06E-07	1.45E-89	93.5	1.30E-07	2.18E-64	108.5	1.20E-08	8.70E-61
63.6	2.02E-05	2.25E-120	78.6	2.29E-07	5.92E-89	93.6	1.27E-07	2.98E-64	108.6	1.22E-08	9.12E-61
63.7	2.02E-05	2.25E-120	78.7	2.32E-07	8.63E-89	93.7	1.24E-07	3.06E-64	108.7	1.23E-08	9.39E-61
63.8	2.02E-05	2.25E-120	78.8	2.17E-07	1.45E-88	93.8	1.24E-07	3.06E-64	108.8	1.18E-08	9.63E-61
63.9	2.01E-05	3.67E-120	78.9	2.20E-07	2.11E-88	93.9	1.21E-07	3.58E-64	108.9	1.19E-08	1.10E-60
64	1.77E-05	2.33E-120	79	2.14E-07	4.77E-88	94	1.21E-07	3.74E-64	109	1.14E-08	1.09E-60
64.1	1.64E-05	3.14E-120	79.1	2.01E-07	1.14E-87	94.1	1.15E-07	3.81E-64	109.1	1.14E-08	1.09E-60
64.2	1.62E-05	3.28E-120	79.2	2.07E-07	2.09E-87	94.2	1.08E-07	4.42E-64	109.2	1.14E-08	1.09E-60
64.3	1.60E-05	4.18E-120	79.3	2.08E-07	3.32E-87	94.3	1.07E-07	4.54E-64	109.3	1.11E-08	1.17E-60
64.4	1.40E-05	5.65E-120	79.4	2.05E-07	5.71E-87	94.4	1.01E-07	5.32E-64	109.4	1.07E-08	1.17E-60
64.5	1.34E-05	8.91E-120	79.5	2.11E-07	9.23E-87	94.5	1.02E-07	6.24E-64	109.5	1.07E-08	1.17E-60
64.6	1.44E-05	1.25E-119	79.6	2.09E-07	1.87E-86	94.6	9.88E-08	7.21E-64	109.6	1.07E-08	1.17E-60
64.7	1.30E-05	1.52E-119	79.7	1.98E-07	3.29E-86	94.7	9.85E-08	8.57E-64	109.7	1.04E-08	1.17E-60
64.8	1.47E-05	3.64E-119	79.8	2.15E-07	8.48E-86	94.8	9.66E-08	8.14E-64	109.8	9.99E-09	1.18E-60
64.9	1.45E-05	4.01E-119	79.9	2.27E-07	1.59E-85	94.9	9.37E-08	9.51E-64	109.9	9.99E-09	1.18E-60
65	1.40E-05	6.89E-119	80	2.26E-07	3.30E-85	95	9.47E-08	9.97E-64	110	9.99E-09	1.18E-60
65.1	1.33E-05	9.59E-119	80.1	2.39E-07	7.58E-85	95.1	9.31E-08	1.16E-63	110.1	9.88E-09	1.22E-60
65.2	1.18E-05	1.12E-118	80.2	2.36E-07	1.09E-84	95.2	8.98E-08	1.24E-63	110.2	9.88E-09	1.22E-60
65.3	1.15E-05	2.44E-118	80.3	2.23E-07	1.93E-84	95.3	9.17E-08	1.48E-63	110.3	9.88E-09	1.22E-60
65.4	1.15E-05	2.68E-118	80.4	2.38E-07	3.98E-84	95.4	9.26E-08	1.88E-63	110.4	9.69E-09	1.33E-60
65.5	1.08E-05	3.94E-118	80.5	2.19E-07	7.40E-84	95.5	8.87E-08	1.88E-63	110.5	9.30E-09	1.30E-60
65.6	1.07E-05	4.93E-118	80.6	2.18E-07	1.17E-83	95.6	8.65E-08	2.44E-63	110.6	9.30E-09	1.30E-60
65.7	1.09E-05	6.48E-118	80.7	2.02E-07	2.11E-83	95.7	8.27E-08	2.50E-63	110.7	9.05E-09	1.36E-60
65.8	1.03E-05	1.27E-117	80.8	2.01E-07	4.37E-83	95.8	7.83E-08	2.94E-63	110.8	9.04E-09	1.38E-60
65.9	9.76E-06	1.91E-117	80.9	2.10E-07	6.96E-83	95.9	7.70E-08	3.56E-63	110.9	8.14E-09	1.42E-60
66	8.31E-06	2.90E-117	81	2.15E-07	1.97E-82	96	7.70E-08	3.56E-63	111	8.14E-09	1.42E-60
66.1	8.64E-06	5.96E-117	81.1	2.13E-07	3.42E-82	96.1	7.47E-08	3.50E-63	111.1	8.05E-09	1.49E-60
66.2	9.21E-06	1.41E-116	81.2	2.19E-07	6.67E-82	96.2	7.04E-08	3.40E-63	111.2	8.09E-09	1.56E-60
66.3	8.72E-06	1.90E-116	81.3	2.20E-07	1.23E-81	96.3	6.78E-08	4.64E-63	111.3	8.48E-09	1.65E-60
66.4	8.54E-06	2.24E-116	81.4	2.21E-07	2.10E-81	96.4	6.78E-08	4.64E-63	111.4	8.45E-09	1.74E-60
66.5	7.77E-06	3.27E-116	81.5	2.17E-07	3.50E-81	96.5	6.75E-08	6.31E-63	111.5	8.45E-09	1.74E-60
66.6	7.77E-06	3.27E-116	81.6	2.24E-07	7.24E-81	96.6	6.50E-08	6.46E-63	111.6	8.22E-09	1.94E-60
66.7	6.65E-06	6.43E-116	81.7	2.38E-07	1.86E-80	96.7	6.42E-08	6.46E-63	111.7	8.22E-09	1.94E-60
66.8	6.82E-06	8.29E-116	81.8	2.66E-07	5.56E-80	96.8	6.07E-08	6.71E-63	111.8	8.16E-09	2.04E-60
66.9	6.48E-06	1.44E-115	81.9	2.30E-07	7.22E-80	96.9	6.13E-08	7.25E-63	111.9	8.24E-09	2.12E-60
67	6.18E-06	1.99E-115	82	2.37E-07	1.71E-79	97	6.13E-08	7.25E-63	112	8.17E-09	2.24E-60
67.1	5.75E-06	2.45E-115	82.1	2.40E-07	2.60E-79	97.1	6.12E-08	8.53E-63	112.1	8.17E-09	2.24E-60
67.2	5.82E-06	3.06E-115	82.2	2.39E-07	3.79E-79	97.2	6.20E-08	9.99E-63	112.2	7.73E-09	2.42E-60
67.3	4.74E-06	5.29E-115	82.3	2.38E-07	6.52E-79	97.3	6.20E-08	9.99E-63	112.3	7.73E-09	2.50E-60
67.4	4.68E-06	6.96E-115	82.4	2.32E-07	1.19E-78	97.4	6.04E-08	1.07E-62	112.4	7.66E-09	2.84E-60
67.5	4.32E-06	1.03E-114	82.5	2.37E-07	1.64E-78	97.5	5.94E-08	1.19E-62	112.5	7.66E-09	2.84E-60
67.6	4.09E-06	1.12E-114	82.6	2.48E-07	2.38E-78	97.6	5.92E-08	1.46E-62	112.6	7.66E-09	2.84E-60
67.7	3.73E-06	2.73E-114	82.7	2.43E-07	4.92E-78	97.7	5.93E-08	1.57E-62	112.7	7.66E-09	2.84E-60
67.8	3.54E-06	4.04E-114	82.8	2.30E-07	1.57E-77	97.8	5.93E-08	1.57E-62	112.8	7.74E-09	2.94E-60
67.9	3.24E-06	6.23E-114	82.9	2.24E-07	2.06E-77	97.9	5.66E-08	1.62E-62	112.9	7.63E-09	3.10E-60
68	2.97E-06	7.90E-114	83	2.20E-07	3.02E-77	98	5.75E-08	1.79E-62	113	7.38E-09	3.16E-60
68.1	3.00E-06	1.18E-113	83.1	2.22E-07	8.96E-77	98.1	5.75E-08	1.79E-62	113.1	7.38E-09	3.16E-60
68.2	2.69E-06	1.46E-113	83.2	2.16E-07	1.50E-76	98.2	5.75E-08	1.79E-62	113.2	7.38E-09	3.16E-60
68.3	2.65E-06	2.15E-113	83.3	2.37E-07	3.69E-76	98.3	5.27E-08	2.13E-62	113.3	7.12E-09	3.21E-60
68.4	2.36E-06	2.50E-113	83.4	2.56E-07	7.90E-76	98.4	5.03E-08	2.05E-62	113.4	6.88E-09	3.21E-60
68.5	2.13E-06	2.96E-113	83.5	2.55E-07	1.37E-75	98.5	4.97E-08	2.22E-62	113.5	6.88E-09	3.21E-60
68.6	1.84E-06	7.97E-113	83.6	2.47E-07	3.06E-75	98.6	4.97E-08	2.22E-62	113.6	6.85E-09	3.31E-60
68.7	1.83E-06	1.60E-112	83.7	2.83E-07	7.85E-75	98.7	4.81E-08	2.21E-62	113.7	6.81E-09	3.34E-60
68.8	1.54E-06	2.14E-112	83.8	2.82E-07	1.15E-74	98.8	4.81E-08	2.49E-62	113.8	6.81E-09	3.34E-60
68.9	1.46E-06	2.30E-112	83.9	2.72E-07	2.90E-74	98.9	4.76E-08	2.46E-62	113.9	6.71E-09	3.54E-60

69	1.22E-06	7.13E-112	84	2.77E-07	5.66E-74	99	4.76E-08	2.46E-62	114	6.45E-09	3.53E-60
69.1	1.19E-06	1.25E-111	84.1	2.69E-07	7.28E-74	99.1	4.70E-08	2.68E-62	114.1	6.17E-09	3.61E-60
69.2	1.18E-06	1.53E-111	84.2	2.93E-07	1.67E-73	99.2	4.67E-08	2.82E-62	114.2	6.13E-09	3.62E-60
69.3	1.19E-06	5.78E-111	84.3	2.91E-07	2.71E-73	99.3	4.49E-08	2.80E-62	114.3	6.21E-09	3.91E-60
69.4	1.18E-06	1.08E-110	84.4	2.99E-07	3.63E-73	99.4	4.41E-08	3.21E-62	114.4	6.21E-09	3.91E-60
69.5	1.18E-06	1.08E-110	84.5	3.12E-07	6.32E-73	99.5	4.41E-08	3.21E-62	114.5	5.94E-09	3.78E-60
69.6	1.15E-06	1.52E-110	84.6	2.85E-07	9.91E-73	99.6	4.41E-08	3.21E-62	114.6	5.83E-09	4.15E-60
69.7	9.89E-07	3.23E-110	84.7	2.84E-07	1.14E-72	99.7	3.91E-08	3.19E-62	114.7	5.93E-09	4.87E-60
69.8	8.97E-07	5.39E-110	84.8	2.76E-07	1.27E-72	99.8	3.83E-08	3.36E-62	114.8	5.73E-09	4.91E-60
69.9	8.78E-07	6.87E-110	84.9	2.76E-07	1.27E-72	99.9	3.83E-08	3.36E-62	114.9	5.56E-09	5.00E-60
70	8.73E-07	1.26E-109	85	2.52E-07	2.29E-72	100	3.90E-08	3.46E-62	115	5.27E-09	5.19E-60
70.1	1.04E-06	4.82E-109	85.1	2.43E-07	4.21E-72	100.1	3.90E-08	3.57E-62	115.1	5.26E-09	5.36E-60
70.2	1.04E-06	4.82E-109	85.2	2.38E-07	6.80E-72	100.2	3.90E-08	3.57E-62	115.2	5.09E-09	5.45E-60
70.3	1.01E-06	7.04E-109	85.3	2.42E-07	7.81E-72	100.3	3.86E-08	3.58E-62	115.3	4.98E-09	5.56E-60
70.4	9.18E-07	8.73E-109	85.4	2.34E-07	1.17E-71	100.4	3.59E-08	3.63E-62	115.4	4.98E-09	5.56E-60
70.5	9.18E-07	1.55E-108	85.5	2.24E-07	1.29E-71	100.5	3.59E-08	3.63E-62	115.5	4.93E-09	6.21E-60
70.6	8.95E-07	3.35E-108	85.6	2.17E-07	2.21E-71	100.6	3.47E-08	3.74E-62	115.6	4.93E-09	6.21E-60
70.7	8.40E-07	5.31E-108	85.7	2.15E-07	2.69E-71	100.7	3.47E-08	3.74E-62	115.7	4.93E-09	6.21E-60
70.8	8.78E-07	7.11E-108	85.8	2.07E-07	3.57E-71	100.8	3.47E-08	3.74E-62	115.8	4.95E-09	6.41E-60
70.9	8.23E-07	9.37E-108	85.9	2.17E-07	5.27E-71	100.9	3.47E-08	3.74E-62	115.9	4.75E-09	6.32E-60
71	7.78E-07	1.69E-107	86	2.24E-07	6.48E-71	101	3.40E-08	3.73E-62	116	4.61E-09	6.58E-60
71.1	8.12E-07	3.03E-107	86.1	2.30E-07	1.40E-70	101.1	3.19E-08	3.45E-62	116.1	4.61E-09	6.58E-60
71.2	7.90E-07	4.82E-107	86.2	2.24E-07	1.52E-70	101.2	3.19E-08	3.45E-62	116.2	4.61E-09	6.22E-60
71.3	7.99E-07	7.31E-107	86.3	2.37E-07	3.24E-70	101.3	3.08E-08	3.67E-62	116.3	4.23E-09	6.69E-60
71.4	8.26E-07	3.22E-106	86.4	2.17E-07	3.50E-70	101.4	3.08E-08	3.67E-62	116.4	4.23E-09	6.69E-60
71.5	7.76E-07	4.59E-106	86.5	2.38E-07	5.24E-70	101.5	3.00E-08	3.66E-62	116.5	4.10E-09	6.77E-60
71.6	7.15E-07	6.38E-106	86.6	2.36E-07	8.98E-70	101.6	2.88E-08	3.76E-62	116.6	3.84E-09	6.79E-60
71.7	7.12E-07	8.09E-106	86.7	2.45E-07	1.20E-69	101.7	2.73E-08	4.23E-62	116.7	3.84E-09	6.79E-60
71.8	7.05E-07	9.74E-106	86.8	2.48E-07	1.50E-69	101.8	2.73E-08	4.23E-62	116.8	3.84E-09	6.79E-60
71.9	7.05E-07	9.74E-106	86.9	2.36E-07	1.97E-69	101.9	2.73E-08	4.28E-62	116.9	3.84E-09	6.79E-60
72	7.02E-07	1.46E-105	87	2.44E-07	2.55E-69	102	2.73E-08	4.28E-62	117	3.78E-09	7.06E-60
72.1	6.60E-07	1.95E-105	87.1	2.53E-07	4.46E-69	102.1	2.62E-08	5.46E-62	117.1	3.71E-09	6.38E-60
72.2	6.24E-07	4.96E-105	87.2	2.54E-07	5.64E-69	102.2	2.60E-08	5.69E-62	117.2	3.60E-09	7.03E-60
72.3	6.09E-07	1.38E-104	87.3	2.65E-07	8.15E-69	102.3	2.70E-08	8.05E-62	117.3	3.63E-09	7.22E-60
72.4	5.80E-07	1.64E-104	87.4	2.57E-07	8.34E-69	102.4	2.60E-08	7.94E-62	117.4	3.63E-09	7.22E-60
72.5	6.85E-07	4.65E-104	87.5	2.55E-07	1.08E-68	102.5	2.49E-08	8.70E-62	117.5	3.51E-09	7.61E-60
72.6	6.62E-07	6.65E-104	87.6	2.69E-07	1.67E-68	102.6	2.49E-08	8.70E-62	117.6	3.46E-09	7.66E-60
72.7	5.90E-07	1.29E-103	87.7	2.65E-07	1.94E-68	102.7	2.49E-08	8.70E-62	117.7	3.46E-09	7.66E-60
72.8	5.95E-07	1.98E-103	87.8	2.75E-07	3.12E-68	102.8	2.41E-08	9.13E-62	117.8	3.46E-09	8.43E-60
72.9	5.64E-07	2.97E-103	87.9	2.75E-07	3.12E-68	102.9	2.41E-08	9.13E-62	117.9	3.46E-09	8.43E-60
73	5.55E-07	5.69E-103	88	2.97E-07	4.64E-68	103	2.45E-08	9.67E-62	118	3.28E-09	9.86E-60
73.1	6.20E-07	1.00E-102	88.1	3.10E-07	6.50E-68	103.1	2.40E-08	9.91E-62	118.1	3.28E-09	9.86E-60
73.2	6.21E-07	2.45E-102	88.2	3.04E-07	8.48E-68	103.2	2.42E-08	1.07E-61	118.2	3.28E-09	9.86E-60
73.3	6.61E-07	4.08E-102	88.3	3.09E-07	1.15E-67	103.3	2.33E-08	1.04E-61	118.3	3.19E-09	1.04E-59
73.4	6.38E-07	6.96E-102	88.4	3.06E-07	1.14E-67	103.4	2.38E-08	1.07E-61	118.4	3.19E-09	1.04E-59
73.5	6.25E-07	1.25E-101	88.5	2.83E-07	1.34E-67	103.5	2.36E-08	1.18E-61	118.5	3.13E-09	1.12E-59
73.6	6.11E-07	1.88E-101	88.6	2.88E-07	1.59E-67	103.6	2.36E-08	1.18E-61	118.6	3.01E-09	1.16E-59
73.7	6.09E-07	3.76E-101	88.7	2.86E-07	2.06E-67	103.7	2.29E-08	1.26E-61	118.7	3.01E-09	1.16E-59
73.8	5.89E-07	5.66E-101	88.8	2.86E-07	2.06E-67	103.8	2.36E-08	1.83E-61	118.8	3.01E-09	1.16E-59
73.9	5.50E-07	8.45E-101	88.9	2.81E-07	2.34E-67	103.9	2.38E-08	2.01E-61	118.9	2.88E-09	1.18E-59
74	5.45E-07	1.16E-100	89	2.58E-07	2.68E-67	104	2.38E-08	2.01E-61	119	2.83E-09	1.15E-59
74.1	5.68E-07	2.20E-100	89.1	2.50E-07	2.94E-67	104.1	2.19E-08	2.15E-61	119.1	2.78E-09	1.19E-59
74.2	5.10E-07	4.07E-100	89.2	2.50E-07	2.94E-67	104.2	2.15E-08	2.24E-61	119.2	2.74E-09	1.29E-59
74.3	5.74E-07	1.23E-99	89.3	2.40E-07	3.79E-67	104.3	2.07E-08	2.24E-61	119.3	2.74E-09	1.29E-59
74.4	5.49E-07	1.48E-99	89.4	2.34E-07	4.73E-67	104.4	2.07E-08	2.24E-61	119.4	2.61E-09	1.32E-59
74.5	5.59E-07	2.49E-99	89.5	2.25E-07	5.21E-67	104.5	2.07E-08	2.24E-61	119.5	2.62E-09	1.38E-59
74.6	4.88E-07	4.94E-99	89.6	2.17E-07	8.02E-67	104.6	1.85E-08	2.30E-61	119.6	2.64E-09	1.51E-59
74.7	4.86E-07	8.38E-99	89.7	2.15E-07	9.17E-67	104.7	1.81E-08	2.44E-61	119.7	2.58E-09	1.52E-59
74.8	4.96E-07	1.25E-98	89.8	2.22E-07	1.26E-66	104.8	1.81E-08	2.44E-61	119.8	2.58E-09	1.52E-59
74.9	4.96E-07	1.25E-98	89.9	2.09E-07	1.32E-66	104.9	1.81E-08	2.44E-61	119.9	2.40E-09	1.55E-59

120	2.40E-09	1.55E-59	135	1.63E-10	5.21E-57	150	4.54E-11	9.41E-53	165	2.80E-11	6.87E-51
120.1	2.36E-09	1.58E-59	135.1	1.62E-10	5.32E-57	150.1	4.54E-11	9.41E-53	165.1	2.63E-11	6.64E-51
120.2	2.36E-09	1.58E-59	135.2	1.47E-10	6.74E-57	150.2	4.53E-11	1.04E-52	165.2	2.63E-11	6.64E-51
120.3	2.36E-09	1.58E-59	135.3	1.48E-10	7.14E-57	150.3	4.53E-11	1.04E-52	165.3	2.63E-11	6.64E-51
120.4	2.29E-09	1.56E-59	135.4	1.48E-10	7.14E-57	150.4	4.47E-11	9.50E-53	165.4	2.63E-11	6.64E-51
120.5	2.25E-09	1.66E-59	135.5	1.49E-10	7.91E-57	150.5	4.45E-11	1.10E-52	165.5	2.62E-11	8.00E-51
120.6	2.25E-09	1.66E-59	135.6	1.47E-10	8.50E-57	150.6	4.34E-11	1.13E-52	165.6	2.62E-11	8.00E-51
120.7	2.18E-09	1.62E-59	135.7	1.47E-10	8.50E-57	150.7	4.21E-11	1.14E-52	165.7	2.62E-11	8.00E-51
120.8	2.21E-09	1.75E-59	135.8	1.46E-10	9.46E-57	150.8	4.21E-11	1.14E-52	165.8	2.63E-11	8.97E-51
120.9	2.12E-09	1.89E-59	135.9	1.44E-10	1.05E-56	150.9	4.21E-11	1.14E-52	165.9	2.63E-11	8.97E-51
121	2.05E-09	1.96E-59	136	1.44E-10	1.05E-56	151	4.06E-11	1.09E-52	166	2.66E-11	1.03E-50
121.1	2.05E-09	1.96E-59	136.1	1.36E-10	1.04E-56	151.1	4.13E-11	1.15E-52	166.1	2.66E-11	1.03E-50
121.2	2.00E-09	2.14E-59	136.2	1.31E-10	1.10E-56	151.2	4.17E-11	1.23E-52	166.2	2.66E-11	1.03E-50
121.3	2.00E-09	2.14E-59	136.3	1.21E-10	1.19E-56	151.3	4.17E-11	1.23E-52	166.3	2.65E-11	9.84E-51
121.4	1.97E-09	2.29E-59	136.4	1.18E-10	1.28E-56	151.4	4.17E-11	1.23E-52	166.4	2.67E-11	1.06E-50
121.5	1.97E-09	2.29E-59	136.5	1.16E-10	1.30E-56	151.5	4.01E-11	1.22E-52	166.5	2.57E-11	1.02E-50
121.6	1.95E-09	2.33E-59	136.6	1.13E-10	1.37E-56	151.6	4.06E-11	1.43E-52	166.6	2.48E-11	9.94E-51
121.7	1.95E-09	2.33E-59	136.7	1.09E-10	1.37E-56	151.7	3.96E-11	1.45E-52	166.7	2.52E-11	1.06E-50
121.8	1.95E-09	2.33E-59	136.8	1.07E-10	1.63E-56	151.8	3.96E-11	1.45E-52	166.8	2.52E-11	1.06E-50
121.9	1.85E-09	2.46E-59	136.9	1.07E-10	1.63E-56	151.9	3.96E-11	1.45E-52	166.9	2.53E-11	1.20E-50
122	1.85E-09	2.46E-59	137	1.06E-10	1.84E-56	152	3.96E-11	1.45E-52	167	2.53E-11	1.20E-50
122.1	1.80E-09	2.61E-59	137.1	1.05E-10	2.26E-56	152.1	3.96E-11	1.45E-52	167.1	2.56E-11	1.35E-50
122.2	1.80E-09	2.61E-59	137.2	1.02E-10	2.40E-56	152.2	3.96E-11	1.45E-52	167.2	2.58E-11	1.44E-50
122.3	1.81E-09	3.05E-59	137.3	1.03E-10	2.61E-56	152.3	3.86E-11	1.55E-52	167.3	2.42E-11	1.42E-50
122.4	1.76E-09	3.00E-59	137.4	1.01E-10	2.98E-56	152.4	3.59E-11	1.82E-52	167.4	2.42E-11	1.42E-50
122.5	1.69E-09	3.28E-59	137.5	1.01E-10	2.98E-56	152.5	3.50E-11	1.73E-52	167.5	2.42E-11	1.42E-50
122.6	1.69E-09	3.28E-59	137.6	9.81E-11	3.33E-56	152.6	3.50E-11	1.73E-52	167.6	2.41E-11	1.45E-50
122.7	1.69E-09	3.28E-59	137.7	1.01E-10	3.29E-56	152.7	3.50E-11	1.73E-52	167.7	2.47E-11	1.59E-50
122.8	1.68E-09	3.75E-59	137.8	1.00E-10	3.33E-56	152.8	3.50E-11	1.73E-52	167.8	2.36E-11	1.66E-50
122.9	1.68E-09	3.75E-59	137.9	1.03E-10	3.45E-56	152.9	3.53E-11	1.79E-52	167.9	2.36E-11	1.66E-50
123	1.68E-09	3.75E-59	138	1.06E-10	3.41E-56	153	3.53E-11	1.79E-52	168	2.25E-11	1.66E-50
123.1	1.56E-09	4.04E-59	138.1	1.03E-10	3.70E-56	153.1	3.46E-11	2.06E-52	168.1	2.15E-11	1.63E-50
123.2	1.56E-09	4.04E-59	138.2	1.04E-10	4.10E-56	153.2	3.42E-11	2.22E-52	168.2	2.15E-11	1.63E-50
123.3	1.49E-09	4.15E-59	138.3	1.05E-10	4.62E-56	153.3	3.42E-11	2.22E-52	168.3	2.15E-11	1.63E-50
123.4	1.49E-09	4.34E-59	138.4	1.05E-10	4.65E-56	153.4	3.42E-11	2.22E-52	168.4	2.15E-11	1.63E-50
123.5	1.49E-09	4.60E-59	138.5	1.03E-10	5.10E-56	153.5	3.42E-11	2.22E-52	168.5	2.15E-11	1.63E-50
123.6	1.48E-09	4.90E-59	138.6	1.01E-10	5.68E-56	153.6	3.40E-11	2.43E-52	168.6	2.20E-11	1.90E-50
123.7	1.43E-09	5.00E-59	138.7	1.02E-10	6.27E-56	153.7	3.41E-11	2.58E-52	168.7	2.20E-11	1.90E-50
123.8	1.43E-09	5.00E-59	138.8	9.90E-11	6.28E-56	153.8	3.42E-11	2.70E-52	168.8	2.09E-11	1.83E-50
123.9	1.43E-09	5.00E-59	138.9	9.68E-11	7.26E-56	153.9	3.41E-11	2.85E-52	168.9	2.09E-11	1.83E-50
124	1.33E-09	5.09E-59	139	9.72E-11	7.92E-56	154	3.50E-11	3.21E-52	169	2.09E-11	1.83E-50
124.1	1.33E-09	5.46E-59	139.1	9.50E-11	9.33E-56	154.1	3.50E-11	3.21E-52	169.1	1.98E-11	1.81E-50
124.2	1.31E-09	5.71E-59	139.2	9.67E-11	1.02E-55	154.2	3.50E-11	3.21E-52	169.2	1.98E-11	1.81E-50
124.3	1.25E-09	5.78E-59	139.3	9.38E-11	1.18E-55	154.3	3.54E-11	3.60E-52	169.3	1.98E-11	1.81E-50
124.4	1.20E-09	5.83E-59	139.4	9.38E-11	1.18E-55	154.4	3.54E-11	3.60E-52	169.4	2.03E-11	1.94E-50
124.5	1.13E-09	5.76E-59	139.5	9.36E-11	1.30E-55	154.5	3.54E-11	3.54E-52	169.5	2.05E-11	1.91E-50
124.6	1.18E-09	6.51E-59	139.6	8.69E-11	1.53E-55	154.6	3.42E-11	3.59E-52	169.6	1.98E-11	2.07E-50
124.7	1.21E-09	7.17E-59	139.7	8.69E-11	1.53E-55	154.7	3.42E-11	3.59E-52	169.7	1.93E-11	2.09E-50
124.8	1.23E-09	7.78E-59	139.8	8.40E-11	1.48E-55	154.8	3.46E-11	3.92E-52	169.8	1.93E-11	2.09E-50
124.9	1.18E-09	8.01E-59	139.9	8.40E-11	1.48E-55	154.9	3.58E-11	4.54E-52	169.9	1.91E-11	2.13E-50
125	1.18E-09	8.01E-59	140	8.32E-11	1.64E-55	155	3.67E-11	5.15E-52	170	1.82E-11	2.09E-50
125.1	1.18E-09	8.46E-59	140.1	7.96E-11	1.83E-55	155.1	3.54E-11	5.18E-52	170.1	1.84E-11	2.13E-50
125.2	1.12E-09	9.19E-59	140.2	7.62E-11	2.00E-55	155.2	3.54E-11	5.18E-52	170.2	1.76E-11	2.12E-50
125.3	1.10E-09	9.63E-59	140.3	7.62E-11	2.00E-55	155.3	3.54E-11	5.18E-52	170.3	1.76E-11	2.12E-50
125.4	1.10E-09	9.63E-59	140.4	7.62E-11	2.00E-55	155.4	3.54E-11	5.18E-52	170.4	1.76E-11	2.38E-50
125.5	1.09E-09	1.00E-58	140.5	7.65E-11	2.24E-55	155.5	3.54E-11	5.18E-52	170.5	1.76E-11	2.38E-50
125.6	1.06E-09	1.03E-58	140.6	7.52E-11	2.55E-55	155.6	3.62E-11	5.85E-52	170.6	1.72E-11	2.37E-50
125.7	1.07E-09	1.14E-58	140.7	7.50E-11	2.43E-55	155.7	3.62E-11	5.85E-52	170.7	1.72E-11	2.37E-50
125.8	1.03E-09	1.11E-58	140.8	7.50E-11	2.43E-55	155.8	3.62E-11	5.85E-52	170.8	1.71E-11	2.53E-50
125.9	1.00E-09	1.07E-58	140.9	7.34E-11	2.64E-55	155.9	3.77E-11	6.79E-52	170.9	1.71E-11	2.53E-50
126	1.00E-09	1.07E-58	141	7.29E-11	3.19E-55	156	3.89E-11	7.50E-52	171	1.65E-11	2.49E-50

126.1	1.00E-09	1.07E-58	141.1	7.34E-11	3.62E-55	156.1	3.89E-11	7.50E-52	171.1	1.65E-11	2.49E-50
126.2	9.77E-10	1.02E-58	141.2	6.82E-11	3.54E-55	156.2	4.01E-11	7.56E-52	171.2	1.68E-11	2.68E-50
126.3	9.72E-10	1.03E-58	141.3	7.01E-11	4.08E-55	156.3	4.01E-11	7.56E-52	171.3	1.63E-11	2.72E-50
126.4	9.72E-10	1.03E-58	141.4	6.78E-11	4.22E-55	156.4	4.09E-11	7.95E-52	171.4	1.65E-11	2.97E-50
126.5	1.03E-09	1.20E-58	141.5	6.68E-11	5.49E-55	156.5	4.09E-11	7.95E-52	171.5	1.56E-11	2.91E-50
126.6	9.94E-10	1.26E-58	141.6	6.60E-11	5.99E-55	156.6	4.11E-11	8.06E-52	171.6	1.56E-11	2.91E-50
126.7	9.58E-10	1.28E-58	141.7	6.50E-11	6.88E-55	156.7	4.11E-11	8.06E-52	171.7	1.56E-11	2.91E-50
126.8	9.58E-10	1.28E-58	141.8	6.25E-11	6.74E-55	156.8	4.15E-11	8.54E-52	171.8	1.57E-11	3.00E-50
126.9	9.56E-10	1.37E-58	141.9	6.44E-11	7.61E-55	156.9	3.94E-11	8.10E-52	171.9	1.52E-11	3.02E-50
127	9.17E-10	1.32E-58	142	6.44E-11	7.61E-55	157	3.82E-11	8.27E-52	172	1.49E-11	2.89E-50
127.1	8.44E-10	1.48E-58	142.1	6.35E-11	9.28E-55	157.1	3.92E-11	8.84E-52	172.1	1.46E-11	3.11E-50
127.2	7.99E-10	1.41E-58	142.2	6.26E-11	9.51E-55	157.2	4.07E-11	1.00E-51	172.2	1.46E-11	3.11E-50
127.3	7.99E-10	1.41E-58	142.3	6.26E-11	9.51E-55	157.3	4.10E-11	1.04E-51	172.3	1.42E-11	3.13E-50
127.4	7.91E-10	1.50E-58	142.4	6.26E-11	9.51E-55	157.4	4.21E-11	1.19E-51	172.4	1.42E-11	3.13E-50
127.5	7.52E-10	1.53E-58	142.5	6.16E-11	1.13E-54	157.5	4.24E-11	1.24E-51	172.5	1.42E-11	3.13E-50
127.6	7.52E-10	1.53E-58	142.6	6.22E-11	1.25E-54	157.6	4.24E-11	1.24E-51	172.6	1.42E-11	3.13E-50
127.7	7.58E-10	1.66E-58	142.7	6.19E-11	1.62E-54	157.7	4.14E-11	1.30E-51	172.7	1.42E-11	3.13E-50
127.8	7.58E-10	1.66E-58	142.8	5.97E-11	1.65E-54	157.8	4.14E-11	1.30E-51	172.8	1.42E-11	3.01E-50
127.9	7.30E-10	1.66E-58	142.9	6.08E-11	1.90E-54	157.9	4.14E-11	1.30E-51	172.9	1.36E-11	2.98E-50
128	7.14E-10	1.71E-58	143	6.11E-11	2.10E-54	158	4.21E-11	1.45E-51	173	1.34E-11	3.05E-50
128.1	7.14E-10	1.71E-58	143.1	6.00E-11	2.17E-54	158.1	4.25E-11	1.54E-51	173.1	1.28E-11	3.04E-50
128.2	7.14E-10	1.71E-58	143.2	5.89E-11	2.43E-54	158.2	4.32E-11	1.60E-51	173.2	1.27E-11	3.04E-50
128.3	6.62E-10	1.96E-58	143.3	5.77E-11	2.98E-54	158.3	4.32E-11	1.60E-51	173.3	1.22E-11	3.09E-50
128.4	6.28E-10	2.19E-58	143.4	5.77E-11	2.98E-54	158.4	4.32E-11	1.60E-51	173.4	1.20E-11	3.30E-50
128.5	6.29E-10	2.55E-58	143.5	5.78E-11	3.05E-54	158.5	4.32E-11	1.60E-51	173.5	1.20E-11	3.30E-50
128.6	6.17E-10	2.73E-58	143.6	5.85E-11	3.59E-54	158.6	4.33E-11	1.85E-51	173.6	1.08E-11	3.24E-50
128.7	6.12E-10	3.10E-58	143.7	5.79E-11	3.65E-54	158.7	4.44E-11	2.14E-51	173.7	1.08E-11	3.24E-50
128.8	5.75E-10	3.18E-58	143.8	5.71E-11	4.11E-54	158.8	4.34E-11	2.16E-51	173.8	1.08E-11	3.24E-50
128.9	5.75E-10	3.18E-58	143.9	5.81E-11	4.74E-54	158.9	4.44E-11	2.33E-51	173.9	1.07E-11	3.05E-50
129	5.70E-10	3.40E-58	144	5.75E-11	5.73E-54	159	4.24E-11	2.27E-51	174	1.07E-11	3.05E-50
129.1	5.70E-10	3.40E-58	144.1	5.75E-11	5.73E-54	159.1	4.24E-11	2.27E-51	174.1	1.02E-11	3.15E-50
129.2	5.70E-10	3.68E-58	144.2	5.75E-11	5.73E-54	159.2	4.24E-11	2.27E-51	174.2	1.01E-11	3.29E-50
129.3	5.65E-10	3.86E-58	144.3	5.59E-11	7.87E-54	159.3	4.28E-11	2.36E-51	174.3	9.87E-12	3.37E-50
129.4	5.49E-10	3.84E-58	144.4	5.59E-11	7.87E-54	159.4	4.28E-11	2.36E-51	174.4	9.65E-12	3.33E-50
129.5	5.25E-10	4.18E-58	144.5	5.50E-11	8.21E-54	159.5	4.28E-11	2.36E-51	174.5	9.41E-12	3.32E-50
129.6	4.84E-10	4.81E-58	144.6	5.44E-11	8.60E-54	159.6	4.11E-11	2.26E-51	174.6	9.41E-12	3.32E-50
129.7	4.78E-10	5.10E-58	144.7	5.44E-11	8.60E-54	159.7	4.04E-11	2.36E-51	174.7	9.78E-12	3.71E-50
129.8	4.85E-10	5.39E-58	144.8	5.43E-11	1.07E-53	159.8	4.04E-11	2.36E-51	174.8	9.78E-12	3.71E-50
129.9	4.76E-10	5.41E-58	144.9	5.43E-11	1.07E-53	159.9	4.04E-11	2.36E-51	174.9	9.78E-12	3.71E-50
130	4.61E-10	5.37E-58	145	5.47E-11	1.12E-53	160	4.04E-11	2.36E-51	175	9.78E-12	3.71E-50
130.1	4.40E-10	5.52E-58	145.1	5.35E-11	1.14E-53	160.1	4.04E-11	2.36E-51	175.1	9.40E-12	3.71E-50
130.2	4.30E-10	5.86E-58	145.2	5.35E-11	1.14E-53	160.2	4.09E-11	2.50E-51	175.2	9.40E-12	3.71E-50
130.3	4.04E-10	6.43E-58	145.3	5.34E-11	1.28E-53	160.3	4.10E-11	2.82E-51	175.3	8.84E-12	4.00E-50
130.4	3.94E-10	6.79E-58	145.4	5.34E-11	1.28E-53	160.4	4.17E-11	3.13E-51	175.4	8.74E-12	4.15E-50
130.5	3.92E-10	7.72E-58	145.5	4.95E-11	1.30E-53	160.5	4.14E-11	3.27E-51	175.5	8.74E-12	4.15E-50
130.6	3.94E-10	8.41E-58	145.6	4.95E-11	1.30E-53	160.6	3.98E-11	3.14E-51	175.6	8.74E-12	4.15E-50
130.7	3.81E-10	9.94E-58	145.7	5.05E-11	1.39E-53	160.7	3.98E-11	3.14E-51	175.7	8.74E-12	4.15E-50
130.8	3.81E-10	9.94E-58	145.8	4.88E-11	1.42E-53	160.8	3.98E-11	3.14E-51	175.8	8.60E-12	4.29E-50
130.9	3.80E-10	1.08E-57	145.9	4.88E-11	1.42E-53	160.9	3.91E-11	2.84E-51	175.9	8.16E-12	4.73E-50
131	3.71E-10	1.07E-57	146	4.97E-11	1.48E-53	161	3.96E-11	2.98E-51	176	8.20E-12	5.13E-50
131.1	3.73E-10	1.21E-57	146.1	5.06E-11	1.67E-53	161.1	3.96E-11	2.98E-51	176.1	8.20E-12	5.13E-50
131.2	3.73E-10	1.21E-57	146.2	5.06E-11	1.67E-53	161.2	3.78E-11	2.90E-51	176.2	8.20E-12	5.13E-50
131.3	3.63E-10	1.23E-57	146.3	5.14E-11	1.87E-53	161.3	3.75E-11	2.92E-51	176.3	7.98E-12	5.21E-50
131.4	3.56E-10	1.20E-57	146.4	5.14E-11	1.87E-53	161.4	3.52E-11	2.98E-51	176.4	7.98E-12	5.21E-50
131.5	3.56E-10	1.20E-57	146.5	5.14E-11	1.93E-53	161.5	3.52E-11	2.98E-51	176.5	7.80E-12	5.37E-50
131.6	3.18E-10	1.43E-57	146.6	5.14E-11	1.93E-53	161.6	3.57E-11	3.04E-51	176.6	7.83E-12	5.63E-50
131.7	3.13E-10	1.50E-57	146.7	5.16E-11	1.95E-53	161.7	3.60E-11	3.18E-51	176.7	7.97E-12	6.43E-50
131.8	3.05E-10	1.77E-57	146.8	4.97E-11	2.01E-53	161.8	3.59E-11	3.61E-51	176.8	7.97E-12	6.43E-50
131.9	3.05E-10	1.77E-57	146.9	5.09E-11	2.35E-53	161.9	3.59E-11	3.61E-51	176.9	7.91E-12	6.71E-50
132	2.99E-10	2.04E-57	147	5.09E-11	2.35E-53	162	3.59E-11	3.61E-51	177	7.79E-12	6.98E-50
132.1	2.99E-10	2.04E-57	147.1	5.16E-11	2.50E-53	162.1	3.59E-11	3.61E-51	177.1	7.79E-12	6.98E-50
132.2	2.78E-10	2.09E-57	147.2	5.03E-11	2.62E-53	162.2	3.59E-11	3.61E-51	177.2	7.79E-12	6.98E-50
132.3	2.67E-10	2.21E-57	147.3	4.89E-11	2.92E-53	162.3	3.41E-11	4.53E-51	177.3	7.65E-12	6.93E-50
132.4	2.63E-10	2.54E-57	147.4	4.89E-11	2.92E-53	162.4	3.41E-11	4.53E-51	177.4	7.65E-12	6.93E-50
132.5	2.52E-10	2.70E-57	147.5	4.82E-11	3.01E-53	162.5	3.17E-11	4.27E-51	177.5	7.54E-12	7.43E-50
132.6	2.36E-10	2.31E-57	147.6	5.01E-11	3.54E-53	162.6	3.17E-11	4.27E-51	177.6	7.54E-12	7.43E-50
132.7	2.27E-10	2.55E-57	147.7	5.02E-11	3.75E-53	162.7	3.17E-11	4.27E-51	177.7	7.61E-12	7.65E-50
132.8	2.27E-10	2.55E-57	147.8	5.02E-11	3.75E-53	162.8	3.19E-11	4.19E-51	177.8	7.46E-12	7.91E-50
132.9	2.21E-10	2.65E-57	147.9	5.02E-11	3.75E-53	162.9	3.21E-11	5.05E-51	177.9	7.46E-12	7.91E-50
133	2.21E-10	2.69E-57	148	5.02E-11	3.75E-53	163	3.21E-11	5.05E-51	178	6.68E-12	8.52E-50
133.1	2.21E-10	2.69E-57	148.1	4.62E-11	3.79E-53	163.1	3.04E-11	4.86E-51	178.1	6.68E-12	8.52E-50
133.2	2.07E-10	3.01E-57	148.2	4.59E-11	3.96E-53	163.2	3.04E-11	4.86E-51	178.2	6.48E-12	8.73E-50
133.3	2.01E-10	3.14E-57	148.3	4.59E-11	3.96E-53	163.3	3.04E-11	4.86E-51	178.3	6.26E-12	9.36E-50
133.4	1.93E-10	3.45E-57	148.4	4.61E-11	4.66E-53	163.4	2.87E-11	4.66E-51	178.4	6.11E-12	9.61E-50
133.5	1.90E-10	3.50E-57	148.5	4.61E-11	4.66E-53	163.5	2.87E-11	5.25E-51	178.5	6.11E-12	9.61E-50
133.6	1.90E-10	3.50E-57	148.6	4.56E-11	4.82E-53	163.6	2.87E-11	5.25E-51	178.6	5.87E-12	9.73E-50
133.7	1.85E-10	3.78E-57	148.7	4.56E-11	4.82E-53	163.7	2.87E-11	5.25E-51	178.7	5.75E-12	1.01E-49
133.8	1.83E-10	4.06E-57	148.8	4.53E-11	5.32E-53	163.8	2.91E-11	5.62E-51	178.8	5.75E-12	1.01E-49
133.9	1.83E-10	4.06E-57	148.9	4.48E-11	5.14E-53	163.9	2.94E-11	6.24E-51	178.9	5.75E-12	1.01E-49
134	1.80E-10	3.62E-57	149	4.48E-11	5.14E-53	164	2.83E-11	6.97E-51	179	5.75E-12	1.01E-49
134.1	1.80E-10	3.62E-57	149.1	4.59E-11	5.78E-53	164.1	2.83E-11	6.97E-51	179.1	5.60E-12	1.07E-49
134.2	1.85E-10	4.01E-57	149.2	4.62E-11	5.99E-53	164.2	2.83E-11	6.97E-51	179.2	5.43E-12	1.10E-49
134.3	1.81E-10	4.72E-57	149.3	4.62E-11	5.99E-53	164.3	2.83E-11	6.97E-51	179.3	5.41E-12	1.24E-49
134.4	1.73E-10	4.80E-57	149.4	4.47E-11	6.25E-53	164.4	2.83E-11	6.97E-51	179.4	5.29E-12	1.28E-49
134.5	1.71E-10	4.55E-57	149.5	4.33E-11	6.47E-53	164.5	2.76E-11	6.80E-51	179.5	5.29E-12	1.28E-49
134.6	1.65E-10	5.19E-57	149.6	4.50E-11	7.40E-53	164.6	2.72E-11	6.92E-51	179.6	5.09E-12	1.32E-49
134.7	1.65E-10	5.19E-57	149.7	4.53E-11	7.79E-53	164.7	2.75E-11	6.77E-51	179.7	5.09E-12	1.32E-49
134.8	1.65E-10	5.19E-57	149.8	4.54E-11	9.41E-53	164.8	2.75E-11	6.77E-51	179.8	5.04E-12	1.27E-49
134.9	1.63E-10	5.21E-57	149.9	4.54E-11	9.41E-53	164.9	2.75E-11	6.77E-51	179.9	4.83E-12	1.35E-49

180	4.78E-12	1.46E-49	195	2.14E-12	2.10E-47	210	1.69E-12	1.49E-47	225	1.63E-12	3.06E-47
180.1	4.78E-12	1.46E-49	195.1	2.20E-12	2.18E-47	210.1	1.69E-12	1.49E-47	225.1	1.63E-12	3.06E-47
180.2	4.61E-12	1.48E-49	195.2	2.23E-12	2.24E-47	210.2	1.69E-12	1.49E-47	225.2	1.63E-12	3.06E-47
180.3	4.61E-12	1.48E-49	195.3	2.23E-12	2.24E-47	210.3	1.69E-12	1.49E-47	225.3	1.63E-12	3.06E-47
180.4	4.75E-12	1.63E-49	195.4	2.23E-12	2.24E-47	210.4	1.69E-12	1.49E-47	225.4	1.63E-12	3.06E-47
180.5	4.53E-12	1.91E-49	195.5	2.23E-12	2.24E-47	210.5	1.69E-12	1.49E-47	225.5	1.63E-12	3.06E-47
180.6	4.44E-12	1.92E-49	195.6	2.26E-12	2.34E-47	210.6	1.69E-12	1.49E-47	225.6	1.63E-12	3.06E-47
180.7	4.44E-12	1.92E-49	195.7	2.26E-12	2.34E-47	210.7	1.69E-12	1.49E-47	225.7	1.66E-12	3.16E-47
180.8	4.30E-12	2.09E-49	195.8	2.26E-12	2.34E-47	210.8	1.69E-12	1.49E-47	225.8	1.66E-12	3.16E-47
180.9	4.30E-12	2.09E-49	195.9	2.26E-12	2.34E-47	210.9	1.69E-12	1.49E-47	225.9	1.66E-12	3.16E-47
181	4.30E-12	2.09E-49	196	2.26E-12	2.34E-47	211	1.69E-12	1.49E-47	226	1.66E-12	3.16E-47
181.1	4.30E-12	2.09E-49	196.1	2.26E-12	2.34E-47	211.1	1.69E-12	1.49E-47	226.1	1.66E-12	3.16E-47
181.2	4.30E-12	2.09E-49	196.2	2.26E-12	2.34E-47	211.2	1.69E-12	1.49E-47	226.2	1.66E-12	3.16E-47
181.3	4.11E-12	2.16E-49	196.3	2.26E-12	2.34E-47	211.3	1.69E-12	1.49E-47	226.3	1.66E-12	3.16E-47
181.4	4.11E-12	2.16E-49	196.4	2.26E-12	2.34E-47	211.4	1.69E-12	1.49E-47	226.4	1.66E-12	3.16E-47
181.5	4.02E-12	2.23E-49	196.5	2.26E-12	2.34E-47	211.5	1.73E-12	1.52E-47	226.5	1.66E-12	3.16E-47
181.6	3.87E-12	2.53E-49	196.6	2.26E-12	2.34E-47	211.6	1.73E-12	1.52E-47	226.6	1.66E-12	3.16E-47
181.7	3.87E-12	2.53E-49	196.7	2.26E-12	2.34E-47	211.7	1.72E-12	1.56E-47	226.7	1.66E-12	3.16E-47
181.8	3.87E-12	2.53E-49	196.8	2.18E-12	2.12E-47	211.8	1.84E-12	1.74E-47	226.8	1.66E-12	3.16E-47
181.9	3.86E-12	2.72E-49	196.9	2.21E-12	2.11E-47	211.9	1.82E-12	1.69E-47	226.9	1.66E-12	3.16E-47
182	3.49E-12	2.84E-49	197	2.21E-12	2.11E-47	212	1.82E-12	1.69E-47	227	1.66E-12	3.29E-47
182.1	3.49E-12	2.84E-49	197.1	2.16E-12	2.15E-47	212.1	1.82E-12	1.69E-47	227.1	1.61E-12	2.99E-47
182.2	3.49E-12	2.84E-49	197.2	2.16E-12	2.15E-47	212.2	1.82E-12	1.69E-47	227.2	1.61E-12	2.99E-47
182.3	3.51E-12	3.08E-49	197.3	2.14E-12	2.19E-47	212.3	1.82E-12	1.69E-47	227.3	1.61E-12	2.99E-47
182.4	3.42E-12	3.22E-49	197.4	2.14E-12	2.19E-47	212.4	1.82E-12	1.69E-47	227.4	1.61E-12	2.99E-47
182.5	3.36E-12	3.41E-49	197.5	2.14E-12	2.19E-47	212.5	1.82E-12	1.69E-47	227.5	1.61E-12	2.99E-47
182.6	3.29E-12	3.60E-49	197.6	2.09E-12	1.97E-47	212.6	1.82E-12	1.69E-47	227.6	1.61E-12	2.99E-47
182.7	3.20E-12	3.77E-49	197.7	2.09E-12	1.97E-47	212.7	1.82E-12	1.69E-47	227.7	1.61E-12	2.99E-47
182.8	3.20E-12	3.77E-49	197.8	2.09E-12	1.97E-47	212.8	1.82E-12	1.69E-47	227.8	1.61E-12	2.99E-47
182.9	3.25E-12	4.32E-49	197.9	2.09E-12	1.97E-47	212.9	1.82E-12	1.69E-47	227.9	1.61E-12	2.99E-47
183	3.10E-12	4.59E-49	198	2.00E-12	1.92E-47	213	1.77E-12	1.76E-47	228	1.62E-12	2.98E-47
183.1	3.10E-12	4.59E-49	198.1	2.00E-12	1.92E-47	213.1	1.76E-12	1.75E-47	228.1	1.72E-12	3.26E-47

183.2	2.99E-12	4.76E-49
183.3	2.90E-12	5.11E-49
183.4	2.90E-12	5.11E-49
183.5	2.90E-12	5.11E-49
183.6	2.85E-12	5.94E-49
183.7	2.89E-12	6.38E-49
183.8	2.91E-12	6.94E-49
183.9	2.91E-12	6.94E-49
184	3.04E-12	8.03E-49
184.1	2.97E-12	7.34E-49
184.2	2.92E-12	6.93E-49
184.3	2.97E-12	7.55E-49
184.4	2.87E-12	8.01E-49
184.5	2.87E-12	8.65E-49
184.6	2.90E-12	9.51E-49
184.7	2.90E-12	9.51E-49
184.8	2.82E-12	1.01E-48
184.9	2.82E-12	1.09E-48
185	2.82E-12	1.09E-48
185.1	2.83E-12	1.16E-48
185.2	2.82E-12	1.24E-48
185.3	2.82E-12	1.24E-48
185.4	2.82E-12	1.24E-48
185.5	2.62E-12	1.29E-48
185.6	2.63E-12	1.40E-48
185.7	2.53E-12	1.45E-48
185.8	2.55E-12	1.65E-48
185.9	2.55E-12	1.65E-48
186	2.50E-12	1.77E-48
186.1	2.50E-12	1.77E-48
186.2	2.50E-12	1.93E-48
186.3	2.50E-12	1.93E-48
186.4	2.49E-12	2.09E-48
186.5	2.53E-12	2.33E-48
186.6	2.53E-12	2.33E-48
186.7	2.63E-12	2.80E-48
186.8	2.64E-12	2.99E-48
186.9	2.64E-12	3.31E-48
187	2.64E-12	3.31E-48
187.1	2.60E-12	3.64E-48
187.2	2.62E-12	3.99E-48
187.3	2.62E-12	4.43E-48
187.4	2.59E-12	4.80E-48
187.5	2.59E-12	4.80E-48
187.6	2.59E-12	4.80E-48
187.7	2.53E-12	5.20E-48
187.8	2.53E-12	5.20E-48
187.9	2.49E-12	4.94E-48
188	2.63E-12	5.72E-48
188.1	2.56E-12	6.23E-48
188.2	2.56E-12	6.23E-48
188.3	2.51E-12	7.63E-48
188.4	2.51E-12	7.63E-48
188.5	2.54E-12	8.57E-48
188.6	2.48E-12	9.31E-48
188.7	2.48E-12	9.31E-48
188.8	2.41E-12	1.01E-47
188.9	2.39E-12	1.00E-47
189	2.41E-12	1.18E-47
189.1	2.41E-12	1.18E-47
189.2	2.35E-12	1.28E-47
189.3	2.43E-12	1.60E-47
189.4	2.43E-12	1.60E-47
189.5	2.43E-12	1.60E-47
189.6	2.35E-12	1.90E-47
189.7	2.35E-12	1.90E-47
189.8	2.37E-12	1.91E-47
189.9	2.37E-12	1.91E-47
190	2.36E-12	2.14E-47
190.1	2.36E-12	2.14E-47
190.2	2.36E-12	2.14E-47
190.3	2.36E-12	2.14E-47
190.4	2.36E-12	2.14E-47
190.5	2.36E-12	2.14E-47
190.6	2.36E-12	2.14E-47
190.7	2.36E-12	2.14E-47
190.8	2.36E-12	2.14E-47
190.9	2.36E-12	2.14E-47
191	2.36E-12	2.14E-47
191.1	2.35E-12	2.18E-47
191.2	2.35E-12	2.18E-47
191.3	2.31E-12	1.98E-47
191.4	2.31E-12	1.98E-47
191.5	2.31E-12	1.98E-47
191.6	2.31E-12	1.98E-47
191.7	2.31E-12	1.98E-47
191.8	2.28E-12	1.95E-47
191.9	2.33E-12	2.11E-47
192	2.31E-12	1.93E-47
192.1	2.20E-12	1.91E-47
192.2	2.20E-12	1.91E-47
192.3	2.20E-12	1.91E-47
192.4	2.20E-12	1.91E-47
192.5	2.20E-12	1.91E-47
192.6	2.20E-12	1.91E-47
192.7	2.20E-12	1.91E-47
192.8	2.20E-12	1.91E-47
192.9	2.20E-12	1.91E-47
193	2.20E-12	1.91E-47
193.1	2.20E-12	1.91E-47
193.2	2.20E-12	1.91E-47
193.3	2.20E-12	1.91E-47
193.4	2.20E-12	1.91E-47
193.5	2.20E-12	1.91E-47
193.6	2.20E-12	1.91E-47
193.7	2.20E-12	1.91E-47
193.8	2.20E-12	1.91E-47
193.9	2.20E-12	1.91E-47
194	2.20E-12	1.91E-47
194.1	2.20E-12	1.91E-47
194.2	2.18E-12	2.12E-47
194.3	2.16E-12	2.09E-47
194.4	2.16E-12	2.09E-47
194.5	2.16E-12	2.09E-47
194.6	2.16E-12	2.09E-47
194.7	2.16E-12	2.09E-47
194.8	2.16E-12	2.09E-47
194.9	2.14E-12	2.10E-47
198.2	1.96E-12	1.74E-47
198.3	1.96E-12	1.74E-47
198.4	1.98E-12	1.76E-47
198.5	1.98E-12	1.76E-47
198.6	1.98E-12	1.76E-47
198.7	1.98E-12	1.76E-47
198.8	1.99E-12	1.68E-47
198.9	1.94E-12	1.65E-47
199	1.93E-12	1.63E-47
199.1	1.93E-12	1.63E-47
199.2	1.90E-12	1.65E-47
199.3	1.90E-12	1.66E-47
199.4	1.93E-12	1.71E-47
199.5	1.94E-12	1.85E-47
199.6	1.94E-12	1.85E-47
199.7	1.94E-12	1.85E-47
199.8	1.93E-12	1.91E-47
199.9	1.93E-12	1.91E-47
200	1.93E-12	1.91E-47
200.1	1.95E-12	2.00E-47
200.2	1.95E-12	2.00E-47
200.3	1.95E-12	2.00E-47
200.4	1.95E-12	2.00E-47
200.5	1.95E-12	2.00E-47
200.6	1.95E-12	2.00E-47
200.7	1.95E-12	1.96E-47
200.8	1.93E-12	1.84E-47
200.9	1.93E-12	1.84E-47
201	1.93E-12	1.84E-47
201.1	1.93E-12	1.84E-47
201.2	1.90E-12	1.66E-47
201.3	1.90E-12	1.66E-47
201.4	1.90E-12	1.66E-47
201.5	1.90E-12	1.66E-47
201.6	1.91E-12	1.82E-47
201.7	1.91E-12	1.82E-47
201.8	1.90E-12	1.77E-47
201.9	1.90E-12	1.77E-47
202	1.90E-12	1.77E-47
202.1	1.90E-12	1.77E-47
202.2	1.86E-12	1.79E-47
202.3	1.83E-12	1.67E-47
202.4	1.83E-12	1.67E-47
202.5	1.83E-12	1.67E-47
202.6	1.83E-12	1.67E-47
202.7	1.83E-12	1.67E-47
202.8	1.83E-12	1.67E-47
202.9	1.83E-12	1.67E-47
203	1.83E-12	1.67E-47
203.1	1.83E-12	1.67E-47
203.2	1.83E-12	1.67E-47
203.3	1.80E-12	1.66E-47
203.4	1.80E-12	1.66E-47
203.5	1.75E-12	1.48E-47
203.6	1.75E-12	1.48E-47
203.7	1.76E-12	1.48E-47
203.8	1.76E-12	1.48E-47
203.9	1.76E-12	1.48E-47
204	1.76E-12	1.48E-47
204.1	1.76E-12	1.48E-47
204.2	1.76E-12	1.48E-47
204.3	1.76E-12	1.48E-47
204.4	1.76E-12	1.48E-47
204.5	1.76E-12	1.48E-47
204.6	1.76E-12	1.48E-47
204.7	1.76E-12	1.48E-47
204.8	1.76E-12	1.48E-47
204.9	1.77E-12	1.50E-47
205	1.77E-12	1.50E-47
205.1	1.77E-12	1.50E-47
205.2	1.77E-12	1.50E-47
205.3	1.77E-12	1.50E-47
205.4	1.77E-12	1.50E-47
205.5	1.77E-12	1.50E-47
205.6	1.77E-12	1.50E-47
205.7	1.77E-12	1.50E-47
205.8	1.77E-12	1.50E-47
205.9	1.77E-12	1.50E-47
206	1.77E-12	1.50E-47
206.1	1.77E-12	1.50E-47
206.2	1.77E-12	1.50E-47
206.3	1.77E-12	1.50E-47
206.4	1.77E-12	1.50E-47
206.5	1.77E-12	1.50E-47
206.6	1.77E-12	1.50E-47
206.7	1.77E-12	1.50E-47
206.8	1.77E-12	1.50E-47
206.9	1.77E-12	1.50E-47
207	1.77E-12	1.50E-47
207.1	1.74E-12	1.38E-47
207.2	1.71E-12	1.36E-47
207.3	1.66E-12	1.34E-47
207.4	1.66E-12	1.34E-47
207.5	1.66E-12	1.34E-47
207.6	1.70E-12	1.38E-47
207.7	1.70E-12	1.38E-47
207.8	1.70E-12	1.38E-47
207.9	1.70E-12	1.38E-47
208	1.70E-12	1.38E-47
208.1	1.70E-12	1.38E-47
208.2	1.70E-12	1.38E-47
208.3	1.70E-12	1.38E-47
208.4	1.70E-12	1.38E-47
208.5	1.65E-12	1.38E-47
208.6	1.65E-12	1.38E-47
208.7	1.65E-12	1.38E-47
208.8	1.65E-12	1.38E-47
208.9	1.65E-12	1.38E-47
209	1.68E-12	1.41E-47
209.1	1.68E-12	1.41E-47
209.2	1.68E-12	1.41E-47
209.3	1.69E-12	1.49E-47
209.4	1.69E-12	1.49E-47
209.5	1.69E-12	1.49E-47
209.6	1.69E-12	1.49E-47
209.7	1.69E-12	1.49E-47
209.8	1.69E-12	1.49E-47
209.9	1.69E-12	1.49E-47
213.2	1.76E-12	1.75E-47
213.3	1.76E-12	1.75E-47
213.4	1.76E-12	1.75E-47
213.5	1.77E-12	1.84E-47
213.6	1.75E-12	1.79E-47
213.7	1.76E-12	1.84E-47
213.8	1.76E-12	1.84E-47
213.9	1.76E-12	1.84E-47
214	1.76E-12	1.84E-47
214.1	1.76E-12	1.84E-47
214.2	1.76E-12	1.84E-47
214.3	1.79E-12	1.99E-47
214.4	1.79E-12	1.99E-47
214.5	1.79E-12	1.95E-47
214.6	1.79E-12	1.95E-47
214.7	1.79E-12	1.95E-47
214.8	1.79E-12	1.95E-47
214.9	1.79E-12	1.95E-47
215	1.79E-12	1.95E-47
215.1	1.79E-12	1.95E-47
215.2	1.79E-12	1.95E-47
215.3	1.79E-12	1.95E-47
215.4	1.78E-12	1.92E-47
215.5	1.78E-12	1.92E-47
215.6	1.71E-12	1.93E-47
215.7	1.71E-12	1.88E-47
215.8	1.71E-12	1.88E-47
215.9	1.71E-12	1.88E-47
216	1.71E-12	1.88E-47
216.1	1.71E-12	1.88E-47
216.2	1.68E-12	1.99E-47
216.3	1.68E-12	1.99E-47
216.4	1.68E-12	1.99E-47
216.5	1.68E-12	1.99E-47
216.6	1.68E-12	1.99E-47
216.7	1.68E-12	1.99E-47
216.8	1.78E-12	2.16E-47
216.9	1.78E-12	2.16E-47
217	1.78E-12	2.16E-47
217.1	1.78E-12	2.16E-47
217.2	1.78E-12	2.16E-47
217.3	1.76E-12	2.08E-47
217.4	1.73E-12	2.14E-47
217.5	1.73E-12	2.14E-47
217.6	1.73E-12	2.14E-47
217.7	1.73E-12	2.14E-47
217.8	1.71E-12	2.16E-47
217.9	1.72E-12	2.15E-47
218	1.72E-12	2.15E-47
218.1	1.72E-12	2.15E-47
218.2	1.72E-12	2.15E-47
218.3	1.72E-12	2.15E-47
218.4	1.72E-12	2.15E-47
218.5	1.72E-12	2.15E-47
218.6	1.72E-12	2.15E-47
218.7	1.72E-12	2.15E-47
218.8	1.72E-12	2.15E-47
218.9	1.72E-12	2.15E-47
219	1.72E-12	2.15E-47
219.1	1.80E-12	2.39E-47
219.2	1.80E-12	2.39E-47
219.3	1.80E-12	2.39E-47
219.4	1.77E-12	2.28E-47
219.5	1.77E-12	2.28E-47
219.6	1.77E-12	2.28E-47
219.7	1.77E-12	2.28E-47
219.8	1.77E-12	2.28E-47
219.9	1.62E-12	2.28E-47
220	1.62E-12	2.41E-47
220.1	1.62E-12	2.41E-47
220.2	1.62E-12	2.41E-47
220.3	1.62E-12	2.41E-47
220.4	1.62E-12	2.41E-47
220.5	1.62E-12	2.41E-47
220.6	1.62E-12	2.41E-47
220.7	1.62E-12	2.41E-47
220.8	1.60E-12	2.32E-47
220.9	1.62E-12	2.48E-47
221	1.62E-12	2.48E-47
221.1	1.62E-12	2.48E-47
221.2	1.62E-12	2.48E-47
221.3	1.63E-12	2.48E-47
221.4	1.63E-12	2.48E-47
221.5	1.63E-12	2.48E-47
221.6	1.63E-12	2.48E-47
221.7	1.67E-12	2.62E-47
221.8	1.67E-12	2.62E-47
221.9	1.67E-12	2.62E-47
222	1.67E-12	2.62E-47
222.1	1.63E-12	2.52E-47
222.2	1.63E-12	2.52E-47
222.3	1.63E-12	2.52E-47
222.4	1.63E-12	2.52E-47
222.5	1.63E-12	2.52E-47
222.6	1.57E-12	2.52E-47
222.7	1.57E-12	2.52E-47
222.8	1.61E-12	2.52E-47
222.9	1.61E-12	2.52E-47
223	1.61E-12	2.52E-47
223.1	1.61E-12	2.52E-47
223.2	1.61E-12	2.52E-47
223.3	1.60E-12	2.52E-47
223.4	1.62E-12	2.76E-47
223.5	1.65E-12	3.06E-47
223.6	1.65E-12	3.06E-47
223.7	1.66E-12	3.00E-47
223.8	1.66E-12	3.00E-47
223.9	1.66E-12	3.00E-47
224	1.67E-12	3.08E-47
224.1	1.67E-12	3.08E-47
224.2	1.67E-12	3.08E-47
224.3	1.62E-12	3.07E-47
224.4	1.62E-12	3.07E-47
224.5	1.62E-12	3.07E-47
224.6	1.62E-12	3.07E-47
224.7	1.62E-12	3.07E-47
224.8	1.64E-12	3.05E-47
224.9	1.64E-12	3.05E-47
228.2	1.72E-12	3.26E-47
228.3	1.74E-12	3.32E-47
228.4	1.70E-12	3.04E-47
228.5	1.70E-12	3.04E-47
228.6	1.70E-12	3.04E-47
228.7	1.70E-12	3.04E-47
228.8	1.70E-12	3.04E-47
228.9	1.70E-12	3.04E-47
229	1.70E-12	3.04E-47
229.1	1.70E-12	3.04E-47
229.2	1.70E-12	3.04E-47
229.3	1.70E-12	3.04E-47
229.4	1.67E-12	3.14E-47
229.5	1.67E-12	3.14E-47
229.6	1.67E-12	3.14E-47
229.7	1.67E-12	3.14E-47
229.8	1.67E-12	3.14E-47
229.9	1.67E-12	3.14E-47
230	1.67E-12	3.14E-47
230.1	1.67E-12	3.14E-47
230.2	1.67E-12	3.14E-47
230.3	1.67E-12	3.14E-47
230.4	1.67E-12	3.14E-47
230.5	1.67E-12	3.14E-47
230.6	1.67E-12	3.14E-47
230.7	1.67E-12	3.14E-47
230.8	1.62E-12	3.16E-47
230.9	1.62E-12	3.16E-47
231	1.62E-12	3.16E-47
231.1	1.62E-12	3.16E-47
231.2	1.62E-12	3.16E-47
231.3	1.62E-12	3.16E-47
231.4	1.62E-12	3.16E-47
231.5	1.64E-12	3.24E-47
231.6	1.65E-12	3.44E-47
231.7	1.63E-12	3.32E-47
231.8	1.63E-12	3.32E-47
231.9	1.63E-12	3.32E-47
232	1.62E-12	3.30E-47
232.1	1.62E-12	3.30E-47
232.2	1.62E-12	3.30E-47
232.3	1.62E-12	3.30E-47
232.4	1.59E-12	3.31E-47
232.5	1.59E-12	3.31E-47
232.6	1.59E-12	3.31E-47
232.7	1.59E-12	3.31E-47
232.8	1.59E-12	3.31E-47
232.9	1.59E-12	3.31E-47
233	1.59E-12	3.31E-47
233.1	1.59E-12	3.31E-47
233.2	1.59E-12	3.31E-47
233.3	1.59E-12	3.31E-47
233.4	1.59E-12	3.31E-47
233.5	1.59E-12	3.31E-47
233.6	1.59E-12	3.31E-47
233.7	1.59E-12	3.31E-47
233.8	1.53E-12	3.22E-47
233.9	1.53E-12	3.22E-47
234	1.53E-12	3.22E-47
234.1	1.56E-12	3.52E-47
234.2	1.56E-12	3.52E-47
234.3	1.56E-12	3.52E-47
234.4	1.52E-12	3.55E-47
234.5	1.52E-12	3.55E-47
234.6	1.52E-12	3.55E-47
234.7	1.52E-12	3.55E-47
234.8	1.48E-12	3.59E-47
234.9	1.48E-12	3.59E-47
235	1.48E-12	3.59E-47
235.1	1.48E-12	3.59E-47
235.2	1.48E-12	3.59E-47
235.3	1.48E-12	3.59E-47
235.4	1.48E-12	3.59E-47
235.5	1.48E-12	3.59E-47
235.6	1.48E-12	3.59E-47
235.7	1.46E-12	3.63E-47
235.8	1.46E-12	3.63E-47
235.9	1.46E-12	3.63E-47
236	1.43E-12	3.64E-47
236.1	1.43E-12	3.64E-47
236.2	1.43E-12	3.64E-47
236.3	1.43E-12	3.64E-47
236.4	1.43E-12	3.64E-47
236.5	1.43E-12	3.64E-47
236.6	1.43E-12	3.64E-47
236.7	1.43E-12	3.64E-47
236.8	1.43E-12	3.64E-47
236.9	1.43E-12	3.64E-47
237	1.38E-12	3.49E-47
237.1	1.38E-12	3.49E-47
237.2	1.38E-12	3.49E-47
237.3	1.38E-12	3.49E-47
237.4	1.36E-12	3.44E-47
237.5	1.36E-12	3.44E-47
237.6	1.36E-12	3.44E-47
237.7	1.36E-12	3.44E-47
237.8	1.37E-12	3.40E-47
237.9	1.37E-12	3.40E-47
238	1.37E-12	3.40E-47
238.1	1.37E-12	3.40E-47
238.2	1.37E-12	3.40E-47
238.3	1.38E-12	3.47E-47
238.4	1.38E-12	3.47E-47
238.5	1.38E-12	3.42E-47
238.6	1.38E-12	3.42E-47
238.7	1.40E-12	3.48E-47
238.8	1.40E-12	3.48E-47
238.9	1.40E-12	3.48E-47
239	1.40E-12	3.48E-47
239.1	1.40E-12	3.48E-47
239.2	1.40E-12	3.48E-47
239.3	1.40E-12	3.48E-47
239.4	1.40E-12	3.48E-47
239.5	1.38E-12	3.44E-47
239.6	1.38E-12	3.44E-47
239.7	1.38E-12	3.44E-47
239.8	1.44E-12	3.48E-47
239.9	1.44E-12	3.48E-47
240	1.41E-12	3.42E-47

Table S8: PRCC analysis data for [LACT] with [G](0)=2 mM: PRCC sensitivity indices and their corresponding p-values

Sensitivity analysis data from PRCC for [LACT] with [G](0)=2 mM (only the parameters to which [LACT]'s level is sensitive to appear)

Time discretization	L_E	[LACT]_0	Time discretization	L_E	[LACT]_0	Time discretization	L_E	[LACT]_0	Time discretization	L_E	[LACT]_0
0	0	1	15	0.27910113	0.9566633	30	0.3327426	0.92828007	45	0.40172353	0.88525255
0.1	0.35885275	0.99530055	15.1	0.27984464	0.9565653	30.1	0.33287573	0.92819922	45.1	0.40272407	0.88519457
0.2	0.32781431	0.98747498	15.2	0.27935348	0.95653173	30.2	0.33277572	0.92808666	45.2	0.40320103	0.88512001
0.3	0.31180973	0.98260605	15.3	0.28071533	0.95649185	30.3	0.33206511	0.92785367	45.3	0.40275586	0.88486022
0.4	0.30401377	0.97965127	15.4	0.28057867	0.9562992	30.4	0.33183875	0.92762631	45.4	0.40280879	0.88477497
0.5	0.30289145	0.97863813	15.5	0.28290232	0.95623061	30.5	0.33352925	0.92745473	45.5	0.40317109	0.88468938
0.6	0.29826456	0.97734045	15.6	0.28271815	0.95620387	30.6	0.33514223	0.92740672	45.6	0.40365836	0.88443641
0.7	0.29532129	0.97669268	15.7	0.28344858	0.95620648	30.7	0.33695369	0.92713713	45.7	0.40373104	0.88410719
0.8	0.29046864	0.97622302	15.8	0.28592302	0.95607405	30.8	0.33904974	0.92656957	45.8	0.40376218	0.88398762
0.9	0.28808942	0.9756646	15.9	0.28443906	0.95590603	30.9	0.33986484	0.92596653	45.9	0.40474523	0.8840711
1	0.28658068	0.97521818	16	0.28531793	0.95583549	31	0.34100344	0.92561467	46	0.40476298	0.88397342
1.1	0.28646545	0.9750018	16.1	0.2866845	0.9558658	31.1	0.34132352	0.92530723	46.1	0.40515169	0.88387902
1.2	0.28168228	0.9747368	16.2	0.28840399	0.95565963	31.2	0.34115843	0.9250555	46.2	0.40556394	0.88381036
1.3	0.27962314	0.97440863	16.3	0.28914251	0.95544672	31.3	0.3431874	0.92482108	46.3	0.40572541	0.88378241
1.4	0.2766894	0.97402614	16.4	0.28839766	0.95518726	31.4	0.34447443	0.92450059	46.4	0.40640727	0.88342804
1.5	0.27752932	0.97390234	16.5	0.28808158	0.95511638	31.5	0.34344843	0.92405215	46.5	0.40688761	0.88349896
1.6	0.27689464	0.9736838	16.6	0.28849343	0.95489787	31.6	0.34276941	0.9238008	46.6	0.40699583	0.88339574
1.7	0.27688827	0.97343091	16.7	0.28862197	0.9548066	31.7	0.34278609	0.9237546	46.7	0.40736613	0.88339017
1.8	0.27633651	0.97319855	16.8	0.28794688	0.95476336	31.8	0.34267855	0.92365204	46.8	0.40788594	0.88323809
1.9	0.27446932	0.97304432	16.9	0.28942027	0.95470395	31.9	0.34316226	0.92346534	46.9	0.40740389	0.88286113
2	0.27006163	0.97285278	17	0.2882014	0.95444173	32	0.34296942	0.92309547	47	0.40790395	0.88275901
2.1	0.26812261	0.97281316	17.1	0.28836881	0.95409014	32.1	0.34216882	0.92277629	47.1	0.40776437	0.88247278
2.2	0.265511	0.97248147	17.2	0.28832046	0.95394831	32.2	0.34213625	0.92249262	47.2	0.40773235	0.88244569
2.3	0.26663337	0.97238699	17.3	0.28861458	0.95382083	32.3	0.3400266	0.92212353	47.3	0.40848122	0.88245573
2.4	0.26697079	0.97235545	17.4	0.2867328	0.95334486	32.4	0.34051751	0.92159726	47.4	0.4101774	0.88213972
2.5	0.26812836	0.97227737	17.5	0.2877117	0.95323691	32.5	0.34063173	0.92145338	47.5	0.41111391	0.88202582
2.6	0.26807176	0.97223386	17.6	0.28614915	0.9529904	32.6	0.33996385	0.92081422	47.6	0.41096081	0.88176706
2.7	0.27112289	0.97216527	17.7	0.28474787	0.95266947	32.7	0.3397811	0.92046395	47.7	0.41210723	0.88177019
2.8	0.2691376	0.97207322	17.8	0.28660235	0.95239235	32.8	0.34025968	0.9202512	47.8	0.41302618	0.88161866
2.9	0.26962206	0.97192977	17.9	0.28687032	0.95216675	32.9	0.33970464	0.9194638	47.9	0.41436056	0.88160656
3	0.26974664	0.97196425	18	0.28666424	0.95198244	33	0.34090093	0.91901371	48	0.41469727	0.88148522
3.1	0.27117777	0.97194477	18.1	0.28705087	0.95173608	33.1	0.34186411	0.91858364	48.1	0.41484599	0.8814282
3.2	0.26977639	0.97190052	18.2	0.28496717	0.95119174	33.2	0.341527	0.9179365	48.2	0.41438883	0.88122604
3.3	0.26953821	0.97187805	18.3	0.28501225	0.95084349	33.3	0.34148862	0.91772497	48.3	0.4150024	0.88094998
3.4	0.27018234	0.97182639	18.4	0.28302825	0.95042039	33.4	0.34181147	0.91717969	48.4	0.41484246	0.8807593
3.5	0.27007328	0.97176037	18.5	0.28220378	0.95029949	33.5	0.34144127	0.91671244	48.5	0.41466075	0.88064063
3.6	0.27338777	0.97165029	18.6	0.28387667	0.95021925	33.6	0.34183049	0.91633736	48.6	0.41515402	0.88036348
3.7	0.27338777	0.97165029	18.7	0.28482046	0.95011065	33.7	0.34268695	0.91594498	48.7	0.41435766	0.88012459
3.8	0.27406102	0.97163841	18.8	0.28485314	0.95002221	33.8	0.3423508	0.91571385	48.8	0.41522702	0.88006199
3.9	0.27533267	0.97159431	18.9	0.28448756	0.94972792	33.9	0.3445633	0.9155476	48.9	0.41595239	0.87996898
4	0.2745025	0.97151767	19	0.28363459	0.94930727	34	0.34565031	0.91505916	49	0.41652666	0.879924
4.1	0.27669707	0.97141693	19.1	0.28228787	0.94910656	34.1	0.34684799	0.91463859	49.1	0.41697178	0.87988221
4.2	0.27788873	0.97129839	19.2	0.28304988	0.94903198	34.2	0.34724891	0.91423274	49.2	0.41720301	0.87958809
4.3	0.2776353	0.97126802	19.3	0.28158743	0.94885983	34.3	0.34710099	0.91376096	49.3	0.41796046	0.87946129
4.4	0.27780932	0.97122128	19.4	0.28110043	0.94867055	34.4	0.34841734	0.91331725	49.4	0.4184266	0.87949846
4.5	0.27818155	0.97109614	19.5	0.28109432	0.94836159	34.5	0.34868759	0.91301776	49.5	0.41874018	0.8789294
4.6	0.27874451	0.9709436	19.6	0.27958203	0.94802197	34.6	0.34823177	0.91268582	49.6	0.41900418	0.87889269
4.7	0.27816775	0.97092728	19.7	0.28036119	0.94790852	34.7	0.34748255	0.91247238	49.7	0.4198836	0.87863196
4.8	0.27827507	0.97078943	19.8	0.28042357	0.94749184	34.8	0.34880511	0.91229988	49.8	0.42097486	0.87852564
4.9	0.27748867	0.97073886	19.9	0.28322976	0.94765198	34.9	0.34997312	0.91219204	49.9	0.4208349	0.87822472
5	0.27751135	0.97057454	20	0.28359498	0.94766738	35	0.35074099	0.91196229	50	0.42226273	0.87816765
5.1	0.27650334	0.97052693	20.1	0.28290734	0.94757369	35.1	0.3536122	0.91156937	50.1	0.42286459	0.87788861
5.2	0.27952391	0.97051059	20.2	0.28436084	0.94747854	35.2	0.35498434	0.9109913	50.2	0.4230642	0.87764335
5.3	0.27787449	0.97027825	20.3	0.28507433	0.94732847	35.3	0.35478362	0.91068097	50.3	0.42309925	0.87764693
5.4	0.27805861	0.97024459	20.4	0.28536975	0.94706286	35.4	0.35502693	0.91004943	50.4	0.42354786	0.87739391
5.5	0.27728898	0.9701115	20.5	0.28565912	0.94689294	35.5	0.35511042	0.90967036	50.5	0.42350327	0.87732644
5.6	0.27706326	0.97006462	20.6	0.28619853	0.94689915	35.6	0.35541167	0.90910448	50.6	0.42442686	0.87730275
5.7	0.2776897	0.97002758	20.7	0.28532325	0.94674989	35.7	0.35526556	0.90882485	50.7	0.42452302	0.87702813
5.8	0.27783958	0.96998521	20.8	0.28459221	0.94650404	35.8	0.35515029	0.90789005	50.8	0.42499327	0.87710849
5.9	0.28091622	0.96985167	20.9	0.28524399	0.94640964	35.9	0.35691333	0.90759597	50.9	0.4253769	0.87667521
6	0.28230433	0.96982619	21	0.28635301	0.94606411	36	0.35653503	0.90693619	51	0.42594354	0.87652703
6.1	0.28409311	0.96977753	21.1	0.28786185	0.94564766	36.1	0.35636686	0.90669649	51.1	0.42639634	0.87623209
6.2	0.28383087	0.96970812	21.2	0.28873904	0.9454501	36.2	0.35621165	0.90642567	51.2	0.42638049	0.87609049
6.3	0.28265744	0.96964447	21.3	0.28975706	0.94525544	36.3	0.35653304	0.90602837	51.3	0.4259089	0.87580621
6.4	0.28241112	0.96960769	21.4	0.28978817	0.94514933	36.4	0.35693281	0.9058653	51.4	0.42583265	0.87558503
6.5	0.28311866	0.96949278	21.5	0.29227046	0.9449386	36.5	0.35646127	0.90538197	51.5	0.42575329	0.87541053
6.6	0.28488114	0.96931882	21.6	0.29275017	0.9449018	36.6	0.35581776	0.90459106	51.6	0.42562002	0.87533681
6.7	0.28573416	0.96913784	21.7	0.29226762	0.94465855	36.7	0.35784174	0.90401315	51.7	0.42540012	0.87507497
6.8	0.28561237	0.96882633	21.8	0.29256233	0.94454582	36.8	0.35736417	0.9036782	51.8	0.42517831	0.87490363
6.9	0.28655334	0.96853704	21.9	0.29371448	0.94414853	36.9	0.35787594	0.90312818	51.9	0.42566529	0.87459521
7	0.28640957	0.9684504	22	0.29421966	0.94372429	37	0.3576055	0.90300055	52	0.42627332	0.87453919
7.1	0.28491426	0.96834442	22.1	0.29455349	0.9437124	37.1	0.35841944	0.90232895	52.1	0.42717096	0.87457155
7.2	0.28392077	0.96814839	22.2	0.29539903	0.94365493	37.2	0.35912651	0.90212651	52.2	0.42686457	0.87439282
7.3	0.28506081	0.9679104	22.3	0.29799679	0.94361817	37.3	0.3577566	0.90175831	52.3	0.42708473	0.87420143
7.4	0.28364265	0.96768067	22.4	0.29632992	0.94339011	37.4	0.35827503	0.90132552	52.4	0.42704611	0.87402472
7.5	0.28497975	0.96768881	22.5	0.29538164	0.94306247	37.5	0.35895909	0.90073746	52.5	0.42713515	0.87379721
7.6	0.28523373	0.96743406	22.6	0.29492285	0.9427284	37.6	0.3585277	0.90037389	52.6	0.42897261	0.87370396
7.7	0.28623199	0.96719156	22.7	0.29496257	0.94259726	37.7	0.3581037	0.90007284	52.7	0.42932714	0.87360821
7.8	0.28738689	0.96717824	22.8	0.29392878	0.94235646	37.8	0.3593992	0.90008475	52.8	0.42902539	0.87351531
7.9	0.28876566	0.96711996	22.9	0.29279233	0.94218341	37.9	0.36021851	0.90012846	52.9	0.4294316	0.87354429
8	0.28767464	0.96694896	23	0.29289755	0.94199743	38	0.36206831	0.90012066	53	0.42968187	0.87348053
8.1	0.28798598	0.96686561	23.1	0.29354972	0.94175808	38.1	0.36213985	0.90003048	53.1	0.42918247	0.87332182
8.2	0.28568175	0.96676153	23.2	0.29406686	0.94159683	38.2	0.36316024	0.89995246	53.2	0.42956993	0.87323075
8.3	0.28447448	0.96655747	23.3	0.2955408	0.94137579	38.3	0.36473831	0.89969257	53.3	0.4295008	0.87292166
8.4	0.28487722	0.96649583	23.4	0.29696148	0.94123125	38.4	0.36464932	0.89930408	53.4	0.43090807	0.87268629
8.5	0.28517602	0.96640589	23.5	0.29762362	0.94119047	38.5	0.3650117	0.89899462	53.5	0.43090393	0.87243015
8.6	0.28444292	0.96623796	23.6	0.29780521	0.94112252	38.6	0.36613142	0.89896774	53.6	0.43084092	0.8719486
8.7	0.28314024	0.96614608	23.7	0.29834858	0.94099912	38.7	0.36728996	0.89907865	53.7	0.43075357	0.87169782
8.8	0.28423776	0.96585758	23.8	0.29907533	0.94089416	38.8	0.36785682	0.89889673	53.8	0.43047664	0.87146636
8.9	0.28221819	0.96562975	23.9	0.29942252	0.94079349	38.9	0.36793505	0.89860774	53.9	0.43125858	0.87126795
9	0.28385858	0.96554971	24	0.29932581	0.94069681	39	0.36908469	0.89844945	54	0.43081486	0.87091958
9.1	0.28532072	0.96552126	24.1	0.29916991	0.94063646	39.1	0.36968977	0.89804903	54.1	0.43095535	0.87056048
9.2	0.28500844	0.96542268	24.2	0.30000445	0.94055524	39.2	0.37206296	0.89813917	54.2	0.43083941	0.87045671
9.3	0.28452164	0.96533453	24.3	0.30095938	0.94045214	39.3	0.37264659	0.89792203	54.3	0.42988183	0.86972009
9.4	0.28428706	0.96518856	24.4	0.30092118	0.94036257	39.4	0.37277547	0.89774177	54.4	0.42984081	0.86943291
9.5	0.2859094	0.96492987	24.5	0.29951715	0.94019574	39.5	0.37293473	0.89771266	54.5	0.42984543	0.86928609
9.6	0.28712171	0.96491858	24.6	0.3004053	0.94001268	39.6	0.37361559	0.89734065	54.6	0.43005011	0.86903143
9.7	0.28799796	0.96480546	24.7	0.30144047	0.939994	39.7	0.37465512	0.89716662	54.7	0.43034337	0.86898343
9.8	0.28906488	0.96471436	24.8	0.30139537	0.93990656	39.8	0.37557093	0.89669786	54.8	0.43038151	0.86874425
9.9	0.288804	0.96468461	24.9	0.30280529	0.93952327	39.9	0.37647866	0.89667494	54.9	0.43100377	0.86866516
10	0.29092203	0.96458702	25	0.30301152	0.93908174	40	0.3763956	0.89663496	55	0.43096874	0.86850486
10.1	0.29149947	0.9643889	25.1	0.30425774	0.93881247	40.1	0.37777619	0.89653834	55.1	0.43081201	0.86818883
10.2	0.29058964	0.96411789	25.2	0.30407442	0.93882872	40.2	0.37872078	0.89621359	55.2	0.43100033	0.86812289
10.3	0.28975435	0.96395702	25.3	0.30569787	0.93877313	40.3	0.3801797	0.89589011	55.3	0.43083305	0.8678467
10.4	0.29081182	0.96391692	25.4	0.30756908	0.93873056	40.4	0.38041578	0.89586925	55.4	0.43084053	0.86755193
10.5	0.28939215	0.96366086	25.5	0.30894839	0.93871321	40.5	0.37998663	0.895681	55.5	0.43132122	0.86744763
10.6	0.29277965	0.96362989	25.6	0.30851357	0.93849482	40.6	0.37998607	0.89545398	55.6	0.43172239	0.86703701
10.7	0.29216859	0.96353628	25.7	0.30741274	0.93816433	40.7	0.38075895	0.89510712	55.7	0.43156499	0.86675992
10.8	0.29073069	0.96346662	25.8	0.30769296	0.9381432	40.8	0.38098949	0.89483373	55.8	0.43172439	0.86674869
10.9	0.29001371	0.96317426	25.9	0.3083971	0.9379583	40.9	0.38136116	0.89466462	55.9	0.43212284	0.86646365
11	0.29145508	0.96295866	26	0.30784315	0.93758112	41	0.38218685	0.89449729	56	0.43230156	0.86613349
11.1	0.29103235	0.96263723	26.1	0.30776539	0.93737191	41.1	0.38200998	0.89435216	56.1	0.43282418	0.86593428
11.2	0.29317029	0.96264801	26.2	0.3088498	0.93686389	41.2	0.3838429	0.89419716	56.2	0.43305624	0.86581183
11.3	0.29342254	0.9626232	26.3	0.30854043	0.93676769	41.3	0.38414486	0.89378861	56.3	0.43421781	0.86560192
11.4	0.29263689	0.96225339	26.4	0.30797446	0.93657415	41.4	0.38419012	0.89367433	56.4	0.43393034	0.86528863
11.5	0.2917621	0.962132	26.5	0.3089447	0.93649555	41.5	0.38413616	0.89356582	56.5	0.43380849	0.86477247
11.6	0.29190873	0.96215169	26.6	0.31036858	0.93647349	41.6	0.38399174	0.89300269	56.6	0.43335934	0.86447405
11.7	0.2925338	0.96199667	26.7	0.31065158	0.93613664	41.7	0.38501482	0.89277858	56.7	0.43384774	0.86422012
11.8	0.29231169	0.96190239	26.8	0.31121388	0.93588013	41.8	0.38564925	0.89235387	56.8	0.43454167	0.86400777
11.9	0.29310204	0.96170443	26.9	0.31173636	0.93563881	41.9	0.38575026	0.89223556	56.9	0.4341606	0.86369775

12	0.29146547	0.96150202
12.1	0.29030131	0.96136941
12.2	0.2867006	0.96095661
12.3	0.28628313	0.9607768
12.4	0.2863951	0.96049943
12.5	0.28595842	0.96032572
12.6	0.28602279	0.9603096
12.7	0.28624495	0.95993002
12.8	0.28455707	0.95972979
12.9	0.28324496	0.95955493
13	0.28287309	0.95948241
13.1	0.28406666	0.9592474
13.2	0.28381642	0.95905812
13.3	0.2819947	0.95887742
13.4	0.28080478	0.95883428
13.5	0.28087253	0.95867924
13.6	0.28290369	0.95831571
13.7	0.28320112	0.95833992
13.8	0.28253997	0.95811606
13.9	0.28142188	0.95794188
14	0.28100131	0.95784717
14.1	0.28078908	0.95765835
14.2	0.28045384	0.95752221
14.3	0.27920554	0.95739694
14.4	0.28025163	0.95731562
14.5	0.2813448	0.95731335
14.6	0.2807653	0.95720274
14.7	0.2796724	0.95703454
14.8	0.27774183	0.95692308
14.9	0.2782418	0.95672452

27	0.31095896	0.93546014
27.1	0.31261092	0.93539621
27.2	0.31272881	0.93534479
27.3	0.31419158	0.93518325
27.4	0.31336496	0.93499983
27.5	0.31418076	0.93472854
27.6	0.31351748	0.93410634
27.7	0.31382137	0.93372476
27.8	0.3137681	0.93344644
27.9	0.31523919	0.93305006
28	0.31444082	0.93280672
28.1	0.31495458	0.93245529
28.2	0.31524746	0.93227698
28.3	0.31520184	0.93197413
28.4	0.31492504	0.93183988
28.5	0.31629517	0.93107689
28.6	0.31760239	0.9309106
28.7	0.3179599	0.93060282
28.8	0.31848413	0.93043365
28.9	0.32038527	0.93027664
29	0.32178947	0.93028104
29.1	0.3231967	0.93010415
29.2	0.32528491	0.92976908
29.3	0.32499776	0.92951621
29.4	0.32679933	0.92928767
29.5	0.32731743	0.92905322
29.6	0.32780447	0.9289306
29.7	0.32815252	0.92866851
29.8	0.3302602	0.92849232
29.9	0.33208456	0.92850528

42	0.38683266	0.89209196
42.1	0.3887039	0.89183043
42.2	0.38883563	0.89141058
42.3	0.38961853	0.89115556
42.4	0.38999844	0.89084204
42.5	0.39016314	0.89064777
42.6	0.39182097	0.89037925
42.7	0.39243159	0.89015678
42.8	0.39278085	0.88967825
42.9	0.3937441	0.88957623
43	0.39404584	0.88932315
43.1	0.39356481	0.88891396
43.2	0.39419211	0.88885022
43.3	0.39493479	0.88870832
43.4	0.39519228	0.88843306
43.5	0.39543397	0.88804325
43.6	0.39613904	0.88767706
43.7	0.39652183	0.88719787
43.8	0.39705193	0.88704667
43.9	0.39760963	0.88676174
44	0.39809784	0.88638623
44.1	0.39872792	0.8860497
44.2	0.3988438	0.88600084
44.3	0.39865324	0.8857076
44.4	0.39893389	0.88566382
44.5	0.39946567	0.88567063
44.6	0.40007652	0.88563598
44.7	0.40003035	0.88564499
44.8	0.40033626	0.88551599
44.9	0.40037545	0.88527137

57	0.43437274	0.86363515
57.1	0.43388375	0.86350372
57.2	0.43453042	0.86331586
57.3	0.43487794	0.8631
57.4	0.43539246	0.86304415
57.5	0.43568936	0.86277793
57.6	0.43563682	0.86256866
57.7	0.43635808	0.86221865
57.8	0.4360921	0.86203658
57.9	0.43660407	0.86184753
58	0.43825612	0.86195175
58.1	0.43847589	0.86188189
58.2	0.43885043	0.86175061
58.3	0.43941904	0.86139774
58.4	0.4394489	0.86123371
58.5	0.43900542	0.86092298
58.6	0.43925626	0.86051596
58.7	0.43985463	0.86025427
58.8	0.43979921	0.86004637
58.9	0.4392389	0.85953875
59	0.43936651	0.85948799
59.1	0.43947399	0.85938783
59.2	0.43957756	0.85923698
59.3	0.43988814	0.85906241
59.4	0.43981137	0.85885841
59.5	0.44010328	0.85879352
59.6	0.44135908	0.8585104
59.7	0.44153168	0.85837072
59.8	0.44164437	0.8583831
59.9	0.44227147	0.85832406

60	0.44309078	0.85785629
60.1	0.44292431	0.85758231
60.2	0.44262671	0.85732449
60.3	0.44303969	0.85714723
60.4	0.44234722	0.8567385
60.5	0.44304993	0.85665439
60.6	0.44354893	0.85649109
60.7	0.44319718	0.85614844
60.8	0.44250415	0.85561088
60.9	0.44253616	0.85522679
61	0.44253222	0.85471173
61.1	0.44307334	0.85458962
61.2	0.44318962	0.85415654
61.3	0.44347198	0.85403168
61.4	0.44395215	0.85386883
61.5	0.44365657	0.85363477
61.6	0.44408976	0.85353414
61.7	0.44560406	0.85317346
61.8	0.44553011	0.85279717
61.9	0.44647896	0.85265804
62	0.44608513	0.85222152
62.1	0.44750414	0.85201718
62.2	0.44715588	0.85153392
62.3	0.44746163	0.85127736
62.4	0.44796712	0.85064764
62.5	0.44798873	0.85015867
62.6	0.44770374	0.84931983
62.7	0.44798432	0.84854322
62.8	0.44816232	0.84763195
62.9	0.44856076	0.84726434
63	0.4490813	0.84666025
63.1	0.44981389	0.84638857
63.2	0.45060942	0.84630266
63.3	0.45161333	0.84615345
63.4	0.45218065	0.84556825
63.5	0.45265061	0.84540681
63.6	0.45373706	0.84529725
63.7	0.45411265	0.84523699
63.8	0.45440564	0.8449712
63.9	0.4543585	0.84470866
64	0.45531863	0.84423903
64.1	0.45664462	0.84353458
64.2	0.45686273	0.84348864
64.3	0.45746185	0.84344602
64.4	0.45830206	0.84314896
64.5	0.45907496	0.84313428
64.6	0.45950802	0.84305356
64.7	0.45980426	0.84245676
64.8	0.45989342	0.84216097
64.9	0.46050483	0.84169708
65	0.46048957	0.84135997
65.1	0.46102014	0.8412914
65.2	0.46234122	0.84129666
65.3	0.46349083	0.84101134
65.4	0.46379077	0.84095873
65.5	0.46431817	0.84096857
65.6	0.46465564	0.84095495
65.7	0.46557009	0.84035427
65.8	0.46626599	0.83987823
65.9	0.46688372	0.8394454
66	0.46726505	0.83903409
66.1	0.46810586	0.83902165
66.2	0.46806311	0.83871422
66.3	0.4684355	0.83811451
66.4	0.46879533	0.83760324
66.5	0.4708158	0.83739961
66.6	0.47144968	0.83735397
66.7	0.47152472	0.83707439
66.8	0.47242763	0.83690365
66.9	0.47348376	0.836641
67	0.47424698	0.83647962
67.1	0.47449721	0.83628494
67.2	0.47517862	0.83628692
67.3	0.47527212	0.83623849
67.4	0.47482901	0.8360127
67.5	0.47522998	0.83574776
67.6	0.47586492	0.8357289
67.7	0.47747844	0.83554338
67.8	0.47773084	0.8353829
67.9	0.47738409	0.83514705
68	0.4772398	0.83502716
68.1	0.47798972	0.8346173
68.2	0.47890794	0.83446924
68.3	0.47906286	0.83384689
68.4	0.47945624	0.83362373
68.5	0.47930193	0.83323899
68.6	0.47955023	0.83308114
68.7	0.48037657	0.83250135
68.8	0.48056561	0.83185623
68.9	0.48082042	0.83164207
69	0.48109943	0.83152002
69.1	0.48131023	0.83113857

75	0.49276937	0.81014435
75.1	0.49322649	0.80979851
75.2	0.49341126	0.80952889
75.3	0.49364022	0.80877973
75.4	0.49381227	0.80855514
75.5	0.49381227	0.80855514
75.6	0.49405268	0.80813058
75.7	0.49434578	0.80793056
75.8	0.49429865	0.807533
75.9	0.49492375	0.80743832
76	0.49475062	0.80689853
76.1	0.49489473	0.80676059
76.2	0.49503054	0.80653455
76.3	0.49487485	0.80637785
76.4	0.49500711	0.80600436
76.5	0.49544754	0.80565766
76.6	0.49595764	0.80550372
76.7	0.49653992	0.80499993
76.8	0.49655535	0.80465009
76.9	0.49641776	0.80400306
77	0.4964773	0.80374878
77.1	0.49639328	0.80362145
77.2	0.49639328	0.80362145
77.3	0.49688956	0.80343603
77.4	0.49691033	0.803384
77.5	0.49697974	0.80319233
77.6	0.49753374	0.80293889
77.7	0.49804317	0.80279415
77.8	0.49824823	0.80222858
77.9	0.49831758	0.80185593
78	0.49797147	0.80135109
78.1	0.49765237	0.80105061
78.2	0.49740011	0.80034824
78.3	0.49785471	0.79995058
78.4	0.49841567	0.79992105
78.5	0.49842615	0.79936017
78.6	0.4984565	0.79885597
78.7	0.49869127	0.79864457
78.8	0.49882357	0.7980902
78.9	0.49844785	0.79791183
79	0.49907319	0.79771758
79.1	0.49865517	0.7970899
79.2	0.4987676	0.79634356
79.3	0.49845226	0.79553981
79.4	0.4983983	0.79443959
79.5	0.49853465	0.79393321
79.6	0.49860139	0.79372512
79.7	0.49831821	0.79269429
79.8	0.49801152	0.79208567
79.9	0.49763158	0.79128979
80	0.49763158	0.79128979
80.1	0.49708136	0.79013642
80.2	0.49699695	0.78926412
80.3	0.49750235	0.78896912
80.4	0.49709893	0.78867981
80.5	0.4966283	0.7878055
80.6	0.49690518	0.78752687
80.7	0.49658726	0.78695739
80.8	0.4968502	0.78700624
80.9	0.49703543	0.78681266
81	0.49707444	0.78670258
81.1	0.49722644	0.78664415
81.2	0.49693829	0.78600567
81.3	0.49636184	0.78515493
81.4	0.49634312	0.78437021
81.5	0.49617529	0.78393158
81.6	0.49618609	0.7836436
81.7	0.49547488	0.78270927
81.8	0.49522128	0.78236991
81.9	0.49481517	0.78153589
82	0.49516198	0.78072404
82.1	0.49530206	0.78031885
82.2	0.49561223	0.77980719
82.3	0.49546202	0.77959388
82.4	0.49574602	0.7791335
82.5	0.49561795	0.77867744
82.6	0.4959162	0.77841396
82.7	0.49603117	0.77799722
82.8	0.49644955	0.77769271
82.9	0.49678499	0.77741679
83	0.49697851	0.77727765
83.1	0.49736342	0.77722757
83.2	0.49740579	0.77699747
83.3	0.49798049	0.77709863
83.4	0.49795939	0.77662768
83.5	0.49800978	0.77587931
83.6	0.49800581	0.77572323
83.7	0.49793679	0.77556519
83.8	0.49781887	0.77486644
83.9	0.49729287	0.7738732
84	0.49778395	0.77358578
84.1	0.49774216	0.77306061

90	0.50456702	0.75388195
90.1	0.50497861	0.75374007
90.2	0.5051952	0.75334542
90.3	0.50528135	0.75317079
90.4	0.50528135	0.75317079
90.5	0.50581008	0.75332939
90.6	0.50582809	0.75323248
90.7	0.50594055	0.75309268
90.8	0.5062857	0.75303439
90.9	0.50660272	0.75301517
91	0.50695427	0.75297266
91.1	0.50714242	0.75300229
91.2	0.50714242	0.75300229
91.3	0.50731883	0.75298153
91.4	0.50778996	0.7528633
91.5	0.50786483	0.75290592
91.6	0.50774767	0.75261351
91.7	0.50763927	0.75251744
91.8	0.50763927	0.75251744
91.9	0.50775391	0.75236437
92	0.50831439	0.75207998
92.1	0.50841549	0.75203039
92.2	0.50874164	0.75213759
92.3	0.50913387	0.75210975
92.4	0.50928506	0.75186225
92.5	0.50946144	0.75171968
92.6	0.50930523	0.7516695
92.7	0.50930523	0.7516695
92.8	0.50982894	0.75145347
92.9	0.50982894	0.75145347
93	0.51010742	0.75144998
93.1	0.51034394	0.75141572
93.2	0.51050297	0.75132915
93.3	0.51079436	0.75129249
93.4	0.51053535	0.75091371
93.5	0.51062276	0.75070346
93.6	0.5114582	0.75035167
93.7	0.51169628	0.75030706
93.8	0.51208989	0.75032095
93.9	0.5127399	0.75025018
94	0.5127399	0.75025018
94.1	0.5126706	0.74995406
94.2	0.51293766	0.74993249
94.3	0.51324481	0.74960348
94.4	0.51270761	0.74942222
94.5	0.5127292	0.74930321
94.6	0.51291676	0.74939223
94.7	0.51271485	0.74909001
94.8	0.51301086	0.74884788
94.9	0.51301086	0.74884788
95	0.51335275	0.74878269
95.1	0.51335284	0.74856897
95.2	0.51314285	0.74843905
95.3	0.51318182	0.74828821
95.4	0.51335592	0.74791574
95.5	0.51342876	0.74786355
95.6	0.51342876	0.74786355
95.7	0.51342876	0.74786355
95.8	0.51394737	0.74812833
95.9	0.51372024	0.74802199
96	0.51407558	0.74786166
96.1	0.51407558	0.74786166
96.2	0.51385988	0.74772113
96.3	0.51408383	0.74776634
96.4	0.51458569	0.7476355
96.5	0.51458569	0.7476355
96.6	0.51460789	0.74733732
96.7	0.51460789	0.74733732
96.8	0.51514843	0.74718287
96.9	0.51491034	0.74677655
97	0.51494687	0.74661419
97.1	0.51493458	0.74640846
97.2	0.51529659	0.74637693
97.3	0.51517754	0.7462737
97.4	0.51537248	0.74633672
97.5	0.51537248	0.74633672
97.6	0.51537248	0.74633672
97.7	0.51549792	0.74615673
97.8	0.51549782	0.74579747
97.9	0.51553043	0.74565083
98	0.51553043	0.74565083
98.1	0.51575634	0.7456151
98.2	0.51554294	0.74542916
98.3	0.51556641	0.74523428
98.4	0.51500842	0.74499361
98.5	0.51496362	0.74497157
98.6	0.51501381	0.7446908
98.7	0.51506126	0.74441847
98.8	0.5149652	0.74422019
98.9	0.51489281	0.74406323
99	0.51498418	0.74386038
99.1	0.51512778	0.7435949

105	0.51431501	0.73817112
105.1	0.51383884	0.73795115
105.2	0.51379543	0.73789882
105.3	0.51388368	0.73784275
105.4	0.51407909	0.73765602
105.5	0.51414401	0.7375648
105.6	0.51414401	0.7375648
105.7	0.51414401	0.7375648
105.8	0.51414715	0.7373376
105.9	0.51412879	0.73720899
106	0.51430387	0.73727041
106.1	0.51389865	0.73712794
106.2	0.51389865	0.73712794
106.3	0.51352408	0.73678571
106.4	0.51336349	0.73653339
106.5	0.51312395	0.73596066
106.6	0.51269712	0.73563231
106.7	0.51172536	0.7348723
106.8	0.51210385	0.73493225
106.9	0.51180874	0.73467636
107	0.51182413	0.73450758
107.1	0.51129955	0.73395971
107.2	0.51129955	0.73395971
107.3	0.51140495	0.73395085
107.4	0.51125576	0.7337386
107.5	0.51144237	0.73373281
107.6	0.51144237	0.73373281
107.7	0.51138968	0.73364409
107.8	0.51147578	0.73331039
107.9	0.5114976	0.73325765
108	0.51109998	0.73295321
108.1	0.51109998	0.73295321
108.2	0.51132793	0.73292076
108.3	0.51143319	0.73270165
108.4	0.51143808	0.73259456
108.5	0.51168575	0.73253881
108.6	0.51178382	0.73225706
108.7	0.51190867	0.732356
108.8	0.51182174	0.73195392
108.9	0.51159277	0.73177539
109	0.51159277	0.73177539
109.1	0.5115234	0.73169141
109.2	0.51135435	0.73133528
109.3	0.51139351	0.73131248
109.4	0.51139351	0.73131248
109.5	0.51164993	0.73127525
109.6	0.51161923	0.73124746
109.7	0.51192401	0.73136074
109.8	0.51192401	0.73136074
109.9	0.51192401	0.73136074
110	0.51192401	0.73136074
110.1	0.51192401	0.73136074
110.2	0.51227617	0.7314775
110.3	0.51227617	0.7314775
110.4	0.51253445	0.73158156
110.5	0.51259266	0.73148932
110.6	0.51272736	0.73098039
110.7	0.51272736	0.73098039
110.8	0.51280776	0.73093483
110.9	0.51321033	0.73099139
111	0.51340066	0.73088554
111.1	0.51348323	0.73070145
111.2	0.51390808	0.73031861
111.3	0.51390808	0.73031861
111.4	0.51375191	0.73020188
111.5	0.51397646	0.73017808
111.6	0.51397646	0.73017808
111.7	0.51397646	0.73017808
111.8	0.51397646	0.73017808
111.9	0.51408694	0.73004546
112	0.51408694	0.73004546
112.1	0.51420965	0.7298889
112.2	0.51438465	0.72989552
112.3	0.51438465	0.72989552
112.4	0.51438465	0.72989552
112.5	0.5144315	0.729478
112.6	0.51466757	0.72920023
112.7	0.5145538	0.72917347
112.8	0.5145616	0.72863901
112.9	0.51456154	0.7285925
113	0.51459171	0.72841257
113.1	0.51487391	0.72813455
113.2	0.51487391	0.72813455
113.3	0.51496418	0.72812302
113.4	0.51496418	0.72812302
113.5	0.51513721	0.7279208
113.6	0.51507801	0.72785428
113.7	0.51507818	0.72766495
113.8	0.51521516	0.72755359
113.9	0.5152809	0.72743279
114	0.51539205	0.72738769
114.1	0.51525694	0.72726735

69.2	0.48171274	0.83080219
69.3	0.48171655	0.83024254
69.4	0.48218267	0.83009474
69.5	0.4822991	0.82954679
69.6	0.48251045	0.82935956
69.7	0.48264265	0.8293209
69.8	0.48296083	0.82920617
69.9	0.48316461	0.82878114
70	0.48368748	0.82865972
70.1	0.48359679	0.8280549
70.2	0.48387002	0.82774253
70.3	0.48421509	0.82743634
70.4	0.48466996	0.82712125
70.5	0.4854783	0.82642178
70.6	0.48551354	0.826213
70.7	0.48535938	0.82587463
70.8	0.48546121	0.82570091
70.9	0.48518479	0.82497733
71	0.48523735	0.82487854
71.1	0.48559103	0.82442734
71.2	0.48574882	0.82402603
71.3	0.48606199	0.82370715
71.4	0.48636977	0.82348619
71.5	0.4862058	0.82311485
71.6	0.48639161	0.8229831
71.7	0.48682502	0.82257634
71.8	0.48698913	0.82235101
71.9	0.48650831	0.82216449
72	0.48721233	0.82183113
72.1	0.48715756	0.82143355
72.2	0.48722094	0.82107197
72.3	0.48748172	0.82066996
72.4	0.48782883	0.82062669
72.5	0.48795869	0.8203033
72.6	0.48828225	0.8199751
72.7	0.48874379	0.81949796
72.8	0.48882525	0.81921
72.9	0.48915314	0.81897557
73	0.48884358	0.81849583
73.1	0.48884358	0.81849583
73.2	0.48917298	0.81813392
73.3	0.48964671	0.81746306
73.4	0.48984302	0.81682493
73.5	0.49007688	0.81644041
73.6	0.489722	0.81596687
73.7	0.49002393	0.81588854
73.8	0.49033075	0.81559731
73.9	0.49099301	0.81549314
74	0.49148345	0.81527996
74.1	0.49169922	0.81477557
74.2	0.4918676	0.81468405
74.3	0.49211745	0.81425891
74.4	0.49195674	0.81368718
74.5	0.49206112	0.81339845
74.6	0.49214234	0.81209409
74.7	0.49209801	0.81147011
74.8	0.49222693	0.81136698
74.9	0.49246047	0.81079998

84.2	0.49762246	0.77294882
84.3	0.49788684	0.77286745
84.4	0.49813643	0.77264066
84.5	0.49791863	0.77229543
84.6	0.49761001	0.77145666
84.7	0.49814166	0.77107077
84.8	0.49856548	0.77002232
84.9	0.49829203	0.76975347
85	0.49815048	0.76956378
85.1	0.49878463	0.76932454
85.2	0.49901967	0.7686969
85.3	0.49904598	0.76833698
85.4	0.49916167	0.76790327
85.5	0.49899549	0.76739478
85.6	0.49956752	0.76734657
85.7	0.49959049	0.76681951
85.8	0.49949699	0.76627185
85.9	0.499235	0.76521092
86	0.4994024	0.76457467
86.1	0.50016199	0.7642013
86.2	0.49974774	0.76353418
86.3	0.50019259	0.76330495
86.4	0.50033963	0.76299553
86.5	0.50073199	0.76281941
86.6	0.50054515	0.76211991
86.7	0.50021963	0.761663
86.8	0.50065718	0.76195476
86.9	0.50061585	0.76164268
87	0.50091108	0.76165541
87.1	0.50081769	0.76153243
87.2	0.5014453	0.76114769
87.3	0.50207034	0.7610012
87.4	0.50207034	0.7610012
87.5	0.50202922	0.76086067
87.6	0.50253581	0.76057677
87.7	0.50252477	0.7604048
87.8	0.50217267	0.75943596
87.9	0.50257293	0.75905613
88	0.50253386	0.75855042
88.1	0.50276226	0.75826505
88.2	0.50247328	0.75779872
88.3	0.50254531	0.75787385
88.4	0.50277004	0.75776736
88.5	0.5034391	0.75727832
88.6	0.50333339	0.75687385
88.7	0.50306213	0.75662355
88.8	0.50355773	0.75631101
88.9	0.5037554	0.75614792
89	0.5037039	0.75590401
89.1	0.50405331	0.75579953
89.2	0.50406074	0.75549334
89.3	0.50455353	0.75568655
89.4	0.5045426	0.755596
89.5	0.50511228	0.75522956
89.6	0.50438553	0.75469315
89.7	0.50415958	0.75449429
89.8	0.50457663	0.75425802
89.9	0.50479952	0.75431333

99.2	0.5150359	0.74336667
99.3	0.51509137	0.74321836
99.4	0.51535829	0.74284881
99.5	0.51535829	0.74284881
99.6	0.51550756	0.74278111
99.7	0.51499585	0.74204544
99.8	0.51502194	0.74141926
99.9	0.51536929	0.74126096
100	0.51536929	0.74126096
100.1	0.51542896	0.74104548
100.2	0.51542896	0.74104548
100.3	0.51492914	0.74028764
100.4	0.51500665	0.74032438
100.5	0.51500665	0.74032438
100.6	0.51500665	0.74032438
100.7	0.51499086	0.73989297
100.8	0.51477238	0.73973928
100.9	0.51477238	0.73973928
101	0.5151585	0.73985799
101.1	0.5151585	0.73985799
101.2	0.51552753	0.73995899
101.3	0.5155778	0.73989639
101.4	0.5155778	0.73989639
101.5	0.5155778	0.73989639
101.6	0.5155778	0.73989639
101.7	0.5155778	0.73989639
101.8	0.51582888	0.73994653
101.9	0.51582888	0.73994653
102	0.51582888	0.73994653
102.1	0.51593661	0.73990401
102.2	0.51593661	0.73990401
102.3	0.51602772	0.73989023
102.4	0.51555562	0.73970655
102.5	0.51547421	0.73969522
102.6	0.51567898	0.73972446
102.7	0.51567898	0.73972446
102.8	0.51577957	0.73969464
102.9	0.51577957	0.73969464
103	0.51577957	0.73969464
103.1	0.51597111	0.73966333
103.2	0.51608516	0.73967087
103.3	0.51595144	0.73950106
103.4	0.51550325	0.73938861
103.5	0.51531127	0.73926116
103.6	0.51540151	0.73913048
103.7	0.51496528	0.73897201
103.8	0.51515914	0.73891229
103.9	0.51515914	0.73891229
104	0.51529267	0.73885806
104.1	0.51529267	0.73885806
104.2	0.51524702	0.73878947
104.3	0.51546443	0.73873907
104.4	0.5148158	0.7384682
104.5	0.5148158	0.7384682
104.6	0.51446513	0.73833404
104.7	0.51446513	0.73833404
104.8	0.51431053	0.73825491
104.9	0.51431053	0.73825491

114.2	0.51532913	0.72718919
114.3	0.51532913	0.72718919
114.4	0.51559214	0.72728016
114.5	0.51581657	0.7273534
114.6	0.51591165	0.72741666
114.7	0.51591165	0.72741666
114.8	0.51583847	0.72687749
114.9	0.51610942	0.7268905
115	0.51638568	0.72655851
115.1	0.51656679	0.72650374
115.2	0.51656679	0.72650374
115.3	0.51698638	0.72658861
115.4	0.51711744	0.72657521
115.5	0.51734981	0.72642388
115.6	0.51770356	0.72650216
115.7	0.51779548	0.72617719
115.8	0.51789192	0.72609345
115.9	0.51845664	0.72611932
116	0.51845664	0.72611932
116.1	0.51845664	0.72611932
116.2	0.51839047	0.72611558
116.3	0.51839707	0.72604143
116.4	0.51840618	0.72589874
116.5	0.51858414	0.72588749
116.6	0.51848724	0.72572212
116.7	0.51878943	0.72581031
116.8	0.51935641	0.72612354
116.9	0.51935641	0.72612354
117	0.51953391	0.72588763
117.1	0.51972906	0.72574682
117.2	0.51972906	0.72574682
117.3	0.51978089	0.72568798
117.4	0.51978089	0.72568798
117.5	0.51966539	0.72554472
117.6	0.51988079	0.72539528
117.7	0.51996794	0.72516645
117.8	0.52016907	0.72512396
117.9	0.52019358	0.72513588
118	0.52027462	0.72500183
118.1	0.52027462	0.72500183
118.2	0.5203552	0.7249531
118.3	0.5204402	0.72485165
118.4	0.52080367	0.72471883
118.5	0.52087359	0.72459996
118.6	0.52086248	0.72464439
118.7	0.52079663	0.72457496
118.8	0.52069405	0.72432447
118.9	0.52100515	0.72408231
119	0.52105539	0.72409591
119.1	0.52082233	0.7238873
119.2	0.52096885	0.72366036
119.3	0.52096885	0.72366036
119.4	0.52124787	0.72352499
119.5	0.52131791	0.72330584
119.6	0.52137709	0.72319063
119.7	0.5215381	0.72334517
119.8	0.52155176	0.72312598
119.9	0.5215412	0.72310608

120	0.52142344	0.72293145
120.1	0.5211973	0.72243791
120.2	0.52099954	0.7221354
120.3	0.52116584	0.72206037
120.4	0.52116584	0.72206037
120.5	0.52097357	0.72192054
120.6	0.52103038	0.72125282
120.7	0.52094267	0.72113846
120.8	0.52107389	0.72080712
120.9	0.5211705	0.72041787
121	0.52080901	0.72027982
121.1	0.52081247	0.72022134
121.2	0.52087684	0.71998927
121.3	0.5205416	0.71946756
121.4	0.52056238	0.71953993
121.5	0.52092318	0.7195648
121.6	0.52076862	0.71962539
121.7	0.52121406	0.71951128
121.8	0.5211418	0.71908991
121.9	0.52090121	0.7189812
122	0.52101304	0.71875136
122.1	0.52129676	0.71884417
122.2	0.52120225	0.71868705
122.3	0.52126525	0.71849397
122.4	0.52114573	0.71819851
122.5	0.52103277	0.71815145
122.6	0.52121501	0.71805717
122.7	0.52126734	0.71793292
122.8	0.52126734	0.71793292
122.9	0.52134979	0.71779815
123	0.52123358	0.71781004
123.1	0.52095965	0.71736063
123.2	0.52112982	0.71709644
123.3	0.52109422	0.71662079
123.4	0.52121012	0.71660863
123.5	0.52173746	0.71668
123.6	0.52201925	0.71670134
123.7	0.52201925	0.71670134
123.8	0.5220433	0.71664039
123.9	0.52204099	0.7164075
124	0.52204099	0.7164075
124.1	0.52182878	0.71634401
124.2	0.52182878	0.71634401
124.3	0.52174316	0.71630578
124.4	0.52171356	0.71627266
124.5	0.52200272	0.71639054
124.6	0.52182862	0.71602431
124.7	0.52196197	0.71599036
124.8	0.52221255	0.71591441
124.9	0.52213629	0.7156003
125	0.52213629	0.7156003
125.1	0.52198786	0.71548799
125.2	0.52216353	0.71542456
125.3	0.52203493	0.71519037
125.4	0.52197604	0.71473973
125.5	0.52198221	0.71446237
125.6	0.52251725	0.7144547
125.7	0.52251725	0.7144547
125.8	0.52257906	0.71405945
125.9	0.52274943	0.71374534
126	0.52268607	0.71354158
126.1	0.52223848	0.71322738
126.2	0.52223848	0.71322738
126.3	0.52223848	0.71322738

135	0.51413898	0.68463008
135.1	0.51428086	0.68460885
135.2	0.51427205	0.68404442
135.3	0.51441414	0.68398166
135.4	0.51451891	0.6836573
135.5	0.51452298	0.68362057
135.6	0.51470295	0.68345034
135.7	0.51470295	0.68345034
135.8	0.5146988	0.68307632
135.9	0.51462031	0.68272282
136	0.51468739	0.68241741
136.1	0.51468739	0.68241741
136.2	0.51456602	0.68177514
136.3	0.51474099	0.68159375
136.4	0.51518505	0.68128576
136.5	0.51523484	0.68103191
136.6	0.51508376	0.68072078
136.7	0.51501801	0.68061758
136.8	0.5149298	0.68056981
136.9	0.51533765	0.68038862
137	0.51533765	0.68038862
137.1	0.51554074	0.68022053
137.2	0.51570496	0.68023634
137.3	0.51568536	0.68005346
137.4	0.51568536	0.68005346
137.5	0.5157193	0.67984758
137.6	0.51609129	0.68005334
137.7	0.51609129	0.68005334
137.8	0.51620288	0.67991633
137.9	0.51623766	0.67963633
138	0.51605809	0.67958023
138.1	0.51605809	0.67958023
138.2	0.51621788	0.67969284
138.3	0.51621788	0.67969284
138.4	0.51648486	0.67966188
138.5	0.51661855	0.67942316
138.6	0.51661855	0.67942316
138.7	0.51671435	0.67931367
138.8	0.51670765	0.67913238
138.9	0.51670765	0.67913238
139	0.51668151	0.67903792
139.1	0.51672513	0.67883702
139.2	0.51691853	0.6788227
139.3	0.51699701	0.67868487
139.4	0.51729044	0.67847615
139.5	0.51744922	0.67802134
139.6	0.51739329	0.67766325
139.7	0.51739329	0.67766325
139.8	0.51739329	0.67766325
139.9	0.51711185	0.67719707
140	0.51696668	0.67689648
140.1	0.51711584	0.67701009
140.2	0.51733329	0.67689586
140.3	0.51698441	0.67639482
140.4	0.51742105	0.6763536
140.5	0.51742105	0.6763536
140.6	0.51738177	0.67610537
140.7	0.51738177	0.67610537
140.8	0.51734626	0.67600699
140.9	0.51713268	0.67584095
141	0.51691351	0.67587882
141.1	0.51721897	0.67599374
141.2	0.51721897	0.67599374
141.3	0.51727345	0.67582341

150	0.51898126	0.66781178
150.1	0.51895871	0.66780535
150.2	0.51895871	0.66780535
150.3	0.51895871	0.66780535
150.4	0.51895871	0.66780535
150.5	0.51895871	0.66780535
150.6	0.51895871	0.66780535
150.7	0.51895871	0.66780535
150.8	0.51895871	0.66780535
150.9	0.51920831	0.66788277
151	0.51920831	0.66788277
151.1	0.51920831	0.66788277
151.2	0.51946089	0.66781115
151.3	0.51953214	0.66768534
151.4	0.51953214	0.66768534
151.5	0.51953214	0.66768534
151.6	0.51980123	0.66766629
151.7	0.51989469	0.66763258
151.8	0.52004732	0.66752189
151.9	0.52004732	0.66752189
152	0.52004732	0.66752189
152.1	0.52004732	0.66752189
152.2	0.52004732	0.66752189
152.3	0.5202094	0.66746012
152.4	0.5202094	0.66746012
152.5	0.52027142	0.66728783
152.6	0.52008208	0.66724855
152.7	0.52008208	0.66724855
152.8	0.52024969	0.66711848
152.9	0.52024969	0.66711848
153	0.52024969	0.66711848
153.1	0.52025329	0.66711716
153.2	0.52017676	0.66696344
153.3	0.52023047	0.66686078
153.4	0.52023047	0.66686078
153.5	0.5205446	0.66659487
153.6	0.5205446	0.66659487
153.7	0.5205446	0.66659487
153.8	0.52093991	0.66667796
153.9	0.52093991	0.66667796
154	0.52093991	0.66667796
154.1	0.52110551	0.66653044
154.2	0.52110551	0.66653044
154.3	0.52119544	0.66633848
154.4	0.52119544	0.66633848
154.5	0.52126865	0.66617493
154.6	0.52126007	0.6659258
154.7	0.52126007	0.6659258
154.8	0.52135405	0.66577272
154.9	0.52135405	0.66577272
155	0.52135405	0.66577272
155.1	0.52132645	0.6657449
155.2	0.52132645	0.6657449
155.3	0.52132645	0.6657449
155.4	0.52132645	0.6657449
155.5	0.52146949	0.6657139
155.6	0.52146949	0.6657139
155.7	0.52146949	0.6657139
155.8	0.52146949	0.6657139
155.9	0.52155793	0.66558155
156	0.52155793	0.66558155
156.1	0.52155793	0.66558155
156.2	0.52155793	0.66558155
156.3	0.52174047	0.66552283

165	0.52501523	0.66317317
165.1	0.52507581	0.66332211
165.2	0.52502071	0.66332344
165.3	0.52502071	0.66332344
165.4	0.52502071	0.66332344
165.5	0.52502071	0.66332344
165.6	0.52502071	0.66332344
165.7	0.52490234	0.66319878
165.8	0.52490234	0.66319878
165.9	0.52512481	0.66321672
166	0.52509751	0.66301136
166.1	0.52509751	0.66301136
166.2	0.52509751	0.66301136
166.3	0.52525077	0.66302229
166.4	0.52525077	0.66302229
166.5	0.52536924	0.66245383
166.6	0.52536924	0.66245383
166.7	0.52536924	0.66245383
166.8	0.52536924	0.66245383
166.9	0.52536924	0.66245383
167	0.52549682	0.66239999
167.1	0.52560142	0.66218643
167.2	0.52560142	0.66218643
167.3	0.52560142	0.66218643
167.4	0.52560142	0.66218643
167.5	0.52560142	0.66218643
167.6	0.52572381	0.66208416
167.7	0.52572381	0.66208416
167.8	0.52591423	0.66192536
167.9	0.52603606	0.66198111
168	0.52614644	0.66188567
168.1	0.52626952	0.66189355
168.2	0.52626952	0.66189355
168.3	0.52650169	0.66191371
168.4	0.52650169	0.66191371
168.5	0.52650169	0.66191371
168.6	0.52662406	0.6617704
168.7	0.52662406	0.6617704
168.8	0.52641857	0.66152414
168.9	0.52641857	0.66152414
169	0.52641857	0.66152414
169.1	0.52641857	0.66152414
169.2	0.52641857	0.66152414
169.3	0.52641857	0.66152414
169.4	0.52641857	0.66152414
169.5	0.52641857	0.66152414
169.6	0.52636825	0.66127357
169.7	0.52636825	0.66127357
169.8	0.52636825	0.66127357
169.9	0.5265362	0.66118693
170	0.5265362	0.66118693
170.1	0.5265362	0.66118693
170.2	0.5265362	0.66118693
170.3	0.5265362	0.66118693
170.4	0.5265362	0.66118693
170.5	0.52629612	0.66116726
170.6	0.52629612	0.66116726
170.7	0.52629612	0.66116726
170.8	0.52629612	0.66116726
170.9	0.52629612	0.66116726
171	0.52641202	0.66112746
171.1	0.52641202	0.66112746
171.2	0.52641202	0.66112746
171.3	0.52662394	0.66111656

126.4	0.52223848	0.71322738
126.5	0.52245166	0.71305647
126.6	0.52211219	0.71281893
126.7	0.52211219	0.71281893
126.8	0.52211219	0.71281893
126.9	0.52191595	0.71252771
127	0.52222343	0.71222849
127.1	0.52232518	0.71190475
127.2	0.52218556	0.71137734
127.3	0.52231604	0.71134533
127.4	0.52228675	0.71089249
127.5	0.52237037	0.71064182
127.6	0.52250593	0.71055469
127.7	0.52224872	0.71055774
127.8	0.52223089	0.71038327
127.9	0.52223089	0.71038327
128	0.52258086	0.71000131
128.1	0.52252363	0.70981969
128.2	0.522175	0.70936863
128.3	0.52268848	0.70910621
128.4	0.52257398	0.70877586
128.5	0.52228606	0.7085654
128.6	0.52249707	0.70854994
128.7	0.52249707	0.70854994
128.8	0.522259	0.70812057
128.9	0.52222134	0.70815288
129	0.52211668	0.70772641
129.1	0.52202654	0.70761145
129.2	0.52188546	0.70751477
129.3	0.52158159	0.70706514
129.4	0.52173172	0.70694475
129.5	0.52173172	0.70694475
129.6	0.52194921	0.70636195
129.7	0.52195391	0.70619025
129.8	0.52231297	0.70618029
129.9	0.52220863	0.70599086
130	0.52226399	0.70541993
130.1	0.52217799	0.70538714
130.2	0.52230781	0.70526118
130.3	0.52200424	0.70500414
130.4	0.52145088	0.70449669
130.5	0.52136839	0.70418722
130.6	0.52070647	0.70328667
130.7	0.52042754	0.70297471
130.8	0.52025802	0.70259064
130.9	0.52032694	0.70216946
131	0.51993759	0.70140925
131.1	0.51979893	0.70124743
131.2	0.51918095	0.70068859
131.3	0.51889052	0.70047486
131.4	0.51882227	0.69987425
131.5	0.51829617	0.69927666
131.6	0.51803535	0.69869129
131.7	0.51794341	0.69822653
131.8	0.51794341	0.69822653
131.9	0.51833207	0.69819129
132	0.51834936	0.69812025
132.1	0.51867248	0.69817854
132.2	0.51871238	0.69809641
132.3	0.51845962	0.69775328
132.4	0.5179429	0.69733126
132.5	0.51775019	0.69661632
132.6	0.5177335	0.69640183
132.7	0.51740895	0.69605424
132.8	0.51717325	0.69563811
132.9	0.51698663	0.69469053
133	0.51674344	0.69398424
133.1	0.51646316	0.69355566
133.2	0.51659254	0.6934131
133.3	0.51639118	0.69278474
133.4	0.51620828	0.6925767
133.5	0.51607573	0.69199516
133.6	0.51619496	0.69151606
133.7	0.51617698	0.6908023
133.8	0.51624241	0.69035004
133.9	0.51608413	0.69014506
134	0.51602006	0.68980484
134.1	0.51545684	0.68901452
134.2	0.51525968	0.68854009
134.3	0.51517521	0.68774607
134.4	0.51516275	0.6876321
134.5	0.51521729	0.68728442
134.6	0.51521366	0.68703325
134.7	0.51456638	0.6861016
134.8	0.51480133	0.68579065
134.9	0.51437376	0.68487222

141.4	0.51777637	0.67600402
141.5	0.51777637	0.67600402
141.6	0.51777637	0.67600402
141.7	0.51777637	0.67600402
141.8	0.51780791	0.67584588
141.9	0.51772901	0.67573199
142	0.51772901	0.67573199
142.1	0.51761272	0.67557909
142.2	0.51763423	0.67535717
142.3	0.51759599	0.67519572
142.4	0.51779319	0.67500216
142.5	0.51779319	0.67500216
142.6	0.51798301	0.67487515
142.7	0.51798301	0.67487515
142.8	0.51799398	0.67483323
142.9	0.51799398	0.67483323
143	0.51814323	0.67466116
143.1	0.51814323	0.67466116
143.2	0.51814323	0.67466116
143.3	0.51819897	0.67459583
143.4	0.51840862	0.67456256
143.5	0.51840862	0.67456256
143.6	0.51831351	0.67441393
143.7	0.51831351	0.67441393
143.8	0.5183166	0.67431376
143.9	0.5183166	0.67431376
144	0.51858004	0.67423779
144.1	0.51846674	0.6740299
144.2	0.51846674	0.6740299
144.3	0.51837086	0.67379898
144.4	0.51854437	0.67362944
144.5	0.51854437	0.67362944
144.6	0.51854437	0.67362944
144.7	0.51854933	0.67359625
144.8	0.51836279	0.6730301
144.9	0.51834697	0.67298266
145	0.51834697	0.67298266
145.1	0.51834697	0.67298266
145.2	0.51828903	0.67277417
145.3	0.51823256	0.67273819
145.4	0.51822753	0.67275388
145.5	0.51815837	0.67266888
145.6	0.51815837	0.67266888
145.7	0.51804194	0.67248998
145.8	0.5180398	0.67236745
145.9	0.51796037	0.67222903
146	0.51790584	0.67191922
146.1	0.51812692	0.67150898
146.2	0.51821775	0.67151456
146.3	0.51787118	0.67107904
146.4	0.51786449	0.67101124
146.5	0.51795323	0.67050215
146.6	0.51767704	0.67020185
146.7	0.51740762	0.66988874
146.8	0.51751205	0.66997981
146.9	0.51736199	0.669782
147	0.51727667	0.66942979
147.1	0.51716229	0.66915317
147.2	0.51756376	0.66880905
147.3	0.51750699	0.66845207
147.4	0.51751734	0.66852013
147.5	0.51777786	0.66851366
147.6	0.51777786	0.66851366
147.7	0.51757514	0.66818129
147.8	0.51779631	0.66805987
147.9	0.51779631	0.66805987
148	0.51777227	0.66791667
148.1	0.51777227	0.66791667
148.2	0.51777227	0.66791667
148.3	0.51777227	0.66791667
148.4	0.51777227	0.66791667
148.5	0.51777227	0.66791667
148.6	0.5179062	0.66776056
148.7	0.5179062	0.66776056
148.8	0.5179062	0.66776056
148.9	0.5179062	0.66776056
149	0.5179062	0.66776056
149.1	0.5179062	0.66776056
149.2	0.5179062	0.66776056
149.3	0.5179062	0.66776056
149.4	0.51795071	0.66767492
149.5	0.51821565	0.66765923
149.6	0.51849896	0.66777367
149.7	0.51872984	0.6678021
149.8	0.51898126	0.66781178
149.9	0.51898126	0.66781178

156.4	0.52174047	0.66552283
156.5	0.52174047	0.66552283
156.6	0.52174047	0.66552283
156.7	0.52174047	0.66552283
156.8	0.52174047	0.66552283
156.9	0.52174047	0.66552283
157	0.52174047	0.66552283
157.1	0.52174047	0.66552283
157.2	0.52189164	0.66556485
157.3	0.52189164	0.66556485
157.4	0.52194219	0.66561173
157.5	0.52207872	0.66554816
157.6	0.52207872	0.66554816
157.7	0.52207872	0.66554816
157.8	0.52224961	0.66543402
157.9	0.52224961	0.66543402
158	0.5223833	0.66537569
158.1	0.5223833	0.66537569
158.2	0.5223833	0.66537569
158.3	0.5223833	0.66537569
158.4	0.52241718	0.66515161
158.5	0.5226066	0.66506303
158.6	0.52220286	0.66491678
158.7	0.52237939	0.66484575
158.8	0.52270571	0.6649817
158.9	0.52270571	0.6649817
159	0.52270571	0.6649817
159.1	0.52270571	0.6649817
159.2	0.52270571	0.6649817
159.3	0.52270571	0.6649817
159.4	0.52270571	0.6649817
159.5	0.52270571	0.6649817
159.6	0.52280262	0.66484191
159.7	0.52284268	0.66463271
159.8	0.52292136	0.66444931
159.9	0.52320767	0.66453283
160	0.52320767	0.66453283
160.1	0.52320767	0.66453283
160.2	0.523469	0.66465264
160.3	0.523469	0.66465264
160.4	0.5235486	0.66470113
160.5	0.5235486	0.66470113
160.6	0.5235486	0.66470113
160.7	0.5235486	0.66470113
160.8	0.52362709	0.66450614
160.9	0.52362709	0.66450614
161	0.52362709	0.66450614
161.1	0.52359346	0.66442507
161.2	0.52359346	0.66442507
161.3	0.52359346	0.66442507
161.4	0.52359346	0.66442507
161.5	0.52374869	0.6643376
161.6	0.52374869	0.6643376
161.7	0.52374869	0.6643376
161.8	0.52390798	0.66423327
161.9	0.52390798	0.66423327
162	0.52397037	0.66415837
162.1	0.52397037	0.66415837
162.2	0.52397037	0.66415837
162.3	0.52413929	0.66415811
162.4	0.52413929	0.66415811
162.5	0.52413929	0.66415811
162.6	0.52413929	0.66415811
162.7	0.52413929	0.66415811
162.8	0.52432234	0.66415312
162.9	0.52432234	0.66415312
163	0.52432234	0.66415312
163.1	0.52423809	0.66389726
163.2	0.52423809	0.66389726
163.3	0.52423809	0.66389726
163.4	0.52436866	0.66378623
163.5	0.52436866	0.66378623
163.6	0.52436866	0.66378623
163.7	0.52460552	0.66373747
163.8	0.52460552	0.66373747
163.9	0.52460552	0.66373747
164	0.52465708	0.66360776
164.1	0.5248037	0.66346255
164.2	0.52492598	0.66330029
164.3	0.52492598	0.66330029
164.4	0.52501523	0.66317317
164.5	0.52501523	0.66317317
164.6	0.52501523	0.66317317
164.7	0.52501523	0.66317317
164.8	0.52501523	0.66317317
164.9	0.52501523	0.66317317

171.4	0.5268466	0.66112762
171.5	0.5268466	0.66112762
171.6	0.5268466	0.66112762
171.7	0.52721886	0.6610448
171.8	0.52729938	0.66099171
171.9	0.52729938	0.66099171
172	0.52717636	0.6609883
172.1	0.52717636	0.6609883
172.2	0.52717636	0.6609883
172.3	0.52717636	0.6609883
172.4	0.52717636	0.6609883
172.5	0.52717636	0.6609883
172.6	0.52717636	0.6609883
172.7	0.52717636	0.6609883
172.8	0.52739135	0.66105875
172.9	0.5276105	0.66116229
173	0.52768715	0.66081075
173.1	0.52787103	0.66092685
173.2	0.52787103	0.66092685
173.3	0.52795404	0.66088393
173.4	0.52825387	0.66091145
173.5	0.52825387	0.66091145
173.6	0.52832936	0.66080189
173.7	0.5283986	0.66064277
173.8	0.5283986	0.66064277
173.9	0.5283986	0.66064277
174	0.5283986	0.66064277
174.1	0.52852459	0.66055832
174.2	0.52852459	0.66055832
174.3	0.52852459	0.66055832
174.4	0.52852459	0.66055832
174.5	0.52852459	0.66055832
174.6	0.52862126	0.66038816
174.7	0.52862126	0.66038816
174.8	0.52862126	0.66038816
174.9	0.52862126	0.66038816
175	0.52865764	0.66025222
175.1	0.52869328	0.66010528
175.2	0.52862766	0.65969482
175.3	0.52862766	0.65969482
175.4	0.52876247	0.6595877
175.5	0.52876247	0.6595877
175.6	0.52876247	0.6595877
175.7	0.52876247	0.6595877
175.8	0.52876247	0.6595877
175.9	0.52876247	0.6595877
176	0.52876247	0.6595877
176.1	0.52876247	0.6595877
176.2	0.52880626	0.6595458
176.3	0.52880626	0.6595458
176.4	0.52896133	0.65940498
176.5	0.52895131	0.65940954
176.6	0.52895131	0.65940954
176.7	0.52895131	0.65940954
176.8	0.52895131	0.65940954
176.9	0.52895131	0.65940954
177	0.52895131	0.65940954
177.1	0.52908713	0.65928532
177.2	0.52901303	0.65919465
177.3	0.52901303	0.65919465
177.4	0.52909476	0.6589128
177.5	0.52909476	0.6589128
177.6	0.52911301	0.65882597
177.7	0.52927121	0.65855884
177.8	0.52927121	0.65855884
177.9	0.52934593	0.65833972
178	0.52934593	0.65833972
178.1	0.52941009	0.65820892
178.2	0.52941009	0.65820892
178.3	0.52941009	0.65820892
178.4	0.52941009	0.65820892
178.5	0.52941009	0.65820892
178.6	0.52941009	0.65820892
178.7	0.52941009	0.65820892
178.8	0.52941009	0.65820892
178.9	0.52952357	0.65820258
179	0.52946836	0.65813581
179.1	0.52968807	0.65804602
179.2	0.52970768	0.6578516
179.3	0.5296854	0.65783663
179.4	0.52974365	0.65772815
179.5	0.52974365	0.65772815
179.6	0.52995316	0.65780507
179.7	0.53019912	0.6577467
179.8	0.53019912	0.6577467
179.9	0.53022922	0.65769841

180	0.53022922	0.65769841
180.1	0.53034549	0.65753566
180.2	0.53034549	0.65753566
180.3	0.53034549	0.65753566
180.4	0.53034549	0.65753566
180.5	0.5303652	0.65732922
180.6	0.5303652	0.65732922
180.7	0.53020386	0.65721077
180.8	0.53020386	0.65721077
180.9	0.53020386	0.65721077
181	0.53020386	0.65721077
181.1	0.53020386	0.65721077
181.2	0.53020386	0.65721077
181.3	0.53020386	0.65721077
181.4	0.53021194	0.65698216
181.5	0.53019217	0.65685419
181.6	0.53019217	0.65685419
181.7	0.53019217	0.65685419
181.8	0.53019217	0.65685419
181.9	0.5304068	0.65663167
182	0.53035082	0.65643139
182.1	0.53035082	0.65643139
182.2	0.53036673	0.65628979
182.3	0.53025723	0.65635842
182.4	0.53022218	0.65621818
182.5	0.53022218	0.65621818
182.6	0.53018813	0.65572153
182.7	0.53018813	0.65572153
182.8	0.53018813	0.65572153
182.9	0.53018813	0.65572153
183	0.53018813	0.65572153
183.1	0.53018813	0.65572153
183.2	0.52990309	0.65518601
183.3	0.52984068	0.65498599
183.4	0.52974781	0.65481757
183.5	0.52978849	0.65462648

195	0.52935473	0.6509838
195.1	0.52935473	0.6509838
195.2	0.52935473	0.6509838
195.3	0.52935473	0.6509838
195.4	0.52935473	0.6509838
195.5	0.52935473	0.6509838
195.6	0.52935473	0.6509838
195.7	0.52935473	0.6509838
195.8	0.52925973	0.65102226
195.9	0.52930453	0.65088278
196	0.52930453	0.65088278
196.1	0.52930453	0.65088278
196.2	0.52930453	0.65088278
196.3	0.52930453	0.65088278
196.4	0.52930453	0.65088278
196.5	0.52934441	0.65091974
196.6	0.52934441	0.65091974
196.7	0.52928768	0.65072561
196.8	0.52928768	0.65072561
196.9	0.52928768	0.65072561
197	0.52922659	0.6504813
197.1	0.52922659	0.6504813
197.2	0.52922659	0.6504813
197.3	0.5290978	0.65046006
197.4	0.52936945	0.65032376
197.5	0.52936945	0.65032376
197.6	0.52937495	0.65020991
197.7	0.52930868	0.65000499
197.8	0.52930868	0.65000499
197.9	0.52930868	0.65000499
198	0.52930868	0.65000499
198.1	0.52941675	0.65011162
198.2	0.52941675	0.65011162
198.3	0.52941675	0.65011162
198.4	0.52941675	0.65011162
198.5	0.52946799	0.64998433

210	0.52866653	0.64777646
210.1	0.52866653	0.64777646
210.2	0.52893506	0.64778245
210.3	0.52893506	0.64778245
210.4	0.52893506	0.64778245
210.5	0.52893506	0.64778245
210.6	0.52893506	0.64778245
210.7	0.52893506	0.64778245
210.8	0.52893506	0.64778245
210.9	0.52893506	0.64778245
211	0.52893506	0.64778245
211.1	0.52893506	0.64778245
211.2	0.52893506	0.64778245
211.3	0.52893506	0.64778245
211.4	0.52893506	0.64778245
211.5	0.52882756	0.64765427
211.6	0.52882756	0.64765427
211.7	0.52882756	0.64765427
211.8	0.52882756	0.64765427
211.9	0.52894945	0.64770809
212	0.52894945	0.64770809
212.1	0.52894945	0.64770809
212.2	0.52894945	0.64770809
212.3	0.52894945	0.64770809
212.4	0.52894945	0.64770809
212.5	0.52894945	0.64770809
212.6	0.52894945	0.64770809
212.7	0.52930139	0.64788399
212.8	0.52930139	0.64788399
212.9	0.52930139	0.64788399
213	0.52930139	0.64788399
213.1	0.52930139	0.64788399
213.2	0.52930139	0.64788399
213.3	0.52930139	0.64788399
213.4	0.52930139	0.64788399
213.5	0.52930139	0.64788399

225	0.52909571	0.64694833
225.1	0.52943703	0.64709341
225.2	0.52943703	0.64709341
225.3	0.52926898	0.64698447
225.4	0.52907333	0.64678722
225.5	0.52927212	0.64674508
225.6	0.52927212	0.64674508
225.7	0.52927212	0.64674508
225.8	0.52927212	0.64674508
225.9	0.52927212	0.64674508
226	0.52895248	0.64662397
226.1	0.52895248	0.64662397
226.2	0.52895248	0.64662397
226.3	0.52895248	0.64662397
226.4	0.52895248	0.64662397
226.5	0.52885848	0.64643216
226.6	0.52881827	0.64631383
226.7	0.52862281	0.64628998
226.8	0.52862281	0.64628998
226.9	0.52836856	0.64602373
227	0.52836856	0.64602373
227.1	0.52828987	0.64588746
227.2	0.52828987	0.64588746
227.3	0.52828987	0.64588746
227.4	0.52828987	0.64588746
227.5	0.52828987	0.64588746
227.6	0.52857291	0.64601621
227.7	0.52857291	0.64601621
227.8	0.52857291	0.64601621
227.9	0.52857291	0.64601621
228	0.52857291	0.64601621
228.1	0.52857291	0.64601621
228.2	0.52857291	0.64601621
228.3	0.52857291	0.64601621
228.4	0.52857291	0.64601621
228.5	0.52857291	0.64601621

183.6	0.52977738	0.65459266
183.7	0.52977738	0.65459266
183.8	0.52977738	0.65459266
183.9	0.52950449	0.6539662
184	0.52939516	0.65375259
184.1	0.52940314	0.65375314
184.2	0.52947129	0.65363817
184.3	0.52948393	0.65327697
184.4	0.52953432	0.65311703
184.5	0.52953432	0.65311703
184.6	0.52953432	0.65311703
184.7	0.52953432	0.65311703
184.8	0.52953432	0.65311703
184.9	0.52949105	0.65312052
185	0.52949105	0.65312052
185.1	0.52949105	0.65312052
185.2	0.52928838	0.65302333
185.3	0.52928838	0.65302333
185.4	0.52928838	0.65302333
185.5	0.52928838	0.65302333
185.6	0.52934749	0.65285977
185.7	0.52934749	0.65285977
185.8	0.52934749	0.65285977
185.9	0.52934749	0.65285977
186	0.52939236	0.65285835
186.1	0.52939236	0.65285835
186.2	0.52939236	0.65285835
186.3	0.52939236	0.65285835
186.4	0.52939236	0.65285835
186.5	0.52939236	0.65285835
186.6	0.52939236	0.65285835
186.7	0.52939236	0.65285835
186.8	0.52930043	0.65288843
186.9	0.52953555	0.65306918
187	0.5295458	0.65295621
187.1	0.52947423	0.65293269
187.2	0.52947423	0.65293269
187.3	0.52947423	0.65293269
187.4	0.52947423	0.65293269
187.5	0.52947423	0.65293269
187.6	0.52947423	0.65293269
187.7	0.5292439	0.65279173
187.8	0.5292439	0.65279173
187.9	0.52949359	0.65286678
188	0.52949359	0.65286678
188.1	0.52949359	0.65286678
188.2	0.52949359	0.65286678
188.3	0.52948606	0.65272411
188.4	0.52948606	0.65272411
188.5	0.52948606	0.65272411
188.6	0.52958617	0.65263563
188.7	0.52958617	0.65263563
188.8	0.52958617	0.65263563
188.9	0.52958617	0.65263563
189	0.52958617	0.65263563
189.1	0.52958617	0.65263563
189.2	0.52958617	0.65263563
189.3	0.52960451	0.65252459
189.4	0.52960451	0.65252459
189.5	0.52969532	0.65238172
189.6	0.52931951	0.65228298
189.7	0.52937898	0.65217765
189.8	0.52937898	0.65217765
189.9	0.52937898	0.65217765
190	0.52937898	0.65217765
190.1	0.52937898	0.65217765
190.2	0.52937898	0.65217765
190.3	0.5293592	0.65223201
190.4	0.5293592	0.65223201
190.5	0.5293592	0.65223201
190.6	0.5293592	0.65223201
190.7	0.5293592	0.65223201
190.8	0.5293592	0.65223201
190.9	0.52897308	0.6521155
191	0.52897308	0.6521155
191.1	0.52897308	0.6521155
191.2	0.52916356	0.65215657
191.3	0.52910963	0.65197494
191.4	0.52910963	0.65197494
191.5	0.52910963	0.65197494
191.6	0.52945754	0.65212267
191.7	0.52937841	0.65193307
191.8	0.52937841	0.65193307
191.9	0.52920221	0.65180968
192	0.52920221	0.65180968
192.1	0.52920221	0.65180968
192.2	0.52920221	0.65180968
192.3	0.52930005	0.65178002
192.4	0.52930005	0.65178002
192.5	0.52930005	0.65178002
192.6	0.52930005	0.65178002
192.7	0.52930005	0.65178002
192.8	0.52938708	0.65169562
192.9	0.52938708	0.65169562
193	0.52905424	0.65142658
193.1	0.52905424	0.65142658
193.2	0.52905424	0.65142658
193.3	0.52905424	0.65142658
193.4	0.52905424	0.65142658
193.5	0.52905424	0.65142658
193.6	0.52914366	0.65130244
193.7	0.52914366	0.65130244
193.8	0.52898787	0.65118986
193.9	0.52898787	0.65118986
194	0.52898787	0.65118986
194.1	0.52898787	0.65118986
194.2	0.52930533	0.65113526
194.3	0.52930533	0.65113526
194.4	0.52922852	0.65090733
194.5	0.52922852	0.65090733
194.6	0.52922852	0.65090733
194.7	0.52925421	0.65089636
194.8	0.52925421	0.65089636
194.9	0.52935473	0.6509838

198.6	0.52942977	0.649815
198.7	0.52960604	0.64973898
198.8	0.52960604	0.64973898
198.9	0.52960604	0.64973898
199	0.52960604	0.64973898
199.1	0.52949866	0.64951047
199.2	0.52949866	0.64951047
199.3	0.52949866	0.64951047
199.4	0.52943714	0.64940766
199.5	0.52939347	0.64916919
199.6	0.52939347	0.64916919
199.7	0.5294129	0.64901026
199.8	0.5294129	0.64901026
199.9	0.52936354	0.64871501
200	0.52931351	0.64861373
200.1	0.52899042	0.64832919
200.2	0.528916	0.64804802
200.3	0.52896483	0.64803637
200.4	0.52896483	0.64803637
200.5	0.52896483	0.64803637
200.6	0.52896483	0.64803637
200.7	0.52896483	0.64803637
200.8	0.52896483	0.64803637
200.9	0.52896483	0.64803637
201	0.52868263	0.6479344
201.1	0.52868263	0.6479344
201.2	0.52868263	0.6479344
201.3	0.52868263	0.6479344
201.4	0.52868263	0.6479344
201.5	0.52868263	0.6479344
201.6	0.52868263	0.6479344
201.7	0.52868263	0.6479344
201.8	0.52868263	0.6479344
201.9	0.52868263	0.6479344
202	0.52868263	0.6479344
202.1	0.52868263	0.6479344
202.2	0.52868263	0.6479344
202.3	0.52868263	0.6479344
202.4	0.52868263	0.6479344
202.5	0.52868263	0.6479344
202.6	0.52860032	0.64788808
202.7	0.52860032	0.64788808
202.8	0.52860032	0.64788808
202.9	0.52860032	0.64788808
203	0.52860032	0.64788808
203.1	0.52860032	0.64788808
203.2	0.52860032	0.64788808
203.3	0.52860032	0.64788808
203.4	0.52860032	0.64788808
203.5	0.52860032	0.64788808
203.6	0.52860032	0.64788808
203.7	0.52860032	0.64788808
203.8	0.52860032	0.64788808
203.9	0.52860032	0.64788808
204	0.52860032	0.64788808
204.1	0.52860032	0.64788808
204.2	0.52860032	0.64788808
204.3	0.52860032	0.64788808
204.4	0.52860032	0.64788808
204.5	0.52860032	0.64788808
204.6	0.52860032	0.64788808
204.7	0.52860032	0.64788808
204.8	0.52860032	0.64788808
204.9	0.52860032	0.64788808
205	0.52860032	0.64788808
205.1	0.52860032	0.64788808
205.2	0.52860032	0.64788808
205.3	0.52860032	0.64788808
205.4	0.52860032	0.64788808
205.5	0.52860032	0.64788808
205.6	0.52857298	0.64795581
205.7	0.52857298	0.64795581
205.8	0.52857298	0.64795581
205.9	0.52825981	0.64780722
206	0.52847374	0.64773899
206.1	0.52847374	0.64773899
206.2	0.52847374	0.64773899
206.3	0.52847374	0.64773899
206.4	0.52847374	0.64773899
206.5	0.5286063	0.64779363
206.6	0.5286063	0.64779363
206.7	0.5286063	0.64779363
206.8	0.5286063	0.64779363
206.9	0.5286063	0.64779363
207	0.5286063	0.64779363
207.1	0.5286063	0.64779363
207.2	0.5286063	0.64779363
207.3	0.5286063	0.64779363
207.4	0.5286063	0.64779363
207.5	0.5286063	0.64779363
207.6	0.5286063	0.64779363
207.7	0.5286063	0.64779363
207.8	0.5286063	0.64779363
207.9	0.5286063	0.64779363
208	0.5286063	0.64779363
208.1	0.5286063	0.64779363
208.2	0.5286063	0.64779363
208.3	0.52882725	0.64786287
208.4	0.52892784	0.64788972
208.5	0.52892784	0.64788972
208.6	0.52892784	0.64788972
208.7	0.52892784	0.64788972
208.8	0.52866653	0.64777646
208.9	0.52866653	0.64777646
209	0.52866653	0.64777646
209.1	0.52866653	0.64777646
209.2	0.52866653	0.64777646
209.3	0.52866653	0.64777646
209.4	0.52866653	0.64777646
209.5	0.52866653	0.64777646
209.6	0.52866653	0.64777646
209.7	0.52866653	0.64777646
209.8	0.52866653	0.64777646
209.9	0.52866653	0.64777646

213.6	0.52930139	0.64788399
213.7	0.52930139	0.64788399
213.8	0.52930139	0.64788399
213.9	0.5290661	0.64780542
214	0.5290661	0.64780542
214.1	0.5289922	0.64774316
214.2	0.5289922	0.64774316
214.3	0.5289922	0.64774316
214.4	0.5289922	0.64774316
214.5	0.5289922	0.64774316
214.6	0.5289922	0.64774316
214.7	0.5289922	0.64774316
214.8	0.5289922	0.64774316
214.9	0.5289937	0.6476208
215	0.5289937	0.6476208
215.1	0.5289937	0.6476208
215.2	0.5289937	0.6476208
215.3	0.5289937	0.6476208
215.4	0.5289937	0.6476208
215.5	0.5289937	0.6476208
215.6	0.5289937	0.6476208
215.7	0.5289937	0.6476208
215.8	0.5289937	0.6476208
215.9	0.5289937	0.6476208
216	0.52891656	0.64759142
216.1	0.52891656	0.64759142
216.2	0.52891656	0.64759142
216.3	0.52891656	0.64759142
216.4	0.52891656	0.64759142
216.5	0.52891656	0.64759142
216.6	0.52891656	0.64759142
216.7	0.52891656	0.64759142
216.8	0.52891656	0.64759142
216.9	0.52912737	0.64764296
217	0.52912737	0.64764296
217.1	0.52927643	0.64757099
217.2	0.52927643	0.64757099
217.3	0.52952874	0.64765232
217.4	0.52952874	0.64765232
217.5	0.52952874	0.64765232
217.6	0.52952874	0.64765232
217.7	0.52952874	0.64765232
217.8	0.52952874	0.64765232
217.9	0.52952874	0.64765232
218	0.52952874	0.64765232
218.1	0.52952874	0.64765232
218.2	0.52952874	0.64765232
218.3	0.52952874	0.64765232
218.4	0.52952874	0.64765232
218.5	0.52952874	0.64765232
218.6	0.52918877	0.64752313
218.7	0.52918877	0.64752313
218.8	0.52918877	0.64752313
218.9	0.52918877	0.64752313
219	0.52918877	0.64752313
219.1	0.52918877	0.64752313
219.2	0.52918877	0.64752313
219.3	0.52918877	0.64752313
219.4	0.52928589	0.64749873
219.5	0.52928589	0.64749873
219.6	0.52928589	0.64749873
219.7	0.52951534	0.64748653
219.8	0.52951534	0.64748653
219.9	0.52942881	0.6474448
220	0.52942881	0.6474448
220.1	0.52942881	0.6474448
220.2	0.52942881	0.6474448
220.3	0.52942881	0.6474448
220.4	0.52942881	0.6474448
220.5	0.52942881	0.6474448
220.6	0.52942881	0.6474448
220.7	0.52942881	0.6474448
220.8	0.52942881	0.6474448
220.9	0.52942881	0.6474448
221	0.52956058	0.64745025
221.1	0.52941785	0.64736776
221.2	0.52941785	0.64736776
221.3	0.52941785	0.64736776
221.4	0.52941785	0.64736776
221.5	0.52941785	0.64736776
221.6	0.52941785	0.64736776
221.7	0.52941785	0.64736776
221.8	0.52941785	0.64736776
221.9	0.52924609	0.64713956
222	0.52924609	0.64713956
222.1	0.52924609	0.64713956
222.2	0.52924609	0.64713956
222.3	0.52924609	0.64713956
222.4	0.52924609	0.64713956
222.5	0.52924609	0.64713956
222.6	0.52924609	0.64713956
222.7	0.52924609	0.64713956
222.8	0.52924609	0.64713956
222.9	0.52944106	0.64708753
223	0.52944106	0.64708753
223.1	0.52944106	0.64708753
223.2	0.52944106	0.64708753
223.3	0.52944106	0.64708753
223.4	0.52944106	0.64708753
223.5	0.52951432	0.6471967
223.6	0.52951432	0.6471967
223.7	0.52951432	0.6471967
223.8	0.52951432	0.6471967
223.9	0.52951432	0.6471967
224	0.52982434	0.64737795
224.1	0.52982434	0.64737795
224.2	0.52982434	0.64737795
224.3	0.52982434	0.64737795
224.4	0.52982434	0.64737795
224.5	0.52982434	0.64737795
224.6	0.52959664	0.64730494
224.7	0.52959664	0.64730494
224.8	0.52959664	0.64730494
224.9	0.52926868	0.64713795

228.6	0.52857291	0.64601621
228.7	0.52857291	0.64601621
228.8	0.52857291	0.64601621
228.9	0.52857291	0.64601621
229	0.52857291	0.64601621
229.1	0.52857291	0.64601621
229.2	0.5287645	0.6460651
229.3	0.5287645	0.6460651
229.4	0.5287645	0.6460651
229.5	0.5287645	0.6460651
229.6	0.5287645	0.6460651
229.7	0.5287645	0.6460651
229.8	0.5287645	0.6460651
229.9	0.5287645	0.6460651
230	0.52876949	0.64601835
230.1	0.52876949	0.64601835
230.2	0.52876949	0.64601835
230.3	0.52876949	0.64601835
230.4	0.52876949	0.64601835
230.5	0.52876949	0.64601835
230.6	0.52876949	0.64601835
230.7	0.52876949	0.64601835
230.8	0.52876949	0.64601835
230.9	0.52876949	0.64601835
231	0.52876949	0.64601835
231.1	0.52876949	0.64601835
231.2	0.52891278	0.64602366
231.3	0.52891278	0.64602366
231.4	0.52899574	0.64590632
231.5	0.52899574	0.64590632
231.6	0.52899574	0.64590632
231.7	0.52882873	0.64583142
231.8	0.52882043	0.64588928
231.9	0.52882043	0.64588928
232	0.52882043	0.64588928
232.1	0.52882043	0.64588928
232.2	0.52882043	0.64588928
232.3	0.52905981	0.64596483
232.4	0.52905981	0.64596483
232.5	0.52905981	0.64596483
232.6	0.52905981	0.64596483
232.7	0.52905981	0.64596483
232.8	0.52905981	0.64596483
232.9	0.52931133	0.6459628
233	0.52931133	0.6459628
233.1	0.52952242	0.64595758
233.2	0.52952242	0.64595758
233.3	0.52952242	0.64595758
233.4	0.52952242	0.64595758
233.5	0.52957585	0.6459351
233.6	0.52973729	0.64587593
233.7	0.52974235	0.64578634
233.8	0.52974235	0.64578634
233.9	0.52974235	0.64578634
234	0.52974235	0.64578634
234.1	0.52974235	0.64578634
234.2	0.5295919	0.64563798
234.3	0.5295919	0.64563798
234.4	0.5295919	0.64563798
234.5	0.5295919	0.64563798
234.6	0.5295919	0.64563798
234.7	0.5295919	0.64563798
234.8	0.5295919	0.64563798
234.9	0.5295919	0.64563798
235	0.5295919	0.64563798
235.1	0.5295919	0.64563798
235.2	0.5295919	0.64563798
235.3	0.5295919	0.64563798
235.4	0.5295919	0.64563798
235.5	0.5295919	0.64563798
235.6	0.5295919	0.64563798
235.7	0.5295919	0.64563798
235.8	0.5295919	0.64563798
235.9	0.52984258	0.64559708
236	0.5300827	0.64567452
236.1	0.5300827	0.64567452
236.2	0.53021097	0.64557705
236.3	0.53034417	0.64551258
236.4	0.53034417	0.64551258
236.5	0.53073772	0.64570721
236.6	0.53073772	0.64570721
236.7	0.5308682	0.64577226
236.8	0.5308682	0.64577226
236.9	0.5308682	0.64577226
237	0.5308682	0.64577226
237.1	0.5308682	0.64577226
237.2	0.5308682	0.64577226
237.3	0.5308682	0.64577226
237.4	0.5308682	0.64577226
237.5	0.5308682	0.64577226
237.6	0.5308682	0.64577226
237.7	0.5308682	0.64577226
237.8	0.5308682	0.64577226
237.9	0.5308682	0.64577226
238	0.53095629	0.64557963
238.1	0.53095629	0.64557963
238.2	0.53095629	0.64557963
238.3	0.53095629	0.64557963
238.4	0.53095629	0.64557963
238.5	0.53095629	0.64557963
238.6	0.53095629	0.64557963
238.7	0.5312419	0.64557485
238.8	0.5312419	0.64557485
238.9	0.53129056	0.64550721
239	0.53129056	0.64550721
239.1	0.53129056	0.64550721
239.2	0.53126979	0.64556212
239.3	0.53126979	0.64556212
239.4	0.53126979	0.64556212
239.5	0.53135888	0.64538842
239.6	0.53135888	0.64538842
239.7	0.53135888	0.64538842
239.8	0.53135888	0.64538842
239.9	0.53135888	0.64538842
240	0.53168381	0.64556285

Corresponding p-values for PRCC sensitivtiy indices

Time discretization	L_E	[LACT]_0
0	0	0
0.1	1.66E-06	2.87E-171
0.2	1.36E-05	7.38E-136
0.3	3.68E-05	4.90E-124
0.4	5.86E-05	2.12E-118
0.5	6.26E-05	1.18E-116
0.6	8.19E-05	1.53E-114
0.7	9.70E-05	1.57E-113
0.8	0.00012762	8.16E-113
0.9	0.00014574	5.54E-112
1	0.00015845	2.48E-111
1.1	0.00015946	5.08E-111
1.2	0.00020717	1.21E-110
1.3	0.00023155	3.51E-110
1.4	0.00027092	1.19E-109
1.5	0.00025906	1.77E-109
1.6	0.00026798	3.51E-109
1.7	0.00026807	7.73E-109
1.8	0.00027606	1.58E-108
1.9	0.00030476	2.54E-108
2	0.00038383	4.55E-108
2.1	0.00042431	5.13E-108
2.2	0.00048509	1.39E-107
2.3	0.00045804	1.85E-107
2.4	0.00045019	2.03E-107
2.5	0.00042419	2.56E-107
2.6	0.00042542	2.91E-107
2.7	0.00036322	3.57E-107
2.8	0.00040265	4.69E-107
2.9	0.00039268	7.15E-107
3	0.00039016	6.46E-107
3.1	0.00036219	6.84E-107
3.2	0.00038955	7.79E-107
3.3	0.00039439	8.32E-107
3.4	0.00038143	9.67E-107
3.5	0.0003836	1.17E-106
3.6	0.00032262	1.62E-106
3.7	0.00032262	1.62E-106
3.8	0.00031139	1.67E-106
3.9	0.00029116	1.90E-106
4	0.00030422	2.37E-106
4.1	0.00027081	3.17E-106
4.2	0.00025413	4.46E-106
4.3	0.0002576	4.87E-106
4.4	0.00025521	5.56E-106
4.5	0.00025018	7.95E-106
4.6	0.00024275	1.23E-105
4.7	0.00025037	1.28E-105
4.8	0.00024893	1.90E-105
4.9	0.00025962	2.19E-105
5	0.00025931	3.46E-105
5.1	0.00027362	3.96E-105
5.2	0.00023279	4.14E-105
5.3	0.00025433	7.90E-105
5.4	0.00025183	8.67E-105
5.5	0.0002624	1.25E-104
5.6	0.00026558	1.42E-104
5.7	0.00025685	1.58E-104
5.8	0.0002548	1.77E-104
5.9	0.00021595	2.55E-104
6	0.00020029	2.73E-104
6.1	0.00018167	3.12E-104
6.2	0.0001843	3.77E-104
6.3	0.00019648	4.48E-104
6.4	0.00019913	4.95E-104
6.5	0.00019161	6.75E-104
6.6	0.00017399	1.08E-103
6.7	0.00016602	1.75E-103
6.8	0.00016714	3.99E-103
6.9	0.00015869	8.53E-103
7	0.00015995	1.07E-102
7.1	0.00017368	1.41E-102
7.2	0.00018339	2.34E-102
7.3	0.00017228	4.31E-102
7.4	0.0001862	7.75E-102
7.5	0.00017305	7.59E-102
7.6	0.00017066	1.45E-101
7.7	0.00016153	2.66E-101
7.8	0.00015154	2.75E-101
7.9	0.00014036	3.18E-101
8	0.00014914	4.88E-101
8.1	0.00014658	6.00E-101
8.2	0.0001665	7.76E-101
8.3	0.00017792	1.28E-100
8.4	0.00017403	1.49E-100
8.5	0.0001712	1.86E-100
8.6	0.00017823	2.80E-100
8.7	0.00019138	3.50E-100
8.8	0.00018024	7.02E-100
8.9	0.00020123	1.21E-99
9	0.00018402	1.47E-99
9.1	0.00016984	1.57E-99
9.2	0.00017278	1.98E-99
9.3	0.00017746	2.45E-99
9.4	0.00017975	3.45E-99
9.5	0.00016443	6.34E-99
9.6	0.00015378	6.51E-99
9.7	0.00014649	8.48E-99
9.8	0.00013804	1.05E-98
9.9	0.00014006	1.12E-98
10	0.00012442	1.41E-98
10.1	0.00012045	2.23E-98
10.2	0.00012676	4.14E-98
10.3	0.00013283	5.98E-98
10.4	0.00012519	6.55E-98
10.5	0.00013554	1.17E-97
10.6	0.00011206	1.25E-97
10.7	0.00011599	1.55E-97
10.8	0.00012576	1.81E-97
10.9	0.00013092	3.48E-97
11	0.00012075	5.62E-97
11.1	0.00012365	1.14E-96
11.2	0.00010961	1.11E-96
11.3	0.00010805	1.18E-96
11.4	0.00011297	2.64E-96
11.5	0.00011868	3.43E-96
11.6	0.0001177	3.29E-96
11.7	0.00011362	4.59E-96
11.8	0.00011506	5.62E-96

Time discretization	L_E	[LACT]_0
15	0.00023815	2.13E-91
15.1	0.00022881	2.56E-91
15.2	0.00023494	2.73E-91
15.3	0.00021831	2.94E-91
15.4	0.00021993	4.22E-91
15.5	0.00019388	4.79E-91
15.6	0.00019583	5.04E-91
15.7	0.00018819	5.01E-91
15.8	0.0001643	6.41E-91
15.9	0.00017826	8.76E-91
16	0.00016987	9.98E-91
16.1	0.00015754	9.44E-91
16.2	0.00014322	1.38E-90
16.3	0.00013745	2.04E-90
16.4	0.00014327	3.28E-90
16.5	0.00014581	3.73E-90
16.6	0.00014251	5.55E-90
16.7	0.00014149	6.54E-90
16.8	0.0001469	7.07E-90
16.9	0.00013533	7.87E-90
17	0.00014484	1.26E-89
17.1	0.0001435	2.36E-89
17.2	0.00014388	3.04E-89
17.3	0.00014155	3.80E-89
17.4	0.00015712	8.78E-89
17.5	0.00014883	1.06E-88
17.6	0.00016227	1.63E-88
17.7	0.00017527	2.83E-88
17.8	0.00015826	4.56E-88
17.9	0.00015593	6.71E-88
18	0.00015772	9.18E-88
18.1	0.00015438	1.39E-87
18.2	0.00017317	3.47E-87
18.3	0.00017274	6.20E-87
18.4	0.00019255	1.25E-86
18.5	0.00020139	1.52E-86
18.6	0.00018384	1.73E-86
18.7	0.00017457	2.07E-86
18.8	0.00017426	2.39E-86
18.9	0.00017779	3.85E-86
19	0.00018629	7.59E-86
19.1	0.00020047	1.05E-85
19.2	0.00019233	1.18E-85
19.3	0.00020824	1.55E-85
19.4	0.00021381	2.10E-85
19.5	0.00021388	3.41E-85
19.6	0.00023207	5.82E-85
19.7	0.00022252	6.95E-85
19.8	0.00022178	1.33E-84
19.9	0.00019045	1.04E-84
20	0.00018669	1.01E-84
20.1	0.00019383	1.17E-84
20.2	0.00017903	1.36E-84
20.3	0.00017216	1.71E-84
20.4	0.00016938	2.57E-84
20.5	0.00016671	3.34E-84
20.6	0.00016183	3.31E-84
20.7	0.00016982	4.15E-84
20.8	0.00017677	6.04E-84
20.9	0.00017056	6.97E-84
21	0.00016045	1.18E-83
21.1	0.0001476	2.20E-83
21.2	0.00014057	2.95E-83
21.3	0.00013281	3.94E-83
21.4	0.00013258	4.61E-83
21.5	0.00011533	6.29E-83
21.6	0.00011224	6.64E-83
21.7	0.00011534	9.49E-83
21.8	0.00011344	1.12E-82
21.9	0.00010628	2.00E-82
22	0.00010328	3.69E-82
22.1	0.00010133	3.76E-82
22.2	9.66E-05	4.08E-82
22.3	8.32E-05	4.30E-82
22.4	9.16E-05	5.97E-82
22.5	9.67E-05	9.54E-82
22.6	9.92E-05	1.53E-81
22.7	9.90E-05	1.85E-81
22.8	0.000105	2.59E-81
22.9	0.00011198	3.30E-81
23	0.00011131	4.29E-81
23.1	0.00010728	5.99E-81
23.2	0.00010418	7.49E-81
23.3	9.58E-05	1.02E-80
23.4	8.83E-05	1.24E-80
23.5	8.50E-05	1.31E-80
23.6	8.41E-05	1.44E-80
23.7	8.15E-05	1.71E-80
23.8	7.82E-05	1.97E-80
23.9	7.66E-05	2.27E-80
24	7.71E-05	2.59E-80
24.1	7.78E-05	2.81E-80
24.2	7.41E-05	3.14E-80
24.3	7.01E-05	3.61E-80
24.4	7.02E-05	4.08E-80
24.5	7.62E-05	5.11E-80
24.6	7.24E-05	6.55E-80
24.7	6.81E-05	6.72E-80
24.8	6.83E-05	7.56E-80
24.9	6.29E-05	1.26E-79
25	6.22E-05	2.28E-79
25.1	5.78E-05	3.26E-79
25.2	5.84E-05	3.19E-79
25.3	5.31E-05	3.43E-79
25.4	4.75E-05	3.63E-79
25.5	4.37E-05	3.71E-79
25.6	4.49E-05	4.95E-79
25.7	4.79E-05	7.64E-79
25.8	4.71E-05	7.85E-79
25.9	4.52E-05	9.99E-79
26	4.67E-05	1.63E-78
26.1	4.69E-05	2.14E-78
26.2	4.40E-05	4.11E-78
26.3	4.48E-05	4.65E-78
26.4	4.63E-05	5.95E-78
26.5	4.37E-05	6.58E-78
26.6	4.01E-05	6.77E-78
26.7	3.95E-05	1.04E-77
26.8	3.81E-05	1.43E-77

Time discretization	L_E	[LACT]_0
30	9.86E-06	1.20E-73
30.1	9.78E-06	1.31E-73
30.2	9.84E-06	1.49E-73
30.3	1.03E-05	1.93E-73
30.4	1.05E-05	2.49E-73
30.5	9.37E-06	3.01E-73
30.6	8.43E-06	3.18E-73
30.7	7.48E-06	4.28E-73
30.8	6.51E-06	7.99E-73
30.9	6.16E-06	1.54E-72
31	5.71E-06	2.26E-72
31.1	5.59E-06	3.15E-72
31.2	5.65E-06	4.12E-72
31.3	4.93E-06	5.30E-72
31.4	4.52E-06	7.45E-72
31.5	4.84E-06	1.20E-71
31.6	5.07E-06	1.56E-71
31.7	5.06E-06	1.64E-71
31.8	5.10E-06	1.83E-71
31.9	4.94E-06	2.22E-71
32	5.00E-06	3.28E-71
32.1	5.28E-06	4.57E-71
32.2	5.29E-06	6.13E-71
32.3	6.10E-06	8.97E-71
32.4	5.90E-06	1.54E-70
32.5	5.85E-06	1.78E-70
32.6	6.12E-06	3.41E-70
32.7	6.20E-06	4.86E-70
32.8	6.00E-06	6.02E-70
32.9	6.23E-06	1.32E-69
33	5.75E-06	2.07E-69
33.1	5.39E-06	3.16E-69
33.2	5.51E-06	5.95E-69
33.3	5.53E-06	7.32E-69
33.4	5.41E-06	1.24E-68
33.5	5.54E-06	1.95E-68
33.6	5.40E-06	2.79E-68
33.7	5.10E-06	4.05E-68
33.8	5.21E-06	5.05E-68
33.9	4.49E-06	5.91E-68
34	4.17E-06	9.36E-68
34.1	3.84E-06	1.39E-67
34.2	3.74E-06	2.03E-67
34.3	3.78E-06	3.14E-67
34.4	3.45E-06	4.74E-67
34.5	3.39E-06	6.24E-67
34.6	3.49E-06	8.45E-67
34.7	3.68E-06	1.03E-66
34.8	3.36E-06	1.20E-66
34.9	3.10E-06	1.33E-66
35	2.94E-06	1.63E-66
35.1	2.40E-06	2.33E-66
35.2	2.18E-06	3.91E-66
35.3	2.21E-06	5.17E-66
35.4	2.18E-06	9.05E-66
35.5	2.16E-06	1.27E-65
35.6	2.12E-06	2.08E-65
35.7	2.14E-06	2.66E-65
35.8	2.16E-06	5.99E-65
35.9	1.90E-06	7.71E-65
36	1.96E-06	1.36E-64
36.1	1.98E-06	1.67E-64
36.2	2.00E-06	2.10E-64
36.3	1.96E-06	2.94E-64
36.4	1.90E-06	3.37E-64
36.5	1.97E-06	5.07E-64
36.6	2.06E-06	9.82E-64
36.7	1.78E-06	1.59E-63
36.8	1.84E-06	2.09E-63
36.9	1.78E-06	3.29E-63
37	1.81E-06	3.65E-63
37.1	1.71E-06	6.31E-63
37.2	1.63E-06	7.43E-63
37.3	1.79E-06	1.00E-62
37.4	1.73E-06	1.42E-62
37.5	1.65E-06	2.27E-62
37.6	1.70E-06	3.03E-62
37.7	1.75E-06	3.85E-62
37.8	1.60E-06	3.82E-62
37.9	1.50E-06	3.69E-62
38	1.32E-06	3.71E-62
38.1	1.31E-06	3.99E-62
38.2	1.22E-06	4.24E-62
38.3	1.09E-06	5.21E-62
38.4	1.09E-06	7.07E-62
38.5	1.06E-06	9.02E-62
38.6	9.81E-07	9.21E-62
38.7	9.01E-07	8.44E-62
38.8	8.64E-07	9.74E-62
38.9	8.59E-07	1.22E-61
39	7.89E-07	1.38E-61
39.1	7.55E-07	1.89E-61
39.2	6.33E-07	1.76E-61
39.3	6.06E-07	2.08E-61
39.4	6.00E-07	2.39E-61
39.5	5.93E-07	2.45E-61
39.6	5.63E-07	3.26E-61
39.7	5.21E-07	3.73E-61
39.8	4.86E-07	5.34E-61
39.9	4.54E-07	5.44E-61
40	4.57E-07	5.60E-61
40.1	4.12E-07	6.03E-61
40.2	3.83E-07	7.73E-61
40.3	3.43E-07	9.89E-61
40.4	3.36E-07	1.00E-60
40.5	3.48E-07	1.16E-60
40.6	3.48E-07	1.38E-60
40.7	3.28E-07	1.79E-60
40.8	3.22E-07	2.19E-60
40.9	3.13E-07	2.49E-60
41	2.94E-07	2.82E-60
41.1	2.98E-07	3.15E-60
41.2	2.58E-07	3.53E-60
41.3	2.52E-07	4.79E-60
41.4	2.51E-07	5.22E-60
41.5	2.52E-07	5.65E-60
41.6	2.55E-07	8.57E-60
41.7	2.36E-07	1.01E-59
41.8	2.24E-07	1.38E-59

Time discretization	L_E	[LACT]_0
45	6.18E-08	2.11E-57
45.1	5.69E-08	2.19E-57
45.2	5.47E-08	2.31E-57
45.3	5.68E-08	2.76E-57
45.4	5.65E-08	2.92E-57
45.5	5.49E-08	3.10E-57
45.6	5.27E-08	3.68E-57
45.7	5.24E-08	4.60E-57
45.8	5.22E-08	4.99E-57
45.9	4.81E-08	4.71E-57
46	4.81E-08	5.04E-57
46.1	4.65E-08	5.37E-57
46.2	4.50E-08	5.62E-57
46.3	4.44E-08	5.73E-57
46.4	4.19E-08	7.28E-57
46.5	4.03E-08	6.94E-57
46.6	3.99E-08	7.44E-57
46.7	3.87E-08	7.46E-57
46.8	3.70E-08	8.27E-57
46.9	3.86E-08	1.06E-56
47	3.70E-08	1.14E-56
47.1	3.74E-08	1.38E-56
47.2	3.75E-08	1.41E-56
47.3	3.52E-08	1.40E-56
47.4	3.05E-08	1.72E-56
47.5	2.82E-08	1.86E-56
47.6	2.86E-08	2.21E-56
47.7	2.59E-08	2.20E-56
47.8	2.40E-08	2.43E-56
47.9	2.14E-08	2.45E-56
48	2.08E-08	2.66E-56
48.1	2.05E-08	2.76E-56
48.2	2.13E-08	3.16E-56
48.3	2.02E-08	3.79E-56
48.4	2.05E-08	4.29E-56
48.5	2.08E-08	4.64E-56
48.6	2.00E-08	5.56E-56
48.7	2.14E-08	6.50E-56
48.8	1.98E-08	6.78E-56
48.9	1.86E-08	7.20E-56
49	1.77E-08	7.41E-56
49.1	1.71E-08	7.62E-56
49.2	1.67E-08	9.23E-56
49.3	1.57E-08	1.00E-55
49.4	1.50E-08	9.78E-56
49.5	1.46E-08	1.41E-55
49.6	1.43E-08	1.45E-55
49.7	1.32E-08	1.71E-55
49.8	1.20E-08	1.83E-55
49.9	1.22E-08	2.23E-55
50	1.08E-08	2.31E-55
50.1	1.02E-08	2.76E-55
50.2	1.00E-08	3.23E-55
50.3	9.99E-09	3.22E-55
50.4	9.60E-09	3.79E-55
50.5	9.64E-09	3.96E-55
50.6	8.88E-09	4.02E-55
50.7	8.81E-09	4.78E-55
50.8	8.45E-09	4.55E-55
50.9	8.16E-09	5.98E-55
51	7.76E-09	6.57E-55
51.1	7.45E-09	7.92E-55
51.2	7.46E-09	8.66E-55
51.3	7.79E-09	1.04E-54
51.4	7.84E-09	1.19E-54
51.5	7.89E-09	1.33E-54
51.6	7.99E-09	1.39E-54
51.7	8.15E-09	1.64E-54
51.8	8.31E-09	1.82E-54
51.9	7.96E-09	2.21E-54
52	7.54E-09	2.29E-54
52.1	6.96E-09	2.24E-54
52.2	7.15E-09	2.51E-54
52.3	7.01E-09	2.82E-54
52.4	7.03E-09	3.15E-54
52.5	6.98E-09	3.63E-54
52.6	5.92E-09	3.84E-54
52.7	5.73E-09	4.07E-54
52.8	5.89E-09	4.31E-54
52.9	5.68E-09	4.24E-54
53	5.55E-09	4.41E-54
53.1	5.81E-09	4.86E-54
53.2	5.61E-09	5.14E-54
53.3	5.64E-09	6.22E-54
53.4	4.97E-09	7.18E-54
53.5	4.97E-09	8.40E-54
53.6	5.00E-09	1.13E-53
53.7	5.04E-09	1.31E-53
53.8	5.17E-09	1.51E-53
53.9	4.81E-09	1.70E-53
54	5.01E-09	2.10E-53
54.1	4.95E-09	2.61E-53
54.2	5.00E-09	2.78E-53
54.3	5.45E-09	4.32E-53
54.4	5.47E-09	5.13E-53
54.5	5.47E-09	5.59E-53
54.6	5.37E-09	6.51E-53
54.7	5.23E-09	6.70E-53
54.8	5.21E-09	7.72E-53
54.9	4.93E-09	8.09E-53
55	4.94E-09	8.89E-53
55.1	5.01E-09	1.07E-52
55.2	4.93E-09	1.11E-52
55.3	5.00E-09	1.31E-52
55.4	5.00E-09	1.56E-52
55.5	4.79E-09	1.66E-52
55.6	4.61E-09	2.11E-52
55.7	4.68E-09	2.48E-52
55.8	4.61E-09	2.49E-52
55.9	4.45E-09	2.94E-52
56	4.38E-09	3.57E-52
56.1	4.17E-09	4.00E-52
56.2	4.09E-09	4.30E-52
56.3	3.68E-09	4.85E-52
56.4	3.77E-09	5.81E-52
56.5	3.82E-09	7.82E-52
56.6	3.98E-09	9.28E-52
56.7	3.80E-09	1.07E-51
56.8	3.57E-09	1.21E-51

11.9	0.00011003	8.60E-96	26.9	3.69E-05	1.94E-77	41.9	2.23E-07	1.51E-59	56.9	3.70E-09	1.45E-51
12	0.00012068	1.32E-95	27	3.87E-05	2.43E-77	42	2.05E-07	1.67E-59	57	3.62E-09	1.50E-51
12.1	0.00012883	1.76E-95	27.1	3.50E-05	2.63E-77	42.1	1.77E-07	2.03E-59	57.1	3.79E-09	1.61E-51
12.2	0.0001574	4.19E-95	27.2	3.48E-05	2.81E-77	42.2	1.75E-07	2.75E-59	57.2	3.57E-09	1.80E-51
12.3	0.00016107	6.11E-95	27.3	3.18E-05	3.43E-77	42.3	1.64E-07	3.31E-59	57.3	3.46E-09	2.03E-51
12.4	0.00016008	1.09E-94	27.4	3.35E-05	4.31E-77	42.4	1.59E-07	4.15E-59	57.4	3.30E-09	2.09E-51
12.5	0.00016398	1.56E-94	27.5	3.18E-05	6.04E-77	42.5	1.57E-07	4.77E-59	57.5	3.21E-09	2.44E-51
12.6	0.0001634	1.61E-94	27.6	3.32E-05	1.30E-76	42.6	1.38E-07	5.79E-59	57.6	3.23E-09	2.74E-51
12.7	0.00016141	3.51E-94	27.7	3.25E-05	2.07E-76	42.7	1.31E-07	6.79E-59	57.7	3.02E-09	3.34E-51
12.8	0.00017711	5.27E-94	27.8	3.27E-05	2.90E-76	42.8	1.28E-07	9.56E-59	57.8	3.09E-09	3.70E-51
12.9	0.00019029	7.52E-94	27.9	2.98E-05	4.69E-76	42.9	1.18E-07	1.03E-58	57.9	2.95E-09	4.11E-51
13	0.00019419	8.70E-94	28	3.13E-05	6.28E-76	43	1.15E-07	1.23E-58	58	2.53E-09	3.88E-51
13.1	0.00018194	1.40E-93	28.1	3.04E-05	9.57E-76	43.1	1.20E-07	1.65E-58	58.1	2.48E-09	4.03E-51
13.2	0.00018444	2.04E-93	28.2	2.98E-05	1.18E-75	43.2	1.14E-07	1.72E-58	58.2	2.40E-09	4.34E-51
13.3	0.00020369	2.93E-93	28.3	2.99E-05	1.70E-75	43.3	1.07E-07	1.91E-58	58.3	2.27E-09	5.29E-51
13.4	0.00021726	3.19E-93	28.4	3.04E-05	1.99E-75	43.4	1.05E-07	2.31E-58	58.4	2.27E-09	5.79E-51
13.5	0.00021646	4.34E-93	28.5	2.80E-05	4.88E-75	43.5	1.03E-07	3.04E-58	58.5	2.36E-09	6.89E-51
13.6	0.00019386	8.87E-93	28.6	2.58E-05	5.92E-75	43.6	9.75E-08	3.94E-58	58.6	2.31E-09	8.64E-51
13.7	0.00019075	8.46E-93	28.7	2.52E-05	8.48E-75	43.7	9.45E-08	5.50E-58	58.7	2.18E-09	9.99E-51
13.8	0.00019774	1.31E-92	28.8	2.44E-05	1.03E-74	43.8	9.06E-08	6.11E-58	58.8	2.20E-09	1.12E-50
13.9	0.00021012	1.84E-92	28.9	2.17E-05	1.24E-74	43.9	8.65E-08	7.45E-58	58.9	2.31E-09	1.48E-50
14	0.00021496	2.21E-92	29	1.99E-05	1.23E-74	44	8.32E-08	9.67E-58	59	2.29E-09	1.52E-50
14.1	0.00021744	3.19E-92	29.1	1.82E-05	1.51E-74	44.1	7.90E-08	1.22E-57	59.1	2.26E-09	1.61E-50
14.2	0.00022141	4.15E-92	29.2	1.59E-05	2.22E-74	44.2	7.83E-08	1.26E-57	59.2	2.24E-09	1.75E-50
14.3	0.00023681	5.27E-92	29.3	1.62E-05	2.96E-74	44.3	7.95E-08	1.54E-57	59.3	2.18E-09	1.93E-50
14.4	0.00022384	6.16E-92	29.4	1.45E-05	3.84E-74	44.4	7.77E-08	1.59E-57	59.4	2.19E-09	2.15E-50
14.5	0.000211	6.19E-92	29.5	1.40E-05	5.02E-74	44.5	7.44E-08	1.58E-57	59.5	2.13E-09	2.23E-50
14.6	0.00021772	7.65E-92	29.6	1.36E-05	5.77E-74	44.6	7.08E-08	1.62E-57	59.6	1.90E-09	2.61E-50
14.7	0.00023094	1.05E-91	29.7	1.33E-05	7.76E-74	44.7	7.10E-08	1.61E-57	59.7	1.87E-09	2.81E-50
14.8	0.00025614	1.30E-91	29.8	1.16E-05	9.46E-74	44.8	6.93E-08	1.76E-57	59.8	1.85E-09	2.79E-50
14.9	0.00024938	1.90E-91	29.9	1.03E-05	9.32E-74	44.9	6.91E-08	2.08E-57	59.9	1.74E-09	2.89E-50

60	1.61E-09	3.72E-50	75	1.01E-11	1.40E-40	90	2.66E-12	2.79E-32	105	8.52E-13	2.35E-30
60.1	1.64E-09	4.32E-50	75.1	9.56E-12	1.61E-40	90.1	2.54E-12	2.91E-32	105.1	9.02E-13	2.50E-30
60.2	1.68E-09	4.97E-50	75.2	9.37E-12	1.79E-40	90.2	2.47E-12	3.26E-32	105.2	9.06E-13	2.54E-30
60.3	1.62E-09	5.47E-50	75.3	9.14E-12	2.40E-40	90.3	2.45E-12	3.43E-32	105.3	8.97E-13	2.57E-30
60.4	1.73E-09	6.82E-50	75.4	8.96E-12	2.62E-40	90.4	2.45E-12	3.43E-32	105.4	8.76E-13	2.71E-30
60.5	1.62E-09	7.13E-50	75.5	8.96E-12	2.62E-40	90.5	2.31E-12	3.28E-32	105.5	8.70E-13	2.78E-30
60.6	1.54E-09	7.79E-50	75.6	8.73E-12	3.10E-40	90.6	2.30E-12	3.37E-32	105.6	8.70E-13	2.78E-30
60.7	1.60E-09	9.36E-50	75.7	8.45E-12	3.35E-40	90.7	2.27E-12	3.51E-32	105.7	8.70E-13	2.78E-30
60.8	1.70E-09	1.25E-49	75.8	8.49E-12	3.91E-40	90.8	2.18E-12	3.57E-32	105.8	8.69E-13	2.95E-30
60.9	1.70E-09	1.53E-49	75.9	7.92E-12	4.05E-40	90.9	2.10E-12	3.59E-32	105.9	8.71E-13	3.06E-30
61	1.70E-09	2.02E-49	76	8.08E-12	5.00E-40	91	2.02E-12	3.64E-32	106	8.53E-13	3.01E-30
61.1	1.62E-09	2.15E-49	76.1	7.95E-12	5.27E-40	91.1	1.98E-12	3.60E-32	106.1	8.95E-13	3.13E-30
61.2	1.60E-09	2.71E-49	76.2	7.83E-12	5.75E-40	91.2	1.98E-12	3.60E-32	106.2	8.95E-13	3.13E-30
61.3	1.56E-09	2.89E-49	76.3	7.96E-12	6.11E-40	91.3	1.94E-12	3.63E-32	106.3	9.36E-13	3.43E-30
61.4	1.49E-09	3.15E-49	76.4	7.85E-12	7.06E-40	91.4	1.83E-12	3.75E-32	106.4	9.54E-13	3.67E-30
61.5	1.53E-09	3.56E-49	76.5	7.47E-12	8.07E-40	91.5	1.82E-12	3.71E-32	106.5	9.81E-13	4.28E-30
61.6	1.47E-09	3.76E-49	76.6	7.06E-12	8.56E-40	91.6	1.84E-12	4.04E-32	106.6	1.03E-12	4.68E-30
61.7	1.27E-09	4.54E-49	76.7	6.61E-12	1.04E-39	91.7	1.87E-12	4.15E-32	106.7	1.16E-12	5.74E-30
61.8	1.28E-09	5.53E-49	76.8	6.60E-12	1.19E-39	91.8	1.87E-12	4.15E-32	106.8	1.11E-12	5.65E-30
61.9	1.17E-09	5.95E-49	76.9	6.70E-12	1.52E-39	91.9	1.84E-12	4.34E-32	106.9	1.15E-12	6.05E-30
62	1.21E-09	7.47E-49	77	6.66E-12	1.67E-39	92	1.72E-12	4.72E-32	107	1.14E-12	6.33E-30
62.1	1.06E-09	8.31E-49	77.1	6.72E-12	1.76E-39	92.1	1.70E-12	4.78E-32	107.1	1.22E-12	7.33E-30
62.2	1.10E-09	1.07E-48	77.2	6.72E-12	1.76E-39	92.2	1.64E-12	4.64E-32	107.2	1.22E-12	7.33E-30
62.3	1.07E-09	1.22E-48	77.3	6.36E-12	1.89E-39	92.3	1.57E-12	4.67E-32	107.3	1.20E-12	7.34E-30
62.4	1.01E-09	1.69E-48	77.4	6.34E-12	1.92E-39	92.4	1.54E-12	5.02E-32	107.4	1.22E-12	7.77E-30
62.5	1.01E-09	2.17E-48	77.5	6.30E-12	2.07E-39	92.5	1.51E-12	5.23E-32	107.5	1.20E-12	7.78E-30
62.6	1.04E-09	3.33E-48	77.6	5.92E-12	2.28E-39	92.6	1.54E-12	5.31E-32	107.6	1.20E-12	7.78E-30
62.7	1.01E-09	4.95E-48	77.7	5.59E-12	2.41E-39	92.7	1.54E-12	5.31E-32	107.7	1.20E-12	7.97E-30
62.8	9.96E-10	7.85E-48	77.8	5.46E-12	2.98E-39	92.8	1.45E-12	5.65E-32	107.8	1.19E-12	8.71E-30
62.9	9.59E-10	9.44E-48	77.9	5.42E-12	3.43E-39	92.9	1.45E-12	5.65E-32	107.9	1.19E-12	8.84E-30
63	9.12E-10	1.28E-47	78	5.63E-12	4.14E-39	93	1.40E-12	5.66E-32	108	1.25E-12	9.58E-30
63.1	8.50E-10	1.46E-47	78.1	5.84E-12	4.64E-39	93.1	1.36E-12	5.72E-32	108.1	1.25E-12	9.58E-30
63.2	7.87E-10	1.53E-47	78.2	6.00E-12	6.03E-39	93.2	1.34E-12	5.86E-32	108.2	1.21E-12	9.66E-30
63.3	7.14E-10	1.65E-47	78.3	5.71E-12	6.99E-39	93.3	1.29E-12	5.92E-32	108.3	1.20E-12	1.02E-29
63.4	6.76E-10	2.20E-47	78.4	5.36E-12	7.07E-39	93.4	1.33E-12	6.61E-32	108.4	1.20E-12	1.05E-29
63.5	6.46E-10	2.39E-47	78.5	5.35E-12	8.71E-39	93.5	1.32E-12	7.02E-32	108.5	1.16E-12	1.07E-29
63.6	5.81E-10	2.52E-47	78.6	5.33E-12	1.05E-38	93.6	1.19E-12	7.77E-32	108.6	1.15E-12	1.15E-29
63.7	5.60E-10	2.60E-47	78.7	5.19E-12	1.14E-38	93.7	1.16E-12	7.87E-32	108.7	1.13E-12	1.12E-29
63.8	5.44E-10	2.96E-47	78.8	5.12E-12	1.39E-38	93.8	1.11E-12	7.84E-32	108.8	1.14E-12	1.25E-29
63.9	5.47E-10	3.37E-47	78.9	5.34E-12	1.49E-38	93.9	1.03E-12	8.00E-32	108.9	1.18E-12	1.31E-29
64	4.98E-10	4.25E-47	79	4.97E-12	1.60E-38	94	1.03E-12	8.00E-32	109	1.18E-12	1.31E-29
64.1	4.37E-10	6.01E-47	79.1	5.21E-12	2.01E-38	94.1	1.04E-12	8.72E-32	109.1	1.18E-12	1.34E-29
64.2	4.28E-10	6.14E-47	79.2	5.15E-12	2.64E-38	94.2	1.00E-12	8.77E-32	109.2	1.21E-12	1.47E-29
64.3	4.03E-10	6.27E-47	79.3	5.33E-12	3.54E-38	94.3	9.67E-13	9.64E-32	109.3	1.20E-12	1.48E-29
64.4	3.71E-10	7.25E-47	79.4	5.37E-12	5.27E-38	94.4	1.03E-12	1.02E-31	109.4	1.20E-12	1.48E-29
64.5	3.44E-10	7.30E-47	79.5	5.29E-12	6.33E-38	94.5	1.03E-12	1.05E-31	109.5	1.17E-12	1.49E-29
64.6	3.29E-10	7.60E-47	79.6	5.25E-12	6.82E-38	94.6	1.01E-12	1.02E-31	109.6	1.17E-12	1.51E-29
64.7	3.20E-10	1.02E-46	79.7	5.42E-12	9.87E-38	94.7	1.03E-12	1.12E-31	109.7	1.13E-12	1.46E-29
64.8	3.17E-10	1.17E-46	79.8	5.61E-12	1.23E-37	94.8	9.94E-13	1.20E-31	109.8	1.13E-12	1.46E-29
64.9	2.98E-10	1.47E-46	79.9	5.85E-12	1.63E-37	94.9	9.94E-13	1.20E-31	109.9	1.13E-12	1.46E-29
65	2.99E-10	1.73E-46	80	5.85E-12	1.63E-37	95	9.55E-13	1.22E-31	110	1.13E-12	1.46E-29
65.1	2.83E-10	1.79E-46	80.1	6.22E-12	2.45E-37	95.1	9.55E-13	1.30E-31	110.1	1.13E-12	1.46E-29
65.2	2.48E-10	1.78E-46	80.2	6.28E-12	3.32E-37	95.2	9.79E-13	1.35E-31	110.2	1.08E-12	1.42E-29
65.3	2.21E-10	2.04E-46	80.3	5.94E-12	3.69E-37	95.3	9.74E-13	1.40E-31	110.3	1.08E-12	1.42E-29
65.4	2.15E-10	2.10E-46	80.4	6.21E-12	4.08E-37	95.4	9.55E-13	1.56E-31	110.4	1.05E-12	1.38E-29
65.5	2.03E-10	2.09E-46	80.5	6.55E-12	5.53E-37	95.5	9.46E-13	1.59E-31	110.5	1.04E-12	1.41E-29
65.6	1.97E-10	2.10E-46	80.6	6.35E-12	6.10E-37	95.6	9.46E-13	1.59E-31	110.6	1.03E-12	1.61E-29
65.7	1.79E-10	2.80E-46	80.7	6.58E-12	7.43E-37	95.7	9.46E-13	1.59E-31	110.7	1.03E-12	1.61E-29
65.8	1.67E-10	3.52E-46	80.8	6.39E-12	7.30E-37	95.8	8.90E-13	1.47E-31	110.8	1.02E-12	1.63E-29
65.9	1.57E-10	4.32E-46	80.9	6.26E-12	7.81E-37	95.9	9.14E-13	1.52E-31	110.9	9.71E-13	1.61E-29
66	1.51E-10	5.26E-46	81	6.23E-12	8.11E-37	96	8.77E-13	1.59E-31	111	9.50E-13	1.66E-29
66.1	1.39E-10	5.29E-46	81.1	6.12E-12	8.28E-37	96.1	8.77E-13	1.59E-31	111.1	9.40E-13	1.74E-29
66.2	1.39E-10	6.12E-46	81.2	6.32E-12	1.03E-36	96.2	8.99E-13	1.65E-31	111.2	8.94E-13	1.92E-29
66.3	1.34E-10	8.12E-46	81.3	6.75E-12	1.38E-36	96.3	8.76E-13	1.63E-31	111.3	8.94E-13	1.92E-29
66.4	1.29E-10	1.03E-45	81.4	6.76E-12	1.81E-36	96.4	8.25E-13	1.69E-31	111.4	9.11E-13	1.98E-29
66.5	1.05E-10	1.14E-45	81.5	6.89E-12	2.10E-36	96.5	8.25E-13	1.69E-31	111.5	8.87E-13	1.99E-29
66.6	9.83E-11	1.16E-45	81.6	6.88E-12	2.32E-36	96.6	8.23E-13	1.84E-31	111.6	8.87E-13	1.99E-29
66.7	9.75E-11	1.32E-45	81.7	7.45E-12	3.18E-36	96.7	8.23E-13	1.84E-31	111.7	8.87E-13	1.99E-29
66.8	8.88E-11	1.43E-45	81.8	7.66E-12	3.57E-36	96.8	7.72E-13	1.92E-31	111.8	8.87E-13	1.99E-29
66.9	7.96E-11	1.62E-45	81.9	8.02E-12	4.73E-36	96.9	7.94E-13	2.16E-31	111.9	8.75E-13	2.07E-29
67	7.35E-11	1.75E-45	82	7.71E-12	6.21E-36	97	7.91E-13	2.26E-31	112	8.75E-13	2.07E-29
67.1	7.16E-11	1.91E-45	82.1	7.59E-12	7.12E-36	97.1	7.92E-13	2.40E-31	112.1	8.63E-13	2.15E-29
67.2	6.67E-11	1.91E-45	82.2	7.34E-12	8.44E-36	97.2	7.58E-13	2.42E-31	112.2	8.45E-13	2.15E-29
67.3	6.61E-11	1.96E-45	82.3	7.46E-12	9.07E-36	97.3	7.69E-13	2.49E-31	112.3	8.45E-13	2.15E-29
67.4	6.92E-11	2.17E-45	82.4	7.23E-12	1.06E-35	97.4	7.51E-13	2.44E-31	112.4	8.45E-13	2.15E-29
67.5	6.63E-11	2.46E-45	82.5	7.33E-12	1.23E-35	97.5	7.51E-13	2.44E-31	112.5	8.40E-13	2.40E-29
67.6	6.21E-11	2.48E-45	82.6	7.09E-12	1.34E-35	97.6	7.51E-13	2.44E-31	112.6	8.17E-13	2.58E-29
67.7	5.24E-11	2.70E-45	82.7	7.00E-12	1.54E-35	97.7	7.40E-13	2.57E-31	112.7	8.28E-13	2.59E-29
67.8	5.10E-11	2.91E-45	82.8	6.68E-12	1.70E-35	97.8	7.40E-13	2.85E-31	112.8	8.28E-13	2.98E-29
67.9	5.29E-11	3.24E-45	82.9	6.43E-12	1.86E-35	97.9	7.37E-13	2.97E-31	112.9	8.28E-13	3.02E-29
68	5.37E-11	3.43E-45	83	6.30E-12	1.95E-35	98	7.37E-13	2.97E-31	113	8.25E-13	3.16E-29
68.1	4.97E-11	4.14E-45	83.1	6.03E-12	1.98E-35	98.1	7.18E-13	3.00E-31	113.1	7.97E-13	3.40E-29
68.2	4.51E-11	4.44E-45	83.2	6.00E-12	2.14E-35	98.2	7.36E-13	3.16E-31	113.2	7.97E-13	3.40E-29
68.3	4.43E-11	5.90E-45	83.3	5.63E-12	2.07E-35	98.3	7.34E-13	3.34E-31	113.3	7.89E-13	3.41E-29
68.4	4.25E-11	6.54E-45	83.4	5.64E-12	2.42E-35	98.4	7.85E-13	3.57E-31	113.4	7.89E-13	3.41E-29
68.5	4.32E-11	7.79E-45	83.5	5.61E-12	3.09E-35	98.5	7.89E-13	3.59E-31	113.5	7.73E-13	3.60E-29
68.6	4.21E-11	8.38E-45	83.6	5.61E-12	3.25E-35	98.6	7.84E-13	3.89E-31	113.6	7.78E-13	3.66E-29
68.7	3.86E-11	1.09E-44	83.7	5.65E-12	3.42E-35	98.7	7.80E-13	4.19E-31	113.7	7.78E-13	3.84E-29
68.8	3.78E-11	1.46E-44	83.8	5.73E-12	4.30E-35	98.8	7.89E-13	4.43E-31	113.8	7.66E-13	3.96E-29
68.9	3.68E-11	1.61E-44	83.9	6.08E-12	5.93E-35	98.9	7.96E-13	4.63E-31	113.9	7.60E-13	4.08E-29

69	3.57E-11	1.70E-44	84	5.75E-12	6.51E-35	99	7.87E-13	4.90E-31	114	7.50E-13	4.13E-29
69.1	3.49E-11	2.02E-44	84.1	5.78E-12	7.71E-35	99.1	7.74E-13	5.28E-31	114.1	7.62E-13	4.26E-29
69.2	3.35E-11	2.35E-44	84.2	5.86E-12	7.99E-35	99.2	7.82E-13	5.63E-31	114.2	7.55E-13	4.35E-29
69.3	3.34E-11	3.02E-44	84.3	5.69E-12	8.20E-35	99.3	7.77E-13	5.86E-31	114.3	7.55E-13	4.35E-29
69.4	3.18E-11	3.22E-44	84.4	5.53E-12	8.82E-35	99.4	7.53E-13	6.50E-31	114.4	7.32E-13	4.25E-29
69.5	3.14E-11	4.12E-44	84.5	5.67E-12	9.86E-35	99.5	7.53E-13	6.50E-31	114.5	7.13E-13	4.17E-29
69.6	3.07E-11	4.48E-44	84.6	5.87E-12	1.29E-34	99.6	7.39E-13	6.62E-31	114.6	7.05E-13	4.10E-29
69.7	3.03E-11	4.55E-44	84.7	5.52E-12	1.46E-34	99.7	7.86E-13	8.12E-31	114.7	7.05E-13	4.10E-29
69.8	2.93E-11	4.79E-44	84.8	5.27E-12	2.04E-34	99.8	7.83E-13	9.66E-31	114.8	7.11E-13	4.71E-29
69.9	2.86E-11	5.79E-44	84.9	5.43E-12	2.22E-34	99.9	7.52E-13	1.01E-30	114.9	6.88E-13	4.70E-29
70	2.71E-11	6.11E-44	85	5.52E-12	2.35E-34	100	7.52E-13	1.01E-30	115	6.66E-13	5.12E-29
70.1	2.73E-11	7.98E-44	85.1	5.14E-12	2.54E-34	100.1	7.46E-13	1.07E-30	115.1	6.52E-13	5.19E-29
70.2	2.66E-11	9.15E-44	85.2	5.00E-12	3.09E-34	100.2	7.46E-13	1.07E-30	115.2	6.52E-13	5.19E-29
70.3	2.56E-11	1.05E-43	85.3	4.99E-12	3.47E-34	100.3	7.92E-13	1.32E-30	115.3	6.20E-13	5.08E-29
70.4	2.44E-11	1.20E-43	85.4	4.92E-12	3.97E-34	100.4	7.85E-13	1.31E-30	115.4	6.10E-13	5.10E-29
70.5	2.23E-11	1.63E-43	85.5	5.02E-12	4.66E-34	100.5	7.85E-13	1.31E-30	115.5	5.93E-13	5.30E-29
70.6	2.22E-11	1.79E-43	85.6	4.70E-12	4.73E-34	100.6	7.85E-13	1.31E-30	115.6	5.69E-13	5.19E-29
70.7	2.26E-11	2.07E-43	85.7	4.69E-12	5.58E-34	100.7	7.86E-13	1.47E-30	115.7	5.63E-13	5.65E-29
70.8	2.24E-11	2.24E-43	85.8	4.74E-12	6.61E-34	100.8	8.07E-13	1.53E-30	115.8	5.56E-13	5.77E-29
70.9	2.30E-11	3.06E-43	85.9	4.88E-12	9.19E-34	100.9	8.07E-13	1.53E-30	115.9	5.20E-13	5.73E-29
71	2.29E-11	3.19E-43	86	4.79E-12	1.12E-33	101	7.71E-13	1.48E-30	116	5.20E-13	5.73E-29
71.1	2.21E-11	3.88E-43	86.1	4.40E-12	1.26E-33	101.1	7.71E-13	1.48E-30	116.1	5.20E-13	5.73E-29
71.2	2.17E-11	4.61E-43	86.2	4.61E-12	1.54E-33	101.2	7.38E-13	1.44E-30	116.2	5.24E-13	5.74E-29
71.3	2.10E-11	5.29E-43	86.3	4.38E-12	1.65E-33	101.3	7.33E-13	1.47E-30	116.3	5.23E-13	5.85E-29
71.4	2.03E-11	5.81E-43	86.4	4.31E-12	1.82E-33	101.4	7.33E-13	1.47E-30	116.4	5.23E-13	6.07E-29
71.5	2.06E-11	6.81E-43	86.5	4.12E-12	1.92E-33	101.5	7.33E-13	1.47E-30	116.5	5.12E-13	6.09E-29
71.6	2.02E-11	7.21E-43	86.6	4.21E-12	2.38E-33	101.6	7.33E-13	1.47E-30	116.6	5.18E-13	6.35E-29
71.7	1.93E-11	8.58E-43	86.7	4.37E-12	2.73E-33	101.7	7.33E-13	1.47E-30	116.7	4.99E-13	6.21E-29
71.8	1.90E-11	9.44E-43	86.8	4.16E-12	2.50E-33	101.8	7.12E-13	1.45E-30	116.8	4.66E-13	5.73E-29
71.9	2.00E-11	1.02E-42	86.9	4.18E-12	2.75E-33	101.9	7.12E-13	1.45E-30	116.9	4.66E-13	5.73E-29
72	1.85E-11	1.18E-42	87	4.04E-12	2.74E-33	102	7.12E-13	1.45E-30	117	4.56E-13	6.09E-29
72.1	1.86E-11	1.39E-42	87.1	4.08E-12	2.84E-33	102.1	7.03E-13	1.47E-30	117.1	4.46E-13	6.31E-29
72.2	1.85E-11	1.62E-42	87.2	3.80E-12	3.19E-33	102.2	7.03E-13	1.47E-30	117.2	4.46E-13	6.31E-29
72.3	1.80E-11	1.92E-42	87.3	3.54E-12	3.34E-33	102.3	6.95E-13	1.47E-30	117.3	4.43E-13	6.41E-29
72.4	1.73E-11	1.96E-42	87.4	3.54E-12	3.34E-33	102.4	7.35E-13	1.55E-30	117.4	4.43E-13	6.41E-29
72.5	1.71E-11	2.24E-42	87.5	3.56E-12	3.48E-33	102.5	7.42E-13	1.55E-30	117.5	4.49E-13	6.65E-29
72.6	1.65E-11	2.57E-42	87.6	3.36E-12	3.80E-33	102.6	7.25E-13	1.54E-30	117.6	4.38E-13	6.91E-29
72.7	1.57E-11	3.14E-42	87.7	3.36E-12	4.00E-33	102.7	7.25E-13	1.54E-30	117.7	4.33E-13	7.33E-29
72.8	1.55E-11	3.54E-42	87.8	3.50E-12	5.36E-33	102.8	7.16E-13	1.55E-30	117.8	4.23E-13	7.41E-29
72.9	1.50E-11	3.91E-42	87.9	3.34E-12	6.01E-33	102.9	7.16E-13	1.55E-30	117.9	4.21E-13	7.38E-29
73	1.55E-11	4.77E-42	88	3.36E-12	6.99E-33	103	7.16E-13	1.55E-30	118	4.17E-13	7.64E-29
73.1	1.55E-11	4.77E-42	88.1	3.27E-12	7.61E-33	103.1	7.00E-13	1.57E-30	118.1	4.17E-13	7.64E-29
73.2	1.49E-11	5.54E-42	88.2	3.38E-12	8.75E-33	103.2	6.90E-13	1.56E-30	118.2	4.13E-13	7.74E-29
73.3	1.42E-11	7.32E-42	88.3	3.35E-12	8.56E-33	103.3	7.01E-13	1.64E-30	118.3	4.09E-13	7.94E-29
73.4	1.39E-11	9.52E-42	88.4	3.27E-12	8.84E-33	103.4	7.40E-13	1.69E-30	118.4	3.91E-13	8.22E-29
73.5	1.35E-11	1.11E-41	88.5	3.03E-12	1.02E-32	103.5	7.57E-13	1.75E-30	118.5	3.88E-13	8.47E-29
73.6	1.41E-11	1.35E-41	88.6	3.06E-12	1.15E-32	103.6	7.49E-13	1.81E-30	118.6	3.89E-13	8.38E-29
73.7	1.36E-11	1.40E-41	88.7	3.16E-12	1.24E-32	103.7	7.89E-13	1.89E-30	118.7	3.92E-13	8.53E-29
73.8	1.32E-11	1.57E-41	88.8	2.99E-12	1.36E-32	103.8	7.71E-13	1.92E-30	118.8	3.97E-13	9.09E-29
73.9	1.22E-11	1.64E-41	88.9	2.92E-12	1.43E-32	103.9	7.71E-13	1.92E-30	118.9	3.82E-13	9.67E-29
74	1.16E-11	1.79E-41	89	2.94E-12	1.54E-32	104	7.59E-13	1.95E-30	119	3.80E-13	9.64E-29
74.1	1.13E-11	2.20E-41	89.1	2.82E-12	1.59E-32	104.1	7.59E-13	1.95E-30	119.1	3.90E-13	1.02E-28
74.2	1.11E-11	2.28E-41	89.2	2.82E-12	1.74E-32	104.2	7.63E-13	1.99E-30	119.2	3.84E-13	1.08E-28
74.3	1.08E-11	2.71E-41	89.3	2.66E-12	1.64E-32	104.3	7.43E-13	2.02E-30	119.3	3.84E-13	1.08E-28
74.4	1.10E-11	3.42E-41	89.4	2.67E-12	1.68E-32	104.4	8.03E-13	2.17E-30	119.4	3.71E-13	1.12E-28
74.5	1.09E-11	3.84E-41	89.5	2.50E-12	1.88E-32	104.5	8.03E-13	2.17E-30	119.5	3.68E-13	1.18E-28
74.6	1.08E-11	6.47E-41	89.6	2.72E-12	2.20E-32	104.6	8.37E-13	2.25E-30	119.6	3.65E-13	1.21E-28
74.7	1.08E-11	8.30E-41	89.7	2.79E-12	2.33E-32	104.7	8.37E-13	2.25E-30	119.7	3.58E-13	1.17E-28
74.8	1.07E-11	8.65E-41	89.8	2.66E-12	2.50E-32	104.8	8.53E-13	2.30E-30	119.8	3.57E-13	1.23E-28
74.9	1.04E-11	1.08E-40	89.9	2.59E-12	2.46E-32	104.9	8.53E-13	2.30E-30	119.9	3.58E-13	1.24E-28

120	3.63E-13	1.30E-28	135	8.70E-13	1.04E-24	150	4.88E-13	3.52E-23	165	2.34E-13	8.95E-23
120.1	3.73E-13	1.47E-28	135.1	8.56E-13	1.04E-24	150.1	4.89E-13	3.53E-23	165.1	2.32E-13	8.69E-23
120.2	3.82E-13	1.59E-28	135.2	8.56E-13	1.18E-24	150.2	4.89E-13	3.53E-23	165.2	2.34E-13	8.69E-23
120.3	3.75E-13	1.62E-28	135.3	8.42E-13	1.19E-24	150.3	4.89E-13	3.53E-23	165.3	2.34E-13	8.69E-23
120.4	3.75E-13	1.62E-28	135.4	8.32E-13	1.28E-24	150.4	4.89E-13	3.53E-23	165.4	2.34E-13	8.69E-23
120.5	3.83E-13	1.68E-28	135.5	8.31E-13	1.29E-24	150.5	4.89E-13	3.53E-23	165.5	2.34E-13	8.69E-23
120.6	3.81E-13	1.98E-28	135.6	8.14E-13	1.34E-24	150.6	4.89E-13	3.53E-23	165.6	2.34E-13	8.69E-23
120.7	3.85E-13	2.04E-28	135.7	8.14E-13	1.34E-24	150.7	4.89E-13	3.53E-23	165.7	2.37E-13	8.90E-23
120.8	3.79E-13	2.22E-28	135.8	8.14E-13	1.45E-24	150.8	4.89E-13	3.53E-23	165.8	2.37E-13	8.90E-23
120.9	3.74E-13	2.45E-28	135.9	8.22E-13	1.57E-24	150.9	4.75E-13	3.47E-23	165.9	2.31E-13	8.87E-23
121	3.91E-13	2.54E-28	136	8.15E-13	1.67E-24	151	4.75E-13	3.47E-23	166	2.32E-13	9.24E-23
121.1	3.91E-13	2.57E-28	136.1	8.15E-13	1.67E-24	151.1	4.75E-13	3.47E-23	166.1	2.32E-13	9.24E-23
121.2	3.88E-13	2.73E-28	136.2	8.27E-13	1.92E-24	151.2	4.60E-13	3.52E-23	166.2	2.32E-13	9.24E-23
121.3	4.04E-13	3.11E-28	136.3	8.10E-13	2.00E-24	151.3	4.56E-13	3.62E-23	166.3	2.27E-13	9.22E-23
121.4	4.03E-13	3.05E-28	136.4	7.68E-13	2.13E-24	151.4	4.56E-13	3.62E-23	166.4	2.27E-13	9.22E-23
121.5	3.86E-13	3.03E-28	136.5	7.64E-13	2.25E-24	151.5	4.56E-13	3.62E-23	166.5	2.24E-13	1.03E-22
121.6	3.93E-13	2.99E-28	136.6	7.78E-13	2.41E-24	151.6	4.42E-13	3.63E-23	166.6	2.24E-13	1.03E-22
121.7	3.72E-13	3.07E-28	136.7	7.84E-13	2.46E-24	151.7	4.37E-13	3.65E-23	166.7	2.24E-13	1.03E-22
121.8	3.76E-13	3.42E-28	136.8	7.92E-13	2.48E-24	151.8	4.29E-13	3.74E-23	166.8	2.24E-13	1.03E-22
121.9	3.87E-13	3.51E-28	136.9	7.55E-13	2.58E-24	151.9	4.29E-13	3.74E-23	166.9	2.24E-13	1.03E-22
122	3.82E-13	3.72E-28	137	7.55E-13	2.58E-24	152	4.29E-13	3.74E-23	167	2.21E-13	1.04E-22
122.1	3.69E-13	3.63E-28	137.1	7.37E-13	2.68E-24	152.1	4.29E-13	3.74E-23	167.1	2.18E-13	1.09E-22
122.2	3.73E-13	3.78E-28	137.2	7.22E-13	2.67E-24	152.2	4.29E-13	3.74E-23	167.2	2.18E-13	1.09E-22
122.3	3.70E-13	3.96E-28	137.3	7.24E-13	2.77E-24	152.3	4.21E-13	3.78E-23	167.3	2.18E-13	1.09E-22
122.4	3.75E-13	4.27E-28	137.4	7.24E-13	2.77E-24	152.4	4.21E-13	3.78E-23	167.4	2.18E-13	1.09E-22
122.5	3.81E-13	4.32E-28	137.5	7.21E-13	2.90E-24	152.5	4.17E-13	3.92E-23	167.5	2.18E-13	1.09E-22
122.6	3.72E-13	4.42E-28	137.6	6.90E-13	2.77E-24	152.6	4.27E-13	3.95E-23	167.6	2.15E-13	1.11E-22
122.7	3.70E-13	4.56E-28	137.7	6.90E-13	2.77E-24	152.7	4.27E-13	3.95E-23	167.7	2.15E-13	1.11E-22
122.8	3.70E-13	4.56E-28	137.8	6.81E-13	2.85E-24	152.8	4.19E-13	4.05E-23	167.8	2.10E-13	1.15E-22
122.9	3.66E-13	4.71E-28	137.9	6.78E-13	3.03E-24	152.9	4.19E-13	4.05E-23	167.9	2.06E-13	1.13E-22
123	3.71E-13	4.70E-28	138	6.93E-13	3.07E-24	153	4.19E-13	4.05E-23	168	2.04E-13	1.16E-22
123.1	3.84E-13	5.26E-28	138.1	6.93E-13	3.07E-24	153.1	4.18E-13	4.06E-23	168.1	2.01E-13	1.15E-22
123.2	3.76E-13	5.61E-28	138.2	6.79E-13	2.99E-24	153.2	4.22E-13	4.18E-23	168.2	2.01E-13	1.15E-22
123.3	3.78E-13	6.31E-28	138.3	6.79E-13	2.99E-24	153.3	4.19E-13	4.27E-23	168.3	1.95E-13	1.15E-22
123.4	3.73E-13	6.33E-28	138.4	6.58E-13	3.01E-24	153.4	4.19E-13	4.27E-23	168.4	1.95E-13	1.15E-22
123.5	3.49E-13	6.22E-28	138.5	6.48E-13	3.17E-24	153.5	4.04E-13	4.51E-23	168.5	1.95E-13	1.15E-22
123.6	3.38E-13	6.19E-28	138.6	6.48E-13	3.17E-24	153.6	4.04E-13	4.51E-23	168.6	1.92E-13	1.18E-22
123.7	3.38E-13	6.19E-28	138.7	6.40E-13	3.24E-24	153.7	4.04E-13	4.51E-23	168.7	1.92E-13	1.18E-22
123.8	3.37E-13	6.28E-28	138.8	6.41E-13	3.37E-24	153.8	3.85E-13	4.43E-23	168.8	1.97E-13	1.24E-22
123.9	3.37E-13	6.66E-28	138.9	6.41E-13	3.37E-24	153.9	3.85E-13	4.43E-23	168.9	1.97E-13	1.24E-22
124	3.37E-13	6.66E-28	139	6.43E-13	3.44E-24	154	3.85E-13	4.43E-23	169	1.97E-13	1.24E-22
124.1	3.46E-13	6.76E-28	139.1	6.39E-13	3.59E-24	154.1	3.77E-13	4.57E-23	169.1	1.97E-13	1.24E-22
124.2	3.46E-13	6.76E-28	139.2	6.25E-13	3.60E-24	154.2	3.77E-13	4.57E-23	169.2	1.97E-13	1.24E-22
124.3	3.49E-13	6.82E-28	139.3	6.19E-13	3.71E-24	154.3	3.73E-13	4.75E-23	169.3	1.97E-13	1.24E-22
124.4	3.50E-13	6.88E-28	139.4	5.98E-13	3.87E-24	154.4	3.73E-13	4.75E-23	169.4	1.97E-13	1.24E-22
124.5	3.38E-13	6.68E-28	139.5	5.86E-13	4.26E-24	154.5	3.70E-13	4.91E-23	169.5	1.97E-13	1.24E-22
124.6	3.46E-13	7.32E-28	139.6	5.90E-13	4.60E-24	154.6	3.70E-13	5.16E-23	169.6	1.98E-13	1.30E-22
124.7	3.40E-13	7.38E-28	139.7	5.90E-13	4.60E-24	154.7	3.70E-13	5.16E-23	169.7	1.98E-13	1.30E-22
124.8	3.30E-13	7.52E-28	139.8	5.90E-13	4.60E-24	154.8	3.66E-13	5.32E-23	169.8	1.98E-13	1.30E-22
124.9	3.33E-13	8.12E-28	139.9	6.11E-13	5.07E-24	154.9	3.66E-13	5.32E-23	169.9	1.94E-13	1.33E-22
125	3.33E-13	8.12E-28	140	6.21E-13	5.40E-24	155	3.66E-13	5.32E-23	170	1.94E-13	1.33E-22
125.1	3.39E-13	8.35E-28	140.1	6.10E-13	5.28E-24	155.1	3.67E-13	5.35E-23	170.1	1.94E-13	1.33E-22
125.2	3.32E-13	8.48E-28	140.2	5.95E-13	5.40E-24	155.2	3.67E-13	5.35E-23	170.2	1.94E-13	1.33E-22
125.3	3.37E-13	8.98E-28	140.3	6.20E-13	6.00E-24	155.3	3.67E-13	5.35E-23	170.3	1.94E-13	1.33E-22
125.4	3.39E-13	1.00E-27	140.4	5.88E-13	6.06E-24	155.4	3.67E-13	5.35E-23	170.4	1.94E-13	1.33E-22
125.5	3.39E-13	1.07E-27	140.5	5.88E-13	6.06E-24	155.5	3.61E-13	5.38E-23	170.5	2.00E-13	1.33E-22
125.6	3.18E-13	1.08E-27	140.6	5.91E-13	6.38E-24	155.6	3.61E-13	5.38E-23	170.6	2.00E-13	1.33E-22
125.7	3.18E-13	1.08E-27	140.7	5.91E-13	6.38E-24	155.7	3.61E-13	5.38E-23	170.7	2.00E-13	1.33E-22
125.8	3.15E-13	1.19E-27	140.8	5.94E-13	6.51E-24	155.8	3.61E-13	5.38E-23	170.8	2.00E-13	1.33E-22
125.9	3.09E-13	1.28E-27	140.9	6.09E-13	6.74E-24	155.9	3.57E-13	5.53E-23	170.9	2.00E-13	1.33E-22
126	3.11E-13	1.35E-27	141	6.25E-13	6.69E-24	156	3.57E-13	5.53E-23	171	1.97E-13	1.34E-22

126.1	3.29E-13	1.45E-27	141.1	6.03E-13	6.53E-24	156.1	3.57E-13	5.53E-23	171.1	1.97E-13	1.34E-22
126.2	3.29E-13	1.45E-27	141.2	6.03E-13	6.53E-24	156.2	3.57E-13	5.53E-23	171.2	1.97E-13	1.34E-22
126.3	3.29E-13	1.45E-27	141.3	5.99E-13	6.77E-24	156.3	3.49E-13	5.59E-23	171.3	1.92E-13	1.35E-22
126.4	3.29E-13	1.45E-27	141.4	5.64E-13	6.52E-24	156.4	3.49E-13	5.59E-23	171.4	1.87E-13	1.34E-22
126.5	3.20E-13	1.51E-27	141.5	5.64E-13	6.52E-24	156.5	3.49E-13	5.59E-23	171.5	1.87E-13	1.34E-22
126.6	3.34E-13	1.60E-27	141.6	5.64E-13	6.52E-24	156.6	3.49E-13	5.59E-23	171.6	1.87E-13	1.34E-22
126.7	3.34E-13	1.60E-27	141.7	5.64E-13	6.52E-24	156.7	3.49E-13	5.59E-23	171.7	1.78E-13	1.37E-22
126.8	3.34E-13	1.60E-27	141.8	5.62E-13	6.74E-24	156.8	3.49E-13	5.59E-23	171.8	1.77E-13	1.38E-22
126.9	3.42E-13	1.72E-27	141.9	5.67E-13	6.90E-24	156.9	3.49E-13	5.59E-23	171.9	1.77E-13	1.38E-22
127	3.29E-13	1.85E-27	142	5.67E-13	6.90E-24	157	3.49E-13	5.59E-23	172	1.79E-13	1.38E-22
127.1	3.25E-13	2.00E-27	142.1	5.75E-13	7.12E-24	157.1	3.49E-13	5.59E-23	172.1	1.79E-13	1.38E-22
127.2	3.31E-13	2.28E-27	142.2	5.74E-13	7.46E-24	157.2	3.43E-13	5.55E-23	172.2	1.79E-13	1.38E-22
127.3	3.26E-13	2.29E-27	142.3	5.76E-13	7.72E-24	157.3	3.43E-13	5.55E-23	172.3	1.79E-13	1.38E-22
127.4	3.27E-13	2.56E-27	142.4	5.63E-13	8.03E-24	157.4	3.41E-13	5.49E-23	172.4	1.79E-13	1.38E-22
127.5	3.24E-13	2.72E-27	142.5	5.63E-13	8.03E-24	157.5	3.35E-13	5.56E-23	172.5	1.79E-13	1.38E-22
127.6	3.18E-13	2.78E-27	142.6	5.50E-13	8.25E-24	157.6	3.35E-13	5.56E-23	172.6	1.79E-13	1.38E-22
127.7	3.28E-13	2.78E-27	142.7	5.50E-13	8.25E-24	157.7	3.35E-13	5.56E-23	172.7	1.79E-13	1.38E-22
127.8	3.29E-13	2.89E-27	142.8	5.49E-13	8.32E-24	157.8	3.28E-13	5.69E-23	172.8	1.75E-13	1.36E-22
127.9	3.29E-13	2.89E-27	142.9	5.49E-13	8.32E-24	157.9	3.28E-13	5.69E-23	172.9	1.70E-13	1.33E-22
128	3.15E-13	3.17E-27	143	5.40E-13	8.63E-24	158	3.23E-13	5.76E-23	173	1.68E-13	1.43E-22
128.1	3.18E-13	3.31E-27	143.1	5.40E-13	8.63E-24	158.1	3.23E-13	5.76E-23	173.1	1.65E-13	1.40E-22
128.2	3.31E-13	3.69E-27	143.2	5.40E-13	8.63E-24	158.2	3.23E-13	5.76E-23	173.2	1.65E-13	1.40E-22
128.3	3.11E-13	3.93E-27	143.3	5.36E-13	8.74E-24	158.3	3.23E-13	5.76E-23	173.3	1.63E-13	1.41E-22
128.4	3.16E-13	4.26E-27	143.4	5.23E-13	8.80E-24	158.4	3.22E-13	6.03E-23	173.4	1.57E-13	1.40E-22
128.5	3.27E-13	4.48E-27	143.5	5.23E-13	8.80E-24	158.5	3.14E-13	6.13E-23	173.5	1.57E-13	1.40E-22
128.6	3.19E-13	4.49E-27	143.6	5.29E-13	9.08E-24	158.6	3.30E-13	6.32E-23	173.6	1.55E-13	1.43E-22
128.7	3.19E-13	4.49E-27	143.7	5.29E-13	9.08E-24	158.7	3.23E-13	6.41E-23	173.7	1.54E-13	1.48E-22
128.8	3.28E-13	4.98E-27	143.8	5.28E-13	9.27E-24	158.8	3.11E-13	6.24E-23	173.8	1.54E-13	1.48E-22
128.9	3.29E-13	4.94E-27	143.9	5.28E-13	9.27E-24	158.9	3.11E-13	6.24E-23	173.9	1.54E-13	1.48E-22
129	3.34E-13	5.47E-27	144	5.12E-13	9.42E-24	159	3.11E-13	6.24E-23	174	1.54E-13	1.48E-22
129.1	3.37E-13	5.62E-27	144.1	5.19E-13	9.84E-24	159.1	3.11E-13	6.24E-23	174.1	1.52E-13	1.50E-22
129.2	3.43E-13	5.75E-27	144.2	5.19E-13	9.84E-24	159.2	3.11E-13	6.24E-23	174.2	1.52E-13	1.50E-22
129.3	3.56E-13	6.40E-27	144.3	5.25E-13	1.03E-23	159.3	3.11E-13	6.24E-23	174.3	1.52E-13	1.50E-22
129.4	3.50E-13	6.59E-27	144.4	5.14E-13	1.07E-23	159.4	3.11E-13	6.24E-23	174.4	1.52E-13	1.50E-22
129.5	3.50E-13	6.59E-27	144.5	5.14E-13	1.07E-23	159.5	3.11E-13	6.24E-23	174.5	1.52E-13	1.50E-22
129.6	3.41E-13	7.56E-27	144.6	5.14E-13	1.07E-23	159.6	3.07E-13	6.41E-23	174.6	1.50E-13	1.55E-22
129.7	3.40E-13	7.88E-27	144.7	5.14E-13	1.08E-23	159.7	3.05E-13	6.69E-23	174.7	1.50E-13	1.55E-22
129.8	3.26E-13	7.89E-27	144.8	5.25E-13	1.21E-23	159.8	3.03E-13	6.94E-23	174.8	1.50E-13	1.55E-22
129.9	3.30E-13	8.26E-27	144.9	5.26E-13	1.22E-23	159.9	2.92E-13	6.82E-23	174.9	1.50E-13	1.55E-22
130	3.28E-13	9.45E-27	145	5.26E-13	1.22E-23	160	2.92E-13	6.82E-23	175	1.49E-13	1.60E-22
130.1	3.31E-13	9.52E-27	145.1	5.26E-13	1.22E-23	160.1	2.92E-13	6.82E-23	175.1	1.49E-13	1.64E-22
130.2	3.26E-13	9.81E-27	145.2	5.30E-13	1.28E-23	160.2	2.83E-13	6.66E-23	175.2	1.50E-13	1.78E-22
130.3	3.38E-13	1.04E-26	145.3	5.34E-13	1.29E-23	160.3	2.83E-13	6.66E-23	175.3	1.50E-13	1.78E-22
130.4	3.62E-13	1.17E-26	145.4	5.34E-13	1.28E-23	160.4	2.80E-13	6.60E-23	175.4	1.47E-13	1.82E-22
130.5	3.65E-13	1.26E-26	145.5	5.39E-13	1.30E-23	160.5	2.80E-13	6.60E-23	175.5	1.47E-13	1.82E-22
130.6	3.96E-13	1.56E-26	145.6	5.39E-13	1.30E-23	160.6	2.80E-13	6.60E-23	175.6	1.47E-13	1.82E-22
130.7	4.10E-13	1.68E-26	145.7	5.46E-13	1.35E-23	160.7	2.80E-13	6.60E-23	175.7	1.47E-13	1.82E-22
130.8	4.18E-13	1.83E-26	145.8	5.46E-13	1.39E-23	160.8	2.78E-13	6.86E-23	175.8	1.47E-13	1.82E-22
130.9	4.15E-13	2.02E-26	145.9	5.51E-13	1.43E-23	160.9	2.78E-13	6.86E-23	175.9	1.47E-13	1.82E-22
131	4.35E-13	2.41E-26	146	5.55E-13	1.52E-23	161	2.78E-13	6.86E-23	176	1.47E-13	1.82E-22
131.1	4.42E-13	2.51E-26	146.1	5.41E-13	1.66E-23	161.1	2.79E-13	6.97E-23	176.1	1.47E-13	1.82E-22
131.2	4.76E-13	2.85E-26	146.2	5.35E-13	1.65E-23	161.2	2.79E-13	6.97E-23	176.2	1.46E-13	1.83E-22
131.3	4.93E-13	3.00E-26	146.3	5.57E-13	1.81E-23	161.3	2.79E-13	6.97E-23	176.3	1.46E-13	1.83E-22
131.4	4.97E-13	3.44E-26	146.4	5.58E-13	1.83E-23	161.4	2.79E-13	6.97E-23	176.4	1.44E-13	1.89E-22
131.5	5.30E-13	3.95E-26	146.5	5.52E-13	2.04E-23	161.5	2.73E-13	7.09E-23	176.5	1.44E-13	1.88E-22
131.6	5.47E-13	4.52E-26	146.6	5.71E-13	2.17E-23	161.6	2.73E-13	7.09E-23	176.6	1.44E-13	1.88E-22
131.7	5.53E-13	5.03E-26	146.7	5.89E-13	2.31E-23	161.7	2.73E-13	7.09E-23	176.7	1.44E-13	1.88E-22
131.8	5.53E-13	5.03E-26	146.8	5.82E-13	2.27E-23	161.8	2.68E-13	7.24E-23	176.8	1.44E-13	1.88E-22
131.9	5.27E-13	5.07E-26	146.9	5.93E-13	2.36E-23	161.9	2.68E-13	7.24E-23	176.9	1.44E-13	1.88E-22
132	5.26E-13	5.15E-26	147	5.99E-13	2.54E-23	162	2.66E-13	7.35E-23	177	1.44E-13	1.88E-22
132.1	5.06E-13	5.08E-26	147.1	6.07E-13	2.68E-23	162.1	2.66E-13	7.35E-23	177.1	1.41E-13	1.93E-22
132.2	5.04E-13	5.18E-26	147.2	5.78E-13	2.88E-23	162.2	2.66E-13	7.35E-23	177.2	1.43E-13	1.96E-22
132.3	5.19E-13	5.60E-26	147.3	5.82E-13	3.09E-23	162.3	2.61E-13	7.35E-23	177.3	1.43E-13	1.96E-22
132.4	5.53E-13	6.17E-26	147.4	5.82E-13	3.05E-23	162.4	2.61E-13	7.35E-23	177.4	1.41E-13	2.08E-22
132.5	5.66E-13	7.26E-26	147.5	5.64E-13	3.06E-23	162.5	2.61E-13	7.35E-23	177.5	1.41E-13	2.08E-22
132.6	5.67E-13	7.62E-26	147.6	5.64E-13	3.06E-23	162.6	2.61E-13	7.35E-23	177.6	1.41E-13	2.11E-22
132.7	5.89E-13	8.25E-26	147.7	5.78E-13	3.27E-23	162.7	2.61E-13	7.35E-23	177.7	1.38E-13	2.23E-22
132.8	6.06E-13	9.06E-26	147.8	5.62E-13	3.35E-23	162.8	2.55E-13	7.36E-23	177.8	1.38E-13	2.23E-22
132.9	6.20E-13	1.12E-25	147.9	5.62E-13	3.35E-23	162.9	2.55E-13	7.36E-23	177.9	1.37E-13	2.32E-22
133	6.38E-13	1.32E-25	148	5.64E-13	3.45E-23	163	2.55E-13	7.36E-23	178	1.37E-13	2.32E-22
133.1	6.60E-13	1.45E-25	148.1	5.64E-13	3.45E-23	163.1	2.58E-13	7.75E-23	178.1	1.36E-13	2.38E-22
133.2	6.50E-13	1.50E-25	148.2	5.64E-13	3.45E-23	163.2	2.58E-13	7.75E-23	178.2	1.36E-13	2.38E-22
133.3	6.66E-13	1.72E-25	148.3	5.64E-13	3.45E-23	163.3	2.58E-13	7.75E-23	178.3	1.36E-13	2.38E-22
133.4	6.80E-13	1.81E-25	148.4	5.64E-13	3.45E-23	163.4	2.53E-13	7.92E-23	178.4	1.36E-13	2.38E-22
133.5	6.91E-13	2.06E-25	148.5	5.64E-13	3.45E-23	163.5	2.53E-13	7.92E-23	178.5	1.36E-13	2.38E-22
133.6	6.81E-13	2.29E-25	148.6	5.55E-13	3.56E-23	163.6	2.53E-13	7.92E-23	178.6	1.36E-13	2.38E-22
133.7	6.83E-13	2.68E-25	148.7	5.55E-13	3.56E-23	163.7	2.46E-13	8.00E-23	178.7	1.36E-13	2.38E-22
133.8	6.77E-13	2.96E-25	148.8	5.55E-13	3.56E-23	163.8	2.46E-13	8.00E-23	178.8	1.36E-13	2.38E-22
133.9	6.90E-13	3.10E-25	148.9	5.55E-13	3.56E-23	163.9	2.46E-13	8.00E-23	178.9	1.34E-13	2.39E-22
134	6.96E-13	3.34E-25	149	5.55E-13	3.56E-23	164	2.45E-13	8.21E-23	179	1.35E-13	2.42E-22
134.1	7.44E-13	3.98E-25	149.1	5.55E-13	3.56E-23	164.1	2.40E-13	8.45E-23	179.1	1.31E-13	2.46E-22
134.2	7.62E-13	4.42E-25	149.2	5.55E-13	3.56E-23	164.2	2.37E-13	8.73E-23	179.2	1.31E-13	2.56E-22
134.3	7.69E-13	5.26E-25	149.3	5.55E-13	3.56E-23	164.3	2.37E-13	8.73E-23	179.3	1.31E-13	2.56E-22
134.4	7.70E-13	5.40E-25	149.4	5.52E-13	3.62E-23	164.4	2.34E-13	8.95E-23	179.4	1.30E-13	2.62E-22
134.5	7.65E-13	5.82E-25	149.5	5.35E-13	3.63E-23	164.5	2.34E-13	8.95E-23	179.5	1.30E-13	2.62E-22
134.6	7.66E-13	6.15E-25	149.6	5.17E-13	3.55E-23	164.6	2.34E-13	8.95E-23	179.6	1.27E-13	2.58E-22
134.7	8.27E-13	7.54E-25	149.7	5.03E-13	3.53E-23	164.7	2.34E-13	8.95E-23	179.7	1.23E-13	2.61E-22
134.8	8.04E-13	8.07E-25	149.8	4.88E-13	3.52E-23	164.8	2.34E-13	8.95E-23	179.8	1.23E-13	2.61E-22
134.9	8.46E-13	9.85E-25	149.9	4.88E-13	3.52E-23	164.9	2.34E-13	8.95E-23	179.9	1.23E-13	2.63E-22

180	1.23E-13	2.63E-22	195	1.37E-13	9.60E-22	210	1.49E-13	1.76E-21	225	1.41E-13	2.06E-21
180.1	1.21E-13	2.72E-22	195.1	1.37E-13	9.60E-22	210.1	1.49E-13	1.76E-21	225.1	1.35E-13	2.00E-21
180.2	1.21E-13	2.72E-22	195.2	1.37E-13	9.60E-22	210.2	1.44E-13	1.76E-21	225.2	1.35E-13	2.00E-21
180.3	1.21E-13	2.72E-22	195.3	1.37E-13	9.60E-22	210.3	1.44E-13	1.76E-21	225.3	1.38E-13	2.04E-21
180.4	1.21E-13	2.72E-22	195.4	1.37E-13	9.60E-22	210.4	1.44E-13	1.76E-21	225.4	1.42E-13	2.12E-21
180.5	1.21E-13	2.83E-22	195.5	1.37E-13	9.60E-22	210.5	1.44E-13	1.76E-21	225.5	1.38E-13	2.13E-21
180.6	1.21E-13	2.83E-22	195.6	1.37E-13	9.60E-22	210.6	1.44E-13	1.76E-21	225.6	1.38E-13	2.13E-21
180.7	1.23E-13	2.90E-22	195.7	1.37E-13	9.60E-22	210.7	1.44E-13	1.76E-21	225.7	1.38E-13	2.13E-21
180.8	1.23E-13	2.90E-22	195.8	1.38E-13	9.53E-22	210.8	1.44E-13	1.76E-21	225.8	1.38E-13	2.13E-21
180.9	1.23E-13	2.90E-22	195.9	1.38E-13	9.78E-22	210.9	1.44E-13	1.76E-21	225.9	1.38E-13	2.13E-21
181	1.23E-13	2.90E-22	196	1.38E-13	9.78E-22	211	1.44E-13	1.76E-21	226	1.44E-13	2.18E-21
181.1	1.23E-13	2.90E-22	196.1	1.38E-13	9.78E-22	211.1	1.44E-13	1.76E-21	226.1	1.44E-13	2.18E-21
181.2	1.23E-13	2.90E-22	196.2	1.38E-13	9.78E-22	211.2	1.44E-13	1.76E-21	226.2	1.44E-13	2.18E-21
181.3	1.23E-13	2.90E-22	196.3	1.38E-13	9.78E-22	211.3	1.44E-13	1.76E-21	226.3	1.44E-13	2.18E-21
181.4	1.23E-13	3.03E-22	196.4	1.38E-13	9.78E-22	211.4	1.44E-13	1.76E-21	226.4	1.44E-13	2.18E-21
181.5	1.23E-13	3.10E-22	196.5	1.37E-13	9.71E-22	211.5	1.46E-13	1.80E-21	226.5	1.46E-13	2.26E-21
181.6	1.23E-13	3.10E-22	196.6	1.37E-13	9.71E-22	211.6	1.46E-13	1.80E-21	226.6	1.46E-13	2.31E-21
181.7	1.23E-13	3.10E-22	196.7	1.38E-13	1.01E-21	211.7	1.46E-13	1.80E-21	226.7	1.50E-13	2.32E-21
181.8	1.23E-13	3.10E-22	196.8	1.38E-13	1.01E-21	211.8	1.46E-13	1.80E-21	226.8	1.50E-13	2.32E-21
181.9	1.20E-13	3.24E-22	196.9	1.38E-13	1.01E-21	211.9	1.44E-13	1.78E-21	226.9	1.55E-13	2.44E-21
182	1.21E-13	3.37E-22	197	1.39E-13	1.06E-21	212	1.44E-13	1.78E-21	227	1.55E-13	2.44E-21
182.1	1.21E-13	3.37E-22	197.1	1.39E-13	1.06E-21	212.1	1.44E-13	1.78E-21	227.1	1.56E-13	2.51E-21
182.2	1.21E-13	3.46E-22	197.2	1.39E-13	1.06E-21	212.2	1.44E-13	1.78E-21	227.2	1.56E-13	2.51E-21
182.3	1.22E-13	3.42E-22	197.3	1.41E-13	1.06E-21	212.3	1.44E-13	1.78E-21	227.3	1.56E-13	2.51E-21
182.4	1.23E-13	3.51E-22	197.4	1.37E-13	1.09E-21	212.4	1.44E-13	1.78E-21	227.4	1.56E-13	2.51E-21
182.5	1.23E-13	3.51E-22	197.5	1.37E-13	1.09E-21	212.5	1.44E-13	1.78E-21	227.5	1.56E-13	2.51E-21
182.6	1.23E-13	3.87E-22	197.6	1.36E-13	1.11E-21	212.6	1.44E-13	1.78E-21	227.6	1.51E-13	2.45E-21
182.7	1.23E-13	3.87E-22	197.7	1.38E-13	1.16E-21	212.7	1.38E-13	1.72E-21	227.7	1.51E-13	2.45E-21
182.8	1.23E-13	3.87E-22	197.8	1.38E-13	1.16E-21	212.8	1.38E-13	1.72E-21	227.8	1.51E-13	2.45E-21
182.9	1.23E-13	3.87E-22	197.9	1.38E-13	1.16E-21	212.9	1.38E-13	1.72E-21	227.9	1.51E-13	2.45E-21
183	1.23E-13	3.87E-22	198	1.38E-13	1.16E-21	213	1.38E-13	1.72E-21	228	1.51E-13	2.45E-21
183.1	1.23E-13	3.87E-22	198.1	1.36E-13	1.13E-21	213.1	1.38E-13	1.72E-21	228.1	1.51E-13	2.45E-21

183.2	1.28E-13	4.29E-22
183.3	1.29E-13	4.46E-22
183.4	1.30E-13	4.60E-22
183.5	1.30E-13	4.78E-22
183.6	1.30E-13	4.81E-22
183.7	1.30E-13	4.81E-22
183.8	1.30E-13	4.81E-22
183.9	1.34E-13	5.42E-22
184	1.36E-13	5.65E-22
184.1	1.36E-13	5.65E-22
184.2	1.35E-13	5.78E-22
184.3	1.35E-13	6.19E-22
184.4	1.34E-13	6.39E-22
184.5	1.34E-13	6.39E-22
184.6	1.34E-13	6.39E-22
184.7	1.34E-13	6.39E-22
184.8	1.34E-13	6.39E-22
184.9	1.35E-13	6.38E-22
185	1.35E-13	6.38E-22
185.1	1.35E-13	6.38E-22
185.2	1.38E-13	6.50E-22
185.3	1.38E-13	6.50E-22
185.4	1.38E-13	6.50E-22
185.5	1.38E-13	6.50E-22
185.6	1.37E-13	6.71E-22
185.7	1.37E-13	6.71E-22
185.8	1.37E-13	6.71E-22
185.9	1.37E-13	6.71E-22
186	1.36E-13	6.71E-22
186.1	1.36E-13	6.71E-22
186.2	1.36E-13	6.71E-22
186.3	1.36E-13	6.71E-22
186.4	1.36E-13	6.71E-22
186.5	1.36E-13	6.71E-22
186.6	1.36E-13	6.71E-22
186.7	1.36E-13	6.71E-22
186.8	1.38E-13	6.67E-22
186.9	1.34E-13	6.44E-22
187	1.34E-13	6.59E-22
187.1	1.35E-13	6.61E-22
187.2	1.35E-13	6.61E-22
187.3	1.35E-13	6.61E-22
187.4	1.35E-13	6.61E-22
187.5	1.35E-13	6.61E-22
187.6	1.35E-13	6.61E-22
187.7	1.39E-13	6.80E-22
187.8	1.39E-13	6.80E-22
187.9	1.34E-13	6.70E-22
188	1.34E-13	6.70E-22
188.1	1.34E-13	6.70E-22
188.2	1.34E-13	6.70E-22
188.3	1.35E-13	6.88E-22
188.4	1.35E-13	6.88E-22
188.5	1.35E-13	6.88E-22
188.6	1.33E-13	7.00E-22
188.7	1.33E-13	7.00E-22
188.8	1.33E-13	7.00E-22
188.9	1.33E-13	7.00E-22
189	1.33E-13	7.00E-22
189.1	1.33E-13	7.00E-22
189.2	1.33E-13	7.00E-22
189.3	1.33E-13	7.15E-22
189.4	1.33E-13	7.15E-22
189.5	1.31E-13	7.35E-22
189.6	1.37E-13	7.49E-22
189.7	1.36E-13	7.64E-22
189.8	1.36E-13	7.64E-22
189.9	1.36E-13	7.64E-22
190	1.36E-13	7.64E-22
190.1	1.36E-13	7.64E-22
190.2	1.36E-13	7.64E-22
190.3	1.37E-13	7.56E-22
190.4	1.37E-13	7.56E-22
190.5	1.37E-13	7.56E-22
190.6	1.37E-13	7.56E-22
190.7	1.37E-13	7.56E-22
190.8	1.37E-13	7.56E-22
190.9	1.43E-13	7.73E-22
191	1.43E-13	7.73E-22
191.1	1.43E-13	7.73E-22
191.2	1.40E-13	7.67E-22
191.3	1.41E-13	7.94E-22
191.4	1.41E-13	7.94E-22
191.5	1.41E-13	7.94E-22
191.6	1.35E-13	7.72E-22
191.7	1.36E-13	8.01E-22
191.8	1.36E-13	8.01E-22
191.9	1.39E-13	8.20E-22
192	1.39E-13	8.20E-22
192.1	1.39E-13	8.20E-22
192.2	1.39E-13	8.20E-22
192.3	1.38E-13	8.25E-22
192.4	1.38E-13	8.25E-22
192.5	1.38E-13	8.25E-22
192.6	1.38E-13	8.25E-22
192.7	1.38E-13	8.25E-22
192.8	1.36E-13	8.38E-22
192.9	1.36E-13	8.38E-22
193	1.42E-13	8.82E-22
193.1	1.42E-13	8.82E-22
193.2	1.42E-13	8.82E-22
193.3	1.42E-13	8.82E-22
193.4	1.42E-13	8.82E-22
193.5	1.42E-13	8.82E-22
193.6	1.40E-13	9.03E-22
193.7	1.40E-13	9.03E-22
193.8	1.43E-13	9.23E-22
193.9	1.43E-13	9.23E-22
194	1.43E-13	9.23E-22
194.1	1.43E-13	9.23E-22
194.2	1.38E-13	9.32E-22
194.3	1.38E-13	9.32E-22
194.4	1.39E-13	9.74E-22
194.5	1.39E-13	9.74E-22
194.6	1.39E-13	9.74E-22
194.7	1.39E-13	9.76E-22
194.8	1.39E-13	9.76E-22
194.9	1.37E-13	9.60E-22
198.2	1.36E-13	1.13E-21
198.3	1.36E-13	1.13E-21
198.4	1.36E-13	1.13E-21
198.5	1.35E-13	1.16E-21
198.6	1.36E-13	1.20E-21
198.7	1.33E-13	1.22E-21
198.8	1.33E-13	1.22E-21
198.9	1.33E-13	1.22E-21
199	1.33E-13	1.22E-21
199.1	1.34E-13	1.27E-21
199.2	1.34E-13	1.27E-21
199.3	1.34E-13	1.27E-21
199.4	1.35E-13	1.29E-21
199.5	1.36E-13	1.35E-21
199.6	1.36E-13	1.35E-21
199.7	1.36E-13	1.39E-21
199.8	1.36E-13	1.39E-21
199.9	1.37E-13	1.47E-21
200	1.38E-13	1.50E-21
200.1	1.43E-13	1.59E-21
200.2	1.45E-13	1.67E-21
200.3	1.44E-13	1.68E-21
200.4	1.44E-13	1.68E-21
200.5	1.44E-13	1.68E-21
200.6	1.44E-13	1.68E-21
200.7	1.44E-13	1.68E-21
200.8	1.44E-13	1.68E-21
200.9	1.44E-13	1.68E-21
201	1.49E-13	1.71E-21
201.1	1.49E-13	1.71E-21
201.2	1.49E-13	1.71E-21
201.3	1.49E-13	1.71E-21
201.4	1.49E-13	1.71E-21
201.5	1.49E-13	1.71E-21
201.6	1.49E-13	1.71E-21
201.7	1.49E-13	1.71E-21
201.8	1.49E-13	1.71E-21
201.9	1.49E-13	1.71E-21
202	1.49E-13	1.71E-21
202.1	1.49E-13	1.71E-21
202.2	1.49E-13	1.71E-21
202.3	1.49E-13	1.71E-21
202.4	1.49E-13	1.71E-21
202.5	1.49E-13	1.71E-21
202.6	1.50E-13	1.72E-21
202.7	1.50E-13	1.72E-21
202.8	1.50E-13	1.72E-21
202.9	1.50E-13	1.72E-21
203	1.50E-13	1.72E-21
203.1	1.50E-13	1.72E-21
203.2	1.50E-13	1.72E-21
203.3	1.50E-13	1.72E-21
203.4	1.50E-13	1.72E-21
203.5	1.50E-13	1.72E-21
203.6	1.50E-13	1.72E-21
203.7	1.50E-13	1.72E-21
203.8	1.50E-13	1.72E-21
203.9	1.50E-13	1.72E-21
204	1.50E-13	1.72E-21
204.1	1.50E-13	1.72E-21
204.2	1.50E-13	1.72E-21
204.3	1.50E-13	1.72E-21
204.4	1.50E-13	1.72E-21
204.5	1.50E-13	1.72E-21
204.6	1.50E-13	1.72E-21
204.7	1.50E-13	1.72E-21
204.8	1.50E-13	1.72E-21
204.9	1.50E-13	1.72E-21
205	1.50E-13	1.72E-21
205.1	1.50E-13	1.72E-21
205.2	1.50E-13	1.72E-21
205.3	1.50E-13	1.72E-21
205.4	1.50E-13	1.72E-21
205.5	1.50E-13	1.72E-21
205.6	1.51E-13	1.70E-21
205.7	1.51E-13	1.70E-21
205.8	1.51E-13	1.70E-21
205.9	1.57E-13	1.75E-21
206	1.53E-13	1.77E-21
206.1	1.53E-13	1.77E-21
206.2	1.53E-13	1.77E-21
206.3	1.53E-13	1.77E-21
206.4	1.53E-13	1.77E-21
206.5	1.50E-13	1.75E-21
206.6	1.50E-13	1.75E-21
206.7	1.50E-13	1.75E-21
206.8	1.50E-13	1.75E-21
206.9	1.50E-13	1.75E-21
207	1.50E-13	1.75E-21
207.1	1.50E-13	1.75E-21
207.2	1.50E-13	1.75E-21
207.3	1.50E-13	1.75E-21
207.4	1.50E-13	1.75E-21
207.5	1.50E-13	1.75E-21
207.6	1.50E-13	1.75E-21
207.7	1.50E-13	1.75E-21
207.8	1.50E-13	1.75E-21
207.9	1.50E-13	1.75E-21
208	1.50E-13	1.75E-21
208.1	1.50E-13	1.75E-21
208.2	1.50E-13	1.75E-21
208.3	1.46E-13	1.73E-21
208.4	1.44E-13	1.72E-21
208.5	1.44E-13	1.72E-21
208.6	1.44E-13	1.72E-21
208.7	1.44E-13	1.72E-21
208.8	1.49E-13	1.76E-21
208.9	1.49E-13	1.76E-21
209	1.49E-13	1.76E-21
209.1	1.49E-13	1.76E-21
209.2	1.49E-13	1.76E-21
209.3	1.49E-13	1.76E-21
209.4	1.49E-13	1.76E-21
209.5	1.49E-13	1.76E-21
209.6	1.49E-13	1.76E-21
209.7	1.49E-13	1.76E-21
209.8	1.49E-13	1.76E-21
209.9	1.49E-13	1.76E-21
213.2	1.38E-13	1.72E-21
213.3	1.38E-13	1.72E-21
213.4	1.38E-13	1.72E-21
213.5	1.38E-13	1.72E-21
213.6	1.38E-13	1.72E-21
213.7	1.38E-13	1.72E-21
213.8	1.38E-13	1.72E-21
213.9	1.42E-13	1.75E-21
214	1.42E-13	1.75E-21
214.1	1.43E-13	1.77E-21
214.2	1.43E-13	1.77E-21
214.3	1.43E-13	1.77E-21
214.4	1.43E-13	1.77E-21
214.5	1.43E-13	1.77E-21
214.6	1.43E-13	1.77E-21
214.7	1.43E-13	1.77E-21
214.8	1.43E-13	1.77E-21
214.9	1.43E-13	1.81E-21
215	1.43E-13	1.81E-21
215.1	1.43E-13	1.81E-21
215.2	1.43E-13	1.81E-21
215.3	1.43E-13	1.81E-21
215.4	1.43E-13	1.81E-21
215.5	1.43E-13	1.81E-21
215.6	1.43E-13	1.81E-21
215.7	1.43E-13	1.81E-21
215.8	1.43E-13	1.81E-21
215.9	1.43E-13	1.81E-21
216	1.45E-13	1.82E-21
216.1	1.45E-13	1.82E-21
216.2	1.45E-13	1.82E-21
216.3	1.45E-13	1.82E-21
216.4	1.45E-13	1.82E-21
216.5	1.45E-13	1.82E-21
216.6	1.45E-13	1.82E-21
216.7	1.45E-13	1.82E-21
216.8	1.45E-13	1.82E-21
216.9	1.41E-13	1.80E-21
217	1.41E-13	1.80E-21
217.1	1.38E-13	1.83E-21
217.2	1.38E-13	1.83E-21
217.3	1.34E-13	1.80E-21
217.4	1.34E-13	1.80E-21
217.5	1.34E-13	1.80E-21
217.6	1.34E-13	1.80E-21
217.7	1.34E-13	1.80E-21
217.8	1.34E-13	1.80E-21
217.9	1.34E-13	1.80E-21
218	1.34E-13	1.80E-21
218.1	1.34E-13	1.80E-21
218.2	1.34E-13	1.80E-21
218.3	1.34E-13	1.80E-21
218.4	1.34E-13	1.80E-21
218.5	1.34E-13	1.80E-21
218.6	1.40E-13	1.85E-21
218.7	1.40E-13	1.85E-21
218.8	1.40E-13	1.85E-21
218.9	1.40E-13	1.85E-21
219	1.40E-13	1.85E-21
219.1	1.40E-13	1.85E-21
219.2	1.40E-13	1.85E-21
219.3	1.40E-13	1.85E-21
219.4	1.38E-13	1.85E-21
219.5	1.38E-13	1.85E-21
219.6	1.38E-13	1.85E-21
219.7	1.34E-13	1.86E-21
219.8	1.34E-13	1.86E-21
219.9	1.36E-13	1.87E-21
220	1.36E-13	1.87E-21
220.1	1.36E-13	1.87E-21
220.2	1.36E-13	1.87E-21
220.3	1.36E-13	1.87E-21
220.4	1.36E-13	1.87E-21
220.5	1.36E-13	1.87E-21
220.6	1.36E-13	1.87E-21
220.7	1.36E-13	1.87E-21
220.8	1.36E-13	1.87E-21
220.9	1.36E-13	1.87E-21
221	1.33E-13	1.87E-21
221.1	1.36E-13	1.90E-21
221.2	1.36E-13	1.90E-21
221.3	1.36E-13	1.90E-21
221.4	1.36E-13	1.90E-21
221.5	1.36E-13	1.90E-21
221.6	1.36E-13	1.90E-21
221.7	1.36E-13	1.90E-21
221.8	1.36E-13	1.90E-21
221.9	1.39E-13	1.98E-21
222	1.39E-13	1.98E-21
222.1	1.39E-13	1.98E-21
222.2	1.39E-13	1.98E-21
222.3	1.39E-13	1.98E-21
222.4	1.39E-13	1.98E-21
222.5	1.39E-13	1.98E-21
222.6	1.39E-13	1.98E-21
222.7	1.39E-13	1.98E-21
222.8	1.39E-13	1.98E-21
222.9	1.35E-13	2.00E-21
223	1.35E-13	2.00E-21
223.1	1.35E-13	2.00E-21
223.2	1.35E-13	2.00E-21
223.3	1.35E-13	2.00E-21
223.4	1.35E-13	2.00E-21
223.5	1.34E-13	1.96E-21
223.6	1.34E-13	1.96E-21
223.7	1.34E-13	1.96E-21
223.8	1.34E-13	1.96E-21
223.9	1.34E-13	1.96E-21
224	1.29E-13	1.90E-21
224.1	1.29E-13	1.90E-21
224.2	1.29E-13	1.90E-21
224.3	1.29E-13	1.90E-21
224.4	1.29E-13	1.90E-21
224.5	1.29E-13	1.90E-21
224.6	1.33E-13	1.92E-21
224.7	1.33E-13	1.92E-21
224.8	1.33E-13	1.92E-21
224.9	1.38E-13	1.98E-21
228.2	1.51E-13	2.45E-21
228.3	1.51E-13	2.45E-21
228.4	1.51E-13	2.45E-21
228.5	1.51E-13	2.45E-21
228.6	1.51E-13	2.45E-21
228.7	1.51E-13	2.45E-21
228.8	1.51E-13	2.45E-21
228.9	1.51E-13	2.45E-21
229	1.51E-13	2.45E-21
229.1	1.51E-13	2.45E-21
229.2	1.47E-13	2.42E-21
229.3	1.47E-13	2.42E-21
229.4	1.47E-13	2.42E-21
229.5	1.47E-13	2.42E-21
229.6	1.47E-13	2.42E-21
229.7	1.47E-13	2.42E-21
229.8	1.47E-13	2.42E-21
229.9	1.47E-13	2.42E-21
230	1.47E-13	2.45E-21
230.1	1.47E-13	2.45E-21
230.2	1.47E-13	2.45E-21
230.3	1.47E-13	2.45E-21
230.4	1.47E-13	2.45E-21
230.5	1.47E-13	2.45E-21
230.6	1.47E-13	2.45E-21
230.7	1.47E-13	2.45E-21
230.8	1.47E-13	2.45E-21
230.9	1.47E-13	2.45E-21
231	1.47E-13	2.45E-21
231.1	1.47E-13	2.45E-21
231.2	1.45E-13	2.44E-21
231.3	1.45E-13	2.44E-21
231.4	1.43E-13	2.50E-21
231.5	1.43E-13	2.50E-21
231.6	1.43E-13	2.50E-21
231.7	1.46E-13	2.53E-21
231.8	1.46E-13	2.51E-21
231.9	1.46E-13	2.51E-21
232	1.46E-13	2.51E-21
232.1	1.46E-13	2.51E-21
232.2	1.46E-13	2.51E-21
232.3	1.42E-13	2.47E-21
232.4	1.42E-13	2.47E-21
232.5	1.42E-13	2.47E-21
232.6	1.42E-13	2.47E-21
232.7	1.42E-13	2.47E-21
232.8	1.42E-13	2.47E-21
232.9	1.38E-13	2.47E-21
233	1.38E-13	2.47E-21
233.1	1.34E-13	2.47E-21
233.2	1.34E-13	2.47E-21
233.3	1.34E-13	2.47E-21
233.4	1.34E-13	2.47E-21
233.5	1.33E-13	2.48E-21
233.6	1.30E-13	2.51E-21
233.7	1.30E-13	2.55E-21
233.8	1.30E-13	2.55E-21
233.9	1.30E-13	2.55E-21
234	1.30E-13	2.55E-21
234.1	1.30E-13	2.55E-21
234.2	1.33E-13	2.63E-21
234.3	1.33E-13	2.63E-21
234.4	1.33E-13	2.63E-21
234.5	1.33E-13	2.63E-21
234.6	1.33E-13	2.63E-21
234.7	1.33E-13	2.63E-21
234.8	1.33E-13	2.63E-21
234.9	1.33E-13	2.63E-21
235	1.33E-13	2.63E-21
235.1	1.33E-13	2.63E-21
235.2	1.33E-13	2.63E-21
235.3	1.33E-13	2.63E-21
235.4	1.33E-13	2.63E-21
235.5	1.33E-13	2.63E-21
235.6	1.33E-13	2.63E-21
235.7	1.33E-13	2.63E-21
235.8	1.33E-13	2.63E-21
235.9	1.29E-13	2.65E-21
236	1.25E-13	2.61E-21
236.1	1.25E-13	2.61E-21
236.2	1.23E-13	2.66E-21
236.3	1.21E-13	2.69E-21
236.4	1.21E-13	2.69E-21
236.5	1.15E-13	2.59E-21
236.6	1.15E-13	2.59E-21
236.7	1.13E-13	2.56E-21
236.8	1.13E-13	2.56E-21
236.9	1.13E-13	2.56E-21
237	1.13E-13	2.56E-21
237.1	1.13E-13	2.56E-21
237.2	1.13E-13	2.56E-21
237.3	1.13E-13	2.56E-21
237.4	1.13E-13	2.56E-21
237.5	1.13E-13	2.56E-21
237.6	1.13E-13	2.56E-21
237.7	1.13E-13	2.56E-21
237.8	1.13E-13	2.56E-21
237.9	1.13E-13	2.56E-21
238	1.12E-13	2.65E-21
238.1	1.12E-13	2.65E-21
238.2	1.12E-13	2.65E-21
238.3	1.12E-13	2.65E-21
238.4	1.12E-13	2.65E-21
238.5	1.12E-13	2.65E-21
238.6	1.12E-13	2.65E-21
238.7	1.08E-13	2.66E-21
238.8	1.08E-13	2.66E-21
238.9	1.07E-13	2.69E-21
239	1.07E-13	2.69E-21
239.1	1.07E-13	2.69E-21
239.2	1.08E-13	2.66E-21
239.3	1.08E-13	2.66E-21
239.4	1.08E-13	2.66E-21
239.5	1.06E-13	2.75E-21
239.6	1.06E-13	2.75E-21
239.7	1.06E-13	2.75E-21
239.8	1.06E-13	2.75E-21
239.9	1.06E-13	2.75E-21
240	1.02E-13	2.66E-21

Table S9: eFAST analysis data for [G] with [G](0)=0.02 mM: eFAST sensitivity indices

Sensitivity analysis data from eFAST for [G] with [G](0)=0.02 mM (only the parameters to which [G]'s variability is sensitive to appear)

Time discretization	lambda	K_{[G]}	K_{[G3P]}	alpha	rho	K_{[ACoA]}	phi	G_E
0	0	0	0	0	0	0	0	0
0.1	0.26364452	0.28942958	0.66823516	0.30125249	0.31865725	0.37913415	0.2653399	0.32419884
0.2	0.25626604	0.27325374	0.6682485	0.28799098	0.3044495	0.36240316	0.24737547	0.31459465
0.3	0.2490055	0.25798017	0.66826889	0.27522175	0.29057923	0.34612074	0.23084339	0.30531647
0.4	0.24208864	0.24420986	0.66824734	0.26344971	0.27768086	0.33095288	0.21624888	0.29671489
0.5	0.2355984	0.23200138	0.66813677	0.25277779	0.26590808	0.31707419	0.20349775	0.2888821
0.6	0.22953637	0.2211928	0.66790794	0.24312591	0.25519844	0.30442346	0.1923234	0.28178563
0.7	0.22387921	0.21159402	0.66754476	0.23438113	0.2454472	0.29288106	0.18245846	0.27536346
0.8	0.2185938	0.20304588	0.66703924	0.22643477	0.23654946	0.2823275	0.17368112	0.26955047
0.9	0.21364761	0.19541779	0.66638929	0.21918924	0.2284058	0.27265331	0.16581501	0.26428763
1	0.20901012	0.18860242	0.66559628	0.2125577	0.22092562	0.26375753	0.15871888	0.25952214
1.1	0.20465328	0.18251205	0.66466343	0.20646506	0.21402873	0.25555086	0.15227911	0.25520635
1.2	0.20055092	0.17707351	0.66359573	0.20084728	0.20764551	0.24795489	0.14640329	0.25129849
1.3	0.19668052	0.17222937	0.66239953	0.19565099	0.20171791	0.24090155	0.1410153	0.24776161
1.4	0.19302259	0.16793439	0.66108144	0.19083048	0.196197	0.23433155	0.13605244	0.24456315
1.5	0.18955929	0.16415237	0.6596481	0.18634672	0.1910403	0.22819467	0.13146315	0.24167464
1.6	0.18627511	0.16085677	0.65810614	0.18216609	0.18621095	0.22244763	0.12720459	0.23907129
1.7	0.18315605	0.1580285	0.65646174	0.17825905	0.18167655	0.21705246	0.12324037	0.23673107
1.8	0.18018913	0.15565579	0.65472104	0.1746002	0.17740875	0.21197611	0.11953954	0.23463481
1.9	0.17736292	0.15373415	0.65288976	0.17116718	0.17338273	0.20718925	0.1160754	0.23276566
2	0.17466731	0.15226555	0.65097288	0.16794016	0.16957639	0.20266613	0.1128248	0.23110891
2.1	0.17209286	0.15126044	0.64897543	0.16490204	0.16597024	0.1983839	0.10976768	0.2296519
2.2	0.1696312	0.15073603	0.64690223	0.16203796	0.1625474	0.19432224	0.1068864	0.22838324
2.3	0.16727473	0.15071644	0.6447574	0.15933471	0.15929289	0.1904632	0.10416546	0.22729315
2.4	0.16501646	0.15123828	0.64254493	0.15678085	0.15619328	0.18679078	0.10159132	0.22637355
2.5	0.16285025	0.15234736	0.64026903	0.15436642	0.1532369	0.18329063	0.09915207	0.22561742
2.6	0.16077053	0.15409853	0.63793348	0.15208275	0.15041309	0.17995006	0.09683697	0.22501874
2.7	0.15877215	0.15656564	0.63554186	0.14992205	0.14771211	0.17675749	0.09463653	0.22457257
2.8	0.15685048	0.15983692	0.63309804	0.14787745	0.1451254	0.17370253	0.09254225	0.22427512
2.9	0.1550013	0.16401211	0.63060623	0.14594331	0.14264515	0.17077591	0.09054643	0.22412384
3	0.15322076	0.16921699	0.6280706	0.14411456	0.1402642	0.16796903	0.08864213	0.22411693
3.1	0.15150537	0.17559704	0.62549549	0.14238669	0.13797607	0.16527404	0.08682309	0.22425335
3.2	0.149852	0.18330885	0.62288591	0.14075615	0.1357749	0.16268398	0.08508361	0.22453336
3.3	0.14825764	0.19253323	0.62024761	0.13922005	0.13365533	0.16019236	0.0834185	0.22495833
3.4	0.14671963	0.20346645	0.61758706	0.13777598	0.13161238	0.15779308	0.081823	0.22553031
3.5	0.14523558	0.21630281	0.61491167	0.13642214	0.12964157	0.15548074	0.08029279	0.22625235
3.6	0.14380326	0.23123315	0.61222997	0.1351573	0.12773876	0.15325035	0.0788239	0.2271289
3.7	0.14242071	0.24843057	0.60955195	0.13398084	0.12590017	0.15109721	0.07741268	0.2281655
3.8	0.14108613	0.26802693	0.60688943	0.13289271	0.12412232	0.14901702	0.07605576	0.22936844
3.9	0.13979784	0.29008879	0.60425627	0.1318934	0.12240198	0.14700584	0.07475007	0.23074535
4	0.13855434	0.31459899	0.60166856	0.13098404	0.12073614	0.14506004	0.07349276	0.23230541
4.1	0.13735432	0.34144024	0.59914493	0.13016635	0.11912202	0.14317617	0.07228118	0.23405897
4.2	0.13619663	0.37037973	0.59670716	0.12944269	0.11755708	0.14135111	0.07111289	0.23601761
4.3	0.13508025	0.40106966	0.59438047	0.12881608	0.11603892	0.13958195	0.06998563	0.23819455
4.4	0.13400429	0.43305474	0.59219388	0.1282901	0.11456533	0.13786597	0.06889732	0.24060436
4.5	0.13296795	0.46580031	0.59018035	0.12786903	0.1131342	0.13620062	0.06784601	0.24326254
4.6	0.13197057	0.49873447	0.5883768	0.12755828	0.11174358	0.1345835	0.06682987	0.24618572
4.7	0.13101169	0.53128068	0.58682492	0.12736434	0.11039166	0.13301242	0.06584717	0.24939175
4.8	0.13009092	0.56290133	0.5855712	0.12729455	0.10907672	0.13148531	0.06489635	0.25290022
4.9	0.12920794	0.59313036	0.58466579	0.12735723	0.10779715	0.13000021	0.06397592	0.25673128
5	0.12836257	0.62159425	0.58416239	0.12756188	0.10655146	0.1285553	0.06308446	0.26090453
5.1	0.12755479	0.64802513	0.58411846	0.12792009	0.10533824	0.12714882	0.06222067	0.26543933
5.2	0.12678474	0.67225747	0.58459341	0.12844552	0.10415613	0.12577916	0.0613833	0.27035503
5.3	0.12605263	0.69421957	0.58564588	0.12915334	0.10300388	0.12444475	0.06057119	0.27567112
5.4	0.12535876	0.71391895	0.58733288	0.13006053	0.10188031	0.12314416	0.05978327	0.2814049
5.5	0.12470362	0.7314247	0.58970754	0.13118635	0.10078431	0.12187604	0.05901848	0.28756969
5.6	0.12408795	0.74685106	0.59281499	0.13255368	0.0997148	0.12063908	0.05827586	0.29417598
5.7	0.12351263	0.76034269	0.59668913	0.13418993	0.09867081	0.11943208	0.05755448	0.30123307
5.8	0.12297859	0.77206112	0.60135009	0.13612613	0.09765137	0.11825385	0.05685347	0.30874591
5.9	0.12248689	0.78217425	0.60679958	0.13839763	0.09665562	0.11710329	0.05617202	0.31671229
6	0.12203871	0.79084908	0.61301835	0.1410455	0.09568268	0.11597937	0.05550936	0.3251229
6.1	0.12163538	0.79824641	0.61996596	0.14411654	0.09473176	0.11488111	0.05486475	0.33396413
6.2	0.12127848	0.8045172	0.62758054	0.14766552	0.09380209	0.11380759	0.0542375	0.34321721
6.3	0.12096997	0.80980063	0.63577827	0.15175679	0.09289294	0.11275794	0.05362694	0.3528553
6.4	0.12071202	0.81422321	0.64445591	0.15646393	0.09200362	0.11173131	0.05303246	0.36284469
6.5	0.1205069	0.81789834	0.6534966	0.16187137	0.09113346	0.11072688	0.05245345	0.37314606
6.6	0.12035706	0.8209269	0.66277469	0.16807836	0.09028185	0.1097439	0.05188937	0.3837145
6.7	0.12026508	0.82339804	0.67216162	0.17519845	0.08944817	0.10878163	0.05133966	0.39449987
6.8	0.12023379	0.82538959	0.68153189	0.18335859	0.08863186	0.10783939	0.05080382	0.40544851
6.9	0.12026636	0.82696908	0.6907684	0.19270269	0.08783236	0.10691652	0.05028136	0.41650324
7	0.12036636	0.82819501	0.69976675	0.20338788	0.08704914	0.10601242	0.04977182	0.42760515
7.1	0.12053746	0.82911804	0.70843875	0.21558115	0.0862817	0.10512647	0.04927475	0.43869584
7.2	0.12078361	0.82978188	0.71671366	0.22945649	0.08552955	0.1042581	0.04878975	0.44971634
7.3	0.12110917	0.83022371	0.72453838	0.24518495	0.08479225	0.10340677	0.04831641	0.46060866
7.4	0.1215187	0.83047521	0.73187651	0.26292543	0.08406935	0.10257193	0.04785433	0.47131902
7.5	0.12201696	0.83056334	0.73870706	0.28280138	0.08336042	0.1017531	0.04740316	0.48179706
7.6	0.12260876	0.83051089	0.74502245	0.30488489	0.08266507	0.1009498	0.04696254	0.49199583
7.7	0.12329911	0.83033731	0.75082622	0.3291822	0.0819829	0.10016157	0.04653213	0.50187438
7.8	0.12409337	0.8300591	0.75613099	0.35560446	0.08131354	0.09938796	0.04611161	0.51139771
7.9	0.12499709	0.82969017	0.76095613	0.38395738	0.08065663	0.09862855	0.04570068	0.520535
8	0.12601608	0.82924223	0.76532612	0.41394305	0.08001181	0.09788296	0.04529904	0.52926147
8.1	0.12715652	0.82872509	0.76926817	0.44515729	0.07937877	0.09715078	0.04490641	0.53755998
8.2	0.12842467	0.82814704	0.77281132	0.47711226	0.07875716	0.09643166	0.04452251	0.54541863
8.3	0.12982619	0.82751518	0.7759856	0.50927268	0.0781467	0.09572522	0.04414709	0.55283019
8.4	0.13136677	0.8268355	0.77882066	0.54109046	0.07754708	0.09503113	0.04377988	0.55979264
8.5	0.13305251	0.8261131	0.78134544	0.57204271	0.07695802	0.09434907	0.04342066	0.56630769
8.6	0.13488954	0.82535241	0.78358738	0.60166865	0.07637925	0.0936787	0.04306919	0.57238041
8.7	0.13688397	0.82455721	0.7855723	0.62959426	0.0758105	0.09301973	0.04272525	0.5780191
8.8	0.13904188	0.82373069	0.78732461	0.6555466	0.07525152	0.09237187	0.04238862	0.58323451
8.9	0.1413688	0.82287569	0.788867	0.67935542	0.07470206	0.09173483	0.04205911	0.58803977
9	0.14386908	0.8219948	0.79022028	0.70094713	0.07416188	0.09110833	0.0417365	0.5924498
9.1	0.14654687	0.82109041	0.79140321	0.72033064	0.07363076	0.09049212	0.04142062	0.59648054
9.2	0.14940661	0.82016466	0.79243265	0.73757852	0.07310847	0.08988593	0.04111127	0.60014813
9.3	0.1524524	0.81921943	0.79332393	0.75281017	0.0725948	0.08928953	0.04080829	0.60346886
9.4	0.15568795	0.81825643	0.79409098	0.76617446	0.07208954	0.08870267	0.0405115	0.60645942
9.5	0.15911657	0.81727733	0.79474618	0.77783529	0.07159249	0.08812515	0.04022074	0.60913603
9.6	0.16274111	0.81628374	0.79530009	0.7879613	0.07110347	0.08755674	0.03993586	0.61151442
9.7	0.16656212	0.81527716	0.79576203	0.79671761	0.07062228	0.08699723	0.03965669	0.61360987
9.8	0.17057886	0.81425895	0.79614058	0.80425962	0.07014875	0.08644642	0.03938309	0.6154366
9.9	0.17479109	0.81323041	0.79644337	0.81073019	0.06968271	0.0859041	0.03911493	0.6170075
10	0.17919798	0.81219285	0.79667673	0.81625845	0.06922397	0.08537009	0.03885206	0.6183344
10.1	0.18379797	0.8111475	0.79684607	0.82095922	0.0687724	0.0848442	0.03859435	0.61942812
10.2	0.18858882	0.81009555	0.79695631	0.82493325	0.06832781	0.08432627	0.03834167	0.62029862
10.3	0.19356759	0.80903814	0.79701152	0.82826853	0.06789007	0.08381612	0.03809391	0.62095481
10.4	0.1987302	0.80797634	0.79701506	0.83104144	0.06745902	0.08331359	0.03785094	0.62140459
10.5	0.20407103	0.80691121	0.79696983	0.83331794	0.06703452	0.08281852	0.03761266	0.62165496
10.6	0.20958341	0.80584377	0.79687826	0.8351553	0.06661644	0.08233076	0.03737894	0.62171255
10.7	0.21526035	0.804775	0.79674242	0.83660325	0.06620463	0.08185017	0.03714969	0.62158389
10.8	0.22109456	0.80370585	0.7965641	0.83770524	0.06579897	0.0813766	0.03692479	0.62127534
10.9	0.22707811	0.80263728	0.79634476	0.83849907	0.06539933	0.08090992	0.03670416	0.62079348
11	0.23320252	0.80157016	0.79608567	0.83901764	0.06500559	0.08044999	0.03648768	0.62014498
11.1	0.23945884	0.8005054	0.79578796	0.83929012	0.06461763	0.07999671	0.03627528	0.61933663
11.2	0.24583751	0.79944385	0.79545261	0.83934251	0.06423533	0.07954994	0.03606685	0.61837569
11.3	0.25232852	0.79838629	0.79508048	0.83919797	0.06385859	0.07910956	0.03586231	0.61726977
11.4	0.25892095	0.79733347	0.79467253	0.838877	0.06348729	0.07867548	0.03566158	0.61602702
11.5	0.26560346	0.79628616	0.79422966	0.838398	0.06312133	0.07824757	0.03546458	0.61465623
11.6	0.27236496	0.79524507	0.79375279	0.83777733	0.06276061	0.07782573	0.03527122	0.61316652
11.7	0.27919432	0.79421084	0.79324294	0.83702955	0.06240502	0.07740987	0.03508144	0.61156748
11.8	0.28608032	0.79318411	0.7927011	0.83616786	0.06205446	0.07699987	0.03489515	0.60986867
11.9	0.29301198	0.79216543	0.79212814	0.83520391	0.06170886	0.07659565	0.03471228	0.60807931
12	0.29997868	0.7911553	0.791525	0.83414786	0.0613681	0.0761971	0.03453277	0.60620868
12.1	0.3069698	0.79015415	0.79089271	0.83300891	0.06103211	0.07580416	0.03435655	0.60426582
12.2	0.31397458	0.78916237	0.79023238	0.83179534	0.0607008	0.07541672	0.03418355	0.60225919
12.3	0.32098226	0.78818033	0.78954516	0.83051431	0.06037409	0.07503473	0.0340137	0.60019683
12.4	0.32798244	0.78720833	0.78883217	0.82917216	0.06005188	0.07465808	0.03384696	0.59808632
12.5	0.33496511	0.78624661	0.78809446	0.82777455	0.05973411	0.07428672	0.03368325	0.59593486
12.6	0.34192066	0.78529534	0.78733307	0.82632654	0.0594207	0.07392058	0.03352253	0.59374898
12.7	0.34884053	0.78435462	0.78654907	0.8248327	0.05911157	0.07355958	0.03336473	0.59153424
12.8	0.35571666	0.78342452	0.78574351	0.82329707	0.05880665	0.07320365	0.03320981	0.58929554
12.9	0.36254095	0.78250506	0.78491748	0.82172334	0.05850587	0.07285275	0.03305772	0.58703727
13	0.36930577	0.78159619	0.78407193	0.82011488	0.05820916	0.0725068	0.0329084	0.58476315
13.1	0.3760039	0.78069782	0.78320775	0.8184746	0.05791646	0.07216576	0.03276181	0.58247621
13.2	0.38262861	0.7798098	0.7823258	0.81680504	0.0576277	0.07182956	0.0326179	0.58017894
13.3	0.38917367	0.77893194	0.78142692	0.81510848	0.05734283	0.07149815	0.03247662	0.5778734
13.4	0.3956332	0.778064	0.7805118	0.813387	0.05706178	0.07117148	0.03233792	0.57556123
13.5	0.40200182	0.77720574	0.77958104	0.81164238	0.05678449	0.07084952	0.03220177	0.5732436
13.6	0.40827473	0.77635689	0.7786352	0.80987619	0.0565109	0.07053221	0.03206812	0.57092132
13.7	0.41444806	0.77551711	0.77767476	0.80808988	0.05624097	0.07021951	0.03193694	0.56859484
13.8	0.42051842	0.77468603	0.77670012	0.80628479	0.05597463	0.06991139	0.03180818	0.56626443
13.9	0.42648259	0.77386324	0.77571158	0.80446212	0.05571184	0.0696078	0.03168181	0.56393015
14	0.43233784	0.77304834	0.77470937	0.80262294	0.05545254	0.06930872	0.03155779	0.56159194
14.1	0.43808166	0.77224093	0.77369363	0.80076834	0.05519668	0.06901411	0.03143609	0.55924962
14.2	0.44371197	0.77144057	0.77266444	0.79889944	0.05494422	0.06872395	0.03131667	0.55690294
14.3	0.44922695	0.77064685	0.77162177	0.79701734	0.05469511	0.0684382	0.03119951	0.5545516
14.4	0.45462514	0.76985929	0.77056556	0.79512335	0.0544493	0.06815686	0.03108456	0.55219522
14.5	0.45990542	0.76907741	0.76949569	0.79321883	0.05420675	0.06787988	0.03097181	0.5498335
14.6	0.46506702	0.76830074	0.76841198	0.79130518	0.05396742	0.06760726	0.03086122	0.54746614
14.7	0.4701097	0.76752884	0.7673142	0.78938387	0.05373126	0.06733898	0.03075277	0.54509295
14.8	0.47503344	0.76676122	0.76620208	0.78745652	0.05349824	0.06707505	0.03064642	0.54271375
14.9	0.47983848	0.76599742	0.7650753	0.7855248	0.05326832	0.06681544	0.03054214	0.54032843
15	0.48452521	0.76523697	0.76393351	0.7835903	0.05304146	0.06656017	0.03043992	0.53793694
15.1	0.48909415	0.7644794	0.76277634	0.78165467	0.05281763	0.06630923	0.03033973	0.53553927
15.2	0.49354599	0.76372426	0.76160341	0.77971969	0.05259679	0.06606262	0.03024154	0.53313547
15.3	0.4978816	0.7629711	0.76041436	0.77778703	0.0523789	0.06582034	0.03014534	0.53072568
15.4	0.502102	0.76221951	0.75920883	0.77585829	0.05216395	0.0655824	0.03005109	0.52831013
15.5	0.5062083	0.76146906	0.75798644	0.77393504	0.0519519	0.06534881	0.02995878	0.52588909
15.6	0.51020179	0.7607193	0.75674688	0.77201885	0.05174271	0.06511959	0.02986839	0.52346293
15.7	0.51408394	0.75996985	0.75548982	0.77011116	0.05153638	0.06489476	0.0297799	0.52103203
15.8	0.51785634	0.75922031	0.75421497	0.76821331	0.05133285	0.06467433	0.02969329	0.51859689
15.9	0.52152066	0.75847032	0.75292209	0.76632658	0.05113212	0.06445832	0.02960854	0.51615815
16	0.52507861	0.75771952	0.75161097	0.76445213	0.05093415	0.06424677	0.02952565	0.51371642
16.1	0.52853195	0.75696756	0.75028146	0.76259097	0.05073892	0.06403969	0.02944459	0.51127237
16.2	0.53188261	0.7562141	0.74893344	0.76074395	0.05054641	0.06383711	0.02936535	0.50882678
16.3	0.5351325	0.75545884	0.74756686	0.75891185	0.0503566	0.06363907	0.02928791	0.50638051
16.4	0.53828352	0.75470147	0.74618172	0.75709539	0.05016945	0.06344563	0.02921227	0.50393445
16.5	0.54133758	0.75394171	0.74477807	0.75529515	0.04998496	0.06325684	0.02913842	0.50148953
16.6	0.54429662	0.75317928	0.74335603	0.75351157	0.04980311	0.06307274	0.02906633	0.49904675
16.7	0.54716266	0.75241394	0.74191579	0.75174497	0.04962387	0.06289338	0.02899601	0.49660718
16.8	0.54993771	0.75164546	0.74045759	0.74999555	0.04944724	0.06271882	0.02892743	0.49417185
16.9	0.55262379	0.75087361	0.73898174	0.74826345	0.04927319	0.06254912	0.0288606	0.49174187
17	0.55522293	0.75009819	0.73748858	0.74654866	0.04910171	0.06238434	0.0287955	0.4893184
17.1	0.55773728	0.74931902	0.73597855	0.74485109	0.04893278	0.06222455	0.02873212	0.48690261
17.2	0.56016895	0.74853593	0.73445212	0.74317059	0.04876639	0.06206984	0.02867046	0.48449566
17.3	0.56251993	0.74774877	0.7329098	0.74150692	0.04860254	0.06192027	0.02861052	0.48209873
17.4	0.5647922	0.74695741	0.73135211	0.73985974	0.04844121	0.06177594	0.02855228	0.47971296
17.5	0.5669877	0.74616175	0.72977962	0.73822865	0.0482824	0.06163694	0.02849574	0.47733952
17.6	0.56910838	0.74536169	0.72819294	0.73661316	0.0481261	0.06150338	0.02844091	0.47497959
17.7	0.5711562	0.74455713	0.72659268	0.7350127	0.04797231	0.0613754	0.02838776	0.4726343
17.8	0.57313307	0.74374803	0.72497949	0.73342663	0.04782104	0.06125317	0.02833631	0.47030473
17.9	0.57504085	0.74293434	0.72335403	0.73185429	0.04767227	0.06113683	0.02828655	0.46799194
18	0.57688148	0.74211602	0.72171697	0.73029499	0.047526	0.06102652	0.02823848	0.46569699
18.1	0.57865684	0.74129307	0.720069	0.72874799	0.04738224	0.06092239	0.0281921	0.46342087
18.2	0.58036873	0.7404655	0.71841081	0.72721251	0.04724099	0.0608246	0.02814741	0.46116451
18.3	0.58201889	0.73963332	0.71674306	0.72568781	0.04710224	0.06073331	0.02810442	0.45892877
18.4	0.58360905	0.73879658	0.71506641	0.72417316	0.046966	0.06064869	0.02806312	0.45671448
18.5	0.58514088	0.73795533	0.71338148	0.72266787	0.04683227	0.06057091	0.02802352	0.45452244
18.6	0.58661601	0.73710962	0.71168888	0.72117128	0.04670106	0.06050016	0.02798563	0.45235338
18.7	0.58803605	0.73625953	0.70998921	0.71968284	0.04657237	0.06043664	0.02794944	0.45020799
18.8	0.58940254	0.73540515	0.70828304	0.71820199	0.04644621	0.06038055	0.02791497	0.44808689
18.9	0.59071702	0.73454657	0.70657092	0.71672825	0.04632259	0.06033213	0.02788222	0.44599065
19	0.591981	0.73368391	0.70485332	0.71526123	0.04620151	0.06029162	0.0278512	0.4439198
19.1	0.59319594	0.73281728	0.70313073	0.71380063	0.04608299	0.06025933	0.02782193	0.44187481
19.2	0.59436328	0.73194683	0.70140359	0.71234625	0.04596703	0.06023558	0.02779441	0.43985609
19.3	0.59548444	0.73107268	0.69967232	0.71089795	0.04585367	0.06022067	0.02776865	0.437864
19.4	0.59656081	0.73019499	0.69793728	0.70945566	0.04574291	0.06021493	0.02774467	0.43589885
19.5	0.59759369	0.72931391	0.69619883	0.70801937	0.0456348	0.06021873	0.02772249	0.43396087
19.6	0.59858438	0.72842958	0.69445726	0.70658913	0.04552937	0.06023244	0.02770211	0.43205028
19.7	0.59953413	0.72754214	0.69271287	0.7051651	0.04542665	0.06025643	0.02768357	0.43016727
19.8	0.60044415	0.72665175	0.69096591	0.70374747	0.04532665	0.06029111	0.02766687	0.42831197
19.9	0.60131561	0.72575859	0.68921659	0.70233646	0.04522942	0.06033692	0.02765203	0.4264845
20	0.6021496	0.72486282	0.68746509	0.70093236	0.04513499	0.06039438	0.02763907	0.42468494
20.1	0.60294724	0.72396461	0.68571159	0.69953544	0.04504338	0.0604641	0.02762802	0.42291331
20.2	0.60370961	0.72306413	0.68395624	0.69814596	0.04495465	0.0605467	0.0276189	0.42116962
20.3	0.60443781	0.72216151	0.68219917	0.69676417	0.04486881	0.0606428	0.02761172	0.41945385
20.4	0.60513291	0.72125692	0.68044048	0.69539036	0.04478592	0.06075307	0.02760651	0.41776592
20.5	0.60579598	0.72035052	0.67868028	0.69402483	0.04470602	0.06087821	0.02760329	0.41610573
20.6	0.60642807	0.71944244	0.67691867	0.69266787	0.04462914	0.06101895	0.02760209	0.41447312
20.7	0.60703021	0.71853283	0.67515571	0.69131977	0.04455534	0.06117606	0.02760294	0.41286796
20.8	0.60760341	0.71762185	0.67339148	0.68998081	0.04448467	0.06135051	0.02760588	0.41129009
20.9	0.60814868	0.71670961	0.67162604	0.68865122	0.04441717	0.06154346	0.02761094	0.40973934
21	0.6086671	0.71579625	0.66985946	0.68733123	0.04435289	0.06175608	0.02761816	0.40821551
21.1	0.60915966	0.71488191	0.66809182	0.68602101	0.04429191	0.06198956	0.02762756	0.40671837
21.2	0.60962736	0.71396669	0.66632319	0.68472076	0.04423431	0.06224518	0.02763921	0.40524768
21.3	0.61007125	0.71305073	0.66455364	0.68343061	0.0441802	0.06252434	0.02765313	0.40380318
21.4	0.61049236	0.71213411	0.66278324	0.68215067	0.04412965	0.06282847	0.02766936	0.40238458
21.5	0.61089177	0.71121695	0.66101205	0.68088105	0.04408275	0.06315914	0.02768796	0.40099159
21.6	0.61127052	0.71029936	0.65924017	0.67962179	0.04403958	0.06351812	0.02770897	0.3996239
21.7	0.6116297	0.70938144	0.6574677	0.6783729	0.04400025	0.06390754	0.02773242	0.3982812
21.8	0.61197037	0.70846326	0.65569477	0.67713431	0.04396484	0.06432978	0.02775838	0.3969632
21.9	0.61229363	0.7075449	0.65392149	0.67590598	0.04393346	0.06478719	0.02778691	0.39566956
22	0.61260055	0.70662645	0.65214794	0.67468781	0.04390621	0.06528235	0.02781805	0.39439995
22.1	0.6128922	0.70570796	0.65037425	0.67347969	0.04388321	0.06581803	0.02785189	0.39315404
22.2	0.61316964	0.70478952	0.64860054	0.67228147	0.04386457	0.0663973	0.02788849	0.39193148
22.3	0.61343395	0.70387119	0.6468269	0.67109298	0.04385041	0.06702407	0.02792792	0.39073189
22.4	0.61368619	0.70295303	0.64505348	0.66991404	0.04384087	0.06770276	0.02797025	0.38955491
22.5	0.6139274	0.70203512	0.64328038	0.66874444	0.04383615	0.0684378	0.02801556	0.38840018
22.6	0.61415857	0.70111751	0.64150774	0.66758395	0.04383641	0.06923414	0.02806392	0.38726734
22.7	0.61438061	0.70020026	0.63973566	0.66643236	0.04384183	0.07009714	0.0281154	0.38615602
22.8	0.61459434	0.6992834	0.63796429	0.66528941	0.04385259	0.0710326	0.02817009	0.38506588
22.9	0.61480051	0.69836699	0.63619372	0.6641549	0.04386887	0.07204734	0.02822807	0.38399653
23	0.61499983	0.69745109	0.63442408	0.6630286	0.0438909	0.07314935	0.02828943	0.38294761
23.1	0.61519289	0.69653576	0.63265546	0.66191029	0.04391889	0.07434721	0.02835427	0.38191876
23.2	0.61538022	0.69562106	0.63088795	0.66079979	0.04395308	0.07565016	0.02842272	0.38090962
23.3	0.61556216	0.69470704	0.62912165	0.65969689	0.04399369	0.07706842	0.02849488	0.3799198
23.4	0.61573886	0.69379375	0.62735665	0.65860144	0.04404098	0.07861366	0.02857088	0.37894895
23.5	0.61591035	0.69288127	0.62559303	0.65751327	0.04409519	0.08029939	0.02865083	0.37799669
23.6	0.61607652	0.69196963	0.62383087	0.65643229	0.04415661	0.08214032	0.02873486	0.37706267
23.7	0.61623709	0.6910589	0.62207023	0.6553584	0.04422551	0.08415224	0.02882311	0.37614653
23.8	0.61639162	0.69014912	0.62031118	0.65429154	0.04430227	0.0863523	0.02891571	0.37524792
23.9	0.61653948	0.68924034	0.61855376	0.65323166	0.04438727	0.08875922	0.02901282	0.37436646
24	0.6166799	0.68833262	0.61679803	0.65217877	0.04448087	0.09139393	0.02911461	0.37350179
24.1	0.61681197	0.687426	0.61504402	0.65113285	0.04458347	0.09427923	0.02922124	0.37265356
24.2	0.61693462	0.68652054	0.61329176	0.65009394	0.04469549	0.09743934	0.02933289	0.37182141
24.3	0.61704657	0.68561629	0.6115413	0.64906206	0.0448174	0.10090025	0.02944976	0.37100501
24.4	0.61714641	0.68471331	0.60979267	0.64803729	0.04494968	0.10468954	0.02957201	0.37020402
24.5	0.61723258	0.68381165	0.60804589	0.64701969	0.04509283	0.10883614	0.02969986	0.36941812
24.6	0.61730345	0.68291138	0.606301	0.64600934	0.04524737	0.11337012	0.02983352	0.36864697
24.7	0.61735733	0.68201257	0.60455802	0.64500633	0.04541394	0.11832207	0.02997321	0.36789026
24.8	0.61739245	0.68111526	0.602817	0.64401075	0.04559321	0.1237219	0.03011921	0.36714767
24.9	0.61740695	0.68021952	0.60107797	0.64302271	0.04578584	0.12959882	0.03027178	0.3664189
25	0.61739901	0.67932542	0.59934098	0.64204232	0.04599256	0.13598159	0.03043118	0.36570363
25.1	0.6173668	0.67843299	0.59760608	0.64106967	0.04621412	0.14289709	0.03059769	0.36500159
25.2	0.6173086	0.67754231	0.59587332	0.64010489	0.04645135	0.15036748	0.03077162	0.36431249
25.3	0.61722272	0.67665342	0.59414276	0.63914808	0.04670515	0.15841055	0.03095328	0.36363603
25.4	0.61710745	0.67576639	0.59241449	0.63819935	0.04697654	0.16704053	0.031143	0.36297193
25.5	0.61696118	0.67488126	0.59068859	0.63725882	0.04726661	0.1762663	0.03134116	0.36231991
25.6	0.61678241	0.6739981	0.58896515	0.6363266	0.04757645	0.18608804	0.03154815	0.36167969
25.7	0.6165698	0.67311695	0.58724428	0.6354028	0.04790727	0.19649792	0.03176438	0.36105097
25.8	0.61632218	0.67223786	0.5855261	0.6344875	0.04826031	0.20748205	0.03199026	0.36043348
25.9	0.61603851	0.67136088	0.58381072	0.63358081	0.04863692	0.21901894	0.03222625	0.35982695
26	0.61571792	0.67048604	0.58209828	0.63268281	0.04903854	0.23107743	0.03247283	0.35923115
26.1	0.61535968	0.6696134	0.58038891	0.63179361	0.04946683	0.24361769	0.03273052	0.35864584
26.2	0.6149632	0.668743	0.57868278	0.63091326	0.04992356	0.25659299	0.03299988	0.35807078
26.3	0.61452811	0.66787487	0.57698004	0.63004185	0.05041057	0.26994961	0.03328153	0.35750576
26.4	0.61405426	0.66700905	0.57528086	0.62917943	0.05092981	0.28362823	0.03357606	0.35695054
26.5	0.6135416	0.66614557	0.57358543	0.62832607	0.05148339	0.29756505	0.0338841	0.35640488
26.6	0.61299027	0.66528446	0.57189393	0.62748183	0.05207357	0.31169246	0.03420633	0.35586857
26.7	0.61240052	0.66442573	0.57020656	0.62664675	0.05270285	0.32594052	0.03454346	0.35534139
26.8	0.61177279	0.66356942	0.56852352	0.62582089	0.05337384	0.34023883	0.03489631	0.35482311
26.9	0.61110769	0.66271553	0.566845	0.62500429	0.05408939	0.35451834	0.03526574	0.35431354
27	0.61040585	0.66186409	0.56517123	0.62419697	0.05485252	0.36871233	0.03565262	0.35381247
27.1	0.60966803	0.6610151	0.56350241	0.62339896	0.05566646	0.38275692	0.03605791	0.35331971
27.2	0.60889515	0.66016857	0.56183875	0.62261028	0.05653472	0.39659215	0.03648256	0.35283505
27.3	0.60808828	0.65932451	0.56018047	0.62183095	0.05746102	0.41016333	0.0369276	0.3523583
27.4	0.6072486	0.65848291	0.55852781	0.62106097	0.05844933	0.4234217	0.03739419	0.35188925
27.5	0.60637731	0.65764378	0.55688096	0.62030036	0.0595039	0.43632421	0.03788355	0.35142772
27.6	0.60547558	0.65680711	0.55524015	0.61954911	0.06062926	0.44883384	0.03839696	0.35097351
27.7	0.60454458	0.65597289	0.55360559	0.61880722	0.06183027	0.46091951	0.03893574	0.35052645
27.8	0.60358561	0.65514111	0.55197749	0.61807469	0.06311207	0.47255625	0.0395013	0.35008638
27.9	0.60260001	0.65431176	0.55035605	0.61735151	0.06448007	0.48372489	0.04009511	0.34965313
28	0.60158914	0.65348482	0.54874149	0.61663766	0.06594002	0.49441147	0.04071881	0.34922653
28.1	0.60055437	0.65266026	0.547134	0.61593311	0.067498	0.50460696	0.04137415	0.34880642
28.2	0.59949706	0.65183806	0.54553379	0.61523786	0.0691604	0.51430676	0.04206288	0.34839263
28.3	0.59841851	0.65101819	0.54394104	0.61455186	0.07093395	0.52351041	0.04278684	0.34798503
28.4	0.59732002	0.65020063	0.54235596	0.61387509	0.07282566	0.53222108	0.04354795	0.34758344
28.5	0.59620287	0.64938533	0.54077874	0.61320751	0.07484289	0.54044503	0.04434825	0.34718771
28.6	0.59506828	0.64857228	0.53920955	0.6125491	0.07699334	0.54819105	0.04518995	0.3467977
28.7	0.59391746	0.64776144	0.53764858	0.61189981	0.07928489	0.55547001	0.04607539	0.34641327
28.8	0.59275153	0.64695277	0.53609598	0.61125961	0.08172567	0.56229448	0.04700694	0.34603426
28.9	0.59157158	0.64614623	0.53455193	0.61062843	0.08432405	0.56867868	0.04798701	0.34566054
29	0.5903787	0.6453418	0.53301657	0.61000623	0.08708864	0.57463797	0.04901812	0.34529199
29.1	0.58917387	0.64453943	0.53149005	0.60939296	0.09002825	0.58018838	0.05010289	0.34492846
29.2	0.58795805	0.64373908	0.52997251	0.60878858	0.09315168	0.58534653	0.05124406	0.34456982
29.3	0.58673213	0.64294072	0.52846408	0.60819302	0.09646769	0.59012967	0.05244445	0.34421594
29.4	0.58549694	0.6421443	0.52696489	0.60760622	0.09998498	0.59455526	0.05370693	0.34386672
29.5	0.58425327	0.6413498	0.52547506	0.60702814	0.10371219	0.59864056	0.05503434	0.34352201
29.6	0.58300188	0.64055716	0.52399468	0.6064587	0.10765779	0.60240274	0.05642959	0.34318172
29.7	0.58174342	0.63976635	0.52252387	0.60589784	0.11182997	0.60585866	0.05789563	0.34284572
29.8	0.5804785	0.63897733	0.52106273	0.60534548	0.11623643	0.60902487	0.05943544	0.3425139
29.9	0.5792077	0.63819005	0.51961133	0.60480157	0.12088435	0.61191751	0.06105193	0.34218616
30	0.57793154	0.63740447	0.51816976	0.60426602	0.12578033	0.61455229	0.06274788	0.34186241
30.1	0.57665051	0.63662057	0.51673811	0.60373877	0.13093023	0.6169444	0.06452603	0.34154255
30.2	0.57536508	0.6358383	0.51531644	0.60321975	0.1363391	0.61910846	0.06638898	0.34122649
30.3	0.57407563	0.63505763	0.5139048	0.60270891	0.14201102	0.6210583	0.0683392	0.34091412
30.4	0.57278254	0.63427852	0.51250327	0.60220615	0.14794898	0.62280712	0.07037895	0.34060535
30.5	0.57148612	0.63350094	0.51111188	0.6017114	0.15415474	0.6243675	0.07251026	0.34030011
30.6	0.57018669	0.63272486	0.50973067	0.60122459	0.16062885	0.6257514	0.07473484	0.33999829
30.7	0.56888451	0.63195025	0.50835968	0.60074565	0.1673705	0.62697026	0.07705406	0.33969981
30.8	0.56757987	0.63117707	0.50699893	0.60027449	0.17437745	0.62803489	0.07946895	0.33940458
30.9	0.56627298	0.6304053	0.50564844	0.59981104	0.18164606	0.62895543	0.0819801	0.33911254
31	0.56496407	0.62963491	0.50430822	0.59935524	0.18917112	0.62974138	0.08458769	0.33882359
31.1	0.56365334	0.62886587	0.50297828	0.598907	0.1969458	0.63040165	0.08729137	0.33853767
31.2	0.56234101	0.62809817	0.50165863	0.59846625	0.20496166	0.63094464	0.09009027	0.33825471
31.3	0.56102726	0.62733177	0.50034926	0.5980329	0.21320871	0.63137827	0.09298294	0.33797462
31.4	0.55971225	0.62656665	0.49905016	0.59760688	0.22167548	0.63170987	0.09596739	0.33769735
31.5	0.55839615	0.62580279	0.49776133	0.59718811	0.23034914	0.63194624	0.099041	0.33742282
31.6	0.55707915	0.62504018	0.49648274	0.59677651	0.23921546	0.63209374	0.10220051	0.33715097
31.7	0.55576146	0.62427878	0.49521437	0.59637199	0.24825891	0.63215821	0.10544202	0.33688174
31.8	0.55444326	0.62351859	0.4939562	0.59597448	0.25746274	0.63214502	0.10876104	0.33661507
31.9	0.55312474	0.62275958	0.49270819	0.59558389	0.26680916	0.63205916	0.11215253	0.3363509
32	0.55180609	0.62200175	0.49147032	0.59520015	0.27627966	0.63190528	0.11561091	0.33608918
32.1	0.55048751	0.62124507	0.49024253	0.59482317	0.28585508	0.63168771	0.11913008	0.33582985
32.2	0.54916921	0.62048954	0.4890248	0.59445288	0.29551568	0.63141046	0.12270338	0.33557286
32.3	0.54785143	0.61973514	0.48781707	0.5940892	0.30524134	0.63107722	0.12632367	0.33531815
32.4	0.54653438	0.61898186	0.48661929	0.59373204	0.31501172	0.63069138	0.12998347	0.33506568
32.5	0.54521831	0.61822968	0.48543141	0.59338133	0.32480651	0.63025608	0.13367507	0.33481539
32.6	0.54390346	0.61747859	0.48425337	0.59303699	0.33460564	0.62977426	0.1373906	0.33456724
32.7	0.54259013	0.61672859	0.48308512	0.59269895	0.34438933	0.62924864	0.14112205	0.33432118
32.8	0.54127859	0.61597965	0.4819266	0.59236711	0.35413823	0.62868169	0.14486126	0.33407718
32.9	0.53996914	0.61523178	0.48077774	0.5920414	0.36383361	0.62807568	0.1486001	0.33383517
33	0.53866207	0.61448496	0.47963847	0.59172172	0.37345752	0.62743283	0.15233051	0.33359512
33.1	0.53735767	0.61373918	0.47850873	0.591408	0.38299282	0.62675515	0.15604467	0.33335699
33.2	0.53605626	0.61299445	0.47738844	0.59110016	0.39242337	0.62604449	0.15973504	0.33312073
33.3	0.53475814	0.61225074	0.47627755	0.59079812	0.40173413	0.62530257	0.16339443	0.3328863
33.4	0.53346364	0.61150805	0.47517597	0.59050181	0.41091117	0.62453108	0.16701601	0.33265367
33.5	0.53217308	0.61076637	0.47408364	0.59021115	0.41994164	0.62373164	0.17059355	0.3324228
33.6	0.53088681	0.61002569	0.47300048	0.58992607	0.42881382	0.62290576	0.17412135	0.33219365
33.7	0.52960516	0.60928602	0.47192641	0.58964649	0.43751717	0.62205481	0.17759431	0.33196621
33.8	0.52832846	0.60854733	0.47086136	0.58937234	0.44604231	0.62118005	0.18100792	0.33174042
33.9	0.52705708	0.60780963	0.46980524	0.58910353	0.45438098	0.62028267	0.18435807	0.33151626
34	0.52579134	0.6070729	0.46875798	0.58884	0.46252611	0.61936385	0.18764103	0.3312937
34.1	0.52453157	0.60633715	0.46771947	0.58858167	0.47047177	0.61842468	0.19085379	0.3310727
34.2	0.52327809	0.60560237	0.46668963	0.58832846	0.47821303	0.61746621	0.19399411	0.33085323
34.3	0.52203122	0.60486855	0.46566838	0.58808028	0.48574599	0.61648948	0.19706032	0.33063526
34.4	0.52079128	0.60413569	0.46465563	0.58783708	0.4930677	0.61549542	0.20005134	0.33041876
34.5	0.51955858	0.60340379	0.46365129	0.58759877	0.50017614	0.6144848	0.20296663	0.33020369
34.6	0.51833342	0.60267285	0.46265526	0.58736527	0.50707013	0.61345839	0.20580619	0.32999002
34.7	0.51711612	0.60194285	0.46166745	0.58713652	0.51374921	0.61241696	0.20857041	0.32977774
34.8	0.51590696	0.60121381	0.46068777	0.58691243	0.5202136	0.61136126	0.21125987	0.3295668
34.9	0.51470624	0.60048573	0.45971612	0.58669292	0.52646422	0.61029195	0.21387537	0.32935719
35	0.51351426	0.59975859	0.4587524	0.58647792	0.53250251	0.60920968	0.21641815	0.32914886
35.1	0.51233126	0.59903241	0.45779652	0.58626736	0.53833052	0.60811502	0.21888989	0.3289418
35.2	0.5111575	0.59830718	0.45684838	0.58606116	0.54395074	0.60700847	0.22129245	0.32873597
35.3	0.50999321	0.5975829	0.45590789	0.58585923	0.54936614	0.60589046	0.22362787	0.32853134
35.4	0.50883863	0.59685957	0.45497493	0.5856615	0.55458004	0.6047614	0.22589833	0.3283279
35.5	0.50769398	0.5961372	0.45404942	0.58546789	0.55959603	0.60362167	0.22810609	0.32812561
35.6	0.50655946	0.59541578	0.45313125	0.58527833	0.56441796	0.60247161	0.23025355	0.32792444
35.7	0.50543529	0.59469532	0.45222031	0.58509274	0.56904992	0.60131154	0.23234313	0.32772438
35.8	0.50432164	0.59397581	0.4513165	0.58491106	0.57349619	0.60014177	0.23437721	0.32752539
35.9	0.50321869	0.59325727	0.45041971	0.58473319	0.57776123	0.59896251	0.23635815	0.32732746
36	0.50212656	0.59253969	0.44952984	0.58455907	0.58184962	0.59777391	0.23828831	0.32713055
36.1	0.50104538	0.59182308	0.44864678	0.58438862	0.58576605	0.59657614	0.24017006	0.32693465
36.2	0.49997527	0.59110745	0.44777043	0.58422177	0.58951521	0.59536936	0.2420057	0.32673972
36.3	0.49891635	0.5903928	0.44690067	0.58405846	0.59310185	0.59415371	0.24379747	0.32654576
36.4	0.4978687	0.58967913	0.4460374	0.5838986	0.59653073	0.5929293	0.24554752	0.32635272
36.5	0.49683241	0.58896646	0.4451805	0.58374213	0.59980663	0.59169622	0.24725792	0.3261606
36.6	0.49580755	0.58825478	0.44432987	0.58358898	0.60293428	0.59045456	0.24893064	0.32596937
36.7	0.49479416	0.5875441	0.44348539	0.58343909	0.60591841	0.5892044	0.25056764	0.325779
36.8	0.4937923	0.58683442	0.44264697	0.58329238	0.6087637	0.58794578	0.25217077	0.32558948
36.9	0.49280197	0.58612577	0.44181448	0.5831488	0.61147478	0.58667879	0.25374178	0.32540078
37	0.49182321	0.58541813	0.44098783	0.58300829	0.61405619	0.58540346	0.25528227	0.32521289
37.1	0.49085602	0.58471153	0.44016691	0.58287078	0.61651239	0.58411986	0.2567938	0.32502578
37.2	0.48990038	0.58400596	0.4393516	0.58273621	0.61884776	0.58282803	0.25827779	0.32483943
37.3	0.48895628	0.58330145	0.4385418	0.58260454	0.62106661	0.58152806	0.25973558	0.32465383
37.4	0.48802366	0.58259799	0.4377374	0.58247571	0.62317315	0.5802201	0.26116841	0.32446895
37.5	0.48710245	0.58189559	0.4369383	0.58234967	0.62517152	0.57890424	0.26257743	0.32428478
37.6	0.48619258	0.58119427	0.43614439	0.58222637	0.62706571	0.5775806	0.26396384	0.32410131
37.7	0.48529397	0.58049404	0.43535558	0.58210576	0.62885958	0.57624929	0.26532872	0.32391851
37.8	0.48440652	0.5797949	0.43457175	0.58198779	0.63055695	0.57491043	0.26667308	0.32373637
37.9	0.48353013	0.57909686	0.43379281	0.58187242	0.63216149	0.57356415	0.26799787	0.32355487
38	0.4826647	0.57839994	0.43301866	0.5817596	0.63367681	0.57221059	0.26930394	0.32337399
38.1	0.4818101	0.57770415	0.43224921	0.5816493	0.63510641	0.57084998	0.27059204	0.32319373
38.2	0.48096621	0.57700949	0.43148436	0.58154147	0.63645365	0.56948257	0.27186285	0.32301407
38.3	0.48013287	0.57631599	0.43072401	0.58143608	0.6377218	0.56810857	0.27311696	0.32283499
38.4	0.47930994	0.57562364	0.42996807	0.58133309	0.63891398	0.56672818	0.27435493	0.32265649
38.5	0.47849725	0.57493247	0.42921646	0.58123246	0.64003325	0.56534163	0.27557744	0.32247854
38.6	0.47769462	0.57424248	0.42846908	0.58113417	0.64108254	0.5639491	0.2767851	0.32230114
38.7	0.47690186	0.57355368	0.42772584	0.58103818	0.64206469	0.56255083	0.27797836	0.32212428
38.8	0.47611881	0.57286609	0.42698667	0.58094445	0.64298241	0.56114711	0.27915759	0.32194795
38.9	0.47534525	0.57217972	0.42625148	0.58085296	0.64383835	0.55973827	0.28032315	0.32177214
39	0.47458098	0.57149458	0.42552019	0.58076367	0.64463504	0.55832458	0.28147531	0.32159684
39.1	0.47382581	0.57081067	0.42479272	0.58067654	0.64537492	0.55690633	0.28261433	0.32142205
39.2	0.47307951	0.57012801	0.42406899	0.58059155	0.64606033	0.55548378	0.28374042	0.32124774
39.3	0.47234188	0.5694466	0.42334892	0.58050866	0.64669353	0.5540572	0.28485374	0.32107393
39.4	0.47161268	0.56876645	0.42263245	0.58042784	0.64727667	0.55262686	0.28595456	0.32090059
39.5	0.4708917	0.56808758	0.4219195	0.58034908	0.64781183	0.55119308	0.28704312	0.32072774
39.6	0.47017869	0.56740999	0.42121	0.58027233	0.64830098	0.54975616	0.28811956	0.32055535
39.7	0.46947344	0.56673369	0.42050388	0.58019757	0.648746	0.5483164	0.28918394	0.32038344
39.8	0.46877571	0.5660587	0.41980109	0.58012478	0.64914868	0.54687409	0.29023641	0.32021198
39.9	0.46808528	0.56538501	0.41910155	0.58005394	0.64951076	0.5454295	0.29127715	0.32004099
40	0.46740192	0.56471265	0.4184052	0.57998502	0.64983393	0.54398289	0.29230627	0.31987045
40.1	0.46672537	0.56404162	0.41771199	0.579918	0.65011975	0.54253454	0.2933238	0.31970035
40.2	0.46605542	0.56337193	0.41702184	0.57985286	0.65036973	0.54108473	0.29432976	0.31953071
40.3	0.46539182	0.56270359	0.41633471	0.57978957	0.65058531	0.53963372	0.29532416	0.3193615
40.4	0.46473434	0.56203661	0.41565054	0.57972811	0.65076784	0.53818179	0.29630698	0.31919274
40.5	0.46408276	0.56137099	0.41496928	0.57966844	0.65091864	0.53672919	0.29727818	0.31902441
40.6	0.46343683	0.56070674	0.41429086	0.57961057	0.65103896	0.53527617	0.29823771	0.31885651
40.7	0.46279635	0.56004388	0.41361524	0.57955444	0.65112998	0.53382298	0.29918553	0.31868904
40.8	0.46216109	0.5593824	0.41294238	0.57950006	0.65119284	0.53236988	0.30012159	0.31852201
40.9	0.46153084	0.55872232	0.41227222	0.57944739	0.65122861	0.53091709	0.30104587	0.31835541
41	0.4609054	0.55806364	0.41160471	0.57939641	0.65123832	0.52946484	0.30195843	0.31818924
41.1	0.46028455	0.55740637	0.41093982	0.5793471	0.65122297	0.52801334	0.3028593	0.3180235
41.2	0.45966811	0.55675052	0.41027751	0.57929944	0.65118349	0.52656283	0.30374855	0.31785819
41.3	0.45905586	0.55609608	0.40961773	0.57925342	0.65112076	0.52511354	0.30462616	0.3176933
41.4	0.45844762	0.55544306	0.40896044	0.579209	0.65103562	0.52366569	0.30549213	0.31752884
41.5	0.45784319	0.55479147	0.40830562	0.57916616	0.65092889	0.52221948	0.30634646	0.3173648
41.6	0.45724238	0.55414131	0.40765322	0.5791249	0.65080136	0.52077513	0.30718914	0.31720119
41.7	0.45664504	0.55349259	0.40700321	0.57908518	0.65065378	0.51933283	0.30802015	0.317038
41.8	0.45605098	0.55284531	0.40635556	0.57904698	0.65048684	0.51789276	0.30883949	0.31687523
41.9	0.45546004	0.55219946	0.40571023	0.5790103	0.65030123	0.51645512	0.30964716	0.31671288
42	0.45487207	0.55155506	0.4050672	0.57897511	0.65009756	0.51502007	0.31044317	0.31655096
42.1	0.45428692	0.55091211	0.40442645	0.57894139	0.64987646	0.51358781	0.3112275	0.31638945
42.2	0.45370444	0.5502706	0.40378794	0.57890911	0.64963853	0.51215851	0.31200017	0.31622837
42.3	0.4531245	0.54963053	0.40315164	0.57887827	0.64938432	0.51073236	0.31276116	0.31606772
42.4	0.45254698	0.54899192	0.40251755	0.57884885	0.64911438	0.5093095	0.3135105	0.31590748
42.5	0.45197176	0.54835474	0.40188563	0.57882081	0.64882922	0.50789011	0.31424827	0.31574766
42.6	0.4513987	0.54771902	0.40125586	0.57879416	0.64852935	0.50647435	0.31497456	0.31558827
42.7	0.45082769	0.54708474	0.40062823	0.57876887	0.64821525	0.50506237	0.31568943	0.3154293
42.8	0.45025862	0.54645191	0.40000273	0.57874494	0.64788739	0.50365432	0.31639295	0.31527075
42.9	0.44969138	0.54582052	0.39937933	0.57872233	0.6475462	0.50225035	0.31708518	0.31511264
43	0.44912588	0.54519058	0.39875802	0.57870103	0.64719211	0.50085058	0.31776615	0.31495496
43.1	0.44856202	0.54456207	0.39813878	0.57868102	0.64682553	0.49945516	0.31843593	0.31479771
43.2	0.44799971	0.543935	0.3975216	0.57866229	0.64644684	0.49806421	0.31909458	0.31464089
43.3	0.44743887	0.54330936	0.39690647	0.5786448	0.64605643	0.49667785	0.31974216	0.31448451
43.4	0.44687941	0.54268515	0.39629337	0.57862856	0.64565467	0.49529619	0.32037874	0.31432857
43.5	0.44632127	0.54206236	0.39568229	0.57861353	0.64524193	0.49391933	0.32100439	0.31417306
43.6	0.4457644	0.54144099	0.39507323	0.57859969	0.64481856	0.49254737	0.3216192	0.314018
43.7	0.44520873	0.54082103	0.39446617	0.57858704	0.64438489	0.49118041	0.32222323	0.31386338
43.8	0.4446542	0.54020248	0.39386111	0.57857556	0.64394126	0.48981854	0.32281656	0.3137092
43.9	0.44410076	0.53958533	0.39325805	0.57856523	0.64348797	0.48846184	0.32339927	0.31355547
44	0.44354835	0.53896957	0.39265695	0.57855602	0.64302534	0.48711038	0.32397144	0.31340217
44.1	0.44299695	0.5383552	0.39205783	0.57854791	0.64255367	0.48576423	0.32453313	0.31324933
44.2	0.4424465	0.53774221	0.39146068	0.5785409	0.64207326	0.48442347	0.32508442	0.31309693
44.3	0.44189695	0.5371306	0.39086548	0.57853495	0.64158441	0.48308817	0.32562538	0.31294498
44.4	0.44134826	0.53652035	0.39027223	0.57853006	0.6410874	0.48175839	0.3261561	0.31279349
44.5	0.44080041	0.53591147	0.38968093	0.57852619	0.64058249	0.4804342	0.32667664	0.31264245
44.6	0.44025336	0.53530393	0.38909157	0.57852334	0.64006995	0.47911564	0.32718709	0.31249187
44.7	0.43970707	0.53469774	0.38850414	0.57852149	0.63955004	0.47780277	0.32768751	0.31234173
44.8	0.43916152	0.53409288	0.38791864	0.57852061	0.63902301	0.47649563	0.32817798	0.31219206
44.9	0.43861669	0.53348935	0.38733507	0.5785207	0.63848909	0.47519426	0.32865856	0.31204284
45	0.43807256	0.53288714	0.38675341	0.57852172	0.63794854	0.4738987	0.32912933	0.31189408
45.1	0.43752912	0.53228624	0.38617367	0.57852367	0.6374016	0.47260898	0.32959034	0.31174577
45.2	0.43698635	0.53168664	0.38559584	0.57852652	0.63684849	0.47132515	0.33004168	0.31159792
45.3	0.43644423	0.53108834	0.38501992	0.57853026	0.63628944	0.47004724	0.33048339	0.31145053
45.4	0.43590275	0.53049132	0.38444589	0.57853486	0.63572468	0.46877525	0.33091556	0.3113036
45.5	0.4353619	0.52989557	0.38387376	0.57854033	0.63515441	0.46750921	0.33133824	0.31115713
45.6	0.43482165	0.52930109	0.38330353	0.57854662	0.63457885	0.46624914	0.3317515	0.31101111
45.7	0.434282	0.52870786	0.38273518	0.57855374	0.63399821	0.46499505	0.33215538	0.31086556
45.8	0.43374294	0.52811587	0.38216872	0.57856166	0.63341269	0.46374698	0.33254993	0.31072047
45.9	0.43320444	0.52752511	0.38160415	0.57857036	0.63282249	0.46250492	0.3329352	0.31057584
46	0.4326665	0.52693557	0.38104145	0.57857983	0.6322278	0.46126891	0.33331123	0.31043167
46.1	0.43212911	0.52634724	0.38048062	0.57859005	0.6316288	0.46003895	0.33367808	0.31028796
46.2	0.43159226	0.52576012	0.37992167	0.578601	0.63102569	0.45881505	0.33403578	0.31014471
46.3	0.43105594	0.52517418	0.37936459	0.57861268	0.63041864	0.45759722	0.33438437	0.31000192
46.4	0.43052014	0.52458943	0.37880938	0.57862507	0.62980783	0.45638547	0.3347239	0.3098596
46.5	0.42998486	0.52400585	0.37825602	0.57863815	0.62919343	0.4551798	0.33505438	0.30971773
46.6	0.4294501	0.52342344	0.37770452	0.5786519	0.62857561	0.45398022	0.33537587	0.30957633
46.7	0.42891583	0.52284218	0.37715488	0.57866632	0.62795454	0.45278671	0.33568839	0.30943539
46.8	0.42838207	0.52226206	0.37660708	0.57868139	0.62733038	0.45159928	0.33599198	0.30929491
46.9	0.42784879	0.52168308	0.37606112	0.5786971	0.62670329	0.45041792	0.33628668	0.3091549
47	0.427316	0.52110523	0.375517	0.57871343	0.62607344	0.44924262	0.33657252	0.30901535
47.1	0.42678368	0.5205285	0.37497471	0.57873037	0.62544097	0.44807337	0.33684954	0.30887627
47.2	0.42625183	0.51995287	0.37443425	0.5787479	0.62480605	0.44691018	0.33711779	0.30873766
47.3	0.42572044	0.51937835	0.37389561	0.57876603	0.62416882	0.44575302	0.3373773	0.30859951
47.4	0.4251895	0.51880492	0.37335879	0.57878473	0.62352942	0.44460188	0.33762811	0.30846182
47.5	0.42465901	0.51823257	0.37282378	0.578804	0.62288801	0.44345675	0.33787024	0.30832461
47.6	0.42412898	0.51766129	0.37229058	0.57882382	0.62224471	0.44231762	0.33810373	0.30818786
47.7	0.42359938	0.51709108	0.3717592	0.57884419	0.62159966	0.44118446	0.33832859	0.30805158
47.8	0.4230702	0.51652192	0.37122961	0.57886509	0.62095299	0.44005727	0.33854487	0.30791577
47.9	0.42254144	0.51595381	0.37070182	0.57888651	0.62030484	0.438936	0.33875257	0.30778042
48	0.42201309	0.51538674	0.37017582	0.57890845	0.61965533	0.43782065	0.33895171	0.30764553
48.1	0.42148515	0.5148207	0.3696516	0.57893089	0.61900459	0.43671117	0.33914232	0.30751111
48.2	0.4209576	0.51425568	0.36912916	0.57895383	0.61835274	0.43560754	0.33932443	0.30737715
48.3	0.42043044	0.51369168	0.36860849	0.57897726	0.61769991	0.43450973	0.33949807	0.30724365
48.4	0.41990367	0.51312868	0.36808959	0.57900117	0.61704622	0.4334177	0.33966325	0.30711061
48.5	0.41937726	0.51256668	0.36757245	0.57902556	0.61639177	0.43233145	0.33982001	0.30697803
48.6	0.4188512	0.51200567	0.36705707	0.5790504	0.61573668	0.43125092	0.33996838	0.30684591
48.7	0.41832549	0.51144564	0.36654344	0.57907571	0.61508106	0.43017611	0.3401084	0.30671424
48.8	0.41780012	0.51088659	0.36603155	0.57910146	0.61442503	0.42910697	0.34024009	0.30658304
48.9	0.41727506	0.51032851	0.3655214	0.57912766	0.61376869	0.42804346	0.34036349	0.30645228
49	0.41675032	0.5097714	0.36501298	0.5791543	0.61311214	0.42698554	0.34047866	0.30632198
49.1	0.41622588	0.50921524	0.36450629	0.57918138	0.61245548	0.42593319	0.34058563	0.30619214
49.2	0.41570173	0.50866004	0.36400132	0.57920889	0.6117988	0.42488636	0.34068445	0.30606274
49.3	0.41517785	0.50810578	0.36349807	0.57923681	0.61114221	0.42384501	0.34077515	0.30593379
49.4	0.41465423	0.50755246	0.36299652	0.57926515	0.61048578	0.42280911	0.34085779	0.30580529
49.5	0.41413088	0.50700008	0.36249668	0.5792939	0.60982961	0.42177861	0.34093246	0.30567724
49.6	0.4136078	0.50644862	0.36199854	0.57932304	0.60917378	0.42075348	0.34099919	0.30554964
49.7	0.41308497	0.50589809	0.36150209	0.57935259	0.60851838	0.41973368	0.34105807	0.30542248
49.8	0.4125624	0.50534848	0.36100732	0.57938252	0.60786348	0.41871917	0.34110914	0.30529578
49.9	0.41204007	0.50479977	0.36051424	0.57941283	0.60720916	0.4177099	0.34115247	0.30516952
50	0.41151797	0.50425198	0.36002284	0.57944352	0.60655551	0.41670584	0.34118812	0.30504372
50.1	0.41099608	0.50370509	0.35953311	0.57947457	0.60590259	0.41570695	0.34121618	0.30491837
50.2	0.41047441	0.50315909	0.35904504	0.57950599	0.60525047	0.41471318	0.3412367	0.30479346
50.3	0.40995294	0.50261399	0.35855863	0.57953776	0.60459922	0.41372448	0.34124978	0.30466901
50.4	0.40943165	0.50206977	0.35807389	0.57956989	0.60394891	0.41274084	0.34125548	0.30454501
50.5	0.40891055	0.50152645	0.35759079	0.57960236	0.6032996	0.41176219	0.34125387	0.30442145
50.6	0.40838961	0.500984	0.35710934	0.57963517	0.60265134	0.41078851	0.34124506	0.30429835
50.7	0.40786882	0.50044243	0.35662953	0.57966831	0.6020042	0.40981974	0.34122911	0.3041757
50.8	0.40734818	0.49990174	0.35615136	0.57970177	0.60135821	0.40885586	0.34120612	0.30405349
50.9	0.40682766	0.49936192	0.35567481	0.57973556	0.60071345	0.40789681	0.34117619	0.30393173
51	0.40630726	0.49882296	0.35519988	0.57976966	0.60006995	0.40694256	0.34113939	0.30381042
51.1	0.40578695	0.49828487	0.35472658	0.57980406	0.59942778	0.40599307	0.34109584	0.30368955
51.2	0.40526674	0.49774765	0.35425488	0.57983875	0.59878697	0.40504829	0.34104562	0.30356914
51.3	0.4047466	0.49721128	0.35378479	0.57987374	0.59814757	0.4041082	0.34098885	0.30344916
51.4	0.40422653	0.49667578	0.3533163	0.57990902	0.59750963	0.40317275	0.34092561	0.30332964
51.5	0.40370651	0.49614112	0.35284941	0.57994457	0.59687318	0.40224191	0.34085601	0.30321055
51.6	0.40318655	0.49560733	0.3523841	0.5799804	0.59623826	0.40131563	0.34078016	0.30309191
51.7	0.40266662	0.49507438	0.35192038	0.58001649	0.59560491	0.40039388	0.34069814	0.30297372
51.8	0.40214674	0.49454228	0.35145824	0.58005285	0.59497316	0.39947663	0.34061006	0.30285596
51.9	0.40162687	0.49401103	0.35099767	0.58008946	0.59434305	0.39856384	0.34051604	0.30273865
52	0.40110703	0.49348062	0.35053866	0.58012632	0.59371461	0.39765546	0.3404162	0.30262177
52.1	0.40058718	0.49295106	0.35008122	0.58016342	0.59308785	0.39675146	0.34031064	0.30250534
52.2	0.40006732	0.49242233	0.34962532	0.58020075	0.59246282	0.39585179	0.34019948	0.30238934
52.3	0.39954746	0.49189444	0.34917098	0.58023832	0.59183952	0.39495643	0.34008282	0.30227379
52.4	0.39902758	0.49136739	0.34871817	0.5802761	0.59121799	0.39406533	0.33996075	0.30215867
52.5	0.39850769	0.49084118	0.34826689	0.5803141	0.59059825	0.39317846	0.33983341	0.30204399
52.6	0.39798776	0.4903158	0.34781714	0.58035231	0.58998032	0.39229579	0.33970088	0.30192976
52.7	0.3974678	0.48979126	0.34736891	0.58039072	0.58936422	0.39141727	0.3395633	0.30181597
52.8	0.39694779	0.48926756	0.3469222	0.58042932	0.58874996	0.39054286	0.33942076	0.30170262
52.9	0.39642772	0.48874469	0.34647698	0.58046811	0.58813758	0.38967255	0.33927339	0.30158971
53	0.39590758	0.48822266	0.34603326	0.58050707	0.58752707	0.38880628	0.33912131	0.30147725
53.1	0.39538737	0.48770145	0.34559103	0.58054621	0.58691846	0.38794402	0.33896461	0.30136524
53.2	0.39486707	0.48718107	0.34515028	0.58058551	0.58631175	0.38708573	0.33880343	0.30125367
53.3	0.39434669	0.48666152	0.34471101	0.58062496	0.58570697	0.38623139	0.33863788	0.30114254
53.4	0.39382619	0.4861428	0.34427319	0.58066456	0.58510411	0.38538095	0.33846807	0.30103187
53.5	0.39330559	0.48562491	0.34383684	0.58070431	0.58450318	0.38453438	0.3382941	0.30092164
53.6	0.39278486	0.48510784	0.34340193	0.58074419	0.5839042	0.38369164	0.33811609	0.30081186
53.7	0.392264	0.48459159	0.34296846	0.58078419	0.58330717	0.38285269	0.33793414	0.30070253
53.8	0.391743	0.48407618	0.34253642	0.58082432	0.58271209	0.3820175	0.33774837	0.30059364
53.9	0.39122186	0.48356159	0.3421058	0.58086457	0.58211897	0.38118603	0.33755889	0.30048521
54	0.39070057	0.48304783	0.3416766	0.58090492	0.58152781	0.38035825	0.33736581	0.30037722
54.1	0.39017912	0.4825349	0.3412488	0.58094537	0.58093862	0.37953412	0.33716923	0.30026968
54.2	0.3896575	0.48202279	0.3408224	0.58098592	0.58035139	0.3787136	0.33696929	0.30016259
54.3	0.3891357	0.48151151	0.34039738	0.58102657	0.57976613	0.37789667	0.33676606	0.30005594
54.4	0.38861371	0.48100105	0.33997374	0.5810673	0.57918283	0.37708328	0.33655965	0.29994974
54.5	0.38809153	0.48049142	0.33955146	0.58110811	0.57860149	0.37627342	0.33635014	0.29984399
54.6	0.38756915	0.47998261	0.33913055	0.581149	0.57802211	0.37546703	0.33613762	0.29973869
54.7	0.38704655	0.47947462	0.33871098	0.58118995	0.57744469	0.3746641	0.33592219	0.29963383
54.8	0.38652374	0.47896745	0.33829275	0.58123096	0.57686922	0.37386457	0.33570394	0.29952943
54.9	0.38600069	0.4784611	0.33787586	0.58127204	0.57629569	0.37306843	0.33548298	0.29942546
55	0.38547741	0.47795557	0.33746028	0.58131317	0.57572411	0.37227563	0.3352594	0.29932194
55.1	0.38495389	0.47745085	0.33704601	0.58135435	0.57515445	0.37148616	0.33503329	0.29921887
55.2	0.38443012	0.47694695	0.33663304	0.58139558	0.57458672	0.37069997	0.33480475	0.29911624
55.3	0.38390611	0.47644387	0.33622137	0.58143685	0.5740209	0.36991704	0.33457387	0.29901406
55.4	0.38338184	0.47594161	0.33581098	0.58147816	0.57345699	0.36913733	0.33434075	0.29891232
55.5	0.38285732	0.47544015	0.33540186	0.58151952	0.57289498	0.36836082	0.33410547	0.29881103
55.6	0.38233253	0.47493951	0.33499401	0.5815609	0.57233486	0.36758748	0.33386812	0.29871018
55.7	0.38180747	0.47443967	0.33458741	0.58160232	0.57177662	0.36681727	0.3336288	0.29860977
55.8	0.38128212	0.47394064	0.33418206	0.58164377	0.57122025	0.36605016	0.33338757	0.29850981
55.9	0.3807565	0.47344241	0.33377794	0.58168525	0.57066572	0.36528612	0.33314454	0.29841029
56	0.38023059	0.47294498	0.33337504	0.58172676	0.57011303	0.36452513	0.33289978	0.29831122
56.1	0.3797044	0.47244834	0.33297336	0.5817683	0.56956215	0.36376715	0.33265338	0.29821258
56.2	0.37917791	0.4719525	0.33257289	0.58180986	0.56901308	0.36301216	0.33240542	0.29811439
56.3	0.37865112	0.47145744	0.3321736	0.58185145	0.5684658	0.36226012	0.33215598	0.29801664
56.4	0.37812403	0.47096317	0.3317755	0.58189306	0.56792029	0.36151102	0.33190515	0.29791934
56.5	0.37759664	0.47046968	0.33137858	0.58193469	0.56737653	0.36076482	0.33165301	0.29782247
56.6	0.37706894	0.46997696	0.33098282	0.58197634	0.56683449	0.36002151	0.33139964	0.29772604
56.7	0.37654093	0.46948502	0.33058821	0.58201801	0.56629418	0.35928104	0.3311451	0.29763005
56.8	0.37601261	0.46899385	0.33019475	0.58205971	0.56575555	0.35854341	0.33088947	0.2975345
56.9	0.37548398	0.46850344	0.32980242	0.58210142	0.5652186	0.35780857	0.33063283	0.29743939
57	0.37495503	0.46801379	0.32941121	0.58214316	0.5646833	0.35707651	0.33037524	0.29734471
57.1	0.37442577	0.4675249	0.32902113	0.58218491	0.56414963	0.3563472	0.33011677	0.29725046
57.2	0.37389619	0.46703675	0.32863214	0.58222669	0.56361757	0.35562062	0.3298575	0.29715665
57.3	0.37336629	0.46654935	0.32824426	0.5822685	0.56308711	0.35489674	0.3295975	0.29706328
57.4	0.37283608	0.46606269	0.32785747	0.58231032	0.56255821	0.35417554	0.32933684	0.29697033
57.5	0.37230555	0.46557676	0.32747176	0.58235217	0.56203085	0.35345699	0.32907558	0.29687782
57.6	0.37177471	0.46509155	0.32708713	0.58239404	0.56150501	0.35274107	0.32881378	0.29678573
57.7	0.37124356	0.46460707	0.32670356	0.58243593	0.56098068	0.35202776	0.32855149	0.29669407
57.8	0.3707121	0.4641233	0.32632106	0.58247785	0.56045782	0.35131702	0.3282888	0.29660284
57.9	0.37018033	0.46364024	0.3259396	0.5825198	0.5599364	0.35060884	0.32802574	0.29651204
58	0.36964826	0.46315789	0.32555919	0.58256177	0.55941642	0.34990319	0.32776238	0.29642166
58.1	0.36911591	0.46267623	0.32517981	0.58260377	0.55889784	0.34920005	0.32749879	0.2963317
58.2	0.36858327	0.46219527	0.32480147	0.58264579	0.55838064	0.3484994	0.32723502	0.29624217
58.3	0.36805036	0.46171499	0.32442414	0.58268784	0.55786478	0.34780121	0.32697114	0.29615305
58.4	0.36751717	0.4612354	0.32404783	0.58272992	0.55735026	0.34710546	0.32670719	0.29606435
58.5	0.36698372	0.46075649	0.32367253	0.58277204	0.55683704	0.34641213	0.32644323	0.29597608
58.6	0.36645001	0.46027824	0.32329824	0.58281418	0.55632509	0.34572119	0.32617933	0.29588822
58.7	0.36591605	0.45980065	0.32292494	0.58285636	0.55581439	0.34503261	0.32591553	0.29580078
58.8	0.36538185	0.45932373	0.32255262	0.58289856	0.55530492	0.34434638	0.3256519	0.29571376
58.9	0.36484741	0.45884746	0.32218129	0.58294081	0.55479665	0.34366247	0.32538848	0.29562715
59	0.36431275	0.45837183	0.32181094	0.58298308	0.55428956	0.34298085	0.32512532	0.29554097
59.1	0.36377786	0.45789685	0.32144155	0.5830254	0.55378363	0.34230151	0.32486248	0.2954552
59.2	0.36324277	0.45742251	0.32107313	0.58306775	0.55327881	0.34162442	0.32460001	0.29536984
59.3	0.36270749	0.45694879	0.32070567	0.58311014	0.5527751	0.34094956	0.32433795	0.2952849
59.4	0.36217201	0.4564757	0.32033915	0.58315257	0.55227246	0.34027691	0.32407634	0.29520038
59.5	0.36163636	0.45600324	0.31997359	0.58319504	0.55177088	0.33960643	0.32381524	0.29511627
59.6	0.36110054	0.45553139	0.31960897	0.58323755	0.55127033	0.33893811	0.32355467	0.29503256
59.7	0.36056456	0.45506015	0.31924528	0.58328011	0.55077078	0.33827193	0.32329469	0.29494927
59.8	0.36002843	0.45458952	0.31888252	0.58332272	0.55027221	0.33760785	0.32303531	0.29486639
59.9	0.35949217	0.45411948	0.31852069	0.58336537	0.54977461	0.33694585	0.3227766	0.29478392

60	0.35895579	0.45365005	0.31815978	0.58340807	0.54927794	0.33628591	0.32251859	0.29470185
60.1	0.35841929	0.4531812	0.31779978	0.58345081	0.54878218	0.33562801	0.32226131	0.29462018
60.2	0.35788269	0.45271294	0.3174407	0.5834936	0.54828733	0.33497213	0.3220048	0.29453892
60.3	0.357346	0.45224527	0.31708252	0.58353643	0.54779335	0.33431824	0.32174909	0.29445807
60.4	0.35680923	0.45177818	0.31672525	0.58357932	0.54730022	0.33366632	0.32149422	0.29437761
60.5	0.35627239	0.45131167	0.31636887	0.58362225	0.54680794	0.33301636	0.32124021	0.29429755
60.6	0.3557355	0.45084574	0.31601338	0.58366523	0.54631647	0.33236833	0.3209871	0.29421789
60.7	0.35519857	0.45038038	0.31565878	0.58370825	0.54582581	0.33172221	0.32073491	0.29413862
60.8	0.35466161	0.4499156	0.31530507	0.58375133	0.54533593	0.33107799	0.32048367	0.29405975
60.9	0.35412464	0.44945138	0.31495224	0.58379446	0.54484681	0.33043564	0.32023341	0.29398127
61	0.35358767	0.44898773	0.31460028	0.58383763	0.54435845	0.32979515	0.31998417	0.29390318
61.1	0.35305071	0.44852465	0.31424919	0.58388085	0.54387082	0.3291565	0.31973597	0.29382548
61.2	0.35251377	0.44806214	0.31389898	0.58392412	0.54338392	0.32851967	0.31948883	0.29374816
61.3	0.35197686	0.4476002	0.31354963	0.58396744	0.54289772	0.32788466	0.31924277	0.29367124
61.4	0.35144001	0.44713882	0.31320114	0.58401081	0.54241222	0.32725144	0.31899783	0.29359469
61.5	0.35090321	0.44667801	0.31285351	0.58405423	0.54192741	0.32662	0.31875402	0.29351852
61.6	0.35036649	0.44621776	0.31250673	0.5840977	0.54144327	0.32599033	0.31851135	0.29344273
61.7	0.34982985	0.44575808	0.31216081	0.58414122	0.5409598	0.32536242	0.31826986	0.29336732
61.8	0.34929331	0.44529897	0.31181573	0.58418479	0.54047698	0.32473625	0.31802955	0.29329228
61.9	0.34875689	0.44484042	0.31147149	0.58422842	0.53999481	0.32411182	0.31779045	0.29321762
62	0.34822059	0.44438244	0.3111281	0.58427209	0.53951328	0.32348911	0.31755258	0.29314332
62.1	0.34768443	0.44392503	0.31078554	0.58431582	0.53903237	0.32286812	0.31731595	0.2930694
62.2	0.34714842	0.44346819	0.31044382	0.5843596	0.53855207	0.32224884	0.31708058	0.29299584
62.3	0.34661256	0.44301191	0.31010293	0.58440342	0.5380724	0.32163125	0.31684648	0.29292264
62.4	0.34607687	0.44255621	0.30976286	0.5844473	0.53759334	0.32101536	0.31661367	0.29284981
62.5	0.34554136	0.44210109	0.30942363	0.58449123	0.53711489	0.32040116	0.31638217	0.29277734
62.6	0.34500605	0.44164654	0.30908521	0.58453521	0.53663705	0.31978863	0.31615198	0.29270522
62.7	0.34447094	0.44119256	0.30874761	0.58457923	0.53615982	0.31917778	0.31592312	0.29263346
62.8	0.34393604	0.44073917	0.30841084	0.5846233	0.53568321	0.3185686	0.31569562	0.29256205
62.9	0.34340137	0.44028636	0.30807487	0.58466743	0.5352072	0.31796108	0.31546947	0.292491
63	0.34286693	0.43983413	0.30773972	0.58471159	0.53473182	0.31735522	0.31524469	0.29242029
63.1	0.34233273	0.43938249	0.30740538	0.58475581	0.53425705	0.31675102	0.31502129	0.29234993
63.2	0.34179879	0.43893143	0.30707185	0.58480007	0.53378291	0.31614848	0.31479929	0.29227992
63.3	0.34126512	0.43848097	0.30673912	0.58484438	0.53330941	0.31554759	0.31457869	0.29221025
63.4	0.34073172	0.43803109	0.30640719	0.58488873	0.53283654	0.31494836	0.31435949	0.29214092
63.5	0.3401986	0.43758182	0.30607605	0.58493313	0.53236433	0.31435079	0.31414172	0.29207193
63.6	0.33966577	0.43713315	0.30574571	0.58497758	0.53189277	0.31375487	0.31392536	0.29200327
63.7	0.33913324	0.43668508	0.30541616	0.58502207	0.53142187	0.31316061	0.31371042	0.29193495
63.8	0.33860102	0.43623762	0.3050874	0.58506661	0.53095166	0.31256802	0.31349692	0.29186695
63.9	0.33806912	0.43579078	0.30475943	0.58511119	0.53048213	0.31197708	0.31328485	0.29179929
64	0.33753754	0.43534455	0.30443224	0.58515582	0.5300133	0.3113878	0.31307422	0.29173195
64.1	0.3370063	0.43489893	0.30410583	0.58520049	0.52954519	0.31080019	0.31286503	0.29166494
64.2	0.3364754	0.43445395	0.3037802	0.5852452	0.52907781	0.31021424	0.31265729	0.29159825
64.3	0.33594485	0.43400959	0.30345534	0.58528996	0.52861116	0.30962995	0.31245098	0.29153188
64.4	0.33541466	0.43356587	0.30313127	0.58533476	0.52814528	0.30904734	0.31224613	0.29146582
64.5	0.33488482	0.43312278	0.30280796	0.58537961	0.52768016	0.3084664	0.31204272	0.29140008
64.6	0.33435536	0.43268034	0.30248543	0.5854245	0.52721582	0.30788713	0.31184077	0.29133465
64.7	0.33382628	0.43223854	0.30216367	0.58546943	0.52675228	0.30730953	0.31164027	0.29126953
64.8	0.33329758	0.43179739	0.30184268	0.5855144	0.52628954	0.30673361	0.31144122	0.29120472
64.9	0.33276927	0.43135689	0.30152245	0.58555942	0.52582763	0.30615937	0.31124362	0.29114021
65	0.33224135	0.43091706	0.30120298	0.58560447	0.52536655	0.30558681	0.31104747	0.29107601
65.1	0.33171384	0.43047788	0.30088427	0.58564957	0.52490633	0.30501593	0.31085278	0.29101211
65.2	0.33118673	0.43003937	0.30056631	0.5856947	0.52444697	0.30444674	0.31065953	0.2909485
65.3	0.33066003	0.42960154	0.30024911	0.58573987	0.52398849	0.30387924	0.31046774	0.29088519
65.4	0.33013375	0.42916438	0.29993266	0.58578508	0.52353091	0.30331343	0.31027739	0.29082218
65.5	0.32960789	0.42872791	0.29961695	0.58583033	0.52307423	0.30274931	0.31008848	0.29075947
65.6	0.32908247	0.42829212	0.29930199	0.58587561	0.52261848	0.30218689	0.30990102	0.29069704
65.7	0.32855747	0.42785702	0.29898777	0.58592093	0.52216367	0.30162617	0.30971499	0.29063491
65.8	0.32803292	0.42742261	0.29867429	0.58596628	0.52170981	0.30106714	0.30953039	0.29057306
65.9	0.32750881	0.42698891	0.29836155	0.58601166	0.52125692	0.30050982	0.30934722	0.2905115
66	0.32698515	0.42655591	0.29804954	0.58605708	0.520805	0.2999542	0.30916547	0.29045022
66.1	0.32646195	0.42612362	0.29773826	0.58610253	0.52035407	0.29940028	0.30898515	0.29038922
66.2	0.3259392	0.42569204	0.29742771	0.586148	0.51990414	0.29884806	0.30880624	0.29032851
66.3	0.32541692	0.42526117	0.29711788	0.58619351	0.51945522	0.29829754	0.30862874	0.29026807
66.4	0.3248951	0.42483103	0.29680878	0.58623904	0.51900731	0.29774872	0.30845265	0.29020791
66.5	0.32437375	0.42440161	0.2965004	0.58628459	0.51856044	0.29720161	0.30827796	0.29014802
66.6	0.32385287	0.42397291	0.29619273	0.58633018	0.51811461	0.2966562	0.30810466	0.2900884
66.7	0.32333247	0.42354495	0.29588578	0.58637579	0.51766983	0.2961125	0.30793275	0.29002906
66.8	0.32281256	0.42311773	0.29557954	0.58642142	0.51722611	0.2955705	0.30776223	0.28996998
66.9	0.32229313	0.42269125	0.29527401	0.58646708	0.51678345	0.2950302	0.30759307	0.28991116
67	0.32177419	0.42226551	0.29496919	0.58651275	0.51634187	0.29449161	0.30742529	0.28985262
67.1	0.32125574	0.42184052	0.29466506	0.58655846	0.51590137	0.29395473	0.30725886	0.28979433
67.2	0.32073779	0.42141629	0.29436164	0.58660418	0.51546196	0.29341955	0.30709379	0.2897363
67.3	0.32022035	0.42099281	0.29405892	0.58664992	0.51502364	0.29288608	0.30693006	0.28967854
67.4	0.3197034	0.4205701	0.29375689	0.58669568	0.51458641	0.29235431	0.30676766	0.28962103
67.5	0.31918697	0.42014815	0.29345555	0.58674146	0.51415028	0.29182424	0.3066066	0.28956377
67.6	0.31867105	0.41972697	0.2931549	0.58678726	0.51371525	0.29129588	0.30644685	0.28950677
67.7	0.31815564	0.41930657	0.29285494	0.58683307	0.51328133	0.29076923	0.30628842	0.28945002
67.8	0.31764076	0.41888694	0.29255566	0.5868789	0.51284851	0.29024427	0.30613129	0.28939352
67.9	0.3171264	0.41846809	0.29225707	0.58692474	0.51241681	0.28972102	0.30597544	0.28933726
68	0.31661256	0.41805003	0.29195916	0.5869706	0.5119862	0.28919947	0.30582088	0.28928126
68.1	0.31609925	0.41763276	0.29166192	0.58701647	0.51155671	0.28867962	0.30566759	0.28922549
68.2	0.31558648	0.41721628	0.29136535	0.58706234	0.51112832	0.28816147	0.30551557	0.28916998
68.3	0.31507424	0.4168006	0.29106946	0.58710822	0.51070104	0.28764501	0.30536479	0.2891147
68.4	0.31456253	0.41638571	0.29077424	0.5871541	0.51027485	0.28713025	0.30521526	0.28905966
68.5	0.31405138	0.41597164	0.29047969	0.58719999	0.50984977	0.28661718	0.30506696	0.28900486
68.6	0.31354076	0.41555836	0.29018581	0.58724589	0.50942578	0.28610581	0.30491988	0.2889503
68.7	0.3130307	0.4151459	0.28989259	0.58729179	0.50900288	0.28559612	0.30477401	0.28889597
68.8	0.31252119	0.41473426	0.28960003	0.58733769	0.50858107	0.28508811	0.30462934	0.28884188
68.9	0.31201224	0.41432343	0.28930813	0.58738359	0.50816033	0.28458178	0.30448587	0.28878801
69	0.31150385	0.41391343	0.28901689	0.5874295	0.50774066	0.28407714	0.30434357	0.28873438
69.1	0.31099602	0.41350425	0.2887263	0.5874754	0.50732206	0.28357417	0.30420245	0.28868098
69.2	0.31048877	0.41309591	0.28843637	0.58752131	0.50690451	0.28307287	0.30406249	0.2886278
69.3	0.30998208	0.4126884	0.28814709	0.58756721	0.506488	0.28257324	0.30392368	0.28857485
69.4	0.30947597	0.41228172	0.28785845	0.58761312	0.50607254	0.28207528	0.30378601	0.28852213
69.5	0.30897044	0.41187588	0.28757046	0.58765902	0.50565811	0.28157898	0.30364947	0.28846962
69.6	0.30846549	0.41147089	0.28728312	0.58770491	0.50524469	0.28108434	0.30351405	0.28841734
69.7	0.30796113	0.41106674	0.28699642	0.5877508	0.50483229	0.28059136	0.30337975	0.28836527
69.8	0.30745737	0.41066344	0.28671036	0.58779669	0.50442089	0.28010003	0.30324654	0.28831343
69.9	0.30695419	0.41026099	0.28642494	0.58784257	0.50401049	0.27961036	0.30311443	0.2882618
70	0.30645162	0.40985939	0.28614015	0.58788844	0.50360106	0.27912233	0.30298341	0.28821038
70.1	0.30594965	0.40945865	0.285856	0.5879343	0.50319259	0.27863594	0.30285346	0.28815918
70.2	0.30544829	0.40905877	0.28557249	0.58798016	0.50278508	0.27815119	0.30272457	0.28810819
70.3	0.30494754	0.40865975	0.2852896	0.588026	0.50237852	0.27766808	0.30259674	0.28805741
70.4	0.3044474	0.4082616	0.28500735	0.58807183	0.50197288	0.2771866	0.30246995	0.28800684
70.5	0.30394789	0.40786431	0.28472572	0.58811764	0.50156817	0.27670675	0.30234421	0.28795648
70.6	0.303449	0.40746789	0.28444472	0.58816344	0.50116437	0.27622852	0.30221949	0.28790633
70.7	0.30295073	0.40707235	0.28416434	0.58820923	0.50076146	0.27575192	0.30209579	0.28785638
70.8	0.3024531	0.40667769	0.28388458	0.58825501	0.50035945	0.27527693	0.30197311	0.28780663
70.9	0.30195611	0.4062839	0.28360545	0.58830076	0.49995831	0.27480356	0.30185143	0.28775708
71	0.30145975	0.40589099	0.28332693	0.58834651	0.49955804	0.27433179	0.30173074	0.28770774
71.1	0.30096404	0.40549897	0.28304903	0.58839223	0.49915863	0.27386163	0.30161104	0.28765859
71.2	0.30046898	0.40510783	0.28277175	0.58843794	0.49876006	0.27339308	0.30149232	0.28760965
71.3	0.29997457	0.40471758	0.28249509	0.58848364	0.49836234	0.27292612	0.30137458	0.2875609
71.4	0.29948083	0.40432822	0.28221903	0.58852931	0.49796544	0.27246076	0.3012578	0.28751234
71.5	0.29898774	0.40393976	0.28194359	0.58857497	0.49756936	0.27199698	0.30114198	0.28746399
71.6	0.29849532	0.4035522	0.28166876	0.5886206	0.4971741	0.27153479	0.30102711	0.28741582
71.7	0.29800358	0.40316553	0.28139453	0.58866622	0.49677964	0.27107418	0.30091318	0.28736785
71.8	0.29751251	0.40277976	0.28112092	0.58871182	0.49638597	0.27061514	0.30080018	0.28732007
71.9	0.29702212	0.4023949	0.28084791	0.5887574	0.4959931	0.27015768	0.3006881	0.28727248
72	0.29653242	0.40201095	0.2805755	0.58880296	0.49560101	0.26970178	0.30057695	0.28722508
72.1	0.29604341	0.4016279	0.2803037	0.58884849	0.49520969	0.26924744	0.30046671	0.28717787
72.2	0.29555509	0.40124577	0.2800325	0.58889401	0.49481915	0.26879466	0.30035737	0.28713085
72.3	0.29506748	0.40086455	0.2797619	0.5889395	0.49442938	0.26834343	0.30024894	0.28708401
72.4	0.29458056	0.40048424	0.2794919	0.58898498	0.49404037	0.26789376	0.30014139	0.28703736
72.5	0.29409436	0.40010485	0.27922249	0.58903043	0.49365212	0.26744563	0.30003472	0.2869909
72.6	0.29360886	0.39972639	0.27895368	0.58907586	0.49326463	0.26699904	0.29992894	0.28694461
72.7	0.29312408	0.39934884	0.27868546	0.58912127	0.49287789	0.26655399	0.29982402	0.28689851
72.8	0.29264003	0.39897221	0.27841783	0.58916665	0.4924919	0.26611047	0.29971997	0.2868526
72.9	0.2921567	0.39859651	0.2781508	0.58921202	0.49210666	0.26566848	0.29961678	0.28680686
73	0.2916741	0.39822174	0.27788435	0.58925736	0.49172217	0.26522801	0.29951444	0.2867613
73.1	0.29119223	0.39784789	0.27761849	0.58930268	0.49133841	0.26478907	0.29941295	0.28671592
73.2	0.2907111	0.39747498	0.27735322	0.58934799	0.49095539	0.26435164	0.2993123	0.28667071
73.3	0.29023071	0.39710299	0.27708854	0.58939327	0.49057311	0.26391573	0.29921248	0.28662568
73.4	0.28975107	0.39673194	0.27682445	0.58943853	0.49019157	0.26348132	0.29911349	0.28658083
73.5	0.28927219	0.39636182	0.27656094	0.58948377	0.48981077	0.26304841	0.29901532	0.28653615
73.6	0.28879405	0.39599264	0.27629801	0.58952899	0.48943071	0.26261701	0.29891797	0.28649164
73.7	0.28831668	0.39562439	0.27603567	0.58957419	0.4890514	0.2621871	0.29882142	0.2864473
73.8	0.28784007	0.39525709	0.27577391	0.58961937	0.48867283	0.26175868	0.29872568	0.28640314
73.9	0.28736423	0.39489073	0.27551274	0.58966453	0.48829501	0.26133175	0.29863074	0.28635914
74	0.28688917	0.39452531	0.27525214	0.58970967	0.48791794	0.26090631	0.29853659	0.28631531
74.1	0.28641488	0.39416083	0.27499213	0.58975479	0.48754162	0.26048234	0.29844323	0.28627165
74.2	0.28594137	0.3937973	0.2747327	0.58979989	0.48716606	0.26005985	0.29835065	0.28622816
74.3	0.28546864	0.39343472	0.27447384	0.58984497	0.48679126	0.25963883	0.29825884	0.28618483
74.4	0.28499671	0.39307309	0.27421555	0.58989002	0.48641722	0.25921927	0.29816781	0.28614167
74.5	0.28452556	0.3927124	0.27395784	0.58993506	0.48604394	0.25880118	0.29807755	0.28609868
74.6	0.28405522	0.39235267	0.2737007	0.58998008	0.48567144	0.25838454	0.29798804	0.28605585
74.7	0.28358567	0.3919939	0.27344413	0.59002507	0.48529971	0.25796936	0.2978993	0.28601318
74.8	0.28311693	0.39163607	0.27318814	0.59007005	0.48492876	0.25755563	0.2978113	0.28597067
74.9	0.282649	0.3912792	0.27293271	0.590115	0.4845586	0.25714334	0.29772405	0.28592833
120	0.16071987	0.30718193	0.19866305	0.6084631	0.38695022	0.16383243	0.30053241	0.27666164
120.1	0.16059592	0.30710674	0.19856031	0.60849741	0.38684219	0.16373946	0.30060128	0.27665469

75	0.28218188	0.39092329	0.27267785	0.59015994	0.48418922	0.25673249	0.29763754	0.28588614
75.1	0.28171558	0.39056833	0.27242356	0.59020486	0.48382063	0.25632308	0.29755177	0.28584412
75.2	0.28125009	0.39021433	0.27216984	0.59024976	0.48345284	0.25591511	0.29746673	0.28580225
75.3	0.28078543	0.38986128	0.27191668	0.59029464	0.48308586	0.25550856	0.29738242	0.28576055
75.4	0.28032159	0.3895092	0.27166409	0.5903395	0.48271968	0.25510344	0.29729883	0.285719
75.5	0.27985859	0.38915807	0.27141206	0.59038435	0.48235431	0.25469973	0.29721596	0.2856776
75.6	0.27939642	0.3888079	0.2711606	0.59042919	0.48198976	0.25429745	0.29713381	0.28563637
75.7	0.2789351	0.3884587	0.2709097	0.59047401	0.48162603	0.25389658	0.29705236	0.28559528
75.8	0.27847461	0.38811045	0.27065937	0.59051881	0.48126313	0.25349712	0.29697163	0.28555436
75.9	0.27801498	0.38776317	0.27040959	0.5905636	0.48090106	0.25309906	0.29689159	0.28551358
76	0.27755619	0.38741685	0.27016038	0.59060837	0.48053982	0.25270241	0.29681225	0.28547296
76.1	0.27709827	0.38707148	0.26991172	0.59065313	0.48017942	0.25230715	0.29673361	0.28543249
76.2	0.2766412	0.38672708	0.26966362	0.59069788	0.47981986	0.25191328	0.29665565	0.28539217
76.3	0.27618499	0.38638364	0.26941608	0.59074261	0.47946116	0.25152081	0.29657838	0.285352
76.4	0.27572965	0.38604117	0.26916909	0.59078733	0.4791033	0.25112972	0.2965018	0.28531199
76.5	0.27527517	0.38569965	0.26892265	0.59083205	0.47874631	0.25074	0.29642588	0.28527212
76.6	0.27482157	0.3853591	0.26867677	0.59087675	0.47839017	0.25035167	0.29635064	0.2852324
76.7	0.27436884	0.38501951	0.26843145	0.59092144	0.4780349	0.24996471	0.29627607	0.28519283
76.8	0.27391698	0.38468089	0.26818667	0.59096612	0.47768049	0.24957911	0.29620216	0.28515341
76.9	0.27346601	0.38434322	0.26794245	0.59101079	0.47732695	0.24919488	0.29612891	0.28511413
77	0.27301591	0.38400653	0.26769878	0.59105545	0.47697429	0.24881201	0.29605631	0.285075
77.1	0.27256671	0.38367079	0.26745566	0.5911001	0.4766225	0.2484305	0.29598437	0.28503602
77.2	0.27211839	0.38333602	0.2672131	0.59114474	0.47627159	0.24805033	0.29591308	0.28499718
77.3	0.27167097	0.38300221	0.26697108	0.59118937	0.47592155	0.24767152	0.29584244	0.28495848
77.4	0.27122444	0.38266936	0.26672961	0.591234	0.47557238	0.24729405	0.29577243	0.28491993
77.5	0.27077881	0.38233748	0.26648869	0.59127861	0.4752241	0.24691792	0.29570307	0.28488152
77.6	0.27033408	0.38200656	0.26624832	0.59132322	0.47487669	0.24654313	0.29563435	0.28484325
77.7	0.26989027	0.3816766	0.2660085	0.59136781	0.47453016	0.24616967	0.29556625	0.28480513
77.8	0.26944736	0.38134761	0.26576922	0.5914124	0.47418451	0.24579754	0.29549879	0.28476714
77.9	0.26900536	0.38101958	0.26553048	0.59145699	0.47383974	0.24542673	0.29543196	0.28472929
78	0.26856428	0.38069251	0.26529229	0.59150156	0.47349585	0.24505724	0.29536575	0.28469159
78.1	0.26812412	0.3803664	0.26505465	0.59154613	0.47315285	0.24468907	0.29530016	0.28465402
78.2	0.26768488	0.38004126	0.26481754	0.5915907	0.47281072	0.24432222	0.29523518	0.28461658
78.3	0.26724657	0.37971707	0.26458098	0.59163525	0.47246948	0.24395667	0.29517083	0.28457929
78.4	0.26680918	0.37939385	0.26434496	0.5916798	0.47212912	0.24359243	0.29510708	0.28454213
78.5	0.26637272	0.37907158	0.26410948	0.59172435	0.47178963	0.24322949	0.29504395	0.28450511
78.6	0.2659372	0.37875027	0.26387454	0.59176888	0.47145102	0.24286785	0.29498142	0.28446823
78.7	0.26550262	0.37842993	0.26364014	0.59181341	0.47111329	0.2425075	0.29491949	0.28443148
78.8	0.26506897	0.37811054	0.26340628	0.59185794	0.47077644	0.24214844	0.29485816	0.28439486
78.9	0.26463627	0.3777921	0.26317295	0.59190246	0.47044046	0.24179067	0.29479743	0.28435838
79	0.26420451	0.37747463	0.26294016	0.59194697	0.47010536	0.24143418	0.29473729	0.28432203
79.1	0.2637737	0.37715811	0.2627079	0.59199148	0.46977112	0.24107897	0.29467774	0.28428581
79.2	0.26334384	0.37684255	0.26247617	0.59203599	0.46943775	0.24072504	0.29461878	0.28424972
79.3	0.26291494	0.37652794	0.26224498	0.59208049	0.46910525	0.24037238	0.2945604	0.28421377
79.4	0.26248699	0.37621428	0.26201431	0.59212498	0.46877361	0.24002098	0.29450261	0.28417794
79.5	0.26205999	0.37590158	0.26178418	0.59216948	0.46844283	0.23967085	0.29444539	0.28414225
79.6	0.26163396	0.37558983	0.26155457	0.59221396	0.46811291	0.23932197	0.29438875	0.28410668
79.7	0.2612089	0.37527904	0.2613255	0.59225845	0.46778384	0.23897435	0.29433269	0.28407125
79.8	0.26078479	0.37496919	0.26109694	0.59230293	0.46745562	0.23862798	0.29427719	0.28403594
79.9	0.26036166	0.37466029	0.26086892	0.5923474	0.46712824	0.23828286	0.29422226	0.28400075
80	0.25993949	0.37435235	0.26064142	0.59239187	0.46680171	0.23793898	0.2941679	0.2839657
80.1	0.2595183	0.37404535	0.26041444	0.59243634	0.46647602	0.23759634	0.2941141	0.28393077
80.2	0.25909807	0.3737393	0.26018798	0.5924808	0.46615117	0.23725494	0.29406086	0.28389596
80.3	0.25867883	0.37343419	0.25996205	0.59252526	0.46582714	0.23691476	0.29400817	0.28386128
80.4	0.25826056	0.37313002	0.25973664	0.59256971	0.46550395	0.23657582	0.29395604	0.28382672
80.5	0.25784327	0.3728268	0.25951174	0.59261416	0.46518158	0.23623809	0.29390446	0.28379229
80.6	0.25742696	0.37252453	0.25928737	0.59265861	0.46486002	0.23590159	0.29385343	0.28375797
80.7	0.25701164	0.37222319	0.25906351	0.59270305	0.46453928	0.2355663	0.29380295	0.28372378
80.8	0.2565973	0.37192279	0.25884017	0.59274749	0.46421935	0.23523223	0.29375301	0.28368971
80.9	0.25618395	0.37162333	0.25861734	0.59279193	0.46390023	0.23489936	0.2937036	0.28365576
81	0.25577159	0.37132481	0.25839503	0.59283636	0.4635819	0.2345677	0.29365474	0.28362192
81.1	0.25536022	0.37102722	0.25817323	0.59288079	0.46326437	0.23423724	0.29360641	0.28358821
81.2	0.25494984	0.37073057	0.25795195	0.59292521	0.46294764	0.23390797	0.29355861	0.28355461
81.3	0.25454045	0.37043486	0.25773117	0.59296963	0.46263169	0.2335799	0.29351134	0.28352113
81.4	0.25413206	0.37014007	0.25751091	0.59301405	0.46231651	0.23325301	0.29346459	0.28348777
81.5	0.25372467	0.36984622	0.25729115	0.59305846	0.46200212	0.23292731	0.29341837	0.28345452
81.6	0.25331828	0.3695533	0.2570719	0.59310287	0.46168849	0.23260279	0.29337266	0.28342139
81.7	0.25291288	0.36926131	0.25685316	0.59314727	0.46137563	0.23227945	0.29332747	0.28338837
81.8	0.25250849	0.36897025	0.25663492	0.59319167	0.46106353	0.23195728	0.29328279	0.28335547
81.9	0.2521051	0.36868011	0.25641719	0.59323607	0.46075218	0.23163629	0.29323862	0.28332268
82	0.25170271	0.3683909	0.25619996	0.59328046	0.46044158	0.23131645	0.29319496	0.28329
82.1	0.25130132	0.36810262	0.25598323	0.59332484	0.46013172	0.23099778	0.29315181	0.28325744
82.2	0.25090094	0.36781525	0.255767	0.59336922	0.45982261	0.23068027	0.29310916	0.28322499
82.3	0.25050157	0.36752881	0.25555127	0.59341359	0.45951423	0.23036391	0.29306701	0.28319265
82.4	0.25010321	0.36724328	0.25533603	0.59345796	0.45920658	0.2300487	0.29302535	0.28316042
82.5	0.24970585	0.36695867	0.25512129	0.59350232	0.45889966	0.22973464	0.29298419	0.2831283
82.6	0.24930951	0.36667497	0.25490705	0.59354667	0.45859346	0.22942173	0.29294353	0.2830963
82.7	0.24891417	0.36639219	0.25469329	0.59359102	0.45828797	0.22910995	0.29290335	0.2830644
82.8	0.24851985	0.36611031	0.25448003	0.59363536	0.4579832	0.22879931	0.29286366	0.28303261
82.9	0.24812654	0.36582935	0.25426726	0.5936797	0.45767914	0.2284898	0.29282445	0.28300092
83	0.24773425	0.36554929	0.25405498	0.59372402	0.45737578	0.22818141	0.29278572	0.28296935
83.1	0.24734296	0.36527014	0.25384318	0.59376834	0.45707312	0.22787415	0.29274747	0.28293788
83.2	0.24695269	0.3649919	0.25363186	0.59381265	0.45677116	0.22756802	0.29270969	0.28290652
83.3	0.24656343	0.36471456	0.25342103	0.59385696	0.4564699	0.22726299	0.29267239	0.28287526
83.4	0.24617519	0.36443811	0.25321069	0.59390125	0.45616932	0.22695908	0.29263557	0.28284411
83.5	0.24578796	0.36416257	0.25300082	0.59394554	0.45586943	0.22665628	0.29259921	0.28281306
83.6	0.24540175	0.36388792	0.25279143	0.59398982	0.45557022	0.22635459	0.29256331	0.28278212
83.7	0.24501655	0.36361417	0.25258251	0.59403409	0.45527169	0.22605399	0.29252788	0.28275129
83.8	0.24463237	0.3633413	0.25237407	0.59407835	0.45497383	0.22575449	0.29249291	0.28272055
83.9	0.2442492	0.36306933	0.25216611	0.59412261	0.45467665	0.22545609	0.2924584	0.28268992
84	0.24386705	0.36279825	0.25195861	0.59416685	0.45438014	0.22515877	0.29242435	0.28265939
84.1	0.24348592	0.36252806	0.25175158	0.59421108	0.4540843	0.22486254	0.29239075	0.28262896
84.2	0.2431058	0.36225875	0.25154503	0.59425531	0.45378913	0.22456739	0.2923576	0.28259863
84.3	0.2427267	0.36199033	0.25133893	0.59429952	0.45349461	0.22427332	0.2923249	0.2825684
84.4	0.24234862	0.36172279	0.25113331	0.59434372	0.45320076	0.22398032	0.29229265	0.28253828
84.5	0.24197156	0.36145612	0.25092814	0.59438791	0.45290756	0.22368839	0.29226084	0.28250825
84.6	0.24159551	0.36119034	0.25072343	0.59443209	0.45261502	0.22339753	0.29222947	0.28247832
84.7	0.24122048	0.36092543	0.25051919	0.59447626	0.45232314	0.22310773	0.29219854	0.28244849
84.8	0.24084647	0.3606614	0.2503154	0.59452042	0.45203191	0.22281898	0.29216805	0.28241876
84.9	0.24047347	0.36039824	0.25011206	0.59456456	0.45174133	0.22253129	0.29213799	0.28238913
85	0.2401015	0.36013596	0.24990918	0.5946087	0.4514514	0.22224465	0.29210836	0.2823596
85.1	0.23973054	0.35987454	0.24970675	0.59465282	0.45116211	0.22195905	0.29207916	0.28233016
85.2	0.23936059	0.35961399	0.24950477	0.59469693	0.45087348	0.2216745	0.29205039	0.28230082
85.3	0.23899167	0.35935431	0.24930323	0.59474102	0.45058549	0.22139098	0.29202205	0.28227158
85.4	0.23862376	0.35909549	0.24910214	0.5947851	0.45029815	0.22110849	0.29199413	0.28224244
85.5	0.23825686	0.35883753	0.24890149	0.59482917	0.45001144	0.22082703	0.29196662	0.28221339
85.6	0.23789099	0.35858043	0.24870129	0.59487323	0.44972538	0.2205466	0.29193954	0.28218444
85.7	0.23752612	0.35832419	0.24850152	0.59491727	0.44943997	0.22026719	0.29191287	0.28215559
85.8	0.23716228	0.35806881	0.24830219	0.5949613	0.44915519	0.21998879	0.29188662	0.28212683
85.9	0.23679945	0.35781428	0.24810329	0.59500532	0.44887105	0.21971141	0.29186078	0.28209817
86	0.23643763	0.35756061	0.24790483	0.59504932	0.44858756	0.21943504	0.29183534	0.2820696
86.1	0.23607683	0.35730778	0.2477068	0.5950933	0.4483047	0.21915968	0.29181031	0.28204112
86.2	0.23571704	0.35705581	0.2475092	0.59513727	0.44802248	0.21888531	0.29178569	0.28201274
86.3	0.23535826	0.35680468	0.24731202	0.59518122	0.4477409	0.21861194	0.29176147	0.28198446
86.4	0.2350005	0.3565544	0.24711527	0.59522516	0.44745997	0.21833957	0.29173764	0.28195627
86.5	0.23464375	0.35630496	0.24691894	0.59526908	0.44717967	0.21806819	0.29171422	0.28192817
86.6	0.23428801	0.35605636	0.24672304	0.59531299	0.44690001	0.21779779	0.29169118	0.28190016
86.7	0.23393329	0.35580861	0.24652755	0.59535688	0.44662099	0.21752837	0.29166854	0.28187224
86.8	0.23357957	0.35556169	0.24633248	0.59540076	0.44634261	0.21725994	0.29164629	0.28184442
86.9	0.23322686	0.3553156	0.24613783	0.59544462	0.44606487	0.21699247	0.29162442	0.28181669
87	0.23287517	0.35507035	0.24594359	0.59548846	0.44578777	0.21672598	0.29160294	0.28178906
87.1	0.23252448	0.35482593	0.24574976	0.59553229	0.44551131	0.21646046	0.29158184	0.28176151
87.2	0.2321748	0.35458234	0.24555634	0.5955761	0.44523549	0.21619589	0.29156113	0.28173405
87.3	0.23182613	0.35433958	0.24536333	0.59561989	0.44496031	0.21593229	0.29154079	0.28170669
87.4	0.23147847	0.35409764	0.24517072	0.59566366	0.44468578	0.21566964	0.29152083	0.28167942
87.5	0.23113181	0.35385653	0.24497852	0.59570742	0.44441189	0.21540795	0.29150124	0.28165224
87.6	0.23078616	0.35361624	0.24478672	0.59575116	0.44413864	0.2151472	0.29148202	0.28162514
87.7	0.23044152	0.35337676	0.24459532	0.59579488	0.44386604	0.21488739	0.29146318	0.28159814
87.8	0.23009788	0.35313811	0.24440432	0.59583858	0.44359408	0.21462853	0.2914447	0.28157123
87.9	0.22975524	0.35290027	0.24421372	0.59588227	0.44332277	0.2143706	0.29142659	0.28154441
88	0.2294136	0.35266325	0.24402351	0.59592593	0.44305211	0.21411361	0.29140884	0.28151768
88.1	0.22907297	0.35242704	0.24383369	0.59596958	0.44278209	0.21385755	0.29139146	0.28149104
88.2	0.22873334	0.35219164	0.24364427	0.59601321	0.44251273	0.21360241	0.29137443	0.28146448
88.3	0.22839471	0.35195704	0.24345524	0.59605682	0.44224401	0.21334819	0.29135777	0.28143802
88.4	0.22805708	0.35172326	0.24326659	0.5961004	0.44197594	0.21309489	0.29134146	0.28141165
88.5	0.22772045	0.35149028	0.24307834	0.59614397	0.44170852	0.2128425	0.2913255	0.28138536
88.6	0.22738481	0.3512581	0.24289046	0.59618752	0.44144175	0.21259103	0.2913099	0.28135916
88.7	0.22705017	0.35102672	0.24270298	0.59623104	0.44117564	0.21234045	0.29129465	0.28133306
88.8	0.22671653	0.35079614	0.24251587	0.59627455	0.44091018	0.21209079	0.29127975	0.28130704
88.9	0.22638388	0.35056635	0.24232915	0.59631803	0.44064537	0.21184202	0.2912652	0.28128111
89	0.22605222	0.35033736	0.24214281	0.59636149	0.44038122	0.21159414	0.29125099	0.28125526
89.1	0.22572155	0.35010916	0.24195684	0.59640493	0.44011772	0.21134715	0.29123713	0.28122951
89.2	0.22539187	0.34988175	0.24177125	0.59644835	0.43985489	0.21110106	0.29122361	0.28120384
89.3	0.22506318	0.34965513	0.24158604	0.59649174	0.43959271	0.21085584	0.29121043	0.28117826
89.4	0.22473548	0.34942929	0.2414012	0.59653511	0.43933119	0.21061151	0.29119759	0.28115277
89.5	0.22440876	0.34920424	0.24121673	0.59657846	0.43907033	0.21036805	0.29118508	0.28112736
89.6	0.22408302	0.34897997	0.24103263	0.59662179	0.43881013	0.21012546	0.29117292	0.28110204
89.7	0.22375826	0.34875648	0.2408489	0.59666509	0.43855059	0.20988375	0.29116108	0.2810768
89.8	0.22343449	0.34853376	0.24066554	0.59670838	0.43829171	0.20964289	0.29114958	0.28105165
89.9	0.22311169	0.34831182	0.24048254	0.59675163	0.4380335	0.2094029	0.29113841	0.28102659
135	0.14550405	0.29884077	0.18519279	0.61330293	0.372771	0.15159418	0.31225567	0.27591199
135.1	0.14542032	0.29880214	0.18511465	0.61333336	0.3726925	0.15152147	0.3123402	0.27590859

90	0.22278987	0.34809066	0.24029991	0.59679487	0.43777595	0.20916377	0.29112757	0.28100161
90.1	0.22246903	0.34787027	0.24011765	0.59683808	0.43751906	0.20892549	0.29111706	0.28097672
90.2	0.22214916	0.34765064	0.23993574	0.59688126	0.43726284	0.20868806	0.29110687	0.28095192
90.3	0.22183027	0.34743179	0.2397542	0.59692443	0.43700729	0.20845147	0.291097	0.2809272
90.4	0.22151234	0.3472137	0.23957301	0.59696756	0.43675241	0.20821573	0.29108746	0.28090256
90.5	0.22119539	0.34699637	0.23939219	0.59701068	0.43649819	0.20798082	0.29107824	0.28087801
90.6	0.2208794	0.3467798	0.23921172	0.59705377	0.43624465	0.20774676	0.29106934	0.28085355
90.7	0.22056439	0.34656399	0.2390316	0.59709683	0.43599178	0.20751352	0.29106075	0.28082917
90.8	0.22025033	0.34634894	0.23885184	0.59713987	0.43573957	0.20728111	0.29105248	0.28080487
90.9	0.21993724	0.34613464	0.23867244	0.59718288	0.43548804	0.20704952	0.29104453	0.28078066
91	0.21962512	0.34592109	0.23849338	0.59722587	0.43523719	0.20681876	0.29103688	0.28075653
91.1	0.21931395	0.3457083	0.23831468	0.59726883	0.434987	0.20658881	0.29102955	0.28073249
91.2	0.21900374	0.34549625	0.23813632	0.59731177	0.43473749	0.20635967	0.29102253	0.28070853
91.3	0.21869449	0.34528495	0.23795831	0.59735468	0.43448866	0.20613135	0.29101581	0.28068466
91.4	0.21838619	0.3450744	0.23778065	0.59739757	0.4342405	0.20590383	0.2910094	0.28066087
91.5	0.21807885	0.34486458	0.23760334	0.59744042	0.43399301	0.20567711	0.2910033	0.28063716
91.6	0.21777246	0.34465551	0.23742637	0.59748326	0.4337462	0.2054512	0.2909975	0.28061354
91.7	0.21746702	0.34444717	0.23724974	0.59752606	0.43350007	0.20522608	0.290992	0.28059
91.8	0.21716252	0.34423957	0.23707346	0.59756884	0.43325461	0.20500175	0.2909868	0.28056655
91.9	0.21685898	0.34403271	0.23689752	0.59761159	0.43300984	0.20477821	0.2909819	0.28054317
92	0.21655637	0.34382657	0.23672191	0.59765432	0.43276574	0.20455546	0.2909773	0.28051988
92.1	0.21625471	0.34362117	0.23654665	0.59769702	0.43252232	0.20433349	0.290973	0.28049667
92.2	0.215954	0.34341649	0.23637173	0.59773969	0.43227958	0.2041123	0.29096899	0.28047355
92.3	0.21565421	0.34321254	0.23619714	0.59778233	0.43203751	0.20389188	0.29096527	0.2804505
92.4	0.21535537	0.34300931	0.23602289	0.59782495	0.43179613	0.20367224	0.29096185	0.28042754
92.5	0.21505746	0.34280681	0.23584897	0.59786754	0.43155543	0.20345336	0.29095871	0.28040466
92.6	0.21476048	0.34260502	0.23567539	0.5979101	0.4313154	0.20323525	0.29095587	0.28038187
92.7	0.21446443	0.34240395	0.23550214	0.59795264	0.43107606	0.20301791	0.29095331	0.28035915
92.8	0.2141693	0.34220359	0.23532923	0.59799514	0.4308374	0.20280132	0.29095104	0.28033652
92.9	0.21387511	0.34200395	0.23515664	0.59803762	0.43059941	0.20258549	0.29094906	0.28031396
93	0.21358183	0.34180502	0.23498439	0.59808007	0.43036211	0.20237041	0.29094736	0.28029149
93.1	0.21328948	0.34160679	0.23481246	0.59812249	0.43012548	0.20215608	0.29094595	0.2802691
93.2	0.21299805	0.34140928	0.23464087	0.59816489	0.42988954	0.20194249	0.29094482	0.28024679
93.3	0.21270754	0.34121246	0.2344696	0.59820725	0.42965427	0.20172965	0.29094397	0.28022457
93.4	0.21241794	0.34101635	0.23429866	0.5982496	0.42941969	0.20151755	0.2909434	0.28020242
93.5	0.21212925	0.34082094	0.23412805	0.59829191	0.42918578	0.20130618	0.29094311	0.28018036
93.6	0.21184148	0.34062623	0.23395776	0.5983342	0.42895255	0.20109555	0.2909431	0.28015838
93.7	0.21155461	0.34043221	0.23378779	0.59837646	0.42872001	0.20088565	0.29094336	0.28013647
93.8	0.21126865	0.34023889	0.23361815	0.59841869	0.42848814	0.20067647	0.29094391	0.28011465
93.9	0.2109836	0.34004626	0.23344884	0.59846089	0.42825695	0.20046802	0.29094473	0.28009291
94	0.21069944	0.33985431	0.23327984	0.59850307	0.42802644	0.20026029	0.29094582	0.28007125
94.1	0.21041619	0.33966306	0.23311117	0.59854522	0.42779661	0.20005328	0.29094719	0.28004967
94.2	0.21013384	0.33947249	0.23294281	0.59858734	0.42756746	0.19984698	0.29094884	0.28002818
94.3	0.20985238	0.33928261	0.23277478	0.59862944	0.42733899	0.19964139	0.29095076	0.28000676
94.4	0.20957181	0.3390934	0.23260707	0.5986715	0.42711119	0.19943651	0.29095295	0.27998542
94.5	0.20929213	0.33890488	0.23243967	0.59871354	0.42688407	0.19923233	0.29095541	0.27996416
94.6	0.20901335	0.33871703	0.2322726	0.59875555	0.42665763	0.19902885	0.29095814	0.27994298
94.7	0.20873545	0.33852986	0.23210584	0.59879754	0.42643187	0.19882608	0.29096115	0.27992188
94.8	0.20845843	0.33834336	0.2319394	0.59883949	0.42620678	0.198624	0.29096442	0.27990086
94.9	0.2081823	0.33815753	0.23177327	0.59888142	0.42598237	0.19842261	0.29096797	0.27987992
95	0.20790704	0.33797237	0.23160746	0.59892332	0.42575863	0.19822191	0.29097178	0.27985906
95.1	0.20763267	0.33778788	0.23144196	0.59896519	0.42553557	0.1980219	0.29097586	0.27983827
95.2	0.20735917	0.33760405	0.23127678	0.59900703	0.42531318	0.19782258	0.29098021	0.27981757
95.3	0.20708655	0.33742087	0.23111191	0.59904885	0.42509147	0.19762393	0.29098483	0.27979694
95.4	0.20681479	0.33723836	0.23094735	0.59909063	0.42487043	0.19742596	0.29098972	0.27977639
95.5	0.20654391	0.33705651	0.23078311	0.59913239	0.42465006	0.19722867	0.29099488	0.27975593
95.6	0.20627389	0.33687531	0.23061918	0.59917412	0.42443036	0.19703206	0.2910003	0.27973553
95.7	0.20600473	0.33669476	0.23045555	0.59921583	0.42421134	0.19683611	0.29100599	0.27971522
95.8	0.20573644	0.33651486	0.23029224	0.5992575	0.42399299	0.19664082	0.29101195	0.27969499
95.9	0.205469	0.33633561	0.23012924	0.59929915	0.4237753	0.19644621	0.29101817	0.27967483
96	0.20520242	0.33615701	0.22996654	0.59934077	0.42355829	0.19625225	0.29102466	0.27965475
96.1	0.2049367	0.33597905	0.22980416	0.59938236	0.42334195	0.19605895	0.29103141	0.27963475
96.2	0.20467182	0.33580174	0.22964208	0.59942392	0.42312627	0.19586631	0.29103843	0.27961482
96.3	0.2044078	0.33562506	0.2294803	0.59946546	0.42291126	0.19567432	0.29104572	0.27959498
96.4	0.20414462	0.33544902	0.22931884	0.59950696	0.42269692	0.19548298	0.29105327	0.2795752
96.5	0.20388229	0.33527361	0.22915768	0.59954844	0.42248324	0.19529228	0.29106109	0.27955551
96.6	0.2036208	0.33509883	0.22899683	0.59958989	0.42227023	0.19510224	0.29106917	0.27953589
96.7	0.20336015	0.33492469	0.22883628	0.59963131	0.42205789	0.19491283	0.29107752	0.27951635
96.8	0.20310034	0.33475117	0.22867603	0.59967271	0.4218462	0.19472406	0.29108614	0.27949689
96.9	0.20284137	0.33457828	0.22851609	0.59971408	0.42163518	0.19453593	0.29109502	0.2794775
97	0.20258322	0.33440601	0.22835645	0.59975541	0.42142482	0.19434843	0.29110417	0.27945819
97.1	0.20232591	0.33423436	0.22819712	0.59979673	0.42121512	0.19416157	0.29111358	0.27943895
97.2	0.20206943	0.33406332	0.22803809	0.59983801	0.42100607	0.19397533	0.29112326	0.27941979
97.3	0.20181377	0.3338929	0.22787935	0.59987926	0.42079769	0.19378972	0.29113321	0.2794007
97.4	0.20155894	0.3337231	0.22772092	0.59992049	0.42058996	0.19360473	0.29114342	0.27938169
97.5	0.20130493	0.3335539	0.22756279	0.59996169	0.42038288	0.19342036	0.29115391	0.27936275
97.6	0.20105174	0.33338531	0.22740496	0.60000286	0.42017646	0.19323662	0.29116466	0.27934389
97.7	0.20079937	0.33321732	0.22724743	0.60004401	0.41997069	0.19305348	0.29117568	0.27932511
97.8	0.20054781	0.33304994	0.2270902	0.60008512	0.41976557	0.19287096	0.29118697	0.2793064
97.9	0.20029707	0.33288315	0.22693326	0.60012621	0.41956111	0.19268905	0.29119853	0.27928776
98	0.20004713	0.33271696	0.22677663	0.60016727	0.41935729	0.19250775	0.29121036	0.2792692
98.1	0.199798	0.33255136	0.22662029	0.6002083	0.41915411	0.19232706	0.29122246	0.27925071
98.2	0.19954968	0.33238636	0.22646425	0.6002493	0.41895158	0.19214696	0.29123483	0.27923229
98.3	0.19930216	0.33222194	0.2263085	0.60029027	0.4187497	0.19196747	0.29124747	0.27921395
98.4	0.19905545	0.3320581	0.22615305	0.60033121	0.41854846	0.19178857	0.29126038	0.27919569
98.5	0.19880953	0.33189485	0.2259979	0.60037212	0.41834786	0.19161027	0.29127356	0.2791775
98.6	0.19856441	0.33173217	0.22584304	0.60041301	0.4181479	0.19143257	0.29128701	0.27915938
98.7	0.19832008	0.33157007	0.22568847	0.60045386	0.41794859	0.19125545	0.29130074	0.27914134
98.8	0.19807654	0.33140855	0.22553419	0.60049468	0.4177499	0.19107892	0.29131474	0.27912336
98.9	0.19783379	0.33124759	0.22538021	0.60053548	0.41755186	0.19090298	0.29132901	0.27910547
99	0.19759183	0.3310872	0.22522652	0.60057625	0.41735445	0.19072762	0.29134355	0.27908764
99.1	0.19735066	0.33092738	0.22507312	0.60061698	0.41715767	0.19055284	0.29135837	0.27906989
99.2	0.19711026	0.33076811	0.22492002	0.60065769	0.41696153	0.19037864	0.29137346	0.27905222
99.3	0.19687064	0.33060941	0.2247672	0.60069837	0.41676602	0.19020502	0.29138883	0.27903461
99.4	0.1966318	0.33045126	0.22461467	0.60073902	0.41657113	0.19003197	0.29140447	0.27901708
99.5	0.19639374	0.33029366	0.22446243	0.60077964	0.41637687	0.18985949	0.29142039	0.27899963
99.6	0.19615644	0.33013661	0.22431048	0.60082023	0.41618324	0.18968758	0.29143659	0.27898224
99.7	0.19591992	0.32998011	0.22415882	0.6008608	0.41599023	0.18951623	0.29145306	0.27896493
99.8	0.19568417	0.32982415	0.22400744	0.60090133	0.41579785	0.18934546	0.29146981	0.27894769
99.9	0.19544917	0.32966873	0.22385636	0.60094183	0.41560608	0.18917524	0.29148684	0.27893053
100	0.19521495	0.32951385	0.22370556	0.60098231	0.41541493	0.18900558	0.29150415	0.27891343
100.1	0.19498148	0.3293595	0.22355504	0.60102275	0.4152244	0.18883649	0.29152174	0.27889641
100.2	0.19474877	0.32920568	0.22340481	0.60106317	0.41503449	0.18866794	0.2915396	0.27887946
100.3	0.19451682	0.32905239	0.22325487	0.60110355	0.41484519	0.18849995	0.29155775	0.27886259
100.4	0.19428562	0.32889963	0.22310521	0.60114391	0.4146565	0.18833251	0.29157618	0.27884578
100.5	0.19405517	0.32874739	0.22295584	0.60118424	0.41446842	0.18816562	0.29159489	0.27882905
100.6	0.19382547	0.32859567	0.22280675	0.60122454	0.41428095	0.18799928	0.29161388	0.27881239
100.7	0.19359651	0.32844446	0.22265794	0.60126481	0.41409409	0.18783348	0.29163316	0.2787958
100.8	0.1933683	0.32829377	0.22250942	0.60130505	0.41390784	0.18766823	0.29165271	0.27877928
100.9	0.19314083	0.32814359	0.22236118	0.60134526	0.41372219	0.18750351	0.29167255	0.27876283
101	0.19291411	0.32799392	0.22221322	0.60138544	0.41353714	0.18733933	0.29169268	0.27874646
101.1	0.19268811	0.32784476	0.22206554	0.60142559	0.41335269	0.18717569	0.29171308	0.27873015
101.2	0.19246286	0.32769609	0.22191815	0.60146572	0.41316884	0.18701258	0.29173377	0.27871392
101.3	0.19223833	0.32754793	0.22177103	0.60150581	0.41298559	0.18685001	0.29175475	0.27869775
101.4	0.19201454	0.32740027	0.22162419	0.60154587	0.41280294	0.18668796	0.29177601	0.27868166
101.5	0.19179148	0.3272531	0.22147764	0.60158591	0.41262087	0.18652644	0.29179756	0.27866564
101.6	0.19156914	0.32710642	0.22133136	0.60162591	0.4124394	0.18636544	0.29181939	0.27864969
101.7	0.19134752	0.32696024	0.22118536	0.60166589	0.41225852	0.18620497	0.29184151	0.27863381
101.8	0.19112662	0.32681454	0.22103963	0.60170583	0.41207823	0.18604502	0.29186392	0.278618
101.9	0.19090645	0.32666933	0.22089419	0.60174575	0.41189852	0.18588559	0.29188662	0.27860226
102	0.19068699	0.3265246	0.22074902	0.60178564	0.41171939	0.18572668	0.2919096	0.27858659
102.1	0.19046824	0.32638035	0.22060413	0.60182549	0.41154085	0.18556828	0.29193288	0.27857099
102.2	0.1902502	0.32623659	0.22045951	0.60186532	0.41136289	0.18541039	0.29195644	0.27855546
102.3	0.19003288	0.3260933	0.22031517	0.60190511	0.4111855	0.18525301	0.29198029	0.27854
102.4	0.18981626	0.32595048	0.2201711	0.60194488	0.4110087	0.18509615	0.29200443	0.27852461
102.5	0.18960034	0.32580814	0.22002731	0.60198461	0.41083246	0.18493978	0.29202886	0.27850928
102.6	0.18938513	0.32566628	0.21988379	0.60202432	0.41065679	0.18478393	0.29205358	0.27849403
102.7	0.18917062	0.32552488	0.21974054	0.60206399	0.4104817	0.18462857	0.29207859	0.27847885
102.8	0.1889568	0.32538395	0.21959757	0.60210364	0.41030717	0.18447372	0.29210389	0.27846373
102.9	0.18874368	0.32524348	0.21945487	0.60214325	0.41013322	0.18431937	0.29212948	0.27844868
103	0.18853126	0.32510349	0.21931244	0.60218283	0.40995982	0.18416551	0.29215536	0.2784337
103.1	0.18831952	0.32496395	0.21917028	0.60222239	0.40978699	0.18401214	0.29218154	0.27841879
103.2	0.18810847	0.32482488	0.21902839	0.60226191	0.40961472	0.18385927	0.292208	0.27840395
103.3	0.18789811	0.32468627	0.21888677	0.6023014	0.409443	0.18370689	0.29223476	0.27838917
103.4	0.18768843	0.32454811	0.21874542	0.60234086	0.40927184	0.183555	0.29226181	0.27837447
103.5	0.18747943	0.32441041	0.21860434	0.60238029	0.40910124	0.18340359	0.29228915	0.27835982
103.6	0.18727111	0.32427317	0.21846353	0.60241969	0.40893119	0.18325267	0.29231678	0.27834525
103.7	0.18706347	0.32413638	0.21832299	0.60245906	0.40876169	0.18310223	0.29234471	0.27833074
103.8	0.1868565	0.32400005	0.21818272	0.60249839	0.40859274	0.18295228	0.29237293	0.2783163
103.9	0.18665021	0.32386416	0.21804271	0.6025377	0.40842433	0.1828028	0.29240144	0.27830192
104	0.18644458	0.32372872	0.21790297	0.60257697	0.40825647	0.1826538	0.29243025	0.27828761
104.1	0.18623962	0.32359374	0.2177635	0.60261622	0.40808915	0.18250527	0.29245935	0.27827337
104.2	0.18603532	0.32345919	0.2176243	0.60265543	0.40792236	0.18235722	0.29248874	0.27825919
104.3	0.18583169	0.3233251	0.21748536	0.60269462	0.40775612	0.18220964	0.29251842	0.27824508
104.4	0.18562872	0.32319145	0.21734668	0.60273377	0.40759041	0.18206253	0.2925484	0.27823103
104.5	0.18542641	0.32305824	0.21720827	0.60277289	0.40742524	0.18191588	0.29257867	0.27821704
104.6	0.18522475	0.32292547	0.21707012	0.60281198	0.4072606	0.18176971	0.29260924	0.27820312
104.7	0.18502375	0.32279314	0.21693224	0.60285104	0.40709649	0.181624	0.2926401	0.27818927
104.8	0.18482339	0.32266126	0.21679461	0.60289007	0.40693291	0.18147874	0.29267125	0.27817547
104.9	0.18462369	0.32252981	0.21665726	0.60292907	0.40676986	0.18133396	0.29270269	0.27816174
150	0.13449727	0.29487206	0.17478715	0.6176569	0.36440306	0.14174704	0.32512696	0.27556514
150.1	0.13443273	0.29485614	0.17472535	0.61768458	0.36437308	0.14168811	0.32521187	0.27556376

105	0.18442464	0.3223988	0.21652016	0.60296804	0.40660733	0.18118962	0.29273443	0.27814807
105.1	0.18422622	0.32226823	0.21638332	0.60300699	0.40644532	0.18104575	0.29276646	0.27813447
105.2	0.18402846	0.32213809	0.21624675	0.6030459	0.40628383	0.18090233	0.29279878	0.27812093
105.3	0.18383133	0.32200838	0.21611044	0.60308478	0.40612287	0.18075937	0.29283139	0.27810745
105.4	0.18363484	0.32187911	0.21597438	0.60312363	0.40596241	0.18061685	0.2928643	0.27809403
105.5	0.18343898	0.32175027	0.21583859	0.60316245	0.40580248	0.18047479	0.2928975	0.27808067
105.6	0.18324376	0.32162186	0.21570306	0.60320124	0.40564305	0.18033318	0.292931	0.27806737
105.7	0.18304917	0.32149389	0.21556779	0.60324	0.40548413	0.18019201	0.29296478	0.27805414
105.8	0.18285521	0.32136634	0.21543277	0.60327873	0.40532572	0.18005128	0.29299886	0.27804096
105.9	0.18266187	0.32123921	0.21529802	0.60331743	0.40516781	0.179911	0.29303323	0.27802785
106	0.18246916	0.32111252	0.21516352	0.6033561	0.40501041	0.17977117	0.2930679	0.27801479
106.1	0.18227707	0.32098625	0.21502927	0.60339474	0.40485351	0.17963177	0.29310285	0.2780018
106.2	0.1820856	0.32086041	0.21489529	0.60343335	0.40469711	0.1794928	0.2931381	0.27798886
106.3	0.18189476	0.32073499	0.21476156	0.60347193	0.4045412	0.17935428	0.29317364	0.27797598
106.4	0.18170452	0.32060999	0.21462809	0.60351048	0.40438579	0.17921619	0.29320948	0.27796317
106.5	0.1815149	0.32048542	0.21449487	0.60354899	0.40423087	0.17907853	0.2932456	0.27795041
106.6	0.18132589	0.32036127	0.21436191	0.60358748	0.40407644	0.17894131	0.29328202	0.2779377
106.7	0.18113749	0.32023753	0.2142292	0.60362594	0.4039225	0.17880451	0.29331873	0.27792506
106.8	0.1809497	0.32011422	0.21409674	0.60366436	0.40376905	0.17866814	0.29335573	0.27791248
106.9	0.18076251	0.31999133	0.21396454	0.60370276	0.40361608	0.1785322	0.29339302	0.27789995
107	0.18057593	0.31986885	0.21383259	0.60374112	0.40346359	0.17839668	0.2934306	0.27788748
107.1	0.18038995	0.31974679	0.2137009	0.60377946	0.40331158	0.17826159	0.29346847	0.27787506
107.2	0.18020456	0.31962515	0.21356946	0.60381776	0.40316005	0.17812691	0.29350663	0.27786271
107.3	0.18001977	0.31950392	0.21343827	0.60385604	0.403009	0.17799266	0.29354509	0.27785041
107.4	0.17983558	0.31938311	0.21330733	0.60389428	0.40285842	0.17785883	0.29358383	0.27783816
107.5	0.17965198	0.3192627	0.21317664	0.60393249	0.40270831	0.17772541	0.29362286	0.27782598
107.6	0.17946896	0.31914272	0.2130462	0.60397067	0.40255867	0.17759241	0.29366218	0.27781384
107.7	0.17928654	0.31902314	0.21291601	0.60400882	0.4024095	0.17745982	0.29370179	0.27780177
107.8	0.1791047	0.31890397	0.21278607	0.60404693	0.4022608	0.17732764	0.29374168	0.27778975
107.9	0.17892345	0.31878521	0.21265638	0.60408502	0.40211256	0.17719588	0.29378187	0.27777778
108	0.17874278	0.31866686	0.21252694	0.60412308	0.40196478	0.17706452	0.29382234	0.27776587
108.1	0.17856268	0.31854891	0.21239775	0.6041611	0.40181745	0.17693357	0.29386309	0.27775401
108.2	0.17838317	0.31843137	0.2122688	0.60419909	0.40167059	0.17680302	0.29390413	0.2777422
108.3	0.17820423	0.31831424	0.2121401	0.60423706	0.40152418	0.17667288	0.29394546	0.27773045
108.4	0.17802586	0.3181975	0.21201165	0.60427499	0.40137823	0.17654315	0.29398707	0.27771875
108.5	0.17784807	0.31808118	0.21188344	0.60431289	0.40123272	0.17641381	0.29402897	0.27770711
108.6	0.17767084	0.31796525	0.21175548	0.60435076	0.40108767	0.17628488	0.29407115	0.27769552
108.7	0.17749418	0.31784972	0.21162776	0.6043886	0.40094306	0.17615634	0.29411361	0.27768398
108.8	0.17731809	0.3177346	0.21150029	0.6044264	0.4007989	0.1760282	0.29415636	0.27767249
108.9	0.17714255	0.31761988	0.21137306	0.60446418	0.40065517	0.17590045	0.29419938	0.27766105
109	0.17696758	0.31750555	0.21124608	0.60450193	0.40051189	0.1757731	0.29424269	0.27764967
109.1	0.17679317	0.31739162	0.21111934	0.60453964	0.40036905	0.17564614	0.29428628	0.27763834
109.2	0.17661932	0.3172781	0.21099284	0.60457732	0.40022665	0.17551957	0.29433015	0.27762706
109.3	0.17644602	0.31716496	0.21086659	0.60461497	0.40008468	0.17539339	0.29437431	0.27761583
109.4	0.17627327	0.31705223	0.21074058	0.60465259	0.39994314	0.1752676	0.29441874	0.27760465
109.5	0.17610107	0.31693989	0.21061481	0.60469018	0.39980204	0.1751422	0.29446345	0.27759352
109.6	0.17592943	0.31682794	0.21048928	0.60472773	0.39966136	0.17501718	0.29450844	0.27758244
109.7	0.17575833	0.31671639	0.21036399	0.60476526	0.39952111	0.17489254	0.2945537	0.27757141
109.8	0.17558777	0.31660523	0.21023894	0.60480275	0.39938128	0.17476828	0.29459925	0.27756043
109.9	0.17541776	0.31649446	0.21011414	0.60484021	0.39924188	0.17464441	0.29464507	0.2775495
110	0.17524829	0.31638409	0.20998957	0.60487764	0.39910289	0.17452091	0.29469116	0.27753861
110.1	0.17507935	0.3162741	0.20986524	0.60491504	0.39896432	0.17439779	0.29473753	0.27752778
110.2	0.17491096	0.31616451	0.20974115	0.60495241	0.39882617	0.17427504	0.29478418	0.277517
110.3	0.1747431	0.31605531	0.2096173	0.60498975	0.39868844	0.17415268	0.2948311	0.27750626
110.4	0.17457577	0.31594649	0.20949369	0.60502705	0.39855111	0.17403068	0.29487829	0.27749558
110.5	0.17440897	0.31583807	0.20937031	0.60506432	0.3984142	0.17390905	0.29492575	0.27748494
110.6	0.17424271	0.31573003	0.20924717	0.60510156	0.39827769	0.1737878	0.29497348	0.27747435
110.7	0.17407696	0.31562238	0.20912427	0.60513878	0.39814159	0.17366692	0.29502149	0.2774638
110.8	0.17391175	0.31551511	0.2090016	0.60517596	0.39800589	0.1735464	0.29506976	0.2774533
110.9	0.17374706	0.31540823	0.20887917	0.6052131	0.3978706	0.17342624	0.29511831	0.27744285
111	0.17358289	0.31530173	0.20875698	0.60525022	0.3977357	0.17330646	0.29516712	0.27743245
111.1	0.17341924	0.31519562	0.20863502	0.60528731	0.3976012	0.17318703	0.2952162	0.27742209
111.2	0.17325611	0.31508989	0.20851329	0.60532436	0.3974671	0.17306797	0.29526554	0.27741178
111.3	0.17309349	0.31498455	0.2083918	0.60536138	0.39733339	0.17294926	0.29531515	0.27740152
111.4	0.17293139	0.31487958	0.20827054	0.60539837	0.39720007	0.17283092	0.29536503	0.2773913
111.5	0.17276979	0.314775	0.20814951	0.60543533	0.39706714	0.17271293	0.29541517	0.27738113
111.6	0.17260871	0.31467079	0.20802871	0.60547226	0.39693459	0.1725953	0.29546557	0.277371
111.7	0.17244814	0.31456696	0.20790815	0.60550916	0.39680244	0.17247802	0.29551624	0.27736091
111.8	0.17228808	0.31446352	0.20778781	0.60554602	0.39667066	0.1723611	0.29556716	0.27735088
111.9	0.17212851	0.31436044	0.20766771	0.60558286	0.39653927	0.17224453	0.29561835	0.27734088
112	0.17196945	0.31425775	0.20754784	0.60561966	0.39640825	0.1721283	0.29566979	0.27733093
112.1	0.17181089	0.31415543	0.2074282	0.60565643	0.39627761	0.17201243	0.2957215	0.27732103
112.2	0.17165283	0.31405348	0.20730879	0.60569317	0.39614735	0.1718969	0.29577346	0.27731116
112.3	0.17149527	0.31395191	0.2071896	0.60572988	0.39601746	0.17178172	0.29582567	0.27730135
112.4	0.1713382	0.31385072	0.20707064	0.60576656	0.39588793	0.17166688	0.29587814	0.27729157
112.5	0.17118163	0.31374989	0.20695192	0.6058032	0.39575878	0.17155239	0.29593087	0.27728184
112.6	0.17102554	0.31364944	0.20683342	0.60583982	0.39562999	0.17143824	0.29598385	0.27727215
112.7	0.17086995	0.31354935	0.20671514	0.6058764	0.39550157	0.17132442	0.29603709	0.27726251
112.8	0.17071484	0.31344964	0.20659709	0.60591295	0.3953735	0.17121095	0.29609058	0.2772529
112.9	0.17056022	0.3133503	0.20647927	0.60594947	0.3952458	0.17109781	0.29614432	0.27724334
113	0.17040608	0.31325132	0.20636167	0.60598596	0.39511845	0.17098501	0.29619831	0.27723382
113.1	0.17025243	0.31315271	0.2062443	0.60602242	0.39499146	0.17087255	0.29625254	0.27722434
113.2	0.17009925	0.31305446	0.20612715	0.60605885	0.39486482	0.17076041	0.29630703	0.2772149
113.3	0.16994656	0.31295658	0.20601023	0.60609524	0.39473853	0.17064861	0.29636176	0.27720551
113.4	0.16979434	0.31285906	0.20589353	0.60613161	0.39461259	0.17053714	0.29641674	0.27719615
113.5	0.16964259	0.3127619	0.20577705	0.60616794	0.39448699	0.17042599	0.29647197	0.27718684
113.6	0.16949132	0.3126651	0.2056608	0.60620425	0.39436174	0.17031518	0.29652743	0.27717757
113.7	0.16934053	0.31256866	0.20554476	0.60624052	0.39423683	0.17020468	0.29658315	0.27716833
113.8	0.1691902	0.31247258	0.20542895	0.60627676	0.39411225	0.17009452	0.2966391	0.27715914
113.9	0.16904034	0.31237686	0.20531337	0.60631297	0.39398801	0.16998467	0.29669529	0.27714999
114	0.16889095	0.3122815	0.205198	0.60634915	0.3938641	0.16987515	0.29675172	0.27714088
114.1	0.16874202	0.31218649	0.20508285	0.6063853	0.39374053	0.16976595	0.29680839	0.2771318
114.2	0.16859355	0.31209184	0.20496792	0.60642141	0.39361728	0.16965706	0.2968653	0.27712277
114.3	0.16844555	0.31199755	0.20485321	0.6064575	0.39349436	0.16954849	0.29692244	0.27711378
114.4	0.16829801	0.31190361	0.20473873	0.60649355	0.39337177	0.16944024	0.29697981	0.27710482
114.5	0.16815092	0.31181002	0.20462445	0.60652957	0.3932495	0.1693323	0.29703742	0.27709591
114.6	0.16800429	0.31171679	0.2045104	0.60656557	0.39312755	0.16922467	0.29709527	0.27708703
114.7	0.16785812	0.31162391	0.20439657	0.60660152	0.39300591	0.16911736	0.29715334	0.2770782
114.8	0.1677124	0.31153138	0.20428295	0.60663745	0.39288459	0.16901035	0.29721164	0.2770694
114.9	0.16756713	0.31143919	0.20416955	0.60667335	0.39276358	0.16890366	0.29727017	0.27706064
115	0.16742231	0.31134736	0.20405637	0.60670921	0.39264288	0.16879726	0.29732893	0.27705192
115.1	0.16727794	0.31125588	0.2039434	0.60674505	0.39252249	0.16869118	0.29738791	0.27704323
115.2	0.16713402	0.31116474	0.20383064	0.60678085	0.39240241	0.16858539	0.29744712	0.27703459
115.3	0.16699054	0.31107396	0.20371811	0.60681662	0.39228263	0.16847991	0.29750655	0.27702598
115.4	0.1668475	0.31098351	0.20360578	0.60685236	0.39216315	0.16837473	0.29756621	0.27701741
115.5	0.16670491	0.31089342	0.20349367	0.60688807	0.39204397	0.16826985	0.29762609	0.27700888
115.6	0.16656276	0.31080366	0.20338178	0.60692375	0.39192508	0.16816527	0.29768619	0.27700039
115.7	0.16642104	0.31071425	0.20327009	0.60695939	0.39180649	0.16806098	0.29774651	0.27699193
115.8	0.16627976	0.31062518	0.20315862	0.606995	0.39168818	0.16795698	0.29780705	0.27698351
115.9	0.16613892	0.31053646	0.20304736	0.60703059	0.39157017	0.16785328	0.29786781	0.27697513
116	0.16599851	0.31044807	0.20293632	0.60706614	0.39145244	0.16774987	0.29792878	0.27696678
116.1	0.16585853	0.31036003	0.20282548	0.60710166	0.391335	0.16764675	0.29798997	0.27695848
116.2	0.16571898	0.31027232	0.20271486	0.60713715	0.39121783	0.16754391	0.29805138	0.2769502
116.3	0.16557986	0.31018495	0.20260444	0.60717261	0.39110095	0.16744136	0.298113	0.27694197
116.4	0.16544117	0.31009792	0.20249424	0.60720804	0.39098434	0.1673391	0.29817483	0.27693377
116.5	0.1653029	0.31001123	0.20238425	0.60724343	0.390868	0.16723711	0.29823688	0.2769256
116.6	0.16516506	0.30992487	0.20227446	0.6072788	0.39075194	0.16713541	0.29829913	0.27691748
116.7	0.16502764	0.30983885	0.20216488	0.60731413	0.39063614	0.16703399	0.29836159	0.27690939
116.8	0.16489064	0.30975316	0.20205552	0.60734944	0.39052061	0.16693285	0.29842426	0.27690133
116.9	0.16475407	0.3096678	0.20194635	0.60738471	0.39040535	0.16683198	0.29848714	0.27689331
117	0.1646179	0.30958277	0.2018374	0.60741996	0.39029034	0.16673138	0.29855022	0.27688533
117.1	0.16448216	0.30949808	0.20172865	0.60745517	0.3901756	0.16663106	0.29861351	0.27687738
117.2	0.16434683	0.30941372	0.20162011	0.60749035	0.39006111	0.16653101	0.298677	0.27686946
117.3	0.16421191	0.30932969	0.20151178	0.6075255	0.38994688	0.16643123	0.29874069	0.27686158
117.4	0.16407741	0.30924598	0.20140365	0.60756062	0.38983289	0.16633172	0.29880458	0.27685374
117.5	0.16394331	0.3091626	0.20129573	0.60759571	0.38971916	0.16623247	0.29886867	0.27684593
117.6	0.16380963	0.30907956	0.20118801	0.60763077	0.38960568	0.16613348	0.29893296	0.27683816
117.7	0.16367635	0.30899683	0.2010805	0.60766579	0.38949244	0.16603476	0.29899745	0.27683042
117.8	0.16354347	0.30891443	0.20097319	0.60770079	0.38937944	0.1659363	0.29906213	0.27682271
117.9	0.163411	0.30883236	0.20086608	0.60773575	0.38926668	0.1658381	0.29912701	0.27681504
118	0.16327894	0.30875061	0.20075918	0.60777069	0.38915416	0.16574015	0.29919208	0.2768074
118.1	0.16314727	0.30866918	0.20065247	0.60780559	0.38904188	0.16564246	0.29925734	0.2767998
118.2	0.16301601	0.30858808	0.20054597	0.60784047	0.38892982	0.16554502	0.2993228	0.27679223
118.3	0.16288514	0.3085073	0.20043968	0.60787531	0.388818	0.16544784	0.29938844	0.27678469
118.4	0.16275467	0.30842683	0.20033358	0.60791012	0.38870641	0.1653509	0.29945428	0.27677719
118.5	0.16262459	0.30834668	0.20022768	0.60794491	0.38859504	0.16525421	0.2995203	0.27676972
118.6	0.16249491	0.30826685	0.20012199	0.60797966	0.3884839	0.16515777	0.2995865	0.27676228
118.7	0.16236562	0.30818733	0.20001649	0.60801438	0.38837297	0.16506158	0.29965289	0.27675488
118.8	0.16223672	0.30810813	0.1999112	0.60804908	0.38826227	0.16496562	0.29971947	0.27674751
118.9	0.16210821	0.30802924	0.1998061	0.60808374	0.38815179	0.16486991	0.29978623	0.27674018
119	0.16198009	0.30795067	0.1997012	0.60811838	0.38804152	0.16477443	0.29985317	0.27673287
119.1	0.16185235	0.3078724	0.1995965	0.60815298	0.38793146	0.1646792	0.2999203	0.2767256
119.2	0.161725	0.30779445	0.199492	0.60818755	0.38782161	0.16458419	0.2999876	0.27671837
119.3	0.16159803	0.30771681	0.19938769	0.6082221	0.38771198	0.16448942	0.30005509	0.27671116
119.4	0.16147144	0.30763948	0.19928358	0.60825661	0.38760255	0.16439489	0.30012275	0.27670399
119.5	0.16134524	0.30756245	0.19917967	0.6082911	0.38749332	0.16430058	0.30019059	0.27669685
119.6	0.16121941	0.30748574	0.19907596	0.60832556	0.3873843	0.1642065	0.30025861	0.27668974
119.7	0.16109396	0.30740933	0.19897244	0.60835998	0.38727548	0.16411265	0.3003268	0.27668267
119.8	0.16096889	0.30733322	0.19886911	0.60839438	0.38716686	0.16401902	0.30039516	0.27667563
119.9	0.16084419	0.30725743	0.19876598	0.60842875	0.38705844	0.16392561	0.3004637	0.27666861
165	0.12587179	0.29362218	0.16634126	0.62161241	0.36314832	0.13388678	0.33719533	0.27546184
165.1	0.12582075	0.29362042	0.16628996	0.62163746	0.36315678	0.13384084	0.33727107	0.27546174

120.2	0.16047234	0.30703186	0.19845776	0.60853169	0.38673435	0.16364671	0.30067033	0.27664777
120.3	0.16034913	0.30695727	0.1983554	0.60856595	0.38662671	0.16355418	0.30073955	0.27664089
120.4	0.16022628	0.30688299	0.19825324	0.60860017	0.38651925	0.16346185	0.30080893	0.27663404
120.5	0.1601038	0.30680901	0.19815127	0.60863437	0.38641198	0.16336974	0.30087848	0.27662721
120.6	0.15998169	0.30673533	0.19804949	0.60866853	0.3863049	0.16327784	0.3009482	0.27662042
120.7	0.15985993	0.30666194	0.19794791	0.60870267	0.38619801	0.16318615	0.30101808	0.27661366
120.8	0.15973854	0.30658886	0.19784651	0.60873678	0.3860913	0.16309466	0.30108812	0.27660693
120.9	0.15961752	0.30651607	0.1977453	0.60877085	0.38598477	0.16300338	0.30115832	0.27660024
121	0.15949684	0.30644358	0.19764429	0.6088049	0.38587842	0.1629123	0.30122868	0.27659357
121.1	0.15937653	0.30637138	0.19754346	0.60883892	0.38577225	0.16282142	0.30129921	0.27658693
121.2	0.15925657	0.30629948	0.19744283	0.60887291	0.38566626	0.16273074	0.30136989	0.27658033
121.3	0.15913697	0.30622787	0.19734238	0.60890687	0.38556045	0.16264026	0.30144073	0.27657375
121.4	0.15901772	0.30615655	0.19724212	0.6089408	0.38545481	0.16254997	0.30151173	0.2765672
121.5	0.15889882	0.30608553	0.19714205	0.6089747	0.38534934	0.16245988	0.30158288	0.27656069
121.6	0.15878028	0.3060148	0.19704217	0.60900857	0.38524405	0.16236998	0.30165419	0.2765542
121.7	0.15866208	0.30594435	0.19694247	0.60904241	0.38513893	0.16228026	0.30172566	0.27654774
121.8	0.15854423	0.3058742	0.19684296	0.60907622	0.38503399	0.16219074	0.30179728	0.27654132
121.9	0.15842672	0.30580434	0.19674364	0.60911	0.38492921	0.1621014	0.30186905	0.27653492
122	0.15830956	0.30573476	0.1966445	0.60914375	0.3848246	0.16201225	0.30194097	0.27652855
122.1	0.15819275	0.30566548	0.19654555	0.60917747	0.38472017	0.16192328	0.30201305	0.27652221
122.2	0.15807627	0.30559647	0.19644679	0.60921116	0.3846159	0.16183449	0.30208527	0.2765159
122.3	0.15796014	0.30552776	0.19634821	0.60924483	0.38451179	0.16174588	0.30215764	0.27650962
122.4	0.15784434	0.30545933	0.19624981	0.60927846	0.38440786	0.16165746	0.30223016	0.27650337
122.5	0.15772889	0.30539118	0.1961516	0.60931206	0.38430409	0.1615692	0.30230283	0.27649715
122.6	0.15761377	0.30532331	0.19605357	0.60934564	0.38420049	0.16148113	0.30237564	0.27649096
122.7	0.15749898	0.30525573	0.19595572	0.60937919	0.38409705	0.16139322	0.3024486	0.2764848
122.8	0.15738453	0.30518843	0.19585806	0.6094127	0.38399378	0.16130549	0.3025217	0.27647866
122.9	0.15727042	0.30512141	0.19576058	0.60944619	0.38389067	0.16121793	0.30259494	0.27647255
123	0.15715663	0.30505467	0.19566328	0.60947965	0.38378773	0.16113054	0.30266833	0.27646647
123.1	0.15704317	0.3049882	0.19556616	0.60951309	0.38368495	0.16104331	0.30274185	0.27646042
123.2	0.15693004	0.30492202	0.19546923	0.60954649	0.38358234	0.16095625	0.30281552	0.2764544
123.3	0.15681724	0.30485611	0.19537247	0.60957986	0.3834799	0.16086936	0.30288932	0.27644841
123.4	0.15670477	0.30479048	0.19527589	0.60961321	0.38337761	0.16078262	0.30296327	0.27644244
123.5	0.15659261	0.30472513	0.1951795	0.60964653	0.38327549	0.16069605	0.30303734	0.27643651
123.6	0.15648079	0.30466004	0.19508328	0.60967982	0.38317354	0.16060964	0.30311156	0.2764306
123.7	0.15636928	0.30459524	0.19498724	0.60971308	0.38307174	0.16052339	0.30318591	0.27642471
123.8	0.15625809	0.3045307	0.19489138	0.60974631	0.38297012	0.1604373	0.30326039	0.27641886
123.9	0.15614723	0.30446644	0.1947957	0.60977951	0.38286865	0.16035136	0.30333501	0.27641303
124	0.15603667	0.30440245	0.19470019	0.60981269	0.38276735	0.16026557	0.30340975	0.27640723
124.1	0.15592644	0.30433873	0.19460487	0.60984583	0.3826662	0.16017994	0.30348463	0.27640146
124.2	0.15581652	0.30427527	0.19450972	0.60987895	0.38256523	0.16009446	0.30355964	0.27639572
124.3	0.15570692	0.30421209	0.19441474	0.60991204	0.38246441	0.16000914	0.30363478	0.27639
124.4	0.15559762	0.30414917	0.19431994	0.6099451	0.38236376	0.15992396	0.30371004	0.27638431
124.5	0.15548864	0.30408653	0.19422532	0.60997813	0.38226327	0.15983893	0.30378543	0.27637864
124.6	0.15537997	0.30402414	0.19413087	0.61001114	0.38216295	0.15975404	0.30386095	0.27637301
124.7	0.1552716	0.30396203	0.1940366	0.61004412	0.38206279	0.15966931	0.3039366	0.27636739
124.8	0.15516354	0.30390018	0.1939425	0.61007706	0.38196279	0.15958471	0.30401237	0.27636181
124.9	0.15505579	0.30383859	0.19384858	0.61010998	0.38186295	0.15950027	0.30408826	0.27635625
125	0.15494834	0.30377726	0.19375483	0.61014287	0.38176328	0.15941596	0.30416428	0.27635072
125.1	0.15484119	0.3037162	0.19366126	0.61017574	0.38166378	0.1593318	0.30424042	0.27634522
125.2	0.15473435	0.3036554	0.19356785	0.61020857	0.38156444	0.15924777	0.30431669	0.27633974
125.3	0.1546278	0.30359486	0.19347462	0.61024138	0.38146527	0.15916389	0.30439307	0.27633428
125.4	0.15452156	0.30353458	0.19338156	0.61027416	0.38136626	0.15908015	0.30446958	0.27632885
125.5	0.15441561	0.30347455	0.19328868	0.61030691	0.38126742	0.15899654	0.30454621	0.27632345
125.6	0.15430996	0.30341479	0.19319596	0.61033963	0.38116874	0.15891307	0.30462295	0.27631807
125.7	0.1542046	0.30335528	0.19310342	0.61037233	0.38107024	0.15882973	0.30469981	0.27631272
125.8	0.15409953	0.30329603	0.19301104	0.610405	0.3809719	0.15874653	0.30477679	0.27630739
125.9	0.15399476	0.30323704	0.19291884	0.61043764	0.38087373	0.15866346	0.30485389	0.27630209
126	0.15389028	0.3031783	0.19282681	0.61047025	0.38077573	0.15858053	0.3049311	0.27629682
126.1	0.15378608	0.30311982	0.19273495	0.61050284	0.3806779	0.15849772	0.30500843	0.27629157
126.2	0.15368218	0.30306158	0.19264325	0.6105354	0.38058024	0.15841505	0.30508587	0.27628634
126.3	0.15357856	0.30300361	0.19255173	0.61056793	0.38048275	0.15833251	0.30516342	0.27628114
126.4	0.15347522	0.30294588	0.19246037	0.61060043	0.38038543	0.1582501	0.30524109	0.27627596
126.5	0.15337217	0.3028884	0.19236918	0.61063291	0.38028829	0.15816781	0.30531887	0.27627081
126.6	0.15326941	0.30283118	0.19227816	0.61066537	0.38019132	0.15808566	0.30539675	0.27626568
126.7	0.15316692	0.3027742	0.1921873	0.61069779	0.38009453	0.15800363	0.30547475	0.27626058
126.8	0.15306471	0.30271747	0.19209662	0.61073019	0.37999791	0.15792173	0.30555285	0.2762555
126.9	0.15296278	0.30266099	0.1920061	0.61076257	0.37990147	0.15783995	0.30563107	0.27625044
127	0.15286113	0.30260476	0.19191574	0.61079491	0.3798052	0.1577583	0.30570939	0.27624541
127.1	0.15275976	0.30254877	0.19182555	0.61082724	0.37970912	0.15767677	0.30578782	0.27624041
127.2	0.15265865	0.30249303	0.19173553	0.61085953	0.37961321	0.15759537	0.30586635	0.27623542
127.3	0.15255782	0.30243753	0.19164567	0.6108918	0.37951749	0.15751409	0.30594499	0.27623046
127.4	0.15245726	0.30238228	0.19155598	0.61092405	0.37942195	0.15743293	0.30602374	0.27622553
127.5	0.15235698	0.30232727	0.19146645	0.61095627	0.37932659	0.1573519	0.30610258	0.27622061
127.6	0.15225696	0.30227251	0.19137709	0.61098846	0.37923141	0.15727098	0.30618154	0.27621573
127.7	0.1521572	0.30221798	0.19128788	0.61102063	0.37913642	0.15719019	0.30626059	0.27621086
127.8	0.15205772	0.3021637	0.19119885	0.61105277	0.37904161	0.15710951	0.30633974	0.27620602
127.9	0.15195849	0.30210965	0.19110997	0.61108488	0.37894699	0.15702896	0.306419	0.2762012
128	0.15185954	0.30205585	0.19102126	0.61111697	0.37885256	0.15694852	0.30649835	0.2761964
128.1	0.15176084	0.30200228	0.19093271	0.61114904	0.37875831	0.15686821	0.30657781	0.27619163
128.2	0.1516624	0.30194895	0.19084432	0.61118108	0.37866426	0.15678801	0.30665736	0.27618688
128.3	0.15156422	0.30189586	0.19075609	0.61121309	0.37857039	0.15670793	0.30673701	0.27618215
128.4	0.1514663	0.30184301	0.19066802	0.61124508	0.37847672	0.15662796	0.30681676	0.27617744
128.5	0.15136864	0.30179039	0.19058011	0.61127704	0.37838323	0.15654811	0.3068966	0.27617276
128.6	0.15127123	0.301738	0.19049237	0.61130898	0.37828995	0.15646838	0.30697653	0.2761681
128.7	0.15117408	0.30168585	0.19040478	0.61134089	0.37819685	0.15638877	0.30705656	0.27616346
128.8	0.15107717	0.30163393	0.19031736	0.61137278	0.37810395	0.15630927	0.30713669	0.27615884
128.9	0.15098052	0.30158225	0.19023009	0.61140464	0.37801125	0.15622988	0.30721691	0.27615425
129	0.15088411	0.30153079	0.19014298	0.61143648	0.37791874	0.15615061	0.30729721	0.27614968
129.1	0.15078796	0.30147957	0.19005603	0.61146829	0.37782644	0.15607145	0.30737761	0.27614513
129.2	0.15069204	0.30142858	0.18996924	0.61150008	0.37773433	0.15599241	0.3074581	0.2761406
129.3	0.15059638	0.30137781	0.1898826	0.61153184	0.37764242	0.15591348	0.30753868	0.27613609
129.4	0.15050096	0.30132728	0.18979612	0.61156358	0.37755072	0.15583466	0.30761935	0.27613161
129.5	0.15040577	0.30127697	0.1897098	0.61159529	0.37745922	0.15575596	0.30770011	0.27612714
129.6	0.15031083	0.30122689	0.18962364	0.61162698	0.37736792	0.15567737	0.30778095	0.2761227
129.7	0.15021613	0.30117704	0.18953763	0.61165864	0.37727682	0.15559888	0.30786188	0.27611828
129.8	0.15012167	0.30112741	0.18945178	0.61169028	0.37718594	0.15552052	0.30794289	0.27611388
129.9	0.15002744	0.30107801	0.18936608	0.61172189	0.37709526	0.15544226	0.30802399	0.2761095
130	0.14993345	0.30102883	0.18928054	0.61175348	0.37700479	0.15536411	0.30810518	0.27610514
130.1	0.14983969	0.30097987	0.18919515	0.61178504	0.37691453	0.15528608	0.30818644	0.2761008
130.2	0.14974617	0.30093114	0.18910991	0.61181658	0.37682447	0.15520815	0.30826779	0.27609649
130.3	0.14965287	0.30088263	0.18902483	0.6118481	0.37673464	0.15513034	0.30834922	0.27609219
130.4	0.14955981	0.30083434	0.18893991	0.61187959	0.37664501	0.15505264	0.30843074	0.27608792
130.5	0.14946697	0.30078627	0.18885513	0.61191105	0.3765556	0.15497504	0.30851233	0.27608366
130.6	0.14937436	0.30073843	0.18877051	0.6119425	0.37646641	0.15489756	0.308594	0.27607943
130.7	0.14928197	0.3006908	0.18868605	0.61197391	0.37637744	0.15482018	0.30867575	0.27607521
130.8	0.14918981	0.30064339	0.18860173	0.61200531	0.37628868	0.15474291	0.30875758	0.27607102
130.9	0.14909787	0.3005962	0.18851756	0.61203667	0.37620014	0.15466576	0.30883948	0.27606684
131	0.14900616	0.30054922	0.18843355	0.61206802	0.37611182	0.15458871	0.30892146	0.27606269
131.1	0.14891466	0.30050246	0.18834969	0.61209934	0.37602373	0.15451177	0.30900352	0.27605856
131.2	0.14882338	0.30045592	0.18826598	0.61213064	0.37593586	0.15443494	0.30908565	0.27605444
131.3	0.14873232	0.30040959	0.18818241	0.61216191	0.37584821	0.15435821	0.30916786	0.27605035
131.4	0.14864147	0.30036348	0.188099	0.61219316	0.37576078	0.1542816	0.30925014	0.27604627
131.5	0.14855084	0.30031758	0.18801574	0.61222438	0.37567358	0.15420509	0.30933249	0.27604222
131.6	0.14846042	0.30027189	0.18793262	0.61225558	0.37558661	0.15412869	0.30941492	0.27603818
131.7	0.14837021	0.30022642	0.18784966	0.61228676	0.37549987	0.1540524	0.30949741	0.27603416
131.8	0.14828022	0.30018116	0.18776684	0.61231791	0.37541335	0.15397621	0.30957998	0.27603017
131.9	0.14819043	0.30013611	0.18768417	0.61234904	0.37532706	0.15390014	0.30966262	0.27602619
132	0.14810085	0.30009127	0.18760165	0.61238015	0.37524101	0.15382417	0.30974533	0.27602223
132.1	0.14801148	0.30004664	0.18751928	0.61241123	0.37515519	0.1537483	0.30982811	0.27601829
132.2	0.14792231	0.30000222	0.18743705	0.61244229	0.3750696	0.15367255	0.30991095	0.27601437
132.3	0.14783335	0.29995801	0.18735497	0.61247333	0.37498424	0.1535969	0.30999386	0.27601046
132.4	0.14774459	0.29991401	0.18727303	0.61250434	0.37489912	0.15352135	0.31007684	0.27600658
132.5	0.14765603	0.29987021	0.18719125	0.61253534	0.37481423	0.15344591	0.31015989	0.27600271
132.6	0.14756767	0.29982663	0.1871096	0.61256631	0.37472958	0.15337058	0.310243	0.27599887
132.7	0.14747951	0.29978324	0.1870281	0.61259725	0.37464517	0.15329536	0.31032618	0.27599504
132.8	0.14739154	0.29974007	0.18694675	0.61262818	0.374561	0.15322024	0.31040942	0.27599123
132.9	0.14730378	0.29969709	0.18686554	0.61265908	0.37447707	0.15314523	0.31049273	0.27598744
133	0.14721621	0.29965432	0.18678447	0.61268996	0.37439337	0.15307032	0.3105761	0.27598366
133.1	0.14712883	0.29961176	0.18670355	0.61272082	0.37430992	0.15299552	0.31065953	0.27597991
133.2	0.14704165	0.29956939	0.18662277	0.61275165	0.37422671	0.15292082	0.31074302	0.27597617
133.3	0.14695466	0.29952723	0.18654213	0.61278246	0.37414375	0.15284623	0.31082657	0.27597246
133.4	0.14686786	0.29948527	0.18646164	0.61281325	0.37406102	0.15277174	0.31091019	0.27596876
133.5	0.14678124	0.29944351	0.18638129	0.61284402	0.37397854	0.15269736	0.31099386	0.27596507
133.6	0.14669482	0.29940195	0.18630108	0.61287477	0.3738963	0.15262308	0.31107759	0.27596141
133.7	0.14660859	0.29936059	0.18622101	0.61290549	0.37381431	0.15254891	0.31116138	0.27595777
133.8	0.14652254	0.29931943	0.18614108	0.61293619	0.37373256	0.15247485	0.31124523	0.27595414
133.9	0.14643667	0.29927847	0.18606129	0.61296687	0.37365106	0.15240088	0.31132914	0.27595053
134	0.14635099	0.2992377	0.18598164	0.61299753	0.37356981	0.15232703	0.3114131	0.27594694
134.1	0.14626549	0.29919713	0.18590213	0.61302817	0.3734888	0.15225327	0.31149712	0.27594336
134.2	0.14618017	0.29915676	0.18582277	0.61305878	0.37340804	0.15217962	0.31158119	0.27593981
134.3	0.14609504	0.29911658	0.18574354	0.61308938	0.37332754	0.15210608	0.31166531	0.27593627
134.4	0.14601008	0.2990766	0.18566444	0.61311995	0.37324728	0.15203264	0.31174949	0.27593275
134.5	0.1459253	0.29903681	0.18558549	0.6131505	0.37316727	0.1519593	0.31183373	0.27592925
134.6	0.1458407	0.29899722	0.18550668	0.61318103	0.37308751	0.15188607	0.31191801	0.27592576
134.7	0.14575627	0.29895782	0.185428	0.61321154	0.373008	0.15181294	0.31200235	0.27592229
134.8	0.14567202	0.29891861	0.18534946	0.61324202	0.37292875	0.15173992	0.31208674	0.27591884
134.9	0.14558795	0.29887959	0.18527105	0.61327249	0.37284975	0.151667	0.31217118	0.27591541
180	0.11905093	0.29406836	0.15931898	0.62518551	0.36545374	0.12783313	0.34784497	0.27549936
180.1	0.1190108	0.29407542	0.15927647	0.62520799	0.36547273	0.12779775	0.34791109	0.27549985
180.2	0.11897074	0.29408253	0.159234	0.62523045	0.36549175	0.12776242	0.34797714	0.27550034
180.3	0.11893074	0.29408968	0.15919159	0.62525288	0.36551078	0.12772715	0.34804313	0.27550084
180.4	0.11889081	0.29409688	0.15914923	0.62527529	0.36552983	0.12769193	0.34810906	0.27550134

135.2	0.14533676	0.29876369	0.18503666	0.61336376	0.37261426	0.15144886	0.31242479	0.27590521
135.3	0.14525337	0.29872544	0.1849588	0.61339414	0.37253628	0.15137635	0.31250942	0.27590185
135.4	0.14517015	0.29868737	0.18488107	0.6134245	0.37245855	0.15130395	0.3125941	0.2758985
135.5	0.1450871	0.29864949	0.18480348	0.61345484	0.37238108	0.15123165	0.31267883	0.27589517
135.6	0.14500422	0.2986118	0.18472602	0.61348516	0.37230386	0.15115945	0.3127636	0.27589185
135.7	0.1449215	0.2985743	0.1846487	0.61351546	0.37222691	0.15108736	0.31284842	0.27588856
135.8	0.14483895	0.29853698	0.18457151	0.61354574	0.37215021	0.15101537	0.31293328	0.27588528
135.9	0.14475656	0.29849985	0.18449445	0.613576	0.37207377	0.15094348	0.31301818	0.27588201
136	0.14467434	0.29846291	0.18441753	0.61360623	0.37199759	0.15087169	0.31310313	0.27587876
136.1	0.14459228	0.29842615	0.18434073	0.61363645	0.37192167	0.15080001	0.31318811	0.27587553
136.2	0.14451039	0.29838957	0.18426407	0.61366665	0.37184601	0.15072842	0.31327314	0.27587232
136.3	0.14442865	0.29835318	0.18418755	0.61369682	0.37177062	0.15065694	0.31335821	0.27586912
136.4	0.14434708	0.29831697	0.18411115	0.61372698	0.37169548	0.15058556	0.31344332	0.27586594
136.5	0.14426566	0.29828094	0.18403488	0.61375711	0.37162062	0.15051428	0.31352847	0.27586277
136.6	0.1441844	0.29824509	0.18395875	0.61378723	0.37154601	0.15044311	0.31361366	0.27585962
136.7	0.1441033	0.29820943	0.18388274	0.61381732	0.37147167	0.15037203	0.31369888	0.27585649
136.8	0.14402236	0.29817395	0.18380686	0.6138474	0.3713976	0.15030105	0.31378415	0.27585337
136.9	0.14394158	0.29813864	0.18373112	0.61387745	0.3713238	0.15023018	0.31386944	0.27585027
137	0.14386095	0.29810352	0.1836555	0.61390748	0.37125027	0.15015941	0.31395478	0.27584719
137.1	0.14378048	0.29806857	0.18358001	0.6139375	0.371177	0.15008873	0.31404015	0.27584412
137.2	0.14370016	0.29803381	0.18350465	0.6139675	0.37110401	0.15001816	0.31412555	0.27584107
137.3	0.14361999	0.29799922	0.18342942	0.61399747	0.37103129	0.14994769	0.31421099	0.27583803
137.4	0.14353998	0.2979648	0.18335432	0.61402743	0.37095884	0.14987731	0.31429646	0.27583501
137.5	0.14346012	0.29793057	0.18327934	0.61405736	0.37088667	0.14980704	0.31438196	0.27583201
137.6	0.14338041	0.29789651	0.18320449	0.61408728	0.37081478	0.14973687	0.31446749	0.27582902
137.7	0.14330085	0.29786263	0.18312977	0.61411718	0.37074316	0.14966679	0.31455306	0.27582604
137.8	0.14322145	0.29782892	0.18305517	0.61414705	0.37067182	0.14959682	0.31463865	0.27582309
137.9	0.14314219	0.29779538	0.1829807	0.61417691	0.37060076	0.14952694	0.31472428	0.27582014
138	0.14306308	0.29776202	0.18290635	0.61420675	0.37052998	0.14945716	0.31480993	0.27581722
138.1	0.14298411	0.29772883	0.18283213	0.61423657	0.37045948	0.14938749	0.31489562	0.27581431
138.2	0.1429053	0.29769582	0.18275804	0.61426637	0.37038926	0.1493179	0.31498133	0.27581141
138.3	0.14282663	0.29766297	0.18268406	0.61429616	0.37031934	0.14924842	0.31506707	0.27580853
138.4	0.1427481	0.2976303	0.18261022	0.61432592	0.37024969	0.14917904	0.31515284	0.27580567
138.5	0.14266973	0.2975978	0.18253649	0.61435566	0.37018034	0.14910975	0.31523863	0.27580281
138.6	0.14259149	0.29756547	0.18246289	0.61438539	0.37011127	0.14904056	0.31532445	0.27579998
138.7	0.1425134	0.29753331	0.18238941	0.6144151	0.3700425	0.14897147	0.31541029	0.27579716
138.8	0.14243545	0.29750132	0.18231606	0.61444479	0.36997401	0.14890248	0.31549616	0.27579435
138.9	0.14235765	0.2974695	0.18224282	0.61447445	0.36990582	0.14883358	0.31558205	0.27579156
139	0.14227998	0.29743784	0.18216971	0.61450411	0.36983793	0.14876478	0.31566796	0.27578879
139.1	0.14220246	0.29740636	0.18209672	0.61453374	0.36977033	0.14869607	0.3157539	0.27578603
139.2	0.14212508	0.29737504	0.18202385	0.61456335	0.36970302	0.14862746	0.31583986	0.27578328
139.3	0.14204784	0.29734388	0.18195111	0.61459295	0.36963602	0.14855895	0.31592584	0.27578055
139.4	0.14197074	0.2973129	0.18187848	0.61462252	0.36956932	0.14849053	0.31601184	0.27577784
139.5	0.14189377	0.29728207	0.18180597	0.61465208	0.36950291	0.14842221	0.31609785	0.27577514
139.6	0.14181695	0.29725142	0.18173358	0.61468162	0.36943682	0.14835398	0.31618389	0.27577245
139.7	0.14174026	0.29722093	0.18166131	0.61471114	0.36937102	0.14828585	0.31626995	0.27576978
139.8	0.14166371	0.2971906	0.18158916	0.61474065	0.36930553	0.14821781	0.31635602	0.27576712
139.9	0.1415873	0.29716043	0.18151713	0.61477013	0.36924035	0.14814986	0.31644212	0.27576448
140	0.14151102	0.29713043	0.18144522	0.6147996	0.36917548	0.14808201	0.31652822	0.27576185
140.1	0.14143487	0.29710059	0.18137342	0.61482905	0.36911091	0.14801425	0.31661435	0.27575924
140.2	0.14135887	0.29707092	0.18130174	0.61485848	0.36904666	0.14794659	0.31670048	0.27575664
140.3	0.14128299	0.2970414	0.18123018	0.61488789	0.36898272	0.14787902	0.31678664	0.27575405
140.4	0.14120725	0.29701205	0.18115873	0.61491729	0.36891909	0.14781154	0.3168728	0.27575148
140.5	0.14113164	0.29698285	0.18108741	0.61494667	0.36885578	0.14774416	0.31695898	0.27574892
140.6	0.14105617	0.29695382	0.18101619	0.61497603	0.36879278	0.14767687	0.31704518	0.27574638
140.7	0.14098083	0.29692494	0.1809451	0.61500537	0.36873011	0.14760967	0.31713138	0.27574385
140.8	0.14090562	0.29689623	0.18087411	0.61503469	0.36866775	0.14754256	0.3172176	0.27574134
140.9	0.14083054	0.29686767	0.18080325	0.615064	0.36860571	0.14747554	0.31730383	0.27573884
141	0.14075559	0.29683927	0.18073249	0.61509329	0.368544	0.14740862	0.31739006	0.27573635
141.1	0.14068077	0.29681103	0.18066186	0.61512256	0.36848261	0.14734179	0.31747631	0.27573388
141.2	0.14060608	0.29678294	0.18059133	0.61515181	0.36842154	0.14727505	0.31756257	0.27573142
141.3	0.14053152	0.29675501	0.18052092	0.61518104	0.3683608	0.1472084	0.31764884	0.27572898
141.4	0.14045709	0.29672724	0.18045062	0.61521026	0.36830038	0.14714184	0.31773512	0.27572655
141.5	0.14038279	0.29669962	0.18038043	0.61523946	0.3682403	0.14707538	0.3178214	0.27572413
141.6	0.14030861	0.29667216	0.18031036	0.61526864	0.36818054	0.147009	0.3179077	0.27572173
141.7	0.14023456	0.29664485	0.1802404	0.6152978	0.36812111	0.14694271	0.317994	0.27571934
141.8	0.14016064	0.29661769	0.18017055	0.61532695	0.36806202	0.14687652	0.3180803	0.27571697
141.9	0.14008685	0.29659069	0.18010081	0.61535608	0.36800326	0.14681041	0.31816661	0.27571461
142	0.14001318	0.29656385	0.18003118	0.61538519	0.36794483	0.1467444	0.31825293	0.27571226
142.1	0.13993964	0.29653715	0.17996166	0.61541428	0.36788673	0.14667847	0.31833925	0.27570993
142.2	0.13986622	0.29651061	0.17989225	0.61544336	0.36782898	0.14661263	0.31842558	0.27570761
142.3	0.13979293	0.29648422	0.17982295	0.61547241	0.36777156	0.14654689	0.31851191	0.2757053
142.4	0.13971976	0.29645798	0.17975376	0.61550145	0.36771447	0.14648123	0.31859824	0.27570301
142.5	0.13964671	0.29643189	0.17968468	0.61553047	0.36765773	0.14641566	0.31868457	0.27570073
142.6	0.13957379	0.29640595	0.1796157	0.61555948	0.36760132	0.14635018	0.31877091	0.27569846
142.7	0.13950099	0.29638016	0.17954684	0.61558847	0.36754526	0.14628479	0.31885725	0.27569621
142.8	0.13942832	0.29635452	0.17947808	0.61561743	0.36748953	0.14621949	0.31894358	0.27569397
142.9	0.13935576	0.29632903	0.17940943	0.61564639	0.36743415	0.14615427	0.31902992	0.27569175
143	0.13928333	0.29630368	0.17934088	0.61567532	0.36737912	0.14608914	0.31911626	0.27568954
143.1	0.13921102	0.29627849	0.17927244	0.61570424	0.36732442	0.14602411	0.3192026	0.27568734
143.2	0.13913883	0.29625344	0.17920411	0.61573314	0.36727007	0.14595916	0.31928893	0.27568515
143.3	0.13906676	0.29622854	0.17913588	0.61576202	0.36721607	0.1458943	0.31937526	0.27568298
143.4	0.13899481	0.29620379	0.17906776	0.61579088	0.36716241	0.14582952	0.3194616	0.27568082
143.5	0.13892298	0.29617918	0.17899974	0.61581973	0.3671091	0.14576484	0.31954792	0.27567867
143.6	0.13885128	0.29615472	0.17893183	0.61584856	0.36705613	0.14570024	0.31963425	0.27567654
143.7	0.13877969	0.2961304	0.17886402	0.61587737	0.36700352	0.14563573	0.31972057	0.27567442
143.8	0.13870822	0.29610623	0.17879631	0.61590617	0.36695125	0.1455713	0.31980688	0.27567231
143.9	0.13863686	0.2960822	0.17872871	0.61593494	0.36689933	0.14550697	0.31989319	0.27567022
144	0.13856563	0.29605832	0.17866121	0.6159637	0.36684776	0.14544272	0.3199795	0.27566813
144.1	0.13849451	0.29603457	0.17859381	0.61599244	0.36679654	0.14537856	0.32006579	0.27566606
144.2	0.13842352	0.29601098	0.17852652	0.61602117	0.36674568	0.14531449	0.32015208	0.27566401
144.3	0.13835264	0.29598752	0.17845932	0.61604987	0.36669516	0.1452505	0.32023837	0.27566196
144.4	0.13828187	0.29596421	0.17839223	0.61607856	0.36664499	0.1451866	0.32032464	0.27565993
144.5	0.13821122	0.29594104	0.17832524	0.61610723	0.36659518	0.14512279	0.32041091	0.27565792
144.6	0.13814069	0.29591801	0.17825834	0.61613588	0.36654572	0.14505907	0.32049717	0.27565591
144.7	0.13807028	0.29589512	0.17819155	0.61616451	0.36649661	0.14499543	0.32058342	0.27565392
144.8	0.13799998	0.29587237	0.17812486	0.61619313	0.36644785	0.14493188	0.32066965	0.27565194
144.9	0.13792979	0.29584976	0.17805827	0.61622173	0.36639946	0.14486841	0.32075588	0.27564997
145	0.13785972	0.29582729	0.17799177	0.61625031	0.36635141	0.14480504	0.3208421	0.27564801
145.1	0.13778977	0.29580496	0.17792538	0.61627887	0.36630372	0.14474175	0.3209283	0.27564607
145.2	0.13771993	0.29578277	0.17785908	0.61630742	0.36625639	0.14467855	0.32101449	0.27564413
145.3	0.1376502	0.29576071	0.17779288	0.61633595	0.36620941	0.14461543	0.32110067	0.27564221
145.4	0.13758059	0.2957388	0.17772678	0.61636446	0.36616279	0.1445524	0.32118684	0.27564031
145.5	0.13751109	0.29571702	0.17766078	0.61639295	0.36611653	0.14448946	0.32127299	0.27563841
145.6	0.13744171	0.29569537	0.17759488	0.61642143	0.36607062	0.1444266	0.32135913	0.27563653
145.7	0.13737244	0.29567387	0.17752907	0.61644988	0.36602507	0.14436383	0.32144525	0.27563466
145.8	0.13730328	0.2956525	0.17746336	0.61647832	0.36597988	0.14430115	0.32153136	0.2756328
145.9	0.13723423	0.29563126	0.17739774	0.61650674	0.36593504	0.14423855	0.32161745	0.27563095
146	0.1371653	0.29561016	0.17733222	0.61653515	0.36589056	0.14417605	0.32170353	0.27562912
146.1	0.13709648	0.2955892	0.1772668	0.61656353	0.36584644	0.14411362	0.32178959	0.27562729
146.2	0.13702777	0.29556837	0.17720147	0.6165919	0.36580268	0.14405129	0.32187563	0.27562548
146.3	0.13695917	0.29554767	0.17713624	0.61662025	0.36575927	0.14398904	0.32196165	0.27562368
146.4	0.13689068	0.29552711	0.1770711	0.61664859	0.36571622	0.14392688	0.32204766	0.27562189
146.5	0.13682231	0.29550667	0.17700606	0.6166769	0.36567353	0.1438648	0.32213364	0.27562012
146.6	0.13675404	0.29548638	0.17694111	0.6167052	0.3656312	0.14380282	0.32221961	0.27561835
146.7	0.13668589	0.29546621	0.17687625	0.61673348	0.36558922	0.14374091	0.32230556	0.2756166
146.8	0.13661784	0.29544617	0.17681149	0.61676175	0.3655476	0.1436791	0.32239149	0.27561486
146.9	0.13654991	0.29542627	0.17674683	0.61678999	0.36550634	0.14361737	0.32247739	0.27561313
147	0.13648208	0.2954065	0.17668225	0.61681822	0.36546544	0.14355573	0.32256328	0.27561141
147.1	0.13641437	0.29538685	0.17661777	0.61684643	0.36542489	0.14349418	0.32264914	0.27560971
147.2	0.13634676	0.29536734	0.17655338	0.61687462	0.3653847	0.14343271	0.32273499	0.27560801
147.3	0.13627926	0.29534796	0.17648909	0.6169028	0.36534486	0.14337133	0.32282081	0.27560633
147.4	0.13621188	0.2953287	0.17642488	0.61693096	0.36530538	0.14331004	0.3229066	0.27560466
147.5	0.1361446	0.29530957	0.17636077	0.6169591	0.36526626	0.14324883	0.32299237	0.275603
147.6	0.13607743	0.29529058	0.17629675	0.61698722	0.3652275	0.14318771	0.32307812	0.27560135
147.7	0.13601036	0.29527171	0.17623282	0.61701533	0.36518909	0.14312668	0.32316384	0.27559971
147.8	0.13594341	0.29525296	0.17616898	0.61704341	0.36515103	0.14306574	0.32324954	0.27559809
147.9	0.13587656	0.29523435	0.17610524	0.61707148	0.36511333	0.14300488	0.32333521	0.27559647
148	0.13580982	0.29521586	0.17604158	0.61709954	0.36507598	0.14294411	0.32342085	0.27559487
148.1	0.13574319	0.29519749	0.17597802	0.61712757	0.36503899	0.14288343	0.32350646	0.27559328
148.2	0.13567666	0.29517926	0.17591454	0.61715559	0.36500235	0.14282283	0.32359205	0.2755917
148.3	0.13561024	0.29516115	0.17585116	0.61718359	0.36496607	0.14276232	0.3236776	0.27559013
148.4	0.13554393	0.29514316	0.17578786	0.61721157	0.36493014	0.1427019	0.32376313	0.27558857
148.5	0.13547773	0.2951253	0.17572466	0.61723953	0.36489456	0.14264156	0.32384863	0.27558702
148.6	0.13541163	0.29510756	0.17566154	0.61726748	0.36485933	0.14258132	0.32393409	0.27558549
148.7	0.13534563	0.29508995	0.17559851	0.61729541	0.36482446	0.14252116	0.32401953	0.27558396
148.8	0.13527975	0.29507245	0.17553557	0.61732332	0.36478994	0.14246109	0.32410493	0.27558245
148.9	0.13521396	0.29505509	0.17547272	0.61735122	0.36475577	0.1424011	0.3241903	0.27558094
149	0.13514829	0.29503784	0.17540996	0.61737909	0.36472196	0.1423412	0.32427563	0.27557945
149.1	0.13508272	0.29502072	0.17534729	0.61740695	0.3646885	0.14228139	0.32436093	0.27557797
149.2	0.13501725	0.29500372	0.1752847	0.61743479	0.36465538	0.14222167	0.3244462	0.2755765
149.3	0.13495189	0.29498684	0.1752222	0.61746262	0.36462262	0.14216203	0.32453142	0.27557504
149.4	0.13488663	0.29497008	0.17515979	0.61749042	0.36459021	0.14210249	0.32461662	0.2755736
149.5	0.13482148	0.29495345	0.17509747	0.61751821	0.36455815	0.14204303	0.32470177	0.27557216
149.6	0.13475643	0.29493693	0.17503523	0.61754599	0.36452643	0.14198365	0.32478689	0.27557073
149.7	0.13469148	0.29492053	0.17497308	0.61757374	0.36449507	0.14192437	0.32487196	0.27556932
149.8	0.13462664	0.29490426	0.17491102	0.61760148	0.36446405	0.14186517	0.324957	0.27556791
149.9	0.1345619	0.2948881	0.17484904	0.6176292	0.36443338	0.14180606	0.325042	0.27556652
195	0.11372225	0.29556394	0.15347724	0.62815525	0.36842989	0.12304924	0.35706223	0.27559764
195.1	0.11369102	0.29557639	0.15344145	0.62817274	0.36845048	0.12302052	0.35711914	0.27559844
195.2	0.11365983	0.29558887	0.15340569	0.62819021	0.36847109	0.12299183	0.357176	0.27559924
195.3	0.1136287	0.29560138	0.15336997	0.62820766	0.3684917	0.12296318	0.3572328	0.27560004
195.4	0.11359762	0.29561392	0.15333429	0.62822508	0.36851233	0.12293457	0.35728954	0.27560085

150.2	0.13436831	0.29484034	0.17466363	0.61771225	0.36434344	0.14162926	0.32529674	0.2755624
150.3	0.13430398	0.29482466	0.17460199	0.6177399	0.36431415	0.1415705	0.32538157	0.27556105
150.4	0.13423976	0.29480909	0.17454045	0.61776753	0.36428521	0.14151183	0.32546636	0.27555971
150.5	0.13417563	0.29479364	0.17447898	0.61779514	0.3642566	0.14145325	0.3255511	0.27555838
150.6	0.13411161	0.29477831	0.1744176	0.61782274	0.36422834	0.14139475	0.3256358	0.27555706
150.7	0.1340477	0.29476309	0.17435631	0.61785032	0.36420042	0.14133635	0.32572045	0.27555575
150.8	0.13398388	0.29474799	0.17429509	0.61787788	0.36417284	0.14127803	0.32580505	0.27555445
150.9	0.13392017	0.29473301	0.17423397	0.61790543	0.3641456	0.1412198	0.3258896	0.27555316
151	0.13385656	0.29471814	0.17417292	0.61793295	0.36411869	0.14116166	0.32597411	0.27555189
151.1	0.13379304	0.29470338	0.17411196	0.61796046	0.36409213	0.1411036	0.32605857	0.27555062
151.2	0.13372963	0.29468874	0.17405108	0.61798795	0.3640659	0.14104564	0.32614298	0.27554936
151.3	0.13366632	0.29467422	0.17399029	0.61801543	0.36404001	0.14098776	0.32622734	0.27554811
151.4	0.13360312	0.29465981	0.17392957	0.61804288	0.36401445	0.14092997	0.32631164	0.27554688
151.5	0.13354001	0.29464551	0.17386894	0.61807032	0.36398922	0.14087227	0.3263959	0.27554565
151.6	0.133477	0.29463133	0.17380839	0.61809774	0.36396433	0.14081465	0.3264801	0.27554444
151.7	0.13341409	0.29461725	0.17374793	0.61812515	0.36393977	0.14075713	0.32656425	0.27554323
151.8	0.13335129	0.29460329	0.17368754	0.61815253	0.36391554	0.14069969	0.32664834	0.27554203
151.9	0.13328858	0.29458945	0.17362724	0.6181799	0.36389164	0.14064234	0.32673238	0.27554085
152	0.13322597	0.29457571	0.17356701	0.61820725	0.36386807	0.14058508	0.32681636	0.27553967
152.1	0.13316347	0.29456209	0.17350687	0.61823459	0.36384483	0.14052791	0.32690029	0.27553851
152.2	0.13310106	0.29454857	0.17344681	0.6182619	0.36382192	0.14047083	0.32698416	0.27553735
152.3	0.13303876	0.29453517	0.17338682	0.6182892	0.36379933	0.14041383	0.32706797	0.27553621
152.4	0.13297655	0.29452188	0.17332692	0.61831648	0.36377707	0.14035693	0.32715173	0.27553507
152.5	0.13291444	0.2945087	0.17326709	0.61834375	0.36375513	0.14030011	0.32723542	0.27553395
152.6	0.13285243	0.29449562	0.17320735	0.61837099	0.36373352	0.14024338	0.32731906	0.27553283
152.7	0.13279053	0.29448266	0.17314768	0.61839822	0.36371223	0.14018674	0.32740263	0.27553173
152.8	0.13272871	0.2944698	0.1730881	0.61842543	0.36369126	0.14013018	0.32748615	0.27553063
152.9	0.132667	0.29445706	0.17302859	0.61845263	0.36367061	0.14007372	0.32756961	0.27552955
153	0.13260539	0.29444442	0.17296916	0.6184798	0.36365029	0.14001734	0.327653	0.27552847
153.1	0.13254387	0.29443189	0.1729098	0.61850696	0.36363029	0.13996106	0.32773633	0.27552741
153.2	0.13248246	0.29441947	0.17285052	0.6185341	0.3636106	0.13990486	0.3278196	0.27552635
153.3	0.13242114	0.29440716	0.17279133	0.61856122	0.36359123	0.13984875	0.32790281	0.2755253
153.4	0.13235992	0.29439495	0.1727322	0.61858833	0.36357218	0.13979273	0.32798595	0.27552427
153.5	0.13229879	0.29438285	0.17267316	0.61861542	0.36355344	0.13973679	0.32806903	0.27552324
153.6	0.13223776	0.29437085	0.17261419	0.61864249	0.36353501	0.13968095	0.32815205	0.27552222
153.7	0.13217683	0.29435896	0.17255529	0.61866954	0.3635169	0.13962519	0.328235	0.27552122
153.8	0.132116	0.29434718	0.17249647	0.61869657	0.36349909	0.13956952	0.32831789	0.27552022
153.9	0.13205527	0.2943355	0.17243773	0.61872359	0.3634816	0.13951394	0.32840071	0.27551923
154	0.13199463	0.29432393	0.17237906	0.61875059	0.36346441	0.13945845	0.32848347	0.27551825
154.1	0.13193408	0.29431246	0.17232047	0.61877757	0.36344753	0.13940305	0.32856617	0.27551728
154.2	0.13187364	0.2943011	0.17226195	0.61880453	0.36343096	0.13934773	0.3286488	0.27551632
154.3	0.13181329	0.29428984	0.1722035	0.61883148	0.3634147	0.13929251	0.32873136	0.27551537
154.4	0.13175303	0.29427868	0.17214513	0.61885841	0.36339873	0.13923737	0.32881386	0.27551443
154.5	0.13169287	0.29426763	0.17208683	0.61888532	0.36338307	0.13918232	0.32889629	0.2755135
154.6	0.13163281	0.29425668	0.1720286	0.61891222	0.36336771	0.13912736	0.32897865	0.27551258
154.7	0.13157284	0.29424583	0.17197045	0.61893909	0.36335265	0.13907249	0.32906095	0.27551166
154.8	0.13151297	0.29423509	0.17191237	0.61896595	0.36333789	0.1390177	0.32914319	0.27551076
154.9	0.13145319	0.29422444	0.17185437	0.61899279	0.36332342	0.13896301	0.32922535	0.27550986
155	0.1313935	0.2942139	0.17179643	0.61901961	0.36330925	0.1389084	0.32930746	0.27550898
155.1	0.13133392	0.29420346	0.17173857	0.61904642	0.36329538	0.13885388	0.32938949	0.2755081
155.2	0.13127443	0.29419312	0.17168078	0.61907321	0.3632818	0.13879945	0.32947146	0.27550723
155.3	0.13121503	0.29418288	0.17162306	0.61909997	0.36326851	0.13874511	0.32955337	0.27550637
155.4	0.13115573	0.29417274	0.17156541	0.61912673	0.36325551	0.13869086	0.32963521	0.27550553
155.5	0.13109651	0.2941627	0.17150783	0.61915346	0.3632428	0.13863669	0.32971698	0.27550469
155.6	0.1310374	0.29415276	0.17145032	0.61918018	0.36323037	0.13858262	0.32979869	0.27550385
155.7	0.13097837	0.29414292	0.17139289	0.61920687	0.36321824	0.13852863	0.32988033	0.27550303
155.8	0.13091944	0.29413318	0.17133552	0.61923356	0.36320638	0.13847473	0.32996191	0.27550222
155.9	0.1308606	0.29412354	0.17127822	0.61926022	0.36319481	0.13842092	0.33004342	0.27550141
156	0.13080186	0.294114	0.171221	0.61928686	0.36318353	0.13836719	0.33012486	0.27550062
156.1	0.1307432	0.29410455	0.17116384	0.61931349	0.36317252	0.13831356	0.33020624	0.27549983
156.2	0.13068464	0.29409521	0.17110675	0.6193401	0.36316179	0.13826001	0.33028755	0.27549905
156.3	0.13062618	0.29408596	0.17104974	0.61936669	0.36315135	0.13820655	0.3303688	0.27549829
156.4	0.1305678	0.2940768	0.17099279	0.61939327	0.36314118	0.13815318	0.33044998	0.27549753
156.5	0.13050951	0.29406775	0.17093591	0.61941982	0.36313128	0.13809989	0.3305311	0.27549677
156.6	0.13045132	0.29405879	0.1708791	0.61944636	0.36312166	0.1380467	0.33061215	0.27549603
156.7	0.13039322	0.29404993	0.17082235	0.61947288	0.36311232	0.13799359	0.33069313	0.2754953
156.8	0.13033521	0.29404116	0.17076567	0.61949938	0.36310324	0.13794057	0.33077405	0.27549457
156.9	0.13027729	0.29403249	0.17070907	0.61952587	0.36309444	0.13788764	0.33085491	0.27549386
157	0.13021947	0.29402391	0.17065253	0.61955234	0.36308591	0.1378348	0.3309357	0.27549315
157.1	0.13016173	0.29401543	0.17059606	0.61957879	0.36307765	0.13778204	0.33101643	0.27549245
157.2	0.13010408	0.29400704	0.17053965	0.61960522	0.36306965	0.13772937	0.33109709	0.27549176
157.3	0.13004653	0.29399875	0.17048331	0.61963163	0.36306192	0.13767679	0.33117768	0.27549107
157.4	0.12998906	0.29399055	0.17042705	0.61965803	0.36305446	0.1376243	0.33125821	0.2754904
157.5	0.12993169	0.29398245	0.17037084	0.6196844	0.36304725	0.13757189	0.33133868	0.27548973
157.6	0.1298744	0.29397444	0.17031471	0.61971076	0.36304031	0.13751957	0.33141908	0.27548908
157.7	0.12981721	0.29396652	0.17025864	0.61973711	0.36303363	0.13746734	0.33149942	0.27548843
157.8	0.1297601	0.2939587	0.17020264	0.61976343	0.36302721	0.1374152	0.3315797	0.27548779
157.9	0.12970308	0.29395097	0.17014671	0.61978974	0.36302104	0.13736314	0.33165991	0.27548715
158	0.12964615	0.29394333	0.17009084	0.61981602	0.36301513	0.13731118	0.33174005	0.27548653
158.1	0.12958931	0.29393578	0.17003504	0.61984229	0.36300947	0.1372593	0.33182013	0.27548591
158.2	0.12953256	0.29392833	0.1699793	0.61986855	0.36300407	0.1372075	0.33190015	0.27548531
158.3	0.1294759	0.29392097	0.16992363	0.61989478	0.36299892	0.1371558	0.3319801	0.27548471
158.4	0.12941933	0.2939137	0.16986803	0.619921	0.36299402	0.13710418	0.33206	0.27548411
158.5	0.12936284	0.29390652	0.16981249	0.6199472	0.36298936	0.13705265	0.33213982	0.27548353
158.6	0.12930644	0.29389943	0.16975702	0.61997338	0.36298495	0.1370012	0.33221959	0.27548296
158.7	0.12925013	0.29389243	0.16970162	0.61999954	0.36298079	0.13694984	0.33229929	0.27548239
158.8	0.12919391	0.29388552	0.16964628	0.62002569	0.36297687	0.13689857	0.33237893	0.27548183
158.9	0.12913777	0.2938787	0.16959101	0.62005182	0.3629732	0.13684739	0.3324585	0.27548128
159	0.12908173	0.29387197	0.1695358	0.62007793	0.36296976	0.13679629	0.33253801	0.27548074
159.1	0.12902576	0.29386533	0.16948066	0.62010402	0.36296657	0.13674528	0.33261746	0.2754802
159.2	0.12896989	0.29385878	0.16942558	0.62013009	0.36296361	0.13669436	0.33269684	0.27547967
159.3	0.1289141	0.29385232	0.16937057	0.62015615	0.36296089	0.13664352	0.33277616	0.27547915
159.4	0.1288584	0.29384595	0.16931563	0.62018219	0.3629584	0.13659277	0.33285542	0.27547864
159.5	0.12880278	0.29383967	0.16926075	0.62020821	0.36295615	0.13654211	0.33293462	0.27547814
159.6	0.12874725	0.29383347	0.16920593	0.62023421	0.36295413	0.13649153	0.33301375	0.27547764
159.7	0.12869181	0.29382736	0.16915118	0.6202602	0.36295234	0.13644104	0.33309283	0.27547716
159.8	0.12863645	0.29382134	0.16909649	0.62028617	0.36295077	0.13639064	0.33317184	0.27547668
159.9	0.12858118	0.29381541	0.16904188	0.62031212	0.36294944	0.13634032	0.33325078	0.2754762
160	0.12852599	0.29380956	0.16898732	0.62033805	0.36294833	0.13629009	0.33332967	0.27547574
160.1	0.12847089	0.2938038	0.16893283	0.62036396	0.36294744	0.13623994	0.33340849	0.27547528
160.2	0.12841587	0.29379812	0.1688784	0.62038986	0.36294678	0.13618989	0.33348725	0.27547483
160.3	0.12836094	0.29379254	0.16882404	0.62041574	0.36294634	0.13613991	0.33356595	0.27547439
160.4	0.12830609	0.29378703	0.16876975	0.6204416	0.36294612	0.13609003	0.33364459	0.27547396
160.5	0.12825132	0.29378162	0.16871552	0.62046745	0.36294612	0.13604022	0.33372317	0.27547353
160.6	0.12819664	0.29377628	0.16866135	0.62049327	0.36294633	0.13599051	0.33380168	0.27547311
160.7	0.12814205	0.29377104	0.16860725	0.62051908	0.36294676	0.13594088	0.33388013	0.2754727
160.8	0.12808754	0.29376588	0.16855321	0.62054488	0.3629474	0.13589133	0.33395852	0.2754723
160.9	0.12803311	0.2937608	0.16849923	0.62057065	0.36294826	0.13584188	0.33403685	0.27547191
161	0.12797876	0.2937558	0.16844532	0.62059641	0.36294933	0.1357925	0.33411512	0.27547152
161.1	0.1279245	0.2937509	0.16839148	0.62062215	0.36295061	0.13574322	0.33419332	0.27547114
161.2	0.12787032	0.29374607	0.1683377	0.62064787	0.36295209	0.13569401	0.33427146	0.27547076
161.3	0.12781623	0.29374133	0.16828398	0.62067357	0.36295378	0.1356449	0.33434955	0.2754704
161.4	0.12776221	0.29373667	0.16823033	0.62069926	0.36295568	0.13559587	0.33442757	0.27547004
161.5	0.12770828	0.29373209	0.16817674	0.62072493	0.36295778	0.13554692	0.33450552	0.27546969
161.6	0.12765444	0.2937276	0.16812322	0.62075058	0.36296008	0.13549806	0.33458342	0.27546934
161.7	0.12760067	0.29372318	0.16806976	0.62077621	0.36296259	0.13544928	0.33466126	0.27546901
161.8	0.12754699	0.29371885	0.16801636	0.62080183	0.36296529	0.13540059	0.33473903	0.27546868
161.9	0.12749339	0.29371461	0.16796303	0.62082743	0.36296819	0.13535198	0.33481674	0.27546836
162	0.12743987	0.29371044	0.16790976	0.62085301	0.36297129	0.13530346	0.33489439	0.27546804
162.1	0.12738644	0.29370635	0.16785655	0.62087857	0.36297458	0.13525502	0.33497198	0.27546774
162.2	0.12733308	0.29370235	0.16780341	0.62090412	0.36297807	0.13520667	0.33504951	0.27546744
162.3	0.12727981	0.29369843	0.16775034	0.62092965	0.36298174	0.1351584	0.33512698	0.27546714
162.4	0.12722662	0.29369458	0.16769732	0.62095516	0.36298561	0.13511022	0.33520438	0.27546686
162.5	0.12717351	0.29369082	0.16764437	0.62098065	0.36298967	0.13506212	0.33528173	0.27546658
162.6	0.12712048	0.29368714	0.16759149	0.62100613	0.36299392	0.1350141	0.33535901	0.27546631
162.7	0.12706753	0.29368354	0.16753867	0.62103159	0.36299835	0.13496617	0.33543623	0.27546604
162.8	0.12701466	0.29368002	0.16748591	0.62105703	0.36300296	0.13491833	0.33551339	0.27546579
162.9	0.12696187	0.29367657	0.16743321	0.62108246	0.36300776	0.13487056	0.33559049	0.27546554
163	0.12690917	0.29367321	0.16738058	0.62110786	0.36301274	0.13482288	0.33566752	0.27546529
163.1	0.12685654	0.29366992	0.16732801	0.62113325	0.36301791	0.13477529	0.3357445	0.27546506
163.2	0.126804	0.29366672	0.16727551	0.62115863	0.36302325	0.13472778	0.33582141	0.27546483
163.3	0.12675153	0.29366359	0.16722307	0.62118398	0.36302876	0.13468035	0.33589826	0.2754646
163.4	0.12669915	0.29366054	0.16717069	0.62120932	0.36303446	0.13463301	0.33597505	0.27546439
163.5	0.12664684	0.29365757	0.16711838	0.62123464	0.36304033	0.13458574	0.33605178	0.27546418
163.6	0.12659462	0.29365467	0.16706613	0.62125995	0.36304637	0.13453857	0.33612845	0.27546398
163.7	0.12654247	0.29365186	0.16701394	0.62128523	0.36305258	0.13449147	0.33620505	0.27546378
163.8	0.12649041	0.29364912	0.16696182	0.6213105	0.36305896	0.13444446	0.3362816	0.27546359
163.9	0.12643842	0.29364646	0.16690976	0.62133576	0.36306551	0.13439753	0.33635808	0.27546341
164	0.12638651	0.29364387	0.16685776	0.62136099	0.36307223	0.13435069	0.3364345	0.27546324
164.1	0.12633469	0.29364136	0.16680583	0.62138621	0.36307912	0.13430393	0.33651086	0.27546307
164.2	0.12628294	0.29363893	0.16675396	0.62141141	0.36308617	0.13425725	0.33658716	0.27546291
164.3	0.12623127	0.29363657	0.16670215	0.6214366	0.36309338	0.13421065	0.3366634	0.27546275
164.4	0.12617968	0.29363429	0.1666504	0.62146176	0.36310076	0.13416414	0.33673957	0.2754626
164.5	0.12612817	0.29363208	0.16659872	0.62148691	0.36310829	0.1341177	0.33681568	0.27546246
164.6	0.12607674	0.29362995	0.1665471	0.62151205	0.36311598	0.13407135	0.33689174	0.27546232
164.7	0.12602538	0.2936279	0.16649555	0.62153716	0.36312383	0.13402509	0.33696773	0.27546219
164.8	0.12597411	0.29362591	0.16644406	0.62156226	0.36313184	0.1339789	0.33704366	0.27546207
164.9	0.12592291	0.29362401	0.16639263	0.62158734	0.36314	0.1339328	0.33711952	0.27546195
210	0.10956644	0.29768579	0.1484556	0.63058834	0.37163125	0.11917414	0.36498732	0.2757247
210.1	0.10954201	0.29770132	0.14842429	0.63060351	0.37165293	0.11915117	0.36503621	0.27572558
210.2	0.10951761	0.29771686	0.148393	0.63061866	0.3716746	0.11912824	0.36508505	0.27572647
210.3	0.10949326	0.29773242	0.14836174	0.6306338	0.37169627	0.11910534	0.36513384	0.27572735
210.4	0.10946894	0.29774799	0.14833051	0.63064893	0.37171792	0.11908248	0.36518258	0.27572824

165.2	0.12576979	0.29361873	0.16623872	0.62166249	0.3631654	0.13379499	0.33734675	0.27546164
165.3	0.12571891	0.29361712	0.16618754	0.6216875	0.36317417	0.13374921	0.33742237	0.27546155
165.4	0.1256681	0.29361559	0.16613643	0.6217125	0.36318308	0.13370352	0.33749793	0.27546147
165.5	0.12561737	0.29361412	0.16608537	0.62173748	0.36319214	0.13365791	0.33757343	0.27546139
165.6	0.12556672	0.29361273	0.16603439	0.62176245	0.36320135	0.13361238	0.33764886	0.27546132
165.7	0.12551615	0.29361141	0.16598346	0.62178739	0.3632107	0.13356693	0.33772423	0.27546125
165.8	0.12546566	0.29361017	0.16593259	0.62181232	0.36322019	0.13352156	0.33779954	0.27546119
165.9	0.12541524	0.29360899	0.16588179	0.62183724	0.36322982	0.13347627	0.33787479	0.27546114
166	0.1253649	0.29360789	0.16583105	0.62186213	0.36323959	0.13343107	0.33794997	0.27546109
166.1	0.12531464	0.29360686	0.16578038	0.62188701	0.3632495	0.13338594	0.33802509	0.27546105
166.2	0.12526445	0.2936059	0.16572976	0.62191188	0.36325954	0.1333409	0.33810015	0.27546101
166.3	0.12521435	0.29360502	0.16567921	0.62193672	0.36326972	0.13329593	0.33817515	0.27546098
166.4	0.12516431	0.2936042	0.16562872	0.62196155	0.36328003	0.13325105	0.33825009	0.27546096
166.5	0.12511436	0.29360346	0.1655783	0.62198636	0.36329048	0.13320625	0.33832496	0.27546094
166.6	0.12506449	0.29360279	0.16552793	0.62201116	0.36330105	0.13316153	0.33839977	0.27546093
166.7	0.12501469	0.29360218	0.16547763	0.62203594	0.36331176	0.13311688	0.33847452	0.27546093
166.8	0.12496497	0.29360165	0.16542739	0.6220607	0.36332259	0.13307232	0.3385492	0.27546093
166.9	0.12491532	0.29360119	0.16537721	0.62208545	0.36333355	0.13302784	0.33862383	0.27546093
167	0.12486576	0.29360079	0.1653271	0.62211018	0.36334463	0.13298344	0.33869839	0.27546094
167.1	0.12481627	0.29360047	0.16527704	0.62213489	0.36335584	0.13293911	0.33877288	0.27546096
167.2	0.12476685	0.29360022	0.16522705	0.62215958	0.36336717	0.13289487	0.33884732	0.27546099
167.3	0.12471752	0.29360003	0.16517712	0.62218426	0.36337862	0.13285071	0.33892169	0.27546102
167.4	0.12466826	0.29359992	0.16512726	0.62220893	0.3633902	0.13280662	0.338996	0.27546105
167.5	0.12461908	0.29359987	0.16507745	0.62223357	0.36340189	0.13276262	0.33907024	0.27546109
167.6	0.12456997	0.29359989	0.16502771	0.6222582	0.3634137	0.13271869	0.33914443	0.27546114
167.7	0.12452095	0.29359998	0.16497803	0.62228281	0.36342563	0.13267484	0.33921855	0.27546119
167.8	0.12447199	0.29360014	0.16492841	0.62230741	0.36343767	0.13263108	0.3392926	0.27546125
167.9	0.12442312	0.29360036	0.16487885	0.62233199	0.36344983	0.13258739	0.3393666	0.27546131
168	0.12437432	0.29360066	0.16482935	0.62235655	0.3634621	0.13254377	0.33944053	0.27546138
168.1	0.1243256	0.29360102	0.16477991	0.6223811	0.36347448	0.13250024	0.33951439	0.27546145
168.2	0.12427695	0.29360144	0.16473054	0.62240562	0.36348697	0.13245679	0.3395882	0.27546153
168.3	0.12422838	0.29360194	0.16468123	0.62243014	0.36349958	0.13241341	0.33966194	0.27546162
168.4	0.12417988	0.2936025	0.16463198	0.62245463	0.36351228	0.13237011	0.33973561	0.27546171
168.5	0.12413147	0.29360313	0.16458279	0.62247911	0.3635251	0.13232689	0.33980923	0.27546181
168.6	0.12408312	0.29360383	0.16453366	0.62250358	0.36353802	0.13228375	0.33988278	0.27546191
168.7	0.12403486	0.29360459	0.16448459	0.62252802	0.36355105	0.13224069	0.33995626	0.27546201
168.8	0.12398667	0.29360541	0.16443559	0.62255246	0.36356418	0.1321977	0.34002969	0.27546212
168.9	0.12393855	0.29360631	0.16438664	0.62257687	0.36357741	0.13215479	0.34010305	0.27546224
169	0.12389051	0.29360726	0.16433776	0.62260127	0.36359074	0.13211196	0.34017634	0.27546236
169.1	0.12384255	0.29360829	0.16428894	0.62262565	0.36360417	0.1320692	0.34024958	0.27546249
169.2	0.12379466	0.29360938	0.16424017	0.62265001	0.3636177	0.13202652	0.34032274	0.27546262
169.3	0.12374685	0.29361053	0.16419147	0.62267436	0.36363133	0.13198392	0.34039585	0.27546276
169.4	0.12369911	0.29361175	0.16414283	0.6226987	0.36364506	0.13194139	0.34046889	0.2754629
169.5	0.12365145	0.29361303	0.16409425	0.62272301	0.36365888	0.13189895	0.34054187	0.27546305
169.6	0.12360386	0.29361438	0.16404574	0.62274731	0.36367279	0.13185657	0.34061478	0.2754632
169.7	0.12355635	0.29361579	0.16399728	0.6227716	0.3636868	0.13181428	0.34068763	0.27546336
169.8	0.12350892	0.29361726	0.16394888	0.62279586	0.3637009	0.13177206	0.34076042	0.27546352
169.9	0.12346156	0.2936188	0.16390054	0.62282012	0.36371509	0.13172991	0.34083314	0.27546369
170	0.12341427	0.2936204	0.16385227	0.62284435	0.36372937	0.13168785	0.3409058	0.27546386
170.1	0.12336706	0.29362207	0.16380405	0.62286857	0.36374374	0.13164585	0.34097839	0.27546404
170.2	0.12331992	0.2936238	0.1637559	0.62289277	0.3637582	0.13160394	0.34105092	0.27546422
170.3	0.12327286	0.29362559	0.1637078	0.62291696	0.36377274	0.13156209	0.34112338	0.27546441
170.4	0.12322588	0.29362744	0.16365977	0.62294113	0.36378737	0.13152033	0.34119578	0.2754646
170.5	0.12317896	0.29362936	0.16361179	0.62296528	0.36380209	0.13147864	0.34126812	0.2754648
170.6	0.12313213	0.29363133	0.16356388	0.62298942	0.36381688	0.13143702	0.34134039	0.275465
170.7	0.12308536	0.29363337	0.16351603	0.62301354	0.36383176	0.13139548	0.3414126	0.2754652
170.8	0.12303867	0.29363548	0.16346823	0.62303765	0.36384673	0.13135401	0.34148474	0.27546541
170.9	0.12299206	0.29363764	0.1634205	0.62306174	0.36386177	0.13131262	0.34155682	0.27546563
171	0.12294552	0.29363986	0.16337282	0.62308581	0.36387689	0.1312713	0.34162884	0.27546584
171.1	0.12289905	0.29364215	0.16332521	0.62310987	0.36389209	0.13123006	0.34170079	0.27546607
171.2	0.12285266	0.2936445	0.16327766	0.62313391	0.36390737	0.13118889	0.34177267	0.27546629
171.3	0.12280634	0.2936469	0.16323016	0.62315794	0.36392272	0.13114779	0.34184449	0.27546652
171.4	0.1227601	0.29364937	0.16318273	0.62318195	0.36393814	0.13110677	0.34191625	0.27546676
171.5	0.12271393	0.2936519	0.16313535	0.62320594	0.36395364	0.13106582	0.34198794	0.275467
171.6	0.12266783	0.29365449	0.16308804	0.62322992	0.36396922	0.13102494	0.34205957	0.27546724
171.7	0.12262181	0.29365714	0.16304078	0.62325388	0.36398486	0.13098414	0.34213113	0.27546749
171.8	0.12257586	0.29365984	0.16299358	0.62327783	0.36400057	0.13094341	0.34220263	0.27546774
171.9	0.12252999	0.29366261	0.16294645	0.62330176	0.36401636	0.13090275	0.34227407	0.275468
172	0.12248419	0.29366544	0.16289937	0.62332567	0.36403221	0.13086217	0.34234543	0.27546826
172.1	0.12243846	0.29366833	0.16285235	0.62334957	0.36404813	0.13082165	0.34241674	0.27546853
172.2	0.1223928	0.29367127	0.16280539	0.62337345	0.36406412	0.13078121	0.34248798	0.27546879
172.3	0.12234722	0.29367428	0.16275849	0.62339732	0.36408018	0.13074085	0.34255916	0.27546907
172.4	0.12230171	0.29367734	0.16271165	0.62342117	0.3640963	0.13070055	0.34263027	0.27546934
172.5	0.12225628	0.29368046	0.16266487	0.623445	0.36411248	0.13066033	0.34270132	0.27546962
172.6	0.12221092	0.29368364	0.16261814	0.62346882	0.36412873	0.13062017	0.3427723	0.27546991
172.7	0.12216563	0.29368688	0.16257148	0.62349262	0.36414504	0.13058009	0.34284321	0.27547019
172.8	0.12212041	0.29369017	0.16252487	0.62351641	0.36416141	0.13054008	0.34291407	0.27547048
172.9	0.12207527	0.29369353	0.16247833	0.62354018	0.36417784	0.13050014	0.34298486	0.27547078
173	0.1220302	0.29369694	0.16243184	0.62356394	0.36419433	0.13046027	0.34305558	0.27547108
173.1	0.1219852	0.29370041	0.16238541	0.62358768	0.36421088	0.13042048	0.34312624	0.27547138
173.2	0.12194028	0.29370393	0.16233904	0.6236114	0.36422749	0.13038075	0.34319683	0.27547169
173.3	0.12189542	0.29370751	0.16229272	0.62363511	0.36424416	0.13034109	0.34326736	0.275472
173.4	0.12185064	0.29371115	0.16224647	0.6236588	0.36426088	0.1303015	0.34333782	0.27547231
173.5	0.12180594	0.29371485	0.16220027	0.62368247	0.36427766	0.13026199	0.34340822	0.27547262
173.6	0.1217613	0.2937186	0.16215413	0.62370613	0.36429449	0.13022254	0.34347856	0.27547294
173.7	0.12171674	0.29372241	0.16210805	0.62372978	0.36431137	0.13018316	0.34354883	0.27547327
173.8	0.12167224	0.29372628	0.16206203	0.62375341	0.36432831	0.13014385	0.34361903	0.27547359
173.9	0.12162783	0.2937302	0.16201607	0.62377702	0.3643453	0.13010461	0.34368917	0.27547392
174	0.12158348	0.29373417	0.16197016	0.62380061	0.36436235	0.13006544	0.34375925	0.27547426
174.1	0.1215392	0.29373821	0.16192431	0.62382419	0.36437944	0.13002634	0.34382926	0.27547459
174.2	0.121495	0.29374229	0.16187852	0.62384776	0.36439658	0.12998731	0.3438992	0.27547493
174.3	0.12145087	0.29374644	0.16183279	0.62387131	0.36441377	0.12994834	0.34396909	0.27547528
174.4	0.12140681	0.29375063	0.16178712	0.62389484	0.36443101	0.12990945	0.3440389	0.27547562
174.5	0.12136282	0.29375489	0.1617415	0.62391835	0.3644483	0.12987062	0.34410865	0.27547597
174.6	0.1213189	0.29375919	0.16169594	0.62394185	0.36446564	0.12983186	0.34417834	0.27547633
174.7	0.12127505	0.29376355	0.16165044	0.62396534	0.36448302	0.12979316	0.34424796	0.27547668
174.8	0.12123128	0.29376797	0.16160499	0.6239888	0.36450044	0.12975454	0.34431752	0.27547704
174.9	0.12118757	0.29377244	0.1615596	0.62401226	0.36451791	0.12971598	0.34438701	0.2754774
175	0.12114394	0.29377696	0.16151427	0.62403569	0.36453543	0.12967748	0.34445643	0.27547777
175.1	0.12110038	0.29378154	0.161469	0.62405911	0.36455299	0.12963906	0.34452579	0.27547813
175.2	0.12105689	0.29378617	0.16142378	0.62408252	0.36457059	0.1296007	0.34459509	0.27547851
175.3	0.12101347	0.29379086	0.16137862	0.6241059	0.36458823	0.12956241	0.34466432	0.27547888
175.4	0.12097012	0.2937956	0.16133352	0.62412927	0.36460592	0.12952418	0.34473349	0.27547926
175.5	0.12092684	0.29380039	0.16128848	0.62415263	0.36462364	0.12948602	0.34480259	0.27547964
175.6	0.12088363	0.29380523	0.16124349	0.62417597	0.3646414	0.12944792	0.34487162	0.27548002
175.7	0.12084049	0.29381013	0.16119855	0.62419929	0.36465921	0.12940989	0.3449406	0.2754804
175.8	0.12079742	0.29381508	0.16115368	0.6242226	0.36467705	0.12937193	0.3450095	0.27548079
175.9	0.12075443	0.29382008	0.16110886	0.62424588	0.36469493	0.12933403	0.34507834	0.27548118
176	0.1207115	0.29382514	0.1610641	0.62426916	0.36471285	0.12929619	0.34514712	0.27548157
176.1	0.12066864	0.29383024	0.16101939	0.62429242	0.3647308	0.12925842	0.34521583	0.27548197
176.2	0.12062586	0.2938354	0.16097474	0.62431566	0.36474879	0.12922071	0.34528448	0.27548237
176.3	0.12058314	0.29384061	0.16093015	0.62433888	0.36476682	0.12918307	0.34535306	0.27548277
176.4	0.12054049	0.29384587	0.16088561	0.62436209	0.36478488	0.12914549	0.34542157	0.27548317
176.5	0.12049791	0.29385119	0.16084113	0.62438528	0.36480297	0.12910797	0.34549003	0.27548358
176.6	0.12045541	0.29385655	0.1607967	0.62440845	0.3648211	0.12907052	0.34555841	0.27548399
176.7	0.12041297	0.29386197	0.16075233	0.62443161	0.36483926	0.12903313	0.34562673	0.2754844
176.8	0.1203706	0.29386744	0.16070802	0.62445475	0.36485745	0.1289958	0.34569499	0.27548482
176.9	0.1203283	0.29387295	0.16066376	0.62447787	0.36487568	0.12895854	0.34576318	0.27548523
177	0.12028607	0.29387852	0.16061956	0.62450098	0.36489393	0.12892134	0.34583131	0.27548565
177.1	0.12024391	0.29388414	0.16057541	0.62452407	0.36491222	0.1288842	0.34589937	0.27548607
177.2	0.12020182	0.29388981	0.16053132	0.62454714	0.36493054	0.12884712	0.34596737	0.2754865
177.3	0.1201598	0.29389553	0.16048729	0.62457019	0.36494888	0.1288101	0.3460353	0.27548693
177.4	0.12011784	0.2939013	0.16044331	0.62459323	0.36496726	0.12877315	0.34610317	0.27548736
177.5	0.12007596	0.29390712	0.16039938	0.62461625	0.36498566	0.12873626	0.34617097	0.27548779
177.6	0.12003414	0.29391299	0.16035551	0.62463925	0.3650041	0.12869942	0.34623871	0.27548822
177.7	0.11999239	0.29391891	0.1603117	0.62466224	0.36502256	0.12866265	0.34630638	0.27548866
177.8	0.11995071	0.29392488	0.16026794	0.6246852	0.36504104	0.12862594	0.34637399	0.2754891
177.9	0.1199091	0.29393089	0.16022423	0.62470815	0.36505956	0.12858929	0.34644153	0.27548954
178	0.11986756	0.29393696	0.16018058	0.62473108	0.36507809	0.1285527	0.34650901	0.27548998
178.1	0.11982609	0.29394308	0.16013699	0.624754	0.36509666	0.12851617	0.34657642	0.27549043
178.2	0.11978468	0.29394924	0.16009345	0.62477689	0.36511525	0.1284797	0.34664377	0.27549088
178.3	0.11974334	0.29395545	0.16004996	0.62479976	0.36513387	0.12844329	0.34671105	0.27549133
178.4	0.11970207	0.29396171	0.16000653	0.62482262	0.36515251	0.12840693	0.34677827	0.27549178
178.5	0.11966087	0.29396802	0.15996316	0.62484546	0.36517117	0.12837064	0.34684542	0.27549224
178.6	0.11961974	0.29397438	0.15991983	0.62486827	0.36518986	0.1283344	0.34691251	0.2754927
178.7	0.11957867	0.29398079	0.15987657	0.62489107	0.36520857	0.12829823	0.34697954	0.27549316
178.8	0.11953767	0.29398724	0.15983335	0.62491385	0.3652273	0.12826211	0.3470465	0.27549362
178.9	0.11949674	0.29399374	0.15979019	0.62493661	0.36524606	0.12822605	0.34711339	0.27549409
179	0.11945588	0.29400029	0.15974709	0.62495935	0.36526483	0.12819004	0.34718022	0.27549455
179.1	0.11941508	0.29400689	0.15970404	0.62498206	0.36528363	0.1281541	0.34724699	0.27549502
179.2	0.11937435	0.29401353	0.15966104	0.62500476	0.36530245	0.12811821	0.34731369	0.2754955
179.3	0.11933369	0.29402022	0.15961809	0.62502743	0.3653213	0.12808238	0.34738033	0.27549597
179.4	0.11929309	0.29402696	0.1595752	0.62505008	0.36534016	0.1280466	0.3474469	0.27549645
179.5	0.11925257	0.29403374	0.15953237	0.62507271	0.36535904	0.12801089	0.3475134	0.27549693
179.6	0.1192121	0.29404057	0.15948958	0.62509532	0.36537794	0.12797522	0.34757985	0.27549741
179.7	0.11917171	0.29404745	0.15944685	0.6251179	0.36539686	0.12793962	0.34764622	0.27549789
179.8	0.11913138	0.29405437	0.15940418	0.62514046	0.3654158	0.12790407	0.34771254	0.27549838
179.9	0.11909112	0.29406134	0.15936155	0.625163	0.36543476	0.12786857	0.34777879	0.27549887
225	0.10630492	0.30013308	0.14402151	0.63273205	0.37471368	0.11611593	0.37179239	0.27586118
225.1	0.10628566	0.30014996	0.14399359	0.63274551	0.37473287	0.116098	0.37183435	0.27586212
225.2	0.10626643	0.30016686	0.1439657	0.63275896	0.37475204	0.1160801	0.37187627	0.27586305
225.3	0.10624723	0.30018375	0.14393783	0.63277241	0.37477119	0.11606223	0.37191814	0.27586399
225.4	0.10622806	0.30020065	0.14390998	0.63278584	0.37479033	0.1160444	0.37195998	0.27586493

180.5	0.11885095	0.29410412	0.15910693	0.62529767	0.36554889	0.12765676	0.34817492	0.27550184
180.6	0.11881115	0.29411141	0.15906468	0.62532002	0.36556798	0.12762165	0.34824072	0.27550235
180.7	0.11877142	0.29411875	0.15902248	0.62534233	0.36558708	0.12758659	0.34830645	0.27550285
180.8	0.11873175	0.29412613	0.15898033	0.62536462	0.36560619	0.12755159	0.34837212	0.27550336
180.9	0.11869215	0.29413355	0.15893823	0.62538688	0.36562532	0.12751664	0.34843772	0.27550387
181	0.11865262	0.29414102	0.15889619	0.62540911	0.36564447	0.12748174	0.34850326	0.27550439
181.1	0.11861315	0.29414853	0.1588542	0.6254313	0.36566363	0.1274469	0.34856873	0.2755049
181.2	0.11857375	0.29415609	0.15881226	0.62545347	0.36568281	0.1274121	0.34863414	0.27550542
181.3	0.11853441	0.29416369	0.15877037	0.6254756	0.365702	0.12737736	0.34869949	0.27550594
181.4	0.11849513	0.29417134	0.15872854	0.62549769	0.36572121	0.12734268	0.34876477	0.27550646
181.5	0.11845593	0.29417903	0.15868675	0.62551975	0.36574043	0.12730804	0.34882998	0.27550698
181.6	0.11841678	0.29418676	0.15864502	0.62554178	0.36575967	0.12727346	0.34889514	0.27550751
181.7	0.11837771	0.29419454	0.15860334	0.62556377	0.36577892	0.12723893	0.34896023	0.27550804
181.8	0.11833869	0.29420236	0.15856172	0.62558572	0.36579818	0.12720445	0.34902525	0.27550857
181.9	0.11829975	0.29421022	0.15852014	0.62560763	0.36581746	0.12717002	0.34909021	0.2755091
182	0.11826086	0.29421813	0.15847861	0.62562951	0.36583675	0.12713564	0.34915511	0.27550964
182.1	0.11822205	0.29422607	0.15843714	0.62565135	0.36585606	0.12710132	0.34921994	0.27551018
182.2	0.11818329	0.29423407	0.15839572	0.62567315	0.36587537	0.12706704	0.3492847	0.27551072
182.3	0.11814461	0.2942421	0.15835435	0.62569491	0.3658947	0.12703282	0.34934941	0.27551126
182.4	0.11810598	0.29425018	0.15831303	0.62571663	0.36591405	0.12699864	0.34941405	0.2755118
182.5	0.11806742	0.2942583	0.15827176	0.62573831	0.3659334	0.12696452	0.34947862	0.27551235
182.6	0.11802893	0.29426646	0.15823054	0.62575995	0.36595277	0.12693044	0.34954313	0.2755129
182.7	0.1179905	0.29427466	0.15818937	0.62578155	0.36597215	0.12689642	0.34960758	0.27551345
182.8	0.11795213	0.29428291	0.15814825	0.62580311	0.36599154	0.12686244	0.34967196	0.275514
182.9	0.11791383	0.2942912	0.15810719	0.62582462	0.36601095	0.12682852	0.34973628	0.27551456
183	0.1178756	0.29429953	0.15806617	0.6258461	0.36603036	0.12679464	0.34980054	0.27551512
183.1	0.11783742	0.2943079	0.1580252	0.62586753	0.36604979	0.12676081	0.34986473	0.27551568
183.2	0.11779931	0.29431631	0.15798429	0.62588893	0.36606923	0.12672703	0.34992886	0.27551624
183.3	0.11776127	0.29432477	0.15794342	0.62591028	0.36608868	0.1266933	0.34999292	0.2755168
183.4	0.11772328	0.29433326	0.15790261	0.62593158	0.36610814	0.12665962	0.35005692	0.27551737
183.5	0.11768536	0.2943418	0.15786184	0.62595285	0.36612762	0.12662599	0.35012085	0.27551794
183.6	0.11764751	0.29435038	0.15782113	0.62597407	0.3661471	0.1265924	0.35018473	0.27551851
183.7	0.11760972	0.29435899	0.15778046	0.62599525	0.3661666	0.12655886	0.35024854	0.27551908
183.8	0.11757199	0.29436765	0.15773985	0.62601639	0.3661861	0.12652537	0.35031228	0.27551966
183.9	0.11753432	0.29437635	0.15769928	0.62603749	0.36620562	0.12649193	0.35037596	0.27552024
184	0.11749672	0.29438509	0.15765876	0.62605854	0.36622515	0.12645854	0.35043958	0.27552082
184.1	0.11745918	0.29439387	0.1576183	0.62607955	0.36624468	0.12642519	0.35050313	0.2755214
184.2	0.1174217	0.29440269	0.15757788	0.62610052	0.36626423	0.12639189	0.35056662	0.27552199
184.3	0.11738429	0.29441155	0.15753751	0.62612144	0.36628379	0.12635863	0.35063005	0.27552257
184.4	0.11734694	0.29442045	0.15749719	0.62614233	0.36630336	0.12632543	0.35069342	0.27552316
184.5	0.11730965	0.29442939	0.15745692	0.62616317	0.36632293	0.12629227	0.35075672	0.27552375
184.6	0.11727242	0.29443837	0.1574167	0.62618396	0.36634252	0.12625915	0.35081995	0.27552435
184.7	0.11723526	0.29444738	0.15737652	0.62620472	0.36636212	0.12622609	0.35088313	0.27552494
184.8	0.11719815	0.29445644	0.1573364	0.62622543	0.36638173	0.12619307	0.35094624	0.27552554
184.9	0.11716111	0.29446554	0.15729632	0.62624611	0.36640135	0.12616009	0.35100929	0.27552614
185	0.11712414	0.29447467	0.1572563	0.62626674	0.36642097	0.12612716	0.35107227	0.27552675
185.1	0.11708722	0.29448385	0.15721632	0.62628733	0.36644061	0.12609428	0.3511352	0.27552735
185.2	0.11705037	0.29449306	0.15717639	0.62630788	0.36646026	0.12606144	0.35119806	0.27552796
185.3	0.11701358	0.29450231	0.1571365	0.62632838	0.36647991	0.12602865	0.35126085	0.27552857
185.4	0.11697684	0.2945116	0.15709667	0.62634885	0.36649958	0.1259959	0.35132359	0.27552918
185.5	0.11694018	0.29452093	0.15705688	0.62636927	0.36651925	0.1259632	0.35138626	0.27552979
185.6	0.11690357	0.2945303	0.15701714	0.62638966	0.36653894	0.12593054	0.35144887	0.27553041
185.7	0.11686702	0.2945397	0.15697745	0.62641	0.36655863	0.12589793	0.35151141	0.27553103
185.8	0.11683054	0.29454914	0.15693781	0.6264303	0.36657833	0.12586536	0.35157389	0.27553165
185.9	0.11679411	0.29455862	0.15689821	0.62645057	0.36659805	0.12583284	0.35163631	0.27553227
186	0.11675775	0.29456814	0.15685866	0.62647079	0.36661777	0.12580036	0.35169867	0.2755329
186.1	0.11672145	0.2945777	0.15681916	0.62649097	0.3666375	0.12576793	0.35176097	0.27553352
186.2	0.11668521	0.29458729	0.15677971	0.62651112	0.36665724	0.12573554	0.3518232	0.27553415
186.3	0.11664903	0.29459692	0.1567403	0.62653122	0.36667698	0.1257032	0.35188537	0.27553478
186.4	0.11661291	0.29460659	0.15670094	0.62655129	0.36669674	0.1256709	0.35194748	0.27553542
186.5	0.11657685	0.29461629	0.15666163	0.62657132	0.3667165	0.12563864	0.35200953	0.27553605
186.6	0.11654085	0.29462603	0.15662236	0.6265913	0.36673628	0.12560643	0.35207151	0.27553669
186.7	0.11650491	0.29463581	0.15658314	0.62661125	0.36675606	0.12557426	0.35213343	0.27553733
186.8	0.11646903	0.29464562	0.15654397	0.62663117	0.36677585	0.12554213	0.35219529	0.27553798
186.9	0.11643322	0.29465548	0.15650484	0.62665104	0.36679565	0.12551005	0.35225709	0.27553862
187	0.11639746	0.29466536	0.15646576	0.62667088	0.36681546	0.12547801	0.35231882	0.27553927
187.1	0.11636176	0.29467529	0.15642672	0.62669068	0.36683527	0.12544601	0.3523805	0.27553992
187.2	0.11632612	0.29468525	0.15638773	0.62671044	0.3668551	0.12541406	0.35244211	0.27554057
187.3	0.11629054	0.29469524	0.15634879	0.62673017	0.36687493	0.12538215	0.35250366	0.27554122
187.4	0.11625502	0.29470528	0.15630989	0.62674985	0.36689477	0.12535029	0.35256515	0.27554188
187.5	0.11621957	0.29471534	0.15627104	0.62676951	0.36691462	0.12531846	0.35262658	0.27554254
187.6	0.11618417	0.29472545	0.15623224	0.62678912	0.36693448	0.12528668	0.35268794	0.2755432
187.7	0.11614883	0.29473559	0.15619347	0.6268087	0.36695435	0.12525495	0.35274925	0.27554386
187.8	0.11611354	0.29474576	0.15615476	0.62682825	0.36697422	0.12522325	0.35281049	0.27554452
187.9	0.11607832	0.29475597	0.15611609	0.62684775	0.36699411	0.1251916	0.35287167	0.27554519
188	0.11604316	0.29476622	0.15607746	0.62686723	0.367014	0.12515999	0.35293279	0.27554586
188.1	0.11600805	0.2947765	0.15603888	0.62688666	0.3670339	0.12512842	0.35299385	0.27554653
188.2	0.11597301	0.29478681	0.15600035	0.62690607	0.36705381	0.12509689	0.35305485	0.27554721
188.3	0.11593802	0.29479716	0.15596185	0.62692543	0.36707373	0.12506541	0.35311579	0.27554788
188.4	0.11590309	0.29480755	0.15592341	0.62694477	0.36709366	0.12503397	0.35317666	0.27554856
188.5	0.11586823	0.29481797	0.155885	0.62696407	0.36711359	0.12500257	0.35323748	0.27554924
188.6	0.11583341	0.29482842	0.15584665	0.62698333	0.36713354	0.12497121	0.35329823	0.27554992
188.7	0.11579866	0.29483891	0.15580833	0.62700256	0.36715349	0.1249399	0.35335892	0.2755506
188.8	0.11576397	0.29484943	0.15577006	0.62702176	0.36717345	0.12490862	0.35341956	0.27555129
188.9	0.11572933	0.29485999	0.15573183	0.62704093	0.36719342	0.12487739	0.35348013	0.27555198
189	0.11569475	0.29487058	0.15569365	0.62706006	0.3672134	0.1248462	0.35354064	0.27555267
189.1	0.11566023	0.2948812	0.15565551	0.62707916	0.36723339	0.12481505	0.35360109	0.27555336
189.2	0.11562577	0.29489186	0.15561742	0.62709823	0.36725339	0.12478395	0.35366148	0.27555406
189.3	0.11559136	0.29490255	0.15557936	0.62711726	0.3672734	0.12475288	0.35372181	0.27555475
189.4	0.11555702	0.29491328	0.15554136	0.62713626	0.36729341	0.12472186	0.35378207	0.27555545
189.5	0.11552273	0.29492404	0.15550339	0.62715523	0.36731344	0.12469087	0.35384228	0.27555615
189.6	0.11548849	0.29493483	0.15546547	0.62717417	0.36733347	0.12465993	0.35390243	0.27555686
189.7	0.11545432	0.29494565	0.15542758	0.62719308	0.36735351	0.12462903	0.35396252	0.27555756
189.8	0.1154202	0.29495651	0.15538975	0.62721195	0.36737356	0.12459817	0.35402254	0.27555827
189.9	0.11538614	0.2949674	0.15535195	0.6272308	0.36739362	0.12456736	0.35408251	0.27555898
190	0.11535213	0.29497833	0.1553142	0.62724961	0.36741369	0.12453658	0.35414241	0.27555969
190.1	0.11531819	0.29498928	0.15527649	0.62726839	0.36743377	0.12450584	0.35420226	0.2755604
190.2	0.1152843	0.29500027	0.15523882	0.62728714	0.36745386	0.12447515	0.35426205	0.27556112
190.3	0.11525046	0.29501129	0.15520119	0.62730587	0.36747396	0.12444449	0.35432177	0.27556184
190.4	0.11521668	0.29502235	0.1551636	0.62732456	0.36749407	0.12441388	0.35438144	0.27556256
190.5	0.11518296	0.29503344	0.15512606	0.62734322	0.36751418	0.12438331	0.35444104	0.27556328
190.6	0.1151493	0.29504455	0.15508856	0.62736185	0.36753431	0.12435278	0.35450059	0.275564
190.7	0.11511569	0.2950557	0.1550511	0.62738046	0.36755445	0.12432229	0.35456007	0.27556473
190.8	0.11508214	0.29506689	0.15501368	0.62739903	0.36757459	0.12429184	0.3546195	0.27556545
190.9	0.11504864	0.2950781	0.1549763	0.62741758	0.36759475	0.12426143	0.35467887	0.27556618
191	0.1150152	0.29508935	0.15493896	0.62743609	0.36761491	0.12423106	0.35473817	0.27556692
191.1	0.11498181	0.29510063	0.15490166	0.62745458	0.36763509	0.12420073	0.35479742	0.27556765
191.2	0.11494848	0.29511193	0.1548644	0.62747304	0.36765527	0.12417044	0.35485661	0.27556838
191.3	0.11491521	0.29512328	0.15482719	0.62749147	0.36767547	0.12414019	0.35491574	0.27556912
191.4	0.11488199	0.29513465	0.15479001	0.62750987	0.36769567	0.12410999	0.35497481	0.27556986
191.5	0.11484883	0.29514605	0.15475287	0.62752825	0.36771589	0.12407982	0.35503381	0.2755706
191.6	0.11481572	0.29515748	0.15471578	0.6275466	0.36773611	0.1240497	0.35509276	0.27557134
191.7	0.11478267	0.29516895	0.15467872	0.62756492	0.36775635	0.12401961	0.35515166	0.27557209
191.8	0.11474967	0.29518045	0.1546417	0.62758321	0.36777659	0.12398956	0.35521049	0.27557284
191.9	0.11471673	0.29519197	0.15460473	0.62760148	0.36779685	0.12395956	0.35526926	0.27557359
192	0.11468384	0.29520353	0.15456779	0.62761972	0.36781712	0.12392959	0.35532797	0.27557434
192.1	0.11465101	0.29521512	0.15453089	0.62763793	0.36783739	0.12389967	0.35538663	0.27557509
192.2	0.11461823	0.29522673	0.15449403	0.62765611	0.36785768	0.12386979	0.35544522	0.27557584
192.3	0.11458551	0.29523838	0.15445721	0.62767427	0.36787797	0.12383994	0.35550376	0.2755766
192.4	0.11455284	0.29525006	0.15442043	0.62769241	0.36789828	0.12381014	0.35556224	0.27557736
192.5	0.11452023	0.29526177	0.15438369	0.62771051	0.3679186	0.12378037	0.35562066	0.27557812
192.6	0.11448767	0.29527351	0.15434698	0.62772859	0.36793892	0.12375065	0.35567902	0.27557888
192.7	0.11445516	0.29528528	0.15431031	0.62774665	0.36795926	0.12372097	0.35573732	0.27557964
192.8	0.11442271	0.29529707	0.15427369	0.62776468	0.36797961	0.12369132	0.35579557	0.2755804
192.9	0.11439031	0.2953089	0.1542371	0.62778269	0.36799996	0.12366172	0.35585375	0.27558117
193	0.11435797	0.29532076	0.15420054	0.62780067	0.36802033	0.12363215	0.35591188	0.27558194
193.1	0.11432568	0.29533265	0.15416403	0.62781862	0.36804071	0.12360263	0.35596995	0.27558271
193.2	0.11429344	0.29534456	0.15412755	0.62783655	0.3680611	0.12357315	0.35602796	0.27558348
193.3	0.11426126	0.29535651	0.15409111	0.62785446	0.36808149	0.1235437	0.35608591	0.27558425
193.4	0.11422913	0.29536848	0.15405471	0.62787234	0.3681019	0.1235143	0.3561438	0.27558503
193.5	0.11419705	0.29538048	0.15401835	0.6278902	0.36812232	0.12348494	0.35620164	0.27558581
193.6	0.11416503	0.29539252	0.15398202	0.62790803	0.36814275	0.12345561	0.35625941	0.27558658
193.7	0.11413306	0.29540458	0.15394573	0.62792584	0.36816319	0.12342633	0.35631713	0.27558736
193.8	0.11410114	0.29541667	0.15390947	0.62794362	0.36818364	0.12339708	0.35637479	0.27558815
193.9	0.11406928	0.29542879	0.15387326	0.62796139	0.3682041	0.12336788	0.3564324	0.27558893
194	0.11403747	0.29544093	0.15383707	0.62797912	0.36822457	0.12333871	0.35648994	0.27558971
194.1	0.11400571	0.29545311	0.15380093	0.62799684	0.36824506	0.12330959	0.35654743	0.2755905
194.2	0.11397401	0.29546531	0.15376482	0.62801453	0.36826555	0.1232805	0.35660486	0.27559129
194.3	0.11394235	0.29547754	0.15372875	0.6280322	0.36828606	0.12325146	0.35666223	0.27559208
194.4	0.11391075	0.2954898	0.15369271	0.62804985	0.36830657	0.12322245	0.35671955	0.27559287
194.5	0.11387921	0.29550209	0.15365671	0.62806747	0.3683271	0.12319348	0.3567768	0.27559366
194.6	0.11384771	0.2955144	0.15362074	0.62808507	0.36834764	0.12316456	0.356834	0.27559445
194.7	0.11381627	0.29552675	0.15358482	0.62810265	0.36836818	0.12313567	0.35689115	0.27559525
194.8	0.11378488	0.29553912	0.15354892	0.62812022	0.36838872	0.1231068	0.35694827	0.27559604
194.9	0.11375354	0.29555152	0.15351306	0.62813774	0.36840931	0.12307801	0.35700526	0.27559684
195.5	0.11356659	0.29562648	0.15329864	0.62824249	0.36853296	0.12290599	0.35734623	0.27560165
195.6	0.11353561	0.29563907	0.15326302	0.62825987	0.36855361	0.12287746	0.35740285	0.27560246
195.7	0.11350468	0.29565168	0.15322744	0.62827723	0.36857427	0.12284897	0.35745943	0.27560327
195.8	0.11347381	0.29566433	0.15319189	0.62829457	0.36859494	0.12282052	0.35751594	0.27560408
195.9	0.11344298	0.295677	0.15315638	0.62831189	0.36861562	0.12279211	0.3575724	0.27560489
196	0.11341221	0.29568969	0.1531209	0.62832919	0.36863631	0.12276373	0.3576288	0.2756057
196.1	0.11338149	0.29570241	0.15308545	0.62834647	0.36865702	0.1227354	0.35768514	0.27560651
196.2	0.11335081	0.29571516	0.15305004	0.62836373	0.36867773	0.1227071	0.35774143	0.27560733
196.3	0.11332019	0.29572794	0.15301466	0.62838097	0.36869846	0.12267885	0.35779766	0.27560814
196.4	0.11328962	0.29574074	0.15297932	0.62839818	0.3687192	0.12265063	0.35785383	0.27560896
196.5	0.1132591	0.29575356	0.15294401	0.62841538	0.36873995	0.12262246	0.35790995	0.27560977
196.6	0.11322863	0.29576642	0.15290873	0.62843256	0.36876071	0.12259432	0.35796601	0.27561059
196.7	0.11319821	0.2957793	0.15287348	0.62844972	0.36878149	0.12256622	0.35802202	0.27561141
196.8	0.11316784	0.2957922	0.15283827	0.62846686	0.36880227	0.12253816	0.35807796	0.27561223
196.9	0.11313752	0.29580513	0.15280309	0.62848398	0.36882307	0.12251014	0.35813385	0.27561305
197	0.11310725	0.29581809	0.15276794	0.62850108	0.36884388	0.12248216	0.35818969	0.27561387
197.1	0.11307703	0.29583107	0.15273283	0.62851816	0.3688647	0.12245422	0.35824547	0.2756147
197.2	0.11304686	0.29584407	0.15269775	0.62853522	0.36888553	0.12242632	0.35830119	0.27561552
197.3	0.11301674	0.29585711	0.1526627	0.62855226	0.36890637	0.12239846	0.35835686	0.27561634
197.4	0.11298667	0.29587016	0.15262769	0.62856928	0.36892723	0.12237064	0.35841247	0.27561717
197.5	0.11295665	0.29588324	0.1525927	0.62858629	0.36894809	0.12234285	0.35846803	0.27561799
197.6	0.11292668	0.29589635	0.15255775	0.62860327	0.36896897	0.12231511	0.35852353	0.27561882
197.7	0.11289676	0.29590948	0.15252283	0.62862024	0.36898986	0.12228741	0.35857897	0.27561965
197.8	0.11286689	0.29592264	0.15248794	0.62863719	0.36901076	0.12225974	0.35863436	0.27562047
197.9	0.11283706	0.29593582	0.15245309	0.62865412	0.36903168	0.12223211	0.35868969	0.2756213
198	0.11280729	0.29594903	0.15241827	0.62867103	0.3690526	0.12220453	0.35874497	0.27562213
198.1	0.11277757	0.29596226	0.15238348	0.62868792	0.36907354	0.12217698	0.35880019	0.27562296
198.2	0.11274789	0.29597551	0.15234872	0.62870479	0.36909449	0.12214947	0.35885535	0.27562379
198.3	0.11271826	0.29598879	0.15231399	0.62872165	0.36911545	0.122122	0.35891046	0.27562462
198.4	0.11268869	0.2960021	0.15227929	0.62873849	0.36913643	0.12209457	0.35896552	0.27562545
198.5	0.11265916	0.29601543	0.15224463	0.62875531	0.36915741	0.12206718	0.35902052	0.27562629
198.6	0.11262968	0.29602878	0.15221	0.62877211	0.36917841	0.12203983	0.35907546	0.27562712
198.7	0.11260025	0.29604215	0.15217539	0.6287889	0.36919942	0.12201251	0.35913035	0.27562795
198.8	0.11257086	0.29605555	0.15214082	0.62880567	0.36922044	0.12198524	0.35918518	0.27562878
198.9	0.11254153	0.29606898	0.15210629	0.62882242	0.36924147	0.12195801	0.35923996	0.27562962
199	0.11251224	0.29608242	0.15207178	0.62883915	0.36926251	0.12193081	0.35929469	0.27563045
199.1	0.112483	0.29609589	0.1520373	0.62885586	0.36928357	0.12190365	0.35934935	0.27563129
199.2	0.11245381	0.29610939	0.15200286	0.62887256	0.36930463	0.12187654	0.35940397	0.27563212
199.3	0.11242467	0.29612291	0.15196844	0.62888924	0.36932571	0.12184946	0.35945852	0.27563296
199.4	0.11239558	0.29613645	0.15193406	0.62890591	0.3693468	0.12182242	0.35951302	0.27563379
199.5	0.11236653	0.29615001	0.15189971	0.62892255	0.36936791	0.12179542	0.35956747	0.27563463
199.6	0.11233753	0.2961636	0.15186538	0.62893918	0.36938902	0.12176846	0.35962186	0.27563546
199.7	0.11230858	0.29617721	0.15183109	0.6289558	0.36941015	0.12174154	0.3596762	0.2756363
199.8	0.11227968	0.29619084	0.15179683	0.62897239	0.36943128	0.12171465	0.35973048	0.27563714
199.9	0.11225082	0.2962045	0.1517626	0.62898897	0.36945243	0.12168781	0.3597847	0.27563797
200	0.11222202	0.29621817	0.1517284	0.62900554	0.3694736	0.121661	0.35983887	0.27563881
200.1	0.11219326	0.29623188	0.15169423	0.62902208	0.36949477	0.12163424	0.35989299	0.27563965
200.2	0.11216454	0.2962456	0.1516601	0.62903861	0.36951596	0.12160751	0.35994705	0.27564049
200.3	0.11213588	0.29625934	0.15162599	0.62905513	0.36953715	0.12158082	0.36000106	0.27564133
200.4	0.11210726	0.29627311	0.15159191	0.62907163	0.36955836	0.12155417	0.36005501	0.27564216
200.5	0.11207868	0.2962869	0.15155786	0.62908811	0.36957958	0.12152756	0.3601089	0.275643
200.6	0.11205016	0.29630072	0.15152385	0.62910457	0.36960081	0.12150099	0.36016275	0.27564384
200.7	0.11202168	0.29631455	0.15148986	0.62912102	0.36962206	0.12147446	0.36021653	0.27564468
200.8	0.11199325	0.29632841	0.1514559	0.62913746	0.36964331	0.12144797	0.36027026	0.27564552
200.9	0.11196486	0.29634229	0.15142197	0.62915388	0.36966458	0.12142151	0.36032394	0.27564636
201	0.11193652	0.29635619	0.15138808	0.62917028	0.36968586	0.12139509	0.36037757	0.2756472
201.1	0.11190823	0.29637011	0.15135421	0.62918666	0.36970715	0.12136872	0.36043114	0.27564804
201.2	0.11187999	0.29638405	0.15132037	0.62920303	0.36972845	0.12134238	0.36048465	0.27564888
201.3	0.11185179	0.29639802	0.15128657	0.62921939	0.36974977	0.12131608	0.36053811	0.27564972
201.4	0.11182363	0.29641201	0.15125279	0.62923573	0.36977109	0.12128982	0.36059152	0.27565056
201.5	0.11179553	0.29642601	0.15121904	0.62925205	0.36979243	0.12126359	0.36064487	0.2756514
201.6	0.11176747	0.29644004	0.15118532	0.62926836	0.36981378	0.12123741	0.36069817	0.27565224
201.7	0.11173945	0.29645409	0.15115164	0.62928466	0.36983514	0.12121127	0.36075141	0.27565308
201.8	0.11171148	0.29646817	0.15111798	0.62930094	0.36985651	0.12118516	0.3608046	0.27565392
201.9	0.11168356	0.29648226	0.15108435	0.6293172	0.36987789	0.12115909	0.36085774	0.27565476
202	0.11165568	0.29649637	0.15105075	0.62933345	0.36989928	0.12113306	0.36091082	0.2756556
202.1	0.11162785	0.29651051	0.15101718	0.62934968	0.36992069	0.12110707	0.36096385	0.27565644
202.2	0.11160006	0.29652466	0.15098364	0.6293659	0.3699421	0.12108112	0.36101682	0.27565729
202.3	0.11157232	0.29653884	0.15095013	0.6293821	0.36996353	0.12105521	0.36106974	0.27565813
202.4	0.11154462	0.29655304	0.15091665	0.62939829	0.36998497	0.12102933	0.36112261	0.27565897
202.5	0.11151697	0.29656725	0.15088319	0.62941446	0.37000642	0.1210035	0.36117542	0.27565981
202.6	0.11148937	0.29658149	0.15084977	0.62943062	0.37002788	0.1209777	0.36122818	0.27566066
202.7	0.11146181	0.29659575	0.15081638	0.62944676	0.37004934	0.12095194	0.36128089	0.2756615
202.8	0.11143429	0.29661003	0.15078301	0.62946289	0.37007082	0.12092622	0.36133354	0.27566235
202.9	0.11140682	0.29662433	0.15074968	0.629479	0.37009231	0.12090054	0.36138614	0.27566319
203	0.1113794	0.29663864	0.15071637	0.6294951	0.3701138	0.12087489	0.36143868	0.27566404
203.1	0.11135202	0.29665298	0.1506831	0.62951119	0.37013531	0.12084929	0.36149118	0.27566489
203.2	0.11132468	0.29666734	0.15064985	0.62952726	0.37015682	0.12082372	0.36154361	0.27566573
203.3	0.11129739	0.29668172	0.15061663	0.62954331	0.37017835	0.12079819	0.361596	0.27566658
203.4	0.11127014	0.29669612	0.15058344	0.62955935	0.37019988	0.1207727	0.36164833	0.27566743
203.5	0.11124294	0.29671053	0.15055028	0.62957538	0.37022142	0.12074725	0.36170061	0.27566828
203.6	0.11121578	0.29672497	0.15051715	0.62959139	0.37024296	0.12072184	0.36175284	0.27566913
203.7	0.11118867	0.29673943	0.15048405	0.62960739	0.37026452	0.12069646	0.36180501	0.27566998
203.8	0.1111616	0.2967539	0.15045097	0.62962338	0.37028608	0.12067113	0.36185713	0.27567083
203.9	0.11113457	0.2967684	0.15041793	0.62963935	0.37030765	0.12064583	0.3619092	0.27567168
204	0.11110759	0.29678291	0.15038491	0.6296553	0.37032923	0.12062057	0.36196122	0.27567253
204.1	0.11108065	0.29679745	0.15035192	0.62967124	0.37035082	0.12059535	0.36201318	0.27567338
204.2	0.11105376	0.296812	0.15031897	0.62968717	0.37037242	0.12057016	0.36206509	0.27567424
204.3	0.11102691	0.29682657	0.15028604	0.62970308	0.37039402	0.12054502	0.36211694	0.27567509
204.4	0.1110001	0.29684116	0.15025313	0.62971898	0.37041563	0.12051991	0.36216875	0.27567595
204.5	0.11097334	0.29685577	0.15022026	0.62973487	0.37043724	0.12049484	0.3622205	0.2756768
204.6	0.11094662	0.2968704	0.15018742	0.62975074	0.37045887	0.12046981	0.3622722	0.27567766
204.7	0.11091995	0.29688505	0.1501546	0.6297666	0.3704805	0.12044482	0.36232384	0.27567851
204.8	0.11089332	0.29689971	0.15012182	0.62978245	0.37050214	0.12041987	0.36237544	0.27567937
204.9	0.11086673	0.2969144	0.15008906	0.62979828	0.37052378	0.12039495	0.36242698	0.27568023
205	0.11084018	0.2969291	0.15005633	0.62981409	0.37054543	0.12037007	0.36247847	0.27568109
205.1	0.11081368	0.29694382	0.15002363	0.6298299	0.37056709	0.12034523	0.36252991	0.27568195
205.2	0.11078722	0.29695856	0.14999096	0.62984569	0.37058875	0.12032043	0.36258129	0.27568281
205.3	0.11076081	0.29697332	0.14995831	0.62986147	0.37061042	0.12029567	0.36263262	0.27568367
205.4	0.11073443	0.29698809	0.1499257	0.62987723	0.37063209	0.12027094	0.3626839	0.27568453
205.5	0.1107081	0.29700288	0.14989311	0.62989298	0.37065377	0.12024625	0.36273513	0.27568539
205.6	0.11068182	0.29701769	0.14986055	0.62990872	0.37067545	0.1202216	0.36278631	0.27568625
205.7	0.11065557	0.29703252	0.14982802	0.62992444	0.37069714	0.12019699	0.36283743	0.27568711
205.8	0.11062937	0.29704737	0.14979552	0.62994015	0.37071884	0.12017241	0.3628885	0.27568798
205.9	0.11060321	0.29706223	0.14976304	0.62995584	0.37074054	0.12014788	0.36293952	0.27568884
206	0.11057709	0.29707711	0.1497306	0.62997153	0.37076224	0.12012338	0.36299049	0.27568971
206.1	0.11055102	0.29709201	0.14969818	0.6299872	0.37078395	0.12009891	0.36304141	0.27569057
206.2	0.11052499	0.29710693	0.14966579	0.63000285	0.37080566	0.12007449	0.36309227	0.27569144
206.3	0.110499	0.29712186	0.14963343	0.6300185	0.37082737	0.12005011	0.36314309	0.2756923
206.4	0.11047305	0.29713681	0.1496011	0.63003413	0.37084909	0.12002576	0.36319385	0.27569317
206.5	0.11044714	0.29715178	0.14956879	0.63004975	0.37087081	0.12000145	0.36324456	0.27569404
206.6	0.11042128	0.29716676	0.14953651	0.63006535	0.37089253	0.11997718	0.36329522	0.2756949
206.7	0.11039546	0.29718177	0.14950427	0.63008094	0.37091426	0.11995294	0.36334582	0.27569577
206.8	0.11036968	0.29719678	0.14947204	0.63009652	0.37093599	0.11992874	0.36339638	0.27569664
206.9	0.11034394	0.29721182	0.14943985	0.63011209	0.37095772	0.11990458	0.36344688	0.27569751
207	0.11031825	0.29722687	0.14940769	0.63012764	0.37097946	0.11988046	0.36349734	0.27569838
207.1	0.11029259	0.29724194	0.14937555	0.63014318	0.37100119	0.11985638	0.36354774	0.27569925
207.2	0.11026698	0.29725703	0.14934344	0.6301587	0.37102293	0.11983233	0.36359809	0.27570012
207.3	0.11024141	0.29727213	0.14931136	0.63017422	0.37104467	0.11980832	0.36364839	0.27570099
207.4	0.11021588	0.29728725	0.14927931	0.63018972	0.37106641	0.11978435	0.36369864	0.27570187
207.5	0.11019039	0.29730238	0.14924728	0.63020521	0.37108815	0.11976042	0.36374883	0.27570274
207.6	0.11016494	0.29731753	0.14921528	0.63022068	0.37110989	0.11973652	0.36379898	0.27570361
207.7	0.11013954	0.2973327	0.14918331	0.63023615	0.37113163	0.11971266	0.36384908	0.27570449
207.8	0.11011417	0.29734788	0.14915137	0.6302516	0.37115338	0.11968884	0.36389912	0.27570536
207.9	0.11008885	0.29736308	0.14911946	0.63026703	0.37117512	0.11966505	0.36394911	0.27570623
208	0.11006357	0.29737829	0.14908757	0.63028246	0.37119686	0.1196413	0.36399906	0.27570711
208.1	0.11003833	0.29739352	0.14905571	0.63029787	0.3712186	0.11961759	0.36404895	0.27570798
208.2	0.11001313	0.29740877	0.14902388	0.63031327	0.37124034	0.11959392	0.36409879	0.27570886
208.3	0.10998797	0.29742403	0.14899208	0.63032866	0.37126208	0.11957028	0.36414858	0.27570974
208.4	0.10996285	0.29743931	0.1489603	0.63034403	0.37128382	0.11954669	0.36419832	0.27571061
208.5	0.10993777	0.2974546	0.14892855	0.6303594	0.37130556	0.11952312	0.36424801	0.27571149
208.6	0.10991274	0.29746991	0.14889683	0.63037475	0.37132729	0.1194996	0.36429765	0.27571237
208.7	0.10988774	0.29748523	0.14886514	0.63039008	0.37134903	0.11947611	0.36434724	0.27571325
208.8	0.10986278	0.29750057	0.14883347	0.63040541	0.37137076	0.11945266	0.36439677	0.27571412
208.9	0.10983787	0.29751592	0.14880183	0.63042072	0.37139249	0.11942925	0.36444626	0.275715
209	0.10981299	0.29753129	0.14877022	0.63043602	0.37141421	0.11940587	0.3644957	0.27571588
209.1	0.10978816	0.29754668	0.14873863	0.63045131	0.37143594	0.11938254	0.36454509	0.27571676
209.2	0.10976336	0.29756207	0.14870708	0.63046658	0.37145765	0.11935923	0.36459442	0.27571764
209.3	0.10973861	0.29757749	0.14867555	0.63048185	0.37147937	0.11933597	0.36464371	0.27571852
209.4	0.10971389	0.29759292	0.14864404	0.6304971	0.37150108	0.11931274	0.36469295	0.2757194
209.5	0.10968922	0.29760836	0.14861257	0.63051234	0.37152279	0.11928955	0.36474213	0.27572028
209.6	0.10966458	0.29762381	0.14858112	0.63052756	0.37154449	0.11926639	0.36479127	0.27572117
209.7	0.10963999	0.29763929	0.1485497	0.63054278	0.37156619	0.11924328	0.36484036	0.27572205
209.8	0.10961543	0.29765477	0.14851831	0.63055798	0.3715879	0.1192	0.36488944	0.27572294
209.9	0.10959092	0.29767027	0.14848694	0.63057317	0.37160957	0.11919715	0.36493838	0.27572381
210.5	0.10944466	0.29776357	0.1482993	0.63066404	0.37173957	0.11905965	0.36523127	0.27572912
210.6	0.10942042	0.29777917	0.14826812	0.63067915	0.37176122	0.11903686	0.36527991	0.27573001
210.7	0.10939622	0.29779478	0.14823697	0.63069424	0.37178285	0.11901411	0.3653285	0.27573089
210.8	0.10937206	0.29781041	0.14820584	0.63070932	0.37180448	0.1189914	0.36537704	0.27573178
210.9	0.10934794	0.29782604	0.14817474	0.63072439	0.3718261	0.11896872	0.36542554	0.27573267
211	0.10932386	0.2978417	0.14814367	0.63073944	0.37184771	0.11894607	0.36547398	0.27573355
211.1	0.10929981	0.29785736	0.14811262	0.63075448	0.37186931	0.11892347	0.36552237	0.27573444
211.2	0.10927581	0.29787304	0.1480816	0.63076952	0.3718909	0.1189009	0.36557072	0.27573533
211.3	0.10925184	0.29788873	0.14805061	0.63078454	0.37191249	0.11887836	0.36561902	0.27573622
211.4	0.10922791	0.29790444	0.14801964	0.63079954	0.37193407	0.11885586	0.36566727	0.27573711
211.5	0.10920402	0.29792016	0.1479887	0.63081454	0.37195563	0.1188334	0.36571546	0.275738
211.6	0.10918017	0.29793589	0.14795779	0.63082952	0.37197719	0.11881097	0.36576361	0.27573889
211.7	0.10915636	0.29795163	0.1479269	0.63084449	0.37199873	0.11878858	0.36581172	0.27573978
211.8	0.10913259	0.29796739	0.14789603	0.63085945	0.37202027	0.11876623	0.36585977	0.27574067
211.9	0.10910885	0.29798316	0.1478652	0.6308744	0.37204179	0.11874391	0.36590777	0.27574156
212	0.10908516	0.29799894	0.14783438	0.63088933	0.3720633	0.11872163	0.36595573	0.27574245
212.1	0.1090615	0.29801474	0.1478036	0.63090426	0.37208481	0.11869938	0.36600363	0.27574334
212.2	0.10903788	0.29803055	0.14777284	0.63091917	0.3721063	0.11867717	0.36605149	0.27574423
212.3	0.10901429	0.29804637	0.1477421	0.63093407	0.37212777	0.118655	0.3660993	0.27574513
212.4	0.10899075	0.2980622	0.14771139	0.63094895	0.37214924	0.11863286	0.36614706	0.27574602
212.5	0.10896724	0.29807804	0.14768071	0.63096383	0.3721707	0.11861076	0.36619478	0.27574691
212.6	0.10894377	0.2980939	0.14765005	0.63097869	0.37219214	0.11858869	0.36624244	0.27574781
212.7	0.10892034	0.29810977	0.14761942	0.63099354	0.37221357	0.11856666	0.36629006	0.2757487
212.8	0.10889695	0.29812565	0.14758881	0.63100838	0.37223499	0.11854466	0.36633763	0.27574959
212.9	0.10887359	0.29814154	0.14755823	0.63102321	0.37225639	0.1185227	0.36638515	0.27575049
213	0.10885027	0.29815745	0.14752767	0.63103803	0.37227778	0.11850077	0.36643262	0.27575138
213.1	0.10882699	0.29817337	0.14749714	0.63105283	0.37229916	0.11847889	0.36648004	0.27575228
213.2	0.10880375	0.2981893	0.14746663	0.63106762	0.37232052	0.11845703	0.36652742	0.27575317
213.3	0.10878054	0.29820524	0.14743614	0.6310824	0.37234187	0.11843521	0.36657475	0.27575407
213.4	0.10875737	0.29822119	0.14740568	0.63109717	0.3723632	0.11841343	0.36662203	0.27575497
213.5	0.10873424	0.29823715	0.14737525	0.63111193	0.37238452	0.11839168	0.36666926	0.27575586
213.6	0.10871115	0.29825313	0.14734484	0.63112667	0.37240583	0.11836997	0.36671644	0.27575676
213.7	0.10868809	0.29826911	0.14731445	0.6311414	0.37242712	0.11834829	0.36676358	0.27575766
213.8	0.10866507	0.29828511	0.14728409	0.63115612	0.37244839	0.11832665	0.36681067	0.27575856
213.9	0.10864209	0.29830112	0.14725375	0.63117083	0.37246965	0.11830504	0.36685771	0.27575945
214	0.10861914	0.29831714	0.14722344	0.63118553	0.3724909	0.11828347	0.3669047	0.27576035
214.1	0.10859623	0.29833317	0.14719315	0.63120021	0.37251213	0.11826194	0.36695164	0.27576125
214.2	0.10857336	0.29834922	0.14716288	0.63121489	0.37253334	0.11824044	0.36699854	0.27576215
214.3	0.10855052	0.29836527	0.14713264	0.63122955	0.37255453	0.11821897	0.36704539	0.27576305
214.4	0.10852772	0.29838133	0.14710242	0.6312442	0.37257571	0.11819754	0.36709219	0.27576395
214.5	0.10850496	0.29839741	0.14707223	0.63125883	0.37259687	0.11817614	0.36713894	0.27576485
214.6	0.10848223	0.2984135	0.14704205	0.63127346	0.37261802	0.11815478	0.36718565	0.27576575
214.7	0.10845954	0.29842959	0.1470119	0.63128807	0.37263915	0.11813346	0.36723231	0.27576665
214.8	0.10843689	0.2984457	0.14698178	0.63130267	0.37266026	0.11811216	0.36727892	0.27576756
214.9	0.10841427	0.29846182	0.14695167	0.63131726	0.37268135	0.11809091	0.36732549	0.27576846
215	0.10839169	0.29847794	0.14692159	0.63133184	0.37270243	0.11806969	0.367372	0.27576936
215.1	0.10836914	0.29849408	0.14689153	0.63134641	0.37272349	0.1180485	0.36741847	0.27577026
215.2	0.10834663	0.29851023	0.1468615	0.63136096	0.37274453	0.11802735	0.3674649	0.27577116
215.3	0.10832416	0.29852639	0.14683149	0.6313755	0.37276556	0.11800623	0.36751127	0.27577207
215.4	0.10830172	0.29854256	0.14680149	0.63139003	0.37278656	0.11798515	0.3675576	0.27577297
215.5	0.10827932	0.29855874	0.14677153	0.63140455	0.37280755	0.1179641	0.36760388	0.27577387
215.6	0.10825696	0.29857493	0.14674158	0.63141906	0.37282852	0.11794308	0.36765012	0.27577478
215.7	0.10823463	0.29859113	0.14671166	0.63143355	0.37284947	0.11792211	0.36769631	0.27577568
215.8	0.10821233	0.29860734	0.14668176	0.63144804	0.37287041	0.11790116	0.36774245	0.27577658
215.9	0.10819007	0.29862356	0.14665188	0.63146251	0.37289132	0.11788025	0.36778854	0.27577749
216	0.10816785	0.29863978	0.14662202	0.63147697	0.37291222	0.11785938	0.36783459	0.27577839
216.1	0.10814566	0.29865602	0.14659218	0.63149142	0.37293309	0.11783853	0.36788059	0.2757793
216.2	0.10812351	0.29867227	0.14656237	0.63150585	0.37295395	0.11781773	0.36792654	0.2757802
216.3	0.10810139	0.29868853	0.14653258	0.63152028	0.37297479	0.11779695	0.36797245	0.27578111
216.4	0.10807931	0.2987048	0.1465028	0.63153469	0.37299561	0.11777622	0.36801831	0.27578202
216.5	0.10805726	0.29872107	0.14647306	0.63154909	0.37301641	0.11775551	0.36806413	0.27578292
216.6	0.10803525	0.29873736	0.14644333	0.63156348	0.37303719	0.11773484	0.3681099	0.27578383
216.7	0.10801328	0.29875365	0.14641362	0.63157785	0.37305795	0.11771421	0.36815562	0.27578474
216.8	0.10799134	0.29876996	0.14638394	0.63159222	0.37307869	0.1176936	0.36820129	0.27578564
216.9	0.10796943	0.29878627	0.14635427	0.63160657	0.37309942	0.11767304	0.36824692	0.27578655
217	0.10794756	0.29880259	0.14632463	0.63162092	0.37312012	0.1176525	0.36829251	0.27578746
217.1	0.10792572	0.29881893	0.14629501	0.63163525	0.37314081	0.117632	0.36833804	0.27578837
217.2	0.10790392	0.29883527	0.14626541	0.63164956	0.37316147	0.11761154	0.36838353	0.27578927
217.3	0.10788215	0.29885162	0.14623583	0.63166387	0.37318211	0.11759111	0.36842898	0.27579018
217.4	0.10786042	0.29886797	0.14620627	0.63167817	0.37320274	0.11757071	0.36847438	0.27579109
217.5	0.10783872	0.29888434	0.14617673	0.63169245	0.37322335	0.11755034	0.36851973	0.275792
217.6	0.10781706	0.29890072	0.14614722	0.63170672	0.37324393	0.11753001	0.36856504	0.27579291
217.7	0.10779543	0.2989171	0.14611772	0.63172098	0.3732645	0.11750971	0.3686103	0.27579382
217.8	0.10777383	0.2989335	0.14608825	0.63173523	0.37328504	0.11748945	0.36865552	0.27579473
217.9	0.10775227	0.2989499	0.1460588	0.63174947	0.37330557	0.11746922	0.36870068	0.27579564
218	0.10773074	0.29896631	0.14602936	0.6317637	0.37332608	0.11744902	0.36874581	0.27579655
218.1	0.10770925	0.29898273	0.14599995	0.63177791	0.37334656	0.11742886	0.36879088	0.27579746
218.2	0.10768779	0.29899915	0.14597056	0.63179211	0.37336703	0.11740873	0.36883592	0.27579838
218.3	0.10766636	0.29901559	0.14594119	0.6318063	0.37338748	0.11738863	0.3688809	0.27579929
218.4	0.10764497	0.29903203	0.14591184	0.63182048	0.3734079	0.11736857	0.36892584	0.2758002
218.5	0.10762362	0.29904849	0.14588251	0.63183465	0.37342831	0.11734854	0.36897073	0.27580111
218.6	0.10760229	0.29906495	0.1458532	0.63184881	0.3734487	0.11732854	0.36901558	0.27580203
218.7	0.107581	0.29908142	0.14582392	0.63186295	0.37346907	0.11730858	0.36906038	0.27580294
218.8	0.10755974	0.29909789	0.14579466	0.63187709	0.37348941	0.11728865	0.36910514	0.27580385
218.9	0.10753852	0.29911438	0.14576541	0.63189121	0.37350974	0.11726875	0.36914984	0.27580477
219	0.10751733	0.29913087	0.14573619	0.63190532	0.37353005	0.11724889	0.36919451	0.27580568
219.1	0.10749617	0.29914737	0.14570698	0.63191942	0.37355033	0.11722906	0.36923913	0.2758066
219.2	0.10747505	0.29916388	0.1456778	0.63193351	0.3735706	0.11720926	0.3692837	0.27580751
219.3	0.10745396	0.29918039	0.14564864	0.63194758	0.37359085	0.1171895	0.36932823	0.27580843
219.4	0.1074329	0.29919692	0.1456195	0.63196165	0.37361108	0.11716976	0.36937271	0.27580934
219.5	0.10741188	0.29921345	0.14559038	0.6319757	0.37363128	0.11715007	0.36941714	0.27581026
219.6	0.10739089	0.29922999	0.14556128	0.63198975	0.37365147	0.1171304	0.36946153	0.27581118
219.7	0.10736993	0.29924654	0.1455322	0.63200378	0.37367164	0.11711077	0.36950588	0.27581209
219.8	0.10734901	0.29926309	0.14550315	0.6320178	0.37369179	0.11709116	0.36955018	0.27581301
219.9	0.10732812	0.29927965	0.14547411	0.63203181	0.37371192	0.1170716	0.36959443	0.27581393
220	0.10730726	0.29929622	0.14544509	0.63204581	0.37373203	0.11705206	0.36963864	0.27581484
220.1	0.10728643	0.2993128	0.1454161	0.6320598	0.37375212	0.11703256	0.36968281	0.27581576
220.2	0.10726564	0.29932938	0.14538713	0.63207377	0.37377219	0.11701309	0.36972692	0.27581668
220.3	0.10724488	0.29934597	0.14535817	0.63208774	0.37379224	0.11699365	0.369771	0.2758176
220.4	0.10722415	0.29936257	0.14532924	0.63210169	0.37381227	0.11697424	0.36981503	0.27581852
220.5	0.10720346	0.29937918	0.14530033	0.63211563	0.37383228	0.11695487	0.36985901	0.27581944
220.6	0.1071828	0.29939579	0.14527144	0.63212957	0.37385227	0.11693553	0.36990295	0.27582036
220.7	0.10716216	0.29941241	0.14524257	0.63214349	0.37387225	0.11691622	0.36994684	0.27582128
220.8	0.10714157	0.29942904	0.14521373	0.6321574	0.3738922	0.11689695	0.36999069	0.2758222
220.9	0.107121	0.29944567	0.1451849	0.6321713	0.37391214	0.1168777	0.37003449	0.27582312
221	0.10710047	0.29946231	0.14515609	0.63218519	0.37393205	0.11685849	0.37007825	0.27582404
221.1	0.10707997	0.29947896	0.14512731	0.63219906	0.37395195	0.11683931	0.37012197	0.27582496
221.2	0.1070595	0.29949561	0.14509855	0.63221293	0.37397182	0.11682017	0.37016564	0.27582589
221.3	0.10703906	0.29951227	0.1450698	0.63222678	0.37399168	0.11680105	0.37020926	0.27582681
221.4	0.10701866	0.29952894	0.14504108	0.63224063	0.37401152	0.11678197	0.37025284	0.27582773
221.5	0.10699828	0.29954561	0.14501238	0.63225446	0.37403134	0.11676292	0.37029638	0.27582866
221.6	0.10697794	0.2995623	0.14498371	0.63226829	0.37405114	0.1167439	0.37033987	0.27582958
221.7	0.10695763	0.29957898	0.14495505	0.6322821	0.37407093	0.11672491	0.37038331	0.2758305
221.8	0.10693736	0.29959568	0.14492641	0.6322959	0.37409069	0.11670595	0.37042672	0.27583143
221.9	0.10691711	0.29961238	0.1448978	0.63230969	0.37411044	0.11668703	0.37047007	0.27583235
222	0.1068969	0.29962908	0.14486921	0.63232347	0.37413016	0.11666814	0.37051339	0.27583328
222.1	0.10687671	0.2996458	0.14484064	0.63233724	0.37414987	0.11664928	0.37055666	0.2758342
222.2	0.10685656	0.29966252	0.14481209	0.632351	0.37416956	0.11663045	0.37059988	0.27583513
222.3	0.10683644	0.29967924	0.14478356	0.63236475	0.37418924	0.11661165	0.37064306	0.27583606
222.4	0.10681635	0.29969598	0.14475505	0.63237849	0.37420889	0.11659289	0.3706862	0.27583698
222.5	0.1067963	0.29971271	0.14472657	0.63239222	0.37422853	0.11657416	0.37072929	0.27583791
222.6	0.10677627	0.29972946	0.1446981	0.63240593	0.37424814	0.11655545	0.37077234	0.27583884
222.7	0.10675628	0.29974621	0.14466966	0.63241964	0.37426774	0.11653678	0.37081534	0.27583976
222.8	0.10673632	0.29976297	0.14464124	0.63243334	0.37428732	0.11651814	0.3708583	0.27584069
222.9	0.10671638	0.29977973	0.14461284	0.63244702	0.37430689	0.11649954	0.37090122	0.27584162
223	0.10669648	0.2997965	0.14458446	0.6324607	0.37432643	0.11648096	0.37094409	0.27584255
223.1	0.10667661	0.29981327	0.14455611	0.63247436	0.37434596	0.11646241	0.37098692	0.27584348
223.2	0.10665678	0.29983006	0.14452777	0.63248802	0.37436547	0.1164439	0.3710297	0.27584441
223.3	0.10663697	0.29984684	0.14449946	0.63250166	0.37438496	0.11642542	0.37107244	0.27584534
223.4	0.10661719	0.29986364	0.14447117	0.63251529	0.37440443	0.11640697	0.37111514	0.27584626
223.5	0.10659745	0.29988043	0.1444429	0.63252892	0.37442389	0.11638854	0.37115779	0.27584719
223.6	0.10657773	0.29989724	0.14441466	0.63254253	0.37444333	0.11637015	0.3712004	0.27584813
223.7	0.10655805	0.29991405	0.14438643	0.63255613	0.37446275	0.1163518	0.37124297	0.27584906
223.8	0.10653839	0.29993086	0.14435823	0.63256973	0.37448216	0.11633347	0.37128549	0.27584999
223.9	0.10651877	0.29994768	0.14433005	0.63258331	0.37450154	0.11631517	0.37132797	0.27585092
224	0.10649918	0.29996451	0.14430189	0.63259688	0.37452091	0.11629691	0.37137041	0.27585185
224.1	0.10647962	0.29998134	0.14427375	0.63261044	0.37454027	0.11627867	0.3714128	0.27585278
224.2	0.10646008	0.29999818	0.14424564	0.632624	0.3745596	0.11626047	0.37145515	0.27585371
224.3	0.10644058	0.30001503	0.14421754	0.63263754	0.37457892	0.11624229	0.37149745	0.27585465
224.4	0.10642111	0.30003187	0.14418947	0.63265107	0.37459822	0.11622415	0.37153972	0.27585558
224.5	0.10640167	0.30004873	0.14416142	0.63266459	0.37461751	0.11620604	0.37158194	0.27585651
224.6	0.10638226	0.30006559	0.1441334	0.6326781	0.37463678	0.11618795	0.37162411	0.27585745
224.7	0.10636288	0.30008245	0.14410539	0.6326916	0.37465603	0.1161699	0.37166625	0.27585838
224.8	0.10634353	0.30009932	0.14407741	0.6327051	0.37467526	0.11615188	0.37170834	0.27585931
224.9	0.10632421	0.3001162	0.14404945	0.63271858	0.37469448	0.11613389	0.37175038	0.27586025
225.5	0.10620892	0.30021756	0.14388215	0.63279926	0.37480945	0.11602659	0.37200177	0.27586586
225.6	0.10618981	0.30023447	0.14385435	0.63281267	0.37482855	0.11600881	0.37204351	0.2758668
225.7	0.10617073	0.30025139	0.14382656	0.63282608	0.37484764	0.11599106	0.37208522	0.27586774
225.8	0.10615168	0.30026831	0.1437988	0.63283947	0.37486671	0.11597334	0.37212688	0.27586867
225.9	0.10613266	0.30028523	0.14377107	0.63285285	0.37488577	0.11595566	0.3721685	0.27586961
226	0.10611367	0.30030216	0.14374335	0.63286623	0.37490481	0.115938	0.37221008	0.27587055
226.1	0.1060947	0.3003191	0.14371566	0.63287959	0.37492384	0.11592037	0.37225161	0.27587149
226.2	0.10607577	0.30033604	0.14368799	0.63289295	0.37494284	0.11590278	0.3722931	0.27587243
226.3	0.10605687	0.30035298	0.14366034	0.63290629	0.37496184	0.11588521	0.37233455	0.27587337
226.4	0.10603799	0.30036993	0.14363272	0.63291962	0.37498082	0.11586767	0.37237596	0.27587431
226.5	0.10601915	0.30038689	0.14360511	0.63293295	0.37499978	0.11585017	0.37241732	0.27587524
226.6	0.10600033	0.30040385	0.14357753	0.63294627	0.37501873	0.11583269	0.37245865	0.27587618
226.7	0.10598154	0.30042081	0.14354997	0.63295957	0.37503766	0.11581524	0.37249993	0.27587713
226.8	0.10596279	0.30043778	0.14352244	0.63297287	0.37505657	0.11579782	0.37254117	0.27587807
226.9	0.10594406	0.30045475	0.14349492	0.63298616	0.37507548	0.11578044	0.37258236	0.27587901
227	0.10592536	0.30047173	0.14346743	0.63299943	0.37509436	0.11576308	0.37262352	0.27587995
227.1	0.10590669	0.30048871	0.14343997	0.6330127	0.37511323	0.11574575	0.37266463	0.27588089
227.2	0.10588804	0.3005057	0.14341252	0.63302596	0.37513209	0.11572845	0.3727057	0.27588183
227.3	0.10586943	0.30052269	0.1433851	0.63303921	0.37515093	0.11571118	0.37274673	0.27588277
227.4	0.10585085	0.30053968	0.1433577	0.63305245	0.37516975	0.11569394	0.37278771	0.27588372
227.5	0.10583229	0.30055668	0.14333032	0.63306568	0.37518856	0.11567673	0.37282866	0.27588466
227.6	0.10581376	0.30057369	0.14330297	0.6330789	0.37520736	0.11565955	0.37286956	0.2758856
227.7	0.10579527	0.30059069	0.14327564	0.63309212	0.37522614	0.11564239	0.37291042	0.27588655
227.8	0.1057768	0.30060771	0.14324833	0.63310532	0.37524491	0.11562527	0.37295124	0.27588749
227.9	0.10575836	0.30062472	0.14322104	0.63311851	0.37526366	0.11560818	0.37299202	0.27588844
228	0.10573994	0.30064174	0.14319378	0.6331317	0.37528239	0.11559111	0.37303275	0.27588938
228.1	0.10572156	0.30065877	0.14316653	0.63314487	0.37530112	0.11557408	0.37307345	0.27589033
228.2	0.1057032	0.3006758	0.14313932	0.63315804	0.37531982	0.11555707	0.3731141	0.27589127
228.3	0.10568488	0.30069283	0.14311212	0.63317119	0.37533852	0.11554009	0.37315471	0.27589222
228.4	0.10566658	0.30070987	0.14308495	0.63318434	0.3753572	0.11552315	0.37319528	0.27589316
228.5	0.10564831	0.30072691	0.1430578	0.63319748	0.37537586	0.11550623	0.37323581	0.27589411
228.6	0.10563007	0.30074395	0.14303067	0.63321061	0.37539451	0.11548934	0.3732763	0.27589506
228.7	0.10561185	0.300761	0.14300357	0.63322372	0.37541315	0.11547248	0.37331674	0.275896
228.8	0.10559367	0.30077806	0.14297648	0.63323684	0.37543177	0.11545564	0.37335715	0.27589695
228.9	0.10557551	0.30079511	0.14294943	0.63324994	0.37545038	0.11543884	0.37339751	0.2758979
229	0.10555738	0.30081217	0.14292239	0.63326303	0.37546897	0.11542206	0.37343783	0.27589885
229.1	0.10553928	0.30082924	0.14289538	0.63327611	0.37548755	0.11540532	0.37347811	0.27589979
229.2	0.1055212	0.30084631	0.14286839	0.63328919	0.37550611	0.1153886	0.37351835	0.27590074
229.3	0.10550316	0.30086338	0.14284142	0.63330225	0.37552467	0.11537191	0.37355855	0.27590169
229.4	0.10548514	0.30088046	0.14281447	0.63331531	0.3755432	0.11535525	0.37359871	0.27590264
229.5	0.10546715	0.30089754	0.14278755	0.63332835	0.37556173	0.11533862	0.37363882	0.27590359
229.6	0.10544919	0.30091462	0.14276065	0.63334139	0.37558024	0.11532202	0.3736789	0.27590454
229.7	0.10543125	0.30093171	0.14273378	0.63335442	0.37559873	0.11530544	0.37371894	0.27590549
229.8	0.10541335	0.3009488	0.14270692	0.63336744	0.37561722	0.11528889	0.37375893	0.27590644
229.9	0.10539547	0.30096589	0.14268009	0.63338045	0.37563569	0.11527238	0.37379888	0.27590739
230	0.10537761	0.30098299	0.14265328	0.63339346	0.37565414	0.11525589	0.37383879	0.27590834
230.1	0.10535979	0.30100009	0.1426265	0.63340645	0.37567258	0.11523942	0.37387867	0.27590929
230.2	0.10534199	0.3010172	0.14259974	0.63341943	0.37569101	0.11522299	0.3739185	0.27591025
230.3	0.10532422	0.30103431	0.142573	0.63343241	0.37570943	0.11520658	0.37395829	0.2759112
230.4	0.10530648	0.30105142	0.14254628	0.63344538	0.37572783	0.1151902	0.37399804	0.27591215
230.5	0.10528877	0.30106854	0.14251959	0.63345833	0.37574621	0.11517386	0.37403775	0.2759131
230.6	0.10527108	0.30108566	0.14249292	0.63347128	0.37576459	0.11515754	0.37407742	0.27591406
230.7	0.10525342	0.30110278	0.14246627	0.63348422	0.37578295	0.11514124	0.37411704	0.27591501
230.8	0.10523579	0.30111991	0.14243965	0.63349716	0.3758013	0.11512498	0.37415663	0.27591596
230.9	0.10521818	0.30113704	0.14241304	0.63351008	0.37581963	0.11510874	0.37419618	0.27591691
231	0.1052006	0.30115417	0.14238647	0.63352299	0.37583796	0.11509253	0.37423569	0.27591787
231.1	0.10518305	0.30117131	0.14235991	0.6335359	0.37585627	0.11507635	0.37427515	0.27591882
231.2	0.10516552	0.30118844	0.14233338	0.6335488	0.37587456	0.1150602	0.37431458	0.27591978
231.3	0.10514803	0.30120559	0.14230687	0.63356168	0.37589285	0.11504407	0.37435396	0.27592073
231.4	0.10513055	0.30122273	0.14228038	0.63357456	0.37591112	0.11502797	0.37439331	0.27592169
231.5	0.10511311	0.30123988	0.14225392	0.63358743	0.37592937	0.1150119	0.37443261	0.27592264
231.6	0.10509569	0.30125704	0.14222747	0.6336003	0.37594762	0.11499586	0.37447188	0.2759236
231.7	0.1050783	0.30127419	0.14220106	0.63361315	0.37596585	0.11497984	0.3745111	0.27592455
231.8	0.10506094	0.30129135	0.14217466	0.63362599	0.37598407	0.11496385	0.37455029	0.27592551
231.9	0.1050436	0.30130851	0.14214829	0.63363883	0.37600227	0.11494789	0.37458943	0.27592647
232	0.10502629	0.30132568	0.14212194	0.63365165	0.37602047	0.11493196	0.37462854	0.27592742
232.1	0.105009	0.30134284	0.14209561	0.63366447	0.37603865	0.11491605	0.3746676	0.27592838
232.2	0.10499175	0.30136001	0.14206931	0.63367728	0.37605682	0.11490017	0.37470663	0.27592934
232.3	0.10497451	0.30137719	0.14204303	0.63369009	0.37607497	0.11488432	0.37474561	0.27593029
232.4	0.10495731	0.30139437	0.14201677	0.63370288	0.37609311	0.1148685	0.37478456	0.27593125
232.5	0.10494013	0.30141155	0.14199053	0.63371566	0.37611124	0.1148527	0.37482346	0.27593221
232.6	0.10492298	0.30142873	0.14196432	0.63372844	0.37612936	0.11483693	0.37486232	0.27593317
232.7	0.10490585	0.30144591	0.14193813	0.63374121	0.37614747	0.11482119	0.37490115	0.27593413
232.8	0.10488875	0.3014631	0.14191197	0.63375397	0.37616556	0.11480547	0.37493993	0.27593509
232.9	0.10487168	0.30148029	0.14188582	0.63376672	0.37618364	0.11478978	0.37497868	0.27593605
233	0.10485463	0.30149749	0.1418597	0.63377946	0.37620171	0.11477412	0.37501739	0.275937
233.1	0.10483761	0.30151468	0.1418336	0.63379219	0.37621977	0.11475849	0.37505605	0.27593796
233.2	0.10482061	0.30153188	0.14180753	0.63380492	0.37623781	0.11474288	0.37509468	0.27593892
233.3	0.10480364	0.30154909	0.14178148	0.63381763	0.37625584	0.1147273	0.37513326	0.27593988
233.4	0.1047867	0.30156629	0.14175545	0.63383034	0.37627386	0.11471174	0.37517181	0.27594084
233.5	0.10476978	0.3015835	0.14172944	0.63384304	0.37629187	0.11469621	0.37521032	0.27594181
233.6	0.10475289	0.30160071	0.14170346	0.63385573	0.37630987	0.11468071	0.37524879	0.27594277
233.7	0.10473602	0.30161792	0.1416775	0.63386842	0.37632785	0.11466524	0.37528721	0.27594373
233.8	0.10471918	0.30163514	0.14165156	0.63388109	0.37634582	0.11464979	0.3753256	0.27594469
233.9	0.10470237	0.30165235	0.14162564	0.63389376	0.37636378	0.11463437	0.37536395	0.27594565
234	0.10468558	0.30166958	0.14159975	0.63390642	0.37638173	0.11461897	0.37540226	0.27594661
234.1	0.10466882	0.3016868	0.14157388	0.63391906	0.37639967	0.1146036	0.37544053	0.27594758
234.2	0.10465208	0.30170402	0.14154804	0.63393171	0.37641759	0.11458826	0.37547876	0.27594854
234.3	0.10463537	0.30172125	0.14152221	0.63394434	0.3764355	0.11457294	0.37551696	0.2759495
234.4	0.10461868	0.30173848	0.14149641	0.63395696	0.3764534	0.11455765	0.37555511	0.27595046
234.5	0.10460202	0.30175572	0.14147063	0.63396958	0.37647129	0.11454239	0.37559322	0.27595143
234.6	0.10458538	0.30177295	0.14144488	0.63398219	0.37648917	0.11452715	0.3756313	0.27595239
234.7	0.10456877	0.30179019	0.14141915	0.63399479	0.37650703	0.11451194	0.37566933	0.27595336
234.8	0.10455218	0.30180743	0.14139344	0.63400738	0.37652488	0.11449676	0.37570733	0.27595432
234.9	0.10453562	0.30182467	0.14136775	0.63401996	0.37654272	0.1144816	0.37574528	0.27595528
235	0.10451909	0.30184192	0.14134209	0.63403254	0.37656055	0.11446647	0.3757832	0.27595625
235.1	0.10450258	0.30185916	0.14131644	0.63404511	0.37657837	0.11445136	0.37582108	0.27595721
235.2	0.10448609	0.30187641	0.14129083	0.63405766	0.37659618	0.11443628	0.37585892	0.27595818
235.3	0.10446963	0.30189366	0.14126523	0.63407022	0.37661397	0.11442123	0.37589672	0.27595914
235.4	0.1044532	0.30191092	0.14123966	0.63408276	0.37663176	0.1144062	0.37593448	0.27596011
235.5	0.10443679	0.30192817	0.14121411	0.63409529	0.37664953	0.11439119	0.3759722	0.27596108
235.6	0.1044204	0.30194543	0.14118858	0.63410782	0.37666729	0.11437622	0.37600988	0.27596204
235.7	0.10440404	0.30196269	0.14116307	0.63412034	0.37668504	0.11436127	0.37604753	0.27596301
235.8	0.10438771	0.30197995	0.14113759	0.63413285	0.37670278	0.11434634	0.37608513	0.27596397
235.9	0.1043714	0.30199722	0.14111213	0.63414535	0.3767205	0.11433144	0.3761227	0.27596494
236	0.10435511	0.30201448	0.1410867	0.63415784	0.37673822	0.11431657	0.37616023	0.27596591
236.1	0.10433885	0.30203175	0.14106128	0.63417033	0.37675592	0.11430172	0.37619772	0.27596688
236.2	0.10432261	0.30204902	0.14103589	0.6341828	0.37677361	0.11428689	0.37623516	0.27596784
236.3	0.1043064	0.3020663	0.14101052	0.63419527	0.37679129	0.1142721	0.37627258	0.27596881
236.4	0.10429022	0.30208357	0.14098517	0.63420773	0.37680896	0.11425732	0.37630995	0.27596978
236.5	0.10427405	0.30210085	0.14095985	0.63422019	0.37682662	0.11424258	0.37634728	0.27597075
236.6	0.10425791	0.30211813	0.14093455	0.63423263	0.37684427	0.11422785	0.37638457	0.27597172
236.7	0.1042418	0.30213541	0.14090927	0.63424507	0.37686191	0.11421316	0.37642183	0.27597269
236.8	0.10422571	0.30215269	0.14088401	0.6342575	0.37687953	0.11419849	0.37645905	0.27597365
236.9	0.10420965	0.30216997	0.14085878	0.63426992	0.37689714	0.11418384	0.37649622	0.27597462
237	0.1041936	0.30218726	0.14083357	0.63428233	0.37691475	0.11416922	0.37653336	0.27597559
237.1	0.10417759	0.30220455	0.14080838	0.63429474	0.37693234	0.11415463	0.37657046	0.27597656
237.2	0.10416159	0.30222184	0.14078322	0.63430713	0.37694992	0.11414006	0.37660753	0.27597753
237.3	0.10414563	0.30223913	0.14075807	0.63431952	0.37696749	0.11412551	0.37664455	0.2759785
237.4	0.10412968	0.30225642	0.14073295	0.6343319	0.37698505	0.11411099	0.37668153	0.27597947
237.5	0.10411376	0.30227372	0.14070785	0.63434428	0.37700259	0.11409649	0.37671848	0.27598044
237.6	0.10409786	0.30229101	0.14068278	0.63435664	0.37702013	0.11408202	0.37675538	0.27598141
237.7	0.10408199	0.30230831	0.14065773	0.634369	0.37703766	0.11406758	0.37679225	0.27598239
237.8	0.10406614	0.30232561	0.14063269	0.63438135	0.37705517	0.11405316	0.37682908	0.27598336
237.9	0.10405032	0.30234291	0.14060769	0.63439369	0.37707268	0.11403876	0.37686587	0.27598433
238	0.10403452	0.30236022	0.1405827	0.63440602	0.37709017	0.11402439	0.37690262	0.2759853
238.1	0.10401874	0.30237752	0.14055774	0.63441835	0.37710765	0.11401004	0.37693933	0.27598627
238.2	0.10400299	0.30239483	0.1405328	0.63443066	0.37712512	0.11399572	0.37697601	0.27598724
238.3	0.10398726	0.30241213	0.14050788	0.63444297	0.37714258	0.11398143	0.37701264	0.27598822
238.4	0.10397155	0.30242944	0.14048298	0.63445527	0.37716003	0.11396715	0.37704924	0.27598919
238.5	0.10395587	0.30244676	0.14045811	0.63446757	0.37717747	0.11395291	0.3770858	0.27599016
238.6	0.10394021	0.30246407	0.14043325	0.63447985	0.3771949	0.11393868	0.37712231	0.27599113
238.7	0.10392458	0.30248138	0.14040843	0.63449213	0.37721232	0.11392448	0.37715879	0.27599211
238.8	0.10390896	0.3024987	0.14038362	0.6345044	0.37722973	0.11391031	0.37719523	0.27599308
238.9	0.10389338	0.30251601	0.14035883	0.63451667	0.37724712	0.11389616	0.37723164	0.27599405
239	0.10387781	0.30253333	0.14033407	0.63452892	0.37726451	0.11388203	0.377268	0.27599503
239.1	0.10386227	0.30255065	0.14030933	0.63454117	0.37728189	0.11386793	0.37730432	0.275996
239.2	0.10384675	0.30256797	0.14028461	0.63455341	0.37729925	0.11385386	0.37734061	0.27599698
239.3	0.10383126	0.3025853	0.14025992	0.63456564	0.37731661	0.1138398	0.37737685	0.27599795
239.4	0.10381578	0.30260262	0.14023524	0.63457786	0.37733395	0.11382578	0.37741306	0.27599893
239.5	0.10380034	0.30261994	0.14021059	0.63459008	0.37735129	0.11381177	0.37744923	0.2759999
239.6	0.10378491	0.30263727	0.14018596	0.63460228	0.37736861	0.11379779	0.37748536	0.27600087
239.7	0.10376951	0.3026546	0.14016136	0.63461448	0.37738592	0.11378384	0.37752145	0.27600185
239.8	0.10375413	0.30267193	0.14013677	0.63462668	0.37740323	0.1137699	0.3775575	0.27600283
239.9	0.10373877	0.30268926	0.14011221	0.63463886	0.37742052	0.113756	0.37759351	0.2760038
240	0.10372344	0.30270659	0.14008767	0.63465104	0.3774378	0.11374211	0.37762949	0.27600478

Table S10: eFAST analysis data for [G] with [G](0)=2 mM: eFAST sensitivity indices

Sensitivity analysis data from eFAST for [G] with [G](0)=2 mM (only the parameters to which [G]'s variability is sensitive to appear)

Time discretization	lambda	V_{[G]}	K_{[G]}	delta	q	V_{[PYR]}	K_{[PYR]}	alpha	K_{[ACoA]}	gamma	G_E	L_E
0	0	0	0	0	0	0	0	0	0	0	0	0
0.1	0.00163216	0.00229277	0.00171567	0.00170386	0.0044354	0.8725938	0.18035136	0.00260556	0.00272274	0.00396057	0.00237327	0.00300781
0.2	0.0016336	0.00228649	0.00171608	0.00170861	0.00444248	0.87203006	0.18142648	0.00259436	0.00272616	0.00395836	0.00238019	0.00300447
0.3	0.00163527	0.00228328	0.0017248	0.00171719	0.00445467	0.8714351	0.18254637	0.00257895	0.00273008	0.00395711	0.00239522	0.00300176
0.4	0.00163711	0.00228725	0.0017477	0.00173043	0.00447202	0.87082042	0.18368797	0.00255994	0.00273415	0.0039561	0.00242009	0.00299979
0.5	0.00163908	0.00230336	0.00179142	0.00174905	0.0044937	0.87019418	0.18483506	0.00253866	0.00273816	0.00395526	0.00245761	0.00299873
0.6	0.00164134	0.00233669	0.00186283	0.0017738	0.00451867	0.86955759	0.1859775	0.00251655	0.00274195	0.00395488	0.00251191	0.00299876
0.7	0.00164428	0.00239188	0.00196853	0.00180542	0.00454661	0.8689083	0.18710894	0.00249495	0.00274548	0.00395529	0.00258671	0.00300006
0.8	0.0016484	0.00247279	0.00211464	0.00184466	0.00457832	0.86824328	0.18822578	0.00247527	0.00274872	0.00395699	0.00268536	0.00300254
0.9	0.00165429	0.0025824	0.00230669	0.00189218	0.00461505	0.86756038	0.18932593	0.00245923	0.00275181	0.00396024	0.00281121	0.00300591
1	0.00166252	0.00272332	0.00254953	0.0019486	0.00465828	0.86685836	0.19040826	0.00244889	0.00275486	0.00396527	0.00296762	0.00300993
1.1	0.00167364	0.00289734	0.00284725	0.0020144	0.00470948	0.86613655	0.19147184	0.00244657	0.00275797	0.00397224	0.00315758	0.00301435
1.2	0.00168812	0.0031056	0.00320324	0.00208996	0.00476998	0.86539477	0.19251582	0.00245483	0.00276125	0.00398118	0.00338384	0.00301899
1.3	0.00170645	0.00334888	0.00362008	0.00217554	0.00484102	0.86463296	0.19353961	0.00247638	0.0027648	0.00399211	0.00364883	0.00302371
1.4	0.0017291	0.00362717	0.00409966	0.00227133	0.00492369	0.86385119	0.19454286	0.00251406	0.0027687	0.00400499	0.00395465	0.00302842
1.5	0.00175646	0.00394005	0.00464326	0.00237744	0.00501896	0.86304952	0.19552513	0.00257075	0.00277302	0.00401971	0.00430302	0.00303306
1.6	0.00178893	0.00428682	0.00525159	0.00249393	0.00512771	0.86222802	0.19648598	0.00264941	0.00277781	0.00403617	0.00469531	0.00303758
1.7	0.00182686	0.00466633	0.00592487	0.0026208	0.0052507	0.8613867	0.19742503	0.00275301	0.00278315	0.00405424	0.00513251	0.00304197
1.8	0.00187058	0.00507723	0.00666286	0.00275801	0.00538857	0.86052557	0.19834197	0.00288442	0.00278908	0.00407377	0.00561517	0.00304623
1.9	0.00192037	0.00551801	0.00746491	0.0029055	0.0055418	0.85964456	0.19923647	0.00304646	0.00279563	0.00409463	0.00614356	0.00305037
2	0.00197649	0.00598696	0.00833012	0.00306319	0.00571081	0.85874356	0.2001082	0.0032419	0.00280286	0.00411668	0.00671764	0.00305443
2.1	0.00203914	0.00648231	0.00925729	0.00323097	0.00589599	0.85782243	0.20095694	0.00347338	0.00281081	0.00413978	0.00733702	0.00305843
2.2	0.00210851	0.00700229	0.01024503	0.00340873	0.00609762	0.856881	0.2017825	0.00374335	0.00281952	0.00416379	0.00800112	0.00306239
2.3	0.00218473	0.00754504	0.01129174	0.00359632	0.00631582	0.85591904	0.20258466	0.0040541	0.00282902	0.00418856	0.00870913	0.00306637
2.4	0.0022679	0.00810873	0.01239566	0.0037936	0.0065507	0.85493635	0.20336323	0.00440781	0.00283936	0.00421397	0.00945997	0.00307038
2.5	0.00235808	0.00869162	0.01355493	0.0040004	0.00680232	0.85393268	0.20411812	0.00480648	0.00285055	0.00423989	0.01025235	0.00307447
2.6	0.0024553	0.00929202	0.01476762	0.00421655	0.00707063	0.85290775	0.2048492	0.00525187	0.00286265	0.00426619	0.01108488	0.00307867
2.7	0.00255951	0.00990821	0.01603172	0.00444187	0.00735549	0.85186129	0.20555636	0.00574555	0.00287569	0.00429276	0.01195598	0.00308302
2.8	0.00267067	0.0105386	0.01734513	0.00467618	0.00765674	0.85079303	0.20623954	0.00628894	0.00288971	0.00431948	0.01286386	0.00308755
2.9	0.00278872	0.01118176	0.01870575	0.00491928	0.00797418	0.84970269	0.20689868	0.00688326	0.00290473	0.00434626	0.01380672	0.00309229
3	0.0029136	0.01183629	0.02011153	0.00517099	0.00830753	0.84858996	0.20753368	0.00752952	0.0029208	0.00437301	0.01478264	0.00309729
3.1	0.00304515	0.01250083	0.02156037	0.0054311	0.00865646	0.84745457	0.20814453	0.00822856	0.00293797	0.00439965	0.01578964	0.00310255
3.2	0.00318322	0.01317416	0.0230502	0.00569944	0.00902063	0.84629622	0.20873124	0.00898105	0.00295626	0.0044261	0.0168257	0.00310813
3.3	0.00332765	0.01385522	0.02457895	0.00597582	0.00939964	0.84511465	0.20929377	0.00978751	0.00297572	0.0044523	0.01788876	0.00311405
3.4	0.00347829	0.01454295	0.02614463	0.00626003	0.00979307	0.84390962	0.20983205	0.01064828	0.00299638	0.00447822	0.01897678	0.00312034
3.5	0.0036349	0.01523636	0.0277453	0.00655184	0.01020046	0.84268084	0.210346	0.01156356	0.00301829	0.0045038	0.02008772	0.00312702
3.6	0.00379729	0.01593462	0.02937903	0.00685108	0.01062137	0.84142808	0.21083557	0.01253339	0.00304149	0.00452899	0.02121958	0.00313412
3.7	0.00396522	0.01663699	0.03104397	0.00715756	0.01105534	0.84015108	0.21130073	0.0135577	0.00306604	0.00455376	0.02237039	0.00314167
3.8	0.00413849	0.01734273	0.03273831	0.00747105	0.01150189	0.83884963	0.21174141	0.01463629	0.00309198	0.00457808	0.02353823	0.00314972
3.9	0.00431685	0.01805126	0.03446035	0.00779136	0.0119605	0.83752354	0.21215753	0.01576887	0.00311935	0.00460197	0.02472123	0.0031583
4	0.00450006	0.01876206	0.03620839	0.00811828	0.01243067	0.83617262	0.21254904	0.01695503	0.00314819	0.0046254	0.02591759	0.00316743
4.1	0.00468787	0.01947465	0.0379808	0.00845166	0.0129119	0.8347967	0.21291582	0.01819426	0.00317857	0.00464838	0.02712553	0.00317713
4.2	0.00488006	0.02018859	0.03977607	0.00879129	0.0134037	0.83339562	0.21325773	0.01948599	0.00321054	0.00467093	0.02834338	0.00318743
4.3	0.0050764	0.02090347	0.04159274	0.00913698	0.01390558	0.83196925	0.21357461	0.02082957	0.00324415	0.00469307	0.02956958	0.00319837
4.4	0.00527667	0.02161896	0.04342942	0.00948853	0.01441704	0.83051751	0.21386627	0.02222431	0.00327945	0.00471484	0.03080263	0.00320996
4.5	0.00548065	0.02233487	0.04528477	0.00984576	0.01493761	0.82904034	0.21413258	0.02366944	0.00331649	0.00473629	0.03204108	0.00322225
4.6	0.00568814	0.02305105	0.0471575	0.01020852	0.01546682	0.82753768	0.2143733	0.02516418	0.00335533	0.00475748	0.03328355	0.00323526
4.7	0.00589896	0.02376736	0.04904638	0.01057665	0.01600425	0.82600948	0.21458809	0.0267077	0.00339601	0.00477847	0.03452878	0.00324902
4.8	0.00611297	0.0244837	0.05095031	0.01095	0.01654945	0.82445576	0.2147766	0.02829915	0.0034386	0.00479935	0.03577559	0.00326356
4.9	0.00633001	0.02519999	0.05286827	0.01132837	0.01710202	0.82287653	0.21493849	0.02993765	0.00348315	0.0048202	0.03702283	0.00327891
5	0.00654996	0.02591624	0.05479929	0.01171165	0.01766156	0.82127185	0.21507345	0.03162236	0.00352972	0.00484113	0.03826943	0.0032951
5.1	0.00677268	0.02663252	0.05674246	0.01209969	0.01822771	0.81964179	0.21518104	0.0333524	0.00357836	0.00486224	0.03951443	0.00331216
5.2	0.00699809	0.02734893	0.05869696	0.01249238	0.01880017	0.81798642	0.21526065	0.03512686	0.00362913	0.00488366	0.04075697	0.00333013
5.3	0.00722612	0.02806556	0.06066201	0.01288961	0.01937863	0.81630584	0.2153116	0.03694481	0.00368207	0.00490551	0.04199625	0.00334906
5.4	0.00745671	0.02878255	0.06263692	0.01329126	0.01996284	0.81460026	0.21533316	0.03880532	0.00373724	0.00492793	0.04323151	0.00336897
5.5	0.00768986	0.02950006	0.0646211	0.01369721	0.02055254	0.81286993	0.21532436	0.04070749	0.00379471	0.00495105	0.04446204	0.0033899
5.6	0.00792553	0.03021829	0.06661408	0.01410738	0.0211475	0.81111508	0.21528412	0.04265059	0.00385452	0.004975	0.0456872	0.00341189
5.7	0.00816368	0.03093743	0.06861545	0.01452165	0.02174751	0.80933591	0.21521108	0.04463407	0.00391673	0.00499993	0.04690648	0.00343497
5.8	0.00840423	0.03165769	0.07062487	0.01493987	0.0223524	0.80753271	0.21510356	0.04665771	0.0039814	0.00502599	0.04811939	0.00345917
5.9	0.00864708	0.03237921	0.07264204	0.01536186	0.02296206	0.80570589	0.21495972	0.04872141	0.00404858	0.00505331	0.04932549	0.00348454
6	0.00889209	0.03310215	0.07466667	0.01578744	0.02357644	0.80385596	0.21477776	0.05082517	0.00411832	0.00508202	0.05052433	0.00351111
6.1	0.00913917	0.0338266	0.07669855	0.0162164	0.02419551	0.80198348	0.21455621	0.05296912	0.00419066	0.00511222	0.05171551	0.00353891
6.2	0.00938827	0.03455263	0.07873753	0.01664848	0.02481926	0.80008901	0.21429411	0.05515329	0.00426564	0.00514399	0.05289872	0.00356799
6.3	0.00963939	0.03528034	0.08078352	0.01708345	0.02544766	0.79817314	0.2139911	0.05737755	0.00434332	0.00517742	0.05407371	0.00359837
6.4	0.0098925	0.03600982	0.08283651	0.01752113	0.02608065	0.79623641	0.21364739	0.0596417	0.00442375	0.00521252	0.05524023	0.00363011
6.5	0.01014788	0.0367412	0.08489659	0.01796139	0.02671812	0.79427928	0.2132637	0.06194533	0.00450699	0.00524925	0.05639808	0.00366322
6.6	0.01040541	0.03747462	0.08696376	0.01840421	0.02735992	0.79230204	0.21284098	0.06428793	0.00459308	0.00528754	0.05754708	0.00369775
6.7	0.01066523	0.03821026	0.089038	0.01884963	0.02800583	0.7903048	0.21238042	0.06666886	0.00468209	0.00532723	0.0586871	0.00373371
6.8	0.01092734	0.03894831	0.0911192	0.01929773	0.02865553	0.78828756	0.21188326	0.06908728	0.00477403	0.0053681	0.05981802	0.00377115
6.9	0.01119174	0.0396889	0.09320719	0.01974862	0.0293086	0.78625031	0.21135062	0.07154219	0.00486894	0.0054098	0.0609397	0.00381007
7	0.01145839	0.04043213	0.0953017	0.02020243	0.02996456	0.78419296	0.21078367	0.07403257	0.00496686	0.00545194	0.06205195	0.00385048
7.1	0.01172726	0.04117808	0.09740234	0.02065927	0.03062293	0.78211538	0.21018353	0.07655744	0.00506784	0.00549415	0.06315471	0.00389241
7.2	0.01199829	0.04192683	0.09950863	0.02111926	0.03128323	0.78001747	0.20955131	0.07911574	0.00517191	0.00553613	0.0642479	0.00393586
7.3	0.01227141	0.04267845	0.10162009	0.02158246	0.03194511	0.77789902	0.20888802	0.08170639	0.00527912	0.00557769	0.06533149	0.00398083
7.4	0.01254651	0.04343299	0.10373623	0.02204896	0.03260833	0.77575977	0.20819455	0.08432827	0.00538953	0.00561879	0.06640543	0.00402732
7.5	0.01282348	0.04419049	0.1058565	0.02251875	0.03327274	0.77359951	0.20747177	0.08698027	0.00550314	0.00565944	0.06746971	0.00407533
7.6	0.0131022	0.04495097	0.10798028	0.02299184	0.03393828	0.77141795	0.20672049	0.0896612	0.00562	0.0056997	0.06852428	0.00412481
7.7	0.01338255	0.04571443	0.11010694	0.02346814	0.03460492	0.76921482	0.2059415	0.09236986	0.00574011	0.00573964	0.06956907	0.00417573
7.8	0.0136644	0.04648085	0.11223583	0.02394756	0.03527264	0.76698986	0.20513558	0.09510495	0.0058635	0.00577935	0.070604	0.00422802
7.9	0.01394762	0.04725021	0.11436619	0.02442992	0.03594144	0.76474283	0.20430349	0.09786517	0.00599021	0.00581893	0.071629	0.00428163
8	0.01423212	0.04802246	0.11649721	0.02491506	0.03661135	0.76247349	0.20344605	0.10064937	0.00612027	0.00585845	0.07264403	0.00433651
8.1	0.01451785	0.04879754	0.11862814	0.02540283	0.03728236	0.76018159	0.20256428	0.10345639	0.00625372	0.00589802	0.07364912	0.00439257
8.2	0.01480471	0.04957536	0.12075824	0.02589304	0.03795447	0.75786696	0.20165927	0.10628511	0.00639057	0.00593771	0.07464428	0.00444977
8.3	0.0150927	0.05035582	0.12288677	0.0263855	0.03862772	0.75552945	0.20073216	0.10913441	0.00653086	0.0059776	0.07562945	0.00450804
8.4	0.0153818	0.05113879	0.12501298	0.02687997	0.03930214	0.75316895	0.19978417	0.11200321	0.00667458	0.00601776	0.07660458	0.00456734
8.5	0.01567204	0.05192416	0.12713613	0.02737626	0.03997775	0.75078534	0.19881661	0.11489038	0.00682175	0.00605825	0.07756961	0.0046276
8.6	0.01596338	0.05271182	0.12925553	0.0278742	0.04065458	0.74837851	0.1978308	0.11779486	0.00697239	0.00609913	0.07852451	0.0046888
8.7	0.01625582	0.0535017	0.13137053	0.02837365	0.04133262	0.74594831	0.19682804	0.12071542	0.0071265	0.00614046	0.07946924	0.00475095
8.8	0.01654937	0.05429371	0.1334805	0.02887447	0.04201181	0.7434946	0.19580966	0.12365082	0.00728411	0.0061823	0.0804038	0.00481404
8.9	0.01684409	0.05508776	0.1355849	0.02937651	0.0426921	0.74101722	0.19477696	0.12660002	0.00744524	0.00622471	0.08132823	0.00487808
9	0.01713999	0.05588375	0.13768326	0.0298797	0.04337342	0.73851604	0.19373119	0.12956205	0.00760989	0.00626772	0.08224265	0.00494309
9.1	0.01743711	0.05668159	0.13977514	0.03038397	0.0440557	0.73599092	0.1926736	0.13253595	0.00777805	0.00631141	0.08314721	0.00500908
9.2	0.01773538	0.0574812	0.14186011	0.03088927	0.04473889	0.73344179	0.19160537	0.13552078	0.0079497	0.00635582	0.08404202	0.00507609
9.3	0.0180347	0.05828248	0.14393777	0.03139555	0.04542291	0.73086865	0.19052767	0.13851565	0.00812483	0.00640099	0.08492719	0.00514413
9.4	0.01833506	0.05908535	0.14600773	0.03190274	0.04610769	0.72827149	0.18944162	0.14151971	0.00830347	0.00644697	0.08580286	0.00521321
9.5	0.01863643	0.05988969	0.1480696	0.03241079	0.04679314	0.7256503	0.18834822	0.14453206	0.0084856	0.00649381	0.08666915	0.00528331
9.6	0.01893882	0.06069541	0.15012298	0.03291964	0.04747918	0.72300512	0.18724846	0.14755174	0.00867124	0.00654154	0.08752618	0.00535444
9.7	0.01924223	0.06150241	0.15216755	0.0334292	0.0481657	0.72033601	0.18614324	0.15057785	0.00886039	0.00659022	0.08837407	0.00542658
9.8	0.01954667	0.0623106	0.15420297	0.03393936	0.0488526	0.71764304	0.18503344	0.15360954	0.00905304	0.00663988	0.08921293	0.00549971
9.9	0.01985214	0.06311986	0.15622893	0.03445003	0.04953978	0.71492633	0.1839199	0.15664602	0.00924919	0.00669056	0.09004289	0.00557383
10	0.02015862	0.06393008	0.15824511	0.03496113	0.05022714	0.71218603	0.18280341	0.15968654	0.00944884	0.00674231	0.09086405	0.00564889
10.1	0.02046613	0.06474114	0.16025119	0.03547256	0.05091453	0.70942231	0.18168468	0.16273037	0.00965199	0.00679516	0.09167648	0.00572488
10.2	0.02077461	0.06555293	0.16224686	0.03598426	0.05160182	0.70663542	0.1805644	0.16577677	0.0098586	0.00684914	0.09248029	0.00580178
10.3	0.02108397	0.06636532	0.16423178	0.03649611	0.05228887	0.70382566	0.1794432	0.16882504	0.01006863	0.0069043	0.09327559	0.00587956
10.4	0.02139408	0.06717819	0.16620565	0.03700804	0.05297553	0.70099331	0.17832168	0.17187446	0.01028203	0.00696067	0.09406247	0.00595819
10.5	0.02170495	0.06799144	0.16816816	0.03751994	0.05366165	0.69813868	0.17720038	0.17492428	0.01049878	0.00701828	0.09484104	0.00603765
10.6	0.02201655	0.06880496	0.17011899	0.03803172	0.05434707	0.6952621	0.17607977	0.17797375	0.01071886	0.00707718	0.09561138	0.0061179
10.7	0.02232885	0.06961866	0.17205787	0.03854323	0.05503163	0.69236388	0.17496029	0.1810222	0.01094225	0.00713738	0.09637361	0.00619893
10.8	0.02264186	0.07043243	0.17398451	0.03905438	0.05571517	0.68944441	0.17384234	0.18406899	0.01116893	0.00719892	0.09712782	0.00628071
10.9	0.02295555	0.07124617	0.1758986	0.03956503	0.05639754	0.68650408	0.17272631	0.18711344	0.01139886	0.00726185	0.09787411	0.00636322
11	0.02326994	0.07205976	0.17779988	0.04007507	0.0570786	0.68354332	0.17161254	0.19015493	0.01163204	0.00732617	0.0986126	0.00644643
11.1	0.02358498	0.07287312	0.17968806	0.04058439	0.05775822	0.68056259	0.17050133	0.19319284	0.01186841	0.00739194	0.0993434	0.00653034
11.2	0.02390068	0.07368614	0.18156289	0.04109287	0.05843626	0.67756237	0.16939298	0.19622659	0.01210795	0.00745917	0.10006661	0.00661492
11.3	0.02421693	0.07449872	0.18342414	0.0416004	0.05911258	0.6745432	0.16828776	0.19925563	0.01235059	0.00752789	0.10078231	0.00670015
11.4	0.02453359	0.07531075	0.18527156	0.04210689	0.05978702	0.6715056	0.16718591	0.20227938	0.01259626	0.00759813	0.10149061	0.00678602
11.5	0.02485061	0.07612215	0.1871049	0.04261225	0.06045942	0.66845012	0.16608769	0.20529723	0.01284489	0.00766992	0.10219158	0.00687251
11.6	0.02516795	0.0769328	0.18892392	0.04311636	0.06112965	0.66537735	0.16499331	0.20830863	0.01309643	0.00774326	0.10288532	0.00695963
11.7	0.02548558	0.07774262	0.19072842	0.04361911	0.06179755	0.66228787	0.163903	0.21131303	0.01335081	0.0078182	0.10357189	0.00704737
11.8	0.02580347	0.07855152	0.19251815	0.0441204	0.06246299	0.65918229	0.16281696	0.21430989	0.01360796	0.00789474	0.10425139	0.00713573
11.9	0.0261216	0.07935939	0.19429292	0.04462013	0.06312586	0.65606126	0.16173543	0.21729869	0.01386784	0.0079729	0.10492389	0.00722472
12	0.02643996	0.08016615	0.19605252	0.0451182	0.06378603	0.65292543	0.16065861	0.22027892	0.01413038	0.00805271	0.10558949	0.00731433
12.1	0.02675853	0.08097171	0.19779679	0.0456145	0.0644434	0.64977549	0.15958674	0.22325011	0.01439551	0.00813418	0.1062483	0.00740457
12.2	0.0270773	0.08177599	0.19952559	0.04610893	0.06509786	0.6466121	0.15852004	0.22621178	0.01466315	0.00821732	0.10690039	0.00749545
12.3	0.02739624	0.08257895	0.20123878	0.04660139	0.06574932	0.64343594	0.15745871	0.2291635	0.01493325	0.00830216	0.10754587	0.00758697
12.4	0.02771529	0.08338051	0.20293622	0.04709178	0.06639767	0.64024773	0.15640295	0.23210482	0.01520572	0.0083887	0.10818484	0.00767914
12.5	0.02803436	0.08418059	0.20461779	0.04758	0.06704281	0.63704818	0.15535295	0.23503532	0.01548048	0.00847696	0.10881739	0.00777196
12.6	0.02835343	0.08497911	0.20628338	0.04806596	0.06768468	0.63383805	0.15430889	0.23795456	0.01575747	0.00856695	0.10944363	0.00786542
12.7	0.02867246	0.08577601	0.20793289	0.04854958	0.06832318	0.6306181	0.15327093	0.24086216	0.01603662	0.00865869	0.11006362	0.00795954
12.8	0.0289914	0.0865712	0.20956623	0.04903078	0.06895824	0.62738908	0.15223925	0.24375779	0.01631789	0.00875219	0.11067747	0.00805431
12.9	0.02931022	0.08736463	0.21118331	0.04950949	0.06958981	0.62415175	0.15121402	0.24664117	0.01660123	0.00884744	0.11128525	0.00814974
13	0.02962885	0.08815621	0.21278406	0.04998564	0.07021784	0.62090686	0.1501954	0.24951198	0.01688661	0.00894447	0.11188703	0.00824582
13.1	0.02994726	0.08894586	0.21436843	0.05045914	0.07084228	0.61765517	0.14918355	0.25236993	0.01717399	0.00904327	0.11248289	0.00834257
13.2	0.03026541	0.08973351	0.21593636	0.05092992	0.07146308	0.61439746	0.14817862	0.25521471	0.01746336	0.00914383	0.11307292	0.00843998
13.3	0.03058326	0.09051911	0.21748781	0.05139792	0.07208019	0.61113452	0.14718073	0.25804603	0.01775471	0.00924617	0.11365718	0.00853806
13.4	0.03090078	0.0913026	0.21902274	0.05186309	0.07269354	0.60786714	0.14619002	0.26086361	0.01804801	0.00935028	0.11423574	0.00863681
13.5	0.03121792	0.09208395	0.22054111	0.05232536	0.0733031	0.60459608	0.14520658	0.2636672	0.01834324	0.00945617	0.1148087	0.00873623
13.6	0.03153464	0.0928631	0.2220429	0.05278468	0.07390881	0.60132212	0.14423054	0.26645654	0.01864037	0.00956383	0.11537612	0.00883633
13.7	0.03185091	0.09364	0.22352811	0.05324097	0.07451063	0.59804603	0.14326199	0.26923139	0.01893938	0.00967328	0.11593806	0.00893711
13.8	0.03216669	0.09441459	0.22499675	0.05369418	0.07510851	0.59476858	0.14230104	0.27199152	0.01924023	0.00978449	0.11649462	0.00903857
13.9	0.03248193	0.09518682	0.22644883	0.05414427	0.07570239	0.59149053	0.14134776	0.27473671	0.01954288	0.00989747	0.11704586	0.00914072
14	0.03279661	0.09595664	0.22788439	0.05459117	0.07629223	0.58821262	0.14040222	0.27746674	0.01984731	0.0100122	0.11759189	0.00924356
14.1	0.03311067	0.096724	0.22930346	0.05503484	0.07687795	0.58493559	0.1394645	0.28018144	0.02015348	0.01012869	0.11813278	0.0093471
14.2	0.03342408	0.09748884	0.23070608	0.05547521	0.07745953	0.5816602	0.13853464	0.28288062	0.02046138	0.01024692	0.1186686	0.00945132
14.3	0.0337368	0.09825111	0.2320923	0.05591224	0.0780369	0.5783872	0.13761271	0.28556412	0.02077097	0.01036689	0.11919941	0.00955622
14.4	0.0340488	0.09901075	0.23346219	0.05634591	0.07861002	0.57511732	0.13669873	0.28823178	0.02108223	0.0104886	0.1197253	0.00966181
14.5	0.03436005	0.09976773	0.23481578	0.05677616	0.07917883	0.57185129	0.13579275	0.2908835	0.02139512	0.01061203	0.12024631	0.00976808
14.6	0.03467053	0.10052198	0.23615314	0.05720296	0.0797433	0.56858985	0.13489481	0.2935192	0.02170963	0.01073718	0.12076251	0.00987504
14.7	0.0349802	0.10127347	0.23747434	0.05762629	0.0803034	0.5653337	0.13400492	0.29613878	0.0220257	0.01086403	0.12127397	0.00998268
14.8	0.03528904	0.10202215	0.23877944	0.05804611	0.08085909	0.56208356	0.13312311	0.29874215	0.02234329	0.01099258	0.12178075	0.010091
14.9	0.035597	0.10276797	0.2400685	0.0584624	0.08141035	0.55884012	0.1322494	0.30132922	0.02266237	0.01112281	0.12228293	0.0102
45	0.06775306	0.20000335	0.32913837	0.08838485	0.13136908	0.21180458	0.05021196	0.61328425	0.09152617	0.0636385	0.1934257	0.05298937
45.1	0.06774755	0.20007576	0.32916279	0.08837341	0.13139687	0.21164467	0.05014605	0.61361208	0.09159849	0.0637804	0.19355308	0.05311016
45.2	0.06774183	0.20014749	0.32918703	0.08836186	0.13142454	0.21148634	0.05008049	0.6139382	0.09167014	0.06392202	0.19368013	0.05323068
45.3	0.0677359	0.20021854	0.32921109	0.08835022	0.13145207	0.21132957	0.0500153	0.61426261	0.09174114	0.06406337	0.19380684	0.05335091
45.4	0.06772977	0.20028891	0.32923496	0.08833849	0.13147947	0.21117435	0.04995045	0.61458533	0.09181148	0.06420444	0.19393323	0.05347085
45.5	0.06772344	0.20035862	0.32925866	0.08832666	0.13150675	0.21102066	0.04988596	0.61490636	0.09188118	0.06434523	0.19405928	0.05359052
45.6	0.0677169	0.20042766	0.32928218	0.08831473	0.1315339	0.21086848	0.04982181	0.61522573	0.09195023	0.06448575	0.19418501	0.0537099
45.7	0.06771017	0.20049605	0.32930553	0.08830272	0.13156094	0.21071779	0.04975801	0.61554343	0.09201865	0.064626	0.19431041	0.05382901
45.8	0.06770324	0.20056378	0.32932871	0.08829062	0.13158786	0.21056859	0.04969455	0.61585949	0.09208644	0.06476598	0.19443549	0.05394783
45.9	0.06769612	0.20063086	0.32935172	0.08827843	0.13161466	0.21042085	0.04963143	0.61617392	0.0921536	0.0649057	0.19456024	0.05406637
46	0.06768881	0.2006973	0.32937457	0.08826615	0.13164134	0.21027457	0.04956865	0.61648672	0.09222013	0.06504514	0.19468467	0.05418463
46.1	0.06768131	0.2007631	0.32939726	0.08825379	0.13166792	0.21012972	0.0495062	0.61679793	0.09228603	0.06518432	0.19480879	0.0543026
46.2	0.06767363	0.20082826	0.32941979	0.08824135	0.13169439	0.20998629	0.04944409	0.61710753	0.09235132	0.06532323	0.19493258	0.0544203
46.3	0.06766576	0.20089279	0.32944217	0.08822882	0.13172074	0.20984427	0.0493823	0.61741555	0.092416	0.06546187	0.19505606	0.05453772
46.4	0.06765771	0.2009567	0.32946439	0.08821622	0.13174699	0.20970365	0.04932084	0.617722	0.09248006	0.06560025	0.19517922	0.05465486
46.5	0.06764949	0.20101999	0.32948646	0.08820354	0.13177314	0.2095644	0.04925971	0.61802689	0.09254352	0.06573835	0.19530206	0.05477172
46.6	0.06764108	0.20108266	0.32950838	0.08819078	0.13179918	0.20942651	0.04919889	0.61833023	0.09260638	0.06587619	0.1954246	0.0548883
46.7	0.0676325	0.20114471	0.32953016	0.08817795	0.13182513	0.20928997	0.0491384	0.61863202	0.09266864	0.06601377	0.19554682	0.0550046
46.8	0.06762374	0.20120616	0.3295518	0.08816504	0.13185097	0.20915476	0.04907822	0.61893229	0.09273031	0.06615107	0.19566873	0.05512062
46.9	0.06761481	0.20126701	0.3295733	0.08815206	0.13187672	0.20902088	0.04901835	0.61923103	0.0927914	0.06628812	0.19579034	0.05523637
47	0.06760571	0.20132726	0.32959465	0.08813901	0.13190237	0.20888831	0.0489588	0.61952827	0.0928519	0.06642491	0.19591164	0.05535184
47.1	0.06759644	0.20138691	0.32961588	0.0881259	0.13192792	0.20875703	0.04889956	0.61982401	0.09291182	0.06656143	0.19603264	0.05546703
47.2	0.067587	0.20144598	0.32963697	0.08811271	0.13195338	0.20862703	0.04884063	0.62011826	0.09297117	0.06669769	0.19615334	0.05558194
47.3	0.0675774	0.20150446	0.32965792	0.08809946	0.13197875	0.2084983	0.048782	0.62041103	0.09302995	0.06683369	0.19627373	0.05569657
47.4	0.06756763	0.20156236	0.32967876	0.08808614	0.13200403	0.20837083	0.04872367	0.62070234	0.09308817	0.06696943	0.19639382	0.05581093
47.5	0.0675577	0.20161968	0.32969946	0.08807276	0.13202923	0.2082446	0.04866565	0.62099219	0.09314582	0.06710491	0.19651362	0.05592501
47.6	0.06754762	0.20167644	0.32972004	0.08805931	0.13205433	0.20811959	0.04860792	0.62128058	0.09320291	0.06724013	0.19663311	0.05603881
47.7	0.06753738	0.20173263	0.3297405	0.08804581	0.13207936	0.20799581	0.04855049	0.62156755	0.09325946	0.06737508	0.19675231	0.05615234
47.8	0.06752698	0.20178826	0.32976083	0.08803224	0.1321043	0.20787323	0.04849336	0.62185308	0.09331545	0.06750977	0.19687122	0.05626559
47.9	0.06751643	0.20184333	0.32978105	0.08801862	0.13212915	0.20775184	0.04843652	0.62213719	0.09337091	0.0676442	0.19698983	0.05637856
48	0.06750574	0.20189785	0.32980116	0.08800493	0.13215393	0.20763163	0.04837997	0.6224199	0.09342582	0.06777837	0.19710815	0.05649126
48.1	0.06749489	0.20195182	0.32982115	0.08799119	0.13217863	0.20751258	0.04832371	0.6227012	0.09348019	0.06791227	0.19722617	0.05660369
48.2	0.0674839	0.20200524	0.32984104	0.0879774	0.13220325	0.2073947	0.04826774	0.62298112	0.09353403	0.06804592	0.19734391	0.05671584

Time discretization	lambda	V_{[G]}	K_{[G]}	delta	q	V_{[PYR]}	K_{[PYR]}	alpha	K_{[ACoA]}	gamma	G_E	L_E
15	0.03590406	0.1035109	0.24134162	0.05887513	0.08195715	0.55560406	0.13138378	0.30389992	0.02298291	0.01125469	0.12278056	0.01030969
15.1	0.03621017	0.10425089	0.24259886	0.05928429	0.08249944	0.55237605	0.13052627	0.30645414	0.02330487	0.01138822	0.12327373	0.01042006
15.2	0.03651529	0.10498792	0.24384032	0.05968984	0.08303718	0.54915674	0.12967686	0.30899183	0.02362821	0.01152338	0.1237625	0.0105311
15.3	0.03681938	0.10572196	0.24506609	0.06009179	0.08357037	0.54594675	0.12883555	0.31151292	0.0239529	0.01166015	0.12424693	0.01064283
15.4	0.03712241	0.106453	0.24627628	0.06049011	0.08409901	0.54274667	0.12800233	0.31401736	0.02427892	0.01179853	0.12472708	0.01075523
15.5	0.03742434	0.10718101	0.24747102	0.06088478	0.08462309	0.53955711	0.1271772	0.3165051	0.02460624	0.01193849	0.12520302	0.0108683
15.6	0.03772513	0.10790594	0.24865039	0.06127579	0.08514263	0.53637864	0.12636014	0.31897613	0.02493481	0.01208	0.12567481	0.01098204
15.7	0.03802475	0.10862776	0.24981451	0.06166312	0.08565763	0.53321181	0.12555116	0.32143041	0.02526459	0.01222305	0.12614252	0.01109645
15.8	0.03832319	0.10934643	0.2509635	0.06204677	0.0861681	0.53005716	0.12475022	0.32386791	0.02559554	0.01236762	0.1266062	0.0112115
15.9	0.0386204	0.11006193	0.25209747	0.06242672	0.08667404	0.52691521	0.1239573	0.32628859	0.02592764	0.01251369	0.12706593	0.01132722
16	0.03891636	0.11077422	0.25321656	0.06280296	0.08717543	0.52378648	0.12317238	0.32869246	0.02626086	0.01266125	0.12752178	0.01144358
16.1	0.03921102	0.11148328	0.25432088	0.06317549	0.08767229	0.52067147	0.12239543	0.33107953	0.02659515	0.01281028	0.12797382	0.0115606
16.2	0.03950435	0.11218908	0.25541057	0.06354431	0.08816462	0.51757065	0.12162642	0.33344983	0.0269305	0.01296077	0.12842209	0.01167827
16.3	0.03979631	0.1128916	0.25648576	0.06390942	0.08865245	0.51448452	0.12086532	0.33580339	0.02726687	0.01311268	0.12886668	0.01179658
16.4	0.04008688	0.1135908	0.25754656	0.0642708	0.08913578	0.5114135	0.1201121	0.33814022	0.02760423	0.01326602	0.12930762	0.01191555
16.5	0.04037603	0.11428667	0.25859309	0.06462847	0.08961464	0.50835805	0.1193667	0.34046034	0.02794255	0.01342076	0.12974498	0.01203517
16.6	0.04066376	0.11497918	0.25962543	0.06498243	0.09008902	0.50531857	0.1186291	0.34276377	0.0282818	0.0135769	0.13017882	0.01215542
16.7	0.04095007	0.11566831	0.26064371	0.06533268	0.09055896	0.50229548	0.11789925	0.34505055	0.02862192	0.01373439	0.1306092	0.01227633
16.8	0.04123494	0.11635404	0.26164808	0.06567922	0.09102444	0.49928918	0.1171771	0.34732069	0.02896287	0.01389322	0.13103616	0.01239787
16.9	0.04151835	0.11703633	0.2626387	0.06602207	0.0914855	0.49630004	0.1164626	0.34957423	0.02930461	0.01405335	0.13145974	0.01252005
17	0.04180028	0.11771518	0.26361569	0.06636122	0.09194214	0.49332844	0.11575573	0.35181123	0.02964709	0.01421476	0.13188	0.01264287
17.1	0.04208071	0.11839057	0.26457922	0.0666967	0.09239439	0.4903747	0.11505642	0.3540317	0.02999028	0.01437743	0.13229698	0.01276631
17.2	0.04235962	0.11906248	0.26552943	0.06702851	0.09284227	0.48743913	0.11436462	0.35623571	0.03033414	0.01454134	0.13271073	0.01289038
17.3	0.04263698	0.11973091	0.26646647	0.06735667	0.09328579	0.48452206	0.11368029	0.3584233	0.03067864	0.01470646	0.13312129	0.01301504
17.4	0.04291277	0.12039586	0.26739048	0.06768118	0.09372497	0.48162379	0.11300338	0.36059452	0.03102373	0.0148727	0.13352872	0.01314031
17.5	0.04318698	0.12105729	0.26830159	0.06800207	0.09415984	0.47874459	0.11233383	0.36274945	0.03136936	0.01504022	0.13393306	0.01326619
17.6	0.04345959	0.1217152	0.26919991	0.06831933	0.0945904	0.47588471	0.11167158	0.36488817	0.03171553	0.01520881	0.13433436	0.01339265
17.7	0.04373057	0.12236958	0.27008555	0.06863299	0.09501669	0.47304441	0.11101659	0.36701076	0.0320622	0.01537852	0.13473266	0.01351971
17.8	0.0439999	0.1230204	0.27095868	0.06894306	0.09543872	0.47022393	0.11036879	0.36911731	0.03240933	0.01554931	0.13512799	0.01364736
17.9	0.04426756	0.12366767	0.27181946	0.06924955	0.09585652	0.46742348	0.10972812	0.37120787	0.0327569	0.01572118	0.13552042	0.01377559
18	0.04453354	0.12431136	0.27266803	0.06955249	0.09627011	0.46464327	0.10909453	0.37328254	0.03310489	0.01589409	0.13590999	0.01390439
18.1	0.04479781	0.12495148	0.27350454	0.06985189	0.09667953	0.46188348	0.10846794	0.37534139	0.03345327	0.01606805	0.13629673	0.01403376
18.2	0.04506035	0.12558801	0.27432916	0.07014777	0.09708478	0.4591443	0.10784829	0.37738449	0.033802	0.01624302	0.13668068	0.01416369
18.3	0.04532114	0.12622094	0.27514202	0.07044015	0.0974859	0.45642589	0.10723553	0.37941193	0.03415105	0.01641898	0.13706189	0.01429419
18.4	0.04558015	0.12685026	0.27594329	0.07072904	0.0978829	0.45372842	0.10662958	0.38142379	0.03450039	0.01659591	0.13744039	0.01442524
18.5	0.04583737	0.12747595	0.27673307	0.07101447	0.09827581	0.45105205	0.10603038	0.38342015	0.03484997	0.01677378	0.13781622	0.01455686
18.6	0.0460928	0.12809802	0.27751147	0.07129646	0.09866464	0.4483969	0.10543785	0.3854011	0.03519975	0.01695256	0.13818941	0.01468902
18.7	0.04634647	0.12871645	0.27827862	0.07157503	0.09904943	0.4457631	0.10485195	0.38736671	0.0355497	0.01713222	0.13856001	0.01482172
18.8	0.04659837	0.12933124	0.27903465	0.07185019	0.09943019	0.44315076	0.10427259	0.3893171	0.0358998	0.01731274	0.13892805	0.01495497
18.9	0.04684849	0.1299424	0.27977973	0.07212198	0.09980695	0.44055997	0.10369973	0.39125235	0.03624998	0.0174941	0.13929355	0.01508874
19	0.04709683	0.13054991	0.28051398	0.07239041	0.10017973	0.43799082	0.10313328	0.39317254	0.03660021	0.01767629	0.13965655	0.01522304
19.1	0.04734338	0.13115379	0.28123757	0.07265552	0.10054857	0.43544338	0.10257319	0.39507777	0.03695045	0.01785927	0.14001708	0.01535786
19.2	0.04758812	0.13175404	0.28195062	0.07291731	0.10091349	0.4329177	0.10201939	0.39696811	0.03730067	0.01804303	0.14037516	0.0154932
19.3	0.04783105	0.13235065	0.28265327	0.07317583	0.10127452	0.43041379	0.10147181	0.39884365	0.03765081	0.01822754	0.14073083	0.01562905
19.4	0.04807216	0.13294363	0.28334568	0.07343107	0.1016317	0.42793169	0.1009304	0.40070451	0.03800085	0.01841278	0.14108412	0.01576539
19.5	0.04831144	0.13353298	0.28402796	0.07368309	0.10198505	0.42547143	0.10039508	0.40255076	0.03835074	0.01859873	0.14143507	0.01590224
19.6	0.04854888	0.13411869	0.28470024	0.07393188	0.1023346	0.42303302	0.09986579	0.40438254	0.03870045	0.01878538	0.14178369	0.01603958
19.7	0.04878448	0.13470077	0.28536264	0.0741775	0.10268038	0.4206165	0.09934245	0.40619997	0.03904996	0.0189727	0.14213004	0.01617742
19.8	0.04901823	0.13527921	0.2860153	0.07441995	0.10302241	0.41822185	0.098825	0.40800315	0.03939923	0.01916067	0.14247413	0.01631573
19.9	0.04925011	0.13585403	0.28665836	0.07465927	0.10336072	0.41584907	0.09831338	0.40979219	0.03974825	0.01934927	0.142816	0.01645451
20	0.04948013	0.13642522	0.28729194	0.07489548	0.10369534	0.41349814	0.09780751	0.41156717	0.04009697	0.01953849	0.14315567	0.01659377
20.1	0.04970828	0.13699279	0.28791617	0.0751261	0.1040263	0.41116907	0.09730734	0.4133282	0.04044538	0.0197283	0.14349318	0.01673349
20.2	0.04993454	0.13755673	0.28853119	0.07535869	0.10435363	0.40886182	0.09681278	0.41507538	0.04079344	0.01991868	0.14382855	0.01687366
20.3	0.05015893	0.13811706	0.28913711	0.07558574	0.10467735	0.40657637	0.09632378	0.41680882	0.04114113	0.02010959	0.14416179	0.01701428
20.4	0.05038144	0.13867378	0.28973405	0.07580979	0.1049975	0.40431267	0.09584026	0.4185286	0.0414884	0.02030102	0.14449295	0.01715534
20.5	0.05060206	0.13922689	0.29032215	0.07603088	0.1053141	0.40207069	0.09536218	0.42023483	0.04183523	0.02049294	0.14482204	0.01729684
20.6	0.05082081	0.1397764	0.29090153	0.07624904	0.10562718	0.39985037	0.09488945	0.42192762	0.04218158	0.02068532	0.14514909	0.01743876
20.7	0.05103769	0.1403223	0.2914723	0.0764643	0.10593678	0.39765163	0.09442201	0.42360706	0.04252744	0.02087816	0.14547412	0.01758111
20.8	0.05125272	0.14086461	0.29203458	0.07667668	0.10624292	0.39547441	0.0939598	0.42527324	0.04287277	0.02107143	0.14579717	0.01772387
20.9	0.05146591	0.14140335	0.29258849	0.07688621	0.10654565	0.39331864	0.09350275	0.42692628	0.04321753	0.0212651	0.14611825	0.01786704
21	0.05167725	0.14193852	0.29313414	0.07709292	0.10684499	0.39118423	0.0930508	0.42856627	0.04356171	0.02145918	0.14643738	0.01801061
21.1	0.05188675	0.14247013	0.29367167	0.07729684	0.10714097	0.38907111	0.09260388	0.4301933	0.04390528	0.02165363	0.14675459	0.01815457
21.2	0.05209441	0.14299818	0.29420117	0.07749798	0.10743362	0.38697918	0.09216193	0.43180748	0.04424822	0.02184845	0.1470699	0.01829893
21.3	0.05230022	0.14352269	0.29472276	0.07769637	0.10772297	0.38490836	0.09172489	0.4334089	0.0445905	0.02204362	0.14738334	0.01844367
21.4	0.05250419	0.14404367	0.29523657	0.07789205	0.10800904	0.38285854	0.09129268	0.43499768	0.04493209	0.02223912	0.14769492	0.0185888
21.5	0.05270631	0.14456113	0.29574269	0.07808503	0.10829187	0.38082963	0.09086524	0.4365739	0.04527297	0.02243493	0.14800467	0.0187343
21.6	0.0529066	0.14507508	0.29624126	0.07827535	0.10857149	0.37882153	0.09044253	0.43813769	0.04561311	0.02263105	0.1483126	0.01888017
21.7	0.05310504	0.14558553	0.29673236	0.07846303	0.10884792	0.37683414	0.09002446	0.43968913	0.04595249	0.02282744	0.14861873	0.0190264
21.8	0.05330165	0.1460925	0.29721611	0.0786481	0.10912119	0.37486734	0.08961099	0.44122833	0.0462911	0.02302412	0.1489231	0.01917298
21.9	0.05349643	0.14659599	0.29769262	0.07883057	0.10939134	0.37292102	0.08920205	0.44275539	0.0466289	0.02322106	0.14922571	0.01931992
22	0.05368938	0.14709601	0.298162	0.07901048	0.10965839	0.37099506	0.08879759	0.44427042	0.04696589	0.02341825	0.14952659	0.01946719
22.1	0.05388051	0.14759258	0.29862434	0.07918784	0.10992238	0.36908929	0.08839753	0.44577351	0.04730204	0.02361568	0.14982576	0.0196148
22.2	0.05406983	0.14808571	0.29907976	0.0793627	0.11018332	0.36720358	0.08800182	0.44726477	0.04763733	0.02381334	0.15012325	0.01976273
22.3	0.05425734	0.14857541	0.29952834	0.07953506	0.11044126	0.36533777	0.0876104	0.44874431	0.04797174	0.0240112	0.15041906	0.01991099
22.4	0.05444304	0.14906168	0.2999702	0.07970496	0.11069622	0.36349174	0.08722321	0.45021222	0.04830525	0.02420926	0.15071322	0.02005956
22.5	0.05462694	0.14954455	0.30040542	0.07987243	0.11094823	0.36166535	0.0868402	0.45166861	0.04863784	0.0244075	0.15100574	0.02020844
22.6	0.05480905	0.15002402	0.30083411	0.08003749	0.11119733	0.35985845	0.08646131	0.45311356	0.04896949	0.0246059	0.15129665	0.02035762
22.7	0.05498936	0.15050011	0.30125636	0.08020017	0.11144354	0.35807091	0.08608648	0.45454718	0.04930019	0.02480445	0.15158596	0.02050711
22.8	0.05516789	0.15097283	0.30167228	0.08036049	0.11168689	0.35630257	0.08571566	0.45596956	0.04962991	0.02500314	0.1518737	0.02065688
22.9	0.05534465	0.15144219	0.30208194	0.08051849	0.1119274	0.35455329	0.08534879	0.4573808	0.04995863	0.02520195	0.15215987	0.02080693
23	0.05551966	0.1519082	0.30248544	0.08067417	0.11216512	0.35282293	0.08498581	0.458781	0.05028633	0.02540087	0.1524445	0.02095727
23.1	0.05569291	0.15237087	0.30288289	0.08082757	0.11240007	0.35111133	0.08462668	0.46017024	0.050613	0.02559988	0.15272759	0.02110787
23.2	0.05586442	0.15283022	0.30327437	0.08097872	0.11263229	0.34941835	0.08427135	0.46154861	0.0509386	0.02579897	0.15300918	0.02125874
23.3	0.0560342	0.15328627	0.30365997	0.08112765	0.11286179	0.34774385	0.08391976	0.46291622	0.05126315	0.02599814	0.15328927	0.02140987
23.4	0.05620227	0.15373901	0.30403978	0.08127437	0.11308863	0.34608767	0.08357188	0.46427315	0.05158661	0.02619735	0.15356788	0.02156124
23.5	0.05636861	0.15418847	0.30441389	0.08141892	0.11331282	0.34444963	0.08322764	0.4656195	0.05190897	0.0263966	0.15384502	0.02171286
23.6	0.05653325	0.15463467	0.30478237	0.08156132	0.1135344	0.3428296	0.08288702	0.46695535	0.05223021	0.02659588	0.15412071	0.02186471
23.7	0.05669619	0.15507763	0.30514531	0.08170161	0.11375338	0.34122741	0.08254995	0.4682808	0.05255033	0.02679517	0.15439496	0.0220168
23.8	0.05685744	0.15551736	0.30550281	0.08183981	0.11396981	0.33964292	0.08221639	0.46959593	0.0528693	0.02699446	0.15466778	0.02216911
23.9	0.05701701	0.15595388	0.30585493	0.08197593	0.1141837	0.33807596	0.0818863	0.47090085	0.05318712	0.02719372	0.1549392	0.02232165
24	0.05717491	0.15638721	0.30620177	0.08211	0.11439508	0.33652639	0.08155964	0.47219562	0.05350375	0.02739297	0.15520921	0.02247439
24.1	0.05733115	0.15681736	0.30654339	0.08224205	0.11460398	0.33499403	0.08123634	0.47348036	0.05381921	0.02759217	0.15547785	0.02262735
24.2	0.05748574	0.15724434	0.30687989	0.08237209	0.11481043	0.33347874	0.08091638	0.47475513	0.05413346	0.02779133	0.15574511	0.02278051
24.3	0.05763868	0.15766818	0.30721133	0.08250015	0.11501444	0.33198035	0.0805997	0.47602004	0.05444652	0.02799043	0.15601101	0.02293387
24.4	0.05778999	0.15808888	0.30753779	0.08262626	0.11521606	0.3304987	0.08028627	0.47727516	0.05475836	0.02818947	0.15627557	0.02308741
24.5	0.05793967	0.15850646	0.30785934	0.08275043	0.1154153	0.32903364	0.07997603	0.47852058	0.05506898	0.02838843	0.15653881	0.02324114
24.6	0.05808772	0.15892094	0.30817607	0.08287269	0.11561219	0.327585	0.07966896	0.47975638	0.05537838	0.02858731	0.15680073	0.02339505
24.7	0.05823416	0.15933232	0.30848804	0.08299305	0.11580675	0.32615263	0.079365	0.48098265	0.05568654	0.0287861	0.15706135	0.02354913
24.8	0.05837901	0.15974064	0.30879534	0.08311155	0.11599902	0.32473636	0.07906412	0.48219946	0.05599346	0.02898478	0.15732069	0.02370338
24.9	0.05852227	0.16014591	0.30909802	0.0832282	0.11618901	0.32333601	0.07876627	0.48340692	0.05629911	0.02918334	0.15757876	0.02385779
25	0.05866395	0.16054813	0.30939617	0.08334303	0.11637675	0.32195142	0.07847142	0.4846051	0.05660349	0.02938179	0.15783556	0.02401236
25.1	0.05880407	0.16094734	0.30968987	0.08345605	0.11656227	0.32058242	0.07817952	0.48579407	0.05690659	0.02958009	0.15809111	0.02416708
25.2	0.05894265	0.16134354	0.30997916	0.08356729	0.11674559	0.31922886	0.07789054	0.48697392	0.0572084	0.02977826	0.15834542	0.02432195
25.3	0.05907968	0.16173675	0.31026413	0.08367676	0.11692674	0.31789056	0.07760445	0.48814473	0.05750891	0.02997629	0.15859851	0.02447696
25.4	0.05921519	0.16212698	0.31054484	0.0837845	0.11710573	0.31656739	0.07732119	0.48930659	0.05780812	0.03017416	0.15885039	0.0246321
25.5	0.05934919	0.16251425	0.31082136	0.08389051	0.1172826	0.31525917	0.07704075	0.49045956	0.058106	0.03037188	0.15910107	0.02478738
25.6	0.05948167	0.16289858	0.31109375	0.08399482	0.11745737	0.31396575	0.07676307	0.49160372	0.05840256	0.03056945	0.15935056	0.02494277
25.7	0.05961266	0.16327999	0.31136208	0.08409745	0.11763006	0.31268698	0.07648813	0.49273916	0.05869778	0.03076687	0.15959887	0.02509828
25.8	0.05974215	0.16365848	0.3116264	0.08419842	0.1178007	0.3114227	0.07621588	0.49386594	0.05899167	0.03096412	0.15984602	0.0252539
25.9	0.05987016	0.16403408	0.31188679	0.08429775	0.1179693	0.31017276	0.07594629	0.49498417	0.05928422	0.03116119	0.16009201	0.02540963
26	0.05999671	0.16440679	0.3121433	0.08439546	0.1181359	0.308937	0.07567934	0.4960939	0.05957541	0.03135809	0.16033685	0.02556546
26.1	0.0601218	0.16477664	0.312396	0.08449158	0.11830052	0.30771527	0.07541498	0.49719523	0.05986524	0.03155481	0.16058056	0.02572138
26.2	0.06024545	0.16514365	0.31264494	0.08458612	0.11846318	0.30650742	0.0751532	0.49828822	0.06015371	0.03175134	0.16082313	0.02587738
26.3	0.06036767	0.16550782	0.31289019	0.08467911	0.11862391	0.30531328	0.07489395	0.49937294	0.06044081	0.03194767	0.16106459	0.02603347
26.4	0.06048846	0.16586917	0.31313181	0.08477056	0.11878275	0.30413272	0.0746372	0.50044946	0.06072653	0.03214381	0.16130494	0.02618965
26.5	0.06060785	0.16622773	0.31336984	0.08486049	0.1189397	0.30296558	0.07438293	0.50151786	0.06101089	0.03233973	0.16154419	0.0263459
26.6	0.06072585	0.16658349	0.31360435	0.08494893	0.11909481	0.30181172	0.0741311	0.5025782	0.06129385	0.03253544	0.16178236	0.02650222
26.7	0.06084245	0.1669365	0.31383539	0.08503588	0.11924809	0.30067099	0.07388168	0.50363056	0.06157543	0.03273094	0.16201944	0.02665862
26.8	0.06095767	0.16728675	0.31406302	0.08512138	0.11939956	0.29954325	0.07363465	0.50467501	0.06185561	0.03292623	0.16225546	0.02681508
26.9	0.06107153	0.16763427	0.31428729	0.08520544	0.11954924	0.29842836	0.07338996	0.50571161	0.06213439	0.03312132	0.16249041	0.0269716
27	0.06118402	0.16797907	0.31450825	0.08528807	0.11969716	0.29732616	0.0731476	0.50674045	0.06241176	0.03331619	0.16272432	0.02712817
27.1	0.06129516	0.16832118	0.31472596	0.0853693	0.11984334	0.29623653	0.07290752	0.50776157	0.06268774	0.03351086	0.16295718	0.0272848
27.2	0.06140497	0.1686606	0.31494047	0.08544915	0.11998779	0.29515932	0.07266971	0.50877506	0.06296231	0.03370531	0.16318901	0.02744147
27.3	0.06151344	0.16899735	0.31515183	0.08552762	0.12013053	0.29409438	0.07243414	0.50978099	0.06323547	0.03389955	0.16341981	0.02759819
27.4	0.0616206	0.16933146	0.31536009	0.08560475	0.1202716	0.29304158	0.07220077	0.51077941	0.06350723	0.03409357	0.16364959	0.02775494
27.5	0.06172646	0.16966293	0.31556529	0.08568054	0.12041099	0.29200077	0.07196957	0.5117704	0.06377757	0.03428737	0.16387837	0.02791172
27.6	0.06183102	0.16999178	0.31576749	0.08575502	0.12054875	0.29097181	0.07174053	0.51275404	0.06404649	0.03448095	0.16410615	0.02806854
27.7	0.06193429	0.17031802	0.31596672	0.08582819	0.12068488	0.28995457	0.07151362	0.51373038	0.06431399	0.03467429	0.16433294	0.02822538
27.8	0.06203629	0.17064169	0.31616304	0.08590008	0.12081941	0.28894889	0.0712888	0.51469949	0.06458006	0.03486739	0.16455875	0.02838224
27.9	0.06213703	0.17096278	0.3163565	0.08597071	0.12095235	0.28795466	0.07106606	0.51566145	0.0648447	0.03506025	0.1647836	0.02853911
28	0.06223651	0.17128133	0.31654713	0.08604008	0.12108373	0.28697173	0.07084537	0.51661632	0.06510791	0.03525287	0.16500748	0.028696
28.1	0.06233476	0.17159734	0.31673498	0.08610822	0.12121356	0.28599997	0.07062669	0.51756416	0.06536969	0.03544523	0.16523041	0.02885289
28.2	0.06243178	0.17191083	0.3169201	0.08617515	0.12134187	0.28503925	0.07041001	0.51850504	0.06563004	0.03563733	0.16545239	0.02900979
28.3	0.06252758	0.17222181	0.31710253	0.08624087	0.12146867	0.28408944	0.0701953	0.51943902	0.06588896	0.03582917	0.16567344	0.02916669
28.4	0.06262216	0.1725303	0.31728231	0.0863054	0.12159398	0.28315041	0.06998254	0.52036617	0.06614643	0.03602074	0.16589355	0.02932358
28.5	0.06271554	0.17283632	0.31745947	0.08636876	0.12171782	0.28222205	0.0697717	0.52128655	0.06640247	0.03621205	0.16611274	0.02948046
28.6	0.06280773	0.17313988	0.31763407	0.08643097	0.12184021	0.28130422	0.06956275	0.52220023	0.06665707	0.03640309	0.16633102	0.02963733
28.7	0.06289874	0.173441	0.31780614	0.08649204	0.12196116	0.28039679	0.06935568	0.52310726	0.06691023	0.03659385	0.16654839	0.02979418
28.8	0.06298857	0.1737397	0.31797572	0.08655198	0.1220807	0.27949964	0.06915046	0.5240077	0.06716195	0.03678434	0.16676486	0.02995101
28.9	0.06307724	0.17403599	0.31814284	0.08661082	0.12219884	0.27861264	0.06894707	0.52490163	0.06741224	0.03697455	0.16698043	0.03010782
29	0.06316476	0.17432988	0.31830756	0.08666856	0.1223156	0.27773568	0.06874548	0.52578909	0.06766108	0.03716448	0.16719511	0.0302646
29.1	0.06325114	0.1746214	0.3184699	0.08672522	0.122431	0.27686863	0.06854567	0.52667014	0.06790848	0.03735412	0.16740892	0.03042135
29.2	0.06333639	0.17491056	0.3186299	0.08678083	0.12254505	0.27601137	0.06834763	0.52754486	0.06815444	0.03754348	0.16762185	0.03057806
29.3	0.06342052	0.17519738	0.3187876	0.08683538	0.12265778	0.2751638	0.06815134	0.52841328	0.06839896	0.03773255	0.16783392	0.03073474
29.4	0.06350354	0.17548187	0.31894304	0.0868889	0.12276921	0.27432579	0.06795676	0.52927548	0.06864203	0.03792132	0.16804512	0.03089138
29.5	0.06358547	0.17576405	0.31909625	0.08694139	0.12287935	0.27349724	0.06776389	0.5301315	0.06888365	0.0381098	0.16825548	0.03104797
29.6	0.0636663	0.17604394	0.31924727	0.08699288	0.12298821	0.27267803	0.0675727	0.53098141	0.06912383	0.03829798	0.16846499	0.03120451
29.7	0.06374606	0.17632155	0.31939613	0.08704336	0.12309582	0.27186806	0.06738317	0.53182525	0.06936257	0.03848585	0.16867366	0.03136099
29.8	0.06382475	0.1765969	0.31954287	0.08709287	0.12320219	0.2710672	0.06719527	0.53266309	0.06959988	0.03867341	0.16888149	0.03151742
29.9	0.06390237	0.17687001	0.31968752	0.08714141	0.12330734	0.27027536	0.067009	0.53349497	0.06983577	0.03886067	0.1690885	0.03167378
60	0.06561066	0.20540341	0.33180337	0.08619983	0.13484838	0.19918511	0.0432178	0.6483375	0.09704679	0.0819558	0.20950997	0.06815302
60.1	0.06559281	0.20541386	0.3318189	0.08618497	0.13487013	0.19914869	0.04318533	0.6485014	0.09705871	0.08205825	0.20960063	0.06823606
60.2	0.06557497	0.20542408	0.33183442	0.08617013	0.13489189	0.19911263	0.043153	0.64866462	0.09707042	0.08216046	0.20969111	0.06831888
60.3	0.06555713	0.20543409	0.33184995	0.08615531	0.13491366	0.19907694	0.04312208	0.64882718	0.09708192	0.08226241	0.20978142	0.06840149
60.4	0.0655393	0.20544387	0.33186548	0.08614051	0.13493543	0.19904162	0.04308874	0.64898908	0.09709321	0.08236412	0.20987155	0.06848389
60.5	0.06552147	0.20545343	0.33188102	0.08612572	0.13495721	0.19900666	0.04305681	0.64915032	0.09710428	0.08246557	0.2099615	0.06856609
60.6	0.06550365	0.20546278	0.33189656	0.08611096	0.13497899	0.19897206	0.04302502	0.6493109	0.09711515	0.08256678	0.21005128	0.06864808
60.7	0.06548585	0.20547191	0.3319121	0.08609622	0.13500078	0.19893781	0.04299336	0.64947083	0.09712582	0.08266773	0.21014088	0.06872986
60.8	0.06546805	0.20548083	0.33192765	0.08608149	0.13502258	0.19890392	0.04296184	0.64963011	0.09713627	0.08276844	0.21023031	0.06881143
60.9	0.06545026	0.20548953	0.3319432	0.08606679	0.13504438	0.19887037	0.04293045	0.64978875	0.09714653	0.0828689	0.21031957	0.0688928
61	0.06543247	0.20549802	0.33195876	0.08605211	0.13506619	0.19883717	0.04289919	0.64994675	0.09715659	0.08296911	0.21040865	0.06897397
61.1	0.0654147	0.2055063	0.33197432	0.08603745	0.135088	0.19880432	0.04286807	0.65010411	0.09716644	0.08306906	0.21049756	0.06905493
61.2	0.06539694	0.20551437	0.33198989	0.08602281	0.13510983	0.1987718	0.04283707	0.65026083	0.0971761	0.08316877	0.2105863	0.06913568
61.3	0.06537918	0.20552223	0.33200546	0.08600819	0.13513166	0.19873962	0.0428062	0.65041693	0.09718556	0.08326824	0.21067487	0.06921623
61.4	0.06536144	0.20552989	0.33202104	0.0859936	0.13515349	0.19870777	0.04277547	0.6505724	0.09719482	0.08336745	0.21076327	0.06929658
61.5	0.06534371	0.20553735	0.33203663	0.08597902	0.13517533	0.19867626	0.04274486	0.65072724	0.0972039	0.08346641	0.2108515	0.06937673
61.6	0.06532598	0.2055446	0.33205222	0.08596447	0.13519719	0.19864507	0.04271439	0.65088147	0.09721278	0.08356512	0.21093955	0.06945668
61.7	0.06530827	0.20555165	0.33206782	0.08594994	0.13521904	0.19861421	0.04268404	0.65103508	0.09722147	0.08366359	0.21102744	0.06953642
61.8	0.06529057	0.2055585	0.33208342	0.08593544	0.13524091	0.19858367	0.04265381	0.65118807	0.09722997	0.08376181	0.21111517	0.06961597
61.9	0.06527288	0.20556515	0.33209903	0.08592095	0.13526278	0.19855344	0.04262372	0.65134046	0.09723828	0.08385977	0.21120272	0.06969531
62	0.06525521	0.20557161	0.33211465	0.08590649	0.13528466	0.19852354	0.04259375	0.65149224	0.09724641	0.08395749	0.21129011	0.06977446
62.1	0.06523755	0.20557787	0.33213028	0.08589205	0.13530655	0.19849395	0.0425639	0.65164342	0.09725436	0.08405496	0.21137733	0.0698534
62.2	0.0652199	0.20558394	0.33214592	0.08587764	0.13532845	0.19846467	0.04253418	0.651794	0.09726212	0.08415218	0.21146439	0.06993215
62.3	0.06520227	0.20558982	0.33216156	0.08586325	0.13535035	0.1984357	0.04250459	0.65194398	0.09726969	0.08424916	0.21155128	0.0700107
62.4	0.06518466	0.2055955	0.33217722	0.08584888	0.13537226	0.19840703	0.04247511	0.65209337	0.09727709	0.08434588	0.21163802	0.07008906
62.5	0.06516706	0.205601	0.33219288	0.08583454	0.13539419	0.19837867	0.04244576	0.65224217	0.09728431	0.08444236	0.21172458	0.07016722
62.6	0.06514947	0.20560631	0.33220855	0.08582022	0.13541612	0.19835062	0.04241654	0.65239039	0.09729135	0.08453858	0.21181099	0.07024518
62.7	0.0651319	0.20561143	0.33222423	0.08580593	0.13543806	0.19832286	0.04238743	0.65253802	0.09729822	0.08463456	0.21189723	0.07032295
62.8	0.06511435	0.20561636	0.33223992	0.08579166	0.13546	0.1982954	0.04235845	0.65268507	0.09730491	0.0847303	0.21198331	0.07040052
62.9	0.06509682	0.20562112	0.33225562	0.08577742	0.13548196	0.19826823	0.04232958	0.65283154	0.09731143	0.08482578	0.21206923	0.0704779
63	0.06507931	0.20562569	0.33227133	0.0857632	0.13550393	0.19824135	0.04230084	0.65297744	0.09731778	0.08492103	0.21215499	0.07055509
63.1	0.06506181	0.20563008	0.33228705	0.08574901	0.1355259	0.19821477	0.04227221	0.65312276	0.09732395	0.08501602	0.21224059	0.07063208
63.2	0.06504434	0.20563429	0.33230277	0.08573484	0.13554788	0.19818847	0.0422437	0.65326752	0.09732996	0.08511077	0.21232603	0.07070888

Time discretization	lambda	V_{[G]}	K_{[G]}	delta	q	V_{[PYR]}	K_{[PYR]}	alpha	K_{[ACoA]}	gamma	G_E	L_E
30	0.06397894	0.17714089	0.31983011	0.087189	0.12341128	0.26949242	0.06682433	0.53432095	0.07007023	0.03904762	0.16929469	0.03183007
30.1	0.06405447	0.17740955	0.31997069	0.08723564	0.12351403	0.26871829	0.06664124	0.53514108	0.07030327	0.03923426	0.16950006	0.0319863
30.2	0.06412896	0.17767601	0.32010927	0.08728135	0.12361561	0.26795285	0.06645972	0.53595542	0.07053489	0.03942059	0.16970462	0.03214244
30.3	0.06420242	0.17794029	0.32024589	0.08732614	0.12371603	0.26719601	0.06627974	0.53676403	0.07076509	0.03960663	0.16990837	0.03229851
30.4	0.06427487	0.1782024	0.32038059	0.08737003	0.1238153	0.26644766	0.06610128	0.53756694	0.07099387	0.03979236	0.17011133	0.0324545
30.5	0.06434631	0.17846235	0.32051338	0.08741303	0.12391344	0.26570769	0.06592433	0.53836422	0.07122125	0.0399778	0.17031349	0.0326104
30.6	0.06441675	0.17872017	0.32064431	0.08745515	0.12401046	0.26497602	0.06574888	0.5391559	0.07144721	0.04016293	0.17051487	0.03276622
30.7	0.06448621	0.17897586	0.3207734	0.0874964	0.12410638	0.26425253	0.06557489	0.53994206	0.07167175	0.04034776	0.17071546	0.03292195
30.8	0.06455469	0.17922944	0.32090067	0.08753679	0.12420121	0.26353713	0.06540236	0.54072272	0.07189489	0.04053228	0.17091528	0.03307758
30.9	0.0646222	0.17948093	0.32102616	0.08757634	0.12429496	0.26282971	0.06523127	0.54149794	0.07211662	0.04071649	0.17111433	0.03323312
31	0.06468875	0.17973035	0.32114989	0.08761506	0.12438765	0.26213018	0.06506159	0.54226778	0.07233694	0.0409004	0.17131262	0.03338856
31.1	0.06475435	0.17997771	0.3212719	0.08765296	0.1244793	0.26143844	0.06489332	0.54303227	0.07255586	0.04108399	0.17151015	0.03354389
31.2	0.064819	0.18022303	0.32139221	0.08769005	0.1245699	0.2607544	0.06472644	0.54379147	0.07277338	0.04126727	0.17170693	0.03369911
31.3	0.0648827	0.18046632	0.32151084	0.08772633	0.12465949	0.26007797	0.06456093	0.54454542	0.07298951	0.04145023	0.17190296	0.03385423
31.4	0.06494548	0.1807076	0.32162782	0.08776183	0.12474807	0.25940904	0.06439678	0.54529416	0.07320424	0.04163287	0.17209825	0.03400922
31.5	0.06500734	0.18094688	0.32174318	0.08779655	0.12483565	0.25874754	0.06423396	0.54603776	0.07341759	0.04181521	0.17229281	0.03416411
31.6	0.06506828	0.18118417	0.32185694	0.0878305	0.12492226	0.25809337	0.06407247	0.54677623	0.07362955	0.04199726	0.17248663	0.03431887
31.7	0.06512832	0.1814195	0.32196913	0.0878637	0.12500789	0.25744645	0.06391229	0.54750965	0.07384012	0.042179	0.17267973	0.0344735
31.8	0.06518747	0.18165288	0.32207977	0.08789615	0.12509257	0.25680669	0.06375341	0.54823804	0.07404931	0.04236044	0.1728721	0.03462801
31.9	0.06524573	0.18188432	0.32218888	0.08792786	0.12517631	0.25617401	0.0635958	0.54896145	0.07425713	0.04254158	0.17306377	0.03478238
32	0.06530311	0.18211383	0.3222965	0.08795886	0.12525911	0.25554832	0.06343946	0.54967995	0.07446356	0.04272241	0.17325471	0.03493662
32.1	0.06535963	0.18234143	0.32240263	0.08798913	0.125341	0.25492954	0.06328437	0.55039357	0.07466863	0.04290294	0.17344496	0.03509072
32.2	0.06541528	0.18256714	0.32250731	0.0880187	0.12542198	0.25431759	0.06313052	0.55110237	0.07487232	0.04308316	0.1736345	0.03524468
32.3	0.06547009	0.18279097	0.32261055	0.08804758	0.12550208	0.25371237	0.06297789	0.55180639	0.07507464	0.04326307	0.17382335	0.03539849
32.4	0.06552405	0.18301292	0.32271238	0.08807577	0.1255813	0.25311382	0.06282647	0.55250566	0.07527561	0.04344268	0.1740115	0.03555215
32.5	0.06557718	0.18323303	0.32281282	0.08810328	0.12565966	0.25252184	0.06267624	0.55320024	0.07547521	0.04362197	0.17419897	0.03570566
32.6	0.06562949	0.1834513	0.32291189	0.08813013	0.12573716	0.25193636	0.0625272	0.55389016	0.07567347	0.04380095	0.17438576	0.03585902
32.7	0.06568098	0.18366775	0.32300961	0.08815632	0.12581382	0.2513573	0.06237932	0.55457545	0.07587038	0.04397962	0.17457187	0.03601221
32.8	0.06573165	0.18388239	0.323106	0.08818186	0.12588966	0.25078458	0.0622326	0.55525617	0.07606594	0.04415798	0.17475731	0.03616525
32.9	0.06578153	0.18409523	0.32320109	0.08820676	0.12596467	0.25021813	0.06208702	0.55593235	0.07626016	0.04433603	0.17494208	0.03631811
33	0.06583061	0.18430629	0.32329489	0.08823104	0.12603887	0.24965787	0.06194257	0.55660402	0.07645305	0.04451376	0.17512619	0.03647081
33.1	0.06587891	0.18451559	0.32338742	0.08825469	0.12611227	0.24910373	0.06179923	0.55727124	0.0766446	0.04469118	0.17530965	0.03662333
33.2	0.06592642	0.18472313	0.32347871	0.08827773	0.12618488	0.24855563	0.061657	0.55793402	0.07683482	0.04486828	0.17549245	0.03677567
33.3	0.06597317	0.18492893	0.32356878	0.08830016	0.12625672	0.2480135	0.06151586	0.55859242	0.07702373	0.04504508	0.1756746	0.03692784
33.4	0.06601914	0.185133	0.32365763	0.08832199	0.12632778	0.24747727	0.0613758	0.55924648	0.07721131	0.04522157	0.17585611	0.03707982
33.5	0.06606436	0.18533535	0.3237453	0.08834324	0.12639809	0.24694687	0.0612368	0.55989622	0.07739758	0.04539774	0.17603698	0.03723162
33.6	0.06610883	0.18553601	0.3238318	0.08836391	0.12646765	0.24642222	0.06109887	0.56054168	0.07758254	0.0455736	0.17621722	0.03738323
33.7	0.06615255	0.18573497	0.32391715	0.088384	0.12653647	0.24590327	0.06096197	0.56118291	0.0777662	0.04574915	0.17639682	0.03753464
33.8	0.06619554	0.18593226	0.32400136	0.08840353	0.12660457	0.24538993	0.06082611	0.56181994	0.07794855	0.04592438	0.1765758	0.03768586
33.9	0.0662378	0.18612789	0.32408446	0.0884225	0.12667195	0.24488215	0.06069128	0.5624528	0.07812962	0.0460993	0.17675415	0.03783688
34	0.06627934	0.18632186	0.32416646	0.08844093	0.12673863	0.24437985	0.06055745	0.56308152	0.07830939	0.04627391	0.17693188	0.0379877
34.1	0.06632017	0.18651419	0.32424738	0.08845881	0.12680461	0.24388298	0.06042463	0.56370615	0.07848787	0.0464482	0.17710899	0.03813831
34.2	0.06636029	0.1867049	0.32432724	0.08847616	0.1268699	0.24339148	0.0602928	0.56432672	0.07866508	0.04662217	0.17728549	0.03828872
34.3	0.06639972	0.186894	0.32440604	0.08849299	0.12693451	0.24290527	0.06016194	0.56494326	0.07884102	0.04679582	0.17746138	0.03843892
34.4	0.06643845	0.1870815	0.32448382	0.0885093	0.12699846	0.24242429	0.06003206	0.56555581	0.07901568	0.04696916	0.17763667	0.0385889
34.5	0.0664765	0.1872674	0.32456057	0.0885251	0.12706174	0.24194849	0.05990313	0.56616439	0.07918909	0.04714218	0.17781136	0.03873868
34.6	0.06651386	0.18745173	0.32463633	0.08854039	0.12712437	0.2414778	0.05977515	0.56676904	0.07936124	0.04731488	0.17798544	0.03888823
34.7	0.06655055	0.1876345	0.32471111	0.08855519	0.12718636	0.24101217	0.05964811	0.5673698	0.07953214	0.04748726	0.17815894	0.03903757
34.8	0.06658657	0.18781571	0.32478491	0.0885695	0.12724771	0.24055153	0.059522	0.56796669	0.0797018	0.04765932	0.17833184	0.03918669
34.9	0.06662194	0.18799538	0.32485776	0.08858333	0.12730843	0.24009582	0.0593968	0.56855976	0.07987021	0.04783107	0.17850416	0.03933558
35	0.06665665	0.18817353	0.32492967	0.08859669	0.12736854	0.23964499	0.05927252	0.56914902	0.0800374	0.04800252	0.17867589	0.03948425
35.1	0.06669072	0.18835016	0.32500065	0.08860957	0.12742804	0.23919898	0.05914913	0.56973452	0.08020336	0.04817365	0.17884705	0.03963269
35.2	0.06672416	0.18852529	0.32507073	0.088622	0.12748693	0.23875773	0.05902664	0.57031628	0.0803681	0.04834449	0.17901763	0.0397809
35.3	0.06675697	0.18869892	0.3251399	0.08863396	0.12754524	0.23832119	0.05890503	0.57089434	0.08053163	0.04851501	0.17918763	0.03992888
35.4	0.06678915	0.18887108	0.32520819	0.08864548	0.12760297	0.2378893	0.05878429	0.57146872	0.08069395	0.04868523	0.17935707	0.04007662
35.5	0.06682071	0.18904178	0.32527561	0.08865656	0.12766012	0.23746202	0.05866441	0.57203946	0.08085507	0.04885514	0.17952595	0.04022412
35.6	0.06685167	0.18921102	0.32534217	0.0886672	0.12771671	0.23703927	0.05854539	0.57260659	0.08101499	0.04902475	0.17969426	0.04037138
35.7	0.06688201	0.18937881	0.32540789	0.08867741	0.12777274	0.23662103	0.05842721	0.57317014	0.08117372	0.04919404	0.17986202	0.04051839
35.8	0.06691176	0.18954517	0.32547278	0.0886872	0.12782822	0.23620722	0.05830987	0.57373013	0.08133126	0.04936302	0.18002922	0.04066515
35.9	0.06694091	0.18971011	0.32553685	0.08869656	0.12788316	0.2357978	0.05819336	0.5742866	0.08148762	0.04953169	0.18019587	0.04081166
36	0.06696947	0.18987364	0.32560011	0.08870552	0.12793756	0.23539272	0.05807766	0.57483958	0.08164282	0.04970005	0.18036198	0.04095792
36.1	0.06699745	0.19003577	0.32566259	0.08871407	0.12799144	0.23499192	0.05796278	0.57538909	0.08179684	0.04986809	0.18052754	0.04110392
36.2	0.06702484	0.19019652	0.32572429	0.08872221	0.1280448	0.23459536	0.0578487	0.57593516	0.0819497	0.05003583	0.18069257	0.04124966
36.3	0.06705167	0.19035588	0.32578522	0.08872996	0.12809765	0.23420297	0.05773541	0.57647782	0.08210141	0.05020326	0.18085706	0.04139515
36.4	0.06707793	0.19051388	0.3258454	0.08873732	0.12815	0.23381473	0.05762291	0.5770171	0.08225197	0.0503704	0.18102102	0.04154038
36.5	0.06710362	0.19067052	0.32590483	0.0887443	0.12820184	0.23343057	0.05751119	0.57755302	0.08240139	0.05053724	0.18118445	0.04168535
36.6	0.06712876	0.19082582	0.32596353	0.08875089	0.1282532	0.23305045	0.05740024	0.57808562	0.08254968	0.05070378	0.18134735	0.04183006
36.7	0.06715335	0.19097978	0.32602152	0.08875712	0.12830408	0.23267433	0.05729006	0.57861494	0.08269683	0.05087002	0.18150973	0.0419745
36.8	0.0671774	0.19113243	0.32607879	0.08876298	0.12835447	0.23230215	0.05718063	0.579141	0.08284286	0.05103595	0.18167159	0.04211869
36.9	0.06720091	0.19128376	0.32613536	0.08876847	0.1284044	0.23193388	0.05707195	0.57966382	0.08298776	0.05120159	0.18183293	0.0422626
37	0.06722389	0.19143379	0.32619125	0.08877361	0.12845386	0.23156946	0.056964	0.58018344	0.08313155	0.05136693	0.18199376	0.04240625
37.1	0.06724635	0.19158253	0.32624646	0.0887784	0.12850287	0.23120886	0.0568568	0.58069989	0.08327421	0.05153197	0.18215408	0.04254964
37.2	0.0672683	0.19173	0.326301	0.08878284	0.12855143	0.23085203	0.05675031	0.58121317	0.08341577	0.05169671	0.18231389	0.04269276
37.3	0.06728973	0.19187619	0.32635488	0.08878694	0.12859954	0.23049892	0.05664455	0.58172333	0.08355623	0.05186114	0.1824732	0.0428356
37.4	0.06731065	0.19202112	0.32640811	0.0887907	0.12864722	0.2301495	0.0565395	0.58223039	0.08369558	0.05202527	0.18263201	0.04297818
37.5	0.06733107	0.19216481	0.32646071	0.08879413	0.12869446	0.22980372	0.05643515	0.58273437	0.08383384	0.0521891	0.18279032	0.04312048
37.6	0.067351	0.19230726	0.32651267	0.08879723	0.12874129	0.22946154	0.05633149	0.58323529	0.08397102	0.05235264	0.18294814	0.0432625
37.7	0.06737044	0.19244847	0.32656401	0.08880001	0.12878769	0.22912293	0.05622853	0.58373319	0.08410712	0.05251586	0.18310547	0.04340426
37.8	0.06738938	0.19258847	0.32661474	0.08880247	0.12883368	0.22878783	0.05612625	0.58422808	0.08424214	0.05267879	0.18326231	0.04354573
37.9	0.06740785	0.19272726	0.32666487	0.08880462	0.12887926	0.22845621	0.05602465	0.58471999	0.08437609	0.05284142	0.18341866	0.04368693
38	0.06742584	0.19286485	0.32671441	0.08880646	0.12892445	0.22812803	0.05592371	0.58520894	0.08450898	0.05300374	0.18357453	0.04382785
38.1	0.06744336	0.19300125	0.32676336	0.088808	0.12896924	0.22780325	0.05582345	0.58569496	0.08464081	0.05316576	0.18372992	0.0439685
38.2	0.06746041	0.19313646	0.32681174	0.08880924	0.12901364	0.22748183	0.05572383	0.58617807	0.08477159	0.05332749	0.18388483	0.04410887
38.3	0.06747699	0.1932705	0.32685956	0.08881018	0.12905765	0.22716373	0.05562487	0.58665829	0.08490133	0.05348891	0.18403927	0.04424896
38.4	0.06749311	0.19340337	0.32690682	0.08881083	0.12910129	0.22684892	0.05552656	0.58713564	0.08503002	0.05365003	0.18419323	0.04438877
38.5	0.06750878	0.1935351	0.32695352	0.08881119	0.12914456	0.22653735	0.05542889	0.58761015	0.08515768	0.05381085	0.18434672	0.0445283
38.6	0.067524	0.19366567	0.32699969	0.08881127	0.12918747	0.226229	0.05533185	0.58808183	0.08528432	0.05397136	0.18449975	0.04466755
38.7	0.06753878	0.19379511	0.32704533	0.08881108	0.12923001	0.22592382	0.05523544	0.58855072	0.08540993	0.05413158	0.18465231	0.04480653
38.8	0.06755312	0.19392342	0.32709044	0.08881061	0.12927219	0.22562179	0.05513966	0.58901683	0.08553454	0.0542915	0.1848044	0.04494522
38.9	0.06756702	0.19405061	0.32713504	0.08880986	0.12931403	0.22532285	0.05504449	0.58948018	0.08565813	0.05445111	0.18495604	0.04508364
39	0.0675805	0.1941767	0.32717913	0.08880886	0.12935552	0.22502699	0.05494993	0.58994079	0.08578073	0.05461043	0.18510721	0.04522178
39.1	0.06759356	0.19430168	0.32722273	0.08880759	0.12939667	0.22473417	0.05485598	0.59039869	0.08590233	0.05476944	0.18525793	0.04535963
39.2	0.0676062	0.19442557	0.32726583	0.08880606	0.12943748	0.22444434	0.05476263	0.5908539	0.08602294	0.05492816	0.1854082	0.0454972
39.3	0.06761842	0.19454837	0.32730845	0.08880428	0.12947797	0.22415749	0.05466987	0.59130644	0.08614258	0.05508657	0.18555802	0.04563449
39.4	0.06763023	0.1946701	0.32735059	0.08880224	0.12951812	0.22387356	0.0545777	0.59175633	0.08626123	0.05524469	0.18570738	0.04577149
39.5	0.06764163	0.19479077	0.32739226	0.08879996	0.12955795	0.22359254	0.05448612	0.59220358	0.08637892	0.05540251	0.18585631	0.04590821
39.6	0.06765263	0.19491037	0.32743347	0.08879744	0.12959747	0.2233144	0.05439511	0.59264823	0.08649564	0.05556003	0.18600479	0.04604465
39.7	0.06766322	0.19502893	0.32747422	0.08879467	0.12963667	0.2230391	0.05430468	0.59309029	0.08661141	0.05571726	0.18615283	0.0461808
39.8	0.06767342	0.19514645	0.32751453	0.08879167	0.12967557	0.2227666	0.05421481	0.59352978	0.08672623	0.0558742	0.18630043	0.04631667
39.9	0.06768323	0.19526293	0.3275544	0.08878844	0.12971416	0.22249688	0.0541255	0.59396672	0.0868401	0.05603084	0.18644759	0.04645226
40	0.06769265	0.19537838	0.32759383	0.08878497	0.12975245	0.22222991	0.05403676	0.59440113	0.08695303	0.05618719	0.18659432	0.04658755
40.1	0.06770169	0.19549282	0.32763283	0.08878128	0.12979045	0.22196565	0.05394856	0.59483303	0.08706503	0.05634324	0.18674061	0.04672257
40.2	0.06771034	0.19560625	0.32767142	0.08877737	0.12982815	0.22170409	0.05386091	0.59526244	0.0871761	0.056499	0.18688648	0.0468573
40.3	0.06771862	0.19571868	0.32770959	0.08877324	0.12986557	0.22144518	0.0537738	0.59568937	0.08728626	0.05665446	0.18703192	0.04699174
40.4	0.06772653	0.19583011	0.32774735	0.08876889	0.1299027	0.2211889	0.05368723	0.59611385	0.0873955	0.05680963	0.18717693	0.0471259
40.5	0.06773407	0.19594056	0.32778471	0.08876433	0.12993956	0.22093522	0.0536012	0.59653589	0.08750383	0.0569645	0.18732153	0.04725977
40.6	0.06774124	0.19605003	0.32782168	0.08875956	0.12997614	0.22068411	0.05351569	0.59695552	0.08761127	0.05711909	0.18746571	0.04739336
40.7	0.06774806	0.19615853	0.32785826	0.08875459	0.13001244	0.22043554	0.0534307	0.59737274	0.0877178	0.05727337	0.18760947	0.04752666
40.8	0.06775453	0.19626606	0.32789445	0.08874941	0.13004849	0.22018949	0.05334623	0.59778758	0.08782345	0.05742737	0.18775281	0.04765968
40.9	0.06776064	0.19637265	0.32793027	0.08874403	0.13008427	0.21994593	0.05326228	0.59820005	0.08792822	0.05758107	0.18789574	0.04779241
41	0.06776641	0.19647828	0.32796572	0.08873846	0.13011979	0.21970482	0.05317883	0.59861018	0.08803211	0.05773448	0.18803827	0.04792485
41.1	0.06777183	0.19658297	0.3280008	0.08873269	0.13015506	0.21946615	0.05309589	0.59901798	0.08813513	0.05788761	0.18818038	0.04805701
41.2	0.06777692	0.19668672	0.32803552	0.08872673	0.13019008	0.21922989	0.05301345	0.59942347	0.08823729	0.05804045	0.18832209	0.04818889
41.3	0.06778167	0.19678955	0.3280699	0.08872058	0.13022485	0.21899601	0.0529315	0.59982667	0.08833858	0.058193	0.1884634	0.04832048
41.4	0.06778608	0.19689146	0.32810392	0.08871425	0.13025938	0.21876448	0.05285005	0.6002276	0.08843902	0.05834527	0.1886043	0.04845179
41.5	0.06779017	0.19699246	0.3281376	0.08870774	0.13029367	0.21853529	0.05276908	0.60062629	0.0885386	0.05849725	0.1887448	0.04858281
41.6	0.06779394	0.19709255	0.32817095	0.08870104	0.13032773	0.2183084	0.05268859	0.60102274	0.08863733	0.05864895	0.1888849	0.04871355
41.7	0.06779739	0.19719174	0.32820396	0.08869418	0.13036155	0.21808378	0.05260858	0.60141697	0.08873522	0.05880036	0.18902461	0.048844
41.8	0.06780051	0.19729004	0.32823664	0.08868713	0.13039515	0.21786143	0.05252905	0.601809	0.08883226	0.05895148	0.18916392	0.04897416
41.9	0.06780333	0.19738746	0.32826901	0.08867992	0.13042852	0.21764131	0.05244998	0.60219884	0.08892847	0.05910232	0.18930284	0.04910404
42	0.06780584	0.19748399	0.32830106	0.08867254	0.13046168	0.21742339	0.05237138	0.60258652	0.08902385	0.05925288	0.18944137	0.04923364
42.1	0.06780804	0.19757966	0.32833279	0.08866499	0.13049461	0.21720766	0.05229324	0.60297204	0.08911841	0.05940315	0.18957952	0.04936295
42.2	0.06780993	0.19767446	0.32836422	0.08865728	0.13052734	0.21699409	0.05221556	0.60335543	0.08921214	0.05955313	0.18971728	0.04949197
42.3	0.06781153	0.1977684	0.32839535	0.08864941	0.13055985	0.21678265	0.05213834	0.6037367	0.08930506	0.05970283	0.18985465	0.04962071
42.4	0.06781282	0.19786149	0.32842619	0.08864137	0.13059216	0.21657333	0.05206156	0.60411587	0.08939717	0.05985224	0.18999165	0.04974917
42.5	0.06781383	0.19795373	0.32845673	0.08863319	0.13062426	0.2163661	0.05198523	0.60449295	0.08948848	0.06000137	0.19012826	0.04987734
42.6	0.06781454	0.19804514	0.32848699	0.08862485	0.13065616	0.21616094	0.05190934	0.60486795	0.08957899	0.06015022	0.1902645	0.05000523
42.7	0.06781497	0.19813571	0.32851697	0.08861636	0.13068786	0.21595782	0.05183389	0.6052409	0.08966871	0.06029877	0.19040037	0.05013283
42.8	0.06781511	0.19822546	0.32854667	0.08860772	0.13071937	0.21575673	0.05175888	0.60561181	0.08975764	0.06044705	0.19053586	0.05026015
42.9	0.06781498	0.19831439	0.3285761	0.08859894	0.13075069	0.21555765	0.05168429	0.60598069	0.0898458	0.06059504	0.19067098	0.05038719
43	0.06781456	0.1984025	0.32860526	0.08859001	0.13078182	0.21536055	0.05161014	0.60634756	0.08993317	0.06074274	0.19080573	0.05051394
43.1	0.06781387	0.19848981	0.32863416	0.08858095	0.13081276	0.21516542	0.05153641	0.60671243	0.09001978	0.06089017	0.19094011	0.05064041
43.2	0.06781292	0.19857631	0.3286628	0.08857175	0.13084352	0.21497223	0.05146311	0.60707531	0.09010563	0.06103732	0.19107413	0.05076659
43.3	0.06781169	0.19866203	0.32869118	0.08856241	0.1308741	0.21478096	0.05139022	0.60743623	0.09019071	0.06118419	0.19120778	0.05089249
43.4	0.0678102	0.19874695	0.32871931	0.08855294	0.13090451	0.21459158	0.05131775	0.6077952	0.09027505	0.06133078	0.19134107	0.05101811
43.5	0.06780846	0.19883109	0.32874719	0.08854334	0.13093474	0.21440409	0.05124569	0.60815223	0.09035863	0.06147709	0.191474	0.05114344
43.6	0.06780645	0.19891446	0.32877484	0.08853361	0.1309648	0.21421845	0.05117404	0.60850733	0.09044148	0.06162313	0.19160657	0.05126849
43.7	0.06780419	0.19899705	0.32880224	0.08852375	0.13099469	0.21403465	0.05110279	0.60886052	0.09052358	0.06176888	0.19173878	0.05139325
43.8	0.06780168	0.19907888	0.32882941	0.08851376	0.13102441	0.21385268	0.05103195	0.60921181	0.09060496	0.06191436	0.19187063	0.05151774
43.9	0.06779892	0.19915995	0.32885634	0.08850366	0.13105398	0.21367251	0.0509615	0.60956123	0.09068561	0.06205955	0.19200213	0.05164193
44	0.06779591	0.19924027	0.32888305	0.08849343	0.13108338	0.21349412	0.05089145	0.60990877	0.09076553	0.06220447	0.19213328	0.05176585
44.1	0.06779266	0.19931985	0.32890954	0.08848308	0.13111262	0.21331751	0.05082179	0.61025445	0.09084474	0.06234911	0.19226408	0.05188948
44.2	0.06778918	0.19939868	0.3289358	0.08847261	0.13114171	0.21314264	0.05075251	0.61059829	0.09092324	0.06249347	0.19239452	0.05201282
44.3	0.06778546	0.19947678	0.32896185	0.08846202	0.13117064	0.2129695	0.05068363	0.6109403	0.09100103	0.06263756	0.19252462	0.05213588
44.4	0.0677815	0.19955415	0.32898768	0.08845133	0.13119942	0.21279808	0.05061512	0.6112805	0.09107811	0.06278137	0.19265437	0.05225866
44.5	0.06777732	0.19963079	0.3290133	0.08844052	0.13122806	0.21262836	0.050547	0.61161889	0.0911545	0.06292491	0.19278377	0.05238115
44.6	0.06777291	0.19970672	0.32903871	0.0884296	0.13125655	0.21246031	0.05047925	0.6119555	0.0912302	0.06306818	0.19291283	0.05250336
44.7	0.06776828	0.19978193	0.32906393	0.08841857	0.13128489	0.21229393	0.05041188	0.61229032	0.09130521	0.06321117	0.19304156	0.05262529
44.8	0.06776342	0.19985644	0.32908893	0.08840743	0.13131309	0.21212919	0.05034487	0.61262338	0.09137954	0.06335389	0.19316994	0.05274693
44.9	0.06775835	0.19993024	0.32911375	0.08839619	0.13134116	0.21196608	0.05027823	0.61295468	0.09145319	0.06349633	0.19329799	0.05286829
75	0.06321413	0.20517796	0.33425158	0.0842697	0.13821513	0.19654607	0.03958381	0.66705972	0.09716527	0.09469832	0.22142406	0.07859135
75.1	0.06320109	0.20516811	0.33426895	0.08425913	0.13823832	0.19654098	0.0395663	0.66715366	0.09715859	0.09476708	0.22149384	0.07864932
75.2	0.06318808	0.2051582	0.33428634	0.0842486	0.1382615	0.196536	0.03954886	0.66724728	0.09715184	0.09483566	0.22156351	0.07870715
75.3	0.06317512	0.2051482	0.33430373	0.0842381	0.13828471	0.19653112	0.0395315	0.66734059	0.09714504	0.09490404	0.22163308	0.07876483
75.4	0.06316221	0.20513814	0.33432114	0.08422762	0.13830792	0.19652634	0.0395142	0.66743358	0.09713817	0.09497223	0.22170254	0.07882236
75.5	0.06314934	0.20512801	0.33433856	0.08421718	0.13833114	0.19652166	0.03949697	0.66752626	0.09713124	0.09504024	0.22177189	0.07887975
75.6	0.06313652	0.2051178	0.334356	0.08420677	0.13835437	0.19651708	0.03947982	0.66761863	0.09712426	0.09510805	0.22184113	0.07893699
75.7	0.06312374	0.20510753	0.33437345	0.0841964	0.1383776	0.1965126	0.03946273	0.66771069	0.09711721	0.09517568	0.22191026	0.07899409
75.8	0.06311101	0.20509718	0.33439092	0.08418605	0.13840084	0.19650822	0.03944572	0.66780245	0.09711011	0.09524311	0.22197929	0.07905104
75.9	0.06309832	0.20508677	0.3344084	0.08417573	0.13842409	0.19650393	0.03942877	0.66789389	0.09710295	0.09531036	0.22204821	0.07910784
76	0.06308568	0.20507629	0.33442589	0.08416545	0.13844735	0.19649974	0.0394119	0.66798503	0.09709573	0.09537742	0.22211703	0.0791645
76.1	0.06307308	0.20506574	0.33444339	0.0841552	0.13847062	0.19649565	0.03939509	0.66807587	0.09708845	0.0954443	0.22218574	0.07922101
76.2	0.06306053	0.20505513	0.33446091	0.08414498	0.13849389	0.19649165	0.03937835	0.6681664	0.09708112	0.09551099	0.22225434	0.07927738
76.3	0.06304802	0.20504445	0.33447845	0.08413478	0.13851717	0.19648775	0.03936168	0.66825664	0.09707373	0.09557749	0.22232284	0.0793336
76.4	0.06303556	0.20503371	0.33449599	0.08412463	0.13854046	0.19648394	0.03934508	0.66834657	0.09706629	0.09564381	0.22239123	0.07938967
76.5	0.06302315	0.2050229	0.33451355	0.0841145	0.13856375	0.19648023	0.03932855	0.6684362	0.09705879	0.09570994	0.22245952	0.0794456
76.6	0.06301078	0.20501203	0.33453112	0.0841044	0.13858705	0.1964766	0.03931208	0.66852554	0.09705124	0.09577589	0.2225277	0.07950139
76.7	0.06299846	0.20500109	0.33454871	0.08409433	0.13861036	0.19647307	0.03929568	0.66861457	0.09704364	0.09584165	0.22259578	0.07955703
76.8	0.06298618	0.20499009	0.33456631	0.0840843	0.13863368	0.19646963	0.03927935	0.66870332	0.09703597	0.09590723	0.22266375	0.07961252
76.9	0.06297394	0.20497903	0.33458392	0.08407429	0.138657	0.19646628	0.03926309	0.66879177	0.09702826	0.09597263	0.22273162	0.07966787
77	0.06296176	0.2049679	0.33460155	0.08406432	0.13868033	0.19646301	0.03924689	0.66887993	0.09702049	0.09603784	0.22279939	0.07972308
77.1	0.06294962	0.20495672	0.33461918	0.08405438	0.13870367	0.19645984	0.03923076	0.66896779	0.09701266	0.09610288	0.22286705	0.07977814
77.2	0.06293752	0.20494547	0.33463684	0.08404447	0.13872701	0.19645675	0.0392147	0.66905537	0.09700478	0.09616773	0.22293461	0.07983306
77.3	0.06292547	0.20493416	0.3346545	0.08403459	0.13875036	0.19645375	0.0391987	0.66914266	0.09699685	0.0962324	0.22300207	0.07988784
77.4	0.06291346	0.20492279	0.33467218	0.08402474	0.13877372	0.19645084	0.03918277	0.66922966	0.09698887	0.09629688	0.22306943	0.07994247
77.5	0.0629015	0.20491136	0.33468987	0.08401492	0.13879708	0.19644801	0.03916691	0.66931637	0.09698083	0.09636119	0.22313668	0.07999697
77.6	0.06288959	0.20489987	0.33470757	0.08400513	0.13882045	0.19644526	0.03915111	0.6694028	0.09697275	0.09642532	0.22320384	0.08005132
77.7	0.06287772	0.20488833	0.33472529	0.08399537	0.13884383	0.1964426	0.03913537	0.66948894	0.09696461	0.09648927	0.22327089	0.08010552
77.8	0.0628659	0.20487672	0.33474302	0.08398564	0.13886721	0.19644002	0.0391197	0.6695748	0.09695643	0.09655304	0.22333784	0.08015959
77.9	0.06285412	0.20486506	0.33476076	0.08397595	0.1388906	0.19643752	0.0391041	0.66966038	0.09694819	0.09661663	0.22340469	0.08021352
78	0.06284239	0.20485334	0.33477852	0.08396628	0.138914	0.19643511	0.03908855	0.66974568	0.09693991	0.09668004	0.22347144	0.0802673
78.1	0.06283071	0.20484156	0.33479628	0.08395665	0.1389374	0.19643277	0.03907307	0.6698307	0.09693158	0.09674328	0.22353809	0.08032095
78.2	0.06281907	0.20482972	0.33481406	0.08394705	0.13896081	0.19643052	0.03905766	0.66991544	0.0969232	0.09680634	0.22360464	0.08037445

48.3	0.06747277	0.20205812	0.32986081	0.08796354	0.1322278	0.20727796	0.04821205	0.62325965	0.09358734	0.06817929	0.19746136	0.05682772
48.4	0.06746149	0.20211046	0.32988049	0.08794964	0.13225227	0.20716236	0.04815664	0.62353682	0.09364013	0.06831241	0.19757852	0.05693932
48.5	0.06745008	0.20216227	0.32990005	0.08793568	0.13227667	0.20704788	0.04810151	0.62381262	0.09369239	0.06844526	0.19769539	0.05705065
48.6	0.06743853	0.20221355	0.32991952	0.08792167	0.132301	0.20693451	0.04804665	0.62408706	0.09374414	0.06857785	0.19781198	0.05716171
48.7	0.06742684	0.2022643	0.32993889	0.08790761	0.13232526	0.20682224	0.04799208	0.62436017	0.09379537	0.06871017	0.19792829	0.05727249
48.8	0.06741501	0.20231454	0.32995816	0.0878935	0.13234945	0.20671107	0.04793778	0.62463193	0.0938461	0.06884223	0.19804432	0.057383
48.9	0.06740306	0.20236425	0.32997733	0.08787935	0.13237358	0.20660097	0.04788375	0.62490237	0.09389632	0.06897403	0.19816006	0.05749324
49	0.06739098	0.20241344	0.32999641	0.08786514	0.13239764	0.20649194	0.04782999	0.62517149	0.09394603	0.06910556	0.19827553	0.0576032
49.1	0.06737877	0.20246213	0.33001539	0.0878509	0.13242163	0.20638396	0.0477765	0.62543931	0.09399525	0.06923682	0.19839071	0.05771289
49.2	0.06736643	0.20251031	0.33003429	0.0878366	0.13244556	0.20627703	0.04772327	0.62570582	0.09404397	0.06936782	0.19850562	0.05782231
49.3	0.06735397	0.20255799	0.33005309	0.08782227	0.13246943	0.20617114	0.04767031	0.62597104	0.0940922	0.06949855	0.19862026	0.05793146
49.4	0.06734138	0.20260516	0.33007181	0.08780789	0.13249324	0.20606627	0.04761761	0.62623498	0.09413994	0.06962901	0.19873461	0.05804034
49.5	0.06732868	0.20265185	0.33009044	0.08779347	0.13251698	0.20596243	0.04756518	0.62649764	0.0941872	0.0697592	0.1988487	0.05814895
49.6	0.06731585	0.20269805	0.33010899	0.08777901	0.13254067	0.20585958	0.047513	0.62675903	0.09423398	0.06988913	0.19896251	0.05825729
49.7	0.06730291	0.20274376	0.33012745	0.08776451	0.13256431	0.20575774	0.04746107	0.62701916	0.09428028	0.07001878	0.19907606	0.05836536
49.8	0.06728985	0.20278898	0.33014584	0.08774998	0.13258788	0.20565688	0.04740941	0.62727804	0.0943261	0.07014817	0.19918933	0.05847317
49.9	0.06727668	0.20283373	0.33016414	0.0877354	0.1326114	0.205557	0.04735799	0.62753568	0.09437146	0.07027729	0.19930234	0.0585807
50	0.0672634	0.202878	0.33018237	0.08772079	0.13263487	0.2054581	0.04730683	0.62779208	0.09441635	0.07040614	0.19941507	0.05868797
50.1	0.06725001	0.2029218	0.33020053	0.08770614	0.13265828	0.20536015	0.04725591	0.62804726	0.09446078	0.07053471	0.19952755	0.05879496
50.2	0.06723651	0.20296513	0.33021861	0.08769146	0.13268164	0.20526316	0.04720524	0.62830122	0.09450475	0.07066302	0.19963975	0.0589017
50.3	0.0672229	0.20300799	0.33023661	0.08767674	0.13270496	0.20516711	0.04715482	0.62855396	0.09454827	0.07079105	0.1997517	0.05900816
50.4	0.06720918	0.20305039	0.33025455	0.08766199	0.13272822	0.20507199	0.04710464	0.6288055	0.09459133	0.07091882	0.19986338	0.05911436
50.5	0.06719536	0.20309233	0.33027241	0.08764721	0.13275144	0.20497779	0.04705471	0.62905584	0.09463393	0.07104631	0.1999748	0.05922029
50.6	0.06718144	0.20313381	0.33029021	0.08763239	0.1327746	0.20488451	0.04700501	0.629305	0.09467609	0.07117353	0.20008596	0.05932596
50.7	0.06716742	0.20317484	0.33030793	0.08761754	0.13279773	0.20479213	0.04695556	0.62955298	0.09471781	0.07130048	0.20019686	0.05943136
50.8	0.0671533	0.20321542	0.33032559	0.08760266	0.1328208	0.20470065	0.04690634	0.62979978	0.09475909	0.07142715	0.2003075	0.0595365
50.9	0.06713908	0.20325556	0.33034319	0.08758776	0.13284384	0.20461006	0.04685735	0.63004542	0.09479992	0.07155356	0.20041789	0.05964138
51	0.06712476	0.20329525	0.33036072	0.08757282	0.13286683	0.20452035	0.0468086	0.63028991	0.09484032	0.07167969	0.20052801	0.05974599
51.1	0.06711035	0.2033345	0.33037819	0.08755785	0.13288978	0.20443151	0.04676008	0.63053324	0.09488029	0.07180554	0.20063789	0.05985034
51.2	0.06709585	0.20337332	0.33039559	0.08754286	0.13291268	0.20434354	0.04671179	0.63077543	0.09491983	0.07193113	0.20074751	0.05995443
51.3	0.06708126	0.2034117	0.33041294	0.08752784	0.13293555	0.20425641	0.04666373	0.63101649	0.09495895	0.07205644	0.20085687	0.06005825
51.4	0.06706658	0.20344965	0.33043023	0.0875128	0.13295838	0.20417014	0.04661589	0.63125641	0.09499764	0.07218148	0.20096599	0.06016181
51.5	0.06705181	0.20348718	0.33044745	0.08749773	0.13298116	0.2040847	0.04656829	0.63149522	0.09503592	0.07230625	0.20107485	0.06026511
51.6	0.06703695	0.20352428	0.33046463	0.08748264	0.13300391	0.20400009	0.0465209	0.63173292	0.09507378	0.07243075	0.20118346	0.06036815
51.7	0.06702201	0.20356096	0.33048174	0.08746753	0.13302663	0.2039163	0.04647374	0.6319695	0.09511122	0.07255499	0.20129182	0.06047093
51.8	0.06700699	0.20359722	0.33049881	0.0874524	0.1330493	0.20383332	0.0464268	0.63220499	0.09514826	0.07267896	0.20139993	0.06057345
51.9	0.06699189	0.20363307	0.33051582	0.08743724	0.13307195	0.20375115	0.04638008	0.63243938	0.09518489	0.07280267	0.2015078	0.06067572
52	0.06697671	0.20366851	0.33053277	0.08742207	0.13309455	0.20366978	0.04633358	0.63267269	0.09522112	0.07292611	0.20161542	0.06077772
52.1	0.06696145	0.20370354	0.33054968	0.08740688	0.13311713	0.20358919	0.04628729	0.63290492	0.09525695	0.07304928	0.20172279	0.06087947
52.2	0.06694611	0.20373816	0.33056654	0.08739167	0.13313967	0.20350939	0.04624122	0.63313607	0.09529238	0.07317219	0.20182992	0.06098096
52.3	0.0669307	0.20377239	0.33058335	0.08737645	0.13316218	0.20343036	0.04619537	0.63336615	0.09532742	0.07329483	0.20193681	0.0610822
52.4	0.06691521	0.20380621	0.33060012	0.0873612	0.13318466	0.2033521	0.04614972	0.63359518	0.09536207	0.07341721	0.20204345	0.06118318
52.5	0.06689966	0.20383964	0.33061683	0.08734595	0.13320711	0.2032746	0.04610429	0.63382315	0.09539632	0.07353932	0.20214985	0.0612839
52.6	0.06688403	0.20387267	0.33063351	0.08733068	0.13322953	0.20319785	0.04605907	0.63405007	0.09543019	0.07366118	0.20225601	0.06138437
52.7	0.06686833	0.20390531	0.33065014	0.08731539	0.13325192	0.20312184	0.04601406	0.63427596	0.09546368	0.07378277	0.20236193	0.06148459
52.8	0.06685257	0.20393757	0.33066672	0.08730009	0.13327428	0.20304658	0.04596925	0.6345008	0.09549678	0.07390411	0.20246761	0.06158455
52.9	0.06683674	0.20396944	0.33068327	0.08728478	0.13329662	0.20297204	0.04592465	0.63472463	0.09552951	0.07402519	0.20257306	0.06168426
53	0.06682085	0.20400093	0.33069978	0.08726946	0.13331893	0.20289824	0.04588025	0.63494743	0.09556186	0.07414601	0.20267827	0.06178371
53.1	0.0668049	0.20403204	0.33071624	0.08725413	0.13334122	0.20282515	0.04583606	0.63516921	0.09559385	0.07426658	0.20278324	0.06188292
53.2	0.06678888	0.20406278	0.33073267	0.08723879	0.13336348	0.20275279	0.04579207	0.63538999	0.09562547	0.07438688	0.20288798	0.06198187
53.3	0.06677281	0.20409314	0.33074906	0.08722343	0.13338571	0.20268112	0.04574829	0.63560976	0.09565672	0.07450693	0.20299249	0.06208057
53.4	0.06675667	0.20412312	0.33076542	0.08720807	0.13340793	0.20261016	0.0457047	0.63582854	0.09568761	0.07462671	0.20309676	0.06217902
53.5	0.06674048	0.20415275	0.33078174	0.08719269	0.13343012	0.20253989	0.04566131	0.63604633	0.09571815	0.07474624	0.20320081	0.06227723
53.6	0.06672423	0.204182	0.33079803	0.08717731	0.13345229	0.20247031	0.04561812	0.63626314	0.09574833	0.07486551	0.20330462	0.06237518
53.7	0.06670792	0.20421089	0.33081428	0.08716192	0.13347443	0.2024014	0.04557513	0.63647897	0.09577815	0.07498451	0.2034082	0.06247289
53.8	0.06669156	0.20423942	0.3308305	0.08714652	0.13349656	0.20233317	0.04553233	0.63669382	0.09580763	0.07510326	0.20351156	0.06257034
53.9	0.06667515	0.20426759	0.33084669	0.08713112	0.13351866	0.2022656	0.04548972	0.63690771	0.09583675	0.07522175	0.20361469	0.06266755
54	0.06665867	0.2042954	0.33086285	0.08711571	0.13354075	0.20219869	0.04544731	0.63712064	0.09586553	0.07533998	0.20371759	0.06276451
54.1	0.06664215	0.20432287	0.33087898	0.0871003	0.13356282	0.20213243	0.04540509	0.63733262	0.09589397	0.07545795	0.20382027	0.06286122
54.2	0.06662557	0.20434998	0.33089508	0.08708488	0.13358487	0.20206682	0.04536306	0.63754365	0.09592207	0.07557566	0.20392272	0.06295769
54.3	0.06660893	0.20437674	0.33091115	0.08706946	0.1336069	0.20200185	0.04532122	0.63775374	0.09594982	0.07569311	0.20402495	0.06305392
54.4	0.06659225	0.20440316	0.3309272	0.08705403	0.13362891	0.20193751	0.04527957	0.63796289	0.09597725	0.07581029	0.20412696	0.0631499
54.5	0.06657552	0.20442923	0.33094323	0.0870386	0.13365091	0.20187379	0.04523811	0.63817111	0.09600434	0.07592722	0.20422874	0.06324563
54.6	0.06655874	0.20445496	0.33095922	0.08702317	0.13367289	0.2018107	0.04519683	0.6383784	0.0960311	0.07604389	0.20433031	0.06334113
54.7	0.06654192	0.20448036	0.3309752	0.08700774	0.13369485	0.20174823	0.04515574	0.63858478	0.09605753	0.07616029	0.20443165	0.06343638
54.8	0.06652505	0.20450542	0.33099115	0.08699231	0.13371681	0.20168636	0.04511484	0.63879024	0.09608364	0.07627644	0.20453278	0.06353139
54.9	0.06650813	0.20453015	0.33100708	0.08697687	0.13373874	0.20162509	0.04507412	0.63899479	0.09610942	0.07639232	0.20463368	0.06362615
55	0.06649117	0.20455455	0.33102298	0.08696144	0.13376066	0.20156443	0.04503358	0.63919843	0.09613488	0.07650794	0.20473437	0.06372068
55.1	0.06647417	0.20457862	0.33103887	0.08694601	0.13378257	0.20150435	0.04499322	0.63940118	0.09616002	0.0766233	0.20483485	0.06381496
55.2	0.06645713	0.20460236	0.33105473	0.08693058	0.13380446	0.20144486	0.04495304	0.63960304	0.09618485	0.0767384	0.2049351	0.06390901
55.3	0.06644005	0.20462578	0.33107058	0.08691515	0.13382635	0.20138595	0.04491304	0.639804	0.09620936	0.07685324	0.20503515	0.06400282
55.4	0.06642293	0.20464889	0.3310864	0.08689972	0.13384822	0.20132762	0.04487322	0.64000409	0.09623356	0.07696781	0.20513498	0.06409639
55.5	0.06640578	0.20467167	0.33110221	0.08688429	0.13387007	0.20126987	0.04483357	0.6402033	0.09625745	0.07708212	0.20523459	0.06418972
55.6	0.06638859	0.20469414	0.331118	0.08686887	0.13389192	0.20121268	0.04479411	0.64040163	0.09628104	0.07719617	0.205334	0.06428281
55.7	0.06637136	0.2047163	0.33113377	0.08685345	0.13391376	0.20115605	0.04475481	0.6405991	0.09630432	0.07730996	0.20543319	0.06437566
55.8	0.06635409	0.20473815	0.33114952	0.08683804	0.13393558	0.20109998	0.0447157	0.64079571	0.09632729	0.07742348	0.20553218	0.06446828
55.9	0.0663368	0.20475969	0.33116526	0.08682263	0.1339574	0.20104447	0.04467675	0.64099146	0.09634997	0.07753674	0.20563095	0.06456067
56	0.06631947	0.20478092	0.33118099	0.08680722	0.1339792	0.2009895	0.04463798	0.64118636	0.09637234	0.07764973	0.20572952	0.06465282
56.1	0.0663021	0.20480185	0.33119669	0.08679182	0.134001	0.20093507	0.04459938	0.64138042	0.09639442	0.07776247	0.20582788	0.06474473
56.2	0.06628471	0.20482248	0.33121239	0.08677643	0.13402279	0.20088117	0.04456095	0.64157363	0.09641621	0.07787493	0.20592603	0.06483642
56.3	0.06626729	0.20484281	0.33122807	0.08676104	0.13404457	0.20082781	0.04452268	0.641766	0.0964377	0.07798713	0.20602397	0.06492786
56.4	0.06624984	0.20486284	0.33124373	0.08674566	0.13406634	0.20077497	0.04448459	0.64195755	0.09645891	0.07809907	0.20612171	0.06501908
56.5	0.06623236	0.20488258	0.33125939	0.08673028	0.1340881	0.20072265	0.04444666	0.64214827	0.09647983	0.07821074	0.20621925	0.06511006
56.6	0.06621485	0.20490202	0.33127503	0.08671492	0.13410986	0.20067084	0.0444089	0.64233816	0.09650046	0.07832215	0.20631658	0.06520082
56.7	0.06619732	0.20492117	0.33129066	0.08669956	0.13413161	0.20061955	0.0443713	0.64252724	0.0965208	0.07843329	0.20641371	0.06529134
56.8	0.06617977	0.20494003	0.33130628	0.08668421	0.13415336	0.20056876	0.04433387	0.64271551	0.09654087	0.07854416	0.20651064	0.06538163
56.9	0.06616219	0.20495861	0.33132189	0.08666887	0.13417509	0.20051847	0.0442966	0.64290297	0.09656066	0.07865477	0.20660736	0.06547169
57	0.06614459	0.2049769	0.33133749	0.08665354	0.13419683	0.20046867	0.0442595	0.64308962	0.09658016	0.07876511	0.20670389	0.06556152
57.1	0.06612697	0.20499491	0.33135308	0.08663822	0.13421856	0.20041936	0.04422255	0.64327548	0.0965994	0.07887519	0.20680021	0.06565113
57.2	0.06610932	0.20501264	0.33136866	0.0866229	0.13424028	0.20037055	0.04418577	0.64346054	0.09661836	0.078985	0.20689634	0.0657405
57.3	0.06609166	0.20503009	0.33138424	0.0866076	0.134262	0.20032221	0.04414915	0.64364481	0.09663705	0.07909456	0.20699226	0.06582965
57.4	0.06607398	0.20504726	0.33139981	0.08659231	0.13428372	0.20027436	0.04411268	0.6438283	0.09665547	0.07920384	0.20708799	0.06591858
57.5	0.06605628	0.20506416	0.33141537	0.08657704	0.13430543	0.20022697	0.04407638	0.644011	0.09667363	0.07931287	0.20718353	0.06600727
57.6	0.06603856	0.20508079	0.33143092	0.08656177	0.13432714	0.20018006	0.04404023	0.64419293	0.09669152	0.07942164	0.20727886	0.06609575
57.7	0.06602083	0.20509715	0.33144647	0.08654652	0.13434885	0.20013361	0.04400424	0.64437409	0.09670916	0.07953015	0.207374	0.06618399
57.8	0.06600308	0.20511324	0.33146201	0.08653128	0.13437056	0.20008763	0.0439684	0.64455447	0.09672653	0.0796384	0.20746895	0.06627202
57.9	0.06598532	0.20512907	0.33147755	0.08651605	0.13439226	0.20004211	0.04393272	0.6447341	0.09674364	0.07974639	0.2075637	0.06635981
58	0.06596754	0.20514463	0.33149308	0.08650083	0.13441397	0.19999704	0.0438972	0.64491297	0.0967605	0.07985412	0.20765826	0.06644739
58.1	0.06594976	0.20515993	0.33150861	0.08648563	0.13443567	0.19995243	0.04386182	0.64509108	0.0967771	0.07996159	0.20775263	0.06653475
58.2	0.06593196	0.20517498	0.33152414	0.08647044	0.13445738	0.19990827	0.0438266	0.64526844	0.09679345	0.08006881	0.20784681	0.06662188
58.3	0.06591415	0.20518976	0.33153966	0.08645527	0.13447908	0.19986454	0.04379153	0.64544506	0.09680955	0.08017578	0.20794079	0.06670879
58.4	0.06589633	0.20520429	0.33155518	0.08644011	0.13450079	0.19982126	0.04375662	0.64562094	0.09682541	0.0802825	0.20803459	0.06679548
58.5	0.0658785	0.20521857	0.3315707	0.08642497	0.13452249	0.19977841	0.04372185	0.64579608	0.09684101	0.08038896	0.20812819	0.06688195
58.6	0.06586067	0.20523259	0.33158621	0.08640984	0.1345442	0.19973598	0.04368723	0.64597049	0.09685637	0.08049517	0.20822161	0.0669682
58.7	0.06584283	0.20524637	0.33160172	0.08639473	0.13456591	0.19969399	0.04365276	0.64614417	0.09687148	0.08060113	0.20831484	0.06705424
58.8	0.06582498	0.20525989	0.33161724	0.08637963	0.13458762	0.19965242	0.04361844	0.64631712	0.09688635	0.08070684	0.20840788	0.06714005
58.9	0.06580713	0.20527317	0.33163275	0.08636455	0.13460933	0.19961127	0.04358426	0.64648936	0.09690099	0.0808123	0.20850073	0.06722565
59	0.06578927	0.2052862	0.33164826	0.08634949	0.13463104	0.19957053	0.04355023	0.64666087	0.09691538	0.08091751	0.2085934	0.06731103
59.1	0.06577141	0.20529898	0.33166376	0.08633444	0.13465276	0.1995302	0.04351635	0.64683168	0.09692954	0.08102247	0.20868588	0.06739619
59.2	0.06575355	0.20531153	0.33167927	0.08631941	0.13467448	0.19949028	0.04348261	0.64700178	0.09694347	0.08112717	0.20877818	0.06748114
59.3	0.06573569	0.20532384	0.33169478	0.0863044	0.1346962	0.19945076	0.04344901	0.64717117	0.09695717	0.08123163	0.20887029	0.06756587
59.4	0.06571782	0.2053359	0.33171029	0.08628941	0.13471793	0.19941165	0.04341556	0.64733986	0.09697065	0.08133583	0.20896222	0.06765039
59.5	0.06569996	0.20534773	0.3317258	0.08627443	0.13473966	0.19937292	0.04338225	0.64750785	0.09698389	0.08143978	0.20905396	0.06773469
59.6	0.0656821	0.20535933	0.33174131	0.08625947	0.1347614	0.19933459	0.04334908	0.64767515	0.09699691	0.08154349	0.20914552	0.06781878
59.7	0.06566423	0.2053707	0.33175682	0.08624454	0.13478313	0.19929665	0.04331605	0.64784176	0.09700971	0.08164694	0.20923691	0.06790266
59.8	0.06564637	0.20538183	0.33177234	0.08622962	0.13480488	0.19925909	0.04328316	0.64800769	0.09702229	0.08175014	0.20932811	0.06798633
59.9	0.06562851	0.20539273	0.33178785	0.08621472	0.13482662	0.19922191	0.04325041	0.64817293	0.09703465	0.0818531	0.20941913	0.06806978

90	0.06174854	0.20313911	0.33697842	0.08301576	0.14174997	0.19657944	0.0376241	0.67825002	0.09571203	0.10312809	0.23082704	0.08582434
90.1	0.06174184	0.20312301	0.33699717	0.08300946	0.1417737	0.19658325	0.03761479	0.67830861	0.09570059	0.1031732	0.23088345	0.08586412
90.2	0.06173518	0.2031069	0.33701591	0.08300318	0.14179742	0.19658708	0.03760552	0.67836704	0.09568914	0.10321817	0.23093978	0.08590381
90.3	0.06172855	0.20309076	0.33703466	0.08299693	0.14182114	0.19659095	0.03759629	0.67842529	0.09567769	0.10326302	0.23099604	0.08594341
90.4	0.06172196	0.2030746	0.33705342	0.0829907	0.14184487	0.19659485	0.0375871	0.67848337	0.09566622	0.10330774	0.23105223	0.08598291
90.5	0.0617154	0.20305843	0.33707217	0.0829845	0.14186859	0.19659877	0.03757796	0.67854127	0.09565474	0.10335234	0.23110834	0.08602232
90.6	0.06170888	0.20304223	0.33709094	0.08297832	0.14189231	0.19660272	0.03756885	0.67859901	0.09564325	0.10339682	0.23116438	0.08606164
90.7	0.06170239	0.20302602	0.3371097	0.08297217	0.14191603	0.1966067	0.03755979	0.67865658	0.09563175	0.10344117	0.23122034	0.08610087
90.8	0.06169594	0.20300979	0.33712847	0.08296604	0.14193976	0.19661071	0.03755077	0.67871399	0.09562024	0.10348539	0.23127623	0.08614
90.9	0.06168952	0.20299354	0.33714725	0.08295993	0.14196348	0.19661475	0.03754178	0.67877122	0.09560872	0.10352949	0.23133205	0.08617905
91	0.06168314	0.20297727	0.33716602	0.08295385	0.1419872	0.19661882	0.03753284	0.67882829	0.09559719	0.10357347	0.23138779	0.086218
91.1	0.0616768	0.20296098	0.3371848	0.08294779	0.14201091	0.19662291	0.03752394	0.67888519	0.09558565	0.10361733	0.23144347	0.08625686
91.2	0.06167048	0.20294468	0.33720359	0.08294176	0.14203463	0.19662703	0.03751507	0.67894193	0.0955741	0.10366107	0.23149906	0.08629563
91.3	0.06166421	0.20292835	0.33722237	0.08293575	0.14205835	0.19663117	0.03750625	0.6789985	0.09556255	0.10370468	0.23155459	0.08633431
91.4	0.06165797	0.20291201	0.33724116	0.08292976	0.14208206	0.19663535	0.03749747	0.6790549	0.09555098	0.10374817	0.23161004	0.0863729
91.5	0.06165176	0.20289566	0.33725996	0.0829238	0.14210578	0.19663954	0.03748872	0.67911114	0.09553941	0.10379154	0.23166542	0.0864114
91.6	0.06164559	0.20287928	0.33727875	0.08291786	0.14212949	0.19664377	0.03748002	0.67916722	0.09552783	0.10383479	0.23172073	0.08644981
91.7	0.06163945	0.20286289	0.33729755	0.08291195	0.1421532	0.19664802	0.03747135	0.67922314	0.09551624	0.10387792	0.23177597	0.08648813
91.8	0.06163334	0.20284648	0.33731635	0.08290606	0.14217691	0.1966523	0.03746273	0.67927889	0.09550464	0.10392093	0.23183114	0.08652637
91.9	0.06162727	0.20283006	0.33733516	0.08290019	0.14220062	0.1966566	0.03745414	0.67933448	0.09549304	0.10396382	0.23188623	0.08656451
92	0.06162124	0.20281362	0.33735396	0.08289434	0.14222433	0.19666093	0.03744559	0.67938991	0.09548142	0.10400659	0.23194125	0.08660256
92.1	0.06161523	0.20279716	0.33737277	0.08288852	0.14224804	0.19666528	0.03743708	0.67944518	0.0954698	0.10404924	0.2319962	0.08664053
92.2	0.06160926	0.20278069	0.33739159	0.08288273	0.14227174	0.19666965	0.03742861	0.67950029	0.09545817	0.10409177	0.23205108	0.08667841
92.3	0.06160333	0.2027642	0.3374104	0.08287696	0.14229544	0.19667406	0.03742018	0.67955524	0.09544653	0.10413418	0.23210589	0.0867162
92.4	0.06159742	0.20274769	0.33742922	0.08287121	0.14231914	0.19667848	0.03741178	0.67961003	0.09543489	0.10417647	0.23216062	0.0867539
92.5	0.06159155	0.20273117	0.33744804	0.08286548	0.14234284	0.19668293	0.03740343	0.67966466	0.09542324	0.10421865	0.23221529	0.08679152
92.6	0.06158572	0.20271464	0.33746686	0.08285978	0.14236654	0.1966874	0.03739511	0.67971914	0.09541158	0.10426071	0.23226988	0.08682905
92.7	0.06157991	0.20269808	0.33748568	0.0828541	0.14239023	0.1966919	0.03738683	0.67977346	0.09539991	0.10430265	0.23232441	0.08686649
92.8	0.06157414	0.20268152	0.33750451	0.08284844	0.14241393	0.19669642	0.03737858	0.67982762	0.09538824	0.10434447	0.23237886	0.08690385
92.9	0.06156841	0.20266494	0.33752333	0.0828428	0.14243762	0.19670097	0.03737038	0.67988163	0.09537656	0.10438617	0.23243324	0.08694111
93	0.0615627	0.20264834	0.33754216	0.08283719	0.14246131	0.19670554	0.03736221	0.67993548	0.09536487	0.10442776	0.23248756	0.0869783
93.1	0.06155703	0.20263173	0.33756099	0.0828316	0.142485	0.19671013	0.03735408	0.67998918	0.09535318	0.10446924	0.2325418	0.08701539
93.2	0.06155139	0.2026151	0.33757983	0.08282604	0.14250868	0.19671474	0.03734599	0.68004272	0.09534148	0.10451059	0.23259597	0.0870524
93.3	0.06154579	0.20259846	0.33759866	0.0828205	0.14253237	0.19671938	0.03733793	0.68009611	0.09532977	0.10455183	0.23265007	0.08708933
93.4	0.06154021	0.2025818	0.33761749	0.08281498	0.14255605	0.19672404	0.03732991	0.68014935	0.09531806	0.10459296	0.23270411	0.08712617
93.5	0.06153467	0.20256514	0.33763633	0.08280948	0.14257973	0.19672872	0.03732193	0.68020243	0.09530634	0.10463397	0.23275807	0.08716293
93.6	0.06152916	0.20254845	0.33765517	0.082804	0.1426034	0.19673342	0.03731399	0.68025536	0.09529461	0.10467486	0.23281196	0.0871996
93.7	0.06152368	0.20253175	0.33767401	0.08279855	0.14262708	0.19673815	0.03730608	0.68030814	0.09528288	0.10471564	0.23286579	0.08723618
93.8	0.06151824	0.20251504	0.33769285	0.08279312	0.14265075	0.19674289	0.03729821	0.68036077	0.09527114	0.10475631	0.23291954	0.08727269
93.9	0.06151283	0.20249832	0.33771169	0.08278771	0.14267442	0.19674766	0.03729037	0.68041325	0.0952594	0.10479686	0.23297323	0.0873091
94	0.06150745	0.20248158	0.33773053	0.08278233	0.14269808	0.19675245	0.03728257	0.68046558	0.09524765	0.1048373	0.23302685	0.08734544
94.1	0.0615021	0.20246483	0.33774938	0.08277697	0.14272175	0.19675726	0.03727481	0.68051776	0.0952359	0.10487763	0.2330804	0.08738169
94.2	0.06149678	0.20244807	0.33776822	0.08277163	0.14274541	0.19676209	0.03726708	0.68056979	0.09522414	0.10491785	0.23313388	0.08741786
94.3	0.06149149	0.20243129	0.33778707	0.08276631	0.14276907	0.19676695	0.03725939	0.68062167	0.09521238	0.10495795	0.23318729	0.08745395
94.4	0.06148623	0.2024145	0.33780591	0.08276101	0.14279272	0.19677182	0.03725174	0.6806734	0.09520061	0.10499794	0.23324064	0.08748995
94.5	0.06148101	0.2023977	0.33782476	0.08275574	0.14281638	0.19677671	0.03724412	0.68072499	0.09518884	0.10503781	0.23329391	0.08752587
94.6	0.06147581	0.20238089	0.33784361	0.08275049	0.14284003	0.19678163	0.03723654	0.68077643	0.09517706	0.10507758	0.23334713	0.08756172
94.7	0.06147065	0.20236406	0.33786246	0.08274526	0.14286368	0.19678656	0.03722899	0.68082772	0.09516528	0.10511724	0.23340027	0.08759748
94.8	0.06146551	0.20234722	0.33788131	0.08274005	0.14288732	0.19679152	0.03722148	0.68087887	0.0951535	0.10515678	0.23345334	0.08763316
94.9	0.06146041	0.20233037	0.33790016	0.08273487	0.14291096	0.19679649	0.037214	0.68092988	0.09514171	0.10519621	0.23350635	0.08766876
95	0.06145534	0.20231351	0.33791901	0.08272971	0.1429346	0.19680148	0.03720656	0.68098073	0.09512991	0.10523554	0.2335593	0.08770428
95.1	0.0614503	0.20229664	0.33793786	0.08272456	0.14295824	0.1968065	0.03719915	0.68103145	0.09511812	0.10527475	0.23361217	0.08773972
95.2	0.06144528	0.20227975	0.33795671	0.08271944	0.14298187	0.19681153	0.03719178	0.68108202	0.09510632	0.10531386	0.23366498	0.08777508
95.3	0.0614403	0.20226286	0.33797557	0.08271434	0.1430055	0.19681658	0.03718444	0.68113244	0.09509451	0.10535286	0.23371773	0.08781036
95.4	0.06143535	0.20224595	0.33799442	0.08270927	0.14302913	0.19682165	0.03717714	0.68118272	0.09508271	0.10539174	0.2337704	0.08784556
95.5	0.06143042	0.20222904	0.33801327	0.08270421	0.14305275	0.19682674	0.03716987	0.68123286	0.09507089	0.10543052	0.23382301	0.08788069
95.6	0.06142553	0.20221211	0.33803212	0.08269918	0.14307637	0.19683185	0.03716264	0.68128286	0.09505908	0.1054692	0.23387556	0.08791573
95.7	0.06142067	0.20219517	0.33805098	0.08269416	0.14309999	0.19683697	0.03715544	0.68133271	0.09504727	0.10550776	0.23392804	0.0879507
95.8	0.06141583	0.20217822	0.33806983	0.08268917	0.1431236	0.19684212	0.03714828	0.68138242	0.09503545	0.10554622	0.23398045	0.08798559
95.9	0.06141103	0.20216126	0.33808869	0.0826842	0.14314722	0.19684728	0.0371415	0.681432	0.09502362	0.10558457	0.2340328	0.0880204
96	0.06140625	0.20214429	0.33810754	0.08267925	0.14317082	0.19685246	0.03713405	0.68148143	0.0950118	0.10562282	0.23408509	0.08805513
96.1	0.0614015	0.20212731	0.3381264	0.08267433	0.14319443	0.19685766	0.03712699	0.68153072	0.09499997	0.10566096	0.23413731	0.08808978
96.2	0.06139679	0.20211032	0.33814525	0.08266942	0.14321803	0.19686288	0.03711996	0.68157988	0.09498814	0.10569899	0.23418946	0.08812436
96.3	0.0613921	0.20209333	0.33816411	0.08266453	0.14324162	0.19686811	0.03711297	0.68162889	0.09497631	0.10573692	0.23424155	0.08815886
96.4	0.06138744	0.20207632	0.33818296	0.08265967	0.14326522	0.19687336	0.03710601	0.68167777	0.09496448	0.10577474	0.23429358	0.08819328
96.5	0.0613828	0.2020593	0.33820182	0.08265482	0.1432888	0.19687863	0.03709908	0.68172651	0.09495264	0.10581246	0.23434554	0.08822763
96.6	0.0613782	0.20204227	0.33822068	0.08265	0.14331239	0.19688392	0.03709219	0.68177511	0.0949408	0.10585008	0.23439744	0.08826189
96.7	0.06137363	0.20202524	0.33823953	0.0826452	0.14333597	0.19688923	0.03708533	0.68182358	0.09492896	0.10588759	0.23444927	0.08829609
96.8	0.06136908	0.20200819	0.33825839	0.08264042	0.14335955	0.19689455	0.0370785	0.68187191	0.09491712	0.10592499	0.23450104	0.0883302

63.3	0.06502688	0.20563832	0.33231851	0.0857207	0.13556988	0.19816245	0.04221531	0.65341171	0.0973358	0.08520527	0.21241131	0.07078549
63.4	0.06500944	0.20564218	0.33233426	0.08570658	0.13559188	0.19813671	0.04218704	0.65355535	0.09734147	0.08529952	0.21249644	0.07086191
63.5	0.06499203	0.20564586	0.33235002	0.08569249	0.13561389	0.19811126	0.04215889	0.65369842	0.09734697	0.08539353	0.2125814	0.07093814
63.6	0.06497463	0.20564936	0.33236579	0.08567843	0.13563592	0.19808608	0.04213085	0.65384093	0.09735232	0.0854873	0.21266621	0.07101418
63.7	0.06495726	0.20565269	0.33238158	0.08566439	0.13565795	0.19806117	0.04210293	0.65398289	0.09735749	0.08558082	0.21275086	0.07109003
63.8	0.06493991	0.20565586	0.33239737	0.08565038	0.13567999	0.19803653	0.04207512	0.6541243	0.09736251	0.08567409	0.21283536	0.07116569
63.9	0.06492258	0.20565885	0.33241317	0.08563639	0.13570204	0.19801217	0.04204743	0.65426516	0.09736737	0.08576713	0.2129197	0.07124117
64	0.06490528	0.20566167	0.33242898	0.08562243	0.13572411	0.19798806	0.04201986	0.65440548	0.09737207	0.08585993	0.21300389	0.07131645
64.1	0.06488799	0.20566432	0.33244481	0.0856085	0.13574618	0.19796422	0.0419924	0.65454525	0.09737661	0.08595249	0.21308792	0.07139156
64.2	0.06487073	0.20566681	0.33246064	0.0855946	0.13576826	0.19794065	0.04196505	0.65468448	0.097381	0.08604482	0.2131718	0.07146647
64.3	0.0648535	0.20566913	0.33247649	0.08558072	0.13579035	0.19791733	0.04193781	0.65482318	0.09738523	0.08613691	0.21325552	0.0715412
64.4	0.06483629	0.20567129	0.33249234	0.08556688	0.13581246	0.19789427	0.04191069	0.65496134	0.0973893	0.08622876	0.2133391	0.07161575
64.5	0.0648191	0.20567328	0.33250821	0.08555306	0.13583457	0.19787146	0.04188368	0.65509898	0.09739323	0.08632038	0.21342252	0.07169011
64.6	0.06480194	0.20567512	0.33252409	0.08553926	0.13585669	0.19784891	0.04185678	0.65523608	0.097397	0.08641176	0.21350579	0.07176428
64.7	0.0647848	0.20567679	0.33253998	0.0855255	0.13587882	0.19782661	0.04182999	0.65537266	0.09740062	0.08650291	0.21358891	0.07183828
64.8	0.0647677	0.2056783	0.33255588	0.08551176	0.13590097	0.19780456	0.04180332	0.65550871	0.09740409	0.08659382	0.21367188	0.07191209
64.9	0.06475061	0.20567966	0.3325718	0.08549806	0.13592312	0.19778276	0.04177675	0.65564425	0.09740742	0.0866845	0.2137547	0.07198572
65	0.06473356	0.20568086	0.33258773	0.08548438	0.13594528	0.1977612	0.04175029	0.65577926	0.09741059	0.08677494	0.21383736	0.07205917
65.1	0.06471653	0.2056819	0.33260366	0.08547073	0.13596746	0.19773988	0.04172394	0.65591376	0.09741363	0.08686515	0.21391988	0.07213244
65.2	0.06469952	0.20568279	0.33261962	0.08545711	0.13598964	0.19771881	0.0416977	0.65604775	0.09741652	0.08695513	0.21400225	0.07220553
65.3	0.06468255	0.20568353	0.33263558	0.08544352	0.13601183	0.19769797	0.04167156	0.65618123	0.09741926	0.08704487	0.21408447	0.07227844
65.4	0.06466561	0.20568411	0.33265155	0.08542995	0.13603404	0.19767737	0.04164554	0.6563142	0.09742187	0.08713438	0.21416654	0.07235117
65.5	0.06464869	0.20568455	0.33266754	0.08541642	0.13605625	0.19765701	0.04161962	0.65644667	0.09742433	0.08722366	0.21424847	0.07242372
65.6	0.0646318	0.20568483	0.33268354	0.08540292	0.13607847	0.19763687	0.04159381	0.65657863	0.09742666	0.08731271	0.21433024	0.0724961
65.7	0.06461495	0.20568496	0.33269956	0.08538944	0.13610071	0.19761697	0.0415681	0.6567101	0.09742885	0.08740152	0.21441187	0.0725683
65.8	0.06459812	0.20568495	0.33271559	0.085376	0.13612295	0.1975973	0.0415425	0.65684106	0.0974309	0.0874901	0.21449336	0.07264032
65.9	0.06458132	0.2056848	0.33273163	0.08536258	0.13614521	0.19757785	0.041517	0.65697154	0.09743282	0.08757845	0.2145747	0.07271217
66	0.06456456	0.20568449	0.33274768	0.0853492	0.13616748	0.19755863	0.04149161	0.65710152	0.0974346	0.08766657	0.21465589	0.07278384
66.1	0.06454782	0.20568405	0.33276375	0.08533584	0.13618975	0.19753963	0.04146632	0.65723101	0.09743625	0.08775446	0.21473694	0.07285534
66.2	0.06453112	0.20568346	0.33277983	0.08532252	0.13621204	0.19752085	0.04144114	0.65736001	0.09743777	0.08784212	0.21481784	0.07292666
66.3	0.06451445	0.20568272	0.33279592	0.08530922	0.13623434	0.19750229	0.04141606	0.65748853	0.09743916	0.08792955	0.2148986	0.07299781
66.4	0.06449781	0.20568185	0.33281203	0.08529595	0.13625665	0.19748395	0.04139108	0.65761657	0.09744042	0.08801675	0.21497922	0.07306879
66.5	0.06448121	0.20568084	0.33282815	0.08528272	0.13627897	0.19746582	0.0413662	0.65774413	0.09744154	0.08810372	0.21505969	0.0731396
66.6	0.06446464	0.20567968	0.33284429	0.08526951	0.1363013	0.1974479	0.04134143	0.65787121	0.09744255	0.08819046	0.21514002	0.07321023
66.7	0.0644481	0.20567839	0.33286044	0.08525633	0.13632364	0.19743019	0.04131676	0.65799782	0.09744342	0.08827697	0.21522022	0.0732807
66.8	0.0644316	0.20567696	0.3328766	0.08524318	0.136346	0.19741269	0.04129219	0.65812396	0.09744417	0.08836325	0.21530027	0.07335099
66.9	0.06441513	0.2056754	0.33289278	0.08523006	0.13636836	0.1973954	0.04126772	0.65824963	0.0974448	0.08844931	0.21538018	0.07342112
67	0.0643987	0.20567369	0.33290897	0.08521697	0.13639074	0.19737831	0.04124335	0.65837483	0.0974453	0.08853513	0.21545995	0.07349107
67.1	0.06438231	0.20567186	0.33292518	0.08520391	0.13641312	0.19736143	0.04121908	0.65849956	0.09744568	0.08862073	0.21553958	0.07356086
67.2	0.06436595	0.20566989	0.3329414	0.08519088	0.13643552	0.19734475	0.04119491	0.65862384	0.09744594	0.0887061	0.21561907	0.07363048
67.3	0.06434963	0.20566779	0.33295764	0.08517788	0.13645793	0.19732827	0.04117084	0.65874765	0.09744608	0.08879124	0.21569842	0.07369994
67.4	0.06433335	0.20566556	0.33297389	0.08516491	0.13648035	0.19731199	0.04114687	0.65887101	0.0974461	0.08887616	0.21577764	0.07376923
67.5	0.0643171	0.20566319	0.33299016	0.08515196	0.13650278	0.19729591	0.04112299	0.65899391	0.097446	0.08896085	0.21585671	0.07383835
67.6	0.06430089	0.2056607	0.33300644	0.08513905	0.13652522	0.19728003	0.04109922	0.65911635	0.09744578	0.08904531	0.21593565	0.07390731
67.7	0.06428471	0.20565808	0.33302273	0.08512617	0.13654767	0.19726433	0.04107554	0.65923835	0.09744545	0.08912955	0.21601446	0.0739761
67.8	0.06426858	0.20565533	0.33303904	0.08511332	0.13657013	0.19724883	0.04105196	0.6593599	0.097445	0.08921356	0.21609312	0.07404473
67.9	0.06425248	0.20565246	0.33305537	0.0851005	0.1365926	0.19723352	0.04102847	0.659481	0.09744444	0.08929735	0.21617165	0.07411319
68	0.06423642	0.20564946	0.33307171	0.08508771	0.13661509	0.19721839	0.04100509	0.65960165	0.09744377	0.08938091	0.21625004	0.07418149
68.1	0.06422039	0.20564633	0.33308806	0.08507494	0.13663758	0.19720346	0.0409818	0.65972187	0.09744298	0.08946424	0.21632829	0.07424963
68.2	0.06420441	0.20564309	0.33310443	0.08506221	0.13666009	0.1971887	0.0409586	0.65984164	0.09744209	0.08954735	0.21640642	0.07431761
68.3	0.06418846	0.20563972	0.33312081	0.08504951	0.13668261	0.19717414	0.0409355	0.65996097	0.09744108	0.08963024	0.2164844	0.07438543
68.4	0.06417254	0.20563622	0.33313721	0.08503684	0.13670514	0.19715975	0.04091249	0.66007987	0.09743996	0.0897129	0.21656225	0.07445308
68.5	0.06415667	0.20563261	0.33315362	0.0850242	0.13672768	0.19714554	0.04088958	0.66019834	0.09743873	0.08979534	0.21663997	0.07452058
68.6	0.06414084	0.20562888	0.33317004	0.08501159	0.13675023	0.19713152	0.04086676	0.66031637	0.0974374	0.08987756	0.21671755	0.07458791
68.7	0.06412504	0.20562503	0.33318648	0.08499901	0.13677279	0.19711767	0.04084403	0.66043398	0.09743596	0.08995955	0.216795	0.07465509
68.8	0.06410928	0.20562106	0.33320294	0.08498646	0.13679536	0.19710399	0.0408214	0.66055115	0.09743441	0.09004133	0.21687232	0.07472211
68.9	0.06409356	0.20561697	0.33321941	0.08497394	0.13681795	0.1970905	0.04079886	0.66066791	0.09743276	0.09012288	0.2169495	0.07478898
69	0.06407788	0.20561277	0.33323589	0.08496145	0.13684055	0.19707717	0.04077641	0.66078423	0.09743101	0.09020421	0.21702655	0.07485568
69.1	0.06406223	0.20560845	0.33325239	0.084949	0.13686316	0.19706402	0.04075405	0.66090014	0.09742915	0.09028532	0.21710347	0.07492223
69.2	0.06404663	0.20560402	0.33326891	0.08493657	0.13688578	0.19705104	0.04073179	0.66101563	0.0974272	0.09036621	0.21718026	0.07498862
69.3	0.06403106	0.20559947	0.33328544	0.08492417	0.13690841	0.19703822	0.04070961	0.6611307	0.09742514	0.09044687	0.21725692	0.07505486
69.4	0.06401554	0.20559482	0.33330198	0.08491181	0.13693105	0.19702558	0.04068753	0.66124535	0.09742298	0.09052732	0.21733345	0.07512095
69.5	0.06400005	0.20559005	0.33331854	0.08489947	0.1369537	0.1970131	0.04066553	0.6613596	0.09742072	0.09060755	0.21740985	0.07518688
69.6	0.0639846	0.20558517	0.33333511	0.08488717	0.13697637	0.19700078	0.04064362	0.66147343	0.09741836	0.09068756	0.21748611	0.07525265
69.7	0.0639692	0.20558018	0.33335169	0.0848749	0.13699904	0.19698863	0.04062181	0.66158685	0.0974159	0.09076735	0.21756225	0.07531828
69.8	0.06395383	0.20557508	0.33336829	0.08486265	0.13702173	0.19697664	0.04060008	0.66169986	0.09741335	0.09084693	0.21763827	0.07538375
69.9	0.06393851	0.20556987	0.33338491	0.08485044	0.13704443	0.19696481	0.04057844	0.66181247	0.0974107	0.0909263	0.21771415	0.07544907
70	0.06392323	0.20556456	0.33340154	0.08483826	0.13706713	0.19695314	0.04055689	0.66192468	0.09740796	0.09100544	0.2177899	0.07551424
70.1	0.06390799	0.20555913	0.33341818	0.08482611	0.13708985	0.19694163	0.04053542	0.66203648	0.09740512	0.09108438	0.21786553	0.07557926
70.2	0.06389279	0.20555361	0.33343484	0.084814	0.13711258	0.19693027	0.04051405	0.66214789	0.09740219	0.09116309	0.21794103	0.07564413
70.3	0.06387763	0.20554797	0.33345152	0.08480191	0.13713532	0.19691907	0.04049276	0.66225889	0.09739917	0.0912416	0.21801641	0.07570885
70.4	0.06386252	0.20554224	0.3334682	0.08478985	0.13715807	0.19690803	0.04047155	0.6623695	0.09739605	0.09131989	0.21809166	0.07577342
70.5	0.06384745	0.2055364	0.3334849	0.08477783	0.13718084	0.19689714	0.04045044	0.66247972	0.09739284	0.09139797	0.21816678	0.07583785
70.6	0.06383242	0.20553045	0.33350162	0.08476584	0.13720361	0.1968864	0.0404294	0.66258954	0.09738955	0.09147583	0.21824178	0.07590213
70.7	0.06381743	0.20552441	0.33351835	0.08475388	0.13722639	0.19687581	0.04040846	0.66269897	0.09738616	0.09155348	0.21831665	0.07596626
70.8	0.06380248	0.20551826	0.3335351	0.08474195	0.13724918	0.19686537	0.0403876	0.66280802	0.09738268	0.09163092	0.2183914	0.07603024
70.9	0.06378758	0.20551201	0.33355186	0.08473005	0.13727199	0.19685507	0.04036682	0.66291667	0.09737912	0.09170815	0.21846603	0.07609409
71	0.06377272	0.20550566	0.33356863	0.08471818	0.1372948	0.19684493	0.04034613	0.66302494	0.09737547	0.09178517	0.21854053	0.07615778
71.1	0.0637579	0.20549921	0.33358542	0.08470635	0.13731762	0.19683493	0.04032552	0.66313283	0.09737173	0.09186197	0.21861491	0.07622134
71.2	0.06374313	0.20549267	0.33360223	0.08469455	0.13734046	0.19682507	0.040305	0.66324034	0.09736791	0.09193857	0.21868916	0.07628475
71.3	0.06372839	0.20548602	0.33361905	0.08468278	0.1373633	0.19681536	0.04028456	0.66334746	0.097364	0.09201496	0.2187633	0.07634801
71.4	0.0637137	0.20547928	0.33363588	0.08467104	0.13738615	0.19680578	0.0402642	0.66345421	0.09736001	0.09209114	0.21883731	0.07641114
71.5	0.06369905	0.20547244	0.33365273	0.08465933	0.13740902	0.19679635	0.04024392	0.66356058	0.09735593	0.09216711	0.21891121	0.07647412
71.6	0.06368445	0.2054655	0.33366959	0.08464766	0.13743189	0.19678706	0.04022373	0.66366658	0.09735177	0.09224288	0.21898498	0.07653696
71.7	0.06366989	0.20545847	0.33368647	0.08463602	0.13745477	0.1967779	0.04020362	0.6637722	0.09734753	0.09231843	0.21905863	0.07659966
71.8	0.06365537	0.20545135	0.33370336	0.08462441	0.13747767	0.19676888	0.04018359	0.66387746	0.0973432	0.09239378	0.21913216	0.07666222
71.9	0.06364089	0.20544413	0.33372027	0.08461283	0.13750057	0.19676	0.04016364	0.66398234	0.0973388	0.09246893	0.21920557	0.07672464
72	0.06362646	0.20543682	0.33373719	0.08460129	0.13752349	0.19675125	0.04014377	0.66408686	0.09733431	0.09254387	0.21927886	0.07678693
72.1	0.06361207	0.20542942	0.33375413	0.08458977	0.13754641	0.19674263	0.04012398	0.66419101	0.09732975	0.0926186	0.21935203	0.07684907
72.2	0.06359773	0.20542193	0.33377108	0.08457829	0.13756934	0.19673415	0.04010428	0.6642948	0.0973251	0.09269312	0.21942509	0.07691107
72.3	0.06358343	0.20541434	0.33378804	0.08456685	0.13759229	0.1967258	0.04008465	0.66439822	0.09732038	0.09276745	0.21949802	0.07697294
72.4	0.06356917	0.20540667	0.33380502	0.08455543	0.13761524	0.1967157	0.0400651	0.66450128	0.09731557	0.09284156	0.21957084	0.07703467
72.5	0.06355495	0.20539891	0.33382202	0.08454404	0.1376382	0.19670948	0.04004563	0.66460399	0.09731069	0.09291548	0.21964354	0.07709626
72.6	0.06354078	0.20539105	0.33383903	0.08453269	0.13766117	0.19670151	0.04002624	0.66470634	0.09730574	0.09298919	0.21971612	0.07715772
72.7	0.06352666	0.20538312	0.33385605	0.08452137	0.13768415	0.19669367	0.04000693	0.66480833	0.0973007	0.09306269	0.21978859	0.07721904
72.8	0.06351258	0.20537509	0.33387309	0.08451008	0.13770714	0.19668596	0.0399877	0.66490997	0.0972956	0.093136	0.21986094	0.07728022
72.9	0.06349854	0.20536698	0.33389014	0.08449882	0.13773014	0.19667837	0.03996854	0.66501126	0.09729041	0.0932091	0.21993318	0.07734126
73	0.06348455	0.20535879	0.33390721	0.0844876	0.13775315	0.1966709	0.03994946	0.66511219	0.09728515	0.093282	0.2200053	0.07740217
73.1	0.0634706	0.20535051	0.33392429	0.0844764	0.13777617	0.19666356	0.03993046	0.66521278	0.09727982	0.0933547	0.2200773	0.07746295
73.2	0.0634567	0.20534215	0.33394138	0.08446524	0.13779919	0.19665633	0.03991154	0.66531302	0.09727442	0.0934272	0.22014919	0.07752358
73.3	0.06344285	0.20533371	0.33395849	0.08445411	0.13782223	0.19664923	0.0398927	0.66541291	0.09726894	0.09349949	0.22022097	0.07758408
73.4	0.06342904	0.20532518	0.33397562	0.08444301	0.13784527	0.19664225	0.03987393	0.66551246	0.09726339	0.09357159	0.22029263	0.07764445
73.5	0.06341527	0.20531657	0.33399276	0.08443195	0.13786832	0.19663538	0.03985524	0.66561167	0.09725776	0.09364349	0.22036418	0.07770468
73.6	0.06340155	0.20530788	0.33400991	0.08442091	0.13789138	0.19662864	0.03983662	0.66571053	0.09725207	0.09371519	0.22043562	0.07776477
73.7	0.06338787	0.20529911	0.33402708	0.08440991	0.13791445	0.19662201	0.03981808	0.66580906	0.09724631	0.09378669	0.22050694	0.07782472
73.8	0.06337424	0.20529026	0.33404427	0.08439893	0.13793753	0.1966155	0.03979961	0.66590724	0.09724048	0.09385799	0.22057816	0.07788453
73.9	0.06336065	0.20528133	0.33406147	0.08438799	0.13796062	0.1966091	0.03978122	0.6660051	0.09723457	0.09392909	0.22064926	0.07794421
74	0.06334711	0.20527231	0.33407868	0.08437708	0.13798372	0.19660282	0.03976291	0.66610261	0.0972286	0.094	0.22072025	0.07800375
74.1	0.06333361	0.20526322	0.3340959	0.0843662	0.13800682	0.19659664	0.03974467	0.6661998	0.09722256	0.09407071	0.22079113	0.07806315
74.2	0.06332015	0.20525406	0.33411315	0.08435536	0.13802993	0.19659059	0.0397265	0.66629665	0.09721646	0.09414122	0.22086189	0.0781224
74.3	0.06330674	0.20524481	0.3341304	0.08434454	0.13805305	0.19658464	0.03970841	0.66639317	0.09721029	0.09421154	0.22093255	0.07818152
74.4	0.06329338	0.20523549	0.33414767	0.08433375	0.13807618	0.1965788	0.03969039	0.66648937	0.09720405	0.09428166	0.22100309	0.0782405
74.5	0.06328006	0.20522609	0.33416495	0.084323	0.13809932	0.19657308	0.03967244	0.66658524	0.09719775	0.09435159	0.22107353	0.07829933
74.6	0.06326678	0.20521661	0.33418225	0.08431228	0.13812247	0.19656746	0.03965457	0.66668078	0.09719138	0.09442132	0.22114385	0.07835802
74.7	0.06325355	0.20520706	0.33419956	0.08430159	0.13814562	0.19656195	0.03963677	0.666776	0.09718495	0.09449086	0.22121407	0.07841657
74.8	0.06324037	0.20519744	0.33421689	0.08429093	0.13816878	0.19655655	0.03961905	0.66687089	0.09717845	0.09456021	0.22128417	0.07847498
74.9	0.06322723	0.20518774	0.33423423	0.0842803	0.13819195	0.19655126	0.03960139	0.66696547	0.09717189	0.09462936	0.22135417	0.07853324

105	0.06108265	0.20059216	0.33980001	0.08231401	0.14527716	0.19737648	0.03662179	0.68541416	0.09394749	0.10866698	0.23853905	0.09088845
105.1	0.06108009	0.2005748	0.33981871	0.08231078	0.14530032	0.19738278	0.03661737	0.68545269	0.09393575	0.10869677	0.2385859	0.09091697
105.2	0.06107755	0.20055744	0.33983741	0.08230756	0.14532347	0.19738908	0.03661298	0.68549111	0.09392402	0.10872647	0.23863269	0.09094543
105.3	0.06107503	0.20054008	0.3398561	0.08230435	0.14534661	0.19739539	0.03660861	0.68552943	0.09391229	0.10875609	0.23867942	0.09097383
105.4	0.06107252	0.20052272	0.3398748	0.08230116	0.14536975	0.19740171	0.03660427	0.68556765	0.09390056	0.10878564	0.2387261	0.09100218
105.5	0.06107004	0.20050536	0.33989348	0.08229799	0.14539288	0.19740804	0.03659995	0.68560577	0.09388884	0.1088151	0.23877273	0.09103046
105.6	0.06106758	0.200488	0.33991217	0.08229484	0.145416	0.19741437	0.03659566	0.68564379	0.09387712	0.10884448	0.2388193	0.09105868
105.7	0.06106514	0.20047064	0.33993085	0.0822917	0.14543911	0.19742071	0.03659139	0.6856817	0.0938654	0.10887379	0.23886581	0.09108685
105.8	0.06106271	0.20045328	0.33994953	0.08228858	0.14546222	0.19742706	0.03658715	0.68571952	0.09385369	0.10890301	0.23891227	0.09111496
105.9	0.06106031	0.20043593	0.3399682	0.08228547	0.14548533	0.19743342	0.03658293	0.68575723	0.09384198	0.10893216	0.23895868	0.09114301
106	0.06105792	0.20041857	0.33998688	0.08228238	0.14550842	0.19743978	0.03657874	0.68579485	0.09383028	0.10896123	0.23900503	0.091171
106.1	0.06105556	0.20040122	0.34000554	0.08227931	0.14553151	0.19744615	0.03657457	0.68583236	0.09381858	0.10899022	0.23905132	0.09119893
106.2	0.06105321	0.20038387	0.34002421	0.08227625	0.14555459	0.19745253	0.03657043	0.68586978	0.09380688	0.10901913	0.23909756	0.09122681
106.3	0.06105088	0.20036651	0.34004287	0.08227321	0.14557767	0.19745891	0.03656631	0.6859071	0.09379519	0.10904796	0.23914375	0.09125462
106.4	0.06104857	0.20034916	0.34006153	0.08227018	0.14560074	0.1974653	0.03656221	0.68594432	0.0937835	0.10907672	0.23918988	0.09128238
106.5	0.06104628	0.20033182	0.34008019	0.08226717	0.1456238	0.1974717	0.03655814	0.68598144	0.09377182	0.1091054	0.23923596	0.09131009
106.6	0.06104401	0.20031447	0.34009884	0.08226417	0.14564685	0.1974781	0.03655409	0.68601846	0.09376014	0.109134	0.23928199	0.09133773
106.7	0.06104176	0.20029712	0.34011749	0.08226119	0.1456699	0.19748451	0.03655007	0.68605539	0.09374847	0.10916252	0.23932796	0.09136532
106.8	0.06103952	0.20027978	0.34013613	0.08225823	0.14569294	0.19749093	0.03654607	0.68609222	0.0937368	0.10919097	0.23937387	0.09139285
106.9	0.0610373	0.20026243	0.34015477	0.08225528	0.14571598	0.19749736	0.0365421	0.68612895	0.09372513	0.10921934	0.23941974	0.09142033
107	0.06103511	0.20024509	0.34017341	0.08225235	0.145739	0.19750379	0.03653814	0.68616559	0.09371347	0.10924764	0.23946555	0.09144775
107.1	0.06103293	0.20022776	0.34019204	0.08224943	0.14576202	0.19751022	0.03653422	0.68620213	0.09370181	0.10927586	0.2395113	0.09147511
107.2	0.06103076	0.20021042	0.34021067	0.08224653	0.14578503	0.19751667	0.03653031	0.68623857	0.09369016	0.109304	0.239557	0.09150242
107.3	0.06102862	0.20019308	0.3402293	0.08224364	0.14580804	0.19752312	0.03652643	0.68627492	0.09367851	0.10933207	0.23960265	0.09152967
107.4	0.06102649	0.20017575	0.34024792	0.08224077	0.14583104	0.19752957	0.03652257	0.68631117	0.09366687	0.10936006	0.23964825	0.09155686
107.5	0.06102438	0.20015842	0.34026653	0.08223792	0.14585403	0.19753603	0.03651874	0.68634733	0.09365523	0.10938798	0.23969379	0.091584
107.6	0.06102229	0.20014109	0.34028515	0.08223508	0.14587701	0.1975425	0.03651493	0.68638339	0.0936436	0.10941583	0.23973928	0.09161109
107.7	0.06102022	0.20012377	0.34030375	0.08223225	0.14589999	0.19754898	0.03651114	0.68641936	0.09363197	0.1094436	0.23978471	0.09163811
107.8	0.06101816	0.20010644	0.34032236	0.08222944	0.14592295	0.19755545	0.03650738	0.68645524	0.09362034	0.10947129	0.2398301	0.09166509
107.9	0.06101613	0.20008912	0.34034096	0.08222665	0.14594591	0.19756194	0.03650364	0.68649102	0.09360872	0.10949892	0.23987543	0.09169201
108	0.06101411	0.2000718	0.34035955	0.08222387	0.14596887	0.19756843	0.03649992	0.68652671	0.0935971	0.10952647	0.2399207	0.09171887
108.1	0.0610121	0.20005449	0.34037815	0.0822211	0.14599181	0.19757493	0.03649623	0.6865623	0.09358549	0.10955394	0.23996593	0.09174568
108.2	0.06101012	0.20003717	0.34039673	0.08221836	0.14601475	0.19758143	0.03649255	0.6865978	0.09357389	0.10958135	0.2400111	0.09177243
108.3	0.06100815	0.20001986	0.34041532	0.08221562	0.14603768	0.19758794	0.0364889	0.68663321	0.09356228	0.10960868	0.24005621	0.09179913
108.4	0.06100619	0.20000255	0.34043389	0.0822129	0.14606061	0.19759445	0.03648528	0.68666853	0.09355069	0.10963593	0.24010128	0.09182577
108.5	0.06100426	0.19998525	0.34045247	0.0822102	0.14608352	0.19760097	0.03648167	0.68670375	0.09353909	0.10966312	0.24014629	0.09185237
108.6	0.06100234	0.19996795	0.34047104	0.08220751	0.14610643	0.19760749	0.03647809	0.68673888	0.09352751	0.10969023	0.24019125	0.0918789
108.7	0.06100044	0.19995065	0.34048961	0.08220484	0.14612933	0.19761402	0.03647453	0.68677393	0.09351592	0.10971727	0.24023615	0.09190539
108.8	0.06099855	0.19993335	0.34050817	0.08220218	0.14615222	0.19762056	0.03647099	0.68680888	0.09350434	0.10974424	0.24028101	0.09193182
108.9	0.06099668	0.19991606	0.34052673	0.08219953	0.14617511	0.1976271	0.03646748	0.68684374	0.09349277	0.10977114	0.24032581	0.09195819
109	0.06099483	0.19989877	0.34054528	0.0821969	0.14619799	0.19763364	0.03646399	0.6868785	0.0934812	0.10979797	0.24037056	0.09198452
109.1	0.06099299	0.19988148	0.34056383	0.08219429	0.14622086	0.19764019	0.03646052	0.68691318	0.09346964	0.10982472	0.24041525	0.09201079
109.2	0.06099117	0.1998642	0.34058238	0.08219169	0.14624372	0.19764674	0.03645707	0.68694777	0.09345808	0.10985141	0.2404599	0.092037
109.3	0.06098937	0.19984692	0.34060092	0.0821891	0.14626657	0.1976533	0.03645364	0.68698227	0.09344653	0.10987802	0.24050449	0.09206317
109.4	0.06098758	0.19982964	0.34061945	0.08218653	0.14628942	0.19765987	0.03645024	0.68701668	0.09343498	0.10990456	0.24054903	0.09208928
109.5	0.06098581	0.19981236	0.34063799	0.08218397	0.14631226	0.19766644	0.03644686	0.687051	0.09342343	0.10993103	0.24059352	0.09211534
109.6	0.06098406	0.19979509	0.34065651	0.08218143	0.14633509	0.19767301	0.0364435	0.68708523	0.0934119	0.10995743	0.24063796	0.09214134
109.7	0.06098232	0.19977783	0.34067504	0.0821789	0.14635791	0.19767959	0.03644016	0.68711937	0.09340036	0.10998376	0.24068234	0.0921673
109.8	0.06098059	0.19976056	0.34069356	0.08217638	0.14638073	0.19768618	0.03643684	0.68715342	0.09338883	0.11001002	0.24072668	0.0921932
109.9	0.06097888	0.1997433	0.34071207	0.08217388	0.14640353	0.19769277	0.03643355	0.68718739	0.09337731	0.11003621	0.24077096	0.09221904
110	0.06097719	0.19972605	0.34073058	0.08217139	0.14642633	0.19769936	0.03643028	0.68722127	0.09336579	0.11006233	0.24081519	0.09224484
110.1	0.06097551	0.19970879	0.34074909	0.08216892	0.14644912	0.19770596	0.03642702	0.68725506	0.09335428	0.11008838	0.24085937	0.09227059
110.2	0.06097385	0.19969155	0.34076759	0.08216646	0.1464719	0.19771256	0.03642379	0.68728876	0.09334277	0.11011436	0.2409035	0.09229628
110.3	0.0609722	0.1996743	0.34078608	0.08216402	0.14649468	0.19771917	0.03642059	0.68732237	0.09333127	0.11014027	0.24094757	0.09232192
110.4	0.06097057	0.19965706	0.34080457	0.08216158	0.14651745	0.19772578	0.0364174	0.6873559	0.09331977	0.11016611	0.2409916	0.09234751
110.5	0.06096896	0.19963982	0.34082306	0.08215917	0.1465402	0.1977324	0.03641423	0.68738934	0.09330828	0.11019189	0.24103557	0.09237305
110.6	0.06096735	0.19962259	0.34084154	0.08215676	0.14656295	0.19773902	0.03641109	0.6874227	0.09329679	0.11021759	0.24107949	0.09239853
110.7	0.06096577	0.19960536	0.34086002	0.08215437	0.1465857	0.19774564	0.03640796	0.68745597	0.09328531	0.11024323	0.24112336	0.09242397
110.8	0.0609642	0.19958814	0.34087849	0.082152	0.14660843	0.19775227	0.03640486	0.68748915	0.09327383	0.1102688	0.24116718	0.09244936
110.9	0.06096264	0.19957092	0.34089696	0.08214963	0.14663115	0.1977589	0.03640178	0.68752225	0.09326236	0.1102943	0.24121095	0.09247469
111	0.0609611	0.1995537	0.34091543	0.08214728	0.14665387	0.19776554	0.03639872	0.68755526	0.09325089	0.11031973	0.24125467	0.09249998
111.1	0.06095957	0.19953649	0.34093388	0.08214495	0.14667658	0.19777218	0.03639568	0.68758819	0.09323943	0.11034509	0.24129834	0.09252521
111.2	0.06095805	0.19951928	0.34095234	0.08214262	0.14669928	0.19777882	0.03639266	0.68762103	0.09322797	0.11037039	0.24134195	0.09255039
111.3	0.06095656	0.19950208	0.34097079	0.08214031	0.14672197	0.19778547	0.03638966	0.68765379	0.09321652	0.11039562	0.24138552	0.09257552
111.4	0.06095507	0.19948488	0.34098923	0.08213802	0.14674466	0.19779212	0.03638668	0.68768647	0.09320508	0.11042079	0.24142903	0.09260061
111.5	0.0609536	0.19946768	0.34100767	0.08213574	0.14676733	0.19779878	0.03638372	0.68771906	0.09319364	0.11044588	0.2414725	0.09262564
111.6	0.06095215	0.19945049	0.3410261	0.08213347	0.14679	0.19780544	0.03638079	0.68775156	0.09318221	0.11047092	0.24151592	0.09265062
111.7	0.0609507	0.19943331	0.34104453	0.08213121	0.14681266	0.1978121	0.03637787	0.68778399	0.09317078	0.11049588	0.24155928	0.09267556
111.8	0.06094928	0.19941613	0.34106295	0.08212897	0.14683531	0.19781877	0.03637498	0.68781633	0.09315936	0.11052078	0.2416026	0.09270044

78.3	0.06280747	0.20481783	0.33483186	0.08393747	0.13898422	0.19642834	0.03904231	0.66999991	0.09691478	0.09686922	0.22367109	0.08042782
78.4	0.06279593	0.20480588	0.33484966	0.08392793	0.13900765	0.19642624	0.03902702	0.6700841	0.09690631	0.09693193	0.22373744	0.08048104
78.5	0.06278442	0.20479388	0.33486748	0.08391842	0.13903107	0.19642422	0.03901179	0.67016802	0.0968978	0.09699446	0.22380369	0.08053413
78.6	0.06277296	0.20478182	0.3348853	0.08390894	0.13905451	0.19642228	0.03899663	0.67025166	0.09688924	0.09705681	0.22386984	0.08058708
78.7	0.06276155	0.20476971	0.33490315	0.08389949	0.13907794	0.19642042	0.03898152	0.67033503	0.09688063	0.09711899	0.2239359	0.08063989
78.8	0.06275019	0.20475755	0.334921	0.08389007	0.13910139	0.19641863	0.03896648	0.67041813	0.09687199	0.09718099	0.22400185	0.08069257
78.9	0.06273886	0.20474533	0.33493886	0.08388068	0.13912484	0.19641692	0.0389515	0.67050096	0.09686329	0.09724283	0.22406771	0.08074511
79	0.06272759	0.20473305	0.33495674	0.08387132	0.1391483	0.19641529	0.03893659	0.67058353	0.09685456	0.09730448	0.22413347	0.08079751
79.1	0.06271636	0.20472073	0.33497463	0.083862	0.13917176	0.19641373	0.03892173	0.67066582	0.09684579	0.09736597	0.22419913	0.08084978
79.2	0.06270517	0.20470835	0.33499253	0.0838527	0.13919523	0.19641224	0.03890693	0.67074785	0.09683697	0.09742728	0.22426469	0.08090191
79.3	0.06269403	0.20469592	0.33501044	0.08384343	0.13921871	0.19641083	0.0388922	0.67082961	0.09682811	0.09748842	0.22433016	0.0809539
79.4	0.06268293	0.20468344	0.33502836	0.0838342	0.13924219	0.19640949	0.03887752	0.67091111	0.09681921	0.09754939	0.22439553	0.08100576
79.5	0.06267188	0.2046709	0.33504629	0.08382499	0.13926567	0.19640822	0.0388629	0.67099235	0.09681028	0.09761019	0.2244608	0.08105749
79.6	0.06266088	0.20465832	0.33506424	0.08381581	0.13928917	0.19640702	0.03884835	0.67107332	0.0968013	0.09767082	0.22452598	0.08110908
79.7	0.06264992	0.20464569	0.3350822	0.08380667	0.13931267	0.1964059	0.03883385	0.67115404	0.09679228	0.09773128	0.22459106	0.08116054
79.8	0.062639	0.20463301	0.33510016	0.08379755	0.13933617	0.19640484	0.03881941	0.67123449	0.09678323	0.09779156	0.22465605	0.08121187
79.9	0.06262813	0.20462029	0.33511814	0.08378846	0.13935968	0.19640385	0.03880504	0.67131469	0.09677413	0.09785168	0.22472094	0.08126306
80	0.0626173	0.20460751	0.33513613	0.08377941	0.1393832	0.19640294	0.03879072	0.67139463	0.096765	0.09791163	0.22478573	0.08131412
80.1	0.06260652	0.20459468	0.33515413	0.08377038	0.13940672	0.19640209	0.03877646	0.67147431	0.09675584	0.09797141	0.22485043	0.08136505
80.2	0.06259579	0.20458181	0.33517214	0.08376138	0.13943024	0.1964013	0.03876226	0.67155374	0.09674663	0.09803102	0.22491503	0.08141585
80.3	0.06258509	0.20456889	0.33519017	0.08375242	0.13945377	0.19640059	0.03874811	0.67163291	0.09673739	0.09809047	0.22497954	0.08146652
80.4	0.06257445	0.20455593	0.3352082	0.08374348	0.13947731	0.19639994	0.03873403	0.67171183	0.0967281	0.09814975	0.22504396	0.08151706
80.5	0.06256384	0.20454291	0.33522624	0.08373457	0.13950085	0.19639936	0.03872	0.6717905	0.09671878	0.09820886	0.22510828	0.08156747
80.6	0.06255329	0.20452985	0.3352443	0.08372569	0.13952439	0.19639884	0.03870603	0.67186892	0.09670943	0.09826781	0.22517251	0.08161775
80.7	0.06254277	0.20451675	0.33526236	0.08371684	0.13954794	0.19639839	0.03869211	0.67194709	0.09670003	0.09832659	0.22523664	0.0816679
80.8	0.0625323	0.2045036	0.33528044	0.08370802	0.1395715	0.19639801	0.03867826	0.67202501	0.0966906	0.0983852	0.22530068	0.08171792
80.9	0.06252188	0.2044904	0.33529852	0.08369923	0.13959505	0.19639769	0.03866446	0.67210268	0.09668113	0.09844365	0.22536463	0.08176782
81	0.0625115	0.20447716	0.33531662	0.08369047	0.13961862	0.19639743	0.03865072	0.67218011	0.09667163	0.09850194	0.22542849	0.08181759
81.1	0.06250116	0.20446387	0.33533473	0.08368174	0.13964219	0.19639723	0.03863703	0.67225729	0.09666209	0.09856006	0.22549225	0.08186723
81.2	0.06249087	0.20445054	0.33535284	0.08367303	0.13966576	0.1963971	0.0386234	0.67233422	0.09665252	0.09861802	0.22555592	0.08191674
81.3	0.06248062	0.20443717	0.33537097	0.08366436	0.13968933	0.19639703	0.03860983	0.67241091	0.09664291	0.09867582	0.2256195	0.08196613
81.4	0.06247041	0.20442375	0.33538911	0.08365571	0.13971291	0.19639702	0.03859631	0.67248735	0.09663326	0.09873345	0.22568299	0.08201539
81.5	0.06246025	0.20441028	0.33540725	0.0836471	0.1397365	0.19639708	0.03858285	0.67256355	0.09662359	0.09879092	0.22574639	0.08206453
81.6	0.06245013	0.20439678	0.33542541	0.08363851	0.13976009	0.19639719	0.03856944	0.67263951	0.09661387	0.09884823	0.22580969	0.08211354
81.7	0.06244006	0.20438323	0.33544358	0.08362995	0.13978368	0.19639736	0.03855609	0.67271523	0.09660413	0.09890538	0.22587291	0.08216243
81.8	0.06243003	0.20436963	0.33546175	0.08362142	0.13980728	0.1963976	0.0385428	0.67279071	0.09659436	0.09896237	0.22593603	0.08221119
81.9	0.06242004	0.204356	0.33547994	0.08361292	0.13983088	0.19639789	0.03852956	0.67286595	0.09658455	0.0990192	0.22599906	0.08225983
82	0.0624101	0.20434232	0.33549813	0.08360444	0.13985448	0.19639824	0.03851638	0.67294095	0.09657471	0.09907586	0.226062	0.08230835
82.1	0.0624002	0.2043286	0.33551634	0.083596	0.13987809	0.19639865	0.03850325	0.67301572	0.09656484	0.09913237	0.22612486	0.08235674
82.2	0.06239035	0.20431484	0.33553455	0.08358758	0.1399017	0.19639912	0.03849017	0.67309025	0.09655494	0.09918872	0.22618762	0.08240501
82.3	0.06238054	0.20430104	0.33555278	0.0835792	0.13992532	0.19639964	0.03847715	0.67316455	0.09654501	0.09924491	0.22625029	0.08245316
82.4	0.06237077	0.20428719	0.33557101	0.08357084	0.13994894	0.19640023	0.03846418	0.67323861	0.09653505	0.09930094	0.22631288	0.08250119
82.5	0.06236105	0.20427331	0.33558925	0.08356251	0.13997256	0.19640086	0.03845127	0.67331245	0.09652506	0.09935681	0.22637538	0.0825491
82.6	0.06235137	0.20425938	0.3356075	0.08355421	0.13999619	0.19640155	0.03843841	0.67338605	0.09651504	0.09941253	0.22643779	0.08259689
82.7	0.06234173	0.20424542	0.33562576	0.08354593	0.14001982	0.1964023	0.0384256	0.67345941	0.09650499	0.09946808	0.22650011	0.08264456
82.8	0.06233213	0.20423141	0.33564403	0.08353769	0.14004345	0.1964031	0.03841285	0.67353255	0.09649491	0.09952349	0.22656234	0.0826921
82.9	0.06232258	0.20421737	0.33566231	0.08352947	0.14006708	0.19640396	0.03840015	0.67360547	0.09648481	0.09957873	0.22662449	0.08273953
83	0.06231307	0.20420329	0.3356806	0.08352128	0.14009072	0.19640487	0.03838751	0.67367815	0.09647467	0.09963382	0.22668655	0.08278684
83.1	0.06230361	0.20418917	0.3356989	0.08351312	0.14011436	0.19640583	0.03837491	0.67375061	0.09646451	0.09968876	0.22674852	0.08283403
83.2	0.06229419	0.20417501	0.3357172	0.08350499	0.14013801	0.19640685	0.03836237	0.67382284	0.09645433	0.09974353	0.2268104	0.0828811
83.3	0.06228481	0.20416081	0.33573551	0.08349689	0.14016166	0.19640792	0.03834988	0.67389484	0.09644411	0.09979816	0.2268722	0.08292805
83.4	0.06227547	0.20414658	0.33575384	0.08348881	0.14018531	0.19640904	0.03833745	0.67396662	0.09643388	0.09985263	0.22693391	0.08297489
83.5	0.06226617	0.20413231	0.33577217	0.08348076	0.14020896	0.19641021	0.03832506	0.67403818	0.09642361	0.09990695	0.22699554	0.08302161
83.6	0.06225692	0.204118	0.33579051	0.08347274	0.14023262	0.19641143	0.03831273	0.67410952	0.09641332	0.09996111	0.22705708	0.08306821
83.7	0.06224771	0.20410365	0.33580885	0.08346475	0.14025628	0.1964127	0.03830045	0.67418064	0.096403	0.10001512	0.22711853	0.08311469
83.8	0.06223855	0.20408927	0.33582721	0.08345679	0.14027994	0.19641403	0.03828822	0.67425153	0.09639266	0.10006898	0.2271799	0.08316106
83.9	0.06222942	0.20407486	0.33584557	0.08344885	0.1403036	0.1964154	0.03827604	0.67432221	0.09638229	0.10012269	0.22724118	0.08320732
84	0.06222034	0.20406041	0.33586394	0.08344094	0.14032727	0.19641682	0.03826392	0.67439266	0.09637189	0.10017624	0.22730238	0.08325345
84.1	0.06221129	0.20404592	0.33588232	0.08343306	0.14035094	0.1964183	0.03825184	0.6744629	0.09636147	0.10022964	0.22736349	0.08329948
84.2	0.06220229	0.2040314	0.33590071	0.08342521	0.14037461	0.19641982	0.03823982	0.67453293	0.09635102	0.1002829	0.22742452	0.08334539
84.3	0.06219334	0.20401684	0.3359191	0.08341738	0.14039828	0.19642139	0.03822785	0.67460273	0.09634055	0.100336	0.22748546	0.08339118
84.4	0.06218442	0.20400225	0.3359375	0.08340959	0.14042196	0.196423	0.03821593	0.67467232	0.09633005	0.10038895	0.22754632	0.08343686
84.5	0.06217554	0.20398762	0.33595591	0.08340182	0.14044564	0.19642467	0.03820406	0.6747417	0.09631953	0.10044175	0.2276071	0.08348243
84.6	0.06216671	0.20397297	0.33597433	0.08339408	0.14046932	0.19642638	0.03819223	0.67481086	0.09630898	0.1004944	0.22766779	0.08352788
84.7	0.06215792	0.20395828	0.33599275	0.08338636	0.140493	0.19642814	0.03818046	0.67487981	0.09629842	0.1005469	0.2277284	0.08357322
84.8	0.06214917	0.20394355	0.33601118	0.08337868	0.14051669	0.19642994	0.03816874	0.67494855	0.09628782	0.10059926	0.22778892	0.08361845
84.9	0.06214046	0.20392879	0.33602962	0.08337102	0.14054037	0.19643179	0.03815707	0.67501707	0.09627721	0.10065147	0.22784937	0.08366357
85	0.06213179	0.203914	0.33604807	0.08336339	0.14056406	0.19643368	0.03814545	0.67508538	0.09626657	0.10070353	0.22790973	0.08370857
85.1	0.06212316	0.20389918	0.33606652	0.08335579	0.14058775	0.19643563	0.03813388	0.67515349	0.09625591	0.10075544	0.22797	0.08375347
85.2	0.06211457	0.20388433	0.33608498	0.08334821	0.14061144	0.19643761	0.03812236	0.67522138	0.09624522	0.10080721	0.2280302	0.08379825
85.3	0.06210602	0.20386944	0.33610344	0.08334066	0.14063514	0.19643964	0.03811088	0.67528907	0.09623452	0.10085883	0.22809031	0.08384292
85.4	0.06209752	0.20385453	0.33612192	0.08333314	0.14065883	0.19644171	0.03809946	0.67535655	0.09622379	0.10091031	0.22815034	0.08388748
85.5	0.06208905	0.20383958	0.3361404	0.08332565	0.14068253	0.19644383	0.03808808	0.67542382	0.09621305	0.10096164	0.22821029	0.08393193
85.6	0.06208062	0.2038246	0.33615888	0.08331818	0.14070623	0.19644599	0.03807676	0.67549089	0.09620228	0.10101282	0.22827016	0.08397628
85.7	0.06207224	0.20380959	0.33617738	0.08331074	0.14072993	0.1964482	0.03806548	0.67555775	0.09619149	0.10106387	0.22832995	0.08402051
85.8	0.06206389	0.20379456	0.33619587	0.08330333	0.14075364	0.19645044	0.03805425	0.67562441	0.09618068	0.10111476	0.22838966	0.08406463
85.9	0.06205559	0.20377949	0.33621438	0.08329595	0.14077734	0.19645273	0.03804307	0.67569086	0.09616985	0.10116552	0.22844928	0.08410865
86	0.06204732	0.20376439	0.33623289	0.08328859	0.14080105	0.19645506	0.03803194	0.67575712	0.09615901	0.10121613	0.22850883	0.08415256
86.1	0.0620391	0.20374926	0.33625141	0.08328126	0.14082475	0.19645744	0.03802086	0.67582317	0.09614814	0.1012666	0.22856829	0.08419636
86.2	0.06203091	0.2037341	0.33626993	0.08327396	0.14084846	0.19645985	0.03800982	0.67588902	0.09613726	0.10131693	0.22862768	0.08424005
86.3	0.06202277	0.20371892	0.33628846	0.08326668	0.14087217	0.19646231	0.03799884	0.67595467	0.09612635	0.10136712	0.22868699	0.08428363
86.4	0.06201466	0.2037037	0.336307	0.08325943	0.14089589	0.1964648	0.0379879	0.67602012	0.09611543	0.10141716	0.22874621	0.08432711
86.5	0.0620066	0.20368846	0.33632554	0.0832522	0.1409196	0.19646734	0.03797701	0.67608538	0.09610449	0.10146707	0.22880536	0.08437048
86.6	0.06199857	0.20367319	0.33634409	0.08324501	0.14094331	0.19646992	0.03796616	0.67615043	0.09609353	0.10151683	0.22886443	0.08441375
86.7	0.06199058	0.20365789	0.33636265	0.08323784	0.14096703	0.19647254	0.03795536	0.67621529	0.09608256	0.10156645	0.22892342	0.08445691
86.8	0.06198263	0.20364257	0.33638122	0.08323069	0.14099075	0.1964752	0.03794461	0.67627996	0.09607157	0.10161594	0.22898233	0.08449996
86.9	0.06197473	0.20362721	0.33639979	0.08322358	0.14101446	0.19647789	0.0379339	0.67634442	0.09606056	0.10166528	0.22904117	0.08454291
87	0.06196686	0.20361183	0.33641836	0.08321649	0.14103818	0.19648063	0.03792325	0.6764087	0.09604953	0.10171449	0.22909992	0.08458575
87.1	0.06195903	0.20359643	0.33643695	0.08320942	0.1410619	0.1964834	0.03791263	0.67647278	0.09603849	0.10176355	0.2291586	0.08462849
87.2	0.06195123	0.20358099	0.33645554	0.08320238	0.14108563	0.19648621	0.03790207	0.67653667	0.09602744	0.10181248	0.2292172	0.08467113
87.3	0.06194348	0.20356553	0.33647414	0.08319537	0.14110935	0.19648906	0.03789154	0.67660036	0.09601636	0.10186127	0.22927572	0.08471366
87.4	0.06193577	0.20355005	0.33649274	0.08318839	0.14113307	0.19649195	0.03788107	0.67666387	0.09600527	0.10190993	0.22933416	0.08475609
87.5	0.06192809	0.20353454	0.33651136	0.08318143	0.14115679	0.19649487	0.03787064	0.67672718	0.09599417	0.10195844	0.22939253	0.08479842
87.6	0.06192046	0.203519	0.33652997	0.08317449	0.14118052	0.19649783	0.03786025	0.6767903	0.09598304	0.10200683	0.22945082	0.08484065
87.7	0.06191286	0.20350344	0.3365486	0.08316759	0.14120424	0.19650083	0.03784991	0.67685324	0.09597191	0.10205507	0.22950903	0.08488277
87.8	0.0619053	0.20348785	0.33656723	0.08316071	0.14122797	0.19650387	0.03783961	0.67691599	0.09596076	0.10210318	0.22956717	0.08492479
87.9	0.06189778	0.20347224	0.33658586	0.08315385	0.1412517	0.19650694	0.03782936	0.67697854	0.09594959	0.10215115	0.22962523	0.08496671
88	0.0618903	0.20345661	0.33660451	0.08314702	0.14127542	0.19651005	0.03781915	0.67704091	0.09593841	0.10219899	0.22968321	0.08500854
88.1	0.06188285	0.20344095	0.33662315	0.08314022	0.14129915	0.19651319	0.03780899	0.6771031	0.09592721	0.1022467	0.22974112	0.08505026
88.2	0.06187545	0.20342526	0.33664181	0.08313344	0.14132288	0.19651637	0.03779887	0.67716509	0.095916	0.10229427	0.22979895	0.08509188
88.3	0.06186808	0.20340956	0.33666047	0.08312668	0.1413466	0.19651959	0.0377888	0.67722691	0.09590478	0.10234171	0.22985671	0.0851334
88.4	0.06186075	0.20339382	0.33667913	0.08311996	0.14137033	0.19652284	0.03777877	0.67728853	0.09589354	0.10238901	0.22991438	0.08517482
88.5	0.06185346	0.20337807	0.3366978	0.08311326	0.14139406	0.19652613	0.03776878	0.67734998	0.09588229	0.10243618	0.22997199	0.08521614
88.6	0.0618462	0.20336229	0.33671648	0.08310658	0.14141779	0.19652945	0.03775883	0.67741124	0.09587102	0.10248322	0.23002951	0.08525737
88.7	0.06183898	0.20334649	0.33673516	0.08309993	0.14144152	0.19653281	0.03774893	0.67747231	0.09585974	0.10253013	0.23008697	0.08529849
88.8	0.0618318	0.20333067	0.33675384	0.0830933	0.14146524	0.1965362	0.03773908	0.67753321	0.09584845	0.10257691	0.23014434	0.08533952
88.9	0.06182466	0.20331482	0.33677253	0.0830867	0.14148897	0.19653963	0.03772926	0.67759392	0.09583715	0.10262355	0.23020164	0.08538045
89	0.06181756	0.20329896	0.33679123	0.08308013	0.1415127	0.19654309	0.03771949	0.67765446	0.09582583	0.10267006	0.23025887	0.08542128
89.1	0.06181049	0.20328307	0.33680993	0.08307358	0.14153643	0.19654658	0.03770976	0.67771481	0.0958145	0.10271645	0.23031602	0.08546202
89.2	0.06180346	0.20326716	0.33682863	0.08306706	0.14156016	0.1965501	0.03770007	0.67777499	0.09580316	0.1027627	0.2303731	0.08550266
89.3	0.06179647	0.20325122	0.33684734	0.08306056	0.14158388	0.19655366	0.03769043	0.67783498	0.09579181	0.10280882	0.23043011	0.0855432
89.4	0.06178951	0.20323527	0.33686605	0.08305408	0.14160761	0.19655725	0.03768083	0.6778948	0.09578045	0.10285482	0.23048703	0.08558365
89.5	0.06178259	0.20321929	0.33688477	0.08304763	0.14163134	0.19656087	0.03767127	0.67795444	0.09576907	0.10290068	0.23054389	0.085624
89.6	0.06177571	0.2032033	0.33690349	0.08304121	0.14165507	0.19656452	0.03766175	0.67801391	0.09575769	0.10294642	0.23060067	0.08566426
89.7	0.06176886	0.20318728	0.33692222	0.08303481	0.14167879	0.19656821	0.03765228	0.6780732	0.09574629	0.10299203	0.23065738	0.08570442
89.8	0.06176205	0.20317125	0.33694095	0.08302843	0.14170252	0.19657192	0.03764284	0.67813231	0.09573488	0.10303751	0.23071401	0.08574449
89.9	0.06175528	0.20315519	0.33695969	0.08302208	0.14172624	0.19657567	0.03763345	0.67819125	0.09572346	0.10308287	0.23077056	0.08578446

120	0.0608733	0.1980282	0.3425545	0.081987	0.14866264	0.19837379	0.03620191	0.69020686	0.09224328	0.11235621	0.24499175	0.09458482
120.1	0.0608728	0.19801157	0.34257243	0.08198574	0.14868454	0.19838063	0.03620053	0.69023308	0.09223237	0.11237629	0.24503118	0.09460604
120.2	0.06087231	0.19799495	0.34259036	0.08198449	0.14870643	0.19838747	0.03619916	0.69025923	0.09222147	0.11239631	0.24507057	0.09462722
120.3	0.06087182	0.19797834	0.34260829	0.08198325	0.14872831	0.19839431	0.0361978	0.69028532	0.09221057	0.11241629	0.24510991	0.09464837
120.4	0.06087135	0.19796174	0.3426262	0.08198202	0.14875018	0.19840115	0.03619647	0.69031134	0.09219969	0.11243621	0.24514921	0.09466947
120.5	0.06087088	0.19794515	0.34264412	0.0819808	0.14877204	0.19840799	0.03619514	0.6903373	0.0921888	0.11245608	0.24518847	0.09469054
120.6	0.06087042	0.19792856	0.34266202	0.08197959	0.14879389	0.19841483	0.03619384	0.6903632	0.09217793	0.11247591	0.24522768	0.09471157
120.7	0.06086997	0.19791198	0.34267992	0.08197839	0.14881573	0.19842167	0.03619255	0.69038903	0.09216706	0.11249568	0.24526686	0.09473256
120.8	0.06086953	0.19789541	0.34269781	0.0819772	0.14883756	0.19842851	0.03619127	0.6904148	0.0921562	0.11251541	0.24530598	0.09475351
120.9	0.0608691	0.19787885	0.34271569	0.08197602	0.14885938	0.19843536	0.03619002	0.6904405	0.09214534	0.11253508	0.24534507	0.09477442
121	0.06086867	0.1978623	0.34273357	0.08197485	0.14888119	0.1984422	0.03618877	0.69046614	0.09213449	0.1125547	0.24538411	0.09479529
121.1	0.06086826	0.19784576	0.34275144	0.08197369	0.14890299	0.19844905	0.03618755	0.69049172	0.09212365	0.11257428	0.2454231	0.09481613
121.2	0.06086785	0.19782922	0.34276931	0.08197254	0.14892478	0.19845589	0.03618634	0.69051724	0.09211282	0.1125938	0.24546206	0.09483693
121.3	0.06086745	0.19781269	0.34278716	0.0819714	0.14894656	0.19846274	0.03618514	0.69054269	0.09210199	0.11261328	0.24550097	0.09485769
121.4	0.06086705	0.19779618	0.34280502	0.08197028	0.14896833	0.19846959	0.03618396	0.69056808	0.09209117	0.1126327	0.24553984	0.09487841
121.5	0.06086667	0.19777967	0.34282286	0.08196916	0.14899009	0.19847644	0.0361828	0.69059341	0.09208035	0.11265208	0.24557866	0.0948991
121.6	0.06086629	0.19776316	0.3428407	0.08196805	0.14901184	0.19848328	0.03618165	0.69061867	0.09206955	0.11267141	0.24561744	0.09491974
121.7	0.06086592	0.19774667	0.34285853	0.08196695	0.14903359	0.19849013	0.03618051	0.69064387	0.09205875	0.11269069	0.24565618	0.09494035
121.8	0.06086556	0.19773019	0.34287635	0.08196586	0.14905532	0.19849698	0.0361794	0.69066901	0.09204795	0.11270992	0.24569488	0.09496093
121.9	0.06086521	0.19771371	0.34289417	0.08196478	0.14907704	0.19850383	0.03617829	0.69069409	0.09203716	0.11272911	0.24573353	0.09498146
122	0.06086487	0.19769724	0.34291198	0.0819637	0.14909875	0.19851068	0.03617721	0.69071911	0.09202638	0.11274824	0.24577214	0.09500196
122.1	0.06086453	0.19768078	0.34292978	0.08196264	0.14912045	0.19851754	0.03617613	0.69074407	0.09201561	0.11276733	0.24581071	0.09502242
122.2	0.0608642	0.19766433	0.34294758	0.08196159	0.14914214	0.19852439	0.03617508	0.69076896	0.09200484	0.11278637	0.24584924	0.09504284
122.3	0.06086388	0.19764789	0.34296537	0.08196055	0.14916382	0.19853124	0.03617404	0.69079379	0.09199408	0.11280536	0.24588772	0.09506323
122.4	0.06086356	0.19763146	0.34298316	0.08195952	0.14918549	0.19853809	0.03617301	0.69081857	0.09198333	0.1128243	0.24592616	0.09508357
122.5	0.06086326	0.19761503	0.34300093	0.08195849	0.14920716	0.19854495	0.036172	0.69084328	0.09197258	0.1128432	0.24596456	0.09510389
122.6	0.06086296	0.19759861	0.3430187	0.08195748	0.14922881	0.1985518	0.036171	0.69086793	0.09196184	0.11286205	0.24600291	0.09512416
122.7	0.06086267	0.19758221	0.34303647	0.08195647	0.14925045	0.19855865	0.03617002	0.69089252	0.09195111	0.11288085	0.24604123	0.0951444
122.8	0.06086238	0.19756581	0.34305422	0.08195548	0.14927208	0.19856551	0.03616906	0.69091705	0.09194038	0.1128996	0.2460795	0.0951646
122.9	0.06086211	0.19754942	0.34307197	0.08195449	0.1492937	0.19857236	0.0361681	0.69094152	0.09192967	0.11291831	0.24611773	0.09518476
123	0.06086184	0.19753304	0.34308972	0.08195352	0.14931531	0.19857922	0.03616717	0.69096593	0.09191895	0.11293697	0.24615592	0.09520489
123.1	0.06086157	0.19751666	0.34310745	0.08195255	0.14933691	0.19858607	0.03616625	0.69099028	0.09190825	0.11295558	0.24619406	0.09522498
123.2	0.06086132	0.1975003	0.34312518	0.08195159	0.1493585	0.19859293	0.03616534	0.69101458	0.09189755	0.11297415	0.24623217	0.09524503
123.3	0.06086107	0.19748394	0.3431429	0.08195064	0.14938008	0.19859978	0.03616445	0.69103881	0.09188686	0.11299267	0.24627023	0.09526505
123.4	0.06086083	0.1974676	0.34316062	0.0819497	0.14940165	0.19860664	0.03616357	0.69106298	0.09187617	0.11301115	0.24630825	0.09528503
123.5	0.0608606	0.19745126	0.34317833	0.08194877	0.14942321	0.19861349	0.03616271	0.69108709	0.09186549	0.11302958	0.24634622	0.09530498
123.6	0.06086037	0.19743493	0.34319603	0.08194785	0.14944476	0.19862035	0.03616186	0.69111115	0.09185482	0.11304796	0.24638416	0.09532488
123.7	0.06086015	0.19741861	0.34321372	0.08194694	0.1494663	0.1986272	0.03616102	0.69113515	0.09184416	0.11306629	0.24642205	0.09534476
123.8	0.06085994	0.1974023	0.34323141	0.08194604	0.14948783	0.19863406	0.03616021	0.69115908	0.0918335	0.11308458	0.24645991	0.09536459
123.9	0.06085973	0.19738599	0.34324909	0.08194514	0.14950935	0.19864091	0.0361594	0.69118296	0.09182285	0.11310283	0.24649772	0.0953844
124	0.06085953	0.1973697	0.34326677	0.08194426	0.14953086	0.19864776	0.03615861	0.69120678	0.09181221	0.11312103	0.24653549	0.09540416
124.1	0.06085934	0.19735341	0.34328443	0.08194338	0.14955236	0.19865462	0.03615783	0.69123054	0.09180157	0.11313918	0.24657321	0.09542389
124.2	0.06085916	0.19733714	0.34330209	0.08194251	0.14957385	0.19866147	0.03615707	0.69125425	0.09179094	0.11315729	0.2466109	0.09544359
124.3	0.06085898	0.19732087	0.34331975	0.08194166	0.14959532	0.19866833	0.03615632	0.6912779	0.09178032	0.11317535	0.24664855	0.09546325
124.4	0.06085881	0.19730461	0.34333739	0.08194081	0.14961679	0.19867518	0.03615559	0.69130148	0.0917697	0.11319337	0.24668615	0.09548287
124.5	0.06085864	0.19728836	0.34335503	0.08193997	0.14963825	0.19868203	0.03615487	0.69132502	0.0917591	0.11321135	0.24672372	0.09550246
124.6	0.06085848	0.19727212	0.34337266	0.08193914	0.1496597	0.19868889	0.03615416	0.69134849	0.09174849	0.11322928	0.24676124	0.09552201
124.7	0.06085833	0.19725589	0.34339029	0.08193832	0.14968113	0.19869574	0.03615347	0.69137191	0.0917379	0.11324716	0.24679872	0.09554153
124.8	0.06085818	0.19723966	0.3434079	0.0819375	0.14970256	0.19870259	0.03615279	0.69139527	0.09172731	0.113265	0.24683616	0.09556101
124.9	0.06085804	0.19722345	0.34342551	0.0819367	0.14972398	0.19870944	0.03615213	0.69141857	0.09171673	0.1132828	0.24687356	0.09558046
125	0.06085791	0.19720725	0.34344312	0.0819359	0.14974538	0.19871629	0.03615148	0.69144182	0.09170616	0.11330055	0.24691092	0.09559988
125.1	0.06085779	0.19719105	0.34346071	0.08193511	0.14976678	0.19872314	0.03615085	0.69146501	0.09169559	0.11331826	0.24694824	0.09561925
125.2	0.06085767	0.19717486	0.3434783	0.08193434	0.14978816	0.19872999	0.03615022	0.69148814	0.09168503	0.11333592	0.24698552	0.0956386
125.3	0.06085755	0.19715869	0.34349589	0.08193357	0.14980954	0.19873684	0.03614962	0.69151122	0.09167448	0.11335354	0.24702276	0.09565791
125.4	0.06085745	0.19714252	0.34351346	0.08193281	0.1498309	0.19874369	0.03614902	0.69153424	0.09166393	0.11337111	0.24705996	0.09567718
125.5	0.06085735	0.19712636	0.34353103	0.08193205	0.14985226	0.19875054	0.03614844	0.6915572	0.09165339	0.11338865	0.24709711	0.09569642
125.6	0.06085725	0.19711021	0.34354859	0.08193131	0.1498736	0.19875739	0.03614787	0.69158011	0.09164286	0.11340614	0.24713423	0.09571563
125.7	0.06085716	0.19709407	0.34356614	0.08193057	0.14989494	0.19876424	0.03614732	0.69160297	0.09163234	0.11342358	0.24717131	0.0957348
125.8	0.06085708	0.19707793	0.34358369	0.08192985	0.14991626	0.19877108	0.03614678	0.69162577	0.09162182	0.11344098	0.24720834	0.09575394
125.9	0.06085701	0.19706181	0.34360123	0.08192913	0.14993757	0.19877793	0.03614625	0.69164851	0.09161131	0.11345834	0.24724534	0.09577304
126	0.06085694	0.1970457	0.34361876	0.08192842	0.14995887	0.19878478	0.03614574	0.6916712	0.09160081	0.11347566	0.2472823	0.09579211
126.1	0.06085687	0.19702959	0.34363628	0.08192772	0.14998017	0.19879162	0.03614524	0.69169383	0.09159031	0.11349293	0.24731921	0.09581115
126.2	0.06085681	0.1970135	0.3436538	0.08192703	0.15000145	0.19879847	0.03614475	0.69171641	0.09157982	0.11351017	0.24735609	0.09583015
126.3	0.06085676	0.19699741	0.34367131	0.08192634	0.15002272	0.19880531	0.03614428	0.69173893	0.09156934	0.11352735	0.24739292	0.09584912
126.4	0.06085672	0.19698133	0.34368882	0.08192567	0.15004398	0.19881215	0.03614382	0.6917614	0.09155887	0.1135445	0.24742972	0.09586806
126.5	0.06085668	0.19696527	0.34370631	0.081925	0.15006523	0.19881899	0.03614337	0.69178381	0.0915484	0.1135616	0.24746647	0.09588696
126.6	0.06085664	0.19694921	0.3437238	0.08192434	0.15008647	0.19882584	0.03614294	0.69180617	0.09153794	0.11357867	0.24750319	0.09590583
126.7	0.06085661	0.19693316	0.34374128	0.08192369	0.1501077	0.19883268	0.03614252	0.69182848	0.09152749	0.11359569	0.24753987	0.09592466
126.8	0.06085659	0.19691712	0.34375875	0.08192305	0.15012892	0.19883952	0.03614212	0.69185073	0.09151704	0.11361266	0.2475765	0.09594346

96.9	0.06136456	0.20199114	0.33827724	0.08263565	0.14338312	0.19689989	0.03707171	0.6819201	0.09490528	0.1059623	0.23455274	0.08836424
97	0.06136007	0.20197407	0.33829609	0.08263091	0.14340669	0.19690524	0.03706495	0.68196816	0.09489343	0.1059995	0.23460438	0.08839821
97.1	0.06135561	0.201957	0.33831495	0.08262619	0.14343026	0.19691062	0.03705822	0.68201609	0.09488159	0.10603659	0.23465596	0.08843209
97.2	0.06135117	0.20193992	0.3383338	0.08262149	0.14345382	0.19691601	0.03705152	0.68206388	0.09486974	0.10607359	0.23470747	0.08846591
97.3	0.06134676	0.20192284	0.33835265	0.08261681	0.14347738	0.19692142	0.03704486	0.68211154	0.09485789	0.10611048	0.23475892	0.08849964
97.4	0.06134239	0.20190574	0.3383715	0.08261216	0.14350094	0.19692684	0.03703823	0.68215906	0.09484605	0.10614727	0.23481031	0.0885333
97.5	0.06133803	0.20188864	0.33839036	0.08260752	0.14352449	0.19693228	0.03703163	0.68220645	0.0948342	0.10618396	0.23486163	0.08856689
97.6	0.06133371	0.20187152	0.33840921	0.0826029	0.14354803	0.19693773	0.03702507	0.68225371	0.09482235	0.10622054	0.23491289	0.08860041
97.7	0.06132941	0.2018544	0.33842806	0.0825983	0.14357158	0.1969432	0.03701853	0.68230084	0.09481049	0.10625703	0.23496409	0.08863384
97.8	0.06132514	0.20183728	0.33844691	0.08259372	0.14359511	0.19694869	0.03701203	0.68234784	0.09479864	0.10629342	0.23501523	0.08866721
97.9	0.0613209	0.20182014	0.33846576	0.08258916	0.14361865	0.19695419	0.03700556	0.68239471	0.09478679	0.1063297	0.2350663	0.0887005
98	0.06131668	0.201803	0.33848461	0.08258462	0.14364218	0.19695971	0.03699912	0.68244144	0.09477494	0.10636588	0.23511731	0.08873371
98.1	0.06131249	0.20178585	0.33850346	0.0825801	0.1436657	0.19696525	0.03699272	0.68248805	0.09476308	0.10640197	0.23516825	0.08876686
98.2	0.06130833	0.20176869	0.3385223	0.0825756	0.14368922	0.1969708	0.03698634	0.68253453	0.09475123	0.10643796	0.23521913	0.08879993
98.3	0.0613042	0.20175153	0.33854115	0.08257113	0.14371274	0.19697636	0.03698	0.68258088	0.09473938	0.10647384	0.23526995	0.08883292
98.4	0.06130009	0.20173436	0.33856	0.08256667	0.14373625	0.19698194	0.03697369	0.6826271	0.09472752	0.10650963	0.23532071	0.08886585
98.5	0.06129601	0.20171718	0.33857885	0.08256223	0.14375976	0.19698753	0.03696741	0.68267319	0.09471567	0.10654532	0.23537141	0.0888987
98.6	0.06129195	0.2017	0.33859769	0.08255781	0.14378326	0.19699314	0.03696116	0.68271916	0.09470381	0.10658091	0.23542204	0.08893148
98.7	0.06128792	0.20168281	0.33861654	0.08255341	0.14380676	0.19699877	0.03695494	0.68276499	0.09469196	0.1066164	0.23547261	0.08896418
98.8	0.06128392	0.20166562	0.33863538	0.08254903	0.14383026	0.1970044	0.03694875	0.68281071	0.0946801	0.1066518	0.23552312	0.08899682
98.9	0.06127994	0.20164841	0.33865422	0.08254467	0.14385375	0.19701006	0.03694259	0.68285629	0.09466825	0.1066871	0.23557357	0.08902938
99	0.06127599	0.20163121	0.33867306	0.08254033	0.14387723	0.19701572	0.03693647	0.68290175	0.0946564	0.1067223	0.23562396	0.08906187
99.1	0.06127207	0.20161399	0.3386919	0.08253601	0.14390071	0.1970214	0.03693037	0.68294709	0.09464454	0.1067574	0.23567428	0.08909429
99.2	0.06126817	0.20159677	0.33871074	0.08253171	0.14392419	0.19702709	0.03692431	0.6829923	0.09463269	0.10679241	0.23572455	0.08912664
99.3	0.0612643	0.20157955	0.33872958	0.08252743	0.14394766	0.1970328	0.03691827	0.68303738	0.09462084	0.10682732	0.23577475	0.08915892
99.4	0.06126045	0.20156232	0.33874842	0.08252316	0.14397113	0.19703852	0.03691227	0.68308234	0.09460898	0.10686214	0.2358249	0.08919113
99.5	0.06125663	0.20154508	0.33876726	0.08251892	0.14399459	0.19704425	0.0369063	0.68312718	0.09459713	0.10689686	0.23587498	0.08922327
99.6	0.06125283	0.20152784	0.33878609	0.0825147	0.14401805	0.19705	0.03690035	0.68317189	0.09458528	0.10693149	0.235925	0.08925533
99.7	0.06124906	0.20151059	0.33880492	0.08251049	0.1440415	0.19705575	0.03689444	0.68321648	0.09457343	0.10696602	0.23597496	0.08928733
99.8	0.06124531	0.20149334	0.33882375	0.08250631	0.14406495	0.19706152	0.03688856	0.68326095	0.09456158	0.10700045	0.23602486	0.08931926
99.9	0.06124159	0.20147608	0.33884258	0.08250215	0.1440884	0.19706731	0.0368827	0.68330529	0.09454973	0.1070348	0.2360747	0.08935111
100	0.0612379	0.20145882	0.33886141	0.082498	0.14411184	0.1970731	0.03687688	0.68334951	0.09453788	0.10706905	0.23612448	0.0893829
100.1	0.06123423	0.20144155	0.33888024	0.08249387	0.14413527	0.19707891	0.03687109	0.68339361	0.09452603	0.1071032	0.2361742	0.08941462
100.2	0.06123058	0.20142428	0.33889907	0.08248977	0.1441587	0.19708473	0.03686532	0.68343759	0.09451419	0.10713726	0.23622386	0.08944627
100.3	0.06122696	0.20140701	0.33891789	0.08248568	0.14418213	0.19709057	0.03685959	0.68348145	0.09450234	0.10717123	0.23627346	0.08947785
100.4	0.06122336	0.20138973	0.33893671	0.08248161	0.14420555	0.19709641	0.03685388	0.68352519	0.09449049	0.10720511	0.236323	0.08950937
100.5	0.06121979	0.20137244	0.33895553	0.08247756	0.14422897	0.19710227	0.03684821	0.68356881	0.09447865	0.10723889	0.23637248	0.08954081
100.6	0.06121624	0.20135515	0.33897435	0.08247352	0.14425238	0.19710814	0.03684256	0.68361231	0.09446681	0.10727258	0.23642191	0.08957219
100.7	0.06121272	0.20133786	0.33899317	0.08246951	0.14427579	0.19711402	0.03683694	0.68365569	0.09445496	0.10730618	0.23647127	0.08960349
100.8	0.06120922	0.20132057	0.33901198	0.08246552	0.14429919	0.19711991	0.03683135	0.68369895	0.09444312	0.10733968	0.23652057	0.08963474
100.9	0.06120575	0.20130327	0.33903079	0.08246154	0.14432258	0.19712582	0.03682579	0.68374209	0.09443128	0.1073731	0.23656981	0.08966591
101	0.0612023	0.20128596	0.3390496	0.08245758	0.14434598	0.19713173	0.03682026	0.68378512	0.09441944	0.10740642	0.236619	0.08969701
101.1	0.06119887	0.20126866	0.33906841	0.08245365	0.14436936	0.19713766	0.03681476	0.68382803	0.0944076	0.10743965	0.23666812	0.08972805
101.2	0.06119546	0.20125135	0.33908722	0.08244973	0.14439274	0.19714359	0.03680929	0.68387082	0.09439576	0.10747279	0.23671719	0.08975902
101.3	0.06119209	0.20123403	0.33910602	0.08244582	0.14441612	0.19714954	0.03680384	0.6839135	0.09438393	0.10750583	0.2367662	0.08978993
101.4	0.06118873	0.20121672	0.33912483	0.08244194	0.14443949	0.1971555	0.03679843	0.68395606	0.09437209	0.10753879	0.23681515	0.08982076
101.5	0.0611854	0.2011994	0.33914363	0.08243808	0.14446286	0.19716147	0.03679304	0.6839985	0.09436026	0.10757165	0.23686404	0.08985154
101.6	0.06118209	0.20118208	0.33916243	0.08243423	0.14448622	0.19716745	0.03678768	0.68404083	0.09434843	0.10760443	0.23691287	0.08988224
101.7	0.0611788	0.20116475	0.33918122	0.0824304	0.14450958	0.19717345	0.03678235	0.68408304	0.0943366	0.10763711	0.23696164	0.08991288
101.8	0.06117554	0.20114742	0.33920002	0.08242659	0.14453293	0.19717945	0.03677705	0.68412514	0.09432477	0.10766971	0.23701035	0.08994345
101.9	0.0611723	0.20113009	0.33921881	0.0824228	0.14455627	0.19718546	0.03677177	0.68416713	0.09431294	0.10770221	0.23705901	0.08997396
102	0.06116908	0.20111276	0.3392376	0.08241903	0.14457961	0.19719148	0.03676653	0.684209	0.09430112	0.10773463	0.23710761	0.09000441
102.1	0.06116588	0.20109543	0.33925638	0.08241527	0.14460295	0.19719752	0.03676131	0.68425076	0.09428929	0.10776696	0.23715615	0.09003479
102.2	0.06116271	0.20107809	0.33927517	0.08241154	0.14462628	0.19720356	0.03675612	0.68429241	0.09427747	0.1077992	0.23720463	0.0900651
102.3	0.06115956	0.20106075	0.33929395	0.08240782	0.1446496	0.19720961	0.03675095	0.68433394	0.09426565	0.10783135	0.23725306	0.09009535
102.4	0.06115643	0.20104341	0.33931273	0.08240412	0.14467292	0.19721568	0.03674582	0.68437536	0.09425383	0.10786341	0.23730142	0.09012554
102.5	0.06115333	0.20102607	0.33933151	0.08240043	0.14469623	0.19722175	0.03674071	0.68441667	0.09424201	0.10789538	0.23734973	0.09015566
102.6	0.06115025	0.20100872	0.33935028	0.08239677	0.14471954	0.19722783	0.03673563	0.68445787	0.0942302	0.10792727	0.23739798	0.09018572
102.7	0.06114719	0.20099138	0.33936906	0.08239312	0.14474284	0.19723393	0.03673058	0.68449896	0.09421838	0.10795907	0.23744618	0.09021571
102.8	0.06114415	0.20097403	0.33938783	0.08238949	0.14476614	0.19724003	0.03672556	0.68453994	0.09420657	0.10799078	0.23749432	0.09024564
102.9	0.06114113	0.20095668	0.33940659	0.08238588	0.14478943	0.19724614	0.03672056	0.68458081	0.09419477	0.10802241	0.2375424	0.09027551
103	0.06113813	0.20093933	0.33942536	0.08238228	0.14481272	0.19725226	0.03671559	0.68462157	0.09418296	0.10805395	0.23759042	0.09030532
103.1	0.06113516	0.20092198	0.33944412	0.0823787	0.144836	0.19725839	0.03671065	0.68466222	0.09417116	0.1080854	0.23763838	0.09033506
103.2	0.06113221	0.20090463	0.33946288	0.08237515	0.14485927	0.19726453	0.03670573	0.68470275	0.09415936	0.10811677	0.23768629	0.09036474
103.3	0.06112928	0.20088727	0.33948163	0.08237161	0.14488254	0.19727068	0.03670085	0.68474319	0.09414756	0.10814805	0.23773415	0.09039435
103.4	0.06112637	0.20086992	0.33950039	0.08236808	0.1449058	0.19727683	0.03669598	0.68478351	0.09413577	0.10817925	0.23778194	0.09042391
103.5	0.06112348	0.20085256	0.33951914	0.08236457	0.14492906	0.197283	0.03669115	0.68482372	0.09412398	0.10821036	0.23782968	0.0904534
103.6	0.06112062	0.2008352	0.33953788	0.08236108	0.14495231	0.19728917	0.03668634	0.68486383	0.09411219	0.10824138	0.23787737	0.09048283
103.7	0.06111777	0.20081785	0.33955663	0.08235761	0.14497555	0.19729536	0.03668156	0.68490383	0.0941004	0.10827232	0.237925	0.0905122
103.8	0.06111495	0.20080049	0.33957537	0.08235416	0.14499879	0.19730155	0.0366768	0.68494372	0.09408862	0.10830318	0.23797257	0.09054151
103.9	0.06111214	0.20078313	0.33959411	0.08235072	0.14502202	0.19730775	0.03667208	0.6849835	0.09407684	0.10833395	0.23802008	0.09057076
104	0.06110936	0.20076577	0.33961284	0.0823473	0.14504525	0.19731395	0.03666737	0.68502318	0.09406507	0.10836464	0.23806754	0.09059994
104.1	0.0611066	0.20074841	0.33963157	0.08234389	0.14506847	0.19732017	0.0366627	0.68506275	0.09405329	0.10839525	0.23811494	0.09062907
104.2	0.06110386	0.20073105	0.3396503	0.0823405	0.14509168	0.1973264	0.03665805	0.68510222	0.09404152	0.10842577	0.23816229	0.09065813
104.3	0.06110113	0.20071369	0.33966903	0.08233713	0.14511489	0.19733263	0.03665343	0.68514157	0.09402976	0.10845621	0.23820958	0.09068713
104.4	0.06109843	0.20069633	0.33968775	0.08233378	0.14513809	0.19733887	0.03664883	0.68518083	0.09401799	0.10848657	0.23825682	0.09071607
104.5	0.06109575	0.20067897	0.33970647	0.08233044	0.14516129	0.19734512	0.03664426	0.68521998	0.09400623	0.10851684	0.238304	0.09074495
104.6	0.06109309	0.20066161	0.33972518	0.08232712	0.14518447	0.19735137	0.03663971	0.68525902	0.09399448	0.10854703	0.23835112	0.09077377
104.7	0.06109045	0.20064424	0.3397439	0.08232382	0.14520766	0.19735764	0.03663519	0.68529796	0.09398272	0.10857714	0.23839819	0.09080253
104.8	0.06108783	0.20062688	0.3397626	0.08232053	0.14523083	0.19736391	0.0366307	0.6853368	0.09397098	0.10860717	0.2384452	0.09083123
104.9	0.06108523	0.20060952	0.33978131	0.08231726	0.145254	0.19737019	0.03662623	0.68537553	0.09395923	0.10863712	0.23849215	0.09085987

135	0.06087197	0.19563423	0.34516635	0.08189663	0.15183288	0.19939675	0.03615047	0.69350463	0.09068504	0.11487197	0.25045071	0.09737994
135.1	0.06087233	0.19561898	0.3451832	0.08189659	0.15185322	0.19940349	0.03615104	0.69352287	0.0906752	0.11488583	0.25048423	0.09739627
135.2	0.0608727	0.19560375	0.34520004	0.08189657	0.15187354	0.19941022	0.03615163	0.69354107	0.09066536	0.11489966	0.25051772	0.09741256
135.3	0.06087307	0.19558853	0.34521688	0.08189655	0.15189386	0.19941696	0.03615222	0.69355923	0.09065553	0.11491346	0.25055116	0.09742884
135.4	0.06087344	0.19557332	0.3452337	0.08189653	0.15191416	0.19942369	0.03615283	0.69357734	0.09064571	0.11492723	0.25058458	0.09744508
135.5	0.06087382	0.19555812	0.34525052	0.08189652	0.15193445	0.19943042	0.03615344	0.69359541	0.0906359	0.11494096	0.25061796	0.0974613
135.6	0.0608742	0.19554293	0.34526733	0.08189652	0.15195473	0.19943715	0.03615407	0.69361344	0.09062609	0.11495466	0.2506513	0.09747749
135.7	0.06087458	0.19552775	0.34528413	0.08189653	0.151975	0.19944388	0.0361547	0.69363143	0.09061629	0.11496832	0.2506846	0.09749365
135.8	0.06087497	0.19551258	0.34530093	0.08189654	0.15199526	0.1994506	0.03615535	0.69364937	0.0906065	0.11498196	0.25071788	0.09750979
135.9	0.06087536	0.19549742	0.34531772	0.08189655	0.15201551	0.19945732	0.036156	0.69366727	0.09059671	0.11499556	0.25075111	0.0975259
136	0.06087575	0.19548227	0.3453345	0.08189657	0.15203575	0.19946404	0.03615667	0.69368513	0.09058693	0.11500913	0.25078431	0.09754198
136.1	0.06087615	0.19546713	0.34535127	0.0818966	0.15205597	0.19947076	0.03615735	0.69370295	0.09057716	0.11502267	0.25081748	0.09755804
136.2	0.06087655	0.195452	0.34536803	0.08189664	0.15207619	0.19947748	0.03615803	0.69372072	0.0905674	0.11503617	0.25085061	0.09757407
136.3	0.06087696	0.19543688	0.34538479	0.08189668	0.15209639	0.1994842	0.03615873	0.69373845	0.09055764	0.11504964	0.2508837	0.09759008
136.4	0.06087736	0.19542177	0.34540154	0.08189673	0.15211659	0.19949091	0.03615944	0.69375615	0.09054789	0.11506308	0.25091676	0.09760605
136.5	0.06087777	0.19540667	0.34541828	0.08189678	0.15213677	0.19949762	0.03616016	0.6937738	0.09053815	0.11507649	0.25094978	0.09762201
136.6	0.06087819	0.19539158	0.34543501	0.08189684	0.15215695	0.19950433	0.03616088	0.6937914	0.09052842	0.11508987	0.25098277	0.09763793
136.7	0.0608786	0.19537651	0.34545174	0.0818969	0.15217711	0.19951104	0.03616162	0.69380897	0.09051869	0.11510321	0.25101573	0.09765383
136.8	0.06087902	0.19536144	0.34546846	0.08189697	0.15219726	0.19951775	0.03616237	0.6938265	0.09050897	0.11511653	0.25104865	0.09766971
136.9	0.06087945	0.19534638	0.34548517	0.08189705	0.1522174	0.19952445	0.03616313	0.69384398	0.09049926	0.11512981	0.25108153	0.09768555
137	0.06087987	0.19533133	0.34550187	0.08189713	0.15223753	0.19953115	0.03616389	0.69386143	0.09048955	0.11514306	0.25111438	0.09770138
137.1	0.0608803	0.1953163	0.34551857	0.08189722	0.15225765	0.19953785	0.03616467	0.69387883	0.09047986	0.11515628	0.2511472	0.09771717
137.2	0.06088073	0.19530127	0.34553525	0.08189732	0.15227775	0.19954455	0.03616546	0.69389619	0.09047017	0.11516947	0.25117998	0.09773294
137.3	0.06088117	0.19528625	0.34555193	0.08189742	0.15229785	0.19955124	0.03616626	0.69391352	0.09046048	0.11518262	0.25121272	0.09774869
137.4	0.06088161	0.19527125	0.34556861	0.08189753	0.15231794	0.19955794	0.03616706	0.6939308	0.09045081	0.11519575	0.25124543	0.09776441
137.5	0.06088205	0.19525625	0.34558527	0.08189764	0.15233801	0.19956463	0.03616788	0.69394804	0.09044114	0.11520884	0.25127811	0.0977801
137.6	0.0608825	0.19524127	0.34560193	0.08189776	0.15235808	0.19957132	0.03616871	0.69396524	0.09043147	0.1152219	0.25131075	0.09779577
137.7	0.06088295	0.19522629	0.34561858	0.08189788	0.15237813	0.199578	0.03616954	0.6939824	0.09042182	0.11523494	0.25134336	0.09781141
137.8	0.0608834	0.19521133	0.34563522	0.08189801	0.15239818	0.19958469	0.03617039	0.69399952	0.09041217	0.11524794	0.25137593	0.09782703
137.9	0.06088386	0.19519637	0.34565185	0.08189815	0.15241821	0.19959137	0.03617125	0.6940166	0.09040253	0.11526091	0.25140847	0.09784262
138	0.06088431	0.19518143	0.34566848	0.08189829	0.15243823	0.19959805	0.03617211	0.69403364	0.0903929	0.11527385	0.25144097	0.09785819
138.1	0.06088477	0.19516649	0.34568509	0.08189844	0.15245824	0.19960473	0.03617299	0.69405064	0.09038328	0.11528675	0.25147344	0.09787373
138.2	0.06088524	0.19515157	0.3457017	0.08189859	0.15247824	0.19961141	0.03617387	0.6940676	0.09037366	0.11529963	0.25150588	0.09788925
138.3	0.06088571	0.19513666	0.34571831	0.08189875	0.15249823	0.19961808	0.03617477	0.69408452	0.09036405	0.11531248	0.25153828	0.09790474
138.4	0.06088618	0.19512176	0.3457349	0.08189891	0.15251821	0.19962476	0.03617567	0.69410141	0.09035444	0.1153253	0.25157065	0.0979202
138.5	0.06088665	0.19510686	0.34575149	0.08189909	0.15253818	0.19963143	0.03617659	0.69411825	0.09034485	0.11533808	0.25160298	0.09793564
138.6	0.06088713	0.19509198	0.34576807	0.08189926	0.15255814	0.1996381	0.03617751	0.69413505	0.09033526	0.11535084	0.25163528	0.09795106
138.7	0.06088761	0.19507711	0.34578464	0.08189944	0.15257808	0.19964476	0.03617844	0.69415182	0.09032568	0.11536356	0.25166755	0.09796645
138.8	0.06088809	0.19506225	0.3458012	0.08189963	0.15259802	0.19965143	0.03617939	0.69416854	0.0903161	0.11537626	0.25169978	0.09798182
138.9	0.06088858	0.1950474	0.34581776	0.08189982	0.15261794	0.19965809	0.03618034	0.69418523	0.09030654	0.11538892	0.25173198	0.09799716
139	0.06088906	0.19503256	0.3458343	0.08190002	0.15263786	0.19966475	0.0361813	0.69420187	0.09029698	0.11540156	0.25176414	0.09801248
139.1	0.06088956	0.19501773	0.34585084	0.08190023	0.15265776	0.19967141	0.03618227	0.69421848	0.09028742	0.11541416	0.25179627	0.09802777
139.2	0.06089005	0.19500291	0.34586737	0.08190044	0.15267765	0.19967806	0.03618325	0.69423505	0.09027788	0.11542674	0.25182837	0.09804304
139.3	0.06089055	0.1949881	0.3458839	0.08190065	0.15269753	0.19968472	0.03618424	0.69425158	0.09026834	0.11543928	0.25186043	0.09805828
139.4	0.06089105	0.1949733	0.34590041	0.08190088	0.1527174	0.19969137	0.03618524	0.69426808	0.09025881	0.1154518	0.25189246	0.0980735
139.5	0.06089155	0.19495852	0.34591692	0.0819011	0.15273726	0.19969802	0.03618625	0.69428453	0.09024928	0.11546428	0.25192446	0.0980887
139.6	0.06089206	0.19494374	0.34593342	0.08190133	0.15275711	0.19970466	0.03618727	0.69430095	0.09023977	0.11547674	0.25195642	0.09810387
139.7	0.06089257	0.19492897	0.34594992	0.08190157	0.15277695	0.19971131	0.03618829	0.69431732	0.09023026	0.11548916	0.25198835	0.09811901
139.8	0.06089308	0.19491421	0.3459664	0.08190181	0.15279678	0.19971795	0.03618933	0.69433366	0.09022076	0.11550156	0.25202025	0.09813414
139.9	0.06089359	0.19489947	0.34598288	0.08190206	0.1528166	0.19972459	0.03619037	0.69434997	0.09021126	0.11551393	0.25205211	0.09814923
140	0.06089411	0.19488473	0.34599935	0.08190232	0.1528364	0.19973123	0.03619143	0.69436623	0.09020177	0.11552626	0.25208394	0.09816431
140.1	0.06089463	0.19487	0.34601581	0.08190258	0.1528562	0.19973787	0.03619249	0.69438246	0.09019229	0.11553857	0.25211574	0.09817936
140.2	0.06089515	0.19485529	0.34603226	0.08190284	0.15287598	0.1997445	0.03619357	0.69439864	0.09018282	0.11555085	0.2521475	0.09819438
140.3	0.06089568	0.19484058	0.3460487	0.08190311	0.15289575	0.19975113	0.03619465	0.69441479	0.09017336	0.1155631	0.25217924	0.09820939
140.4	0.06089621	0.19482588	0.34606514	0.08190339	0.15291552	0.19975776	0.03619574	0.69443091	0.0901639	0.11557532	0.25221093	0.09822436
140.5	0.06089674	0.19481119	0.34608157	0.08190367	0.15293527	0.19976439	0.03619684	0.69444698	0.09015445	0.11558752	0.2522426	0.09823932
140.6	0.06089728	0.19479652	0.34609799	0.08190395	0.15295501	0.19977101	0.03619795	0.69446302	0.090145	0.11559968	0.25227423	0.09825425
140.7	0.06089782	0.19478185	0.3461144	0.08190424	0.15297474	0.19977764	0.03619907	0.69447902	0.09013556	0.11561181	0.25230583	0.09826915
140.8	0.06089836	0.19476719	0.34613081	0.08190454	0.15299446	0.19978426	0.0362002	0.69449499	0.09012613	0.11562392	0.25233739	0.09828404
140.9	0.0608989	0.19475255	0.3461472	0.08190484	0.15301417	0.19979088	0.03620133	0.69451091	0.09011671	0.115636	0.25236892	0.0982989
141	0.06089945	0.19473791	0.34616359	0.08190515	0.15303386	0.19979749	0.03620248	0.6945268	0.0901073	0.11564805	0.25240042	0.09831373
141.1	0.06089999	0.19472328	0.34617997	0.08190546	0.15305355	0.19980411	0.03620363	0.69454266	0.09009789	0.11566007	0.25243189	0.09832854
141.2	0.06090055	0.19470866	0.34619635	0.08190578	0.15307323	0.19981072	0.0362048	0.69455847	0.09008849	0.11567206	0.25246332	0.09834333
141.3	0.0609011	0.19469406	0.34621271	0.0819061	0.15309289	0.19981733	0.03620597	0.69457425	0.09007909	0.11568402	0.25249472	0.0983581
141.4	0.06090166	0.19467946	0.34622907	0.08190643	0.15311254	0.19982393	0.03620715	0.69459	0.09006971	0.11569596	0.25252608	0.09837284
141.5	0.06090222	0.19466487	0.34624542	0.08190676	0.15313219	0.19983054	0.03620834	0.6946057	0.09006033	0.11570786	0.25255742	0.09838756
141.6	0.06090278	0.1946503	0.34626176	0.0819071	0.15315182	0.19983714	0.03620954	0.69462137	0.09005095	0.11571974	0.25258872	0.09840225
141.7	0.06090334	0.19463573	0.34627809	0.08190744	0.15317144	0.19984374	0.03621075	0.69463701	0.09004159	0.11573159	0.25261998	0.09841692
141.8	0.06090391	0.19462117	0.34629442	0.08190779	0.15319105	0.19985034	0.03621197	0.6946526	0.09003223	0.11574341	0.25265122	0.09843157
141.9	0.06090448	0.19460663	0.34631073	0.08190814	0.15321065	0.19985693	0.03621319	0.69466817	0.09002288	0.1157552	0.25268242	0.0984462
142	0.06090506	0.19459209	0.34632704	0.0819085	0.15323024	0.19986353	0.03621443	0.69468369	0.09001354	0.11576697	0.25271358	0.0984608
142.1	0.06090563	0.19457756	0.34634335	0.08190886	0.15324982	0.19987012	0.03621567	0.69469918	0.0900042	0.11577871	0.25274472	0.09847538
142.2	0.06090621	0.19456305	0.34635964	0.08190923	0.15326938	0.1998767	0.03621692	0.69471463	0.08999487	0.11579042	0.25277582	0.09848994
142.3	0.06090679	0.19454854	0.34637592	0.08190961	0.15328894	0.19988329	0.03621818	0.69473005	0.08998555	0.1158021	0.25280689	0.09850447
142.4	0.06090737	0.19453404	0.3463922	0.08190998	0.15330848	0.19988987	0.03621945	0.69474543	0.08997624	0.11581375	0.25283793	0.09851898
142.5	0.06090796	0.19451956	0.34640847	0.08191037	0.15332802	0.19989645	0.03622073	0.69476078	0.08996693	0.11582538	0.25286893	0.09853347
142.6	0.06090855	0.19450508	0.34642473	0.08191076	0.15334754	0.19990303	0.03622201	0.69477609	0.08995763	0.11583698	0.25289991	0.09854794
142.7	0.06090914	0.19449061	0.34644099	0.08191115	0.15336705	0.19990961	0.03622331	0.69479137	0.08994834	0.11584855	0.25293084	0.09856238
142.8	0.06090973	0.19447616	0.34645723	0.08191155	0.15338655	0.19991618	0.03622461	0.69480661	0.08993905	0.11586009	0.25296175	0.0985768
142.9	0.06091032	0.19446171	0.34647347	0.08191195	0.15340604	0.19992275	0.03622592	0.69482181	0.08992977	0.11587161	0.25299263	0.0985912
143	0.06091092	0.19444728	0.3464897	0.08191236	0.15342552	0.19992932	0.03622724	0.69483698	0.0899205	0.1158831	0.25302347	0.09860558
143.1	0.06091152	0.19443285	0.34650592	0.08191277	0.15344499	0.19993589	0.03622857	0.69485212	0.08991124	0.11589456	0.25305428	0.09861993
143.2	0.06091213	0.19441843	0.34652214	0.08191319	0.15346445	0.19994245	0.03622991	0.69486722	0.08990198	0.115906	0.25308505	0.09863426
143.3	0.06091273	0.19440403	0.34653834	0.08191361	0.1534839	0.19994901	0.03623126	0.69488229	0.08989273	0.1159174	0.2531158	0.09864857
143.4	0.06091334	0.19438963	0.34655454	0.08191404	0.15350333	0.19995557	0.03623261	0.69489732	0.08988349	0.11592878	0.25314651	0.09866285
143.5	0.06091395	0.19437525	0.34657073	0.08191447	0.15352276	0.19996213	0.03623397	0.69491231	0.08987426	0.11594014	0.25317719	0.09867712
143.6	0.06091456	0.19436087	0.34658691	0.08191491	0.15354217	0.19996868	0.03623535	0.69492728	0.08986503	0.11595146	0.25320784	0.09869136
143.7	0.06091518	0.19434651	0.34660309	0.08191535	0.15356157	0.19997524	0.03623672	0.6949422	0.08985581	0.11596276	0.25323846	0.09870558
143.8	0.0609158	0.19433215	0.34661925	0.08191579	0.15358097	0.19998179	0.03623811	0.6949571	0.08984659	0.11597404	0.25326904	0.09871978
143.9	0.06091642	0.19431781	0.34663541	0.08191625	0.15360035	0.19998833	0.03623951	0.69497196	0.08983739	0.11598528	0.25329959	0.09873395
144	0.06091704	0.19430348	0.34665156	0.0819167	0.15361972	0.19999488	0.03624091	0.69498678	0.08982819	0.1159965	0.25333011	0.0987481
144.1	0.06091766	0.19428915	0.3466677	0.08191716	0.15363908	0.20000142	0.03624233	0.69500157	0.089819	0.11600769	0.2533606	0.09876224
144.2	0.06091829	0.19427484	0.34668384	0.08191763	0.15365843	0.20000796	0.03624375	0.69501633	0.08980981	0.11601886	0.25339106	0.09877634
144.3	0.06091892	0.19426053	0.34669996	0.0819181	0.15367777	0.2000145	0.03624518	0.69503105	0.08980064	0.11603	0.25342149	0.09879043
144.4	0.06091955	0.19424624	0.34671608	0.08191857	0.15369709	0.20002104	0.03624661	0.69504574	0.08979147	0.11604111	0.25345188	0.0988045
144.5	0.06092018	0.19423196	0.34673219	0.08191905	0.15371641	0.20002757	0.03624806	0.6950604	0.0897823	0.1160522	0.25348224	0.09881854
144.6	0.06092082	0.19421769	0.3467483	0.08191954	0.15373571	0.2000341	0.03624951	0.69507502	0.08977315	0.11606326	0.25351257	0.09883257
144.7	0.06092146	0.19420342	0.34676439	0.08192003	0.15375501	0.20004063	0.03625098	0.69508961	0.089764	0.11607429	0.25354287	0.09884657
144.8	0.0609221	0.19418917	0.34678048	0.08192052	0.15377429	0.20004716	0.03625245	0.69510416	0.08975486	0.1160853	0.25357314	0.09886055
144.9	0.06092274	0.19417493	0.34679656	0.08192102	0.15379357	0.20005368	0.03625392	0.69511868	0.08974572	0.11609628	0.25360337	0.0988745
145	0.06092339	0.1941607	0.34681263	0.08192152	0.15381283	0.20006021	0.03625541	0.69513317	0.0897366	0.11610724	0.25363358	0.09888844
145.1	0.06092404	0.19414647	0.34682869	0.08192203	0.15383208	0.20006672	0.03625691	0.69514762	0.08972748	0.11611817	0.25366375	0.09890235
145.2	0.06092468	0.19413226	0.34684475	0.08192254	0.15385132	0.20007324	0.03625841	0.69516205	0.08971837	0.11612907	0.25369389	0.09891625
145.3	0.06092534	0.19411806	0.3468608	0.08192306	0.15387055	0.20007976	0.03625992	0.69517643	0.08970926	0.11613995	0.253724	0.09893012
145.4	0.06092599	0.19410387	0.34687684	0.08192358	0.15388977	0.20008627	0.03626144	0.69519079	0.08970016	0.1161508	0.25375408	0.09894397

111.9	0.06094786	0.19939895	0.34108137	0.08212674	0.14685795	0.19782543	0.0363721	0.68784858	0.09314794	0.11054561	0.24164586	0.09272527
112	0.06094646	0.19938178	0.34109978	0.08212453	0.14688058	0.19783211	0.03636925	0.68788076	0.09313653	0.11057038	0.24168908	0.09275006
112.1	0.06094508	0.19936461	0.34111819	0.08212232	0.14690321	0.19783878	0.03636641	0.68791285	0.09312513	0.11059508	0.24173225	0.0927748
112.2	0.06094371	0.19934745	0.34113659	0.08212013	0.14692582	0.19784546	0.0363636	0.68794486	0.09311373	0.11061972	0.24177536	0.09279948
112.3	0.06094235	0.1993303	0.34115499	0.08211796	0.14694843	0.19785214	0.0363608	0.68797679	0.09310234	0.11064429	0.24181843	0.09282412
112.4	0.060941	0.19931315	0.34117338	0.08211579	0.14697103	0.19785883	0.03635803	0.68800863	0.09309095	0.11066879	0.24186145	0.09284871
112.5	0.06093967	0.199296	0.34119177	0.08211364	0.14699362	0.19786551	0.03635528	0.6880404	0.09307957	0.11069324	0.24190442	0.09287325
112.6	0.06093835	0.19927886	0.34121015	0.08211151	0.14701621	0.19787221	0.03635254	0.68807208	0.09306819	0.11071761	0.24194734	0.09289774
112.7	0.06093705	0.19926173	0.34122853	0.08210938	0.14703878	0.1978789	0.03634983	0.68810368	0.09305682	0.11074193	0.24199021	0.09292219
112.8	0.06093576	0.1992446	0.3412469	0.08210727	0.14706135	0.1978856	0.03634713	0.68813521	0.09304546	0.11076618	0.24203303	0.09294658
112.9	0.06093448	0.19922747	0.34126527	0.08210517	0.1470839	0.1978923	0.03634446	0.68816665	0.0930341	0.11079036	0.2420758	0.09297093
113	0.06093322	0.19921035	0.34128363	0.08210309	0.14710645	0.197899	0.03634181	0.68819801	0.09302275	0.11081448	0.24211853	0.09299523
113.1	0.06093196	0.19919324	0.34130198	0.08210102	0.14712899	0.19790571	0.03633917	0.68822929	0.0930114	0.11083854	0.2421612	0.09301948
113.2	0.06093073	0.19917613	0.34132033	0.08209896	0.14715153	0.19791241	0.03633656	0.68826049	0.09300006	0.11086254	0.24220383	0.09304368
113.3	0.0609295	0.19915903	0.34133868	0.08209691	0.14717405	0.19791913	0.03633396	0.68829161	0.09298872	0.11088647	0.2422464	0.09306784
113.4	0.06092829	0.19914193	0.34135701	0.08209488	0.14719656	0.19792584	0.03633138	0.68832266	0.0929774	0.11091034	0.24228893	0.09309195
113.5	0.06092709	0.19912484	0.34137535	0.08209286	0.14721907	0.19793256	0.03632883	0.68835362	0.09296607	0.11093414	0.24233141	0.09311601
113.6	0.0609259	0.19910776	0.34139367	0.08209085	0.14724157	0.19793928	0.03632629	0.6883845	0.09295476	0.11095789	0.24237384	0.09314002
113.7	0.06092473	0.19909068	0.341412	0.08208885	0.14726406	0.197946	0.03632377	0.68841531	0.09294344	0.11098157	0.24241622	0.09316399
113.8	0.06092356	0.19907361	0.34143031	0.08208687	0.14728654	0.19795273	0.03632127	0.68844604	0.09293214	0.11100519	0.24245855	0.09318791
113.9	0.06092241	0.19905654	0.34144862	0.0820849	0.14730901	0.19795945	0.03631879	0.68847668	0.09292084	0.11102874	0.24250084	0.09321178
114	0.06092128	0.19903948	0.34146693	0.08208294	0.14733147	0.19796619	0.03631633	0.68850726	0.09290955	0.11105224	0.24254308	0.09323561
114.1	0.06092015	0.19902242	0.34148523	0.082081	0.14735393	0.19797292	0.03631389	0.68853775	0.09289826	0.11107567	0.24258527	0.09325939
114.2	0.06091904	0.19900537	0.34150352	0.08207906	0.14737637	0.19797966	0.03631147	0.68856816	0.09288698	0.11109905	0.24262741	0.09328313
114.3	0.06091794	0.19898833	0.34152181	0.08207714	0.14739881	0.1979864	0.03630907	0.6885985	0.0928757	0.11112236	0.2426695	0.09330681
114.4	0.06091685	0.19897129	0.3415401	0.08207524	0.14742124	0.19799314	0.03630669	0.68862876	0.09286443	0.11114561	0.24271154	0.09333046
114.5	0.06091577	0.19895426	0.34155837	0.08207334	0.14744366	0.19799988	0.03630432	0.68865894	0.09285317	0.1111688	0.24275354	0.09335405
114.6	0.06091471	0.19893724	0.34157664	0.08207146	0.14746607	0.19800663	0.03630197	0.68868905	0.09284191	0.11119193	0.24279549	0.0933776
114.7	0.06091366	0.19892022	0.34159491	0.08206959	0.14748847	0.19801338	0.03629965	0.68871908	0.09283066	0.111215	0.24283739	0.09340111
114.8	0.06091262	0.19890321	0.34161317	0.08206773	0.14751086	0.19802013	0.03629734	0.68874904	0.09281941	0.11123801	0.24287924	0.09342457
114.9	0.06091159	0.1988862	0.34163143	0.08206588	0.14753325	0.19802689	0.03629505	0.68877891	0.09280817	0.11126096	0.24292105	0.09344798
115	0.06091057	0.1988692	0.34164968	0.08206404	0.14755562	0.19803364	0.03629278	0.68880872	0.09279694	0.11128385	0.24296281	0.09347135
115.1	0.06090957	0.19885221	0.34166792	0.08206222	0.14757799	0.1980404	0.03629053	0.68883844	0.09278571	0.11130668	0.24300452	0.09349468
115.2	0.06090858	0.19883522	0.34168616	0.08206041	0.14760035	0.19804716	0.03628829	0.68886809	0.09277449	0.11132946	0.24304618	0.09351795
115.3	0.06090759	0.19881824	0.34170439	0.08205861	0.1476227	0.19805393	0.03628608	0.68889767	0.09276328	0.11135217	0.2430878	0.09354119
115.4	0.06090662	0.19880127	0.34172262	0.08205682	0.14764504	0.19806069	0.03628388	0.68892717	0.09275207	0.11137482	0.24312937	0.09356438
115.5	0.06090567	0.1987843	0.34174084	0.08205505	0.14766737	0.19806746	0.0362817	0.68895659	0.09274086	0.11139742	0.24317089	0.09358752
115.6	0.06090472	0.19876734	0.34175905	0.08205329	0.14768969	0.19807423	0.03627954	0.68898594	0.09272967	0.11141995	0.24321236	0.09361062
115.7	0.06090378	0.19875039	0.34177726	0.08205154	0.147712	0.198081	0.0362774	0.68901522	0.09271848	0.11144243	0.24325379	0.09363368
115.8	0.06090286	0.19873344	0.34179546	0.0820498	0.1477343	0.19808778	0.03627528	0.68904442	0.09270729	0.11146485	0.24329517	0.09365669
115.9	0.06090194	0.1987165	0.34181366	0.08204807	0.1477566	0.19809455	0.03627317	0.68907355	0.09269612	0.11148721	0.2433365	0.09367966
116	0.06090104	0.19869957	0.34183185	0.08204635	0.14777888	0.19810133	0.03627108	0.68910261	0.09268494	0.11150952	0.24337779	0.09370258
116.1	0.06090015	0.19868264	0.34185004	0.08204465	0.14780116	0.19810811	0.03626901	0.68913159	0.09267378	0.11153176	0.24341903	0.09372546
116.2	0.06089927	0.19866572	0.34186822	0.08204296	0.14782342	0.19811489	0.03626696	0.6891605	0.09266262	0.11155395	0.24346022	0.0937483
116.3	0.0608984	0.19864881	0.34188639	0.08204128	0.14784568	0.19812168	0.03626493	0.68918933	0.09265147	0.11157608	0.24350137	0.09377109
116.4	0.06089754	0.1986319	0.34190456	0.08203961	0.14786793	0.19812846	0.03626292	0.68921809	0.09264032	0.11159816	0.24354247	0.09379384
116.5	0.06089669	0.198615	0.34192272	0.08203795	0.14789017	0.19813525	0.03626092	0.68924678	0.09262918	0.11162018	0.24358353	0.09381654
116.6	0.06089586	0.19859811	0.34194088	0.0820363	0.1479124	0.19814204	0.03625894	0.6892754	0.09261804	0.11164214	0.24362453	0.0938392
116.7	0.06089503	0.19858123	0.34195903	0.08203467	0.14793462	0.19814883	0.03625698	0.68930394	0.09260692	0.11166404	0.2436655	0.09386182
116.8	0.06089422	0.19856435	0.34197717	0.08203305	0.14795683	0.19815563	0.03625504	0.68933242	0.09259579	0.11168589	0.24370641	0.0938844
116.9	0.06089341	0.19854748	0.34199531	0.08203144	0.14797904	0.19816242	0.03625311	0.68936082	0.09258468	0.11170768	0.24374728	0.09390693
117	0.06089261	0.19853062	0.34201344	0.08202984	0.14800123	0.19816922	0.0362512	0.68938915	0.09257357	0.11172942	0.2437881	0.09392942
117.1	0.06089183	0.19851376	0.34203156	0.08202825	0.14802341	0.19817602	0.03624931	0.68941741	0.09256247	0.1117511	0.24382888	0.09395187
117.2	0.06089106	0.19849691	0.34204968	0.08202667	0.14804559	0.19818282	0.03624744	0.68944559	0.09255137	0.11177272	0.24386961	0.09397427
117.3	0.06089029	0.19848007	0.34206779	0.0820251	0.14806775	0.19818962	0.03624558	0.68947371	0.09254028	0.11179429	0.2439103	0.09399664
117.4	0.06088954	0.19846324	0.3420859	0.08202355	0.14808991	0.19819643	0.03624374	0.68950176	0.0925292	0.1118158	0.24395094	0.09401896
117.5	0.06088879	0.19844641	0.342104	0.08202201	0.14811205	0.19820323	0.03624192	0.68952973	0.09251812	0.11183726	0.24399154	0.09404123
117.6	0.06088806	0.19842959	0.3421221	0.08202047	0.14813419	0.19821004	0.03624012	0.68955764	0.09250705	0.11185866	0.24403208	0.09406347
117.7	0.06088734	0.19841278	0.34214019	0.08201895	0.14815632	0.19821685	0.03623833	0.68958547	0.09249598	0.11188001	0.24407259	0.09408566
117.8	0.06088662	0.19839597	0.34215827	0.08201745	0.14817844	0.19822366	0.03623657	0.68961324	0.09248493	0.1119013	0.24411305	0.09410781
117.9	0.06088592	0.19837918	0.34217634	0.08201595	0.14820055	0.19823047	0.03623481	0.68964094	0.09247387	0.11192254	0.24415346	0.09412992
118	0.06088522	0.19836239	0.34219441	0.08201446	0.14822265	0.19823729	0.03623308	0.68966856	0.09246283	0.11194373	0.24419382	0.09415199
118.1	0.06088454	0.1983456	0.34221248	0.08201298	0.14824474	0.1982441	0.03623136	0.68969612	0.09245179	0.11196486	0.24423415	0.09417402
118.2	0.06088386	0.19832883	0.34223054	0.08201152	0.14826682	0.19825092	0.03622966	0.68972361	0.09244076	0.11198593	0.24427442	0.09419601
118.3	0.0608832	0.19831206	0.34224859	0.08201007	0.14828889	0.19825773	0.03622798	0.68975103	0.09242973	0.11200695	0.24431465	0.09421795
118.4	0.06088254	0.1982953	0.34226663	0.08200862	0.14831095	0.19826455	0.03622631	0.68977838	0.09241871	0.11202792	0.24435484	0.09423985
118.5	0.0608819	0.19827855	0.34228467	0.08200719	0.148333	0.19827137	0.03622466	0.68980567	0.0924077	0.11204883	0.24439498	0.09426172
118.6	0.06088126	0.1982618	0.3423027	0.08200577	0.14835505	0.19827819	0.03622303	0.68983288	0.09239669	0.11206969	0.24443508	0.09428354
118.7	0.06088063	0.19824507	0.34232073	0.08200436	0.14837708	0.19828501	0.03622141	0.68986003	0.09238569	0.1120905	0.24447513	0.09430532
118.8	0.06088001	0.19822834	0.34233875	0.08200296	0.1483991	0.19829183	0.03621982	0.68988711	0.0923747	0.11211125	0.24451513	0.09432706
118.9	0.0608794	0.19821162	0.34235676	0.08200157	0.14842112	0.19829866	0.03621823	0.68991412	0.09236371	0.11213195	0.2445551	0.09434876
119	0.0608788	0.1981949	0.34237477	0.08200019	0.14844312	0.19830548	0.03621667	0.68994106	0.09235273	0.1121526	0.24459501	0.09437042
119.1	0.06087821	0.1981782	0.34239277	0.08199883	0.14846512	0.19831231	0.03621512	0.68996794	0.09234176	0.1121732	0.24463489	0.09439204
119.2	0.06087763	0.1981615	0.34241077	0.08199747	0.1484871	0.19831914	0.03621359	0.68999475	0.09233079	0.11219374	0.24467471	0.09441362
119.3	0.06087706	0.19814481	0.34242876	0.08199613	0.14850908	0.19832597	0.03621207	0.6900215	0.09231983	0.11221423	0.2447145	0.09443516
119.4	0.0608765	0.19812812	0.34244674	0.08199479	0.14853105	0.19833279	0.03621057	0.69004817	0.09230887	0.11223467	0.24475424	0.09445666
119.5	0.06087594	0.19811145	0.34246471	0.08199347	0.148553	0.19833963	0.03620909	0.69007479	0.09229792	0.11225505	0.24479393	0.09447812
119.6	0.0608754	0.19809478	0.34248268	0.08199215	0.14857495	0.19834646	0.03620762	0.69010133	0.09228698	0.11227539	0.24483359	0.09449954
119.7	0.06087486	0.19807812	0.34250065	0.08199085	0.14859689	0.19835329	0.03620617	0.69012781	0.09227605	0.11229567	0.24487319	0.09452092
119.8	0.06087433	0.19806147	0.3425186	0.08198956	0.14861881	0.19836012	0.03620473	0.69015422	0.09226512	0.1123159	0.24491276	0.09454226
119.9	0.06087381	0.19804483	0.34253655	0.08198827	0.14864073	0.19836696	0.03620332	0.69018057	0.0922542	0.11233608	0.24495227	0.09456356

150	0.06095814	0.19346235	0.34760612	0.08195236	0.15476217	0.20038317	0.03633951	0.69581694	0.08928935	0.11662354	0.25510453	0.09955893
150.1	0.06095888	0.19344865	0.34762179	0.08195309	0.15478088	0.20038957	0.03634138	0.69582984	0.08928059	0.11663327	0.25513318	0.09957184
150.2	0.06095962	0.19343496	0.34763745	0.08195382	0.15479958	0.20039596	0.03634325	0.69584271	0.08927183	0.11664297	0.2551618	0.09958472
150.3	0.06096036	0.19342127	0.3476531	0.08195455	0.15481827	0.20040235	0.03634514	0.69585556	0.08926307	0.11665266	0.25519039	0.09959759
150.4	0.0609611	0.1934076	0.34766874	0.08195529	0.15483696	0.20040874	0.03634703	0.69586837	0.08925433	0.11666231	0.25521896	0.09961043
150.5	0.06096185	0.19339394	0.34768438	0.08195603	0.15485563	0.20041512	0.03634892	0.69588115	0.08924559	0.11667195	0.25524749	0.09962326
150.6	0.0609626	0.19338029	0.3477	0.08195677	0.15487429	0.2004215	0.03635083	0.69589391	0.08923686	0.11668157	0.25527599	0.09963607
150.7	0.06096334	0.19336665	0.34771562	0.08195752	0.15489294	0.20042788	0.03635274	0.69590663	0.08922814	0.11669116	0.25530447	0.09964886
150.8	0.06096409	0.19335302	0.34773123	0.08195827	0.15491158	0.20043426	0.03635466	0.69591933	0.08921942	0.11670073	0.25533291	0.09966163
150.9	0.06096485	0.1933394	0.34774684	0.08195903	0.1549302	0.20044063	0.03635658	0.695932	0.08921071	0.11671028	0.25536133	0.09967438
151	0.0609656	0.19332579	0.34776243	0.08195979	0.15494882	0.200447	0.03635851	0.69594464	0.089202	0.1167198	0.25538971	0.09968711
151.1	0.06096635	0.19331219	0.34777802	0.08196055	0.15496743	0.20045337	0.03636045	0.69595726	0.08919331	0.11672931	0.25541807	0.09969983
151.2	0.06096711	0.1932986	0.3477936	0.08196132	0.15498603	0.20045973	0.03636239	0.69596984	0.08918462	0.11673879	0.2554464	0.09971253
151.3	0.06096786	0.19328503	0.34780917	0.08196209	0.15500461	0.2004661	0.03636435	0.6959824	0.08917593	0.11674825	0.25547469	0.0997252
151.4	0.06096862	0.19327146	0.34782474	0.08196286	0.15502319	0.20047246	0.0363663	0.69599492	0.08916726	0.11675769	0.25550296	0.09973786
151.5	0.06096938	0.1932579	0.34784029	0.08196364	0.15504175	0.20047881	0.03636827	0.69600742	0.08915859	0.11676711	0.2555312	0.0997505
151.6	0.06097014	0.19324435	0.34785584	0.08196442	0.15506031	0.20048517	0.03637024	0.6960199	0.08914993	0.11677651	0.25555941	0.09976312
151.7	0.0609709	0.19323081	0.34787138	0.08196521	0.15507885	0.20049152	0.03637222	0.69603234	0.08914127	0.11678588	0.25558759	0.09977573
151.8	0.06097167	0.19321728	0.34788691	0.081966	0.15509739	0.20049787	0.03637421	0.69604476	0.08913262	0.11679523	0.25561574	0.09978831
151.9	0.06097243	0.19320377	0.34790244	0.08196679	0.15511591	0.20050421	0.0363762	0.69605715	0.08912398	0.11680456	0.25564386	0.09980088
152	0.0609732	0.19319026	0.34791795	0.08196759	0.15513442	0.20051056	0.0363782	0.69606951	0.08911535	0.11681387	0.25567196	0.09981343
152.1	0.06097396	0.19317676	0.34793346	0.08196839	0.15515292	0.2005169	0.0363802	0.69608184	0.08910672	0.11682316	0.25570002	0.09982595
152.2	0.06097473	0.19316327	0.34794896	0.0819692	0.15517142	0.20052324	0.03638221	0.69609415	0.0890981	0.11683243	0.25572806	0.09983847
152.3	0.0609755	0.1931498	0.34796445	0.08197001	0.1551899	0.20052957	0.03638423	0.69610642	0.08908948	0.11684168	0.25575606	0.09985096
152.4	0.06097627	0.19313633	0.34797994	0.08197082	0.15520837	0.2005359	0.03638626	0.69611867	0.08908088	0.1168509	0.25578404	0.09986343
152.5	0.06097705	0.19312287	0.34799541	0.08197163	0.15522683	0.20054223	0.03638829	0.6961309	0.08907228	0.11686011	0.25581199	0.09987589
152.6	0.06097782	0.19310943	0.34801088	0.08197245	0.15524528	0.20054856	0.03639033	0.69614309	0.08906368	0.11686929	0.25583991	0.09988833
152.7	0.0609786	0.19309599	0.34802634	0.08197328	0.15526372	0.20055488	0.03639238	0.69615526	0.0890551	0.11687845	0.2558678	0.09990075
152.8	0.06097937	0.19308256	0.3480418	0.0819741	0.15528214	0.20056121	0.03639443	0.6961674	0.08904652	0.11688759	0.25589566	0.09991315
152.9	0.06098015	0.19306915	0.34805724	0.08197494	0.15530056	0.20056752	0.03639649	0.69617952	0.08903794	0.11689671	0.25592349	0.09992553
153	0.06098093	0.19305574	0.34807268	0.08197577	0.15531897	0.20057384	0.03639855	0.69619161	0.08902938	0.11690581	0.25595129	0.0999379
153.1	0.06098171	0.19304235	0.34808811	0.08197661	0.15533737	0.20058015	0.03640062	0.69620367	0.08902082	0.11691489	0.25597907	0.09995024
153.2	0.06098249	0.19302896	0.34810353	0.08197745	0.15535575	0.20058646	0.0364027	0.6962157	0.08901227	0.11692395	0.25600682	0.09996257
153.3	0.06098328	0.19301559	0.34811894	0.08197829	0.15537413	0.20059277	0.03640478	0.69622771	0.08900372	0.11693298	0.25603453	0.09997488
153.4	0.06098406	0.19300222	0.34813434	0.08197914	0.15539249	0.20059908	0.03640688	0.69623969	0.08899518	0.116942	0.25606222	0.09998718
153.5	0.06098485	0.19298887	0.34814974	0.08197999	0.15541085	0.20060538	0.03640897	0.69625164	0.08898665	0.116951	0.25608988	0.09999945
153.6	0.06098564	0.19297552	0.34816513	0.08198085	0.15542919	0.20061168	0.03641108	0.69626357	0.08897812	0.11695997	0.25611752	0.10001171
153.7	0.06098642	0.19296219	0.34818051	0.08198171	0.15544753	0.20061797	0.03641319	0.69627547	0.08896961	0.11696893	0.25614512	0.10002395
153.8	0.06098721	0.19294886	0.34819588	0.08198257	0.15546585	0.20062427	0.0364153	0.69628735	0.08896109	0.11697786	0.25617269	0.10003617
153.9	0.06098801	0.19293555	0.34821125	0.08198344	0.15548416	0.20063056	0.03641743	0.6962992	0.08895259	0.11698677	0.25620024	0.10004838
154	0.0609888	0.19292225	0.34822661	0.08198431	0.15550247	0.20063685	0.03641956	0.69631102	0.08894409	0.11699567	0.25622776	0.10006056
154.1	0.06098959	0.19290895	0.34824196	0.08198518	0.15552076	0.20064313	0.03642169	0.69632281	0.0889356	0.11700454	0.25625525	0.10007273
154.2	0.06099039	0.19289567	0.3482573	0.08198606	0.15553904	0.20064941	0.03642383	0.69633458	0.08892711	0.11701339	0.25628271	0.10008489
154.3	0.06099118	0.1928824	0.34827263	0.08198694	0.15555731	0.20065569	0.03642598	0.69634632	0.08891864	0.11702223	0.25631014	0.10009702
154.4	0.06099198	0.19286913	0.34828796	0.08198782	0.15557557	0.20066197	0.03642814	0.69635804	0.08891017	0.11703104	0.25633755	0.10010914
154.5	0.06099278	0.19285588	0.34830328	0.08198871	0.15559382	0.20066824	0.0364303	0.69636973	0.0889017	0.11703983	0.25636493	0.10012123
154.6	0.06099358	0.19284264	0.34831859	0.0819896	0.15561206	0.20067451	0.03643247	0.6963814	0.08889324	0.11704861	0.25639228	0.10013332
154.7	0.06099438	0.19282941	0.34833389	0.08199049	0.15563029	0.20068078	0.03643464	0.69639304	0.08888479	0.11705736	0.2564196	0.10014538
154.8	0.06099518	0.19281619	0.34834918	0.08199139	0.15564851	0.20068705	0.03643682	0.69640465	0.08887635	0.11706609	0.25644689	0.10015743
154.9	0.06099598	0.19280297	0.34836447	0.08199229	0.15566672	0.20069331	0.03643901	0.69641624	0.08886791	0.11707481	0.25647415	0.10016946
155	0.06099679	0.19278977	0.34837975	0.08199319	0.15568492	0.20069957	0.0364412	0.6964278	0.08885948	0.1170835	0.25650139	0.10018147
155.1	0.06099759	0.19277658	0.34839502	0.0819941	0.15570311	0.20070583	0.0364434	0.69643934	0.08885106	0.11709218	0.2565286	0.10019346
155.2	0.0609984	0.1927634	0.34841028	0.08199501	0.15572128	0.20071208	0.0364456	0.69645085	0.08884264	0.11710083	0.25655578	0.10020544
155.3	0.0609992	0.19275023	0.34842554	0.08199592	0.15573945	0.20071833	0.03644781	0.69646234	0.08883423	0.11710946	0.25658293	0.1002174
155.4	0.06100001	0.19273707	0.34844078	0.08199684	0.15575761	0.20072458	0.03645003	0.6964738	0.08882583	0.11711808	0.25661005	0.10022934
155.5	0.06100082	0.19272392	0.34845602	0.08199776	0.15577575	0.20073083	0.03645225	0.69648523	0.08881743	0.11712667	0.25663715	0.10024127
155.6	0.06100163	0.19271077	0.34847125	0.08199868	0.15579389	0.20073707	0.03645448	0.69649664	0.08880904	0.11713524	0.25666422	0.10025318
155.7	0.06100245	0.19269764	0.34848648	0.08199961	0.15581202	0.20074331	0.03645671	0.69650803	0.08880066	0.1171438	0.25669126	0.10026507
155.8	0.06100326	0.19268452	0.34850169	0.08200054	0.15583013	0.20074955	0.03645896	0.69651939	0.08879228	0.11715233	0.25671828	0.10027694
155.9	0.06100407	0.19267141	0.3485169	0.08200147	0.15584824	0.20075578	0.0364612	0.69653072	0.08878391	0.11716085	0.25674526	0.1002888
156	0.06100489	0.19265831	0.3485321	0.08200241	0.15586633	0.20076202	0.03646346	0.69654203	0.08877555	0.11716935	0.25677222	0.10030064
156.1	0.0610057	0.19264522	0.34854729	0.08200335	0.15588441	0.20076824	0.03646572	0.69655332	0.08876719	0.11717782	0.25679915	0.10031246
156.2	0.06100652	0.19263214	0.34856248	0.08200429	0.15590249	0.20077447	0.03646798	0.69656458	0.08875884	0.11718628	0.25682605	0.10032427
156.3	0.06100734	0.19261907	0.34857765	0.08200524	0.15592055	0.20078069	0.03647025	0.6965758	0.0887505	0.11719472	0.25685293	0.10033606
156.4	0.06100816	0.19260601	0.34859282	0.08200619	0.1559386	0.20078692	0.03647253	0.69658702	0.08874216	0.11720314	0.25687978	0.10034783
156.5	0.06100898	0.19259296	0.34860798	0.08200714	0.15595665	0.20079313	0.03647481	0.69659821	0.08873383	0.11721153	0.2569066	0.10035959
156.6	0.0610098	0.19257992	0.34862313	0.08200809	0.15597468	0.20079935	0.0364771	0.69660937	0.08872551	0.11721991	0.25693339	0.10037133
156.7	0.06101062	0.19256688	0.34863828	0.08200905	0.1559927	0.20080556	0.0364794	0.69662051	0.08871719	0.11722828	0.25696016	0.10038305
156.8	0.06101144	0.19255386	0.34865341	0.08201001	0.15601071	0.20081177	0.0364817	0.69663162	0.08870888	0.11723662	0.2569869	0.10039475
156.9	0.06101227	0.19254085	0.34866854	0.08201098	0.15602871	0.20081798	0.036484	0.69664271	0.08870057	0.11724494	0.25701361	0.10040644
157	0.06101309	0.19252785	0.34868366	0.08201195	0.15604671	0.20082418	0.03648632	0.69665377	0.08869228	0.11725324	0.25704029	0.10041812
157.1	0.06101392	0.19251486	0.34869877	0.08201292	0.15606469	0.20083038	0.03648864	0.69666481	0.08868399	0.11726153	0.25706695	0.10042977
157.2	0.06101475	0.19250188	0.34871388	0.08201389	0.15608266	0.20083658	0.03649096	0.69667582	0.0886757	0.11726979	0.25709358	0.10044141
157.3	0.06101557	0.1924889	0.34872897	0.08201487	0.15610062	0.20084277	0.03649329	0.69668682	0.08866743	0.11727804	0.25712018	0.10045303
157.4	0.0610164	0.19247594	0.34874406	0.08201585	0.15611857	0.20084896	0.03649563	0.69669778	0.08865916	0.11728627	0.25714676	0.10046464
157.5	0.06101723	0.19246299	0.34875914	0.08201683	0.15613651	0.20085515	0.03649797	0.69670873	0.08865089	0.11729447	0.25717331	0.10047623
157.6	0.06101806	0.19245005	0.34877422	0.08201782	0.15615444	0.20086134	0.03650032	0.69671965	0.08864264	0.11730266	0.25719983	0.1004878
157.7	0.0610189	0.19243712	0.34878928	0.08201881	0.15617236	0.20086752	0.03650267	0.69673054	0.08863438	0.11731083	0.25722633	0.10049936
157.8	0.06101973	0.19242419	0.34880434	0.0820198	0.15619027	0.2008737	0.03650503	0.69674141	0.08862614	0.11731899	0.25725279	0.1005109
157.9	0.06102056	0.19241128	0.34881939	0.0820208	0.15620817	0.20087988	0.03650739	0.69675226	0.0886179	0.11732712	0.25727924	0.10052243
158	0.0610214	0.19239838	0.34883443	0.0820218	0.15622605	0.20088605	0.03650976	0.69676308	0.08860967	0.11733524	0.25730565	0.10053393
158.1	0.06102223	0.19238548	0.34884946	0.0820228	0.15624393	0.20089223	0.03651214	0.69677388	0.08860145	0.11734333	0.25733204	0.10054543
158.2	0.06102307	0.1923726	0.34886448	0.0820238	0.1562618	0.20089839	0.03651452	0.69678466	0.08859323	0.11735141	0.2573584	0.1005569
158.3	0.0610239	0.19235973	0.3488795	0.08202481	0.15627966	0.20090456	0.03651691	0.69679541	0.08858502	0.11735947	0.25738473	0.10056836
158.4	0.06102474	0.19234687	0.34889451	0.08202582	0.15629751	0.20091072	0.0365193	0.69680614	0.08857682	0.11736751	0.25741104	0.1005798
158.5	0.06102558	0.19233401	0.34890951	0.08202683	0.15631534	0.20091688	0.0365217	0.69681685	0.08856862	0.11737553	0.25743732	0.10059123
158.6	0.06102642	0.19232117	0.34892451	0.08202785	0.15633317	0.20092304	0.03652411	0.69682753	0.08856043	0.11738354	0.25746358	0.10060264
158.7	0.06102726	0.19230833	0.34893949	0.08202887	0.15635099	0.2009292	0.03652652	0.69683819	0.08855224	0.11739152	0.2574898	0.10061404
158.8	0.0610281	0.19229551	0.34895447	0.08202989	0.15636879	0.20093535	0.03652894	0.69684883	0.08854406	0.11739949	0.25751601	0.10062542
158.9	0.06102894	0.1922827	0.34896944	0.08203091	0.15638659	0.2009415	0.03653136	0.69685944	0.08853589	0.11740744	0.25754218	0.10063678
159	0.06102979	0.19226989	0.3489844	0.08203194	0.15640438	0.20094764	0.03653379	0.69687003	0.08852773	0.11741537	0.25756833	0.10064813
159.1	0.06103063	0.1922571	0.34899935	0.08203297	0.15642215	0.20095379	0.03653622	0.6968806	0.08851957	0.11742329	0.25759445	0.10065946
159.2	0.06103148	0.19224431	0.3490143	0.082034	0.15643992	0.20095993	0.03653866	0.69689115	0.08851142	0.11743118	0.25762054	0.10067078
159.3	0.06103232	0.19223154	0.34902923	0.08203504	0.15645767	0.20096606	0.0365411	0.69690167	0.08850327	0.11743906	0.25764661	0.10068207
159.4	0.06103317	0.19221878	0.34904416	0.08203608	0.15647542	0.2009722	0.03654355	0.69691217	0.08849513	0.11744692	0.25767265	0.10069336
159.5	0.06103401	0.19220602	0.34905909	0.08203712	0.15649315	0.20097833	0.03654601	0.69692264	0.088487	0.11745476	0.25769867	0.10070463
159.6	0.06103486	0.19219328	0.349074	0.08203816	0.15651088	0.20098446	0.03654847	0.69693309	0.08847888	0.11746258	0.25772465	0.10071588
159.7	0.06103571	0.19218054	0.34908891	0.08203921	0.15652859	0.20099058	0.03655093	0.69694352	0.08847076	0.11747039	0.25775062	0.10072712
159.8	0.06103656	0.19216782	0.3491038	0.08204026	0.1565463	0.20099671	0.03655341	0.69695393	0.08846264	0.11747818	0.25777655	0.10073834
159.9	0.06103741	0.1921551	0.34911869	0.08204131	0.15656399	0.20100283	0.03655588	0.69696432	0.08845454	0.11748595	0.25780246	0.10074954
160	0.06103826	0.1921424	0.34913358	0.08204237	0.15658168	0.20100894	0.03655837	0.69697468	0.08844644	0.1174937	0.25782834	0.10076073
160.1	0.06103912	0.1921297	0.34914845	0.08204343	0.15659935	0.20101506	0.03656085	0.69698502	0.08843835	0.11750144	0.2578542	0.10077191
160.2	0.06103997	0.19211702	0.34916332	0.08204449	0.15661702	0.20102117	0.03656335	0.69699534	0.08843026	0.11750915	0.25788003	0.10078307
160.3	0.06104082	0.19210434	0.34917818	0.08204555	0.15663467	0.20102728	0.03656585	0.69700563	0.08842218	0.11751685	0.25790583	0.10079421
160.4	0.06104168	0.19209167	0.34919303	0.08204662	0.15665231	0.20103339	0.03656835	0.69701591	0.08841411	0.11752454	0.25793161	0.10080534

126.9	0.06085658	0.19690109	0.34377622	0.08192242	0.15015013	0.19884636	0.03614172	0.69187293	0.0915066	0.1136296	0.2476131	0.09596223
127	0.06085656	0.19688507	0.34379368	0.08192179	0.15017133	0.1988532	0.03614134	0.69189507	0.09149617	0.11364649	0.24764966	0.09598097
127.1	0.06085656	0.19686906	0.34381113	0.08192118	0.15019252	0.19886003	0.03614097	0.69191716	0.09148574	0.11366334	0.24768618	0.09599967
127.2	0.06085656	0.19685306	0.34382858	0.08192057	0.15021369	0.19886687	0.03614062	0.6919392	0.09147533	0.11368016	0.24772265	0.09601834
127.3	0.06085657	0.19683706	0.34384602	0.08191997	0.15023486	0.19887371	0.03614028	0.69196118	0.09146491	0.11369692	0.24775909	0.09603698
127.4	0.06085658	0.19682108	0.34386345	0.08191938	0.15025602	0.19888054	0.03613995	0.69198311	0.09145451	0.11371365	0.24779549	0.09605558
127.5	0.0608566	0.19680511	0.34388087	0.08191879	0.15027717	0.19888738	0.03613964	0.69200499	0.09144411	0.11373034	0.24783185	0.09607415
127.6	0.06085662	0.19678914	0.34389828	0.08191822	0.1502983	0.19889421	0.03613933	0.69202682	0.09143373	0.11374698	0.24786818	0.09609269
127.7	0.06085665	0.19677319	0.34391569	0.08191765	0.15031943	0.19890104	0.03613904	0.69204859	0.09142334	0.11376359	0.24790446	0.0961112
127.8	0.06085668	0.19675724	0.34393309	0.08191709	0.15034054	0.19890788	0.03613877	0.69207031	0.09141297	0.11378015	0.2479407	0.09612967
127.9	0.06085672	0.1967413	0.34395049	0.08191654	0.15036165	0.19891471	0.0361385	0.69209197	0.0914026	0.11379667	0.24797691	0.09614811
128	0.06085677	0.19672538	0.34396787	0.081916	0.15038274	0.19892154	0.03613825	0.69211359	0.09139224	0.11381315	0.24801307	0.09616652
128.1	0.06085682	0.19670946	0.34398525	0.08191547	0.15040383	0.19892837	0.03613801	0.69213515	0.09138189	0.11382959	0.2480492	0.0961849
128.2	0.06085688	0.19669355	0.34400262	0.08191494	0.1504249	0.19893519	0.03613779	0.69215666	0.09137154	0.11384599	0.24808528	0.09620324
128.3	0.06085694	0.19667765	0.34401999	0.08191442	0.15044596	0.19894202	0.03613757	0.69217812	0.0913612	0.11386235	0.24812133	0.09622156
128.4	0.06085701	0.19666176	0.34403735	0.08191391	0.15046701	0.19894885	0.03613737	0.69219952	0.09135087	0.11387867	0.24815734	0.09623984
128.5	0.06085708	0.19664589	0.3440547	0.08191341	0.15048806	0.19895567	0.03613718	0.69222088	0.09134054	0.11389495	0.24819332	0.09625809
128.6	0.06085716	0.19663002	0.34407204	0.08191292	0.15050909	0.1989625	0.03613701	0.69224218	0.09133023	0.11391119	0.24822925	0.09627631
128.7	0.06085725	0.19661416	0.34408937	0.08191243	0.15053011	0.19896932	0.03613685	0.69226343	0.09131991	0.11392739	0.24826514	0.09629449
128.8	0.06085733	0.1965983	0.3441067	0.08191195	0.15055112	0.19897614	0.0361367	0.69228464	0.09130961	0.11394355	0.248301	0.09631265
128.9	0.06085743	0.19658246	0.34412402	0.08191148	0.15057212	0.19898296	0.03613656	0.69230579	0.09129932	0.11395967	0.24833682	0.09633077
129	0.06085753	0.19656663	0.34414133	0.08191102	0.15059311	0.19898978	0.03613643	0.69232688	0.09128903	0.11397575	0.24837259	0.09634886
129.1	0.06085763	0.19655081	0.34415864	0.08191057	0.15061408	0.1989966	0.03613632	0.69234793	0.09127874	0.11399179	0.24840834	0.09636692
129.2	0.06085774	0.196535	0.34417594	0.08191012	0.15063505	0.19900342	0.03613622	0.69236893	0.09126847	0.11400779	0.24844404	0.09638495
129.3	0.06085786	0.19651919	0.34419323	0.08190968	0.15065601	0.19901024	0.03613613	0.69238988	0.0912582	0.11402375	0.2484797	0.09640295
129.4	0.06085798	0.1965034	0.34421051	0.08190925	0.15067696	0.19901706	0.03613605	0.69241077	0.09124794	0.11403967	0.24851533	0.09642092
129.5	0.06085811	0.19648762	0.34422779	0.08190883	0.15069789	0.19902387	0.03613599	0.69243162	0.09123769	0.11405555	0.24855091	0.09643885
129.6	0.06085824	0.19647184	0.34424506	0.08190841	0.15071882	0.19903069	0.03613594	0.69245241	0.09122744	0.1140714	0.24858646	0.09645676
129.7	0.06085837	0.19645608	0.34426232	0.081908	0.15073973	0.1990375	0.0361359	0.69247316	0.0912172	0.1140872	0.24862197	0.09647463
129.8	0.06085851	0.19644033	0.34427957	0.0819076	0.15076064	0.19904431	0.03613587	0.69249385	0.09120697	0.11410297	0.24865745	0.09649248
129.9	0.06085866	0.19642458	0.34429682	0.08190721	0.15078153	0.19905112	0.03613585	0.6925145	0.09119675	0.11411869	0.24869288	0.09651029
130	0.06085881	0.19640884	0.34431406	0.08190682	0.15080241	0.19905793	0.03613585	0.6925351	0.09118653	0.11413438	0.24872828	0.09652807
130.1	0.06085897	0.19639312	0.34433129	0.08190645	0.15082329	0.19906474	0.03613586	0.69255564	0.09117632	0.11415003	0.24876364	0.09654582
130.2	0.06085913	0.1963774	0.34434851	0.08190608	0.15084415	0.19907155	0.03613588	0.69257614	0.09116612	0.11416564	0.24879896	0.09656354
130.3	0.06085929	0.19636169	0.34436573	0.08190571	0.150865	0.19907836	0.03613592	0.69259659	0.09115592	0.11418121	0.24883425	0.09658123
130.4	0.06085946	0.19634599	0.34438294	0.08190536	0.15088584	0.19908516	0.03613596	0.69261699	0.09114574	0.11419675	0.24886949	0.09659889
130.5	0.06085964	0.1963303	0.34440014	0.08190501	0.15090667	0.19909197	0.03613602	0.69263734	0.09113556	0.11421224	0.2489047	0.09661652
130.6	0.06085982	0.19631462	0.34441734	0.08190467	0.15092749	0.19909877	0.03613609	0.69265764	0.09112538	0.1142277	0.24893987	0.09663412
130.7	0.06086	0.19629895	0.34443453	0.08190434	0.1509483	0.19910557	0.03613617	0.69267789	0.09111522	0.11424312	0.248975	0.09665169
130.8	0.06086019	0.19628329	0.34445171	0.08190401	0.15096909	0.19911237	0.03613626	0.69269809	0.09110506	0.1142585	0.2490101	0.09666924
130.9	0.06086039	0.19626764	0.34446888	0.08190369	0.15098988	0.19911917	0.03613637	0.69271825	0.09109491	0.11427385	0.24904516	0.09668675
131	0.06086059	0.19625199	0.34448604	0.08190338	0.15101066	0.19912597	0.03613649	0.69273836	0.09108476	0.11428916	0.24908017	0.09670423
131.1	0.06086079	0.19623636	0.3445032	0.08190308	0.15103142	0.19913277	0.03613662	0.69275842	0.09107463	0.11430442	0.24911516	0.09672168
131.2	0.060861	0.19622074	0.34452035	0.08190278	0.15105218	0.19913956	0.03613676	0.69277843	0.0910645	0.11431966	0.2491501	0.0967391
131.3	0.06086121	0.19620512	0.34453749	0.08190249	0.15107292	0.19914636	0.03613691	0.69279839	0.09105438	0.11433485	0.24918501	0.09675649
131.4	0.06086143	0.19618952	0.34455463	0.08190221	0.15109365	0.19915315	0.03613707	0.69281831	0.09104426	0.11435001	0.24921988	0.09677385
131.5	0.06086165	0.19617392	0.34457175	0.08190193	0.15111438	0.19915994	0.03613725	0.69283818	0.09103416	0.11436513	0.24925471	0.09679119
131.6	0.06086187	0.19615834	0.34458887	0.08190167	0.15113509	0.19916673	0.03613744	0.692858	0.09102406	0.11438021	0.24928951	0.09680849
131.7	0.0608621	0.19614276	0.34460598	0.08190141	0.15115579	0.19917352	0.03613764	0.69287777	0.09101397	0.11439526	0.24932426	0.09682577
131.8	0.06086234	0.1961272	0.34462309	0.08190115	0.15117648	0.19918031	0.03613785	0.6928975	0.09100388	0.11441027	0.24935898	0.09684301
131.9	0.06086257	0.19611164	0.34464018	0.0819009	0.15119716	0.1991871	0.03613807	0.69291718	0.0909938	0.11442524	0.24939367	0.09686023
132	0.06086282	0.19609609	0.34465727	0.08190067	0.15121783	0.19919388	0.0361383	0.69293681	0.09098373	0.11444018	0.24942831	0.09687742
132.1	0.06086307	0.19608055	0.34467435	0.08190043	0.15123848	0.19920067	0.03613855	0.6929564	0.09097367	0.11445508	0.24946292	0.09689458
132.2	0.06086332	0.19606503	0.34469143	0.08190021	0.15125913	0.19920745	0.0361388	0.69297594	0.09096362	0.11446995	0.2494975	0.09691171
132.3	0.06086357	0.19604951	0.34470849	0.08189999	0.15127977	0.19921423	0.03613907	0.69299543	0.09095357	0.11448478	0.24953203	0.09692881
132.4	0.06086383	0.196034	0.34472555	0.08189978	0.15130039	0.19922101	0.03613935	0.69301488	0.09094353	0.11449957	0.24956653	0.09694588
132.5	0.0608641	0.1960185	0.3447426	0.08189957	0.15132101	0.19922779	0.03613964	0.69303428	0.0909335	0.11451433	0.24960099	0.09696293
132.6	0.06086436	0.19600301	0.34475964	0.08189937	0.15134161	0.19923457	0.03613994	0.69305364	0.09092347	0.11452905	0.24963542	0.09697994
132.7	0.06086464	0.19598753	0.34477668	0.08189918	0.15136221	0.19924134	0.03614025	0.69307295	0.09091345	0.11454374	0.24966981	0.09699693
132.8	0.06086491	0.19597206	0.34479371	0.081899	0.15138279	0.19924812	0.03614058	0.69309221	0.09090344	0.11455839	0.24970416	0.09701389
132.9	0.06086519	0.1959566	0.34481073	0.08189882	0.15140336	0.19925489	0.03614091	0.69311143	0.09089344	0.11457301	0.24973848	0.09703082
133	0.06086548	0.19594115	0.34482774	0.08189865	0.15142392	0.19926166	0.03614126	0.6931306	0.09088344	0.11458759	0.24977276	0.09704772
133.1	0.06086577	0.19592571	0.34484474	0.08189849	0.15144447	0.19926843	0.03614162	0.69314972	0.09087345	0.11460213	0.249807	0.0970646
133.2	0.06086606	0.19591028	0.34486174	0.08189833	0.15146501	0.1992752	0.03614198	0.6931688	0.09086347	0.11461665	0.2498412	0.09708144
133.3	0.06086636	0.19589486	0.34487873	0.08189818	0.15148554	0.19928197	0.03614236	0.69318784	0.09085349	0.11463112	0.24987537	0.09709826
133.4	0.06086666	0.19587945	0.34489571	0.08189803	0.15150606	0.19928873	0.03614275	0.69320683	0.09084353	0.11464556	0.24990951	0.09711505
133.5	0.06086696	0.19586405	0.34491268	0.0818979	0.15152657	0.19929549	0.03614315	0.69322577	0.09083357	0.11465997	0.2499436	0.09713181
133.6	0.06086727	0.19584866	0.34492965	0.08189777	0.15154706	0.19930226	0.03614357	0.69324467	0.09082362	0.11467434	0.24997766	0.09714855
133.7	0.06086758	0.19583327	0.3449466	0.08189764	0.15156755	0.19930902	0.03614399	0.69326353	0.09081367	0.11468868	0.25001169	0.09716525
133.8	0.0608679	0.1958179	0.34496355	0.08189753	0.15158802	0.19931577	0.03614442	0.69328234	0.09080373	0.11470298	0.25004567	0.09718193
133.9	0.06086822	0.19580254	0.3449805	0.08189742	0.15160849	0.19932253	0.03614487	0.69330111	0.0907938	0.11471725	0.25007962	0.09719858
134	0.06086854	0.19578719	0.34499743	0.08189731	0.15162894	0.19932929	0.03614532	0.69331983	0.09078388	0.11473149	0.25011354	0.09721521
134.1	0.06086887	0.19577185	0.34501436	0.08189721	0.15164938	0.19933604	0.03614579	0.69333851	0.09077396	0.11474569	0.25014742	0.0972318
134.2	0.0608692	0.19575652	0.34503128	0.08189712	0.15166982	0.19934279	0.03614627	0.69335714	0.09076405	0.11475985	0.25018126	0.09724837
134.3	0.06086953	0.1957412	0.34504819	0.08189704	0.15169024	0.19934954	0.03614676	0.69337573	0.09075415	0.11477398	0.25021507	0.09726491
134.4	0.06086987	0.19572588	0.34506509	0.08189696	0.15171065	0.19935629	0.03614726	0.69339427	0.09074426	0.11478808	0.25024884	0.09728143
134.5	0.06087021	0.19571058	0.34508199	0.08189689	0.15173105	0.19936304	0.03614777	0.69341278	0.09073437	0.11480215	0.25028257	0.09729791
134.6	0.06087056	0.19569529	0.34509888	0.08189682	0.15175144	0.19936978	0.03614829	0.69343123	0.09072449	0.11481618	0.25031627	0.09731437
134.7	0.0608709	0.19568001	0.34511576	0.08189677	0.15177181	0.19937653	0.03614882	0.69344965	0.09071462	0.11483018	0.25034994	0.09733081
134.8	0.06087126	0.19566474	0.34513263	0.08189671	0.15179218	0.19938327	0.03614936	0.69346802	0.09070475	0.11484414	0.25038356	0.09734721
134.9	0.06087161	0.19564948	0.34514949	0.08189667	0.15181254	0.19939001	0.03614991	0.69348635	0.09069489	0.11485807	0.25041715	0.09736359

165	0.06108175	0.19151951	0.34986762	0.08209846	0.15745298	0.20131109	0.03668918	0.69746526	0.08804964	0.11785979	0.25908952	0.10130103
165.1	0.06108264	0.1915073	0.3498821	0.08209965	0.15747015	0.20131705	0.03669192	0.69747454	0.08804186	0.1178667	0.2591141	0.10131147
165.2	0.06108352	0.19149509	0.34989657	0.08210083	0.15748731	0.20132302	0.03669467	0.69748381	0.08803409	0.1178736	0.25913865	0.10132189
165.3	0.06108441	0.1914829	0.34991103	0.08210202	0.15750446	0.20132898	0.03669743	0.69749305	0.08802633	0.11788047	0.25916318	0.1013323
165.4	0.0610853	0.19147072	0.34992549	0.08210322	0.1575216	0.20133494	0.03670018	0.69750227	0.08801858	0.11788734	0.25918769	0.10134269
165.5	0.06108619	0.19145854	0.34993993	0.08210441	0.15753873	0.2013409	0.03670295	0.69751148	0.08801083	0.11789419	0.25921217	0.10135307
165.6	0.06108708	0.19144638	0.34995437	0.08210561	0.15755585	0.20134685	0.03670571	0.69752066	0.08800308	0.11790102	0.25923662	0.10136344
165.7	0.06108796	0.19143422	0.34996881	0.08210681	0.15757296	0.2013528	0.03670848	0.69752983	0.08799534	0.11790783	0.25926105	0.10137379
165.8	0.06108885	0.19142207	0.34998323	0.08210801	0.15759006	0.20135875	0.03671126	0.69753897	0.08798761	0.11791464	0.25928546	0.10138413
165.9	0.06108974	0.19140994	0.34999765	0.08210921	0.15760715	0.20136469	0.03671404	0.6975481	0.08797989	0.11792142	0.25930984	0.10139445
166	0.06109063	0.19139781	0.35001205	0.08211042	0.15762424	0.20137063	0.03671683	0.6975572	0.08797217	0.11792819	0.2593342	0.10140476
166.1	0.06109153	0.19138569	0.35002645	0.08211162	0.15764131	0.20137657	0.03671962	0.69756629	0.08796446	0.11793495	0.25935854	0.10141506
166.2	0.06109242	0.19137358	0.35004085	0.08211283	0.15765837	0.2013825	0.03672241	0.69757536	0.08795675	0.11794169	0.25938285	0.10142534
166.3	0.06109331	0.19136148	0.35005523	0.08211405	0.15767542	0.20138844	0.03672521	0.69758441	0.08794905	0.11794842	0.25940713	0.10143561
166.4	0.0610942	0.19134939	0.35006961	0.08211526	0.15769246	0.20139437	0.03672802	0.69759344	0.08794136	0.11795513	0.2594314	0.10144587
166.5	0.06109509	0.19133731	0.35008398	0.08211648	0.15770949	0.20140029	0.03673083	0.69760244	0.08793367	0.11796182	0.25945563	0.10145611
166.6	0.06109599	0.19132524	0.35009834	0.0821177	0.15772651	0.20140622	0.03673364	0.69761144	0.08792599	0.11796851	0.25947985	0.10146634
166.7	0.06109688	0.19131318	0.35011269	0.08211892	0.15774353	0.20141214	0.03673646	0.69762041	0.08791831	0.11797517	0.25950403	0.10147655
166.8	0.06109778	0.19130113	0.35012704	0.08212015	0.15776053	0.20141805	0.03673928	0.69762936	0.08791064	0.11798182	0.2595282	0.10148675
166.9	0.06109867	0.19128909	0.35014137	0.08212137	0.15777752	0.20142397	0.03674211	0.69763829	0.08790298	0.11798846	0.25955234	0.10149694
167	0.06109957	0.19127705	0.3501557	0.0821226	0.1577945	0.20142988	0.03674494	0.69764721	0.08789532	0.11799508	0.25957646	0.10150712
167.1	0.06110046	0.19126503	0.35017002	0.08212383	0.15781148	0.20143579	0.03674778	0.6976561	0.08788767	0.11800169	0.25960055	0.10151728
167.2	0.06110136	0.19125301	0.35018434	0.08212506	0.15782844	0.20144169	0.03675062	0.69766498	0.08788003	0.11800828	0.25962462	0.10152742
167.3	0.06110225	0.19124101	0.35019864	0.0821263	0.15784539	0.2014476	0.03675347	0.69767384	0.08787239	0.11801486	0.25964867	0.10153756
167.4	0.06110315	0.19122901	0.35021294	0.08212754	0.15786233	0.20145349	0.03675632	0.69768268	0.08786475	0.11802142	0.25967269	0.10154768
167.5	0.06110405	0.19121703	0.35022723	0.08212878	0.15787927	0.20145939	0.03675917	0.6976915	0.08785713	0.11802797	0.25969668	0.10155778
167.6	0.06110495	0.19120505	0.35024151	0.08213002	0.15789619	0.20146528	0.03676203	0.6977003	0.08784951	0.1180345	0.25972066	0.10156788
167.7	0.06110584	0.19119308	0.35025579	0.08213126	0.15791311	0.20147117	0.03676489	0.69770908	0.08784189	0.11804102	0.25974461	0.10157796
167.8	0.06110674	0.19118113	0.35027005	0.08213251	0.15793001	0.20147706	0.03676776	0.69771785	0.08783429	0.11804753	0.25976853	0.10158802
167.9	0.06110764	0.19116918	0.35028431	0.08213376	0.1579469	0.20148294	0.03677063	0.69772659	0.08782668	0.11805402	0.25979244	0.10159808
168	0.06110854	0.19115724	0.35029856	0.08213501	0.15796379	0.20148882	0.03677351	0.69773532	0.08781909	0.11806049	0.25981631	0.10160812
168.1	0.06110944	0.19114531	0.3503128	0.08213626	0.15798066	0.2014947	0.03677639	0.69774403	0.0878115	0.11806695	0.25984017	0.10161814
168.2	0.06111034	0.19113339	0.35032704	0.08213751	0.15799753	0.20150057	0.03677928	0.69775272	0.08780392	0.1180734	0.259864	0.10162816
168.3	0.06111124	0.19112148	0.35034126	0.08213877	0.15801438	0.20150644	0.03678217	0.69776139	0.08779634	0.11807983	0.25988781	0.10163816
168.4	0.06111214	0.19110957	0.35035548	0.08214003	0.15803123	0.20151231	0.03678506	0.69777005	0.08778877	0.11808625	0.25991159	0.10164814
168.5	0.06111304	0.19109768	0.35036969	0.08214129	0.15804806	0.20151817	0.03678796	0.69777868	0.0877812	0.11809266	0.25993535	0.10165812
168.6	0.06111395	0.1910858	0.3503839	0.08214255	0.15806489	0.20152403	0.03679086	0.6977873	0.08777364	0.11809905	0.25995909	0.10166808
168.7	0.06111485	0.19107392	0.35039809	0.08214382	0.1580817	0.20152989	0.03679377	0.6977959	0.08776609	0.11810542	0.2599828	0.10167803
168.8	0.06111575	0.19106206	0.35041228	0.08214509	0.15809851	0.20153575	0.03679668	0.69780448	0.08775854	0.11811178	0.26000649	0.10168796
168.9	0.06111665	0.1910502	0.35042646	0.08214636	0.15811531	0.2015416	0.03679959	0.69781304	0.087751	0.11811813	0.26003016	0.10169788
169	0.06111756	0.19103836	0.35044063	0.08214763	0.15813209	0.20154744	0.03680251	0.69782159	0.08774347	0.11812446	0.2600538	0.10170779
169.1	0.06111846	0.19102652	0.35045479	0.0821489	0.15814887	0.20155329	0.03680544	0.69783012	0.08773594	0.11813078	0.26007742	0.10171769
169.2	0.06111936	0.19101469	0.35046895	0.08215018	0.15816564	0.20155913	0.03680837	0.69783863	0.08772842	0.11813709	0.26010101	0.10172757
169.3	0.06112027	0.19100287	0.35048309	0.08215145	0.1581824	0.20156497	0.0368113	0.69784712	0.0877209	0.11814338	0.26012458	0.10173744
169.4	0.06112117	0.19099106	0.35049723	0.08215273	0.15819914	0.20157081	0.03681424	0.69785559	0.08771339	0.11814966	0.26014813	0.10174729
169.5	0.06112207	0.19097926	0.35051137	0.08215402	0.15821588	0.20157664	0.03681718	0.69786405	0.08770588	0.11815592	0.26017166	0.10175714
169.6	0.06112298	0.19096747	0.35052549	0.0821553	0.15823261	0.20158247	0.03682012	0.69787248	0.08769839	0.11816217	0.26019516	0.10176697
169.7	0.06112388	0.19095568	0.35053961	0.08215659	0.15824933	0.20158829	0.03682307	0.6978809	0.08769089	0.11816841	0.26021864	0.10177678
169.8	0.06112479	0.19094391	0.35055371	0.08215787	0.15826604	0.20159412	0.03682603	0.69788931	0.08768341	0.11817463	0.26024209	0.10178659
169.9	0.0611257	0.19093214	0.35056781	0.08215916	0.15828274	0.20159994	0.03682898	0.69789769	0.08767593	0.11818084	0.26026552	0.10179638
170	0.0611266	0.19092039	0.35058191	0.08216045	0.15829943	0.20160575	0.03683195	0.69790606	0.08766845	0.11818703	0.26028893	0.10180616
170.1	0.06112751	0.19090864	0.35059599	0.08216175	0.15831611	0.20161157	0.03683491	0.69791441	0.08766098	0.11819322	0.26031232	0.10181593
170.2	0.06112841	0.19089691	0.35061007	0.08216304	0.15833278	0.20161738	0.03683788	0.69792274	0.08765352	0.11819938	0.26033568	0.10182568
170.3	0.06112932	0.19088518	0.35062414	0.08216434	0.15834944	0.20162319	0.03684086	0.69793106	0.08764607	0.11820554	0.26035902	0.10183542
170.4	0.06113023	0.19087346	0.3506382	0.08216564	0.15836609	0.20162899	0.03684384	0.69793936	0.08763862	0.11821168	0.26038234	0.10184515
170.5	0.06113114	0.19086175	0.35065225	0.08216694	0.15838273	0.20163479	0.03684682	0.69794764	0.08763117	0.11821781	0.26040563	0.10185486
170.6	0.06113204	0.19085005	0.3506663	0.08216825	0.15839937	0.20164059	0.0368498	0.6979559	0.08762373	0.11822392	0.2604289	0.10186457
170.7	0.06113295	0.19083835	0.35068033	0.08216955	0.15841599	0.20164638	0.0368528	0.69796415	0.0876163	0.11823002	0.26045215	0.10187425
170.8	0.06113386	0.19082667	0.35069436	0.08217086	0.1584326	0.20165218	0.03685579	0.69797237	0.08760887	0.11823611	0.26047537	0.10188393
170.9	0.06113477	0.190815	0.35070838	0.08217217	0.1584492	0.20165797	0.03685879	0.69798059	0.08760145	0.11824218	0.26049857	0.1018936
171	0.06113568	0.19080333	0.3507224	0.08217348	0.1584658	0.20166375	0.03686179	0.69798878	0.08759404	0.11824824	0.26052175	0.10190325
171.1	0.06113658	0.19079168	0.3507364	0.08217479	0.15848238	0.20166953	0.0368648	0.69799696	0.08758663	0.11825429	0.2605449	0.10191289
171.2	0.06113749	0.19078003	0.3507504	0.08217611	0.15849896	0.20167531	0.03686781	0.69800512	0.08757923	0.11826032	0.26056804	0.10192252
171.3	0.0611384	0.19076839	0.35076439	0.08217742	0.15851552	0.20168109	0.03687083	0.69801326	0.08757183	0.11826634	0.26059115	0.10193213
171.4	0.06113931	0.19075676	0.35077838	0.08217874	0.15853208	0.20168686	0.03687384	0.69802139	0.08756444	0.11827235	0.26061423	0.10194173
171.5	0.06114022	0.19074514	0.35079235	0.08218006	0.15854862	0.20169263	0.03687687	0.6980295	0.08755706	0.11827834	0.2606373	0.10195132
171.6	0.06114113	0.19073353	0.35080632	0.08218138	0.15856516	0.2016984	0.03687989	0.69803759	0.08754968	0.11828432	0.26066034	0.1019609
171.7	0.06114204	0.19072193	0.35082028	0.08218271	0.15858168	0.20170417	0.03688293	0.69804566	0.08754231	0.11829029	0.26068336	0.10197046
171.8	0.06114295	0.19071034	0.35083423	0.0821840	0.1585982	0.20170993	0.03688596	0.69805372	0.08753494	0.11829624	0.26070635	0.10198001
171.9	0.06114386	0.19069875	0.35084817	0.08218536	0.15861471	0.20171568	0.036889	0.69806177	0.08752758	0.11830218	0.26072932	0.10198955
172	0.06114477	0.19068718	0.35086211	0.08218669	0.15863121	0.20172144	0.03689204	0.69806979	0.08752023	0.11830811	0.26075227	0.10199908
172.1	0.06114568	0.19067561	0.35087603	0.08218802	0.1586477	0.20172719	0.03689509	0.6980778	0.08751288	0.11831403	0.2607752	0.1020086
172.2	0.06114659	0.19066406	0.35088995	0.08218935	0.15866417	0.20173294	0.03689814	0.69808579	0.08750553	0.11831993	0.26079811	0.1020181
172.3	0.0611475	0.19065251	0.35090386	0.08219069	0.15868064	0.20173869	0.0369012	0.69809377	0.0874982	0.11832582	0.26082099	0.10202759
172.4	0.06114842	0.19064097	0.35091777	0.08219202	0.1586971	0.20174443	0.03690425	0.69810173	0.08749087	0.11833169	0.26084385	0.10203707
172.5	0.06114933	0.19062944	0.35093167	0.08219336	0.15871355	0.20175017	0.03690732	0.69810967	0.08748354	0.11833756	0.26086668	0.10204654
172.6	0.06115024	0.19061792	0.35094555	0.0821947	0.15872999	0.20175591	0.03691038	0.6981176	0.08747622	0.11834341	0.2608895	0.10205599
172.7	0.06115115	0.1906064	0.35095943	0.08219604	0.15874643	0.20176164	0.03691345	0.69812551	0.08746891	0.11834924	0.26091229	0.10206543
172.8	0.06115206	0.1905949	0.35097331	0.08219739	0.15876285	0.20176737	0.03691653	0.6981334	0.0874616	0.11835507	0.26093506	0.10207486
172.9	0.06115297	0.19058341	0.35098717	0.08219873	0.15877926	0.2017731	0.03691961	0.69814128	0.0874543	0.11836088	0.26095781	0.10208428
173	0.06115389	0.19057192	0.35100103	0.08220008	0.15879566	0.20177883	0.03692269	0.69814914	0.087447	0.11836668	0.26098053	0.10209368
173.1	0.0611548	0.19056044	0.35101488	0.08220143	0.15881206	0.20178455	0.03692577	0.69815698	0.08743971	0.11837247	0.26100324	0.10210308
173.2	0.06115571	0.19054898	0.35102872	0.08220278	0.15882844	0.20179027	0.03692886	0.69816481	0.08743243	0.11837824	0.26102592	0.10211246
173.3	0.06115662	0.19053752	0.35104255	0.08220413	0.15884482	0.20179599	0.03693196	0.69817262	0.08742515	0.118384	0.26104857	0.10212183
173.4	0.06115754	0.19052607	0.35105638	0.08220548	0.15886118	0.2018017	0.03693506	0.69818042	0.08741788	0.11838975	0.26107121	0.10213119
173.5	0.06115845	0.19051462	0.3510702	0.08220684	0.15887754	0.20180741	0.03693816	0.6981882	0.08741061	0.11839549	0.26109382	0.10214053
173.6	0.06115936	0.19050319	0.35108401	0.08220819	0.15889388	0.20181312	0.03694126	0.69819596	0.08740335	0.11840121	0.26111642	0.10214986
173.7	0.06116027	0.19049177	0.35109781	0.08220955	0.15891022	0.20181882	0.03694437	0.69820371	0.08739609	0.11840692	0.26113898	0.10215919
173.8	0.06116119	0.19048035	0.3511116	0.08221091	0.15892655	0.20182453	0.03694748	0.69821144	0.08738885	0.11841262	0.26116153	0.10216849
173.9	0.0611621	0.19046895	0.35112539	0.08221227	0.15894286	0.20183023	0.0369506	0.69821916	0.0873816	0.11841831	0.26118406	0.10217779
174	0.06116301	0.19045755	0.35113917	0.08221364	0.15895917	0.20183592	0.03695372	0.69822686	0.08737436	0.11842398	0.26120656	0.10218708
174.1	0.06116393	0.19044616	0.35115294	0.082215	0.15897547	0.20184161	0.03695684	0.69823455	0.08736713	0.11842964	0.26122904	0.10219635
174.2	0.06116484	0.19043478	0.35116671	0.08221637	0.15899176	0.20184731	0.03695997	0.69824222	0.08735991	0.11843529	0.2612515	0.10220561
174.3	0.06116575	0.19042341	0.35118046	0.08221774	0.15900804	0.20185299	0.0369631	0.69824987	0.08735269	0.11844092	0.26127393	0.10221486
174.4	0.06116667	0.19041205	0.35119421	0.08221911	0.15902431	0.20185868	0.03696624	0.69825751	0.08734547	0.11844655	0.26129635	0.1022241
174.5	0.06116758	0.1904007	0.35120795	0.08222048	0.15904057	0.20186436	0.03696938	0.69826513	0.08733826	0.11845216	0.26131874	0.10223333
174.6	0.06116849	0.19038935	0.35122168	0.08222185	0.15905683	0.20187004	0.03697252	0.69827274	0.08733106	0.11845776	0.26134111	0.10224254
174.7	0.06116941	0.19037802	0.35123541	0.08222323	0.15907307	0.20187571	0.03697566	0.69828033	0.08732387	0.11846334	0.26136346	0.10225174
174.8	0.06117032	0.19036669	0.35124912	0.0822246	0.1590893	0.20188139	0.03697881	0.6982879	0.08731667	0.11846892	0.26138578	0.10226094
174.9	0.06117124	0.19035537	0.35126283	0.08222598	0.15910553	0.20188706	0.03698197	0.69829546	0.08730949	0.11847448	0.26140809	0.10227011
175	0.06117215	0.19034406	0.35127653	0.08222736	0.15912174	0.20189273	0.03698513	0.69830301	0.08730231	0.11848003	0.26143037	0.10227928
175.1	0.06117306	0.19033276	0.35129023	0.08222874	0.15913795	0.20189839	0.03698829	0.69831053	0.08729514	0.11848557	0.26145263	0.10228844
175.2	0.06117398	0.19032147	0.35130391	0.08223013	0.15915414	0.20190405	0.03699145	0.69831805	0.08728797	0.11849109	0.26147487	0.10229758
175.3	0.06117489	0.19031019	0.35131759	0.08223151	0.15917033	0.20190971	0.03699462	0.69832555	0.08728081	0.11849661	0.26149708	0.10230671
175.4	0.06117581	0.19029891	0.35133126	0.0822329	0.15918651	0.20191537	0.03699779	0.69833303	0.08727365	0.11850211	0.26151928	0.10231584

145.5	0.06092665	0.19408969	0.34689287	0.08192411	0.15390897	0.20009278	0.03626296	0.69520511	0.08969107	0.11616163	0.25378413	0.0989578
145.6	0.0609273	0.19407552	0.34690889	0.08192464	0.15392817	0.20009929	0.0362645	0.6952194	0.08968199	0.11617243	0.25381414	0.09897161
145.7	0.06092796	0.19406136	0.34692491	0.08192518	0.15394736	0.20010579	0.03626604	0.69523366	0.08967291	0.1161832	0.25384413	0.0989854
145.8	0.06092863	0.19404721	0.34694092	0.08192572	0.15396653	0.20011229	0.03626759	0.69524788	0.08966384	0.11619395	0.25387408	0.09899916
145.9	0.06092929	0.19403307	0.34695692	0.08192626	0.1539857	0.20011879	0.03626915	0.69526208	0.08965478	0.11620468	0.253904	0.09901291
146	0.06092996	0.19401894	0.34697291	0.08192681	0.15400485	0.20012529	0.03627071	0.69527623	0.08964573	0.11621538	0.25393389	0.09902663
146.1	0.06093063	0.19400483	0.3469889	0.08192736	0.15402399	0.20013179	0.03627229	0.69529036	0.08963668	0.11622605	0.25396375	0.09904034
146.2	0.0609313	0.19399072	0.34700487	0.08192792	0.15404313	0.20013828	0.03627387	0.69530446	0.08962764	0.1162367	0.25399358	0.09905402
146.3	0.06093197	0.19397662	0.34702084	0.08192849	0.15406225	0.20014477	0.03627546	0.69531852	0.08961861	0.11624733	0.25402338	0.09906768
146.4	0.06093264	0.19396253	0.3470368	0.08192905	0.15408136	0.20015126	0.03627705	0.69533255	0.08960958	0.11625793	0.25405315	0.09908132
146.5	0.06093332	0.19394846	0.34705276	0.08192963	0.15410046	0.20015775	0.03627866	0.69534655	0.08960056	0.11626851	0.25408288	0.09909494
146.6	0.060934	0.19393439	0.3470687	0.0819302	0.15411955	0.20016423	0.03628027	0.69536051	0.08959155	0.11627906	0.25411259	0.09910854
146.7	0.06093468	0.19392034	0.34708464	0.08193078	0.15413862	0.20017071	0.03628189	0.69537445	0.08958255	0.11628958	0.25414226	0.09912212
146.8	0.06093536	0.19390629	0.34710057	0.08193137	0.15415769	0.20017719	0.03628352	0.69538835	0.08957355	0.11630008	0.2541719	0.09913568
146.9	0.06093604	0.19389225	0.34711649	0.08193196	0.15417675	0.20018366	0.03628515	0.69540222	0.08956456	0.11631056	0.25420152	0.09914922
147	0.06093673	0.19387823	0.3471324	0.08193255	0.15419579	0.20019014	0.03628679	0.69541606	0.08955558	0.11632101	0.2542311	0.09916273
147.1	0.06093742	0.19386422	0.34714831	0.08193315	0.15421483	0.20019661	0.03628844	0.69542987	0.0895466	0.11633144	0.25426065	0.09917623
147.2	0.06093811	0.19385021	0.34716421	0.08193375	0.15423385	0.20020308	0.0362901	0.69544364	0.08953763	0.11634185	0.25429017	0.09918971
147.3	0.0609388	0.19383622	0.3471801	0.08193436	0.15425287	0.20020954	0.03629177	0.69545739	0.08952867	0.11635223	0.25431966	0.09920316
147.4	0.06093949	0.19382223	0.34719598	0.08193497	0.15427187	0.20021601	0.03629344	0.6954711	0.08951971	0.11636258	0.25434912	0.0992166
147.5	0.06094019	0.19380826	0.34721186	0.08193559	0.15429086	0.20022247	0.03629512	0.69548479	0.08951077	0.11637291	0.25437855	0.09923002
147.6	0.06094089	0.1937943	0.34722772	0.08193621	0.15430984	0.20022893	0.03629681	0.69549844	0.08950183	0.11638322	0.25440795	0.09924341
147.7	0.06094158	0.19378035	0.34724358	0.08193683	0.15432881	0.20023538	0.0362985	0.69551206	0.08949289	0.1163935	0.25443732	0.09925679
147.8	0.06094228	0.19376641	0.34725943	0.08193746	0.15434777	0.20024184	0.03630021	0.69552565	0.08948397	0.11640376	0.25446666	0.09927014
147.9	0.06094299	0.19375248	0.34727527	0.0819381	0.15436672	0.20024829	0.03630192	0.6955392	0.08947505	0.116414	0.25449596	0.09928347
148	0.06094369	0.19373856	0.34729111	0.08193873	0.15438566	0.20025474	0.03630363	0.69555273	0.08946613	0.11642421	0.25452524	0.09929679
148.1	0.0609444	0.19372465	0.34730693	0.08193937	0.15440458	0.20026118	0.03630536	0.69556623	0.08945723	0.1164344	0.25455449	0.09931008
148.2	0.06094511	0.19371075	0.34732275	0.08194002	0.1544235	0.20026763	0.03630709	0.6955797	0.08944833	0.11644456	0.25458371	0.09932336
148.3	0.06094582	0.19369686	0.34733856	0.08194067	0.15444241	0.20027407	0.03630883	0.69559313	0.08943944	0.1164547	0.2546129	0.09933661
148.4	0.06094653	0.19368298	0.34735436	0.08194133	0.1544613	0.20028051	0.03631058	0.69560654	0.08943055	0.11646482	0.25464206	0.09934985
148.5	0.06094724	0.19366911	0.34737016	0.08194198	0.15448019	0.20028694	0.03631233	0.69561991	0.08942168	0.11647491	0.25467118	0.09936306
148.6	0.06094796	0.19365525	0.34738594	0.08194265	0.15449906	0.20029338	0.03631409	0.69563326	0.08941281	0.11648498	0.25470028	0.09937626
148.7	0.06094867	0.19364141	0.34740172	0.08194331	0.15451792	0.20029981	0.03631586	0.69564657	0.08940394	0.11649503	0.25472935	0.09938943
148.8	0.06094939	0.19362757	0.34741749	0.08194398	0.15453677	0.20030624	0.03631764	0.69565986	0.08939509	0.11650505	0.25475839	0.09940259
148.9	0.06095011	0.19361374	0.34743326	0.08194466	0.15455562	0.20031266	0.03631942	0.69567311	0.08938624	0.11651505	0.2547874	0.09941572
149	0.06095083	0.19359993	0.34744901	0.08194534	0.15457445	0.20031908	0.03632121	0.69568633	0.08937739	0.11652503	0.25481638	0.09942884
149.1	0.06095156	0.19358612	0.34746476	0.08194602	0.15459327	0.20032551	0.03632301	0.69569953	0.08936856	0.11653499	0.25484533	0.09944194
149.2	0.06095228	0.19357233	0.3474805	0.08194671	0.15461208	0.20033192	0.03632481	0.69571269	0.08935973	0.11654492	0.25487424	0.09945501
149.3	0.06095301	0.19355854	0.34749623	0.0819474	0.15463088	0.20033834	0.03632663	0.69572583	0.08935091	0.11655482	0.25490313	0.09946807
149.4	0.06095374	0.19354477	0.34751195	0.0819481	0.15464966	0.20034475	0.03632845	0.69573893	0.08934209	0.11656471	0.25493199	0.09948111
149.5	0.06095446	0.19353101	0.34752766	0.0819488	0.15466844	0.20035116	0.03633027	0.69575201	0.08933329	0.11657457	0.25496082	0.09949413
149.6	0.0609552	0.19351726	0.34754337	0.0819495	0.15468721	0.20035757	0.03633211	0.69576505	0.08932448	0.11658441	0.25498963	0.09950713
149.7	0.06095593	0.19350351	0.34755907	0.08195021	0.15470596	0.20036397	0.03633395	0.69577807	0.08931569	0.11659423	0.2550184	0.09952011
149.8	0.06095666	0.19348978	0.34757476	0.08195093	0.15472471	0.20037038	0.03633579	0.69579106	0.0893069	0.11660402	0.25504714	0.09953307
149.9	0.0609574	0.19347606	0.34759044	0.08195164	0.15474344	0.20037678	0.03633765	0.69580402	0.08929812	0.11661379	0.25507585	0.09954601
160.5	0.06104253	0.19207902	0.34920787	0.08204769	0.15666995	0.20103949	0.03657086	0.69702616	0.08840604	0.1175322	0.25795736	0.10081645
160.6	0.06104339	0.19206637	0.3492227	0.08204876	0.15668757	0.20104559	0.03657338	0.69703638	0.08839798	0.11753985	0.25798309	0.10082755
160.7	0.06104424	0.19205373	0.34923753	0.08204984	0.15670518	0.20105169	0.0365759	0.69704659	0.08838993	0.11754748	0.25800879	0.10083863
160.8	0.0610451	0.19204111	0.34925235	0.08205091	0.15672278	0.20105778	0.03657842	0.69705677	0.08838188	0.11755509	0.25803446	0.10084969
160.9	0.06104596	0.19202849	0.34926716	0.08205199	0.15674038	0.20106387	0.03658095	0.69706694	0.08837384	0.11756269	0.25806011	0.10086074
161	0.06104682	0.19201588	0.34928196	0.08205307	0.15675796	0.20106996	0.03658349	0.69707708	0.0883658	0.11757027	0.25808573	0.10087178
161.1	0.06104768	0.19200328	0.34929676	0.08205416	0.15677553	0.20107605	0.03658603	0.6970872	0.08835777	0.11757783	0.25811132	0.1008828
161.2	0.06104854	0.1919907	0.34931154	0.08205525	0.15679309	0.20108213	0.03658858	0.69709729	0.08834975	0.11758538	0.25813689	0.1008938
161.3	0.0610494	0.19197812	0.34932632	0.08205634	0.15681065	0.20108821	0.03659113	0.69710737	0.08834174	0.1175929	0.25816243	0.10090479
161.4	0.06105026	0.19196555	0.34934109	0.08205743	0.15682819	0.20109428	0.03659369	0.69711742	0.08833373	0.11760041	0.25818795	0.10091577
161.5	0.06105113	0.19195299	0.34935586	0.08205853	0.15684572	0.20110036	0.03659626	0.69712745	0.08832573	0.11760791	0.25821344	0.10092673
161.6	0.06105199	0.19194044	0.34937061	0.08205962	0.15686324	0.20110643	0.03659882	0.69713746	0.08831773	0.11761538	0.2582389	0.10093767
161.7	0.06105285	0.1919279	0.34938536	0.08206073	0.15688075	0.2011125	0.0366014	0.69714745	0.08830974	0.11762284	0.25826434	0.1009486
161.8	0.06105372	0.19191537	0.3494001	0.08206183	0.15689825	0.20111856	0.03660398	0.69715741	0.08830176	0.11763029	0.25828975	0.10095951
161.9	0.06105458	0.19190285	0.34941483	0.08206293	0.15691575	0.20112462	0.03660656	0.69716736	0.08829378	0.11763771	0.25831514	0.10097041
162	0.06105545	0.19189034	0.34942956	0.08206404	0.15693323	0.20113068	0.03660915	0.69717728	0.08828581	0.11764512	0.2583405	0.1009813
162.1	0.06105632	0.19187784	0.34944427	0.08206515	0.1569507	0.20113674	0.03661175	0.69718718	0.08827784	0.11765251	0.25836584	0.10099217
162.2	0.06105719	0.19186535	0.34945898	0.08206627	0.15696816	0.20114279	0.03661435	0.69719706	0.08826989	0.11765989	0.25839115	0.10100302
162.3	0.06105806	0.19185287	0.34947368	0.08206738	0.15698561	0.20114884	0.03661695	0.69720692	0.08826194	0.11766725	0.25841643	0.10101386
162.4	0.06105892	0.1918404	0.34948838	0.0820685	0.15700305	0.20115489	0.03661956	0.69721676	0.08825399	0.11767459	0.25844169	0.10102469
162.5	0.06105979	0.19182794	0.34950306	0.08206962	0.15702048	0.20116093	0.03662218	0.69722658	0.08824605	0.11768192	0.25846692	0.1010355
162.6	0.06106067	0.19181548	0.34951774	0.08207075	0.1570379	0.20116698	0.0366248	0.69723637	0.08823812	0.11768922	0.25849213	0.10104629
162.7	0.06106154	0.19180304	0.34953241	0.08207187	0.15705531	0.20117301	0.03662742	0.69724615	0.08823019	0.11769652	0.25851731	0.10105707
162.8	0.06106241	0.19179061	0.34954707	0.082073	0.15707271	0.20117905	0.03663005	0.6972559	0.08822227	0.11770379	0.25854247	0.10106784
162.9	0.06106328	0.19177818	0.34956172	0.08207413	0.1570901	0.20118508	0.03663269	0.69726564	0.08821436	0.11771105	0.2585676	0.10107859
163	0.06106416	0.19176577	0.34957637	0.08207527	0.15710748	0.20119111	0.03663533	0.69727535	0.08820645	0.1177183	0.2585927	0.10108933
163.1	0.06106503	0.19175337	0.349591	0.0820764	0.15712485	0.20119714	0.03663797	0.69728504	0.08819855	0.11772552	0.25861778	0.10110005
163.2	0.0610659	0.19174097	0.34960563	0.08207754	0.15714221	0.20120316	0.03664062	0.69729471	0.08819066	0.11773274	0.25864284	0.10111076
163.3	0.06106678	0.19172859	0.34962026	0.08207868	0.15715956	0.20120918	0.03664328	0.69730436	0.08818277	0.11773993	0.25866787	0.10112145
163.4	0.06106766	0.19171621	0.34963487	0.08207983	0.1571769	0.2012152	0.03664594	0.69731399	0.08817489	0.11774711	0.25869287	0.10113213
163.5	0.06106853	0.19170385	0.34964948	0.08208097	0.15719423	0.20122121	0.03664861	0.69732359	0.08816701	0.11775427	0.25871785	0.10114279
163.6	0.06106941	0.19169149	0.34966407	0.08208212	0.15721155	0.20122723	0.03665128	0.69733318	0.08815914	0.11776142	0.2587428	0.10115344
163.7	0.06107029	0.19167914	0.34967866	0.08208327	0.15722886	0.20123324	0.03665395	0.69734275	0.08815128	0.11776855	0.25876773	0.10116408
163.8	0.06107117	0.19166681	0.34969325	0.08208443	0.15724616	0.20123924	0.03665663	0.69735229	0.08814342	0.11777566	0.25879263	0.1011747
163.9	0.06107205	0.19165448	0.34970782	0.08208558	0.15726345	0.20124524	0.03665932	0.69736182	0.08813557	0.11778276	0.25881751	0.1011853
164	0.06107293	0.19164216	0.34972239	0.08208674	0.15728073	0.20125124	0.03666201	0.69737132	0.08812773	0.11778984	0.25884236	0.10119589
164.1	0.06107381	0.19162985	0.34973695	0.0820879	0.157298	0.20125724	0.0366647	0.69738081	0.08811989	0.11779691	0.25886719	0.10120647
164.2	0.06107469	0.19161756	0.3497515	0.08208907	0.15731526	0.20126324	0.0366674	0.69739027	0.08811206	0.11780396	0.25889199	0.10121704
164.3	0.06107557	0.19160527	0.34976604	0.08209023	0.15733251	0.20126923	0.03667011	0.69739972	0.08810424	0.11781099	0.25891677	0.10122759
164.4	0.06107645	0.19159299	0.34978057	0.0820914	0.15734975	0.20127522	0.03667282	0.69740914	0.08809642	0.11781801	0.25894152	0.10123812
164.5	0.06107733	0.19158072	0.3497951	0.08209257	0.15736698	0.2012812	0.03667553	0.69741854	0.08808861	0.11782501	0.25896625	0.10124864
164.6	0.06107822	0.19156846	0.34980962	0.08209374	0.1573842	0.20128718	0.03667825	0.69742793	0.0880808	0.117832	0.25899096	0.10125915
164.7	0.0610791	0.19155621	0.34982413	0.08209492	0.15740141	0.20129316	0.03668098	0.69743729	0.088073	0.11783897	0.25901563	0.10126964
164.8	0.06107998	0.19154396	0.34983863	0.0820961	0.15741861	0.20129914	0.03668371	0.69744663	0.08806521	0.11784593	0.25904029	0.10128012
164.9	0.06108087	0.19153173	0.34985313	0.08209728	0.1574358	0.20130511	0.03668644	0.69745596	0.08805742	0.11785287	0.25906491	0.10129058
175.5	0.06117672	0.19028765	0.35134492	0.08223428	0.15920267	0.20192102	0.03700097	0.6983405	0.0872665	0.1185076	0.26154145	0.10232494
175.6	0.06117764	0.19027639	0.35135858	0.08223567	0.15921883	0.20192667	0.03700415	0.69834795	0.08725935	0.11851308	0.2615636	0.10233404
175.7	0.06117855	0.19026514	0.35137222	0.08223706	0.15923498	0.20193232	0.03700733	0.69835539	0.08725221	0.11851854	0.26158573	0.10234313
175.8	0.06117946	0.1902539	0.35138586	0.08223845	0.15925112	0.20193797	0.03701051	0.69836281	0.08724508	0.118524	0.26160784	0.1023522
175.9	0.06118038	0.19024267	0.35139949	0.08223985	0.15926725	0.20194361	0.0370137	0.69837021	0.08723795	0.11852944	0.26162992	0.10236127
176	0.06118129	0.19023145	0.35141311	0.08224124	0.15928337	0.20194925	0.0370169	0.69837761	0.08723083	0.11853487	0.26165199	0.10237032
176.1	0.06118221	0.19022024	0.35142673	0.08224264	0.15929949	0.20195488	0.03702009	0.69838498	0.08722372	0.11854029	0.26167403	0.10237936
176.2	0.06118312	0.19020903	0.35144034	0.08224403	0.15931559	0.20196052	0.03702329	0.69839234	0.0872166	0.11854569	0.26169605	0.10238839
176.3	0.06118404	0.19019784	0.35145394	0.08224543	0.15933168	0.20196615	0.0370265	0.69839969	0.0872095	0.11855109	0.26171805	0.1023974
176.4	0.06118495	0.19018665	0.35146753	0.08224683	0.15934777	0.20197178	0.03702971	0.69840702	0.0872024	0.11855647	0.26174003	0.10240641
176.5	0.06118587	0.19017547	0.35148111	0.08224823	0.15936384	0.2019774	0.03703292	0.69841434	0.08719531	0.11856184	0.26176199	0.10241541
176.6	0.06118678	0.1901643	0.35149469	0.08224964	0.15937991	0.20198303	0.03703613	0.69842164	0.08718822	0.1185672	0.26178392	0.10242439
176.7	0.0611877	0.19015314	0.35150826	0.08225104	0.15939596	0.20198865	0.03703935	0.69842893	0.08718114	0.11857255	0.26180583	0.10243336
176.8	0.06118862	0.19014199	0.35152182	0.08225245	0.15941201	0.20199426	0.03704257	0.6984362	0.08717406	0.11857788	0.26182773	0.10244232
176.9	0.06118953	0.19013084	0.35153538	0.08225386	0.15942805	0.20199988	0.0370458	0.69844346	0.08716699	0.11858321	0.2618496	0.10245127
177	0.06119045	0.19011971	0.35154892	0.08225526	0.15944408	0.20200549	0.03704902	0.6984507	0.08715992	0.11858852	0.26187145	0.10246021
177.1	0.06119136	0.19010858	0.35156246	0.08225667	0.1594601	0.2020111	0.03705226	0.69845793	0.08715287	0.11859382	0.26189327	0.10246914
177.2	0.06119228	0.19009746	0.35157599	0.08225809	0.15947611	0.20201671	0.03705549	0.69846514	0.08714581	0.11859911	0.26191508	0.10247805
177.3	0.06119319	0.19008635	0.35158951	0.0822595	0.15949211	0.20202231	0.03705873	0.69847234	0.08713876	0.11860439	0.26193687	0.10248696
177.4	0.06119411	0.19007525	0.35160303	0.08226091	0.1595081	0.20202791	0.03706197	0.69847952	0.08713172	0.11860965	0.26195863	0.10249585
177.5	0.06119502	0.19006416	0.35161653	0.08226233	0.15952408	0.20203351	0.03706522	0.69848669	0.08712468	0.11861491	0.26198037	0.10250473
177.6	0.06119594	0.19005308	0.35163003	0.08226375	0.15954005	0.2020391	0.03706847	0.69849385	0.08711765	0.11862015	0.26200209	0.1025136
177.7	0.06119686	0.190042	0.35164353	0.08226517	0.15955602	0.2020447	0.03707172	0.69850099	0.08711063	0.11862538	0.26202379	0.10252246
177.8	0.06119777	0.19003093	0.35165701	0.08226659	0.15957197	0.20205029	0.03707498	0.69850812	0.08710361	0.1186306	0.26204547	0.10253131
177.9	0.06119869	0.19001988	0.35167049	0.08226801	0.15958792	0.20205587	0.03707824	0.69851523	0.08709659	0.11863581	0.26206713	0.10254015
178	0.0611996	0.19000883	0.35168395	0.08226943	0.15960386	0.20206146	0.0370815	0.69852232	0.08708959	0.11864101	0.26208877	0.10254898
178.1	0.06120052	0.18999778	0.35169742	0.08227085	0.15961978	0.20206704	0.03708477	0.69852941	0.08708258	0.1186462	0.26211038	0.10255779
178.2	0.06120144	0.18998675	0.35171087	0.08227228	0.1596357	0.20207261	0.03708804	0.69853648	0.08707559	0.11865137	0.26213198	0.1025666
178.3	0.06120235	0.18997573	0.35172431	0.0822737	0.15965161	0.20207819	0.03709131	0.69854353	0.08706859	0.11865654	0.26215355	0.10257539
178.4	0.06120327	0.18996471	0.35173775	0.08227513	0.15966751	0.20208376	0.03709459	0.69855057	0.08706161	0.11866169	0.26217511	0.10258418
178.5	0.06120418	0.1899537	0.35175118	0.08227656	0.1596834	0.20208933	0.03709787	0.69855759	0.08705463	0.11866683	0.26219664	0.10259295
178.6	0.0612051	0.1899427	0.35176461	0.08227799	0.15969928	0.2020949	0.03710115	0.69856461	0.08704765	0.11867196	0.26221815	0.10260171
178.7	0.06120602	0.18993171	0.35177802	0.08227942	0.15971515	0.20210046	0.03710443	0.6985716	0.08704068	0.11867708	0.26223964	0.10261046
178.8	0.06120693	0.18992073	0.35179143	0.08228086	0.15973102	0.20210602	0.03710772	0.69857859	0.08703372	0.11868219	0.26226111	0.1026192
178.9	0.06120785	0.18990976	0.35180483	0.08228229	0.15974687	0.20211158	0.03711102	0.69858556	0.08702676	0.11868729	0.26228255	0.10262793
179	0.06120876	0.18989879	0.35181822	0.08228373	0.15976272	0.20211713	0.03711431	0.69859251	0.08701981	0.11869237	0.26230398	0.10263665
179.1	0.06120968	0.18988784	0.3518316	0.08228517	0.15977855	0.20212269	0.03711761	0.69859945	0.08701286	0.11869745	0.26232539	0.10264536
179.2	0.0612106	0.18987689	0.35184498	0.0822866	0.15979438	0.20212823	0.03712092	0.69860638	0.08700592	0.11870251	0.26234677	0.10265405
179.3	0.06121151	0.18986595	0.35185835	0.08228804	0.1598102	0.20213378	0.03712422	0.69861329	0.08699898	0.11870757	0.26236814	0.10266274
179.4	0.06121243	0.18985502	0.35187171	0.08228948	0.159826	0.20213932	0.03712753	0.69862019	0.08699205	0.11871261	0.26238948	0.10267141
179.5	0.06121334	0.18984409	0.35188507	0.08229093	0.1598418	0.20214486	0.03713084	0.69862708	0.08698513	0.11871764	0.2624108	0.10268008
179.6	0.06121426	0.18983318	0.35189841	0.08229237	0.15985759	0.2021504	0.03713416	0.69863395	0.08697821	0.11872266	0.26243211	0.10268873
179.7	0.06121518	0.18982227	0.35191175	0.08229381	0.15987338	0.20215594	0.03713748	0.69864081	0.0869713	0.11872767	0.26245339	0.10269737
179.8	0.06121609	0.18981137	0.35192508	0.08229526	0.15988915	0.20216147	0.0371408	0.69864765	0.08696439	0.11873267	0.26247465	0.10270601
179.9	0.06121701	0.18980048	0.35193841	0.08229671	0.15990491	0.202167	0.03714413	0.69865448	0.08695749	0.11873766	0.26249589	0.10271463
180	0.06121792	0.1897896	0.35195172	0.08229816	0.15992066	0.20217252	0.03714746	0.6986613	0.08695059	0.11874263	0.26251711	0.10272324
180.1	0.06121884	0.18977873	0.35196503	0.0822996	0.15993641	0.20217805	0.03715079	0.6986681	0.0869437	0.1187476	0.26253831	0.10273184
180.2	0.06121976	0.18976786	0.35197833	0.08230106	0.15995214	0.20218357	0.03715412	0.69867489	0.08693681	0.11875255	0.26255948	0.10274043
180.3	0.06122067	0.18975701	0.35199162	0.08230251	0.15996787	0.20218909	0.03715746	0.69868166	0.08692993	0.1187575	0.26258064	0.10274901
180.4	0.06122159	0.18974616	0.35200491	0.08230396	0.15998359	0.2021946	0.0371608	0.69868843	0.08692306	0.11876243	0.26260178	0.10275758
180.5	0.0612225	0.18973532	0.35201819	0.08230542	0.1599993	0.20220011	0.03716415	0.69869518	0.08691619	0.11876736	0.26262289	0.10276614
180.6	0.06122342	0.18972449	0.35203146	0.08230687	0.160015	0.20220562	0.0371675	0.69870191	0.08690932	0.11877227	0.26264399	0.10277469
180.7	0.06122433	0.18971367	0.35204472	0.08230833	0.16003069	0.20221113	0.03717085	0.69870863	0.08690247	0.11877717	0.26266506	0.10278322
180.8	0.06122525	0.18970285	0.35205797	0.08230978	0.16004637	0.20221663	0.0371742	0.69871534	0.08689561	0.11878206	0.26268612	0.10279175
180.9	0.06122616	0.18969205	0.35207122	0.08231124	0.16006204	0.20222213	0.03717756	0.69872203	0.08688877	0.11878694	0.26270715	0.10280027
181	0.06122708	0.18968125	0.35208446	0.0823127	0.1600777	0.20222763	0.03718092	0.69872872	0.08688192	0.11879181	0.26272817	0.10280877
181.1	0.06122799	0.18967046	0.35209769	0.08231417	0.16009336	0.20223312	0.03718429	0.69873538	0.08687509	0.11879667	0.26274916	0.10281727
181.2	0.06122891	0.18965968	0.35211092	0.08231563	0.160109	0.20223862	0.03718765	0.69874204	0.08686826	0.11880152	0.26277013	0.10282576
181.3	0.06122983	0.1896489	0.35212413	0.08231709	0.16012464	0.20224411	0.03719102	0.69874868	0.08686143	0.11880636	0.26279109	0.10283423
181.4	0.06123074	0.18963814	0.35213734	0.08231856	0.16014026	0.20224959	0.0371944	0.69875531	0.08685461	0.11881119	0.26281202	0.1028427
181.5	0.06123166	0.18962738	0.35215055	0.08232002	0.16015588	0.20225508	0.03719777	0.69876192	0.0868478	0.11881601	0.26283293	0.10285115
181.6	0.06123257	0.18961663	0.35216374	0.08232149	0.16017149	0.20226056	0.03720115	0.69876852	0.08684099	0.11882081	0.26285382	0.1028596
181.7	0.06123348	0.18960589	0.35217693	0.08232296	0.16018709	0.20226604	0.03720454	0.69877511	0.08683418	0.11882561	0.26287469	0.10286803
181.8	0.0612344	0.18959516	0.35219011	0.08232443	0.16020268	0.20227151	0.03720792	0.69878169	0.08682739	0.1188304	0.26289554	0.10287645
181.9	0.06123531	0.18958444	0.35220328	0.0823259	0.16021826	0.20227698	0.03721131	0.69878825	0.08682059	0.11883517	0.26291637	0.10288487
182	0.06123623	0.18957372	0.35221644	0.08232737	0.16023384	0.20228245	0.0372147	0.6987948	0.08681381	0.11883994	0.26293718	0.10289327
182.1	0.06123714	0.18956301	0.3522296	0.08232884	0.1602494	0.20228792	0.0372181	0.69880133	0.08680702	0.11884469	0.26295798	0.10290167
182.2	0.06123806	0.18955232	0.35224275	0.08233032	0.16026496	0.20229339	0.03722149	0.69880786	0.08680025	0.11884944	0.26297875	0.10291005
182.3	0.06123897	0.18954162	0.35225589	0.08233179	0.1602805	0.20229885	0.0372249	0.69881437	0.08679348	0.11885418	0.2629995	0.10291842
182.4	0.06123989	0.18953094	0.35226902	0.08233327	0.16029604	0.20230431	0.0372283	0.69882086	0.08678671	0.1188589	0.26302023	0.10292679
182.5	0.0612408	0.18952027	0.35228215	0.08233475	0.16031157	0.20230976	0.03723171	0.69882735	0.08677995	0.11886361	0.26304094	0.10293514
182.6	0.06124172	0.1895096	0.35229527	0.08233622	0.16032709	0.20231521	0.03723512	0.69883382	0.08677319	0.11886832	0.26306163	0.10294348
182.7	0.06124263	0.18949894	0.35230838	0.0823377	0.1603426	0.20232067	0.03723853	0.69884028	0.08676645	0.11887301	0.2630823	0.10295182
182.8	0.06124354	0.18948829	0.35232148	0.08233918	0.1603581	0.20232611	0.03724195	0.69884672	0.0867597	0.1188777	0.26310294	0.10296014
182.9	0.06124446	0.18947765	0.35233458	0.08234067	0.16037359	0.20233156	0.03724537	0.69885315	0.08675296	0.11888237	0.26312357	0.10296845
183	0.06124537	0.18946701	0.35234767	0.08234215	0.16038907	0.202337	0.03724879	0.69885957	0.08674623	0.11888704	0.26314418	0.10297676
183.1	0.06124628	0.18945639	0.35236075	0.08234363	0.16040455	0.20234244	0.03725221	0.69886598	0.0867395	0.11889169	0.26316477	0.10298505
183.2	0.0612472	0.18944577	0.35237382	0.08234512	0.16042001	0.20234788	0.03725564	0.69887237	0.08673278	0.11889633	0.26318534	0.10299334
183.3	0.06124811	0.18943516	0.35238689	0.0823466	0.16043547	0.20235331	0.03725907	0.69887876	0.08672606	0.11890097	0.26320589	0.10300161
183.4	0.06124902	0.18942455	0.35239995	0.08234809	0.16045092	0.20235874	0.03726251	0.69888513	0.08671935	0.11890559	0.26322642	0.10300987
183.5	0.06124994	0.18941396	0.352413	0.08234958	0.16046636	0.20236417	0.03726594	0.69889148	0.08671264	0.11891021	0.26324693	0.10301813
183.6	0.06125085	0.18940337	0.35242605	0.08235107	0.16048179	0.2023696	0.03726938	0.69889783	0.08670594	0.11891481	0.26326742	0.10302637
183.7	0.06125176	0.1893928	0.35243908	0.08235256	0.16049721	0.20237502	0.03727283	0.69890416	0.08669925	0.1189194	0.2632879	0.1030346
183.8	0.06125268	0.18938222	0.35245211	0.08235405	0.16051262	0.20238044	0.03727627	0.69891048	0.08669256	0.11892399	0.26330835	0.10304283
183.9	0.06125359	0.18937166	0.35246513	0.08235554	0.16052802	0.20238586	0.03727972	0.69891678	0.08668587	0.11892856	0.26332878	0.10305104
184	0.0612545	0.18936111	0.35247815	0.08235704	0.16054341	0.20239127	0.03728317	0.69892308	0.08667919	0.11893313	0.26334919	0.10305925
184.1	0.06125541	0.18935056	0.35249116	0.08235853	0.1605588	0.20239668	0.03728663	0.69892936	0.08667252	0.11893768	0.26336958	0.10306744
184.2	0.06125632	0.18934002	0.35250416	0.08236003	0.16057418	0.20240209	0.03729008	0.69893563	0.08666585	0.11894223	0.26338995	0.10307563
184.3	0.06125724	0.18932949	0.35251715	0.08236153	0.16058954	0.2024075	0.03729354	0.69894188	0.08665918	0.11894676	0.2634103	0.1030838
184.4	0.06125815	0.18931897	0.35253013	0.08236302	0.1606049	0.2024129	0.03729701	0.69894813	0.08665253	0.11895129	0.26343064	0.10309197
184.5	0.06125906	0.18930846	0.35254311	0.08236452	0.16062025	0.2024183	0.03730047	0.69895436	0.08664587	0.1189558	0.26345095	0.10310013
184.6	0.06125997	0.18929795	0.35255608	0.08236602	0.16063559	0.2024237	0.03730394	0.69896058	0.08663922	0.11896031	0.26347124	0.10310827
184.7	0.06126088	0.18928745	0.35256904	0.08236752	0.16065092	0.2024291	0.03730741	0.69896679	0.08663258	0.1189648	0.26349152	0.10311641
184.8	0.06126179	0.18927696	0.352582	0.08236903	0.16066625	0.20243449	0.03731089	0.69897298	0.08662595	0.11896929	0.26351177	0.10312454
184.9	0.0612627	0.18926648	0.35259495	0.08237053	0.16068156	0.20243988	0.03731437	0.69897916	0.08661931	0.11897377	0.26353201	0.10313265
185	0.06126361	0.189256	0.35260789	0.08237203	0.16069687	0.20244527	0.03731785	0.69898533	0.08661269	0.11897823	0.26355222	0.10314076
185.1	0.06126452	0.18924554	0.35262082	0.08237354	0.16071216	0.20245066	0.03732133	0.69899149	0.08660607	0.11898269	0.26357242	0.10314886
185.2	0.06126543	0.18923508	0.35263375	0.08237505	0.16072745	0.20245604	0.03732482	0.69899764	0.08659945	0.11898714	0.26359259	0.10315695
185.3	0.06126634	0.18922463	0.35264667	0.08237655	0.16074273	0.20246142	0.0373283	0.69900377	0.08659284	0.11899158	0.26361275	0.10316502
185.4	0.06126725	0.18921419	0.35265958	0.08237806	0.160758	0.20246679	0.0373318	0.6990099	0.08658623	0.11899601	0.26363289	0.10317309
185.5	0.06126816	0.18920375	0.35267248	0.08237957	0.16077326	0.20247217	0.03733529	0.69901601	0.08657963	0.11900043	0.26365301	0.10318115
185.6	0.06126907	0.18919332	0.35268538	0.08238108	0.16078852	0.20247754	0.03733879	0.6990221	0.08657304	0.11900484	0.26367311	0.1031892
185.7	0.06126998	0.1891829	0.35269827	0.08238259	0.16080376	0.20248291	0.03734229	0.69902819	0.08656645	0.11900924	0.26369319	0.10319724
185.8	0.06127089	0.18917249	0.35271115	0.0823841	0.16081899	0.20248828	0.03734579	0.69903426	0.08655987	0.11901363	0.26371325	0.10320527
185.9	0.0612718	0.18916209	0.35272403	0.08238562	0.16083422	0.20249364	0.0373493	0.69904033	0.08655329	0.11901801	0.26373329	0.10321329
186	0.06127271	0.18915169	0.35273689	0.08238713	0.16084944	0.202499	0.0373528	0.69904638	0.08654671	0.11902238	0.26375331	0.10322131
186.1	0.06127362	0.18914131	0.35274975	0.08238865	0.16086465	0.20250436	0.03735632	0.69905242	0.08654014	0.11902674	0.26377331	0.10322931
186.2	0.06127453	0.18913093	0.35276261	0.08239016	0.16087985	0.20250972	0.03735983	0.69905844	0.08653358	0.1190311	0.26379329	0.1032373
186.3	0.06127543	0.18912055	0.35277545	0.08239168	0.16089504	0.20251507	0.03736335	0.69906446	0.08652702	0.11903544	0.26381326	0.10324528
186.4	0.06127634	0.18911019	0.35278829	0.0823932	0.16091022	0.20252042	0.03736686	0.69907046	0.08652047	0.11903978	0.2638332	0.10325326
186.5	0.06127725	0.18909983	0.35280112	0.08239472	0.16092539	0.20252577	0.03737039	0.69907645	0.08651392	0.1190441	0.26385313	0.10326122
186.6	0.06127816	0.18908949	0.35281395	0.08239624	0.16094056	0.20253111	0.03737391	0.69908243	0.08650738	0.11904842	0.26387303	0.10326918
186.7	0.06127906	0.18907915	0.35282676	0.08239776	0.16095572	0.20253646	0.03737744	0.6990884	0.08650084	0.11905273	0.26389292	0.10327712
186.8	0.06127997	0.18906881	0.35283957	0.08239928	0.16097086	0.2025418	0.03738097	0.69909435	0.08649431	0.11905702	0.26391279	0.10328506
186.9	0.06128088	0.18905849	0.35285237	0.0824008	0.160986	0.20254714	0.0373845	0.6991003	0.08648778	0.11906131	0.26393264	0.10329299
187	0.06128178	0.18904817	0.35286517	0.08240232	0.16100113	0.20255247	0.03738804	0.69910623	0.08648126	0.11906559	0.26395247	0.1033009
187.1	0.06128269	0.18903787	0.35287795	0.08240385	0.16101625	0.2025578	0.03739157	0.69911215	0.08647475	0.11906986	0.26397228	0.10330881
187.2	0.0612836	0.18902757	0.35289073	0.08240537	0.16103137	0.20256313	0.03739511	0.69911806	0.08646824	0.11907412	0.26399207	0.10331671
187.3	0.0612845	0.18901727	0.35290351	0.0824069	0.16104647	0.20256846	0.03739866	0.69912396	0.08646173	0.11907838	0.26401185	0.1033246
187.4	0.06128541	0.18900699	0.35291627	0.08240843	0.16106157	0.20257378	0.0374022	0.69912984	0.08645523	0.11908262	0.2640316	0.10333248
187.5	0.06128631	0.18899671	0.35292903	0.08240995	0.16107665	0.20257911	0.03740575	0.69913572	0.08644873	0.11908685	0.26405134	0.10334035
187.6	0.06128722	0.18898644	0.35294178	0.08241148	0.16109173	0.20258443	0.0374093	0.69914158	0.08644224	0.11909108	0.26407105	0.10334821
187.7	0.06128812	0.18897618	0.35295452	0.08241301	0.1611068	0.20258974	0.03741286	0.69914743	0.08643576	0.1190953	0.26409075	0.10335606
187.8	0.06128903	0.18896593	0.35296726	0.08241454	0.16112186	0.20259506	0.03741641	0.69915327	0.08642928	0.1190995	0.26411043	0.10336391
187.9	0.06128993	0.18895568	0.35297999	0.08241608	0.16113691	0.20260037	0.03741997	0.6991591	0.0864228	0.1191037	0.26413009	0.10337174
188	0.06129084	0.18894545	0.35299271	0.08241761	0.16115196	0.20260568	0.03742353	0.69916492	0.08641633	0.11910789	0.26414973	0.10337957
188.1	0.06129174	0.18893522	0.35300543	0.08241914	0.16116699	0.20261099	0.0374271	0.69917073	0.08640987	0.11911207	0.26416935	0.10338738
188.2	0.06129265	0.188925	0.35301813	0.08242068	0.16118202	0.20261629	0.03743067	0.69917652	0.08640341	0.11911624	0.26418895	0.10339519
188.3	0.06129355	0.18891478	0.35303083	0.08242221	0.16119704	0.20262159	0.03743423	0.69918231	0.08639696	0.1191204	0.26420854	0.10340298
188.4	0.06129445	0.18890458	0.35304353	0.08242375	0.16121205	0.20262689	0.03743781	0.69918808	0.08639051	0.11912456	0.26422811	0.10341077
188.5	0.06129536	0.18889438	0.35305621	0.08242528	0.16122705	0.20263219	0.03744138	0.69919384	0.08638406	0.1191287	0.26424765	0.10341855
188.6	0.06129626	0.18888419	0.35306889	0.08242682	0.16124204	0.20263748	0.03744496	0.69919959	0.08637762	0.11913284	0.26426718	0.10342632
188.7	0.06129716	0.18887401	0.35308156	0.08242836	0.16125702	0.20264277	0.03744854	0.69920533	0.08637119	0.11913696	0.26428669	0.10343408
188.8	0.06129806	0.18886383	0.35309422	0.0824299	0.161272	0.20264806	0.03745212	0.69921105	0.08636476	0.11914108	0.26430618	0.10344183
188.9	0.06129896	0.18885366	0.35310688	0.08243144	0.16128696	0.20265335	0.0374557	0.69921677	0.08635834	0.11914519	0.26432565	0.10344958
189	0.06129987	0.1888435	0.35311953	0.08243298	0.16130192	0.20265863	0.03745929	0.69922248	0.08635192	0.11914929	0.26434511	0.10345731
189.1	0.06130077	0.18883335	0.35313217	0.08243452	0.16131687	0.20266391	0.03746288	0.69922817	0.08634551	0.11915338	0.26436454	0.10346503
189.2	0.06130167	0.18882321	0.35314481	0.08243606	0.16133181	0.20266919	0.03746647	0.69923385	0.0863391	0.11915747	0.26438396	0.10347275
189.3	0.06130257	0.18881307	0.35315744	0.08243761	0.16134674	0.20267446	0.03747007	0.69923952	0.0863327	0.11916154	0.26440336	0.10348046
189.4	0.06130347	0.18880295	0.35317006	0.08243915	0.16136167	0.20267973	0.03747367	0.69924518	0.0863263	0.11916561	0.26442274	0.10348815
189.5	0.06130437	0.18879282	0.35318267	0.0824407	0.16137658	0.202685	0.03747727	0.69925083	0.08631991	0.11916966	0.2644421	0.10349584
189.6	0.06130527	0.18878271	0.35319528	0.08244224	0.16139149	0.20269027	0.03748087	0.69925647	0.08631352	0.11917371	0.26446144	0.10350352
189.7	0.06130617	0.18877261	0.35320788	0.08244379	0.16140639	0.20269553	0.03748447	0.6992621	0.08630714	0.11917775	0.26448077	0.10351119
189.8	0.06130707	0.18876251	0.35322047	0.08244534	0.16142127	0.20270079	0.03748808	0.69926772	0.08630076	0.11918178	0.26450007	0.10351886
189.9	0.06130797	0.18875242	0.35323305	0.08244688	0.16143616	0.20270605	0.03749169	0.69927332	0.08629439	0.1191858	0.26451936	0.10352651
190	0.06130887	0.18874234	0.35324563	0.08244843	0.16145103	0.20271131	0.0374953	0.69927892	0.08628802	0.11918982	0.26453863	0.10353415
190.1	0.06130977	0.18873226	0.3532582	0.08244998	0.16146589	0.20271656	0.03749892	0.6992845	0.08628166	0.11919382	0.26455788	0.10354179
190.2	0.06131067	0.1887222	0.35327077	0.08245153	0.16148075	0.20272181	0.03750253	0.69929007	0.0862753	0.11919782	0.26457711	0.10354942
190.3	0.06131157	0.18871214	0.35328332	0.08245309	0.16149559	0.20272706	0.03750615	0.69929564	0.08626895	0.1192018	0.26459633	0.10355703
190.4	0.06131246	0.18870208	0.35329587	0.08245464	0.16151043	0.20273231	0.03750978	0.69930119	0.08626261	0.11920578	0.26461553	0.10356464
190.5	0.06131336	0.18869204	0.35330841	0.08245619	0.16152526	0.20273755	0.0375134	0.69930673	0.08625626	0.11920975	0.2646347	0.10357224
190.6	0.06131426	0.188682	0.35332095	0.08245774	0.16154009	0.20274279	0.03751703	0.69931226	0.08624993	0.11921372	0.26465386	0.10357983
190.7	0.06131516	0.18867198	0.35333348	0.0824593	0.1615549	0.20274803	0.03752066	0.69931778	0.0862436	0.11921767	0.26467301	0.10358742
190.8	0.06131605	0.18866195	0.353346	0.08246085	0.1615697	0.20275326	0.03752429	0.69932329	0.08623727	0.11922162	0.26469213	0.10359499
190.9	0.06131695	0.18865194	0.35335851	0.08246241	0.1615845	0.2027585	0.03752792	0.69932879	0.08623095	0.11922555	0.26471124	0.10360256
191	0.06131785	0.18864194	0.35337102	0.08246397	0.16159929	0.20276373	0.03753156	0.69933427	0.08622463	0.11922948	0.26473032	0.10361011
191.1	0.06131874	0.18863194	0.35338352	0.08246552	0.16161407	0.20276895	0.0375352	0.69933975	0.08621832	0.1192334	0.26474939	0.10361766
191.2	0.06131964	0.18862195	0.35339601	0.08246708	0.16162884	0.20277418	0.03753884	0.69934522	0.08621201	0.11923731	0.26476844	0.1036252
191.3	0.06132053	0.18861196	0.35340849	0.08246864	0.1616436	0.2027794	0.03754248	0.69935067	0.08620571	0.11924121	0.26478748	0.10363273
191.4	0.06132143	0.18860199	0.35342097	0.0824702	0.16165835	0.20278462	0.03754613	0.69935612	0.08619942	0.11924511	0.26480649	0.10364025
191.5	0.06132233	0.18859202	0.35343344	0.08247176	0.1616731	0.20278984	0.03754978	0.69936155	0.08619313	0.119249	0.26482549	0.10364777
191.6	0.06132322	0.18858206	0.3534459	0.08247332	0.16168784	0.20279505	0.03755343	0.69936697	0.08618684	0.11925287	0.26484447	0.10365527
191.7	0.06132412	0.18857211	0.35345836	0.08247489	0.16170257	0.20280026	0.03755708	0.69937239	0.08618056	0.11925674	0.26486343	0.10366277
191.8	0.06132501	0.18856216	0.35347081	0.08247645	0.16171729	0.20280547	0.03756074	0.69937779	0.08617428	0.1192606	0.26488238	0.10367025
191.9	0.0613259	0.18855222	0.35348325	0.08247801	0.161732	0.20281068	0.03756439	0.69938318	0.08616801	0.11926446	0.2649013	0.10367773
192	0.0613268	0.18854229	0.35349569	0.08247958	0.1617467	0.20281588	0.03756805	0.69938857	0.08616174	0.1192683	0.26492021	0.1036852
192.1	0.06132769	0.18853237	0.35350812	0.08248114	0.1617614	0.20282108	0.03757172	0.69939394	0.08615548	0.11927214	0.2649391	0.10369267
192.2	0.06132858	0.18852246	0.35352054	0.0824827	0.16177609	0.20282628	0.03757538	0.6993993	0.08614923	0.11927597	0.26495797	0.10370012
192.3	0.06132948	0.18851255	0.35353295	0.08248427	0.16179076	0.20283148	0.03757905	0.69940465	0.08614298	0.11927979	0.26497683	0.10370756
192.4	0.06133037	0.18850265	0.35354536	0.08248584	0.16180543	0.20283667	0.03758272	0.69940999	0.08613673	0.1192836	0.26499566	0.103715
192.5	0.06133126	0.18849275	0.35355776	0.0824874	0.1618201	0.20284186	0.03758639	0.69941532	0.08613049	0.1192874	0.26501448	0.10372243
192.6	0.06133216	0.18848287	0.35357015	0.08248897	0.16183475	0.20284705	0.03759006	0.69942064	0.08612425	0.1192912	0.26503328	0.10372985
192.7	0.06133305	0.18847299	0.35358254	0.08249054	0.1618494	0.20285224	0.03759374	0.69942595	0.08611802	0.11929498	0.26505207	0.10373726
192.8	0.06133394	0.18846312	0.35359492	0.08249211	0.16186403	0.20285742	0.03759742	0.69943125	0.08611179	0.11929876	0.26507083	0.10374466
192.9	0.06133483	0.18845326	0.35360729	0.08249368	0.16187866	0.2028626	0.0376011	0.69943654	0.08610557	0.11930254	0.26508958	0.10375206
193	0.06133572	0.1884434	0.35361965	0.08249525	0.16189328	0.20286778	0.03760478	0.69944182	0.08609936	0.1193063	0.26510831	0.10375944
193.1	0.06133661	0.18843356	0.35363201	0.08249682	0.16190789	0.20287295	0.03760847	0.69944708	0.08609314	0.11931005	0.26512702	0.10376682
193.2	0.0613375	0.18842372	0.35364436	0.08249839	0.1619225	0.20287813	0.03761215	0.69945234	0.08608694	0.1193138	0.26514572	0.10377419
193.3	0.06133839	0.18841388	0.35365671	0.08249997	0.16193709	0.2028833	0.03761584	0.69945759	0.08608074	0.11931754	0.2651644	0.10378155
193.4	0.06133928	0.18840406	0.35366904	0.08250154	0.16195168	0.20288847	0.03761953	0.69946283	0.08607454	0.11932127	0.26518306	0.1037889
193.5	0.06134017	0.18839424	0.35368137	0.08250311	0.16196626	0.20289363	0.03762323	0.69946806	0.08606835	0.119325	0.2652017	0.10379625
193.6	0.06134106	0.18838443	0.35369369	0.08250469	0.16198083	0.2028988	0.03762692	0.69947328	0.08606216	0.11932871	0.26522032	0.10380358
193.7	0.06134195	0.18837463	0.35370601	0.08250626	0.16199539	0.20290396	0.03763062	0.69947848	0.08605598	0.11933242	0.26523893	0.10381091
193.8	0.06134284	0.18836483	0.35371832	0.08250784	0.16200994	0.20290912	0.03763432	0.69948368	0.0860498	0.11933612	0.26525752	0.10381823
193.9	0.06134373	0.18835504	0.35373062	0.08250941	0.16202449	0.20291427	0.03763803	0.69948887	0.08604363	0.11933981	0.26527609	0.10382554
194	0.06134462	0.18834526	0.35374292	0.08251099	0.16203903	0.20291942	0.03764173	0.69949405	0.08603746	0.11934349	0.26529465	0.10383284
195	0.06135348	0.18824786	0.35386548	0.08252679	0.16218394	0.20297082	0.0376789	0.69954528	0.08597606	0.1193799	0.26547924	0.10390542
195.1	0.06135437	0.18823816	0.3538777	0.08252837	0.16219839	0.20297595	0.03768263	0.69955034	0.08596994	0.1193835	0.2654976	0.10391263
195.2	0.06135525	0.18822846	0.35388991	0.08252996	0.16221283	0.20298107	0.03768636	0.6995554	0.08596384	0.11938709	0.26551595	0.10391984
195.3	0.06135614	0.18821878	0.35390211	0.08253154	0.16222726	0.20298619	0.03769009	0.69956045	0.08595773	0.11939067	0.26553428	0.10392703
195.4	0.06135702	0.1882091	0.35391431	0.08253313	0.16224168	0.20299131	0.03769383	0.69956549	0.08595163	0.11939424	0.26555259	0.10393422
195.5	0.06135791	0.18819943	0.3539265	0.08253471	0.16225609	0.20299643	0.03769756	0.69957052	0.08594554	0.11939781	0.26557088	0.1039414
195.6	0.06135879	0.18818977	0.35393868	0.0825363	0.1622705	0.20300154	0.0377013	0.69957554	0.08593945	0.11940137	0.26558916	0.10394857
195.7	0.06135967	0.18818011	0.35395086	0.08253788	0.1622849	0.20300666	0.03770504	0.69958055	0.08593337	0.11940492	0.26560742	0.10395574
195.8	0.06136056	0.18817046	0.35396303	0.08253947	0.16229928	0.20301176	0.03770879	0.69958555	0.08592729	0.11940846	0.26562566	0.10396289
195.9	0.06136144	0.18816082	0.35397519	0.08254106	0.16231366	0.20301687	0.03771253	0.69959054	0.08592121	0.119412	0.26564389	0.10397004
196	0.06136232	0.18815119	0.35398735	0.08254265	0.16232804	0.20302198	0.03771628	0.69959552	0.08591514	0.11941552	0.2656621	0.10397718
196.1	0.0613632	0.18814156	0.3539995	0.08254423	0.1623424	0.20302708	0.03772003	0.6996005	0.08590908	0.11941904	0.26568029	0.10398431
196.2	0.06136408	0.18813194	0.35401164	0.08254582	0.16235676	0.20303218	0.03772378	0.69960546	0.08590302	0.11942256	0.26569847	0.10399143
196.3	0.06136497	0.18812233	0.35402377	0.08254741	0.16237111	0.20303727	0.03772753	0.69961041	0.08589696	0.11942606	0.26571662	0.10399855
196.4	0.06136585	0.18811272	0.3540359	0.082549	0.16238545	0.20304237	0.03773129	0.69961535	0.08589091	0.11942956	0.26573476	0.10400566
196.5	0.06136673	0.18810312	0.35404802	0.08255059	0.16239978	0.20304746	0.03773505	0.69962029	0.08588486	0.11943305	0.26575289	0.10401276
196.6	0.06136761	0.18809353	0.35406014	0.08255219	0.1624141	0.20305255	0.03773881	0.69962521	0.08587882	0.11943653	0.265771	0.10401985
196.7	0.06136849	0.18808395	0.35407224	0.08255378	0.16242842	0.20305764	0.03774257	0.69963012	0.08587279	0.11944	0.26578909	0.10402693
196.8	0.06136937	0.18807437	0.35408435	0.08255537	0.16244272	0.20306272	0.03774633	0.69963503	0.08586676	0.11944347	0.26580716	0.10403401
196.9	0.06137025	0.1880648	0.35409644	0.08255696	0.16245702	0.20306781	0.0377501	0.69963992	0.08586073	0.11944693	0.26582522	0.10404108
197	0.06137113	0.18805524	0.35410853	0.08255856	0.16247131	0.20307289	0.03775387	0.69964481	0.08585471	0.11945038	0.26584325	0.10404814
197.1	0.06137201	0.18804569	0.35412061	0.08256015	0.1624856	0.20307797	0.03775764	0.69964968	0.08584869	0.11945382	0.26586128	0.10405519
197.2	0.06137288	0.18803614	0.35413268	0.08256175	0.16249987	0.20308304	0.03776141	0.69965455	0.08584268	0.11945726	0.26587928	0.10406223
197.3	0.06137376	0.1880266	0.35414475	0.08256334	0.16251414	0.20308811	0.03776518	0.69965941	0.08583667	0.11946069	0.26589727	0.10406927
197.4	0.06137464	0.18801706	0.35415681	0.08256494	0.1625284	0.20309319	0.03776896	0.69966425	0.08583067	0.11946411	0.26591524	0.1040763
197.5	0.06137552	0.18800754	0.35416886	0.08256654	0.16254265	0.20309825	0.03777274	0.69966909	0.08582467	0.11946752	0.2659332	0.10408332
197.6	0.0613764	0.18799802	0.3541809	0.08256813	0.16255689	0.20310332	0.03777652	0.69967392	0.08581868	0.11947093	0.26595114	0.10409033
197.7	0.06137727	0.1879885	0.35419294	0.08256973	0.16257112	0.20310838	0.0377803	0.69967874	0.08581269	0.11947433	0.26596906	0.10409733
197.8	0.06137815	0.187979	0.35420497	0.08257133	0.16258535	0.20311345	0.03778408	0.69968355	0.0858067	0.11947772	0.26598696	0.10410433
197.9	0.06137903	0.1879695	0.354217	0.08257293	0.16259957	0.20311851	0.03778787	0.69968835	0.08580073	0.1194811	0.26600485	0.10411132
198	0.0613799	0.18796001	0.35422902	0.08257453	0.16261378	0.20312356	0.03779166	0.69969314	0.08579475	0.11948448	0.26602273	0.1041183
198.1	0.06138078	0.18795053	0.35424103	0.08257613	0.16262798	0.20312862	0.03779544	0.69969792	0.08578878	0.11948785	0.26604058	0.10412527
198.2	0.06138165	0.18794105	0.35425303	0.08257773	0.16264217	0.20313367	0.03779924	0.6997027	0.08578282	0.11949121	0.26605842	0.10413224
198.3	0.06138253	0.18793158	0.35426503	0.08257933	0.16265636	0.20313872	0.03780303	0.69970746	0.08577686	0.11949457	0.26607624	0.1041392
198.4	0.0613834	0.18792212	0.35427702	0.08258093	0.16267054	0.20314377	0.03780683	0.69971221	0.0857709	0.11949791	0.26609405	0.10414615
198.5	0.06138428	0.18791266	0.35428901	0.08258253	0.16268471	0.20314881	0.03781062	0.69971696	0.08576495	0.11950126	0.26611183	0.10415309
198.6	0.06138515	0.18790321	0.35430099	0.08258413	0.16269887	0.20315386	0.03781442	0.69972169	0.08575901	0.11950459	0.26612961	0.10416002
198.7	0.06138603	0.18789377	0.35431296	0.08258574	0.16271302	0.2031589	0.03781822	0.69972642	0.08575307	0.11950791	0.26614736	0.10416695
198.8	0.0613869	0.18788434	0.35432492	0.08258734	0.16272717	0.20316394	0.03782203	0.69973113	0.08574713	0.11951123	0.2661651	0.10417387
198.9	0.06138777	0.18787491	0.35433688	0.08258894	0.16274131	0.20316897	0.03782583	0.69973584	0.0857412	0.11951454	0.26618282	0.10418078
199	0.06138864	0.18786549	0.35434883	0.08259055	0.16275544	0.20317401	0.03782964	0.69974054	0.08573527	0.11951785	0.26620053	0.10418769
199.1	0.06138952	0.18785608	0.35436077	0.08259215	0.16276956	0.20317904	0.03783345	0.69974523	0.08572935	0.11952115	0.26621822	0.10419458
199.2	0.06139039	0.18784667	0.35437271	0.08259376	0.16278367	0.20318407	0.03783726	0.69974991	0.08572343	0.11952444	0.26623589	0.10420147
199.3	0.06139126	0.18783727	0.35438464	0.08259537	0.16279778	0.20318909	0.03784107	0.69975458	0.08571752	0.11952772	0.26625355	0.10420835
199.4	0.06139213	0.18782788	0.35439656	0.08259697	0.16281188	0.20319412	0.03784489	0.69975924	0.08571161	0.11953099	0.26627119	0.10421522
199.5	0.061393	0.1878185	0.35440848	0.08259858	0.16282597	0.20319914	0.0378487	0.69976389	0.08570571	0.11953426	0.26628881	0.10422209
199.6	0.06139387	0.18780912	0.35442039	0.08260019	0.16284005	0.20320416	0.03785252	0.69976854	0.08569981	0.11953752	0.26630642	0.10422895
199.7	0.06139474	0.18779975	0.35443229	0.08260179	0.16285412	0.20320918	0.03785634	0.69977317	0.08569392	0.11954078	0.26632401	0.1042358
199.8	0.06139561	0.18779039	0.35444419	0.0826034	0.16286819	0.20321419	0.03786016	0.69977779	0.08568803	0.11954403	0.26634158	0.10424264
199.9	0.06139648	0.18778103	0.35445608	0.08260501	0.16288225	0.20321921	0.03786399	0.69978241	0.08568214	0.11954727	0.26635914	0.10424948
200	0.06139735	0.18777168	0.35446796	0.08260662	0.1628963	0.20322422	0.03786781	0.69978702	0.08567626	0.1195505	0.26637668	0.10425631
200.1	0.06139822	0.18776234	0.35447984	0.08260823	0.16291034	0.20322923	0.03787164	0.69979161	0.08567039	0.11955373	0.26639421	0.10426313
200.2	0.06139909	0.187753	0.35449171	0.08260984	0.16292438	0.20323423	0.03787547	0.6997962	0.08566452	0.11955695	0.26641172	0.10426994
200.3	0.06139996	0.18774367	0.35450357	0.08261145	0.1629384	0.20323924	0.0378793	0.69980078	0.08565865	0.11956016	0.26642921	0.10427675
200.4	0.06140083	0.18773435	0.35451542	0.08261306	0.16295242	0.20324424	0.03788314	0.69980535	0.08565279	0.11956337	0.26644668	0.10428354
200.5	0.0614017	0.18772504	0.35452727	0.08261467	0.16296643	0.20324924	0.03788697	0.69980991	0.08564694	0.11956657	0.26646414	0.10429033
200.6	0.06140256	0.18771573	0.35453912	0.08261629	0.16298044	0.20325423	0.03789081	0.69981447	0.08564108	0.11956976	0.26648159	0.10429712
200.7	0.06140343	0.18770643	0.35455095	0.0826179	0.16299443	0.20325923	0.03789465	0.69981901	0.08563524	0.11957294	0.26649902	0.10430389
200.8	0.0614043	0.18769713	0.35456278	0.08261951	0.16300842	0.20326422	0.03789849	0.69982355	0.0856294	0.11957612	0.26651643	0.10431066
200.9	0.06140517	0.18768785	0.3545746	0.08262113	0.1630224	0.20326921	0.03790233	0.69982807	0.08562356	0.11957929	0.26653382	0.10431742
201	0.06140603	0.18767857	0.35458642	0.08262274	0.16303637	0.2032742	0.03790617	0.69983259	0.08561773	0.11958246	0.2665512	0.10432418
201.1	0.0614069	0.1876693	0.35459823	0.08262435	0.16305033	0.20327918	0.03791002	0.69983709	0.0856119	0.11958562	0.26656856	0.10433092
201.2	0.06140776	0.18766003	0.35461003	0.08262597	0.16306429	0.20328417	0.03791387	0.69984159	0.08560607	0.11958877	0.26658591	0.10433766
201.3	0.06140863	0.18765077	0.35462183	0.08262758	0.16307824	0.20328915	0.03791772	0.69984608	0.08560025	0.11959191	0.26660324	0.10434439
201.4	0.0614095	0.18764152	0.35463362	0.0826292	0.16309218	0.20329413	0.03792157	0.69985056	0.08559444	0.11959505	0.26662056	0.10435112
201.5	0.06141036	0.18763228	0.3546454	0.08263082	0.16310611	0.2032991	0.03792542	0.69985504	0.08558863	0.11959818	0.26663785	0.10435783
201.6	0.06141122	0.18762304	0.35465717	0.08263243	0.16312003	0.20330408	0.03792928	0.6998595	0.08558283	0.11960131	0.26665514	0.10436454
201.7	0.06141209	0.18761381	0.35466894	0.08263405	0.16313395	0.20330905	0.03793313	0.69986395	0.08557702	0.11960443	0.2666724	0.10437124
201.8	0.06141295	0.18760458	0.35468071	0.08263567	0.16314786	0.20331402	0.03793699	0.6998684	0.08557123	0.11960754	0.26668965	0.10437794
201.9	0.06141382	0.18759537	0.35469246	0.08263728	0.16316176	0.20331899	0.03794085	0.69987284	0.08556544	0.11961064	0.26670689	0.10438462
202	0.06141468	0.18758616	0.35470421	0.0826389	0.16317565	0.20332395	0.03794471	0.69987726	0.08555965	0.11961374	0.26672411	0.1043913
202.1	0.06141554	0.18757695	0.35471595	0.08264052	0.16318954	0.20332892	0.03794858	0.69988168	0.08555387	0.11961683	0.26674131	0.10439798
202.2	0.06141641	0.18756776	0.35472769	0.08264214	0.16320341	0.20333388	0.03795244	0.69988609	0.08554809	0.11961992	0.2667585	0.10440464
202.3	0.06141727	0.18755857	0.35473942	0.08264376	0.16321728	0.20333883	0.03795631	0.6998905	0.08554232	0.11962299	0.26677567	0.1044113
202.4	0.06141813	0.18754939	0.35475114	0.08264538	0.16323114	0.20334379	0.03796018	0.69989489	0.08553655	0.11962607	0.26679282	0.10441795
202.5	0.06141899	0.18754021	0.35476286	0.082647	0.163245	0.20334874	0.03796405	0.69989927	0.08553079	0.11962913	0.26680996	0.1044246
202.6	0.06141985	0.18753104	0.35477457	0.08264862	0.16325884	0.2033537	0.03796792	0.69990365	0.08552503	0.11963219	0.26682708	0.10443123
202.7	0.06142071	0.18752188	0.35478627	0.08265024	0.16327268	0.20335864	0.0379718	0.69990802	0.08551927	0.11963524	0.26684419	0.10443786
202.8	0.06142157	0.18751273	0.35479797	0.08265186	0.16328651	0.20336359	0.03797567	0.69991237	0.08551352	0.11963829	0.26686128	0.10444448
202.9	0.06142243	0.18750358	0.35480966	0.08265349	0.16330034	0.20336854	0.03797955	0.69991672	0.08550778	0.11964133	0.26687836	0.1044511
203	0.06142329	0.18749444	0.35482134	0.08265511	0.16331415	0.20337348	0.03798343	0.69992106	0.08550204	0.11964436	0.26689542	0.10445771
203.1	0.06142415	0.1874853	0.35483302	0.08265673	0.16332796	0.20337842	0.03798731	0.6999254	0.0854963	0.11964738	0.26691246	0.10446431
203.2	0.06142501	0.18747618	0.35484469	0.08265835	0.16334176	0.20338336	0.03799119	0.69992972	0.08549057	0.1196504	0.26692949	0.1044709
203.3	0.06142587	0.18746706	0.35485635	0.08265998	0.16335555	0.20338829	0.03799508	0.69993403	0.08548484	0.11965342	0.2669465	0.10447749
203.4	0.06142673	0.18745794	0.35486801	0.0826616	0.16336933	0.20339323	0.03799896	0.69993834	0.08547912	0.11965642	0.2669635	0.10448407
203.5	0.06142759	0.18744884	0.35487966	0.08266323	0.16338311	0.20339816	0.03800285	0.69994264	0.0854734	0.11965942	0.26698048	0.10449064
203.6	0.06142844	0.18743974	0.35489131	0.08266485	0.16339688	0.20340309	0.03800674	0.69994693	0.08546769	0.11966242	0.26699745	0.10449721
203.7	0.0614293	0.18743064	0.35490295	0.08266647	0.16341064	0.20340801	0.03801063	0.69995121	0.08546198	0.1196654	0.2670144	0.10450376
203.8	0.06143016	0.18742156	0.35491458	0.0826681	0.16342439	0.20341294	0.03801452	0.69995548	0.08545628	0.11966839	0.26703133	0.10451032
203.9	0.06143101	0.18741248	0.3549262	0.08266973	0.16343814	0.20341786	0.03801842	0.69995974	0.08545058	0.11967136	0.26704825	0.10451686
204	0.06143187	0.1874034	0.35493782	0.08267135	0.16345188	0.20342278	0.03802231	0.699964	0.08544488	0.11967433	0.26706515	0.1045234
204.1	0.06143272	0.18739434	0.35494943	0.08267298	0.16346561	0.2034277	0.03802621	0.69996824	0.08543919	0.11967729	0.26708204	0.10452993
204.2	0.06143358	0.18738528	0.35496104	0.0826746	0.16347933	0.20343262	0.03803011	0.69997248	0.0854335	0.11968025	0.26709891	0.10453645
204.3	0.06143444	0.18737623	0.35497264	0.08267623	0.16349304	0.20343753	0.03803401	0.69997671	0.08542782	0.1196832	0.26711577	0.10454297
204.4	0.06143529	0.18736718	0.35498423	0.08267786	0.16350675	0.20344244	0.03803791	0.69998093	0.08542215	0.11968614	0.26713261	0.10454948
204.5	0.06143614	0.18735814	0.35499581	0.08267949	0.16352045	0.20344735	0.03804182	0.69998515	0.08541647	0.11968908	0.26714944	0.10455598
204.6	0.061437	0.18734911	0.35500739	0.08268112	0.16353414	0.20345226	0.03804572	0.69998935	0.08541081	0.11969201	0.26716625	0.10456247
204.7	0.06143785	0.18734009	0.35501897	0.08268274	0.16354783	0.20345716	0.03804963	0.69999354	0.08540514	0.11969493	0.26718304	0.10456896
204.8	0.0614387	0.18733107	0.35503053	0.08268437	0.1635615	0.20346206	0.03805354	0.69999773	0.08539948	0.11969785	0.26719982	0.10457544
204.9	0.06143956	0.18732205	0.35504209	0.082686	0.16357517	0.20346697	0.03805745	0.70000191	0.08539383	0.11970076	0.26721658	0.10458192
205	0.06144041	0.18731305	0.35505365	0.08268763	0.16358883	0.20347186	0.03806136	0.70000608	0.08538818	0.11970367	0.26723333	0.10458839
205.1	0.06144126	0.18730405	0.35506519	0.08268926	0.16360249	0.20347676	0.03806528	0.70001024	0.08538253	0.11970657	0.26725006	0.10459485
205.2	0.06144211	0.18729506	0.35507673	0.08269089	0.16361613	0.20348165	0.03806919	0.7000144	0.08537689	0.11970946	0.26726678	0.1046013
205.3	0.06144296	0.18728607	0.35508827	0.08269252	0.16362977	0.20348655	0.03807311	0.70001854	0.08537126	0.11971235	0.26728348	0.10460775
205.4	0.06144381	0.1872771	0.3550998	0.08269415	0.1636434	0.20349143	0.03807703	0.70002268	0.08536562	0.11971523	0.26730017	0.10461419
205.5	0.06144466	0.18726813	0.35511132	0.08269578	0.16365702	0.20349632	0.03808095	0.70002681	0.08536	0.1197181	0.26731684	0.10462062
205.6	0.06144551	0.18725916	0.35512283	0.08269741	0.16367064	0.20350121	0.03808487	0.70003093	0.08535437	0.11972097	0.2673335	0.10462705
205.7	0.06144636	0.1872502	0.35513434	0.08269905	0.16368425	0.20350609	0.03808879	0.70003504	0.08534875	0.11972383	0.26735014	0.10463347
205.8	0.06144721	0.18724125	0.35514584	0.08270068	0.16369785	0.20351097	0.03809271	0.70003914	0.08534314	0.11972669	0.26736676	0.10463988
205.9	0.06144806	0.18723231	0.35515734	0.08270231	0.16371144	0.20351585	0.03809664	0.70004324	0.08533753	0.11972954	0.26738337	0.10464629
206	0.06144891	0.18722337	0.35516883	0.08270394	0.16372502	0.20352073	0.03810057	0.70004733	0.08533193	0.11973238	0.26739997	0.10465269
206.1	0.06144976	0.18721444	0.35518031	0.08270558	0.1637386	0.2035256	0.0381045	0.70005141	0.08532633	0.11973522	0.26741655	0.10465908
206.2	0.0614506	0.18720551	0.35519179	0.08270721	0.16375217	0.20353047	0.03810843	0.70005548	0.08532073	0.11973805	0.26743311	0.10466547
206.3	0.06145145	0.18719659	0.35520326	0.08270884	0.16376573	0.20353534	0.03811236	0.70005954	0.08531514	0.11974088	0.26744966	0.10467185
206.4	0.0614523	0.18718768	0.35521472	0.08271048	0.16377929	0.20354021	0.03811629	0.70006359	0.08530955	0.1197437	0.2674662	0.10467822
206.5	0.06145314	0.18717878	0.35522618	0.08271211	0.16379284	0.20354508	0.03812023	0.70006764	0.08530397	0.11974651	0.26748272	0.10468459
206.6	0.06145399	0.18716988	0.35523763	0.08271375	0.16380638	0.20354994	0.03812417	0.70007168	0.08529839	0.11974932	0.26749922	0.10469094
206.7	0.06145484	0.18716099	0.35524907	0.08271538	0.16381991	0.2035548	0.0381281	0.70007571	0.08529282	0.11975212	0.26751571	0.1046973
206.8	0.06145568	0.1871521	0.35526051	0.08271702	0.16383343	0.20355966	0.03813204	0.70007973	0.08528725	0.11975491	0.26753218	0.10470364
206.9	0.06145653	0.18714323	0.35527195	0.08271865	0.16384695	0.20356452	0.03813599	0.70008374	0.08528168	0.1197577	0.26754864	0.10470998
207	0.06145737	0.18713436	0.35528337	0.08272029	0.16386046	0.20356937	0.03813993	0.70008775	0.08527612	0.11976048	0.26756509	0.10471631
207.1	0.06145821	0.18712549	0.35529479	0.08272192	0.16387396	0.20357423	0.03814387	0.70009174	0.08527056	0.11976326	0.26758152	0.10472264
207.2	0.06145906	0.18711663	0.3553062	0.08272356	0.16388746	0.20357908	0.03814782	0.70009573	0.08526501	0.11976603	0.26759793	0.10472896
207.3	0.0614599	0.18710778	0.35531761	0.0827252	0.16390094	0.20358393	0.03815177	0.70009971	0.08525947	0.11976879	0.26761433	0.10473527
207.4	0.06146074	0.18709894	0.35532901	0.08272683	0.16391442	0.20358877	0.03815571	0.70010369	0.08525392	0.11977155	0.26763071	0.10474158
207.5	0.06146159	0.1870901	0.3553404	0.08272847	0.16392789	0.20359362	0.03815966	0.70010765	0.08524838	0.1197743	0.26764708	0.10474788
207.6	0.06146243	0.18708126	0.35535179	0.08273011	0.16394136	0.20359846	0.03816362	0.70011161	0.08524285	0.11977705	0.26766343	0.10475417
207.7	0.06146327	0.18707244	0.35536317	0.08273174	0.16395482	0.2036033	0.03816757	0.70011556	0.08523732	0.11977979	0.26767977	0.10476045
207.8	0.06146411	0.18706362	0.35537455	0.08273338	0.16396827	0.20360814	0.03817152	0.7001195	0.0852318	0.11978252	0.26769609	0.10476673
207.9	0.06146495	0.18705481	0.35538592	0.08273502	0.16398171	0.20361297	0.03817548	0.70012343	0.08522627	0.11978525	0.2677124	0.10477301
208	0.06146579	0.187046	0.35539728	0.08273666	0.16399514	0.20361781	0.03817944	0.70012735	0.08522076	0.11978797	0.2677287	0.10477927
208.1	0.06146663	0.1870372	0.35540864	0.0827383	0.16400857	0.20362264	0.0381834	0.70013127	0.08521525	0.11979068	0.26774498	0.10478553
208.2	0.06146747	0.18702841	0.35541999	0.08273994	0.16402199	0.20362747	0.03818736	0.70013518	0.08520974	0.11979339	0.26776124	0.10479178
208.3	0.06146831	0.18701962	0.35543133	0.08274158	0.1640354	0.2036323	0.03819132	0.70013908	0.08520424	0.1197961	0.26777749	0.10479803
208.4	0.06146915	0.18701084	0.35544267	0.08274322	0.16404881	0.20363712	0.03819528	0.70014297	0.08519874	0.11979879	0.26779372	0.10480427
208.5	0.06146999	0.18700207	0.355454	0.08274486	0.1640622	0.20364195	0.03819925	0.70014686	0.08519324	0.11980148	0.26780994	0.1048105
208.6	0.06147083	0.1869933	0.35546533	0.0827465	0.16407559	0.20364677	0.03820321	0.70015074	0.08518775	0.11980417	0.26782615	0.10481673
208.7	0.06147167	0.18698454	0.35547664	0.08274814	0.16408898	0.20365159	0.03820718	0.7001546	0.08518227	0.11980685	0.26784234	0.10482295
208.8	0.0614725	0.18697579	0.35548796	0.08274978	0.16410235	0.20365641	0.03821115	0.70015846	0.08517679	0.11980952	0.26785851	0.10482917
208.9	0.06147334	0.18696704	0.35549926	0.08275142	0.16411572	0.20366122	0.03821512	0.70016232	0.08517131	0.11981219	0.26787468	0.10483537
209	0.06147418	0.1869583	0.35551056	0.08275306	0.16412908	0.20366603	0.03821909	0.70016616	0.08516584	0.11981485	0.26789082	0.10484157
210	0.06148252	0.18687125	0.35562322	0.08276949	0.16426227	0.20371405	0.03825888	0.70020418	0.08511135	0.11984114	0.26805147	0.10490323
210.1	0.06148335	0.18686258	0.35563445	0.08277113	0.16427555	0.20371884	0.03826287	0.70020794	0.08510593	0.11984374	0.26806746	0.10490936
210.2	0.06148419	0.18685392	0.35564568	0.08277277	0.16428882	0.20372363	0.03826686	0.70021169	0.08510051	0.11984633	0.26808343	0.10491548
210.3	0.06148502	0.18684526	0.35565689	0.08277442	0.16430209	0.20372841	0.03827085	0.70021543	0.08509509	0.11984892	0.26809939	0.1049216
210.4	0.06148585	0.18683661	0.35566811	0.08277606	0.16431535	0.2037332	0.03827484	0.70021917	0.08508968	0.1198515	0.26811533	0.10492771
210.5	0.06148668	0.18682796	0.35567931	0.08277771	0.16432859	0.20373798	0.03827883	0.7002229	0.08508427	0.11985407	0.26813126	0.10493381
210.6	0.06148751	0.18681932	0.35569051	0.08277935	0.16434184	0.20374276	0.03828282	0.70022662	0.08507887	0.11985664	0.26814717	0.10493991
210.7	0.06148834	0.18681069	0.35570171	0.082781	0.16435507	0.20374754	0.03828682	0.70023033	0.08507347	0.1198592	0.26816307	0.104946
210.8	0.06148917	0.18680207	0.3557129	0.08278264	0.1643683	0.20375231	0.03829081	0.70023404	0.08506808	0.11986176	0.26817895	0.10495209
210.9	0.06149	0.18679345	0.35572408	0.08278429	0.16438152	0.20375709	0.03829481	0.70023773	0.08506269	0.11986431	0.26819482	0.10495817
211	0.06149083	0.18678484	0.35573525	0.08278594	0.16439473	0.20376186	0.03829881	0.70024142	0.0850573	0.11986686	0.26821067	0.10496424
211.1	0.06149166	0.18677623	0.35574642	0.08278758	0.16440794	0.20376663	0.03830281	0.7002451	0.08505192	0.1198694	0.26822651	0.10497031
211.2	0.06149249	0.18676763	0.35575759	0.08278923	0.16442114	0.20377139	0.03830681	0.70024878	0.08504654	0.11987194	0.26824234	0.10497637
211.3	0.06149331	0.18675904	0.35576874	0.08279088	0.16443433	0.20377616	0.03831081	0.70025244	0.08504117	0.11987447	0.26825815	0.10498242
211.4	0.06149414	0.18675045	0.35577989	0.08279252	0.16444751	0.20378092	0.03831482	0.7002561	0.0850358	0.11987699	0.26827395	0.10498847
211.5	0.06149497	0.18674187	0.35579104	0.08279417	0.16446069	0.20378568	0.03831882	0.70025975	0.08503044	0.11987951	0.26828973	0.10499451
211.6	0.0614958	0.18673329	0.35580218	0.08279582	0.16447386	0.20379044	0.03832283	0.7002634	0.08502508	0.11988202	0.2683055	0.10500055
211.7	0.06149662	0.18672472	0.35581331	0.08279747	0.16448702	0.2037952	0.03832684	0.70026703	0.08501972	0.11988453	0.26832125	0.10500657
211.8	0.06149745	0.18671616	0.35582443	0.08279911	0.16450017	0.20379996	0.03833085	0.70027066	0.08501437	0.11988703	0.26833699	0.1050126
211.9	0.06149827	0.18670761	0.35583555	0.08280076	0.16451332	0.20380471	0.03833486	0.70027428	0.08500902	0.11988953	0.26835272	0.10501861
212	0.0614991	0.18669906	0.35584667	0.08280241	0.16452646	0.20380946	0.03833887	0.7002779	0.08500368	0.11989202	0.26836843	0.10502462
212.1	0.06149992	0.18669051	0.35585778	0.08280406	0.1645396	0.20381421	0.03834288	0.7002815	0.08499834	0.11989451	0.26838413	0.10503063
212.2	0.06150075	0.18668198	0.35586888	0.08280571	0.16455272	0.20381895	0.03834689	0.7002851	0.08499301	0.11989699	0.26839981	0.10503663
212.3	0.06150157	0.18667344	0.35587997	0.08280736	0.16456584	0.2038237	0.03835091	0.70028869	0.08498768	0.11989946	0.26841548	0.10504262
212.4	0.06150239	0.18666492	0.35589106	0.082809	0.16457895	0.20382844	0.03835493	0.70029228	0.08498235	0.11990193	0.26843113	0.1050486
212.5	0.06150322	0.1866564	0.35590214	0.08281065	0.16459206	0.20383318	0.03835894	0.70029585	0.08497703	0.1199044	0.26844677	0.10505458
212.6	0.06150404	0.18664789	0.35591322	0.0828123	0.16460515	0.20383792	0.03836296	0.70029942	0.08497172	0.11990686	0.2684624	0.10506056
212.7	0.06150486	0.18663938	0.35592429	0.08281395	0.16461824	0.20384266	0.03836698	0.70030298	0.0849664	0.11990931	0.26847801	0.10506652
212.8	0.06150568	0.18663088	0.35593536	0.0828156	0.16463133	0.20384739	0.038371	0.70030653	0.08496109	0.11991176	0.26849361	0.10507249
212.9	0.06150651	0.18662239	0.35594641	0.08281725	0.1646444	0.20385212	0.03837503	0.70031008	0.08495579	0.1199142	0.26850919	0.10507844
213	0.06150733	0.1866139	0.35595747	0.0828189	0.16465747	0.20385685	0.03837905	0.70031362	0.08495049	0.11991664	0.26852476	0.10508439
213.1	0.06150815	0.18660542	0.35596851	0.08282055	0.16467053	0.20386158	0.03838308	0.70031715	0.0849452	0.11991907	0.26854032	0.10509033
213.2	0.06150897	0.18659695	0.35597955	0.0828222	0.16468358	0.20386631	0.0383871	0.70032067	0.0849399	0.1199215	0.26855586	0.10509627
213.3	0.06150979	0.18658848	0.35599059	0.08282385	0.16469663	0.20387103	0.03839113	0.70032419	0.08493462	0.11992392	0.26857139	0.1051022
213.4	0.06151061	0.18658002	0.35600161	0.0828255	0.16470967	0.20387575	0.03839516	0.7003277	0.08492933	0.11992634	0.2685869	0.10510812
213.5	0.06151143	0.18657156	0.35601264	0.08282716	0.1647227	0.20388047	0.03839919	0.7003312	0.08492405	0.11992875	0.2686024	0.10511404
213.6	0.06151225	0.18656311	0.35602365	0.08282881	0.16473573	0.20388519	0.03840322	0.7003347	0.08491878	0.11993116	0.26861789	0.10511995
213.7	0.06151307	0.18655467	0.35603466	0.08283046	0.16474875	0.2038899	0.03840726	0.70033818	0.08491351	0.11993356	0.26863336	0.10512586
213.8	0.06151388	0.18654623	0.35604566	0.08283211	0.16476176	0.20389462	0.03841129	0.70034166	0.08490824	0.11993595	0.26864882	0.10513176
213.9	0.0615147	0.1865378	0.35605666	0.08283376	0.16477476	0.20389933	0.03841532	0.70034514	0.08490298	0.11993834	0.26866426	0.10513766
214	0.06151552	0.18652937	0.35606765	0.08283541	0.16478776	0.20390404	0.03841936	0.7003486	0.08489772	0.11994073	0.26867969	0.10514354
214.1	0.06151634	0.18652095	0.35607864	0.08283706	0.16480075	0.20390875	0.0384234	0.70035206	0.08489247	0.11994311	0.26869511	0.10514943
214.2	0.06151715	0.18651254	0.35608962	0.08283872	0.16481373	0.20391345	0.03842744	0.70035551	0.08488722	0.11994548	0.26871051	0.1051553
214.3	0.06151797	0.18650413	0.35610059	0.08284037	0.16482671	0.20391815	0.03843148	0.70035895	0.08488198	0.11994785	0.2687259	0.10516117
214.4	0.06151878	0.18649573	0.35611156	0.08284202	0.16483968	0.20392285	0.03843552	0.70036239	0.08487674	0.11995022	0.26874127	0.10516704
214.5	0.0615196	0.18648734	0.35612252	0.08284367	0.16485264	0.20392755	0.03843956	0.70036582	0.0848715	0.11995258	0.26875663	0.10517289
214.6	0.06152041	0.18647895	0.35613347	0.08284533	0.16486559	0.20393225	0.0384436	0.70036924	0.08486627	0.11995493	0.26877198	0.10517875
214.7	0.06152123	0.18647057	0.35614442	0.08284698	0.16487854	0.20393695	0.03844765	0.70037265	0.08486104	0.11995728	0.26878731	0.10518459
214.8	0.06152204	0.1864622	0.35615536	0.08284863	0.16489148	0.20394164	0.0384517	0.70037606	0.08485582	0.11995963	0.26880263	0.10519043
214.9	0.06152286	0.18645383	0.3561663	0.08285028	0.16490441	0.20394633	0.03845574	0.70037946	0.0848506	0.11996197	0.26881794	0.10519627
215	0.06152367	0.18644546	0.35617723	0.08285194	0.16491734	0.20395102	0.03845979	0.70038286	0.08484538	0.1199643	0.26883323	0.1052021
215.1	0.06152448	0.18643711	0.35618815	0.08285359	0.16493025	0.2039557	0.03846384	0.70038624	0.08484017	0.11996663	0.26884851	0.10520792
215.2	0.0615253	0.18642876	0.35619907	0.08285524	0.16494317	0.20396039	0.03846789	0.70038962	0.08483496	0.11996896	0.26886377	0.10521374
215.3	0.06152611	0.18642041	0.35620998	0.0828569	0.16495607	0.20396507	0.03847194	0.70039299	0.08482976	0.11997128	0.26887902	0.10521955
215.4	0.06152692	0.18641207	0.35622089	0.08285855	0.16496897	0.20396975	0.03847599	0.70039636	0.08482456	0.11997359	0.26889426	0.10522535
215.5	0.06152773	0.18640374	0.35623179	0.08286021	0.16498186	0.20397443	0.03848005	0.70039971	0.08481937	0.1199759	0.26890948	0.10523115
215.6	0.06152854	0.18639542	0.35624268	0.08286186	0.16499474	0.20397911	0.0384841	0.70040306	0.08481418	0.11997821	0.26892469	0.10523695
215.7	0.06152936	0.1863871	0.35625357	0.08286351	0.16500762	0.20398378	0.03848816	0.70040641	0.08480899	0.11998051	0.26893988	0.10524273
215.8	0.06153017	0.18637878	0.35626445	0.08286517	0.16502049	0.20398846	0.03849222	0.70040974	0.08480381	0.1199828	0.26895507	0.10524851
215.9	0.06153098	0.18637048	0.35627533	0.08286682	0.16503335	0.20399313	0.03849628	0.70041307	0.08479863	0.11998509	0.26897023	0.10525429
216	0.06153179	0.18636218	0.3562862	0.08286848	0.16504621	0.20399779	0.03850034	0.70041639	0.08479346	0.11998738	0.26898539	0.10526006
216.1	0.0615326	0.18635388	0.35629706	0.08287013	0.16505905	0.20400246	0.0385044	0.70041971	0.08478829	0.11998966	0.26900053	0.10526582
216.2	0.0615334	0.18634559	0.35630792	0.08287179	0.16507189	0.20400713	0.03850846	0.70042302	0.08478312	0.11999194	0.26901566	0.10527158
216.3	0.06153421	0.18633731	0.35631877	0.08287344	0.16508473	0.20401179	0.03851252	0.70042632	0.08477796	0.11999421	0.26903077	0.10527734
216.4	0.06153502	0.18632904	0.35632962	0.0828751	0.16509756	0.20401645	0.03851658	0.70042961	0.0847728	0.11999647	0.26904587	0.10528308
216.5	0.06153583	0.18632077	0.35634046	0.08287675	0.16511038	0.20402111	0.03852065	0.7004329	0.08476765	0.11999873	0.26906096	0.10528882
216.6	0.06153664	0.1863125	0.35635129	0.08287841	0.16512319	0.20402576	0.03852472	0.70043618	0.0847625	0.12000099	0.26907603	0.10529456
216.7	0.06153744	0.18630425	0.35636212	0.08288006	0.165136	0.20403042	0.03852878	0.70043945	0.08475736	0.12000324	0.26909109	0.10530029
216.8	0.06153825	0.18629599	0.35637294	0.08288172	0.1651488	0.20403507	0.03853285	0.70044272	0.08475222	0.12000548	0.26910614	0.10530601
216.9	0.06153906	0.18628775	0.35638376	0.08288337	0.16516159	0.20403972	0.03853692	0.70044598	0.08474708	0.12000772	0.26912117	0.10531173
217	0.06153986	0.18627951	0.35639457	0.08288503	0.16517437	0.20404437	0.03854099	0.70044923	0.08474195	0.12000996	0.26913619	0.10531744
217.1	0.06154067	0.18627128	0.35640537	0.08288669	0.16518715	0.20404902	0.03854506	0.70045248	0.08473682	0.12001219	0.2691512	0.10532315
217.2	0.06154147	0.18626305	0.35641617	0.08288834	0.16519992	0.20405366	0.03854914	0.70045571	0.08473169	0.12001442	0.26916619	0.10532885
217.3	0.06154228	0.18625483	0.35642696	0.08289	0.16521269	0.2040583	0.03855321	0.70045895	0.08472657	0.12001664	0.26918117	0.10533454
217.4	0.06154308	0.18624662	0.35643774	0.08289165	0.16522545	0.20406294	0.03855728	0.70046217	0.08472146	0.12001885	0.26919614	0.10534023
217.5	0.06154389	0.18623841	0.35644852	0.08289331	0.1652382	0.20406758	0.03856136	0.70046539	0.08471635	0.12002106	0.26921109	0.10534592
217.6	0.06154469	0.1862302	0.3564593	0.08289497	0.16525094	0.20407222	0.03856544	0.7004686	0.08471124	0.12002327	0.26922603	0.10535159
217.7	0.0615455	0.18622201	0.35647007	0.08289662	0.16526368	0.20407685	0.03856952	0.7004718	0.08470614	0.12002547	0.26924096	0.10535727
217.8	0.0615463	0.18621382	0.35648083	0.08289828	0.16527641	0.20408149	0.03857359	0.700475	0.08470104	0.12002767	0.26925587	0.10536293
217.9	0.0615471	0.18620563	0.35649158	0.08289993	0.16528913	0.20408612	0.03857767	0.70047819	0.08469594	0.12002986	0.26927077	0.10536859
218	0.0615479	0.18619746	0.35650233	0.08290159	0.16530185	0.20409074	0.03858176	0.70048138	0.08469085	0.12003205	0.26928565	0.10537425
218.1	0.06154871	0.18618928	0.35651308	0.08290325	0.16531455	0.20409537	0.03858584	0.70048455	0.08468576	0.12003423	0.26930053	0.1053799
218.2	0.06154951	0.18618112	0.35652382	0.0829049	0.16532726	0.2041	0.03858992	0.70048772	0.08468068	0.12003641	0.26931539	0.10538554
218.3	0.06155031	0.18617296	0.35653455	0.08290656	0.16533995	0.20410462	0.03859401	0.70049089	0.0846756	0.12003858	0.26933023	0.10539118
218.4	0.06155111	0.18616481	0.35654528	0.08290822	0.16535264	0.20410924	0.03859809	0.70049404	0.08467053	0.12004075	0.26934507	0.10539681
218.5	0.06155191	0.18615666	0.356556	0.08290987	0.16536532	0.20411386	0.03860218	0.70049719	0.08466546	0.12004291	0.26935989	0.10540244
218.6	0.06155271	0.18614852	0.35656671	0.08291153	0.16537799	0.20411847	0.03860626	0.70050034	0.08466039	0.12004507	0.26937469	0.10540806
218.7	0.06155351	0.18614038	0.35657742	0.08291319	0.16539066	0.20412309	0.03861035	0.70050347	0.08465533	0.12004722	0.26938949	0.10541367
218.8	0.06155431	0.18613225	0.35658812	0.08291484	0.16540332	0.2041277	0.03861444	0.7005066	0.08465027	0.12004937	0.26940427	0.10541928
218.9	0.06155511	0.18612413	0.35659882	0.0829165	0.16541598	0.20413231	0.03861853	0.70050973	0.08464521	0.12005152	0.26941903	0.10542489
219	0.06155591	0.18611601	0.35660951	0.08291816	0.16542862	0.20413692	0.03862262	0.70051284	0.08464016	0.12005366	0.26943379	0.10543049
219.1	0.06155671	0.1861079	0.35662019	0.08291982	0.16544126	0.20414153	0.03862672	0.70051595	0.08463512	0.12005579	0.26944853	0.10543608
219.2	0.06155751	0.18609979	0.35663087	0.08292147	0.1654539	0.20414613	0.03863081	0.70051905	0.08463008	0.12005792	0.26946326	0.10544167
219.3	0.06155831	0.18609169	0.35664155	0.08292313	0.16546652	0.20415074	0.0386349	0.70052215	0.08462504	0.12006005	0.26947797	0.10544725
219.4	0.0615591	0.1860836	0.35665221	0.08292479	0.16547914	0.20415534	0.038639	0.70052524	0.08462	0.12006217	0.26949267	0.10545282
219.5	0.0615599	0.18607551	0.35666287	0.08292645	0.16549175	0.20415993	0.03864309	0.70052832	0.08461497	0.12006428	0.26950736	0.10545839
219.6	0.0615607	0.18606743	0.35667353	0.0829281	0.16550436	0.20416453	0.03864719	0.7005314	0.08460995	0.1200664	0.26952204	0.10546396
219.7	0.0615615	0.18605936	0.35668418	0.08292976	0.16551696	0.20416913	0.03865129	0.70053447	0.08460493	0.1200685	0.2695367	0.10546952
219.8	0.06156229	0.18605129	0.35669482	0.08293142	0.16552955	0.20417372	0.03865539	0.70053753	0.08459991	0.1200706	0.26955135	0.10547507
219.9	0.06156309	0.18604322	0.35670546	0.08293308	0.16554214	0.20417831	0.03865949	0.70054059	0.08459489	0.1200727	0.26956598	0.10548062
220	0.06156388	0.18603517	0.35671609	0.08293474	0.16555471	0.2041829	0.03866359	0.70054364	0.08458989	0.1200748	0.26958061	0.10548616
220.1	0.06156468	0.18602711	0.35672672	0.08293639	0.16556729	0.20418749	0.03866769	0.70054668	0.08458488	0.12007688	0.26959522	0.1054917
220.2	0.06156547	0.18601907	0.35673734	0.08293805	0.16557985	0.20419207	0.0386718	0.70054972	0.08457988	0.12007897	0.26960981	0.10549723
220.3	0.06156627	0.18601103	0.35674795	0.08293971	0.16559241	0.20419665	0.0386759	0.70055275	0.08457488	0.12008105	0.2696244	0.10550276
220.4	0.06156706	0.186003	0.35675856	0.08294137	0.16560496	0.20420124	0.03868	0.70055578	0.08456989	0.12008312	0.26963897	0.10550828
220.5	0.06156786	0.18599497	0.35676916	0.08294303	0.1656175	0.20420581	0.03868411	0.70055879	0.0845649	0.12008519	0.26965353	0.10551379
220.6	0.06156865	0.18598694	0.35677976	0.08294469	0.16563004	0.20421039	0.03868822	0.7005618	0.08455991	0.12008725	0.26966807	0.1055193
220.7	0.06156945	0.18597893	0.35679035	0.08294634	0.16564257	0.20421497	0.03869232	0.70056481	0.08455493	0.12008931	0.26968261	0.10552481
220.8	0.06157024	0.18597092	0.35680093	0.082948	0.16565509	0.20421954	0.03869643	0.70056781	0.08454995	0.12009137	0.26969713	0.10553031
220.9	0.06157103	0.18596291	0.35681151	0.08294966	0.16566761	0.20422411	0.03870054	0.7005708	0.08454498	0.12009342	0.26971164	0.1055358
221	0.06157182	0.18595492	0.35682209	0.08295132	0.16568012	0.20422868	0.03870465	0.70057378	0.08454001	0.12009547	0.26972613	0.10554129
221.1	0.06157262	0.18594692	0.35683265	0.08295298	0.16569262	0.20423325	0.03870877	0.70057676	0.08453505	0.12009751	0.26974061	0.10554677
221.2	0.06157341	0.18593894	0.35684322	0.08295464	0.16570512	0.20423781	0.03871288	0.70057974	0.08453008	0.12009955	0.26975508	0.10555225
221.3	0.0615742	0.18593095	0.35685377	0.0829563	0.16571761	0.20424238	0.03871699	0.7005827	0.08452513	0.12010158	0.26976954	0.10555772
221.4	0.06157499	0.18592298	0.35686432	0.08295796	0.16573009	0.20424694	0.03872111	0.70058566	0.08452017	0.12010361	0.26978398	0.10556319
221.5	0.06157578	0.18591501	0.35687486	0.08295962	0.16574257	0.2042515	0.03872522	0.70058861	0.08451522	0.12010563	0.26979841	0.10556865
221.6	0.06157657	0.18590705	0.3568854	0.08296128	0.16575504	0.20425606	0.03872934	0.70059156	0.08451028	0.12010765	0.26981283	0.1055741
221.7	0.06157736	0.18589909	0.35689594	0.08296294	0.1657675	0.20426061	0.03873345	0.7005945	0.08450534	0.12010967	0.26982723	0.10557955
221.8	0.06157815	0.18589114	0.35690646	0.0829646	0.16577996	0.20426517	0.03873757	0.70059744	0.0845004	0.12011168	0.26984162	0.105585
221.9	0.06157894	0.18588319	0.35691698	0.08296626	0.16579241	0.20426972	0.03874169	0.70060036	0.08449547	0.12011368	0.269856	0.10559043
222	0.06157973	0.18587525	0.3569275	0.08296792	0.16580485	0.20427427	0.03874581	0.70060329	0.08449054	0.12011569	0.26987037	0.10559587
222.1	0.06158052	0.18586732	0.35693801	0.08296958	0.16581729	0.20427882	0.03874993	0.7006062	0.08448561	0.12011768	0.26988473	0.1056013
222.2	0.06158131	0.18585939	0.35694851	0.08297124	0.16582971	0.20428337	0.03875405	0.70060911	0.08448069	0.12011968	0.26989907	0.10560672
222.3	0.06158209	0.18585147	0.35695901	0.0829729	0.16584214	0.20428791	0.03875818	0.70061201	0.08447577	0.12012166	0.2699134	0.10561214
222.4	0.06158288	0.18584355	0.3569695	0.08297456	0.16585455	0.20429245	0.0387623	0.70061491	0.08447086	0.12012365	0.26992771	0.10561755
222.5	0.06158367	0.18583564	0.35697998	0.08297622	0.16586696	0.20429699	0.03876642	0.7006178	0.08446595	0.12012563	0.26994202	0.10562296
222.6	0.06158446	0.18582773	0.35699046	0.08297788	0.16587936	0.20430153	0.03877055	0.70062068	0.08446105	0.1201276	0.26995631	0.10562836
222.7	0.06158524	0.18581983	0.35700094	0.08297954	0.16589176	0.20430607	0.03877467	0.70062356	0.08445614	0.12012957	0.26997059	0.10563375
222.8	0.06158603	0.18581194	0.3570114	0.0829812	0.16590415	0.2043106	0.0387788	0.70062643	0.08445125	0.12013154	0.26998485	0.10563914
222.9	0.06158682	0.18580405	0.35702187	0.08298286	0.16591653	0.20431514	0.03878293	0.7006293	0.08444635	0.1201335	0.2699991	0.10564453
223	0.0615876	0.18579617	0.35703232	0.08298452	0.1659289	0.20431967	0.03878706	0.70063216	0.08444146	0.12013546	0.27001335	0.10564991
223.1	0.06158839	0.18578829	0.35704277	0.08298618	0.16594127	0.2043242	0.03879119	0.70063501	0.08443658	0.12013742	0.27002757	0.10565529
223.2	0.06158917	0.18578042	0.35705322	0.08298784	0.16595363	0.20432872	0.03879532	0.70063785	0.0844317	0.12013936	0.27004179	0.10566066
223.3	0.06158996	0.18577256	0.35706366	0.08298951	0.16596599	0.20433325	0.03879945	0.7006407	0.08442682	0.12014131	0.27005599	0.10566602
223.4	0.06159074	0.1857647	0.35707409	0.08299117	0.16597834	0.20433777	0.03880358	0.70064353	0.08442194	0.12014325	0.27007018	0.10567138
223.5	0.06159152	0.18575685	0.35708452	0.08299283	0.16599068	0.20434229	0.03880771	0.70064636	0.08441707	0.12014519	0.27008436	0.10567673
223.6	0.06159231	0.185749	0.35709494	0.08299449	0.16600302	0.20434681	0.03881185	0.70064918	0.08441221	0.12014712	0.27009853	0.10568208
223.7	0.06159309	0.18574116	0.35710536	0.08299615	0.16601534	0.20435133	0.03881598	0.700652	0.08440735	0.12014905	0.27011268	0.10568742
223.8	0.06159387	0.18573332	0.35711577	0.08299781	0.16602767	0.20435585	0.03882012	0.70065481	0.08440249	0.12015097	0.27012682	0.10569276
223.9	0.06159466	0.18572549	0.35712617	0.08299947	0.16603998	0.20436036	0.03882425	0.70065761	0.08439764	0.12015289	0.27014095	0.1056981
224	0.06159544	0.18571766	0.35713657	0.08300114	0.16605229	0.20436487	0.03882839	0.70066041	0.08439278	0.12015481	0.27015507	0.10570342

194.1	0.06134551	0.18833549	0.3537552	0.08251257	0.16205356	0.20292458	0.03764544	0.69949921	0.0860313	0.11934717	0.26531319	0.10384014
194.2	0.06134639	0.18832572	0.35376748	0.08251415	0.16206808	0.20292972	0.03764915	0.69950437	0.08602514	0.11935084	0.26533171	0.10384742
194.3	0.06134728	0.18831596	0.35377976	0.08251573	0.16208259	0.20293487	0.03765286	0.69950952	0.08601899	0.1193545	0.26535021	0.1038547
194.4	0.06134817	0.18830621	0.35379202	0.0825173	0.16209709	0.20294001	0.03765657	0.69951466	0.08601284	0.11935815	0.26536869	0.10386197
194.5	0.06134905	0.18829647	0.35380428	0.08251888	0.16211159	0.20294515	0.03766029	0.69951978	0.0860067	0.11936179	0.26538716	0.10386923
194.6	0.06134994	0.18828673	0.35381654	0.08252046	0.16212608	0.20295029	0.03766401	0.6995249	0.08600056	0.11936543	0.26540561	0.10387649
194.7	0.06135083	0.188277	0.35382878	0.08252204	0.16214056	0.20295543	0.03766773	0.69953001	0.08599443	0.11936906	0.26542404	0.10388373
194.8	0.06135171	0.18826728	0.35384102	0.08252363	0.16215503	0.20296056	0.03767145	0.69953511	0.0859883	0.11937268	0.26544246	0.10389097
194.9	0.0613526	0.18825756	0.35385325	0.08252521	0.16216949	0.20296569	0.03767517	0.6995402	0.08598218	0.11937629	0.26546086	0.1038982

225	0.06160324	0.18563973	0.35724023	0.08301776	0.16617501	0.20440989	0.03886981	0.70068805	0.0843445	0.12017373	0.27029555	0.10575641
225.1	0.06160402	0.18563196	0.35725057	0.08301942	0.16618725	0.20441438	0.03887395	0.70069078	0.08433969	0.1201756	0.27030953	0.10576168
225.2	0.0616048	0.18562421	0.3572609	0.08302108	0.16619948	0.20441887	0.0388781	0.70069351	0.08433489	0.12017747	0.2703235	0.10576695
225.3	0.06160557	0.18561645	0.35727122	0.08302274	0.1662117	0.20442336	0.03888225	0.70069623	0.08433009	0.12017933	0.27033746	0.10577221
225.4	0.06160635	0.18560871	0.35728154	0.08302441	0.16622392	0.20442784	0.0388864	0.70069894	0.0843253	0.12018119	0.2703514	0.10577747
225.5	0.06160713	0.18560097	0.35729185	0.08302607	0.16623613	0.20443233	0.03889055	0.70070165	0.0843205	0.12018304	0.27036534	0.10578272
225.6	0.06160791	0.18559323	0.35730216	0.08302773	0.16624833	0.20443681	0.0388947	0.70070435	0.08431572	0.12018489	0.27037926	0.10578796
225.7	0.06160868	0.1855855	0.35731246	0.08302939	0.16626053	0.20444129	0.03889885	0.70070705	0.08431093	0.12018674	0.27039316	0.1057932
225.8	0.06160946	0.18557778	0.35732275	0.08303106	0.16627272	0.20444577	0.038903	0.70070974	0.08430615	0.12018858	0.27040706	0.10579844
225.9	0.06161023	0.18557006	0.35733304	0.08303272	0.1662849	0.20445024	0.03890715	0.70071242	0.08430138	0.12019042	0.27042094	0.10580367
226	0.06161101	0.18556235	0.35734332	0.08303438	0.16629708	0.20445472	0.0389113	0.7007151	0.08429661	0.12019225	0.27043481	0.10580889
226.1	0.06161178	0.18555464	0.3573536	0.08303604	0.16630925	0.20445919	0.03891546	0.70071777	0.08429184	0.12019408	0.27044867	0.10581411
226.2	0.06161256	0.18554694	0.35736387	0.08303771	0.16632141	0.20446366	0.03891961	0.70072044	0.08428707	0.12019591	0.27046252	0.10581933
226.3	0.06161333	0.18553925	0.35737414	0.08303937	0.16633357	0.20446813	0.03892377	0.7007231	0.08428231	0.12019773	0.27047636	0.10582454
226.4	0.06161411	0.18553155	0.3573844	0.08304103	0.16634572	0.2044726	0.03892792	0.70072576	0.08427756	0.12019954	0.27049018	0.10582974
226.5	0.06161488	0.18552387	0.35739465	0.0830427	0.16635787	0.20447706	0.03893208	0.70072841	0.0842728	0.12020136	0.27050399	0.10583494
226.6	0.06161565	0.18551619	0.3574049	0.08304436	0.16637001	0.20448153	0.03893624	0.70073105	0.08426806	0.12020316	0.27051779	0.10584013
226.7	0.06161643	0.18550852	0.35741514	0.08304602	0.16638214	0.20448599	0.0389404	0.70073369	0.08426331	0.12020497	0.27053158	0.10584532
226.8	0.0616172	0.18550085	0.35742538	0.08304769	0.16639426	0.20449045	0.03894455	0.70073632	0.08425857	0.12020677	0.27054535	0.10585051
226.9	0.06161797	0.18549319	0.35743561	0.08304935	0.16640638	0.20449491	0.03894871	0.70073895	0.08425383	0.12020857	0.27055911	0.10585569
227	0.06161875	0.18548553	0.35744584	0.08305101	0.1664185	0.20449936	0.03895287	0.70074157	0.0842491	0.12021036	0.27057286	0.10586086
227.1	0.06161952	0.18547788	0.35745606	0.08305268	0.1664306	0.20450382	0.03895704	0.70074418	0.08424437	0.12021215	0.2705866	0.10586603
227.2	0.06162029	0.18547023	0.35746627	0.08305434	0.1664427	0.20450827	0.0389612	0.70074679	0.08423964	0.12021393	0.27060033	0.1058712
227.3	0.06162106	0.18546259	0.35747648	0.083056	0.1664548	0.20451272	0.03896536	0.7007494	0.08423492	0.12021571	0.27061405	0.10587636
227.4	0.06162183	0.18545496	0.35748668	0.08305767	0.16646688	0.20451717	0.03896952	0.70075199	0.0842302	0.12021749	0.27062775	0.10588151
227.5	0.0616226	0.18544733	0.35749688	0.08305933	0.16647896	0.20452161	0.03897369	0.70075459	0.08422549	0.12021926	0.27064144	0.10588666
227.6	0.06162337	0.1854397	0.35750707	0.083061	0.16649104	0.20452606	0.03897785	0.70075717	0.08422078	0.12022103	0.27065512	0.1058918
227.7	0.06162414	0.18543209	0.35751725	0.08306266	0.1665031	0.2045305	0.03898202	0.70075975	0.08421607	0.12022279	0.27066879	0.10589694
227.8	0.06162491	0.18542447	0.35752743	0.08306432	0.16651516	0.20453494	0.03898619	0.70076233	0.08421137	0.12022455	0.27068244	0.10590208
227.9	0.06162568	0.18541687	0.35753761	0.08306599	0.16652722	0.20453938	0.03899035	0.7007649	0.08420667	0.12022631	0.27069609	0.10590721
228	0.06162645	0.18540926	0.35754778	0.08306765	0.16653927	0.20454382	0.03899452	0.70076746	0.08420198	0.12022806	0.27070972	0.10591233
228.1	0.06162722	0.18540167	0.35755794	0.08306931	0.16655131	0.20454826	0.03899869	0.70077002	0.08419729	0.12022981	0.27072334	0.10591745
228.2	0.06162799	0.18539408	0.3575681	0.08307098	0.16656335	0.20455269	0.03900286	0.70077257	0.0841926	0.12023156	0.27073695	0.10592257
228.3	0.06162875	0.18538649	0.35757825	0.08307264	0.16657537	0.20455712	0.03900703	0.70077512	0.08418791	0.1202333	0.27075055	0.10592768
228.4	0.06162952	0.18537891	0.35758839	0.08307431	0.1665874	0.20456155	0.0390112	0.70077766	0.08418323	0.12023504	0.27076413	0.10593279
228.5	0.06163029	0.18537134	0.35759853	0.08307597	0.16659941	0.20456598	0.03901537	0.7007802	0.08417856	0.12023677	0.2707777	0.10593789
228.6	0.06163105	0.18536377	0.35760867	0.08307763	0.16661142	0.20457041	0.03901954	0.70078273	0.08417389	0.1202385	0.27079127	0.10594298
228.7	0.06163182	0.1853562	0.35761879	0.0830793	0.16662343	0.20457483	0.03902371	0.70078526	0.08416922	0.12024022	0.27080482	0.10594807
228.8	0.06163259	0.18534865	0.35762892	0.08308096	0.16663542	0.20457925	0.03902789	0.70078778	0.08416455	0.12024195	0.27081835	0.10595316
228.9	0.06163335	0.18534109	0.35763903	0.08308263	0.16664741	0.20458368	0.03903206	0.70079029	0.08415989	0.12024366	0.27083188	0.10595824
229	0.06163412	0.18533355	0.35764914	0.08308429	0.1666594	0.20458809	0.03903624	0.7007928	0.08415524	0.12024538	0.27084539	0.10596332
229.1	0.06163488	0.185326	0.35765925	0.08308596	0.16667138	0.20459251	0.03904041	0.7007953	0.08415058	0.12024709	0.2708589	0.10596839
229.2	0.06163565	0.18531847	0.35766935	0.08308762	0.16668335	0.20459693	0.03904459	0.7007978	0.08414593	0.12024879	0.27087239	0.10597346
229.3	0.06163641	0.18531094	0.35767944	0.08308928	0.16669531	0.20460134	0.03904876	0.70080029	0.08414129	0.1202505	0.27088587	0.10597852
229.4	0.06163718	0.18530341	0.35768953	0.08309095	0.16670727	0.20460575	0.03905294	0.70080278	0.08413665	0.1202522	0.27089934	0.10598357
229.5	0.06163794	0.18529589	0.35769962	0.08309261	0.16671923	0.20461016	0.03905712	0.70080526	0.08413201	0.12025389	0.27091279	0.10598863
229.6	0.06163871	0.18528838	0.35770969	0.08309428	0.16673117	0.20461457	0.0390613	0.70080774	0.08412737	0.12025558	0.27092624	0.10599367
229.7	0.06163947	0.18528087	0.35771976	0.08309594	0.16674311	0.20461898	0.03906548	0.70081021	0.08412274	0.12025727	0.27093967	0.10599872
229.8	0.06164023	0.18527336	0.35772983	0.08309761	0.16675505	0.20462338	0.03906966	0.70081267	0.08411812	0.12025895	0.27095309	0.10600375
229.9	0.06164099	0.18526587	0.35773989	0.08309927	0.16676697	0.20462779	0.03907384	0.70081513	0.08411349	0.12026063	0.2709665	0.10600879
230	0.06164176	0.18525837	0.35774994	0.08310094	0.16677889	0.20463219	0.03907802	0.70081759	0.08410887	0.12026231	0.2709799	0.10601382
230.1	0.06164252	0.18525088	0.35775999	0.0831026	0.16679081	0.20463659	0.0390822	0.70082004	0.08410426	0.12026398	0.27099329	0.10601884
230.2	0.06164328	0.1852434	0.35777004	0.08310426	0.16680272	0.20464098	0.03908638	0.70082248	0.08409965	0.12026565	0.27100666	0.10602386
230.3	0.06164404	0.18523593	0.35778007	0.08310593	0.16681462	0.20464538	0.03909057	0.70082492	0.08409504	0.12026732	0.27102003	0.10602887
230.4	0.0616448	0.18522845	0.35779011	0.08310759	0.16682652	0.20464977	0.03909475	0.70082735	0.08409043	0.12026898	0.27103338	0.10603388
230.5	0.06164556	0.18522099	0.35780013	0.08310926	0.16683841	0.20465417	0.03909894	0.70082978	0.08408583	0.12027064	0.27104672	0.10603889
230.6	0.06164632	0.18521353	0.35781015	0.08311092	0.16685029	0.20465856	0.03910312	0.7008322	0.08408124	0.12027229	0.27106005	0.10604389
230.7	0.06164708	0.18520607	0.35782017	0.08311259	0.16686217	0.20466295	0.03910731	0.70083462	0.08407664	0.12027394	0.27107337	0.10604888
230.8	0.06164784	0.18519862	0.35783018	0.08311425	0.16687404	0.20466733	0.03911149	0.70083703	0.08407206	0.12027559	0.27108667	0.10605387
230.9	0.0616486	0.18519118	0.35784018	0.08311592	0.1668859	0.20467172	0.03911568	0.70083944	0.08406747	0.12027723	0.27109997	0.10605886
231	0.06164936	0.18518374	0.35785018	0.08311758	0.16689776	0.2046761	0.03911987	0.70084184	0.08406289	0.12027887	0.27111325	0.10606384
231.1	0.06165012	0.1851763	0.35786017	0.08311925	0.16690961	0.20468048	0.03912406	0.70084423	0.08405831	0.12028051	0.27112652	0.10606882
231.2	0.06165088	0.18516887	0.35787016	0.08312091	0.16692146	0.20468486	0.03912825	0.70084663	0.08405374	0.12028214	0.27113978	0.10607379
231.3	0.06165164	0.18516145	0.35788014	0.08312258	0.1669333	0.20468924	0.03913244	0.70084901	0.08404916	0.12028377	0.27115303	0.10607876
231.4	0.06165239	0.18515403	0.35789011	0.08312424	0.16694513	0.20469362	0.03913663	0.70085139	0.0840446	0.12028539	0.27116627	0.10608372
231.5	0.06165315	0.18514662	0.35790008	0.08312591	0.16695696	0.20469799	0.03914082	0.70085377	0.08404003	0.12028701	0.2711795	0.10608868
231.6	0.06165391	0.18513921	0.35791005	0.08312757	0.16696878	0.20470236	0.03914501	0.70085614	0.08403548	0.12028863	0.27119271	0.10609363
231.7	0.06165467	0.18513181	0.35792	0.08312923	0.16698059	0.20470673	0.0391492	0.7008585	0.08403092	0.12029025	0.27120592	0.10609858
231.8	0.06165542	0.18512441	0.35792996	0.0831309	0.1669924	0.2047111	0.03915339	0.70086086	0.08402637	0.12029186	0.27121911	0.10610352
231.9	0.06165618	0.18511702	0.3579399	0.08313256	0.1670042	0.20471547	0.03915759	0.70086321	0.08402182	0.12029346	0.27123229	0.10610846
232	0.06165693	0.18510964	0.35794985	0.08313423	0.167016	0.20471983	0.03916178	0.70086556	0.08401727	0.12029507	0.27124546	0.10611339
232.1	0.06165769	0.18510226	0.35795978	0.08313589	0.16702778	0.2047242	0.03916598	0.70086791	0.08401273	0.12029667	0.27125862	0.10611832
232.2	0.06165845	0.18509488	0.35796971	0.08313756	0.16703957	0.20472856	0.03917017	0.70087025	0.0840082	0.12029826	0.27127176	0.10612325
232.3	0.0616592	0.18508751	0.35797964	0.08313922	0.16705134	0.20473292	0.03917437	0.70087258	0.08400366	0.12029986	0.2712849	0.10612817
232.4	0.06165996	0.18508014	0.35798956	0.08314089	0.16706312	0.20473728	0.03917856	0.70087491	0.08399913	0.12030145	0.27129802	0.10613309
232.5	0.06166071	0.18507279	0.35799947	0.08314255	0.16707488	0.20474164	0.03918276	0.70087723	0.08399461	0.12030303	0.27131114	0.106138
232.6	0.06166146	0.18506543	0.35800938	0.08314421	0.16708664	0.20474599	0.03918696	0.70087955	0.08399008	0.12030461	0.27132424	0.10614291
232.7	0.06166222	0.18505808	0.35801928	0.08314588	0.16709839	0.20475034	0.03919116	0.70088186	0.08398557	0.12030619	0.27133733	0.10614781
232.8	0.06166297	0.18505074	0.35802918	0.08314754	0.16711014	0.20475469	0.03919535	0.70088417	0.08398105	0.12030777	0.27135041	0.10615271
232.9	0.06166372	0.1850434	0.35803907	0.08314921	0.16712188	0.20475904	0.03919955	0.70088648	0.08397654	0.12030934	0.27136348	0.1061576
233	0.06166448	0.18503607	0.35804895	0.08315087	0.16713361	0.20476339	0.03920375	0.70088877	0.08397203	0.12031091	0.27137653	0.10616249
233.1	0.06166523	0.18502874	0.35805883	0.08315254	0.16714534	0.20476774	0.03920795	0.70089107	0.08396753	0.12031247	0.27138958	0.10616737
233.2	0.06166598	0.18502141	0.35806871	0.0831542	0.16715706	0.20477208	0.03921216	0.70089336	0.08396303	0.12031404	0.27140261	0.10617225
233.3	0.06166673	0.1850141	0.35807857	0.08315586	0.16716877	0.20477642	0.03921636	0.70089564	0.08395853	0.12031559	0.27141564	0.10617713
233.4	0.06166748	0.18500678	0.35808844	0.08315753	0.16718048	0.20478077	0.03922056	0.70089792	0.08395404	0.12031715	0.27142865	0.106182
233.5	0.06166824	0.18499948	0.35809829	0.08315919	0.16719218	0.2047851	0.03922476	0.70090019	0.08394955	0.1203187	0.27144165	0.10618686
233.6	0.06166899	0.18499217	0.35810815	0.08316086	0.16720388	0.20478944	0.03922897	0.70090246	0.08394506	0.12032025	0.27145464	0.10619173
233.7	0.06166974	0.18498488	0.35811799	0.08316252	0.16721557	0.20479378	0.03923317	0.70090472	0.08394058	0.12032179	0.27146762	0.10619658
233.8	0.06167049	0.18497759	0.35812783	0.08316418	0.16722726	0.20479811	0.03923737	0.70090698	0.0839361	0.12032333	0.27148059	0.10620144
233.9	0.06167124	0.1849703	0.35813767	0.08316585	0.16723893	0.20480244	0.03924158	0.70090923	0.08393163	0.12032487	0.27149354	0.10620628
234	0.06167199	0.18496302	0.3581475	0.08316751	0.16725061	0.20480677	0.03924578	0.70091148	0.08392715	0.12032641	0.27150649	0.10621113
234.1	0.06167274	0.18495574	0.35815732	0.08316917	0.16726227	0.2048111	0.03924999	0.70091372	0.08392269	0.12032794	0.27151942	0.10621597
234.2	0.06167349	0.18494847	0.35816714	0.08317084	0.16727393	0.20481543	0.0392542	0.70091596	0.08391822	0.12032946	0.27153235	0.1062208
234.3	0.06167423	0.18494121	0.35817695	0.0831725	0.16728558	0.20481975	0.0392584	0.70091819	0.08391376	0.12033099	0.27154526	0.10622563
234.4	0.06167498	0.18493395	0.35818676	0.08317416	0.16729723	0.20482408	0.03926261	0.70092042	0.08390931	0.12033251	0.27155816	0.10623046
234.5	0.06167573	0.18492669	0.35819656	0.08317583	0.16730887	0.2048284	0.03926682	0.70092265	0.08390485	0.12033403	0.27157105	0.10623528
234.6	0.06167648	0.18491944	0.35820636	0.08317749	0.16732051	0.20483272	0.03927103	0.70092487	0.0839004	0.12033554	0.27158393	0.10624009
234.7	0.06167723	0.1849122	0.35821615	0.08317915	0.16733214	0.20483704	0.03927524	0.70092708	0.08389596	0.12033705	0.2715968	0.10624491
234.8	0.06167797	0.18490496	0.35822593	0.08318082	0.16734376	0.20484135	0.03927945	0.70092929	0.08389152	0.12033856	0.27160965	0.10624971
234.9	0.06167872	0.18489772	0.35823571	0.08318248	0.16735538	0.20484567	0.03928366	0.70093149	0.08388708	0.12034006	0.2716225	0.10625452
235	0.06167947	0.18489049	0.35824549	0.08318414	0.16736699	0.20484998	0.03928787	0.70093369	0.08388264	0.12034156	0.27163533	0.10625932
235.1	0.06168021	0.18488327	0.35825526	0.08318581	0.16737859	0.20485429	0.03929208	0.70093589	0.08387821	0.12034306	0.27164816	0.10626411
235.2	0.06168096	0.18487605	0.35826502	0.08318747	0.16739019	0.2048586	0.03929629	0.70093808	0.08387378	0.12034456	0.27166097	0.1062689
235.3	0.06168171	0.18486884	0.35827478	0.08318913	0.16740178	0.20486291	0.03930051	0.70094026	0.08386936	0.12034605	0.27167377	0.10627369
235.4	0.06168245	0.18486163	0.35828453	0.0831908	0.16741337	0.20486721	0.03930472	0.70094244	0.08386494	0.12034753	0.27168657	0.10627847
235.5	0.0616832	0.18485442	0.35829428	0.08319246	0.16742495	0.20487152	0.03930893	0.70094462	0.08386052	0.12034902	0.27169935	0.10628324
235.6	0.06168394	0.18484722	0.35830402	0.08319412	0.16743652	0.20487582	0.03931315	0.70094679	0.08385611	0.1203505	0.27171212	0.10628802
235.7	0.06168469	0.18484003	0.35831375	0.08319579	0.16744809	0.20488012	0.03931736	0.70094895	0.0838517	0.12035198	0.27172487	0.10629279
235.8	0.06168543	0.18483284	0.35832348	0.08319745	0.16745965	0.20488442	0.03932158	0.70095111	0.08384729	0.12035345	0.27173762	0.10629755
235.9	0.06168617	0.18482566	0.3583332	0.08319911	0.16747121	0.20488872	0.03932579	0.70095327	0.08384289	0.12035492	0.27175036	0.10630231
236	0.06168692	0.18481848	0.35834292	0.08320077	0.16748276	0.20489301	0.03933001	0.70095542	0.08383849	0.12035639	0.27176308	0.10630706
236.1	0.06168766	0.18481131	0.35835264	0.08320244	0.1674943	0.20489731	0.03933422	0.70095757	0.0838341	0.12035786	0.2717758	0.10631181
236.2	0.0616884	0.18480414	0.35836234	0.0832041	0.16750584	0.2049016	0.03933844	0.70095971	0.0838297	0.12035932	0.2717885	0.10631656
236.3	0.06168915	0.18479698	0.35837205	0.08320576	0.16751737	0.20490589	0.03934266	0.70096184	0.08382532	0.12036078	0.2718012	0.1063213
236.4	0.06168989	0.18478982	0.35838174	0.08320742	0.1675289	0.20491018	0.03934688	0.70096398	0.08382093	0.12036223	0.27181388	0.10632604
236.5	0.06169063	0.18478267	0.35839143	0.08320908	0.16754042	0.20491446	0.03935109	0.7009661	0.08381655	0.12036368	0.27182655	0.10633077
236.6	0.06169137	0.18477552	0.35840112	0.08321075	0.16755193	0.20491875	0.03935531	0.70096823	0.08381217	0.12036513	0.27183921	0.1063355
236.7	0.06169212	0.18476838	0.3584108	0.08321241	0.16756344	0.20492303	0.03935953	0.70097035	0.0838078	0.12036658	0.27185186	0.10634023
236.8	0.06169286	0.18476124	0.35842047	0.08321407	0.16757494	0.20492731	0.03936375	0.70097246	0.08380343	0.12036802	0.2718645	0.10634495
236.9	0.0616936	0.18475411	0.35843014	0.08321573	0.16758643	0.20493159	0.03936797	0.70097457	0.08379906	0.12036946	0.27187713	0.10634966
237	0.06169434	0.18474699	0.35843981	0.08321739	0.16759792	0.20493587	0.03937219	0.70097667	0.0837947	0.12037089	0.27188975	0.10635437
237.1	0.06169508	0.18473986	0.35844946	0.08321906	0.16760941	0.20494015	0.03937642	0.70097877	0.08379034	0.12037233	0.27190236	0.10635908
237.2	0.06169582	0.18473275	0.35845912	0.08322072	0.16762088	0.20494442	0.03938064	0.70098087	0.08378598	0.12037376	0.27191495	0.10636378
237.3	0.06169656	0.18472564	0.35846876	0.08322238	0.16763235	0.2049487	0.03938486	0.70098296	0.08378163	0.12037518	0.27192754	0.10636848
237.4	0.0616973	0.18471853	0.3584784	0.08322404	0.16764382	0.20495297	0.03938908	0.70098504	0.08377728	0.1203766	0.27194011	0.10637318
237.5	0.06169804	0.18471143	0.35848804	0.0832257	0.16765528	0.20495724	0.03939331	0.70098713	0.08377294	0.12037802	0.27195268	0.10637787
237.6	0.06169878	0.18470433	0.35849767	0.08322736	0.16766673	0.20496151	0.03939753	0.7009892	0.08376859	0.12037944	0.27196523	0.10638255
237.7	0.06169952	0.18469724	0.3585073	0.08322902	0.16767818	0.20496577	0.03940175	0.70099127	0.08376425	0.12038085	0.27197778	0.10638724
237.8	0.06170026	0.18469015	0.35851692	0.08323069	0.16768962	0.20497004	0.03940598	0.70099334	0.08375992	0.12038226	0.27199031	0.10639191
237.9	0.06170099	0.18468307	0.35852653	0.08323235	0.16770106	0.2049743	0.0394102	0.7009954	0.08375559	0.12038367	0.27200283	0.10639659
238	0.06170173	0.184676	0.35853614	0.08323401	0.16771248	0.20497856	0.03941443	0.70099746	0.08375126	0.12038507	0.27201534	0.10640126
238.1	0.06170247	0.18466892	0.35854574	0.08323567	0.16772391	0.20498282	0.03941866	0.70099952	0.08374694	0.12038647	0.27202784	0.10640592
238.2	0.06170321	0.18466186	0.35855534	0.08323733	0.16773533	0.20498708	0.03942288	0.70100157	0.08374262	0.12038787	0.27204033	0.10641058
238.3	0.06170394	0.1846548	0.35856493	0.08323899	0.16774674	0.20499134	0.03942711	0.70100361	0.0837383	0.12038927	0.27205281	0.10641524
238.4	0.06170468	0.18464774	0.35857452	0.08324065	0.16775814	0.20499559	0.03943134	0.70100565	0.08373398	0.12039066	0.27206528	0.10641989
238.5	0.06170542	0.18464069	0.3585841	0.08324231	0.16776954	0.20499984	0.03943556	0.70100769	0.08372967	0.12039204	0.27207774	0.10642454
238.6	0.06170615	0.18463364	0.35859368	0.08324397	0.16778093	0.20500409	0.03943979	0.70100972	0.08372537	0.12039343	0.27209018	0.10642918
238.7	0.06170689	0.1846266	0.35860325	0.08324563	0.16779232	0.20500834	0.03944402	0.70101175	0.08372106	0.12039481	0.27210262	0.10643382
238.8	0.06170762	0.18461957	0.35861281	0.08324729	0.1678037	0.20501259	0.03944825	0.70101377	0.08371676	0.12039619	0.27211505	0.10643846
238.9	0.06170836	0.18461253	0.35862237	0.08324895	0.16781508	0.20501684	0.03945248	0.70101579	0.08371247	0.12039756	0.27212746	0.10644309
239	0.0617091	0.18460551	0.35863192	0.08325061	0.16782645	0.20502108	0.03945671	0.7010178	0.08370817	0.12039894	0.27213987	0.10644771
239.1	0.06170983	0.18459849	0.35864147	0.08325227	0.16783781	0.20502532	0.03946094	0.70101981	0.08370389	0.12040031	0.27215226	0.10645234
239.2	0.06171057	0.18459147	0.35865102	0.08325393	0.16784917	0.20502956	0.03946517	0.70102181	0.0836996	0.12040167	0.27216465	0.10645696
239.3	0.0617113	0.18458446	0.35866056	0.08325559	0.1678605	0.2050338	0.0394694	0.70102381	0.08369532	0.12040304	0.27217702	0.10646157
239.4	0.06171203	0.18457745	0.35867009	0.08325725	0.16787187	0.20503804	0.03947363	0.7010258	0.08369104	0.1204044	0.27218938	0.10646618
239.5	0.06171277	0.18457045	0.35867961	0.08325891	0.16788321	0.20504228	0.03947787	0.7010278	0.08368676	0.12040575	0.27220174	0.10647079
239.6	0.0617135	0.18456346	0.35868914	0.08326057	0.16789454	0.20504651	0.0394821	0.70102979	0.08368249	0.12040711	0.27221408	0.10647539
239.7	0.06171423	0.18455646	0.35869865	0.08326223	0.16790587	0.20505074	0.03948633	0.70103177	0.08367822	0.12040846	0.27222641	0.10647999
239.8	0.06171497	0.18454948	0.35870816	0.08326388	0.16791719	0.20505497	0.03949056	0.70103375	0.08367396	0.1204098	0.27223873	0.10648458
239.9	0.0617157	0.1845425	0.35871767	0.08326554	0.16792851	0.2050592	0.0394948	0.70103572	0.0836697	0.12041115	0.27225104	0.10648917
240	0.06171643	0.18453552	0.35872717	0.0832672	0.16793982	0.20506343	0.03949903	0.70103769	0.08366544	0.12041249	0.27226334	0.10649375

209.1	0.06147501	0.18694957	0.35552186	0.0827547	0.16414243	0.20367084	0.03822306	0.70017	0.08516037	0.11981751	0.26790695	0.10484777
209.2	0.06147585	0.18694084	0.35553314	0.08275634	0.16415578	0.20367565	0.03822704	0.70017383	0.08515491	0.11982016	0.26792307	0.10485396
209.3	0.06147668	0.18693212	0.35554442	0.08275798	0.16416911	0.20368046	0.03823101	0.70017765	0.08514945	0.1198228	0.26793917	0.10486014
209.4	0.06147752	0.1869234	0.3555557	0.08275963	0.16418245	0.20368526	0.03823499	0.70018146	0.08514399	0.11982544	0.26795526	0.10486631
209.5	0.06147835	0.1869147	0.35556697	0.08276127	0.16419577	0.20369007	0.03823897	0.70018527	0.08513854	0.11982807	0.26797133	0.10487248
209.6	0.06147919	0.18690599	0.35557823	0.08276291	0.16420908	0.20369487	0.03824295	0.70018907	0.08513309	0.11983069	0.26798739	0.10487864
209.7	0.06148002	0.1868973	0.35558949	0.08276455	0.16422239	0.20369967	0.03824693	0.70019286	0.08512765	0.11983332	0.26800343	0.1048848
209.8	0.06148086	0.18688861	0.35560074	0.0827662	0.16423569	0.20370446	0.03825091	0.70019664	0.08512221	0.11983593	0.26801946	0.10489095
209.9	0.06148169	0.18687993	0.35561198	0.08276784	0.16424899	0.20370926	0.0382549	0.70020041	0.08511678	0.11983854	0.26803548	0.10489709

224.1	0.06159622	0.18570984	0.35714696	0.0830028	0.16606459	0.20436938	0.03883253	0.7006632	0.08438794	0.12015672	0.27016917	0.10570875
224.2	0.061597	0.18570203	0.35715735	0.08300446	0.16607689	0.20437389	0.03883667	0.70066598	0.0843831	0.12015863	0.27018326	0.10571406
224.3	0.06159778	0.18569422	0.35716773	0.08300612	0.16608918	0.2043784	0.03884081	0.70066876	0.08437826	0.12016053	0.27019734	0.10571938
224.4	0.06159856	0.18568642	0.3571781	0.08300778	0.16610146	0.2043829	0.03884495	0.70067154	0.08437342	0.12016243	0.27021141	0.10572468
224.5	0.06159934	0.18567862	0.35718847	0.08300944	0.16611373	0.20438741	0.03884909	0.7006743	0.08436859	0.12016432	0.27022546	0.10572998
224.6	0.06160012	0.18567083	0.35719884	0.08301111	0.166126	0.20439191	0.03885323	0.70067707	0.08436377	0.12016621	0.27023951	0.10573528
224.7	0.0616009	0.18566305	0.3572092	0.08301277	0.16613827	0.2043964	0.03885737	0.70067982	0.08435894	0.1201681	0.27025354	0.10574057
224.8	0.06160168	0.18565527	0.35721955	0.08301443	0.16615052	0.2044009	0.03886152	0.70068257	0.08435413	0.12016998	0.27026755	0.10574586
224.9	0.06160246	0.18564749	0.35722989	0.08301609	0.16616277	0.2044054	0.03886566	0.70068531	0.08434931	0.12017186	0.27028156	0.10575114

Table S11: eFAST analysis data for [G3P] with [G](0)=0.02 mM: eFAST sensitivity indices

Sensitivity analysis data from eFAST for [G3P] with [G](0)=0.02 mM (only the parameters to which [G3P]'s variability is sensitive to appear)

Time discretization	lambda	K_{[G]}	q	K_{[G3P]}	K_{[PYR]}	alpha	rho	K_{[ACoA]}	phi	V_{[CIT]}
0	0	0	0	0	0	0	0	0	0	0
0.1	0.06178358	0.06836141	0.2510534	0.8982346	0.06298496	0.05999327	0.05673605	0.05985366	0.06761566	0.06174887
0.2	0.06097344	0.06680862	0.24846811	0.89821957	0.06193371	0.05641903	0.0547048	0.05859645	0.0655944	0.06055409
0.3	0.0600853	0.06514535	0.24572565	0.89807398	0.06080251	0.05299811	0.05270308	0.05723308	0.06341866	0.05927892
0.4	0.05914094	0.06342298	0.24292828	0.89777999	0.05963877	0.04995162	0.050816	0.05581734	0.06117224	0.05796763
0.5	0.05816487	0.06168847	0.24013211	0.89733094	0.05847616	0.04739517	0.04906582	0.05438677	0.05891209	0.05665073
0.6	0.05717348	0.05997405	0.23736582	0.89672627	0.0573345	0.04537572	0.04745126	0.05295966	0.05667323	0.05534775
0.7	0.05617645	0.05830325	0.23465184	0.8959675	0.05622938	0.04391199	0.04596024	0.05154604	0.05448135	0.05406986
0.8	0.05518101	0.05669284	0.23200297	0.89505539	0.05517454	0.04300909	0.04457939	0.05015322	0.0523539	0.05282372
0.9	0.05419237	0.05515772	0.22942916	0.89399303	0.05418103	0.04266647	0.04329843	0.0487871	0.05030301	0.05161779
1	0.05321552	0.05371077	0.22693919	0.89278498	0.05325613	0.04287746	0.04210818	0.0474525	0.04833817	0.05045639
1.1	0.05225426	0.0523614	0.22453795	0.89143579	0.05240515	0.04363075	0.04100038	0.04615264	0.04646529	0.04934314
1.2	0.05131112	0.05111743	0.22222825	0.88995003	0.05163143	0.04491134	0.03996746	0.04489062	0.04468724	0.0482817
1.3	0.05038891	0.04998568	0.22001233	0.88833269	0.05093596	0.04670117	0.03900309	0.04366892	0.04300573	0.04727386
1.4	0.04949015	0.04897174	0.21789163	0.88658847	0.05031856	0.04898058	0.03810135	0.04248877	0.04142067	0.04632108
1.5	0.04861647	0.0480815	0.21586605	0.884722	0.04977766	0.05172775	0.03725673	0.04135141	0.03993038	0.0454239
1.6	0.0477692	0.04732089	0.21393476	0.88273837	0.04931053	0.05492004	0.03646474	0.04025763	0.03853282	0.04458247
1.7	0.04694986	0.0466954	0.21209737	0.88064245	0.04891396	0.05853394	0.03572126	0.03920728	0.037225	0.04379658
1.8	0.04615893	0.04621169	0.21035312	0.87843902	0.04858403	0.06254542	0.03502244	0.03820026	0.03600332	0.0430658
1.9	0.04539674	0.04587756	0.20870063	0.87613303	0.0483164	0.06693109	0.03436492	0.03723634	0.03486416	0.04238983
2	0.04466412	0.04570167	0.20713876	0.87372958	0.04810654	0.07166716	0.03374565	0.03631471	0.0338035	0.04176784
2.1	0.04396122	0.04569397	0.20566626	0.87123378	0.0479497	0.07672972	0.03316169	0.03543446	0.03281736	0.04119887
2.2	0.04328807	0.04586622	0.20428178	0.86865064	0.04784133	0.0820957	0.03261022	0.03459476	0.03190177	0.04068228
2.3	0.04264497	0.04623166	0.2029842	0.86598544	0.04777696	0.08774291	0.03208866	0.03379466	0.03105258	0.04021733
2.4	0.04203176	0.04680526	0.20177235	0.86324371	0.04775224	0.09364962	0.03159474	0.03303304	0.03026584	0.03980315
2.5	0.0414483	0.04760403	0.20064506	0.86043094	0.04776303	0.0997947	0.03112641	0.03230861	0.02953773	0.03943907
2.6	0.04089457	0.04864657	0.19960133	0.85755254	0.04780541	0.10615811	0.03068171	0.03162001	0.02886455	0.03912445
2.7	0.0403703	0.04995276	0.19864045	0.85461411	0.04787568	0.11272096	0.03025886	0.03096599	0.02824279	0.03885852
2.8	0.03987525	0.05154423	0.19776191	0.85162135	0.04797031	0.11946511	0.02985632	0.03034533	0.02766907	0.03864071
2.9	0.03940935	0.05344375	0.1969652	0.84858003	0.04808604	0.1263732	0.02947268	0.02975668	0.02714018	0.03847055
3	0.0389724	0.05567345	0.19625004	0.84549588	0.04821989	0.13342904	0.02910656	0.02919862	0.0266531	0.03834744
3.1	0.03856416	0.05825564	0.19561644	0.8423746	0.04836903	0.14061761	0.02875675	0.02866993	0.02620502	0.03827091
3.2	0.03818445	0.06121285	0.19506472	0.83922206	0.04853085	0.14792459	0.02842215	0.02816948	0.02579328	0.0382407
3.3	0.03783307	0.06456354	0.19459536	0.83604424	0.04870304	0.15533636	0.02810181	0.02769596	0.02541536	0.03825648
3.4	0.03750984	0.0683231	0.19420911	0.83284717	0.04888346	0.16284026	0.02779479	0.02724813	0.02506898	0.03831794
3.5	0.03721461	0.07250586	0.19390697	0.8296368	0.04907012	0.17042476	0.02750019	0.02682491	0.02475199	0.038425
3.6	0.03694734	0.07711946	0.19369017	0.82641903	0.04926128	0.17807923	0.02721724	0.02642522	0.02446237	0.03857767
3.7	0.03670785	0.08216532	0.19356028	0.82319995	0.04945538	0.18579351	0.02694525	0.0260479	0.02419825	0.03877595
3.8	0.03649597	0.08764169	0.19351898	0.81998572	0.04965098	0.19355819	0.0266836	0.0256919	0.02395793	0.03901987
3.9	0.03631162	0.09354036	0.19356814	0.81678264	0.04984685	0.20136478	0.02643166	0.02535628	0.02373981	0.03930955
4	0.03615479	0.09984679	0.19370982	0.81359686	0.0500419	0.20920569	0.02618888	0.02504013	0.0235424	0.03964519
4.1	0.03602542	0.10654254	0.19394614	0.81043437	0.05023519	0.21707404	0.02595479	0.0247425	0.02336433	0.04002704
4.2	0.03592343	0.11360512	0.19427952	0.80730126	0.0504259	0.22496348	0.02572899	0.02446248	0.02320434	0.04045548
4.3	0.03584879	0.12100806	0.1947125	0.80420376	0.05061332	0.23286827	0.02551111	0.02419925	0.02306128	0.04093097
4.4	0.03580153	0.12872288	0.19524747	0.80114805	0.05079685	0.24078343	0.02530087	0.02395206	0.02293409	0.041454
4.5	0.03578168	0.1367197	0.19588689	0.79814011	0.05097598	0.24870452	0.025098	0.02372016	0.02282179	0.04202507
4.6	0.03578928	0.14496699	0.19663318	0.7951857	0.05115028	0.25662773	0.02490225	0.0235028	0.02272347	0.04264466
4.7	0.03582439	0.15343312	0.19748837	0.7922906	0.05131942	0.26454982	0.02471337	0.02329925	0.0226383	0.04331335
4.8	0.03588714	0.16208634	0.19845459	0.78946057	0.05148312	0.27246805	0.02453115	0.02310886	0.02256551	0.04403177
4.9	0.03597768	0.17089512	0.19953432	0.78670113	0.05164119	0.28038014	0.02435545	0.02293105	0.0225044	0.04480065
5	0.03609617	0.17982821	0.20072974	0.78401755	0.05179347	0.28828428	0.02418616	0.02276523	0.02245434	0.04562079
5.1	0.03624279	0.18885381	0.20204245	0.78141474	0.05193984	0.29617926	0.02402313	0.02261083	0.02241477	0.04649284
5.2	0.0364177	0.19793999	0.20347414	0.77889715	0.05208022	0.3040644	0.02386624	0.02246729	0.02238517	0.04741742
5.3	0.03662117	0.2070554	0.20502679	0.77646887	0.0522146	0.31193948	0.02371541	0.02233411	0.02236506	0.04839522
5.4	0.03685347	0.21616915	0.20670192	0.7741335	0.05234302	0.31980469	0.02357056	0.02221086	0.022354	0.04942705
5.5	0.03711487	0.22525077	0.20850094	0.77189386	0.05246551	0.32766058	0.02343163	0.0220971	0.02235157	0.05051374
5.6	0.03740566	0.23427093	0.21042561	0.76975196	0.05258213	0.33550827	0.02329857	0.02199243	0.02235743	0.05165616
5.7	0.03772613	0.24320211	0.21247776	0.76770912	0.05269299	0.3433495	0.02317133	0.02189643	0.02237125	0.05285509
5.8	0.03807666	0.25201867	0.21465893	0.76576582	0.05279823	0.35118652	0.02304988	0.02180875	0.02239276	0.05411121
5.9	0.03845761	0.26069709	0.21697074	0.76392175	0.05289798	0.35902218	0.0229342	0.02172904	0.02242168	0.05542536
6	0.03886938	0.26921628	0.21941486	0.7621758	0.05299239	0.36685989	0.02282429	0.02165701	0.02245779	0.05679851
6.1	0.03931236	0.27755768	0.2219926	0.76052587	0.0530816	0.37470347	0.02272014	0.02159236	0.02250086	0.05823163
6.2	0.03978691	0.28570528	0.22470427	0.75896892	0.05316582	0.38255737	0.02262174	0.02153481	0.0225507	0.05972576
6.3	0.04029326	0.29364583	0.22754963	0.75750145	0.05324527	0.39042669	0.02252911	0.02148409	0.02260716	0.06128201
6.4	0.04083168	0.30136855	0.2305277	0.75611977	0.05332018	0.39831698	0.02244227	0.02143996	0.02267008	0.06290162
6.5	0.0414025	0.30886475	0.23363604	0.75481965	0.0533908	0.40623476	0.02236123	0.02140221	0.02273933	0.06458573
6.6	0.04200612	0.31612805	0.23687073	0.75359658	0.05345742	0.41418751	0.02228602	0.02137066	0.02281477	0.06633524
6.7	0.04264288	0.32315408	0.24022591	0.7524461	0.0535203	0.42218299	0.02221669	0.02134514	0.02289631	0.06815112
6.8	0.04331313	0.32993997	0.24369379	0.75136333	0.05357971	0.43022917	0.02215326	0.02132546	0.02298385	0.07003458
6.9	0.04401701	0.33648427	0.24726479	0.75034333	0.05363592	0.43833421	0.02209577	0.02131147	0.02307732	0.07198684
7	0.04475446	0.34278706	0.25092772	0.74938156	0.05368922	0.44650615	0.02204425	0.02130303	0.02317663	0.07400833
7.1	0.04552552	0.34884981	0.25466984	0.7484736	0.05373992	0.45475307	0.02199873	0.02129999	0.02328172	0.07609905
7.2	0.04633024	0.35467509	0.2584772	0.74761518	0.0537883	0.46308258	0.02195925	0.02130224	0.02339254	0.07825903
7.3	0.04716863	0.3602661	0.26233506	0.74680222	0.05383466	0.47150155	0.02192585	0.02130967	0.02350902	0.08048797
7.4	0.04804066	0.36562685	0.26622807	0.74603066	0.05387931	0.48001593	0.02189856	0.02132217	0.02363113	0.08278474
7.5	0.04894628	0.37076205	0.27014072	0.74529644	0.05392253	0.48862951	0.02187744	0.02133965	0.02375882	0.08514724
7.6	0.04988511	0.37567687	0.2740575	0.74459577	0.05396453	0.49734355	0.02186254	0.02136204	0.02389205	0.08757294
7.7	0.05085663	0.38037696	0.27796286	0.74392515	0.05400557	0.50615691	0.02185389	0.02138926	0.0240308	0.09005847
7.8	0.05186042	0.3848683	0.28184145	0.74328115	0.05404591	0.5150658	0.02185154	0.02142125	0.02417502	0.09259923
7.9	0.05289603	0.38915714	0.28567812	0.7426605	0.05408579	0.52406353	0.02185554	0.02145795	0.0243247	0.09518999
8	0.05396292	0.39324999	0.28945778	0.74206013	0.05412542	0.53314034	0.02186591	0.02149931	0.02447981	0.09782495
8.1	0.05506036	0.39715354	0.29316554	0.74147688	0.05416494	0.54228408	0.02188267	0.02154528	0.02464034	0.10049762
8.2	0.05618752	0.40087449	0.29678681	0.74090768	0.05420446	0.55147957	0.02190588	0.02159582	0.02480627	0.10320074
8.3	0.05734349	0.40441951	0.30030747	0.74034973	0.05424407	0.56070784	0.02193555	0.02165089	0.02497758	0.10592662
8.4	0.05852724	0.40779527	0.30371417	0.7398004	0.05428386	0.5699473	0.02197174	0.02171045	0.02515425	0.10866763
8.5	0.05973775	0.41100841	0.30699434	0.73925721	0.0543239	0.5791743	0.02201448	0.02177447	0.02533628	0.11141633
8.6	0.06097393	0.41406557	0.31013654	0.73871767	0.05436422	0.58836332	0.02206381	0.02184293	0.02552364	0.11416503
8.7	0.06223463	0.41697343	0.31313077	0.73817921	0.0544049	0.59748784	0.02211977	0.02191583	0.02571632	0.11690583
8.8	0.06351864	0.41973855	0.31596822	0.73763951	0.05444597	0.60652087	0.02218238	0.02199315	0.0259143	0.11963147
8.9	0.06482461	0.42236738	0.31864165	0.73709647	0.05448741	0.61543539	0.02225168	0.02207489	0.02611758	0.12233505
9	0.06615129	0.42486612	0.32114559	0.73654821	0.05452922	0.62420505	0.02232769	0.02216103	0.02632612	0.12500975
9.1	0.06749748	0.42724071	0.3234762	0.7359929	0.0545714	0.63280503	0.02241044	0.02225158	0.02653993	0.12764951
9.2	0.06886182	0.42949711	0.32563133	0.73542861	0.05461402	0.64121241	0.02249995	0.02234652	0.02675897	0.13024886
9.3	0.07024292	0.43164122	0.32761045	0.73485352	0.05465715	0.64940642	0.02259624	0.02244587	0.02698325	0.13280273
9.4	0.07163935	0.43367875	0.32941444	0.73426603	0.05470083	0.65736882	0.02269935	0.02254962	0.02721272	0.13530674
9.5	0.07304967	0.43561503	0.33104542	0.73366468	0.05474512	0.66508397	0.02280931	0.02265778	0.02744738	0.13775706
9.6	0.07447238	0.43745517	0.33250667	0.73304794	0.05479009	0.67253905	0.02292613	0.02277035	0.02768723	0.14015007
9.7	0.07590625	0.43920403	0.33380258	0.73241434	0.05483588	0.67972416	0.02304985	0.02288735	0.02793224	0.14248268
9.8	0.07735002	0.44086623	0.33493841	0.73176256	0.05488265	0.68663195	0.02318048	0.02300878	0.02818241	0.14475264
9.9	0.07880225	0.44244622	0.33591999	0.73109145	0.05493061	0.69325758	0.02331804	0.02313466	0.02843772	0.14695812
10	0.08026153	0.44394822	0.33675363	0.7304	0.05498005	0.69959876	0.02346255	0.02326501	0.02869815	0.14909749
10.1	0.08172645	0.44537618	0.33744597	0.72968735	0.05503127	0.70565538	0.02361403	0.02339985	0.02896368	0.15116962
10.2	0.08319561	0.44673382	0.33800391	0.72895273	0.0550846	0.71142906	0.02377249	0.02353919	0.02923429	0.1531739
10.3	0.08466759	0.44802464	0.33843462	0.72819561	0.05514037	0.7169232	0.02393795	0.02368305	0.02950995	0.15510991
10.4	0.08614113	0.44925191	0.33874517	0.7274157	0.05519897	0.72214293	0.02411043	0.02383145	0.02979066	0.15697731
10.5	0.08761519	0.45041867	0.33894244	0.72661262	0.0552608	0.72709457	0.02428995	0.0239844	0.03007638	0.15877606
10.6	0.08908876	0.4515277	0.33903326	0.72578619	0.05532631	0.7317855	0.02447651	0.02414192	0.0303671	0.16050655
10.7	0.09056074	0.4525816	0.33902424	0.72493652	0.05539599	0.73622416	0.02467014	0.02430403	0.03066279	0.16216934
10.8	0.09203003	0.45358282	0.33892165	0.72406383	0.05547032	0.74041958	0.02487085	0.02447076	0.03096343	0.16376505
10.9	0.09349553	0.4545336	0.33873153	0.72316846	0.05554982	0.7443812	0.02507864	0.02464212	0.03126901	0.16529445
11	0.09495619	0.45543603	0.3384597	0.72225094	0.05563503	0.74811887	0.02529352	0.02481815	0.03157949	0.16675857
11.1	0.09641098	0.45629197	0.33811167	0.72131187	0.05572652	0.75164255	0.02551551	0.02499887	0.03189486	0.1681585
11.2	0.09785903	0.45710311	0.33769253	0.72035186	0.05582487	0.75496218	0.02574462	0.02518429	0.03221509	0.16949539
11.3	0.09929954	0.45787106	0.33720716	0.71937157	0.05593065	0.75808753	0.02598087	0.02537444	0.03254015	0.17077047
11.4	0.10073183	0.45859728	0.33666012	0.71837174	0.05604448	0.76102814	0.02622426	0.02556936	0.03287001	0.17198506
11.5	0.10215536	0.45928311	0.3360557	0.71735319	0.05616695	0.76379316	0.02647481	0.02576906	0.03320465	0.17314074
11.6	0.10356948	0.45992977	0.33539796	0.71631674	0.05629867	0.7663914	0.02673254	0.02597358	0.03354404	0.17423924
11.7	0.10497358	0.46053836	0.33469072	0.7152632	0.05644024	0.76883137	0.02699745	0.02618295	0.03388816	0.17528233
11.8	0.10636708	0.46110988	0.33393753	0.71419331	0.05659227	0.77112113	0.02726955	0.02639719	0.03423697	0.1762717
11.9	0.10774946	0.46164529	0.33314172	0.71310774	0.05675537	0.77326836	0.02754886	0.02661634	0.03459045	0.17720909
12	0.10912021	0.46214551	0.33230651	0.71200709	0.05693016	0.77528038	0.02783537	0.02684043	0.03494858	0.17809631
12.1	0.11047889	0.46261136	0.33143491	0.71089198	0.05711727	0.77716408	0.02812909	0.02706948	0.03531133	0.17893533
12.2	0.1118252	0.46304358	0.33052978	0.70976295	0.05731732	0.77892597	0.02843003	0.02730353	0.03567867	0.17972834
12.3	0.11315902	0.46344286	0.32959382	0.70862049	0.05753092	0.78057229	0.02873819	0.02754261	0.03605058	0.18047749
12.4	0.1144802	0.46380987	0.32862955	0.70746496	0.0577587	0.78210895	0.02905356	0.02778675	0.03642704	0.18118484
12.5	0.11578858	0.46414529	0.32763937	0.70629666	0.0580013	0.78354152	0.02937616	0.02803597	0.03680801	0.18185249
12.6	0.11708401	0.46444977	0.32662555	0.70511583	0.05825934	0.78487528	0.02970601	0.0282903	0.03719346	0.18248263
12.7	0.11836634	0.4647239	0.32559026	0.70392266	0.0585334	0.7861153	0.0300431	0.02854979	0.03758338	0.18307749
12.8	0.11963543	0.46496823	0.32453553	0.70271722	0.05882407	0.78726642	0.03038745	0.02881445	0.03797773	0.18363935
12.9	0.12089124	0.46518328	0.32346332	0.70149957	0.0591319	0.78833325	0.03073909	0.02908434	0.03837648	0.18417047
13	0.12213378	0.4653696	0.32237545	0.70026969	0.05945742	0.78932017	0.03109802	0.02935948	0.03877959	0.18467306
13.1	0.12336319	0.46552776	0.32127368	0.69902753	0.05980117	0.79023144	0.03146426	0.02963992	0.03918704	0.18514931
13.2	0.12457963	0.46565826	0.32015965	0.697773	0.06016369	0.79107114	0.03183782	0.02992568	0.0395988	0.18560134
13.3	0.12578324	0.46576164	0.31903491	0.69650592	0.06054548	0.79184324	0.03221874	0.03021681	0.04001484	0.18603121
13.4	0.12697419	0.46583842	0.31790094	0.69522608	0.06094705	0.79255163	0.03260701	0.03051333	0.04043512	0.18644084
13.5	0.12815267	0.46588914	0.31675914	0.69393325	0.0613689	0.79320012	0.03300266	0.03081529	0.04085961	0.18683209
13.6	0.1293189	0.46591431	0.31561086	0.69262713	0.06181151	0.79379247	0.03340568	0.03112272	0.04128828	0.18720663
13.7	0.13047304	0.46591445	0.31445738	0.69130739	0.06227533	0.79433238	0.03381611	0.03143566	0.04172112	0.18756602
13.8	0.1316153	0.46589012	0.3132999	0.68997375	0.06276072	0.79482356	0.03423394	0.03175415	0.04215808	0.1879117
[illegible]	[illegible]	[illegible]	[illegible]	[illegible]	[illegible]	[illegible]	[illegible]	[illegible]	[illegible]	[illegible]
[illegible]	[illegible]	[illegible]	[illegible]	[illegible]	[illegible]	[illegible]	[illegible]	[illegible]	[illegible]	[illegible]
[illegible]	[illegible]	[illegible]	[illegible]	[illegible]	[illegible]	[illegible]	[illegible]	[illegible]	[illegible]	[illegible]
[illegible]	[illegible]	[illegible]	[illegible]	[illegible]	[illegible]	[illegible]	[illegible]	[illegible]	[illegible]	[illegible]
[illegible]	[illegible]	[illegible]	[illegible]	[illegible]	[illegible]	[illegible]	[illegible]	[illegible]	[illegible]	[illegible]
[illegible]	[illegible]	[illegible]	[illegible]	[illegible]	[illegible]	[illegible]	[illegible]	[illegible]	[illegible]	[illegible]
[illegible]	[illegible]	[illegible]	[illegible]	[illegible]	[illegible]	[illegible]	[illegible]	[illegible]	[illegible]	[illegible]
[illegible]	[illegible]	[illegible]	[illegible]	[illegible]	[illegible]	[illegible]	[illegible]	[illegible]	[illegible]	[illegible]
[illegible]	[illegible]	[illegible]	[illegible]	[illegible]	[illegible]	[illegible]	[illegible]	[illegible]	[illegible]	[illegible]
[illegible]	[illegible]	[illegible]	[illegible]	[illegible]	[illegible]	[illegible]	[illegible]	[illegible]	[illegible]	[illegible]
[illegible]	[illegible]	[illegible]	[illegible]	[illegible]	[illegible]	[illegible]	[illegible]	[illegible]	[illegible]	[illegible]
15	0.14450336	0.46388981	0.29944283	0.6727971	0.07036079	0.79815774	0.03983238	0.03602801	0.04771531	0.19133539
15.1	0.14551847	0.46359794	0.29831238	0.67126393	0.07114622	0.79831039	0.04034828	0.0364235	0.04820379	0.19157686
15.2	0.14652595	0.4632894	0.29718792	0.66971525	0.07195502	0.79845408	0.04087191	0.03682531	0.04869606	0.19181286
15.3	0.14752606	0.46296473	0.2960697	0.66815128	0.07278705	0.79859031	0.04140331	0.0372335	0.04919207	0.19204349
15.4	0.14851908	0.46262446	0.29495796	0.66657232	0.07364214	0.79872036	0.04194253	0.03764814	0.0496918	0.19226886
15.5	0.14950531	0.46226913	0.29385285	0.66497873	0.0745201	0.79884533	0.04248965	0.03806933	0.05019521	0.19248908
15.6	0.15048502	0.46189924	0.29275451	0.66337094	0.07542071	0.79896609	0.04304471	0.03849717	0.05070231	0.19270421
15.7	0.15145849	0.4615153	0.29166305	0.66174942	0.07634369	0.79908341	0.04360776	0.03893174	0.05121309	0.19291433
15.8	0.15242596	0.46111781	0.29057851	0.6601147	0.07728872	0.7991979	0.04417885	0.03937317	0.05172754	0.19311952
15.9	0.15338764	0.46070727	0.28950096	0.65846736	0.07825548	0.79931003	0.04475802	0.03982157	0.05224563	0.19331989
16	0.15434375	0.46028418	0.28843042	0.65680796	0.07924362	0.79942019	0.04534532	0.04027702	0.05276734	0.19351552
16.1	0.15529454	0.459849	0.28736686	0.65513711	0.08025277	0.79952865	0.0459408	0.04073964	0.05329268	0.19370649
16.2	0.15624023	0.45940218	0.2863103	0.65345548	0.08128251	0.79963556	0.04654452	0.0412095	0.05382162	0.19389291
16.3	0.15718107	0.45894419	0.28526072	0.65176369	0.08233236	0.79974098	0.04715652	0.04168671	0.05435417	0.19407489
16.4	0.15811726	0.4584755	0.28421813	0.65006241	0.08340186	0.79984491	0.04777685	0.04217136	0.05489029	0.19425255
16.5	0.15904902	0.45799658	0.28318247	0.64835233	0.08449053	0.79994728	0.04840557	0.04266356	0.05542998	0.19442601
16.6	0.15997658	0.45750787	0.28215374	0.64663414	0.08559787	0.80004792	0.04904273	0.0431634	0.05597322	0.19459539
16.7	0.16090012	0.45700984	0.28113189	0.64490848	0.08672337	0.80014666	0.04968838	0.04367099	0.05652	0.1947609
16.8	0.16181985	0.45650293	0.2801169	0.64317599	0.08786651	0.80024327	0.05034258	0.04418645	0.0570703	0.19492274
16.9	0.16273596	0.45598761	0.27910873	0.64143729	0.08902673	0.80033751	0.05100539	0.04470986	0.0576241	0.19508107
17	0.1636487	0.45546432	0.27810735	0.63969297	0.09020345	0.80042911	0.05167687	0.04524135	0.0581814	0.19523606
17.1	0.16455837	0.45493351	0.27711274	0.63794358	0.09139608	0.80051777	0.05235708	0.04578103	0.05874217	0.19538789
17.2	0.16546527	0.45439564	0.27612489	0.63618966	0.09260403	0.80060322	0.05304608	0.04632899	0.05930642	0.19553674
17.3	0.16636969	0.45385114	0.27514381	0.63443167	0.09382672	0.80068521	0.05374398	0.04688535	0.05987417	0.19568279
17.4	0.16727192	0.45330045	0.27416945	0.63267006	0.09506354	0.80076348	0.0544509	0.04745028	0.0604454	0.19582623
17.5	0.16817225	0.45274402	0.2732018	0.63090524	0.0963139	0.80083781	0.05516693	0.04802397	0.06102011	0.19596725
17.6	0.16907101	0.45218225	0.27224083	0.62913752	0.09757718	0.80090803	0.05589218	0.0486066	0.0615983	0.19610606
17.7	0.16996851	0.45161556	0.27128652	0.62736716	0.09885275	0.80097403	0.05662683	0.04919835	0.06217995	0.19624285
17.8	0.17086511	0.45104434	0.27033885	0.62559439	0.10014003	0.80103574	0.05737098	0.04979948	0.06276506	0.19637783
17.9	0.17176117	0.45046896	0.2693978	0.62381942	0.10143839	0.80109317	0.05812473	0.05041024	0.06335364	0.19651121
18	0.17265716	0.44988979	0.26846335	0.6220424	0.10274726	0.8011464	0.0588882	0.05103081	0.06394569	0.19664319
18.1	0.17355364	0.44930717	0.26753548	0.62026346	0.10406602	0.80119556	0.05966149	0.05166139	0.0645412	0.19677398
18.2	0.17445109	0.44872139	0.26661416	0.61848267	0.10539409	0.80124088	0.06044471	0.05230215	0.06514019	0.1969038
18.3	0.17535007	0.44813277	0.26569934	0.61670007	0.10673087	0.80128264	0.06123797	0.05295329	0.06574264	0.19703294
18.4	0.17625116	0.44754159	0.264791	0.61491567	0.10807577	0.80132118	0.06204139	0.053615	0.06634856	0.19716167
18.5	0.17715498	0.44694811	0.26388908	0.61312943	0.10942817	0.80135694	0.06285509	0.05428747	0.06695795	0.19729027
18.6	0.17806221	0.44635256	0.26299355	0.6113413	0.11078747	0.80139039	0.06367918	0.0549709	0.06757081	0.19741899
18.7	0.17897353	0.44575519	0.26210437	0.60955119	0.11215309	0.80142202	0.06451378	0.0556655	0.06818715	0.1975481
18.8	0.17988968	0.44515619	0.26122149	0.607759	0.11352447	0.80145237	0.06535902	0.05637148	0.06880699	0.19767787
18.9	0.18081147	0.44455575	0.26034485	0.60596457	0.11490107	0.80148198	0.06621502	0.05708904	0.06943035	0.19780861
19	0.18173986	0.44395405	0.25947438	0.60416776	0.11628235	0.80151142	0.06708189	0.0578184	0.07005724	0.19794061
19.1	0.18267583	0.44335124	0.25861003	0.60236841	0.11766779	0.80154121	0.06795978	0.05855979	0.07068769	0.19807415
19.2	0.1836204	0.44274748	0.25775172	0.60056636	0.11905686	0.8015719	0.0688488	0.05931343	0.07132171	0.19820955
19.3	0.18457465	0.44214287	0.25689938	0.59876148	0.12044908	0.801604	0.06974911	0.06007956	0.07195933	0.19834712
19.4	0.18553967	0.44153754	0.25605292	0.59695361	0.12184396	0.80163803	0.07066095	0.06085851	0.0726006	0.19848719
19.5	0.18651667	0.44093159	0.25521228	0.59514263	0.12324102	0.80167442	0.07158463	0.06165065	0.07324556	0.19863017
19.6	0.18750688	0.44032509	0.25437737	0.5933284	0.1246398	0.8017136	0.07252041	0.06245625	0.07389421	0.19877653
19.7	0.1885116	0.43971811	0.2535481	0.59151083	0.12603985	0.80175594	0.07346852	0.06327562	0.07454659	0.19892666
19.8	0.18953218	0.43911072	0.2527244	0.58968982	0.12744074	0.80180176	0.07442916	0.06410918	0.07520271	0.19908097
19.9	0.19057005	0.43850297	0.25190617	0.5878653	0.12884205	0.80185135	0.07540255	0.06495734	0.07586261	0.19923989
20	0.19162665	0.43789487	0.25109332	0.58603719	0.13024335	0.80190496	0.07638889	0.06582045	0.07652634	0.19940384
20.1	0.1927035	0.43728649	0.25028576	0.58420548	0.13164426	0.8019628	0.07738842	0.0666989	0.07719396	0.19957329
20.2	0.19380213	0.43667784	0.24948342	0.58237017	0.13304437	0.80202505	0.07840136	0.06759308	0.07786546	0.19974871
20.3	0.19492413	0.43606898	0.24868618	0.5805313	0.13444325	0.80209186	0.07942792	0.06850334	0.07854087	0.1999306
20.4	0.19607109	0.43545993	0.24789398	0.5786889	0.13584052	0.80216331	0.08046834	0.0694301	0.07922023	0.20011947
20.5	0.19724463	0.4348507	0.2471067	0.57684306	0.13723582	0.80223945	0.08152284	0.07037375	0.07990357	0.20031585
20.6	0.1984464	0.43424131	0.24632425	0.57499386	0.1386288	0.8023203	0.08259165	0.07133468	0.08059091	0.20052031
20.7	0.19967804	0.43363179	0.24554654	0.57314144	0.14001911	0.80240583	0.083675	0.07231331	0.08128232	0.20073353
20.8	0.20094111	0.43302213	0.2447735	0.57128592	0.1414064	0.80249604	0.08477313	0.07331023	0.08197788	0.20095622
20.9	0.20223718	0.43241237	0.24400502	0.56942744	0.14279035	0.80259085	0.08588627	0.07432606	0.08267764	0.20118909
21	0.20356778	0.43180254	0.24324104	0.56756617	0.14417068	0.80269018	0.08701466	0.07536134	0.08338167	0.20143284
21.1	0.20493438	0.43119266	0.24248146	0.56570228	0.14554708	0.8027939	0.08815862	0.0764166	0.08409001	0.20168824
21.2	0.20633837	0.43058277	0.2417262	0.56383596	0.14691928	0.80290189	0.0893186	0.07749254	0.08480276	0.20195608
21.3	0.20778104	0.42997292	0.2409752	0.56196742	0.14828701	0.803014	0.09049511	0.07858991	0.08552001	0.20223719
21.4	0.20926358	0.42936314	0.24022837	0.56009686	0.14965001	0.80313007	0.09168855	0.07970932	0.08624182	0.20253244
21.5	0.21078703	0.42875348	0.23948565	0.55822449	0.15100806	0.80324991	0.09289928	0.08085145	0.08696825	0.20284283
21.6	0.2123523	0.42814397	0.23874696	0.5563505	0.1523609	0.80337332	0.09412765	0.08201698	0.08769937	0.20316944
21.7	0.21396014	0.42753466	0.23801227	0.55447511	0.15370832	0.80350012	0.09537405	0.08320661	0.08843526	0.20351335
21.8	0.21561112	0.4269256	0.2372815	0.55259855	0.15505011	0.80363013	0.09663885	0.0844211	0.08917599	0.20387571
21.9	0.21730562	0.42631683	0.2365546	0.55072102	0.15638604	0.80376315	0.09792243	0.08566118	0.08992168	0.20425771
22	0.21904377	0.42570841	0.23583154	0.54884274	0.15771589	0.803899	0.09922518	0.08692761	0.09067241	0.20466058
22.1	0.2208255	0.42510039	0.23511225	0.54696391	0.15903946	0.80403751	0.10054751	0.08822118	0.09142828	0.20508565
22.2	0.22265059	0.42449284	0.2343967	0.54508472	0.16035658	0.8041785	0.10188981	0.08954281	0.09218944	0.20553438
22.3	0.22451862	0.42388582	0.23368486	0.54320537	0.16166708	0.80432184	0.10325246	0.09089351	0.09295599	0.20600829
22.4	0.22642894	0.42327939	0.23297669	0.54132605	0.16297078	0.8044674	0.10463596	0.09227428	0.09372805	0.20650895
22.5	0.22838066	0.4226736	0.23227216	0.53944693	0.16426754	0.80461506	0.10604091	0.09368606	0.09450576	0.20703801
22.6	0.23037274	0.42206852	0.23157125	0.5375682	0.16555721	0.80476476	0.10746791	0.09512998	0.09528922	0.20759718
22.7	0.23240392	0.4214642	0.23087393	0.53569004	0.16683966	0.80491642	0.10891747	0.09660724	0.09607854	0.20818826
22.8	0.23447273	0.4208607	0.23018018	0.53381262	0.16811476	0.80507002	0.11039009	0.09811898	0.09687384	0.20881312
22.9	0.23657757	0.42025808	0.22948999	0.5319361	0.1693824	0.80522553	0.11188634	0.09966632	0.09767523	0.20947378
23	0.23871663	0.41965639	0.22880333	0.53006064	0.17064249	0.80538296	0.11340691	0.10125042	0.09848285	0.2101723
23.1	0.24088796	0.41905567	0.2281202	0.52818643	0.17189493	0.80554231	0.11495243	0.10287248	0.09929687	0.21091085
23.2	0.24308942	0.41845597	0.22744058	0.52631363	0.17313963	0.80570363	0.11652351	0.10453376	0.10011746	0.21169165
23.3	0.24531877	0.41785734	0.22676446	0.52444242	0.1743765	0.80586696	0.11812078	0.10623564	0.10094481	0.212517
23.4	0.24757365	0.41725982	0.22609183	0.52257296	0.17560548	0.80603236	0.11974482	0.10797949	0.10177904	0.2133892
23.5	0.2498516	0.41666346	0.22542268	0.52070545	0.17682651	0.80619989	0.12139625	0.10976656	0.10262033	0.21431067
23.6	0.25215011	0.41606831	0.22475701	0.51884009	0.17803954	0.80636963	0.12307568	0.11159816	0.10346886	0.21528388
23.7	0.25446661	0.41547438	0.22409481	0.51697706	0.1792445	0.80654163	0.12478377	0.11347566	0.1043248	0.21631142
23.8	0.25679847	0.41488172	0.22343608	0.51511661	0.18044135	0.80671596	0.1265213	0.11540044	0.10518832	0.21739583
23.9	0.25914301	0.41429035	0.22278083	0.51325894	0.18163006	0.80689268	0.12828909	0.11737379	0.10605963	0.21853966
24	0.26149758	0.41370028	0.22212905	0.51140429	0.18281058	0.80707185	0.13008785	0.11939682	0.10693899	0.21974553
24.1	0.26385954	0.41311154	0.22148076	0.50955293	0.1839829	0.80725351	0.13191831	0.1214706	0.10782665	0.22101609
24.2	0.26622629	0.41252416	0.22083594	0.50770512	0.18514698	0.80743771	0.13378134	0.12359614	0.10872283	0.22235395
24.3	0.26859526	0.41193815	0.22019461	0.50586114	0.18630281	0.80762449	0.1356778	0.1257744	0.10962777	0.22376159
24.4	0.27096392	0.41135354	0.21955677	0.50402128	0.18745038	0.80781388	0.13760854	0.12800611	0.11054169	0.22524141
24.5	0.27332973	0.41077035	0.21892242	0.50218584	0.1885897	0.80800591	0.13957436	0.13029186	0.11146479	0.2267958
24.6	0.27569018	0.41018859	0.21829156	0.50035516	0.18972077	0.80820057	0.14157611	0.13263217	0.1123973	0.22842702
24.7	0.27804286	0.40960827	0.21766422	0.49852955	0.1908436	0.80839788	0.14361467	0.13502737	0.11333949	0.23013706
24.8	0.28038548	0.40902941	0.21704038	0.49670935	0.19195823	0.80859784	0.145691	0.13747757	0.11429169	0.2319278
24.9	0.28271584	0.40845201	0.21642007	0.49489492	0.19306465	0.80880043	0.14780599	0.13998267	0.11525421	0.23380094
25	0.28503181	0.40787608	0.21580328	0.4930866	0.19416291	0.80900565	0.14996051	0.14254242	0.11622732	0.23575793
25.1	0.28733136	0.40730162	0.21519003	0.49128476	0.19525302	0.80921346	0.15215548	0.14515635	0.11721129	0.23779985
25.2	0.28961258	0.40672863	0.21458031	0.48948975	0.19633502	0.80942385	0.15439186	0.14782381	0.11820644	0.23992749
25.3	0.29187366	0.40615712	0.21397413	0.48770195	0.19740894	0.80963677	0.15667064	0.15054396	0.11921305	0.24214136
25.4	0.29411286	0.40558709	0.21337151	0.48592169	0.19847481	0.80985219	0.15899281	0.15331574	0.12023142	0.24444165
25.5	0.29632853	0.40501854	0.21277244	0.48414934	0.19953267	0.81007006	0.16135932	0.15613797	0.12126186	0.24682812
25.6	0.29851919	0.40445144	0.21217694	0.48238525	0.20058256	0.81029034	0.16377113	0.15900935	0.12230472	0.24930026
25.7	0.30068343	0.40388579	0.21158501	0.48062978	0.20162452	0.81051297	0.16622917	0.16192848	0.12336032	0.2518572
25.8	0.30281995	0.40332159	0.21099665	0.47888326	0.20265861	0.8107379	0.16873433	0.16489371	0.12442901	0.25449764
25.9	0.30492752	0.4027588	0.21041188	0.47714603	0.20368489	0.81096507	0.17128753	0.16790317	0.12551118	0.25721985
26	0.30700506	0.40219742	0.20983072	0.47541839	0.20470341	0.81119441	0.17388967	0.17095493	0.12660719	0.26002175
26.1	0.30905157	0.40163741	0.20925316	0.47370065	0.20571423	0.81142588	0.17654164	0.17404713	0.12771742	0.26290095
26.2	0.31106612	0.40107877	0.20867921	0.47199309	0.2067174	0.8116594	0.17924426	0.17717778	0.1288423	0.26585466
26.3	0.31304791	0.40052145	0.2081089	0.47029601	0.20771299	0.81189493	0.18199835	0.18034484	0.1299822	0.26887975
26.4	0.31499623	0.39996545	0.20754223	0.46860968	0.20870105	0.81213239	0.18480467	0.18354614	0.13113746	0.27197277
26.5	0.31691046	0.39941074	0.20697921	0.46693437	0.20968165	0.81237171	0.18766392	0.18677942	0.1323084	0.27512993
26.6	0.31879006	0.3988573	0.20641984	0.46527035	0.21065486	0.81261283	0.19057669	0.1900424	0.13349536	0.27834715
26.7	0.32063456	0.39830508	0.20586415	0.46361783	0.21162076	0.81285567	0.19354344	0.19333282	0.13469868	0.28162011
26.8	0.32244355	0.39775407	0.20531215	0.46197703	0.2125794	0.81310016	0.19656459	0.19664842	0.13591873	0.28494421
26.9	0.32421671	0.39720424	0.20476384	0.46034816	0.21353087	0.81334625	0.19964053	0.19998677	0.13715587	0.28831466
27	0.32595379	0.39665551	0.20421924	0.45873139	0.21447524	0.81359385	0.20277156	0.20334534	0.13841042	0.29172663
27.1	0.32765461	0.3961079	0.20367835	0.45712692	0.21541258	0.8138429	0.20595776	0.20672157	0.13968269	0.29517512
27.2	0.32931906	0.39556137	0.20314119	0.45553489	0.21634295	0.81409333	0.20919905	0.21011289	0.14097295	0.2986551
27.3	0.3309471	0.39501586	0.20260776	0.45395549	0.21726642	0.81434507	0.21249527	0.21351672	0.14228145	0.30216143
27.4	0.33253873	0.39447136	0.20207807	0.45238885	0.21818307	0.81459804	0.21584621	0.21693047	0.1436084	0.30568897
27.5	0.33409402	0.39392782	0.20155214	0.45083512	0.21909295	0.81485219	0.21925152	0.22035149	0.14495399	0.30923259
27.6	0.33561305	0.3933852	0.20102998	0.44929441	0.21999614	0.81510744	0.22271068	0.22377712	0.14631839	0.31278721
27.7	0.33709592	0.39284349	0.20051158	0.44776683	0.2208927	0.81536372	0.22622294	0.22720466	0.14770176	0.31634778
27.8	0.33854284	0.39230264	0.19999697	0.44625249	0.22178272	0.81562098	0.22978737	0.23063132	0.14910419	0.31990934
27.9	0.33995405	0.39176263	0.19948616	0.44475146	0.22266625	0.81587914	0.23340298	0.23405435	0.15052573	0.323467
28	0.34132982	0.39122344	0.19897915	0.44326384	0.22354338	0.81613815	0.23706861	0.23747107	0.15196636	0.32701595
28.1	0.34267043	0.39068501	0.19847596	0.44178968	0.22441416	0.81639794	0.24078291	0.24087871	0.15342598	0.33055152
28.2	0.3439762	0.39014734	0.1979766	0.44032906	0.22527867	0.81665845	0.24454419	0.24427452	0.15490443	0.33406927
28.3	0.34524749	0.3896104	0.19748107	0.43888204	0.22613699	0.81691961	0.24835063	0.24765583	0.1564015	0.33756487
28.4	0.34648472	0.38907416	0.19698938	0.43744865	0.22698919	0.81718137	0.25220032	0.25101997	0.15791689	0.34103416
28.5	0.34768829	0.38853859	0.19650155	0.43602895	0.22783534	0.81744367	0.25609117	0.25436436	0.15945025	0.3444732
28.6	0.34885867	0.38800367	0.19601759	0.43462295	0.22867551	0.81770644	0.26002092	0.25768644	0.16100106	0.34787826
28.7	0.34999633	0.38746939	0.19553752	0.43323069	0.22950978	0.81796964	0.26398709	0.26098375	0.16256867	0.35124581
28.8	0.35110177	0.38693571	0.19506134	0.43185218	0.23033821	0.81823319	0.26798706	0.26425388	0.16415235	0.35457252
28.9	0.3521755	0.38640262	0.19458907	0.43048744	0.23116088	0.81849706	0.27201809	0.26749453	0.16575134	0.35785529
29	0.35321802	0.38587011	0.19412073	0.42913646	0.23197785	0.81876119	0.27607731	0.27070346	0.1673648	0.36109118
29.1	0.35422983	0.38533815	0.19365633	0.42779924	0.23278919	0.81902552	0.28016168	0.27387853	0.16899175	0.36427747
29.2	0.35521142	0.38480673	0.19319589	0.42647578	0.23359498	0.81929001	0.28426808	0.27701764	0.17063104	0.36741173
29.3	0.35616334	0.38427583	0.19273943	0.42516605	0.23439528	0.81955459	0.28839329	0.28011885	0.17228136	0.37049173
29.4	0.35708618	0.38374542	0.19228696	0.42387004	0.23519017	0.81981923	0.29253397	0.28318031	0.17394133	0.37351538
29.5	0.35798052	0.3832155	0.19183851	0.42258772	0.23597969	0.82008386	0.29668667	0.28620029	0.17560948	0.37648078
29.6	0.35884696	0.38268605	0.19139408	0.42131906	0.23676393	0.82034844	0.30084784	0.2891772	0.17728428	0.37938623
29.7	0.35968606	0.38215705	0.19095371	0.42006404	0.23754295	0.82061293	0.30501393	0.29210953	0.17896404	0.38223024
[illegible]	[illegible]	[illegible]	[illegible]	[illegible]	[illegible]	[illegible]	[illegible]	[illegible]	[illegible]	[illegible]
[illegible]	[illegible]	[illegible]	[illegible]	[illegible]	[illegible]	[illegible]	[illegible]	[illegible]	[illegible]	[illegible]
30	0.36204498	0.38057262	0.18965707	0.41638031	0.23984935	0.8214054	0.31750689	0.30062531	0.1840146	0.39038147
30.1	0.36278044	0.38004527	0.18923308	0.41517935	0.24060815	0.82166907	0.32165774	0.30336607	0.18569533	0.39296838
30.2	0.36349151	0.3795183	0.18881324	0.41399177	0.24136205	0.82193243	0.32579602	0.30605622	0.18737124	0.395489
30.3	0.3641788	0.37899169	0.18839756	0.41281751	0.24211113	0.82219544	0.32991833	0.30869506	0.18904021	0.39794274
30.4	0.36484288	0.37846542	0.18798606	0.4116565	0.24285545	0.82245805	0.33402133	0.31128192	0.19070006	0.40032917
30.5	0.36548435	0.37793947	0.18757876	0.41050868	0.24359507	0.82272024	0.33810177	0.3138162	0.19234863	0.40264797
30.6	0.36610376	0.37741384	0.18717567	0.40937398	0.24433006	0.82298196	0.34215649	0.31629739	0.19398379	0.40489892
30.7	0.36670175	0.3768885	0.18677681	0.40825231	0.24506049	0.82324318	0.34618245	0.31872507	0.1956034	0.40708197
30.8	0.36727892	0.37636345	0.18638218	0.40714362	0.24578642	0.82350387	0.35017672	0.32109895	0.19720529	0.40919718
30.9	0.36783581	0.37583865	0.18599181	0.40604781	0.24650792	0.82376399	0.3541365	0.3234188	0.19878738	0.41124469
31	0.368373	0.37531409	0.1856057	0.4049648	0.24722504	0.8240235	0.35805915	0.32568455	0.20034763	0.41322476
31.1	0.36889104	0.37478975	0.18522386	0.40389451	0.24793785	0.82428238	0.36194216	0.32789624	0.2018841	0.41513771
31.2	0.36939051	0.37426561	0.18484631	0.40283685	0.24864639	0.8245406	0.36578313	0.33005398	0.20339503	0.41698392
31.3	0.36987194	0.37374165	0.18447306	0.40179173	0.24935073	0.82479812	0.36957978	0.33215791	0.20487872	0.41876387
31.4	0.37033584	0.37321786	0.18410412	0.40075907	0.25005094	0.82505491	0.37332997	0.33420827	0.20633358	0.42047806
31.5	0.37078273	0.37269421	0.18373949	0.39973877	0.25074707	0.82531094	0.37703172	0.33620533	0.20775814	0.42212709
31.6	0.37121311	0.37217068	0.18337918	0.39873073	0.25143918	0.82556619	0.38068322	0.33814947	0.20915108	0.42371157
31.7	0.37162751	0.37164726	0.18302319	0.39773485	0.25212734	0.82582062	0.38428283	0.34004106	0.21051128	0.42523221
31.8	0.37202642	0.37112394	0.18267154	0.39675104	0.2528116	0.82607421	0.38782902	0.34188052	0.21183775	0.42668974
31.9	0.37241033	0.37060068	0.18232421	0.39577919	0.25349203	0.82632695	0.39132044	0.34366841	0.2131297	0.42808494
32	0.3727797	0.37007748	0.18198121	0.39481921	0.25416868	0.82657879	0.39475585	0.3454053	0.21438648	0.42941862
32.1	0.373135	0.36955433	0.18164254	0.39387097	0.25484161	0.82682974	0.39813416	0.34709182	0.21560764	0.43069161
32.2	0.37347663	0.36903119	0.18130819	0.39293438	0.25551088	0.82707975	0.40145442	0.34872862	0.21679291	0.43190482
32.3	0.37380502	0.36850807	0.18097818	0.39200932	0.25617653	0.82732881	0.40471582	0.35031636	0.21794209	0.43305917
32.4	0.37412057	0.36798495	0.18065248	0.39109569	0.25683863	0.8275769	0.40791768	0.3518557	0.21905516	0.43415555
32.5	0.37442365	0.36746182	0.18033109	0.39019337	0.25749723	0.82782399	0.41105942	0.35334735	0.22013233	0.43519484
32.6	0.37471463	0.36693867	0.18001401	0.38930224	0.25815239	0.82807008	0.4141406	0.35479205	0.22117395	0.43617797
32.7	0.37499386	0.3664155	0.17970122	0.3884222	0.25880417	0.82831514	0.4171608	0.35619059	0.22218051	0.43710594
32.8	0.3752617	0.36589231	0.17939271	0.38755312	0.2594526	0.82855915	0.4201197	0.35754378	0.22315257	0.43797978
32.9	0.37551848	0.3653691	0.17908848	0.3866949	0.26009775	0.8288021	0.42301712	0.35885243	0.22409084	0.43880053
33	0.37576448	0.36484586	0.1787885	0.38584741	0.26073967	0.82904397	0.425853	0.36011732	0.22499608	0.43956925
33.1	0.37600002	0.3643226	0.17849276	0.38501053	0.26137842	0.82928475	0.42862736	0.3613392	0.22586918	0.44028703
33.2	0.37622536	0.36379933	0.17820125	0.38418415	0.26201405	0.82952441	0.43134033	0.36251887	0.22671109	0.440955
33.3	0.37644075	0.36327605	0.17791395	0.38336815	0.2626466	0.82976295	0.4339921	0.36365714	0.22752287	0.44157426
33.4	0.37664641	0.36275279	0.17763084	0.3825624	0.26327614	0.83000036	0.43658295	0.3647548	0.22830564	0.44214594
33.5	0.37684257	0.36222956	0.1773519	0.38176678	0.26390271	0.83023661	0.43911319	0.36581265	0.22906054	0.44267117
33.6	0.37702947	0.36170637	0.1770771	0.38098118	0.26452635	0.8304717	0.44158325	0.36683147	0.22978874	0.44315113
33.7	0.37720732	0.36118325	0.17680642	0.38020546	0.26514712	0.83070561	0.44399359	0.36781204	0.23049143	0.443587
33.8	0.37737633	0.36066024	0.17653984	0.37943951	0.26576504	0.83093832	0.44634472	0.36875512	0.23116985	0.44397992
33.9	0.37753666	0.36013734	0.17627733	0.3786832	0.26638017	0.83116984	0.44863716	0.36966149	0.23182527	0.44433103
34	0.37768846	0.3596146	0.17601886	0.37793642	0.26699255	0.83140013	0.45087151	0.37053188	0.23245894	0.44464153
34.1	0.37783187	0.35909205	0.17576441	0.37719903	0.26760222	0.8316292	0.4530484	0.37136702	0.23307209	0.44491261
34.2	0.37796701	0.35856972	0.17551393	0.37647092	0.26820923	0.83185702	0.45516853	0.37216763	0.23366597	0.44514546
34.3	0.378094	0.35804766	0.1752674	0.37575196	0.26881361	0.83208358	0.45723263	0.37293441	0.23424179	0.44534125
34.4	0.37821297	0.35752591	0.17502478	0.37504204	0.26941541	0.83230887	0.45924147	0.37366805	0.23480079	0.44550117
34.5	0.37832401	0.35700451	0.17478603	0.37434103	0.27001467	0.83253288	0.46119582	0.37436923	0.23534411	0.44562639
34.6	0.37842722	0.35648351	0.17455112	0.37364881	0.27061141	0.8327556	0.46309649	0.37503862	0.23587292	0.44571805
34.7	0.37852269	0.35596296	0.17432001	0.37296526	0.27120569	0.83297701	0.46494427	0.37567686	0.23638833	0.44577728
34.8	0.37861049	0.35544291	0.17409266	0.37229027	0.27179754	0.8331971	0.46673999	0.37628462	0.23689139	0.44580523
34.9	0.37869069	0.35492341	0.17386902	0.37162372	0.27238699	0.83341587	0.4684845	0.37686253	0.23738316	0.445803
35	0.37876335	0.35440452	0.17364907	0.37096549	0.27297406	0.8336333	0.47017862	0.3774112	0.23786461	0.44577168
35.1	0.37882851	0.3538863	0.17343276	0.37031546	0.27355878	0.83384939	0.47182323	0.37793126	0.23833671	0.44571235
35.2	0.3788862	0.35336879	0.17322005	0.36967353	0.27414118	0.83406413	0.4734192	0.37842329	0.23880036	0.44562608
35.3	0.37893645	0.35285207	0.17301089	0.36903957	0.2747213	0.83427752	0.47496741	0.3788879	0.23925643	0.44551391
35.4	0.37897929	0.35233619	0.17280526	0.36841349	0.27529915	0.83448956	0.47646878	0.37932567	0.23970578	0.44537685
35.5	0.37901472	0.35182122	0.1726031	0.36779517	0.27587477	0.83470026	0.47792418	0.37973714	0.2401492	0.44521592
35.6	0.37904277	0.35130722	0.17240438	0.3671845	0.27644818	0.83490961	0.47933452	0.38012285	0.24058743	0.44503209
35.7	0.37906341	0.35079424	0.17220906	0.36658138	0.27701941	0.83511762	0.48070067	0.38048336	0.24102119	0.44482629
35.8	0.37907665	0.35028236	0.17201708	0.36598571	0.27758848	0.8353243	0.48202352	0.38081921	0.24145111	0.44459943
35.9	0.37908247	0.34977164	0.17182842	0.36539738	0.27815541	0.83552966	0.48330394	0.38113095	0.24187782	0.44435237
36	0.37908083	0.34926215	0.17164303	0.36481631	0.27872022	0.83573372	0.48454282	0.38141911	0.24230192	0.44408594
36.1	0.37907172	0.34875395	0.17146087	0.36424239	0.27928293	0.83593649	0.48574102	0.38168422	0.24272398	0.44380095
36.2	0.37905513	0.3482471	0.17128189	0.36367553	0.27984356	0.83613799	0.48689939	0.38192677	0.24314451	0.44349819
36.3	0.37903104	0.34774168	0.17110606	0.36311565	0.28040213	0.83633823	0.48801877	0.38214727	0.24356402	0.44317843
36.4	0.37899943	0.34723775	0.17093335	0.36256264	0.28095864	0.83653724	0.48909998	0.38234623	0.24398296	0.4428424
36.5	0.37896028	0.34673536	0.1707637	0.36201642	0.28151311	0.83673505	0.49014382	0.38252416	0.24440177	0.4424908
36.6	0.37891356	0.34623459	0.1705971	0.36147691	0.28206555	0.83693167	0.4911511	0.38268158	0.24482083	0.44212429
36.7	0.37885925	0.34573549	0.17043349	0.36094404	0.28261597	0.83712713	0.49212263	0.38281897	0.24524047	0.44174352
36.8	0.37879733	0.34523813	0.17027286	0.36041771	0.28316438	0.83732146	0.49305919	0.38293682	0.24566102	0.4413491
36.9	0.37872776	0.34474257	0.17011516	0.35989784	0.2837108	0.83751468	0.49396155	0.3830356	0.24608278	0.44094162
37	0.37865052	0.34424888	0.16996036	0.35938438	0.28425523	0.83770682	0.49483046	0.38311577	0.24650605	0.44052164
37.1	0.37856557	0.34375711	0.16980844	0.35887724	0.28479767	0.8378979	0.49566665	0.38317779	0.24693106	0.44008968
37.2	0.37847288	0.34326732	0.16965936	0.35837635	0.28533813	0.83808796	0.49647086	0.38322214	0.24735804	0.43964623
37.3	0.37837244	0.34277958	0.16951309	0.35788164	0.28587661	0.83827701	0.4972438	0.38324928	0.2477872	0.43919176
37.4	0.37826424	0.34229394	0.16936962	0.35739304	0.28641311	0.8384651	0.49798619	0.38325968	0.24821873	0.43872674
37.5	0.37814829	0.34181046	0.1692289	0.3569105	0.28694765	0.83865223	0.49869869	0.38325377	0.24865281	0.43825158
37.6	0.37802458	0.3413292	0.16909092	0.35643394	0.28748022	0.83883843	0.49938196	0.38323198	0.24908959	0.4377667
37.7	0.37789311	0.3408502	0.16895565	0.35596331	0.28801081	0.83902374	0.50003664	0.38319474	0.24952918	0.43727247
37.8	0.37775386	0.34037352	0.16882307	0.35549855	0.28853944	0.83920817	0.5006634	0.38314243	0.24997169	0.43676928
37.9	0.37760683	0.3398992	0.16869316	0.35503959	0.28906609	0.83939175	0.50126285	0.38307549	0.25041717	0.43625746
38	0.37745204	0.33942731	0.16856589	0.35458638	0.28959078	0.8395745	0.50183561	0.38299436	0.25086573	0.43573735
38.1	0.3772895	0.33895788	0.16844125	0.35413887	0.29011349	0.83975643	0.50238226	0.38289944	0.2513174	0.43520924
38.2	0.37711926	0.33849097	0.16831921	0.35369699	0.29063423	0.83993759	0.5029034	0.38279112	0.25177224	0.43467342
38.3	0.37694133	0.33802662	0.16819976	0.35326069	0.29115299	0.84011797	0.5033996	0.3826698	0.25223026	0.43413013
38.4	0.37675573	0.33756487	0.16808288	0.35282991	0.29166977	0.8402976	0.50387145	0.38253586	0.2526915	0.43357966
38.5	0.3765625	0.33710576	0.16796854	0.35240461	0.29218457	0.8404765	0.50431949	0.38238969	0.25315595	0.43302226
38.6	0.37636168	0.33664935	0.16785673	0.35198473	0.29269737	0.84065469	0.50474426	0.38223167	0.2536236	0.43245816
38.7	0.37615334	0.33619566	0.16774744	0.35157023	0.29320818	0.84083218	0.50514626	0.38206219	0.25409445	0.4318876
38.8	0.37593752	0.33574473	0.16764064	0.35116104	0.29371698	0.841009	0.505526	0.38188165	0.25456849	0.43131078
38.9	0.37571431	0.3352966	0.16753632	0.35075713	0.29422378	0.84118515	0.505884	0.38169041	0.25504569	0.43072793
39	0.37548377	0.3348513	0.16743446	0.35035844	0.29472856	0.84136065	0.50622076	0.38148883	0.25552602	0.43013924
39.1	0.37524599	0.33440886	0.16733504	0.34996493	0.29523132	0.84153551	0.50653679	0.38127729	0.25600943	0.4295449
39.2	0.37500107	0.33396932	0.16723805	0.34957656	0.29573206	0.84170976	0.50683255	0.38105615	0.25649588	0.42894511
39.3	0.37474912	0.3335327	0.16714347	0.34919327	0.29623075	0.84188339	0.50710853	0.38082575	0.2569853	0.42834003
39.4	0.37449024	0.33309902	0.16705129	0.34881503	0.2967274	0.84205642	0.50736521	0.38058646	0.25747763	0.42772986
39.5	0.37422454	0.33266831	0.16696149	0.34844179	0.29722199	0.84222887	0.50760305	0.38033864	0.2579728	0.42711477
39.6	0.37395214	0.33224059	0.16687406	0.3480735	0.29771452	0.84240074	0.50782251	0.3800826	0.25847075	0.42649495
39.7	0.37367319	0.33181589	0.16678897	0.34771013	0.29820498	0.84257204	0.50802404	0.3798187	0.25897139	0.42587057
39.8	0.37338783	0.33139421	0.16670623	0.34735164	0.29869336	0.84274279	0.50820805	0.37954726	0.25947466	0.4252418
39.9	0.37309621	0.33097559	0.16662581	0.34699799	0.29917965	0.84291298	0.50837499	0.3792686	0.25998047	0.42460881
40	0.37279845	0.33056004	0.1665477	0.34664912	0.29966385	0.84308264	0.5085253	0.37898306	0.26048875	0.42397176
40.1	0.3724947	0.33014756	0.16647188	0.34630502	0.30014594	0.84325175	0.5086594	0.37869095	0.2609994	0.42333082
40.2	0.37218512	0.32973817	0.16639835	0.34596563	0.30062592	0.84342035	0.50877771	0.37839258	0.26151235	0.42268614
40.3	0.37186986	0.3293319	0.16632708	0.34563092	0.30110378	0.84358842	0.50888066	0.37808826	0.2620275	0.42203789
40.4	0.3715491	0.32892873	0.16625808	0.34530086	0.3015795	0.84375597	0.50896865	0.37777828	0.26254477	0.42138625
40.5	0.37122301	0.32852869	0.16619132	0.34497541	0.3020531	0.84392301	0.50904207	0.37746294	0.26306406	0.42073139
40.6	0.37089176	0.32813178	0.1661268	0.34465453	0.30252455	0.84408954	0.50910134	0.37714252	0.26358527	0.42007347
40.7	0.37055552	0.32773801	0.1660645	0.34433818	0.30299385	0.84425557	0.50914682	0.37681729	0.26410832	0.41941265
40.8	0.37021447	0.32734737	0.16600442	0.34402634	0.303461	0.84442109	0.50917891	0.37648754	0.26463312	0.41874911
40.9	0.36986879	0.32695988	0.16594653	0.34371897	0.30392598	0.84458611	0.50919799	0.37615354	0.26515959	0.418083
41	0.36951868	0.32657553	0.16589085	0.34341604	0.3043888	0.84475063	0.50920441	0.37581554	0.26568764	0.41741449
41.1	0.36916433	0.32619431	0.16583734	0.34311752	0.30484943	0.84491465	0.50919856	0.37547381	0.26621716	0.41674376
41.2	0.36880591	0.32581624	0.16578602	0.34282337	0.30530789	0.84507817	0.50918079	0.37512856	0.26674806	0.41607097
41.3	0.36844363	0.3254413	0.16573686	0.34253356	0.30576416	0.84524119	0.50915146	0.37478002	0.26728025	0.41539629
41.4	0.36807765	0.32506948	0.16568986	0.34224806	0.30621823	0.84540372	0.50911092	0.37442843	0.26781362	0.41471989
41.5	0.36770816	0.32470079	0.16564502	0.34196683	0.3066701	0.84556574	0.5090595	0.37407399	0.26834808	0.41404191
41.6	0.36733536	0.32433522	0.16560232	0.34168986	0.30711976	0.84572727	0.50899754	0.37371693	0.26888356	0.41336251
41.7	0.36695942	0.32397276	0.16556177	0.3414171	0.3075672	0.8458883	0.50892535	0.37335745	0.26941996	0.41268186
41.8	0.36658051	0.3236134	0.16552336	0.34114853	0.30801242	0.84604882	0.50884326	0.37299575	0.2699572	0.41200013
41.9	0.36619883	0.32325713	0.16548708	0.34088411	0.30845541	0.84620884	0.50875158	0.37263202	0.27049518	0.41131745
42	0.36581455	0.32290394	0.16545293	0.34062382	0.30889618	0.84636836	0.50865061	0.37226644	0.27103382	0.41063399
42.1	0.36542785	0.32255381	0.16542092	0.34036762	0.30933473	0.84652737	0.50854067	0.3718992	0.27157304	0.40994989
42.2	0.3650389	0.32220674	0.16539103	0.34011549	0.30977104	0.84668587	0.50842202	0.37153045	0.27211274	0.40926531
42.3	0.36464788	0.32186271	0.16536326	0.33986739	0.31020511	0.84684385	0.50829496	0.37116033	0.27265285	0.40858042
42.4	0.36425494	0.3215217	0.16533762	0.3396233	0.31063695	0.84700132	0.50815976	0.37078897	0.27319329	0.40789535
42.5	0.36386026	0.3211837	0.16531411	0.33938318	0.31106655	0.84715827	0.5080167	0.37041653	0.27373397	0.40721025
42.6	0.36346399	0.32084869	0.16529273	0.33914701	0.3114939	0.84731469	0.50786604	0.37004314	0.27427481	0.40652528
42.7	0.36306629	0.32051665	0.16527347	0.33891477	0.31191901	0.84747059	0.50770804	0.3696689	0.27481573	0.40584056
42.8	0.36266731	0.32018758	0.16525633	0.33868641	0.31234188	0.84762595	0.50754295	0.36929395	0.27535665	0.40515624
42.9	0.36226722	0.31986144	0.16524133	0.33846191	0.3127625	0.84778078	0.50737102	0.3689184	0.27589749	0.40447247
43	0.36186616	0.31953823	0.16522847	0.33824124	0.31318087	0.84793507	0.50719247	0.36854237	0.27643818	0.40378937
43.1	0.36146426	0.31921793	0.16521774	0.33802438	0.313597	0.84808881	0.50700753	0.36816596	0.27697866	0.40310707
43.2	0.36106169	0.31890051	0.16520915	0.3378113	0.31401089	0.84824202	0.50681643	0.36778925	0.27751886	0.40242572
43.3	0.36065856	0.31858596	0.16520271	0.33760197	0.31442254	0.84839467	0.5066194	0.36741235	0.2780587	0.40174543
43.4	0.36025501	0.31827426	0.16519841	0.33739636	0.31483196	0.84854677	0.50641666	0.36703533	0.27859813	0.40106634
43.5	0.35985115	0.31796539	0.16519627	0.33719444	0.31523915	0.84869832	0.50620842	0.36665827	0.27913707	0.40038857
43.6	0.35944713	0.31765934	0.16519628	0.3369962	0.31564411	0.84884931	0.50599489	0.36628127	0.27967547	0.39971224
43.7	0.35904305	0.31735608	0.16519846	0.33680159	0.31604685	0.84899974	0.50577626	0.36590439	0.28021326	0.39903747
43.8	0.35863902	0.31705559	0.1652028	0.33661059	0.31644737	0.8491496	0.50555274	0.3655277	0.2807504	0.39836435
43.9	0.35823516	0.31675787	0.16520932	0.33642318	0.31684567	0.8492989	0.50532453	0.36515126	0.28128684	0.39769301
44	0.35783156	0.31646288	0.16521801	0.33623933	0.31724176	0.84944763	0.50509183	0.36477515	0.28182254	0.39702354
44.1	0.35742834	0.31617061	0.1652289	0.33605901	0.31763564	0.84959579	0.50485481	0.36439941	0.28235746	0.39635605
44.2	0.35702558	0.31588105	0.16524197	0.3358822	0.31802732	0.84974338	0.50461368	0.36402408	0.28289155	0.39569064
44.3	0.35662338	0.31559417	0.16525725	0.33570886	0.31841681	0.84989039	0.50436859	0.36364922	0.28342479	0.39502741
44.4	0.35622181	0.31530996	0.16527473	0.33553897	0.3188041	0.85003683	0.50411974	0.36327487	0.28395713	0.39436643
44.5	0.35582096	0.3150284	0.16529442	0.3353725	0.31918922	0.85018269	0.50386728	0.36290108	0.28448855	0.3937078
44.6	0.35542091	0.31474948	0.16531633	0.33520944	0.31957216	0.85032798	0.5036114	0.36252789	0.28501901	0.3930516
44.7	0.35502173	0.31447317	0.16534047	0.33504974	0.31995294	0.85047269	0.50335227	0.36215534	0.28554851	0.3923979
44.8	0.35462349	0.31419946	0.16536683	0.33489339	0.32033156	0.85061682	0.50309006	0.36178345	0.28607702	0.39174679
44.9	0.35422625	0.31392834	0.16539544	0.33474037	0.32070803	0.85076039	0.50282493	0.36141227	0.28660455	0.39109832
45	0.35383009	0.31365979	0.16542628	0.33459064	0.32108237	0.85090338	0.50255704	0.36104182	0.28713108	0.39045255
45.1	0.35343507	0.31339379	0.16545937	0.33444418	0.32145457	0.85104581	0.50228655	0.36067212	0.2876566	0.38980955
45.2	0.35304122	0.31313032	0.16549471	0.33430097	0.32182465	0.85118766	0.50201362	0.36030321	0.28818111	0.38916937
45.3	0.35264861	0.31286938	0.16553231	0.33416097	0.32219262	0.85132896	0.50173839	0.35993511	0.28870463	0.38853206
45.4	0.35225729	0.31261094	0.16557217	0.33402417	0.32255849	0.85146969	0.50146102	0.35956782	0.28922714	0.38789766
45.5	0.35186731	0.312355	0.1656143	0.33389055	0.32292227	0.85160987	0.50118165	0.35920137	0.28974866	0.38726623
45.6	0.35147871	0.31210154	0.16565869	0.33376007	0.32328398	0.8517495	0.50090043	0.35883577	0.2902692	0.38663781
45.7	0.35109154	0.31185053	0.16570536	0.33363272	0.32364362	0.85188858	0.50061748	0.35847104	0.29078879	0.38601242
45.8	0.35070582	0.31160198	0.1657543	0.33350848	0.3240012	0.85202713	0.50033296	0.35810718	0.29130744	0.38539009
45.9	0.3503216	0.31135587	0.16580553	0.33338731	0.32435674	0.85216513	0.50004698	0.35774422	0.2918252	0.38477087
46	0.34993891	0.31111218	0.16585904	0.33326919	0.32471025	0.85230261	0.49975969	0.35738218	0.29234207	0.38415476
46.1	0.34955776	0.3108709	0.16591484	0.3331541	0.32506174	0.85243956	0.4994712	0.35702105	0.2928581	0.38354181
46.2	0.3491782	0.31063202	0.16597292	0.33304202	0.32541122	0.85257599	0.49918166	0.35666084	0.29337331	0.38293202
46.3	0.34880025	0.31039552	0.1660333	0.33293291	0.3257587	0.85271191	0.49889117	0.35630156	0.29388775	0.38232542
46.4	0.34842394	0.3101614	0.16609597	0.33282676	0.3261042	0.85284732	0.49859987	0.35594323	0.29440144	0.38172203
46.5	0.34804928	0.30992963	0.16616093	0.33272354	0.32644773	0.85298223	0.49830786	0.35558583	0.29491443	0.38112185
46.6	0.34767629	0.30970022	0.16622819	0.33262322	0.3267893	0.85311664	0.49801526	0.35522938	0.29542674	0.38052491
46.7	0.347305	0.30947313	0.16629774	0.33252579	0.32712893	0.85325057	0.49772218	0.35487389	0.29593844	0.37993121
46.8	0.34693542	0.30924838	0.16636958	0.33243122	0.32746663	0.85338401	0.49742871	0.35451937	0.29644956	0.37934077
46.9	0.34656758	0.30902594	0.16644371	0.33233948	0.32780241	0.85351698	0.49713498	0.35416581	0.29696014	0.37875359
47	0.34620148	0.30880579	0.16652014	0.33225055	0.32813628	0.85364947	0.49684106	0.35381324	0.29747024	0.37816968
47.1	0.34583714	0.30858794	0.16659885	0.33216441	0.32846827	0.8537815	0.49654707	0.35346164	0.29797989	0.37758904
47.2	0.34547458	0.30837237	0.16667985	0.33208103	0.32879837	0.85391307	0.49625308	0.35311102	0.29848915	0.37701166
47.3	0.34511381	0.30815907	0.16676313	0.3320004	0.3291266	0.85404419	0.49595919	0.3527614	0.29899804	0.37643754
47.4	0.34475485	0.30794803	0.1668487	0.33192248	0.32945299	0.85417485	0.49566547	0.35241276	0.29950663	0.3758667
47.5	0.34439771	0.30773923	0.16693654	0.33184726	0.32977753	0.85430507	0.495372	0.35206513	0.30001495	0.37529912
47.6	0.3440424	0.30753268	0.16702666	0.33177472	0.33010025	0.85443485	0.49507887	0.35171852	0.30052305	0.37473482
47.7	0.34368893	0.30732835	0.16711905	0.33170482	0.33042115	0.8545642	0.49478615	0.35137292	0.30103099	0.37417379
47.8	0.34333732	0.30712624	0.1672137	0.33163756	0.33074026	0.85469312	0.4944939	0.35102835	0.3015388	0.37361605
47.9	0.34298757	0.30692634	0.16731062	0.3315729	0.33105758	0.8548216	0.49420219	0.35068482	0.30204653	0.3730616
48	0.3426397	0.30672865	0.16740979	0.33151082	0.33137313	0.85494967	0.49391109	0.35034233	0.30255423	0.37251044
48.1	0.34229373	0.30653314	0.1675112	0.3314513	0.33168693	0.85507732	0.49362066	0.35000089	0.30306194	0.37196258
48.2	0.34194966	0.30633982	0.16761486	0.33139433	0.33199899	0.85520455	0.49333097	0.3496605	0.3035697	0.37141802
48.3	0.3416075	0.30614868	0.16772075	0.33133987	0.33230933	0.85533137	0.49304206	0.34932119	0.30407755	0.37087677
48.4	0.34126726	0.30595969	0.16782887	0.33128791	0.33261795	0.85545778	0.492754	0.34898296	0.30458552	0.37033882
48.5	0.34092895	0.30577286	0.1679392	0.33123843	0.33292487	0.85558379	0.49246682	0.34864582	0.30509367	0.36980418
48.6	0.34059258	0.30558818	0.16805175	0.3311914	0.3332301	0.85570939	0.4921806	0.34830979	0.30560201	0.36927287
48.7	0.34025817	0.30540563	0.16816649	0.3311468	0.33353367	0.85583459	0.49189536	0.34797486	0.30611059	0.36874488
48.8	0.33992571	0.30522521	0.16828342	0.33110461	0.33383557	0.8559594	0.49161116	0.34764105	0.30661945	0.36822022
48.9	0.33959522	0.30504691	0.16840252	0.33106481	0.33413583	0.85608381	0.49132803	0.34730838	0.3071286	0.36769892
49	0.33926672	0.30487071	0.1685238	0.33102738	0.33443445	0.85620783	0.49104601	0.34697685	0.30763808	0.36718096
49.1	0.3389402	0.30469662	0.16864722	0.3309923	0.33473145	0.85633147	0.49076515	0.34664648	0.30814792	0.36666638
49.2	0.33861568	0.30452461	0.16877279	0.33095954	0.33502685	0.85645471	0.49048547	0.34631728	0.30865813	0.36615517
49.3	0.33829316	0.30435468	0.16890049	0.33092909	0.33532066	0.85657757	0.490207	0.34598926	0.30916875	0.36564736
49.4	0.33797264	0.30418682	0.1690303	0.33090092	0.33561289	0.85670004	0.48992976	0.34566243	0.3096798	0.36514296
49.5	0.33765414	0.30402102	0.1691622	0.33087501	0.33590355	0.85682213	0.4896538	0.34533681	0.3101913	0.36464197
49.6	0.33733766	0.30385728	0.1692962	0.33085134	0.33619266	0.85694385	0.48937912	0.3450124	0.31070326	0.36414442
49.7	0.33702321	0.30369557	0.16943226	0.33082989	0.33648023	0.85706518	0.48910575	0.34468923	0.31121571	0.3636503
49.8	0.33671079	0.3035359	0.16957038	0.33081064	0.33676626	0.85718613	0.48883372	0.34436729	0.31172866	0.36315965
49.9	0.33640042	0.30337824	0.16971054	0.33079357	0.33705078	0.8573067	0.48856304	0.3440466	0.31224212	0.36267248
50	0.33609209	0.3032226	0.16985272	0.33077866	0.3373338	0.8574269	0.48829372	0.34372717	0.31275611	0.36218879
50.1	0.33578581	0.30306895	0.1699969	0.33076589	0.33761532	0.85754672	0.48802578	0.34340901	0.31327063	0.3617086
50.2	0.33548159	0.3029173	0.17014308	0.33075523	0.33789536	0.85766617	0.48775922	0.34309213	0.31378571	0.36123193
50.3	0.33517943	0.30276762	0.17029122	0.33074668	0.33817394	0.85778524	0.48749406	0.34277655	0.31430134	0.36075878
50.4	0.33487934	0.3026199	0.17044132	0.3307402	0.33845106	0.85790394	0.4872303	0.34246226	0.31481754	0.36028918
50.5	0.33458132	0.30247415	0.17059334	0.33073577	0.33872673	0.85802226	0.48696797	0.34214929	0.3153343	0.35982312
50.6	0.33428537	0.30233034	0.17074729	0.33073339	0.33900097	0.85814022	0.48670705	0.34183764	0.31585164	0.35936062
50.7	0.3339915	0.30218846	0.17090313	0.33073302	0.33927379	0.8582578	0.48644755	0.34152732	0.31636955	0.35890169
50.8	0.33369971	0.3020485	0.17106085	0.33073465	0.3395452	0.858375	0.48618948	0.34121834	0.31688804	0.35844634
50.9	0.33341001	0.30191046	0.17122043	0.33073826	0.33981521	0.85849183	0.48593284	0.34091071	0.31740711	0.35799456
51	0.33312238	0.30177432	0.17138186	0.33074382	0.34008383	0.8586083	0.48567762	0.34060445	0.31792674	0.35754637
51.1	0.33283684	0.30164006	0.1715451	0.33075132	0.34035107	0.85872438	0.48542383	0.34029957	0.31844695	0.35710178
51.2	0.33255339	0.30150768	0.17171015	0.33076073	0.34061695	0.8588401	0.48517146	0.33999607	0.31896772	0.35666078
51.3	0.33227204	0.30137716	0.17187699	0.33077205	0.34088147	0.85895544	0.48492052	0.33969398	0.31948907	0.35622337
51.4	0.33199278	0.3012485	0.17204559	0.33078525	0.34114465	0.85907041	0.484671	0.33939328	0.32001097	0.35578957
51.5	0.33171562	0.30112168	0.17221594	0.33080031	0.34140649	0.85918501	0.48442289	0.33909401	0.32053344	0.35535937
51.6	0.33144055	0.30099668	0.17238802	0.33081721	0.34166701	0.85929923	0.48417619	0.33879615	0.32105645	0.35493277
51.7	0.33116758	0.3008735	0.1725618	0.33083593	0.34192622	0.85941308	0.4839309	0.33849972	0.32157999	0.35450976
51.8	0.3308967	0.30075212	0.17273728	0.33085645	0.34218414	0.85952655	0.48368701	0.33820472	0.32210407	0.35409035
51.9	0.33062791	0.30063253	0.17291442	0.33087876	0.34244076	0.85963965	0.4834445	0.33791117	0.32262866	0.35367453
52	0.33036123	0.30051471	0.1730932	0.33090283	0.3426961	0.85975237	0.48320338	0.33761907	0.32315376	0.3532623
52.1	0.33009664	0.30039865	0.17327362	0.33092865	0.34295017	0.85986472	0.48296362	0.33732844	0.32367935	0.35285365
52.2	0.32983415	0.30028434	0.17345564	0.33095619	0.34320298	0.8599767	0.48272523	0.33703929	0.32420542	0.35244856
52.3	0.32957375	0.30017176	0.17363926	0.33098544	0.34345454	0.8600883	0.48248817	0.33675163	0.32473195	0.35204704
52.4	0.32931544	0.30006089	0.17382443	0.33101638	0.34370486	0.86019952	0.48225243	0.33646546	0.32525895	0.35164907
52.5	0.32905923	0.29995173	0.17401116	0.33104898	0.34395395	0.86031037	0.482018	0.33618079	0.32578638	0.35125463
52.6	0.32880511	0.29984426	0.17419941	0.33108324	0.34420182	0.86042084	0.48178486	0.33589764	0.32631425	0.35086371
52.7	0.32855309	0.29973847	0.17438917	0.33111914	0.34444848	0.86053093	0.481553	0.33561601	0.32684252	0.35047629
52.8	0.32830317	0.29963433	0.17458042	0.33115665	0.34469393	0.86064065	0.4813224	0.33533592	0.32737119	0.35009237
52.9	0.32805534	0.29953184	0.17477313	0.33119575	0.34493819	0.86074999	0.48109304	0.33505738	0.32790024	0.34971192
53	0.32780961	0.29943097	0.17496728	0.33123644	0.34518127	0.86085896	0.48086492	0.33478039	0.32842965	0.34933493
53.1	0.32756597	0.29933172	0.17516286	0.33127869	0.34542317	0.86096755	0.48063801	0.33450496	0.3289594	0.34896137
53.2	0.32732443	0.29923406	0.17535985	0.33132248	0.3456639	0.86107577	0.48041229	0.33423112	0.32948948	0.34859123
53.3	0.32708498	0.29913798	0.17555822	0.33136779	0.34590348	0.86118361	0.48018774	0.33395886	0.33001986	0.34822449
53.4	0.32684762	0.29904346	0.17575795	0.33141462	0.34614192	0.86129108	0.47996435	0.33368819	0.33055053	0.34786112
53.5	0.32661235	0.29895049	0.17595902	0.33146294	0.34637921	0.86139818	0.47974211	0.33341914	0.33108147	0.34750111
53.6	0.32637916	0.29885905	0.17616142	0.33151273	0.34661538	0.8615049	0.47952098	0.3331517	0.33161266	0.34714443
53.7	0.32614807	0.29876912	0.17636512	0.33156399	0.34685042	0.86161125	0.47930095	0.33288588	0.33214408	0.34679105
53.8	0.32591906	0.29868069	0.17657009	0.33161668	0.34708436	0.86171723	0.479082	0.3326217	0.33267571	0.34644096
53.9	0.32569214	0.29859373	0.17677633	0.3316708	0.34731719	0.86182284	0.47886411	0.33235917	0.33320753	0.34609412
54	0.32546731	0.29850824	0.1769838	0.33172633	0.34754894	0.86192808	0.47864727	0.33209829	0.33373952	0.3457505
54.1	0.32524456	0.29842419	0.17719249	0.33178325	0.3477796	0.86203295	0.47843144	0.33183908	0.33427167	0.34541009
54.2	0.32502391	0.29834157	0.17740238	0.33184155	0.34800919	0.86213746	0.47821662	0.33158155	0.33480394	0.34507286
54.3	0.32480535	0.29826036	0.17761344	0.33190122	0.34823771	0.86224161	0.47800278	0.33132571	0.33533632	0.34473878
54.4	0.32458887	0.29818054	0.17782565	0.33196223	0.34846519	0.86234539	0.4777899	0.33107156	0.33586879	0.34440782
54.5	0.32437449	0.29810209	0.17803899	0.33202457	0.34869161	0.86244881	0.47757796	0.33081912	0.33640134	0.34407996
54.6	0.3241622	0.298025	0.17825345	0.33208823	0.348917	0.86255187	0.47736692	0.3305684	0.33693394	0.34375516
54.7	0.323952	0.29794924	0.178469	0.3321532	0.34914136	0.86265457	0.47715678	0.33031941	0.33746658	0.34343339
54.8	0.3237439	0.29787481	0.17868562	0.33221946	0.3493647	0.86275692	0.4769475	0.33007216	0.33799924	0.34311463
54.9	0.3235379	0.29780167	0.17890329	0.33228699	0.34958704	0.86285891	0.47673906	0.32982665	0.3385319	0.34279885
55	0.32333399	0.29772983	0.17912198	0.33235578	0.34980836	0.86296054	0.47653144	0.3295829	0.33906453	0.34248601
55.1	0.32313218	0.29765924	0.17934169	0.33242582	0.3500287	0.86306183	0.47632463	0.32934092	0.33959712	0.34217609
55.2	0.32293248	0.29758991	0.17956239	0.3324971	0.35024805	0.86316276	0.47611859	0.32910071	0.34012965	0.34186906
55.3	0.32273488	0.2975218	0.17978405	0.33256961	0.35046642	0.86326334	0.47591332	0.3288623	0.34066209	0.34156489
55.4	0.32253938	0.29745491	0.18000666	0.33264332	0.35068382	0.86336358	0.4757088	0.32862568	0.34119442	0.34126355
55.5	0.322346	0.29738921	0.18023019	0.33271823	0.35090027	0.86346347	0.47550499	0.32839087	0.34172663	0.34096501
55.6	0.32215473	0.29732469	0.18045463	0.33279432	0.35111576	0.86356302	0.47530189	0.32815786	0.3422587	0.34066924
55.7	0.32196557	0.29726134	0.18067995	0.33287159	0.35133031	0.86366222	0.47509947	0.32792668	0.34279059	0.34037621
55.8	0.32177853	0.29719912	0.18090613	0.33295001	0.35154393	0.86376109	0.47489773	0.32769731	0.3433223	0.34008591
55.9	0.32159361	0.29713803	0.18113316	0.33302958	0.35175662	0.86385961	0.47469665	0.32746978	0.3438538	0.33979829
56	0.32141081	0.29707805	0.18136101	0.33311029	0.35196839	0.8639578	0.4744962	0.32724409	0.34438508	0.33951333
56.1	0.32123015	0.29701917	0.18158966	0.33319212	0.35217926	0.86405565	0.47429638	0.32702023	0.3449161	0.339231
56.2	0.32105162	0.29696137	0.1818191	0.33327506	0.35238923	0.86415316	0.47409716	0.32679821	0.34544685	0.33895128
56.3	0.32087522	0.29690464	0.1820493	0.3333591	0.35259831	0.86425034	0.47389852	0.32657804	0.34597731	0.33867415
56.4	0.32070096	0.29684896	0.18228024	0.33344424	0.35280651	0.86434719	0.47370044	0.32635972	0.34650747	0.33839957
56.5	0.32052884	0.29679431	0.18251191	0.33353044	0.35301383	0.8644437	0.47350292	0.32614325	0.34703728	0.33812752
56.6	0.32035886	0.29674069	0.18274429	0.33361772	0.3532203	0.86453989	0.47330592	0.32592863	0.34756675	0.33785798
56.7	0.32019103	0.29668808	0.18297735	0.33370605	0.35342591	0.86463575	0.47310945	0.32571587	0.34809585	0.33759092
56.8	0.32002533	0.29663646	0.18321108	0.33379542	0.35363068	0.86473128	0.47291349	0.32550496	0.34862456	0.33732631
56.9	0.31986179	0.29658583	0.18344546	0.33388583	0.35383461	0.86482648	0.47271803	0.32529591	0.34915286	0.33706415
57	0.31970039	0.29653617	0.18368047	0.33397725	0.35403772	0.86492136	0.47252305	0.32508872	0.34968073	0.33680439
57.1	0.31954115	0.29648748	0.18391609	0.3340697	0.35424	0.86501592	0.47232855	0.32488339	0.35020816	0.33654703
57.2	0.31938406	0.29643973	0.1841523	0.33416314	0.35444148	0.86511015	0.47213452	0.32467991	0.35073512	0.33629205
57.3	0.31922913	0.29639293	0.18438908	0.33425758	0.35464216	0.86520406	0.47194094	0.32447829	0.3512616	0.33603941
57.4	0.31907635	0.29634706	0.18462642	0.33435299	0.35484205	0.86529765	0.47174781	0.32427853	0.35178757	0.3357891
57.5	0.31892572	0.29630211	0.1848643	0.33444938	0.35504116	0.86539093	0.47155514	0.32408062	0.35231303	0.3355411
57.6	0.31877724	0.29625808	0.18510269	0.33454674	0.3552395	0.86548388	0.4713629	0.32388456	0.35283794	0.3352954
57.7	0.31863092	0.29621495	0.18534158	0.33464504	0.35543708	0.86557652	0.4711711	0.32369035	0.3533623	0.33505197
57.8	0.31848675	0.29617272	0.18558096	0.3347443	0.35563391	0.86566884	0.47097974	0.32349799	0.35388609	0.33481081
57.9	0.31834473	0.29613138	0.1858208	0.33484448	0.35583	0.86576085	0.47078881	0.32330748	0.35440928	0.33457188
58	0.31820486	0.29609093	0.18606109	0.3349456	0.35602535	0.86585255	0.47059831	0.3231188	0.35493186	0.33433519
58.1	0.31806713	0.29605135	0.18630181	0.33504763	0.35621997	0.86594393	0.47040823	0.32293195	0.35545382	0.33410071
58.2	0.31793155	0.29601265	0.18654294	0.33515057	0.3564138	0.866035	0.47021858	0.32274693	0.35597513	0.33386843
58.3	0.31779812	0.29597481	0.18678448	0.33525441	0.35660709	0.86612576	0.47002934	0.32256373	0.35649578	0.33363834
58.4	0.31766682	0.29593783	0.18702639	0.33535914	0.3567996	0.86621621	0.46984053	0.32238235	0.35701575	0.33341042
58.5	0.31753765	0.29590172	0.18726866	0.33546475	0.35699143	0.86630636	0.46965214	0.32220277	0.35753503	0.33318467
58.6	0.31741062	0.29586646	0.18751129	0.33557124	0.35718258	0.86639619	0.46946417	0.322025	0.3580536	0.33296107
58.7	0.31728572	0.29583205	0.18775424	0.33567858	0.35737306	0.86648572	0.46927662	0.32184902	0.35857144	0.33273961
58.8	0.31716294	0.29579849	0.1879975	0.33578679	0.35756289	0.86657495	0.46908952	0.32167484	0.35908853	0.33252029
58.9	0.31704227	0.29576577	0.18824107	0.33589583	0.35775207	0.86666387	0.46890285	0.32150244	0.35960486	0.33230309
59	0.31692371	0.2957339	0.18848491	0.33600572	0.35794061	0.86675248	0.46871662	0.32133181	0.36012042	0.33208801
59.1	0.31680725	0.29570286	0.18872902	0.33611644	0.35812853	0.8668408	0.46853085	0.32116295	0.36063518	0.33187504
59.2	0.31669289	0.29567267	0.18897339	0.33622797	0.35831584	0.86692881	0.46834554	0.32099586	0.36114914	0.33166417
59.3	0.31658061	0.29564331	0.18921798	0.33634032	0.35850254	0.86701653	0.46816069	0.32083052	0.36166227	0.3314554
59.4	0.31647041	0.29561478	0.1894628	0.33645348	0.35868864	0.86710394	0.46797631	0.32066693	0.36217456	0.33124872
59.5	0.31636229	0.29558708	0.18970782	0.33656743	0.35887416	0.86719106	0.4677924	0.32050508	0.362686	0.33104413
59.6	0.31625623	0.29556021	0.18995304	0.33668217	0.35905911	0.86727788	0.46760897	0.32034496	0.36319658	0.33084162
59.7	0.31615223	0.29553418	0.19019843	0.33679769	0.3592435	0.8673644	0.46742603	0.32018657	0.36370626	0.33064119
59.8	0.31605028	0.29550897	0.19044398	0.33691399	0.35942734	0.86745063	0.46724358	0.32002989	0.36421506	0.33044284
59.9	0.31595037	0.29548458	0.19068968	0.33703105	0.35961063	0.86753657	0.46706163	0.31987492	0.36472294	0.33024655

60	0.31585249	0.29546102	0.19093552	0.33714887	0.3597934	0.86762221	0.4668802	0.31972165	0.36522989	0.33005234
60.1	0.31575663	0.29543828	0.19118148	0.33726743	0.35997564	0.86770756	0.4666993	0.31957006	0.36573591	0.32986021
60.2	0.31566278	0.29541636	0.19142754	0.33738674	0.36015738	0.86779262	0.46651892	0.31942016	0.36624097	0.32967014
60.3	0.31557093	0.29539527	0.1916737	0.33750679	0.36033862	0.86787739	0.4663391	0.31927193	0.36674508	0.32948215
60.4	0.31548106	0.29537499	0.19191994	0.33762757	0.36051937	0.86796188	0.46615983	0.31912536	0.36724821	0.32929623
60.5	0.31539317	0.29535554	0.19216625	0.33774906	0.36069965	0.86804607	0.46598113	0.31898044	0.36775035	0.32911239
60.6	0.31530725	0.2953369	0.19241261	0.33787127	0.36087946	0.86812998	0.46580301	0.31883717	0.3682515	0.32893063
60.7	0.31522329	0.29531908	0.19265901	0.33799419	0.36105882	0.86821361	0.46562548	0.31869552	0.36875164	0.32875094
60.8	0.31514128	0.29530207	0.19290544	0.3381178	0.36123774	0.86829695	0.46544853	0.3185555	0.36925076	0.32857334
60.9	0.3150612	0.29528588	0.19315188	0.33824211	0.36141622	0.86838001	0.46527219	0.3184171	0.36974886	0.32839782
61	0.31498305	0.2952705	0.19339833	0.33836711	0.36159429	0.86846278	0.46509645	0.3182803	0.37024591	0.3282244
61.1	0.31490681	0.29525593	0.19364478	0.33849278	0.36177195	0.86854528	0.46492132	0.31814509	0.37074191	0.32805306
61.2	0.31483247	0.29524217	0.1938912	0.33861912	0.36194921	0.86862749	0.46474682	0.31801147	0.37123685	0.32788383
61.3	0.31476003	0.29522921	0.1941376	0.33874613	0.36212608	0.86870943	0.46457295	0.31787941	0.37173073	0.3277167
61.4	0.31468946	0.29521706	0.19438395	0.3388738	0.36230258	0.86879109	0.46439973	0.31774892	0.37222352	0.32755168
61.5	0.31462076	0.29520571	0.19463024	0.33900213	0.36247872	0.86887247	0.46422715	0.31761998	0.37271522	0.32738877
61.6	0.31455391	0.29519516	0.19487647	0.3391311	0.3626545	0.86895358	0.46405524	0.31749258	0.37320583	0.32722798
61.7	0.31448892	0.29518542	0.19512262	0.33926071	0.36282995	0.86903441	0.46388399	0.31736671	0.37369532	0.32706932
61.8	0.31442576	0.29517647	0.19536869	0.33939095	0.36300506	0.86911497	0.4637134	0.31724236	0.3741837	0.3269128
61.9	0.31436442	0.29516831	0.19561466	0.33952182	0.36317986	0.86919526	0.46354348	0.31711951	0.37467095	0.32675841
62	0.3143049	0.29516095	0.19586051	0.33965332	0.36335435	0.86927528	0.46337423	0.31699817	0.37515707	0.32660617
62.1	0.31424717	0.29515438	0.19610625	0.33978543	0.36352854	0.86935502	0.46320566	0.31687831	0.37564204	0.32645609
62.2	0.31419124	0.2951486	0.19635186	0.33991815	0.36370245	0.86943451	0.46303778	0.31675993	0.37612586	0.32630816

[illegible]

120.3	0.40836387	0.35745894	0.29701066	0.43151959	0.49994902	0.89368863	0.43429645	0.37968791	0.51363651	0.4076904
120.4	0.40853118	0.35759616	0.29714312	0.43166859	0.50015605	0.89370576	0.43433325	0.37982481	0.51375044	0.40789813
120.5	0.40869819	0.35773333	0.29727549	0.43181757	0.50036282	0.89372284	0.43437013	0.37996149	0.51386416	0.40810581
120.6	0.40886491	0.35787047	0.29740774	0.43196653	0.50056934	0.89373989	0.43440708	0.38009795	0.51397769	0.40831345
120.7	0.40903132	0.35800757	0.2975399	0.43211547	0.50077559	0.89375689	0.43444411	0.38023421	0.51409102	0.40852103
120.8	0.40919744	0.35814462	0.29767195	0.43226439	0.50098159	0.89377386	0.43448121	0.38037026	0.51420415	0.40872856
120.9	0.40936327	0.35828164	0.29780391	0.43241329	0.50118734	0.89379078	0.43451839	0.38050609	0.51431709	0.40893604
121	0.40952879	0.35841861	0.29793575	0.43256217	0.50139283	0.89380767	0.43455565	0.38064172	0.51442983	0.40914347
121.1	0.40969402	0.35855554	0.2980675	0.43271102	0.50159806	0.89382452	0.43459299	0.38077715	0.51454238	0.40935085
121.2	0.40985895	0.35869243	0.29819914	0.43285985	0.50180304	0.89384133	0.4346304	0.38091236	0.51465473	0.40955817
121.3	0.41002358	0.35882928	0.29833068	0.43300866	0.50200776	0.8938581	0.4346679	0.38104738	0.5147669	0.40976545
121.4	0.41018791	0.35896609	0.29846211	0.43315745	0.50221224	0.89387483	0.43470547	0.38118219	0.51487887	0.40997266
121.5	0.41035194	0.35910285	0.29859344	0.43330621	0.50241646	0.89389153	0.43474312	0.3813168	0.51499065	0.41017983
121.6	0.41051567	0.35923957	0.29872467	0.43345495	0.50262043	0.89390818	0.43478085	0.38145121	0.51510224	0.41038693
121.7	0.41067911	0.35937625	0.2988558	0.43360367	0.50282414	0.8939248	0.43481866	0.38158542	0.51521364	0.41059398
121.8	0.41084224	0.35951288	0.29898682	0.43375236	0.50302761	0.89394138	0.43485655	0.38171944	0.51532485	0.41080098
121.9	0.41100507	0.35964947	0.29911773	0.43390103	0.50323083	0.89395793	0.43489453	0.38185325	0.51543588	0.41100792
122	0.4111676	0.35978602	0.29924855	0.43404967	0.5034338	0.89397443	0.43493258	0.38198688	0.51554671	0.4112148
122.1	0.41132982	0.35992252	0.29937925	0.43419829	0.50363652	0.8939909	0.43497072	0.3821203	0.51565737	0.41142162
122.2	0.41149175	0.36005898	0.29950986	0.43434688	0.50383899	0.89400734	0.43500893	0.38225354	0.51576783	0.41162838
122.3	0.41165337	0.36019539	0.29964036	0.43449545	0.50404121	0.89402373	0.43504723	0.38238658	0.51587812	0.41183509
122.4	0.4118147	0.36033176	0.29977075	0.43464399	0.50424319	0.89404009	0.43508561	0.38251943	0.51598822	0.41204173
122.5	0.41197571	0.36046809	0.29990105	0.43479251	0.50444492	0.89405641	0.43512408	0.38265209	0.51609814	0.41224831
122.6	0.41213643	0.36060436	0.30003123	0.434941	0.5046464	0.8940727	0.43516263	0.38278457	0.51620787	0.41245484
122.7	0.41229684	0.3607406	0.30016131	0.43508947	0.50484764	0.89408895	0.43520127	0.38291686	0.51631743	0.4126613
122.8	0.41245695	0.36087679	0.30029129	0.43523791	0.50504864	0.89410516	0.43523998	0.38304896	0.51642681	0.41286769
122.9	0.41261675	0.36101293	0.30042116	0.43538632	0.50524939	0.89412134	0.43527879	0.38318088	0.51653601	0.41307403
123	0.41277625	0.36114902	0.30055093	0.4355347	0.5054499	0.89413748	0.43531768	0.38331262	0.51664503	0.4132803
123.1	0.41293544	0.36128507	0.3006806	0.43568306	0.50565016	0.89415359	0.43535665	0.38344418	0.51675387	0.41348651
123.2	0.41309433	0.36142107	0.30081015	0.43583139	0.50585019	0.89416966	0.43539572	0.38357556	0.51686254	0.41369266
123.3	0.41325291	0.36155703	0.30093961	0.43597969	0.50604997	0.8941857	0.43543487	0.38370675	0.51697103	0.41389874
123.4	0.41341119	0.36169294	0.30106895	0.43612797	0.50624951	0.8942017	0.4354741	0.38383777	0.51707935	0.41410475
123.5	0.41356915	0.3618288	0.3011982	0.43627622	0.50644881	0.89421766	0.43551343	0.38396861	0.5171875	0.4143107
123.6	0.41372682	0.36196461	0.30132734	0.43642443	0.50664787	0.89423359	0.43555284	0.38409928	0.51729547	0.41451658
123.7	0.41388417	0.36210038	0.30145637	0.43657262	0.50684669	0.89424949	0.43559234	0.38422977	0.51740327	0.4147224
123.8	0.41404122	0.3622361	0.3015853	0.43672078	0.50704527	0.89426535	0.43563193	0.38436009	0.5175109	0.41492814
123.9	0.41419796	0.36237177	0.30171412	0.43686891	0.50724361	0.89428118	0.43567161	0.38449023	0.51761836	0.41513382
124	0.4143544	0.36250739	0.30184284	0.43701701	0.50744171	0.89429697	0.43571137	0.38462021	0.51772565	0.41533943
124.1	0.41451052	0.36264297	0.30197145	0.43716508	0.50763958	0.89431273	0.43575123	0.38475002	0.51783277	0.41554498
124.2	0.41466634	0.36277849	0.30209996	0.43731312	0.50783721	0.89432846	0.43579117	0.38487965	0.51793973	0.41575045
124.3	0.41482185	0.36291397	0.30222837	0.43746114	0.5080346	0.89434415	0.4358312	0.38500912	0.51804652	0.41595585
124.4	0.41497705	0.3630494	0.30235667	0.43760912	0.50823176	0.89435981	0.43587132	0.38513843	0.51815314	0.41616118
124.5	0.41513194	0.36318478	0.30248486	0.43775707	0.50842868	0.89437543	0.43591153	0.38526757	0.5182596	0.41636644
124.6	0.41528652	0.36332011	0.30261295	0.43790499	0.50862537	0.89439102	0.43595183	0.38539654	0.5183659	0.41657162
124.7	0.41544079	0.36345539	0.30274094	0.43805288	0.50882182	0.89440658	0.43599222	0.38552535	0.51847203	0.41677674
124.8	0.41559475	0.36359062	0.30286882	0.43820073	0.50901804	0.8944221	0.4360327	0.385654	0.518578	0.41698178
124.9	0.4157484	0.3637258	0.30299659	0.43834856	0.50921403	0.89443759	0.43607327	0.38578248	0.51868381	0.41718675
125	0.41590174	0.36386093	0.30312427	0.43849635	0.50940978	0.89445305	0.43611393	0.38591081	0.51878946	0.41739164
125.1	0.41605477	0.36399601	0.30325183	0.43864412	0.5096053	0.89446848	0.43615468	0.38603898	0.51889496	0.41759647
125.2	0.41620749	0.36413104	0.3033793	0.43879185	0.50980059	0.89448387	0.43619551	0.38616698	0.51900029	0.41780121
125.3	0.4163599	0.36426602	0.30350666	0.43893954	0.50999565	0.89449923	0.43623644	0.38629483	0.51910546	0.41800588
125.4	0.416512	0.36440095	0.30363391	0.43908721	0.51019047	0.89451455	0.43627746	0.38642253	0.51921048	0.41821048
125.5	0.41666378	0.36453582	0.30376106	0.43923484	0.51038507	0.89452985	0.43631857	0.38655006	0.51931535	0.41841499
125.6	0.41681525	0.36467065	0.30388811	0.43938244	0.51057943	0.89454511	0.43635977	0.38667745	0.51942005	0.41861944
125.7	0.41696641	0.36480542	0.30401505	0.43953	0.51077357	0.89456034	0.43640106	0.38680468	0.51952461	0.4188238
125.8	0.41711726	0.36494015	0.30414188	0.43967754	0.51096748	0.89457553	0.43644244	0.38693175	0.51962901	0.41902809
125.9	0.41726779	0.36507482	0.30426862	0.43982503	0.51116116	0.8945907	0.4364839	0.38705868	0.51973326	0.41923229
126	0.41741802	0.36520944	0.30439524	0.4399725	0.51135461	0.89460583	0.43652546	0.38718545	0.51983736	0.41943642
126.1	0.41756793	0.365344	0.30452177	0.44011993	0.51154783	0.89462093	0.43656711	0.38731208	0.5199413	0.41964048
126.2	0.41771753	0.36547852	0.30464819	0.44026732	0.51174083	0.89463599	0.43660886	0.38743855	0.5200451	0.41984445
126.3	0.41786681	0.36561298	0.3047745	0.44041468	0.5119336	0.89465103	0.43665069	0.38756488	0.52014875	0.42004834
126.4	0.41801579	0.36574739	0.30490072	0.440562	0.51212614	0.89466603	0.43669261	0.38769105	0.52025225	0.42025215
126.5	0.41816445	0.36588175	0.30502683	0.44070929	0.51231846	0.89468101	0.43673462	0.38781708	0.5203556	0.42045588
126.6	0.4183128	0.36601605	0.30515284	0.44085655	0.51251055	0.89469595	0.43677673	0.38794297	0.52045881	0.42065953
126.7	0.41846084	0.3661503	0.30527875	0.44100376	0.51270242	0.89471085	0.43681893	0.3880687	0.52056187	0.4208631
126.8	0.41860857	0.3662845	0.30540456	0.44115094	0.51289406	0.89472573	0.43686121	0.3881943	0.52066478	0.42106658
126.9	0.41875598	0.36641864	0.30553026	0.44129809	0.51308548	0.89474058	0.43690359	0.38831974	0.52076756	0.42126999
127	0.41890309	0.36655273	0.30565587	0.44144519	0.51327667	0.89475539	0.43694606	0.38844505	0.52087019	0.42147331
127.1	0.41904988	0.36668677	0.30578137	0.44159226	0.51346764	0.89477017	0.43698862	0.38857021	0.52097267	0.42167655
127.2	0.41919636	0.36682075	0.30590677	0.44173929	0.51365839	0.89478493	0.43703127	0.38869523	0.52107502	0.4218797
127.3	0.41934253	0.36695468	0.30603207	0.44188629	0.51384892	0.89479965	0.43707402	0.38882011	0.52117722	0.42208277
127.4	0.41948839	0.36708855	0.30615728	0.44203324	0.51403922	0.89481433	0.43711685	0.38894485	0.52127929	0.42228575
127.5	0.41963394	0.36722237	0.30628238	0.44218016	0.5142293	0.89482899	0.43715978	0.38906944	0.52138121	0.42248865
127.6	0.41977918	0.36735613	0.30640739	0.44232704	0.51441916	0.89484362	0.43720279	0.3891939	0.521483	0.42269147
127.7	0.41992411	0.36748984	0.30653229	0.44247388	0.5146088	0.89485822	0.4372459	0.38931822	0.52158465	0.42289419
127.8	0.42006874	0.3676235	0.3066571	0.44262068	0.51479822	0.89487278	0.4372891	0.3894424	0.52168616	0.42309684
127.9	0.42021305	0.36775709	0.30678181	0.44276745	0.51498742	0.89488731	0.43733238	0.38956645	0.52178754	0.42329939
128	0.42035706	0.36789064	0.30690642	0.44291417	0.5151764	0.89490182	0.43737576	0.38969035	0.52188878	0.42350186
128.1	0.42050076	0.36802413	0.30703094	0.44306086	0.51536517	0.89491629	0.43741923	0.38981412	0.52198989	0.42370424
128.2	0.42064415	0.36815756	0.30715535	0.4432075	0.51555371	0.89493073	0.43746279	0.38993776	0.52209086	0.42390653
128.3	0.42078723	0.36829094	0.30727967	0.44335411	0.51574203	0.89494514	0.43750644	0.39006126	0.5221917	0.42410874
128.4	0.42093001	0.36842426	0.3074039	0.44350067	0.51593014	0.89495952	0.43755017	0.39018462	0.52229241	0.42431085
128.5	0.42107249	0.36855752	0.30752802	0.4436472	0.51611803	0.89497387	0.437594	0.39030785	0.52239299	0.42451288
128.6	0.42121465	0.36869073	0.30765205	0.44379368	0.5163057	0.89498819	0.43763792	0.39043094	0.52249343	0.42471482
128.7	0.42135652	0.36882388	0.30777598	0.44394013	0.51649315	0.89500248	0.43768192	0.39055391	0.52259375	0.42491666
128.8	0.42149807	0.36895698	0.30789982	0.44408653	0.51668039	0.89501674	0.43772602	0.39067674	0.52269394	0.42511842
128.9	0.42163933	0.36909002	0.30802356	0.44423289	0.51686741	0.89503097	0.4377702	0.39079943	0.52279399	0.42532009
129	0.42178028	0.369223	0.3081472	0.44437921	0.51705422	0.89504517	0.43781447	0.39092199	0.52289392	0.42552166
129.1	0.42192093	0.36935592	0.30827074	0.44452549	0.51724081	0.89505933	0.43785883	0.39104443	0.52299373	0.42572315
129.2	0.42206128	0.36948879	0.30839419	0.44467173	0.51742719	0.89507347	0.43790328	0.39116673	0.5230934	0.42592454
129.3	0.42220132	0.3696216	0.30851755	0.44481792	0.51761335	0.89508758	0.43794781	0.3912889	0.52319295	0.42612584
129.4	0.42234107	0.36975436	0.3086408	0.44496408	0.5177993	0.89510165	0.43799244	0.39141093	0.52329238	0.42632705
129.5	0.42248052	0.36988705	0.30876396	0.44511019	0.51798503	0.8951157	0.43803715	0.39153284	0.52339168	0.42652817
129.6	0.42261966	0.37001969	0.30888703	0.44525625	0.51817056	0.89512972	0.43808194	0.39165462	0.52349085	0.42672919
129.7	0.42275851	0.37015227	0.30900999	0.44540228	0.51835586	0.89514371	0.43812683	0.39177627	0.52358991	0.42693013
129.8	0.42289705	0.37028479	0.30913286	0.44554826	0.51854096	0.89515766	0.4381718	0.39189778	0.52368884	0.42713096
129.9	0.4230353	0.37041726	0.30925563	0.4456942	0.51872584	0.89517159	0.43821685	0.39201917	0.52378765	0.42733171
130	0.42317326	0.37054966	0.3093783	0.4458401	0.51891051	0.89518549	0.438262	0.39214043	0.52388634	0.42753236
130.1	0.42331091	0.37068201	0.30950087	0.44598595	0.51909497	0.89519935	0.43830722	0.39226156	0.5239849	0.42773291
130.2	0.42344827	0.3708143	0.30962334	0.44613176	0.51927922	0.89521319	0.43835254	0.39238256	0.52408335	0.42793338
130.3	0.42358534	0.37094653	0.30974571	0.44627753	0.51946326	0.895227	0.43839794	0.39250344	0.52418168	0.42813374
130.4	0.42372211	0.3710787	0.30986798	0.44642325	0.51964708	0.89524078	0.43844342	0.39262418	0.52427989	0.42833401
130.5	0.42385859	0.37121082	0.30999016	0.44656892	0.5198307	0.89525453	0.43848899	0.3927448	0.52437799	0.42853419
130.6	0.42399478	0.37134287	0.31011223	0.44671456	0.52001411	0.89526825	0.43853464	0.39286529	0.52447596	0.42873427
130.7	0.42413067	0.37147487	0.31023421	0.44686015	0.52019731	0.89528194	0.43858038	0.39298565	0.52457382	0.42893425
130.8	0.42426628	0.37160681	0.31035608	0.44700569	0.5203803	0.8952956	0.4386262	0.39310588	0.52467156	0.42913414
130.9	0.4244016	0.37173868	0.31047786	0.44715119	0.52056308	0.89530923	0.43867211	0.39322599	0.52476919	0.42933393
131	0.42453663	0.3718705	0.31059953	0.44729665	0.52074565	0.89532283	0.4387181	0.39334597	0.5248667	0.42953362
131.1	0.42467137	0.37200226	0.3107211	0.44744206	0.52092801	0.8953364	0.43876417	0.39346582	0.5249641	0.42973322
131.2	0.42480583	0.37213396	0.31084257	0.44758742	0.52111017	0.89534994	0.43881033	0.39358555	0.52506139	0.42993272
131.3	0.42494	0.3722656	0.31096395	0.44773274	0.52129212	0.89536345	0.43885657	0.39370515	0.52515856	0.43013212
131.4	0.42507389	0.37239718	0.31108521	0.44787802	0.52147386	0.89537694	0.43890289	0.39382462	0.52525562	0.43033142
131.5	0.42520749	0.3725287	0.31120638	0.44802325	0.52165539	0.89539039	0.4389493	0.39394397	0.52535257	0.43053062
131.6	0.42534082	0.37266016	0.31132745	0.44816843	0.52183672	0.89540382	0.43899578	0.39406319	0.5254494	0.43072972
131.7	0.42547386	0.37279156	0.31144841	0.44831357	0.52201785	0.89541721	0.43904235	0.39418229	0.52554613	0.43092873
131.8	0.42560663	0.37292291	0.31156927	0.44845866	0.52219877	0.89543058	0.439089	0.39430125	0.52564274	0.43112763
131.9	0.42573911	0.37305419	0.31169003	0.4486037	0.52237948	0.89544392	0.43913574	0.3944201	0.52573925	0.43132644
132	0.42587132	0.37318541	0.31181068	0.4487487	0.52255999	0.89545723	0.43918255	0.39453881	0.52583564	0.43152514
132.1	0.42600325	0.37331657	0.31193123	0.44889365	0.52274029	0.89547051	0.43922944	0.39465741	0.52593193	0.43172375
132.2	0.42613491	0.37344767	0.31205168	0.44903855	0.52292039	0.89548376	0.43927642	0.39477587	0.52602811	0.43192225
132.3	0.4262663	0.37357871	0.31217202	0.44918341	0.52310029	0.89549698	0.43932348	0.39489421	0.52612419	0.43212066
132.4	0.42639741	0.37370969	0.31229226	0.44932822	0.52327998	0.89551017	0.43937061	0.39501242	0.52622015	0.43231896
132.5	0.42652825	0.37384061	0.31241239	0.44947298	0.52345947	0.89552334	0.43941783	0.39513051	0.52631601	0.43251716
132.6	0.42665882	0.37397147	0.31253242	0.44961769	0.52363875	0.89553647	0.43946512	0.39524847	0.52641176	0.43271526
132.7	0.42678912	0.37410227	0.31265234	0.44976235	0.52381784	0.89554958	0.4395125	0.39536631	0.52650741	0.43291326
132.8	0.42691916	0.374233	0.31277216	0.44990697	0.52399672	0.89556266	0.43955995	0.39548402	0.52660295	0.43311115
132.9	0.42704893	0.37436368	0.31289187	0.45005154	0.5241754	0.89557571	0.43960748	0.3956016	0.52669839	0.43330894
133	0.42717843	0.37449429	0.31301148	0.45019606	0.52435387	0.89558873	0.43965509	0.39571906	0.52679373	0.43350663
133.1	0.42730768	0.37462485	0.31313098	0.45034053	0.52453215	0.89560172	0.43970278	0.39583639	0.52688896	0.43370422
133.2	0.42743666	0.37475534	0.31325037	0.45048495	0.52471023	0.89561468	0.43975055	0.3959536	0.52698409	0.4339017
133.3	0.42756538	0.37488577	0.31336966	0.45062932	0.5248881	0.89562762	0.43979839	0.39607068	0.52707911	0.43409908
133.4	0.42769384	0.37501614	0.31348883	0.45077365	0.52506578	0.89564052	0.43984631	0.39618764	0.52717403	0.43429636
133.5	0.42782204	0.37514645	0.31360791	0.45091792	0.52524325	0.8956534	0.43989431	0.39630447	0.52726886	0.43449353
133.6	0.42794999	0.3752767	0.31372687	0.45106214	0.52542052	0.89566625	0.43994238	0.39642117	0.52736358	0.4346906
133.7	0.42807768	0.37540688	0.31384573	0.45120631	0.5255976	0.89567907	0.43999053	0.39653775	0.5274582	0.43488756
133.8	0.42820511	0.375537	0.31396447	0.45135044	0.52577448	0.89569186	0.44003875	0.3966542	0.52755271	0.43508442
133.9	0.4283323	0.37566707	0.31408311	0.45149451	0.52595115	0.89570463	0.44008705	0.39677053	0.52764713	0.43528118
134	0.42845923	0.37579707	0.31420164	0.45163853	0.52612763	0.89571736	0.44013543	0.39688673	0.52774146	0.43547782
134.1	0.42858591	0.375927	0.31432006	0.4517825	0.52630391	0.89573007	0.44018388	0.3970028	0.52783568	0.43567437
134.2	0.42871234	0.37605688	0.31443837	0.45192642	0.52647999	0.89574275	0.44023241	0.39711875	0.5279298	0.4358708
134.3	0.42883852	0.37618669	0.31455656	0.45207029	0.52665588	0.8957554	0.44028101	0.39723457	0.52802383	0.43606713
134.4	0.42896446	0.37631644	0.31467465	0.4522141	0.52683157	0.89576802	0.44032968	0.39735027	0.52811776	0.43626336
134.5	0.42909015	0.37644613	0.31479262	0.45235787	0.52700706	0.89578061	0.44037843	0.39746583	0.52821159	0.43645948
134.6	0.42921559	0.37657576	0.31491049	0.45250158	0.52718235	0.89579318	0.44042725	0.39758128	0.52830532	0.43665549
134.7	0.4293408	0.37670532	0.31502824	0.45264525	0.52735745	0.89580572	0.44047615	0.39769659	0.52839896	0.43685139
134.8	0.42946576	0.37683483	0.31514588	0.45278886	0.52753235	0.89581822	0.44052511	0.39781178	0.52849251	0.43704719
134.9	0.42959048	0.37696426	0.31526341	0.45293242	0.52770706	0.8958307	0.44057415	0.39792684	0.52858595	0.43724288
180	0.47147839	0.42864591	0.35698407	0.51099613	0.59010062	0.89908789	0.46416983	0.44087835	0.56336278	0.51291044
180.1	0.47154888	0.42874604	0.3570533	0.51110817	0.59020934	0.89909113	0.46421783	0.44095657	0.56342618	0.51304887
180.2	0.47161929	0.42884612	0.35712242	0.51122012	0.59031795	0.89909436	0.46426581	0.44103469	0.56348954	0.51318718
180.3	0.47168962	0.42894614	0.35719145	0.511332	0.59042645	0.89909757	0.46431377	0.44111274	0.56355285	0.51332536
180.4	0.47175988	0.42904609	0.35726039	0.5114438	0.59053485	0.89910075	0.4643617	0.44119069	0.56361612	0.5134634
180.5	0.47183005	0.42914599	0.35732924	0.51155551	0.59064315	0.89910392	0.46440961	0.44126857	0.56367934	0.51360132
135.3	[illegible]	[illegible]	[illegible]	[illegible]	[illegible]	[illegible]	[illegible]	[illegible]	[illegible]	[illegible]
135.4	[illegible]	[illegible]	[illegible]	[illegible]	[illegible]	[illegible]	[illegible]	[illegible]	[illegible]	[illegible]
135.5	[illegible]	[illegible]	[illegible]	[illegible]	[illegible]	[illegible]	[illegible]	[illegible]	[illegible]	[illegible]
135.6	[illegible]	[illegible]	[illegible]	[illegible]	[illegible]	[illegible]	[illegible]	[illegible]	[illegible]	[illegible]
135.7	[illegible]	[illegible]	[illegible]	[illegible]	[illegible]	[illegible]	[illegible]	[illegible]	[illegible]	[illegible]
135.8	0.43070235	0.37812639	0.31631612	0.45422208	0.52927064	0.89594176	0.44101864	0.3989567	0.52942276	0.43899923
135.9	0.43082474	0.37825519	0.31643252	0.45436511	0.5294434	0.89595396	0.44106837	0.39907049	0.52951528	0.43919384
136	0.4309469	0.37838394	0.31654881	0.45450809	0.52961597	0.89596613	0.44111816	0.39918416	0.52960771	0.43938833
136.1	0.43106884	0.37851262	0.31666498	0.45465101	0.52978835	0.89597827	0.44116802	0.3992977	0.52970004	0.43958272
136.2	0.43119055	0.37864124	0.31678105	0.45479388	0.52996053	0.89599038	0.44121795	0.39941112	0.52979229	0.43977699
136.3	0.43131204	0.37876979	0.316897	0.4549367	0.53013252	0.89600247	0.44126795	0.39952441	0.52988444	0.43997115
136.4	0.4314333	0.37889829	0.31701283	0.45507946	0.53030432	0.89601453	0.44131801	0.39963757	0.52997651	0.44016518
136.5	0.43155435	0.37902671	0.31712855	0.45522217	0.53047593	0.89602656	0.44136813	0.3997506	0.53006848	0.4403591
136.6	0.43167518	0.37915508	0.31724416	0.45536482	0.53064735	0.89603856	0.44141832	0.39986351	0.53016037	0.4405529
136.7	0.43179579	0.37928338	0.31735965	0.45550742	0.53081858	0.89605053	0.44146857	0.39997629	0.53025217	0.44074659
136.8	0.43191618	0.37941161	0.31747503	0.45564996	0.53098962	0.89606248	0.44151888	0.40008895	0.53034388	0.44094015
136.9	0.43203636	0.37953979	0.31759029	0.45579245	0.53116046	0.89607439	0.44156925	0.40020148	0.5304355	0.4411336
137	0.43215632	0.37966789	0.31770544	0.45593488	0.53133112	0.89608628	0.44161969	0.40031388	0.53052704	0.44132693
137.1	0.43227607	0.37979594	0.31782048	0.45607726	0.53150159	0.89609815	0.44167019	0.40042616	0.53061849	0.44152014
137.2	0.43239561	0.37992392	0.3179354	0.45621959	0.53167186	0.89610998	0.44172075	0.40053831	0.53070985	0.44171323
137.3	0.43251494	0.38005183	0.31805021	0.45636186	0.53184195	0.89612178	0.44177137	0.40065034	0.53080112	0.44190621
137.4	0.43263406	0.38017968	0.3181649	0.45650407	0.53201185	0.89613356	0.44182205	0.40076224	0.53089231	0.44209907
137.5	0.43275297	0.38030747	0.31827947	0.45664623	0.53218156	0.89614531	0.44187278	0.40087402	0.53098341	0.44229181
137.6	0.43287167	0.38043519	0.31839394	0.45678833	0.53235108	0.89615703	0.44192358	0.40098567	0.53107443	0.44248444
137.7	0.43299017	0.38056285	0.31850828	0.45693037	0.53252042	0.89616872	0.44197444	0.4010972	0.53116536	0.44267695
137.8	0.43310846	0.38069045	0.31862252	0.45707236	0.53268956	0.89618039	0.44202535	0.4012086	0.5312562	0.44286934
137.9	0.43322655	0.38081798	0.31873663	0.4572143	0.53285852	0.89619202	0.44207632	0.40131988	0.53134697	0.44306161
138	0.43334443	0.38094544	0.31885063	0.45735618	0.53302729	0.89620363	0.44212735	0.40143103	0.53143764	0.44325377
138.1	0.43346212	0.38107284	0.31896452	0.457498	0.53319587	0.89621521	0.44217844	0.40154206	0.53152824	0.44344581
138.2	0.4335796	0.38120018	0.31907829	0.45763976	0.53336427	0.89622677	0.44222958	0.40165297	0.53161874	0.44363773
138.3	0.43369688	0.38132745	0.31919195	0.45778147	0.53353248	0.89623829	0.44228078	0.40176376	0.53170917	0.44382953
138.4	0.43381396	0.38145465	0.31930549	0.45792312	0.5337005	0.89624979	0.44233203	0.40187442	0.53179951	0.44402122
138.5	0.43393085	0.38158179	0.31941891	0.45806472	0.53386834	0.89626126	0.44238334	0.40198496	0.53188977	0.4442128
138.6	0.43404754	0.38170887	0.31953222	0.45820626	0.53403599	0.8962727	0.44243471	0.40209538	0.53197995	0.44440425
138.7	0.43416403	0.38183588	0.31964541	0.45834774	0.53420346	0.89628411	0.44248613	0.40220568	0.53207004	0.44459559
138.8	0.43428033	0.38196283	0.31975849	0.45848916	0.53437074	0.8962955	0.4425376	0.40231585	0.53216005	0.44478681
138.9	0.43439644	0.38208971	0.31987145	0.45863053	0.53453783	0.89630685	0.44258912	0.40242591	0.53224998	0.44497792
139	0.43451235	0.38221652	0.31998429	0.45877184	0.53470474	0.89631818	0.4426407	0.40253585	0.53233983	0.44516891
139.1	0.43462807	0.38234327	0.32009702	0.4589131	0.53487147	0.89632949	0.44269233	0.40264567	0.5324296	0.44535978
139.2	0.4347436	0.38246996	0.32020963	0.45905429	0.53503801	0.89634076	0.44274401	0.40275537	0.53251928	0.44555054
139.3	0.43485895	0.38259658	0.32032212	0.45919543	0.53520437	0.89635201	0.44279574	0.40286495	0.53260889	0.44574118
139.4	0.4349741	0.38272313	0.3204345	0.45933651	0.53537054	0.89636322	0.44284753	0.40297442	0.53269842	0.44593171
139.5	0.43508906	0.38284962	0.32054677	0.45947753	0.53553653	0.89637441	0.44289936	0.40308376	0.53278786	0.44612211
139.6	0.43520384	0.38297604	0.32065891	0.45961849	0.53570234	0.89638558	0.44295124	0.40319299	0.53287723	0.44631241
139.7	0.43531844	0.3831024	0.32077094	0.4597594	0.53586796	0.89639671	0.44300317	0.40330211	0.53296651	0.44650258
139.8	0.43543285	0.38322869	0.32088286	0.45990024	0.5360334	0.89640782	0.44305516	0.4034111	0.53305572	0.44669264
139.9	0.43554707	0.38335492	0.32099466	0.46004103	0.53619866	0.8964189	0.44310719	0.40351999	0.53314484	0.44688259
140	0.43566111	0.38348108	0.32110634	0.46018176	0.53636374	0.89642995	0.44315926	0.40362875	0.53323389	0.44707242
140.1	0.43577497	0.38360718	0.3212179	0.46032243	0.53652863	0.89644097	0.44321139	0.40373741	0.53332286	0.44726213
140.2	0.43588865	0.38373321	0.32132935	0.46046305	0.53669334	0.89645197	0.44326356	0.40384595	0.53341175	0.44745173
140.3	0.43600214	0.38385917	0.32144068	0.4606036	0.53685788	0.89646294	0.44331578	0.40395437	0.53350056	0.44764121
140.4	0.43611546	0.38398507	0.3215519	0.46074409	0.53702222	0.89647388	0.44336805	0.40406269	0.53358929	0.44783058
140.5	0.4362286	0.3841109	0.321663	0.46088453	0.53718639	0.89648479	0.44342036	0.40417089	0.53367795	0.44801983
140.6	0.43634156	0.38423667	0.32177398	0.46102491	0.53735038	0.89649568	0.44347271	0.40427898	0.53376652	0.44820896
140.7	0.43645434	0.38436237	0.32188485	0.46116522	0.53751419	0.89650654	0.44352511	0.40438697	0.53385502	0.44839797
140.8	0.43656695	0.38448801	0.3219956	0.46130548	0.53767781	0.89651737	0.44357756	0.40449484	0.53394344	0.44858685
140.9	0.43667939	0.38461358	0.32210624	0.46144568	0.53784126	0.89652817	0.44363005	0.4046026	0.53403179	0.44877561
141	0.43679164	0.38473908	0.32221675	0.46158582	0.53800453	0.89653895	0.44368258	0.40471025	0.53412005	0.44896424
141.1	0.43690373	0.38486452	0.32232716	0.4617259	0.53816761	0.89654969	0.44373516	0.4048178	0.53420824	0.44915275
141.2	0.43701564	0.38498989	0.32243744	0.46186592	0.53833052	0.89656041	0.44378777	0.40492524	0.53429636	0.44934114
141.3	0.43712738	0.38511519	0.32254761	0.46200588	0.53849325	0.89657111	0.44384044	0.40503257	0.5343844	0.4495294
141.4	0.43723895	0.38524043	0.32265766	0.46214579	0.5386558	0.89658177	0.44389314	0.4051398	0.53447236	0.44971754
141.5	0.43735034	0.38536561	0.3227676	0.46228563	0.53881817	0.89659241	0.44394588	0.40524692	0.53456024	0.44990556
141.6	0.43746157	0.38549071	0.32287742	0.46242541	0.53898036	0.89660302	0.44399867	0.40535393	0.53464805	0.45009345
141.7	0.43757263	0.38561575	0.32298712	0.46256513	0.53914238	0.89661361	0.44405149	0.40546084	0.53473579	0.45028122
141.8	0.43768352	0.38574073	0.32309671	0.46270479	0.53930421	0.89662416	0.44410435	0.40556765	0.53482344	0.45046886
141.9	0.43779425	0.38586564	0.32320618	0.4628444	0.53946587	0.89663469	0.44415726	0.40567435	0.53491103	0.45065639
142	0.4379048	0.38599048	0.32331553	0.46298394	0.53962735	0.89664519	0.4442102	0.40578095	0.53499853	0.45084379
142.1	0.43801519	0.38611525	0.32342477	0.46312342	0.53978866	0.89665567	0.44426318	0.40588745	0.53508597	0.45103107
142.2	0.43812542	0.38623996	0.32353389	0.46326284	0.53994979	0.89666612	0.4443162	0.40599385	0.53517333	0.45121822
142.3	0.43823548	0.38636461	0.3236429	0.4634022	0.54011074	0.89667654	0.44436926	0.40610015	0.53526061	0.45140526
142.4	0.43834538	0.38648918	0.32375179	0.4635415	0.54027151	0.89668693	0.44442236	0.40620635	0.53534782	0.45159217
142.5	0.43845511	0.38661369	0.32386056	0.46368074	0.54043211	0.89669729	0.44447549	0.40631245	0.53543495	0.45177895
142.6	0.43856468	0.38673814	0.32396921	0.46381992	0.54059253	0.89670763	0.44452866	0.40641845	0.53552201	0.45196562
142.7	0.4386741	0.38686251	0.32407775	0.46395904	0.54075278	0.89671794	0.44458187	0.40652436	0.535609	0.45215216
142.8	0.43878335	0.38698682	0.32418618	0.4640981	0.54091285	0.89672823	0.44463511	0.40663016	0.53569591	0.45233859
142.9	0.43889243	0.38711107	0.32429449	0.46423709	0.54107274	0.89673849	0.44468839	0.40673587	0.53578275	0.45252489
143	0.43900136	0.38723525	0.32440268	0.46437603	0.54123246	0.89674872	0.4447417	0.40684149	0.53586952	0.45271106
143.1	0.43911014	0.38735936	0.32451075	0.4645149	0.54139201	0.89675892	0.44479504	0.40694701	0.53595621	0.45289712
143.2	0.43921875	0.3874834	0.32461871	0.46465372	0.54155138	0.8967691	0.44484842	0.40705243	0.53604283	0.45308305
143.3	0.4393272	0.38760738	0.32472655	0.46479247	0.54171058	0.89677925	0.44490184	0.40715776	0.53612937	0.45326887
143.4	0.4394355	0.38773129	0.32483428	0.46493116	0.5418696	0.89678937	0.44495529	0.407263	0.53621584	0.45345456
143.5	0.43954365	0.38785514	0.32494189	0.46506979	0.54202845	0.89679947	0.44500877	0.40736814	0.53630224	0.45364013
143.6	0.43965163	0.38797891	0.32504939	0.46520836	0.54218712	0.89680954	0.44506228	0.40747319	0.53638856	0.45382558
143.7	0.43975946	0.38810262	0.32515677	0.46534686	0.54234562	0.89681958	0.44511583	0.40757814	0.53647482	0.45401091
143.8	0.43986714	0.38822627	0.32526403	0.4654853	0.54250394	0.8968296	0.4451694	0.40768301	0.536561	0.45419611
143.9	0.43997467	0.38834985	0.32537118	0.46562368	0.54266209	0.89683959	0.44522301	0.40778779	0.53664711	0.4543812
144	0.44008204	0.38847336	0.32547821	0.465762	0.54282006	0.89684955	0.44527665	0.40789247	0.53673314	0.45456616
144.1	0.44018926	0.3885968	0.32558513	0.46590026	0.54297787	0.89685949	0.44533032	0.40799706	0.5368191	0.45475101
144.2	0.44029633	0.38872018	0.32569193	0.46603846	0.5431355	0.8968694	0.44538402	0.40810157	0.53690499	0.45493573
144.3	0.44040325	0.38884349	0.32579861	0.46617659	0.54329295	0.89687928	0.44543775	0.40820598	0.53699081	0.45512033
144.4	0.44051001	0.38896673	0.32590518	0.46631466	0.54345024	0.89688914	0.44549151	0.40831031	0.53707656	0.45530482
144.5	0.44061663	0.38908991	0.32601164	0.46645267	0.54360735	0.89689897	0.44554529	0.40841455	0.53716223	0.45548918
144.6	0.4407231	0.38921302	0.32611798	0.46659061	0.54376429	0.89690877	0.44559911	0.4085187	0.53724784	0.45567342
144.7	0.44082942	0.38933606	0.3262242	0.46672849	0.54392106	0.89691855	0.44565295	0.40862276	0.53733337	0.45585755
144.8	0.4409356	0.38945904	0.32633031	0.46686632	0.54407765	0.8969283	0.44570682	0.40872674	0.53741883	0.45604155
144.9	0.44104162	0.38958195	0.3264363	0.46700407	0.54423408	0.89693802	0.44576072	0.40883063	0.53750421	0.45622544
145	0.4411475	0.38970479	0.32654218	0.46714177	0.54439033	0.89694772	0.44581464	0.40893444	0.53758953	0.4564092
145.1	0.44125324	0.38982756	0.32664794	0.4672794	0.54454642	0.8969574	0.44586859	0.40903816	0.53767477	0.45659283
145.2	0.44135883	0.38995027	0.32675358	0.46741697	0.54470233	0.89696704	0.44592257	0.40914179	0.53775995	0.45677633
145.3	0.44146427	0.39007291	0.32685912	0.46755447	0.54485807	0.89697666	0.44597657	0.40924535	0.53784505	0.45695971
145.4	0.44156957	0.39019548	0.32696453	0.46769192	0.54501365	0.89698626	0.4460306	0.40934881	0.53793008	0.45714296
145.5	0.44167473	0.39031799	0.32706983	0.4678293	0.54516905	0.89699583	0.44608465	0.4094522	0.53801504	0.45732609
145.6	0.44177975	0.39044043	0.32717502	0.46796661	0.54532429	0.89700537	0.44613872	0.4095555	0.53809993	0.45750909
145.7	0.44188462	0.3905628	0.32728009	0.46810386	0.54547936	0.89701488	0.44619282	0.40965872	0.53818475	0.45769196
145.8	0.44198935	0.39068511	0.32738505	0.46824105	0.54563425	0.89702438	0.44624694	0.40976185	0.5382695	0.45787471
145.9	0.44209394	0.39080735	0.32748989	0.46837818	0.54578899	0.89703384	0.44630109	0.40986491	0.53835417	0.45805733
146	0.44219839	0.39092952	0.32759462	0.46851524	0.54594355	0.89704328	0.44635526	0.40996788	0.53843878	0.45823982
146.1	0.4423027	0.39105162	0.32769923	0.46865223	0.54609794	0.89705269	0.44640945	0.41007077	0.53852331	0.45842219
146.2	0.44240686	0.39117366	0.32780373	0.46878916	0.54625217	0.89706208	0.44646366	0.41017358	0.53860778	0.45860443
146.3	0.44251089	0.39129563	0.32790811	0.46892603	0.54640623	0.89707144	0.44651789	0.41027631	0.53869217	0.45878655
146.4	0.44261478	0.39141753	0.32801238	0.46906283	0.54656013	0.89708078	0.44657215	0.41037896	0.5387765	0.45896854
146.5	0.44271854	0.39153937	0.32811654	0.46919957	0.54671385	0.89709009	0.44662642	0.41048153	0.53886075	0.45915041
146.6	0.44282215	0.39166114	0.32822058	0.46933625	0.54686741	0.89709937	0.44668072	0.41058402	0.53894494	0.45933215
146.7	0.44292563	0.39178284	0.3283245	0.46947285	0.54702081	0.89710863	0.44673503	0.41068643	0.53902905	0.45951377
146.8	0.44302897	0.39190447	0.32842831	0.4696094	0.54717404	0.89711787	0.44678936	0.41078877	0.53911309	0.45969526
146.9	0.44313217	0.39202604	0.32853201	0.46974588	0.5473271	0.89712708	0.44684372	0.41089102	0.53919707	0.45987662
147	0.44323524	0.39214754	0.32863559	0.46988229	0.54748	0.89713626	0.44689809	0.4109932	0.53928097	0.46005786
147.1	0.44333818	0.39226897	0.32873906	0.47001864	0.54763273	0.89714542	0.44695248	0.4110953	0.5393648	0.46023898
147.2	0.44344098	0.39239034	0.32884242	0.47015493	0.54778529	0.89715455	0.44700688	0.41119732	0.53944856	0.46041997
147.3	0.44354364	0.39251163	0.32894566	0.47029114	0.54793769	0.89716366	0.44706131	0.41129927	0.53953226	0.46060084
147.4	0.44364617	0.39263286	0.32904879	0.4704273	0.54808993	0.89717274	0.44711575	0.41140113	0.53961588	0.46078158
147.5	0.44374857	0.39275403	0.3291518	0.47056338	0.548242	0.8971818	0.44717021	0.41150293	0.53969943	0.4609622
147.6	0.44385084	0.39287512	0.3292547	0.47069941	0.5483939	0.89719083	0.44722468	0.41160464	0.53978292	0.46114269
147.7	0.44395297	0.39299615	0.32935748	0.47083536	0.54854564	0.89719984	0.44727917	0.41170628	0.53986633	0.46132306
147.8	0.44405497	0.39311711	0.32946016	0.47097125	0.54869721	0.89720882	0.44733368	0.41180784	0.53994967	0.46150331
147.9	0.44415684	0.39323801	0.32956271	0.47110708	0.54884862	0.89721778	0.4473882	0.41190933	0.54003295	0.46168343
148	0.44425858	0.39335884	0.32966516	0.47124283	0.54899987	0.89722671	0.44744274	0.41201075	0.54011615	0.46186343
148.1	0.44436019	0.3934796	0.32976749	0.47137853	0.54915095	0.89723562	0.44749729	0.41211208	0.54019929	0.4620433
148.2	0.44446166	0.39360029	0.32986971	0.47151415	0.54930187	0.8972445	0.44755185	0.41221335	0.54028235	0.46222305
148.3	0.44456301	0.39372091	0.32997181	0.47164971	0.54945262	0.89725336	0.44760643	0.41231454	0.54036535	0.46240268
148.4	0.44466423	0.39384147	0.3300738	0.4717852	0.54960321	0.89726219	0.44766102	0.41241565	0.54044827	0.46258218
148.5	0.44476532	0.39396196	0.33017568	0.47192063	0.54975364	0.897271	0.44771562	0.41251669	0.54053113	0.46276157
148.6	0.44486628	0.39408238	0.33027744	0.47205599	0.54990391	0.89727979	0.44777023	0.41261766	0.54061391	0.46294082
148.7	0.44496712	0.39420274	0.3303791	0.47219128	0.55005401	0.89728855	0.44782486	0.41271855	0.54069663	0.46311996
148.8	0.44506782	0.39432303	0.33048063	0.47232651	0.55020395	0.89729728	0.4478795	0.41281937	0.54077928	0.46329897
148.9	0.4451684	0.39444325	0.33058206	0.47246167	0.55035373	0.897306	0.44793415	0.41292012	0.54086186	0.46347786
149	0.44526886	0.3945634	0.33068337	0.47259676	0.55050335	0.89731468	0.44798881	0.41302079	0.54094437	0.46365663
149.1	0.44536918	0.39468349	0.33078457	0.47273178	0.5506528	0.89732335	0.44804348	0.41312139	0.54102681	0.46383527
149.2	0.44546938	0.39480351	0.33088566	0.47286674	0.5508021	0.89733198	0.44809816	0.41322192	0.54110918	0.46401379
149.3	0.44556946	0.39492346	0.33098664	0.47300163	0.55095123	0.8973406	0.44815285	0.41332238	0.54119148	0.46419219
149.4	0.44566941	0.39504334	0.3310875	0.47313646	0.5511002	0.89734919	0.44820755	0.41342276	0.54127371	0.46437046
149.5	0.44576924	0.39516316	0.33118825	0.47327121	0.55124902	0.89735776	0.44826226	0.41352306	0.54135587	0.46454861
149.6	0.44586894	0.39528291	0.33128889	0.4734059	0.55139767	0.8973663	0.44831698	0.4136233	0.54143796	0.46472663
149.7	0.44596852	0.39540259	0.33138941	0.47354052	0.55154616	0.89737481	0.4483717	0.41372346	0.54151999	0.46490453
149.8	0.44606798	0.3955222	0.33148983	0.47367508	0.5516945	0.89738331	0.44842643	0.41382355	0.54160194	0.4650823
149.9	0.44616731	0.39564175	0.33159013	0.47380957	0.55184267	0.89739178	0.44848117	0.41392356	0.54168383	0.46525995
195	0.48121688	0.44300863	0.36641169	0.52683658	0.60529306	0.89943523	0.47113624	0.45168865	0.57240067	0.53223886
195.1	0.48127655	0.44310008	0.36646868	0.52693526	0.60538729	0.89943688	0.47118131	0.4517551	0.57245787	0.53235832
195.2	[illegible]	[illegible]	[illegible]	[illegible]	[illegible]	[illegible]	[illegible]	[illegible]	[illegible]	[illegible]
195.3	[illegible]	[illegible]	[illegible]	[illegible]	[illegible]	[illegible]	[illegible]	[illegible]	[illegible]	[illegible]
195.4	[illegible]	[illegible]	[illegible]	[illegible]	[illegible]	[illegible]	[illegible]	[illegible]	[illegible]	[illegible]
195.5	[illegible]	[illegible]	[illegible]	[illegible]	[illegible]	[illegible]	[illegible]	[illegible]	[illegible]	[illegible]
150.3	0.44656343	0.39611926	0.33199022	0.47434684	0.55243378	0.89742542	0.4487002	0.41432291	0.54201069	0.46596928
150.4	0.44666216	0.39623847	0.33208996	0.47448099	0.55258116	0.89743377	0.44875497	0.41442256	0.54209223	0.46614629
150.5	0.44676077	0.39635761	0.33218959	0.47461507	0.55272838	0.8974421	0.44880975	0.41452215	0.54217371	0.46632319
150.6	0.44685926	0.39647669	0.33228911	0.47474908	0.55287545	0.89745041	0.44886453	0.41462167	0.54225511	0.46649995
150.7	0.44695762	0.39659569	0.33238851	0.47488302	0.55302236	0.89745869	0.44891931	0.41472111	0.54233645	0.46667659
150.8	0.44705587	0.39671463	0.33248781	0.4750169	0.55316911	0.89746694	0.4489741	0.41482048	0.54241772	0.46685311
150.9	0.447154	0.39683351	0.332587	0.47515071	0.5533157	0.89747518	0.44902889	0.41491979	0.54249892	0.4670295
151	0.44725202	0.39695231	0.33268607	0.47528445	0.55346214	0.89748339	0.44908369	0.41501902	0.54258005	0.46720576
151.1	0.44734991	0.39707105	0.33278503	0.47541812	0.55360842	0.89749158	0.44913848	0.41511818	0.54266111	0.4673819
151.2	0.44744769	0.39718972	0.33288388	0.47555173	0.55375454	0.89749974	0.44919328	0.41521727	0.5427421	0.46755791
151.3	0.44754535	0.39730833	0.33298262	0.47568527	0.55390051	0.89750788	0.44924809	0.4153163	0.54282303	0.4677338
151.4	0.44764289	0.39742686	0.33308125	0.47581873	0.55404631	0.897516	0.44930289	0.41541525	0.54290389	0.46790956
151.5	0.44774032	0.39754533	0.33317977	0.47595213	0.55419197	0.8975241	0.4493577	0.41551414	0.54298468	0.4680852
151.6	0.44783763	0.39766373	0.33327818	0.47608546	0.55433746	0.89753217	0.4494125	0.41561295	0.5430654	0.46826071
151.7	0.44793483	0.39778207	0.33337647	0.47621873	0.5544828	0.89754022	0.44946731	0.4157117	0.54314605	0.46843609
151.8	0.44803191	0.39790033	0.33347466	0.47635192	0.55462798	0.89754824	0.44952212	0.41581037	0.54322664	0.46861135
151.9	0.44812887	0.39801853	0.33357274	0.47648505	0.55477301	0.89755625	0.44957693	0.41590898	0.54330716	0.46878648
152	0.44822572	0.39813667	0.3336707	0.47661811	0.55491788	0.89756423	0.44963173	0.41600752	0.54338761	0.46896149
152.1	0.44832246	0.39825473	0.33376855	0.4767511	0.5550626	0.89757218	0.44968654	0.416106	0.54346799	0.46913637
152.2	0.44841909	0.39837273	0.3338663	0.47688402	0.55520716	0.89758012	0.44974134	0.4162044	0.5435483	0.46931112
152.3	0.4485156	0.39849066	0.33396393	0.47701687	0.55535157	0.89758803	0.44979615	0.41630274	0.54362855	0.46948575
152.4	0.44861199	0.39860852	0.33406146	0.47714965	0.55549582	0.89759592	0.44985095	0.416401	0.54370873	0.46966026
152.5	0.44870828	0.39872632	0.33415887	0.47728237	0.55563992	0.89760379	0.44990575	0.4164992	0.54378884	0.46983464
152.6	0.44880445	0.39884405	0.33425618	0.47741501	0.55578386	0.89761164	0.44996054	0.41659734	0.54386888	0.47000889
152.7	0.44890051	0.39896171	0.33435337	0.47754759	0.55592765	0.89761946	0.45001534	0.4166954	0.54394886	0.47018302
152.8	0.44899646	0.3990793	0.33445046	0.4776801	0.55607129	0.89762726	0.45007013	0.4167934	0.54402877	0.47035702
152.9	0.4490923	0.39919683	0.33454743	0.47781254	0.55621477	0.89763504	0.45012491	0.41689133	0.54410861	0.4705309
153	0.44918803	0.39931429	0.3346443	0.47794491	0.55635811	0.89764279	0.4501797	0.41698919	0.54418838	0.47070465
153.1	0.44928364	0.39943169	0.33474106	0.47807721	0.55650128	0.89765053	0.45023447	0.41708699	0.54426809	0.47087827
153.2	0.44937915	0.39954901	0.33483771	0.47820944	0.55664431	0.89765824	0.45028925	0.41718471	0.54434772	0.47105177
153.3	0.44947455	0.39966627	0.33493424	0.4783416	0.55678718	0.89766593	0.45034402	0.41728237	0.54442729	0.47122515
153.4	0.44956984	0.39978346	0.33503067	0.4784737	0.5569299	0.89767359	0.45039878	0.41737996	0.5445068	0.4713984
153.5	0.44966501	0.39990059	0.33512699	0.47860572	0.55707247	0.89768124	0.45045354	0.41747748	0.54458623	0.47157152
153.6	0.44976008	0.40001764	0.3352232	0.47873768	0.55721489	0.89768886	0.45050829	0.41757493	0.5446656	0.47174452
153.7	0.44985504	0.40013463	0.33531931	0.47886956	0.55735716	0.89769647	0.45056303	0.41767231	0.5447449	0.4719174
153.8	0.4499499	0.40025156	0.3354153	0.47900138	0.55749927	0.89770405	0.45061777	0.41776963	0.54482414	0.47209015
153.9	0.45004464	0.40036841	0.33551119	0.47913312	0.55764124	0.8977116	0.45067251	0.41786688	0.54490331	0.47226277
154	0.45013928	0.4004852	0.33560696	0.4792648	0.55778305	0.89771914	0.45072724	0.41796406	0.54498241	0.47243528
154.1	0.45023381	0.40060193	0.33570263	0.47939641	0.55792471	0.89772666	0.45078196	0.41806117	0.54506144	0.47260766
154.2	0.45032823	0.40071858	0.33579819	0.47952795	0.55806622	0.89773415	0.45083667	0.41815822	0.5451404	0.47277991
154.3	0.45042255	0.40083517	0.33589364	0.47965942	0.55820759	0.89774162	0.45089138	0.41825519	0.5452193	0.47295204
154.4	0.45051676	0.40095169	0.33598898	0.47979081	0.5583488	0.89774907	0.45094608	0.4183521	0.54529813	0.47312405
154.5	0.45061086	0.40106815	0.33608422	0.47992214	0.55848986	0.8977565	0.45100077	0.41844895	0.5453769	0.47329593
154.6	0.45070486	0.40118453	0.33617934	0.4800534	0.55863078	0.89776391	0.45105545	0.41854573	0.54545559	0.47346769
154.7	0.45079875	0.40130085	0.33627436	0.48018459	0.55877154	0.89777129	0.45111012	0.41864244	0.54553422	0.47363932
154.8	0.45089254	0.40141711	0.33636927	0.48031571	0.55891216	0.89777866	0.45116478	0.41873908	0.54561279	0.47381083
154.9	0.45098622	0.40153329	0.33646407	0.48044675	0.55905263	0.897786	0.45121944	0.41883566	0.54569128	0.47398222
155	0.4510798	0.40164941	0.33655876	0.48057773	0.55919294	0.89779333	0.45127408	0.41893216	0.54576971	0.47415348
155.1	0.45117328	0.40176547	0.33665335	0.48070864	0.55933311	0.89780063	0.45132872	0.41902861	0.54584807	0.47432462
155.2	0.45126665	0.40188145	0.33674783	0.48083948	0.55947313	0.89780791	0.45138334	0.41912498	0.54592637	0.47449564
155.3	0.45135992	0.40199737	0.3368422	0.48097025	0.55961301	0.89781517	0.45143795	0.41922129	0.5460046	0.47466653
155.4	0.45145308	0.40211322	0.33693646	0.48110094	0.55975273	0.89782241	0.45149256	0.41931754	0.54608276	0.4748373
155.5	0.45154614	0.40222901	0.33703062	0.48123157	0.55989231	0.89782962	0.45154715	0.41941371	0.54616085	0.47500794
155.6	0.4516391	0.40234473	0.33712466	0.48136212	0.56003174	0.89783682	0.45160173	0.41950982	0.54623888	0.47517846
155.7	0.45173196	0.40246038	0.33721861	0.48149261	0.56017102	0.897844	0.45165629	0.41960587	0.54631684	0.47534886
155.8	0.45182471	0.40257597	0.33731244	0.48162302	0.56031016	0.89785115	0.45171085	0.41970185	0.54639473	0.47551913
155.9	0.45191736	0.40269149	0.33740616	0.48175336	0.56044914	0.89785829	0.45176539	0.41979776	0.54647255	0.47568928
156	0.45200991	0.40280694	0.33749978	0.48188364	0.56058799	0.8978654	0.45181992	0.4198936	0.54655031	0.4758593
156.1	0.45210236	0.40292232	0.33759329	0.48201384	0.56072668	0.8978725	0.45187444	0.41998938	0.546628	0.4760292
156.2	0.4521947	0.40303764	0.3376867	0.48214397	0.56086523	0.89787957	0.45192894	0.4200851	0.54670563	0.47619898
156.3	0.45228695	0.40315289	0.33778	0.48227403	0.56100363	0.89788662	0.45198343	0.42018074	0.54678319	0.47636863
156.4	0.45237909	0.40326808	0.33787319	0.48240401	0.56114189	0.89789366	0.45203791	0.42027633	0.54686068	0.47653816
156.5	0.45247113	0.4033832	0.33796627	0.48253393	0.56128	0.89790067	0.45209237	0.42037184	0.5469381	0.47670757
156.6	0.45256308	0.40349825	0.33805925	0.48266377	0.56141796	0.89790766	0.45214682	0.42046729	0.54701546	0.47687685
156.7	0.45265492	0.40361323	0.33815212	0.48279355	0.56155578	0.89791463	0.45220125	0.42056268	0.54709275	0.47704601
156.8	0.45274666	0.40372815	0.33824488	0.48292325	0.56169346	0.89792159	0.45225567	0.42065799	0.54716997	0.47721504
156.9	0.4528383	0.403843	0.33833753	0.48305288	0.56183098	0.89792852	0.45231007	0.42075324	0.54724712	0.47738395
157	0.45292984	0.40395779	0.33843008	0.48318243	0.56196837	0.89793543	0.45236446	0.42084843	0.54732421	0.47755273
157.1	0.45302129	0.40407251	0.33852253	0.48331192	0.56210561	0.89794232	0.45241883	0.42094354	0.54740123	0.47772139
157.2	0.45311263	0.40418716	0.33861486	0.48344133	0.56224271	0.89794919	0.45247319	0.42103859	0.54747819	0.47788993
157.3	0.45320387	0.40430175	0.33870709	0.48357068	0.56237966	0.89795605	0.45252753	0.42113357	0.54755508	0.47805834
157.4	0.45329502	0.40441627	0.33879922	0.48369995	0.56251646	0.89796288	0.45258185	0.42122848	0.5476319	0.47822663
157.5	0.45338606	0.40453072	0.33889123	0.48382914	0.56265313	0.89796969	0.45263615	0.42132333	0.54770865	0.47839479
157.6	0.45347701	0.40464511	0.33898315	0.48395827	0.56278965	0.89797649	0.45269044	0.42141811	0.54778534	0.47856283
157.7	0.45356785	0.40475943	0.33907495	0.48408732	0.56292603	0.89798326	0.45274471	0.42151282	0.54786195	0.47873074
157.8	0.4536586	0.40487368	0.33916665	0.4842163	0.56306226	0.89799001	0.45279897	0.42160747	0.54793851	0.47889853
157.9	0.45374925	0.40498787	0.33925824	0.48434521	0.56319835	0.89799675	0.4528532	0.42170204	0.54801499	0.4790662
158	0.4538398	0.40510199	0.33934973	0.48447405	0.5633343	0.89800346	0.45290742	0.42179656	0.54809141	0.47923374
158.1	0.45393026	0.40521604	0.33944111	0.48460281	0.56347011	0.89801016	0.45296162	0.421891	0.54816776	0.47940115
158.2	0.45402061	0.40533003	0.33953239	0.4847315	0.56360577	0.89801684	0.4530158	0.42198538	0.54824404	0.47956844
158.3	0.45411087	0.40544395	0.33962355	0.48486012	0.56374129	0.89802349	0.45306996	0.42207969	0.54832026	0.4797356
158.4	0.45420103	0.40555781	0.33971462	0.48498867	0.56387667	0.89803013	0.4531241	0.42217393	0.54839641	0.47990264
158.5	0.45429109	0.4056716	0.33980558	0.48511714	0.56401191	0.89803675	0.45317823	0.42226811	0.54847249	0.48006955
158.6	0.45438105	0.40578532	0.33989643	0.48524554	0.56414701	0.89804335	0.45323233	0.42236222	0.5485485	0.48023634
158.7	0.45447091	0.40589898	0.33998717	0.48537387	0.56428196	0.89804993	0.45328641	0.42245627	0.54862445	0.480403
158.8	0.45456068	0.40601257	0.34007782	0.48550212	0.56441678	0.8980565	0.45334048	0.42255025	0.54870033	0.48056954
158.9	0.45465035	0.40612609	0.34016835	0.48563031	0.56455145	0.89806304	0.45339452	0.42264416	0.54877614	0.48073595
159	0.45473992	0.40623955	0.34025878	0.48575841	0.56468598	0.89806956	0.45344854	0.42273801	0.54885189	0.48090223
159.1	0.4548294	0.40635295	0.34034911	0.48588645	0.56482037	0.89807607	0.45350255	0.42283178	0.54892757	0.48106839
159.2	0.45491877	0.40646627	0.34043933	0.48601442	0.56495462	0.89808256	0.45355653	0.4229255	0.54900318	0.48123442
159.3	0.45500805	0.40657953	0.34052944	0.48614231	0.56508873	0.89808902	0.45361049	0.42301914	0.54907872	0.48140032
159.4	0.45509724	0.40669273	0.34061945	0.48627013	0.56522271	0.89809547	0.45366443	0.42311272	0.5491542	0.4815661
159.5	0.45518632	0.40680585	0.34070935	0.48639787	0.56535654	0.8981019	0.45371834	0.42320624	0.5492296	0.48173175
159.6	0.45527531	0.40691892	0.34079915	0.48652554	0.56549023	0.89810832	0.45377224	0.42329969	0.54930494	0.48189728
159.7	0.4553642	0.40703191	0.34088885	0.48665314	0.56562378	0.89811471	0.45382611	0.42339307	0.54938022	0.48206268
159.8	0.455453	0.40714484	0.34097843	0.48678067	0.56575719	0.89812109	0.45387996	0.42348638	0.54945542	0.48222795
159.9	0.45554169	0.40725771	0.34106792	0.48690812	0.56589047	0.89812744	0.45393379	0.42357963	0.54953056	0.48239309
160	0.45563029	0.4073705	0.3411573	0.4870355	0.5660236	0.89813378	0.4539876	0.42367281	0.54960563	0.48255811
160.1	0.4557188	0.40748324	0.34124657	0.48716281	0.5661566	0.8981401	0.45404138	0.42376593	0.54968064	0.48272299
160.2	0.4558072	0.4075959	0.34133574	0.48729004	0.56628946	0.8981464	0.45409514	0.42385898	0.54975557	0.48288776
160.3	0.45589551	0.40770851	0.3414248	0.4874172	0.56642218	0.89815269	0.45414888	0.42395197	0.54983044	0.48305239
160.4	0.45598372	0.40782104	0.34151376	0.48754428	0.56655476	0.89815895	0.45420259	0.42404488	0.54990524	0.4832169
160.5	0.45607184	0.40793351	0.34160262	0.4876713	0.56668721	0.8981652	0.45425628	0.42413774	0.54997997	0.48338127
160.6	0.45615986	0.40804591	0.34169137	0.48779824	0.56681951	0.89817143	0.45430995	0.42423052	0.55005464	0.48354552
160.7	0.45624778	0.40815825	0.34178001	0.4879251	0.56695168	0.89817764	0.45436359	0.42432324	0.55012924	0.48370964
160.8	0.45633561	0.40827052	0.34186855	0.48805189	0.56708372	0.89818384	0.45441721	0.42441589	0.55020377	0.48387364
160.9	0.45642334	0.40838273	0.34195699	0.48817861	0.56721561	0.89819002	0.4544708	0.42450847	0.55027823	0.4840375
161	0.45651097	0.40849487	0.34204532	0.48830525	0.56734737	0.89819617	0.45452437	0.42460099	0.55035263	0.48420124
161.1	0.4565985	0.40860695	0.34213355	0.48843183	0.56747899	0.89820231	0.45457791	0.42469344	0.55042695	0.48436484
161.2	0.45668594	0.40871896	0.34222167	0.48855832	0.56761048	0.89820844	0.45463143	0.42478582	0.55050121	0.48452832
161.3	0.45677329	0.4088309	0.34230969	0.48868474	0.56774183	0.89821454	0.45468493	0.42487814	0.55057541	0.48469167
161.4	0.45686053	0.40894278	0.34239761	0.48881109	0.56787305	0.89822063	0.4547384	0.42497038	0.55064953	0.48485489
161.5	0.45694768	0.40905459	0.34248542	0.48893737	0.56800413	0.8982267	0.45479184	0.42506256	0.55072359	0.48501798
161.6	0.45703474	0.40916634	0.34257313	0.48906357	0.56813507	0.89823276	0.45484526	0.42515468	0.55079758	0.48518094
161.7	0.45712169	0.40927803	0.34266073	0.4891897	0.56826588	0.89823879	0.45489865	0.42524672	0.5508715	0.48534376
161.8	0.45720855	0.40938964	0.34274823	0.48931575	0.56839655	0.89824481	0.45495202	0.4253387	0.55094536	0.48550646
161.9	0.45729532	0.40950119	0.34283562	0.48944173	0.56852709	0.89825081	0.45500536	0.42543061	0.55101914	0.48566903
162	0.45738198	0.40961268	0.34292291	0.48956763	0.5686575	0.8982568	0.45505868	0.42552246	0.55109286	0.48583147
162.1	0.45746856	0.4097241	0.3430101	0.48969346	0.56878777	0.89826276	0.45511197	0.42561423	0.55116652	0.48599378
162.2	0.45755503	0.40983546	0.34309718	0.48981922	0.5689179	0.89826871	0.45516523	0.42570594	0.5512401	0.48615596
162.3	0.45764141	0.40994675	0.34318416	0.4899449	0.5690479	0.89827464	0.45521847	0.42579758	0.55131362	0.48631801
162.4	0.45772769	0.41005798	0.34327104	0.49007051	0.56917777	0.89828056	0.45527168	0.42588916	0.55138707	0.48647992
162.5	0.45781388	0.41016914	0.34335781	0.49019604	0.5693075	0.89828646	0.45532486	0.42598066	0.55146045	0.48664171
162.6	0.45789997	0.41028023	0.34344448	0.4903215	0.5694371	0.89829234	0.45537802	0.4260721	0.55153376	0.48680337
162.7	0.45798596	0.41039126	0.34353104	0.49044688	0.56956657	0.89829821	0.45543115	0.42616347	0.55160701	0.48696489
162.8	0.45807186	0.41050223	0.3436175	0.49057219	0.5696959	0.89830405	0.45548425	0.42625477	0.55168019	0.48712628
162.9	0.45815766	0.41061313	0.34370386	0.49069743	0.5698251	0.89830988	0.45553732	0.42634601	0.55175331	0.48728755
163	0.45824336	0.41072396	0.34379011	0.49082259	0.56995417	0.8983157	0.45559037	0.42643717	0.55182635	0.48744868
163.1	0.45832897	0.41083473	0.34387626	0.49094767	0.5700831	0.8983215	0.45564339	0.42652827	0.55189933	0.48760968
163.2	0.45841448	0.41094543	0.34396231	0.49107269	0.5702119	0.89832728	0.45569638	0.4266193	0.55197224	0.48777055
163.3	0.4584999	0.41105607	0.34404825	0.49119762	0.57034057	0.89833304	0.45574935	0.42671026	0.55204508	0.48793129
163.4	0.45858522	0.41116665	0.34413409	0.49132249	0.5704691	0.89833879	0.45580229	0.42680116	0.55211786	0.4880919
163.5	0.45867044	0.41127716	0.34421983	0.49144727	0.57059751	0.89834452	0.45585519	0.42689198	0.55219057	0.48825238
163.6	0.45875557	0.4113876	0.34430546	0.49157199	0.57072578	0.89835024	0.45590808	0.42698274	0.55226321	0.48841272
163.7	0.4588406	0.41149798	0.34439099	0.49169663	0.57085392	0.89835594	0.45596093	0.42707343	0.55233579	0.48857294
163.8	0.45892554	0.4116083	0.34447641	0.49182119	0.57098192	0.89836162	0.45601375	0.42716405	0.5524083	0.48873302
163.9	0.45901038	0.41171855	0.34456174	0.49194568	0.5711098	0.89836729	0.45606655	0.42725461	0.55248074	0.48889297
164	0.45909512	0.41182874	0.34464696	0.4920701	0.57123755	0.89837294	0.45611931	0.42734509	0.55255311	0.48905279
164.1	0.45917977	0.41193886	0.34473207	0.49219444	0.57136516	0.89837857	0.45617205	0.42743551	0.55262542	0.48921248
164.2	0.45926433	0.41204891	0.34481709	0.4923187	0.57149264	0.89838419	0.45622476	0.42752585	0.55269766	0.48937204
164.3	0.45934879	0.41215891	0.344902	0.49244289	0.57162	0.89838979	0.45627744	0.42761613	0.55276984	0.48953146
164.4	0.45943315	0.41226883	0.3449868	0.49256701	0.57174722	0.89839538	0.45633009	0.42770634	0.55284194	0.48969076
164.5	0.45951741	0.4123787	0.34507151	0.49269105	0.57187431	0.89840094	0.45638271	0.42779648	0.55291398	0.48984992
164.6	0.45960159	0.41248849	0.34515611	0.49281502	0.57200127	0.8984065	0.4564353	0.42788656	0.55298596	0.49000895
164.7	0.45968566	0.41259823	0.34524061	0.49293891	0.5721281	0.89841204	0.45648786	0.42797656	0.55305787	0.49016785
164.8	0.45976964	0.4127079	0.345325	0.49306273	0.5722548	0.89841756	0.45654039	0.4280665	0.55312971	0.49032661
164.9	0.45985353	0.4128175	0.3454093	0.49318647	0.57238137	0.89842306	0.45659289	0.42815636	0.55320148	0.49048525
210	0.48950235	0.45612957	0.37409829	0.5407649	0.6185122	0.89960769	0.47770161	0.46107738	0.58055171	0.54899581
210.1	0.48955339	0.45621317	0.3741439	0.54085267	0.61859455	0.89960839	0.477744	0.46113666	0.58060321	0.5491004
[illegible]	[illegible]	[illegible]	[illegible]	[illegible]	[illegible]	[illegible]	[illegible]	[illegible]	[illegible]	[illegible]
165.3	0.46018811	0.41325528	0.34574544	0.49368068	0.57288636	0.89844493	0.4568026	0.42851514	0.55348793	0.49111845
165.4	0.46027152	0.41336456	0.34582922	0.49380405	0.57301228	0.89845036	0.45685496	0.42860466	0.55355937	0.49127643
165.5	0.46035484	0.41347378	0.3459129	0.49392734	0.57313808	0.89845577	0.45690728	0.42869411	0.55363075	0.49143426
165.6	0.46043806	0.41358294	0.34599648	0.49405055	0.57326375	0.89846117	0.45695957	0.42878349	0.55370206	0.49159197
165.7	0.46052118	0.41369203	0.34607995	0.4941737	0.57338928	0.89846655	0.45701183	0.4288728	0.55377331	0.49174954
165.8	0.46060421	0.41380105	0.34616332	0.49429676	0.57351469	0.89847192	0.45706406	0.42896204	0.5538445	0.49190698
165.9	0.46068714	0.41391002	0.34624659	0.49441975	0.57363997	0.89847727	0.45711626	0.42905122	0.55391561	0.49206429
166	0.46076998	0.41401891	0.34632975	0.49454267	0.57376513	0.8984826	0.45716843	0.42914032	0.55398667	0.49222146
166.1	0.46085273	0.41412775	0.34641281	0.49466551	0.57389015	0.89848792	0.45722057	0.42922935	0.55405765	0.49237851
166.2	0.46093538	0.41423652	0.34649576	0.49478827	0.57401505	0.89849323	0.45727268	0.42931831	0.55412857	0.49253541
166.3	0.46101794	0.41434523	0.34657862	0.49491096	0.57413982	0.89849852	0.45732476	0.4294072	0.55419943	0.49269219
166.4	0.4611004	0.41445387	0.34666137	0.49503357	0.57426447	0.89850379	0.45737681	0.42949602	0.55427022	0.49284883
166.5	0.46118277	0.41456245	0.34674401	0.49515611	0.57438898	0.89850905	0.45742883	0.42958477	0.55434095	0.49300534
166.6	0.46126504	0.41467096	0.34682656	0.49527857	0.57451337	0.8985143	0.45748081	0.42967346	0.55441161	0.49316172
166.7	0.46134722	0.41477941	0.346909	0.49540096	0.57463763	0.89851953	0.45753277	0.42976207	0.55448221	0.49331796
166.8	0.4614293	0.4148878	0.34699134	0.49552327	0.57476177	0.89852474	0.45758469	0.42985061	0.55455275	0.49347407
166.9	0.4615113	0.41499613	0.34707358	0.4956455	0.57488578	0.89852994	0.45763658	0.42993907	0.55462322	0.49363005
167	0.46159319	0.41510439	0.34715571	0.49576766	0.57500966	0.89853513	0.45768844	0.43002747	0.55469362	0.49378589
167.1	0.461675	0.41521258	0.34723774	0.49588975	0.57513342	0.8985403	0.45774027	0.4301158	0.55476397	0.4939416
167.2	0.46175671	0.41532072	0.34731968	0.49601175	0.57525705	0.89854545	0.45779207	0.43020406	0.55483424	0.49409718
167.3	0.46183832	0.41542878	0.34740151	0.49613369	0.57538055	0.89855059	0.45784384	0.43029224	0.55490446	0.49425262
167.4	0.46191985	0.41553679	0.34748323	0.49625554	0.57550393	0.89855572	0.45789558	0.43038036	0.55497461	0.49440793
167.5	0.46200128	0.41564473	0.34756486	0.49637732	0.57562719	0.89856083	0.45794728	0.4304684	0.5550447	0.49456311
167.6	0.46208261	0.41575261	0.34764638	0.49649903	0.57575032	0.89856592	0.45799896	0.43055637	0.55511472	0.49471815
167.7	0.46216385	0.41586042	0.34772781	0.49662066	0.57587332	0.898571	0.4580506	0.43064427	0.55518469	0.49487306
167.8	0.462245	0.41596818	0.34780913	0.49674221	0.5759962	0.89857607	0.45810221	0.4307321	0.55525459	0.49502784
167.9	0.46232606	0.41607586	0.34789035	0.49686369	0.57611896	0.89858112	0.45815379	0.43081986	0.55532442	0.49518248
168	0.46240702	0.41618349	0.34797147	0.49698509	0.57624159	0.89858616	0.45820533	0.43090754	0.5553942	0.49533699
168.1	0.46248789	0.41629105	0.34805249	0.49710642	0.5763641	0.89859118	0.45825685	0.43099516	0.55546391	0.49549137
168.2	0.46256867	0.41639855	0.3481334	0.49722767	0.57648648	0.89859619	0.45830833	0.4310827	0.55553356	0.49564561
168.3	0.46264936	0.41650598	0.34821422	0.49734884	0.57660874	0.89860118	0.45835979	0.43117017	0.55560315	0.49579972
168.4	0.46272995	0.41661335	0.34829493	0.49746994	0.57673087	0.89860616	0.45841121	0.43125757	0.55567267	0.49595369
168.5	0.46281045	0.41672066	0.34837554	0.49759096	0.57685289	0.89861113	0.45846259	0.43134489	0.55574214	0.49610753
168.6	0.46289086	0.41682791	0.34845604	0.49771191	0.57697478	0.89861608	0.45851395	0.43143215	0.55581154	0.49626124
168.7	0.46297117	0.41693509	0.34853645	0.49783278	0.57709654	0.89862101	0.45856528	0.43151933	0.55588088	0.49641481
168.8	0.4630514	0.41704221	0.34861675	0.49795357	0.57721818	0.89862593	0.45861657	0.43160644	0.55595016	0.49656825
168.9	0.46313153	0.41714926	0.34869696	0.49807429	0.5773397	0.89863084	0.45866783	0.43169348	0.55601938	0.49672156
169	0.46321157	0.41725626	0.34877706	0.49819493	0.5774611	0.89863573	0.45871906	0.43178044	0.55608854	0.49687473
169.1	0.46329151	0.41736319	0.34885706	0.4983155	0.57758237	0.89864061	0.45877026	0.43186734	0.55615764	0.49702777
169.2	0.46337137	0.41747005	0.34893696	0.49843598	0.57770353	0.89864548	0.45882142	0.43195416	0.55622668	0.49718068
169.3	0.46345113	0.41757686	0.34901675	0.4985564	0.57782456	0.89865033	0.45887255	0.4320409	0.55629565	0.49733345
169.4	0.4635308	0.4176836	0.34909645	0.49867674	0.57794546	0.89865516	0.45892366	0.43212758	0.55636457	0.49748609
169.5	0.46361038	0.41779027	0.34917605	0.498797	0.57806625	0.89865998	0.45897473	0.43221418	0.55643343	0.49763859
169.6	0.46368987	0.41789689	0.34925554	0.49891718	0.57818691	0.89866479	0.45902576	0.43230071	0.55650223	0.49779096
169.7	0.46376927	0.41800344	0.34933494	0.49903729	0.57830746	0.89866958	0.45907677	0.43238716	0.55657096	0.4979432
169.8	0.46384858	0.41810993	0.34941423	0.49915732	0.57842788	0.89867436	0.45912775	0.43247355	0.55663964	0.4980953
169.9	0.46392779	0.41821636	0.34949343	0.49927728	0.57854818	0.89867913	0.45917869	0.43255985	0.55670826	0.49824727
170	0.46400692	0.41832272	0.34957253	0.49939716	0.57866836	0.89868388	0.45922961	0.43264609	0.55677682	0.4983991
170.1	0.46408595	0.41842902	0.34965152	0.49951696	0.57878841	0.89868862	0.45928049	0.43273225	0.55684532	0.49855081
170.2	0.46416489	0.41853526	0.34973042	0.49963669	0.57890835	0.89869334	0.45933134	0.43281834	0.55691377	0.49870237
170.3	0.46424374	0.41864143	0.34980921	0.49975634	0.57902817	0.89869805	0.45938216	0.43290435	0.55698215	0.49885381
170.4	0.4643225	0.41874755	0.3498879	0.49987591	0.57914786	0.89870274	0.45943295	0.43299029	0.55705048	0.49900511
170.5	0.46440118	0.4188536	0.3499665	0.49999541	0.57926744	0.89870743	0.4594837	0.43307616	0.55711874	0.49915628
170.6	0.46447976	0.41895958	0.35004499	0.50011483	0.5793869	0.89871209	0.45953443	0.43316195	0.55718695	0.49930731
170.7	0.46455825	0.41906551	0.35012338	0.50023418	0.57950623	0.89871675	0.45958513	0.43324767	0.5572551	0.49945821
170.8	0.46463665	0.41917137	0.35020167	0.50035345	0.57962545	0.89872139	0.45963579	0.43333331	0.5573232	0.49960897
170.9	0.46471495	0.41927717	0.35027986	0.50047264	0.57974454	0.89872601	0.45968642	0.43341888	0.55739123	0.49975961
171	0.46479317	0.4193829	0.35035795	0.50059176	0.57986352	0.89873062	0.45973703	0.43350437	0.55745921	0.4999101
171.1	0.4648713	0.41948858	0.35043594	0.50071079	0.57998238	0.89873522	0.4597876	0.43358979	0.55752713	0.50006047
171.2	0.46494934	0.41959419	0.35051383	0.50082976	0.58010111	0.8987398	0.45983814	0.43367514	0.557595	0.5002107
171.3	0.4650273	0.41969974	0.35059162	0.50094864	0.58021973	0.89874437	0.45988865	0.43376041	0.55766281	0.5003608
171.4	0.46510516	0.41980523	0.35066931	0.50106745	0.58033823	0.89874893	0.45993912	0.4338456	0.55773056	0.50051076
171.5	0.46518293	0.41991065	0.3507469	0.50118618	0.58045661	0.89875347	0.45998957	0.43393072	0.55779825	0.50066059
171.6	0.46526061	0.42001601	0.3508244	0.50130484	0.58057487	0.898758	0.46003999	0.43401576	0.55786589	0.50081029
171.7	0.4653382	0.42012131	0.35090179	0.50142342	0.58069302	0.89876252	0.46009037	0.43410073	0.55793347	0.50095985
171.8	0.46541571	0.42022655	0.35097908	0.50154192	0.58081104	0.89876702	0.46014073	0.43418563	0.558001	0.50110928
171.9	0.46549312	0.42033172	0.35105628	0.50166035	0.58092895	0.8987715	0.46019105	0.43427044	0.55806847	0.50125858
172	0.46557045	0.42043684	0.35113337	0.50177869	0.58104674	0.89877598	0.46024134	0.43435518	0.55813589	0.50140774
172.1	0.46564768	0.42054189	0.35121037	0.50189697	0.58116441	0.89878044	0.4602916	0.43443985	0.55820325	0.50155677
172.2	0.46572483	0.42064688	0.35128726	0.50201516	0.58128196	0.89878488	0.46034183	0.43452444	0.55827055	0.50170566
172.3	0.46580189	0.4207518	0.35136406	0.50213328	0.5813994	0.89878932	0.46039203	0.43460895	0.5583378	0.50185443
172.4	0.46587886	0.42085666	0.35144076	0.50225132	0.58151672	0.89879373	0.4604422	0.43469339	0.558405	0.50200306
172.5	0.46595575	0.42096147	0.35151737	0.50236929	0.58163392	0.89879814	0.46049234	0.43477775	0.55847214	0.50215155
172.6	0.46603254	0.4210662	0.35159387	0.50248718	0.581751	0.89880253	0.46054245	0.43486204	0.55853922	0.50229991
172.7	0.46610925	0.42117088	0.35167027	0.50260499	0.58186797	0.89880691	0.46059253	0.43494625	0.55860625	0.50244814
172.8	0.46618586	0.4212755	0.35174658	0.50272272	0.58198482	0.89881127	0.46064257	0.43503038	0.55867323	0.50259624
172.9	0.4662624	0.42138005	0.35182279	0.50284038	0.58210155	0.89881562	0.46069259	0.43511443	0.55874015	0.5027442
173	0.46633884	0.42148454	0.35189889	0.50295797	0.58221817	0.89881996	0.46074258	0.43519841	0.55880702	0.50289203
173.1	0.46641519	0.42158897	0.3519749	0.50307547	0.58233466	0.89882428	0.46079253	0.43528231	0.55887384	0.50303973
173.2	0.46649146	0.42169334	0.35205082	0.5031929	0.58245105	0.89882859	0.46084246	0.43536614	0.5589406	0.50318729
173.3	0.46656764	0.42179764	0.35212663	0.50331025	0.58256731	0.89883289	0.46089235	0.43544988	0.55900731	0.50333472
173.4	0.46664373	0.42190188	0.35220234	0.50342753	0.58268347	0.89883717	0.46094221	0.43553355	0.55907396	0.50348202
173.5	0.46671974	0.42200606	0.35227796	0.50354473	0.5827995	0.89884144	0.46099205	0.43561714	0.55914057	0.50362918
173.6	0.46679566	0.42211018	0.35235348	0.50366185	0.58291542	0.89884569	0.46104185	0.43570066	0.55920712	0.50377621
173.7	0.46687149	0.42221424	0.3524289	0.5037789	0.58303122	0.89884993	0.46109163	0.43578409	0.55927361	0.50392311
173.8	0.46694724	0.42231824	0.35250422	0.50389587	0.58314691	0.89885416	0.46114137	0.43586745	0.55934006	0.50406987
173.9	0.46702289	0.42242217	0.35257944	0.50401276	0.58326248	0.89885837	0.46119109	0.43595073	0.55940645	0.50421651
174	0.46709847	0.42252604	0.35265457	0.50412957	0.58337794	0.89886257	0.46124077	0.43603393	0.55947279	0.504363
174.1	0.46717395	0.42262985	0.3527296	0.50424631	0.58349329	0.89886676	0.46129042	0.43611705	0.55953908	0.50450937
174.2	0.46724935	0.4227336	0.35280452	0.50436297	0.58360852	0.89887093	0.46134005	0.4362001	0.55960531	0.5046556
174.3	0.46732466	0.42283728	0.35287936	0.50447955	0.58372363	0.89887509	0.46138964	0.43628306	0.5596715	0.5048017
174.4	0.46739989	0.42294091	0.35295409	0.50459606	0.58383863	0.89887923	0.46143921	0.43636595	0.55973763	0.50494766
174.5	0.46747503	0.42304447	0.35302872	0.50471249	0.58395352	0.89888336	0.46148874	0.43644876	0.55980371	0.5050935
174.6	0.46755009	0.42314797	0.35310326	0.50482884	0.58406829	0.89888748	0.46153825	0.43653149	0.55986974	0.5052392
174.7	0.46762506	0.42325141	0.3531777	0.50494512	0.58418295	0.89889159	0.46158772	0.43661414	0.55993572	0.50538476
174.8	0.46769994	0.42335479	0.35325204	0.50506132	0.58429749	0.89889567	0.46163717	0.43669671	0.56000164	0.5055302
174.9	0.46777474	0.42345811	0.35332629	0.50517744	0.58441192	0.89889975	0.46168659	0.4367792	0.56006752	0.5056755
175	0.46784945	0.42356136	0.35340044	0.50529348	0.58452624	0.89890381	0.46173598	0.43686161	0.56013335	0.50582067
175.1	0.46792408	0.42366455	0.35347448	0.50540945	0.58464045	0.89890786	0.46178534	0.43694395	0.56019912	0.5059657
175.2	0.46799862	0.42376768	0.35354844	0.50552534	0.58475454	0.8989119	0.46183467	0.4370262	0.56026484	0.5061106
175.3	0.46807308	0.42387075	0.35362229	0.50564115	0.58486852	0.89891592	0.46188397	0.43710837	0.56033052	0.50625537
175.4	0.46814745	0.42397376	0.35369605	0.50575688	0.58498238	0.89891992	0.46193324	0.43719047	0.56039614	0.50640001
175.5	0.46822174	0.42407671	0.35376971	0.50587254	0.58509614	0.89892392	0.46198248	0.43727248	0.56046172	0.50654452
175.6	0.46829594	0.42417959	0.35384327	0.50598812	0.58520978	0.89892789	0.4620317	0.43735441	0.56052724	0.50668889
175.7	0.46837006	0.42428242	0.35391673	0.50610362	0.5853233	0.89893186	0.46208088	0.43743627	0.56059272	0.50683313
175.8	0.46844409	0.42438518	0.3539901	0.50621904	0.58543672	0.89893581	0.46213004	0.43751804	0.56065814	0.50697723
175.9	0.46851804	0.42448788	0.35406337	0.50633439	0.58555002	0.89893975	0.46217916	0.43759973	0.56072352	0.50712121
176	0.4685919	0.42459052	0.35413654	0.50644966	0.58566322	0.89894367	0.46222826	0.43768134	0.56078884	0.50726505
176.1	0.46866568	0.4246931	0.35420961	0.50656485	0.5857763	0.89894758	0.46227733	0.43776287	0.56085412	0.50740876
176.2	0.46873938	0.42479561	0.35428259	0.50667996	0.58588926	0.89895147	0.46232638	0.43784432	0.56091935	0.50755234
176.3	0.46881299	0.42489807	0.35435547	0.506795	0.58600212	0.89895535	0.46237539	0.43792569	0.56098453	0.50769578
176.4	0.46888652	0.42500046	0.35442825	0.50690996	0.58611487	0.89895922	0.46242438	0.43800698	0.56104966	0.50783909
176.5	0.46895996	0.42510279	0.35450094	0.50702484	0.5862275	0.89896307	0.46247334	0.43808818	0.56111474	0.50798227
176.6	0.46903332	0.42520506	0.35457353	0.50713964	0.58634002	0.8989669	0.46252227	0.43816931	0.56117977	0.50812532
176.7	0.46910659	0.42530727	0.35464602	0.50725436	0.58645243	0.89897072	0.46257117	0.43825035	0.56124476	0.50826823
176.8	0.46917979	0.42540942	0.35471841	0.50736901	0.58656473	0.89897453	0.46262004	0.43833131	0.56130969	0.50841102
176.9	0.46925289	0.42551151	0.35479071	0.50748358	0.58667692	0.89897832	0.46266889	0.43841219	0.56137458	0.50855367
177	0.46932592	0.42561353	0.35486291	0.50759806	0.586789	0.8989821	0.46271771	0.43849299	0.56143942	0.50869619
177.1	0.46939886	0.4257155	0.35493501	0.50771248	0.58690097	0.89898586	0.4627665	0.43857371	0.56150421	0.50883857
177.2	0.46947172	0.4258174	0.35500702	0.50782681	0.58701283	0.89898961	0.46281526	0.43865434	0.56156895	0.50898083
177.3	0.46954449	0.42591924	0.35507893	0.50794106	0.58712458	0.89899334	0.462864	0.43873489	0.56163365	0.50912295
177.4	0.46961719	0.42602102	0.35515074	0.50805524	0.58723622	0.89899706	0.4629127	0.43881536	0.5616983	0.50926494
177.5	0.46968979	0.42612274	0.35522246	0.50816934	0.58734775	0.89900076	0.46296138	0.43889575	0.5617629	0.5094068
177.6	0.46976232	0.4262244	0.35529408	0.50828336	0.58745916	0.89900445	0.46301004	0.43897605	0.56182745	0.50954852
177.7	0.46983476	0.426326	0.3553656	0.5083973	0.58757047	0.89900812	0.46305867	0.43905627	0.56189196	0.50969011
177.8	0.46990712	0.42642753	0.35543703	0.50851116	0.58768167	0.89901178	0.46310727	0.43913641	0.56195642	0.50983158
177.9	0.4699794	0.42652901	0.35550835	0.50862494	0.58779276	0.89901542	0.46315584	0.43921647	0.56202083	0.50997291
178	0.4700516	0.42663042	0.35557959	0.50873865	0.58790374	0.89901904	0.46320439	0.43929644	0.5620852	0.5101141
178.1	0.47012371	0.42673177	0.35565072	0.50885227	0.58801462	0.89902265	0.4632529	0.43937633	0.56214951	0.51025517
178.2	0.47019574	0.42683307	0.35572176	0.50896582	0.58812538	0.89902624	0.4633014	0.43945614	0.56221379	0.51039611
178.3	0.47026769	0.4269343	0.35579271	0.50907929	0.58823603	0.89902982	0.46334986	0.43953586	0.56227801	0.51053691
178.4	0.47033956	0.42703547	0.35586356	0.50919268	0.58834658	0.89903337	0.4633983	0.4396155	0.56234219	0.51067758
178.5	0.47041135	0.42713657	0.35593431	0.50930599	0.58845701	0.89903692	0.46344672	0.43969506	0.56240632	0.51081812
178.6	0.47048305	0.42723762	0.35600496	0.50941922	0.58856734	0.89904044	0.46349511	0.43977453	0.56247041	0.51095853
178.7	0.47055467	0.42733861	0.35607552	0.50953238	0.58867756	0.89904395	0.46354347	0.43985392	0.56253444	0.5110988
178.8	0.47062621	0.42743953	0.35614598	0.50964545	0.58878767	0.89904744	0.4635918	0.43993323	0.56259844	0.51123895
178.9	0.47069767	0.4275404	0.35621635	0.50975844	0.58889768	0.89905091	0.46364011	0.44001245	0.56266238	0.51137896
179	0.47076905	0.4276412	0.35628662	0.50987136	0.58900757	0.89905436	0.46368839	0.44009159	0.56272629	0.51151884
179.1	0.47084035	0.42774195	0.3563568	0.50998419	0.58911736	0.8990578	0.46373665	0.44017065	0.56279014	0.51165859
179.2	0.47091157	0.42784263	0.35642688	0.51009695	0.58922704	0.89906122	0.46378488	0.44024962	0.56285395	0.51179821
179.3	0.4709827	0.42794325	0.35649686	0.51020963	0.58933661	0.89906462	0.46383309	0.4403285	0.56291771	0.5119377
179.4	0.47105376	0.42804381	0.35656675	0.51032222	0.58944608	0.899068	0.46388127	0.44040731	0.56298143	0.51207706
179.5	0.47112473	0.42814431	0.35663654	0.51043474	0.58955544	0.89907136	0.46392943	0.44048603	0.5630451	0.51221628
179.6	0.47119562	0.42824475	0.35670624	0.51054718	0.58966469	0.89907471	0.46397756	0.44056466	0.56310873	0.51235537
179.7	0.47126643	0.42834513	0.35677584	0.51065953	0.58977383	0.89907803	0.46402566	0.44064321	0.56317231	0.51249434
179.8	0.47133717	0.42844545	0.35684535	0.51077181	0.58988287	0.89908134	0.46407374	0.44072168	0.56323585	0.51263317
179.9	0.47140782	0.42854571	0.35691476	0.51088401	0.5899918	0.89908462	0.4641218	0.44080006	0.56329934	0.51277187
225	0.49660766	0.46813761	0.38019239	0.55332676	0.63011004	0.89966268	0.48393064	0.46958978	0.58786981	0.56377078
225.1	0.49665151	0.4682142	0.3802285	0.55340668	0.63018257	0.89966275	0.4839719	0.46964416	0.58791586	0.56386346
[illegible]	[illegible]	[illegible]	[illegible]	[illegible]	[illegible]	[illegible]	[illegible]	[illegible]	[illegible]	[illegible]

180.6	0.47190014	0.42924582	0.35739799	0.51166715	0.59075133	0.89910707	0.46445749	0.44134635	0.56374252	0.5137391
180.7	0.47197015	0.4293456	0.35746664	0.51177871	0.59085941	0.8991102	0.46450535	0.44142405	0.56380565	0.51387675
180.8	0.47204009	0.42944531	0.3575352	0.51189018	0.59096739	0.8991133	0.46455318	0.44150167	0.56386874	0.51401427
180.9	0.47210994	0.42954496	0.35760367	0.51200157	0.59107526	0.89911639	0.46460099	0.4415792	0.56393178	0.51415166
181	0.47217972	0.42964456	0.35767205	0.51211289	0.59118303	0.89911946	0.46464878	0.44165665	0.56399478	0.51428893
181.1	0.47224941	0.42974409	0.35774033	0.51222412	0.59129069	0.89912251	0.46469654	0.44173401	0.56405774	0.51442605
181.2	0.47231903	0.42984356	0.35780852	0.51233527	0.59139824	0.89912554	0.46474428	0.44181129	0.56412065	0.51456305
181.3	0.47238857	0.42994298	0.35787661	0.51244634	0.59150569	0.89912855	0.46479199	0.44188848	0.56418351	0.51469992
181.4	0.47245803	0.43004233	0.35794461	0.51255732	0.59161304	0.89913154	0.46483968	0.44196558	0.56424634	0.51483666
181.5	0.47252741	0.43014162	0.35801252	0.51266823	0.59172028	0.89913451	0.46488735	0.4420426	0.56430912	0.51497327
181.6	0.47259671	0.43024085	0.35808033	0.51277906	0.59182742	0.89913747	0.46493499	0.44211954	0.56437185	0.51510974
181.7	0.47266594	0.43034003	0.35814806	0.5128898	0.59193445	0.89914041	0.46498261	0.44219638	0.56443454	0.51524609
181.8	0.47273508	0.43043914	0.35821569	0.51300046	0.59204138	0.89914333	0.4650302	0.44227315	0.56449719	0.51538231
181.9	0.47280415	0.43053819	0.35828323	0.51311104	0.59214821	0.89914623	0.46507778	0.44234982	0.56455979	0.51551839
182	0.47287314	0.43063718	0.35835067	0.51322154	0.59225493	0.89914912	0.46512533	0.44242642	0.56462235	0.51565435
182.1	0.47294206	0.43073612	0.35841803	0.51333195	0.59236155	0.89915199	0.46517285	0.44250292	0.56468487	0.51579017
182.2	0.47301089	0.43083499	0.35848529	0.51344229	0.59246806	0.89915484	0.46522036	0.44257934	0.56474734	0.51592587
182.3	0.47307965	0.4309338	0.35855246	0.51355254	0.59257447	0.89915768	0.46526784	0.44265568	0.56480977	0.51606143
182.4	0.47314833	0.43103255	0.35861954	0.5136627	0.59268078	0.8991605	0.46531529	0.44273193	0.56487216	0.51619687
182.5	0.47321693	0.43113125	0.35868653	0.51377279	0.59278698	0.8991633	0.46536273	0.44280809	0.5649345	0.51633217
182.6	0.47328545	0.43122988	0.35875343	0.51388279	0.59289308	0.8991661	0.46541014	0.44288417	0.5649968	0.51646735
182.7	0.4733539	0.43132845	0.35882023	0.51399271	0.59299908	0.89916887	0.46545753	0.44296016	0.56505906	0.51660239
182.8	0.47342227	0.43142697	0.35888695	0.51410255	0.59310497	0.89917163	0.46550489	0.44303607	0.56512127	0.51673731
182.9	0.47349056	0.43152542	0.35895358	0.51421231	0.59321077	0.89917438	0.46555224	0.44311189	0.56518344	0.51687209
183	0.47355878	0.43162382	0.35902011	0.51432198	0.59331646	0.89917712	0.46559956	0.44318762	0.56524557	0.51700675
183.1	0.47362692	0.43172215	0.35908656	0.51443157	0.59342204	0.89917984	0.46564686	0.44326327	0.56530765	0.51714128
183.2	0.47369498	0.43182043	0.35915292	0.51454107	0.59352753	0.89918254	0.46569414	0.44333884	0.56536969	0.51727567
183.3	0.47376297	0.43191864	0.35921918	0.51465049	0.59363291	0.89918524	0.46574139	0.44341432	0.56543169	0.51740994
183.4	0.47383088	0.4320168	0.35928536	0.51475983	0.59373819	0.89918792	0.46578862	0.44348971	0.56549364	0.51754408
183.5	0.47389871	0.43211489	0.35935145	0.51486908	0.59384337	0.89919059	0.46583583	0.44356502	0.56555555	0.51767808
183.6	0.47396647	0.43221293	0.35941745	0.51497825	0.59394845	0.89919324	0.46588302	0.44364024	0.56561742	0.51781196
183.7	0.47403415	0.43231091	0.35948336	0.51508734	0.59405342	0.89919589	0.46593019	0.44371537	0.56567925	0.51794571
183.8	0.47410175	0.43240883	0.35954918	0.51519634	0.5941583	0.89919852	0.46597733	0.44379042	0.56574103	0.51807933
183.9	0.47416928	0.43250669	0.35961492	0.51530526	0.59426307	0.89920114	0.46602446	0.44386539	0.56580277	0.51821282
184	0.47423673	0.43260449	0.35968056	0.51541409	0.59436774	0.89920375	0.46607156	0.44394027	0.56586447	0.51834618
184.1	0.47430411	0.43270223	0.35974612	0.51552284	0.59447231	0.89920635	0.46611864	0.44401506	0.56592613	0.51847941
184.2	0.47437141	0.43279991	0.35981159	0.5156315	0.59457678	0.89920893	0.4661657	0.44408977	0.56598774	0.51861252
184.3	0.47443863	0.43289753	0.35987698	0.51574008	0.59468115	0.89921151	0.46621273	0.44416439	0.56604932	0.51874549
184.4	0.47450578	0.43299509	0.35994227	0.51584857	0.59478542	0.89921407	0.46625975	0.44423893	0.56611084	0.51887834
184.5	0.47457285	0.4330926	0.36000748	0.51595698	0.59488959	0.89921662	0.46630674	0.44431338	0.56617233	0.51901105
184.6	0.47463985	0.43319004	0.36007261	0.5160653	0.59499365	0.89921917	0.46635372	0.44438775	0.56623378	0.51914364
184.7	0.47470678	0.43328743	0.36013764	0.51617354	0.59509762	0.8992217	0.46640067	0.44446203	0.56629518	0.5192761
184.8	0.47477363	0.43338476	0.3602026	0.51628169	0.59520149	0.89922422	0.4664476	0.44453623	0.56635654	0.51940843
184.9	0.4748404	0.43348202	0.36026746	0.51638976	0.59530526	0.89922673	0.46649451	0.44461034	0.56641786	0.51954063
185	0.4749071	0.43357923	0.36033224	0.51649773	0.59540892	0.89922923	0.4665414	0.44468437	0.56647914	0.5196727
185.1	0.47497372	0.43367638	0.36039694	0.51660563	0.59551249	0.89923172	0.46658827	0.44475832	0.56654037	0.51980464
185.2	0.47504027	0.43377347	0.36046155	0.51671344	0.59561596	0.8992342	0.46663511	0.44483217	0.56660156	0.51993646
185.3	0.47510675	0.43387051	0.36052607	0.51682116	0.59571933	0.89923667	0.46668194	0.44490595	0.56666272	0.52006814
185.4	0.47517315	0.43396748	0.36059051	0.51692879	0.5958226	0.89923913	0.46672875	0.44497964	0.56672383	0.5201997
185.5	0.47523947	0.4340644	0.36065487	0.51703634	0.59592577	0.89924159	0.46677553	0.44505324	0.56678489	0.52033112
185.6	0.47530572	0.43416125	0.36071914	0.5171438	0.59602884	0.89924403	0.4668223	0.44512676	0.56684592	0.52046242
185.7	0.4753719	0.43425805	0.36078333	0.51725117	0.59613181	0.89924646	0.46686904	0.4452002	0.5669069	0.52059359
185.8	0.475438	0.43435479	0.36084743	0.51735846	0.59623468	0.89924888	0.46691577	0.44527355	0.56696785	0.52072464
185.9	0.47550403	0.43445147	0.36091145	0.51746565	0.59633746	0.8992513	0.46696247	0.44534682	0.56702875	0.52085555
186	0.47556999	0.43454809	0.36097539	0.51757276	0.59644013	0.8992537	0.46700916	0.44542001	0.56708961	0.52098633
186.1	0.47563587	0.43464465	0.36103925	0.51767979	0.59654271	0.8992561	0.46705583	0.44549311	0.56715042	0.52111699
186.2	0.47570168	0.43474116	0.36110302	0.51778672	0.59664519	0.89925848	0.46710247	0.44556613	0.5672112	0.52124752
186.3	0.47576742	0.4348376	0.36116671	0.51789357	0.59674757	0.89926086	0.4671491	0.44563906	0.56727194	0.52137792
186.4	0.47583308	0.43493399	0.36123032	0.51800033	0.59684985	0.89926323	0.46719571	0.44571191	0.56733263	0.52150819
186.5	0.47589867	0.43503032	0.36129385	0.518107	0.59695204	0.89926558	0.4672423	0.44578468	0.56739328	0.52163833
186.6	0.47596418	0.43512659	0.36135729	0.51821358	0.59705413	0.89926793	0.46728886	0.44585737	0.56745389	0.52176834
186.7	0.47602962	0.4352228	0.36142065	0.51832007	0.59715611	0.89927027	0.46733541	0.44592997	0.56751446	0.52189823
186.8	0.47609499	0.43531895	0.36148394	0.51842647	0.59725801	0.8992726	0.46738194	0.44600249	0.56757499	0.52202798
186.9	0.47616029	0.43541505	0.36154714	0.51853279	0.5973598	0.89927493	0.46742846	0.44607493	0.56763548	0.52215761
187	0.47622551	0.43551109	0.36161026	0.51863901	0.5974615	0.89927724	0.46747495	0.44614729	0.56769593	0.52228711
187.1	0.47629066	0.43560707	0.3616733	0.51874515	0.5975631	0.89927954	0.46752142	0.44621956	0.56775633	0.52241649
187.2	0.47635574	0.43570299	0.36173625	0.51885119	0.5976646	0.89928184	0.46756788	0.44629175	0.56781669	0.52254573
187.3	0.47642075	0.43579885	0.36179913	0.51895715	0.59776601	0.89928413	0.46761431	0.44636386	0.56787702	0.52267485
187.4	0.47648568	0.43589466	0.36186193	0.51906301	0.59786732	0.89928641	0.46766073	0.44643589	0.5679373	0.52280384
187.5	0.47655055	0.4359904	0.36192465	0.51916879	0.59796853	0.89928867	0.46770713	0.44650784	0.56799754	0.5229327
187.6	0.47661534	0.43608609	0.36198729	0.51927447	0.59806965	0.89929094	0.4677535	0.44657971	0.56805774	0.52306143
187.7	0.47668005	0.43618172	0.36204985	0.51938007	0.59817067	0.89929319	0.46779986	0.44665149	0.5681179	0.52319003
187.8	0.4767447	0.4362773	0.36211233	0.51948557	0.59827159	0.89929543	0.46784621	0.4467232	0.56817802	0.52331851
187.9	0.47680927	0.43637281	0.36217473	0.51959099	0.59837242	0.89929767	0.46789253	0.44679482	0.56823809	0.52344686
188	0.47687378	0.43646827	0.36223706	0.51969631	0.59847315	0.89929989	0.46793883	0.44686636	0.56829813	0.52357508
188.1	0.47693821	0.43656367	0.3622993	0.51980154	0.59857378	0.89930211	0.46798512	0.44693783	0.56835813	0.52370317
188.2	0.47700257	0.43665901	0.36236147	0.51990668	0.59867432	0.89930432	0.46803139	0.44700921	0.56841808	0.52383114
188.3	0.47706685	0.43675429	0.36242356	0.52001173	0.59877477	0.89930652	0.46807764	0.44708051	0.568478	0.52395898
188.4	0.47713107	0.43684952	0.36248557	0.52011668	0.59887512	0.89930871	0.46812387	0.44715174	0.56853787	0.52408669
188.5	0.47719522	0.43694469	0.3625475	0.52022155	0.59897537	0.8993109	0.46817008	0.44722288	0.5685977	0.52421427
188.6	0.47725929	0.4370398	0.36260935	0.52032632	0.59907553	0.89931307	0.46821627	0.44729394	0.56865749	0.52434173
188.7	0.4773233	0.43713485	0.36267113	0.520431	0.59917559	0.89931524	0.46826245	0.44736493	0.56871725	0.52446906
188.8	0.47738723	0.43722985	0.36273283	0.52053558	0.59927556	0.8993174	0.46830861	0.44743584	0.56877696	0.52459626
188.9	0.47745109	0.43732479	0.36279445	0.52064008	0.59937543	0.89931955	0.46835474	0.44750667	0.56883663	0.52472333
189	0.47751488	0.43741967	0.362856	0.52074448	0.59947521	0.89932169	0.46840087	0.44757742	0.56889626	0.52485028
189.1	0.47757861	0.43751449	0.36291747	0.52084879	0.59957489	0.89932382	0.46844697	0.44764809	0.56895585	0.5249771
189.2	0.47764226	0.43760926	0.36297886	0.52095301	0.59967448	0.89932594	0.46849305	0.44771868	0.5690154	0.52510379
189.3	0.47770584	0.43770396	0.36304018	0.52105713	0.59977397	0.89932806	0.46853912	0.4477892	0.5690749	0.52523036
189.4	0.47776935	0.43779862	0.36310142	0.52116116	0.59987337	0.89933017	0.46858517	0.44785964	0.56913437	0.52535679
189.5	0.47783279	0.43789321	0.36316258	0.5212651	0.59997268	0.89933227	0.4686312	0.44793	0.5691938	0.52548311
189.6	0.47789616	0.43798775	0.36322367	0.52136894	0.60007189	0.89933436	0.46867721	0.44800029	0.56925318	0.52560929
189.7	0.47795946	0.43808223	0.36328468	0.52147269	0.60017101	0.89933644	0.46872321	0.4480705	0.56931253	0.52573535
189.8	0.47802269	0.43817665	0.36334561	0.52157635	0.60027003	0.89933852	0.46876919	0.44814063	0.56937184	0.52586128
189.9	0.47808585	0.43827101	0.36340647	0.52167991	0.60036897	0.89934058	0.46881515	0.44821069	0.5694311	0.52598709
190	0.47814894	0.43836532	0.36346726	0.52178338	0.6004678	0.89934264	0.46886109	0.44828067	0.56949033	0.52611277
190.1	0.47821196	0.43845957	0.36352797	0.52188675	0.60056655	0.89934469	0.46890701	0.44835057	0.56954951	0.52623832
190.2	0.47827491	0.43855376	0.3635886	0.52199004	0.6006652	0.89934673	0.46895292	0.4484204	0.56960866	0.52636375
190.3	0.47833779	0.4386479	0.36364916	0.52209322	0.60076375	0.89934876	0.46899881	0.44849016	0.56966776	0.52648905
190.4	0.47840061	0.43874198	0.36370964	0.52219631	0.60086222	0.89935079	0.46904468	0.44855983	0.56972682	0.52661422
190.5	0.47846335	0.438836	0.36377005	0.52229931	0.60096059	0.8993528	0.46909054	0.44862944	0.56978585	0.52673927
190.6	0.47852603	0.43892997	0.36383038	0.52240222	0.60105887	0.89935481	0.46913637	0.44869897	0.56984483	0.52686419
190.7	0.47858863	0.43902387	0.36389064	0.52250502	0.60115705	0.89935681	0.46918219	0.44876843	0.56990377	0.52698899
190.8	0.47865117	0.43911773	0.36395082	0.52260774	0.60125515	0.8993588	0.46922799	0.44883781	0.56996268	0.52711367
190.9	0.47871364	0.43921152	0.36401093	0.52271036	0.60135315	0.89936079	0.46927378	0.44890712	0.57002154	0.52723821
191	0.47877604	0.43930526	0.36407097	0.52281288	0.60145106	0.89936276	0.46931954	0.44897635	0.57008036	0.52736263
191.1	0.47883837	0.43939894	0.36413092	0.52291532	0.60154887	0.89936473	0.46936529	0.44904551	0.57013915	0.52748693
191.2	0.47890063	0.43949256	0.36419081	0.52301765	0.6016466	0.89936669	0.46941102	0.4491146	0.57019789	0.5276111
191.3	0.47896282	0.43958613	0.36425062	0.52311989	0.60174423	0.89936864	0.46945674	0.44918362	0.57025659	0.52773515
191.4	0.47902495	0.43967964	0.36431036	0.52322204	0.60184177	0.89937058	0.46950244	0.44925256	0.57031525	0.52785907
191.5	0.47908701	0.4397731	0.36437002	0.52332409	0.60193922	0.89937252	0.46954812	0.44932143	0.57037388	0.52798287
191.6	0.479149	0.4398665	0.36442961	0.52342605	0.60203658	0.89937445	0.46959378	0.44939024	0.57043246	0.52810654
191.7	0.47921092	0.43995984	0.36448913	0.52352791	0.60213384	0.89937636	0.46963943	0.44945896	0.570491	0.52823009
191.8	0.47927277	0.44005312	0.36454857	0.52362968	0.60223102	0.89937827	0.46968506	0.44952762	0.5705495	0.52835352
191.9	0.47933456	0.44014635	0.36460793	0.52373135	0.6023281	0.89938018	0.46973067	0.44959621	0.57060797	0.52847682
192	0.47939628	0.44023952	0.36466723	0.52383293	0.60242509	0.89938207	0.46977626	0.44966472	0.57066639	0.52859999
192.1	0.47945793	0.44033264	0.36472645	0.52393441	0.60252199	0.89938396	0.46982184	0.44973317	0.57072477	0.52872305
192.2	0.47951951	0.4404257	0.36478559	0.5240358	0.6026188	0.89938584	0.4698674	0.44980155	0.57078312	0.52884598
192.3	0.47958103	0.4405187	0.36484466	0.52413709	0.60271552	0.89938771	0.46991294	0.44986985	0.57084142	0.52896879
192.4	0.47964248	0.44061165	0.36490366	0.52423829	0.60281215	0.89938957	0.46995847	0.44993809	0.57089969	0.52909147
192.5	0.47970386	0.44070454	0.36496259	0.5243394	0.60290869	0.89939142	0.47000398	0.45000625	0.57095791	0.52921403
192.6	0.47976517	0.44079737	0.36502144	0.52444041	0.60300514	0.89939327	0.47004947	0.45007435	0.5710161	0.52933647
192.7	0.47982642	0.44089015	0.36508021	0.52454133	0.60310149	0.89939511	0.47009495	0.45014238	0.57107424	0.52945879
192.8	0.4798876	0.44098287	0.36513892	0.52464215	0.60319776	0.89939694	0.47014041	0.45021034	0.57113235	0.52958098
192.9	0.47994871	0.44107554	0.36519755	0.52474287	0.60329394	0.89939876	0.47018585	0.45027823	0.57119041	0.52970306
193	0.48000976	0.44116815	0.3652561	0.52484351	0.60339003	0.89940058	0.47023127	0.45034605	0.57124844	0.52982501
193.1	0.48007074	0.4412607	0.36531459	0.52494405	0.60348602	0.89940238	0.47027668	0.45041381	0.57130642	0.52994684
193.2	0.48013166	0.4413532	0.36537299	0.52504449	0.60358193	0.89940418	0.47032207	0.4504815	0.57136437	0.53006855
193.3	0.48019251	0.44144564	0.36543133	0.52514484	0.60367775	0.89940597	0.47036745	0.45054912	0.57142228	0.53019014
193.4	0.48025329	0.44153803	0.36548959	0.5252451	0.60377348	0.89940776	0.4704128	0.45061667	0.57148015	0.5303116
193.5	0.480314	0.44163036	0.36554778	0.52534526	0.60386912	0.89940953	0.47045814	0.45068416	0.57153797	0.53043295
193.6	0.48037465	0.44172263	0.36560589	0.52544533	0.60396467	0.8994113	0.47050347	0.45075158	0.57159576	0.53055418
193.7	0.48043524	0.44181485	0.36566393	0.52554531	0.60406013	0.89941306	0.47054877	0.45081894	0.57165351	0.53067528
193.8	0.48049576	0.44190701	0.3657219	0.52564519	0.6041555	0.89941481	0.47059406	0.45088623	0.57171122	0.53079627
193.9	0.48055621	0.44199912	0.36577979	0.52574499	0.60425078	0.89941655	0.47063934	0.45095345	0.57176889	0.53091714
194	0.4806166	0.44209117	0.3658376	0.52584468	0.60434598	0.89941829	0.47068459	0.45102061	0.57182653	0.53103788
194.1	0.48067692	0.44218317	0.36589535	0.52594429	0.60444108	0.89942002	0.47072983	0.4510877	0.57188412	0.53115851
194.2	0.48073717	0.44227511	0.36595302	0.5260438	0.6045361	0.89942174	0.47077506	0.45115473	0.57194167	0.53127902
194.3	0.48079736	0.44236699	0.36601061	0.52614322	0.60463103	0.89942345	0.47082026	0.45122169	0.57199919	0.53139941
194.4	0.48085749	0.44245882	0.36606813	0.52624255	0.60472587	0.89942516	0.47086545	0.45128859	0.57205666	0.53151969
194.5	0.48091755	0.44255059	0.36612558	0.52634178	0.60482062	0.89942686	0.47091062	0.45135542	0.5721141	0.53163984
194.6	0.48097754	0.44264231	0.36618295	0.52644092	0.60491528	0.89942855	0.47095578	0.4514222	0.57217149	0.53175988
194.7	0.48103748	0.44273398	0.36624024	0.52653997	0.60500986	0.89943023	0.47100092	0.4514889	0.57222884	0.5318798
194.8	0.48109734	0.44282558	0.36629747	0.52663893	0.60510435	0.8994319	0.47104604	0.45155555	0.57228616	0.5319996
194.9	0.48115714	0.44291714	0.36635461	0.5267378	0.60519875	0.89943357	0.47109115	0.45162213	0.57234344	0.53211929

195.6	0.48157395	0.44355646	0.36675254	0.52742734	0.6058571	0.89944503	0.47140642	0.45208646	0.57274328	0.53295387
195.7	0.48163323	0.44364757	0.36680909	0.52752548	0.60595081	0.89944664	0.47145139	0.45215254	0.57280025	0.53307263
195.8	0.48169246	0.44373863	0.36686556	0.52762353	0.60604442	0.89944824	0.47149634	0.45221857	0.57285717	0.53319128
195.9	0.48175162	0.44382963	0.36692195	0.5277215	0.60613795	0.89944983	0.47154128	0.45228454	0.57291406	0.53330982
196	0.48181071	0.44392058	0.36697827	0.52781938	0.6062314	0.89945141	0.4715862	0.45235044	0.5729709	0.53342825
196.1	0.48186974	0.44401147	0.36703452	0.52791716	0.60632475	0.89945299	0.47163111	0.45241629	0.57302771	0.53354656
196.2	0.48192871	0.44410231	0.36709068	0.52801486	0.60641802	0.89945456	0.471676	0.45248207	0.57308448	0.53366476
196.3	0.48198762	0.44419309	0.36714677	0.52811248	0.60651121	0.89945612	0.47172087	0.4525478	0.57314121	0.53378284
196.4	0.48204646	0.44428382	0.36720278	0.52821	0.60660431	0.89945768	0.47176572	0.45261347	0.5731979	0.53390081
196.5	0.48210524	0.44437449	0.36725872	0.52830744	0.60669732	0.89945922	0.47181056	0.45267908	0.57325455	0.53401868
196.6	0.48216396	0.44446511	0.36731458	0.52840479	0.60679025	0.89946076	0.47185538	0.45274463	0.57331116	0.53413643
196.7	0.48222261	0.44455567	0.36737037	0.52850205	0.60688309	0.8994623	0.47190019	0.45281012	0.57336774	0.53425406
196.8	0.4822812	0.44464618	0.36742607	0.52859923	0.60697584	0.89946382	0.47194497	0.45287555	0.57342427	0.53437159
196.9	0.48233973	0.44473664	0.3674817	0.52869632	0.60706851	0.89946534	0.47198975	0.45294093	0.57348076	0.53448901
197	0.4823982	0.44482704	0.36753725	0.52879332	0.6071611	0.89946685	0.4720345	0.45300625	0.57353722	0.53460631
197.1	0.4824566	0.44491738	0.36759273	0.52889024	0.6072536	0.89946836	0.47207924	0.45307151	0.57359364	0.5347235
197.2	0.48251494	0.44500767	0.36764813	0.52898707	0.60734601	0.89946985	0.47212396	0.45313671	0.57365002	0.53484059
197.3	0.48257322	0.44509791	0.36770345	0.52908382	0.60743834	0.89947134	0.47216866	0.45320186	0.57370636	0.53495756
197.4	0.48263144	0.44518809	0.36775869	0.52918049	0.60753059	0.89947282	0.47221335	0.45326695	0.57376266	0.53507443
197.5	0.48268959	0.44527822	0.36781386	0.52927706	0.60762275	0.8994743	0.47225802	0.45333198	0.57381892	0.53519118
197.6	0.48274769	0.4453683	0.36786894	0.52937356	0.60771482	0.89947577	0.47230267	0.45339696	0.57387514	0.53530783
197.7	0.48280572	0.44545832	0.36792395	0.52946997	0.60780682	0.89947723	0.47234731	0.45346188	0.57393133	0.53542436
197.8	0.48286369	0.44554828	0.36797888	0.5295663	0.60789872	0.89947868	0.47239193	0.45352674	0.57398747	0.53554079
197.9	0.4829216	0.44563819	0.36803374	0.52966254	0.60799055	0.89948013	0.47243653	0.45359155	0.57404358	0.53565711
198	0.48297944	0.44572805	0.36808851	0.5297587	0.60808228	0.89948157	0.47248112	0.4536563	0.57409965	0.53577332
198.1	0.48303723	0.44581785	0.36814321	0.52985478	0.60817394	0.899483	0.47252569	0.453721	0.57415568	0.53588943
198.2	0.48309495	0.4459076	0.36819783	0.52995077	0.60826551	0.89948442	0.47257024	0.45378564	0.57421167	0.53600542
198.3	0.48315262	0.4459973	0.36825237	0.53004668	0.608357	0.89948584	0.47261477	0.45385023	0.57426762	0.53612131
198.4	0.48321022	0.44608694	0.36830683	0.53014251	0.6084484	0.89948725	0.47265929	0.45391477	0.57432353	0.53623709
198.5	0.48326776	0.44617653	0.36836121	0.53023826	0.60853972	0.89948866	0.47270379	0.45397925	0.57437941	0.53635277
198.6	0.48332524	0.44626606	0.36841551	0.53033393	0.60863096	0.89949005	0.47274828	0.45404367	0.57443525	0.53646833
198.7	0.48338266	0.44635554	0.36846974	0.53042951	0.60872211	0.89949144	0.47279274	0.45410804	0.57449104	0.53658379
198.8	0.48344002	0.44644497	0.36852388	0.53052501	0.60881318	0.89949283	0.47283719	0.45417236	0.5745468	0.53669915
198.9	0.48349732	0.44653434	0.36857795	0.53062044	0.60890417	0.8994942	0.47288163	0.45423662	0.57460252	0.5368144
199	0.48355456	0.44662366	0.36863194	0.53071578	0.60899507	0.89949557	0.47292604	0.45430083	0.5746582	0.53692954
199.1	0.48361174	0.44671293	0.36868585	0.53081104	0.60908589	0.89949693	0.47297044	0.45436499	0.57471385	0.53704458
199.2	0.48366885	0.44680214	0.36873967	0.53090622	0.60917663	0.89949829	0.47301482	0.4544291	0.57476945	0.53715951
199.3	0.48372591	0.44689129	0.36879342	0.53100132	0.60926729	0.89949964	0.47305919	0.45449315	0.57482502	0.53727434
199.4	0.48378291	0.4469804	0.36884709	0.53109635	0.60935786	0.89950098	0.47310353	0.45455715	0.57488054	0.53738906
199.5	0.48383985	0.44706945	0.36890069	0.53119129	0.60944835	0.89950231	0.47314786	0.4546211	0.57493603	0.53750368
199.6	0.48389673	0.44715845	0.3689542	0.53128615	0.60953876	0.89950364	0.47319217	0.45468499	0.57499148	0.5376182
199.7	0.48395355	0.44724739	0.36900763	0.53138094	0.60962909	0.89950496	0.47323647	0.45474883	0.5750469	0.53773261
199.8	0.48401031	0.44733628	0.36906098	0.53147565	0.60971933	0.89950628	0.47328075	0.45481262	0.57510227	0.53784691
199.9	0.48406701	0.44742511	0.36911426	0.53157028	0.60980949	0.89950758	0.47332501	0.45487636	0.57515761	0.53796112
200	0.48412365	0.4475139	0.36916745	0.53166483	0.60989957	0.89950889	0.47336925	0.45494005	0.5752129	0.53807521
200.1	0.48418023	0.44760263	0.36922056	0.5317593	0.60998957	0.89951018	0.47341348	0.45500369	0.57526816	0.53818921
200.2	0.48423675	0.4476913	0.3692736	0.5318537	0.61007949	0.89951147	0.47345768	0.45506728	0.57532338	0.5383031
200.3	0.48429322	0.44777993	0.36932655	0.53194802	0.61016932	0.89951275	0.47350188	0.45513081	0.57537857	0.53841689
200.4	0.48434962	0.4478685	0.36937943	0.53204226	0.61025908	0.89951402	0.47354605	0.4551943	0.57543371	0.53853058
200.5	0.48440597	0.44795701	0.36943223	0.53213642	0.61034875	0.89951529	0.4735902	0.45525773	0.57548882	0.53864417
200.6	0.48446225	0.44804548	0.36948495	0.53223051	0.61043834	0.89951655	0.47363434	0.45532112	0.57554389	0.53875765
200.7	0.48451848	0.44813389	0.36953758	0.53232452	0.61052785	0.8995178	0.47367846	0.45538445	0.57559892	0.53887103
200.8	0.48457465	0.44822224	0.36959014	0.53241846	0.61061728	0.89951905	0.47372257	0.45544773	0.57565391	0.53898431
200.9	0.48463076	0.44831055	0.36964262	0.53251232	0.61070663	0.89952029	0.47376665	0.45551097	0.57570886	0.53909749
201	0.48468681	0.4483988	0.36969502	0.53260611	0.6107959	0.89952153	0.47381072	0.45557415	0.57576378	0.53921057
201.1	0.48474281	0.448487	0.36974734	0.53269982	0.61088508	0.89952275	0.47385477	0.45563729	0.57581865	0.53932354
201.2	0.48479874	0.44857514	0.36979959	0.53279346	0.61097419	0.89952398	0.4738988	0.45570038	0.57587349	0.53943642
201.3	0.48485462	0.44866324	0.36985175	0.53288702	0.61106321	0.89952519	0.47394282	0.45576341	0.57592829	0.5395492
201.4	0.48491044	0.44875128	0.36990383	0.5329805	0.61115216	0.8995264	0.47398681	0.4558264	0.57598305	0.53966187
201.5	0.4849662	0.44883926	0.36995584	0.53307392	0.61124103	0.8995276	0.47403079	0.45588934	0.57603778	0.53977444
201.6	0.4850219	0.4489272	0.37000777	0.53316725	0.61132981	0.89952879	0.47407475	0.45595224	0.57609247	0.53988692
201.7	0.48507755	0.44901508	0.37005961	0.53326052	0.61141852	0.89952998	0.47411869	0.45601508	0.57614711	0.53999929
201.8	0.48513314	0.44910291	0.37011138	0.53335371	0.61150714	0.89953117	0.47416262	0.45607787	0.57620173	0.54011157
201.9	0.48518867	0.44919068	0.37016307	0.53344683	0.61159569	0.89953234	0.47420653	0.45614062	0.5762563	0.54022375
202	0.48524414	0.44927841	0.37021468	0.53353987	0.61168415	0.89953351	0.47425042	0.45620332	0.57631083	0.54033582
202.1	0.48529956	0.44936608	0.37026621	0.53363285	0.61177254	0.89953467	0.47429429	0.45626597	0.57636533	0.5404478
202.2	0.48535492	0.4494537	0.37031767	0.53372575	0.61186084	0.89953583	0.47433814	0.45632858	0.57641979	0.54055968
202.3	0.48541022	0.44954126	0.37036904	0.53381857	0.61194907	0.89953698	0.47438198	0.45639114	0.57647421	0.54067146
202.4	0.48546546	0.44962878	0.37042034	0.53391133	0.61203722	0.89953812	0.4744258	0.45645365	0.57652859	0.54078314
202.5	0.48552065	0.44971624	0.37047156	0.53400401	0.61212529	0.89953926	0.4744696	0.45651611	0.57658294	0.54089472
202.6	0.48557578	0.44980365	0.3705227	0.53409663	0.61221328	0.89954039	0.47451338	0.45657853	0.57663724	0.54100621
202.7	0.48563085	0.449891	0.37057376	0.53418917	0.61230119	0.89954152	0.47455714	0.4566409	0.57669151	0.5411176
202.8	0.48568587	0.44997831	0.37062475	0.53428164	0.61238902	0.89954264	0.47460089	0.45670322	0.57674574	0.54122889
202.9	0.48574083	0.45006556	0.37067565	0.53437404	0.61247677	0.89954375	0.47464462	0.4567655	0.57679994	0.54134008
203	0.48579573	0.45015276	0.37072648	0.53446636	0.61256445	0.89954485	0.47468833	0.45682773	0.57685409	0.54145118
203.1	0.48585058	0.45023991	0.37077723	0.53455862	0.61265204	0.89954595	0.47473202	0.45688991	0.57690821	0.54156217
203.2	0.48590537	0.450327	0.37082791	0.53465081	0.61273956	0.89954705	0.47477569	0.45695205	0.57696229	0.54167308
203.3	0.4859601	0.45041405	0.3708785	0.53474292	0.612827	0.89954814	0.47481935	0.45701415	0.57701633	0.54178388
203.4	0.48601478	0.45050104	0.37092902	0.53483497	0.61291436	0.89954922	0.47486299	0.4570762	0.57707033	0.54189459
203.5	0.4860694	0.45058798	0.37097946	0.53492695	0.61300164	0.89955029	0.47490661	0.4571382	0.5771243	0.5420052
203.6	0.48612397	0.45067486	0.37102983	0.53501886	0.61308885	0.89955136	0.47495021	0.45720016	0.57717823	0.54211572
203.7	0.48617848	0.4507617	0.37108011	0.53511069	0.61317598	0.89955242	0.47499379	0.45726207	0.57723212	0.54222613
203.8	0.48623293	0.45084848	0.37113032	0.53520246	0.61326302	0.89955348	0.47503735	0.45732394	0.57728597	0.54233646
203.9	0.48628733	0.45093522	0.37118046	0.53529417	0.61335	0.89955453	0.4750809	0.45738576	0.57733979	0.54244669
204	0.48634167	0.4510219	0.37123052	0.5353858	0.61343689	0.89955557	0.47512442	0.45744754	0.57739357	0.54255682
204.1	0.48639596	0.45110853	0.3712805	0.53547736	0.61352371	0.89955661	0.47516793	0.45750927	0.57744731	0.54266685
204.2	0.48645019	0.4511951	0.3713304	0.53556886	0.61361044	0.89955765	0.47521142	0.45757096	0.57750101	0.5427768
204.3	0.48650436	0.45128163	0.37138023	0.53566028	0.61369711	0.89955867	0.47525489	0.4576326	0.57755467	0.54288664
204.4	0.48655848	0.4513681	0.37142998	0.53575164	0.61378369	0.89955969	0.47529835	0.45769421	0.5776083	0.5429964
204.5	0.48661255	0.45145453	0.37147966	0.53584293	0.6138702	0.89956071	0.47534178	0.45775576	0.57766189	0.54310605
204.6	0.48666655	0.4515409	0.37152926	0.53593416	0.61395663	0.89956171	0.4753852	0.45781728	0.57771544	0.54321562
204.7	0.48672051	0.45162722	0.37157878	0.53602532	0.61404298	0.89956272	0.47542859	0.45787875	0.57776896	0.54332508
204.8	0.48677441	0.45171348	0.37162823	0.5361164	0.61412926	0.89956371	0.47547197	0.45794017	0.57782243	0.54343446
204.9	0.48682825	0.4517997	0.3716776	0.53620743	0.61421546	0.8995647	0.47551533	0.45800155	0.57787587	0.54354374
205	0.48688204	0.45188587	0.3717269	0.53629838	0.61430158	0.89956569	0.47555867	0.45806289	0.57792927	0.54365292
205.1	0.48693577	0.45197198	0.37177612	0.53638927	0.61438762	0.89956667	0.47560199	0.45812419	0.57798264	0.54376202
205.2	0.48698945	0.45205804	0.37182527	0.5364801	0.61447359	0.89956764	0.4756453	0.45818544	0.57803596	0.54387102
205.3	0.48704307	0.45214405	0.37187434	0.53657085	0.61455949	0.89956861	0.47568858	0.45824665	0.57808925	0.54397992
205.4	0.48709664	0.45223001	0.37192334	0.53666155	0.61464531	0.89956957	0.47573185	0.45830782	0.5781425	0.54408873
205.5	0.48715015	0.45231592	0.37197227	0.53675217	0.61473105	0.89957052	0.47577509	0.45836895	0.57819571	0.54419745
205.6	0.48720361	0.45240178	0.37202111	0.53684273	0.61481671	0.89957147	0.47581832	0.45843003	0.57824889	0.54430608
205.7	0.48725701	0.45248759	0.37206989	0.53693323	0.6149023	0.89957241	0.47586153	0.45849107	0.57830203	0.54441461
205.8	0.48731036	0.45257334	0.37211859	0.53702365	0.61498782	0.89957335	0.47590472	0.45855207	0.57835513	0.54452305
205.9	0.48736365	0.45265905	0.37216721	0.53711402	0.61507325	0.89957428	0.47594789	0.45861302	0.57840819	0.5446314
206	0.48741689	0.4527447	0.37221576	0.53720432	0.61515862	0.89957521	0.47599104	0.45867394	0.57846122	0.54473966
206.1	0.48747007	0.45283031	0.37226424	0.53729455	0.6152439	0.89957613	0.47603418	0.45873481	0.57851421	0.54484782
206.2	0.4875232	0.45291586	0.37231264	0.53738472	0.61532911	0.89957704	0.47607729	0.45879564	0.57856716	0.54495589
206.3	0.48757628	0.45300136	0.37236097	0.53747483	0.61541425	0.89957795	0.47612039	0.45885643	0.57862007	0.54506387
206.4	0.4876293	0.45308681	0.37240922	0.53756487	0.61549931	0.89957886	0.47616346	0.45891718	0.57867295	0.54517176
206.5	0.48768227	0.45317221	0.37245741	0.53765485	0.6155843	0.89957975	0.47620652	0.45897788	0.57872579	0.54527956
206.6	0.48773518	0.45325756	0.37250551	0.53774476	0.61566921	0.89958064	0.47624956	0.45903855	0.57877859	0.54538726
206.7	0.48778804	0.45334286	0.37255355	0.53783461	0.61575404	0.89958153	0.47629258	0.45909917	0.57883136	0.54549487
206.8	0.48784084	0.45342811	0.37260151	0.53792439	0.6158388	0.89958241	0.47633558	0.45915975	0.57888408	0.5456024
206.9	0.48789359	0.4535133	0.3726494	0.53801412	0.61592349	0.89958329	0.47637856	0.4592203	0.57893677	0.54570983
207	0.48794629	0.45359845	0.37269721	0.53810378	0.6160081	0.89958416	0.47642152	0.4592808	0.57898942	0.54581717
207.1	0.48799893	0.45368355	0.37274495	0.53819337	0.61609264	0.89958502	0.47646446	0.45934126	0.57904204	0.54592442
207.2	0.48805152	0.45376859	0.37279262	0.53828291	0.6161771	0.89958588	0.47650739	0.45940168	0.57909462	0.54603158
207.3	0.48810406	0.45385359	0.37284022	0.53837238	0.61626149	0.89958673	0.47655029	0.45946206	0.57914716	0.54613865
207.4	0.48815654	0.45393853	0.37288774	0.53846179	0.6163458	0.89958758	0.47659318	0.45952239	0.57919966	0.54624563
207.5	0.48820896	0.45402343	0.37293519	0.53855113	0.61643004	0.89958842	0.47663605	0.45958269	0.57925213	0.54635251
207.6	0.48826134	0.45410827	0.37298257	0.53864041	0.61651421	0.89958925	0.4766789	0.45964295	0.57930456	0.54645931
207.7	0.48831366	0.45419307	0.37302988	0.53872964	0.6165983	0.89959008	0.47672172	0.45970317	0.57935695	0.54656602
207.8	0.48836593	0.45427781	0.37307711	0.53881879	0.61668232	0.89959091	0.47676453	0.45976335	0.5794093	0.54667264
207.9	0.48841814	0.45436251	0.37312428	0.53890789	0.61676626	0.89959173	0.47680732	0.45982349	0.57946162	0.54677917
208	0.4884703	0.45444715	0.37317137	0.53899693	0.61685013	0.89959254	0.4768501	0.45988359	0.5795139	0.54688561
208.1	0.4885224	0.45453174	0.37321839	0.5390859	0.61693393	0.89959335	0.47689285	0.45994364	0.57956614	0.54699196
208.2	0.48857446	0.45461629	0.37326533	0.53917481	0.61701765	0.89959415	0.47693558	0.46000366	0.57961835	0.54709822
208.3	0.48862646	0.45470078	0.37331221	0.53926366	0.6171013	0.89959495	0.4769783	0.46006364	0.57967052	0.54720439
208.4	0.4886784	0.45478522	0.37335901	0.53935246	0.61718488	0.89959574	0.477021	0.46012359	0.57972265	0.54731048
208.5	0.4887303	0.45486962	0.37340575	0.53944118	0.61726838	0.89959653	0.47706367	0.46018349	0.57977474	0.54741647
208.6	0.48878213	0.45495396	0.37345241	0.53952985	0.61735181	0.89959731	0.47710633	0.46024335	0.5798268	0.54752237
208.7	0.48883392	0.45503826	0.373499	0.53961846	0.61743517	0.89959808	0.47714897	0.46030317	0.57987882	0.54762819
208.8	0.48888566	0.4551225	0.37354552	0.53970701	0.61751845	0.89959886	0.47719159	0.46036296	0.5799308	0.54773392
208.9	0.48893734	0.4552067	0.37359197	0.53979549	0.61760166	0.89959962	0.4772342	0.46042271	0.57998275	0.54783956
209	0.48898896	0.45529084	0.37363835	0.53988392	0.6176848	0.89960038	0.47727678	0.46048241	0.58003466	0.54794511
209.1	0.48904054	0.45537494	0.37368466	0.53997229	0.61776787	0.89960114	0.47731935	0.46054208	0.58008653	0.54805058
209.2	0.48909206	0.45545898	0.3737309	0.54006059	0.61785086	0.89960188	0.4773619	0.46060171	0.58013836	0.54815595
209.3	0.48914353	0.45554298	0.37377707	0.54014884	0.61793378	0.89960263	0.47740442	0.4606613	0.58019016	0.54826124
209.4	0.48919495	0.45562693	0.37382316	0.54023702	0.61801663	0.89960337	0.47744694	0.46072086	0.58024192	0.54836644
209.5	0.48924631	0.45571082	0.37386919	0.54032515	0.6180994	0.8996041	0.47748943	0.46078037	0.58029364	0.54847155
209.6	0.48929762	0.45579467	0.37391515	0.54041322	0.61818211	0.89960483	0.4775319	0.46083985	0.58034533	0.54857658
209.7	0.48934888	0.45587847	0.37396104	0.54050123	0.61826474	0.89960555	0.47757436	0.46089929	0.58039698	0.54868152
209.8	0.48940009	0.45596222	0.37400686	0.54058918	0.6183473	0.89960627	0.47761679	0.46095869	0.58044859	0.54878637
209.9	0.48945124	0.45604592	0.37405261	0.54067707	0.61842979	0.89960698	0.47765921	0.46101805	0.58050017	0.54889113

210.6	0.48980786	0.45663043	0.37437093	0.54129065	0.61900519	0.89961183	0.47795565	0.46143254	0.58086017	0.54962205
210.7	0.48985859	0.45671374	0.37441613	0.54137807	0.61908711	0.8996125	0.47799793	0.4614916	0.58091145	0.54972612
210.8	0.48990928	0.45679699	0.37446126	0.54146544	0.61916895	0.89961316	0.47804019	0.46155063	0.58096269	0.54983011
210.9	0.48995991	0.4568802	0.37450632	0.54155274	0.61925072	0.89961382	0.47808243	0.46160961	0.5810139	0.549934
211	0.49001049	0.45696336	0.37455132	0.54163999	0.61933243	0.89961448	0.47812465	0.46166857	0.58106507	0.55003782
211.1	0.49006102	0.45704647	0.37459624	0.54172718	0.61941406	0.89961513	0.47816686	0.46172748	0.5811162	0.55014154
211.2	0.49011149	0.45712953	0.3746411	0.54181431	0.61949562	0.89961578	0.47820905	0.46178636	0.5811673	0.55024519
211.3	0.49016192	0.45721254	0.37468589	0.54190139	0.61957711	0.89961642	0.47825123	0.4618452	0.58121836	0.55034874
211.4	0.49021229	0.4572955	0.37473062	0.54198841	0.61965853	0.89961706	0.47829338	0.46190401	0.58126938	0.55045221
211.5	0.49026261	0.45737842	0.37477527	0.54207537	0.61973987	0.89961769	0.47833552	0.46196277	0.58132037	0.5505556
211.6	0.49031288	0.45746128	0.37481986	0.54216228	0.61982115	0.89961831	0.47837765	0.46202151	0.58137132	0.5506589
211.7	0.4903631	0.4575441	0.37486437	0.54224912	0.61990236	0.89961894	0.47841976	0.4620802	0.58142223	0.55076211
211.8	0.49041327	0.45762687	0.37490883	0.54233591	0.6199835	0.89961955	0.47846185	0.46213886	0.58147311	0.55086524
211.9	0.49046338	0.45770959	0.37495321	0.54242265	0.62006456	0.89962016	0.47850392	0.46219748	0.58152395	0.55096829
212	0.49051345	0.45779226	0.37499752	0.54250933	0.62014556	0.89962077	0.47854598	0.46225607	0.58157475	0.55107125
212.1	0.49056346	0.45787488	0.37504177	0.54259595	0.62022649	0.89962137	0.47858802	0.46231462	0.58162552	0.55117412
212.2	0.49061342	0.45795746	0.37508595	0.54268251	0.62030735	0.89962197	0.47863005	0.46237314	0.58167625	0.55127691
212.3	0.49066333	0.45803998	0.37513006	0.54276902	0.62038813	0.89962256	0.47867206	0.46243162	0.58172694	0.55137962
212.4	0.49071319	0.45812246	0.37517411	0.54285548	0.62046885	0.89962315	0.47871406	0.46249006	0.58177759	0.55148224
212.5	0.490763	0.45820489	0.37521809	0.54294187	0.6205495	0.89962373	0.47875604	0.46254847	0.58182821	0.55158478
212.6	0.49081276	0.45828727	0.375262	0.54302822	0.62063008	0.89962431	0.478798	0.46260684	0.5818788	0.55168723
212.7	0.49086246	0.4583696	0.37530584	0.5431145	0.62071059	0.89962488	0.47883995	0.46266518	0.58192934	0.5517896
212.8	0.49091212	0.45845188	0.37534961	0.54320073	0.62079103	0.89962545	0.47888188	0.46272348	0.58197985	0.55189189
212.9	0.49096173	0.45853411	0.37539332	0.54328691	0.6208714	0.89962601	0.4789238	0.46278175	0.58203033	0.55199409
213	0.49101128	0.4586163	0.37543696	0.54337303	0.6209517	0.89962657	0.47896571	0.46283998	0.58208076	0.55209621
213.1	0.49106078	0.45869844	0.37548054	0.54345909	0.62103193	0.89962713	0.4790076	0.46289818	0.58213116	0.55219825
213.2	0.49111024	0.45878053	0.37552404	0.5435451	0.6211121	0.89962767	0.47904947	0.46295634	0.58218152	0.5523002
213.3	0.49115964	0.45886257	0.37556748	0.54363105	0.62119219	0.89962822	0.47909133	0.46301446	0.58223185	0.55240207
213.4	0.49120899	0.45894456	0.37561085	0.54371695	0.62127222	0.89962876	0.47913317	0.46307255	0.58228214	0.55250385
213.5	0.4912583	0.45902651	0.37565415	0.5438028	0.62135217	0.89962929	0.47917501	0.46313061	0.58233239	0.55260555
213.6	0.49130755	0.45910841	0.37569739	0.54388859	0.62143206	0.89962982	0.47921682	0.46318863	0.58238261	0.55270717
213.7	0.49135675	0.45919025	0.37574055	0.54397432	0.62151188	0.89963035	0.47925863	0.46324662	0.58243279	0.55280871
213.8	0.4914059	0.45927205	0.37578365	0.54406	0.62159163	0.89963087	0.47930042	0.46330457	0.58248294	0.55291016
213.9	0.491455	0.45935381	0.37582669	0.54414562	0.62167132	0.89963138	0.47934219	0.46336249	0.58253304	0.55301153
214	0.49150405	0.45943551	0.37586965	0.5442312	0.62175093	0.8996319	0.47938395	0.46342037	0.58258311	0.55311281
214.1	0.49155306	0.45951717	0.37591255	0.54431671	0.62183048	0.8996324	0.4794257	0.46347822	0.58263315	0.55321402
214.2	0.49160201	0.45959878	0.37595538	0.54440218	0.62190996	0.89963291	0.47946744	0.46353604	0.58268315	0.55331514
214.3	0.49165091	0.45968034	0.37599814	0.54448758	0.62198937	0.8996334	0.47950916	0.46359382	0.58273311	0.55341618
214.4	0.49169976	0.45976185	0.37604083	0.54457294	0.62206871	0.8996339	0.47955087	0.46365157	0.58278303	0.55351714
214.5	0.49174856	0.45984331	0.37608346	0.54465824	0.62214798	0.89963438	0.47959256	0.46370928	0.58283292	0.55361801
214.6	0.49179732	0.45992473	0.37612602	0.54474348	0.62222719	0.89963487	0.47963425	0.46376696	0.58288277	0.55371881
214.7	0.49184602	0.4600061	0.37616851	0.54482868	0.62230633	0.89963535	0.47967592	0.4638246	0.58293259	0.55381952
214.8	0.49189467	0.46008742	0.37621093	0.54491382	0.6223854	0.89963582	0.47971758	0.46388221	0.58298237	0.55392014
214.9	0.49194328	0.4601687	0.37625328	0.5449989	0.62246441	0.89963629	0.47975922	0.46393979	0.58303211	0.55402069
215	0.49199183	0.46024992	0.37629557	0.54508393	0.62254334	0.89963676	0.47980086	0.46399733	0.58308182	0.55412116
215.1	0.49204034	0.4603311	0.37633778	0.54516891	0.62262221	0.89963722	0.47984248	0.46405484	0.58313149	0.55422154
215.2	0.49208879	0.46041223	0.37637993	0.54525384	0.62270101	0.89963768	0.47988409	0.46411231	0.58318112	0.55432184
215.3	0.4921372	0.46049331	0.37642201	0.54533871	0.62277975	0.89963813	0.47992569	0.46416976	0.58323072	0.55442206
215.4	0.49218556	0.46057435	0.37646402	0.54542353	0.62285842	0.89963858	0.47996728	0.46422716	0.58328028	0.5545222
215.5	0.49223387	0.46065534	0.37650597	0.54550829	0.62293702	0.89963902	0.48000885	0.46428454	0.58332981	0.55462226
215.6	0.49228213	0.46073628	0.37654784	0.545593	0.62301555	0.89963946	0.48005042	0.46434188	0.5833793	0.55472224
215.7	0.49233034	0.46081717	0.37658964	0.54567766	0.62309402	0.89963989	0.48009197	0.46439919	0.58342875	0.55482213
215.8	0.4923785	0.46089802	0.37663138	0.54576227	0.62317242	0.89964032	0.48013351	0.46445646	0.58347816	0.55492195
215.9	0.49242661	0.46097882	0.37667305	0.54584682	0.62325075	0.89964075	0.48017504	0.4645137	0.58352754	0.55502168
216	0.49247467	0.46105957	0.37671465	0.54593132	0.62332902	0.89964117	0.48021656	0.46457091	0.58357689	0.55512134
216.1	0.49252269	0.46114027	0.37675617	0.54601577	0.62340722	0.89964159	0.48025808	0.46462808	0.58362619	0.55522091
216.2	0.49257066	0.46122093	0.37679763	0.54610016	0.62348535	0.899642	0.48029958	0.46468522	0.58367547	0.5553204
216.3	0.49261857	0.46130154	0.37683903	0.5461845	0.62356342	0.89964241	0.48034107	0.46474233	0.5837247	0.55541981
216.4	0.49266644	0.4613821	0.37688035	0.54626879	0.62364142	0.89964281	0.48038255	0.46479941	0.5837739	0.55551914
216.5	0.49271426	0.46146261	0.3769216	0.54635303	0.62371935	0.89964321	0.48042402	0.46485645	0.58382306	0.55561839
216.6	0.49276203	0.46154308	0.37696278	0.54643721	0.62379722	0.89964361	0.48046548	0.46491346	0.58387219	0.55571756
216.7	0.49280976	0.4616235	0.37700389	0.54652134	0.62387502	0.899644	0.48050693	0.46497044	0.58392128	0.55581666
216.8	0.49285743	0.46170387	0.37704494	0.54660542	0.62395276	0.89964438	0.48054837	0.46502738	0.58397033	0.55591567
216.9	0.49290506	0.4617842	0.37708591	0.54668945	0.62403043	0.89964477	0.4805898	0.46508429	0.58401935	0.5560146
217	0.49295264	0.46186448	0.37712682	0.54677342	0.62410803	0.89964514	0.48063123	0.46514117	0.58406833	0.55611345
217.1	0.49300017	0.46194471	0.37716765	0.54685735	0.62418557	0.89964552	0.48067264	0.46519801	0.58411728	0.55621222
217.2	0.49304765	0.4620249	0.37720842	0.54694122	0.62426304	0.89964589	0.48071404	0.46525483	0.58416619	0.55631091
217.3	0.49309509	0.46210503	0.37724912	0.54702503	0.62434045	0.89964625	0.48075544	0.46531161	0.58421506	0.55640952
217.4	0.49314247	0.46218513	0.37728974	0.5471088	0.62441779	0.89964662	0.48079683	0.46536836	0.5842639	0.55650805
217.5	0.49318981	0.46226517	0.3773303	0.54719251	0.62449507	0.89964697	0.48083821	0.46542507	0.5843127	0.5566065
217.6	0.4932371	0.46234517	0.37737079	0.54727617	0.62457228	0.89964733	0.48087958	0.46548176	0.58436146	0.55670487
217.7	0.49328434	0.46242512	0.37741121	0.54735978	0.62464942	0.89964767	0.48092095	0.46553841	0.58441019	0.55680317
217.8	0.49333154	0.46250502	0.37745156	0.54744334	0.6247265	0.89964802	0.4809623	0.46559502	0.58445888	0.55690138
217.9	0.49337869	0.46258488	0.37749184	0.54752685	0.62480352	0.89964836	0.48100365	0.46565161	0.58450754	0.55699951
218	0.49342579	0.46266469	0.37753205	0.5476103	0.62488047	0.8996487	0.48104499	0.46570817	0.58455616	0.55709757
218.1	0.49347284	0.46274446	0.37757219	0.54769371	0.62495735	0.89964903	0.48108633	0.46576469	0.58460474	0.55719555
218.2	0.49351984	0.46282417	0.37761226	0.54777706	0.62503417	0.89964936	0.48112765	0.46582118	0.58465329	0.55729345
218.3	0.4935668	0.46290385	0.37765227	0.54786036	0.62511093	0.89964968	0.48116897	0.46587764	0.5847018	0.55739126
218.4	0.49361371	0.46298347	0.3776922	0.5479436	0.62518762	0.89965	0.48121028	0.46593406	0.58475028	0.557489
218.5	0.49366057	0.46306305	0.37773207	0.5480268	0.62526425	0.89965032	0.48125159	0.46599046	0.58479872	0.55758667
218.6	0.49370739	0.46314258	0.37777187	0.54810995	0.62534081	0.89965063	0.48129289	0.46604682	0.58484712	0.55768425
218.7	0.49375416	0.46322207	0.37781161	0.54819304	0.6254173	0.89965094	0.48133418	0.46610315	0.58489549	0.55778176
218.8	0.49380088	0.4633015	0.37785127	0.54827608	0.62549374	0.89965124	0.48137547	0.46615945	0.58494382	0.55787918
218.9	0.49384755	0.4633809	0.37789087	0.54835907	0.62557011	0.89965154	0.48141675	0.46621572	0.58499211	0.55797653
219	0.49389418	0.46346024	0.37793039	0.54844201	0.62564641	0.89965183	0.48145803	0.46627195	0.58504037	0.5580738
219.1	0.49394076	0.46353954	0.37796986	0.5485249	0.62572265	0.89965213	0.4814993	0.46632816	0.58508859	0.558171
219.2	0.49398729	0.4636188	0.37800925	0.54860774	0.62579883	0.89965241	0.48154056	0.46638433	0.58513678	0.55826811
219.3	0.49403378	0.46369801	0.37804858	0.54869052	0.62587494	0.8996527	0.48158182	0.46644047	0.58518493	0.55836515
219.4	0.49408022	0.46377717	0.37808784	0.54877326	0.62595099	0.89965298	0.48162307	0.46649658	0.58523305	0.55846211
219.5	0.49412661	0.46385628	0.37812704	0.54885594	0.62602697	0.89965325	0.48166432	0.46655266	0.58528113	0.558559
219.6	0.49417296	0.46393535	0.37816617	0.54893858	0.62610289	0.89965353	0.48170556	0.46660871	0.58532917	0.5586558
219.7	0.49421926	0.46401438	0.37820523	0.54902116	0.62617875	0.89965379	0.4817468	0.46666472	0.58537717	0.55875253
219.8	0.49426551	0.46409335	0.37824423	0.54910369	0.62625454	0.89965406	0.48178804	0.46672071	0.58542514	0.55884919
219.9	0.49431172	0.46417229	0.37828317	0.54918617	0.62633027	0.89965432	0.48182927	0.46677666	0.58547308	0.55894576
220	0.49435788	0.46425117	0.37832204	0.5492686	0.62640594	0.89965457	0.4818705	0.46683258	0.58552098	0.55904226
220.1	0.49440399	0.46433001	0.37836085	0.54935098	0.62648154	0.89965483	0.48191172	0.46688847	0.58556884	0.55913868
220.2	0.49445006	0.4644088	0.37839959	0.54943331	0.62655708	0.89965508	0.48195294	0.46694433	0.58561666	0.55923503
220.3	0.49449608	0.46448755	0.37843827	0.54951559	0.62663256	0.89965532	0.48199415	0.46700016	0.58566445	0.55933129
220.4	0.49454205	0.46456625	0.37847689	0.54959781	0.62670797	0.89965556	0.48203536	0.46705596	0.58571221	0.55942749
220.5	0.49458798	0.46464491	0.37851544	0.54967999	0.62678332	0.8996558	0.48207657	0.46711172	0.58575993	0.5595236
220.6	0.49463386	0.46472352	0.37855394	0.54976212	0.62685861	0.89965603	0.48211778	0.46716746	0.58580761	0.55961964
220.7	0.4946797	0.46480209	0.37859237	0.5498442	0.62693384	0.89965626	0.48215898	0.46722317	0.58585525	0.5597156
220.8	0.49472549	0.46488061	0.37863074	0.54992622	0.627009	0.89965649	0.48220018	0.46727884	0.58590286	0.55981148
220.9	0.49477123	0.46495908	0.37866905	0.5500082	0.6270841	0.89965671	0.48224138	0.46733448	0.58595044	0.55990729
221	0.49481693	0.46503751	0.3787073	0.55009013	0.62715913	0.89965693	0.48228258	0.4673901	0.58599797	0.56000303
221.1	0.49486258	0.46511589	0.37874549	0.550172	0.62723411	0.89965714	0.48232377	0.46744568	0.58604547	0.56009868
221.2	0.49490819	0.46519423	0.37878361	0.55025383	0.62730902	0.89965735	0.48236496	0.46750123	0.58609294	0.56019426
221.3	0.49495375	0.46527252	0.37882168	0.55033561	0.62738387	0.89965756	0.48240615	0.46755675	0.58614037	0.56028977
221.4	0.49499927	0.46535077	0.3788597	0.55041734	0.62745865	0.89965776	0.48244734	0.46761224	0.58618776	0.5603852
221.5	0.49504474	0.46542897	0.37889765	0.55049901	0.62753338	0.89965796	0.48248853	0.4676677	0.58623512	0.56048055
221.6	0.49509016	0.46550712	0.37893554	0.55058064	0.62760804	0.89965816	0.48252971	0.46772313	0.58628244	0.56057583
221.7	0.49513554	0.46558523	0.37897338	0.55066222	0.62768264	0.89965835	0.4825709	0.46777852	0.58632972	0.56067103
221.8	0.49518087	0.4656633	0.37901116	0.55074375	0.62775718	0.89965854	0.48261208	0.46783389	0.58637697	0.56076616
221.9	0.49522616	0.46574131	0.37904889	0.55082523	0.62783165	0.89965872	0.48265326	0.46788923	0.58642418	0.56086121
222	0.4952714	0.46581929	0.37908655	0.55090666	0.62790607	0.8996589	0.48269445	0.46794453	0.58647136	0.56095619
222.1	0.49531659	0.46589722	0.37912416	0.55098804	0.62798042	0.89965908	0.48273563	0.46799981	0.5865185	0.56105109
222.2	0.49536174	0.4659751	0.37916172	0.55106937	0.62805471	0.89965925	0.48277681	0.46805506	0.5865656	0.56114591
222.3	0.49540685	0.46605294	0.37919922	0.55115065	0.62812894	0.89965942	0.48281799	0.46811027	0.58661267	0.56124066
222.4	0.49545191	0.46613073	0.37923667	0.55123188	0.62820311	0.89965959	0.48285918	0.46816546	0.5866597	0.56133534
222.5	0.49549692	0.46620848	0.37927406	0.55131307	0.62827721	0.89965975	0.48290036	0.46822061	0.5867067	0.56142994
222.6	0.49554189	0.46628618	0.3793114	0.5513942	0.62835126	0.89965991	0.48294154	0.46827574	0.58675365	0.56152446
222.7	0.49558682	0.46636384	0.37934868	0.55147529	0.62842524	0.89966007	0.48298273	0.46833083	0.58680058	0.56161892
222.8	0.49563169	0.46644145	0.37938592	0.55155632	0.62849916	0.89966022	0.48302391	0.4683859	0.58684746	0.56171329
222.9	0.49567653	0.46651902	0.3794231	0.55163731	0.62857302	0.89966037	0.4830651	0.46844093	0.58689432	0.56180759
223	0.49572132	0.46659654	0.37946022	0.55171824	0.62864682	0.89966051	0.48310629	0.46849594	0.58694113	0.56190182
223.1	0.49576606	0.46667402	0.3794973	0.55179913	0.62872056	0.89966065	0.48314748	0.46855091	0.58698791	0.56199597
223.2	0.49581076	0.46675146	0.37953432	0.55187997	0.62879423	0.89966079	0.48318867	0.46860586	0.58703465	0.56209005
223.3	0.49585541	0.46682884	0.3795713	0.55196076	0.62886785	0.89966092	0.48322987	0.46866077	0.58708136	0.56218405
223.4	0.49590002	0.46690619	0.37960822	0.5520415	0.6289414	0.89966105	0.48327106	0.46871566	0.58712803	0.56227798
223.5	0.49594458	0.46698349	0.37964509	0.5521222	0.6290149	0.89966118	0.48331226	0.46877051	0.58717466	0.56237183
223.6	0.4959891	0.46706074	0.37968191	0.55220284	0.62908833	0.89966131	0.48335346	0.46882534	0.58722126	0.56246561
223.7	0.49603357	0.46713795	0.37971869	0.55228344	0.6291617	0.89966143	0.48339467	0.46888013	0.58726782	0.56255932
223.8	0.496078	0.46721511	0.37975541	0.55236398	0.62923501	0.89966154	0.48343587	0.4689349	0.58731434	0.56265295
223.9	0.49612239	0.46729223	0.37979209	0.55244448	0.62930826	0.89966166	0.48347708	0.46898964	0.58736083	0.56274651
224	0.49616673	0.46736931	0.37982871	0.55252493	0.62938145	0.89966177	0.48351829	0.46904434	0.58740728	0.56283999
224.1	0.49621102	0.46744634	0.37986529	0.55260533	0.62945458	0.89966187	0.48355951	0.46909902	0.5874537	0.5629334
224.2	0.49625527	0.46752333	0.37990182	0.55268568	0.62952765	0.89966197	0.48360073	0.46915367	0.58750008	0.56302674
224.3	0.49629947	0.46760027	0.3799383	0.55276599	0.62960066	0.89966207	0.48364195	0.46920828	0.58754642	0.56312
224.4	0.49634363	0.46767717	0.37997474	0.55284624	0.62967361	0.89966217	0.48368318	0.46926287	0.58759273	0.56321319
224.5	0.49638775	0.46775402	0.38001113	0.55292645	0.6297465	0.89966226	0.48372441	0.46931743	0.587639	0.56330631
224.6	0.49643182	0.46783083	0.38004747	0.55300661	0.62981933	0.89966235	0.48376565	0.46937196	0.58768524	0.56339935
224.7	0.49647585	0.46790759	0.38008377	0.55308672	0.6298921	0.89966244	0.48380689	0.46942646	0.58773143	0.56349232
224.8	0.49651983	0.46798431	0.38012002	0.55316678	0.62996481	0.89966252	0.48384813	0.46948093	0.5877776	0.56358521
224.9	0.49656377	0.46806098	0.38015623	0.5532468	0.63003746	0.8996626	0.48388938	0.46953537	0.58782372	0.56367803

225.6	0.49687009	0.46859646	0.38040841	0.55380555	0.63054433	0.89966306	0.48417829	0.46991563	0.58814558	0.56432574
225.7	0.49691367	0.46867278	0.38044427	0.55388518	0.6306165	0.89966311	0.48421959	0.46996984	0.58819142	0.56441798
225.8	0.49695721	0.46874906	0.38048008	0.55396476	0.63068861	0.89966316	0.48426089	0.47002402	0.58823721	0.56451014
225.9	0.49700071	0.46882529	0.38051584	0.55404429	0.63076067	0.89966321	0.4843022	0.47007817	0.58828298	0.56460224
226	0.49704416	0.46890148	0.38055157	0.55412378	0.63083266	0.89966325	0.48434352	0.47013229	0.5883287	0.56469426
226.1	0.49708756	0.46897762	0.38058725	0.55420322	0.6309046	0.89966329	0.48438484	0.47018638	0.58837439	0.5647862
226.2	0.49713093	0.46905372	0.38062289	0.55428261	0.63097647	0.89966333	0.48442617	0.47024044	0.58842004	0.56487808
226.3	0.49717425	0.46912978	0.38065849	0.55436195	0.63104829	0.89966336	0.48446751	0.47029447	0.58846565	0.56496988
226.4	0.49721752	0.46920579	0.38069404	0.55444125	0.63112005	0.89966339	0.48450885	0.47034847	0.58851123	0.56506161
226.5	0.49726075	0.46928176	0.38072956	0.5545205	0.63119175	0.89966341	0.4845502	0.47040245	0.58855677	0.56515327
226.6	0.49730394	0.46935768	0.38076504	0.5545997	0.63126339	0.89966344	0.48459156	0.47045639	0.58860228	0.56524486
226.7	0.49734708	0.46943356	0.38080047	0.55467885	0.63133497	0.89966346	0.48463293	0.47051031	0.58864775	0.56533637
226.8	0.49739018	0.4695094	0.38083586	0.55475796	0.6314065	0.89966347	0.4846743	0.4705642	0.58869318	0.56542781
226.9	0.49743324	0.46958519	0.38087122	0.55483702	0.63147796	0.89966349	0.48471569	0.47061806	0.58873857	0.56551918
227	0.49747625	0.46966094	0.38090653	0.55491603	0.63154937	0.8996635	0.48475708	0.47067189	0.58878393	0.56561048
227.1	0.49751922	0.46973664	0.38094181	0.55499499	0.63162072	0.89966351	0.48479847	0.47072569	0.58882925	0.5657017
227.2	0.49756215	0.46981231	0.38097704	0.55507391	0.63169201	0.89966351	0.48483988	0.47077946	0.58887454	0.56579286
227.3	0.49760503	0.46988792	0.38101224	0.55515278	0.63176324	0.89966351	0.4848813	0.47083321	0.58891979	0.56588394
227.4	0.49764786	0.4699635	0.38104739	0.5552316	0.63183441	0.89966351	0.48492272	0.47088692	0.588965	0.56597495
227.5	0.49769066	0.47003903	0.38108251	0.55531037	0.63190553	0.8996635	0.48496416	0.47094061	0.58901017	0.56606589
227.6	0.49773341	0.47011452	0.38111759	0.5553891	0.63197658	0.89966349	0.4850056	0.47099427	0.58905531	0.56615676
227.7	0.49777612	0.47018996	0.38115263	0.55546778	0.63204758	0.89966348	0.48504705	0.4710479	0.58910041	0.56624755
227.8	0.49781878	0.47026536	0.38118763	0.55554642	0.63211852	0.89966347	0.48508852	0.4711015	0.58914547	0.56633828
227.9	0.4978614	0.47034072	0.3812226	0.555625	0.63218941	0.89966345	0.48512999	0.47115508	0.5891905	0.56642893
228	0.49790398	0.47041603	0.38125752	0.55570354	0.63226023	0.89966343	0.48517147	0.47120862	0.58923549	0.56651951
228.1	0.49794651	0.4704913	0.38129241	0.55578203	0.632331	0.89966341	0.48521297	0.47126214	0.58928044	0.56661002
228.2	0.497989	0.47056653	0.38132727	0.55586048	0.63240171	0.89966338	0.48525447	0.47131563	0.58932535	0.56670046
228.3	0.49803145	0.47064171	0.38136208	0.55593888	0.63247236	0.89966335	0.48529598	0.47136909	0.58937023	0.56679083
228.4	0.49807385	0.47071685	0.38139686	0.55601723	0.63254296	0.89966332	0.48533751	0.47142252	0.58941507	0.56688113
228.5	0.49811621	0.47079195	0.3814316	0.55609554	0.6326135	0.89966328	0.48537905	0.47147592	0.58945987	0.56697136
228.6	0.49815853	0.470867	0.3814663	0.55617379	0.63268398	0.89966324	0.48542059	0.4715293	0.58950464	0.56706151
228.7	0.49820081	0.47094201	0.38150097	0.55625201	0.6327544	0.8996632	0.48546215	0.47158264	0.58954937	0.5671516
228.8	0.49824304	0.47101698	0.3815356	0.55633017	0.63282476	0.89966315	0.48550372	0.47163596	0.58959406	0.56724161
228.9	0.49828523	0.4710919	0.3815702	0.55640829	0.63289507	0.89966311	0.48554531	0.47168925	0.58963871	0.56733155
229	0.49832737	0.47116679	0.38160475	0.55648636	0.63296532	0.89966305	0.4855869	0.47174252	0.58968333	0.56742142
229.1	0.49836948	0.47124162	0.38163928	0.55656438	0.63303552	0.899663	0.48562851	0.47179575	0.58972791	0.56751122
229.2	0.49841154	0.47131642	0.38167376	0.55664236	0.63310566	0.89966294	0.48567013	0.47184896	0.58977245	0.56760095
229.3	0.49845355	0.47139117	0.38170822	0.55672029	0.63317574	0.89966288	0.48571176	0.47190213	0.58981695	0.56769061
229.4	0.49849553	0.47146588	0.38174263	0.55679818	0.63324576	0.89966282	0.48575341	0.47195528	0.58986142	0.5677802
229.5	0.49853746	0.47154055	0.38177701	0.55687602	0.63331573	0.89966275	0.48579507	0.47200841	0.58990585	0.56786972
229.6	0.49857935	0.47161517	0.38181136	0.55695381	0.63338563	0.89966268	0.48583674	0.4720615	0.58995024	0.56795917
229.7	0.49862119	0.47168975	0.38184567	0.55703156	0.63345549	0.89966261	0.48587842	0.47211457	0.5899946	0.56804855
229.8	0.498663	0.47176429	0.38187995	0.55710926	0.63352528	0.89966254	0.48592012	0.47216761	0.59003891	0.56813786
229.9	0.49870476	0.47183878	0.38191419	0.55718691	0.63359502	0.89966246	0.48596183	0.47222062	0.59008319	0.56822709
230	0.49874648	0.47191324	0.38194839	0.55726452	0.63366471	0.89966238	0.48600356	0.4722736	0.59012743	0.56831626
230.1	0.49878815	0.47198765	0.38198256	0.55734208	0.63373433	0.8996623	0.48604529	0.47232655	0.59017163	0.56840536
230.2	0.49882979	0.47206201	0.3820167	0.55741959	0.6338039	0.89966221	0.48608705	0.47237948	0.5902158	0.56849438
230.3	0.49887138	0.47213634	0.38205081	0.55749706	0.63387342	0.89966212	0.48612882	0.47243238	0.59025992	0.56858334
230.4	0.49891293	0.47221062	0.38208488	0.55757448	0.63394288	0.89966203	0.4861706	0.47248525	0.59030401	0.56867223
230.5	0.49895443	0.47228486	0.38211891	0.55765186	0.63401228	0.89966193	0.48621239	0.4725381	0.59034806	0.56876104
230.6	0.4989959	0.47235905	0.38215291	0.55772919	0.63408162	0.89966183	0.4862542	0.47259091	0.59039208	0.56884979
230.7	0.49903732	0.47243321	0.38218688	0.55780647	0.63415091	0.89966173	0.48629603	0.4726437	0.59043605	0.56893847
230.8	0.4990787	0.47250732	0.38222081	0.55788371	0.63422015	0.89966163	0.48633787	0.47269646	0.59047999	0.56902707
230.9	0.49912004	0.47258139	0.38225472	0.5579609	0.63428932	0.89966152	0.48637973	0.4727492	0.59052388	0.56911561
231	0.49916133	0.47265541	0.38228858	0.55803805	0.63435845	0.89966141	0.4864216	0.4728019	0.59056774	0.56920408
231.1	0.49920258	0.4727294	0.38232242	0.55811515	0.63442751	0.8996613	0.48646349	0.47285458	0.59061156	0.56929247
231.2	0.49924379	0.47280334	0.38235622	0.5581922	0.63449652	0.89966118	0.48650539	0.47290723	0.59065534	0.5693808
231.3	0.49928496	0.47287724	0.38238999	0.55826921	0.63456548	0.89966107	0.48654731	0.47295986	0.59069909	0.56946906
231.4	0.49932609	0.4729511	0.38242372	0.55834617	0.63463437	0.89966095	0.48658925	0.47301245	0.59074279	0.56955725
231.5	0.49936717	0.47302491	0.38245742	0.55842309	0.63470322	0.89966082	0.4866312	0.47306502	0.59078646	0.56964537
231.6	0.49940822	0.47309868	0.38249109	0.55849996	0.634772	0.8996607	0.48667316	0.47311756	0.59083009	0.56973342
231.7	0.49944922	0.47317242	0.38252473	0.55857679	0.63484074	0.89966057	0.48671515	0.47317008	0.59087368	0.5698214
231.8	0.49949018	0.4732461	0.38255833	0.55865357	0.63490942	0.89966044	0.48675715	0.47322256	0.59091723	0.56990931
231.9	0.49953109	0.47331975	0.3825919	0.5587303	0.63497804	0.8996603	0.48679917	0.47327502	0.59096074	0.56999715
232	0.49957197	0.47339336	0.38262544	0.55880699	0.6350466	0.89966016	0.4868412	0.47332745	0.59100421	0.57008493
232.1	0.4996128	0.47346692	0.38265895	0.55888363	0.63511511	0.89966002	0.48688325	0.47337986	0.59104764	0.57017263
232.2	0.4996536	0.47354044	0.38269243	0.55896023	0.63518357	0.89965988	0.48692532	0.47343224	0.59109104	0.57026026
232.3	0.49969435	0.47361392	0.38272587	0.55903678	0.63525197	0.89965973	0.48696741	0.47348459	0.5911344	0.57034783
232.4	0.49973506	0.47368735	0.38275928	0.55911329	0.63532032	0.89965959	0.48700951	0.47353691	0.59117771	0.57043532
232.5	0.49977572	0.47376075	0.38279266	0.55918975	0.63538861	0.89965943	0.48705163	0.4735892	0.59122098	0.57052275
232.6	0.49981635	0.4738341	0.38282601	0.55926616	0.63545685	0.89965928	0.48709377	0.47364147	0.59126422	0.57061011
232.7	0.49985693	0.47390741	0.38285933	0.55934253	0.63552503	0.89965912	0.48713593	0.47369371	0.59130741	0.5706974
232.8	0.49989748	0.47398068	0.38289261	0.55941886	0.63559315	0.89965896	0.4871781	0.47374593	0.59135057	0.57078462
232.9	0.49993798	0.47405391	0.38292587	0.55949514	0.63566123	0.8996588	0.4872203	0.47379811	0.59139369	0.57087177
233	0.49997844	0.4741271	0.38295909	0.55957137	0.63572924	0.89965864	0.48726251	0.47385027	0.59143676	0.57095885
233.1	0.50001886	0.47420024	0.38299228	0.55964756	0.63579721	0.89965847	0.48730474	0.4739024	0.5914798	0.57104586
233.2	0.50005924	0.47427334	0.38302544	0.5597237	0.63586511	0.8996583	0.48734699	0.47395451	0.5915228	0.57113281
233.3	0.50009958	0.4743464	0.38305857	0.5597998	0.63593297	0.89965813	0.48738926	0.47400659	0.59156576	0.57121969
233.4	0.50013987	0.47441942	0.38309166	0.55987585	0.63600077	0.89965795	0.48743154	0.47405864	0.59160867	0.57130649
233.5	0.50018013	0.4744924	0.38312473	0.55995186	0.63606851	0.89965777	0.48747385	0.47411067	0.59165155	0.57139323
233.6	0.50022034	0.47456534	0.38315777	0.56002782	0.6361362	0.89965759	0.48751617	0.47416266	0.59169439	0.5714799
233.7	0.50026051	0.47463823	0.38319077	0.56010374	0.63620384	0.89965741	0.48755852	0.47421463	0.59173718	0.5715665
233.8	0.50030064	0.47471108	0.38322375	0.56017961	0.63627142	0.89965722	0.48760088	0.47426658	0.59177994	0.57165304
233.9	0.50034073	0.4747839	0.38325669	0.56025544	0.63633895	0.89965704	0.48764326	0.47431849	0.59182265	0.5717395
234	0.50038078	0.47485667	0.3832896	0.56033122	0.63640642	0.89965684	0.48768566	0.47437038	0.59186532	0.5718259
234.1	0.50042079	0.47492939	0.38332248	0.56040696	0.63647384	0.89965665	0.48772808	0.47442225	0.59190796	0.57191222
234.2	0.50046076	0.47500208	0.38335533	0.56048265	0.63654121	0.89965645	0.48777053	0.47447408	0.59195055	0.57199848
234.3	0.50050069	0.47507473	0.38338815	0.5605583	0.63660852	0.89965626	0.48781299	0.47452589	0.5919931	0.57208467
234.4	0.50054057	0.47514733	0.38342094	0.5606339	0.63667578	0.89965605	0.48785547	0.47457767	0.59203561	0.57217079
234.5	0.50058042	0.4752199	0.3834537	0.56070946	0.63674298	0.89965585	0.48789797	0.47462943	0.59207808	0.57225685
234.6	0.50062023	0.47529242	0.38348643	0.56078497	0.63681013	0.89965564	0.48794049	0.47468116	0.5921205	0.57234283
234.7	0.50065999	0.4753649	0.38351913	0.56086044	0.63687723	0.89965543	0.48798304	0.47473286	0.59216289	0.57242875
234.8	0.50069971	0.47543734	0.3835518	0.56093587	0.63694427	0.89965522	0.4880256	0.47478453	0.59220523	0.5725146
234.9	0.5007394	0.47550974	0.38358444	0.56101124	0.63701126	0.89965501	0.48806818	0.47483618	0.59224753	0.57260038
235	0.50077904	0.4755821	0.38361705	0.56108658	0.6370782	0.89965479	0.48811079	0.4748878	0.59228979	0.57268609
235.1	0.50081864	0.47565442	0.38364962	0.56116187	0.63714508	0.89965457	0.48815341	0.4749394	0.59233201	0.57277174
235.2	0.50085821	0.47572669	0.38368217	0.56123711	0.63721191	0.89965435	0.48819606	0.47499097	0.59237418	0.57285731
235.3	0.50089773	0.47579893	0.38371469	0.56131231	0.63727869	0.89965412	0.48823872	0.47504251	0.59241631	0.57294282
235.4	0.50093721	0.47587112	0.38374718	0.56138747	0.63734541	0.8996539	0.48828141	0.47509402	0.5924584	0.57302826
235.5	0.50097665	0.47594327	0.38377963	0.56146258	0.63741208	0.89965367	0.48832412	0.47514551	0.59250045	0.57311363
235.6	0.50101605	0.47601539	0.38381206	0.56153765	0.6374787	0.89965343	0.48836686	0.47519697	0.59254245	0.57319894
235.7	0.50105541	0.47608746	0.38384446	0.56161267	0.63754526	0.8996532	0.4884096	0.47524841	0.59258441	0.57328417
235.8	0.50109473	0.47615949	0.38387683	0.56168765	0.63761177	0.89965296	0.48845237	0.47529982	0.59262633	0.57336934
235.9	0.50113402	0.47623148	0.38390917	0.56176258	0.63767823	0.89965272	0.48849517	0.4753512	0.59266821	0.57345444
236	0.50117326	0.47630343	0.38394147	0.56183747	0.63774464	0.89965248	0.48853798	0.47540256	0.59271004	0.57353947
236.1	0.50121246	0.47637533	0.38397375	0.56191231	0.63781099	0.89965223	0.48858082	0.47545388	0.59275182	0.57362444
236.2	0.50125162	0.4764472	0.384006	0.56198711	0.63787729	0.89965199	0.48862368	0.47550519	0.59279357	0.57370933
236.3	0.50129074	0.47651903	0.38403822	0.56206187	0.63794353	0.89965174	0.48866656	0.47555646	0.59283527	0.57379416
236.4	0.50132982	0.47659082	0.38407041	0.56213658	0.63800973	0.89965148	0.48870947	0.47560771	0.59287692	0.57387892
236.5	0.50136886	0.47666256	0.38410257	0.56221125	0.63807587	0.89965123	0.48875239	0.47565894	0.59291853	0.57396362
236.6	0.50140786	0.47673427	0.3841347	0.56228587	0.63814196	0.89965097	0.48879534	0.47571013	0.5929601	0.57404824
236.7	0.50144682	0.47680593	0.3841668	0.56236045	0.63820799	0.89965071	0.48883831	0.4757613	0.59300162	0.5741328
236.8	0.50148574	0.47687755	0.38419887	0.56243498	0.63827398	0.89965045	0.4888813	0.47581245	0.59304309	0.57421729
236.9	0.50152463	0.47694914	0.38423091	0.56250947	0.63833991	0.89965019	0.48892431	0.47586356	0.59308453	0.57430171
237	0.50156347	0.47702068	0.38426292	0.56258392	0.63840579	0.89964992	0.48896735	0.47591466	0.59312591	0.57438606
237.1	0.50160227	0.47709218	0.38429491	0.56265832	0.63847162	0.89964965	0.4890104	0.47596572	0.59316725	0.57447035
237.2	0.50164104	0.47716365	0.38432686	0.56273268	0.63853739	0.89964938	0.48905348	0.47601676	0.59320855	0.57455457
237.3	0.50167976	0.47723507	0.38435878	0.56280699	0.63860311	0.8996491	0.48909658	0.47606777	0.5932498	0.57463872
237.4	0.50171844	0.47730645	0.38439068	0.56288126	0.63866878	0.89964883	0.48913971	0.47611876	0.593291	0.5747228
237.5	0.50175709	0.47737779	0.38442254	0.56295549	0.6387344	0.89964855	0.48918286	0.47616972	0.59333215	0.57480682
237.6	0.5017957	0.47744909	0.38445438	0.56302967	0.63879997	0.89964827	0.48922602	0.47622065	0.59337326	0.57489077
237.7	0.50183426	0.47752035	0.38448618	0.56310381	0.63886548	0.89964798	0.48926922	0.47627156	0.59341433	0.57497465
237.8	0.50187279	0.47759157	0.38451796	0.56317791	0.63893094	0.89964769	0.48931243	0.47632244	0.59345534	0.57505846
237.9	0.50191128	0.47766275	0.38454971	0.56325196	0.63899636	0.89964741	0.48935567	0.47637329	0.59349631	0.5751422
238	0.50194973	0.47773389	0.38458142	0.56332596	0.63906171	0.89964711	0.48939893	0.47642412	0.59353723	0.57522588
238.1	0.50198813	0.47780499	0.38461311	0.56339993	0.63912702	0.89964682	0.48944221	0.47647493	0.5935781	0.57530949
238.2	0.5020265	0.47787605	0.38464477	0.56347384	0.63919228	0.89964653	0.48948552	0.4765257	0.59361893	0.57539303
238.3	0.50206484	0.47794707	0.3846764	0.56354772	0.63925748	0.89964623	0.48952885	0.47657645	0.5936597	0.57547651
238.4	0.50210313	0.47801805	0.384708	0.56362155	0.63932264	0.89964593	0.4895722	0.47662718	0.59370043	0.57555992
238.5	0.50214138	0.47808899	0.38473957	0.56369534	0.63938774	0.89964562	0.48961558	0.47667788	0.59374111	0.57564326
238.6	0.5021796	0.47815989	0.38477112	0.56376908	0.63945279	0.89964532	0.48965898	0.47672855	0.59378174	0.57572653
238.7	0.50221777	0.47823075	0.38480263	0.56384278	0.63951779	0.89964501	0.4897024	0.47677919	0.59382232	0.57580973
238.8	0.50225591	0.47830157	0.38483411	0.56391644	0.63958273	0.8996447	0.48974584	0.47682981	0.59386285	0.57589287
238.9	0.50229401	0.47837235	0.38486557	0.56399006	0.63964763	0.89964439	0.48978931	0.47688041	0.59390333	0.57597594
239	0.50233206	0.47844309	0.38489699	0.56406363	0.63971247	0.89964407	0.4898328	0.47693097	0.59394376	0.57605894
239.1	0.50237008	0.47851379	0.38492839	0.56413715	0.63977727	0.89964376	0.48987632	0.47698152	0.59398414	0.57614188
239.2	0.50240807	0.47858445	0.38495976	0.56421063	0.63984201	0.89964344	0.48991986	0.47703203	0.59402447	0.57622474
239.3	0.50244601	0.47865507	0.3849911	0.56428407	0.6399067	0.89964311	0.48996342	0.47708252	0.59406475	0.57630754
239.4	0.50248391	0.47872565	0.3850224	0.56435747	0.63997134	0.89964279	0.490007	0.47713298	0.59410497	0.57639028
239.5	0.50252178	0.47879619	0.38505368	0.56443082	0.64003593	0.89964246	0.49005061	0.47718342	0.59414515	0.57647294
239.6	0.50255961	0.47886669	0.38508494	0.56450413	0.64010047	0.89964213	0.49009424	0.47723383	0.59418527	0.57655554
239.7	0.50259739	0.47893715	0.38511616	0.5645774	0.64016496	0.8996418	0.4901379	0.47728422	0.59422534	0.57663807
239.8	0.50263514	0.47900757	0.38514735	0.56465062	0.6402294	0.89964147	0.49018158	0.47733458	0.59426535	0.57672053
239.9	0.50267286	0.47907795	0.38517851	0.5647238	0.64029379	0.89964113	0.49022528	0.47738491	0.59430532	0.57680293
240	0.50271053	0.4791483	0.38520965	0.56479694	0.64035812	0.8996408	0.49026901	0.47743522	0.59434523	0.57688525

Table S12: eFAST analysis data for [G3P] with [G](0)=2 mM: eFAST sensitivity indices

Sensitivity analysis data from eFAST for [G3P] with [G](0)=2 mM (only the parameters to which [G3P]'s variability is sensitive to appear)

Time discretization	K_{[G]}	q	V_{[G3P]}	K_{[PYR]}	alpha	V_{[ACoA]}
0	0	0	0	0	0	0
0.1	0.00258988	0.91958708	0.09367251	0.00189433	0.00386007	0.00218882
0.2	0.00260515	0.91838293	0.09300872	0.00195226	0.00676909	0.00212192
0.3	0.00262009	0.91612857	0.09224086	0.00201443	0.01101822	0.00205509
0.4	0.00263512	0.91272881	0.09137575	0.00207835	0.01665745	0.00199038
0.5	0.00265012	0.90822196	0.09042359	0.00214212	0.02359343	0.00192904
0.6	0.0026647	0.90268736	0.08939996	0.00220498	0.03170736	0.0018717
0.7	0.00267852	0.89622408	0.08832281	0.00226699	0.04087437	0.00181871
0.8	0.00269147	0.88894053	0.08720705	0.00232816	0.05096895	0.0017702
0.9	0.0027035	0.88094508	0.08606571	0.00238854	0.06186712	0.00172615
1	0.00271471	0.87234217	0.08490894	0.00244824	0.07345131	0.00168656
1.1	0.00272522	0.86322922	0.08374486	0.00250733	0.0856102	0.00165135
1.2	0.00273526	0.85369524	0.08258028	0.00256597	0.09823959	0.00162047
1.3	0.00274506	0.84382209	0.08142079	0.00262429	0.11124418	0.00159388
1.4	0.00275483	0.83368431	0.08027103	0.00268243	0.12453685	0.0015716
1.5	0.00276474	0.82334886	0.07913502	0.00274054	0.13803811	0.00155361
1.6	0.00277499	0.81287655	0.0780158	0.00279885	0.15167698	0.0015399
1.7	0.0027859	0.80232186	0.07691599	0.00285759	0.16538963	0.00153048
1.8	0.00279775	0.79173212	0.07583803	0.00291692	0.17911955	0.00152534
1.9	0.00281072	0.78114911	0.07478367	0.00297708	0.19281763	0.00152449
2	0.00282507	0.77060951	0.0737542	0.00303837	0.20644005	0.00152793
2.1	0.00284109	0.76014425	0.07275089	0.00310092	0.21994913	0.00153566
2.2	0.00285896	0.74978003	0.07177466	0.00316486	0.23331351	0.00154767
2.3	0.0028789	0.73953974	0.07082609	0.00323042	0.246506	0.00156396
2.4	0.00290112	0.72944187	0.06990562	0.00329775	0.25950324	0.00158452
2.5	0.00292582	0.71950181	0.06901357	0.00336696	0.27228662	0.00160933
2.6	0.00295315	0.70973234	0.06814997	0.00343823	0.28484144	0.00163838
2.7	0.00298328	0.70014333	0.06731473	0.00351171	0.2971557	0.00167165
2.8	0.00301636	0.69074261	0.06650768	0.0035875	0.30921989	0.00170914
2.9	0.00305251	0.68153628	0.06572857	0.00366574	0.3210274	0.00175081
3	0.00309187	0.67252863	0.06497701	0.00374653	0.33257375	0.00179667
3.1	0.00313455	0.66372246	0.06425259	0.00382996	0.34385606	0.0018467
3.2	0.00318063	0.65511956	0.06355491	0.00391614	0.35487303	0.00190087
3.3	0.00323021	0.64672059	0.06288348	0.00400518	0.3656248	0.00195918
3.4	0.00328337	0.63852516	0.06223769	0.0040972	0.37611262	0.00202161
3.5	0.00334021	0.63053214	0.06161695	0.00419225	0.38633849	0.00208816
3.6	0.00340081	0.62273979	0.06102075	0.00429041	0.39630541	0.00215881
3.7	0.00346522	0.61514576	0.06044857	0.00439174	0.40601727	0.00223354
3.8	0.00353348	0.60774711	0.05989978	0.00449633	0.41547841	0.00231234
3.9	0.00360566	0.60054055	0.05937379	0.00460425	0.42469339	0.0023952
4	0.00368181	0.59352258	0.05887001	0.00471555	0.43366702	0.0024821
4.1	0.00376198	0.58668944	0.0583879	0.00483027	0.4424046	0.00257304
4.2	0.00384617	0.58003702	0.05792688	0.00494847	0.4509118	0.002668
4.3	0.00393443	0.57356106	0.05748638	0.00507021	0.45919435	0.00276697
4.4	0.00402677	0.56725732	0.05706586	0.00519551	0.46725788	0.00286992
4.5	0.00412321	0.5611215	0.05666478	0.00532443	0.47510793	0.00297686
4.6	0.00422378	0.55514918	0.05628259	0.00545702	0.48275022	0.00308776
4.7	0.00432848	0.5493359	0.05591879	0.00559331	0.4901906	0.00320262
4.8	0.00443731	0.54367722	0.05557286	0.00573338	0.49743481	0.00332142
4.9	0.00455028	0.53816883	0.05524431	0.00587726	0.50448837	0.00344416
5	0.00466739	0.53280648	0.05493266	0.00602501	0.51135664	0.00357081
5.1	0.00478864	0.52758592	0.05463742	0.0061767	0.51804483	0.00370137
5.2	0.00491403	0.52250294	0.05435813	0.00633242	0.52455806	0.00383583
5.3	0.00504356	0.51755343	0.05409436	0.00649227	0.5309012	0.00397419
5.4	0.00517722	0.5127334	0.05384565	0.00665636	0.5370788	0.00411645
5.5	0.00531501	0.50803899	0.05361159	0.00682489	0.54309505	0.00426259
5.6	0.00545693	0.50346641	0.05339174	0.00699807	0.54895385	0.00441264
5.7	0.00560298	0.49901196	0.05318571	0.00717619	0.55465899	0.00456662
5.8	0.00575316	0.49467191	0.05299307	0.00735958	0.56021439	0.00472454
5.9	0.00590748	0.49044254	0.05281342	0.0075486	0.5656241	0.00488647
6	0.00606597	0.48632013	0.05264628	0.00774367	0.57089228	0.00505244
6.1	0.0062287	0.482301	0.05249121	0.0079452	0.57602316	0.00522253
6.2	0.00639577	0.47838142	0.05234773	0.00815365	0.58102093	0.00539679
6.3	0.0065674	0.47455762	0.05221541	0.00836942	0.58588975	0.00557524
6.4	0.0067439	0.47082581	0.05209389	0.00859294	0.5906337	0.0057579
6.5	0.00692558	0.46718226	0.05198286	0.00882461	0.5952568	0.00594476
6.6	0.00711275	0.46362348	0.05188206	0.0090648	0.59976298	0.00613578
6.7	0.0073056	0.4601464	0.05179127	0.00931384	0.60415604	0.00633092
6.8	0.00750424	0.45674839	0.05171025	0.00957204	0.60843962	0.00653011
6.9	0.0077087	0.45342715	0.0516388	0.00983968	0.61261726	0.00673331
7	0.00791892	0.4501806	0.05157672	0.01011699	0.61669237	0.00694043
7.1	0.00813481	0.4470068	0.05152381	0.01040416	0.62066829	0.00715142
7.2	0.00835623	0.44390392	0.05147992	0.01070133	0.62454826	0.0073662
7.3	0.00858303	0.44087018	0.05144486	0.01100864	0.62833537	0.0075847
7.4	0.00881508	0.43790382	0.05141846	0.01132622	0.63203261	0.00780686
7.5	0.0090522	0.43500321	0.05140056	0.0116542	0.63564288	0.00803262
7.6	0.00929427	0.43216674	0.05139101	0.01199272	0.63916895	0.00826192
7.7	0.00954119	0.42939284	0.05138964	0.01234191	0.64261348	0.00849471
7.8	0.00979284	0.42667996	0.05139631	0.0127019	0.64597904	0.00873093
7.9	0.01004917	0.4240266	0.05141086	0.0130728	0.64926813	0.00897053
8	0.01031012	0.4214313	0.05143317	0.01345469	0.65248317	0.00921347
8.1	0.01057568	0.41889261	0.0514631	0.01384755	0.65562648	0.00945972
8.2	0.01084584	0.41640911	0.0515005	0.01425133	0.6587003	0.00970923
8.3	0.01112065	0.4139794	0.05154526	0.0146659	0.66170677	0.00996197
8.4	0.01140019	0.41160215	0.05159722	0.01509107	0.66464794	0.0102179
8.5	0.01168459	0.40927607	0.05165628	0.01552662	0.66752582	0.010477
8.6	0.01197407	0.40699992	0.05172229	0.01597227	0.67034232	0.01073922
8.7	0.01226888	0.40477249	0.05179516	0.01642773	0.6730993	0.01100454
8.8	0.01256933	0.4025926	0.05187477	0.01689268	0.67579856	0.01127293
8.9	0.01287576	0.40045908	0.051961	0.01736676	0.6784418	0.01154437
9	0.01318859	0.39837083	0.05205374	0.01784963	0.6810307	0.01181882
9.1	0.01350829	0.39632676	0.05215289	0.01834092	0.68356686	0.01209628
9.2	0.01383532	0.3943258	0.05225836	0.01884024	0.68605183	0.0123767
9.3	0.01417016	0.39236691	0.05237007	0.01934724	0.68848711	0.01266007
9.4	0.01451319	0.39044906	0.05248791	0.01986155	0.69087413	0.01294637
9.5	0.01486477	0.3885713	0.05261182	0.02038281	0.69321426	0.01323557
9.6	0.01522514	0.38673271	0.05274171	0.02091067	0.69550885	0.01352765
9.7	0.0155945	0.38493239	0.05287753	0.02144479	0.69775917	0.01382258
9.8	0.01597298	0.38316947	0.05301922	0.02198485	0.69996647	0.01412035
9.9	0.01636066	0.3814431	0.0531667	0.02253049	0.70213193	0.01442094
10	0.01675754	0.37975248	0.05331993	0.02308142	0.70425674	0.01472432
10.1	0.01716361	0.37809685	0.05347886	0.02363731	0.70634201	0.01503048
10.2	0.01757882	0.37647544	0.05364343	0.02419789	0.70838883	0.01533939
10.3	0.01800308	0.37488751	0.05381357	0.02476287	0.71039824	0.01565104
10.4	0.01843625	0.37333234	0.05398924	0.02533199	0.71237124	0.0159654
10.5	0.01887821	0.37180924	0.05417036	0.02590497	0.7143088	0.01628245
10.6	0.01932878	0.37031752	0.05435687	0.02648161	0.71621185	0.01660218
10.7	0.01978779	0.36885658	0.0545487	0.02706167	0.71808129	0.01692456
10.8	0.02025505	0.36742582	0.05474579	0.02764497	0.71991796	0.01724957
10.9	0.02073037	0.36602465	0.05494806	0.0282313	0.72172272	0.01757719
11	0.02121354	0.36465251	0.05515544	0.0288205	0.72349635	0.01790739
11.1	0.02170436	0.36330886	0.05536784	0.02941239	0.72523965	0.01824016
11.2	0.02220263	0.36199315	0.05558518	0.03000682	0.72695337	0.01857547
11.3	0.02270816	0.3607049	0.05580739	0.03060363	0.72863824	0.01891331
11.4	0.02322074	0.35944361	0.05603438	0.03120268	0.73029499	0.01925365
11.5	0.02374018	0.35820883	0.05626606	0.0318038	0.73192429	0.01959647
11.6	0.02426628	0.35700011	0.05650235	0.03240686	0.73352681	0.01994175
11.7	0.02479886	0.35581703	0.05674315	0.0330117	0.7351032	0.02028948
11.8	0.02533774	0.35465917	0.05698837	0.03361817	0.73665408	0.02063963
15	0.04496638	0.3284919	0.06666867	0.05317352	0.77579693	0.03299162
15.1	0.04562947	0.32794299	0.06701605	0.05377334	0.77676607	0.03340941
15.2	0.04629434	0.32740655	0.06736559	0.05437187	0.7777233	0.03382889
15.3	0.0469609	0.32688227	0.06771726	0.05496909	0.77866885	0.03425004
15.4	0.04762905	0.32636985	0.06807101	0.05556497	0.77960295	0.03467283
15.5	0.04829869	0.32586899	0.06842681	0.05615949	0.78052582	0.03509726
15.6	0.04896974	0.32537942	0.06878461	0.05675261	0.78143768	0.0355233
15.7	0.04964211	0.32490085	0.06914439	0.05734431	0.78233874	0.03595092
15.8	0.0503157	0.324433	0.06950612	0.05793459	0.78322919	0.03638012
15.9	0.05099044	0.32397563	0.06986976	0.05852341	0.78410923	0.03681085
16	0.05166623	0.3235285	0.07023529	0.05911078	0.78497905	0.03724312
16.1	0.05234299	0.32309137	0.07060267	0.05969668	0.78583883	0.03767689
16.2	0.05302064	0.322664	0.07097188	0.0602811	0.78668874	0.03811216
16.3	0.05369911	0.32224617	0.07134288	0.06086403	0.78752896	0.03854889
16.4	0.0543783	0.32183766	0.07171565	0.06144547	0.78835964	0.03898708
16.5	0.05505816	0.32143827	0.07209016	0.06202541	0.78918094	0.03942669
16.6	0.0557386	0.32104781	0.07246637	0.06260385	0.78999302	0.03986773
16.7	0.05641956	0.32066608	0.07284425	0.0631808	0.79079602	0.04031015
16.8	0.05710097	0.3202929	0.07322379	0.06375624	0.79159008	0.04075395
16.9	0.05778276	0.31992812	0.07360494	0.06433019	0.79237534	0.0411991
17	0.05846485	0.31957156	0.07398768	0.06490265	0.79315194	0.04164559
17.1	0.05914719	0.31922308	0.07437196	0.0654736	0.79392002	0.0420934
17.2	0.05982969	0.31888251	0.07475778	0.06604307	0.7946797	0.0425425
17.3	0.06051231	0.31854973	0.07514509	0.06661104	0.79543112	0.04299288
17.4	0.06119497	0.31822458	0.07553387	0.06717752	0.7961744	0.04344453
17.5	0.06187762	0.31790694	0.07592409	0.06774251	0.79690966	0.04389743
17.6	0.0625602	0.31759668	0.07631572	0.06830602	0.79763704	0.04435155
17.7	0.06324267	0.31729369	0.07670874	0.06886806	0.79835665	0.0448069
17.8	0.06392497	0.31699786	0.07710312	0.06942862	0.79906861	0.04526344
17.9	0.06460703	0.31670906	0.07749883	0.06998774	0.79977304	0.04572117
18	0.06528882	0.31642721	0.07789586	0.0705454	0.80047005	0.04618006
18.1	0.06597027	0.31615219	0.07829417	0.07110162	0.80115977	0.04664011
18.2	0.06665133	0.3158839	0.07869373	0.07165642	0.8018423	0.04710129
18.3	0.06733197	0.31562224	0.07909453	0.0722098	0.80251775	0.04756359
18.4	0.06801212	0.31536713	0.07949653	0.07276178	0.80318623	0.04802699
18.5	0.06869175	0.31511846	0.07989971	0.07331236	0.80384784	0.04849148
18.6	0.06937081	0.31487614	0.08030404	0.07386157	0.8045027	0.04895703
18.7	0.07004928	0.31464008	0.08070949	0.07440941	0.80515089	0.04942364
18.8	0.0707271	0.31441021	0.08111604	0.07495588	0.80579252	0.04989129
18.9	0.07140424	0.31418642	0.08152366	0.07550101	0.8064277	0.05035996
19	0.07208067	0.31396864	0.08193232	0.07604481	0.80705651	0.05082964
19.1	0.07275633	0.31375678	0.082342	0.07658729	0.80767906	0.05130032
19.2	0.0734312	0.31355076	0.08275268	0.07712845	0.80829542	0.05177198
19.3	0.07410523	0.3133505	0.08316433	0.07766831	0.80890571	0.0522446
19.4	0.0747784	0.31315592	0.08357693	0.07820688	0.80950999	0.05271817
19.5	0.07545068	0.31296695	0.08399046	0.07874417	0.81010836	0.05319267
19.6	0.07612203	0.3127835	0.08440489	0.0792802	0.81070091	0.0536681
19.7	0.07679242	0.3126055	0.0848202	0.07981498	0.81128771	0.05414445
19.8	0.07746183	0.31243289	0.08523638	0.08034851	0.81186886	0.05462169
19.9	0.07813024	0.31226558	0.08565339	0.08088082	0.81244442	0.05509981
20	0.0787976	0.3121035	0.08607122	0.08141192	0.81301449	0.0555788
20.1	0.07946391	0.31194659	0.08648984	0.08194181	0.81357913	0.05605864
20.2	0.08012913	0.31179476	0.08690923	0.08247052	0.81413842	0.05653932
20.3	0.08079324	0.31164795	0.08732937	0.08299805	0.81469244	0.05702082
20.4	0.08145622	0.3115061	0.08775024	0.08352442	0.81524125	0.05750314
20.5	0.08211804	0.31136914	0.08817181	0.08404963	0.81578494	0.05798625
20.6	0.0827787	0.311237	0.08859406	0.0845737	0.81632357	0.05847014
20.7	0.08343816	0.31110963	0.08901697	0.08509665	0.81685722	0.05895479
20.8	0.08409642	0.31098694	0.08944053	0.08561847	0.81738595	0.0594402
20.9	0.08475344	0.31086889	0.08986472	0.0861392	0.81790982	0.05992634
21	0.08540922	0.3107554	0.09028951	0.08665883	0.81842892	0.0604132
21.1	0.08606373	0.31064641	0.09071488	0.08717738	0.81894329	0.06090077
21.2	0.08671695	0.31054186	0.09114082	0.08769485	0.81945301	0.06138903
21.3	0.08736888	0.31044169	0.09156732	0.08821127	0.81995813	0.06187797
21.4	0.0880195	0.31034584	0.09199435	0.08872665	0.82045872	0.06236758
21.5	0.08866879	0.31025424	0.0924219	0.08924098	0.82095484	0.06285784
21.6	0.08931674	0.31016685	0.09284995	0.08975429	0.82144654	0.06334874
21.7	0.08996335	0.3100836	0.09327848	0.09026658	0.82193389	0.06384025
21.8	0.09060859	0.31000443	0.09370749	0.09077786	0.82241694	0.06433238
21.9	0.09125246	0.3099293	0.09413696	0.09128814	0.82289575	0.06482511
22	0.09189494	0.30985813	0.09456687	0.09179743	0.82337038	0.06531841
22.1	0.09253602	0.30979089	0.09499721	0.09230574	0.82384087	0.06581229
22.2	0.09317569	0.30972751	0.09542796	0.09281308	0.8243073	0.06630672
22.3	0.09381395	0.30966793	0.09585912	0.09331945	0.8247697	0.06680169
22.4	0.09445079	0.30961212	0.09629068	0.09382487	0.82522814	0.06729719
22.5	0.09508619	0.30956001	0.09672261	0.09432934	0.82568266	0.0677932
22.6	0.09572016	0.30951156	0.09715491	0.09483286	0.82613332	0.06828971
22.7	0.09635268	0.3094667	0.09758756	0.09533546	0.82658016	0.0687867
22.8	0.09698375	0.3094254	0.09802056	0.09583713	0.82702323	0.06928416
22.9	0.09761336	0.3093876	0.09845389	0.09633788	0.82746259	0.06978208
23	0.0982415	0.30935325	0.09888753	0.09683772	0.82789828	0.07028044
23.1	0.09886818	0.3093223	0.09932148	0.09733665	0.82833035	0.07077923
23.2	0.09949338	0.30929471	0.09975573	0.0978347	0.82875884	0.07127843
23.3	0.10011711	0.30927043	0.10019026	0.09833185	0.82918382	0.07177803
23.4	0.10073936	0.30924942	0.10062506	0.09882813	0.82960531	0.07227802
23.5	0.10136013	0.30923162	0.10106012	0.09932353	0.83002337	0.07277839
23.6	0.10197941	0.309217	0.10149543	0.09981806	0.83043804	0.07327911
23.7	0.1025972	0.30920552	0.10193097	0.10031173	0.83084936	0.07378019
23.8	0.10321351	0.30919713	0.10236675	0.10080454	0.83125739	0.0742816
23.9	0.10382831	0.30919178	0.10280274	0.10129649	0.83166216	0.07478333
24	0.10444162	0.30918943	0.10323893	0.1017876	0.83206372	0.07528537
24.1	0.10505344	0.30919004	0.10367532	0.10227787	0.83246212	0.0757877
24.2	0.10566375	0.30919356	0.10411189	0.1027673	0.8328574	0.07629032
24.3	0.10627256	0.30919996	0.10454863	0.1032559	0.8332496	0.07679321
24.4	0.10687986	0.30920919	0.10498555	0.10374367	0.83363876	0.07729635
24.5	0.10748567	0.30922122	0.10542261	0.10423062	0.83402492	0.07779974
24.6	0.10808997	0.309236	0.10585982	0.10471676	0.83440813	0.07830336
24.7	0.10869277	0.30925349	0.10629717	0.10520207	0.83478844	0.07880719
24.8	0.10929407	0.30927366	0.10673465	0.10568658	0.83516587	0.07931124
24.9	0.10989387	0.30929647	0.10717224	0.10617028	0.83554048	0.07981548
25	0.11049218	0.30932188	0.10760993	0.10665318	0.8359123	0.0803199
25.1	0.11108898	0.30934986	0.10804773	0.10713528	0.83628138	0.0808245
25.2	0.11168429	0.30938036	0.10848561	0.10761659	0.83664776	0.08132926
25.3	0.11227811	0.30941336	0.10892357	0.10809711	0.83701147	0.08183417
25.4	0.11287043	0.30944881	0.10936161	0.10857685	0.83737255	0.08233922
25.5	0.11346126	0.30948669	0.10979971	0.1090558	0.83773105	0.0828444
25.6	0.1140506	0.30952695	0.11023786	0.10953397	0.838087	0.0833497
25.7	0.11463846	0.30956957	0.11067607	0.11001137	0.83844044	0.08385511
25.8	0.11522482	0.3096145	0.11111431	0.11048799	0.83879139	0.08436061
25.9	0.11580971	0.30966172	0.11155259	0.11096384	0.83913989	0.0848662
26	0.11639311	0.30971119	0.1119909	0.11143893	0.83948598	0.08537187
26.1	0.11697504	0.30976288	0.11242922	0.11191324	0.83982968	0.0858776
26.2	0.11755548	0.30981676	0.11286756	0.1123868	0.84017102	0.08638339
26.3	0.11813446	0.3098728	0.1133059	0.11285959	0.84051003	0.08688922
26.4	0.11871196	0.30993096	0.11374424	0.11333163	0.84084673	0.08739509
26.5	0.11928799	0.30999121	0.11418257	0.11380292	0.84118114	0.08790098
26.6	0.11986256	0.31005353	0.11462089	0.11427346	0.84151328	0.08840688
26.7	0.12043567	0.31011789	0.11505918	0.11474325	0.84184319	0.08891279
26.8	0.12100732	0.31018425	0.11549744	0.1152123	0.84217087	0.08941869
30	0.13855521	0.31320909	0.12946776	0.12984372	0.85157194	0.10556512
30.1	0.13908113	0.31332746	0.12990157	0.13028962	0.85183445	0.10606748
30.2	0.13960573	0.31344705	0.13033516	0.13073485	0.85209521	0.10656965
30.3	0.14012904	0.31356783	0.13076852	0.13117943	0.85235425	0.10707164
30.4	0.14065105	0.3136898	0.13120164	0.13162335	0.85261158	0.10757344
30.5	0.14117176	0.31381292	0.13163453	0.13206662	0.8528672	0.10807504
30.6	0.14169119	0.31393719	0.13206718	0.13250923	0.85312114	0.10857644
30.7	0.14220933	0.31406259	0.13249958	0.1329512	0.8533734	0.10907764
30.8	0.1427262	0.31418909	0.13293173	0.13339251	0.853624	0.10957863
30.9	0.14324179	0.3143167	0.13336363	0.13383319	0.85387295	0.1100794
31	0.14375612	0.31444538	0.13379526	0.13427322	0.85412026	0.11057996
31.1	0.14426918	0.31457512	0.13422664	0.1347126	0.85436596	0.11108029
31.2	0.14478099	0.3147059	0.13465775	0.13515135	0.85461004	0.1115804
31.3	0.14529155	0.31483772	0.13508859	0.13558947	0.85485253	0.11208028
31.4	0.14580086	0.31497055	0.13551915	0.13602694	0.85509343	0.11257992
31.5	0.14630893	0.31510439	0.13594944	0.13646378	0.85533276	0.11307932
31.6	0.14681577	0.3152392	0.13637944	0.13689998	0.85557055	0.11357848
31.7	0.14732137	0.31537499	0.13680916	0.13733555	0.85580678	0.11407739
31.8	0.14782575	0.31551174	0.13723859	0.13777049	0.85604149	0.11457604
31.9	0.1483289	0.31564942	0.13766773	0.1382048	0.85627469	0.11507443
32	0.14883084	0.31578804	0.13809657	0.13863848	0.85650638	0.11557255
32.1	0.14933157	0.31592757	0.13852511	0.13907154	0.85673659	0.11607041
32.2	0.1498311	0.316068	0.13895335	0.13950398	0.85696532	0.116568
32.3	0.15032942	0.31620931	0.13938128	0.13993579	0.85719259	0.1170653
32.4	0.15082655	0.3163515	0.1398089	0.14036698	0.8574184	0.11756232
32.5	0.15132249	0.31649454	0.14023621	0.14079755	0.85764279	0.11805906
32.6	0.15181725	0.31663843	0.1406632	0.14122751	0.85786574	0.11855551
32.7	0.15231083	0.31678316	0.14108987	0.14165684	0.85808729	0.11905166
32.8	0.15280324	0.3169287	0.14151622	0.14208557	0.85830744	0.11954752
32.9	0.15329449	0.31707504	0.14194225	0.14251368	0.85852621	0.12004307
33	0.15378457	0.31722218	0.14236794	0.14294117	0.8587436	0.12053832
33.1	0.15427349	0.3173701	0.1427933	0.14336805	0.85895964	0.12103326
33.2	0.15476127	0.31751879	0.14321832	0.14379433	0.85917433	0.12152789
33.3	0.1552479	0.31766824	0.143643	0.14422	0.85938769	0.1220222
33.4	0.15573339	0.31781843	0.14406734	0.14464506	0.85959973	0.1225162
33.5	0.15621774	0.31796936	0.14449134	0.14506952	0.85981046	0.12300987
33.6	0.15670096	0.31812101	0.14491498	0.14549338	0.86001991	0.12350321
33.7	0.15718306	0.31827336	0.14533828	0.14591663	0.86022807	0.12399623
33.8	0.15766404	0.31842642	0.14576122	0.14633929	0.86043498	0.12448892
33.9	0.1581439	0.31858016	0.14618381	0.14676135	0.86064064	0.12498127
34	0.15862265	0.31873457	0.14660604	0.14718281	0.86084506	0.12547328
34.1	0.15910029	0.31888965	0.14702791	0.14760368	0.86104825	0.12596496
34.2	0.15957684	0.31904538	0.14744941	0.14802395	0.86125024	0.1264563
34.3	0.16005229	0.31920175	0.14787055	0.14844363	0.86145103	0.12694729
34.4	0.16052665	0.31935875	0.14829132	0.14886272	0.86165064	0.12743793
34.5	0.16099992	0.31951636	0.14871172	0.14928122	0.86184908	0.12792823
34.6	0.16147211	0.31967459	0.14913175	0.14969913	0.86204637	0.12841818
34.7	0.16194323	0.31983341	0.1495514	0.15011645	0.86224251	0.12890778
34.8	0.16241328	0.31999282	0.14997067	0.15053318	0.86243752	0.12939702
34.9	0.16288226	0.32015281	0.15038956	0.15094933	0.86263141	0.12988591
35	0.16335018	0.32031336	0.15080807	0.1513649	0.8628242	0.13037445
35.1	0.16381705	0.32047447	0.15122619	0.15177988	0.86301589	0.13086262
35.2	0.16428287	0.32063612	0.15164393	0.15219429	0.8632065	0.13135044
35.3	0.16474765	0.32079831	0.15206128	0.15260811	0.86339605	0.1318379
35.4	0.16521139	0.32096102	0.15247824	0.15302136	0.86358453	0.132325
35.5	0.1656741	0.32112425	0.15289481	0.15343404	0.86377197	0.13281173
35.6	0.16613577	0.32128799	0.15331098	0.15384614	0.86395838	0.13329811
35.7	0.16659643	0.32145222	0.15372676	0.15425767	0.86414376	0.13378412
35.8	0.16705606	0.32161694	0.15414214	0.15466863	0.86432814	0.13426976
35.9	0.16751468	0.32178215	0.15455713	0.15507902	0.86451151	0.13475504
36	0.1679723	0.32194781	0.15497171	0.15548885	0.86469389	0.13523996
36.1	0.1684289	0.32211394	0.15538588	0.15589812	0.8648753	0.13572452
36.2	0.16888451	0.32228052	0.15579965	0.15630682	0.86505574	0.1362087
36.3	0.16933912	0.32244755	0.15621301	0.15671496	0.86523522	0.13669252
36.4	0.16979274	0.322615	0.15662597	0.15712254	0.86541375	0.13717598
36.5	0.17024537	0.32278288	0.15703851	0.15752957	0.86559134	0.13765907
36.6	0.17069702	0.32295118	0.15745064	0.15793604	0.865768	0.13814179
36.7	0.17114769	0.32311989	0.15786235	0.15834196	0.86594374	0.13862414
36.8	0.17159738	0.323289	0.15827365	0.15874734	0.86611857	0.13910612
36.9	0.17204611	0.32345849	0.15868453	0.15915217	0.86629249	0.13958774
37	0.17249386	0.32362837	0.15909499	0.15955645	0.86646552	0.14006898
37.1	0.17294066	0.32379863	0.15950503	0.15996019	0.86663767	0.14054986
37.2	0.1733865	0.32396925	0.15991465	0.1603634	0.86680893	0.14103037
37.3	0.17383139	0.32414023	0.16032385	0.16076607	0.86697932	0.14151051
37.4	0.17427533	0.32431157	0.16073262	0.1611682	0.86714884	0.14199027
37.5	0.17471833	0.32448324	0.16114097	0.1615698	0.86731751	0.14246967
37.6	0.17516038	0.32465525	0.16154889	0.16197088	0.86748533	0.1429487
37.7	0.1756015	0.32482759	0.16195638	0.16237142	0.8676523	0.14342736
37.8	0.17604169	0.32500025	0.16236344	0.16277145	0.86781843	0.14390566
37.9	0.17648094	0.32517322	0.16277007	0.16317095	0.86798373	0.14438358
38	0.17691928	0.32534651	0.16317627	0.16356994	0.8681482	0.14486113
38.1	0.1773567	0.32552009	0.16358203	0.1639684	0.86831185	0.1453383
38.2	0.17779319	0.32569397	0.16398736	0.16436635	0.86847469	0.14581511
38.3	0.17822878	0.32586813	0.16439225	0.16476377	0.86863672	0.14629154
38.4	0.17866346	0.32604258	0.16479671	0.16516067	0.86879795	0.1467676
38.5	0.17909724	0.3262173	0.16520073	0.16555705	0.86895838	0.14724328
38.6	0.17953012	0.32639229	0.16560431	0.1659529	0.86911802	0.14771859
38.7	0.1799621	0.32656754	0.16600745	0.16634824	0.86927688	0.14819352
38.8	0.18039318	0.32674305	0.16641016	0.16674307	0.86943496	0.14866807
38.9	0.18082338	0.32691881	0.16681242	0.16713739	0.86959226	0.14914224
39	0.1812527	0.32709481	0.16721424	0.16753121	0.8697488	0.14961604
39.1	0.18168113	0.32727105	0.16761562	0.16792453	0.86990458	0.15008946
39.2	0.18210869	0.32744753	0.16801656	0.16831735	0.8700596	0.1505625
39.3	0.18253538	0.32762423	0.16841706	0.16870967	0.87021388	0.15103515
39.4	0.18296119	0.32780116	0.16881711	0.16910151	0.87036741	0.15150743
39.5	0.18338614	0.32797831	0.16921672	0.16949286	0.8705202	0.15197933
39.6	0.18381024	0.32815567	0.16961589	0.16988372	0.87067227	0.15245084
39.7	0.18423347	0.32833324	0.1700146	0.17027411	0.8708236	0.15292197
39.8	0.18465585	0.32851102	0.17041288	0.17066402	0.87097422	0.15339271
39.9	0.18507738	0.32868899	0.17081071	0.17105345	0.87112412	0.15386307
40	0.18549806	0.32886716	0.17120809	0.17144242	0.87127332	0.15433305
40.1	0.18591789	0.32904552	0.17160503	0.17183091	0.8714218	0.15480263
40.2	0.18633689	0.32922407	0.17200152	0.17221895	0.87156959	0.15527183
40.3	0.18675505	0.32940281	0.17239756	0.17260651	0.87171668	0.15574063
40.4	0.18717238	0.32958172	0.17279316	0.1729936	0.87186308	0.15620905
40.5	0.18758887	0.32976081	0.17318831	0.17338022	0.87200879	0.15667708
40.6	0.18800455	0.32994006	0.17358301	0.17376637	0.87215381	0.15714471
40.7	0.1884194	0.33011949	0.17397726	0.17415206	0.87229816	0.15761195
40.8	0.18883343	0.33029908	0.17437106	0.17453729	0.87244183	0.15807879
40.9	0.18924664	0.33047884	0.17476441	0.17492207	0.87258483	0.15854524
41	0.18965905	0.33065875	0.1751573	0.17530639	0.87272717	0.1590113
41.1	0.19007065	0.33083881	0.17554975	0.17569026	0.87286884	0.15947695
41.2	0.19048144	0.33101903	0.17594175	0.17607368	0.87300986	0.15994221
41.3	0.19089143	0.33119939	0.17633329	0.17645666	0.87315022	0.16040706
41.4	0.19130062	0.33137991	0.17672438	0.1768392	0.87328993	0.16087152
41.5	0.19170901	0.33156056	0.17711502	0.1772213	0.873429	0.16133557
41.6	0.19211661	0.33174136	0.1775052	0.17760297	0.87356743	0.16179922
41.7	0.19252342	0.33192229	0.17789492	0.17798421	0.87370521	0.16226246
41.8	0.19292944	0.33210336	0.17828419	0.17836501	0.87384237	0.1627253
45	0.20552492	0.33795575	0.19049586	0.19033364	0.87791496	0.17731712
45.1	0.20590665	0.33814008	0.19086971	0.19070115	0.87803283	0.17776614
45.2	0.2062877	0.33832448	0.19124308	0.19106827	0.87815014	0.17821472
45.3	0.20666806	0.33850894	0.19161598	0.19143501	0.87826691	0.17866288
45.4	0.20704774	0.33869346	0.1919884	0.19180137	0.87838315	0.1791106
45.5	0.20742674	0.33887805	0.19236034	0.19216735	0.87849884	0.1795579
45.6	0.20780506	0.33906269	0.1927318	0.19253295	0.87861399	0.18000476
45.7	0.20818271	0.33924738	0.19310279	0.19289816	0.8787286	0.18045119
45.8	0.20855968	0.33943213	0.1934733	0.193263	0.87884269	0.18089719
45.9	0.20893598	0.33961693	0.19384333	0.19362746	0.87895624	0.18134276
46	0.20931162	0.33980179	0.19421287	0.19399154	0.87906926	0.18178789
46.1	0.20968659	0.33998669	0.19458194	0.19435524	0.87918176	0.18223259
46.2	0.2100609	0.34017163	0.19495053	0.19471857	0.87929373	0.18267686
46.3	0.21043455	0.34035662	0.19531864	0.19508151	0.87940517	0.18312069
46.4	0.21080755	0.34054166	0.19568627	0.19544408	0.8795161	0.1835641
46.5	0.21117989	0.34072673	0.19605342	0.19580627	0.87962651	0.18400707
46.6	0.21155159	0.34091184	0.19642008	0.19616808	0.8797364	0.1844496
46.7	0.21192263	0.34109699	0.19678627	0.19652952	0.87984577	0.18489171
46.8	0.21229303	0.34128218	0.19715197	0.19689058	0.87995464	0.18533338
46.9	0.21266279	0.3414674	0.1975172	0.19725126	0.880063	0.18577461
47	0.21303191	0.34165265	0.19788193	0.19761157	0.88017085	0.18621541
47.1	0.21340039	0.34183793	0.19824619	0.1979715	0.88027819	0.18665577
47.2	0.21376824	0.34202325	0.19860997	0.19833106	0.88038504	0.1870957
47.3	0.21413545	0.34220859	0.19897326	0.19869024	0.88049138	0.18753519
47.4	0.21450204	0.34239395	0.19933607	0.19904904	0.88059723	0.18797425
47.5	0.21486801	0.34257934	0.1996984	0.19940747	0.88070259	0.18841286
47.6	0.21523335	0.34276475	0.20006024	0.19976552	0.88080746	0.18885104
47.7	0.21559808	0.34295018	0.2004216	0.2001232	0.88091184	0.18928877
47.8	0.21596219	0.34313563	0.20078248	0.2004805	0.88101573	0.18972607
47.9	0.21632568	0.34332109	0.20114288	0.20083743	0.88111915	0.19016292
48	0.21668857	0.34350657	0.2015028	0.20119398	0.88122208	0.19059934
48.1	0.21705085	0.34369206	0.20186223	0.20155015	0.88132454	0.19103532
48.2	0.21741253	0.34387756	0.20222118	0.20190595	0.88142653	0.19147086
48.3	0.2177736	0.34406307	0.20257964	0.20226137	0.88152805	0.19190596
48.4	0.21813408	0.34424858	0.20293763	0.20261642	0.8816291	0.19234062
48.5	0.21849397	0.3444341	0.20329513	0.20297109	0.8817297	0.19277485
48.6	0.21885326	0.34461963	0.20365216	0.20332538	0.88182983	0.19320863
48.7	0.21921196	0.34480515	0.2040087	0.2036793	0.8819295	0.19364198
48.8	0.21957008	0.34499067	0.20436476	0.20403285	0.88202873	0.19407489
48.9	0.21992761	0.34517619	0.20472034	0.20438602	0.8821275	0.19450735
49	0.22028456	0.34536171	0.20507544	0.20473881	0.88222583	0.19493938
49.1	0.22064094	0.34554721	0.20543006	0.20509122	0.88232372	0.19537096
49.2	0.22099674	0.34573271	0.2057842	0.20544326	0.88242117	0.19580211
49.3	0.22135196	0.3459182	0.20613786	0.20579493	0.88251818	0.19623281
49.4	0.22170662	0.34610368	0.20649104	0.20614622	0.88261477	0.19666307
49.5	0.22206071	0.34628914	0.20684374	0.20649713	0.88271092	0.1970929
49.6	0.22241423	0.34647459	0.20719596	0.20684766	0.88280666	0.19752227
49.7	0.22276719	0.34666002	0.2075477	0.20719782	0.88290197	0.19795121
49.8	0.22311959	0.34684543	0.20789897	0.2075476	0.88299687	0.19837971
49.9	0.22347143	0.34703082	0.20824975	0.20789701	0.88309135	0.19880776
50	0.22382272	0.34721619	0.20860006	0.20824603	0.88318543	0.19923538
50.1	0.22417345	0.34740153	0.2089499	0.20859469	0.8832791	0.19966256
50.2	0.22452363	0.34758684	0.20929925	0.20894297	0.88337237	0.20008929
50.3	0.22487327	0.34777213	0.20964813	0.20929087	0.88346524	0.20051559
50.4	0.22522236	0.34795739	0.20999653	0.2096384	0.88355772	0.20094144
50.5	0.2255709	0.34814262	0.21034446	0.20998555	0.88364981	0.20136685
50.6	0.22591891	0.34832781	0.21069191	0.21033233	0.88374151	0.20179183
50.7	0.22626637	0.34851298	0.21103888	0.21067874	0.88383284	0.20221636
50.8	0.2266133	0.3486981	0.21138538	0.21102478	0.88392378	0.20264045
50.9	0.2269597	0.34888319	0.2117314	0.21137044	0.88401436	0.20306411
51	0.22730556	0.34906823	0.21207696	0.21171573	0.88410456	0.20348732
51.1	0.22765089	0.34925324	0.21242203	0.21206065	0.88419439	0.2039101
51.2	0.2279957	0.3494382	0.21276664	0.2124052	0.88428386	0.20433243
51.3	0.22833998	0.34962312	0.21311077	0.21274938	0.88437298	0.20475433
51.4	0.22868374	0.34980799	0.21345442	0.21309319	0.88446173	0.20517579
51.5	0.22902697	0.34999282	0.21379761	0.21343664	0.88455014	0.20559681
51.6	0.22936969	0.3501776	0.21414032	0.21377972	0.88463819	0.2060174
51.7	0.22971189	0.35036232	0.21448257	0.21412243	0.88472591	0.20643754
51.8	0.23005358	0.350547	0.21482434	0.21446477	0.88481328	0.20685725
51.9	0.23039475	0.35073162	0.21516564	0.21480676	0.88490031	0.20727651
52	0.23073541	0.35091619	0.21550648	0.21514838	0.88498701	0.20769534
52.1	0.23107557	0.3511007	0.21584684	0.21548963	0.88507338	0.20811374
52.2	0.23141522	0.35128515	0.21618674	0.21583052	0.88515943	0.2085317
52.3	0.23175437	0.35146955	0.21652617	0.21617106	0.88524515	0.20894922
52.4	0.23209302	0.35165388	0.21686513	0.21651123	0.88533055	0.20936631
52.5	0.23243116	0.35183815	0.21720363	0.21685104	0.88541563	0.20978297
52.6	0.23276881	0.35202236	0.21754165	0.21719049	0.88550041	0.21019919
52.7	0.23310597	0.35220651	0.21787921	0.21752959	0.88558487	0.21061498
52.8	0.23344263	0.35239058	0.21821631	0.21786832	0.88566903	0.21103033
52.9	0.23377881	0.35257459	0.21855294	0.2182067	0.88575288	0.21144526
53	0.23411449	0.35275854	0.2188891	0.21854472	0.88583644	0.21185975
53.1	0.23444969	0.35294241	0.2192248	0.21888239	0.8859197	0.21227381
53.2	0.2347844	0.35312621	0.21956004	0.2192197	0.88600267	0.21268744
53.3	0.23511863	0.35330994	0.21989481	0.21955666	0.88608535	0.21310064
53.4	0.23545237	0.35349359	0.22022912	0.21989327	0.88616774	0.21351341
53.5	0.23578564	0.35367718	0.22056297	0.22022952	0.88624985	0.21392575
53.6	0.23611843	0.35386068	0.22089635	0.22056542	0.88633168	0.21433765
53.7	0.23645074	0.35404411	0.22122928	0.22090097	0.88641323	0.21474913
53.8	0.23678258	0.35422747	0.22156174	0.22123618	0.8864945	0.21516018
53.9	0.23711394	0.35441075	0.22189374	0.22157103	0.88657551	0.2155708
54	0.23744484	0.35459394	0.22222528	0.22190553	0.88665624	0.21598098
54.1	0.23777526	0.35477706	0.22255636	0.22223969	0.88673671	0.21639075
54.2	0.23810522	0.3549601	0.22288699	0.2225735	0.88681691	0.21680008
54.3	0.23843471	0.35514305	0.22321715	0.22290696	0.88689686	0.21720899
54.4	0.23876374	0.35532593	0.22354686	0.22324008	0.88697654	0.21761746
54.5	0.23909231	0.35550871	0.22387611	0.22357286	0.88705597	0.21802552
54.6	0.23942042	0.35569142	0.2242049	0.22390529	0.88713514	0.21843314
54.7	0.23974807	0.35587403	0.22453323	0.22423738	0.88721406	0.21884034
54.8	0.24007526	0.35605656	0.22486111	0.22456913	0.88729273	0.21924711
54.9	0.24040199	0.35623901	0.22518854	0.22490054	0.88737116	0.21965346
55	0.24072828	0.35642136	0.22551551	0.22523161	0.88744933	0.22005938
55.1	0.24105411	0.35660362	0.22584202	0.22556234	0.88752727	0.22046488
55.2	0.24137949	0.3567858	0.22616808	0.22589273	0.88760496	0.22086995
55.3	0.24170442	0.35696788	0.22649369	0.22622279	0.88768241	0.2212746
55.4	0.2420289	0.35714987	0.22681884	0.2265525	0.88775962	0.22167883
55.5	0.24235293	0.35733176	0.22714354	0.22688188	0.88783659	0.22208263
55.6	0.24267652	0.35751357	0.22746778	0.22721092	0.88791333	0.22248602
55.7	0.24299967	0.35769528	0.22779158	0.22753963	0.88798984	0.22288898
55.8	0.24332238	0.35787689	0.22811492	0.227868	0.88806611	0.22329152
55.9	0.24364464	0.3580584	0.22843781	0.22819603	0.88814215	0.22369365
56	0.24396647	0.35823982	0.22876025	0.22852374	0.88821796	0.22409535
56.1	0.24428786	0.35842114	0.22908225	0.2288511	0.88829354	0.22449663
56.2	0.24460881	0.35860237	0.22940379	0.22917814	0.8883689	0.2248975
56.3	0.24492932	0.35878349	0.22972488	0.22950484	0.88844402	0.22529794
56.4	0.2452494	0.35896451	0.23004552	0.22983122	0.88851893	0.22569797
56.5	0.24556905	0.35914543	0.23036572	0.23015726	0.8885936	0.22609757
56.6	0.24588827	0.35932625	0.23068546	0.23048297	0.88866806	0.22649676
56.7	0.24620705	0.35950697	0.23100476	0.23080835	0.88874229	0.22689554
56.8	0.24652541	0.35968759	0.23132361	0.2311334	0.8888163	0.2272939

11.9	0.02588274	0.35352612	0.05723792	0.03422612	0.73818006	0.02099217
12	0.02643368	0.35241749	0.0574917	0.03483541	0.73968174	0.0213471
12.1	0.0269904	0.35133286	0.05774963	0.03544589	0.7411597	0.02170438
12.2	0.02755272	0.35027181	0.0580116	0.03605744	0.74261449	0.02206401
12.3	0.02812049	0.34923393	0.05827753	0.03666991	0.74404666	0.02242594
12.4	0.02869355	0.34821882	0.05854732	0.03728317	0.74545672	0.02279016
12.5	0.02927171	0.34722605	0.05882089	0.0378971	0.7468452	0.02315664
12.6	0.02985483	0.34625521	0.05909813	0.03851156	0.7482126	0.02352536
12.7	0.03044274	0.34530588	0.05937897	0.03912645	0.7495594	0.02389629
12.8	0.03103527	0.34437764	0.05966333	0.03974164	0.75088609	0.02426943
12.9	0.03163228	0.34347005	0.05995112	0.04035704	0.75219313	0.02464474
13	0.03223362	0.34258272	0.06024227	0.04097253	0.75348096	0.02502221
13.1	0.03283914	0.34171522	0.0605367	0.04158801	0.75475001	0.02540182
13.2	0.0334487	0.34086716	0.06083434	0.04220338	0.75600072	0.02578354
13.3	0.03406214	0.34003811	0.06113512	0.04281855	0.7572335	0.02616736
13.4	0.03467933	0.33922769	0.06143896	0.04343344	0.75844874	0.02655325
13.5	0.03530016	0.33843549	0.0617458	0.04404795	0.75964684	0.02694121
13.6	0.03592448	0.33766113	0.06205558	0.04466201	0.76082818	0.02733119
13.7	0.03655217	0.33690423	0.06236822	0.04527552	0.76199313	0.02772317
13.8	0.03718311	0.33616441	0.06268366	0.04588843	0.76314204	0.02811714
13.9	0.03781717	0.33544131	0.06300184	0.04650066	0.76427527	0.02851308
14	0.03845425	0.33473457	0.06332271	0.04711213	0.76539315	0.02891095
14.1	0.03909422	0.33404383	0.06364622	0.0477228	0.76649603	0.02931075
14.2	0.03973699	0.33336875	0.06397231	0.04833259	0.76758422	0.02971244
14.3	0.04038243	0.33270897	0.06430093	0.04894145	0.76865804	0.030116
14.4	0.04103043	0.33206417	0.06463205	0.04954933	0.76971779	0.03052142
14.5	0.04168088	0.33143403	0.06496561	0.05015619	0.77076377	0.03092866
14.6	0.04233368	0.33081821	0.06530158	0.05076196	0.77179627	0.03133772
14.7	0.04298872	0.33021641	0.06563991	0.05136663	0.77281556	0.03174856
14.8	0.04364591	0.3296283	0.06598056	0.05197013	0.77382193	0.03216117
14.9	0.04430517	0.32905357	0.0663235	0.05257244	0.77481563	0.03257553

60	0.25649181	0.36541026	0.24129362	0.24136363	0.8910702	0.2398252
60.1	0.25679652	0.36558723	0.24159799	0.24167805	0.89113708	0.24021017
60.2	0.25710084	0.36576409	0.24190193	0.24199215	0.89120374	0.24059474
60.3	0.25740475	0.36594082	0.24220543	0.24230593	0.89127019	0.24097891
60.4	0.25770827	0.36611745	0.24250851	0.2426194	0.89133642	0.24136269
60.5	0.25801139	0.36629395	0.24281116	0.24293256	0.89140243	0.24174608
60.6	0.25831412	0.36647033	0.24311339	0.2432454	0.89146823	0.24212908
60.7	0.25861645	0.3666466	0.24341518	0.24355793	0.89153381	0.24251168
60.8	0.25891839	0.36682275	0.24371655	0.24387015	0.89159917	0.24289388
60.9	0.25921994	0.36699877	0.24401749	0.24418206	0.89166432	0.2432757
61	0.25952109	0.36717468	0.244318	0.24449366	0.89172924	0.24365712
61.1	0.25982185	0.36735046	0.24461809	0.24480494	0.89179395	0.24403815
61.2	0.26012223	0.36752613	0.24491775	0.24511591	0.89185844	0.24441879
61.3	0.26042221	0.36770167	0.24521699	0.24542658	0.89192271	0.24479904
61.4	0.2607218	0.3678771	0.2455158	0.24573693	0.89198677	0.2451789
61.5	0.261021	0.3680524	0.24581419	0.24604698	0.8920506	0.24555837
61.6	0.26131982	0.36822758	0.24611216	0.24635671	0.89211422	0.24593745
61.7	0.26161825	0.36840264	0.2464097	0.24666614	0.89217762	0.24631614
61.8	0.26191629	0.36857757	0.24670683	0.24697525	0.8922408	0.24669445
61.9	0.26221395	0.36875238	0.24700353	0.24728406	0.89230375	0.24707236
62	0.26251122	0.36892707	0.24729981	0.24759256	0.89236649	0.24744989
62.1	0.26280811	0.36910164	0.24759567	0.24790076	0.89242901	0.24782703
62.2	0.26310462	0.36927608	0.24789111	0.24820864	0.89249131	0.24820379
62.3	0.26340074	0.3694504	0.24818613	0.24851622	0.89255339	0.24858016
62.4	0.26369649	0.36962459	0.24848073	0.2488235	0.89261525	0.24895614
62.5	0.26399185	0.36979866	0.24877491	0.24913046	0.89267689	0.24933174
62.6	0.26428683	0.36997261	0.24906868	0.24943712	0.89273832	0.24970695
62.7	0.26458143	0.37014643	0.24936203	0.24974348	0.89279952	0.25008178
62.8	0.26487566	0.37032013	0.24965496	0.25004953	0.8928605	0.25045623
62.9	0.26516951	0.37049371	0.24994747	0.25035527	0.89292127	0.25083029
63	0.26546298	0.37066716	0.25023957	0.25066071	0.89298182	0.25120397
63.1	0.26575607	0.37084048	0.25053125	0.25096585	0.89304214	0.25157726
63.2	0.26604879	0.37101368	0.25082252	0.25127068	0.89310225	0.25195018
63.3	0.26634114	0.37118675	0.25111338	0.25157521	0.89316214	0.25232271
63.4	0.26663311	0.3713597	0.25140382	0.25187943	0.89322181	0.25269487
63.5	0.26692472	0.37153252	0.25169384	0.25218335	0.89328127	0.25306664
63.6	0.26721595	0.37170522	0.25198346	0.25248697	0.8933405	0.25343803
63.7	0.26750681	0.37187779	0.25227266	0.25279028	0.89339952	0.25380905
63.8	0.2677973	0.37205023	0.25256145	0.2530933	0.89345832	0.25417969
63.9	0.26808742	0.37222255	0.25284983	0.25339601	0.89351691	0.25454995
64	0.26837718	0.37239474	0.2531378	0.25369842	0.89357528	0.25491983
64.1	0.26866657	0.3725668	0.25342536	0.25400052	0.89363343	0.25528933
64.2	0.26895559	0.37273873	0.25371252	0.25430233	0.89369137	0.25565846
64.3	0.26924425	0.37291054	0.25399926	0.25460384	0.89374909	0.25602722
64.4	0.26953254	0.37308222	0.25428559	0.25490505	0.89380659	0.2563956
64.5	0.26982048	0.37325377	0.25457152	0.25520596	0.89386389	0.2567636
64.6	0.27010805	0.37342519	0.25485704	0.25550657	0.89392097	0.25713123
64.7	0.27039526	0.37359649	0.25514215	0.25580689	0.89397783	0.25749849
64.8	0.2706821	0.37376766	0.25542685	0.2561069	0.89403449	0.25786537
64.9	0.27096859	0.37393869	0.25571116	0.25640662	0.89409093	0.25823189
65	0.27125473	0.3741096	0.25599505	0.25670604	0.89414716	0.25859803
65.1	0.2715405	0.37428038	0.25627854	0.25700516	0.89420318	0.25896379
65.2	0.27182592	0.37445103	0.25656163	0.25730399	0.89425899	0.25932919
65.3	0.27211098	0.37462156	0.25684431	0.25760252	0.89431459	0.25969422
65.4	0.27239569	0.37479195	0.2571266	0.25790075	0.89436999	0.26005888
65.5	0.27268005	0.37496221	0.25740847	0.25819869	0.89442517	0.26042316
65.6	0.27296405	0.37513235	0.25768995	0.25849633	0.89448015	0.26078708
65.7	0.2732477	0.37530235	0.25797103	0.25879368	0.89453492	0.26115063
65.8	0.273531	0.37547223	0.2582517	0.25909073	0.89458948	0.26151381
65.9	0.27381396	0.37564198	0.25853198	0.25938748	0.89464384	0.26187663
66	0.27409656	0.37581159	0.25881185	0.25968395	0.89469799	0.26223908
66.1	0.27437882	0.37598108	0.25909133	0.25998011	0.89475194	0.26260116
66.2	0.27466072	0.37615044	0.25937041	0.26027599	0.89480568	0.26296287
66.3	0.27494229	0.37631967	0.25964909	0.26057157	0.89485923	0.26332422
66.4	0.2752235	0.37648877	0.25992737	0.26086685	0.89491257	0.26368521
66.5	0.27550438	0.37665774	0.26020526	0.26116185	0.89496571	0.26404583
66.6	0.27578491	0.37682658	0.26048275	0.26145655	0.89501865	0.26440609
66.7	0.27606509	0.37699529	0.26075984	0.26175096	0.89507138	0.26476598
66.8	0.27634494	0.37716387	0.26103654	0.26204507	0.89512392	0.26512551
66.9	0.27662444	0.37733232	0.26131284	0.2623389	0.89517627	0.26548468
67	0.2769036	0.37750064	0.26158876	0.26263243	0.89522841	0.26584349
67.1	0.27718243	0.37766883	0.26186427	0.26292568	0.89528036	0.26620193
67.2	0.27746091	0.37783689	0.2621394	0.26321863	0.89533211	0.26656002
67.3	0.27773906	0.37800482	0.26241413	0.2635113	0.89538367	0.26691774
67.4	0.27801687	0.37817262	0.26268848	0.26380367	0.89543503	0.26727511
67.5	0.27829435	0.37834029	0.26296243	0.26409576	0.89548619	0.26763211
67.6	0.27857149	0.37850782	0.26323599	0.26438755	0.89553717	0.26798876
67.7	0.2788483	0.37867523	0.26350916	0.26467906	0.89558795	0.26834505
67.8	0.27912477	0.3788425	0.26378194	0.26497028	0.89563854	0.26870098
67.9	0.27940091	0.37900964	0.26405434	0.26526121	0.89568893	0.26905655
68	0.27967672	0.37917665	0.26432634	0.26555185	0.89573914	0.26941177
68.1	0.27995219	0.37934353	0.26459796	0.26584221	0.89578915	0.26976663
68.2	0.28022734	0.37951028	0.2648692	0.26613228	0.89583898	0.27012113
68.3	0.28050215	0.3796769	0.26514004	0.26642206	0.89588861	0.27047528
68.4	0.28077664	0.37984339	0.2654105	0.26671155	0.89593806	0.27082907
68.5	0.2810508	0.38000975	0.26568058	0.26700076	0.89598732	0.27118251
68.6	0.28132463	0.38017598	0.26595027	0.26728969	0.89603639	0.27153559
68.7	0.28159814	0.38034207	0.26621958	0.26757833	0.89608528	0.27188832
68.8	0.28187131	0.38050804	0.2664885	0.26786668	0.89613398	0.2722407
68.9	0.28214417	0.38067387	0.26675705	0.26815475	0.89618249	0.27259273

26.9	0.12157752	0.31025258	0.11593566	0.1156806	0.84249634	0.08992457
27	0.12214626	0.31032287	0.11637384	0.11614816	0.84281963	0.09043043
27.1	0.12271356	0.31039507	0.11681197	0.11661499	0.84314074	0.09093626
27.2	0.12327941	0.31046916	0.11725004	0.11708108	0.8434597	0.09144205
27.3	0.12384383	0.31054511	0.11768805	0.11754644	0.84377651	0.09194779
27.4	0.12440681	0.31062289	0.11812598	0.11801106	0.84409119	0.09245348
27.5	0.12496837	0.31070248	0.11856384	0.11847497	0.84440376	0.0929591
27.6	0.1255285	0.31078385	0.11900162	0.11893815	0.84471423	0.09346466
27.7	0.1260872	0.31086698	0.11943931	0.1194006	0.84502262	0.09397014
27.8	0.12664449	0.31095184	0.1198769	0.11986234	0.84532893	0.09447555
27.9	0.12720037	0.3110384	0.12031439	0.12032336	0.84563319	0.09498087
28	0.12775484	0.31112664	0.12075176	0.12078367	0.8459354	0.0954861
28.1	0.1283079	0.31121654	0.12118903	0.12124326	0.84623558	0.09599123
28.2	0.12885957	0.31130807	0.12162618	0.12170214	0.84653375	0.09649627
28.3	0.12940984	0.3114012	0.12206319	0.12216032	0.84682991	0.0970012
28.4	0.12995871	0.31149593	0.12250008	0.12261779	0.84712409	0.09750603
28.5	0.13050621	0.31159221	0.12293683	0.12307456	0.84741629	0.09801074
28.6	0.13105232	0.31169003	0.12337343	0.12353063	0.84770654	0.09851533
28.7	0.13159705	0.31178937	0.12380988	0.123986	0.84799483	0.0990198
28.8	0.13214041	0.3118902	0.12424618	0.12444069	0.8482812	0.09952415
28.9	0.13268241	0.31199251	0.12468232	0.12489468	0.84856565	0.10002837
29	0.13322303	0.31209626	0.12511829	0.12534798	0.84884819	0.10053245
29.1	0.1337623	0.31220145	0.12555409	0.12580059	0.84912885	0.1010364
29.2	0.13430021	0.31230805	0.12598971	0.12625253	0.84940762	0.10154021
29.3	0.13483677	0.31241603	0.12642516	0.12670378	0.84968454	0.10204387
29.4	0.13537198	0.31252538	0.12686041	0.12715436	0.8499596	0.10254738
29.5	0.13590585	0.31263608	0.12729548	0.12760426	0.85023283	0.10305075
29.6	0.13643838	0.31274811	0.12773035	0.12805349	0.85050424	0.10355395
29.7	0.13696957	0.31286144	0.12816502	0.12850205	0.85077384	0.104057
29.8	0.13749944	0.31297606	0.12859948	0.12894994	0.85104165	0.10455987
29.9	0.13802798	0.31309195	0.12903373	0.12939717	0.85130767	0.10506258

75	0.2981979	0.39053975	0.28243825	0.28519972	0.89881341	0.29341937
75.1	0.2984518	0.39069734	0.28268423	0.28547068	0.89885155	0.2937505
75.2	0.29870542	0.3908548	0.28292986	0.28574138	0.89888955	0.2940813
75.3	0.29895875	0.39101213	0.28317514	0.28601182	0.8989274	0.29441178
75.4	0.29921179	0.39116932	0.28342008	0.28628199	0.8989651	0.29474194
75.5	0.29946455	0.39132638	0.28366468	0.28655189	0.89900266	0.29507178
75.6	0.29971703	0.3914833	0.28390893	0.28682152	0.89904007	0.2954013
75.7	0.29996922	0.39164009	0.28415284	0.28709089	0.89907733	0.2957305
75.8	0.30022113	0.39179674	0.28439642	0.28735999	0.89911445	0.29605938
75.9	0.30047276	0.39195325	0.28463965	0.28762883	0.89915143	0.29638793
76	0.3007241	0.39210964	0.28488254	0.28789741	0.89918827	0.29671617
76.1	0.30097516	0.39226588	0.28512509	0.28816571	0.89922496	0.29704409
76.2	0.30122595	0.392422	0.2853673	0.28843376	0.89926151	0.2973717
76.3	0.30147645	0.39257797	0.28560917	0.28870154	0.89929792	0.29769898
76.4	0.30172667	0.39273381	0.28585071	0.28896905	0.89933419	0.29802595
76.5	0.30197662	0.39288952	0.28609191	0.28923631	0.89937031	0.2983526
76.6	0.30222628	0.39304509	0.28633277	0.2895033	0.8994063	0.29867893
76.7	0.30247567	0.39320052	0.2865733	0.28977002	0.89944215	0.29900495
76.8	0.30272478	0.39335582	0.28681349	0.29003648	0.89947786	0.29933065
76.9	0.30297362	0.39351099	0.28705335	0.29030268	0.89951343	0.29965603
77	0.30322217	0.39366601	0.28729287	0.29056862	0.89954886	0.2999811
77.1	0.30347046	0.39382091	0.28753206	0.29083429	0.89958415	0.30030586
77.2	0.30371846	0.39397566	0.28777092	0.2910997	0.89961931	0.3006303
77.3	0.30396619	0.39413029	0.28800945	0.29136485	0.89965433	0.30095443
77.4	0.30421365	0.39428477	0.28824764	0.29162974	0.89968922	0.30127824
77.5	0.30446083	0.39443912	0.28848551	0.29189436	0.89972396	0.30160174
77.6	0.30470775	0.39459333	0.28872304	0.29215872	0.89975858	0.30192493
77.7	0.30495438	0.39474741	0.28896025	0.29242283	0.89979305	0.3022478
77.8	0.30520075	0.39490135	0.28919712	0.29268667	0.8998274	0.30257037
77.9	0.30544684	0.39505516	0.28943367	0.29295025	0.8998616	0.30289262
78	0.30569267	0.39520883	0.28966989	0.29321357	0.89989568	0.30321456
78.1	0.30593822	0.39536236	0.28990579	0.29347663	0.89992962	0.30353619
78.2	0.30618351	0.39551576	0.29014135	0.29373942	0.89996342	0.30385751
78.3	0.30642852	0.39566902	0.2903766	0.29400196	0.89999709	0.30417852
78.4	0.30667327	0.39582215	0.29061151	0.29426424	0.90003063	0.30449922
78.5	0.30691774	0.39597514	0.29084611	0.29452626	0.90006404	0.30481962
78.6	0.30716195	0.39612799	0.29108038	0.29478802	0.90009731	0.3051397
78.7	0.3074059	0.39628071	0.29131433	0.29504952	0.90013046	0.30545948
78.8	0.30764957	0.3964333	0.29154795	0.29531076	0.90016347	0.30577894
78.9	0.30789298	0.39658574	0.29178125	0.29557174	0.90019635	0.30609811
79	0.30813613	0.39673806	0.29201424	0.29583247	0.90022909	0.30641696
79.1	0.30837901	0.39689024	0.2922469	0.29609293	0.90026171	0.30673551
79.2	0.30862163	0.39704228	0.29247924	0.29635314	0.9002942	0.30705375
79.3	0.30886398	0.39719419	0.29271127	0.29661309	0.90032655	0.30737169
79.4	0.30910607	0.39734596	0.29294297	0.29687278	0.90035878	0.30768932
79.5	0.30934789	0.3974976	0.29317436	0.29713222	0.90039088	0.30800664
79.6	0.30958946	0.3976491	0.29340543	0.2973914	0.90042285	0.30832366
79.7	0.30983076	0.39780047	0.29363618	0.29765032	0.90045469	0.30864038
79.8	0.3100718	0.39795171	0.29386662	0.29790898	0.9004864	0.30895679
79.9	0.31031258	0.3981028	0.29409674	0.29816739	0.90051798	0.3092729
80	0.31055309	0.39825377	0.29432654	0.29842554	0.90054944	0.30958871
80.1	0.31079335	0.3984046	0.29455604	0.29868344	0.90058076	0.30990422
80.2	0.31103335	0.39855529	0.29478521	0.29894107	0.90061196	0.31021942
80.3	0.31127309	0.39870585	0.29501408	0.29919846	0.90064304	0.31053432
80.4	0.31151257	0.39885627	0.29524264	0.29945559	0.90067398	0.31084892
80.5	0.31175179	0.39900656	0.29547088	0.29971246	0.90070481	0.31116321
80.6	0.31199076	0.39915672	0.29569881	0.29996908	0.9007355	0.31147721
80.7	0.31222947	0.39930673	0.29592643	0.30022544	0.90076607	0.3117909
80.8	0.31246792	0.39945662	0.29615374	0.30048155	0.90079652	0.3121043
80.9	0.31270611	0.39960637	0.29638075	0.3007374	0.90082684	0.31241739
81	0.31294405	0.39975598	0.29660744	0.300993	0.90085703	0.31273019
81.1	0.31318174	0.39990546	0.29683383	0.30124835	0.9008871	0.31304269
81.2	0.31341917	0.40005481	0.29705991	0.30150344	0.90091705	0.31335489
81.3	0.31365634	0.40020402	0.29728568	0.30175828	0.90094688	0.31366679
81.4	0.31389327	0.40035309	0.29751115	0.30201286	0.90097658	0.31397839
81.5	0.31412993	0.40050203	0.29773631	0.3022672	0.90100616	0.31428969
81.6	0.31436635	0.40065084	0.29796116	0.30252128	0.90103562	0.3146007
81.7	0.31460251	0.40079951	0.29818572	0.30277511	0.90106495	0.31491141
81.8	0.31483842	0.40094805	0.29840997	0.30302868	0.90109417	0.31522182
81.9	0.31507408	0.40109645	0.29863391	0.30328201	0.90112326	0.31553194
82	0.31530949	0.40124471	0.29885756	0.30353508	0.90115224	0.31584176
82.1	0.31554465	0.40139285	0.2990809	0.30378791	0.90118109	0.31615129
82.2	0.31577956	0.40154085	0.29930394	0.30404048	0.90120982	0.31646052
82.3	0.31601422	0.40168871	0.29952668	0.3042928	0.90123844	0.31676945
82.4	0.31624863	0.40183644	0.29974912	0.30454487	0.90126693	0.31707809
82.5	0.31648279	0.40198404	0.29997126	0.30479669	0.90129531	0.31738644
82.6	0.3167167	0.4021315	0.3001931	0.30504826	0.90132357	0.31769449
82.7	0.31695036	0.40227882	0.30041465	0.30529958	0.90135171	0.31800225
82.8	0.31718378	0.40242602	0.30063589	0.30555066	0.90137973	0.31830972
82.9	0.31741694	0.40257308	0.30085684	0.30580148	0.90140764	0.31861689
83	0.31764987	0.40272	0.30107749	0.30605205	0.90143542	0.31892377
83.1	0.31788254	0.40286679	0.30129785	0.30630237	0.9014631	0.31923036
83.2	0.31811497	0.40301345	0.30151791	0.30655245	0.90149065	0.31953666
83.3	0.31834715	0.40315997	0.30173768	0.30680228	0.90151809	0.31984267
83.4	0.31857909	0.40330636	0.30195715	0.30705185	0.90154542	0.32014838
83.5	0.31881079	0.40345262	0.30217633	0.30730118	0.90157263	0.3204538
83.6	0.31904224	0.40359874	0.30239521	0.30755027	0.90159972	0.32075894
83.7	0.31927344	0.40374473	0.3026138	0.3077991	0.9016267	0.32106378
83.8	0.31950441	0.40389058	0.3028321	0.30804769	0.90165357	0.32136833
83.9	0.31973513	0.4040363	0.30305011	0.30829603	0.90168032	0.32167259

41.9	0.19333468	0.33228457	0.178673	0.17874539	0.87397889	0.16318774
42	0.19373914	0.3324659	0.17906136	0.17912534	0.87411479	0.16364977
42.1	0.19414282	0.33264736	0.17944925	0.17950488	0.87425006	0.16411138
42.2	0.19454573	0.33282894	0.17983669	0.179884	0.87438471	0.16457259
42.3	0.19494787	0.33301065	0.18022366	0.1802627	0.87451874	0.16503339
42.4	0.19534923	0.33319248	0.18061018	0.18064097	0.87465216	0.16549378
42.5	0.19574983	0.33337442	0.18099623	0.18101882	0.87478497	0.16595376
42.6	0.19614967	0.33355649	0.18138182	0.18139625	0.87491717	0.16641332
42.7	0.19654875	0.33373866	0.18176695	0.18177326	0.87504876	0.16687247
42.8	0.19694708	0.33392095	0.18215162	0.18214985	0.87517976	0.16733121
42.9	0.19734465	0.33410335	0.18253582	0.18252603	0.87531016	0.16778954
43	0.19774147	0.33428586	0.18291956	0.18290179	0.87543997	0.16824744
43.1	0.19813754	0.33446847	0.18330283	0.18327715	0.87556918	0.16870494
43.2	0.19853288	0.33465119	0.18368564	0.18365209	0.8756978	0.16916201
43.3	0.19892747	0.33483401	0.18406798	0.18402664	0.87582584	0.16961867
43.4	0.19932132	0.33501693	0.18444985	0.18440077	0.87595329	0.1700749
43.5	0.19971444	0.33519995	0.18483126	0.18477451	0.87608016	0.17053072
43.6	0.20010682	0.33538307	0.1852122	0.18514786	0.87620646	0.17098612
43.7	0.20049848	0.33556628	0.18559267	0.1855208	0.87633217	0.17144109
43.8	0.20088941	0.33574959	0.18597267	0.18589335	0.87645731	0.17189565
43.9	0.20127962	0.33593298	0.1863522	0.18626551	0.87658188	0.17234978
44	0.20166911	0.33611647	0.18673125	0.18663728	0.87670588	0.17280348
44.1	0.20205787	0.33630004	0.18710984	0.18700866	0.87682931	0.17325676
44.2	0.20244593	0.33648369	0.18748796	0.18737965	0.87695217	0.17370962
44.3	0.20283327	0.33666743	0.1878656	0.18775026	0.87707448	0.17416205
44.4	0.20321989	0.33685125	0.18824278	0.18812048	0.87719621	0.17461405
44.5	0.20360582	0.33703515	0.18861948	0.1884903	0.87731739	0.17506563
44.6	0.20399104	0.33721912	0.1889957	0.18885975	0.87743802	0.17551678
44.7	0.20437555	0.33740317	0.18937145	0.1892288	0.87755808	0.17596751
44.8	0.20475937	0.33758729	0.18974673	0.18959746	0.87767759	0.17641781
44.9	0.20514249	0.33777149	0.19012153	0.18996574	0.87779655	0.17686768

90	0.33336726	0.41267546	0.31582126	0.32298443	0.90311159	0.33969747
90.1	0.33358375	0.41281305	0.31602236	0.32321781	0.90313198	0.33998439
90.2	0.33380001	0.4129505	0.31622319	0.32345095	0.90315227	0.34027103
90.3	0.33401606	0.41308783	0.31642378	0.32368385	0.90317246	0.3405574
90.4	0.33423189	0.41322503	0.31662411	0.32391652	0.90319257	0.34084351
90.5	0.33444751	0.4133621	0.31682418	0.32414896	0.90321258	0.34112934
90.6	0.33466291	0.41349904	0.317024	0.32438117	0.9032325	0.34141491
90.7	0.33487809	0.41363586	0.31722357	0.32461314	0.90325233	0.34170021
90.8	0.33509306	0.41377255	0.31742289	0.32484488	0.90327207	0.34198523
90.9	0.33530781	0.41390911	0.31762196	0.32507639	0.90329171	0.34226999
91	0.33552235	0.41404554	0.31782077	0.32530766	0.90331127	0.34255449
91.1	0.33573668	0.41418185	0.31801934	0.3255387	0.90333073	0.34283871
91.2	0.33595079	0.41431803	0.31821765	0.32576951	0.9033501	0.34312267
91.3	0.33616468	0.41445408	0.31841572	0.32600009	0.90336939	0.34340636
91.4	0.33637837	0.41459001	0.31861353	0.32623044	0.90338858	0.34368979
91.5	0.33659184	0.41472581	0.3188111	0.32646055	0.90340768	0.34397294
91.6	0.33680509	0.41486148	0.31900842	0.32669044	0.9034267	0.34425584
91.7	0.33701814	0.41499703	0.31920549	0.32692009	0.90344562	0.34453846
91.8	0.33723097	0.41513245	0.31940231	0.32714951	0.90346446	0.34482082
91.9	0.3374436	0.41526774	0.31959889	0.32737871	0.90348321	0.34510292
92	0.33765601	0.41540291	0.31979522	0.32760767	0.90350187	0.34538475
92.1	0.33786821	0.41553795	0.3199913	0.3278364	0.90352044	0.34566632
92.2	0.3380802	0.41567287	0.32018715	0.32806491	0.90353892	0.34594762
92.3	0.33829198	0.41580766	0.32038274	0.32829318	0.90355732	0.34622866
92.4	0.33850355	0.41594232	0.32057809	0.32852123	0.90357563	0.34650943
92.5	0.33871491	0.41607686	0.3207732	0.32874904	0.90359385	0.34678994
92.6	0.33892606	0.41621128	0.32096806	0.32897663	0.90361199	0.34707019
92.7	0.33913701	0.41634556	0.32116268	0.32920399	0.90363004	0.34735017
92.8	0.33934774	0.41647973	0.32135706	0.32943113	0.903648	0.3476299
92.9	0.33955827	0.41661377	0.32155119	0.32965803	0.90366588	0.34790936
93	0.33976859	0.41674768	0.32174509	0.32988471	0.90368367	0.34818855
93.1	0.3399787	0.41688147	0.32193874	0.33011116	0.90370137	0.34846749
93.2	0.34018861	0.41701513	0.32213215	0.33033739	0.903719	0.34874616
93.3	0.34039831	0.41714867	0.32232533	0.33056339	0.90373653	0.34902458
93.4	0.3406078	0.41728208	0.32251826	0.33078916	0.90375399	0.34930273
93.5	0.34081709	0.41741537	0.32271095	0.33101471	0.90377135	0.34958062
93.6	0.34102617	0.41754854	0.3229034	0.33124003	0.90378864	0.34985825
93.7	0.34123505	0.41768158	0.32309562	0.33146513	0.90380584	0.35013562
93.8	0.34144372	0.41781449	0.3232876	0.33169	0.90382296	0.35041273
93.9	0.34165218	0.41794728	0.32347934	0.33191465	0.90383999	0.35068958
94	0.34186045	0.41807995	0.32367084	0.33213908	0.90385694	0.35096618
94.1	0.34206851	0.4182125	0.32386211	0.33236328	0.90387381	0.35124251
94.2	0.34227636	0.41834492	0.32405314	0.33258725	0.9038906	0.35151858
94.3	0.34248402	0.41847722	0.32424393	0.332811	0.9039073	0.3517944
94.4	0.34269146	0.41860939	0.32443449	0.33303453	0.90392393	0.35206995
94.5	0.34289871	0.41874144	0.32462481	0.33325784	0.90394047	0.35234525
94.6	0.34310575	0.41887337	0.3248149	0.33348092	0.90395693	0.35262029
94.7	0.34331258	0.41900517	0.32500475	0.33370378	0.90397331	0.35289508
94.8	0.34351921	0.41913686	0.32519437	0.33392642	0.9039896	0.3531696
94.9	0.34372564	0.41926841	0.32538376	0.33414884	0.90400582	0.35344387
95	0.34393187	0.41939985	0.32557291	0.33437104	0.90402196	0.35371788
95.1	0.34413789	0.41953116	0.32576183	0.33459301	0.90403802	0.35399164
95.2	0.34434371	0.41966236	0.32595052	0.33481476	0.904054	0.35426514
95.3	0.34454932	0.41979343	0.32613898	0.33503629	0.9040699	0.35453838
95.4	0.34475474	0.41992437	0.32632721	0.33525761	0.90408572	0.35481137
95.5	0.34495995	0.4200552	0.3265152	0.3354787	0.90410146	0.3550841
95.6	0.34516496	0.4201859	0.32670297	0.33569957	0.90411713	0.35535658
95.7	0.34536977	0.42031648	0.3268905	0.33592022	0.90413271	0.3556288
95.8	0.34557438	0.42044694	0.32707781	0.33614066	0.90414822	0.35590077
95.9	0.34577879	0.42057728	0.32726488	0.33636087	0.90416365	0.35617248
96	0.345983	0.4207075	0.32745173	0.33658087	0.904179	0.35644394
96.1	0.34618701	0.42083759	0.32763835	0.33680065	0.90419428	0.35671514
96.2	0.34639082	0.42096757	0.32782474	0.3370202	0.90420948	0.35698609
96.3	0.34659443	0.42109742	0.32801091	0.33723954	0.9042246	0.35725679
96.4	0.34679785	0.42122715	0.32819684	0.33745867	0.90423965	0.35752724
96.5	0.34700106	0.42135676	0.32838255	0.33767757	0.90425462	0.35779743
96.6	0.34720408	0.42148626	0.32856804	0.33789626	0.90426952	0.35806737
96.7	0.34740689	0.42161563	0.3287533	0.33811473	0.90428434	0.35833705
96.8	0.34760952	0.42174488	0.32893833	0.33833299	0.90429909	0.35860649
96.9	0.34781194	0.42187401	0.32912314	0.33855103	0.90431376	0.35887567
97	0.34801417	0.42200302	0.32930772	0.33876885	0.90432836	0.3591446
97.1	0.3482162	0.42213191	0.32949208	0.33898646	0.90434288	0.35941329
97.2	0.34841803	0.42226068	0.32967621	0.33920385	0.90435733	0.35968171
97.3	0.34861967	0.42238933	0.32986012	0.33942102	0.90437171	0.35994989
97.4	0.34882112	0.42251786	0.33004381	0.33963799	0.90438602	0.36021782
97.5	0.34902237	0.42264628	0.33022727	0.33985473	0.90440025	0.3604855
97.6	0.34922342	0.42277457	0.33041051	0.34007127	0.90441441	0.36075293
97.7	0.34942428	0.42290274	0.33059353	0.34028759	0.9044285	0.36102011
97.8	0.34962494	0.4230308	0.33077633	0.34050369	0.90444251	0.36128704
97.9	0.34982541	0.42315874	0.33095891	0.34071958	0.90445646	0.36155372
98	0.35002569	0.42328656	0.33114126	0.34093526	0.90447033	0.36182015
98.1	0.35022578	0.42341426	0.3313234	0.34115073	0.90448413	0.36208633
98.2	0.35042567	0.42354184	0.33150532	0.34136598	0.90449787	0.36235226
98.3	0.35062536	0.4236693	0.33168701	0.34158102	0.90451153	0.36261795
98.4	0.35082487	0.42379665	0.33186849	0.34179585	0.90452512	0.36288339
98.5	0.35102418	0.42392387	0.33204975	0.34201047	0.90453864	0.36314858
98.6	0.35122331	0.42405098	0.33223079	0.34222487	0.9045521	0.36341352
98.7	0.35142224	0.42417797	0.33241161	0.34243907	0.90456548	0.36367822
98.8	0.35162098	0.42430485	0.33259221	0.34265305	0.9045788	0.36394266
98.9	0.35181953	0.4244316	0.33277259	0.34286682	0.90459205	0.36420687

56.9	0.24684333	0.3598681	0.23164202	0.23145813	0.88889009	0.22769184
57	0.24716083	0.36004851	0.23195998	0.23178252	0.88896366	0.22808937
57.1	0.2474779	0.36022881	0.23227749	0.23210658	0.88903701	0.22848648
57.2	0.24779455	0.36040901	0.23259456	0.23243032	0.88911014	0.22888318
57.3	0.24811077	0.3605891	0.23291118	0.23275373	0.88918306	0.22927946
57.4	0.24842657	0.36076909	0.23322736	0.23307681	0.88925575	0.22967533
57.5	0.24874195	0.36094897	0.23354309	0.23339957	0.88932823	0.23007079
57.6	0.2490569	0.36112874	0.23385838	0.233722	0.88940049	0.23046584
57.7	0.24937144	0.36130841	0.23417323	0.23404411	0.88947254	0.23086048
57.8	0.24968555	0.36148797	0.23448763	0.23436589	0.88954437	0.2312547
57.9	0.24999925	0.36166742	0.23480159	0.23468735	0.88961599	0.23164852
58	0.25031253	0.36184676	0.23511511	0.23500848	0.88968739	0.23204193
58.1	0.2506254	0.36202599	0.23542819	0.23532929	0.88975857	0.23243493
58.2	0.25093784	0.36220512	0.23574083	0.23564977	0.88982954	0.23282752
58.3	0.25124988	0.36238414	0.23605303	0.23596994	0.8899029	0.2332197
58.4	0.2515615	0.36256304	0.23636479	0.23628978	0.88997083	0.23361148
58.5	0.2518727	0.36274184	0.23667611	0.23660929	0.89004115	0.23400285
58.6	0.2521835	0.36292052	0.23698699	0.23692849	0.89011126	0.23439382
58.7	0.25249388	0.3630991	0.23729744	0.23724736	0.89018116	0.23478438
58.8	0.25280385	0.36327756	0.23760745	0.23756592	0.89025084	0.23517454
58.9	0.25311341	0.36345591	0.23791702	0.23788415	0.8903203	0.2355643
59	0.25342257	0.36363415	0.23822615	0.23820206	0.89038955	0.23595366
59.1	0.25373131	0.36381227	0.23853485	0.23851965	0.89045858	0.23634261
59.2	0.25403965	0.36399029	0.23884312	0.23883692	0.8905274	0.23673116
59.3	0.25434759	0.36416818	0.23915095	0.23915387	0.890596	0.23711931
59.4	0.25465511	0.36434597	0.23945834	0.2394705	0.89066439	0.23750706
59.5	0.25496224	0.36452364	0.2397653	0.23978682	0.89073257	0.23789441
59.6	0.25526895	0.36470119	0.24007183	0.24010282	0.89080052	0.23828137
59.7	0.25557527	0.36487863	0.24037793	0.2404185	0.89086826	0.23866792
59.8	0.25588118	0.36505596	0.24068359	0.24073386	0.89093579	0.23905408
59.9	0.2561867	0.36523316	0.24098882	0.2410489	0.8910031	0.23943984

105	0.36358009	0.43194697	0.34337919	0.35552037	0.90528362	0.37986724
105.1	0.3637673	0.4320667	0.34354678	0.35572164	0.90529326	0.38011666
105.2	0.36395434	0.43218633	0.34371418	0.35592272	0.90530285	0.38036584
105.3	0.3641412	0.43230585	0.34388137	0.35612361	0.90531238	0.3806148
105.4	0.36432789	0.43242526	0.34404838	0.3563243	0.90532187	0.38086352
105.5	0.3645144	0.43254456	0.34421518	0.3565248	0.90533132	0.38111202
105.6	0.36470074	0.43266375	0.34438179	0.35672511	0.90534071	0.38136028
105.7	0.36488691	0.43278283	0.34454821	0.35692522	0.90535006	0.38160832
105.8	0.36507291	0.43290181	0.34471443	0.35712515	0.90535936	0.38185612
105.9	0.36525873	0.43302067	0.34488046	0.35732488	0.90536862	0.3821037
106	0.36544438	0.43313943	0.34504629	0.35752442	0.90537783	0.38235105
106.1	0.36562986	0.43325808	0.34521193	0.35772376	0.90538699	0.38259817
106.2	0.36581517	0.43337662	0.34537738	0.35792292	0.90539611	0.38284506
106.3	0.3660003	0.43349505	0.34554263	0.35812188	0.90540518	0.38309172
106.4	0.36618527	0.43361338	0.34570769	0.35832066	0.90541421	0.38333816
106.5	0.36637006	0.4337316	0.34587255	0.35851924	0.90542319	0.38358436
106.6	0.36655468	0.43384971	0.34603723	0.35871763	0.90543212	0.38383034
106.7	0.36673913	0.43396772	0.34620171	0.35891584	0.90544102	0.38407609
106.8	0.36692341	0.43408561	0.346366	0.35911385	0.90544987	0.38432162
106.9	0.36710752	0.4342034	0.34653009	0.35931168	0.90545867	0.38456692
107	0.36729146	0.43432109	0.346694	0.35950931	0.90546743	0.38481199
107.1	0.36747523	0.43443866	0.34685771	0.35970676	0.90547615	0.38505684
107.2	0.36765883	0.43455613	0.34702123	0.35990401	0.90548482	0.38530146
107.3	0.36784226	0.4346735	0.34718456	0.36010108	0.90549345	0.38554585
107.4	0.36802552	0.43479075	0.3473477	0.36029796	0.90550204	0.38579002
107.5	0.36820861	0.43490791	0.34751065	0.36049465	0.90551058	0.38603396
107.6	0.36839153	0.43502495	0.34767341	0.36069116	0.90551909	0.38627768
107.7	0.36857428	0.43514189	0.34783598	0.36088747	0.90552755	0.38652117
107.8	0.36875686	0.43525872	0.34799836	0.3610836	0.90553597	0.38676444
107.9	0.36893928	0.43537545	0.34816055	0.36127954	0.90554434	0.38700748
108	0.36912153	0.43549207	0.34832255	0.3614753	0.90555268	0.3872503
108.1	0.36930361	0.43560859	0.34848437	0.36167086	0.90556097	0.3874929
108.2	0.36948552	0.435725	0.34864599	0.36186624	0.90556922	0.38773527
108.3	0.36966726	0.43584131	0.34880743	0.36206144	0.90557743	0.38797742
108.4	0.36984884	0.43595751	0.34896868	0.36225644	0.9055856	0.38821934
108.5	0.37003025	0.4360736	0.34912975	0.36245126	0.90559373	0.38846104
108.6	0.3702115	0.43618959	0.34929062	0.3626459	0.90560182	0.38870252
108.7	0.37039258	0.43630548	0.34945131	0.36284035	0.90560987	0.38894378
108.8	0.37057349	0.43642126	0.34961181	0.36303461	0.90561788	0.38918481
108.9	0.37075423	0.43653694	0.34977213	0.36322869	0.90562585	0.38942562
109	0.37093482	0.43665251	0.34993226	0.36342258	0.90563378	0.38966621
109.1	0.37111523	0.43676798	0.3500922	0.36361629	0.90564166	0.38990658
109.2	0.37129548	0.43688334	0.35025196	0.36380982	0.90564951	0.39014672
109.3	0.37147557	0.4369986	0.35041153	0.36400315	0.90565732	0.39038665
109.4	0.37165549	0.43711376	0.35057091	0.36419631	0.90566509	0.39062635
109.5	0.37183524	0.43722881	0.35073011	0.36438928	0.90567283	0.39086583
109.6	0.37201483	0.43734376	0.35088913	0.36458207	0.90568052	0.39110509
109.7	0.37219426	0.43745861	0.35104796	0.36477467	0.90568817	0.39134413
109.8	0.37237353	0.43757335	0.35120661	0.36496709	0.90569579	0.39158296
109.9	0.37255263	0.43768799	0.35136508	0.36515933	0.90570336	0.39182156
110	0.37273156	0.43780252	0.35152336	0.36535138	0.9057109	0.39205994
110.1	0.37291034	0.43791696	0.35168146	0.36554325	0.9057184	0.3922981
110.2	0.37308895	0.43803129	0.35183937	0.36573494	0.90572586	0.39253604
110.3	0.3732674	0.43814552	0.3519971	0.36592645	0.90573329	0.39277377
110.4	0.37344569	0.43825964	0.35215465	0.36611778	0.90574067	0.39301127
110.5	0.37362381	0.43837366	0.35231202	0.36630892	0.90574802	0.39324856
110.6	0.37380177	0.43848758	0.3524692	0.36649988	0.90575533	0.39348562
110.7	0.37397957	0.4386014	0.35262621	0.36669066	0.9057626	0.39372247
110.8	0.37415721	0.43871512	0.35278303	0.36688126	0.90576984	0.3939591
110.9	0.37433469	0.43882873	0.35293967	0.36707168	0.90577704	0.39419552
111	0.37451201	0.43894224	0.35309612	0.36726191	0.9057842	0.39443171
111.1	0.37468916	0.43905565	0.3532524	0.36745197	0.90579132	0.39466769
111.2	0.37486616	0.43916896	0.3534085	0.36764184	0.90579841	0.39490345
111.3	0.37504299	0.43928217	0.35356441	0.36783154	0.90580546	0.395139
111.4	0.37521967	0.43939527	0.35372015	0.36802105	0.90581247	0.39537433
111.5	0.37539618	0.43950828	0.3538757	0.36821039	0.90581944	0.39560944
111.6	0.37557254	0.43962118	0.35403108	0.36839954	0.90582638	0.39584433
111.7	0.37574873	0.43973399	0.35418628	0.36858852	0.90583328	0.39607901
111.8	0.37592477	0.43984669	0.35434129	0.36877732	0.90584015	0.39631347
111.9	0.37610065	0.43995929	0.35449613	0.36896593	0.90584698	0.39654772
112	0.37627637	0.44007179	0.35465079	0.36915437	0.90585377	0.39678176
112.1	0.37645193	0.44018419	0.35480528	0.36934263	0.90586053	0.39701557
112.2	0.37662733	0.44029649	0.35495958	0.36953071	0.90586725	0.39724917
112.3	0.37680257	0.44040869	0.35511371	0.36971861	0.90587394	0.39748256
112.4	0.37697766	0.44052079	0.35526766	0.36990634	0.90588059	0.39771574
112.5	0.37715259	0.44063279	0.35542143	0.37009388	0.9058872	0.3979487
112.6	0.37732736	0.44074469	0.35557503	0.37028125	0.90589378	0.39818144
112.7	0.37750197	0.44085649	0.35572845	0.37046844	0.90590032	0.39841397
112.8	0.37767642	0.44096819	0.35588169	0.37065546	0.90590682	0.39864629
112.9	0.37785072	0.44107979	0.35603476	0.37084229	0.90591329	0.39887839
113	0.37802487	0.44119129	0.35618765	0.37102895	0.90591973	0.39911028
113.1	0.37819885	0.44130269	0.35634036	0.37121543	0.90592613	0.39934196
113.2	0.37837268	0.44141399	0.3564929	0.37140174	0.90593249	0.39957343
113.3	0.37854635	0.4415252	0.35664526	0.37158787	0.90593882	0.39980468
113.4	0.37871987	0.4416363	0.35679745	0.37177382	0.90594511	0.40003572
113.5	0.37889323	0.44174731	0.35694946	0.3719596	0.90595137	0.40026655
113.6	0.37906644	0.44185822	0.3571013	0.3721452	0.9059576	0.40049717
113.7	0.37923949	0.44196902	0.35725296	0.37233062	0.90596378	0.40072757
113.8	0.37941238	0.44207974	0.35740445	0.37251587	0.90596994	0.40095777
113.9	0.37958512	0.44219035	0.35755577	0.37270095	0.90597606	0.40118775

69	0.28241669	0.38083958	0.26702521	0.26844253	0.89623082	0.2729444
69.1	0.2826889	0.38100515	0.26729299	0.26873003	0.89627897	0.27329572
69.2	0.28296077	0.3811706	0.26756039	0.26901725	0.89632693	0.27364669
69.3	0.28323233	0.38133591	0.26782741	0.26930419	0.8963747	0.27399731
69.4	0.28350356	0.38150109	0.26809405	0.26959084	0.8964223	0.27434758
69.5	0.28377448	0.38166614	0.26836031	0.2698772	0.89646971	0.27469751
69.6	0.28404507	0.38183106	0.26862619	0.27016329	0.89651694	0.27504708
69.7	0.28431534	0.38199585	0.26889169	0.27044909	0.89656399	0.2753963
69.8	0.28458529	0.38216051	0.26915682	0.27073461	0.89661085	0.27574518
69.9	0.28485493	0.38232503	0.26942157	0.27101985	0.89665754	0.27609371
70	0.28512424	0.38248943	0.26968595	0.2713048	0.89670405	0.27644189
70.1	0.28539324	0.38265369	0.26994995	0.27158948	0.89675038	0.27678972
70.2	0.28566192	0.38281782	0.27021357	0.27187387	0.89679653	0.27713721
70.3	0.28593028	0.38298182	0.27047682	0.27215798	0.8968425	0.27748436
70.4	0.28619833	0.38314569	0.2707397	0.27244182	0.8968883	0.27783116
70.5	0.28646606	0.38330942	0.2710022	0.27272537	0.89693392	0.27817761
70.6	0.28673348	0.38347302	0.27126433	0.27300864	0.89697936	0.27852372
70.7	0.28700059	0.38363649	0.27152609	0.27329163	0.89702463	0.27886949
70.8	0.28726738	0.38379983	0.27178748	0.27357435	0.89706972	0.27921491
70.9	0.28753386	0.38396304	0.2720485	0.27385678	0.89711464	0.27955999
71	0.28780002	0.38412611	0.27230914	0.27413894	0.89715938	0.27990473
71.1	0.28806588	0.38428905	0.27256942	0.27442081	0.89720395	0.28024913
71.2	0.28833142	0.38445186	0.27282933	0.27470241	0.89724835	0.28059319
71.3	0.28859666	0.38461454	0.27308887	0.27498373	0.89729257	0.2809369
71.4	0.28886158	0.38477708	0.27334804	0.27526477	0.89733662	0.28128028
71.5	0.2891262	0.38493949	0.27360685	0.27554554	0.8973805	0.28162331
71.6	0.2893905	0.38510176	0.27386529	0.27582603	0.89742422	0.28196601
71.7	0.2896545	0.38526391	0.27412336	0.27610624	0.89746776	0.28230837
71.8	0.28991819	0.38542592	0.27438107	0.27638617	0.89751113	0.28265039
71.9	0.29018158	0.3855878	0.27463841	0.27666583	0.89755433	0.28299207
72	0.29044466	0.38574954	0.27489539	0.27694521	0.89759737	0.28333342
72.1	0.29070744	0.38591115	0.27515201	0.27722432	0.89764023	0.28367443
72.2	0.29096991	0.38607263	0.27540826	0.27750315	0.89768293	0.2840151
72.3	0.29123207	0.38623398	0.27566415	0.2777817	0.89772547	0.28435544
72.4	0.29149393	0.38639519	0.27591968	0.27805998	0.89776784	0.28469544
72.5	0.2917555	0.38655627	0.27617485	0.27833798	0.89781004	0.28503511
72.6	0.29201675	0.38671722	0.27642965	0.27861571	0.89785208	0.28537444
72.7	0.29227771	0.38687803	0.2766841	0.27889317	0.89789395	0.28571344
72.8	0.29253837	0.38703871	0.27693819	0.27917035	0.89793567	0.28605211
72.9	0.29279872	0.38719925	0.27719191	0.27944725	0.89797721	0.28639044
73	0.29305878	0.38735966	0.27744528	0.27972389	0.8980186	0.28672844
73.1	0.29331854	0.38751994	0.27769829	0.28000025	0.89805983	0.28706611
73.2	0.29357799	0.38768009	0.27795095	0.28027634	0.8981009	0.28740345
73.3	0.29383715	0.3878401	0.27820324	0.28055216	0.8981418	0.28774046
73.4	0.29409602	0.38799997	0.27845518	0.28082771	0.89818255	0.28807713
73.5	0.29435458	0.38815971	0.27870677	0.28110298	0.89822314	0.28841348
73.6	0.29461285	0.38831932	0.278958	0.28137799	0.89826357	0.28874949
73.7	0.29487083	0.3884788	0.27920887	0.28165272	0.89830385	0.28908518
73.8	0.29512851	0.38863814	0.27945939	0.28192718	0.89834396	0.28942054
73.9	0.2953859	0.38879734	0.27970956	0.28220137	0.89838393	0.28975557
74	0.29564299	0.38895641	0.27995938	0.28247529	0.89842373	0.29009027
74.1	0.29589979	0.38911535	0.28020884	0.28274895	0.89846339	0.29042465
74.2	0.29615629	0.38927415	0.28045795	0.28302233	0.89850288	0.2907587
74.3	0.29641251	0.38943282	0.28070671	0.28329544	0.89854223	0.29109242
74.4	0.29666843	0.38959136	0.28095512	0.28356829	0.89858142	0.29142581
74.5	0.29692407	0.38974976	0.28120318	0.28384086	0.89862046	0.29175888
74.6	0.29717941	0.38990802	0.28145089	0.28411317	0.89865935	0.29209163
74.7	0.29743447	0.39006616	0.28169825	0.28438521	0.89869809	0.29242405
74.8	0.29768923	0.39022415	0.28194527	0.28465698	0.89873668	0.29275615
74.9	0.29794371	0.39038202	0.28219193	0.28492848	0.89877512	0.29308792
120	0.38983583	0.44875656	0.36646597	0.38366722	0.90628656	0.41482589
120.1	0.3899993	0.44886131	0.36660694	0.38384183	0.90629068	0.4150432
120.2	0.39016263	0.44896597	0.36674776	0.38401628	0.90629477	0.4152603
120.3	0.39032581	0.44907053	0.36688841	0.38419056	0.90629883	0.41547721
120.4	0.39048886	0.44917501	0.36702891	0.38436469	0.90630286	0.41569393
120.5	0.39065176	0.44927939	0.36716924	0.38453865	0.90630687	0.41591044
120.6	0.39081452	0.44938369	0.36730942	0.38471245	0.90631084	0.41612676
120.7	0.39097714	0.44948789	0.36744944	0.38488609	0.90631479	0.41634288
120.8	0.39113961	0.449592	0.3675893	0.38505957	0.9063187	0.41655881
120.9	0.39130195	0.44969603	0.36772901	0.38523289	0.90632259	0.41677454
121	0.39146414	0.44979996	0.36786855	0.38540605	0.90632645	0.41699007
121.1	0.39162619	0.4499038	0.36800794	0.38557905	0.90633028	0.41720541
121.2	0.3917881	0.45000755	0.36814718	0.38575189	0.90633409	0.41742055
121.3	0.39194987	0.45011122	0.36828625	0.38592457	0.90633786	0.41763549
121.4	0.39211151	0.45021479	0.36842517	0.38609708	0.90634161	0.41785024
121.5	0.392273	0.45031827	0.36856394	0.38626944	0.90634533	0.4180648
121.6	0.39243435	0.45042166	0.36870254	0.38644164	0.90634902	0.41827916
121.7	0.39259556	0.45052497	0.36884099	0.38661368	0.90635269	0.41849332
121.8	0.39275663	0.45062818	0.36897929	0.38678556	0.90635633	0.41870729
121.9	0.39291756	0.45073131	0.36911743	0.38695729	0.90635994	0.41892107
122	0.39307835	0.45083434	0.36925542	0.38712885	0.90636352	0.41913465
122.1	0.39323901	0.45093729	0.36939325	0.38730025	0.90636708	0.41934804
122.2	0.39339952	0.45104015	0.36953092	0.3874715	0.9063706	0.41956123
122.3	0.3935599	0.45114292	0.36966844	0.38764259	0.90637411	0.41977423
122.4	0.39372014	0.4512456	0.36980581	0.38781352	0.90637758	0.41998703
122.5	0.39388024	0.45134819	0.36994302	0.38798429	0.90638103	0.42019965
122.6	0.3940402	0.45145069	0.37008008	0.38815491	0.90638445	0.42041207
122.7	0.39420003	0.4515531	0.37021699	0.38832536	0.90638785	0.42062429
122.8	0.39435972	0.45165543	0.37035374	0.38849566	0.90639122	0.42083633
122.9	0.39451927	0.45175767	0.37049034	0.38866581	0.90639456	0.42104817
123	0.39467869	0.45185982	0.37062678	0.38883579	0.90639788	0.42125982
123.1	0.39483797	0.45196188	0.37076307	0.38900562	0.90640117	0.42147127
123.2	0.39499711	0.45206385	0.37089921	0.3891753	0.90640444	0.42168254
123.3	0.39515611	0.45216574	0.3710352	0.38934481	0.90640768	0.42189361
123.4	0.39531498	0.45226754	0.37117104	0.38951417	0.9064109	0.42210449
123.5	0.39547372	0.45236925	0.37130672	0.38968338	0.90641409	0.42231518
123.6	0.39563231	0.45247087	0.37144225	0.38985243	0.90641725	0.42252568
123.7	0.39579078	0.4525724	0.37157763	0.39002132	0.90642039	0.42273599
123.8	0.3959491	0.45267385	0.37171286	0.39019006	0.90642351	0.42294611
123.9	0.3961073	0.45277521	0.37184794	0.39035864	0.9064266	0.42315603
124	0.39626535	0.45287648	0.37198286	0.39052706	0.90642966	0.42336577
124.1	0.39642328	0.45297767	0.37211764	0.39069534	0.90643271	0.42357531
124.2	0.39658107	0.45307877	0.37225226	0.39086345	0.90643572	0.42378467
124.3	0.39673872	0.45317978	0.37238674	0.39103142	0.90643871	0.42399384
124.4	0.39689624	0.45328071	0.37252106	0.39119923	0.90644168	0.42420281
124.5	0.39705363	0.45338154	0.37265524	0.39136688	0.90644463	0.4244116
124.6	0.39721088	0.4534823	0.37278926	0.39153438	0.90644755	0.4246202
124.7	0.397368	0.45358296	0.37292314	0.39170173	0.90645044	0.42482862
124.8	0.39752498	0.45368354	0.37305686	0.39186893	0.90645332	0.42503682
124.9	0.39768184	0.45378403	0.37319044	0.39203597	0.90645617	0.42524485
125	0.39783856	0.45388444	0.37332387	0.39220286	0.90645899	0.4254527
125.1	0.39799514	0.45398476	0.37345715	0.3923696	0.90646179	0.42566035
125.2	0.3981516	0.454085	0.37359028	0.39253618	0.90646457	0.42586782
125.3	0.39830792	0.45418514	0.37372326	0.39270261	0.90646733	0.42607509
125.4	0.39846411	0.4542852	0.3738561	0.39286889	0.90647007	0.42628219
125.5	0.39862017	0.45438518	0.37398879	0.39303502	0.90647278	0.42648909
125.6	0.39877609	0.45448507	0.37412133	0.393201	0.90647547	0.4266958
125.7	0.39893189	0.45458488	0.37425372	0.39336682	0.90647813	0.42690233
125.8	0.39908755	0.4546846	0.37438596	0.3935325	0.90648078	0.42710867
125.9	0.39924308	0.45478423	0.37451806	0.39369802	0.9064834	0.42731483
126	0.39939848	0.45488378	0.37465001	0.39386339	0.906486	0.4275208

84	0.3199656	0.40418189	0.30326783	0.30854412	0.90170696	0.32197657
84.1	0.32019584	0.40432735	0.30348526	0.30879197	0.90173349	0.32228025
84.2	0.32042584	0.40447267	0.3037024	0.30903956	0.9017599	0.32258365
84.3	0.32065559	0.40461785	0.30391925	0.30928692	0.90178621	0.32288676
84.4	0.3208851	0.40476291	0.30413581	0.30953402	0.9018124	0.32318958
84.5	0.32111438	0.40490783	0.30435208	0.30978088	0.90183848	0.32349211
84.6	0.32134341	0.40505262	0.30456807	0.31002749	0.90186445	0.32379436
84.7	0.3215722	0.40519727	0.30478377	0.31027386	0.90189031	0.32409631
84.8	0.32180076	0.40534179	0.30499919	0.31051998	0.90191606	0.32439799
84.9	0.32202907	0.40548618	0.30521432	0.31076586	0.90194169	0.32469937
85	0.32225715	0.40563044	0.30542916	0.31101149	0.90196722	0.32500047
85.1	0.32248499	0.40577456	0.30564372	0.31125688	0.90199264	0.32530128
85.2	0.3227126	0.40591855	0.305858	0.31150202	0.90201796	0.32560181
85.3	0.32293997	0.40606241	0.30607199	0.31174692	0.90204316	0.32590205
85.4	0.3231671	0.40620614	0.30628571	0.31199157	0.90206825	0.32620201
85.5	0.32339399	0.40634973	0.30649914	0.31223598	0.90209324	0.32650169
85.6	0.32362065	0.40649319	0.30671228	0.31248014	0.90211812	0.32680108
85.7	0.32384708	0.40663652	0.30692515	0.31272406	0.9021429	0.32710018
85.8	0.32407327	0.40677972	0.30713774	0.31296773	0.90216756	0.327399
85.9	0.32429923	0.40692278	0.30735005	0.31321117	0.90219212	0.32769754
86	0.32452496	0.40706572	0.30756208	0.31345436	0.90221658	0.3279958
86.1	0.32475045	0.40720852	0.30777383	0.3136973	0.90224093	0.32829377
86.2	0.32497571	0.40735119	0.30798531	0.31394	0.90226517	0.32859146
86.3	0.32520074	0.40749372	0.30819651	0.31418246	0.90228931	0.32888887
86.4	0.32542553	0.40763613	0.30840743	0.31442468	0.90231335	0.32918599
86.5	0.3256501	0.4077784	0.30861807	0.31466665	0.90233728	0.32948284
86.6	0.32587443	0.40792054	0.30882844	0.31490838	0.9023611	0.3297794
86.7	0.32609854	0.40806255	0.30903854	0.31514987	0.90238483	0.33007569
86.8	0.32632241	0.40820442	0.30924836	0.31539112	0.90240844	0.33037169
86.9	0.32654606	0.40834617	0.3094579	0.31563213	0.90243196	0.33066741
87	0.32676948	0.40848778	0.30966718	0.31587289	0.90245537	0.33096285
87.1	0.32699266	0.40862926	0.30987618	0.31611341	0.90247869	0.33125802
87.2	0.32721562	0.40877062	0.31008491	0.31635369	0.9025019	0.3315529
87.3	0.32743836	0.40891183	0.31029336	0.31659373	0.902525	0.33184751
87.4	0.32766086	0.40905292	0.31050155	0.31683353	0.90254801	0.33214183
87.5	0.32788314	0.40919388	0.31070946	0.31707309	0.90257092	0.33243588
87.6	0.32810519	0.40933471	0.31091711	0.31731241	0.90259372	0.33272965
87.7	0.32832702	0.4094754	0.31112448	0.31755149	0.90261642	0.33302314
87.8	0.32854862	0.40961596	0.31133159	0.31779032	0.90263903	0.33331636
87.9	0.32877	0.4097564	0.31153843	0.31802892	0.90266153	0.33360929
88	0.32899115	0.4098967	0.31174501	0.31826728	0.90268394	0.33390195
88.1	0.32921207	0.41003687	0.31195131	0.3185054	0.90270624	0.33419434
88.2	0.32943278	0.41017692	0.31215735	0.31874328	0.90272845	0.33448644
88.3	0.32965326	0.41031683	0.31236313	0.31898092	0.90275056	0.33477828
88.4	0.32987351	0.41045661	0.31256864	0.31921832	0.90277257	0.33506983
88.5	0.33009354	0.41059626	0.31277389	0.31945548	0.90279448	0.33536111
88.6	0.33031335	0.41073578	0.31297887	0.31969241	0.90281629	0.33565212
88.7	0.33053293	0.41087517	0.31318359	0.31992909	0.90283801	0.33594285
88.8	0.33075228	0.41101443	0.31338805	0.32016554	0.90285963	0.3362333
88.9	0.33097142	0.41115356	0.31359225	0.32040175	0.90288115	0.33652349
89	0.33119033	0.41129256	0.31379618	0.32063772	0.90290258	0.33681339
89.1	0.33140902	0.41143143	0.31399986	0.32087346	0.90292391	0.33710303
89.2	0.33162748	0.41157017	0.31420327	0.32110896	0.90294514	0.33739239
89.3	0.33184573	0.41170879	0.31440642	0.32134422	0.90296628	0.33768148
89.4	0.33206375	0.41184727	0.31460932	0.32157924	0.90298732	0.33797029
89.5	0.33228155	0.41198562	0.31481195	0.32181403	0.90300827	0.33825883
89.6	0.33249913	0.41212385	0.31501433	0.32204858	0.90302912	0.3385471
89.7	0.33271649	0.41226195	0.31521645	0.3222829	0.90304988	0.3388351
89.8	0.33293363	0.41239991	0.31541831	0.32251698	0.90307055	0.33912283
89.9	0.33315056	0.41253775	0.31561991	0.32275082	0.90309112	0.33941029
135	0.41286609	0.46350666	0.38594936	0.40814914	0.90664791	0.4453175
135.1	0.41301017	0.46359886	0.38606878	0.40830146	0.9066491	0.44550727
135.2	0.41315413	0.46369098	0.38618807	0.40845363	0.90665029	0.44569687
135.3	0.41329798	0.46378303	0.38630723	0.40860568	0.90665146	0.4458863
135.4	0.41344171	0.463875	0.38642626	0.40875758	0.90665263	0.44607556
135.5	0.41358532	0.4639669	0.38654517	0.40890935	0.90665378	0.44626465
135.6	0.41372881	0.46405872	0.38666395	0.40906099	0.90665493	0.44645357
135.7	0.41387219	0.46415046	0.3867826	0.40921249	0.90665606	0.44664232
135.8	0.41401545	0.46424213	0.38690112	0.40936385	0.90665719	0.44683091
135.9	0.41415859	0.46433372	0.38701951	0.40951508	0.9066583	0.44701932
136	0.41430162	0.46442523	0.38713778	0.40966617	0.90665941	0.44720757
136.1	0.41444452	0.46451667	0.38725592	0.40981713	0.90666051	0.44739565
136.2	0.41458732	0.46460803	0.38737393	0.40996795	0.90666159	0.44758356
136.3	0.41472999	0.46469932	0.38749182	0.41011864	0.90666267	0.4477713
136.4	0.41487255	0.46479053	0.38760958	0.41026919	0.90666374	0.44795887
136.5	0.41501499	0.46488167	0.38772721	0.41041961	0.9066648	0.44814628
136.6	0.41515732	0.46497273	0.38784472	0.4105699	0.90666585	0.44833352
136.7	0.41529953	0.46506371	0.3879621	0.41072005	0.90666689	0.44852059
136.8	0.41544162	0.46515462	0.38807936	0.41087007	0.90666792	0.4487075
136.9	0.4155836	0.46524546	0.38819649	0.41101995	0.90666894	0.44889424
137	0.41572547	0.46533622	0.38831349	0.4111697	0.90666995	0.44908081
137.1	0.41586721	0.4654269	0.38843037	0.41131932	0.90667095	0.44926722
137.2	0.41600884	0.46551751	0.38854712	0.4114688	0.90667194	0.44945346
137.3	0.41615036	0.46560804	0.38866375	0.41161815	0.90667293	0.44963953
137.4	0.41629176	0.4656985	0.38878026	0.41176736	0.9066739	0.44982544
137.5	0.41643305	0.46578889	0.38889664	0.41191645	0.90667486	0.45001118
137.6	0.41657422	0.4658792	0.38901289	0.4120654	0.90667582	0.45019675
137.7	0.41671528	0.46596944	0.38912902	0.41221421	0.90667676	0.45038216
137.8	0.41685622	0.4660596	0.38924503	0.4123629	0.9066777	0.45056741
137.9	0.41699705	0.46614968	0.38936091	0.41251145	0.90667862	0.45075249
138	0.41713776	0.4662397	0.38947667	0.41265987	0.90667954	0.4509374
138.1	0.41727836	0.46632964	0.38959231	0.41280816	0.90668044	0.45112215
138.2	0.41741885	0.4664195	0.38970783	0.41295632	0.90668133	0.45130674
138.3	0.41755922	0.46650929	0.38982322	0.41310434	0.90668222	0.45149116
138.4	0.41769948	0.46659901	0.38993849	0.41325223	0.90668309	0.45167542
138.5	0.41783962	0.46668865	0.39005364	0.41339999	0.90668396	0.45185951
138.6	0.41797965	0.46677822	0.39016866	0.41354762	0.90668481	0.45204344
138.7	0.41811957	0.46686771	0.39028357	0.41369512	0.90668566	0.4522272
138.8	0.41825937	0.46695714	0.39039835	0.41384249	0.90668649	0.4524108
138.9	0.41839906	0.46704648	0.39051301	0.41398972	0.90668731	0.45259424
139	0.41853864	0.46713576	0.39062755	0.41413683	0.90668813	0.45277752
139.1	0.4186781	0.46722496	0.39074197	0.41428381	0.90668893	0.45296063
139.2	0.41881745	0.46731409	0.39085627	0.41443065	0.90668972	0.45314357
139.3	0.41895669	0.46740314	0.39097045	0.41457737	0.9066905	0.45332636
139.4	0.41909581	0.46749212	0.39108451	0.41472396	0.90669128	0.45350898
139.5	0.41923483	0.46758103	0.39119845	0.41487041	0.90669204	0.45369144
139.6	0.41937373	0.46766987	0.39131226	0.41501674	0.90669279	0.45387374
139.7	0.41951252	0.46775863	0.39142596	0.41516294	0.90669352	0.45405587
139.8	0.41965119	0.46784732	0.39153954	0.41530901	0.90669425	0.45423785
139.9	0.41978976	0.46793594	0.391653	0.41545495	0.90669497	0.45441966
140	0.41992821	0.46802448	0.39176634	0.41560077	0.90669567	0.45460131
140.1	0.42006655	0.46811295	0.39187956	0.41574645	0.90669637	0.4547828
140.2	0.42020478	0.46820135	0.39199266	0.41589201	0.90669705	0.45496412
140.3	0.42034289	0.46828968	0.39210564	0.41603744	0.90669772	0.45514529
140.4	0.4204809	0.46837793	0.3922185	0.41618274	0.90669838	0.45532629
140.5	0.42061879	0.46846611	0.39233124	0.41632791	0.90669903	0.45550713
140.6	0.42075658	0.46855422	0.39244387	0.41647295	0.90669966	0.45568782
140.7	0.42089425	0.46864226	0.39255638	0.41661787	0.90670029	0.45586834
140.8	0.42103181	0.46873023	0.39266877	0.41676266	0.9067009	0.4560487
140.9	0.42116926	0.46881812	0.39278104	0.41690732	0.9067015	0.4562289
141	0.4213066	0.46890594	0.39289319	0.41705185	0.90670209	0.45640894

99	0.35201789	0.42455824	0.33295276	0.34308039	0.90460523	0.36447082
99.1	0.35221606	0.42468476	0.33313271	0.34329374	0.90461834	0.36473453
99.2	0.35241404	0.42481117	0.33331245	0.34350688	0.90463138	0.364998
99.3	0.35261183	0.42493746	0.33349197	0.34371982	0.90464436	0.36526122
99.4	0.35280943	0.42506363	0.33367127	0.34393254	0.90465727	0.36552419
99.5	0.35300684	0.42518968	0.33385036	0.34414506	0.90467012	0.36578692
99.6	0.35320407	0.42531562	0.33402923	0.34435737	0.90468289	0.3660494
99.7	0.35340111	0.42544144	0.33420789	0.34456947	0.90469561	0.36631164
99.8	0.35359795	0.42556715	0.33438634	0.34478136	0.90470825	0.36657364
99.9	0.35379461	0.42569273	0.33456457	0.34499305	0.90472083	0.36683539
100	0.35399109	0.42581821	0.33474259	0.34520452	0.90473335	0.3670969
100.1	0.35418737	0.42594357	0.33492039	0.34541579	0.9047458	0.36735816
100.2	0.35438347	0.42606881	0.33509798	0.34562686	0.90475819	0.36761919
100.3	0.35457939	0.42619393	0.33527536	0.34583772	0.90477051	0.36787996
100.4	0.35477511	0.42631894	0.33545253	0.34604837	0.90478277	0.3681405
100.5	0.35497065	0.42644384	0.33562948	0.34625881	0.90479497	0.36840079
100.6	0.355166	0.42656862	0.33580623	0.34646905	0.90480711	0.36866084
100.7	0.35536116	0.42669328	0.33598276	0.34667909	0.90481918	0.36892065
100.8	0.35555614	0.42681783	0.33615908	0.34688892	0.90483119	0.36918022
100.9	0.35575093	0.42694227	0.3363352	0.34709855	0.90484313	0.36943955
101	0.35594553	0.42706659	0.3365111	0.34730797	0.90485502	0.36969863
101.1	0.35613994	0.4271908	0.3366868	0.34751718	0.90486685	0.36995748
101.2	0.35633417	0.42731489	0.33686228	0.3477262	0.90487861	0.37021608
101.3	0.35652822	0.42743886	0.33703756	0.34793501	0.90489031	0.37047445
101.4	0.35672207	0.42756273	0.33721263	0.34814362	0.90490195	0.37073257
101.5	0.35691575	0.42768648	0.33738749	0.34835202	0.90491354	0.37099045
101.6	0.35710924	0.42781011	0.33756214	0.34856022	0.90492506	0.3712481
101.7	0.35730254	0.42793364	0.33773659	0.34876822	0.90493652	0.3715055
101.8	0.35749566	0.42805704	0.33791083	0.34897602	0.90494793	0.37176267
101.9	0.3576886	0.42818034	0.33808486	0.34918361	0.90495928	0.37201959
102	0.35788135	0.42830352	0.33825869	0.349391	0.90497056	0.37227628
102.1	0.35807392	0.42842659	0.33843231	0.3495982	0.90498179	0.37253273
102.2	0.35826631	0.42854955	0.33860573	0.34980519	0.90499296	0.37278894
102.3	0.35845851	0.42867239	0.33877894	0.35001198	0.90500408	0.37304492
102.4	0.35865053	0.42879512	0.33895194	0.35021857	0.90501514	0.37330066
102.5	0.35884237	0.42891774	0.33912475	0.35042496	0.90502614	0.37355615
102.6	0.35903403	0.42904024	0.33929734	0.35063114	0.90503708	0.37381142
102.7	0.35922551	0.42916263	0.33946974	0.35083713	0.90504797	0.37406644
102.8	0.35941681	0.42928491	0.33964192	0.35104292	0.9050588	0.37432123
102.9	0.35960792	0.42940708	0.33981391	0.35124852	0.90506958	0.37457578
103	0.35979886	0.42952914	0.33998569	0.35145391	0.9050803	0.3748301
103.1	0.35998961	0.42965108	0.34015727	0.3516591	0.90509097	0.37508418
103.2	0.36018019	0.42977292	0.34032865	0.3518641	0.90510158	0.37533803
103.3	0.36037058	0.42989464	0.34049983	0.35206889	0.90511214	0.37559164
103.4	0.3605608	0.43001625	0.3406708	0.35227349	0.90512264	0.37584501
103.5	0.36075083	0.43013775	0.34084157	0.35247789	0.90513309	0.37609815
103.6	0.36094069	0.43025914	0.34101214	0.3526821	0.90514349	0.37635106
103.7	0.36113037	0.43038041	0.34118251	0.3528861	0.90515383	0.37660373
103.8	0.36131987	0.43050158	0.34135268	0.35308992	0.90516412	0.37685617
103.9	0.3615092	0.43062263	0.34152265	0.35329353	0.90517436	0.37710837
104	0.36169834	0.43074358	0.34169242	0.35349695	0.90518455	0.37736034
104.1	0.36188731	0.43086441	0.34186199	0.35370017	0.90519468	0.37761208
104.2	0.3620761	0.43098514	0.34203136	0.35390319	0.90520477	0.37786358
104.3	0.36226472	0.43110575	0.34220054	0.35410602	0.9052148	0.37811485
104.4	0.36245315	0.43122625	0.34236951	0.35430866	0.90522478	0.37836589
104.5	0.36264142	0.43134665	0.34253828	0.3545111	0.90523471	0.37861669
104.6	0.3628295	0.43146693	0.34270686	0.35471334	0.9052446	0.37886727
104.7	0.36301741	0.43158711	0.34287524	0.35491539	0.90525443	0.37911761
104.8	0.36320515	0.43170717	0.34304342	0.35511724	0.90526421	0.37936772
104.9	0.36339271	0.43182712	0.3432114	0.3553189	0.90527394	0.3796176
150	0.4332284	0.47652573	0.40252404	0.4295563	0.90669865	0.47197705
150.1	0.43335615	0.47660735	0.40262611	0.42968982	0.90669796	0.47214319
150.2	0.4334838	0.4766889	0.40272808	0.42982323	0.90669726	0.47230918
150.3	0.43361135	0.47677039	0.40282994	0.42995653	0.90669654	0.47247502
150.4	0.4337388	0.47685181	0.4029317	0.43008971	0.9066958	0.47264072
150.5	0.43386614	0.47693317	0.40303336	0.43022277	0.90669506	0.47280627
150.6	0.43399339	0.47701446	0.40313491	0.43035573	0.90669429	0.47297167
150.7	0.43412054	0.47709569	0.40323636	0.43048856	0.90669352	0.47313694
150.8	0.43424759	0.47717686	0.40333771	0.43062129	0.90669273	0.47330205
150.9	0.43437454	0.47725796	0.40343895	0.43075389	0.90669193	0.47346702
151	0.43450139	0.477339	0.40354009	0.43088639	0.90669111	0.47363185
151.1	0.43462814	0.47741997	0.40364113	0.43101877	0.90669028	0.47379653
151.2	0.43475479	0.47750088	0.40374207	0.43115104	0.90668943	0.47396107
151.3	0.43488134	0.47758172	0.4038429	0.43128319	0.90668857	0.47412547
151.4	0.43500779	0.47766251	0.40394363	0.43141523	0.9066877	0.47428972
151.5	0.43513415	0.47774322	0.40404426	0.43154715	0.90668682	0.47445382
151.6	0.4352604	0.47782388	0.40414478	0.43167897	0.90668592	0.47461778
151.7	0.43538656	0.47790447	0.40424521	0.43181067	0.90668501	0.4747816
151.8	0.43551262	0.477985	0.40434553	0.43194225	0.90668408	0.47494528
151.9	0.43563858	0.47806546	0.40444575	0.43207373	0.90668314	0.47510881
152	0.43576444	0.47814586	0.40454587	0.43220509	0.90668219	0.4752722
152.1	0.4358902	0.4782262	0.40464589	0.43233633	0.90668122	0.47543544
152.2	0.43601586	0.47830647	0.40474581	0.43246747	0.90668024	0.47559854
152.3	0.43614143	0.47838669	0.40484562	0.43259849	0.90667925	0.4757615
152.4	0.4362669	0.47846683	0.40494534	0.4327294	0.90667824	0.47592432
152.5	0.43639227	0.47854692	0.40504495	0.4328602	0.90667722	0.476087
152.6	0.43651754	0.47862694	0.40514447	0.43299089	0.90667618	0.47624953
152.7	0.43664271	0.4787069	0.40524388	0.43312146	0.90667514	0.47641192
152.8	0.43676779	0.4787868	0.4053432	0.43325192	0.90667408	0.47657417
152.9	0.43689277	0.47886663	0.40544241	0.43338227	0.906673	0.47673627
153	0.43701765	0.4789464	0.40554152	0.43351251	0.90667192	0.47689824
153.1	0.43714244	0.47902611	0.40564054	0.43364264	0.90667082	0.47706006
153.2	0.43726713	0.47910576	0.40573945	0.43377265	0.90666971	0.47722174
153.3	0.43739172	0.47918534	0.40583827	0.43390256	0.90666858	0.47738328
153.4	0.43751621	0.47926486	0.40593698	0.43403235	0.90666744	0.47754468
153.5	0.43764061	0.47934432	0.4060356	0.43416203	0.90666629	0.47770593
153.6	0.43776491	0.47942372	0.40613412	0.4342916	0.90666513	0.47786705
153.7	0.43788911	0.47950305	0.40623253	0.43442106	0.90666395	0.47802803
153.8	0.43801322	0.47958233	0.40633085	0.43455041	0.90666276	0.47818886
153.9	0.43813723	0.47966154	0.40642907	0.43467965	0.90666155	0.47834955
154	0.43826114	0.47974069	0.4065272	0.43480879	0.90666034	0.47851011
154.1	0.43838496	0.47981977	0.40662522	0.43493781	0.90665911	0.47867052
154.2	0.43850868	0.4798988	0.40672314	0.43506672	0.90665787	0.47883079
154.3	0.43863231	0.47997776	0.40682097	0.43519552	0.90665661	0.47899093
154.4	0.43875584	0.48005667	0.4069187	0.43532421	0.90665535	0.47915092
154.5	0.43887927	0.48013551	0.40701633	0.43545279	0.90665407	0.47931077
154.6	0.43900261	0.48021428	0.40711387	0.43558126	0.90665278	0.47947049
154.7	0.43912585	0.480293	0.4072113	0.43570963	0.90665147	0.47963006
154.8	0.43924899	0.48037166	0.40730864	0.43583788	0.90665016	0.4797895
154.9	0.43937205	0.48045025	0.40740588	0.43596603	0.90664883	0.47994879
155	0.439495	0.48052879	0.40750302	0.43609406	0.90664748	0.48010795
155.1	0.43961786	0.48060726	0.40760007	0.43622199	0.90664613	0.48026697
155.2	0.43974063	0.48068567	0.40769702	0.43634981	0.90664476	0.48042585
155.3	0.4398633	0.48076402	0.40779387	0.43647752	0.90664339	0.48058459
155.4	0.43998587	0.48084231	0.40789063	0.43660512	0.906642	0.48074319
155.5	0.44010835	0.48092054	0.40798729	0.43673262	0.90664059	0.48090165
155.6	0.44023074	0.4809987	0.40808385	0.43686	0.90663918	0.48105998
155.7	0.44035303	0.48107681	0.40818032	0.43698728	0.90663775	0.48121817
155.8	0.44047523	0.48115485	0.40827669	0.43711445	0.90663631	0.48137622
155.9	0.44059733	0.48123284	0.40837296	0.43724152	0.90663486	0.48153413
156	0.44071934	0.48131076	0.40846914	0.43736847	0.9066334	0.4816919

114	0.37975771	0.44230086	0.35770691	0.37288585	0.90598214	0.40141752
114.1	0.37993014	0.44241128	0.35785788	0.37307057	0.90598819	0.40164708
114.2	0.38010241	0.4425216	0.35800868	0.37325512	0.9059942	0.40187643
114.3	0.38027453	0.44263182	0.3581593	0.3734395	0.90600019	0.40210557
114.4	0.3804465	0.44274194	0.35830975	0.3736237	0.90600613	0.4023345
114.5	0.38061831	0.44285196	0.35846003	0.37380772	0.90601204	0.40256322
114.6	0.38078997	0.44296189	0.35861013	0.37399158	0.90601792	0.40279173
114.7	0.38096147	0.44307172	0.35876006	0.37417526	0.90602376	0.40302003
114.8	0.38113282	0.44318145	0.35890983	0.37435876	0.90602957	0.40324812
114.9	0.38130402	0.44329109	0.35905941	0.3745421	0.90603534	0.40347601
115	0.38147507	0.44340063	0.35920883	0.37472526	0.90604109	0.40370368
115.1	0.38164596	0.44351007	0.35935808	0.37490824	0.90604679	0.40393114
115.2	0.3818167	0.44361942	0.35950716	0.37509106	0.90605246	0.4041584
115.3	0.38198728	0.44372867	0.35965606	0.3752737	0.9060581	0.40438545
115.4	0.38215772	0.44383782	0.35980479	0.37545617	0.90606371	0.40461229
115.5	0.382328	0.44394688	0.35995336	0.37563847	0.90606928	0.40483892
115.6	0.38249813	0.44405584	0.36010175	0.3758206	0.90607482	0.40506535
115.7	0.3826681	0.4441647	0.36024998	0.37600255	0.90608032	0.40529157
115.8	0.38283793	0.44427347	0.36039803	0.37618434	0.90608579	0.40551758
115.9	0.3830076	0.44438214	0.36054592	0.37636595	0.90609123	0.40574339
116	0.38317712	0.44449071	0.36069363	0.37654739	0.90609664	0.40596898
116.1	0.38334649	0.44459919	0.36084118	0.37672866	0.90610201	0.40619438
116.2	0.38351571	0.44470758	0.36098856	0.37690976	0.90610735	0.40641956
116.3	0.38368478	0.44481587	0.36113577	0.37709069	0.90611265	0.40664454
116.4	0.3838537	0.44492406	0.36128281	0.37727145	0.90611792	0.40686931
116.5	0.38402246	0.44503216	0.36142968	0.37745204	0.90612316	0.40709388
116.6	0.38419108	0.44514017	0.36157638	0.37763246	0.90612837	0.40731825
116.7	0.38435954	0.44524808	0.36172292	0.37781271	0.90613354	0.4075424
116.8	0.38452786	0.44535589	0.36186929	0.37799279	0.90613869	0.40776636
116.9	0.38469602	0.44546361	0.36201549	0.3781727	0.9061438	0.4079901
117	0.38486404	0.44557124	0.36216152	0.37835244	0.90614887	0.40821365
117.1	0.3850319	0.44567877	0.36230739	0.37853202	0.90615392	0.40843699
117.2	0.38519962	0.4457862	0.36245309	0.37871142	0.90615893	0.40866012
117.3	0.38536718	0.44589354	0.36259863	0.37889066	0.90616391	0.40888305
117.4	0.3855346	0.44600079	0.36274399	0.37906973	0.90616886	0.40910578
117.5	0.38570187	0.44610795	0.3628892	0.37924863	0.90617377	0.40932831
117.6	0.38586899	0.44621501	0.36303423	0.37942737	0.90617866	0.40955063
117.7	0.38603596	0.44632197	0.3631791	0.37960593	0.90618351	0.40977275
117.8	0.38620278	0.44642885	0.36332381	0.37978433	0.90618833	0.40999466
117.9	0.38636946	0.44653562	0.36346835	0.37996256	0.90619312	0.41021637
118	0.38653599	0.44664231	0.36361272	0.38014063	0.90619788	0.41043788
118.1	0.38670237	0.4467489	0.36375693	0.38031853	0.9062026	0.41065919
118.2	0.3868686	0.4468554	0.36390098	0.38049626	0.9062073	0.4108803
118.3	0.38703468	0.44696181	0.36404486	0.38067383	0.90621196	0.4111012
118.4	0.38720062	0.44706812	0.36418858	0.38085123	0.90621659	0.4113219
118.5	0.38736641	0.44717434	0.36433213	0.38102846	0.90622119	0.41154241
118.6	0.38753206	0.44728047	0.36447552	0.38120553	0.90622576	0.41176271
118.7	0.38769756	0.4473865	0.36461875	0.38138243	0.9062303	0.4119828
118.8	0.38786291	0.44749244	0.36476182	0.38155917	0.90623481	0.4122027
118.9	0.38802812	0.44759829	0.36490472	0.38173574	0.90623929	0.4124224
119	0.38819318	0.44770405	0.36504745	0.38191215	0.90624374	0.4126419
119.1	0.38835809	0.44780971	0.36519003	0.3820884	0.90624816	0.41286119
119.2	0.38852287	0.44791529	0.36533244	0.38226448	0.90625254	0.41308029
119.3	0.38868749	0.44802077	0.3654747	0.38244039	0.9062569	0.41329919
119.4	0.38885197	0.44812616	0.36561679	0.38261614	0.90626123	0.41351788
119.5	0.38901631	0.44823145	0.36575872	0.38279173	0.90626553	0.41373638
119.6	0.3891805	0.44833666	0.36590049	0.38296715	0.90626979	0.41395468
119.7	0.38934455	0.44844177	0.3660421	0.38314242	0.90627403	0.41417278
119.8	0.38950845	0.44854679	0.36618355	0.38331751	0.90627824	0.41439068
119.9	0.38967221	0.44865172	0.36632484	0.38349245	0.90628241	0.41460838
165	0.45132563	0.48808319	0.41674914	0.44836745	0.90645795	0.49534444
165.1	0.45143945	0.48815585	0.41683713	0.44848507	0.90645557	0.49549024
165.2	0.45155318	0.48822845	0.41692504	0.44860259	0.90645318	0.49563592
165.3	0.45166682	0.48830099	0.41701286	0.44872001	0.90645079	0.49578147
165.4	0.45178038	0.48837348	0.4171006	0.44883734	0.90644838	0.49592689
165.5	0.45189385	0.48844592	0.41718826	0.44895457	0.90644597	0.49607219
165.6	0.45200724	0.4885183	0.41727583	0.4490717	0.90644355	0.49621737
165.7	0.45212054	0.48859062	0.41736331	0.44918873	0.90644112	0.49636242
165.8	0.45223376	0.48866289	0.41745072	0.44930567	0.90643868	0.49650735
165.9	0.45234689	0.48873511	0.41753803	0.44942251	0.90643623	0.49665215
166	0.45245994	0.48880727	0.41762527	0.44953925	0.90643378	0.49679683
166.1	0.4525729	0.48887937	0.41771242	0.4496559	0.90643131	0.49694138
166.2	0.45268577	0.48895142	0.41779949	0.44977244	0.90642884	0.49708581
166.3	0.45279857	0.48902341	0.41788648	0.4498889	0.90642636	0.49723012
166.4	0.45291127	0.48909536	0.41797338	0.45000525	0.90642388	0.4973743
166.5	0.45302389	0.48916724	0.4180602	0.45012151	0.90642138	0.49751836
166.6	0.45313643	0.48923907	0.41814693	0.45023767	0.90641888	0.49766229
166.7	0.45324888	0.48931085	0.41823359	0.45035374	0.90641637	0.49780611
166.8	0.45336125	0.48938257	0.41832016	0.45046971	0.90641386	0.49794979
166.9	0.45347354	0.48945424	0.41840664	0.45058559	0.90641133	0.49809336
167	0.45358573	0.48952585	0.41849305	0.45070136	0.9064088	0.4982368
167.1	0.45369785	0.48959741	0.41857937	0.45081705	0.90640626	0.49838012
167.2	0.45380988	0.48966892	0.41866561	0.45093263	0.90640371	0.49852332
167.3	0.45392183	0.48974037	0.41875177	0.45104812	0.90640115	0.49866639
167.4	0.45403369	0.48981177	0.41883785	0.45116352	0.90639859	0.49880934
167.5	0.45414547	0.48988311	0.41892384	0.45127882	0.90639602	0.49895217
167.6	0.45425717	0.4899544	0.41900975	0.45139402	0.90639344	0.49909488
167.7	0.45436878	0.49002563	0.41909559	0.45150913	0.90639086	0.49923747
167.8	0.4544803	0.49009681	0.41918133	0.45162414	0.90638826	0.49937993
167.9	0.45459175	0.49016794	0.419267	0.45173906	0.90638566	0.49952227
168	0.45470311	0.49023901	0.41935259	0.45185389	0.90638305	0.49966449
168.1	0.45481439	0.49031003	0.41943809	0.45196861	0.90638044	0.49980659
168.2	0.45492558	0.490381	0.41952352	0.45208325	0.90637782	0.49994857
168.3	0.45503669	0.49045191	0.41960886	0.45219779	0.90637519	0.50009043
168.4	0.45514772	0.49052277	0.41969412	0.45231223	0.90637255	0.50023216
168.5	0.45525867	0.49059357	0.4197793	0.45242659	0.90636991	0.50037378
168.6	0.45536953	0.49066432	0.4198644	0.45254084	0.90636725	0.50051527
168.7	0.45548031	0.49073502	0.41994942	0.45265501	0.9063646	0.50065665
168.8	0.455591	0.49080567	0.42003436	0.45276908	0.90636193	0.5007979
168.9	0.45570162	0.49087626	0.42011922	0.45288305	0.90635926	0.50093903
169	0.45581215	0.4909468	0.420204	0.45299694	0.90635658	0.50108005
169.1	0.45592259	0.49101728	0.42028869	0.45311073	0.90635389	0.50122094
169.2	0.45603296	0.49108771	0.42037331	0.45322442	0.9063512	0.50136171
169.3	0.45614324	0.49115809	0.42045785	0.45333803	0.9063485	0.50150237
169.4	0.45625344	0.49122842	0.42054231	0.45345154	0.90634579	0.5016429
169.5	0.45636356	0.49129869	0.42062668	0.45356495	0.90634308	0.50178331
169.6	0.4564736	0.49136891	0.42071098	0.45367828	0.90634036	0.50192361
169.7	0.45658355	0.49143907	0.4207952	0.45379151	0.90633763	0.50206378
169.8	0.45669343	0.49150919	0.42087934	0.45390464	0.90633489	0.50220384
169.9	0.45680322	0.49157925	0.4209634	0.45401769	0.90633215	0.50234378
170	0.45691292	0.49164926	0.42104738	0.45413064	0.9063294	0.5024836
170.1	0.45702255	0.49171921	0.42113128	0.4542435	0.90632665	0.5026233
170.2	0.4571321	0.49178911	0.4212151	0.45435627	0.90632389	0.50276288
170.3	0.45724156	0.49185896	0.42129884	0.45446895	0.90632112	0.50290234
170.4	0.45735094	0.49192876	0.42138251	0.45458153	0.90631835	0.50304169
170.5	0.45746024	0.49199851	0.42146609	0.45469402	0.90631556	0.50318092
170.6	0.45756946	0.4920682	0.4215496	0.45480642	0.90631278	0.50332002
170.7	0.4576786	0.49213784	0.42163303	0.45491873	0.90630998	0.50345902
170.8	0.45778766	0.49220743	0.42171638	0.45503094	0.90630718	0.50359789
170.9	0.45789664	0.49227696	0.42179965	0.45514307	0.90630438	0.50373665
171	0.45800054	0.49234645	0.42188285	0.4552551	0.90630156	0.50387528

126.1	0.39955375	0.45498325	0.37478181	0.39402861	0.90648858	0.42772658
126.2	0.39970889	0.45508263	0.37491347	0.39419368	0.90649113	0.42793217
126.3	0.3998639	0.45518192	0.37504498	0.3943586	0.90649367	0.42813758
126.4	0.40001877	0.45528113	0.37517635	0.39452337	0.90649618	0.42834281
126.5	0.40017352	0.45538026	0.37530757	0.39468799	0.90649867	0.42854785
126.6	0.40032814	0.4554793	0.37543864	0.39485246	0.90650114	0.4287527
126.7	0.40048263	0.45557825	0.37556957	0.39501678	0.90650359	0.42895737
126.8	0.40063698	0.45567712	0.37570035	0.39518094	0.90650602	0.42916185
126.9	0.40079121	0.45577591	0.37583099	0.39534496	0.90650843	0.42936615
127	0.40094531	0.45587462	0.37596148	0.39550883	0.90651082	0.42957026
127.1	0.40109927	0.45597323	0.37609183	0.39567256	0.90651318	0.42977419
127.2	0.40125311	0.45607177	0.37622204	0.39583613	0.90651553	0.42997793
127.3	0.40140682	0.45617022	0.3763521	0.39599955	0.90651786	0.43018149
127.4	0.4015604	0.45626859	0.37648201	0.39616282	0.90652016	0.43038487
127.5	0.40171385	0.45636687	0.37661178	0.39632595	0.90652245	0.43058806
127.6	0.40186718	0.45646507	0.37674141	0.39648893	0.90652472	0.43079107
127.7	0.40202037	0.45656319	0.3768709	0.39665176	0.90652697	0.43099389
127.8	0.40217344	0.45666122	0.37700024	0.39681444	0.90652919	0.43119653
127.9	0.40232637	0.45675917	0.37712944	0.39697697	0.9065314	0.43139899
128	0.40247918	0.45685704	0.37725849	0.39713935	0.90653359	0.43160127
128.1	0.40263186	0.45695482	0.37738741	0.39730159	0.90653576	0.43180336
128.2	0.40278442	0.45705252	0.37751618	0.39746368	0.90653791	0.43200528
128.3	0.40293684	0.45715014	0.37764481	0.39762562	0.90654005	0.43220701
128.4	0.40308914	0.45724767	0.37777329	0.39778742	0.90654216	0.43240855
128.5	0.40324131	0.45734512	0.37790164	0.39794907	0.90654426	0.43260992
128.6	0.40339336	0.45744249	0.37802984	0.39811057	0.90654633	0.4328111
128.7	0.40354527	0.45753978	0.37815791	0.39827193	0.90654839	0.43301211
128.8	0.40369706	0.45763698	0.37828583	0.39843314	0.90655043	0.43321293
128.9	0.40384872	0.4577341	0.37841361	0.3985942	0.90655246	0.43341357
129	0.40400026	0.45783114	0.37854125	0.39875512	0.90655446	0.43361403
129.1	0.40415167	0.4579281	0.37866875	0.39891589	0.90655645	0.43381431
129.2	0.40430295	0.45802498	0.3787961	0.39907651	0.90655842	0.43401441
129.3	0.40445411	0.45812177	0.37892332	0.39923699	0.90656038	0.43421432
129.4	0.40460514	0.45821848	0.3790504	0.39939733	0.90656231	0.43441406
129.5	0.40475604	0.45831511	0.37917734	0.39955752	0.90656423	0.43461362
129.6	0.40490682	0.45841166	0.37930414	0.39971756	0.90656613	0.434813
129.7	0.40505748	0.45850813	0.3794308	0.39987746	0.90656802	0.4350122
129.8	0.405208	0.45860451	0.37955732	0.40003721	0.90656989	0.43521122
129.9	0.4053584	0.45870082	0.3796837	0.40019683	0.90657174	0.43541006
130	0.40550868	0.45879704	0.37980994	0.40035629	0.90657358	0.43560872
130.1	0.40565883	0.45889318	0.37993604	0.40051562	0.9065754	0.43580721
130.2	0.40580886	0.45898924	0.38006201	0.40067479	0.9065772	0.43600551
130.3	0.40595876	0.45908522	0.38018784	0.40083383	0.90657899	0.43620364
130.4	0.40610854	0.45918112	0.38031353	0.40099272	0.90658076	0.43640158
130.5	0.40625819	0.45927694	0.38043908	0.40115147	0.90658252	0.43659935
130.6	0.40640772	0.45937268	0.38056449	0.40131007	0.90658426	0.43679694
130.7	0.40655712	0.45946833	0.38068977	0.40146853	0.90658599	0.43699436
130.8	0.4067064	0.45956391	0.38081491	0.40162685	0.9065877	0.43719159
130.9	0.40685556	0.45965941	0.38093991	0.40178503	0.9065894	0.43738865
131	0.40700459	0.45975482	0.38106477	0.40194306	0.90659108	0.43758553
131.1	0.4071535	0.45985016	0.3811895	0.40210095	0.90659275	0.43778224
131.2	0.40730228	0.45994541	0.38131409	0.4022587	0.9065944	0.43797876
131.3	0.40745094	0.46004059	0.38143855	0.40241631	0.90659604	0.43817512
131.4	0.40759948	0.46013569	0.38156287	0.40257377	0.90659767	0.43837129
131.5	0.4077479	0.4602307	0.38168705	0.40273109	0.90659928	0.43856729
131.6	0.40789619	0.46032564	0.3818111	0.40288827	0.90660087	0.43876311
131.7	0.40804436	0.4604205	0.38193501	0.40304531	0.90660246	0.43895876
131.8	0.40819241	0.46051527	0.38205879	0.40320221	0.90660403	0.43915423
131.9	0.40834034	0.46060997	0.38218243	0.40335896	0.90660558	0.43934952
132	0.40848814	0.46070459	0.38230593	0.40351558	0.90660713	0.43954464
132.1	0.40863582	0.46079913	0.38242931	0.40367205	0.90660866	0.43973958
132.2	0.40878338	0.46089359	0.38255254	0.40382839	0.90661018	0.43993435
132.3	0.40893082	0.46098797	0.38267565	0.40398458	0.90661168	0.44012895
132.4	0.40907814	0.46108227	0.38279862	0.40414063	0.90661317	0.44032337
132.5	0.40922533	0.46117649	0.38292145	0.40429654	0.90661465	0.44051761
132.6	0.40937241	0.46127064	0.38304415	0.40445231	0.90661612	0.44071168
132.7	0.40951936	0.4613647	0.38316672	0.40460794	0.90661757	0.44090558
132.8	0.40966619	0.46145869	0.38328916	0.40476344	0.90661902	0.4410993
132.9	0.4098129	0.46155259	0.38341146	0.40491879	0.90662045	0.44129285
133	0.40995949	0.46164642	0.38353363	0.405074	0.90662186	0.44148622
133.1	0.41010596	0.46174017	0.38365566	0.40522907	0.90662327	0.44167942
133.2	0.4102523	0.46183385	0.38377756	0.405384	0.90662467	0.44187245
133.3	0.41039853	0.46192744	0.38389934	0.4055388	0.90662605	0.4420653
133.4	0.41054464	0.46202096	0.38402097	0.40569345	0.90662742	0.44225799
133.5	0.41069062	0.46211439	0.38414248	0.40584797	0.90662878	0.44245049
133.6	0.41083649	0.46220775	0.38426385	0.40600234	0.90663013	0.44264283
133.7	0.41098224	0.46230104	0.3843851	0.40615658	0.90663147	0.44283499
133.8	0.41112786	0.46239424	0.38450621	0.40631068	0.9066328	0.44302699
133.9	0.41127337	0.46248737	0.38462719	0.40646464	0.90663412	0.4432188
134	0.41141876	0.46258042	0.38474804	0.40661847	0.90663542	0.44341045
134.1	0.41156403	0.46267339	0.38486876	0.40677215	0.90663672	0.44360193
134.2	0.41170917	0.46276628	0.38498934	0.4069257	0.906638	0.44379323
134.3	0.4118542	0.4628591	0.3851098	0.40707911	0.90663928	0.44398436
134.4	0.41199912	0.46295184	0.38523013	0.40723238	0.90664054	0.44417532
134.5	0.41214391	0.4630445	0.38535032	0.40738552	0.90664179	0.44436611
134.6	0.41228858	0.46313709	0.38547039	0.40753852	0.90664304	0.44455673
134.7	0.41243314	0.46322959	0.38559032	0.40769138	0.90664427	0.44474718
134.8	0.41257757	0.46332203	0.38571013	0.4078441	0.90664549	0.44493746
134.9	0.41272189	0.46341438	0.38582981	0.40799669	0.90664671	0.44512757
180	0.46748498	0.49839235	0.42906201	0.46497432	0.90602572	0.51588846
180.1	0.46758684	0.49845729	0.42913848	0.46507841	0.90602244	0.51601696
180.2	0.46768863	0.49852218	0.42921489	0.46518241	0.90601915	0.51614535
180.3	0.46779035	0.49858702	0.42929122	0.46528633	0.90601586	0.51627364
180.4	0.46789199	0.49865181	0.42936749	0.46539016	0.90601257	0.51640182
180.5	0.46799356	0.49871656	0.42944368	0.46549392	0.90600928	0.5165299
180.6	0.46809506	0.49878126	0.42951981	0.46559759	0.90600598	0.51665786
180.7	0.46819648	0.49884591	0.42959587	0.46570117	0.90600267	0.51678573
180.8	0.46829783	0.49891052	0.42967185	0.46580468	0.90599936	0.51691349
180.9	0.46839911	0.49897507	0.42974777	0.4659081	0.90599605	0.51704114
181	0.46850031	0.49903958	0.42982362	0.46601144	0.90599274	0.51716869
181.1	0.46860144	0.49910405	0.42989941	0.4661147	0.90598942	0.51729614
181.2	0.4687025	0.49916846	0.42997512	0.46621788	0.90598609	0.51742348
181.3	0.46880348	0.49923283	0.43005076	0.46632097	0.90598277	0.51755071
181.4	0.46890439	0.49929715	0.43012634	0.46642399	0.90597944	0.51767785
181.5	0.46900523	0.49936142	0.43020185	0.46652692	0.9059761	0.51780487
181.6	0.469106	0.49942565	0.43027729	0.46662977	0.90597276	0.5179318
181.7	0.46920669	0.49948983	0.43035266	0.46673253	0.90596942	0.51805862
181.8	0.46930731	0.49955396	0.43042797	0.46683522	0.90596608	0.51818533
181.9	0.46940786	0.49961805	0.4305032	0.46693783	0.90596273	0.51831194
182	0.46950833	0.49968209	0.43057837	0.46704035	0.90595937	0.51843845
182.1	0.46960874	0.49974608	0.43065347	0.46714279	0.90595602	0.51856485
182.2	0.46970907	0.49981003	0.4307285	0.46724515	0.90595266	0.51869115
182.3	0.46980933	0.49987392	0.43080347	0.46734743	0.9059493	0.51881735
182.4	0.46990951	0.49993778	0.43087837	0.46744963	0.90594593	0.51894345
182.5	0.47000963	0.50000158	0.4309532	0.46755175	0.90594256	0.51906944
182.6	0.47010967	0.50006534	0.43102796	0.46765379	0.90593919	0.51919532
182.7	0.47020964	0.50012905	0.43110266	0.46775575	0.90593581	0.51932111
182.8	0.47030954	0.50019272	0.43117729	0.46785762	0.90593243	0.51944679
182.9	0.47040936	0.50025634	0.43125185	0.46795942	0.90592904	0.51957237
183	0.47050912	0.50031991	0.43132634	0.46806113	0.90592566	0.51969785
183.1	0.4706088	0.50038344	0.43140077	0.46816277	0.90592227	0.51982322

141.1	0.42144383	0.46899369	0.39300523	0.41719626	0.90670267	0.45658882
141.2	0.42158094	0.46908137	0.39311715	0.41734054	0.90670323	0.45676854
141.3	0.42171795	0.46916898	0.39322895	0.4174847	0.90670378	0.4569481
141.4	0.42185485	0.46925651	0.39334063	0.41762872	0.90670432	0.45712751
141.5	0.42199164	0.46934398	0.3934522	0.41777262	0.90670485	0.45730675
141.6	0.42212831	0.46943137	0.39356365	0.4179164	0.90670536	0.45748583
141.7	0.42226488	0.46951869	0.39367498	0.41806005	0.90670586	0.45766476
141.8	0.42240134	0.46960594	0.3937862	0.41820357	0.90670635	0.45784352
141.9	0.42253769	0.46969312	0.3938973	0.41834697	0.90670683	0.45802213
142	0.42267393	0.46978023	0.39400829	0.41849024	0.90670729	0.45820058
142.1	0.42281006	0.46986726	0.39411915	0.41863338	0.90670774	0.45837887
142.2	0.42294608	0.46995423	0.39422991	0.4187764	0.90670817	0.458557
142.3	0.42308199	0.47004112	0.39434054	0.41891929	0.9067086	0.45873497
142.4	0.42321779	0.47012795	0.39445106	0.41906206	0.90670901	0.45891279
142.5	0.42335348	0.4702147	0.39456147	0.41920471	0.90670941	0.45909045
142.6	0.42348906	0.47030139	0.39467176	0.41934723	0.90670979	0.45926795
142.7	0.42362454	0.470388	0.39478193	0.41948962	0.90671016	0.45944529
142.8	0.42375991	0.47047454	0.39489199	0.41963189	0.90671052	0.45962248
142.9	0.42389516	0.47056101	0.39500194	0.41977403	0.90671086	0.45979951
143	0.42403031	0.47064741	0.39511177	0.41991605	0.90671119	0.45997638
143.1	0.42416535	0.47073374	0.39522149	0.42005795	0.9067115	0.46015309
143.2	0.42430029	0.47082	0.39533109	0.42019972	0.90671181	0.46032965
143.3	0.42443511	0.4709062	0.39544058	0.42034137	0.90671209	0.46050606
143.4	0.42456983	0.47099232	0.39554995	0.42048289	0.90671237	0.4606823
143.5	0.42470444	0.47107837	0.39565921	0.42062429	0.90671263	0.46085839
143.6	0.42483894	0.47116435	0.39576836	0.42076557	0.90671287	0.46103433
143.7	0.42497333	0.47125026	0.39587739	0.42090672	0.90671311	0.46121011
143.8	0.42510762	0.4713361	0.39598631	0.42104775	0.90671333	0.46138573
143.9	0.4252418	0.47142188	0.39609512	0.42118866	0.90671353	0.4615612
144	0.42537587	0.47150758	0.39620381	0.42132945	0.90671372	0.46173651
144.1	0.42550984	0.47159321	0.39631239	0.42147011	0.9067139	0.46191167
144.2	0.42564369	0.47167878	0.39642086	0.42161065	0.90671406	0.46208667
144.3	0.42577744	0.47176427	0.39652921	0.42175106	0.9067142	0.46226152
144.4	0.42591109	0.4718497	0.39663746	0.42189135	0.90671434	0.46243622
144.5	0.42604463	0.47193506	0.39674559	0.42203152	0.90671446	0.46261076
144.6	0.42617806	0.47202035	0.3968536	0.42217157	0.90671456	0.46278514
144.7	0.42631138	0.47210557	0.39696151	0.4223115	0.90671465	0.46295937
144.8	0.4264446	0.47219072	0.39706931	0.4224513	0.90671473	0.46313345
144.9	0.42657772	0.4722758	0.39717699	0.42259099	0.90671479	0.46330737
145	0.42671072	0.47236081	0.39728456	0.42273055	0.90671484	0.46348114
145.1	0.42684362	0.47244576	0.39739202	0.42286999	0.90671487	0.46365476
145.2	0.42697642	0.47253064	0.39749937	0.42300931	0.90671489	0.46382822
145.3	0.42710911	0.47261544	0.39760661	0.4231485	0.9067149	0.46400153
145.4	0.42724169	0.47270018	0.39771374	0.42328758	0.90671488	0.46417469
145.5	0.42737417	0.47278486	0.39782075	0.42342653	0.90671486	0.46434769
145.6	0.42750655	0.47286946	0.39792766	0.42356537	0.90671482	0.46452054
145.7	0.42763881	0.472954	0.39803446	0.42370408	0.90671477	0.46469324
145.8	0.42777098	0.47303846	0.39814114	0.42384267	0.9067147	0.46486578
145.9	0.42790304	0.47312286	0.39824771	0.42398114	0.90671462	0.46503818
146	0.42803499	0.47320719	0.39835418	0.42411949	0.90671452	0.46521042
146.1	0.42816684	0.47329146	0.39846053	0.42425772	0.90671441	0.46538251
146.2	0.42829858	0.47337566	0.39856677	0.42439583	0.90671428	0.46555444
146.3	0.42843022	0.47345979	0.39867291	0.42453382	0.90671414	0.46572623
146.4	0.42856176	0.47354385	0.39877893	0.4246717	0.90671398	0.46589786
146.5	0.42869319	0.47362784	0.39888484	0.42480945	0.90671381	0.46606935
146.6	0.42882451	0.47371177	0.39899065	0.42494708	0.90671363	0.46624068
146.7	0.42895574	0.47379563	0.39909634	0.42508459	0.90671343	0.46641186
146.8	0.42908685	0.47387942	0.39920193	0.42522198	0.90671321	0.46658289
146.9	0.42921787	0.47396314	0.39930741	0.42535926	0.90671298	0.46675377
147	0.42934878	0.4740468	0.39941278	0.42549641	0.90671274	0.4669245
147.1	0.42947959	0.47413039	0.39951804	0.42563345	0.90671248	0.46709507
147.2	0.42961029	0.47421392	0.39962319	0.42577036	0.90671221	0.4672655
147.3	0.42974089	0.47429738	0.39972824	0.42590716	0.90671192	0.46743578
147.4	0.42987139	0.47438077	0.39983317	0.42604384	0.90671162	0.46760591
147.5	0.43000178	0.47446409	0.399938	0.4261804	0.9067113	0.46777588
147.6	0.43013207	0.47454735	0.40004272	0.42631684	0.90671097	0.46794571
147.7	0.43026226	0.47463054	0.40014734	0.42645317	0.90671062	0.46811539
147.8	0.43039235	0.47471366	0.40025184	0.42658937	0.90671026	0.46828492
147.9	0.43052233	0.47479672	0.40035624	0.42672546	0.90670988	0.4684543
148	0.43065221	0.47487971	0.40046053	0.42686143	0.90670949	0.46862353
148.1	0.43078198	0.47496264	0.40056471	0.42699729	0.90670909	0.46879261
148.2	0.43091166	0.4750455	0.40066879	0.42713302	0.90670867	0.46896154
148.3	0.43104123	0.47512829	0.40077276	0.42726864	0.90670824	0.46913033
148.4	0.4311707	0.47521102	0.40087662	0.42740414	0.90670779	0.46929896
148.5	0.43130007	0.47529368	0.40098038	0.42753952	0.90670733	0.46946745
148.6	0.43142933	0.47537627	0.40108403	0.42767479	0.90670685	0.46963579
148.7	0.4315585	0.4754588	0.40118757	0.42780994	0.90670636	0.46980398
148.8	0.43168756	0.47554127	0.40129101	0.42794497	0.90670585	0.46997202
148.9	0.43181652	0.47562366	0.40139434	0.42807989	0.90670533	0.47013992
149	0.43194538	0.475706	0.40149757	0.42821468	0.90670479	0.47030766
149.1	0.43207414	0.47578826	0.40160069	0.42834937	0.90670424	0.47047526
149.2	0.43220279	0.47587046	0.4017037	0.42848393	0.90670368	0.47064272
149.3	0.43233135	0.4759526	0.40180661	0.42861838	0.9067031	0.47081002
149.4	0.4324598	0.47603467	0.40190942	0.42875272	0.90670251	0.47097718
149.5	0.43258815	0.47611668	0.40201212	0.42888694	0.9067019	0.47114419
149.6	0.43271641	0.47619862	0.40211471	0.42902104	0.90670128	0.47131106
149.7	0.43284456	0.47628049	0.4022172	0.42915503	0.90670064	0.47147778
149.8	0.43297261	0.47636231	0.40231959	0.4292889	0.90669999	0.47164435
149.9	0.43310056	0.47644405	0.40242187	0.42942265	0.90669933	0.47181077
195	0.48197865	0.50762406	0.43980451	0.4797057	0.90550012	0.53404154
195.1	0.48207021	0.50768233	0.43987151	0.47979826	0.90549645	0.53415538
195.2	0.48216171	0.50774055	0.43993845	0.47989074	0.90549279	0.53426913
195.3	0.48225315	0.50779873	0.44000533	0.47998315	0.90548912	0.53438279
195.4	0.48234452	0.50785687	0.44007216	0.48007549	0.90548545	0.53449635
195.5	0.48243583	0.50791497	0.44013893	0.48016777	0.90548179	0.53460983
195.6	0.48252707	0.50797302	0.44020564	0.48025997	0.90547811	0.53472322
195.7	0.48261825	0.50803104	0.44027229	0.4803521	0.90547444	0.53483652
195.8	0.48270937	0.50808901	0.44033889	0.48044416	0.90547077	0.53494973
195.9	0.48280042	0.50814695	0.44040543	0.48053615	0.90546709	0.53506286
196	0.48289141	0.50820484	0.44047192	0.48062807	0.90546342	0.53517589
196.1	0.48298234	0.50826269	0.44053835	0.48071992	0.90545974	0.53528883
196.2	0.48307321	0.5083205	0.44060472	0.4808117	0.90545606	0.53540169
196.3	0.48316401	0.50837827	0.44067103	0.48090342	0.90545238	0.53551445
196.4	0.48325475	0.508436	0.44073729	0.48099506	0.9054487	0.53562713
196.5	0.48334542	0.50849368	0.44080349	0.48108663	0.90544502	0.53573972
196.6	0.48343603	0.50855133	0.44086964	0.48117813	0.90544134	0.53585221
196.7	0.48352659	0.50860893	0.44093573	0.48126956	0.90543765	0.53596463
196.8	0.48361707	0.5086665	0.44100176	0.48136092	0.90543397	0.53607695
196.9	0.4837075	0.50872402	0.44106774	0.48145222	0.90543028	0.53618918
197	0.48379786	0.50878151	0.44113366	0.48154344	0.90542659	0.53630133
197.1	0.48388816	0.50883895	0.44119953	0.4816346	0.9054229	0.53641338
197.2	0.4839784	0.50889635	0.44126533	0.48172568	0.90541921	0.53652535
197.3	0.48406857	0.50895371	0.44133109	0.4818167	0.90541552	0.53663724
197.4	0.48415868	0.50901103	0.44139679	0.48190765	0.90541183	0.53674903
197.5	0.48424873	0.50906831	0.44146243	0.48199852	0.90540813	0.53686073
197.6	0.48433872	0.50912555	0.44152802	0.48208933	0.90540444	0.53697235
197.7	0.48442864	0.50918275	0.44159355	0.48218007	0.90540074	0.53708388
197.8	0.48451851	0.50923991	0.44165902	0.48227074	0.90539705	0.53719533
197.9	0.48460831	0.50929703	0.44172444	0.48236135	0.90539335	0.53730668
198	0.48469805	0.50935411	0.44178981	0.48245188	0.90538965	0.53741795
198.1	0.48478772	0.50941115	0.44185512	0.48254234	0.90538595	0.53752913

156.1	0.44084125	0.48138862	0.40856522	0.43749532	0.90663192	0.48184954
156.2	0.44096307	0.48146643	0.40866121	0.43762206	0.90663044	0.48200704
156.3	0.44108479	0.48154417	0.4087571	0.43774869	0.90662894	0.4821644
156.4	0.44120642	0.48162185	0.4088529	0.43787521	0.90662743	0.48232162
156.5	0.44132796	0.48169947	0.4089486	0.43800163	0.90662591	0.48247871
156.6	0.4414494	0.48177703	0.4090442	0.43812794	0.90662437	0.48263566
156.7	0.44157075	0.48185453	0.40913971	0.43825415	0.90662283	0.48279248
156.8	0.44169201	0.48193198	0.40923513	0.43838024	0.90662127	0.48294915
156.9	0.44181317	0.48200936	0.40933045	0.43850624	0.9066197	0.48310569
157	0.44193424	0.48208668	0.40942567	0.43863212	0.90661812	0.4832621
157.1	0.44205522	0.48216394	0.40952081	0.4387579	0.90661653	0.48341837
157.2	0.4421761	0.48224114	0.40961584	0.43888357	0.90661493	0.4835745
157.3	0.44229689	0.48231828	0.40971079	0.43900913	0.90661331	0.4837305
157.4	0.44241758	0.48239536	0.40980564	0.43913459	0.90661169	0.48388636
157.5	0.44253819	0.48247238	0.40990039	0.43925995	0.90661005	0.48404208
157.6	0.4426587	0.48254934	0.40999506	0.43938519	0.9066084	0.48419767
157.7	0.44277912	0.48262625	0.41008963	0.43951033	0.90660674	0.48435313
157.8	0.44289944	0.48270309	0.4101841	0.43963537	0.90660507	0.48450844
157.9	0.44301968	0.48277987	0.41027849	0.4397603	0.90660339	0.48466363
158	0.44313982	0.4828566	0.41037278	0.43988513	0.9066017	0.48481868
158.1	0.44325987	0.48293326	0.41046697	0.44000985	0.90659999	0.48497359
158.2	0.44337982	0.48300987	0.41056108	0.44013446	0.90659828	0.48512837
158.3	0.44349969	0.48308641	0.41065509	0.44025897	0.90659655	0.48528301
158.4	0.44361946	0.4831629	0.41074901	0.44038337	0.90659482	0.48543752
158.5	0.44373914	0.48323933	0.41084284	0.44050767	0.90659307	0.4855919
158.6	0.44385873	0.4833157	0.41093657	0.44063187	0.90659131	0.48574614
158.7	0.44397823	0.48339201	0.41103021	0.44075596	0.90658954	0.48590025
158.8	0.44409763	0.48346826	0.41112376	0.44087994	0.90658776	0.48605422
158.9	0.44421695	0.48354445	0.41121722	0.44100382	0.90658597	0.48620806
159	0.44433617	0.48362058	0.41131059	0.4411276	0.90658417	0.48636177
159.1	0.4444553	0.48369666	0.41140386	0.44125127	0.90658235	0.48651534
159.2	0.44457434	0.48377267	0.41149705	0.44137484	0.90658053	0.48666878
159.3	0.44469329	0.48384863	0.41159014	0.4414983	0.9065787	0.48682208
159.4	0.44481215	0.48392453	0.41168314	0.44162167	0.90657685	0.48697526
159.5	0.44493091	0.48400037	0.41177605	0.44174492	0.906575	0.4871283
159.6	0.44504959	0.48407615	0.41186887	0.44186807	0.90657313	0.4872812
159.7	0.44516817	0.48415187	0.41196159	0.44199112	0.90657126	0.48743398
159.8	0.44528667	0.48422754	0.41205423	0.44211407	0.90656937	0.48758662
159.9	0.44540507	0.48430315	0.41214677	0.44223691	0.90656748	0.48773913
160	0.44552339	0.48437869	0.41223923	0.44235965	0.90656557	0.4878915
160.1	0.44564161	0.48445418	0.41233159	0.44248229	0.90656365	0.48804375
160.2	0.44575974	0.48452962	0.41242387	0.44260482	0.90656172	0.48819586
160.3	0.44587778	0.48460499	0.41251605	0.44272725	0.90655979	0.48834784
160.4	0.44599574	0.48468031	0.41260815	0.44284958	0.90655784	0.48849968
160.5	0.4461136	0.48475557	0.41270015	0.44297181	0.90655588	0.4886514
160.6	0.44623137	0.48483077	0.41279206	0.44309393	0.90655391	0.48880298
160.7	0.44634905	0.48490591	0.41288389	0.44321595	0.90655194	0.48895444
160.8	0.44646665	0.484981	0.41297562	0.44333787	0.90654995	0.48910576
160.9	0.44658415	0.48505603	0.41306727	0.44345968	0.90654795	0.48925695
161	0.44670156	0.485131	0.41315882	0.4435814	0.90654594	0.48940801
161.1	0.44681889	0.48520591	0.41325029	0.44370301	0.90654393	0.48955894
161.2	0.44693612	0.48528076	0.41334167	0.44382452	0.9065419	0.48970973
161.3	0.44705327	0.48535556	0.41343296	0.44394592	0.90653986	0.4898604
161.4	0.44717032	0.4854303	0.41352416	0.44406723	0.90653781	0.49001094
161.5	0.44728729	0.48550498	0.41361527	0.44418844	0.90653576	0.49016134
161.6	0.44740417	0.48557961	0.41370629	0.44430954	0.90653369	0.49031162
161.7	0.44752095	0.48565418	0.41379722	0.44443054	0.90653162	0.49046176
161.8	0.44763765	0.48572869	0.41388807	0.44455144	0.90652953	0.49061178
161.9	0.44775426	0.48580315	0.41397883	0.44467224	0.90652743	0.49076166
162	0.44787079	0.48587754	0.41406949	0.44479294	0.90652533	0.49091142
162.1	0.44798722	0.48595188	0.41416008	0.44491354	0.90652322	0.49106105
162.2	0.44810356	0.48602617	0.41425057	0.44503403	0.90652109	0.49121054
162.3	0.44821982	0.48610039	0.41434097	0.44515443	0.90651896	0.49135991
162.4	0.44833599	0.48617456	0.41443129	0.44527473	0.90651682	0.49150915
162.5	0.44845207	0.48624867	0.41452152	0.44539492	0.90651467	0.49165826
162.6	0.44856806	0.48632273	0.41461166	0.44551502	0.9065125	0.49180724
162.7	0.44868396	0.48639673	0.41470172	0.44563501	0.90651033	0.49195609
162.8	0.44879978	0.48647067	0.41479169	0.4457549	0.90650816	0.49210481
162.9	0.44891551	0.48654456	0.41488157	0.4458747	0.90650597	0.49225341
163	0.44903115	0.48661839	0.41497136	0.44599439	0.90650377	0.49240187
163.1	0.4491467	0.48669216	0.41506107	0.44611399	0.90650156	0.49255021
163.2	0.44926216	0.48676588	0.41515069	0.44623348	0.90649935	0.49269842
163.3	0.44937754	0.48683954	0.41524022	0.44635288	0.90649712	0.4928465
163.4	0.44949283	0.48691314	0.41532967	0.44647217	0.90649489	0.49299445
163.5	0.44960803	0.48698669	0.41541903	0.44659137	0.90649264	0.49314228
163.6	0.44972314	0.48706018	0.4155083	0.44671047	0.90649039	0.49328998
163.7	0.44983817	0.48713362	0.41559749	0.44682946	0.90648813	0.49343755
163.8	0.44995311	0.487207	0.41568659	0.44694836	0.90648586	0.49358499
163.9	0.45006796	0.48728032	0.41577561	0.44706716	0.90648358	0.49373231
164	0.45018273	0.48735359	0.41586454	0.44718586	0.9064813	0.4938795
164.1	0.45029741	0.4874268	0.41595338	0.44730446	0.906479	0.49402656
164.2	0.450412	0.48749995	0.41604214	0.44742297	0.9064767	0.4941735
164.3	0.45052651	0.48757305	0.41613082	0.44754137	0.90647438	0.49432031
164.4	0.45064093	0.4876461	0.4162194	0.44765968	0.90647206	0.49446699
164.5	0.45075526	0.48771909	0.41630791	0.44777788	0.90646973	0.49461355
164.6	0.45086951	0.48779202	0.41639632	0.44789599	0.90646739	0.49475998
164.7	0.45098367	0.4878649	0.41648465	0.448014	0.90646505	0.49490628
164.8	0.45109774	0.48793772	0.4165729	0.44813192	0.90646269	0.49505246
164.9	0.45121173	0.48801048	0.41666106	0.44824973	0.90646033	0.49519851
210	0.49503345	0.51592123	0.44924983	0.49283411	0.90493929	0.55016305
210.1	0.49511611	0.51597369	0.44930895	0.49291677	0.90493549	0.55026442
210.2	0.49519871	0.51602611	0.44936802	0.49299937	0.90493169	0.5503657
210.3	0.49528126	0.5160785	0.44942705	0.49308191	0.9049279	0.55046691
210.4	0.49536375	0.51613084	0.44948603	0.49316439	0.9049241	0.55056804
210.5	0.49544619	0.51618316	0.44954496	0.49324681	0.9049203	0.5506691
210.6	0.49552857	0.51623543	0.44960384	0.49332917	0.9049165	0.55077007
210.7	0.49561089	0.51628767	0.44966268	0.49341146	0.9049127	0.55087098
210.8	0.49569316	0.51633988	0.44972147	0.49349369	0.9049089	0.5509718
210.9	0.49577538	0.51639205	0.44978021	0.49357587	0.9049051	0.55107255
211	0.49585754	0.51644418	0.4498389	0.49365798	0.9049013	0.55117323
211.1	0.49593965	0.51649628	0.44989755	0.49374003	0.9048975	0.55127382
211.2	0.4960217	0.51654834	0.44995614	0.49382202	0.90489369	0.55137434
211.3	0.4961037	0.51660036	0.4500147	0.49390395	0.90488989	0.55147479
211.4	0.49618564	0.51665235	0.4500732	0.49398582	0.90488609	0.55157516
211.5	0.49626753	0.51670431	0.45013166	0.49406763	0.90488228	0.55167545
211.6	0.49634936	0.51675623	0.45019006	0.49414938	0.90487848	0.55177567
211.7	0.49643114	0.51680811	0.45024843	0.49423107	0.90487467	0.55187581
211.8	0.49651287	0.51685996	0.45030674	0.4943127	0.90487087	0.55197588
211.9	0.49659454	0.51691177	0.45036501	0.49439426	0.90486706	0.55207587
212	0.49667616	0.51696355	0.45042323	0.49447577	0.90486326	0.55217579
212.1	0.49675772	0.51701529	0.45048141	0.49455722	0.90485945	0.55227563
212.2	0.49683923	0.51706699	0.45053953	0.4946386	0.90485564	0.55237539
212.3	0.49692068	0.51711866	0.45059761	0.49471993	0.90485183	0.55247508
212.4	0.49700208	0.5171703	0.45065565	0.4948012	0.90484802	0.5525747
212.5	0.49708343	0.5172219	0.45071363	0.4948824	0.90484421	0.55267424
212.6	0.49716472	0.51727346	0.45077157	0.49496355	0.90484041	0.5527737
212.7	0.49724596	0.51732499	0.45082947	0.49504464	0.9048366	0.5528731
212.8	0.49732714	0.51737648	0.45088732	0.49512567	0.90483278	0.55297241
212.9	0.49740827	0.51742794	0.45094512	0.49520663	0.90482897	0.55307165
213	0.49748935	0.51747937	0.45100287	0.49528754	0.90482516	0.55317082
213.1	0.49757037	0.51753075	0.45106058	0.49536839	0.90482135	0.55326991

171.1	0.45811435	0.49241588	0.42196596	0.45536704	0.90629874	0.50401381
171.2	0.45822309	0.49248526	0.422049	0.45547889	0.90629592	0.50415221
171.3	0.45833174	0.49255458	0.42213196	0.45559065	0.90629308	0.5042905
171.4	0.45844031	0.49262386	0.42221484	0.45570231	0.90629025	0.50442867
171.5	0.45854881	0.49269308	0.42229764	0.45581389	0.9062874	0.50456672
171.6	0.45865722	0.49276225	0.42238037	0.45592537	0.90628455	0.50470466
171.7	0.45876555	0.49283137	0.42246302	0.45603676	0.90628169	0.50484248
171.8	0.45887381	0.49290044	0.42254559	0.45614806	0.90627883	0.50498018
171.9	0.45898198	0.49296945	0.42262808	0.45625928	0.90627596	0.50511777
172	0.45909007	0.49303842	0.4227105	0.4563704	0.90627308	0.50525524
172.1	0.45919808	0.49310733	0.42279284	0.45648143	0.9062702	0.5053926
172.2	0.45930601	0.49317619	0.4228751	0.45659237	0.90626732	0.50552984
172.3	0.45941387	0.493245	0.42295729	0.45670321	0.90626442	0.50566696
172.4	0.45952164	0.49331376	0.4230394	0.45681397	0.90626152	0.50580397
172.5	0.45962933	0.49338246	0.42312143	0.45692464	0.90625862	0.50594086
172.6	0.45973695	0.49345112	0.42320339	0.45703522	0.9062557	0.50607764
172.7	0.45984448	0.49351972	0.42328526	0.45714571	0.90625279	0.5062143
172.8	0.45995194	0.49358827	0.42336707	0.4572561	0.90624986	0.50635085
172.9	0.46005931	0.49365677	0.42344879	0.45736641	0.90624693	0.50648729
173	0.46016661	0.49372522	0.42353044	0.45747663	0.906244	0.5066236
173.1	0.46027382	0.49379362	0.42361202	0.45758676	0.90624106	0.50675981
173.2	0.46038096	0.49386197	0.42369351	0.4576968	0.90623811	0.5068959
173.3	0.46048802	0.49393026	0.42377493	0.45780674	0.90623516	0.50703187
173.4	0.460595	0.49399851	0.42385628	0.4579166	0.9062322	0.50716773
173.5	0.4607019	0.4940667	0.42393755	0.45802637	0.90622924	0.50730348
173.6	0.46080873	0.49413485	0.42401874	0.45813605	0.90622627	0.50743911
173.7	0.46091547	0.49420294	0.42409986	0.45824565	0.90622329	0.50757463
173.8	0.46102213	0.49427098	0.4241809	0.45835515	0.90622031	0.50771004
173.9	0.46112872	0.49433897	0.42426187	0.45846456	0.90621733	0.50784533
174	0.46123523	0.49440691	0.42434276	0.45857389	0.90621434	0.50798051
174.1	0.46134166	0.4944748	0.42442358	0.45868312	0.90621134	0.50811557
174.2	0.46144801	0.49454264	0.42450432	0.45879227	0.90620834	0.50825053
174.3	0.46155428	0.49461043	0.42458499	0.45890133	0.90620533	0.50838537
174.4	0.46166048	0.49467817	0.42466558	0.4590103	0.90620232	0.50852009
174.5	0.4617666	0.49474585	0.4247461	0.45911918	0.9061993	0.5086547
174.6	0.46187263	0.49481349	0.42482654	0.45922797	0.90619627	0.50878921
174.7	0.46197859	0.49488108	0.4249069	0.45933668	0.90619324	0.50892359
174.8	0.46208448	0.49494861	0.4249872	0.45944529	0.90619021	0.50905787
174.9	0.46219028	0.4950161	0.42506741	0.45955382	0.90618717	0.50919203
175	0.46229601	0.49508354	0.42514756	0.45966226	0.90618412	0.50932608
175.1	0.46240166	0.49515092	0.42522763	0.45977061	0.90618107	0.50946002
175.2	0.46250723	0.49521826	0.42530762	0.45987888	0.90617802	0.50959385
175.3	0.46261273	0.49528554	0.42538754	0.45998706	0.90617496	0.50972757
175.4	0.46271815	0.49535278	0.42546739	0.46009515	0.90617189	0.50986117
175.5	0.46282349	0.49541996	0.42554716	0.46020315	0.90616882	0.50999467
175.6	0.46292875	0.4954871	0.42562686	0.46031106	0.90616574	0.51012805
175.7	0.46303393	0.49555418	0.42570649	0.46041889	0.90616266	0.51026132
175.8	0.46313904	0.49562122	0.42578604	0.46052663	0.90615958	0.51039448
175.9	0.46324408	0.49568821	0.42586552	0.46063429	0.90615648	0.51052752
176	0.46334903	0.49575514	0.42594492	0.46074185	0.90615339	0.51066046
176.1	0.46345391	0.49582203	0.42602426	0.46084933	0.90615029	0.51079329
176.2	0.46355871	0.49588887	0.42610351	0.46095673	0.90614718	0.510926
176.3	0.46366343	0.49595565	0.4261827	0.46106403	0.90614407	0.51105861
176.4	0.46376808	0.49602239	0.42626181	0.46117125	0.90614095	0.5111911
176.5	0.46387265	0.49608908	0.42634085	0.46127839	0.90613783	0.51132349
176.6	0.46397715	0.49615572	0.42641982	0.46138543	0.90613471	0.51145576
176.7	0.46408156	0.49622231	0.42649872	0.46149239	0.90613158	0.51158793
176.8	0.46418591	0.49628885	0.42657754	0.46159927	0.90612844	0.51171998
176.9	0.46429017	0.49635534	0.42665629	0.46170606	0.9061253	0.51185193
177	0.46439436	0.49642178	0.42673497	0.46181276	0.90612216	0.51198377
177.1	0.46449847	0.49648817	0.42681357	0.46191938	0.90611901	0.51211549
177.2	0.46460251	0.49655451	0.4268921	0.46202591	0.90611585	0.51224711
177.3	0.46470647	0.49662081	0.42697057	0.46213235	0.90611269	0.51237862
177.4	0.46481036	0.49668705	0.42704895	0.46223871	0.90610953	0.51251001
177.5	0.46491417	0.49675325	0.42712727	0.46234499	0.90610636	0.5126413
177.6	0.4650179	0.4968194	0.42720552	0.46245117	0.90610319	0.51277248
177.7	0.46512156	0.49688549	0.42728369	0.46255728	0.90610001	0.51290356
177.8	0.46522514	0.49695154	0.42736179	0.4626633	0.90609683	0.51303452
177.9	0.46532865	0.49701754	0.42743982	0.46276923	0.90609364	0.51316537
178	0.46543208	0.49708349	0.42751778	0.46287508	0.90609045	0.51329612
178.1	0.46553544	0.4971494	0.42759567	0.46298084	0.90608726	0.51342676
178.2	0.46563872	0.49721525	0.42767348	0.46308652	0.90608406	0.51355729
178.3	0.46574192	0.49728106	0.42775123	0.46319211	0.90608085	0.51368771
178.4	0.46584505	0.49734681	0.4278289	0.46329762	0.90607764	0.51381802
178.5	0.46594811	0.49741252	0.4279065	0.46340304	0.90607443	0.51394823
178.6	0.46605109	0.49747818	0.42798403	0.46350838	0.90607121	0.51407833
178.7	0.46615399	0.49754379	0.42806148	0.46361364	0.90606799	0.51420832
178.8	0.46625682	0.49760935	0.42813887	0.46371881	0.90606476	0.5143382
178.9	0.46635958	0.49767487	0.42821619	0.4638239	0.90606153	0.51446798
179	0.46646226	0.49774033	0.42829343	0.4639289	0.9060583	0.51459765
179.1	0.46656486	0.49780575	0.42837061	0.46403382	0.90605506	0.51472721
179.2	0.4666674	0.49787112	0.42844771	0.46413865	0.90605181	0.51485666
179.3	0.46676985	0.49793644	0.42852474	0.46424341	0.90604857	0.51498601
179.4	0.46687224	0.49800172	0.42860171	0.46434807	0.90604531	0.51511525
179.5	0.46697454	0.49806694	0.4286786	0.46445266	0.90604206	0.51524439
179.6	0.46707678	0.49813212	0.42875542	0.46455716	0.9060388	0.51537341
179.7	0.46717894	0.49819725	0.42883217	0.46466157	0.90603553	0.51550234
179.8	0.46728103	0.49826233	0.42890886	0.46476591	0.90603227	0.51563115
179.9	0.46738304	0.49832736	0.42898547	0.46487016	0.90602899	0.51575986
225	0.50684249	0.52340327	0.45761105	0.50458275	0.9043655	0.56455013
225.1	0.50691742	0.52345064	0.45766355	0.5046569	0.90436166	0.56464079
225.2	0.5069923	0.52349798	0.45771601	0.504731	0.90435782	0.56473138
225.3	0.50706714	0.52354529	0.45776843	0.50480506	0.90435398	0.56482191
225.4	0.50714192	0.52359257	0.45782081	0.50487905	0.90435014	0.56491237
225.5	0.50721666	0.52363982	0.45787315	0.504953	0.9043463	0.56500276
225.6	0.50729135	0.52368703	0.45792545	0.50502689	0.90434246	0.56509309
225.7	0.50736599	0.52373422	0.45797771	0.50510074	0.90433862	0.56518336
225.8	0.50744058	0.52378137	0.45802992	0.50517453	0.90433478	0.56527355
225.9	0.50751513	0.52382849	0.4580821	0.50524826	0.90433094	0.56536368
226	0.50758963	0.52387557	0.45813424	0.50532195	0.9043271	0.56545375
226.1	0.50766408	0.52392263	0.45818633	0.50539558	0.90432326	0.56554375
226.2	0.50773848	0.52396965	0.45823839	0.50546916	0.90431942	0.56563369
226.3	0.50781284	0.52401665	0.45829041	0.50554269	0.90431558	0.56572356
226.4	0.50788715	0.52406361	0.45834238	0.50561617	0.90431174	0.56581336
226.5	0.50796141	0.52411054	0.45839432	0.5056896	0.9043079	0.5659031
226.6	0.50803562	0.52415743	0.45844622	0.50576297	0.90430406	0.56599277
226.7	0.50810979	0.5242043	0.45849807	0.50583629	0.90430022	0.56608238
226.8	0.50818391	0.52425114	0.45854989	0.50590956	0.90429638	0.56617192
226.9	0.50825798	0.52429794	0.45860167	0.50598278	0.90429254	0.5662614
227	0.508332	0.52434471	0.4586534	0.50605595	0.9042887	0.56635081
227.1	0.50840598	0.52439145	0.4587051	0.50612907	0.90428486	0.56644016
227.2	0.50847991	0.52443816	0.45875676	0.50620213	0.90428102	0.56652944
227.3	0.50855379	0.52448484	0.45880838	0.50627514	0.90427718	0.56661866
227.4	0.50862763	0.52453148	0.45885995	0.50634811	0.90427334	0.56670781
227.5	0.50870141	0.5245781	0.45891149	0.50642102	0.9042695	0.5667969
227.6	0.50877516	0.52462468	0.45896299	0.50649387	0.90426566	0.56688593
227.7	0.50884885	0.52467123	0.45901445	0.50656668	0.90426182	0.56697488
227.8	0.5089225	0.52471775	0.45906587	0.50663944	0.90425798	0.56706378
227.9	0.5089961	0.52476424	0.45911725	0.50671214	0.90425414	0.56715261
228	0.50906965	0.5248107	0.45916859	0.5067848	0.90425031	0.56724137
228.1	0.50914316	0.52485712	0.45921989	0.5068574	0.90424647	0.56733008

183.2	0.47070841	0.50044692	0.43147513	0.46826432	0.90591887	0.51994849
183.3	0.47080795	0.50051035	0.43154943	0.4683658	0.90591547	0.52007366
183.4	0.47090742	0.50057374	0.43162365	0.46846719	0.90591207	0.52019873
183.5	0.47100682	0.50063708	0.43169782	0.46856851	0.90590867	0.52032369
183.6	0.47110615	0.50070037	0.43177191	0.46866974	0.90590526	0.52044856
183.7	0.4712054	0.50076362	0.43184594	0.4687709	0.90590185	0.52057332
183.8	0.47130459	0.50082683	0.4319199	0.46887197	0.90589844	0.52069798
183.9	0.4714037	0.50088998	0.4319938	0.46897297	0.90589502	0.52082254
184	0.47150274	0.50095309	0.43206763	0.46907388	0.9058916	0.52094699
184.1	0.47160171	0.50101616	0.4321414	0.46917472	0.90588818	0.52107135
184.2	0.47170061	0.50107918	0.4322151	0.46927547	0.90588475	0.5211956
184.3	0.47179944	0.50114215	0.43228873	0.46937615	0.90588132	0.52131976
184.4	0.4718982	0.50120508	0.4323623	0.46947675	0.90587789	0.52144381
184.5	0.47199689	0.50126796	0.4324358	0.46957727	0.90587445	0.52156776
184.6	0.4720955	0.50133079	0.43250924	0.46967771	0.90587101	0.52169161
184.7	0.47219405	0.50139358	0.43258261	0.46977807	0.90586757	0.52181536
184.8	0.47229253	0.50145633	0.43265591	0.46987835	0.90586413	0.52193901
184.9	0.47239093	0.50151903	0.43272916	0.46997855	0.90586068	0.52206256
185	0.47248927	0.50158168	0.43280233	0.47007868	0.90585723	0.522186
185.1	0.47258753	0.50164429	0.43287544	0.47017872	0.90585377	0.52230935
185.2	0.47268573	0.50170685	0.43294849	0.47027869	0.90585032	0.5224326
185.3	0.47278385	0.50176937	0.43302147	0.47037858	0.90584686	0.52255575
185.4	0.47288191	0.50183184	0.43309439	0.47047839	0.90584339	0.52267879
185.5	0.47297989	0.50189426	0.43316724	0.47057812	0.90583993	0.52280174
185.6	0.47307781	0.50195665	0.43324002	0.47067777	0.90583646	0.52292459
185.7	0.47317565	0.50201898	0.43331275	0.47077735	0.90583299	0.52304734
185.8	0.47327343	0.50208127	0.4333854	0.47087685	0.90582952	0.52316998
185.9	0.47337113	0.50214352	0.433458	0.47097626	0.90582604	0.52329253
186	0.47346877	0.50220572	0.43353053	0.47107561	0.90582256	0.52341498
186.1	0.47356633	0.50226787	0.43360299	0.47117487	0.90581908	0.52353733
186.2	0.47366383	0.50232998	0.43367539	0.47127405	0.90581559	0.52365958
186.3	0.47376126	0.50239205	0.43374773	0.47137316	0.9058121	0.52378173
186.4	0.47385861	0.50245406	0.43382	0.47147219	0.90580861	0.52390379
186.5	0.4739559	0.50251604	0.43389221	0.47157114	0.90580512	0.52402574
186.6	0.47405312	0.50257797	0.43396436	0.47167002	0.90580162	0.5241476
186.7	0.47415027	0.50263985	0.43403644	0.47176882	0.90579812	0.52426935
186.8	0.47424735	0.5027017	0.43410846	0.47186754	0.90579462	0.52439101
186.9	0.47434436	0.50276349	0.43418041	0.47196618	0.90579112	0.52451257
187	0.47444131	0.50282524	0.4342523	0.47206474	0.90578761	0.52463403
187.1	0.47453818	0.50288695	0.43432413	0.47216323	0.9057841	0.5247554
187.2	0.47463498	0.50294861	0.4343959	0.47226164	0.90578059	0.52487666
187.3	0.47473172	0.50301023	0.4344676	0.47235998	0.90577708	0.52499783
187.4	0.47482839	0.5030718	0.43453924	0.47245823	0.90577356	0.5251189
187.5	0.47492498	0.50313333	0.43461081	0.47255641	0.90577004	0.52523987
187.6	0.47502151	0.50319481	0.43468232	0.47265452	0.90576652	0.52536075
187.7	0.47511798	0.50325625	0.43475377	0.47275255	0.90576299	0.52548152
187.8	0.47521437	0.50331764	0.43482516	0.4728505	0.90575947	0.5256022
187.9	0.47531069	0.50337899	0.43489648	0.47294837	0.90575594	0.52572278
188	0.47540695	0.5034403	0.43496775	0.47304617	0.90575241	0.52584327
188.1	0.47550314	0.50350156	0.43503895	0.47314389	0.90574887	0.52596365
188.2	0.47559925	0.50356278	0.43511008	0.47324153	0.90574534	0.52608394
188.3	0.47569531	0.50362395	0.43518116	0.47333909	0.9057418	0.52620414
188.4	0.47579129	0.50368508	0.43525217	0.47343658	0.90573826	0.52632423
188.5	0.4758872	0.50374617	0.43532312	0.473534	0.90573471	0.52644423
188.6	0.47598305	0.50380721	0.43539401	0.47363133	0.90573117	0.52656414
188.7	0.47607883	0.50386821	0.43546483	0.47372859	0.90572762	0.52668394
188.8	0.47617454	0.50392916	0.4355356	0.47382578	0.90572407	0.52680365
188.9	0.47627018	0.50399007	0.4356063	0.47392288	0.90572051	0.52692326
189	0.47636576	0.50405093	0.43567694	0.47401991	0.90571696	0.52704278
189.1	0.47646127	0.50411176	0.43574751	0.47411687	0.9057134	0.5271622
189.2	0.47655671	0.50417253	0.43581803	0.47421375	0.90570984	0.52728153
189.3	0.47665208	0.50423327	0.43588848	0.47431055	0.90570628	0.52740076
189.4	0.47674738	0.50429396	0.43595887	0.47440728	0.90570272	0.52751989
189.5	0.47684262	0.5043546	0.4360292	0.47450393	0.90569915	0.52763893
189.6	0.47693779	0.50441521	0.43609947	0.47460051	0.90569558	0.52775787
189.7	0.47703289	0.50447577	0.43616967	0.47469701	0.90569201	0.52787672
189.8	0.47712793	0.50453628	0.43623982	0.47479343	0.90568844	0.52799547
189.9	0.4772229	0.50459675	0.4363099	0.47488978	0.90568486	0.52811412
190	0.4773178	0.50465718	0.43637992	0.47498605	0.90568129	0.52823268
190.1	0.47741263	0.50471757	0.43644988	0.47508225	0.90567771	0.52835115
190.2	0.4775074	0.50477791	0.43651978	0.47517837	0.90567413	0.52846952
190.3	0.4776021	0.50483821	0.43658962	0.47527442	0.90567054	0.52858779
190.4	0.47769673	0.50489847	0.4366594	0.47537039	0.90566696	0.52870597
190.5	0.4777913	0.50495868	0.43672911	0.47546629	0.90566337	0.52882406
190.6	0.4778858	0.50501885	0.43679877	0.47556212	0.90565978	0.52894205
190.7	0.47798023	0.50507897	0.43686836	0.47565786	0.90565619	0.52905995
190.8	0.4780746	0.50513905	0.4369379	0.47575354	0.9056526	0.52917775
190.9	0.4781689	0.50519909	0.43700737	0.47584914	0.905649	0.52929546
191	0.47826313	0.50525909	0.43707678	0.47594466	0.90564541	0.52941307
191.1	0.4783573	0.50531904	0.43714613	0.47604011	0.90564181	0.52953059
191.2	0.4784514	0.50537895	0.43721542	0.47613549	0.90563821	0.52964802
191.3	0.47854543	0.50543882	0.43728465	0.47623079	0.90563461	0.52976535
191.4	0.4786394	0.50549865	0.43735382	0.47632602	0.905631	0.52988259
191.5	0.4787333	0.50555843	0.43742294	0.47642117	0.9056274	0.52999973
191.6	0.47882713	0.50561817	0.43749199	0.47651625	0.90562379	0.53011678
191.7	0.4789209	0.50567786	0.43756098	0.47661125	0.90562018	0.53023374
191.8	0.47901461	0.50573752	0.43762991	0.47670618	0.90561657	0.5303506
191.9	0.47910824	0.50579713	0.43769878	0.47680104	0.90561295	0.53046737
192	0.47920182	0.50585669	0.43776759	0.47689583	0.90560934	0.53058405
192.1	0.47929532	0.50591622	0.43783634	0.47699054	0.90560572	0.53070063
192.2	0.47938876	0.5059757	0.43790503	0.47708517	0.9056021	0.53081712
192.3	0.47948214	0.50603514	0.43797366	0.47717974	0.90559848	0.53093352
192.4	0.47957545	0.50609454	0.43804224	0.47727423	0.90559486	0.53104983
192.5	0.47966869	0.50615389	0.43811075	0.47736865	0.90559124	0.53116604
192.6	0.47976187	0.50621321	0.4381792	0.477463	0.90558761	0.53128216
192.7	0.47985498	0.50627248	0.4382476	0.47755727	0.90558398	0.53139819
192.8	0.47994802	0.5063317	0.43831593	0.47765147	0.90558035	0.53151412
192.9	0.48004101	0.50639089	0.43838421	0.4777456	0.90557672	0.53162997
193	0.48013392	0.50645003	0.43845243	0.47783966	0.90557309	0.53174572
193.1	0.48022677	0.50650913	0.43852059	0.47793364	0.90556946	0.53186138
193.2	0.48031956	0.50656819	0.43858869	0.47802755	0.90556582	0.53197694
193.3	0.48041228	0.50662721	0.43865673	0.47812139	0.90556218	0.53209242
193.4	0.48050494	0.50668618	0.43872471	0.47821516	0.90555855	0.5322078
193.5	0.48059753	0.50674511	0.43879263	0.47830886	0.9055549	0.53232309
193.6	0.48069005	0.506804	0.4388605	0.47840248	0.90555126	0.53243829
193.7	0.48078251	0.50686285	0.43892831	0.47849603	0.90554762	0.5325534
193.8	0.48087491	0.50692166	0.43899605	0.47858951	0.90554397	0.53266842
193.9	0.48096724	0.50698042	0.43906375	0.47868292	0.90554033	0.53278335
194	0.48105951	0.50703914	0.43913138	0.47877626	0.90553668	0.53289818
194.1	0.48115171	0.50709782	0.43919895	0.47886952	0.90553303	0.53301293
194.2	0.48124385	0.50715646	0.43926647	0.47896271	0.90552938	0.53312758
194.3	0.48133592	0.50721506	0.43933392	0.47905584	0.90552573	0.53324214
194.4	0.48142793	0.50727361	0.43940132	0.47914889	0.90552207	0.53335661
194.5	0.48151988	0.50733212	0.43946867	0.47924187	0.90551842	0.53347099
194.6	0.48161176	0.50739059	0.43953595	0.47933478	0.90551476	0.53358528
194.7	0.48170358	0.50744902	0.43960318	0.47942761	0.9055111	0.53369948
194.8	0.48179533	0.50750741	0.43967035	0.47952038	0.90550744	0.53381359
194.9	0.48188702	0.50756576	0.43973746	0.47961308	0.90550378	0.53392761
198.2	0.48487734	0.50946815	0.44192037	0.48263274	0.90538224	0.53764023
198.3	0.48496689	0.5095251	0.44198557	0.48272307	0.90537854	0.53775123
198.4	0.48505638	0.50958202	0.44205072	0.48281332	0.90537484	0.53786215
198.5	0.48514581	0.5096389	0.44211581	0.48290351	0.90537113	0.53797299
198.6	0.48523518	0.50969574	0.44218084	0.48299363	0.90536743	0.53808373
198.7	0.48532449	0.50975253	0.44224582	0.48308368	0.90536372	0.53819439
198.8	0.48541373	0.50980929	0.44231075	0.48317366	0.90536001	0.53830497
198.9	0.48550291	0.50986601	0.44237562	0.48326358	0.9053563	0.53841545
199	0.48559204	0.50992269	0.44244044	0.48335342	0.90535259	0.53852585
199.1	0.4856811	0.50997932	0.4425052	0.4834432	0.90534888	0.53863617
199.2	0.4857701	0.51003592	0.4425699	0.48353291	0.90534517	0.53874639
199.3	0.48585903	0.51009248	0.44263456	0.48362255	0.90534146	0.53885654
199.4	0.48594791	0.510149	0.44269916	0.48371212	0.90533774	0.53896659
199.5	0.48603672	0.51020548	0.4427637	0.48380162	0.90533403	0.53907656
199.6	0.48612548	0.51026191	0.44282819	0.48389106	0.90533031	0.53918645
199.7	0.48621417	0.51031831	0.44289263	0.48398043	0.90532659	0.53929625
199.8	0.4863028	0.51037467	0.44295701	0.48406972	0.90532287	0.53940596
199.9	0.48639138	0.51043099	0.44302133	0.48415896	0.90531915	0.53951559
200	0.48647989	0.51048727	0.44308561	0.48424812	0.90531543	0.53962513
200.1	0.48656834	0.51054351	0.44314983	0.48433722	0.90531171	0.53973459
200.2	0.48665672	0.51059971	0.44321399	0.48442624	0.90530799	0.53984396
200.3	0.48674505	0.51065587	0.4432781	0.48451521	0.90530427	0.53995324
200.4	0.48683332	0.510712	0.44334215	0.4846041	0.90530054	0.54006244
200.5	0.48692153	0.51076808	0.44340616	0.48469293	0.90529682	0.54017156
200.6	0.48700967	0.51082412	0.4434701	0.48478168	0.90529309	0.54028059
200.7	0.48709776	0.51088012	0.443534	0.48487038	0.90528937	0.54038954
200.8	0.48718578	0.51093609	0.44359784	0.484959	0.90528564	0.5404984
200.9	0.48727375	0.51099201	0.44366163	0.48504756	0.90528191	0.54060718
201	0.48736165	0.5110479	0.44372536	0.48513605	0.90527818	0.54071587
201.1	0.4874495	0.51110375	0.44378904	0.48522447	0.90527445	0.54082448
201.2	0.48753728	0.51115955	0.44385266	0.48531283	0.90527072	0.540933
201.3	0.48762501	0.51121532	0.44391624	0.48540111	0.90526699	0.54104144
201.4	0.48771267	0.51127105	0.44397975	0.48548934	0.90526325	0.5411498
201.5	0.48780027	0.51132674	0.44404322	0.48557749	0.90525952	0.54125807
201.6	0.48788782	0.51138239	0.44410663	0.48566558	0.90525579	0.54136626
201.7	0.4879753	0.511438	0.44416999	0.4857536	0.90525205	0.54147436
201.8	0.48806273	0.51149358	0.4442333	0.48584156	0.90524831	0.54158238
201.9	0.48815009	0.51154911	0.44429655	0.48592945	0.90524458	0.54169032
202	0.4882374	0.51160461	0.44435975	0.48601727	0.90524084	0.54179817
202.1	0.48832464	0.51166006	0.4444229	0.48610503	0.9052371	0.54190594
202.2	0.48841183	0.51171548	0.44448599	0.48619272	0.90523336	0.54201362
202.3	0.48849895	0.51177086	0.44454903	0.48628034	0.90522962	0.54212123
202.4	0.48858602	0.5118262	0.44461202	0.4863679	0.90522587	0.54222875
202.5	0.48867303	0.5118815	0.44467496	0.4864554	0.90522213	0.54233618
202.6	0.48875997	0.51193677	0.44473784	0.48654282	0.90521839	0.54244353
202.7	0.48884686	0.51199199	0.44480067	0.48663018	0.90521464	0.5425508
202.8	0.48893369	0.51204717	0.44486345	0.48671748	0.9052109	0.54265799
202.9	0.48902046	0.51210232	0.44492618	0.48680471	0.90520715	0.54276509
203	0.48910717	0.51215743	0.44498885	0.48689187	0.90520341	0.54287212
203.1	0.48919383	0.5122125	0.44505147	0.48697897	0.90519966	0.54297905
203.2	0.48928042	0.51226753	0.44511404	0.487066	0.90519591	0.54308591
203.3	0.48936695	0.51232252	0.44517656	0.48715297	0.90519216	0.54319268
203.4	0.48945343	0.51237748	0.44523902	0.48723987	0.90518841	0.54329937
203.5	0.48953984	0.5124324	0.44530144	0.48732671	0.90518466	0.54340598
203.6	0.4896262	0.51248727	0.4453638	0.48741348	0.90518091	0.54351251
203.7	0.4897125	0.51254211	0.44542611	0.48750018	0.90517715	0.54361895
203.8	0.48979874	0.51259692	0.44548836	0.48758682	0.9051734	0.54372532
203.9	0.48988492	0.51265168	0.44555057	0.4876734	0.90516965	0.5438316
204	0.48997105	0.5127064	0.44561272	0.48775991	0.90516589	0.5439378
204.1	0.49005711	0.51276109	0.44567483	0.48784636	0.90516214	0.54404391
204.2	0.49014312	0.51281574	0.44573688	0.48793274	0.90515838	0.54414995
204.3	0.49022907	0.51287035	0.44579888	0.48801906	0.90515462	0.5442559
204.4	0.49031496	0.51292492	0.44586083	0.48810531	0.90515086	0.54436177
204.5	0.49040079	0.51297946	0.44592272	0.48819149	0.9051471	0.54446756
204.6	0.49048656	0.51303395	0.44598457	0.48827762	0.90514334	0.54457327
204.7	0.49057228	0.51308841	0.44604636	0.48836368	0.90513958	0.5446789
204.8	0.49065794	0.51314283	0.4461081	0.48844967	0.90513582	0.54478445
204.9	0.49074354	0.51319721	0.4461698	0.4885356	0.90513206	0.54488991
205	0.49082908	0.51325156	0.44623144	0.48862146	0.9051283	0.5449953
205.1	0.49091456	0.51330587	0.44629303	0.48870727	0.90512454	0.5451006
205.2	0.49099999	0.51336014	0.44635457	0.488793	0.90512077	0.54520582
205.3	0.49108536	0.51341437	0.44641606	0.48887868	0.90511701	0.54531097
205.4	0.49117067	0.51346856	0.44647749	0.48896428	0.90511324	0.54541603
205.5	0.49125592	0.51352272	0.44653888	0.48904983	0.90510947	0.54552101
205.6	0.49134111	0.51357683	0.44660022	0.48913531	0.90510571	0.54562591
205.7	0.49142625	0.51363092	0.4466615	0.48922073	0.90510194	0.54573073
205.8	0.49151133	0.51368496	0.44672274	0.48930608	0.90509817	0.54583547
205.9	0.49159635	0.51373896	0.44678392	0.48939137	0.9050944	0.54594013
206	0.49168132	0.51379293	0.44684506	0.4894766	0.90509063	0.5460447
206.1	0.49176623	0.51384686	0.44690614	0.48956176	0.90508686	0.5461492
206.2	0.49185108	0.51390075	0.44696717	0.48964686	0.90508309	0.54625362
206.3	0.49193587	0.51395461	0.44702816	0.48973189	0.90507932	0.54635796
206.4	0.49202061	0.51400843	0.44708909	0.48981687	0.90507554	0.54646222
206.5	0.49210528	0.51406221	0.44714997	0.48990178	0.90507177	0.5465664
206.6	0.49218991	0.51411595	0.44721081	0.48998662	0.905068	0.5466705
206.7	0.49227447	0.51416966	0.44727159	0.4900714	0.90506422	0.54677452
206.8	0.49235898	0.51422333	0.44733232	0.49015612	0.90506045	0.54687846
206.9	0.49244343	0.51427696	0.44739301	0.49024078	0.90505667	0.54698232
207	0.49252782	0.51433055	0.44745364	0.49032537	0.90505289	0.5470861
207.1	0.49261216	0.51438411	0.44751423	0.49040991	0.90504912	0.54718981
207.2	0.49269644	0.51443763	0.44757476	0.49049437	0.90504534	0.54729343
207.3	0.49278066	0.51449111	0.44763525	0.49057878	0.90504156	0.54739697
207.4	0.49286483	0.51454456	0.44769568	0.49066312	0.90503778	0.54750044
207.5	0.49294894	0.51459796	0.44775607	0.4907474	0.905034	0.54760382
207.6	0.493033	0.51465134	0.4478164	0.49083162	0.90503022	0.54770713
207.7	0.49311699	0.51470467	0.44787669	0.49091577	0.90502644	0.54781036
207.8	0.49320093	0.51475797	0.44793693	0.49099986	0.90502265	0.54791351
207.9	0.49328482	0.51481123	0.44799712	0.49108389	0.90501887	0.54801658
208	0.49336865	0.51486445	0.44805726	0.49116786	0.90501509	0.54811957
208.1	0.49345242	0.51491764	0.44811735	0.49125177	0.9050113	0.54822249
208.2	0.49353613	0.51497079	0.44817739	0.49133561	0.90500752	0.54832532
208.3	0.49361979	0.5150239	0.44823738	0.49141939	0.90500373	0.54842808
208.4	0.4937034	0.51507698	0.44829733	0.4915031	0.90499995	0.54853076
208.5	0.49378694	0.51513002	0.44835722	0.49158676	0.90499616	0.54863336
208.6	0.49387043	0.51518302	0.44841707	0.49167035	0.90499237	0.54873588
208.7	0.49395387	0.51523599	0.44847687	0.49175388	0.90498859	0.54883833
208.8	0.49403725	0.51528892	0.44853662	0.49183734	0.9049848	0.5489407
208.9	0.49412057	0.51534181	0.44859632	0.49192075	0.90498101	0.54904299
209	0.49420384	0.51539467	0.44865597	0.49200409	0.90497722	0.5491452
209.1	0.49428705	0.51544749	0.44871557	0.49208737	0.90497343	0.54924733
209.2	0.49437021	0.51550027	0.44877513	0.49217059	0.90496964	0.54934939
209.3	0.49445331	0.51555302	0.44883463	0.49225375	0.90496585	0.54945137
209.4	0.49453635	0.51560573	0.44889409	0.49233684	0.90496205	0.54955327
209.5	0.49461934	0.51565841	0.4489535	0.49241987	0.90495826	0.54965509
209.6	0.49470227	0.51571104	0.44901286	0.49250284	0.90495447	0.54975684
209.7	0.49478515	0.51576365	0.44907218	0.49258575	0.90495067	0.54985851
209.8	0.49486797	0.51581621	0.44913144	0.4926686	0.90494688	0.5499601
209.9	0.49495074	0.51586874	0.44919066	0.49275138	0.90494308	0.55006162
213.2	0.49765134	0.51758211	0.45111824	0.49544918	0.90481754	0.55336893
213.3	0.49773225	0.51763343	0.45117585	0.49552991	0.90481373	0.55346787
213.4	0.49781311	0.51768471	0.45123342	0.49561058	0.90480991	0.55356674
213.5	0.49789392	0.51773596	0.45129095	0.49569119	0.9048061	0.55366553
213.6	0.49797467	0.51778717	0.45134842	0.49577174	0.90480228	0.55376425
213.7	0.49805538	0.51783835	0.45140585	0.49585223	0.90479847	0.5538629
213.8	0.49813602	0.51788949	0.45146324	0.49593266	0.90479465	0.55396147
213.9	0.49821662	0.5179406	0.45152058	0.49601304	0.90479084	0.55405997
214	0.49829716	0.51799167	0.45157787	0.49609335	0.90478702	0.55415839
214.1	0.49837764	0.51804271	0.45163511	0.49617361	0.90478321	0.55425674
214.2	0.49845808	0.51809371	0.45169232	0.49625381	0.90477939	0.55435502
214.3	0.49853846	0.51814468	0.45174947	0.49633395	0.90477557	0.55445322
214.4	0.49861879	0.51819562	0.45180658	0.49641402	0.90477175	0.55455135
214.5	0.49869906	0.51824652	0.45186364	0.49649405	0.90476794	0.5546494
214.6	0.49877928	0.51829738	0.45192066	0.49657401	0.90476412	0.55474738
214.7	0.49885945	0.51834821	0.45197763	0.49665391	0.9047603	0.55484529
214.8	0.49893957	0.51839901	0.45203456	0.49673376	0.90475648	0.55494313
214.9	0.49901963	0.51844977	0.45209144	0.49681354	0.90475266	0.55504089
215	0.49909964	0.51850049	0.45214828	0.49689327	0.90474884	0.55513858
215.1	0.4991796	0.51855118	0.45220507	0.49697294	0.90474502	0.55523619
215.2	0.4992595	0.51860184	0.45226181	0.49705255	0.9047412	0.55533374
215.3	0.49933936	0.51865246	0.45231851	0.49713211	0.90473738	0.5554312
215.4	0.49941916	0.51870305	0.45237517	0.4972116	0.90473356	0.5555286
215.5	0.4994989	0.51875361	0.45243178	0.49729104	0.90472973	0.55562593
215.6	0.4995786	0.51880413	0.45248834	0.49737042	0.90472591	0.55572318
215.7	0.49965824	0.51885461	0.45254486	0.49744974	0.90472209	0.55582035
215.8	0.49973783	0.51890506	0.45260134	0.497529	0.90471827	0.55591746
215.9	0.49981736	0.51895548	0.45265777	0.49760821	0.90471444	0.55601449
216	0.49989685	0.51900586	0.45271415	0.49768736	0.90471062	0.55611145
216.1	0.49997628	0.51905621	0.45277049	0.49776645	0.90470679	0.55620834
216.2	0.50005566	0.51910652	0.45282679	0.49784548	0.90470297	0.55630516
216.3	0.50013499	0.5191568	0.45288304	0.49792446	0.90469914	0.5564019
216.4	0.50021427	0.51920705	0.45293924	0.49800338	0.90469532	0.55649858
216.5	0.50029349	0.51925726	0.4529954	0.49808224	0.90469149	0.55659518
216.6	0.50037266	0.51930743	0.45305152	0.49816105	0.90468767	0.5566917
216.7	0.50045178	0.51935758	0.45310759	0.4982398	0.90468384	0.55678816
216.8	0.50053085	0.51940768	0.45316362	0.49831849	0.90468002	0.55688454
216.9	0.50060986	0.51945776	0.4532196	0.49839712	0.90467619	0.55698085
217	0.50068883	0.5195078	0.45327554	0.4984757	0.90467236	0.5570771
217.1	0.50076774	0.51955781	0.45333143	0.49855422	0.90466853	0.55717326
217.2	0.5008466	0.51960778	0.45338728	0.49863269	0.90466471	0.55726936
217.3	0.50092541	0.51965772	0.45344309	0.4987111	0.90466088	0.55736539
217.4	0.50100417	0.51970762	0.45349885	0.49878945	0.90465705	0.55746134
217.5	0.50108287	0.51975749	0.45355457	0.49886774	0.90465322	0.55755722
217.6	0.50116152	0.51980733	0.45361024	0.49894598	0.90464939	0.55765304
217.7	0.50124013	0.51985713	0.45366587	0.49902417	0.90464556	0.55774878
217.8	0.50131868	0.5199069	0.45372146	0.49910229	0.90464173	0.55784445
217.9	0.50139718	0.51995664	0.453777	0.49918036	0.9046379	0.55794004
218	0.50147562	0.52000634	0.45383249	0.49925838	0.90463407	0.55803557
218.1	0.50155402	0.52005601	0.45388795	0.49933633	0.90463024	0.55813103
218.2	0.50163237	0.52010564	0.45394336	0.49941424	0.90462641	0.55822641
218.3	0.50171066	0.52015524	0.45399872	0.49949208	0.90462258	0.55832173
218.4	0.50178891	0.52020481	0.45405405	0.49956987	0.90461875	0.55841697
218.5	0.5018671	0.52025434	0.45410932	0.4996476	0.90461492	0.55851215
218.6	0.50194524	0.52030384	0.45416456	0.49972528	0.90461109	0.55860725
218.7	0.50202333	0.52035331	0.45421975	0.4998029	0.90460725	0.55870228
218.8	0.50210137	0.52040274	0.4542749	0.49988047	0.90460342	0.55879724
218.9	0.50217936	0.52045214	0.45433	0.49995798	0.90459959	0.55889213
219	0.5022573	0.52050151	0.45438506	0.50003543	0.90459576	0.55898696
219.1	0.50233519	0.52055084	0.45444008	0.50011283	0.90459192	0.55908171
219.2	0.50241302	0.52060014	0.45449506	0.50019017	0.90458809	0.55917639
219.3	0.50249081	0.52064941	0.45454999	0.50026746	0.90458426	0.559271
219.4	0.50256855	0.52069864	0.45460488	0.50034469	0.90458042	0.55936554
219.5	0.50264623	0.52074784	0.45465972	0.50042187	0.90457659	0.55946001
219.6	0.50272387	0.520797	0.45471452	0.50049899	0.90457276	0.55955441
219.7	0.50280145	0.52084614	0.45476928	0.50057606	0.90456892	0.55964874
219.8	0.50287899	0.52089524	0.454824	0.50065307	0.90456509	0.559743
219.9	0.50295647	0.5209443	0.45487867	0.50073002	0.90456125	0.55983719
220	0.5030339	0.52099334	0.4549333	0.50080692	0.90455742	0.55993132
220.1	0.50311129	0.52104234	0.45498789	0.50088377	0.90455358	0.56002537
220.2	0.50318862	0.5210913	0.45504243	0.50096056	0.90454975	0.56011935
220.3	0.5032659	0.52114024	0.45509693	0.50103729	0.90454591	0.56021326
220.4	0.50334314	0.52118914	0.45515139	0.50111397	0.90454208	0.56030711
220.5	0.50342032	0.521238	0.4552058	0.5011906	0.90453824	0.56040088
220.6	0.50349745	0.52128684	0.45526018	0.50126717	0.9045344	0.56049459
220.7	0.50357454	0.52133564	0.45531451	0.50134368	0.90453057	0.56058823
220.8	0.50365157	0.52138441	0.4553688	0.50142014	0.90452673	0.56068179
220.9	0.50372855	0.52143314	0.45542304	0.50149655	0.90452289	0.56077529
221	0.50380549	0.52148185	0.45547724	0.5015729	0.90451906	0.56086872
221.1	0.50388237	0.52153052	0.45553141	0.5016492	0.90451522	0.56096208
221.2	0.5039592	0.52157915	0.45558552	0.50172544	0.90451138	0.56105537
221.3	0.50403599	0.52162776	0.4556396	0.50180163	0.90450755	0.5611486
221.4	0.50411272	0.52167633	0.45569363	0.50187776	0.90450371	0.56124175
221.5	0.50418941	0.52172487	0.45574762	0.50195384	0.90449987	0.56133484
221.6	0.50426604	0.52177338	0.45580157	0.50202986	0.90449603	0.56142785
221.7	0.50434263	0.52182185	0.45585548	0.50210583	0.9044922	0.5615208
221.8	0.50441917	0.52187029	0.45590934	0.50218175	0.90448836	0.56161368
221.9	0.50449565	0.5219187	0.45596317	0.50225761	0.90448452	0.5617065
222	0.50457209	0.52196707	0.45601695	0.50233342	0.90448068	0.56179924
222.1	0.50464848	0.52201542	0.45607069	0.50240917	0.90447684	0.56189192
222.2	0.50472482	0.52206373	0.45612438	0.50248487	0.90447301	0.56198452
222.3	0.50480111	0.522112	0.45617804	0.50256052	0.90446917	0.56207706
222.4	0.50487735	0.52216025	0.45623165	0.50263611	0.90446533	0.56216953
222.5	0.50495354	0.52220846	0.45628522	0.50271165	0.90446149	0.56226194
222.6	0.50502968	0.52225664	0.45633875	0.50278713	0.90445765	0.56235427
222.7	0.50510578	0.52230479	0.45639224	0.50286256	0.90445381	0.56244654
222.8	0.50518182	0.5223529	0.45644568	0.50293794	0.90444997	0.56253874
222.9	0.50525782	0.52240099	0.45649909	0.50301326	0.90444613	0.56263087
223	0.50533377	0.52244904	0.45655245	0.50308853	0.90444229	0.56272294
223.1	0.50540966	0.52249705	0.45660577	0.50316375	0.90443846	0.56281493
223.2	0.50548551	0.52254504	0.45665905	0.50323891	0.90443462	0.56290686
223.3	0.50556131	0.52259299	0.45671229	0.50331402	0.90443078	0.56299873
223.4	0.50563706	0.52264091	0.45676549	0.50338907	0.90442694	0.56309052
223.5	0.50571277	0.5226888	0.45681865	0.50346408	0.9044231	0.56318225
223.6	0.50578842	0.52273666	0.45687176	0.50353903	0.90441926	0.56327391
223.7	0.50586403	0.52278448	0.45692483	0.50361392	0.90441542	0.5633655
223.8	0.50593958	0.52283227	0.45697787	0.50368876	0.90441158	0.56345703
223.9	0.50601509	0.52288003	0.45703086	0.50376355	0.90440774	0.56354849
224	0.50609055	0.52292776	0.45708381	0.50383829	0.9044039	0.56363988
224.1	0.50616596	0.52297546	0.45713672	0.50391297	0.90440006	0.56373121
224.2	0.50624133	0.52302312	0.4571896	0.5039876	0.90439622	0.56382246
224.3	0.50631664	0.52307075	0.45724241	0.50406218	0.90439238	0.56391365
224.4	0.50639191	0.52311835	0.4572952	0.50413671	0.90438854	0.56400478
224.5	0.50646712	0.52316592	0.45734794	0.50421118	0.9043847	0.56409583
224.6	0.50654229	0.52321345	0.45740064	0.5042856	0.90438086	0.56418683
224.7	0.50661742	0.52326095	0.45745331	0.50435996	0.90437702	0.56427775
224.8	0.50669249	0.52330842	0.45750593	0.50443428	0.90437318	0.56436861
224.9	0.50676751	0.52335586	0.45755851	0.50450854	0.90436934	0.5644594
228.2	0.50921662	0.52490352	0.45927116	0.50692995	0.90424263	0.56741871
228.3	0.50929003	0.52494988	0.45932238	0.50700245	0.90423879	0.56750728
228.4	0.5093634	0.52499622	0.45937357	0.5070749	0.90423495	0.56759579
228.5	0.50943672	0.52504252	0.45942471	0.5071473	0.90423111	0.56768424
228.6	0.50950999	0.52508879	0.45947582	0.50721965	0.90422727	0.56777262
228.7	0.50958322	0.52513503	0.45952688	0.50729195	0.90422344	0.56786093
228.8	0.5096564	0.52518124	0.45957791	0.5073642	0.9042196	0.56794918
228.9	0.50972953	0.52522742	0.4596289	0.50743639	0.90421576	0.56803737
229	0.50980262	0.52527356	0.45967985	0.50750854	0.90421192	0.5681255
229.1	0.50987566	0.52531968	0.45973076	0.50758063	0.90420808	0.56821356
229.2	0.50994866	0.52536576	0.45978163	0.50765268	0.90420425	0.56830155
229.3	0.51002161	0.52541182	0.45983247	0.50772467	0.90420041	0.56838949
229.4	0.51009451	0.52545784	0.45988326	0.50779662	0.90419657	0.56847736
229.5	0.51016736	0.52550384	0.45993402	0.50786851	0.90419273	0.56856516
229.6	0.51024017	0.5255498	0.45998474	0.50794035	0.9041889	0.56865291
229.7	0.51031294	0.52559573	0.46003541	0.50801215	0.90418506	0.56874058
229.8	0.51038565	0.52564163	0.46008605	0.50808389	0.90418122	0.5688282
229.9	0.51045833	0.5256875	0.46013666	0.50815558	0.90417739	0.56891575
230	0.51053095	0.52573334	0.46018722	0.50822722	0.90417355	0.56900324
230.1	0.51060353	0.52577915	0.46023774	0.50829881	0.90416971	0.56909067
230.2	0.51067606	0.52582492	0.46028823	0.50837035	0.90416588	0.56917803
230.3	0.51074855	0.52587067	0.46033868	0.50844185	0.90416204	0.56926533
230.4	0.51082099	0.52591639	0.46038909	0.50851329	0.90415821	0.56935257
230.5	0.51089339	0.52596207	0.46043946	0.50858468	0.90415437	0.56943974
230.6	0.51096574	0.52600773	0.46048979	0.50865602	0.90415054	0.56952685
230.7	0.51103804	0.52605335	0.46054008	0.50872731	0.9041467	0.5696139
230.8	0.5111103	0.52609895	0.46059034	0.50879855	0.90414287	0.56970088
230.9	0.51118251	0.52614451	0.46064056	0.50886974	0.90413903	0.56978781
231	0.51125468	0.52619005	0.46069074	0.50894089	0.9041352	0.56987467
231.1	0.5113268	0.52623555	0.46074088	0.50901198	0.90413136	0.56996146
231.2	0.51139887	0.52628102	0.46079098	0.50908302	0.90412753	0.5700482
231.3	0.5114709	0.52632646	0.46084105	0.50915402	0.90412369	0.57013487
231.4	0.51154289	0.52637188	0.46089108	0.50922496	0.90411986	0.57022148
231.5	0.51161483	0.52641726	0.46094107	0.50929585	0.90411603	0.57030803
231.6	0.51168672	0.52646261	0.46099102	0.5093667	0.90411219	0.57039451
231.7	0.51175857	0.52650793	0.46104094	0.50943749	0.90410836	0.57048093
231.8	0.51183037	0.52655322	0.46109081	0.50950824	0.90410453	0.57056729
231.9	0.51190213	0.52659848	0.46114065	0.50957894	0.9041007	0.57065359
232	0.51197384	0.52664372	0.46119045	0.50964958	0.90409686	0.57073983
232.1	0.51204551	0.52668892	0.46124022	0.50972018	0.90409303	0.570826
232.2	0.51211713	0.52673409	0.46128994	0.50979073	0.9040892	0.57091212
232.3	0.5121887	0.52677923	0.46133963	0.50986123	0.90408537	0.57099817
232.4	0.51226024	0.52682434	0.46138928	0.50993168	0.90408154	0.57108415
232.5	0.51233172	0.52686942	0.46143889	0.51000208	0.9040777	0.57117008
232.6	0.51240316	0.52691447	0.46148847	0.51007243	0.90407387	0.57125595
232.7	0.51247456	0.52695949	0.46153801	0.51014274	0.90407004	0.57134175
232.8	0.51254591	0.52700448	0.46158751	0.51021299	0.90406621	0.57142749
232.9	0.51261722	0.52704944	0.46163697	0.51028319	0.90406238	0.57151317
233	0.51268848	0.52709437	0.4616864	0.51035335	0.90405855	0.57159879
233.1	0.51275969	0.52713928	0.46173579	0.51042346	0.90405472	0.57168435
233.2	0.51283086	0.52718415	0.46178514	0.51049352	0.90405089	0.57176984
233.3	0.51290199	0.52722899	0.46183446	0.51056353	0.90404706	0.57185528
233.4	0.51297307	0.5272738	0.46188373	0.51063349	0.90404324	0.57194065
233.5	0.51304411	0.52731858	0.46193298	0.5107034	0.90403941	0.57202596
233.6	0.5131151	0.52736334	0.46198218	0.51077326	0.90403558	0.57211121
233.7	0.51318605	0.52740806	0.46203135	0.51084308	0.90403175	0.5721964
233.8	0.51325695	0.52745275	0.46208047	0.51091285	0.90402792	0.57228153
233.9	0.51332781	0.52749742	0.46212957	0.51098256	0.9040241	0.5723666
234	0.51339863	0.52754205	0.46217862	0.51105223	0.90402027	0.5724516
234.1	0.5134694	0.52758665	0.46222764	0.51112186	0.90401644	0.57253655
234.2	0.51354012	0.52763123	0.46227662	0.51119143	0.90401262	0.57262143
234.3	0.5136108	0.52767577	0.46232557	0.51126095	0.90400879	0.57270626
234.4	0.51368144	0.52772029	0.46237448	0.51133043	0.90400496	0.57279102
234.5	0.51375203	0.52776478	0.46242335	0.51139986	0.90400114	0.57287572
234.6	0.51382258	0.52780923	0.46247219	0.51146924	0.90399731	0.57296037
234.7	0.51389308	0.52785366	0.46252098	0.51153857	0.90399349	0.57304495
234.8	0.51396354	0.52789806	0.46256975	0.51160785	0.90398966	0.57312947
234.9	0.51403396	0.52794243	0.46261847	0.51167709	0.90398584	0.57321393
235	0.51410433	0.52798677	0.46266716	0.51174628	0.90398201	0.57329833
235.1	0.51417466	0.52803108	0.46271581	0.51181541	0.90397819	0.57338267
235.2	0.51424494	0.52807536	0.46276443	0.51188451	0.90397437	0.57346695
235.3	0.51431518	0.52811961	0.46281301	0.51195355	0.90397054	0.57355117
235.4	0.51438537	0.52816383	0.46286155	0.51202255	0.90396672	0.57363533
235.5	0.51445552	0.52820802	0.46291006	0.51209149	0.9039629	0.57371943
235.6	0.51452563	0.52825219	0.46295853	0.51216039	0.90395908	0.57380347
235.7	0.51459569	0.52829632	0.46300697	0.51222924	0.90395526	0.57388744
235.8	0.51466571	0.52834043	0.46305537	0.51229805	0.90395143	0.57397136
235.9	0.51473569	0.5283845	0.46310373	0.51236681	0.90394761	0.57405522
236	0.51480562	0.52842855	0.46315205	0.51243551	0.90394379	0.57413902
236.1	0.51487551	0.52847257	0.46320034	0.51250418	0.90393997	0.57422276
236.2	0.51494535	0.52851656	0.4632486	0.51257279	0.90393615	0.57430644
236.3	0.51501515	0.52856052	0.46329682	0.51264136	0.90393233	0.57439006
236.4	0.51508491	0.52860445	0.463345	0.51270987	0.90392851	0.57447362
236.5	0.51515462	0.52864835	0.46339315	0.51277835	0.90392469	0.57455712
236.6	0.51522429	0.52869223	0.46344126	0.51284677	0.90392088	0.57464056
236.7	0.51529392	0.52873607	0.46348933	0.51291515	0.90391706	0.57472395
236.8	0.5153635	0.52877989	0.46353737	0.51298348	0.90391324	0.57480727
236.9	0.51543304	0.52882367	0.46358537	0.51305176	0.90390942	0.57489053
237	0.51550254	0.52886743	0.46363334	0.51311999	0.90390561	0.57497374
237.1	0.51557199	0.52891116	0.46368127	0.51318818	0.90390179	0.57505688
237.2	0.5156414	0.52895486	0.46372917	0.51325632	0.90389797	0.57513997
237.3	0.51571076	0.52899853	0.46377703	0.51332441	0.90389416	0.57522299
237.4	0.51578009	0.52904217	0.46382485	0.51339246	0.90389034	0.57530596
237.5	0.51584937	0.52908579	0.46387264	0.51346046	0.90388653	0.57538887
237.6	0.5159186	0.52912937	0.4639204	0.51352841	0.90388271	0.57547172
237.7	0.5159878	0.52917293	0.46396812	0.51359631	0.9038789	0.57555451
237.8	0.51605695	0.52921646	0.4640158	0.51366417	0.90387509	0.57563724
237.9	0.51612605	0.52925996	0.46406345	0.51373198	0.90387127	0.57571991
238	0.51619512	0.52930343	0.46411106	0.51379975	0.90386746	0.57580253
238.1	0.51626414	0.52934687	0.46415864	0.51386746	0.90386365	0.57588508
238.2	0.51633312	0.52939028	0.46420618	0.51393513	0.90385983	0.57596758
238.3	0.51640205	0.52943367	0.46425368	0.51400276	0.90385602	0.57605002
238.4	0.51647094	0.52947703	0.46430115	0.51407034	0.90385221	0.57613239
238.5	0.51653979	0.52952035	0.46434859	0.51413787	0.9038484	0.57621472
238.6	0.5166086	0.52956365	0.46439599	0.51420535	0.90384459	0.57629698
238.7	0.51667736	0.52960693	0.46444336	0.51427279	0.90384078	0.57637918
238.8	0.51674608	0.52965017	0.46449069	0.51434018	0.90383697	0.57646133
238.9	0.51681476	0.52969338	0.46453798	0.51440752	0.90383316	0.57654342
239	0.5168834	0.52973657	0.46458524	0.51447482	0.90382935	0.57662545
239.1	0.51695199	0.52977973	0.46463247	0.51454207	0.90382554	0.57670742
239.2	0.51702054	0.52982286	0.46467966	0.51460928	0.90382174	0.57678933
239.3	0.51708905	0.52986596	0.46472682	0.51467643	0.90381793	0.57687118
239.4	0.51715751	0.52990903	0.46477394	0.51474355	0.90381412	0.57695298
239.5	0.51722594	0.52995208	0.46482102	0.51481061	0.90381032	0.57703472
239.6	0.51729432	0.5299951	0.46486808	0.51487763	0.90380651	0.5771164
239.7	0.51736266	0.53003808	0.46491509	0.51494461	0.9038027	0.57719802
239.8	0.51743095	0.53008104	0.46496208	0.51501153	0.9037989	0.57727959
239.9	0.5174992	0.53012398	0.46500902	0.51507842	0.90379509	0.5773611
240	0.51756742	0.53016688	0.46505594	0.51514525	0.90379129	0.57744255

Table S13: eFAST analysis data for [PYR] with [G](0)=0.02 mM: eFAST sensitivity indices

Sensitivity analysis data from eFAST for [PYR] with [G](0)=0.02 mM (only the parameters to which [PYR]'s variability is sensitive to appear)

Time discretization	K_{[G]}	V_{[PYR]}	K_{[PYR]}	alpha	rho	K_{[ACoA]}	L_E
0	0	0	0	0	0	0	0
0.1	0.56232552	0.53248372	0.91232363	0.54680901	0.56961769	0.56743376	0.5748556
0.2	0.55028488	0.52710283	0.91331852	0.53714696	0.55914575	0.55648273	0.56297913
0.3	0.53815739	0.52159478	0.91429932	0.5273905	0.54845231	0.5453361	0.5510171
0.4	0.5266208	0.51617523	0.91520476	0.51804899	0.5381202	0.53457936	0.53959521
0.5	0.51587558	0.5109417	0.91601623	0.50926392	0.52832139	0.52440984	0.52889782
0.6	0.50589618	0.50591754	0.91673187	0.5010202	0.51904694	0.51483884	0.51892147
0.7	0.49660461	0.50110258	0.91735442	0.49327635	0.51025458	0.50582198	0.50960594
0.8	0.48793025	0.49648662	0.9178882	0.48598902	0.50190494	0.49731501	0.50088234
0.9	0.47980924	0.49205563	0.91833827	0.47911532	0.49395808	0.48927878	0.49268651
1	0.47218331	0.48779452	0.91870972	0.47261407	0.48637492	0.48167333	0.48496363
1.1	0.46500152	0.48368936	0.91900736	0.46644768	0.47911957	0.47446216	0.47766737
1.2	0.45821709	0.47972807	0.91923594	0.46058345	0.47216024	0.46761149	0.47075628
1.3	0.45179141	0.47589957	0.91940006	0.4549953	0.46547274	0.46109049	0.46419414
1.4	0.44569163	0.47219416	0.91950414	0.44966129	0.45903782	0.45487142	0.45794866
1.5	0.43988787	0.46860276	0.9195526	0.44456161	0.45283825	0.44893141	0.45199211
1.6	0.43435518	0.46511703	0.91954958	0.43967894	0.44685925	0.44325096	0.44630043
1.7	0.42907167	0.46172941	0.91949897	0.43499788	0.4410875	0.4378125	0.44085194
1.8	0.42401771	0.45843359	0.91940457	0.43050436	0.43551107	0.432601	0.43562773
1.9	0.41917627	0.45522396	0.91926996	0.42618591	0.43011972	0.42760234	0.43061096
2	0.41453164	0.45209525	0.91909854	0.42203075	0.42490388	0.42280371	0.42578676
2.1	0.41007006	0.44904334	0.91889359	0.41802783	0.41985508	0.41819363	0.4211419
2.2	0.40577961	0.44606379	0.91865814	0.41416824	0.41496636	0.41376138	0.41666416
2.3	0.40164898	0.44315237	0.91839503	0.41044393	0.41023148	0.40949732	0.4123425
2.4	0.39766822	0.44030616	0.91810695	0.40684721	0.40564485	0.40539263	0.40816736
2.5	0.39382839	0.43752214	0.9177964	0.4033714	0.40120177	0.40143918	0.4041299
2.6	0.39012113	0.43479724	0.91746568	0.40001056	0.39689762	0.39763008	0.40022213
2.7	0.38653899	0.43212895	0.91711695	0.39675879	0.39272818	0.39395863	0.39643687
2.8	0.38307515	0.42951524	0.91675219	0.39361061	0.38868971	0.39041861	0.3927676
2.9	0.37972321	0.42695388	0.91637323	0.39056125	0.38477844	0.38700485	0.38920836
3	0.37647764	0.42444261	0.91598175	0.38760585	0.38099079	0.38371217	0.38575357
3.1	0.37333324	0.42197982	0.91557925	0.38473977	0.37732347	0.38053557	0.38239818
3.2	0.37028503	0.41956407	0.91516715	0.3819591	0.37377322	0.37747078	0.37913767
3.3	0.36732859	0.41719378	0.91474675	0.37925997	0.37033693	0.37451362	0.37596785
3.4	0.36445992	0.41486753	0.9143192	0.37663851	0.36701156	0.37165989	0.37288471
3.5	0.36167517	0.41258409	0.91388554	0.37409127	0.36379415	0.36890577	0.36988451
3.6	0.35897075	0.4103423	0.91344678	0.37161505	0.3606818	0.36624769	0.36696393
3.7	0.35634347	0.40814105	0.91300379	0.3692066	0.35767167	0.36368221	0.36411986
3.8	0.3537904	0.40597931	0.91255737	0.36686298	0.35476102	0.36120597	0.3613493
3.9	0.35130878	0.40385607	0.91210822	0.36458152	0.35194706	0.35881582	0.35864937
4	0.34889609	0.40177036	0.91165699	0.36235975	0.34922695	0.35650875	0.35601744
4.1	0.34655009	0.39972123	0.91120429	0.36019511	0.34659789	0.35428186	0.35345112
4.2	0.34426886	0.39770786	0.91075063	0.35808508	0.34405721	0.3521324	0.35094808
4.3	0.34205085	0.39572955	0.91029647	0.3560274	0.34160223	0.35005766	0.3485061
4.4	0.33989491	0.39378555	0.90984226	0.35401995	0.33923022	0.34805503	0.34612304
4.5	0.33780043	0.39187506	0.90938839	0.35206064	0.3369385	0.34612203	0.3437969
4.6	0.33576746	0.38999729	0.90893518	0.35014737	0.33472443	0.34425631	0.34152586
4.7	0.33379688	0.38815157	0.90848292	0.34827812	0.33258545	0.34245555	0.33930813
4.8	0.33189076	0.38633729	0.90803187	0.346451	0.33051901	0.34071743	0.33714196
4.9	0.3300526	0.38455386	0.90758227	0.34466425	0.32852259	0.33903976	0.33502569
5	0.32828787	0.3828006	0.90713433	0.34291611	0.32659371	0.33742047	0.33295771
5.1	0.3266045	0.38107685	0.90668823	0.34120482	0.32472994	0.33585753	0.33093653
5.2	0.32501366	0.37938203	0.90624412	0.33952867	0.32292894	0.33434891	0.32896071
5.3	0.32353075	0.3777156	0.90580211	0.33788615	0.32118842	0.33289261	0.32702888
5.4	0.32217664	0.37607701	0.90536233	0.33627584	0.31950613	0.33148676	0.3251397
5.5	0.32097928	0.37446572	0.90492489	0.33469639	0.31787988	0.33012957	0.32329189
5.6	0.31997596	0.37288116	0.90448988	0.33314656	0.31630752	0.32881925	0.32148419
5.7	0.3192158	0.37132281	0.90405739	0.33162525	0.31478701	0.32755405	0.31971539
5.8	0.31876317	0.36979018	0.90362746	0.33013156	0.31331634	0.32633226	0.31798431
5.9	0.3187019	0.36828277	0.90320015	0.32866482	0.31189355	0.32515225	0.31628984
6	0.31914007	0.36680007	0.9027755	0.32722468	0.31051675	0.32401249	0.31463092
6.1	0.32021539	0.36534157	0.90235358	0.32581119	0.3091841	0.32291146	0.3130065
6.2	0.32210117	0.36390679	0.90193441	0.32442501	0.30789381	0.32184765	0.31141559
6.3	0.32501156	0.36249529	0.90151802	0.32306759	0.30664417	0.32081959	0.30985719
6.4	0.32920461	0.36110661	0.90110443	0.32174141	0.30543354	0.31982597	0.30833035
6.5	0.33498144	0.3597403	0.90069364	0.32045038	0.30426032	0.31886549	0.30683413
6.6	0.34267963	0.35839589	0.90028568	0.31920054	0.30312295	0.31793685	0.30536764
6.7	0.35265657	0.35707294	0.89988054	0.31800076	0.30201993	0.31703878	0.30392999
6.8	0.36526066	0.35577101	0.89947823	0.31686377	0.30094983	0.31617004	0.30252036
6.9	0.38078914	0.35448966	0.89907874	0.31580803	0.29991126	0.31532948	0.30113793
7	0.39943799	0.35322847	0.89868207	0.31485997	0.29890289	0.31451599	0.29978192
7.1	0.42124865	0.35198702	0.8982882	0.31405663	0.29792346	0.31372854	0.29845155
7.2	0.44605914	0.35076489	0.89789711	0.31345074	0.29697174	0.31296606	0.29714608
7.3	0.47348291	0.3495617	0.89750878	0.3131165	0.29604656	0.31222755	0.29586476
7.4	0.50292151	0.34837702	0.89712317	0.31315616	0.2951468	0.31151203	0.29460688
7.5	0.53361244	0.34721047	0.89674025	0.31371093	0.29427138	0.31081853	0.29337175
7.6	0.56470962	0.34606168	0.89635995	0.31497288	0.29341925	0.31014614	0.29215871
7.7	0.59537694	0.34493025	0.89598222	0.31719683	0.29258943	0.309494	0.2909671
7.8	0.62487164	0.34381583	0.89560698	0.3207114	0.29178095	0.30886131	0.28979632
7.9	0.65260422	0.34271804	0.89523415	0.3259275	0.29099291	0.30824724	0.28864575
8	0.67816657	0.34163652	0.89486362	0.33333891	0.29022445	0.30765101	0.28751481
8.1	0.70132864	0.3405709	0.89449529	0.34348649	0.28947474	0.30707191	0.28640292
8.2	0.72201585	0.33952083	0.89412899	0.35691606	0.28874297	0.30650925	0.28530953
8.3	0.74027438	0.33848597	0.89376456	0.3741207	0.28802841	0.30596236	0.2842341
8.4	0.75623403	0.33746601	0.89340181	0.39541443	0.28733033	0.30543056	0.28317609
8.5	0.77007641	0.33646062	0.8930405	0.42081921	0.28664801	0.30491323	0.28213501
8.6	0.78200829	0.33546948	0.89268036	0.44999986	0.28598081	0.30440976	0.28111035
8.7	0.79224228	0.33449229	0.89232107	0.48223593	0.28532807	0.30391962	0.28010166
8.8	0.80098426	0.33352873	0.89196227	0.51649149	0.28468919	0.30344226	0.27910847
8.9	0.80842563	0.33257851	0.89160353	0.55156871	0.28406359	0.30297713	0.27813034
9	0.81473941	0.33164131	0.89124436	0.5862812	0.28345072	0.30252373	0.27716682
9.1	0.82007878	0.33071686	0.8908842	0.6195988	0.28285006	0.30208158	0.27621751
9.2	0.82457764	0.32980489	0.89052241	0.65075519	0.28226109	0.30165023	0.27528198
9.3	0.82835171	0.32890511	0.89015824	0.67926951	0.28168334	0.30122922	0.27435986
9.4	0.83150018	0.32801727	0.88979084	0.70491401	0.28111635	0.30081813	0.27345075
9.5	0.83410773	0.32714111	0.88941924	0.72766727	0.28055969	0.30041654	0.27255429
9.6	0.83624652	0.32627639	0.88904232	0.7476463	0.28001293	0.30002405	0.27167012
9.7	0.83797764	0.32542284	0.88865885	0.76505072	0.27947568	0.2996403	0.27079789
9.8	0.83935291	0.32458024	0.88826741	0.78012145	0.27894756	0.29926494	0.26993726
9.9	0.84041641	0.32374834	0.8878664	0.79310994	0.2784282	0.29889762	0.26908789
10	0.84120556	0.32292691	0.88745399	0.80426155	0.27791727	0.29853804	0.26824948
10.1	0.84175222	0.32211574	0.88702814	0.81380274	0.27741443	0.29818587	0.26742171
10.2	0.84208356	0.32131461	0.88658658	0.82193736	0.27691936	0.29784084	0.26660429
10.3	0.84222305	0.3205233	0.88612676	0.82884587	0.27643176	0.29750263	0.26579692
10.4	0.84219083	0.31974161	0.88564581	0.8346856	0.27595134	0.29717098	0.26499934
10.5	0.84200414	0.31896934	0.88514053	0.8395937	0.27547783	0.29684562	0.26421127
10.6	0.84167797	0.31820629	0.88460739	0.84368839	0.27501096	0.29652631	0.26343244
10.7	0.84122545	0.31745227	0.88404245	0.84707016	0.27455049	0.2962128	0.26266262
10.8	0.84065812	0.3167071	0.88344143	0.84982629	0.27409619	0.29590487	0.26190153
10.9	0.83998627	0.31597059	0.88279958	0.85203212	0.27364783	0.2956023	0.26114896
11	0.83921913	0.31524257	0.88211168	0.85375067	0.27320521	0.29530489	0.26040466
11.1	0.83836496	0.31452287	0.88137201	0.85503594	0.2727681	0.29501243	0.25966842
11.2	0.83743114	0.31381132	0.8805743	0.85593528	0.27233632	0.29472474	0.25894004
11.3	0.83642434	0.31310777	0.87971171	0.85648927	0.27190967	0.29444163	0.25821929
11.4	0.83535066	0.31241204	0.87877685	0.8567322	0.27148795	0.29416292	0.25750598
11.5	0.83421581	0.31172399	0.87776171	0.85669444	0.271071	0.29388846	0.25679993
11.6	0.83302522	0.31104348	0.87665767	0.85640309	0.27065865	0.29361807	0.25610094
11.7	0.83178396	0.31037035	0.87545543	0.85588154	0.27025076	0.29335161	0.25540885
11.8	0.83049675	0.30970447	0.87414513	0.85515073	0.26984718	0.29308892	0.25472345

Time discretization	K_{[G]}	V_{[PYR]}	K_{[PYR]}	alpha	rho	K_{[ACoA]}	L_E
15	0.78637583	0.29187978	0.70385111	0.78395559	0.25859425	0.28607216	0.23567791
15.1	0.78535262	0.29147098	0.69341278	0.7810805	0.25828114	0.28588315	0.23516737
15.2	0.78436046	0.2910836	0.68276674	0.77819215	0.25796982	0.28569543	0.23466294
15.3	0.78339865	0.29072127	0.67194013	0.77529316	0.25766024	0.28550897	0.23416491
15.4	0.78246637	0.29038838	0.66096108	0.77238659	0.25735238	0.28532374	0.23367359
15.5	0.78156271	0.29009022	0.6498584	0.76947594	0.25704619	0.2851397	0.23318936
15.6	0.78068666	0.28983309	0.63866129	0.76656476	0.25674166	0.28495684	0.23271264
15.7	0.77983711	0.2896245	0.62739929	0.76365668	0.25643874	0.28477512	0.2322439
15.8	0.77901297	0.28947331	0.61610193	0.76075572	0.25613742	0.28459451	0.23178369
15.9	0.77821306	0.28939002	0.60479834	0.75786599	0.25583767	0.284415	0.23133265
16	0.77743616	0.289387	0.59351689	0.75499149	0.25553946	0.28423655	0.23089146
16.1	0.77668099	0.28947869	0.58228525	0.75213615	0.25524276	0.28405915	0.23046096
16.2	0.77594627	0.28968192	0.57113004	0.74930389	0.25494756	0.28388276	0.23004207
16.3	0.77523072	0.29001619	0.56007622	0.74649866	0.25465383	0.28370736	0.22963588
16.4	0.77453304	0.29050402	0.549147	0.7437242	0.25436156	0.28353293	0.22924369
16.5	0.77385194	0.2911712	0.53836383	0.74098406	0.25407072	0.28335945	0.22886697
16.6	0.77318619	0.29204711	0.52774644	0.73828165	0.25378129	0.28318689	0.2285075
16.7	0.77253455	0.293165	0.51731308	0.73561996	0.25349325	0.28301524	0.22816738
16.8	0.77189582	0.2945622	0.50708041	0.7330017	0.25320659	0.28284447	0.22784907
16.9	0.77126886	0.29628024	0.49706341	0.73042938	0.25292128	0.28267457	0.22755558
17	0.77065254	0.29836487	0.48727569	0.72790532	0.25263731	0.28250552	0.22729053
17.1	0.77004577	0.30086592	0.47772925	0.72543149	0.25235466	0.28233729	0.22705825
17.2	0.76944752	0.30383679	0.46843432	0.72300958	0.25207332	0.28216987	0.22686401
17.3	0.76885685	0.30733392	0.45939962	0.72064097	0.25179325	0.28200325	0.22671435
17.4	0.7682728	0.31141586	0.45063227	0.71832675	0.25151446	0.2818374	0.22661722
17.5	0.76769449	0.31614209	0.44213796	0.71606773	0.25123693	0.28167231	0.22658226
17.6	0.76712107	0.32157119	0.43392129	0.71386438	0.25096063	0.28150798	0.22662156
17.7	0.76655182	0.327758	0.42598578	0.71171688	0.25068557	0.28134437	0.22674993
17.8	0.76598608	0.33475119	0.41833387	0.70962515	0.25041171	0.28118149	0.22698524
17.9	0.76542318	0.34259101	0.41096708	0.70758889	0.25013906	0.2810193	0.22734976
18	0.76486248	0.35130598	0.40388601	0.70560759	0.24986759	0.28085781	0.22787073
18.1	0.76430341	0.36090941	0.39709057	0.70368046	0.24959729	0.28069699	0.22858068
18.2	0.76374544	0.37139651	0.39058019	0.70180642	0.24932816	0.28053684	0.22951935
18.3	0.76318808	0.38274069	0.38435394	0.69998414	0.24906018	0.28037734	0.23073461
18.4	0.76263088	0.39489231	0.37841012	0.69821206	0.24879334	0.28021848	0.23228263
18.5	0.76207341	0.40777897	0.3727464	0.69648849	0.24852763	0.28006025	0.2342295
18.6	0.7615153	0.42130585	0.36736004	0.69481156	0.24826303	0.27990263	0.23665199
18.7	0.76095624	0.43535779	0.36224802	0.69317924	0.24799953	0.27974563	0.23963713
18.8	0.76039591	0.44980225	0.35740683	0.69158933	0.24773714	0.27958922	0.24328177
18.9	0.75983405	0.46449286	0.35283268	0.69003951	0.24747583	0.27943339	0.24769131
19	0.75927041	0.47927608	0.34852163	0.6885274	0.24721561	0.27927814	0.25297708
19.1	0.75870478	0.49399792	0.34446939	0.68705065	0.24695646	0.27912345	0.2592521
19.2	0.75813695	0.50850942	0.34067158	0.68560681	0.24669838	0.27896931	0.26662371
19.3	0.75756674	0.52267174	0.33712379	0.68419339	0.24644135	0.27881572	0.27518688
19.4	0.75699397	0.53635924	0.33382159	0.68280789	0.24618538	0.27866267	0.28501635
19.5	0.75641846	0.54946366	0.33076016	0.68144798	0.24593044	0.27851014	0.29615331
19.6	0.75584005	0.56189831	0.3279342	0.6801114	0.24567654	0.27835814	0.30859623
19.7	0.7552586	0.57359805	0.32533829	0.67879597	0.24542366	0.27820665	0.32229496
19.8	0.75467398	0.58451818	0.32296708	0.67749969	0.2451718	0.27805567	0.33714226
19.9	0.75408611	0.59463314	0.32081509	0.67622076	0.24492096	0.27790519	0.35297203
20	0.75349491	0.60393447	0.31887676	0.67495747	0.24467112	0.27775521	0.36956631
20.1	0.75290028	0.61242757	0.3171464	0.67370825	0.24442228	0.27760571	0.38666489
20.2	0.75230214	0.62012826	0.31561806	0.67247163	0.24417442	0.27745669	0.40397713
20.3	0.75170042	0.62706263	0.31428548	0.67124635	0.24392756	0.27730814	0.42120172
20.4	0.75109507	0.63326545	0.31314241	0.67003134	0.24368167	0.27716005	0.43804588
20.5	0.750486	0.63877669	0.31218265	0.66882574	0.24343675	0.27701242	0.4542412
20.6	0.74987314	0.64363943	0.31139994	0.6676288	0.24319279	0.27686523	0.46955791
20.7	0.74925645	0.64789821	0.31078794	0.66643989	0.24294979	0.27671849	0.48381416
20.8	0.74863587	0.65159782	0.31034026	0.6652585	0.24270774	0.27657217	0.49687921
20.9	0.74801136	0.65478112	0.31005045	0.66408426	0.24246664	0.27642629	0.50867191
21	0.7473829	0.65748961	0.3099119	0.66291688	0.24222647	0.27628083	0.51915652
21.1	0.74675047	0.65976391	0.30991796	0.66175619	0.24198724	0.27613578	0.5283371
21.2	0.74611402	0.66164268	0.310062	0.66060208	0.24174894	0.27599114	0.53624918
21.3	0.74547353	0.66316218	0.3103374	0.65945449	0.24151156	0.27584691	0.54295104
21.4	0.74482901	0.66435592	0.31073751	0.65831349	0.2412751	0.27570308	0.5485169
21.5	0.74418046	0.66525473	0.31125574	0.65717921	0.24103955	0.27555964	0.55303103
21.6	0.74352788	0.66588642	0.31188572	0.65605181	0.24080491	0.2754166	0.55658229
21.7	0.74287127	0.66627617	0.31262116	0.6549314	0.24057118	0.27527395	0.55925977
21.8	0.74221065	0.66644718	0.31345581	0.65381817	0.24033835	0.27513168	0.5611501
21.9	0.74154604	0.66642096	0.31438347	0.65271233	0.24010642	0.27498978	0.56233556
22	0.74087743	0.66621724	0.31539798	0.65161409	0.23987538	0.27484827	0.56289375
22.1	0.74020486	0.66585367	0.31649329	0.65052367	0.23964523	0.27470713	0.56289609
22.2	0.73952835	0.66534605	0.31766341	0.64944124	0.23941596	0.27456636	0.56240704
22.3	0.73884793	0.6647085	0.31890237	0.648367	0.23918758	0.27442596	0.56148501
22.4	0.73816367	0.66395358	0.32020459	0.64730111	0.23896007	0.27428594	0.5601823
22.5	0.73747562	0.66309265	0.32156484	0.64624374	0.23873345	0.27414628	0.5585457
22.6	0.73678384	0.66213619	0.32297797	0.64519495	0.23850769	0.27400701	0.55661688
22.7	0.73608837	0.66109397	0.32443893	0.64415483	0.23828281	0.27386811	0.5544324
22.8	0.73538926	0.65997466	0.32594279	0.64312341	0.23805879	0.27372959	0.55202463
22.9	0.73468658	0.6587859	0.32748485	0.64210066	0.23783564	0.27359146	0.54942264
23	0.73398038	0.6575345	0.32906055	0.64108655	0.23761335	0.27345372	0.54665207
23.1	0.73327072	0.65622646	0.33066541	0.64008099	0.23739192	0.2733164	0.54373516
23.2	0.73255764	0.65486721	0.33229525	0.63908386	0.23717136	0.27317949	0.54069126
23.3	0.73184125	0.65346193	0.33394634	0.63809497	0.23695165	0.27304303	0.53753734
23.4	0.73112162	0.65201556	0.33561511	0.63711414	0.23673279	0.27290701	0.53428847
23.5	0.73039883	0.65053254	0.33729806	0.63614115	0.23651479	0.27277148	0.53095804
23.6	0.72967294	0.64901663	0.33899188	0.63517575	0.23629765	0.27263646	0.52755784
23.7	0.72894403	0.64747121	0.34069341	0.63421766	0.23608136	0.27250199	0.524098
23.8	0.72821219	0.64589935	0.34239969	0.63326661	0.23586593	0.27236809	0.52058767
23.9	0.72747751	0.6443038	0.34410791	0.63232233	0.23565135	0.27223481	0.51703513
24	0.72674007	0.64268717	0.34581541	0.63138452	0.23543762	0.27210222	0.51344752
24.1	0.72599995	0.64105236	0.34751981	0.63045289	0.23522476	0.27197038	0.50983136
24.2	0.72525722	0.63940208	0.34921895	0.62952719	0.23501276	0.27183935	0.5061924
24.3	0.72451198	0.63773829	0.35091073	0.62860713	0.23480164	0.27170925	0.50253545
24.4	0.72376434	0.63606273	0.3525932	0.62769247	0.23459138	0.27158017	0.49886479
24.5	0.72301438	0.63437694	0.35426456	0.62678298	0.234382	0.27145225	0.49518433
24.6	0.72226222	0.63268235	0.35592317	0.62587849	0.23417351	0.27132566	0.49149759
24.7	0.72150794	0.63098021	0.35756752	0.62497883	0.23396591	0.27120057	0.48780776
24.8	0.72075165	0.62927171	0.3591962	0.6240839	0.23375921	0.27107724	0.4841179
24.9	0.7199934	0.62755811	0.36080798	0.62319358	0.23355342	0.27095593	0.48043105
25	0.71923329	0.62584059	0.36240173	0.62230784	0.23334856	0.27083696	0.47674981
25.1	0.7184714	0.62412047	0.36397643	0.6214266	0.23314464	0.27072074	0.47307624
25.2	0.71770781	0.62239908	0.36553118	0.62054984	0.23294167	0.27060772	0.4694121
25.3	0.71694263	0.62067725	0.36706521	0.61967757	0.23273968	0.27049849	0.4657589
25.4	0.71617595	0.61895568	0.3685778	0.61880984	0.23253868	0.2703937	0.46211806
25.5	0.71540788	0.61723501	0.37006835	0.61794671	0.2323387	0.27029415	0.45849109
25.6	0.71463852	0.61551579	0.37153633	0.61708826	0.23213977	0.27020078	0.45487974
25.7	0.71386797	0.61379868	0.37298129	0.61623459	0.23194191	0.27011469	0.45128543
25.8	0.71309632	0.6120843	0.37440285	0.6153858	0.23174517	0.27003717	0.4477092
25.9	0.71232367	0.6103731	0.37580072	0.61454203	0.23154958	0.26996975	0.4441519
26	0.71155012	0.60866547	0.37717463	0.61370343	0.23135519	0.2699142	0.44061427
26.1	0.71077576	0.60696216	0.37852437	0.61287018	0.23116204	0.26987257	0.43709697
26.2	0.71000071	0.60526394	0.37984982	0.61204244	0.23097021	0.26984726	0.43360065
26.3	0.70922506	0.60357118	0.38115091	0.61122038	0.23077975	0.269841	0.4301261
26.4	0.70844892	0.60188417	0.3824276	0.61040419	0.23059075	0.26985695	0.42667411
26.5	0.70767238	0.60020319	0.38367991	0.6095941	0.23040328	0.26989867	0.42324525
26.6	0.70689556	0.59852857	0.38490791	0.60879031	0.23021746	0.26997021	0.41983989
26.7	0.70611856	0.59686056	0.3861117	0.60799301	0.23003339	0.27007617	0.41645832
26.8	0.70534149	0.59519939	0.38729142	0.6072024	0.2298512	0.27022171	0.4131008

Time discretization	K_{[G]}	V_{[PYR]}	K_{[PYR]}	alpha	rho	K_{[ACoA]}	L_E
30	0.68099221	0.54685947	0.41416706	0.58641878	0.2290352	0.35392328	0.31916187
30.1	0.68026151	0.54552392	0.41473816	0.58593123	0.22957168	0.36090085	0.31664711
30.2	0.67953325	0.54419991	0.41529733	0.58545409	0.23020535	0.36817731	0.31415678
30.3	0.67880744	0.54288753	0.41584494	0.58498734	0.23094836	0.37574052	0.31169066
30.4	0.67808412	0.54158684	0.41638134	0.58453098	0.23181405	0.3835767	0.30924855
30.5	0.67736331	0.5402979	0.41690689	0.58408497	0.23281709	0.39167069	0.30683024
30.6	0.67664502	0.53902079	0.41742192	0.5836493	0.23397363	0.40000553	0.30443556
30.7	0.67592927	0.53775555	0.4179268	0.58322394	0.23530121	0.4085629	0.30206433
30.8	0.67521609	0.53650228	0.41842188	0.58280886	0.23681884	0.41732326	0.29971636
30.9	0.67450547	0.53526106	0.41890748	0.582404	0.2385467	0.42626583	0.29739147
31	0.67379743	0.53403206	0.41938395	0.58200934	0.24050623	0.43536895	0.29508949
31.1	0.67309198	0.53281539	0.41985161	0.58162483	0.24272025	0.44461022	0.29281022
31.2	0.67238911	0.53161113	0.42031079	0.58125042	0.2452127	0.45396676	0.29055342
31.3	0.67168883	0.53041936	0.4207618	0.58088604	0.2480085	0.46341554	0.28831885
31.4	0.67099114	0.52924014	0.42120495	0.58053163	0.2511331	0.47293337	0.28610631
31.5	0.67029603	0.52807355	0.42164052	0.58018714	0.25461202	0.48249714	0.28391561
31.6	0.6696035	0.52691964	0.4220688	0.5798525	0.25847072	0.49208405	0.28174655
31.7	0.66891355	0.5257785	0.42249007	0.57952765	0.26273422	0.50167173	0.27959894
31.8	0.66822617	0.52465013	0.4229046	0.57921249	0.26742654	0.51123831	0.27747256
31.9	0.66754134	0.52353452	0.42331267	0.57890696	0.27257004	0.52076239	0.27536724
32	0.66685905	0.52243164	0.42371454	0.57861097	0.27818458	0.5302233	0.27328275
32.1	0.66617929	0.52134154	0.42411048	0.57832445	0.28428682	0.53960138	0.27121889
32.2	0.66550207	0.52026425	0.42450073	0.57804729	0.29088986	0.54887769	0.26917546
32.3	0.66482735	0.51919978	0.42488556	0.57777942	0.29800254	0.55803426	0.26715226
32.4	0.66415513	0.51814818	0.4252652	0.57752074	0.30562868	0.56705411	0.26514912
32.5	0.66348538	0.51710947	0.42563988	0.57727115	0.31376639	0.57592117	0.26316584
32.6	0.66281809	0.51608368	0.42600983	0.57703056	0.32240752	0.58462058	0.26120224
32.7	0.66215322	0.5150708	0.42637527	0.57679888	0.33153743	0.59313865	0.25925814
32.8	0.66149077	0.51407083	0.42673639	0.57657601	0.34113494	0.60146275	0.25733336
32.9	0.66083071	0.51308377	0.4270934	0.57636183	0.35117235	0.60958122	0.25542769
33	0.66017301	0.5121096	0.4274465	0.57615625	0.36161557	0.61748346	0.25354092
33.1	0.65951764	0.51114835	0.42779585	0.57595918	0.37242444	0.62516014	0.25167287
33.2	0.65886457	0.51020004	0.42814165	0.57577052	0.38355329	0.63260336	0.24982338
33.3	0.65821379	0.50926467	0.42848406	0.57559017	0.39495202	0.6398062	0.24799225
33.4	0.65756526	0.50834222	0.42882324	0.57541803	0.40656697	0.64676236	0.24617933
33.5	0.65691895	0.50743266	0.42915935	0.575254	0.41834169	0.65346663	0.24438445
33.6	0.65627483	0.50653593	0.42949251	0.57509795	0.43021818	0.65991565	0.24260745
33.7	0.65563286	0.50565198	0.42982287	0.57494979	0.44213786	0.66610752	0.24084819
33.8	0.65499302	0.50478074	0.43015054	0.57480939	0.4540428	0.67204144	0.23910652
33.9	0.65435525	0.50392211	0.43047566	0.57467665	0.46587691	0.67771784	0.23738231
34	0.65371954	0.50307602	0.43079833	0.57455146	0.47758751	0.68313818	0.23567541
34.1	0.65308583	0.5022424	0.43111867	0.57443371	0.48912568	0.68830433	0.23398571
34.2	0.6524541	0.50142114	0.43143678	0.57432331	0.50044617	0.69321796	0.23231307
34.3	0.65182431	0.50061214	0.43175276	0.57422014	0.5115083	0.69788197	0.23065738
34.4	0.65119642	0.4998153	0.43206669	0.5741241	0.52227647	0.70230088	0.22901851
34.5	0.6505704	0.49903049	0.43237865	0.57403508	0.53272035	0.70647966	0.22739637
34.6	0.64994619	0.4982576	0.43268873	0.57395296	0.54281477	0.7104238	0.2257908
34.7	0.64932377	0.49749651	0.43299697	0.57387766	0.55253992	0.71413943	0.22420167
34.8	0.64870309	0.49674709	0.43330343	0.57380905	0.5618811	0.71763297	0.22262886
34.9	0.64808412	0.49600919	0.43360819	0.57374703	0.57082795	0.72091005	0.22107225
35	0.64746681	0.49528267	0.4339113	0.5736915	0.57937413	0.72397665	0.21953177
35.1	0.64685112	0.49456738	0.43421281	0.57364236	0.58751712	0.7268399	0.21800731
35.2	0.64623701	0.49386318	0.43451278	0.57359949	0.59525783	0.72950698	0.21649881
35.3	0.64562443	0.49316992	0.43481124	0.57356278	0.60259988	0.73198486	0.21500619
35.4	0.64501334	0.49248747	0.43510823	0.57353214	0.60954923	0.73428058	0.21352938
35.5	0.64440369	0.49181569	0.43540379	0.57350745	0.61611415	0.73640127	0.2120683
35.6	0.64379544	0.49115439	0.43569794	0.5734886	0.62230456	0.73835343	0.21062286
35.7	0.64318854	0.49050339	0.43599072	0.57347551	0.62813167	0.74014346	0.20919298
35.8	0.64258294	0.48986249	0.43628214	0.57346805	0.63360757	0.74177809	0.20777861
35.9	0.64197861	0.48923153	0.43657223	0.57346614	0.63874509	0.74326393	0.20637969
36	0.6413755	0.48861033	0.43686102	0.57346966	0.64355757	0.74460743	0.20499618
36.1	0.64077356	0.48799871	0.43714854	0.57347851	0.64805859	0.7458146	0.20362805
36.2	0.64017274	0.48739649	0.43743479	0.57349261	0.65226185	0.74689132	0.20227527
36.3	0.63957301	0.4868035	0.4377198	0.57351184	0.65618097	0.74784331	0.20093779
36.4	0.63897431	0.48621954	0.43800357	0.57353611	0.65982934	0.74867584	0.19961556
36.5	0.63837661	0.48564445	0.43828612	0.57356532	0.66322004	0.74939414	0.19830857
36.6	0.63777986	0.48507804	0.43856746	0.57359938	0.66636579	0.75000335	0.19701681
36.7	0.63718402	0.48452013	0.4388476	0.57363819	0.66927892	0.75050834	0.19574026
36.8	0.63658905	0.48397056	0.43912656	0.57368166	0.67197125	0.75091372	0.19447894
36.9	0.6359949	0.48342918	0.43940435	0.57372969	0.67445417	0.75122386	0.19323285
37	0.63540153	0.48289584	0.439681	0.5737822	0.67673862	0.75144289	0.192002
37.1	0.6348089	0.48237038	0.43995652	0.57383907	0.67883498	0.7515747	0.19078642
37.2	0.63421696	0.48185265	0.44023094	0.57390021	0.68075302	0.75162304	0.18958614
37.3	0.63362568	0.48134249	0.4405043	0.57396554	0.68250199	0.75159152	0.18840117
37.4	0.63303502	0.48083975	0.44077659	0.57403495	0.68409067	0.75148348	0.18723156
37.5	0.63244493	0.48034429	0.4410478	0.57410836	0.68552732	0.75130211	0.18607732
37.6	0.6318554	0.47985597	0.44131792	0.57418568	0.6868198	0.75105047	0.18493848
37.7	0.63126638	0.47937463	0.44158697	0.57426681	0.68797553	0.75073152	0.1838151
37.8	0.63067784	0.47890014	0.44185499	0.57435166	0.68900142	0.750348	0.18270723
37.9	0.63008974	0.47843236	0.44212198	0.57444014	0.68990401	0.7499025	0.18161494
38	0.62950206	0.47797115	0.44238798	0.57453215	0.69068947	0.7493974	0.18053828
38.1	0.62891475	0.47751637	0.442653	0.57462762	0.6913636	0.74883486	0.17947731
38.2	0.62832778	0.47706792	0.44291709	0.57472645	0.69193199	0.74821688	0.17843209
38.3	0.62774113	0.4766257	0.44318025	0.57482855	0.69239983	0.74754547	0.1774027
38.4	0.62715476	0.47618959	0.44344253	0.57493383	0.69277202	0.74682251	0.17638922
38.5	0.62656865	0.47575949	0.44370396	0.57504221	0.69305321	0.74604977	0.17539173
38.6	0.62598277	0.47533529	0.44396456	0.57515359	0.69324776	0.74522904	0.17441031
38.7	0.6253971	0.47491691	0.44422436	0.57526789	0.6933598	0.74436194	0.17344504
38.8	0.62481161	0.47450423	0.4444834	0.57538502	0.69339332	0.74344988	0.17249602
38.9	0.62422629	0.47409718	0.44474171	0.5755049	0.69335205	0.74249424	0.17156333
39	0.62364111	0.47369565	0.44499933	0.57562744	0.69323949	0.74149633	0.17064708
39.1	0.62305604	0.47329955	0.44525631	0.57575256	0.69305898	0.74045736	0.16974735
39.2	0.62247106	0.47290879	0.44551268	0.57588018	0.69281373	0.7393785	0.16886424
39.3	0.62188615	0.47252328	0.4457685	0.57601022	0.69250681	0.73826084	0.16799782
39.4	0.6213013	0.47214293	0.4460238	0.5761426	0.69214114	0.73710534	0.16714818
39.5	0.62071649	0.4717677	0.44627863	0.57627726	0.69171945	0.73591307	0.16631541
39.6	0.6201317	0.47139749	0.44653303	0.57641411	0.69124437	0.73468511	0.16549958
39.7	0.61954693	0.47103226	0.44678703	0.57655308	0.69071843	0.73342232	0.16470078
39.8	0.61896215	0.47067196	0.44704068	0.5766941	0.69014403	0.73212549	0.16391909
39.9	0.61837736	0.47031651	0.44729402	0.57683711	0.68952349	0.73079538	0.16315459
40	0.61779254	0.46996585	0.4475471	0.57698202	0.68885894	0.72943274	0.16240735
40.1	0.6172077	0.4696199	0.44779995	0.57712878	0.68815247	0.72803825	0.16167743
40.2	0.61662281	0.4692786	0.44805263	0.57727731	0.68740609	0.72661258	0.16096492
40.3	0.61603788	0.46894186	0.44830517	0.57742757	0.68662172	0.72515644	0.16026987
40.4	0.6154529	0.46860961	0.4485576	0.57757947	0.68580119	0.7236706	0.15959235
40.5	0.61486786	0.46828173	0.44880998	0.57773297	0.68494623	0.72215574	0.15893243
40.6	0.61428276	0.46795817	0.44906234	0.577888	0.68405847	0.72061243	0.15829017
40.7	0.6136976	0.46763885	0.44931469	0.57804449	0.68313954	0.71904123	0.15766561
40.8	0.61311237	0.46732371	0.44956706	0.5782024	0.682191	0.71744269	0.15705881
40.9	0.61252708	0.46701267	0.44981946	0.57836168	0.68121429	0.71581737	0.1564698
41	0.61194172	0.46670568	0.45007194	0.57852226	0.68021074	0.71416577	0.15589863
41.1	0.6113563	0.46640266	0.45032453	0.57868409	0.67918165	0.71248838	0.1553453
41.2	0.61077081	0.46610354	0.45057725	0.57884713	0.67812829	0.71078577	0.15480985
41.3	0.61018526	0.46580826	0.45083014	0.57901132	0.67705185	0.70905855	0.15429229
41.4	0.60959965	0.46551678	0.45108322	0.57917663	0.67595349	0.70730723	0.15379265
41.5	0.60901399	0.46522902	0.45133652	0.57934299	0.67483431	0.70553236	0.15331095
41.6	0.60842827	0.46494492	0.45159009	0.57951037	0.67369536	0.70373449	0.15284718
41.7	0.6078425	0.46466441	0.45184396	0.57967873	0.67253761	0.70191412	0.15240135
41.8	0.60725669	0.46438742	0.45209817	0.57984802	0.67136198	0.70007175	0.15197346

Time discretization	K_{[G]}	V_{[PYR]}	K_{[PYR]}	alpha	rho	K_{[ACoA]}	L_E
45	0.58854879	0.45698395	0.46045203	0.58556638	0.62897387	0.63303017	0.14745855
45.1	0.58796779	0.45678579	0.4607191	0.58574992	0.62763702	0.63080497	0.14758727
45.2	0.58738719	0.45658889	0.46098634	0.58593355	0.62630771	0.62857969	0.14773082
45.3	0.58680698	0.4563932	0.46125372	0.58611725	0.62498667	0.62635496	0.14788902
45.4	0.5862272	0.45619864	0.46152121	0.586301	0.62367452	0.62413141	0.14806171
45.5	0.58564784	0.45600514	0.46178881	0.5864848	0.62237187	0.62190965	0.14824868
45.6	0.58506894	0.45581266	0.46205648	0.58666863	0.62107932	0.61969032	0.14844976
45.7	0.5844905	0.45562112	0.46232421	0.58685248	0.6197975	0.61747405	0.14866476
45.8	0.58391254	0.45543047	0.46259198	0.58703633	0.61852698	0.61526148	0.14889347
45.9	0.58333508	0.45524064	0.46285976	0.58722018	0.61726836	0.61305321	0.14913569
46	0.58275812	0.45505157	0.46312752	0.58740401	0.61602218	0.6108498	0.14939122
46.1	0.5821817	0.4548632	0.46339524	0.58758781	0.61478899	0.60865182	0.14965986
46.2	0.58160581	0.45467547	0.46366288	0.58777158	0.61356929	0.60645982	0.14994142
46.3	0.58103048	0.4544836	0.4639304	0.5879553	0.61236352	0.60427437	0.1502357
46.4	0.58045571	0.4543018	0.46419778	0.58813895	0.61117213	0.60209606	0.1505425
46.5	0.57988153	0.45411575	0.46446496	0.58832254	0.60999554	0.59992543	0.15086163
46.6	0.57930795	0.45393016	0.46473193	0.58850606	0.60883414	0.597763	0.15119286
46.7	0.57873498	0.45374499	0.46499865	0.58868948	0.60768831	0.59560926	0.15153598
46.8	0.57816263	0.45356017	0.4652651	0.58887281	0.60655839	0.59346468	0.15189079
46.9	0.57759093	0.45337567	0.46553123	0.58905603	0.60544469	0.5913297	0.15225705
47	0.57701988	0.45319143	0.46579702	0.58923914	0.60434749	0.58920479	0.15263451
47.1	0.5764495	0.45300741	0.46606242	0.58942212	0.60326702	0.58709034	0.15302297
47.2	0.57587981	0.45282358	0.46632741	0.58960496	0.60220354	0.58498677	0.1534222
47.3	0.57531081	0.4526399	0.46659197	0.58978766	0.60115725	0.58289444	0.15383197
47.4	0.57474253	0.45245633	0.46685605	0.5899702	0.60012836	0.58081376	0.15425208
47.5	0.57417497	0.45227283	0.46711963	0.59015259	0.59911705	0.57874508	0.15468231
47.6	0.57360814	0.45208938	0.46738267	0.59033479	0.59812346	0.57668877	0.15512245
47.7	0.57304207	0.45190593	0.46764514	0.59051681	0.59714774	0.57464515	0.15557228
47.8	0.57247676	0.45172246	0.46790701	0.59069864	0.59619	0.57261453	0.15603159
47.9	0.57191221	0.45153894	0.46816824	0.59088027	0.59525037	0.57059718	0.15650015
48	0.57134846	0.45135535	0.4684288	0.59106168	0.59432893	0.5685934	0.15697777
48.1	0.5707855	0.45117167	0.46868866	0.59124288	0.59342577	0.56660343	0.15746422
48.2	0.57022335	0.45098787	0.46894777	0.59142384	0.59254095	0.56462754	0.1579593
48.3	0.56966201	0.45080393	0.4692061	0.59160457	0.59167452	0.56266595	0.1584628
48.4	0.56910151	0.45061982	0.46946362	0.59178505	0.59082656	0.56071889	0.15897451
48.5	0.56854185	0.45043552	0.46972029	0.59196527	0.58999711	0.55878656	0.15949424
48.6	0.56798304	0.45025102	0.46997607	0.59214523	0.5891862	0.55686914	0.16002178
48.7	0.56742509	0.45006629	0.47023091	0.59232492	0.58839386	0.55496683	0.1605569
48.8	0.56686801	0.44988132	0.4704848	0.59250432	0.58762011	0.55307976	0.16109937
48.9	0.56631182	0.44969608	0.47073768	0.59268344	0.58686496	0.55120806	0.16164899
49	0.56575652	0.44951057	0.47098952	0.59286225	0.58612843	0.54935185	0.16220554
49.1	0.56520212	0.44932475	0.47124029	0.59304076	0.58541053	0.54751126	0.16276882
49.2	0.56464863	0.44913864	0.47148995	0.59321896	0.58471125	0.54568638	0.16333864
49.3	0.56409607	0.4489522	0.47173848	0.59339683	0.58403061	0.5438773	0.16391481
49.4	0.56354444	0.44876543	0.47198582	0.59357437	0.58336861	0.5420841	0.16449713
49.5	0.56299375	0.44857831	0.47223196	0.59375157	0.58272525	0.54030683	0.16508542
49.6	0.562444	0.44839084	0.47247684	0.59392843	0.58210054	0.53854556	0.1656795
49.7	0.56189522	0.44820301	0.47272044	0.59410492	0.58149448	0.53680031	0.16627917
49.8	0.56134741	0.44801479	0.47296272	0.59428105	0.58090708	0.53507113	0.16688425
49.9	0.56080056	0.44782619	0.47320365	0.59445681	0.58033836	0.53335802	0.16749456
50	0.5602547	0.44763718	0.47344317	0.59463219	0.5797883	0.53166099	0.16810992
50.1	0.55970983	0.44744777	0.47368126	0.59480718	0.5792569	0.52998005	0.16873015
50.2	0.55916595	0.44725795	0.47391788	0.59498178	0.57874415	0.52831517	0.1693508
50.3	0.55862308	0.4470677	0.47415299	0.59515598	0.57825005	0.52666636	0.16998455
50.4	0.55808122	0.44687702	0.47438654	0.59532976	0.57777461	0.52503359	0.17061839
50.5	0.55754037	0.4466859	0.47461851	0.59550313	0.57731782	0.52341683	0.17125644
50.6	0.55700054	0.44649433	0.47484882	0.59567607	0.57687968	0.52181601	0.17189852
50.7	0.55646174	0.44630231	0.47507746	0.59584857	0.5764602	0.5202311	0.17254446
50.8	0.55592396	0.44610981	0.47530438	0.59602064	0.57605942	0.51866204	0.1731941
50.9	0.55538722	0.44591683	0.47552954	0.59619225	0.57567731	0.51710877	0.1738473
51	0.55485152	0.44572337	0.4757529	0.59636342	0.57531385	0.51557121	0.1745039
51.1	0.55431686	0.4455294	0.47597443	0.59653412	0.57496906	0.5140493	0.17516374
51.2	0.55378325	0.44533493	0.47619407	0.59670435	0.5746429	0.51254294	0.17582668
51.3	0.5532507	0.44513994	0.47641179	0.5968741	0.57433539	0.51105206	0.17649258
51.4	0.5527192	0.44494444	0.47662755	0.59704337	0.57404648	0.50957654	0.17716129
51.5	0.55218876	0.44474842	0.47684131	0.59721215	0.57377616	0.5081163	0.17783267
51.6	0.55165939	0.44455186	0.47705301	0.59738043	0.57352439	0.50667121	0.17850659
51.7	0.55113109	0.44435477	0.47726262	0.59754821	0.57329115	0.50524119	0.17918291
51.8	0.55060386	0.44415714	0.47747009	0.59771548	0.57307638	0.50382611	0.1798615
51.9	0.5500777	0.44395895	0.47767537	0.59788224	0.57288003	0.50242587	0.18054222
52	0.54955262	0.44376021	0.47787842	0.59804849	0.57270205	0.50104035	0.18122496
52.1	0.54902861	0.4435609	0.4780792	0.59821421	0.5725424	0.49966942	0.18190958
52.2	0.5485057	0.44336103	0.47827765	0.59837939	0.57240104	0.49831297	0.18259596
52.3	0.54798386	0.44316059	0.47847374	0.59854405	0.5722779	0.49697086	0.183284
52.4	0.54746312	0.44295957	0.47866741	0.59870816	0.5721729	0.49564297	0.18397357
52.5	0.54694347	0.44275798	0.47885861	0.59887172	0.57208596	0.49432915	0.18466455
52.6	0.54642491	0.4425558	0.47904732	0.59903474	0.57201699	0.49302928	0.18535683
52.7	0.54590745	0.44235304	0.47923349	0.59919719	0.57196587	0.49174322	0.18605031
52.8	0.54539109	0.4421497	0.47941707	0.59935909	0.57193251	0.49047082	0.18674488
52.9	0.54487583	0.44194578	0.47959801	0.59952041	0.57191679	0.48921196	0.18744044
53	0.54436168	0.44174127	0.47977628	0.59968117	0.57191859	0.48796649	0.18813688
53.1	0.54384862	0.44153619	0.47995183	0.59984135	0.57193779	0.48673427	0.18883411
53.2	0.54333667	0.44133053	0.4801246	0.60000095	0.57197423	0.48551515	0.18953203
53.3	0.54282583	0.4411243	0.48029454	0.60015996	0.57202779	0.484309	0.19023055
53.4	0.54231609	0.44091751	0.48046162	0.60031838	0.5720983	0.48311567	0.19092955
53.5	0.54180746	0.44071017	0.48062576	0.60047622	0.57218562	0.481935	0.19162896
53.6	0.54129994	0.44050227	0.48078692	0.60063345	0.57228959	0.48076684	0.19232869
53.7	0.54079352	0.44029383	0.48094502	0.60079008	0.57241002	0.47961106	0.19302863
53.8	0.54028821	0.44008485	0.48110002	0.60094609	0.57254675	0.47846748	0.1937287
53.9	0.53978401	0.43987536	0.48125187	0.6011015	0.57269959	0.47733597	0.19442882
54	0.53928092	0.43966535	0.4814005	0.60125629	0.57286835	0.47621638	0.19512891
54.1	0.53877893	0.43945484	0.48154586	0.60141046	0.57305284	0.47510854	0.19582889
54.2	0.53827806	0.43924384	0.4816879	0.60156399	0.57325285	0.47401231	0.19652867
54.3	0.53777829	0.43903238	0.48182657	0.6017169	0.5734682	0.47292753	0.19722819
54.4	0.53727964	0.43882046	0.48196181	0.60186917	0.57369873	0.47185404	0.19792737
54.5	0.53678209	0.4386081	0.48209358	0.60202079	0.57394424	0.4707917	0.19862615
54.6	0.53628566	0.43839532	0.48222181	0.60217177	0.57420451	0.46974034	0.19932446
54.7	0.53579033	0.43818215	0.48234647	0.60232209	0.57447932	0.46869982	0.20002223
54.8	0.53529611	0.43796858	0.48246748	0.60247175	0.57476846	0.46766998	0.20071938
54.9	0.534803	0.43775465	0.4825848	0.60262075	0.57507168	0.46665067	0.20141586
55	0.534311	0.43754038	0.48269837	0.60276907	0.57538877	0.46564174	0.20211159
55.1	0.53382011	0.43732579	0.48280814	0.60291673	0.57571948	0.46464302	0.20280651
55.2	0.53333033	0.4371109	0.48291405	0.6030637	0.57606358	0.46365437	0.20350056
55.3	0.53284165	0.43689574	0.48301606	0.60320999	0.57642082	0.46267562	0.20419368
55.4	0.53235408	0.43668033	0.48311411	0.60335559	0.57679098	0.46170662	0.20488581
55.5	0.53186761	0.4364647	0.48320816	0.6035005	0.57717378	0.46074724	0.20557688
55.6	0.53138225	0.43624887	0.48329816	0.6036447	0.57756899	0.45979731	0.20626683
55.7	0.53089799	0.43603288	0.48338406	0.6037882	0.57797634	0.45885672	0.20695563
55.8	0.53041484	0.43581674	0.48346581	0.603931	0.57839559	0.4579253	0.20764321
55.9	0.52993279	0.43560048	0.48354336	0.60407308	0.57882647	0.45700293	0.20832952
56	0.52945184	0.43538413	0.48361667	0.60421444	0.57926872	0.45608944	0.2090145
56.1	0.528972	0.43516771	0.48368569	0.60435509	0.57972208	0.45518471	0.20969812
56.2	0.52849325	0.43495126	0.48375039	0.60449501	0.5801863	0.45428857	0.21038031
56.3	0.52801561	0.43473479	0.48381072	0.6046342	0.58066109	0.45340089	0.21106105
56.4	0.52753906	0.43451835	0.48386665	0.60477265	0.58114621	0.45252154	0.21174027
56.5	0.5270636	0.43430195	0.48391814	0.60491037	0.58164141	0.45165035	0.21241794
56.6	0.52658924	0.43408562	0.48396516	0.60504735	0.58214647	0.45078721	0.21309402
56.7	0.52611597	0.4338694	0.48400765	0.60518358	0.58266116	0.44993195	0.21376845
56.8	0.52564379	0.43365331	0.48404557	0.60531907	0.58318521	0.44908446	0.21444119

11.9	0.82916809	0.30904571	0.87271623	0.85422994	0.26944779	0.29282988	0.2540446
12	0.82780225	0.30839394	0.87115752	0.85313662	0.26905244	0.29257435	0.25337213
12.1	0.82640332	0.30774904	0.86945711	0.85188657	0.26866103	0.29232222	0.25270589
12.2	0.82497527	0.30711088	0.86760263	0.85049432	0.26827341	0.29207336	0.25204573
12.3	0.8235221	0.30647936	0.86558111	0.84897343	0.26788947	0.29182766	0.25139151
12.4	0.82204766	0.30585436	0.86337903	0.84733622	0.26750907	0.291585	0.25074312
12.5	0.82055565	0.30523579	0.86098233	0.84559369	0.26713211	0.29134528	0.25010041
12.6	0.81904972	0.30462353	0.85837655	0.84375573	0.26675849	0.29110837	0.24946327
12.7	0.81753354	0.30401751	0.85554696	0.8418314	0.26638811	0.29087419	0.2488316
12.8	0.81601069	0.30341763	0.8524788	0.83982894	0.26602087	0.29064266	0.24820529
12.9	0.8144846	0.30282382	0.84915739	0.83775551	0.26565671	0.29041367	0.24758424
13	0.81295858	0.30223601	0.84556806	0.83561702	0.26529553	0.29018715	0.24696834
13.1	0.81143594	0.30165415	0.84169633	0.83341845	0.26493727	0.28996302	0.24635754
13.2	0.80991993	0.30107818	0.83752821	0.83116415	0.26458183	0.28974119	0.24575174
13.3	0.80841365	0.3005081	0.83305031	0.82885785	0.26422916	0.28952161	0.24515088
13.4	0.80692003	0.29994389	0.82825011	0.82650307	0.26387915	0.28930419	0.2445549
13.5	0.80544179	0.29938557	0.82311604	0.82410297	0.26353176	0.28908886	0.24396374
13.6	0.80398152	0.29883316	0.81763789	0.82165978	0.2631869	0.28887556	0.24337738
13.7	0.80254161	0.29828674	0.81180695	0.81917542	0.26284452	0.28866422	0.24279578
13.8	0.80112422	0.29774641	0.80561627	0.81665204	0.26250455	0.28845479	0.24221893
13.9	0.79973136	0.29721232	0.79906101	0.81409181	0.26216693	0.28824722	0.24164681
14	0.79836485	0.29668468	0.79213841	0.81149656	0.26183161	0.28804144	0.24107943
14.1	0.79702622	0.29616374	0.78484793	0.80886785	0.26149854	0.28783741	0.24051681
14.2	0.79571675	0.29564986	0.77719159	0.80620705	0.26116767	0.28763508	0.23995898
14.3	0.79443752	0.2951435	0.76917383	0.80351561	0.26083894	0.2874344	0.23940599
14.4	0.7931894	0.29464521	0.76080168	0.80079522	0.26051232	0.28723533	0.23885791
14.5	0.79197302	0.29415572	0.75208479	0.79804748	0.26018775	0.28703782	0.23831481
14.6	0.7907888	0.2936759	0.74303551	0.79527388	0.25986519	0.28684182	0.23777678
14.7	0.78963697	0.29320687	0.73366888	0.79247596	0.2595446	0.28664729	0.23724394
14.8	0.78851762	0.29274999	0.72400232	0.78965561	0.25922594	0.28645419	0.2367164
14.9	0.78743067	0.29230694	0.71405571	0.7868148	0.25890917	0.2862625	0.23619432

60	0.51110292	0.4269636	0.48267784	0.60925656	0.60342033	0.42530921	0.23486945
60.1	0.510666	0.42676577	0.48255084	0.60936743	0.60411768	0.42464999	0.23546815
60.2	0.51023011	0.42656882	0.48241861	0.60947761	0.60481657	0.42399475	0.23606417
60.3	0.50979527	0.42637276	0.48228114	0.60958708	0.6055168	0.4233434	0.2366575
60.4	0.50936145	0.42617762	0.48213844	0.60969586	0.60621819	0.42269587	0.23724813
60.5	0.50892867	0.42598339	0.4819905	0.60980395	0.60692055	0.42205208	0.23783603
60.6	0.50849692	0.4257901	0.48183732	0.60991135	0.60762371	0.42141194	0.23842118
60.7	0.50806619	0.42559776	0.48167891	0.61001807	0.60832747	0.42077539	0.23900358
60.8	0.50763649	0.42540637	0.48151527	0.61012412	0.60903166	0.42014235	0.2395832
60.9	0.50720782	0.42521595	0.48134639	0.61022949	0.60973612	0.41951273	0.24016003
61	0.50678016	0.4250265	0.48117228	0.6103342	0.61044068	0.41888648	0.24073406
61.1	0.50635353	0.42483804	0.48099295	0.61043824	0.61114518	0.41826351	0.24130527
61.2	0.50592791	0.42465057	0.4808084	0.61054163	0.61184947	0.41764377	0.24187365
61.3	0.50550331	0.42446411	0.48061865	0.61064437	0.61255336	0.41702718	0.24243919
61.4	0.50507972	0.42427865	0.4804237	0.61074646	0.61325672	0.41641368	0.24300187
61.5	0.50465714	0.42409422	0.48022355	0.6108479	0.61395938	0.41580321	0.24356167
61.6	0.50423556	0.42391081	0.48001822	0.61094871	0.61466119	0.4151957	0.24411861
61.7	0.503815	0.42372843	0.47980773	0.61104889	0.615362	0.41459109	0.24467266
61.8	0.50339543	0.42354709	0.47959208	0.61114845	0.61606166	0.41398932	0.24522381
61.9	0.50297687	0.4233668	0.47937128	0.61124738	0.61676003	0.4133903	0.24577207
62	0.50255931	0.42318755	0.47914536	0.6113457	0.61745698	0.41279399	0.24631741
62.1	0.50214274	0.42300936	0.47891433	0.61144341	0.61815237	0.41220032	0.24685983
62.2	0.50172716	0.42283223	0.47867822	0.61154051	0.61884607	0.41160924	0.24739932
62.3	0.50131258	0.42265615	0.47843703	0.61163702	0.61953796	0.41102069	0.24793586
62.4	0.50089898	0.42248115	0.4781908	0.61173293	0.62022789	0.41043461	0.24846946
62.5	0.50048637	0.42230721	0.47793954	0.61182825	0.62091575	0.40985096	0.2490001
62.6	0.50007474	0.42213434	0.47768328	0.61192298	0.62160141	0.40926966	0.24952778
62.7	0.49966409	0.42196255	0.47742204	0.61201714	0.62228476	0.40869068	0.2500525
62.8	0.49925442	0.42179183	0.47715584	0.61211072	0.62296569	0.40811395	0.25057424
62.9	0.49884572	0.42162219	0.47688471	0.61220373	0.62364408	0.40753942	0.25109302
63	0.498438	0.42145364	0.47660869	0.61229618	0.62431982	0.40696705	0.25160883
63.1	0.49803126	0.42128616	0.47632778	0.61238806	0.62499281	0.40639679	0.25212167
63.2	0.49762548	0.42111977	0.47604204	0.61247939	0.62566296	0.40582858	0.25263154
63.3	0.49722067	0.42095446	0.47575147	0.61257018	0.62633016	0.40526238	0.25313843
63.4	0.49681683	0.42079024	0.47545613	0.61266042	0.62699433	0.40469814	0.25364236
63.5	0.49641395	0.4206271	0.47515603	0.61275012	0.62765537	0.40413581	0.25414331
63.6	0.49601203	0.42046505	0.47485122	0.61283928	0.62831319	0.40357533	0.25464129
63.7	0.49561107	0.42030408	0.47454173	0.61292792	0.6289677	0.40301668	0.25513629
63.8	0.49521107	0.4201442	0.47422759	0.61301604	0.62961882	0.40245979	0.25562832
63.9	0.49481202	0.41998541	0.47390884	0.61310363	0.63026646	0.40190463	0.25611739
64	0.49441392	0.41982769	0.47358552	0.61319072	0.63091055	0.40135116	0.25660348
64.1	0.49401678	0.41967106	0.47325766	0.6132773	0.63155099	0.40079934	0.2570866
64.2	0.49362058	0.41951551	0.47292531	0.61336337	0.63218773	0.40024912	0.25756675
64.3	0.49322532	0.41936103	0.47258851	0.61344895	0.63282067	0.39970047	0.25804393
64.4	0.49283101	0.41920763	0.4722473	0.61353404	0.63344977	0.39915335	0.25851815
64.5	0.49243764	0.4190553	0.47190171	0.61361864	0.63407495	0.39860772	0.2589894
64.6	0.49204522	0.41890404	0.4715518	0.61370275	0.63469615	0.39806355	0.2594577
64.7	0.49165372	0.41875385	0.47119761	0.61378639	0.63531332	0.39752079	0.25992305
64.8	0.49126317	0.41860472	0.47083918	0.61386956	0.6359264	0.39697942	0.26038544
64.9	0.49087354	0.41845665	0.47047657	0.61395226	0.63653534	0.3964394	0.2608449
65	0.49048485	0.41830964	0.47010981	0.61403449	0.63714008	0.3959007	0.26130142
65.1	0.49009709	0.41816368	0.46973895	0.61411626	0.63774057	0.39536327	0.26175501
65.2	0.48971026	0.41801877	0.46936405	0.61419758	0.63833675	0.39482708	0.26220569
65.3	0.48932435	0.41787491	0.46898515	0.61427845	0.63892858	0.39429211	0.26265347
65.4	0.48893936	0.4177321	0.46860231	0.61435888	0.63951602	0.39375831	0.26309835
65.5	0.4885553	0.41759032	0.46821557	0.61443886	0.64009902	0.39322567	0.26354033
65.6	0.48817216	0.41744958	0.46782498	0.61451841	0.64067753	0.39269414	0.26397944
65.7	0.48778994	0.41730988	0.4674306	0.61459752	0.64125153	0.3921637	0.26441567
65.8	0.48740864	0.4171712	0.46703247	0.61467621	0.64182097	0.39163434	0.26484903
65.9	0.48702825	0.41703354	0.46663065	0.61475447	0.64238583	0.39110601	0.26527954
66	0.48664878	0.4168969	0.46622519	0.61483232	0.64294605	0.39057871	0.26570721
66.1	0.48627022	0.41676128	0.46581613	0.61490975	0.64350163	0.3900524	0.26613204
66.2	0.48589257	0.41662666	0.46540352	0.61498677	0.64405252	0.38952707	0.26655405
66.3	0.48551583	0.41649304	0.46498742	0.61506338	0.6445987	0.38900268	0.26697325
66.4	0.48514	0.41636043	0.46456788	0.6151396	0.64514015	0.38847923	0.26738965
66.5	0.48476507	0.41622881	0.46414495	0.61521541	0.64567683	0.38795669	0.26780326
66.6	0.48439105	0.41609817	0.46371868	0.61529083	0.64620873	0.38743504	0.26821408
66.7	0.48401793	0.41596852	0.46328914	0.61536586	0.64673581	0.38691426	0.26862214
66.8	0.48364572	0.41583985	0.46285637	0.6154405	0.64725805	0.38639433	0.26902745
66.9	0.4832744	0.41571216	0.46242043	0.61551476	0.64777545	0.38587524	0.26943001
67	0.48290398	0.41558543	0.46198137	0.61558864	0.64828797	0.38535696	0.26982985
67.1	0.48253445	0.41545966	0.46153924	0.61566215	0.64879562	0.38483949	0.27022698
67.2	0.48216582	0.41533485	0.4610941	0.61573529	0.6492984	0.38432281	0.27062141
67.3	0.48179807	0.415211	0.46064601	0.61580807	0.6497963	0.3838069	0.27101317
67.4	0.48143122	0.41508808	0.46019501	0.61588048	0.65028931	0.38329175	0.27140226
67.5	0.48106526	0.41496611	0.45974117	0.61595253	0.65077742	0.38277735	0.2717887
67.6	0.48070018	0.41484508	0.45928453	0.61602423	0.65126064	0.38226368	0.2721725
67.7	0.48033599	0.41472497	0.45882515	0.61609558	0.65173895	0.38175074	0.27255368
67.8	0.47997268	0.41460578	0.45836308	0.61616658	0.65221235	0.38123852	0.27293225
67.9	0.47961026	0.41448751	0.45789839	0.61623724	0.65268082	0.380727	0.27330821
68	0.47924872	0.41437015	0.45743112	0.61630756	0.65314439	0.38021617	0.2736816
68.1	0.47888805	0.4142537	0.45696131	0.61637754	0.65360303	0.37970603	0.27405242
68.2	0.47852827	0.41413815	0.45648903	0.61644719	0.65405677	0.37919658	0.27442069
68.3	0.47816935	0.41402349	0.45601432	0.61651651	0.65450558	0.37868779	0.27478642
68.4	0.47781132	0.41390972	0.45553723	0.61658551	0.65494949	0.37817968	0.27514962
68.5	0.47745415	0.41379683	0.45505783	0.61665419	0.65538849	0.37767222	0.27551033
68.6	0.47709786	0.41368481	0.45457615	0.61672254	0.65582259	0.37716542	0.27586854
68.7	0.47674243	0.41357367	0.45409226	0.61679058	0.65625179	0.37665928	0.27622428
68.8	0.47638787	0.41346339	0.4536062	0.61685832	0.65667611	0.37615379	0.27657756
68.9	0.47603417	0.41335398	0.45311803	0.61692574	0.65709556	0.37564894	0.2769284

26.9	0.70456444	0.59354526	0.38844724	0.60641868	0.22967106	0.27041252	0.40976761
27	0.70378753	0.59189834	0.38957935	0.60564204	0.22949311	0.27065493	0.40645912
27.1	0.70301087	0.59025911	0.39068798	0.60487268	0.22931755	0.27095593	0.40317564
27.2	0.70223454	0.58862807	0.39177336	0.6041108	0.22914462	0.27132321	0.39991738
27.3	0.70145865	0.5870054	0.39283576	0.60335657	0.22897457	0.27176512	0.39668458
27.4	0.7006833	0.58539126	0.39387547	0.60261017	0.22880768	0.27229069	0.39347738
27.5	0.69990858	0.58378579	0.39489281	0.60187179	0.22864429	0.27290964	0.39029582
27.6	0.69913458	0.58218917	0.39588812	0.6011416	0.22848478	0.2736324	0.38713996
27.7	0.69836139	0.58060158	0.39686173	0.60041977	0.2283296	0.27447003	0.38400987
27.8	0.69758913	0.57902317	0.39781401	0.59970646	0.22817926	0.27543421	0.38090567
27.9	0.69681788	0.57745406	0.39874532	0.59900182	0.22803434	0.27653715	0.37782743
28	0.69604773	0.57589434	0.39965606	0.59830602	0.22789548	0.27779161	0.3747752
28.1	0.69527878	0.57434425	0.40054662	0.5976192	0.22776344	0.27921081	0.37174903
28.2	0.6945111	0.57280399	0.40141739	0.59694151	0.22763911	0.28080825	0.36874893
28.3	0.69374477	0.57127354	0.40226879	0.59627308	0.22752351	0.28259769	0.36577488
28.4	0.69297987	0.56975292	0.40310123	0.59561404	0.2274178	0.28459297	0.36282684
28.5	0.69221647	0.56824222	0.40391513	0.59496452	0.22732328	0.28680798	0.35990481
28.6	0.69145466	0.56674151	0.40471085	0.59432463	0.22724143	0.2892565	0.35700877
28.7	0.6906945	0.56525096	0.40548877	0.59369447	0.227174	0.29195204	0.35413871
28.8	0.68993607	0.56377077	0.4062493	0.59307413	0.22712301	0.29490755	0.35129459
28.9	0.68917944	0.56230112	0.40699286	0.59246367	0.22709074	0.29813527	0.34847638
29	0.68842467	0.5608421	0.40771984	0.59186317	0.22707976	0.30164664	0.34568404
29.1	0.68767183	0.55939383	0.40843066	0.5912727	0.22709299	0.30545202	0.34291749
29.2	0.68692098	0.55795641	0.40912572	0.59069232	0.22713375	0.30956039	0.34017663
29.3	0.68617217	0.55652994	0.40980542	0.59012211	0.2272059	0.31397955	0.33746139
29.4	0.68542548	0.55511452	0.41047015	0.5895621	0.22731377	0.3187161	0.33477166
29.5	0.68468095	0.55371023	0.41112035	0.58901236	0.22746228	0.32377484	0.33210732
29.6	0.68393862	0.55231717	0.41175641	0.58847292	0.22765699	0.3291585	0.32946822
29.7	0.68319855	0.55093544	0.41237872	0.58794382	0.22790417	0.33486766	0.32685424
29.8	0.68246076	0.5495652	0.41298769	0.58742509	0.22821084	0.34090071	0.32426528
29.9	0.68172531	0.54820654	0.41358368	0.58691674	0.22858489	0.34725423	0.32170121

75	0.45600522	0.40809991	0.42111027	0.62055822	0.6740398	0.3461554	0.29434568
75.1	0.45570052	0.40803418	0.42057307	0.62061101	0.67418465	0.34569619	0.29457569
75.2	0.45539654	0.40796903	0.42003609	0.6206636	0.67432545	0.34523788	0.2948042
75.3	0.45509325	0.40790446	0.41949936	0.620716	0.67446221	0.34478045	0.29503121
75.4	0.45479065	0.40784046	0.41896289	0.62076821	0.67459494	0.34432392	0.29525674
75.5	0.45448875	0.40777703	0.41842671	0.62082023	0.67472366	0.3438683	0.29548079
75.6	0.45418754	0.40771417	0.41789083	0.62087206	0.67484837	0.34341357	0.29570338
75.7	0.45388702	0.40765187	0.41735529	0.62092369	0.67496912	0.34295975	0.29592451
75.8	0.45358719	0.40759013	0.41682011	0.62097514	0.6750859	0.34250685	0.2961442
75.9	0.45328803	0.40752895	0.41628529	0.62102641	0.67519875	0.34205485	0.29636246
76	0.45298956	0.40746833	0.41575087	0.62107748	0.67530767	0.34160378	0.2965793
76.1	0.45269176	0.40740826	0.41521686	0.62112837	0.67541269	0.34115361	0.29679473
76.2	0.45239464	0.40734874	0.41468329	0.62117908	0.67551383	0.34070436	0.29700877
76.3	0.45209819	0.40728977	0.41415018	0.6212296	0.6756111	0.34025603	0.29722141
76.4	0.45180241	0.40723134	0.41361754	0.62127994	0.67570453	0.33980862	0.29743267
76.5	0.45150729	0.40717345	0.4130854	0.6213301	0.67579414	0.33936213	0.29764256
76.6	0.45121284	0.4071161	0.41255378	0.62138008	0.67587995	0.33891657	0.2978511
76.7	0.45091905	0.40705928	0.41202268	0.62142988	0.675962	0.33847193	0.29805828
76.8	0.45062591	0.40700299	0.41149214	0.6214795	0.67604029	0.33802822	0.29826413
76.9	0.45033344	0.40694724	0.41096216	0.62152895	0.67611485	0.33758544	0.29846864
77	0.45004161	0.40689201	0.41043278	0.62157822	0.67618571	0.33714358	0.29867182
77.1	0.44975043	0.4068373	0.409904	0.62162731	0.67625288	0.33670266	0.2988737
77.2	0.4494599	0.40678312	0.40937584	0.62167623	0.6763164	0.33626267	0.29907426
77.3	0.44917002	0.40672945	0.40884832	0.62172498	0.67637629	0.3358236	0.29927353
77.4	0.44888078	0.4066763	0.40832146	0.62177356	0.67643257	0.33538547	0.29947151
77.5	0.44859218	0.40662366	0.40779528	0.62182197	0.67648528	0.33494827	0.29966821
77.6	0.44830422	0.40657153	0.40726978	0.62187021	0.67653444	0.334512	0.29986363
77.7	0.44801689	0.40651991	0.40674499	0.62191828	0.67658008	0.33407667	0.30005779
77.8	0.44773019	0.4064688	0.40622092	0.62196619	0.67662224	0.33364227	0.3002507
77.9	0.44744413	0.40641818	0.40569759	0.62201393	0.67666094	0.3332088	0.30044235
78	0.44715869	0.40636807	0.40517501	0.6220615	0.67669621	0.33277627	0.30063277
78.1	0.44687388	0.40631846	0.40465319	0.62210891	0.67672808	0.33234468	0.30082195
78.2	0.44658969	0.40626934	0.40413216	0.62215616	0.67675658	0.33191402	0.3010099
78.3	0.44630612	0.40622072	0.40361193	0.62220325	0.67678175	0.3314843	0.30119663
78.4	0.44602317	0.40617258	0.4030925	0.62225018	0.67680362	0.33105551	0.30138214
78.5	0.44574084	0.40612494	0.4025739	0.62229696	0.67682221	0.33062766	0.30156644
78.6	0.44545912	0.40607778	0.40205613	0.62234357	0.67683757	0.33020075	0.30174953
78.7	0.44517801	0.4060311	0.4015392	0.62239003	0.67684973	0.32977477	0.30193143
78.8	0.44489752	0.40598491	0.40102314	0.62243634	0.67685872	0.32934972	0.30211213
78.9	0.44461763	0.40593919	0.40050794	0.62248249	0.67686457	0.32892561	0.30229165
79	0.44433835	0.40589395	0.39999363	0.62252849	0.67686733	0.32850243	0.30246998
79.1	0.44405967	0.40584918	0.3994802	0.62257434	0.67686701	0.32808019	0.30264714
79.2	0.44378159	0.40580489	0.39896769	0.62262004	0.67686365	0.32765888	0.30282313
79.3	0.44350411	0.40576106	0.39845609	0.62266559	0.6768573	0.3272385	0.30299795
79.4	0.44322723	0.4057177	0.39794543	0.622711	0.67684797	0.32681906	0.3031716
79.5	0.44295095	0.40567481	0.39743571	0.62275626	0.6768357	0.32640054	0.30334411
79.6	0.44267526	0.40563237	0.39692693	0.62280137	0.67682053	0.32598296	0.30351545
79.7	0.44240017	0.4055904	0.39641913	0.62284635	0.6768025	0.32556631	0.30368565
79.8	0.44212566	0.40554889	0.39591229	0.62289118	0.67678162	0.32515059	0.3038547
79.9	0.44185174	0.40550784	0.39540644	0.62293587	0.67675795	0.32473579	0.3040226
80	0.44157841	0.40546723	0.39490158	0.62298042	0.67673151	0.32432192	0.30418936
80.1	0.44130567	0.40542708	0.39439772	0.62302483	0.67670234	0.32390897	0.30435499
80.2	0.4410335	0.40538738	0.39389488	0.6230691	0.67667046	0.32349693	0.30451948
80.3	0.44076192	0.40534813	0.39339307	0.62311324	0.67663592	0.32308582	0.30468283
80.4	0.44049092	0.40530932	0.39289228	0.62315725	0.67659875	0.32267563	0.30484505
80.5	0.44022049	0.40527096	0.39239255	0.62320112	0.67655898	0.32226635	0.30500615
80.6	0.43995064	0.40523304	0.39189386	0.62324486	0.67651664	0.321858	0.30516612
80.7	0.43968136	0.40519556	0.39139623	0.62328847	0.67647177	0.32145057	0.30532497
80.8	0.43941266	0.40515851	0.39089968	0.62333195	0.67642439	0.32104405	0.30548269
80.9	0.43914453	0.4051219	0.3904042	0.6233753	0.67637455	0.32063846	0.30563929
81	0.43887696	0.40508572	0.38990981	0.62341852	0.67632227	0.32023379	0.30579476
81.1	0.43860996	0.40504997	0.38941651	0.62346162	0.67626758	0.31983003	0.30594911
81.2	0.43834353	0.40501465	0.38892432	0.62350459	0.67621052	0.3194272	0.30610235
81.3	0.43807767	0.40497975	0.38843323	0.62354744	0.67615111	0.31902528	0.30625445
81.4	0.43781237	0.40494528	0.38794327	0.62359017	0.6760894	0.31862427	0.30640544
81.5	0.43754763	0.40491123	0.38745443	0.62363277	0.6760254	0.31822418	0.30655531
81.6	0.43728345	0.40487759	0.38696673	0.62367526	0.67595915	0.31782501	0.30670405
81.7	0.43701982	0.40484438	0.38648017	0.62371762	0.67589069	0.31742675	0.30685168
81.8	0.43675676	0.40481158	0.38599476	0.62375987	0.67582004	0.31702941	0.30699818
81.9	0.43649425	0.4047792	0.3855105	0.623802	0.67574723	0.31663297	0.30714355
82	0.43623229	0.40474723	0.3850274	0.62384401	0.67567229	0.31623746	0.3072878
82.1	0.43597089	0.40471567	0.38454547	0.62388591	0.67559526	0.31584285	0.30743092
82.2	0.43571004	0.40468451	0.38406472	0.6239277	0.67551617	0.31544916	0.30757291
82.3	0.43544973	0.40465376	0.38358515	0.62396938	0.67543504	0.31505637	0.30771377
82.4	0.43518998	0.40462342	0.38310676	0.62401094	0.6753519	0.3146645	0.30785349
82.5	0.43493077	0.40459347	0.38262956	0.62405239	0.67526678	0.31427354	0.30799208
82.6	0.43467211	0.40456393	0.38215356	0.62409374	0.67517971	0.31388348	0.30812953
82.7	0.43441399	0.40453478	0.38167876	0.62413497	0.67509072	0.31349433	0.30826584
82.8	0.43415641	0.40450603	0.38120516	0.6241761	0.67499984	0.31310608	0.308401
82.9	0.43389938	0.40447768	0.38073278	0.62421712	0.67490709	0.31271874	0.30853502
83	0.43364288	0.40444972	0.38026161	0.62425804	0.6748125	0.31233229	0.30866788
83.1	0.43338693	0.40442214	0.37979166	0.62429886	0.67471609	0.31194675	0.3087996
83.2	0.43313151	0.40439496	0.37932293	0.62433957	0.6746179	0.31156211	0.30893015
83.3	0.43287662	0.40436816	0.37885544	0.62438017	0.67451794	0.31117837	0.30905955
83.4	0.43262227	0.40434175	0.37838918	0.62442068	0.67441625	0.31079553	0.30918778
83.5	0.43236845	0.40431572	0.37792415	0.62446109	0.67431285	0.31041359	0.30931484
83.6	0.43211517	0.40429008	0.37746037	0.6245014	0.67420777	0.31003254	0.30944073
83.7	0.43186241	0.40426481	0.37699784	0.62454161	0.67410102	0.30965234	0.30956545
83.8	0.43161018	0.40423992	0.37653655	0.62458172	0.67399264	0.30927315	0.30968898
83.9	0.43135848	0.40421541	0.37607662	0.62462174	0.67388265	0.3088948	0.30981132

41.9	0.60667085	0.46411389	0.45235273	0.5800182	0.67016941	0.69820789	0.15156348
42	0.60608497	0.46384375	0.45260765	0.58018924	0.66896077	0.69632302	0.15117141
42.1	0.60549908	0.46357693	0.45286295	0.5803611	0.66773692	0.69441769	0.15079721
42.2	0.60491316	0.46331336	0.45311863	0.58053374	0.66649869	0.69249245	0.15044085
42.3	0.60432725	0.46305298	0.45337472	0.58070713	0.6652469	0.69054787	0.1501023
42.4	0.60374134	0.46279573	0.45363122	0.58088123	0.6639823	0.68858456	0.14978156
42.5	0.60315544	0.46254154	0.45388813	0.581056	0.66270563	0.68660313	0.14947857
42.6	0.60256956	0.46229033	0.45414549	0.58123141	0.66141757	0.68460416	0.1491933
42.7	0.60198372	0.46204205	0.45440329	0.58140744	0.66011889	0.68258828	0.14892569
42.8	0.60139793	0.46179663	0.45466155	0.58158404	0.65881035	0.68055609	0.14867569
42.9	0.6008122	0.46155398	0.45492027	0.5817612	0.65749267	0.6785082	0.14844325
43	0.60022654	0.46131406	0.45517944	0.58193888	0.65616657	0.67644521	0.14822829
43.1	0.59964096	0.4610768	0.45543907	0.58211706	0.65483273	0.67436776	0.14803073
43.2	0.59905547	0.46084213	0.45569917	0.5822957	0.65349182	0.67227647	0.14785051
43.3	0.59847009	0.46060998	0.45595973	0.5824748	0.65214455	0.67017197	0.14768755
43.4	0.59788484	0.46038027	0.45622075	0.58265432	0.65079168	0.66805491	0.14754177
43.5	0.59729971	0.46015294	0.45648222	0.58283423	0.64943395	0.66592593	0.14741308
43.6	0.59671474	0.45992793	0.45674417	0.58301452	0.64807205	0.66378567	0.14730139
43.7	0.59612993	0.45970517	0.45700657	0.58319516	0.64670671	0.66163484	0.1472066
43.8	0.59554529	0.4594846	0.4572694	0.58337614	0.64533861	0.65947416	0.1471286
43.9	0.59496085	0.45926615	0.45753265	0.58355742	0.64396846	0.65730432	0.14706729
44	0.59437661	0.45904976	0.4577963	0.58373899	0.64259702	0.65512602	0.14702256
44.1	0.59379259	0.45883534	0.45806032	0.58392083	0.64122504	0.65293995	0.14699429
44.2	0.59320881	0.45862283	0.45832472	0.58410291	0.63985324	0.65074677	0.14698236
44.3	0.59262527	0.45841217	0.4585895	0.58428522	0.63848236	0.64854714	0.14698664
44.4	0.592042	0.45820329	0.45885465	0.58446775	0.63711312	0.64634179	0.147007
44.5	0.59145901	0.45799612	0.45912014	0.58465046	0.63574624	0.64413145	0.14704329
44.6	0.59087631	0.45779061	0.45938596	0.58483336	0.63438248	0.64191683	0.14709538
44.7	0.59029393	0.45758671	0.45965208	0.58501641	0.63302261	0.63969865	0.1471631
44.8	0.58971187	0.45738434	0.45991849	0.58519961	0.63166739	0.63747768	0.14724631
44.9	0.58913015	0.45718344	0.46018514	0.58538294	0.63031757	0.63525462	0.14734484

90	0.41695697	0.40336294	0.35046883	0.62691282	0.66494052	0.28749326	0.3149285
90.1	0.41673577	0.40335843	0.3500896	0.62694834	0.66476903	0.28716959	0.31497451
90.2	0.41651503	0.40335419	0.34971166	0.62698382	0.66459708	0.28684679	0.3150194
90.3	0.41629474	0.40335022	0.34933501	0.62701923	0.66442469	0.28652486	0.31506319
90.4	0.41607491	0.40334652	0.34895965	0.6270546	0.66425187	0.28620379	0.31510588
90.5	0.41585554	0.40334309	0.34858557	0.62708991	0.66407863	0.28588358	0.31514747
90.6	0.41563662	0.40333992	0.34821277	0.62712517	0.663905	0.28556424	0.31518798
90.7	0.41541815	0.40333701	0.34784126	0.62716038	0.66373098	0.28524576	0.31522742
90.8	0.41520013	0.40333437	0.34747102	0.62719553	0.66355658	0.28492815	0.3152658
90.9	0.41498257	0.40333199	0.34710206	0.62723064	0.66338184	0.28461139	0.31530312
91	0.41476545	0.40332987	0.34673438	0.62726569	0.66320675	0.28429549	0.31533939
91.1	0.41454878	0.40332801	0.34636796	0.62730069	0.66303133	0.28398046	0.31537462
91.2	0.41433256	0.40332641	0.34600282	0.62733565	0.6628556	0.28366628	0.31540883
91.3	0.41411679	0.40332506	0.34563894	0.62737055	0.66267957	0.28335295	0.31544202
91.4	0.41390146	0.40332396	0.34527632	0.62740541	0.66250325	0.28304049	0.3154742
91.5	0.41368658	0.40332312	0.34491497	0.62744021	0.66232665	0.28272887	0.31550538
91.6	0.41347214	0.40332253	0.34455487	0.62747497	0.6621498	0.28241812	0.31553557
91.7	0.41325814	0.40332219	0.34419603	0.62750968	0.6619727	0.28210822	0.31556477
91.8	0.41304458	0.4033221	0.34383845	0.62754434	0.66179537	0.28179917	0.315593
91.9	0.41283146	0.40332225	0.34348211	0.62757896	0.66161781	0.28149098	0.31562026
92	0.41261879	0.40332265	0.34312703	0.62761353	0.66144005	0.28118364	0.31564656
92.1	0.41240655	0.40332329	0.34277318	0.62764805	0.6612621	0.28087715	0.31567191
92.2	0.41219474	0.40332418	0.34242058	0.62768252	0.66108397	0.28057151	0.31569632
92.3	0.41198338	0.40332531	0.34206922	0.62771695	0.66090566	0.28026673	0.3157198
92.4	0.41177245	0.40332668	0.34171909	0.62775133	0.66072721	0.27996279	0.31574235
92.5	0.41156195	0.40332828	0.34137019	0.62778567	0.66054861	0.2796597	0.31576399
92.6	0.41135189	0.40333013	0.34102252	0.62781996	0.66036988	0.27935747	0.31578473
92.7	0.41114226	0.40333221	0.34067608	0.62785421	0.66019103	0.27905608	0.31580458
92.8	0.41093306	0.40333452	0.34033086	0.62788841	0.66001208	0.27875554	0.31582354
92.9	0.41072429	0.40333707	0.33998686	0.62792257	0.65983304	0.27845584	0.31584161
93	0.41051595	0.40333985	0.33964407	0.62795668	0.65965392	0.27815699	0.31585882
93.1	0.41030804	0.40334286	0.3393025	0.62799075	0.65947473	0.27785899	0.31587516
93.2	0.41010056	0.4033461	0.33896214	0.62802478	0.65929548	0.27756183	0.31589066
93.3	0.4098935	0.40334956	0.33862298	0.62805877	0.65911618	0.27726551	0.3159053
93.4	0.40968687	0.40335325	0.33828502	0.62809271	0.65893686	0.27697004	0.31591911
93.5	0.40948067	0.40335717	0.33794826	0.62812661	0.65875751	0.27667541	0.31593209
93.6	0.40927488	0.40336131	0.3376127	0.62816046	0.65857814	0.27638162	0.31594426
93.7	0.40906952	0.40336567	0.33727832	0.62819428	0.65839878	0.27608868	0.31595561
93.8	0.40886458	0.40337025	0.33694514	0.62822806	0.65821943	0.27579657	0.31596616
93.9	0.40866007	0.40337505	0.33661314	0.62826179	0.65804011	0.27550531	0.31597591
94	0.40845597	0.40338007	0.33628232	0.62829548	0.65786081	0.27521489	0.31598488
94.1	0.40825229	0.4033853	0.33595268	0.62832913	0.65768156	0.2749253	0.31599308
94.2	0.40804902	0.40339075	0.3356242	0.62836275	0.65750235	0.27463655	0.3160005
94.3	0.40784618	0.40339641	0.3352969	0.62839632	0.65732321	0.27434864	0.31600715
94.4	0.40764375	0.40340229	0.33497077	0.62842985	0.65714413	0.27406157	0.31601305
94.5	0.40744173	0.40340837	0.33464579	0.62846334	0.65696513	0.27377533	0.3160182
94.6	0.40724013	0.40341467	0.33432197	0.62849679	0.65678621	0.27348993	0.3160226
94.7	0.40703895	0.40342118	0.33399931	0.62853021	0.65660739	0.27320537	0.31602627
94.8	0.40683817	0.40342789	0.3336778	0.62856358	0.65642868	0.27292163	0.31602921
94.9	0.40663781	0.4034348	0.33335743	0.62859692	0.65625008	0.27263874	0.31603144
95	0.40643785	0.40344193	0.3330382	0.62863022	0.6560716	0.27235667	0.31603295
95.1	0.40623831	0.40344925	0.33272012	0.62866348	0.65589326	0.27207544	0.31603376
95.2	0.40603917	0.40345678	0.33240316	0.62869671	0.65571505	0.27179504	0.31603387
95.3	0.40584044	0.40346451	0.33208734	0.62872989	0.655537	0.27151548	0.3160333
95.4	0.40564212	0.40347243	0.33177264	0.62876304	0.6553591	0.27123674	0.31603204
95.5	0.40544421	0.40348056	0.33145907	0.62879616	0.65518137	0.27095884	0.31603011
95.6	0.4052467	0.40348888	0.33114661	0.62882923	0.65500382	0.27068176	0.31602751
95.7	0.40504959	0.40349739	0.33083526	0.62886227	0.65482645	0.27040551	0.31602425
95.8	0.40485288	0.4035061	0.33052503	0.62889528	0.65464927	0.27013009	0.31602034
95.9	0.40465658	0.403515	0.3302159	0.62892825	0.65447229	0.26985549	0.31601578
96	0.40446068	0.4035241	0.32990788	0.62896118	0.65429552	0.26958172	0.31601058
96.1	0.40426518	0.40353338	0.32960095	0.62899408	0.65411897	0.26930877	0.31600475
96.2	0.40407008	0.40354286	0.32929511	0.62902694	0.65394263	0.26903665	0.3159983
96.3	0.40387538	0.40355252	0.32899037	0.62905977	0.65376653	0.26876534	0.31599122
96.4	0.40368107	0.40356237	0.32868671	0.62909256	0.65359067	0.26849486	0.31598354
96.5	0.40348716	0.4035724	0.32838413	0.62912532	0.65341505	0.26822519	0.31597524
96.6	0.40329365	0.40358262	0.32808263	0.62915805	0.65323968	0.26795635	0.31596635
96.7	0.40310053	0.40359302	0.3277822	0.62919074	0.65306458	0.26768832	0.31595687
96.8	0.40290781	0.4036036	0.32748284	0.6292234	0.65288974	0.26742111	0.3159468
96.9	0.40271548	0.40361436	0.32718454	0.62925603	0.65271517	0.26715471	0.31593615
97	0.40252354	0.4036253	0.32688731	0.62928862	0.65254088	0.26688913	0.31592493
97.1	0.40233199	0.40363642	0.32659113	0.62932119	0.65236688	0.26662436	0.31591314
97.2	0.40214084	0.40364772	0.326296	0.62935372	0.65219317	0.26636041	0.31590079
97.3	0.40195007	0.40365919	0.32600192	0.62938621	0.65201976	0.26609726	0.31588789
97.4	0.40175969	0.40367083	0.32570888	0.62941868	0.65184666	0.26583493	0.31587443
97.5	0.40156971	0.40368265	0.32541688	0.62945111	0.65167385	0.2655734	0.31586044
97.6	0.40138011	0.40369464	0.32512591	0.62948352	0.65150136	0.26531269	0.31584591
97.7	0.40119089	0.40370679	0.32483598	0.62951589	0.65132919	0.26505278	0.31583085
97.8	0.40100206	0.40371912	0.32454707	0.62954823	0.65115734	0.26479368	0.31581527
97.9	0.40081362	0.40373162	0.32425918	0.62958054	0.65098582	0.26453538	0.31579917
98	0.40062556	0.40374428	0.3239723	0.62961282	0.65081463	0.26427789	0.31578255
98.1	0.40043789	0.40375711	0.32368644	0.62964507	0.65064378	0.2640212	0.31576544
98.2	0.4002506	0.4037701	0.32340159	0.62967729	0.65047327	0.26376531	0.31574782
98.3	0.40006369	0.40378325	0.32311774	0.62970948	0.6503031	0.26351022	0.31572971
98.4	0.39987716	0.40379657	0.32283488	0.62974164	0.65013329	0.26325593	0.31571111
98.5	0.39969101	0.40381004	0.32255302	0.62977378	0.64996384	0.26300244	0.31569202
98.6	0.39950524	0.40382368	0.32227215	0.62980588	0.64979474	0.26274975	0.31567246
98.7	0.39931927	0.40383747	0.32199189	0.62983792	0.64962534	0.2624974	0.31565258
98.8	0.39913484	0.40385142	0.32171335	0.62987	0.64945766	0.26224675	0.31563192
98.9	0.39895021	0.40386553	0.32143542	0.62990201	0.64928967	0.26199644	0.31561096

56.9	0.5251727	0.43343739	0.48407889	0.60545381	0.58371836	0.4482446	0.21511221
57	0.5247027	0.43322165	0.48410757	0.60558779	0.58426038	0.44741223	0.21578145
57.1	0.52423379	0.43300614	0.48413158	0.60572103	0.58481099	0.44658721	0.21644889
57.2	0.52376597	0.43279086	0.48415087	0.6058535	0.58536996	0.44576942	0.21711448
57.3	0.52329923	0.43257586	0.48416542	0.60598522	0.58593702	0.44495871	0.21777819
57.4	0.52283358	0.43236116	0.4841752	0.60611619	0.58651193	0.44415496	0.21843997
57.5	0.52236901	0.43214678	0.48418018	0.60624639	0.58709442	0.44335804	0.2190998
57.6	0.52190552	0.43193275	0.48418034	0.60637583	0.58768426	0.44256782	0.21975763
57.7	0.52144312	0.43171911	0.48417563	0.60650451	0.58828119	0.44178418	0.22041344
57.8	0.52098179	0.43150586	0.48416603	0.60663243	0.58888496	0.44100697	0.22106718
57.9	0.52052154	0.43129304	0.48415152	0.60675958	0.58949532	0.44023608	0.22171883
58	0.52006237	0.43108067	0.48413206	0.60688598	0.59011204	0.43947139	0.22236835
58.1	0.51960428	0.43086879	0.48410764	0.60701161	0.59073485	0.43871277	0.22301571
58.2	0.51914727	0.4306574	0.48407823	0.60713649	0.59136351	0.43796012	0.22366088
58.3	0.51869132	0.43044655	0.48404382	0.6072606	0.59199779	0.4372133	0.22430384
58.4	0.51823646	0.43023624	0.48400439	0.60738396	0.59263744	0.43647222	0.22494456
58.5	0.51778266	0.43002651	0.48395992	0.60750656	0.59328223	0.43573675	0.225583
58.6	0.51732993	0.42981738	0.48391039	0.60762841	0.59393191	0.4350068	0.22621914
58.7	0.51687827	0.42960887	0.48385578	0.6077495	0.59458627	0.43428225	0.22685295
58.8	0.51642768	0.429401	0.48379608	0.60786985	0.5952451	0.433563	0.22748441
58.9	0.51597815	0.42919379	0.48373128	0.60798945	0.59590817	0.43284894	0.22811349
59	0.51552968	0.42898726	0.48366136	0.6081083	0.59657526	0.43213998	0.22874017
59.1	0.51508227	0.42878143	0.4835863	0.60822641	0.59724616	0.43143601	0.22936442
59.2	0.51463591	0.42857632	0.4835061	0.60834378	0.59792065	0.43073694	0.22998622
59.3	0.51419061	0.42837196	0.48342073	0.60846042	0.59859852	0.43004267	0.23060553
59.4	0.51374637	0.42816835	0.4833302	0.60857632	0.59927954	0.42935309	0.23122235
59.5	0.51330318	0.42796552	0.48323448	0.6086915	0.59996351	0.42866812	0.23183664
59.6	0.51286103	0.42776349	0.48313357	0.60880595	0.60065021	0.42798767	0.23244838
59.7	0.51241994	0.42756226	0.48302746	0.60891967	0.60133945	0.42731164	0.23305755
59.8	0.51197989	0.42736186	0.48291614	0.60903268	0.60203101	0.42663995	0.23366414
59.9	0.51154088	0.4271623	0.4827996	0.60914498	0.6027247	0.4259725	0.23426811

105	0.38837375	0.40497616	0.30615802	0.63180754	0.63986796	0.24816373	0.3136599
105.1	0.38821119	0.40499785	0.3059325	0.63183807	0.6397282	0.2479593	0.31361977
105.2	0.38804897	0.40501964	0.30570771	0.63186857	0.63958894	0.24775554	0.31357945
105.3	0.38788708	0.40504153	0.30548364	0.63189906	0.63945017	0.24755245	0.31353895
105.4	0.38772553	0.4050635	0.3052603	0.63192952	0.63931188	0.24735003	0.31349827
105.5	0.38756431	0.40508558	0.30503768	0.63195996	0.63917409	0.24714828	0.31345742
105.6	0.38740343	0.40510774	0.30481578	0.63199038	0.63903679	0.2469472	0.3134164
105.7	0.38724288	0.40513	0.30459459	0.63202078	0.63889997	0.24674679	0.31337522
105.8	0.38708266	0.40515235	0.30437412	0.63205116	0.63876364	0.24654703	0.31333387
105.9	0.38692278	0.40517479	0.30415435	0.63208152	0.6386278	0.24634794	0.31329236
106	0.38676322	0.40519732	0.30393529	0.63211186	0.63849245	0.2461495	0.31325069
106.1	0.386604	0.40521994	0.30371694	0.63214217	0.63835759	0.24595171	0.31320887
106.2	0.38644511	0.40524264	0.30349928	0.63217247	0.63822321	0.24575458	0.3131669
106.3	0.38628655	0.40526544	0.30328233	0.63220274	0.63808932	0.2455581	0.31312478
106.4	0.38612831	0.40528832	0.30306606	0.63223299	0.63795592	0.24536226	0.31308252
106.5	0.38597041	0.4053113	0.30285049	0.63226322	0.637823	0.24516706	0.31304011
106.6	0.38581283	0.40533435	0.30263561	0.63229343	0.63769057	0.24497251	0.31299757
106.7	0.38565558	0.4053575	0.30242142	0.63232361	0.63755863	0.2447786	0.31295489
106.8	0.38549865	0.40538072	0.3022079	0.63235378	0.63742717	0.24458532	0.31291208
106.9	0.38534206	0.40540404	0.30199508	0.63238392	0.63729619	0.24439268	0.31286913
107	0.38518578	0.40542743	0.30178292	0.63241404	0.6371657	0.24420067	0.31282606
107.1	0.38502983	0.40545091	0.30157145	0.63244414	0.6370357	0.24400929	0.31278286
107.2	0.38487421	0.40547448	0.30136065	0.63247422	0.63690617	0.24381853	0.31273954
107.3	0.38471891	0.40549812	0.30115052	0.63250428	0.63677713	0.2436284	0.3126961
107.4	0.38456393	0.40552185	0.30094106	0.63253431	0.63664857	0.24343889	0.31265254
107.5	0.38440928	0.40554565	0.30073227	0.63256433	0.6365205	0.24325	0.31260887
107.6	0.38425494	0.40556954	0.30052414	0.63259432	0.6363929	0.24306172	0.31256509
107.7	0.38410093	0.40559351	0.30031667	0.63262429	0.63626579	0.24287406	0.31252119
107.8	0.38394724	0.40561755	0.30010985	0.63265424	0.63613915	0.24268701	0.31247719
107.9	0.38379387	0.40564168	0.2999037	0.63268417	0.636013	0.24250057	0.31243309
108	0.38364081	0.40566588	0.2996982	0.63271408	0.63588732	0.24231474	0.31238889
108.1	0.38348808	0.40569016	0.29949335	0.63274396	0.63576212	0.2421295	0.31234458
108.2	0.38333567	0.40571452	0.29928915	0.63277383	0.6356374	0.24194487	0.31230018
108.3	0.38318357	0.40573895	0.2990856	0.63280367	0.63551316	0.24176084	0.31225568
108.4	0.38303179	0.40576346	0.29888269	0.6328335	0.63538939	0.2415774	0.3122111
108.5	0.38288032	0.40578804	0.29868042	0.6328633	0.6352661	0.24139455	0.31216642
108.6	0.38272917	0.4058127	0.29847879	0.63289308	0.63514328	0.2412123	0.31212165
108.7	0.38257834	0.40583744	0.2982778	0.63292284	0.63502094	0.24103063	0.31207681
108.8	0.38242782	0.40586224	0.29807745	0.63295258	0.63489907	0.24084955	0.31203187
108.9	0.38227761	0.40588712	0.29787773	0.6329823	0.63477767	0.24066905	0.31198686
109	0.38212772	0.40591208	0.29767863	0.63301199	0.63465673	0.24048914	0.31194177
109.1	0.38197814	0.4059371	0.29748017	0.63304167	0.63453627	0.2403098	0.3118966
109.2	0.38182888	0.4059622	0.29728233	0.63307132	0.63441627	0.24013103	0.31185136
109.3	0.38167992	0.40598737	0.29708512	0.63310095	0.63429674	0.23995284	0.31180605
109.4	0.38153128	0.40601261	0.29688852	0.63313056	0.63417768	0.23977521	0.31176067
109.5	0.38138295	0.40603792	0.29669255	0.63316015	0.63405908	0.23959816	0.31171522
109.6	0.38123492	0.40606329	0.2964972	0.63318972	0.63394094	0.23942167	0.31166971
109.7	0.38108721	0.40608874	0.29630246	0.63321926	0.63382327	0.23924574	0.31162413
109.8	0.38093981	0.40611426	0.29610833	0.63324879	0.63370605	0.23907037	0.31157849
109.9	0.38079271	0.40613985	0.29591481	0.63327829	0.6335893	0.23889556	0.31153279
110	0.38064592	0.4061655	0.29572191	0.63330777	0.63347301	0.2387213	0.31148704
110.1	0.38049944	0.40619123	0.29552961	0.63333723	0.63335717	0.2385476	0.31144123
110.2	0.38035326	0.40621702	0.29533791	0.63336666	0.63324179	0.23837445	0.31139537
110.3	0.38020739	0.40624287	0.29514682	0.63339608	0.63312687	0.23820184	0.31134945
110.4	0.38006183	0.4062688	0.29495633	0.63342547	0.6330124	0.23802978	0.31130349
110.5	0.37991657	0.40629479	0.29476644	0.63345485	0.63289839	0.23785826	0.31125748
110.6	0.37977161	0.40632084	0.29457715	0.6334842	0.63278483	0.23768728	0.31121142
110.7	0.37962696	0.40634696	0.29438845	0.63351353	0.63267172	0.23751683	0.31116532
110.8	0.37948261	0.40637314	0.29420034	0.63354284	0.63255906	0.23734693	0.31111918
110.9	0.37933857	0.40639939	0.29401283	0.63357213	0.63244685	0.23717755	0.311073
111	0.37919482	0.4064257	0.2938259	0.63360139	0.63233508	0.23700871	0.31102678
111.1	0.37905138	0.40645208	0.29363957	0.63363064	0.63222377	0.23684039	0.31098052
111.2	0.37890824	0.40647851	0.29345381	0.63365987	0.63211289	0.2366726	0.31093423
111.3	0.37876539	0.40650501	0.29326864	0.63368907	0.63200247	0.23650533	0.31088791
111.4	0.37862285	0.40653157	0.29308406	0.63371825	0.63189248	0.23633858	0.31084155
111.5	0.37848061	0.40655819	0.29290005	0.63374741	0.63178294	0.23617235	0.31079517
111.6	0.37833866	0.40658488	0.29271662	0.63377655	0.63167384	0.23600663	0.31074876
111.7	0.37819701	0.40661162	0.29253377	0.63380567	0.63156518	0.23584143	0.31070232
111.8	0.37805566	0.40663843	0.29235149	0.63383476	0.63145696	0.23567674	0.31065585
111.9	0.37791461	0.40666529	0.29216978	0.63386383	0.63134917	0.23551256	0.31060937
112	0.37777385	0.40669222	0.29198865	0.63389289	0.63124182	0.23534889	0.31056286
112.1	0.37763338	0.4067192	0.29180808	0.63392192	0.63113491	0.23518572	0.31051634
112.2	0.37749322	0.40674624	0.29162808	0.63395092	0.63102843	0.23502305	0.31046979
112.3	0.37735334	0.40677334	0.29144864	0.63397991	0.63092238	0.23486089	0.31042323
112.4	0.37721376	0.4068005	0.29126977	0.63400887	0.63081676	0.23469922	0.31037666
112.5	0.37707447	0.40682772	0.29109145	0.63403781	0.63071158	0.23453804	0.31033007
112.6	0.37693548	0.40685499	0.2909137	0.63406673	0.63060682	0.23437736	0.31028346
112.7	0.37679678	0.40688233	0.29073651	0.63409563	0.63050249	0.23421718	0.31023685
112.8	0.37665837	0.40690972	0.29055987	0.6341245	0.63039858	0.23405747	0.31019023
112.9	0.37652025	0.40693716	0.29038378	0.63415336	0.6302951	0.23389826	0.31014361
113	0.37638242	0.40696466	0.29020825	0.63418219	0.63019204	0.23373953	0.31009697
113.1	0.37624488	0.40699222	0.29003326	0.634211	0.63008941	0.23358129	0.31005033
113.2	0.37610762	0.40701984	0.28985883	0.63423979	0.6299872	0.23342352	0.31000369
113.3	0.37597066	0.4070475	0.28968494	0.63426855	0.6298854	0.23326623	0.30995705
113.4	0.37583399	0.40707523	0.2895116	0.6342973	0.62978403	0.23310942	0.3099104
113.5	0.3756976	0.40710301	0.2893388	0.63432602	0.62968306	0.23295309	0.30986376
113.6	0.3755615	0.40713084	0.28916655	0.63435472	0.62958252	0.23279722	0.30981712
113.7	0.37542569	0.40715868	0.28899483	0.6343834	0.62948238	0.23264183	0.30977048
113.8	0.37529016	0.40718664	0.28882365	0.63441205	0.62938266	0.2324869	0.30972384
113.9	0.37515492	0.40721465	0.28865301	0.63444069	0.62928233	0.23233244	0.30967721

69	0.47568134	0.41324541	0.4526278	0.61699286	0.65751013	0.37514473	0.27727681
69.1	0.47532936	0.4131377	0.45213555	0.61705968	0.65791984	0.37464117	0.27762282
69.2	0.47497825	0.41303082	0.45164134	0.6171262	0.65832469	0.37413825	0.27796642
69.3	0.47462799	0.41292479	0.45114522	0.61719242	0.65872471	0.37363596	0.27830765
69.4	0.47427858	0.41281958	0.45064724	0.61725836	0.65911989	0.3731343	0.27864651
69.5	0.47393003	0.4127152	0.45014746	0.61732401	0.65951025	0.37263329	0.27898303
69.6	0.47358233	0.41261164	0.44964591	0.61738938	0.65989581	0.3721329	0.27931722
69.7	0.47323548	0.41250889	0.44914266	0.61745446	0.66027658	0.37163316	0.2796491
69.8	0.47288948	0.41240695	0.44863774	0.61751926	0.66065258	0.37113405	0.27997868
69.9	0.47254432	0.41230582	0.44813121	0.61758379	0.66102382	0.37063558	0.28030598
70	0.47220001	0.41220548	0.44762311	0.61764805	0.66139034	0.37013776	0.28063101
70.1	0.47185653	0.41210594	0.4471135	0.61771203	0.66175214	0.36964058	0.2809538
70.2	0.47151389	0.41200719	0.4466024	0.61777575	0.66210924	0.36914404	0.28127436
70.3	0.47117209	0.41190921	0.44608987	0.6178392	0.66246167	0.36864816	0.2815927
70.4	0.47083113	0.41181202	0.44557594	0.61790239	0.66280944	0.36815293	0.28190885
70.5	0.47049099	0.41171559	0.44506067	0.61796532	0.66315258	0.36765835	0.28222282
70.6	0.47015169	0.41161993	0.4445441	0.61802799	0.66349109	0.36716443	0.28253462
70.7	0.46981321	0.41152503	0.44402627	0.61809041	0.663825	0.36667117	0.28284427
70.8	0.46947556	0.41143089	0.44350723	0.61815257	0.66415433	0.36617857	0.2831518
70.9	0.46913873	0.4113375	0.44298701	0.61821449	0.66447909	0.36568664	0.2834572
71	0.46880273	0.41124485	0.44246566	0.61827615	0.6647993	0.36519538	0.28376051
71.1	0.46846754	0.41115295	0.44194322	0.61833757	0.66511498	0.3647048	0.28406173
71.2	0.46813316	0.41106178	0.44141974	0.61839875	0.66542616	0.3642149	0.28436088
71.3	0.4677996	0.41097135	0.44089524	0.61845968	0.66573285	0.36372568	0.28465798
71.4	0.46746686	0.41088164	0.44036979	0.61852038	0.66603507	0.36323716	0.28495304
71.5	0.46713492	0.41079266	0.43984341	0.61858083	0.66633283	0.36274934	0.28524609
71.6	0.46680378	0.41070439	0.43931614	0.61864105	0.66662615	0.36226222	0.28553712
71.7	0.46647346	0.41061683	0.43878803	0.61870104	0.66691505	0.3617758	0.28582617
71.8	0.46614393	0.41052998	0.43825911	0.6187608	0.66719953	0.3612901	0.28611325
71.9	0.4658152	0.41044383	0.43772942	0.61882032	0.66747962	0.3608051	0.28639836
72	0.46548726	0.41035839	0.437199	0.61887961	0.66775532	0.36032083	0.28668154
72.1	0.46516012	0.41027363	0.43666788	0.61893868	0.66802665	0.35983729	0.28696278
72.2	0.46483377	0.41018957	0.43613609	0.61899752	0.66829362	0.35935448	0.28724212
72.3	0.46450821	0.4101062	0.43560368	0.61905614	0.66855624	0.3588724	0.28751956
72.4	0.46418343	0.4100235	0.43507068	0.61911453	0.66881453	0.35839107	0.28779511
72.5	0.46385943	0.40994148	0.43453711	0.6191727	0.66906849	0.35791048	0.2880688
72.6	0.46353622	0.40986014	0.43400302	0.61923065	0.66931814	0.35743065	0.28834064
72.7	0.46321378	0.40977947	0.43346844	0.61928838	0.66956349	0.35695158	0.28861063
72.8	0.46289211	0.40969946	0.43293341	0.6193459	0.66980454	0.35647327	0.28887881
72.9	0.46257122	0.40962011	0.43239795	0.6194032	0.67004131	0.35599574	0.28914518
73	0.46225109	0.40954143	0.4318621	0.61946028	0.67027381	0.35551898	0.28940975
73.1	0.46193173	0.40946339	0.4313259	0.61951714	0.67050205	0.355043	0.28967255
73.2	0.46161313	0.409386	0.43078937	0.6195738	0.67072602	0.35456781	0.28993358
73.3	0.4612953	0.40930926	0.43025255	0.61963024	0.67094575	0.35409342	0.29019285
73.4	0.46097821	0.40923316	0.42971547	0.61968647	0.67116124	0.35361981	0.2904504
73.5	0.46066188	0.4091577	0.42917815	0.61974249	0.67137249	0.35314702	0.29070621
73.6	0.4603463	0.40908288	0.42864064	0.6197983	0.67157952	0.35267503	0.29096032
73.7	0.46003147	0.40900868	0.42810296	0.6198539	0.67178233	0.35220386	0.29121274
73.8	0.45971738	0.40893511	0.42756513	0.6199093	0.67198093	0.35173351	0.29146347
73.9	0.45940403	0.40886217	0.42702719	0.61996449	0.67217534	0.351264	0.29171253
74	0.45909142	0.40878984	0.42648917	0.62001947	0.67236557	0.35079531	0.29195993
74.1	0.45877954	0.40871813	0.42595109	0.62007425	0.67255162	0.35032747	0.2922057
74.2	0.45846839	0.40864703	0.42541299	0.62012883	0.67273351	0.34986047	0.29244983
74.3	0.45815798	0.40857654	0.42487488	0.6201832	0.67291124	0.34939432	0.29269234
74.4	0.45784829	0.40850666	0.42433681	0.62023737	0.67308482	0.34892902	0.29293325
74.5	0.45753932	0.40843738	0.42379878	0.62029134	0.67325427	0.34846458	0.29317257
74.6	0.45723107	0.4083687	0.42326083	0.62034512	0.67341959	0.34800101	0.2934103
74.7	0.45692354	0.40830062	0.42272298	0.62039869	0.67358079	0.34753829	0.29364647
74.8	0.45661672	0.40823313	0.42218525	0.62045206	0.67373788	0.34707645	0.29388108
74.9	0.45631061	0.40816623	0.42164767	0.62050524	0.67389088	0.34661549	0.29411414
120	0.36742213	0.40901234	0.27919657	0.63614391	0.62395381	0.22372068	0.30688458
120.1	0.36730348	0.40904313	0.27905618	0.63617111	0.62387772	0.22359177	0.30684018
120.2	0.36718508	0.40907397	0.27891623	0.63619829	0.62380197	0.22346323	0.30679583
120.3	0.36706693	0.40910484	0.27877673	0.63622545	0.62372657	0.22333505	0.30675154
120.4	0.36694903	0.40913576	0.27863766	0.63625259	0.62365152	0.22320724	0.3067073
120.5	0.36683137	0.40916671	0.27849903	0.6362797	0.6235768	0.22307979	0.30666313
120.6	0.36671397	0.40919769	0.27836084	0.63630679	0.62350244	0.2229527	0.30661901
120.7	0.36659681	0.40922872	0.27822308	0.63633386	0.62342841	0.22282597	0.30657496
120.8	0.3664799	0.40925978	0.27808575	0.6363609	0.62335473	0.2226996	0.30653096
120.9	0.36636324	0.40929088	0.27794886	0.63638792	0.62328139	0.2225736	0.30648702
121	0.36624682	0.40932201	0.2778124	0.63641492	0.62320839	0.22244794	0.30644315
121.1	0.36613065	0.40935319	0.27767637	0.63644189	0.62313574	0.22232265	0.30639933
121.2	0.36601472	0.4093844	0.27754076	0.63646884	0.62306342	0.22219771	0.30635558
121.3	0.36589904	0.40941564	0.27740559	0.63649577	0.62299144	0.22207313	0.30631189
121.4	0.3657836	0.40944692	0.27727083	0.63652267	0.62291981	0.22194889	0.30626825
121.5	0.3656684	0.40947824	0.2771365	0.63654955	0.62284851	0.22182501	0.30622469
121.6	0.36555345	0.40950959	0.2770026	0.6365764	0.62277755	0.22170148	0.30618118
121.7	0.36543874	0.40954098	0.27686911	0.63660323	0.62270692	0.2215783	0.30613774
121.8	0.36532427	0.40957241	0.27673605	0.63663004	0.62263664	0.22145547	0.30609436
121.9	0.36521004	0.40960387	0.2766034	0.63665682	0.62256668	0.22133299	0.30605104
122	0.36509606	0.40963536	0.27647117	0.63668358	0.62249707	0.22121086	0.30600778
122.1	0.36498231	0.40966689	0.27633936	0.63671031	0.62242779	0.22108906	0.30596459
122.2	0.3648688	0.40969845	0.27620796	0.63673703	0.62235885	0.22096762	0.30592147
122.3	0.36475554	0.40973005	0.27607697	0.63676371	0.62229024	0.22084652	0.3058784
122.4	0.36464251	0.40976168	0.2759464	0.63679038	0.62222196	0.22072576	0.30583541
122.5	0.36452972	0.40979335	0.27581623	0.63681702	0.62215402	0.22060534	0.30579247
122.6	0.36441717	0.40982505	0.27568648	0.63684363	0.62208642	0.22048526	0.3057496
122.7	0.36430485	0.40985679	0.27555713	0.63687022	0.62201914	0.22036552	0.3057068
122.8	0.36419277	0.40988855	0.27542819	0.63689679	0.6219522	0.22024612	0.30566406
122.9	0.36408093	0.40992036	0.27529965	0.63692334	0.62188559	0.22012706	0.30562139
123	0.36396932	0.40995219	0.27517152	0.63694986	0.62181932	0.22000834	0.30557878
123.1	0.36385795	0.40998406	0.27504379	0.63697636	0.62175338	0.21988994	0.30553623
123.2	0.36374681	0.41001596	0.27491646	0.63700283	0.62168777	0.21977189	0.30549376
123.3	0.3636359	0.41004789	0.27478953	0.63702929	0.62162249	0.21965417	0.30545135
123.4	0.36352523	0.41007986	0.274663	0.63705571	0.62155754	0.21953678	0.305409
123.5	0.36341479	0.41011186	0.27453686	0.63708212	0.62149293	0.21941972	0.30536672
123.6	0.36330459	0.41014389	0.27441113	0.6371085	0.62142865	0.21930299	0.30532451
123.7	0.36319461	0.41017595	0.27428578	0.63713485	0.62136469	0.21918659	0.30528237
123.8	0.36308487	0.41020805	0.27416083	0.63716119	0.62130107	0.21907052	0.30524029
123.9	0.36297536	0.41024018	0.27403627	0.63718749	0.62123778	0.21895478	0.30519827
124	0.36286608	0.41027233	0.27391211	0.63721378	0.62117481	0.21883937	0.30515633
124.1	0.36275702	0.41030452	0.27378833	0.63724004	0.62111218	0.21872428	0.30511445
124.2	0.3626482	0.41033675	0.27366494	0.63726628	0.62104987	0.21860951	0.30507264
124.3	0.36253961	0.410369	0.27354194	0.63729249	0.62098789	0.21849507	0.30503089
124.4	0.36243124	0.41040128	0.27341932	0.63731868	0.62092624	0.21838095	0.30498922
124.5	0.36232311	0.4104336	0.27329709	0.63734484	0.62086492	0.21826716	0.3049476
124.6	0.3622152	0.41046594	0.27317524	0.63737098	0.62080392	0.21815368	0.30490606
124.7	0.36210751	0.41049832	0.27305377	0.6373971	0.62074325	0.21804053	0.30486458
124.8	0.36200006	0.41053072	0.27293268	0.63742319	0.62068291	0.21792769	0.30482317
124.9	0.36189282	0.41056316	0.27281197	0.63744926	0.62062289	0.21781517	0.30478183
125	0.36178582	0.41059562	0.27269164	0.63747531	0.62056321	0.21770297	0.30474056
125.1	0.36167904	0.41062812	0.27257169	0.63750133	0.62050384	0.21759109	0.30469935
125.2	0.36157248	0.41066065	0.27245211	0.63752732	0.62044481	0.21747952	0.30465821
125.3	0.36146615	0.4106932	0.2723329	0.6375533	0.62038609	0.21736826	0.30461714
125.4	0.36136004	0.41072578	0.27221407	0.63757924	0.62032771	0.21725732	0.30457613
125.5	0.36125415	0.4107584	0.27209561	0.63760517	0.62026965	0.2171467	0.3045352
125.6	0.36114848	0.41079104	0.27197752	0.63763107	0.62021191	0.21703638	0.30449432
125.7	0.36104304	0.41082371	0.2718598	0.63765694	0.6201545	0.21692637	0.30445352
125.8	0.36093782	0.41085641	0.27174245	0.6376828	0.62009742	0.21681668	0.30441278
125.9	0.36083282	0.41088914	0.27162546	0.63770862	0.62004066	0.21670729	0.30437211
126	0.36072803	0.41092189	0.27150884	0.63773443	0.61998422	0.21659822	0.30433151

84	0.43110731	0.40419127	0.37561774	0.62466166	0.67377107	0.30851735	0.30993248
84.1	0.43085667	0.4041675	0.37516023	0.62470149	0.67365793	0.30814079	0.31005244
84.2	0.43060654	0.4041441	0.37470398	0.62474122	0.67354325	0.30776514	0.3101712
84.3	0.43035694	0.40412108	0.374249	0.62478086	0.67342705	0.30739037	0.31028876
84.4	0.43010786	0.40409841	0.37379529	0.62482041	0.67330936	0.30701651	0.31040512
84.5	0.4298593	0.40407612	0.37334286	0.62485987	0.6731902	0.30664353	0.31052026
84.6	0.42961127	0.40405419	0.3728917	0.62489924	0.67306959	0.30627146	0.31063419
84.7	0.42936374	0.40403262	0.37244183	0.62493852	0.67294755	0.30590027	0.3107469
84.8	0.42911674	0.40401141	0.37199324	0.62497772	0.67282412	0.30552998	0.31085838
84.9	0.42887025	0.40399055	0.37154593	0.62501683	0.67269929	0.30516058	0.31096864
85	0.42862428	0.40397006	0.37109992	0.62505585	0.6725731	0.30479208	0.31107766
85.1	0.42837882	0.40394992	0.37065519	0.62509478	0.67244557	0.30442447	0.31118544
85.2	0.42813387	0.40393013	0.37021176	0.62513363	0.67231672	0.30405774	0.31129198
85.3	0.42788943	0.4039107	0.36976963	0.6251724	0.67218656	0.30369191	0.31139728
85.4	0.4276455	0.40389161	0.3693288	0.62521108	0.67205513	0.30332697	0.31150132
85.5	0.42740208	0.40387288	0.36888927	0.62524969	0.67192243	0.30296292	0.31160412
85.6	0.42715917	0.40385449	0.36845103	0.62528821	0.67178849	0.30259976	0.31170566
85.7	0.42691676	0.40383644	0.36801411	0.62532665	0.67165333	0.30223748	0.31180595
85.8	0.42667487	0.40381874	0.36757849	0.62536501	0.67151697	0.30187609	0.31190498
85.9	0.42643347	0.40380138	0.36714417	0.62540329	0.67137943	0.30151559	0.31200275
86	0.42619258	0.40378436	0.36671117	0.62544149	0.67124073	0.30115597	0.31209926
86.1	0.42595219	0.40376767	0.36627947	0.62547962	0.67110088	0.30079724	0.31219449
86.2	0.4257123	0.40375133	0.36584908	0.62551767	0.6709599	0.3004394	0.31228846
86.3	0.42547291	0.40373531	0.36542	0.62555564	0.67081782	0.30008244	0.31238116
86.4	0.42523402	0.40371963	0.36499224	0.62559354	0.67067465	0.29972637	0.31247257
86.5	0.42499563	0.40370428	0.36456579	0.62563136	0.6705304	0.29937118	0.31256271
86.6	0.42475773	0.40368926	0.36414066	0.62566911	0.6703851	0.29901687	0.31265158
86.7	0.42452033	0.40367457	0.36371684	0.62570678	0.67023876	0.29866345	0.31273917
86.8	0.42428343	0.40366021	0.36329434	0.62574438	0.6700914	0.29831091	0.31282549
86.9	0.42404701	0.40364617	0.36287315	0.62578191	0.66994304	0.29795925	0.31291054
87	0.42381109	0.40363245	0.36245328	0.62581937	0.6697937	0.29760848	0.31299431
87.1	0.42357566	0.40361905	0.36203473	0.62585676	0.6696434	0.29725859	0.31307681
87.2	0.42334072	0.40360597	0.3616175	0.62589408	0.66949214	0.29690957	0.31315804
87.3	0.42310627	0.40359321	0.36120158	0.62593132	0.66933996	0.29656144	0.31323799
87.4	0.42287231	0.40358076	0.36078699	0.6259685	0.66918686	0.2962142	0.31331668
87.5	0.42263884	0.40356863	0.36037371	0.62600561	0.66903286	0.29586783	0.31339409
87.6	0.42240585	0.40355682	0.35996175	0.62604266	0.66887799	0.29552234	0.31347024
87.7	0.42217334	0.40354531	0.3595511	0.62607963	0.66872226	0.29517773	0.31354511
87.8	0.42194132	0.40353412	0.35914178	0.62611654	0.66856568	0.294834	0.31361873
87.9	0.42170978	0.40352323	0.35873377	0.62615339	0.66840827	0.29449115	0.31369108
88	0.42147872	0.40351265	0.35832708	0.62619017	0.66825005	0.29414918	0.31376218
88.1	0.42124814	0.40350237	0.35792171	0.62622688	0.66809103	0.29380809	0.31383204
88.2	0.42101804	0.4034924	0.35751765	0.62626353	0.66793124	0.29346788	0.31390064
88.3	0.42078842	0.40348273	0.35711491	0.62630011	0.66777068	0.29312854	0.31396801
88.4	0.42055928	0.40347336	0.35671349	0.62633664	0.66760938	0.29279008	0.31403414
88.5	0.42033061	0.40346428	0.35631338	0.6263731	0.66744735	0.29245249	0.31409903
88.6	0.42010242	0.40345551	0.35591459	0.62640949	0.66728461	0.29211578	0.3141627
88.7	0.4198747	0.40344703	0.35551711	0.62644583	0.66712117	0.29177993	0.31422514
88.8	0.41964745	0.40343884	0.35512094	0.6264821	0.66695706	0.29144497	0.31428637
88.9	0.41942068	0.40343095	0.35472609	0.62651832	0.66679228	0.29111087	0.31434638
89	0.41919437	0.40342335	0.35433255	0.62655447	0.66662685	0.29077765	0.31440519
89.1	0.41896854	0.40341603	0.35394031	0.62659057	0.66646079	0.2904453	0.3144628
89.2	0.41874317	0.40340901	0.35354938	0.6266266	0.66629411	0.29011381	0.31451922
89.3	0.41851827	0.40340226	0.35315975	0.62666258	0.66612684	0.2897832	0.31457445
89.4	0.41829383	0.40339581	0.35277144	0.6266985	0.66595898	0.28945346	0.31462851
89.5	0.41806987	0.40338963	0.35238442	0.62673436	0.66579056	0.28912459	0.31468139
89.6	0.41784636	0.40338374	0.3519987	0.62677016	0.66562159	0.28879659	0.31473311
89.7	0.41762332	0.40337813	0.35161429	0.62680591	0.66545208	0.28846945	0.31478368
89.8	0.41740074	0.40337279	0.35123117	0.6268416	0.66528206	0.28814319	0.31483309
89.9	0.41717862	0.40336773	0.35084935	0.62687724	0.66511153	0.28781779	0.31488136
135	0.35213778	0.41396939	0.26236219	0.63996396	0.61617373	0.20794453	0.30091363
135.1	0.35205101	0.41400418	0.26227402	0.63998772	0.61614449	0.20786027	0.30087764
135.2	0.35196441	0.41403898	0.26218611	0.64001146	0.61611551	0.20777626	0.30084168
135.3	0.35187799	0.4140738	0.26209847	0.64003518	0.61608677	0.20769249	0.30080575
135.4	0.35179175	0.41410864	0.26201108	0.64005887	0.61605829	0.20760895	0.30076984
135.5	0.35170568	0.4141435	0.26192396	0.64008255	0.61603005	0.20752566	0.30073395
135.6	0.35161979	0.41417837	0.2618371	0.6401062	0.61600207	0.2074426	0.30069809
135.7	0.35153407	0.41421326	0.2617505	0.64012983	0.61597432	0.20735978	0.30066225
135.8	0.35144852	0.41424816	0.26166415	0.64015343	0.61594683	0.2072772	0.30062644
135.9	0.35136315	0.41428308	0.26157807	0.64017702	0.61591957	0.20719485	0.30059064
136	0.35127795	0.41431802	0.26149224	0.64020058	0.61589256	0.20711274	0.30055487
136.1	0.35119293	0.41435297	0.26140666	0.64022412	0.61586578	0.20703086	0.30051912
136.2	0.35110807	0.41438794	0.26132134	0.64024763	0.61583924	0.20694922	0.30048339
136.3	0.35102339	0.41442292	0.26123627	0.64027113	0.61581294	0.20686781	0.30044768
136.4	0.35093888	0.41445792	0.26115146	0.6402946	0.61578687	0.20678664	0.30041198
136.5	0.35085455	0.41449293	0.2610669	0.64031805	0.61576102	0.2067057	0.30037631
136.6	0.35077038	0.41452796	0.26098259	0.64034148	0.61573541	0.20662498	0.30034066
136.7	0.35068638	0.414563	0.26089853	0.64036489	0.61571002	0.2065445	0.30030502
136.8	0.35060255	0.41459806	0.26081471	0.64038827	0.61568486	0.20646425	0.3002694
136.9	0.3505189	0.41463313	0.26073115	0.64041163	0.61565991	0.20638423	0.30023379
137	0.35043541	0.41466822	0.26064783	0.64043497	0.61563519	0.20630444	0.30019821
137.1	0.35035209	0.41470332	0.26056476	0.64045829	0.61561068	0.20622488	0.30016264
137.2	0.35026894	0.41473844	0.26048194	0.64048158	0.61558638	0.20614555	0.30012708
137.3	0.35018596	0.41477357	0.26039936	0.64050486	0.6155623	0.20606645	0.30009154
137.4	0.35010314	0.41480872	0.26031702	0.64052811	0.61553842	0.20598757	0.30005601
137.5	0.35002049	0.41484388	0.26023493	0.64055134	0.61551475	0.20590891	0.3000205
137.6	0.34993801	0.41487905	0.26015307	0.64057455	0.61549128	0.20583049	0.29998499
137.7	0.3498557	0.41491424	0.26007146	0.64059773	0.61546801	0.20575229	0.29994951
137.8	0.34977355	0.41494944	0.25999009	0.64062089	0.61544493	0.20567431	0.29991403
137.9	0.34969156	0.41498465	0.25990896	0.64064404	0.61542205	0.20559656	0.29987857
138	0.34960974	0.41501988	0.25982807	0.64066716	0.61539936	0.20551903	0.29984312
138.1	0.34952809	0.41505512	0.25974741	0.64069026	0.61537686	0.20544173	0.29980768
138.2	0.3494466	0.41509037	0.25966699	0.64071333	0.61535454	0.20536464	0.29977225
138.3	0.34936527	0.41512564	0.25958681	0.64073639	0.61533241	0.20528778	0.29973683
138.4	0.34928411	0.41516092	0.25950686	0.64075942	0.61531045	0.20521114	0.29970143
138.5	0.34920311	0.41519622	0.25942715	0.64078243	0.61528867	0.20513473	0.29966603
138.6	0.34912228	0.41523152	0.25934767	0.64080542	0.61526706	0.20505853	0.29963064
138.7	0.3490416	0.41526684	0.25926843	0.64082839	0.61524562	0.20498255	0.29959527
138.8	0.34896109	0.41530218	0.25918941	0.64085134	0.61522434	0.20490679	0.2995599
138.9	0.34888074	0.41533752	0.25911063	0.64087427	0.61520323	0.20483125	0.29952454
139	0.34880055	0.41537288	0.25903207	0.64089717	0.61518227	0.20475592	0.29948919
139.1	0.34872052	0.41540825	0.25895375	0.64092005	0.61516147	0.20468082	0.29945385
139.2	0.34864066	0.41544363	0.25887565	0.64094291	0.61514082	0.20460593	0.29941852
139.3	0.34856095	0.41547903	0.25879778	0.64096574	0.61512031	0.20453126	0.29938319
139.4	0.3484814	0.41551444	0.25872014	0.64098856	0.61509995	0.2044568	0.29934788
139.5	0.34840201	0.41554986	0.25864272	0.64101135	0.61507973	0.20438256	0.29931257
139.6	0.34832278	0.41558529	0.25856553	0.64103413	0.61505964	0.20430853	0.29927726
139.7	0.34824371	0.41562073	0.25848857	0.64105688	0.61503969	0.20423472	0.29924197
139.8	0.3481648	0.41565619	0.25841182	0.6410796	0.61501987	0.20416112	0.29920668
139.9	0.34808604	0.41569165	0.2583353	0.64110231	0.61500016	0.20408773	0.2991714
140	0.34800744	0.41572713	0.258259	0.641125	0.61498058	0.20401456	0.29913613
140.1	0.347929	0.41576263	0.25818293	0.64114766	0.61496112	0.20394159	0.29910086
140.2	0.34785072	0.41579813	0.25810707	0.6411703	0.61494177	0.20386884	0.2990656
140.3	0.34777259	0.41583364	0.25803144	0.64119292	0.61492253	0.2037963	0.29903035
140.4	0.34769462	0.41586917	0.25795602	0.64121552	0.61490339	0.20372397	0.2989951
140.5	0.3476168	0.41590471	0.25788082	0.6412381	0.61488435	0.20365185	0.29895986
140.6	0.34753914	0.41594025	0.25780584	0.64126066	0.61486541	0.20357994	0.29892463
140.7	0.34746163	0.41597581	0.25773107	0.64128319	0.61484657	0.20350823	0.2988894
140.8	0.34738428	0.41601138	0.25765652	0.64130571	0.61482781	0.20343674	0.29885418
140.9	0.34730708	0.41604696	0.25758219	0.6413282	0.61480913	0.20336545	0.29881897
141	0.34723004	0.41608256	0.25750807	0.64135067	0.61479054	0.20329437	0.29878376

99	0.39876595	0.40387979	0.32115846	0.629934	0.64912207	0.26174692	0.31558955
99.1	0.39858207	0.4038942	0.32088247	0.62996596	0.64895484	0.26149819	0.31556768
99.2	0.39839857	0.40390876	0.32060744	0.6299979	0.64878801	0.26125026	0.31554537
99.3	0.39821544	0.40392347	0.32033336	0.6300298	0.64862156	0.26100311	0.31552262
99.4	0.39803268	0.40393834	0.32006024	0.63006168	0.64845551	0.26075675	0.31549944
99.5	0.3978503	0.40395335	0.31978807	0.63009353	0.64828985	0.26051117	0.31547583
99.6	0.39766828	0.40396851	0.31951685	0.63012535	0.6481246	0.26026637	0.3154518
99.7	0.39748664	0.40398381	0.31924656	0.63015715	0.64795975	0.26002236	0.31542735
99.8	0.39730537	0.40399926	0.31897721	0.63018892	0.64779532	0.25977913	0.31540249
99.9	0.39712447	0.40401485	0.31870879	0.63022066	0.64763129	0.25953667	0.31537722
100	0.39694394	0.40403059	0.3184413	0.63025238	0.64746768	0.259295	0.31535156
100.1	0.39676378	0.40404647	0.31817473	0.63028407	0.64730449	0.2590541	0.31532549
100.2	0.39658399	0.40406248	0.31790908	0.63031573	0.64714172	0.25881397	0.31529903
100.3	0.39640456	0.40407864	0.31764435	0.63034737	0.64697938	0.25857461	0.31527219
100.4	0.3962255	0.40409493	0.31738052	0.63037899	0.64681747	0.25833603	0.31524497
100.5	0.39604681	0.40411136	0.3171176	0.63041058	0.64665598	0.25809822	0.31521737
100.6	0.39586848	0.40412793	0.31685558	0.63044214	0.64649493	0.25786117	0.31518939
100.7	0.39569052	0.40414463	0.31659446	0.63047368	0.64633432	0.25762489	0.31516106
100.8	0.39551292	0.40416146	0.31633423	0.63050519	0.64617414	0.25738938	0.31513236
100.9	0.39533568	0.40417843	0.31607489	0.63053668	0.64601441	0.25715463	0.3151033
101	0.39515881	0.40419553	0.31581643	0.63056814	0.64585512	0.25692063	0.31507389
101.1	0.3949823	0.40421275	0.31555885	0.63059958	0.64569628	0.2566874	0.31504414
101.2	0.39480615	0.40423011	0.31530215	0.63063099	0.64553788	0.25645493	0.31501404
101.3	0.39463036	0.40424759	0.31504632	0.63066238	0.64537994	0.25622321	0.31498361
101.4	0.39445493	0.4042652	0.31479136	0.63069375	0.64522245	0.25599224	0.31495284
101.5	0.39427986	0.40428294	0.31453727	0.63072509	0.64506542	0.25576203	0.31492175
101.6	0.39410514	0.4043008	0.31428403	0.63075641	0.64490884	0.25553257	0.31489033
101.7	0.39393079	0.40431879	0.31403165	0.6307877	0.64475272	0.25530386	0.31485859
101.8	0.39375679	0.4043369	0.31378012	0.63081897	0.64459707	0.25507589	0.31482654
101.9	0.39358315	0.40435513	0.31352944	0.63085021	0.64444187	0.25484867	0.31479418
102	0.39340987	0.40437349	0.3132796	0.63088144	0.64428715	0.2546222	0.31476151
102.1	0.39323694	0.40439196	0.3130306	0.63091264	0.64413289	0.25439646	0.31472854
102.2	0.39306436	0.40441055	0.31278244	0.63094381	0.6439791	0.25417146	0.31469528
102.3	0.39289214	0.40442926	0.31253511	0.63097497	0.64382578	0.25394721	0.31466172
102.4	0.39272028	0.40444809	0.3122886	0.63100609	0.64367293	0.25372369	0.31462787
102.5	0.39254876	0.40446704	0.31204293	0.6310372	0.64352055	0.2535009	0.31459374
102.6	0.3923776	0.4044861	0.31179807	0.63106829	0.64336865	0.25327884	0.31455933
102.7	0.39220679	0.40450528	0.31155403	0.63109935	0.64321723	0.25305752	0.31452465
102.8	0.39203633	0.40452457	0.3113108	0.63113039	0.64306628	0.25283692	0.31448969
102.9	0.39186622	0.40454397	0.31106838	0.6311614	0.64291581	0.25261705	0.31445447
103	0.39169646	0.40456349	0.31082676	0.63119239	0.64276582	0.2523979	0.31441898
103.1	0.39152705	0.40458311	0.31058595	0.63122336	0.64261631	0.25217948	0.31438323
103.2	0.39135799	0.40460285	0.31034594	0.63125431	0.64246729	0.25196177	0.31434723
103.3	0.39118927	0.4046227	0.31010672	0.63128524	0.64231875	0.25174478	0.31431098
103.4	0.39102091	0.40464265	0.30986829	0.63131614	0.64217069	0.25152851	0.31427448
103.5	0.39085288	0.40466272	0.30963064	0.63134702	0.64202311	0.25131295	0.31423773
103.6	0.39068521	0.40468289	0.30939378	0.63137788	0.64187602	0.2510981	0.31420075
103.7	0.39051788	0.40470317	0.3091577	0.63140871	0.64172942	0.25088396	0.31416353
103.8	0.3903509	0.40472356	0.30892239	0.63143952	0.6415833	0.25067053	0.31412608
103.9	0.39018425	0.40474405	0.30868786	0.63147031	0.64143767	0.25045781	0.3140884
104	0.39001796	0.40476464	0.3084541	0.63150108	0.64129252	0.25024578	0.3140505
104.1	0.389852	0.40478534	0.3082211	0.63153183	0.64114786	0.25003446	0.31401237
104.2	0.38968639	0.40480614	0.30798886	0.63156255	0.64100369	0.24982384	0.31397403
104.3	0.38952112	0.40482704	0.30775739	0.63159325	0.64086001	0.24961391	0.31393548
104.4	0.38935619	0.40484805	0.30752666	0.63162393	0.64071681	0.24940468	0.31389671
104.5	0.3891916	0.40486915	0.3072967	0.63165459	0.64057411	0.24919614	0.31385774
104.6	0.38902735	0.40489036	0.30706748	0.63168522	0.6404319	0.24898829	0.31381857
104.7	0.38886344	0.40491166	0.306839	0.63171583	0.64029017	0.24878112	0.31377919
104.8	0.38869987	0.40493306	0.30661127	0.63174642	0.64014894	0.24857464	0.31373962
104.9	0.38853664	0.40495456	0.30638428	0.63177699	0.6400082	0.24836885	0.31369986
150	0.34088461	0.41931892	0.25162954	0.64328668	0.61306667	0.19767978	0.29567548
150.1	0.34082021	0.41935514	0.2515722	0.64330725	0.61304417	0.19762542	0.29564214
150.2	0.34075595	0.41939137	0.25151501	0.64332779	0.61302156	0.19757122	0.29560885
150.3	0.3406918	0.4194276	0.25145799	0.64334831	0.61299886	0.19751718	0.2955756
150.4	0.34062778	0.41946383	0.25140112	0.64336881	0.61297604	0.1974633	0.29554238
150.5	0.34056389	0.41950006	0.25134441	0.64338929	0.61295312	0.19740958	0.2955092
150.6	0.34050012	0.4195363	0.25128785	0.64340974	0.61293009	0.19735603	0.29547606
150.7	0.34043648	0.41957254	0.25123146	0.64343018	0.61290696	0.19730263	0.29544297
150.8	0.34037296	0.41960879	0.25117522	0.6434506	0.61288372	0.1972494	0.29540991
150.9	0.34030956	0.41964503	0.25111913	0.643471	0.61286038	0.19719632	0.29537689
151	0.34024629	0.41968128	0.2510632	0.64349137	0.61283693	0.19714341	0.29534392
151.1	0.34018314	0.41971754	0.25100743	0.64351173	0.61281337	0.19709065	0.29531098
151.2	0.34012011	0.41975379	0.25095181	0.64353206	0.6127897	0.19703805	0.29527809
151.3	0.3400572	0.41979005	0.25089635	0.64355238	0.61276593	0.1969856	0.29524524
151.4	0.33999442	0.41982631	0.25084104	0.64357267	0.61274204	0.19693332	0.29521243
151.5	0.33993176	0.41986257	0.25078588	0.64359295	0.61271806	0.19688119	0.29517966
151.6	0.33986922	0.41989884	0.25073087	0.6436132	0.61269396	0.19682922	0.29514694
151.7	0.33980681	0.41993511	0.25067602	0.64363343	0.61266976	0.19677741	0.29511426
151.8	0.33974451	0.41997138	0.25062132	0.64365365	0.61264544	0.19672575	0.29508162
151.9	0.33968234	0.42000765	0.25056677	0.64367384	0.61262103	0.19667424	0.29504903
152	0.33962029	0.42004393	0.25051237	0.64369401	0.6125965	0.19662289	0.29501648
152.1	0.33955835	0.42008021	0.25045812	0.64371416	0.61257186	0.1965717	0.29498398
152.2	0.33949654	0.42011649	0.25040403	0.64373429	0.61254712	0.19652066	0.29495152
152.3	0.33943485	0.42015277	0.25035008	0.6437544	0.61252227	0.19646978	0.2949191
152.4	0.33937328	0.42018906	0.25029628	0.64377449	0.61249731	0.19641904	0.29488673
152.5	0.33931183	0.42022535	0.25024263	0.64379456	0.61247225	0.19636846	0.2948544
152.6	0.33925049	0.42026164	0.25018913	0.64381461	0.61244708	0.19631804	0.29482212
152.7	0.33918928	0.42029793	0.25013578	0.64383464	0.61242179	0.19626776	0.29478989
152.8	0.33912819	0.42033422	0.25008258	0.64385465	0.6123964	0.19621764	0.2947577
152.9	0.33906721	0.42037052	0.25002952	0.64387464	0.61237091	0.19616767	0.29472555
153	0.33900635	0.42040681	0.24997661	0.64389461	0.6123453	0.19611785	0.29469346
153.1	0.33894561	0.42044311	0.24992385	0.64391456	0.61231959	0.19606818	0.2946614
153.2	0.33888499	0.42047942	0.24987123	0.64393449	0.61229377	0.19601866	0.2946294
153.3	0.33882449	0.42051572	0.24981876	0.6439544	0.61226784	0.1959693	0.29459744
153.4	0.3387641	0.42055202	0.24976643	0.64397429	0.61224181	0.19592008	0.29456553
153.5	0.33870383	0.42058833	0.24971425	0.64399416	0.61221567	0.195871	0.29453367
153.6	0.33864368	0.42062464	0.24966222	0.64401402	0.61218942	0.19582208	0.29450186
153.7	0.33858364	0.42066095	0.24961033	0.64403385	0.61216306	0.19577331	0.29447009
153.8	0.33852372	0.42069726	0.24955858	0.64405366	0.6121366	0.19572468	0.29443837
153.9	0.33846392	0.42073357	0.24950698	0.64407345	0.61211003	0.1956762	0.2944067
154	0.33840423	0.42076988	0.24945551	0.64409322	0.61208335	0.19562787	0.29437507
154.1	0.33834466	0.42080619	0.2494042	0.64411297	0.61205657	0.19557968	0.2943435
154.2	0.3382852	0.42084251	0.24935302	0.6441327	0.61202968	0.19553165	0.29431198
154.3	0.33822585	0.42087883	0.24930199	0.64415241	0.61200268	0.19548375	0.2942805
154.4	0.33816663	0.42091514	0.24925109	0.6441721	0.61197558	0.195436	0.29424907
154.5	0.33810751	0.42095146	0.24920034	0.64419178	0.61194838	0.1953884	0.29421769
154.6	0.33804852	0.42098778	0.24914973	0.64421143	0.61192107	0.19534094	0.29418637
154.7	0.33798963	0.4210241	0.24909926	0.64423106	0.61189365	0.19529363	0.29415509
154.8	0.33793086	0.42106042	0.24904893	0.64425067	0.61186613	0.19524646	0.29412386
154.9	0.3378722	0.42109675	0.24899874	0.64427026	0.6118385	0.19519944	0.29409268
155	0.33781366	0.42113307	0.24894869	0.64428983	0.61181077	0.19515255	0.29406155
155.1	0.33775523	0.42116939	0.24889878	0.64430938	0.61178294	0.19510581	0.29403048
155.2	0.33769691	0.42120571	0.24884901	0.64432891	0.611755	0.19505922	0.29399945
155.3	0.33763871	0.42124204	0.24879937	0.64434843	0.61172695	0.19501276	0.29396847
155.4	0.33758061	0.42127836	0.24874987	0.64436792	0.61169881	0.19496645	0.29393755
155.5	0.33752263	0.42131469	0.24870051	0.64438739	0.61167055	0.19492027	0.29390668
155.6	0.33746477	0.42135101	0.24865129	0.64440684	0.6116422	0.19487424	0.29387585
155.7	0.33740701	0.42138734	0.24860221	0.64442627	0.61161374	0.19482835	0.29384508
155.8	0.33734937	0.42142366	0.24855326	0.64444568	0.61158517	0.1947826	0.29381436
155.9	0.33729183	0.42145999	0.24850444	0.64446507	0.6115565	0.19473699	0.2937837
156	0.33723441	0.42149632	0.24845576	0.64448444	0.61152773	0.19469152	0.29375308

114	0.37501996	0.4072427	0.28848291	0.6344693	0.62918445	0.23217844	0.30963059
114.1	0.37488528	0.40727079	0.28831334	0.63449789	0.62908596	0.23202491	0.30958398
114.2	0.37475089	0.40729894	0.2881443	0.63452645	0.62898788	0.23187183	0.30953737
114.3	0.37461678	0.40732714	0.28797579	0.634555	0.62889019	0.23171922	0.30949078
114.4	0.37448296	0.40735539	0.28780781	0.63458352	0.62879292	0.23156706	0.3094442
114.5	0.37434941	0.4073837	0.28764035	0.63461202	0.62869604	0.23141535	0.30939763
114.6	0.37421615	0.40741205	0.28747343	0.63464049	0.62859957	0.2312641	0.30935108
114.7	0.37408317	0.40744046	0.28730702	0.63466895	0.62850349	0.2311133	0.30930454
114.8	0.37395047	0.40746891	0.28714114	0.63469738	0.62840782	0.23096294	0.30925801
114.9	0.37381805	0.40749742	0.28697578	0.63472578	0.62831254	0.23081303	0.30921151
115	0.3736859	0.40752598	0.28681094	0.63475417	0.62821765	0.23066357	0.30916502
115.1	0.37355404	0.40755458	0.28664661	0.63478253	0.62812316	0.23051455	0.30911855
115.2	0.37342245	0.40758324	0.2864828	0.63481087	0.62802907	0.23036597	0.30907211
115.3	0.37329114	0.40761195	0.28631951	0.63483919	0.62793536	0.23021783	0.30902568
115.4	0.37316011	0.4076407	0.28615673	0.63486748	0.62784205	0.23007012	0.30897928
115.5	0.37302935	0.40766951	0.28599445	0.63489575	0.62774913	0.22992286	0.3089329
115.6	0.37289887	0.40769836	0.28583269	0.634924	0.62765659	0.22977602	0.30888655
115.7	0.37276866	0.40772727	0.28567144	0.63495222	0.62756444	0.22962962	0.30884022
115.8	0.37263873	0.40775622	0.28551069	0.63498042	0.62747268	0.22948365	0.30879393
115.9	0.37250908	0.40778521	0.28535044	0.6350086	0.62738131	0.22933811	0.30874766
116	0.37237969	0.40781426	0.2851907	0.63503676	0.62729031	0.229193	0.30870141
116.1	0.37225058	0.40784335	0.28503147	0.63506489	0.6271997	0.22904831	0.3086552
116.2	0.37212174	0.40787249	0.28487273	0.635093	0.62710948	0.22890404	0.30860902
116.3	0.37199317	0.40790168	0.28471449	0.63512108	0.62701963	0.2287602	0.30856287
116.4	0.37186488	0.40793091	0.28455674	0.63514915	0.62693016	0.22861678	0.30851675
116.5	0.37173685	0.40796019	0.2843995	0.63517719	0.62684107	0.22847377	0.30847067
116.6	0.3716091	0.40798951	0.28424274	0.63520521	0.62675236	0.22833118	0.30842462
116.7	0.37148161	0.40801888	0.28408648	0.6352332	0.62666403	0.22818901	0.30837861
116.8	0.3713544	0.4080483	0.28393071	0.63526117	0.62657607	0.22804725	0.30833263
116.9	0.37122745	0.40807776	0.28377543	0.63528912	0.62648848	0.22790591	0.30828668
117	0.37110077	0.40810726	0.28362064	0.63531704	0.62640127	0.22776497	0.30824078
117.1	0.37097435	0.40813681	0.28346634	0.63534495	0.62631443	0.22762445	0.30819491
117.2	0.37084821	0.40816641	0.28331252	0.63537282	0.62622796	0.22748433	0.30814909
117.3	0.37072233	0.40819605	0.28315918	0.63540068	0.62614186	0.22734462	0.3081033
117.4	0.37059671	0.40822573	0.28300633	0.63542851	0.62605613	0.22720531	0.30805755
117.5	0.37047136	0.40825546	0.28285395	0.63545632	0.62597077	0.2270664	0.30801185
117.6	0.37034628	0.40828523	0.28270206	0.6354841	0.62588577	0.2269279	0.30796618
117.7	0.37022146	0.40831505	0.28255064	0.63551186	0.62580115	0.2267898	0.30792056
117.8	0.3700969	0.40834491	0.2823997	0.6355396	0.62571688	0.22665209	0.30787499
117.9	0.3699726	0.40837481	0.28224924	0.63556731	0.62563299	0.22651479	0.30782946
118	0.36984857	0.40840476	0.28209925	0.635595	0.62554945	0.22637788	0.30778397
118.1	0.3697248	0.40843474	0.28194973	0.63562266	0.62546628	0.22624136	0.30773853
118.2	0.36960129	0.40846477	0.28180068	0.63565031	0.62538347	0.22610524	0.30769313
118.3	0.36947804	0.40849485	0.2816521	0.63567793	0.62530102	0.22596951	0.30764778
118.4	0.36935505	0.40852496	0.28150398	0.63570552	0.62521893	0.22583417	0.30760248
118.5	0.36923232	0.40855512	0.28135634	0.63573309	0.6251372	0.22569922	0.30755722
118.6	0.36910985	0.40858532	0.28120916	0.63576064	0.62505583	0.22556465	0.30751202
118.7	0.36898764	0.40861556	0.28106244	0.63578817	0.62497481	0.22543047	0.30746686
118.8	0.36886568	0.40864584	0.28091618	0.63581567	0.62489416	0.22529668	0.30742176
118.9	0.36874398	0.40867616	0.28077038	0.63584315	0.62481385	0.22516327	0.3073767
119	0.36862254	0.40870652	0.28062504	0.63587061	0.62473391	0.22503024	0.3073317
119.1	0.36850136	0.40873692	0.28048016	0.63589804	0.62465432	0.22489759	0.30728674
119.2	0.36838043	0.40876736	0.28033573	0.63592545	0.62457508	0.22476532	0.30724184
119.3	0.36825975	0.40879785	0.28019176	0.63595284	0.6244962	0.22463343	0.307197
119.4	0.36813933	0.40882837	0.28004824	0.6359802	0.62441766	0.22450192	0.3071522
119.5	0.36801917	0.40885893	0.27990518	0.63600754	0.62433948	0.22437078	0.30710746
119.6	0.36789926	0.40888953	0.27976256	0.63603486	0.62426165	0.22424002	0.30706278
119.7	0.3677796	0.40892018	0.27962039	0.63606215	0.62418417	0.22410963	0.30701814
119.8	0.36766019	0.40895086	0.27947867	0.63608943	0.62410703	0.22397961	0.30697357
119.9	0.36754104	0.40898158	0.2793374	0.63611668	0.62403025	0.22384996	0.30692905
165	0.33249537	0.4247599	0.24458904	0.64614455	0.60853277	0.19112392	0.29122181
165.1	0.3324472	0.42479603	0.24455137	0.64616209	0.60849518	0.1910896	0.29119625
165.2	0.33239912	0.42483216	0.2445138	0.64617961	0.60845749	0.19105539	0.29117074
165.3	0.33235113	0.42486829	0.24447635	0.64619711	0.60841972	0.19102128	0.29114529
165.4	0.33230323	0.42490441	0.244439	0.64621459	0.60838186	0.19098727	0.29111989
165.5	0.33225543	0.42494053	0.24440175	0.64623205	0.60834392	0.19095338	0.29109455
165.6	0.33220772	0.42497664	0.24436462	0.64624949	0.60830588	0.19091958	0.29106927
165.7	0.3321601	0.42501275	0.24432759	0.64626691	0.60826776	0.19088589	0.29104405
165.8	0.33211258	0.42504886	0.24429066	0.64628432	0.60822955	0.1908523	0.29101888
165.9	0.33206514	0.42508496	0.24425385	0.6463017	0.60819126	0.19081882	0.29099377
166	0.3320178	0.42512105	0.24421713	0.64631906	0.60815288	0.19078544	0.29096871
166.1	0.33197055	0.42515715	0.24418053	0.64633641	0.60811441	0.19075216	0.29094371
166.2	0.33192338	0.42519324	0.24414403	0.64635373	0.60807586	0.19071899	0.29091877
166.3	0.33187631	0.42522932	0.24410763	0.64637104	0.60803722	0.19068592	0.29089389
166.4	0.33182933	0.4252654	0.24407133	0.64638832	0.6079985	0.19065295	0.29086906
166.5	0.33178244	0.42530147	0.24403515	0.64640559	0.60795969	0.19062008	0.29084428
166.6	0.33173564	0.42533754	0.24399906	0.64642284	0.6079208	0.19058731	0.29081957
166.7	0.33168894	0.42537361	0.24396308	0.64644007	0.60788183	0.19055464	0.29079491
166.8	0.33164232	0.42540967	0.2439272	0.64645727	0.60784278	0.19052208	0.2907703
166.9	0.33159579	0.42544573	0.24389143	0.64647446	0.60780364	0.19048961	0.29074575
167	0.33154935	0.42548178	0.24385576	0.64649164	0.60776442	0.19045724	0.29072126
167.1	0.331503	0.42551783	0.24382019	0.64650879	0.60772512	0.19042497	0.29069683
167.2	0.33145673	0.42555387	0.24378472	0.64652592	0.60768574	0.19039281	0.29067245
167.3	0.33141056	0.42558991	0.24374936	0.64654303	0.60764627	0.19036074	0.29064812
167.4	0.33136448	0.42562594	0.2437141	0.64656013	0.60760673	0.19032877	0.29062386
167.5	0.33131848	0.42566197	0.24367894	0.64657721	0.60756711	0.19029689	0.29059964
167.6	0.33127258	0.42569799	0.24364388	0.64659426	0.60752741	0.19026512	0.29057549
167.7	0.33122676	0.42573401	0.24360892	0.6466113	0.60748763	0.19023344	0.29055139
167.8	0.33118103	0.42577002	0.24357406	0.64662832	0.60744777	0.19020186	0.29052734
167.9	0.33113539	0.42580603	0.2435393	0.64664532	0.60740783	0.19017038	0.29050335
168	0.33108983	0.42584203	0.24350465	0.6466623	0.60736782	0.19013899	0.29047942
168.1	0.33104437	0.42587803	0.24347009	0.64667926	0.60732773	0.1901077	0.29045554
168.2	0.33099899	0.42591402	0.24343563	0.64669621	0.60728757	0.19007651	0.29043172
168.3	0.3309537	0.42595001	0.24340127	0.64671313	0.60724733	0.19004541	0.29040796
168.4	0.33090849	0.42598599	0.24336702	0.64673004	0.60720702	0.19001441	0.29038425
168.5	0.33086338	0.42602197	0.24333286	0.64674692	0.60716663	0.1899835	0.29036059
168.6	0.33081835	0.42605794	0.2432988	0.64676379	0.60712617	0.18995269	0.29033699
168.7	0.3307734	0.42609391	0.24326484	0.64678064	0.60708564	0.18992197	0.29031345
168.8	0.33072854	0.42612987	0.24323097	0.64679747	0.60704503	0.18989135	0.29028996
168.9	0.33068377	0.42616582	0.24319721	0.64681428	0.60700436	0.18986082	0.29026653
169	0.33063909	0.42620177	0.24316354	0.64683108	0.60696361	0.18983038	0.29024315
169.1	0.33059449	0.42623772	0.24312997	0.64684785	0.60692279	0.18980004	0.29021983
169.2	0.33054998	0.42627365	0.2430965	0.64686461	0.60688191	0.18976979	0.29019656
169.3	0.33050555	0.42630959	0.24306312	0.64688135	0.60684095	0.18973963	0.29017335
169.4	0.33046121	0.42634552	0.24302984	0.64689806	0.60679993	0.18970957	0.29015019
169.5	0.33041695	0.42638144	0.24299666	0.64691476	0.60675885	0.1896796	0.29012709
169.6	0.33037278	0.42641735	0.24296358	0.64693145	0.60671769	0.18964972	0.29010405
169.7	0.3303287	0.42645326	0.24293059	0.64694811	0.60667647	0.18961993	0.29008106
169.8	0.3302847	0.42648917	0.2428977	0.64696476	0.60663519	0.18959024	0.29005812
169.9	0.33024078	0.42652507	0.2428649	0.64698138	0.60659384	0.18956064	0.29003524
170	0.33019695	0.42656096	0.2428322	0.64699799	0.60655243	0.18953113	0.29001241
170.1	0.3301532	0.42659685	0.24279959	0.64701458	0.60651095	0.18950171	0.28998964
170.2	0.33010954	0.42663273	0.24276708	0.64703115	0.60646942	0.18947238	0.28996693
170.3	0.33006596	0.42666861	0.24273467	0.6470477	0.60642782	0.18944314	0.28994427
170.4	0.33002246	0.42670448	0.24270235	0.64706424	0.60638616	0.18941399	0.28992166
170.5	0.32997905	0.42674034	0.24267012	0.64708075	0.60634445	0.18938493	0.28989911
170.6	0.32993572	0.4267762	0.24263799	0.64709725	0.60630267	0.18935596	0.28987661
170.7	0.32989248	0.42681205	0.24260595	0.64711373	0.60626084	0.18932708	0.28985416
170.8	0.32984932	0.42684789	0.24257401	0.64713019	0.60621895	0.18929828	0.28983178
170.9	0.32980624	0.42688373	0.24254216	0.64714664	0.60617701	0.18926958	0.28980944
171	0.32976324	0.42691957	0.24251041	0.64716306	0.60613501	0.18924096	0.28978716

126.1	0.36062347	0.41095468	0.27139258	0.63776021	0.6199281	0.21648945	0.30429097
126.2	0.36051913	0.41098749	0.27127669	0.63778597	0.61987231	0.21638098	0.3042505
126.3	0.360415	0.41102033	0.27116116	0.63781171	0.61981684	0.21627283	0.30421009
126.4	0.3603111	0.4110532	0.27104598	0.63783742	0.61976169	0.21616497	0.30416975
126.5	0.36020741	0.41108609	0.27093117	0.63786311	0.61970687	0.21605743	0.30412948
126.6	0.36010394	0.41111901	0.27081672	0.63788877	0.61965236	0.21595018	0.30408927
126.7	0.36000068	0.41115197	0.27070262	0.63791442	0.61959818	0.21584324	0.30404913
126.8	0.35989764	0.41118494	0.27058887	0.63794004	0.61954432	0.2157366	0.30400906
126.9	0.35979482	0.41121795	0.27047548	0.63796563	0.61949078	0.21563026	0.30396904
127	0.35969221	0.41125098	0.27036245	0.63799121	0.61943756	0.21552422	0.3039291
127.1	0.35958982	0.41128404	0.27024976	0.63801676	0.61938466	0.21541848	0.30388922
127.2	0.35948764	0.41131712	0.27013743	0.63804229	0.61933208	0.21531304	0.3038494
127.3	0.35938568	0.41135024	0.27002545	0.6380678	0.61927982	0.2152079	0.30380965
127.4	0.35928393	0.41138337	0.26991381	0.63809328	0.61922788	0.21510305	0.30376997
127.5	0.35918239	0.41141654	0.26980252	0.63811874	0.61917626	0.2149985	0.30373034
127.6	0.35908106	0.41144973	0.26969158	0.63814418	0.61912496	0.21489424	0.30369078
127.7	0.35897994	0.41148295	0.26958098	0.63816959	0.61907398	0.21479028	0.30365129
127.8	0.35887904	0.41151619	0.26947073	0.63819499	0.61902331	0.21468661	0.30361186
127.9	0.35877835	0.41154946	0.26936082	0.63822035	0.61897297	0.21458324	0.30357249
128	0.35867786	0.41158275	0.26925125	0.6382457	0.61892294	0.21448015	0.30353318
128.1	0.35857759	0.41161607	0.26914201	0.63827102	0.61887323	0.21437736	0.30349394
128.2	0.35847753	0.41164942	0.26903312	0.63829633	0.61882383	0.21427486	0.30345476
128.3	0.35837767	0.41168279	0.26892457	0.6383216	0.61877475	0.21417264	0.30341564
128.4	0.35827802	0.41171618	0.26881635	0.63834686	0.61872599	0.21407072	0.30337658
128.5	0.35817859	0.41174961	0.26870847	0.63837209	0.61867754	0.21396908	0.30333759
128.6	0.35807935	0.41178305	0.26860092	0.6383973	0.6186294	0.21386773	0.30329865
128.7	0.35798033	0.41181652	0.2684937	0.63842249	0.61858158	0.21376667	0.30325978
128.8	0.35788151	0.41185002	0.26838682	0.63844765	0.61853408	0.21366589	0.30322097
128.9	0.3577829	0.41188354	0.26828027	0.63847279	0.61848689	0.2135654	0.30318221
129	0.35768449	0.41191708	0.26817405	0.63849791	0.61844001	0.21346519	0.30314352
129.1	0.35758629	0.41195065	0.26806815	0.63852301	0.61839344	0.21336526	0.30310489
129.2	0.35748829	0.41198424	0.26796258	0.63854808	0.61834718	0.21326562	0.30306631
129.3	0.35739049	0.41201786	0.26785734	0.63857313	0.61830124	0.21316626	0.30302779
129.4	0.3572929	0.4120515	0.26775243	0.63859816	0.61825561	0.21306718	0.30298934
129.5	0.35719551	0.41208516	0.26764784	0.63862316	0.61821029	0.21296838	0.30295094
129.6	0.35709833	0.41211885	0.26754357	0.63864814	0.61816527	0.21286986	0.30291259
129.7	0.35700134	0.41215256	0.26743962	0.6386731	0.61812057	0.21277161	0.30287431
129.8	0.35690456	0.41218629	0.267336	0.63869804	0.61807617	0.21267365	0.30283608
129.9	0.35680798	0.41222005	0.26723269	0.63872295	0.61803209	0.21257596	0.3027979
130	0.3567116	0.41225383	0.2671297	0.63874784	0.61798831	0.21247855	0.30275979
130.1	0.35661542	0.41228763	0.26702703	0.63877271	0.61794483	0.21238141	0.30272172
130.2	0.35651944	0.41232146	0.26692468	0.63879755	0.61790166	0.21228455	0.30268372
130.3	0.35642365	0.4123553	0.26682264	0.63882238	0.6178588	0.21218797	0.30264576
130.4	0.35632807	0.41238918	0.26672091	0.63884718	0.61781624	0.21209165	0.30260786
130.5	0.35623268	0.41242307	0.2666195	0.63887195	0.61777398	0.21199561	0.30257002
130.6	0.3561375	0.41245698	0.2665184	0.63889671	0.61773203	0.21189984	0.30253223
130.7	0.3560425	0.41249092	0.26641761	0.63892144	0.61769037	0.21180435	0.30249449
130.8	0.35594771	0.41252488	0.26631713	0.63894615	0.61764902	0.21170912	0.3024568
130.9	0.35585311	0.41255886	0.26621695	0.63897083	0.61760798	0.21161416	0.30241916
131	0.35575871	0.41259286	0.26611709	0.63899549	0.61756723	0.21151947	0.30238158
131.1	0.3556645	0.41262689	0.26601753	0.63902014	0.61752678	0.21142506	0.30234404
131.2	0.35557049	0.41266093	0.26591827	0.63904475	0.61748663	0.2113309	0.30230656
131.3	0.35547667	0.412695	0.26581932	0.63906935	0.61744678	0.21123702	0.30226912
131.4	0.35538305	0.41272909	0.26572067	0.63909392	0.61740723	0.2111434	0.30223174
131.5	0.35528962	0.4127632	0.26562232	0.63911847	0.61736798	0.21105005	0.3021944
131.6	0.35519638	0.41279733	0.26552428	0.639143	0.61732902	0.21095696	0.30215711
131.7	0.35510333	0.41283148	0.26542653	0.63916751	0.61729036	0.21086414	0.30211987
131.8	0.35501048	0.41286565	0.26532908	0.63919199	0.61725199	0.21077158	0.30208267
131.9	0.35491782	0.41289985	0.26523193	0.63921645	0.61721391	0.21067928	0.30204552
132	0.35482534	0.41293406	0.26513508	0.63924089	0.61717613	0.21058724	0.30200842
132.1	0.35473306	0.41296829	0.26503852	0.63926531	0.61713864	0.21049547	0.30197136
132.2	0.35464097	0.41300255	0.26494226	0.6392897	0.61710145	0.21040395	0.30193435
132.3	0.35454907	0.41303682	0.26484629	0.63931408	0.61706454	0.2103127	0.30189738
132.4	0.35445736	0.41307112	0.26475061	0.63933843	0.61702792	0.2102217	0.30186045
132.5	0.35436584	0.41310543	0.26465522	0.63936276	0.61699159	0.21013097	0.30182357
132.6	0.3542745	0.41313976	0.26456013	0.63938707	0.61695554	0.21004049	0.30178673
132.7	0.35418336	0.41317412	0.26446532	0.63941135	0.61691978	0.20995026	0.30174993
132.8	0.3540924	0.41320849	0.2643708	0.63943562	0.61688431	0.2098603	0.30171318
132.9	0.35400163	0.41324288	0.26427657	0.63945986	0.61684911	0.20977059	0.30167646
133	0.35391104	0.41327729	0.26418262	0.63948409	0.61681421	0.20968113	0.30163978
133.1	0.35382064	0.41331172	0.26408896	0.63950829	0.61677958	0.20959193	0.30160314
133.2	0.35373042	0.41334617	0.26399558	0.63953246	0.61674523	0.20950298	0.30156654
133.3	0.3536404	0.41338064	0.26390249	0.63955662	0.61671116	0.20941429	0.30152998
133.4	0.35355055	0.41341513	0.26380967	0.63958076	0.61667738	0.20932584	0.30149346
133.5	0.35346089	0.41344963	0.26371714	0.63960487	0.61664387	0.20923765	0.30145697
133.6	0.35337141	0.41348416	0.26362489	0.63962896	0.61661063	0.20914971	0.30142052
133.7	0.35328212	0.4135187	0.26353292	0.63965303	0.61657768	0.20906202	0.3013841
133.8	0.35319301	0.41355326	0.26344122	0.63967708	0.616545	0.20897458	0.30134772
133.9	0.35310408	0.41358784	0.2633498	0.63970111	0.61651259	0.20888739	0.30131137
134	0.35301533	0.41362244	0.26325866	0.63972511	0.61648045	0.20880045	0.30127506
134.1	0.35292677	0.41365706	0.26316779	0.63974909	0.61644858	0.20871375	0.30123877
134.2	0.35283838	0.41369169	0.2630772	0.63977305	0.61641699	0.2086273	0.30120253
134.3	0.35275018	0.41372634	0.26298688	0.63979699	0.61638566	0.2085411	0.30116631
134.4	0.35266216	0.41376101	0.26289683	0.63982091	0.61635459	0.20845514	0.30113012
134.5	0.35257432	0.4137957	0.26280705	0.63984481	0.6163238	0.20836943	0.30109397
134.6	0.35248665	0.4138304	0.26271755	0.63986868	0.61629326	0.20828397	0.30105784
134.7	0.35239917	0.41386512	0.26262831	0.63989253	0.61626299	0.20819874	0.30102175
134.8	0.35231186	0.41389986	0.26253934	0.63991636	0.61623298	0.20811376	0.30098568
134.9	0.35222473	0.41393461	0.26245063	0.63994017	0.61620323	0.20802902	0.30094964

180	0.3262133	0.43011583	0.24000819	0.64856899	0.60223209	0.18699327	0.28799554
180.1	0.32617721	0.43015099	0.23998405	0.64858384	0.60218849	0.1869716	0.2879779
180.2	0.32614118	0.43018615	0.23995998	0.64859867	0.6021449	0.18695	0.28796031
180.3	0.32610523	0.4302213	0.23993599	0.64861348	0.60210134	0.18692847	0.28794276
180.4	0.32606934	0.43025645	0.23991207	0.64862828	0.60205779	0.186907	0.28792527
180.5	0.32603353	0.43029158	0.23988822	0.64864306	0.60201426	0.1868856	0.28790781
180.6	0.32599778	0.43032671	0.23986445	0.64865783	0.60197076	0.18686427	0.28789041
180.7	0.3259621	0.43036183	0.23984075	0.64867258	0.60192727	0.186843	0.28787305
180.8	0.3259265	0.43039694	0.23981712	0.64868731	0.60188381	0.18682179	0.28785574
180.9	0.32589096	0.43043204	0.23979357	0.64870202	0.60184037	0.18680066	0.28783848
181	0.32585548	0.43046714	0.23977009	0.64871672	0.60179695	0.18677958	0.28782126
181.1	0.32582008	0.43050223	0.23974668	0.64873139	0.60175355	0.18675858	0.28780409
181.2	0.32578475	0.43053731	0.23972335	0.64874605	0.60171018	0.18673763	0.28778696
181.3	0.32574948	0.43057238	0.23970008	0.6487607	0.60166683	0.18671676	0.28776988
181.4	0.32571428	0.43060744	0.23967689	0.64877532	0.60162351	0.18669594	0.28775285
181.5	0.32567915	0.4306425	0.23965377	0.64878993	0.60158022	0.18667519	0.28773586
181.6	0.32564409	0.43067755	0.23963073	0.64880452	0.60153695	0.18665451	0.28771892
181.7	0.32560909	0.43071259	0.23960775	0.64881909	0.60149371	0.18663389	0.28770203
181.8	0.32557416	0.43074762	0.23958485	0.64883365	0.60145049	0.18661333	0.28768518
181.9	0.3255393	0.43078264	0.23956201	0.64884818	0.60140731	0.18659284	0.28766838
182	0.32550451	0.43081766	0.23953925	0.6488627	0.60136415	0.18657241	0.28765162
182.1	0.32546979	0.43085267	0.23951656	0.6488772	0.60132102	0.18655205	0.28763491
182.2	0.32543513	0.43088767	0.23949394	0.64889168	0.60127792	0.18653174	0.28761825
182.3	0.32540053	0.43092266	0.23947139	0.64890614	0.60123486	0.18651151	0.28760163
182.4	0.32536601	0.43095764	0.23944891	0.64892058	0.60119182	0.18649133	0.28758505
182.5	0.32533155	0.43099262	0.2394265	0.648935	0.60114881	0.18647122	0.28756852
182.6	0.32529716	0.43102759	0.23940416	0.6489494	0.60110584	0.18645117	0.28755204
182.7	0.32526283	0.43106255	0.23938189	0.64896377	0.6010629	0.18643119	0.2875356
182.8	0.32522858	0.4310975	0.23935969	0.64897813	0.60101999	0.18641127	0.28751921
182.9	0.32519438	0.43113244	0.23933755	0.64899247	0.60097712	0.18639141	0.28750286
183	0.32516026	0.43116738	0.23931549	0.64900679	0.60093428	0.18637161	0.28748655
183.1	0.32512619	0.43120231	0.2392935	0.64902108	0.60089147	0.18635188	0.28747029

141.1	0.34715315	0.41611816	0.25743416	0.64137312	0.61477203	0.20322349	0.29874856
141.2	0.34707641	0.41615377	0.25736046	0.64139556	0.61475359	0.20315282	0.29871337
141.3	0.34699983	0.4161894	0.25728698	0.64141796	0.61473522	0.20308236	0.29867818
141.4	0.34692339	0.41622503	0.25721371	0.64144035	0.61471692	0.2030121	0.298643
141.5	0.34684711	0.41626067	0.25714065	0.64146272	0.61469867	0.20294204	0.29860783
141.6	0.34677099	0.41629633	0.2570678	0.64148506	0.61468049	0.20287219	0.29857266
141.7	0.34669501	0.41633199	0.25699516	0.64150738	0.61466236	0.20280254	0.2985375
141.8	0.34661918	0.41636767	0.25692272	0.64152968	0.61464428	0.2027331	0.29850235
141.9	0.34654351	0.41640335	0.2568505	0.64155196	0.61462625	0.20266385	0.29846721
142	0.34646798	0.41643905	0.25677848	0.64157422	0.61460826	0.20259481	0.29843208
142.1	0.34639261	0.41647475	0.25670667	0.64159646	0.61459031	0.20252597	0.29839695
142.2	0.34631738	0.41651046	0.25663506	0.64161867	0.6145724	0.20245733	0.29836183
142.3	0.34624231	0.41654618	0.25656366	0.64164086	0.61455452	0.20238888	0.29832672
142.4	0.34616738	0.41658191	0.25649246	0.64166303	0.61453666	0.20232064	0.29829161
142.5	0.3460926	0.41661765	0.25642146	0.64168518	0.61451884	0.2022526	0.29825652
142.6	0.34601797	0.4166534	0.25635067	0.64170731	0.61450103	0.20218476	0.29822143
142.7	0.34594349	0.41668916	0.25628008	0.64172942	0.61448325	0.20211711	0.29818636
142.8	0.34586915	0.41672493	0.25620969	0.6417515	0.61446547	0.20204966	0.29815129
142.9	0.34579496	0.41676071	0.2561395	0.64177357	0.61444771	0.20198241	0.29811623
143	0.34572092	0.41679649	0.25606951	0.64179561	0.61442996	0.20191535	0.29808118
143.1	0.34564703	0.41683229	0.25599972	0.64181763	0.61441221	0.20184849	0.29804614
143.2	0.34557328	0.41686809	0.25593013	0.64183963	0.61439447	0.20178183	0.29801111
143.3	0.34549968	0.4169039	0.25586074	0.64186161	0.61437672	0.20171536	0.2979761
143.4	0.34542623	0.41693972	0.25579154	0.64188357	0.61435897	0.20164908	0.29794109
143.5	0.34535292	0.41697555	0.25572254	0.64190551	0.61434121	0.201583	0.29790609
143.6	0.34527975	0.41701139	0.25565373	0.64192742	0.61432344	0.20151711	0.29787111
143.7	0.34520673	0.41704723	0.25558512	0.64194932	0.61430566	0.20145141	0.29783614
143.8	0.34513385	0.41708308	0.25551671	0.64197119	0.61428786	0.20138591	0.29780118
143.9	0.34506112	0.41711895	0.25544849	0.64199305	0.61427005	0.2013206	0.29776623
144	0.34498853	0.41715481	0.25538046	0.64201488	0.61425221	0.20125548	0.2977313
144.1	0.34491609	0.41719069	0.25531262	0.64203669	0.61423434	0.20119055	0.29769638
144.2	0.34484378	0.41722658	0.25524498	0.64205848	0.61421645	0.20112581	0.29766148
144.3	0.34477162	0.41726247	0.25517752	0.64208025	0.61419853	0.20106126	0.29762659
144.4	0.34469961	0.41729837	0.25511026	0.64210199	0.61418058	0.2009969	0.29759171
144.5	0.34462773	0.41733428	0.25504319	0.64212372	0.61416259	0.20093272	0.29755685
144.6	0.344556	0.41737019	0.2549763	0.64214543	0.61414456	0.20086874	0.29752201
144.7	0.34448441	0.41740612	0.25490961	0.64216711	0.6141265	0.20080494	0.29748718
144.8	0.34441296	0.41744205	0.2548431	0.64218877	0.61410839	0.20074133	0.29745237
144.9	0.34434165	0.41747799	0.25477678	0.64221042	0.61409024	0.20067791	0.29741758
145	0.34427048	0.41751393	0.25471065	0.64223204	0.61407204	0.20061467	0.2973828
145.1	0.34419945	0.41754988	0.2546447	0.64225364	0.61405379	0.20055162	0.29734804
145.2	0.34412856	0.41758584	0.25457894	0.64227522	0.61403549	0.20048876	0.2973133
145.3	0.34405781	0.41762181	0.25451336	0.64229678	0.61401714	0.20042608	0.29727857
145.4	0.3439872	0.41765778	0.25444797	0.64231832	0.61399873	0.20036358	0.29724387
145.5	0.34391673	0.41769376	0.25438276	0.64233983	0.61398027	0.20030127	0.29720918
145.6	0.3438464	0.41772975	0.25431773	0.64236133	0.61396175	0.20023914	0.29717452
145.7	0.34377621	0.41776575	0.25425289	0.64238281	0.61394317	0.20017719	0.29713987
145.8	0.34370615	0.41780175	0.25418823	0.64240426	0.61392452	0.20011543	0.29710525
145.9	0.34363623	0.41783775	0.25412375	0.6424257	0.61390581	0.20005384	0.29707065
146	0.34356645	0.41787377	0.25405945	0.64244711	0.61388704	0.19999244	0.29703607
146.1	0.34349681	0.41790979	0.25399534	0.64246851	0.61386819	0.19993122	0.29700151
146.2	0.3434273	0.41794581	0.2539314	0.64248988	0.61384928	0.19987018	0.29696697
146.3	0.34335794	0.41798185	0.25386764	0.64251123	0.61383029	0.19980932	0.29693246
146.4	0.3432887	0.41801789	0.25380406	0.64253257	0.61381124	0.19974864	0.29689797
146.5	0.34321961	0.41805393	0.25374066	0.64255388	0.6137921	0.19968814	0.2968635
146.6	0.34315064	0.41808998	0.25367743	0.64257517	0.61377289	0.19962782	0.29682906
146.7	0.34308182	0.41812604	0.25361438	0.64259644	0.61375361	0.19956767	0.29679464
146.8	0.34301313	0.41816211	0.25355151	0.64261769	0.61373424	0.1995077	0.29676025
146.9	0.34294457	0.41819817	0.25348882	0.64263892	0.6137148	0.19944791	0.29672588
147	0.34287615	0.41823425	0.25342629	0.64266013	0.61369527	0.1993883	0.29669154
147.1	0.34280786	0.41827033	0.25336395	0.64268132	0.61367566	0.19932886	0.29665723
147.2	0.34273971	0.41830642	0.25330178	0.64270249	0.61365597	0.1992696	0.29662294
147.3	0.34267169	0.41834251	0.25323978	0.64272364	0.61363619	0.19921051	0.29658868
147.4	0.3426038	0.41837861	0.25317796	0.64274477	0.61361633	0.1991516	0.29655445
147.5	0.34253605	0.41841471	0.2531163	0.64276587	0.61359638	0.19909286	0.29652024
147.6	0.34246843	0.41845082	0.25305482	0.64278696	0.61357633	0.1990343	0.29648606
147.7	0.34240094	0.41848694	0.25299351	0.64280803	0.61355621	0.19897591	0.29645192
147.8	0.34233358	0.41852306	0.25293238	0.64282907	0.61353598	0.19891769	0.2964178
147.9	0.34226636	0.41855919	0.25287141	0.64285009	0.61351567	0.19885965	0.29638371
148	0.34219927	0.41859532	0.25281061	0.64287109	0.61349527	0.19880178	0.29634965
148.1	0.3421323	0.41863146	0.25274998	0.64289207	0.61347477	0.19874408	0.29631563
148.2	0.34206547	0.4186676	0.25268952	0.64291303	0.61345418	0.19868655	0.29628163
148.3	0.34199877	0.41870374	0.25262923	0.64293397	0.61343349	0.19862919	0.29624767
148.4	0.3419322	0.4187399	0.2525691	0.64295489	0.61341271	0.198572	0.29621374
148.5	0.34186576	0.41877605	0.25250915	0.64297578	0.61339183	0.19851498	0.29617984
148.6	0.34179946	0.41881222	0.25244936	0.64299666	0.61337086	0.19845813	0.29614597
148.7	0.34173328	0.41884838	0.25238973	0.64301751	0.61334978	0.19840145	0.29611214
148.8	0.34166723	0.41888455	0.25233028	0.64303834	0.61332861	0.19834494	0.29607834
148.9	0.3416013	0.41892073	0.25227098	0.64305915	0.61330734	0.1982886	0.29604457
149	0.34153551	0.41895691	0.25221186	0.64307994	0.61328597	0.19823242	0.29601084
149.1	0.34146985	0.41899309	0.25215289	0.6431007	0.6132645	0.19817641	0.29597714
149.2	0.34140431	0.41902928	0.25209409	0.64312145	0.61324292	0.19812057	0.29594348
149.3	0.3413389	0.41906547	0.25203546	0.64314218	0.61322125	0.1980649	0.29590985
149.4	0.34127362	0.41910167	0.25197698	0.64316288	0.61319948	0.19800939	0.29587626
149.5	0.34120847	0.41913787	0.25191867	0.64318357	0.6131776	0.19795404	0.2958427
149.6	0.34114344	0.41917407	0.25186053	0.64320423	0.61315562	0.19789886	0.29580918
149.7	0.34107855	0.41921028	0.25180254	0.64322488	0.61313354	0.19784384	0.2957757
149.8	0.34101377	0.41924649	0.25174471	0.6432455	0.61311135	0.19778899	0.29574226
149.9	0.34094913	0.4192827	0.25168705	0.6432661	0.61308906	0.1977343	0.29570885

195	0.32151017	0.43529927	0.23712389	0.6504288	0.59615059	0.18440749	0.28583108
195.1	0.32148318	0.43533317	0.23710906	0.65043734	0.59611429	0.18439423	0.28581959
195.2	0.32145625	0.43536706	0.23709427	0.65044582	0.59607805	0.18438102	0.28580814
195.3	0.32142937	0.43540095	0.23707954	0.65045423	0.59604188	0.18436785	0.28579672
195.4	0.32140254	0.43543483	0.23706485	0.65046258	0.59600577	0.18435473	0.28578533
195.5	0.32137576	0.43546869	0.23705022	0.65047087	0.59596972	0.18434165	0.28577398
195.6	0.32134903	0.43550255	0.23703564	0.6504791	0.59593374	0.18432862	0.28576266
195.7	0.32132236	0.4355364	0.23702111	0.65048727	0.59589782	0.18431564	0.28575138
195.8	0.32129574	0.43557024	0.23700663	0.65049537	0.59586197	0.1843027	0.28574013
195.9	0.32126917	0.43560407	0.2369922	0.65050342	0.59582618	0.18428981	0.28572892
196	0.32124265	0.4356379	0.23697782	0.6505114	0.59579046	0.18427696	0.28571774
196.1	0.32121618	0.43567171	0.23696349	0.65051932	0.5957548	0.18426416	0.2857066
196.2	0.32118977	0.43570551	0.23694921	0.65052717	0.5957192	0.1842514	0.28569549
196.3	0.3211634	0.43573931	0.23693498	0.65053497	0.59568367	0.18423868	0.28568441
196.4	0.32113709	0.4357731	0.2369208	0.6505427	0.5956482	0.18422602	0.28567337
196.5	0.32111083	0.43580687	0.23690667	0.65055037	0.5956128	0.18421339	0.28566236
196.6	0.32108462	0.43584064	0.23689258	0.65055798	0.59557746	0.18420081	0.28565138
196.7	0.32105846	0.4358744	0.23687855	0.65056553	0.59554218	0.18418827	0.28564044
196.8	0.32103235	0.43590815	0.23686457	0.65057302	0.59550697	0.18417578	0.28562953
196.9	0.32100629	0.43594189	0.23685063	0.65058044	0.59547183	0.18416333	0.28561866
197	0.32098029	0.43597563	0.23683675	0.65058781	0.59543675	0.18415093	0.28560781
197.1	0.32095433	0.43600935	0.23682291	0.65059511	0.59540173	0.18413857	0.28559701
197.2	0.32092842	0.43604307	0.23680912	0.65060235	0.59536678	0.18412625	0.28558623
197.3	0.32090257	0.43607677	0.23679538	0.65060952	0.59533189	0.18411398	0.28557549
197.4	0.32087676	0.43611047	0.23678169	0.65061664	0.59529707	0.18410175	0.28556478
197.5	0.32085101	0.43614416	0.23676805	0.65062369	0.59526231	0.18408956	0.2855541
197.6	0.32082531	0.43617784	0.23675445	0.65063069	0.59522761	0.18407741	0.28554346
197.7	0.32079965	0.43621151	0.23674091	0.65063762	0.59519298	0.18406531	0.28553285
197.8	0.32077405	0.43624518	0.23672741	0.65064449	0.59515842	0.18405325	0.28552227
197.9	0.32074849	0.43627883	0.23671396	0.6506513	0.59512391	0.18404123	0.28551173
198	0.32072299	0.43631247	0.23670055	0.65065804	0.59508948	0.18402926	0.28550122
198.1	0.32069754	0.43634611	0.2366872	0.65066473	0.5950551	0.18401733	0.28549074

156.1	0.3371771	0.42153264	0.24840722	0.64450379	0.61149886	0.19464619	0.29372252
156.2	0.3371199	0.42156897	0.24835881	0.64452311	0.61146988	0.194601	0.29369201
156.3	0.33706281	0.42160529	0.24831054	0.64454242	0.61144079	0.19455595	0.29366155
156.4	0.33700583	0.42164162	0.2482624	0.64456171	0.61141161	0.19451103	0.29363114
156.5	0.33694896	0.42167795	0.2482144	0.64458098	0.61138232	0.19446625	0.29360079
156.6	0.3368922	0.42171427	0.24816653	0.64460023	0.61135293	0.19442161	0.29357049
156.7	0.33683555	0.4217506	0.24811879	0.64461945	0.61132344	0.19437711	0.29354024
156.8	0.33677901	0.42178692	0.24807118	0.64463866	0.61129385	0.19433274	0.29351004
156.9	0.33672258	0.42182325	0.24802371	0.64465785	0.61126415	0.19428851	0.2934799
157	0.33666626	0.42185957	0.24797637	0.64467701	0.61123435	0.19424441	0.29344981
157.1	0.33661005	0.42189589	0.24792916	0.64469616	0.61120445	0.19420045	0.29341977
157.2	0.33655394	0.42193222	0.24788209	0.64471528	0.61117445	0.19415663	0.29338979
157.3	0.33649795	0.42196854	0.24783514	0.64473439	0.61114435	0.19411294	0.29335986
157.4	0.33644206	0.42200486	0.24778833	0.64475347	0.61111415	0.19406938	0.29332999
157.5	0.33638628	0.42204118	0.24774164	0.64477254	0.61108384	0.19402595	0.29330017
157.6	0.33633061	0.4220775	0.24769509	0.64479158	0.61105344	0.19398266	0.2932704
157.7	0.33627504	0.42211382	0.24764866	0.64481061	0.61102294	0.19393951	0.29324068
157.8	0.33621958	0.42215014	0.24760237	0.64482961	0.61099233	0.19389648	0.29321102
157.9	0.33616423	0.42218646	0.2475562	0.64484859	0.61096163	0.19385359	0.29318142
158	0.33610899	0.42222278	0.24751016	0.64486755	0.61093082	0.19381083	0.29315187
158.1	0.33605386	0.42225909	0.24746425	0.6448865	0.61089992	0.1937682	0.29312237
158.2	0.33599883	0.42229541	0.24741847	0.64490542	0.61086892	0.19372571	0.29309293
158.3	0.3359439	0.42233172	0.24737282	0.64492432	0.61083782	0.19368334	0.29306354
158.4	0.33588908	0.42236803	0.24732729	0.6449432	0.61080661	0.19364111	0.2930342
158.5	0.33583437	0.42240434	0.2472819	0.64496206	0.61077531	0.193599	0.29300493
158.6	0.33577977	0.42244065	0.24723662	0.6449809	0.61074391	0.19355703	0.2929757
158.7	0.33572527	0.42247696	0.24719148	0.64499972	0.61071241	0.19351518	0.29294653
158.8	0.33567087	0.42251327	0.24714646	0.64501852	0.61068081	0.19347346	0.29291742
158.9	0.33561658	0.42254957	0.24710157	0.64503729	0.61064911	0.19343187	0.29288836
159	0.3355624	0.42258588	0.2470568	0.64505605	0.61061731	0.19339042	0.29285936
159.1	0.33550831	0.42262218	0.24701216	0.64507479	0.61058541	0.19334908	0.29283041
159.2	0.33545434	0.42265848	0.24696764	0.64509351	0.61055341	0.19330788	0.29280151
159.3	0.33540047	0.42269478	0.24692325	0.64511221	0.61052132	0.1932668	0.29277267
159.4	0.3353467	0.42273108	0.24687898	0.64513088	0.61048913	0.19322585	0.29274389
159.5	0.33529303	0.42276738	0.24683483	0.64514954	0.61045683	0.19318503	0.29271516
159.6	0.33523947	0.42280367	0.24679081	0.64516817	0.61042444	0.19314433	0.29268649
159.7	0.33518601	0.42283997	0.24674692	0.64518679	0.61039195	0.19310376	0.29265788
159.8	0.33513266	0.42287626	0.24670314	0.64520538	0.61035937	0.19306332	0.29262931
159.9	0.33507941	0.42291255	0.24665949	0.64522395	0.61032668	0.193023	0.29260081
160	0.33502626	0.42294883	0.24661596	0.6452425	0.6102939	0.1929828	0.29257236
160.1	0.33497321	0.42298512	0.24657255	0.64526104	0.61026102	0.19294273	0.29254397
160.2	0.33492027	0.4230214	0.24652927	0.64527955	0.61022804	0.19290278	0.29251563
160.3	0.33486743	0.42305769	0.2464861	0.64529804	0.61019497	0.19286296	0.29248735
160.4	0.33481469	0.42309397	0.24644306	0.64531651	0.6101618	0.19282326	0.29245912
160.5	0.33476205	0.42313024	0.24640014	0.64533496	0.61012853	0.19278368	0.29243095
160.6	0.33470951	0.42316652	0.24635734	0.64535339	0.61009516	0.19274423	0.29240284
160.7	0.33465708	0.42320279	0.24631466	0.6453718	0.6100617	0.1927049	0.29237478
160.8	0.33460474	0.42323907	0.24627209	0.64539019	0.61002814	0.19266568	0.29234678
160.9	0.33455251	0.42327534	0.24622965	0.64540856	0.60999449	0.1926266	0.29231883
161	0.33450038	0.4233116	0.24618733	0.64542691	0.60996074	0.19258763	0.29229094
161.1	0.33444834	0.42334787	0.24614512	0.64544524	0.60992689	0.19254878	0.29226311
161.2	0.33439641	0.42338413	0.24610304	0.64546355	0.60989294	0.19251005	0.29223534
161.3	0.33434458	0.42342039	0.24606107	0.64548183	0.6098589	0.19247144	0.29220762
161.4	0.33429285	0.42345665	0.24601922	0.6455001	0.60982476	0.19243296	0.29217995
161.5	0.33424122	0.4234929	0.24597749	0.64551835	0.60979053	0.19239459	0.29215235
161.6	0.33418968	0.42352916	0.24593588	0.64553658	0.6097562	0.19235634	0.29212479
161.7	0.33413825	0.42356541	0.24589438	0.64555479	0.60972177	0.19231821	0.2920973
161.8	0.33408692	0.42360165	0.245853	0.64557298	0.60968724	0.19228019	0.29206986
161.9	0.33403568	0.4236379	0.24581174	0.64559114	0.60965262	0.1922423	0.29204248
162	0.33398454	0.42367414	0.2457706	0.64560929	0.60961791	0.19220452	0.29201516
162.1	0.3339335	0.42371038	0.24572957	0.64562742	0.6095831	0.19216686	0.29198789
162.2	0.33388256	0.42374662	0.24568865	0.64564553	0.60954819	0.19212931	0.29196068
162.3	0.33383172	0.42378285	0.24564786	0.64566362	0.60951319	0.19209188	0.29193352
162.4	0.33378098	0.42381908	0.24560717	0.64568169	0.60947809	0.19205457	0.29190642
162.5	0.33373033	0.42385531	0.24556661	0.64569973	0.6094429	0.19201737	0.29187938
162.6	0.33367978	0.42389153	0.24552616	0.64571776	0.60940761	0.19198029	0.2918524
162.7	0.33362933	0.42392775	0.24548582	0.64573577	0.60937223	0.19194332	0.29182547
162.8	0.33357898	0.42396397	0.2454456	0.64575376	0.60933675	0.19190647	0.2917986
162.9	0.33352872	0.42400019	0.24540549	0.64577173	0.60930118	0.19186973	0.29177179
163	0.33347856	0.4240364	0.24536549	0.64578968	0.60926552	0.1918331	0.29174503
163.1	0.3334285	0.42407261	0.24532561	0.64580761	0.60922976	0.19179659	0.29171833
163.2	0.33337853	0.42410882	0.24528584	0.64582552	0.60919391	0.19176019	0.29169168
163.3	0.33332866	0.42414502	0.24524618	0.64584341	0.60915796	0.19172391	0.2916651
163.4	0.33327888	0.42418122	0.24520664	0.64586128	0.60912192	0.19168773	0.29163857
163.5	0.3332292	0.42421741	0.24516721	0.64587913	0.60908579	0.19165167	0.29161209
163.6	0.33317962	0.4242536	0.24512789	0.64589696	0.60904957	0.19161572	0.29158568
163.7	0.33313013	0.42428979	0.24508869	0.64591478	0.60901325	0.19157988	0.29155932
163.8	0.33308073	0.42432598	0.24504959	0.64593257	0.60897684	0.19154415	0.29153301
163.9	0.33303143	0.42436216	0.24501061	0.64595034	0.60894033	0.19150853	0.29150677
164	0.33298223	0.42439834	0.24497173	0.64596809	0.60890374	0.19147303	0.29148058
164.1	0.33293312	0.42443451	0.24493297	0.64598583	0.60886705	0.19143763	0.29145445
164.2	0.33288411	0.42447068	0.24489432	0.64600354	0.60883027	0.19140234	0.29142837
164.3	0.33283519	0.42450685	0.24485577	0.64602124	0.6087934	0.19136716	0.29140235
164.4	0.33278636	0.42454301	0.24481734	0.64603891	0.60875644	0.19133209	0.29137639
164.5	0.33273763	0.42457917	0.24477902	0.64605657	0.60871939	0.19129712	0.29135048
164.6	0.33268899	0.42461532	0.2447408	0.6460742	0.60868225	0.19126227	0.29132464
164.7	0.33264044	0.42465147	0.2447027	0.64609182	0.60864501	0.19122752	0.29129884
164.8	0.33259199	0.42468762	0.2446647	0.64610942	0.60860769	0.19119288	0.29127311
164.9	0.33254363	0.42472376	0.24462682	0.646127	0.60857027	0.19115835	0.29124743

210	0.31799948	0.44028391	0.2354044	0.65105217	0.59141378	0.18286526	0.28445576
210.1	0.31797938	0.44031643	0.23539595	0.6510523	0.59138683	0.18285759	0.28444868
210.2	0.31795933	0.44034894	0.23538754	0.65105238	0.59135993	0.18284995	0.28444163
210.3	0.31793932	0.44038143	0.23537916	0.65105241	0.59133309	0.18284234	0.2844346
210.4	0.31791935	0.44041392	0.23537081	0.6510524	0.5913063	0.18283476	0.2844276
210.5	0.31789942	0.4404464	0.2353625	0.65105234	0.59127958	0.18282721	0.28442062
210.6	0.31787952	0.44047887	0.23535422	0.65105223	0.59125291	0.18281969	0.28441367
210.7	0.31785967	0.44051133	0.23534598	0.65105208	0.5912263	0.18281219	0.28440673
210.8	0.31783986	0.44054378	0.23533777	0.65105189	0.59119975	0.18280473	0.28439983
210.9	0.31782008	0.44057622	0.2353296	0.65105165	0.59117325	0.1827973	0.28439294
211	0.31780035	0.44060865	0.23532146	0.65105136	0.59114681	0.1827899	0.28438609
211.1	0.31778065	0.44064107	0.23531336	0.65105103	0.59112043	0.18278252	0.28437925
211.2	0.31776099	0.44067348	0.23530529	0.65105065	0.59109411	0.18277518	0.28437244
211.3	0.31774138	0.44070587	0.23529725	0.65105023	0.59106785	0.18276786	0.28436565
211.4	0.3177218	0.44073826	0.23528925	0.65104976	0.59104164	0.18276058	0.28435889
211.5	0.31770226	0.44077064	0.23528128	0.65104925	0.59101549	0.18275332	0.28435215
211.6	0.31768276	0.44080301	0.23527334	0.6510487	0.59098939	0.18274609	0.28434543
211.7	0.31766329	0.44083537	0.23526544	0.6510481	0.59096335	0.18273889	0.28433874
211.8	0.31764387	0.44086772	0.23525757	0.65104745	0.59093737	0.18273171	0.28433207
211.9	0.31762449	0.44090006	0.23524974	0.65104676	0.59091145	0.18272457	0.28432542
212	0.31760514	0.44093239	0.23524194	0.65104603	0.59088558	0.18271745	0.28431879
212.1	0.31758583	0.44096471	0.23523417	0.65104525	0.59085977	0.18271037	0.28431219
212.2	0.31756656	0.44099702	0.23522643	0.65104443	0.59083402	0.18270331	0.28430562
212.3	0.31754733	0.44102932	0.23521873	0.65104357	0.59080832	0.18269628	0.28429906
212.4	0.31752814	0.44106161	0.23521106	0.65104266	0.59078268	0.18268927	0.28429253
212.5	0.31750899	0.44109389	0.23520343	0.65104171	0.59075709	0.1826823	0.28428602
212.6	0.31748987	0.44112616	0.23519583	0.65104072	0.59073157	0.18267535	0.28427954
212.7	0.31747079	0.44115842	0.23518826	0.65103968	0.59070609	0.18266843	0.28427308
212.8	0.31745176	0.44119066	0.23518072	0.6510386	0.59068068	0.18266154	0.28426664
212.9	0.31743275	0.4412229	0.23517322	0.65103748	0.59065531	0.18265468	0.28426022
213	0.31741379	0.44125513	0.23516575	0.65103631	0.59063001	0.18264784	0.28425383
213.1	0.31739487	0.44128735	0.23515831	0.65103511	0.59060476	0.18264103	0.28424746

171.1	0.32972033	0.4269554	0.24247875	0.64717947	0.60609295	0.18921244	0.28976493
171.2	0.3296775	0.42699122	0.24244718	0.64719586	0.60605084	0.189184	0.28974276
171.3	0.32963475	0.42702703	0.2424157	0.64721223	0.60600868	0.18915564	0.28972065
171.4	0.32959208	0.42706284	0.24238432	0.64722858	0.60596647	0.18912738	0.28969858
171.5	0.3295495	0.42709865	0.24235303	0.64724491	0.60592421	0.1890992	0.28967657
171.6	0.329507	0.42713444	0.24232184	0.64726123	0.60588189	0.18907111	0.28965462
171.7	0.32946458	0.42717023	0.24229073	0.64727752	0.60583953	0.1890431	0.28963271
171.8	0.32942224	0.42720602	0.24225972	0.6472938	0.60579711	0.18901518	0.28961086
171.9	0.32937998	0.4272418	0.2422288	0.64731006	0.60575465	0.18898735	0.28958907
172	0.3293378	0.42727757	0.24219797	0.6473263	0.60571214	0.1889596	0.28956733
172.1	0.32929571	0.42731334	0.24216723	0.64734252	0.60566959	0.18893193	0.28954564
172.2	0.32925369	0.4273491	0.24213658	0.64735873	0.60562699	0.18890435	0.28952401
172.3	0.32921176	0.42738485	0.24210602	0.64737491	0.60558434	0.18887686	0.28950243
172.4	0.3291699	0.4274206	0.24207556	0.64739108	0.60554165	0.18884944	0.2894809
172.5	0.32912813	0.42745634	0.24204518	0.64740723	0.60549892	0.18882212	0.28945942
172.6	0.32908644	0.42749207	0.24201489	0.64742336	0.60545614	0.18879487	0.289438
172.7	0.32904482	0.4275278	0.24198469	0.64743948	0.60541333	0.18876772	0.28941664
172.8	0.32900329	0.42756352	0.24195459	0.64745557	0.60537047	0.18874064	0.28939532
172.9	0.32896184	0.42759923	0.24192457	0.64747165	0.60532757	0.18871365	0.28937406
173	0.32892046	0.42763494	0.24189464	0.64748771	0.60528463	0.18868673	0.28935285
173.1	0.32887917	0.42767064	0.2418648	0.64750375	0.60524166	0.18865991	0.2893317
173.2	0.32883795	0.42770633	0.24183505	0.64751977	0.60519864	0.18863316	0.2893106
173.3	0.32879682	0.42774201	0.24180538	0.64753577	0.6051556	0.1886065	0.28928955
173.4	0.32875576	0.42777769	0.24177581	0.64755176	0.60511251	0.18857991	0.28926855
173.5	0.32871478	0.42781336	0.24174632	0.64756773	0.60506939	0.18855341	0.28924761
173.6	0.32867388	0.42784902	0.24171692	0.64758368	0.60502624	0.18852699	0.28922671
173.7	0.32863306	0.42788468	0.24168761	0.64759961	0.60498305	0.18850065	0.28920588
173.8	0.32859232	0.42792033	0.24165838	0.64761553	0.60493983	0.18847439	0.28918509
173.9	0.32855165	0.42795597	0.24162925	0.64763143	0.60489658	0.18844821	0.28916436
174	0.32851106	0.42799161	0.24160019	0.64764731	0.6048533	0.18842211	0.28914367
174.1	0.32847056	0.42802723	0.24157123	0.64766317	0.60480999	0.18839609	0.28912304
174.2	0.32843012	0.42806285	0.24154235	0.64767901	0.60476665	0.18837015	0.28910247
174.3	0.32838977	0.42809847	0.24151356	0.64769484	0.60472328	0.18834429	0.28908194
174.4	0.3283495	0.42813407	0.24148486	0.64771065	0.60467989	0.18831851	0.28906147
174.5	0.3283093	0.42816967	0.24145624	0.64772645	0.60463647	0.1882928	0.28904104
174.6	0.32826918	0.42820526	0.2414277	0.64774222	0.60459302	0.18826718	0.28902067
174.7	0.32822913	0.42824085	0.24139925	0.64775798	0.60454955	0.18824163	0.28900036
174.8	0.32818916	0.42827642	0.24137089	0.64777372	0.60450606	0.18821616	0.28898009
174.9	0.32814927	0.42831199	0.24134261	0.64778945	0.60446254	0.18819077	0.28895988
175	0.32810946	0.42834756	0.24131442	0.64780515	0.604419	0.18816545	0.28893971
175.1	0.32806972	0.42838311	0.24128631	0.64782084	0.60437544	0.18814022	0.2889196
175.2	0.32803006	0.42841866	0.24125829	0.64783652	0.60433186	0.18811506	0.28889954
175.3	0.32799047	0.4284542	0.24123035	0.64785217	0.60428826	0.18808997	0.28887953
175.4	0.32795097	0.42848973	0.24120249	0.64786781	0.60424465	0.18806497	0.28885957
175.5	0.32791153	0.42852525	0.24117472	0.64788343	0.60420101	0.18804004	0.28883967
175.6	0.32787217	0.42856077	0.24114703	0.64789904	0.60415736	0.18801518	0.28881981
175.7	0.32783289	0.42859628	0.24111942	0.64791463	0.60411369	0.1879904	0.28880001
175.8	0.32779369	0.42863178	0.2410919	0.6479302	0.60407001	0.1879657	0.28878025
175.9	0.32775455	0.42866727	0.24106446	0.64794575	0.60402631	0.18794107	0.28876055
176	0.3277155	0.42870276	0.2410371	0.64796129	0.6039826	0.18791652	0.2887409
176.1	0.32767652	0.42873824	0.24100982	0.64797681	0.60393888	0.18789204	0.2887213
176.2	0.32763761	0.42877371	0.24098263	0.64799231	0.60389514	0.18786763	0.28870175
176.3	0.32759878	0.42880917	0.24095552	0.6480078	0.6038514	0.1878433	0.28868225
176.4	0.32756002	0.42884462	0.24092849	0.64802326	0.60380764	0.18781905	0.2886628
176.5	0.32752134	0.42888007	0.24090154	0.64803872	0.60376388	0.18779487	0.2886434
176.6	0.32748273	0.42891551	0.24087467	0.64805415	0.60372011	0.18777076	0.28862405
176.7	0.3274442	0.42895094	0.24084788	0.64806957	0.60367633	0.18774672	0.28860475
176.8	0.32740574	0.42898637	0.24082118	0.64808497	0.60363254	0.18772276	0.28858551
176.9	0.32736735	0.42902178	0.24079455	0.64810036	0.60358874	0.18769887	0.28856631
177	0.32732904	0.42905719	0.24076801	0.64811572	0.60354494	0.18767505	0.28854716
177.1	0.3272908	0.42909259	0.24074154	0.64813108	0.60350114	0.18765131	0.28852806
177.2	0.32725264	0.42912799	0.24071516	0.64814641	0.60345733	0.18762764	0.28850901
177.3	0.32721455	0.42916337	0.24068886	0.64816173	0.60341352	0.18760404	0.28849002
177.4	0.32717653	0.42919875	0.24066263	0.64817703	0.6033697	0.18758051	0.28847107
177.5	0.32713858	0.42923412	0.24063649	0.64819231	0.60332589	0.18755705	0.28845217
177.6	0.32710071	0.42926948	0.24061042	0.64820758	0.60328207	0.18753367	0.28843332
177.7	0.32706291	0.42930483	0.24058444	0.64822283	0.60323825	0.18751035	0.28841452
177.8	0.32702518	0.42934018	0.24055853	0.64823806	0.60319443	0.18748711	0.28839577
177.9	0.32698753	0.42937552	0.2405327	0.64825328	0.60315062	0.18746393	0.28837706
178	0.32694995	0.42941085	0.24050695	0.64826848	0.6031068	0.18744083	0.28835841
178.1	0.32691244	0.42944617	0.24048128	0.64828367	0.60306299	0.1874178	0.28833981
178.2	0.326875	0.42948149	0.24045569	0.64829883	0.60301918	0.18739484	0.28832125
178.3	0.32683763	0.4295168	0.24043018	0.64831398	0.60297538	0.18737194	0.28830275
178.4	0.32680034	0.42955209	0.24040474	0.64832912	0.60293158	0.18734912	0.28828429
178.5	0.32676312	0.42958739	0.24037938	0.64834423	0.60288779	0.18732637	0.28826588
178.6	0.32672597	0.42962267	0.2403541	0.64835934	0.602844	0.18730368	0.28824752
178.7	0.32668889	0.42965795	0.2403289	0.64837442	0.60280022	0.18728107	0.28822921
178.8	0.32665188	0.42969322	0.24030377	0.64838949	0.60275645	0.18725852	0.28821095
178.9	0.32661494	0.42972848	0.24027872	0.64840454	0.60271269	0.18723605	0.28819273
179	0.32657808	0.42976373	0.24025375	0.64841957	0.60266894	0.18721364	0.28817457
179.1	0.32654128	0.42979897	0.24022885	0.64843459	0.6026252	0.1871913	0.28815645
179.2	0.32650456	0.42983421	0.24020403	0.64844959	0.60258146	0.18716902	0.28813838
179.3	0.32646791	0.42986944	0.24017928	0.64846457	0.60253774	0.18714682	0.28812036
179.4	0.32643132	0.42990466	0.24015461	0.64847954	0.60249404	0.18712468	0.28810238
179.5	0.32639481	0.42993988	0.24013002	0.64849449	0.60245034	0.18710261	0.28808446
179.6	0.32635837	0.42997508	0.2401055	0.64850942	0.60240666	0.18708061	0.28806658
179.7	0.326322	0.43001028	0.24008106	0.64852434	0.602363	0.18705867	0.28804875
179.8	0.32628569	0.43004547	0.2400567	0.64853924	0.60231934	0.18703681	0.28803097
179.9	0.32624946	0.43008065	0.2400324	0.64855412	0.60227571	0.187015	0.28801323

225	0.31539541	0.44505313	0.2344834	0.6506248	0.58797906	0.18200869	0.28363421
225.1	0.31538059	0.44508421	0.23447933	0.6506194	0.58796014	0.18200473	0.28363013
225.2	0.3153658	0.44511528	0.23447529	0.65061396	0.58794127	0.18200079	0.28362607
225.3	0.31535103	0.44514634	0.23447127	0.6506085	0.58792245	0.18199687	0.28362203
225.4	0.3153363	0.4451774	0.23446727	0.65060301	0.5879037	0.18199297	0.283618
225.5	0.3153216	0.44520844	0.2344633	0.65059749	0.58788499	0.18198909	0.28361398
225.6	0.31530693	0.44523948	0.23445935	0.65059194	0.58786634	0.18198524	0.28360998
225.7	0.31529229	0.4452705	0.23445543	0.65058637	0.58784775	0.1819814	0.283606
225.8	0.31527769	0.44530152	0.23445153	0.65058077	0.58782921	0.18197758	0.28360203
225.9	0.31526311	0.44533252	0.23444765	0.65057514	0.58781073	0.18197378	0.28359808
226	0.31524856	0.44536352	0.23444379	0.65056948	0.58779231	0.18197	0.28359414
226.1	0.31523404	0.44539451	0.23443996	0.6505638	0.58777394	0.18196624	0.28359022
226.2	0.31521955	0.44542549	0.23443616	0.65055809	0.58775563	0.18196249	0.28358631
226.3	0.3152051	0.44545646	0.23443237	0.65055235	0.58773738	0.18195877	0.28358242
226.4	0.31519067	0.44548742	0.23442862	0.65054658	0.58771918	0.18195507	0.28357854
226.5	0.31517627	0.44551837	0.23442488	0.65054079	0.58770104	0.18195139	0.28357468
226.6	0.3151619	0.44554931	0.23442117	0.65053497	0.58768296	0.18194773	0.28357083
226.7	0.31514757	0.44558024	0.23441748	0.65052912	0.58766493	0.18194408	0.283567
226.8	0.31513326	0.44561117	0.23441381	0.65052325	0.58764697	0.18194046	0.28356318
226.9	0.31511898	0.44564208	0.23441017	0.65051735	0.58762906	0.18193685	0.28355937
227	0.31510473	0.44567298	0.23440655	0.65051143	0.58761121	0.18193327	0.28355558
227.1	0.31509051	0.44570388	0.23440295	0.65050548	0.58759342	0.1819297	0.28355181
227.2	0.31507632	0.44573477	0.23439937	0.6504995	0.58757568	0.18192615	0.28354805
227.3	0.31506217	0.44576564	0.23439582	0.6504935	0.58755801	0.18192263	0.2835443
227.4	0.31504804	0.44579651	0.23439229	0.65048747	0.58754039	0.18191912	0.28354057
227.5	0.31503394	0.44582737	0.23438879	0.65048141	0.58752284	0.18191563	0.28353686
227.6	0.31501986	0.44585822	0.2343853	0.65047533	0.58750534	0.18191216	0.28353315
227.7	0.31500582	0.44588906	0.23438184	0.65046923	0.58748791	0.1819087	0.28352947
227.8	0.31499181	0.44591989	0.23437841	0.65046309	0.58747053	0.18190527	0.28352579
227.9	0.31497783	0.44595071	0.23437499	0.65045694	0.58745322	0.18190186	0.28352213
228	0.31496388	0.44598152	0.2343716	0.65045075	0.58743597	0.18189846	0.28351849
228.1	0.31494995	0.44601233	0.23436823	0.65044455	0.58741878	0.18189508	0.28351485

183.2	0.3250922	0.43123723	0.23927158	0.64903535	0.6008487	0.18633221	0.28745408
183.3	0.32505827	0.43127214	0.23924972	0.6490496	0.60080597	0.1863126	0.28743791
183.4	0.32502441	0.43130704	0.23922794	0.64906383	0.60076327	0.18629305	0.28742178
183.5	0.32499061	0.43134194	0.23920622	0.64907803	0.6007206	0.18627356	0.2874057
183.6	0.32495688	0.43137683	0.23918457	0.64909221	0.60067798	0.18625414	0.28738967
183.7	0.32492321	0.43141171	0.23916299	0.64910636	0.60063539	0.18623478	0.28737367
183.8	0.32488961	0.43144658	0.23914147	0.64912049	0.60059284	0.18621548	0.28735772
183.9	0.32485607	0.43148144	0.23912003	0.64913459	0.60055033	0.18619624	0.28734182
184	0.3248226	0.4315163	0.23909865	0.64914867	0.60050786	0.18617706	0.28732596
184.1	0.32478919	0.43155115	0.23907734	0.64916271	0.60046543	0.18615794	0.28731014
184.2	0.32475585	0.43158599	0.2390561	0.64917674	0.60042304	0.18613888	0.28729437
184.3	0.32472257	0.43162082	0.23903492	0.64919073	0.60038068	0.18611988	0.28727864
184.4	0.32468936	0.43165564	0.23901381	0.6492047	0.60033837	0.18610095	0.28726296
184.5	0.32465621	0.43169046	0.23899277	0.64921863	0.6002961	0.18608207	0.28724732
184.6	0.32462312	0.43172527	0.2389718	0.64923254	0.60025387	0.18606325	0.28723172
184.7	0.3245901	0.43176007	0.23895089	0.64924642	0.60021169	0.18604449	0.28721616
184.8	0.32455714	0.43179486	0.23893005	0.64926026	0.60016954	0.1860258	0.28720065
184.9	0.32452425	0.43182964	0.23890927	0.64927408	0.60012744	0.18600716	0.28718519
185	0.32449142	0.43186441	0.23888856	0.64928786	0.60008539	0.18598858	0.28716976
185.1	0.32445865	0.43189918	0.23886792	0.64930161	0.60004337	0.18597006	0.28715438
185.2	0.32442595	0.43193394	0.23884734	0.64931533	0.60000141	0.1859516	0.28713904
185.3	0.32439331	0.43196869	0.23882683	0.64932901	0.59995948	0.1859332	0.28712375
185.4	0.32436074	0.43200343	0.23880638	0.64934267	0.5999176	0.18591486	0.28710849
185.5	0.32432822	0.43203817	0.238786	0.64935628	0.59987577	0.18589658	0.28709328
185.6	0.32429577	0.43207289	0.23876568	0.64936986	0.59983398	0.18587835	0.28707812
185.7	0.32426339	0.43210761	0.23874543	0.64938341	0.59979224	0.18586019	0.28706299
185.8	0.32423106	0.43214232	0.23872524	0.64939692	0.59975055	0.18584208	0.28704791
185.9	0.3241988	0.43217702	0.23870512	0.64941039	0.5997089	0.18582403	0.28703287
186	0.3241666	0.43221172	0.23868506	0.64942382	0.5996673	0.18580604	0.28701787
186.1	0.32413447	0.4322464	0.23866507	0.64943722	0.59962575	0.18578811	0.28700292
186.2	0.32410239	0.43228108	0.23864514	0.64945058	0.59958424	0.18577023	0.28698801
186.3	0.32407038	0.43231575	0.23862527	0.6494639	0.59954279	0.18575241	0.28697314
186.4	0.32403843	0.43235041	0.23860547	0.64947718	0.59950138	0.18573465	0.28695831
186.5	0.32400654	0.43238506	0.23858573	0.64949042	0.59946002	0.18571695	0.28694352
186.6	0.32397472	0.43241971	0.23856605	0.64950362	0.59941871	0.1856993	0.28692878
186.7	0.32394295	0.43245434	0.23854644	0.64951677	0.59937745	0.18568171	0.28691407
186.8	0.32391125	0.43248897	0.23852689	0.64952989	0.59933624	0.18566418	0.28689941
186.9	0.32387961	0.43252359	0.23850741	0.64954296	0.59929509	0.18564671	0.28688479
187	0.32384803	0.43255821	0.23848798	0.649556	0.59925398	0.18562929	0.28687021
187.1	0.32381651	0.43259281	0.23846862	0.64956898	0.59921292	0.18561193	0.28685568
187.2	0.32378506	0.43262741	0.23844932	0.64958193	0.59917192	0.18559462	0.28684118
187.3	0.32375366	0.43266199	0.23843008	0.64959483	0.59913096	0.18557737	0.28682673
187.4	0.32372233	0.43269657	0.23841091	0.64960768	0.59909006	0.18556018	0.28681231
187.5	0.32369105	0.43273114	0.23839179	0.64962049	0.59904921	0.18554304	0.28679794
187.6	0.32365984	0.43276571	0.23837274	0.64963326	0.59900841	0.18552596	0.28678361
187.7	0.32362868	0.43280026	0.23835375	0.64964597	0.59896767	0.18550893	0.28676932
187.8	0.32359759	0.43283481	0.23833482	0.64965865	0.59892698	0.18549196	0.28675507
187.9	0.32356656	0.43286935	0.23831595	0.64967127	0.59888634	0.18547504	0.28674086
188	0.32353559	0.43290388	0.23829715	0.64968385	0.59884575	0.18545818	0.28672669
188.1	0.32350468	0.4329384	0.2382784	0.64969638	0.59880522	0.18544138	0.28671257
188.2	0.32347383	0.43297291	0.23825972	0.64970886	0.59876475	0.18542463	0.28669848
188.3	0.32344303	0.43300742	0.23824109	0.64972129	0.59872433	0.18540793	0.28668443
188.4	0.3234123	0.43304192	0.23822253	0.64973368	0.59868396	0.18539129	0.28667043
188.5	0.32338163	0.43307641	0.23820402	0.64974602	0.59864365	0.1853747	0.28665646
188.6	0.32335101	0.43311089	0.23818558	0.6497583	0.59860339	0.18535817	0.28664253
188.7	0.32332046	0.43314536	0.2381672	0.64977054	0.59856319	0.18534169	0.28662865
188.8	0.32328997	0.43317983	0.23814887	0.64978273	0.59852305	0.18532527	0.2866148
188.9	0.32325953	0.43321428	0.23813061	0.64979486	0.59848296	0.1853089	0.286601
189	0.32322915	0.43324873	0.2381124	0.64980695	0.59844292	0.18529258	0.28658723
189.1	0.32319883	0.43328317	0.23809426	0.64981898	0.59840295	0.18527632	0.2865735
189.2	0.32316857	0.4333176	0.23807617	0.64983096	0.59836303	0.18526011	0.28655982
189.3	0.32313837	0.43335202	0.23805815	0.64984289	0.59832317	0.18524395	0.28654617
189.4	0.32310823	0.43338643	0.23804018	0.64985477	0.59828336	0.18522785	0.28653256
189.5	0.32307814	0.43342083	0.23802227	0.64986659	0.59824361	0.1852118	0.286519
189.6	0.32304812	0.43345523	0.23800442	0.64987836	0.59820392	0.1851958	0.28650547
189.7	0.32301815	0.43348961	0.23798663	0.64989008	0.59816429	0.18517986	0.28649198
189.8	0.32298824	0.43352399	0.2379689	0.64990175	0.59812471	0.18516397	0.28647853
189.9	0.32295838	0.43355835	0.23795123	0.64991336	0.5980852	0.18514813	0.28646512
190	0.32292859	0.43359271	0.23793361	0.64992491	0.59804574	0.18513234	0.28645174
190.1	0.32289885	0.43362706	0.23791605	0.64993641	0.59800634	0.18511661	0.28643841
190.2	0.32286917	0.4336614	0.23789855	0.64994786	0.597967	0.18510093	0.28642512
190.3	0.32283955	0.43369573	0.23788111	0.64995925	0.59792772	0.1850853	0.28641186
190.4	0.32280998	0.43373006	0.23786373	0.64997059	0.5978885	0.18506972	0.28639864
190.5	0.32278048	0.43376437	0.2378464	0.64998187	0.59784933	0.1850542	0.28638547
190.6	0.32275103	0.43379868	0.23782913	0.64999309	0.59781023	0.18503872	0.28637233
190.7	0.32272163	0.43383297	0.23781192	0.65000426	0.59777119	0.1850233	0.28635923
190.8	0.3226923	0.43386726	0.23779476	0.65001538	0.5977322	0.18500793	0.28634616
190.9	0.32266302	0.43390154	0.23777766	0.65002643	0.59769328	0.18499261	0.28633314
191	0.32263379	0.43393581	0.23776062	0.65003743	0.59765442	0.18497734	0.28632015
191.1	0.32260463	0.43397007	0.23774364	0.65004837	0.59761561	0.18496213	0.2863072
191.2	0.32257552	0.43400432	0.23772671	0.65005925	0.59757687	0.18494696	0.28629429
191.3	0.32254646	0.43403856	0.23770984	0.65007008	0.59753819	0.18493185	0.28628142
191.4	0.32251747	0.43407279	0.23769302	0.65008085	0.59749957	0.18491679	0.28626858
191.5	0.32248852	0.43410702	0.23767626	0.65009156	0.59746101	0.18490178	0.28625579
191.6	0.32245964	0.43414123	0.23765956	0.65010221	0.59742251	0.18488681	0.28624303
191.7	0.32243081	0.43417544	0.23764291	0.65011281	0.59738407	0.1848719	0.28623031
191.8	0.32240204	0.43420964	0.23762632	0.65012334	0.59734569	0.18485704	0.28621762
191.9	0.32237332	0.43424383	0.23760979	0.65013382	0.59730738	0.18484224	0.28620498
192	0.32234466	0.43427801	0.23759331	0.65014423	0.59726912	0.18482748	0.28619237
192.1	0.32231605	0.43431218	0.23757688	0.65015459	0.59723093	0.18481277	0.2861798
192.2	0.3222875	0.43434634	0.23756051	0.65016489	0.5971928	0.18479811	0.28616726
192.3	0.322259	0.4343805	0.23754419	0.65017513	0.59715473	0.1847835	0.28615476
192.4	0.32223056	0.43441464	0.23752793	0.65018532	0.59711672	0.18476894	0.2861423
192.5	0.32220218	0.43444878	0.23751173	0.65019544	0.59707878	0.18475443	0.28612988
192.6	0.32217385	0.4344829	0.23749557	0.6502055	0.5970409	0.18473996	0.28611749
192.7	0.32214557	0.43451702	0.23747948	0.6502155	0.59700308	0.18472555	0.28610514
192.8	0.32211735	0.43455113	0.23746343	0.65022545	0.59696532	0.18471119	0.28609283
192.9	0.32208919	0.43458523	0.23744744	0.65023533	0.59692762	0.18469688	0.28608055
193	0.32206107	0.43461932	0.23743151	0.65024515	0.59688999	0.18468261	0.28606831
193.1	0.32203302	0.4346534	0.23741562	0.65025491	0.59685242	0.1846684	0.28605611
193.2	0.32200502	0.43468748	0.23739979	0.65026461	0.59681491	0.18465424	0.28604394
193.3	0.32197707	0.43472154	0.23738402	0.65027425	0.59677747	0.18464012	0.28603181
193.4	0.32194917	0.4347556	0.2373683	0.65028383	0.59674009	0.18462605	0.28601972
193.5	0.32192134	0.43478964	0.23735263	0.65029335	0.59670277	0.18461203	0.28600766
193.6	0.32189355	0.43482368	0.23733701	0.65030281	0.59666551	0.18459806	0.28599564
193.7	0.32186582	0.43485771	0.23732145	0.65031221	0.59662832	0.18458414	0.28598365
193.8	0.32183814	0.43489173	0.23730594	0.65032155	0.59659119	0.18457027	0.2859717
193.9	0.32181052	0.43492574	0.23729048	0.65033082	0.59655412	0.18455644	0.28595979
194	0.32178295	0.43495974	0.23727508	0.65034004	0.59651712	0.18454266	0.28594791
194.1	0.32175543	0.43499373	0.23725973	0.65034919	0.59648018	0.18452893	0.28593607
194.2	0.32172797	0.43502772	0.23724443	0.65035828	0.59644331	0.18451525	0.28592426
194.3	0.32170056	0.43506169	0.23722918	0.65036731	0.59640649	0.18450162	0.28591249
194.4	0.3216732	0.43509566	0.23721398	0.65037628	0.59636974	0.18448803	0.28590076
194.5	0.3216459	0.43512962	0.23719884	0.65038519	0.59633306	0.18447449	0.28588905
194.6	0.32161864	0.43516356	0.23718375	0.65039403	0.59629644	0.184461	0.28587739
194.7	0.32159145	0.4351975	0.23716871	0.65040282	0.59625988	0.18444755	0.28586576
194.8	0.3215643	0.43523143	0.23715372	0.65041154	0.59622339	0.18443415	0.28585416
194.9	0.32153721	0.43526535	0.23713878	0.6504202	0.59618696	0.1844208	0.2858426
198.2	0.32067213	0.43637974	0.23667389	0.65067135	0.5950208	0.18400544	0.28548029
198.3	0.32064678	0.43641336	0.23666063	0.65067792	0.59498655	0.18399359	0.28546988
198.4	0.32062147	0.43644697	0.23664741	0.65068442	0.59495237	0.18398178	0.28545949
198.5	0.32059622	0.43648057	0.23663425	0.65069086	0.59491826	0.18397002	0.28544914
198.6	0.32057101	0.43651416	0.23662112	0.65069724	0.59488421	0.18395829	0.28543883
198.7	0.32054585	0.43654775	0.23660805	0.65070356	0.59485022	0.18394661	0.28542854
198.8	0.32052074	0.43658132	0.23659502	0.65070982	0.5948163	0.18393497	0.28541829
198.9	0.32049568	0.43661489	0.23658204	0.65071601	0.59478244	0.18392337	0.28540806
199	0.32047067	0.43664845	0.23656911	0.65072215	0.59474865	0.18391181	0.28539787
199.1	0.32044571	0.436682	0.23655622	0.65072823	0.59471492	0.18390029	0.28538772
199.2	0.3204208	0.43671554	0.23654337	0.65073425	0.59468125	0.18388882	0.28537759
199.3	0.32039594	0.43674907	0.23653058	0.6507402	0.59464765	0.18387738	0.2853675
199.4	0.32037112	0.43678259	0.23651783	0.6507461	0.59461411	0.18386598	0.28535743
199.5	0.32034636	0.4368161	0.23650512	0.65075193	0.59458064	0.18385463	0.2853474
199.6	0.32032164	0.43684961	0.23649246	0.65075771	0.59454723	0.18384331	0.2853374
199.7	0.32029697	0.4368831	0.23647985	0.65076342	0.59451389	0.18383204	0.28532743
199.8	0.32027235	0.43691659	0.23646728	0.65076908	0.59448061	0.1838208	0.2853175
199.9	0.32024778	0.43695007	0.23645476	0.65077467	0.59444739	0.18380961	0.28530759
200	0.32022325	0.43698354	0.23644228	0.65078021	0.59441424	0.18379845	0.28529771
200.1	0.32019878	0.437017	0.23642985	0.65078568	0.59438115	0.18378733	0.28528787
200.2	0.32017435	0.43705045	0.23641746	0.6507911	0.59434813	0.18377626	0.28527806
200.3	0.32014997	0.43708389	0.23640512	0.65079645	0.59431517	0.18376522	0.28526828
200.4	0.32012564	0.43711732	0.23639282	0.65080175	0.59428227	0.18375422	0.28525852
200.5	0.32010135	0.43715075	0.23638056	0.65080699	0.59424944	0.18374326	0.2852488
200.6	0.32007712	0.43718416	0.23636835	0.65081217	0.59421667	0.18373234	0.28523911
200.7	0.32005293	0.43721757	0.23635619	0.65081728	0.59418396	0.18372146	0.28522946
200.8	0.32002879	0.43725096	0.23634407	0.65082234	0.59415132	0.18371062	0.28521983
200.9	0.32000469	0.43728435	0.23633199	0.65082735	0.59411874	0.18369982	0.28521023
201	0.31998065	0.43731773	0.23631996	0.65083229	0.59408623	0.18368905	0.28520066
201.1	0.31995665	0.4373511	0.23630797	0.65083717	0.59405378	0.18367832	0.28519112
201.2	0.31993269	0.43738446	0.23629603	0.650842	0.59402139	0.18366763	0.28518162
201.3	0.31990879	0.43741781	0.23628413	0.65084676	0.59398907	0.18365698	0.28517214
201.4	0.31988493	0.43745116	0.23627227	0.65085147	0.59395681	0.18364637	0.28516269
201.5	0.31986112	0.43748449	0.23626046	0.65085612	0.59392461	0.18363579	0.28515328
201.6	0.31983735	0.43751782	0.23624869	0.65086071	0.59389247	0.18362526	0.28514389
201.7	0.31981363	0.43755113	0.23623696	0.65086525	0.5938604	0.18361476	0.28513454
201.8	0.31978996	0.43758444	0.23622528	0.65086972	0.5938284	0.1836043	0.28512521
201.9	0.31976633	0.43761774	0.23621364	0.65087414	0.59379645	0.18359387	0.28511591
202	0.31974275	0.43765102	0.23620204	0.6508785	0.59376457	0.18358349	0.28510665
202.1	0.31971922	0.4376843	0.23619049	0.65088281	0.59373275	0.18357314	0.28509741
202.2	0.31969573	0.43771758	0.23617898	0.65088705	0.593701	0.18356282	0.2850882
202.3	0.31967229	0.43775084	0.23616751	0.65089124	0.59366931	0.18355255	0.28507902
202.4	0.31964889	0.43778409	0.23615609	0.65089537	0.59363768	0.18354231	0.28506987
202.5	0.31962555	0.43781733	0.2361447	0.65089945	0.59360611	0.18353211	0.28506075
202.6	0.31960224	0.43785057	0.23613336	0.65090347	0.59357461	0.18352195	0.28505166
202.7	0.31957898	0.4378838	0.23612206	0.65090743	0.59354317	0.18351182	0.2850426
202.8	0.31955577	0.43791701	0.2361108	0.65091134	0.59351179	0.18350173	0.28503357
202.9	0.31953261	0.43795022	0.23609959	0.65091519	0.59348048	0.18349167	0.28502457
203	0.31950948	0.43798342	0.23608842	0.65091898	0.59344923	0.18348165	0.2850156
203.1	0.31948641	0.43801661	0.23607728	0.65092272	0.59341804	0.18347167	0.28500665
203.2	0.31946338	0.43804979	0.23606619	0.6509264	0.59338691	0.18346172	0.28499774
203.3	0.31944039	0.43808296	0.23605515	0.65093002	0.59335585	0.18345181	0.28498885
203.4	0.31941745	0.43811612	0.23604414	0.65093359	0.59332485	0.18344194	0.28497999
203.5	0.31939456	0.43814928	0.23603317	0.6509371	0.59329391	0.1834321	0.28497116
203.6	0.31937171	0.43818242	0.23602225	0.65094056	0.59326303	0.1834223	0.28496236
203.7	0.31934891	0.43821556	0.23601137	0.65094396	0.59323221	0.18341253	0.28495359
203.8	0.31932615	0.43824868	0.23600052	0.65094731	0.59320146	0.1834028	0.28494485
203.9	0.31930344	0.4382818	0.23598972	0.6509506	0.59317077	0.18339311	0.28493613
204	0.31928077	0.43831491	0.23597896	0.65095384	0.59314014	0.18338345	0.28492745
204.1	0.31925814	0.438348	0.23596824	0.65095702	0.59310958	0.18337383	0.28491879
204.2	0.31923556	0.43838109	0.23595757	0.65096015	0.59307907	0.18336424	0.28491016
204.3	0.31921303	0.43841417	0.23594693	0.65096322	0.59304863	0.18335469	0.28490156
204.4	0.31919054	0.43844725	0.23593633	0.65096624	0.59301825	0.18334517	0.28489298
204.5	0.31916809	0.43848031	0.23592577	0.6509692	0.59298793	0.18333569	0.28488444
204.6	0.31914569	0.43851336	0.23591525	0.65097211	0.59295767	0.18332624	0.28487592
204.7	0.31912333	0.4385464	0.23590478	0.65097496	0.59292748	0.18331683	0.28486743
204.8	0.31910102	0.43857944	0.23589434	0.65097776	0.59289734	0.18330745	0.28485897
204.9	0.31907875	0.43861246	0.23588394	0.65098051	0.59286727	0.18329811	0.28485054
205	0.31905652	0.43864548	0.23587358	0.6509832	0.59283726	0.1832888	0.28484213
205.1	0.31903434	0.43867848	0.23586327	0.65098584	0.59280731	0.18327952	0.28483375
205.2	0.3190122	0.43871148	0.23585299	0.65098842	0.59277742	0.18327028	0.2848254
205.3	0.31899011	0.43874447	0.23584275	0.65099095	0.5927476	0.18326108	0.28481708
205.4	0.31896806	0.43877745	0.23583255	0.65099343	0.59271783	0.18325191	0.28480878
205.5	0.31894605	0.43881041	0.23582239	0.65099586	0.59268813	0.18324277	0.28480051
205.6	0.31892409	0.43884337	0.23581227	0.65099823	0.59265849	0.18323366	0.28479227
205.7	0.31890217	0.43887632	0.23580219	0.65100055	0.5926289	0.18322459	0.28478406
205.8	0.31888029	0.43890926	0.23579215	0.65100281	0.59259938	0.18321556	0.28477587
205.9	0.31885846	0.43894219	0.23578214	0.65100503	0.59256992	0.18320656	0.28476771
206	0.31883667	0.43897511	0.23577218	0.65100719	0.59254052	0.18319759	0.28475958
206.1	0.31881492	0.43900802	0.23576225	0.6510093	0.59251119	0.18318865	0.28475148
206.2	0.31879322	0.43904092	0.23575237	0.65101135	0.59248191	0.18317975	0.2847434
206.3	0.31877156	0.43907381	0.23574252	0.65101336	0.59245269	0.18317088	0.28473535
206.4	0.31874994	0.43910669	0.23573271	0.65101531	0.59242354	0.18316204	0.28472732
206.5	0.31872836	0.43913956	0.23572294	0.65101721	0.59239444	0.18315324	0.28471932
206.6	0.31870683	0.43917243	0.2357132	0.65101906	0.59236541	0.18314447	0.28471135
206.7	0.31868534	0.43920528	0.23570351	0.65102086	0.59233643	0.18313573	0.28470341
206.8	0.31866389	0.43923812	0.23569385	0.65102261	0.59230752	0.18312703	0.28469549
206.9	0.31864248	0.43927095	0.23568423	0.6510243	0.59227867	0.18311835	0.2846876
207	0.31862112	0.43930378	0.23567465	0.65102595	0.59224987	0.18310971	0.28467973
207.1	0.31859979	0.43933659	0.23566511	0.65102754	0.59222114	0.18310111	0.28467189
207.2	0.31857852	0.43936939	0.2356556	0.65102908	0.59219247	0.18309253	0.28466408
207.3	0.31855728	0.43940219	0.23564614	0.65103057	0.59216385	0.18308399	0.2846563
207.4	0.31853608	0.43943497	0.23563671	0.65103201	0.5921353	0.18307548	0.28464854
207.5	0.31851493	0.43946774	0.23562731	0.65103341	0.59210681	0.183067	0.2846408
207.6	0.31849382	0.43950051	0.23561796	0.65103475	0.59207838	0.18305855	0.28463309
207.7	0.31847275	0.43953326	0.23560864	0.65103604	0.59205	0.18305014	0.28462541
207.8	0.31845172	0.43956601	0.23559936	0.65103728	0.59202169	0.18304175	0.28461776
207.9	0.31843073	0.43959874	0.23559012	0.65103847	0.59199344	0.1830334	0.28461013
208	0.31840979	0.43963147	0.23558091	0.65103961	0.59196524	0.18302508	0.28460252
208.1	0.31838888	0.43966418	0.23557174	0.6510407	0.59193711	0.18301679	0.28459494
208.2	0.31836802	0.43969689	0.23556261	0.65104174	0.59190903	0.18300853	0.28458739
208.3	0.3183472	0.43972959	0.23555351	0.65104273	0.59188102	0.18300031	0.28457986
208.4	0.31832642	0.43976227	0.23554445	0.65104367	0.59185306	0.18299212	0.28457236
208.5	0.31830568	0.43979495	0.23553543	0.65104457	0.59182516	0.18298396	0.28456489
208.6	0.31828498	0.43982761	0.23552644	0.65104541	0.59179733	0.18297583	0.28455744
208.7	0.31826432	0.43986027	0.23551749	0.65104621	0.59176955	0.18296773	0.28455001
208.8	0.31824371	0.43989292	0.23550858	0.65104695	0.59174183	0.18295966	0.28454261
208.9	0.31822313	0.43992555	0.2354997	0.65104765	0.59171417	0.18295162	0.28453524
209	0.3182026	0.43995818	0.23549086	0.6510483	0.59168657	0.18294362	0.28452789
209.1	0.3181821	0.4399908	0.23548205	0.6510489	0.59165903	0.18293564	0.28452056
209.2	0.31816165	0.44002341	0.23547328	0.65104946	0.59163154	0.1829277	0.28451326
209.3	0.31814124	0.440056	0.23546455	0.65104996	0.59160412	0.18291979	0.28450599
209.4	0.31812086	0.44008859	0.23545585	0.65105042	0.59157675	0.18291191	0.28449874
209.5	0.31810053	0.44012117	0.23544719	0.65105083	0.59154944	0.18290406	0.28449151
209.6	0.31808024	0.44015374	0.23543856	0.65105119	0.5915222	0.18289623	0.28448431
209.7	0.31805999	0.44018629	0.23542997	0.65105151	0.59149501	0.18288844	0.28447714
209.8	0.31803978	0.44021884	0.23542141	0.65105177	0.59146787	0.18288068	0.28446999
209.9	0.31801961	0.44025138	0.23541289	0.65105199	0.5914408	0.18287296	0.28446286
213.2	0.31737598	0.44131956	0.2351509	0.65103386	0.59057957	0.18263425	0.28424111
213.3	0.31735713	0.44135176	0.23514353	0.65103257	0.59055443	0.18262749	0.28423478
213.4	0.31733832	0.44138395	0.23513618	0.65103123	0.59052935	0.18262077	0.28422848
213.5	0.31731955	0.44141613	0.23512887	0.65102986	0.59050432	0.18261407	0.2842222
213.6	0.31730081	0.4414483	0.2351216	0.65102844	0.59047935	0.18260739	0.28421594
213.7	0.31728211	0.44148046	0.23511435	0.65102698	0.59045443	0.18260075	0.2842097
213.8	0.31726345	0.44151261	0.23510714	0.65102548	0.59042957	0.18259413	0.28420349
213.9	0.31724483	0.44154475	0.23509995	0.65102394	0.59040476	0.18258754	0.2841973
214	0.31722624	0.44157688	0.2350928	0.65102236	0.59038001	0.18258097	0.28419113
214.1	0.31720769	0.441609	0.23508569	0.65102074	0.59035532	0.18257443	0.28418498
214.2	0.31718918	0.44164111	0.2350786	0.65101907	0.59033067	0.18256792	0.28417885
214.3	0.3171707	0.44167321	0.23507154	0.65101737	0.59030609	0.18256144	0.28417275
214.4	0.31715226	0.44170531	0.23506452	0.65101562	0.59028155	0.18255498	0.28416667
214.5	0.31713386	0.44173739	0.23505753	0.65101383	0.59025708	0.18254855	0.28416061
214.6	0.3171155	0.44176946	0.23505056	0.65101201	0.59023265	0.18254214	0.28415457
214.7	0.31709717	0.44180152	0.23504363	0.65101014	0.59020828	0.18253577	0.28414855
214.8	0.31707888	0.44183357	0.23503673	0.65100823	0.59018397	0.18252941	0.28414256
214.9	0.31706063	0.44186562	0.23502987	0.65100628	0.59015971	0.18252309	0.28413658
215	0.31704241	0.44189765	0.23502303	0.6510043	0.5901355	0.18251679	0.28413063
215.1	0.31702423	0.44192967	0.23501622	0.65100227	0.59011135	0.18251051	0.2841247
215.2	0.31700609	0.44196168	0.23500945	0.6510002	0.59008725	0.18250427	0.28411879
215.3	0.31698798	0.44199369	0.2350027	0.6509981	0.59006321	0.18249804	0.28411291
215.4	0.31696991	0.44202568	0.23499599	0.65099595	0.59003922	0.18249185	0.28410704
215.5	0.31695187	0.44205766	0.2349893	0.65099376	0.59001528	0.18248568	0.28410119
215.6	0.31693388	0.44208964	0.23498265	0.65099154	0.5899914	0.18247953	0.28409537
215.7	0.31691591	0.4421216	0.23497603	0.65098928	0.58996757	0.18247341	0.28408957
215.8	0.31689799	0.44215356	0.23496943	0.65098697	0.58994379	0.18246732	0.28408379
215.9	0.3168801	0.4421855	0.23496287	0.65098463	0.58992007	0.18246125	0.28407802
216	0.31686224	0.44221744	0.23495634	0.65098225	0.5898964	0.18245521	0.28407228
216.1	0.31684442	0.44224936	0.23494984	0.65097983	0.58987278	0.1824492	0.28406657
216.2	0.31682664	0.44228128	0.23494336	0.65097737	0.58984922	0.18244321	0.28406087
216.3	0.31680889	0.44231318	0.23493692	0.65097488	0.58982571	0.18243724	0.28405519
216.4	0.31679118	0.44234508	0.23493051	0.65097235	0.58980225	0.1824313	0.28404953
216.5	0.3167735	0.44237696	0.23492413	0.65096977	0.58977885	0.18242538	0.2840439
216.6	0.31675586	0.44240884	0.23491777	0.65096717	0.5897555	0.18241949	0.28403828
216.7	0.31673826	0.4424407	0.23491145	0.65096452	0.5897322	0.18241363	0.28403269
216.8	0.31672069	0.44247256	0.23490516	0.65096183	0.58970895	0.18240779	0.28402711
216.9	0.31670315	0.44250441	0.23489889	0.65095911	0.58968576	0.18240197	0.28402156
217	0.31668565	0.44253624	0.23489266	0.65095635	0.58966262	0.18239618	0.28401603
217.1	0.31666819	0.44256807	0.23488645	0.65095355	0.58963953	0.18239042	0.28401051
217.2	0.31665076	0.44259989	0.23488028	0.65095072	0.5896165	0.18238467	0.28400502
217.3	0.31663337	0.4426317	0.23487413	0.65094785	0.58959351	0.18237896	0.28399955
217.4	0.31661601	0.4426635	0.23486801	0.65094494	0.58957058	0.18237326	0.2839941
217.5	0.31659869	0.44269529	0.23486192	0.650942	0.58954771	0.1823676	0.28398866
217.6	0.3165814	0.44272707	0.23485586	0.65093902	0.58952488	0.18236195	0.28398325
217.7	0.31656415	0.44275884	0.23484983	0.650936	0.58950211	0.18235633	0.28397786
217.8	0.31654693	0.44279061	0.23484383	0.65093294	0.58947938	0.18235074	0.28397249
217.9	0.31652975	0.44282236	0.23483786	0.65092985	0.58945672	0.18234517	0.28396713
218	0.3165126	0.44285411	0.23483192	0.65092673	0.5894341	0.18233962	0.2839618
218.1	0.31649549	0.44288584	0.234826	0.65092356	0.58941153	0.1823341	0.28395649
218.2	0.31647841	0.44291757	0.23482011	0.65092037	0.58938902	0.1823286	0.28395119
218.3	0.31646137	0.44294929	0.23481426	0.65091713	0.58936656	0.18232313	0.28394592
218.4	0.31644436	0.44298099	0.23480843	0.65091386	0.58934415	0.18231768	0.28394066
218.5	0.31642739	0.44301269	0.23480263	0.65091055	0.58932179	0.18231225	0.28393543
218.6	0.31641045	0.44304438	0.23479685	0.65090721	0.58929948	0.18230685	0.28393021
218.7	0.31639354	0.44307606	0.23479111	0.65090384	0.58927722	0.18230148	0.28392502
218.8	0.31637667	0.44310773	0.23478539	0.65090043	0.58925502	0.18229612	0.28391984
218.9	0.31635984	0.44313939	0.2347797	0.65089698	0.58923287	0.1822908	0.28391468
219	0.31634304	0.44317104	0.23477404	0.6508935	0.58921077	0.18228549	0.28390955
219.1	0.31632627	0.44320269	0.23476841	0.65088998	0.58918872	0.18228021	0.28390443
219.2	0.31630954	0.44323432	0.23476281	0.65088643	0.58916672	0.18227495	0.28389933
219.3	0.31629284	0.44326594	0.23475723	0.65088284	0.58914477	0.18226972	0.28389425
219.4	0.31627617	0.44329756	0.23475168	0.65087922	0.58912288	0.18226451	0.28388918
219.5	0.31625954	0.44332917	0.23474616	0.65087557	0.58910104	0.18225932	0.28388414
219.6	0.31624295	0.44336076	0.23474067	0.65087188	0.58907924	0.18225416	0.28387912
219.7	0.31622638	0.44339235	0.23473521	0.65086815	0.5890575	0.18224902	0.28387411
219.8	0.31620985	0.44342393	0.23472977	0.6508644	0.58903581	0.18224391	0.28386912
219.9	0.31619336	0.4434555	0.23472436	0.6508606	0.58901417	0.18223882	0.28386416
220	0.3161769	0.44348706	0.23471898	0.65085678	0.58899259	0.18223375	0.28385921
220.1	0.31616047	0.44351861	0.23471362	0.65085292	0.58897105	0.1822287	0.28385428
220.2	0.31614407	0.44355015	0.23470829	0.65084903	0.58894957	0.18222368	0.28384936
220.3	0.31612771	0.44358168	0.23470299	0.6508451	0.58892813	0.18221868	0.28384447
220.4	0.31611138	0.4436132	0.23469772	0.65084114	0.58890675	0.18221371	0.28383959
220.5	0.31609509	0.44364472	0.23469247	0.65083715	0.58888542	0.18220875	0.28383474
220.6	0.31607883	0.44367622	0.23468725	0.65083312	0.58886414	0.18220382	0.2838299
220.7	0.3160626	0.44370771	0.23468206	0.65082906	0.58884291	0.18219892	0.28382508
220.8	0.31604641	0.4437392	0.2346769	0.65082497	0.58882173	0.18219403	0.28382027
220.9	0.31603024	0.44377068	0.23467176	0.65082085	0.58880061	0.18218917	0.28381549
221	0.31601412	0.44380214	0.23466665	0.65081669	0.58877953	0.18218433	0.28381072
221.1	0.31599802	0.4438336	0.23466156	0.6508125	0.58875851	0.18217951	0.28380598
221.2	0.31598196	0.44386505	0.2346565	0.65080828	0.58873753	0.18217472	0.28380125
221.3	0.31596593	0.44389649	0.23465147	0.65080402	0.58871661	0.18216995	0.28379653
221.4	0.31594993	0.44392792	0.23464646	0.65079973	0.58869574	0.1821652	0.28379184
221.5	0.31593397	0.44395934	0.23464149	0.65079541	0.58867493	0.18216047	0.28378716
221.6	0.31591804	0.44399075	0.23463653	0.65079106	0.58865416	0.18215577	0.2837825
221.7	0.31590214	0.44402215	0.23463161	0.65078668	0.58863344	0.18215109	0.28377786
221.8	0.31588627	0.44405354	0.23462671	0.65078226	0.58861278	0.18214643	0.28377324
221.9	0.31587044	0.44408493	0.23462183	0.65077782	0.58859217	0.18214179	0.28376863
222	0.31585464	0.4441163	0.23461699	0.65077334	0.5885716	0.18213718	0.28376404
222.1	0.31583887	0.44414766	0.23461216	0.65076883	0.5885511	0.18213258	0.28375947
222.2	0.31582313	0.44417902	0.23460737	0.65076429	0.58853064	0.18212801	0.28375492
222.3	0.31580743	0.44421037	0.2346026	0.65075971	0.58851023	0.18212346	0.28375038
222.4	0.31579176	0.4442417	0.23459786	0.65075511	0.58848988	0.18211894	0.28374586
222.5	0.31577612	0.44427303	0.23459314	0.65075048	0.58846957	0.18211443	0.28374136
222.6	0.31576051	0.44430434	0.23458845	0.65074581	0.58844932	0.18210995	0.28373687
222.7	0.31574493	0.44433565	0.23458378	0.65074111	0.58842912	0.18210548	0.2837324
222.8	0.31572939	0.44436695	0.23457914	0.65073638	0.58840897	0.18210104	0.28372795
222.9	0.31571388	0.44439824	0.23457452	0.65073163	0.58838888	0.18209662	0.28372352
223	0.3156984	0.44442952	0.23456993	0.65072684	0.58836884	0.18209223	0.2837191
223.1	0.31568295	0.44446079	0.23456537	0.65072202	0.58834884	0.18208785	0.2837147
223.2	0.31566753	0.44449205	0.23456083	0.65071717	0.58832891	0.1820835	0.28371031
223.3	0.31565215	0.4445233	0.23455632	0.65071229	0.58830902	0.18207916	0.28370595
223.4	0.31563679	0.44455454	0.23455183	0.65070738	0.58828919	0.18207485	0.2837016
223.5	0.31562147	0.44458578	0.23454737	0.65070244	0.5882694	0.18207056	0.28369726
223.6	0.31560618	0.444617	0.23454293	0.65069747	0.58824967	0.18206629	0.28369295
223.7	0.31559093	0.44464821	0.23453852	0.65069247	0.58823	0.18206204	0.28368864
223.8	0.3155757	0.44467942	0.23453413	0.65068744	0.58821038	0.18205781	0.28368436
223.9	0.3155605	0.44471061	0.23452976	0.65068238	0.58819081	0.1820536	0.28368009
224	0.31554534	0.4447418	0.23452543	0.65067729	0.58817129	0.18204942	0.28367584
224.1	0.3155302	0.44477297	0.23452111	0.65067217	0.58815182	0.18204525	0.2836716
224.2	0.3155151	0.44480414	0.23451683	0.65066702	0.58813241	0.18204111	0.28366738
224.3	0.31550003	0.4448353	0.23451256	0.65066185	0.58811306	0.18203698	0.28366318
224.4	0.31548499	0.44486644	0.23450832	0.65065664	0.58809375	0.18203288	0.28365899
224.5	0.31546999	0.44489758	0.23450411	0.65065141	0.5880745	0.1820288	0.28365482
224.6	0.31545501	0.44492871	0.23449992	0.65064614	0.58805531	0.18202474	0.28365067
224.7	0.31544006	0.44495983	0.23449575	0.65064085	0.58803616	0.1820207	0.28364653
224.8	0.31542515	0.44499094	0.23449161	0.65063553	0.58801708	0.18201667	0.28364241
224.9	0.31541026	0.44502204	0.23448749	0.65063018	0.58799804	0.18201267	0.2836383
228.2	0.31493606	0.44604312	0.23436488	0.65043831	0.58740165	0.18189172	0.28351124
228.3	0.31492219	0.4460739	0.23436156	0.65043206	0.58738458	0.18188838	0.28350763
228.4	0.31490835	0.44610468	0.23435826	0.65042577	0.58736757	0.18188506	0.28350404
228.5	0.31489455	0.44613545	0.23435497	0.65041946	0.58735063	0.18188176	0.28350047
228.6	0.31488077	0.4461662	0.23435172	0.65041313	0.58733375	0.18187848	0.2834969
228.7	0.31486702	0.44619695	0.23434848	0.65040678	0.58731693	0.18187521	0.28349336
228.8	0.3148533	0.44622769	0.23434527	0.65040039	0.58730018	0.18187197	0.28348982
228.9	0.3148396	0.44625842	0.23434208	0.65039399	0.58728349	0.18186874	0.2834863
229	0.31482594	0.44628914	0.23433891	0.65038756	0.58726686	0.18186553	0.28348279
229.1	0.3148123	0.44631985	0.23433576	0.6503811	0.5872503	0.18186234	0.2834793
229.2	0.3147987	0.44635055	0.23433264	0.65037462	0.5872338	0.18185917	0.28347582
229.3	0.31478512	0.44638124	0.23432953	0.65036812	0.58721737	0.18185602	0.28347235
229.4	0.31477157	0.44641192	0.23432645	0.6503616	0.587201	0.18185288	0.2834689
229.5	0.31475805	0.4464426	0.23432339	0.65035504	0.5871847	0.18184977	0.28346546
229.6	0.31474456	0.44647326	0.23432035	0.65034847	0.58716847	0.18184667	0.28346203
229.7	0.3147311	0.44650392	0.23431734	0.65034187	0.5871523	0.18184359	0.28345862
229.8	0.31471766	0.44653456	0.23431434	0.65033525	0.58713619	0.18184053	0.28345522
229.9	0.31470425	0.4465652	0.23431137	0.65032861	0.58712016	0.18183748	0.28345183
230	0.31469087	0.44659583	0.23430842	0.65032194	0.58710419	0.18183446	0.28344846
230.1	0.31467752	0.44662645	0.23430549	0.65031525	0.58708829	0.18183145	0.2834451
230.2	0.3146642	0.44665706	0.23430259	0.65030853	0.58707245	0.18182846	0.28344175
230.3	0.31465091	0.44668766	0.2342997	0.6503018	0.58705668	0.18182549	0.28343842
230.4	0.31463764	0.44671825	0.23429683	0.65029504	0.58704099	0.18182254	0.2834351
230.5	0.3146244	0.44674883	0.23429399	0.65028825	0.58702536	0.18181961	0.28343179
230.6	0.31461119	0.44677941	0.23429117	0.65028145	0.5870098	0.18181669	0.28342849
230.7	0.31459801	0.44680997	0.23428837	0.65027462	0.58699431	0.18181379	0.28342521
230.8	0.31458485	0.44684053	0.23428559	0.65026777	0.58697889	0.18181091	0.28342194
230.9	0.31457172	0.44687107	0.23428283	0.6502609	0.58696354	0.18180804	0.28341868
231	0.31455862	0.44690161	0.23428009	0.650254	0.58694826	0.1818052	0.28341544
231.1	0.31454555	0.44693214	0.23427738	0.65024708	0.58693305	0.18180237	0.28341221
231.2	0.31453251	0.44696265	0.23427468	0.65024014	0.58691791	0.18179956	0.28340899
231.3	0.31451949	0.44699316	0.23427201	0.65023318	0.58690284	0.18179677	0.28340578
231.4	0.3145065	0.44702366	0.23426936	0.65022619	0.58688785	0.18179399	0.28340259
231.5	0.31449354	0.44705415	0.23426673	0.65021919	0.58687293	0.18179123	0.28339941
231.6	0.31448061	0.44708464	0.23426411	0.65021216	0.58685808	0.18178849	0.28339624
231.7	0.3144677	0.44711511	0.23426152	0.65020511	0.5868433	0.18178577	0.28339308
231.8	0.31445482	0.44714557	0.23425895	0.65019804	0.5868286	0.18178306	0.28338994
231.9	0.31444197	0.44717602	0.2342564	0.65019094	0.58681397	0.18178037	0.28338681
232	0.31442915	0.44720647	0.23425388	0.65018383	0.58679941	0.1817777	0.28338369
232.1	0.31441635	0.44723691	0.23425137	0.65017669	0.58678493	0.18177504	0.28338058
232.2	0.31440358	0.44726733	0.23424888	0.65016954	0.58677053	0.18177241	0.28337749
232.3	0.31439084	0.44729775	0.23424641	0.65016236	0.5867562	0.18176979	0.28337441
232.4	0.31437813	0.44732816	0.23424397	0.65015516	0.58674195	0.18176718	0.28337134
232.5	0.31436544	0.44735856	0.23424154	0.65014794	0.58672777	0.18176459	0.28336828
232.6	0.31435278	0.44738895	0.23423914	0.65014069	0.58671367	0.18176202	0.28336523
232.7	0.31434015	0.44741933	0.23423675	0.65013343	0.58669965	0.18175947	0.2833622
232.8	0.31432754	0.4474497	0.23423439	0.65012615	0.5866857	0.18175694	0.28335918
232.9	0.31431496	0.44748006	0.23423204	0.65011884	0.58667183	0.18175442	0.28335617
233	0.31430241	0.44751042	0.23422972	0.65011152	0.58665804	0.18175191	0.28335317
233.1	0.31428988	0.44754076	0.23422741	0.65010417	0.58664433	0.18174943	0.28335019
233.2	0.31427738	0.4475711	0.23422513	0.65009681	0.58663069	0.18174696	0.28334721
233.3	0.31426491	0.44760143	0.23422287	0.65008942	0.58661714	0.18174451	0.28334425
233.4	0.31425247	0.44763175	0.23422062	0.65008201	0.58660366	0.18174207	0.2833413
233.5	0.31424005	0.44766205	0.2342184	0.65007459	0.58659026	0.18173965	0.28333836
233.6	0.31422766	0.44769236	0.23421619	0.65006714	0.58657695	0.18173725	0.28333544
233.7	0.31421529	0.44772265	0.23421401	0.65005967	0.58656371	0.18173486	0.28333252
233.8	0.31420295	0.44775293	0.23421184	0.65005219	0.58655056	0.18173249	0.28332962
233.9	0.31419064	0.4477832	0.2342097	0.65004468	0.58653748	0.18173014	0.28332673
234	0.31417835	0.44781347	0.23420757	0.65003716	0.58652449	0.1817278	0.28332385
234.1	0.3141661	0.44784372	0.23420547	0.65002961	0.58651158	0.18172548	0.28332098
234.2	0.31415386	0.44787397	0.23420338	0.65002204	0.58649875	0.18172317	0.28331812
234.3	0.31414166	0.4479042	0.23420132	0.65001446	0.58648601	0.18172088	0.28331528
234.4	0.31412948	0.44793443	0.23419927	0.65000686	0.58647334	0.18171861	0.28331245
234.5	0.31411732	0.44796465	0.23419724	0.64999923	0.58646077	0.18171635	0.28330962
234.6	0.31410519	0.44799486	0.23419524	0.64999159	0.58644827	0.18171411	0.28330681
234.7	0.31409309	0.44802506	0.23419325	0.64998393	0.58643586	0.18171189	0.28330401
234.8	0.31408102	0.44805525	0.23419128	0.64997625	0.58642353	0.18170968	0.28330123
234.9	0.31406897	0.44808544	0.23418933	0.64996855	0.58641129	0.18170749	0.28329845
235	0.31405694	0.44811561	0.2341874	0.64996083	0.58639914	0.18170531	0.28329568
235.1	0.31404495	0.44814577	0.23418549	0.64995309	0.58638707	0.18170315	0.28329293
235.2	0.31403297	0.44817593	0.2341836	0.64994534	0.58637508	0.181701	0.28329019
235.3	0.31402103	0.44820608	0.23418172	0.64993756	0.58636319	0.18169888	0.28328745
235.4	0.31400911	0.44823621	0.23417987	0.64992977	0.58635137	0.18169676	0.28328473
235.5	0.31399722	0.44826634	0.23417803	0.64992196	0.58633965	0.18169466	0.28328202
235.6	0.31398535	0.44829646	0.23417622	0.64991413	0.58632802	0.18169258	0.28327933
235.7	0.31397351	0.44832657	0.23417442	0.64990628	0.58631647	0.18169051	0.28327664
235.8	0.31396169	0.44835667	0.23417265	0.64989841	0.58630501	0.18168846	0.28327396
235.9	0.3139499	0.44838677	0.23417089	0.64989052	0.58629364	0.18168643	0.2832713
236	0.31393813	0.44841685	0.23416915	0.64988262	0.58628235	0.18168441	0.28326864
236.1	0.31392639	0.44844692	0.23416743	0.6498747	0.58627116	0.1816824	0.283266
236.2	0.31391468	0.44847699	0.23416572	0.64986676	0.58626006	0.18168041	0.28326337
236.3	0.31390299	0.44850704	0.23416404	0.6498588	0.58624904	0.18167844	0.28326074
236.4	0.31389133	0.44853709	0.23416238	0.64985083	0.58623812	0.18167648	0.28325813
236.5	0.31387969	0.44856713	0.23416073	0.64984284	0.58622729	0.18167454	0.28325553
236.6	0.31386808	0.44859716	0.2341591	0.64983483	0.58621654	0.18167261	0.28325294
236.7	0.31385649	0.44862718	0.23415749	0.6498268	0.58620589	0.1816707	0.28325037
236.8	0.31384493	0.44865719	0.2341559	0.64981875	0.58619533	0.1816688	0.2832478
236.9	0.3138334	0.44868719	0.23415433	0.64981069	0.58618486	0.18166692	0.28324524
237	0.31382188	0.44871718	0.23415278	0.64980261	0.58617449	0.18166505	0.28324269
237.1	0.3138104	0.44874717	0.23415124	0.64979451	0.58616421	0.1816632	0.28324016
237.2	0.31379894	0.44877714	0.23414972	0.6497864	0.58615402	0.18166136	0.28323763
237.3	0.3137875	0.44880711	0.23414823	0.64977827	0.58614392	0.18165954	0.28323512
237.4	0.31377609	0.44883706	0.23414675	0.64977012	0.58613392	0.18165773	0.28323261
237.5	0.31376471	0.44886701	0.23414528	0.64976195	0.58612401	0.18165594	0.28323012
237.6	0.31375335	0.44889695	0.23414384	0.64975377	0.58611419	0.18165416	0.28322764
237.7	0.31374201	0.44892688	0.23414241	0.64974557	0.58610447	0.1816524	0.28322516
237.8	0.3137307	0.4489568	0.234141	0.64973736	0.58609484	0.18165065	0.2832227
237.9	0.31371941	0.44898671	0.23413962	0.64972912	0.58608531	0.18164892	0.28322025
238	0.31370815	0.44901661	0.23413824	0.64972087	0.58607588	0.1816472	0.28321781
238.1	0.31369691	0.44904651	0.23413689	0.64971261	0.58606654	0.1816455	0.28321538
238.2	0.3136857	0.44907639	0.23413555	0.64970433	0.58605729	0.18164381	0.28321296
238.3	0.31367451	0.44910627	0.23413424	0.64969603	0.58604815	0.18164213	0.28321055
238.4	0.31366335	0.44913613	0.23413294	0.64968771	0.5860391	0.18164047	0.28320815
238.5	0.31365221	0.44916599	0.23413165	0.64967938	0.58603014	0.18163883	0.28320576
238.6	0.3136411	0.44919584	0.23413039	0.64967104	0.58602128	0.1816372	0.28320338
238.7	0.31363001	0.44922568	0.23412914	0.64966267	0.58601253	0.18163558	0.28320101
238.8	0.31361895	0.44925551	0.23412791	0.64965429	0.58600386	0.18163398	0.28319865
238.9	0.31360791	0.44928533	0.2341267	0.6496459	0.5859953	0.18163239	0.2831963
239	0.31359689	0.44931515	0.23412551	0.64963749	0.58598684	0.18163082	0.28319396
239.1	0.3135859	0.44934495	0.23412433	0.64962906	0.58597847	0.18162926	0.28319163
239.2	0.31357493	0.44937475	0.23412317	0.64962062	0.5859702	0.18162772	0.28318931
239.3	0.31356399	0.44940453	0.23412203	0.64961216	0.58596203	0.18162619	0.283187
239.4	0.31355307	0.44943431	0.23412091	0.64960369	0.58595397	0.18162468	0.2831847
239.5	0.31354218	0.44946408	0.2341198	0.6495952	0.585946	0.18162318	0.28318242
239.6	0.31353131	0.44949384	0.23411871	0.6495867	0.58593813	0.18162169	0.28318014
239.7	0.31352046	0.44952359	0.23411764	0.64957818	0.58593036	0.18162022	0.28317787
239.8	0.31350964	0.44955333	0.23411658	0.64956964	0.58592269	0.18161877	0.28317561
239.9	0.31349884	0.44958306	0.23411554	0.64956109	0.58591513	0.18161732	0.28317336
240	0.31348807	0.44961278	0.23411452	0.64955253	0.58590766	0.18161589	0.28317112

Table S14: eFAST analysis data for [PYR] with [G](0)=2 mM: eFAST sensitivity indices

Sensitivity analysis data from eFAST for [PYR] with [G](0)=2 mM (only the parameters to which [PYR]'s variability is sensitive to appear)

Time discretization	V_{[G]}	K_{[G]}	q	V_{[PYR]}	K_{[PYR]}	beta	alpha	K_{[LACT]}	V_{[ACoA]}	phi	gamma	G_E	L_E
0	0	0	0	0	0	0	0	0	0	0	0	0	0
0.1	0.0020548	0.00190671	0.01058361	0.83325759	0.19148828	0.00200629	0.00232413	0.00177507	0.00312286	0.00144017	0.00423254	0.00252084	0.00301151
0.2	0.00201894	0.00207538	0.01064673	0.83235091	0.19288782	0.0022221	0.00248634	0.00213712	0.00330548	0.00150824	0.00447307	0.00270182	0.00317694
0.3	0.00199777	0.00227473	0.01075535	0.83150811	0.19430779	0.00246953	0.00270201	0.0026139	0.00354399	0.00159012	0.00477596	0.00291428	0.0033862
0.4	0.00198971	0.00249433	0.0108995	0.83070035	0.19571558	0.00272969	0.00295288	0.00318711	0.00382153	0.00167973	0.00511473	0.00314763	0.00362314
0.5	0.00199156	0.00272341	0.01106495	0.82989713	0.19710042	0.00298844	0.00322096	0.00383144	0.00412006	0.00177159	0.00546546	0.00339018	0.00387253
0.6	0.00200079	0.00295503	0.01123975	0.82907957	0.19846144	0.00323944	0.00349458	0.00452757	0.00442779	0.00186247	0.00581536	0.00363403	0.00412512
0.7	0.00201531	0.00318505	0.01141574	0.82823739	0.1997994	0.00347977	0.0037666	0.0052612	0.00473695	0.00195059	0.00615747	0.00387464	0.00437509
0.8	0.00203348	0.00341065	0.01158848	0.82736619	0.20111656	0.00370806	0.00403273	0.00602206	0.00504251	0.00203498	0.00648804	0.00410912	0.00461895
0.9	0.00205413	0.00362993	0.01175584	0.82646461	0.20241534	0.00392385	0.00429039	0.00680248	0.00534126	0.00211506	0.00680533	0.00433582	0.00485479
1	0.00207638	0.00384173	0.01191668	0.82553316	0.20369831	0.00412746	0.0045382	0.00759674	0.0056312	0.0021906	0.00710871	0.00455363	0.00508142
1.1	0.00209966	0.00404543	0.0120705	0.82457356	0.204968	0.00431947	0.00477568	0.00840042	0.00591116	0.00226162	0.00739807	0.00476196	0.00529827
1.2	0.00212353	0.00424072	0.0122171	0.82358789	0.20622646	0.00450066	0.00500284	0.00921012	0.00618049	0.00232818	0.00767382	0.00496058	0.00550518
1.3	0.00214766	0.0044275	0.0123563	0.82257826	0.2074753	0.00467177	0.0052199	0.01002317	0.00643901	0.0023904	0.00793633	0.0051494	0.00570214
1.4	0.00217185	0.00460591	0.01248816	0.8215467	0.20871591	0.00483336	0.00542717	0.01083743	0.00668672	0.00244849	0.00818601	0.00532861	0.0058894
1.5	0.00219594	0.00477613	0.0126128	0.8204952	0.2099495	0.00498607	0.00562515	0.01165133	0.00692371	0.00250267	0.00842341	0.00549847	0.00606724
1.6	0.00221978	0.00493839	0.01273029	0.81942549	0.21117706	0.0051305	0.00581431	0.01246365	0.00715016	0.00255311	0.00864904	0.00565931	0.00623607
1.7	0.00224328	0.00509297	0.01284083	0.8183391	0.21239942	0.00526718	0.00599512	0.01327338	0.00736636	0.00259997	0.00886347	0.00581154	0.00639626
1.8	0.00226637	0.00524007	0.01294463	0.81723748	0.2136173	0.00539667	0.00616812	0.01407983	0.00757263	0.00264346	0.00906726	0.00595555	0.00654821
1.9	0.00228901	0.00538001	0.01304195	0.81612192	0.21483136	0.00551951	0.00633381	0.01488242	0.00776935	0.00268373	0.00926095	0.00609174	0.00669235
2	0.00231116	0.0055131	0.01313303	0.81499353	0.2160421	0.00563617	0.0064927	0.01568062	0.00795688	0.00272094	0.00944513	0.00622051	0.00682906
[illegible]	[illegible]	[illegible]	[illegible]	[illegible]	[illegible]	[illegible]	[illegible]	[illegible]	[illegible]	[illegible]	[illegible]	[illegible]	[illegible]

Time discretization	V_{[G]}	K_{[G]}	q	V_{[PYR]}	K_{[PYR]}	beta	alpha	K_{[LACT]}	V_{[ACoA]}	phi	gamma	G_E	L_E
15	0.00965378	0.02924619	0.02609669	0.69148358	0.24549609	0.01433076	0.05231921	0.10174174	0.02110648	0.00475092	0.01604282	0.01982831	0.01461997
15.1	0.00983185	0.02975674	0.02637862	0.690755	0.24489675	0.01440023	0.05334202	0.1021963	0.02120374	0.00476754	0.01607557	0.02011667	0.01469451
15.2	0.01001322	0.03027236	0.02666283	0.69002325	0.24429496	0.0144704	0.05438108	0.10264263	0.02130065	0.0047842	0.01610874	0.02040806	0.01476963
15.3	0.01019793	0.03079297	0.02694927	0.68928827	0.24369084	0.01454132	0.05543646	0.10308071	0.02139725	0.00480091	0.01614236	0.02070245	0.01484534
15.4	0.01038599	0.03131847	0.02723792	0.68854999	0.24308449	0.014613	0.05650821	0.10351052	0.02149356	0.00481768	0.01617644	0.02099983	0.01492165
15.5	0.01057743	0.03184878	0.02752873	0.68780833	0.24247603	0.01468546	0.0575964	0.10393204	0.02158961	0.00483452	0.016211	0.02130018	0.01499858
15.6	0.01077227	0.03238382	0.02782167	0.68706323	0.24186557	0.01475875	0.05870105	0.10434528	0.02168542	0.00485144	0.01624605	0.02160345	0.01507614
15.7	0.01097053	0.0329235	0.02811671	0.68631462	0.2412532	0.01483288	0.05982222	0.10475024	0.02178103	0.00486845	0.01628161	0.02190964	0.01515435
15.8	0.01117223	0.03346772	0.02841382	0.68556243	0.24063904	0.01490789	0.06095994	0.10514693	0.02187645	0.00488556	0.01631769	0.02221871	0.01523321
15.9	0.0113774	0.03401642	0.02871296	0.68480659	0.24002317	0.0149838	0.06211423	0.10553537	0.02197171	0.00490278	0.01635431	0.02253063	0.01531273
16	0.01158605	0.0345695	0.02901411	0.68404704	0.23940571	0.01506064	0.06328511	0.10591557	0.02206684	0.00492012	0.01639149	0.02284536	0.01539294
16.1	0.01179821	0.03512687	0.02931723	0.68328371	0.23878674	0.01513845	0.0644726	0.10628755	0.02216185	0.0049376	0.01642923	0.02316288	0.01547383
16.2	0.0120139	0.03568846	0.02962229	0.68251655	0.23816638	0.01521725	0.06567671	0.10665133	0.02225679	0.00495523	0.01646756	0.02348314	0.01555543
16.3	0.01223313	0.03625418	0.02992926	0.68174551	0.23754472	0.01529708	0.06689745	0.10700694	0.02235165	0.00497301	0.01650648	0.02380611	0.01563775
[illegible]	[illegible]	[illegible]	[illegible]	[illegible]	[illegible]	[illegible]	[illegible]	[illegible]	[illegible]	[illegible]	[illegible]	[illegible]	[illegible]

Time discretization	V_{[G]}	K_{[G]}	q	V_{[PYR]}	K_{[PYR]}	beta	alpha	K_{[LACT]}	V_{[ACoA]}	phi	gamma	G_E	L_E
[illegible]	[illegible]	[illegible]	[illegible]	[illegible]	[illegible]	[illegible]	[illegible]	[illegible]	[illegible]	[illegible]	[illegible]	[illegible]	[illegible]

48.2	0.1752217	0.20176947	0.145181	0.42656971	0.12913646	0.13929984	0.58002076	0.0903217	0.06963061	0.05291041	0.05195428	0.11428702	0.08016608
48.3	0.17565517	0.20207489	0.14542183	0.42617983	0.12911347	0.13973088	0.58086703	0.09025896	0.0697814	0.05319468	0.05210355	0.11442865	0.08039351
48.4	0.17608732	0.20237927	0.14566161	0.42579234	0.12909151	0.14016054	0.58170908	0.09019657	0.06993195	0.05347919	0.05225288	0.11456975	0.08062059
48.5	0.17651814	0.20268262	0.14590036	0.42540722	0.12907056	0.14058882	0.58254691	0.09013452	0.07008228	0.0537639	0.05240227	0.11471032	0.08084734
48.6	0.17694764	0.20298494	0.14613807	0.42502447	0.12905063	0.14101572	0.58338056	0.09007281	0.07023238	0.05404883	0.0525517	0.11485036	0.08107374
48.7	0.17737582	0.20328625	0.14637474	0.42464407	0.12903169	0.14144125	0.58421005	0.09001143	0.07038224	0.05433396	0.05270119	0.11498987	0.08129981
48.8	0.17780269	0.20358653	0.14661038	0.42426601	0.12901375	0.1418654	0.58503541	0.08995039	0.07053188	0.05461929	0.05285073	0.11512887	0.08152553
48.9	0.17822825	0.20388581	0.146845	0.42389027	0.12899679	0.14228817	0.58585667	0.08988969	0.07068129	0.0549048	0.05300031	0.11526735	0.08175091
49	0.1786525	0.20418408	0.14707859	0.42351684	0.12898079	0.14270957	0.58667383	0.08982931	0.07083047	0.05519049	0.05314994	0.11540531	0.08197594
49.1	0.17907546	0.20448134	0.14731117	0.42314572	0.12896576	0.1431296	0.58748695	0.08976927	0.07097943	0.05547636	0.05329962	0.11554277	0.08220062
49.2	0.17949712	0.20477761	0.14754272	0.42277688	0.12895169	0.14354825	0.58829602	0.08970955	0.07112815	0.05576239	0.05344933	0.11567972	0.08242496
49.3	0.17991748	0.20507289	0.14777327	0.42241031	0.12893855	0.14396552	0.58910109	0.08965017	0.07127665	0.05604859	0.05359908	0.11581616	0.08264895
49.4	0.18033656	0.20536718	0.14800281	0.422046	0.12892636	0.14438143	0.58990217	0.0895911	0.07142492	0.05633494	0.05374888	0.11595211	0.08287259
49.5	0.18075435	0.20566048	0.14823134	0.42168394	0.12891508	0.14479596	0.59069929	0.08953237	0.07157295	0.05662144	0.0538987	0.11608756	0.08309588
49.6	0.18117087	0.2059528	0.14845887	0.42132412	0.12890473	0.14520912	0.59149247	0.08947395	0.07172075	0.05690808	0.05404856	0.11622251	0.08331882
49.7	0.18158611	0.20624415	0.1486854	0.42096652	0.12889528	0.14562091	0.59228174	0.08941585	0.07186832	0.05719485	0.05419845	0.11635698	0.08354141
49.8	0.18200008	0.20653453	0.14891094	0.42061113	0.12888673	0.14603134	0.59306712	0.08935808	0.07201565	0.05748175	0.05434837	0.11649095	0.08376365
49.9	0.18241279	0.20682395	0.14913549	0.42025794	0.12887907	0.1464404	0.59384864	0.08930062	0.07216275	0.05776878	0.05449832	0.11662445	0.08398553
50	0.18282423	0.2071124	0.14935906	0.41990694	0.1288723	0.14684809	0.59462631	0.08924347	0.07230962	0.05805591	0.0546483	0.11675746	0.08420706
50.1	0.18323442	0.20739989	0.14958164	0.41955811	0.1288664	0.14725442	0.59540016	0.08918664	0.07245626	0.05834316	0.0547983	0.11689	0.08442824
50.2	0.18364336	0.20768644	0.14980324	0.41921144	0.12886136	0.14765939	0.59617021	0.08913012	0.07260266	0.05863052	0.05494833	0.11702206	0.08464906
50.3	0.18405104	0.20797203	0.15002387	0.41886692	0.12885719	0.14806299	0.5969365	0.08907392	0.07274884	0.05891797	0.05509837	0.11715364	0.08486952
50.4	0.18445749	0.20825668	0.15024352	0.41852454	0.12885386	0.14846524	0.59769903	0.08901802	0.07289479	0.05920552	0.05524844	0.11728477	0.08508963
50.5	0.18486269	0.20854039	0.15046221	0.41818429	0.12885138	0.14886613	0.59845783	0.08896243	0.07304051	0.05949316	0.05539853	0.11741543	0.08530939
50.6	0.18526666	0.20882317	0.15067993	0.41784616	0.12884974	0.14926567	0.59921293	0.08890714	0.07318599	0.05978088	0.05554863	0.11754562	0.08552879
50.7	0.1856694	0.20910501	0.1508967	0.41751012	0.12884891	0.14966386	0.59996435	0.08885216	0.07333125	0.06006867	0.05569875	0.11767536	0.08574783
50.8	0.18607091	0.20938593	0.1511125	0.41717618	0.12884891	0.1500607	0.6007121	0.08879748	0.07347629	0.06035653	0.05584888	0.11780465	0.08596651
50.9	0.1864712	0.20966593	0.15132736	0.41684432	0.12884972	0.1504562	0.60145622	0.0887431	0.07362109	0.06064446	0.05599902	0.11793348	0.08618484
51	0.18687027	0.20994501	0.15154126	0.41651452	0.12885133	0.15085035	0.60219672	0.08868903	0.07376566	0.06093244	0.05614918	0.11806186	0.08640281
51.1	0.18726812	0.21022317	0.15175422	0.41618678	0.12885373	0.15124317	0.60293363	0.08863525	0.07391001	0.06122048	0.05629934	0.1181898	0.08662042
51.2	0.18766477	0.21050042	0.15196624	0.41586108	0.12885693	0.15163465	0.60366697	0.08858176	0.07405413	0.06150856	0.05644952	0.11831729	0.08683767
51.3	0.18806021	0.21077677	0.15217732	0.41553741	0.1288609	0.15202479	0.60439676	0.08852857	0.07419803	0.06179668	0.0565997	0.11844435	0.08705457
51.4	0.18845445	0.21105222	0.15238746	0.41521577	0.12886564	0.15241361	0.60512301	0.08847567	0.07434169	0.06208484	0.05674989	0.11857097	0.0872711
51.5	0.1888475	0.21132676	0.15259668	0.41489613	0.12887114	0.1528011	0.60584576	0.08842307	0.07448512	0.06237302	0.05690008	0.11869715	0.08748728
51.6	0.18923935	0.21160042	0.15280497	0.41457849	0.1288774	0.15318727	0.60656502	0.08837075	0.07462832	0.06266122	0.05705028	0.11882291	0.0877031
51.7	0.18963001	0.21187318	0.15301234	0.41426283	0.12888442	0.15357212	0.60728082	0.08831873	0.07477129	0.06294944	0.05720049	0.11894824	0.08791856
51.8	0.19001949	0.21214506	0.15321878	0.41394915	0.12889217	0.15395565	0.60799317	0.08826699	0.07491403	0.06323768	0.05735069	0.11907315	0.08813366
51.9	0.19040779	0.21241606	0.15342432	0.41363743	0.12890066	0.15433787	0.60870209	0.08821553	0.07505654	0.06352591	0.0575009	0.11919764	0.0883484
52	0.19079491	0.21268618	0.15362894	0.41332766	0.12890988	0.15471878	0.60940761	0.08816436	0.07519882	0.06381415	0.05765111	0.11932171	0.08856279
52.1	0.19118087	0.21295543	0.15383266	0.41301984	0.12891983	0.15509838	0.61010975	0.08811347	0.07534087	0.06410238	0.05780133	0.11944536	0.08877681
52.2	0.19156565	0.2132238	0.15403547	0.41271394	0.12893048	0.15547668	0.61080853	0.08806286	0.0754827	0.0643906	0.05795154	0.11956861	0.08899047
52.3	0.19194927	0.21349131	0.15423738	0.41240996	0.12894185	0.15585368	0.61150397	0.08801253	0.0756243	0.0646788	0.05810175	0.11969144	0.08920378
52.4	0.19233173	0.21375796	0.15443839	0.41210789	0.12895392	0.15622938	0.61219609	0.08796248	0.07576567	0.06496698	0.05825197	0.11981388	0.08941672
52.5	0.19271303	0.21402375	0.15463851	0.41180771	0.12896668	0.15660379	0.61288491	0.0879127	0.07590682	0.06525514	0.05840218	0.1199359	0.08962931
52.6	0.19309318	0.21428868	0.15483774	0.41150942	0.12898013	0.15697692	0.61357044	0.08786319	0.07604774	0.06554326	0.0585524	0.12005753	0.08984154
52.7	0.19347219	0.21455277	0.15503609	0.411213	0.12899426	0.15734875	0.61425272	0.08781396	0.07618843	0.06583134	0.05870263	0.12017876	0.0900534
52.8	0.19385005	0.214816	0.15523355	0.41091845	0.12900907	0.15771931	0.61493176	0.087765	0.07632891	0.06611939	0.05885285	0.12029959	0.09026491
52.9	0.19422677	0.21507839	0.15543013	0.41062575	0.12902455	0.15808858	0.61560758	0.08771631	0.07646916	0.06640738	0.05900308	0.12042003	0.09047605
53	0.19460235	0.21533995	0.15562584	0.41033489	0.12904069	0.15845658	0.6162802	0.08766788	0.07660918	0.06669532	0.0591533	0.12054008	0.09068684
53.1	0.1949768	0.21560066	0.15582068	0.41004587	0.12905749	0.15882331	0.61694963	0.08761972	0.07674899	0.06698321	0.05930353	0.12065975	0.09089726
53.2	0.19535012	0.21586055	0.15601465	0.40975868	0.12907493	0.15918878	0.61761591	0.08757183	0.07688857	0.06727103	0.05945377	0.12077903	0.09110733
53.3	0.19572231	0.2161196	0.15620776	0.4094733	0.12909303	0.15955298	0.61827904	0.0875242	0.07702793	0.06755879	0.059604	0.12089792	0.09131703
53.4	0.19609338	0.21637783	0.15640001	0.40918972	0.12911176	0.15991593	0.61893906	0.08747683	0.07716707	0.06784648	0.05975424	0.12101644	0.09152638
53.5	0.19646333	0.21663524	0.1565914	0.40890794	0.12913112	0.16027761	0.61959596	0.08742973	0.07730598	0.06813409	0.05990447	0.12113458	0.09173537
53.6	0.19683217	0.21689184	0.15678193	0.40862794	0.12915111	0.16063805	0.62024979	0.08738288	0.07744468	0.06842162	0.06005472	0.12125234	0.09194399
53.7	0.1971999	0.21714761	0.15697162	0.40834972	0.12917171	0.16099725	0.62090054	0.08733629	0.07758315	0.06870906	0.06020496	0.12136973	0.09215226
53.8	0.19756652	0.21740258	0.15716047	0.40807326	0.12919293	0.16135519	0.62154825	0.08728995	0.0777214	0.06899641	0.06035521	0.12148675	0.09236017
53.9	0.19793203	0.21765674	0.15734847	0.40779855	0.12921476	0.16171191	0.62219293	0.08724387	0.07785943	0.06928367	0.06050546	0.1216034	0.09256772
54	0.19829644	0.2179101	0.15753563	0.40752559	0.12923719	0.16206738	0.6228346	0.08719804	0.07799724	0.06957083	0.06065571	0.12171969	0.09277491
54.1	0.19865976	0.21816266	0.15772196	0.40725436	0.12926022	0.16242162	0.62347327	0.08715247	0.07813483	0.06985789	0.06080597	0.12183562	0.09298174
54.2	0.19902198	0.21841443	0.15790745	0.40698485	0.12928384	0.16277464	0.62410898	0.08710714	0.0782722	0.07014484	0.06095624	0.12195118	0.09318821
54.3	0.19938311	0.2186654	0.15809212	0.40671707	0.12930804	0.16312644	0.62474172	0.08706207	0.07840935	0.07043168	0.06110651	0.12206639	0.09339433
54.4	0.19974316	0.21891558	0.15827596	0.40645099	0.12933282	0.16347701	0.62537153	0.08701724	0.07854628	0.07071841	0.06125679	0.12218125	0.09360008
54.5	0.20010213	0.21916498	0.15845898	0.4061866	0.12935817	0.16382638	0.62599842	0.08697266	0.078683	0.07100501	0.06140707	0.12229575	0.09380548
54.6	0.20046002	0.2194136	0.15864119	0.40592391	0.12938409	0.16417453	0.62662241	0.08692832	0.0788195	0.07129149	0.06155736	0.1224099	0.09401053
54.7	0.20081683	0.21966144	0.15882258	0.40566289	0.12941057	0.16452147	0.62724351	0.08688422	0.07895578	0.07157785	0.06170765	0.1225237	0.09421521
54.8	0.20117258	0.2199085	0.15900316	0.40540355	0.12943761	0.16486722	0.62786175	0.08684037	0.07909185	0.07186407	0.06185796	0.12263716	0.09441954
54.9	0.20152726	0.2201548	0.15918293	0.40514587	0.1294652	0.16521177	0.62847713	0.08679676	0.07922771	0.07215015	0.06200827	0.12275027	0.09462352
55	0.20188087	0.22040032	0.15936191	0.40488985	0.12949333	0.16555512	0.62908969	0.08675339	0.07936335	0.0724361	0.06215859	0.12286304	0.09482714
55.1	0.20223343	0.22064509	0.15954008	0.40463547	0.12952201	0.16589728	0.62969943	0.08671026	0.07949877	0.0727219	0.06230892	0.12297547	0.0950304
55.2	0.20258493	0.22088909	0.15971746	0.40438273	0.12955122	0.16623826	0.63030638	0.08666736	0.07963398	0.07300756	0.06245927	0.12308757	0.09523331
55.3	0.20293538	0.22113233	0.15989405	0.40413161	0.12958096	0.16657805	0.63091055	0.0866247	0.07976898	0.07329306	0.06260963	0.12319933	0.09543586
55.4	0.20328478	0.22137482	0.16006986	0.40388212	0.12961122	0.16691667	0.63151195	0.08658227	0.07990377	0.07357842	0.06276	0.12331076	0.09563806
55.5	0.20363314	0.22161656	0.16024488	0.40363424	0.12964201	0.16725411	0.63211061	0.08654007	0.08003835	0.07386361	0.06291038	0.12342186	0.09583991
55.6	0.20398046	0.22185756	0.16041912	0.40338797	0.1296733	0.16759039	0.63270653	0.08649811	0.08017271	0.07414865	0.06306078	0.12353263	0.0960414
55.7	0.20432674	0.22209781	0.16059258	0.4031433	0.12970511	0.1679255	0.63329975	0.08645637	0.08030687	0.07443352	0.0632112	0.12364307	0.09624253
55.8	0.20467199	0.22233731	0.16076528	0.40290021	0.12973742	0.16825945	0.63389027	0.08641487	0.08044081	0.07471822	0.06336163	0.12375319	0.09644332
55.9	0.20501621	0.22257608	0.16093721	0.40265872	0.12977023	0.16859224	0.6344781	0.08637359	0.08057455	0.07500275	0.06351208	0.12386299	0.09664375
56	0.2053594	0.22281412	0.16110837	0.40241879	0.12980354	0.16892389	0.63506328	0.08633253	0.08070808	0.0752871	0.06366255	0.12397247	0.09684382
56.1	0.20570157	0.22305143	0.16127878	0.40218044	0.12983733	0.16925438	0.6356458	0.0862917	0.0808414	0.07557127	0.06381303	0.12408162	0.09704355
56.2	0.20604272	0.22328801	0.16144843	0.40194365	0.12987161	0.16958374	0.63622569	0.08625109	0.08097452	0.07585526	0.06396354	0.12419047	0.09724292
56.3	0.20638286	0.22352386	0.16161733	0.40170841	0.12990637	0.16991196	0.63680297	0.08621071	0.08110742	0.07613907	0.06411406	0.124299	0.09744194
56.4	0.20672198	0.223759	0.16178549	0.40147472	0.1299416	0.17023904	0.63737764	0.08617054	0.08124013	0.07642269	0.06426461	0.12440721	0.09764061
56.5	0.20706009	0.22399341	0.1619529	0.40124257	0.1299773	0.170565	0.63794973	0.0861306	0.08137263	0.07670611	0.06441518	0.12451512	0.09783893
56.6	0.20739719	0.22422711	0.16211957	0.40101196	0.13001347	0.17088984	0.63851925	0.08609087	0.08150492	0.07698934	0.06456577	0.12462272	0.09803689
56.7	0.2077333	0.22446011	0.16228551	0.40078286	0.1300501	0.17121355	0.63908622	0.08605136	0.08163701	0.07727237	0.06471639	0.12473001	0.09823451
56.8	0.2080684	0.22469239	0.16245072	0.40055529	0.13008718	0.17153614	0.63965065	0.08601206	0.0817689	0.07755519	0.06486703	0.124837	0.09843177
56.9	0.20840251	0.22492397	0.16261521	0.40032922	0.13012471	0.17185761	0.64021255	0.08597298	0.08190058	0.07783782	0.0650177	0.12494368	0.09862869
57	0.20873562	0.22515484	0.16277897	0.40010466	0.13016269	0.17217798	0.64077195	0.08593411	0.08203206	0.07812023	0.06516839	0.12505007	0.09882525
57.1	0.20906775	0.22538502	0.16294201	0.39988159	0.13020111	0.17249724	0.64132886	0.08589545	0.08216335	0.07840243	0.06531911	0.12515615	0.09902147
57.2	0.20939889	0.2256145	0.16310434	0.39966002	0.13023996	0.17281541	0.64188329	0.085857	0.08229443	0.07868442	0.06546986	0.12526195	0.09921734
57.3	0.20972905	0.22584329	0.16326595	0.39943992	0.13027925	0.17313248	0.64243525	0.08581876	0.08242531	0.07896619	0.06562064	0.12536744	0.09941285
57.4	0.21005824	0.22607139	0.16342686	0.39922131	0.13031896	0.17344846	0.64298477	0.08578073	0.082556	0.07924774	0.06577145	0.12547265	0.09960802
57.5	0.21038644	0.2262988	0.16358707	0.39900416	0.1303591	0.17376336	0.64353186	0.08574291	0.08268648	0.07952907	0.06592228	0.12557757	0.09980284
57.6	0.21071368	0.22652553	0.16374658	0.39878847	0.13039965	0.17407718	0.64407652	0.08570529	0.08281677	0.07981017	0.06607315	0.1256822	0.09999732
57.7	0.21103995	0.22675158	0.16390539	0.39857423	0.13044062	0.17438994	0.64461879	0.08566787	0.08294686	0.08009104	0.06622405	0.12578654	0.10019144
57.8	0.21136525	0.22697695	0.16406351	0.39836144	0.13048199	0.17470162	0.64515866	0.08563066	0.08307675	0.08037169	0.06637498	0.1258906	0.10038522
57.9	0.21168959	0.22720165	0.16422094	0.39815009	0.13052377	0.17501225	0.64569616	0.08559364	0.08320645	0.08065209	0.06652594	0.12599438	0.10057866
58	0.21201298	0.22742567	0.16437769	0.39794018	0.13056595	0.17532182	0.6462313	0.08555683	0.08333595	0.08093227	0.06667694	0.12609787	0.10077174
58.1	0.21233541	0.22764903	0.16453376	0.39773168	0.13060853	0.17563034	0.64676409	0.08552022	0.08346525	0.0812122	0.06682797	0.1262011	0.10096449

[illegible]

96.8	0.29115883	0.28192758	0.1967025	0.37579874	0.1563715	0.24761601	0.75678945	0.07906145	0.1237321	0.16608898	0.12710721	0.15473136	0.15598846
96.9	0.29128781	0.28201273	0.19674363	0.37581897	0.15643918	0.24772961	0.75693794	0.07905404	0.12381845	0.16625411	0.12725939	0.15478773	0.15609803
97	0.29141656	0.2820977	0.19678461	0.37583937	0.15650683	0.24784302	0.75708604	0.07904665	0.12390473	0.16641903	0.12741151	0.15484403	0.15620751
97.1	0.29154507	0.28218247	0.19682543	0.37585995	0.15657444	0.24795623	0.75723375	0.07903928	0.12399095	0.16658372	0.12756358	0.15490028	0.1563169
97.2	0.29167334	0.28226706	0.1968661	0.37588069	0.15664202	0.24806923	0.75738109	0.07903192	0.12407711	0.1667482	0.12771559	0.15495647	0.15642619
97.3	0.29180139	0.28235146	0.19690661	0.3759016	0.15670955	0.24818204	0.75752804	0.07902459	0.1241632	0.16691247	0.12786754	0.1550126	0.1565354
97.4	0.2919292	0.28243568	0.19694698	0.37592268	0.15677705	0.24829464	0.75767462	0.07901728	0.12424922	0.16707652	0.12801944	0.15506866	0.15664451
97.5	0.29205679	0.28251971	0.19698718	0.37594393	0.15684452	0.24840705	0.75782082	0.07900998	0.12433519	0.16724036	0.12817128	0.15512467	0.15675354
97.6	0.29218414	0.28260355	0.19702724	0.37596534	0.15691194	0.24851926	0.75796664	0.07900271	0.12442108	0.16740398	0.12832306	0.15518062	0.15686247
97.7	0.29231126	0.28268721	0.19706715	0.37598691	0.15697933	0.24863127	0.75811209	0.07899545	0.12450692	0.16756739	0.12847478	0.15523652	0.15697131
97.8	0.29243816	0.28277069	0.1971069	0.37600865	0.15704668	0.24874309	0.75825716	0.07898821	0.12459269	0.16773059	0.12862645	0.15529235	0.15708006
97.9	0.29256483	0.28285398	0.1971465	0.37603055	0.157114	0.24885471	0.75840186	0.07898098	0.1246784	0.16789358	0.12877805	0.15534812	0.15718873
98	0.29269127	0.2829371	0.19718596	0.37605261	0.15718127	0.24896613	0.75854619	0.07897378	0.12476404	0.16805636	0.1289296	0.15540383	0.1572973
98.1	0.29281749	0.28302002	0.19722526	0.37607483	0.15724851	0.24907736	0.75869015	0.0789666	0.12484962	0.16821893	0.12908109	0.15545949	0.15740578
98.2	0.29294347	0.28310277	0.19726441	0.37609721	0.15731571	0.2491884	0.75883375	0.07895943	0.12493513	0.16838129	0.12923252	0.15551508	0.15751418
98.3	0.29306924	0.28318534	0.19730342	0.37611975	0.15738287	0.24929924	0.75897697	0.07895228	0.12502059	0.16854344	0.12938389	0.15557062	0.15762249
98.4	0.29319478	0.28326773	0.19734227	0.37614244	0.15744999	0.24940989	0.75911983	0.07894515	0.12510598	0.16870539	0.12953521	0.1556261	0.15773071
98.5	0.2933201	0.28334993	0.19738098	0.3761653	0.15751707	0.24952035	0.75926232	0.07893803	0.12519131	0.16886713	0.12968646	0.15568151	0.15783884
98.6	0.29344519	0.28343196	0.19741954	0.37618831	0.15758412	0.24963062	0.75940445	0.07893093	0.12527657	0.16902866	0.12983765	0.15573688	0.15794688
98.7	0.29357006	0.28351381	0.19745795	0.37621147	0.15765112	0.2497407	0.75954622	0.07892385	0.12536177	0.16918998	0.12998879	0.15579218	0.15805484
98.8	0.29369471	0.28359548	0.19749621	0.37623479	0.15771809	0.24985059	0.75968762	0.07891679	0.12544691	0.1693511	0.13013986	0.15584742	0.1581627
98.9	0.29381914	0.28367698	0.19753433	0.37625826	0.15778502	0.2499603	0.75982867	0.07890975	0.12553199	0.16951201	0.13029088	0.15590261	0.15827048
99	0.29394335	0.28375829	0.19757231	0.37628189	0.15785191	0.25006981	0.75996935	0.07890272	0.12561701	0.16967272	0.13044183	0.15595774	0.15837818
99.1	0.29406734	0.28383943	0.19761013	0.37630566	0.15791876	0.25017914	0.76010968	0.07889571	0.12570196	0.16983323	0.13059273	0.15601281	0.15848579
99.2	0.29419111	0.2839204	0.19764782	0.37632959	0.15798558	0.25028828	0.76024965	0.07888872	0.12578685	0.16999353	0.13074356	0.15606783	0.15859331
99.3	0.29431466	0.28400119	0.19768535	0.37635367	0.15805235	0.25039724	0.76038927	0.07888174	0.12587168	0.17015364	0.13089434	0.15612279	0.15870075
99.4	0.29443799	0.2840818	0.19772275	0.37637789	0.15811908	0.25050601	0.76052853	0.07887478	0.12595645	0.17031354	0.13104505	0.15617769	0.1588081
99.5	0.29456111	0.28416224	0.19776	0.37640227	0.15818578	0.2506146	0.76066744	0.07886784	0.12604116	0.17047323	0.13119571	0.15623254	0.15891536
99.6	0.29468401	0.28424251	0.19779711	0.37642679	0.15825243	0.25072301	0.760806	0.07886091	0.1261258	0.17063273	0.1313463	0.15628733	0.15902254
99.7	0.2948067	0.2843226	0.19783407	0.37645145	0.15831905	0.25083123	0.76094421	0.078854	0.12621039	0.17079203	0.13149683	0.15634206	0.15912964
99.8	0.29492917	0.28440252	0.1978709	0.37647627	0.15838563	0.25093927	0.76108207	0.07884711	0.12629491	0.17095112	0.1316473	0.15639674	0.15923665
99.9	0.29505142	0.28448226	0.19790758	0.37650122	0.15845217	0.25104712	0.76121958	0.07884023	0.12637937	0.17111002	0.13179771	0.15645136	0.15934358
100	0.29517347	0.28456183	0.19794412	0.37652633	0.15851867	0.2511548	0.76135674	0.07883337	0.12646377	0.17126872	0.13194806	0.15650593	0.15945042
100.1	0.2952953	0.28464124	0.19798052	0.37655157	0.15858512	0.25126229	0.76149356	0.07882653	0.12654811	0.17142722	0.13209835	0.15656044	0.15955718
100.2	0.29541691	0.28472047	0.19801678	0.37657696	0.15865154	0.25136961	0.76163003	0.0788197	0.12663239	0.17158553	0.13224858	0.15661489	0.15966386
100.3	0.29553832	0.28479953	0.19805291	0.37660248	0.15871792	0.25147674	0.76176616	0.07881289	0.12671661	0.17174364	0.13239874	0.15666929	0.15977045
100.4	0.29565951	0.28487842	0.19808889	0.37662815	0.15878426	0.2515837	0.76190194	0.0788061	0.12680077	0.17190155	0.13254884	0.15672364	0.15987696
100.5	0.2957805	0.28495714	0.19812474	0.37665396	0.15885056	0.25169048	0.76203739	0.07879932	0.12688486	0.17205926	0.13269888	0.15677793	0.15998339
100.6	0.29590127	0.28503569	0.19816044	0.37667991	0.15891682	0.25179708	0.76217249	0.07879256	0.1269689	0.17221678	0.13284886	0.15683217	0.16008973
100.7	0.29602183	0.28511408	0.19819602	0.376706	0.15898304	0.2519035	0.76230726	0.07878581	0.12705288	0.17237411	0.13299878	0.15688635	0.16019599
100.8	0.29614219	0.28519229	0.19823145	0.37673222	0.15904922	0.25200974	0.76244168	0.07877908	0.1271368	0.17253124	0.13314863	0.15694048	0.16030217
100.9	0.29626234	0.28527034	0.19826675	0.37675858	0.15911536	0.25211581	0.76257577	0.07877236	0.12722065	0.17268818	0.13329843	0.15699455	0.16040827
101	0.29638227	0.28534823	0.19830191	0.37678508	0.15918146	0.25222171	0.76270953	0.07876566	0.12730445	0.17284493	0.13344816	0.15704857	0.16051429
[illegible]													

145.4	0.33495356	0.30888649	0.20592537	0.3953039	0.18337273	0.28719075	0.80025069	0.07651091	0.15962484	0.22815185	0.19345349	0.17736349	0.20118341
145.5	0.33501525	0.3089226	0.205932	0.39535193	0.18341449	0.28725063	0.80030326	0.07650648	0.15968847	0.2282508	0.19357403	0.17740297	0.20126268
145.6	0.33507686	0.30895865	0.20593861	0.39539996	0.18345619	0.28731046	0.80035573	0.07650205	0.15975208	0.22834966	0.1936945	0.17744242	0.20134191
145.7	0.33513838	0.30899466	0.2059452	0.39544801	0.18349783	0.28737023	0.80040811	0.07649762	0.15981564	0.22844842	0.19381492	0.17748185	0.20142108
145.8	0.33519981	0.30903061	0.20595176	0.39549605	0.18353942	0.28742994	0.8004604	0.07649319	0.15987917	0.22854709	0.19393526	0.17752126	0.20150021
145.9	0.33526115	0.3090665	0.20595829	0.3955441	0.18358095	0.28748959	0.80051259	0.07648876	0.15994267	0.22864567	0.19405554	0.17756064	0.20157929
146	0.3353224	0.30910235	0.20596479	0.39559216	0.18362241	0.28754919	0.8005647	0.07648433	0.16000613	0.22874415	0.19417576	0.17759999	0.20165832
146.1	0.33538357	0.30913814	0.20597127	0.39564022	0.18366382	0.28760872	0.80061671	0.0764799	0.16006956	0.22884254	0.19429592	0.17763933	0.2017373
146.2	0.33544465	0.30917388	0.20597773	0.39568829	0.18370518	0.2876682	0.80066864	0.07647548	0.16013295	0.22894084	0.19441601	0.17767864	0.20181623
146.3	0.33550564	0.30920957	0.20598416	0.39573636	0.18374647	0.28772762	0.80072047	0.07647105	0.16019631	0.22903904	0.19453604	0.17771793	0.20189512
146.4	0.33556655	0.3092452	0.20599056	0.39578444	0.18378771	0.28778699	0.80077222	0.07646663	0.16025963	0.22913715	0.194656	0.17775719	0.20197395
146.5	0.33562737	0.30928079	0.20599694	0.39583253	0.18382889	0.2878463	0.80082387	0.0764622	0.16032292	0.22923517	0.1947759	0.17779643	0.20205274
146.6	0.3356881	0.30931632	0.20600329	0.39588061	0.18387001	0.28790555	0.80087544	0.07645778	0.16038618	0.2293331	0.19489574	0.17783564	0.20213148
146.7	0.33574875	0.3093518	0.20600961	0.39592871	0.18391107	0.28796474	0.80092691	0.07645336	0.1604494	0.22943093	0.19501551	0.17787484	0.20221017
146.8	0.33580931	0.30938723	0.20601591	0.3959768	0.18395207	0.28802388	0.8009783	0.07644894	0.16051259	0.22952868	0.19513522	0.177914	0.20228881
146.9	0.33586979	0.3094226	0.20602219	0.39602491	0.18399302	0.28808295	0.8010296	0.07644452	0.16057574	0.22962633	0.19525486	0.17795315	0.20236741
147	0.33593018	0.30945793	0.20602844	0.39607301	0.18403391	0.28814198	0.8010808	0.0764401	0.16063886	0.22972388	0.19537445	0.17799227	0.20244595
147.1	0.33599048	0.3094932	0.20603466	0.39612112	0.18407474	0.28820094	0.80113192	0.07643568	0.16070194	0.22982135	0.19549396	0.17803137	0.20252445
147.2	0.3360507	0.30952843	0.20604086	0.39616924	0.18411552	0.28825985	0.80118296	0.07643126	0.16076499	0.22991872	0.19561342	0.17807044	0.2026029
147.3	0.33611084	0.3095636	0.20604704	0.39621736	0.18415624	0.28831871	0.8012339	0.07642685	0.16082801	0.230016	0.19573281	0.17810949	0.2026813
147.4	0.33617088	0.30959872	0.20605319	0.39626548	0.1841969	0.28837751	0.80128475	0.07642243	0.16089099	0.2301132	0.19585214	0.17814852	0.20275965
147.5	0.33623085	0.30963379	0.20605932	0.39631361	0.1842375	0.28843625	0.80133552	0.07641801	0.16095394	0.23021029	0.1959714	0.17818753	0.20283796
147.6	0.33629073	0.30966881	0.20606542	0.39636174	0.18427805	0.28849493	0.8013862	0.0764136	0.16101685	0.2303073	0.1960906	0.17822651	0.20291621
147.7	0.33635053	0.30970377	0.20607149	0.39640988	0.18431854	0.28855356	0.8014368	0.07640919	0.16107973	0.23040422	0.19620974	0.17826547	0.20299442
147.8	0.33641024	0.30973869	0.20607755	0.39645802	0.18435897	0.28861214	0.8014873	0.07640477	0.16114258	0.23050104	0.19632882	0.1783044	0.20307258
147.9	0.33646987	0.30977356	0.20608357	0.39650617	0.18439935	0.28867065	0.80153772	0.07640036	0.16120539	0.23059778	0.19644783	0.17834331	0.2031507
148	0.33652941	0.30980837	0.20608958	0.39655431	0.18443967	0.28872912	0.80158805	0.07639595	0.16126817	0.23069442	0.19656678	0.1783822	0.20322876
148.1	0.33658887	0.30984314	0.20609556	0.39660247	0.18447993	0.28878752	0.8016383	0.07639154	0.16133092	0.23079097	0.19668566	0.17842107	0.20330678
148.2	0.33664825	0.30987786	0.20610151	0.39665062	0.18452014	0.28884588	0.80168846	0.07638713	0.16139363	0.23088743	0.19680448	0.17845991	0.20338475
148.3	0.33670755	0.30991252	0.20610744	0.39669878	0.18456029	0.28890417	0.80173854	0.07638272	0.16145631	0.23098381	0.19692324	0.17849873	0.20346267
148.4	0.33676676	0.30994714	0.20611335	0.39674694	0.18460038	0.28896242	0.80178853	0.07637831	0.16151895	0.23108009	0.19704193	0.17853753	0.20354054
148.5	0.33682589	0.3099817	0.20611923	0.39679511	0.18464042	0.2890206	0.80183843	0.0763739	0.16158156	0.23117628	0.19716056	0.1785763	0.20361837
148.6	0.33688493	0.31001622	0.20612509	0.39684328	0.1846804	0.28907873	0.80188825	0.0763695	0.16164414	0.23127238	0.19727913	0.17861505	0.20369615
148.7	0.3369439	0.31005069	0.20613093	0.39689145	0.18472033	0.28913681	0.80193798	0.07636509	0.16170668	0.23136839	0.19739763	0.17865378	0.20377388
148.8	0.33700278	0.3100851	0.20613674	0.39693963	0.1847602	0.28919484	0.80198763	0.07636069	0.16176919	0.23146431	0.19751608	0.17869249	0.20385156
148.9	0.33706158	0.31011947	0.20614253	0.39698781	0.18480002	0.2892528	0.80203719	0.07635628	0.16183167	0.23156014	0.19763445	0.17873117	0.2039292
[illegible]													

160.4	0.34332394	0.31376926	0.20667136	0.40253115	0.18903114	0.29558144	0.80722339	0.07585369	0.16880129	0.24201137	0.21083159	0.18303161	0.21254583
160.5	0.34337442	0.31379858	0.20667492	0.40257923	0.18906515	0.29563375	0.80726442	0.07584935	0.1688601	0.24209754	0.21094277	0.18306776	0.21261809
160.6	0.34342484	0.31382787	0.20667847	0.40262732	0.18909913	0.29568601	0.80730539	0.07584501	0.16891887	0.24218364	0.21105389	0.18310388	0.2126903
160.7	0.3434752	0.31385711	0.206682	0.4026754	0.18913306	0.29573822	0.8073463	0.07584067	0.16897762	0.24226966	0.21116495	0.18313998	0.21276247
160.8	0.34352549	0.31388632	0.20668551	0.40272347	0.18916694	0.2957904	0.80738714	0.07583633	0.16903633	0.2423556	0.21127595	0.18317606	0.21283459
160.9	0.34357572	0.31391549	0.20668901	0.40277154	0.18920079	0.29584253	0.80742793	0.07583199	0.16909502	0.24244146	0.21138689	0.18321212	0.21290666
161	0.3436259	0.31394462	0.20669249	0.40281961	0.18923459	0.29589461	0.80746864	0.07582765	0.16915368	0.24252725	0.21149776	0.18324815	0.21297869
161.1	0.343676	0.31397372	0.20669596	0.40286767	0.18926834	0.29594666	0.8075093	0.07582331	0.1692123	0.24261296	0.21160858	0.18328417	0.21305068
161.2	0.34372605	0.31400277	0.20669941	0.40291573	0.18930206	0.29599866	0.80754989	0.07581898	0.1692709	0.2426986	0.21171933	0.18332017	0.21312262
161.3	0.34377604	0.31403179	0.20670285	0.40296378	0.18933573	0.29605061	0.80759042	0.07581464	0.16932947	0.24278416	0.21183003	0.18335614	0.21319451
161.4	0.34382596	0.31406078	0.20670627	0.40301183	0.18936936	0.29610253	0.80763088	0.0758103	0.16938801	0.24286964	0.21194066	0.18339209	0.21326636
161.5	0.34387583	0.31408972	0.20670968	0.40305987	0.18940294	0.2961544	0.80767129	0.07580597	0.16944652	0.24295505	0.21205124	0.18342803	0.21333816
161.6	0.34392563	0.31411863	0.20671307	0.40310791	0.18943649	0.29620623	0.80771163	0.07580163	0.169505	0.24304038	0.21216175	0.18346394	0.21340992
161.7	0.34397537	0.3141475	0.20671645	0.40315594	0.18946999	0.29625801	0.80775191	0.0757973	0.16956345	0.24312563	0.2122722	0.18349983	0.21348163
161.8	0.34402505	0.31417633	0.20671981	0.40320397	0.18950345	0.29630975	0.80779212	0.07579297	0.16962187	0.24321081	0.21238259	0.1835357	0.2135533
161.9	0.34407467	0.31420512	0.20672316	0.403252	0.18953686	0.29636145	0.80783228	0.07578863	0.16968026	0.24329591	0.21249293	0.18357155	0.21362492
162	0.34412423	0.31423388	0.20672649	0.40330001	0.18957024	0.29641311	0.80787237	0.0757843	0.16973862	0.24338094	0.2126032	0.18360738	0.2136965
162.1	0.34417372	0.3142626	0.20672981	0.40334803	0.18960357	0.29646473	0.8079124	0.07577997	0.16979696	0.24346589	0.21271341	0.18364319	0.21376803
162.2	0.34422316	0.31429129	0.20673312	0.40339604	0.18963686	0.2965163	0.80795237	0.07577563	0.16985526	0.24355076	0.21282356	0.18367897	0.21383952
162.3	0.34427254	0.31431993	0.2067364	0.40344404	0.18967011	0.29656783	0.80799228	0.0757713	0.16991354	0.24363556	0.21293365	0.18371474	0.21391096
162.4	0.34432186	0.31434855	0.20673968	0.40349204	0.18970332	0.29661932	0.80803213	0.07576697	0.16997178	0.24372028	0.21304368	0.18375048	0.21398235
162.5	0.34437111	0.31437712	0.20674294	0.40354003	0.18973648	0.29667076	0.80807191	0.07576264	0.17003	0.24380493	0.21315364	0.18378621	0.2140537
162.6	0.34442031	0.31440566	0.20674618	0.40358802	0.18976961	0.29672216	0.80811164	0.07575831	0.17008819	0.2438895	0.21326355	0.18382191	0.21412501
162.7	0.34446945	0.31443416	0.20674941	0.403636	0.18980269	0.29677353	0.8081513	0.07575398	0.17014635	0.243974	0.2133734	0.1838576	0.21419627
162.8	0.34451853	0.31446262	0.20675263	0.40368398	0.18983573	0.29682484	0.80819091	0.07574965	0.17020448	0.24405842	0.21348319	0.18389326	0.21426748
162.9	0.34456754	0.31449105	0.20675583	0.40373195	0.18986873	0.29687612	0.80823045	0.07574533	0.17026258	0.24414277	0.21359292	0.1839289	0.21433865
163	0.3446165	0.31451944	0.20675902	0.40377992	0.18990169	0.29692736	0.80826994	0.075741	0.17032066	0.24422704	0.21370258	0.18396452	0.21440978
163.1	0.3446654	0.3145478	0.20676219	0.40382788	0.18993461	0.29697855	0.80830936	0.07573667	0.1703787	0.24431124	0.21381219	0.18400012	0.21448085
163.2	0.34471424	0.31457612	0.20676535	0.40387583	0.18996749	0.2970297	0.80834872	0.07573234	0.17043671	0.24439536	0.21392174	0.1840357	0.21455189
163.3	0.34476302	0.3146044	0.20676849	0.40392378	0.19000032	0.29708081	0.80838802	0.07572802	0.1704947	0.24447941	0.21403123	0.18407126	0.21462288
163.4	0.34481175	0.31463265	0.20677162	0.40397173	0.19003312	0.29713188	0.80842727	0.07572369	0.17055266	0.24456338	0.21414065	0.1841068	0.21469382
163.5	0.34486041	0.31466086	0.20677474	0.40401966	0.19006587	0.29718291	0.80846645	0.07571937	0.17061059	0.24464728	0.21425002	0.18414232	0.21476472
163.6	0.34490901	0.31468903	0.20677784	0.4040676	0.19009859	0.29723389	0.80850557	0.07571504	0.17066848	0.2447311	0.21435933	0.18417781	0.21483558
163.7	0.34495756	0.31471717	0.20678093	0.40411552	0.19013126	0.29728484	0.80854464	0.07571072	0.17072635	0.24481485	0.21446857	0.18421329	0.21490638
163.8	0.34500605	0.31474527	0.20678401	0.40416344	0.1901639	0.29733574	0.80858364	0.07570639	0.1707842	0.24489853	0.21457776	0.18424875	0.21497715
163.9	0.34505448	0.31477334	0.20678707	0.40421136	0.19019649	0.2973866	0.80862259	0.07570207	0.17084201	0.24498213	0.21468688	0.18428418	0.21504787
[illegible]													

194	0.35746907	0.32186393	0.20724328	0.41818941	0.19851587	0.31108677	0.81815268	0.07441338	0.18706213	0.26714934	0.24494281	0.19408945	0.2344476
194.1	0.35750445	0.32188374	0.20724368	0.41823395	0.19853946	0.3111277	0.81817841	0.07440913	0.18711248	0.26721417	0.24503519	0.1941194	0.23450627
194.2	0.3575398	0.32190352	0.20724408	0.41827849	0.19856302	0.3111686	0.81820411	0.07440488	0.18716281	0.26727895	0.24512752	0.19414934	0.2345649
194.3	0.35757511	0.32192329	0.20724448	0.41832301	0.19858656	0.31120948	0.81822977	0.07440062	0.18721312	0.26734368	0.24521979	0.19417927	0.2346235
194.4	0.3576104	0.32194304	0.20724487	0.41836751	0.19861009	0.31125033	0.8182554	0.07439637	0.18726341	0.26740835	0.24531202	0.19420918	0.23468206
194.5	0.35764565	0.32196277	0.20724525	0.41841201	0.19863358	0.31129115	0.818281	0.07439212	0.18731367	0.26747298	0.24540419	0.19423907	0.23474058
194.6	0.35768087	0.32198247	0.20724563	0.41845648	0.19865706	0.31133195	0.81830657	0.07438787	0.18736392	0.26753755	0.24549632	0.19426894	0.23479907
194.7	0.35771606	0.32200216	0.207246	0.41850095	0.19868052	0.31137272	0.81833211	0.07438362	0.18741414	0.26760207	0.24558839	0.19429881	0.23485752
194.8	0.35775122	0.32202183	0.20724637	0.4185454	0.19870395	0.31141347	0.81835761	0.07437938	0.18746435	0.26766654	0.24568041	0.19432865	0.23491594
194.9	0.35778635	0.32204147	0.20724673	0.41858984	0.19872736	0.31145419	0.81838309	0.07437513	0.18751453	0.26773095	0.24577239	0.19435848	0.23497432
225	0.36714745	0.32717875	0.2071885	0.43135458	0.20494103	0.32268661	0.82483715	0.07311883	0.20174932	0.28503988	0.2713045	0.20268076	0.25101728
225.1	0.36717512	0.32719366	0.20718791	0.43139502	0.20495931	0.32272093	0.82485519	0.07311475	0.20179398	0.2850912	0.27138263	0.20270641	0.25106593
225.2	0.36720277	0.32720856	0.20718732	0.43143544	0.20497758	0.32275524	0.82487321	0.07311067	0.20183861	0.28514249	0.27146072	0.20273205	0.25111455
225.3	0.36723041	0.32722344	0.20718673	0.43147585	0.20499583	0.32278953	0.8248912	0.0731066	0.20188324	0.28519375	0.27153877	0.20275767	0.25116314
225.4	0.36725802	0.32723831	0.20718614	0.43151625	0.20501407	0.3228238	0.82490918	0.07310252	0.20192784	0.28524497	0.27161678	0.20278328	0.2512117
225.5	0.36728561	0.32725317	0.20718555	0.43155663	0.2050323	0.32285805	0.82492714	0.07309844	0.20197244	0.28529615	0.27169474	0.20280888	0.25126024
225.6	0.36731319	0.32726802	0.20718495	0.431597	0.20505052	0.32289229	0.82494508	0.07309437	0.20201701	0.28534729	0.27177267	0.20283447	0.25130875
225.7	0.36734074	0.32728285	0.20718435	0.43163736	0.20506872	0.32292651	0.824963	0.07309029	0.20206157	0.2853984	0.27185055	0.20286004	0.25135723
225.8	0.36736828	0.32729768	0.20718375	0.43167771	0.20508691	0.32296071	0.82498091	0.07308622	0.20210611	0.28544947	0.2719284	0.2028856	0.25140568
225.9	0.3673958	0.32731249	0.20718315	0.43171804	0.20510509	0.3229949	0.82499879	0.07308214	0.20215064	0.28550051	0.2720062	0.20291116	0.2514541
226	0.36742329	0.32732729	0.20718254	0.43175836	0.20512325	0.32302906	0.82501665	0.07307807	0.20219515	0.28555151	0.27208396	0.20293669	0.2515025
226.1	0.36745077	0.32734208	0.20718193	0.43179867	0.2051414	0.32306322	0.8250345	0.073074	0.20223965	0.28560247	0.27216168	0.20296222	0.25155087
226.2	0.36747823	0.32735685	0.20718132	0.43183897	0.20515954	0.32309735	0.82505233	0.07306993	0.20228413	0.2856534	0.27223935	0.20298773	0.25159921
226.3	0.36750567	0.32737162	0.20718071	0.43187925	0.20517766	0.32313146	0.82507014	0.07306586	0.20232859	0.28570429	0.27231699	0.20301324	0.25164753
226.4	0.36753309	0.32738637	0.2071801	0.43191952	0.20519577	0.32316556	0.82508793	0.07306179	0.20237304	0.28575514	0.27239459	0.20303873	0.25169581
226.5	0.36756049	0.32740112	0.20717948	0.43195978	0.20521387	0.32319964	0.8251057	0.07305772	0.20241747	0.28580596	0.27247214	0.20306421	0.25174407
226.6	0.36758788	0.32741585	0.20717886	0.43200003	0.20523195	0.32323371	0.82512345	0.07305365	0.20246189	0.28585675	0.27254966	0.20308967	0.2517923
226.7	0.36761524	0.32743056	0.20717824	0.43204026	0.20525003	0.32326775	0.82514118	0.07304959	0.20250629	0.28590749	0.27262713	0.20311513	0.25184051
226.8	0.36764258	0.32744527	0.20717762	0.43208048	0.20526809	0.32330178	0.8251589	0.07304552	0.20255067	0.2859582	0.27270456	0.20314057	0.25188868
226.9	0.36766991	0.32745997	0.20717699	0.43212069	0.20528613	0.3233358	0.8251766	0.07304145	0.20259504	0.28600888	0.27278195	0.203166	0.25193683
227	0.36769722	0.32747465	0.20717637	0.43216088	0.20530416	0.32336979	0.82519428	0.07303739	0.20263939	0.28605952	0.2728593	0.20319142	0.25198495
227.1	0.3677245	0.32748932	0.20717574	0.43220107	0.20532218	0.32340377	0.82521194	0.07303332	0.20268373	0.28611012	0.27293661	0.20321682	0.25203305
227.2	0.36775177	0.32750398	0.2071751	0.43224124	0.20534019	0.32343773	0.82522958	0.07302926	0.20272805	0.28616069	0.27301388	0.20324222	0.25208111
227.3	0.36777902	0.32751863	0.20717447	0.43228139	0.20535818	0.32347167	0.8252472	0.0730252	0.20277236	0.28621122	0.2730911	0.2032676	0.25212915
227.4	0.36780626	0.32753327	0.20717383	0.43232154	0.20537616	0.3235056	0.8252648	0.07302114	0.20281665	0.28626172	0.27316829	0.20329297	0.25217717
227.5	0.36783347	0.3275479	0.2071732	0.43236167	0.20539413	0.32353951	0.82528239	0.07301707	0.20286093	0.28631218	0.27324544	0.20331833	0.25222515
227.6	0.36786066	0.32756251	0.20717256	0.43240179	0.20541209	0.3235734	0.82529996	0.07301301	0.20290519	0.28636261	0.27332254	0.20334368	0.25227311
227.7	0.36788784	0.32757711	0.20717191	0.4324419	0.20543003	0.32360728	0.82531751	0.07300895	0.20294943	0.28641299	0.27339961	0.20336901	0.25232104
227.8	0.36791499	0.3275917	0.20717127	0.432482	0.20544796	0.32364114	0.82533504	0.07300489	0.20299366	0.28646335	0.27347663	0.20339433	0.25236894
227.9	0.36794213	0.32760628	0.20717062	0.43252208	0.20546587	0.32367498	0.82535255	0.07300084	0.20303787	0.28651367	0.27355361	0.20341964	0.25241682
228	0.36796925	0.32762085	0.20716997	0.43256215	0.20548378	0.3237088	0.82537005	0.07299678	0.20308207	0.28656395	0.27363055	0.20344494	0.25246467
228.1	0.36799635	0.32763541	0.20716932	0.43260221	0.20550167	0.32374261	0.82538752	0.07299272	0.20312625	0.2866142	0.27370746	0.20347023	0.25251249
228.2	0.36802343	0.32764996	0.20716867	0.43264225	0.20551955	0.3237764	0.82540498	0.07298867	0.20317042	0.28666441	0.27378432	0.2034955	0.25256028
228.3	0.36805049	0.32766449	0.20716801	0.43268228	0.20553741	0.32381018	0.82542242	0.07298461	0.20321457	0.28671459	0.27386114	0.20352077	0.25260805
228.4	0.36807754	0.32767901	0.20716736	0.4327223	0.20555526	0.32384393	0.82543984	0.07298056	0.20325871	0.28676473	0.27393792	0.20354602	0.25265579
228.5	0.36810457	0.32769353	0.2071667	0.43276231	0.2055731	0.32387767	0.82545725	0.0729765	0.20330283	0.28681484	0.27401466	0.20357126	0.2527035
228.6	0.36813157	0.32770803	0.20716604	0.43280231	0.20559093	0.3239114	0.82547463	0.07297245	0.20334693	0.28686491	0.27409135	0.20359648	0.25275119
228.7	0.36815856	0.32772252	0.20716537	0.43284229	0.20560874	0.3239451	0.825492	0.0729684	0.20339102	0.28691494	0.27416801	0.2036217	0.25279885
228.8	0.36818553	0.32773699	0.20716471	0.43288226	0.20562654	0.32397879	0.82550935	0.07296435	0.20343509	0.28696494	0.27424463	0.2036469	0.25284648
228.9	0.36821249	0.32775146	0.20716404	0.43292222	0.20564433	0.32401247	0.82552668	0.0729603	0.20347915	0.28701491	0.27432121	0.2036721	0.25289408
229	0.36823942	0.32776592	0.20716337	0.43296216	0.20566211	0.32404612	0.82554399	0.07295625	0.2035232	0.28706484	0.27439775	0.20369728	0.25294166
229.1	0.36826634	0.32778036	0.2071627	0.4330021	0.20567987	0.32407976	0.82556129	0.0729522	0.20356722	0.28711474	0.27447424	0.20372244	0.25298921
229.2	0.36829324	0.32779479	0.20716202	0.43304202	0.20569762	0.32411338	0.82557857	0.07294815	0.20361124	0.2871646	0.2745507	0.2037476	0.25303674
229.3	0.36832012	0.32780921	0.20716135	0.43308192	0.20571536	0.32414699	0.82559583	0.0729441	0.20365523	0.28721442	0.27462712	0.20377275	0.25308423
229.4	0.36834698	0.32782362	0.20716067	0.43312182	0.20573309	0.32418058	0.82561307	0.07294006	0.20369922	0.28726421	0.27470349	0.20379788	0.2531317
229.5	0.36837382	0.32783802	0.20715999	0.4331617	0.2057508	0.32421415	0.82563029	0.07293601	0.20374318	0.28731397	0.27477983	0.203823	0.25317915
229.6	0.36840065	0.32785241	0.20715931	0.43320157	0.2057685	0.32424771	0.8256475	0.07293196	0.20378713	0.28736369	0.27485612	0.20384811	0.25322656
229.7	0.36842746	0.32786679	0.20715862	0.43324143	0.20578619	0.32428125	0.82566469	0.07292792	0.20383107	0.28741338	0.27493238	0.20387321	0.25327395
229.8	0.36845424	0.32788115	0.20715794	0.43328128	0.20580386	0.32431477	0.82568186	0.07292388	0.20387499	0.28746303	0.27500859	0.20389829	0.25332132
229.9	0.36848102	0.32789551	0.20715725	0.43332111	0.20582152	0.32434828	0.82569901	0.07291983	0.2039189	0.28751265	0.27508477	0.20392337	0.25336865
230	0.36850777	0.32790985	0.20715656	0.43336093	0.20583917	0.32438177	0.82571614	0.07291579	0.20396279	0.28756223	0.2751609	0.20394843	0.25341596
230.1	0.36853451	0.32792418	0.20715587	0.43340074	0.20585681	0.32441524	0.82573326	0.07291175	0.20400666	0.28761178	0.275237	0.20397348	0.25346325
230.2	0.36856122	0.3279385	0.20715517	0.43344054	0.20587444	0.3244487	0.82575036	0.07290771	0.20405052	0.28766129	0.27531305	0.20399852	0.2535105
230.3	0.36858792	0.32795281	0.20715448	0.43348032	0.20589205	0.32448214	0.82576744	0.07290367	0.20409436	0.28771077	0.27538907	0.20402355	0.25355773
230.4	0.36861461	0.32796711	0.20715378	0.43352009	0.20590965	0.32451556	0.82578451	0.07289963	0.20413819	0.28776021	0.27546504	0.20404856	0.25360494
230.5	0.36864127	0.3279814	0.20715308	0.43355985	0.20592724	0.32454897	0.82580156	0.07289559	0.20418201	0.28780962	0.27554098	0.20407357	0.25365211
230.6	0.36866792	0.32799567	0.20715238	0.4335996	0.20594481	0.32458236	0.82581859	0.07289156	0.20422581	0.28785899	0.27561687	0.20409856	0.25369926
230.7	0.36869454	0.32800994	0.20715167	0.43363933	0.20596237	0.32461573	0.8258356	0.07288752	0.20426959	0.28790833	0.27569273	0.20412354	0.25374638
230.8	0.36872116	0.32802419	0.20715097	0.43367905	0.20597992	0.32464909	0.82585259	0.07288348	0.20431336	0.28795764	0.27576854	0.20414851	0.25379348
230.9	0.36874775	0.32803844	0.20715026	0.43371876	0.20599746	0.32468243	0.82586957	0.07287945	0.20435711	0.28800691	0.27584432	0.20417347	0.25384055
231	0.36877432	0.32805267	0.20714955	0.43375846	0.20601499	0.32471575	0.82588653	0.07287541	0.20440085	0.28805615	0.27592005	0.20419841	0.25388759
231.1	0.36880088	0.32806689	0.20714884	0.43379815	0.2060325	0.32474906	0.82590347	0.07287138	0.20444457	0.28810535	0.27599575	0.20422335	0.25393461
231.2	0.36882742	0.3280811	0.20714813	0.43383782	0.20605	0.32478235	0.82592039	0.07286735	0.20448828	0.28815452	0.27607141	0.20424827	0.2539816
231.3	0.36885394	0.3280953	0.20714741	0.43387748	0.20606749	0.32481563	0.8259373	0.07286332	0.20453197	0.28820365	0.27614702	0.20427318	0.25402857
231.4	0.36888045	0.32810949	0.20714669	0.43391713	0.20608497	0.32484889	0.82595419	0.07285929	0.20457565	0.28825275	0.2762226	0.20429808	0.2540755
231.5	0.36890693	0.32812367	0.20714597	0.43395676	0.20610243	0.32488213	0.82597106	0.07285526	0.20461931	0.28830182	0.27629814	0.20432297	0.25412242
231.6	0.3689334	0.32813783	0.20714525	0.43399638	0.20611988	0.32491536	0.82598792	0.07285123	0.20466296	0.28835085	0.27637363	0.20434784	0.2541693
231.7	0.36895986	0.32815199	0.20714453	0.434036	0.20613732	0.32494857	0.82600476	0.0728472	0.20470659	0.28839985	0.27644909	0.20437271	0.25421616
231.8	0.36898629	0.32816613	0.20714381	0.43407559	0.20615475	0.32498176	0.82602158	0.07284317	0.20475021	0.28844881	0.27652451	0.20439756	0.25426299
231.9	0.36901271	0.32818027	0.20714308	0.43411518	0.20617216	0.32501494	0.82603838	0.07283914	0.20479381	0.28849774	0.27659989	0.2044224	0.2543098
232	0.36903911	0.32819439	0.20714235	0.43415475	0.20618957	0.3250481	0.82605517	0.07283512	0.2048374	0.28854664	0.27667523	0.20444723	0.25435658
232.1	0.36906549	0.3282085	0.20714162	0.43419432	0.20620696	0.32508125	0.82607194	0.07283109	0.20488097	0.2885955	0.27675053	0.20447205	0.25440333
232.2	0.36909185	0.3282226	0.20714089	0.43423387	0.20622433	0.32511438	0.82608869	0.07282707	0.20492453	0.28864433	0.27682579	0.20449686	0.25445006
232.3	0.3691182	0.32823669	0.20714016	0.4342734	0.2062417	0.32514749	0.82610542	0.07282304	0.20496807	0.28869312	0.27690101	0.20452165	0.25449676
232.4	0.36914453	0.32825077	0.20713942	0.43431293	0.20625905	0.32518059	0.82612214	0.07281902	0.2050116	0.28874188	0.27697619	0.20454644	0.25454343
232.5	0.36917084	0.32826484	0.20713868	0.43435244	0.2062764	0.32521367	0.82613884	0.072815	0.20505511	0.28879061	0.27705133	0.20457121	0.25459008
232.6	0.36919714	0.3282789	0.20713794	0.43439194	0.20629373	0.32524673	0.82615553	0.07281097	0.20509861	0.2888393	0.27712643	0.20459597	0.25463671
232.7	0.36922341	0.32829295	0.2071372	0.43443143	0.20631104	0.32527978	0.82617219	0.07280695	0.20514209	0.28888796	0.27720149	0.20462072	0.2546833
232.8	0.36924967	0.32830698	0.20713646	0.4344709	0.20632835	0.32531281	0.82618884	0.07280293	0.20518556	0.28893658	0.27727652	0.20464546	0.25472987
232.9	0.36927592	0.32832101	0.20713572	0.43451037	0.20634564	0.32534583	0.82620548	0.07279891	0.20522901	0.28898517	0.2773515	0.20467019	0.25477642
233	0.36930214	0.32833502	0.20713497	0.43454982	0.20636292	0.32537883	0.82622209	0.0727949	0.20527245	0.28903373	0.27742645	0.2046949	0.25482294
233.1	0.36932835	0.32834903	0.20713422	0.43458926	0.20638019	0.32541181	0.82623869	0.07279088	0.20531587	0.28908226	0.27750135	0.2047196	0.25486943
233.2	0.36935454	0.32836302	0.20713347	0.43462868	0.20639745	0.32544478	0.82625527	0.07278686	0.20535928	0.28913075	0.27757622	0.2047443	0.25491589
233.3	0.36938072	0.32837701	0.20713272	0.4346681	0.20641469	0.32547773	0.82627184	0.07278284	0.20540267	0.2891792	0.27765105	0.20476898	0.25496233
233.4	0.36940687	0.32839098	0.20713197	0.4347075	0.20643193	0.32551067	0.82628839	0.07277883	0.20544605	0.28922763	0.27772584	0.20479365	0.25500875
233.5	0.36943301	0.32840494	0.20713121	0.43474689	0.20644915	0.32554359	0.82630492	0.07277482	0.20548942	0.28927602	0.27780059	0.2048183	0.25505514
233.6	0.36945913	0.32841889	0.20713045	0.43478626	0.20646636	0.32557649	0.82632143	0.0727708	0.20553276	0.28932437	0.2778753	0.20484295	0.2551015
233.7	0.36948524	0.32843283	0.2071297	0.43482563	0.20648355	0.32560938	0.82633793	0.07276679	0.2055761	0.2893727	0.27794997	0.20486759	0.25514783
233.8	0.36951133	0.32844676	0.20712894	0.43486498	0.20650074	0.32564226	0.82635441	0.07276278	0.20561942	0.28942099	0.2780246	0.20489221	0.25519415
233.9	0.3695374	0.32846068	0.20712817	0.43490432	0.20651791	0.32567511	0.82637088	0.07275877	0.20566272	0.28946924	0.27809919	0.20491682	0.25524043
234	0.36956345	0.32847458	0.20712741	0.43494365	0.20653507	0.32570795	0.82638732	0.07275475	0.20570601	0.28951747	0.27817375	0.20494142	0.25528669
234.1	0.36958949	0.32848848	0.20712665	0.43498297	0.20655222	0.32574078	0.82640376	0.07275075	0.20574928	0.28956566	0.27824826	0.20496601	0.25533292
234.2	0.36961551	0.32850237	0.20712588	0.43502227	0.20656936	0.32577359	0.82642017	0.07274674	0.20579254	0.28961381	0.27832274	0.20499059	0.25537913
234.3	0.36964152	0.32851624	0.20712511	0.43506156	0.20658649	0.32580638	0.82643657	0.07274273	0.20583579	0.28966194	0.27839717	0.20501516	0.25542531
234.4	0.3696675	0.32853011	0.20712434	0.43510084	0.2066036	0.32583916	0.82645295	0.07273872	0.20587902	0.28971003	0.27847157	0.20503971	0.25547147
234.5	0.36969347	0.32854396	0.20712357	0.43514011	0.2066207	0.32587192	0.82646931	0.07273471	0.20592223	0.28975809	0.27854593	0.20506426	0.2555176
234.6	0.36971942	0.32855781	0.20712279	0.43517937	0.20663779	0.32590466	0.82648566	0.07273071	0.20596544	0.28980611	0.27862025	0.20508879	0.2555637
234.7	0.36974536	0.32857164	0.20712202	0.43521861	0.20665487	0.32593739	0.82650199	0.0727267	0.20600862	0.2898541	0.27869453	0.20511331	0.25560978
234.8	0.36977128	0.32858546	0.20712124	0.43525784	0.20667194	0.32597011	0.82651831	0.0727227	0.20605179	0.28990206	0.27876878	0.20513782	0.25565583
234.9	0.36979718	0.32859928	0.20712046	0.43529706	0.20668899	0.32600281	0.82653461	0.0727187	0.20609495	0.28994999	0.27884298	0.20516232	0.25570186
235	0.36982307	0.32861308	0.20711968	0.43533627	0.20670603	0.32603549	0.82655089	0.07271469	0.20613809	0.28999788	0.27891715	0.20518681	0.25574786
235.1	0.36984894	0.32862687	0.2071189	0.43537546	0.20672306	0.32606816	0.82656716	0.07271069	0.20618122	0.29004574	0.27899127	0.20521129	0.25579384
235.2	0.36987479	0.32864065	0.20711812	0.43541464	0.20674008	0.32610081	0.8265834	0.07270669	0.20622433	0.29009356	0.27906536	0.20523575	0.25583979
235.3	0.36990062	0.32865442	0.20711733	0.43545381	0.20675709	0.32613344	0.82659964	0.07270269	0.20626743	0.29014136	0.27913941	0.20526021	0.25588571
235.4	0.36992644	0.32866818	0.20711655	0.43549297	0.20677409	0.32616606	0.82661585	0.07269869	0.20631051	0.29018912	0.27921342	0.20528465	0.25593161
235.5	0.36995224	0.32868193	0.20711576	0.43553212	0.20679107	0.32619867	0.82663205	0.07269469	0.20635358	0.29023685	0.2792874	0.20530908	0.25597749
235.6	0.36997803	0.32869567	0.20711497	0.43557125	0.20680804	0.32623126	0.82664824	0.0726907	0.20639664	0.29028454	0.27936133	0.2053335	0.25602334
235.7	0.3700038	0.3287094	0.20711418	0.43561037	0.206825	0.32626383	0.82666441	0.0726867	0.20643967	0.29033221	0.27943523	0.20535791	0.25606916
235.8	0.37002955	0.32872312	0.20711339	0.43564948	0.20684195	0.32629639	0.82668056	0.0726827	0.2064827	0.29037984	0.27950908	0.20538231	0.25611496
235.9	0.37005528	0.32873682	0.20711259	0.43568858	0.20685889	0.32632893	0.82669669	0.07267871	0.20652571	0.29042743	0.2795829	0.2054067	0.25616073
236	0.370081	0.32875052	0.20711179	0.43572766	0.20687581	0.32636146	0.82671281	0.07267471	0.20656871	0.290475	0.27965668	0.20543107	0.25620648
236.1	0.3701067	0.32876421	0.207111	0.43576674	0.20689273	0.32639397	0.82672891	0.07267072	0.20661169	0.29052253	0.27973042	0.20545544	0.2562522
236.2	0.37013239	0.32877789	0.2071102	0.4358058	0.20690963	0.32642646	0.826745	0.07266673	0.20665465	0.29057003	0.27980413	0.20547979	0.2562979
236.3	0.37015806	0.32879155	0.2071094	0.43584485	0.20692652	0.32645894	0.82676107	0.07266273	0.20669761	0.2906175	0.27987779	0.20550413	0.25634357
236.4	0.37018371	0.32880521	0.2071086	0.43588388	0.2069434	0.32649141	0.82677713	0.07265874	0.20674054	0.29066493	0.27995142	0.20552846	0.25638922
236.5	0.37020935	0.32881885	0.20710779	0.43592291	0.20696026	0.32652385	0.82679316	0.07265475	0.20678347	0.29071234	0.28002501	0.20555278	0.25643484
236.6	0.37023497	0.32883249	0.20710699	0.43596192	0.20697712	0.32655629	0.82680919	0.07265076	0.20682638	0.29075971	0.28009856	0.20557709	0.25648044
236.7	0.37026057	0.32884611	0.20710618	0.43600092	0.20699396	0.32658871	0.82682519	0.07264677	0.20686927	0.29080704	0.28017207	0.20560139	0.25652601
236.8	0.37028616	0.32885973	0.20710537	0.43603991	0.2070108	0.32662111	0.82684118	0.07264279	0.20691215	0.29085435	0.28024554	0.20562567	0.25657155
236.9	0.37031173	0.32887333	0.20710456	0.43607889	0.20702762	0.3266535	0.82685716	0.0726388	0.20695501	0.29090162	0.28031898	0.20564995	0.25661707
237	0.37033728	0.32888693	0.20710375	0.43611785	0.20704443	0.32668587	0.82687311	0.07263481	0.20699787	0.29094886	0.28039237	0.20567421	0.25666257
237.1	0.37036282	0.32890051	0.20710294	0.4361568	0.20706123	0.32671822	0.82688906	0.07263083	0.2070407	0.29099607	0.28046573	0.20569847	0.25670804
237.2	0.37038834	0.32891408	0.20710212	0.43619574	0.20707801	0.32675057	0.82690498	0.07262684	0.20708352	0.29104325	0.28053905	0.20572271	0.25675348
237.3	0.37041384	0.32892765	0.20710131	0.43623467	0.20709479	0.32678289	0.82692089	0.07262286	0.20712633	0.29109039	0.28061233	0.20574694	0.2567989
237.4	0.37043933	0.3289412	0.20710049	0.43627359	0.20711155	0.3268152	0.82693679	0.07261888	0.20716913	0.2911375	0.28068558	0.20577116	0.2568443
237.5	0.37046481	0.32895474	0.20709967	0.43631249	0.2071283	0.3268475	0.82695267	0.07261489	0.2072119	0.29118458	0.28075879	0.20579537	0.25688967
237.6	0.37049026	0.32896827	0.20709885	0.43635139	0.20714504	0.32687978	0.82696853	0.07261091	0.20725467	0.29123163	0.28083195	0.20581957	0.25693501
237.7	0.3705157	0.3289818	0.20709803	0.43639027	0.20716177	0.32691204	0.82698437	0.07260693	0.20729742	0.29127865	0.28090508	0.20584375	0.25698033
237.8	0.37054113	0.32899531	0.20709721	0.43642913	0.20717849	0.32694429	0.82700021	0.07260295	0.20734015	0.29132563	0.28097818	0.20586793	0.25702563
237.9	0.37056653	0.32900881	0.20709638	0.43646799	0.2071952	0.32697652	0.82701602	0.07259897	0.20738288	0.29137258	0.28105123	0.2058921	0.2570709
238	0.37059193	0.3290223	0.20709556	0.43650683	0.20721189	0.32700874	0.82703182	0.072595	0.20742558	0.2914195	0.28112425	0.20591625	0.25711614
238.1	0.3706173	0.32903578	0.20709473	0.43654567	0.20722858	0.32704095	0.8270476	0.07259102	0.20746828	0.29146639	0.28119723	0.20594039	0.25716136
238.2	0.37064266	0.32904925	0.2070939	0.43658449	0.20724525	0.32707314	0.82706337	0.07258704	0.20751096	0.29151325	0.28127017	0.20596452	0.25720656
238.3	0.370668	0.32906272	0.20709307	0.43662329	0.20726191	0.32710531	0.82707912	0.07258307	0.20755362	0.29156007	0.28134307	0.20598865	0.25725173
238.4	0.37069333	0.32907617	0.20709224	0.43666209	0.20727856	0.32713747	0.82709486	0.07257909	0.20759627	0.29160687	0.28141594	0.20601276	0.25729687
238.5	0.37071864	0.32908961	0.20709141	0.43670087	0.2072952	0.32716961	0.82711058	0.07257512	0.20763891	0.29165363	0.28148876	0.20603685	0.25734199
238.6	0.37074394	0.32910304	0.20709057	0.43673965	0.20731183	0.32720174	0.82712629	0.07257114	0.20768153	0.29170036	0.28156155	0.20606094	0.25738709
238.7	0.37076922	0.32911646	0.20708974	0.43677841	0.20732844	0.32723385	0.82714198	0.07256717	0.20772413	0.29174705	0.2816343	0.20608502	0.25743216
238.8	0.37079448	0.32912987	0.2070889	0.43681715	0.20734505	0.32726595	0.82715765	0.0725632	0.20776673	0.29179372	0.28170702	0.20610909	0.2574772
238.9	0.37081973	0.32914327	0.20708806	0.43685589	0.20736164	0.32729803	0.82717331	0.07255923	0.20780931	0.29184035	0.28177969	0.20613314	0.25752223
239	0.37084496	0.32915666	0.20708722	0.43689461	0.20737822	0.3273301	0.82718895	0.07255526	0.20785187	0.29188695	0.28185233	0.20615719	0.25756722
239.1	0.37087018	0.32917004	0.20708638	0.43693333	0.20739479	0.32736215	0.82720458	0.07255129	0.20789442	0.29193353	0.28192493	0.20618122	0.25761219
239.2	0.37089537	0.32918341	0.20708554	0.43697203	0.20741135	0.32739419	0.82722019	0.07254732	0.20793696	0.29198006	0.2819975	0.20620524	0.25765714
239.3	0.37092056	0.32919677	0.2070847	0.43701071	0.2074279	0.32742621	0.82723579	0.07254335	0.20797948	0.29202657	0.28207002	0.20622925	0.25770206
239.4	0.37094573	0.32921012	0.20708385	0.43704939	0.20744444	0.32745822	0.82725137	0.07253939	0.20802199	0.29207305	0.28214251	0.20625325	0.25774696
239.5	0.37097088	0.32922346	0.20708301	0.43708805	0.20746096	0.32749021	0.82726693	0.07253542	0.20806448	0.29211949	0.28221496	0.20627724	0.25779183
239.6	0.37099601	0.3292368	0.20708216	0.43712671	0.20747748	0.32752219	0.82728249	0.07253146	0.20810696	0.29216591	0.28228738	0.20630122	0.25783668
239.7	0.37102114	0.32925012	0.20708131	0.43716535	0.20749398	0.32755415	0.82729802	0.07252749	0.20814942	0.29221229	0.28235975	0.20632519	0.25788151
239.8	0.37104624	0.32926343	0.20708046	0.43720398	0.20751048	0.3275861	0.82731354	0.07252353	0.20819188	0.29225864	0.28243209	0.20634915	0.2579263
239.9	0.37107133	0.32927673	0.20707962	0.43724259	0.20752696	0.32761803	0.82732904	0.07251957	0.20823431	0.29230496	0.28250439	0.20637309	0.25797108
240	0.3710964	0.32929002	0.20707875	0.4372812	0.20754343	0.32764995	0.82734453	0.0725156	0.20827674	0.29235125	0.28257666	0.20639703	0.25801583

209	0.36244926	0.32462496	0.20725436	0.42471909	0.20182917	0.31695443	0.82168374	0.07378028	0.19438916	0.27632829	0.2582502	0.19841322	0.24285503
209.1	0.36248044	0.32464207	0.20725415	0.42476161	0.20184987	0.31699183	0.82170525	0.0737761	0.19443655	0.27638601	0.25833532	0.19844095	0.24290851
209.2	0.36251159	0.32465916	0.20725394	0.42480411	0.20187056	0.31702922	0.82172674	0.07377192	0.19448393	0.27644368	0.2584204	0.19846867	0.24296195
209.3	0.36254272	0.32467624	0.20725373	0.42484659	0.20189123	0.31706658	0.8217482	0.07376773	0.19453128	0.27650131	0.25850543	0.19849637	0.24301537
209.4	0.36257382	0.3246933	0.20725352	0.42488907	0.20191188	0.31710393	0.82176963	0.07376355	0.19457862	0.27655889	0.25859042	0.19852406	0.24306875
209.5	0.36260489	0.32471034	0.2072533	0.42493153	0.20193252	0.31714125	0.82179104	0.07375937	0.19462594	0.27661643	0.25867536	0.19855173	0.2431221
209.6	0.36263595	0.32472737	0.20725308	0.42497397	0.20195314	0.31717855	0.82181243	0.07375519	0.19467324	0.27667393	0.25876025	0.1985794	0.24317541
209.7	0.36266697	0.32474439	0.20725285	0.42501641	0.20197374	0.31721583	0.82183379	0.07375101	0.19472052	0.27673138	0.2588451	0.19860704	0.24322869
209.8	0.36269797	0.32476138	0.20725262	0.42505883	0.20199432	0.31725309	0.82185513	0.07374683	0.19476778	0.27678879	0.2589299	0.19863468	0.24328195
209.9	0.36272895	0.32477837	0.20725239	0.42510123	0.20201489	0.31729032	0.82187644	0.07374265	0.19481503	0.27684616	0.25901466	0.1986623	0.24333516

224	0.36686965	0.32702903	0.20719425	0.43094954	0.20475747	0.3223424	0.82465575	0.07315966	0.20130189	0.28452459	0.27052089	0.20242361	0.25052928
224.1	0.36689752	0.32704406	0.20719368	0.4309901	0.20477589	0.3223769	0.82467397	0.07315558	0.2013467	0.28457628	0.27059944	0.20244938	0.25057821
224.2	0.36692537	0.32705907	0.20719312	0.43103065	0.20479429	0.32241138	0.82469218	0.07315149	0.2013915	0.28462794	0.27067794	0.20247514	0.2506271
224.3	0.3669532	0.32707407	0.20719255	0.43107118	0.20481268	0.32244584	0.82471037	0.07314741	0.20143629	0.28467956	0.27075641	0.20250089	0.25067597
224.4	0.36698101	0.32708906	0.20719197	0.43111171	0.20483105	0.32248029	0.82472854	0.07314332	0.20148105	0.28473114	0.27083483	0.20252662	0.25072482
224.5	0.3670088	0.32710404	0.2071914	0.43115222	0.20484942	0.32251472	0.82474669	0.07313924	0.2015258	0.28478269	0.27091321	0.20255234	0.25077363
224.6	0.36703657	0.32711901	0.20719082	0.43119272	0.20486776	0.32254913	0.82476482	0.07313516	0.20157054	0.2848342	0.27099155	0.20257805	0.25082242
224.7	0.36706432	0.32713396	0.20719025	0.4312332	0.2048861	0.32258353	0.82478293	0.07313107	0.20161526	0.28488568	0.27106985	0.20260375	0.25087118
224.8	0.36709205	0.32714891	0.20718967	0.43127368	0.20490442	0.3226179	0.82480103	0.07312699	0.20165996	0.28493711	0.27114811	0.20262943	0.25091991
224.9	0.36711976	0.32716384	0.20718908	0.43131414	0.20492273	0.32265226	0.8248191	0.07312291	0.20170465	0.28498851	0.27122632	0.2026551	0.25096861

Table S15: eFAST analysis data for [LACT] with [G](0)=0.02 mM: eFAST sensitivity indices

Sensitivity analysis data from eFAST for [LACT] with [G](0)=0.02 mM (only the parameters to which [LACT]'s variability is sensitive to appear)

Time discretization	V_{[G]}	K_{[G]}	V_{[PYR]}	alpha	rho	K_{[LACT]}	G_E	L_E
0	0	0	0	0	0	0	0	0
0.1	0.0247112	0.02479256	0.02580558	0.02513702	0.02549426	0.02551571	0.02505049	0.99864453
0.2	0.02468473	0.02481756	0.02570011	0.02506605	0.02541587	0.02540167	0.0250309	0.99906425
0.3	0.02477332	0.0249485	0.0257118	0.02512791	0.02545776	0.02542337	0.02512721	0.99938409
0.4	0.02489024	0.02511544	0.0257606	0.02523719	0.02553403	0.02549494	0.02524611	0.99957246
0.5	0.02495008	0.02523818	0.02577062	0.02529802	0.0255658	0.02553353	0.02530611	0.99968021
0.6	0.02495521	0.02530507	0.02575004	0.02530612	0.02556253	0.02554275	0.02531659	0.99974449
0.7	0.02493383	0.02533807	0.02572217	0.02528805	0.02554964	0.0255417	0.02530675	0.99978541
0.8	0.02490376	0.02535726	0.02569984	0.02526175	0.02554019	0.02554206	0.02529344	0.99981295
0.9	0.02487305	0.02537371	0.02568747	0.0252355	0.02553814	0.02554812	0.02528343	0.99983225
1	0.02484483	0.02539256	0.02568564	0.0252126	0.02554334	0.02556042	0.02527873	0.99984616
1.1	0.02482009	0.02541588	0.02569348	0.02519413	0.02555426	0.02557809	0.02527945	0.99985636
1.2	0.02479902	0.02544412	0.02570964	0.02518026	0.02556923	0.02559994	0.025285	0.99986391
1.3	0.02478145	0.02547682	0.02573273	0.02517072	0.02558679	0.02562482	0.02529462	0.99986952
1.4	0.02476711	0.02551303	0.02576143	0.02516504	0.02560586	0.02565178	0.02530749	0.99987364
1.5	0.02475578	0.02555158	0.02579459	0.02516262	0.02562576	0.02568003	0.02532295	0.99987663
1.6	0.02474725	0.02559125	0.02583121	0.0251629	0.02564617	0.02570896	0.02534045	0.9998787
1.7	0.02474141	0.02563102	0.02587041	0.02516528	0.02566696	0.02573806	0.02535959	0.99988005
1.8	0.02473819	0.02567006	0.0259115	0.02516928	0.02568812	0.02576703	0.02538008	0.99988077
1.9	0.02473752	0.02570781	0.02595387	0.0251745	0.02570969	0.02579567	0.02540173	0.99988097
2	0.02473937	0.02574399	0.02599701	0.02518064	0.02573165	0.02582384	0.02542441	0.9998807
2.1	0.02474366	0.0257785	0.02604043	0.02518752	0.02575393	0.02585145	0.02544801	0.99988001
2.2	0.0247502	0.02581142	0.02608367	0.02519497	0.02577643	0.02587842	0.02547248	0.99987896
2.3	0.02475867	0.02584296	0.02612625	0.0252029	0.02579896	0.02590471	0.02549775	0.9998776
2.4	0.02476865	0.02587336	0.02616779	0.02521126	0.02582137	0.02593028	0.0255237	0.999876
2.5	0.02477969	0.02590285	0.02620799	0.02521998	0.02584352	0.02595506	0.02555016	0.99987423
2.6	0.02479143	0.02593167	0.0262466	0.02522898	0.02586529	0.02597903	0.025577	0.99987237
2.7	0.02480364	0.02595994	0.02628349	0.02523821	0.02588658	0.02600226	0.02560406	0.99987046
2.8	0.02481615	0.02598776	0.02631858	0.02524762	0.02590729	0.0260249	0.02563119	0.99986856
2.9	0.02482888	0.02601517	0.02635186	0.02525716	0.02592736	0.02604706	0.02565817	0.99986669
3	0.02484187	0.02604219	0.02638337	0.02526679	0.02594675	0.0260688	0.02568479	0.99986485
3.1	0.02485519	0.02606878	0.02641318	0.02527652	0.02596544	0.02609018	0.02571085	0.99986303
3.2	0.02486897	0.0260949	0.02644139	0.02528637	0.02598342	0.02611133	0.0257363	0.99986122
3.3	0.02488337	0.0261205	0.0264681	0.02529638	0.02600072	0.02613241	0.02576114	0.99985941
3.4	0.02489858	0.0261455	0.02649343	0.0253066	0.02601739	0.02615353	0.02578535	0.99985755
3.5	0.02491472	0.02616979	0.02651751	0.02531712	0.02603351	0.02617474	0.02580899	0.99985565
3.6	0.02493194	0.02619332	0.02654048	0.02532802	0.02604913	0.026196	0.02583211	0.99985367
3.7	0.02495037	0.02621602	0.02656248	0.0253394	0.02606436	0.02621719	0.02585477	0.9998516
3.8	0.02497009	0.02623787	0.02658362	0.02535136	0.02607932	0.02623817	0.02587694	0.99984942
3.9	0.0249912	0.02625886	0.02660402	0.02536402	0.0260941	0.02625882	0.02589857	0.99984713
4	0.02501376	0.02627896	0.02662381	0.0253775	0.02610882	0.02627902	0.02591958	0.99984472
4.1	0.02503786	0.02629821	0.02664309	0.02539193	0.02612358	0.02629873	0.02593992	0.9998422
4.2	0.02506347	0.02631666	0.02666198	0.02540744	0.0261385	0.02631791	0.02595954	0.99983958
4.3	0.02509049	0.02633436	0.02668056	0.02542418	0.02615367	0.0263366	0.02597834	0.99983687
4.4	0.02511867	0.02635135	0.0266989	0.02544224	0.02616921	0.02635484	0.02599629	0.99983409
4.5	0.02514764	0.02636772	0.02671706	0.02546174	0.02618523	0.02637271	0.02601342	0.99983127
4.6	0.02517701	0.02638356	0.02673509	0.02548279	0.02620183	0.02639029	0.02602974	0.99982842
4.7	0.02520634	0.02639896	0.02675303	0.02550547	0.02621904	0.02640771	0.02604526	0.99982555
4.8	0.02523528	0.02641401	0.02677093	0.02552984	0.02623683	0.02642505	0.02606005	0.99982269
4.9	0.02526357	0.0264288	0.02678879	0.0255559	0.02625511	0.0264424	0.02607414	0.99981983
5	0.02529103	0.02644345	0.0268066	0.02558363	0.02627375	0.02645988	0.02608759	0.99981697
5.1	0.02531751	0.02645805	0.02682436	0.02561294	0.02629263	0.02647761	0.02610044	0.99981412
5.2	0.02534294	0.02647269	0.02684206	0.02564372	0.0263116	0.02649567	0.02611273	0.9998113
5.3	0.02536733	0.02648746	0.02685968	0.02567584	0.02633052	0.02651418	0.02612451	0.99980852
5.4	0.02539075	0.02650245	0.02687724	0.02570912	0.02634927	0.0265332	0.02613581	0.99980581
5.5	0.02541325	0.02651774	0.02689474	0.02574342	0.02636774	0.02655278	0.02614665	0.9998032
5.6	0.02543491	0.02653343	0.02691218	0.02577855	0.02638585	0.02657293	0.02615705	0.99980069
5.7	0.02545578	0.02654954	0.02692955	0.02581431	0.02640352	0.02659366	0.02616704	0.99979827
5.8	0.02547588	0.02656601	0.02694687	0.02585049	0.02642071	0.02661495	0.02617664	0.99979594
5.9	0.02549529	0.0265827	0.02696417	0.02588685	0.02643743	0.02663681	0.02618587	0.99979369
6	0.02551405	0.02659941	0.02698145	0.02592324	0.02645366	0.02665921	0.02619476	0.99979152
6.1	0.0255322	0.02661595	0.02699875	0.02595951	0.0264694	0.02668212	0.02620332	0.99978943
6.2	0.02554977	0.02663219	0.02701607	0.02599553	0.02648464	0.02670543	0.02621158	0.99978741
6.3	0.02556679	0.02664804	0.02703336	0.02603121	0.02649939	0.02672903	0.02621955	0.99978545
6.4	0.02558329	0.02666349	0.02705054	0.02606648	0.02651366	0.02675277	0.02622725	0.99978355
6.5	0.02559929	0.02667854	0.02706752	0.02610125	0.02652748	0.0267765	0.0262347	0.99978171
6.6	0.02561483	0.02669317	0.02708422	0.02613548	0.02654086	0.02680003	0.02624192	0.99977992
6.7	0.02562993	0.02670737	0.02710058	0.02616915	0.02655381	0.0268232	0.02624892	0.99977818
6.8	0.02564461	0.02672113	0.02711654	0.02620221	0.02656636	0.02684586	0.02625572	0.99977649
6.9	0.02565891	0.02673446	0.02713208	0.02623459	0.02657853	0.02686794	0.02626234	0.99977484
7	0.02567283	0.02674737	0.02714717	0.02626625	0.02659035	0.02688941	0.02626878	0.99977324
7.1	0.02568642	0.02675989	0.02716183	0.02629714	0.02660182	0.02691026	0.02627507	0.99977167
7.2	0.02569969	0.02677203	0.02717606	0.02632722	0.02661297	0.02693051	0.02628122	0.99977013
7.3	0.02571268	0.02678382	0.02718987	0.02635649	0.02662382	0.02695021	0.02628725	0.99976863
7.4	0.0257254	0.02679529	0.02720327	0.02638496	0.02663438	0.02696939	0.02629317	0.99976715
7.5	0.02573788	0.02680644	0.02721628	0.02641264	0.02664467	0.02698806	0.02629901	0.9997657
7.6	0.02575014	0.0268173	0.02722891	0.02643955	0.02665471	0.02700626	0.02630478	0.99976427
7.7	0.02576221	0.02682788	0.02724118	0.02646569	0.02666451	0.02702402	0.0263105	0.99976285
7.8	0.0257741	0.02683819	0.02725311	0.02649112	0.0266741	0.02704135	0.02631618	0.99976144
7.9	0.02578585	0.02684823	0.02726471	0.02651587	0.02668349	0.02705826	0.02632186	0.99976004
8	0.0257975	0.026858	0.02727599	0.02653997	0.0266927	0.02707478	0.02632755	0.99975864
8.1	0.02580908	0.02686753	0.02728697	0.02656344	0.02670174	0.02709093	0.02633327	0.99975724
8.2	0.02582065	0.02687681	0.02729766	0.0265863	0.02671064	0.02710672	0.02633904	0.99975582
8.3	0.02583223	0.02688586	0.02730807	0.0266086	0.0267194	0.02712219	0.0263449	0.99975439
8.4	0.02584387	0.02689468	0.02731821	0.02663034	0.02672805	0.02713736	0.02635085	0.99975294
8.5	0.02585558	0.02690328	0.02732809	0.02665153	0.02673661	0.02715225	0.02635694	0.99975147
8.6	0.02586739	0.02691167	0.02733772	0.02667221	0.02674509	0.02716691	0.02636319	0.99974999
8.7	0.02587928	0.02691986	0.0273471	0.02669238	0.02675353	0.02718136	0.02636963	0.99974849
8.8	0.02589125	0.02692785	0.02735626	0.02671206	0.02676195	0.02719564	0.0263763	0.99974698
8.9	0.02590328	0.02693567	0.02736519	0.02673127	0.02677038	0.02720978	0.02638322	0.99974547
9	0.02591534	0.02694332	0.0273739	0.02675002	0.02677884	0.02722381	0.02639044	0.99974397
9.1	0.02592741	0.02695081	0.02738241	0.02676834	0.02678737	0.02723779	0.02639799	0.99974248
9.2	0.02593941	0.02695814	0.02739073	0.02678624	0.02679601	0.02725177	0.02640584	0.99974101
9.3	0.02595133	0.02696532	0.02739885	0.02680372	0.02680479	0.0272658	0.02641398	0.99973956
9.4	0.02596314	0.02697237	0.02740678	0.02682082	0.02681375	0.02727994	0.02642236	0.99973814
9.5	0.02597485	0.02697929	0.02741454	0.02683753	0.02682295	0.02729426	0.02643093	0.99973675
9.6	0.02598645	0.02698608	0.02742213	0.02685387	0.02683243	0.02730881	0.02643961	0.99973539
9.7	0.02599796	0.02699276	0.02742956	0.02686986	0.02684221	0.0273236	0.02644833	0.99973406
9.8	0.02600939	0.02699934	0.02743683	0.0268855	0.02685232	0.02733863	0.02645705	0.99973276
9.9	0.02602076	0.02700581	0.02744396	0.0269008	0.02686273	0.02735387	0.02646572	0.99973148
10	0.0260321	0.0270122	0.02745094	0.02691578	0.0268734	0.0273693	0.02647432	0.99973024
10.1	0.02604341	0.02701851	0.02745778	0.02693044	0.02688427	0.02738489	0.02648283	0.99972902
10.2	0.02605471	0.02702475	0.0274645	0.02694479	0.02689523	0.02740057	0.02649123	0.99972783
10.3	0.026066	0.02703094	0.0274711	0.02695885	0.02690621	0.02741623	0.02649953	0.99972667
10.4	0.02607726	0.02703707	0.02747758	0.02697262	0.02691713	0.02743174	0.02650774	0.99972552
10.5	0.02608845	0.02704316	0.02748395	0.02698611	0.02692794	0.02744705	0.02651588	0.99972439
10.6	0.02609954	0.02704923	0.02749022	0.02699933	0.02693859	0.02746217	0.02652398	0.99972329
10.7	0.02611048	0.02705527	0.0274964	0.02701228	0.0269491	0.02747706	0.02653204	0.99972221
10.8	0.02612125	0.02706132	0.0275025	0.02702498	0.02695946	0.02749169	0.02654009	0.99972114
10.9	0.02613183	0.02706737	0.02750852	0.02703742	0.02696969	0.02750607	0.02654816	0.99972009
11	0.02614221	0.02707344	0.02751446	0.02704962	0.02697984	0.0275202	0.02655629	0.99971906
11.1	0.02615238	0.02707955	0.02752035	0.02706159	0.02698994	0.02753411	0.02656449	0.99971804
11.2	0.02616233	0.02708572	0.02752617	0.02707333	0.02699999	0.02754781	0.02657277	0.99971704
11.3	0.02617208	0.02709194	0.02753196	0.02708484	0.02701	0.02756131	0.02658117	0.99971605
11.4	0.02618161	0.02709825	0.02753773	0.02709613	0.02701996	0.02757464	0.02658967	0.99971508
11.5	0.02619094	0.02710464	0.02754349	0.02710722	0.02702988	0.02758781	0.02659828	0.99971412
11.6	0.02620008	0.02711112	0.02754927	0.02711809	0.02703976	0.02760085	0.026607	0.99971318
11.7	0.02620904	0.02711768	0.02755507	0.02712876	0.02704962	0.02761379	0.0266158	0.99971224
11.8	0.02621781	0.02712434	0.02756091	0.02713924	0.02705946	0.02762662	0.02662467	0.99971132
11.9	0.0262264	0.02713106	0.02756683	0.02714952	0.02706929	0.02763937	0.02663357	0.99971041
12	0.02623482	0.02713783	0.02757284	0.02715962	0.02707911	0.02765206	0.02664247	0.99970951
12.1	0.02624308	0.02714462	0.02757897	0.02716953	0.02708893	0.0276647	0.02665134	0.99970862
12.2	0.02625119	0.02715141	0.02758524	0.02717927	0.02709874	0.02767733	0.02666015	0.99970774
12.3	0.02625915	0.02715817	0.0275917	0.02718882	0.02710854	0.02768996	0.02666888	0.99970687
12.4	0.02626696	0.02716486	0.02759836	0.02719821	0.02711832	0.02770261	0.0266775	0.999706
12.5	0.02627463	0.02717145	0.02760526	0.02720743	0.02712805	0.02771533	0.02668599	0.99970515
12.6	0.02628216	0.02717793	0.02761241	0.02721649	0.02713773	0.02772814	0.02669437	0.9997043
12.7	0.02628956	0.0271843	0.02761982	0.02722539	0.02714734	0.02774106	0.02670262	0.99970345
12.8	0.02629683	0.02719055	0.02762748	0.02723414	0.02715687	0.0277541	0.02671077	0.99970261
12.9	0.02630398	0.02719666	0.02763534	0.02724273	0.0271663	0.02776728	0.02671882	0.99970178
13	0.02631103	0.02720266	0.02764338	0.02725117	0.02717563	0.02778058	0.02672677	0.99970094
13.1	0.02631797	0.02720853	0.02765153	0.02725947	0.02718483	0.02779399	0.02673465	0.99970011
13.2	0.02632481	0.0272143	0.02765972	0.02726762	0.02719391	0.02780746	0.02674247	0.99969928
13.3	0.02633157	0.02721997	0.0276679	0.02727563	0.02720284	0.02782095	0.02675024	0.99969845
13.4	0.02633825	0.02722554	0.02767602	0.02728351	0.02721164	0.02783443	0.02675795	0.99969762
13.5	0.02634486	0.02723102	0.02768405	0.02729126	0.0272203	0.02784784	0.02676562	0.99969678
13.6	0.02635141	0.02723642	0.02769195	0.02729887	0.02722882	0.02786115	0.02677325	0.99969593
13.7	0.02635792	0.02724174	0.02769972	0.02730636	0.0272372	0.02787431	0.02678083	0.99969508
13.8	0.02636439	0.02724697	0.02770735	0.02731372	0.02724543	0.02788733	0.02678836	0.99969422
13.9	0.02637083	0.02725211	0.02771486	0.02732095	0.02725353	0.02790019	0.02679585	0.99969335
14	0.02637726	0.02725717	0.02772223	0.02732807	0.0272615	0.02791288	0.0268033	0.99969246
14.1	0.02638369	0.02726215	0.02772947	0.02733507	0.02726933	0.02792541	0.02681072	0.99969156
14.2	0.02639012	0.02726704	0.02773659	0.02734195	0.02727704	0.02793777	0.02681813	0.99969063
14.3	0.02639657	0.02727186	0.02774361	0.02734872	0.02728463	0.02794997	0.02682554	0.99968969
14.4	0.02640305	0.02727659	0.02775053	0.02735537	0.02729209	0.02796201	0.02683297	0.99968872
14.5	0.02640955	0.02728124	0.02775734	0.02736192	0.02729944	0.02797389	0.02684046	0.99968772
14.6	0.02641607	0.02728581	0.02776406	0.02736836	0.02730668	0.02798561	0.02684804	0.99968668
14.7	0.02642262	0.02729029	0.02777069	0.0273747	0.0273138	0.02799717	0.02685572	0.99968562
14.8	0.02642917	0.02729469	0.02777723	0.02738093	0.02732082	0.02800861	0.02686356	0.99968451
14.9	0.02643569	0.02729901	0.02778368	0.02738706	0.02732772	0.02801992	0.02687157	0.99968338
15	0.02644218	0.02730325	0.02779005	0.02739309	0.02733453	0.02803111	0.02687978	0.9996822
15.1	0.02644862	0.02730741	0.02779632	0.02739903	0.02734122	0.02804219	0.02688824	0.99968099
15.2	0.02645498	0.02731151	0.0278025	0.02740487	0.02734782	0.02805315	0.02689697	0.99967974
15.3	0.02646126	0.02731552	0.02780859	0.02741061	0.02735433	0.028064	0.026906	0.99967845
15.4	0.02646745	0.02731947	0.02781459	0.02741627	0.02736073	0.02807475	0.02691537	0.99967713
15.5	0.02647353	0.02732336	0.02782051	0.02742183	0.02736704	0.02808541	0.02692512	0.99967576
15.6	0.0264795	0.02732717	0.02782633	0.02742731	0.02737326	0.02809597	0.02693527	0.99967434
15.7	0.02648537	0.02733093	0.02783207	0.0274327	0.02737938	0.02810645	0.02694583	0.99967287
15.8	0.02649113	0.02733463	0.02783773	0.027438	0.02738542	0.02811684	0.02695681	0.99967134
15.9	0.02649677	0.02733828	0.0278433	0.02744322	0.02739137	0.02812715	0.02696813	0.99966974
16	0.02650231	0.02734187	0.02784879	0.02744836	0.02739723	0.02813738	0.02697975	0.99966806
16.1	0.02650773	0.0273454	0.02785421	0.02745342	0.02740301	0.02814752	0.02699158	0.99966631
16.2	0.02651305	0.02734889	0.02785954	0.02745841	0.02740871	0.02815759	0.02700357	0.99966448
16.3	0.02651827	0.02735232	0.02786481	0.02746331	0.02741432	0.02816759	0.02701565	0.99966257
16.4	0.02652338	0.02735571	0.02786999	0.02746814	0.02741986	0.02817752	0.02702776	0.9996606
16.5	0.02652839	0.02735905	0.02787511	0.0274729	0.02742531	0.02818739	0.02703985	0.99965857
16.6	0.02653331	0.02736234	0.02788016	0.02747759	0.02743069	0.02819719	0.02705187	0.9996565
16.7	0.02653814	0.02736559	0.02788513	0.02748221	0.027436	0.02820693	0.02706383	0.9996544
16.8	0.02654287	0.0273688	0.02789004	0.02748675	0.02744123	0.02821661	0.02707571	0.99965228
16.9	0.02654752	0.02737197	0.02789488	0.02749123	0.02744639	0.02822624	0.0270875	0.99965016
17	0.02655208	0.0273751	0.02789965	0.02749565	0.02745147	0.02823581	0.02709922	0.99964806
17.1	0.02655655	0.0273782	0.02790436	0.0275	0.02745649	0.02824534	0.02711085	0.99964597
17.2	0.02656094	0.02738127	0.02790901	0.02750429	0.02746143	0.02825483	0.02712241	0.99964391
17.3	0.02656524	0.02738431	0.02791359	0.02750852	0.02746631	0.02826428	0.02713392	0.99964187
17.4	0.02656947	0.02738733	0.02791811	0.02751269	0.02747112	0.02827369	0.02714536	0.99963986
17.5	0.02657362	0.02739032	0.02792257	0.0275168	0.02747586	0.02828307	0.02715675	0.99963788
17.6	0.02657769	0.0273933	0.02792697	0.02752085	0.02748054	0.02829241	0.0271681	0.99963593
17.7	0.02658168	0.02739627	0.02793131	0.02752485	0.02748516	0.02830174	0.02717941	0.99963401
17.8	0.0265856	0.02739922	0.02793559	0.0275288	0.02748971	0.02831103	0.02719072	0.9996321
17.9	0.02658944	0.02740217	0.02793982	0.02753269	0.0274942	0.02832031	0.027202	0.99963021
18	0.02659322	0.02740512	0.027944	0.02753654	0.02749863	0.02832957	0.02721326	0.99962834
18.1	0.02659693	0.02740808	0.02794811	0.02754033	0.02750301	0.02833881	0.02722445	0.99962649
18.2	0.02660057	0.02741105	0.02795218	0.02754408	0.02750732	0.02834804	0.02723556	0.99962465
18.3	0.02660415	0.02741404	0.02795619	0.02754778	0.02751157	0.02835726	0.02724656	0.99962283
18.4	0.02660767	0.02741705	0.02796015	0.02755144	0.02751577	0.02836648	0.02725743	0.99962103
18.5	0.02661113	0.0274201	0.02796406	0.02755506	0.02751992	0.02837569	0.02726816	0.99961924
18.6	0.02661453	0.02742319	0.02796792	0.02755864	0.02752401	0.0283849	0.02727875	0.99961748
18.7	0.02661787	0.02742633	0.02797173	0.02756217	0.02752804	0.02839411	0.02728917	0.99961573
18.8	0.02662117	0.02742955	0.02797549	0.02756567	0.02753202	0.02840333	0.02729941	0.999614
18.9	0.02662441	0.02743284	0.0279792	0.02756914	0.02753595	0.02841255	0.02730947	0.99961228
19	0.0266276	0.02743623	0.02798286	0.02757257	0.02753984	0.02842178	0.02731935	0.99961059
19.1	0.02663074	0.02743973	0.02798648	0.02757596	0.02754367	0.02843103	0.02732906	0.99960891
19.2	0.02663384	0.02744335	0.02799005	0.02757932	0.02754745	0.02844028	0.02733859	0.99960726
19.3	0.0266369	0.02744712	0.02799358	0.02758266	0.02755118	0.02844956	0.02734794	0.99960562
19.4	0.02663992	0.02745104	0.02799706	0.02758596	0.02755487	0.02845885	0.02735713	0.99960401
19.5	0.02664291	0.02745513	0.02800049	0.02758924	0.02755851	0.02846816	0.02736616	0.99960241
19.6	0.02664587	0.02745941	0.02800389	0.0275925	0.02756211	0.0284775	0.02737505	0.99960083
19.7	0.02664881	0.02746386	0.02800724	0.02759573	0.02756566	0.02848686	0.02738377	0.99959927
19.8	0.02665172	0.02746848	0.02801054	0.02759893	0.02756917	0.02849625	0.02739234	0.99959773
19.9	0.02665462	0.02747325	0.02801381	0.02760212	0.02757263	0.02850567	0.02740076	0.99959621
20	0.0266575	0.02747816	0.02801703	0.02760529	0.02757605	0.02851512	0.02740902	0.9995947
20.1	0.02666038	0.02748316	0.02802022	0.02760844	0.02757943	0.02852461	0.02741713	0.9995932
20.2	0.02666325	0.02748824	0.02802336	0.02761157	0.02758277	0.02853414	0.0274251	0.99959172
20.3	0.02666613	0.02749336	0.02802647	0.02761469	0.02758607	0.0285437	0.02743291	0.99959026
20.4	0.02666901	0.02749849	0.02802953	0.0276178	0.02758933	0.0285533	0.02744059	0.99958881
20.5	0.02667191	0.02750361	0.02803256	0.0276209	0.02759255	0.02856295	0.02744812	0.99958737
20.6	0.02667483	0.02750873	0.02803554	0.02762399	0.02759574	0.02857265	0.02745552	0.99958594
20.7	0.02667778	0.02751384	0.02803849	0.02762706	0.02759888	0.02858239	0.02746277	0.99958452
20.8	0.02668076	0.02751894	0.02804141	0.02763014	0.02760199	0.02859218	0.0274699	0.99958311
20.9	0.02668379	0.02752401	0.02804428	0.0276332	0.02760506	0.02860202	0.02747689	0.99958172
21	0.02668687	0.02752907	0.02804713	0.02763627	0.0276081	0.02861192	0.02748375	0.99958034
21.1	0.02669002	0.02753409	0.02804993	0.02763933	0.0276111	0.02862187	0.02749048	0.99957896
21.2	0.02669324	0.0275391	0.0280527	0.02764239	0.02761407	0.02863189	0.02749708	0.9995776
21.3	0.02669655	0.02754409	0.02805543	0.02764545	0.027617	0.02864196	0.02750357	0.99957625
21.4	0.02669994	0.02754906	0.02805813	0.02764852	0.0276199	0.02865209	0.02750993	0.99957491
21.5	0.02670342	0.02755401	0.0280608	0.02765159	0.02762277	0.02866229	0.02751617	0.99957358
21.6	0.02670699	0.02755896	0.02806343	0.02765467	0.0276256	0.02867255	0.0275223	0.99957226
21.7	0.02671065	0.02756389	0.02806603	0.02765775	0.0276284	0.02868288	0.02752832	0.99957096
21.8	0.02671438	0.02756883	0.0280686	0.02766084	0.02763117	0.02869327	0.02753423	0.99956967
21.9	0.02671818	0.02757377	0.02807113	0.02766395	0.02763391	0.02870374	0.02754003	0.99956838
22	0.02672204	0.02757871	0.02807364	0.02766706	0.02763662	0.02871429	0.02754573	0.99956711
22.1	0.02672593	0.02758367	0.02807611	0.0276702	0.0276393	0.0287249	0.02755133	0.99956585
22.2	0.02672985	0.02758865	0.02807855	0.02767334	0.02764196	0.0287356	0.02755683	0.9995646
22.3	0.02673378	0.02759367	0.02808096	0.02767651	0.02764458	0.02874637	0.02756223	0.99956336
22.4	0.02673771	0.02759872	0.02808334	0.02767969	0.02764718	0.02875722	0.02756755	0.99956213
22.5	0.02674162	0.02760381	0.02808568	0.02768289	0.02764975	0.02876815	0.02757277	0.9995609
22.6	0.0267455	0.02760896	0.02808801	0.02768612	0.02765229	0.02877916	0.02757791	0.99955969
22.7	0.02674935	0.02761418	0.0280903	0.02768937	0.0276548	0.02879026	0.02758297	0.99955848
22.8	0.02675316	0.02761948	0.02809256	0.02769264	0.0276573	0.02880144	0.02758794	0.99955727
22.9	0.02675694	0.02762487	0.0280948	0.02769594	0.02765976	0.02881272	0.02759284	0.99955607
23	0.02676069	0.02763033	0.028097	0.02769927	0.02766221	0.02882408	0.02759767	0.99955488
23.1	0.0267644	0.02763588	0.02809919	0.02770264	0.02766463	0.02883553	0.02760242	0.99955368
23.2	0.0267681	0.02764151	0.02810134	0.02770603	0.02766702	0.02884708	0.0276071	0.99955249
23.3	0.02677178	0.02764719	0.02810347	0.02770946	0.0276694	0.02885872	0.02761171	0.9995513
23.4	0.02677545	0.0276529	0.02810557	0.02771292	0.02767175	0.02887045	0.02761626	0.99955011
23.5	0.02677913	0.02765864	0.02810765	0.02771642	0.02767409	0.02888228	0.02762075	0.99954891
23.6	0.02678282	0.02766438	0.02810971	0.02771996	0.0276764	0.02889421	0.02762517	0.99954771
23.7	0.02678653	0.02767011	0.02811174	0.02772354	0.0276787	0.02890624	0.02762955	0.99954651
23.8	0.02679026	0.02767581	0.02811375	0.02772716	0.02768098	0.02891837	0.02763386	0.99954531
23.9	0.02679402	0.02768149	0.02811573	0.02773083	0.02768324	0.02893061	0.02763813	0.9995441
24	0.02679782	0.02768714	0.0281177	0.02773454	0.02768549	0.02894295	0.02764235	0.99954289
24.1	0.02680167	0.02769275	0.02811964	0.0277383	0.02768772	0.0289554	0.02764652	0.99954168
24.2	0.02680557	0.02769832	0.02812156	0.02774211	0.02768994	0.02896795	0.02765065	0.99954048
24.3	0.02680953	0.02770385	0.02812346	0.02774597	0.02769215	0.02898062	0.02765475	0.99953927
24.4	0.02681357	0.02770934	0.02812534	0.02774989	0.02769435	0.02899339	0.02765881	0.99953808
24.5	0.02681768	0.02771479	0.0281272	0.02775386	0.02769654	0.02900628	0.02766283	0.99953689
24.6	0.02682187	0.0277202	0.02812905	0.02775788	0.02769872	0.02901929	0.02766683	0.9995357
24.7	0.02682612	0.02772559	0.02813088	0.02776196	0.02770089	0.02903241	0.02767079	0.99953453
24.8	0.02683043	0.02773095	0.02813269	0.02776611	0.02770306	0.02904564	0.02767474	0.99953336
24.9	0.02683478	0.02773629	0.02813449	0.02777031	0.02770523	0.029059	0.02767866	0.9995322
25	0.02683913	0.02774161	0.02813627	0.02777458	0.02770739	0.02907248	0.02768257	0.99953106
25.1	0.02684348	0.02774691	0.02813804	0.02777891	0.02770956	0.02908608	0.02768646	0.99952992
25.2	0.02684781	0.02775219	0.0281398	0.02778332	0.02771173	0.0290998	0.02769034	0.9995288
25.3	0.02685211	0.02775746	0.02814155	0.02778779	0.0277139	0.02911365	0.02769421	0.99952768
25.4	0.02685637	0.02776271	0.0281433	0.02779233	0.02771609	0.02912763	0.02769807	0.99952658
25.5	0.02686059	0.02776797	0.02814503	0.02779695	0.02771829	0.02914173	0.02770193	0.99952549
25.6	0.02686476	0.02777321	0.02814676	0.02780164	0.02772052	0.02915597	0.02770579	0.9995244
25.7	0.0268689	0.02777846	0.02814849	0.0278064	0.02772277	0.02917034	0.02770965	0.99952333
25.8	0.02687298	0.0277837	0.02815022	0.02781125	0.02772506	0.02918485	0.02771352	0.99952227
25.9	0.02687701	0.02778895	0.02815196	0.02781618	0.02772738	0.02919949	0.02771739	0.99952122
26	0.026881	0.0277942	0.02815369	0.02782119	0.02772975	0.02921427	0.02772128	0.99952017
26.1	0.02688493	0.02779946	0.02815544	0.02782628	0.02773218	0.02922919	0.02772518	0.99951914
26.2	0.02688881	0.02780473	0.0281572	0.02783147	0.02773467	0.02924425	0.0277291	0.99951812
26.3	0.02689265	0.02781001	0.02815898	0.02783674	0.02773724	0.02925946	0.02773304	0.9995171
26.4	0.02689643	0.02781532	0.02816079	0.0278421	0.02773989	0.02927481	0.02773701	0.9995161
26.5	0.02690018	0.02782065	0.02816262	0.02784756	0.02774265	0.02929031	0.027741	0.9995151
26.6	0.02690388	0.02782601	0.02816448	0.02785311	0.02774553	0.02930595	0.02774502	0.99951411
26.7	0.02690754	0.02783141	0.0281664	0.02785877	0.02774855	0.02932175	0.02774907	0.99951313
26.8	0.02691116	0.02783685	0.02816836	0.02786452	0.02775173	0.0293377	0.02775316	0.99951216
26.9	0.02691476	0.02784235	0.02817039	0.02787038	0.02775507	0.02935381	0.02775728	0.99951119
27	0.02691831	0.02784791	0.02817249	0.02787634	0.02775859	0.02937007	0.02776145	0.99951024
27.1	0.02692183	0.02785354	0.02817469	0.02788241	0.02776227	0.0293865	0.02776567	0.99950929
27.2	0.02692531	0.02785926	0.02817698	0.02788859	0.0277661	0.02940308	0.02776993	0.99950835
27.3	0.02692875	0.02786508	0.0281794	0.02789489	0.02777005	0.02941984	0.02777424	0.99950742
27.4	0.02693216	0.02787101	0.02818195	0.0279013	0.0277741	0.02943676	0.02777861	0.99950649
27.5	0.02693553	0.02787707	0.02818465	0.02790783	0.02777822	0.02945385	0.02778303	0.99950557
27.6	0.02693887	0.02788327	0.02818751	0.02791448	0.02778237	0.02947112	0.02778752	0.99950466
27.7	0.02694218	0.02788961	0.02819053	0.02792126	0.02778653	0.02948857	0.02779207	0.99950376
27.8	0.02694546	0.0278961	0.02819369	0.02792817	0.02779069	0.0295062	0.0277967	0.99950286
27.9	0.02694871	0.02790274	0.02819697	0.02793521	0.02779483	0.02952401	0.02780139	0.99950198
28	0.02695193	0.0279095	0.02820035	0.02794238	0.02779894	0.02954202	0.02780617	0.99950109
28.1	0.02695512	0.02791638	0.02820378	0.02794969	0.02780302	0.02956022	0.02781102	0.99950022
28.2	0.02695828	0.02792335	0.02820725	0.02795714	0.02780709	0.02957862	0.02781596	0.99949935
28.3	0.02696141	0.02793039	0.02821071	0.02796475	0.02781113	0.02959723	0.0278210	0.99949849
28.4	0.02696452	0.02793749	0.02821415	0.02797251	0.02781514	0.02961605	0.02782612	0.99949763
28.5	0.0269676	0.02794461	0.02821757	0.02798043	0.02781913	0.02963509	0.02783135	0.99949678
28.6	0.02697066	0.02795174	0.02822095	0.02798852	0.02782309	0.02965436	0.02783668	0.99949594
28.7	0.02697369	0.02795887	0.02822429	0.02799678	0.02782704	0.02967387	0.02784212	0.9994951
28.8	0.02697669	0.02796599	0.02822761	0.02800522	0.02783096	0.02969363	0.02784768	0.99949427
28.9	0.02697967	0.02797309	0.0282309	0.02801385	0.02783487	0.02971364	0.02785336	0.99949344
29	0.02698263	0.02798018	0.02823417	0.02802266	0.02783876	0.02973393	0.02785917	0.99949262
29.1	0.02698556	0.02798724	0.02823743	0.02803168	0.02784264	0.02975448	0.02786513	0.9994918
29.2	0.02698847	0.02799428	0.02824066	0.02804091	0.02784651	0.02977533	0.02787125	0.99949099
29.3	0.02699136	0.02800131	0.02824387	0.02805035	0.02785036	0.02979646	0.02787754	0.99949019
29.4	0.02699424	0.02800833	0.02824705	0.02806002	0.02785421	0.02981787	0.0278840	0.99948939
29.5	0.02699709	0.02801533	0.02825021	0.02806992	0.02785806	0.02983957	0.02789063	0.99948859
29.6	0.02699993	0.02802234	0.02825335	0.02808007	0.0278619	0.02986154	0.02789742	0.9994878
29.7	0.02700275	0.02802934	0.02825646	0.02809048	0.02786575	0.02988376	0.02790437	0.99948702
29.8	0.02700556	0.02803634	0.02825954	0.02810115	0.02786959	0.02990622	0.02791148	0.99948623
29.9	0.02700836	0.02804335	0.0282626	0.0281121	0.02787345	0.02992891	0.02791876	0.99948545
30	0.02701116	0.02805036	0.02826563	0.02812331	0.02787732	0.02995179	0.02792623	0.99948468
30.1	0.02701395	0.02805737	0.02826863	0.02813479	0.02788117	0.02997487	0.0279339	0.99948391
30.2	0.02701674	0.02806439	0.0282716	0.02814651	0.0278851	0.02999813	0.02794175	0.99948314
30.3	0.02701954	0.02807142	0.02827455	0.02815848	0.02788903	0.03002157	0.02794975	0.99948238
30.4	0.02702234	0.02807845	0.02827748	0.02817067	0.02789299	0.03004519	0.02795789	0.99948162
30.5	0.02702515	0.02808548	0.02828038	0.0281831	0.02789697	0.03006899	0.02796614	0.99948086
30.6	0.02702799	0.02809251	0.02828326	0.02819573	0.027901	0.03009297	0.02797451	0.99948011
30.7	0.02703085	0.02809955	0.02828612	0.02820858	0.02790507	0.03011712	0.02798299	0.99947936
30.8	0.02703375	0.02810659	0.02828897	0.02822162	0.02790919	0.03014146	0.02799157	0.99947861
30.9	0.02703668	0.02811363	0.02829179	0.02823486	0.02791336	0.03016598	0.02800026	0.99947787
31	0.02703966	0.02812068	0.02829459	0.0282483	0.0279176	0.03019067	0.02800905	0.99947712
31.1	0.02704267	0.02812774	0.02829738	0.02826194	0.0279219	0.03021555	0.02801795	0.99947638
31.2	0.02704572	0.0281348	0.02830015	0.02827578	0.02792629	0.03024062	0.02802695	0.99947564
31.3	0.02704879	0.02814188	0.0283029	0.02828983	0.02793075	0.03026586	0.02803606	0.99947491
31.4	0.02705187	0.02814897	0.02830564	0.02830408	0.0279353	0.03029129	0.02804528	0.99947417
31.5	0.02705496	0.02815606	0.02830836	0.02831853	0.02793993	0.03031691	0.02805462	0.99947344
31.6	0.02705804	0.02816318	0.02831107	0.02833321	0.02794464	0.03034271	0.02806408	0.9994727
31.7	0.0270611	0.0281703	0.02831376	0.0283481	0.02794942	0.0303687	0.02807365	0.99947197
31.8	0.02706413	0.02817745	0.02831645	0.02836321	0.02795424	0.03039487	0.02808335	0.99947124
31.9	0.02706713	0.02818461	0.02831912	0.02837855	0.02795909	0.03042124	0.02809317	0.99947051
32	0.0270701	0.02819179	0.02832178	0.02839412	0.02796395	0.0304478	0.02810313	0.99946977
32.1	0.02707304	0.02819899	0.02832443	0.02840993	0.0279688	0.03047455	0.02811321	0.99946904
32.2	0.02707595	0.02820621	0.02832707	0.02842598	0.02797363	0.0305015	0.02812344	0.99946831
32.3	0.02707884	0.02821346	0.0283297	0.02844227	0.02797842	0.03052863	0.02813379	0.99946758
32.4	0.02708172	0.02822074	0.02833233	0.02845881	0.02798318	0.03055597	0.0281443	0.99946684
32.5	0.02708457	0.02822804	0.02833494	0.0284756	0.0279879	0.0305835	0.02815494	0.99946611
32.6	0.02708741	0.02823537	0.02833756	0.02849265	0.02799258	0.03061123	0.02816573	0.99946537
32.7	0.02709023	0.02824273	0.02834016	0.02850996	0.02799724	0.03063915	0.02817668	0.99946463
32.8	0.02709303	0.02825012	0.02834276	0.02852753	0.02800187	0.03066728	0.02818777	0.9994639
32.9	0.02709581	0.02825754	0.02834536	0.02854537	0.02800649	0.03069561	0.02819903	0.99946315
33	0.02709858	0.028265	0.02834795	0.02856349	0.0280111	0.03072414	0.02821045	0.99946241
33.1	0.02710132	0.02827249	0.02835055	0.02858188	0.02801569	0.03075287	0.02822203	0.99946166
33.2	0.02710405	0.02828003	0.02835313	0.02860055	0.02802027	0.03078181	0.02823378	0.99946091
33.3	0.02710677	0.0282876	0.02835572	0.02861951	0.02802483	0.03081095	0.02824571	0.99946016
33.4	0.02710946	0.02829521	0.02835831	0.02863876	0.02802937	0.0308403	0.02825781	0.9994594
33.5	0.02711213	0.02830287	0.02836089	0.0286583	0.02803388	0.03086986	0.0282701	0.99945864
33.6	0.02711479	0.02831057	0.02836348	0.02867814	0.02803837	0.03089963	0.02828257	0.99945787
33.7	0.02711743	0.02831831	0.02836607	0.02869829	0.02804284	0.03092961	0.02829523	0.9994571
33.8	0.02712005	0.02832611	0.02836867	0.02871874	0.02804727	0.0309598	0.02830809	0.99945633
33.9	0.02712266	0.02833396	0.02837126	0.02873951	0.02805168	0.03099021	0.02832116	0.99945555
34	0.02712526	0.02834186	0.02837386	0.02876059	0.02805607	0.03102083	0.02833443	0.99945476
34.1	0.02712784	0.02834981	0.02837647	0.028782	0.02806043	0.03105168	0.02834792	0.99945397
34.2	0.02713041	0.02835782	0.02837908	0.02880373	0.02806477	0.03108274	0.02836163	0.99945317
34.3	0.02713297	0.0283659	0.0283817	0.0288258	0.0280691	0.03111402	0.02837557	0.99945237
34.4	0.02713551	0.02837404	0.02838432	0.0288482	0.0280734	0.03114553	0.02838976	0.99945155
34.5	0.02713804	0.02838225	0.02838696	0.02887095	0.02807769	0.03117727	0.0284042	0.99945073
34.6	0.02714055	0.02839053	0.0283896	0.02889404	0.02808197	0.03120924	0.0284189	0.99944991
34.7	0.02714306	0.02839889	0.02839225	0.02891749	0.02808624	0.03124144	0.02843389	0.99944907
34.8	0.02714555	0.02840733	0.02839492	0.0289413	0.02809049	0.03127387	0.02844917	0.99944823
34.9	0.02714803	0.02841586	0.02839759	0.02896547	0.02809474	0.03130654	0.02846475	0.99944738
35	0.0271505	0.02842448	0.02840028	0.02899002	0.02809897	0.03133945	0.02848066	0.99944651
35.1	0.02715296	0.0284332	0.02840298	0.02901494	0.0281032	0.03137261	0.0284969	0.99944564
35.2	0.02715541	0.02844202	0.0284057	0.02904025	0.02810742	0.03140602	0.0285135	0.99944476
35.3	0.02715785	0.02845096	0.02840843	0.02906594	0.02811164	0.03143968	0.02853045	0.99944387
35.4	0.02716028	0.02846003	0.02841117	0.02909204	0.02811585	0.0314736	0.02854776	0.99944297
35.5	0.0271627	0.02846924	0.02841394	0.02911853	0.02812006	0.03150779	0.02856539	0.99944205
35.6	0.02716511	0.0284786	0.02841672	0.02914545	0.02812427	0.03154225	0.02858334	0.99944113
35.7	0.02716751	0.02848813	0.02841952	0.02917279	0.02812847	0.03157698	0.02860157	0.99944019
35.8	0.0271699	0.02849784	0.02842234	0.02920056	0.02813267	0.03161201	0.02862006	0.99943924
35.9	0.02717229	0.02850774	0.02842518	0.02922877	0.02813688	0.03164733	0.02863879	0.99943828
36	0.02717467	0.02851781	0.02842804	0.02925744	0.02814109	0.03168295	0.02865773	0.9994373
36.1	0.02717705	0.02852804	0.02843092	0.02928658	0.02814531	0.03171889	0.02867687	0.99943631
36.2	0.02717941	0.02853843	0.02843383	0.0293162	0.02814953	0.03175515	0.0286962	0.9994353
36.3	0.02718178	0.02854894	0.02843676	0.02934633	0.02815375	0.03179174	0.02871571	0.99943429
36.4	0.02718414	0.02855955	0.02843972	0.02937697	0.02815799	0.03182867	0.02873541	0.99943325
36.5	0.02718649	0.02857022	0.02844271	0.02940816	0.02816224	0.03186594	0.02875529	0.9994322
36.6	0.02718884	0.02858095	0.02844572	0.02943992	0.0281665	0.03190354	0.02877537	0.99943113
36.7	0.02719119	0.02859172	0.02844876	0.02947227	0.02817077	0.03194146	0.02879565	0.99943005
36.8	0.02719354	0.02860251	0.02845183	0.02950524	0.02817506	0.0319797	0.02881615	0.99942895
36.9	0.02719588	0.02861334	0.02845493	0.02953887	0.02817937	0.03201823	0.02883687	0.99942784
37	0.02719822	0.02862421	0.02845807	0.02957317	0.02818369	0.03205704	0.02885781	0.9994267
37.1	0.02720057	0.02863512	0.02846123	0.02960816	0.02818804	0.03209611	0.02887899	0.99942555
37.2	0.02720291	0.02864606	0.02846443	0.02964382	0.02819241	0.03213544	0.02890041	0.99942437
37.3	0.02720525	0.02865704	0.02846767	0.02968013	0.0281968	0.032175	0.02892208	0.99942318
37.4	0.02720759	0.02866806	0.02847094	0.02971703	0.02820122	0.0322148	0.02894401	0.99942197
37.5	0.02720993	0.02867913	0.02847424	0.02975449	0.02820566	0.03225483	0.02896619	0.99942074
37.6	0.02721227	0.02869026	0.02847759	0.02979245	0.02821014	0.03229509	0.02898863	0.99941948
37.7	0.02721461	0.02870144	0.02848098	0.0298309	0.02821464	0.03233557	0.02901133	0.99941821
37.8	0.02721696	0.02871268	0.0284844	0.02986984	0.02821918	0.03237627	0.0290343	0.99941691
37.9	0.0272193	0.02872398	0.02848787	0.02990925	0.02822375	0.0324172	0.02905756	0.99941559
38	0.02722165	0.02873534	0.02849138	0.02994912	0.02822836	0.03245834	0.0290811	0.99941424
38.1	0.02722401	0.02874676	0.02849493	0.02998945	0.02823301	0.03249971	0.02910494	0.99941288
38.2	0.02722637	0.02875823	0.02849853	0.03003025	0.0282377	0.0325413	0.02912909	0.99941148
38.3	0.02722873	0.02876976	0.02850217	0.03007151	0.02824243	0.03258313	0.02915356	0.99941007
38.4	0.0272311	0.02878134	0.02850586	0.03011323	0.0282472	0.03262519	0.02917836	0.99940862
38.5	0.02723347	0.02879297	0.0285096	0.03015542	0.02825202	0.03266748	0.02920352	0.99940715
38.6	0.02723584	0.02880465	0.02851339	0.03019807	0.02825689	0.03271	0.02922903	0.99940566
38.7	0.02723822	0.02881638	0.02851723	0.0302412	0.02826181	0.03275275	0.0292549	0.99940414
38.8	0.02724061	0.02882816	0.02852113	0.03028481	0.02826678	0.03279574	0.02928115	0.99940259
38.9	0.02724301	0.02883999	0.02852507	0.0303289	0.02827181	0.03283897	0.02930776	0.99940101
39	0.02724541	0.02885187	0.02852907	0.03037347	0.02827689	0.03288244	0.02933472	0.9993994
39.1	0.02724782	0.0288638	0.02853313	0.03041852	0.02828203	0.03292614	0.02936203	0.99939776
39.2	0.02725024	0.02887578	0.02853724	0.03046407	0.02828723	0.03297009	0.02938967	0.99939609
39.3	0.02725266	0.02888781	0.02854141	0.03051012	0.02829249	0.03301428	0.02941762	0.99939439
39.4	0.0272551	0.02889989	0.02854565	0.03055666	0.02829782	0.03305871	0.02944587	0.99939266
39.5	0.02725754	0.02891203	0.02854994	0.03060371	0.02830321	0.03310338	0.02947442	0.9993909
39.6	0.02726	0.02892423	0.0285543	0.03065126	0.02830867	0.03314829	0.02950325	0.9993891
39.7	0.02726246	0.02893647	0.02855872	0.03069932	0.0283142	0.03319344	0.02953235	0.99938727
39.8	0.02726494	0.02894878	0.0285632	0.0307479	0.0283198	0.03323883	0.02956173	0.99938541
39.9	0.02726743	0.02896114	0.02856775	0.03079699	0.02832547	0.03328447	0.02959139	0.99938351
40	0.02726993	0.02897356	0.02857237	0.03084661	0.02833122	0.03333035	0.02962133	0.99938157
40.1	0.02727245	0.02898604	0.02857706	0.03089675	0.02833705	0.03337647	0.02965155	0.9993796
40.2	0.02727499	0.02899857	0.02858182	0.03094742	0.02834295	0.03342283	0.02968207	0.9993776
40.3	0.02727754	0.02901117	0.02858665	0.03099862	0.02834894	0.03346944	0.02971288	0.99937555
40.4	0.02728011	0.02902382	0.02859156	0.03105036	0.02835502	0.0335163	0.029744	0.99937347
40.5	0.0272827	0.02903654	0.02859654	0.03110264	0.02836118	0.0335634	0.02977542	0.99937134
40.6	0.02728532	0.02904931	0.0286016	0.03115546	0.02836742	0.03361075	0.02980716	0.99936918
40.7	0.02728796	0.02906215	0.02860673	0.03120883	0.02837376	0.03365834	0.02983922	0.99936698
40.8	0.02729064	0.02907504	0.02861195	0.03126275	0.02838019	0.03370619	0.02987161	0.99936474
40.9	0.02729335	0.029088	0.02861725	0.03131723	0.02838671	0.03375428	0.02990433	0.99936245
41	0.0272961	0.02910103	0.02862263	0.03137226	0.02839333	0.03380263	0.02993739	0.99936012
41.1	0.02729889	0.02911411	0.02862809	0.03142786	0.02840004	0.03385123	0.02997079	0.99935775
41.2	0.02730174	0.02912726	0.02863364	0.03148402	0.02840686	0.03390008	0.03000454	0.99935533
41.3	0.02730465	0.02914048	0.02863928	0.03154075	0.02841378	0.03394919	0.03003866	0.99935287
41.4	0.02730761	0.02915376	0.02864501	0.03159805	0.0284208	0.03399855	0.03007315	0.99935037
41.5	0.02731063	0.02916711	0.02865082	0.03165593	0.02842792	0.03404816	0.03010803	0.99934781
41.6	0.02731371	0.02918053	0.02865673	0.03171439	0.02843516	0.03409804	0.0301433	0.99934521
41.7	0.02731683	0.02919401	0.02866274	0.03177343	0.0284425	0.03414817	0.03017898	0.99934256
41.8	0.02732	0.02920756	0.02866884	0.03183306	0.02844996	0.03419856	0.03021509	0.99933987
41.9	0.02732321	0.02922119	0.02867503	0.03189328	0.02845753	0.03424921	0.03025163	0.99933712
42	0.02732646	0.02923488	0.02868133	0.03195409	0.02846521	0.03430012	0.03028863	0.99933432
42.1	0.02732975	0.02924864	0.02868772	0.03201551	0.02847301	0.0343513	0.0303261	0.99933147
42.2	0.02733308	0.02926248	0.02869422	0.03207752	0.02848093	0.03440273	0.03036405	0.99932857
42.3	0.02733647	0.02927638	0.02870082	0.03214013	0.02848898	0.03445443	0.03040248	0.99932562
42.4	0.0273399	0.02929036	0.02870753	0.03220336	0.02849714	0.0345064	0.03044135	0.99932261
42.5	0.02734338	0.02930442	0.02871434	0.03226719	0.02850544	0.03455862	0.03048064	0.99931955
42.6	0.02734693	0.02931855	0.02872127	0.03233164	0.02851386	0.03461112	0.03052032	0.99931643
42.7	0.02735055	0.02933275	0.0287283	0.03239671	0.0285224	0.03466388	0.03056035	0.99931326
42.8	0.02735425	0.02934704	0.02873545	0.0324624	0.02853109	0.03471691	0.03060071	0.99931003
42.9	0.02735804	0.0293614	0.02874271	0.03252872	0.0285399	0.03477021	0.03064134	0.99930674
43	0.02736192	0.02937584	0.02875009	0.03259567	0.02854885	0.03482378	0.03068222	0.99930339
43.1	0.02736593	0.02939035	0.02875758	0.03266325	0.02855794	0.03487762	0.03072335	0.99929998
43.2	0.02737006	0.02940495	0.0287652	0.03273147	0.02856717	0.03493173	0.03076472	0.99929651
43.3	0.02737433	0.02941963	0.02877293	0.03280032	0.02857654	0.03498612	0.03080632	0.99929298
43.4	0.02737875	0.02943439	0.02878079	0.03286983	0.02858606	0.03504077	0.03084815	0.99928939
43.5	0.02738333	0.02944923	0.02878877	0.03293998	0.02859572	0.0350957	0.03089022	0.99928573
43.6	0.02738807	0.02946416	0.02879689	0.03301077	0.02860553	0.03515091	0.03093254	0.99928201
43.7	0.02739298	0.02947918	0.02880512	0.03308223	0.02861549	0.03520638	0.03097511	0.99927823
43.8	0.02739803	0.02949427	0.0288135	0.03315434	0.0286256	0.03526214	0.03101793	0.99927437
43.9	0.02740321	0.02950946	0.028822	0.03322711	0.02863587	0.03531817	0.03106102	0.99927045
44	0.02740848	0.02952473	0.02883064	0.03330055	0.0286463	0.03537448	0.03110438	0.99926646
44.1	0.02741382	0.02954009	0.02883941	0.03337466	0.02865689	0.03543107	0.03114801	0.99926241
44.2	0.02741921	0.02955554	0.02884832	0.03344944	0.02866765	0.03548794	0.0311919	0.99925828
44.3	0.02742465	0.02957108	0.02885737	0.0335249	0.02867857	0.03554509	0.03123606	0.99925408
44.4	0.02743012	0.02958672	0.02886657	0.03360103	0.02868966	0.03560251	0.0312805	0.99924981
44.5	0.02743562	0.02960244	0.0288759	0.03367785	0.02870092	0.03566022	0.03132521	0.99924546
44.6	0.02744114	0.02961826	0.02888539	0.03375535	0.02871236	0.03571821	0.03137019	0.99924104
44.7	0.02744669	0.02963417	0.02889502	0.03383354	0.02872398	0.03577648	0.03141546	0.99923655
44.8	0.02745225	0.02965018	0.0289048	0.03391243	0.02873578	0.03583504	0.031461	0.99923198
44.9	0.02745784	0.02966628	0.02891473	0.03399202	0.02874776	0.03589388	0.03150683	0.99922733
45	0.02746345	0.02968248	0.02892482	0.0340723	0.02875993	0.035953	0.03155294	0.9992226
45.1	0.02746908	0.02969878	0.02893506	0.03415329	0.02877229	0.03601241	0.03159933	0.9992178
45.2	0.02747474	0.02971518	0.02894546	0.03423498	0.02878484	0.0360721	0.03164601	0.99921291
45.3	0.02748042	0.02973168	0.02895602	0.03431739	0.02879759	0.03613208	0.03169298	0.99920794
45.4	0.02748613	0.02974828	0.02896674	0.03440051	0.02881055	0.03619235	0.03174024	0.99920289
45.5	0.02749187	0.02976498	0.02897763	0.03448436	0.02882372	0.0362529	0.0317878	0.99919776
45.6	0.02749764	0.02978178	0.02898867	0.03456892	0.02883711	0.03631374	0.03183564	0.99919254
45.7	0.02750344	0.02979869	0.02899989	0.03465421	0.02885073	0.03637487	0.03188379	0.99918723
45.8	0.02750928	0.0298157	0.02901127	0.03474024	0.02886458	0.03643628	0.03193223	0.99918184
45.9	0.02751515	0.02983282	0.02902283	0.03482699	0.02887867	0.03649799	0.03198098	0.99917636
46	0.02752107	0.02985005	0.02903456	0.03491449	0.02889301	0.03655999	0.03203003	0.99917079
46.1	0.02752702	0.02986738	0.02904647	0.03500272	0.02890761	0.03662227	0.03207939	0.99916513
46.2	0.02753301	0.02988482	0.02905855	0.0350917	0.02892247	0.03668485	0.03212905	0.99915938
46.3	0.02753905	0.02990237	0.02907081	0.03518143	0.0289376	0.03674772	0.03217903	0.99915353
46.4	0.02754513	0.02992003	0.02908325	0.03527191	0.02895297	0.03681088	0.03222933	0.99914759
46.5	0.02755126	0.0299378	0.02909587	0.03536315	0.0289686	0.03687434	0.03227994	0.99914156
46.6	0.02755745	0.02995568	0.02910868	0.03545514	0.02898446	0.03693809	0.03233087	0.99913543
46.7	0.02756369	0.02997367	0.02912168	0.0355479	0.02900056	0.03700213	0.03238212	0.9991292
46.8	0.02757	0.02999178	0.02913486	0.03564142	0.02901686	0.03706646	0.0324337	0.99912287
46.9	0.02757637	0.03001	0.02914824	0.03573572	0.02903337	0.03713109	0.03248561	0.99911645
47	0.02758281	0.03002833	0.02916181	0.03583078	0.02905008	0.03719602	0.03253785	0.99910992
47.1	0.02758932	0.03004678	0.02917557	0.03592663	0.02906699	0.03726124	0.03259043	0.99910329
47.2	0.02759589	0.03006535	0.02918953	0.03602326	0.02908408	0.03732675	0.03264335	0.99909656
47.3	0.02760253	0.03008404	0.02920369	0.03612068	0.02910137	0.03739257	0.03269662	0.99908972
47.4	0.02760923	0.03010284	0.02921805	0.03621888	0.02911884	0.03745867	0.03275024	0.99908278
47.5	0.02761599	0.03012175	0.02923261	0.03631789	0.0291365	0.03752508	0.03280422	0.99907573
47.6	0.02762279	0.03014079	0.02924737	0.03641769	0.02915436	0.03759178	0.03285856	0.99906857
47.7	0.02762964	0.03015994	0.02926234	0.0365183	0.02917242	0.03765878	0.03291328	0.9990613
47.8	0.02763653	0.03017921	0.02927752	0.03661972	0.02919068	0.03772607	0.03296837	0.99905392
47.9	0.02764347	0.0301986	0.02929291	0.03672195	0.02920915	0.03779367	0.03302386	0.99904642
48	0.02765043	0.03021811	0.02930851	0.036825	0.02922782	0.03786156	0.03307974	0.99903882
48.1	0.02765743	0.03023775	0.02932433	0.03692887	0.02924672	0.03792975	0.03313602	0.9990311
48.2	0.02766446	0.03025751	0.02934036	0.03703357	0.02926582	0.03799824	0.03319271	0.99902326
48.3	0.0276715	0.0302774	0.02935661	0.0371391	0.02928514	0.03806703	0.0332498	0.99901531
48.4	0.02767857	0.03029742	0.02937308	0.03724547	0.02930467	0.03813612	0.03330729	0.99900723
48.5	0.02768567	0.03031758	0.02938977	0.03735268	0.02932441	0.03820551	0.03336517	0.99899904
48.6	0.02769279	0.03033788	0.02940669	0.03746074	0.02934437	0.03827519	0.03342343	0.99899072
48.7	0.02769993	0.03035832	0.02942383	0.03756964	0.02936454	0.03834518	0.03348205	0.99898229
48.8	0.0277071	0.03037892	0.02944119	0.03767941	0.02938492	0.03841547	0.03354104	0.99897372
48.9	0.02771429	0.03039966	0.02945879	0.03779003	0.02940552	0.03848605	0.03360037	0.99896504
49	0.02772149	0.03042057	0.02947661	0.03790153	0.02942633	0.03855694	0.03366004	0.99895622
49.1	0.02772872	0.03044162	0.02949467	0.0380139	0.02944737	0.03862812	0.03372005	0.99894728
49.2	0.02773597	0.03046283	0.02951297	0.03812714	0.02946862	0.03869961	0.03378039	0.99893821
49.3	0.02774325	0.0304842	0.0295315	0.03824125	0.02949009	0.0387714	0.03384108	0.99892901
49.4	0.02775055	0.03050572	0.02955027	0.03835624	0.02951178	0.03884348	0.03390211	0.99891967
49.5	0.02775788	0.0305274	0.02956928	0.0384721	0.0295337	0.03891587	0.03396348	0.9989102
49.6	0.02776524	0.03054923	0.02958853	0.03858884	0.02955583	0.03898855	0.03402519	0.9989006
49.7	0.02777263	0.03057122	0.02960802	0.03870644	0.02957819	0.03906154	0.03408726	0.99889085
49.8	0.02778005	0.03059337	0.02962776	0.03882491	0.02960078	0.03913483	0.03414967	0.99888097
49.9	0.0277875	0.03061567	0.02964775	0.03894424	0.02962359	0.03920842	0.03421244	0.99887095
50	0.027795	0.03063814	0.02966798	0.03906444	0.02964663	0.0392823	0.03427555	0.99886079
50.1	0.02780253	0.03066076	0.02968847	0.03918551	0.02966989	0.03935649	0.03433903	0.99885049
50.2	0.0278101	0.03068355	0.02970921	0.03930745	0.02969339	0.03943098	0.03440285	0.99884004
50.3	0.02781772	0.0307065	0.0297302	0.03943026	0.02971712	0.03950577	0.03446704	0.99882945
50.4	0.0278254	0.03072961	0.02975145	0.03955394	0.02974108	0.03958086	0.03453158	0.9988187
50.5	0.02783312	0.03075289	0.02977296	0.03967849	0.02976527	0.03965626	0.03459648	0.99880781
50.6	0.02784089	0.03077633	0.02979473	0.03980391	0.0297897	0.03973195	0.03466173	0.99879678
50.7	0.0278487	0.03079994	0.02981675	0.03993022	0.02981436	0.03980794	0.03472735	0.99878558
50.8	0.02785656	0.03082372	0.02983904	0.04005742	0.02983926	0.03988423	0.03479333	0.99877424
50.9	0.02786447	0.03084766	0.02986159	0.04018549	0.02986439	0.03996082	0.03485966	0.99876274
51	0.02787241	0.03087178	0.02988441	0.04031446	0.02988977	0.04003772	0.03492636	0.99875108
51.1	0.02788039	0.03089607	0.0299075	0.04044431	0.02991538	0.04011491	0.03499343	0.99873927
51.2	0.02788842	0.03092053	0.02993085	0.04057507	0.02994123	0.0401924	0.03506085	0.9987273
51.3	0.02789648	0.03094516	0.02995448	0.04070671	0.02996733	0.04027019	0.03512865	0.99871517
51.4	0.02790458	0.03096997	0.02997837	0.04083926	0.02999366	0.04034828	0.03519681	0.99870287
51.5	0.02791272	0.03099495	0.03000254	0.04097272	0.03002024	0.04042667	0.03526534	0.99869041
51.6	0.02792091	0.03102011	0.03002699	0.04110708	0.03004706	0.04050536	0.03533423	0.99867779
51.7	0.02792913	0.03104545	0.03005171	0.04124235	0.03007413	0.04058435	0.0354035	0.99866499
51.8	0.0279374	0.03107096	0.03007671	0.04137853	0.03010144	0.04066363	0.03547314	0.99865204
51.9	0.02794571	0.03109666	0.03010199	0.04151563	0.030129	0.04074322	0.03554315	0.99863891
52	0.02795407	0.03112254	0.03012754	0.04165364	0.03015681	0.0408231	0.03561354	0.9986256
52.1	0.02796247	0.0311486	0.03015339	0.04179258	0.03018486	0.04090328	0.0356843	0.99861213
52.2	0.02797091	0.03117484	0.03017951	0.04193244	0.03021317	0.04098376	0.03575544	0.99859848
52.3	0.02797941	0.03120128	0.03020592	0.04207322	0.03024172	0.04106454	0.03582695	0.99858465
52.4	0.02798794	0.0312279	0.03023261	0.04221494	0.03027053	0.04114561	0.03589884	0.99857065
52.5	0.02799653	0.03125472	0.0302596	0.04235758	0.03029959	0.04122698	0.03597112	0.99855647
52.6	0.02800516	0.03128174	0.03028687	0.04250117	0.0303289	0.04130865	0.03604377	0.9985421
52.7	0.02801384	0.03130896	0.03031443	0.04264568	0.03035846	0.04139061	0.0361168	0.99852755
52.8	0.02802256	0.0313364	0.03034228	0.04279114	0.03038828	0.04147287	0.03619022	0.99851282
52.9	0.02803134	0.03136404	0.03037043	0.04293754	0.03041836	0.04155542	0.03626402	0.99849791
53	0.02804016	0.03139191	0.03039886	0.04308489	0.03044869	0.04163826	0.03633821	0.9984828
53.1	0.02804904	0.03142	0.0304276	0.04323318	0.03047928	0.04172141	0.03641278	0.99846751
53.2	0.02805796	0.0314483	0.03045663	0.04338242	0.03051013	0.04180484	0.03648774	0.99845202
53.3	0.02806694	0.03147681	0.03048595	0.04353261	0.03054124	0.04188857	0.0365631	0.99843634
53.4	0.02807597	0.03150553	0.03051558	0.04368376	0.0305726	0.04197259	0.03663884	0.99842047
53.5	0.02808505	0.03153446	0.0305455	0.04383586	0.03060423	0.0420569	0.03671498	0.9984044
53.6	0.02809419	0.03156359	0.03057573	0.04398893	0.03063612	0.04214151	0.03679152	0.99838813
53.7	0.02810337	0.03159292	0.03060625	0.04414295	0.03066827	0.04222641	0.03686845	0.99837167
53.8	0.02811262	0.03162244	0.03063708	0.04429794	0.03070069	0.0423116	0.03694578	0.998355
53.9	0.02812191	0.03165216	0.03066822	0.0444539	0.03073336	0.04239708	0.03702351	0.99833813
54	0.02813126	0.03168209	0.03069966	0.04461082	0.03076631	0.04248285	0.03710165	0.99832106
54.1	0.02814067	0.03171221	0.0307314	0.04476871	0.03079952	0.04256891	0.03718019	0.99830378
54.2	0.02815014	0.03174253	0.03076345	0.04492758	0.030833	0.04265526	0.03725914	0.9982863
54.3	0.02815966	0.03177306	0.03079581	0.04508742	0.03086674	0.0427419	0.03733851	0.9982686
54.4	0.02816923	0.03180379	0.03082849	0.04524824	0.03090076	0.04282882	0.0374183	0.9982507
54.5	0.02817887	0.03183472	0.03086147	0.04541003	0.03093504	0.04291604	0.03749851	0.99823258
54.6	0.02818857	0.03186586	0.03089476	0.04557281	0.03096959	0.04300354	0.03757915	0.99821425
54.7	0.02819832	0.0318972	0.03092837	0.04573657	0.03100442	0.04309133	0.03766023	0.9981957
54.8	0.02820814	0.03192875	0.03096229	0.04590132	0.03103952	0.0431794	0.03774174	0.99817694
54.9	0.02821801	0.03196051	0.03099653	0.04606705	0.03107489	0.04326776	0.03782369	0.99815795
55	0.02822795	0.03199247	0.03103108	0.04623377	0.03111053	0.0433564	0.03790609	0.99813875
55.1	0.02823795	0.03202465	0.03106595	0.04640149	0.03114645	0.04344533	0.03798895	0.99811933
55.2	0.02824801	0.03205703	0.03110115	0.04657019	0.03118265	0.04353454	0.03807226	0.99809968
55.3	0.02825813	0.03208963	0.03113666	0.04673989	0.03121912	0.04362403	0.03815602	0.9980798
55.4	0.02826832	0.03212244	0.0311725	0.04691059	0.03125587	0.0437138	0.03824024	0.9980597
55.5	0.02827857	0.03215546	0.03120866	0.04708229	0.0312929	0.04380386	0.03832493	0.99803937
55.6	0.02828889	0.0321887	0.03124514	0.04725498	0.0313302	0.04389419	0.03841008	0.99801882
55.7	0.02829927	0.03222215	0.03128196	0.04742868	0.03136779	0.04398481	0.0384957	0.99799803
55.8	0.02830972	0.03225582	0.0313191	0.04760339	0.03140566	0.0440757	0.03858179	0.99797701
55.9	0.02832023	0.03228971	0.03135659	0.04777912	0.03144381	0.04416687	0.03866836	0.99795576
56	0.02833081	0.03232382	0.03139441	0.04795588	0.03148225	0.04425832	0.03875541	0.99793427
56.1	0.02834146	0.03235815	0.03143256	0.04813368	0.03152097	0.04435005	0.03884295	0.99791254
56.2	0.02835217	0.03239271	0.03147107	0.04831253	0.03155997	0.04444205	0.03893097	0.99789058
56.3	0.02836296	0.03242749	0.03150992	0.04849242	0.03159926	0.04453433	0.03901949	0.99786838
56.4	0.02837381	0.0324625	0.03154912	0.04867337	0.03163884	0.04462688	0.03910849	0.99784594
56.5	0.02838473	0.03249774	0.03158869	0.04885539	0.03167871	0.04471971	0.03919798	0.99782326
56.6	0.02839572	0.03253321	0.03162862	0.04903847	0.03171886	0.04481281	0.03928794	0.99780034
56.7	0.02840679	0.03256891	0.03166891	0.04922263	0.03175931	0.04490618	0.03937832	0.99777716
56.8	0.02841792	0.03260486	0.03170959	0.04940788	0.03180004	0.04499982	0.0394691	0.99775375
56.9	0.02842913	0.03264105	0.03175065	0.04959421	0.03184107	0.04509373	0.03956024	0.99773008
57	0.0284404	0.03267748	0.03179209	0.04978164	0.03188239	0.04518791	0.03965171	0.99770616
57.1	0.02845175	0.03271416	0.03183392	0.04997019	0.03192401	0.04528236	0.0397435	0.99768198
57.2	0.02846318	0.0327511	0.03187613	0.05015984	0.03196592	0.04537707	0.0398356	0.99765754
57.3	0.02847468	0.03278829	0.03191872	0.05035062	0.03200813	0.04547206	0.03992802	0.99763283
57.4	0.02848625	0.03282576	0.03196168	0.05054254	0.03205063	0.0455673	0.04002072	0.99760784
57.5	0.0284979	0.03286351	0.032005	0.05073565	0.03209344	0.04566282	0.04011371	0.99758256
57.6	0.02850962	0.03290154	0.03204867	0.05092995	0.03213655	0.0457586	0.04020698	0.997557
57.7	0.02852142	0.03293986	0.03209268	0.0511255	0.03217996	0.04585464	0.04030054	0.99753113
57.8	0.0285333	0.03297848	0.03213703	0.05132233	0.03222367	0.04595094	0.04039438	0.99750496
57.9	0.02854525	0.03301739	0.03218171	0.05152049	0.03226769	0.0460475	0.04048885	0.99747847
58	0.02855728	0.03305659	0.03222673	0.05172007	0.03231202	0.04614433	0.0405829	0.99745165
58.1	0.02856939	0.03309607	0.03227208	0.05192115	0.03235665	0.04624141	0.04067758	0.99742451
58.2	0.02858157	0.03313582	0.03231777	0.05212379	0.0324016	0.04633875	0.04077254	0.99739703
58.3	0.02859384	0.03317583	0.03236379	0.0523281	0.03244686	0.04643635	0.04086779	0.9973692
58.4	0.02860618	0.0332161	0.03241014	0.0525342	0.03249243	0.04653421	0.04096331	0.99734103
58.5	0.0286186	0.03325661	0.03245683	0.05274217	0.03253832	0.04663232	0.04105912	0.9973125
58.6	0.02863111	0.03329737	0.03250387	0.05295204	0.03258453	0.04673068	0.04115521	0.99728361
58.7	0.02864369	0.03333839	0.03255124	0.05316372	0.03263107	0.0468293	0.04125159	0.99725435
58.8	0.02865636	0.03337966	0.03259896	0.05337702	0.03267793	0.04692817	0.04134824	0.99722471
58.9	0.02866911	0.0334212	0.03264702	0.05359168	0.03272512	0.04702729	0.04144518	0.99719466
59	0.02868194	0.033463	0.03269543	0.05380745	0.03277264	0.04712667	0.0415424	0.99716418
59.1	0.02869485	0.03350509	0.03274418	0.0540241	0.0328205	0.04722629	0.0416399	0.99713328
59.2	0.02870785	0.03354747	0.03279328	0.05424144	0.03286687	0.04732615	0.04173768	0.99710192
59.3	0.02872092	0.03359015	0.03284273	0.05445928	0.03291725	0.04742627	0.04183573	0.99707009
59.4	0.02873409	0.03363314	0.03289253	0.05467757	0.03296615	0.04752663	0.04193407	0.99703776
59.5	0.02874734	0.03367645	0.03294268	0.05489627	0.03301541	0.04762724	0.04203269	0.9970049
59.6	0.02876067	0.03372009	0.03299317	0.05511532	0.03306504	0.04772809	0.04213158	0.9969715
59.7	0.02877409	0.03376408	0.03304402	0.05533471	0.03311504	0.04782918	0.04223075	0.99693751
59.8	0.02878759	0.03380842	0.03309522	0.05555448	0.03316542	0.04793051	0.0423302	0.99690292
59.9	0.02880118	0.0338531	0.03314677	0.05577462	0.03321617	0.04803209	0.04242993	0.9968677

60	0.02881486	0.0338981	0.03319868	0.05599514	0.03326731	0.0481339	0.04252993	0.99683184
60.1	0.02882862	0.03394339	0.03325094	0.05621605	0.03331882	0.04823595	0.04263021	0.99679535
60.2	0.02884247	0.03398895	0.03330356	0.05643738	0.03337071	0.04833824	0.04273077	0.99675828
60.3	0.02885641	0.03403476	0.03335654	0.05665912	0.03342296	0.04844076	0.0428316	0.99672066
60.4	0.02887043	0.03408078	0.03340988	0.05688127	0.03347557	0.04854352	0.04293271	0.99668258
60.5	0.02888455	0.03412701	0.03346357	0.05710384	0.03352853	0.04864652	0.04303409	0.99664408
60.6	0.02889875	0.03417344	0.03351763	0.05732683	0.03358183	0.04874974	0.04313575	0.9966052
60.7	0.02891304	0.03422007	0.03357205	0.05755023	0.03363547	0.0488532	0.04323769	0.99656596
60.8	0.02892741	0.03426689	0.03362684	0.05777406	0.03368944	0.04895688	0.04333989	0.9965264
60.9	0.02894187	0.0343139	0.03368199	0.05799831	0.03374373	0.0490608	0.04344237	0.99648656
61	0.02895643	0.03436111	0.0337375	0.05822299	0.03379833	0.04916494	0.04354513	0.99644647
61.1	0.02897106	0.03440851	0.03379339	0.05844809	0.03385325	0.04926931	0.04364815	0.99640615
61.2	0.02898579	0.03445611	0.03384964	0.05867362	0.03390849	0.0493739	0.04375145	0.99636558
61.3	0.0290006	0.0345039	0.03390626	0.05889958	0.03396404	0.04947871	0.04385502	0.99632477
61.4	0.02901549	0.03455188	0.03396325	0.05912597	0.03401991	0.04958375	0.04395885	0.99628372
61.5	0.02903047	0.03460006	0.03402062	0.0593528	0.03407608	0.04968901	0.04406296	0.99624243
61.6	0.02904554	0.03464844	0.03407835	0.05958006	0.03413257	0.04979449	0.04416733	0.99620091
61.7	0.02906068	0.03469705	0.03413646	0.05980776	0.03418937	0.04990019	0.04427197	0.99615913
61.8	0.02907591	0.03474588	0.03419495	0.06003591	0.03424649	0.05000611	0.04437688	0.9961171
61.9	0.02909123	0.03479494	0.03425382	0.06026449	0.03430392	0.05011224	0.04448205	0.99607482
62	0.02910662	0.03484423	0.03431306	0.06049353	0.03436166	0.05021858	0.04458749	0.99603228
62.1	0.02912209	0.03489376	0.03437268	0.06072301	0.03441972	0.05032515	0.0446932	0.99598947
62.2	0.02913765	0.03494353	0.03443268	0.06095294	0.0344781	0.05043192	0.04479917	0.99594639
62.3	0.02915328	0.03499357	0.03449306	0.06118333	0.0345368	0.0505389	0.0449054	0.99590305
62.4	0.029169	0.03504387	0.03455382	0.06141417	0.03459582	0.0506461	0.0450119	0.99585944
62.5	0.0291848	0.03509446	0.03461497	0.06164547	0.03465516	0.0507535	0.04511866	0.99581556
62.6	0.0292007	0.03514535	0.03467651	0.06187724	0.03471481	0.05086111	0.04522568	0.9957714
62.7	0.02921669	0.03519657	0.03473843	0.06210948	0.03477479	0.05096893	0.04533296	0.99572697
62.8	0.02923279	0.03524811	0.03480073	0.06234219	0.03483509	0.05107695	0.0454405	0.99568227
62.9	0.029249	0.03530001	0.03486343	0.06257538	0.03489571	0.05118517	0.0455483	0.99563729
63	0.02926532	0.03535225	0.03492651	0.06280905	0.03495665	0.0512936	0.04565636	0.99559203
63.1	0.02928177	0.03540487	0.03498999	0.0630432	0.03501791	0.05140223	0.04576468	0.99554649
63.2	0.02929834	0.03545788	0.03505385	0.06327785	0.03507949	0.05151105	0.04587326	0.99550066
63.3	0.02931503	0.03551127	0.03511811	0.06351301	0.0351414	0.05162008	0.04598209	0.99545456
63.4	0.02933185	0.03556504	0.03518277	0.06374866	0.03520363	0.0517293	0.04609118	0.99540816
63.5	0.02934881	0.03561914	0.03524782	0.06398483	0.03526618	0.05183871	0.04620053	0.99536148
63.6	0.02936591	0.03567352	0.03531326	0.06422152	0.03532905	0.05194832	0.04631013	0.99531451
63.7	0.02938315	0.03572812	0.0353791	0.06445871	0.03539225	0.05205813	0.04641998	0.99526725
63.8	0.02940053	0.03578292	0.03544534	0.06469641	0.03545577	0.05216812	0.04653008	0.99521969
63.9	0.02941805	0.03583787	0.03551198	0.06493461	0.03551962	0.05227831	0.04664044	0.99517184
64	0.02943572	0.03589296	0.03557903	0.0651733	0.03558379	0.05238868	0.04675105	0.99512369
64.1	0.02945354	0.03594816	0.03564647	0.06541247	0.03564828	0.05249924	0.04686191	0.99507525
64.2	0.02947152	0.03600347	0.03571431	0.06565211	0.0357131	0.05260999	0.04697302	0.99502651
64.3	0.02948964	0.03605889	0.03578256	0.06589221	0.03577824	0.05272092	0.04708438	0.99497747
64.4	0.02950793	0.03611442	0.03585122	0.06613278	0.03584371	0.05283204	0.04719598	0.99492812
64.5	0.02952638	0.03617006	0.03592028	0.06637381	0.0359095	0.05294333	0.04730784	0.99487848
64.6	0.02954499	0.03622582	0.03598975	0.06661531	0.03597561	0.05305481	0.04741994	0.99482853
64.7	0.02956377	0.0362817	0.03605963	0.06685726	0.03604205	0.05316647	0.04753229	0.99477828
64.8	0.02958273	0.03633769	0.03612992	0.06709968	0.03610882	0.0532783	0.04764488	0.99472772
64.9	0.02960186	0.03639381	0.03620062	0.06734257	0.03617591	0.05339031	0.04775771	0.99467685
65	0.02962118	0.03645004	0.03627173	0.06758591	0.03624332	0.0535025	0.04787079	0.99462568
65.1	0.02964068	0.03650639	0.03634325	0.06782972	0.03631106	0.05361485	0.04798411	0.99457419
65.2	0.02966038	0.03656287	0.03641519	0.06807399	0.03637913	0.05372738	0.04809768	0.99452239
65.3	0.02968028	0.03661946	0.03648755	0.06831873	0.03644752	0.05384008	0.04821148	0.99447027
65.4	0.02970038	0.03667617	0.03656032	0.06856393	0.03651623	0.05395295	0.04832552	0.99441784
65.5	0.0297207	0.03673299	0.03663351	0.06880959	0.03658527	0.05406599	0.04843981	0.9943651
65.6	0.02974124	0.03678994	0.03670712	0.06905572	0.03665464	0.05417919	0.04855432	0.99431203
65.7	0.02976201	0.03684701	0.03678115	0.06930232	0.03672433	0.05429255	0.04866908	0.99425865
65.8	0.02978301	0.03690421	0.0368556	0.06954938	0.03679434	0.05440608	0.04878407	0.99420494
65.9	0.02980425	0.03696153	0.03693048	0.06979692	0.03686468	0.05451977	0.0488993	0.99415091
66	0.02982572	0.03701898	0.03700577	0.07004492	0.03693534	0.05463362	0.04901476	0.99409656
66.1	0.02984742	0.03707656	0.03708149	0.07029339	0.03700633	0.05474763	0.04913045	0.99404189
66.2	0.02986933	0.03713427	0.03715764	0.07054232	0.03707765	0.0548618	0.04924638	0.99398688
66.3	0.02989144	0.03719211	0.03723421	0.07079173	0.03714929	0.05497612	0.04936253	0.99393155
66.4	0.02991375	0.03725007	0.03731121	0.07104161	0.03722125	0.05509059	0.04947891	0.99387589
66.5	0.02993623	0.03730816	0.03738864	0.07129196	0.03729354	0.05520522	0.04959553	0.99381989
66.6	0.02995889	0.03736637	0.0374665	0.07154278	0.03736615	0.05532	0.04971237	0.99376357
66.7	0.02998172	0.0374247	0.03754479	0.07179407	0.03743908	0.05543493	0.04982944	0.99370691
66.8	0.03000471	0.03748316	0.03762351	0.07204583	0.03751234	0.05555	0.04994673	0.99364991
66.9	0.03002785	0.03754174	0.03770266	0.07229807	0.03758593	0.05566522	0.05006424	0.99359258
67	0.03005113	0.03760044	0.03778225	0.07255078	0.03765984	0.05578059	0.05018199	0.99353491
67.1	0.03007454	0.03765926	0.03786227	0.07280396	0.03773407	0.0558961	0.05029995	0.9934769
67.2	0.03009809	0.0377182	0.03794273	0.07305761	0.03780862	0.05601175	0.05041813	0.99341854
67.3	0.03012177	0.03777725	0.03802363	0.07331174	0.0378835	0.05612755	0.05053654	0.99335985
67.4	0.03014561	0.03783642	0.03810497	0.07356634	0.0379587	0.05624348	0.05065516	0.99330081
67.5	0.03016958	0.03789569	0.03818674	0.07382142	0.03803422	0.05635955	0.050774	0.99324142
67.6	0.03019371	0.03795508	0.03826896	0.07407697	0.03811006	0.05647575	0.05089306	0.99318169
67.7	0.03021798	0.03801458	0.03835162	0.07433299	0.03818623	0.05659209	0.05101233	0.99312161
67.8	0.03024239	0.03807419	0.03843473	0.07458949	0.03826272	0.05670856	0.05113182	0.99306117
67.9	0.03026696	0.03813392	0.03851828	0.07484647	0.03833953	0.05682516	0.05125152	0.99300039
68	0.03029168	0.03819376	0.03860228	0.07510392	0.03841666	0.0569419	0.05137144	0.99293925
68.1	0.03031655	0.03825373	0.03868672	0.07536185	0.03849411	0.05705876	0.05149157	0.99287775
68.2	0.03034156	0.03831382	0.03877162	0.07562025	0.03857188	0.05717574	0.0516119	0.9928159
68.3	0.0303667	0.03837403	0.03885697	0.07587913	0.03864998	0.05729285	0.05173245	0.99275369
68.4	0.03039197	0.03843436	0.03894278	0.07613849	0.03872839	0.05741009	0.05185321	0.99269113
68.5	0.03041736	0.03849482	0.03902904	0.07639833	0.03880712	0.05752744	0.05197417	0.9926282
68.6	0.03044288	0.0385554	0.03911575	0.07665864	0.03888618	0.05764492	0.05209534	0.9925649
68.7	0.03046853	0.03861612	0.03920293	0.07691943	0.03896555	0.05776251	0.05221671	0.99250125
68.8	0.03049432	0.03867696	0.03929058	0.0771807	0.03904524	0.05788023	0.05233829	0.99243722
68.9	0.03052024	0.03873793	0.03937869	0.07744245	0.03912525	0.05799805	0.05246007	0.99237283
69	0.03054631	0.03879902	0.03946728	0.07770467	0.03920557	0.05811599	0.05258206	0.99230808
69.1	0.03057251	0.03886025	0.03955635	0.07796737	0.03928622	0.05823404	0.05270424	0.99224295
69.2	0.03059887	0.03892161	0.0396459	0.07823056	0.03936718	0.05835221	0.05282663	0.99217744
69.3	0.03062536	0.03898309	0.03973594	0.07849422	0.03944846	0.05847048	0.05294921	0.99211157
69.4	0.030652	0.03904471	0.03982649	0.07875836	0.03953006	0.05858886	0.05307199	0.99204532
69.5	0.03067879	0.03910645	0.03991754	0.07902298	0.03961197	0.05870734	0.05319496	0.99197869
69.6	0.03070572	0.03916832	0.0400091	0.07928807	0.0396942	0.05882593	0.05331814	0.99191169
69.7	0.03073281	0.03923032	0.04010117	0.07955365	0.03977674	0.05894462	0.0534415	0.9918443
69.8	0.03076005	0.03929245	0.04019374	0.07981971	0.0398596	0.05906341	0.05356506	0.99177654
69.9	0.03078744	0.03935471	0.04028681	0.08008624	0.03994277	0.0591823	0.05368882	0.99170839
70	0.03081499	0.0394171	0.04038035	0.08035326	0.04002626	0.05930129	0.05381276	0.99163985
70.1	0.03084269	0.03947961	0.04047437	0.08062076	0.04011006	0.05942037	0.05393689	0.99157093
70.2	0.03087056	0.03954226	0.04056885	0.08088874	0.04019418	0.05953955	0.05406121	0.99150162
70.3	0.0308986	0.03960503	0.04066378	0.08115721	0.04027861	0.05965882	0.05418572	0.99143191
70.4	0.0309268	0.03966794	0.04075917	0.08142615	0.04036335	0.05977818	0.05431042	0.99136181
70.5	0.03095517	0.03973097	0.040855	0.08169558	0.0404484	0.05989763	0.0544353	0.99129132
70.6	0.03098372	0.03979413	0.04095127	0.08196549	0.04053377	0.06001717	0.05456037	0.99122043
70.7	0.03101247	0.03985742	0.04104798	0.08223588	0.04061945	0.0601368	0.05468562	0.99114915
70.8	0.03104141	0.03992085	0.04114514	0.08250676	0.04070544	0.0602565	0.05481106	0.99107746
70.9	0.03107057	0.03998439	0.04124274	0.08277812	0.04079174	0.06037629	0.05493667	0.99100537
71	0.03109994	0.04004807	0.04134078	0.08304996	0.04087835	0.06049616	0.05506247	0.99093287
71.1	0.03112954	0.04011186	0.04143927	0.08332229	0.04096527	0.06061611	0.05518845	0.99085996
71.2	0.03115938	0.04017579	0.0415382	0.08359511	0.0410525	0.06073614	0.05531461	0.99078664
71.3	0.03118944	0.04023983	0.04163758	0.08386841	0.04114004	0.06085624	0.05544094	0.99071291
71.4	0.0312197	0.040304	0.0417374	0.08414221	0.04122788	0.06097642	0.05556746	0.99063876
71.5	0.03125016	0.04036829	0.04183767	0.0844165	0.04131604	0.06109667	0.05569415	0.99056419
71.6	0.03128077	0.04043271	0.04193839	0.0846913	0.0414045	0.06121699	0.05582101	0.99048919
71.7	0.03131153	0.04049726	0.04203955	0.08496659	0.04149327	0.06133737	0.05594805	0.99041377
71.8	0.03134242	0.04056193	0.04214117	0.08524239	0.04158234	0.06145783	0.05607526	0.99033791
71.9	0.03137343	0.04062673	0.04224323	0.0855187	0.04167172	0.06157835	0.05620265	0.99026162
72	0.03140455	0.04069165	0.04234574	0.08579552	0.0417614	0.06169893	0.0563302	0.99018489
72.1	0.03143578	0.04075671	0.0424487	0.08607286	0.04185139	0.06181958	0.05645793	0.99010772
72.2	0.03146712	0.04082189	0.04255211	0.08635073	0.04194168	0.06194029	0.05658583	0.9900301
72.3	0.03149857	0.04088721	0.04265597	0.08662912	0.04203228	0.06206106	0.0567139	0.98995202
72.4	0.03153013	0.04095266	0.04276029	0.08690804	0.04212317	0.06218188	0.05684213	0.98987348
72.5	0.0315618	0.04101825	0.04286505	0.0871875	0.04221437	0.06230276	0.05697053	0.98979448
72.6	0.0315936	0.04108397	0.04297026	0.0874675	0.04230587	0.06242369	0.0570991	0.989715
72.7	0.03162551	0.04114984	0.04307593	0.08774806	0.04239767	0.06254468	0.05722783	0.98963504
72.8	0.03165754	0.04121585	0.04318205	0.08802918	0.04248976	0.06266571	0.05735673	0.98955459
72.9	0.03168969	0.041282	0.04328862	0.08831087	0.04258216	0.0627868	0.05748579	0.98947365
73	0.03172196	0.0413483	0.04339564	0.08859314	0.04267486	0.06290793	0.05761502	0.98939222
73.1	0.03175436	0.04141475	0.04350312	0.08887601	0.04276785	0.06302911	0.0577444	0.9893103
73.2	0.03178688	0.04148135	0.04361105	0.08915948	0.04286114	0.06315033	0.05787395	0.98922789
73.3	0.03181952	0.04154809	0.04371943	0.08944358	0.04295472	0.06327159	0.05800366	0.98914499
73.4	0.03185229	0.04161498	0.04382827	0.08972832	0.0430486	0.0633929	0.05813353	0.9890616
73.5	0.03188518	0.04168201	0.04393757	0.09001371	0.04314277	0.06351424	0.05826356	0.98897773
73.6	0.03191821	0.04174918	0.04404732	0.09029977	0.04323723	0.06363562	0.05839375	0.98889339
73.7	0.03195136	0.04181649	0.04415752	0.0905865	0.04333199	0.06375703	0.0585241	0.98880859
73.8	0.03198464	0.04188394	0.04426819	0.09087387	0.04342703	0.06387848	0.0586546	0.98872333
73.9	0.03201805	0.04195152	0.0443793	0.09116188	0.04352237	0.06399996	0.05878527	0.98863763
74	0.0320516	0.04201924	0.04449088	0.09145047	0.04361799	0.06412146	0.05891609	0.98855149
74.1	0.03208529	0.0420871	0.04460292	0.09173961	0.04371391	0.064243	0.05904708	0.98846491
74.2	0.03211912	0.0421551	0.04471542	0.09202925	0.04381011	0.06436456	0.05917822	0.98837789
74.3	0.03215308	0.04222323	0.04482838	0.09231935	0.04390659	0.06448615	0.05930951	0.98829041
74.4	0.03218718	0.04229149	0.0449418	0.09260985	0.04400336	0.06460776	0.05944097	0.98820248
74.5	0.03222143	0.04235988	0.04505569	0.09290074	0.04410042	0.0647294	0.05957258	0.98811407
74.6	0.03225583	0.04242841	0.04517004	0.09319197	0.04419775	0.06485105	0.05970435	0.98802518
74.7	0.03229038	0.04249707	0.04528487	0.09348353	0.04429537	0.06497272	0.05983628	0.98793579
74.8	0.03232508	0.04256587	0.04540016	0.09377542	0.04439327	0.0650944	0.05996837	0.98784587
74.9	0.03235994	0.0426348	0.04551593	0.09406764	0.04449145	0.06521611	0.06010062	0.98775543

120	0.06228999	0.08669082	0.11344342	0.22137775	0.09603735	0.08972493	0.11585388	0.90976752
120.1	0.0623931	0.08681157	0.11355644	0.22159963	0.09612133	0.08969842	0.11595559	0.90954193
120.2	0.0624967	0.08693248	0.11366908	0.22182136	0.09620497	0.08967166	0.1160574	0.9093163

75	0.03239496	0.04270387	0.04563217	0.09436018	0.04458991	0.06533782	0.06023303	0.98766444
75.1	0.03243016	0.04277307	0.0457489	0.09465304	0.04468864	0.06545954	0.0603656	0.98757289
75.2	0.03246553	0.04284241	0.0458661	0.09494622	0.04478765	0.06558128	0.06049834	0.98748078
75.3	0.03250107	0.04291189	0.0459838	0.09523974	0.04488693	0.06570302	0.06063124	0.98738814
75.4	0.0325368	0.04298151	0.04610198	0.0955336	0.04498649	0.06582476	0.06076431	0.987295
75.5	0.03257272	0.04305127	0.04622065	0.09582781	0.04508631	0.06594651	0.06089755	0.98720138
75.6	0.03260882	0.04312116	0.04633983	0.09612235	0.04518641	0.06606827	0.06103096	0.98710732
75.7	0.03264512	0.04319119	0.0464595	0.09641723	0.04528678	0.06619002	0.06116455	0.98701284
75.8	0.03268162	0.04326137	0.04657969	0.09671245	0.04538741	0.06631177	0.06129832	0.98691796
75.9	0.03271831	0.04333168	0.04670038	0.09700801	0.04548831	0.06643352	0.06143228	0.98682271
76	0.03275521	0.04340213	0.04682159	0.09730389	0.04558948	0.06655527	0.06156643	0.98672709
76.1	0.03279232	0.04347272	0.04694331	0.0976001	0.04569091	0.06667701	0.06170077	0.98663111
76.2	0.03282964	0.04354345	0.04706553	0.09789663	0.0457926	0.06679874	0.06183531	0.98653477
76.3	0.03286717	0.04361432	0.04718826	0.09819346	0.04589455	0.06692046	0.06197006	0.98643807
76.4	0.03290493	0.04368533	0.04731149	0.0984906	0.04599676	0.06704217	0.06210502	0.98634101
76.5	0.0329429	0.04375647	0.04743521	0.09878805	0.04609923	0.06716387	0.06224019	0.98624359
76.6	0.03298108	0.04382776	0.04755941	0.0990858	0.04620195	0.06728556	0.06237559	0.98614582
76.7	0.03301946	0.04389918	0.04768409	0.09938386	0.04630493	0.06740722	0.06251121	0.98604767
76.8	0.03305802	0.04397074	0.04780923	0.09968221	0.04640817	0.06752887	0.06264707	0.98594916
76.9	0.03309674	0.04404244	0.04793483	0.09998087	0.04651165	0.0676505	0.06278316	0.98585029
77	0.03313558	0.04411428	0.04806088	0.10027983	0.04661539	0.06777211	0.06291946	0.98575104
77.1	0.03317454	0.04418625	0.04818737	0.10057908	0.04671937	0.0678937	0.06305596	0.98565142
77.2	0.03321359	0.04425837	0.04831431	0.10087864	0.0468236	0.06801526	0.06319265	0.98555144
77.3	0.03325272	0.04433062	0.04844169	0.10117848	0.04692808	0.0681368	0.0633295	0.98545108
77.4	0.03329194	0.04440302	0.04856951	0.1014786	0.0470328	0.06825831	0.06346652	0.98535036
77.5	0.03333125	0.04447555	0.04869777	0.10177901	0.04713777	0.06837979	0.06360369	0.98524926
77.6	0.03337064	0.04454822	0.04882647	0.1020797	0.04724297	0.06850124	0.06374101	0.98514779
77.7	0.03341012	0.04462103	0.04895561	0.10238068	0.04734842	0.06862266	0.06387847	0.98504595
77.8	0.03344969	0.04469398	0.04908519	0.10268195	0.0474541	0.06874405	0.06401605	0.98494373
77.9	0.03348934	0.04476708	0.04921521	0.10298351	0.04756002	0.0688654	0.06415374	0.98484114
78	0.03352908	0.04484031	0.04934568	0.10328538	0.04766618	0.06898671	0.06429152	0.98473817
78.1	0.03356891	0.04491368	0.04947659	0.10358755	0.04777256	0.06910799	0.06442938	0.98463482
78.2	0.03360884	0.04498719	0.04960796	0.10389001	0.04787918	0.06922923	0.06456732	0.9845311
78.3	0.03364885	0.04506083	0.04973978	0.10419276	0.04798603	0.06935042	0.06470532	0.98442699
78.4	0.03368896	0.04513462	0.04987207	0.1044958	0.04809311	0.06947158	0.0648434	0.98432251
78.5	0.03372916	0.04520855	0.05000482	0.10479912	0.04820042	0.06959269	0.06498154	0.98421764
78.6	0.03376946	0.04528262	0.05013806	0.10510273	0.04830795	0.06971376	0.06511977	0.98411239
78.7	0.03380986	0.04535682	0.05027178	0.10540662	0.04841571	0.06983479	0.06525807	0.98400676
78.8	0.03385035	0.04543117	0.05040598	0.1057108	0.04852369	0.06995576	0.06539646	0.98390075
78.9	0.03389094	0.04550565	0.05054066	0.10601526	0.04863189	0.07007669	0.06553494	0.98379436
79	0.03393163	0.04558028	0.0506758	0.10632001	0.04874031	0.07019757	0.06567353	0.98368758
79.1	0.03397242	0.04565504	0.05081139	0.10662504	0.04884895	0.07031841	0.06581223	0.98358042
79.2	0.03401331	0.04572994	0.05094741	0.10693036	0.0489578	0.0704392	0.06595106	0.98347288
79.3	0.0340543	0.04580499	0.05108385	0.10723595	0.04906687	0.07055995	0.06609001	0.98336495
79.4	0.03409539	0.04588017	0.0512207	0.10754181	0.04917615	0.07068066	0.06622909	0.98325663
79.5	0.03413659	0.04595549	0.05135795	0.10784795	0.04928564	0.07080132	0.06636828	0.98314793
79.6	0.03417789	0.04603095	0.05149558	0.10815435	0.04939535	0.07092194	0.06650757	0.98303883
79.7	0.0342193	0.04610656	0.05163361	0.10846103	0.04950526	0.07104251	0.06664695	0.98292936
79.8	0.03426081	0.0461823	0.05177201	0.10876797	0.04961538	0.07116304	0.06678638	0.98281949
79.9	0.03430243	0.04625818	0.05191079	0.10907517	0.0497257	0.07128354	0.06692585	0.98270923
80	0.03434416	0.04633421	0.05204995	0.10938263	0.04983623	0.07140399	0.06706535	0.98259858
80.1	0.03438601	0.04641037	0.05218949	0.10969035	0.04994696	0.07152441	0.06720487	0.98248755
80.2	0.03442796	0.04648667	0.0523294	0.10999833	0.0500579	0.07164479	0.06734442	0.98237612
80.3	0.03447003	0.04656311	0.05246969	0.11030657	0.05016903	0.07176514	0.06748398	0.9822643
80.4	0.03451221	0.0466397	0.05261035	0.11061506	0.05028036	0.07188546	0.06762356	0.98215209
80.5	0.03455451	0.04671642	0.05275139	0.11092381	0.05039188	0.07200575	0.06776317	0.98203949
80.6	0.03459692	0.04679328	0.05289281	0.11123281	0.0505036	0.07212603	0.06790279	0.98192649
80.7	0.03463946	0.04687029	0.05303461	0.11154206	0.05061552	0.07224629	0.06804242	0.9818131
80.8	0.03468211	0.04694743	0.05317678	0.11185156	0.05072763	0.07236654	0.06818208	0.98169932
80.9	0.03472488	0.04702471	0.05331933	0.1121613	0.05083992	0.07248678	0.06832177	0.98158514
81	0.03476777	0.04710213	0.05346226	0.11247129	0.05095241	0.07260702	0.06846148	0.98147057
81.1	0.03481078	0.0471797	0.05360556	0.11278153	0.05106508	0.07272727	0.0686012	0.9813556
81.2	0.03485391	0.0472574	0.05374924	0.11309201	0.05117794	0.07284754	0.06874094	0.98124023
81.3	0.03489717	0.04733524	0.0538933	0.11340273	0.05129099	0.07296784	0.0688807	0.98112447
81.4	0.03494056	0.04741322	0.05403773	0.11371369	0.05140422	0.07308818	0.06902047	0.98100831
81.5	0.03498407	0.04749135	0.05418255	0.11402489	0.05151762	0.07320857	0.06916025	0.98089176
81.6	0.03502771	0.04756961	0.05432775	0.11433633	0.05163121	0.073329	0.06930004	0.9807748
81.7	0.03507148	0.04764801	0.05447333	0.114648	0.05174498	0.07344948	0.06943984	0.98065745
81.8	0.03511538	0.04772656	0.05461931	0.11495991	0.05185893	0.07357002	0.06957966	0.98053969
81.9	0.03515941	0.04780524	0.05476568	0.11527205	0.05197305	0.07369065	0.06971949	0.98042154
82	0.03520358	0.04788406	0.05491244	0.11558442	0.05208735	0.07381136	0.06985933	0.98030299
82.1	0.03524787	0.04796303	0.05505961	0.11589703	0.05220182	0.07393216	0.06999918	0.98018403
82.2	0.03529231	0.04804213	0.0552072	0.11620987	0.05231646	0.07405301	0.07013905	0.98006467
82.3	0.03533688	0.04812137	0.05535521	0.11652295	0.05243128	0.07417384	0.07027893	0.97994491
82.4	0.03538159	0.04820076	0.05550365	0.11683625	0.05254626	0.07429459	0.07041883	0.97982475
82.5	0.03542644	0.04828028	0.05565253	0.11714978	0.05266141	0.07441519	0.07055874	0.97970418
82.6	0.03547144	0.04835994	0.05580187	0.11746354	0.05277673	0.07453559	0.07069867	0.97958321
82.7	0.0355166	0.04843975	0.05595168	0.11777753	0.05289221	0.07465573	0.07083862	0.97946184
82.8	0.03556191	0.04851969	0.05610196	0.11809175	0.05300786	0.07477555	0.07097859	0.97934005
82.9	0.03560738	0.04859978	0.05625274	0.11840619	0.05312367	0.074895	0.07111857	0.97921787
83	0.03565302	0.04868	0.05640402	0.11872086	0.05323964	0.07501405	0.07125858	0.97909527
83.1	0.03569883	0.04876036	0.05655584	0.11903576	0.05335577	0.07513272	0.07139862	0.97897227
83.2	0.03574481	0.04884087	0.05670819	0.11935087	0.05347206	0.07525107	0.07153868	0.97884885
83.3	0.03579098	0.04892152	0.05686111	0.11966621	0.0535885	0.07536916	0.07167878	0.97872503
83.4	0.03583735	0.04900231	0.05701462	0.11998178	0.0537051	0.07548706	0.07181892	0.9786008
83.5	0.03588394	0.04908324	0.05716871	0.12029756	0.05382186	0.07560473	0.0719591	0.97847616
83.6	0.03593075	0.04916431	0.05732336	0.12061357	0.05393877	0.07572214	0.07209932	0.97835111
83.7	0.0359778	0.04924552	0.05747852	0.12092979	0.05405583	0.07583925	0.07223961	0.97822565
83.8	0.0360251	0.04932688	0.05763413	0.12124623	0.05417305	0.07595605	0.07237997	0.97809978
83.9	0.03607267	0.04940838	0.0577901	0.12156288	0.05429041	0.07607252	0.07252039	0.97797349
84	0.03612052	0.04949003	0.05794637	0.12187975	0.05440792	0.07618867	0.07266091	0.97784679
84.1	0.03616865	0.04957181	0.05810287	0.12219682	0.05452558	0.07630452	0.07280152	0.97771968
84.2	0.03621708	0.04965374	0.05825958	0.1225141	0.05464338	0.07642008	0.07294223	0.97759215
84.3	0.0362658	0.04973582	0.0584165	0.1228316	0.05476133	0.07653534	0.07308305	0.9774642
84.4	0.03631479	0.04981803	0.05857363	0.12314932	0.05487942	0.07665031	0.07322397	0.97733584
84.5	0.036364	0.0499004	0.05873096	0.12346725	0.05499766	0.076765	0.07336497	0.97720705
84.6	0.0364134	0.0499829	0.0588885	0.1237854	0.05511603	0.0768794	0.07350605	0.97707785
84.7	0.03646294	0.05006556	0.05904623	0.12410376	0.05523454	0.07699349	0.0736472	0.97694822
84.8	0.03651258	0.05014836	0.05920416	0.12442234	0.05535319	0.07710727	0.0737884	0.97681818
84.9	0.03656229	0.0502313	0.05936229	0.12474113	0.05547198	0.07722076	0.07392966	0.97668771
85	0.03661205	0.05031439	0.05952063	0.12506014	0.05559091	0.07733393	0.07407096	0.97655681
85.1	0.03666185	0.05039763	0.05967917	0.12537936	0.05570996	0.07744681	0.07421229	0.97642549
85.2	0.0367117	0.05048103	0.05983793	0.1256988	0.05582916	0.0775594	0.07435365	0.97629375
85.3	0.03676161	0.05056457	0.0599969	0.12601846	0.05594848	0.07767171	0.07449502	0.97616158
85.4	0.03681157	0.05064826	0.06015611	0.12633833	0.05606793	0.07778374	0.07463641	0.97602897
85.5	0.03686158	0.05073211	0.06031556	0.12665842	0.05618752	0.0778955	0.07477779	0.97589594
85.6	0.03691167	0.05081611	0.06047529	0.12697872	0.05630723	0.07800699	0.07491917	0.97576248
85.7	0.03696184	0.05090027	0.06063529	0.12729924	0.05642706	0.07811823	0.07506054	0.97562857
85.8	0.0370121	0.05098459	0.0607956	0.12761998	0.05654703	0.07822922	0.07520191	0.97549422
85.9	0.03706244	0.05106907	0.06095621	0.12794093	0.05666712	0.07833997	0.07534328	0.97535942
86	0.03711286	0.05115371	0.06111712	0.12826208	0.05678733	0.07845049	0.07548466	0.97522417
86.1	0.03716336	0.05123852	0.0612783	0.12858342	0.05690766	0.07856079	0.07562604	0.97508847
86.2	0.03721394	0.05132348	0.0614397	0.12890495	0.05702812	0.0786709	0.07576744	0.97495231
86.3	0.0372646	0.05140861	0.06160127	0.12922667	0.05714869	0.07878082	0.07590887	0.97481568
86.4	0.03731534	0.05149391	0.06176296	0.12954858	0.05726938	0.07889057	0.07605033	0.9746786
86.5	0.03736614	0.05157936	0.06192475	0.12987068	0.05739019	0.07900018	0.07619183	0.97454104
86.6	0.03741702	0.05166496	0.06208662	0.13019301	0.05751112	0.07910966	0.07633339	0.97440301
86.7	0.03746796	0.05175072	0.06224855	0.1305156	0.05763216	0.07921905	0.07647502	0.97426451
86.8	0.03751898	0.05183662	0.06241052	0.13083845	0.05775331	0.07932835	0.07661671	0.97412553
86.9	0.03757007	0.05192267	0.06257255	0.13116157	0.05787458	0.07943754	0.07675846	0.97398606
87	0.03762124	0.05200887	0.06273463	0.13148498	0.05799595	0.07954658	0.07690029	0.9738461
87.1	0.03767247	0.0520952	0.06289677	0.13180867	0.05811744	0.07965542	0.07704218	0.97370565
87.2	0.03772379	0.05218167	0.06305895	0.13213267	0.05823904	0.07976401	0.07718413	0.9735647
87.3	0.03777518	0.05226827	0.0632212	0.13245698	0.05836074	0.07987233	0.07732613	0.97342325
87.4	0.03782665	0.05235502	0.0633835	0.13278162	0.05848255	0.07998038	0.07746819	0.97328132
87.5	0.03787819	0.05244189	0.06354587	0.13310661	0.05860446	0.08008813	0.07761029	0.97313892
87.6	0.03792981	0.05252889	0.0637083	0.13343194	0.05872648	0.08019557	0.07775247	0.97299606
87.7	0.03798152	0.05261603	0.06387079	0.13375765	0.0588486	0.08030267	0.07789472	0.97285278
87.8	0.0380333	0.05270329	0.06403335	0.13408375	0.05897082	0.08040948	0.07803705	0.97270907
87.9	0.03808517	0.05279068	0.06419597	0.13441024	0.05909314	0.08051605	0.07817944	0.97256497
88	0.03813712	0.0528782	0.06435866	0.13473714	0.05921557	0.08062239	0.07832183	0.97242047
88.1	0.03818916	0.05296586	0.06452141	0.13506448	0.05933809	0.08072854	0.07846416	0.97227558
88.2	0.03824129	0.05305363	0.06468424	0.13539226	0.0594607	0.08083455	0.07860637	0.97213031
88.3	0.03829351	0.05314154	0.06484713	0.1357205	0.05958341	0.08094044	0.07874842	0.97198466
88.4	0.03834582	0.05322958	0.0650101	0.13604922	0.05970622	0.08104622	0.07889031	0.97183864
88.5	0.03839822	0.05331774	0.06517316	0.13637844	0.05982912	0.08115186	0.07903204	0.97169225
88.6	0.03845071	0.05340604	0.06533629	0.13670817	0.05995211	0.08125731	0.07917362	0.97154548
88.7	0.03850329	0.05349446	0.06549952	0.13703843	0.06007519	0.08136249	0.07931506	0.97139834
88.8	0.03855596	0.05358301	0.06566284	0.13736913	0.06019836	0.08146736	0.07945636	0.97125083
88.9	0.03860873	0.05367168	0.06582625	0.13770003	0.06032162	0.08157185	0.07959753	0.97110296
89	0.03866159	0.05376049	0.06598978	0.13803085	0.06044496	0.08167592	0.07973855	0.97095471
89.1	0.03871454	0.05384942	0.06615342	0.13836138	0.06056839	0.08177951	0.0798794	0.97080609
89.2	0.03876759	0.05393849	0.06631717	0.13869135	0.06069191	0.08188262	0.08002007	0.9706571
89.3	0.03882074	0.05402768	0.06648106	0.13902049	0.06081551	0.08198526	0.08016056	0.97050773
89.4	0.03887398	0.05411699	0.06664509	0.13934868	0.06093919	0.08208742	0.08030085	0.970358
89.5	0.03892733	0.05420644	0.06680927	0.13967596	0.06106295	0.08218909	0.08044094	0.97020789
89.6	0.03898076	0.05429601	0.06697362	0.14000246	0.06118679	0.08229031	0.08058084	0.97005741
89.7	0.0390343	0.05438571	0.06713813	0.14032833	0.06131072	0.08239112	0.08072055	0.96990656
89.8	0.03908794	0.05447553	0.06730282	0.14065369	0.06143472	0.08249154	0.08086008	0.96975534
89.9	0.03914167	0.05456548	0.0674677	0.14097861	0.06155879	0.08259162	0.08099942	0.96960375

135	0.07853091	0.1042364	0.12742569	0.25360821	0.10587178	0.08422507	0.12801853	0.87558798
135.1	0.07863677	0.10434501	0.12750715	0.25381101	0.10592391	0.08418567	0.12808115	0.87536009
135.2	0.0787426	0.10445352	0.12758854	0.25401347	0.10597592	0.08414635	0.12814359	0.87513223

90	0.0391955	0.05465556	0.06763279	0.1413031	0.06168294	0.08269141	0.08113858	0.96945179
90.1	0.03924944	0.05474577	0.06779809	0.14162715	0.06180717	0.08279091	0.08127758	0.96929945
90.2	0.03930347	0.0548361	0.06796363	0.1419508	0.06193146	0.08289009	0.08141643	0.96914674
90.3	0.03935761	0.05492655	0.06812941	0.14227407	0.06205583	0.08298888	0.08155512	0.96899366
90.4	0.03941185	0.05501714	0.06829544	0.14259694	0.06218027	0.08308718	0.08169365	0.96884021
90.5	0.03946619	0.05510785	0.0684617	0.14291937	0.06230479	0.08318487	0.08183203	0.96868639
90.6	0.03952064	0.05519868	0.06862813	0.14324135	0.06242936	0.08328184	0.08197026	0.9685322
90.7	0.0395752	0.05528964	0.06879468	0.14356293	0.06255401	0.08337805	0.08210833	0.96837763
90.8	0.03962986	0.05538073	0.06896127	0.14388408	0.06267872	0.08347347	0.08224626	0.9682227
90.9	0.03968462	0.05547194	0.06912785	0.14420479	0.0628035	0.08356804	0.08238404	0.96806739
91	0.0397395	0.05556327	0.06929438	0.14452507	0.06292834	0.08366173	0.08252168	0.96791171
91.1	0.03979448	0.05565473	0.06946084	0.14484493	0.06305325	0.08375451	0.08265918	0.96775566
91.2	0.03984958	0.05574632	0.06962724	0.14516437	0.06317822	0.08384636	0.08279655	0.96759924
91.3	0.03990478	0.05583803	0.06979359	0.1454834	0.06330324	0.08393729	0.08293378	0.96744244
91.4	0.0399601	0.05592986	0.06995992	0.14580203	0.06342833	0.08402734	0.08307088	0.96728528
91.5	0.04001553	0.05602182	0.07012621	0.14612027	0.06355348	0.08411654	0.08320785	0.96712774
91.6	0.04007107	0.0561139	0.07029247	0.14643812	0.06367868	0.0842049	0.08334469	0.96696983
91.7	0.04012672	0.05620611	0.07045869	0.14675558	0.06380394	0.08429241	0.08348141	0.96681155
91.8	0.04018249	0.05629844	0.07062487	0.14707266	0.06392926	0.08437909	0.08361803	0.9666529
91.9	0.04023838	0.05639089	0.07079102	0.14738937	0.06405463	0.08446495	0.08375456	0.96649388
92	0.04029437	0.05648347	0.07095714	0.14770571	0.06418005	0.08455004	0.08389102	0.96633448
92.1	0.04035049	0.05657617	0.07112321	0.14802169	0.06430552	0.08463434	0.08402742	0.96617472
92.2	0.04040672	0.05666899	0.07128926	0.14833729	0.06443105	0.08471787	0.08416376	0.96601458
92.3	0.04046307	0.05676194	0.07145526	0.14865254	0.06455663	0.08480062	0.08430003	0.96585407
92.4	0.04051954	0.056855	0.07162123	0.14896744	0.06468225	0.08488259	0.08443622	0.9656932
92.5	0.04057613	0.05694819	0.07178716	0.14928198	0.06480793	0.0849638	0.08457233	0.96553195
92.6	0.04063284	0.05704151	0.07195305	0.14959619	0.06493365	0.08504424	0.08470833	0.96537032
92.7	0.04068967	0.05713494	0.07211891	0.14991006	0.06505941	0.0851239	0.08484424	0.96520833
92.8	0.04074662	0.0572285	0.07228475	0.1502236	0.06518522	0.08520279	0.08498004	0.96504597
92.9	0.04080369	0.05732218	0.07245055	0.15053682	0.06531108	0.08528091	0.08511576	0.96488324
93	0.04086088	0.05741598	0.07261633	0.15084974	0.06543698	0.08535825	0.08525139	0.96472013
93.1	0.0409182	0.05750991	0.0727821	0.15116235	0.06556292	0.08543481	0.08538693	0.96455666
93.2	0.04097565	0.05760396	0.07294786	0.15147469	0.0656889	0.0855106	0.08552235	0.96439281
93.3	0.04103321	0.05769813	0.0731136	0.15178674	0.06581492	0.08558562	0.0856576	0.9642286
93.4	0.04109091	0.05779242	0.07327934	0.15209853	0.06594097	0.08565988	0.08579264	0.96406401
93.5	0.04114873	0.05788684	0.07344508	0.15241007	0.06606707	0.08573338	0.08592744	0.96389906
93.6	0.04120667	0.05798137	0.07361081	0.15272135	0.0661932	0.08580613	0.08606195	0.96373373
93.7	0.04126475	0.05807603	0.07377655	0.15303237	0.06631937	0.08587813	0.08619614	0.96356803
93.8	0.04132295	0.05817082	0.07394228	0.15334309	0.06644557	0.08594941	0.08632999	0.96340197
93.9	0.04138129	0.05826572	0.07410803	0.15365349	0.06657181	0.08601996	0.08646348	0.96323553
94	0.04143975	0.05836075	0.07427379	0.15396355	0.06669808	0.08608979	0.08659661	0.96306872
94.1	0.04149835	0.0584559	0.07443957	0.15427326	0.06682438	0.0861589	0.0867294	0.96290154
94.2	0.04155708	0.05855117	0.07460538	0.15458259	0.06695071	0.08622731	0.08686185	0.96273399
94.3	0.04161594	0.05864656	0.07477122	0.15489156	0.06707708	0.08629501	0.08699397	0.96256606
94.4	0.04167493	0.05874207	0.07493709	0.15520018	0.06720347	0.08636203	0.08712576	0.96239777
94.5	0.04173406	0.05883771	0.07510301	0.15550845	0.06732989	0.08642836	0.08725722	0.96222911
94.6	0.04179333	0.05893347	0.07526899	0.15581639	0.06745633	0.086494	0.08738836	0.96206007
94.7	0.04185273	0.05902935	0.07543502	0.15612399	0.06758281	0.08655897	0.08751919	0.96189067
94.8	0.04191226	0.05912535	0.07560112	0.15643125	0.06770931	0.08662328	0.08764971	0.96172089
94.9	0.04197194	0.05922147	0.07576729	0.15673818	0.06783583	0.08668692	0.08777994	0.96155075
95	0.04203175	0.05931771	0.07593352	0.15704479	0.06796237	0.08674991	0.08790987	0.96138023
95.1	0.04209171	0.05941408	0.0760998	0.15735108	0.06808894	0.08681225	0.0880395	0.96120934
95.2	0.04215181	0.05951056	0.07626611	0.15765707	0.06821553	0.08687395	0.08816885	0.96103809
95.3	0.04221205	0.05960717	0.07643243	0.15796276	0.06834215	0.08693502	0.08829791	0.96086646
95.4	0.04227243	0.0597039	0.07659876	0.15826815	0.06846878	0.08699547	0.08842669	0.96069447
95.5	0.04233295	0.05980075	0.07676508	0.15857323	0.06859543	0.08705531	0.08855519	0.9605221
95.6	0.04239363	0.05989772	0.07693139	0.15887796	0.0687221	0.08711455	0.08868341	0.96034937
95.7	0.04245445	0.05999482	0.07709768	0.15918234	0.06884879	0.08717319	0.08881136	0.96017626
95.8	0.04251541	0.06009203	0.07726395	0.15948635	0.06897549	0.08723125	0.08893903	0.96000279
95.9	0.04257653	0.06018937	0.0774302	0.15978996	0.06910221	0.08728874	0.08906644	0.95982894
96	0.04263779	0.06028683	0.07759643	0.16009317	0.06922895	0.08734565	0.08919358	0.95965473
96.1	0.04269921	0.06038441	0.07776265	0.16039598	0.0693557	0.08740199	0.08932046	0.95948015
96.2	0.04276077	0.06048212	0.07792886	0.16069842	0.06948246	0.08745776	0.08944708	0.9593052
96.3	0.0428225	0.06057994	0.07809505	0.1610005	0.06960924	0.08751295	0.08957345	0.95912988
96.4	0.04288437	0.0606779	0.07826123	0.16130226	0.06973602	0.08756758	0.08969958	0.95895419
96.5	0.0429464	0.06077597	0.07842741	0.16160371	0.06986282	0.08762163	0.08982546	0.95877814
96.6	0.04300859	0.06087417	0.07859357	0.16190486	0.06998963	0.08767511	0.08995111	0.95860171
96.7	0.04307094	0.06097249	0.07875973	0.16220571	0.07011645	0.08772801	0.09007653	0.95842492
96.8	0.04313345	0.06107094	0.07892587	0.16250622	0.07024328	0.08778033	0.09020171	0.95824776
96.9	0.04319612	0.06116951	0.07909201	0.16280638	0.07037012	0.08783207	0.09032668	0.95807023
97	0.04325895	0.06126821	0.07925815	0.16310615	0.07049696	0.08788324	0.09045142	0.95789234
97.1	0.04332196	0.06136704	0.07942428	0.16340552	0.07062381	0.08793384	0.09057594	0.95771407
97.2	0.04338513	0.06146599	0.0795904	0.16370446	0.07075066	0.08798386	0.09070025	0.95753544
97.3	0.04344848	0.06156507	0.07975652	0.16400297	0.07087752	0.08803332	0.09082436	0.95735644
97.4	0.04351201	0.06166428	0.07992265	0.16430104	0.07100439	0.0880822	0.09094828	0.95717707
97.5	0.04357573	0.06176362	0.08008877	0.16459868	0.07113126	0.08813052	0.091072	0.95699734
97.6	0.04363962	0.06186309	0.08025489	0.16489592	0.07125813	0.08817828	0.09119553	0.95681724
97.7	0.04370371	0.06196269	0.08042102	0.16519277	0.071385	0.08822548	0.09131889	0.95663677
97.8	0.04376799	0.06206243	0.08058715	0.16548925	0.07151187	0.08827214	0.09144208	0.95645594
97.9	0.04383248	0.0621623	0.08075328	0.16578537	0.07163875	0.08831826	0.09156511	0.95627474
98	0.04389716	0.06226231	0.08091943	0.16608113	0.07176562	0.08836384	0.09168797	0.95609317
98.1	0.04396206	0.06236245	0.08108558	0.16637655	0.0718925	0.08840889	0.09181065	0.95591123
98.2	0.04402718	0.06246274	0.08125174	0.16667164	0.07201937	0.08845341	0.09193314	0.95572893
98.3	0.04409251	0.06256318	0.08141791	0.1669664	0.07214624	0.08849743	0.09205544	0.95554627
98.4	0.04415807	0.06266376	0.08158409	0.16726085	0.07227311	0.08854094	0.09217753	0.95536323
98.5	0.04422385	0.06276449	0.0817503	0.16755501	0.07239998	0.08858395	0.0922994	0.95517983
98.6	0.04428985	0.06286537	0.08191652	0.16784887	0.07252685	0.08862649	0.09242106	0.95499607
98.7	0.04435607	0.0629664	0.08208277	0.16814247	0.07265371	0.08866855	0.09254251	0.95481193
98.8	0.04442249	0.0630676	0.08224904	0.1684358	0.07278056	0.08871015	0.09266375	0.95462744
98.9	0.04448912	0.06316896	0.08241535	0.1687289	0.07290742	0.0887513	0.09278479	0.95444257
99	0.04455593	0.06327049	0.08258169	0.16902178	0.07303426	0.08879199	0.09290563	0.95425734
99.1	0.04462293	0.0633722	0.08274807	0.16931444	0.07316111	0.08883224	0.09302627	0.95407175
99.2	0.0446901	0.0634741	0.0829145	0.16960691	0.07328794	0.08887205	0.09314674	0.95388579
99.3	0.04475744	0.06357618	0.08308099	0.16989921	0.07341477	0.0889114	0.09326702	0.95369946
99.4	0.04482495	0.06367846	0.08324754	0.17019137	0.07354159	0.0889503	0.09338712	0.95351277
99.5	0.04489261	0.06378095	0.08341416	0.1704834	0.07366841	0.08898873	0.09350704	0.95332571
99.6	0.04496043	0.06388365	0.08358087	0.17077534	0.07379521	0.0890267	0.09362677	0.95313828
99.7	0.04502841	0.06398657	0.0837477	0.17106721	0.07392201	0.08906421	0.09374633	0.95295049
99.8	0.04509654	0.06408971	0.08391466	0.17135904	0.0740488	0.08910124	0.09386572	0.95276233
99.9	0.04516484	0.06419308	0.08408179	0.1716508	0.07417557	0.08913782	0.09398494	0.9525738
100	0.04523329	0.06429666	0.08424909	0.1719424	0.07430234	0.08917393	0.094104	0.95238491
100.1	0.04530191	0.06440045	0.0844166	0.17223372	0.0744291	0.08920959	0.0942229	0.95219565
100.2	0.04537069	0.06450442	0.08458431	0.17252463	0.07455585	0.08924481	0.09434165	0.95200602
100.3	0.04543963	0.06460857	0.08475224	0.17281501	0.07468259	0.08927958	0.09446026	0.95181602
100.4	0.04550874	0.06471287	0.08492036	0.17310478	0.07480932	0.08931392	0.09457872	0.95162566
100.5	0.04557802	0.0648173	0.08508862	0.17339389	0.07493604	0.08934782	0.09469705	0.95143492
100.6	0.04564746	0.06492184	0.08525697	0.1736823	0.07506275	0.08938129	0.09481526	0.95124382
100.7	0.04571707	0.06502647	0.08542539	0.17396997	0.07518945	0.08941433	0.09493335	0.95105234
100.8	0.04578685	0.0651312	0.08559383	0.17425687	0.07531615	0.08944694	0.09505132	0.95086048
100.9	0.0458568	0.06523601	0.08576228	0.174543	0.07544285	0.08947913	0.09516919	0.95066824
101	0.04592692	0.0653409	0.08593076	0.17482839	0.07556954	0.08951088	0.09528698	0.95047562
101.1	0.04599721	0.06544586	0.08609926	0.1751131	0.07569624	0.08954221	0.09540467	0.95028261
101.2	0.04606767	0.0655509	0.08626776	0.17539713	0.07582294	0.08957311	0.09552228	0.95008922
101.3	0.0461383	0.06565601	0.08643629	0.17568043	0.07594965	0.08960359	0.0956398	0.94989543
101.4	0.04620909	0.0657612	0.08660483	0.17596304	0.07607637	0.08963366	0.09575722	0.94970125
101.5	0.04628006	0.06586646	0.0867734	0.17624496	0.0762031	0.08966333	0.09587454	0.94950669
101.6	0.0463512	0.0659718	0.086942	0.17652622	0.07632987	0.08969259	0.09599173	0.94931175
101.7	0.04642251	0.06607722	0.08711064	0.17680685	0.07645667	0.08972145	0.09610879	0.94911642
101.8	0.04649399	0.06618273	0.08727932	0.17708684	0.07658352	0.08974993	0.09622571	0.94892072
101.9	0.04656564	0.06628832	0.08744805	0.17736617	0.07671044	0.08977803	0.09634247	0.94872462
102	0.04663747	0.06639398	0.08761684	0.17764484	0.07683743	0.08980576	0.09645907	0.94852813
102.1	0.04670947	0.06649973	0.0877857	0.17792287	0.07696451	0.08983313	0.09657551	0.94833125
102.2	0.04678164	0.06660557	0.08795465	0.17820024	0.0770917	0.08986015	0.09669179	0.94813396
102.3	0.04685399	0.06671149	0.08812372	0.17847696	0.077219	0.0898868	0.0968079	0.94793626
102.4	0.04692651	0.06681748	0.0882929	0.17875303	0.07734645	0.08991308	0.09692385	0.94773813
102.5	0.04699921	0.06692356	0.08846215	0.17902844	0.07747405	0.08993898	0.09703964	0.94753958
102.6	0.04707208	0.06702971	0.08863143	0.17930319	0.07760179	0.08996447	0.09715527	0.94734056
102.7	0.04714513	0.06713594	0.08880063	0.17957729	0.07772964	0.08998954	0.09727075	0.94714107
102.8	0.04721836	0.06724224	0.08896965	0.17985073	0.07785755	0.09001419	0.09738607	0.94694108
102.9	0.04729176	0.06734861	0.08913842	0.18012351	0.07798547	0.09003841	0.09750124	0.94674057
103	0.04736534	0.06745506	0.08930684	0.18039564	0.07811337	0.09006219	0.09761626	0.94653952
103.1	0.0474391	0.06756158	0.08947489	0.18066714	0.07824118	0.09008554	0.09773114	0.94633791
103.2	0.04751305	0.06766817	0.08964256	0.18093801	0.07836884	0.09010845	0.09784587	0.94613572
103.3	0.04758717	0.06777483	0.08980983	0.18120827	0.07849634	0.09013094	0.09796047	0.94593294
103.4	0.04766147	0.06788156	0.08997671	0.18147793	0.07862368	0.090153	0.09807493	0.94572959
103.5	0.04773596	0.06798836	0.09014325	0.18174702	0.07875083	0.09017464	0.09818924	0.94552572
103.6	0.04781064	0.06809523	0.09030949	0.18201554	0.07887777	0.09019587	0.09830343	0.94532138
103.7	0.0478855	0.06820217	0.09047541	0.18228349	0.07900448	0.09021669	0.09841749	0.94511665
103.8	0.04796056	0.06830918	0.09064104	0.18255089	0.07913098	0.09023711	0.09853142	0.94491161
103.9	0.0480358	0.06841627	0.09080638	0.18281775	0.07925726	0.09025714	0.09864522	0.9447063
104	0.04811124	0.06852343	0.09097144	0.18308406	0.07938334	0.09027676	0.09875889	0.94450075
104.1	0.04818687	0.06863065	0.09113619	0.18334985	0.07950924	0.09029599	0.09887244	0.94429498
104.2	0.04826271	0.06873795	0.09130062	0.1836151	0.07963498	0.09031483	0.09898586	0.94408899
104.3	0.04833875	0.06884532	0.09146474	0.18387984	0.07976058	0.09033329	0.09909914	0.94388279
104.4	0.04841501	0.06895276	0.09162855	0.18414406	0.07988603	0.09035137	0.0992123	0.94367636
104.5	0.04849148	0.06906027	0.09179203	0.18440777	0.08001137	0.09036906	0.09932532	0.94346969
104.6	0.04856816	0.06916785	0.09195519	0.18467097	0.08013662	0.09038637	0.09943821	0.94326275
104.7	0.04864508	0.0692755	0.09211805	0.18493367	0.0802618	0.0904033	0.09955096	0.94305555
104.8	0.04872222	0.06938322	0.09228062	0.18519587	0.08038692	0.09041986	0.09966359	0.94284807
104.9	0.0487996	0.06949102	0.09244292	0.18545758	0.08051201	0.09043604	0.09977609	0.9426403

150	0.09408863	0.11951548	0.13727079	0.28122663	0.11305307	0.07857652	0.13530781	0.84384858
150.1	0.09418699	0.11961121	0.13732228	0.28139754	0.11309972	0.07854404	0.13534719	0.843653
150.2	0.09428523	0.11970683	0.13737361	0.28156836	0.11314638	0.07851164	0.13538649	0.84345759

105	0.04887723	0.06959888	0.09260495	0.1857188	0.08063707	0.09045186	0.09988847	0.94243222
105.1	0.0489551	0.06970681	0.09276675	0.18597955	0.08076211	0.09046731	0.10000072	0.94222382
105.2	0.04903324	0.06981481	0.09292829	0.18623981	0.08088709	0.09048239	0.10011284	0.94201511
105.3	0.04911165	0.06992288	0.09308959	0.18649961	0.08101194	0.09049712	0.10022485	0.94180606
105.4	0.04919034	0.07003101	0.09325062	0.18675895	0.08113659	0.09051148	0.10033675	0.94159667
105.5	0.04926931	0.07013922	0.09341139	0.18701783	0.08126097	0.09052549	0.10044853	0.94138692
105.6	0.04934859	0.07024749	0.09357189	0.18727627	0.08138502	0.09053915	0.10056021	0.94117679
105.7	0.04942817	0.07035583	0.09373211	0.18753426	0.0815087	0.09055247	0.10067178	0.94096627
105.8	0.04950806	0.07046424	0.09389205	0.18779182	0.08163201	0.09056544	0.10078325	0.9407554
105.9	0.04958826	0.07057272	0.0940517	0.18804896	0.08175491	0.09057807	0.10089462	0.94054418
106	0.04966875	0.07068126	0.09421104	0.18830567	0.08187742	0.09059036	0.10100589	0.94033266
106.1	0.0497495	0.07078987	0.09437008	0.18856198	0.08199954	0.09060232	0.10111706	0.94012089
106.2	0.04983047	0.07089854	0.0945288	0.18881789	0.08212129	0.09061394	0.10122812	0.93990896
106.3	0.04991163	0.07100728	0.09468721	0.18907342	0.08224265	0.09062524	0.10133908	0.93969691
106.4	0.04999294	0.07111609	0.09484532	0.18932857	0.08236362	0.09063621	0.10144992	0.93948479
106.5	0.05007437	0.07122496	0.09500311	0.18958334	0.08248423	0.09064686	0.10156066	0.93927261
106.6	0.05015591	0.0713339	0.0951606	0.18983776	0.08260447	0.0906572	0.10167129	0.93906039
106.7	0.05023755	0.0714429	0.0953178	0.19009181	0.08272435	0.09066721	0.1017818	0.93884813
106.8	0.05031929	0.07155196	0.0954747	0.19034551	0.08284387	0.09067691	0.10189221	0.9386358
106.9	0.05040114	0.07166109	0.09563131	0.19059884	0.08296301	0.09068631	0.1020025	0.93842338
107	0.05048311	0.07177029	0.09578763	0.19085181	0.0830818	0.0906954	0.10211267	0.93821087
107.1	0.0505652	0.07187954	0.09594368	0.19110443	0.08320022	0.09070419	0.10222273	0.93799826
107.2	0.05064739	0.07198886	0.09609944	0.19135668	0.08331828	0.09071269	0.10233268	0.93778552
107.3	0.0507297	0.07209825	0.09625493	0.19160859	0.08343598	0.0907209	0.10244251	0.93757265
107.4	0.05081213	0.07220769	0.09641016	0.19186015	0.08355331	0.09072882	0.10255224	0.93735964
107.5	0.05089467	0.0723172	0.09656512	0.19211138	0.0836703	0.09073645	0.10266186	0.9371465
107.6	0.05097735	0.07242677	0.09671982	0.19236229	0.08378695	0.09074381	0.10277137	0.93693324
107.7	0.05106015	0.0725364	0.09687428	0.19261287	0.08390326	0.0907509	0.10288078	0.93671987
107.8	0.05114307	0.0726461	0.0970285	0.19286313	0.08401923	0.09075771	0.10299008	0.9365064
107.9	0.05122611	0.07275586	0.09718249	0.19311304	0.08413487	0.09076426	0.10309928	0.93629283
108	0.05130928	0.07286569	0.09733626	0.19336258	0.08425016	0.09077055	0.10320838	0.93607917
108.1	0.05139257	0.07297558	0.09748984	0.19361175	0.0843651	0.09077659	0.10331738	0.93586542
108.2	0.05147599	0.07308553	0.09764322	0.1938605	0.08447969	0.09078238	0.10342629	0.93565161
108.3	0.05155953	0.07319555	0.09779643	0.19410883	0.08459393	0.09078795	0.10353512	0.93543771
108.4	0.05164319	0.07330564	0.09794948	0.19435673	0.08470782	0.09079328	0.10364385	0.93522373
108.5	0.05172697	0.07341578	0.09810241	0.19460419	0.08482135	0.0907984	0.10375251	0.93500967
108.6	0.05181088	0.07352599	0.09825523	0.19485121	0.08493453	0.0908033	0.10386109	0.93479551
108.7	0.05189491	0.07363627	0.09840798	0.19509779	0.08504737	0.09080799	0.10396959	0.93458127
108.8	0.05197905	0.07374661	0.09856067	0.19534392	0.08515986	0.09081249	0.10407802	0.93436693
108.9	0.05206332	0.07385702	0.09871333	0.1955896	0.085272	0.0908168	0.10418638	0.9341525
109	0.05214771	0.07396749	0.09886598	0.19583484	0.0853838	0.09082093	0.10429467	0.93393797
109.1	0.05223222	0.07407802	0.0990186	0.19607962	0.08549527	0.09082488	0.1044029	0.93372334
109.2	0.05231686	0.07418862	0.09917114	0.19632396	0.0856064	0.09082868	0.10451107	0.93350863
109.3	0.05240162	0.07429928	0.09932353	0.19656785	0.08571719	0.09083232	0.10461918	0.93329382
109.4	0.0524865	0.07441001	0.09947569	0.19681129	0.08582766	0.09083583	0.10472724	0.93307891
109.5	0.0525715	0.0745208	0.09962753	0.19705429	0.08593781	0.09083921	0.10483524	0.93286392
109.6	0.05265663	0.07463166	0.09977897	0.19729687	0.08604763	0.09084248	0.10494318	0.93264884
109.7	0.05274189	0.07474258	0.09992996	0.19753904	0.08615713	0.09084563	0.10505107	0.93243366
109.8	0.05282727	0.07485357	0.1000805	0.19778081	0.08626631	0.09084866	0.1051589	0.93221839
109.9	0.05291278	0.07496462	0.1002306	0.1980222	0.08637518	0.09085155	0.10526667	0.93200302
110	0.05299841	0.07507574	0.10038024	0.19826322	0.08648373	0.09085426	0.10537438	0.93178755
110.1	0.05308417	0.07518692	0.10052942	0.19850388	0.08659198	0.09085676	0.10548202	0.93157198
110.2	0.05317005	0.07529817	0.10067814	0.19874421	0.08669992	0.09085904	0.1055896	0.93135632
110.3	0.05325606	0.07540948	0.1008264	0.19898419	0.08680757	0.09086108	0.10569712	0.93114056
110.4	0.0533422	0.07552086	0.10097421	0.19922385	0.08691493	0.09086288	0.10580457	0.9309247
110.5	0.05342847	0.07563231	0.10112157	0.19946317	0.08702199	0.09086443	0.10591196	0.93070875
110.6	0.05351486	0.07574382	0.10126848	0.19970219	0.08712877	0.09086575	0.10601929	0.93049271
110.7	0.05360138	0.0758554	0.10141494	0.19994089	0.08723527	0.09086683	0.10612657	0.93027658
110.8	0.05368804	0.07596704	0.10156095	0.2001793	0.08734148	0.0908677	0.10623379	0.93006036
110.9	0.05377483	0.07607876	0.10170652	0.20041743	0.08744743	0.09086836	0.10634095	0.92984404
111	0.05386175	0.07619054	0.10185166	0.20065531	0.08755309	0.0908688	0.10644807	0.92962765
111.1	0.0539488	0.07630238	0.10199637	0.20089295	0.08765849	0.09086903	0.10655514	0.92941117
111.2	0.05403599	0.0764143	0.10214066	0.20113036	0.08776362	0.09086904	0.10666217	0.92919461
111.3	0.05412332	0.07652629	0.10228453	0.20136755	0.0878685	0.09086884	0.10676915	0.92897797
111.4	0.05421077	0.07663834	0.102428	0.20160452	0.08797311	0.09086841	0.10687609	0.92876125
111.5	0.05429836	0.07675047	0.10257106	0.20184129	0.08807747	0.09086776	0.106983	0.92854443
111.6	0.05438609	0.07686266	0.10271373	0.20207784	0.08818158	0.09086688	0.10708987	0.92832752
111.7	0.05447394	0.07697493	0.102856	0.2023142	0.08828544	0.09086581	0.10719671	0.92811053
111.8	0.05456193	0.07708727	0.10299789	0.20255035	0.08838906	0.09086454	0.10730353	0.92789344
111.9	0.05465006	0.07719968	0.10313941	0.2027863	0.08849244	0.09086311	0.10741032	0.92767626
112	0.05473832	0.07731216	0.10328055	0.20302206	0.08859561	0.09086152	0.1075171	0.92745899
112.1	0.05482672	0.07742472	0.10342133	0.20325764	0.08869856	0.0908598	0.10762387	0.92724163
112.2	0.05491525	0.07753736	0.10356176	0.20349303	0.08880132	0.09085795	0.10773066	0.92702418
112.3	0.05500392	0.07765007	0.10370183	0.20372824	0.08890389	0.09085597	0.10783749	0.92680664
112.4	0.05509272	0.07776286	0.10384156	0.20396328	0.08900628	0.09085387	0.10794436	0.92658902
112.5	0.05518167	0.07787573	0.10398095	0.20419814	0.08910848	0.09085163	0.10805127	0.9263713
112.6	0.05527075	0.07798869	0.10412002	0.20443285	0.08921046	0.09084925	0.10815819	0.9261535
112.7	0.05535997	0.07810174	0.10425878	0.2046674	0.08931222	0.09084669	0.10826508	0.92593561
112.8	0.05544932	0.07821487	0.10439724	0.20490179	0.08941373	0.09084392	0.10837192	0.92571763
112.9	0.05553882	0.07832809	0.10453542	0.20513605	0.08951502	0.0908409	0.1084787	0.92549957
113	0.05562845	0.07844141	0.10467334	0.20537016	0.08961606	0.09083757	0.1085854	0.92528141
113.1	0.05571822	0.07855483	0.10481099	0.20560414	0.08971678	0.0908339	0.108692	0.92506317
113.2	0.05580813	0.07866834	0.10494839	0.205838	0.0898171	0.09082986	0.1087985	0.92484484
113.3	0.05589818	0.07878196	0.10508551	0.20607175	0.089917	0.0908254	0.10890488	0.92462642
113.4	0.05598837	0.07889568	0.10522231	0.20630538	0.09001651	0.09082052	0.10901116	0.9244079
113.5	0.0560787	0.07900951	0.10535877	0.20653892	0.09011569	0.09081522	0.10911733	0.9241893
113.6	0.05616917	0.07912347	0.10549485	0.20677238	0.09021456	0.09080949	0.10922339	0.9239706
113.7	0.05625978	0.07923755	0.10563054	0.20700578	0.09031315	0.09080331	0.10932934	0.92375181
113.8	0.05635053	0.07935176	0.10576582	0.20723912	0.09041147	0.0907967	0.1094352	0.92353294
113.9	0.05644142	0.07946611	0.10590068	0.20747243	0.09050953	0.09078966	0.10954097	0.92331397
114	0.05653245	0.07958062	0.10603512	0.20770571	0.09060733	0.0907822	0.10964665	0.9230949
114.1	0.05662363	0.07969528	0.10616914	0.207939	0.09070486	0.09077432	0.10975224	0.92287575
114.2	0.05671494	0.0798101	0.10630273	0.20817229	0.09080211	0.09076603	0.10985775	0.92265651
114.3	0.0568064	0.0799251	0.10643589	0.20840562	0.09089908	0.09075735	0.10996319	0.92243717
114.4	0.056898	0.08004028	0.10656863	0.208639	0.09099574	0.09074828	0.11006854	0.92221774
114.5	0.05698975	0.08015566	0.10670095	0.20887245	0.09109209	0.09073881	0.11017381	0.92199822
114.6	0.05708164	0.08027125	0.10683286	0.20910595	0.09118815	0.09072896	0.110279	0.9217786
114.7	0.05717367	0.08038705	0.10696436	0.20933948	0.09128391	0.09071874	0.11038409	0.92155889
114.8	0.05726584	0.08050309	0.10709545	0.20957299	0.09137938	0.09070814	0.11048909	0.92133909
114.9	0.05735816	0.08061937	0.10722614	0.2098064	0.09147456	0.09069718	0.110594	0.9211192
115	0.05745063	0.0807359	0.10735643	0.21003964	0.09156946	0.09068586	0.11069882	0.92089921
115.1	0.05754324	0.08085267	0.10748633	0.21027266	0.09166407	0.09067418	0.11080355	0.92067914
115.2	0.05763599	0.08096968	0.10761585	0.2105054	0.09175839	0.09066214	0.11090819	0.92045897
115.3	0.05772889	0.08108693	0.10774497	0.21073782	0.09185245	0.09064974	0.11101275	0.92023871
115.4	0.05782194	0.0812044	0.10787372	0.2109699	0.09194622	0.090637	0.11111722	0.92001835
115.5	0.05791514	0.08132208	0.10800208	0.21120164	0.09203973	0.09062391	0.11122161	0.91979791
115.6	0.05800848	0.08143997	0.10813009	0.21143304	0.09213297	0.09061047	0.11132593	0.91957737
115.7	0.05810198	0.08155806	0.10825772	0.21166408	0.09222595	0.0905967	0.11143017	0.91935674
115.8	0.05819562	0.08167631	0.108385	0.21189478	0.09231866	0.09058258	0.11153435	0.91913602
115.9	0.05828942	0.08179471	0.10851192	0.21212515	0.09241111	0.09056813	0.11163847	0.9189152
116	0.05838337	0.08191327	0.1086385	0.21235521	0.09250331	0.09055335	0.11174255	0.91869429
116.1	0.05847747	0.08203195	0.10876473	0.21258498	0.09259525	0.09053826	0.11184659	0.91847329
116.2	0.05857172	0.08215076	0.10889063	0.21281447	0.09268693	0.09052285	0.11195059	0.91825219
116.3	0.05866613	0.08226968	0.1090162	0.2130437	0.09277837	0.09050714	0.11205457	0.91803099
116.4	0.05876069	0.08238871	0.10914146	0.21327268	0.09286957	0.09049111	0.11215851	0.91780971
116.5	0.05885541	0.08250781	0.1092664	0.21350142	0.09296055	0.09047479	0.11226242	0.91758832
116.6	0.05895029	0.08262696	0.10939105	0.21372993	0.09305131	0.09045816	0.11236628	0.91736684
116.7	0.05904533	0.08274611	0.10951541	0.21395821	0.09314185	0.09044124	0.11247009	0.91714527
116.8	0.05914053	0.08286521	0.10963949	0.21418626	0.09323219	0.09042403	0.11257384	0.9169236
116.9	0.05923589	0.08298425	0.1097633	0.21441408	0.09332232	0.09040655	0.11267753	0.91670183
117	0.05933141	0.08310323	0.10988686	0.21464168	0.09341226	0.09038881	0.11278115	0.91647996
117.1	0.0594271	0.0832222	0.11001018	0.21486906	0.093502	0.09037081	0.1128847	0.916258
117.2	0.05952296	0.08334118	0.11013326	0.21509622	0.09359156	0.09035255	0.11298817	0.91603594
117.3	0.05961898	0.08346019	0.1102561	0.21532317	0.09368094	0.09033403	0.11309158	0.91581377
117.4	0.05971517	0.08357924	0.1103787	0.2155499	0.09377014	0.09031527	0.11319492	0.9155915
117.5	0.05981154	0.08369835	0.11050103	0.21577642	0.09385917	0.09029625	0.1132982	0.91536911
117.6	0.05990808	0.08381751	0.11062308	0.21600272	0.09394804	0.09027696	0.11340144	0.91514662
117.7	0.0600048	0.08393673	0.1107448	0.21622882	0.09403675	0.09025739	0.11350464	0.91492402
117.8	0.06010169	0.08405599	0.11086618	0.21645472	0.09412532	0.09023754	0.11360779	0.9147013
117.9	0.06019877	0.08417529	0.11098719	0.21668041	0.09421374	0.0902174	0.1137109	0.91447846
118	0.06029604	0.08429463	0.11110781	0.2169059	0.09430203	0.09019694	0.11381395	0.91425551
118.1	0.06039349	0.08441399	0.11122804	0.2171312	0.09439019	0.09017617	0.11391692	0.91403243
118.2	0.06049113	0.08453338	0.11134788	0.2173563	0.09447823	0.09015508	0.11401979	0.91380922
118.3	0.06058897	0.0846528	0.11146734	0.21758121	0.09456615	0.09013367	0.11412255	0.91358589
118.4	0.060687	0.08477224	0.11158642	0.21780593	0.09465396	0.09011196	0.1142252	0.91336242
118.5	0.06078524	0.08489172	0.11170512	0.21803046	0.09474166	0.09008994	0.11432772	0.91313882
118.6	0.0608837	0.08501123	0.11182345	0.21825481	0.09482926	0.09006761	0.11443012	0.91291508
118.7	0.06098238	0.08513078	0.11194141	0.21847898	0.09491675	0.09004499	0.11453238	0.91269119
118.8	0.06108128	0.08525037	0.11205902	0.21870297	0.09500415	0.09002206	0.11463451	0.91246716
118.9	0.06118041	0.08537	0.11217629	0.21892678	0.09509145	0.08999884	0.11473651	0.91224298
119	0.06127979	0.08548967	0.11229321	0.21915042	0.09517867	0.08997533	0.11483839	0.91201864
119.1	0.06137942	0.0856094	0.1124098	0.21937389	0.09526579	0.08995153	0.11494016	0.91179414
119.2	0.06147931	0.0857292	0.11252605	0.21959719	0.09535274	0.08992745	0.11504182	0.91156947
119.3	0.06157948	0.08584906	0.11264196	0.21982032	0.09543948	0.08990309	0.1151434	0.91134463
119.4	0.06167994	0.08596901	0.11275753	0.22004328	0.09552594	0.08987845	0.11524492	0.91111965
119.5	0.06178071	0.08608904	0.11287275	0.22026609	0.09561209	0.08985353	0.11534638	0.91089452
119.6	0.06188181	0.08620917	0.11298762	0.22048873	0.09569787	0.08982834	0.11544783	0.91066928
119.7	0.06198327	0.0863294	0.11310212	0.22071122	0.09578329	0.08980288	0.11554927	0.91044394
119.8	0.0620851	0.08644975	0.11321626	0.22093355	0.09586834	0.08977716	0.11565074	0.91021854
119.9	0.06218733	0.08657022	0.11333003	0.22115573	0.09595302	0.08975117	0.11575226	0.90999306

165	0.10775521	0.13241324	0.14369494	0.30528312	0.12009812	0.07440744	0.14028128	0.81611681
165.1	0.10784032	0.13248862	0.14372993	0.30543302	0.12014299	0.07438571	0.14031043	0.81594324
165.2	0.10792537	0.132564	0.14376486	0.30558278	0.12018784	0.07436411	0.14033954	0.81576982

120.3	0.0626008	0.08705358	0.11378136	0.22204295	0.09628828	0.08964465	0.11615922	0.90909063
120.4	0.06270536	0.08717487	0.11389326	0.22226439	0.09637125	0.08961739	0.11626101	0.90886492
120.5	0.06281035	0.08729637	0.1140048	0.2224857	0.09645389	0.08958988	0.11636271	0.90863918
120.6	0.06291574	0.0874181	0.11411598	0.22270686	0.0965362	0.08956213	0.11646421	0.9084134
120.7	0.06302147	0.08754008	0.1142268	0.22292789	0.09661817	0.08953414	0.11656541	0.90818758
120.8	0.06312749	0.08766232	0.11433725	0.22314879	0.09669982	0.08950591	0.11666625	0.90796173
120.9	0.06323377	0.08778485	0.11444736	0.22336955	0.09678115	0.08947745	0.11676672	0.90773583
121	0.0633403	0.08790768	0.1145571	0.22359019	0.09686215	0.08944875	0.11686678	0.90750991
121.1	0.06344708	0.08803083	0.1146665	0.22381069	0.09694283	0.08941983	0.11696641	0.90728395
121.2	0.06355412	0.08815429	0.11477556	0.22403108	0.0970232	0.08939068	0.11706561	0.90705796
121.3	0.06366143	0.08827801	0.11488428	0.22425133	0.09710326	0.0893613	0.11716442	0.90683194
121.4	0.06376904	0.08840188	0.11499265	0.22447147	0.097183	0.08933171	0.11726283	0.90660588
121.5	0.0638769	0.08852584	0.11510069	0.22469148	0.09726244	0.0893019	0.11736086	0.90637979
121.6	0.063985	0.08864982	0.11520839	0.22491138	0.09734157	0.08927187	0.11745852	0.90615367
121.7	0.06409329	0.08877376	0.11531576	0.22513116	0.09742039	0.08924163	0.11755583	0.90592752
121.8	0.06420172	0.08889764	0.1154228	0.22535083	0.09749892	0.08921117	0.1176528	0.90570134
121.9	0.06431024	0.08902145	0.11552951	0.22557039	0.09757714	0.08918051	0.11774943	0.90547512
122	0.06441884	0.08914517	0.1156359	0.22578983	0.09765507	0.08914964	0.11784573	0.90524888
122.1	0.06452751	0.0892688	0.11574196	0.22600917	0.0977327	0.08911857	0.11794169	0.9050226
122.2	0.06463625	0.08939233	0.1158477	0.22622841	0.09781003	0.0890873	0.11803733	0.90479629
122.3	0.06474506	0.08951577	0.11595311	0.22644754	0.09788708	0.08905583	0.11813265	0.90456994
122.4	0.06485393	0.08963912	0.11605821	0.22666657	0.09796383	0.08902416	0.11822766	0.90434356
122.5	0.06496291	0.08976237	0.11616299	0.2268855	0.0980403	0.0889923	0.11832236	0.90411715
122.6	0.065072	0.08988551	0.11626745	0.22710433	0.09811648	0.08896025	0.11841677	0.9038907
122.7	0.06518124	0.09000855	0.11637161	0.22732307	0.09819237	0.08892801	0.11851087	0.90366421
122.8	0.06529065	0.09013148	0.11647545	0.22754171	0.09826799	0.08889559	0.11860468	0.90343769
122.9	0.06540024	0.0902543	0.11657898	0.22776027	0.09834333	0.08886298	0.1186982	0.90321113
123	0.06551002	0.090377	0.1166822	0.22797874	0.09841839	0.0888302	0.11879142	0.90298453
123.1	0.06562001	0.09049957	0.11678512	0.22819712	0.09849317	0.08879724	0.11888436	0.90275789
123.2	0.06573019	0.09062203	0.11688774	0.22841543	0.09856769	0.08876411	0.118977	0.90253121
123.3	0.06584054	0.09074437	0.11699006	0.22863365	0.09864193	0.08873081	0.11906935	0.90230448
123.4	0.065951	0.09086661	0.11709208	0.2288518	0.09871591	0.08869734	0.11916141	0.90207771
123.5	0.06606153	0.09098872	0.1171938	0.22906989	0.09878963	0.08866371	0.11925318	0.90185088
123.6	0.06617209	0.09111072	0.11729524	0.2292879	0.09886308	0.08862992	0.11934467	0.90162401
123.7	0.06628268	0.0912326	0.11739638	0.22950585	0.09893628	0.08859597	0.1194359	0.90139708
123.8	0.06639326	0.09135437	0.11749724	0.22972375	0.09900921	0.08856187	0.11952687	0.90117009
123.9	0.06650381	0.09147603	0.11759782	0.22994159	0.0990819	0.08852762	0.11961759	0.90094303
124	0.06661429	0.0915976	0.11769811	0.23015938	0.09915433	0.08849321	0.11970807	0.90071591
124.1	0.06672469	0.09171907	0.11779813	0.23037712	0.09922651	0.08845865	0.1197983	0.90048871
124.2	0.066835	0.09184043	0.11789788	0.23059484	0.09929845	0.08842393	0.11988831	0.90026143
124.3	0.06694524	0.09196169	0.11799735	0.23081253	0.09937015	0.08838905	0.11997811	0.90003408
124.4	0.06705539	0.09208284	0.11809655	0.2310302	0.09944161	0.08835402	0.12006771	0.89980663
124.5	0.06716545	0.09220387	0.11819549	0.23124787	0.09951283	0.08831883	0.12015711	0.8995791
124.6	0.06727541	0.09232478	0.11829416	0.23146553	0.09958382	0.08828348	0.12024633	0.89935147
124.7	0.06738531	0.09244555	0.11839256	0.23168321	0.09965458	0.08824797	0.12033536	0.89912374
124.8	0.06749513	0.09256618	0.1184907	0.23190091	0.09972512	0.08821229	0.1204242	0.89889591
124.9	0.0676049	0.09268665	0.11858858	0.23211866	0.09979543	0.08817645	0.12051284	0.89866798
125	0.06771461	0.09280696	0.1186862	0.23233648	0.09986552	0.08814045	0.12060127	0.89843993
125.1	0.06782427	0.09292712	0.11878355	0.23255438	0.0999354	0.08810428	0.12068946	0.89821179
125.2	0.06793388	0.09304713	0.11888065	0.23277241	0.10000506	0.08806795	0.12077739	0.89798357
125.3	0.06804345	0.093167	0.11897749	0.23299057	0.10007452	0.08803145	0.12086504	0.89775529
125.4	0.06815298	0.09328674	0.11907408	0.23320889	0.10014378	0.0879948	0.12095237	0.89752697
125.5	0.06826248	0.09340636	0.11917041	0.23342741	0.10021284	0.08795799	0.12103937	0.89729863
125.6	0.06837194	0.09352585	0.11926649	0.23364613	0.10028171	0.08792105	0.12112603	0.89707027
125.7	0.06848136	0.09364524	0.11936232	0.23386507	0.10035038	0.08788397	0.12121236	0.89684192
125.8	0.06859076	0.0937645	0.1194579	0.2340842	0.10041886	0.08784677	0.12129836	0.89661356
125.9	0.06870013	0.09388366	0.11955324	0.23430346	0.10048714	0.08780946	0.12138402	0.89638522
126	0.06880948	0.09400269	0.11964833	0.23452277	0.10055521	0.08777205	0.12146934	0.89615689
126.1	0.0689188	0.09412161	0.11974317	0.23474205	0.10062306	0.08773455	0.12155432	0.89592857
126.2	0.06902811	0.09424041	0.11983778	0.23496121	0.10069068	0.08769695	0.12163897	0.89570027
126.3	0.06913741	0.09435909	0.11993214	0.2351802	0.10075806	0.08765927	0.12172328	0.89547199
126.4	0.0692467	0.09447764	0.12002627	0.23539898	0.1008252	0.08762149	0.12180726	0.89524371
126.5	0.06935598	0.09459606	0.12012016	0.23561753	0.1008921	0.08758363	0.1218909	0.89501544
126.6	0.06946526	0.09471436	0.12021382	0.23583584	0.10095874	0.08754568	0.12197423	0.89478718
126.7	0.06957455	0.09483253	0.12030725	0.23605391	0.10102515	0.08750764	0.12205722	0.89455893
126.8	0.06968387	0.09495059	0.12040045	0.23627171	0.10109132	0.08746952	0.1221399	0.89433069
126.9	0.06979321	0.09506852	0.12049341	0.23648926	0.10115725	0.08743132	0.12222226	0.89410246
127	0.06990259	0.09518633	0.12058615	0.23670655	0.10122296	0.08739304	0.1223043	0.89387423
127.1	0.07001201	0.09530402	0.12067867	0.23692362	0.10128844	0.08735469	0.12238603	0.89364601
127.2	0.07012149	0.09542159	0.12077096	0.23714047	0.1013537	0.08731625	0.12246745	0.89341779
127.3	0.07023105	0.09553903	0.12086303	0.23735712	0.10141873	0.08727774	0.12254857	0.89318959
127.4	0.07034068	0.09565636	0.12095487	0.23757355	0.10148354	0.08723916	0.12262937	0.89296139
127.5	0.07045041	0.09577356	0.1210465	0.23778977	0.10154813	0.08720051	0.12270988	0.89273321
127.6	0.07056023	0.09589064	0.12113792	0.23800578	0.1016125	0.08716178	0.12279008	0.89250503
127.7	0.07067012	0.09600761	0.12122911	0.23822159	0.10167665	0.08712299	0.12286999	0.89227686
127.8	0.07078007	0.09612446	0.12132009	0.23843722	0.10174058	0.08708414	0.1229496	0.8920487
127.9	0.07089004	0.0962412	0.12141086	0.23865265	0.10180429	0.08704523	0.12302891	0.89182055
128	0.071	0.09635782	0.12150142	0.23886789	0.10186779	0.08700625	0.12310793	0.89159241
128.1	0.07110992	0.09647432	0.12159177	0.23908295	0.10193108	0.08696722	0.12318666	0.89136427
128.2	0.07121977	0.09659071	0.12168192	0.23929781	0.10199415	0.08692813	0.12326509	0.89113614
128.3	0.07132954	0.09670698	0.12177185	0.23951249	0.10205702	0.08688899	0.12334324	0.89090802
128.4	0.07143922	0.09682313	0.12186159	0.23972698	0.10211967	0.08684979	0.12342111	0.8906799
128.5	0.07154881	0.09693917	0.12195112	0.23994129	0.10218212	0.08681054	0.12349869	0.89045178
128.6	0.0716583	0.09705509	0.12204044	0.24015541	0.10224436	0.08677125	0.12357599	0.89022366
128.7	0.07176769	0.09717089	0.12212957	0.24036936	0.1023064	0.0867319	0.12365301	0.88999553
128.8	0.07187699	0.09728658	0.12221851	0.24058311	0.10236823	0.08669251	0.12372976	0.8897674
128.9	0.07198621	0.09740215	0.12230724	0.24079669	0.10242987	0.08665307	0.12380623	0.88953927
129	0.07209537	0.0975176	0.12239578	0.2410101	0.10249131	0.08661359	0.12388243	0.88931112
129.1	0.07220445	0.09763294	0.12248413	0.24122334	0.10255255	0.08657407	0.12395837	0.88908297
129.2	0.07231347	0.09774817	0.12257229	0.24143642	0.1026136	0.08653451	0.12403403	0.88885479
129.3	0.07242242	0.09786327	0.12266025	0.24164934	0.10267445	0.08649491	0.12410943	0.8886266
129.4	0.0725313	0.09797826	0.12274803	0.2418621	0.10273511	0.08645528	0.12418457	0.88839838
129.5	0.07264012	0.09809314	0.12283562	0.24207472	0.10279558	0.08641561	0.12425945	0.88817012
129.6	0.07274887	0.09820789	0.12292303	0.2422872	0.10285587	0.08637592	0.12433406	0.88794183
129.7	0.07285756	0.09832253	0.12301025	0.24249953	0.10291596	0.08633619	0.12440842	0.88771348
129.8	0.07296617	0.09843706	0.12309729	0.24271173	0.10297587	0.08629643	0.12448251	0.88748508
129.9	0.0730747	0.09855147	0.12318414	0.2429238	0.10303559	0.08625665	0.12455635	0.88725662
130	0.07318317	0.09866576	0.12327082	0.24313573	0.10309514	0.08621685	0.12462992	0.88702808
130.1	0.07329155	0.09877994	0.12335732	0.24334755	0.1031545	0.08617702	0.12470324	0.88679947
130.2	0.07339985	0.098894	0.12344365	0.24355925	0.10321368	0.08613717	0.1247763	0.88657076
130.3	0.07350809	0.09900794	0.1235298	0.24377083	0.10327268	0.08609731	0.1248491	0.88634195
130.4	0.07361624	0.09912177	0.12361578	0.2439823	0.10333151	0.08605744	0.12492166	0.88611303
130.5	0.07372433	0.09923549	0.12370158	0.24419366	0.10339016	0.08601756	0.12499397	0.88588401
130.6	0.07383236	0.09934909	0.12378722	0.24440492	0.10344864	0.08597767	0.12506603	0.8856549
130.7	0.07394032	0.09946258	0.12387269	0.24461609	0.10350695	0.08593778	0.12513786	0.88542569
130.8	0.07404822	0.09957595	0.12395799	0.24482716	0.10356508	0.08589789	0.12520944	0.88519641
130.9	0.07415607	0.0996892	0.12404313	0.24503816	0.10362305	0.08585801	0.12528078	0.88496709
131	0.07426385	0.09980235	0.1241281	0.24524913	0.10368085	0.08581813	0.12535189	0.88473774
131.1	0.07437157	0.09991538	0.12421291	0.2454601	0.10373849	0.08577827	0.12542277	0.88450838
131.2	0.07447923	0.10002829	0.12429756	0.24567111	0.10379596	0.08573843	0.12549341	0.88427903
131.3	0.07458682	0.10014109	0.12438206	0.2458822	0.10385327	0.0856986	0.12556383	0.88404968
131.4	0.07469434	0.10025378	0.12446639	0.24609339	0.10391041	0.08565879	0.12563401	0.88382034
131.5	0.07480181	0.10036636	0.12455056	0.24630473	0.1039674	0.08561901	0.12570396	0.88359098
131.6	0.07490921	0.10047882	0.12463459	0.24651624	0.10402423	0.08557923	0.12577369	0.88336161
131.7	0.07501656	0.10059117	0.12471846	0.24672794	0.1040809	0.08553946	0.12584319	0.8831322
131.8	0.07512385	0.10070341	0.12480217	0.24693982	0.10413741	0.08549969	0.12591247	0.88290276
131.9	0.07523108	0.10081553	0.12488574	0.24715182	0.10419377	0.0854599	0.12598153	0.88267329
132	0.07533826	0.10092754	0.12496916	0.2473639	0.10424998	0.08542009	0.12605036	0.88244379
132.1	0.07544537	0.10103944	0.12505244	0.24757601	0.10430604	0.08538027	0.12611897	0.88221428
132.2	0.07555244	0.10115123	0.12513557	0.24778813	0.10436194	0.08534042	0.12618736	0.88198478
132.3	0.07565944	0.1012629	0.12521857	0.24800025	0.1044177	0.08530056	0.12625554	0.88175532
132.4	0.07576639	0.10137446	0.12530143	0.2482123	0.10447331	0.08526068	0.12632349	0.88152591
132.5	0.07587329	0.10148591	0.12538415	0.2484242	0.10452877	0.08522078	0.12639123	0.88129656
132.6	0.07598013	0.10159725	0.12546674	0.24863587	0.10458409	0.08518086	0.12645876	0.8810673
132.7	0.07608692	0.10170848	0.1255492	0.24884724	0.10463927	0.08514094	0.12652607	0.88083811
132.8	0.07619366	0.10181959	0.12563154	0.24905831	0.10469431	0.08510101	0.12659317	0.88060902
132.9	0.07630034	0.1019306	0.12571376	0.24926903	0.1047492	0.08506107	0.12666006	0.88038001
133	0.07640698	0.10204149	0.12579586	0.24947938	0.10480395	0.08502112	0.12672674	0.88015109
133.1	0.07651357	0.10215227	0.12587785	0.24968933	0.10485857	0.08498117	0.12679321	0.87992226
133.2	0.07662012	0.10226295	0.12595973	0.24989889	0.10491305	0.08494122	0.12685948	0.87969352
133.3	0.07672661	0.10237351	0.12604151	0.25010804	0.10496739	0.08490128	0.12692554	0.87946488
133.4	0.07683306	0.10248396	0.1261232	0.25031679	0.1050216	0.08486134	0.12699139	0.87923632
133.5	0.07693947	0.1025943	0.12620481	0.25052515	0.10507568	0.08482141	0.12705705	0.87900784
133.6	0.07704583	0.10270453	0.12628634	0.25073314	0.10512963	0.08478148	0.1271225	0.87877944
133.7	0.07715215	0.10281465	0.1263678	0.25094076	0.10518344	0.08474157	0.12718775	0.87855113
133.8	0.07725843	0.10292466	0.1264492	0.25114803	0.10523713	0.08470167	0.12725281	0.87832289
133.9	0.07736466	0.10303457	0.12653056	0.25135493	0.10529069	0.08466178	0.12731766	0.87809473
134	0.07747086	0.10314436	0.12661188	0.25156148	0.10534412	0.0846219	0.12738233	0.87786664
134.1	0.07757701	0.10325405	0.12669317	0.25176768	0.10539743	0.08458205	0.12744679	0.87763863
134.2	0.07768313	0.10336363	0.12677446	0.25197354	0.10545061	0.08454223	0.12751107	0.87741069
134.3	0.07778921	0.1034731	0.12685575	0.25217907	0.10550367	0.08450243	0.12757515	0.87718281
134.4	0.07789526	0.10358246	0.12693705	0.25238426	0.10555661	0.08446267	0.12763904	0.87695499
134.5	0.07800128	0.10369172	0.12701839	0.2525891	0.10560943	0.08442295	0.12770275	0.87672721
134.6	0.07810726	0.10380087	0.12709978	0.25279361	0.10566214	0.08438326	0.12776627	0.87649943
134.7	0.07821321	0.10390991	0.12718122	0.25299777	0.10571472	0.08434363	0.12782961	0.87627162
134.8	0.07831914	0.10401885	0.1272627	0.25320159	0.10576719	0.08430405	0.12789276	0.87604378
134.9	0.07842504	0.10412768	0.1273442	0.25340507	0.10581954	0.08426453	0.12795574	0.87581589
180	0.12011795	0.14182063	0.14782166	0.32643344	0.12676875	0.07162794	0.14437857	0.79204126
180.1	0.12019618	0.14187052	0.14784344	0.32656441	0.12681385	0.07161187	0.14440054	0.7918969
180.2	0.12027432	0.14192025	0.14786517	0.32669526	0.12685896	0.07159583	0.14442241	0.79175271
180.3	0.12035237	0.14196985	0.14788685	0.32682597	0.12690409	0.07157982	0.14444419	0.79160869
180.4	0.12043032	0.1420193	0.1479085	0.32695655	0.12694923	0.07156384	0.14446587	0.79146483
180.5	0.12050817	0.1420686	0.14793009	0.32708701	0.12699439	0.07154789	0.14448747	0.79132115
135.3	0.07884842	0.10456193	0.12766984	0.2542156	0.10602783	0.08410711	0.12820584	0.87490444
135.4	0.07895422	0.10467023	0.12775103	0.25441739	0.10607963	0.08406794	0.12826793	0.87467673
135.5	0.07906002	0.10477842	0.12783209	0.25461885	0.10613131	0.08402885	0.12832983	0.87444912
135.6	0.07916581	0.10488651	0.12791299	0.25481998	0.1061829	0.08398981	0.12839157	0.87422162
135.7	0.0792716	0.1049945	0.12799372	0.25502078	0.10623438	0.08395082	0.12845313	0.87399421
135.8	0.07937739	0.10510238	0.12807424	0.25522126	0.10628575	0.08391186	0.12851452	0.8737669
135.9	0.07948318	0.10521016	0.12815453	0.25542141	0.10633702	0.08387292	0.12857573	0.87353968
136	0.07958899	0.10531784	0.12823457	0.25562125	0.10638819	0.08383399	0.12863678	0.87331253
136.1	0.0796948	0.10542542	0.12831438	0.25582077	0.10643926	0.08379506	0.12869766	0.87308545
136.2	0.07980065	0.10553289	0.12839399	0.25601997	0.10649023	0.08375611	0.12875838	0.87285844
136.3	0.07990652	0.10564026	0.12847342	0.25621885	0.1065411	0.08371715	0.12881893	0.87263152
136.4	0.08001243	0.10574754	0.12855268	0.25641743	0.10659188	0.08367818	0.12887932	0.87240473
136.5	0.08011839	0.10585471	0.12863176	0.25661569	0.10664256	0.08363919	0.12893955	0.87217809
136.6	0.08022442	0.10596178	0.12871062	0.25681365	0.10669314	0.08360018	0.12899962	0.87195165
136.7	0.08033051	0.10606876	0.12878926	0.2570113	0.10674363	0.08356115	0.12905953	0.87172547
136.8	0.0804367	0.10617564	0.12886767	0.25720865	0.10679403	0.08352209	0.12911929	0.87149955
136.9	0.08054297	0.10628242	0.12894585	0.25740569	0.10684434	0.08348301	0.1291789	0.87127392
137	0.08064936	0.10638911	0.12902378	0.25760244	0.10689456	0.08344393	0.12923835	0.87104856
137.1	0.08075588	0.10649571	0.12910145	0.25779888	0.10694469	0.08340484	0.12929766	0.87082349
137.2	0.08086254	0.10660221	0.12917885	0.25799503	0.10699473	0.08336576	0.12935682	0.87059869
137.3	0.08096936	0.10670863	0.12925597	0.25819089	0.10704469	0.08332669	0.12941583	0.87037418
137.4	0.08107633	0.10681496	0.12933281	0.25838645	0.10709456	0.08328764	0.1294747	0.87014995
137.5	0.08118344	0.10692121	0.12940937	0.25858172	0.10714434	0.08324861	0.12953343	0.86992602
137.6	0.08129063	0.10702737	0.12948566	0.2587767	0.10719405	0.0832096	0.12959202	0.86970235
137.7	0.08139784	0.10713345	0.12956168	0.25897139	0.10724367	0.08317062	0.12965049	0.86947896
137.8	0.08150504	0.10723945	0.12963742	0.2591658	0.10729321	0.08313166	0.12970883	0.86925583
137.9	0.08161219	0.10734537	0.12971289	0.25935993	0.10734267	0.08309271	0.12976705	0.86903297
138	0.08171927	0.10745121	0.12978809	0.25955377	0.10739205	0.08305376	0.12982515	0.86881038
138.1	0.08182626	0.10755697	0.129863	0.25974733	0.10744136	0.08301481	0.12988314	0.86858805
138.2	0.08193318	0.10766264	0.12993765	0.25994062	0.10749058	0.08297583	0.12994101	0.86836599
138.3	0.08204001	0.10776823	0.13001201	0.26013362	0.10753974	0.08293683	0.12999877	0.86814418
138.4	0.08214676	0.10787373	0.13008611	0.26032635	0.10758882	0.08289779	0.13005642	0.86792263
138.5	0.08225343	0.10797914	0.13015993	0.26051881	0.10763782	0.08285871	0.13011397	0.86770135
138.6	0.08236003	0.10808445	0.13023348	0.260711	0.10768675	0.08281958	0.13017142	0.86748032
138.7	0.08246655	0.10818967	0.13030677	0.26090291	0.10773562	0.08278042	0.13022877	0.86725956
138.8	0.082573	0.10829479	0.13037979	0.26109456	0.10778441	0.08274121	0.13028603	0.86703905
138.9	0.08267937	0.10839981	0.13045256	0.26128594	0.10783313	0.08270195	0.13034319	0.8668188
139	0.08278567	0.10850474	0.13052506	0.26147706	0.10788179	0.08266266	0.13040027	0.8665988
139.1	0.0828919	0.10860957	0.1305973	0.26166791	0.10793038	0.08262333	0.13045726	0.86637906
139.2	0.08299806	0.10871429	0.13066929	0.2618585	0.10797891	0.08258396	0.13051416	0.86615957
139.3	0.08310414	0.10881891	0.13074103	0.26204883	0.10802737	0.08254455	0.13057099	0.86594033
139.4	0.08321017	0.10892343	0.13081251	0.2622389	0.10807576	0.08250509	0.13062774	0.86572135
139.5	0.08331613	0.10902784	0.13088374	0.26242872	0.1081241	0.08246559	0.13068442	0.86550261
139.6	0.08342203	0.10913215	0.13095473	0.26261827	0.10817237	0.08242604	0.13074103	0.86528413
139.7	0.08352788	0.10923636	0.13102546	0.26280758	0.10822058	0.08238645	0.13079758	0.8650659
139.8	0.08363368	0.10934046	0.13109596	0.26299663	0.10826874	0.0823468	0.13085406	0.86484793
139.9	0.08373943	0.10944446	0.13116621	0.26318543	0.10831684	0.08230712	0.13091049	0.86463022
140	0.08384515	0.10954835	0.13123622	0.26337399	0.10836488	0.0822674	0.13096687	0.86441278
140.1	0.08395083	0.10965214	0.131306	0.26356229	0.10841286	0.08222766	0.1310232	0.86419561
140.2	0.08405648	0.10975583	0.13137554	0.26375035	0.10846079	0.0821879	0.13107948	0.86397871
140.3	0.08416211	0.10985941	0.13144485	0.26393817	0.10850866	0.08214814	0.13113572	0.86376208
140.4	0.08426773	0.1099629	0.13151393	0.26412574	0.10855649	0.08210839	0.13119192	0.86354572
140.5	0.08437335	0.11006628	0.13158278	0.26431307	0.10860426	0.08206864	0.13124808	0.86332963
140.6	0.08447898	0.11016955	0.13165141	0.26450016	0.10865198	0.08202891	0.13130422	0.86311383
140.7	0.08458464	0.11027273	0.13171981	0.26468701	0.10869965	0.08198919	0.13136028	0.8628983
140.8	0.08469032	0.11037581	0.13178799	0.26487363	0.10874727	0.08194949	0.13141618	0.86268306
140.9	0.08479604	0.11047879	0.13185595	0.26506001	0.10879485	0.08190979	0.13147182	0.86246809
141	0.08490181	0.11058168	0.1319237	0.26524616	0.10884238	0.0818701	0.1315271	0.8622534
141.1	0.08500765	0.11068447	0.13199124	0.26543207	0.10888986	0.0818304	0.13158193	0.86203899
141.2	0.08511356	0.11078716	0.13205857	0.26561776	0.1089373	0.08179069	0.13163625	0.86182486
141.3	0.08521954	0.11088976	0.1321257	0.26580321	0.1089847	0.08175099	0.13168998	0.86161101
141.4	0.08532557	0.11099226	0.13219264	0.26598844	0.10903205	0.0817113	0.13174305	0.86139744
141.5	0.08543164	0.11109467	0.13225938	0.26617344	0.10907937	0.08167164	0.13179536	0.86118413
141.6	0.08553772	0.11119699	0.13232594	0.26635821	0.10912664	0.08163203	0.13184688	0.86097109
141.7	0.08564376	0.11129922	0.13239231	0.26654277	0.10917388	0.08159246	0.1318976	0.86075831
141.8	0.08574975	0.11140135	0.1324585	0.2667271	0.10922108	0.08155295	0.13194748	0.86054578
141.9	0.08585565	0.11150339	0.13252452	0.26691121	0.10926824	0.0815135	0.13199651	0.8603335
142	0.08596145	0.11160535	0.13259037	0.2670951	0.10931537	0.08147412	0.13204468	0.86012146
142.1	0.08606712	0.11170721	0.13265606	0.26727877	0.10936246	0.0814348	0.13209204	0.85990966
142.2	0.08617268	0.11180899	0.13272159	0.26746223	0.10940952	0.08139555	0.13213862	0.8596981
142.3	0.0862781	0.11191067	0.13278696	0.26764548	0.10945654	0.08135638	0.13218447	0.85948678
142.4	0.08638339	0.11201227	0.13285217	0.26782851	0.10950354	0.08131728	0.13222966	0.85927568
142.5	0.08648855	0.11211379	0.13291722	0.26801133	0.1095505	0.08127825	0.13227426	0.85906481
142.6	0.08659358	0.11221522	0.13298211	0.26819394	0.10959744	0.0812393	0.13231835	0.85885418
142.7	0.08669847	0.11231657	0.13304684	0.26837635	0.10964435	0.08120043	0.13236204	0.85864376
142.8	0.08680324	0.11241784	0.1331114	0.26855855	0.10969124	0.08116164	0.13240543	0.85843358
142.9	0.08690788	0.11251903	0.13317579	0.26874054	0.1097381	0.08112293	0.13244854	0.85822362
143	0.0870124	0.11262015	0.13323999	0.26892233	0.10978495	0.0810843	0.13249134	0.85801388
143.1	0.0871168	0.11272119	0.13330402	0.26910392	0.10983177	0.08104576	0.13253388	0.85780437
143.2	0.08722107	0.11282218	0.13336785	0.26928532	0.10987858	0.08100731	0.13257621	0.85759508
143.3	0.08732522	0.11292309	0.13343149	0.26946651	0.10992537	0.08096895	0.13261837	0.85738602
143.4	0.08742926	0.11302395	0.13349492	0.26964751	0.10997215	0.08093068	0.1326604	0.85717717
143.5	0.08753317	0.11312476	0.13355816	0.26982832	0.11001892	0.0808925	0.13270232	0.85696855
143.6	0.08763697	0.11322552	0.13362122	0.27000894	0.11006567	0.08085443	0.13274411	0.85676015
143.7	0.08774065	0.11332623	0.13368406	0.27018937	0.11011242	0.08081645	0.1327858	0.85655198
143.8	0.08784421	0.11342691	0.13374668	0.27036961	0.11015916	0.08077857	0.1328274	0.85634402
143.9	0.08794765	0.11352756	0.13380905	0.27054967	0.1102059	0.08074079	0.13286894	0.85613628
144	0.08805098	0.11362819	0.13387116	0.27072954	0.11025264	0.0807031	0.1329104	0.85592875
144.1	0.08815419	0.11372879	0.13393304	0.27090923	0.11029938	0.08066548	0.13295178	0.85572145
144.2	0.08825729	0.11382937	0.13399471	0.27108873	0.11034612	0.08062795	0.1329931	0.85551436
144.3	0.08836027	0.11392991	0.13405618	0.27126805	0.11039287	0.08059048	0.13303434	0.85530749
144.4	0.08846313	0.1140304	0.13411746	0.27144718	0.11043963	0.08055308	0.13307552	0.85510083
144.5	0.08856589	0.11413082	0.13417857	0.27162613	0.1104864	0.08051574	0.13311664	0.85489438
144.6	0.08866853	0.11423115	0.1342395	0.2718049	0.11053318	0.08047847	0.13315768	0.85468815
144.7	0.08877106	0.11433139	0.13430025	0.27198348	0.11057998	0.08044126	0.13319866	0.85448213
144.8	0.08887348	0.11443152	0.13436081	0.27216189	0.1106268	0.08040411	0.13323957	0.85427632
144.9	0.08897579	0.11453154	0.13442118	0.27234011	0.11067365	0.08036703	0.13328041	0.85407073
145	0.08907799	0.11463146	0.13448135	0.27251815	0.11072052	0.08033001	0.13332118	0.85386534
145.1	0.08918009	0.11473126	0.13454134	0.27269602	0.1107674	0.08029305	0.13336189	0.85366016
145.2	0.08928207	0.11483094	0.13460113	0.27287371	0.11081429	0.08025616	0.13340252	0.8534552
145.3	0.08938396	0.11493052	0.13466074	0.27305122	0.11086118	0.08021934	0.13344309	0.85325044
145.4	0.08948573	0.11502998	0.13472016	0.27322856	0.11090806	0.08018258	0.13348359	0.85304588
145.5	0.08958741	0.11512933	0.13477939	0.27340574	0.11095491	0.0801459	0.13352402	0.85284154
145.6	0.08968898	0.11522857	0.13483844	0.27358274	0.11100174	0.08010928	0.13356438	0.8526374
145.7	0.08979045	0.1153277	0.13489731	0.27375958	0.11104854	0.08007273	0.13360467	0.85243346
145.8	0.08989181	0.11542673	0.134956	0.27393626	0.11109532	0.08003625	0.13364488	0.85222973
145.9	0.08999308	0.11552564	0.13501451	0.27411277	0.11114208	0.07999984	0.13368503	0.85202621
146	0.09009426	0.11562445	0.13507285	0.27428912	0.11118884	0.0799635	0.13372511	0.85182288
146.1	0.09019534	0.11572315	0.13513101	0.27446532	0.11123558	0.07992723	0.13376511	0.85161976
146.2	0.09029632	0.11582175	0.135189	0.27464136	0.11128231	0.07989103	0.13380506	0.85141684
146.3	0.09039721	0.11592025	0.13524683	0.27481724	0.11132903	0.0798549	0.13384494	0.85121412
146.4	0.09049802	0.11601866	0.13530449	0.27499297	0.11137574	0.07981885	0.13388477	0.8510116
146.5	0.09059874	0.11611697	0.13536198	0.27516856	0.11142244	0.07978286	0.13392455	0.85080928
146.6	0.09069937	0.11621519	0.13541932	0.275344	0.11146912	0.07974694	0.13396429	0.85060716
146.7	0.09079992	0.11631332	0.1354765	0.27551929	0.11151579	0.0797111	0.13400399	0.85040524
146.8	0.0909004	0.11641136	0.13553352	0.27569444	0.11156245	0.07967533	0.13404366	0.85020351
146.9	0.0910008	0.11650931	0.13559039	0.27586945	0.11160909	0.07963963	0.1340833	0.85000198
147	0.09110113	0.11660718	0.1356471	0.27604432	0.11165572	0.079604	0.1341229	0.84980065
147.1	0.0912014	0.11670497	0.13570367	0.27621904	0.11170234	0.07956844	0.13416247	0.84959951
147.2	0.09130161	0.11680269	0.1357601	0.27639363	0.11174894	0.07953297	0.13420201	0.84939857
147.3	0.09140176	0.11690032	0.13581637	0.27656806	0.11179554	0.07949756	0.13424151	0.84919782
147.4	0.09150187	0.11699789	0.13587251	0.27674235	0.11184213	0.07946224	0.13428099	0.84899727
147.5	0.09160194	0.11709538	0.13592848	0.2769165	0.11188871	0.079427	0.13432044	0.84879691
147.6	0.09170198	0.11719281	0.1359843	0.27709049	0.11193529	0.07939183	0.13435988	0.84859674
147.7	0.091802	0.11729018	0.13603996	0.27726435	0.11198185	0.07935674	0.13439933	0.84839676
147.8	0.091902	0.11738748	0.13609544	0.27743806	0.11202842	0.07932174	0.13443878	0.84819697
147.9	0.09200199	0.11748472	0.13615074	0.27761163	0.11207498	0.07928682	0.13447822	0.84799738
148	0.09210197	0.11758191	0.13620586	0.27778506	0.11212153	0.07925198	0.13451767	0.84779797
148.1	0.09220193	0.11767905	0.1362608	0.27795835	0.11216809	0.07921723	0.13455712	0.84759875
148.2	0.09230187	0.11777614	0.13631555	0.2781315	0.11221464	0.07918257	0.13459657	0.84739972
148.3	0.09240177	0.11787319	0.13637013	0.27830451	0.11226119	0.07914799	0.13463603	0.84720088
148.4	0.09250163	0.1179702	0.13642451	0.27847739	0.11230774	0.07911349	0.13467549	0.84700222
148.5	0.09260142	0.11806717	0.13647871	0.27865014	0.11235429	0.07907909	0.13471496	0.84680375
148.6	0.09270115	0.11816411	0.13653272	0.27882276	0.11240084	0.07904478	0.13475443	0.84660547
148.7	0.09280082	0.11826101	0.13658654	0.27899524	0.1124474	0.07901057	0.13479391	0.84640737
148.8	0.09290041	0.1183579	0.13664019	0.2791676	0.11249395	0.07897645	0.13483339	0.84620946
148.9	0.09299992	0.11845476	0.13669366	0.27933983	0.11254051	0.07894244	0.13487289	0.84601172
149	0.09309935	0.11855159	0.13674696	0.27951193	0.11258708	0.07890853	0.13491239	0.84581417
149.1	0.0931987	0.11864838	0.13680009	0.27968392	0.11263364	0.07887473	0.13495191	0.84561681
149.2	0.09329796	0.11874512	0.13685306	0.27985578	0.11268022	0.07884106	0.13499143	0.84541962
149.3	0.09339713	0.11884179	0.13690585	0.28002752	0.1127268	0.0788075	0.13503097	0.84522262
149.4	0.09349622	0.11893838	0.13695848	0.28019915	0.11277338	0.07877408	0.13507052	0.84502579
149.5	0.09359521	0.11903487	0.13701094	0.28037066	0.11281997	0.0787408	0.13511009	0.84482914
149.6	0.0936941	0.11913124	0.13706323	0.28054206	0.11286658	0.07870766	0.13514967	0.84463268
149.7	0.09379289	0.1192275	0.13711536	0.28071336	0.11291318	0.07867467	0.13518926	0.84443639
149.8	0.09389158	0.11932362	0.13716733	0.28088455	0.1129598	0.07864182	0.13522883	0.84424027
149.9	0.09399016	0.11941961	0.13721914	0.28105564	0.11300643	0.07860911	0.13526835	0.84404434
195	0.13096567	0.14800803	0.15044232	0.34482624	0.13378881	0.06960159	0.1467959	0.77270383
195.1	0.13103375	0.14804275	0.15045453	0.3449403	0.1338379	0.06959079	0.14680744	0.77259034
195.2	0.13110177	0.14807734	0.15046668	0.34505427	0.13388702	0.06958002	0.14681893	0.77247704
195.3	0.13116973	0.1481118	0.15047879	0.34516814	0.13393618	0.06956928	0.14683038	0.77236391
195.4	0.13123764	0.14814613	0.15049084	0.34528192	0.13398538	0.06955858	0.14684179	0.77225097
195.5	0.13130548	0.14818035	0.15050284	0.34539561	0.13403462	0.06954792	0.14685314	0.7721382
150.3	0.09438337	0.11980233	0.13742479	0.28173912	0.11319306	0.07847931	0.13542568	0.84326235
150.4	0.09448139	0.11989773	0.13747581	0.28190982	0.11323974	0.07844705	0.13546477	0.84306729
150.5	0.0945793	0.11999303	0.13752668	0.28208046	0.11328645	0.07841485	0.13550375	0.8428724
150.6	0.09467709	0.12008823	0.13757739	0.28225106	0.11333316	0.07838269	0.13554262	0.84267768
150.7	0.09477478	0.12018334	0.13762795	0.28242163	0.1133799	0.07835057	0.13558137	0.84248314
150.8	0.09487235	0.12027836	0.13767836	0.28259219	0.11342664	0.07831849	0.13562001	0.84228876
150.9	0.09496981	0.12037329	0.13772862	0.28276275	0.11347341	0.07828645	0.13565853	0.84209456
151	0.09506715	0.12046814	0.13777873	0.28293332	0.11352019	0.07825445	0.13569694	0.84190052
151.1	0.09516439	0.12056293	0.13782869	0.2831039	0.11356699	0.07822247	0.13573524	0.84170666
151.2	0.09526151	0.12065764	0.13787851	0.2832745	0.11361381	0.07819053	0.13577343	0.84151296
151.3	0.09535853	0.1207523	0.13792818	0.28344509	0.11366065	0.07815863	0.1358115	0.84131943
151.4	0.09545543	0.12084691	0.13797771	0.28361565	0.11370751	0.07812676	0.13584946	0.84112607
151.5	0.09555223	0.12094147	0.13802709	0.28378614	0.11375439	0.07809491	0.13588731	0.84093287
151.6	0.09564891	0.12103598	0.13807634	0.28395653	0.11380129	0.0780631	0.13592506	0.84073984
151.7	0.09574549	0.12113046	0.13812545	0.28412679	0.11384822	0.07803132	0.13596269	0.84054697
151.8	0.09584196	0.12122488	0.13817442	0.28429693	0.11389516	0.07799956	0.13600021	0.84035427
151.9	0.09593831	0.12131926	0.13822326	0.2844669	0.11394213	0.07796783	0.13603763	0.84016172
152	0.09603456	0.12141358	0.13827196	0.28463671	0.11398913	0.07793612	0.13607493	0.83996934
152.1	0.09613071	0.12150783	0.13832054	0.28480631	0.11403615	0.07790443	0.13611214	0.83977712
152.2	0.09622674	0.121602	0.13836898	0.28497571	0.11408319	0.07787276	0.13614923	0.83958506
152.3	0.09632267	0.12169609	0.1384173	0.28514493	0.11413026	0.0778411	0.13618622	0.83939315
152.4	0.09641849	0.12179007	0.13846549	0.28531395	0.11417736	0.07780947	0.13622311	0.83920141
152.5	0.09651421	0.12188393	0.13851356	0.28548278	0.11422448	0.07777785	0.13625989	0.83900981
152.6	0.09660982	0.12197767	0.13856151	0.28565143	0.11427164	0.07774628	0.13629657	0.83881838
152.7	0.09670532	0.12207128	0.13860934	0.28581988	0.11431882	0.07771474	0.13633315	0.8386271
152.8	0.09680071	0.12216474	0.13865706	0.28598815	0.11436603	0.07768326	0.13636963	0.83843597
152.9	0.09689601	0.12225804	0.13870467	0.28615625	0.11441327	0.07765184	0.13640601	0.83824499
153	0.09699119	0.12235119	0.13875217	0.28632419	0.11446054	0.07762049	0.1364423	0.83805416
153.1	0.09708628	0.12244417	0.13879957	0.28649197	0.11450785	0.0775892	0.13647848	0.83786347
153.2	0.09718125	0.122537	0.13884688	0.28665961	0.11455519	0.07755799	0.13651456	0.83767294
153.3	0.09727613	0.12262966	0.13889409	0.28682711	0.11460256	0.07752684	0.13655055	0.83748255
153.4	0.0973709	0.12272216	0.1389412	0.28699448	0.11464997	0.07749576	0.13658644	0.8372923
153.5	0.09746557	0.12281449	0.13898824	0.28716172	0.11469742	0.07746476	0.13662224	0.8371022
153.6	0.09756014	0.12290667	0.13903519	0.28732885	0.1147449	0.07743383	0.13665794	0.83691223
153.7	0.0976546	0.12299868	0.13908208	0.28749586	0.11479242	0.07740296	0.13669355	0.83672241
153.8	0.09774896	0.12309053	0.13912889	0.28766276	0.11483999	0.07737215	0.13672906	0.83653271
153.9	0.09784322	0.12318223	0.13917564	0.28782953	0.1148876	0.0773414	0.13676448	0.83634315
154	0.09793738	0.12327376	0.13922232	0.28799616	0.11493525	0.07731071	0.13679981	0.83615372
154.1	0.09803144	0.12336514	0.13926893	0.28816265	0.11498296	0.07728008	0.13683505	0.83596441
154.2	0.0981254	0.12345636	0.13931547	0.28832898	0.11503072	0.07724952	0.1368702	0.83577523
154.3	0.09821926	0.12354742	0.13936192	0.28849516	0.11507853	0.07721902	0.13690525	0.83558617
154.4	0.09831302	0.12363833	0.13940828	0.28866118	0.11512641	0.07718859	0.13694022	0.83539723
154.5	0.09840668	0.12372908	0.13945453	0.28882705	0.11517435	0.07715822	0.1369751	0.8352084
154.6	0.09850024	0.12381967	0.13950067	0.28899275	0.11522236	0.07712792	0.1370099	0.83501969
154.7	0.0985937	0.12391011	0.13954668	0.28915829	0.11527046	0.0770977	0.1370446	0.83483108
154.8	0.09868706	0.12400039	0.13959255	0.28932365	0.11531864	0.07706756	0.13707922	0.83464258
154.9	0.09878032	0.12409052	0.13963829	0.28948883	0.11536692	0.07703749	0.13711376	0.83445419
155	0.09887348	0.1241805	0.13968389	0.28965385	0.11541531	0.07700751	0.13714821	0.8342659
155.1	0.09896655	0.12427033	0.13972935	0.28981868	0.11546383	0.0769776	0.13718257	0.83407773
155.2	0.09905952	0.12436	0.13977468	0.28998333	0.11551246	0.07694777	0.13721685	0.83388966
155.3	0.0991524	0.12444953	0.13981988	0.29014779	0.11556123	0.07691803	0.13725105	0.83370172
155.4	0.09924517	0.1245389	0.13986496	0.29031204	0.11561013	0.07688837	0.13728517	0.8335139
155.5	0.09933785	0.12462813	0.13990991	0.29047609	0.11565912	0.07685879	0.13731921	0.83332622
155.6	0.09943044	0.1247172	0.13995475	0.29063993	0.11570818	0.07682929	0.13735317	0.83313867
155.7	0.09952293	0.12480613	0.13999949	0.29080358	0.11575727	0.07679987	0.13738704	0.83295127
155.8	0.09961532	0.12489491	0.14004412	0.29096701	0.11580634	0.07677053	0.13742085	0.83276402
155.9	0.09970762	0.12498355	0.14008866	0.29113024	0.11585539	0.07674127	0.13745457	0.83257692
156	0.09979983	0.12507203	0.14013309	0.29129326	0.11590439	0.07671209	0.13748822	0.83238998
156.1	0.09989194	0.12516038	0.14017743	0.29145608	0.11595333	0.07668298	0.1375218	0.83220318
156.2	0.09998396	0.12524858	0.14022168	0.29161869	0.11600219	0.07665396	0.1375553	0.83201653
156.3	0.10007589	0.12533663	0.14026583	0.2917811	0.11605097	0.07662501	0.13758873	0.83183004
156.4	0.10016772	0.12542454	0.1403099	0.2919433	0.11609968	0.07659614	0.13762209	0.83164369
156.5	0.10025946	0.12551232	0.14035387	0.2921053	0.11614831	0.07656735	0.13765537	0.8314575
156.6	0.10035112	0.12559994	0.14039775	0.2922671	0.11619687	0.07653864	0.13768859	0.83127146
156.7	0.10044267	0.12568743	0.14044153	0.29242871	0.11624538	0.07651001	0.13772173	0.83108556
156.8	0.10053414	0.12577478	0.14048523	0.29259011	0.11629383	0.07648145	0.13775481	0.83089981
156.9	0.10062552	0.12586199	0.14052883	0.29275133	0.11634222	0.07645297	0.13778782	0.8307142
157	0.10071681	0.12594908	0.14057235	0.29291235	0.11639056	0.07642457	0.13782075	0.83052874
157.1	0.100808	0.12603606	0.14061576	0.29307318	0.11643884	0.07639625	0.13785363	0.83034342
157.2	0.10089911	0.12612293	0.14065907	0.29323382	0.11648707	0.076368	0.13788643	0.83015824
157.3	0.10099012	0.12620969	0.14070228	0.29339427	0.11653525	0.07633983	0.13791917	0.82997319
157.4	0.10108105	0.12629637	0.14074537	0.29355453	0.11658337	0.07631175	0.13795184	0.82978828
157.5	0.10117189	0.12638294	0.14078834	0.2937146	0.11663142	0.07628374	0.13798444	0.82960349
157.6	0.10126264	0.12646943	0.14083118	0.29387449	0.11667942	0.0762558	0.13801698	0.82941884
157.7	0.10135329	0.12655583	0.14087388	0.2940342	0.11672736	0.07622795	0.13804945	0.82923431
157.8	0.10144387	0.12664215	0.14091645	0.29419371	0.11677523	0.07620018	0.13808186	0.82904991
157.9	0.10153435	0.12672838	0.14095889	0.29435305	0.11682304	0.07617248	0.13811421	0.82886563
158	0.10162474	0.12681451	0.14100119	0.29451221	0.1168708	0.07614486	0.13814649	0.82868146
158.1	0.10171505	0.12690053	0.14104335	0.29467118	0.11691849	0.07611733	0.13817871	0.8284974
158.2	0.10180527	0.12698641	0.14108538	0.29482998	0.11696612	0.07608987	0.13821087	0.82831345
158.3	0.10189541	0.12707215	0.14112727	0.29498859	0.1170137	0.07606248	0.13824296	0.8281296
158.4	0.10198546	0.12715771	0.14116903	0.29514703	0.11706122	0.07603518	0.138275	0.82794584
158.5	0.10207542	0.12724309	0.14121067	0.2953053	0.11710868	0.07600796	0.13830697	0.82776217
158.6	0.1021653	0.12732829	0.14125218	0.29546339	0.11715609	0.07598081	0.13833888	0.82757858
158.7	0.10225509	0.12741329	0.14129358	0.29562131	0.11720344	0.07595374	0.13837074	0.82739507
158.8	0.1023448	0.12749809	0.14133487	0.29577905	0.11725074	0.07592675	0.13840253	0.82721163
158.9	0.10243442	0.1275827	0.14137604	0.29593663	0.11729798	0.07589984	0.13843427	0.82702825
159	0.10252395	0.12766711	0.14141711	0.29609404	0.11734517	0.075873	0.13846595	0.82684493
159.1	0.10261341	0.12775132	0.14145808	0.29625128	0.11739231	0.07584625	0.13849757	0.82666165
159.2	0.10270278	0.12783533	0.14149896	0.29640835	0.11743939	0.07581957	0.13852914	0.82647842
159.3	0.10279206	0.12791915	0.14153974	0.29656525	0.11748643	0.07579297	0.13856065	0.82629524
159.4	0.10288127	0.12800278	0.14158043	0.29672199	0.11753341	0.07576645	0.1385921	0.82611211
159.5	0.10297039	0.12808623	0.14162104	0.29687856	0.11758034	0.07574001	0.1386235	0.82592905
159.6	0.10305942	0.12816948	0.14166157	0.29703497	0.11762722	0.07571365	0.13865484	0.82574606
159.7	0.10314838	0.12825256	0.14170203	0.29719122	0.11767405	0.07568736	0.13868613	0.82556316
159.8	0.10323725	0.12833544	0.14174241	0.2973473	0.11772084	0.07566116	0.13871737	0.82538036
159.9	0.10332604	0.12841814	0.14178273	0.29750323	0.11776757	0.07563503	0.13874855	0.82519768
160	0.10341475	0.12850066	0.14182298	0.297659	0.11781426	0.07560897	0.13877969	0.82501513
160.1	0.10350338	0.128583	0.14186315	0.29781462	0.1178609	0.075583	0.13881077	0.82483273
160.2	0.10359193	0.12866514	0.14190326	0.29797009	0.11790749	0.0755571	0.1388418	0.82465047
160.3	0.1036804	0.12874711	0.14194328	0.2981254	0.11795404	0.07553128	0.13887277	0.82446837
160.4	0.10376878	0.12882889	0.14198321	0.29828056	0.11800054	0.07550553	0.1389037	0.82428645
160.5	0.10385709	0.12891049	0.14202304	0.29843557	0.11804699	0.07547986	0.13893458	0.8241047
160.6	0.10394532	0.1289919	0.14206276	0.29859044	0.1180934	0.07545427	0.13896541	0.82392314
160.7	0.10403347	0.12907313	0.14210236	0.29874516	0.11813977	0.07542876	0.13899619	0.82374177
160.8	0.10412154	0.12915418	0.14214184	0.29889973	0.11818609	0.07540332	0.13902693	0.82356058
160.9	0.10420953	0.12923506	0.14218119	0.29905416	0.11823237	0.07537796	0.13905762	0.82337958
161	0.10429744	0.12931576	0.1422204	0.29920845	0.1182786	0.07535268	0.13908826	0.82319877
161.1	0.10438528	0.12939628	0.14225948	0.29936259	0.11832479	0.07532747	0.13911885	0.82301815
161.2	0.10447304	0.12947663	0.14229843	0.2995166	0.11837094	0.07530234	0.1391494	0.82283772
161.3	0.10456072	0.1295568	0.14233725	0.29967047	0.11841705	0.07527729	0.13917991	0.82265749
161.4	0.10464832	0.1296368	0.14237593	0.29982421	0.11846312	0.07525231	0.13921037	0.82247746
161.5	0.10473585	0.12971662	0.14241448	0.29997781	0.11850914	0.07522742	0.13924078	0.82229762
161.6	0.1048233	0.12979627	0.1424529	0.30013128	0.11855513	0.0752026	0.13927115	0.82211798
161.7	0.10491068	0.12987574	0.1424912	0.30028462	0.11860108	0.07517785	0.13930148	0.82193854
161.8	0.10499799	0.12995504	0.14252937	0.30043783	0.11864699	0.07515319	0.13933177	0.82175929
161.9	0.10508522	0.13003416	0.14256742	0.30059091	0.11869285	0.0751286	0.13936201	0.82158023
162	0.10517238	0.13011311	0.14260534	0.30074388	0.11873869	0.07510408	0.13939222	0.82140137
162.1	0.10525946	0.13019188	0.14264315	0.30089672	0.11878448	0.07507965	0.13942238	0.82122269
162.2	0.10534647	0.13027048	0.14268083	0.30104944	0.11883023	0.07505529	0.1394525	0.82104419
162.3	0.10543342	0.13034891	0.14271841	0.30120204	0.11887595	0.07503101	0.13948258	0.82086587
162.4	0.10552028	0.13042716	0.14275586	0.30135453	0.11892164	0.07500681	0.13951262	0.82068773
162.5	0.10560708	0.13050523	0.14279321	0.3015069	0.11896728	0.07498269	0.13954262	0.82050976
162.6	0.10569381	0.13058314	0.14283044	0.30165916	0.11901289	0.07495865	0.13957258	0.82033196
162.7	0.10578047	0.13066087	0.14286757	0.30181131	0.11905847	0.07493469	0.13960251	0.82015434
162.8	0.10586706	0.13073844	0.14290459	0.30196333	0.11910401	0.0749108	0.13963239	0.81997689
162.9	0.10595357	0.13081584	0.14294151	0.30211524	0.11914952	0.074887	0.13966224	0.81979961
163	0.10604002	0.13089307	0.14297832	0.30226703	0.11919499	0.07486328	0.13969206	0.81962251
163.1	0.1061264	0.13097014	0.14301504	0.3024187	0.11924044	0.07483964	0.13972183	0.81944559
163.2	0.10621272	0.13104706	0.14305166	0.30257025	0.11928584	0.07481609	0.13975157	0.81926884
163.3	0.10629896	0.13112383	0.14308818	0.30272168	0.11933122	0.07479262	0.13978128	0.81909228
163.4	0.10638514	0.13120045	0.1431246	0.302873	0.11937657	0.07476923	0.13981095	0.81891588
163.5	0.10647125	0.13127694	0.14316095	0.30302421	0.11942188	0.07474592	0.13984058	0.81873967
163.6	0.10655729	0.13135331	0.14319719	0.30317531	0.11946716	0.0747227	0.13987018	0.81856363
163.7	0.10664327	0.13142955	0.14323334	0.30332633	0.11951241	0.07469957	0.13989975	0.81838777
163.8	0.10672918	0.13150568	0.14326939	0.30347725	0.11955764	0.07467652	0.13992929	0.81821208
163.9	0.10681503	0.13158171	0.14330534	0.3036281	0.11960283	0.07465356	0.13995879	0.81803656
164	0.10690082	0.13165764	0.1433412	0.30377888	0.11964799	0.07463069	0.13998826	0.81786121
164.1	0.10698653	0.13173348	0.14337696	0.30392959	0.11969313	0.07460792	0.1400177	0.81768604
164.2	0.10707219	0.13180924	0.14341263	0.30408025	0.11973823	0.07458524	0.1400471	0.81751103
164.3	0.10715778	0.13188492	0.1434482	0.30423086	0.11978331	0.07456265	0.14007648	0.81733619
164.4	0.10724331	0.13196053	0.14348368	0.30438142	0.11982836	0.07454016	0.14010582	0.81716151
164.5	0.10732878	0.13203608	0.14351908	0.30453191	0.11987339	0.07451778	0.14013514	0.816987
164.6	0.10741419	0.13211157	0.14355439	0.30468234	0.11991839	0.07449549	0.14016442	0.81681264
164.7	0.10749953	0.13218703	0.14358963	0.30483269	0.11996336	0.07447331	0.14019368	0.81663845
164.8	0.10758482	0.13226245	0.14362479	0.30498294	0.1200083	0.07445124	0.14022291	0.81646442
164.9	0.10767004	0.13233785	0.14365989	0.30513309	0.12005323	0.07442928	0.14025211	0.81629054
210	0.14065845	0.15220365	0.15170765	0.36087606	0.14162068	0.06838431	0.14810776	0.75754964
210.1	0.14072067	0.15222515	0.15171321	0.36097524	0.1416764	0.06837864	0.14811446	0.75746026
210.2	0.14078283	0.1522466	0.15171873	0.36107432	0.14173216	0.068373	0.14812115	0.75737103
210.3	0.14084494	0.152268	0.15172424	0.36117331	0.14178798	0.0683674	0.14812783	0.75728192
210.4	0.140907	0.15228935	0.15172971	0.3612722	0.14184384	0.06836184	0.14813449	0.75719296
210.5	0.14096901	0.15231065	0.15173516	0.36137101	0.14189976	0.06835631	0.14814113	0.75710413
165.3	0.10801036	0.1326394	0.14379973	0.3057324	0.12023267	0.07434263	0.14036864	0.81559654
165.4	0.1080953	0.13271482	0.14383454	0.30588188	0.12027747	0.07432129	0.1403977	0.81542342
165.5	0.10818018	0.13279026	0.14386925	0.30603121	0.12032225	0.07430006	0.14042675	0.81525043
165.6	0.108265	0.1328657	0.14390387	0.30618039	0.12036701	0.07427895	0.14045576	0.81507759
165.7	0.10834977	0.13294112	0.14393839	0.30632944	0.12041174	0.07425796	0.14048476	0.81490489
165.8	0.10843449	0.13301647	0.1439728	0.30647834	0.12045645	0.07423708	0.14051373	0.81473233
165.9	0.10851915	0.13309173	0.1440071	0.3066271	0.12050115	0.0742163	0.14054268	0.81455989
166	0.10860377	0.13316685	0.14404126	0.30677573	0.12054582	0.07419564	0.14057161	0.81438759
166.1	0.10868833	0.1332418	0.1440753	0.30692422	0.12059047	0.07417507	0.14060052	0.81421541
166.2	0.10877284	0.13331657	0.14410921	0.30707258	0.1206351	0.07415458	0.1406294	0.81404336
166.3	0.1088573	0.13339112	0.144143	0.3072208	0.12067972	0.07413415	0.14065827	0.81387142
166.4	0.10894171	0.13346545	0.14417666	0.30736889	0.12072431	0.07411378	0.14068711	0.81369958
166.5	0.10902607	0.13353956	0.14421019	0.30751685	0.12076889	0.07409346	0.14071594	0.81352785
166.6	0.10911038	0.13361346	0.14424359	0.30766469	0.12081345	0.07407318	0.14074474	0.81335621
166.7	0.10919465	0.13368713	0.14427687	0.30781239	0.12085799	0.07405293	0.14077353	0.81318465
166.8	0.10927887	0.13376058	0.14431002	0.30795997	0.12090251	0.0740327	0.1408023	0.81301316
166.9	0.10936305	0.1338338	0.14434305	0.30810742	0.12094701	0.07401249	0.14083105	0.81284174
167	0.10944718	0.13390682	0.14437597	0.30825474	0.1209915	0.07399231	0.14085978	0.81267037
167.1	0.10953128	0.13397961	0.14440877	0.30840194	0.12103598	0.07397216	0.1408885	0.81249907
167.2	0.10961533	0.13405219	0.14444145	0.30854902	0.12108043	0.07395202	0.1409172	0.81232783
167.3	0.10969936	0.13412456	0.14447402	0.30869598	0.12112488	0.0739319	0.14094589	0.81215667
167.4	0.10978335	0.13419672	0.14450647	0.30884282	0.1211693	0.0739118	0.14097456	0.81198559
167.5	0.10986732	0.13426867	0.14453882	0.30898954	0.12121372	0.07389172	0.14100321	0.81181462
167.6	0.10995126	0.13434042	0.14457107	0.30913615	0.12125812	0.07387168	0.14103185	0.81164377
167.7	0.11003519	0.13441196	0.14460321	0.30928265	0.1213025	0.07385166	0.14106047	0.81147306
167.8	0.11011911	0.13448329	0.14463525	0.30942904	0.12134687	0.07383167	0.14108908	0.8113025
167.9	0.11020302	0.13455442	0.1446672	0.30957533	0.12139123	0.07381171	0.14111768	0.8111321
168	0.11028694	0.13462534	0.14469906	0.30972151	0.12143558	0.07379178	0.14114626	0.81096187
168.1	0.11037086	0.13469605	0.14473083	0.3098676	0.12147991	0.07377189	0.14117483	0.81079183
168.2	0.1104548	0.13476656	0.14476252	0.31001359	0.12152424	0.07375203	0.14120339	0.81062196
168.3	0.11053877	0.13483686	0.14479413	0.31015949	0.12156855	0.0737322	0.14123194	0.81045227
168.4	0.11062278	0.13490696	0.14482567	0.31030531	0.12161285	0.0737124	0.14126047	0.81028274
168.5	0.11070685	0.13497685	0.14485714	0.31045104	0.12165714	0.07369264	0.141289	0.81011337
168.6	0.11079097	0.13504654	0.14488855	0.31059669	0.12170141	0.07367291	0.14131751	0.80994417
168.7	0.11087517	0.13511602	0.14491988	0.31074227	0.12174568	0.07365321	0.14134602	0.80977512
168.8	0.11095943	0.13518531	0.14495114	0.31088777	0.12178994	0.07363354	0.14137451	0.80960622
168.9	0.11104375	0.13525439	0.14498231	0.31103321	0.12183419	0.0736139	0.141403	0.80943746
169	0.11112812	0.13532327	0.14501339	0.31117857	0.12187843	0.0735943	0.14143148	0.80926882
169.1	0.11121254	0.13539195	0.14504436	0.31132387	0.12192267	0.07357472	0.14145994	0.80910031
169.2	0.11129698	0.13546044	0.14507522	0.31146909	0.12196689	0.07355518	0.14148841	0.80893193
169.3	0.11138144	0.13552872	0.14510597	0.31161422	0.12201111	0.07353566	0.14151686	0.80876368
169.4	0.11146589	0.13559682	0.14513659	0.31175925	0.12205532	0.07351618	0.14154531	0.80859556
169.5	0.11155034	0.13566471	0.14516708	0.31190417	0.12209952	0.07349673	0.14157375	0.80842759
169.6	0.11163478	0.13573241	0.14519746	0.31204898	0.12214372	0.0734773	0.14160218	0.80825978
169.7	0.1117192	0.13579991	0.14522771	0.31219368	0.12218791	0.07345791	0.14163061	0.80809214
169.8	0.1118036	0.13586722	0.14525783	0.31233827	0.12223209	0.07343855	0.14165904	0.8079247
169.9	0.11188796	0.13593434	0.14528783	0.31248275	0.12227627	0.07341921	0.14168746	0.80775747
170	0.11197229	0.13600126	0.1453177	0.31262712	0.12232044	0.07339991	0.14171588	0.80759046
170.1	0.11205656	0.13606799	0.14534746	0.31277139	0.12236461	0.07338064	0.14174429	0.80742367
170.2	0.11214078	0.13613453	0.14537709	0.31291555	0.12240878	0.07336139	0.1417727	0.80725711
170.3	0.11222494	0.13620088	0.14540661	0.3130596	0.12245293	0.07334218	0.14180111	0.80709079
170.4	0.11230903	0.13626704	0.145436	0.31320356	0.12249709	0.073323	0.14182952	0.80692469
170.5	0.11239304	0.13633302	0.14546528	0.31334742	0.12254124	0.07330385	0.14185793	0.80675882
170.6	0.11247695	0.1363988	0.14549444	0.31349119	0.12258539	0.07328472	0.14188635	0.80659317
170.7	0.11256079	0.1364644	0.14552349	0.31363487	0.12262954	0.07326563	0.14191478	0.80642775
170.8	0.11264454	0.13652982	0.14555243	0.31377846	0.12267368	0.07324657	0.14194321	0.80626254
170.9	0.11272821	0.13659505	0.14558126	0.31392197	0.12271782	0.07322754	0.14197165	0.80609755
171	0.1128118	0.1366601	0.14560997	0.31406539	0.12276196	0.07320853	0.1420001	0.80593278
171.1	0.11289531	0.13672497	0.14563857	0.31420874	0.1228061	0.07318956	0.14202857	0.80576822
171.2	0.11297874	0.13678967	0.14566707	0.314352	0.12285024	0.07317062	0.14205707	0.80560386
171.3	0.1130621	0.13685418	0.14569546	0.31449516	0.12289437	0.07315171	0.1420856	0.80543972
171.4	0.11314539	0.13691851	0.14572374	0.31463823	0.12293851	0.07313283	0.14211417	0.80527578
171.5	0.1132286	0.13698267	0.14575192	0.31478118	0.12298265	0.07311398	0.14214278	0.80511204
171.6	0.11331174	0.13704664	0.14577999	0.31492402	0.12302678	0.07309516	0.14217146	0.8049485
171.7	0.11339482	0.13711044	0.14580796	0.31506673	0.12307092	0.07307637	0.1422002	0.80478515
171.8	0.11347783	0.13717406	0.14583583	0.31520931	0.12311506	0.07305761	0.14222902	0.80462199
171.9	0.11356077	0.13723751	0.1458636	0.31535178	0.1231592	0.07303888	0.14225793	0.80445902
172	0.11364365	0.13730078	0.14589126	0.31549413	0.12320334	0.07302018	0.14228694	0.80429622
172.1	0.11372648	0.13736388	0.14591883	0.31563637	0.12324748	0.07300151	0.14231606	0.80413361
172.2	0.11380924	0.13742681	0.14594628	0.31577852	0.12329162	0.07298287	0.14234532	0.80397117
172.3	0.11389196	0.13748956	0.14597363	0.31592057	0.12333577	0.07296427	0.14237469	0.80380894
172.4	0.11397463	0.13755215	0.14600088	0.31606254	0.12337992	0.07294569	0.14240417	0.80364692
172.5	0.11405727	0.13761456	0.14602801	0.31620443	0.12342408	0.07292714	0.14243374	0.80348511
172.6	0.11413987	0.13767682	0.14605503	0.31634625	0.12346824	0.07290863	0.14246336	0.80332354
172.7	0.11422244	0.13773891	0.14608194	0.316488	0.1235124	0.07289014	0.142493	0.80316221
172.8	0.11430499	0.13780085	0.14610873	0.31662966	0.12355657	0.07287169	0.14252264	0.80300113
172.9	0.11438752	0.13786264	0.14613541	0.31677122	0.12360074	0.07285326	0.14255225	0.80284031
173	0.11447005	0.13792428	0.14616197	0.31691266	0.12364491	0.07283487	0.14258182	0.80267975
173.1	0.11455257	0.13798577	0.14618842	0.31705397	0.1236891	0.07281651	0.14261134	0.80251945
173.2	0.1146351	0.13804712	0.14621475	0.31719514	0.12373329	0.07279818	0.1426408	0.80235941
173.3	0.11471764	0.13810833	0.14624097	0.31733615	0.12377748	0.07277987	0.14267019	0.80219963
173.4	0.11480021	0.13816941	0.14626709	0.31747699	0.12382168	0.0727616	0.1426995	0.8020401
173.5	0.11488281	0.13823035	0.14629309	0.31761767	0.12386589	0.07274336	0.14272871	0.80188084
173.6	0.11496543	0.13829115	0.146319	0.31775817	0.12391011	0.07272515	0.14275782	0.80172183
173.7	0.11504806	0.13835183	0.14634481	0.31789851	0.12395433	0.07270697	0.14278682	0.80156309
173.8	0.11513069	0.13841236	0.14637052	0.31803866	0.12399856	0.07268882	0.1428157	0.80140461
173.9	0.11521331	0.13847274	0.14639614	0.31817865	0.1240428	0.07267071	0.14284447	0.80124639
174	0.11529589	0.13853295	0.14642167	0.31831847	0.12408705	0.07265262	0.1428731	0.80108844
174.1	0.11537843	0.13859299	0.14644711	0.31845811	0.12413131	0.07263456	0.14290161	0.80093075
174.2	0.11546091	0.13865283	0.14647246	0.31859759	0.12417558	0.07261654	0.14292999	0.80077331
174.3	0.11554331	0.13871248	0.14649773	0.3187369	0.12421986	0.07259855	0.14295825	0.80061613
174.4	0.11562564	0.13877193	0.14652291	0.31887604	0.12426414	0.07258058	0.14298637	0.80045919
174.5	0.11570789	0.13883119	0.14654801	0.31901503	0.12430845	0.07256265	0.14301435	0.8003025
174.6	0.11579006	0.13889025	0.14657302	0.31915385	0.12435276	0.07254475	0.1430422	0.80014607
174.7	0.11587216	0.13894912	0.14659795	0.31929251	0.12439708	0.07252688	0.14306991	0.7999899
174.8	0.11595418	0.13900779	0.14662279	0.319431	0.12444142	0.07250904	0.14309749	0.79983399
174.9	0.11603613	0.13906627	0.14664756	0.31956934	0.12448577	0.07249124	0.14312493	0.79967834
175	0.11611801	0.13912456	0.14667224	0.31970752	0.12453014	0.07247346	0.14315224	0.79952294
175.1	0.11619983	0.13918267	0.14669683	0.31984554	0.12457452	0.07245572	0.14317941	0.79936779
175.2	0.11628157	0.13924059	0.14672135	0.31998341	0.12461892	0.07243801	0.14320646	0.79921289
175.3	0.11636324	0.13929833	0.14674579	0.32012111	0.12466333	0.07242032	0.14323338	0.79905824
175.4	0.11644484	0.13935588	0.14677015	0.32025867	0.12470777	0.07240268	0.14326019	0.79890382
175.5	0.11652637	0.13941326	0.14679442	0.32039607	0.12475222	0.07238506	0.14328688	0.79874965
175.6	0.11660783	0.13947045	0.14681862	0.32053332	0.1247967	0.07236747	0.14331346	0.79859572
175.7	0.1166892	0.13952746	0.14684275	0.32067043	0.1248412	0.07234992	0.14333992	0.79844202
175.8	0.11677048	0.13958429	0.14686679	0.32080738	0.12488573	0.0723324	0.14336627	0.79828855
175.9	0.11685168	0.13964095	0.14689076	0.32094419	0.12493028	0.07231491	0.14339251	0.79813532
176	0.11693278	0.13969742	0.14691466	0.32108085	0.12497486	0.07229746	0.14341865	0.79798233
176.1	0.1170138	0.13975372	0.14693848	0.32121737	0.12501946	0.07228004	0.14344467	0.79782956
176.2	0.11709473	0.13980985	0.14696223	0.32135375	0.12506409	0.07226265	0.14347058	0.79767702
176.3	0.11717556	0.1398658	0.1469859	0.32148999	0.12510875	0.0722453	0.14349639	0.7975247
176.4	0.11725631	0.13992158	0.1470095	0.32162608	0.12515342	0.07222798	0.14352208	0.79737262
176.5	0.11733697	0.13997719	0.14703303	0.32176205	0.12519811	0.07221069	0.14354768	0.79722075
176.6	0.11741756	0.14003263	0.14705649	0.32189788	0.12524282	0.07219345	0.14357316	0.79706912
176.7	0.11749806	0.1400879	0.14707988	0.32203358	0.12528753	0.07217623	0.14359854	0.79691771
176.8	0.11757848	0.14014299	0.14710321	0.32216915	0.12533225	0.07215905	0.14362381	0.79676652
176.9	0.11765883	0.14019792	0.14712646	0.32230459	0.12537699	0.07214191	0.14364898	0.79661556
177	0.11773911	0.14025268	0.14714964	0.3224399	0.12542173	0.07212481	0.14367404	0.79646481
177.1	0.11781932	0.14030727	0.14717276	0.32257508	0.12546647	0.07210774	0.143699	0.79631429
177.2	0.11789946	0.1403617	0.14719582	0.32271013	0.12551123	0.07209071	0.14372385	0.79616399
177.3	0.11797954	0.14041595	0.1472188	0.32284503	0.12555599	0.07207372	0.1437486	0.79601391
177.4	0.11805956	0.14047005	0.14724173	0.32297979	0.12560076	0.07205677	0.14377325	0.79586405
177.5	0.11813953	0.14052397	0.1472646	0.32311441	0.12564554	0.07203985	0.14379779	0.79571441
177.6	0.11821944	0.14057774	0.1472874	0.32324889	0.12569032	0.07202298	0.14382223	0.79556498
177.7	0.1182993	0.14063134	0.14731015	0.32338322	0.12573512	0.07200614	0.14384656	0.79541576
177.8	0.11837911	0.14068477	0.14733284	0.3235174	0.12577993	0.07198934	0.14387079	0.79526676
177.9	0.11845887	0.14073805	0.14735547	0.32365143	0.12582475	0.07197257	0.14389492	0.79511797
178	0.11853857	0.14079116	0.14737805	0.32378532	0.12586958	0.07195584	0.14391894	0.7949694
178.1	0.1186182	0.14084411	0.14740058	0.32391907	0.12591442	0.07193915	0.14394287	0.79482104
178.2	0.11869777	0.14089691	0.14742306	0.32405266	0.12595927	0.07192249	0.14396669	0.79467289
178.3	0.11877726	0.14094954	0.14744549	0.32418612	0.12600413	0.07190586	0.14399041	0.79452495
178.4	0.11885669	0.14100202	0.14746788	0.32431943	0.12604901	0.07188927	0.14401403	0.79437722
178.5	0.11893603	0.14105433	0.14749022	0.3244526	0.12609389	0.0718727	0.14403755	0.7942297
178.6	0.1190153	0.1411065	0.14751253	0.32458563	0.12613879	0.07185617	0.14406097	0.79408239
178.7	0.11909449	0.1411585	0.1475348	0.32471851	0.12618371	0.07183967	0.14408429	0.79393529
178.8	0.11917361	0.14121035	0.14755704	0.32485126	0.12622863	0.0718232	0.14410751	0.79378839
178.9	0.11925265	0.14126204	0.14757925	0.32498386	0.12627357	0.07180676	0.14413063	0.7936417
179	0.11933163	0.14131358	0.14760144	0.32511632	0.12631852	0.07179035	0.14415366	0.79349521
179.1	0.11941054	0.14136496	0.14762361	0.32524865	0.12636349	0.07177397	0.14417658	0.79334893
179.2	0.11948939	0.1414162	0.14764575	0.32538084	0.12640846	0.07175762	0.14419941	0.79320284
179.3	0.11956817	0.14146728	0.14766788	0.32551289	0.12645345	0.07174131	0.14422214	0.79305696
179.4	0.11964691	0.1415182	0.14768997	0.3256448	0.12649845	0.07172502	0.14424478	0.79291127
179.5	0.11972558	0.14156898	0.14771202	0.32577658	0.12654347	0.07170876	0.14426731	0.79276579
179.6	0.11980419	0.14161961	0.14773404	0.32590822	0.1265885	0.07169254	0.14428976	0.7926205
179.7	0.11988274	0.14167009	0.14775602	0.32603972	0.12663354	0.07167634	0.1443121	0.79247541
179.8	0.11996122	0.14172042	0.14777795	0.3261711	0.1266786	0.07166018	0.14433435	0.79233051
179.9	0.12003963	0.1417706	0.14779983	0.32630233	0.12672367	0.07164405	0.14435651	0.7921858
225	0.14936058	0.15477646	0.15235586	0.37493675	0.15061162	0.0678001	0.14900162	0.74550682
225.1	0.14941478	0.15478847	0.15235872	0.37502455	0.15067445	0.0677978	0.14900696	0.74543479
225.2	0.14946892	0.15480041	0.15236155	0.37511221	0.15073724	0.06779551	0.14901229	0.74536285
225.3	0.14952299	0.15481228	0.15236436	0.37519971	0.1508	0.06779323	0.14901762	0.74529101
225.4	0.149577	0.15482407	0.15236714	0.37528707	0.15086273	0.06779095	0.14902294	0.74521925
225.5	0.14963095	0.1548358	0.1523699	0.37537428	0.15092542	0.06778868	0.14902825	0.74514758

180.6	0.12058592	0.14211776	0.14795165	0.32721733	0.12703956	0.07153197	0.14450896	0.79117765
180.7	0.12066357	0.14216677	0.14797316	0.32734753	0.12708475	0.07151608	0.14453037	0.79103433
180.8	0.12074113	0.14221565	0.14799464	0.3274776	0.12712995	0.07150022	0.14455169	0.7908912
180.9	0.12081858	0.14226438	0.14801609	0.32760755	0.12717517	0.07148439	0.14457291	0.79074828
181	0.12089594	0.14231297	0.1480375	0.32773737	0.12722041	0.07146859	0.14459404	0.79060557
181.1	0.1209732	0.14236142	0.14805887	0.32786706	0.12726566	0.07145281	0.14461508	0.79046307
181.2	0.12105037	0.14240973	0.14808019	0.32799664	0.12731093	0.07143707	0.14463603	0.79032079
181.3	0.12112745	0.1424579	0.14810147	0.32812609	0.12735621	0.07142136	0.14465688	0.79017874
181.4	0.12120443	0.14250594	0.14812271	0.32825542	0.12740152	0.07140567	0.14467765	0.79003692
181.5	0.12128133	0.14255383	0.14814391	0.32838464	0.12744683	0.07139002	0.14469833	0.78989533
181.6	0.12135813	0.14260159	0.14816507	0.32851373	0.12749217	0.0713744	0.14471892	0.78975398
181.7	0.12143484	0.14264921	0.14818618	0.3286427	0.12753752	0.07135882	0.14473941	0.78961286
181.8	0.12151146	0.1426967	0.14820726	0.32877157	0.1275829	0.07134326	0.14475982	0.78947197
181.9	0.12158799	0.14274405	0.1482283	0.32890031	0.12762828	0.07132774	0.14478014	0.78933131
182	0.12166443	0.14279127	0.14824931	0.32902895	0.12767369	0.07131226	0.14480037	0.78919088
182.1	0.12174078	0.14283836	0.14827029	0.32915747	0.12771912	0.07129681	0.14482051	0.78905068
182.2	0.12181704	0.14288531	0.14829125	0.32928589	0.12776456	0.07128139	0.14484057	0.7889107
182.3	0.1218932	0.14293213	0.14831219	0.32941419	0.12781002	0.07126601	0.14486053	0.78877095
182.4	0.12196928	0.14297882	0.14833311	0.32954239	0.1278555	0.07125066	0.14488041	0.78863142
182.5	0.12204527	0.14302538	0.14835402	0.32967049	0.127901	0.07123534	0.1449002	0.78849211
182.6	0.12212117	0.14307181	0.14837494	0.32979849	0.12794652	0.07122006	0.14491991	0.78835303
182.7	0.12219698	0.14311811	0.14839585	0.32992639	0.12799206	0.07120482	0.14493952	0.78821417
182.8	0.1222727	0.14316429	0.14841677	0.33005419	0.12803761	0.07118961	0.14495905	0.78807553
182.9	0.12234833	0.14321034	0.1484377	0.3301819	0.12808319	0.07117444	0.14497849	0.7879371
183	0.12242388	0.14325626	0.14845862	0.33030952	0.12812878	0.07115929	0.14499785	0.7877989
183.1	0.12249934	0.14330205	0.14847954	0.33043704	0.1281744	0.07114419	0.14501711	0.7876609
183.2	0.12257471	0.14334772	0.14850045	0.33056448	0.12822003	0.07112912	0.1450363	0.78752312
183.3	0.12264999	0.14339326	0.14852132	0.33069182	0.12826569	0.07111408	0.14505539	0.78738555
183.4	0.12272519	0.14343868	0.14854216	0.33081907	0.12831136	0.07109908	0.1450744	0.78724818
183.5	0.1228003	0.14348397	0.14856294	0.33094622	0.12835706	0.07108411	0.14509333	0.78711102
183.6	0.12287532	0.14352913	0.14858365	0.33107326	0.12840278	0.07106918	0.14511216	0.78697407
183.7	0.12295026	0.14357418	0.14860429	0.33120019	0.12844851	0.07105428	0.14513092	0.78683732
183.8	0.12302512	0.14361909	0.14862484	0.33132702	0.12849427	0.07103942	0.14514958	0.78670077
183.9	0.12309989	0.14366388	0.1486453	0.33145372	0.12854005	0.07102459	0.14516816	0.78656442
184	0.12317457	0.14370855	0.14866567	0.33158032	0.12858585	0.0710098	0.14518666	0.78642827
184.1	0.12324918	0.14375309	0.14868595	0.3317068	0.12863168	0.07099504	0.14520507	0.78629231
184.2	0.1233237	0.14379751	0.14870613	0.33183316	0.12867752	0.07098032	0.14522339	0.78615654
184.3	0.12339813	0.14384181	0.14872622	0.33195942	0.12872339	0.07096563	0.14524163	0.78602097
184.4	0.12347249	0.14388599	0.1487462	0.33208556	0.12876927	0.07095097	0.14525978	0.78588559
184.5	0.12354676	0.14393005	0.1487661	0.33221158	0.12881518	0.07093635	0.14527784	0.78575038
184.6	0.12362095	0.143974	0.1487859	0.3323375	0.12886111	0.07092177	0.14529581	0.78561535
184.7	0.12369506	0.14401782	0.14880562	0.33246331	0.12890707	0.07090722	0.1453137	0.78548049
184.8	0.12376908	0.14406153	0.14882524	0.332589	0.12895305	0.07089271	0.1453315	0.78534579
184.9	0.12384303	0.14410513	0.14884478	0.3327146	0.12899905	0.07087823	0.14534922	0.78521126
185	0.12391689	0.14414861	0.14886424	0.33284009	0.12904507	0.07086379	0.14536685	0.78507688
185.1	0.12399068	0.14419198	0.14888361	0.33296549	0.12909111	0.07084938	0.14538439	0.78494265
185.2	0.12406438	0.14423524	0.1489029	0.3330908	0.12913718	0.070835	0.14540184	0.78480857
185.3	0.12413801	0.14427839	0.14892211	0.33321602	0.12918328	0.07082066	0.14541921	0.78467465
185.4	0.12421156	0.14432144	0.14894124	0.33334116	0.12922939	0.07080636	0.14543649	0.78454087
185.5	0.12428502	0.14436438	0.14896028	0.33346622	0.12927553	0.07079209	0.14545369	0.78440724
185.6	0.12435841	0.14440722	0.14897926	0.3335912	0.12932169	0.07077786	0.1454708	0.78427378
185.7	0.12443172	0.14444996	0.14899815	0.33371612	0.12936788	0.07076366	0.14548783	0.7841405
185.8	0.12450496	0.14449261	0.14901697	0.33384097	0.12941409	0.0707495	0.14550479	0.78400742
185.9	0.12457811	0.14453516	0.14903571	0.33396576	0.12946033	0.07073537	0.14552166	0.78387454
186	0.12465119	0.14457761	0.14905438	0.33409049	0.12950659	0.07072128	0.14553846	0.78374188
186.1	0.12472419	0.14461997	0.14907298	0.33421517	0.12955288	0.07070723	0.14555518	0.78360945
186.2	0.12479711	0.14466224	0.14909152	0.33433981	0.12959919	0.0706932	0.14557184	0.78347724
186.3	0.12486996	0.14470441	0.14910998	0.3344644	0.12964552	0.07067922	0.14558841	0.78334526
186.4	0.12494273	0.14474648	0.14912839	0.33458891	0.12969188	0.07066527	0.14560492	0.78321352
186.5	0.12501543	0.14478845	0.14914673	0.33471335	0.12973827	0.07065135	0.14562136	0.78308202
186.6	0.12508805	0.14483032	0.149165	0.33483768	0.12978468	0.07063747	0.14563773	0.78295076
186.7	0.1251606	0.14487209	0.14918323	0.33496189	0.12983112	0.07062363	0.14565402	0.78281973
186.8	0.12523307	0.14491375	0.14920139	0.33508597	0.12987758	0.07060982	0.14567025	0.78268894
186.9	0.12530546	0.1449553	0.1492195	0.33520991	0.12992407	0.07059605	0.14568641	0.78255839
187	0.12537779	0.14499672	0.14923757	0.33533371	0.12997058	0.07058231	0.1457025	0.78242807
187.1	0.12545004	0.14503802	0.14925559	0.33545738	0.13001712	0.07056861	0.14571852	0.78229799
187.2	0.12552221	0.14507921	0.14927355	0.33558089	0.13006369	0.07055494	0.14573448	0.78216814
187.3	0.12559431	0.14512028	0.14929146	0.33570427	0.13011028	0.07054131	0.14575036	0.78203852
187.4	0.12566634	0.14516124	0.14930931	0.33582751	0.1301569	0.07052772	0.14576618	0.78190914
187.5	0.1257383	0.14520207	0.14932711	0.33595062	0.13020355	0.07051416	0.14578192	0.78177998
187.6	0.12581019	0.1452428	0.14934485	0.33607359	0.13025023	0.07050064	0.14579761	0.78165106
187.7	0.125882	0.14528341	0.14936255	0.33619642	0.13029693	0.07048715	0.14581322	0.78152236
187.8	0.12595374	0.1453239	0.14938018	0.33631912	0.13034366	0.0704737	0.14582877	0.78139389
187.9	0.12602542	0.14536429	0.14939775	0.33644168	0.13039041	0.07046028	0.14584426	0.78126565
188	0.12609702	0.14540457	0.14941524	0.33656411	0.1304372	0.0704469	0.14585968	0.78113764
188.1	0.12616856	0.14544473	0.14943265	0.3366864	0.13048401	0.07043356	0.14587503	0.78100985
188.2	0.12624003	0.14548479	0.14944996	0.33680856	0.13053085	0.07042025	0.14589033	0.78088229
188.3	0.12631143	0.14552475	0.14946718	0.33693059	0.13057771	0.07040697	0.14590556	0.78075495
188.4	0.12638276	0.14556459	0.14948431	0.33705248	0.13062461	0.07039374	0.14592073	0.78062784
188.5	0.12645403	0.14560433	0.14950135	0.33717424	0.13067153	0.07038054	0.14593583	0.78050095
188.6	0.12652523	0.14564397	0.1495183	0.33729587	0.13071849	0.07036737	0.14595088	0.78037429
188.7	0.12659636	0.14568349	0.14953516	0.33741737	0.13076547	0.07035424	0.14596586	0.78024785
188.8	0.12666743	0.14572292	0.14955193	0.33753874	0.13081248	0.07034115	0.14598078	0.78012163
188.9	0.12673844	0.14576223	0.14956862	0.33765998	0.13085952	0.07032809	0.14599564	0.77999563
189	0.12680938	0.14580145	0.14958522	0.33778109	0.13090658	0.07031507	0.14601044	0.77986985
189.1	0.12688026	0.14584056	0.14960173	0.33790207	0.13095368	0.07030209	0.14602517	0.7797443
189.2	0.12695107	0.14587956	0.14961816	0.33802292	0.13100081	0.07028914	0.14603985	0.77961896
189.3	0.12702182	0.14591847	0.1496345	0.33814364	0.13104796	0.07027623	0.14605446	0.77949384
189.4	0.1270925	0.14595727	0.14965075	0.33826423	0.13109515	0.07026336	0.14606902	0.77936894
189.5	0.12716313	0.14599597	0.14966692	0.33838468	0.13114236	0.07025052	0.14608351	0.77924426
189.6	0.12723369	0.14603458	0.149683	0.33850501	0.13118961	0.07023771	0.14609795	0.77911979
189.7	0.12730419	0.14607309	0.14969899	0.33862521	0.13123688	0.07022495	0.14611232	0.77899554
189.8	0.12737463	0.1461115	0.1497149	0.33874529	0.13128419	0.07021222	0.14612664	0.77887151
189.9	0.127445	0.14614982	0.14973072	0.33886524	0.13133152	0.07019953	0.1461409	0.77874769
190	0.12751532	0.14618804	0.14974647	0.33898506	0.13137889	0.07018687	0.1461551	0.77862408
190.1	0.12758558	0.14622617	0.14976213	0.33910476	0.13142628	0.07017425	0.14616924	0.77850069
190.2	0.12765578	0.14626421	0.14977771	0.33922433	0.13147371	0.07016167	0.14618332	0.77837751
190.3	0.12772591	0.14630217	0.14979322	0.33934377	0.13152117	0.07014913	0.14619735	0.77825455
190.4	0.12779599	0.14634004	0.14980864	0.3394631	0.13156865	0.07013662	0.14621131	0.77813179
190.5	0.12786602	0.14637783	0.14982399	0.33958229	0.13161617	0.07012415	0.14622522	0.77800925
190.6	0.12793598	0.14641554	0.14983926	0.33970137	0.13166372	0.07011171	0.14623907	0.77788692
190.7	0.12800589	0.14645317	0.14985445	0.33982032	0.13171131	0.07009932	0.14625287	0.7777648
190.8	0.12807574	0.14649072	0.14986956	0.33993915	0.13175892	0.07008696	0.14626661	0.77764289
190.9	0.12814553	0.14652821	0.14988461	0.34005786	0.13180657	0.07007464	0.14628029	0.77752119
191	0.12821527	0.14656563	0.14989957	0.34017644	0.13185424	0.07006237	0.14629392	0.77739969
191.1	0.12828495	0.14660298	0.14991447	0.34029491	0.13190195	0.07005012	0.14630749	0.77727841
191.2	0.12835458	0.14664027	0.14992929	0.34041325	0.13194969	0.07003792	0.146321	0.77715733
191.3	0.12842415	0.14667751	0.14994403	0.34053148	0.13199746	0.07002576	0.14633446	0.77703646
191.4	0.12849367	0.14671468	0.14995871	0.34064958	0.13204527	0.07001364	0.14634787	0.77691579
191.5	0.12856314	0.1467518	0.14997332	0.34076757	0.13209311	0.07000157	0.14636122	0.77679533
191.6	0.12863255	0.14678886	0.14998786	0.34088544	0.13214098	0.06998953	0.14637452	0.77667508
191.7	0.12870191	0.14682584	0.15000233	0.34100319	0.13218888	0.06997753	0.14638776	0.77655502
191.8	0.12877122	0.14686275	0.15001674	0.34112082	0.13223682	0.06996558	0.14640094	0.77643518
191.9	0.12884047	0.14689957	0.15003109	0.34123834	0.13228478	0.06995367	0.14641408	0.77631553
192	0.12890968	0.14693631	0.15004538	0.34135574	0.13233278	0.06994179	0.14642716	0.77619609
192.1	0.12897883	0.14697295	0.1500596	0.34147302	0.13238082	0.06992995	0.14644019	0.77607685
192.2	0.12904793	0.14700949	0.15007377	0.34159019	0.13242889	0.06991815	0.14645316	0.77595781
192.3	0.12911699	0.14704594	0.15008787	0.34170725	0.13247699	0.06990638	0.14646608	0.77583897
192.4	0.12918599	0.14708228	0.15010191	0.34182419	0.13252512	0.06989464	0.14647895	0.77572033
192.5	0.12925495	0.14711853	0.15011589	0.34194101	0.13257329	0.06988294	0.14649177	0.77560188
192.6	0.12932386	0.14715469	0.15012979	0.34205772	0.13262149	0.06987128	0.14650453	0.77548364
192.7	0.12939271	0.14719076	0.15014362	0.34217432	0.13266973	0.06985964	0.14651725	0.7753656
192.8	0.12946153	0.14722676	0.15015739	0.34229081	0.132718	0.06984804	0.14652991	0.77524775
192.9	0.12953029	0.14726268	0.15017108	0.34240718	0.1327663	0.06983648	0.14654252	0.7751301
193	0.12959901	0.14729853	0.1501847	0.34252345	0.13281464	0.06982495	0.14655508	0.77501265
193.1	0.12966769	0.14733431	0.15019825	0.3426396	0.13286302	0.06981345	0.14656758	0.77489539
193.2	[illegible]	[illegible]	[illegible]	[illegible]	[illegible]	[illegible]	[illegible]	[illegible]
193.3	[illegible]	[illegible]	[illegible]	[illegible]	[illegible]	[illegible]	[illegible]	[illegible]
193.4	[illegible]	[illegible]	[illegible]	[illegible]	[illegible]	[illegible]	[illegible]	[illegible]
193.5	[illegible]	[illegible]	[illegible]	[illegible]	[illegible]	[illegible]	[illegible]	[illegible]
193.6	[illegible]	[illegible]	[illegible]	[illegible]	[illegible]	[illegible]	[illegible]	[illegible]
193.7	[illegible]	[illegible]	[illegible]	[illegible]	[illegible]	[illegible]	[illegible]	[illegible]
193.8	[illegible]	[illegible]	[illegible]	[illegible]	[illegible]	[illegible]	[illegible]	[illegible]
193.9	[illegible]	[illegible]	[illegible]	[illegible]	[illegible]	[illegible]	[illegible]	[illegible]
194	[illegible]	[illegible]	[illegible]	[illegible]	[illegible]	[illegible]	[illegible]	[illegible]
194.1	[illegible]	[illegible]	[illegible]	[illegible]	[illegible]	[illegible]	[illegible]	[illegible]
194.2	[illegible]	[illegible]	[illegible]	[illegible]	[illegible]	[illegible]	[illegible]	[illegible]
194.3	[illegible]	[illegible]	[illegible]	[illegible]	[illegible]	[illegible]	[illegible]	[illegible]
194.4	[illegible]	[illegible]	[illegible]	[illegible]	[illegible]	[illegible]	[illegible]	[illegible]
194.5	[illegible]	[illegible]	[illegible]	[illegible]	[illegible]	[illegible]	[illegible]	[illegible]
194.6	[illegible]	[illegible]	[illegible]	[illegible]	[illegible]	[illegible]	[illegible]	[illegible]
194.7	[illegible]	[illegible]	[illegible]	[illegible]	[illegible]	[illegible]	[illegible]	[illegible]
194.8	[illegible]	[illegible]	[illegible]	[illegible]	[illegible]	[illegible]	[illegible]	[illegible]
194.9	[illegible]	[illegible]	[illegible]	[illegible]	[illegible]	[illegible]	[illegible]	[illegible]
195.6	0.13137326	0.14821446	0.15051479	0.34550921	0.13408389	0.06953729	0.14686446	0.7720256
195.7	0.13144098	0.14824849	0.15052669	0.34562271	0.1341332	0.0695267	0.14687572	0.77191318
195.8	0.13150863	0.14828245	0.15053853	0.34573613	0.13418255	0.06951614	0.14688695	0.77180093
195.9	0.13157622	0.14831635	0.15055032	0.34584946	0.13423193	0.06950561	0.14689813	0.77168885
196	0.13164375	0.1483502	0.15056204	0.3459627	0.13428136	0.06949512	0.14690926	0.77157694
196.1	0.13171123	0.14838402	0.1505737	0.34607586	0.13433082	0.06948467	0.14692035	0.77146519
196.2	0.13177864	0.14841782	0.15058529	0.34618894	0.13438032	0.06947425	0.1469314	0.77135362
196.3	0.131846	0.14845161	0.15059682	0.34630193	0.13442986	0.06946387	0.1469424	0.7712422
196.4	0.1319133	0.14848539	0.15060828	0.34641485	0.13447943	0.06945352	0.14695336	0.77113094
196.5	0.13198055	0.14851918	0.15061967	0.3465277	0.13452905	0.06944321	0.14696428	0.77101984
196.6	0.13204774	0.14855296	0.15063099	0.34664048	0.1345787	0.06943293	0.14697515	0.7709089
196.7	0.13211488	0.14858674	0.15064225	0.34675319	0.13462839	0.06942269	0.14698598	0.77079811
196.8	0.13218197	0.14862051	0.15065345	0.34686584	0.13467812	0.06941249	0.14699677	0.77068747
196.9	0.13224901	0.14865426	0.15066458	0.34697843	0.13472789	0.06940232	0.14700752	0.77057698
197	0.132316	0.14868797	0.15067564	0.34709098	0.1347777	0.06939218	0.14701822	0.77046664
197.1	0.13238294	0.14872162	0.15068665	0.34720349	0.13482755	0.06938208	0.14702888	0.77035644
197.2	0.13244983	0.14875519	0.15069759	0.34731595	0.13487743	0.06937202	0.14703951	0.7702464
197.3	0.13251667	0.14878866	0.15070848	0.34742839	0.13492736	0.06936199	0.14705009	0.77013652
197.4	0.13258347	0.14882202	0.1507193	0.34754079	0.13497732	0.069352	0.14706062	0.77002681
197.5	0.13265022	0.14885527	0.15073006	0.34765315	0.13502733	0.06934205	0.14707112	0.76991727
197.6	0.13271693	0.14888842	0.15074076	0.34776546	0.13507737	0.06933213	0.14708158	0.76980791
197.7	0.13278359	0.14892144	0.1507514	0.34787771	0.13512746	0.06932224	0.147092	0.76969873
197.8	0.13285021	0.14895435	0.15076197	0.34798989	0.13517758	0.0693124	0.14710237	0.76958974
197.9	0.13291679	0.14898714	0.15077249	0.34810198	0.13522774	0.06930258	0.14711271	0.76948093
198	0.13298334	0.1490198	0.15078295	0.34821399	0.13527795	0.06929281	0.147123	0.76937231
198.1	0.13304984	0.14905235	0.15079335	0.3483259	0.13532819	0.06928307	0.14713326	0.76926387
198.2	0.13311631	0.14908477	0.15080368	0.34843772	0.13537848	0.06927337	0.14714348	0.76915562
198.3	0.13318275	0.14911708	0.15081396	0.34854942	0.1354288	0.0692637	0.14715366	0.76904755
198.4	0.13324915	0.14914927	0.15082418	0.34866103	0.13547916	0.06925407	0.1471638	0.76893966
198.5	0.13331552	0.14918134	0.15083435	0.34877254	0.13552957	0.06924448	0.14717391	0.76883195
198.6	0.13338186	0.14921329	0.15084446	0.34888394	0.13558002	0.06923492	0.14718397	0.76872442
198.7	0.13344816	0.14924512	0.15085451	0.34899525	0.1356305	0.0692254	0.14719401	0.76861707
198.8	0.13351443	0.14927684	0.1508645	0.34910645	0.13568103	0.06921592	0.14720401	0.7685099
198.9	0.13358066	0.14930844	0.15087444	0.34921756	0.1357316	0.06920648	0.14721397	0.76840291
199	0.13364685	0.14933993	0.15088432	0.34932858	0.13578221	0.06919707	0.1472239	0.7682961
199.1	0.13371299	0.1493713	0.15089415	0.34943951	0.13583286	0.0691877	0.1472338	0.76818947
199.2	0.13377909	0.14940256	0.15090393	0.34955035	0.13588355	0.06917836	0.14724367	0.76808302
199.3	0.13384513	0.14943371	0.15091365	0.34966111	0.13593428	0.06916907	0.1472535	0.76797674
199.4	0.13391113	0.14946474	0.15092333	0.34977179	0.13598506	0.06915981	0.1472633	0.76787065
199.5	0.13397707	0.14949566	0.15093295	0.34988238	0.13603587	0.06915059	0.14727306	0.76776473
199.6	0.13404295	0.14952646	0.15094252	0.34999289	0.13608673	0.06914141	0.14728278	0.76765899
199.7	0.13410879	0.14955716	0.15095204	0.3501033	0.13613763	0.06913227	0.14729247	0.76755343
199.8	0.13417456	0.14958774	0.15096152	0.35021362	0.13618857	0.06912317	0.14730212	0.76744805
199.9	0.13424028	0.14961821	0.15097094	0.35032385	0.13623956	0.06911411	0.14731173	0.76734284
200	0.13430594	0.14964857	0.15098033	0.35043399	0.13629058	0.06910508	0.1473213	0.7672378
200.1	0.13437155	0.14967882	0.15098966	0.35054402	0.13634165	0.0690961	0.14733084	0.76713294
200.2	0.1344371	0.14970897	0.15099896	0.35065395	0.13639276	0.06908716	0.14734033	0.76702826
200.3	0.13450259	0.149739	0.15100822	0.35076376	0.13644391	0.06907826	0.14734979	0.76692375
200.4	0.13456803	0.14976892	0.15101744	0.35087346	0.13649511	0.06906941	0.1473592	0.76681941
200.5	0.1346334	0.14979874	0.15102662	0.35098304	0.13654634	0.0690606	0.14736858	0.76671525
200.6	0.13469873	0.14982845	0.15103577	0.35109251	0.13659762	0.06905183	0.14737792	0.76661126
200.7	0.13476399	0.14985805	0.15104489	0.35120187	0.13664895	0.06904311	0.14738722	0.76650744
200.8	0.1348292	0.14988755	0.15105398	0.35131112	0.13670031	0.06903444	0.14739648	0.76640379
200.9	0.13489435	0.14991694	0.15106304	0.35142025	0.13675172	0.06902582	0.1474057	0.76630032
201	0.13495945	0.14994622	0.15107207	0.35152927	0.13680317	0.06901724	0.14741488	0.76619702
201.1	0.1350245	0.1499754	0.15108105	0.35163816	0.13685466	0.06900872	0.14742403	0.76609388
201.2	0.13508948	0.15000447	0.15108999	0.35174694	0.1369062	0.06900025	0.14743313	0.76599092
201.3	0.13515442	0.15003344	0.15109888	0.3518556	0.13695778	0.06899184	0.1474422	0.76588813
201.4	0.1352193	0.15006231	0.15110772	0.35196414	0.13700941	0.06898347	0.14745123	0.7657855
201.5	0.13528413	0.15009107	0.1511165	0.35207256	0.13706107	0.06897515	0.14746022	0.76568305
201.6	0.1353489	0.15011973	0.15112523	0.35218085	0.13711278	0.06896688	0.14746917	0.76558076
201.7	0.13541363	0.15014829	0.1511339	0.35228902	0.13716454	0.06895866	0.14747808	0.76547864
201.8	0.1354783	0.15017674	0.15114251	0.35239707	0.13721634	0.06895046	0.14748696	0.76537669
201.9	0.13554292	0.15020509	0.15115105	0.35250499	0.13726818	0.06894231	0.1474958	0.76527491
202	0.13560749	0.15023335	0.15115954	0.3526128	0.13732006	0.06893418	0.14750459	0.76517329
202.1	0.13567201	0.1502615	0.15116796	0.35272048	0.13737199	0.06892608	0.14751335	0.76507184
202.2	0.13573648	0.15028955	0.15117633	0.35282804	0.13742397	0.06891801	0.14752207	0.76497056
202.3	0.13580089	0.1503175	0.15118464	0.35293548	0.13747599	0.06890996	0.14753075	0.76486944
202.4	0.13586526	0.15034535	0.1511929	0.35304279	0.13752805	0.06890195	0.14753939	0.76476848
202.5	0.13592958	0.1503731	0.1512011	0.35314999	0.13758016	0.06889397	0.14754799	0.76466769
202.6	0.13599385	0.15040075	0.15120925	0.35325707	0.13763231	0.06888602	0.14755656	0.76456707
202.7	0.13605807	0.15042831	0.15121735	0.35336403	0.1376845	0.0688781	0.14756508	0.76446661
202.8	0.13612224	0.15045577	0.1512254	0.35347087	0.13773674	0.06887022	0.14757355	0.76436631
202.9	0.13618636	0.15048313	0.1512334	0.35357759	0.13778903	0.06886237	0.14758199	0.76426617
203	0.13625044	0.15051039	0.15124136	0.35368419	0.13784136	0.06885456	0.14759039	0.7641662
203.1	0.13631447	0.15053756	0.15124927	0.35379068	0.13789373	0.06884678	0.14759874	0.76406639
203.2	0.13637845	0.15056463	0.15125714	0.35389705	0.13794615	0.06883903	0.14760705	0.76396674
203.3	0.13644238	0.1505916	0.15126496	0.35400331	0.13799862	0.06883133	0.14761532	0.76386726
203.4	0.13650627	0.15061848	0.15127274	0.35410945	0.13805113	0.06882367	0.14762354	0.76376793
203.5	0.13657011	0.15064527	0.15128047	0.35421548	0.13810368	0.06881604	0.14763171	0.76366876
203.6	0.1366339	0.15067196	0.15128816	0.35432138	0.13815628	0.06880845	0.14763983	0.76356976
203.7	0.13669765	0.15069856	0.15129581	0.35442718	0.13820893	0.06880091	0.14764791	0.76347091
203.8	0.13676136	0.15072506	0.15130341	0.35453286	0.13826162	0.0687934	0.14765595	0.76337223
203.9	0.13682502	0.15075147	0.15131097	0.35463842	0.13831436	0.06878592	0.14766395	0.7632737
204	0.13688863	0.15077779	0.15131849	0.35474387	0.13836714	0.06877849	0.14767191	0.76317533
204.1	0.13695221	0.15080402	0.15132597	0.35484921	0.13841997	0.06877108	0.14767983	0.76307712
204.2	0.13701574	0.15083015	0.1513334	0.35495443	0.13847284	0.06876371	0.14768771	0.76297907
204.3	0.13707923	0.1508562	0.1513408	0.35505954	0.13852576	0.06875637	0.14769555	0.76288118
204.4	0.13714268	0.15088215	0.15134815	0.35516454	0.13857873	0.06874905	0.14770336	0.76278344
204.5	0.13720608	0.15090801	0.15135546	0.35526943	0.13863174	0.06874177	0.14771114	0.76268586
204.6	0.13726945	0.15093378	0.15136274	0.3553742	0.1386848	0.06873451	0.14771889	0.76258844
204.7	0.13733277	0.15095946	0.15136997	0.35547886	0.13873791	0.06872729	0.14772662	0.76249117
204.8	0.13739606	0.15098505	0.15137716	0.35558341	0.13879106	0.06872008	0.14773433	0.76239406
204.9	0.13745931	0.15101056	0.15138432	0.35568785	0.13884425	0.06871291	0.14774201	0.7622971
205	0.13752252	0.15103597	0.15139143	0.35579219	0.1388975	0.06870577	0.14774968	0.7622003
205.1	0.13758569	0.1510613	0.15139851	0.35589641	0.13895079	0.06869865	0.14775733	0.76210365
205.2	0.13764882	0.15108654	0.15140555	0.35600052	0.13900413	0.06869156	0.14776496	0.76200716
205.3	0.13771192	0.1511117	0.15141256	0.35610452	0.13905751	0.06868449	0.14777257	0.76191081
205.4	0.13777499	0.15113676	0.15141952	0.35620842	0.13911094	0.06867746	0.14778016	0.76181463
205.5	0.13783802	0.15116174	0.15142645	0.3563122	0.13916442	0.06867045	0.14778773	0.76171859
205.6	0.13790102	0.15118664	0.15143335	0.35641588	0.13921794	0.06866347	0.14779527	0.76162271
205.7	0.13796399	0.15121145	0.15144021	0.35651945	0.13927152	0.06865651	0.1478028	0.76152698
205.8	0.13802692	0.15123618	0.15144703	0.35662291	0.13932514	0.06864959	0.1478103	0.76143141
205.9	0.13808983	0.15126082	0.15145383	0.35672627	0.1393788	0.06864269	0.14781778	0.76133598
206	0.13815272	0.15128538	0.15146058	0.35682952	0.13943251	0.06863582	0.14782524	0.76124071
206.1	0.13821558	0.15130986	0.15146731	0.35693266	0.13948628	0.06862898	0.14783268	0.76114558
206.2	0.13827841	0.15133426	0.151474	0.3570357	0.13954008	0.06862216	0.14784009	0.76105061
206.3	0.13834123	0.15135858	0.15148066	0.35713863	0.13959394	0.06861537	0.14784749	0.76095579
206.4	0.13840403	0.15138282	0.15148729	0.35724146	0.13964784	0.06860861	0.14785486	0.76086111
206.5	0.1384668	0.15140699	0.15149389	0.35734418	0.13970179	0.06860188	0.14786221	0.76076659
206.6	0.13852955	0.15143107	0.15150046	0.3574468	0.13975579	0.06859517	0.14786955	0.76067221
206.7	0.13859229	0.15145508	0.15150701	0.35754931	0.13980984	0.06858849	0.14787686	0.76057799
206.8	0.138655	0.15147901	0.15151353	0.35765172	0.13986393	0.06858184	0.14788415	0.76048391
206.9	0.13871771	0.15150286	0.15152002	0.35775403	0.13991808	0.06857522	0.14789142	0.76038998
207	0.1387804	0.15152663	0.1515265	0.35785623	0.13997227	0.06856863	0.14789867	0.7602962
207.1	0.13884308	0.15155032	0.15153295	0.35795833	0.14002651	0.06856206	0.1479059	0.76020256
207.2	0.13890573	0.15157393	0.15153938	0.35806033	0.14008079	0.06855552	0.14791311	0.76010907
207.3	0.13896836	0.15159746	0.15154579	0.35816223	0.14013513	0.06854901	0.1479203	0.76001573
207.4	0.13903096	0.1516209	0.15155219	0.35826402	0.14018951	0.06854253	0.14792748	0.75992253
207.5	0.13909355	0.15164427	0.15155856	0.35836571	0.14024394	0.06853607	0.14793463	0.75982948
207.6	0.13915612	0.15166755	0.15156491	0.35846731	0.14029843	0.06852965	0.14794177	0.75973658
207.7	0.13921868	0.15169076	0.15157124	0.3585688	0.14035295	0.06852325	0.14794889	0.75964382
207.8	0.13928124	0.15171388	0.15157753	0.35867019	0.14040753	0.06851688	0.14795599	0.75955121
207.9	0.1393438	0.15173693	0.1515838	0.35877148	0.14046216	0.06851055	0.14796307	0.75945874
208	0.13940637	0.15175989	0.15159003	0.35887267	0.14051683	0.06850424	0.14797013	0.75936641
208.1	0.13946894	0.15178277	0.15159623	0.35897376	0.14057156	0.06849796	0.14797718	0.75927423
208.2	[illegible]	[illegible]	[illegible]	[illegible]	[illegible]	[illegible]	[illegible]	[illegible]
208.3	[illegible]	[illegible]	[illegible]	[illegible]	[illegible]	[illegible]	[illegible]	[illegible]
208.4	[illegible]	[illegible]	[illegible]	[illegible]	[illegible]	[illegible]	[illegible]	[illegible]
208.5	[illegible]	[illegible]	[illegible]	[illegible]	[illegible]	[illegible]	[illegible]	[illegible]
208.6	[illegible]	[illegible]	[illegible]	[illegible]	[illegible]	[illegible]	[illegible]	[illegible]
208.7	[illegible]	[illegible]	[illegible]	[illegible]	[illegible]	[illegible]	[illegible]	[illegible]
208.8	[illegible]	[illegible]	[illegible]	[illegible]	[illegible]	[illegible]	[illegible]	[illegible]
208.9	[illegible]	[illegible]	[illegible]	[illegible]	[illegible]	[illegible]	[illegible]	[illegible]
209	[illegible]	[illegible]	[illegible]	[illegible]	[illegible]	[illegible]	[illegible]	[illegible]
209.1	[illegible]	[illegible]	[illegible]	[illegible]	[illegible]	[illegible]	[illegible]	[illegible]
209.2	[illegible]	[illegible]	[illegible]	[illegible]	[illegible]	[illegible]	[illegible]	[illegible]
209.3	[illegible]	[illegible]	[illegible]	[illegible]	[illegible]	[illegible]	[illegible]	[illegible]
209.4	[illegible]	[illegible]	[illegible]	[illegible]	[illegible]	[illegible]	[illegible]	[illegible]
209.5	[illegible]	[illegible]	[illegible]	[illegible]	[illegible]	[illegible]	[illegible]	[illegible]
209.6	[illegible]	[illegible]	[illegible]	[illegible]	[illegible]	[illegible]	[illegible]	[illegible]
209.7	[illegible]	[illegible]	[illegible]	[illegible]	[illegible]	[illegible]	[illegible]	[illegible]
209.8	[illegible]	[illegible]	[illegible]	[illegible]	[illegible]	[illegible]	[illegible]	[illegible]
209.9	[illegible]	[illegible]	[illegible]	[illegible]	[illegible]	[illegible]	[illegible]	[illegible]
210.6	0.14103096	0.15233191	0.15174058	0.36146972	0.14195572	0.06835083	0.14814776	0.75701543
210.7	0.14109287	0.15235313	0.15174597	0.36156834	0.14201174	0.06834538	0.14815437	0.75692687
210.8	0.14115473	0.1523743	0.15175134	0.36166687	0.14206781	0.06833997	0.14816097	0.75683845
210.9	0.14121654	0.15239544	0.15175668	0.36176532	0.14212392	0.06833461	0.14816755	0.75675016
211	0.1412783	0.15241655	0.151762	0.36186367	0.14218009	0.06832929	0.14817412	0.756662
211.1	0.14134001	0.15243762	0.15176729	0.36196193	0.14223631	0.06832401	0.14818068	0.75657398
211.2	0.14140168	0.15245866	0.15177255	0.3620601	0.14229258	0.06831878	0.14818722	0.75648609
211.3	0.14146329	0.15247966	0.1517778	0.36215819	0.1423489	0.0683136	0.14819374	0.75639834
211.4	0.14152486	0.15250062	0.15178302	0.36225619	0.14240527	0.06830847	0.14820025	0.75631072
211.5	0.14158638	0.15252153	0.15178822	0.36235409	0.14246169	0.06830339	0.14820675	0.75622323
211.6	0.14164785	0.1525424	0.15179339	0.36245191	0.14251817	0.06829837	0.14821324	0.75613587
211.7	0.14170928	0.15256322	0.15179854	0.36254965	0.14257469	0.06829341	0.14821971	0.75604865
211.8	0.14177066	0.15258398	0.15180367	0.36264729	0.14263127	0.0682885	0.14822616	0.75596156
211.9	0.14183199	0.15260467	0.15180878	0.36274485	0.1426879	0.06828363	0.14823261	0.7558746
212	0.14189328	0.1526253	0.15181386	0.36284233	0.14274458	0.0682788	0.14823904	0.75578777
212.1	0.14195452	0.15264585	0.15181893	0.36293971	0.14280131	0.06827398	0.14824545	0.75570107
212.2	0.14201572	0.15266634	0.15182397	0.36303702	0.1428581	0.0682692	0.14825186	0.75561451
212.3	0.14207687	0.15268675	0.151829	0.36313423	0.14291494	0.06826443	0.14825825	0.75552807
212.4	0.14213798	0.15270709	0.151834	0.36323137	0.14297184	0.06825968	0.14826463	0.75544176
212.5	0.14219905	0.15272736	0.15183899	0.36332841	0.14302879	0.06825494	0.148271	0.75535559
212.6	0.14226008	0.15274757	0.15184395	0.36342538	0.14308579	0.06825021	0.14827735	0.75526954
212.7	0.14232106	0.1527677	0.1518489	0.36352226	0.14314285	0.0682455	0.14828369	0.75518362
212.8	0.142382	0.15278778	0.15185383	0.36361906	0.14319996	0.0682408	0.14829003	0.75509783
212.9	0.1424429	0.15280779	0.15185874	0.36371577	0.14325713	0.06823612	0.14829634	0.75501217
213	0.14250375	0.15282773	0.15186363	0.3638124	0.14331435	0.06823144	0.14830265	0.75492664
213.1	0.14256457	0.15284762	0.15186851	0.36390895	0.14337162	0.06822678	0.14830895	0.75484124
213.2	0.14262534	0.15286746	0.15187337	0.36400542	0.14342896	0.06822213	0.14831523	0.75475596
213.3	0.14268608	0.15288723	0.15187822	0.36410181	0.14348634	0.0682175	0.14832151	0.75467081
213.4	0.14274677	0.15290694	0.15188304	0.36419812	0.14354378	0.06821288	0.14832778	0.75458579
213.5	0.14280743	0.15292658	0.15188786	0.36429434	0.14360128	0.06820828	0.14833403	0.75450089
213.6	0.14286804	0.15294616	0.15189265	0.36439049	0.14365884	0.06820368	0.14834028	0.75441612
213.7	0.14292862	0.15296568	0.15189744	0.36448655	0.14371645	0.06819911	0.14834652	0.75433148
213.8	0.14298916	0.15298513	0.15190221	0.36458254	0.14377411	0.06819454	0.14835275	0.75424696
213.9	0.14304966	0.15300452	0.15190697	0.36467845	0.14383184	0.06819	0.14835897	0.75416257
214	0.14311013	0.15302384	0.15191172	0.36477428	0.14388962	0.06818546	0.14836518	0.7540783
214.1	0.14317056	0.1530431	0.15191647	0.36487004	0.14394746	0.06818094	0.14837139	0.75399415
214.2	0.14323095	0.15306229	0.15192122	0.36496572	0.14400536	0.06817643	0.14837759	0.75391014
214.3	0.14329131	0.15308142	0.15192597	0.36506132	0.14406331	0.06817194	0.14838378	0.75382624
214.4	0.14335164	0.15310049	0.15193073	0.36515685	0.14412132	0.06816747	0.14838998	0.75374247
214.5	0.14341193	0.15311951	0.15193551	0.3652523	0.14417939	0.068163	0.14839616	0.75365882
214.6	0.14347219	0.15313848	0.1519403	0.36534768	0.14423752	0.06815856	0.14840235	0.7535753
214.7	0.14353241	0.15315742	0.15194511	0.36544298	0.14429571	0.06815412	0.14840854	0.7534919
214.8	0.1435926	0.15317631	0.15194995	0.36553821	0.14435396	0.0681497	0.14841472	0.75340862
214.9	0.14365277	0.15319516	0.15195483	0.36563337	0.14441227	0.0681453	0.1484209	0.75332546
215	0.1437129	0.15321399	0.15195974	0.36572846	0.14447063	0.06814091	0.14842708	0.75324243
215.1	0.143773	0.15323279	0.15196469	0.36582348	0.14452906	0.06813654	0.14843326	0.75315952
215.2	0.14383307	0.15325157	0.15196969	0.36591842	0.14458755	0.06813218	0.14843945	0.75307673
215.3	0.14389311	0.15327033	0.15197473	0.3660133	0.1446461	0.06812783	0.14844563	0.75299406
215.4	0.14395312	0.15328908	0.15197982	0.36610811	0.14470471	0.0681235	0.14845181	0.75291151
215.5	0.1440131	0.15330781	0.15198494	0.36620285	0.14476338	0.06811919	0.14845799	0.75282908
215.6	0.14407305	0.15332651	0.15199008	0.36629753	0.14482211	0.06811489	0.14846416	0.75274678
215.7	0.14413298	0.1533452	0.15199522	0.36639214	0.14488091	0.0681106	0.14847033	0.75266459
215.8	0.14419288	0.15336385	0.15200035	0.36648669	0.14493977	0.06810633	0.14847649	0.75258252
215.9	0.14425276	0.15338246	0.15200547	0.36658119	0.14499869	0.06810208	0.14848265	0.75250057
216	0.14431261	0.15340101	0.15201055	0.36667562	0.14505767	0.06809784	0.14848879	0.75241875
216.1	0.14437243	0.15341951	0.15201558	0.36677	0.14511672	0.06809362	0.14849493	0.75233704
216.2	0.14443223	0.15343794	0.15202058	0.36686433	0.14517583	0.06808941	0.14850106	0.75225545
216.3	0.144492	0.1534563	0.15202554	0.3669586	0.14523501	0.06808522	0.14850718	0.75217398
216.4	0.14455175	0.1534746	0.15203047	0.36705282	0.14529425	0.06808104	0.14851328	0.75209263
216.5	0.14461148	0.15349284	0.15203535	0.36714699	0.14535357	0.06807688	0.14851938	0.75201139
216.6	0.14467119	0.15351099	0.1520402	0.36724112	0.14541296	0.06807274	0.14852546	0.75193028
216.7	0.14473087	0.15352907	0.15204501	0.36733519	0.14547244	0.06806861	0.14853154	0.75184928
216.8	0.14479054	0.15354705	0.15204979	0.36742923	0.145532	0.06806449	0.1485376	0.7517684
216.9	0.14485018	0.15356496	0.15205454	0.36752323	0.14559165	0.0680604	0.14854365	0.75168764
217	0.1449098	0.15358279	0.15205926	0.36761718	0.14565138	0.06805632	0.14854969	0.75160699
217.1	0.1449694	0.15360055	0.15206395	0.3677111	0.14571122	0.06805225	0.14855572	0.75152646
217.2	0.14502898	0.15361823	0.15206863	0.36780499	0.14577115	0.06804821	0.14856173	0.75144604
217.3	0.14508849	0.15363585	0.15207327	0.36789885	0.14583118	0.06804418	0.14856774	0.75136574
217.4	0.1451479	0.15365338	0.15207789	0.3679927	0.14589133	0.06804017	0.14857374	0.75128555
217.5	0.14520716	0.15367084	0.15208248	0.36808653	0.14595159	0.06803618	0.14857973	0.75120547
217.6	0.14526623	0.15368822	0.15208704	0.36818037	0.14601197	0.0680322	0.1485857	0.75112551
217.7	0.14532509	0.15370554	0.15209157	0.3682742	0.14607248	0.06802824	0.14859167	0.75104566
217.8	0.1453837	0.15372278	0.15209608	0.36836802	0.14613312	0.0680243	0.14859762	0.75096592
217.9	0.14544206	0.15373996	0.15210055	0.36846182	0.14619389	0.06802038	0.14860357	0.75088629
218	0.14550012	0.15375706	0.15210499	0.36855558	0.14625478	0.06801648	0.1486095	0.75080677
218.1	0.14555786	0.15377411	0.1521094	0.3686493	0.14631577	0.0680126	0.14861543	0.75072736
218.2	0.14561529	0.15379108	0.15211377	0.36874298	0.14637683	0.06800873	0.14862134	0.75064806
218.3	0.14567241	0.153808	0.15211812	0.36883663	0.14643795	0.06800488	0.14862724	0.75056887
218.4	0.1457292	0.15382485	0.15212244	0.36893028	0.1464991	0.06800105	0.14863314	0.75048978
218.5	0.14578568	0.15384165	0.15212673	0.36902395	0.14656028	0.06799725	0.14863902	0.75041081
218.6	0.14584186	0.15385839	0.15213099	0.36911766	0.14662147	0.06799346	0.14864489	0.75033193
218.7	0.14589776	0.15387507	0.15213523	0.36921141	0.14668268	0.06798968	0.14865076	0.75025317
218.8	0.14595341	0.15389168	0.15213944	0.36930519	0.14674391	0.06798593	0.14865661	0.7501745
218.9	0.14600883	0.15390823	0.15214362	0.36939897	0.14680515	0.0679822	0.14866245	0.75009594
219	0.14606406	0.15392471	0.15214779	0.36949271	0.1468664	0.06797849	0.14866829	0.75001748
219.1	0.14611912	0.15394113	0.15215193	0.36958639	0.14692768	0.0679748	0.14867411	0.74993913
219.2	0.14617406	0.15395746	0.15215605	0.36968001	0.14698896	0.06797113	0.14867993	0.74986087
219.3	0.14622892	0.15397373	0.15216015	0.36977355	0.14705027	0.06796747	0.14868573	0.74978272
219.4	0.14628375	0.15398992	0.15216424	0.36986699	0.1471116	0.06796384	0.14869153	0.74970466
219.5	0.14633852	0.15400603	0.15216831	0.36996033	0.14717296	0.06796023	0.14869731	0.7496267
219.6	0.14639322	0.15402206	0.15217236	0.37005355	0.14723434	0.06795664	0.14870308	0.74954885
219.7	0.14644783	0.15403801	0.1521764	0.37014666	0.14729576	0.06795307	0.14870885	0.74947109
219.8	0.14650239	0.15405388	0.15218043	0.37023966	0.14735721	0.06794952	0.1487146	0.74939344
219.9	0.14655691	0.15406968	0.15218445	0.37033255	0.14741869	0.06794599	0.14872034	0.74931589
220	0.14661138	0.15408539	0.15218845	0.37042534	0.14748021	0.06794249	0.14872608	0.74923845
220.1	0.14666584	0.15410103	0.15219243	0.37051802	0.14754175	0.067939	0.1487318	0.74916112
220.2	0.14672028	0.15411659	0.15219639	0.37061059	0.14760334	0.06793554	0.14873752	0.74908389
220.3	0.14677469	0.15413206	0.15220032	0.37070308	0.14766496	0.06793209	0.14874322	0.74900678
220.4	0.14682909	0.15414747	0.15220423	0.37079546	0.14772661	0.06792867	0.14874892	0.74892978
220.5	0.14688348	0.15416279	0.1522081	0.37088775	0.14778831	0.06792527	0.1487546	0.74885289
220.6	0.14693786	0.15417804	0.15221195	0.37097994	0.14785005	0.0679219	0.14876028	0.74877611
220.7	0.14699224	0.15419321	0.15221576	0.37107203	0.14791183	0.06791854	0.14876595	0.74869945
220.8	0.14704662	0.1542083	0.15221954	0.37116402	0.14797365	0.06791521	0.1487716	0.7486229
220.9	0.14710102	0.15422332	0.15222329	0.37125591	0.14803552	0.0679119	0.14877724	0.74854646
221	0.14715544	0.15423826	0.15222701	0.3713477	0.14809743	0.06790862	0.14878287	0.74847013
221.1	0.14720988	0.15425312	0.15223071	0.37143938	0.14815939	0.06790535	0.14878849	0.74839391
221.2	0.14726435	0.15426791	0.15223437	0.37153094	0.1482214	0.06790211	0.1487941	0.74831781
221.3	0.14731887	0.15428263	0.15223801	0.37162239	0.14828346	0.0678989	0.1487997	0.74824181
221.4	0.14737344	0.15429727	0.15224162	0.37171372	0.14834557	0.06789571	0.14880528	0.74816593
221.5	0.14742806	0.15431184	0.1522452	0.37180492	0.14840773	0.06789254	0.14881086	0.74809015
221.6	0.14748275	0.15432633	0.15224875	0.37189599	0.14846995	0.06788939	0.14881642	0.74801449
221.7	0.14753752	0.15434075	0.15225228	0.37198693	0.14853223	0.06788627	0.14882198	0.74793893
221.8	0.14759237	0.1543551	0.15225577	0.37207774	0.14859456	0.06788318	0.14882753	0.74786349
221.9	0.14764731	0.15436937	0.15225924	0.37216844	0.14865696	0.06788011	0.14883306	0.74778816
222	0.14770236	0.15438357	0.15226268	0.37225902	0.14871942	0.06787706	0.14883859	0.74771294
222.1	0.14775753	0.15439769	0.1522661	0.37234949	0.14878194	0.06787404	0.14884411	0.74763783
222.2	0.1478128	0.15441175	0.15226949	0.37243986	0.14884453	0.06787104	0.14884962	0.74756284
222.3	0.14786819	0.15442573	0.15227284	0.37253012	0.14890719	0.06786807	0.14885512	0.74748796
222.4	0.14792367	0.15443964	0.15227618	0.37262029	0.14896992	0.06786514	0.14886062	0.74741319
222.5	0.14797924	0.15445347	0.15227949	0.37271036	0.14903272	0.06786224	0.1488661	0.74733854
222.6	0.14803489	0.15446724	0.15228277	0.37280033	0.14909561	0.06785937	0.14887159	0.74726401
222.7	0.14809062	0.15448093	0.15228603	0.37289021	0.14915859	0.06785655	0.14887706	0.74718959
222.8	0.14814641	0.15449455	0.15228926	0.37298	0.14922167	0.06785377	0.14888253	0.74711529
222.9	0.14820226	0.15450811	0.15229247	0.37306971	0.14928485	0.06785103	0.14888801	0.7470411
223	0.14825814	0.15452158	0.15229566	0.37315934	0.14934812	0.06784833	0.14889347	0.74696704
223.1	0.14831402	0.15453499	0.15229882	0.37324888	0.14941146	0.06784567	0.14889894	0.74689309
223.2	[illegible]	[illegible]	[illegible]	[illegible]	[illegible]	[illegible]	[illegible]	[illegible]
223.3	[illegible]	[illegible]	[illegible]	[illegible]	[illegible]	[illegible]	[illegible]	[illegible]
223.4	[illegible]	[illegible]	[illegible]	[illegible]	[illegible]	[illegible]	[illegible]	[illegible]
223.5	[illegible]	[illegible]	[illegible]	[illegible]	[illegible]	[illegible]	[illegible]	[illegible]
223.6	[illegible]	[illegible]	[illegible]	[illegible]	[illegible]	[illegible]	[illegible]	[illegible]
223.7	[illegible]	[illegible]	[illegible]	[illegible]	[illegible]	[illegible]	[illegible]	[illegible]
223.8	[illegible]	[illegible]	[illegible]	[illegible]	[illegible]	[illegible]	[illegible]	[illegible]
223.9	[illegible]	[illegible]	[illegible]	[illegible]	[illegible]	[illegible]	[illegible]	[illegible]
224	[illegible]	[illegible]	[illegible]	[illegible]	[illegible]	[illegible]	[illegible]	[illegible]
224.1	[illegible]	[illegible]	[illegible]	[illegible]	[illegible]	[illegible]	[illegible]	[illegible]
224.2	[illegible]	[illegible]	[illegible]	[illegible]	[illegible]	[illegible]	[illegible]	[illegible]
224.3	[illegible]	[illegible]	[illegible]	[illegible]	[illegible]	[illegible]	[illegible]	[illegible]
224.4	[illegible]	[illegible]	[illegible]	[illegible]	[illegible]	[illegible]	[illegible]	[illegible]
224.5	[illegible]	[illegible]	[illegible]	[illegible]	[illegible]	[illegible]	[illegible]	[illegible]
224.6	[illegible]	[illegible]	[illegible]	[illegible]	[illegible]	[illegible]	[illegible]	[illegible]
224.7	[illegible]	[illegible]	[illegible]	[illegible]	[illegible]	[illegible]	[illegible]	[illegible]
224.8	[illegible]	[illegible]	[illegible]	[illegible]	[illegible]	[illegible]	[illegible]	[illegible]
224.9	[illegible]	[illegible]	[illegible]	[illegible]	[illegible]	[illegible]	[illegible]	[illegible]
225.6	0.14968484	0.15484745	0.15237263	0.37546134	0.15098807	0.06778642	0.14903355	0.74507601
225.7	0.14973868	0.15485902	0.15237534	0.37554826	0.15105069	0.06778417	0.14903885	0.74500452
225.8	0.14979245	0.15487053	0.15237802	0.37563504	0.15111327	0.06778192	0.14904414	0.74493312
225.9	0.14984616	0.15488195	0.15238068	0.37572167	0.15117582	0.06777968	0.14904942	0.7448618
226	0.1498998	0.1548933	0.15238331	0.37580817	0.15123834	0.06777745	0.1490547	0.74479057
226.1	0.14995334	0.15490457	0.15238592	0.37589452	0.15130081	0.06777522	0.14905997	0.74471943
226.2	0.15000679	0.15491576	0.15238849	0.37598074	0.15136326	0.067773	0.14906524	0.74464837
226.3	0.15006013	0.15492686	0.15239105	0.37606681	0.15142567	0.06777079	0.14907049	0.74457739
226.4	0.15011335	0.15493789	0.15239358	0.37615275	0.15148804	0.06776859	0.14907575	0.7445065
226.5	0.15016645	0.15494884	0.15239608	0.37623856	0.15155037	0.06776639	0.14908099	0.74443569
226.6	0.15021941	0.1549597	0.15239856	0.37632423	0.15161265	0.0677642	0.14908623	0.74436496
226.7	0.15027226	0.15497049	0.15240102	0.37640978	0.1516749	0.06776202	0.14909146	0.74429432
226.8	0.15032497	0.15498119	0.15240345	0.37649519	0.1517371	0.06775984	0.14909669	0.74422375
226.9	0.15037756	0.15499182	0.15240585	0.37658046	0.15179927	0.06775768	0.14910191	0.74415326
227	0.15043003	0.15500237	0.15240823	0.3766656	0.15186139	0.06775552	0.14910712	0.74408285
227.1	0.15048239	0.15501287	0.15241059	0.37675061	0.15192348	0.06775337	0.14911233	0.74401252
227.2	0.15053462	0.1550233	0.15241293	0.37683548	0.15198554	0.06775122	0.14911753	0.74394226
227.3	0.15058674	0.15503368	0.15241524	0.37692022	0.15204756	0.06774908	0.14912273	0.74387208
227.4	0.15063874	0.155044	0.15241752	0.37700482	0.15210956	0.06774695	0.14912792	0.74380197
227.5	0.15069063	0.15505426	0.15241978	0.37708929	0.15217153	0.06774483	0.14913311	0.74373193
227.6	0.15074239	0.15506448	0.15242202	0.37717362	0.15223347	0.06774271	0.1491383	0.74366196
227.7	0.15079403	0.15507466	0.15242424	0.37725782	0.15229541	0.06774061	0.14914348	0.74359206
227.8	0.15084556	0.15508479	0.15242643	0.37734189	0.15235732	0.06773851	0.14914865	0.74352223
227.9	0.15089697	0.15509487	0.1524286	0.37742582	0.15241924	0.06773641	0.14915383	0.74345245
228	0.15094826	0.15510491	0.15243075	0.37750961	0.15248114	0.06773433	0.14915899	0.74338274
228.1	0.15099943	0.15511491	0.15243288	0.37759327	0.15254304	0.06773225	0.14916416	0.74331309
228.2	0.15105049	0.15512486	0.15243498	0.3776768	0.15260493	0.06773019	0.14916932	0.74324351
228.3	0.15110143	0.15513477	0.15243706	0.3777602	0.15266681	0.06772813	0.14917448	0.74317398
228.4	0.15115226	0.15514464	0.15243912	0.37784347	0.15272867	0.06772607	0.14917963	0.74310451
228.5	0.15120298	0.15515447	0.15244115	0.37792661	0.15279052	0.06772403	0.14918477	0.74303511
228.6	0.15125358	0.15516425	0.15244317	0.37800961	0.15285234	0.067722	0.14918991	0.74296578
228.7	0.15130407	0.155174	0.15244516	0.37809248	0.15291414	0.06771997	0.14919505	0.74289652
228.8	0.15135445	0.1551837	0.15244713	0.37817523	0.15297591	0.06771795	0.14920018	0.74282732
228.9	0.15140472	0.15519337	0.15244908	0.37825784	0.15303764	0.06771594	0.1492053	0.7427582
229	0.15145488	0.15520299	0.15245101	0.37834033	0.15309933	0.06771394	0.14921042	0.74268916
229.1	0.15150493	0.15521258	0.15245292	0.37842268	0.15316098	0.06771194	0.14921553	0.74262019
229.2	0.15155487	0.15522212	0.1524548	0.37850491	0.15322259	0.06770996	0.14922063	0.7425513
229.3	0.1516047	0.15523163	0.15245667	0.37858701	0.15328416	0.06770798	0.14922573	0.74248249
229.4	0.15165442	0.15524109	0.15245851	0.37866897	0.15334569	0.06770602	0.14923082	0.74241376
229.5	0.15170403	0.15525052	0.15246034	0.37875082	0.15340718	0.06770406	0.14923591	0.74234511
229.6	0.15175353	0.15525991	0.15246214	0.37883253	0.15346863	0.06770211	0.14924099	0.74227654
229.7	0.15180292	0.15526926	0.15246392	0.37891412	0.15353005	0.06770017	0.14924606	0.74220806
229.8	0.15185221	0.15527857	0.15246569	0.37899558	0.15359144	0.06769824	0.14925113	0.74213966
229.9	0.15190139	0.15528785	0.15246743	0.37907691	0.15365279	0.06769632	0.14925619	0.74207136
230	0.15195046	0.15529709	0.15246915	0.37915811	0.1537141	0.0676944	0.14926124	0.74200316
230.1	0.15199943	0.1553063	0.15247086	0.37923919	0.15377539	0.0676925	0.14926629	0.74193505
230.2	0.1520483	0.15531548	0.15247254	0.37932014	0.15383664	0.06769061	0.14927133	0.74186703
230.3	0.15209706	0.15532462	0.1524742	0.37940097	0.15389786	0.06768872	0.14927637	0.74179911
230.4	0.15214572	0.15533373	0.15247585	0.37948167	0.15395905	0.06768685	0.1492814	0.74173129
230.5	0.15219428	0.15534282	0.15247748	0.37956224	0.15402021	0.06768498	0.14928642	0.74166356
230.6	0.15224273	0.15535187	0.15247908	0.37964269	0.15408133	0.06768312	0.14929144	0.74159593
230.7	0.15229109	0.15536091	0.15248067	0.37972301	0.15414242	0.06768128	0.14929645	0.74152839
230.8	0.15233935	0.15536992	0.15248224	0.37980321	0.15420348	0.06767944	0.14930146	0.74146094
230.9	0.15238751	0.1553789	0.1524838	0.37988328	0.1542645	0.06767761	0.14930646	0.74139358
231	0.15243558	0.15538787	0.15248533	0.37996323	0.15432549	0.06767579	0.14931146	0.74132632
231.1	0.15248355	0.15539683	0.15248684	0.38004306	0.15438645	0.06767398	0.14931645	0.74125914
231.2	0.15253142	0.15540576	0.15248834	0.38012276	0.15444737	0.06767218	0.14932143	0.74119206
231.3	0.1525792	0.15541469	0.15248982	0.38020234	0.15450826	0.0676704	0.14932641	0.74112508
231.4	0.15262688	0.1554236	0.15249128	0.3802818	0.15456912	0.06766862	0.14933138	0.74105818
231.5	0.15267447	0.15543251	0.15249273	0.38036113	0.15462994	0.06766685	0.14933635	0.74099137
231.6	0.15272198	0.1554414	0.15249416	0.38044035	0.15469074	0.06766509	0.14934131	0.74092466
231.7	0.15276939	0.15545028	0.15249557	0.38051944	0.1547515	0.06766334	0.14934626	0.74085803
231.8	0.15281672	0.15545915	0.15249696	0.38059841	0.15481222	0.0676616	0.14935121	0.7407915
231.9	0.15286396	0.15546799	0.15249834	0.38067725	0.15487292	0.06765987	0.14935616	0.74072506
232	0.15291113	0.15547681	0.1524997	0.38075598	0.15493358	0.06765815	0.1493611	0.74065871
232.1	0.15295822	0.1554856	0.15250104	0.38083459	0.1549942	0.06765644	0.14936603	0.74059245
232.2	0.15300524	0.15549435	0.15250237	0.38091307	0.1550548	0.06765474	0.14937096	0.74052628
232.3	0.1530522	0.15550306	0.15250368	0.38099144	0.15511537	0.06765305	0.14937588	0.7404602
232.4	0.15309908	0.15551172	0.15250498	0.38106968	0.1551759	0.06765138	0.1493808	0.74039421
232.5	0.15314591	0.15552034	0.15250626	0.38114781	0.1552364	0.06764971	0.14938571	0.74032831
232.6	0.15319267	0.15552891	0.15250752	0.38122581	0.15529687	0.06764805	0.14939062	0.7402625
232.7	0.15323939	0.15553742	0.15250877	0.3813037	0.15535731	0.0676464	0.14939552	0.74019678
232.8	0.15328605	0.15554589	0.15251001	0.38138147	0.15541771	0.06764477	0.14940041	0.74013114
232.9	0.15333265	0.1555543	0.15251123	0.38145912	0.15547809	0.06764314	0.1494053	0.7400656
233	0.15337918	0.15556266	0.15251243	0.38153665	0.15553843	0.06764153	0.14941019	0.74000014
233.1	0.15342564	0.15557096	0.15251362	0.38161406	0.15559875	0.06763992	0.14941506	0.73993477
233.2	0.153472	0.15557921	0.1525148	0.38169135	0.15565903	0.06763833	0.14941994	0.73986949
233.3	0.15351828	0.1555874	0.15251597	0.38176853	0.15571929	0.06763674	0.14942481	0.7398043
233.4	0.15356445	0.15559553	0.15251712	0.38184559	0.15577951	0.06763517	0.14942967	0.73973919
233.5	0.15361052	0.15560361	0.15251826	0.38192253	0.1558397	0.06763361	0.14943453	0.73967417
233.6	0.15365648	0.15561162	0.15251939	0.38199936	0.15589986	0.06763206	0.14943938	0.73960924
233.7	0.15370232	0.15561959	0.1525205	0.38207607	0.15596	0.06763052	0.14944423	0.73954439
233.8	0.15374805	0.15562749	0.1525216	0.38215266	0.1560201	0.06762899	0.14944907	0.73947963
233.9	0.15379367	0.15563535	0.1525227	0.38222914	0.15608017	0.06762747	0.14945391	0.73941496
234	0.15383917	0.15564316	0.15252378	0.3823055	0.15614022	0.06762597	0.14945874	0.73935037
234.1	0.15388455	0.15565092	0.15252485	0.38238175	0.15620023	0.06762447	0.14946357	0.73928587
234.2	0.15392981	0.15565864	0.15252591	0.38245789	0.15626022	0.06762298	0.14946839	0.73922145
234.3	0.15397495	0.15566631	0.15252696	0.38253391	0.15632018	0.06762151	0.14947321	0.73915711
234.4	0.15401998	0.15567394	0.152528	0.38260981	0.15638011	0.06762005	0.14947802	0.73909286
234.5	0.1540649	0.15568153	0.15252903	0.3826856	0.15644001	0.0676186	0.14948283	0.7390287
234.6	0.1541097	0.15568908	0.15253006	0.38276128	0.15649989	0.06761716	0.14948764	0.73896462
234.7	0.15415438	0.15569658	0.15253108	0.38283685	0.15655975	0.06761574	0.14949243	0.73890062
234.8	0.15419896	0.15570406	0.1525321	0.3829123	0.15661958	0.06761432	0.14949723	0.7388367
234.9	0.15424341	0.15571149	0.15253311	0.38298765	0.15667939	0.06761292	0.14950201	0.73877287
235	0.15428776	0.15571888	0.15253411	0.38306288	0.15673918	0.06761153	0.1495068	0.73870912
235.1	0.15433199	0.15572623	0.15253512	0.383138	0.15679895	0.06761016	0.14951158	0.73864545
235.2	0.15437612	0.15573355	0.15253612	0.38321301	0.15685869	0.06760879	0.14951635	0.73858187
235.3	0.15442012	0.15574083	0.15253713	0.38328791	0.15691841	0.06760744	0.14952112	0.73851836
235.4	0.15446402	0.15574807	0.15253813	0.3833627	0.15697811	0.06760611	0.14952588	0.73845494
235.5	0.15450781	0.15575527	0.15253914	0.38343738	0.15703779	0.06760478	0.14953064	0.73839159
235.6	0.15455149	0.15576244	0.15254015	0.38351196	0.15709745	0.06760347	0.14953539	0.73832833
235.7	0.15459505	0.15576957	0.15254116	0.38358643	0.15715709	0.06760217	0.14954014	0.73826514
235.8	0.15463851	0.15577665	0.15254218	0.38366079	0.15721672	0.06760089	0.14954489	0.73820204
235.9	0.15468185	0.15578371	0.15254321	0.38373505	0.15727632	0.06759962	0.14954963	0.73813901
236	0.15472509	0.15579072	0.15254424	0.3838092	0.15733591	0.06759836	0.14955437	0.73807606
236.1	0.15476822	0.1557977	0.15254527	0.38388324	0.15739547	0.06759711	0.1495591	0.73801319
236.2	0.15481124	0.15580464	0.1525463	0.38395719	0.15745503	0.06759588	0.14956382	0.7379504
236.3	0.15485415	0.15581154	0.15254733	0.38403103	0.15751456	0.06759467	0.14956855	0.73788768
236.4	0.15489695	0.15581841	0.15254835	0.38410476	0.15757408	0.06759347	0.14957326	0.73782504
236.5	0.15493964	0.15582524	0.15254937	0.3841784	0.15763358	0.06759228	0.14957798	0.73776247
236.6	0.15498223	0.15583203	0.15255039	0.38425194	0.15769307	0.0675911	0.14958268	0.73769998
236.7	0.15502471	0.15583879	0.1525514	0.38432538	0.15775254	0.06758995	0.14958739	0.73763756
236.8	0.15506708	0.15584551	0.15255241	0.38439872	0.157812	0.0675888	0.14959209	0.73757522
236.9	0.15510935	0.15585219	0.15255341	0.38447197	0.15787145	0.06758767	0.14959678	0.73751295
237	0.1551515	0.15585884	0.1525544	0.38454511	0.15793089	0.06758656	0.14960147	0.73745074
237.1	0.15519356	0.15586545	0.1525554	0.38461817	0.15799031	0.06758546	0.14960616	0.73738861
237.2	0.15523551	0.15587203	0.15255639	0.38469112	0.15804972	0.06758437	0.14961084	0.73732654
237.3	0.15527735	0.15587857	0.15255737	0.38476398	0.15810912	0.0675833	0.14961552	0.73726454
237.4	0.15531908	0.15588507	0.15255835	0.38483674	0.1581685	0.06758225	0.14962019	0.73720261
237.5	0.15536072	0.15589154	0.15255932	0.3849094	0.15822788	0.06758121	0.14962486	0.73714074
237.6	0.15540224	0.15589798	0.15256028	0.38498196	0.15828725	0.06758019	0.14962952	0.73707893
237.7	0.15544367	0.15590438	0.15256122	0.38505441	0.15834661	0.06757918	0.14963418	0.73701719
237.8	0.15548498	0.15591074	0.15256215	0.38512676	0.15840596	0.06757819	0.14963884	0.73695551
237.9	0.1555262	0.15591707	0.15256307	0.38519901	0.15846531	0.06757721	0.14964349	0.73689388
238	0.15556731	0.15592337	0.15256397	0.38527115	0.15852465	0.06757625	0.14964813	0.73683232
238.1	0.15560832	0.15592963	0.15256485	0.38534319	0.15858398	0.06757531	0.14965278	0.73677081
238.2	0.15564923	0.15593585	0.15256571	0.38541511	0.15864331	0.06757438	0.14965742	0.73670936
238.3	0.15569003	0.15594205	0.15256656	0.38548694	0.15870263	0.06757347	0.14966205	0.73664796
238.4	0.15573073	0.1559482	0.15256738	0.38555865	0.15876195	0.06757258	0.14966668	0.73658661
238.5	0.15577133	0.15595433	0.15256819	0.38563026	0.15882126	0.0675717	0.14967131	0.73652532
238.6	0.15581183	0.15596042	0.15256898	0.38570177	0.15888057	0.06757084	0.14967593	0.73646408
238.7	0.15585222	0.15596647	0.15256976	0.38577316	0.15893988	0.06757	0.14968055	0.73640289
238.8	0.15589252	0.15597249	0.15257052	0.38584445	0.15899919	0.06756919	0.14968516	0.73634177
238.9	0.15593271	0.15597848	0.15257126	0.38591563	0.15905851	0.0675684	0.14968977	0.7362807
239	0.15597281	0.15598444	0.15257198	0.3859867	0.15911782	0.06756763	0.14969437	0.7362197
239.1	0.1560128	0.15599036	0.15257269	0.38605767	0.15917714	0.0675669	0.14969898	0.73615876
239.2	0.1560527	0.15599625	0.15257338	0.38612853	0.15923645	0.06756619	0.14970357	0.73609789
239.3	0.15609249	0.1560021	0.15257405	0.38619928	0.15929575	0.06756551	0.14970817	0.73603709
239.4	0.15613218	0.15600793	0.15257471	0.38626993	0.15935502	0.06756486	0.14971276	0.73597637
239.5	0.15617178	0.15601372	0.15257535	0.38634047	0.15941427	0.06756425	0.14971734	0.73591573
239.6	0.15621128	0.15601948	0.15257597	0.38641091	0.15947349	0.06756367	0.14972192	0.73585517
239.7	0.15625068	0.1560252	0.15257658	0.38648124	0.15953267	0.06756314	0.1497265	0.73579469
239.8	0.15628998	0.15603089	0.15257717	0.38655146	0.15959181	0.06756264	0.14973107	0.7357343
239.9	0.15632918	0.15603655	0.15257775	0.38662158	0.1596509	0.06756218	0.14973564	0.73567399
240	0.15636829	0.15604218	0.15257831	0.38669159	0.15970994	0.06756177	0.14974021	0.73561376

Table S16: eFAST analysis data for [LACT] with [G](0)=2 mM: eFAST sensitivity indices

Sensitivity analysis data from eFAST for [LACT] with [G](0)=2 mM (only the parameters to which [LACT]'s variability is sensitive to appear)

Time discretization	K_{[G]}	q	V_{[PYR]}	alpha	K_{[LACT]}	G_E	L_E
0	0	0	0	0	0	0	0
0.1	0.02518701	0.02390633	0.02473207	0.02528177	0.02454414	0.02489018	0.99856702
0.2	0.0251855	0.02415145	0.02484326	0.02527446	0.02466936	0.02494426	0.99899621
0.3	0.02525913	0.02445758	0.02500919	0.0253463	0.02484782	0.02507868	0.99932455
0.4	0.02533178	0.02473311	0.02513962	0.02541753	0.02500294	0.02520226	0.99951801
0.5	0.02534947	0.02491857	0.02519205	0.0254311	0.02508322	0.025256	0.99962835
0.6	0.02533012	0.0250239	0.02519583	0.02540778	0.02510205	0.02525961	0.99969313
0.7	0.02529996	0.02508348	0.02518335	0.02537581	0.02509026	0.02524346	0.99973284
0.8	0.02527141	0.02512135	0.02517006	0.02534727	0.02506657	0.02522421	0.99975789
0.9	0.02524855	0.02514982	0.02516176	0.02532546	0.02503967	0.02520889	0.99977379
1	0.02523206	0.02517453	0.02516005	0.02531032	0.0250132	0.02519994	0.99978373
1.1	0.02522153	0.02519784	0.02516485	0.02530092	0.02498855	0.02519771	0.99978966
1.2	0.02521626	0.02522054	0.02517535	0.02529623	0.02496619	0.02520164	0.99979285
1.3	0.02521548	0.02524272	0.02519028	0.02529541	0.02494616	0.02521068	0.99979416
1.4	0.02521846	0.02526428	0.02520834	0.02529774	0.02492841	0.02522365	0.99979415
1.5	0.02522449	0.02528509	0.02522827	0.02530265	0.02491281	0.02523939	0.99979318
1.6	0.02523285	0.02530507	0.02524889	0.02530964	0.02489925	0.02525696	0.99979149
1.7	0.02524292	0.02532417	0.02526922	0.02531826	0.02488767	0.02527572	0.99978921
1.8	0.02525414	0.02534236	0.02528849	0.02532817	0.02487804	0.02529531	0.99978643
1.9	0.02526603	0.02535956	0.02530625	0.02533914	0.02487039	0.02531561	0.99978315
2	0.02527821	0.02537568	0.02532232	0.025351	0.02486478	0.02533656	0.99977939
2.1	0.02529041	0.02539059	0.0253367	0.02536364	0.02486132	0.02535811	0.99977513
2.2	0.02530244	0.02540417	0.0253496	0.02537679	0.02486015	0.0253802	0.99977035
2.3	0.02531415	0.02541626	0.02536126	0.02539006	0.02486146	0.02540269	0.99976503
2.4	0.0253254	0.02542671	0.02537198	0.02540294	0.02486547	0.02542534	0.99975915
2.5	0.02533604	0.02543541	0.02538206	0.02541491	0.02487245	0.0254478	0.99975267
2.6	0.02534601	0.0254423	0.02539177	0.02542551	0.02488272	0.02546967	0.99974558
2.7	0.0253553	0.02544741	0.02540141	0.0254345	0.02489663	0.02549042	0.99973785
2.8	0.02536379	0.02545082	0.02541126	0.02544176	0.02491449	0.02550968	0.99972947
2.9	0.02537136	0.02545259	0.02542159	0.02544731	0.02493649	0.02552723	0.99972041
3	0.02537789	0.02545272	0.02543264	0.02545128	0.02496268	0.02554286	0.99971067
3.1	0.02538326	0.02545116	0.02544455	0.02545388	0.02499279	0.02555652	0.99970023
3.2	0.02538741	0.02544788	0.02545738	0.0254553	0.02502621	0.02556828	0.99968906
3.3	0.02539038	0.02544281	0.02547099	0.02545571	0.02506215	0.02557831	0.99967715
3.4	0.02539221	0.02543593	0.0254852	0.02545523	0.02509972	0.02558681	0.99966447
3.5	0.025393	0.02542725	0.02549975	0.02545381	0.0251382	0.02559405	0.999651
3.6	0.02539289	0.02541681	0.02551445	0.02545128	0.02517701	0.02560031	0.99963672
3.7	0.02539204	0.02540466	0.02552905	0.02544739	0.02521561	0.02560585	0.9996216
3.8	0.02539066	0.02539089	0.02554338	0.02544194	0.02525371	0.02561089	0.99960563
3.9	0.02538893	0.02537564	0.02555745	0.02543477	0.02529112	0.02561558	0.99958877
4	0.025387	0.02535903	0.02557132	0.0254258	0.02532777	0.02562002	0.99957102
4.1	0.02538498	0.0253412	0.02558506	0.025415	0.02536369	0.0256242	0.99955236
4.2	0.0253828	0.0253223	0.02559876	0.02540245	0.02539899	0.02562802	0.99953278
4.3	0.02538034	0.02530251	0.02561247	0.02538823	0.02543375	0.02563133	0.99951228
4.4	0.02537733	0.025282	0.02562617	0.02537246	0.02546815	0.02563401	0.99949084
4.5	0.02537349	0.02526091	0.0256398	0.02535527	0.0255023	0.02563593	0.99946846
4.6	0.02536857	0.02523933	0.02565322	0.02533679	0.02553635	0.02563699	0.99944514
4.7	0.02536241	0.02521725	0.02566633	0.02531711	0.0255704	0.02563716	0.99942086
4.8	0.0253549	0.02519455	0.02567904	0.02529631	0.02560452	0.02563644	0.99939561
4.9	0.02534604	0.02517108	0.02569132	0.02527449	0.02563873	0.02563485	0.99936938
5	0.02533583	0.02514668	0.02570314	0.02525171	0.02567307	0.0256324	0.99934215
5.1	0.02532428	0.0251213	0.02571457	0.02522804	0.0257076	0.02562922	0.9993139
5.2	0.02531142	0.02509497	0.02572568	0.02520358	0.02574237	0.02562545	0.99928461
5.3	0.02529729	0.02506777	0.02573655	0.02517839	0.02577741	0.02562118	0.99925426
5.4	0.02528195	0.02503981	0.02574721	0.02515256	0.02581271	0.02561648	0.99922283
5.5	0.02526545	0.02501116	0.02575767	0.02512615	0.02584829	0.02561142	0.99919029
5.6	0.02524789	0.02498188	0.02576793	0.02509925	0.02588416	0.02560603	0.99915662
5.7	0.02522936	0.02495204	0.02577795	0.02507194	0.02592032	0.02560029	0.99912177
5.8	0.02520995	0.02492169	0.0257877	0.02504431	0.0259568	0.02559416	0.99908572
5.9	0.0251897	0.02489084	0.02579714	0.02501644	0.02599364	0.02558759	0.99904843
6	0.02516868	0.0248595	0.02580626	0.02498845	0.02603088	0.02558048	0.99900986
6.1	0.0251469	0.02482765	0.02581505	0.02496043	0.0260686	0.0255727	0.99896998
6.2	0.02512439	0.02479524	0.02582354	0.0249325	0.02610683	0.02556411	0.99892876
6.3	0.02510113	0.02476226	0.02583174	0.02490474	0.02614562	0.02555461	0.99888619
6.4	0.02507712	0.02472869	0.02583968	0.02487724	0.02618497	0.02554414	0.99884231
6.5	0.02505236	0.0246945	0.02584742	0.02485004	0.02622488	0.02553261	0.99879717
6.6	0.02502685	0.02465971	0.02585502	0.02482313	0.02626533	0.02551999	0.99875084
6.7	0.0250006	0.02462434	0.02586253	0.0247965	0.02630629	0.02550625	0.99870342
6.8	0.02497363	0.02458846	0.02587004	0.02477009	0.02634772	0.02549141	0.99865498
6.9	0.02494595	0.02455212	0.02587762	0.02474387	0.02638957	0.0254755	0.99860557
7	0.02491763	0.02451539	0.02588532	0.02471779	0.02643177	0.02545855	0.99855519
7.1	0.02488871	0.02447835	0.02589323	0.02469186	0.02647431	0.0254406	0.99850386
7.2	0.02485926	0.02444107	0.02590143	0.02466604	0.02651715	0.02542169	0.99845157
7.3	0.02482933	0.02440359	0.02590999	0.02464025	0.02656028	0.02540186	0.99839835
7.4	0.024799	0.02436593	0.025919	0.02461437	0.02660367	0.02538115	0.99834419
7.5	0.02476829	0.02432813	0.02592851	0.02458825	0.0266473	0.02535959	0.99828911
7.6	0.02473722	0.02429019	0.02593856	0.02456173	0.0266912	0.02533723	0.9982331
7.7	0.02470578	0.02425211	0.02594916	0.02453466	0.02673537	0.02531407	0.99817617
7.8	0.02467393	0.02421391	0.02596022	0.02450697	0.02677982	0.02529015	0.99811834
7.9	0.02464158	0.02417558	0.02597167	0.0244786	0.02682458	0.02526549	0.99805959
8	0.02460867	0.02413713	0.0259834	0.02444955	0.02686965	0.02524012	0.99799994
8.1	0.02457512	0.02409855	0.02599529	0.0244198	0.02691506	0.02521407	0.99793939
8.2	0.02454085	0.02405981	0.02600723	0.02438939	0.02696084	0.02518734	0.99787793
8.3	0.02450584	0.02402089	0.02601916	0.02435835	0.02700701	0.02515997	0.99781557
8.4	0.02447007	0.02398176	0.02603103	0.02432671	0.02705356	0.02513197	0.99775231
8.5	0.02443357	0.02394239	0.02604284	0.02429448	0.02710053	0.02510337	0.99768815
8.6	0.02439637	0.02390272	0.02605459	0.02426171	0.02714792	0.02507418	0.99762308
8.7	0.02435848	0.02386268	0.02606632	0.02422844	0.02719575	0.02504444	0.99755711
8.8	0.02431992	0.02382227	0.02607803	0.0241947	0.02724404	0.02501415	0.99749024
8.9	0.02428072	0.02378149	0.02608974	0.02416052	0.0272928	0.02498334	0.99742247
9	0.0242409	0.02374037	0.0261015	0.02412593	0.02734204	0.02495202	0.9973538
9.1	0.02420048	0.0236989	0.02611336	0.02409094	0.02739178	0.02492022	0.99728422
9.2	0.02415949	0.0236571	0.02612536	0.0240556	0.02744204	0.02488795	0.99721375
9.3	0.02411795	0.023615	0.0261375	0.02401992	0.02749283	0.02485523	0.99714238
9.4	0.02407588	0.02357265	0.02614982	0.02398394	0.02754418	0.02482208	0.99707012
9.5	0.0240333	0.02353002	0.02616236	0.02394768	0.0275961	0.02478852	0.99699696
9.6	0.02399023	0.0234871	0.02617515	0.02391116	0.02764862	0.02475456	0.99692291
9.7	0.0239467	0.02344387	0.02618821	0.0238744	0.02770176	0.02472022	0.99684798
9.8	0.02390273	0.02340028	0.02620157	0.02383739	0.02775557	0.02468551	0.99677216
9.9	0.02385834	0.02335634	0.02621526	0.02380013	0.02781006	0.02465047	0.99669546
10	0.02381357	0.02331202	0.02622932	0.02376261	0.02786529	0.0246151	0.99661788
10.1	0.02376845	0.02326733	0.02624381	0.0237248	0.02792128	0.02457943	0.99653943
10.2	0.02372302	0.02322225	0.02625877	0.02368671	0.02797807	0.02454348	0.99646012
10.3	0.02367732	0.02317682	0.02627427	0.02364835	0.02803571	0.02450727	0.99637994
10.4	0.0236314	0.02313107	0.02629033	0.02360973	0.02809421	0.02447081	0.9962989
10.5	0.02358534	0.02308505	0.02630698	0.02357086	0.02815361	0.02443415	0.996217
10.6	0.02353919	0.0230388	0.02632418	0.02353177	0.0282139	0.02439729	0.99613426
10.7	0.02349303	0.02299235	0.02634193	0.02349247	0.02827506	0.02436028	0.99605066
10.8	0.02344694	0.02294576	0.02636025	0.02345295	0.02833704	0.02432315	0.99596623
10.9	0.02340101	0.0228991	0.02637912	0.02341322	0.02839976	0.02428593	0.99588096
11	0.02335534	0.02285244	0.0263985	0.02337326	0.02846305	0.02424867	0.99579486
11.1	0.02330998	0.02280582	0.02641831	0.02333307	0.02852677	0.02421141	0.99570793
11.2	0.02326497	0.02275929	0.02643854	0.02329265	0.02859081	0.02417419	0.99562018
11.3	0.0232203	0.02271287	0.02645918	0.02325201	0.02865509	0.02413704	0.99553162
11.4	0.02317593	0.02266658	0.02648019	0.02321119	0.02871955	0.02409999	0.99544224
11.5	0.02313183	0.0226204	0.02650154	0.02317018	0.02878417	0.02406306	0.99535205
11.6	0.0230879	0.02257428	0.02652323	0.02312903	0.02884893	0.02402626	0.99526106
11.7	0.02304403	0.02252819	0.02654523	0.02308774	0.02891388	0.02398957	0.99516929
11.8	0.02300012	0.02248209	0.02656752	0.02304636	0.02897903	0.023953	0.99507672
15	0.02155108	0.02104399	0.02737714	0.02168458	0.03118454	0.02271795	0.9916971
15.1	0.02150745	0.02099849	0.02740464	0.02164076	0.03125795	0.02267681	0.99157699
15.2	0.02146395	0.02095291	0.0274323	0.02159696	0.03133152	0.02263559	0.99145588
15.3	0.02142053	0.02090727	0.02746013	0.02155317	0.03140525	0.02259428	0.99133382
15.4	0.02137718	0.02086157	0.02748812	0.02150942	0.03147913	0.02255291	0.99121088
15.5	0.02133389	0.02081585	0.02751628	0.02146572	0.03155316	0.02251146	0.99108714
15.6	0.02129061	0.02077012	0.02754461	0.02142207	0.03162735	0.02246996	0.99096269
15.7	0.0212473	0.02072442	0.02757312	0.02137849	0.03170171	0.0224284	0.9908376
15.8	0.02120396	0.02067876	0.0276018	0.02133498	0.03177631	0.02238679	0.99071192
15.9	0.02116058	0.02063319	0.02763067	0.02129158	0.03185117	0.02234515	0.9905857
16	0.02111714	0.02058775	0.02765972	0.02124829	0.03192635	0.02230347	0.99045895
16.1	0.02107363	0.02054245	0.02768898	0.02120513	0.03200191	0.02226176	0.99033169
16.2	0.02103005	0.02049736	0.02771844	0.02116212	0.03207791	0.02222004	0.99020392
16.3	0.02098643	0.0204525	0.02774813	0.02111929	0.03215442	0.02217831	0.99007566
16.4	0.02094276	0.02040792	0.02777803	0.02107667	0.03223147	0.02213657	0.98994689
16.5	0.02089907	0.02036365	0.02780817	0.02103427	0.03230904	0.02209484	0.9898176
16.6	0.02085534	0.02031971	0.02783856	0.02099213	0.03238707	0.02205313	0.98968779
16.7	0.02081159	0.02027612	0.02786919	0.02095028	0.03246547	0.02201143	0.98955744
16.8	0.02076782	0.02023288	0.02790008	0.02090875	0.03254411	0.02196977	0.98942654
16.9	0.02072404	0.02018999	0.02793124	0.02086756	0.03262292	0.02192815	0.98929508
17	0.02068027	0.02014742	0.02796268	0.02082672	0.03270182	0.02188657	0.98916304
17.1	0.0206365	0.02010517	0.02799441	0.02078626	0.0327807	0.02184506	0.98903041
17.2	0.02059275	0.02006322	0.02802644	0.02074622	0.03285951	0.02180362	0.98889717
17.3	0.02054902	0.02002157	0.02805879	0.02070662	0.0329382	0.02176227	0.9887633
17.4	0.02050532	0.01998022	0.02809146	0.02066751	0.03301669	0.02172102	0.98862879
17.5	0.02046166	0.01993916	0.02812448	0.02062892	0.03309489	0.0216799	0.98849359
17.6	0.02041805	0.01989838	0.02815785	0.02059085	0.03317273	0.02163892	0.98835771
17.7	0.02037448	0.01985789	0.02819158	0.02055328	0.03325019	0.0215981	0.98822114
17.8	0.02033098	0.01981768	0.02822568	0.02051615	0.03332719	0.02155747	0.98808388
17.9	0.02028755	0.01977773	0.02826013	0.02047942	0.03340371	0.02151706	0.98794596
18	0.02024419	0.01973803	0.02829492	0.02044302	0.03347973	0.0214769	0.98780738
18.1	0.02020093	0.01969855	0.02833003	0.02040693	0.03355524	0.021437	0.98766816
18.2	0.02015777	0.01965927	0.02836545	0.02037112	0.03363024	0.0213974	0.9875283
18.3	0.02011473	0.01962021	0.02840118	0.02033555	0.03370474	0.0213581	0.98738781
18.4	0.02007183	0.01958138	0.02843725	0.02030018	0.03377875	0.02131913	0.9872467
18.5	0.02002908	0.01954278	0.02847366	0.02026502	0.03385228	0.0212805	0.987105
18.6	0.01998652	0.01950442	0.02851044	0.02023008	0.03392533	0.0212422	0.98696272
18.7	0.01994416	0.01946632	0.02854762	0.02019536	0.03399791	0.02120425	0.98681989
18.8	0.01990204	0.01942852	0.02858522	0.02016087	0.03407005	0.02116666	0.98667653
18.9	0.0198602	0.01939105	0.02862329	0.0201266	0.03414174	0.02112944	0.98653267
19	0.01981868	0.01935393	0.02866184	0.02009257	0.034213	0.02109254	0.98638833
19.1	0.01977752	0.01931718	0.02870089	0.02005881	0.03428383	0.02105594	0.98624352
19.2	0.01973674	0.01928083	0.02874046	0.02002531	0.03435424	0.0210196	0.98609822
19.3	0.01969637	0.01924488	0.02878058	0.01999211	0.03442424	0.02098345	0.98595246
19.4	0.0196564	0.0192093	0.02882125	0.0199592	0.03449383	0.02094744	0.98580622
19.5	0.01961682	0.01917409	0.02886246	0.0199266	0.03456304	0.0209115	0.98565954
19.6	0.01957757	0.0191392	0.02890419	0.01989434	0.03463188	0.02087561	0.98551244
19.7	0.01953862	0.01910463	0.02894641	0.01986242	0.03470037	0.02083974	0.98536492
19.8	0.0194999	0.01907034	0.02898907	0.01983086	0.03476854	0.02080389	0.98521703
19.9	0.01946137	0.01903634	0.0290321	0.01979969	0.03483639	0.02076805	0.9850688
20	0.019423	0.01900261	0.02907546	0.01976891	0.03490395	0.02073224	0.98492028
20.1	0.01938479	0.01896916	0.02911911	0.01973855	0.03497122	0.02069647	0.98477149
20.2	0.01934673	0.01893597	0.02916301	0.01970863	0.0350382	0.02066075	0.98462242
20.3	0.01930883	0.01890303	0.02920711	0.01967916	0.0351049	0.02062509	0.98447309
20.4	0.01927107	0.01887029	0.02925139	0.01965017	0.03517131	0.0205895	0.9843235
20.5	0.01923348	0.01883772	0.02929584	0.01962168	0.03523745	0.02055399	0.98417366
20.6	0.01919604	0.01880527	0.02934045	0.01959373	0.03530333	0.02051856	0.98402357
20.7	0.01915876	0.01877291	0.02938521	0.01956633	0.03536895	0.02048323	0.98387325
20.8	0.01912164	0.01874059	0.02943011	0.01953952	0.03543435	0.020448	0.98372271
20.9	0.01908468	0.01870827	0.02947516	0.0195133	0.03549954	0.02041288	0.98357198
21	0.01904788	0.01867593	0.02952035	0.01948768	0.03556454	0.02037788	0.98342109
21.1	0.01901124	0.01864355	0.02956571	0.01946267	0.03562937	0.02034302	0.98327005
21.2	0.01897476	0.01861114	0.02961124	0.01943825	0.03569406	0.02030828	0.98311888
21.3	0.01893843	0.0185787	0.02965696	0.01941442	0.03575866	0.02027368	0.98296758
21.4	0.01890226	0.01854624	0.02970288	0.01939117	0.03582323	0.0202392	0.98281618
21.5	0.01886622	0.01851378	0.02974904	0.01936849	0.03588779	0.02020485	0.98266467
21.6	0.01883035	0.01848133	0.02979547	0.01934635	0.03595239	0.02017061	0.98251308
21.7	0.01879462	0.01844889	0.02984218	0.01932472	0.03601706	0.02013651	0.9823614
21.8	0.01875904	0.01841645	0.02988921	0.01930359	0.03608187	0.02010254	0.98220962
21.9	0.01872362	0.01838402	0.02993657	0.01928293	0.03614688	0.0200687	0.98205775
22	0.01868835	0.01835161	0.02998428	0.01926271	0.03621213	0.02003502	0.98190576
22.1	0.01865324	0.01831925	0.03003233	0.01924291	0.03627769	0.02000148	0.98175367
22.2	0.01861829	0.01828694	0.0300807	0.01922354	0.03634361	0.01996808	0.98160146
22.3	0.0185835	0.01825469	0.03012938	0.01920461	0.03640991	0.01993481	0.98144914
22.4	0.01854889	0.0182225	0.03017837	0.01918612	0.03647653	0.01990165	0.98129669
22.5	0.01851444	0.01819037	0.03022762	0.01916806	0.0365434	0.01986859	0.98114412
22.6	0.01848016	0.01815831	0.03027714	0.01915044	0.03661037	0.01983562	0.98099141
22.7	0.01844607	0.01812632	0.03032691	0.01913325	0.03667722	0.01980275	0.98083857
22.8	0.01841215	0.01809441	0.03037692	0.01911651	0.0367438	0.01976995	0.98068558
22.9	0.01837841	0.01806258	0.03042715	0.01910019	0.03680994	0.01973725	0.98053246
23	0.01834486	0.01803084	0.03047761	0.01908431	0.03687552	0.01970463	0.98037919
23.1	0.0183115	0.0179992	0.03052827	0.01906884	0.03694049	0.0196721	0.98022577
23.2	0.01827833	0.01796765	0.0305791	0.0190538	0.03700484	0.01963965	0.98007219
23.3	0.01824536	0.0179362	0.03063005	0.01903917	0.03706857	0.01960728	0.97991846
23.4	0.01821259	0.0179487	0.0306811	0.01902497	0.0371317	0.01957498	0.97976456
23.5	0.01818002	0.01787364	0.03073223	0.01901118	0.03719423	0.01954275	0.9796105
23.6	0.01814765	0.01784254	0.0307834	0.01899783	0.03725616	0.01951059	0.97945627
23.7	0.0181155	0.01781157	0.03083461	0.01898491	0.03731752	0.01947849	0.97930186
23.8	0.01808357	0.01778074	0.03088583	0.01897242	0.03737836	0.01944645	0.97914728
23.9	0.01805186	0.01775007	0.03093708	0.01896039	0.03743874	0.01941447	0.97899249
24	0.01802039	0.01771956	0.03098835	0.01894882	0.03749865	0.01938253	0.97883751
24.1	0.01798916	0.01768922	0.03103965	0.01893772	0.03755809	0.01935065	0.97868232
24.2	0.01795819	0.01765907	0.03109099	0.01892709	0.0376171	0.0193188	0.97852692
24.3	0.01792749	0.01762913	0.03114237	0.01891695	0.03767571	0.019287	0.97837129
24.4	0.01789708	0.01759941	0.0311938	0.01890729	0.03773393	0.01925525	0.97821542
24.5	0.01786697	0.01756993	0.03124529	0.01889813	0.03779176	0.01922355	0.97805931
24.6	0.01783719	0.01754073	0.03129683	0.01888948	0.03784921	0.01919189	0.97790291
24.7	0.01780776	0.01751182	0.03134843	0.01888135	0.03790625	0.01916029	0.97774619
24.8	0.01777869	0.01748324	0.03140008	0.01887376	0.03796287	0.01912874	0.97758912
24.9	0.01775	0.01745502	0.03145179	0.0188667	0.03801903	0.01909725	0.97743167
25	0.01772169	0.01742717	0.03150356	0.01886018	0.03807474	0.01906583	0.97727381
25.1	0.01769377	0.01739972	0.0315554	0.01885422	0.03812995	0.01903449	0.97711558
25.2	0.01766621	0.01737268	0.0316073	0.01884883	0.03818468	0.01900322	0.97695699
25.3	0.01763899	0.01734605	0.03165926	0.01884402	0.03823892	0.01897204	0.97679805
25.4	0.0176121	0.01731981	0.03171128	0.01883981	0.03829268	0.01894096	0.97663878
25.5	0.01758552	0.01729398	0.03176336	0.01883621	0.03834597	0.01890997	0.97647922
25.6	0.01755925	0.01726853	0.0318155	0.01883325	0.03839879	0.0188791	0.97631947
25.7	0.01753328	0.01724347	0.03186771	0.01883095	0.03845117	0.01884834	0.97615959
25.8	0.01750761	0.01721877	0.03191998	0.01882932	0.0385031	0.01881772	0.97599958
25.9	0.01748225	0.01719442	0.03197232	0.0188284	0.0385546	0.01878724	0.97583941
26	0.01745723	0.01717039	0.03202472	0.0188282	0.03860567	0.0187569	0.97567909
26.1	0.01743256	0.01714667	0.03207718	0.01882873	0.03865633	0.01872674	0.9755186
26.2	0.01740823	0.01712325	0.0321297	0.01883001	0.03870656	0.01869676	0.97535794
26.3	0.01738425	0.01710008	0.03218229	0.01883207	0.03875637	0.01866699	0.9751971
26.4	0.01736063	0.01707718	0.03223496	0.01883491	0.03880578	0.01863744	0.97503609
26.5	0.01733734	0.01705453	0.0322877	0.01883857	0.03885477	0.01860814	0.97487493
26.6	0.01731438	0.01703214	0.03234052	0.01884308	0.03890336	0.01857911	0.97471365
26.7	0.01729173	0.01701001	0.03239344	0.01884846	0.03895155	0.01855037	0.97455226
26.8	0.01726939	0.01698814	0.03244646	0.01885473	0.03899934	0.01852196	0.97439081
30	0.01665026	0.01637589	0.03420926	0.01943677	0.04035278	0.01772369	0.96921267
30.1	0.01663322	0.01635827	0.03426559	0.01946633	0.04039127	0.01770136	0.96904968
30.2	0.01661635	0.01634073	0.034322	0.01949657	0.04042966	0.01767916	0.96888657
30.3	0.01659962	0.01632326	0.0343785	0.01952748	0.04046797	0.01765711	0.96872335
30.4	0.01658303	0.01630586	0.03443514	0.01955907	0.04050619	0.01763522	0.96856005
30.5	0.01656656	0.01628855	0.03449194	0.01959135	0.04054433	0.0176135	0.96839669
30.6	0.0165502	0.01627132	0.03454894	0.01962434	0.04058236	0.01759194	0.96823328
30.7	0.01653395	0.01625417	0.03460615	0.01965804	0.04062028	0.01757057	0.96806985
30.8	0.01651782	0.01623711	0.03466358	0.01969248	0.04065806	0.01754938	0.9679064
30.9	0.01650179	0.01622014	0.03472125	0.01972768	0.04069568	0.01752838	0.96774293
31	0.01648586	0.01620326	0.03477914	0.01976363	0.04073313	0.01750758	0.96757945
31.1	0.01647005	0.01618648	0.03483726	0.01980035	0.04077041	0.01748699	0.96741594
31.2	0.01645434	0.0161698	0.03489562	0.01983784	0.04080751	0.01746659	0.96725242
31.3	0.01643874	0.01615321	0.0349542	0.01987611	0.04084444	0.0174464	0.96708887
31.4	0.01642325	0.01613674	0.03501301	0.01991517	0.04088119	0.01742641	0.96692529
31.5	0.01640787	0.01612036	0.03507207	0.01995499	0.04091777	0.01740659	0.96676167
31.6	0.01639261	0.0161041	0.03513139	0.01999557	0.0409542	0.01738694	0.96659801
31.7	0.01637747	0.01608796	0.03519099	0.02003691	0.04099049	0.01736746	0.96643428
31.8	0.01636245	0.01607193	0.03525088	0.02007899	0.04102663	0.01734812	0.96627051
31.9	0.01634754	0.01605602	0.03531108	0.02012182	0.04106264	0.01732892	0.96610667
32	0.01633277	0.01604024	0.03537162	0.0201654	0.04109853	0.01730986	0.96594278
32.1	0.01631811	0.01602458	0.03543252	0.02020975	0.04113431	0.01729093	0.96577882
32.2	0.01630358	0.01600905	0.03549378	0.02025487	0.04116999	0.01727212	0.96561479
32.3	0.01628918	0.01599367	0.0355554	0.02030076	0.04120559	0.01725345	0.96545067
32.4	0.01627491	0.01597843	0.03561738	0.02034742	0.04124111	0.01723491	0.96528644
32.5	0.01626078	0.01596334	0.03567969	0.02039487	0.04127658	0.01721651	0.9651221
32.6	0.01624678	0.01594841	0.03574231	0.02044313	0.04131199	0.01719825	0.96495764
32.7	0.01623291	0.01593365	0.0358052	0.02049219	0.04134734	0.01718015	0.96479305
32.8	0.01621919	0.01591905	0.03586831	0.02054207	0.04138261	0.0171622	0.96462831
32.9	0.01620562	0.01590464	0.03593162	0.02059279	0.04141777	0.0171444	0.96446341
33	0.01619219	0.01589041	0.0359951	0.02064434	0.04145278	0.01712676	0.96429835
33.1	0.01617891	0.01587637	0.03605874	0.02069674	0.04148763	0.01710928	0.9641331
33.2	0.01616579	0.01586254	0.03612254	0.02074998	0.0415223	0.01709195	0.96396766
33.3	0.01615283	0.01584892	0.0361865	0.02080408	0.0415568	0.01707479	0.963802
33.4	0.01614003	0.01583553	0.0362506	0.02085902	0.04159115	0.0170578	0.96363611
33.5	0.0161274	0.01582238	0.03631485	0.0209148	0.04162536	0.01704096	0.96346999
33.6	0.01611495	0.01580949	0.03637926	0.02097145	0.04165944	0.01702429	0.9633036
33.7	0.01610268	0.01579688	0.03644382	0.02102895	0.04169339	0.01700778	0.96313695
33.8	0.01609058	0.01578458	0.03650853	0.02108732	0.04172721	0.01699143	0.96297001
33.9	0.01607867	0.0157726	0.0365734	0.02114657	0.0417609	0.01697526	0.96280283
34	0.01606694	0.01576097	0.03663843	0.02120671	0.04179448	0.01695925	0.96263544
34.1	0.01605539	0.0157497	0.03670363	0.02126776	0.04182795	0.01694342	0.9624679
34.2	0.01604402	0.01573882	0.03676899	0.02132973	0.0418613	0.01692776	0.96230025
34.3	0.01603284	0.01572831	0.03683452	0.02139264	0.04189453	0.01691229	0.96213251
34.4	0.01602185	0.01571816	0.03690023	0.02145649	0.04192765	0.01689701	0.96196472
34.5	0.01601104	0.01570832	0.03696611	0.02152131	0.04196067	0.0168819	0.9617969
34.6	0.01600043	0.01569875	0.03703217	0.02158713	0.04199358	0.01686707	0.96162908
34.7	0.01599002	0.0156894	0.03709841	0.02165398	0.0420264	0.01685242	0.96146126
34.8	0.01597981	0.01568024	0.03716484	0.02172188	0.04205911	0.016838	0.96129347
34.9	0.01596982	0.01567122	0.03723146	0.02179086	0.04209173	0.0168238	0.96112572
35	0.01596004	0.01566232	0.03729827	0.02186094	0.04212423	0.01680984	0.96095802
35.1	0.01595048	0.0156535	0.03736527	0.02193212	0.04215663	0.0167961	0.96079037
35.2	0.01594117	0.01564475	0.03743248	0.02200441	0.04218893	0.01678259	0.96062277
35.3	0.0159321	0.01563606	0.0374999	0.02207785	0.04222111	0.01676929	0.96045522
35.4	0.0159233	0.01562741	0.03756753	0.02215247	0.04225319	0.01675619	0.96028772
35.5	0.01591479	0.01561881	0.03763538	0.02222833	0.04228516	0.01674329	0.96012026
35.6	0.01590659	0.01561025	0.03770345	0.02230547	0.04231701	0.01673058	0.95995283
35.7	0.01589873	0.01560174	0.03777176	0.0223839	0.04234876	0.01671806	0.95978544
35.8	0.01589124	0.01559328	0.03784029	0.02246354	0.0423804	0.01670573	0.95961807
35.9	0.01588414	0.01558487	0.03790905	0.02254426	0.04241194	0.0166936	0.95945073
36	0.01587746	0.01557653	0.03797803	0.02262586	0.04244337	0.01668167	0.9592834
36.1	0.01587122	0.01556825	0.03804722	0.02270819	0.0424747	0.01666994	0.95911608
36.2	0.01586542	0.01556004	0.03811661	0.02279112	0.04250592	0.01665842	0.95894878
36.3	0.01586007	0.01555189	0.0381862	0.02287459	0.04253705	0.01664711	0.95878148
36.4	0.01585514	0.0155438	0.03825595	0.02295856	0.04256808	0.01663602	0.95861418
36.5	0.0158506	0.01553579	0.03832588	0.02304302	0.042599	0.01662513	0.95844689
36.6	0.01584644	0.01552785	0.03839598	0.02312797	0.04262984	0.01661444	0.95827958
36.7	0.01584261	0.01552	0.03846624	0.0232134	0.04266058	0.01660395	0.95811226
36.8	0.0158391	0.01551223	0.03853668	0.02329935	0.04269123	0.01659365	0.95794493
36.9	0.01583585	0.01550455	0.03860729	0.02338579	0.04272179	0.01658353	0.95777758
37	0.01583284	0.01549696	0.03867807	0.02347275	0.04275227	0.01657358	0.9576102
37.1	0.01583002	0.01548947	0.03874904	0.02356021	0.04278266	0.0165638	0.95744279
37.2	0.01582736	0.01548207	0.0388202	0.02364819	0.04281296	0.01655419	0.95727535
37.3	0.01582483	0.01547477	0.03889155	0.02373668	0.04284319	0.01654474	0.95710788
37.4	0.01582241	0.01546758	0.03896309	0.02382568	0.04287334	0.01653547	0.95694036
37.5	0.01582009	0.0154605	0.03903484	0.02391519	0.0429034	0.01652635	0.95677279
37.6	0.01581788	0.01545353	0.03910681	0.02400522	0.04293339	0.01651741	0.95660517
37.7	0.01581577	0.01544668	0.03917899	0.02409577	0.04296331	0.01650863	0.95643748
37.8	0.01581376	0.01543996	0.0392514	0.02418686	0.04299316	0.01650003	0.95626974
37.9	0.01581186	0.01543336	0.03932404	0.02427848	0.04302294	0.01649159	0.95610192
38	0.01581007	0.0154269	0.03939692	0.02437064	0.04305265	0.01648332	0.95593402
38.1	0.0158084	0.01542058	0.03947005	0.02446333	0.0430823	0.01647523	0.95576605
38.2	0.01580685	0.0154144	0.03954343	0.02455658	0.04311189	0.0164673	0.95559797
38.3	0.01580541	0.01540837	0.03961708	0.02465037	0.04314142	0.01645956	0.9554298
38.4	0.01580408	0.0154025	0.03969102	0.02474471	0.04317089	0.01645198	0.95526151
38.5	0.01580286	0.01539677	0.03976525	0.02483961	0.04320031	0.01644458	0.9550931
38.6	0.01580176	0.01539118	0.03983981	0.02493506	0.04322968	0.01643736	0.95492456
38.7	0.01580076	0.01538572	0.03991471	0.02503108	0.04325901	0.01643031	0.95475589
38.8	0.01579989	0.01538036	0.03998997	0.02512765	0.04328828	0.01642343	0.9545871
38.9	0.01579913	0.01537512	0.04006559	0.0252248	0.04331752	0.01641674	0.95441818
39	0.0157985	0.01536997	0.04014155	0.02532253	0.04334672	0.01641022	0.95424916
39.1	0.01579799	0.01536493	0.04021785	0.02542083	0.04337589	0.01640389	0.95408005
39.2	0.01579763	0.01535998	0.04029446	0.02551972	0.04340504	0.01639773	0.95391084
39.3	0.0157974	0.01535513	0.04037139	0.02561919	0.04343416	0.01639175	0.95374159
39.4	0.01579732	0.01535038	0.04044862	0.02571925	0.04346326	0.01638595	0.95357232
39.5	0.0157974	0.01534573	0.04052605	0.0258199	0.04349235	0.01638033	0.95340304
39.6	0.01579762	0.01534119	0.04060359	0.02592116	0.04352144	0.0163749	0.95323373
39.7	0.01579798	0.01533676	0.04068119	0.02602301	0.04355052	0.01636965	0.95306434
39.8	0.01579848	0.01533244	0.04075887	0.02612547	0.04357961	0.01636458	0.95289487
39.9	0.0157991	0.01532824	0.04083668	0.02622853	0.0436087	0.01635969	0.95272538
40	0.01579983	0.01532416	0.04091465	0.02633221	0.04363782	0.01635499	0.95255589
40.1	0.01580067	0.01532021	0.0409928	0.0264365	0.04366695	0.01635048	0.9523864
40.2	0.01580161	0.01531639	0.04107113	0.02654141	0.04369611	0.01634615	0.95221693
40.3	0.01580265	0.0153127	0.04114964	0.02664694	0.04372529	0.016342	0.95204747
40.4	0.01580379	0.01530915	0.0412283	0.02675309	0.0437545	0.01633805	0.951878
40.5	0.01580503	0.01530575	0.04130711	0.02685987	0.04378373	0.01633428	0.95170852
40.6	0.01580636	0.01530249	0.04138605	0.02696728	0.04381297	0.0163307	0.95153902
40.7	0.01580778	0.01529939	0.04146512	0.02707533	0.04384223	0.01632731	0.95136949
40.8	0.01580929	0.01529645	0.04154434	0.02718401	0.04387148	0.01632412	0.95119991
40.9	0.0158109	0.01529367	0.04162369	0.02729334	0.04390074	0.01632111	0.95103031
41	0.01581261	0.01529105	0.04170319	0.0274033	0.04393	0.0163183	0.95086066
41.1	0.01581442	0.01528861	0.04178284	0.02751391	0.04395926	0.01631568	0.95069098
41.2	0.01581633	0.01528633	0.04186262	0.02762518	0.04398855	0.01631326	0.95052126
41.3	0.01581833	0.01528422	0.04194256	0.02773709	0.04401785	0.01631103	0.95035149
41.4	0.01582044	0.01528228	0.04202264	0.02784967	0.0440472	0.016309	0.95018167
41.5	0.01582265	0.01528051	0.04210288	0.0279629	0.04407659	0.01630717	0.95001182
41.6	0.01582496	0.01527892	0.04218328	0.0280768	0.04410604	0.01630554	0.94984192
41.7	0.01582738	0.0152775	0.04226384	0.02819136	0.04413558	0.01630411	0.94967197
41.8	0.0158299	0.01527628	0.04234457	0.02830659	0.0441652	0.01630288	0.94950197
45	0.01596761	0.01533262	0.04502818	0.0323707	0.04511048	0.01639283	0.9440271
45.1	0.01597378	0.01533658	0.04511421	0.03251023	0.04513997	0.01639973	0.94385463
45.2	0.01598007	0.01534073	0.04520029	0.03265054	0.04516957	0.01640681	0.94368206
45.3	0.01598648	0.01534506	0.04528643	0.03279162	0.04519931	0.01641407	0.94350939
45.4	0.01599301	0.0153496	0.04537261	0.03293347	0.04522919	0.01642152	0.94333661
45.5	0.01599966	0.01535432	0.04545886	0.0330761	0.04525925	0.01642915	0.94316373
45.6	0.01600643	0.01535925	0.04554516	0.0332195	0.04528949	0.01643696	0.94299075
45.7	0.01601331	0.01536436	0.04563154	0.03336368	0.04531987	0.01644497	0.94281765
45.8	0.01602032	0.01536967	0.045718	0.03350865	0.04535039	0.01645316	0.94264445
45.9	0.01602745	0.01537517	0.04580454	0.03365441	0.04538102	0.01646154	0.94247113
46	0.0160347	0.01538086	0.04589115	0.03380098	0.04541174	0.01647012	0.9422977
46.1	0.01604207	0.01538675	0.04597782	0.03394836	0.04544251	0.01647889	0.94212416
46.2	0.01604956	0.01539283	0.04606456	0.03409656	0.0454733	0.01648785	0.9419505
46.3	0.01605717	0.01539908	0.04615136	0.0342456	0.04550406	0.01649702	0.94177672
46.4	0.01606491	0.01540549	0.04623823	0.03439549	0.04553476	0.01650638	0.94160282
46.5	0.01607277	0.01541206	0.04632516	0.03454623	0.04556539	0.01651594	0.9414288
46.6	0.01608075	0.01541878	0.04641218	0.03469784	0.04559592	0.01652571	0.94125464
46.7	0.01608885	0.01542566	0.04649927	0.03485033	0.04562632	0.01653568	0.94108036
46.8	0.01609708	0.01543269	0.04658644	0.03500367	0.04565658	0.01654586	0.94090595
46.9	0.01610543	0.01543989	0.04667368	0.03515788	0.0456867	0.01655625	0.9407314
47	0.01611391	0.01544725	0.046761	0.03531296	0.04571668	0.01656685	0.94055672
47.1	0.01612251	0.01545476	0.04684839	0.0354689	0.04574652	0.01657768	0.9403819
47.2	0.01613124	0.01546241	0.04693587	0.03562571	0.04577622	0.01658872	0.94020693
47.3	0.01614009	0.01547023	0.04702343	0.03578341	0.04580577	0.01659999	0.94003182
47.4	0.01614906	0.0154782	0.04711107	0.03594199	0.04583519	0.01661149	0.93985656
47.5	0.01615816	0.01548633	0.04719881	0.03610147	0.04586449	0.01662322	0.93968115
47.6	0.01616739	0.0154946	0.04728664	0.03626184	0.04589365	0.0166352	0.93950558
47.7	0.01617675	0.01550299	0.04737456	0.0364231	0.0459227	0.01664743	0.93932985
47.8	0.01618623	0.01551151	0.04746257	0.03658522	0.04595162	0.01665991	0.93915394
47.9	0.01619583	0.01552015	0.04755069	0.0367482	0.04598041	0.01667265	0.93897786
48	0.01620557	0.01552892	0.0476389	0.03691202	0.04600908	0.01668567	0.93880161
48.1	0.01621543	0.01553781	0.04772722	0.03707664	0.04603763	0.01669896	0.93862517
48.2	0.01622542	0.01554683	0.04781565	0.03724204	0.04606606	0.01671253	0.93844855
48.3	0.01623554	0.01555598	0.04790419	0.03740821	0.04609437	0.01672638	0.93827174
48.4	0.01624579	0.01556526	0.04799284	0.03757511	0.04612257	0.01674051	0.93809476
48.5	0.01625617	0.01557465	0.0480816	0.03774275	0.04615066	0.01675491	0.93791759
48.6	0.01626667	0.01558415	0.0481705	0.03791109	0.04617864	0.0167696	0.93774025
48.7	0.01627731	0.01559375	0.04825952	0.03808015	0.0462065	0.01678456	0.93756275
48.8	0.01628807	0.01560343	0.04834868	0.03824991	0.04623426	0.01679979	0.9373851
48.9	0.01629897	0.01561321	0.04843798	0.03842037	0.04626192	0.0168153	0.9372073
49	0.01630999	0.01562306	0.04852744	0.03859154	0.04628947	0.01683108	0.93702938
49.1	0.01632115	0.015633	0.04861707	0.03876341	0.04631692	0.01684715	0.93685133
49.2	0.01633244	0.01564301	0.04870686	0.03893599	0.04634428	0.0168635	0.93667318
49.3	0.01634385	0.01565309	0.04879684	0.03910928	0.04637154	0.01688014	0.93649491
49.4	0.0163554	0.01566324	0.04888702	0.03928328	0.04639871	0.01689709	0.93631655
49.5	0.01636709	0.01567347	0.0489774	0.039458	0.0464258	0.01691436	0.93613808
49.6	0.0163789	0.01568378	0.04906801	0.03963344	0.0464528	0.01693196	0.93595952
49.7	0.01639085	0.01569418	0.04915885	0.03980961	0.04647973	0.0169499	0.93578085
49.8	0.01640293	0.01570468	0.04924995	0.0399865	0.04650657	0.01696818	0.93560207
49.9	0.01641514	0.01571528	0.04934131	0.04016411	0.04653335	0.01698681	0.9354232
50	0.01642749	0.01572598	0.04943296	0.04034246	0.04656005	0.01700578	0.93524421
50.1	0.01643997	0.01573679	0.0495249	0.04052153	0.04658668	0.01702509	0.93506513
50.2	0.01645259	0.01574772	0.04961715	0.04070133	0.04661323	0.01704474	0.93488593
50.3	0.01646534	0.01575878	0.04970973	0.04088185	0.04663972	0.01706474	0.93470663
50.4	0.01647822	0.01576997	0.04980265	0.04106312	0.04666613	0.01708509	0.93452721
50.5	0.01649124	0.01578131	0.04989593	0.04124511	0.04669247	0.01710579	0.93434767
50.6	0.01650439	0.0157928	0.04998958	0.04142784	0.04671873	0.01712685	0.93416802
50.7	0.01651768	0.01580445	0.0500836	0.0416113	0.04674493	0.01714825	0.93398826
50.8	0.0165311	0.01581629	0.05017796	0.0417955	0.04677105	0.01717	0.93380838
50.9	0.01654466	0.01582833	0.05027264	0.04198044	0.04679711	0.01719208	0.93362838
51	0.01655835	0.01584057	0.05036757	0.04216611	0.0468231	0.01721446	0.93344825
51.1	0.01657218	0.01585305	0.0504627	0.04235251	0.04684902	0.01723713	0.93326801
51.2	0.01658615	0.01586576	0.05055796	0.04253966	0.04687488	0.01726007	0.93308763
51.3	0.01660026	0.01587873	0.05065331	0.04272754	0.04690067	0.01728325	0.93290712
51.4	0.0166145	0.01589193	0.0507487	0.04291615	0.0469264	0.01730666	0.93272648
51.5	0.01662888	0.01590535	0.0508441	0.04310551	0.04695208	0.0173303	0.93254571
51.6	0.0166434	0.01591894	0.05093948	0.04329561	0.04697769	0.01735415	0.9323648
51.7	0.01665805	0.01593269	0.05103483	0.04348644	0.04700325	0.01737821	0.93218375
51.8	0.01667285	0.01594654	0.05113014	0.04367802	0.04702876	0.01740247	0.93200256
51.9	0.01668778	0.01596048	0.05122542	0.04387033	0.04705421	0.01742692	0.93182123
52	0.01670285	0.01597448	0.05132067	0.04406339	0.04707961	0.01745155	0.93163975
52.1	0.01671807	0.01598854	0.0514159	0.04425719	0.04710496	0.01747636	0.93145811
52.2	0.01673342	0.01600264	0.05151112	0.04445173	0.04713027	0.01750133	0.93127632
52.3	0.01674892	0.01601675	0.05160632	0.04464702	0.04715552	0.01752646	0.93109438
52.4	0.01676456	0.01603089	0.0517015	0.04484305	0.04718074	0.01755174	0.93091227
52.5	0.01678035	0.01604505	0.05179666	0.04503982	0.04720591	0.01757716	0.93073001
52.6	0.01679629	0.01605922	0.05189182	0.04523734	0.04723106	0.01760271	0.93054758
52.7	0.01681237	0.01607341	0.05198698	0.0454356	0.04725618	0.01762838	0.93036499
52.8	0.0168286	0.01608761	0.05208216	0.04563461	0.04728128	0.01765417	0.93018224
52.9	0.01684499	0.01610184	0.05217736	0.04583437	0.04730637	0.01768009	0.92999931
53	0.01686154	0.0161161	0.05227256	0.04603487	0.04733147	0.01770613	0.92981622
53.1	0.01687824	0.01613039	0.05236776	0.04623612	0.04735658	0.0177323	0.92963295
53.2	0.01689511	0.01614471	0.05246298	0.04643813	0.04738173	0.01775859	0.9294495
53.3	0.01691215	0.01615907	0.0525582	0.04664088	0.04740694	0.01778501	0.92926587
53.4	0.01692935	0.01617346	0.05265344	0.04684438	0.04743222	0.01781156	0.92908206
53.5	0.01694673	0.0161879	0.05274869	0.04704864	0.04745761	0.01783824	0.92889807
53.6	0.01696428	0.01620238	0.05284394	0.04725364	0.04748311	0.01786505	0.92871389
53.7	0.01698201	0.01621691	0.05293921	0.0474594	0.04750878	0.01789201	0.92852953
53.8	0.01699992	0.01623149	0.05303449	0.04766591	0.04753464	0.0179191	0.92834499
53.9	0.01701801	0.01624612	0.05312977	0.04787317	0.04756074	0.01794633	0.92816032
54	0.01703629	0.0162608	0.05322506	0.04808118	0.0475871	0.0179737	0.92797553
54.1	0.01705476	0.01627554	0.05332036	0.04828995	0.04761373	0.01800122	0.92779066
54.2	0.01707342	0.01629035	0.05341568	0.04849947	0.04764058	0.01802888	0.92760574
54.3	0.01709226	0.01630522	0.05351101	0.04870974	0.0476676	0.01805667	0.92742077
54.4	0.01711128	0.01632017	0.05360636	0.04892077	0.0476947	0.01808461	0.92723579
54.5	0.01713049	0.01633519	0.05370173	0.04913255	0.04772184	0.01811268	0.92705082
54.6	0.01714987	0.0163503	0.05379712	0.04934508	0.04774895	0.01814089	0.92686587
54.7	0.01716941	0.0163655	0.05389253	0.04955837	0.04777599	0.01816923	0.92668096
54.8	0.01718912	0.0163808	0.05398798	0.04977242	0.04780291	0.01819771	0.92649609
54.9	0.01720898	0.01639621	0.05408346	0.04998722	0.04782965	0.01822633	0.92631126
55	0.017229	0.01641174	0.05417898	0.05020278	0.04785619	0.01825508	0.92612648
55.1	0.01724917	0.0164274	0.05427455	0.0504191	0.04788256	0.01828397	0.92594174
55.2	0.01726948	0.0164432	0.05437017	0.05063617	0.04790877	0.018313	0.92575703
55.3	0.01728993	0.01645915	0.05446585	0.050854	0.0479348	0.01834216	0.92557235
55.4	0.01731053	0.01647526	0.05456159	0.05107258	0.04796065	0.01837146	0.92538768
55.5	0.01733127	0.01649153	0.0546574	0.05129193	0.04798631	0.01840089	0.92520302
55.6	0.01735216	0.01650796	0.05475329	0.05151203	0.04801179	0.01843047	0.92501833
55.7	0.01737318	0.01652455	0.05484926	0.05173289	0.0480371	0.01846019	0.92483363
55.8	0.01739434	0.01654128	0.05494531	0.0519545	0.04806223	0.01849005	0.92464888
55.9	0.01741564	0.01655815	0.05504144	0.05217688	0.04808717	0.01852006	0.92446408
56	0.01743708	0.01657514	0.05513764	0.05240001	0.04811192	0.01855021	0.92427922
56.1	0.01745866	0.01659225	0.05523391	0.0526239	0.04813648	0.01858051	0.92409429
56.2	0.01748038	0.01660947	0.05533024	0.05284855	0.04816084	0.01861096	0.92390926
56.3	0.01750224	0.01662679	0.0554266	0.05307396	0.04818501	0.01864155	0.92372412
56.4	0.01752424	0.0166442	0.055523	0.05330013	0.048209	0.0186723	0.92353888
56.5	0.01754639	0.01666173	0.05561942	0.05352706	0.0482328	0.0187032	0.92335352
56.6	0.0175687	0.01667935	0.05571588	0.05375475	0.04825643	0.01873426	0.92316806
56.7	0.01759115	0.01669706	0.05581236	0.0539832	0.04827988	0.01876549	0.92298252
56.8	0.01761375	0.01671484	0.05590887	0.05421241	0.04830318	0.01879687	0.92279694

11.9	0.02295609	0.02243599	0.02659004	0.02300492	0.02904436	0.0239165	0.99498338
12	0.0229119	0.02238986	0.02661278	0.02296344	0.02910988	0.02388003	0.99488927
12.1	0.02286754	0.0223437	0.02663575	0.02292195	0.02917559	0.02384358	0.99479439
12.2	0.02282298	0.02229748	0.02665892	0.02288048	0.02924147	0.02380714	0.99469876
12.3	0.02277817	0.02225116	0.02668231	0.02283903	0.02930752	0.02377066	0.99460237
12.4	0.02273312	0.02220468	0.02670595	0.02279761	0.02937374	0.02373411	0.99450523
12.5	0.02268789	0.02215811	0.02672984	0.0227562	0.02944014	0.02369748	0.99440735
12.6	0.02264249	0.02211155	0.02675402	0.02271477	0.0295067	0.02366072	0.99430872
12.7	0.02259693	0.02206513	0.02677845	0.0226733	0.02957345	0.0236238	0.99420934
12.8	0.02255126	0.02201893	0.02680313	0.02263175	0.02964038	0.02358669	0.99410919
12.9	0.02250548	0.02197303	0.02682803	0.02259007	0.0297075	0.02354937	0.99400828
13	0.02245963	0.02192752	0.0268531	0.02254824	0.02977482	0.02351181	0.99390658
13.1	0.02241373	0.02188246	0.02687832	0.02250624	0.02984235	0.02347399	0.99380409
13.2	0.02236777	0.0218379	0.02690366	0.02246406	0.02991012	0.02343593	0.9937008
13.3	0.02232178	0.02179384	0.02692909	0.02242171	0.02997812	0.02339763	0.99359671
13.4	0.02227574	0.02175021	0.02695463	0.0223792	0.03004641	0.02335908	0.99349181
13.5	0.02222966	0.02170688	0.02698026	0.02233653	0.03011499	0.0233203	0.99338608
13.6	0.02218356	0.02166372	0.02700596	0.02229373	0.03018391	0.02328129	0.99327953
13.7	0.02213745	0.02162057	0.02703175	0.02225078	0.03025318	0.02324208	0.99317216
13.8	0.02209135	0.02157736	0.02705764	0.0222077	0.03032282	0.02320269	0.99306395
13.9	0.02204531	0.02153402	0.02708362	0.02216451	0.03039284	0.0231631	0.99295491
14	0.02199934	0.02149048	0.02710971	0.02212121	0.03046325	0.02312333	0.99284503
14.1	0.02195348	0.02144674	0.02713589	0.02207782	0.03053403	0.02308339	0.9927343
14.2	0.02190778	0.02140277	0.02716219	0.02203434	0.03060517	0.02304329	0.99262271
14.3	0.02186229	0.02135859	0.02718861	0.02199079	0.03067665	0.02300305	0.99251025
14.4	0.02181705	0.02131417	0.02721514	0.02194717	0.03074843	0.02296268	0.9923969
14.5	0.02177209	0.02126955	0.0272418	0.02190349	0.03082051	0.02292218	0.99228264
14.6	0.02172741	0.02122475	0.02726859	0.02185976	0.03089286	0.02288155	0.99216746
14.7	0.02168301	0.02117977	0.02729551	0.021816	0.03096545	0.02284081	0.99205134
14.8	0.02163884	0.02113464	0.02732258	0.02177221	0.03103828	0.02279996	0.99193426
14.9	0.02159487	0.02108937	0.02734978	0.0217284	0.03111131	0.022759	0.99181618
60	0.01843323	0.01731074	0.0590136	0.06195665	0.04897207	0.0198803	0.91691739
60.1	0.01846185	0.01733075	0.05911121	0.06221189	0.04899195	0.0199164	0.91673548
60.2	0.01849062	0.01735092	0.05920886	0.06246794	0.04901168	0.01995264	0.91655367
60.3	0.01851953	0.01737128	0.05930655	0.06272479	0.0490312	0.01998901	0.91637197
60.4	0.01854859	0.01739184	0.05940428	0.06298245	0.04905047	0.02002552	0.91619037
60.5	0.01857778	0.01741263	0.05950205	0.0632409	0.04906946	0.02006216	0.91600887
60.6	0.0186071	0.01743365	0.05959986	0.06350016	0.04908818	0.02009894	0.91582747
60.7	0.01863656	0.01745493	0.05969771	0.06376022	0.04910662	0.02013585	0.91564618
60.8	0.01866616	0.01747646	0.05979561	0.06402109	0.04912477	0.0201729	0.91546498
60.9	0.01869588	0.01749824	0.05989354	0.06428276	0.04914263	0.02021008	0.91528387
61	0.01872574	0.01752024	0.05999152	0.06454526	0.04916021	0.0202474	0.91510287
61.1	0.01875572	0.01754242	0.06008954	0.0648086	0.04917752	0.02028486	0.91492195
61.2	0.01878584	0.01756476	0.06018761	0.06507277	0.04919455	0.02032245	0.91474113
61.3	0.01881609	0.01758722	0.06028572	0.06533779	0.04921131	0.02036017	0.91456041
61.4	0.01884647	0.01760979	0.06038389	0.06560369	0.0492278	0.02039804	0.91437977
61.5	0.01887699	0.01763245	0.0604821	0.06587045	0.04924403	0.02043604	0.91419923
61.6	0.01890764	0.01765515	0.06058037	0.06613807	0.04926	0.02047417	0.91401878
61.7	0.01893843	0.01767788	0.0606787	0.06640653	0.04927571	0.02051244	0.91383843
61.8	0.01896937	0.01770064	0.0607771	0.06667583	0.04929115	0.02055085	0.91365816
61.9	0.01900044	0.01772342	0.06087558	0.06694593	0.04930632	0.02058939	0.91347797
62	0.01903167	0.0177462	0.06097415	0.06721682	0.04932124	0.02062807	0.91329788
62.1	0.01906304	0.01776897	0.06107282	0.06748847	0.04933589	0.02066688	0.91311786
62.2	0.01909456	0.01779174	0.06117159	0.06776088	0.04935028	0.02070584	0.91293792
62.3	0.01912623	0.0178145	0.06127047	0.06803404	0.04936442	0.02074492	0.91275805
62.4	0.01915807	0.01783725	0.06136948	0.06830793	0.04937831	0.02078415	0.91257824
62.5	0.01919006	0.01785999	0.06146861	0.06858253	0.04939195	0.02082351	0.9123985
62.6	0.01922222	0.01788272	0.06156787	0.06885783	0.04940535	0.02086301	0.91221883
62.7	0.01925454	0.01790544	0.06166727	0.06913381	0.04941851	0.02090264	0.91203921
62.8	0.01928705	0.01792814	0.06176682	0.06941046	0.04943144	0.02094242	0.91185964
62.9	0.01931973	0.01795084	0.06186652	0.06968779	0.04944415	0.02098232	0.91168013
63	0.0193526	0.01797353	0.06196637	0.06996578	0.04945663	0.02102237	0.91150067
63.1	0.01938567	0.0179962	0.06206638	0.07024443	0.0494689	0.02106255	0.91132126
63.2	0.01941894	0.01801887	0.06216657	0.07052375	0.04948096	0.02110287	0.9111419
63.3	0.01945242	0.01804153	0.06226693	0.07080375	0.04949281	0.02114333	0.91096259
63.4	0.0194861	0.01806418	0.06236747	0.07108445	0.04950446	0.02118393	0.91078332
63.5	0.01952	0.01808683	0.0624682	0.07136586	0.04951591	0.02122466	0.9106041
63.6	0.01955412	0.01810947	0.06256914	0.07164798	0.04952717	0.02126553	0.91042491
63.7	0.01958847	0.0181321	0.06267029	0.07193082	0.04953823	0.02130654	0.91024577
63.8	0.01962304	0.01815474	0.06277168	0.0722144	0.04954909	0.02134769	0.91006666
63.9	0.01965784	0.01817737	0.06287327	0.07249872	0.04955976	0.02138898	0.90988757
64	0.01969287	0.0182	0.06297503	0.07278378	0.04957024	0.02143041	0.90970851
64.1	0.01972816	0.01822263	0.06307694	0.07306956	0.04958054	0.02147197	0.90952948
64.2	0.0197637	0.01824526	0.06317898	0.07335603	0.04959065	0.02151368	0.90935045
64.3	0.01979949	0.0182679	0.06328111	0.07364315	0.04960058	0.02155552	0.90917143
64.4	0.01983556	0.01829054	0.0633833	0.0739309	0.04961034	0.02159751	0.9089924
64.5	0.0198719	0.01831318	0.06348554	0.07421924	0.04961992	0.02163964	0.90881337
64.6	0.0199085	0.01833583	0.06358779	0.07450816	0.04962934	0.0216819	0.90863431
64.7	0.01994534	0.01835849	0.06369005	0.07479766	0.04963861	0.02172431	0.90845522
64.8	0.01998241	0.01838116	0.06379231	0.07508772	0.04964772	0.02176687	0.90827609
64.9	0.02001969	0.01840383	0.06389453	0.07537834	0.04965668	0.02180956	0.90809691
65	0.02005717	0.01842652	0.06399666	0.07566953	0.04966551	0.0218524	0.90791766
65.1	0.02009482	0.01844922	0.06409866	0.07596128	0.04967421	0.02189538	0.90773835
65.2	0.02013265	0.01847194	0.06420049	0.07625361	0.04968278	0.02193851	0.90755898
65.3	0.02017064	0.01849467	0.06430212	0.07654654	0.04969124	0.02198178	0.90737958
65.4	0.0202088	0.01851742	0.06440352	0.07684007	0.04969959	0.02202519	0.90720017
65.5	0.02024713	0.01854019	0.06450464	0.07713421	0.04970785	0.02206876	0.90702078
65.6	0.02028561	0.01856299	0.06460552	0.07742899	0.04971602	0.02211247	0.90684144
65.7	0.02032427	0.01858581	0.06470618	0.07772441	0.04972413	0.02215634	0.90666217
65.8	0.0203631	0.01860866	0.06480663	0.07802051	0.04973218	0.02220036	0.90648301
65.9	0.02040212	0.01863154	0.06490685	0.0783173	0.04974019	0.02224454	0.90630395
66	0.02044136	0.01865446	0.06500684	0.0786148	0.04974816	0.02228887	0.90612501
66.1	0.02048083	0.01867742	0.06510661	0.07891303	0.04975609	0.02233337	0.90594619
66.2	0.02052055	0.01870043	0.0652062	0.079212	0.049764	0.02237803	0.90576748
66.3	0.02056052	0.01872349	0.06530559	0.07951165	0.04977187	0.02242287	0.90558887
66.4	0.02060075	0.0187466	0.06540479	0.07981192	0.04977972	0.02246788	0.90541037
66.5	0.02064122	0.01876979	0.06550379	0.08011271	0.04978752	0.02251307	0.90523197
66.6	0.0206819	0.01879304	0.06560259	0.08041393	0.04979526	0.02255845	0.90505367
66.7	0.02072276	0.01881637	0.06570121	0.08071549	0.04980295	0.02260403	0.90487547
66.8	0.02076379	0.01883977	0.06579964	0.08101735	0.04981054	0.02264981	0.90469736
66.9	0.02080497	0.01886324	0.06589788	0.08131948	0.04981802	0.02269579	0.90451936
67	0.0208463	0.01888677	0.06599592	0.0816219	0.04982536	0.02274199	0.90434146
67.1	0.02088776	0.01891035	0.06609377	0.08192463	0.04983253	0.0227884	0.90416367
67.2	0.02092934	0.01893395	0.06619143	0.08222764	0.04983951	0.02283501	0.90398597
67.3	0.02097103	0.01895756	0.06628891	0.08253091	0.04984631	0.02288182	0.90380837
67.4	0.02101283	0.01898119	0.06638621	0.08283446	0.0498529	0.0229288	0.90363088
67.5	0.02105472	0.01900482	0.06648333	0.0831383	0.0498593	0.02297596	0.90345347
67.6	0.0210967	0.01902845	0.06658028	0.08344248	0.0498655	0.02302327	0.90327617
67.7	0.02113876	0.01905207	0.06667707	0.083747	0.0498715	0.02307073	0.90309895
67.8	0.02118088	0.0190757	0.0667737	0.08405182	0.0498773	0.02311832	0.90292183
67.9	0.02122307	0.01909932	0.06687017	0.08435697	0.04988293	0.02316603	0.9027448
68	0.02126533	0.01912294	0.06696651	0.08466243	0.04988838	0.02321387	0.90256784
68.1	0.02130764	0.01914656	0.06706271	0.08496824	0.04989368	0.02326183	0.90239095
68.2	0.02135002	0.01917017	0.06715876	0.08527437	0.04989883	0.0233099	0.90221413
68.3	0.02139246	0.01919379	0.06725469	0.08558084	0.04990385	0.02335808	0.90203737
68.4	0.02143497	0.0192174	0.06735049	0.08588763	0.04990874	0.02340636	0.90186067
68.5	0.02147755	0.01924102	0.06744616	0.08619474	0.04991353	0.02345476	0.90168402
68.6	0.02152021	0.01926463	0.06754171	0.08650217	0.04991823	0.02350328	0.90150741
68.7	0.02156297	0.01928825	0.06763715	0.08680992	0.04992287	0.0235519	0.90133085
68.8	0.02160583	0.01931187	0.06773249	0.08711799	0.04992746	0.02360064	0.90115434
68.9	0.02164882	0.0193355	0.06782774	0.08742639	0.04993204	0.02364949	0.90097788

26.9	0.01724734	0.01696656	0.03249961	0.01886187	0.03904674	0.01849387	0.97422933
27	0.01722559	0.0169453	0.03255288	0.01886989	0.03909374	0.0184661	0.97406784
27.1	0.01720413	0.01692436	0.03260629	0.01887876	0.03914037	0.01843866	0.97390635
27.2	0.01718296	0.0169037	0.03265985	0.01888846	0.03918661	0.01841152	0.97374487
27.3	0.01716206	0.01688332	0.03271356	0.01889895	0.03923248	0.01838464	0.97358339
27.4	0.01714142	0.01686317	0.03276742	0.01891018	0.03927798	0.018358	0.97342191
27.5	0.01712101	0.01684319	0.03282142	0.01892211	0.03932312	0.01833157	0.97326042
27.6	0.0171008	0.01682332	0.03287555	0.01893475	0.0393679	0.01830531	0.97309893
27.7	0.01708074	0.01680355	0.03292981	0.01894807	0.03941233	0.01827922	0.97293741
27.8	0.01706083	0.01678386	0.03298419	0.01896204	0.03945641	0.01825328	0.97277587
27.9	0.01704102	0.01676426	0.0330387	0.01897661	0.03950015	0.01822749	0.97261431
28	0.01702133	0.01674474	0.03309334	0.0189918	0.03954355	0.01820185	0.97245273
28.1	0.01700173	0.01672532	0.03314813	0.01900759	0.03958661	0.01817634	0.97229112
28.2	0.01698222	0.016706	0.03320306	0.019024	0.03962933	0.01815099	0.97212948
28.3	0.0169628	0.01668678	0.03325816	0.01904103	0.03967173	0.01812579	0.97196783
28.4	0.01694347	0.01666768	0.03331343	0.01905869	0.03971381	0.01810076	0.97180615
28.5	0.01692423	0.0166487	0.03336889	0.019077	0.03975557	0.01807591	0.97164446
28.6	0.0169051	0.01662985	0.03342454	0.01909597	0.03979703	0.01805125	0.97148275
28.7	0.01688607	0.01661113	0.03348035	0.01911562	0.03983818	0.01802679	0.97132102
28.8	0.01686714	0.01659255	0.03353629	0.01913598	0.03987905	0.01800251	0.97115927
28.9	0.01684831	0.01657409	0.03359233	0.01915707	0.03991965	0.01797842	0.97099749
29	0.0168296	0.01655573	0.03364842	0.01917888	0.03995999	0.01795452	0.97083567
29.1	0.01681102	0.01653746	0.03370453	0.01920143	0.0400001	0.01793078	0.9706738
29.2	0.01679257	0.01651926	0.03376062	0.01922474	0.04003999	0.01790721	0.97051187
29.3	0.01677424	0.01650112	0.03381669	0.01924878	0.04007968	0.01788379	0.97034986
29.4	0.01675606	0.01648304	0.03387274	0.01927356	0.04011917	0.01786051	0.97018776
29.5	0.01673801	0.01646502	0.03392877	0.01929906	0.04015849	0.01783738	0.97002556
29.6	0.01672012	0.01644706	0.03398478	0.01932526	0.04019764	0.01781438	0.96986325
29.7	0.01670239	0.01642917	0.0340408	0.01935213	0.04023663	0.01779151	0.96970081
29.8	0.01668484	0.01641134	0.03409686	0.01937968	0.04027548	0.01776877	0.96953823
29.9	0.01666746	0.01639358	0.03415301	0.01940789	0.04031419	0.01774616	0.96937552
75	0.02437687	0.02080679	0.07345465	0.10692151	0.04986396	0.02685564	0.89038856
75.1	0.02442213	0.02083177	0.07354423	0.10724969	0.04985502	0.02691174	0.89021771
75.2	0.02446741	0.02085679	0.07363373	0.10757788	0.04984582	0.02696794	0.89004692
75.3	0.02451271	0.02088185	0.07372314	0.10790599	0.04983637	0.02702423	0.88987622
75.4	0.02455803	0.02090695	0.07381248	0.10823394	0.04982667	0.02708061	0.88970558
75.5	0.02460337	0.0209321	0.07390175	0.10856167	0.04981674	0.02713709	0.88953502
75.6	0.02464872	0.02095728	0.07399096	0.10888914	0.04980656	0.02719366	0.88936452
75.7	0.02469411	0.02098251	0.07408011	0.10921631	0.04979615	0.02725033	0.88919408
75.8	0.02473951	0.02100779	0.07416921	0.10954317	0.04978551	0.02730709	0.88902371
75.9	0.02478495	0.02103311	0.07425826	0.1098697	0.04977464	0.02736394	0.88885337
76	0.02483042	0.02105847	0.07434727	0.11019588	0.04976355	0.02742089	0.88868308
76.1	0.02487592	0.02108388	0.07443624	0.11052168	0.04975225	0.02747793	0.88851281
76.2	0.02492146	0.02110934	0.07452517	0.11084711	0.04974073	0.02753506	0.88834256
76.3	0.02496702	0.02113484	0.07461408	0.11117219	0.04972899	0.02759228	0.88817233
76.4	0.02501261	0.0211604	0.07470297	0.11149694	0.04971705	0.0276496	0.8880021
76.5	0.02505824	0.021186	0.07479188	0.11182139	0.04970491	0.02770701	0.88783187
76.6	0.02510392	0.02121166	0.0748808	0.11214555	0.04969257	0.02776451	0.88766162
76.7	0.02514966	0.02123736	0.07496976	0.1124694	0.04968004	0.0278221	0.88749134
76.8	0.02519546	0.02126312	0.07505875	0.11279296	0.04966731	0.02787978	0.88732101
76.9	0.02524129	0.02128893	0.07514778	0.11311625	0.04965439	0.02793756	0.88715064
77	0.02528715	0.0213148	0.07523683	0.11343932	0.0496413	0.02799542	0.88698019
77.1	0.025333	0.02134072	0.07532588	0.11376218	0.04962804	0.02805338	0.88680968
77.2	0.02537882	0.02136669	0.07541491	0.11408484	0.04961462	0.02811143	0.88663911
77.3	0.0254246	0.02139273	0.0755039	0.11440732	0.04960106	0.02816957	0.88646852
77.4	0.02547032	0.02141882	0.07559281	0.11472963	0.04958735	0.0282278	0.88629793
77.5	0.02551598	0.02144497	0.07568162	0.11505178	0.04957352	0.02828612	0.88612739
77.6	0.02556159	0.02147119	0.0757703	0.1153738	0.04955955	0.02834453	0.88595693
77.7	0.02560715	0.02149747	0.07585883	0.1156957	0.04954547	0.02840304	0.88578657
77.8	0.02565266	0.02152381	0.0759472	0.1160175	0.04953127	0.02846163	0.88561633
77.9	0.02569814	0.02155022	0.07603541	0.1163392	0.04951696	0.02852032	0.8854462
78	0.02574357	0.0215767	0.07612344	0.11666081	0.04950255	0.0285791	0.88527616
78.1	0.02578897	0.02160325	0.07621127	0.11698233	0.04948803	0.02863797	0.8851062
78.2	0.02583433	0.02162988	0.0762989	0.11730375	0.04947343	0.02869693	0.88493633
78.3	0.02587965	0.0216566	0.07638634	0.11762505	0.04945872	0.02875599	0.88476657
78.4	0.02592494	0.02168339	0.07647356	0.11794621	0.04944394	0.02881514	0.88459688
78.5	0.02597018	0.02171028	0.07656056	0.11826723	0.04942908	0.02887439	0.88442726
78.6	0.0260154	0.02173726	0.07664734	0.11858811	0.04941415	0.02893373	0.88425769
78.7	0.02606058	0.02176434	0.0767339	0.11890887	0.04939916	0.02899317	0.88408819
78.8	0.02610573	0.02179152	0.07682024	0.11922951	0.04938412	0.02905271	0.88391879
78.9	0.02615085	0.02181882	0.07690636	0.11955007	0.04936902	0.02911235	0.88374953
79	0.02619596	0.02184624	0.07699227	0.11987055	0.04935387	0.02917208	0.88358043
79.1	0.02624104	0.02187379	0.07707796	0.12019098	0.04933867	0.02923192	0.8834115
79.2	0.02628609	0.02190148	0.07716346	0.12051138	0.04932338	0.02929186	0.88324275
79.3	0.02633112	0.02192931	0.07724874	0.12083167	0.04930801	0.02935189	0.88307417
79.4	0.02637613	0.02195729	0.07733382	0.12115172	0.04929253	0.02941201	0.88290577
79.5	0.0264211	0.02198544	0.0774187	0.12147146	0.04927695	0.02947223	0.88273757
79.6	0.02646606	0.02201374	0.07750338	0.12179079	0.04926124	0.02953254	0.88256957
79.7	0.02651099	0.02204219	0.07758786	0.12210962	0.04924541	0.02959293	0.88240181
79.8	0.0265559	0.02207079	0.07767215	0.12242784	0.04922947	0.02965342	0.88223431
79.9	0.02660079	0.02209952	0.07775626	0.12274537	0.04921342	0.02971399	0.8820671
80	0.02664567	0.02212838	0.07784018	0.12306221	0.04919725	0.02977465	0.88190018
80.1	0.02669053	0.02215738	0.07792393	0.12337838	0.04918099	0.0298354	0.88173358
80.2	0.02673539	0.0221865	0.0780075	0.12369395	0.04916461	0.02989623	0.88156729
80.3	0.02678025	0.02221577	0.07809089	0.12400897	0.04914814	0.02995715	0.88140132
80.4	0.02682511	0.02224519	0.07817411	0.12432352	0.04913158	0.03001816	0.88123566
80.5	0.02686998	0.02227478	0.07825716	0.12463767	0.04911491	0.03007925	0.88107033
80.6	0.02691485	0.02230455	0.07834004	0.12495142	0.04909816	0.03014044	0.88090532
80.7	0.02695975	0.02233449	0.07842276	0.12526475	0.04908133	0.03020171	0.88074063
80.8	0.02700467	0.02236459	0.07850531	0.12557756	0.04906441	0.03026306	0.88057625
80.9	0.02704962	0.02239483	0.0785877	0.12588976	0.04904742	0.03032451	0.88041219
81	0.0270946	0.02242518	0.07866993	0.1262013	0.04903035	0.03038604	0.88024844
81.1	0.02713963	0.02245563	0.078752	0.12651213	0.04901322	0.03044766	0.88008499
81.2	0.02718471	0.02248617	0.07883392	0.12682224	0.04899602	0.03050936	0.87992184
81.3	0.02722984	0.02251678	0.07891568	0.12713163	0.04897876	0.03057115	0.879759
81.4	0.02727503	0.02254746	0.0789973	0.12744025	0.04896144	0.03063303	0.87959645
81.5	0.02732029	0.02257819	0.07907878	0.12774808	0.04894407	0.030695	0.87943419
81.6	0.02736561	0.02260894	0.07916011	0.12805512	0.04892665	0.03075705	0.87927222
81.7	0.02741097	0.02263971	0.0792413	0.12836141	0.04890919	0.03081919	0.87911054
81.8	0.02745637	0.02267048	0.07932237	0.12866698	0.04889169	0.03088142	0.87894914
81.9	0.02750178	0.02270123	0.07940331	0.12897189	0.04887416	0.03094373	0.87878803
82	0.02754721	0.02273194	0.07948414	0.12927613	0.04885659	0.03100614	0.8786272
82.1	0.02759263	0.02276261	0.07956485	0.12957968	0.04883897	0.03106863	0.87846664
82.2	0.02763806	0.02279322	0.07964545	0.12988253	0.0488213	0.03113121	0.87830637
82.3	0.02768349	0.02282379	0.07972596	0.13018471	0.04880355	0.03119387	0.87814638
82.4	0.02772894	0.02285432	0.07980638	0.13048623	0.04878573	0.03125663	0.87798667
82.5	0.0277744	0.02288482	0.07988672	0.13078713	0.04876783	0.03131947	0.87782725
82.6	0.02781989	0.0229153	0.07996699	0.1310874	0.04874983	0.0313824	0.87766811
82.7	0.02786539	0.02294576	0.08004721	0.13138705	0.04873174	0.03144542	0.87750928
82.8	0.02791093	0.0229762	0.08012737	0.13168606	0.04871356	0.03150853	0.87735074
82.9	0.02795648	0.02300663	0.08020751	0.13198444	0.04869529	0.03157173	0.87719251
83	0.02800206	0.02303705	0.08028763	0.13228221	0.04867692	0.03163502	0.87703458
83.1	0.02804766	0.02306745	0.08036774	0.13257935	0.04865846	0.0316984	0.87687695
83.2	0.02809327	0.02309784	0.08044786	0.13287588	0.04863991	0.03176188	0.87671962
83.3	0.0281389	0.02312823	0.08052798	0.1331718	0.04862128	0.03182544	0.8765626
83.4	0.02818454	0.0231586	0.0806081	0.13346711	0.04860256	0.0318891	0.87640586
83.5	0.02823019	0.02318896	0.08068824	0.13376181	0.04858377	0.03195286	0.87624942
83.6	0.02827586	0.02321931	0.08076839	0.13405589	0.04856489	0.03201672	0.87609326
83.7	0.02832153	0.02324965	0.08084854	0.13434936	0.04854594	0.03208068	0.87593738
83.8	0.02836722	0.02327998	0.08092869	0.13464223	0.04852692	0.03214474	0.87578177
83.9	0.02841292	0.02331029	0.0810088	0.13493449	0.04850782	0.03220892	0.87562645

41.9	0.01583252	0.01527524	0.04242546	0.0284225	0.04419494	0.01630186	0.94933192
42	0.01583525	0.01527441	0.04250654	0.02853908	0.0442248	0.01630105	0.94916183
42.1	0.01583809	0.0152738	0.04258779	0.02865635	0.04425477	0.01630045	0.94899167
42.2	0.01584103	0.01527341	0.04266924	0.02877429	0.04428484	0.01630006	0.94882147
42.3	0.01584407	0.01527327	0.04275088	0.02889292	0.04431497	0.01629989	0.9486512
42.4	0.01584722	0.01527339	0.04283273	0.02901224	0.0443451	0.01629994	0.94848088
42.5	0.01585048	0.01527378	0.0429148	0.02913225	0.04437517	0.01630021	0.9483105
42.6	0.01585384	0.01527446	0.04299709	0.02925296	0.04440518	0.01630071	0.94814006
42.7	0.01585731	0.01527542	0.04307962	0.02937437	0.04443509	0.01630144	0.94796956
42.8	0.01586088	0.01527664	0.04316238	0.02949648	0.0444649	0.01630241	0.94779899
42.9	0.01586456	0.01527806	0.04324539	0.0296193	0.04449462	0.01630362	0.94762836
43	0.01586836	0.01527967	0.04332863	0.02974284	0.04452426	0.01630508	0.94745765
43.1	0.01587225	0.01528142	0.04341211	0.02986709	0.04455384	0.0163068	0.94728688
43.2	0.01587626	0.01528329	0.0434958	0.02999206	0.04458336	0.01630878	0.94711604
43.3	0.01588038	0.01528525	0.04357969	0.03011776	0.04461284	0.01631104	0.94694513
43.4	0.01588461	0.01528727	0.04366376	0.0302442	0.04464228	0.01631359	0.94677414
43.5	0.01588894	0.01528937	0.04374801	0.03037136	0.04467168	0.01631642	0.94660307
43.6	0.01589339	0.01529154	0.04383241	0.03049927	0.04470105	0.01631956	0.94643193
43.7	0.01589795	0.01529378	0.04391697	0.03062793	0.04473039	0.01632299	0.94626072
43.8	0.01590263	0.01529609	0.04400168	0.03075734	0.0447597	0.01632672	0.94608942
43.9	0.01590741	0.01529849	0.04408654	0.0308875	0.04478896	0.01633075	0.94591804
44	0.01591231	0.01530099	0.04417156	0.03101843	0.0448182	0.01633508	0.94574658
44.1	0.01591732	0.0153036	0.04425674	0.03115012	0.04484741	0.01633972	0.94557503
44.2	0.01592245	0.01530632	0.04434207	0.03128259	0.04487659	0.01634466	0.9454034
44.3	0.01592769	0.01530915	0.04442752	0.03141584	0.04490575	0.01634989	0.94523168
44.4	0.01593304	0.0153121	0.0445131	0.03154987	0.04493492	0.01635539	0.94505988
44.5	0.01593851	0.01531518	0.04459876	0.0316847	0.04496409	0.01636112	0.94488798
44.6	0.0159441	0.01531839	0.04468451	0.03182032	0.04499328	0.01636708	0.94471599
44.7	0.0159498	0.01532173	0.04477034	0.03195673	0.0450225	0.01637324	0.94454391
44.8	0.01595562	0.01532521	0.04485624	0.03209394	0.04505176	0.01637959	0.94437174
44.9	0.01596156	0.01532883	0.04494218	0.03223193	0.04508108	0.01638612	0.94419946
90	0.03129462	0.02515445	0.08554833	0.15181244	0.04727009	0.03636412	0.86659794
90.1	0.03134397	0.02518481	0.08561743	0.15207651	0.04724965	0.0364343	0.86645704
90.2	0.03139341	0.02521517	0.08568639	0.15234034	0.04722925	0.03650448	0.86631635
90.3	0.03144294	0.02524555	0.08575521	0.15260395	0.04720889	0.03657466	0.86617586
90.4	0.03149256	0.02527594	0.0858239	0.15286735	0.04718856	0.03664484	0.86603558
90.5	0.03154226	0.02530633	0.08589245	0.15313055	0.04716828	0.03671502	0.8658955
90.6	0.03159205	0.02533674	0.08596087	0.15339356	0.04714804	0.03678519	0.86575563
90.7	0.03164191	0.02536716	0.08602916	0.15365637	0.04712786	0.03685536	0.86561595
90.8	0.03169186	0.0253976	0.08609732	0.15391897	0.04710774	0.03692553	0.86547647
90.9	0.03174189	0.02542804	0.08616536	0.15418136	0.04708768	0.03699569	0.8653372
91	0.031792	0.0254585	0.08623326	0.15444352	0.04706769	0.03706584	0.86519811
91.1	0.03184218	0.02548897	0.08630105	0.15470544	0.04704777	0.03713599	0.86505923
91.2	0.03189245	0.02551945	0.0863687	0.15496712	0.04702794	0.03720613	0.86492053
91.3	0.03194279	0.02554995	0.08643624	0.15522854	0.04700819	0.03727627	0.86478203
91.4	0.03199322	0.02558046	0.08650365	0.15548971	0.04698852	0.0373464	0.86464372
91.5	0.03204373	0.02561098	0.08657095	0.15575062	0.04696893	0.03741653	0.86450559
91.6	0.03209432	0.02564152	0.08663812	0.15601126	0.0469494	0.03748665	0.86436765
91.7	0.032145	0.02567207	0.08670518	0.15627164	0.04692991	0.03755676	0.86422989
91.8	0.03219577	0.02570264	0.08677212	0.15653176	0.04691045	0.03762686	0.86409231
91.9	0.03224662	0.02573322	0.08683895	0.15679163	0.04689101	0.03769696	0.86395491
92	0.03229756	0.02576381	0.08690567	0.15705124	0.04687158	0.03776706	0.86381767
92.1	0.03234858	0.02579443	0.08697227	0.15731061	0.04685216	0.03783715	0.8636806
92.2	0.0323997	0.02582505	0.08703876	0.15756975	0.04683276	0.03790724	0.86354369
92.3	0.0324509	0.0258557	0.08710515	0.15782865	0.04681338	0.03797732	0.86340694
92.4	0.03250219	0.02588636	0.08717142	0.15808728	0.04679402	0.03804741	0.86327036
92.5	0.03255357	0.02591703	0.08723759	0.15834562	0.04677469	0.03811749	0.86313392
92.6	0.03260505	0.02594773	0.08730366	0.15860364	0.0467554	0.03818757	0.86299764
92.7	0.03265662	0.02597844	0.08736963	0.15886128	0.04673615	0.03825765	0.8628615
92.8	0.03270828	0.02600917	0.08743549	0.15911852	0.04671693	0.03832774	0.86272551
92.9	0.03276003	0.02603991	0.08750126	0.15937533	0.04669775	0.03839782	0.86258965
93	0.03281189	0.02607068	0.08756693	0.15963167	0.04667861	0.03846791	0.86245394
93.1	0.03286383	0.02610146	0.0876325	0.15988755	0.04665949	0.038538	0.86231836
93.2	0.03291588	0.02613226	0.08769798	0.16014294	0.0466404	0.0386081	0.86218291
93.3	0.03296802	0.02616308	0.08776337	0.16039786	0.04662133	0.0386782	0.86204758
93.4	0.03302027	0.02619393	0.08782867	0.1606523	0.04660227	0.03874831	0.86191239
93.5	0.03307261	0.02622479	0.08789388	0.16090625	0.04658322	0.03881844	0.86177735
93.6	0.03312506	0.02625567	0.087959	0.16115972	0.0465642	0.03888857	0.86164248
93.7	0.03317762	0.02628657	0.08802404	0.16141269	0.04654519	0.03895872	0.8615078
93.8	0.03323028	0.02631749	0.08808901	0.16166519	0.04652621	0.03902889	0.86137331
93.9	0.03328305	0.02634844	0.08815389	0.1619172	0.04650726	0.03909907	0.86123902
94	0.03333593	0.0263794	0.08821869	0.16216874	0.04648832	0.03916928	0.86110492
94.1	0.03338893	0.02641039	0.08828342	0.16241981	0.04646942	0.03923951	0.86097102
94.2	0.03344204	0.0264414	0.08834808	0.16267042	0.04645054	0.03930977	0.86083732
94.3	0.03349527	0.02647243	0.08841266	0.16292057	0.04643169	0.03938007	0.86070382
94.4	0.03354862	0.02650348	0.08847716	0.16317028	0.04641286	0.03945041	0.86057051
94.5	0.0336021	0.02653456	0.08854159	0.16341954	0.04639406	0.03952081	0.8604374
94.6	0.03365572	0.02656566	0.08860593	0.16366836	0.04637527	0.03959125	0.86030449
94.7	0.03370949	0.02659678	0.08867019	0.16391674	0.04635648	0.03966176	0.86017177
94.8	0.03376341	0.02662793	0.08873437	0.16416467	0.04633767	0.03973234	0.86003924
94.9	0.03381749	0.0266591	0.08879847	0.16441217	0.04631882	0.039803	0.8599069
95	0.03387174	0.0266903	0.08886249	0.16465923	0.04629993	0.03987374	0.85977475
95.1	0.03392616	0.02672152	0.08892642	0.16490586	0.04628099	0.03994457	0.8596428
95.2	0.03398076	0.02675277	0.08899026	0.16515207	0.04626199	0.04001551	0.85951103
95.3	0.03403556	0.02678404	0.08905403	0.16539785	0.04624293	0.04008654	0.85937945
95.4	0.03409054	0.02681534	0.08911771	0.16564322	0.04622381	0.04015765	0.85924806
95.5	0.03414571	0.02684666	0.08918132	0.16588817	0.04620464	0.04022882	0.85911686
95.6	0.03420107	0.02687801	0.08924484	0.16613272	0.04618541	0.04030003	0.85898584
95.7	0.03425658	0.02690939	0.08930828	0.16637686	0.04616614	0.04037125	0.858855
95.8	0.03431224	0.02694079	0.08937165	0.1666206	0.04614683	0.04044245	0.85872435
95.9	0.03436802	0.02697223	0.08943494	0.16686393	0.04612747	0.04051362	0.85859388
96	0.0344239	0.02700369	0.08949815	0.16710688	0.04610806	0.04058474	0.85846359
96.1	0.03447985	0.02703518	0.08956129	0.16734942	0.04608862	0.04065579	0.85833348
96.2	0.03453587	0.0270667	0.08962436	0.16759158	0.04606914	0.04072677	0.85820356
96.3	0.03459195	0.02709825	0.08968736	0.16783336	0.04604963	0.04079768	0.85807381
96.4	0.03464808	0.02712984	0.08975028	0.16807475	0.0460301	0.04086853	0.85794424
96.5	0.03470427	0.02716145	0.08981314	0.16831577	0.04601054	0.04093933	0.85781485
96.6	0.03476052	0.0271931	0.08987594	0.16855641	0.04599095	0.04101006	0.85768564
96.7	0.03481684	0.02722478	0.08993866	0.16879668	0.04597133	0.04108073	0.8575566
96.8	0.03487323	0.02725649	0.09000132	0.16903658	0.04595169	0.04115135	0.85742774
96.9	0.03492969	0.02728824	0.09006392	0.16927612	0.04593203	0.04122191	0.85729905
97	0.03498622	0.02732003	0.09012646	0.1695153	0.04591235	0.04129241	0.85717054
97.1	0.03504281	0.02735185	0.09018893	0.16975413	0.04589264	0.04136285	0.8570422
97.2	0.03509946	0.02738371	0.09025135	0.16999261	0.04587292	0.04143324	0.85691403
97.3	0.03515617	0.02741561	0.09031371	0.17023075	0.04585318	0.04150357	0.85678603
97.4	0.03521293	0.02744756	0.09037601	0.17046857	0.04583342	0.04157385	0.8566582
97.5	0.03526974	0.02747954	0.09043825	0.17070606	0.04581364	0.04164407	0.85653054
97.6	0.03532659	0.02751157	0.09050044	0.17094323	0.04579385	0.04171424	0.85640305
97.7	0.0353835	0.02754365	0.09056258	0.1711801	0.04577405	0.04178436	0.85627573
97.8	0.03544047	0.02757578	0.09062466	0.17141667	0.04575423	0.04185443	0.85614858
97.9	0.03549748	0.02760795	0.09068669	0.17165294	0.0457344	0.04192446	0.85602159
98	0.03555455	0.02764018	0.09074867	0.17188893	0.04571456	0.04199445	0.85589477
98.1	0.03561167	0.02767247	0.09081061	0.17212464	0.04569471	0.0420644	0.85576811
98.2	0.03566886	0.02770482	0.09087249	0.17236009	0.04567485	0.04213431	0.85564162
98.3	0.0357261	0.02773725	0.09093434	0.17259527	0.04565499	0.04220419	0.85551529
98.4	0.0357834	0.02776974	0.09099613	0.17283019	0.04563512	0.04227404	0.85538912
98.5	0.03584076	0.02780231	0.09105788	0.17306486	0.04561526	0.04234388	0.85526312
98.6	0.03589818	0.02783496	0.09111959	0.17329927	0.04559539	0.04241369	0.85513727
98.7	0.03595567	0.0278677	0.09118125	0.17353341	0.04557553	0.0424835	0.85501159
98.8	0.03601322	0.02790053	0.09124286	0.1737673	0.04555567	0.04255331	0.85488606
98.9	0.03607084	0.02793346	0.09130443	0.17400092	0.04553582	0.04262312	0.8547607

56.9	0.0176365	0.01673268	0.05600541	0.05444238	0.04832632	0.01882842	0.92261137
57	0.01765941	0.01675055	0.05610197	0.05467312	0.0483493	0.01886014	0.92242583
57.1	0.01768248	0.01676846	0.05619856	0.05490461	0.0483721	0.01889203	0.92224037
57.2	0.01770571	0.0167864	0.05629517	0.05513687	0.04839473	0.0189241	0.92205503
57.3	0.01772911	0.01680438	0.05639182	0.0553699	0.04841718	0.01895634	0.92186982
57.4	0.01775267	0.0168224	0.05648849	0.05560368	0.04843946	0.01898875	0.92168476
57.5	0.0177764	0.01684045	0.0565852	0.05583823	0.04846157	0.01902133	0.92149984
57.6	0.01780031	0.01685855	0.05668193	0.05607354	0.0484835	0.01905406	0.92131508
57.7	0.01782439	0.0168767	0.05677869	0.05630962	0.04850525	0.01908693	0.92113045
57.8	0.01784866	0.01689489	0.05687549	0.05654647	0.04852684	0.01911995	0.92094596
57.9	0.0178731	0.01691312	0.05697232	0.05678409	0.04854825	0.0191531	0.92076161
58	0.01789774	0.01693141	0.05706918	0.05702248	0.0485695	0.01918639	0.92057742
58.1	0.01792257	0.01694974	0.05716607	0.05726164	0.04859058	0.01921982	0.92039338
58.2	0.0179476	0.01696813	0.05726299	0.05750158	0.04861151	0.01925337	0.92020948
58.3	0.01797284	0.01698657	0.05735995	0.0577423	0.04863228	0.01928706	0.92002569
58.4	0.01799829	0.01700506	0.05745694	0.05798379	0.04865291	0.01932088	0.919842
58.5	0.01802396	0.01702361	0.05755396	0.05822607	0.04867341	0.01935484	0.91965842
58.6	0.01804986	0.01704221	0.05765102	0.05846913	0.04869378	0.01938893	0.91947495
58.7	0.01807599	0.01706088	0.05774811	0.05871298	0.04871403	0.01942316	0.9192916
58.8	0.01810235	0.0170796	0.05784524	0.05895762	0.04873416	0.01945752	0.91910835
58.9	0.01812893	0.01709839	0.0579424	0.05920306	0.0487542	0.01949202	0.91892521
59	0.01815571	0.01711725	0.0580396	0.05944931	0.04877413	0.01952665	0.91874218
59.1	0.01818269	0.01713618	0.05813684	0.05969636	0.04879398	0.01956141	0.91855924
59.2	0.01820986	0.01715519	0.05823411	0.05994422	0.04881375	0.01959631	0.91837639
59.3	0.0182372	0.01717428	0.05833141	0.06019289	0.04883346	0.01963134	0.91819366
59.4	0.01826473	0.01719346	0.05842876	0.06044238	0.04885313	0.01966651	0.91801102
59.5	0.01829242	0.01721272	0.05852614	0.06069269	0.04887281	0.0197018	0.91782848
59.6	0.01832027	0.01723209	0.05862355	0.06094383	0.04889254	0.01973724	0.91764605
59.7	0.01834828	0.01725157	0.05872101	0.0611958	0.04891234	0.0197728	0.91746373
59.8	0.01837644	0.01727116	0.0588185	0.06144859	0.04893222	0.0198085	0.91728151
59.9	0.01840476	0.01729088	0.05891603	0.06170221	0.04895214	0.01984433	0.9170994
105	0.03973451	0.03000387	0.09498465	0.18772636	0.04436688	0.04676417	0.84737045
105.1	0.03979843	0.03003825	0.09504356	0.18794028	0.04434892	0.04682948	0.84725301
105.2	0.0398625	0.03007264	0.09510242	0.18815385	0.04433099	0.04689473	0.84713571
105.3	0.03992672	0.03010705	0.09516123	0.18836708	0.04431312	0.04695993	0.84701853
105.4	0.03999107	0.03014148	0.09522	0.18857995	0.04429529	0.04702506	0.84690147
105.5	0.04005556	0.03017593	0.09527873	0.18879248	0.0442775	0.04709015	0.84678454
105.6	0.0401202	0.03021039	0.09533742	0.18900466	0.04425975	0.04715518	0.84666774
105.7	0.04018496	0.03024487	0.09539608	0.1892165	0.04424205	0.04722017	0.84655107
105.8	0.04024984	0.03027936	0.0954547	0.189428	0.04422438	0.04728513	0.84643454
105.9	0.04031478	0.03031387	0.09551329	0.18963916	0.04420676	0.04735004	0.84631813
106	0.04037976	0.0303484	0.09557186	0.18984998	0.0441892	0.04741491	0.84620186
106.1	0.04044476	0.03038295	0.0956304	0.19006047	0.0441717	0.04747975	0.84608573
106.2	0.04050976	0.03041751	0.09568891	0.19027063	0.04415426	0.04754454	0.84596974
106.3	0.04057476	0.0304521	0.09574741	0.19048045	0.04413687	0.04760927	0.84585388
106.4	0.04063972	0.03048669	0.0958059	0.19068994	0.04411954	0.04767395	0.84573816
106.5	0.04070464	0.03052131	0.09586437	0.1908991	0.04410228	0.04773858	0.84562257
106.6	0.04076953	0.03055595	0.09592284	0.19110794	0.0440851	0.04780315	0.84550713
106.7	0.04083436	0.0305906	0.09598131	0.19131645	0.04406801	0.04786767	0.84539182
106.8	0.04089914	0.03062527	0.09603978	0.19152464	0.044051	0.04793214	0.84527665
106.9	0.04096387	0.03065997	0.09609826	0.19173251	0.0440341	0.04799655	0.84516161
107	0.04102855	0.03069468	0.09615673	0.19194006	0.04401729	0.0480609	0.84504671
107.1	0.04109318	0.0307294	0.09621519	0.19214729	0.04400058	0.04812519	0.84493195
107.2	0.04115777	0.03076415	0.09627363	0.19235421	0.04398396	0.04818941	0.84481732
107.3	0.0412223	0.03079892	0.09633203	0.19256081	0.04396745	0.04825355	0.84470282
107.4	0.0412868	0.0308337	0.09639039	0.19276711	0.04395103	0.04831761	0.84458846
107.5	0.04135125	0.03086851	0.0964487	0.1929731	0.04393471	0.04838157	0.84447423
107.6	0.04141567	0.03090333	0.09650694	0.19317878	0.04391848	0.04844543	0.84436013
107.7	0.04148005	0.03093818	0.09656512	0.19338415	0.04390236	0.04850918	0.84424616
107.8	0.04154439	0.03097304	0.09662324	0.19358922	0.04388633	0.04857284	0.84413232
107.9	0.0416087	0.03100793	0.09668128	0.19379399	0.0438704	0.04863639	0.84401861
108	0.04167297	0.03104283	0.09673927	0.19399846	0.04385458	0.04869984	0.84390503
108.1	0.04173721	0.03107776	0.0967972	0.19420263	0.04383886	0.04876321	0.84379159
108.2	0.04180142	0.0311127	0.09685508	0.19440651	0.04382324	0.04882648	0.84367827
108.3	0.04186559	0.03114767	0.09691291	0.19461009	0.04380773	0.04888967	0.84356508
108.4	0.04192974	0.03118266	0.09697069	0.19481337	0.04379231	0.04895277	0.84345201
108.5	0.04199386	0.03121766	0.09702843	0.19501637	0.04377698	0.04901578	0.84333908
108.6	0.04205795	0.03125269	0.09708612	0.19521907	0.04376175	0.04907869	0.84322628
108.7	0.04212201	0.03128774	0.09714377	0.19542149	0.04374659	0.04914151	0.8431136
108.8	0.04218606	0.03132281	0.09720138	0.19562361	0.04373153	0.04920423	0.84300105
108.9	0.04225008	0.03135791	0.09725896	0.19582546	0.04371654	0.04926685	0.84288863
109	0.04231409	0.03139302	0.09731649	0.19602702	0.04370164	0.04932938	0.84277633
109.1	0.04237808	0.03142815	0.097374	0.1962283	0.04368681	0.04939181	0.84266416
109.2	0.04244206	0.03146331	0.09743147	0.19642929	0.04367206	0.04945414	0.84255211
109.3	0.04250603	0.03149849	0.09748891	0.19663001	0.04365738	0.04951639	0.84244019
109.4	0.04256999	0.03153369	0.09754633	0.19683045	0.04364278	0.04957854	0.84232838
109.5	0.04263395	0.03156892	0.09760372	0.19703062	0.04362825	0.04964061	0.8422167
109.6	0.0426979	0.03160416	0.09766109	0.19723051	0.04361378	0.04970259	0.84210515
109.7	0.04276185	0.03163943	0.09771843	0.19743013	0.04359939	0.04976449	0.84199371
109.8	0.04282579	0.03167472	0.09777576	0.19762948	0.04358508	0.04982631	0.84188239
109.9	0.04288972	0.03171004	0.09783308	0.19782856	0.04357085	0.04988805	0.84177119
110	0.04295364	0.03174537	0.09789038	0.19802737	0.04355673	0.04994971	0.84166011
110.1	0.04301753	0.03178073	0.09794766	0.19822591	0.04354271	0.05001131	0.84154914
110.2	0.0430814	0.03181612	0.09800489	0.19842419	0.0435288	0.05007283	0.8414383
110.3	0.04314524	0.03185152	0.09806206	0.1986222	0.04351501	0.05013429	0.84132757
110.4	0.04320906	0.03188695	0.09811915	0.19881996	0.04350135	0.05019569	0.84121695
110.5	0.04327284	0.03192241	0.09817613	0.19901745	0.04348781	0.05025702	0.84110645
110.6	0.04333658	0.03195788	0.098233	0.19921468	0.04347441	0.05031831	0.84099607
110.7	0.04340029	0.03199338	0.09828973	0.19941166	0.04346114	0.05037953	0.8408858
110.8	0.04346396	0.03202891	0.09834633	0.19960838	0.04344802	0.05044071	0.84077564
110.9	0.0435276	0.03206446	0.09840279	0.19980484	0.04343503	0.05050185	0.84066559
111	0.0435912	0.03210003	0.09845911	0.20000105	0.04342219	0.05056295	0.84055566
111.1	0.04365476	0.03213563	0.09851531	0.20019701	0.04340949	0.05062402	0.84044584
111.2	0.04371829	0.03217125	0.09857137	0.20039272	0.04339694	0.05068506	0.84033613
111.3	0.04378179	0.03220689	0.09862731	0.20058818	0.04338453	0.05074608	0.84022653
111.4	0.04384525	0.03224256	0.09868311	0.20078339	0.04337226	0.05080708	0.84011703
111.5	0.04390868	0.03227826	0.09873877	0.20097836	0.04336015	0.05086806	0.84000765
111.6	0.04397208	0.03231398	0.09879432	0.20117308	0.04334819	0.05092901	0.83989838
111.7	0.04403545	0.03234972	0.09884975	0.20136756	0.04333638	0.05098993	0.83978921
111.8	0.04409879	0.03238549	0.09890505	0.20156179	0.04332472	0.05105083	0.83968015
111.9	0.0441621	0.03242129	0.09896024	0.20175579	0.04331322	0.05111171	0.83957119
112	0.04422539	0.03245711	0.09901531	0.20194955	0.04330188	0.05117255	0.83946235
112.1	0.04428865	0.03249296	0.09907025	0.20214306	0.04329071	0.05123336	0.8393536
112.2	0.04435188	0.03252883	0.09912508	0.20233635	0.04327971	0.05129412	0.83924496
112.3	0.04441508	0.03256472	0.09917979	0.20252939	0.04326888	0.05135481	0.83913643
112.4	0.04447826	0.03260065	0.09923438	0.20272221	0.04325823	0.05141544	0.839028
112.5	0.04454141	0.03263659	0.09928886	0.20291479	0.04324776	0.051476	0.83891967
112.6	0.04460454	0.03267257	0.09934322	0.20310714	0.04323749	0.05153646	0.83881144
112.7	0.04466764	0.03270857	0.09939745	0.20329926	0.04322741	0.05159684	0.83870331
112.8	0.04473071	0.0327446	0.09945158	0.20349115	0.04321752	0.05165713	0.83859529
112.9	0.04479376	0.03278065	0.09950558	0.20368282	0.04320779	0.05171733	0.83848735
113	0.04485679	0.03281673	0.09955946	0.20387425	0.0431982	0.05177744	0.83837951
113.1	0.04491979	0.03285283	0.09961323	0.20406547	0.04318874	0.05183746	0.83827175
113.2	0.04498277	0.03288897	0.09966689	0.20425646	0.0431794	0.05189738	0.83816407
113.3	0.04504572	0.03292513	0.09972042	0.20444723	0.04317015	0.05195721	0.83805647
113.4	0.04510866	0.03296131	0.09977385	0.20463778	0.04316099	0.05201695	0.83794897
113.5	0.04517157	0.03299753	0.09982715	0.20482811	0.04315192	0.05207658	0.83784155
113.6	0.04523446	0.03303377	0.09988035	0.20501822	0.04314294	0.05213611	0.83773423
113.7	0.04529733	0.03307003	0.09993343	0.20520811	0.04313403	0.05219554	0.837627
113.8	0.04536018	0.03310633	0.0999864	0.20539779	0.04312521	0.05225487	0.83751985
113.9	0.04542302	0.03314265	0.10003926	0.20558725	0.04311646	0.0523141	0.83741278

69	0.02169195	0.01935912	0.0679229	0.0877351	0.04993664	0.02369846	0.90080147
69.1	0.02173523	0.01938276	0.06801799	0.08804414	0.04994127	0.02374754	0.90062514
69.2	0.02177868	0.0194064	0.06811299	0.08835351	0.04994599	0.02379673	0.90044888
69.3	0.02182231	0.01943004	0.0682079	0.0886632	0.0499508	0.02384603	0.9002727
69.4	0.02186611	0.01945369	0.06830271	0.08897324	0.04995573	0.02389544	0.90009662
69.5	0.02191012	0.01947735	0.06839743	0.08928363	0.04996074	0.02394497	0.89992063
69.6	0.02195432	0.01950101	0.06849204	0.08959438	0.04996577	0.02399462	0.89974474
69.7	0.02199872	0.01952469	0.06858653	0.08990549	0.04997075	0.02404438	0.89956895
69.8	0.0220433	0.01954837	0.06868088	0.09021697	0.04997562	0.02409426	0.89939327
69.9	0.02208806	0.01957206	0.0687751	0.09052884	0.04998028	0.02414425	0.8992177
70	0.02213295	0.01959577	0.06886919	0.09084109	0.04998467	0.02419435	0.89904223
70.1	0.02217794	0.01961948	0.06896315	0.09115375	0.04998876	0.02424457	0.89886687
70.2	0.02222301	0.01964321	0.06905698	0.09146683	0.04999253	0.0242949	0.89869161
70.3	0.02226812	0.01966694	0.0691507	0.09178035	0.04999594	0.02434536	0.89851645
70.4	0.02231325	0.01969069	0.0692443	0.09209432	0.049999	0.02439592	0.8983414
70.5	0.02235837	0.01971446	0.06933781	0.09240874	0.05000171	0.0244466	0.89816645
70.6	0.02240347	0.01973824	0.06943123	0.09272362	0.05000409	0.0244974	0.89799159
70.7	0.02244854	0.01976203	0.06952455	0.09303894	0.05000614	0.02454832	0.89781684
70.8	0.02249357	0.01978583	0.06961778	0.09335467	0.05000789	0.02459934	0.89764218
70.9	0.02253857	0.01980966	0.06971091	0.09367079	0.05000933	0.02465049	0.89746762
71	0.02258354	0.0198335	0.06980393	0.09398728	0.05001049	0.02470175	0.89729316
71.1	0.02262848	0.01985735	0.06989682	0.09430411	0.05001138	0.02475313	0.89711878
71.2	0.02267338	0.01988123	0.0699896	0.09462126	0.05001199	0.02480463	0.89694451
71.3	0.02271827	0.01990512	0.07008224	0.09493871	0.05001234	0.02485624	0.89677033
71.4	0.02276312	0.01992903	0.07017475	0.09525644	0.05001242	0.02490798	0.89659624
71.5	0.02280795	0.01995296	0.07026712	0.09557444	0.05001226	0.02495983	0.89642224
71.6	0.02285276	0.01997691	0.07035936	0.0958927	0.05001184	0.02501181	0.89624833
71.7	0.02289756	0.02000087	0.07045147	0.09621125	0.05001119	0.02506391	0.89607452
71.8	0.02294235	0.02002486	0.07054345	0.09653008	0.05001032	0.02511614	0.89590079
71.9	0.02298712	0.02004887	0.07063532	0.0968492	0.05000924	0.0251685	0.89572716
72	0.02303188	0.02007291	0.07072708	0.09716861	0.05000797	0.02522099	0.89555361
72.1	0.02307663	0.02009696	0.07081874	0.09748833	0.05000651	0.02527361	0.89538016
72.2	0.02312138	0.02012104	0.07091032	0.09780836	0.05000487	0.02532638	0.89520679
72.3	0.02316611	0.02014514	0.07100182	0.09812873	0.05000305	0.02537928	0.89503352
72.4	0.02321084	0.02016927	0.07109324	0.09844945	0.05000105	0.02543234	0.89486033
72.5	0.02325555	0.02019342	0.07118461	0.09877054	0.04999885	0.02548555	0.89468723
72.6	0.02330026	0.02021759	0.07127593	0.09909202	0.04999646	0.02553891	0.89451422
72.7	0.02334497	0.02024179	0.0713672	0.0994139	0.04999385	0.02559243	0.8943413
72.8	0.02338967	0.02026602	0.07145845	0.09973621	0.04999103	0.02564611	0.89416847
72.9	0.02343436	0.02029027	0.07154968	0.10005896	0.049988	0.02569994	0.89399573
73	0.02347906	0.02031456	0.0716409	0.10038215	0.04998478	0.02575391	0.89382308
73.1	0.02352376	0.02033886	0.07173214	0.10070578	0.04998136	0.02580802	0.89365052
73.2	0.02356845	0.0203632	0.07182339	0.10102985	0.04997772	0.02586225	0.89347805
73.3	0.02361316	0.02038757	0.07191466	0.10135437	0.04997384	0.0259166	0.89330568
73.4	0.02365787	0.02041197	0.07200594	0.10167935	0.0499697	0.02597106	0.89313339
73.5	0.02370259	0.02043639	0.07209722	0.1020048	0.04996529	0.02602561	0.89296119
73.6	0.02374733	0.02046085	0.07218847	0.10233073	0.04996058	0.02608027	0.89278908
73.7	0.02379208	0.02048534	0.07227967	0.10265713	0.04995557	0.02613502	0.89261706
73.8	0.02383685	0.02050986	0.07237078	0.10298401	0.04995026	0.02618986	0.89244513
73.9	0.02388164	0.02053441	0.07246178	0.10331136	0.04994464	0.0262448	0.89227328
74	0.02392646	0.020559	0.07255268	0.10363912	0.04993873	0.02629985	0.89210152
74.1	0.02397131	0.02058362	0.07264345	0.1039672	0.04993251	0.02635499	0.89192985
74.2	0.0240162	0.02060827	0.07273409	0.10429548	0.049926	0.02641023	0.89175827
74.3	0.02406113	0.02063296	0.07282459	0.10462387	0.0499192	0.02646557	0.89158677
74.4	0.0241061	0.02065769	0.07291496	0.10495225	0.04991213	0.026521	0.89141535
74.5	0.02415111	0.02068244	0.0730052	0.10528057	0.04990478	0.02657654	0.89124401
74.6	0.02419617	0.02070724	0.07309532	0.10560882	0.04989715	0.02663217	0.89107276
74.7	0.02424128	0.02073207	0.07318531	0.10593702	0.04988924	0.02668789	0.89090159
74.8	0.02428644	0.02075694	0.07327519	0.10626519	0.04988108	0.02674371	0.8907305
74.9	0.02433164	0.02078184	0.07336497	0.10659335	0.04987265	0.02679963	0.89055949
120	0.04924477	0.03541381	0.10309858	0.21682338	0.04275379	0.05577491	0.83109973
120.1	0.04930756	0.03545201	0.1031469	0.21700356	0.04275109	0.05582998	0.83099968
120.2	0.04937034	0.03549024	0.10319514	0.21718349	0.04274849	0.05588499	0.83089973
120.3	0.0494331	0.03552851	0.10324328	0.2173632	0.042746	0.05593995	0.83079989
120.4	0.04949585	0.03556681	0.10329134	0.2175427	0.04274362	0.05599485	0.83070016
120.5	0.04955859	0.03560514	0.10333931	0.217722	0.04274134	0.05604971	0.83060053
120.6	0.04962131	0.0356435	0.1033872	0.21790111	0.04273916	0.05610452	0.83050101
120.7	0.04968402	0.0356819	0.103435	0.21808004	0.0427371	0.05615929	0.83040158
120.8	0.04974672	0.03572034	0.10348273	0.2182588	0.04273513	0.05621401	0.83030226
120.9	0.04980942	0.03575881	0.10353039	0.21843739	0.04273328	0.0562687	0.83020304
121	0.04987211	0.03579731	0.10357798	0.2186158	0.04273153	0.05632336	0.83010392
121.1	0.04993479	0.03583584	0.10362552	0.21879401	0.04272988	0.05637799	0.83000489
121.2	0.04999747	0.03587441	0.10367298	0.21897203	0.04272834	0.0564326	0.82990596
121.3	0.05006015	0.03591302	0.10372038	0.21914983	0.0427269	0.05648718	0.82980712
121.4	0.05012284	0.03595166	0.10376768	0.2193274	0.04272557	0.05654173	0.82970838
121.5	0.05018553	0.03599033	0.1038149	0.21950473	0.04272435	0.05659625	0.82960973
121.6	0.05024822	0.03602904	0.10386202	0.21968178	0.04272323	0.05665074	0.82951116
121.7	0.05031092	0.03606778	0.10390902	0.21985857	0.04272221	0.05670522	0.82941269
121.8	0.05037363	0.03610655	0.10395591	0.22003508	0.0427213	0.05675968	0.82931431
121.9	0.05043636	0.03614537	0.10400267	0.22021131	0.0427205	0.05681415	0.82921603
122	0.05049909	0.03618421	0.1040493	0.22038725	0.0427198	0.05686864	0.82911785
122.1	0.05056184	0.03622309	0.10409581	0.22056291	0.04271921	0.05692318	0.82901977
122.2	0.0506246	0.03626201	0.10414218	0.22073827	0.04271873	0.05697778	0.82892181
122.3	0.05068737	0.03630096	0.10418842	0.22091336	0.04271835	0.05703244	0.82882395
122.4	0.05075015	0.03633995	0.10423452	0.22108817	0.04271809	0.05708719	0.82872621
122.5	0.05081293	0.03637897	0.10428047	0.2212627	0.04271792	0.05714202	0.82862858
122.6	0.05087571	0.03641803	0.10432629	0.22143695	0.04271787	0.05719693	0.82853107
122.7	0.05093849	0.03645712	0.10437198	0.22161093	0.04271792	0.05725194	0.82843368
122.8	0.05100126	0.03649625	0.10441753	0.22178465	0.04271808	0.05730703	0.82833641
122.9	0.05106404	0.03653541	0.10446295	0.22195809	0.04271835	0.05736219	0.82823926
123	0.05112681	0.03657462	0.10450823	0.22213127	0.04271873	0.05741741	0.82814222
123.1	0.05118959	0.03661385	0.10455337	0.22230419	0.04271921	0.05747267	0.82804531
123.2	0.05125237	0.03665313	0.10459838	0.22247684	0.0427198	0.05752796	0.82794851
123.3	0.05131516	0.03669243	0.10464326	0.22264923	0.04272051	0.05758327	0.82785183
123.4	0.05137796	0.03673178	0.10468801	0.22282136	0.04272132	0.05763856	0.82775527
123.5	0.05144077	0.03677116	0.10473262	0.22299322	0.04272224	0.05769381	0.82765883
123.6	0.0515036	0.03681058	0.1047771	0.22316483	0.04272327	0.05774899	0.8275625
123.7	0.05156645	0.03685003	0.10482146	0.22333617	0.04272441	0.05780407	0.82746629
123.8	0.05162932	0.03688953	0.10486568	0.22350727	0.04272567	0.05785902	0.8273702
123.9	0.05169222	0.03692905	0.10490977	0.22367811	0.04272703	0.05791385	0.82727422
124	0.05175515	0.03696862	0.10495374	0.22384871	0.04272851	0.05796853	0.82717835
124.1	0.0518181	0.03700822	0.10499758	0.22401906	0.04273011	0.05802305	0.82708259
124.2	0.05188109	0.03704786	0.10504129	0.22418916	0.04273181	0.05807743	0.82698695
124.3	0.05194411	0.03708754	0.10508488	0.22435902	0.04273364	0.05813165	0.82689142
124.4	0.05200717	0.03712725	0.10512835	0.22452863	0.04273558	0.05818572	0.82679599
124.5	0.05207027	0.037167	0.10517169	0.22469801	0.04273763	0.05823965	0.82670068
124.6	0.05213341	0.03720679	0.1052149	0.22486714	0.04273981	0.05829342	0.82660548
124.7	0.05219659	0.03724662	0.105258	0.22503603	0.0427421	0.05834705	0.82651038
124.8	0.05225982	0.03728649	0.10530097	0.22520468	0.04274451	0.05840052	0.82641539
124.9	0.05232309	0.03732639	0.10534382	0.2253731	0.04274704	0.05845385	0.82632051
125	0.05238638	0.03736634	0.10538656	0.22554128	0.04274968	0.05850704	0.82622573
125.1	0.0524497	0.03740632	0.10542917	0.22570922	0.04275245	0.05856009	0.82613106
125.2	0.05251304	0.03744634	0.10547166	0.22587693	0.04275533	0.058613	0.82603649
125.3	0.05257638	0.03748641	0.10551404	0.22604441	0.04275832	0.05866578	0.82594202
125.4	0.05263972	0.03752651	0.1055563	0.22621167	0.04276142	0.05871841	0.82584764
125.5	0.05270306	0.03756666	0.10559844	0.22637872	0.04276464	0.0587709	0.82575336
125.6	0.05276639	0.03760684	0.10564047	0.22654554	0.04276796	0.05882326	0.82565918
125.7	0.05282969	0.03764707	0.10568238	0.22671216	0.04277138	0.05887549	0.82556509
125.8	0.05289297	0.03768734	0.10572418	0.22687858	0.0427749	0.05892758	0.82547109
125.9	0.05295622	0.03772765	0.10576586	0.22704479	0.04277853	0.05897954	0.82537717
126	0.05301945	0.03776801	0.10580743	0.22721082	0.04278226	0.05903137	0.82528335

84	0.02845863	0.0233406	0.08108886	0.13522616	0.04848866	0.0322732	0.8754714
84.1	0.02850436	0.0233709	0.08116884	0.13551723	0.04846942	0.03233758	0.87531661
84.2	0.02855011	0.02340119	0.08124871	0.13580772	0.04845012	0.03240208	0.87516209
84.3	0.02859589	0.02343147	0.08132847	0.13609763	0.04843075	0.0324667	0.87500783
84.4	0.0286417	0.02346175	0.08140809	0.13638695	0.04841132	0.03253143	0.87485383
84.5	0.02868753	0.02349201	0.08148755	0.13667571	0.04839182	0.03259628	0.87470009
84.6	0.02873339	0.02352227	0.08156684	0.13696391	0.04837227	0.03266125	0.8745466
84.7	0.02877926	0.02355252	0.08164592	0.13725154	0.04835265	0.03272634	0.87439336
84.8	0.02882515	0.02358276	0.08172477	0.13753862	0.04833297	0.03279157	0.87424037
84.9	0.02887104	0.023613	0.0818034	0.13782515	0.04831323	0.03285692	0.87408763
85	0.02891694	0.02364323	0.08188181	0.13811113	0.04829344	0.03292241	0.87393511
85.1	0.02896284	0.02367346	0.08196	0.13839657	0.04827359	0.03298804	0.87378283
85.2	0.02900874	0.02370368	0.08203797	0.13868148	0.04825369	0.0330538	0.87363076
85.3	0.02905466	0.02373389	0.0821157	0.13896585	0.04823373	0.03311969	0.87347891
85.4	0.02910061	0.02376411	0.08219319	0.1392497	0.04821373	0.03318572	0.87332726
85.5	0.02914659	0.02379431	0.08227047	0.13953302	0.04819368	0.03325188	0.87317582
85.6	0.02919261	0.02382451	0.08234752	0.13981583	0.04817358	0.03331817	0.87302457
85.7	0.02923868	0.02385471	0.08242436	0.14009812	0.04815344	0.03338459	0.87287351
85.8	0.0292848	0.02388491	0.08250097	0.14037991	0.04813325	0.03345115	0.87272264
85.9	0.02933099	0.0239151	0.08257738	0.14066119	0.04811302	0.03351785	0.87257196
86	0.02937723	0.02394529	0.08265356	0.14094198	0.04809275	0.0335847	0.87242147
86.1	0.02942354	0.02397548	0.08272954	0.14122228	0.04807244	0.03365168	0.8722712
86.2	0.02946992	0.02400567	0.0828053	0.1415021	0.0480521	0.03371882	0.87212113
86.3	0.02951637	0.02403585	0.08288086	0.14178144	0.04803172	0.03378612	0.8719713
86.4	0.02956291	0.02406603	0.08295621	0.14206031	0.04801131	0.03385357	0.8718217
86.5	0.02960952	0.02409622	0.08303135	0.14233871	0.04799086	0.03392119	0.87167234
86.6	0.02965623	0.0241264	0.0831063	0.14261665	0.04797038	0.03398899	0.87152322
86.7	0.02970303	0.02415658	0.08318104	0.14289414	0.04794988	0.03405699	0.87137435
86.8	0.02974992	0.02418676	0.08325557	0.14317117	0.04792935	0.0341252	0.87122574
86.9	0.02979691	0.02421694	0.08332992	0.14344777	0.0479088	0.03419364	0.87107737
87	0.02984401	0.02424712	0.08340406	0.14372392	0.04788822	0.03426231	0.87092927
87.1	0.02989123	0.02427731	0.08347801	0.14399963	0.04786762	0.03433121	0.87078142
87.2	0.02993856	0.02430749	0.08355177	0.14427489	0.04784701	0.03440034	0.87063382
87.3	0.02998604	0.02433768	0.08362534	0.14454971	0.04782638	0.03446968	0.87048648
87.4	0.03003364	0.02436787	0.08369872	0.14482408	0.04780574	0.03453921	0.87033939
87.5	0.03008139	0.02439806	0.08377192	0.14509801	0.0477851	0.03460891	0.87019255
87.6	0.03012927	0.02442825	0.08384494	0.1453715	0.04776444	0.03467875	0.87004596
87.7	0.03017728	0.02445845	0.08391778	0.14564454	0.04774379	0.0347487	0.86989961
87.8	0.03022538	0.02448865	0.08399045	0.14591714	0.04772313	0.03481875	0.86975351
87.9	0.03027357	0.02451885	0.08406294	0.1461893	0.04770248	0.03488888	0.86960765
88	0.03032182	0.02454906	0.08413526	0.14646103	0.04768183	0.03495907	0.86946204
88.1	0.03037012	0.02457927	0.08420741	0.14673232	0.04766119	0.03502932	0.86931666
88.2	0.03041843	0.02460948	0.0842794	0.14700318	0.04764055	0.0350996	0.86917151
88.3	0.03046678	0.0246397	0.08435122	0.14727361	0.04761992	0.03516991	0.86902661
88.4	0.03051515	0.02466993	0.08442288	0.14754362	0.04759929	0.03524023	0.86888193
88.5	0.03056354	0.02470016	0.08449438	0.14781322	0.04757867	0.03531056	0.86873749
88.6	0.03061196	0.02473039	0.08456571	0.14808241	0.04755805	0.03538089	0.86859328
88.7	0.03066039	0.02476063	0.08463689	0.1483512	0.04753743	0.03545121	0.8684493
88.8	0.03070885	0.02479088	0.0847079	0.1486196	0.04751681	0.0355215	0.86830555
88.9	0.03075734	0.02482114	0.08477877	0.1488876	0.0474962	0.03559178	0.86816203
89	0.03080586	0.0248514	0.08484947	0.14915522	0.04747558	0.03566205	0.86801874
89.1	0.03085442	0.02488167	0.08492003	0.14942246	0.04745498	0.03573229	0.86787567
89.2	0.03090303	0.02491194	0.08499043	0.14968935	0.04743437	0.03580253	0.86773282
89.3	0.0309517	0.02494223	0.08506068	0.14995587	0.04741378	0.03587275	0.8675902
89.4	0.03100044	0.02497252	0.08513078	0.15022205	0.0473932	0.03594297	0.8674478
89.5	0.03104925	0.02500282	0.08520073	0.15048789	0.04737263	0.03601318	0.86730562
89.6	0.03109814	0.02503313	0.08527054	0.15075341	0.04735208	0.03608338	0.86716365
89.7	0.03114712	0.02506344	0.0853402	0.15101861	0.04733155	0.03615357	0.8670219
89.8	0.03119619	0.02509377	0.08540972	0.1512835	0.04731104	0.03622376	0.86688037
89.9	0.03124536	0.02512411	0.0854791	0.15154811	0.04729055	0.03629394	0.86673905
135	0.05863252	0.0415756	0.10916867	0.24138003	0.04354018	0.06325434	0.81726712
135.1	0.05869449	0.04161924	0.10920263	0.24152868	0.04355314	0.06329743	0.81718303
135.2	0.05875646	0.04166291	0.10923655	0.24167716	0.04356617	0.06334044	0.81709906
135.3	0.05881845	0.0417066	0.10927041	0.24182547	0.04357926	0.06338336	0.81701521
135.4	0.05888045	0.04175032	0.10930424	0.24197361	0.04359242	0.0634262	0.81693148
135.5	0.05894246	0.04179407	0.10933803	0.24212158	0.04360564	0.06346897	0.81684787
135.6	0.05900448	0.04183785	0.10937178	0.24226939	0.04361893	0.06351166	0.81676438
135.7	0.05906653	0.04188165	0.1094055	0.24241702	0.04363228	0.06355428	0.81668101
135.8	0.05912859	0.04192548	0.10943919	0.24256448	0.0436457	0.06359683	0.81659776
135.9	0.05919067	0.04196934	0.10947286	0.24271173	0.04365918	0.06363931	0.81651462
136	0.05925278	0.04201322	0.1095065	0.24285878	0.04367273	0.06368172	0.81643159
136.1	0.05931491	0.04205713	0.10954013	0.24300559	0.04368634	0.06372406	0.81634868
136.2	0.05937706	0.04210107	0.10957375	0.24315217	0.04370001	0.06376633	0.81626589
136.3	0.05943925	0.04214504	0.10960736	0.2432985	0.04371376	0.06380852	0.8161832
136.4	0.05950146	0.04218904	0.10964098	0.24344458	0.04372756	0.06385064	0.81610063
136.5	0.05956371	0.04223306	0.1096746	0.2435904	0.04374144	0.06389268	0.81601817
136.6	0.05962599	0.04227711	0.1097082	0.24373596	0.04375537	0.06393465	0.81593581
136.7	0.0596883	0.04232119	0.10974178	0.24388126	0.04376938	0.06397653	0.81585356
136.8	0.05975064	0.0423653	0.10977533	0.2440263	0.04378344	0.06401833	0.8157714
136.9	0.05981301	0.04240943	0.10980884	0.24417107	0.04379758	0.06406005	0.81568935
137	0.05987539	0.04245359	0.10984228	0.24431557	0.04381178	0.06410169	0.81560739
137.1	0.05993779	0.04249779	0.10987565	0.24445982	0.04382604	0.06414326	0.81552553
137.2	0.0600002	0.04254201	0.10990893	0.2446038	0.04384037	0.06418475	0.81544375
137.3	0.06006261	0.04258625	0.10994211	0.24474752	0.04385476	0.06422617	0.81536207
137.4	0.06012503	0.04263053	0.10997519	0.24489098	0.04386922	0.06426751	0.81528047
137.5	0.06018744	0.04267483	0.11000817	0.24503418	0.04388375	0.06430879	0.81519896
137.6	0.06024985	0.04271917	0.11004104	0.24517712	0.04389834	0.06435	0.81511753
137.7	0.06031224	0.04276353	0.11007381	0.2453198	0.04391299	0.06439114	0.81503619
137.8	0.06037463	0.04280792	0.11010648	0.24546222	0.04392772	0.06443223	0.81495496
137.9	0.060437	0.04285234	0.11013905	0.24560439	0.0439425	0.06447325	0.81487383
138	0.06049936	0.04289679	0.11017151	0.2457463	0.04395735	0.06451423	0.81479281
138.1	0.0605617	0.04294126	0.11020388	0.24588796	0.04397227	0.06455515	0.81471191
138.2	0.06062404	0.04298577	0.11023615	0.24602936	0.04398725	0.06459603	0.81463113
138.3	0.06068636	0.04303031	0.11026832	0.24617051	0.0440023	0.06463688	0.81455047
138.4	0.06074867	0.04307487	0.11030039	0.24631141	0.04401742	0.06467769	0.81446994
138.5	0.06081098	0.04311946	0.11033236	0.24645206	0.0440326	0.06471848	0.81438953
138.6	0.06087327	0.04316409	0.11036424	0.24659246	0.04404784	0.06475926	0.81430924
138.7	0.06093555	0.04320874	0.11039602	0.24673262	0.04406316	0.06480002	0.81422908
138.8	0.06099782	0.04325342	0.11042772	0.24687253	0.04407853	0.06484078	0.81414904
138.9	0.06106008	0.04329813	0.11045932	0.24701219	0.04409398	0.06488155	0.81406911
139	0.06112234	0.04334287	0.11049085	0.2471516	0.04410949	0.06492232	0.81398931
139.1	0.06118459	0.04338764	0.11052229	0.24729078	0.04412507	0.06496312	0.81390963
139.2	0.06124684	0.04343244	0.11055365	0.24742971	0.04414071	0.06500393	0.81383008
139.3	0.06130908	0.04347727	0.11058495	0.24756839	0.04415642	0.06504477	0.81375067
139.4	0.06137131	0.04352214	0.11061617	0.24770684	0.0441722	0.06508562	0.8136714
139.5	0.06143354	0.04356703	0.11064733	0.24784505	0.04418804	0.06512649	0.81359229
139.6	0.06149577	0.04361195	0.1106784	0.24798302	0.04420395	0.06516735	0.81351332
139.7	0.06155798	0.0436569	0.1107094	0.24812075	0.04421992	0.0652082	0.8134345
139.8	0.0616202	0.04370189	0.11074033	0.24825824	0.04423597	0.06524902	0.81335584
139.9	0.06168241	0.0437469	0.11077119	0.2483955	0.04425208	0.06528978	0.81327733
140	0.06174462	0.04379195	0.11080198	0.24853252	0.04426826	0.06533048	0.81319897
140.1	0.06180682	0.04383702	0.1108327	0.24866931	0.0442845	0.0653711	0.81312074
140.2	0.06186902	0.04388213	0.11086335	0.24880586	0.04430082	0.06541165	0.81304269
140.3	0.06193122	0.04392727	0.11089395	0.24894218	0.0443172	0.06545212	0.81296476
140.4	0.06199342	0.04397244	0.11092449	0.24907827	0.04433365	0.0654925	0.81288698
140.5	0.06205561	0.04401765	0.11095498	0.24921413	0.04435017	0.06553281	0.81280933
140.6	0.06211781	0.04406288	0.11098541	0.24934977	0.04436677	0.06557303	0.81273181
140.7	0.06218001	0.04410815	0.11101579	0.24948517	0.04438343	0.06561315	0.81265443
140.8	0.0622422	0.04415346	0.11104611	0.24962034	0.04440016	0.06565317	0.81257719
140.9	0.0623044	0.04419879	0.11107636	0.24975529	0.04441696	0.06569308	0.81250007
141	0.0623666	0.04424416	0.11110654	0.24989001	0.04443384	0.06573288	0.8124231

99	0.03612853	0.02796649	0.09136595	0.17423428	0.04551598	0.04269295	0.85463549
99.1	0.03618628	0.02799963	0.09142743	0.17446738	0.04549615	0.04276281	0.85451044
99.2	0.0362441	0.02803287	0.09148886	0.17470022	0.04547633	0.04283269	0.85438555
99.3	0.03630199	0.02806622	0.09155024	0.17493282	0.04545653	0.04290262	0.85426081
99.4	0.03635994	0.02809966	0.09161159	0.17516519	0.04543675	0.04297258	0.85413623
99.5	0.03641796	0.02813319	0.09167289	0.17539732	0.045417	0.04304258	0.8540118
99.6	0.03647605	0.02816678	0.09173415	0.17562925	0.04539727	0.04311258	0.85388752
99.7	0.0365342	0.02820043	0.09179536	0.17586098	0.04537756	0.04318258	0.85376339
99.8	0.03659241	0.02823413	0.09185653	0.17609253	0.04535788	0.04325256	0.85363942
99.9	0.03665069	0.02826785	0.09191767	0.17632389	0.04533823	0.04332249	0.8535156
100	0.03670903	0.0283016	0.09197876	0.17655509	0.0453186	0.04339234	0.85339192
100.1	0.03676743	0.02833536	0.09203981	0.17678612	0.04529899	0.04346211	0.85326839
100.2	0.0368259	0.02836914	0.09210081	0.17701699	0.04527939	0.04353178	0.85314501
100.3	0.03688443	0.02840294	0.09216178	0.17724769	0.04525981	0.04360135	0.85302178
100.4	0.03694303	0.02843674	0.09222271	0.17747821	0.04524023	0.0436708	0.8528987
100.5	0.0370017	0.02847056	0.0922836	0.17770857	0.04522066	0.04374014	0.85277575
100.6	0.03706043	0.02850439	0.09234445	0.17793876	0.04520111	0.04380938	0.85265295
100.7	0.03711924	0.02853823	0.09240526	0.17816879	0.04518156	0.04387851	0.8525303
100.8	0.0371781	0.02857207	0.09246603	0.17839866	0.04516201	0.04394754	0.85240778
100.9	0.03723704	0.02860592	0.09252676	0.17862839	0.04514247	0.04401647	0.85228541
101	0.03729605	0.02863979	0.09258746	0.17885794	0.04512294	0.0440853	0.85216317
101.1	0.03735513	0.02867366	0.09264812	0.17908727	0.04510343	0.04415403	0.85204107
101.2	0.03741428	0.02870755	0.09270877	0.17931631	0.04508393	0.04422266	0.85191911
101.3	0.03747351	0.02874144	0.09276941	0.17954503	0.04506444	0.0442912	0.85179729
101.4	0.03753281	0.02877534	0.09283007	0.17977338	0.04504497	0.04435963	0.8516756
101.5	0.03759219	0.02880926	0.09289075	0.18000133	0.04502552	0.04442798	0.85155404
101.6	0.03765165	0.02884319	0.09295143	0.18022886	0.04500609	0.04449623	0.85143261
101.7	0.03771119	0.02887713	0.0930121	0.18045597	0.04498667	0.04456439	0.85131132
101.8	0.03777081	0.02891108	0.09307274	0.18068267	0.04496728	0.04463245	0.85119015
101.9	0.0378305	0.02894504	0.09313335	0.18090895	0.0449479	0.04470042	0.8510691
102	0.03789028	0.02897902	0.0931939	0.18113481	0.04492854	0.0447683	0.85094818
102.1	0.03795014	0.02901301	0.09325439	0.18136025	0.0449092	0.04483609	0.85082738
102.2	0.03801009	0.029047	0.09331482	0.18158527	0.04488989	0.04490379	0.85070669
102.3	0.03807012	0.02908101	0.09337517	0.18180988	0.04487061	0.0449714	0.85058612
102.4	0.03813024	0.02911503	0.09343546	0.18203409	0.04485136	0.04503891	0.85046566
102.5	0.03819044	0.02914906	0.09349567	0.18225788	0.04483214	0.04510634	0.85034532
102.6	0.03825074	0.02918311	0.09355582	0.18248127	0.04481295	0.04517368	0.85022508
102.7	0.03831112	0.02921716	0.09361592	0.18270426	0.04479381	0.04524092	0.85010495
102.8	0.0383716	0.02925122	0.09367596	0.18292685	0.04477472	0.04530808	0.84998491
102.9	0.03843218	0.0292853	0.09373594	0.18314904	0.04475569	0.04537514	0.84986498
103	0.03849286	0.02931938	0.09379588	0.18337083	0.0447367	0.04544212	0.84974515
103.1	0.03855364	0.02935348	0.09385578	0.18359222	0.04471776	0.04550901	0.8496254
103.2	0.03861453	0.02938759	0.09391564	0.18381322	0.04469887	0.04557581	0.84950575
103.3	0.03867553	0.02942171	0.09397545	0.18403382	0.04468001	0.04564252	0.84938618
103.4	0.03873664	0.02945585	0.09403521	0.18425404	0.0446612	0.04570915	0.8492667
103.5	0.03879786	0.02948999	0.09409492	0.18447387	0.04464244	0.04577569	0.84914731
103.6	0.0388592	0.02952415	0.09415459	0.18469332	0.04462372	0.04584214	0.84902802
103.7	0.03892065	0.02955833	0.09421421	0.18491238	0.04460504	0.04590851	0.84890883
103.8	0.03898224	0.02959251	0.09427378	0.18513106	0.04458642	0.0459748	0.84878974
103.9	0.03904396	0.02962671	0.0943333	0.18534937	0.04456784	0.046041	0.84867076
104	0.03910582	0.02966093	0.09439278	0.1855673	0.04454932	0.04610712	0.84855191
104.1	0.03916784	0.02969516	0.0944522	0.18578485	0.04453085	0.04617317	0.84843317
104.2	0.03923002	0.0297294	0.09451156	0.18600204	0.04451243	0.04623913	0.84831456
104.3	0.03929237	0.02976365	0.09457088	0.18621885	0.04449406	0.04630502	0.84819608
104.4	0.03935492	0.02979792	0.09463015	0.1864353	0.04447574	0.04637083	0.84807773
104.5	0.03941767	0.02983221	0.09468937	0.18665138	0.04445747	0.04643657	0.84795952
104.6	0.03948062	0.02986651	0.09474853	0.18686709	0.04443925	0.04650223	0.84784145
104.7	0.03954379	0.02990083	0.09480764	0.18708245	0.04442109	0.04656782	0.84772351
104.8	0.03960717	0.02993516	0.09486669	0.18729744	0.04440297	0.04663334	0.84760569
104.9	0.03967075	0.0299695	0.0949257	0.18751208	0.0443849	0.04669879	0.84748801
150	0.0680137	0.04846222	0.1135371	0.26119755	0.04627451	0.06887635	0.80598894
150.1	0.06807659	0.04851035	0.11356022	0.26131515	0.04629842	0.06890711	0.80592252
150.2	0.06813945	0.04855851	0.11358325	0.2614326	0.04632233	0.0689378	0.80585621
150.3	0.06820228	0.04860669	0.11360619	0.2615499	0.04634622	0.06896841	0.80579
150.4	0.06826509	0.0486549	0.11362903	0.26166705	0.0463701	0.06899896	0.8057239
150.5	0.06832787	0.04870314	0.11365179	0.26178405	0.04639398	0.06902943	0.80565791
150.6	0.06839062	0.04875141	0.11367446	0.2619009	0.04641784	0.06905983	0.80559203
150.7	0.06845335	0.0487997	0.11369703	0.26201759	0.0464417	0.06909016	0.80552625
150.8	0.06851605	0.04884802	0.11371952	0.26213414	0.04646553	0.06912042	0.80546058
150.9	0.06857873	0.04889637	0.11374192	0.26225053	0.04648936	0.06915061	0.80539501
151	0.06864138	0.04894475	0.11376423	0.26236676	0.04651318	0.06918073	0.80532954
151.1	0.06870401	0.04899315	0.11378645	0.26248283	0.046537	0.06921078	0.80526417
151.2	0.06876662	0.04904158	0.11380858	0.26259874	0.04656082	0.06924077	0.8051989
151.3	0.0688292	0.04909004	0.11383063	0.2627145	0.04658465	0.06927069	0.80513373
151.4	0.06889176	0.04913852	0.11385259	0.2628301	0.04660847	0.06930054	0.80506866
151.5	0.0689543	0.04918704	0.11387446	0.26294553	0.0466323	0.06933033	0.80500369
151.6	0.06901681	0.04923558	0.11389625	0.26306081	0.04665612	0.06936005	0.80493882
151.7	0.0690793	0.04928414	0.11391795	0.26317592	0.04667994	0.06938971	0.80487404
151.8	0.06914177	0.04933274	0.11393957	0.26329088	0.04670376	0.0694193	0.80480936
151.9	0.06920422	0.04938136	0.1139611	0.26340568	0.04672758	0.06944884	0.80474477
152	0.06926664	0.04943001	0.11398255	0.26352032	0.04675141	0.06947831	0.80468028
152.1	0.06932905	0.04947869	0.11400391	0.2636348	0.04677524	0.06950773	0.80461587
152.2	0.06939143	0.0495274	0.11402519	0.26374913	0.04679907	0.06953709	0.80455156
152.3	0.06945379	0.04957613	0.11404639	0.26386331	0.0468229	0.06956639	0.80448733
152.4	0.06951614	0.04962489	0.1140675	0.26397733	0.04684674	0.06959563	0.80442319
152.5	0.06957846	0.04967368	0.11408853	0.2640912	0.04687057	0.06962482	0.80435914
152.6	0.06964076	0.0497225	0.11410948	0.26420491	0.04689441	0.06965396	0.80429517
152.7	0.06970304	0.04977135	0.11413035	0.26431847	0.04691826	0.06968305	0.80423128
152.8	0.06976531	0.04982022	0.11415113	0.26443189	0.0469421	0.06971208	0.80416748
152.9	0.06982755	0.04986912	0.11417184	0.26454515	0.04696595	0.06974106	0.80410378
153	0.06988978	0.04991805	0.11419246	0.26465826	0.04698981	0.06976999	0.80404016
153.1	0.06995198	0.049967	0.11421301	0.26477123	0.04701367	0.06979887	0.80397663
153.2	0.07001417	0.05001599	0.11423347	0.26488404	0.04703754	0.06982772	0.8039132
153.3	0.07007635	0.050065	0.11425386	0.26499671	0.04706142	0.06985652	0.80384987
153.4	0.0701385	0.05011404	0.11427416	0.26510924	0.0470853	0.06988528	0.80378664
153.5	0.07020064	0.0501631	0.11429439	0.26522163	0.04710919	0.069914	0.80372352
153.6	0.07026276	0.0502122	0.11431454	0.26533387	0.04713309	0.06994269	0.8036605
153.7	0.07032487	0.05026132	0.11433461	0.26544597	0.047157	0.06997135	0.8035976
153.8	0.07038696	0.05031047	0.11435461	0.26555793	0.04718093	0.06999998	0.8035348
153.9	0.07044903	0.05035965	0.11437453	0.26566976	0.04720487	0.07002858	0.80347212
154	0.07051109	0.05040886	0.11439437	0.26578144	0.04722883	0.07005716	0.80340955
154.1	0.07057313	0.05045809	0.11441414	0.26589299	0.0472528	0.0700857	0.80334709
154.2	0.07063516	0.05050736	0.11443383	0.26600441	0.0472768	0.07011421	0.80328474
154.3	0.07069718	0.05055665	0.11445345	0.26611569	0.04730083	0.07014269	0.8032225
154.4	0.07075918	0.05060596	0.11447299	0.26622684	0.04732488	0.07017113	0.80316036
154.5	0.07082117	0.05065531	0.11449246	0.26633785	0.04734896	0.07019953	0.80309834
154.6	0.07088315	0.05070469	0.11451186	0.26644874	0.04737307	0.0702279	0.80303643
154.7	0.07094511	0.05075409	0.11453118	0.2665595	0.04739722	0.07025622	0.80297462
154.8	0.07100705	0.05080352	0.11455043	0.26667013	0.04742139	0.0702845	0.80291291
154.9	0.07106898	0.05085298	0.11456961	0.26678064	0.04744559	0.07031273	0.80285132
155	0.0711309	0.05090246	0.11458872	0.26689103	0.04746982	0.07034092	0.80278983
155.1	0.07119281	0.05095198	0.11460775	0.26700131	0.04749405	0.07036907	0.80272845
155.2	0.07125471	0.05100152	0.11462672	0.26711147	0.0475183	0.07039717	0.80266717
155.3	0.07131659	0.05105109	0.11464562	0.26722153	0.04754255	0.0704252	0.802606
155.4	0.07137847	0.05110069	0.11466445	0.26733148	0.0475668	0.07045327	0.80254493
155.5	0.07144034	0.05115032	0.11468321	0.26744133	0.04759104	0.07048127	0.80248397
155.6	0.0715022	0.05119998	0.1147019	0.26755108	0.04761528	0.07050924	0.80242312
155.7	0.07156405	0.05124966	0.11472053	0.26766073	0.04763952	0.07053718	0.80236237
155.8	0.0716259	0.05129937	0.11473909	0.26777029	0.04766374	0.07056508	0.80230172
155.9	0.07168774	0.05134911	0.11475758	0.26787976	0.04768797	0.07059296	0.80224118
156	0.07174958	0.05139888	0.11477602	0.26798914	0.04771219	0.07062081	0.80218074

114	0.04548583	0.033179	0.10009201	0.20577651	0.04310781	0.05237322	0.83730579
114.1	0.04554863	0.03321538	0.10014466	0.20596555	0.04309923	0.05243224	0.83719892
114.2	0.0456114	0.03325178	0.10019719	0.20615439	0.04309075	0.05249114	0.83709215
114.3	0.04567416	0.03328821	0.10024961	0.20634302	0.04308236	0.05254995	0.83698551
114.4	0.04573691	0.03332467	0.10030192	0.20653145	0.04307405	0.05260864	0.83687899
114.5	0.04579963	0.03336116	0.10035412	0.20671967	0.04306584	0.05266723	0.83677261
114.6	0.04586234	0.03339768	0.10040621	0.20690769	0.04305772	0.05272572	0.83666636
114.7	0.04592504	0.03343422	0.1004582	0.20709551	0.04304969	0.05278409	0.83656024
114.8	0.04598772	0.03347079	0.10051007	0.20728313	0.04304174	0.05284237	0.83645425
114.9	0.04605038	0.0335074	0.10056184	0.20747055	0.04303388	0.05290055	0.83634839
115	0.04611303	0.03354403	0.10061349	0.20765777	0.0430261	0.05295863	0.83624266
115.1	0.04617567	0.03358068	0.10066504	0.2078448	0.04301839	0.05301661	0.83613705
115.2	0.04623829	0.03361737	0.10071649	0.20803164	0.04301075	0.05307449	0.83603156
115.3	0.0463009	0.03365409	0.10076782	0.20821829	0.04300318	0.05313229	0.83592621
115.4	0.04636349	0.03369083	0.10081905	0.20840474	0.04299567	0.05318998	0.83582097
115.5	0.04642608	0.0337276	0.10087018	0.20859101	0.04298822	0.05324758	0.83571586
115.6	0.04648865	0.0337644	0.10092121	0.20877709	0.04298083	0.05330509	0.83561087
115.7	0.04655121	0.03380124	0.10097214	0.20896298	0.04297348	0.0533625	0.835506
115.8	0.04661376	0.0338381	0.10102298	0.20914869	0.0429662	0.05341982	0.83540125
115.9	0.0466763	0.03387499	0.10107372	0.20933422	0.04295896	0.05347705	0.83529662
116	0.04673883	0.03391191	0.10112438	0.20951957	0.04295178	0.05353419	0.83519211
116.1	0.04680135	0.03394885	0.10117494	0.20970474	0.04294468	0.05359125	0.83508771
116.2	0.04686387	0.03398583	0.10122542	0.20988973	0.04293765	0.05364822	0.83498342
116.3	0.04692638	0.03402284	0.10127581	0.21007454	0.0429307	0.05370511	0.83487926
116.4	0.04698888	0.03405988	0.10132613	0.21025918	0.04292385	0.05376192	0.8347752
116.5	0.04705137	0.03409695	0.10137636	0.21044365	0.04291711	0.05381866	0.83467125
116.6	0.04711386	0.03413404	0.1014265	0.21062794	0.04291046	0.05387532	0.83456742
116.7	0.04717635	0.03417117	0.10147657	0.21081206	0.04290394	0.05393191	0.83446369
116.8	0.04723883	0.03420833	0.10152657	0.21099602	0.04289753	0.05398844	0.83436008
116.9	0.04730131	0.03424552	0.10157648	0.2111798	0.04289124	0.05404489	0.83425657
117	0.04736379	0.03428273	0.10162633	0.21136343	0.04288507	0.05410128	0.83415317
117.1	0.04742626	0.03431998	0.10167609	0.21154689	0.04287902	0.05415761	0.83404987
117.2	0.04748874	0.03435726	0.10172579	0.2117302	0.04287309	0.05421388	0.83394668
117.3	0.04755123	0.03439457	0.10177541	0.21191335	0.04286729	0.05427009	0.8338436
117.4	0.04761372	0.03443191	0.10182497	0.21209636	0.04286161	0.05432625	0.83374061
117.5	0.04767622	0.03446928	0.10187445	0.21227923	0.04285606	0.05438236	0.83363773
117.6	0.04773872	0.03450669	0.10192388	0.21246196	0.04285063	0.05443842	0.83353495
117.7	0.04780124	0.03454412	0.10197324	0.21264455	0.04284532	0.05449445	0.83343226
117.8	0.04786378	0.03458158	0.10202255	0.21282701	0.04284014	0.05455045	0.83332968
117.9	0.04792633	0.03461908	0.1020718	0.21300934	0.04283507	0.05460642	0.83322719
118	0.0479889	0.0346566	0.10212099	0.21319156	0.04283012	0.05466237	0.83312479
118.1	0.0480515	0.03469416	0.10217013	0.21337369	0.04282528	0.05471829	0.83302249
118.2	0.04811411	0.03473175	0.10221923	0.21355572	0.04282055	0.0547742	0.83292029
118.3	0.04817676	0.03476937	0.10226829	0.21373768	0.04281594	0.05483009	0.83281818
118.4	0.04823943	0.03480702	0.10231731	0.21391958	0.04281143	0.05488595	0.83271617
118.5	0.04830214	0.03484471	0.10236631	0.21410142	0.04280703	0.0549418	0.83261427
118.6	0.04836488	0.03488242	0.10241528	0.21428322	0.04280274	0.05499763	0.83251248
118.7	0.04842766	0.03492017	0.10246424	0.214465	0.04279856	0.05505345	0.8324108
118.8	0.04849047	0.03495795	0.10251318	0.21464677	0.04279448	0.05510923	0.83230924
118.9	0.04855331	0.03499576	0.10256212	0.21482855	0.04279051	0.05516499	0.83220779
119	0.04861618	0.0350336	0.10261106	0.21501033	0.04278664	0.05522071	0.83210647
119.1	0.04867905	0.03507148	0.10266	0.21519212	0.04278288	0.05527638	0.83200526
119.2	0.04874193	0.03510938	0.10270894	0.2153739	0.04277922	0.05533201	0.83190418
119.3	0.04880481	0.03514732	0.10275786	0.21555564	0.04277567	0.05538758	0.83180322
119.4	0.04886769	0.03518529	0.10280674	0.2157373	0.04277223	0.05544309	0.83170238
119.5	0.04893057	0.0352233	0.10285558	0.21591884	0.04276889	0.05549856	0.83160166
119.6	0.04899345	0.03526133	0.10290435	0.2161002	0.04276566	0.05555396	0.83150105
119.7	0.0490563	0.0352994	0.10295303	0.21628136	0.04276253	0.0556093	0.83140055
119.8	0.04911914	0.03533751	0.10300164	0.21646229	0.04275951	0.05566457	0.83130016
119.9	0.04918196	0.03537564	0.10305015	0.21664296	0.0427566	0.05571977	0.83119989
165	0.07730062	0.05599897	0.11620587	0.27728556	0.04988245	0.07295666	0.79713439
165.1	0.07736096	0.05605165	0.11621915	0.27738239	0.04990625	0.07298119	0.79708234
165.2	0.0774212	0.05610438	0.11623238	0.27747911	0.04993008	0.07300565	0.79703036
165.3	0.07748136	0.05615716	0.11624555	0.27757574	0.04995393	0.07303006	0.79697846
165.4	0.07754143	0.05620999	0.11625865	0.27767226	0.04997782	0.07305441	0.79692664
165.5	0.0776014	0.05626286	0.1162717	0.2777687	0.05000174	0.0730787	0.7968749
165.6	0.07766129	0.05631576	0.11628469	0.27786505	0.0500257	0.07310293	0.79682323
165.7	0.0777211	0.05636871	0.11629763	0.2779613	0.05004971	0.07312711	0.79677163
165.8	0.07778081	0.05642168	0.1163105	0.27805745	0.05007377	0.07315124	0.79672011
165.9	0.07784044	0.05647469	0.11632332	0.27815348	0.05009788	0.07317531	0.79666867
166	0.07789998	0.05652771	0.11633608	0.27824941	0.05012206	0.07319932	0.7966173
166.1	0.07795944	0.05658076	0.11634879	0.27834521	0.05014629	0.07322329	0.796566
166.2	0.07801881	0.05663382	0.11636144	0.27844089	0.05017059	0.0732472	0.79651478
166.3	0.0780781	0.05668691	0.11637403	0.27853644	0.05019493	0.07327105	0.79646364
166.4	0.07813731	0.05674001	0.11638656	0.27863186	0.05021932	0.07329486	0.79641256
166.5	0.07819643	0.05679315	0.11639904	0.27872714	0.05024375	0.07331861	0.79636156
166.6	0.07825546	0.05684631	0.11641146	0.27882228	0.0502682	0.07334232	0.79631063
166.7	0.07831442	0.05689951	0.11642383	0.27891729	0.05029266	0.07336597	0.79625977
166.8	0.07837329	0.05695274	0.11643614	0.27901217	0.05031713	0.07338957	0.79620898
166.9	0.07843208	0.057006	0.1164484	0.27910691	0.05034158	0.07341313	0.79615826
167	0.07849079	0.05705929	0.11646061	0.27920152	0.050366	0.07343663	0.79610762
167.1	0.07854942	0.0571126	0.11647276	0.27929599	0.05039038	0.07346009	0.79605704
167.2	0.07860796	0.05716594	0.11648485	0.27939032	0.05041472	0.0734835	0.79600653
167.3	0.07866643	0.05721931	0.11649689	0.27948453	0.050439	0.07350686	0.7959561
167.4	0.07872481	0.0572727	0.11650888	0.2795786	0.05046322	0.07353018	0.79590572
167.5	0.07878312	0.05732612	0.11652081	0.27967255	0.05048739	0.07355345	0.79585542
167.6	0.07884135	0.05737956	0.11653269	0.27976636	0.0505115	0.07357668	0.79580518
167.7	0.0788995	0.05743302	0.11654452	0.27986004	0.05053558	0.07359987	0.79575501
167.8	0.07895757	0.05748652	0.11655629	0.2799536	0.0505596	0.07362301	0.79570489
167.9	0.07901556	0.05754004	0.11656802	0.28004703	0.05058357	0.07364611	0.79565485
168	0.07907348	0.05759359	0.11657969	0.28014034	0.0506075	0.07366917	0.79560486
168.1	0.07913132	0.05764716	0.1165913	0.28023351	0.05063138	0.0736922	0.79555493
168.2	0.07918908	0.05770076	0.11660287	0.28032657	0.05065521	0.07371518	0.79550507
168.3	0.07924677	0.05775439	0.11661438	0.2804195	0.050679	0.07373814	0.79545526
168.4	0.07930439	0.05780805	0.11662585	0.28051231	0.05070274	0.07376105	0.79540551
168.5	0.07936192	0.05786174	0.11663726	0.28060499	0.05072643	0.07378394	0.79535581
168.6	0.07941939	0.05791545	0.11664862	0.28069755	0.05075006	0.07380679	0.79530617
168.7	0.07947678	0.05796919	0.11665993	0.28079	0.05077364	0.07382962	0.79525659
168.8	0.07953409	0.05802296	0.11667119	0.28088233	0.05079717	0.07385242	0.79520706
168.9	0.07959133	0.05807675	0.11668239	0.28097455	0.05082064	0.0738752	0.79515758
169	0.0796485	0.05813057	0.11669355	0.28106666	0.05084406	0.07389796	0.79510814
169.1	0.0797056	0.05818442	0.11670466	0.28115865	0.05086742	0.0739207	0.79505876
169.2	0.07976263	0.05823829	0.11671572	0.28125054	0.05089073	0.07394342	0.79500943
169.3	0.07981959	0.0582922	0.11672673	0.28134233	0.05091399	0.07396612	0.79496015
169.4	0.07987647	0.05834613	0.11673769	0.28143401	0.0509372	0.0739888	0.79491092
169.5	0.07993329	0.05840009	0.1167486	0.28152559	0.05096035	0.07401145	0.79486175
169.6	0.07999003	0.05845408	0.11675947	0.28161708	0.05098345	0.07403408	0.79481264
169.7	0.08004671	0.05850811	0.11677028	0.28170847	0.0510065	0.07405668	0.7947636
169.8	0.08010331	0.05856216	0.11678105	0.28179978	0.05102949	0.07407923	0.79471462
169.9	0.08015984	0.05861625	0.11679176	0.281891	0.05105242	0.07410176	0.79466571
170	0.0802163	0.05867037	0.11680243	0.28198214	0.05107529	0.07412424	0.79461687
170.1	0.08027268	0.05872453	0.11681306	0.28207321	0.05109812	0.07414667	0.79456811
170.2	0.080329	0.05877872	0.11682363	0.28216421	0.05112089	0.07416906	0.79451943
170.3	0.08038523	0.05883295	0.11683416	0.28225514	0.0511436	0.07419141	0.79447082
170.4	0.0804414	0.05888721	0.11684464	0.28234601	0.05116628	0.0742137	0.79442229
170.5	0.08049749	0.0589415	0.11685507	0.28243682	0.0511889	0.07423595	0.79437385
170.6	0.08055351	0.05899584	0.11686546	0.28252757	0.05121149	0.07425816	0.79432548
170.7	0.08060946	0.05905021	0.1168758	0.28261827	0.05123404	0.07428032	0.79427719
170.8	0.08066533	0.05910462	0.1168861	0.28270889	0.05125654	0.07430244	0.79422898
170.9	0.08072112	0.05915907	0.11689635	0.28279945	0.051279	0.07432451	0.79418085
171	0.08077684	0.05921357	0.11690655	0.28288993	0.05130141	0.07434653	0.7941328

126.1	0.05308264	0.03780841	0.10584889	0.22737665	0.04278609	0.05908307	0.8251896
126.2	0.0531458	0.03784885	0.10589023	0.22754231	0.04279002	0.05913464	0.82509593
126.3	0.05320893	0.03788934	0.10593146	0.22770779	0.04279406	0.05918607	0.82500234
126.4	0.05327202	0.03792988	0.10597259	0.2278731	0.04279819	0.05923738	0.82490882
126.5	0.05333509	0.03797046	0.1060136	0.22803826	0.04280242	0.05928855	0.8248154
126.6	0.05339813	0.0380111	0.1060545	0.22820325	0.04280676	0.05933958	0.82472206
126.7	0.05346114	0.03805178	0.1060953	0.22836807	0.04281119	0.05939049	0.82462882
126.8	0.05352411	0.03809251	0.10613599	0.22853271	0.04281573	0.05944126	0.82453568
126.9	0.05358707	0.0381333	0.10617657	0.22869716	0.04282036	0.0594919	0.82444266
127	0.05364999	0.03817414	0.10621704	0.22886142	0.0428251	0.05954241	0.82434975
127.1	0.0537129	0.03821504	0.10625741	0.22902547	0.04282994	0.05959279	0.82425695
127.2	0.05377579	0.038256	0.10629767	0.22918931	0.04283487	0.05964304	0.82416428
127.3	0.05383866	0.03829701	0.10633783	0.22935293	0.04283991	0.05969317	0.82407172
127.4	0.05390151	0.03833809	0.10637789	0.22951633	0.04284505	0.05974317	0.82397928
127.5	0.05396434	0.03837922	0.10641784	0.22967952	0.0428503	0.05979304	0.82388697
127.6	0.05402714	0.03842043	0.10645769	0.22984248	0.04285564	0.05984279	0.82379477
127.7	0.05408993	0.0384617	0.10649744	0.23000522	0.04286108	0.05989242	0.8237027
127.8	0.0541527	0.03850304	0.10653709	0.23016775	0.04286663	0.05994192	0.82361075
127.9	0.05421545	0.03854445	0.10657663	0.23033006	0.04287227	0.0599913	0.82351891
128	0.05427818	0.03858594	0.10661608	0.23049216	0.04287801	0.06004056	0.8234272
128.1	0.05434089	0.0386275	0.10665543	0.23065406	0.04288386	0.06008971	0.82333561
128.2	0.05440358	0.03866915	0.10669468	0.23081576	0.0428898	0.06013873	0.82324413
128.3	0.05446625	0.03871087	0.10673383	0.23097726	0.04289585	0.06018763	0.82315278
128.4	0.05452891	0.03875268	0.10677289	0.23113857	0.04290199	0.06023642	0.82306154
128.5	0.05459154	0.03879455	0.10681185	0.23129969	0.04290824	0.0602851	0.82297042
128.6	0.05465415	0.03883649	0.10685071	0.23146062	0.04291458	0.06033365	0.82287941
128.7	0.05471674	0.03887848	0.10688948	0.23162138	0.04292103	0.0603821	0.82278852
128.8	0.05477931	0.03892051	0.10692816	0.23178195	0.04292757	0.06043043	0.82269775
128.9	0.05484186	0.03896258	0.10696674	0.23194236	0.04293421	0.06047864	0.82260709
129	0.05490439	0.03900467	0.10700523	0.2321026	0.04294096	0.06052675	0.82251654
129.1	0.0549669	0.0390468	0.10704362	0.23226267	0.0429478	0.06057474	0.82242611
129.2	0.05502939	0.03908894	0.10708193	0.23242257	0.04295474	0.06062263	0.8223358
129.3	0.05509186	0.03913111	0.10712014	0.2325823	0.04296178	0.0606704	0.82224559
129.4	0.05515432	0.03917331	0.10715827	0.23274185	0.04296892	0.06071807	0.8221555
129.5	0.05521675	0.03921552	0.1071963	0.23290121	0.04297616	0.06076563	0.82206552
129.6	0.05527917	0.03925776	0.10723425	0.23306039	0.04298351	0.06081309	0.82197566
129.7	0.05534157	0.03930002	0.10727211	0.23321936	0.04299095	0.06086043	0.8218859
129.8	0.05540396	0.0393423	0.10730988	0.23337814	0.04299849	0.06090768	0.82179625
129.9	0.05546632	0.03938461	0.10734757	0.23353672	0.04300614	0.06095482	0.82170671
130	0.05552868	0.03942694	0.10738517	0.23369509	0.04301388	0.06100185	0.82161727
130.1	0.05559101	0.03946929	0.10742269	0.23385326	0.04302173	0.06104879	0.82152794
130.2	0.05565333	0.03951166	0.10746012	0.23401123	0.04302968	0.06109562	0.82143872
130.3	0.05571564	0.03955406	0.10749747	0.23416901	0.04303772	0.06114236	0.82134959
130.4	0.05577793	0.03959649	0.10753475	0.23432659	0.04304587	0.061189	0.82126057
130.5	0.05584021	0.03963894	0.10757194	0.23448398	0.04305412	0.06123554	0.82117165
130.6	0.05590247	0.03968141	0.10760905	0.2346412	0.04306248	0.06128199	0.82108283
130.7	0.05596472	0.03972392	0.10764609	0.23479825	0.04307093	0.06132834	0.8209941
130.8	0.05602695	0.03976644	0.10768305	0.23495515	0.04307948	0.06137459	0.82090547
130.9	0.05608917	0.03980899	0.10771994	0.23511188	0.04308814	0.06142076	0.82081693
131	0.05615138	0.03985157	0.10775675	0.23526847	0.04309689	0.06146684	0.82072848
131.1	0.05621358	0.03989418	0.10779349	0.23542493	0.04310575	0.06151282	0.82064011
131.2	0.05627576	0.03993681	0.10783016	0.23558125	0.04311471	0.06155872	0.82055183
131.3	0.05633794	0.03997946	0.10786674	0.23573745	0.04312377	0.06160453	0.82046362
131.4	0.0564001	0.04002214	0.10790325	0.23589352	0.04313293	0.06165026	0.82037549
131.5	0.05646224	0.04006485	0.10793969	0.23604947	0.0431422	0.06169591	0.82028743
131.6	0.05652438	0.04010758	0.10797604	0.23620527	0.04315158	0.06174147	0.82019944
131.7	0.05658651	0.04015033	0.10801232	0.2363609	0.04316107	0.06178695	0.82011151
131.8	0.05664862	0.04019311	0.10804852	0.23651635	0.04317067	0.06183236	0.82002364
131.9	0.05671073	0.04023591	0.10808464	0.23667161	0.04318039	0.06187769	0.81993584
132	0.05677282	0.04027874	0.10812068	0.23682667	0.04319022	0.06192296	0.8198481
132.1	0.05683491	0.0403216	0.10815665	0.23698153	0.04320016	0.06196816	0.81976044
132.2	0.05689698	0.04036448	0.10819253	0.23713618	0.04321023	0.06201331	0.81967286
132.3	0.05695905	0.04040738	0.10822834	0.23729061	0.04322042	0.0620584	0.81958537
132.4	0.05702111	0.04045031	0.10826407	0.23744482	0.04323074	0.06210345	0.81949797
132.5	0.05708316	0.04049327	0.10829973	0.23759879	0.04324119	0.06214845	0.81941066
132.6	0.05714519	0.04053625	0.10833531	0.23775252	0.04325178	0.06219342	0.81932345
132.7	0.05720723	0.04057925	0.10837082	0.237906	0.0432625	0.06223836	0.81923633
132.8	0.05726925	0.04062229	0.10840626	0.23805925	0.04327338	0.06228326	0.81914932
132.9	0.05733126	0.04066534	0.10844162	0.23821228	0.04328441	0.06232814	0.81906242
133	0.05739327	0.04070843	0.10847691	0.23836508	0.0432956	0.06237298	0.81897563
133.1	0.05745527	0.04075154	0.10851213	0.23851767	0.04330694	0.06241779	0.81888897
133.2	0.05751727	0.04079467	0.10854728	0.23867005	0.04331842	0.06246256	0.81880243
133.3	0.05757925	0.04083783	0.10858236	0.23882222	0.04333001	0.06250728	0.81871603
133.4	0.05764123	0.04088102	0.10861737	0.2389742	0.04334171	0.06255194	0.81862975
133.5	0.05770321	0.04092423	0.1086523	0.23912599	0.04335355	0.06259653	0.81854361
133.6	0.05776518	0.04096747	0.10868717	0.23927759	0.0433655	0.06264105	0.8184576
133.7	0.05782714	0.04101074	0.10872198	0.23942902	0.04337754	0.06268549	0.81837172
133.8	0.0578891	0.04105403	0.10875671	0.23958028	0.04338966	0.06272984	0.81828598
133.9	0.05795106	0.04109735	0.10879138	0.23973135	0.04340184	0.0627741	0.81820038
134	0.05801301	0.04114069	0.10882598	0.23988223	0.0434141	0.06281825	0.81811491
134.1	0.05807496	0.04118406	0.10886052	0.24003294	0.04342642	0.06286231	0.81802956
134.2	0.05813691	0.04122746	0.108895	0.24018346	0.0434388	0.06290626	0.81794435
134.3	0.05819886	0.04127088	0.10892942	0.24033379	0.04345125	0.06295011	0.81785925
134.4	0.05826081	0.04131433	0.10896377	0.24048392	0.04346376	0.06299386	0.81777429
134.5	0.05832276	0.04135781	0.10899806	0.24063382	0.04347634	0.06303752	0.81768945
134.6	0.05838471	0.04140132	0.1090323	0.24078349	0.04348897	0.06308107	0.81760473
134.7	0.05844665	0.04144485	0.10906648	0.24093293	0.04350168	0.06312452	0.81752014
134.8	0.05850861	0.04148841	0.1091006	0.24108216	0.04351445	0.06316789	0.81743568
134.9	0.05857056	0.04153199	0.10913466	0.24123119	0.04352728	0.06321116	0.81735134
180	0.08552782	0.0642606	0.11766404	0.2905249	0.05317131	0.07620771	0.79010233
180.1	0.08557754	0.06431633	0.1176711	0.29060446	0.05319043	0.07622687	0.79006058
180.2	0.0856272	0.06437204	0.11767816	0.29068391	0.0532095	0.07624599	0.7900189
180.3	0.08567679	0.06442773	0.11768521	0.29076327	0.05322853	0.07626509	0.78997728
180.4	0.08572631	0.06448342	0.11769226	0.29084252	0.05324752	0.07628414	0.78993572
180.5	0.08577576	0.06453908	0.1176993	0.29092167	0.05326647	0.07630316	0.78989423
180.6	0.08582514	0.06459474	0.11770633	0.29100072	0.05328537	0.07632215	0.7898528
180.7	0.08587446	0.06465038	0.11771334	0.29107966	0.05330423	0.0763411	0.78981143
180.8	0.08592371	0.064706	0.11772034	0.29115851	0.05332306	0.07636002	0.78977012
180.9	0.08597289	0.06476161	0.11772732	0.29123726	0.05334184	0.07637891	0.78972887
181	0.086022	0.06481721	0.11773428	0.29131591	0.05336058	0.07639777	0.78968769
181.1	0.08607105	0.06487279	0.11774122	0.29139446	0.05337928	0.07641659	0.78964656
181.2	0.08612003	0.06492836	0.11774814	0.29147292	0.05339794	0.07643538	0.7896055
181.3	0.08616895	0.06498392	0.11775502	0.29155127	0.05341655	0.07645414	0.7895645
181.4	0.0862178	0.06503946	0.11776188	0.29162954	0.05343513	0.07647287	0.78952357
181.5	0.08626658	0.06509499	0.1177687	0.29170771	0.05345367	0.07649158	0.78948269
181.6	0.0863153	0.06515051	0.11777548	0.29178578	0.05347216	0.07651025	0.78944188
181.7	0.08636396	0.06520601	0.11778223	0.29186377	0.05349062	0.0765289	0.78940113
181.8	0.08641255	0.0652615	0.11778895	0.29194166	0.05350904	0.07654752	0.78936044
181.9	0.08646107	0.06531698	0.11779563	0.29201947	0.05352741	0.07656612	0.78931982
182	0.08650953	0.06537245	0.11780227	0.29209719	0.05354575	0.07658469	0.78927925
182.1	0.08655792	0.0654279	0.11780888	0.29217482	0.05356405	0.07660324	0.78923876
182.2	0.08660625	0.06548334	0.11781545	0.29225237	0.0535823	0.07662177	0.78919832
182.3	0.08665452	0.06553877	0.11782199	0.29232983	0.05360052	0.07664028	0.78915795
182.4	0.08670272	0.06559419	0.11782849	0.29240721	0.0536187	0.07665877	0.78911764
182.5	0.08675086	0.0656496	0.11783497	0.29248452	0.05363684	0.07667724	0.7890774
182.6	0.08679893	0.065705	0.1178414	0.29256174	0.05365495	0.07669569	0.78903722
182.7	0.08684695	0.06576039	0.11784781	0.29263888	0.05367301	0.07671413	0.78899709
182.8	0.08689489	0.06581577	0.11785418	0.29271595	0.05369103	0.07673255	0.78895703
182.9	0.08694278	0.06587114	0.11786052	0.29279294	0.05370902	0.07675094	0.78891703
183	0.0869906	0.0659265	0.11786682	0.29286986	0.05372697	0.07676932	0.78887708
183.1	0.08703837	0.06598186	0.1178731	0.2929467	0.05374488	0.07678767	0.78883719

141.1	0.0624288	0.04428957	0.11113665	0.25002451	0.04445078	0.06577256	0.81234625
141.2	0.06249099	0.04433501	0.11116668	0.25015878	0.04446779	0.06581213	0.81226954
141.3	0.06255319	0.04438048	0.11119663	0.25029282	0.04448486	0.06585158	0.81219295
141.4	0.06261538	0.04442599	0.11122652	0.25042665	0.04450201	0.06589092	0.8121165
141.5	0.06267757	0.04447154	0.11125634	0.25056025	0.04451922	0.06593013	0.81204018
141.6	0.06273976	0.04451712	0.1112861	0.25069363	0.0445365	0.06596923	0.811964
141.7	0.06280196	0.04456275	0.11131579	0.25082679	0.04455384	0.06600822	0.81188794
141.8	0.06286415	0.04460841	0.11134541	0.25095973	0.04457125	0.06604709	0.81181201
141.9	0.06292634	0.0446541	0.11137497	0.25109245	0.04458873	0.06608586	0.81173622
142	0.06298853	0.04469984	0.11140446	0.25122496	0.04460627	0.06612451	0.81166055
142.1	0.06305072	0.04474562	0.11143387	0.25135725	0.04462388	0.06616306	0.81158501
142.2	0.06311292	0.04479144	0.11146322	0.25148932	0.04464156	0.0662015	0.8115096
142.3	0.06317512	0.04483731	0.11149249	0.25162118	0.0446593	0.06623984	0.81143432
142.4	0.06323731	0.04488322	0.11152169	0.25175282	0.04467711	0.06627807	0.81135916
142.5	0.06329952	0.04492918	0.1115508	0.25188425	0.04469498	0.06631619	0.81128414
142.6	0.06336172	0.04497519	0.11157984	0.25201547	0.04471292	0.0663542	0.81120924
142.7	0.06342393	0.04502125	0.1116088	0.25214647	0.04473093	0.06639211	0.81113447
142.8	0.06348614	0.04506735	0.11163768	0.25227727	0.04474901	0.06642989	0.81105982
142.9	0.06354836	0.0451135	0.11166648	0.25240785	0.04476715	0.06646756	0.8109853
143	0.06361058	0.0451597	0.1116952	0.25253822	0.04478535	0.06650512	0.8109109
143.1	0.06367281	0.04520594	0.11172384	0.25266839	0.04480363	0.06654256	0.81083663
143.2	0.06373504	0.04525221	0.1117524	0.25279835	0.04482197	0.06657987	0.81076249
143.3	0.06379728	0.04529852	0.11178089	0.2529281	0.04484038	0.06661707	0.81068847
143.4	0.06385953	0.04534487	0.1118093	0.25305764	0.04485885	0.06665415	0.81061457
143.5	0.06392178	0.04539124	0.11183765	0.25318698	0.0448774	0.06669111	0.81054079
143.6	0.06398404	0.04543765	0.11186593	0.25331612	0.04489601	0.06672796	0.81046714
143.7	0.06404632	0.04548408	0.11189414	0.25344505	0.04491469	0.06676469	0.81039362
143.8	0.0641086	0.04553054	0.1119223	0.25357378	0.04493344	0.0668013	0.81032021
143.9	0.06417091	0.04557703	0.11195039	0.2537023	0.04495226	0.0668378	0.81024693
144	0.06423323	0.04562354	0.11197844	0.25383063	0.04497114	0.06687418	0.81017377
144.1	0.06429556	0.04567008	0.11200644	0.25395876	0.0449901	0.06691046	0.81010073
144.2	0.06435792	0.04571664	0.1120344	0.25408669	0.04500913	0.06694661	0.81002781
144.3	0.0644203	0.04576323	0.11206232	0.25421442	0.04502823	0.06698266	0.80995501
144.4	0.06448271	0.04580984	0.11209021	0.25434195	0.0450474	0.0670186	0.80988232
144.5	0.06454515	0.04585648	0.11211807	0.25446929	0.04506664	0.06705443	0.80980976
144.6	0.06460763	0.04590314	0.1121459	0.25459644	0.04508595	0.06709014	0.80973732
144.7	0.06467014	0.04594983	0.11217371	0.25472339	0.04510534	0.06712575	0.80966499
144.8	0.0647327	0.04599655	0.11220149	0.25485015	0.0451248	0.06716126	0.80959279
144.9	0.0647953	0.04604329	0.11222925	0.25497672	0.04514433	0.06719665	0.8095207
145	0.06485795	0.04609006	0.11225697	0.25510309	0.04516394	0.06723194	0.80944872
145.1	0.06492066	0.04613685	0.11228467	0.25522928	0.04518363	0.06726712	0.80937686
145.2	0.06498343	0.04618367	0.11231232	0.25535528	0.0452034	0.0673022	0.80930512
145.3	0.06504626	0.04623052	0.11233994	0.25548109	0.04522325	0.06733717	0.8092335
145.4	0.06510917	0.04627739	0.11236751	0.25560672	0.0452432	0.06737204	0.80916199
145.5	0.06517216	0.04632429	0.11239503	0.25573216	0.04526323	0.0674068	0.80909059
145.6	0.06523522	0.04637121	0.1124225	0.25585742	0.04528336	0.06744147	0.80901931
145.7	0.06529835	0.04641816	0.11244991	0.2559825	0.04530358	0.06747603	0.80894814
145.8	0.06536155	0.04646514	0.11247725	0.2561074	0.0453239	0.06751049	0.80887709
145.9	0.06542481	0.04651214	0.11250451	0.25623212	0.04534432	0.06754485	0.80880615
146	0.06548811	0.04655917	0.11253169	0.25635667	0.04536485	0.06757911	0.80873532
146.1	0.06555144	0.04660623	0.11255878	0.25648104	0.04538548	0.06761328	0.8086646
146.2	0.06561479	0.04665331	0.11258578	0.25660525	0.04540623	0.06764734	0.80859399
146.3	0.06567814	0.04670042	0.11261269	0.25672928	0.04542709	0.06768131	0.80852349
146.4	0.0657415	0.04674755	0.11263951	0.25685314	0.04544807	0.06771519	0.8084531
146.5	0.06580486	0.04679471	0.11266622	0.25697684	0.04546918	0.06774896	0.80838282
146.6	0.06586822	0.0468419	0.11269284	0.25710038	0.04549042	0.06778265	0.80831265
146.7	0.06593157	0.04688912	0.11271935	0.25722375	0.0455118	0.06781624	0.80824258
146.8	0.0659949	0.04693636	0.11274577	0.25734695	0.04553332	0.06784973	0.80817261
146.9	0.06605821	0.04698362	0.11277208	0.25746998	0.04555498	0.06788314	0.80810275
147	0.06612149	0.04703092	0.1127983	0.25759284	0.04557679	0.06791646	0.80803299
147.1	0.06618474	0.04707824	0.1128244	0.25771553	0.04559875	0.06794968	0.80796333
147.2	0.06624797	0.04712559	0.1128504	0.25783804	0.04562086	0.06798282	0.80789376
147.3	0.06631119	0.04717296	0.1128763	0.25796037	0.04564311	0.06801587	0.80782429
147.4	0.06637438	0.04722036	0.11290208	0.25808252	0.04566549	0.06804884	0.80775492
147.5	0.06643755	0.04726779	0.11292777	0.2582045	0.045688	0.06808171	0.80768564
147.6	0.0665007	0.04731524	0.11295334	0.2583263	0.04571061	0.0681145	0.80761646
147.7	0.06656382	0.04736272	0.11297882	0.25844791	0.04573331	0.0681472	0.80754737
147.8	0.06662693	0.04741023	0.11300419	0.25856935	0.04575609	0.06817982	0.80747839
147.9	0.06669002	0.04745776	0.11302946	0.25869061	0.04577895	0.06821235	0.8074095
148	0.0667531	0.04750533	0.11305462	0.25881169	0.04580188	0.06824479	0.80734073
148.1	0.06681617	0.04755291	0.11307969	0.2589326	0.04582489	0.06827714	0.80727206
148.2	0.06687923	0.04760053	0.11310465	0.25905332	0.04584797	0.06830941	0.8072035
148.3	0.06694228	0.04764817	0.11312951	0.25917387	0.04587111	0.0683416	0.80713506
148.4	0.06700532	0.04769584	0.11315427	0.25929425	0.04589434	0.0683737	0.80706674
148.5	0.06706837	0.04774354	0.11317892	0.25941445	0.04591764	0.06840571	0.80699853
148.6	0.06713142	0.04779126	0.11320348	0.25953448	0.04594103	0.06843765	0.80693043
148.7	0.06719447	0.04783901	0.11322794	0.25965434	0.0459645	0.0684695	0.80686245
148.8	0.06725752	0.04788678	0.1132523	0.25977403	0.04598805	0.06850127	0.80679458
148.9	0.06732058	0.04793459	0.11327656	0.25989355	0.04601168	0.06853295	0.80672683
149	0.06738364	0.04798242	0.11330072	0.26001291	0.0460354	0.06856456	0.80665919
149.1	0.06744671	0.04803028	0.11332479	0.2601321	0.04605918	0.06859609	0.80659167
149.2	0.06750977	0.04807816	0.11334876	0.26025112	0.04608302	0.06862754	0.80652425
149.3	0.06757282	0.04812607	0.11337263	0.26036999	0.04610691	0.06865891	0.80645695
149.4	0.06763586	0.04817401	0.11339641	0.26048868	0.04613083	0.0686902	0.80638976
149.5	0.06769889	0.04822198	0.11342009	0.26060722	0.04615478	0.06872142	0.80632268
149.6	0.0677619	0.04826997	0.11344368	0.2607256	0.04617874	0.06875256	0.80625571
149.7	0.06782488	0.04831799	0.11346717	0.26084382	0.04620269	0.06878362	0.80618885
149.8	0.06788785	0.04836604	0.11349057	0.26096189	0.04622664	0.0688146	0.80612211
149.9	0.06795079	0.04841412	0.11351388	0.2610798	0.04625058	0.06884551	0.80605547
195	0.09232719	0.07256137	0.11843857	0.30141707	0.05563944	0.07878945	0.78446754
195.1	0.09236868	0.07261623	0.11844207	0.30148289	0.05565366	0.07880468	0.78443398
195.2	0.09241013	0.07267108	0.11844556	0.30154864	0.05566786	0.07881989	0.78440047
195.3	0.09245154	0.0727259	0.11844904	0.3016143	0.05568203	0.07883506	0.78436701
195.4	0.09249291	0.0727807	0.1184525	0.30167988	0.05569617	0.0788502	0.7843336
195.5	0.09253423	0.07283546	0.11845596	0.30174538	0.0557103	0.0788653	0.78430024
195.6	0.0925755	0.07289019	0.1184594	0.3018108	0.0557244	0.07888037	0.78426693
195.7	0.09261674	0.07294488	0.11846283	0.30187615	0.05573848	0.07889541	0.78423366
195.8	0.09265793	0.07299953	0.11846625	0.30194141	0.05575254	0.07891041	0.78420045
195.9	0.09269908	0.07305413	0.11846966	0.30200659	0.05576657	0.07892538	0.78416728
196	0.09274019	0.07310869	0.11847306	0.3020717	0.05578059	0.07894032	0.78413416
196.1	0.09278126	0.07316318	0.11847645	0.30213673	0.05579458	0.07895522	0.78410109
196.2	0.09282228	0.07321762	0.11847984	0.30220168	0.05580855	0.0789701	0.78406806
196.3	0.09286326	0.073272	0.11848321	0.30226655	0.0558225	0.07898494	0.78403509
196.4	0.0929042	0.07332634	0.11848656	0.30233134	0.05583642	0.07899974	0.78400216
196.5	0.0929451	0.07338063	0.11848991	0.30239606	0.05585033	0.07901452	0.78396928
196.6	0.09298595	0.07343486	0.11849324	0.3024607	0.05586422	0.07902926	0.78393645
196.7	0.09302677	0.07348906	0.11849656	0.30252526	0.05587808	0.07904397	0.78390366
196.8	0.09306754	0.0735432	0.11849987	0.30258974	0.05589193	0.07905865	0.78387092
196.9	0.09310827	0.07359731	0.11850316	0.30265415	0.05590576	0.0790733	0.78383823
197	0.09314896	0.07365137	0.11850644	0.30271848	0.05591956	0.07908792	0.78380559
197.1	0.09318961	0.07370539	0.11850972	0.30278274	0.05593335	0.07910251	0.78377299
197.2	0.09323022	0.07375938	0.11851299	0.30284692	0.05594712	0.07911706	0.78374045
197.3	0.09327079	0.07381333	0.11851625	0.30291102	0.05596088	0.07913159	0.78370794
197.4	0.09331132	0.07386724	0.11851951	0.30297505	0.05597461	0.07914608	0.78367549
197.5	0.0933518	0.07392113	0.11852276	0.303039	0.05598833	0.07916054	0.78364308
197.6	0.09339225	0.07397498	0.11852601	0.30310288	0.05600203	0.07917498	0.78361072
197.7	0.09343266	0.0740288	0.11852926	0.30316668	0.05601571	0.07918938	0.7835784
197.8	0.09347302	0.0740826	0.11853251	0.30323041	0.05602938	0.07920375	0.78354613
197.9	0.09351335	0.07413637	0.11853575	0.30329407	0.05604304	0.0792181	0.78351391
198	0.09355363	0.07419012	0.11853899	0.30335765	0.05605667	0.07923241	0.78348174
198.1	0.09359388	0.07424384	0.11854223	0.30342115	0.05607029	0.0792467	0.78344961

156.1	0.07181142	0.05144868	0.11479439	0.26809844	0.04773641	0.07064863	0.80212041
156.2	0.07187325	0.0514985	0.11481269	0.26820766	0.04776062	0.07067642	0.80206018
156.3	0.07193509	0.05154835	0.11483094	0.26831681	0.04778483	0.07070418	0.80200006
156.4	0.07199694	0.05159823	0.11484913	0.26842588	0.04780903	0.07073191	0.80194004
156.5	0.0720588	0.05164814	0.11486726	0.26853489	0.04783324	0.07075962	0.80188012
156.6	0.07212067	0.05169808	0.11488532	0.26864384	0.04785744	0.07078729	0.8018203
156.7	0.07218256	0.05174805	0.11490334	0.26875273	0.04788164	0.07081494	0.80176059
156.8	0.07224446	0.05179804	0.11492129	0.26886157	0.04790584	0.07084255	0.80170097
156.9	0.07230639	0.05184807	0.1149392	0.26897036	0.04793004	0.07087012	0.80164146
157	0.07236834	0.05189812	0.11495705	0.2690791	0.04795424	0.07089765	0.80158205
157.1	0.07243032	0.0519482	0.11497485	0.26918777	0.04797844	0.07092513	0.80152275
157.2	0.07249233	0.0519983	0.11499261	0.26929637	0.04800264	0.07095257	0.80146354
157.3	0.07255438	0.05204844	0.11501032	0.26940486	0.04802683	0.07097996	0.80140444
157.4	0.07261646	0.0520986	0.11502799	0.26951324	0.04805103	0.07100729	0.80134543
157.5	0.07267859	0.0521488	0.11504561	0.26962148	0.04807523	0.07103458	0.80128653
157.6	0.07274076	0.05219902	0.11506319	0.26972958	0.04809944	0.07106183	0.80122773
157.7	0.07280296	0.05224927	0.11508073	0.26983752	0.04812364	0.07108902	0.80116903
157.8	0.07286519	0.05229955	0.1150982	0.26994531	0.04814785	0.07111617	0.80111043
157.9	0.07292743	0.05234986	0.11511562	0.27005294	0.04817206	0.07114328	0.80105192
158	0.07298967	0.05240019	0.11513298	0.2701604	0.04819628	0.07117034	0.80099352
158.1	0.07305189	0.05245056	0.11515029	0.27026768	0.0482205	0.07119737	0.80093522
158.2	0.0731141	0.05250095	0.11516755	0.2703748	0.04824472	0.07122435	0.80087702
158.3	0.07317626	0.05255137	0.11518477	0.27048174	0.04826896	0.0712513	0.80081891
158.4	0.07323838	0.05260182	0.11520194	0.27058851	0.04829322	0.07127821	0.80076091
158.5	0.07330046	0.0526523	0.11521907	0.27069511	0.0483175	0.07130509	0.800703
158.6	0.07336249	0.05270281	0.11523614	0.27080153	0.04834182	0.07133193	0.8006452
158.7	0.07342447	0.05275335	0.11525314	0.27090778	0.04836617	0.07135873	0.80058748
158.8	0.07348642	0.05280391	0.11527006	0.27101387	0.04839056	0.07138549	0.80052987
158.9	0.07354832	0.05285451	0.11528691	0.27111978	0.048415	0.0714122	0.80047235
159	0.0736102	0.05290513	0.11530368	0.27122552	0.04843946	0.07143887	0.80041493
159.1	0.07367205	0.05295578	0.11532037	0.27133109	0.04846395	0.07146547	0.8003576
159.2	0.07373385	0.05300646	0.11533698	0.2714365	0.04848846	0.07149202	0.80030037
159.3	0.07379562	0.05305717	0.11535352	0.27154174	0.04851296	0.0715185	0.80024323
159.4	0.07385735	0.05310791	0.11536999	0.27164681	0.04853746	0.07154491	0.80018619
159.5	0.07391905	0.05315868	0.11538639	0.27175172	0.04856195	0.07157127	0.80012923
159.6	0.07398073	0.05320948	0.11540273	0.27185646	0.04858642	0.07159756	0.80007237
159.7	0.07404239	0.0532603	0.11541899	0.27196103	0.04861088	0.0716238	0.80001561
159.8	0.07410403	0.05331116	0.11543519	0.27206545	0.04863531	0.07164998	0.79995893
159.9	0.07416567	0.05336204	0.11545133	0.2721697	0.04865972	0.07167611	0.79990235
160	0.0742273	0.05341296	0.11546741	0.27227378	0.04868411	0.07170218	0.79984586
160.1	0.07428892	0.0534639	0.11548343	0.27237771	0.04870848	0.07172819	0.79978947
160.2	0.07435053	0.05351487	0.11549939	0.27248147	0.04873282	0.07175416	0.79973316
160.3	0.07441214	0.05356588	0.1155153	0.27258507	0.04875714	0.07178007	0.79967694
160.4	0.07447374	0.05361691	0.11553115	0.27268852	0.04878143	0.07180594	0.79962082
160.5	0.07453535	0.05366797	0.11554694	0.2727918	0.04880571	0.07183175	0.79956478
160.6	0.07459696	0.05371906	0.11556269	0.27289492	0.04882996	0.07185751	0.79950884
160.7	0.07465858	0.05377018	0.11557838	0.27299788	0.0488542	0.07188322	0.79945299
160.8	0.0747202	0.05382133	0.11559403	0.27310069	0.04887842	0.07190888	0.79939722
160.9	0.07478183	0.05387251	0.11560962	0.27320333	0.04890261	0.07193449	0.79934155
161	0.07484345	0.05392372	0.11562517	0.27330583	0.04892679	0.07196005	0.79928596
161.1	0.07490506	0.05397496	0.11564068	0.27340816	0.04895096	0.07198556	0.79923047
161.2	0.07496666	0.05402623	0.11565615	0.27351034	0.0489751	0.07201102	0.79917506
161.3	0.07502825	0.05407753	0.11567158	0.27361236	0.04899922	0.07203644	0.79911975
161.4	0.07508982	0.05412886	0.11568697	0.27371423	0.04902333	0.07206181	0.79906452
161.5	0.07515138	0.05418023	0.11570233	0.27381595	0.04904741	0.07208714	0.79900938
161.6	0.07521293	0.05423162	0.11571766	0.27391751	0.04907148	0.07211242	0.79895433
161.7	0.07527446	0.05428304	0.11573296	0.27401892	0.04909553	0.07213766	0.79889937
161.8	0.07533597	0.0543345	0.11574822	0.27412018	0.04911957	0.07216285	0.79884449
161.9	0.07539748	0.05438598	0.11576344	0.27422129	0.04914358	0.07218801	0.7987897
162	0.07545897	0.0544375	0.11577862	0.27432225	0.04916758	0.07221312	0.798735
162.1	0.07552046	0.05448904	0.11579377	0.27442306	0.04919157	0.07223819	0.79868039
162.2	0.07558195	0.05454062	0.11580887	0.27452372	0.04921554	0.07226323	0.79862587
162.3	0.07564344	0.05459223	0.11582391	0.27462423	0.04923949	0.07228823	0.79857143
162.4	0.07570494	0.05464387	0.1158389	0.27472459	0.04926343	0.07231319	0.79851708
162.5	0.07576646	0.05469555	0.11585382	0.27482481	0.04928736	0.07233812	0.79846281
162.6	0.075828	0.05474725	0.11586867	0.27492488	0.04931127	0.07236302	0.79840863
162.7	0.07588957	0.05479899	0.11588346	0.27502481	0.04933517	0.07238789	0.79835454
162.8	0.07595119	0.05485076	0.11589817	0.27512459	0.04935906	0.07241272	0.79830054
162.9	0.07601284	0.05490257	0.11591282	0.27522423	0.04938293	0.07243754	0.79824663
163	0.07607454	0.0549544	0.1159274	0.27532373	0.04940679	0.07246232	0.7981928
163.1	0.07613629	0.05500627	0.11594192	0.27542308	0.04943065	0.07248709	0.79813905
163.2	0.07619809	0.05505818	0.11595637	0.2755223	0.04945449	0.07251184	0.7980854
163.3	0.07625994	0.05511012	0.11597076	0.27562137	0.04947832	0.07253657	0.79803183
163.4	0.07632182	0.05516209	0.11598508	0.27572031	0.04950214	0.07256129	0.79797835
163.5	0.07638369	0.0552141	0.11599934	0.27581911	0.04952595	0.07258601	0.79792496
163.6	0.07644552	0.05526615	0.11601354	0.27591777	0.04954975	0.07261072	0.79787165
163.7	0.07650729	0.05531823	0.11602767	0.2760163	0.04957354	0.07263543	0.79781843
163.8	0.07656898	0.05537035	0.11604175	0.2761147	0.04959733	0.07266015	0.7977653
163.9	0.07663056	0.05542251	0.11605576	0.27621296	0.04962111	0.07268489	0.79771225
164	0.07669202	0.05547471	0.11606971	0.27631108	0.04964488	0.07270964	0.7976593
164.1	0.07675336	0.05552694	0.1160836	0.27640908	0.04966864	0.07273441	0.79760643
164.2	0.07681458	0.05557922	0.11609743	0.27650695	0.0496924	0.07275917	0.79755364
164.3	0.07687568	0.05563153	0.1161112	0.27660469	0.04971615	0.07278391	0.79750095
164.4	0.07693668	0.05568389	0.1161249	0.27670231	0.0497399	0.07280863	0.79744834
164.5	0.07699757	0.05573629	0.11613855	0.27679981	0.04976364	0.07283334	0.79739581
164.6	0.07705837	0.05578873	0.11615213	0.27689718	0.04978739	0.07285805	0.79734337
164.7	0.07711907	0.05584122	0.11616566	0.27699445	0.04981114	0.07288277	0.797291
164.8	0.07717968	0.05589375	0.11617912	0.2770916	0.0498349	0.07290745	0.79723872
164.9	0.0772402	0.05594633	0.11619252	0.27718863	0.04985867	0.07293208	0.79718651
210	0.09813048	0.08026865	0.11879468	0.31051524	0.05757555	0.08079949	0.77992975
210.1	0.09816673	0.08031607	0.11879564	0.31057115	0.05758724	0.08081128	0.7799024
210.2	0.09820295	0.08036344	0.11879658	0.310627	0.05759891	0.08082305	0.77987509
210.3	0.09823915	0.08041077	0.11879751	0.31068279	0.05761056	0.08083479	0.77984781
210.4	0.09827531	0.08045805	0.11879842	0.31073853	0.0576222	0.0808465	0.77982058
210.5	0.09831146	0.08050528	0.11879931	0.31079421	0.05763383	0.08085818	0.77979338
210.6	0.09834757	0.08055246	0.11880018	0.31084984	0.05764543	0.08086984	0.77976622
210.7	0.09838366	0.0805996	0.11880104	0.31090542	0.05765703	0.08088148	0.7797391
210.8	0.09841972	0.08064669	0.11880188	0.31096094	0.0576686	0.08089308	0.77971201
210.9	0.09845576	0.08069374	0.11880271	0.3110164	0.05768016	0.08090466	0.77968497
211	0.09849177	0.08074074	0.11880352	0.31107182	0.05769171	0.08091622	0.77965796
211.1	0.09852775	0.08078769	0.11880431	0.31112717	0.05770324	0.08092775	0.77963099
211.2	0.09856371	0.0808346	0.11880509	0.31118247	0.05771475	0.08093925	0.77960406
211.3	0.09859964	0.08088146	0.11880585	0.31123772	0.05772625	0.08095073	0.77957717
211.4	0.09863554	0.08092827	0.1188066	0.3112929	0.05773774	0.08096218	0.77955032
211.5	0.09867142	0.08097504	0.11880733	0.31134803	0.05774921	0.08097361	0.7795235
211.6	0.09870728	0.08102177	0.11880804	0.3114031	0.05776066	0.08098501	0.77949672
211.7	0.09874311	0.08106845	0.11880874	0.31145811	0.0577721	0.08099638	0.77946998
211.8	0.09877891	0.08111508	0.11880943	0.31151307	0.05778352	0.08100773	0.77944328
211.9	0.09881469	0.08116167	0.1188101	0.31156796	0.05779493	0.08101906	0.77941661
212	0.09885044	0.08120822	0.11881076	0.3116228	0.05780633	0.08103036	0.77938998
212.1	0.09888617	0.08125472	0.1188114	0.31167758	0.05781771	0.08104163	0.77936339
212.2	0.09892188	0.08130117	0.11881203	0.3117323	0.05782907	0.08105288	0.77933683
212.3	0.09895756	0.08134758	0.11881265	0.31178697	0.05784042	0.08106411	0.77931032
212.4	0.09899321	0.08139395	0.11881326	0.31184157	0.05785176	0.08107531	0.77928384
212.5	0.09902885	0.08144028	0.11881385	0.31189612	0.05786308	0.08108649	0.77925739
212.6	0.09906445	0.08148656	0.11881443	0.31195061	0.05787438	0.08109764	0.77923099
212.7	0.09910004	0.08153279	0.118815	0.31200505	0.05788567	0.08110877	0.77920462
212.8	0.0991356	0.08157899	0.11881556	0.31205942	0.05789695	0.08111987	0.77917828
212.9	0.09917113	0.08162514	0.1188161	0.31211374	0.05790821	0.08113095	0.77915199
213	0.09920665	0.08167124	0.11881665	0.31216801	0.05791946	0.08114201	0.77912573
213.1	0.09924214	0.08171731	0.11881718	0.31222222	0.0579307	0.08115304	0.77909951

171.1	0.08083249	0.05926811	0.11691671	0.28298033	0.05132377	0.07436851	0.79408482
171.2	0.08088807	0.05932271	0.11692682	0.28307062	0.05134608	0.07439045	0.79403692
171.3	0.08094357	0.05937736	0.11693688	0.28316082	0.05136835	0.07441235	0.7939891
171.4	0.080999	0.05943206	0.1169469	0.2832509	0.05139058	0.07443421	0.79394135
171.5	0.08105436	0.05948682	0.11695688	0.28334085	0.05141277	0.07445603	0.79389368
171.6	0.08110965	0.05954163	0.11696681	0.28343068	0.05143491	0.0744778	0.79384609
171.7	0.08116486	0.05959652	0.1169767	0.28352037	0.05145701	0.07449954	0.79379857
171.8	0.08122001	0.05965146	0.11698654	0.28360993	0.05147906	0.07452124	0.79375113
171.9	0.08127508	0.05970647	0.11699634	0.28369934	0.05150108	0.0745429	0.79370376
172	0.08133008	0.05976156	0.1170061	0.28378861	0.05152305	0.07456452	0.79365646
172.1	0.08138502	0.05981671	0.11701581	0.28387775	0.05154498	0.07458611	0.79360925
172.2	0.08143988	0.05987195	0.11702548	0.28396675	0.05156687	0.07460766	0.7935621
172.3	0.08149467	0.05992726	0.11703511	0.28405561	0.05158872	0.07462917	0.79351503
172.4	0.0815494	0.05998266	0.11704469	0.28414433	0.05161052	0.07465065	0.79346804
172.5	0.08160406	0.06003815	0.11705423	0.28423292	0.05163228	0.07467209	0.79342112
172.6	0.08165865	0.06009375	0.11706373	0.28432137	0.051654	0.0746935	0.79337427
172.7	0.08171317	0.06014946	0.11707318	0.28440968	0.05167567	0.07471488	0.7933275
172.8	0.08176763	0.06020529	0.11708259	0.28449785	0.0516973	0.07473622	0.7932808
172.9	0.08182202	0.06026126	0.11709196	0.28458588	0.05171889	0.07475754	0.79323418
173	0.08187635	0.06031737	0.11710129	0.28467378	0.05174044	0.07477882	0.79318763
173.1	0.08193061	0.06037361	0.11711058	0.28476155	0.05176194	0.07480007	0.79314115
173.2	0.0819848	0.06042999	0.11711983	0.2848492	0.05178341	0.0748213	0.79309475
173.3	0.08203893	0.06048647	0.11712903	0.28493672	0.05180483	0.0748425	0.79304842
173.4	0.082093	0.06054304	0.1171382	0.28502412	0.05182622	0.07486368	0.79300217
173.5	0.082147	0.06059969	0.11714732	0.28511139	0.05184757	0.07488484	0.79295598
173.6	0.08220095	0.06065639	0.1171564	0.28519853	0.05186887	0.07490597	0.79290987
173.7	0.08225483	0.06071315	0.11716544	0.28528554	0.05189014	0.07492709	0.79286384
173.8	0.08230865	0.06076994	0.11717444	0.28537243	0.05191138	0.0749482	0.79281788
173.9	0.08236241	0.06082675	0.11718341	0.28545919	0.05193257	0.07496929	0.79277199
174	0.08241612	0.06088358	0.11719233	0.28554583	0.05195373	0.07499037	0.79272617
174.1	0.08246977	0.06094042	0.11720121	0.28563234	0.05197485	0.07501145	0.79268043
174.2	0.08252336	0.06099726	0.11721005	0.28571873	0.05199594	0.07503253	0.79263475
174.3	0.0825769	0.06105413	0.11721886	0.285805	0.05201699	0.07505361	0.79258915
174.4	0.0826304	0.06111101	0.11722762	0.28589114	0.05203801	0.07507469	0.79254362
174.5	0.08268384	0.0611679	0.11723635	0.28597716	0.052059	0.07509577	0.79249816
174.6	0.08273725	0.06122478	0.11724504	0.28606306	0.05207996	0.07511684	0.79245277
174.7	0.0827906	0.06128163	0.11725368	0.28614883	0.05210089	0.07513791	0.79240746
174.8	0.08284392	0.06133844	0.11726229	0.28623448	0.05212179	0.07515897	0.79236221
174.9	0.08289719	0.06139521	0.11727087	0.28632	0.05214267	0.07518	0.79231703
175	0.08295042	0.06145194	0.1172794	0.2864054	0.05216352	0.075201	0.79227193
175.1	0.08300362	0.06150862	0.1172879	0.28649067	0.05218435	0.07522198	0.79222689
175.2	0.08305678	0.06156527	0.11729636	0.28657582	0.05220516	0.07524292	0.79218192
175.3	0.08310991	0.06162189	0.11730478	0.28666085	0.05222595	0.07526383	0.79213702
175.4	0.08316299	0.06167848	0.11731317	0.28674576	0.05224672	0.0752847	0.7920922
175.5	0.08321604	0.06173505	0.11732152	0.28683054	0.05226748	0.07530554	0.79204744
175.6	0.08326903	0.0617916	0.11732983	0.2869152	0.05228822	0.07532635	0.79200274
175.7	0.08332197	0.06184812	0.11733811	0.28699973	0.05230894	0.07534712	0.79195812
175.8	0.08337484	0.06190463	0.11734635	0.28708415	0.05232966	0.07536785	0.79191357
175.9	0.08342764	0.06196113	0.11735455	0.28716844	0.05235036	0.07538856	0.79186908
176	0.08348036	0.06201761	0.11736272	0.28725262	0.05237107	0.07540923	0.79182467
176.1	0.08353301	0.06207407	0.11737085	0.28733667	0.05239176	0.07542988	0.79178032
176.2	0.08358559	0.06213051	0.11737895	0.2874206	0.05241246	0.0754505	0.79173604
176.3	0.08363808	0.06218693	0.11738702	0.28750441	0.05243314	0.07547109	0.79169183
176.4	0.08369048	0.06224331	0.11739504	0.2875881	0.05245382	0.07549165	0.79164769
176.5	0.0837428	0.06229967	0.11740304	0.28767167	0.05247448	0.07551219	0.79160361
176.6	0.08379503	0.06235601	0.117411	0.28775513	0.05249512	0.07553271	0.7915596
176.7	0.08384717	0.06241233	0.11741893	0.28783847	0.05251573	0.07555321	0.79151566
176.8	0.08389924	0.06246862	0.11742682	0.28792168	0.0525363	0.07557368	0.79147179
176.9	0.08395123	0.0625249	0.11743468	0.28800479	0.05255685	0.07559412	0.79142798
177	0.08400315	0.06258116	0.1174425	0.28808777	0.05257735	0.07561453	0.79138424
177.1	0.084055	0.0626374	0.1174503	0.28817064	0.05259781	0.0756349	0.79134056
177.2	0.08410679	0.06269362	0.11745806	0.2882534	0.05261823	0.07565523	0.79129696
177.3	0.08415852	0.06274983	0.11746579	0.28833603	0.05263859	0.07567553	0.79125342
177.4	0.08421018	0.06280601	0.11747349	0.28841856	0.05265892	0.07569578	0.79120994
177.5	0.08426177	0.06286217	0.11748116	0.28850096	0.0526792	0.07571599	0.79116653
177.6	0.08431329	0.06291832	0.11748879	0.28858325	0.05269943	0.07573616	0.79112319
177.7	0.08436474	0.06297445	0.1174964	0.28866543	0.05271961	0.07575628	0.79107991
177.8	0.08441611	0.06303056	0.11750397	0.28874749	0.05273974	0.07577636	0.7910367
177.9	0.0844674	0.06308665	0.11751152	0.28882944	0.05275983	0.0757964	0.79099356
178	0.08451862	0.06314273	0.11751904	0.28891128	0.05277987	0.0758164	0.79095047
178.1	0.08456976	0.06319878	0.11752652	0.288993	0.05279987	0.07583635	0.79090746
178.2	0.08462083	0.06325482	0.11753398	0.28907461	0.05281982	0.07585626	0.79086451
178.3	0.08467182	0.06331084	0.11754141	0.2891561	0.05283972	0.07587613	0.79082162
178.4	0.08472275	0.06336684	0.11754881	0.28923749	0.05285958	0.07589595	0.7907788
178.5	0.0847736	0.06342282	0.11755619	0.28931877	0.0528794	0.07591573	0.79073604
178.6	0.08482438	0.06347879	0.11756354	0.28939993	0.05289917	0.07593547	0.79069335
178.7	0.08487508	0.06353474	0.11757086	0.28948099	0.05291889	0.07595517	0.79065072
178.8	0.08492572	0.06359067	0.11757815	0.28956194	0.05293857	0.07597483	0.79060816
178.9	0.08497628	0.06364659	0.11758542	0.28964277	0.05295821	0.07599445	0.79056565
179	0.08502677	0.06370249	0.11759266	0.2897235	0.0529778	0.07601403	0.79052322
179.1	0.08507719	0.06375837	0.11759989	0.28980412	0.05299734	0.07603357	0.79048084
179.2	0.08512754	0.06381424	0.11760708	0.28988463	0.05301685	0.07605308	0.79043853
179.3	0.08517782	0.06387009	0.11761426	0.28996504	0.05303631	0.07607254	0.79039629
179.4	0.08522803	0.06392592	0.11762142	0.29004534	0.05305572	0.07609196	0.7903541
179.5	0.08527816	0.06398174	0.11762856	0.29012553	0.05307509	0.07611135	0.79031198
179.6	0.08532823	0.06403755	0.11763568	0.29020561	0.05309442	0.07613069	0.79026993
179.7	0.08537823	0.06409333	0.11764279	0.29028559	0.05311371	0.07615	0.79022793
179.8	0.08542817	0.0641491	0.11764988	0.29036546	0.05313295	0.07616927	0.790186
179.9	0.08547803	0.06420486	0.11765696	0.29044523	0.05315215	0.07618851	0.79014413
225	0.10331812	0.08694391	0.11877299	0.31831445	0.05917974	0.08233728	0.77622298
225.1	0.10335118	0.08698549	0.11877185	0.31836275	0.05918961	0.08234613	0.77620074
225.2	0.10338423	0.08702702	0.11877071	0.318411	0.05919947	0.08235496	0.77617852
225.3	0.10341726	0.08706853	0.11876958	0.3184592	0.05920932	0.08236377	0.77615634
225.4	0.10345028	0.08710999	0.11876843	0.31850735	0.05921917	0.08237255	0.77613418
225.5	0.10348328	0.08715141	0.11876729	0.31855545	0.059229	0.08238132	0.77611205
225.6	0.10351626	0.08719279	0.11876614	0.3186035	0.05923882	0.08239006	0.77608995
225.7	0.10354923	0.08723414	0.11876498	0.31865151	0.05924864	0.08239878	0.77606788
225.8	0.10358219	0.08727545	0.11876382	0.31869946	0.05925845	0.08240748	0.77604583
225.9	0.10361513	0.08731671	0.11876265	0.31874737	0.05926825	0.08241616	0.77602382
226	0.10364805	0.08735794	0.11876147	0.31879523	0.05927804	0.08242481	0.77600182
226.1	0.10368096	0.08739914	0.11876029	0.31884305	0.05928782	0.08243345	0.77597986
226.2	0.10371386	0.08744029	0.1187591	0.31889081	0.0592976	0.08244206	0.77595792
226.3	0.10374674	0.08748141	0.11875791	0.31893853	0.05930736	0.08245066	0.77593601
226.4	0.10377961	0.08752248	0.11875671	0.3189862	0.05931712	0.08245923	0.77591413
226.5	0.10381247	0.08756352	0.1187555	0.31903383	0.05932687	0.08246778	0.77589227
226.6	0.10384531	0.08760453	0.11875429	0.3190814	0.05933661	0.08247632	0.77587044
226.7	0.10387814	0.08764549	0.11875308	0.31912893	0.05934634	0.08248483	0.77584864
226.8	0.10391096	0.08768642	0.11875186	0.31917642	0.05935607	0.08249332	0.77582686
226.9	0.10394376	0.08772731	0.11875064	0.31922385	0.05936578	0.08250179	0.77580511
227	0.10397656	0.08776817	0.11874942	0.31927124	0.05937549	0.08251024	0.77578338
227.1	0.10400934	0.08780898	0.11874819	0.31931859	0.05938519	0.08251867	0.77576168
227.2	0.10404211	0.08784976	0.11874697	0.31936588	0.05939489	0.08252707	0.77574001
227.3	0.10407488	0.08789051	0.11874575	0.31941314	0.05940457	0.08253546	0.77571837
227.4	0.10410763	0.08793121	0.11874453	0.31946034	0.05941425	0.08254383	0.77569675
227.5	0.10414038	0.08797188	0.11874331	0.3195075	0.05942392	0.08255218	0.77567515
227.6	0.10417312	0.08801252	0.1187421	0.31955462	0.05943358	0.08256051	0.77565358
227.7	0.10420586	0.08805312	0.11874088	0.31960169	0.05944323	0.08256882	0.77563204
227.8	0.10423859	0.08809368	0.11873966	0.31964872	0.05945288	0.0825771	0.77561053
227.9	0.10427131	0.0881342	0.11873844	0.3196957	0.05946252	0.08258537	0.77558904
228	0.10430404	0.0881747	0.11873722	0.31974263	0.05947215	0.08259362	0.77556758
228.1	0.10433676	0.08821515	0.11873599	0.31978952	0.05948177	0.08260185	0.77554614

183.2	0.08708606	0.06603721	0.11787934	0.29302347	0.05376275	0.076806	0.78879736
183.3	0.0871337	0.06609255	0.11788555	0.29310016	0.05378058	0.07682431	0.78875758
183.4	0.08718128	0.06614788	0.11789173	0.29317677	0.05379838	0.0768426	0.78871786
183.5	0.08722879	0.06620321	0.11789788	0.29325331	0.05381614	0.07686086	0.7886782
183.6	0.08727624	0.06625854	0.11790399	0.29332978	0.05383386	0.0768791	0.78863858
183.7	0.08732364	0.06631385	0.11791008	0.29340617	0.05385154	0.07689731	0.78859903
183.8	0.08737097	0.06636917	0.11791613	0.29348249	0.05386919	0.0769155	0.78855952
183.9	0.08741824	0.06642447	0.11792215	0.29355873	0.0538868	0.07693368	0.78852007
184	0.08746544	0.06647978	0.11792813	0.29363488	0.05390437	0.07695184	0.78848068
184.1	0.08751259	0.06653508	0.11793409	0.29371095	0.0539219	0.07696998	0.78844134
184.2	0.08755968	0.06659038	0.11794002	0.29378692	0.0539394	0.07698811	0.78840205
184.3	0.08760671	0.06664568	0.11794591	0.29386279	0.05395686	0.07700624	0.78836281
184.4	0.08765368	0.06670097	0.11795178	0.29393856	0.05397429	0.07702435	0.78832362
184.5	0.08770059	0.06675627	0.11795761	0.29401424	0.05399168	0.07704247	0.78828449
184.6	0.08774744	0.06681156	0.11796342	0.29408981	0.05400903	0.07706058	0.78824541
184.7	0.08779423	0.06686686	0.11796919	0.29416527	0.05402635	0.07707869	0.78820638
184.8	0.08784096	0.06692215	0.11797494	0.29424063	0.05404363	0.0770968	0.7881674
184.9	0.08788764	0.06697746	0.11798066	0.29431588	0.05406088	0.0771149	0.78812847
185	0.08793425	0.06703277	0.11798634	0.29439102	0.05407809	0.07713301	0.78808959
185.1	0.08798081	0.06708808	0.11799201	0.29446606	0.05409526	0.07715109	0.78805075
185.2	0.08802731	0.06714341	0.11799764	0.29454099	0.0541124	0.07716916	0.78801197
185.3	0.08807375	0.06719875	0.11800325	0.29461581	0.0541295	0.0771872	0.78797323
185.4	0.08812013	0.0672541	0.11800883	0.29469053	0.05414657	0.07720522	0.78793453
185.5	0.08816646	0.06730948	0.11801438	0.29476514	0.0541636	0.0772232	0.78789588
185.6	0.08821272	0.06736487	0.11801991	0.29483964	0.0541806	0.07724116	0.78785728
185.7	0.08825893	0.06742029	0.11802542	0.29491405	0.05419757	0.07725909	0.78781871
185.8	0.08830509	0.06747574	0.1180309	0.29498834	0.0542145	0.077277	0.78778019
185.9	0.08835118	0.06753122	0.11803636	0.29506253	0.05423139	0.07729487	0.78774171
186	0.08839722	0.06758673	0.1180418	0.29513662	0.05424825	0.07731271	0.78770327
186.1	0.08844321	0.06764229	0.11804721	0.29521061	0.05426507	0.07733052	0.78766487
186.2	0.08848913	0.06769788	0.1180526	0.29528449	0.05428187	0.0773483	0.78762651
186.3	0.088535	0.06775352	0.11805797	0.29535827	0.05429862	0.07736605	0.7875882
186.4	0.08858082	0.06780922	0.11806332	0.29543195	0.05431535	0.07738377	0.78754993
186.5	0.08862657	0.06786496	0.11806865	0.29550553	0.05433204	0.07740146	0.78751172
186.6	0.08867228	0.06792076	0.11807396	0.295579	0.05434869	0.07741912	0.78747356
186.7	0.08871792	0.0679766	0.11807925	0.29565238	0.05436531	0.07743676	0.78743545
186.8	0.08876351	0.06803247	0.11808452	0.29572565	0.0543819	0.07745435	0.78739741
186.9	0.08880905	0.06808836	0.11808976	0.29579882	0.05439846	0.07747191	0.78735942
187	0.08885453	0.06814425	0.11809498	0.29587189	0.05441498	0.07748944	0.78732148
187.1	0.08889995	0.06820014	0.11810017	0.29594486	0.05443147	0.07750692	0.78728361
187.2	0.08894533	0.068256	0.11810533	0.29601772	0.05444793	0.07752437	0.78724579
187.3	0.08899064	0.06831184	0.11811047	0.29609049	0.05446435	0.07754178	0.78720804
187.4	0.0890359	0.06836765	0.11811558	0.29616316	0.05448074	0.07755915	0.78717034
187.5	0.08908111	0.06842343	0.11812067	0.29623573	0.0544971	0.07757649	0.7871327
187.6	0.08912626	0.06847918	0.11812573	0.29630821	0.05451342	0.07759379	0.78709512
187.7	0.08917136	0.06853488	0.11813076	0.29638058	0.05452972	0.07761105	0.7870576
187.8	0.08921641	0.06859056	0.11813577	0.29645285	0.05454598	0.07762828	0.78702014
187.9	0.0892614	0.06864619	0.11814074	0.29652503	0.05456221	0.07764547	0.78698274
188	0.08930634	0.06870181	0.1181457	0.29659711	0.0545784	0.07766262	0.78694539
188.1	0.08935123	0.06875739	0.11815063	0.29666909	0.05459457	0.07767974	0.78690811
188.2	0.08939606	0.06881294	0.11815553	0.29674098	0.0546107	0.07769682	0.78687088
188.3	0.08944084	0.06886847	0.1181604	0.29681276	0.0546268	0.07771387	0.7868337
188.4	0.08948557	0.06892396	0.11816525	0.29688446	0.05464287	0.07773088	0.78679659
188.5	0.08953024	0.06897943	0.11817008	0.29695605	0.05465891	0.07774785	0.78675953
188.6	0.08957486	0.06903487	0.11817488	0.29702755	0.05467492	0.07776479	0.78672253
188.7	0.08961943	0.06909029	0.11817966	0.29709896	0.05469089	0.07778169	0.78668559
188.8	0.08966394	0.06914568	0.11818441	0.29717027	0.05470684	0.07779856	0.7866487
188.9	0.08970841	0.06920105	0.11818914	0.29724148	0.05472275	0.07781539	0.78661187
189	0.08975282	0.06925639	0.11819385	0.2973126	0.05473863	0.07783218	0.7865751
189.1	0.08979717	0.0693117	0.11819853	0.29738363	0.05475449	0.07784894	0.78653838
189.2	0.08984148	0.06936699	0.11820319	0.29745456	0.05477031	0.07786567	0.78650173
189.3	0.08988573	0.06942225	0.11820783	0.29752539	0.0547861	0.07788236	0.78646512
189.4	0.08992994	0.06947749	0.11821244	0.29759614	0.05480186	0.07789902	0.78642857
189.5	0.08997409	0.0695327	0.11821703	0.29766679	0.05481759	0.07791564	0.78639208
189.6	0.09001818	0.06958789	0.1182216	0.29773734	0.05483329	0.07793223	0.78635565
189.7	0.09006223	0.06964304	0.11822615	0.29780781	0.05484896	0.07794879	0.78631927
189.8	0.09010623	0.06969818	0.11823068	0.29787818	0.0548646	0.07796531	0.78628294
189.9	0.09015017	0.06975329	0.11823519	0.29794846	0.05488021	0.0779818	0.78624668
190	0.09019407	0.06980838	0.11823968	0.29801865	0.05489579	0.07799825	0.78621046
190.1	0.09023791	0.06986344	0.11824414	0.29808875	0.05491134	0.07801467	0.7861743
190.2	0.0902817	0.06991849	0.11824859	0.29815875	0.05492686	0.07803106	0.7861382
190.3	0.09032545	0.06997351	0.11825302	0.29822867	0.05494235	0.07804741	0.78610215
190.4	0.09036914	0.07002852	0.11825743	0.2982985	0.05495782	0.07806373	0.78606616
190.5	0.09041278	0.07008351	0.11826181	0.29836823	0.05497325	0.07808002	0.78603022
190.6	0.09045637	0.0701385	0.11826618	0.29843788	0.05498865	0.07809628	0.78599434
190.7	0.09049992	0.07019347	0.11827052	0.29850744	0.05500403	0.0781125	0.78595851
190.8	0.09054341	0.07024843	0.11827484	0.29857691	0.05501938	0.0781287	0.78592273
190.9	0.09058685	0.07030339	0.11827913	0.2986463	0.05503469	0.07814486	0.78588701
191	0.09063025	0.07035835	0.11828341	0.29871559	0.05504998	0.07816099	0.78585134
191.1	0.09067359	0.0704133	0.11828766	0.2987848	0.05506524	0.0781771	0.78581573
191.2	0.09071689	0.07046826	0.11829189	0.29885393	0.05508048	0.07819317	0.78578017
191.3	0.09076013	0.07052321	0.1182961	0.29892296	0.05509568	0.07820922	0.78574467
191.4	0.09080333	0.07057818	0.11830028	0.29899191	0.05511086	0.07822524	0.78570921
191.5	0.09084648	0.07063315	0.11830445	0.29906078	0.05512601	0.07824123	0.78567381
191.6	0.09088958	0.07068813	0.1183086	0.29912956	0.05514113	0.0782572	0.78563847
191.7	0.09093264	0.07074313	0.11831272	0.29919825	0.05515622	0.07827315	0.78560318
191.8	0.09097564	0.07079814	0.11831683	0.29926686	0.05517128	0.07828907	0.78556794
191.9	0.0910186	0.07085318	0.11832092	0.29933538	0.05518632	0.07830497	0.78553275
192	0.0910615	0.07090824	0.11832499	0.29940381	0.05520133	0.07832085	0.78549761
192.1	0.09110436	0.07096333	0.11832904	0.29947215	0.05521632	0.0783367	0.78546253
192.2	0.09114718	0.07101847	0.11833308	0.29954041	0.05523127	0.07835254	0.7854275
192.3	0.09118994	0.07107364	0.1183371	0.29960858	0.0552462	0.07836836	0.78539253
192.4	0.09123266	0.07112885	0.11834111	0.29967667	0.0552611	0.07838417	0.7853576
192.5	0.09127533	0.0711841	0.11834509	0.29974466	0.05527598	0.07839995	0.78532273
192.6	0.09131796	0.07123938	0.11834907	0.29981257	0.05529082	0.07841573	0.78528791
192.7	0.09136053	0.07129468	0.11835302	0.2998804	0.05530564	0.07843149	0.78525314
192.8	0.09140306	0.07134998	0.11835696	0.29994814	0.05532044	0.07844724	0.78521843
192.9	0.09144555	0.07140528	0.11836087	0.30001579	0.05533521	0.07846298	0.78518376
193	0.09148798	0.07146057	0.11836477	0.30008336	0.05534995	0.07847871	0.78514915
193.1	0.09153037	0.07151585	0.11836865	0.30015084	0.05536467	0.07849444	0.78511459
193.2	0.09157272	0.07157112	0.11837251	0.30021823	0.05537935	0.07851016	0.78508008
193.3	0.09161502	0.07162637	0.11837634	0.30028555	0.05539402	0.07852586	0.78504562
193.4	0.09165727	0.07168159	0.11838016	0.30035277	0.05540866	0.07854156	0.78501121
193.5	0.09169948	0.07173678	0.11838395	0.30041991	0.05542327	0.07855724	0.78497685
193.6	0.09174164	0.07179192	0.11838773	0.30048697	0.05543786	0.0785729	0.78494255
193.7	0.09178375	0.07184703	0.11839148	0.30055395	0.05545242	0.07858854	0.78490829
193.8	0.09182582	0.07190212	0.11839522	0.30062084	0.05546695	0.07860416	0.78487409
193.9	0.09186785	0.07195717	0.11839893	0.30068764	0.05548146	0.07861976	0.78483993
194	0.09190983	0.0720122	0.11840262	0.30075437	0.05549595	0.07863533	0.78480583
194.1	0.09195177	0.0720672	0.1184063	0.30082101	0.05551041	0.07865087	0.78477178
194.2	0.09199366	0.07212216	0.11840995	0.30088756	0.05552484	0.07866639	0.78473777
194.3	0.0920355	0.0721771	0.11841359	0.30095404	0.05553925	0.07868188	0.78470382
194.4	0.09207731	0.07223202	0.11841721	0.30102043	0.05555364	0.07869733	0.78466991
194.5	0.09211906	0.07228693	0.11842081	0.30108674	0.055568	0.07871276	0.78463606
194.6	0.09216078	0.07234183	0.1184244	0.30115297	0.05558234	0.07872816	0.78460226
194.7	0.09220245	0.07239672	0.11842796	0.30121912	0.05559665	0.07874353	0.7845685
194.8	0.09224407	0.07245161	0.11843151	0.30128518	0.05561094	0.07875887	0.7845348
194.9	0.09228565	0.07250649	0.11843505	0.30135117	0.0556252	0.07877417	0.78450114
198.2	0.09363409	0.07429153	0.11854547	0.30348459	0.0560839	0.07926096	0.78341752
198.3	0.09367425	0.0743512	0.1185487	0.30354795	0.05609748	0.07927519	0.78338548
198.4	0.09371438	0.07440484	0.11855192	0.30361123	0.05611105	0.07928939	0.78335349
198.5	0.09375447	0.07445846	0.11855512	0.30367445	0.0561246	0.07930356	0.78332155
198.6	0.09379452	0.07451205	0.11855831	0.30373759	0.05613813	0.0793177	0.78328965
198.7	0.09383453	0.07456563	0.11856148	0.30380066	0.05615164	0.07933181	0.78325779
198.8	0.0938745	0.07461919	0.11856463	0.30386366	0.05616513	0.0793459	0.78322599
198.9	0.09391443	0.07467274	0.11856775	0.30392659	0.0561786	0.07935996	0.78319422
199	0.09395432	0.07472626	0.11857085	0.30398945	0.05619205	0.07937399	0.78316251
199.1	0.09399418	0.07477976	0.11857393	0.30405224	0.05620547	0.07938799	0.78313083
199.2	0.09403399	0.07483323	0.11857699	0.30411496	0.05621888	0.07940197	0.78309921
199.3	0.09407377	0.07488667	0.11858001	0.3041776	0.05623226	0.07941592	0.78306763
199.4	0.09411351	0.07494006	0.11858302	0.30424018	0.05624563	0.07942984	0.78303609
199.5	0.09415321	0.07499341	0.118586	0.30430269	0.05625897	0.07944374	0.7830046
199.6	0.09419287	0.07504672	0.11858896	0.30436513	0.0562723	0.07945761	0.78297315
199.7	0.0942325	0.07509997	0.11859189	0.3044275	0.0562856	0.07947146	0.78294175
199.8	0.09427209	0.07515316	0.1185948	0.3044898	0.05629888	0.07948528	0.7829104
199.9	0.09431164	0.0752063	0.11859768	0.30455203	0.05631215	0.07949907	0.78287909
200	0.09435115	0.07525936	0.11860055	0.30461419	0.05632539	0.07951284	0.78284782
200.1	0.09439062	0.07531236	0.11860339	0.30467628	0.05633861	0.07952659	0.7828166
200.2	0.09443006	0.0753653	0.11860621	0.3047383	0.05635182	0.07954031	0.78278543
200.3	0.09446946	0.07541817	0.11860901	0.30480025	0.05636501	0.07955401	0.7827543
200.4	0.09450882	0.07547099	0.11861179	0.30486214	0.05637818	0.07956768	0.78272321
200.5	0.09454815	0.07552373	0.11861455	0.30492395	0.05639133	0.07958133	0.78269217
200.6	0.09458744	0.07557641	0.11861729	0.30498569	0.05640446	0.07959496	0.78266118
200.7	0.09462669	0.07562902	0.11862	0.30504737	0.05641757	0.07960857	0.78263023
200.8	0.0946659	0.07568157	0.1186227	0.30510898	0.05643067	0.07962215	0.78259932
200.9	0.09470508	0.07573405	0.11862538	0.30517052	0.05644375	0.07963572	0.78256846
201	0.09474422	0.07578647	0.11862804	0.30523199	0.05645681	0.07964926	0.78253764
201.1	0.09478333	0.07583882	0.11863069	0.30529339	0.05646985	0.07966278	0.78250687
201.2	0.0948224	0.07589111	0.11863331	0.30535473	0.05648288	0.07967629	0.78247615
201.3	0.09486143	0.07594334	0.11863592	0.305416	0.05649589	0.07968978	0.78244547
201.4	0.09490043	0.0759955	0.11863851	0.3054772	0.05650889	0.07970325	0.78241483
201.5	0.09493939	0.0760476	0.11864108	0.30553834	0.05652187	0.07971671	0.78238424
201.6	0.09497831	0.07609964	0.11864364	0.30559941	0.05653484	0.07973015	0.7823537
201.7	0.0950172	0.07615162	0.11864619	0.30566042	0.05654779	0.07974357	0.7823232
201.8	0.09505605	0.07620354	0.11864872	0.30572136	0.05656073	0.07975699	0.78229275
201.9	0.09509487	0.07625539	0.11865125	0.30578223	0.05657365	0.0797704	0.78226235
202	0.09513365	0.07630719	0.11865376	0.30584304	0.05658656	0.0797838	0.78223199
202.1	0.0951724	0.07635892	0.11865626	0.30590379	0.05659946	0.0797972	0.78220167
202.2	0.09521111	0.07641059	0.11865875	0.30596447	0.05661235	0.0798106	0.78217141
202.3	0.09524979	0.07646221	0.11866124	0.30602509	0.05662522	0.07982399	0.78214119
202.4	0.09528843	0.07651376	0.11866372	0.30608565	0.05663809	0.07983739	0.78211102
202.5	0.09532704	0.07656525	0.11866619	0.30614614	0.05665094	0.0798508	0.7820809
202.6	0.09536561	0.07661669	0.11866866	0.30620657	0.05666379	0.0798642	0.78205082
202.7	0.09540415	0.07666806	0.11867112	0.30626694	0.05667663	0.0798776	0.78202079
202.8	0.09544265	0.07671938	0.11867358	0.30632725	0.05668946	0.079891	0.7819908
202.9	0.09548112	0.07677063	0.11867601	0.3063875	0.05670228	0.07990439	0.78196087
203	0.09551955	0.07682183	0.11867844	0.30644769	0.0567151	0.07991777	0.78193097
203.1	0.09555795	0.07687296	0.11868084	0.30650782	0.05672792	0.07993114	0.78190112
203.2	0.09559632	0.07692404	0.11868322	0.3065679	0.05674073	0.07994449	0.78187132
203.3	0.09563465	0.07697506	0.11868557	0.30662792	0.05675354	0.07995781	0.78184155
203.4	0.09567295	0.07702602	0.11868791	0.30668788	0.05676634	0.07997112	0.78181183
203.5	0.09571121	0.07707693	0.11869022	0.30674779	0.05677914	0.0799844	0.78178215
203.6	0.09574944	0.07712777	0.1186925	0.30680765	0.05679193	0.07999765	0.78175251
203.7	0.09578764	0.07717856	0.11869476	0.30686745	0.05680472	0.08001088	0.7817229
203.8	0.0958258	0.07722929	0.118697	0.30692721	0.05681749	0.08002407	0.78169334
203.9	0.09586393	0.07727996	0.11869921	0.30698691	0.05683026	0.08003724	0.78166382
204	0.09590202	0.07733057	0.1187014	0.30704657	0.05684301	0.08005038	0.78163433
204.1	0.09594009	0.07738113	0.11870357	0.30710618	0.05685576	0.08006349	0.78160488
204.2	0.09597811	0.07743163	0.11870571	0.30716575	0.05686848	0.08007658	0.78157547
204.3	0.09601611	0.07748208	0.11870784	0.30722527	0.05688119	0.08008963	0.7815461
204.4	0.09605407	0.07753246	0.11870994	0.30728474	0.05689388	0.08010266	0.78151677
204.5	0.096092	0.07758279	0.11871203	0.30734418	0.05690656	0.08011566	0.78148748
204.6	0.0961299	0.07763307	0.11871409	0.30740356	0.05691921	0.08012864	0.78145822
204.7	0.09616777	0.07768328	0.11871613	0.3074629	0.05693185	0.08014159	0.78142901
204.8	0.0962056	0.07773345	0.11871816	0.30752219	0.05694446	0.08015451	0.78139983
204.9	0.0962434	0.07778355	0.11872016	0.30758142	0.05695706	0.08016741	0.78137069
205	0.09628117	0.0778336	0.11872215	0.30764059	0.05696964	0.08018028	0.78134159
205.1	0.0963189	0.07788359	0.11872411	0.3076997	0.0569822	0.08019313	0.78131253
205.2	0.0963566	0.07793353	0.11872604	0.30775874	0.05699474	0.08020596	0.7812835
205.3	0.09639428	0.07798342	0.11872796	0.30781772	0.05700726	0.08021877	0.78125451
205.4	0.09643192	0.07803325	0.11872985	0.30787662	0.05701976	0.08023155	0.78122556
205.5	0.09646952	0.07808302	0.11873173	0.30793546	0.05703225	0.08024432	0.78119664
205.6	0.0965071	0.07813274	0.11873358	0.30799423	0.05704471	0.08025706	0.78116777
205.7	0.09654464	0.0781824	0.11873541	0.30805292	0.05705716	0.08026979	0.78113893
205.8	0.09658215	0.07823201	0.11873722	0.30811155	0.05706958	0.08028249	0.78111012
205.9	0.09661964	0.07828157	0.118739	0.30817011	0.05708199	0.08029518	0.78108135
206	0.09665709	0.07833107	0.11874077	0.30822861	0.05709438	0.08030785	0.78105262
206.1	0.0966945	0.07838052	0.11874251	0.30828704	0.05710675	0.08032051	0.78102393
206.2	0.09673189	0.07842991	0.11874423	0.30834539	0.05711911	0.08033315	0.78099526
206.3	0.09676925	0.07847925	0.11874593	0.30840369	0.05713144	0.08034577	0.78096664
206.4	0.09680657	0.07852854	0.11874761	0.30846191	0.05714376	0.08035838	0.78093804
206.5	0.09684387	0.07857778	0.11874927	0.30852007	0.05715606	0.08037098	0.78090948
206.6	0.09688113	0.07862696	0.11875091	0.30857817	0.05716834	0.08038357	0.78088095
206.7	0.09691836	0.07867608	0.11875252	0.30863619	0.0571806	0.08039614	0.78085246
206.8	0.09695556	0.07872516	0.11875411	0.30869416	0.05719284	0.08040869	0.780824
206.9	0.09699274	0.07877418	0.11875569	0.30875205	0.05720507	0.08042123	0.78079556
207	0.09702988	0.07882315	0.11875724	0.30880988	0.05721728	0.08043376	0.78076717
207.1	0.09706699	0.07887207	0.11875877	0.30886764	0.05722947	0.08044626	0.7807388
207.2	0.09710407	0.07892094	0.11876028	0.30892534	0.05724164	0.08045874	0.78071046
207.3	0.09714112	0.07896975	0.11876177	0.30898298	0.0572538	0.08047121	0.78068215
207.4	0.09717814	0.07901851	0.11876324	0.30904055	0.05726593	0.08048366	0.78065387
207.5	0.09721513	0.07906722	0.11876469	0.30909805	0.05727806	0.08049609	0.78062563
207.6	0.09725209	0.07911588	0.11876612	0.30915549	0.05729016	0.0805085	0.78059741
207.7	0.09728902	0.07916449	0.11876753	0.30921286	0.05730224	0.08052089	0.78056922
207.8	0.09732593	0.07921305	0.11876892	0.30927017	0.05731431	0.08053326	0.78054106
207.9	0.0973628	0.07926155	0.11877029	0.30932742	0.05732636	0.08054562	0.78051292
208	0.09739964	0.07931001	0.11877164	0.3093846	0.0573384	0.08055795	0.78048481
208.1	0.09743645	0.07935841	0.11877297	0.30944172	0.05735041	0.08057026	0.78045673
208.2	0.09747324	0.07940676	0.11877429	0.30949877	0.05736241	0.08058256	0.78042868
208.3	0.09750999	0.07945506	0.11877558	0.30955577	0.0573744	0.08059483	0.78040065
208.4	0.09754672	0.07950332	0.11877685	0.3096127	0.05738636	0.08060708	0.78037266
208.5	0.09758342	0.07955152	0.11877811	0.30966956	0.05739831	0.0806193	0.78034469
208.6	0.09762009	0.07959967	0.11877934	0.30972637	0.05741024	0.08063151	0.78031676
208.7	0.09765673	0.07964777	0.11878056	0.30978311	0.05742216	0.08064368	0.78028887
208.8	0.09769334	0.07969583	0.11878175	0.30983979	0.05743405	0.08065583	0.78026101
208.9	0.09772992	0.07974383	0.11878293	0.30989641	0.05744594	0.08066796	0.78023319
209	0.09776647	0.07979179	0.11878409	0.30995297	0.0574578	0.08068006	0.7802054
209.1	0.097803	0.07983969	0.11878524	0.31000947	0.05746965	0.08069212	0.78017766
209.2	0.0978395	0.07988755	0.11878636	0.3100659	0.05748148	0.08070417	0.78014996
209.3	0.09787597	0.07993535	0.11878746	0.31012228	0.0574933	0.08071618	0.78012229
209.4	0.09791241	0.07998311	0.11878855	0.31017859	0.0575051	0.08072816	0.78009467
209.5	0.09794882	0.08003082	0.11878962	0.31023485	0.05751688	0.08074012	0.78006708
209.6	0.09798521	0.08007848	0.11879067	0.31029104	0.05752865	0.08075205	0.78003954
209.7	0.09802157	0.0801261	0.1187917	0.31034718	0.0575404	0.08076395	0.78001203
209.8	0.0980579	0.08017366	0.11879271	0.31040326	0.05755213	0.08077583	0.77998457
209.9	0.0980942	0.08022118	0.1187937	0.31045928	0.05756385	0.08078767	0.77995714
213.2	0.09927761	0.08176333	0.1188177	0.31227637	0.05794192	0.08116405	0.77907332
213.3	0.09931305	0.08180931	0.11881822	0.31233046	0.05795312	0.08117504	0.77904717
213.4	0.09934848	0.08185525	0.11881874	0.3123845	0.05796431	0.081186	0.77902106
213.5	0.09938388	0.08190115	0.11881925	0.31243849	0.05797549	0.08119694	0.77899498
213.6	0.09941926	0.081947	0.11881976	0.31249242	0.05798665	0.08120786	0.77896894
213.7	0.09945462	0.08199282	0.11882027	0.31254629	0.0579978	0.08121875	0.77894293
213.8	0.09948996	0.08203859	0.11882078	0.31260011	0.05800894	0.08122962	0.77891696
213.9	0.09952527	0.08208432	0.11882129	0.31265388	0.05802006	0.08124047	0.77889103
214	0.09956057	0.08213001	0.11882179	0.31270759	0.05803117	0.0812513	0.77886514
214.1	0.09959585	0.08217566	0.11882229	0.31276125	0.05804226	0.08126211	0.77883927
214.2	0.09963111	0.08222127	0.11882278	0.31281485	0.05805334	0.0812729	0.77881345
214.3	0.09966635	0.08226684	0.11882326	0.3128684	0.05806441	0.08128366	0.77878766
214.4	0.09970158	0.08231237	0.11882373	0.31292189	0.05807547	0.0812944	0.77876191
214.5	0.09973678	0.08235786	0.11882418	0.31297533	0.0580865	0.08130512	0.77873619
214.6	0.09977197	0.08240331	0.11882462	0.31302872	0.05809753	0.08131582	0.77871051
214.7	0.09980715	0.08244872	0.11882504	0.31308205	0.05810854	0.0813265	0.77868486
214.8	0.09984231	0.08249409	0.11882545	0.31313533	0.05811954	0.08133716	0.77865925
214.9	0.09987745	0.08253943	0.11882583	0.31318856	0.05813053	0.0813478	0.77863367
215	0.09991258	0.08258472	0.1188262	0.31324173	0.0581415	0.08135841	0.77860813
215.1	0.09994769	0.08262998	0.11882655	0.31329485	0.05815246	0.08136901	0.77858262
215.2	0.09998279	0.0826752	0.11882687	0.31334792	0.05816341	0.08137958	0.77855715
215.3	0.10001787	0.08272039	0.11882718	0.31340094	0.05817434	0.08139014	0.77853171
215.4	0.10005293	0.08276553	0.11882748	0.3134539	0.05818526	0.08140068	0.77850631
215.5	0.10008798	0.08281064	0.11882775	0.31350681	0.05819617	0.08141119	0.77848095
215.6	0.100123	0.08285572	0.118828	0.31355967	0.05820706	0.08142169	0.77845561
215.7	0.10015801	0.08290076	0.11882823	0.31361248	0.05821794	0.08143216	0.77843032
215.8	0.10019299	0.08294576	0.11882845	0.31366524	0.05822881	0.08144262	0.77840506
215.9	0.10022795	0.08299074	0.11882865	0.31371794	0.05823967	0.08145306	0.77837983
216	0.1002629	0.08303567	0.11882883	0.3137706	0.05825051	0.08146347	0.77835463
216.1	0.10029781	0.08308058	0.118829	0.3138232	0.05826134	0.08147387	0.77832947
216.2	0.10033271	0.08312545	0.11882914	0.31387575	0.05827216	0.08148425	0.77830435
216.3	0.10036758	0.0831703	0.11882928	0.31392825	0.05828296	0.08149461	0.77827926
216.4	0.10040243	0.08321511	0.11882939	0.31398071	0.05829375	0.08150495	0.7782542
216.5	0.10043726	0.0832599	0.11882949	0.31403311	0.05830453	0.08151528	0.77822918
216.6	0.10047206	0.08330466	0.11882956	0.31408546	0.0583153	0.08152558	0.77820419
216.7	0.10050684	0.08334939	0.11882962	0.31413776	0.05832605	0.08153587	0.77817924
216.8	0.10054159	0.08339409	0.11882967	0.31419001	0.0583368	0.08154614	0.77815432
216.9	0.10057632	0.08343878	0.11882969	0.31424221	0.05834752	0.08155639	0.77812943
217	0.10061102	0.08348344	0.1188297	0.31429436	0.05835824	0.08156662	0.77810458
217.1	0.1006457	0.08352808	0.11882968	0.31434646	0.05836895	0.08157684	0.77807976
217.2	0.10068036	0.08357271	0.11882965	0.31439852	0.05837964	0.08158704	0.77805498
217.3	0.100715	0.08361732	0.1188296	0.31445052	0.05839032	0.08159722	0.77803023
217.4	0.10074961	0.08366192	0.11882953	0.31450248	0.05840099	0.08160738	0.77800551
217.5	0.1007842	0.08370651	0.11882944	0.31455438	0.05841165	0.08161753	0.77798083
217.6	0.10081876	0.08375109	0.11882933	0.31460624	0.05842229	0.08162766	0.77795618
217.7	0.10085331	0.08379567	0.1188292	0.31465805	0.05843292	0.08163777	0.77793156
217.8	0.10088783	0.08384023	0.11882906	0.31470981	0.05844354	0.08164787	0.77790697
217.9	0.10092232	0.08388479	0.11882889	0.31476152	0.05845415	0.08165795	0.77788242
218	0.1009568	0.08392933	0.11882871	0.31481319	0.05846475	0.08166801	0.77785791
218.1	0.10099125	0.08397385	0.1188285	0.31486481	0.05847533	0.08167806	0.77783342
218.2	0.10102568	0.08401835	0.11882828	0.31491638	0.05848591	0.08168809	0.77780897
218.3	0.10106009	0.08406283	0.11882804	0.3149679	0.05849647	0.0816981	0.77778455
218.4	0.10109448	0.08410727	0.11882777	0.31501937	0.05850702	0.0817081	0.77776017
218.5	0.10112884	0.08415167	0.11882749	0.3150708	0.05851756	0.08171809	0.77773582
218.6	0.10116318	0.08419605	0.11882719	0.31512218	0.05852808	0.08172805	0.7777115
218.7	0.1011975	0.08424038	0.11882687	0.31517352	0.0585386	0.08173801	0.77768721
218.8	0.10123179	0.08428467	0.11882653	0.3152248	0.0585491	0.08174795	0.77766296
218.9	0.10126607	0.08432892	0.11882618	0.31527604	0.05855959	0.08175787	0.77763874
219	0.10130032	0.08437312	0.1188258	0.31532724	0.05857007	0.08176778	0.77761455
219.1	0.10133455	0.08441727	0.1188254	0.31537839	0.05858054	0.08177767	0.7775904
219.2	0.10136876	0.08446137	0.11882498	0.31542949	0.058591	0.08178755	0.77756627
219.3	0.10140294	0.08450543	0.11882454	0.31548055	0.05860145	0.08179742	0.77754218
219.4	0.10143711	0.08454944	0.11882408	0.31553156	0.05861189	0.08180727	0.77751813
219.5	0.10147125	0.08459341	0.1188236	0.31558253	0.05862231	0.08181711	0.7774941
219.6	0.10150537	0.08463733	0.1188231	0.31563345	0.05863272	0.08182693	0.77747011
219.7	0.10153947	0.08468119	0.11882258	0.31568432	0.05864313	0.08183675	0.77744615
219.8	0.10157355	0.08472502	0.11882204	0.31573515	0.05865352	0.08184656	0.77742222
219.9	0.1016076	0.08476879	0.11882147	0.31578594	0.0586639	0.08185637	0.77739833
220	0.10164164	0.08481252	0.11882088	0.31583668	0.05867427	0.08186618	0.77737447
220.1	0.10167565	0.0848562	0.11882027	0.31588737	0.05868463	0.08187599	0.77735064
220.2	0.10170964	0.08489984	0.11881964	0.31593803	0.05869497	0.08188581	0.77732684
220.3	0.10174362	0.08494343	0.11881898	0.31598863	0.05870531	0.08189564	0.77730308
220.4	0.10177757	0.08498698	0.1188183	0.3160392	0.05871564	0.08190547	0.77727934
220.5	0.1018115	0.08503048	0.1188176	0.31608972	0.05872595	0.08191532	0.77725564
220.6	0.10184541	0.08507394	0.11881687	0.31614019	0.05873626	0.08192516	0.77723197
220.7	0.10187929	0.08511735	0.11881612	0.31619063	0.05874655	0.081935	0.77720834
220.8	0.10191316	0.08516072	0.11881535	0.31624102	0.05875683	0.08194483	0.77718473
220.9	0.10194701	0.08520404	0.11881456	0.31629136	0.05876711	0.08195465	0.77716116
221	0.10198084	0.08524732	0.11881376	0.31634166	0.05877737	0.08196445	0.77713763
221.1	0.10201464	0.08529056	0.11881293	0.31639192	0.05878762	0.08197424	0.77711412
221.2	0.10204843	0.08533375	0.11881209	0.31644214	0.05879786	0.08198402	0.77709065
221.3	0.10208219	0.0853769	0.11881124	0.31649232	0.05880809	0.08199377	0.7770672
221.4	0.10211594	0.08542001	0.11881037	0.31654245	0.05881832	0.0820035	0.7770438
221.5	0.10214967	0.08546308	0.11880949	0.31659253	0.05882853	0.0820132	0.77702042
221.6	0.10218337	0.0855061	0.1188086	0.31664258	0.05883873	0.08202288	0.77699708
221.7	0.10221706	0.08554907	0.1188077	0.31669258	0.05884892	0.08203252	0.77697377
221.8	0.10225072	0.08559201	0.1188068	0.31674253	0.0588591	0.08204215	0.77695049
221.9	0.10228437	0.0856349	0.11880588	0.31679243	0.05886927	0.08205174	0.77692725
222	0.102318	0.08567775	0.11880496	0.31684229	0.05887943	0.08206131	0.77690404
222.1	0.10235161	0.08572056	0.11880402	0.3168921	0.05888958	0.08207086	0.77688086
222.2	0.10238519	0.08576332	0.11880307	0.31694186	0.05889972	0.08208038	0.77685772
222.3	0.10241876	0.08580604	0.11880211	0.31699158	0.05890985	0.08208988	0.77683461
222.4	0.10245231	0.08584872	0.11880114	0.31704124	0.05891996	0.08209934	0.77681154
222.5	0.10248584	0.08589136	0.11880015	0.31709085	0.05893007	0.08210878	0.7767885
222.6	0.10251935	0.08593395	0.11879916	0.31714041	0.05894018	0.0821182	0.7767655
222.7	0.10255285	0.08597651	0.11879815	0.31718991	0.05895027	0.08212759	0.77674254
222.8	0.10258632	0.08601902	0.11879713	0.31723937	0.05896035	0.08213696	0.77671961
222.9	0.10261978	0.08606149	0.11879611	0.31728877	0.05897042	0.08214631	0.77669671
223	0.10265321	0.08610392	0.11879507	0.31733812	0.05898048	0.08215563	0.77667386
223.1	0.10268663	0.0861463	0.11879403	0.31738742	0.05899053	0.08216493	0.77665103
223.2	0.10272003	0.08618865	0.11879298	0.31743666	0.05900057	0.0821742	0.77662824
223.3	0.10275341	0.08623095	0.11879192	0.31748585	0.05901061	0.08218345	0.77660547
223.4	0.10278677	0.08627322	0.11879085	0.317535	0.05902063	0.08219268	0.77658274
223.5	0.10282011	0.08631544	0.11878977	0.31758409	0.05903064	0.08220189	0.77656004
223.6	0.10285344	0.08635762	0.11878869	0.31763312	0.05904065	0.08221107	0.77653737
223.7	0.10288674	0.08639976	0.1187876	0.31768211	0.05905064	0.08222023	0.77651472
223.8	0.10292003	0.08644186	0.1187865	0.31773105	0.05906063	0.08222937	0.77649211
223.9	0.1029533	0.08648392	0.1187854	0.31777994	0.0590706	0.08223848	0.77646953
224	0.10298656	0.08652593	0.11878428	0.31782878	0.05908057	0.08224758	0.77644697
224.1	0.10301979	0.08656791	0.11878317	0.31787756	0.05909053	0.08225665	0.77642444
224.2	0.10305301	0.08660985	0.11878205	0.3179263	0.05910048	0.0822657	0.77640194
224.3	0.10308621	0.08665175	0.11878092	0.31797499	0.05911042	0.08227472	0.77637947
224.4	0.10311939	0.0866936	0.11877979	0.31802364	0.05912035	0.08228373	0.77635703
224.5	0.10315255	0.08673542	0.11877866	0.31807223	0.05913027	0.08229271	0.77633462
224.6	0.1031857	0.0867772	0.11877753	0.31812077	0.05914018	0.08230167	0.77631223
224.7	0.10321883	0.08681893	0.11877639	0.31816927	0.05915009	0.0823106	0.77628988
224.8	0.10325194	0.08686063	0.11877526	0.31821771	0.05915998	0.08231952	0.77626755
224.9	0.10328504	0.08690229	0.11877412	0.31826611	0.05916987	0.08232841	0.77624525
228.2	0.10436948	0.08825557	0.11873476	0.31983637	0.05949138	0.08261006	0.77552473
228.3	0.1044022	0.08829596	0.11873353	0.31988317	0.05950099	0.08261825	0.77550334
228.4	0.10443492	0.08833631	0.1187323	0.31992993	0.05951059	0.08262642	0.77548198
228.5	0.10446765	0.08837662	0.11873105	0.31997664	0.05952018	0.08263457	0.77546065
228.6	0.10450038	0.0884169	0.11872981	0.32002331	0.05952977	0.0826427	0.77543934
228.7	0.1045331	0.08845715	0.11872855	0.32006993	0.05953935	0.08265081	0.77541806
228.8	0.10456583	0.08849736	0.11872729	0.32011652	0.05954892	0.0826589	0.7753968
228.9	0.10459855	0.08853754	0.11872601	0.32016305	0.05955848	0.08266697	0.77537557
229	0.10463126	0.08857768	0.11872473	0.32020955	0.05956804	0.08267503	0.77535436
229.1	0.10466396	0.08861779	0.11872343	0.320256	0.05957759	0.08268306	0.77533318
229.2	0.10469665	0.08865787	0.11872213	0.3203024	0.05958713	0.08269108	0.77531202
229.3	0.10472933	0.08869791	0.11872082	0.32034877	0.05959666	0.08269907	0.77529089
229.4	0.10476199	0.08873792	0.11871949	0.32039509	0.05960619	0.08270705	0.77526979
229.5	0.10479464	0.0887779	0.11871816	0.32044136	0.05961571	0.08271501	0.77524871
229.6	0.10482727	0.08881785	0.11871682	0.3204876	0.05962522	0.08272295	0.77522765
229.7	0.10485988	0.08885777	0.11871547	0.32053379	0.05963473	0.08273087	0.77520662
229.8	0.10489247	0.08889765	0.11871411	0.32057994	0.05964422	0.08273877	0.77518562
229.9	0.10492505	0.08893751	0.11871274	0.32062605	0.05965372	0.08274666	0.77516464
230	0.10495762	0.08897733	0.11871136	0.32067211	0.0596632	0.08275452	0.77514368
230.1	0.10499016	0.08901713	0.11870997	0.32071814	0.05967268	0.08276237	0.77512275
230.2	0.10502269	0.08905689	0.11870858	0.32076412	0.05968215	0.0827702	0.77510185
230.3	0.1050552	0.08909663	0.11870717	0.32081006	0.05969162	0.08277801	0.77508096
230.4	0.1050877	0.08913633	0.11870577	0.32085596	0.05970108	0.0827858	0.77506011
230.5	0.10512018	0.08917601	0.11870435	0.32090181	0.05971053	0.08279357	0.77503928
230.6	0.10515265	0.08921567	0.11870293	0.32094763	0.05971998	0.08280133	0.77501847
230.7	0.10518511	0.08925529	0.11870151	0.3209934	0.05972943	0.08280907	0.77499769
230.8	0.10521754	0.08929489	0.11870008	0.32103914	0.05973886	0.08281678	0.77497693
230.9	0.10524997	0.08933446	0.11869864	0.32108483	0.05974829	0.08282449	0.7749562
231	0.10528238	0.089374	0.1186972	0.32113048	0.05975772	0.08283217	0.77493549
231.1	0.10531478	0.08941352	0.11869575	0.32117609	0.05976714	0.08283983	0.7749148
231.2	0.10534717	0.08945302	0.1186943	0.32122166	0.05977655	0.08284748	0.77489414
231.3	0.10537955	0.08949249	0.11869284	0.32126719	0.05978596	0.08285511	0.77487351
231.4	0.10541192	0.08953194	0.11869138	0.32131268	0.05979537	0.08286272	0.7748529
231.5	0.10544428	0.08957138	0.11868991	0.32135813	0.05980476	0.08287032	0.77483231
231.6	0.10547663	0.08961079	0.11868843	0.32140354	0.05981416	0.0828779	0.77481175
231.7	0.10550897	0.08965018	0.11868695	0.32144891	0.05982354	0.08288545	0.77479121
231.8	0.10554131	0.08968955	0.11868546	0.32149425	0.05983293	0.082893	0.77477069
231.9	0.10557364	0.0897289	0.11868396	0.32153954	0.0598423	0.08290052	0.7747502
232	0.10560597	0.08976823	0.11868245	0.3215848	0.05985168	0.08290803	0.77472974
232.1	0.1056383	0.08980753	0.11868094	0.32163001	0.05986104	0.08291552	0.77470929
232.2	0.10567063	0.08984682	0.11867942	0.32167519	0.0598704	0.08292299	0.77468887
232.3	0.10570295	0.08988608	0.11867789	0.32172033	0.05987976	0.08293045	0.77466848
232.4	0.10573528	0.08992532	0.11867635	0.32176543	0.05988911	0.08293788	0.77464811
232.5	0.10576761	0.08996454	0.11867481	0.3218105	0.05989846	0.08294531	0.77462776
232.6	0.10579994	0.09000373	0.11867325	0.32185553	0.05990781	0.08295271	0.77460744
232.7	0.10583227	0.0900429	0.1186717	0.32190052	0.05991715	0.0829601	0.77458713
232.8	0.10586459	0.09008204	0.11867013	0.32194547	0.05992648	0.08296747	0.77456686
232.9	0.10589691	0.09012115	0.11866856	0.32199039	0.05993581	0.08297482	0.7745466
233	0.10592921	0.09016023	0.11866697	0.32203527	0.05994514	0.08298216	0.77452637
233.1	0.1059615	0.09019928	0.11866539	0.32208012	0.05995446	0.08298948	0.77450617
233.2	0.10599378	0.0902383	0.11866379	0.32212493	0.05996377	0.08299678	0.77448599
233.3	0.10602604	0.09027728	0.1186622	0.32216971	0.05997309	0.08300407	0.77446583
233.4	0.10605828	0.09031622	0.1186606	0.32221445	0.0599824	0.08301134	0.77444569
233.5	0.1060905	0.09035513	0.11865899	0.32225915	0.0599917	0.08301859	0.77442558
233.6	0.1061227	0.09039401	0.11865739	0.32230383	0.060001	0.08302583	0.77440549
233.7	0.10615488	0.09043284	0.1186558	0.32234847	0.0600103	0.08303305	0.77438542
233.8	0.10618705	0.09047165	0.1186542	0.32239307	0.06001959	0.08304025	0.77436538
233.9	0.10621919	0.09051042	0.11865262	0.32243765	0.06002888	0.08304744	0.77434536
234	0.10625131	0.09054915	0.11865104	0.32248219	0.06003817	0.08305461	0.77432536
234.1	0.10628341	0.09058785	0.11864947	0.3225267	0.06004745	0.08306177	0.77430539
234.2	0.10631549	0.09062652	0.11864791	0.32257118	0.06005673	0.08306891	0.77428543
234.3	0.10634755	0.09066515	0.11864636	0.32261563	0.06006601	0.08307603	0.77426551
234.4	0.10637959	0.09070374	0.11864481	0.32266005	0.06007528	0.08308314	0.7742456
234.5	0.10641162	0.09074231	0.11864328	0.32270444	0.06008455	0.08309023	0.77422572
234.6	0.10644362	0.09078084	0.11864174	0.32274881	0.06009381	0.08309731	0.77420586
234.7	0.1064756	0.09081933	0.11864021	0.32279314	0.06010308	0.08310437	0.77418602
234.8	0.10650756	0.09085779	0.11863867	0.32283745	0.06011234	0.08311141	0.77416621
234.9	0.1065395	0.09089622	0.11863713	0.32288174	0.06012159	0.08311844	0.77414641
235	0.10657142	0.09093461	0.11863558	0.322926	0.06013085	0.08312546	0.77412665
235.1	0.10660333	0.09097297	0.11863402	0.32297023	0.0601401	0.08313245	0.7741069
235.2	0.10663521	0.0910113	0.11863245	0.32301445	0.06014935	0.08313943	0.77408717
235.3	0.10666707	0.09104959	0.11863086	0.32305864	0.0601586	0.0831464	0.77406747
235.4	0.10669892	0.09108785	0.11862927	0.32310281	0.06016784	0.08315335	0.77404779
235.5	0.10673074	0.09112608	0.11862766	0.32314696	0.06017708	0.08316029	0.77402814
235.6	0.10676255	0.09116427	0.11862604	0.3231911	0.06018632	0.08316721	0.7740085
235.7	0.10679434	0.09120243	0.11862441	0.32323522	0.06019556	0.08317411	0.77398889
235.8	0.10682611	0.09124055	0.11862276	0.32327932	0.06020479	0.083181	0.7739693
235.9	0.10685786	0.09127865	0.1186211	0.32332342	0.06021402	0.08318787	0.77394973
236	0.10688959	0.09131671	0.11861943	0.32336751	0.06022325	0.08319473	0.77393019
236.1	0.1069213	0.09135473	0.11861774	0.3234116	0.06023248	0.08320158	0.77391067
236.2	0.10695299	0.09139272	0.11861604	0.32345568	0.06024171	0.08320841	0.77389116
236.3	0.10698467	0.09143068	0.11861433	0.32349977	0.06025093	0.08321522	0.77387169
236.4	0.10701632	0.09146861	0.1186126	0.32354385	0.06026016	0.08322202	0.77385223
236.5	0.10704796	0.0915065	0.11861086	0.32358795	0.06026938	0.0832288	0.77383279
236.6	0.10707958	0.09154437	0.11860911	0.32363205	0.0602786	0.08323557	0.77381338
236.7	0.10711118	0.09158219	0.11860735	0.32367616	0.06028782	0.08324233	0.77379399
236.8	0.10714276	0.09161999	0.11860558	0.32372029	0.06029703	0.08324907	0.77377462
236.9	0.10717433	0.09165775	0.11860379	0.32376443	0.06030625	0.08325579	0.77375527
237	0.10720588	0.09169548	0.11860199	0.3238086	0.06031546	0.0832625	0.77373595
237.1	0.1072374	0.09173318	0.11860018	0.32385279	0.06032468	0.0832692	0.77371664
237.2	0.10726891	0.09177085	0.11859835	0.32389701	0.06033389	0.08327588	0.77369736
237.3	0.10730041	0.09180848	0.11859651	0.32394125	0.0603431	0.08328255	0.7736781
237.4	0.10733188	0.09184608	0.11859466	0.3239855	0.06035231	0.0832892	0.77365886
237.5	0.10736334	0.09188365	0.1185928	0.32402976	0.06036152	0.08329584	0.77363964
237.6	0.10739477	0.09192119	0.11859093	0.32407403	0.06037074	0.08330246	0.77362045
237.7	0.1074262	0.0919587	0.11858904	0.32411828	0.06037995	0.08330907	0.77360127
237.8	0.1074576	0.09199617	0.11858715	0.32416253	0.06038917	0.08331566	0.77358212
237.9	0.10748898	0.09203361	0.11858524	0.32420674	0.0603984	0.08332225	0.77356299
238	0.10752035	0.09207102	0.11858331	0.32425093	0.06040763	0.08332881	0.77354388
238.1	0.1075517	0.0921084	0.11858138	0.32429506	0.06041687	0.08333537	0.77352479
238.2	0.10758303	0.09214574	0.11857943	0.32433916	0.06042612	0.0833419	0.77350572
238.3	0.10761435	0.09218306	0.11857747	0.3243832	0.06043538	0.08334843	0.77348667
238.4	0.10764565	0.09222034	0.1185755	0.32442719	0.06044465	0.08335494	0.77346765
238.5	0.10767693	0.09225759	0.11857352	0.32447112	0.06045394	0.08336144	0.77344864
238.6	0.10770819	0.09229482	0.11857153	0.324515	0.06046325	0.08336792	0.77342966
238.7	0.10773944	0.092332	0.11856952	0.32455882	0.06047257	0.08337439	0.7734107
238.8	0.10777067	0.09236916	0.1185675	0.32460259	0.06048191	0.08338084	0.77339176
238.9	0.10780188	0.09240629	0.11856547	0.3246463	0.06049127	0.08338729	0.77337284
239	0.10783308	0.09244339	0.11856343	0.32468997	0.06050066	0.08339371	0.77335394
239.1	0.10786426	0.09248045	0.11856137	0.32473358	0.06051006	0.08340013	0.77333506
239.2	0.10789542	0.09251749	0.1185593	0.32477713	0.06051949	0.08340653	0.7733162
239.3	0.10792657	0.09255449	0.11855722	0.32482064	0.06052894	0.08341292	0.77329736
239.4	0.1079577	0.09259146	0.11855513	0.3248641	0.0605384	0.08341929	0.77327855
239.5	0.10798881	0.0926284	0.11855303	0.32490751	0.06054787	0.08342565	0.77325975
239.6	0.10801991	0.09266531	0.11855091	0.32495089	0.06055735	0.083432	0.77324098
239.7	0.10805099	0.09270219	0.11854878	0.32499421	0.06056682	0.08343833	0.77322222
239.8	0.10808205	0.09273904	0.11854664	0.32503749	0.06057629	0.08344465	0.77320349
239.9	0.1081131	0.09277586	0.11854449	0.32508073	0.06058576	0.08345096	0.77318478
240	0.10814413	0.09281265	0.11854233	0.32512392	0.06059521	0.08345726	0.77316609
